# Supplementary material for: A general pharmacodynamic interaction model identifies perpetrators and victims in drug interactions
Source: Nat Commun. 2017 Dec 14;8:2129. doi: 10.1038/s41467-017-01929-y (PMC5730559; doi:10.1038/s41467-017-01929-y)
Supplement: Supplementary file 1 — Supplementary Information [file 41467_2017_1929_MOESM1_ESM.pdf]

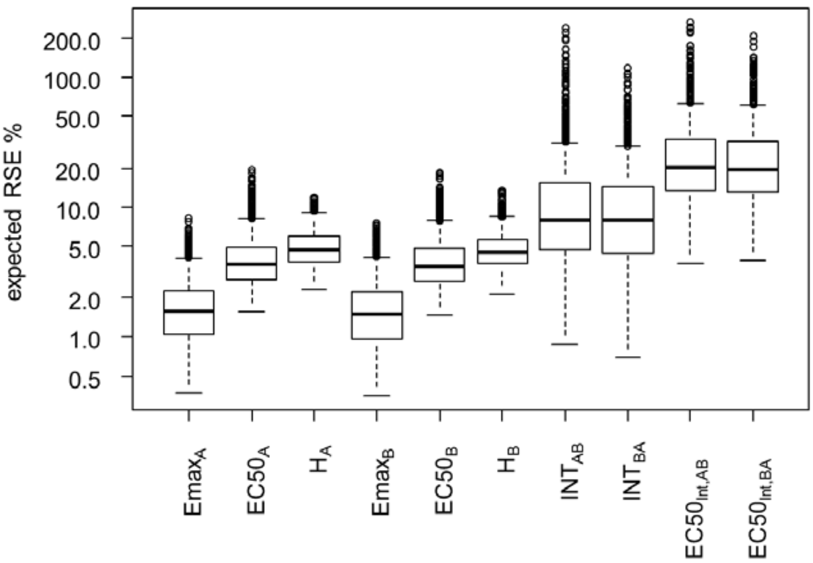

Supplementary Figure 1: Distribution of the expected relative standard errors (RSE) for the mono drug effects (E_{max}, EC50, H) and the GPDI interaction parameters (INT, $EC50_{INT}$) for the combined drug effects.

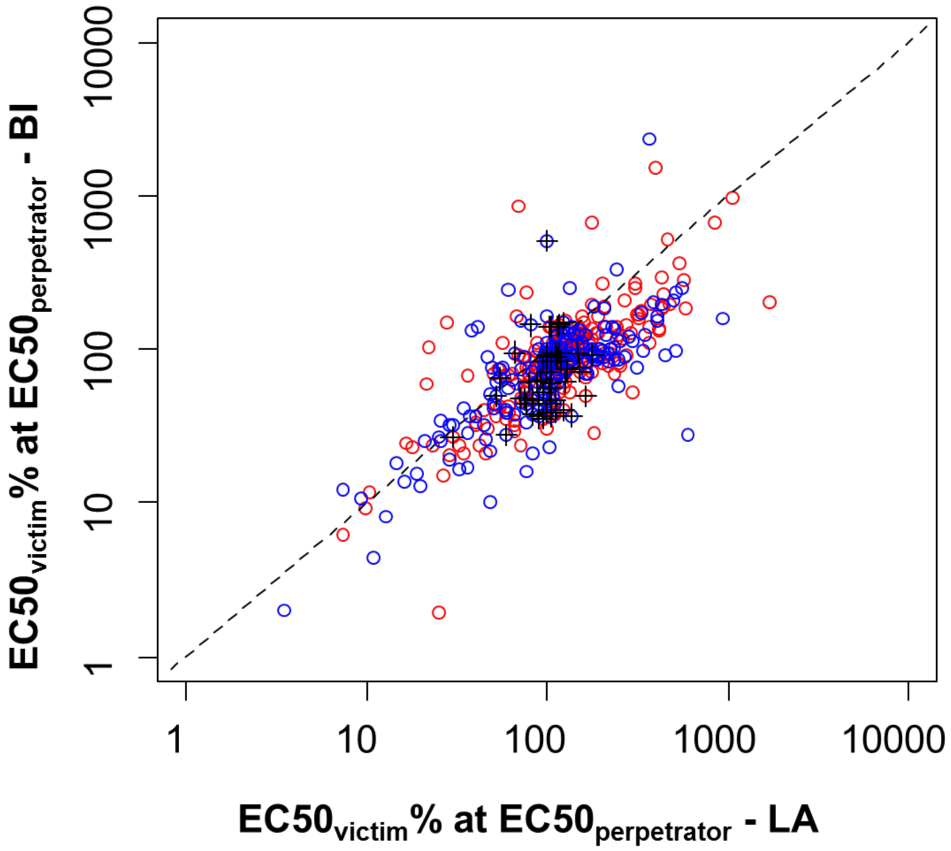

Supplementary Figure 2: Comparison of the estimated $EC50_{victim}$% at $EC50_{perpetrator}$ for Loewe Additivity (LA) and Bliss Independence (BI); estimated EC50B% at EC50A (red), estimated EC50A% at EC50B (blue) from the Cokol dataset; estimates from sham combinations (crossed).

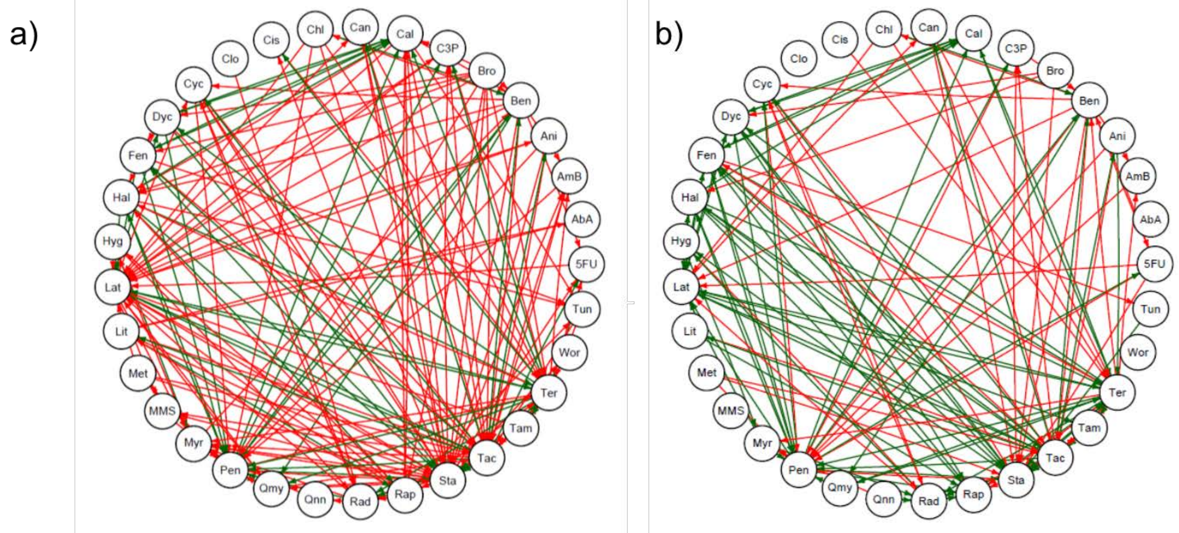

Supplementary Figure 3: Interaction network between the significant interactions from the unjoined Loewe Additivity (a) and Bliss Independence-based GPDI analyses (b). Note the overall higher synergy rate in case of Bliss Independence; arrows visualize direction of the PD interaction from the perpetrator drug to the victim drug, i.e. decrease of the victims EC50 resulting in antagonism (red) or increase of the victims EC50 resulting in synergism (green); note that interactions can be mono-directional or bi-directional of same polarity (joint antagonism or joint synergy) or opposite polarity leading to asymmetric bidirectional interactions; frequency of perpetrator and victim behaviour in the network is presented in b) to d). Abbreviations of the compounds: Aureobasidin A (AbA), Amphotericin B (AmB), Anisomycin (Ani), Benomyl (Ben), Bromopyruvate (Bro), CCCP (C3P), Calyculin A (Cal), Cantharidin (Can), Chlorzoxazone (Chl), Cisplatin (Cis), Clo (Clozapine), Cycloheximide (Cyc), Dyclonine (Dyc), Fenpropimorph (Fen), Haloperidol (Hal), Hygromycin (Hyg), Latrunculin B (Lat), Lithium (Lit), Methotrexate (Met), Methyl methanesulfonate (MMS), Myriocin (Myr), Pentamidine (Pen), Quinine (Qnn), Quinomycin (QMY), Radicicol (Rad), Rapamycin (Rap), Staurosporine (Sta), Tacrolimus (Tac), Tamoxifen (Tam), Tunicamycin (Tun) and Wortmannin (Wor).

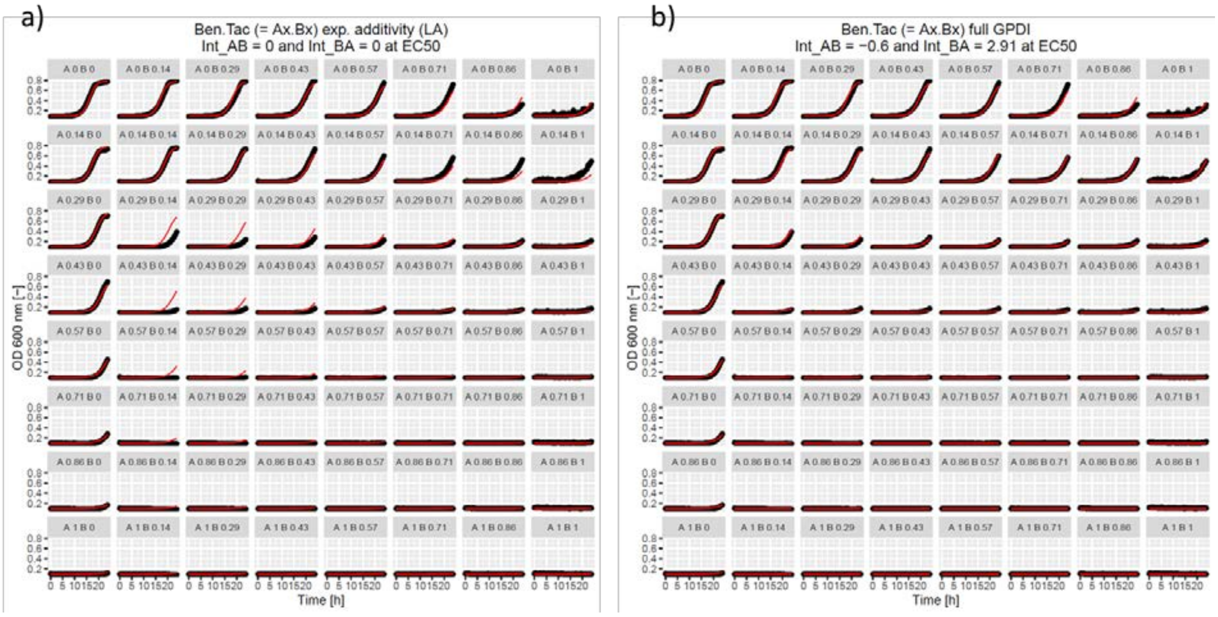

Supplementary Figure 4: Individual predictions (red) and observed time-courses (black) for the combination of Benomyl (Ben, drug A) and Tacrolimus (Tac, drug B) at concentrations AxBx (relative minimal inhibitory concentration[23]); Predicted Loewe Additivity from the single drug effects with both INT parameters set to zero reveals areas of synergy (e.g. A0.29 B0.24, observed response higher than pure additive response) and areas of antagonism (e.g. A0.14 B1, observed response lower than pure additive response) (a); Predicted response using estimated GPDI parameters indicates 'spot-on' fit of the data and quantified that Ben decreases the EC50 of Ben is decreased by Tac (INT_{AB} of -0.6) and the EC50 of TAC is increased by Ben (INT_{BA} of 2.91) capturing this asymmetric bidirectional interaction.

Supplementary Table 1: Parameter values of the Loewe-Additivity (LA)-based and Bliss-Independence (BI)-based GPDI models used for simulating the scenarios presented in Figure 1.

**Scenario / Parameterisation**	**$Emax_A$**	**$EC50_A$**	**H_A**	**$Emax_B$**	**$EC50_B$**	**H_B**	**Int_{AB}**	**Int_{BA}**	**$EC50_{INT,AB}$**	**$EC50_{INT,BA}$**	**$H_{INT,AB}$**	**$H_{INT,BA}$**
LA	1	50	4	1	50	4	0	0	25	25	1	1
LA - bidirectional synergy	1	50	4	1	50	4	-0.9	-0.9	25	25	1	1
LA - bidirectional antagonism	1	50	4	1	50	4	1	1	25	25	1	1
LA - bidirectional antagonism	1	50	4	1	50	4	-0.99	4	25	25	1	1
LA - monodirectional synergy	1	50	4	1	50	4	0	-0.9	25	25	1	1
LA - monodirectional antagonism	1	50	4	1	50	4	0	4	25	25	1	1
BI	1	50	4	1	50	4	0	0	25	25	1	1
BI - bidirectional synergy	1	50	4	1	50	4	-0.9	-0.9	25	25	1	1
BI - bidirectional antagonism	1	50	4	1	50	4	1	1	25	25	1	1
BI - bidirectional antagonism	1	50	4	1	50	4	-0.99	4	25	25	1	1
BI - monodirectional synergy	1	50	4	1	50	4	0	-0.9	25	25	1	1
BI - monodirectional antagonism	1	50	4	1	50	4	0	4	25	25	1	1
BI - different Emax	0.5	50	4	1	50	4	0	0	25	25	4	4
BI - buffering	0.5	50	4	1	50	4	0	-0.9999	50	50	4	4

BI - allosteric inhibition	0	50	4	1	50	4	0	-0.9999	50	50	4	4
BI - allosteric potentiation	0	0	4	0.5	50	4	0	1	50	50	4	4
BI - coalism	0.001	50	4	0.001	50	4	1000	1000	50	50	4	4

Supplementary Table 2: Comparison of directions in suppressive drug interactions analysed in Cokol 2014[30] with the directions derived from the GPDI model; > direction from drug A to B; < direction from drug B to A; derived by Cokol assuming the less potent drug antagonizes the more potent drug, or using the GPDI approach where INT_{AB} indicated a fractional change of EC50 of A (victim) at EC50 of B (perpetrator) resulting in < and/or INT_{BA} indicated a fractional change of EC50 of B (victim) at EC50 of B (perpetrator) resulting in >; significant interaction and resulting direction was concluded for the GPDI model if outside the additivity margin of -0.5 to 0.5.

Drug A	Drug B	Direction Cokol 2014	INT_AB	INT_BA	Direction GPDI	Comment
5FU	STA	>	1.24	0.27	<	different direction in Cokol 2014
AMB	PEN	>	-0.17	3.32	>	
ANI	LAT	><	0.74	0.76	><	
BEN	LAT	>	-0.04	2.12	>	
BEN	STA	>	0.09	0.92	>	
AMB	BEN	<	2.32	0.36	<	
5FU	BEN	<	1.07	-0.33	<	
BEN	HAL	>	0.04	3.19	>	
BEN	TUN	><	0.25	0.5	none	directions correct, but within additivity margin
BEN	CYC	>	-0.01	0.6	>	
ANI	BEN	<	0.4	0.23	none	directions correct, but within additivity margin
BEN	TAC	>	-0.6	2.91	> < (Synergy!)	asymmetric bidirectional with antagonism and synergy
BRO	STA	>	-0.02	15.92	>	
BRO	TAC	>	0.06	3.12	>	

BRO	RAP	>	0.43	1.58	>	
BRO	HAL	>	0.01	3.71	>	
CAL	TAC	>	0	0.64	>	
CAL	RAP	>	0.81	0.69	><	two directions, only one in Cokol 2014
CAN	STA	><	0.87	0.54	><	
CHL	LAT	>	0.06	3.24	>	
CHL	STA	>	0.27	0.77	>	
CIS	STA	>	1.15	0.49	<	different direction in Cokol 2014
CYC	LAT	>	1.02	0.46	<	different direction in Cokol 2014
CYC	STA	>	0.55	0.76	><	different direction in Cokol 2014
DYC	TUN	>	0.1	0.39	none	one direction correct, but within additivity margin
DYC	FEN	>	-0.03	1.86	>	
FEN	TUN	>	-0.04	3.43	>	
HAL	TUN	>	0.08	1.49	>	
LAT	QMY	>	0.94	0.91	><	two directions, only one in Cokol 2014
CIS	LAT	<	0.37	0.21	none	directions correct, but within additivity margin
CAN	LAT	>	3.97	-0.4	>	
MET	STA	>	0.46	4.34	>	
MET	TAC	>	0.04	2.27	>	
MET	PEN	><	0.51	2.08	><	
MYR	STA	><	1.14	1.14	><	
MYR	QNN	><	3.11	0.18	<	one direction only, two in Cokol 2014
QMY	STA	>	0.77	0.87	><	two directions, only one in Cokol 2014

LAT	RAD	<	3.06	0.13	<	
RAD	STA	>	1.38	0.38	<	different direction in Cokol 2014
CYC	RAD	<	0.8	2.07	><	two directions, only one in Cokol 2014
STA	WOR	>	0.36	0.7	>	
C3P	TAC	<	1.43	0.12	<	
RAP	TAC	<	2.85	0.37	<	
AMB	TAC	><	1.3	0.88	><	
QMY	TAC	<	0.32	0.53	<	
LAT	TAM	<	0.62	-0.41	<	
FEN	TER	<	0.32	-0.49	none	Within additivity margin, but rather asymmetric bidirectional
MYR	TER	<	4.5	0.16	<	
CHL	TER	><	0.11	7.47	>	two directions, only one in Cokol 2014
AMB	TER	<	3.32	0.3	<	

Supplementary Table 3: Combinations of terbinafin (Ter) with other drugs with targets outside the ergosterol pathway (Abbreviations see Figure 5); INT_{AB} represents the fractional change of the EC50 of drug A caused by drug B, i.e. quantifying the effect of drug B on A; INT_{BA} represents the fractional change of the EC50 of drug B caused by drug A, i.e. quantifying the effect of drug A on B.

**Drug A**	**Drug B**	**Int_{AB}**	**INT_{BA}**	**Effect of A on B**	**Effect of B on A**
Sta	Ter	-0.7	-0.02	-	SYN
Tac	Ter	2.69[§]	-0.93	SYN	ANT
Rap	Ter	-0.39	0.83	ANT	-
Rad	Ter	-0.75	-0.15	-	SYN
Qmy	Ter	-0.93	-0.35	-	SYN
Pen	Ter	-0.74	1.75	ANT	SYN
Myr	Ter	4.5	0.16	-	ANT
MMS	Ter	0.08	0.45	-	-
Met	Ter	0.28	0.49	-	-
Lat	Ter	-0.79	-0.54	SYN	SYN
Tun	Ter	-0.44	1.94	ANT	-

Supplementary Table 4: Combinations of bromopyruvate (Bro) with other drugs of the Cokol dataset (Abbreviations see Figure 5); INT_{AB} represents the fractional change of the EC50 of drug A caused by drug B, i.e. quantifying the effect of drug B on A; INT_{BA} represents the fractional change of the EC50 of drug B caused by drug A, i.e. quantifying the effect of drug A on B.

**Drug A**	**Drug B**	**Int_{AB}**	**INT_{BA}**	**Effect of A on B**	**Effect of B on A**
Bro	Ben	0.27	0.69	ANT	-
Bro	Cal	0.32	1.38	ANT	-
Bro	Dyc	-0.04	2.18	ANT	-
Bro	Fen	0.17	1.15	ANT	-
Bro	Hal	-0.01	3.71	ANT	-
Bro	Lat	0.24	1.22	ANT	-
Bro	Pen	0.19	1.18	ANT	-
Bro	Rap	0.43	1.58	ANT	-
Bro	Sta	-0.02	15.92	ANT	-
Bro	Tac	0.06	3.12	ANT	-
Bro	Ter	-0.11	1.56	ANT	-
Bro	Tun	0.15	0.32	-	-
