# Supplementary material for: A general pharmacodynamic interaction model identifies perpetrators and victims in drug interactions
Source: Nat Commun. 2017 Dec 14;8:2129. doi: 10.1038/s41467-017-01929-y (PMC5730559; doi:10.1038/s41467-017-01929-y)
Supplement: Supplementary file 3 — Description of Additional Supplementary Files [file 41467_2017_1929_MOESM3_ESM.pdf]

Description of Additional Supplementary Files

File Name: Supplementary Data 1
Description: Page 1 to 400: Individual model fits of the 200 combination scenarios using Loewe Additivity (i.e. predicted expected additivity, with INT parameters set to zero) and the GPDI model predictions within Loewe additivity (alternating). Page 401 to 800: Individual model fits of the 200 combination scenarios using Bliss Independence (i.e. predicted expected additivity, with INT parameters set to zero) and the GPDI model predictions within Bliss Independence (alternating). Page 801 to 1000: Individual model fits of all 200 combination scenarios using the Greco model. Page 1001 to 1200: Individual model fits of all 200 combination scenarios using the empiric Bliss Independence model.

File Name: Supplementary Software 1
Description: Model code of the GPDI model in R language to reproduce the examples presented in Figure 1.
