# Supplementary material for: A general pharmacodynamic interaction model identifies perpetrators and victims in drug interactions
Source: Nat Commun. 2017 Dec 14;8:2129. doi: 10.1038/s41467-017-01929-y (PMC5730559; doi:10.1038/s41467-017-01929-y)
Supplement: Supplementary file 5 — Supplementary Software 1 [file 41467_2017_1929_MOESM5_ESM.docx]

require(ggplot2)

#- Prepare dataset containing single drug and combination data ----------------------

#- define concentration vector of drug A

c.drug.A = seq(1,100,1)

#- define concentration vector of drug A

c.drug.B = seq(1,100,1)

#- setup dataset

dummy = NULL

df = NULL

for(i in 1:length(unique(c.drug.A))){

dummy = cbind(c.drug.A[i],c.drug.B)

df = rbind(df,dummy)

}

colnames(df) = c("DRUG_A","DRUG_B")

df = as.data.frame(df)

#- LA derived interaction model implicit solution ---------------------------

#-for LA, drugs A and B need to have the same Emax value!

LA_GPDI_EC50 = function(C_A,C_B,

parms){

Emax_A = parms["Emax_A"]

EC50_A = parms["EC50_A"]

H_A = parms["H_A"]

Emax_B = parms["Emax_B"]

EC50_B = parms["EC50_B"]

H_B = parms["H_B"]

Int_AB = parms["Int_AB"]

Int_BA = parms["Int_BA"]

EC50_Int_AB = parms["EC50_Int_AB"]

EC50_Int_BA = parms["EC50_Int_BA"]

E_comb=rep(0,length(C_A))

for(i in 1:length(C_A)){

if(C_A[i] == 0 & C_B[i] == 0){E_comb[i]=0}else{

E_comb[i]=optim(0.0001,

fn=function(E){

abs((C_A[i] / (EC50_A * (1 + C_B[i]/(EC50_Int_AB + C_B[i]) * Int_AB) * ((E/(Emax_A-E)) ^ (1/H_A))) +

C_B[i] / (EC50_B * (1 + C_A[i]/(EC50_Int_BA + C_A[i]) * Int_BA) * ((E/(Emax_B-E)) ^ (1/H_B))) - 1 ))},

lower=0,

upper=1,

method="Brent",

control=list(trace=F,

reltol=1e-30))$par

}}

return(E_comb)

}

parset = list(

"LA" =

c("Emax_A" = 1,

"EC50_A" = 50,

"H_A" = 4,

"Emax_B" = 1,

"EC50_B" = 50,

"H_B" = 4,

"Int_AB" = 0,

"Int_BA" = 0,

"EC50_Int_AB" = 25,

"EC50_Int_BA" = 25),

#- joint EC50 decrease

"LA - bidirectional synergy" =

c("Emax_A" = 1,

"EC50_A" = 50,

"H_A" = 4,

"Emax_B" = 1,

"EC50_B" = 50,

"H_B" = 4,

"Int_AB" = -.9,

"Int_BA" = -.9,

"EC50_Int_AB" = 25,

"EC50_Int_BA" = 25),

#- joint EC50 increase

"LA - bidirectional antagonism" =

c("Emax_A" = 1,

"EC50_A" = 50,

"H_A" = 4,

"Emax_B" = 1,

"EC50_B" = 50,

"H_B" = 4,

"Int_AB" = 1,

"Int_BA" = 1,

"EC50_Int_AB" = 25,

"EC50_Int_BA" = 25),

#- A decreases EC50B and B increases EC50A

"LA - bidirectional asymmetric interaction" =

c("Emax_A" = 1,

"EC50_A" = 50,

"H_A" = 4,

"Emax_B" = 1,

"EC50_B" = 50,

"H_B" = 4,

"Int_AB" = -.99,

"Int_BA" = 4,

"EC50_Int_AB" = 25,

"EC50_Int_BA" = 25),

#- A decreases EC50B

"LA - monodirectional synergy" =

c("Emax_A" = 1,

"EC50_A" = 50,

"H_A" = 4,

"Emax_B" = 1,

"EC50_B" = 50,

"H_B" = 4,

"Int_AB" = 0,

"Int_BA" = -0.9,

"EC50_Int_AB" = 25,

"EC50_Int_BA" = 25),

#- A increases EC50B

"LA - monodirectional antagonism" =

c("Emax_A" = 1,

"EC50_A" = 50,

"H_A" = 4,

"Emax_B" = 1,

"EC50_B" = 50,

"H_B" = 4,

"Int_AB" = 0,

"Int_BA" = 4,

"EC50_Int_AB" = 25,

"EC50_Int_BA" = 25))

dummy = NULL

for (i in 1:length(parset)){

df$E = LA_GPDI_EC50(df$DRUG_A,df$DRUG_B,parms=parset[[i]])

df$GROUP = names(parset[i])

dummy = rbind(dummy,df)

}

dummy$GROUP<- factor(dummy$GROUP, levels=c("LA",

"LA - monodirectional antagonism",

"LA - monodirectional synergy",

"LA - bidirectional antagonism",

"LA - bidirectional synergy",

"LA - bidirectional asymmetric interaction"))

plotset_LA_EC50 = dummy

#- Plot Heatmap ---------------------------------------------------------------------

ggplot(data=plotset_LA_EC50, aes(DRUG_A,DRUG_B,E)) +

geom_raster(aes(fill = E), interpolate = T) +

stat_contour(aes(z = E),colour="black", alpha=.2) +

scale_fill_gradient2(high="darkturquoise",mid="yellow", low="magenta", midpoint=0.5, name="Effect", breaks=seq(.1,1,by=.1)) +

theme(axis.text.x = element_text(colour = "black")) +

theme(axis.text.y = element_text(colour = "black")) +

theme(axis.title.x = element_text(face='bold')) +

theme(axis.title.y = element_text(face='bold',angle=90,vjust=0.25)) +

theme(strip.text.x = element_text(size = 9)) +

theme(legend.position = "right") +

scale_x_continuous(name="DRUG A [µM]") +

scale_y_continuous(name="DRUG B [µM]") +

facet_wrap(~GROUP)

#- BI derived interaction model explicit solution ---------------------------

BI_GPDI_EC50 = function(C_A,C_B,

parms){

Emax_A = parms["Emax_A"]

EC50_A = parms["EC50_A"]

H_A = parms["H_A"]

Emax_B = parms["Emax_B"]

EC50_B = parms["EC50_B"]

H_B = parms["H_B"]

Int_AB = parms["Int_AB"]

Int_BA = parms["Int_BA"]

EC50_Int_AB = parms["EC50_Int_AB"]

EC50_Int_BA = parms["EC50_Int_BA"]

E_A = (Emax_A * C_A^H_A) / ((EC50_A * (1 + C_B/(EC50_Int_AB + C_B) * Int_AB))^H_A + C_A^H_A)

E_B = (Emax_B * C_B^H_B) / ((EC50_B * (1 + C_A/(EC50_Int_BA + C_A) * Int_BA))^H_B + C_B^H_B)

E_comb = E_A + E_B - E_A * E_B

return(E_comb)

}

parset = list(

"BI" =

c("Emax_A" = 1,

"EC50_A" = 50,

"H_A" = 4,

"Emax_B" = 1,

"EC50_B" = 50,

"H_B" = 4,

"Int_AB" = 0,

"Int_BA" = 0,

"EC50_Int_AB" = 25,

"EC50_Int_BA" = 25),

#- joint EC50 decrease

"BI - bidirectional synergy" =

c("Emax_A" = 1,

"EC50_A" = 50,

"H_A" = 4,

"Emax_B" = 1,

"EC50_B" = 50,

"H_B" = 4,

"Int_AB" = -.9,

"Int_BA" = -.9,

"EC50_Int_AB" = 25,

"EC50_Int_BA" = 25),

#- joint EC50 increase

"BI - bidirectional antagonism" =

c("Emax_A" = 1,

"EC50_A" = 50,

"H_A" = 4,

"Emax_B" = 1,

"EC50_B" = 50,

"H_B" = 4,

"Int_AB" = 1,

"Int_BA" = 1,

"EC50_Int_AB" = 25,

"EC50_Int_BA" = 25),

#- A decreases EC50B and B increases EC50A

"BI - bidirectional asymmetric interaction" =

c("Emax_A" = 1,

"EC50_A" = 50,

"H_A" = 4,

"Emax_B" = 1,

"EC50_B" = 50,

"H_B" = 4,

"Int_AB" = -.99,

"Int_BA" = 4,

"EC50_Int_AB" = 25,

"EC50_Int_BA" = 25),

#- A decreases EC50B

"BI - monodirectional synergy" =

c("Emax_A" = 1,

"EC50_A" = 50,

"H_A" = 4,

"Emax_B" = 1,

"EC50_B" = 50,

"H_B" = 4,

"Int_AB" = 0,

"Int_BA" = -0.9,

"EC50_Int_AB" = 25,

"EC50_Int_BA" = 25),

#- A increases EC50B

"BI - monodirectional antagonism" =

c("Emax_A" = 1,

"EC50_A" = 50,

"H_A" = 4,

"Emax_B" = 1,

"EC50_B" = 50,

"H_B" = 4,

"Int_AB" = 0,

"Int_BA" = 4,

"EC50_Int_AB" = 25,

"EC50_Int_BA" = 25))

dummy = NULL

for (i in 1:length(parset)){

df$E = BI_GPDI_EC50(df$DRUG_A,df$DRUG_B,parms=parset[[i]])

df$GROUP = names(parset[i])

dummy = rbind(dummy,df)

}

dummy$GROUP<- factor(dummy$GROUP, levels=c("BI",

"BI - monodirectional antagonism",

"BI - monodirectional synergy",

"BI - bidirectional antagonism",

"BI - bidirectional synergy",

"BI - bidirectional asymmetric interaction"))

plotset_BI_EC50 = dummy

#- Plot Heatmap ---------------------------------------------------------------------

ggplot(data=plotset_BI_EC50, aes(DRUG_A,DRUG_B,E)) +

geom_raster(aes(fill = E), interpolate = T) +

stat_contour(aes(z = E),colour="black", alpha=.2) +

scale_fill_gradient2(high="darkturquoise",mid="yellow", low="magenta", midpoint=0.5, name="Effect", breaks=seq(.1,1,by=.1)) +

theme(axis.text.x = element_text(colour = "black")) +

theme(axis.text.y = element_text(colour = "black")) +

theme(axis.title.x = element_text(face='bold')) +

theme(axis.title.y = element_text(face='bold',angle=90,vjust=0.25)) +

theme(strip.text.x = element_text(size = 9)) +

theme(legend.position = "right") +

scale_x_continuous(name="DRUG A [µM]") +

scale_y_continuous(name="DRUG B [µM]") +

facet_wrap(~GROUP)

#- BI derived interaction model explicit solution ---------------------------

BI_GPDI_EMAX = function(C_A,C_B,

parms){

Emax_A = parms["Emax_A"]

EC50_A = parms["EC50_A"]

H_A = parms["H_A"]

Emax_B = parms["Emax_B"]

EC50_B = parms["EC50_B"]

H_B = parms["H_B"]

Int_AB = parms["Int_AB"]

Int_BA = parms["Int_BA"]

EC50_Int_AB = parms["EC50_Int_AB"]

EC50_Int_BA = parms["EC50_Int_BA"]

H_Int_AB = parms["H_Int_AB"]

H_Int_BA = parms["H_Int_BA"]

E_A = (Emax_A * (1 + C_B^H_Int_AB/(EC50_Int_AB^H_Int_AB + C_B^H_Int_AB) * Int_AB) * C_A^H_A) / ((EC50_A)^H_A + C_A^H_A)

E_B = (Emax_B * (1 + C_A^H_Int_BA/(EC50_Int_BA^H_Int_BA + C_A^H_Int_BA) * Int_BA) * C_B^H_B) / ((EC50_B)^H_B + C_B^H_B)

E_comb = E_A + E_B - E_A * E_B

return(E_comb)

}

parset = list(

"BI - different Emax" =

c("Emax_A" = .5,

"EC50_A" = 50,

"H_A" = 4,

"Emax_B" = 1,

"EC50_B" = 50,

"H_B" = 4,

"Int_AB" = 0,

"Int_BA" = 0,

"EC50_Int_AB" = 25,

"EC50_Int_BA" = 25,

"H_Int_AB" = 4,

"H_Int_BA" = 4),

#- A decreases Emax of B, active B

"BI - buffering" =

c("Emax_A" = 0.5,

"EC50_A" = 50,

"H_A" = 4,

"Emax_B" = 1,

"EC50_B" = 50,

"H_B" = 4,

"Int_AB" = 0,

"Int_BA" = -0.9999,

"EC50_Int_AB" = 50,

"EC50_Int_BA" = 50,

"H_Int_AB" = 4,

"H_Int_BA" = 4),

#- A decreases Emax of B, inactive B

"BI - allosteric inhibition" =

c("Emax_A" = 0,

"EC50_A" = 50,

"H_A" = 4,

"Emax_B" = 1,

"EC50_B" = 50,

"H_B" = 4,

"Int_AB" = 0,

"Int_BA" = -0.9999,

"EC50_Int_AB" = 50,

"EC50_Int_BA" = 50,

"H_Int_AB" = 4,

"H_Int_BA" = 4),

#- inactive A increases Emax of B -

"BI - allosteric potentiation" =

c("Emax_A" = 0,

"EC50_A" = 0,

"H_A" = 4,

"Emax_B" = .5,

"EC50_B" = 50,

"H_B" = 4,

"Int_AB" = 0,

"Int_BA" = 1,

"EC50_Int_AB" = 50,

"EC50_Int_BA" = 50,

"H_Int_AB" = 4,

"H_Int_BA" = 4),

#- inactive drugs only active in combination

"BI - coalism" =

c("Emax_A" = 0.001,

"EC50_A" = 50,

"H_A" = 4,

"Emax_B" = 0.001,

"EC50_B" = 50,

"H_B" = 4,

"Int_AB" = 1000,

"Int_BA" = 1000,

"EC50_Int_AB" = 50,

"EC50_Int_BA" = 50,

"H_Int_AB" = 4,

"H_Int_BA" = 4))

dummy = NULL

for (i in 1:length(parset)){

df$E = BI_GPDI_EMAX(df$DRUG_A,df$DRUG_B,parms=parset[[i]])

df$GROUP = names(parset[i])

dummy = rbind(dummy,df)

}

dummy$GROUP<- factor(dummy$GROUP, levels=c("BI - different Emax",

"BI - allosteric potentiation",

"BI - allosteric inhibition",

"BI - buffering",

"BI - coalism"))

plotset_BI_Emax = dummy

#- Plot Heatmap ---------------------------------------------------------------------

ggplot(data=plotset_BI_Emax, aes(DRUG_A,DRUG_B,E)) +

geom_raster(aes(fill = E), interpolate = T) +

stat_contour(aes(z = E),colour="black", alpha=.2) +

scale_fill_gradient2(high="darkturquoise",mid="yellow", low="magenta", midpoint=0.5, name="Effect", breaks=seq(.1,1,by=.1)) +

theme(axis.text.x = element_text(colour = "black")) +

theme(axis.text.y = element_text(colour = "black")) +

theme(axis.title.x = element_text(face='bold')) +

theme(axis.title.y = element_text(face='bold',angle=90,vjust=0.25)) +

theme(strip.text.x = element_text(size = 9)) +

theme(legend.position = "right") +

scale_x_continuous(name="DRUG A [µM]") +

scale_y_continuous(name="DRUG B [µM]") +

facet_wrap(~GROUP)
