# Supplementary material for: A general pharmacodynamic interaction model identifies perpetrators and victims in drug interactions
Source: Nat Commun. 2017 Dec 14;8:2129. doi: 10.1038/s41467-017-01929-y (PMC5730559; doi:10.1038/s41467-017-01929-y)
Supplement: Supplementary file 4 — Supplementary Data 1 [file 41467_2017_1929_MOESM4_ESM.pdf]

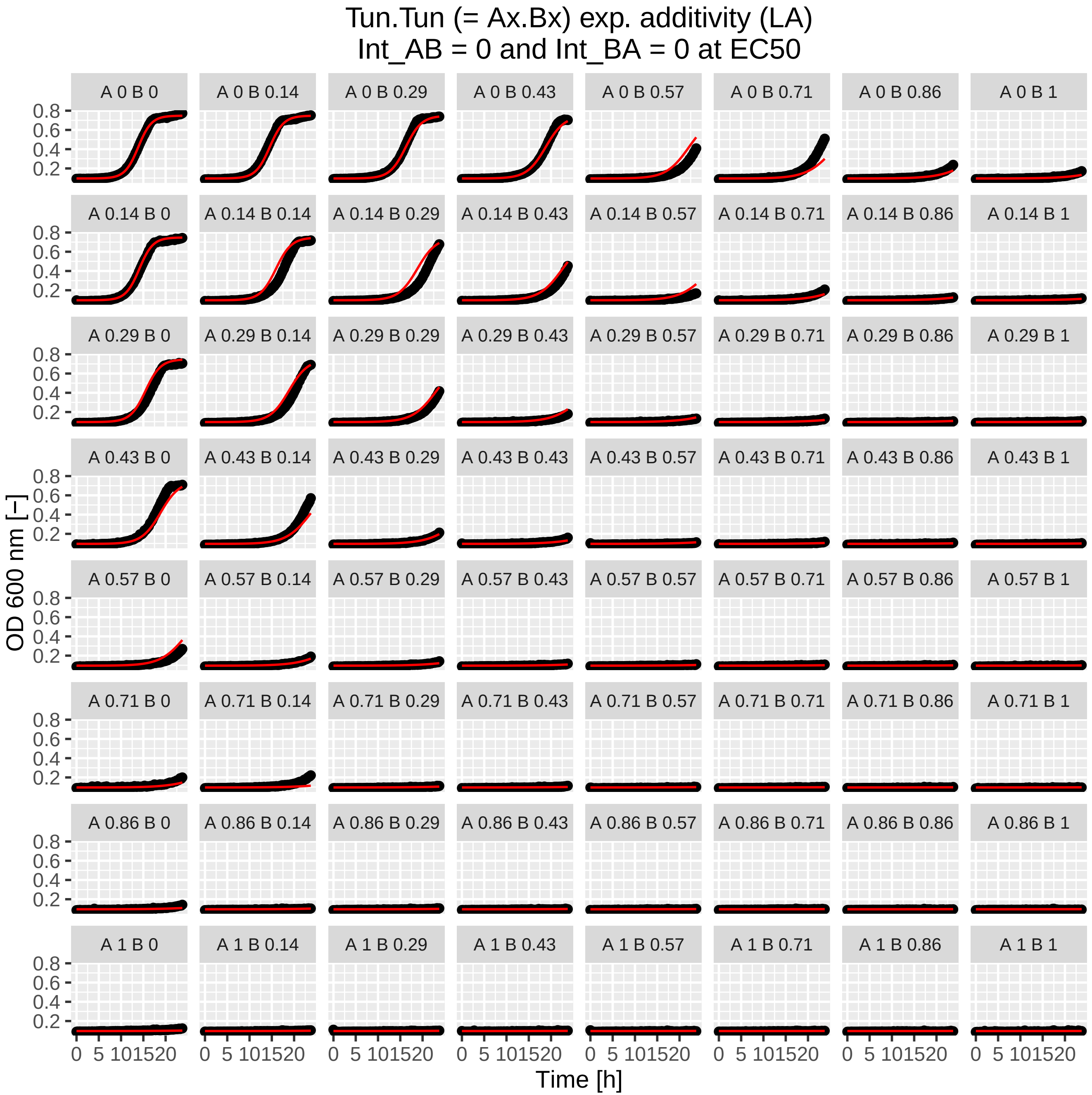
Tun.Tun (= Ax.Bx) exp. additivity (LA)
Int_AB = 0 and Int_BA = 0 at EC50
A 0 B 0
A 0 B 0.14
A 0 B 0.29
A 0 B 0.43
A 0 B 0.57
A 0 B 0.71
A 0 B 0.86
A 0 B 1
A 0.14 B 0
A 0.14 B 0.14
A 0.14 B 0.29
A 0.14 B 0.43
A 0.14 B 0.57
A 0.14 B 0.71
A 0.14 B 0.86
A 0.14 B 1
A 0.29 B 0
A 0.29 B 0.14
A 0.29 B 0.29
A 0.29 B 0.43
A 0.29 B 0.57
A 0.29 B 0.71
A 0.29 B 0.86
A 0.29 B 1
A 0.43 B 0
A 0.43 B 0.14
A 0.43 B 0.29
A 0.43 B 0.43
A 0.43 B 0.57
A 0.43 B 0.71
A 0.43 B 0.86
A 0.43 B 1
A 0.57 B 0
A 0.57 B 0.14
A 0.57 B 0.29
A 0.57 B 0.43
A 0.57 B 0.57
A 0.57 B 0.71
A 0.57 B 0.86
A 0.57 B 1
A 0.71 B 0
A 0.71 B 0.14
A 0.71 B 0.29
A 0.71 B 0.43
A 0.71 B 0.57
A 0.71 B 0.71
A 0.71 B 0.86
A 0.71 B 1
A 0.86 B 0
A 0.86 B 0.14
A 0.86 B 0.29
A 0.86 B 0.43
A 0.86 B 0.57
A 0.86 B 0.71
A 0.86 B 0.86
A 0.86 B 1
A 1 B 0
A 1 B 0.14
A 1 B 0.29
A 1 B 0.43
A 1 B 0.57
A 1 B 0.71
A 1 B 0.86
A 1 B 1
OD 600 nm [–]
0.8
0.6
0.4
0.2
0 5 101520
Time [h]

Tun.Tun (= Ax.Bx) full GPDI
Int_AB = 0.79 and Int_BA = −0.29 at EC50

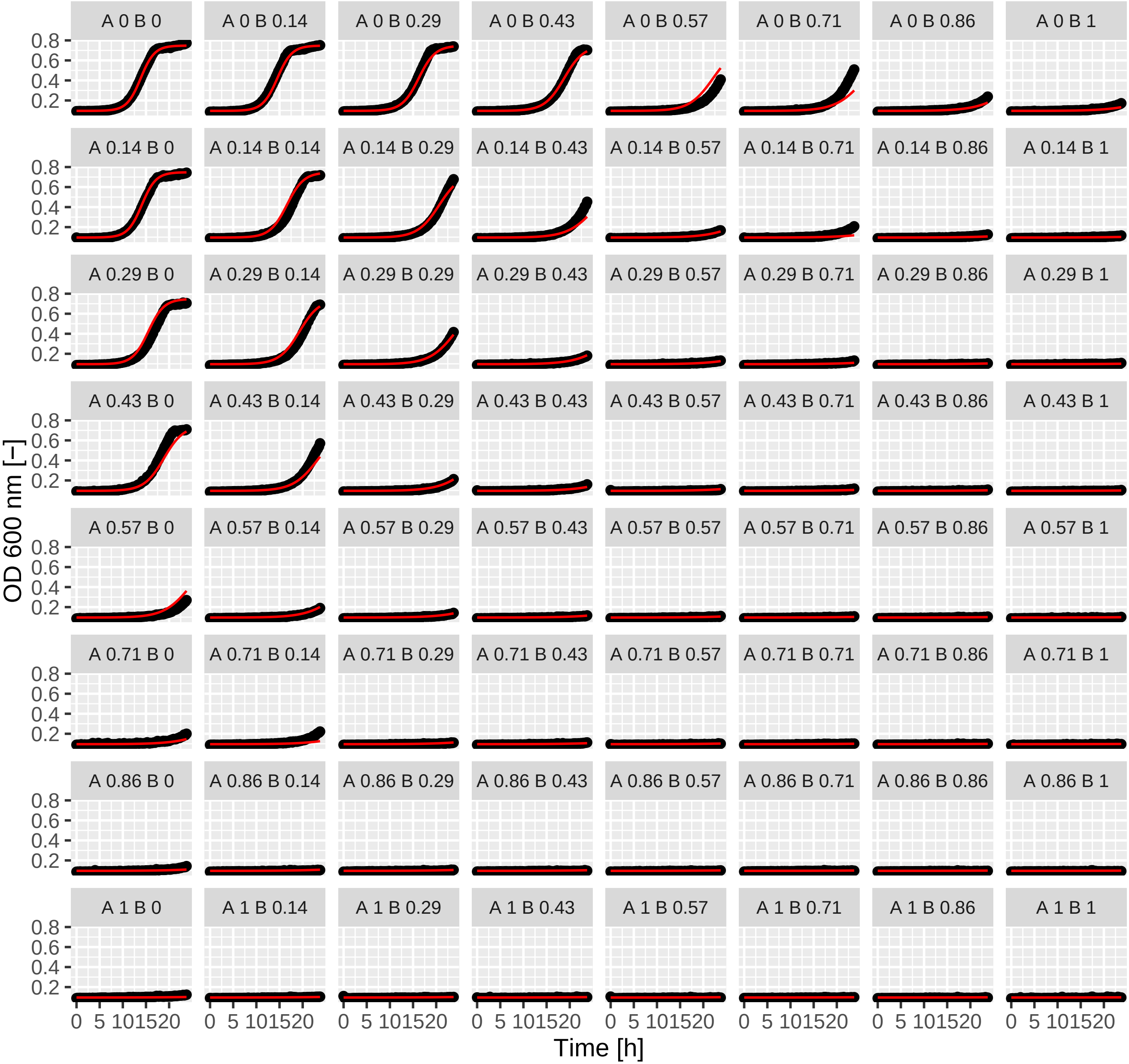

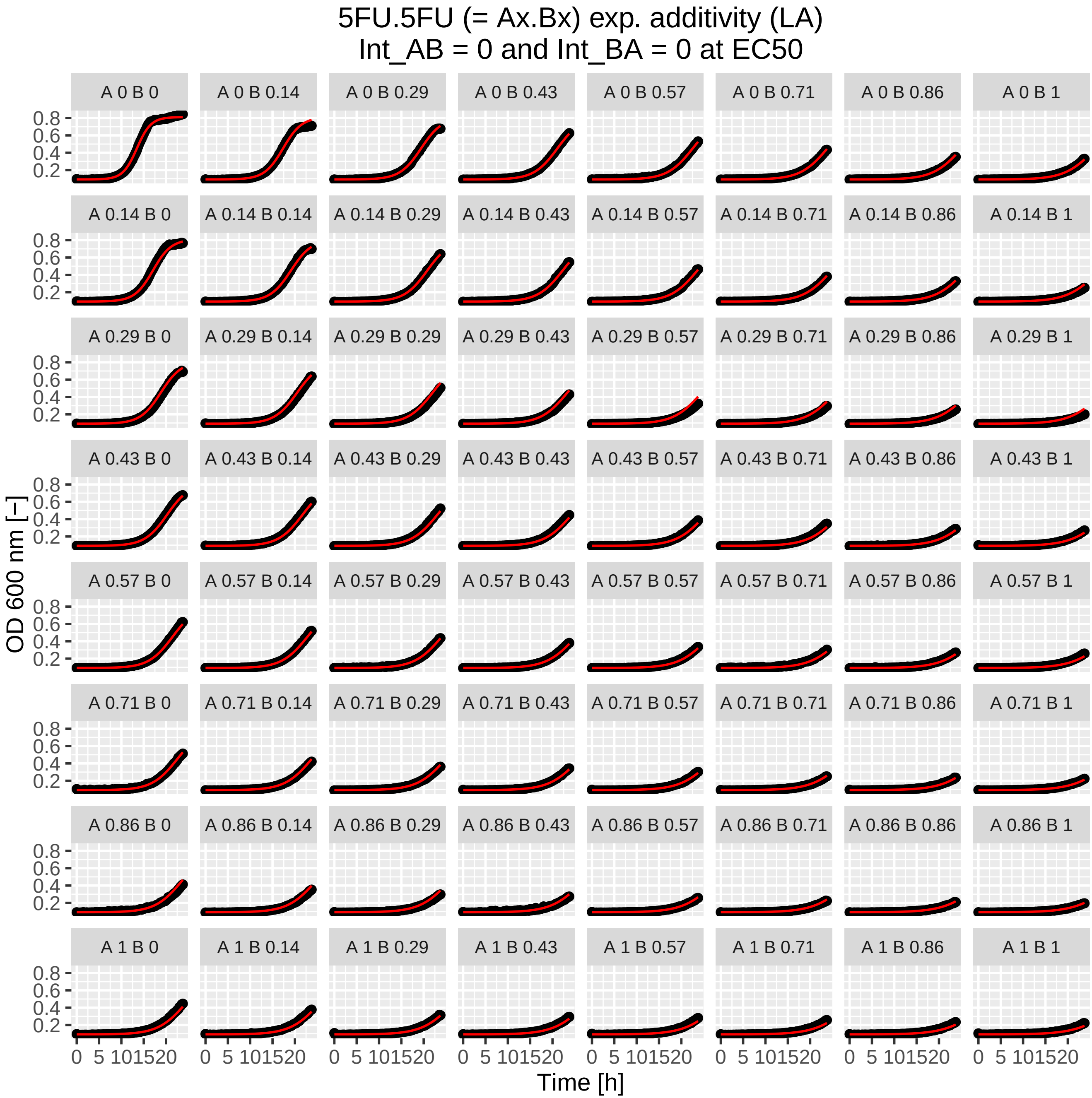

5FU.5FU (= Ax.Bx) exp. additivity (LA)
Int_AB = 0 and Int_BA = 0 at EC50
A 0 B 0
A 0 B 0.14
A 0 B 0.29
A 0 B 0.43
A 0 B 0.57
A 0 B 0.71
A 0 B 0.86
A 0 B 1
A 0.14 B 0
A 0.14 B 0.14
A 0.14 B 0.29
A 0.14 B 0.43
A 0.14 B 0.57
A 0.14 B 0.71
A 0.14 B 0.86
A 0.14 B 1
A 0.29 B 0
A 0.29 B 0.14
A 0.29 B 0.29
A 0.29 B 0.43
A 0.29 B 0.57
A 0.29 B 0.71
A 0.29 B 0.86
A 0.29 B 1
A 0.43 B 0
A 0.43 B 0.14
A 0.43 B 0.29
A 0.43 B 0.43
A 0.43 B 0.57
A 0.43 B 0.71
A 0.43 B 0.86
A 0.43 B 1
A 0.57 B 0
A 0.57 B 0.14
A 0.57 B 0.29
A 0.57 B 0.43
A 0.57 B 0.57
A 0.57 B 0.71
A 0.57 B 0.86
A 0.57 B 1
A 0.71 B 0
A 0.71 B 0.14
A 0.71 B 0.29
A 0.71 B 0.43
A 0.71 B 0.57
A 0.71 B 0.71
A 0.71 B 0.86
A 0.71 B 1
A 0.86 B 0
A 0.86 B 0.14
A 0.86 B 0.29
A 0.86 B 0.43
A 0.86 B 0.57
A 0.86 B 0.71
A 0.86 B 0.86
A 0.86 B 1
A 1 B 0
A 1 B 0.14
A 1 B 0.29
A 1 B 0.43
A 1 B 0.57
A 1 B 0.71
A 1 B 0.86
A 1 B 1
OD 600 nm [–]
0.8
0.6
0.4
0.2
0 5 101520
Time [h]

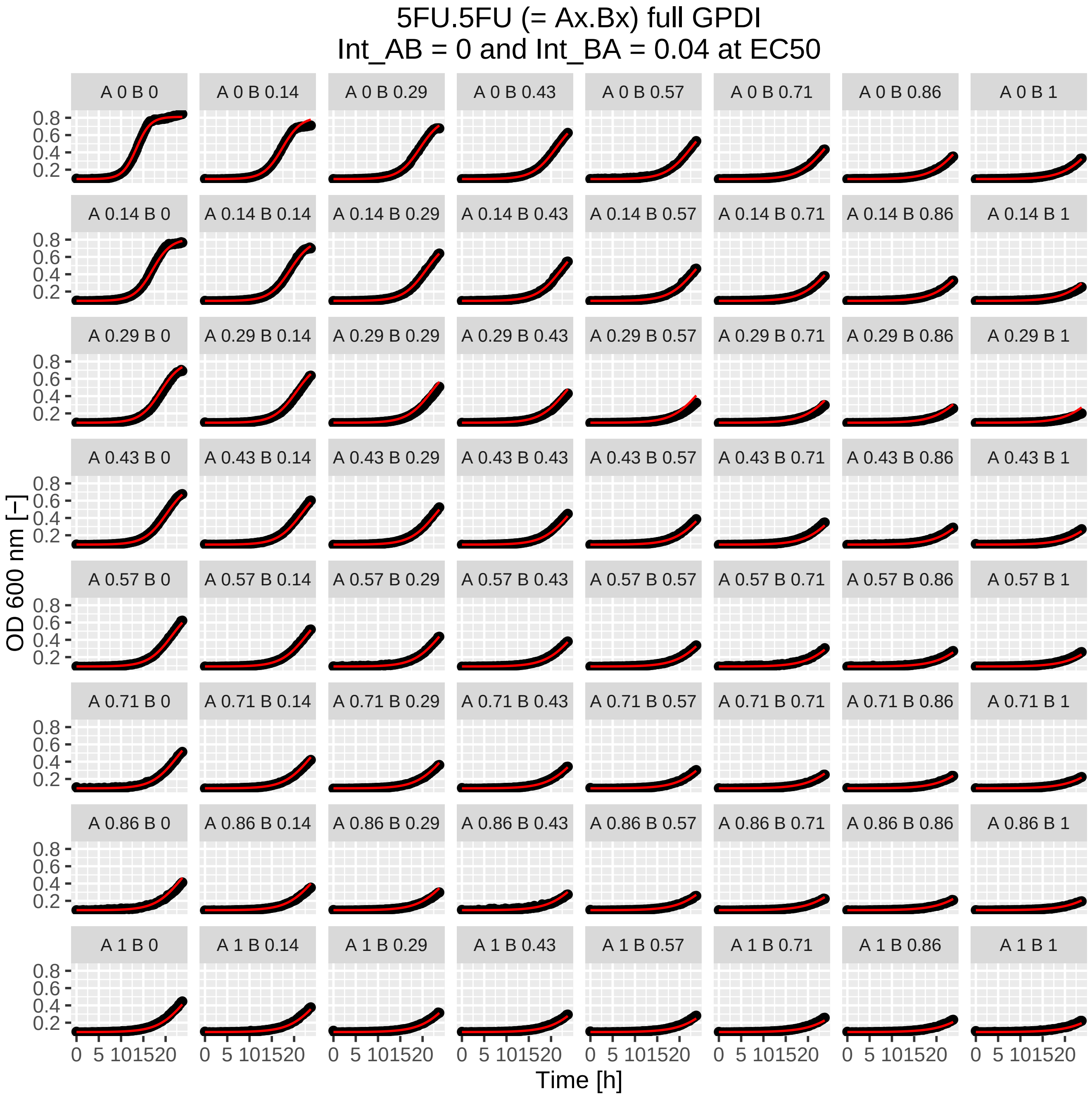
5FU.5FU (= Ax.Bx) full GPDI
Int_AB = 0 and Int_BA = 0.04 at EC50
A 0 B 0
A 0 B 0.14
A 0 B 0.29
A 0 B 0.43
A 0 B 0.57
A 0 B 0.71
A 0 B 0.86
A 0 B 1
A 0.14 B 0
A 0.14 B 0.14
A 0.14 B 0.29
A 0.14 B 0.43
A 0.14 B 0.57
A 0.14 B 0.71
A 0.14 B 0.86
A 0.14 B 1
A 0.29 B 0
A 0.29 B 0.14
A 0.29 B 0.29
A 0.29 B 0.43
A 0.29 B 0.57
A 0.29 B 0.71
A 0.29 B 0.86
A 0.29 B 1
A 0.43 B 0
A 0.43 B 0.14
A 0.43 B 0.29
A 0.43 B 0.43
A 0.43 B 0.57
A 0.43 B 0.71
A 0.43 B 0.86
A 0.43 B 1
A 0.57 B 0
A 0.57 B 0.14
A 0.57 B 0.29
A 0.57 B 0.43
A 0.57 B 0.57
A 0.57 B 0.71
A 0.57 B 0.86
A 0.57 B 1
A 0.71 B 0
A 0.71 B 0.14
A 0.71 B 0.29
A 0.71 B 0.43
A 0.71 B 0.57
A 0.71 B 0.71
A 0.71 B 0.86
A 0.71 B 1
A 0.86 B 0
A 0.86 B 0.14
A 0.86 B 0.29
A 0.86 B 0.43
A 0.86 B 0.57
A 0.86 B 0.71
A 0.86 B 0.86
A 0.86 B 1
A 1 B 0
A 1 B 0.14
A 1 B 0.29
A 1 B 0.43
A 1 B 0.57
A 1 B 0.71
A 1 B 0.86
A 1 B 1
OD 600 nm [–]
0.8
0.6
0.4
0.2
0 5 101520
Time [h]

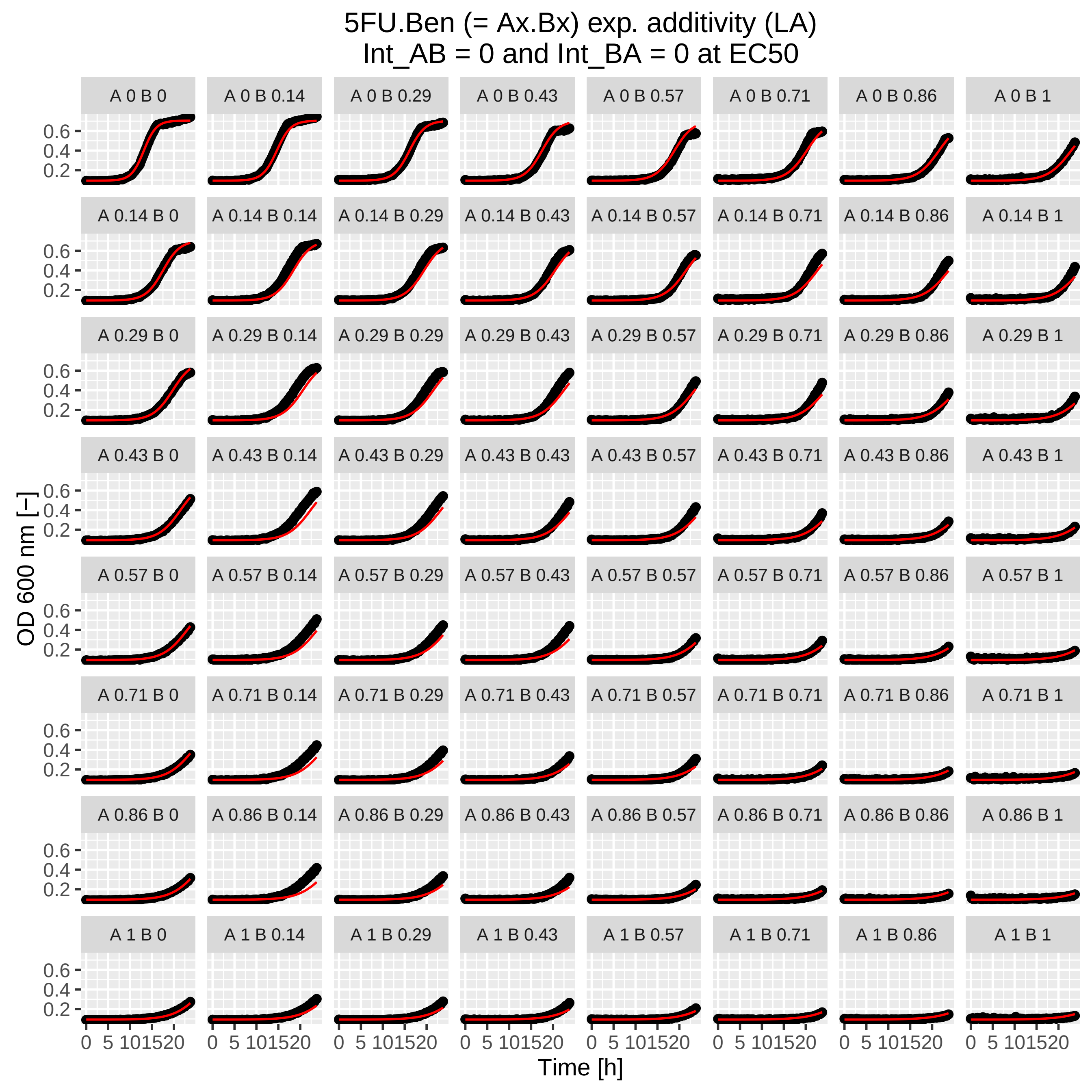
5FU.Ben (= Ax.Bx) exp. additivity (LA)
Int_AB = 0 and Int_BA = 0 at EC50
OD 600 nm [–]
Time [h]
A 0 B 0
A 0 B 0.14
A 0 B 0.29
A 0 B 0.43
A 0 B 0.57
A 0 B 0.71
A 0 B 0.86
A 0 B 1
A 1 B 0
A 1 B 0.14
A 1 B 0.29
A 1 B 0.43
A 1 B 0.57
A 1 B 0.71
A 1 B 0.86
A 1 B 1

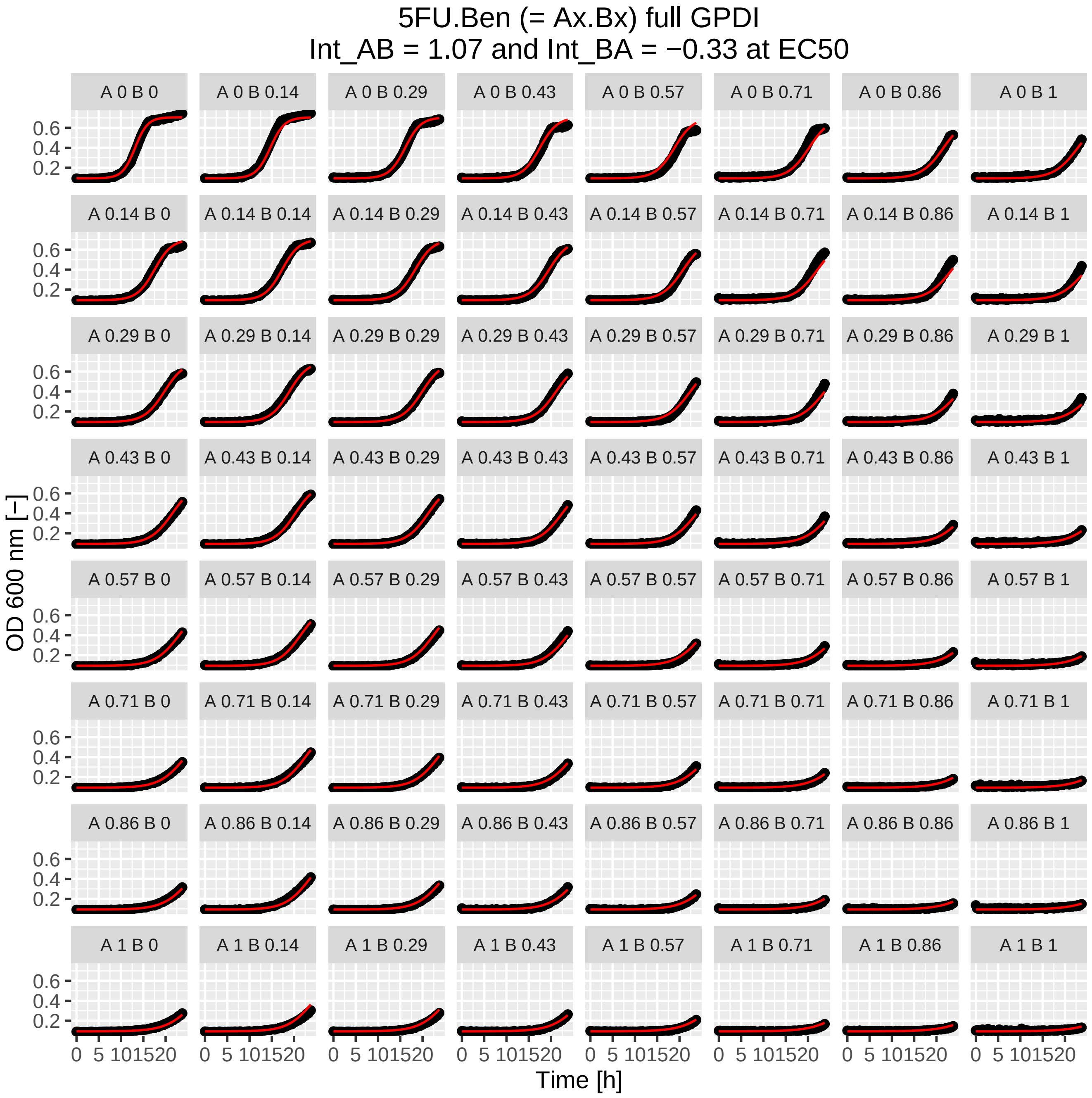
5FU.Ben (= Ax.Bx) full GPDI
Int_AB = 1.07 and Int_BA = −0.33 at EC50
A 0 B 0
A 0 B 0.14
A 0 B 0.29
A 0 B 0.43
A 0 B 0.57
A 0 B 0.71
A 0 B 0.86
A 0 B 1
A 0.14 B 0
A 0.14 B 0.14
A 0.14 B 0.29
A 0.14 B 0.43
A 0.14 B 0.57
A 0.14 B 0.71
A 0.14 B 0.86
A 0.14 B 1
A 0.29 B 0
A 0.29 B 0.14
A 0.29 B 0.29
A 0.29 B 0.43
A 0.29 B 0.57
A 0.29 B 0.71
A 0.29 B 0.86
A 0.29 B 1
A 0.43 B 0
A 0.43 B 0.14
A 0.43 B 0.29
A 0.43 B 0.43
A 0.43 B 0.57
A 0.43 B 0.71
A 0.43 B 0.86
A 0.43 B 1
A 0.57 B 0
A 0.57 B 0.14
A 0.57 B 0.29
A 0.57 B 0.43
A 0.57 B 0.57
A 0.57 B 0.71
A 0.57 B 0.86
A 0.57 B 1
A 0.71 B 0
A 0.71 B 0.14
A 0.71 B 0.29
A 0.71 B 0.43
A 0.71 B 0.57
A 0.71 B 0.71
A 0.71 B 0.86
A 0.71 B 1
A 0.86 B 0
A 0.86 B 0.14
A 0.86 B 0.29
A 0.86 B 0.43
A 0.86 B 0.57
A 0.86 B 0.71
A 0.86 B 0.86
A 0.86 B 1
A 1 B 0
A 1 B 0.14
A 1 B 0.29
A 1 B 0.43
A 1 B 0.57
A 1 B 0.71
A 1 B 0.86
A 1 B 1
0.6
0.4
0.2
OD 600 nm [−]
0 5 101520
Time [h]

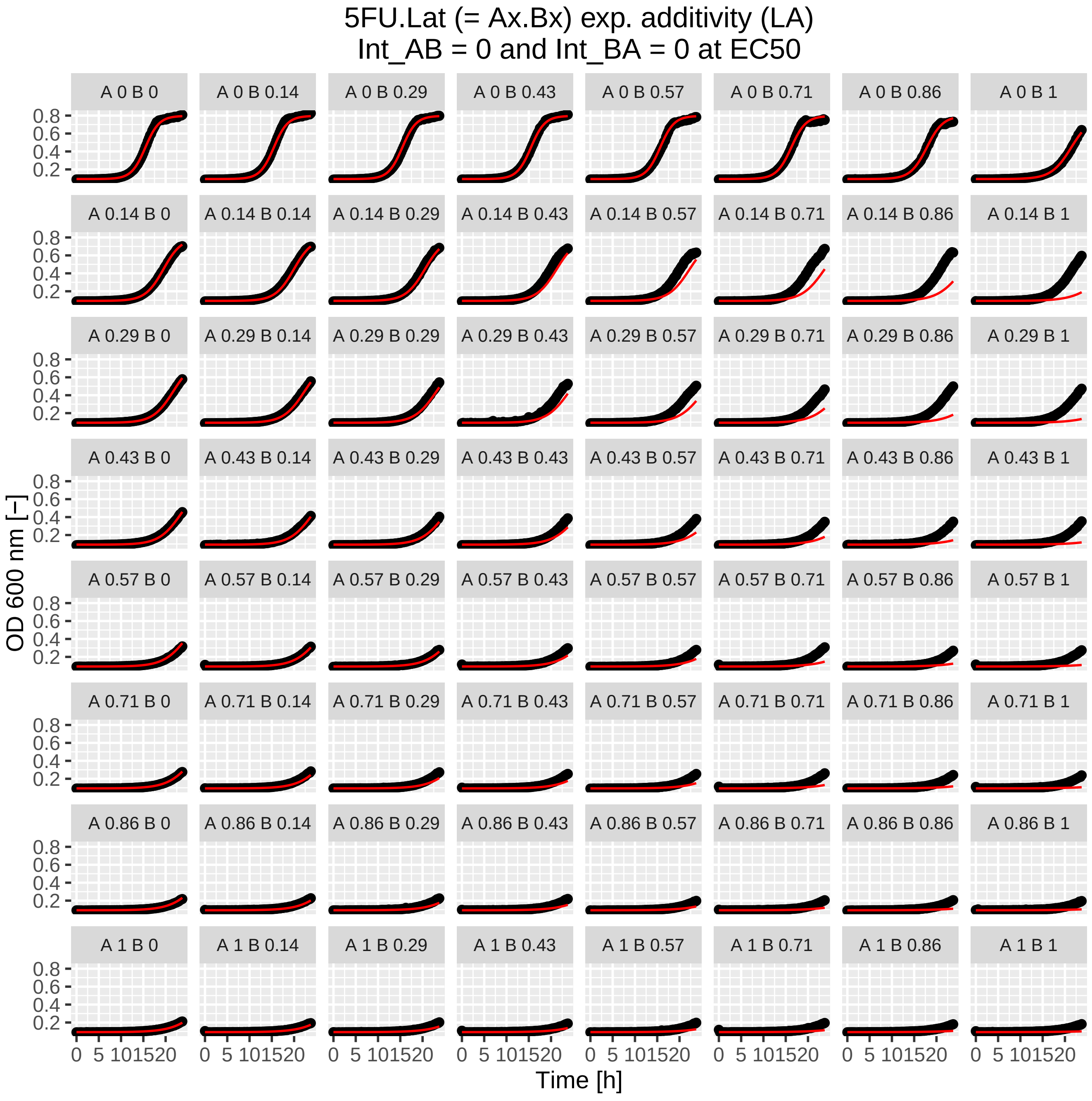

5FU.Lat (= Ax.Bx) exp. additivity (LA)
Int_AB = 0 and Int_BA = 0 at EC50
A 0 B 0
A 0 B 0.14
A 0 B 0.29
A 0 B 0.43
A 0 B 0.57
A 0 B 0.71
A 0 B 0.86
A 0 B 1
A 0.14 B 0
A 0.14 B 0.14
A 0.14 B 0.29
A 0.14 B 0.43
A 0.14 B 0.57
A 0.14 B 0.71
A 0.14 B 0.86
A 0.14 B 1
A 0.29 B 0
A 0.29 B 0.14
A 0.29 B 0.29
A 0.29 B 0.43
A 0.29 B 0.57
A 0.29 B 0.71
A 0.29 B 0.86
A 0.29 B 1
A 0.43 B 0
A 0.43 B 0.14
A 0.43 B 0.29
A 0.43 B 0.43
A 0.43 B 0.57
A 0.43 B 0.71
A 0.43 B 0.86
A 0.43 B 1
A 0.57 B 0
A 0.57 B 0.14
A 0.57 B 0.29
A 0.57 B 0.43
A 0.57 B 0.57
A 0.57 B 0.71
A 0.57 B 0.86
A 0.57 B 1
A 0.71 B 0
A 0.71 B 0.14
A 0.71 B 0.29
A 0.71 B 0.43
A 0.71 B 0.57
A 0.71 B 0.71
A 0.71 B 0.86
A 0.71 B 1
A 0.86 B 0
A 0.86 B 0.14
A 0.86 B 0.29
A 0.86 B 0.43
A 0.86 B 0.57
A 0.86 B 0.71
A 0.86 B 0.86
A 0.86 B 1
A 1 B 0
A 1 B 0.14
A 1 B 0.29
A 1 B 0.43
A 1 B 0.57
A 1 B 0.71
A 1 B 0.86
A 1 B 1
OD 600 nm [–]
0.2
0.4
0.6
0.8
0
5
10
15
20
Time [h]

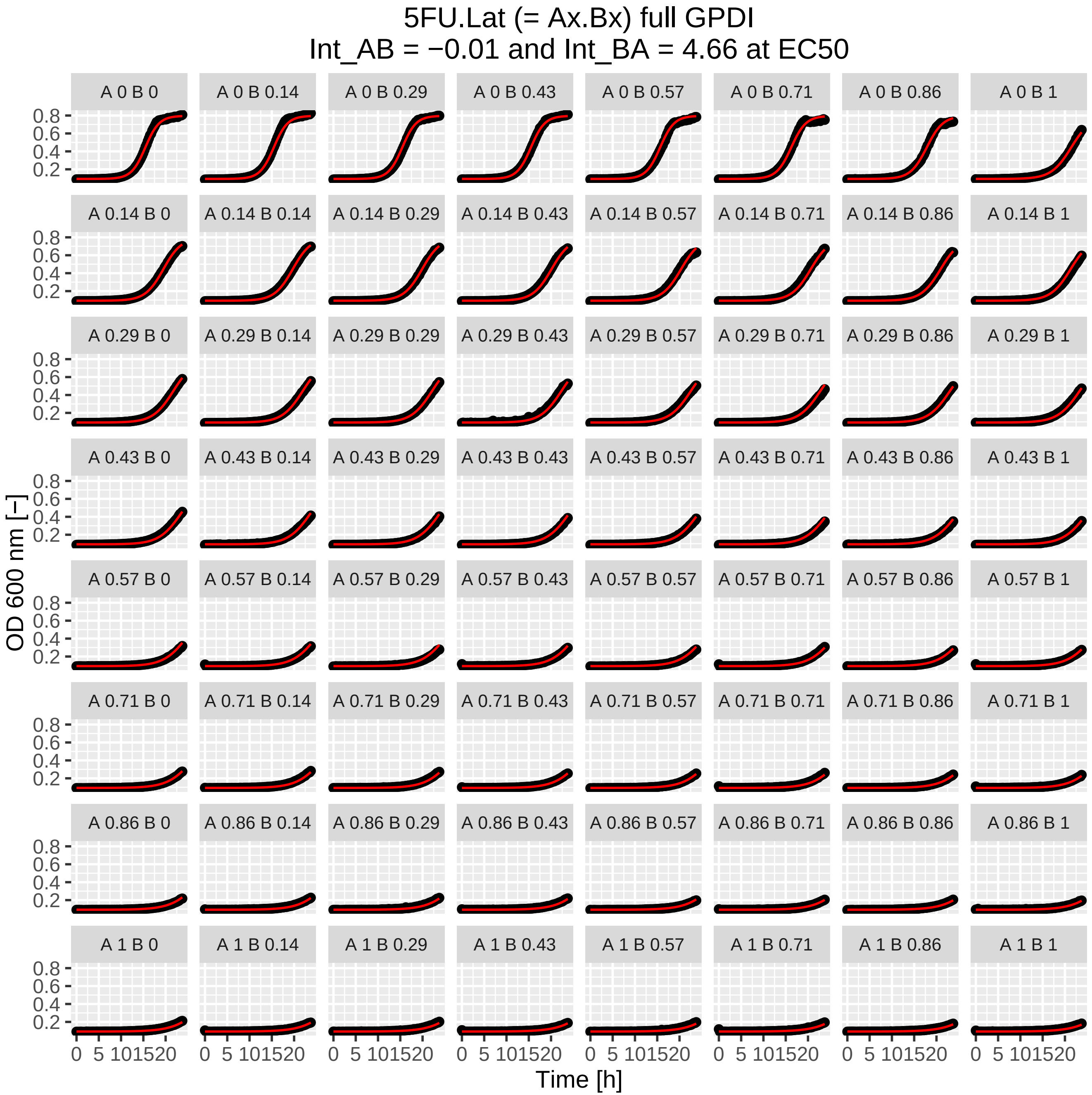
5FU.Lat (= Ax.Bx) full GPDI
Int_AB = −0.01 and Int_BA = 4.66 at EC50
A 0 B 0
A 0 B 0.14
A 0 B 0.29
A 0 B 0.43
A 0 B 0.57
A 0 B 0.71
A 0 B 0.86
A 0 B 1
A 0.14 B 0
A 0.14 B 0.14
A 0.14 B 0.29
A 0.14 B 0.43
A 0.14 B 0.57
A 0.14 B 0.71
A 0.14 B 0.86
A 0.14 B 1
A 0.29 B 0
A 0.29 B 0.14
A 0.29 B 0.29
A 0.29 B 0.43
A 0.29 B 0.57
A 0.29 B 0.71
A 0.29 B 0.86
A 0.29 B 1
A 0.43 B 0
A 0.43 B 0.14
A 0.43 B 0.29
A 0.43 B 0.43
A 0.43 B 0.57
A 0.43 B 0.71
A 0.43 B 0.86
A 0.43 B 1
A 0.57 B 0
A 0.57 B 0.14
A 0.57 B 0.29
A 0.57 B 0.43
A 0.57 B 0.57
A 0.57 B 0.71
A 0.57 B 0.86
A 0.57 B 1
A 0.71 B 0
A 0.71 B 0.14
A 0.71 B 0.29
A 0.71 B 0.43
A 0.71 B 0.57
A 0.71 B 0.71
A 0.71 B 0.86
A 0.71 B 1
A 0.86 B 0
A 0.86 B 0.14
A 0.86 B 0.29
A 0.86 B 0.43
A 0.86 B 0.57
A 0.86 B 0.71
A 0.86 B 0.86
A 0.86 B 1
A 1 B 0
A 1 B 0.14
A 1 B 0.29
A 1 B 0.43
A 1 B 0.57
A 1 B 0.71
A 1 B 0.86
A 1 B 1
0.8
0.6
0.4
0.2
0 5 101520
OD 600 nm [−]
Time [h]

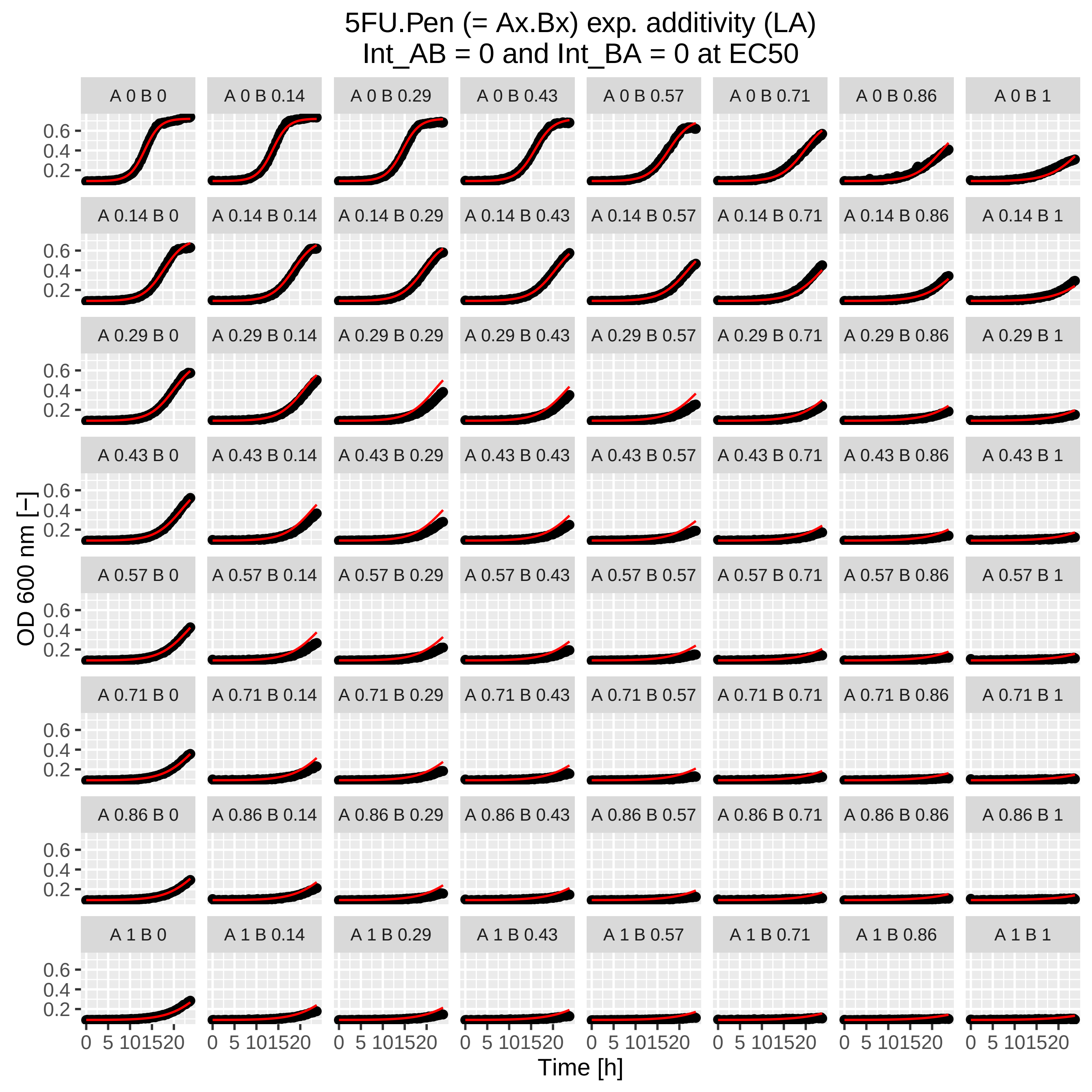
5FU.Pen (= Ax.Bx) exp. additivity (LA)
Int_AB = 0 and Int_BA = 0 at EC50
A 0 B 0
A 0 B 0.14
A 0 B 0.29
A 0 B 0.43
A 0 B 0.57
A 0 B 0.71
A 0 B 0.86
A 0 B 1
A 0.14 B 0
A 0.14 B 0.14
A 0.14 B 0.29
A 0.14 B 0.43
A 0.14 B 0.57
A 0.14 B 0.71
A 0.14 B 0.86
A 0.14 B 1
A 0.29 B 0
A 0.29 B 0.14
A 0.29 B 0.29
A 0.29 B 0.43
A 0.29 B 0.57
A 0.29 B 0.71
A 0.29 B 0.86
A 0.29 B 1
A 0.43 B 0
A 0.43 B 0.14
A 0.43 B 0.29
A 0.43 B 0.43
A 0.43 B 0.57
A 0.43 B 0.71
A 0.43 B 0.86
A 0.43 B 1
A 0.57 B 0
A 0.57 B 0.14
A 0.57 B 0.29
A 0.57 B 0.43
A 0.57 B 0.57
A 0.57 B 0.71
A 0.57 B 0.86
A 0.57 B 1
A 0.71 B 0
A 0.71 B 0.14
A 0.71 B 0.29
A 0.71 B 0.43
A 0.71 B 0.57
A 0.71 B 0.71
A 0.71 B 0.86
A 0.71 B 1
A 0.86 B 0
A 0.86 B 0.14
A 0.86 B 0.29
A 0.86 B 0.43
A 0.86 B 0.57
A 0.86 B 0.71
A 0.86 B 0.86
A 0.86 B 1
A 1 B 0
A 1 B 0.14
A 1 B 0.29
A 1 B 0.43
A 1 B 0.57
A 1 B 0.71
A 1 B 0.86
A 1 B 1
OD 600 nm [–]
0.2
0.4
0.6
0 5 101520
Time [h]

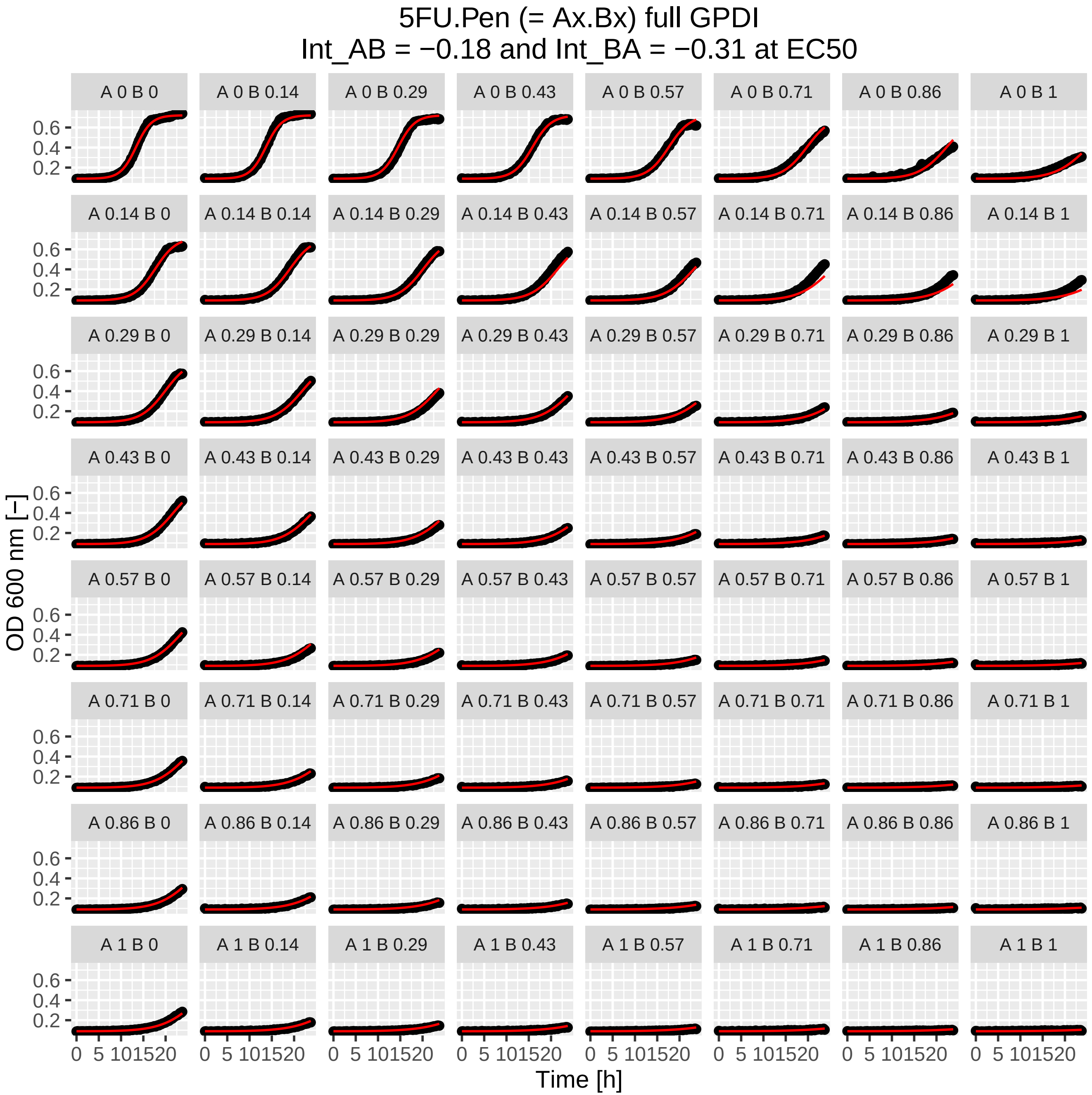
5FU.Pen (= Ax.Bx) full GPDI
Int_AB = −0.18 and Int_BA = −0.31 at EC50
OD 600 nm [–]
Time [h]
A 0 B 0
A 0 B 0.14
A 0 B 0.29
A 0 B 0.43
A 0 B 0.57
A 0 B 0.71
A 0 B 0.86
A 0 B 1
A 0.14 B 0
A 0.14 B 0.14
A 0.14 B 0.29
A 0.14 B 0.43
A 0.14 B 0.57
A 0.14 B 0.71
A 0.14 B 0.86
A 0.14 B 1
A 0.29 B 0
A 0.29 B 0.14
A 0.29 B 0.29
A 0.29 B 0.43
A 0.29 B 0.57
A 0.29 B 0.71
A 0.29 B 0.86
A 0.29 B 1
A 0.43 B 0
A 0.43 B 0.14
A 0.43 B 0.29
A 0.43 B 0.43
A 0.43 B 0.57
A 0.43 B 0.71
A 0.43 B 0.86
A 0.43 B 1
A 0.57 B 0
A 0.57 B 0.14
A 0.57 B 0.29
A 0.57 B 0.43
A 0.57 B 0.57
A 0.57 B 0.71
A 0.57 B 0.86
A 0.57 B 1
A 0.71 B 0
A 0.71 B 0.14
A 0.71 B 0.29
A 0.71 B 0.43
A 0.71 B 0.57
A 0.71 B 0.71
A 0.71 B 0.86
A 0.71 B 1
A 0.86 B 0
A 0.86 B 0.14
A 0.86 B 0.29
A 0.86 B 0.43
A 0.86 B 0.57
A 0.86 B 0.71
A 0.86 B 0.86
A 0.86 B 1
A 1 B 0
A 1 B 0.14
A 1 B 0.29
A 1 B 0.43
A 1 B 0.57
A 1 B 0.71
A 1 B 0.86
A 1 B 1
0.6
0.4
0.2
0 5 101520

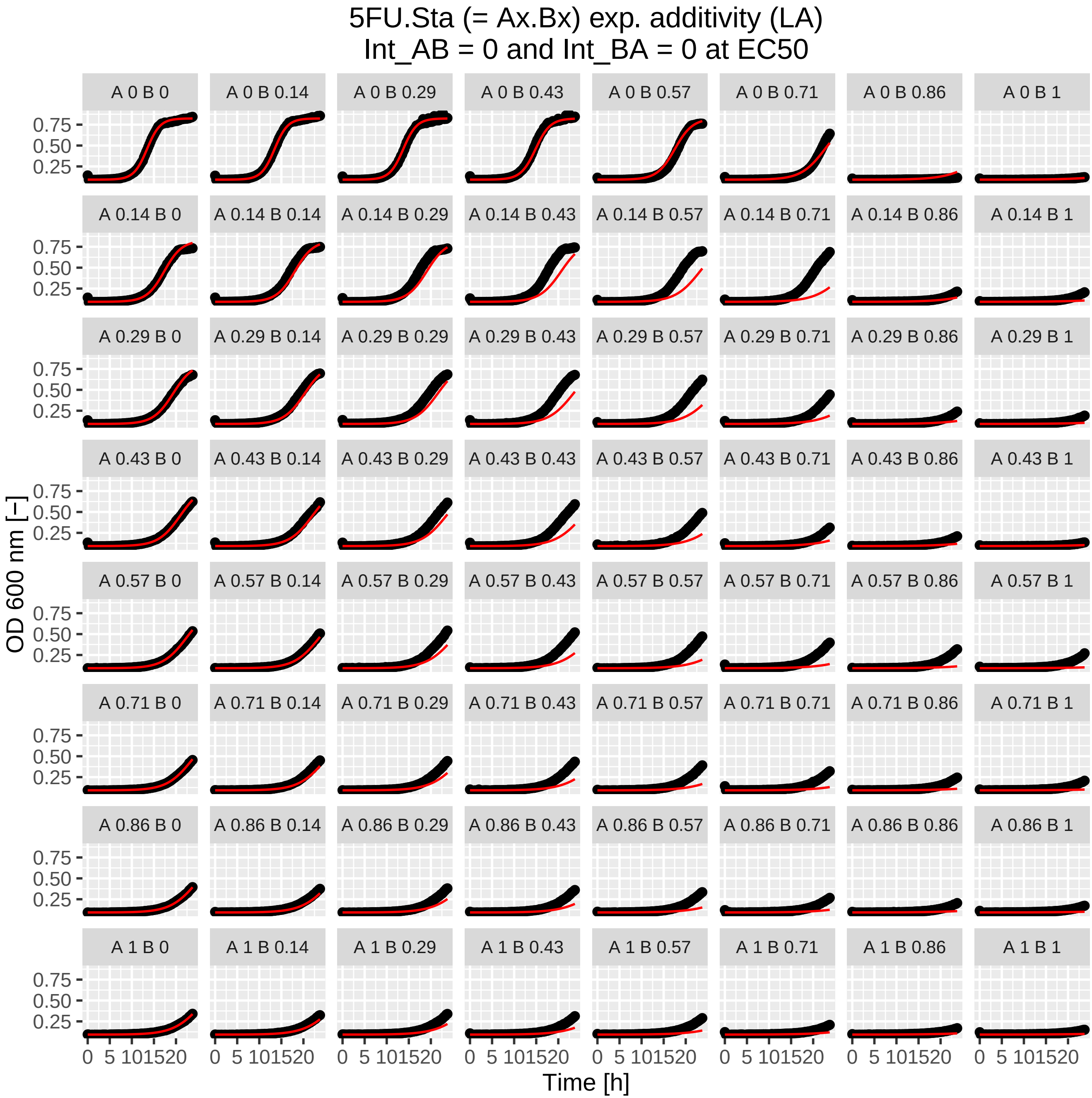

5FU.Sta (= Ax.Bx) exp. additivity (LA)
Int_AB = 0 and Int_BA = 0 at EC50
A 0 B 0
A 0 B 0.14
A 0 B 0.29
A 0 B 0.43
A 0 B 0.57
A 0 B 0.71
A 0 B 0.86
A 0 B 1
A 0.14 B 0
A 0.14 B 0.14
A 0.14 B 0.29
A 0.14 B 0.43
A 0.14 B 0.57
A 0.14 B 0.71
A 0.14 B 0.86
A 0.14 B 1
A 0.29 B 0
A 0.29 B 0.14
A 0.29 B 0.29
A 0.29 B 0.43
A 0.29 B 0.57
A 0.29 B 0.71
A 0.29 B 0.86
A 0.29 B 1
A 0.43 B 0
A 0.43 B 0.14
A 0.43 B 0.29
A 0.43 B 0.43
A 0.43 B 0.57
A 0.43 B 0.71
A 0.43 B 0.86
A 0.43 B 1
A 0.57 B 0
A 0.57 B 0.14
A 0.57 B 0.29
A 0.57 B 0.43
A 0.57 B 0.57
A 0.57 B 0.71
A 0.57 B 0.86
A 0.57 B 1
A 0.71 B 0
A 0.71 B 0.14
A 0.71 B 0.29
A 0.71 B 0.43
A 0.71 B 0.57
A 0.71 B 0.71
A 0.71 B 0.86
A 0.71 B 1
A 0.86 B 0
A 0.86 B 0.14
A 0.86 B 0.29
A 0.86 B 0.43
A 0.86 B 0.57
A 0.86 B 0.71
A 0.86 B 0.86
A 0.86 B 1
A 1 B 0
A 1 B 0.14
A 1 B 0.29
A 1 B 0.43
A 1 B 0.57
A 1 B 0.71
A 1 B 0.86
A 1 B 1
0.75
0.50
0.25
OD 600 nm [–]
0 5 101520
Time [h]

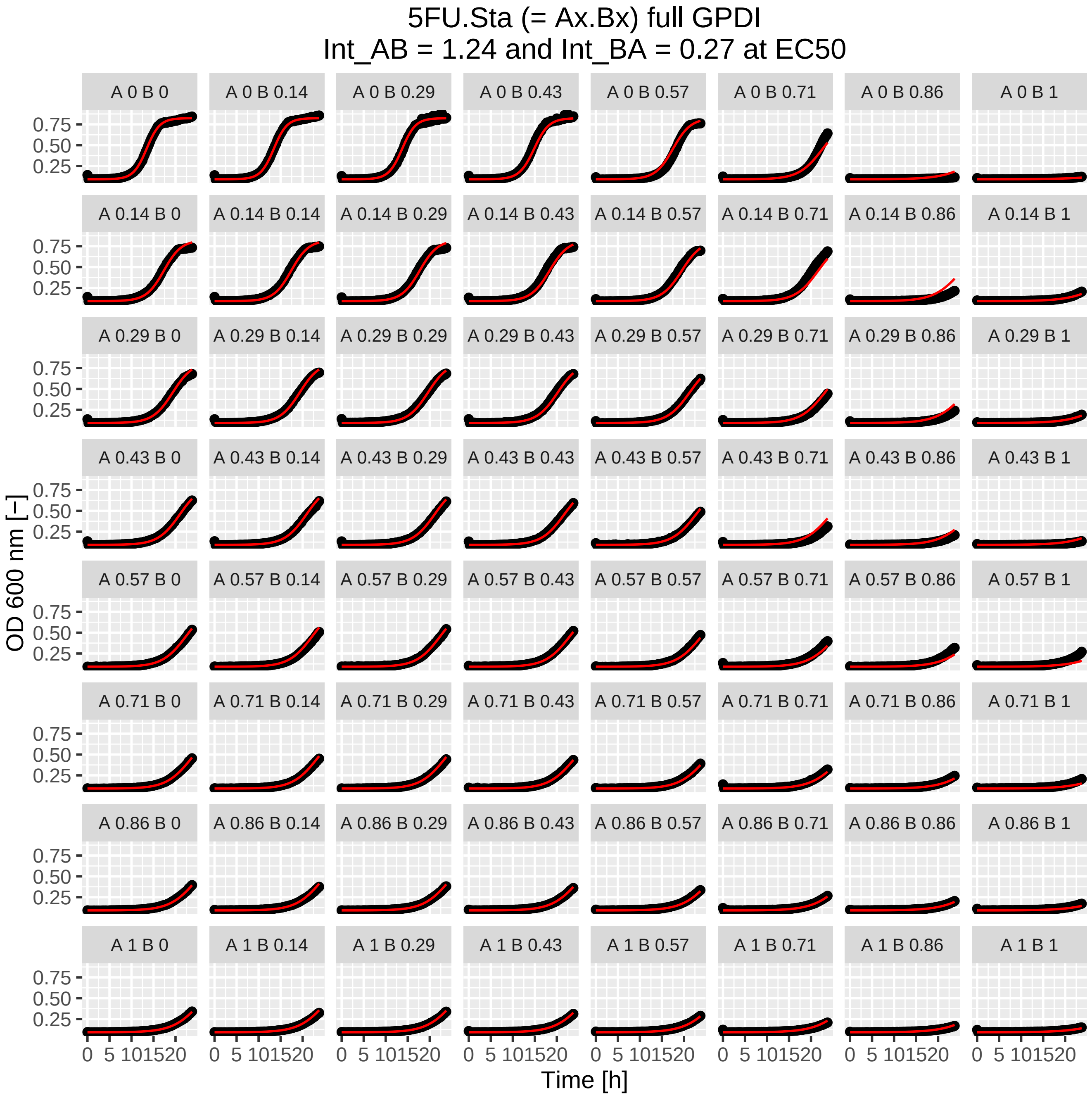
5FU.Sta (= Ax.Bx) full GPDI
Int_AB = 1.24 and Int_BA = 0.27 at EC50
A 0 B 0
A 0 B 0.14
A 0 B 0.29
A 0 B 0.43
A 0 B 0.57
A 0 B 0.71
A 0 B 0.86
A 0 B 1
A 0.14 B 0
A 0.14 B 0.14
A 0.14 B 0.29
A 0.14 B 0.43
A 0.14 B 0.57
A 0.14 B 0.71
A 0.14 B 0.86
A 0.14 B 1
A 0.29 B 0
A 0.29 B 0.14
A 0.29 B 0.29
A 0.29 B 0.43
A 0.29 B 0.57
A 0.29 B 0.71
A 0.29 B 0.86
A 0.29 B 1
A 0.43 B 0
A 0.43 B 0.14
A 0.43 B 0.29
A 0.43 B 0.43
A 0.43 B 0.57
A 0.43 B 0.71
A 0.43 B 0.86
A 0.43 B 1
A 0.57 B 0
A 0.57 B 0.14
A 0.57 B 0.29
A 0.57 B 0.43
A 0.57 B 0.57
A 0.57 B 0.71
A 0.57 B 0.86
A 0.57 B 1
A 0.71 B 0
A 0.71 B 0.14
A 0.71 B 0.29
A 0.71 B 0.43
A 0.71 B 0.57
A 0.71 B 0.71
A 0.71 B 0.86
A 0.71 B 1
A 0.86 B 0
A 0.86 B 0.14
A 0.86 B 0.29
A 0.86 B 0.43
A 0.86 B 0.57
A 0.86 B 0.71
A 0.86 B 0.86
A 0.86 B 1
A 1 B 0
A 1 B 0.14
A 1 B 0.29
A 1 B 0.43
A 1 B 0.57
A 1 B 0.71
A 1 B 0.86
A 1 B 1
0.75
0.50
0.25
0 5 101520
OD 600 nm [–]
Time [h]

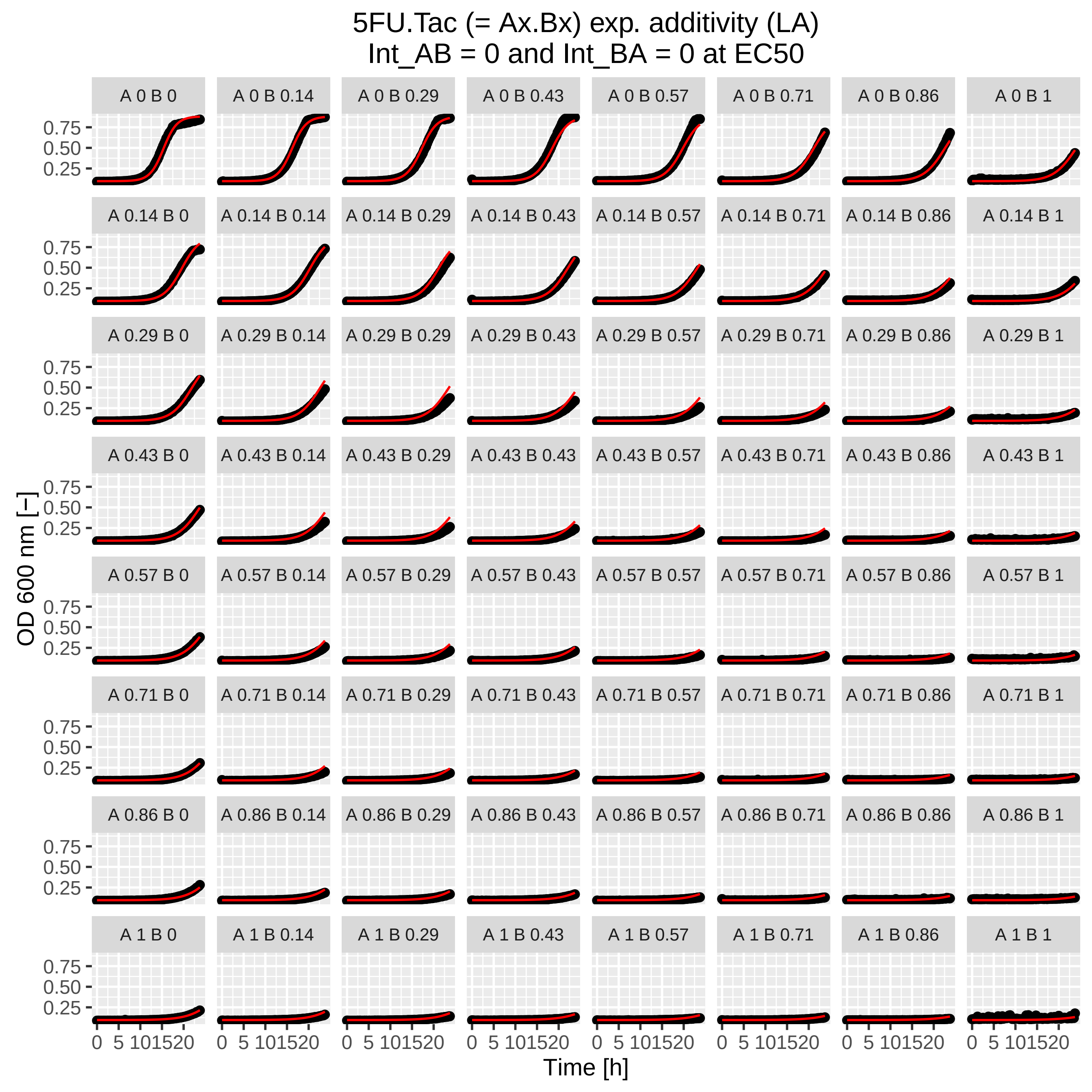
5FU.Tac (= Ax.Bx) exp. additivity (LA)
Int_AB = 0 and Int_BA = 0 at EC50
A 0 B 0
A 0 B 0.14
A 0 B 0.29
A 0 B 0.43
A 0 B 0.57
A 0 B 0.71
A 0 B 0.86
A 0 B 1
A 0.14 B 0
A 0.14 B 0.14
A 0.14 B 0.29
A 0.14 B 0.43
A 0.14 B 0.57
A 0.14 B 0.71
A 0.14 B 0.86
A 0.14 B 1
A 0.29 B 0
A 0.29 B 0.14
A 0.29 B 0.29
A 0.29 B 0.43
A 0.29 B 0.57
A 0.29 B 0.71
A 0.29 B 0.86
A 0.29 B 1
A 0.43 B 0
A 0.43 B 0.14
A 0.43 B 0.29
A 0.43 B 0.43
A 0.43 B 0.57
A 0.43 B 0.71
A 0.43 B 0.86
A 0.43 B 1
A 0.57 B 0
A 0.57 B 0.14
A 0.57 B 0.29
A 0.57 B 0.43
A 0.57 B 0.57
A 0.57 B 0.71
A 0.57 B 0.86
A 0.57 B 1
A 0.71 B 0
A 0.71 B 0.14
A 0.71 B 0.29
A 0.71 B 0.43
A 0.71 B 0.57
A 0.71 B 0.71
A 0.71 B 0.86
A 0.71 B 1
A 0.86 B 0
A 0.86 B 0.14
A 0.86 B 0.29
A 0.86 B 0.43
A 0.86 B 0.57
A 0.86 B 0.71
A 0.86 B 0.86
A 0.86 B 1
A 1 B 0
A 1 B 0.14
A 1 B 0.29
A 1 B 0.43
A 1 B 0.57
A 1 B 0.71
A 1 B 0.86
A 1 B 1
0.75
0.50
0.25
0 5 101520
OD 600 nm [−]
Time [h]

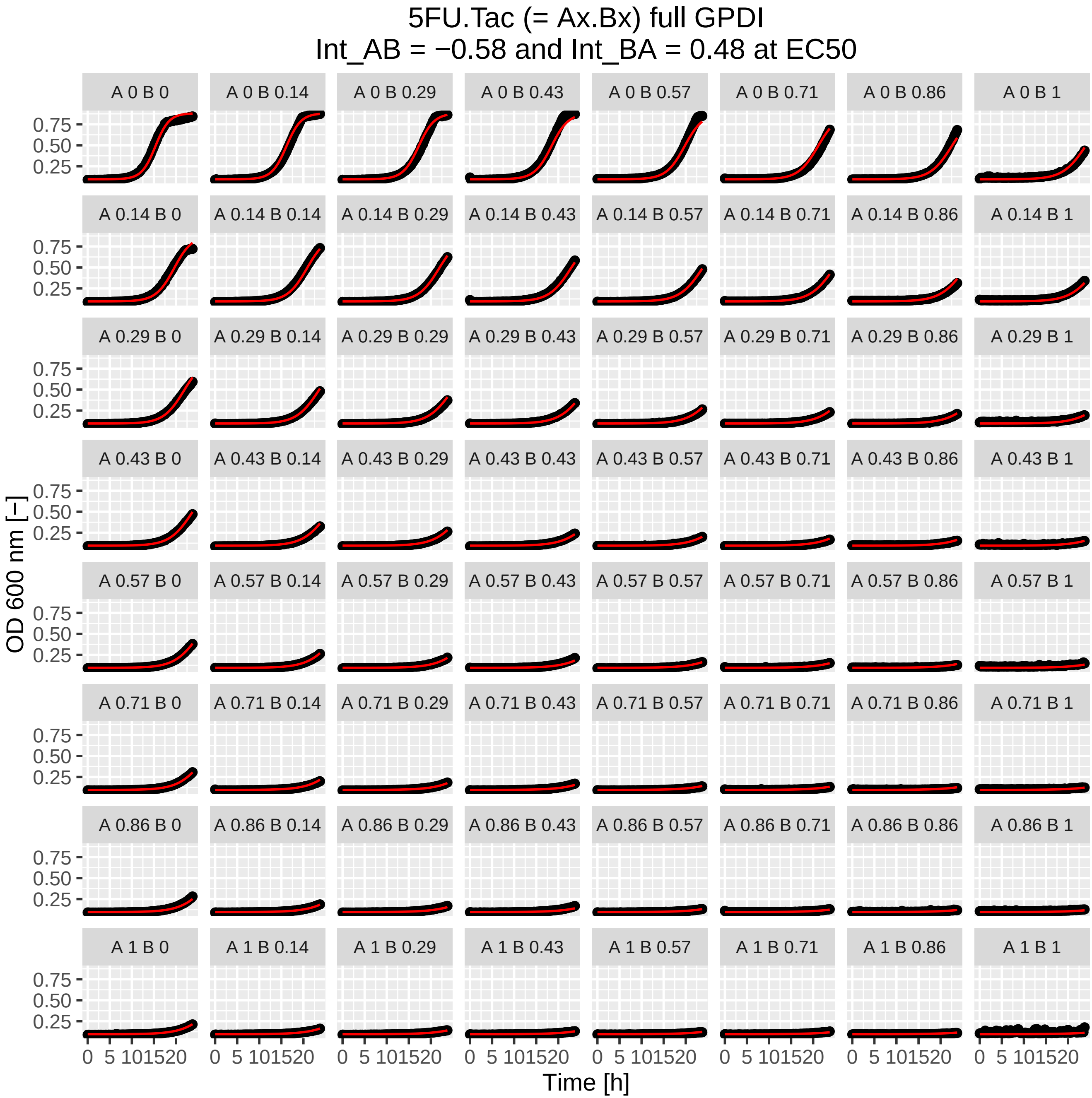

5FU.Tac (= Ax.Bx) full GPDI
Int_AB = −0.58 and Int_BA = 0.48 at EC50
A 0 B 0
A 0 B 0.14
A 0 B 0.29
A 0 B 0.43
A 0 B 0.57
A 0 B 0.71
A 0 B 0.86
A 0 B 1
A 0.14 B 0
A 0.14 B 0.14
A 0.14 B 0.29
A 0.14 B 0.43
A 0.14 B 0.57
A 0.14 B 0.71
A 0.14 B 0.86
A 0.14 B 1
A 0.29 B 0
A 0.29 B 0.14
A 0.29 B 0.29
A 0.29 B 0.43
A 0.29 B 0.57
A 0.29 B 0.71
A 0.29 B 0.86
A 0.29 B 1
A 0.43 B 0
A 0.43 B 0.14
A 0.43 B 0.29
A 0.43 B 0.43
A 0.43 B 0.57
A 0.43 B 0.71
A 0.43 B 0.86
A 0.43 B 1
A 0.57 B 0
A 0.57 B 0.14
A 0.57 B 0.29
A 0.57 B 0.43
A 0.57 B 0.57
A 0.57 B 0.71
A 0.57 B 0.86
A 0.57 B 1
A 0.71 B 0
A 0.71 B 0.14
A 0.71 B 0.29
A 0.71 B 0.43
A 0.71 B 0.57
A 0.71 B 0.71
A 0.71 B 0.86
A 0.71 B 1
A 0.86 B 0
A 0.86 B 0.14
A 0.86 B 0.29
A 0.86 B 0.43
A 0.86 B 0.57
A 0.86 B 0.71
A 0.86 B 0.86
A 0.86 B 1
A 1 B 0
A 1 B 0.14
A 1 B 0.29
A 1 B 0.43
A 1 B 0.57
A 1 B 0.71
A 1 B 0.86
A 1 B 1
0.75
0.50
0.25
0 5 101520
OD 600 nm [−]
Time [h]

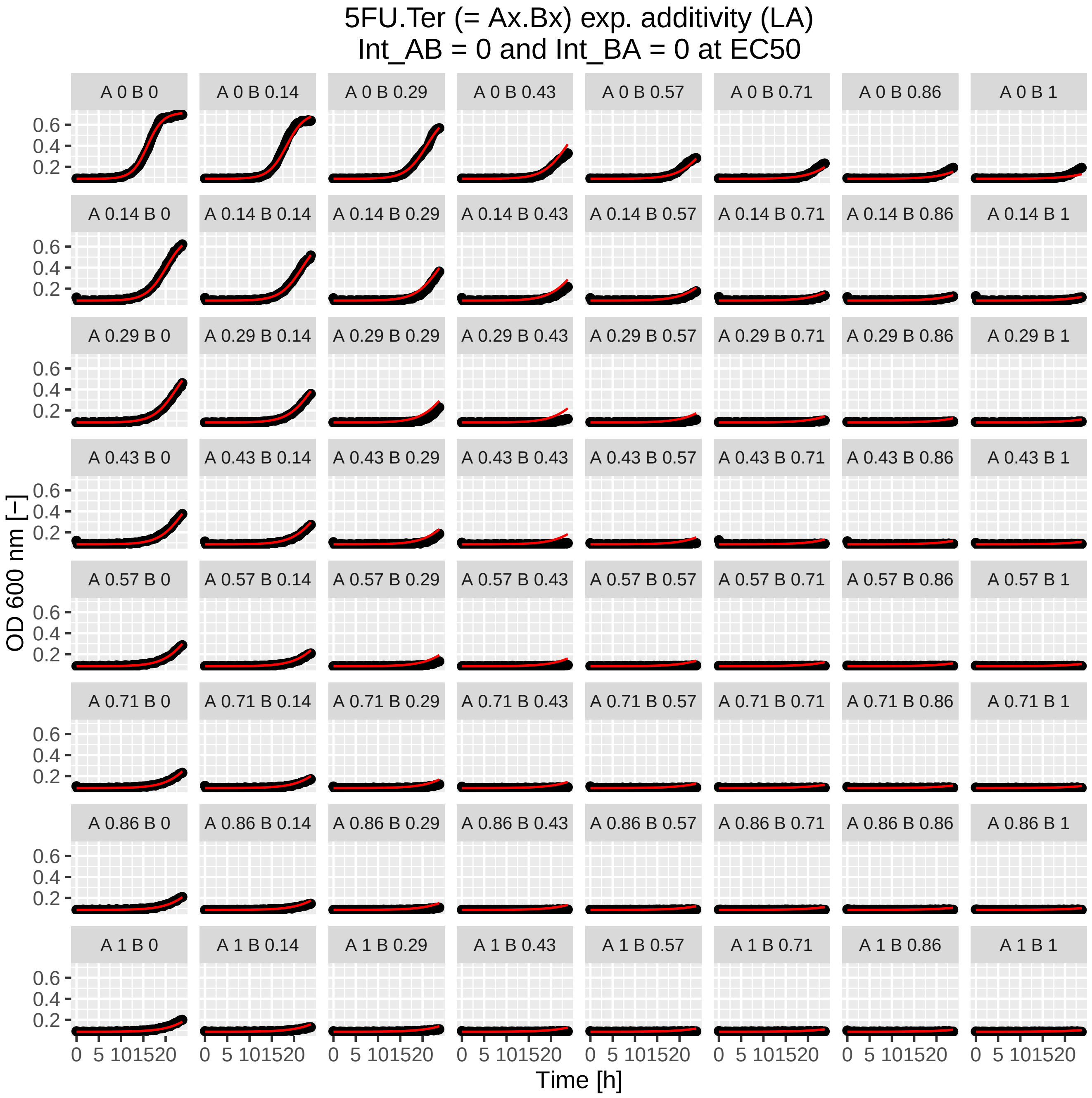
5FU.Ter (= Ax.Bx) exp. additivity (LA)
Int_AB = 0 and Int_BA = 0 at EC50
OD 600 nm [–]
Time [h]
A 0 B 0
A 0 B 0.14
A 0 B 0.29
A 0 B 0.43
A 0 B 0.57
A 0 B 0.71
A 0 B 0.86
A 0 B 1
A 0.14 B 0
A 0.29 B 0
A 0.43 B 0
A 0.57 B 0
A 0.71 B 0
A 0.86 B 0
A 1 B 0
A 1 B 1
0.2
0.4
0.6
0
5
10
15
20

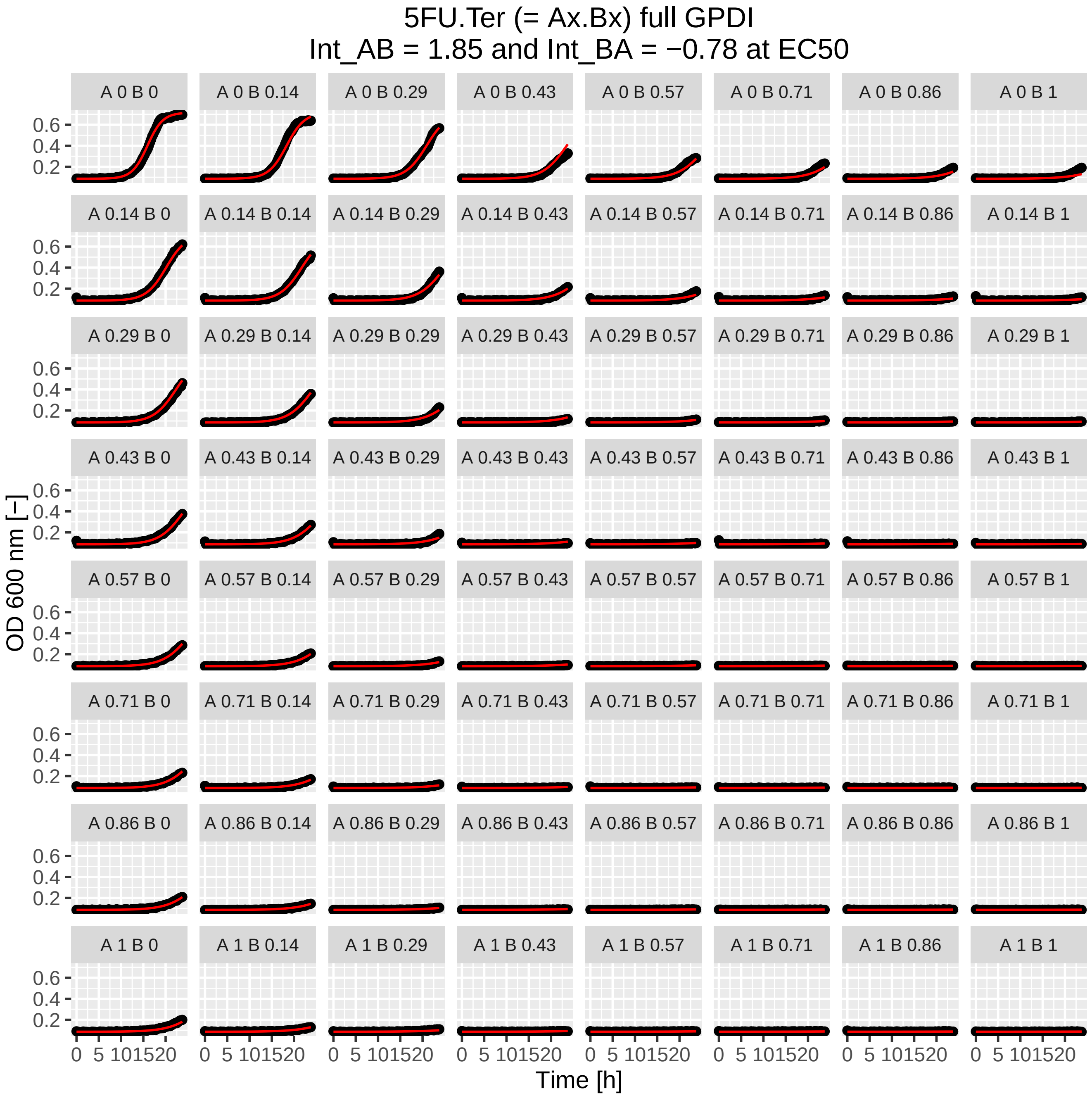

5FU.Ter (= Ax.Bx) full GPDI
Int_AB = 1.85 and Int_BA = −0.78 at EC50
A 0 B 0
A 0 B 0.14
A 0 B 0.29
A 0 B 0.43
A 0 B 0.57
A 0 B 0.71
A 0 B 0.86
A 0 B 1
A 0.14 B 0
A 0.14 B 0.14
A 0.14 B 0.29
A 0.14 B 0.43
A 0.14 B 0.57
A 0.14 B 0.71
A 0.14 B 0.86
A 0.14 B 1
A 0.29 B 0
A 0.29 B 0.14
A 0.29 B 0.29
A 0.29 B 0.43
A 0.29 B 0.57
A 0.29 B 0.71
A 0.29 B 0.86
A 0.29 B 1
A 0.43 B 0
A 0.43 B 0.14
A 0.43 B 0.29
A 0.43 B 0.43
A 0.43 B 0.57
A 0.43 B 0.71
A 0.43 B 0.86
A 0.43 B 1
A 0.57 B 0
A 0.57 B 0.14
A 0.57 B 0.29
A 0.57 B 0.43
A 0.57 B 0.57
A 0.57 B 0.71
A 0.57 B 0.86
A 0.57 B 1
A 0.71 B 0
A 0.71 B 0.14
A 0.71 B 0.29
A 0.71 B 0.43
A 0.71 B 0.57
A 0.71 B 0.71
A 0.71 B 0.86
A 0.71 B 1
A 0.86 B 0
A 0.86 B 0.14
A 0.86 B 0.29
A 0.86 B 0.43
A 0.86 B 0.57
A 0.86 B 0.71
A 0.86 B 0.86
A 0.86 B 1
A 1 B 0
A 1 B 0.14
A 1 B 0.29
A 1 B 0.43
A 1 B 0.57
A 1 B 0.71
A 1 B 0.86
A 1 B 1
0.6
0.4
0.2
OD 600 nm [−]
0 5 101520
Time [h]

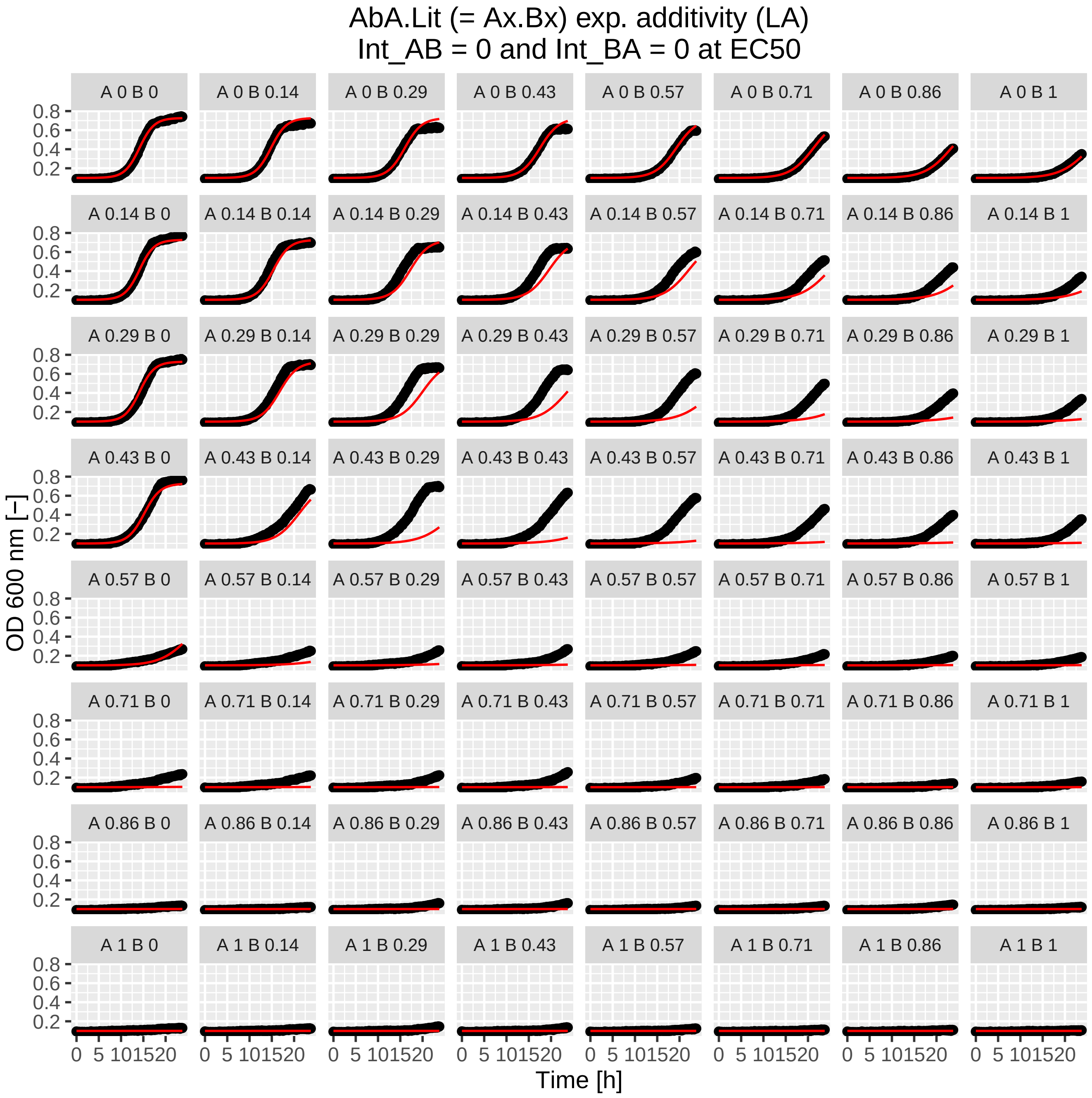
AbA.Lit (= Ax.Bx) exp. additivity (LA)
Int_AB = 0 and Int_BA = 0 at EC50
A 0 B 0
A 0 B 0.14
A 0 B 0.29
A 0 B 0.43
A 0 B 0.57
A 0 B 0.71
A 0 B 0.86
A 0 B 1
A 0.14 B 0
A 0.14 B 0.14
A 0.14 B 0.29
A 0.14 B 0.43
A 0.14 B 0.57
A 0.14 B 0.71
A 0.14 B 0.86
A 0.14 B 1
A 0.29 B 0
A 0.29 B 0.14
A 0.29 B 0.29
A 0.29 B 0.43
A 0.29 B 0.57
A 0.29 B 0.71
A 0.29 B 0.86
A 0.29 B 1
A 0.43 B 0
A 0.43 B 0.14
A 0.43 B 0.29
A 0.43 B 0.43
A 0.43 B 0.57
A 0.43 B 0.71
A 0.43 B 0.86
A 0.43 B 1
A 0.57 B 0
A 0.57 B 0.14
A 0.57 B 0.29
A 0.57 B 0.43
A 0.57 B 0.57
A 0.57 B 0.71
A 0.57 B 0.86
A 0.57 B 1
A 0.71 B 0
A 0.71 B 0.14
A 0.71 B 0.29
A 0.71 B 0.43
A 0.71 B 0.57
A 0.71 B 0.71
A 0.71 B 0.86
A 0.71 B 1
A 0.86 B 0
A 0.86 B 0.14
A 0.86 B 0.29
A 0.86 B 0.43
A 0.86 B 0.57
A 0.86 B 0.71
A 0.86 B 0.86
A 0.86 B 1
A 1 B 0
A 1 B 0.14
A 1 B 0.29
A 1 B 0.43
A 1 B 0.57
A 1 B 0.71
A 1 B 0.86
A 1 B 1
OD 600 nm [–]
0.8
0.6
0.4
0.2
0 5 101520
Time [h]

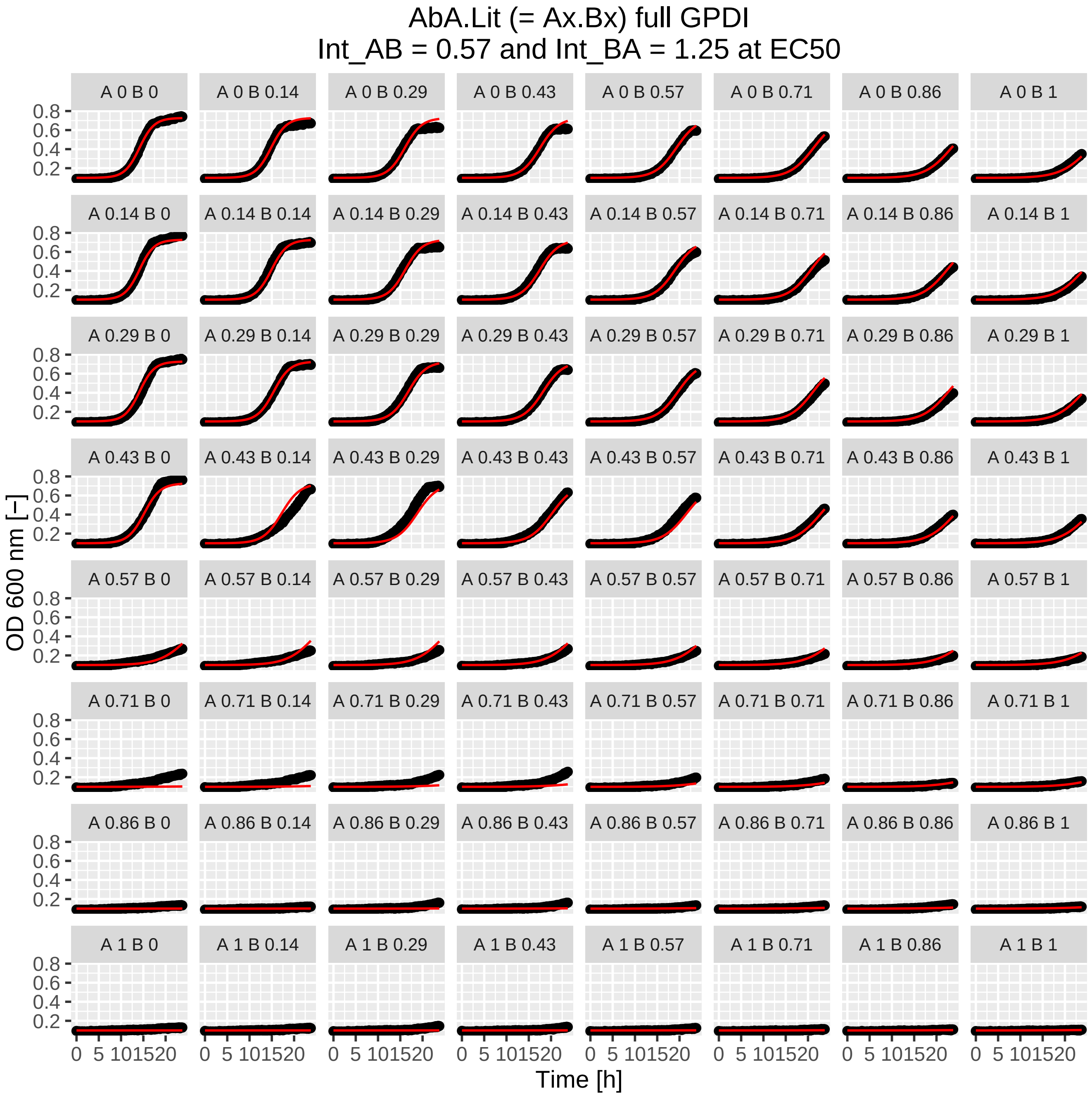

AbA.Lit (= Ax.Bx) full GPDI
Int_AB = 0.57 and Int_BA = 1.25 at EC50
A 0 B 0
A 0 B 0.14
A 0 B 0.29
A 0 B 0.43
A 0 B 0.57
A 0 B 0.71
A 0 B 0.86
A 0 B 1
A 0.14 B 0
A 0.14 B 0.14
A 0.14 B 0.29
A 0.14 B 0.43
A 0.14 B 0.57
A 0.14 B 0.71
A 0.14 B 0.86
A 0.14 B 1
A 0.29 B 0
A 0.29 B 0.14
A 0.29 B 0.29
A 0.29 B 0.43
A 0.29 B 0.57
A 0.29 B 0.71
A 0.29 B 0.86
A 0.29 B 1
A 0.43 B 0
A 0.43 B 0.14
A 0.43 B 0.29
A 0.43 B 0.43
A 0.43 B 0.57
A 0.43 B 0.71
A 0.43 B 0.86
A 0.43 B 1
A 0.57 B 0
A 0.57 B 0.14
A 0.57 B 0.29
A 0.57 B 0.43
A 0.57 B 0.57
A 0.57 B 0.71
A 0.57 B 0.86
A 0.57 B 1
A 0.71 B 0
A 0.71 B 0.14
A 0.71 B 0.29
A 0.71 B 0.43
A 0.71 B 0.57
A 0.71 B 0.71
A 0.71 B 0.86
A 0.71 B 1
A 0.86 B 0
A 0.86 B 0.14
A 0.86 B 0.29
A 0.86 B 0.43
A 0.86 B 0.57
A 0.86 B 0.71
A 0.86 B 0.86
A 0.86 B 1
A 1 B 0
A 1 B 0.14
A 1 B 0.29
A 1 B 0.43
A 1 B 0.57
A 1 B 0.71
A 1 B 0.86
A 1 B 1
OD 600 nm [–]
0.2
0.4
0.6
0.8
0
5
10
15
20
Time [h]

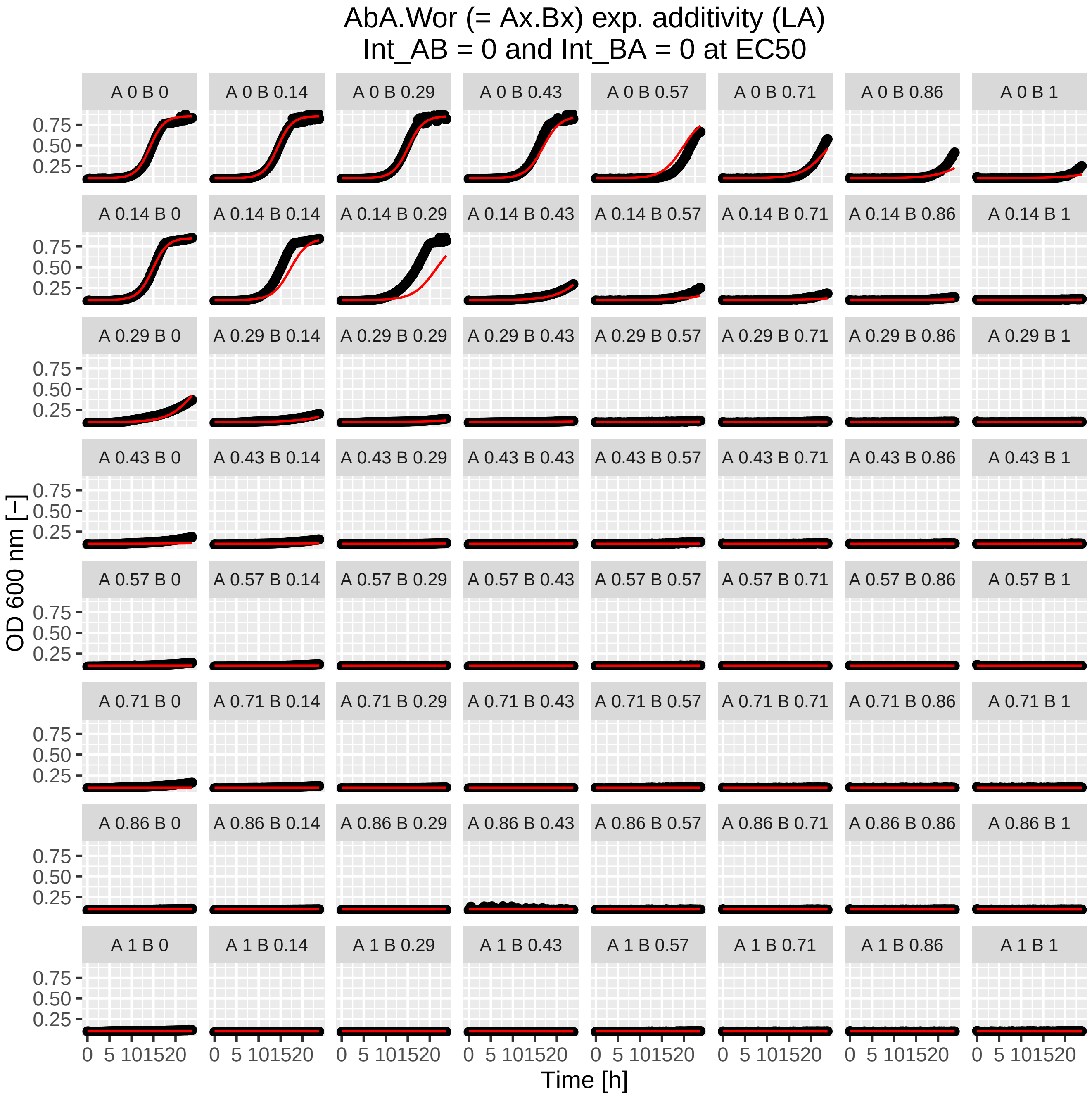

AbA.Wor (= Ax.Bx) exp. additivity (LA)
Int_AB = 0 and Int_BA = 0 at EC50
A 0 B 0
A 0 B 0.14
A 0 B 0.29
A 0 B 0.43
A 0 B 0.57
A 0 B 0.71
A 0 B 0.86
A 0 B 1
A 0.14 B 0
A 0.14 B 0.14
A 0.14 B 0.29
A 0.14 B 0.43
A 0.14 B 0.57
A 0.14 B 0.71
A 0.14 B 0.86
A 0.14 B 1
A 0.29 B 0
A 0.29 B 0.14
A 0.29 B 0.29
A 0.29 B 0.43
A 0.29 B 0.57
A 0.29 B 0.71
A 0.29 B 0.86
A 0.29 B 1
A 0.43 B 0
A 0.43 B 0.14
A 0.43 B 0.29
A 0.43 B 0.43
A 0.43 B 0.57
A 0.43 B 0.71
A 0.43 B 0.86
A 0.43 B 1
A 0.57 B 0
A 0.57 B 0.14
A 0.57 B 0.29
A 0.57 B 0.43
A 0.57 B 0.57
A 0.57 B 0.71
A 0.57 B 0.86
A 0.57 B 1
A 0.71 B 0
A 0.71 B 0.14
A 0.71 B 0.29
A 0.71 B 0.43
A 0.71 B 0.57
A 0.71 B 0.71
A 0.71 B 0.86
A 0.71 B 1
A 0.86 B 0
A 0.86 B 0.14
A 0.86 B 0.29
A 0.86 B 0.43
A 0.86 B 0.57
A 0.86 B 0.71
A 0.86 B 0.86
A 0.86 B 1
A 1 B 0
A 1 B 0.14
A 1 B 0.29
A 1 B 0.43
A 1 B 0.57
A 1 B 0.71
A 1 B 0.86
A 1 B 1
0.75
0.50
0.25
OD 600 nm [–]
0 5 101520
Time [h]

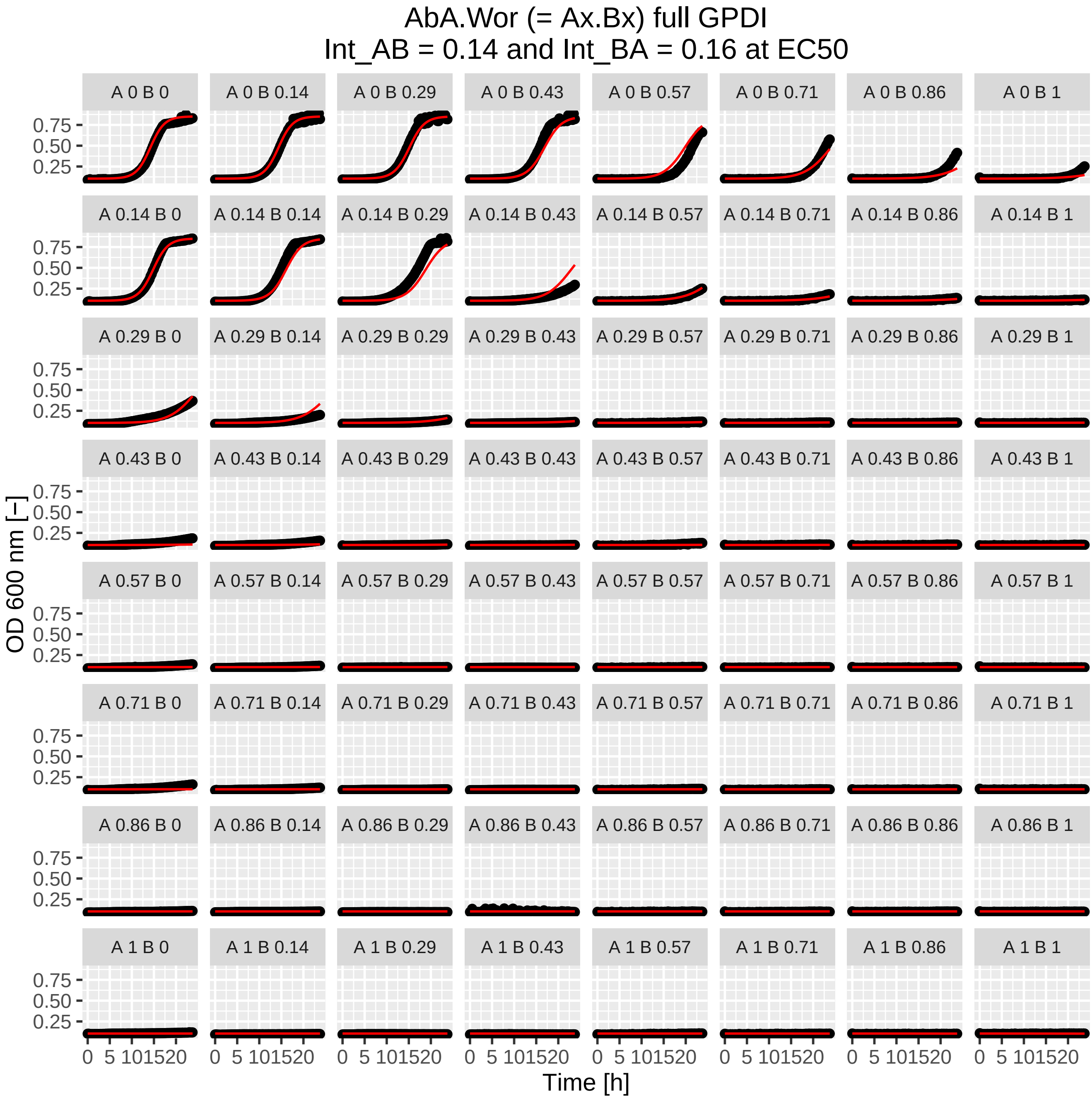
AbA.Wor (= Ax.Bx) full GPDI
Int_AB = 0.14 and Int_BA = 0.16 at EC50
A 0 B 0
A 0 B 0.14
A 0 B 0.29
A 0 B 0.43
A 0 B 0.57
A 0 B 0.71
A 0 B 0.86
A 0 B 1
A 0.14 B 0
A 0.14 B 0.14
A 0.14 B 0.29
A 0.14 B 0.43
A 0.14 B 0.57
A 0.14 B 0.71
A 0.14 B 0.86
A 0.14 B 1
A 0.29 B 0
A 0.29 B 0.14
A 0.29 B 0.29
A 0.29 B 0.43
A 0.29 B 0.57
A 0.29 B 0.71
A 0.29 B 0.86
A 0.29 B 1
A 0.43 B 0
A 0.43 B 0.14
A 0.43 B 0.29
A 0.43 B 0.43
A 0.43 B 0.57
A 0.43 B 0.71
A 0.43 B 0.86
A 0.43 B 1
A 0.57 B 0
A 0.57 B 0.14
A 0.57 B 0.29
A 0.57 B 0.43
A 0.57 B 0.57
A 0.57 B 0.71
A 0.57 B 0.86
A 0.57 B 1
A 0.71 B 0
A 0.71 B 0.14
A 0.71 B 0.29
A 0.71 B 0.43
A 0.71 B 0.57
A 0.71 B 0.71
A 0.71 B 0.86
A 0.71 B 1
A 0.86 B 0
A 0.86 B 0.14
A 0.86 B 0.29
A 0.86 B 0.43
A 0.86 B 0.57
A 0.86 B 0.71
A 0.86 B 0.86
A 0.86 B 1
A 1 B 0
A 1 B 0.14
A 1 B 0.29
A 1 B 0.43
A 1 B 0.57
A 1 B 0.71
A 1 B 0.86
A 1 B 1
0.75
0.50
0.25
OD 600 nm [–]
0 5 101520
Time [h]

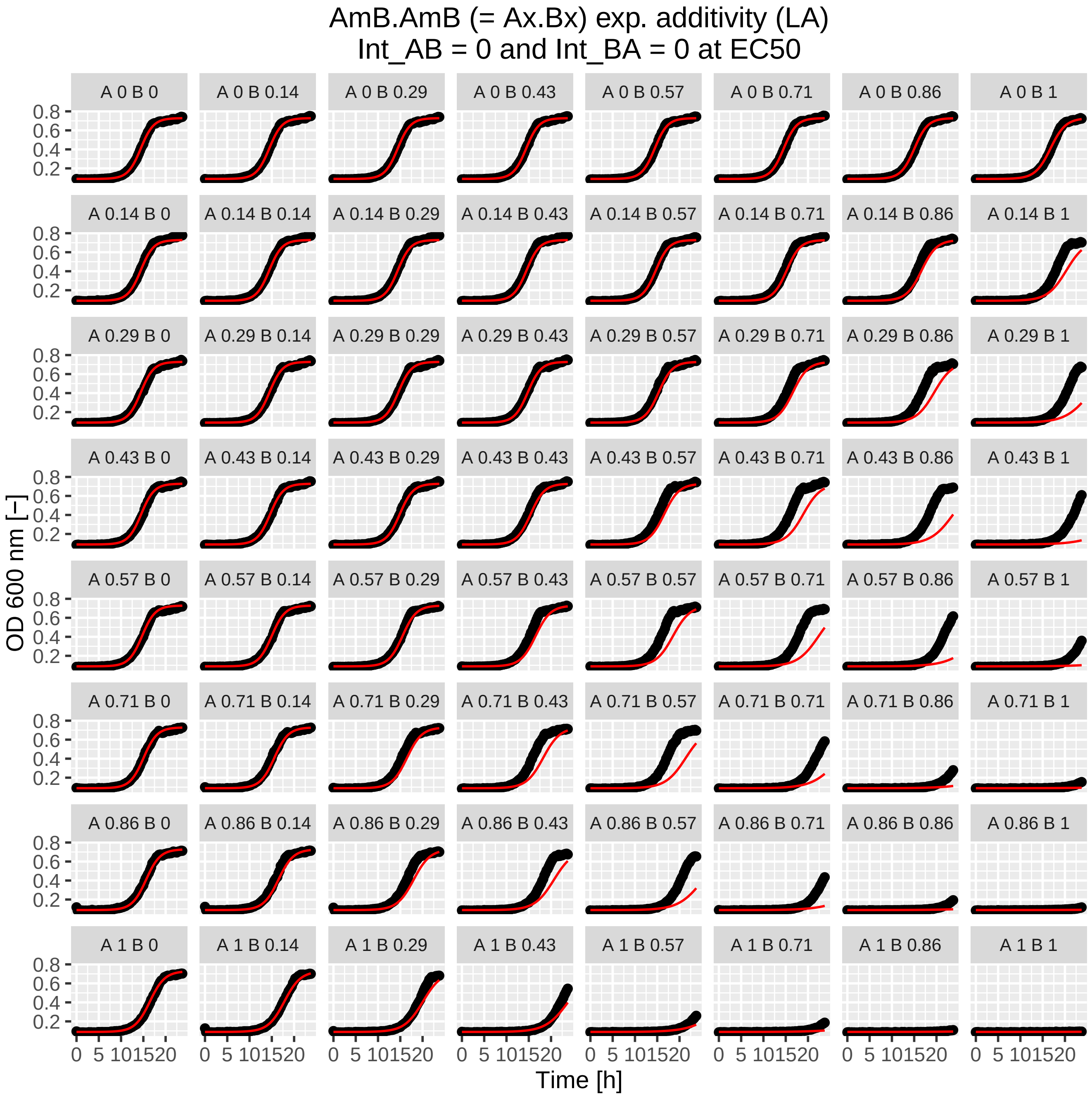
AmB.AmB (= Ax.Bx) exp. additivity (LA)
Int_AB = 0 and Int_BA = 0 at EC50
A 0 B 0
A 0 B 0.14
A 0 B 0.29
A 0 B 0.43
A 0 B 0.57
A 0 B 0.71
A 0 B 0.86
A 0 B 1
A 0.14 B 0
A 0.14 B 0.14
A 0.14 B 0.29
A 0.14 B 0.43
A 0.14 B 0.57
A 0.14 B 0.71
A 0.14 B 0.86
A 0.14 B 1
A 0.29 B 0
A 0.29 B 0.14
A 0.29 B 0.29
A 0.29 B 0.43
A 0.29 B 0.57
A 0.29 B 0.71
A 0.29 B 0.86
A 0.29 B 1
A 0.43 B 0
A 0.43 B 0.14
A 0.43 B 0.29
A 0.43 B 0.43
A 0.43 B 0.57
A 0.43 B 0.71
A 0.43 B 0.86
A 0.43 B 1
A 0.57 B 0
A 0.57 B 0.14
A 0.57 B 0.29
A 0.57 B 0.43
A 0.57 B 0.57
A 0.57 B 0.71
A 0.57 B 0.86
A 0.57 B 1
A 0.71 B 0
A 0.71 B 0.14
A 0.71 B 0.29
A 0.71 B 0.43
A 0.71 B 0.57
A 0.71 B 0.71
A 0.71 B 0.86
A 0.71 B 1
A 0.86 B 0
A 0.86 B 0.14
A 0.86 B 0.29
A 0.86 B 0.43
A 0.86 B 0.57
A 0.86 B 0.71
A 0.86 B 0.86
A 0.86 B 1
A 1 B 0
A 1 B 0.14
A 1 B 0.29
A 1 B 0.43
A 1 B 0.57
A 1 B 0.71
A 1 B 0.86
A 1 B 1
OD 600 nm [–]
0.2
0.4
0.6
0.8
0 5 101520
Time [h]

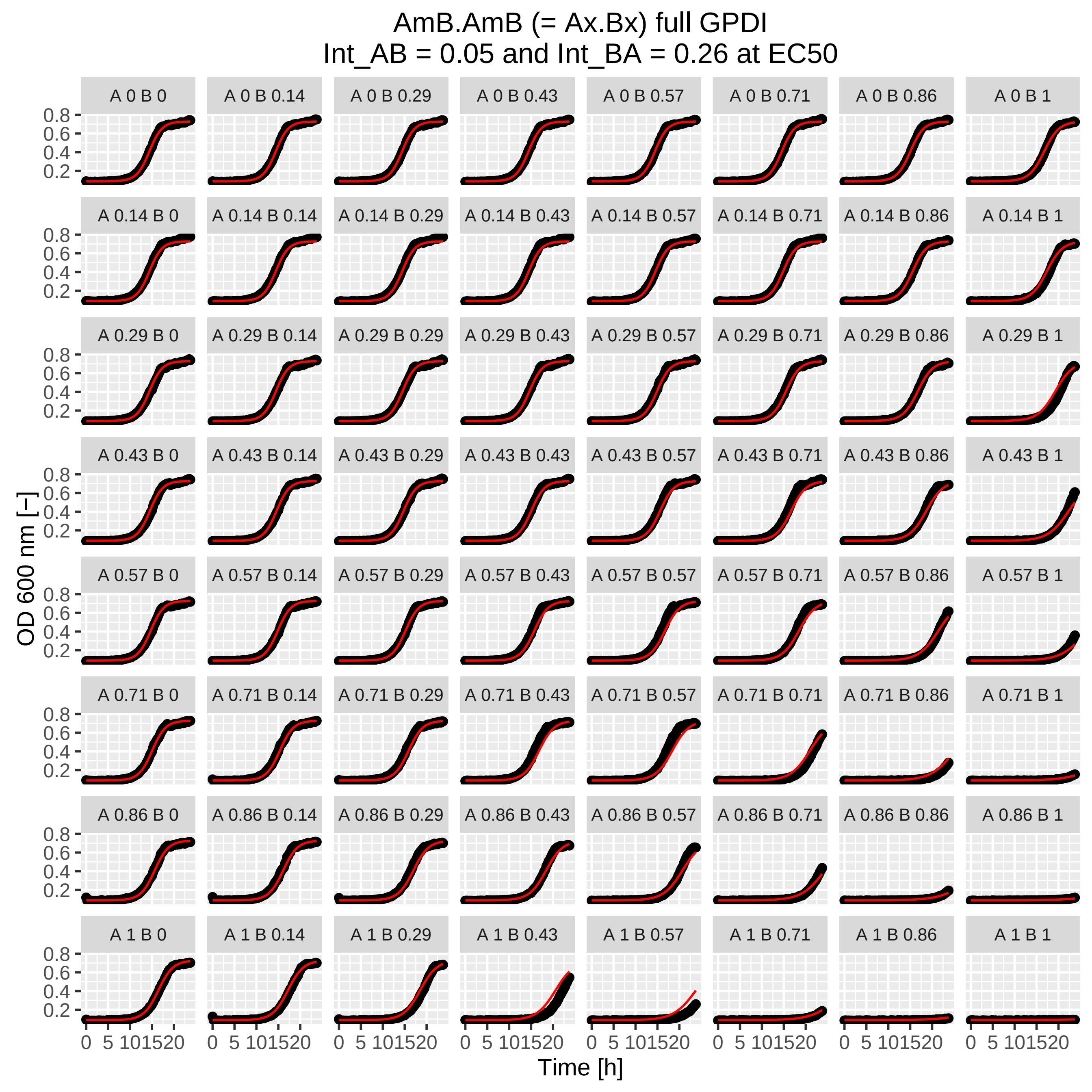
AmB.AmB (= Ax.Bx) full GPDI
Int_AB = 0.05 and Int_BA = 0.26 at EC50
OD 600 nm [–]
Time [h]
A 0 B 0
A 0 B 0.14
A 0 B 0.29
A 0 B 0.43
A 0 B 0.57
A 0 B 0.71
A 0 B 0.86
A 0 B 1
A 0.14 B 0
A 0.14 B 0.14
A 0.14 B 0.29
A 0.14 B 0.43
A 0.14 B 0.57
A 0.14 B 0.71
A 0.14 B 0.86
A 0.14 B 1
A 0.29 B 0
A 0.29 B 0.14
A 0.29 B 0.29
A 0.29 B 0.43
A 0.29 B 0.57
A 0.29 B 0.71
A 0.29 B 0.86
A 0.29 B 1
A 0.43 B 0
A 0.43 B 0.14
A 0.43 B 0.29
A 0.43 B 0.43
A 0.43 B 0.57
A 0.43 B 0.71
A 0.43 B 0.86
A 0.43 B 1
A 0.57 B 0
A 0.57 B 0.14
A 0.57 B 0.29
A 0.57 B 0.43
A 0.57 B 0.57
A 0.57 B 0.71
A 0.57 B 0.86
A 0.57 B 1
A 0.71 B 0
A 0.71 B 0.14
A 0.71 B 0.29
A 0.71 B 0.43
A 0.71 B 0.57
A 0.71 B 0.71
A 0.71 B 0.86
A 0.71 B 1
A 0.86 B 0
A 0.86 B 0.14
A 0.86 B 0.29
A 0.86 B 0.43
A 0.86 B 0.57
A 0.86 B 0.71
A 0.86 B 0.86
A 0.86 B 1
A 1 B 0
A 1 B 0.14
A 1 B 0.29
A 1 B 0.43
A 1 B 0.57
A 1 B 0.71
A 1 B 0.86
A 1 B 1
0.8
0.6
0.4
0.2
0 5 101520

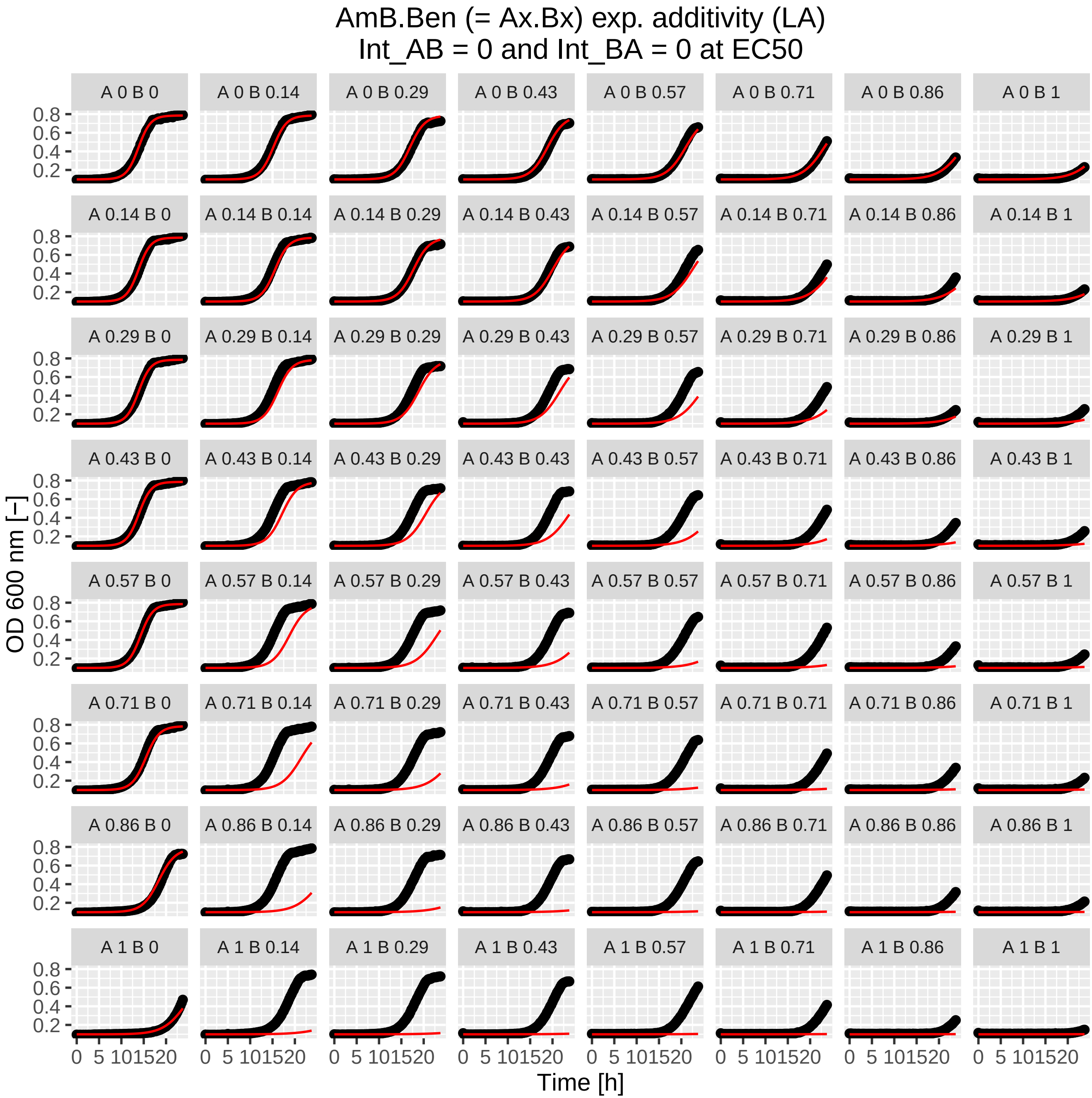

AmB.Ben (= Ax.Bx) exp. additivity (LA)
Int_AB = 0 and Int_BA = 0 at EC50
OD 600 nm [–]
Time [h]
A 0 B 0
A 0 B 0.14
A 0 B 0.29
A 0 B 0.43
A 0 B 0.57
A 0 B 0.71
A 0 B 0.86
A 0 B 1
A 0.14 B 0
A 0.14 B 0.14
A 0.14 B 0.29
A 0.14 B 0.43
A 0.14 B 0.57
A 0.14 B 0.71
A 0.14 B 0.86
A 0.14 B 1
A 0.29 B 0
A 0.29 B 0.14
A 0.29 B 0.29
A 0.29 B 0.43
A 0.29 B 0.57
A 0.29 B 0.71
A 0.29 B 0.86
A 0.29 B 1
A 0.43 B 0
A 0.43 B 0.14
A 0.43 B 0.29
A 0.43 B 0.43
A 0.43 B 0.57
A 0.43 B 0.71
A 0.43 B 0.86
A 0.43 B 1
A 0.57 B 0
A 0.57 B 0.14
A 0.57 B 0.29
A 0.57 B 0.43
A 0.57 B 0.57
A 0.57 B 0.71
A 0.57 B 0.86
A 0.57 B 1
A 0.71 B 0
A 0.71 B 0.14
A 0.71 B 0.29
A 0.71 B 0.43
A 0.71 B 0.57
A 0.71 B 0.71
A 0.71 B 0.86
A 0.71 B 1
A 0.86 B 0
A 0.86 B 0.14
A 0.86 B 0.29
A 0.86 B 0.43
A 0.86 B 0.57
A 0.86 B 0.71
A 0.86 B 0.86
A 0.86 B 1
A 1 B 0
A 1 B 0.14
A 1 B 0.29
A 1 B 0.43
A 1 B 0.57
A 1 B 0.71
A 1 B 0.86
A 1 B 1
0.8
0.6
0.4
0.2
0 5 101520

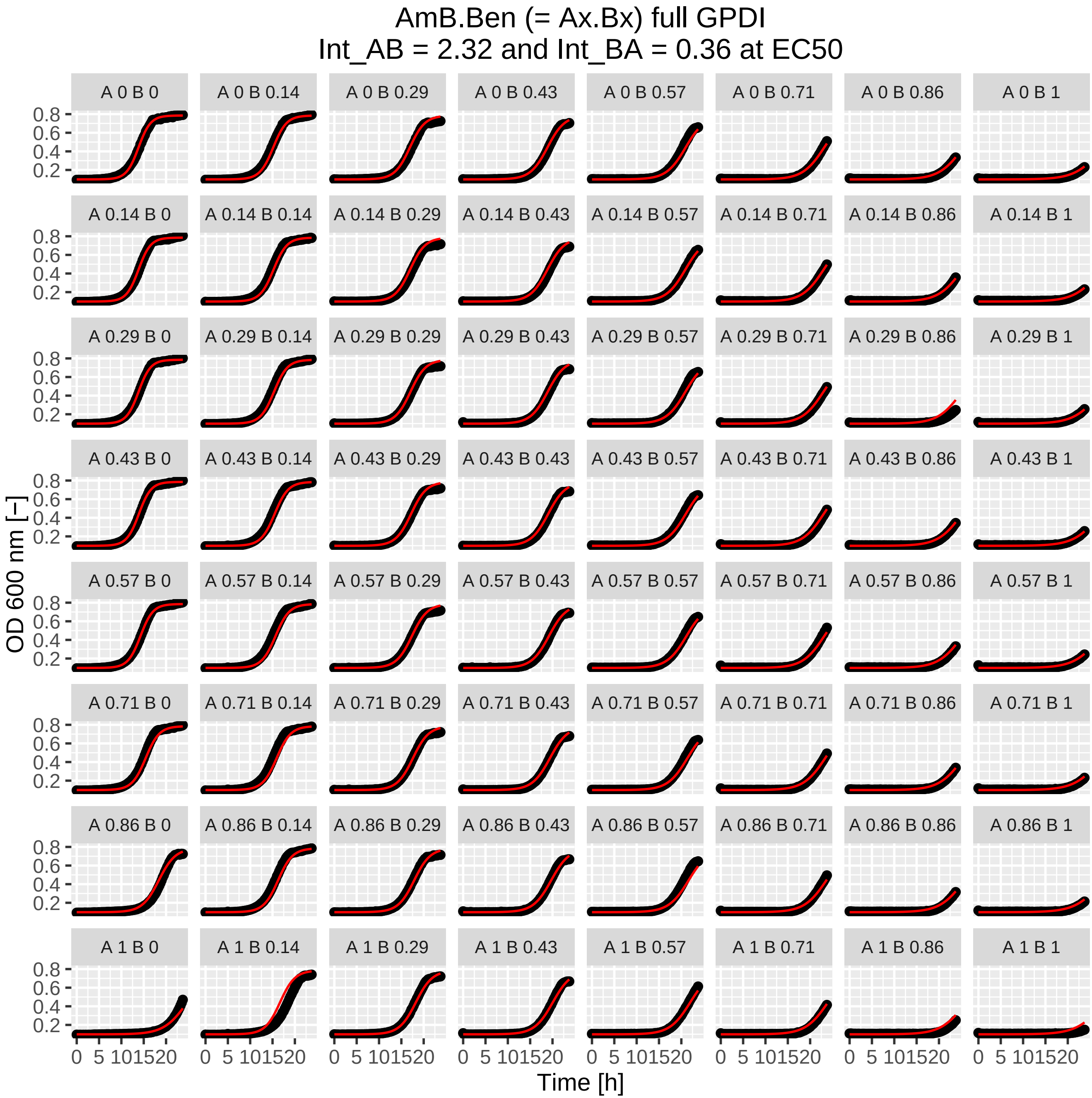

AmB.Ben (= Ax.Bx) full GPDI
Int_AB = 2.32 and Int_BA = 0.36 at EC50
A 0 B 0
A 0 B 0.14
A 0 B 0.29
A 0 B 0.43
A 0 B 0.57
A 0 B 0.71
A 0 B 0.86
A 0 B 1
A 0.14 B 0
A 0.14 B 0.14
A 0.14 B 0.29
A 0.14 B 0.43
A 0.14 B 0.57
A 0.14 B 0.71
A 0.14 B 0.86
A 0.14 B 1
A 0.29 B 0
A 0.29 B 0.14
A 0.29 B 0.29
A 0.29 B 0.43
A 0.29 B 0.57
A 0.29 B 0.71
A 0.29 B 0.86
A 0.29 B 1
A 0.43 B 0
A 0.43 B 0.14
A 0.43 B 0.29
A 0.43 B 0.43
A 0.43 B 0.57
A 0.43 B 0.71
A 0.43 B 0.86
A 0.43 B 1
A 0.57 B 0
A 0.57 B 0.14
A 0.57 B 0.29
A 0.57 B 0.43
A 0.57 B 0.57
A 0.57 B 0.71
A 0.57 B 0.86
A 0.57 B 1
A 0.71 B 0
A 0.71 B 0.14
A 0.71 B 0.29
A 0.71 B 0.43
A 0.71 B 0.57
A 0.71 B 0.71
A 0.71 B 0.86
A 0.71 B 1
A 0.86 B 0
A 0.86 B 0.14
A 0.86 B 0.29
A 0.86 B 0.43
A 0.86 B 0.57
A 0.86 B 0.71
A 0.86 B 0.86
A 0.86 B 1
A 1 B 0
A 1 B 0.14
A 1 B 0.29
A 1 B 0.43
A 1 B 0.57
A 1 B 0.71
A 1 B 0.86
A 1 B 1
OD 600 nm [–]
0.8
0.6
0.4
0.2
0 5 101520
Time [h]

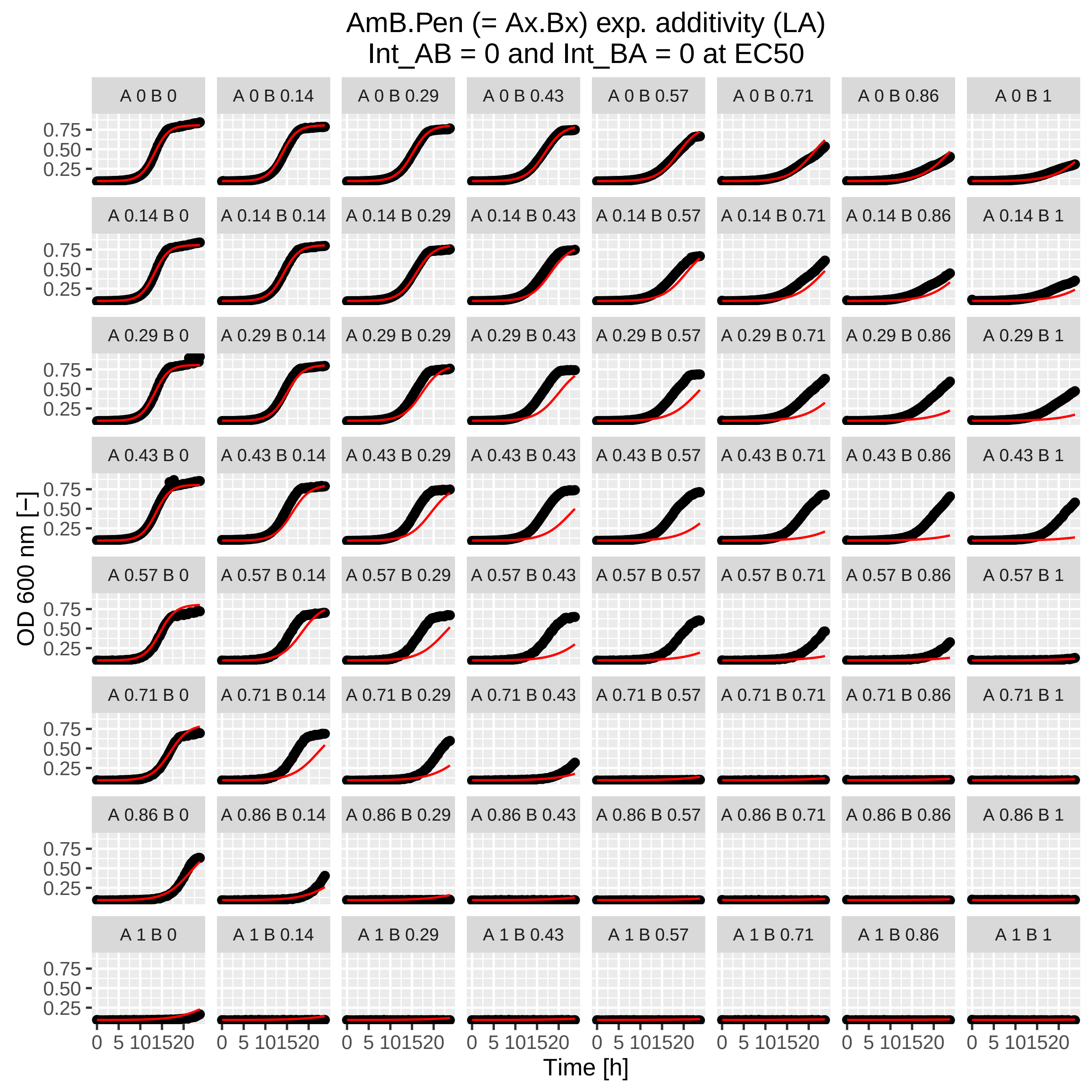
AmB.Pen (= Ax.Bx) exp. additivity (LA)
Int_AB = 0 and Int_BA = 0 at EC50
A 0 B 0
A 0 B 0.14
A 0 B 0.29
A 0 B 0.43
A 0 B 0.57
A 0 B 0.71
A 0 B 0.86
A 0 B 1
A 0.14 B 0
A 0.14 B 0.14
A 0.14 B 0.29
A 0.14 B 0.43
A 0.14 B 0.57
A 0.14 B 0.71
A 0.14 B 0.86
A 0.14 B 1
A 0.29 B 0
A 0.29 B 0.14
A 0.29 B 0.29
A 0.29 B 0.43
A 0.29 B 0.57
A 0.29 B 0.71
A 0.29 B 0.86
A 0.29 B 1
A 0.43 B 0
A 0.43 B 0.14
A 0.43 B 0.29
A 0.43 B 0.43
A 0.43 B 0.57
A 0.43 B 0.71
A 0.43 B 0.86
A 0.43 B 1
A 0.57 B 0
A 0.57 B 0.14
A 0.57 B 0.29
A 0.57 B 0.43
A 0.57 B 0.57
A 0.57 B 0.71
A 0.57 B 0.86
A 0.57 B 1
A 0.71 B 0
A 0.71 B 0.14
A 0.71 B 0.29
A 0.71 B 0.43
A 0.71 B 0.57
A 0.71 B 0.71
A 0.71 B 0.86
A 0.71 B 1
A 0.86 B 0
A 0.86 B 0.14
A 0.86 B 0.29
A 0.86 B 0.43
A 0.86 B 0.57
A 0.86 B 0.71
A 0.86 B 0.86
A 0.86 B 1
A 1 B 0
A 1 B 0.14
A 1 B 0.29
A 1 B 0.43
A 1 B 0.57
A 1 B 0.71
A 1 B 0.86
A 1 B 1
0.75
0.50
0.25
0 5 101520
OD 600 nm [–]
Time [h]

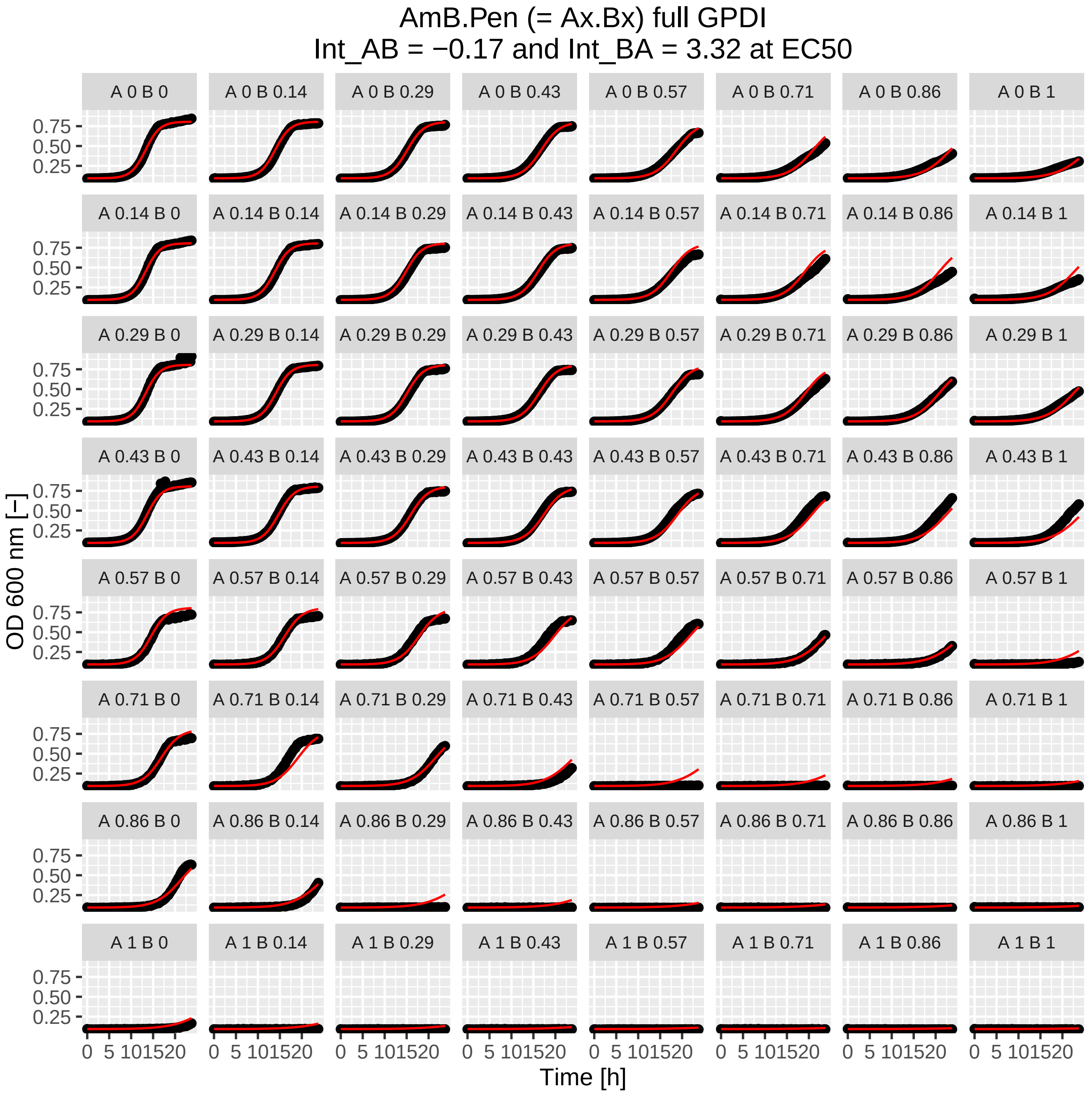

AmB.Pen (= Ax.Bx) full GPDI
Int_AB = −0.17 and Int_BA = 3.32 at EC50
A 0 B 0
A 0 B 0.14
A 0 B 0.29
A 0 B 0.43
A 0 B 0.57
A 0 B 0.71
A 0 B 0.86
A 0 B 1
A 0.14 B 0
A 0.14 B 0.14
A 0.14 B 0.29
A 0.14 B 0.43
A 0.14 B 0.57
A 0.14 B 0.71
A 0.14 B 0.86
A 0.14 B 1
A 0.29 B 0
A 0.29 B 0.14
A 0.29 B 0.29
A 0.29 B 0.43
A 0.29 B 0.57
A 0.29 B 0.71
A 0.29 B 0.86
A 0.29 B 1
A 0.43 B 0
A 0.43 B 0.14
A 0.43 B 0.29
A 0.43 B 0.43
A 0.43 B 0.57
A 0.43 B 0.71
A 0.43 B 0.86
A 0.43 B 1
A 0.57 B 0
A 0.57 B 0.14
A 0.57 B 0.29
A 0.57 B 0.43
A 0.57 B 0.57
A 0.57 B 0.71
A 0.57 B 0.86
A 0.57 B 1
A 0.71 B 0
A 0.71 B 0.14
A 0.71 B 0.29
A 0.71 B 0.43
A 0.71 B 0.57
A 0.71 B 0.71
A 0.71 B 0.86
A 0.71 B 1
A 0.86 B 0
A 0.86 B 0.14
A 0.86 B 0.29
A 0.86 B 0.43
A 0.86 B 0.57
A 0.86 B 0.71
A 0.86 B 0.86
A 0.86 B 1
A 1 B 0
A 1 B 0.14
A 1 B 0.29
A 1 B 0.43
A 1 B 0.57
A 1 B 0.71
A 1 B 0.86
A 1 B 1
OD 600 nm [−]
0.75
0.50
0.25
0 5 101520
Time [h]

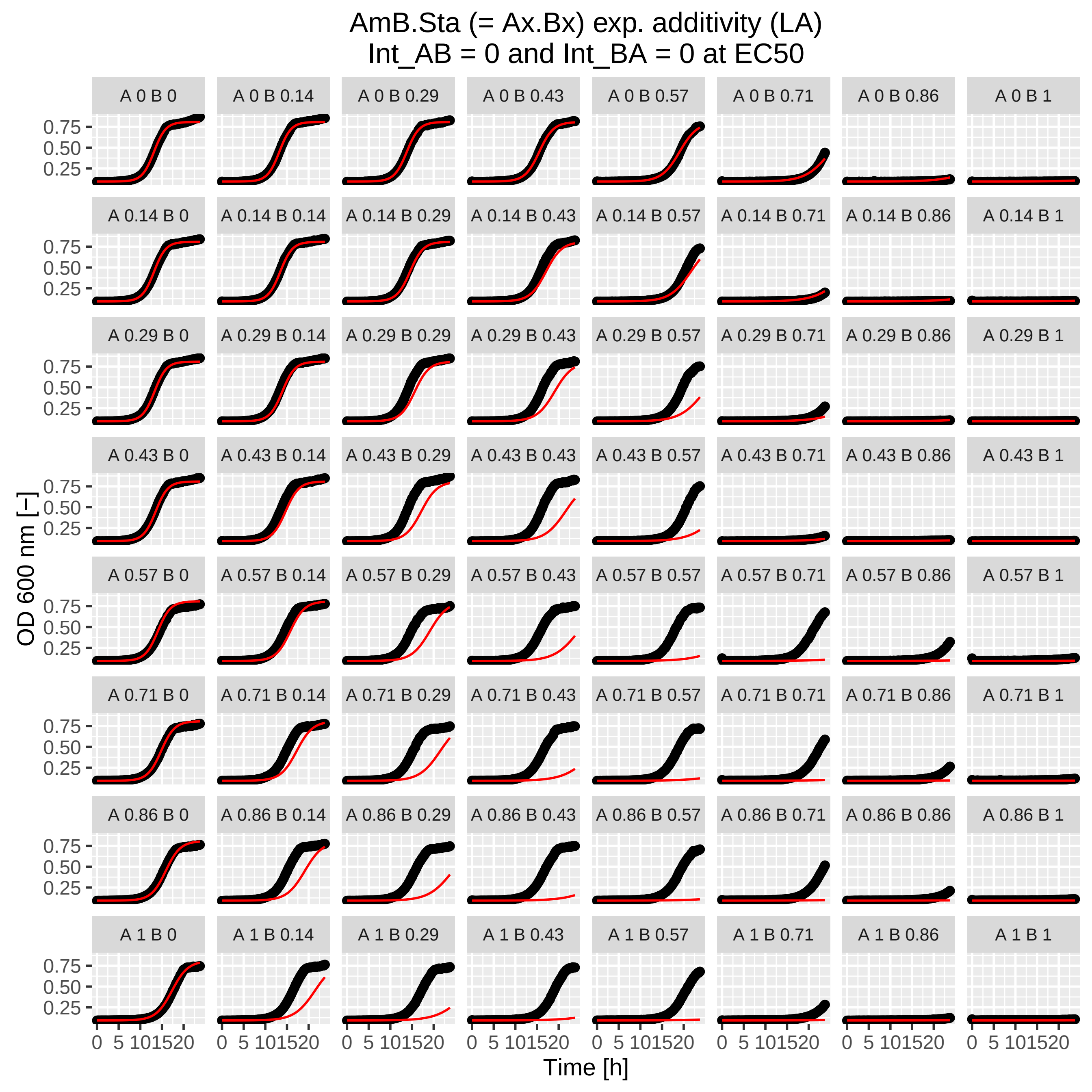
AmB.Sta (= Ax.Bx) exp. additivity (LA)
Int_AB = 0 and Int_BA = 0 at EC50
A 0 B 0
A 0 B 0.14
A 0 B 0.29
A 0 B 0.43
A 0 B 0.57
A 0 B 0.71
A 0 B 0.86
A 0 B 1
A 0.14 B 0
A 0.14 B 0.14
A 0.14 B 0.29
A 0.14 B 0.43
A 0.14 B 0.57
A 0.14 B 0.71
A 0.14 B 0.86
A 0.14 B 1
A 0.29 B 0
A 0.29 B 0.14
A 0.29 B 0.29
A 0.29 B 0.43
A 0.29 B 0.57
A 0.29 B 0.71
A 0.29 B 0.86
A 0.29 B 1
A 0.43 B 0
A 0.43 B 0.14
A 0.43 B 0.29
A 0.43 B 0.43
A 0.43 B 0.57
A 0.43 B 0.71
A 0.43 B 0.86
A 0.43 B 1
A 0.57 B 0
A 0.57 B 0.14
A 0.57 B 0.29
A 0.57 B 0.43
A 0.57 B 0.57
A 0.57 B 0.71
A 0.57 B 0.86
A 0.57 B 1
A 0.71 B 0
A 0.71 B 0.14
A 0.71 B 0.29
A 0.71 B 0.43
A 0.71 B 0.57
A 0.71 B 0.71
A 0.71 B 0.86
A 0.71 B 1
A 0.86 B 0
A 0.86 B 0.14
A 0.86 B 0.29
A 0.86 B 0.43
A 0.86 B 0.57
A 0.86 B 0.71
A 0.86 B 0.86
A 0.86 B 1
A 1 B 0
A 1 B 0.14
A 1 B 0.29
A 1 B 0.43
A 1 B 0.57
A 1 B 0.71
A 1 B 0.86
A 1 B 1
OD 600 nm [–]
0.75
0.50
0.25
0 5 101520
Time [h]

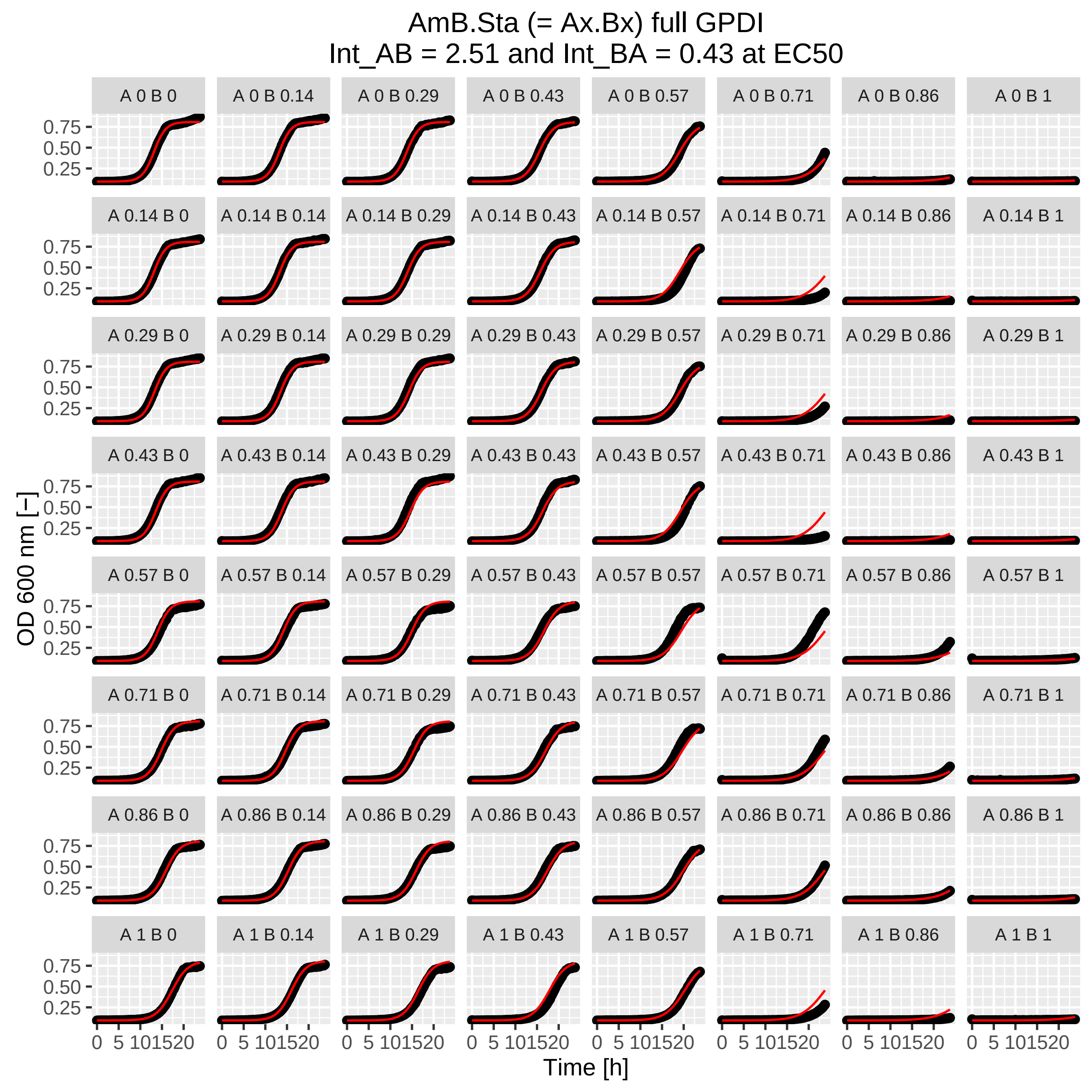
AmB.Sta (= Ax.Bx) full GPDI
Int_AB = 2.51 and Int_BA = 0.43 at EC50
OD 600 nm [−]
Time [h]
0.75
0.50
0.25
0 5 101520
A 0 B 0
A 0 B 0.14
A 0 B 0.29
A 0 B 0.43
A 0 B 0.57
A 0 B 0.71
A 0 B 0.86
A 0 B 1
A 0.14 B 0
A 0.14 B 0.14
A 0.14 B 0.29
A 0.14 B 0.43
A 0.14 B 0.57
A 0.14 B 0.71
A 0.14 B 0.86
A 0.14 B 1
A 0.29 B 0
A 0.29 B 0.14
A 0.29 B 0.29
A 0.29 B 0.43
A 0.29 B 0.57
A 0.29 B 0.71
A 0.29 B 0.86
A 0.29 B 1
A 0.43 B 0
A 0.43 B 0.14
A 0.43 B 0.29
A 0.43 B 0.43
A 0.43 B 0.57
A 0.43 B 0.71
A 0.43 B 0.86
A 0.43 B 1
A 0.57 B 0
A 0.57 B 0.14
A 0.57 B 0.29
A 0.57 B 0.43
A 0.57 B 0.57
A 0.57 B 0.71
A 0.57 B 0.86
A 0.57 B 1
A 0.71 B 0
A 0.71 B 0.14
A 0.71 B 0.29
A 0.71 B 0.43
A 0.71 B 0.57
A 0.71 B 0.71
A 0.71 B 0.86
A 0.71 B 1
A 0.86 B 0
A 0.86 B 0.14
A 0.86 B 0.29
A 0.86 B 0.43
A 0.86 B 0.57
A 0.86 B 0.71
A 0.86 B 0.86
A 0.86 B 1
A 1 B 0
A 1 B 0.14
A 1 B 0.29
A 1 B 0.43
A 1 B 0.57
A 1 B 0.71
A 1 B 0.86
A 1 B 1

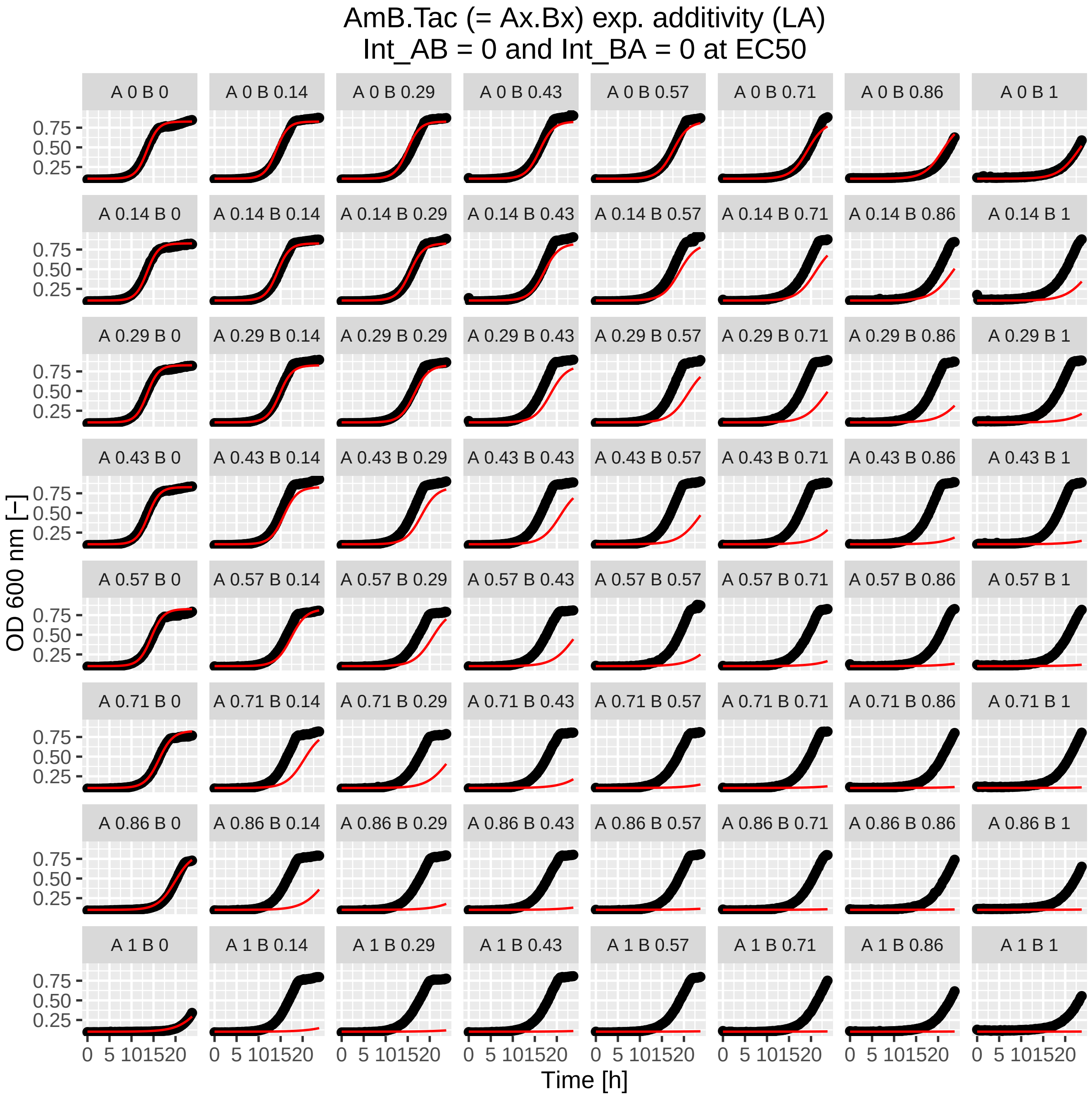
AmB.Tac (= Ax.Bx) exp. additivity (LA)
Int_AB = 0 and Int_BA = 0 at EC50
OD 600 nm [−]
Time [h]
0.25
0.50
0.75
0 5 10 15 20
A 0 B 0
A 0 B 0.14
A 0 B 0.29
A 0 B 0.43
A 0 B 0.57
A 0 B 0.71
A 0 B 0.86
A 0 B 1
A 0.14 B 0
A 0.14 B 0.14
A 0.14 B 0.29
A 0.14 B 0.43
A 0.14 B 0.57
A 0.14 B 0.71
A 0.14 B 0.86
A 0.14 B 1
A 0.29 B 0
A 0.29 B 0.14
A 0.29 B 0.29
A 0.29 B 0.43
A 0.29 B 0.57
A 0.29 B 0.71
A 0.29 B 0.86
A 0.29 B 1
A 0.43 B 0
A 0.43 B 0.14
A 0.43 B 0.29
A 0.43 B 0.43
A 0.43 B 0.57
A 0.43 B 0.71
A 0.43 B 0.86
A 0.43 B 1
A 0.57 B 0
A 0.57 B 0.14
A 0.57 B 0.29
A 0.57 B 0.43
A 0.57 B 0.57
A 0.57 B 0.71
A 0.57 B 0.86
A 0.57 B 1
A 0.71 B 0
A 0.71 B 0.14
A 0.71 B 0.29
A 0.71 B 0.43
A 0.71 B 0.57
A 0.71 B 0.71
A 0.71 B 0.86
A 0.71 B 1
A 0.86 B 0
A 0.86 B 0.14
A 0.86 B 0.29
A 0.86 B 0.43
A 0.86 B 0.57
A 0.86 B 0.71
A 0.86 B 0.86
A 0.86 B 1
A 1 B 0
A 1 B 0.14
A 1 B 0.29
A 1 B 0.43
A 1 B 0.57
A 1 B 0.71
A 1 B 0.86
A 1 B 1

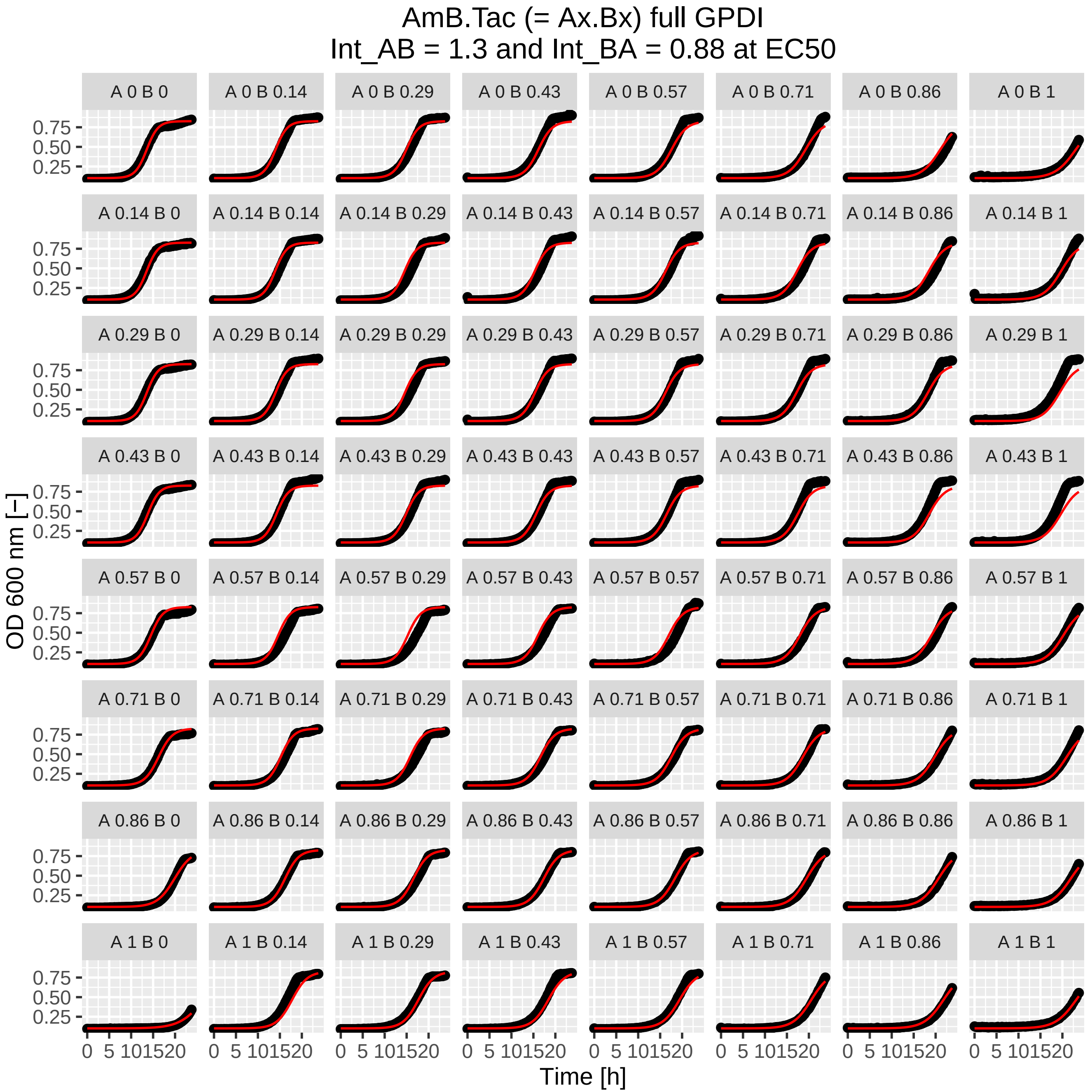
AmB.Tac (= Ax.Bx) full GPDI
Int_AB = 1.3 and Int_BA = 0.88 at EC50
A 0 B 0
A 0 B 0.14
A 0 B 0.29
A 0 B 0.43
A 0 B 0.57
A 0 B 0.71
A 0 B 0.86
A 0 B 1
A 0.14 B 0
A 0.14 B 0.14
A 0.14 B 0.29
A 0.14 B 0.43
A 0.14 B 0.57
A 0.14 B 0.71
A 0.14 B 0.86
A 0.14 B 1
A 0.29 B 0
A 0.29 B 0.14
A 0.29 B 0.29
A 0.29 B 0.43
A 0.29 B 0.57
A 0.29 B 0.71
A 0.29 B 0.86
A 0.29 B 1
A 0.43 B 0
A 0.43 B 0.14
A 0.43 B 0.29
A 0.43 B 0.43
A 0.43 B 0.57
A 0.43 B 0.71
A 0.43 B 0.86
A 0.43 B 1
A 0.57 B 0
A 0.57 B 0.14
A 0.57 B 0.29
A 0.57 B 0.43
A 0.57 B 0.57
A 0.57 B 0.71
A 0.57 B 0.86
A 0.57 B 1
A 0.71 B 0
A 0.71 B 0.14
A 0.71 B 0.29
A 0.71 B 0.43
A 0.71 B 0.57
A 0.71 B 0.71
A 0.71 B 0.86
A 0.71 B 1
A 0.86 B 0
A 0.86 B 0.14
A 0.86 B 0.29
A 0.86 B 0.43
A 0.86 B 0.57
A 0.86 B 0.71
A 0.86 B 0.86
A 0.86 B 1
A 1 B 0
A 1 B 0.14
A 1 B 0.29
A 1 B 0.43
A 1 B 0.57
A 1 B 0.71
A 1 B 0.86
A 1 B 1
0.75
0.50
0.25
OD 600 nm [–]
0 5 101520
Time [h]

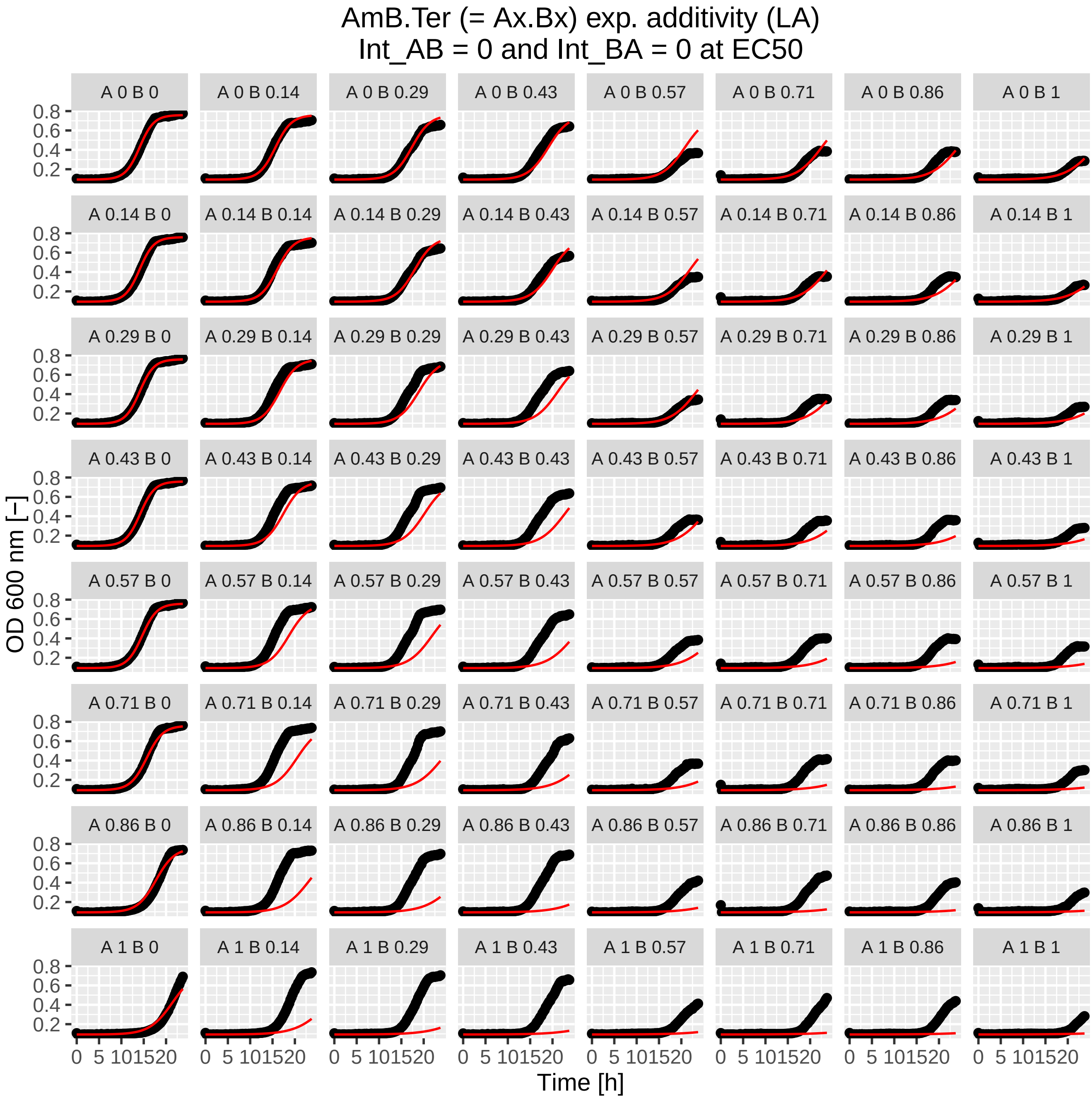

AmB.Ter (= Ax.Bx) exp. additivity (LA)
Int_AB = 0 and Int_BA = 0 at EC50
A 0 B 0
A 0 B 0.14
A 0 B 0.29
A 0 B 0.43
A 0 B 0.57
A 0 B 0.71
A 0 B 0.86
A 0 B 1
A 0.14 B 0
A 0.14 B 0.14
A 0.14 B 0.29
A 0.14 B 0.43
A 0.14 B 0.57
A 0.14 B 0.71
A 0.14 B 0.86
A 0.14 B 1
A 0.29 B 0
A 0.29 B 0.14
A 0.29 B 0.29
A 0.29 B 0.43
A 0.29 B 0.57
A 0.29 B 0.71
A 0.29 B 0.86
A 0.29 B 1
A 0.43 B 0
A 0.43 B 0.14
A 0.43 B 0.29
A 0.43 B 0.43
A 0.43 B 0.57
A 0.43 B 0.71
A 0.43 B 0.86
A 0.43 B 1
A 0.57 B 0
A 0.57 B 0.14
A 0.57 B 0.29
A 0.57 B 0.43
A 0.57 B 0.57
A 0.57 B 0.71
A 0.57 B 0.86
A 0.57 B 1
A 0.71 B 0
A 0.71 B 0.14
A 0.71 B 0.29
A 0.71 B 0.43
A 0.71 B 0.57
A 0.71 B 0.71
A 0.71 B 0.86
A 0.71 B 1
A 0.86 B 0
A 0.86 B 0.14
A 0.86 B 0.29
A 0.86 B 0.43
A 0.86 B 0.57
A 0.86 B 0.71
A 0.86 B 0.86
A 0.86 B 1
A 1 B 0
A 1 B 0.14
A 1 B 0.29
A 1 B 0.43
A 1 B 0.57
A 1 B 0.71
A 1 B 0.86
A 1 B 1
OD 600 nm [–]
0.2
0.4
0.6
0.8
0
5
10
15
20
Time [h]

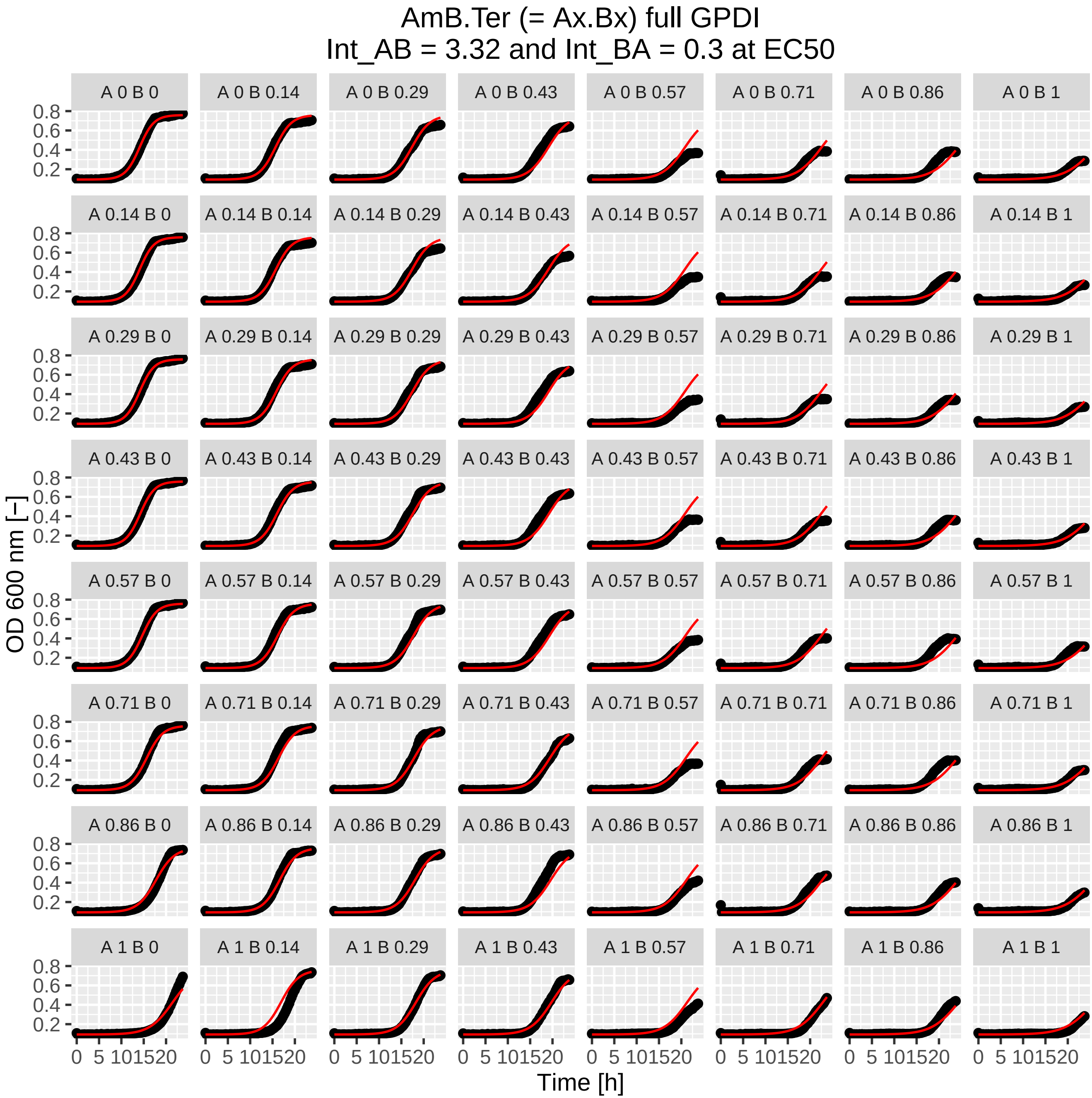
AmB.Ter (= Ax.Bx) full GPDI
Int_AB = 3.32 and Int_BA = 0.3 at EC50
A 0 B 0
A 0 B 0.14
A 0 B 0.29
A 0 B 0.43
A 0 B 0.57
A 0 B 0.71
A 0 B 0.86
A 0 B 1
A 0.14 B 0
A 0.14 B 0.14
A 0.14 B 0.29
A 0.14 B 0.43
A 0.14 B 0.57
A 0.14 B 0.71
A 0.14 B 0.86
A 0.14 B 1
A 0.29 B 0
A 0.29 B 0.14
A 0.29 B 0.29
A 0.29 B 0.43
A 0.29 B 0.57
A 0.29 B 0.71
A 0.29 B 0.86
A 0.29 B 1
A 0.43 B 0
A 0.43 B 0.14
A 0.43 B 0.29
A 0.43 B 0.43
A 0.43 B 0.57
A 0.43 B 0.71
A 0.43 B 0.86
A 0.43 B 1
A 0.57 B 0
A 0.57 B 0.14
A 0.57 B 0.29
A 0.57 B 0.43
A 0.57 B 0.57
A 0.57 B 0.71
A 0.57 B 0.86
A 0.57 B 1
A 0.71 B 0
A 0.71 B 0.14
A 0.71 B 0.29
A 0.71 B 0.43
A 0.71 B 0.57
A 0.71 B 0.71
A 0.71 B 0.86
A 0.71 B 1
A 0.86 B 0
A 0.86 B 0.14
A 0.86 B 0.29
A 0.86 B 0.43
A 0.86 B 0.57
A 0.86 B 0.71
A 0.86 B 0.86
A 0.86 B 1
A 1 B 0
A 1 B 0.14
A 1 B 0.29
A 1 B 0.43
A 1 B 0.57
A 1 B 0.71
A 1 B 0.86
A 1 B 1
OD 600 nm [–]
0.2
0.4
0.6
0.8
0
5
10
15
20
Time [h]

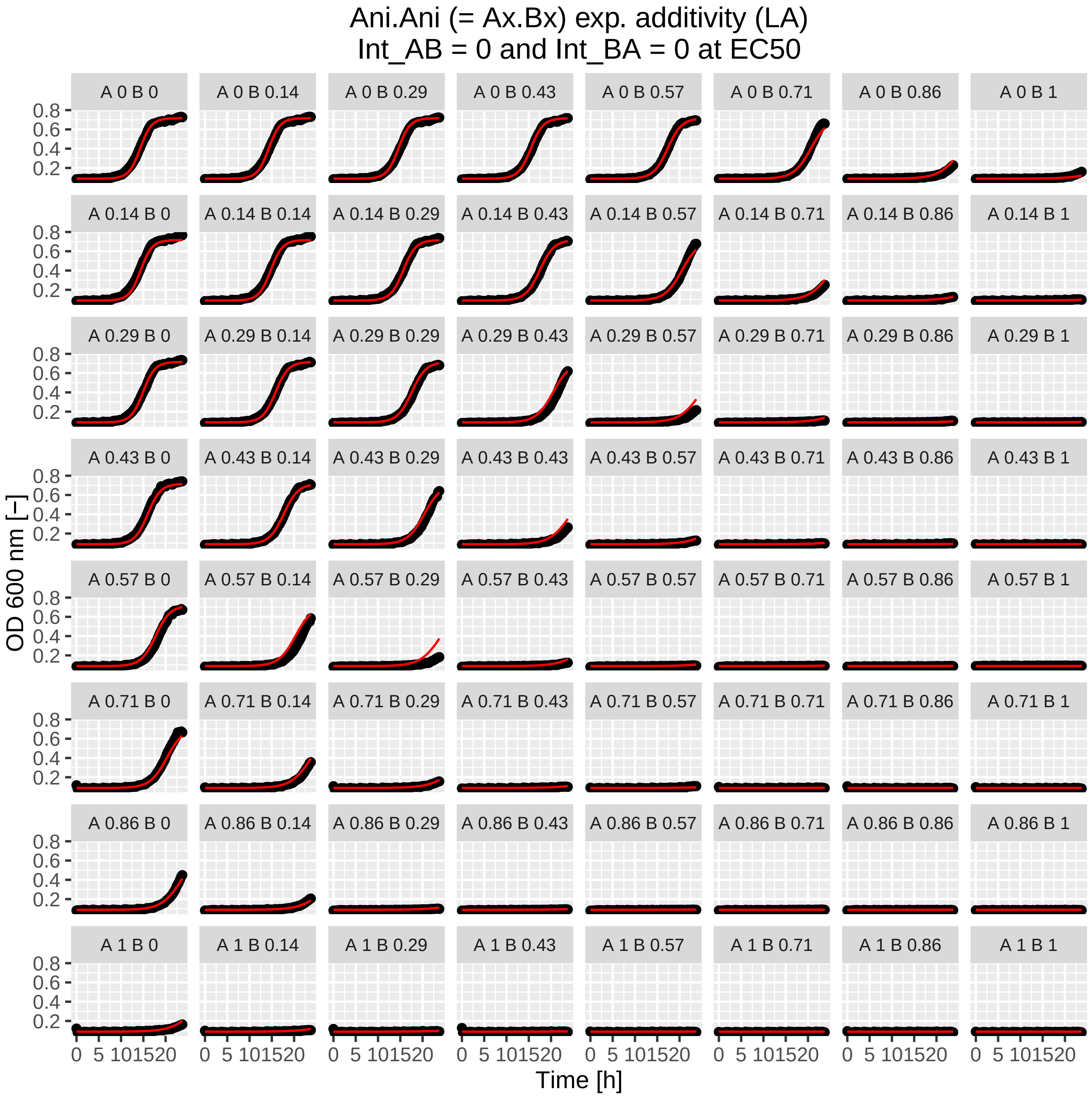

Ani.Ani (= Ax.Bx) exp. additivity (LA)
Int_AB = 0 and Int_BA = 0 at EC50
A 0 B 0
A 0 B 0.14
A 0 B 0.29
A 0 B 0.43
A 0 B 0.57
A 0 B 0.71
A 0 B 0.86
A 0 B 1
A 0.14 B 0
A 0.14 B 0.14
A 0.14 B 0.29
A 0.14 B 0.43
A 0.14 B 0.57
A 0.14 B 0.71
A 0.14 B 0.86
A 0.14 B 1
A 0.29 B 0
A 0.29 B 0.14
A 0.29 B 0.29
A 0.29 B 0.43
A 0.29 B 0.57
A 0.29 B 0.71
A 0.29 B 0.86
A 0.29 B 1
A 0.43 B 0
A 0.43 B 0.14
A 0.43 B 0.29
A 0.43 B 0.43
A 0.43 B 0.57
A 0.43 B 0.71
A 0.43 B 0.86
A 0.43 B 1
A 0.57 B 0
A 0.57 B 0.14
A 0.57 B 0.29
A 0.57 B 0.43
A 0.57 B 0.57
A 0.57 B 0.71
A 0.57 B 0.86
A 0.57 B 1
A 0.71 B 0
A 0.71 B 0.14
A 0.71 B 0.29
A 0.71 B 0.43
A 0.71 B 0.57
A 0.71 B 0.71
A 0.71 B 0.86
A 0.71 B 1
A 0.86 B 0
A 0.86 B 0.14
A 0.86 B 0.29
A 0.86 B 0.43
A 0.86 B 0.57
A 0.86 B 0.71
A 0.86 B 0.86
A 0.86 B 1
A 1 B 0
A 1 B 0.14
A 1 B 0.29
A 1 B 0.43
A 1 B 0.57
A 1 B 0.71
A 1 B 0.86
A 1 B 1
OD 600 nm [–]
0.2
0.4
0.6
0.8
0 5 101520
Time [h]

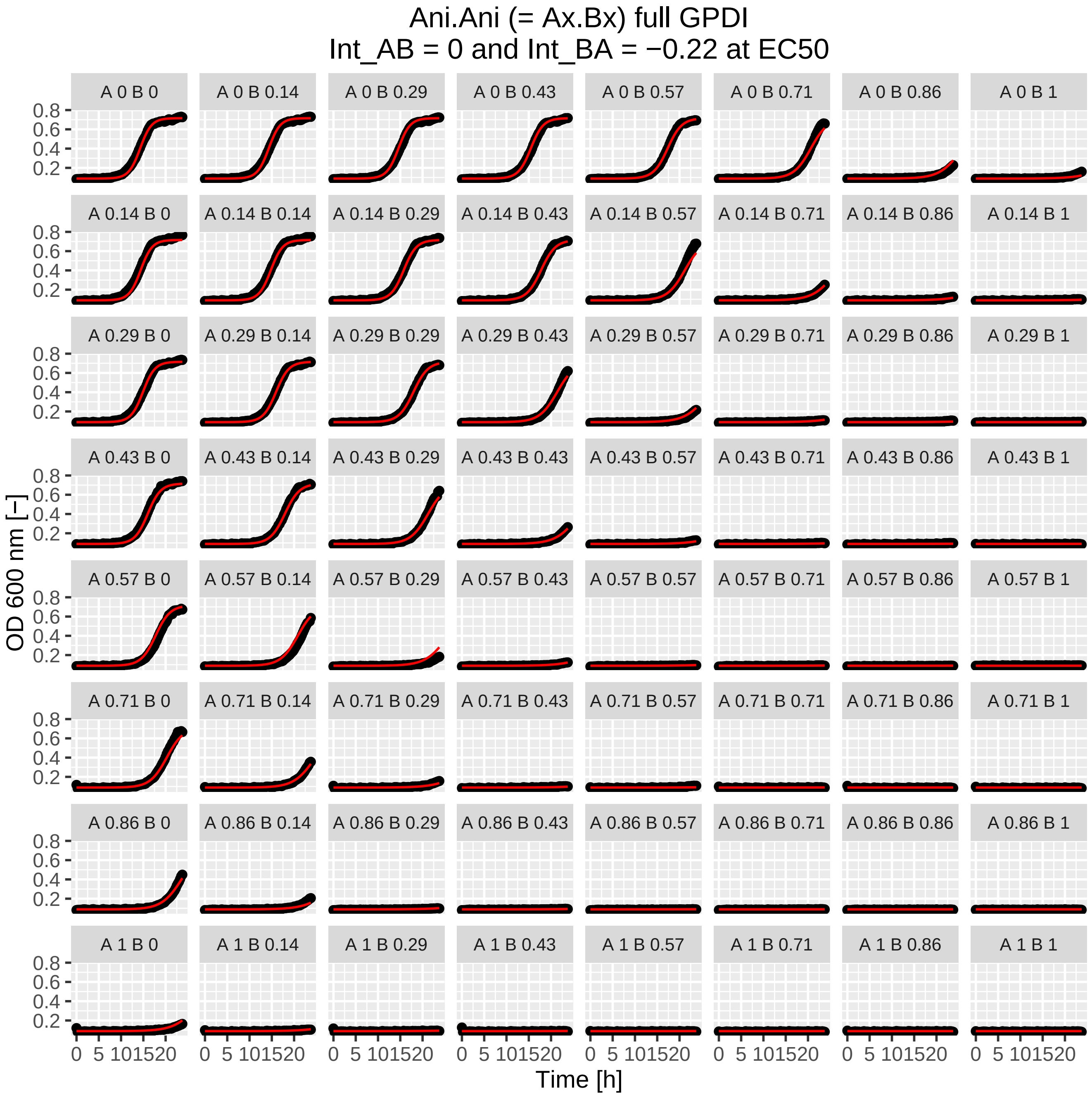
Ani.Ani (= Ax.Bx) full GPDI
Int_AB = 0 and Int_BA = −0.22 at EC50
OD 600 nm [–]
Time [h]
A 0 B 0
A 0 B 0.14
A 0 B 0.29
A 0 B 0.43
A 0 B 0.57
A 0 B 0.71
A 0 B 0.86
A 0 B 1
A 0.14 B 0
A 0.29 B 0
A 0.43 B 0
A 0.57 B 0
A 0.71 B 0
A 0.86 B 0
A 1 B 0
A 1 B 1

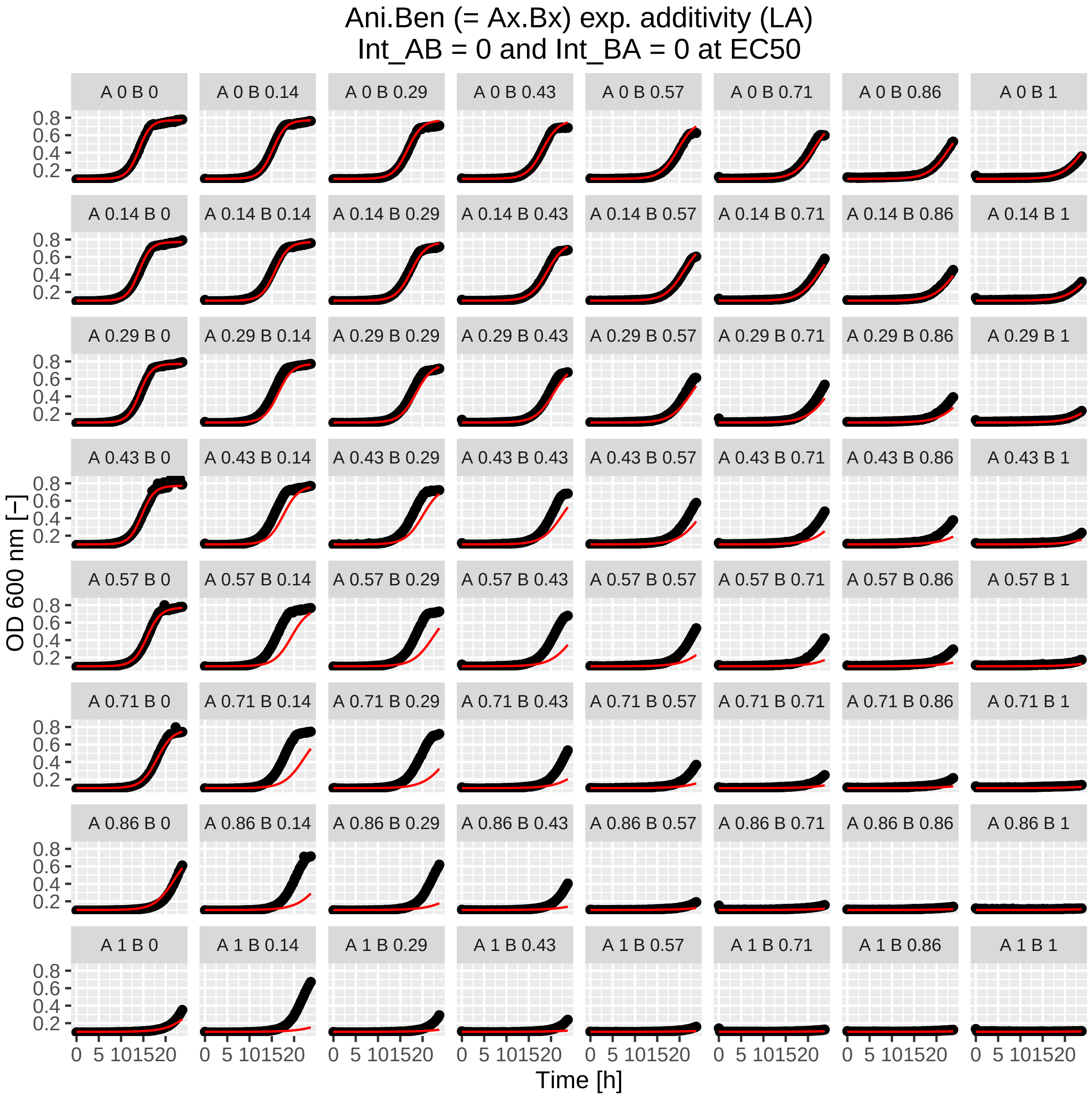

Ani.Ben (= Ax.Bx) exp. additivity (LA)
Int_AB = 0 and Int_BA = 0 at EC50
OD 600 nm [–]
Time [h]
A 0 B 0
A 0 B 0.14
A 0 B 0.29
A 0 B 0.43
A 0 B 0.57
A 0 B 0.71
A 0 B 0.86
A 0 B 1
A 1 B 0
A 1 B 0.14
A 1 B 0.29
A 1 B 0.43
A 1 B 0.57
A 1 B 0.71
A 1 B 0.86
A 1 B 1

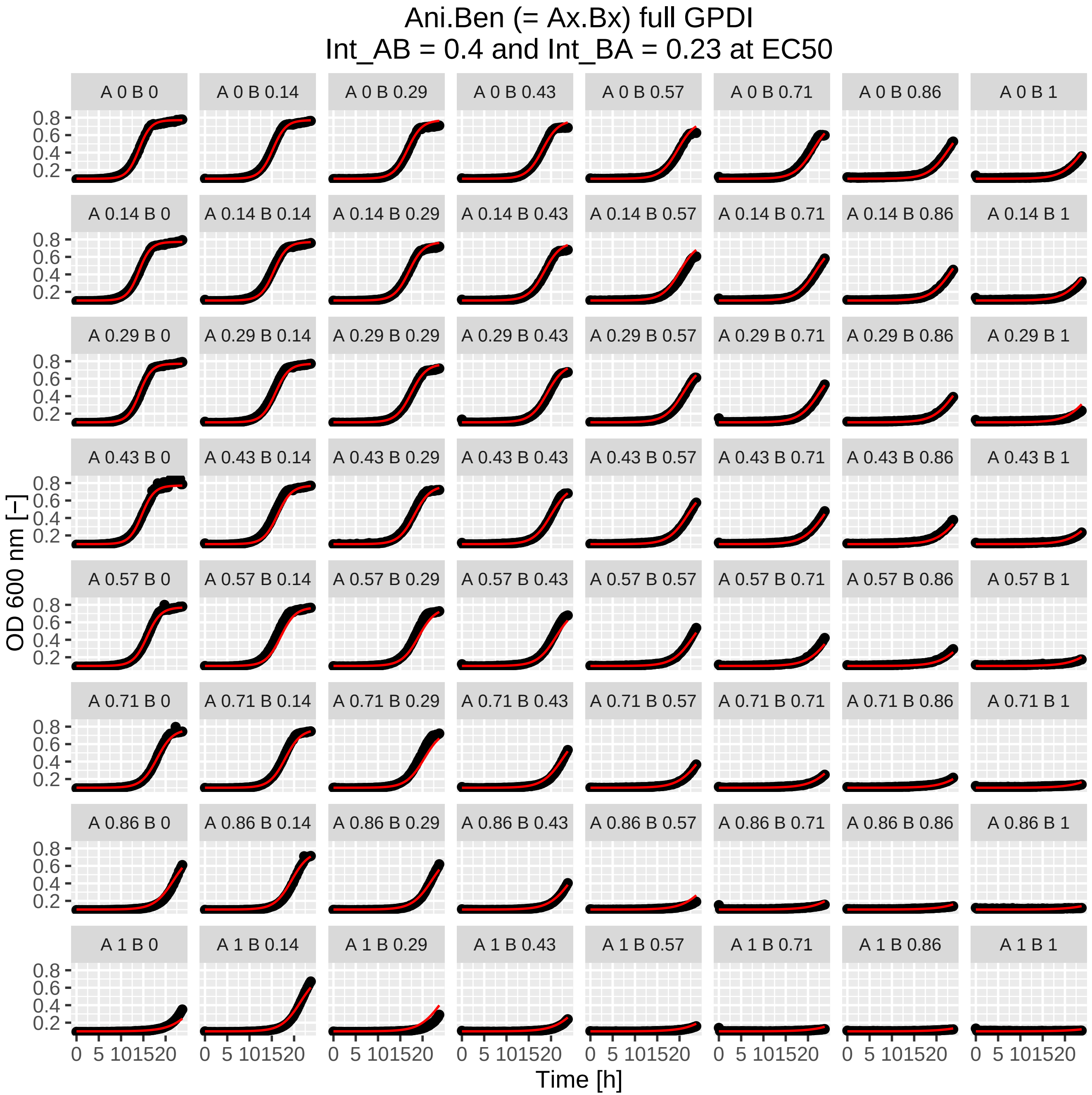

Ani.Ben (= Ax.Bx) full GPDI
Int_AB = 0.4 and Int_BA = 0.23 at EC50
A 0 B 0
A 0 B 0.14
A 0 B 0.29
A 0 B 0.43
A 0 B 0.57
A 0 B 0.71
A 0 B 0.86
A 0 B 1
A 0.14 B 0
A 0.14 B 0.14
A 0.14 B 0.29
A 0.14 B 0.43
A 0.14 B 0.57
A 0.14 B 0.71
A 0.14 B 0.86
A 0.14 B 1
A 0.29 B 0
A 0.29 B 0.14
A 0.29 B 0.29
A 0.29 B 0.43
A 0.29 B 0.57
A 0.29 B 0.71
A 0.29 B 0.86
A 0.29 B 1
A 0.43 B 0
A 0.43 B 0.14
A 0.43 B 0.29
A 0.43 B 0.43
A 0.43 B 0.57
A 0.43 B 0.71
A 0.43 B 0.86
A 0.43 B 1
A 0.57 B 0
A 0.57 B 0.14
A 0.57 B 0.29
A 0.57 B 0.43
A 0.57 B 0.57
A 0.57 B 0.71
A 0.57 B 0.86
A 0.57 B 1
A 0.71 B 0
A 0.71 B 0.14
A 0.71 B 0.29
A 0.71 B 0.43
A 0.71 B 0.57
A 0.71 B 0.71
A 0.71 B 0.86
A 0.71 B 1
A 0.86 B 0
A 0.86 B 0.14
A 0.86 B 0.29
A 0.86 B 0.43
A 0.86 B 0.57
A 0.86 B 0.71
A 0.86 B 0.86
A 0.86 B 1
A 1 B 0
A 1 B 0.14
A 1 B 0.29
A 1 B 0.43
A 1 B 0.57
A 1 B 0.71
A 1 B 0.86
A 1 B 1
OD 600 nm [–]
0.2
0.4
0.6
0.8
0
5
10
15
20
Time [h]

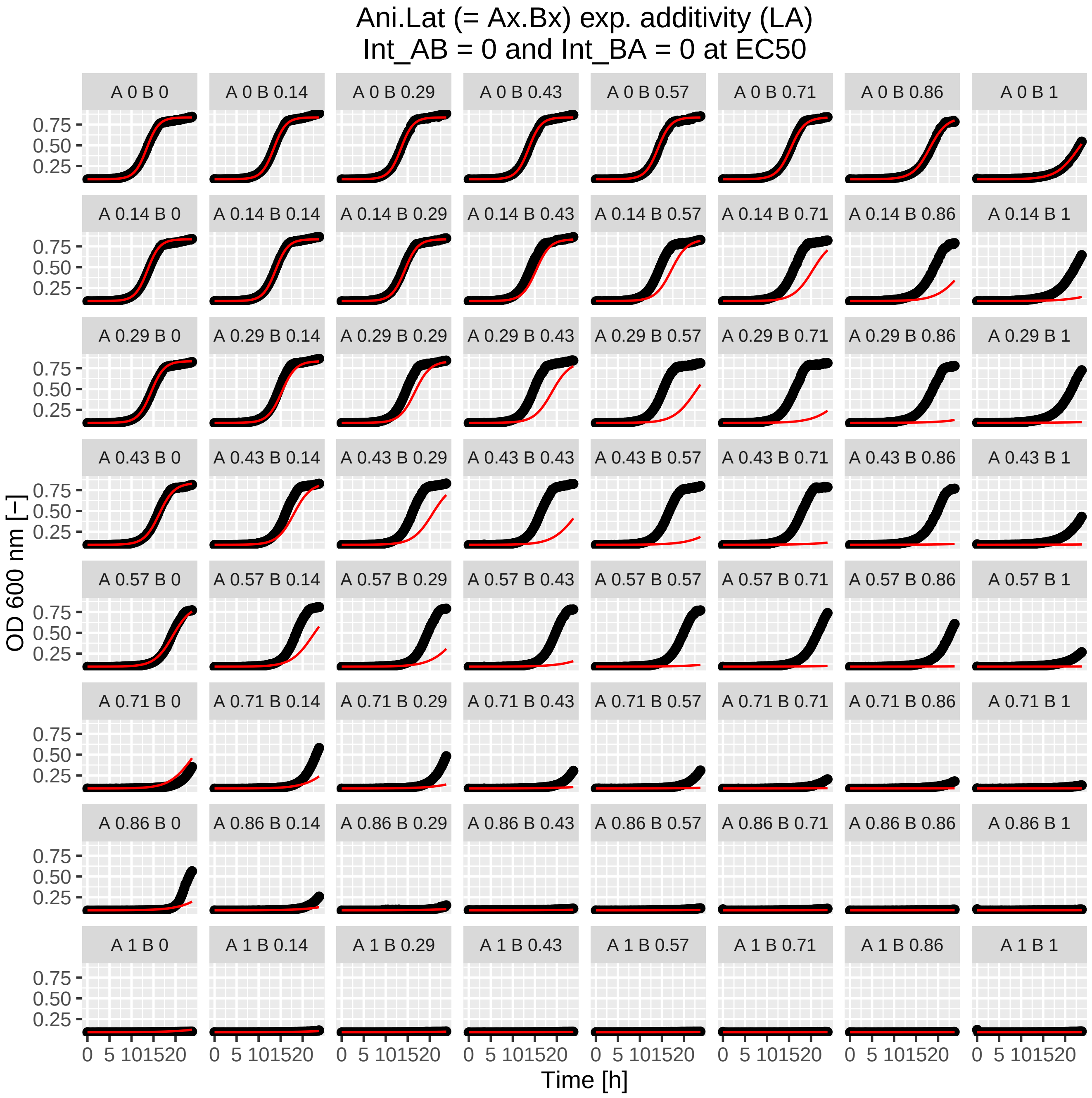
Ani.Lat (= Ax.Bx) exp. additivity (LA)
Int_AB = 0 and Int_BA = 0 at EC50
A 0 B 0
A 0 B 0.14
A 0 B 0.29
A 0 B 0.43
A 0 B 0.57
A 0 B 0.71
A 0 B 0.86
A 0 B 1
A 0.14 B 0
A 0.14 B 0.14
A 0.14 B 0.29
A 0.14 B 0.43
A 0.14 B 0.57
A 0.14 B 0.71
A 0.14 B 0.86
A 0.14 B 1
A 0.29 B 0
A 0.29 B 0.14
A 0.29 B 0.29
A 0.29 B 0.43
A 0.29 B 0.57
A 0.29 B 0.71
A 0.29 B 0.86
A 0.29 B 1
A 0.43 B 0
A 0.43 B 0.14
A 0.43 B 0.29
A 0.43 B 0.43
A 0.43 B 0.57
A 0.43 B 0.71
A 0.43 B 0.86
A 0.43 B 1
A 0.57 B 0
A 0.57 B 0.14
A 0.57 B 0.29
A 0.57 B 0.43
A 0.57 B 0.57
A 0.57 B 0.71
A 0.57 B 0.86
A 0.57 B 1
A 0.71 B 0
A 0.71 B 0.14
A 0.71 B 0.29
A 0.71 B 0.43
A 0.71 B 0.57
A 0.71 B 0.71
A 0.71 B 0.86
A 0.71 B 1
A 0.86 B 0
A 0.86 B 0.14
A 0.86 B 0.29
A 0.86 B 0.43
A 0.86 B 0.57
A 0.86 B 0.71
A 0.86 B 0.86
A 0.86 B 1
A 1 B 0
A 1 B 0.14
A 1 B 0.29
A 1 B 0.43
A 1 B 0.57
A 1 B 0.71
A 1 B 0.86
A 1 B 1
OD 600 nm [–]
0.75
0.50
0.25
0 5 101520
Time [h]

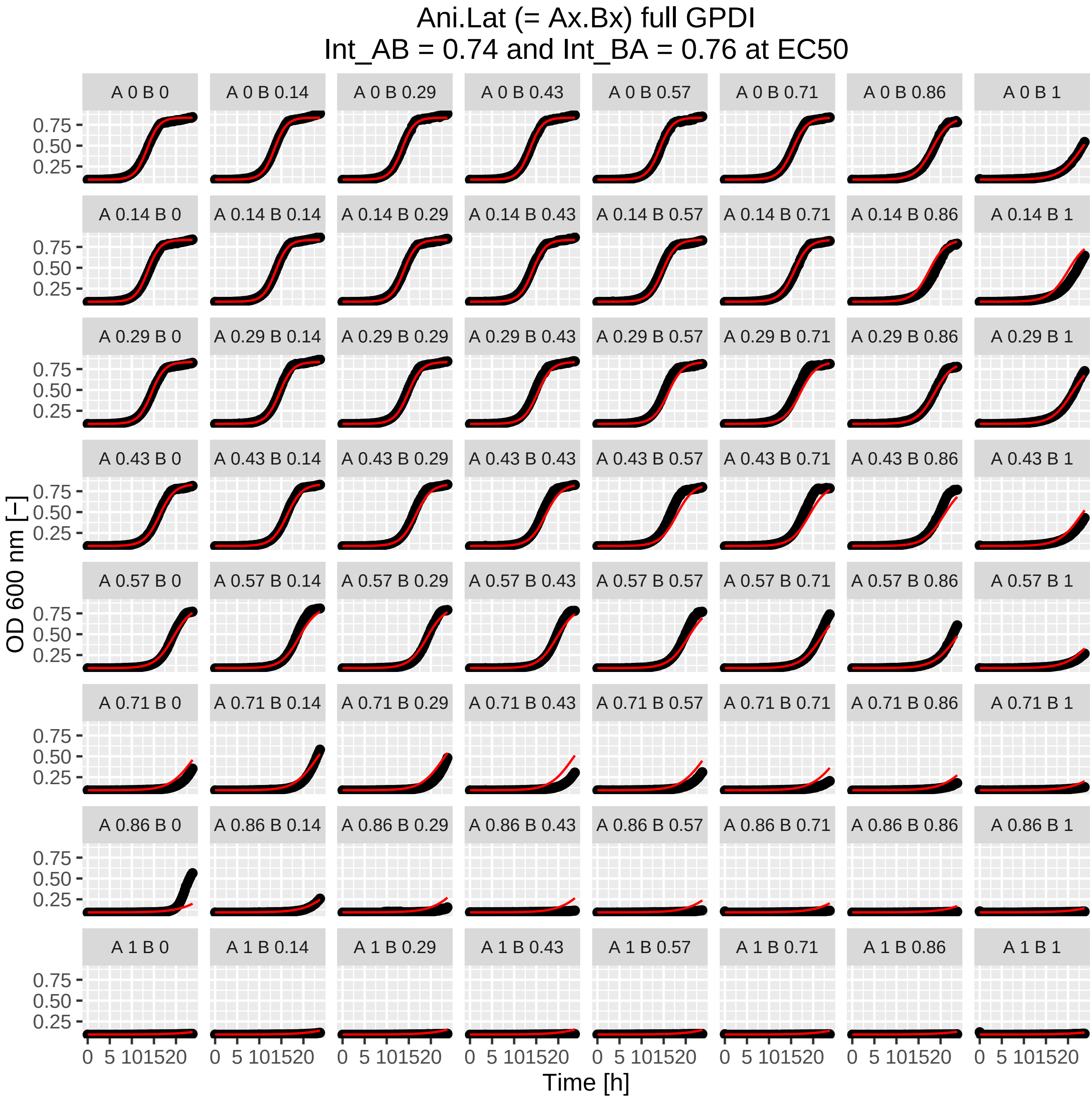

Ani.Lat (= Ax.Bx) full GPDI
Int_AB = 0.74 and Int_BA = 0.76 at EC50
A 0 B 0
A 0 B 0.14
A 0 B 0.29
A 0 B 0.43
A 0 B 0.57
A 0 B 0.71
A 0 B 0.86
A 0 B 1
A 0.14 B 0
A 0.14 B 0.14
A 0.14 B 0.29
A 0.14 B 0.43
A 0.14 B 0.57
A 0.14 B 0.71
A 0.14 B 0.86
A 0.14 B 1
A 0.29 B 0
A 0.29 B 0.14
A 0.29 B 0.29
A 0.29 B 0.43
A 0.29 B 0.57
A 0.29 B 0.71
A 0.29 B 0.86
A 0.29 B 1
A 0.43 B 0
A 0.43 B 0.14
A 0.43 B 0.29
A 0.43 B 0.43
A 0.43 B 0.57
A 0.43 B 0.71
A 0.43 B 0.86
A 0.43 B 1
A 0.57 B 0
A 0.57 B 0.14
A 0.57 B 0.29
A 0.57 B 0.43
A 0.57 B 0.57
A 0.57 B 0.71
A 0.57 B 0.86
A 0.57 B 1
A 0.71 B 0
A 0.71 B 0.14
A 0.71 B 0.29
A 0.71 B 0.43
A 0.71 B 0.57
A 0.71 B 0.71
A 0.71 B 0.86
A 0.71 B 1
A 0.86 B 0
A 0.86 B 0.14
A 0.86 B 0.29
A 0.86 B 0.43
A 0.86 B 0.57
A 0.86 B 0.71
A 0.86 B 0.86
A 0.86 B 1
A 1 B 0
A 1 B 0.14
A 1 B 0.29
A 1 B 0.43
A 1 B 0.57
A 1 B 0.71
A 1 B 0.86
A 1 B 1
0.75
0.50
0.25
OD 600 nm [–]
0 5 101520
Time [h]

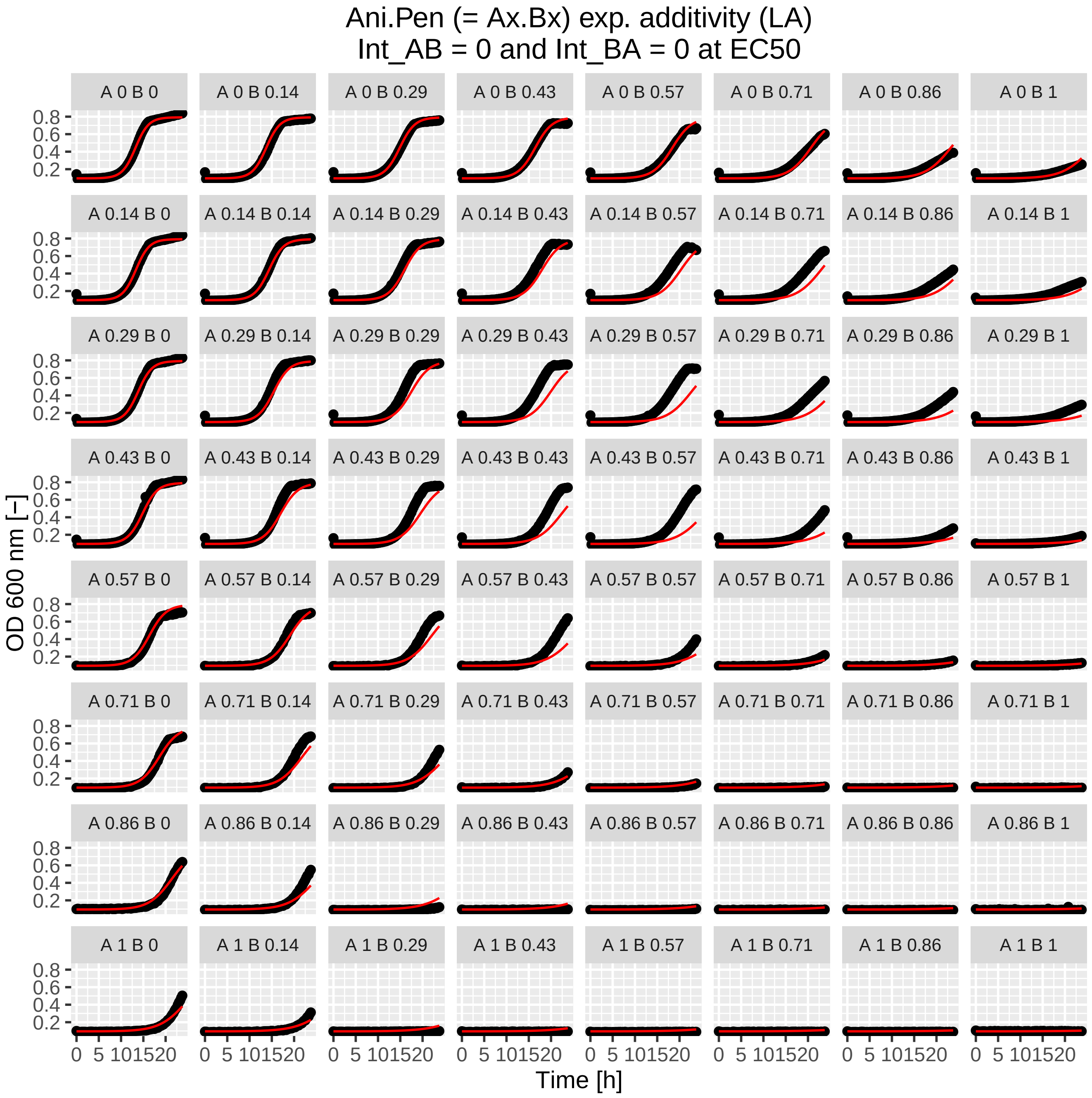

Ani.Pen (= Ax.Bx) exp. additivity (LA)
Int_AB = 0 and Int_BA = 0 at EC50
A 0 B 0
A 0 B 0.14
A 0 B 0.29
A 0 B 0.43
A 0 B 0.57
A 0 B 0.71
A 0 B 0.86
A 0 B 1
A 0.14 B 0
A 0.14 B 0.14
A 0.14 B 0.29
A 0.14 B 0.43
A 0.14 B 0.57
A 0.14 B 0.71
A 0.14 B 0.86
A 0.14 B 1
A 0.29 B 0
A 0.29 B 0.14
A 0.29 B 0.29
A 0.29 B 0.43
A 0.29 B 0.57
A 0.29 B 0.71
A 0.29 B 0.86
A 0.29 B 1
A 0.43 B 0
A 0.43 B 0.14
A 0.43 B 0.29
A 0.43 B 0.43
A 0.43 B 0.57
A 0.43 B 0.71
A 0.43 B 0.86
A 0.43 B 1
A 0.57 B 0
A 0.57 B 0.14
A 0.57 B 0.29
A 0.57 B 0.43
A 0.57 B 0.57
A 0.57 B 0.71
A 0.57 B 0.86
A 0.57 B 1
A 0.71 B 0
A 0.71 B 0.14
A 0.71 B 0.29
A 0.71 B 0.43
A 0.71 B 0.57
A 0.71 B 0.71
A 0.71 B 0.86
A 0.71 B 1
A 0.86 B 0
A 0.86 B 0.14
A 0.86 B 0.29
A 0.86 B 0.43
A 0.86 B 0.57
A 0.86 B 0.71
A 0.86 B 0.86
A 0.86 B 1
A 1 B 0
A 1 B 0.14
A 1 B 0.29
A 1 B 0.43
A 1 B 0.57
A 1 B 0.71
A 1 B 0.86
A 1 B 1
OD 600 nm [–]
0.8
0.6
0.4
0.2
0 5 101520
Time [h]

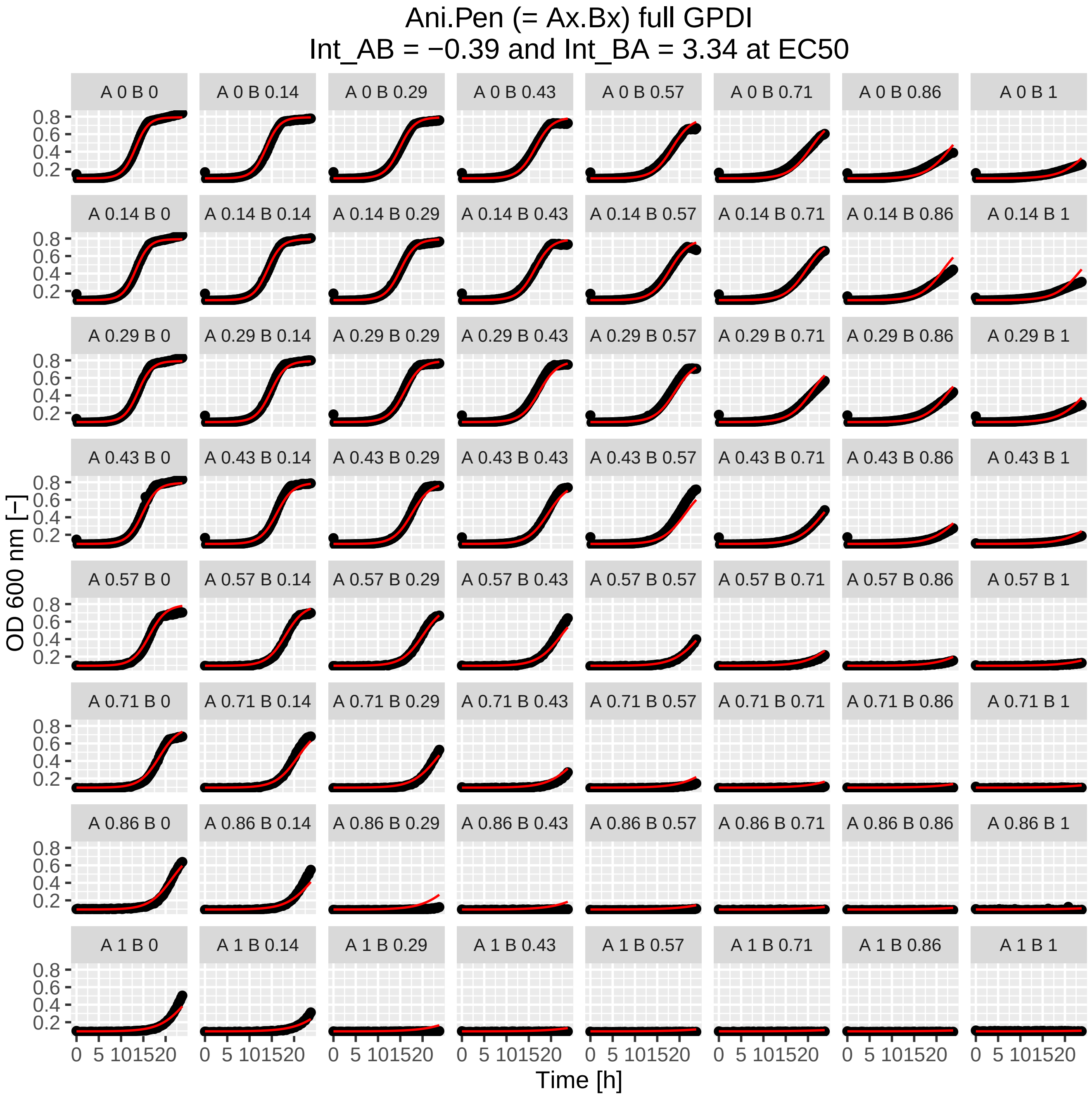
Ani.Pen (= Ax.Bx) full GPDI
Int_AB = −0.39 and Int_BA = 3.34 at EC50
OD 600 nm [−]
Time [h]
0 5 101520
0.2
0.4
0.6
0.8
A 0 B 0
A 0 B 0.14
A 0 B 0.29
A 0 B 0.43
A 0 B 0.57
A 0 B 0.71
A 0 B 0.86
A 0 B 1
A 0.14 B 0
A 0.14 B 0.14
A 0.14 B 0.29
A 0.14 B 0.43
A 0.14 B 0.57
A 0.14 B 0.71
A 0.14 B 0.86
A 0.14 B 1
A 0.29 B 0
A 0.29 B 0.14
A 0.29 B 0.29
A 0.29 B 0.43
A 0.29 B 0.57
A 0.29 B 0.71
A 0.29 B 0.86
A 0.29 B 1
A 0.43 B 0
A 0.43 B 0.14
A 0.43 B 0.29
A 0.43 B 0.43
A 0.43 B 0.57
A 0.43 B 0.71
A 0.43 B 0.86
A 0.43 B 1
A 0.57 B 0
A 0.57 B 0.14
A 0.57 B 0.29
A 0.57 B 0.43
A 0.57 B 0.57
A 0.57 B 0.71
A 0.57 B 0.86
A 0.57 B 1
A 0.71 B 0
A 0.71 B 0.14
A 0.71 B 0.29
A 0.71 B 0.43
A 0.71 B 0.57
A 0.71 B 0.71
A 0.71 B 0.86
A 0.71 B 1
A 0.86 B 0
A 0.86 B 0.14
A 0.86 B 0.29
A 0.86 B 0.43
A 0.86 B 0.57
A 0.86 B 0.71
A 0.86 B 0.86
A 0.86 B 1
A 1 B 0
A 1 B 0.14
A 1 B 0.29
A 1 B 0.43
A 1 B 0.57
A 1 B 0.71
A 1 B 0.86
A 1 B 1

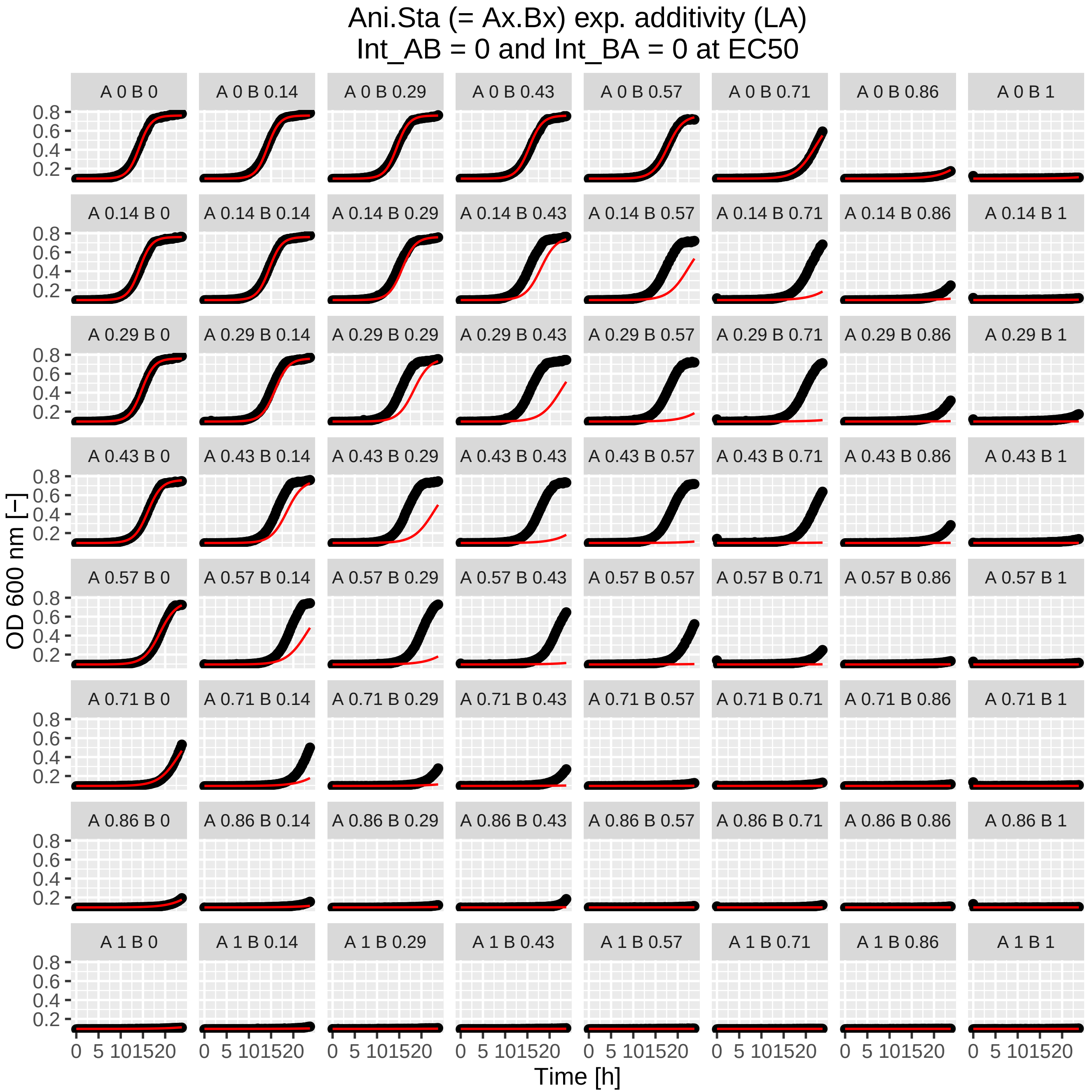

Ani.Sta (= Ax.Bx) exp. additivity (LA)
Int_AB = 0 and Int_BA = 0 at EC50
A 0 B 0
A 0 B 0.14
A 0 B 0.29
A 0 B 0.43
A 0 B 0.57
A 0 B 0.71
A 0 B 0.86
A 0 B 1
A 0.14 B 0
A 0.14 B 0.14
A 0.14 B 0.29
A 0.14 B 0.43
A 0.14 B 0.57
A 0.14 B 0.71
A 0.14 B 0.86
A 0.14 B 1
A 0.29 B 0
A 0.29 B 0.14
A 0.29 B 0.29
A 0.29 B 0.43
A 0.29 B 0.57
A 0.29 B 0.71
A 0.29 B 0.86
A 0.29 B 1
A 0.43 B 0
A 0.43 B 0.14
A 0.43 B 0.29
A 0.43 B 0.43
A 0.43 B 0.57
A 0.43 B 0.71
A 0.43 B 0.86
A 0.43 B 1
A 0.57 B 0
A 0.57 B 0.14
A 0.57 B 0.29
A 0.57 B 0.43
A 0.57 B 0.57
A 0.57 B 0.71
A 0.57 B 0.86
A 0.57 B 1
A 0.71 B 0
A 0.71 B 0.14
A 0.71 B 0.29
A 0.71 B 0.43
A 0.71 B 0.57
A 0.71 B 0.71
A 0.71 B 0.86
A 0.71 B 1
A 0.86 B 0
A 0.86 B 0.14
A 0.86 B 0.29
A 0.86 B 0.43
A 0.86 B 0.57
A 0.86 B 0.71
A 0.86 B 0.86
A 0.86 B 1
A 1 B 0
A 1 B 0.14
A 1 B 0.29
A 1 B 0.43
A 1 B 0.57
A 1 B 0.71
A 1 B 0.86
A 1 B 1
OD 600 nm [−]
0.2
0.4
0.6
0.8
0 5 101520
Time [h]

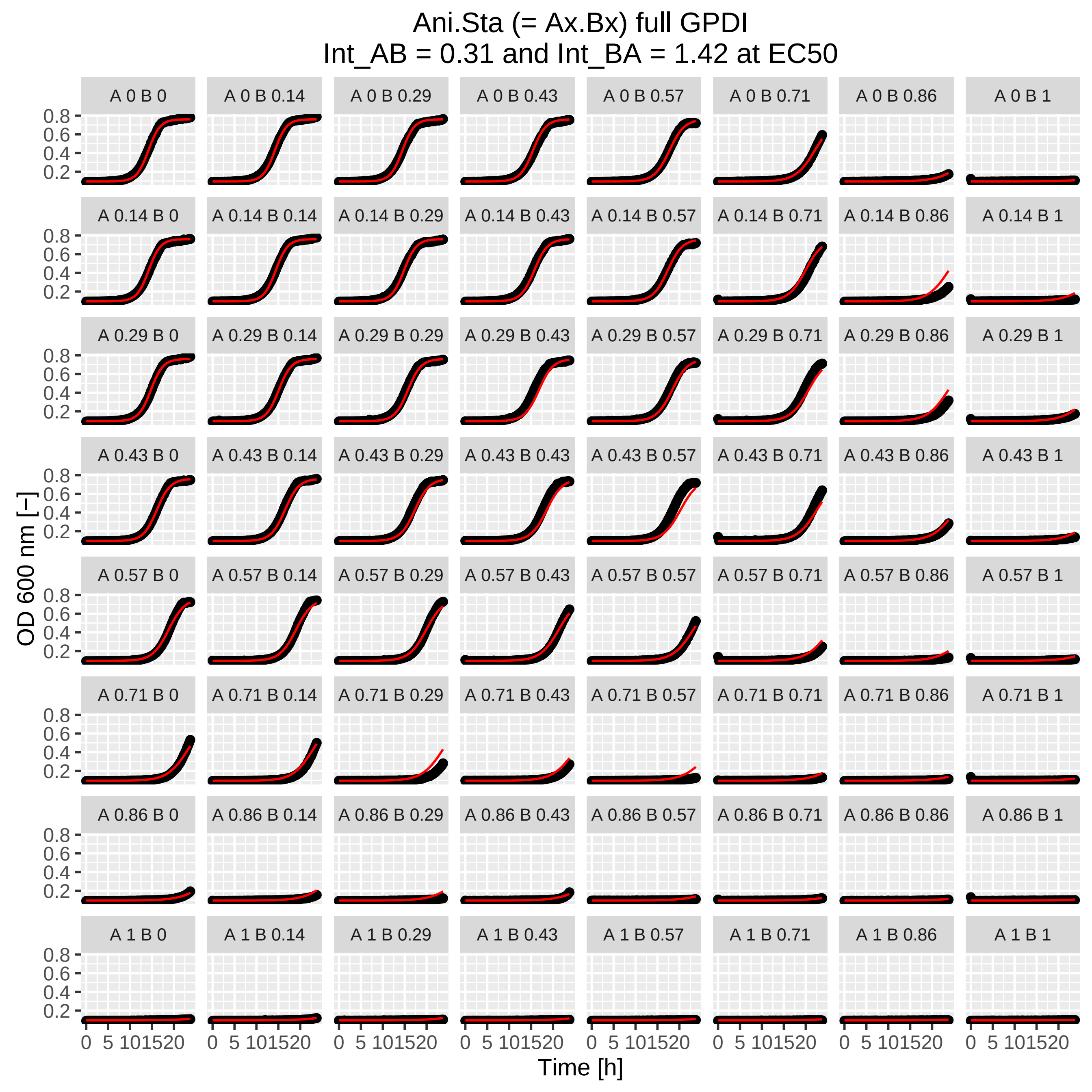

Ani.Sta (= Ax.Bx) full GPDI
Int_AB = 0.31 and Int_BA = 1.42 at EC50
OD 600 nm [−]
Time [h]

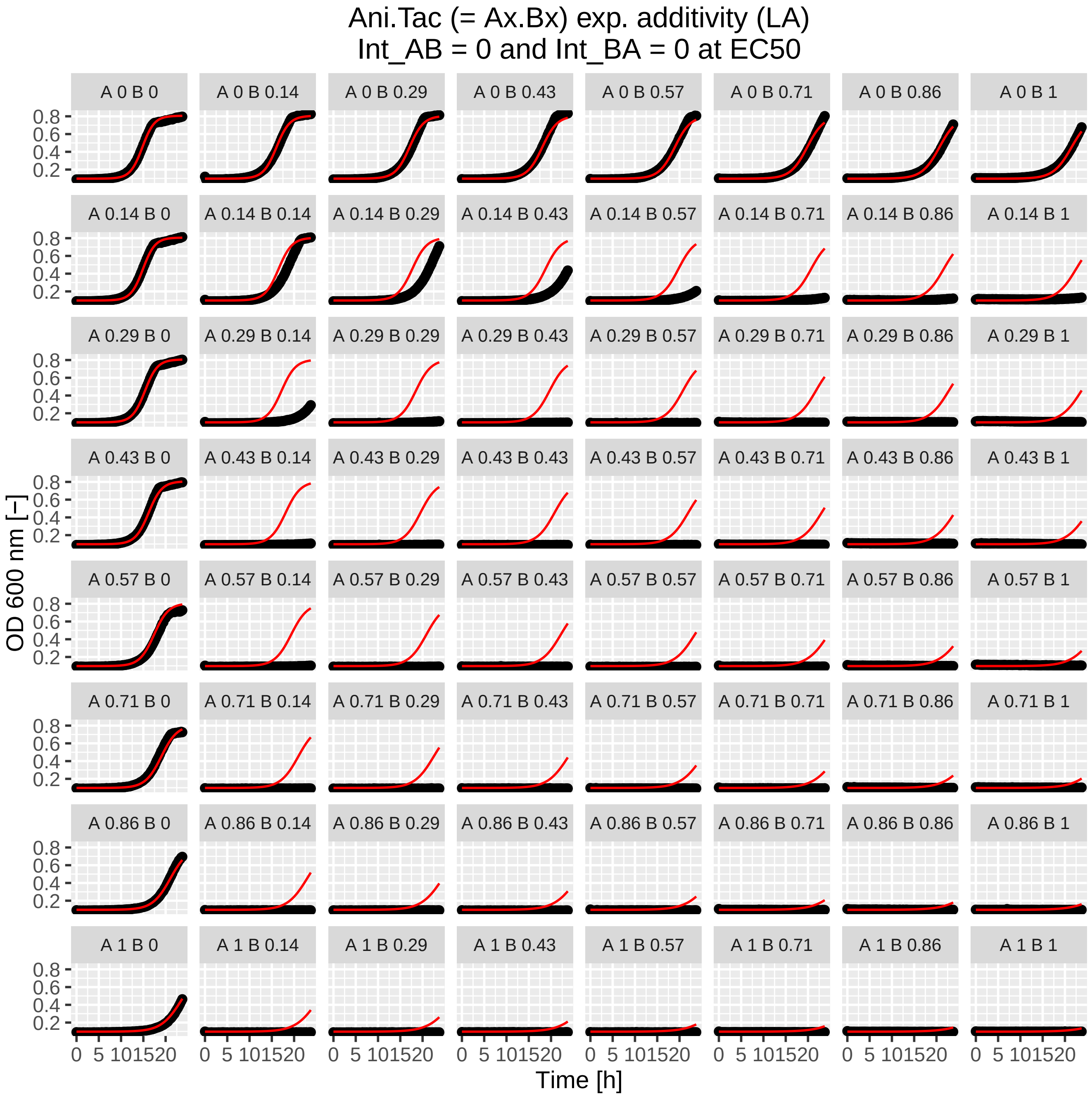

Ani.Tac (= Ax.Bx) exp. additivity (LA)
Int_AB = 0 and Int_BA = 0 at EC50
A 0 B 0
A 0 B 0.14
A 0 B 0.29
A 0 B 0.43
A 0 B 0.57
A 0 B 0.71
A 0 B 0.86
A 0 B 1
A 0.14 B 0
A 0.14 B 0.14
A 0.14 B 0.29
A 0.14 B 0.43
A 0.14 B 0.57
A 0.14 B 0.71
A 0.14 B 0.86
A 0.14 B 1
A 0.29 B 0
A 0.29 B 0.14
A 0.29 B 0.29
A 0.29 B 0.43
A 0.29 B 0.57
A 0.29 B 0.71
A 0.29 B 0.86
A 0.29 B 1
A 0.43 B 0
A 0.43 B 0.14
A 0.43 B 0.29
A 0.43 B 0.43
A 0.43 B 0.57
A 0.43 B 0.71
A 0.43 B 0.86
A 0.43 B 1
A 0.57 B 0
A 0.57 B 0.14
A 0.57 B 0.29
A 0.57 B 0.43
A 0.57 B 0.57
A 0.57 B 0.71
A 0.57 B 0.86
A 0.57 B 1
A 0.71 B 0
A 0.71 B 0.14
A 0.71 B 0.29
A 0.71 B 0.43
A 0.71 B 0.57
A 0.71 B 0.71
A 0.71 B 0.86
A 0.71 B 1
A 0.86 B 0
A 0.86 B 0.14
A 0.86 B 0.29
A 0.86 B 0.43
A 0.86 B 0.57
A 0.86 B 0.71
A 0.86 B 0.86
A 0.86 B 1
A 1 B 0
A 1 B 0.14
A 1 B 0.29
A 1 B 0.43
A 1 B 0.57
A 1 B 0.71
A 1 B 0.86
A 1 B 1
OD 600 nm [–]
0.8
0.6
0.4
0.2
0
5
10
15
20
Time [h]

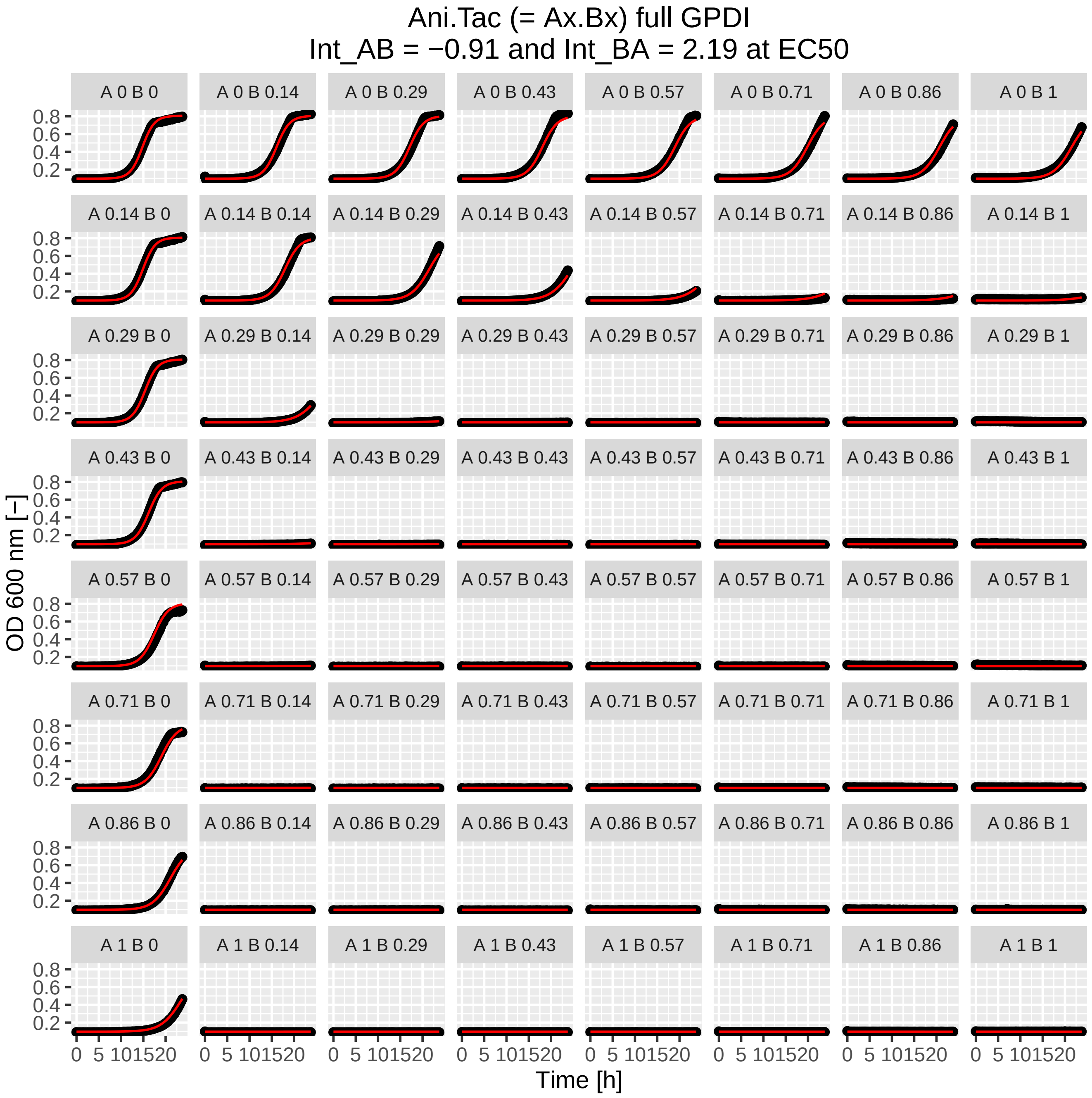
Ani.Tac (= Ax.Bx) full GPDI
Int_AB = −0.91 and Int_BA = 2.19 at EC50
A 0 B 0
A 0 B 0.14
A 0 B 0.29
A 0 B 0.43
A 0 B 0.57
A 0 B 0.71
A 0 B 0.86
A 0 B 1
A 0.14 B 0
A 0.14 B 0.14
A 0.14 B 0.29
A 0.14 B 0.43
A 0.14 B 0.57
A 0.14 B 0.71
A 0.14 B 0.86
A 0.14 B 1
A 0.29 B 0
A 0.29 B 0.14
A 0.29 B 0.29
A 0.29 B 0.43
A 0.29 B 0.57
A 0.29 B 0.71
A 0.29 B 0.86
A 0.29 B 1
A 0.43 B 0
A 0.43 B 0.14
A 0.43 B 0.29
A 0.43 B 0.43
A 0.43 B 0.57
A 0.43 B 0.71
A 0.43 B 0.86
A 0.43 B 1
A 0.57 B 0
A 0.57 B 0.14
A 0.57 B 0.29
A 0.57 B 0.43
A 0.57 B 0.57
A 0.57 B 0.71
A 0.57 B 0.86
A 0.57 B 1
A 0.71 B 0
A 0.71 B 0.14
A 0.71 B 0.29
A 0.71 B 0.43
A 0.71 B 0.57
A 0.71 B 0.71
A 0.71 B 0.86
A 0.71 B 1
A 0.86 B 0
A 0.86 B 0.14
A 0.86 B 0.29
A 0.86 B 0.43
A 0.86 B 0.57
A 0.86 B 0.71
A 0.86 B 0.86
A 0.86 B 1
A 1 B 0
A 1 B 0.14
A 1 B 0.29
A 1 B 0.43
A 1 B 0.57
A 1 B 0.71
A 1 B 0.86
A 1 B 1
OD 600 nm [−]
0.8
0.6
0.4
0.2
0 5 101520
Time [h]

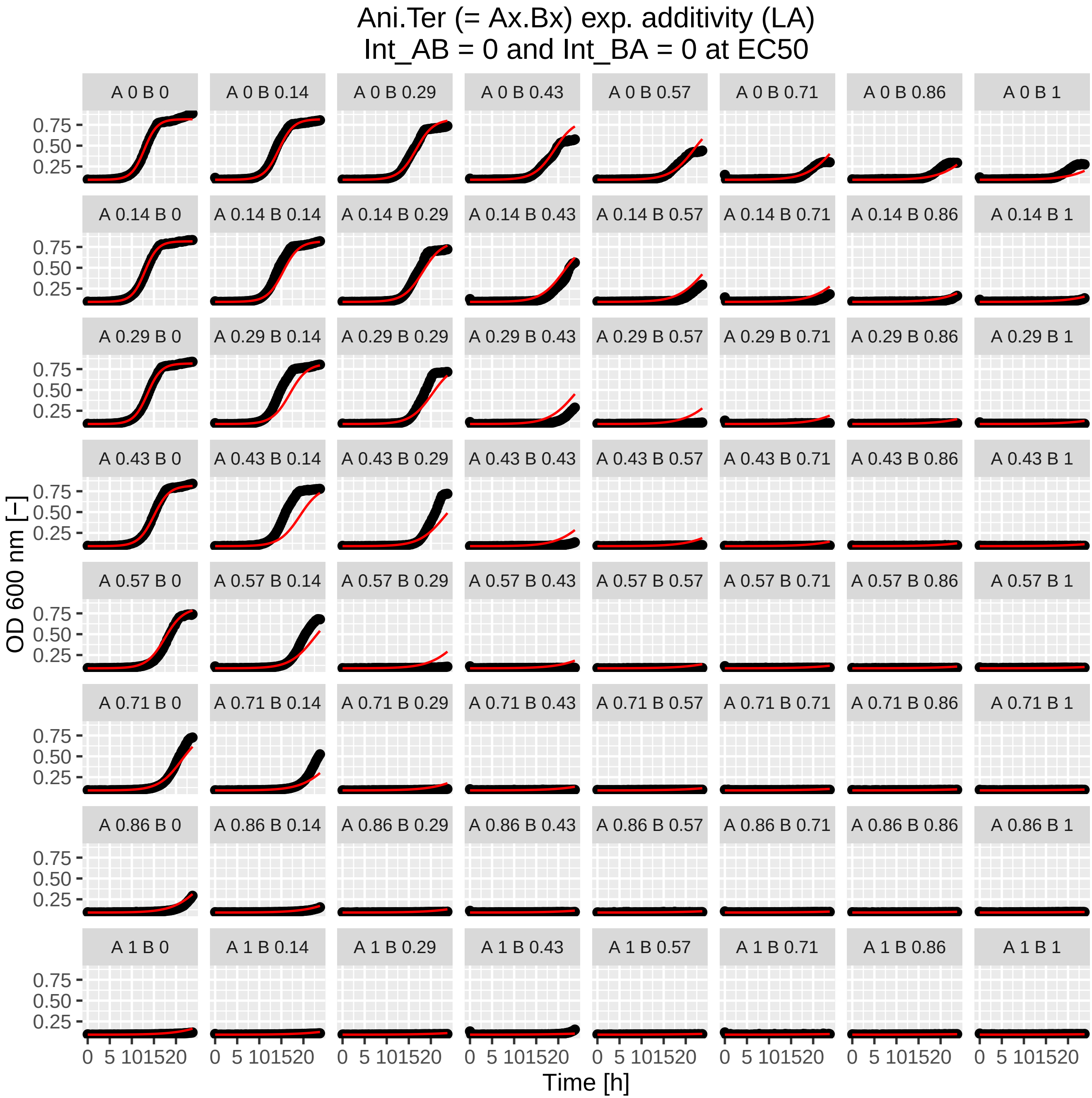

Ani.Ter (= Ax.Bx) exp. additivity (LA)
Int_AB = 0 and Int_BA = 0 at EC50
A 0 B 0
A 0 B 0.14
A 0 B 0.29
A 0 B 0.43
A 0 B 0.57
A 0 B 0.71
A 0 B 0.86
A 0 B 1
A 0.14 B 0
A 0.14 B 0.14
A 0.14 B 0.29
A 0.14 B 0.43
A 0.14 B 0.57
A 0.14 B 0.71
A 0.14 B 0.86
A 0.14 B 1
A 0.29 B 0
A 0.29 B 0.14
A 0.29 B 0.29
A 0.29 B 0.43
A 0.29 B 0.57
A 0.29 B 0.71
A 0.29 B 0.86
A 0.29 B 1
A 0.43 B 0
A 0.43 B 0.14
A 0.43 B 0.29
A 0.43 B 0.43
A 0.43 B 0.57
A 0.43 B 0.71
A 0.43 B 0.86
A 0.43 B 1
A 0.57 B 0
A 0.57 B 0.14
A 0.57 B 0.29
A 0.57 B 0.43
A 0.57 B 0.57
A 0.57 B 0.71
A 0.57 B 0.86
A 0.57 B 1
A 0.71 B 0
A 0.71 B 0.14
A 0.71 B 0.29
A 0.71 B 0.43
A 0.71 B 0.57
A 0.71 B 0.71
A 0.71 B 0.86
A 0.71 B 1
A 0.86 B 0
A 0.86 B 0.14
A 0.86 B 0.29
A 0.86 B 0.43
A 0.86 B 0.57
A 0.86 B 0.71
A 0.86 B 0.86
A 0.86 B 1
A 1 B 0
A 1 B 0.14
A 1 B 0.29
A 1 B 0.43
A 1 B 0.57
A 1 B 0.71
A 1 B 0.86
A 1 B 1
0.75
0.50
0.25
OD 600 nm [−]
0 5 101520
Time [h]

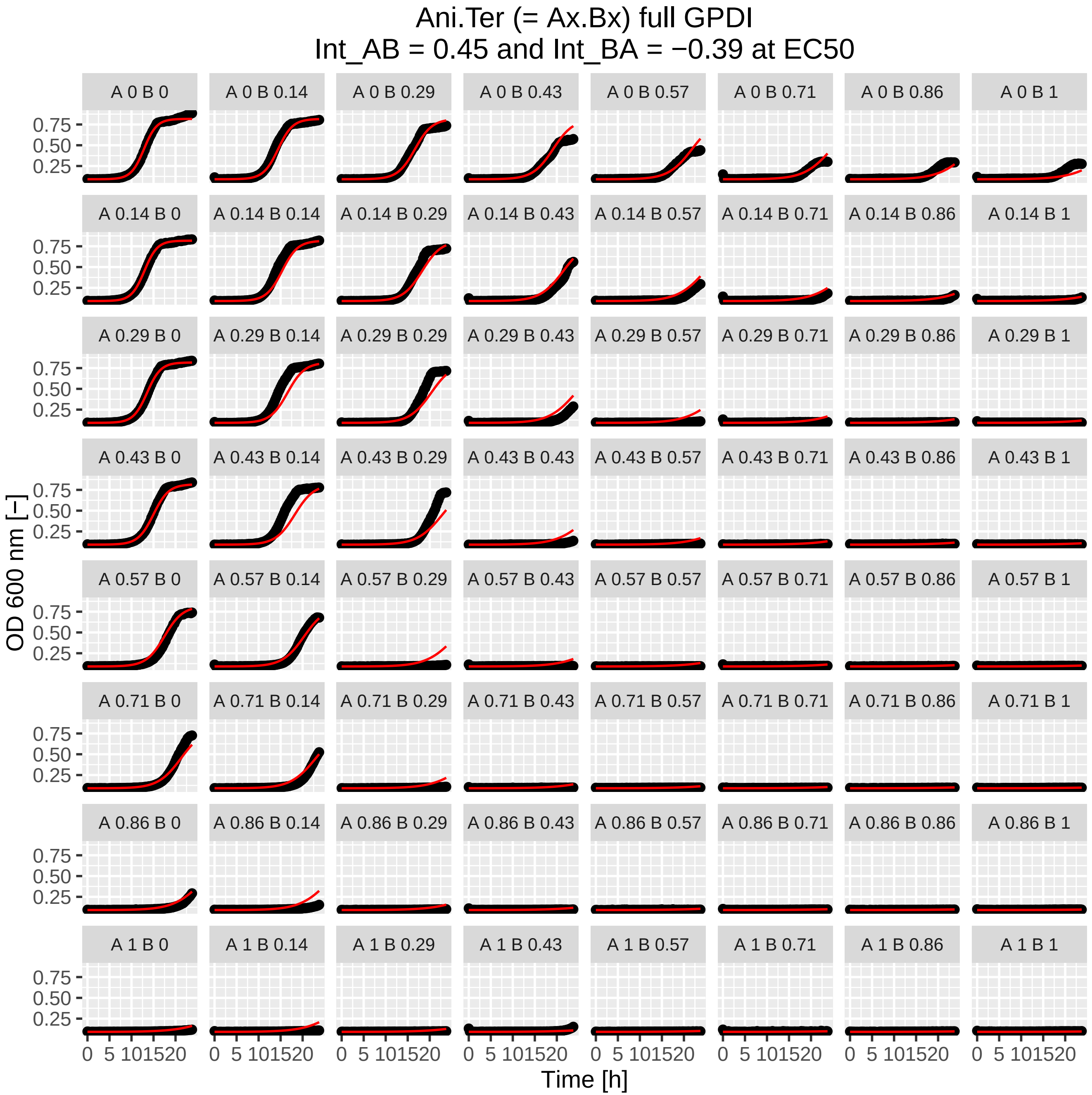
Ani.Ter (= Ax.Bx) full GPDI
Int_AB = 0.45 and Int_BA = −0.39 at EC50
OD 600 nm [−]
Time [h]
0.75
0.50
0.25
0 5 101520
A 0 B 0
A 0 B 0.14
A 0 B 0.29
A 0 B 0.43
A 0 B 0.57
A 0 B 0.71
A 0 B 0.86
A 0 B 1
A 0.14 B 0
A 0.14 B 0.14
A 0.14 B 0.29
A 0.14 B 0.43
A 0.14 B 0.57
A 0.14 B 0.71
A 0.14 B 0.86
A 0.14 B 1
A 0.29 B 0
A 0.29 B 0.14
A 0.29 B 0.29
A 0.29 B 0.43
A 0.29 B 0.57
A 0.29 B 0.71
A 0.29 B 0.86
A 0.29 B 1
A 0.43 B 0
A 0.43 B 0.14
A 0.43 B 0.29
A 0.43 B 0.43
A 0.43 B 0.57
A 0.43 B 0.71
A 0.43 B 0.86
A 0.43 B 1
A 0.57 B 0
A 0.57 B 0.14
A 0.57 B 0.29
A 0.57 B 0.43
A 0.57 B 0.57
A 0.57 B 0.71
A 0.57 B 0.86
A 0.57 B 1
A 0.71 B 0
A 0.71 B 0.14
A 0.71 B 0.29
A 0.71 B 0.43
A 0.71 B 0.57
A 0.71 B 0.71
A 0.71 B 0.86
A 0.71 B 1
A 0.86 B 0
A 0.86 B 0.14
A 0.86 B 0.29
A 0.86 B 0.43
A 0.86 B 0.57
A 0.86 B 0.71
A 0.86 B 0.86
A 0.86 B 1
A 1 B 0
A 1 B 0.14
A 1 B 0.29
A 1 B 0.43
A 1 B 0.57
A 1 B 0.71
A 1 B 0.86
A 1 B 1

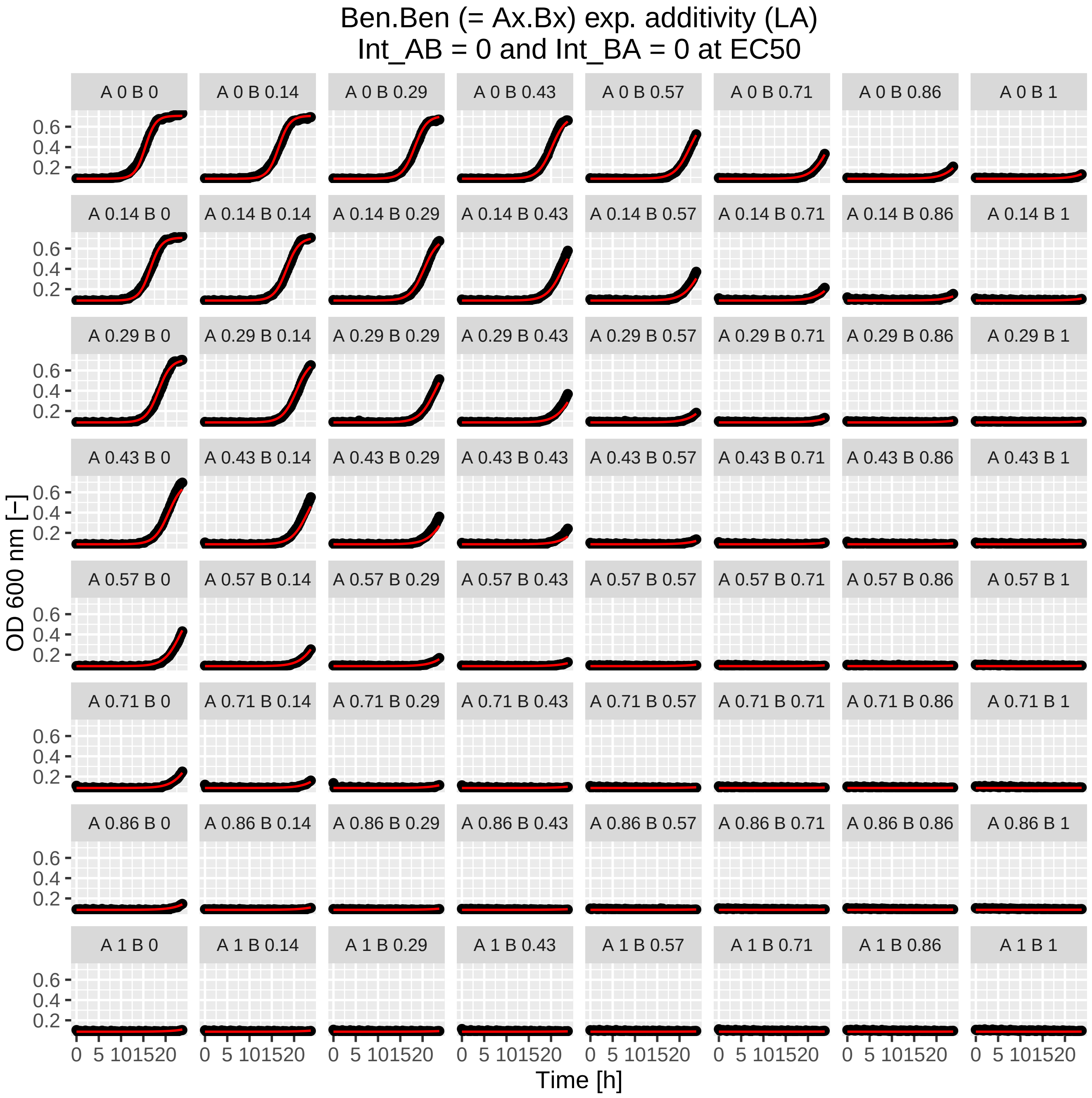

Ben.Ben (= Ax.Bx) exp. additivity (LA)
Int_AB = 0 and Int_BA = 0 at EC50
OD 600 nm [–]
Time [h]

Ben.Ben (= Ax.Bx) full GPDI
Int_AB = 0.1 and Int_BA = 0.16 at EC50

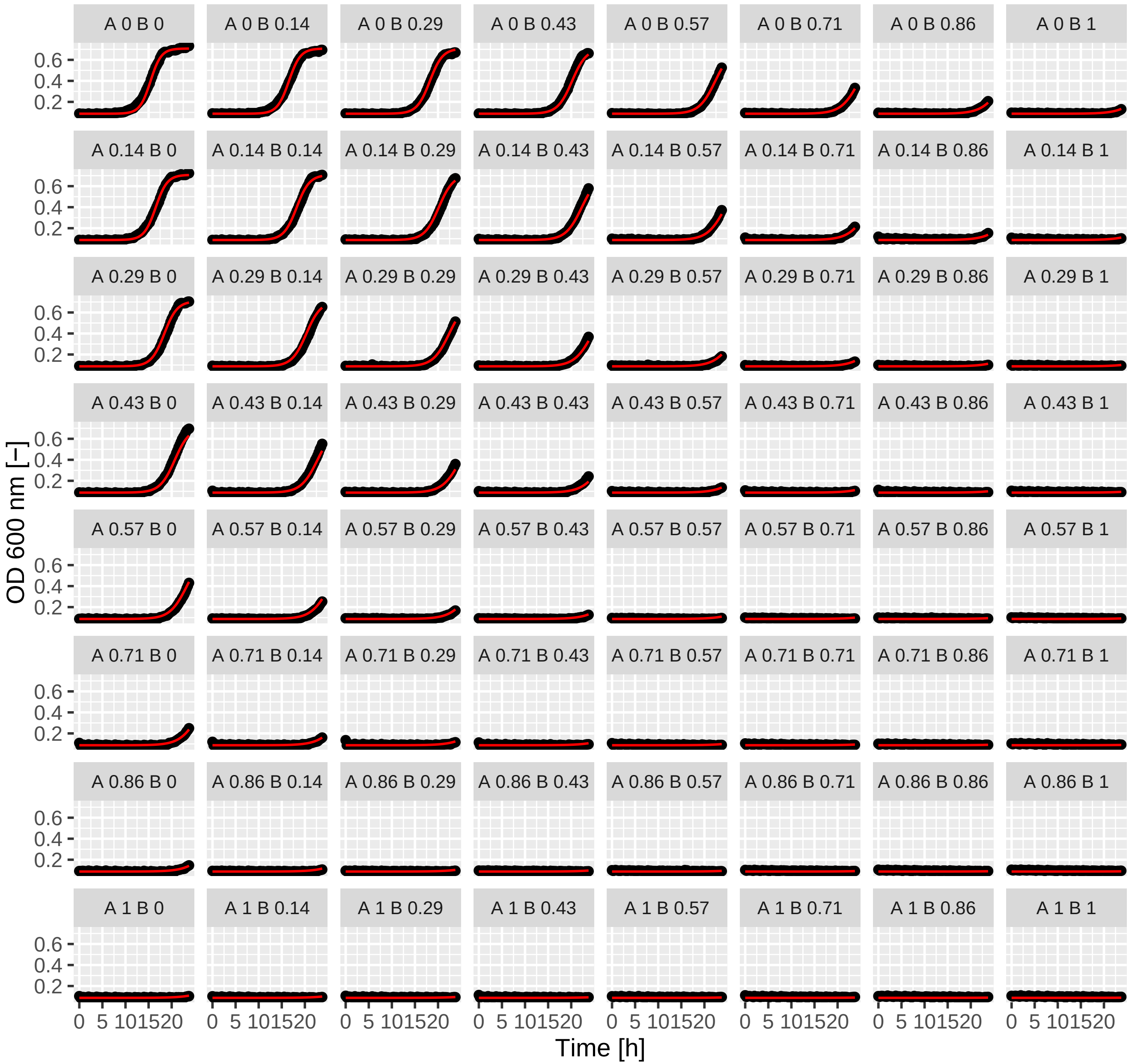

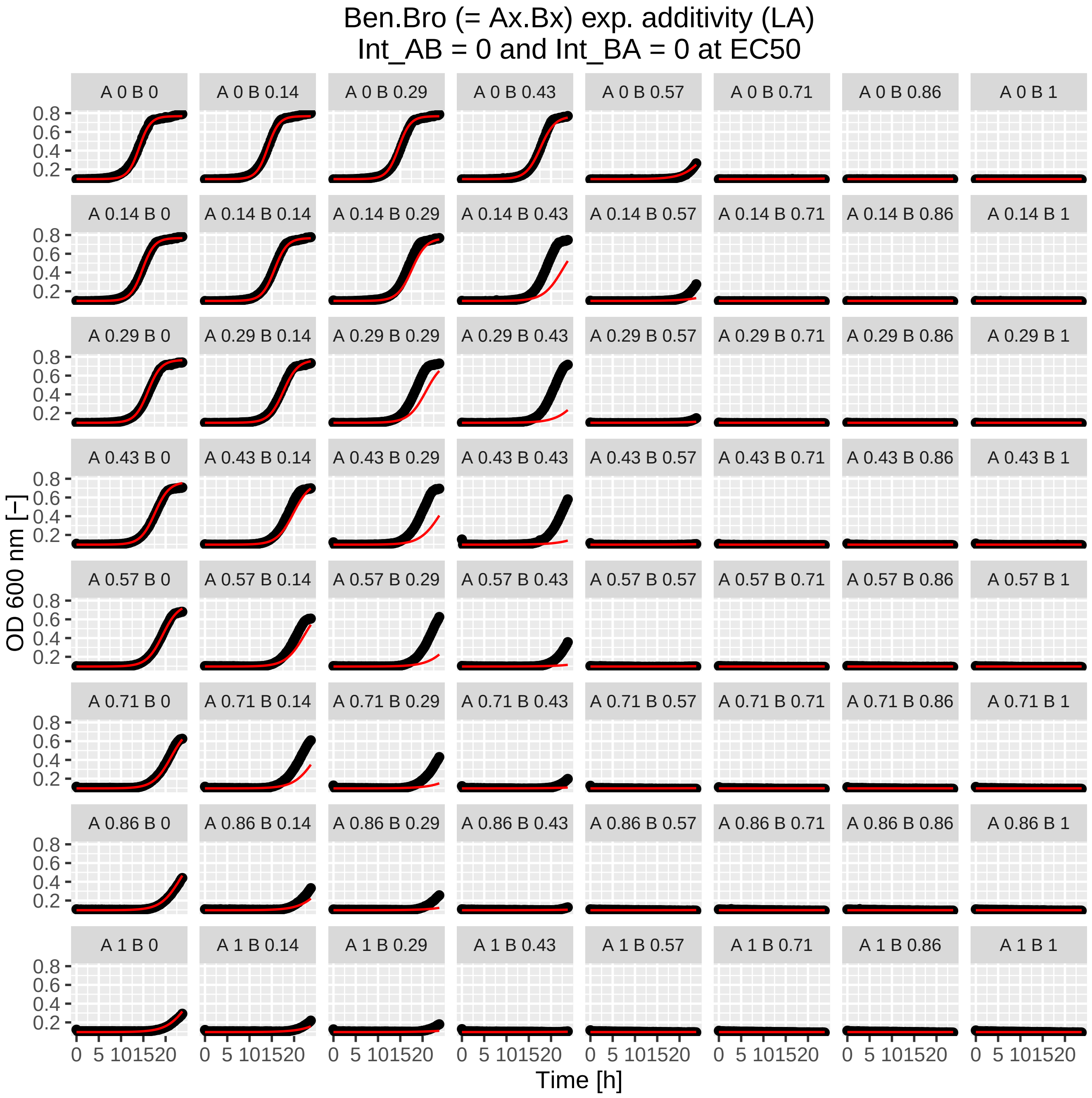
Ben.Bro (= Ax.Bx) exp. additivity (LA)
Int_AB = 0 and Int_BA = 0 at EC50
OD 600 nm [−]
Time [h]
0.2
0.4
0.6
0.8
0 5 101520
A 0 B 0
A 0 B 0.14
A 0 B 0.29
A 0 B 0.43
A 0 B 0.57
A 0 B 0.71
A 0 B 0.86
A 0 B 1
A 0.14 B 0
A 0.14 B 0.14
A 0.14 B 0.29
A 0.14 B 0.43
A 0.14 B 0.57
A 0.14 B 0.71
A 0.14 B 0.86
A 0.14 B 1
A 0.29 B 0
A 0.29 B 0.14
A 0.29 B 0.29
A 0.29 B 0.43
A 0.29 B 0.57
A 0.29 B 0.71
A 0.29 B 0.86
A 0.29 B 1
A 0.43 B 0
A 0.43 B 0.14
A 0.43 B 0.29
A 0.43 B 0.43
A 0.43 B 0.57
A 0.43 B 0.71
A 0.43 B 0.86
A 0.43 B 1
A 0.57 B 0
A 0.57 B 0.14
A 0.57 B 0.29
A 0.57 B 0.43
A 0.57 B 0.57
A 0.57 B 0.71
A 0.57 B 0.86
A 0.57 B 1
A 0.71 B 0
A 0.71 B 0.14
A 0.71 B 0.29
A 0.71 B 0.43
A 0.71 B 0.57
A 0.71 B 0.71
A 0.71 B 0.86
A 0.71 B 1
A 0.86 B 0
A 0.86 B 0.14
A 0.86 B 0.29
A 0.86 B 0.43
A 0.86 B 0.57
A 0.86 B 0.71
A 0.86 B 0.86
A 0.86 B 1
A 1 B 0
A 1 B 0.14
A 1 B 0.29
A 1 B 0.43
A 1 B 0.57
A 1 B 0.71
A 1 B 0.86
A 1 B 1

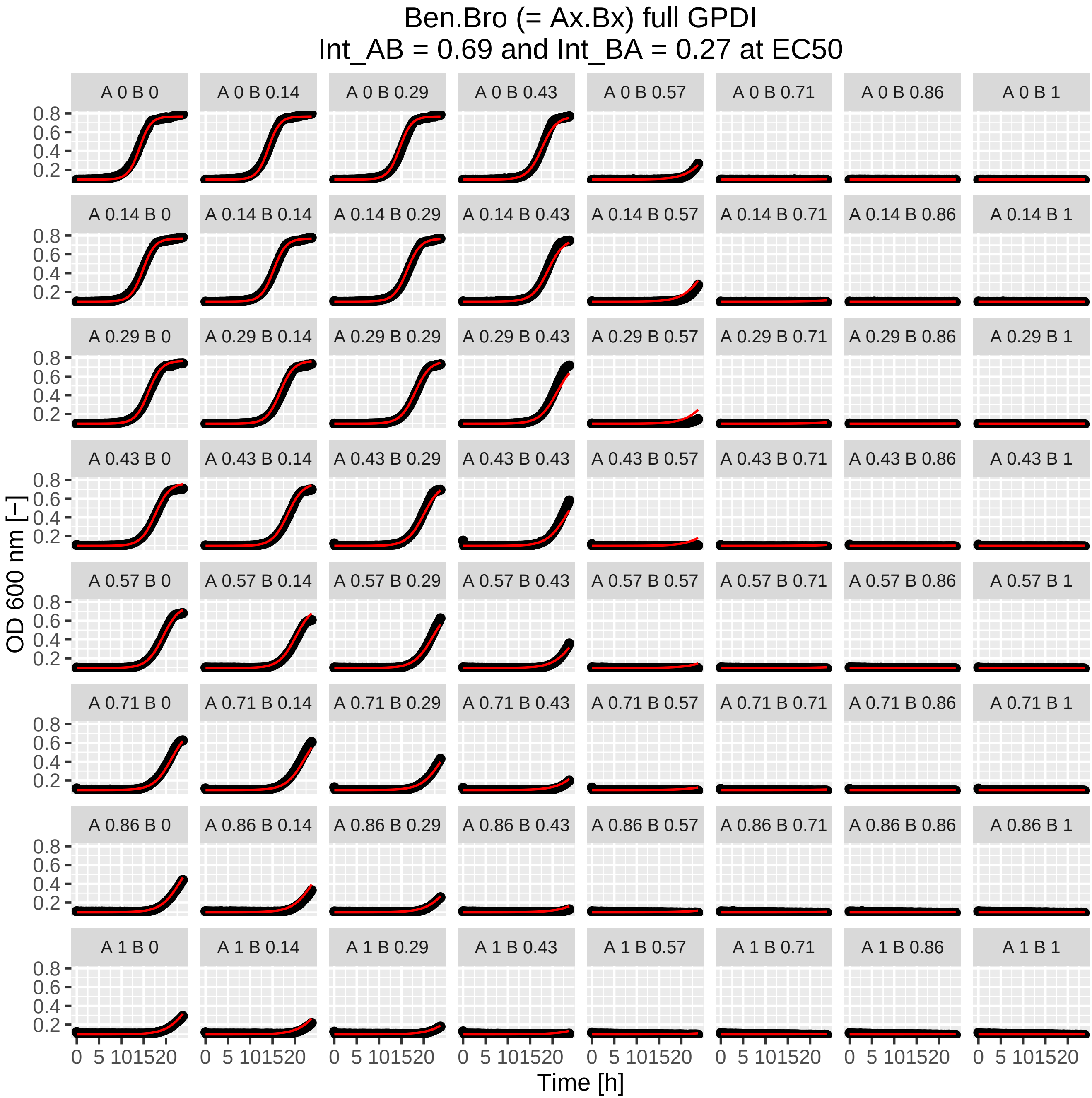

Ben.Bro (= Ax.Bx) full GPDI
Int_AB = 0.69 and Int_BA = 0.27 at EC50
A 0 B 0
A 0 B 0.14
A 0 B 0.29
A 0 B 0.43
A 0 B 0.57
A 0 B 0.71
A 0 B 0.86
A 0 B 1
A 0.14 B 0
A 0.14 B 0.14
A 0.14 B 0.29
A 0.14 B 0.43
A 0.14 B 0.57
A 0.14 B 0.71
A 0.14 B 0.86
A 0.14 B 1
A 0.29 B 0
A 0.29 B 0.14
A 0.29 B 0.29
A 0.29 B 0.43
A 0.29 B 0.57
A 0.29 B 0.71
A 0.29 B 0.86
A 0.29 B 1
A 0.43 B 0
A 0.43 B 0.14
A 0.43 B 0.29
A 0.43 B 0.43
A 0.43 B 0.57
A 0.43 B 0.71
A 0.43 B 0.86
A 0.43 B 1
A 0.57 B 0
A 0.57 B 0.14
A 0.57 B 0.29
A 0.57 B 0.43
A 0.57 B 0.57
A 0.57 B 0.71
A 0.57 B 0.86
A 0.57 B 1
A 0.71 B 0
A 0.71 B 0.14
A 0.71 B 0.29
A 0.71 B 0.43
A 0.71 B 0.57
A 0.71 B 0.71
A 0.71 B 0.86
A 0.71 B 1
A 0.86 B 0
A 0.86 B 0.14
A 0.86 B 0.29
A 0.86 B 0.43
A 0.86 B 0.57
A 0.86 B 0.71
A 0.86 B 0.86
A 0.86 B 1
A 1 B 0
A 1 B 0.14
A 1 B 0.29
A 1 B 0.43
A 1 B 0.57
A 1 B 0.71
A 1 B 0.86
A 1 B 1
0.8
0.6
0.4
0.2
OD 600 nm [–]
0 5 101520
Time [h]

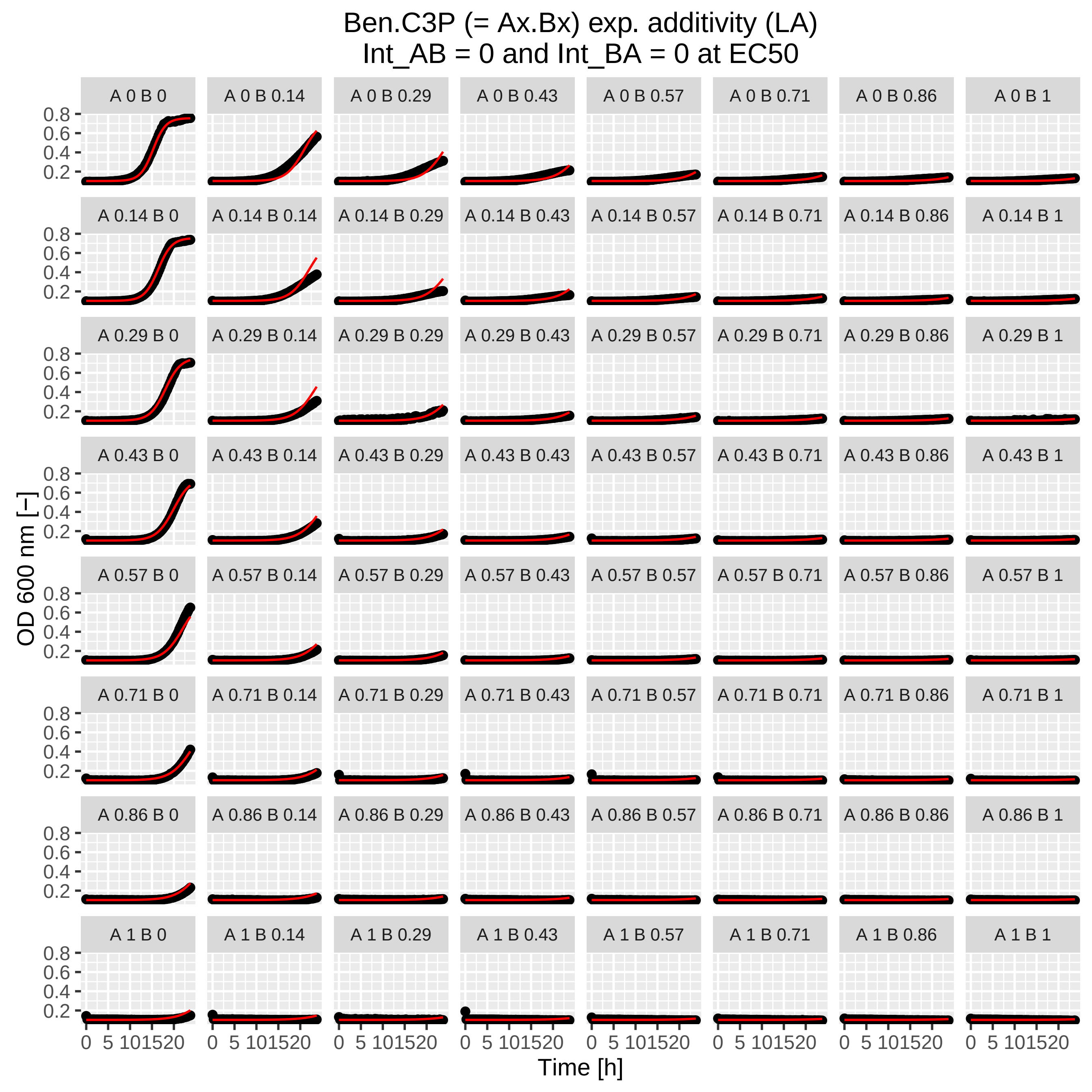

Ben.C3P (= Ax.Bx) exp. additivity (LA)
Int_AB = 0 and Int_BA = 0 at EC50
A 0 B 0
A 0 B 0.14
A 0 B 0.29
A 0 B 0.43
A 0 B 0.57
A 0 B 0.71
A 0 B 0.86
A 0 B 1
A 0.14 B 0
A 0.14 B 0.14
A 0.14 B 0.29
A 0.14 B 0.43
A 0.14 B 0.57
A 0.14 B 0.71
A 0.14 B 0.86
A 0.14 B 1
A 0.29 B 0
A 0.29 B 0.14
A 0.29 B 0.29
A 0.29 B 0.43
A 0.29 B 0.57
A 0.29 B 0.71
A 0.29 B 0.86
A 0.29 B 1
A 0.43 B 0
A 0.43 B 0.14
A 0.43 B 0.29
A 0.43 B 0.43
A 0.43 B 0.57
A 0.43 B 0.71
A 0.43 B 0.86
A 0.43 B 1
A 0.57 B 0
A 0.57 B 0.14
A 0.57 B 0.29
A 0.57 B 0.43
A 0.57 B 0.57
A 0.57 B 0.71
A 0.57 B 0.86
A 0.57 B 1
A 0.71 B 0
A 0.71 B 0.14
A 0.71 B 0.29
A 0.71 B 0.43
A 0.71 B 0.57
A 0.71 B 0.71
A 0.71 B 0.86
A 0.71 B 1
A 0.86 B 0
A 0.86 B 0.14
A 0.86 B 0.29
A 0.86 B 0.43
A 0.86 B 0.57
A 0.86 B 0.71
A 0.86 B 0.86
A 0.86 B 1
A 1 B 0
A 1 B 0.14
A 1 B 0.29
A 1 B 0.43
A 1 B 0.57
A 1 B 0.71
A 1 B 0.86
A 1 B 1
OD 600 nm [–]
0.2
0.4
0.6
0.8
0
5
10
15
20
Time [h]

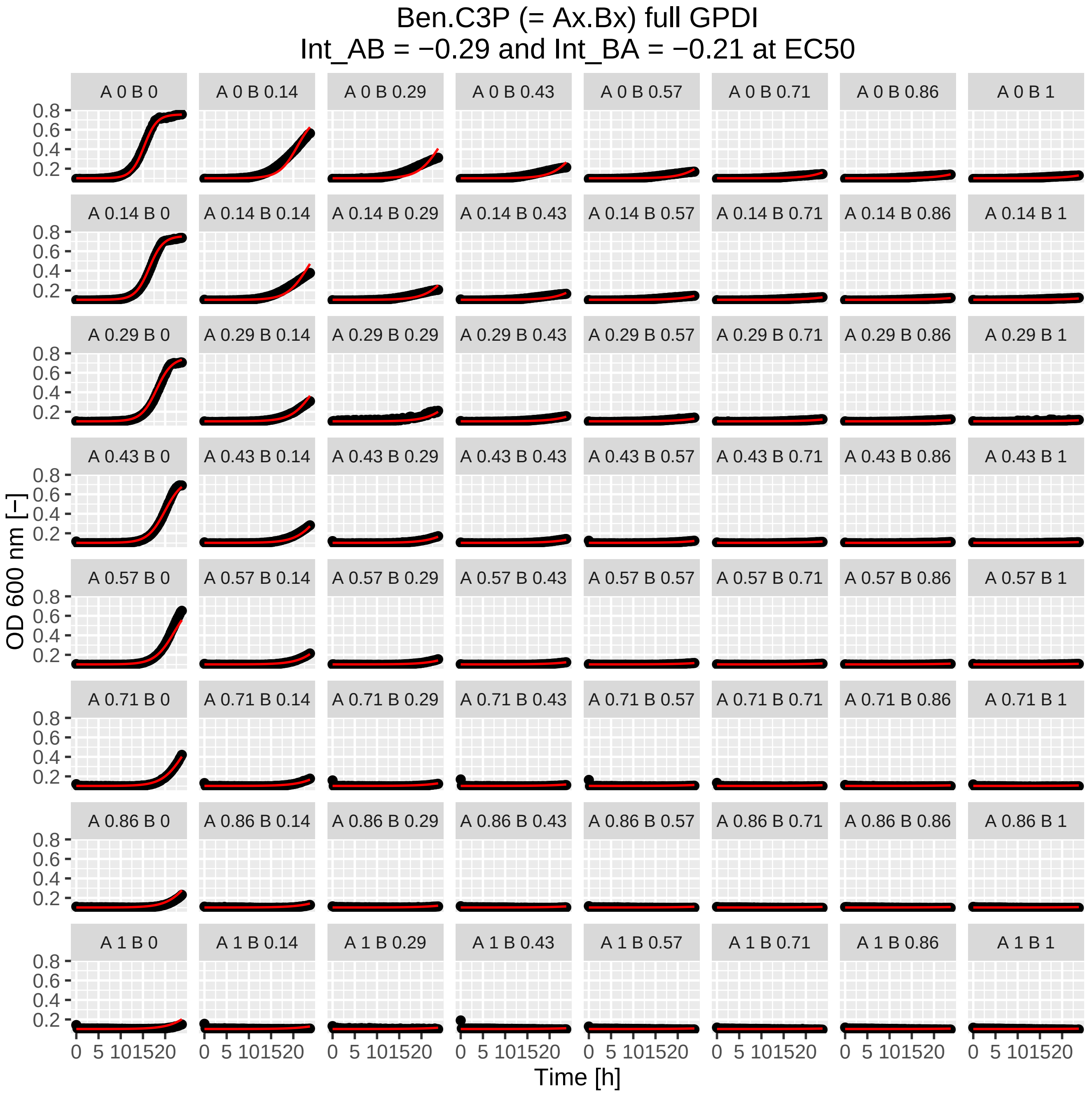
Ben.C3P (= Ax.Bx) full GPDI
Int_AB = −0.29 and Int_BA = −0.21 at EC50
OD 600 nm [−]
Time [h]
A 0 B 0
A 0 B 0.14
A 0 B 0.29
A 0 B 0.43
A 0 B 0.57
A 0 B 0.71
A 0 B 0.86
A 0 B 1
A 0.14 B 0
A 0.14 B 0.14
A 0.14 B 0.29
A 0.14 B 0.43
A 0.14 B 0.57
A 0.14 B 0.71
A 0.14 B 0.86
A 0.14 B 1
A 0.29 B 0
A 0.29 B 0.14
A 0.29 B 0.29
A 0.29 B 0.43
A 0.29 B 0.57
A 0.29 B 0.71
A 0.29 B 0.86
A 0.29 B 1
A 0.43 B 0
A 0.43 B 0.14
A 0.43 B 0.29
A 0.43 B 0.43
A 0.43 B 0.57
A 0.43 B 0.71
A 0.43 B 0.86
A 0.43 B 1
A 0.57 B 0
A 0.57 B 0.14
A 0.57 B 0.29
A 0.57 B 0.43
A 0.57 B 0.57
A 0.57 B 0.71
A 0.57 B 0.86
A 0.57 B 1
A 0.71 B 0
A 0.71 B 0.14
A 0.71 B 0.29
A 0.71 B 0.43
A 0.71 B 0.57
A 0.71 B 0.71
A 0.71 B 0.86
A 0.71 B 1
A 0.86 B 0
A 0.86 B 0.14
A 0.86 B 0.29
A 0.86 B 0.43
A 0.86 B 0.57
A 0.86 B 0.71
A 0.86 B 0.86
A 0.86 B 1
A 1 B 0
A 1 B 0.14
A 1 B 0.29
A 1 B 0.43
A 1 B 0.57
A 1 B 0.71
A 1 B 0.86
A 1 B 1
0.8
0.6
0.4
0.2
0 5 101520

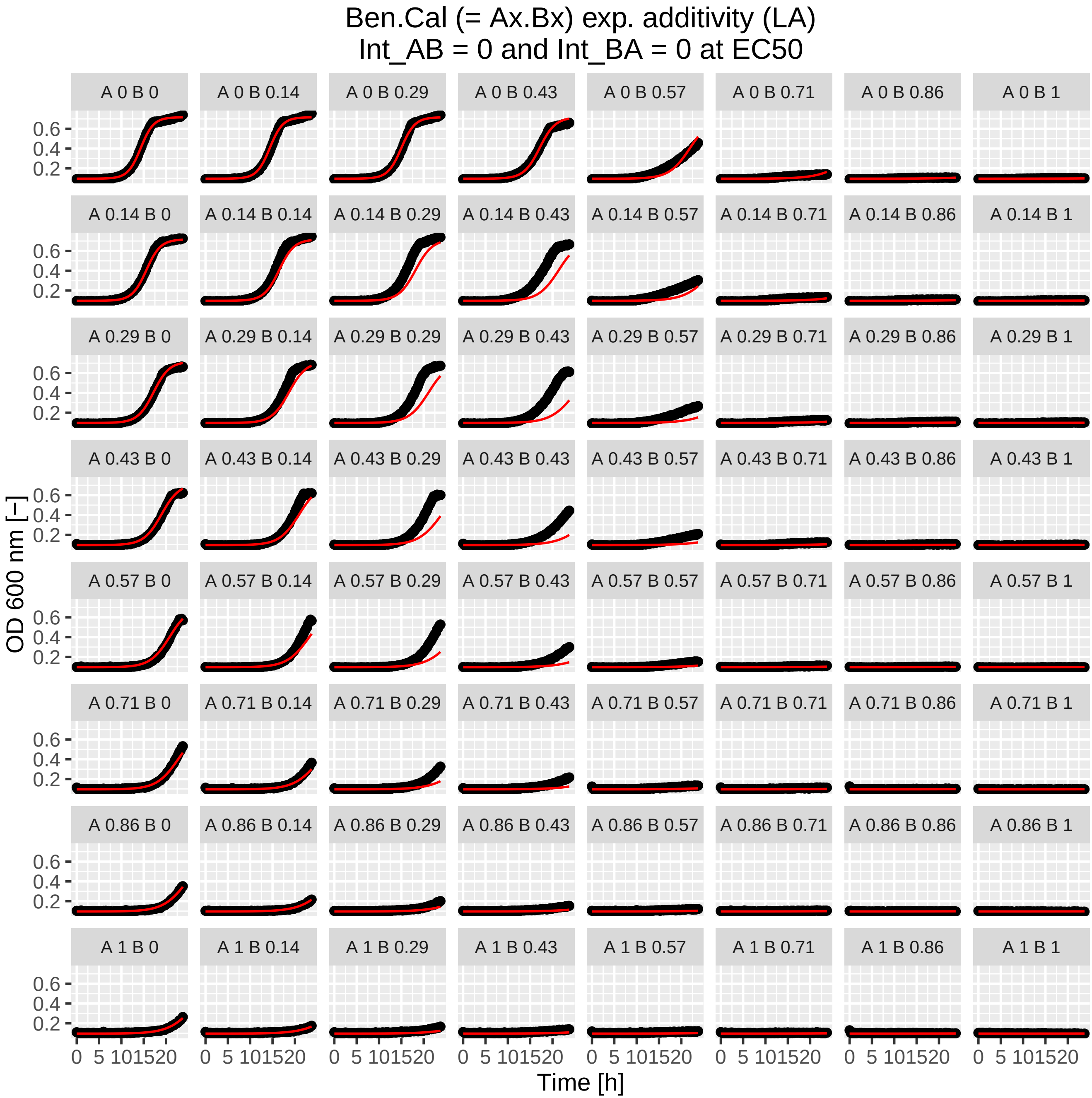
Ben.Cal (= Ax.Bx) exp. additivity (LA)
Int_AB = 0 and Int_BA = 0 at EC50
OD 600 nm [−]
Time [h]
A 0 B 0
A 0 B 0.14
A 0 B 0.29
A 0 B 0.43
A 0 B 0.57
A 0 B 0.71
A 0 B 0.86
A 0 B 1
A 0.14 B 0
A 0.14 B 0.14
A 0.14 B 0.29
A 0.14 B 0.43
A 0.14 B 0.57
A 0.14 B 0.71
A 0.14 B 0.86
A 0.14 B 1
A 0.29 B 0
A 0.29 B 0.14
A 0.29 B 0.29
A 0.29 B 0.43
A 0.29 B 0.57
A 0.29 B 0.71
A 0.29 B 0.86
A 0.29 B 1
A 0.43 B 0
A 0.43 B 0.14
A 0.43 B 0.29
A 0.43 B 0.43
A 0.43 B 0.57
A 0.43 B 0.71
A 0.43 B 0.86
A 0.43 B 1
A 0.57 B 0
A 0.57 B 0.14
A 0.57 B 0.29
A 0.57 B 0.43
A 0.57 B 0.57
A 0.57 B 0.71
A 0.57 B 0.86
A 0.57 B 1
A 0.71 B 0
A 0.71 B 0.14
A 0.71 B 0.29
A 0.71 B 0.43
A 0.71 B 0.57
A 0.71 B 0.71
A 0.71 B 0.86
A 0.71 B 1
A 0.86 B 0
A 0.86 B 0.14
A 0.86 B 0.29
A 0.86 B 0.43
A 0.86 B 0.57
A 0.86 B 0.71
A 0.86 B 0.86
A 0.86 B 1
A 1 B 0
A 1 B 0.14
A 1 B 0.29
A 1 B 0.43
A 1 B 0.57
A 1 B 0.71
A 1 B 0.86
A 1 B 1
0.6
0.4
0.2
0 5 10 15 20

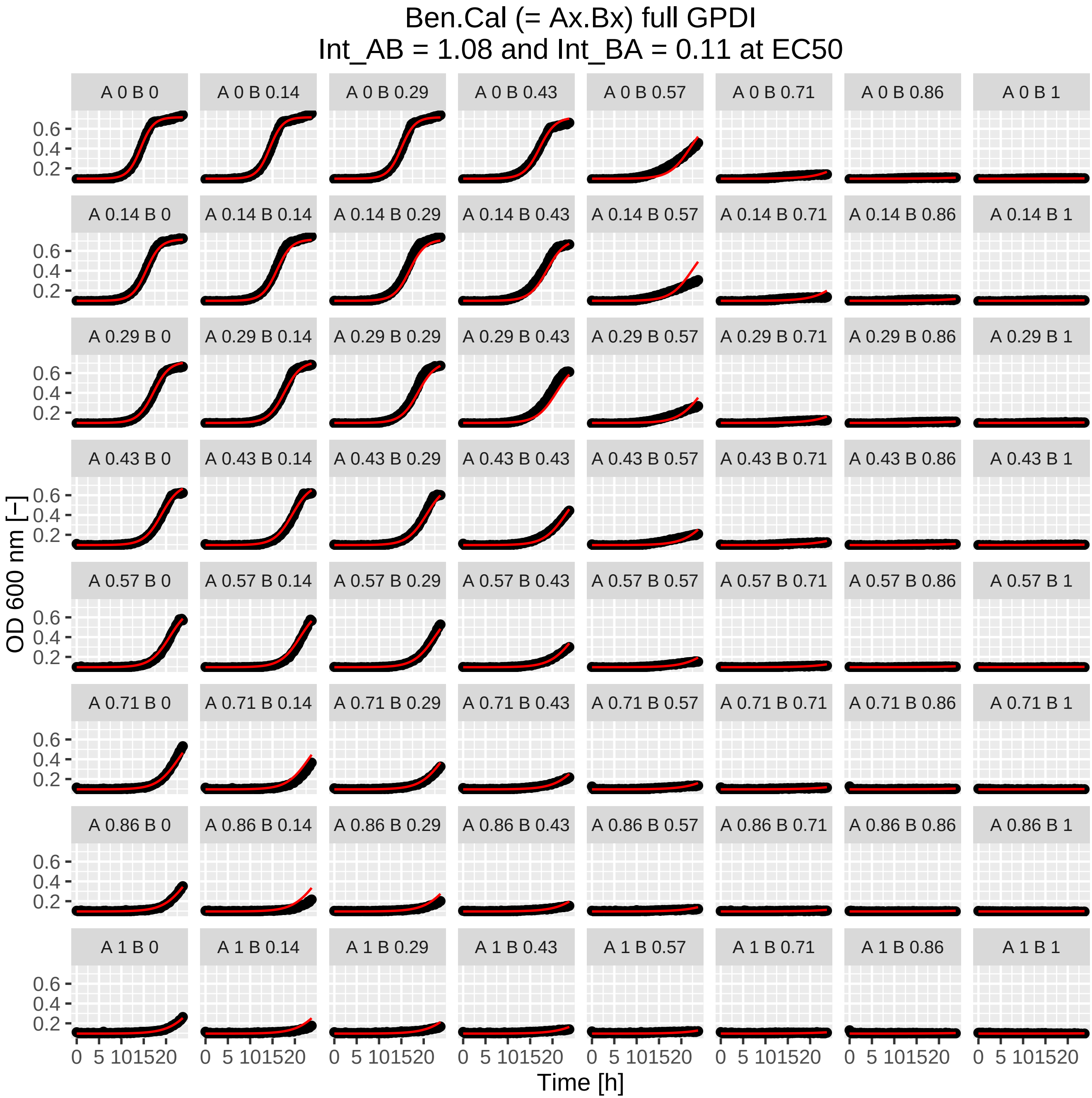
Ben.Cal (= Ax.Bx) full GPDI
Int_AB = 1.08 and Int_BA = 0.11 at EC50
OD 600 nm [–]
Time [h]
A 0 B 0
A 0 B 0.14
A 0 B 0.29
A 0 B 0.43
A 0 B 0.57
A 0 B 0.71
A 0 B 0.86
A 0 B 1
A 0.14 B 0
A 0.14 B 0.14
A 0.14 B 0.29
A 0.14 B 0.43
A 0.14 B 0.57
A 0.14 B 0.71
A 0.14 B 0.86
A 0.14 B 1
A 0.29 B 0
A 0.29 B 0.14
A 0.29 B 0.29
A 0.29 B 0.43
A 0.29 B 0.57
A 0.29 B 0.71
A 0.29 B 0.86
A 0.29 B 1
A 0.43 B 0
A 0.43 B 0.14
A 0.43 B 0.29
A 0.43 B 0.43
A 0.43 B 0.57
A 0.43 B 0.71
A 0.43 B 0.86
A 0.43 B 1
A 0.57 B 0
A 0.57 B 0.14
A 0.57 B 0.29
A 0.57 B 0.43
A 0.57 B 0.57
A 0.57 B 0.71
A 0.57 B 0.86
A 0.57 B 1
A 0.71 B 0
A 0.71 B 0.14
A 0.71 B 0.29
A 0.71 B 0.43
A 0.71 B 0.57
A 0.71 B 0.71
A 0.71 B 0.86
A 0.71 B 1
A 0.86 B 0
A 0.86 B 0.14
A 0.86 B 0.29
A 0.86 B 0.43
A 0.86 B 0.57
A 0.86 B 0.71
A 0.86 B 0.86
A 0.86 B 1
A 1 B 0
A 1 B 0.14
A 1 B 0.29
A 1 B 0.43
A 1 B 0.57
A 1 B 0.71
A 1 B 0.86
A 1 B 1
0.2
0.4
0.6
0 5 101520

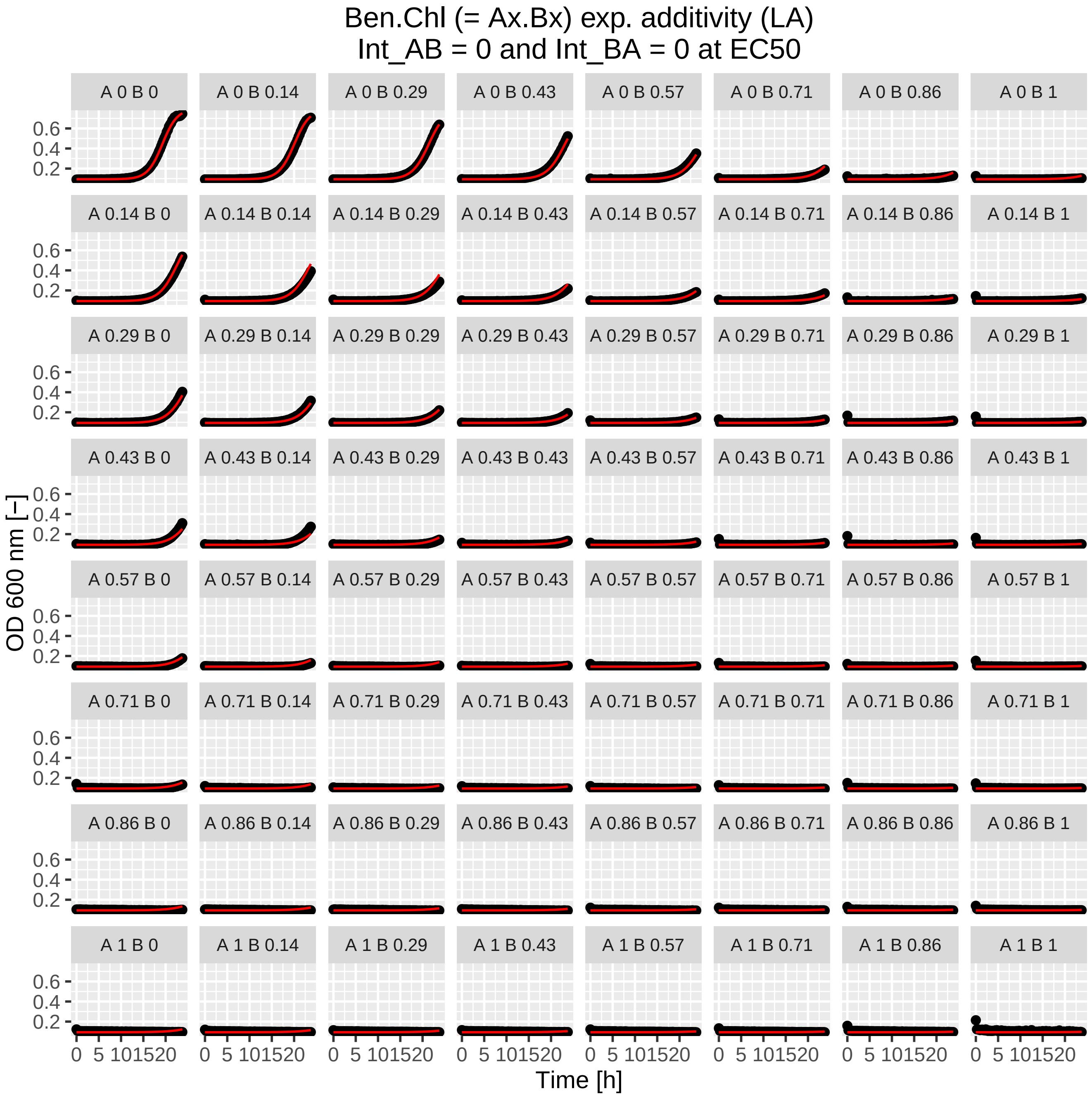
Ben.Chl (= Ax.Bx) exp. additivity (LA)
Int_AB = 0 and Int_BA = 0 at EC50
A 0 B 0
A 0 B 0.14
A 0 B 0.29
A 0 B 0.43
A 0 B 0.57
A 0 B 0.71
A 0 B 0.86
A 0 B 1
A 0.14 B 0
A 0.14 B 0.14
A 0.14 B 0.29
A 0.14 B 0.43
A 0.14 B 0.57
A 0.14 B 0.71
A 0.14 B 0.86
A 0.14 B 1
A 0.29 B 0
A 0.29 B 0.14
A 0.29 B 0.29
A 0.29 B 0.43
A 0.29 B 0.57
A 0.29 B 0.71
A 0.29 B 0.86
A 0.29 B 1
A 0.43 B 0
A 0.43 B 0.14
A 0.43 B 0.29
A 0.43 B 0.43
A 0.43 B 0.57
A 0.43 B 0.71
A 0.43 B 0.86
A 0.43 B 1
A 0.57 B 0
A 0.57 B 0.14
A 0.57 B 0.29
A 0.57 B 0.43
A 0.57 B 0.57
A 0.57 B 0.71
A 0.57 B 0.86
A 0.57 B 1
A 0.71 B 0
A 0.71 B 0.14
A 0.71 B 0.29
A 0.71 B 0.43
A 0.71 B 0.57
A 0.71 B 0.71
A 0.71 B 0.86
A 0.71 B 1
A 0.86 B 0
A 0.86 B 0.14
A 0.86 B 0.29
A 0.86 B 0.43
A 0.86 B 0.57
A 0.86 B 0.71
A 0.86 B 0.86
A 0.86 B 1
A 1 B 0
A 1 B 0.14
A 1 B 0.29
A 1 B 0.43
A 1 B 0.57
A 1 B 0.71
A 1 B 0.86
A 1 B 1
0.6
0.4
0.2
0 5 101520
OD 600 nm [–]
Time [h]

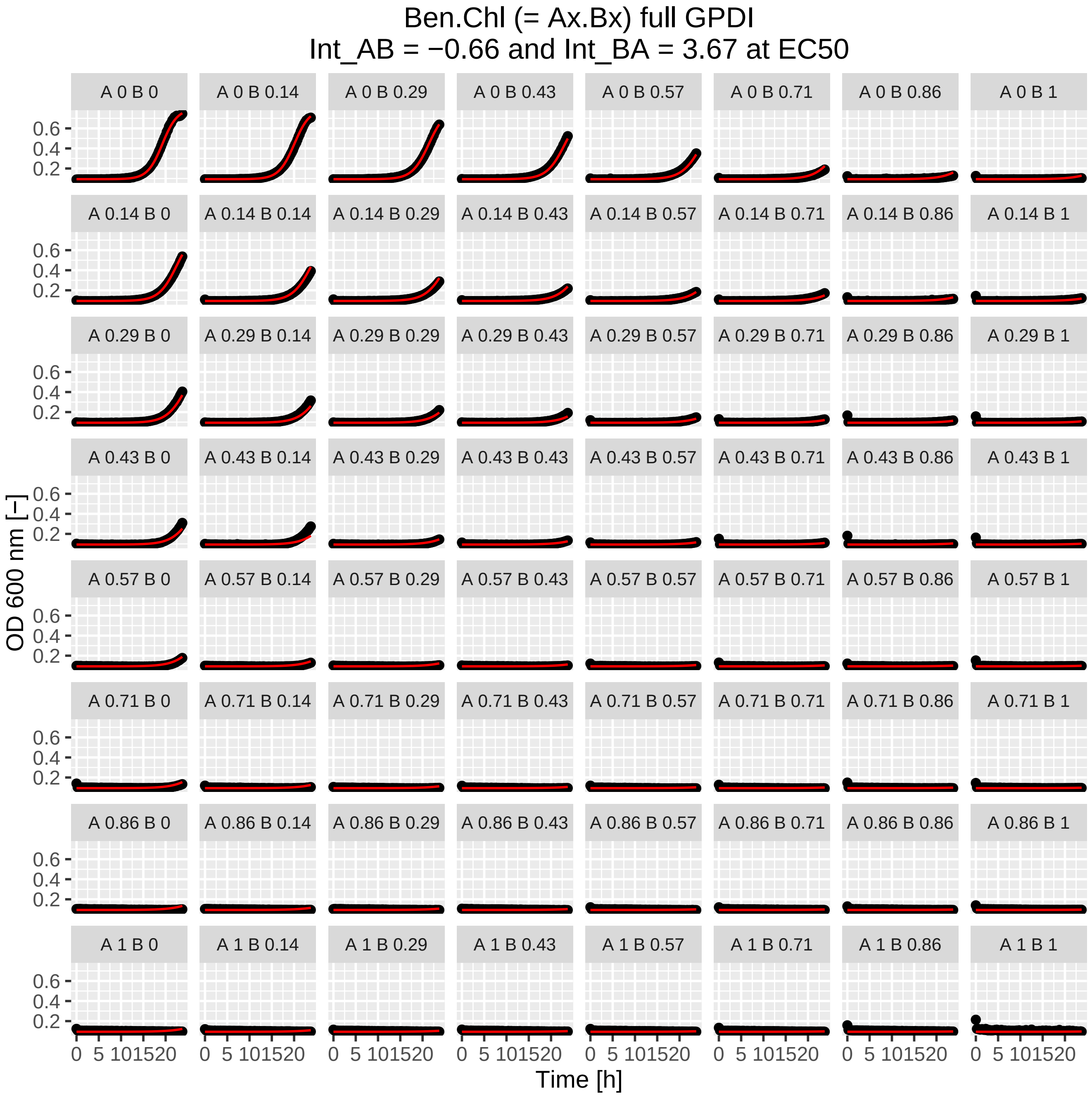

Ben.Chl (= Ax.Bx) full GPDI
Int_AB = −0.66 and Int_BA = 3.67 at EC50
A 0 B 0
A 0 B 0.14
A 0 B 0.29
A 0 B 0.43
A 0 B 0.57
A 0 B 0.71
A 0 B 0.86
A 0 B 1
A 0.14 B 0
A 0.14 B 0.14
A 0.14 B 0.29
A 0.14 B 0.43
A 0.14 B 0.57
A 0.14 B 0.71
A 0.14 B 0.86
A 0.14 B 1
A 0.29 B 0
A 0.29 B 0.14
A 0.29 B 0.29
A 0.29 B 0.43
A 0.29 B 0.57
A 0.29 B 0.71
A 0.29 B 0.86
A 0.29 B 1
A 0.43 B 0
A 0.43 B 0.14
A 0.43 B 0.29
A 0.43 B 0.43
A 0.43 B 0.57
A 0.43 B 0.71
A 0.43 B 0.86
A 0.43 B 1
A 0.57 B 0
A 0.57 B 0.14
A 0.57 B 0.29
A 0.57 B 0.43
A 0.57 B 0.57
A 0.57 B 0.71
A 0.57 B 0.86
A 0.57 B 1
A 0.71 B 0
A 0.71 B 0.14
A 0.71 B 0.29
A 0.71 B 0.43
A 0.71 B 0.57
A 0.71 B 0.71
A 0.71 B 0.86
A 0.71 B 1
A 0.86 B 0
A 0.86 B 0.14
A 0.86 B 0.29
A 0.86 B 0.43
A 0.86 B 0.57
A 0.86 B 0.71
A 0.86 B 0.86
A 0.86 B 1
A 1 B 0
A 1 B 0.14
A 1 B 0.29
A 1 B 0.43
A 1 B 0.57
A 1 B 0.71
A 1 B 0.86
A 1 B 1
OD 600 nm [−]
0.2
0.4
0.6
0 5 101520
Time [h]

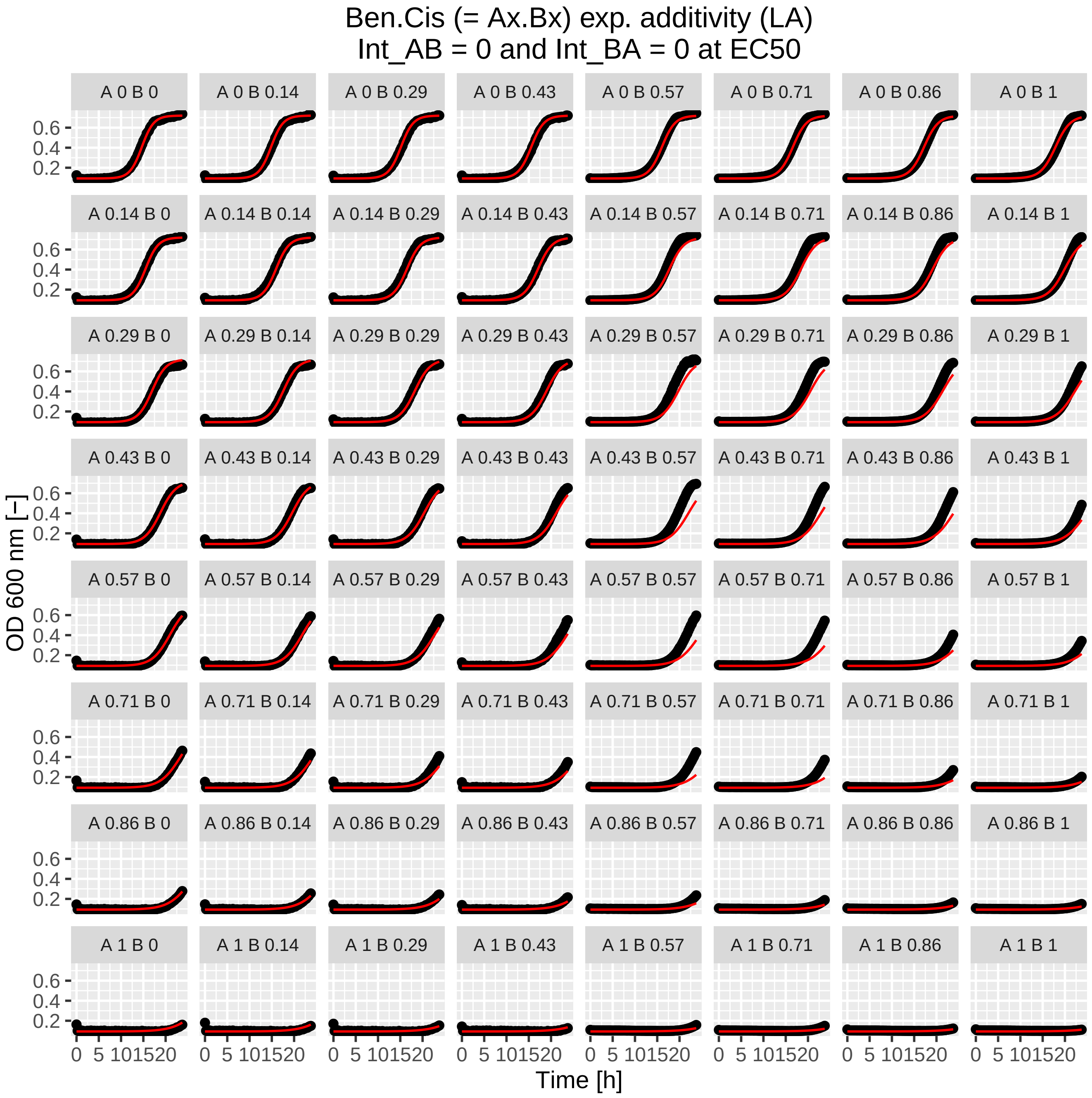
Ben.Cis (= Ax.Bx) exp. additivity (LA)
Int_AB = 0 and Int_BA = 0 at EC50
A 0 B 0
A 0 B 0.14
A 0 B 0.29
A 0 B 0.43
A 0 B 0.57
A 0 B 0.71
A 0 B 0.86
A 0 B 1
A 0.14 B 0
A 0.14 B 0.14
A 0.14 B 0.29
A 0.14 B 0.43
A 0.14 B 0.57
A 0.14 B 0.71
A 0.14 B 0.86
A 0.14 B 1
A 0.29 B 0
A 0.29 B 0.14
A 0.29 B 0.29
A 0.29 B 0.43
A 0.29 B 0.57
A 0.29 B 0.71
A 0.29 B 0.86
A 0.29 B 1
A 0.43 B 0
A 0.43 B 0.14
A 0.43 B 0.29
A 0.43 B 0.43
A 0.43 B 0.57
A 0.43 B 0.71
A 0.43 B 0.86
A 0.43 B 1
A 0.57 B 0
A 0.57 B 0.14
A 0.57 B 0.29
A 0.57 B 0.43
A 0.57 B 0.57
A 0.57 B 0.71
A 0.57 B 0.86
A 0.57 B 1
A 0.71 B 0
A 0.71 B 0.14
A 0.71 B 0.29
A 0.71 B 0.43
A 0.71 B 0.57
A 0.71 B 0.71
A 0.71 B 0.86
A 0.71 B 1
A 0.86 B 0
A 0.86 B 0.14
A 0.86 B 0.29
A 0.86 B 0.43
A 0.86 B 0.57
A 0.86 B 0.71
A 0.86 B 0.86
A 0.86 B 1
A 1 B 0
A 1 B 0.14
A 1 B 0.29
A 1 B 0.43
A 1 B 0.57
A 1 B 0.71
A 1 B 0.86
A 1 B 1
0.6
0.4
0.2
OD 600 nm [−]
0 5 101520
Time [h]

Ben.Cis (= Ax.Bx) full GPDI
Int_AB = 0.28 and Int_BA = 0.26 at EC50

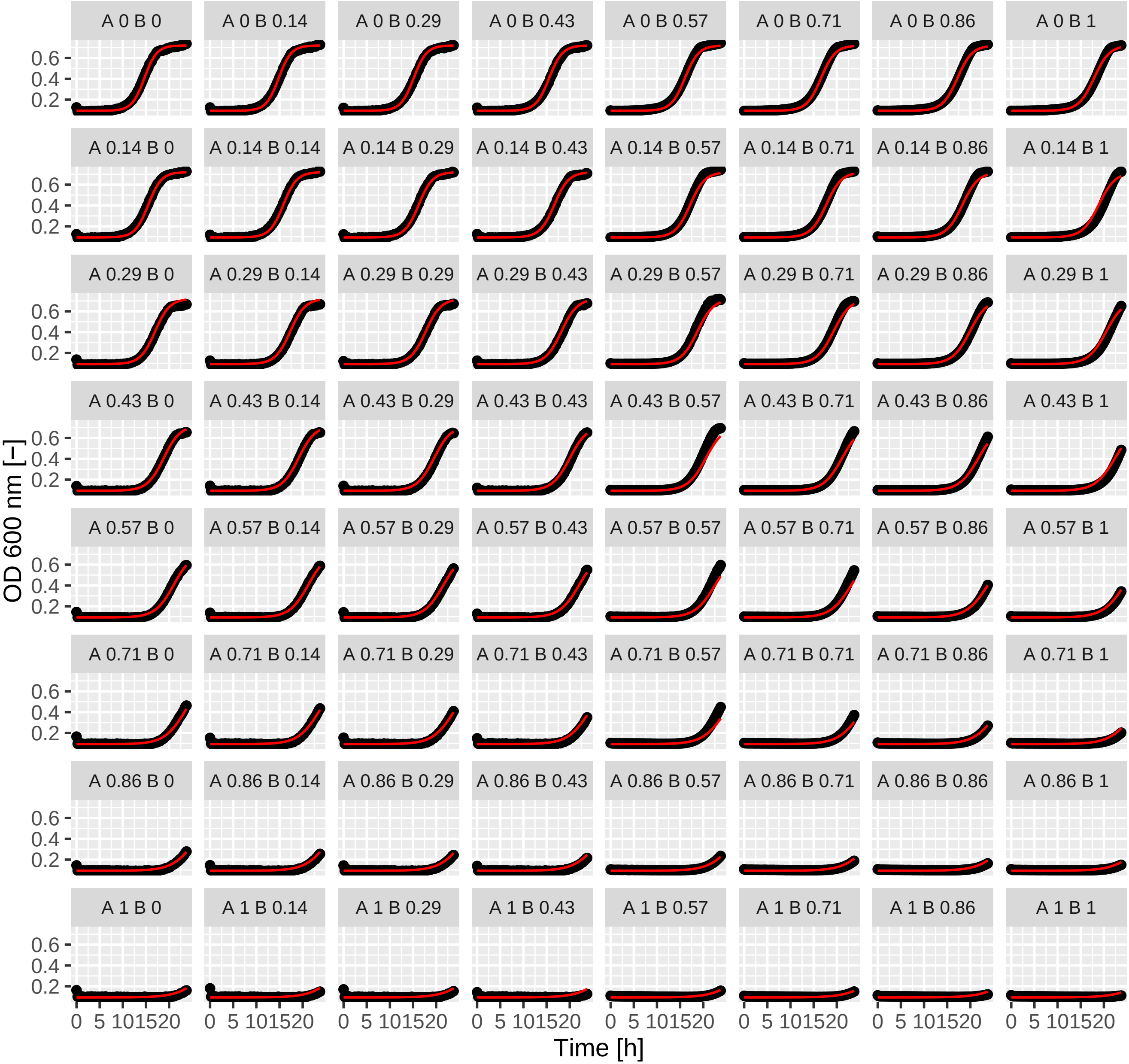

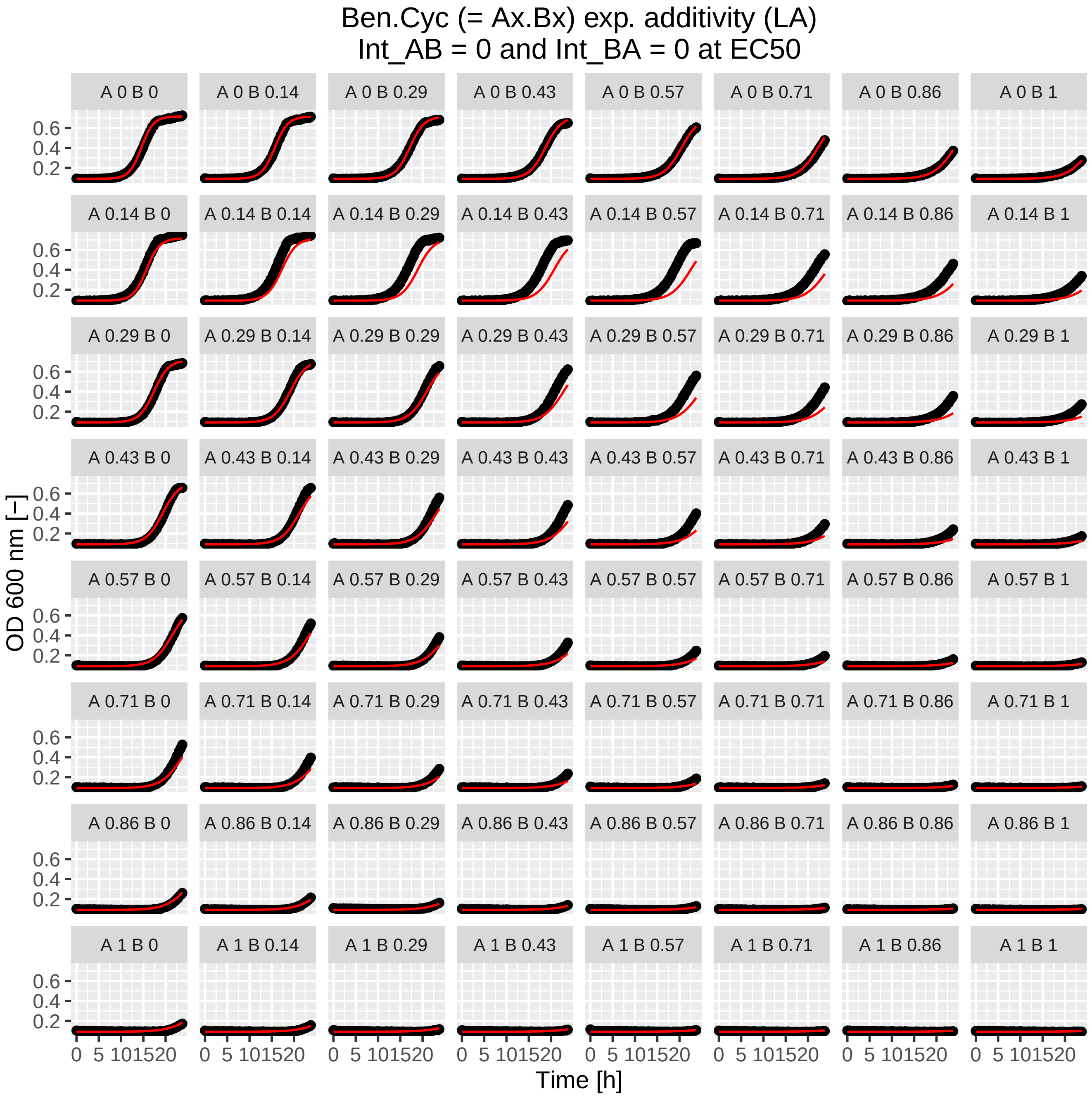

Ben.Cyc (= Ax.Bx) exp. additivity (LA)
Int_AB = 0 and Int_BA = 0 at EC50
OD 600 nm [–]
Time [h]
A 0 B 0
A 0 B 0.14
A 0 B 0.29
A 0 B 0.43
A 0 B 0.57
A 0 B 0.71
A 0 B 0.86
A 0 B 1
A 0.14 B 0
A 0.14 B 0.14
A 0.14 B 0.29
A 0.14 B 0.43
A 0.14 B 0.57
A 0.14 B 0.71
A 0.14 B 0.86
A 0.14 B 1
A 0.29 B 0
A 0.29 B 0.14
A 0.29 B 0.29
A 0.29 B 0.43
A 0.29 B 0.57
A 0.29 B 0.71
A 0.29 B 0.86
A 0.29 B 1
A 0.43 B 0
A 0.43 B 0.14
A 0.43 B 0.29
A 0.43 B 0.43
A 0.43 B 0.57
A 0.43 B 0.71
A 0.43 B 0.86
A 0.43 B 1
A 0.57 B 0
A 0.57 B 0.14
A 0.57 B 0.29
A 0.57 B 0.43
A 0.57 B 0.57
A 0.57 B 0.71
A 0.57 B 0.86
A 0.57 B 1
A 0.71 B 0
A 0.71 B 0.14
A 0.71 B 0.29
A 0.71 B 0.43
A 0.71 B 0.57
A 0.71 B 0.71
A 0.71 B 0.86
A 0.71 B 1
A 0.86 B 0
A 0.86 B 0.14
A 0.86 B 0.29
A 0.86 B 0.43
A 0.86 B 0.57
A 0.86 B 0.71
A 0.86 B 0.86
A 0.86 B 1
A 1 B 0
A 1 B 0.14
A 1 B 0.29
A 1 B 0.43
A 1 B 0.57
A 1 B 0.71
A 1 B 0.86
A 1 B 1
0.2
0.4
0.6
0
5
10
15
20

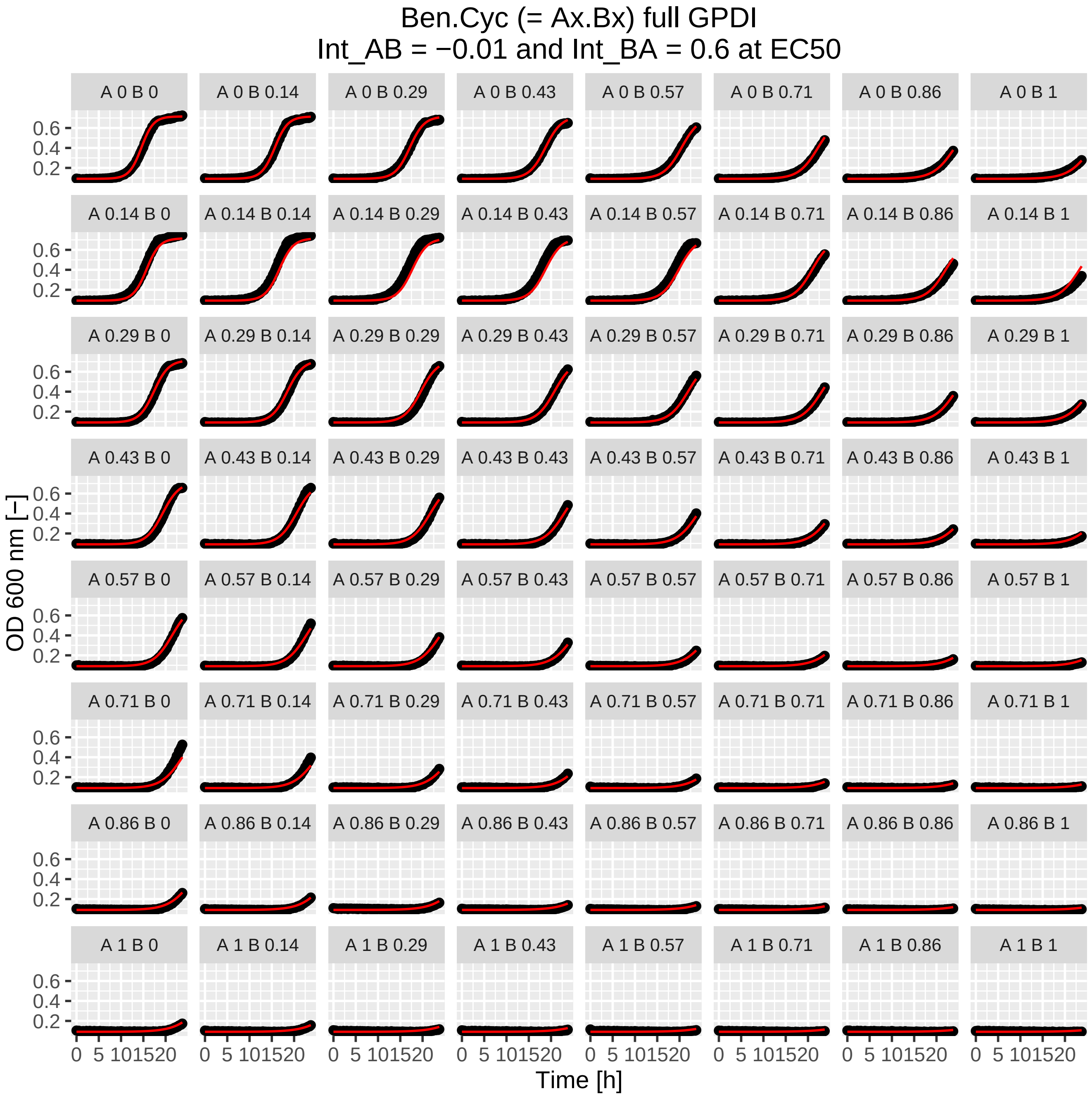
Ben.Cyc (= Ax.Bx) full GPDI
Int_AB = −0.01 and Int_BA = 0.6 at EC50
A 0 B 0
A 0 B 0.14
A 0 B 0.29
A 0 B 0.43
A 0 B 0.57
A 0 B 0.71
A 0 B 0.86
A 0 B 1
A 0.14 B 0
A 0.14 B 0.14
A 0.14 B 0.29
A 0.14 B 0.43
A 0.14 B 0.57
A 0.14 B 0.71
A 0.14 B 0.86
A 0.14 B 1
A 0.29 B 0
A 0.29 B 0.14
A 0.29 B 0.29
A 0.29 B 0.43
A 0.29 B 0.57
A 0.29 B 0.71
A 0.29 B 0.86
A 0.29 B 1
A 0.43 B 0
A 0.43 B 0.14
A 0.43 B 0.29
A 0.43 B 0.43
A 0.43 B 0.57
A 0.43 B 0.71
A 0.43 B 0.86
A 0.43 B 1
A 0.57 B 0
A 0.57 B 0.14
A 0.57 B 0.29
A 0.57 B 0.43
A 0.57 B 0.57
A 0.57 B 0.71
A 0.57 B 0.86
A 0.57 B 1
A 0.71 B 0
A 0.71 B 0.14
A 0.71 B 0.29
A 0.71 B 0.43
A 0.71 B 0.57
A 0.71 B 0.71
A 0.71 B 0.86
A 0.71 B 1
A 0.86 B 0
A 0.86 B 0.14
A 0.86 B 0.29
A 0.86 B 0.43
A 0.86 B 0.57
A 0.86 B 0.71
A 0.86 B 0.86
A 0.86 B 1
A 1 B 0
A 1 B 0.14
A 1 B 0.29
A 1 B 0.43
A 1 B 0.57
A 1 B 0.71
A 1 B 0.86
A 1 B 1
0.6
0.4
0.2
0 5 101520
OD 600 nm [−]
Time [h]

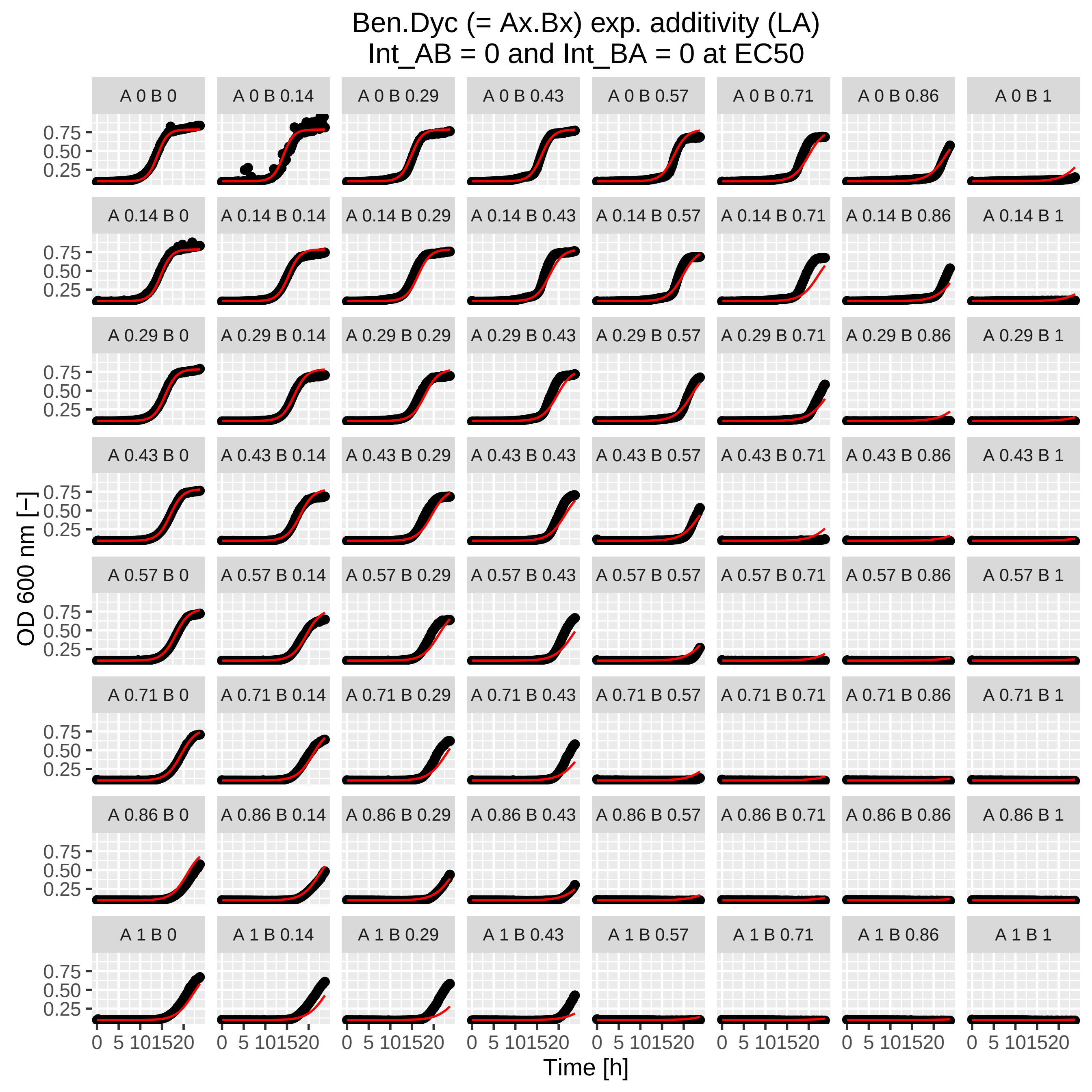

Ben.Dyc (= Ax.Bx) exp. additivity (LA)
Int_AB = 0 and Int_BA = 0 at EC50
A 0 B 0
A 0 B 0.14
A 0 B 0.29
A 0 B 0.43
A 0 B 0.57
A 0 B 0.71
A 0 B 0.86
A 0 B 1
A 0.14 B 0
A 0.14 B 0.14
A 0.14 B 0.29
A 0.14 B 0.43
A 0.14 B 0.57
A 0.14 B 0.71
A 0.14 B 0.86
A 0.14 B 1
A 0.29 B 0
A 0.29 B 0.14
A 0.29 B 0.29
A 0.29 B 0.43
A 0.29 B 0.57
A 0.29 B 0.71
A 0.29 B 0.86
A 0.29 B 1
A 0.43 B 0
A 0.43 B 0.14
A 0.43 B 0.29
A 0.43 B 0.43
A 0.43 B 0.57
A 0.43 B 0.71
A 0.43 B 0.86
A 0.43 B 1
A 0.57 B 0
A 0.57 B 0.14
A 0.57 B 0.29
A 0.57 B 0.43
A 0.57 B 0.57
A 0.57 B 0.71
A 0.57 B 0.86
A 0.57 B 1
A 0.71 B 0
A 0.71 B 0.14
A 0.71 B 0.29
A 0.71 B 0.43
A 0.71 B 0.57
A 0.71 B 0.71
A 0.71 B 0.86
A 0.71 B 1
A 0.86 B 0
A 0.86 B 0.14
A 0.86 B 0.29
A 0.86 B 0.43
A 0.86 B 0.57
A 0.86 B 0.71
A 0.86 B 0.86
A 0.86 B 1
A 1 B 0
A 1 B 0.14
A 1 B 0.29
A 1 B 0.43
A 1 B 0.57
A 1 B 0.71
A 1 B 0.86
A 1 B 1
0.75
0.50
0.25
OD 600 nm [–]
0 5 101520
Time [h]

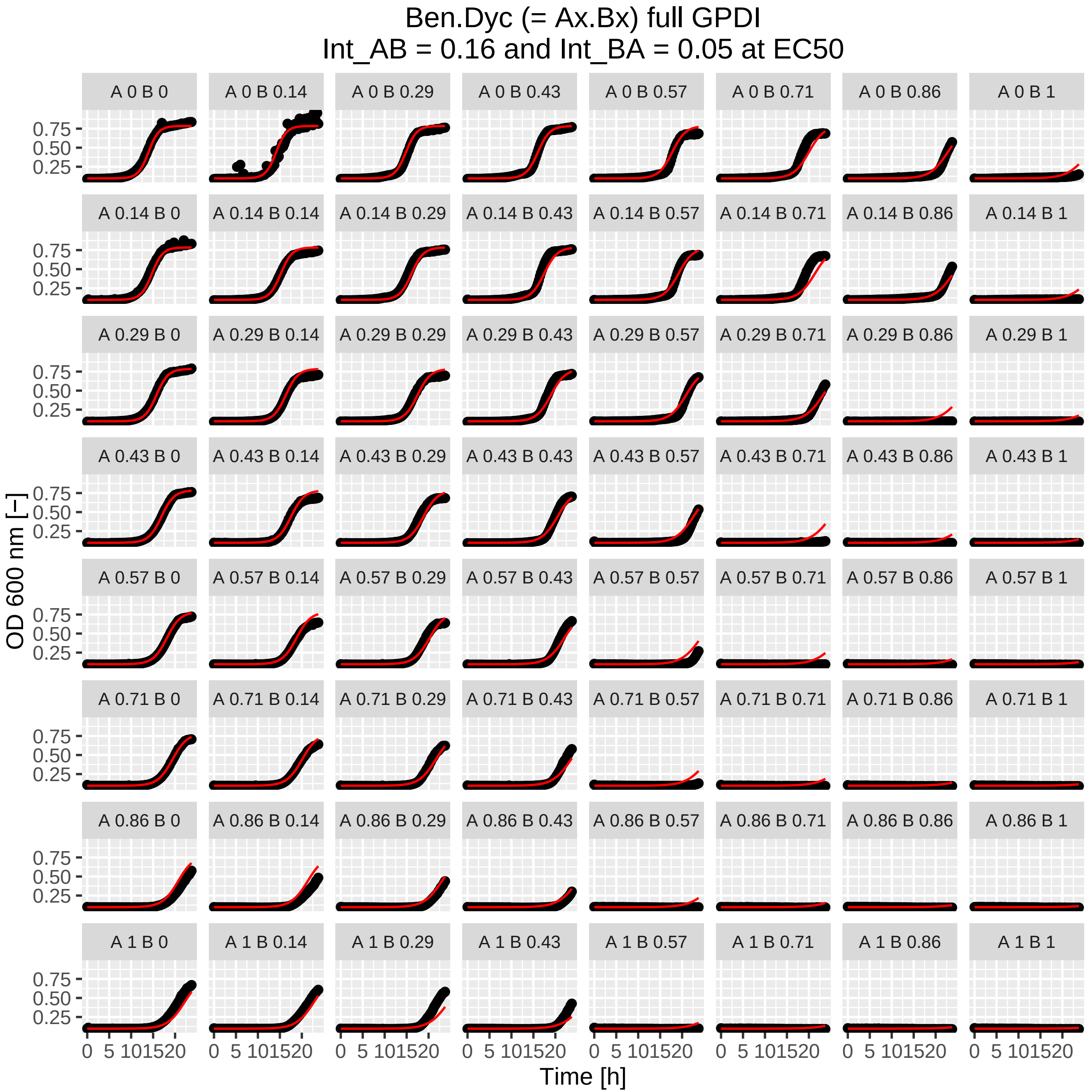
Ben.Dyc (= Ax.Bx) full GPDI
Int_AB = 0.16 and Int_BA = 0.05 at EC50
A 0 B 0
A 0 B 0.14
A 0 B 0.29
A 0 B 0.43
A 0 B 0.57
A 0 B 0.71
A 0 B 0.86
A 0 B 1
A 0.14 B 0
A 0.14 B 0.14
A 0.14 B 0.29
A 0.14 B 0.43
A 0.14 B 0.57
A 0.14 B 0.71
A 0.14 B 0.86
A 0.14 B 1
A 0.29 B 0
A 0.29 B 0.14
A 0.29 B 0.29
A 0.29 B 0.43
A 0.29 B 0.57
A 0.29 B 0.71
A 0.29 B 0.86
A 0.29 B 1
A 0.43 B 0
A 0.43 B 0.14
A 0.43 B 0.29
A 0.43 B 0.43
A 0.43 B 0.57
A 0.43 B 0.71
A 0.43 B 0.86
A 0.43 B 1
A 0.57 B 0
A 0.57 B 0.14
A 0.57 B 0.29
A 0.57 B 0.43
A 0.57 B 0.57
A 0.57 B 0.71
A 0.57 B 0.86
A 0.57 B 1
A 0.71 B 0
A 0.71 B 0.14
A 0.71 B 0.29
A 0.71 B 0.43
A 0.71 B 0.57
A 0.71 B 0.71
A 0.71 B 0.86
A 0.71 B 1
A 0.86 B 0
A 0.86 B 0.14
A 0.86 B 0.29
A 0.86 B 0.43
A 0.86 B 0.57
A 0.86 B 0.71
A 0.86 B 0.86
A 0.86 B 1
A 1 B 0
A 1 B 0.14
A 1 B 0.29
A 1 B 0.43
A 1 B 0.57
A 1 B 0.71
A 1 B 0.86
A 1 B 1
0.75
0.50
0.25
0 5 101520
OD 600 nm [–]
Time [h]

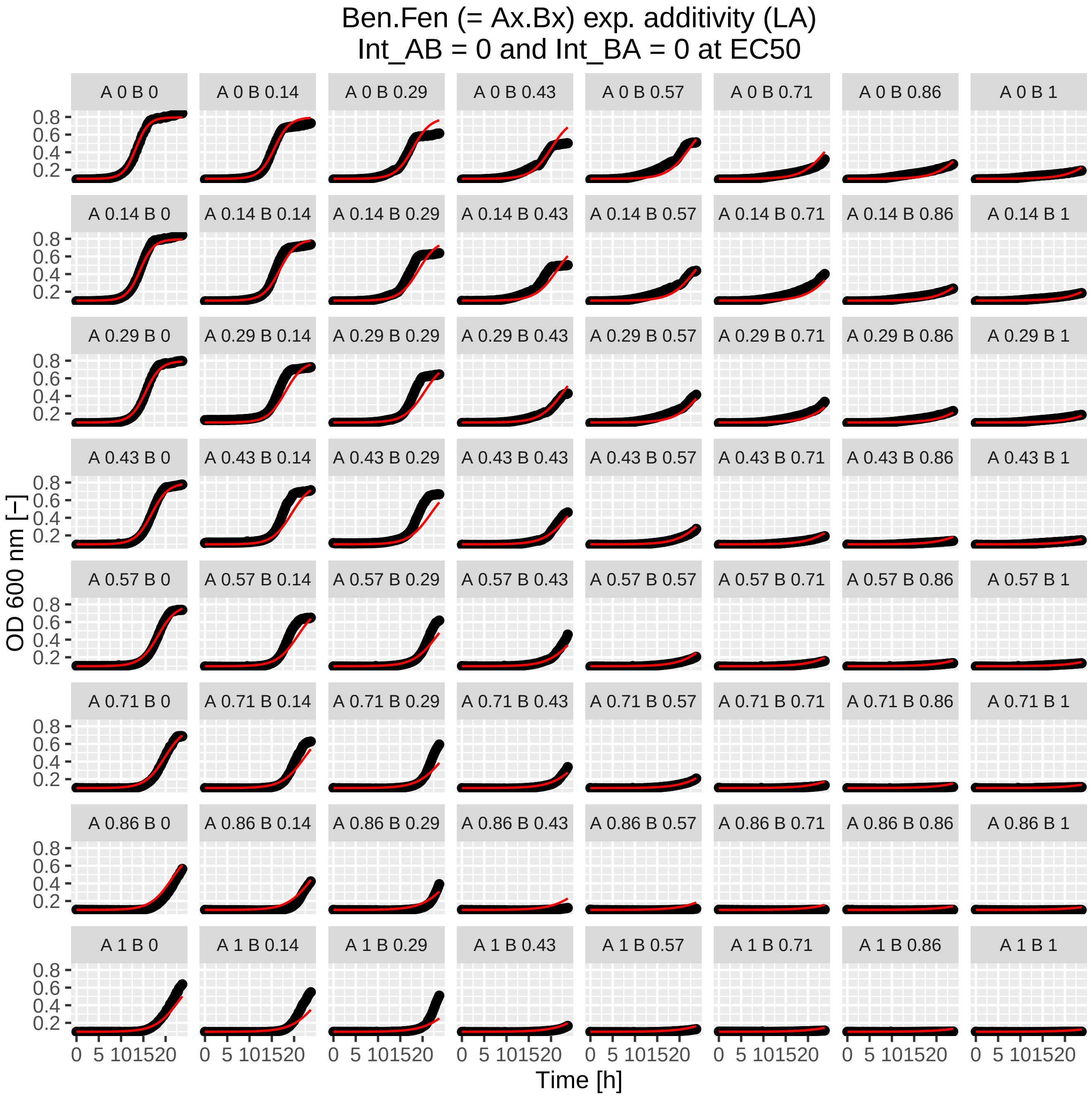
Ben.Fen (= Ax.Bx) exp. additivity (LA)
Int_AB = 0 and Int_BA = 0 at EC50
OD 600 nm [–]
Time [h]
A 0 B 0
A 0 B 0.14
A 0 B 0.29
A 0 B 0.43
A 0 B 0.57
A 0 B 0.71
A 0 B 0.86
A 0 B 1
A 0.14 B 0
A 0.14 B 0.14
A 0.14 B 0.29
A 0.14 B 0.43
A 0.14 B 0.57
A 0.14 B 0.71
A 0.14 B 0.86
A 0.14 B 1
A 0.29 B 0
A 0.29 B 0.14
A 0.29 B 0.29
A 0.29 B 0.43
A 0.29 B 0.57
A 0.29 B 0.71
A 0.29 B 0.86
A 0.29 B 1
A 0.43 B 0
A 0.43 B 0.14
A 0.43 B 0.29
A 0.43 B 0.43
A 0.43 B 0.57
A 0.43 B 0.71
A 0.43 B 0.86
A 0.43 B 1
A 0.57 B 0
A 0.57 B 0.14
A 0.57 B 0.29
A 0.57 B 0.43
A 0.57 B 0.57
A 0.57 B 0.71
A 0.57 B 0.86
A 0.57 B 1
A 0.71 B 0
A 0.71 B 0.14
A 0.71 B 0.29
A 0.71 B 0.43
A 0.71 B 0.57
A 0.71 B 0.71
A 0.71 B 0.86
A 0.71 B 1
A 0.86 B 0
A 0.86 B 0.14
A 0.86 B 0.29
A 0.86 B 0.43
A 0.86 B 0.57
A 0.86 B 0.71
A 0.86 B 0.86
A 0.86 B 1
A 1 B 0
A 1 B 0.14
A 1 B 0.29
A 1 B 0.43
A 1 B 0.57
A 1 B 0.71
A 1 B 0.86
A 1 B 1
0.8
0.6
0.4
0.2
0 5 101520

Ben.Fen (= Ax.Bx) full GPDI
Int_AB = 0.14 and Int_BA = 0.09 at EC50

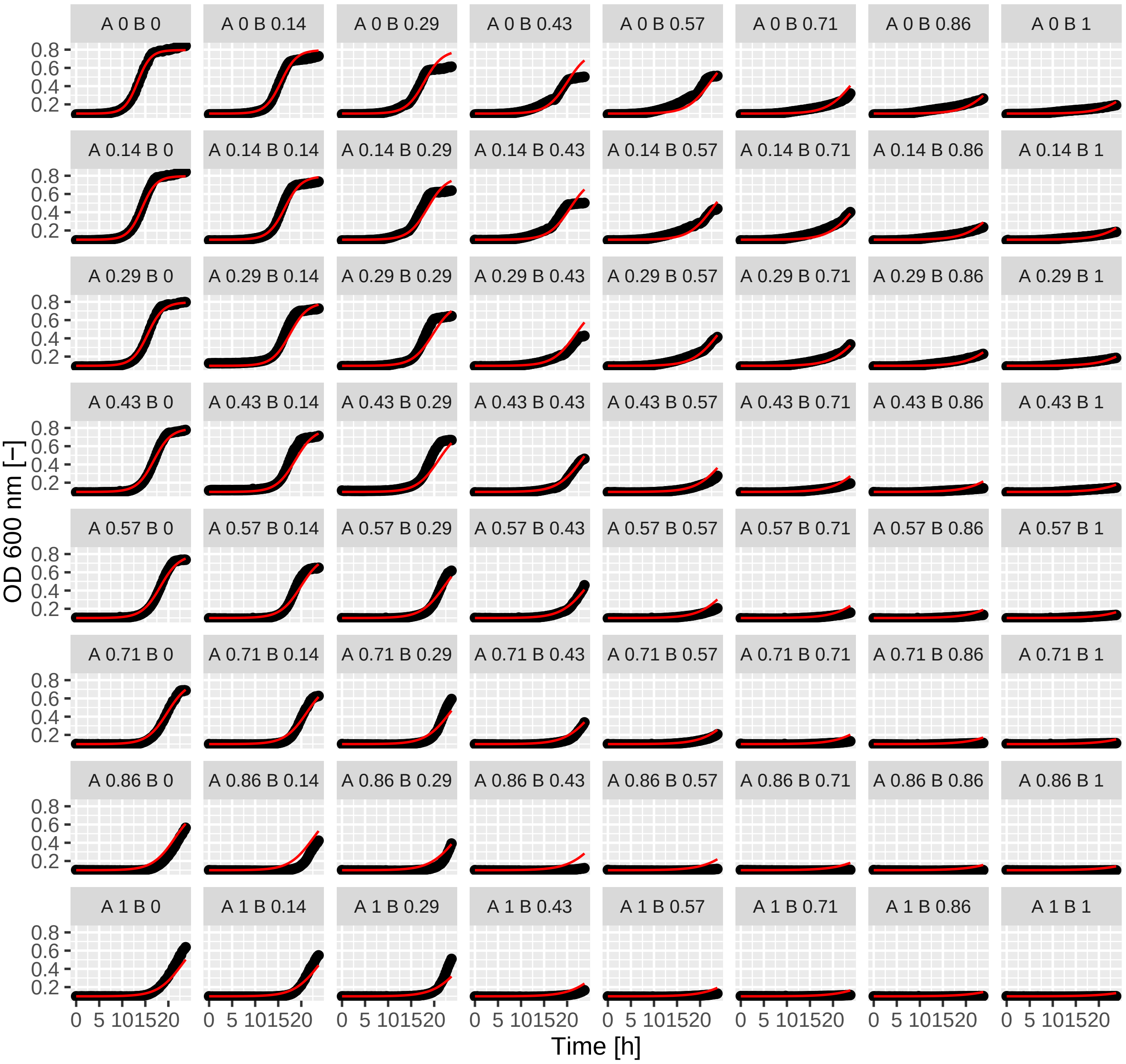

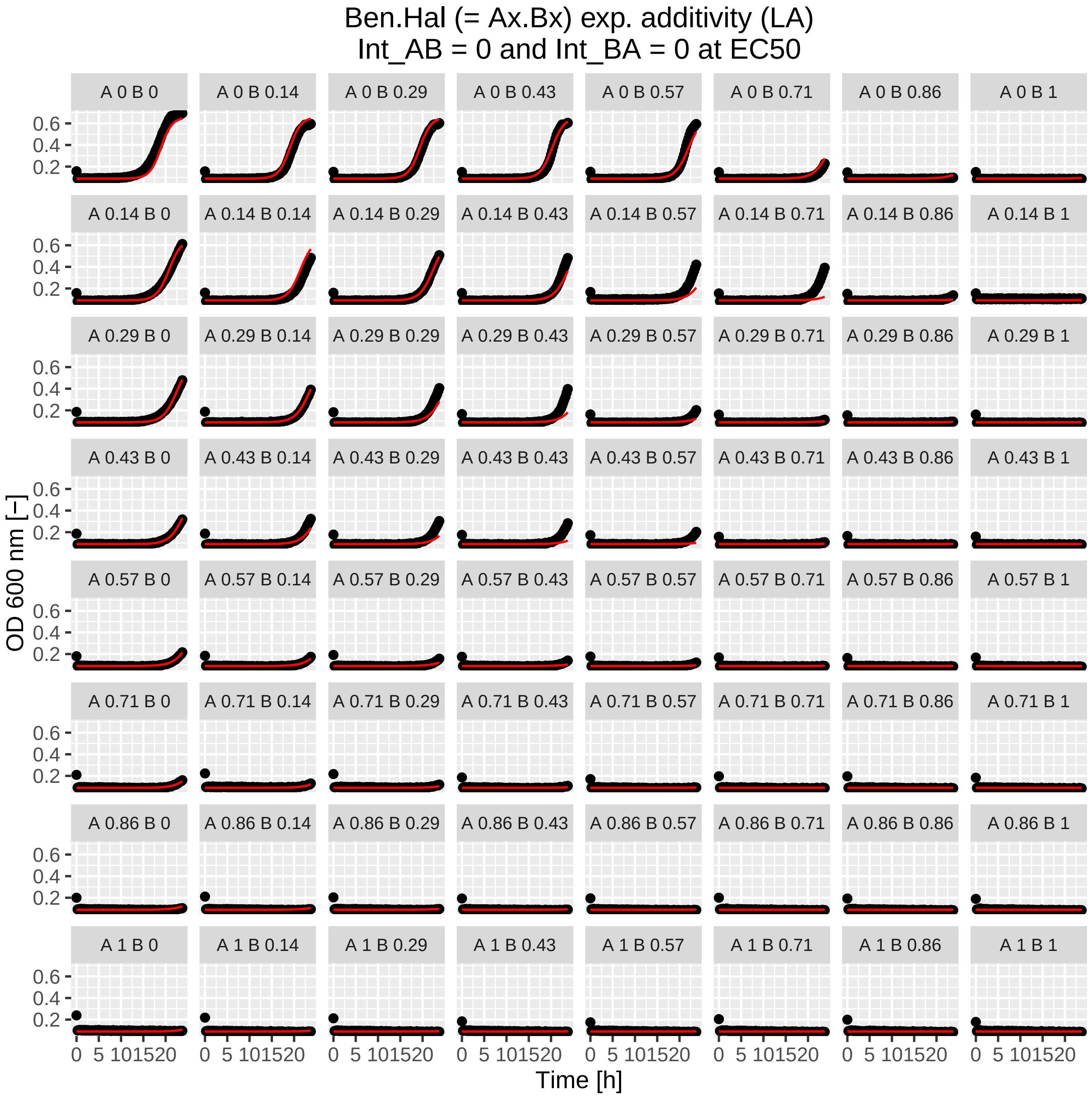
Ben.Hal (= Ax.Bx) exp. additivity (LA)
Int_AB = 0 and Int_BA = 0 at EC50
A 0 B 0
A 0 B 0.14
A 0 B 0.29
A 0 B 0.43
A 0 B 0.57
A 0 B 0.71
A 0 B 0.86
A 0 B 1
A 0.14 B 0
A 0.14 B 0.14
A 0.14 B 0.29
A 0.14 B 0.43
A 0.14 B 0.57
A 0.14 B 0.71
A 0.14 B 0.86
A 0.14 B 1
A 0.29 B 0
A 0.29 B 0.14
A 0.29 B 0.29
A 0.29 B 0.43
A 0.29 B 0.57
A 0.29 B 0.71
A 0.29 B 0.86
A 0.29 B 1
A 0.43 B 0
A 0.43 B 0.14
A 0.43 B 0.29
A 0.43 B 0.43
A 0.43 B 0.57
A 0.43 B 0.71
A 0.43 B 0.86
A 0.43 B 1
A 0.57 B 0
A 0.57 B 0.14
A 0.57 B 0.29
A 0.57 B 0.43
A 0.57 B 0.57
A 0.57 B 0.71
A 0.57 B 0.86
A 0.57 B 1
A 0.71 B 0
A 0.71 B 0.14
A 0.71 B 0.29
A 0.71 B 0.43
A 0.71 B 0.57
A 0.71 B 0.71
A 0.71 B 0.86
A 0.71 B 1
A 0.86 B 0
A 0.86 B 0.14
A 0.86 B 0.29
A 0.86 B 0.43
A 0.86 B 0.57
A 0.86 B 0.71
A 0.86 B 0.86
A 0.86 B 1
A 1 B 0
A 1 B 0.14
A 1 B 0.29
A 1 B 0.43
A 1 B 0.57
A 1 B 0.71
A 1 B 0.86
A 1 B 1
OD 600 nm [–]
0.2
0.4
0.6
0 5 101520
Time [h]

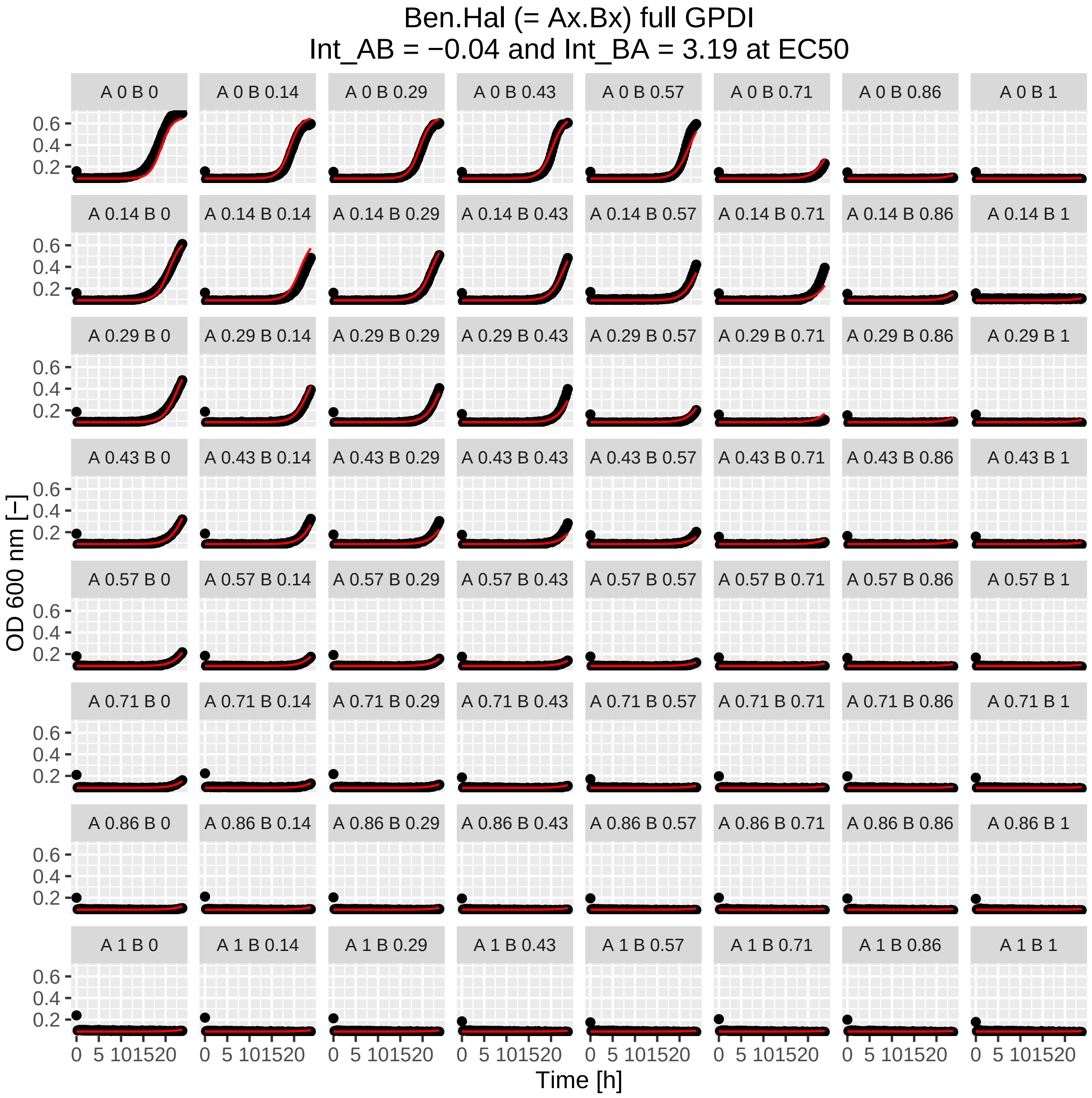

Ben.Hal (= Ax.Bx) full GPDI
Int_AB = −0.04 and Int_BA = 3.19 at EC50
A 0 B 0
A 0 B 0.14
A 0 B 0.29
A 0 B 0.43
A 0 B 0.57
A 0 B 0.71
A 0 B 0.86
A 0 B 1
A 0.14 B 0
A 0.14 B 0.14
A 0.14 B 0.29
A 0.14 B 0.43
A 0.14 B 0.57
A 0.14 B 0.71
A 0.14 B 0.86
A 0.14 B 1
A 0.29 B 0
A 0.29 B 0.14
A 0.29 B 0.29
A 0.29 B 0.43
A 0.29 B 0.57
A 0.29 B 0.71
A 0.29 B 0.86
A 0.29 B 1
A 0.43 B 0
A 0.43 B 0.14
A 0.43 B 0.29
A 0.43 B 0.43
A 0.43 B 0.57
A 0.43 B 0.71
A 0.43 B 0.86
A 0.43 B 1
A 0.57 B 0
A 0.57 B 0.14
A 0.57 B 0.29
A 0.57 B 0.43
A 0.57 B 0.57
A 0.57 B 0.71
A 0.57 B 0.86
A 0.57 B 1
A 0.71 B 0
A 0.71 B 0.14
A 0.71 B 0.29
A 0.71 B 0.43
A 0.71 B 0.57
A 0.71 B 0.71
A 0.71 B 0.86
A 0.71 B 1
A 0.86 B 0
A 0.86 B 0.14
A 0.86 B 0.29
A 0.86 B 0.43
A 0.86 B 0.57
A 0.86 B 0.71
A 0.86 B 0.86
A 0.86 B 1
A 1 B 0
A 1 B 0.14
A 1 B 0.29
A 1 B 0.43
A 1 B 0.57
A 1 B 0.71
A 1 B 0.86
A 1 B 1
OD 600 nm [−]
0.6
0.4
0.2
0 5 101520
Time [h]

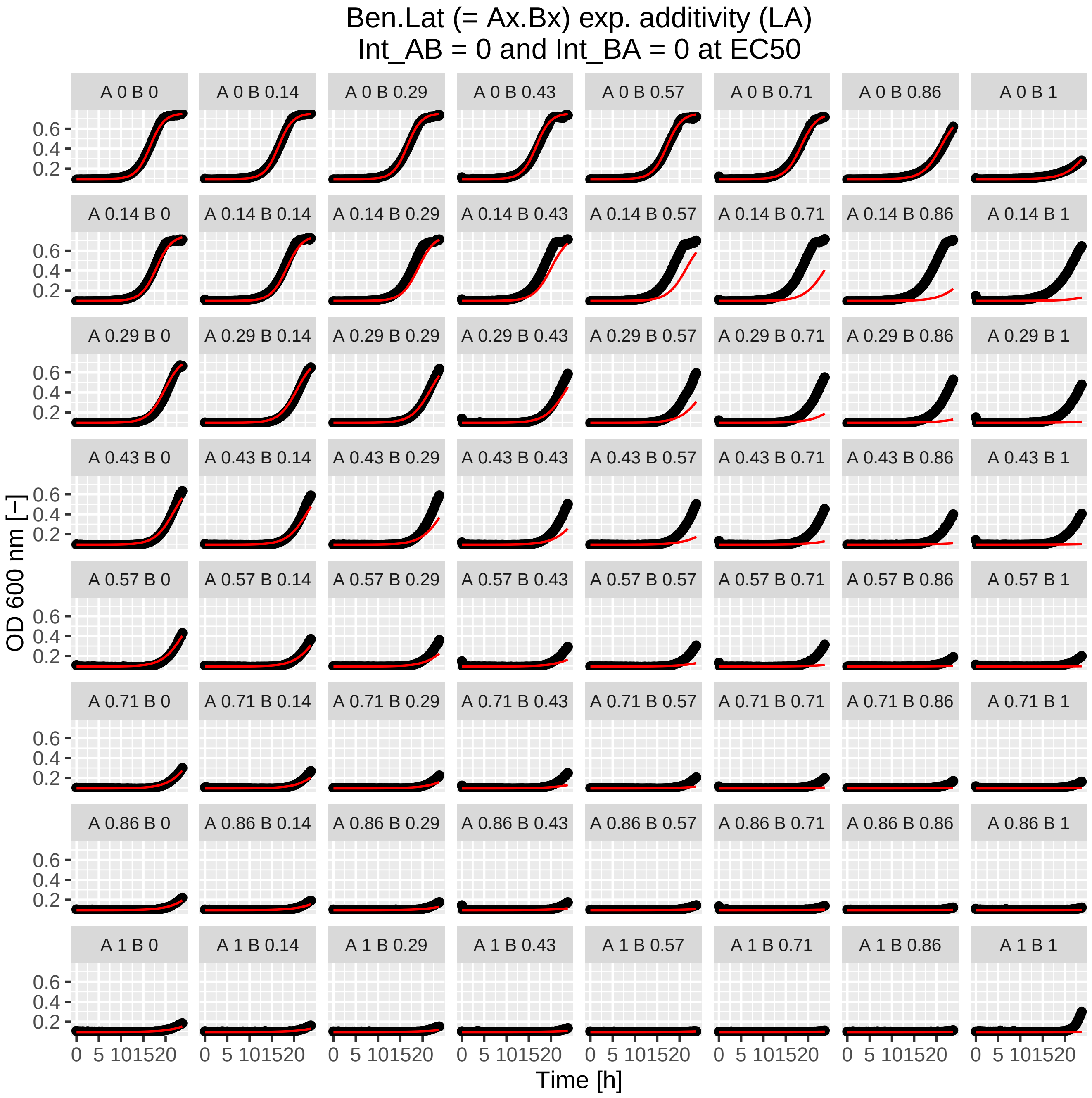
Ben.Lat (= Ax.Bx) exp. additivity (LA)
Int_AB = 0 and Int_BA = 0 at EC50
OD 600 nm [–]
Time [h]
0.6
0.4
0.2
0 5 101520
A 0 B 0
A 0 B 0.14
A 0 B 0.29
A 0 B 0.43
A 0 B 0.57
A 0 B 0.71
A 0 B 0.86
A 0 B 1
A 0.14 B 0
A 0.14 B 0.14
A 0.14 B 0.29
A 0.14 B 0.43
A 0.14 B 0.57
A 0.14 B 0.71
A 0.14 B 0.86
A 0.14 B 1
A 0.29 B 0
A 0.29 B 0.14
A 0.29 B 0.29
A 0.29 B 0.43
A 0.29 B 0.57
A 0.29 B 0.71
A 0.29 B 0.86
A 0.29 B 1
A 0.43 B 0
A 0.43 B 0.14
A 0.43 B 0.29
A 0.43 B 0.43
A 0.43 B 0.57
A 0.43 B 0.71
A 0.43 B 0.86
A 0.43 B 1
A 0.57 B 0
A 0.57 B 0.14
A 0.57 B 0.29
A 0.57 B 0.43
A 0.57 B 0.57
A 0.57 B 0.71
A 0.57 B 0.86
A 0.57 B 1
A 0.71 B 0
A 0.71 B 0.14
A 0.71 B 0.29
A 0.71 B 0.43
A 0.71 B 0.57
A 0.71 B 0.71
A 0.71 B 0.86
A 0.71 B 1
A 0.86 B 0
A 0.86 B 0.14
A 0.86 B 0.29
A 0.86 B 0.43
A 0.86 B 0.57
A 0.86 B 0.71
A 0.86 B 0.86
A 0.86 B 1
A 1 B 0
A 1 B 0.14
A 1 B 0.29
A 1 B 0.43
A 1 B 0.57
A 1 B 0.71
A 1 B 0.86
A 1 B 1

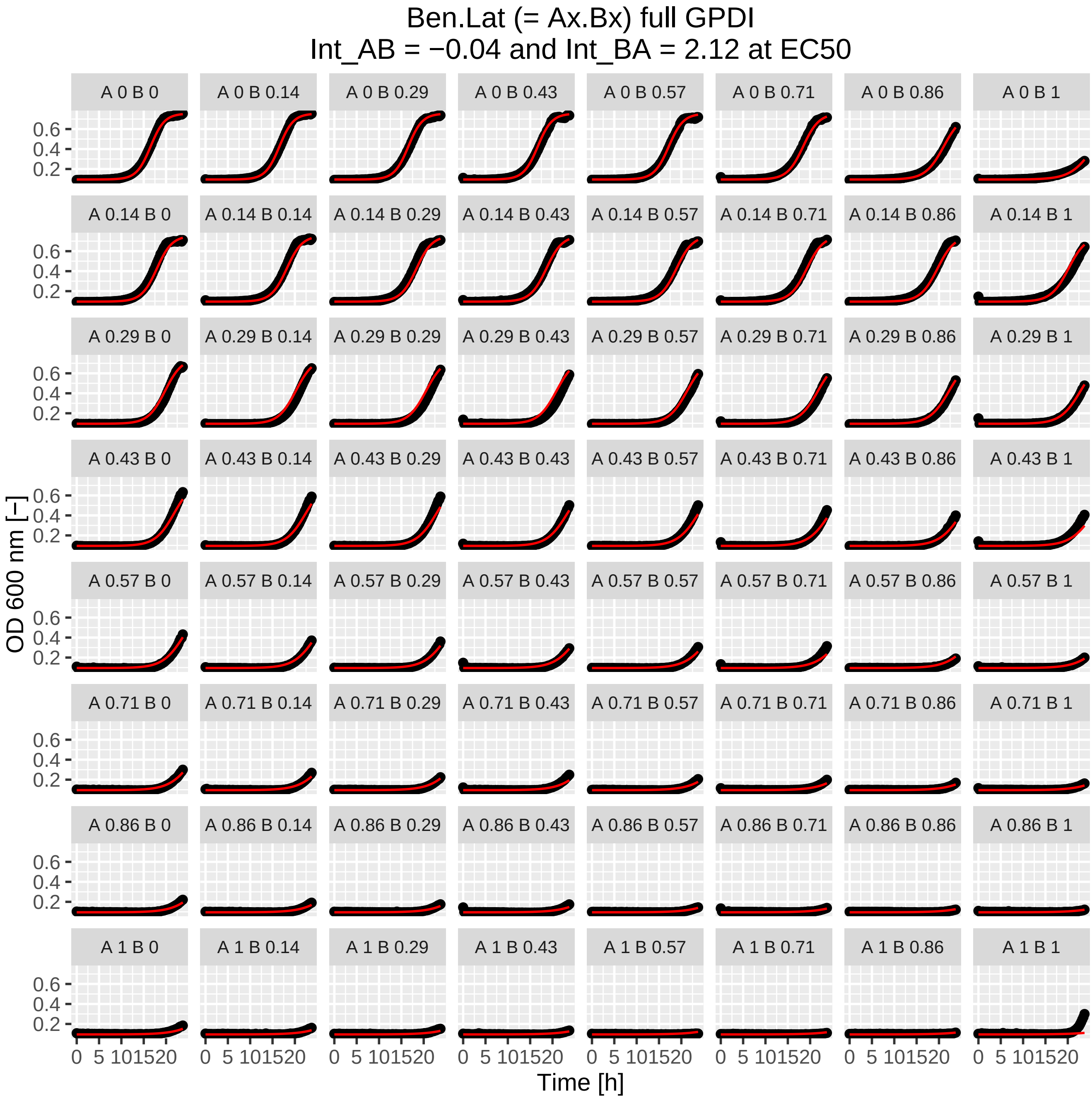

Ben.Lat (= Ax.Bx) full GPDI
Int_AB = −0.04 and Int_BA = 2.12 at EC50
A 0 B 0
A 0 B 0.14
A 0 B 0.29
A 0 B 0.43
A 0 B 0.57
A 0 B 0.71
A 0 B 0.86
A 0 B 1
A 0.14 B 0
A 0.14 B 0.14
A 0.14 B 0.29
A 0.14 B 0.43
A 0.14 B 0.57
A 0.14 B 0.71
A 0.14 B 0.86
A 0.14 B 1
A 0.29 B 0
A 0.29 B 0.14
A 0.29 B 0.29
A 0.29 B 0.43
A 0.29 B 0.57
A 0.29 B 0.71
A 0.29 B 0.86
A 0.29 B 1
A 0.43 B 0
A 0.43 B 0.14
A 0.43 B 0.29
A 0.43 B 0.43
A 0.43 B 0.57
A 0.43 B 0.71
A 0.43 B 0.86
A 0.43 B 1
A 0.57 B 0
A 0.57 B 0.14
A 0.57 B 0.29
A 0.57 B 0.43
A 0.57 B 0.57
A 0.57 B 0.71
A 0.57 B 0.86
A 0.57 B 1
A 0.71 B 0
A 0.71 B 0.14
A 0.71 B 0.29
A 0.71 B 0.43
A 0.71 B 0.57
A 0.71 B 0.71
A 0.71 B 0.86
A 0.71 B 1
A 0.86 B 0
A 0.86 B 0.14
A 0.86 B 0.29
A 0.86 B 0.43
A 0.86 B 0.57
A 0.86 B 0.71
A 0.86 B 0.86
A 0.86 B 1
A 1 B 0
A 1 B 0.14
A 1 B 0.29
A 1 B 0.43
A 1 B 0.57
A 1 B 0.71
A 1 B 0.86
A 1 B 1
OD 600 nm [−]
0.2
0.4
0.6
0 5 101520
Time [h]

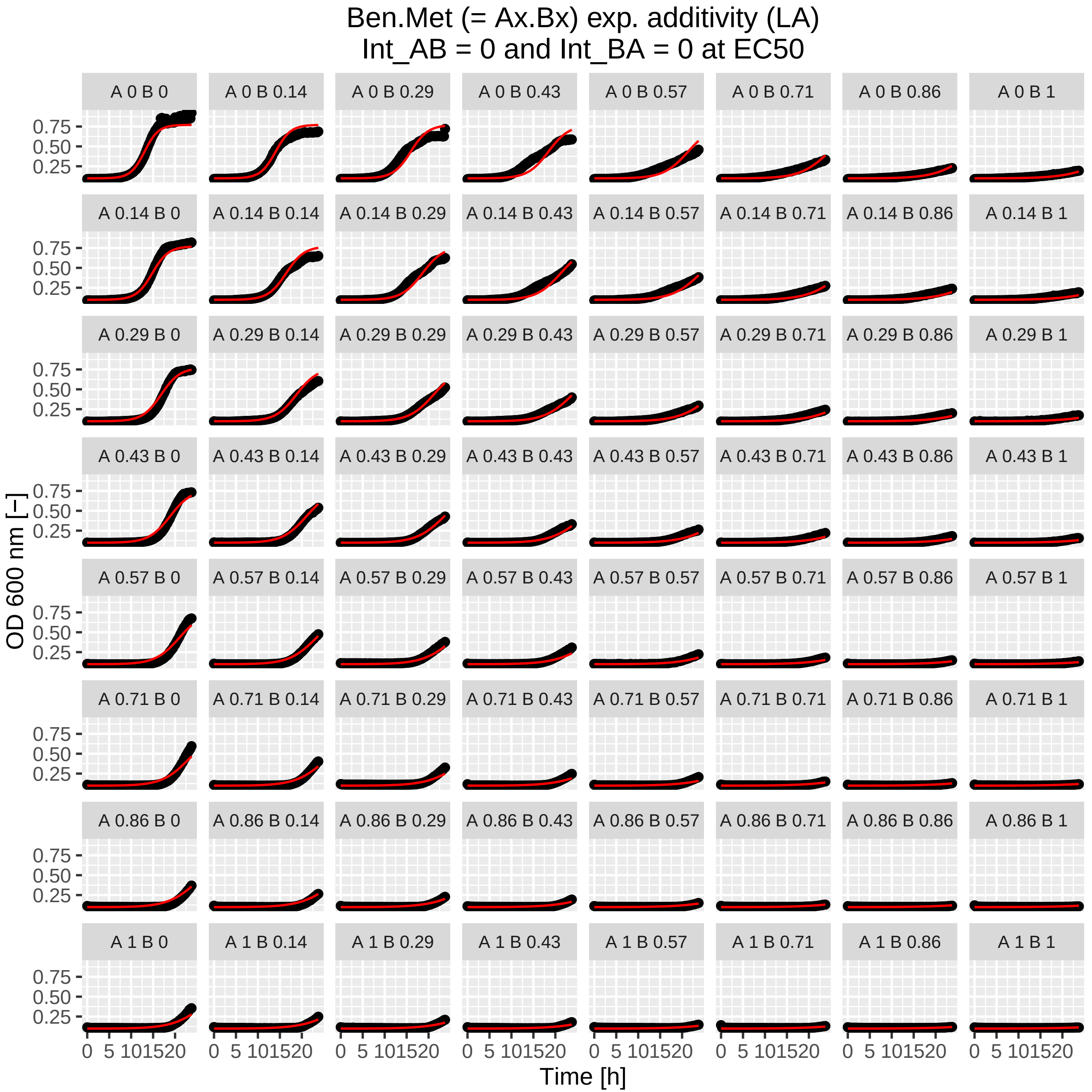
Ben.Met (= Ax.Bx) exp. additivity (LA)
Int_AB = 0 and Int_BA = 0 at EC50
A 0 B 0
A 0 B 0.14
A 0 B 0.29
A 0 B 0.43
A 0 B 0.57
A 0 B 0.71
A 0 B 0.86
A 0 B 1
A 0.14 B 0
A 0.14 B 0.14
A 0.14 B 0.29
A 0.14 B 0.43
A 0.14 B 0.57
A 0.14 B 0.71
A 0.14 B 0.86
A 0.14 B 1
A 0.29 B 0
A 0.29 B 0.14
A 0.29 B 0.29
A 0.29 B 0.43
A 0.29 B 0.57
A 0.29 B 0.71
A 0.29 B 0.86
A 0.29 B 1
A 0.43 B 0
A 0.43 B 0.14
A 0.43 B 0.29
A 0.43 B 0.43
A 0.43 B 0.57
A 0.43 B 0.71
A 0.43 B 0.86
A 0.43 B 1
A 0.57 B 0
A 0.57 B 0.14
A 0.57 B 0.29
A 0.57 B 0.43
A 0.57 B 0.57
A 0.57 B 0.71
A 0.57 B 0.86
A 0.57 B 1
A 0.71 B 0
A 0.71 B 0.14
A 0.71 B 0.29
A 0.71 B 0.43
A 0.71 B 0.57
A 0.71 B 0.71
A 0.71 B 0.86
A 0.71 B 1
A 0.86 B 0
A 0.86 B 0.14
A 0.86 B 0.29
A 0.86 B 0.43
A 0.86 B 0.57
A 0.86 B 0.71
A 0.86 B 0.86
A 0.86 B 1
A 1 B 0
A 1 B 0.14
A 1 B 0.29
A 1 B 0.43
A 1 B 0.57
A 1 B 0.71
A 1 B 0.86
A 1 B 1
OD 600 nm [–]
0.25
0.50
0.75
0 5 101520
Time [h]

Ben.Met (= Ax.Bx) full GPDI
Int_AB = 0 and Int_BA = 0.18 at EC50

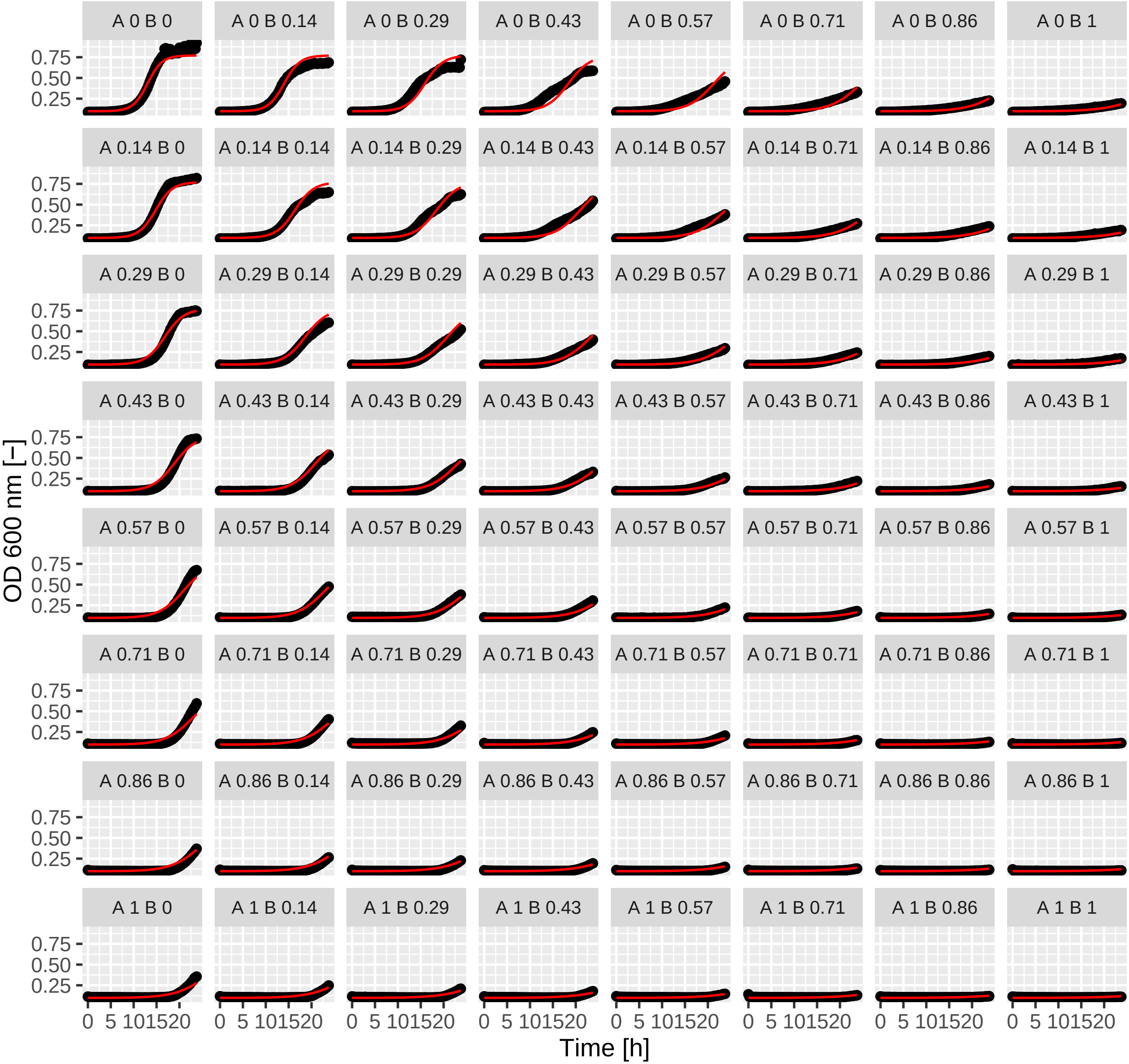

Ben.MMS (= Ax.Bx) exp. additivity (LA)
Int_AB = 0 and Int_BA = 0 at EC50

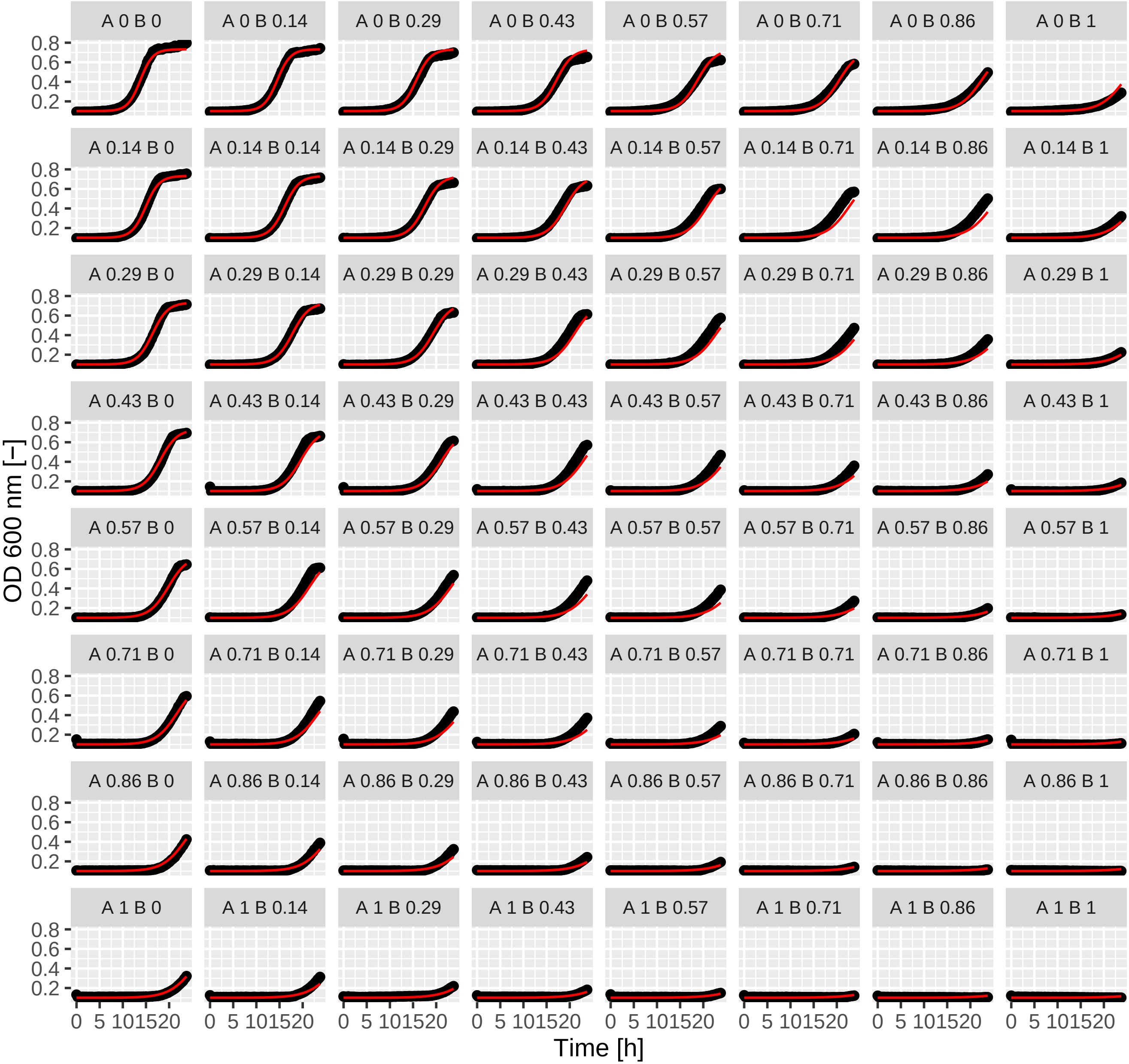

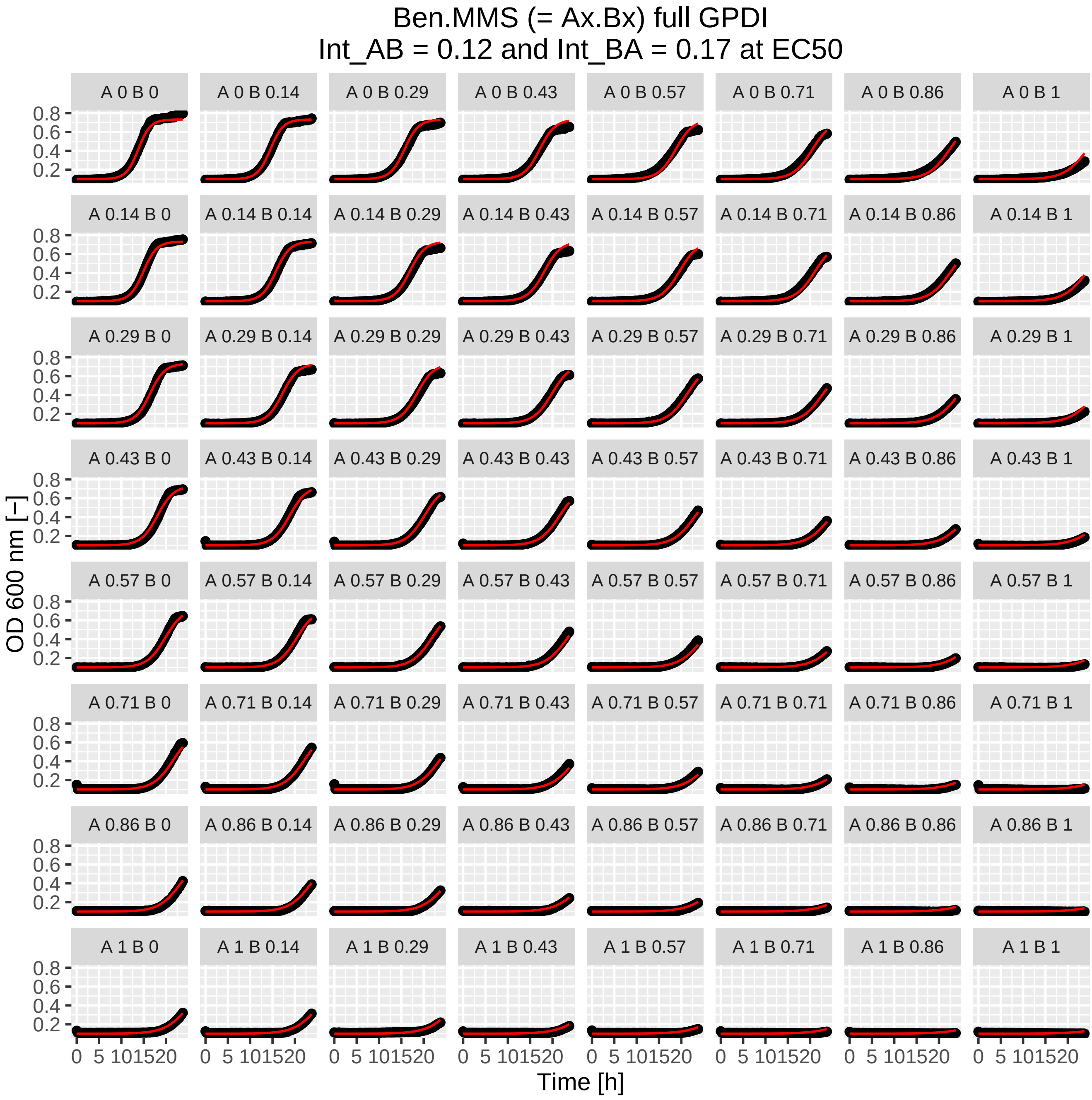

Ben.MMS (= Ax.Bx) full GPDI
Int_AB = 0.12 and Int_BA = 0.17 at EC50
OD 600 nm [−]
Time [h]
A 0 B 0
A 0 B 0.14
A 0 B 0.29
A 0 B 0.43
A 0 B 0.57
A 0 B 0.71
A 0 B 0.86
A 0 B 1
A 0.14 B 0
A 0.14 B 0.14
A 0.14 B 0.29
A 0.14 B 0.43
A 0.14 B 0.57
A 0.14 B 0.71
A 0.14 B 0.86
A 0.14 B 1
A 0.29 B 0
A 0.29 B 0.14
A 0.29 B 0.29
A 0.29 B 0.43
A 0.29 B 0.57
A 0.29 B 0.71
A 0.29 B 0.86
A 0.29 B 1
A 0.43 B 0
A 0.43 B 0.14
A 0.43 B 0.29
A 0.43 B 0.43
A 0.43 B 0.57
A 0.43 B 0.71
A 0.43 B 0.86
A 0.43 B 1
A 0.57 B 0
A 0.57 B 0.14
A 0.57 B 0.29
A 0.57 B 0.43
A 0.57 B 0.57
A 0.57 B 0.71
A 0.57 B 0.86
A 0.57 B 1
A 0.71 B 0
A 0.71 B 0.14
A 0.71 B 0.29
A 0.71 B 0.43
A 0.71 B 0.57
A 0.71 B 0.71
A 0.71 B 0.86
A 0.71 B 1
A 0.86 B 0
A 0.86 B 0.14
A 0.86 B 0.29
A 0.86 B 0.43
A 0.86 B 0.57
A 0.86 B 0.71
A 0.86 B 0.86
A 0.86 B 1
A 1 B 0
A 1 B 0.14
A 1 B 0.29
A 1 B 0.43
A 1 B 0.57
A 1 B 0.71
A 1 B 0.86
A 1 B 1

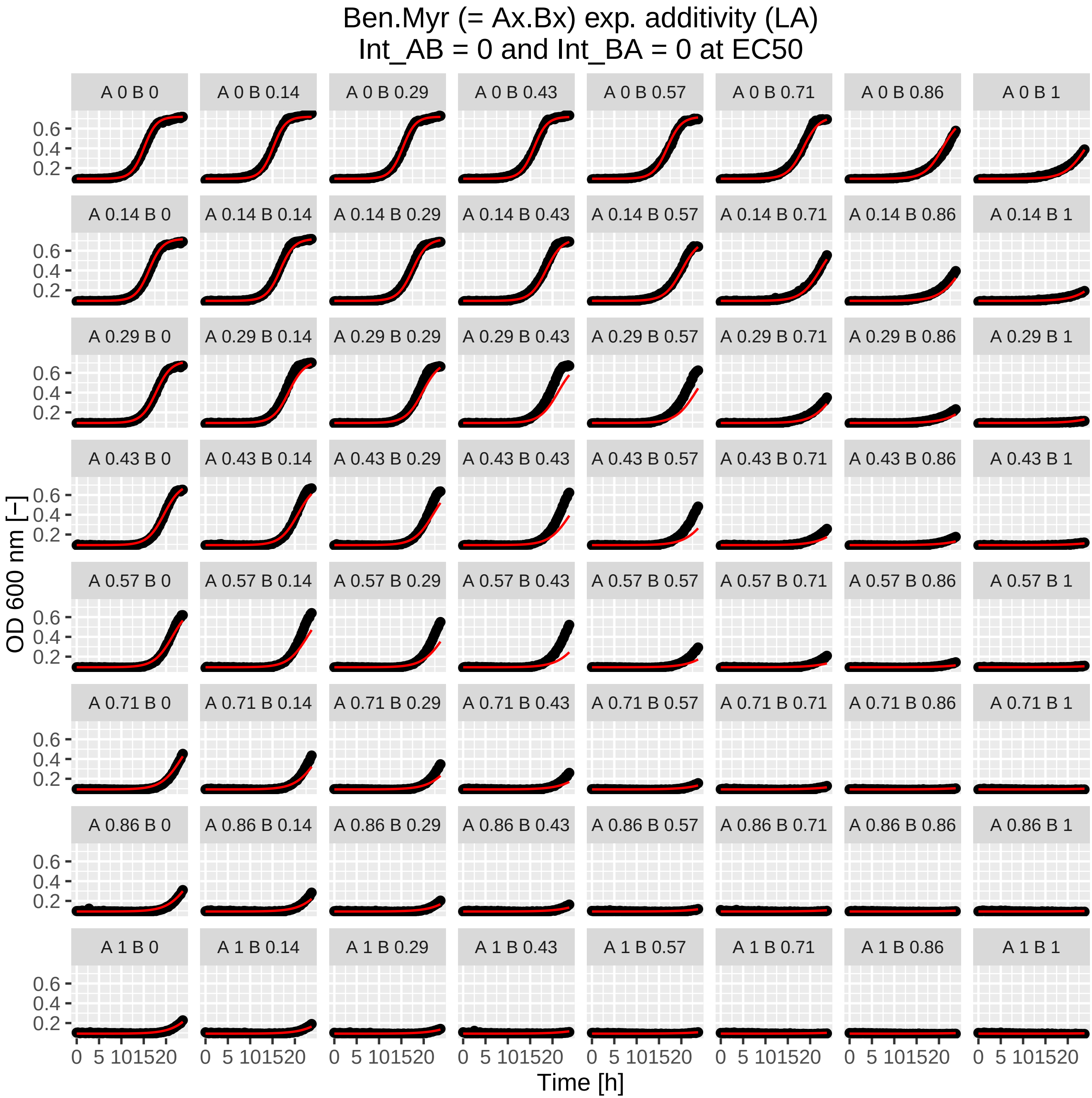

Ben.Myr (= Ax.Bx) exp. additivity (LA)
Int_AB = 0 and Int_BA = 0 at EC50
A 0 B 0
A 0 B 0.14
A 0 B 0.29
A 0 B 0.43
A 0 B 0.57
A 0 B 0.71
A 0 B 0.86
A 0 B 1
A 0.14 B 0
A 0.14 B 0.14
A 0.14 B 0.29
A 0.14 B 0.43
A 0.14 B 0.57
A 0.14 B 0.71
A 0.14 B 0.86
A 0.14 B 1
A 0.29 B 0
A 0.29 B 0.14
A 0.29 B 0.29
A 0.29 B 0.43
A 0.29 B 0.57
A 0.29 B 0.71
A 0.29 B 0.86
A 0.29 B 1
A 0.43 B 0
A 0.43 B 0.14
A 0.43 B 0.29
A 0.43 B 0.43
A 0.43 B 0.57
A 0.43 B 0.71
A 0.43 B 0.86
A 0.43 B 1
A 0.57 B 0
A 0.57 B 0.14
A 0.57 B 0.29
A 0.57 B 0.43
A 0.57 B 0.57
A 0.57 B 0.71
A 0.57 B 0.86
A 0.57 B 1
A 0.71 B 0
A 0.71 B 0.14
A 0.71 B 0.29
A 0.71 B 0.43
A 0.71 B 0.57
A 0.71 B 0.71
A 0.71 B 0.86
A 0.71 B 1
A 0.86 B 0
A 0.86 B 0.14
A 0.86 B 0.29
A 0.86 B 0.43
A 0.86 B 0.57
A 0.86 B 0.71
A 0.86 B 0.86
A 0.86 B 1
A 1 B 0
A 1 B 0.14
A 1 B 0.29
A 1 B 0.43
A 1 B 0.57
A 1 B 0.71
A 1 B 0.86
A 1 B 1
OD 600 nm [–]
0.6
0.4
0.2
0 5 101520
Time [h]

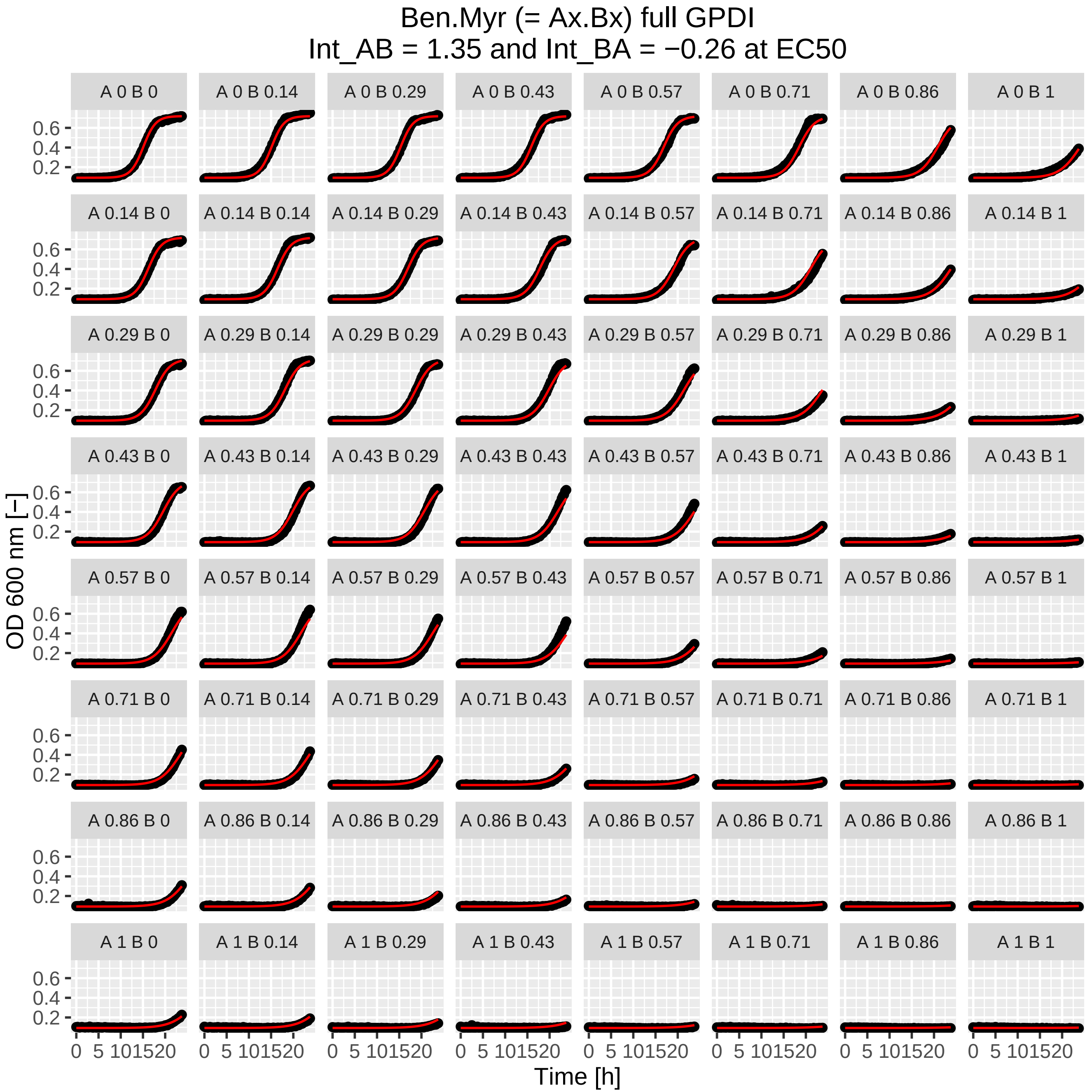
Ben.Myr (= Ax.Bx) full GPDI
Int_AB = 1.35 and Int_BA = −0.26 at EC50
OD 600 nm [−]
Time [h]
A 0 B 0
A 0 B 0.14
A 0 B 0.29
A 0 B 0.43
A 0 B 0.57
A 0 B 0.71
A 0 B 0.86
A 0 B 1
A 0.14 B 0
A 0.14 B 0.14
A 0.14 B 0.29
A 0.14 B 0.43
A 0.14 B 0.57
A 0.14 B 0.71
A 0.14 B 0.86
A 0.14 B 1
A 0.29 B 0
A 0.29 B 0.14
A 0.29 B 0.29
A 0.29 B 0.43
A 0.29 B 0.57
A 0.29 B 0.71
A 0.29 B 0.86
A 0.29 B 1
A 0.43 B 0
A 0.43 B 0.14
A 0.43 B 0.29
A 0.43 B 0.43
A 0.43 B 0.57
A 0.43 B 0.71
A 0.43 B 0.86
A 0.43 B 1
A 0.57 B 0
A 0.57 B 0.14
A 0.57 B 0.29
A 0.57 B 0.43
A 0.57 B 0.57
A 0.57 B 0.71
A 0.57 B 0.86
A 0.57 B 1
A 0.71 B 0
A 0.71 B 0.14
A 0.71 B 0.29
A 0.71 B 0.43
A 0.71 B 0.57
A 0.71 B 0.71
A 0.71 B 0.86
A 0.71 B 1
A 0.86 B 0
A 0.86 B 0.14
A 0.86 B 0.29
A 0.86 B 0.43
A 0.86 B 0.57
A 0.86 B 0.71
A 0.86 B 0.86
A 0.86 B 1
A 1 B 0
A 1 B 0.14
A 1 B 0.29
A 1 B 0.43
A 1 B 0.57
A 1 B 0.71
A 1 B 0.86
A 1 B 1
0.2
0.4
0.6
0 5 101520

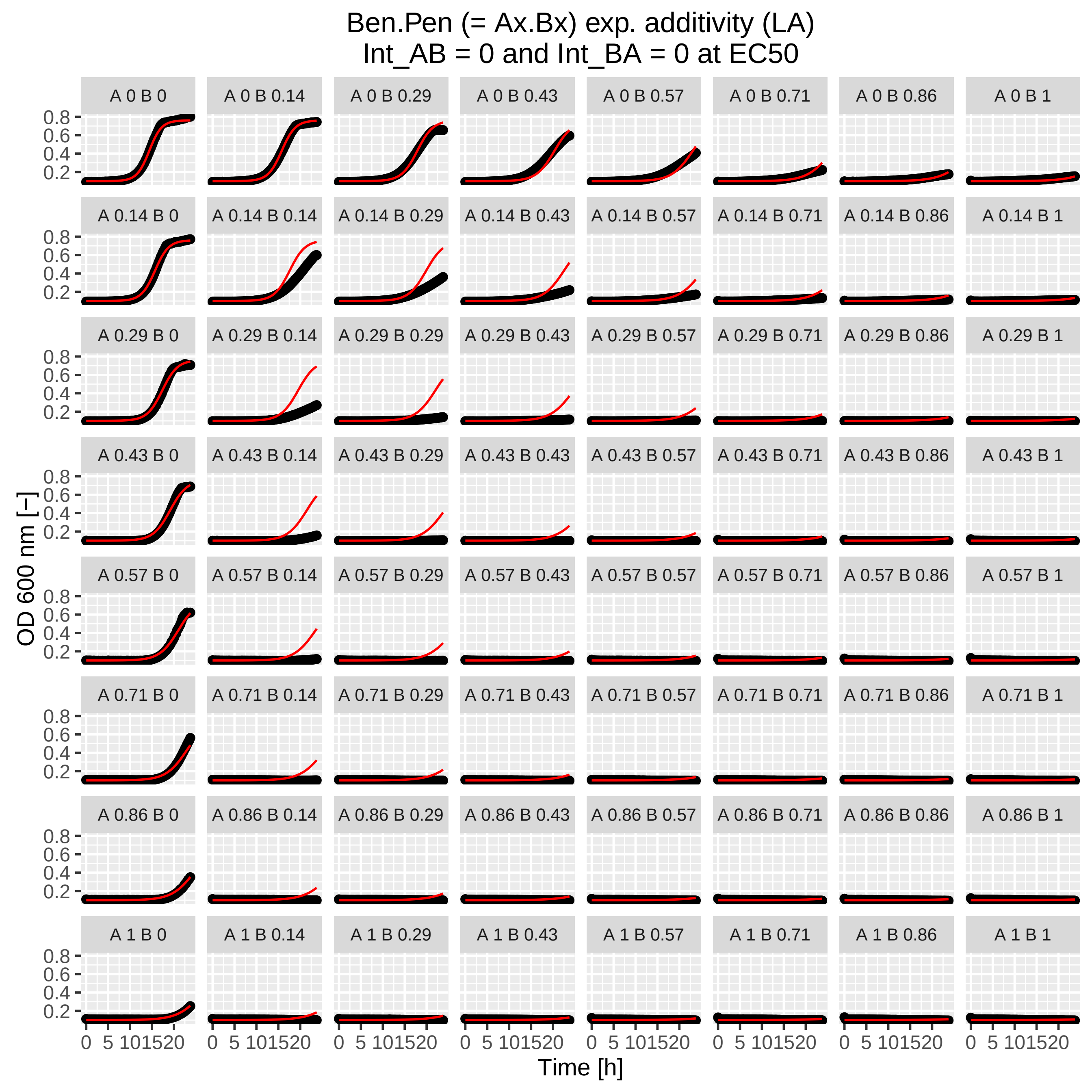

Ben.Pen (= Ax.Bx) exp. additivity (LA)
Int_AB = 0 and Int_BA = 0 at EC50
A 0 B 0
A 0 B 0.14
A 0 B 0.29
A 0 B 0.43
A 0 B 0.57
A 0 B 0.71
A 0 B 0.86
A 0 B 1
A 0.14 B 0
A 0.14 B 0.14
A 0.14 B 0.29
A 0.14 B 0.43
A 0.14 B 0.57
A 0.14 B 0.71
A 0.14 B 0.86
A 0.14 B 1
A 0.29 B 0
A 0.29 B 0.14
A 0.29 B 0.29
A 0.29 B 0.43
A 0.29 B 0.57
A 0.29 B 0.71
A 0.29 B 0.86
A 0.29 B 1
A 0.43 B 0
A 0.43 B 0.14
A 0.43 B 0.29
A 0.43 B 0.43
A 0.43 B 0.57
A 0.43 B 0.71
A 0.43 B 0.86
A 0.43 B 1
A 0.57 B 0
A 0.57 B 0.14
A 0.57 B 0.29
A 0.57 B 0.43
A 0.57 B 0.57
A 0.57 B 0.71
A 0.57 B 0.86
A 0.57 B 1
A 0.71 B 0
A 0.71 B 0.14
A 0.71 B 0.29
A 0.71 B 0.43
A 0.71 B 0.57
A 0.71 B 0.71
A 0.71 B 0.86
A 0.71 B 1
A 0.86 B 0
A 0.86 B 0.14
A 0.86 B 0.29
A 0.86 B 0.43
A 0.86 B 0.57
A 0.86 B 0.71
A 0.86 B 0.86
A 0.86 B 1
A 1 B 0
A 1 B 0.14
A 1 B 0.29
A 1 B 0.43
A 1 B 0.57
A 1 B 0.71
A 1 B 0.86
A 1 B 1
OD 600 nm [–]
0.2
0.4
0.6
0.8
0
5
10
15
20
Time [h]

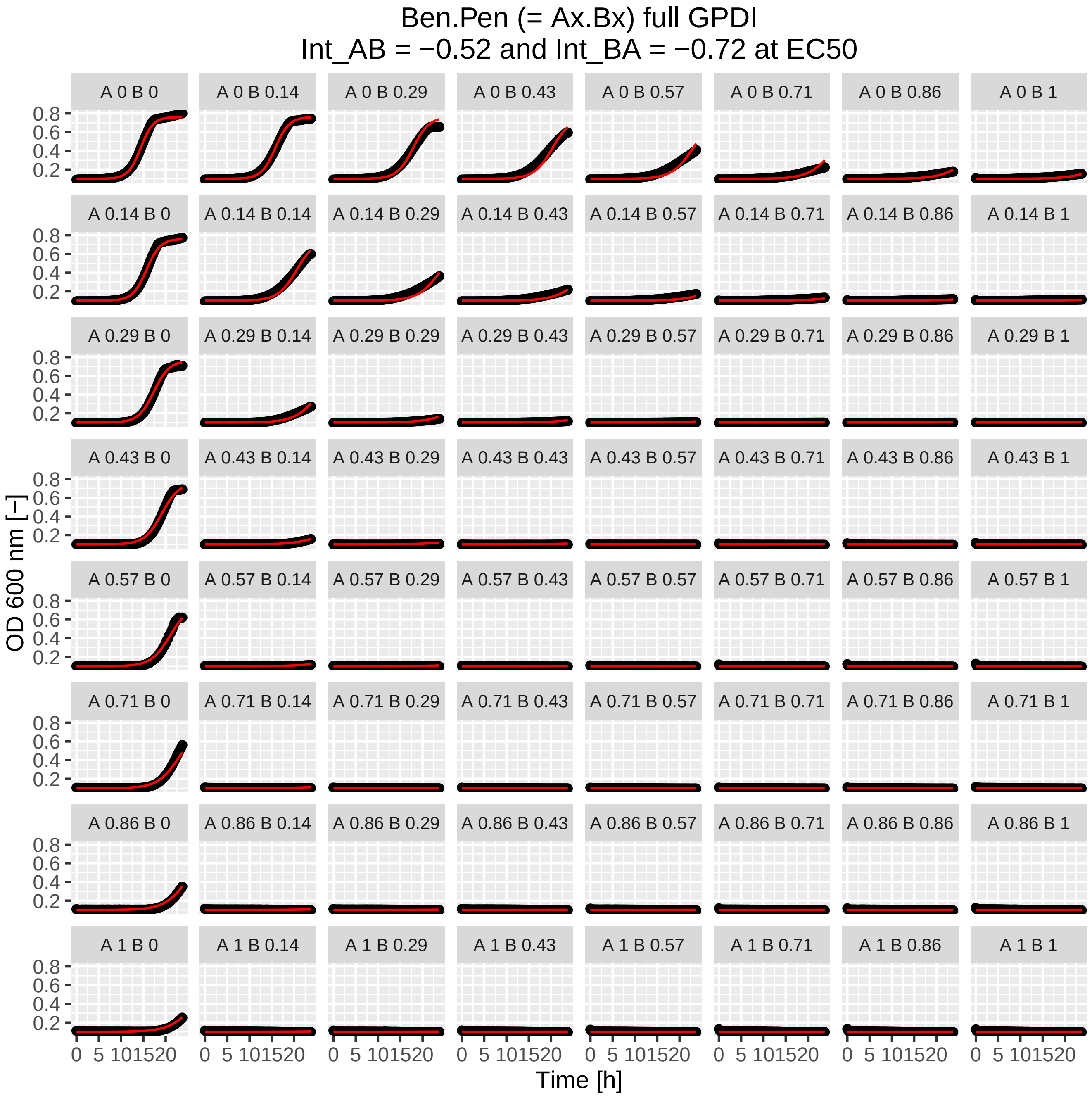

Ben.Pen (= Ax.Bx) full GPDI
Int_AB = −0.52 and Int_BA = −0.72 at EC50
OD 600 nm [−]
Time [h]
A 0 B 0
A 0 B 0.14
A 0 B 0.29
A 0 B 0.43
A 0 B 0.57
A 0 B 0.71
A 0 B 0.86
A 0 B 1
A 0.14 B 0
A 0.29 B 0
A 0.43 B 0
A 0.57 B 0
A 0.71 B 0
A 0.86 B 0
A 1 B 0
A 1 B 1

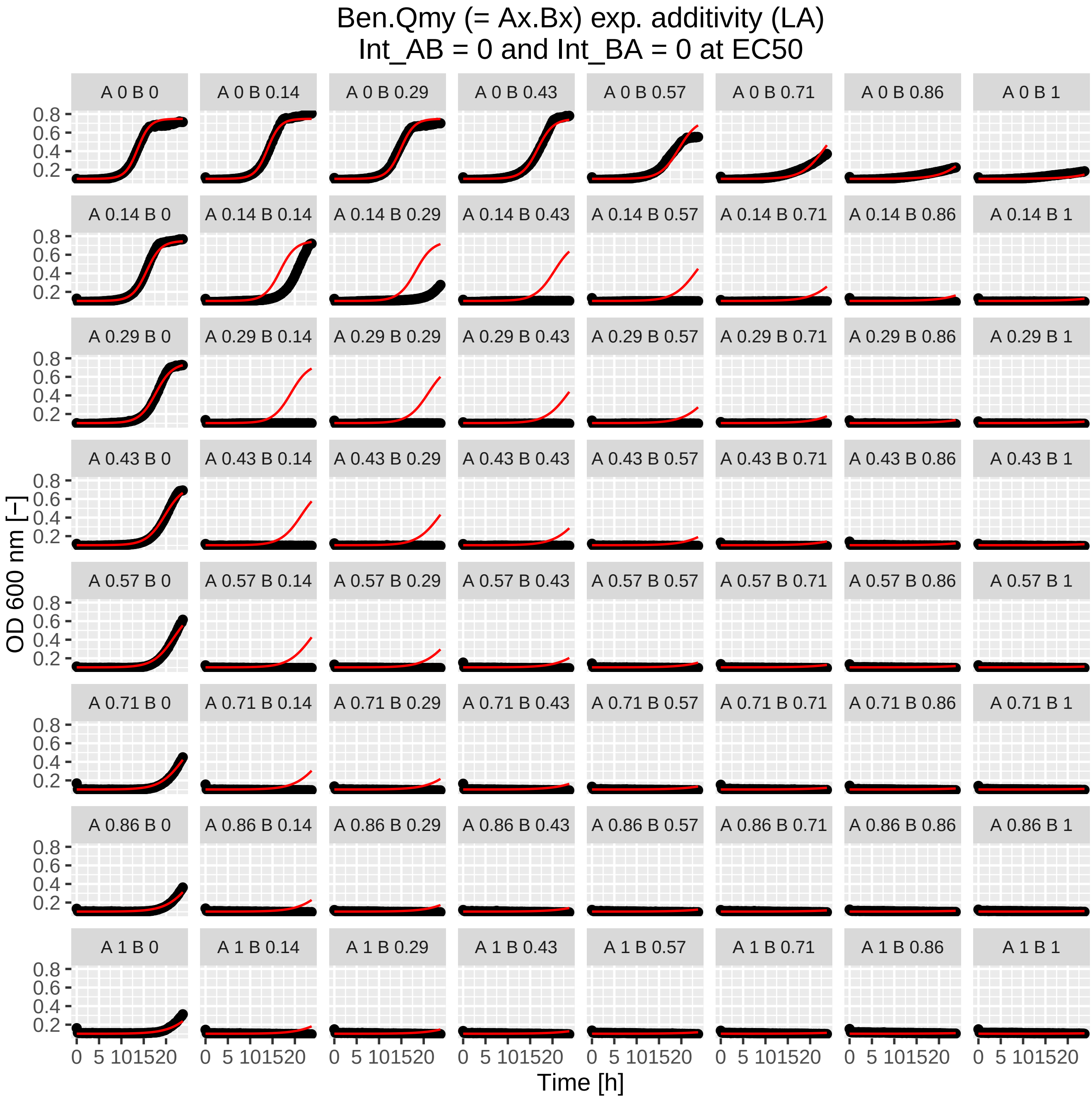
Ben.Qmy (= Ax.Bx) exp. additivity (LA)
Int_AB = 0 and Int_BA = 0 at EC50
A 0 B 0
A 0 B 0.14
A 0 B 0.29
A 0 B 0.43
A 0 B 0.57
A 0 B 0.71
A 0 B 0.86
A 0 B 1
A 0.14 B 0
A 0.14 B 0.14
A 0.14 B 0.29
A 0.14 B 0.43
A 0.14 B 0.57
A 0.14 B 0.71
A 0.14 B 0.86
A 0.14 B 1
A 0.29 B 0
A 0.29 B 0.14
A 0.29 B 0.29
A 0.29 B 0.43
A 0.29 B 0.57
A 0.29 B 0.71
A 0.29 B 0.86
A 0.29 B 1
A 0.43 B 0
A 0.43 B 0.14
A 0.43 B 0.29
A 0.43 B 0.43
A 0.43 B 0.57
A 0.43 B 0.71
A 0.43 B 0.86
A 0.43 B 1
A 0.57 B 0
A 0.57 B 0.14
A 0.57 B 0.29
A 0.57 B 0.43
A 0.57 B 0.57
A 0.57 B 0.71
A 0.57 B 0.86
A 0.57 B 1
A 0.71 B 0
A 0.71 B 0.14
A 0.71 B 0.29
A 0.71 B 0.43
A 0.71 B 0.57
A 0.71 B 0.71
A 0.71 B 0.86
A 0.71 B 1
A 0.86 B 0
A 0.86 B 0.14
A 0.86 B 0.29
A 0.86 B 0.43
A 0.86 B 0.57
A 0.86 B 0.71
A 0.86 B 0.86
A 0.86 B 1
A 1 B 0
A 1 B 0.14
A 1 B 0.29
A 1 B 0.43
A 1 B 0.57
A 1 B 0.71
A 1 B 0.86
A 1 B 1
OD 600 nm [–]
0.2
0.4
0.6
0.8
0 5 101520
Time [h]

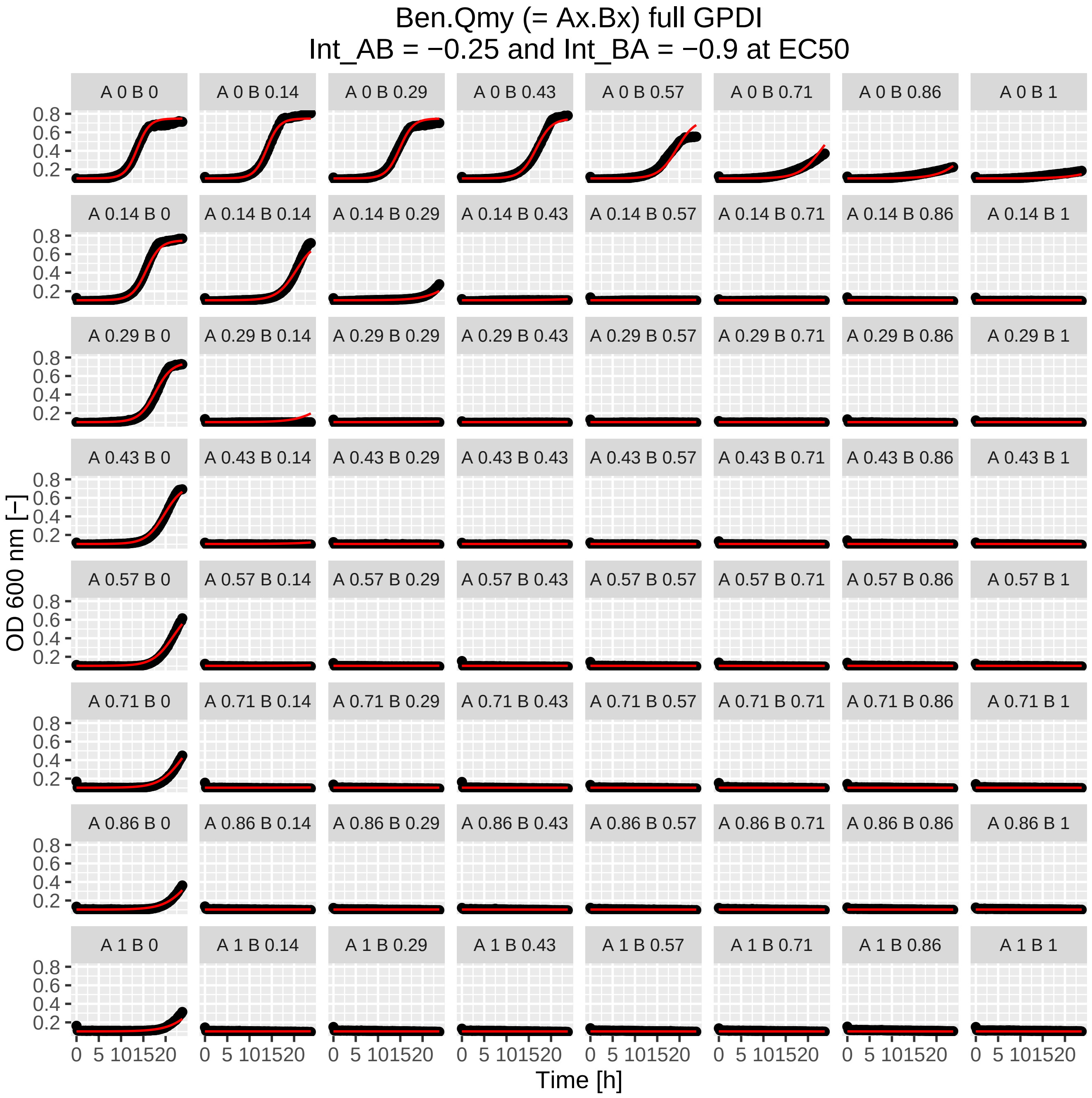
Ben.Qmy (= Ax.Bx) full GPDI
Int_AB = −0.25 and Int_BA = −0.9 at EC50
A 0 B 0
A 0 B 0.14
A 0 B 0.29
A 0 B 0.43
A 0 B 0.57
A 0 B 0.71
A 0 B 0.86
A 0 B 1
A 0.14 B 0
A 0.14 B 0.14
A 0.14 B 0.29
A 0.14 B 0.43
A 0.14 B 0.57
A 0.14 B 0.71
A 0.14 B 0.86
A 0.14 B 1
A 0.29 B 0
A 0.29 B 0.14
A 0.29 B 0.29
A 0.29 B 0.43
A 0.29 B 0.57
A 0.29 B 0.71
A 0.29 B 0.86
A 0.29 B 1
A 0.43 B 0
A 0.43 B 0.14
A 0.43 B 0.29
A 0.43 B 0.43
A 0.43 B 0.57
A 0.43 B 0.71
A 0.43 B 0.86
A 0.43 B 1
A 0.57 B 0
A 0.57 B 0.14
A 0.57 B 0.29
A 0.57 B 0.43
A 0.57 B 0.57
A 0.57 B 0.71
A 0.57 B 0.86
A 0.57 B 1
A 0.71 B 0
A 0.71 B 0.14
A 0.71 B 0.29
A 0.71 B 0.43
A 0.71 B 0.57
A 0.71 B 0.71
A 0.71 B 0.86
A 0.71 B 1
A 0.86 B 0
A 0.86 B 0.14
A 0.86 B 0.29
A 0.86 B 0.43
A 0.86 B 0.57
A 0.86 B 0.71
A 0.86 B 0.86
A 0.86 B 1
A 1 B 0
A 1 B 0.14
A 1 B 0.29
A 1 B 0.43
A 1 B 0.57
A 1 B 0.71
A 1 B 0.86
A 1 B 1
0.8
0.6
0.4
0.2
0 5 101520
OD 600 nm [−]
Time [h]

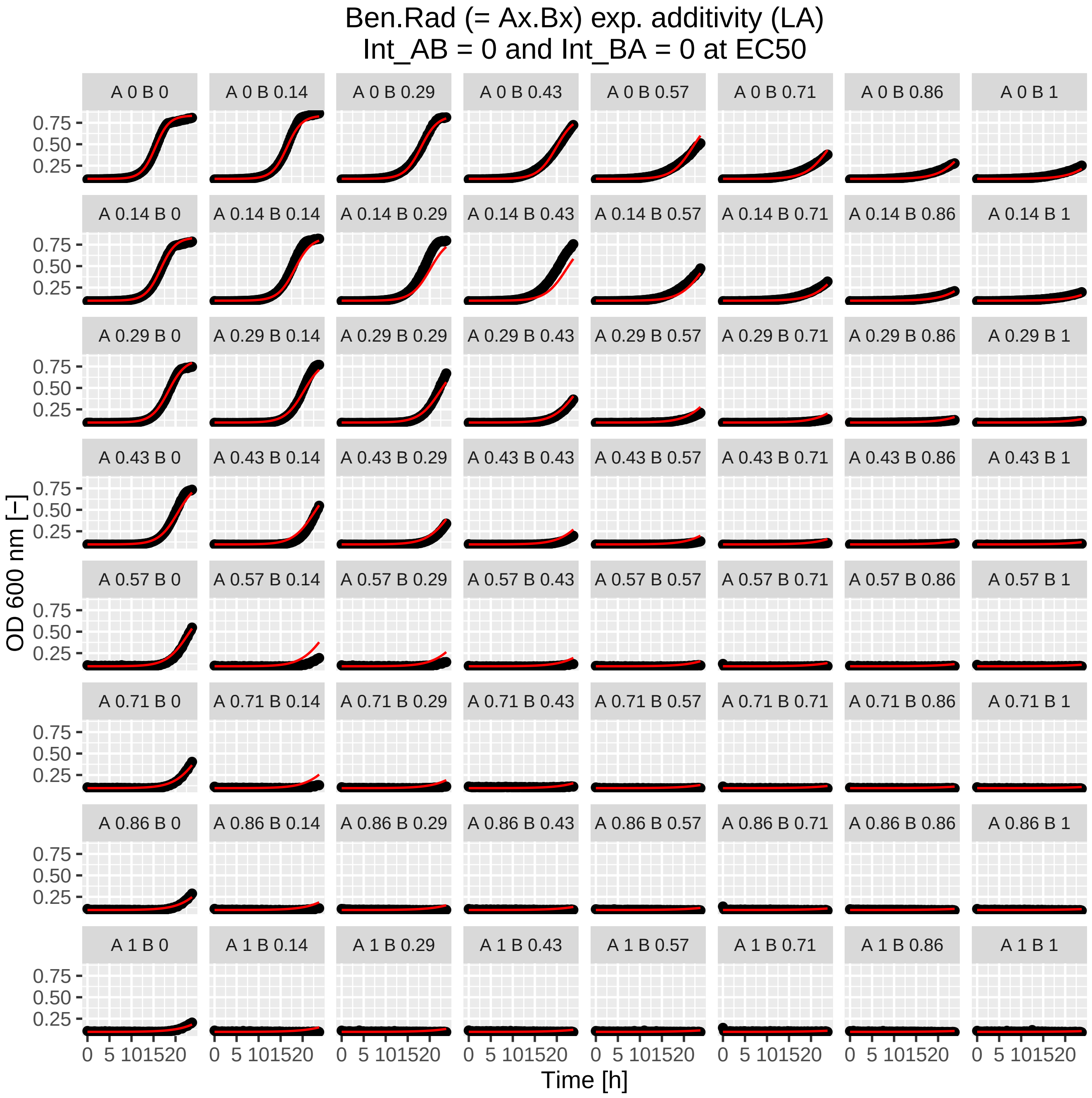
Ben.Rad (= Ax.Bx) exp. additivity (LA)
Int_AB = 0 and Int_BA = 0 at EC50
A 0 B 0
A 0 B 0.14
A 0 B 0.29
A 0 B 0.43
A 0 B 0.57
A 0 B 0.71
A 0 B 0.86
A 0 B 1
A 0.14 B 0
A 0.14 B 0.14
A 0.14 B 0.29
A 0.14 B 0.43
A 0.14 B 0.57
A 0.14 B 0.71
A 0.14 B 0.86
A 0.14 B 1
A 0.29 B 0
A 0.29 B 0.14
A 0.29 B 0.29
A 0.29 B 0.43
A 0.29 B 0.57
A 0.29 B 0.71
A 0.29 B 0.86
A 0.29 B 1
A 0.43 B 0
A 0.43 B 0.14
A 0.43 B 0.29
A 0.43 B 0.43
A 0.43 B 0.57
A 0.43 B 0.71
A 0.43 B 0.86
A 0.43 B 1
A 0.57 B 0
A 0.57 B 0.14
A 0.57 B 0.29
A 0.57 B 0.43
A 0.57 B 0.57
A 0.57 B 0.71
A 0.57 B 0.86
A 0.57 B 1
A 0.71 B 0
A 0.71 B 0.14
A 0.71 B 0.29
A 0.71 B 0.43
A 0.71 B 0.57
A 0.71 B 0.71
A 0.71 B 0.86
A 0.71 B 1
A 0.86 B 0
A 0.86 B 0.14
A 0.86 B 0.29
A 0.86 B 0.43
A 0.86 B 0.57
A 0.86 B 0.71
A 0.86 B 0.86
A 0.86 B 1
A 1 B 0
A 1 B 0.14
A 1 B 0.29
A 1 B 0.43
A 1 B 0.57
A 1 B 0.71
A 1 B 0.86
A 1 B 1
OD 600 nm [–]
0.75
0.50
0.25
Time [h]
0 5 101520

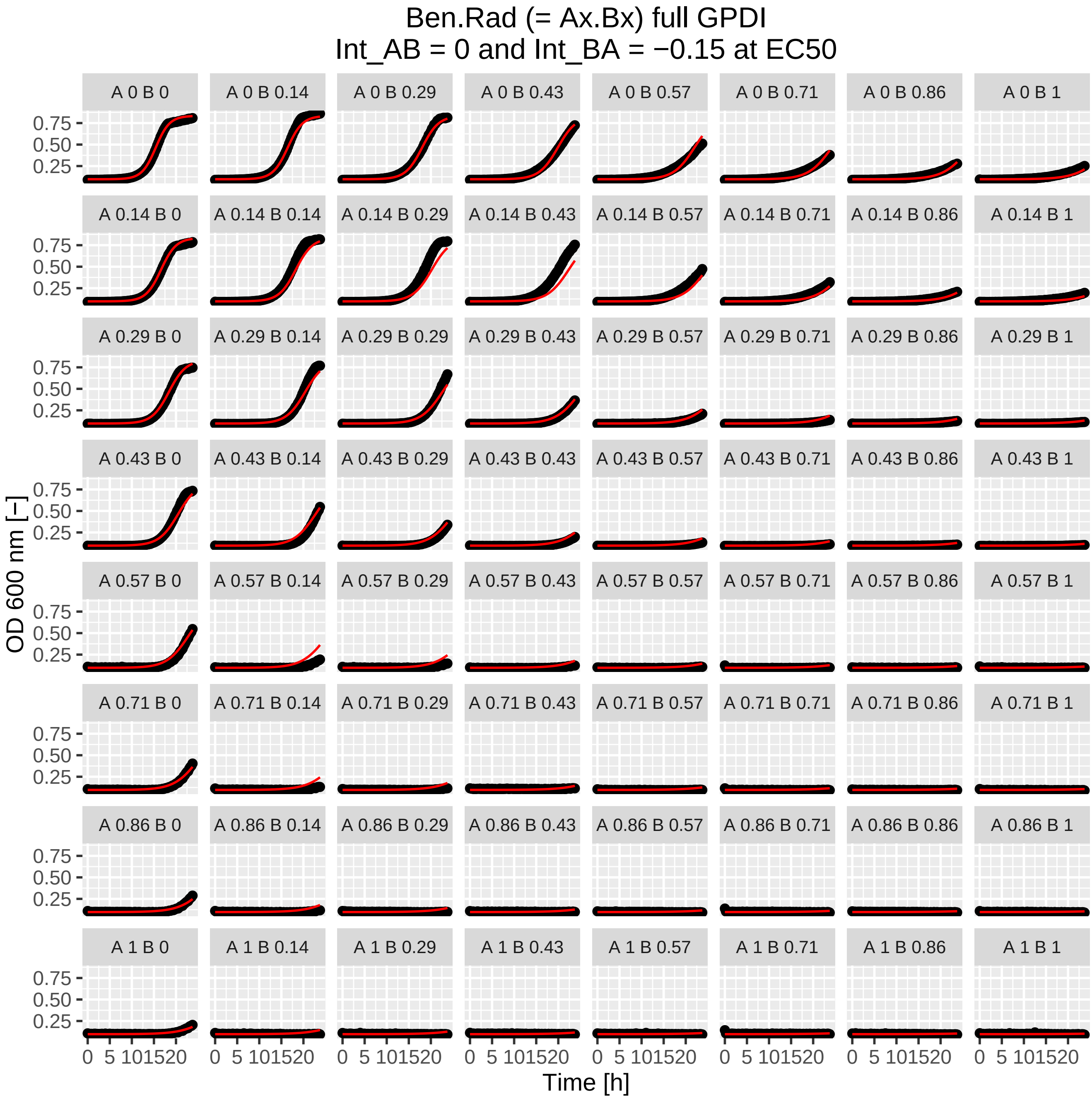

Ben.Rad (= Ax.Bx) full GPDI
Int_AB = 0 and Int_BA = −0.15 at EC50
A 0 B 0
A 0 B 0.14
A 0 B 0.29
A 0 B 0.43
A 0 B 0.57
A 0 B 0.71
A 0 B 0.86
A 0 B 1
A 0.14 B 0
A 0.14 B 0.14
A 0.14 B 0.29
A 0.14 B 0.43
A 0.14 B 0.57
A 0.14 B 0.71
A 0.14 B 0.86
A 0.14 B 1
A 0.29 B 0
A 0.29 B 0.14
A 0.29 B 0.29
A 0.29 B 0.43
A 0.29 B 0.57
A 0.29 B 0.71
A 0.29 B 0.86
A 0.29 B 1
A 0.43 B 0
A 0.43 B 0.14
A 0.43 B 0.29
A 0.43 B 0.43
A 0.43 B 0.57
A 0.43 B 0.71
A 0.43 B 0.86
A 0.43 B 1
A 0.57 B 0
A 0.57 B 0.14
A 0.57 B 0.29
A 0.57 B 0.43
A 0.57 B 0.57
A 0.57 B 0.71
A 0.57 B 0.86
A 0.57 B 1
A 0.71 B 0
A 0.71 B 0.14
A 0.71 B 0.29
A 0.71 B 0.43
A 0.71 B 0.57
A 0.71 B 0.71
A 0.71 B 0.86
A 0.71 B 1
A 0.86 B 0
A 0.86 B 0.14
A 0.86 B 0.29
A 0.86 B 0.43
A 0.86 B 0.57
A 0.86 B 0.71
A 0.86 B 0.86
A 0.86 B 1
A 1 B 0
A 1 B 0.14
A 1 B 0.29
A 1 B 0.43
A 1 B 0.57
A 1 B 0.71
A 1 B 0.86
A 1 B 1
OD 600 nm [−]
0.75
0.50
0.25
0 5 101520
Time [h]

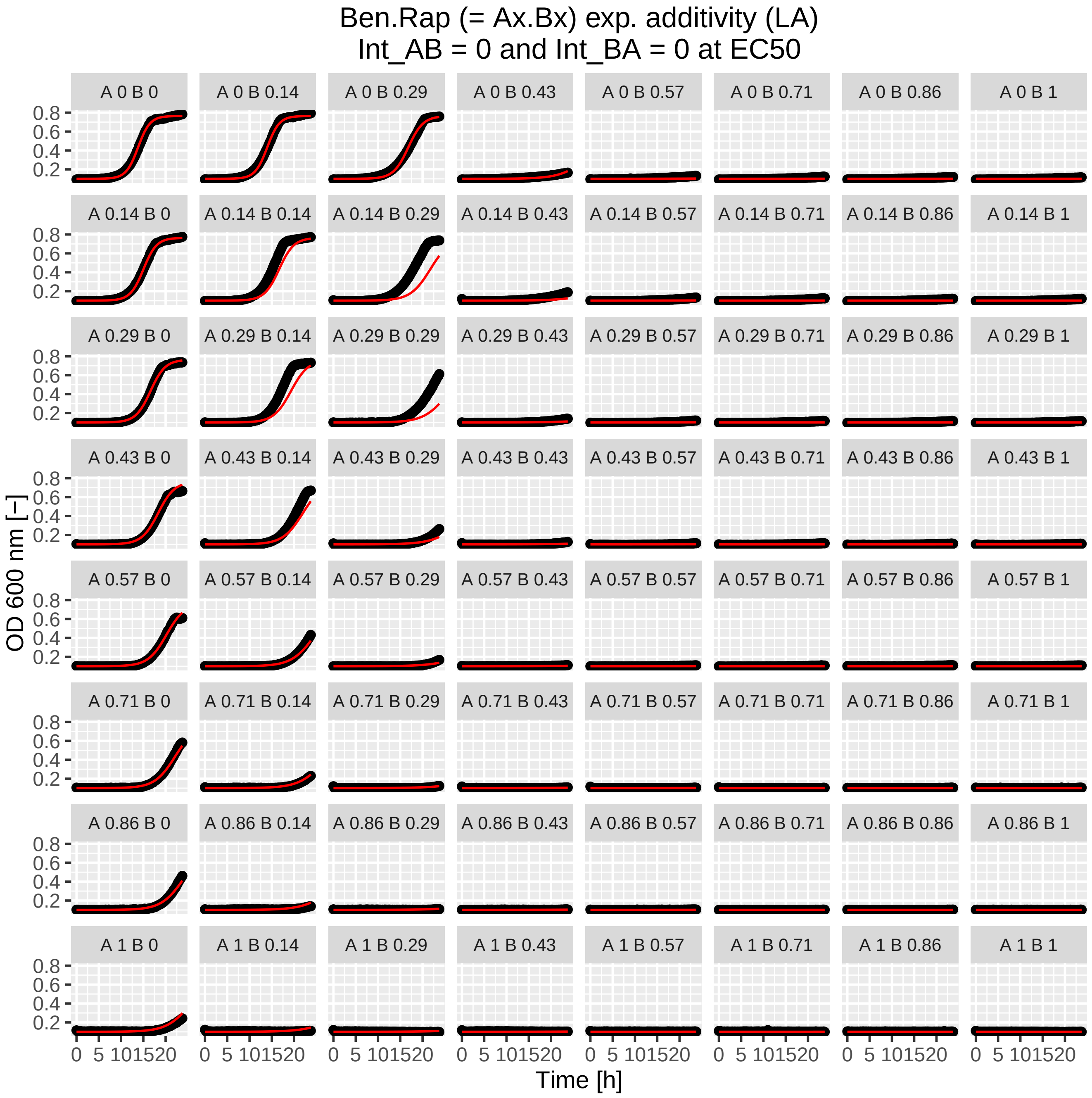
Ben.Rap (= Ax.Bx) exp. additivity (LA)
Int_AB = 0 and Int_BA = 0 at EC50
A 0 B 0
A 0 B 0.14
A 0 B 0.29
A 0 B 0.43
A 0 B 0.57
A 0 B 0.71
A 0 B 0.86
A 0 B 1
A 0.14 B 0
A 0.14 B 0.14
A 0.14 B 0.29
A 0.14 B 0.43
A 0.14 B 0.57
A 0.14 B 0.71
A 0.14 B 0.86
A 0.14 B 1
A 0.29 B 0
A 0.29 B 0.14
A 0.29 B 0.29
A 0.29 B 0.43
A 0.29 B 0.57
A 0.29 B 0.71
A 0.29 B 0.86
A 0.29 B 1
A 0.43 B 0
A 0.43 B 0.14
A 0.43 B 0.29
A 0.43 B 0.43
A 0.43 B 0.57
A 0.43 B 0.71
A 0.43 B 0.86
A 0.43 B 1
A 0.57 B 0
A 0.57 B 0.14
A 0.57 B 0.29
A 0.57 B 0.43
A 0.57 B 0.57
A 0.57 B 0.71
A 0.57 B 0.86
A 0.57 B 1
A 0.71 B 0
A 0.71 B 0.14
A 0.71 B 0.29
A 0.71 B 0.43
A 0.71 B 0.57
A 0.71 B 0.71
A 0.71 B 0.86
A 0.71 B 1
A 0.86 B 0
A 0.86 B 0.14
A 0.86 B 0.29
A 0.86 B 0.43
A 0.86 B 0.57
A 0.86 B 0.71
A 0.86 B 0.86
A 0.86 B 1
A 1 B 0
A 1 B 0.14
A 1 B 0.29
A 1 B 0.43
A 1 B 0.57
A 1 B 0.71
A 1 B 0.86
A 1 B 1
OD 600 nm [−]
0.2
0.4
0.6
0.8
0
5
10
15
20
Time [h]

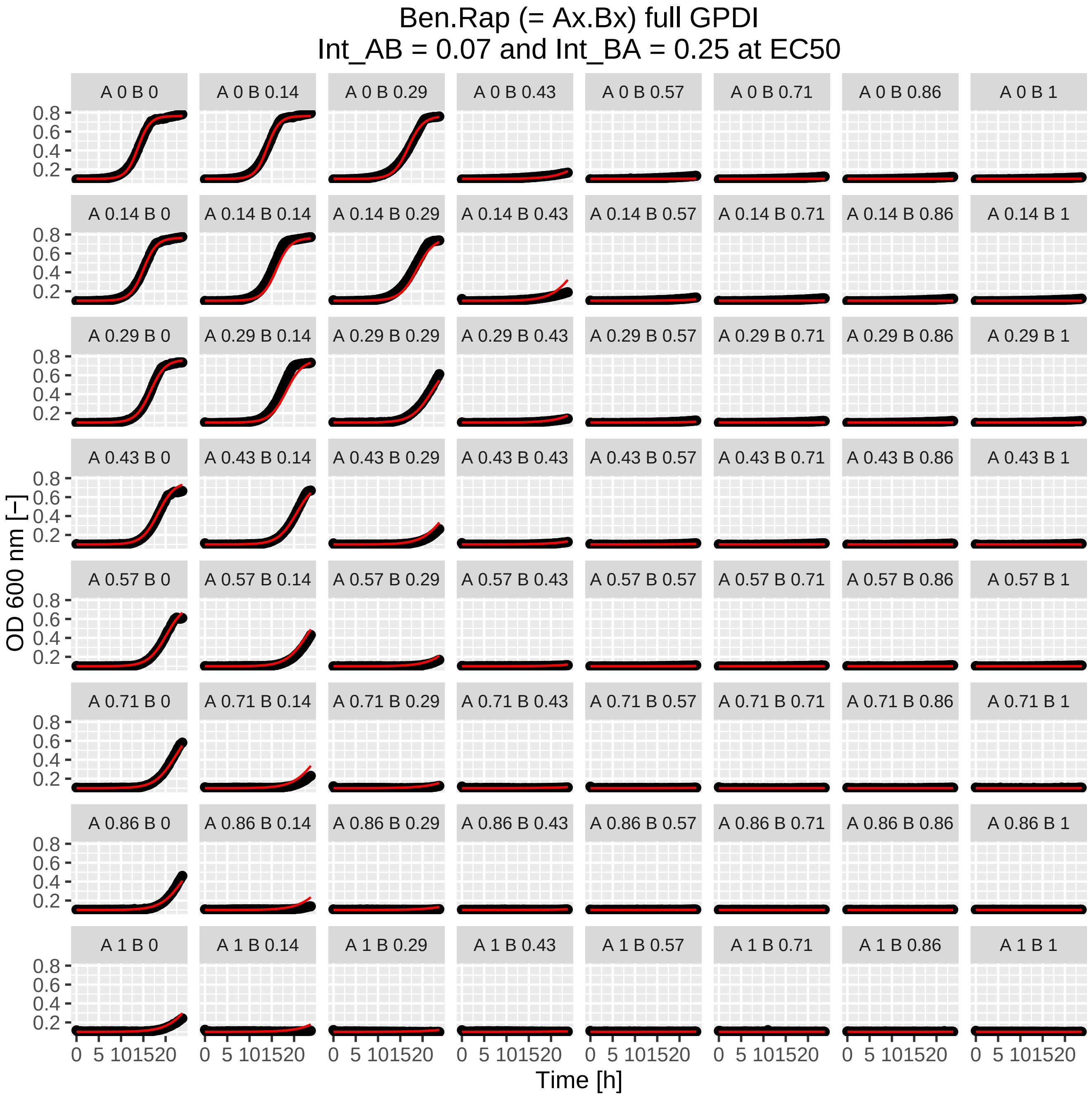
Ben.Rap (= Ax.Bx) full GPDI
Int_AB = 0.07 and Int_BA = 0.25 at EC50
OD 600 nm [–]
Time [h]
A 0 B 0
A 0 B 0.14
A 0 B 0.29
A 0 B 0.43
A 0 B 0.57
A 0 B 0.71
A 0 B 0.86
A 0 B 1
A 0.14 B 0
A 0.14 B 0.14
A 0.14 B 0.29
A 0.14 B 0.43
A 0.14 B 0.57
A 0.14 B 0.71
A 0.14 B 0.86
A 0.14 B 1
A 0.29 B 0
A 0.29 B 0.14
A 0.29 B 0.29
A 0.29 B 0.43
A 0.29 B 0.57
A 0.29 B 0.71
A 0.29 B 0.86
A 0.29 B 1
A 0.43 B 0
A 0.43 B 0.14
A 0.43 B 0.29
A 0.43 B 0.43
A 0.43 B 0.57
A 0.43 B 0.71
A 0.43 B 0.86
A 0.43 B 1
A 0.57 B 0
A 0.57 B 0.14
A 0.57 B 0.29
A 0.57 B 0.43
A 0.57 B 0.57
A 0.57 B 0.71
A 0.57 B 0.86
A 0.57 B 1
A 0.71 B 0
A 0.71 B 0.14
A 0.71 B 0.29
A 0.71 B 0.43
A 0.71 B 0.57
A 0.71 B 0.71
A 0.71 B 0.86
A 0.71 B 1
A 0.86 B 0
A 0.86 B 0.14
A 0.86 B 0.29
A 0.86 B 0.43
A 0.86 B 0.57
A 0.86 B 0.71
A 0.86 B 0.86
A 0.86 B 1
A 1 B 0
A 1 B 0.14
A 1 B 0.29
A 1 B 0.43
A 1 B 0.57
A 1 B 0.71
A 1 B 0.86
A 1 B 1
0.8
0.6
0.4
0.2
0 5 101520

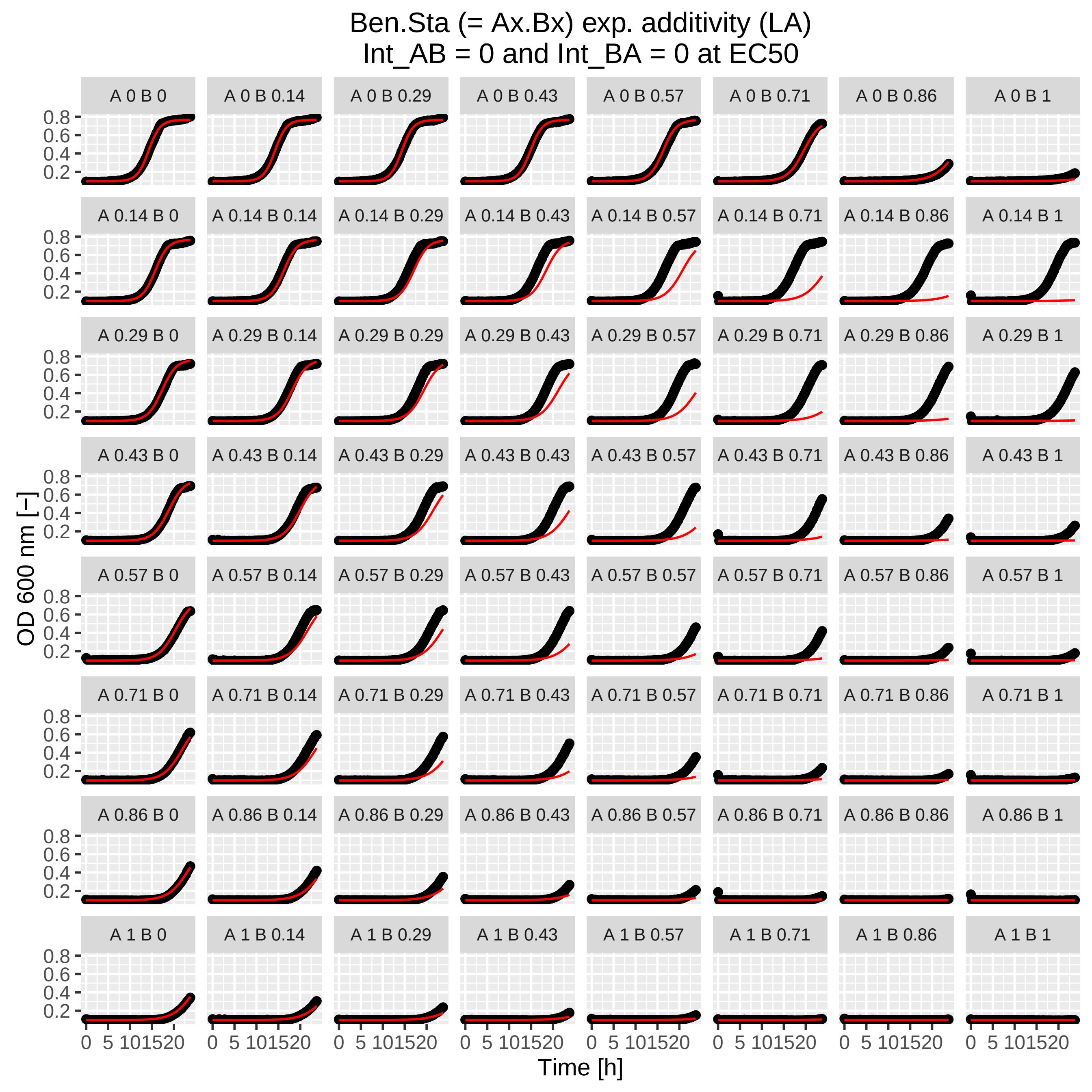

Ben.Sta (= Ax.Bx) exp. additivity (LA)
Int_AB = 0 and Int_BA = 0 at EC50
A 0 B 0
A 0 B 0.14
A 0 B 0.29
A 0 B 0.43
A 0 B 0.57
A 0 B 0.71
A 0 B 0.86
A 0 B 1
A 0.14 B 0
A 0.14 B 0.14
A 0.14 B 0.29
A 0.14 B 0.43
A 0.14 B 0.57
A 0.14 B 0.71
A 0.14 B 0.86
A 0.14 B 1
A 0.29 B 0
A 0.29 B 0.14
A 0.29 B 0.29
A 0.29 B 0.43
A 0.29 B 0.57
A 0.29 B 0.71
A 0.29 B 0.86
A 0.29 B 1
A 0.43 B 0
A 0.43 B 0.14
A 0.43 B 0.29
A 0.43 B 0.43
A 0.43 B 0.57
A 0.43 B 0.71
A 0.43 B 0.86
A 0.43 B 1
A 0.57 B 0
A 0.57 B 0.14
A 0.57 B 0.29
A 0.57 B 0.43
A 0.57 B 0.57
A 0.57 B 0.71
A 0.57 B 0.86
A 0.57 B 1
A 0.71 B 0
A 0.71 B 0.14
A 0.71 B 0.29
A 0.71 B 0.43
A 0.71 B 0.57
A 0.71 B 0.71
A 0.71 B 0.86
A 0.71 B 1
A 0.86 B 0
A 0.86 B 0.14
A 0.86 B 0.29
A 0.86 B 0.43
A 0.86 B 0.57
A 0.86 B 0.71
A 0.86 B 0.86
A 0.86 B 1
A 1 B 0
A 1 B 0.14
A 1 B 0.29
A 1 B 0.43
A 1 B 0.57
A 1 B 0.71
A 1 B 0.86
A 1 B 1
0.8
0.6
0.4
0.2
OD 600 nm [–]
0 5 101520
Time [h]

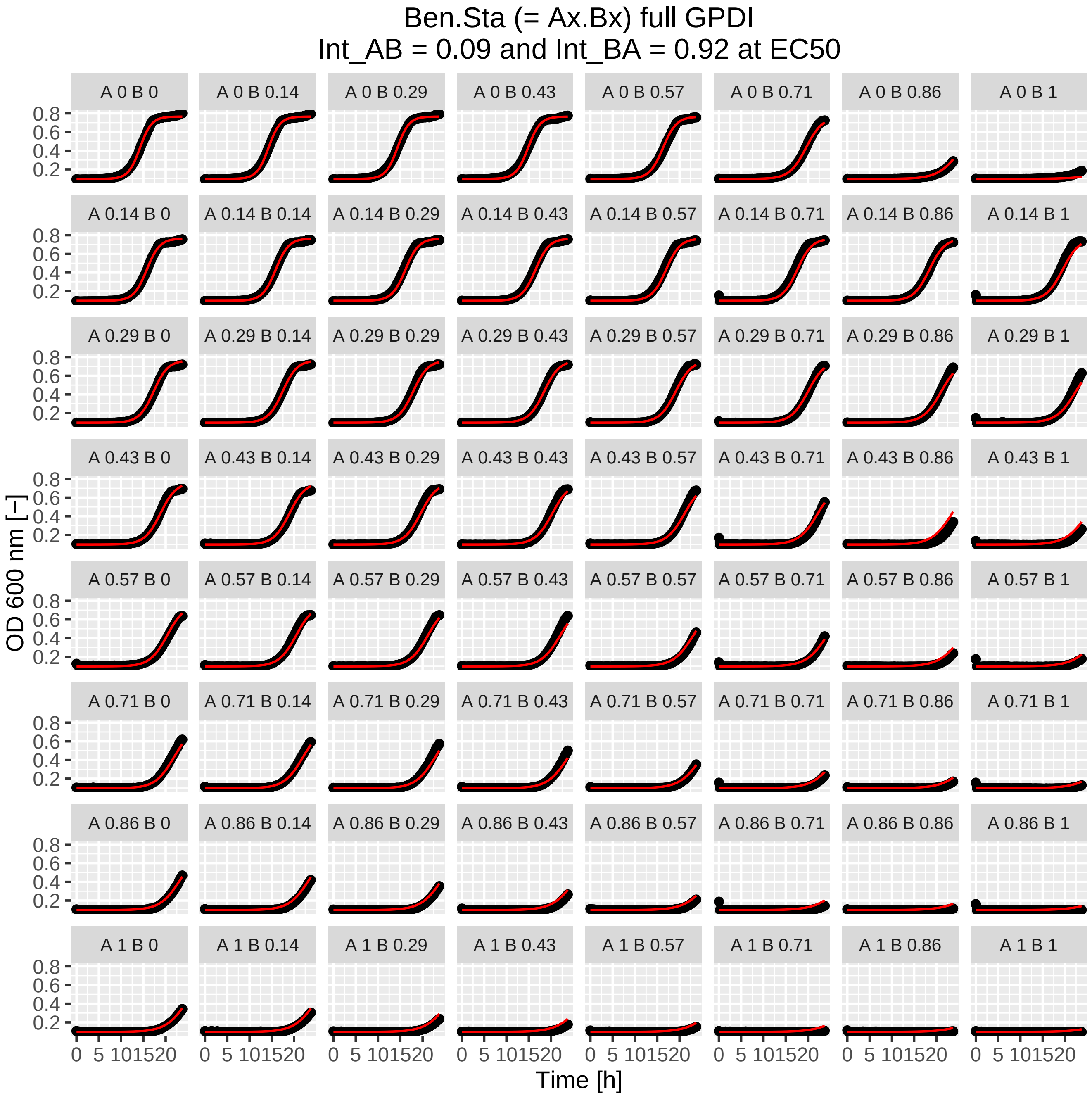
Ben.Sta (= Ax.Bx) full GPDI
Int_AB = 0.09 and Int_BA = 0.92 at EC50
OD 600 nm [–]
Time [h]
A 0 B 0
A 0 B 0.14
A 0 B 0.29
A 0 B 0.43
A 0 B 0.57
A 0 B 0.71
A 0 B 0.86
A 0 B 1
A 0.14 B 0
A 0.29 B 0
A 0.43 B 0
A 0.57 B 0
A 0.71 B 0
A 0.86 B 0
A 1 B 0
A 1 B 1

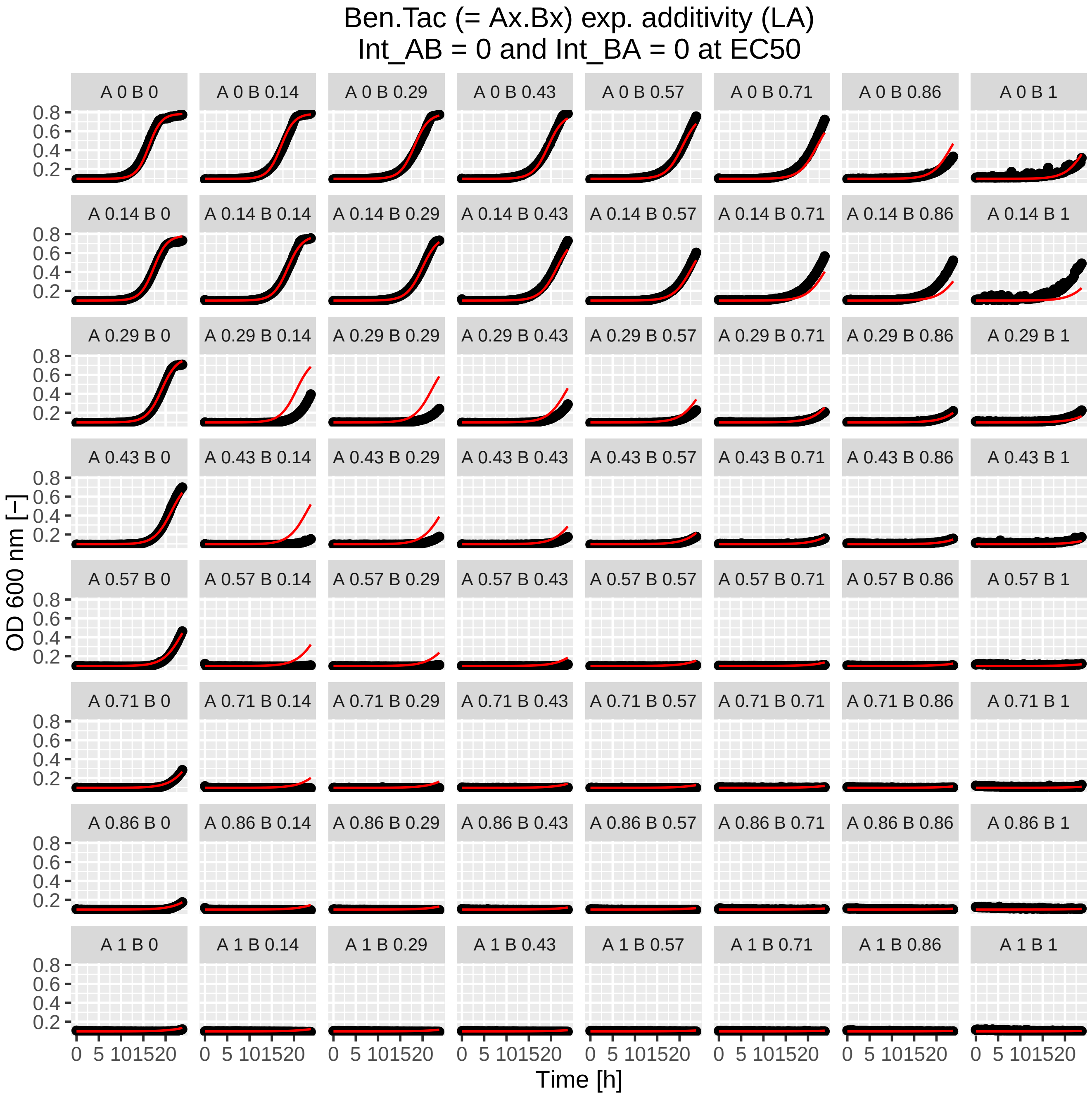

Ben.Tac (= Ax.Bx) exp. additivity (LA)
Int_AB = 0 and Int_BA = 0 at EC50
A 0 B 0
A 0 B 0.14
A 0 B 0.29
A 0 B 0.43
A 0 B 0.57
A 0 B 0.71
A 0 B 0.86
A 0 B 1
A 0.14 B 0
A 0.14 B 0.14
A 0.14 B 0.29
A 0.14 B 0.43
A 0.14 B 0.57
A 0.14 B 0.71
A 0.14 B 0.86
A 0.14 B 1
A 0.29 B 0
A 0.29 B 0.14
A 0.29 B 0.29
A 0.29 B 0.43
A 0.29 B 0.57
A 0.29 B 0.71
A 0.29 B 0.86
A 0.29 B 1
A 0.43 B 0
A 0.43 B 0.14
A 0.43 B 0.29
A 0.43 B 0.43
A 0.43 B 0.57
A 0.43 B 0.71
A 0.43 B 0.86
A 0.43 B 1
A 0.57 B 0
A 0.57 B 0.14
A 0.57 B 0.29
A 0.57 B 0.43
A 0.57 B 0.57
A 0.57 B 0.71
A 0.57 B 0.86
A 0.57 B 1
A 0.71 B 0
A 0.71 B 0.14
A 0.71 B 0.29
A 0.71 B 0.43
A 0.71 B 0.57
A 0.71 B 0.71
A 0.71 B 0.86
A 0.71 B 1
A 0.86 B 0
A 0.86 B 0.14
A 0.86 B 0.29
A 0.86 B 0.43
A 0.86 B 0.57
A 0.86 B 0.71
A 0.86 B 0.86
A 0.86 B 1
A 1 B 0
A 1 B 0.14
A 1 B 0.29
A 1 B 0.43
A 1 B 0.57
A 1 B 0.71
A 1 B 0.86
A 1 B 1
OD 600 nm [–]
0.2
0.4
0.6
0.8
0 5 101520
Time [h]

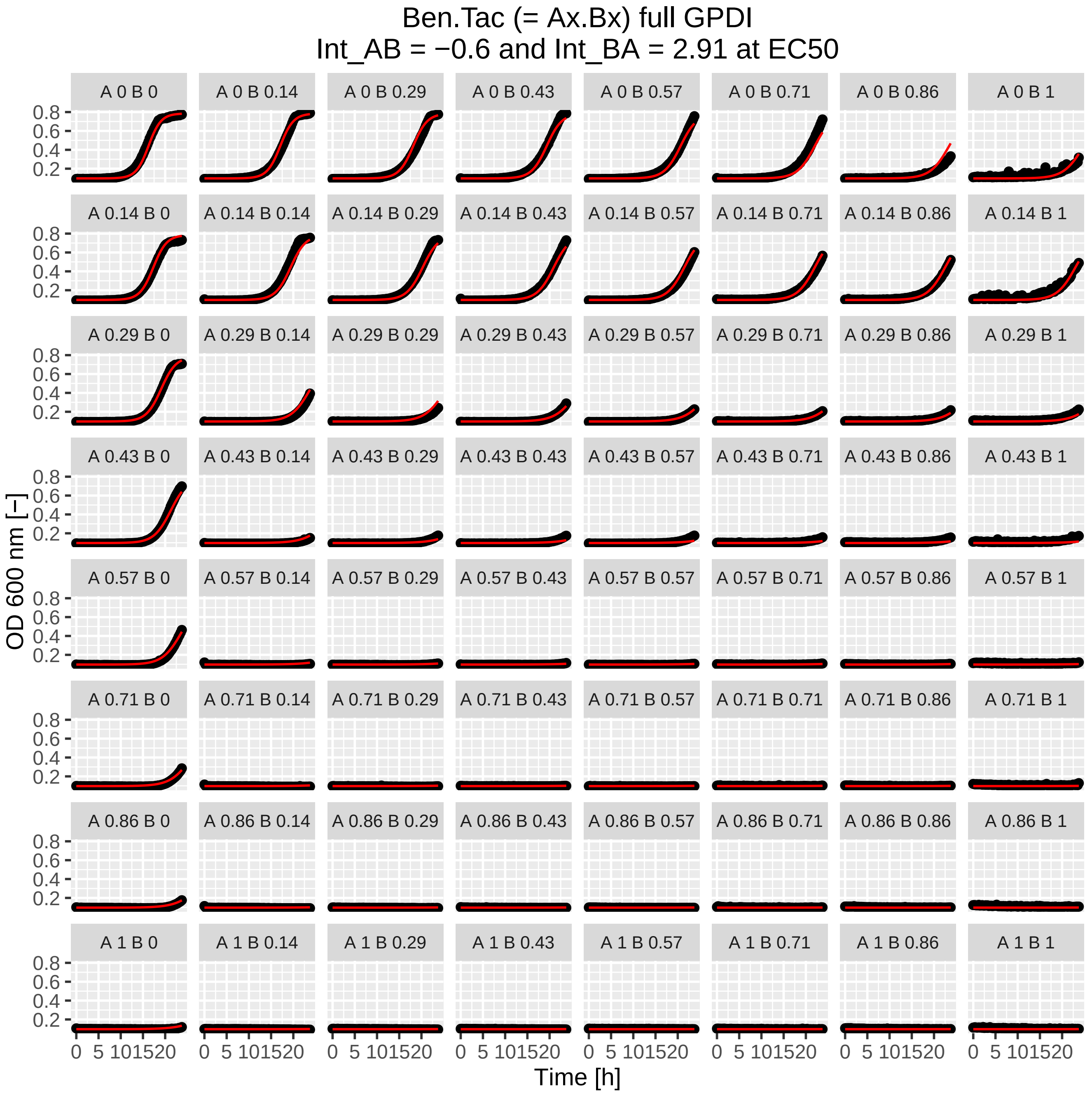

Ben.Tac (= Ax.Bx) full GPDI
Int_AB = −0.6 and Int_BA = 2.91 at EC50
A 0 B 0
A 0 B 0.14
A 0 B 0.29
A 0 B 0.43
A 0 B 0.57
A 0 B 0.71
A 0 B 0.86
A 0 B 1
A 0.14 B 0
A 0.14 B 0.14
A 0.14 B 0.29
A 0.14 B 0.43
A 0.14 B 0.57
A 0.14 B 0.71
A 0.14 B 0.86
A 0.14 B 1
A 0.29 B 0
A 0.29 B 0.14
A 0.29 B 0.29
A 0.29 B 0.43
A 0.29 B 0.57
A 0.29 B 0.71
A 0.29 B 0.86
A 0.29 B 1
A 0.43 B 0
A 0.43 B 0.14
A 0.43 B 0.29
A 0.43 B 0.43
A 0.43 B 0.57
A 0.43 B 0.71
A 0.43 B 0.86
A 0.43 B 1
A 0.57 B 0
A 0.57 B 0.14
A 0.57 B 0.29
A 0.57 B 0.43
A 0.57 B 0.57
A 0.57 B 0.71
A 0.57 B 0.86
A 0.57 B 1
A 0.71 B 0
A 0.71 B 0.14
A 0.71 B 0.29
A 0.71 B 0.43
A 0.71 B 0.57
A 0.71 B 0.71
A 0.71 B 0.86
A 0.71 B 1
A 0.86 B 0
A 0.86 B 0.14
A 0.86 B 0.29
A 0.86 B 0.43
A 0.86 B 0.57
A 0.86 B 0.71
A 0.86 B 0.86
A 0.86 B 1
A 1 B 0
A 1 B 0.14
A 1 B 0.29
A 1 B 0.43
A 1 B 0.57
A 1 B 0.71
A 1 B 0.86
A 1 B 1
OD 600 nm [−]
0.2
0.4
0.6
0.8
0
5
10
15
20
Time [h]

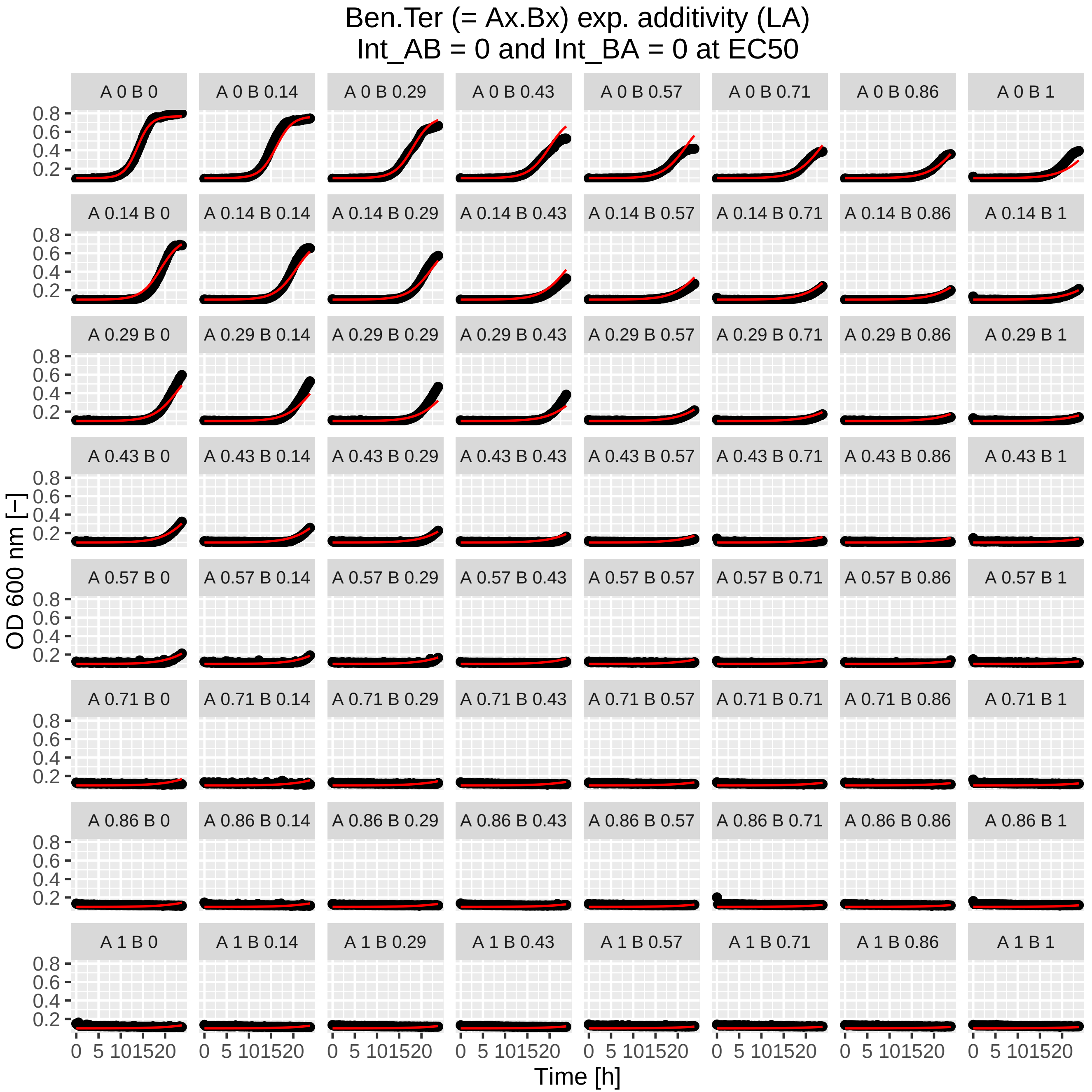
Ben.Ter (= Ax.Bx) exp. additivity (LA)
Int_AB = 0 and Int_BA = 0 at EC50
A 0 B 0
A 0 B 0.14
A 0 B 0.29
A 0 B 0.43
A 0 B 0.57
A 0 B 0.71
A 0 B 0.86
A 0 B 1
A 0.14 B 0
A 0.14 B 0.14
A 0.14 B 0.29
A 0.14 B 0.43
A 0.14 B 0.57
A 0.14 B 0.71
A 0.14 B 0.86
A 0.14 B 1
A 0.29 B 0
A 0.29 B 0.14
A 0.29 B 0.29
A 0.29 B 0.43
A 0.29 B 0.57
A 0.29 B 0.71
A 0.29 B 0.86
A 0.29 B 1
A 0.43 B 0
A 0.43 B 0.14
A 0.43 B 0.29
A 0.43 B 0.43
A 0.43 B 0.57
A 0.43 B 0.71
A 0.43 B 0.86
A 0.43 B 1
A 0.57 B 0
A 0.57 B 0.14
A 0.57 B 0.29
A 0.57 B 0.43
A 0.57 B 0.57
A 0.57 B 0.71
A 0.57 B 0.86
A 0.57 B 1
A 0.71 B 0
A 0.71 B 0.14
A 0.71 B 0.29
A 0.71 B 0.43
A 0.71 B 0.57
A 0.71 B 0.71
A 0.71 B 0.86
A 0.71 B 1
A 0.86 B 0
A 0.86 B 0.14
A 0.86 B 0.29
A 0.86 B 0.43
A 0.86 B 0.57
A 0.86 B 0.71
A 0.86 B 0.86
A 0.86 B 1
A 1 B 0
A 1 B 0.14
A 1 B 0.29
A 1 B 0.43
A 1 B 0.57
A 1 B 0.71
A 1 B 0.86
A 1 B 1
OD 600 nm [−]
0.8
0.6
0.4
0.2
0 5 101520
Time [h]

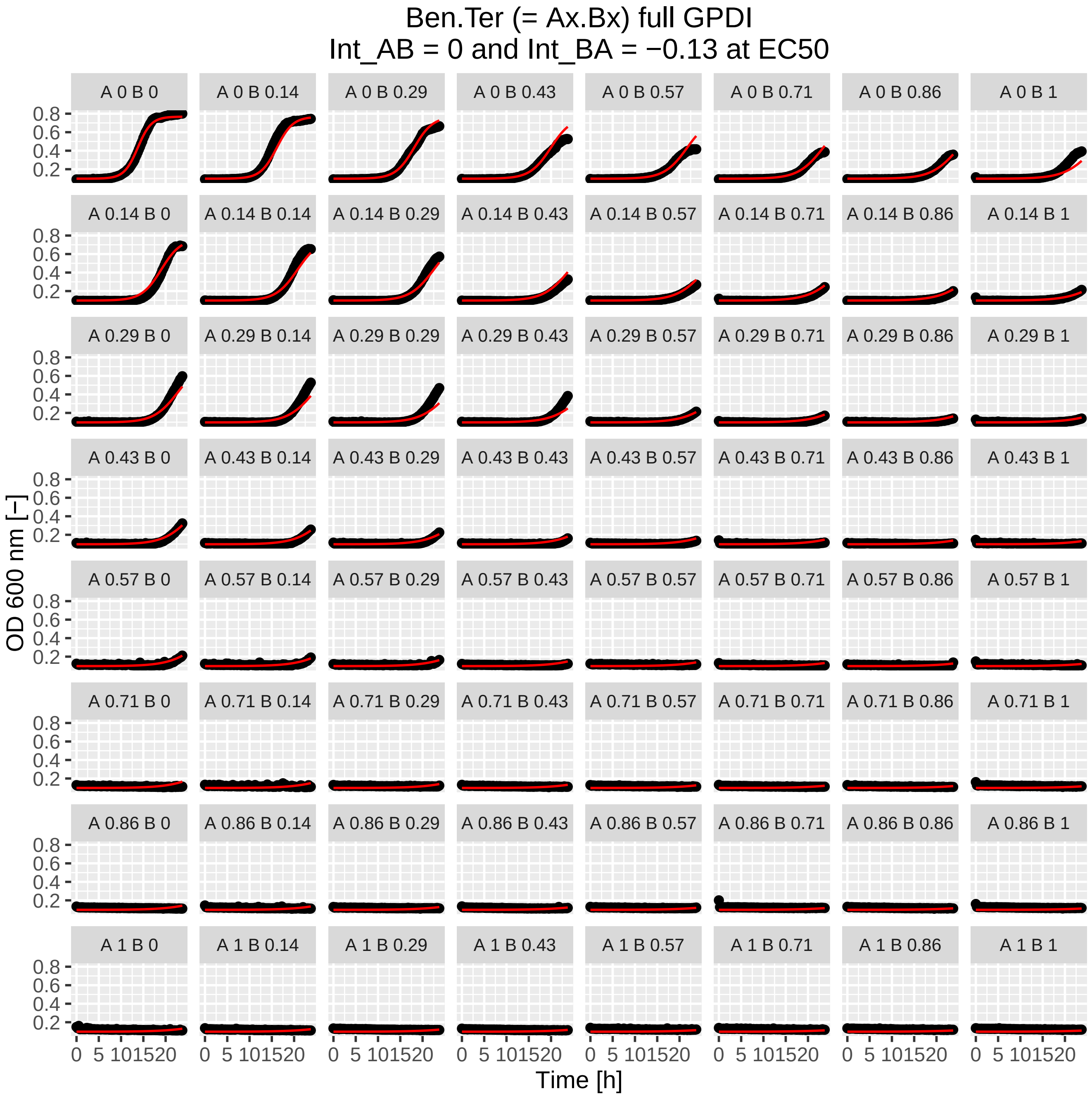
Ben.Ter (= Ax.Bx) full GPDI
Int_AB = 0 and Int_BA = −0.13 at EC50
OD 600 nm [−]
Time [h]
A 0 B 0
A 0 B 0.14
A 0 B 0.29
A 0 B 0.43
A 0 B 0.57
A 0 B 0.71
A 0 B 0.86
A 0 B 1
A 1 B 0
A 1 B 1
0.2
0.4
0.6
0.8
0
5
10
15
20

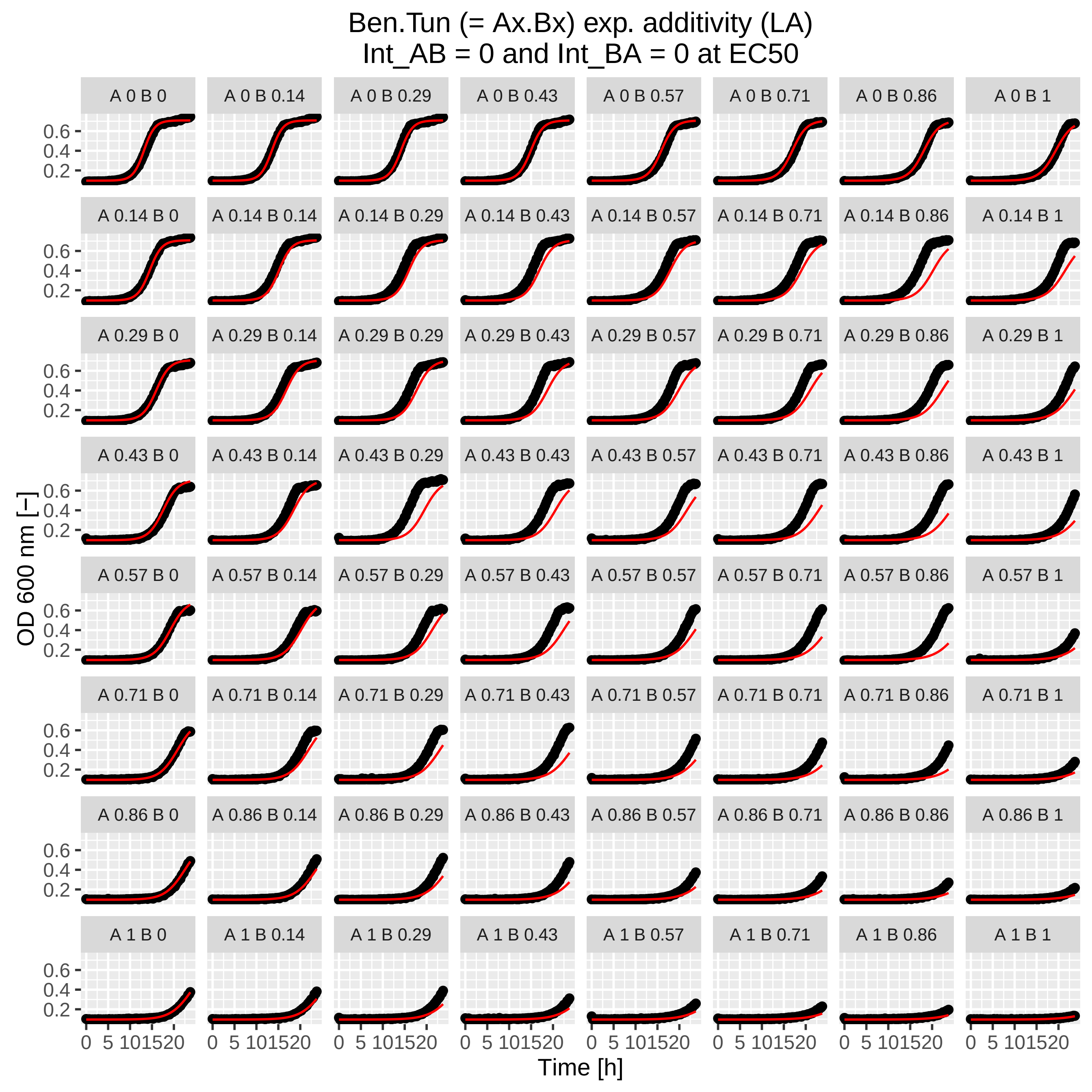

Ben.Tun (= Ax.Bx) exp. additivity (LA)
Int_AB = 0 and Int_BA = 0 at EC50
A 0 B 0
A 0 B 0.14
A 0 B 0.29
A 0 B 0.43
A 0 B 0.57
A 0 B 0.71
A 0 B 0.86
A 0 B 1
A 0.14 B 0
A 0.14 B 0.14
A 0.14 B 0.29
A 0.14 B 0.43
A 0.14 B 0.57
A 0.14 B 0.71
A 0.14 B 0.86
A 0.14 B 1
A 0.29 B 0
A 0.29 B 0.14
A 0.29 B 0.29
A 0.29 B 0.43
A 0.29 B 0.57
A 0.29 B 0.71
A 0.29 B 0.86
A 0.29 B 1
A 0.43 B 0
A 0.43 B 0.14
A 0.43 B 0.29
A 0.43 B 0.43
A 0.43 B 0.57
A 0.43 B 0.71
A 0.43 B 0.86
A 0.43 B 1
A 0.57 B 0
A 0.57 B 0.14
A 0.57 B 0.29
A 0.57 B 0.43
A 0.57 B 0.57
A 0.57 B 0.71
A 0.57 B 0.86
A 0.57 B 1
A 0.71 B 0
A 0.71 B 0.14
A 0.71 B 0.29
A 0.71 B 0.43
A 0.71 B 0.57
A 0.71 B 0.71
A 0.71 B 0.86
A 0.71 B 1
A 0.86 B 0
A 0.86 B 0.14
A 0.86 B 0.29
A 0.86 B 0.43
A 0.86 B 0.57
A 0.86 B 0.71
A 0.86 B 0.86
A 0.86 B 1
A 1 B 0
A 1 B 0.14
A 1 B 0.29
A 1 B 0.43
A 1 B 0.57
A 1 B 0.71
A 1 B 0.86
A 1 B 1
OD 600 nm [–]
0.2
0.4
0.6
0 5 101520
Time [h]

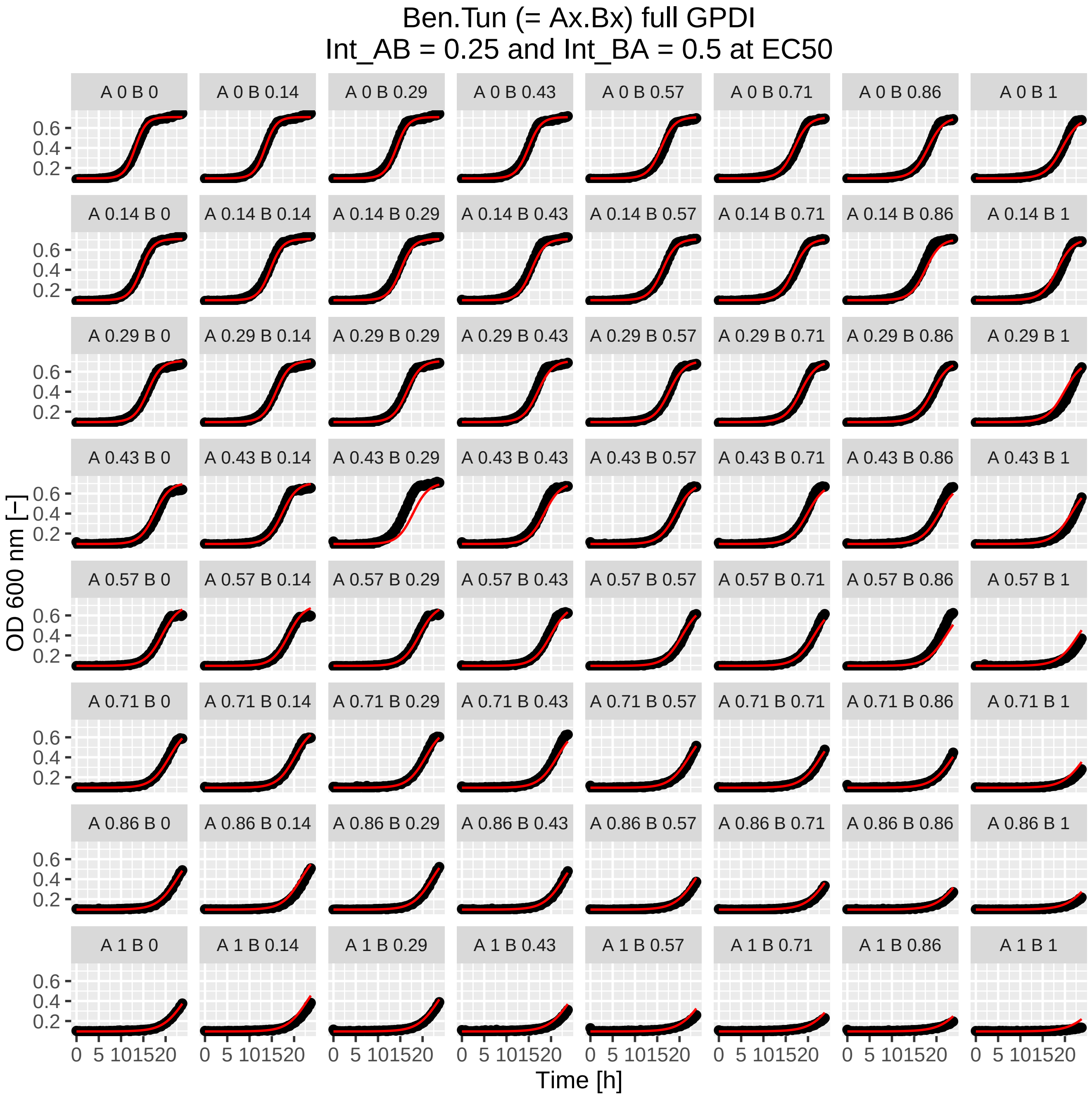

Ben.Tun (= Ax.Bx) full GPDI
Int_AB = 0.25 and Int_BA = 0.5 at EC50
A 0 B 0
A 0 B 0.14
A 0 B 0.29
A 0 B 0.43
A 0 B 0.57
A 0 B 0.71
A 0 B 0.86
A 0 B 1
A 0.14 B 0
A 0.14 B 0.14
A 0.14 B 0.29
A 0.14 B 0.43
A 0.14 B 0.57
A 0.14 B 0.71
A 0.14 B 0.86
A 0.14 B 1
A 0.29 B 0
A 0.29 B 0.14
A 0.29 B 0.29
A 0.29 B 0.43
A 0.29 B 0.57
A 0.29 B 0.71
A 0.29 B 0.86
A 0.29 B 1
A 0.43 B 0
A 0.43 B 0.14
A 0.43 B 0.29
A 0.43 B 0.43
A 0.43 B 0.57
A 0.43 B 0.71
A 0.43 B 0.86
A 0.43 B 1
A 0.57 B 0
A 0.57 B 0.14
A 0.57 B 0.29
A 0.57 B 0.43
A 0.57 B 0.57
A 0.57 B 0.71
A 0.57 B 0.86
A 0.57 B 1
A 0.71 B 0
A 0.71 B 0.14
A 0.71 B 0.29
A 0.71 B 0.43
A 0.71 B 0.57
A 0.71 B 0.71
A 0.71 B 0.86
A 0.71 B 1
A 0.86 B 0
A 0.86 B 0.14
A 0.86 B 0.29
A 0.86 B 0.43
A 0.86 B 0.57
A 0.86 B 0.71
A 0.86 B 0.86
A 0.86 B 1
A 1 B 0
A 1 B 0.14
A 1 B 0.29
A 1 B 0.43
A 1 B 0.57
A 1 B 0.71
A 1 B 0.86
A 1 B 1
0.6
0.4
0.2
OD 600 nm [–]
0 5 101520
Time [h]

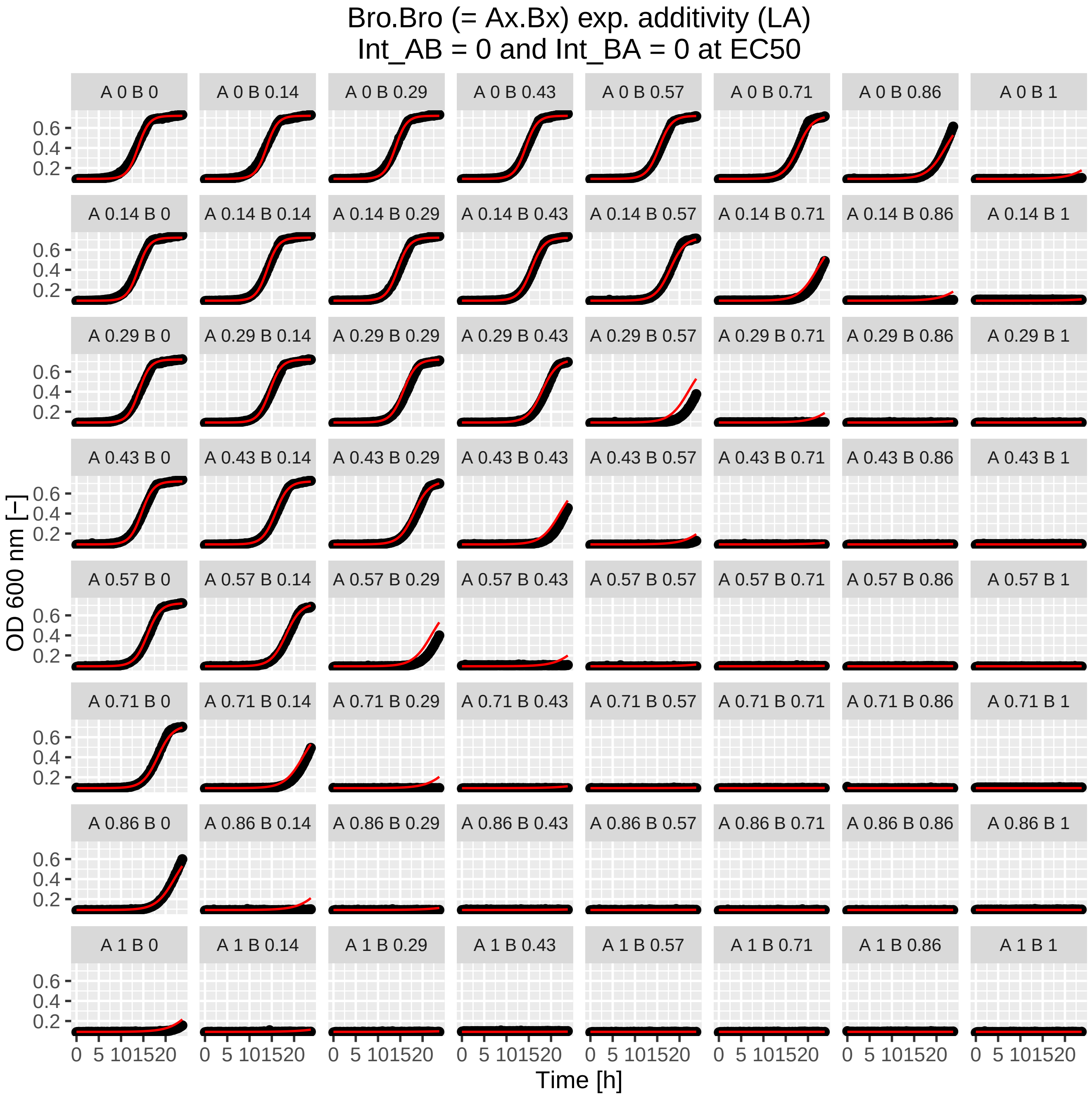

Bro.Bro (= Ax.Bx) exp. additivity (LA)
Int_AB = 0 and Int_BA = 0 at EC50
A 0 B 0
A 0 B 0.14
A 0 B 0.29
A 0 B 0.43
A 0 B 0.57
A 0 B 0.71
A 0 B 0.86
A 0 B 1
A 0.14 B 0
A 0.14 B 0.14
A 0.14 B 0.29
A 0.14 B 0.43
A 0.14 B 0.57
A 0.14 B 0.71
A 0.14 B 0.86
A 0.14 B 1
A 0.29 B 0
A 0.29 B 0.14
A 0.29 B 0.29
A 0.29 B 0.43
A 0.29 B 0.57
A 0.29 B 0.71
A 0.29 B 0.86
A 0.29 B 1
A 0.43 B 0
A 0.43 B 0.14
A 0.43 B 0.29
A 0.43 B 0.43
A 0.43 B 0.57
A 0.43 B 0.71
A 0.43 B 0.86
A 0.43 B 1
A 0.57 B 0
A 0.57 B 0.14
A 0.57 B 0.29
A 0.57 B 0.43
A 0.57 B 0.57
A 0.57 B 0.71
A 0.57 B 0.86
A 0.57 B 1
A 0.71 B 0
A 0.71 B 0.14
A 0.71 B 0.29
A 0.71 B 0.43
A 0.71 B 0.57
A 0.71 B 0.71
A 0.71 B 0.86
A 0.71 B 1
A 0.86 B 0
A 0.86 B 0.14
A 0.86 B 0.29
A 0.86 B 0.43
A 0.86 B 0.57
A 0.86 B 0.71
A 0.86 B 0.86
A 0.86 B 1
A 1 B 0
A 1 B 0.14
A 1 B 0.29
A 1 B 0.43
A 1 B 0.57
A 1 B 0.71
A 1 B 0.86
A 1 B 1
OD 600 nm [–]
0.6
0.4
0.2
0 5 101520
Time [h]

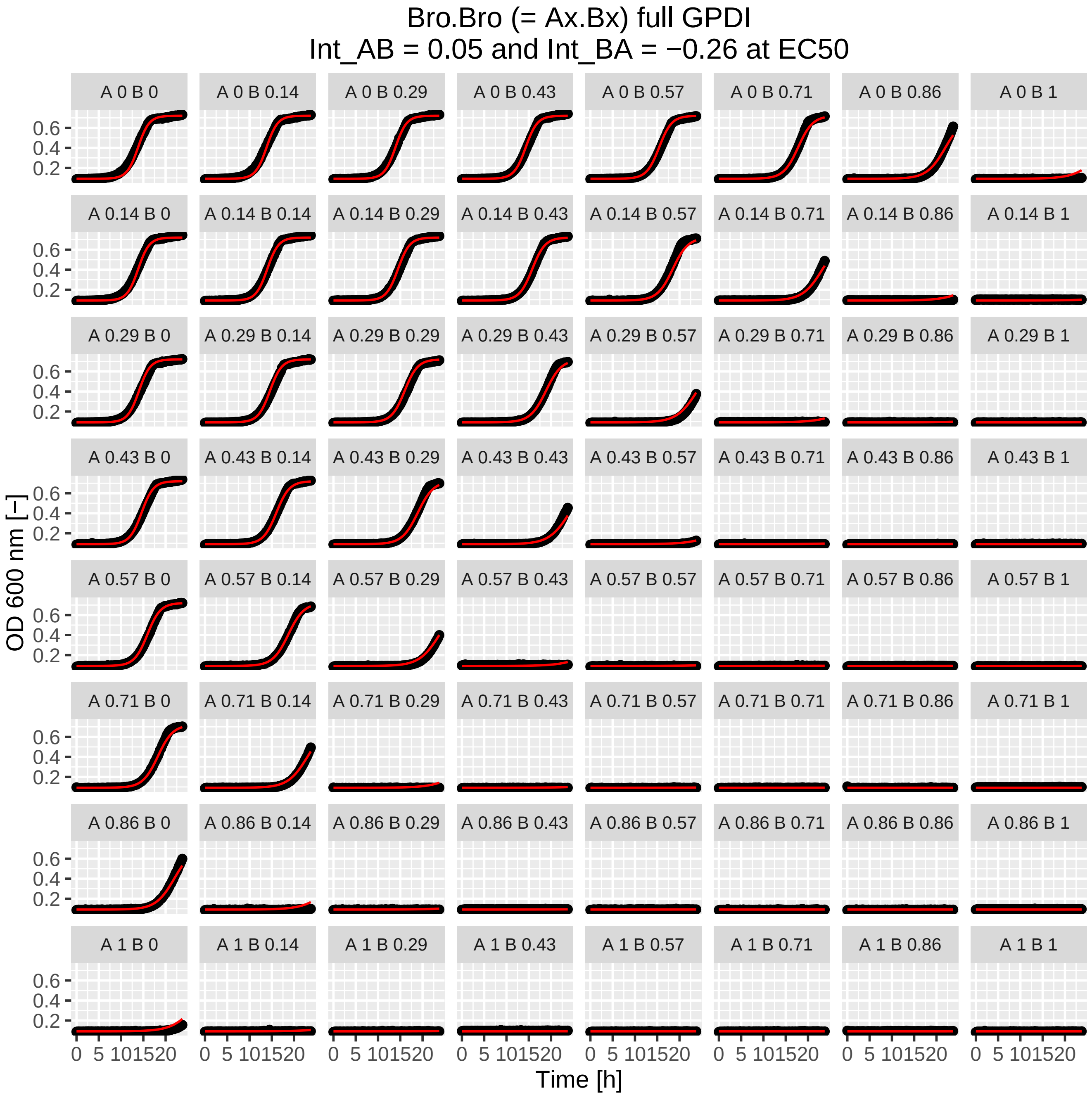
Bro.Bro (= Ax.Bx) full GPDI
Int_AB = 0.05 and Int_BA = −0.26 at EC50
OD 600 nm [−]
Time [h]
0.6
0.4
0.2
0 5 101520
A 0 B 0
A 0 B 0.14
A 0 B 0.29
A 0 B 0.43
A 0 B 0.57
A 0 B 0.71
A 0 B 0.86
A 0 B 1
A 0.14 B 0
A 0.14 B 0.14
A 0.14 B 0.29
A 0.14 B 0.43
A 0.14 B 0.57
A 0.14 B 0.71
A 0.14 B 0.86
A 0.14 B 1
A 0.29 B 0
A 0.29 B 0.14
A 0.29 B 0.29
A 0.29 B 0.43
A 0.29 B 0.57
A 0.29 B 0.71
A 0.29 B 0.86
A 0.29 B 1
A 0.43 B 0
A 0.43 B 0.14
A 0.43 B 0.29
A 0.43 B 0.43
A 0.43 B 0.57
A 0.43 B 0.71
A 0.43 B 0.86
A 0.43 B 1
A 0.57 B 0
A 0.57 B 0.14
A 0.57 B 0.29
A 0.57 B 0.43
A 0.57 B 0.57
A 0.57 B 0.71
A 0.57 B 0.86
A 0.57 B 1
A 0.71 B 0
A 0.71 B 0.14
A 0.71 B 0.29
A 0.71 B 0.43
A 0.71 B 0.57
A 0.71 B 0.71
A 0.71 B 0.86
A 0.71 B 1
A 0.86 B 0
A 0.86 B 0.14
A 0.86 B 0.29
A 0.86 B 0.43
A 0.86 B 0.57
A 0.86 B 0.71
A 0.86 B 0.86
A 0.86 B 1
A 1 B 0
A 1 B 0.14
A 1 B 0.29
A 1 B 0.43
A 1 B 0.57
A 1 B 0.71
A 1 B 0.86
A 1 B 1

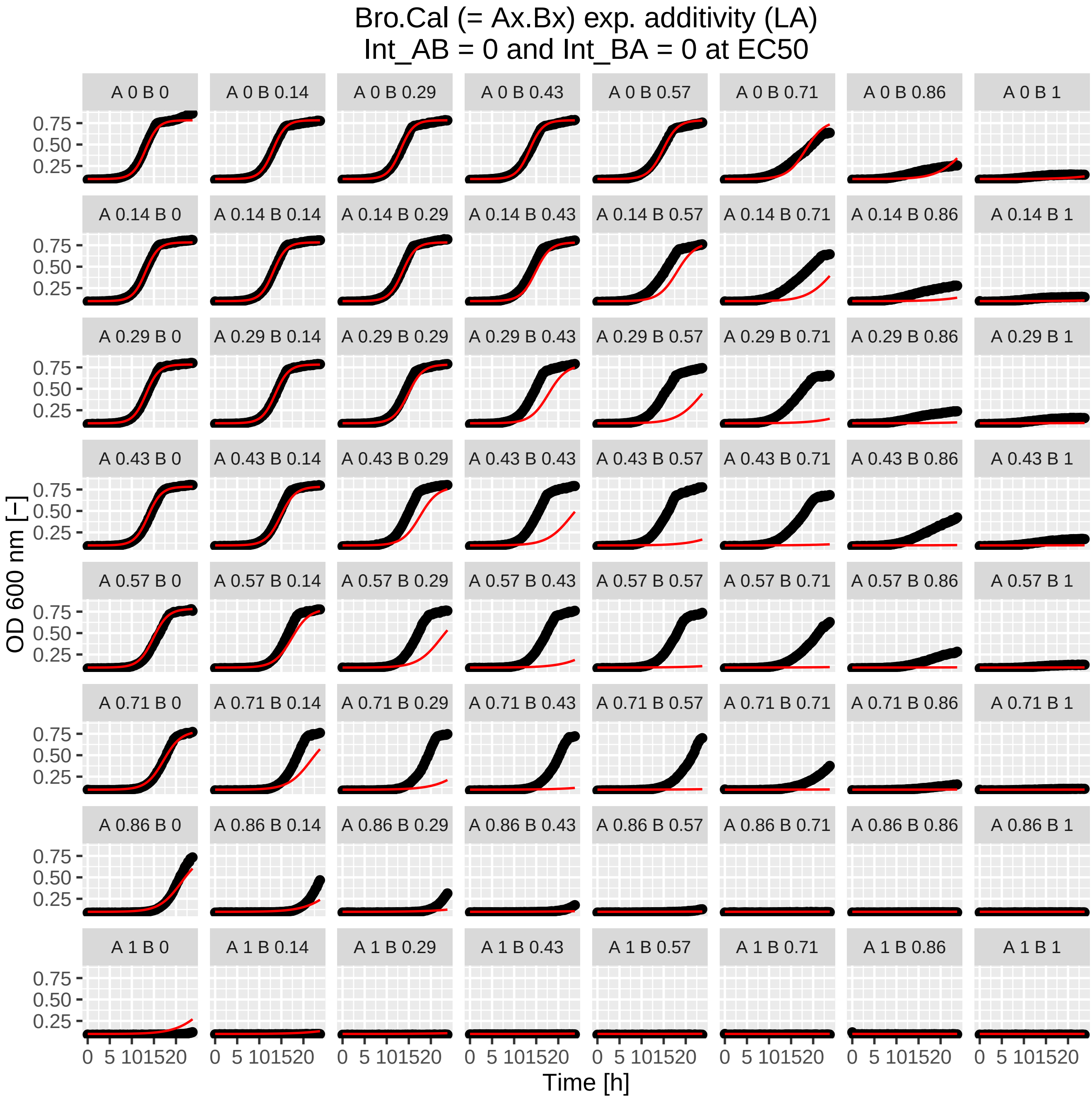

Bro.Cal (= Ax.Bx) exp. additivity (LA)
Int_AB = 0 and Int_BA = 0 at EC50
A 0 B 0
A 0 B 0.14
A 0 B 0.29
A 0 B 0.43
A 0 B 0.57
A 0 B 0.71
A 0 B 0.86
A 0 B 1
A 0.14 B 0
A 0.14 B 0.14
A 0.14 B 0.29
A 0.14 B 0.43
A 0.14 B 0.57
A 0.14 B 0.71
A 0.14 B 0.86
A 0.14 B 1
A 0.29 B 0
A 0.29 B 0.14
A 0.29 B 0.29
A 0.29 B 0.43
A 0.29 B 0.57
A 0.29 B 0.71
A 0.29 B 0.86
A 0.29 B 1
A 0.43 B 0
A 0.43 B 0.14
A 0.43 B 0.29
A 0.43 B 0.43
A 0.43 B 0.57
A 0.43 B 0.71
A 0.43 B 0.86
A 0.43 B 1
A 0.57 B 0
A 0.57 B 0.14
A 0.57 B 0.29
A 0.57 B 0.43
A 0.57 B 0.57
A 0.57 B 0.71
A 0.57 B 0.86
A 0.57 B 1
A 0.71 B 0
A 0.71 B 0.14
A 0.71 B 0.29
A 0.71 B 0.43
A 0.71 B 0.57
A 0.71 B 0.71
A 0.71 B 0.86
A 0.71 B 1
A 0.86 B 0
A 0.86 B 0.14
A 0.86 B 0.29
A 0.86 B 0.43
A 0.86 B 0.57
A 0.86 B 0.71
A 0.86 B 0.86
A 0.86 B 1
A 1 B 0
A 1 B 0.14
A 1 B 0.29
A 1 B 0.43
A 1 B 0.57
A 1 B 0.71
A 1 B 0.86
A 1 B 1
0.75
0.50
0.25
OD 600 nm [−]
0 5 101520
Time [h]

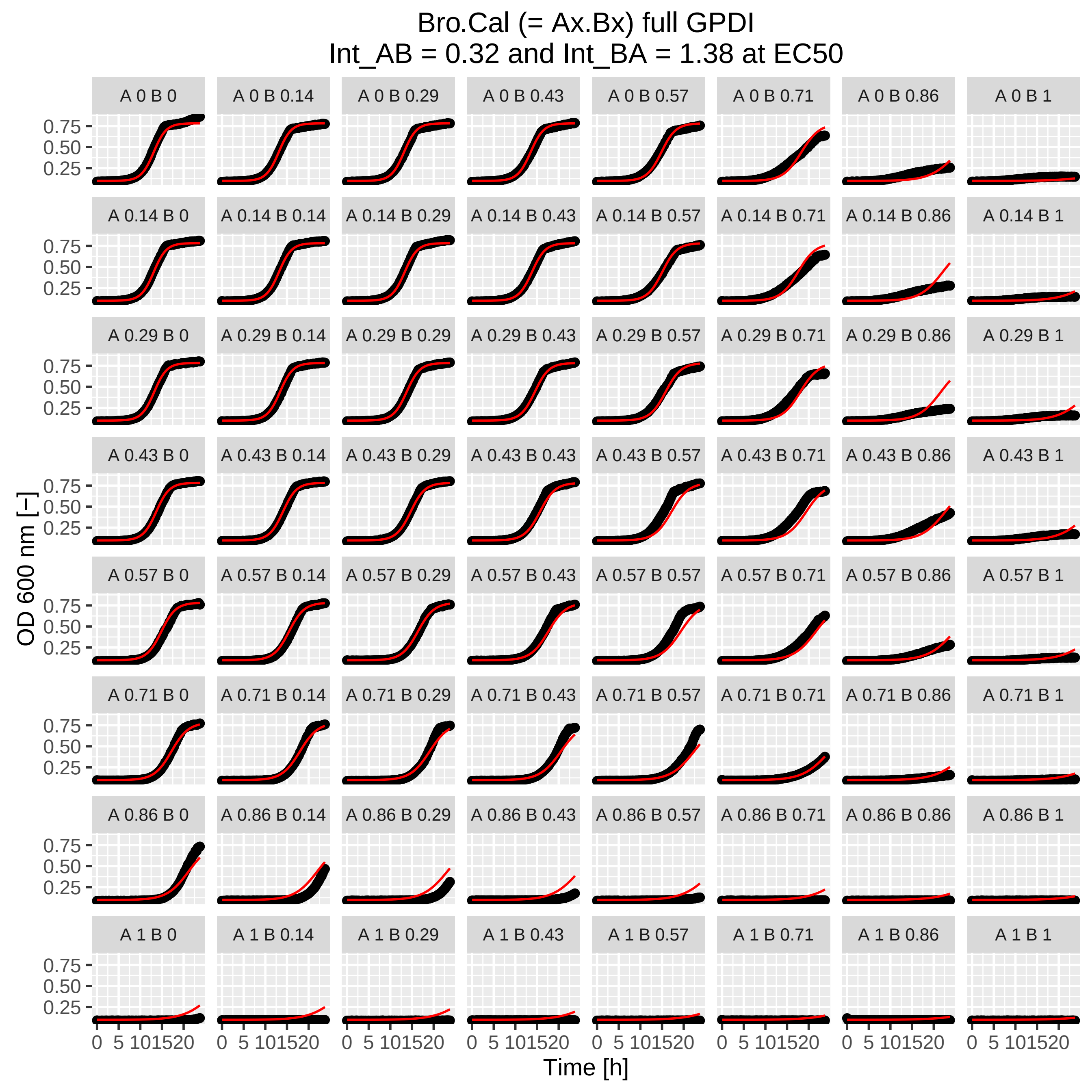

Bro.Cal (= Ax.Bx) full GPDI
Int_AB = 0.32 and Int_BA = 1.38 at EC50
A 0 B 0
A 0 B 0.14
A 0 B 0.29
A 0 B 0.43
A 0 B 0.57
A 0 B 0.71
A 0 B 0.86
A 0 B 1
A 0.14 B 0
A 0.14 B 0.14
A 0.14 B 0.29
A 0.14 B 0.43
A 0.14 B 0.57
A 0.14 B 0.71
A 0.14 B 0.86
A 0.14 B 1
A 0.29 B 0
A 0.29 B 0.14
A 0.29 B 0.29
A 0.29 B 0.43
A 0.29 B 0.57
A 0.29 B 0.71
A 0.29 B 0.86
A 0.29 B 1
A 0.43 B 0
A 0.43 B 0.14
A 0.43 B 0.29
A 0.43 B 0.43
A 0.43 B 0.57
A 0.43 B 0.71
A 0.43 B 0.86
A 0.43 B 1
A 0.57 B 0
A 0.57 B 0.14
A 0.57 B 0.29
A 0.57 B 0.43
A 0.57 B 0.57
A 0.57 B 0.71
A 0.57 B 0.86
A 0.57 B 1
A 0.71 B 0
A 0.71 B 0.14
A 0.71 B 0.29
A 0.71 B 0.43
A 0.71 B 0.57
A 0.71 B 0.71
A 0.71 B 0.86
A 0.71 B 1
A 0.86 B 0
A 0.86 B 0.14
A 0.86 B 0.29
A 0.86 B 0.43
A 0.86 B 0.57
A 0.86 B 0.71
A 0.86 B 0.86
A 0.86 B 1
A 1 B 0
A 1 B 0.14
A 1 B 0.29
A 1 B 0.43
A 1 B 0.57
A 1 B 0.71
A 1 B 0.86
A 1 B 1
0.75
0.50
0.25
0 5 101520
OD 600 nm [–]
Time [h]

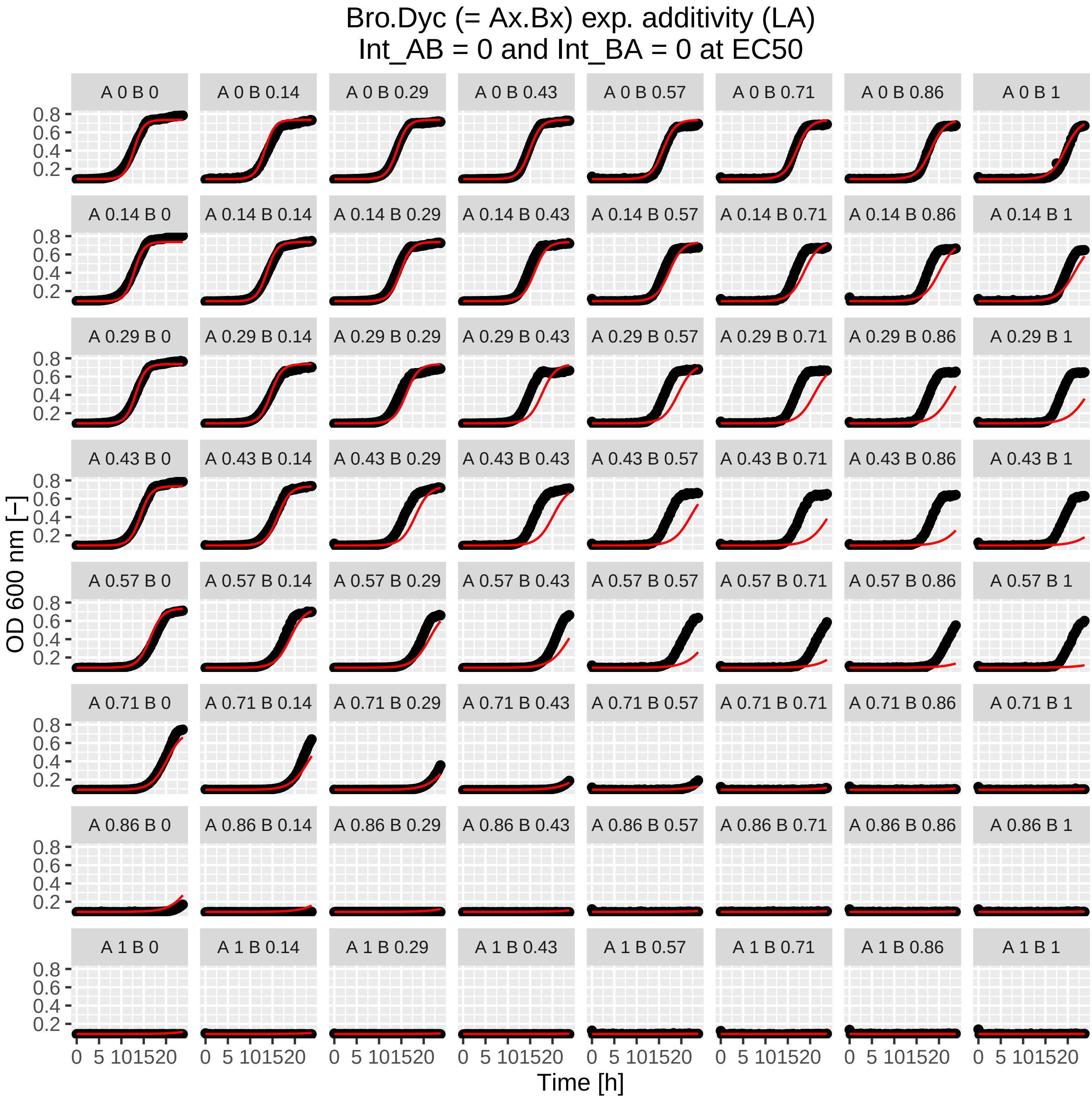

Bro.Dyc (= Ax.Bx) exp. additivity (LA)
Int_AB = 0 and Int_BA = 0 at EC50
A 0 B 0
A 0 B 0.14
A 0 B 0.29
A 0 B 0.43
A 0 B 0.57
A 0 B 0.71
A 0 B 0.86
A 0 B 1
A 0.14 B 0
A 0.14 B 0.14
A 0.14 B 0.29
A 0.14 B 0.43
A 0.14 B 0.57
A 0.14 B 0.71
A 0.14 B 0.86
A 0.14 B 1
A 0.29 B 0
A 0.29 B 0.14
A 0.29 B 0.29
A 0.29 B 0.43
A 0.29 B 0.57
A 0.29 B 0.71
A 0.29 B 0.86
A 0.29 B 1
A 0.43 B 0
A 0.43 B 0.14
A 0.43 B 0.29
A 0.43 B 0.43
A 0.43 B 0.57
A 0.43 B 0.71
A 0.43 B 0.86
A 0.43 B 1
A 0.57 B 0
A 0.57 B 0.14
A 0.57 B 0.29
A 0.57 B 0.43
A 0.57 B 0.57
A 0.57 B 0.71
A 0.57 B 0.86
A 0.57 B 1
A 0.71 B 0
A 0.71 B 0.14
A 0.71 B 0.29
A 0.71 B 0.43
A 0.71 B 0.57
A 0.71 B 0.71
A 0.71 B 0.86
A 0.71 B 1
A 0.86 B 0
A 0.86 B 0.14
A 0.86 B 0.29
A 0.86 B 0.43
A 0.86 B 0.57
A 0.86 B 0.71
A 0.86 B 0.86
A 0.86 B 1
A 1 B 0
A 1 B 0.14
A 1 B 0.29
A 1 B 0.43
A 1 B 0.57
A 1 B 0.71
A 1 B 0.86
A 1 B 1
0.8
0.6
0.4
0.2
OD 600 nm [–]
0 5 101520
Time [h]

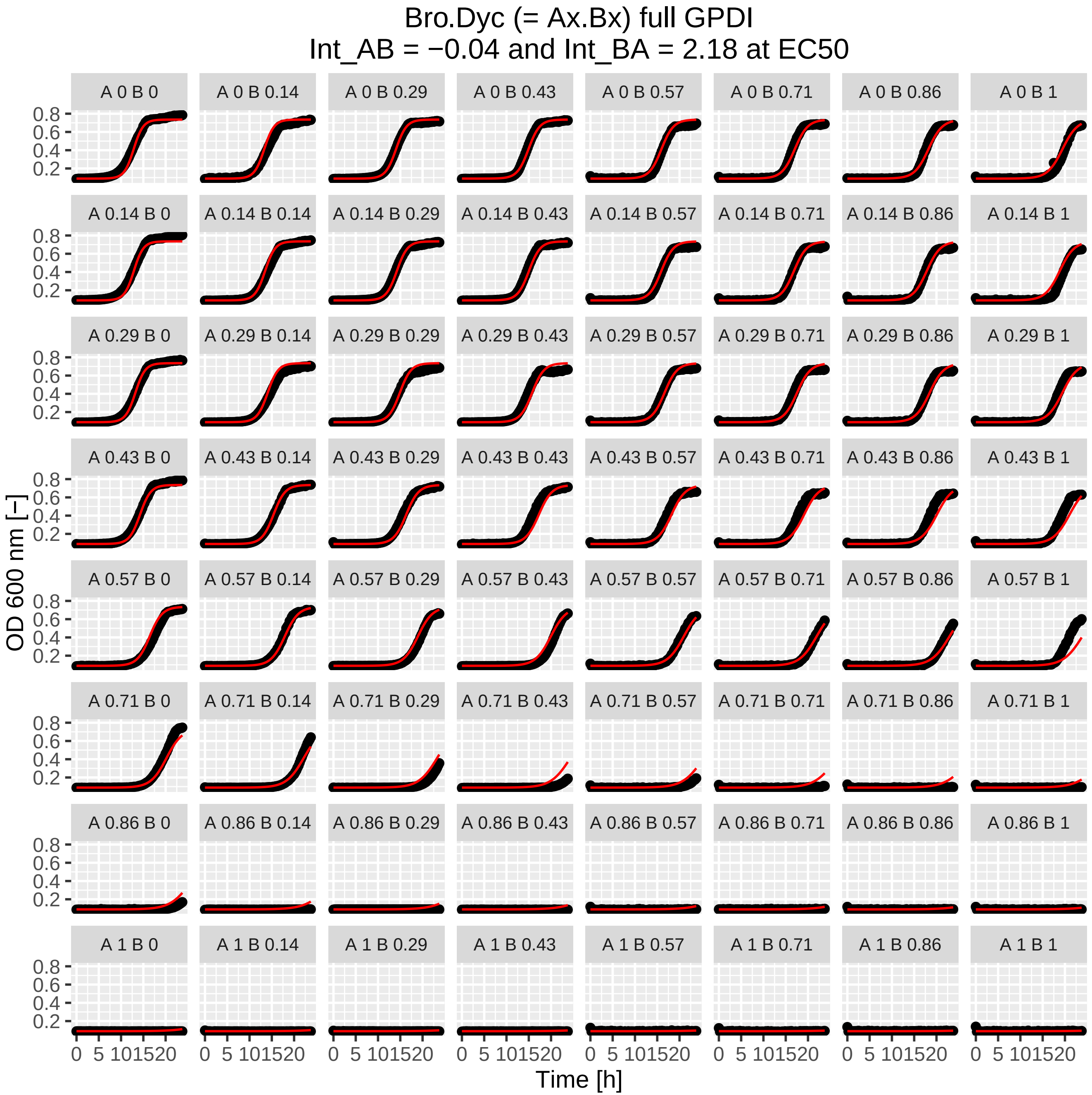
Bro.Dyc (= Ax.Bx) full GPDI
Int_AB = −0.04 and Int_BA = 2.18 at EC50
A 0 B 0
A 0 B 0.14
A 0 B 0.29
A 0 B 0.43
A 0 B 0.57
A 0 B 0.71
A 0 B 0.86
A 0 B 1
A 0.14 B 0
A 0.14 B 0.14
A 0.14 B 0.29
A 0.14 B 0.43
A 0.14 B 0.57
A 0.14 B 0.71
A 0.14 B 0.86
A 0.14 B 1
A 0.29 B 0
A 0.29 B 0.14
A 0.29 B 0.29
A 0.29 B 0.43
A 0.29 B 0.57
A 0.29 B 0.71
A 0.29 B 0.86
A 0.29 B 1
A 0.43 B 0
A 0.43 B 0.14
A 0.43 B 0.29
A 0.43 B 0.43
A 0.43 B 0.57
A 0.43 B 0.71
A 0.43 B 0.86
A 0.43 B 1
A 0.57 B 0
A 0.57 B 0.14
A 0.57 B 0.29
A 0.57 B 0.43
A 0.57 B 0.57
A 0.57 B 0.71
A 0.57 B 0.86
A 0.57 B 1
A 0.71 B 0
A 0.71 B 0.14
A 0.71 B 0.29
A 0.71 B 0.43
A 0.71 B 0.57
A 0.71 B 0.71
A 0.71 B 0.86
A 0.71 B 1
A 0.86 B 0
A 0.86 B 0.14
A 0.86 B 0.29
A 0.86 B 0.43
A 0.86 B 0.57
A 0.86 B 0.71
A 0.86 B 0.86
A 0.86 B 1
A 1 B 0
A 1 B 0.14
A 1 B 0.29
A 1 B 0.43
A 1 B 0.57
A 1 B 0.71
A 1 B 0.86
A 1 B 1
OD 600 nm [−]
0.2
0.4
0.6
0.8
0
5
10
15
20
Time [h]

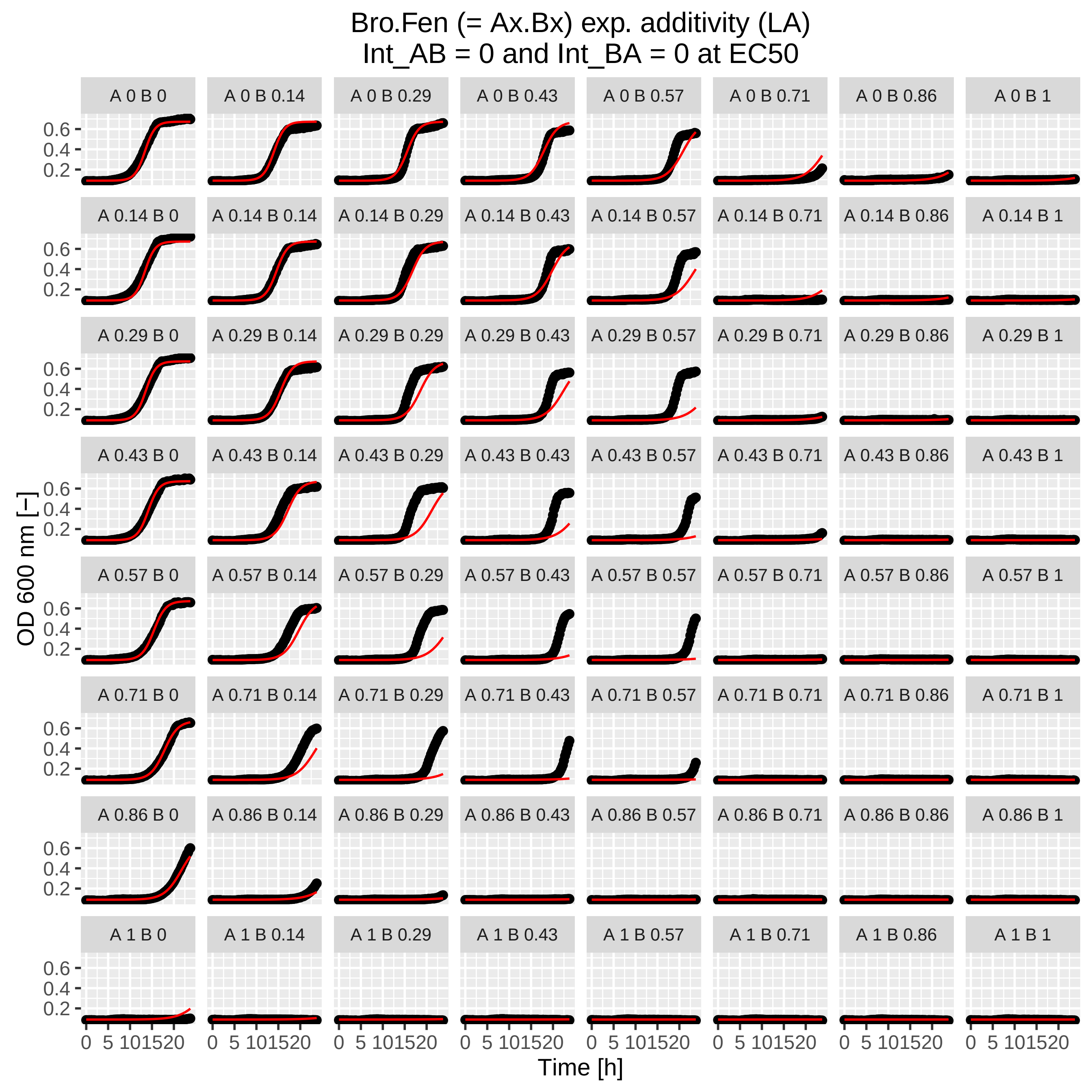

Bro.Fen (= Ax.Bx) exp. additivity (LA)
Int_AB = 0 and Int_BA = 0 at EC50
OD 600 nm [–]
Time [h]
0.6
0.4
0.2
0 5 101520
A 0 B 0
A 0 B 0.14
A 0 B 0.29
A 0 B 0.43
A 0 B 0.57
A 0 B 0.71
A 0 B 0.86
A 0 B 1
A 0.14 B 0
A 0.14 B 0.14
A 0.14 B 0.29
A 0.14 B 0.43
A 0.14 B 0.57
A 0.14 B 0.71
A 0.14 B 0.86
A 0.14 B 1
A 0.29 B 0
A 0.29 B 0.14
A 0.29 B 0.29
A 0.29 B 0.43
A 0.29 B 0.57
A 0.29 B 0.71
A 0.29 B 0.86
A 0.29 B 1
A 0.43 B 0
A 0.43 B 0.14
A 0.43 B 0.29
A 0.43 B 0.43
A 0.43 B 0.57
A 0.43 B 0.71
A 0.43 B 0.86
A 0.43 B 1
A 0.57 B 0
A 0.57 B 0.14
A 0.57 B 0.29
A 0.57 B 0.43
A 0.57 B 0.57
A 0.57 B 0.71
A 0.57 B 0.86
A 0.57 B 1
A 0.71 B 0
A 0.71 B 0.14
A 0.71 B 0.29
A 0.71 B 0.43
A 0.71 B 0.57
A 0.71 B 0.71
A 0.71 B 0.86
A 0.71 B 1
A 0.86 B 0
A 0.86 B 0.14
A 0.86 B 0.29
A 0.86 B 0.43
A 0.86 B 0.57
A 0.86 B 0.71
A 0.86 B 0.86
A 0.86 B 1
A 1 B 0
A 1 B 0.14
A 1 B 0.29
A 1 B 0.43
A 1 B 0.57
A 1 B 0.71
A 1 B 0.86
A 1 B 1

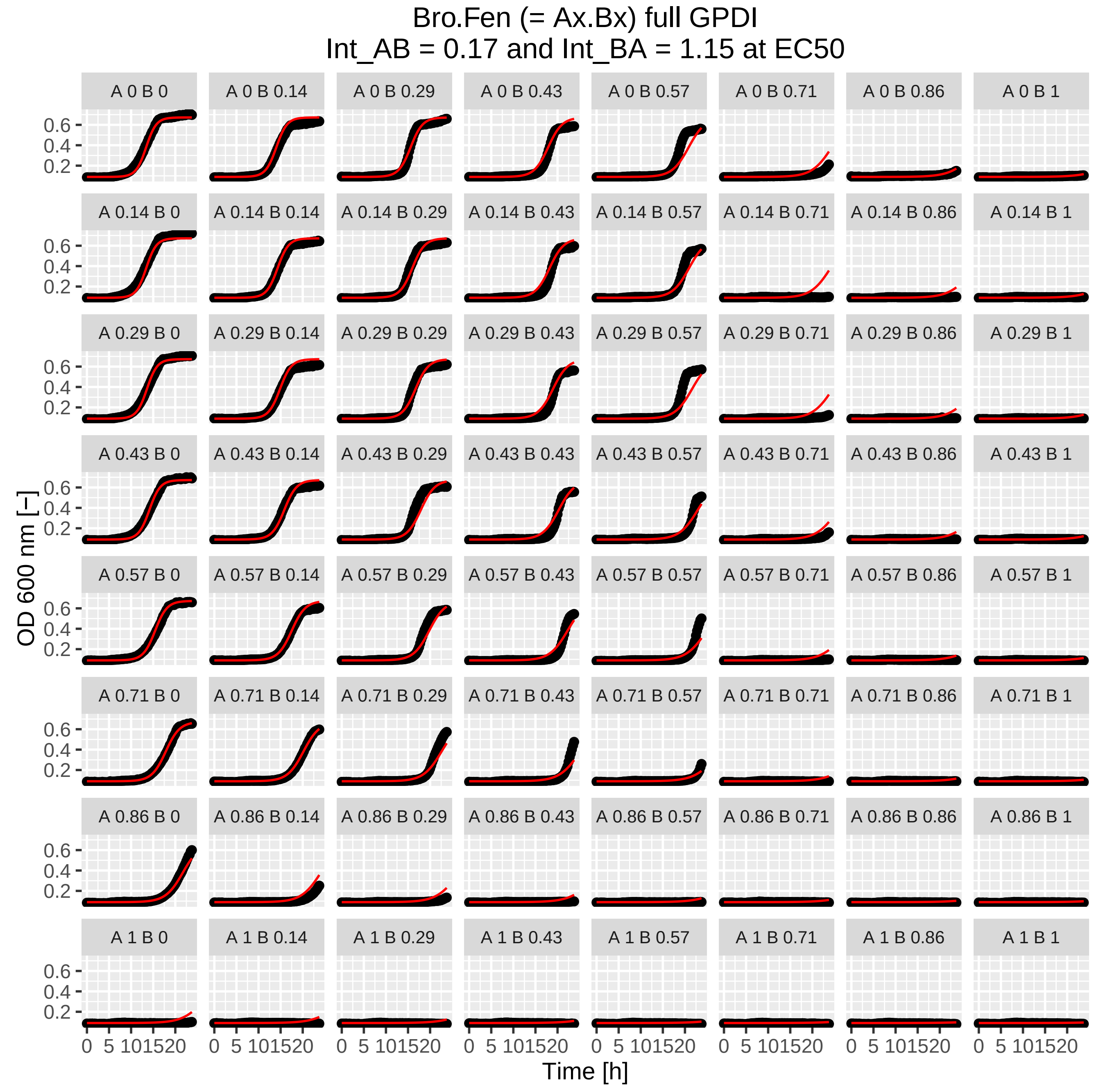
Bro.Fen (= Ax.Bx) full GPDI
Int_AB = 0.17 and Int_BA = 1.15 at EC50
OD 600 nm [–]
Time [h]
A 0 B 0
A 0 B 0.14
A 0 B 0.29
A 0 B 0.43
A 0 B 0.57
A 0 B 0.71
A 0 B 0.86
A 0 B 1
A 1 B 0
A 1 B 1
0.6
0.4
0.2
0 5 101520

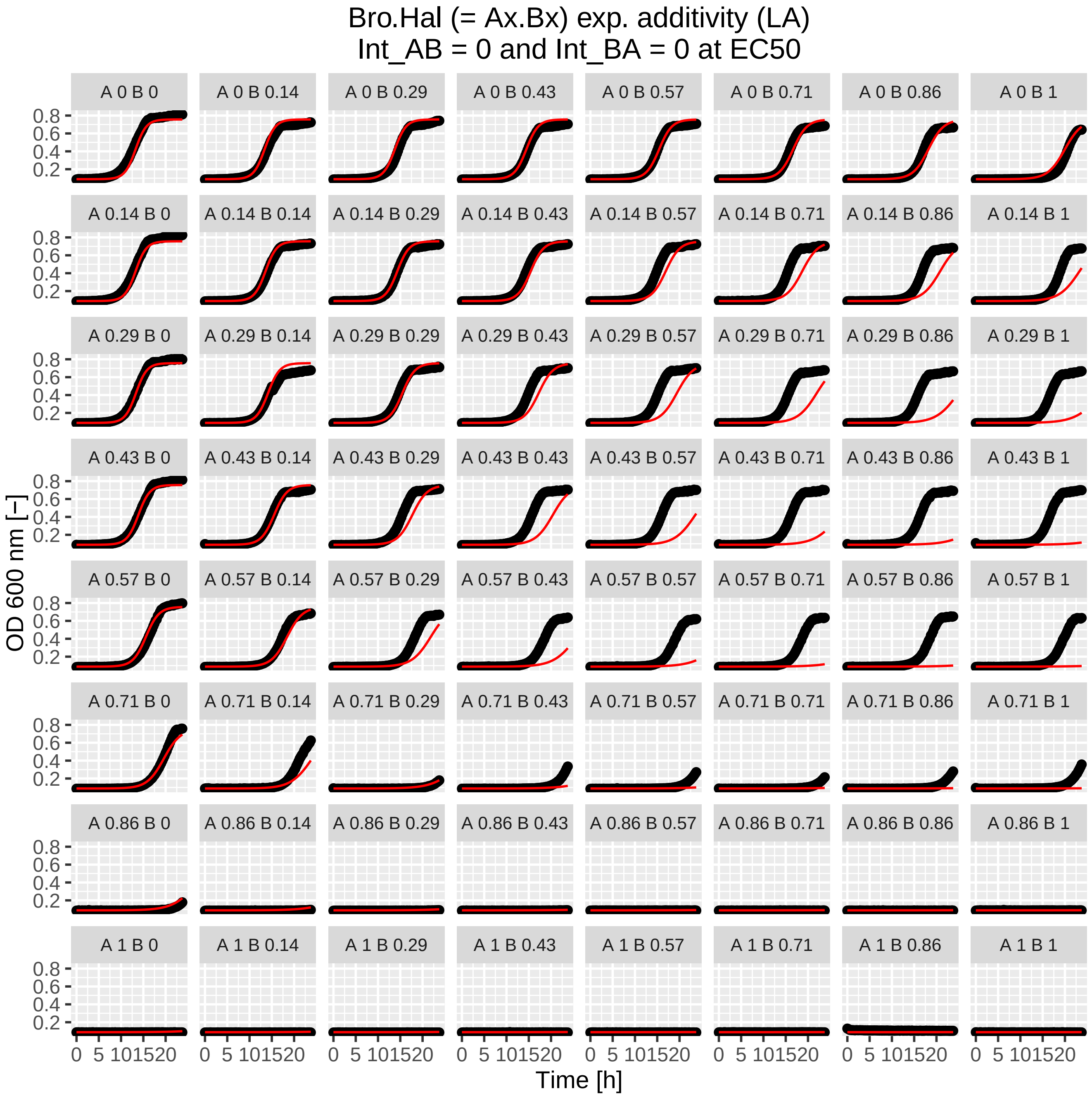
Bro.Hal (= Ax.Bx) exp. additivity (LA)
Int_AB = 0 and Int_BA = 0 at EC50
OD 600 nm [−]
Time [h]
A 0 B 0
A 0 B 0.14
A 0 B 0.29
A 0 B 0.43
A 0 B 0.57
A 0 B 0.71
A 0 B 0.86
A 0 B 1
A 0.14 B 0
A 0.14 B 0.14
A 0.14 B 0.29
A 0.14 B 0.43
A 0.14 B 0.57
A 0.14 B 0.71
A 0.14 B 0.86
A 0.14 B 1
A 0.29 B 0
A 0.29 B 0.14
A 0.29 B 0.29
A 0.29 B 0.43
A 0.29 B 0.57
A 0.29 B 0.71
A 0.29 B 0.86
A 0.29 B 1
A 0.43 B 0
A 0.43 B 0.14
A 0.43 B 0.29
A 0.43 B 0.43
A 0.43 B 0.57
A 0.43 B 0.71
A 0.43 B 0.86
A 0.43 B 1
A 0.57 B 0
A 0.57 B 0.14
A 0.57 B 0.29
A 0.57 B 0.43
A 0.57 B 0.57
A 0.57 B 0.71
A 0.57 B 0.86
A 0.57 B 1
A 0.71 B 0
A 0.71 B 0.14
A 0.71 B 0.29
A 0.71 B 0.43
A 0.71 B 0.57
A 0.71 B 0.71
A 0.71 B 0.86
A 0.71 B 1
A 0.86 B 0
A 0.86 B 0.14
A 0.86 B 0.29
A 0.86 B 0.43
A 0.86 B 0.57
A 0.86 B 0.71
A 0.86 B 0.86
A 0.86 B 1
A 1 B 0
A 1 B 0.14
A 1 B 0.29
A 1 B 0.43
A 1 B 0.57
A 1 B 0.71
A 1 B 0.86
A 1 B 1
0.8
0.6
0.4
0.2
0 5 101520

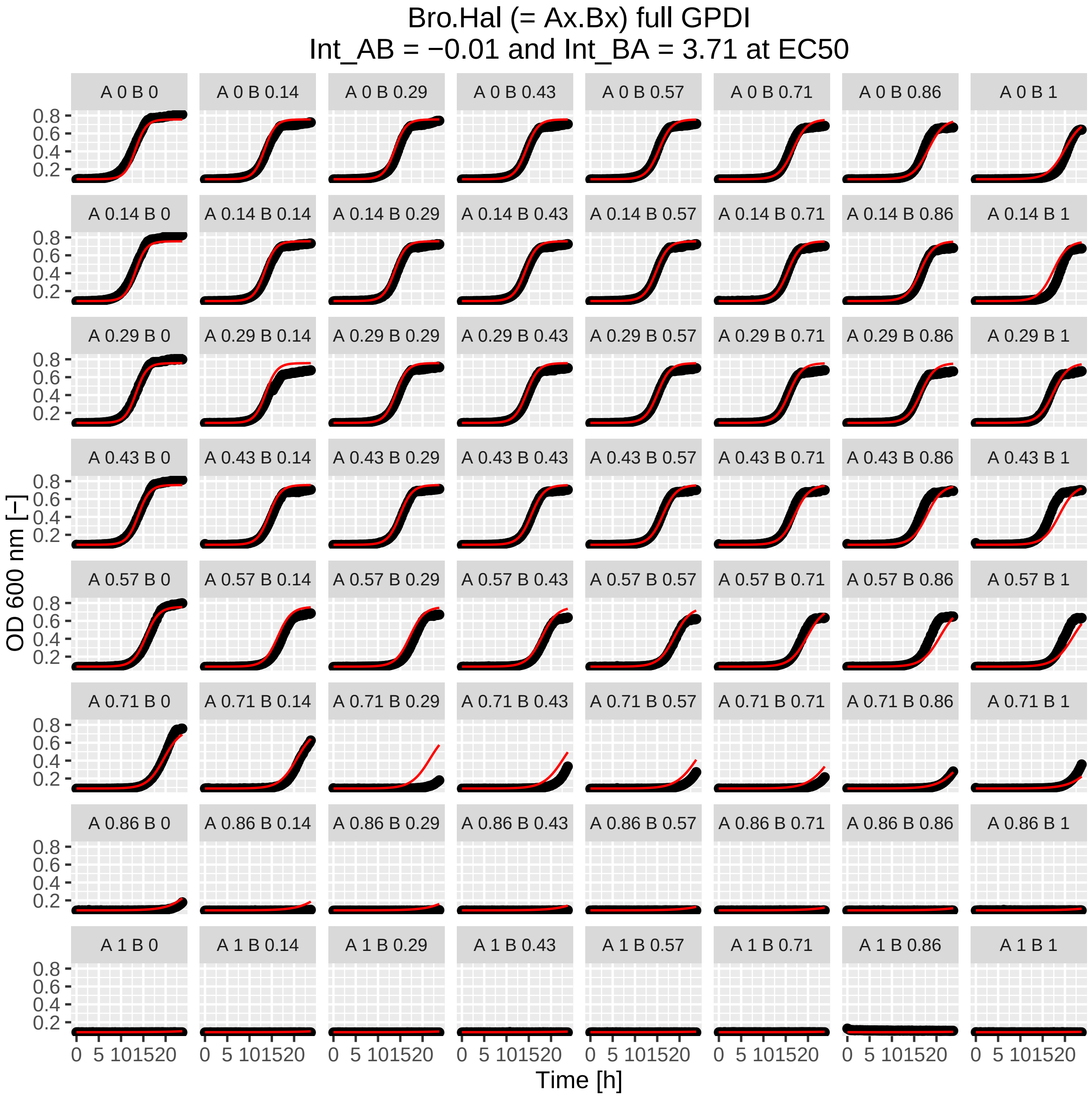

Bro.Hal (= Ax.Bx) full GPDI
Int_AB = −0.01 and Int_BA = 3.71 at EC50
A 0 B 0
A 0 B 0.14
A 0 B 0.29
A 0 B 0.43
A 0 B 0.57
A 0 B 0.71
A 0 B 0.86
A 0 B 1
A 0.14 B 0
A 0.14 B 0.14
A 0.14 B 0.29
A 0.14 B 0.43
A 0.14 B 0.57
A 0.14 B 0.71
A 0.14 B 0.86
A 0.14 B 1
A 0.29 B 0
A 0.29 B 0.14
A 0.29 B 0.29
A 0.29 B 0.43
A 0.29 B 0.57
A 0.29 B 0.71
A 0.29 B 0.86
A 0.29 B 1
A 0.43 B 0
A 0.43 B 0.14
A 0.43 B 0.29
A 0.43 B 0.43
A 0.43 B 0.57
A 0.43 B 0.71
A 0.43 B 0.86
A 0.43 B 1
A 0.57 B 0
A 0.57 B 0.14
A 0.57 B 0.29
A 0.57 B 0.43
A 0.57 B 0.57
A 0.57 B 0.71
A 0.57 B 0.86
A 0.57 B 1
A 0.71 B 0
A 0.71 B 0.14
A 0.71 B 0.29
A 0.71 B 0.43
A 0.71 B 0.57
A 0.71 B 0.71
A 0.71 B 0.86
A 0.71 B 1
A 0.86 B 0
A 0.86 B 0.14
A 0.86 B 0.29
A 0.86 B 0.43
A 0.86 B 0.57
A 0.86 B 0.71
A 0.86 B 0.86
A 0.86 B 1
A 1 B 0
A 1 B 0.14
A 1 B 0.29
A 1 B 0.43
A 1 B 0.57
A 1 B 0.71
A 1 B 0.86
A 1 B 1
0.8
0.6
0.4
0.2
OD 600 nm [−]
0 5 101520
Time [h]

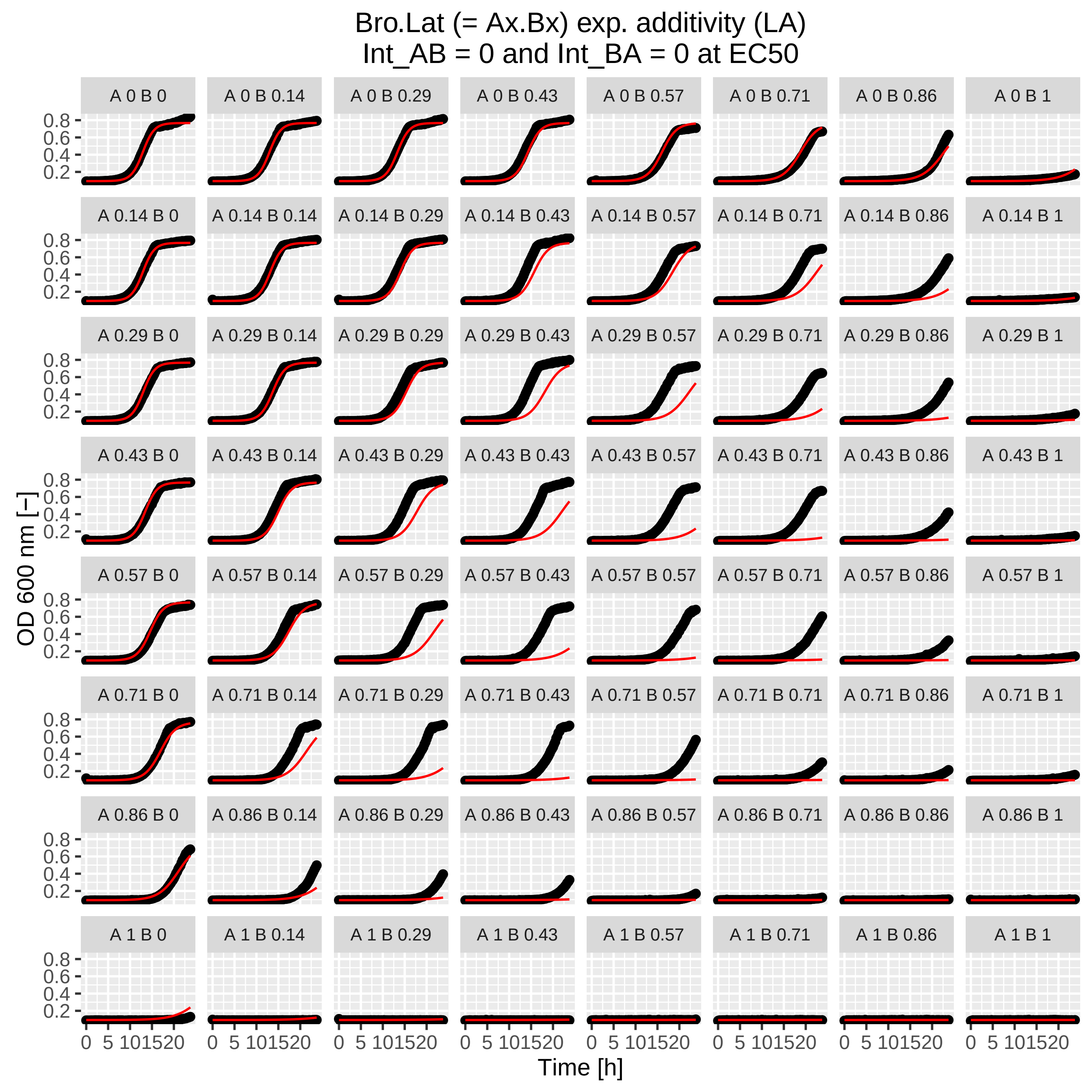
Bro.Lat (= Ax.Bx) exp. additivity (LA)
Int_AB = 0 and Int_BA = 0 at EC50
OD 600 nm [−]
Time [h]
A 0 B 0
A 0 B 0.14
A 0 B 0.29
A 0 B 0.43
A 0 B 0.57
A 0 B 0.71
A 0 B 0.86
A 0 B 1
A 0.14 B 0
A 0.14 B 0.14
A 0.14 B 0.29
A 0.14 B 0.43
A 0.14 B 0.57
A 0.14 B 0.71
A 0.14 B 0.86
A 0.14 B 1
A 0.29 B 0
A 0.29 B 0.14
A 0.29 B 0.29
A 0.29 B 0.43
A 0.29 B 0.57
A 0.29 B 0.71
A 0.29 B 0.86
A 0.29 B 1
A 0.43 B 0
A 0.43 B 0.14
A 0.43 B 0.29
A 0.43 B 0.43
A 0.43 B 0.57
A 0.43 B 0.71
A 0.43 B 0.86
A 0.43 B 1
A 0.57 B 0
A 0.57 B 0.14
A 0.57 B 0.29
A 0.57 B 0.43
A 0.57 B 0.57
A 0.57 B 0.71
A 0.57 B 0.86
A 0.57 B 1
A 0.71 B 0
A 0.71 B 0.14
A 0.71 B 0.29
A 0.71 B 0.43
A 0.71 B 0.57
A 0.71 B 0.71
A 0.71 B 0.86
A 0.71 B 1
A 0.86 B 0
A 0.86 B 0.14
A 0.86 B 0.29
A 0.86 B 0.43
A 0.86 B 0.57
A 0.86 B 0.71
A 0.86 B 0.86
A 0.86 B 1
A 1 B 0
A 1 B 0.14
A 1 B 0.29
A 1 B 0.43
A 1 B 0.57
A 1 B 0.71
A 1 B 0.86
A 1 B 1
0.8
0.6
0.4
0.2
0 5 101520

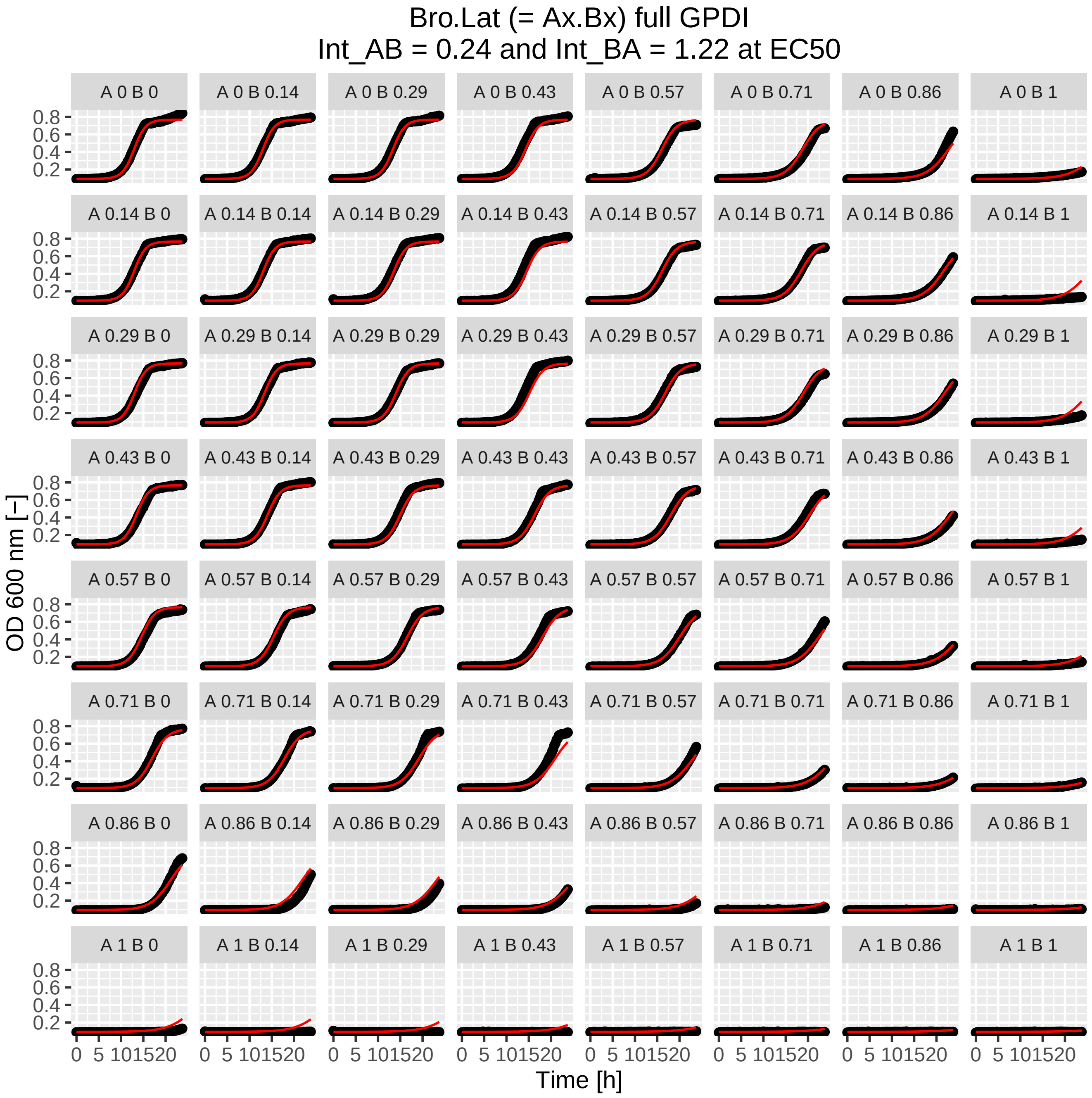
Bro.Lat (= Ax.Bx) full GPDI
Int_AB = 0.24 and Int_BA = 1.22 at EC50
OD 600 nm [–]
Time [h]
A 0 B 0
A 0 B 0.14
A 0 B 0.29
A 0 B 0.43
A 0 B 0.57
A 0 B 0.71
A 0 B 0.86
A 0 B 1
A 1 B 0
A 1 B 0.14
A 1 B 0.29
A 1 B 0.43
A 1 B 0.57
A 1 B 0.71
A 1 B 0.86
A 1 B 1
0.8
0.6
0.4
0.2
0 5 101520

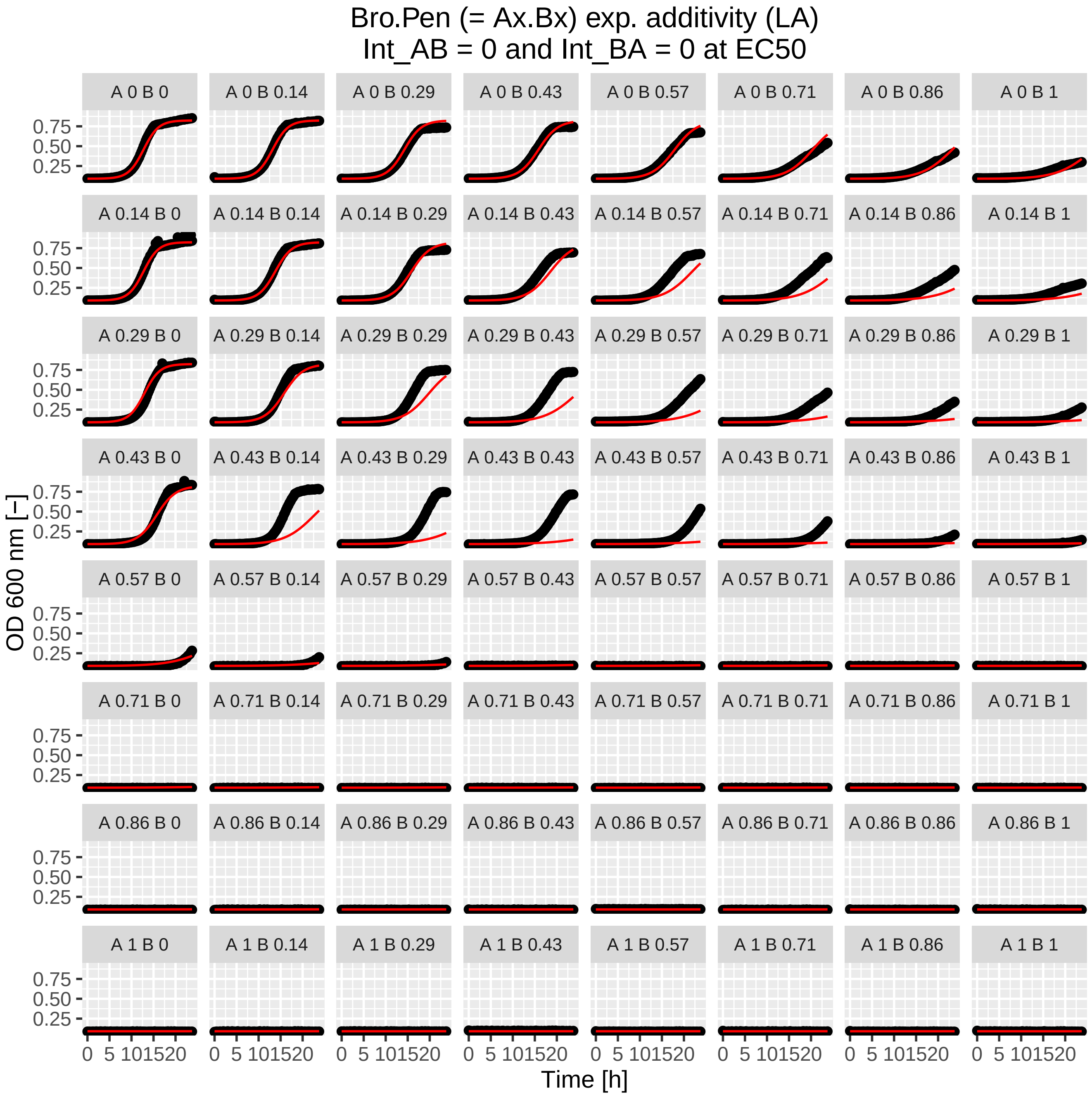
Bro.Pen (= Ax.Bx) exp. additivity (LA)
Int_AB = 0 and Int_BA = 0 at EC50
OD 600 nm [–]
Time [h]
A 0 B 0
A 0 B 0.14
A 0 B 0.29
A 0 B 0.43
A 0 B 0.57
A 0 B 0.71
A 0 B 0.86
A 0 B 1
A 0.14 B 0
A 0.14 B 0.14
A 0.14 B 0.29
A 0.14 B 0.43
A 0.14 B 0.57
A 0.14 B 0.71
A 0.14 B 0.86
A 0.14 B 1
A 0.29 B 0
A 0.29 B 0.14
A 0.29 B 0.29
A 0.29 B 0.43
A 0.29 B 0.57
A 0.29 B 0.71
A 0.29 B 0.86
A 0.29 B 1
A 0.43 B 0
A 0.43 B 0.14
A 0.43 B 0.29
A 0.43 B 0.43
A 0.43 B 0.57
A 0.43 B 0.71
A 0.43 B 0.86
A 0.43 B 1
A 0.57 B 0
A 0.57 B 0.14
A 0.57 B 0.29
A 0.57 B 0.43
A 0.57 B 0.57
A 0.57 B 0.71
A 0.57 B 0.86
A 0.57 B 1
A 0.71 B 0
A 0.71 B 0.14
A 0.71 B 0.29
A 0.71 B 0.43
A 0.71 B 0.57
A 0.71 B 0.71
A 0.71 B 0.86
A 0.71 B 1
A 0.86 B 0
A 0.86 B 0.14
A 0.86 B 0.29
A 0.86 B 0.43
A 0.86 B 0.57
A 0.86 B 0.71
A 0.86 B 0.86
A 0.86 B 1
A 1 B 0
A 1 B 0.14
A 1 B 0.29
A 1 B 0.43
A 1 B 0.57
A 1 B 0.71
A 1 B 0.86
A 1 B 1
0.75
0.50
0.25
0 5 101520

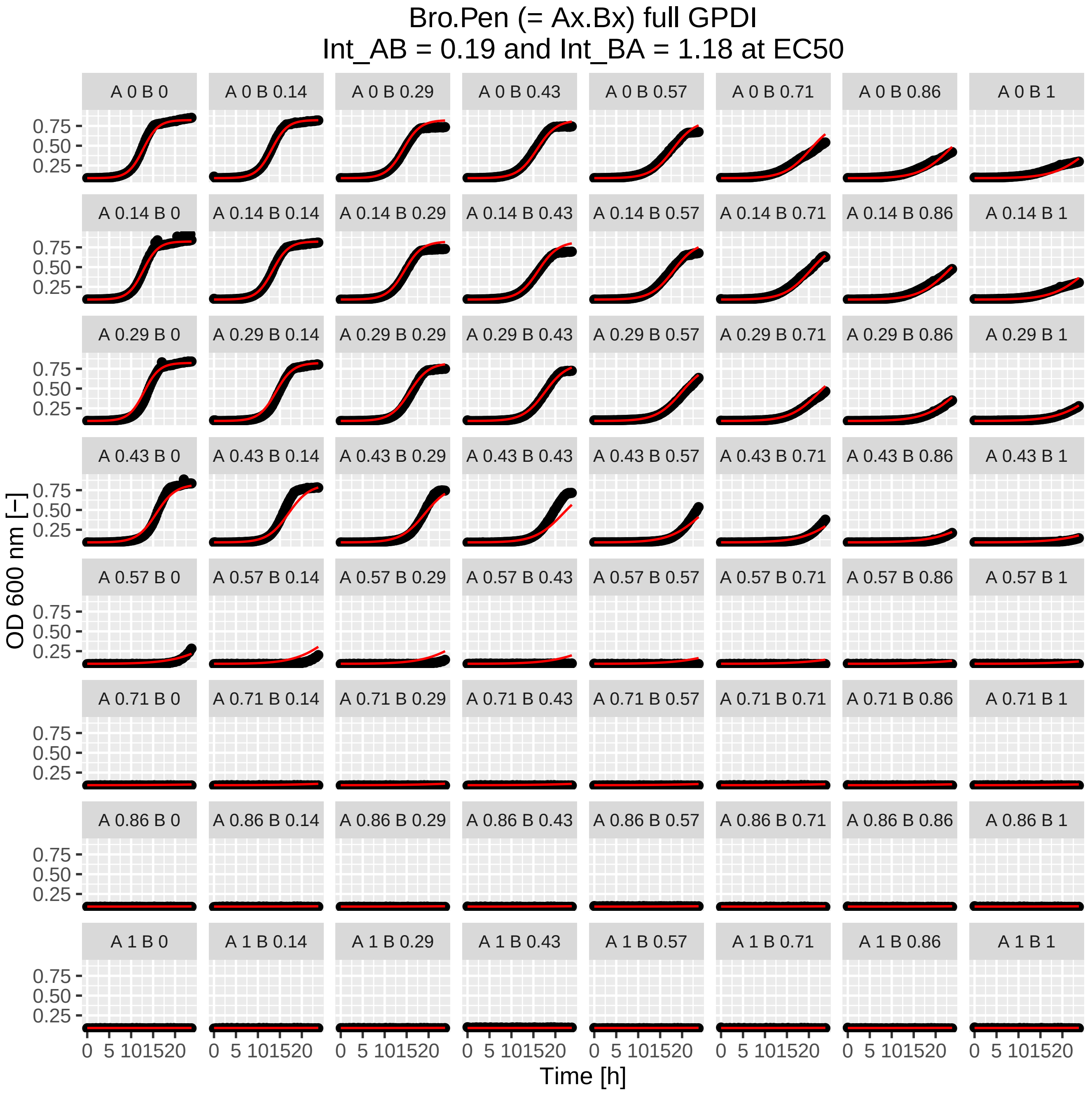
Bro.Pen (= Ax.Bx) full GPDI
Int_AB = 0.19 and Int_BA = 1.18 at EC50
OD 600 nm [−]
Time [h]
0.75
0.50
0.25
0 5 101520
A 0 B 0
A 0 B 0.14
A 0 B 0.29
A 0 B 0.43
A 0 B 0.57
A 0 B 0.71
A 0 B 0.86
A 0 B 1
A 0.14 B 0
A 0.14 B 0.14
A 0.14 B 0.29
A 0.14 B 0.43
A 0.14 B 0.57
A 0.14 B 0.71
A 0.14 B 0.86
A 0.14 B 1
A 0.29 B 0
A 0.29 B 0.14
A 0.29 B 0.29
A 0.29 B 0.43
A 0.29 B 0.57
A 0.29 B 0.71
A 0.29 B 0.86
A 0.29 B 1
A 0.43 B 0
A 0.43 B 0.14
A 0.43 B 0.29
A 0.43 B 0.43
A 0.43 B 0.57
A 0.43 B 0.71
A 0.43 B 0.86
A 0.43 B 1
A 0.57 B 0
A 0.57 B 0.14
A 0.57 B 0.29
A 0.57 B 0.43
A 0.57 B 0.57
A 0.57 B 0.71
A 0.57 B 0.86
A 0.57 B 1
A 0.71 B 0
A 0.71 B 0.14
A 0.71 B 0.29
A 0.71 B 0.43
A 0.71 B 0.57
A 0.71 B 0.71
A 0.71 B 0.86
A 0.71 B 1
A 0.86 B 0
A 0.86 B 0.14
A 0.86 B 0.29
A 0.86 B 0.43
A 0.86 B 0.57
A 0.86 B 0.71
A 0.86 B 0.86
A 0.86 B 1
A 1 B 0
A 1 B 0.14
A 1 B 0.29
A 1 B 0.43
A 1 B 0.57
A 1 B 0.71
A 1 B 0.86
A 1 B 1

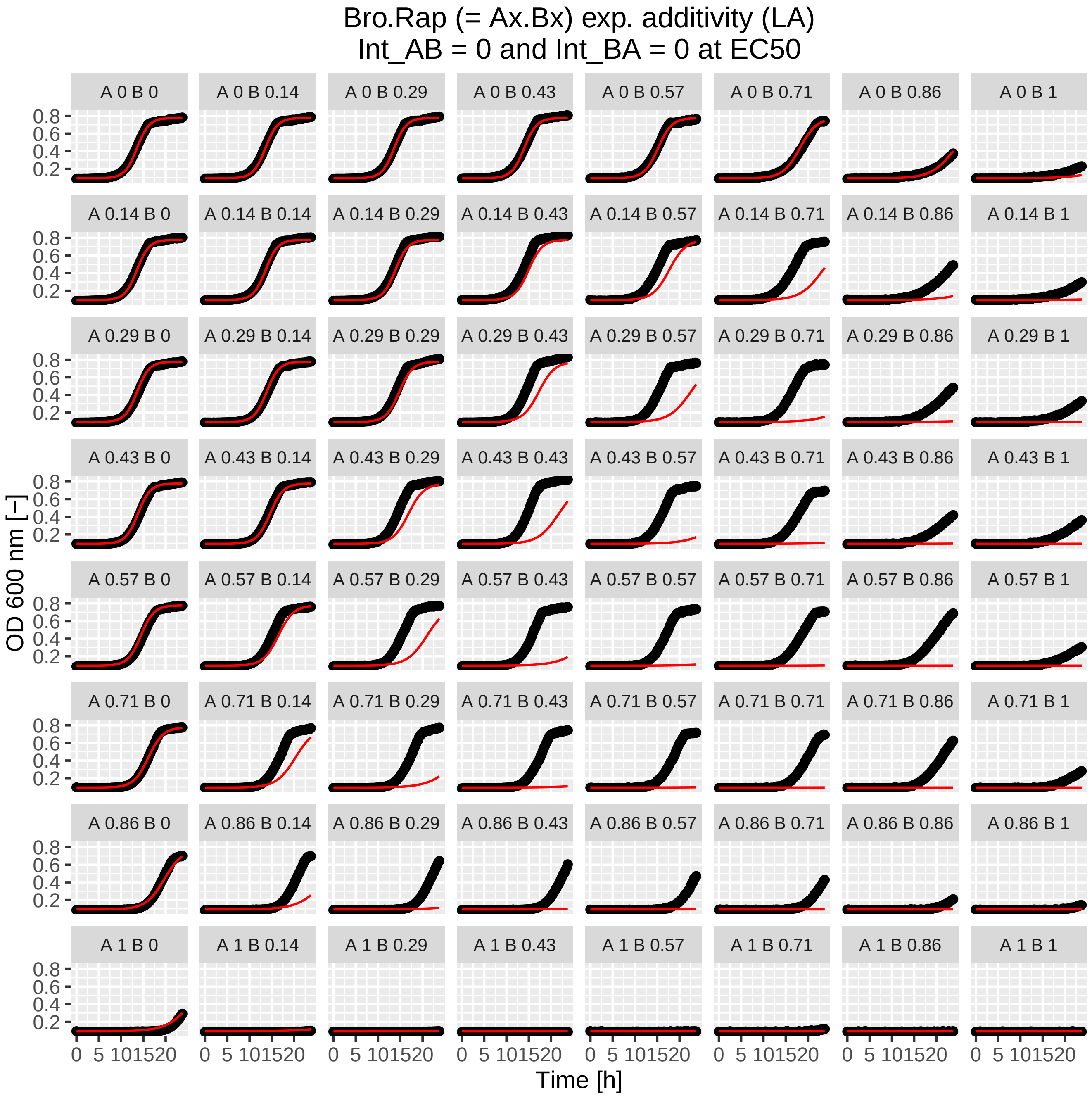

Bro.Rap (= Ax.Bx) exp. additivity (LA)
Int_AB = 0 and Int_BA = 0 at EC50
A 0 B 0
A 0 B 0.14
A 0 B 0.29
A 0 B 0.43
A 0 B 0.57
A 0 B 0.71
A 0 B 0.86
A 0 B 1
A 0.14 B 0
A 0.14 B 0.14
A 0.14 B 0.29
A 0.14 B 0.43
A 0.14 B 0.57
A 0.14 B 0.71
A 0.14 B 0.86
A 0.14 B 1
A 0.29 B 0
A 0.29 B 0.14
A 0.29 B 0.29
A 0.29 B 0.43
A 0.29 B 0.57
A 0.29 B 0.71
A 0.29 B 0.86
A 0.29 B 1
A 0.43 B 0
A 0.43 B 0.14
A 0.43 B 0.29
A 0.43 B 0.43
A 0.43 B 0.57
A 0.43 B 0.71
A 0.43 B 0.86
A 0.43 B 1
A 0.57 B 0
A 0.57 B 0.14
A 0.57 B 0.29
A 0.57 B 0.43
A 0.57 B 0.57
A 0.57 B 0.71
A 0.57 B 0.86
A 0.57 B 1
A 0.71 B 0
A 0.71 B 0.14
A 0.71 B 0.29
A 0.71 B 0.43
A 0.71 B 0.57
A 0.71 B 0.71
A 0.71 B 0.86
A 0.71 B 1
A 0.86 B 0
A 0.86 B 0.14
A 0.86 B 0.29
A 0.86 B 0.43
A 0.86 B 0.57
A 0.86 B 0.71
A 0.86 B 0.86
A 0.86 B 1
A 1 B 0
A 1 B 0.14
A 1 B 0.29
A 1 B 0.43
A 1 B 0.57
A 1 B 0.71
A 1 B 0.86
A 1 B 1
OD 600 nm [–]
0.2
0.4
0.6
0.8
0
5
10
15
20
Time [h]

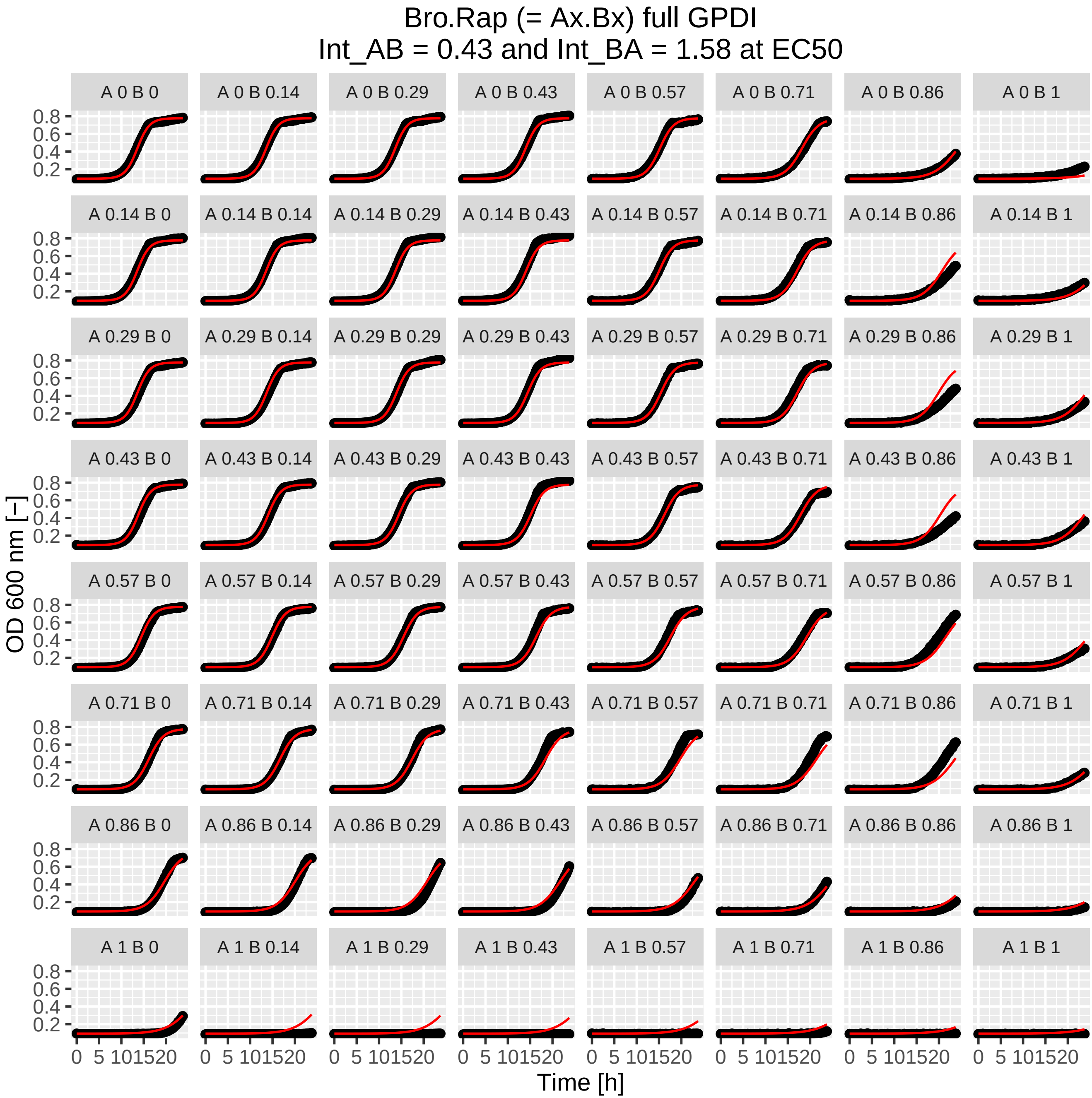
Bro.Rap (= Ax.Bx) full GPDI
Int_AB = 0.43 and Int_BA = 1.58 at EC50
OD 600 nm [−]
Time [h]
A 0 B 0
A 0 B 0.14
A 0 B 0.29
A 0 B 0.43
A 0 B 0.57
A 0 B 0.71
A 0 B 0.86
A 0 B 1
A 1 B 0
A 1 B 0.14
A 1 B 0.29
A 1 B 0.43
A 1 B 0.57
A 1 B 0.71
A 1 B 0.86
A 1 B 1

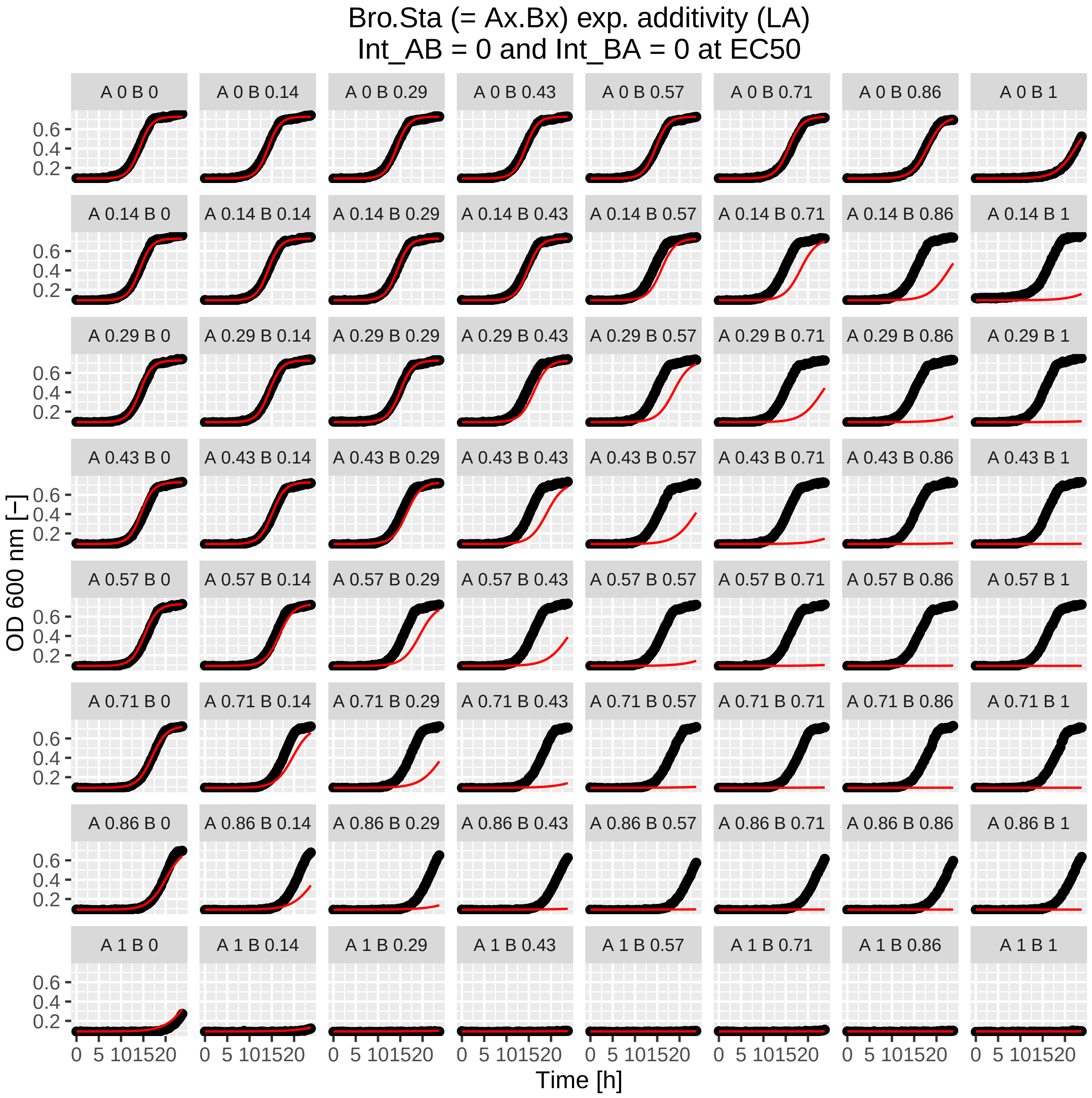

Bro.Sta (= Ax.Bx) exp. additivity (LA)
Int_AB = 0 and Int_BA = 0 at EC50
A 0 B 0
A 0 B 0.14
A 0 B 0.29
A 0 B 0.43
A 0 B 0.57
A 0 B 0.71
A 0 B 0.86
A 0 B 1
A 0.14 B 0
A 0.14 B 0.14
A 0.14 B 0.29
A 0.14 B 0.43
A 0.14 B 0.57
A 0.14 B 0.71
A 0.14 B 0.86
A 0.14 B 1
A 0.29 B 0
A 0.29 B 0.14
A 0.29 B 0.29
A 0.29 B 0.43
A 0.29 B 0.57
A 0.29 B 0.71
A 0.29 B 0.86
A 0.29 B 1
A 0.43 B 0
A 0.43 B 0.14
A 0.43 B 0.29
A 0.43 B 0.43
A 0.43 B 0.57
A 0.43 B 0.71
A 0.43 B 0.86
A 0.43 B 1
A 0.57 B 0
A 0.57 B 0.14
A 0.57 B 0.29
A 0.57 B 0.43
A 0.57 B 0.57
A 0.57 B 0.71
A 0.57 B 0.86
A 0.57 B 1
A 0.71 B 0
A 0.71 B 0.14
A 0.71 B 0.29
A 0.71 B 0.43
A 0.71 B 0.57
A 0.71 B 0.71
A 0.71 B 0.86
A 0.71 B 1
A 0.86 B 0
A 0.86 B 0.14
A 0.86 B 0.29
A 0.86 B 0.43
A 0.86 B 0.57
A 0.86 B 0.71
A 0.86 B 0.86
A 0.86 B 1
A 1 B 0
A 1 B 0.14
A 1 B 0.29
A 1 B 0.43
A 1 B 0.57
A 1 B 0.71
A 1 B 0.86
A 1 B 1
0.6
0.4
0.2
0 5 101520
OD 600 nm [–]
Time [h]

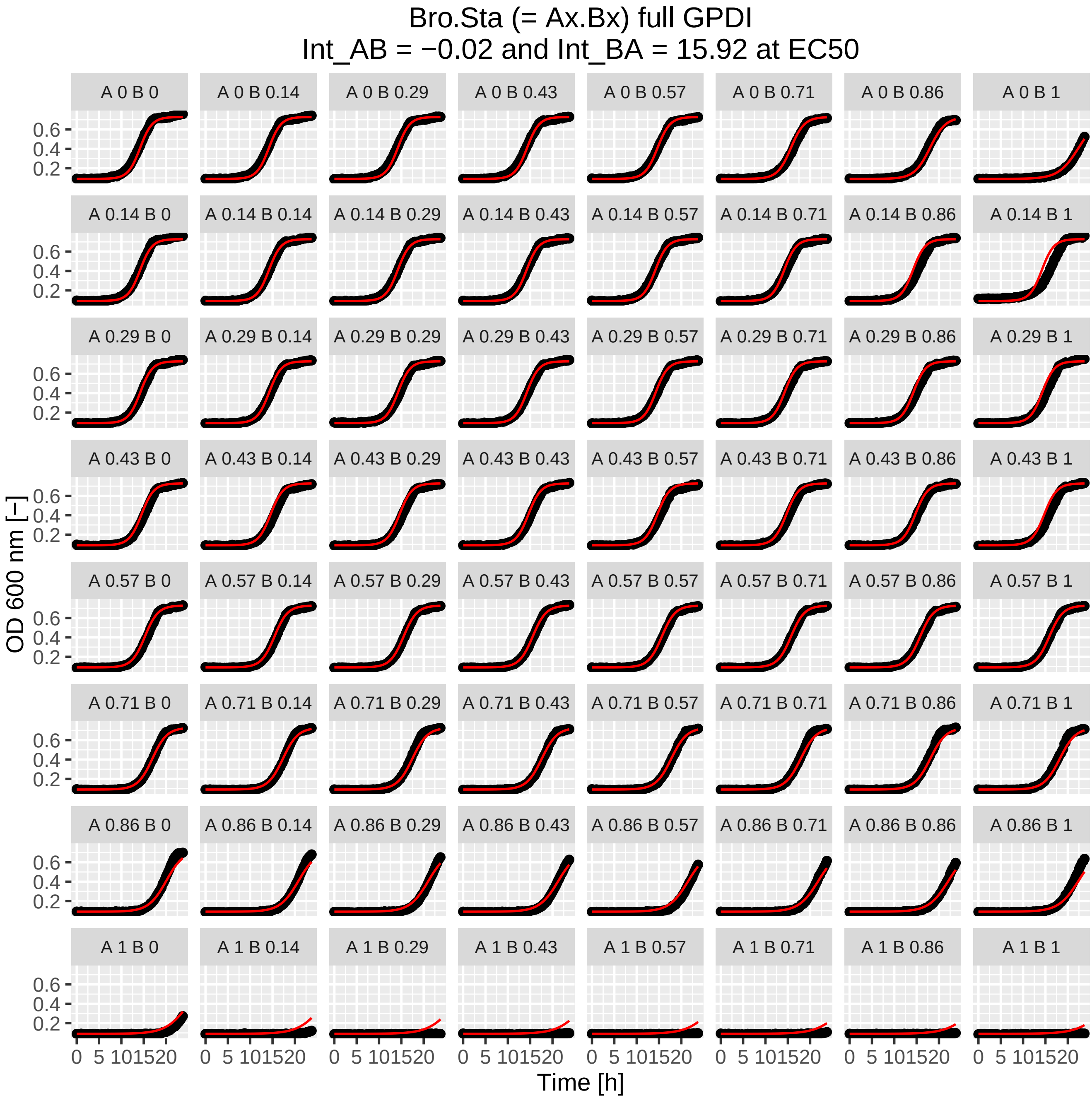

Bro.Sta (= Ax.Bx) full GPDI
Int_AB = −0.02 and Int_BA = 15.92 at EC50
A 0 B 0
A 0 B 0.14
A 0 B 0.29
A 0 B 0.43
A 0 B 0.57
A 0 B 0.71
A 0 B 0.86
A 0 B 1
A 0.14 B 0
A 0.14 B 0.14
A 0.14 B 0.29
A 0.14 B 0.43
A 0.14 B 0.57
A 0.14 B 0.71
A 0.14 B 0.86
A 0.14 B 1
A 0.29 B 0
A 0.29 B 0.14
A 0.29 B 0.29
A 0.29 B 0.43
A 0.29 B 0.57
A 0.29 B 0.71
A 0.29 B 0.86
A 0.29 B 1
A 0.43 B 0
A 0.43 B 0.14
A 0.43 B 0.29
A 0.43 B 0.43
A 0.43 B 0.57
A 0.43 B 0.71
A 0.43 B 0.86
A 0.43 B 1
A 0.57 B 0
A 0.57 B 0.14
A 0.57 B 0.29
A 0.57 B 0.43
A 0.57 B 0.57
A 0.57 B 0.71
A 0.57 B 0.86
A 0.57 B 1
A 0.71 B 0
A 0.71 B 0.14
A 0.71 B 0.29
A 0.71 B 0.43
A 0.71 B 0.57
A 0.71 B 0.71
A 0.71 B 0.86
A 0.71 B 1
A 0.86 B 0
A 0.86 B 0.14
A 0.86 B 0.29
A 0.86 B 0.43
A 0.86 B 0.57
A 0.86 B 0.71
A 0.86 B 0.86
A 0.86 B 1
A 1 B 0
A 1 B 0.14
A 1 B 0.29
A 1 B 0.43
A 1 B 0.57
A 1 B 0.71
A 1 B 0.86
A 1 B 1
OD 600 nm [−]
0.2
0.4
0.6
Time [h]
0
5
10
15
20

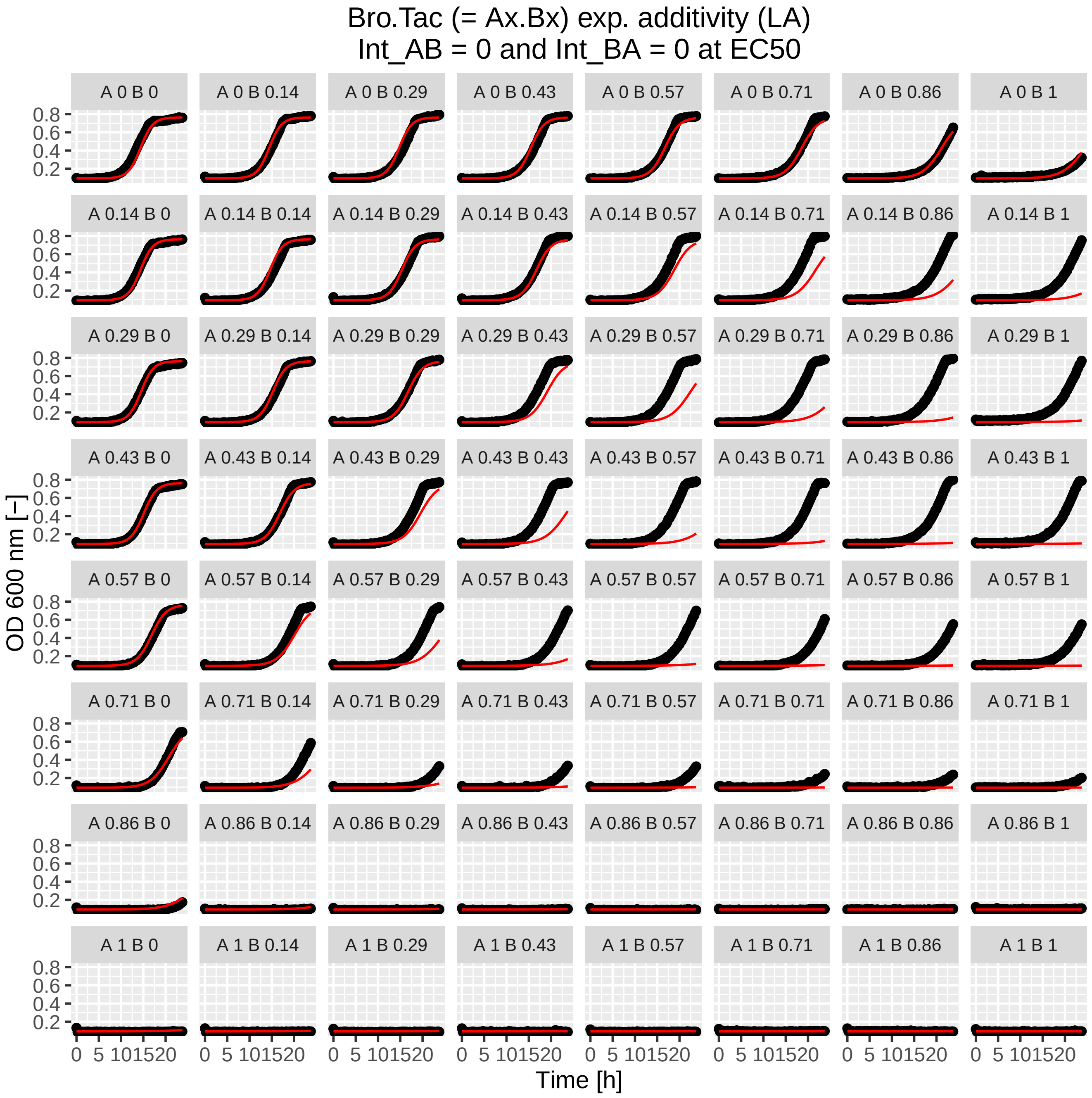

Bro.Tac (= Ax.Bx) exp. additivity (LA)
Int_AB = 0 and Int_BA = 0 at EC50
A 0 B 0
A 0 B 0.14
A 0 B 0.29
A 0 B 0.43
A 0 B 0.57
A 0 B 0.71
A 0 B 0.86
A 0 B 1
A 0.14 B 0
A 0.14 B 0.14
A 0.14 B 0.29
A 0.14 B 0.43
A 0.14 B 0.57
A 0.14 B 0.71
A 0.14 B 0.86
A 0.14 B 1
A 0.29 B 0
A 0.29 B 0.14
A 0.29 B 0.29
A 0.29 B 0.43
A 0.29 B 0.57
A 0.29 B 0.71
A 0.29 B 0.86
A 0.29 B 1
A 0.43 B 0
A 0.43 B 0.14
A 0.43 B 0.29
A 0.43 B 0.43
A 0.43 B 0.57
A 0.43 B 0.71
A 0.43 B 0.86
A 0.43 B 1
A 0.57 B 0
A 0.57 B 0.14
A 0.57 B 0.29
A 0.57 B 0.43
A 0.57 B 0.57
A 0.57 B 0.71
A 0.57 B 0.86
A 0.57 B 1
A 0.71 B 0
A 0.71 B 0.14
A 0.71 B 0.29
A 0.71 B 0.43
A 0.71 B 0.57
A 0.71 B 0.71
A 0.71 B 0.86
A 0.71 B 1
A 0.86 B 0
A 0.86 B 0.14
A 0.86 B 0.29
A 0.86 B 0.43
A 0.86 B 0.57
A 0.86 B 0.71
A 0.86 B 0.86
A 0.86 B 1
A 1 B 0
A 1 B 0.14
A 1 B 0.29
A 1 B 0.43
A 1 B 0.57
A 1 B 0.71
A 1 B 0.86
A 1 B 1
0.8
0.6
0.4
0.2
OD 600 nm [–]
0 5 101520
Time [h]

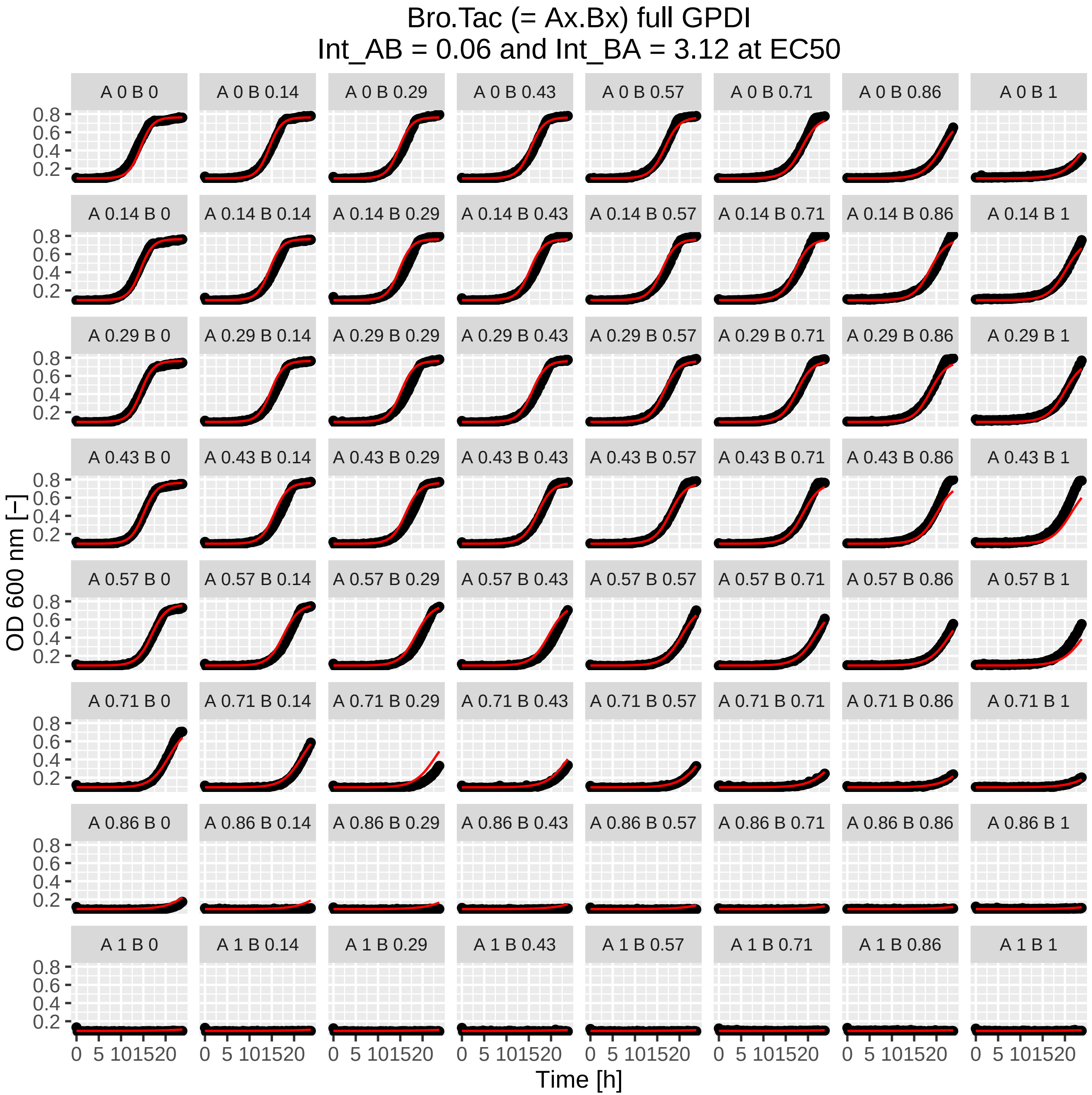

Bro.Tac (= Ax.Bx) full GPDI
Int_AB = 0.06 and Int_BA = 3.12 at EC50
OD 600 nm [–]
Time [h]

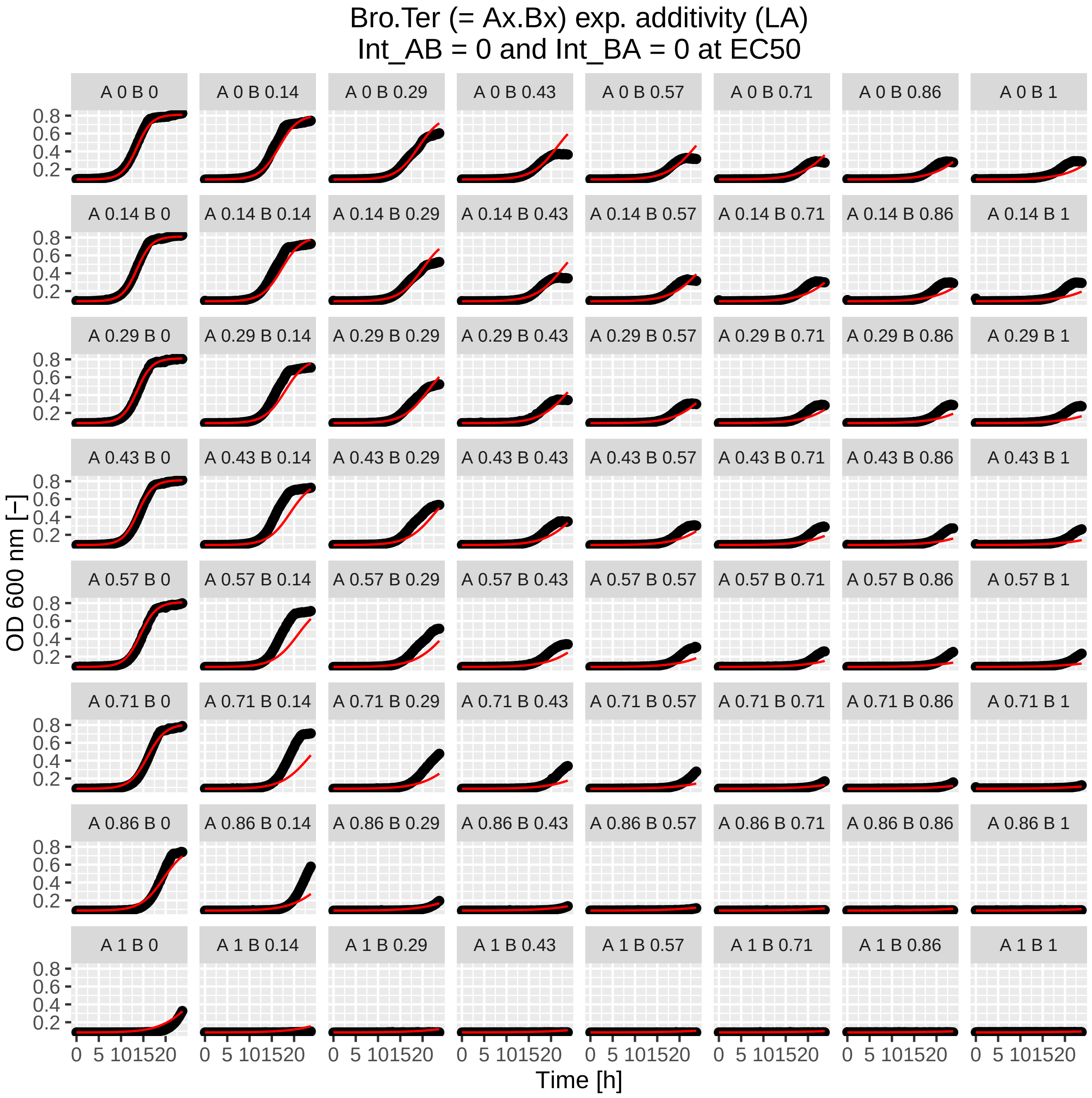
Bro.Ter (= Ax.Bx) exp. additivity (LA)
Int_AB = 0 and Int_BA = 0 at EC50
A 0 B 0
A 0 B 0.14
A 0 B 0.29
A 0 B 0.43
A 0 B 0.57
A 0 B 0.71
A 0 B 0.86
A 0 B 1
A 0.14 B 0
A 0.14 B 0.14
A 0.14 B 0.29
A 0.14 B 0.43
A 0.14 B 0.57
A 0.14 B 0.71
A 0.14 B 0.86
A 0.14 B 1
A 0.29 B 0
A 0.29 B 0.14
A 0.29 B 0.29
A 0.29 B 0.43
A 0.29 B 0.57
A 0.29 B 0.71
A 0.29 B 0.86
A 0.29 B 1
A 0.43 B 0
A 0.43 B 0.14
A 0.43 B 0.29
A 0.43 B 0.43
A 0.43 B 0.57
A 0.43 B 0.71
A 0.43 B 0.86
A 0.43 B 1
A 0.57 B 0
A 0.57 B 0.14
A 0.57 B 0.29
A 0.57 B 0.43
A 0.57 B 0.57
A 0.57 B 0.71
A 0.57 B 0.86
A 0.57 B 1
A 0.71 B 0
A 0.71 B 0.14
A 0.71 B 0.29
A 0.71 B 0.43
A 0.71 B 0.57
A 0.71 B 0.71
A 0.71 B 0.86
A 0.71 B 1
A 0.86 B 0
A 0.86 B 0.14
A 0.86 B 0.29
A 0.86 B 0.43
A 0.86 B 0.57
A 0.86 B 0.71
A 0.86 B 0.86
A 0.86 B 1
A 1 B 0
A 1 B 0.14
A 1 B 0.29
A 1 B 0.43
A 1 B 0.57
A 1 B 0.71
A 1 B 0.86
A 1 B 1
OD 600 nm [–]
0.8
0.6
0.4
0.2
0 5 101520
Time [h]

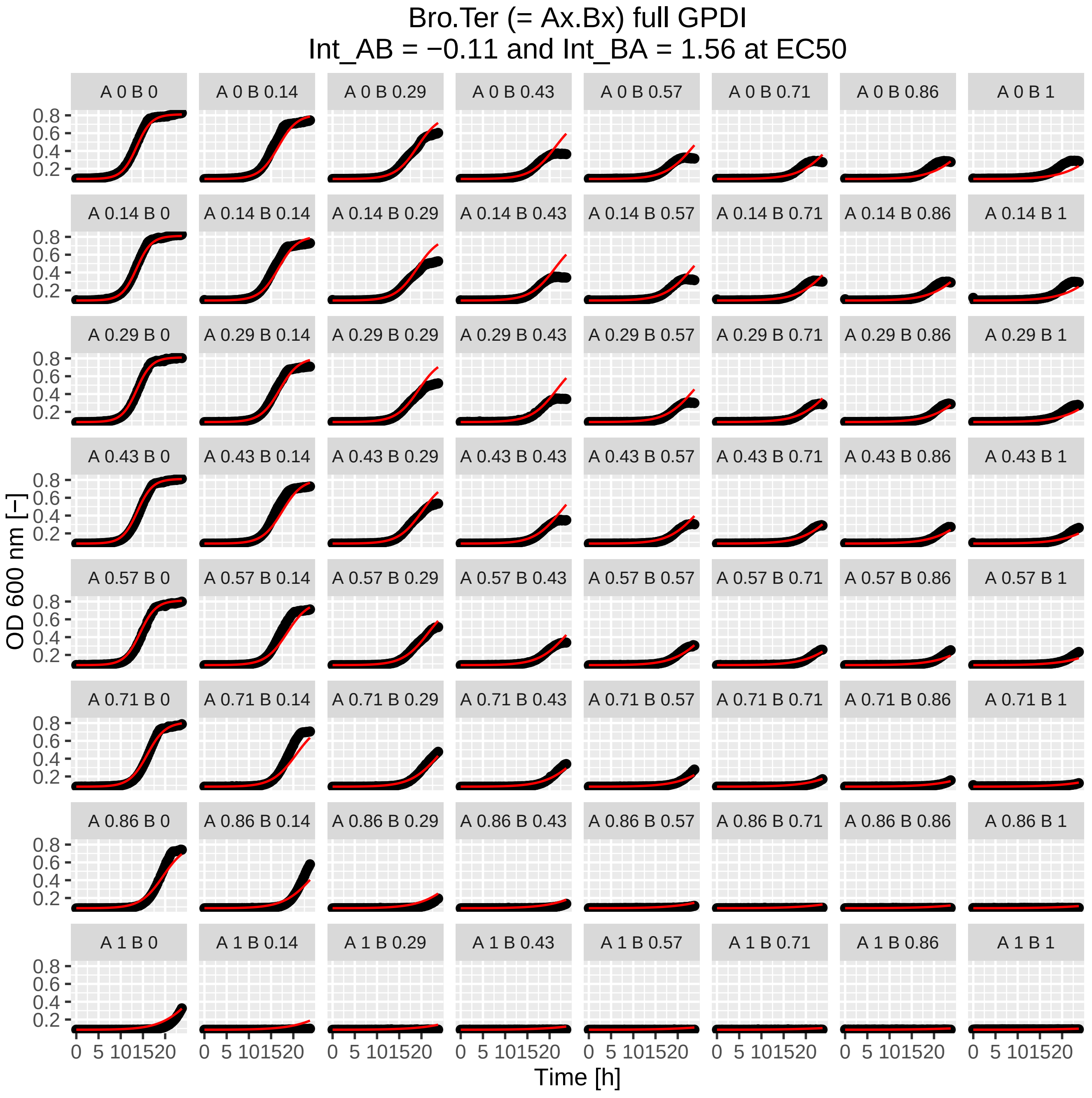
Bro.Ter (= Ax.Bx) full GPDI
Int_AB = −0.11 and Int_BA = 1.56 at EC50
A 0 B 0
A 0 B 0.14
A 0 B 0.29
A 0 B 0.43
A 0 B 0.57
A 0 B 0.71
A 0 B 0.86
A 0 B 1
A 0.14 B 0
A 0.14 B 0.14
A 0.14 B 0.29
A 0.14 B 0.43
A 0.14 B 0.57
A 0.14 B 0.71
A 0.14 B 0.86
A 0.14 B 1
A 0.29 B 0
A 0.29 B 0.14
A 0.29 B 0.29
A 0.29 B 0.43
A 0.29 B 0.57
A 0.29 B 0.71
A 0.29 B 0.86
A 0.29 B 1
A 0.43 B 0
A 0.43 B 0.14
A 0.43 B 0.29
A 0.43 B 0.43
A 0.43 B 0.57
A 0.43 B 0.71
A 0.43 B 0.86
A 0.43 B 1
A 0.57 B 0
A 0.57 B 0.14
A 0.57 B 0.29
A 0.57 B 0.43
A 0.57 B 0.57
A 0.57 B 0.71
A 0.57 B 0.86
A 0.57 B 1
A 0.71 B 0
A 0.71 B 0.14
A 0.71 B 0.29
A 0.71 B 0.43
A 0.71 B 0.57
A 0.71 B 0.71
A 0.71 B 0.86
A 0.71 B 1
A 0.86 B 0
A 0.86 B 0.14
A 0.86 B 0.29
A 0.86 B 0.43
A 0.86 B 0.57
A 0.86 B 0.71
A 0.86 B 0.86
A 0.86 B 1
A 1 B 0
A 1 B 0.14
A 1 B 0.29
A 1 B 0.43
A 1 B 0.57
A 1 B 0.71
A 1 B 0.86
A 1 B 1
OD 600 nm [−]
0.8
0.6
0.4
0.2
0 5 101520
Time [h]

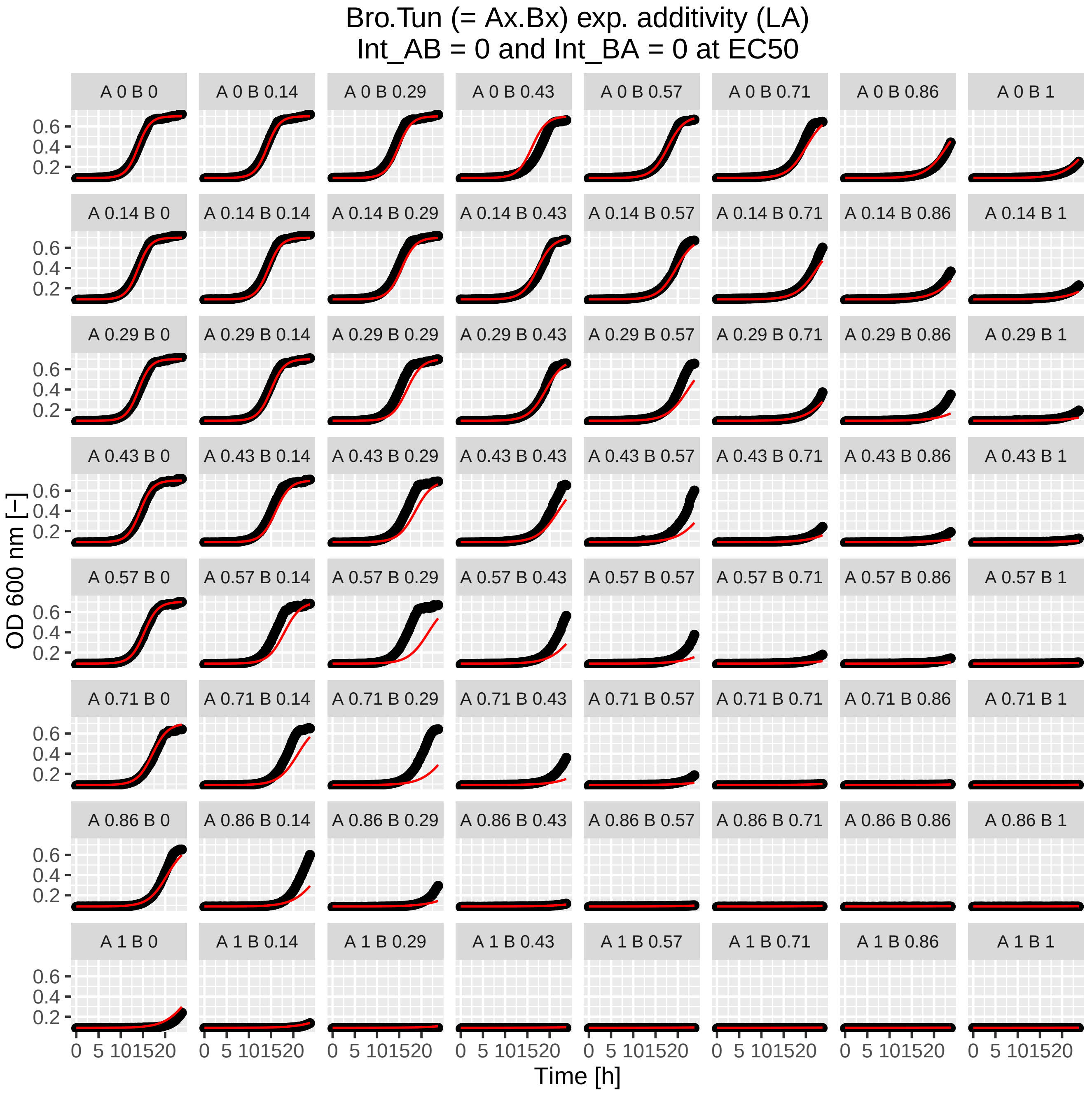

Bro.Tun (= Ax.Bx) exp. additivity (LA)
Int_AB = 0 and Int_BA = 0 at EC50
OD 600 nm [–]
Time [h]
A 0 B 0
A 0 B 0.14
A 0 B 0.29
A 0 B 0.43
A 0 B 0.57
A 0 B 0.71
A 0 B 0.86
A 0 B 1
A 1 B 0
A 1 B 0.14
A 1 B 0.29
A 1 B 0.43
A 1 B 0.57
A 1 B 0.71
A 1 B 0.86
A 1 B 1

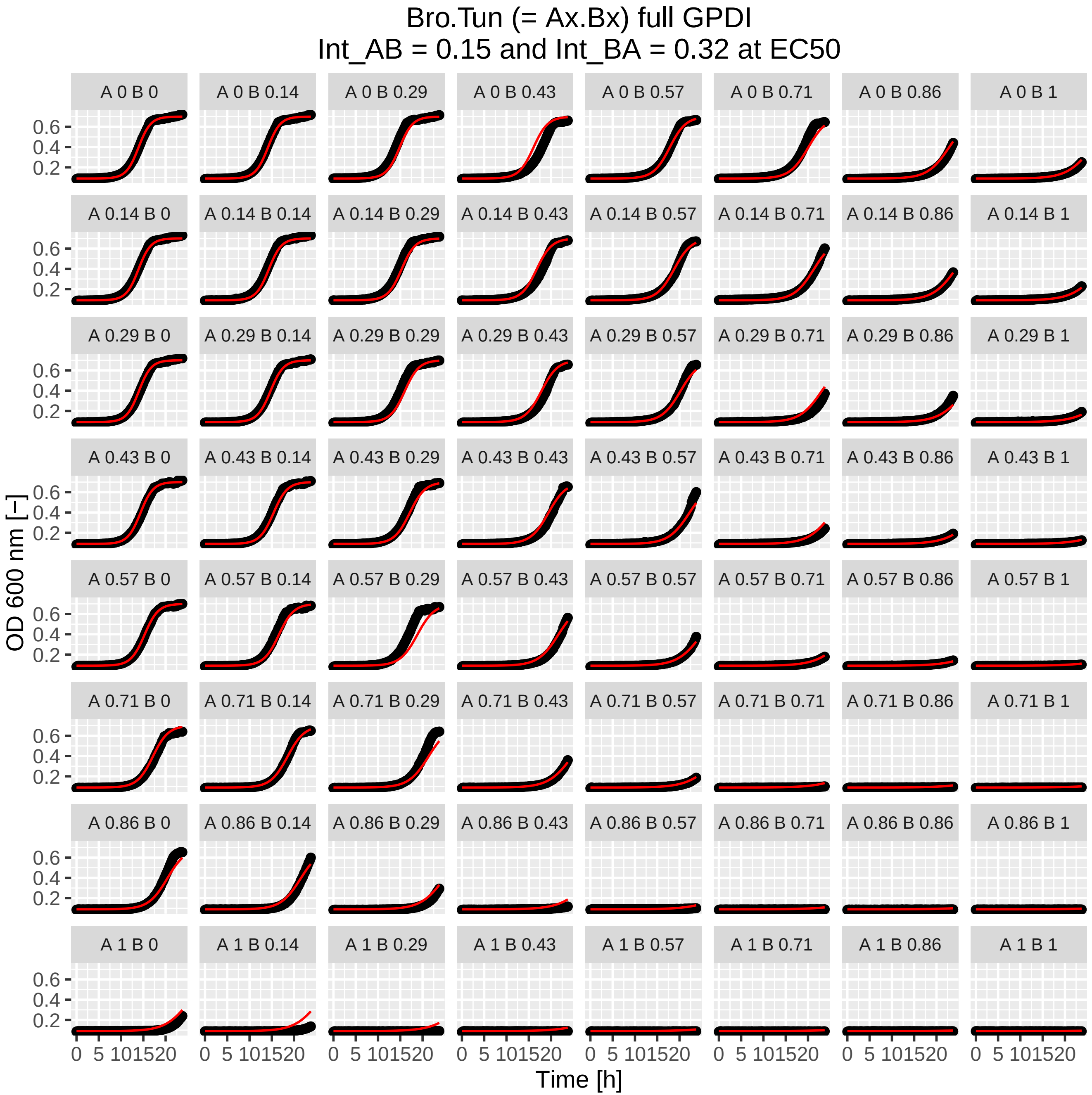

Bro.Tun (= Ax.Bx) full GPDI
Int_AB = 0.15 and Int_BA = 0.32 at EC50
A 0 B 0
A 0 B 0.14
A 0 B 0.29
A 0 B 0.43
A 0 B 0.57
A 0 B 0.71
A 0 B 0.86
A 0 B 1
A 0.14 B 0
A 0.14 B 0.14
A 0.14 B 0.29
A 0.14 B 0.43
A 0.14 B 0.57
A 0.14 B 0.71
A 0.14 B 0.86
A 0.14 B 1
A 0.29 B 0
A 0.29 B 0.14
A 0.29 B 0.29
A 0.29 B 0.43
A 0.29 B 0.57
A 0.29 B 0.71
A 0.29 B 0.86
A 0.29 B 1
A 0.43 B 0
A 0.43 B 0.14
A 0.43 B 0.29
A 0.43 B 0.43
A 0.43 B 0.57
A 0.43 B 0.71
A 0.43 B 0.86
A 0.43 B 1
A 0.57 B 0
A 0.57 B 0.14
A 0.57 B 0.29
A 0.57 B 0.43
A 0.57 B 0.57
A 0.57 B 0.71
A 0.57 B 0.86
A 0.57 B 1
A 0.71 B 0
A 0.71 B 0.14
A 0.71 B 0.29
A 0.71 B 0.43
A 0.71 B 0.57
A 0.71 B 0.71
A 0.71 B 0.86
A 0.71 B 1
A 0.86 B 0
A 0.86 B 0.14
A 0.86 B 0.29
A 0.86 B 0.43
A 0.86 B 0.57
A 0.86 B 0.71
A 0.86 B 0.86
A 0.86 B 1
A 1 B 0
A 1 B 0.14
A 1 B 0.29
A 1 B 0.43
A 1 B 0.57
A 1 B 0.71
A 1 B 0.86
A 1 B 1
OD 600 nm [−]
0.6
0.4
0.2
0 5 101520
Time [h]

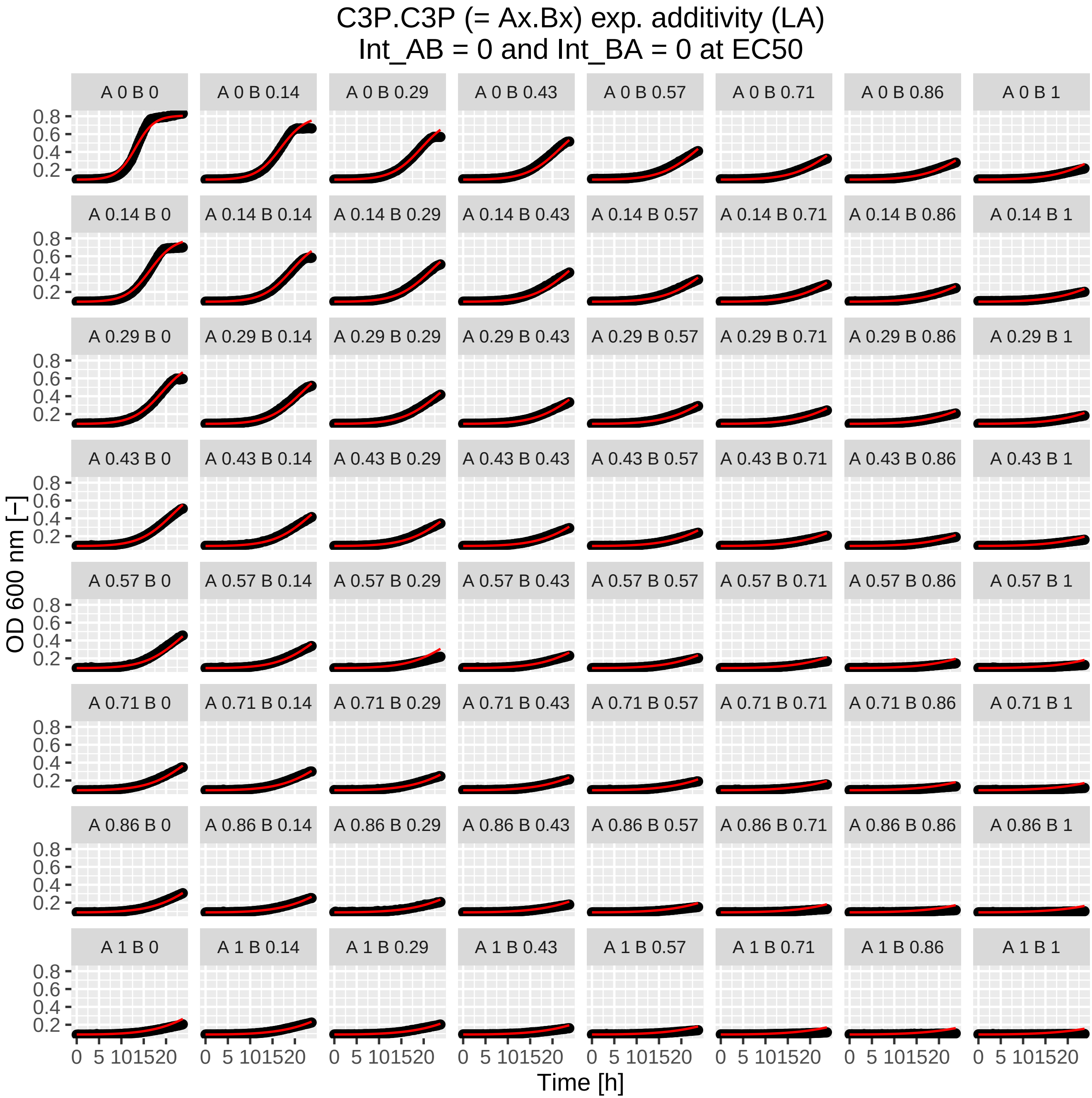

C3P.C3P (= Ax.Bx) exp. additivity (LA)
Int_AB = 0 and Int_BA = 0 at EC50
OD 600 nm [–]
Time [h]
A 0 B 0
A 0 B 0.14
A 0 B 0.29
A 0 B 0.43
A 0 B 0.57
A 0 B 0.71
A 0 B 0.86
A 0 B 1
A 0.14 B 0
A 0.14 B 0.14
A 0.14 B 0.29
A 0.14 B 0.43
A 0.14 B 0.57
A 0.14 B 0.71
A 0.14 B 0.86
A 0.14 B 1
A 0.29 B 0
A 0.29 B 0.14
A 0.29 B 0.29
A 0.29 B 0.43
A 0.29 B 0.57
A 0.29 B 0.71
A 0.29 B 0.86
A 0.29 B 1
A 0.43 B 0
A 0.43 B 0.14
A 0.43 B 0.29
A 0.43 B 0.43
A 0.43 B 0.57
A 0.43 B 0.71
A 0.43 B 0.86
A 0.43 B 1
A 0.57 B 0
A 0.57 B 0.14
A 0.57 B 0.29
A 0.57 B 0.43
A 0.57 B 0.57
A 0.57 B 0.71
A 0.57 B 0.86
A 0.57 B 1
A 0.71 B 0
A 0.71 B 0.14
A 0.71 B 0.29
A 0.71 B 0.43
A 0.71 B 0.57
A 0.71 B 0.71
A 0.71 B 0.86
A 0.71 B 1
A 0.86 B 0
A 0.86 B 0.14
A 0.86 B 0.29
A 0.86 B 0.43
A 0.86 B 0.57
A 0.86 B 0.71
A 0.86 B 0.86
A 0.86 B 1
A 1 B 0
A 1 B 0.14
A 1 B 0.29
A 1 B 0.43
A 1 B 0.57
A 1 B 0.71
A 1 B 0.86
A 1 B 1
0.8
0.6
0.4
0.2
0
5
10
15
20

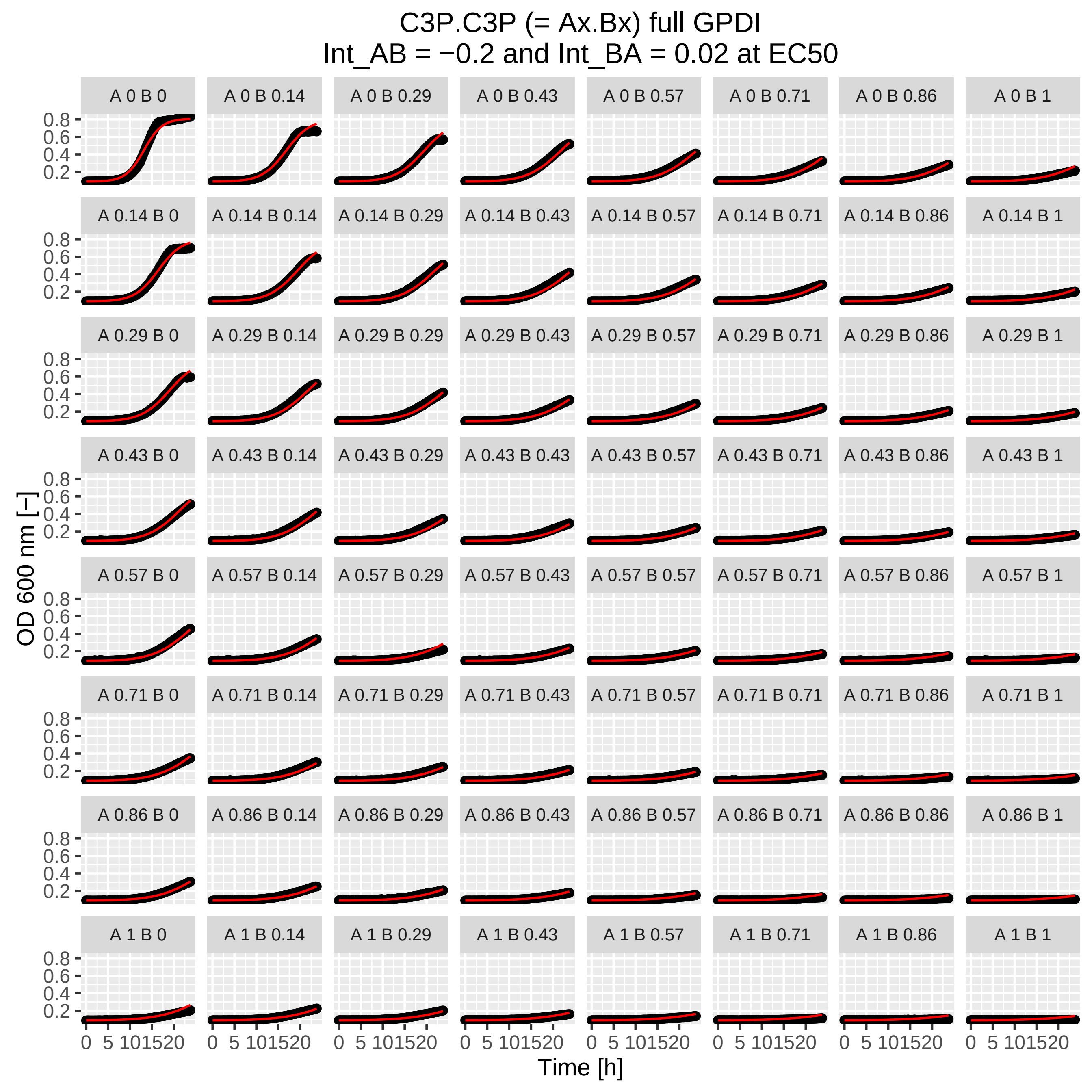

C3P.C3P (= Ax.Bx) full GPDI
Int_AB = −0.2 and Int_BA = 0.02 at EC50
A 0 B 0
A 0 B 0.14
A 0 B 0.29
A 0 B 0.43
A 0 B 0.57
A 0 B 0.71
A 0 B 0.86
A 0 B 1
A 0.14 B 0
A 0.14 B 0.14
A 0.14 B 0.29
A 0.14 B 0.43
A 0.14 B 0.57
A 0.14 B 0.71
A 0.14 B 0.86
A 0.14 B 1
A 0.29 B 0
A 0.29 B 0.14
A 0.29 B 0.29
A 0.29 B 0.43
A 0.29 B 0.57
A 0.29 B 0.71
A 0.29 B 0.86
A 0.29 B 1
A 0.43 B 0
A 0.43 B 0.14
A 0.43 B 0.29
A 0.43 B 0.43
A 0.43 B 0.57
A 0.43 B 0.71
A 0.43 B 0.86
A 0.43 B 1
A 0.57 B 0
A 0.57 B 0.14
A 0.57 B 0.29
A 0.57 B 0.43
A 0.57 B 0.57
A 0.57 B 0.71
A 0.57 B 0.86
A 0.57 B 1
A 0.71 B 0
A 0.71 B 0.14
A 0.71 B 0.29
A 0.71 B 0.43
A 0.71 B 0.57
A 0.71 B 0.71
A 0.71 B 0.86
A 0.71 B 1
A 0.86 B 0
A 0.86 B 0.14
A 0.86 B 0.29
A 0.86 B 0.43
A 0.86 B 0.57
A 0.86 B 0.71
A 0.86 B 0.86
A 0.86 B 1
A 1 B 0
A 1 B 0.14
A 1 B 0.29
A 1 B 0.43
A 1 B 0.57
A 1 B 0.71
A 1 B 0.86
A 1 B 1
OD 600 nm [−]
0.2
0.4
0.6
0.8
0
5
10
15
20
Time [h]

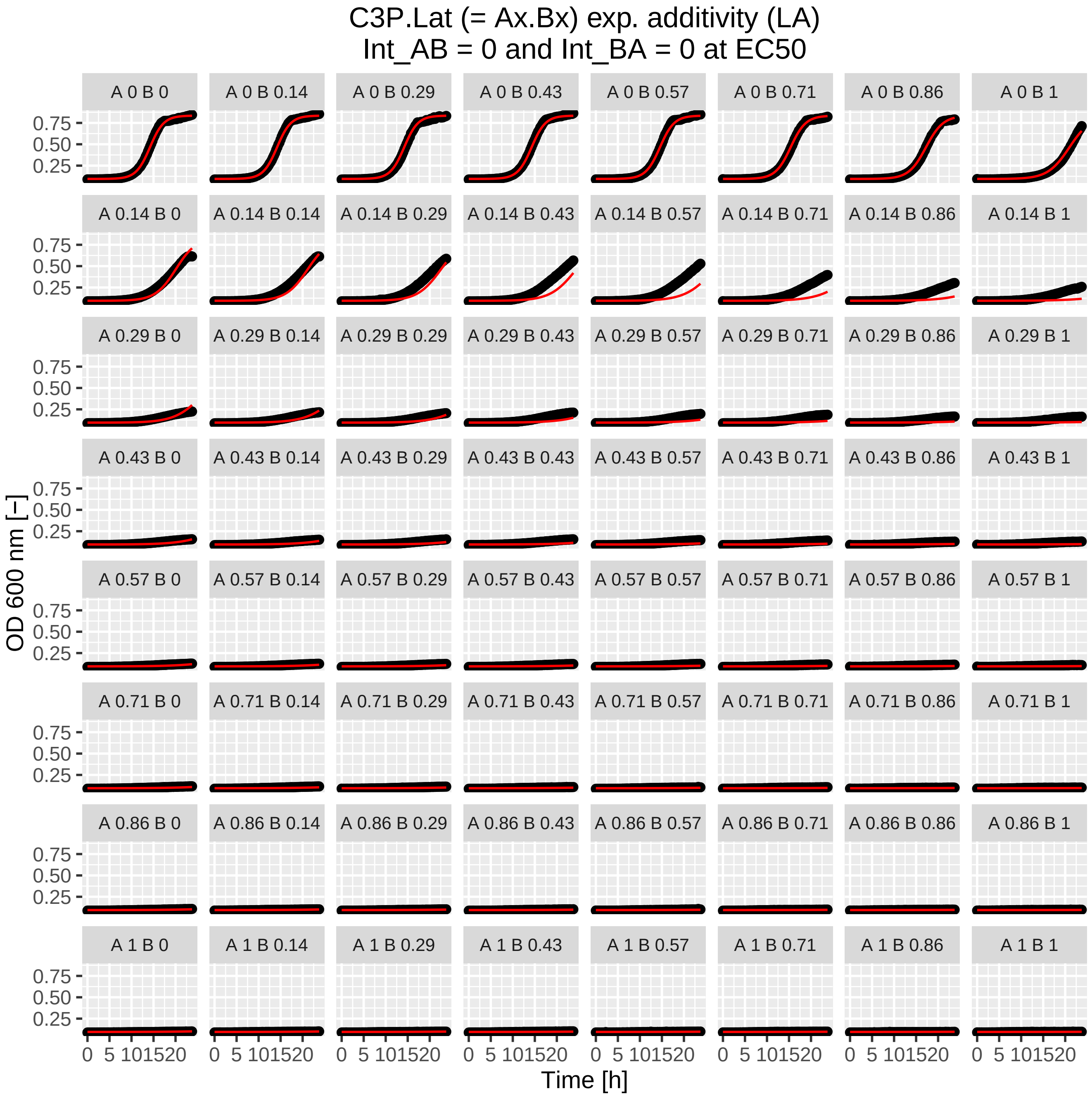

C3P.Lat (= Ax.Bx) exp. additivity (LA)
Int_AB = 0 and Int_BA = 0 at EC50
A 0 B 0
A 0 B 0.14
A 0 B 0.29
A 0 B 0.43
A 0 B 0.57
A 0 B 0.71
A 0 B 0.86
A 0 B 1
A 0.14 B 0
A 0.14 B 0.14
A 0.14 B 0.29
A 0.14 B 0.43
A 0.14 B 0.57
A 0.14 B 0.71
A 0.14 B 0.86
A 0.14 B 1
A 0.29 B 0
A 0.29 B 0.14
A 0.29 B 0.29
A 0.29 B 0.43
A 0.29 B 0.57
A 0.29 B 0.71
A 0.29 B 0.86
A 0.29 B 1
A 0.43 B 0
A 0.43 B 0.14
A 0.43 B 0.29
A 0.43 B 0.43
A 0.43 B 0.57
A 0.43 B 0.71
A 0.43 B 0.86
A 0.43 B 1
A 0.57 B 0
A 0.57 B 0.14
A 0.57 B 0.29
A 0.57 B 0.43
A 0.57 B 0.57
A 0.57 B 0.71
A 0.57 B 0.86
A 0.57 B 1
A 0.71 B 0
A 0.71 B 0.14
A 0.71 B 0.29
A 0.71 B 0.43
A 0.71 B 0.57
A 0.71 B 0.71
A 0.71 B 0.86
A 0.71 B 1
A 0.86 B 0
A 0.86 B 0.14
A 0.86 B 0.29
A 0.86 B 0.43
A 0.86 B 0.57
A 0.86 B 0.71
A 0.86 B 0.86
A 0.86 B 1
A 1 B 0
A 1 B 0.14
A 1 B 0.29
A 1 B 0.43
A 1 B 0.57
A 1 B 0.71
A 1 B 0.86
A 1 B 1
0.75
0.50
0.25
OD 600 nm [–]
0 5 101520
Time [h]

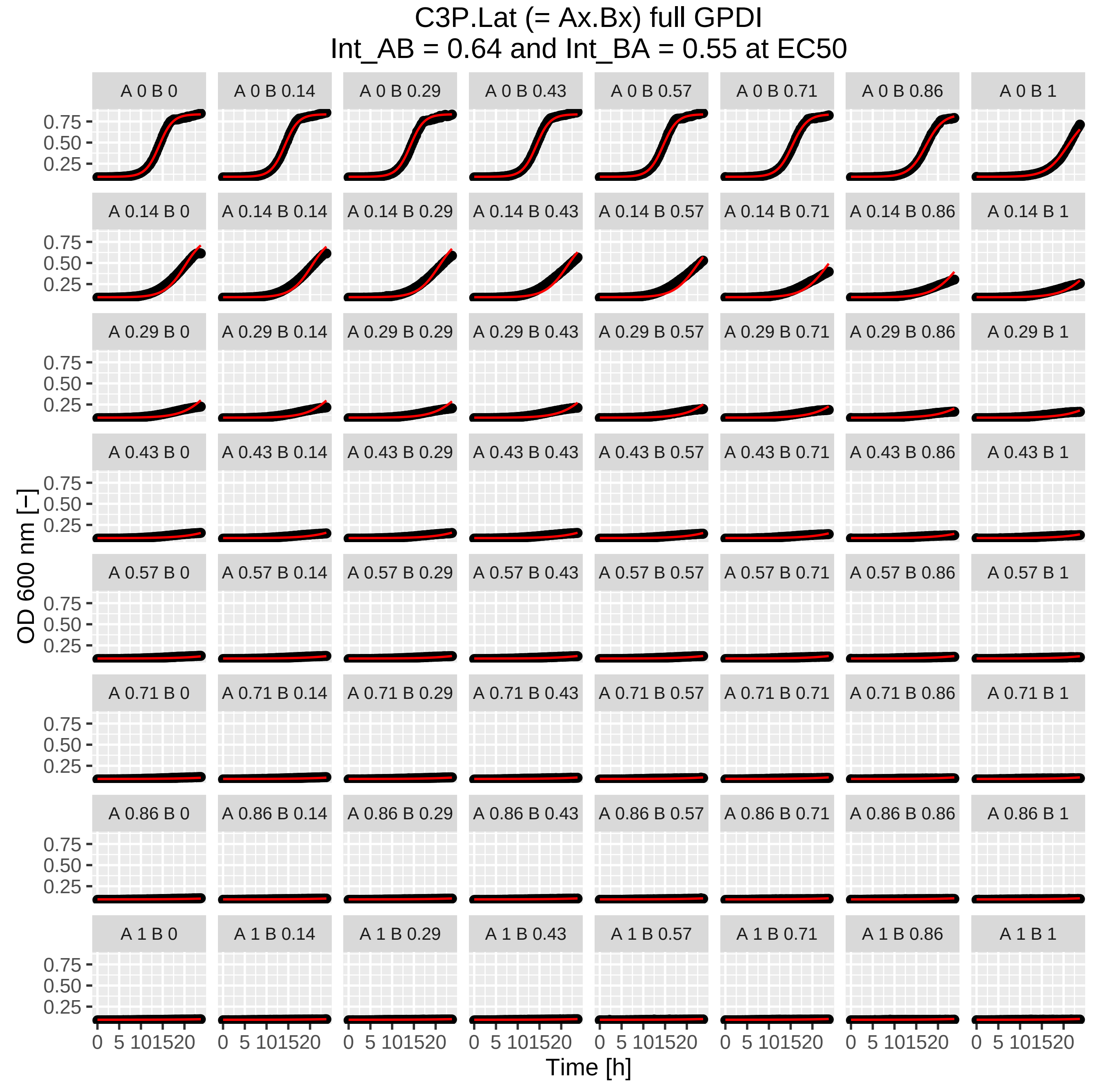
C3P.Lat (= Ax.Bx) full GPDI
Int_AB = 0.64 and Int_BA = 0.55 at EC50
OD 600 nm [−]
Time [h]

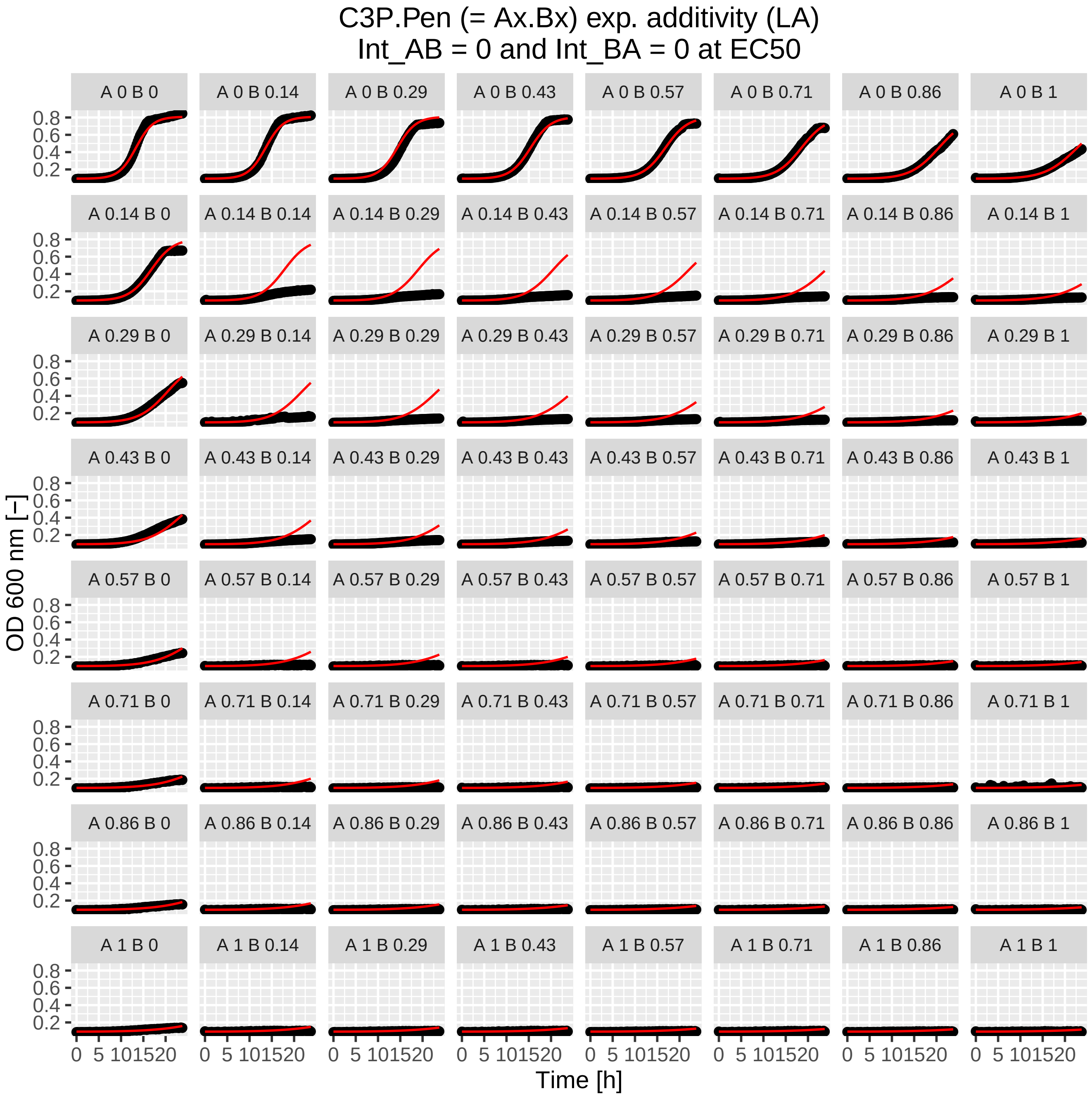
C3P.Pen (= Ax.Bx) exp. additivity (LA)
Int_AB = 0 and Int_BA = 0 at EC50
OD 600 nm [−]
Time [h]
A 0 B 0
A 0 B 0.14
A 0 B 0.29
A 0 B 0.43
A 0 B 0.57
A 0 B 0.71
A 0 B 0.86
A 0 B 1
A 0.14 B 0
A 0.29 B 0
A 0.43 B 0
A 0.57 B 0
A 0.71 B 0
A 0.86 B 0
A 1 B 0
A 1 B 1
0.8
0.6
0.4
0.2
0 5 10 15 20

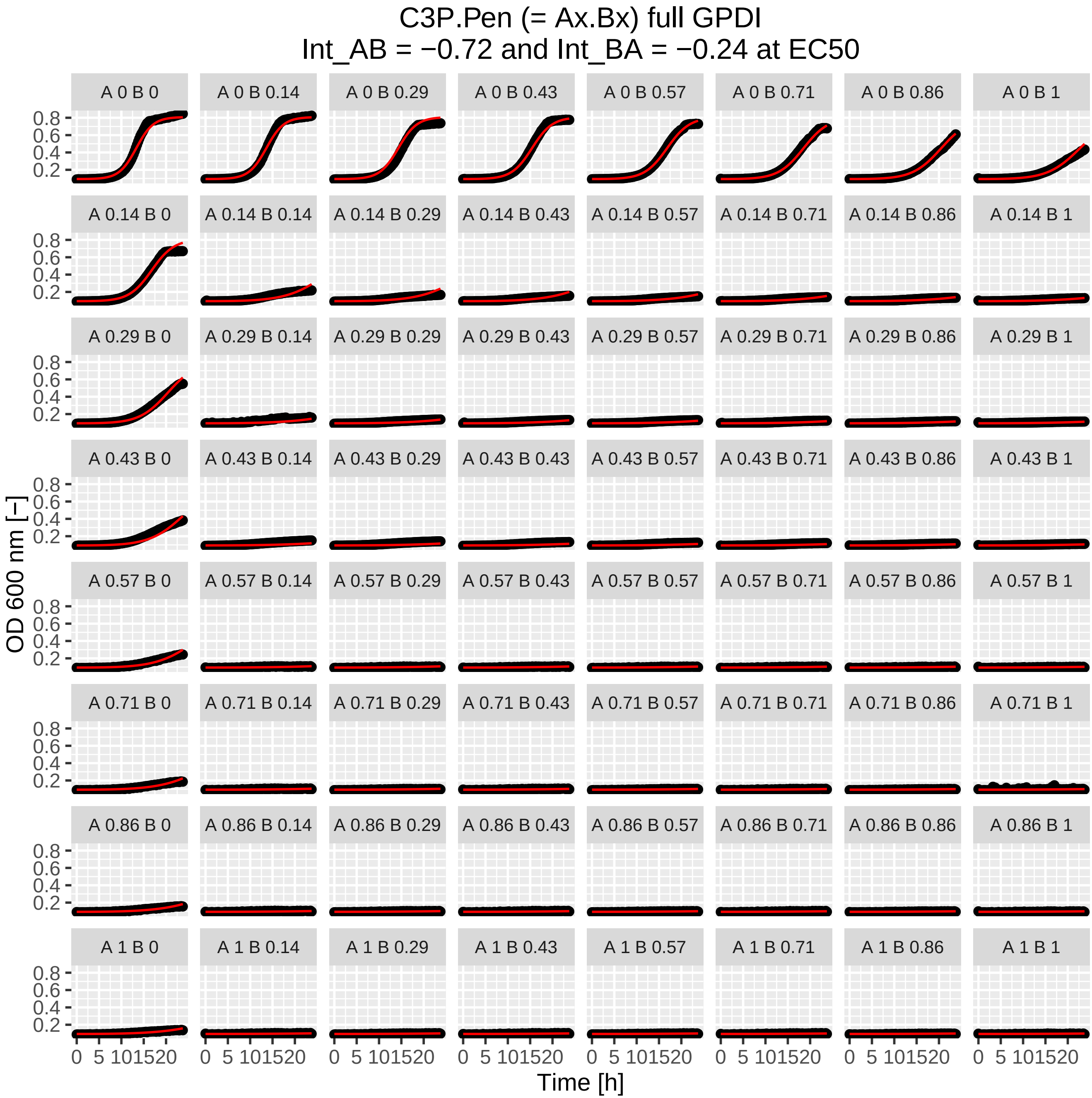

C3P.Pen (= Ax.Bx) full GPDI
Int_AB = −0.72 and Int_BA = −0.24 at EC50
A 0 B 0
A 0 B 0.14
A 0 B 0.29
A 0 B 0.43
A 0 B 0.57
A 0 B 0.71
A 0 B 0.86
A 0 B 1
A 0.14 B 0
A 0.14 B 0.14
A 0.14 B 0.29
A 0.14 B 0.43
A 0.14 B 0.57
A 0.14 B 0.71
A 0.14 B 0.86
A 0.14 B 1
A 0.29 B 0
A 0.29 B 0.14
A 0.29 B 0.29
A 0.29 B 0.43
A 0.29 B 0.57
A 0.29 B 0.71
A 0.29 B 0.86
A 0.29 B 1
A 0.43 B 0
A 0.43 B 0.14
A 0.43 B 0.29
A 0.43 B 0.43
A 0.43 B 0.57
A 0.43 B 0.71
A 0.43 B 0.86
A 0.43 B 1
A 0.57 B 0
A 0.57 B 0.14
A 0.57 B 0.29
A 0.57 B 0.43
A 0.57 B 0.57
A 0.57 B 0.71
A 0.57 B 0.86
A 0.57 B 1
A 0.71 B 0
A 0.71 B 0.14
A 0.71 B 0.29
A 0.71 B 0.43
A 0.71 B 0.57
A 0.71 B 0.71
A 0.71 B 0.86
A 0.71 B 1
A 0.86 B 0
A 0.86 B 0.14
A 0.86 B 0.29
A 0.86 B 0.43
A 0.86 B 0.57
A 0.86 B 0.71
A 0.86 B 0.86
A 0.86 B 1
A 1 B 0
A 1 B 0.14
A 1 B 0.29
A 1 B 0.43
A 1 B 0.57
A 1 B 0.71
A 1 B 0.86
A 1 B 1
OD 600 nm [−]
0.2
0.4
0.6
0.8
0 5 101520
Time [h]

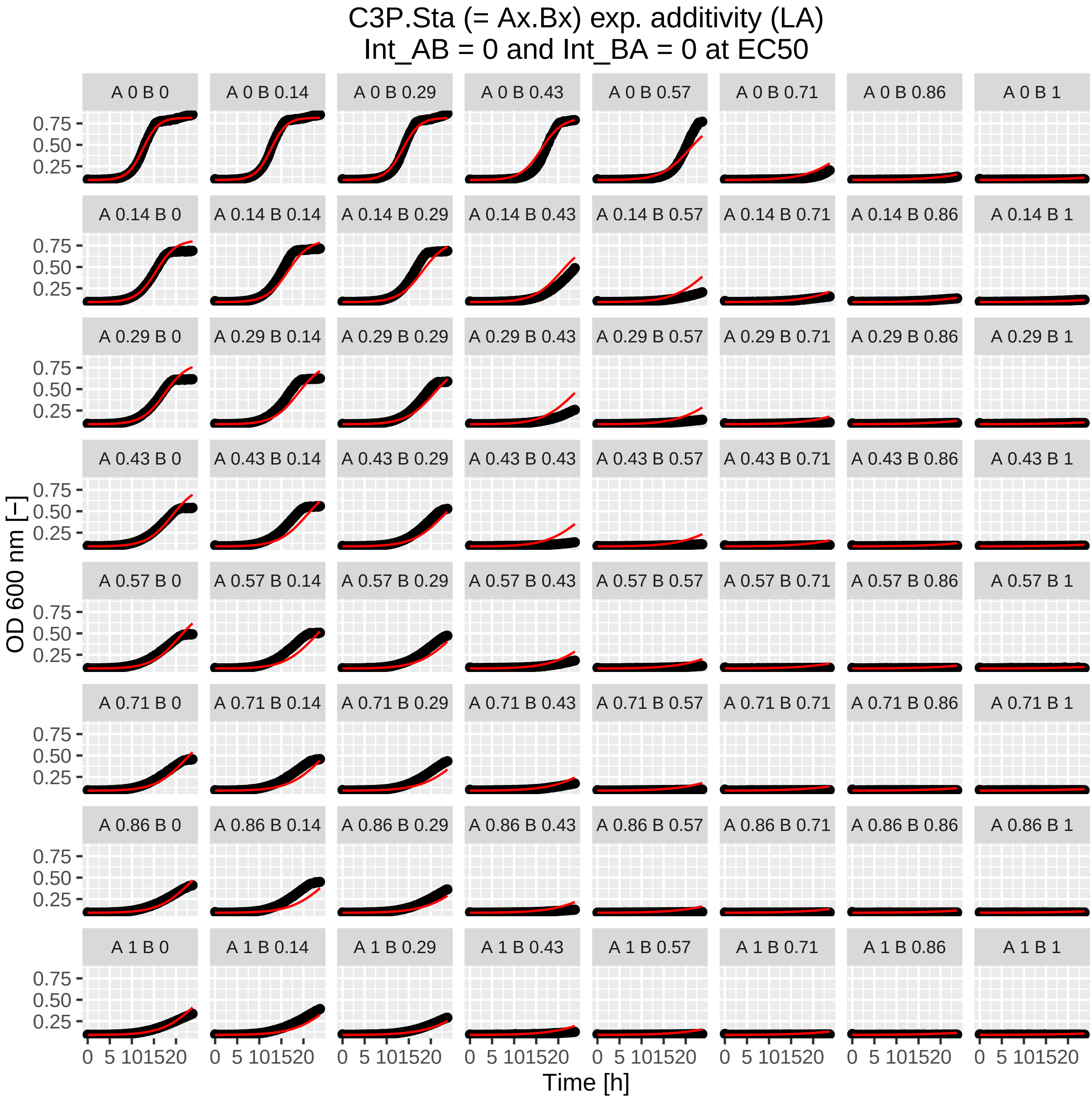

C3P.Sta (= Ax.Bx) exp. additivity (LA)
Int_AB = 0 and Int_BA = 0 at EC50
OD 600 nm [–]
Time [h]
A 0 B 0
A 0 B 0.14
A 0 B 0.29
A 0 B 0.43
A 0 B 0.57
A 0 B 0.71
A 0 B 0.86
A 0 B 1
A 0.14 B 0
A 0.14 B 0.14
A 0.14 B 0.29
A 0.14 B 0.43
A 0.14 B 0.57
A 0.14 B 0.71
A 0.14 B 0.86
A 0.14 B 1
A 0.29 B 0
A 0.29 B 0.14
A 0.29 B 0.29
A 0.29 B 0.43
A 0.29 B 0.57
A 0.29 B 0.71
A 0.29 B 0.86
A 0.29 B 1
A 0.43 B 0
A 0.43 B 0.14
A 0.43 B 0.29
A 0.43 B 0.43
A 0.43 B 0.57
A 0.43 B 0.71
A 0.43 B 0.86
A 0.43 B 1
A 0.57 B 0
A 0.57 B 0.14
A 0.57 B 0.29
A 0.57 B 0.43
A 0.57 B 0.57
A 0.57 B 0.71
A 0.57 B 0.86
A 0.57 B 1
A 0.71 B 0
A 0.71 B 0.14
A 0.71 B 0.29
A 0.71 B 0.43
A 0.71 B 0.57
A 0.71 B 0.71
A 0.71 B 0.86
A 0.71 B 1
A 0.86 B 0
A 0.86 B 0.14
A 0.86 B 0.29
A 0.86 B 0.43
A 0.86 B 0.57
A 0.86 B 0.71
A 0.86 B 0.86
A 0.86 B 1
A 1 B 0
A 1 B 0.14
A 1 B 0.29
A 1 B 0.43
A 1 B 0.57
A 1 B 0.71
A 1 B 0.86
A 1 B 1
0.75
0.50
0.25
0 5 101520

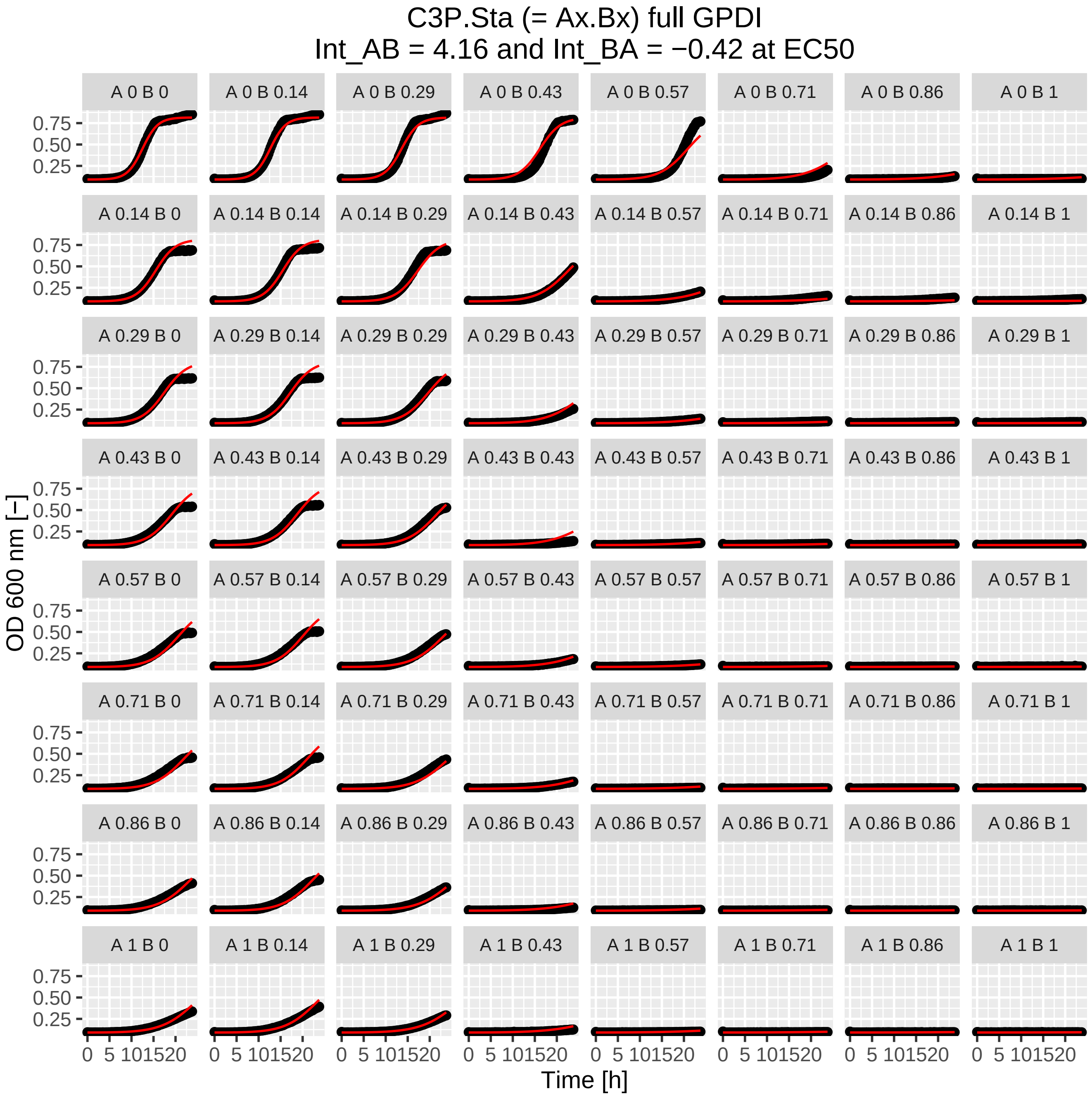

C3P.Sta (= Ax.Bx) full GPDI
Int_AB = 4.16 and Int_BA = −0.42 at EC50
A 0 B 0
A 0 B 0.14
A 0 B 0.29
A 0 B 0.43
A 0 B 0.57
A 0 B 0.71
A 0 B 0.86
A 0 B 1
A 0.14 B 0
A 0.14 B 0.14
A 0.14 B 0.29
A 0.14 B 0.43
A 0.14 B 0.57
A 0.14 B 0.71
A 0.14 B 0.86
A 0.14 B 1
A 0.29 B 0
A 0.29 B 0.14
A 0.29 B 0.29
A 0.29 B 0.43
A 0.29 B 0.57
A 0.29 B 0.71
A 0.29 B 0.86
A 0.29 B 1
A 0.43 B 0
A 0.43 B 0.14
A 0.43 B 0.29
A 0.43 B 0.43
A 0.43 B 0.57
A 0.43 B 0.71
A 0.43 B 0.86
A 0.43 B 1
A 0.57 B 0
A 0.57 B 0.14
A 0.57 B 0.29
A 0.57 B 0.43
A 0.57 B 0.57
A 0.57 B 0.71
A 0.57 B 0.86
A 0.57 B 1
A 0.71 B 0
A 0.71 B 0.14
A 0.71 B 0.29
A 0.71 B 0.43
A 0.71 B 0.57
A 0.71 B 0.71
A 0.71 B 0.86
A 0.71 B 1
A 0.86 B 0
A 0.86 B 0.14
A 0.86 B 0.29
A 0.86 B 0.43
A 0.86 B 0.57
A 0.86 B 0.71
A 0.86 B 0.86
A 0.86 B 1
A 1 B 0
A 1 B 0.14
A 1 B 0.29
A 1 B 0.43
A 1 B 0.57
A 1 B 0.71
A 1 B 0.86
A 1 B 1
0.75
0.50
0.25
OD 600 nm [−]
0 5 101520
Time [h]

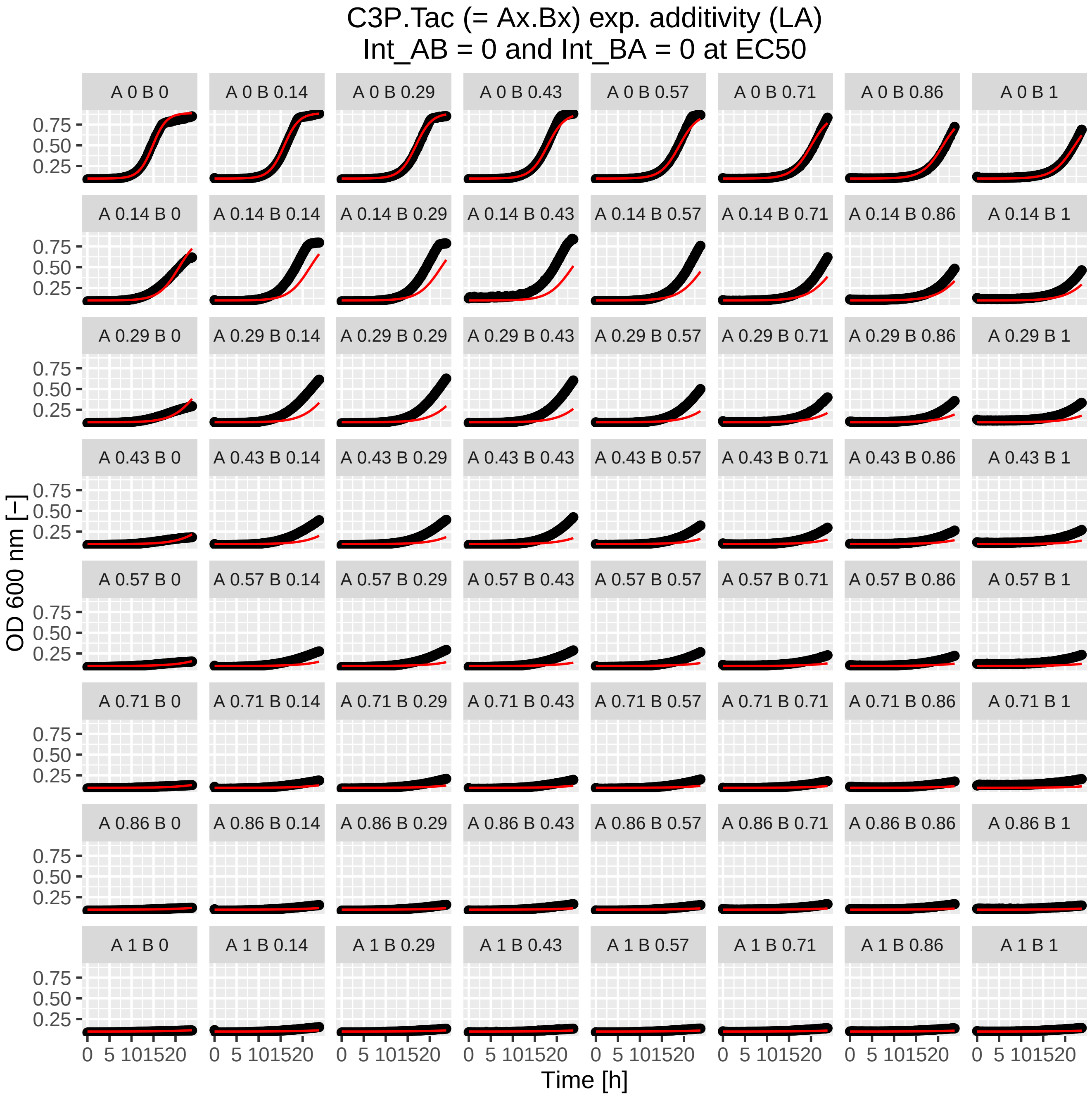

C3P.Tac (= Ax.Bx) exp. additivity (LA)
Int_AB = 0 and Int_BA = 0 at EC50
A 0 B 0
A 0 B 0.14
A 0 B 0.29
A 0 B 0.43
A 0 B 0.57
A 0 B 0.71
A 0 B 0.86
A 0 B 1
A 0.14 B 0
A 0.14 B 0.14
A 0.14 B 0.29
A 0.14 B 0.43
A 0.14 B 0.57
A 0.14 B 0.71
A 0.14 B 0.86
A 0.14 B 1
A 0.29 B 0
A 0.29 B 0.14
A 0.29 B 0.29
A 0.29 B 0.43
A 0.29 B 0.57
A 0.29 B 0.71
A 0.29 B 0.86
A 0.29 B 1
A 0.43 B 0
A 0.43 B 0.14
A 0.43 B 0.29
A 0.43 B 0.43
A 0.43 B 0.57
A 0.43 B 0.71
A 0.43 B 0.86
A 0.43 B 1
A 0.57 B 0
A 0.57 B 0.14
A 0.57 B 0.29
A 0.57 B 0.43
A 0.57 B 0.57
A 0.57 B 0.71
A 0.57 B 0.86
A 0.57 B 1
A 0.71 B 0
A 0.71 B 0.14
A 0.71 B 0.29
A 0.71 B 0.43
A 0.71 B 0.57
A 0.71 B 0.71
A 0.71 B 0.86
A 0.71 B 1
A 0.86 B 0
A 0.86 B 0.14
A 0.86 B 0.29
A 0.86 B 0.43
A 0.86 B 0.57
A 0.86 B 0.71
A 0.86 B 0.86
A 0.86 B 1
A 1 B 0
A 1 B 0.14
A 1 B 0.29
A 1 B 0.43
A 1 B 0.57
A 1 B 0.71
A 1 B 0.86
A 1 B 1
0.75
0.50
0.25
0 5 101520
OD 600 nm [–]
Time [h]

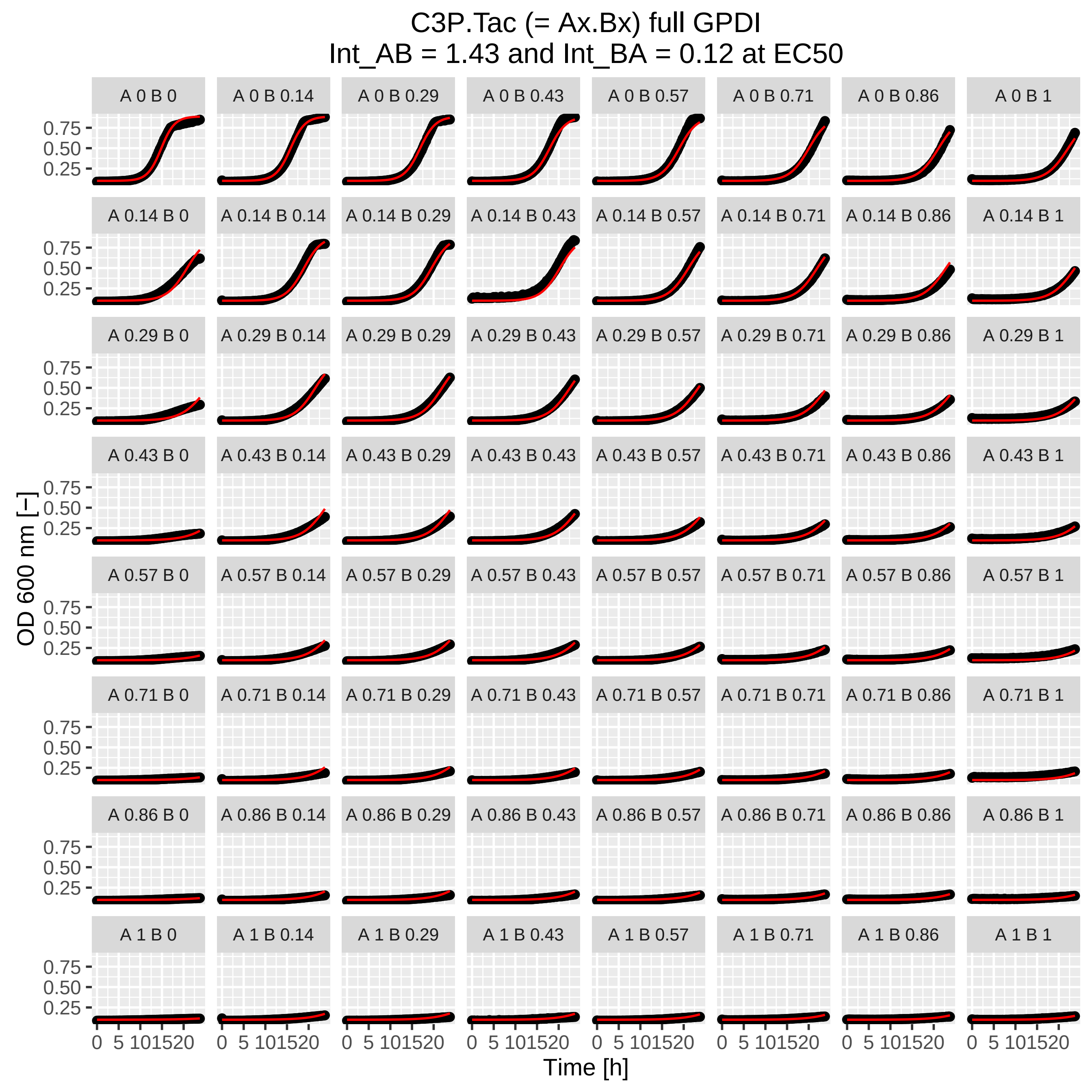

C3P.Tac (= Ax.Bx) full GPDI
Int_AB = 1.43 and Int_BA = 0.12 at EC50
A 0 B 0
A 0 B 0.14
A 0 B 0.29
A 0 B 0.43
A 0 B 0.57
A 0 B 0.71
A 0 B 0.86
A 0 B 1
A 0.14 B 0
A 0.14 B 0.14
A 0.14 B 0.29
A 0.14 B 0.43
A 0.14 B 0.57
A 0.14 B 0.71
A 0.14 B 0.86
A 0.14 B 1
A 0.29 B 0
A 0.29 B 0.14
A 0.29 B 0.29
A 0.29 B 0.43
A 0.29 B 0.57
A 0.29 B 0.71
A 0.29 B 0.86
A 0.29 B 1
A 0.43 B 0
A 0.43 B 0.14
A 0.43 B 0.29
A 0.43 B 0.43
A 0.43 B 0.57
A 0.43 B 0.71
A 0.43 B 0.86
A 0.43 B 1
A 0.57 B 0
A 0.57 B 0.14
A 0.57 B 0.29
A 0.57 B 0.43
A 0.57 B 0.57
A 0.57 B 0.71
A 0.57 B 0.86
A 0.57 B 1
A 0.71 B 0
A 0.71 B 0.14
A 0.71 B 0.29
A 0.71 B 0.43
A 0.71 B 0.57
A 0.71 B 0.71
A 0.71 B 0.86
A 0.71 B 1
A 0.86 B 0
A 0.86 B 0.14
A 0.86 B 0.29
A 0.86 B 0.43
A 0.86 B 0.57
A 0.86 B 0.71
A 0.86 B 0.86
A 0.86 B 1
A 1 B 0
A 1 B 0.14
A 1 B 0.29
A 1 B 0.43
A 1 B 0.57
A 1 B 0.71
A 1 B 0.86
A 1 B 1
0.75
0.50
0.25
OD 600 nm [–]
0 5 101520
Time [h]

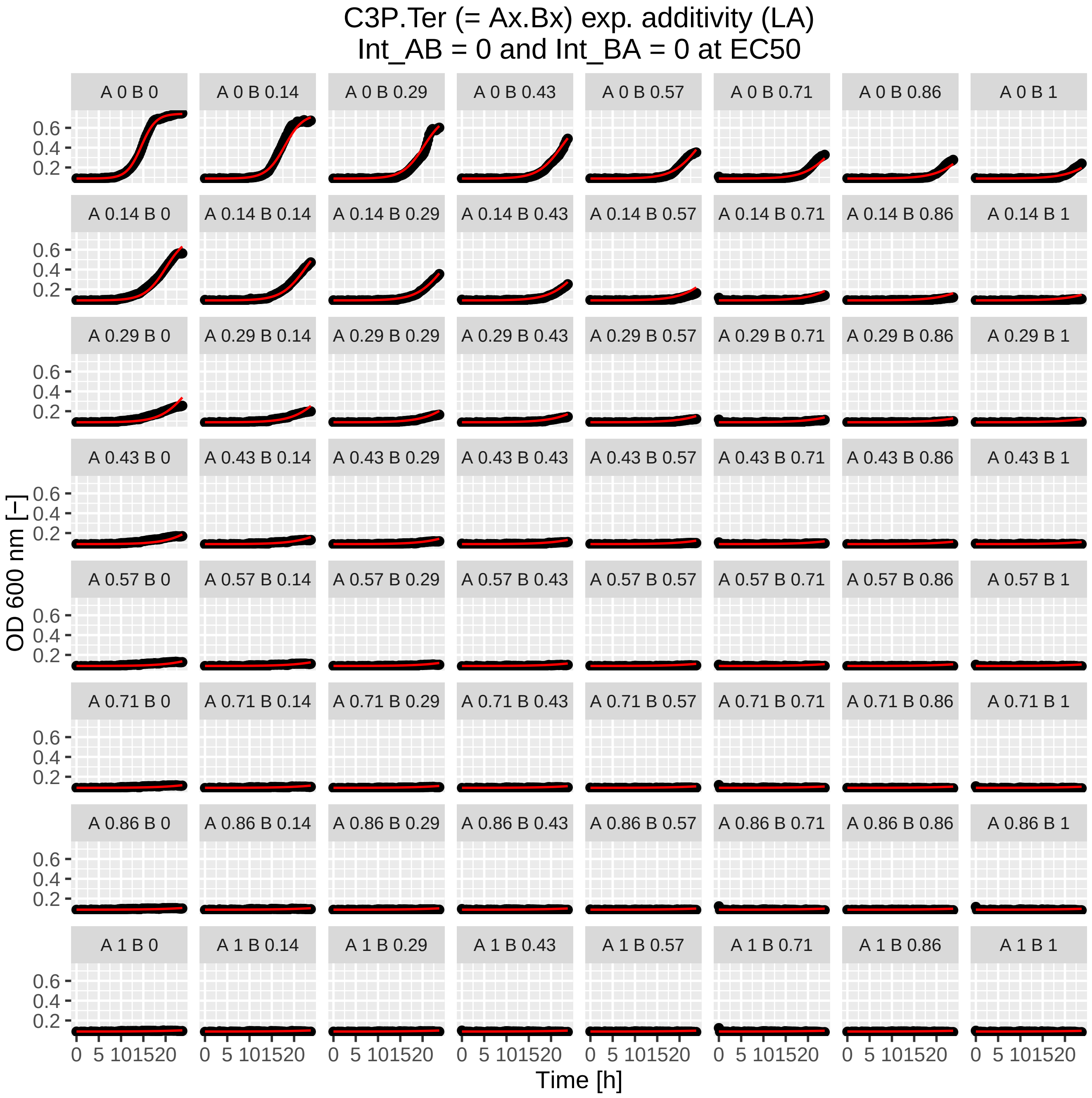
C3P.Ter (= Ax.Bx) exp. additivity (LA)
Int_AB = 0 and Int_BA = 0 at EC50
OD 600 nm [–]
Time [h]
0.6
0.4
0.2
0 5 101520
A 0 B 0
A 0 B 0.14
A 0 B 0.29
A 0 B 0.43
A 0 B 0.57
A 0 B 0.71
A 0 B 0.86
A 0 B 1
A 0.14 B 0
A 0.14 B 0.14
A 0.14 B 0.29
A 0.14 B 0.43
A 0.14 B 0.57
A 0.14 B 0.71
A 0.14 B 0.86
A 0.14 B 1
A 0.29 B 0
A 0.29 B 0.14
A 0.29 B 0.29
A 0.29 B 0.43
A 0.29 B 0.57
A 0.29 B 0.71
A 0.29 B 0.86
A 0.29 B 1
A 0.43 B 0
A 0.43 B 0.14
A 0.43 B 0.29
A 0.43 B 0.43
A 0.43 B 0.57
A 0.43 B 0.71
A 0.43 B 0.86
A 0.43 B 1
A 0.57 B 0
A 0.57 B 0.14
A 0.57 B 0.29
A 0.57 B 0.43
A 0.57 B 0.57
A 0.57 B 0.71
A 0.57 B 0.86
A 0.57 B 1
A 0.71 B 0
A 0.71 B 0.14
A 0.71 B 0.29
A 0.71 B 0.43
A 0.71 B 0.57
A 0.71 B 0.71
A 0.71 B 0.86
A 0.71 B 1
A 0.86 B 0
A 0.86 B 0.14
A 0.86 B 0.29
A 0.86 B 0.43
A 0.86 B 0.57
A 0.86 B 0.71
A 0.86 B 0.86
A 0.86 B 1
A 1 B 0
A 1 B 0.14
A 1 B 0.29
A 1 B 0.43
A 1 B 0.57
A 1 B 0.71
A 1 B 0.86
A 1 B 1

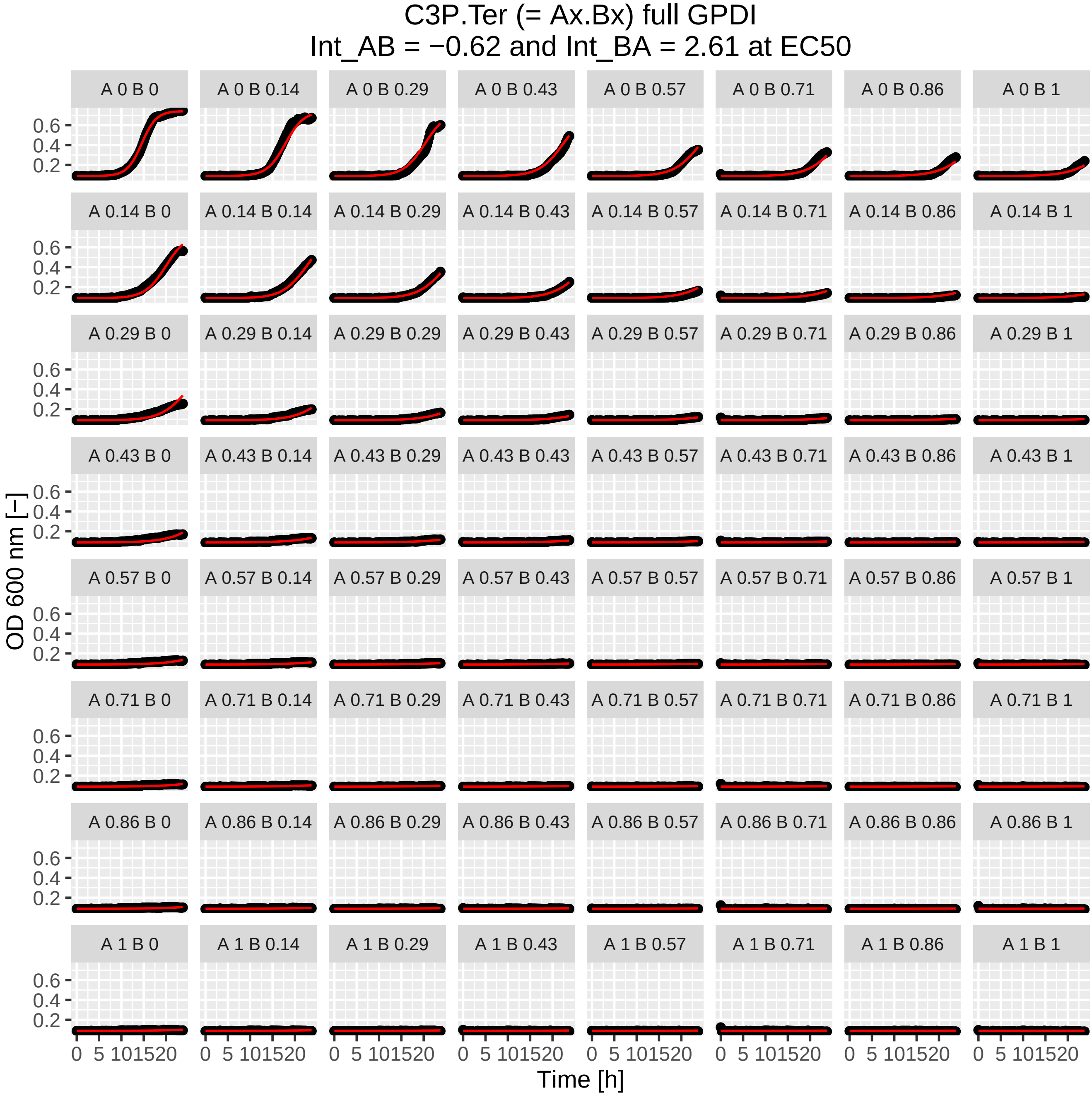
C3P.Ter (= Ax.Bx) full GPDI
Int_AB = −0.62 and Int_BA = 2.61 at EC50
A 0 B 0
A 0 B 0.14
A 0 B 0.29
A 0 B 0.43
A 0 B 0.57
A 0 B 0.71
A 0 B 0.86
A 0 B 1
A 0.14 B 0
A 0.29 B 0
A 0.43 B 0
A 0.57 B 0
A 0.71 B 0
A 0.86 B 0
A 1 B 0
A 1 B 1
0.6
0.4
0.2
0 5 101520
OD 600 nm [–]
Time [h]

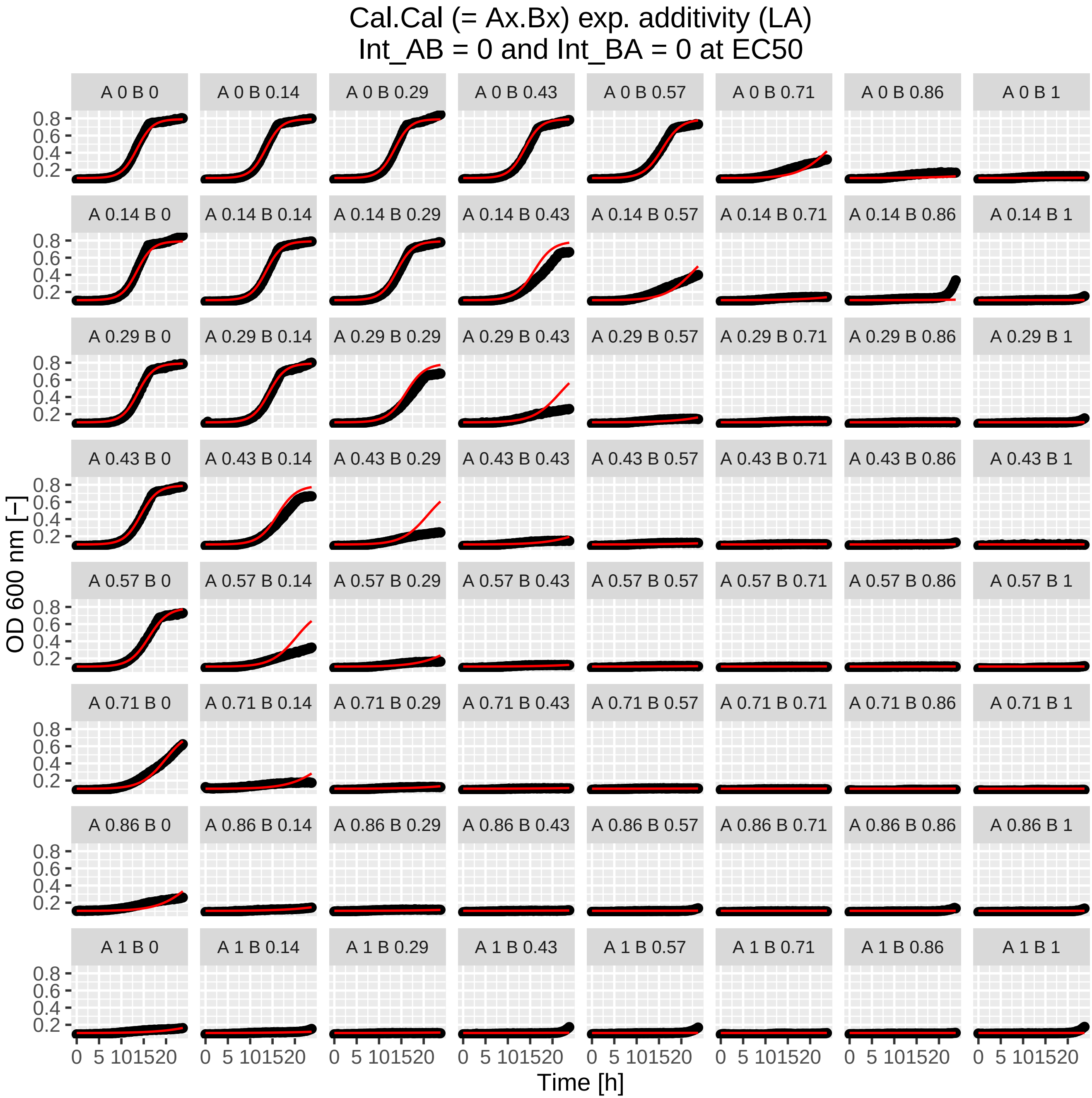

Cal.Cal (= Ax.Bx) exp. additivity (LA)
Int_AB = 0 and Int_BA = 0 at EC50
A 0 B 0
A 0 B 0.14
A 0 B 0.29
A 0 B 0.43
A 0 B 0.57
A 0 B 0.71
A 0 B 0.86
A 0 B 1
A 0.14 B 0
A 0.14 B 0.14
A 0.14 B 0.29
A 0.14 B 0.43
A 0.14 B 0.57
A 0.14 B 0.71
A 0.14 B 0.86
A 0.14 B 1
A 0.29 B 0
A 0.29 B 0.14
A 0.29 B 0.29
A 0.29 B 0.43
A 0.29 B 0.57
A 0.29 B 0.71
A 0.29 B 0.86
A 0.29 B 1
A 0.43 B 0
A 0.43 B 0.14
A 0.43 B 0.29
A 0.43 B 0.43
A 0.43 B 0.57
A 0.43 B 0.71
A 0.43 B 0.86
A 0.43 B 1
A 0.57 B 0
A 0.57 B 0.14
A 0.57 B 0.29
A 0.57 B 0.43
A 0.57 B 0.57
A 0.57 B 0.71
A 0.57 B 0.86
A 0.57 B 1
A 0.71 B 0
A 0.71 B 0.14
A 0.71 B 0.29
A 0.71 B 0.43
A 0.71 B 0.57
A 0.71 B 0.71
A 0.71 B 0.86
A 0.71 B 1
A 0.86 B 0
A 0.86 B 0.14
A 0.86 B 0.29
A 0.86 B 0.43
A 0.86 B 0.57
A 0.86 B 0.71
A 0.86 B 0.86
A 0.86 B 1
A 1 B 0
A 1 B 0.14
A 1 B 0.29
A 1 B 0.43
A 1 B 0.57
A 1 B 0.71
A 1 B 0.86
A 1 B 1
OD 600 nm [–]
0.8
0.6
0.4
0.2
0 5 101520
Time [h]

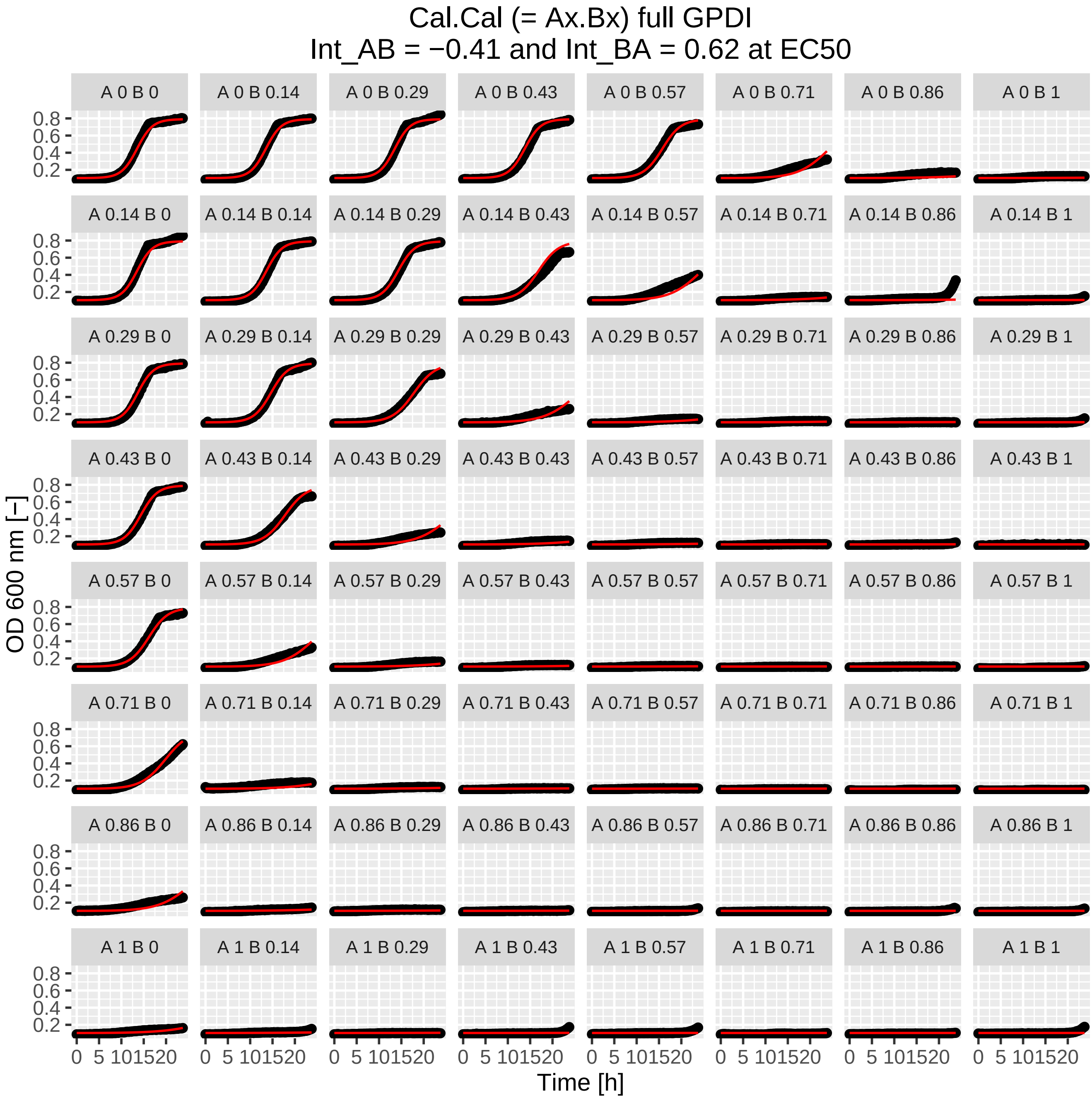
Cal.Cal (= Ax.Bx) full GPDI
Int_AB = −0.41 and Int_BA = 0.62 at EC50
A 0 B 0
A 0 B 0.14
A 0 B 0.29
A 0 B 0.43
A 0 B 0.57
A 0 B 0.71
A 0 B 0.86
A 0 B 1
A 0.14 B 0
A 0.14 B 0.14
A 0.14 B 0.29
A 0.14 B 0.43
A 0.14 B 0.57
A 0.14 B 0.71
A 0.14 B 0.86
A 0.14 B 1
A 0.29 B 0
A 0.29 B 0.14
A 0.29 B 0.29
A 0.29 B 0.43
A 0.29 B 0.57
A 0.29 B 0.71
A 0.29 B 0.86
A 0.29 B 1
A 0.43 B 0
A 0.43 B 0.14
A 0.43 B 0.29
A 0.43 B 0.43
A 0.43 B 0.57
A 0.43 B 0.71
A 0.43 B 0.86
A 0.43 B 1
A 0.57 B 0
A 0.57 B 0.14
A 0.57 B 0.29
A 0.57 B 0.43
A 0.57 B 0.57
A 0.57 B 0.71
A 0.57 B 0.86
A 0.57 B 1
A 0.71 B 0
A 0.71 B 0.14
A 0.71 B 0.29
A 0.71 B 0.43
A 0.71 B 0.57
A 0.71 B 0.71
A 0.71 B 0.86
A 0.71 B 1
A 0.86 B 0
A 0.86 B 0.14
A 0.86 B 0.29
A 0.86 B 0.43
A 0.86 B 0.57
A 0.86 B 0.71
A 0.86 B 0.86
A 0.86 B 1
A 1 B 0
A 1 B 0.14
A 1 B 0.29
A 1 B 0.43
A 1 B 0.57
A 1 B 0.71
A 1 B 0.86
A 1 B 1
OD 600 nm [−]
0.2
0.4
0.6
0.8
0 5 101520
Time [h]

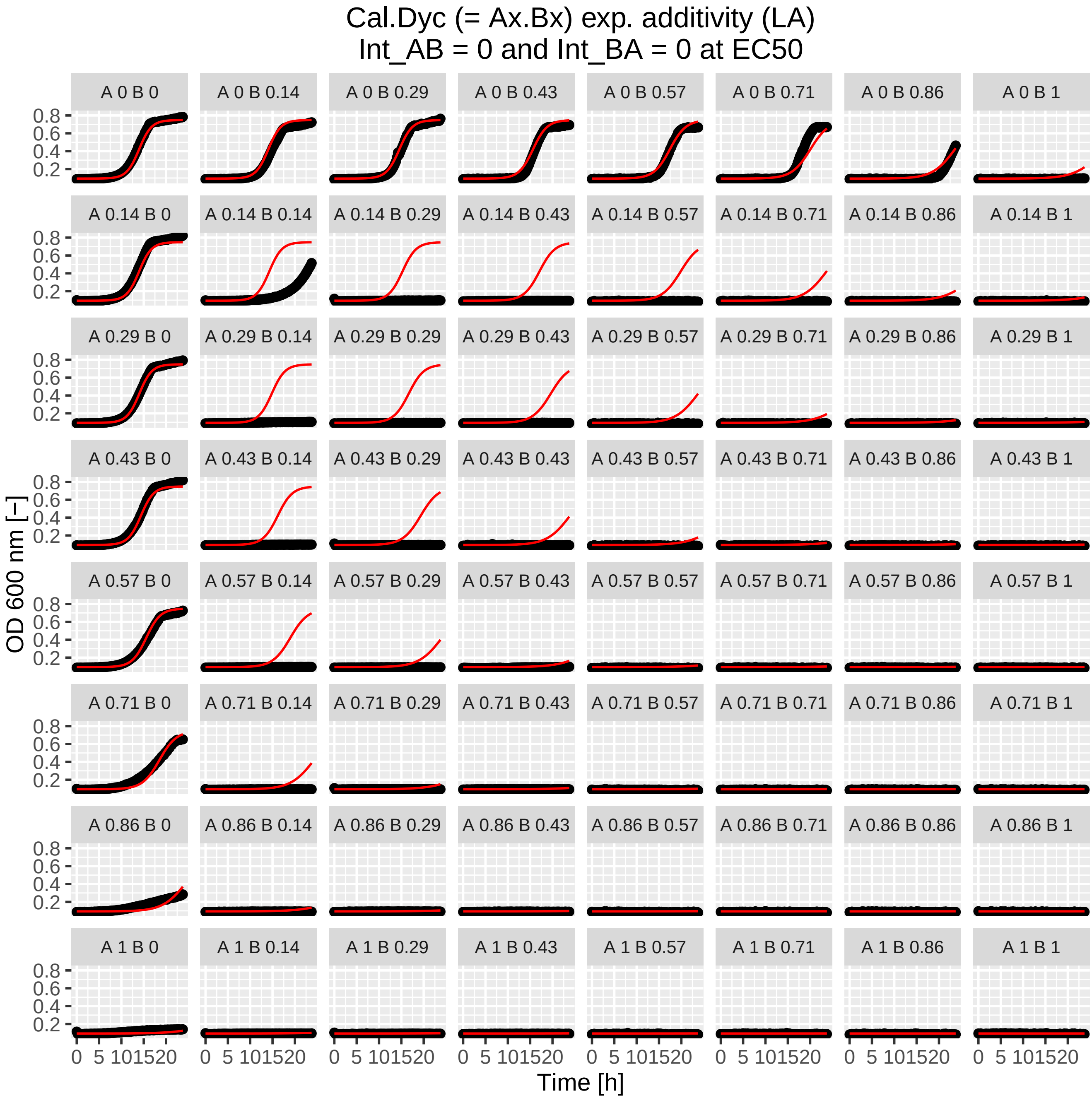
Cal.Dyc (= Ax.Bx) exp. additivity (LA)
Int_AB = 0 and Int_BA = 0 at EC50
OD 600 nm [–]
Time [h]
A 0 B 0
A 0 B 0.14
A 0 B 0.29
A 0 B 0.43
A 0 B 0.57
A 0 B 0.71
A 0 B 0.86
A 0 B 1
A 0.14 B 0
A 0.14 B 0.14
A 0.14 B 0.29
A 0.14 B 0.43
A 0.14 B 0.57
A 0.14 B 0.71
A 0.14 B 0.86
A 0.14 B 1
A 0.29 B 0
A 0.29 B 0.14
A 0.29 B 0.29
A 0.29 B 0.43
A 0.29 B 0.57
A 0.29 B 0.71
A 0.29 B 0.86
A 0.29 B 1
A 0.43 B 0
A 0.43 B 0.14
A 0.43 B 0.29
A 0.43 B 0.43
A 0.43 B 0.57
A 0.43 B 0.71
A 0.43 B 0.86
A 0.43 B 1
A 0.57 B 0
A 0.57 B 0.14
A 0.57 B 0.29
A 0.57 B 0.43
A 0.57 B 0.57
A 0.57 B 0.71
A 0.57 B 0.86
A 0.57 B 1
A 0.71 B 0
A 0.71 B 0.14
A 0.71 B 0.29
A 0.71 B 0.43
A 0.71 B 0.57
A 0.71 B 0.71
A 0.71 B 0.86
A 0.71 B 1
A 0.86 B 0
A 0.86 B 0.14
A 0.86 B 0.29
A 0.86 B 0.43
A 0.86 B 0.57
A 0.86 B 0.71
A 0.86 B 0.86
A 0.86 B 1
A 1 B 0
A 1 B 0.14
A 1 B 0.29
A 1 B 0.43
A 1 B 0.57
A 1 B 0.71
A 1 B 0.86
A 1 B 1
0.8
0.6
0.4
0.2
0 5 101520

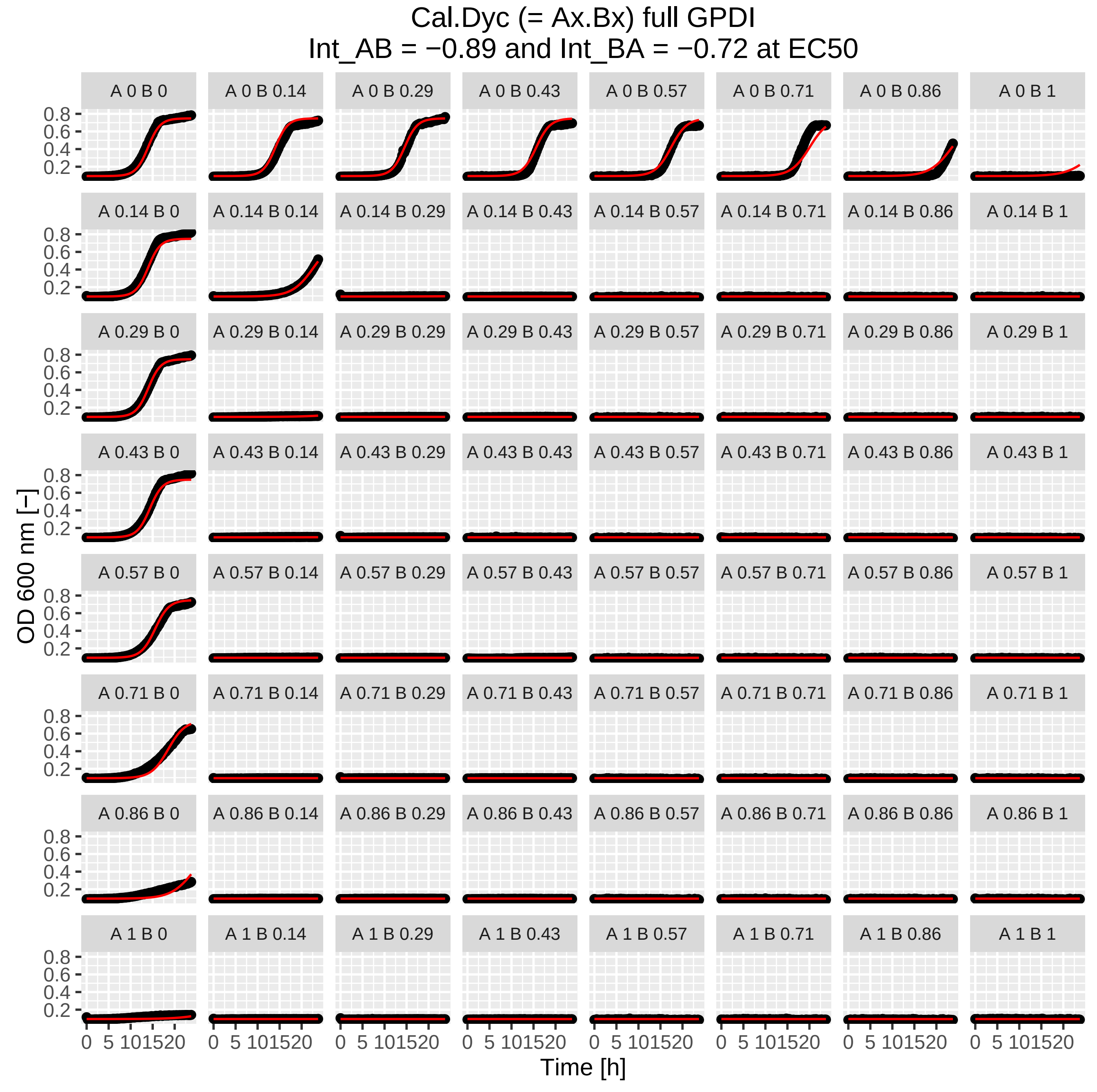

Cal.Dyc (= Ax.Bx) full GPDI
Int_AB = −0.89 and Int_BA = −0.72 at EC50
A 0 B 0
A 0 B 0.14
A 0 B 0.29
A 0 B 0.43
A 0 B 0.57
A 0 B 0.71
A 0 B 0.86
A 0 B 1
A 0.14 B 0
A 0.14 B 0.14
A 0.14 B 0.29
A 0.14 B 0.43
A 0.14 B 0.57
A 0.14 B 0.71
A 0.14 B 0.86
A 0.14 B 1
A 0.29 B 0
A 0.29 B 0.14
A 0.29 B 0.29
A 0.29 B 0.43
A 0.29 B 0.57
A 0.29 B 0.71
A 0.29 B 0.86
A 0.29 B 1
A 0.43 B 0
A 0.43 B 0.14
A 0.43 B 0.29
A 0.43 B 0.43
A 0.43 B 0.57
A 0.43 B 0.71
A 0.43 B 0.86
A 0.43 B 1
A 0.57 B 0
A 0.57 B 0.14
A 0.57 B 0.29
A 0.57 B 0.43
A 0.57 B 0.57
A 0.57 B 0.71
A 0.57 B 0.86
A 0.57 B 1
A 0.71 B 0
A 0.71 B 0.14
A 0.71 B 0.29
A 0.71 B 0.43
A 0.71 B 0.57
A 0.71 B 0.71
A 0.71 B 0.86
A 0.71 B 1
A 0.86 B 0
A 0.86 B 0.14
A 0.86 B 0.29
A 0.86 B 0.43
A 0.86 B 0.57
A 0.86 B 0.71
A 0.86 B 0.86
A 0.86 B 1
A 1 B 0
A 1 B 0.14
A 1 B 0.29
A 1 B 0.43
A 1 B 0.57
A 1 B 0.71
A 1 B 0.86
A 1 B 1
OD 600 nm [−]
0.2
0.4
0.6
0.8
0 5 101520
Time [h]

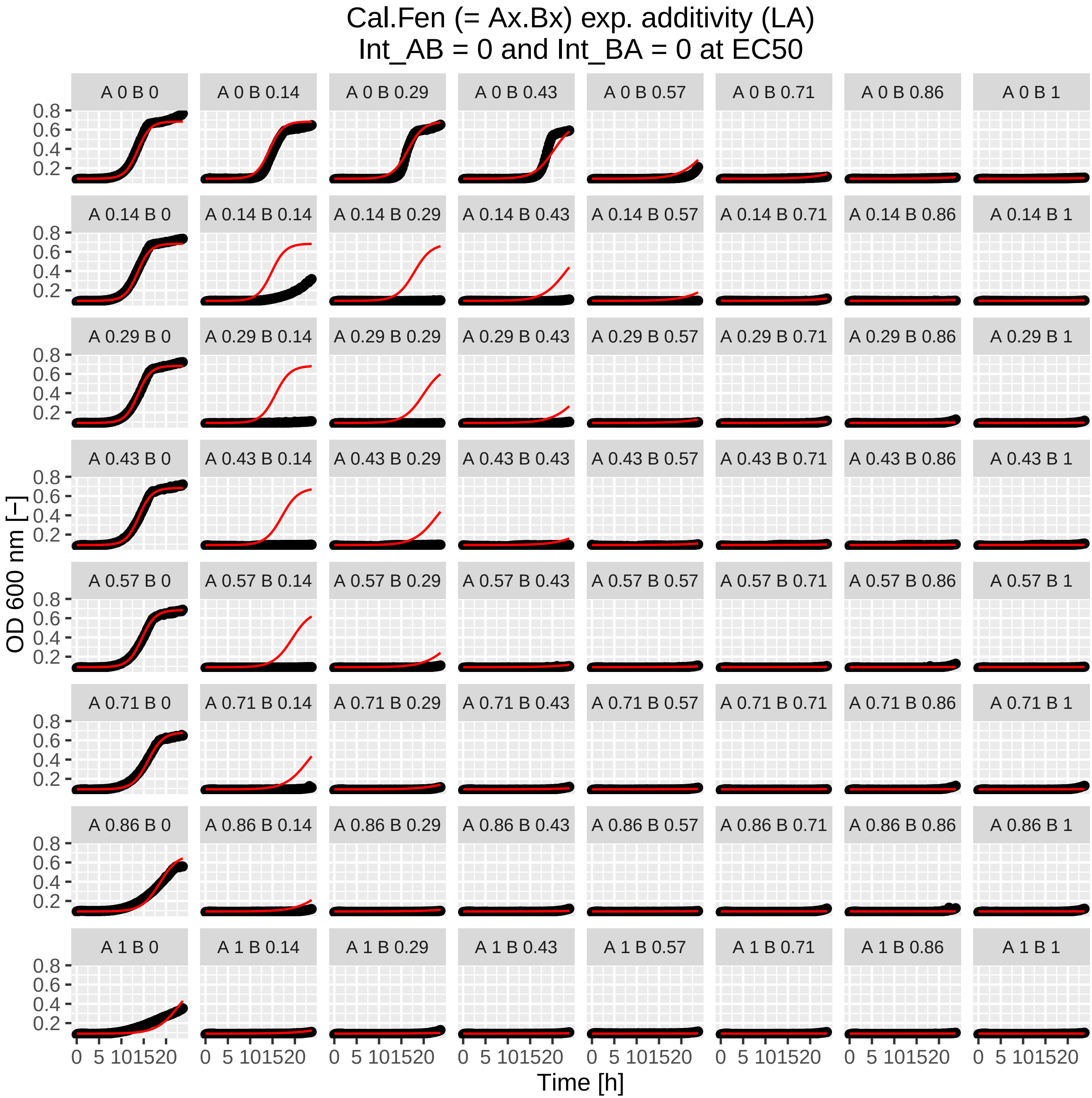

Cal.Fen (= Ax.Bx) exp. additivity (LA)
Int_AB = 0 and Int_BA = 0 at EC50
A 0 B 0
A 0 B 0.14
A 0 B 0.29
A 0 B 0.43
A 0 B 0.57
A 0 B 0.71
A 0 B 0.86
A 0 B 1
A 0.14 B 0
A 0.14 B 0.14
A 0.14 B 0.29
A 0.14 B 0.43
A 0.14 B 0.57
A 0.14 B 0.71
A 0.14 B 0.86
A 0.14 B 1
A 0.29 B 0
A 0.29 B 0.14
A 0.29 B 0.29
A 0.29 B 0.43
A 0.29 B 0.57
A 0.29 B 0.71
A 0.29 B 0.86
A 0.29 B 1
A 0.43 B 0
A 0.43 B 0.14
A 0.43 B 0.29
A 0.43 B 0.43
A 0.43 B 0.57
A 0.43 B 0.71
A 0.43 B 0.86
A 0.43 B 1
A 0.57 B 0
A 0.57 B 0.14
A 0.57 B 0.29
A 0.57 B 0.43
A 0.57 B 0.57
A 0.57 B 0.71
A 0.57 B 0.86
A 0.57 B 1
A 0.71 B 0
A 0.71 B 0.14
A 0.71 B 0.29
A 0.71 B 0.43
A 0.71 B 0.57
A 0.71 B 0.71
A 0.71 B 0.86
A 0.71 B 1
A 0.86 B 0
A 0.86 B 0.14
A 0.86 B 0.29
A 0.86 B 0.43
A 0.86 B 0.57
A 0.86 B 0.71
A 0.86 B 0.86
A 0.86 B 1
A 1 B 0
A 1 B 0.14
A 1 B 0.29
A 1 B 0.43
A 1 B 0.57
A 1 B 0.71
A 1 B 0.86
A 1 B 1
0.8
0.6
0.4
0.2
OD 600 nm [–]
0 5 101520
Time [h]

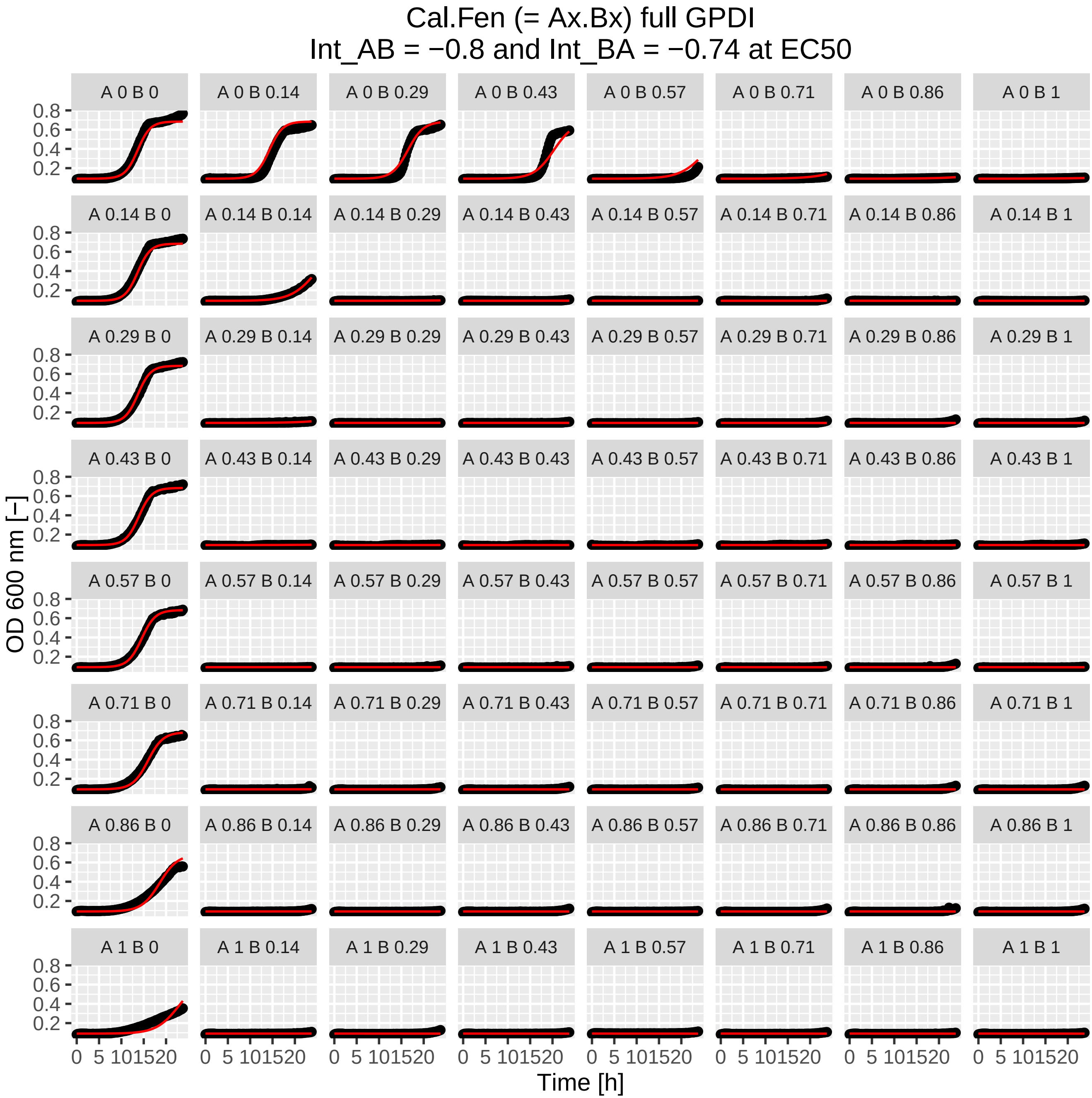

Cal.Fen (= Ax.Bx) full GPDI
Int_AB = −0.8 and Int_BA = −0.74 at EC50
A 0 B 0
A 0 B 0.14
A 0 B 0.29
A 0 B 0.43
A 0 B 0.57
A 0 B 0.71
A 0 B 0.86
A 0 B 1
A 0.14 B 0
A 0.14 B 0.14
A 0.14 B 0.29
A 0.14 B 0.43
A 0.14 B 0.57
A 0.14 B 0.71
A 0.14 B 0.86
A 0.14 B 1
A 0.29 B 0
A 0.29 B 0.14
A 0.29 B 0.29
A 0.29 B 0.43
A 0.29 B 0.57
A 0.29 B 0.71
A 0.29 B 0.86
A 0.29 B 1
A 0.43 B 0
A 0.43 B 0.14
A 0.43 B 0.29
A 0.43 B 0.43
A 0.43 B 0.57
A 0.43 B 0.71
A 0.43 B 0.86
A 0.43 B 1
A 0.57 B 0
A 0.57 B 0.14
A 0.57 B 0.29
A 0.57 B 0.43
A 0.57 B 0.57
A 0.57 B 0.71
A 0.57 B 0.86
A 0.57 B 1
A 0.71 B 0
A 0.71 B 0.14
A 0.71 B 0.29
A 0.71 B 0.43
A 0.71 B 0.57
A 0.71 B 0.71
A 0.71 B 0.86
A 0.71 B 1
A 0.86 B 0
A 0.86 B 0.14
A 0.86 B 0.29
A 0.86 B 0.43
A 0.86 B 0.57
A 0.86 B 0.71
A 0.86 B 0.86
A 0.86 B 1
A 1 B 0
A 1 B 0.14
A 1 B 0.29
A 1 B 0.43
A 1 B 0.57
A 1 B 0.71
A 1 B 0.86
A 1 B 1
OD 600 nm [−]
0.8
0.6
0.4
0.2
0 5 101520
Time [h]

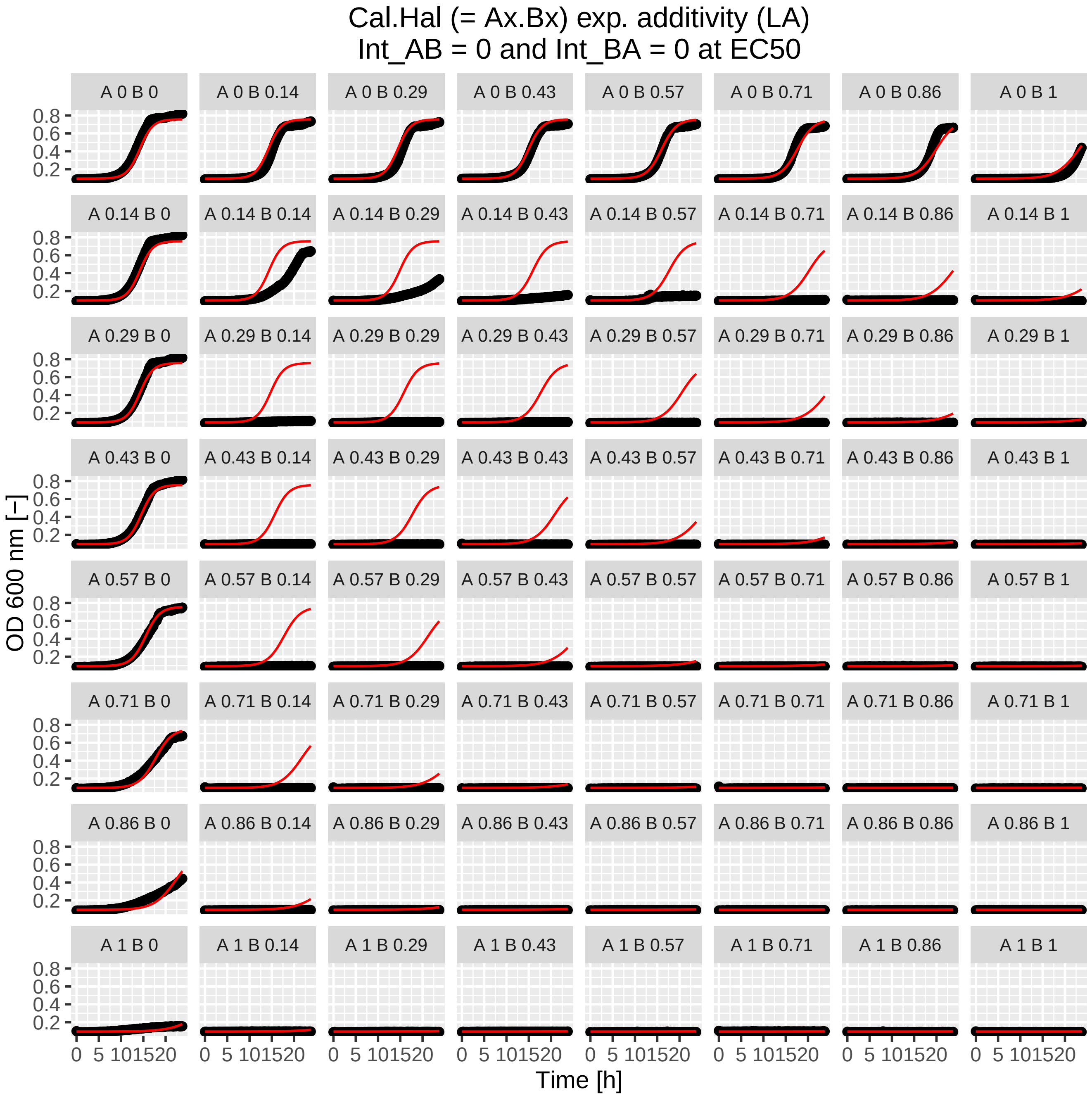

Cal.Hal (= Ax.Bx) exp. additivity (LA)
Int_AB = 0 and Int_BA = 0 at EC50
A 0 B 0
A 0 B 0.14
A 0 B 0.29
A 0 B 0.43
A 0 B 0.57
A 0 B 0.71
A 0 B 0.86
A 0 B 1
A 0.14 B 0
A 0.14 B 0.14
A 0.14 B 0.29
A 0.14 B 0.43
A 0.14 B 0.57
A 0.14 B 0.71
A 0.14 B 0.86
A 0.14 B 1
A 0.29 B 0
A 0.29 B 0.14
A 0.29 B 0.29
A 0.29 B 0.43
A 0.29 B 0.57
A 0.29 B 0.71
A 0.29 B 0.86
A 0.29 B 1
A 0.43 B 0
A 0.43 B 0.14
A 0.43 B 0.29
A 0.43 B 0.43
A 0.43 B 0.57
A 0.43 B 0.71
A 0.43 B 0.86
A 0.43 B 1
A 0.57 B 0
A 0.57 B 0.14
A 0.57 B 0.29
A 0.57 B 0.43
A 0.57 B 0.57
A 0.57 B 0.71
A 0.57 B 0.86
A 0.57 B 1
A 0.71 B 0
A 0.71 B 0.14
A 0.71 B 0.29
A 0.71 B 0.43
A 0.71 B 0.57
A 0.71 B 0.71
A 0.71 B 0.86
A 0.71 B 1
A 0.86 B 0
A 0.86 B 0.14
A 0.86 B 0.29
A 0.86 B 0.43
A 0.86 B 0.57
A 0.86 B 0.71
A 0.86 B 0.86
A 0.86 B 1
A 1 B 0
A 1 B 0.14
A 1 B 0.29
A 1 B 0.43
A 1 B 0.57
A 1 B 0.71
A 1 B 0.86
A 1 B 1
OD 600 nm [–]
0.8
0.6
0.4
0.2
0 5 101520
Time [h]

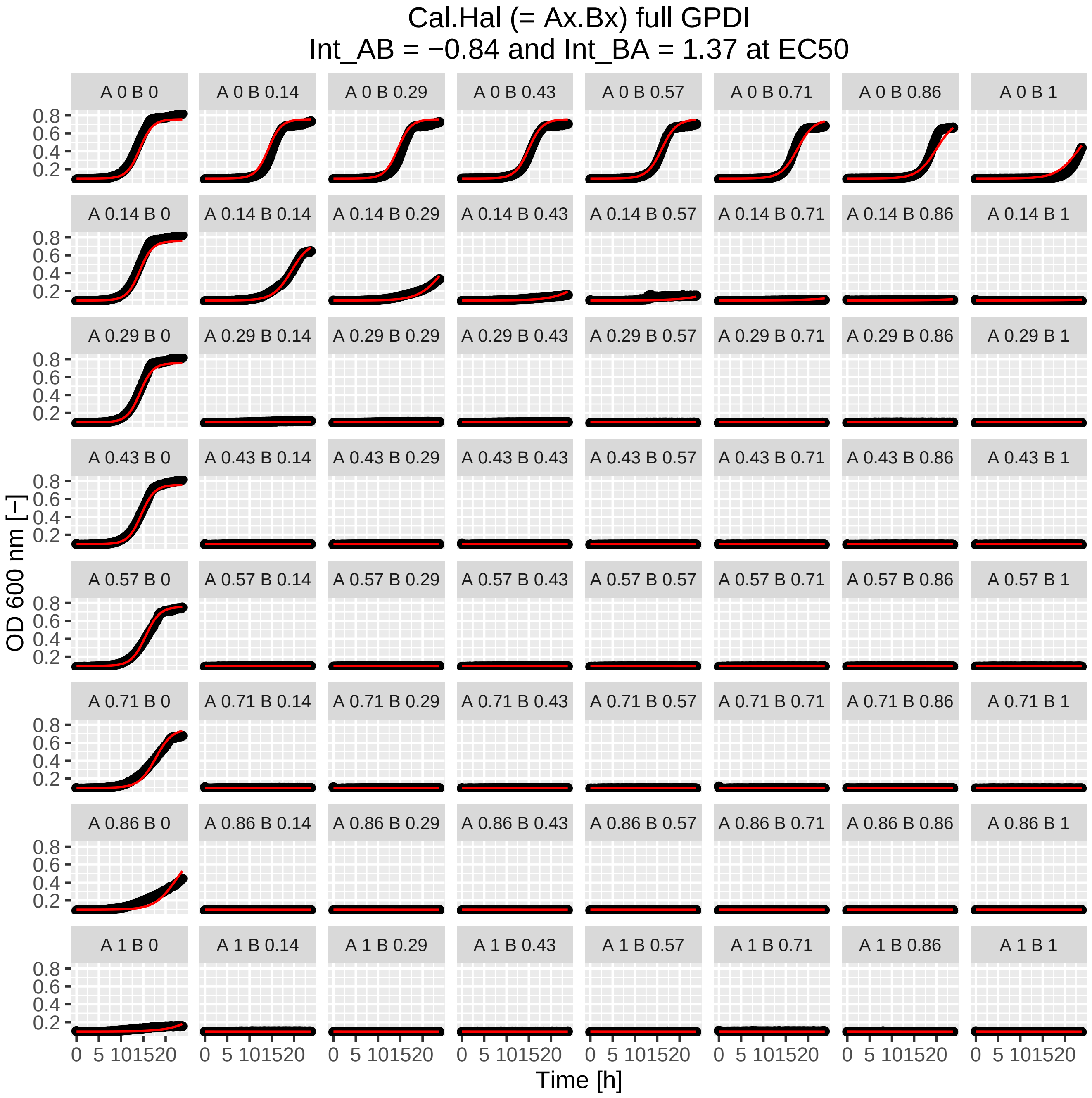

Cal.Hal (= Ax.Bx) full GPDI
Int_AB = −0.84 and Int_BA = 1.37 at EC50
A 0 B 0
A 0 B 0.14
A 0 B 0.29
A 0 B 0.43
A 0 B 0.57
A 0 B 0.71
A 0 B 0.86
A 0 B 1
A 0.14 B 0
A 0.14 B 0.14
A 0.14 B 0.29
A 0.14 B 0.43
A 0.14 B 0.57
A 0.14 B 0.71
A 0.14 B 0.86
A 0.14 B 1
A 0.29 B 0
A 0.29 B 0.14
A 0.29 B 0.29
A 0.29 B 0.43
A 0.29 B 0.57
A 0.29 B 0.71
A 0.29 B 0.86
A 0.29 B 1
A 0.43 B 0
A 0.43 B 0.14
A 0.43 B 0.29
A 0.43 B 0.43
A 0.43 B 0.57
A 0.43 B 0.71
A 0.43 B 0.86
A 0.43 B 1
A 0.57 B 0
A 0.57 B 0.14
A 0.57 B 0.29
A 0.57 B 0.43
A 0.57 B 0.57
A 0.57 B 0.71
A 0.57 B 0.86
A 0.57 B 1
A 0.71 B 0
A 0.71 B 0.14
A 0.71 B 0.29
A 0.71 B 0.43
A 0.71 B 0.57
A 0.71 B 0.71
A 0.71 B 0.86
A 0.71 B 1
A 0.86 B 0
A 0.86 B 0.14
A 0.86 B 0.29
A 0.86 B 0.43
A 0.86 B 0.57
A 0.86 B 0.71
A 0.86 B 0.86
A 0.86 B 1
A 1 B 0
A 1 B 0.14
A 1 B 0.29
A 1 B 0.43
A 1 B 0.57
A 1 B 0.71
A 1 B 0.86
A 1 B 1
0.8
0.6
0.4
0.2
OD 600 nm [−]
0 5 101520
Time [h]

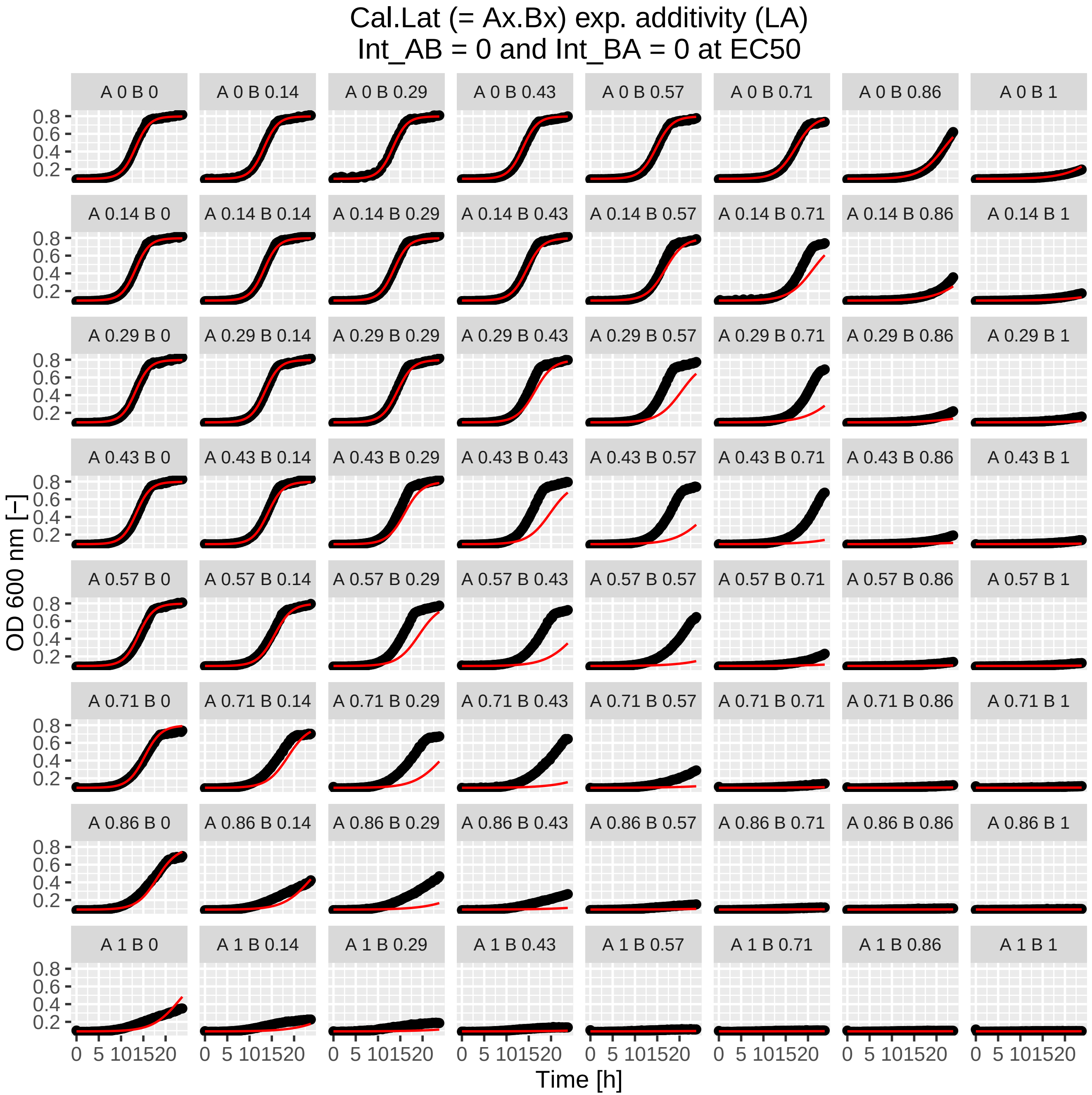
Cal.Lat (= Ax.Bx) exp. additivity (LA)
Int_AB = 0 and Int_BA = 0 at EC50
OD 600 nm [–]
Time [h]
A 0 B 0
A 0 B 0.14
A 0 B 0.29
A 0 B 0.43
A 0 B 0.57
A 0 B 0.71
A 0 B 0.86
A 0 B 1
A 0.14 B 0
A 0.14 B 0.14
A 0.14 B 0.29
A 0.14 B 0.43
A 0.14 B 0.57
A 0.14 B 0.71
A 0.14 B 0.86
A 0.14 B 1
A 0.29 B 0
A 0.29 B 0.14
A 0.29 B 0.29
A 0.29 B 0.43
A 0.29 B 0.57
A 0.29 B 0.71
A 0.29 B 0.86
A 0.29 B 1
A 0.43 B 0
A 0.43 B 0.14
A 0.43 B 0.29
A 0.43 B 0.43
A 0.43 B 0.57
A 0.43 B 0.71
A 0.43 B 0.86
A 0.43 B 1
A 0.57 B 0
A 0.57 B 0.14
A 0.57 B 0.29
A 0.57 B 0.43
A 0.57 B 0.57
A 0.57 B 0.71
A 0.57 B 0.86
A 0.57 B 1
A 0.71 B 0
A 0.71 B 0.14
A 0.71 B 0.29
A 0.71 B 0.43
A 0.71 B 0.57
A 0.71 B 0.71
A 0.71 B 0.86
A 0.71 B 1
A 0.86 B 0
A 0.86 B 0.14
A 0.86 B 0.29
A 0.86 B 0.43
A 0.86 B 0.57
A 0.86 B 0.71
A 0.86 B 0.86
A 0.86 B 1
A 1 B 0
A 1 B 0.14
A 1 B 0.29
A 1 B 0.43
A 1 B 0.57
A 1 B 0.71
A 1 B 0.86
A 1 B 1
0.8
0.6
0.4
0.2
0 5 101520

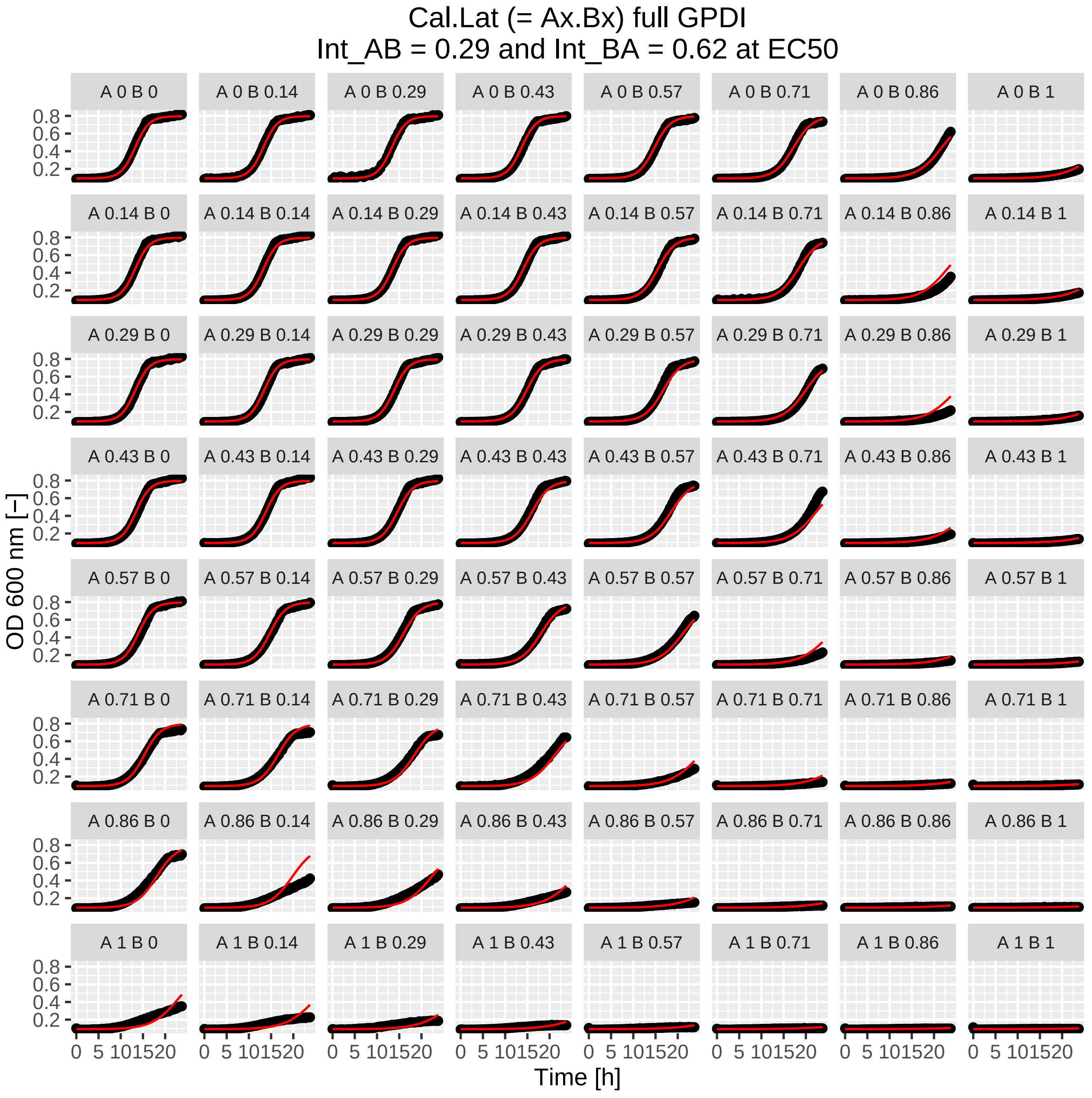
Cal.Lat (= Ax.Bx) full GPDI
Int_AB = 0.29 and Int_BA = 0.62 at EC50
OD 600 nm [−]
Time [h]

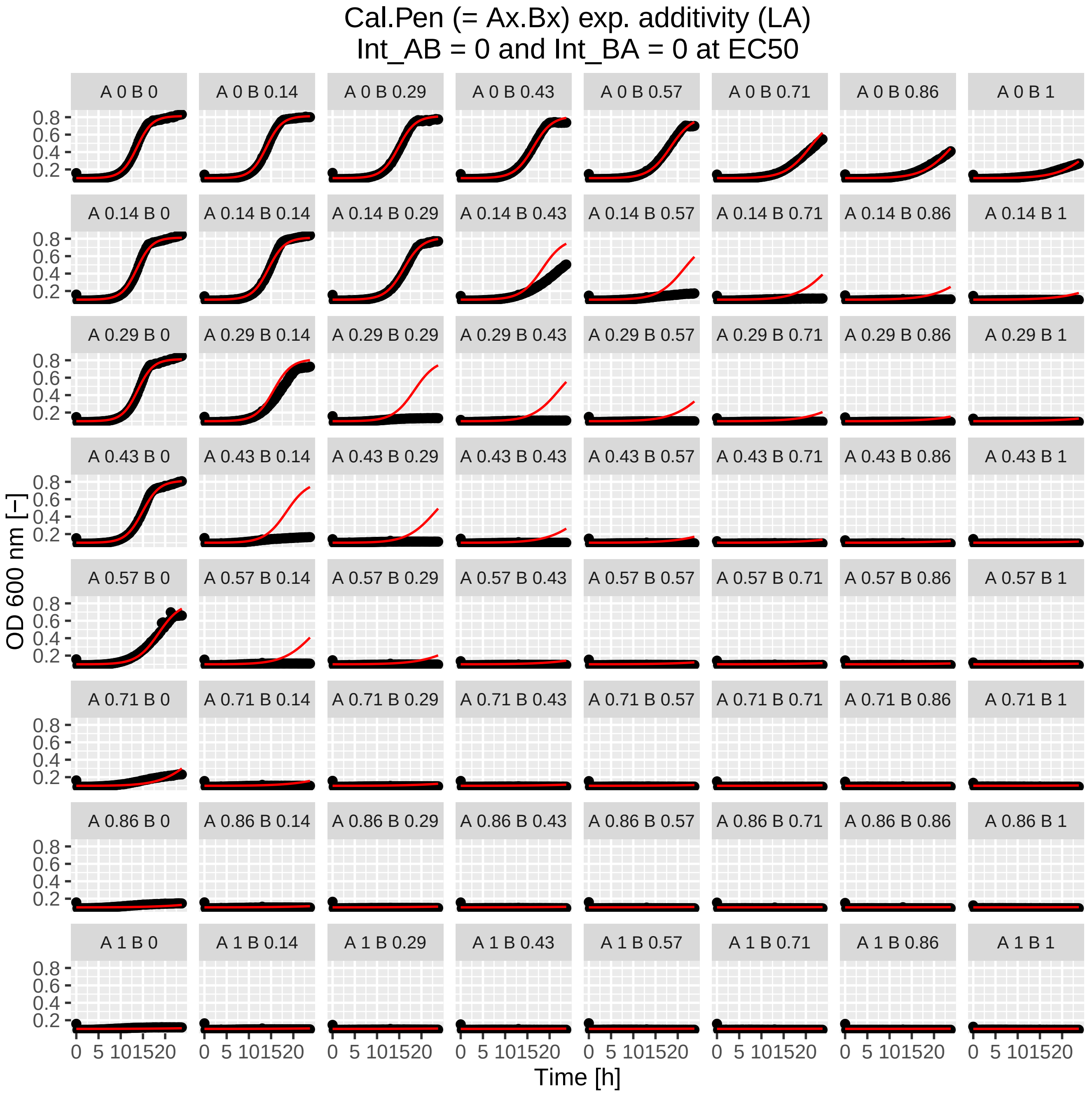

Cal.Pen (= Ax.Bx) exp. additivity (LA)
Int_AB = 0 and Int_BA = 0 at EC50
OD 600 nm [–]
Time [h]
A 0 B 0
A 0 B 0.14
A 0 B 0.29
A 0 B 0.43
A 0 B 0.57
A 0 B 0.71
A 0 B 0.86
A 0 B 1
A 0.14 B 0
A 0.14 B 0.14
A 0.14 B 0.29
A 0.14 B 0.43
A 0.14 B 0.57
A 0.14 B 0.71
A 0.14 B 0.86
A 0.14 B 1
A 0.29 B 0
A 0.29 B 0.14
A 0.29 B 0.29
A 0.29 B 0.43
A 0.29 B 0.57
A 0.29 B 0.71
A 0.29 B 0.86
A 0.29 B 1
A 0.43 B 0
A 0.43 B 0.14
A 0.43 B 0.29
A 0.43 B 0.43
A 0.43 B 0.57
A 0.43 B 0.71
A 0.43 B 0.86
A 0.43 B 1
A 0.57 B 0
A 0.57 B 0.14
A 0.57 B 0.29
A 0.57 B 0.43
A 0.57 B 0.57
A 0.57 B 0.71
A 0.57 B 0.86
A 0.57 B 1
A 0.71 B 0
A 0.71 B 0.14
A 0.71 B 0.29
A 0.71 B 0.43
A 0.71 B 0.57
A 0.71 B 0.71
A 0.71 B 0.86
A 0.71 B 1
A 0.86 B 0
A 0.86 B 0.14
A 0.86 B 0.29
A 0.86 B 0.43
A 0.86 B 0.57
A 0.86 B 0.71
A 0.86 B 0.86
A 0.86 B 1
A 1 B 0
A 1 B 0.14
A 1 B 0.29
A 1 B 0.43
A 1 B 0.57
A 1 B 0.71
A 1 B 0.86
A 1 B 1
0.8
0.6
0.4
0.2
0
5
10
15
20

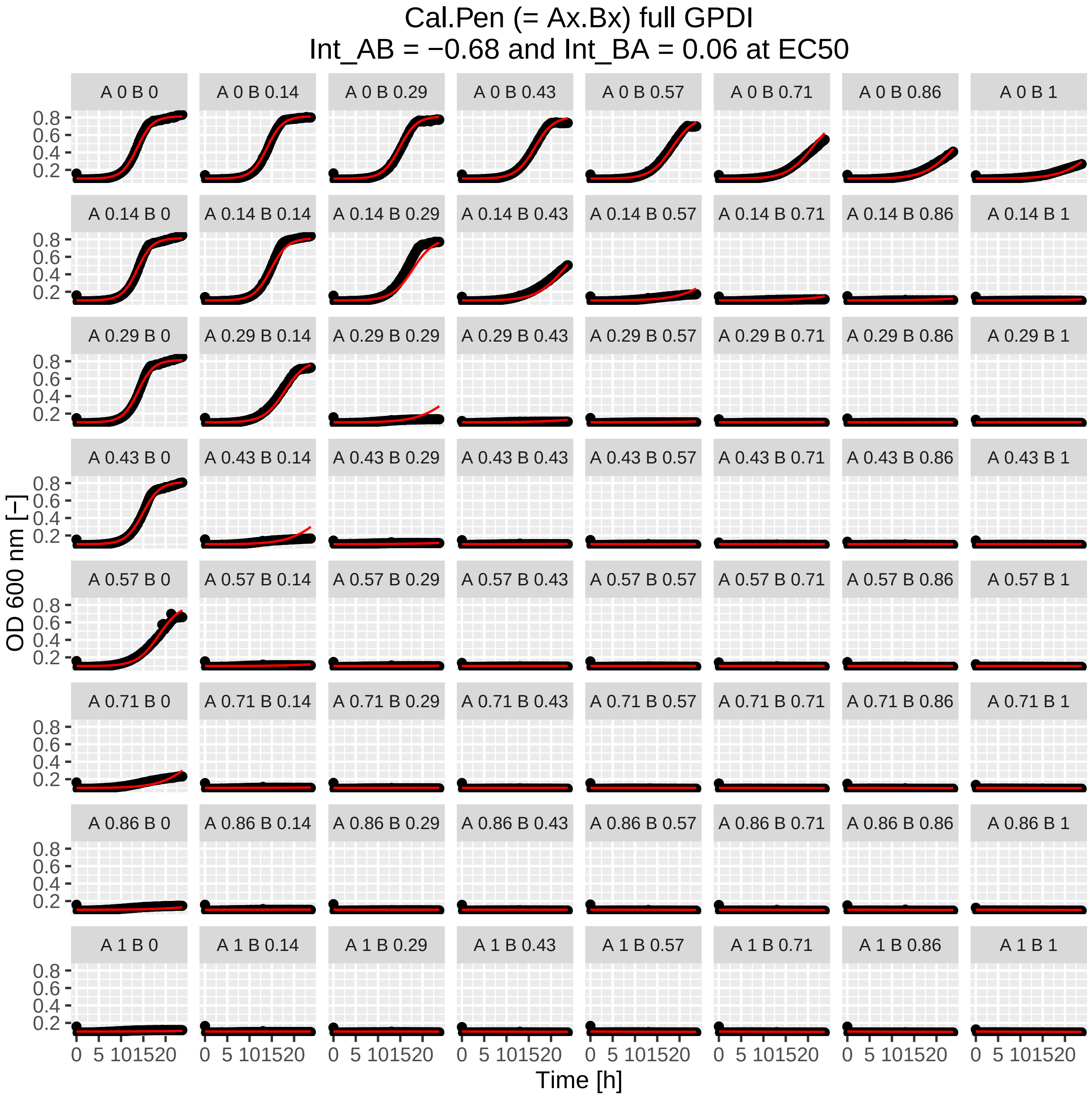

Cal.Pen (= Ax.Bx) full GPDI
Int_AB = −0.68 and Int_BA = 0.06 at EC50
A 0 B 0
A 0 B 0.14
A 0 B 0.29
A 0 B 0.43
A 0 B 0.57
A 0 B 0.71
A 0 B 0.86
A 0 B 1
A 0.14 B 0
A 0.14 B 0.14
A 0.14 B 0.29
A 0.14 B 0.43
A 0.14 B 0.57
A 0.14 B 0.71
A 0.14 B 0.86
A 0.14 B 1
A 0.29 B 0
A 0.29 B 0.14
A 0.29 B 0.29
A 0.29 B 0.43
A 0.29 B 0.57
A 0.29 B 0.71
A 0.29 B 0.86
A 0.29 B 1
A 0.43 B 0
A 0.43 B 0.14
A 0.43 B 0.29
A 0.43 B 0.43
A 0.43 B 0.57
A 0.43 B 0.71
A 0.43 B 0.86
A 0.43 B 1
A 0.57 B 0
A 0.57 B 0.14
A 0.57 B 0.29
A 0.57 B 0.43
A 0.57 B 0.57
A 0.57 B 0.71
A 0.57 B 0.86
A 0.57 B 1
A 0.71 B 0
A 0.71 B 0.14
A 0.71 B 0.29
A 0.71 B 0.43
A 0.71 B 0.57
A 0.71 B 0.71
A 0.71 B 0.86
A 0.71 B 1
A 0.86 B 0
A 0.86 B 0.14
A 0.86 B 0.29
A 0.86 B 0.43
A 0.86 B 0.57
A 0.86 B 0.71
A 0.86 B 0.86
A 0.86 B 1
A 1 B 0
A 1 B 0.14
A 1 B 0.29
A 1 B 0.43
A 1 B 0.57
A 1 B 0.71
A 1 B 0.86
A 1 B 1
OD 600 nm [−]
0.8
0.6
0.4
0.2
0 5 101520
Time [h]

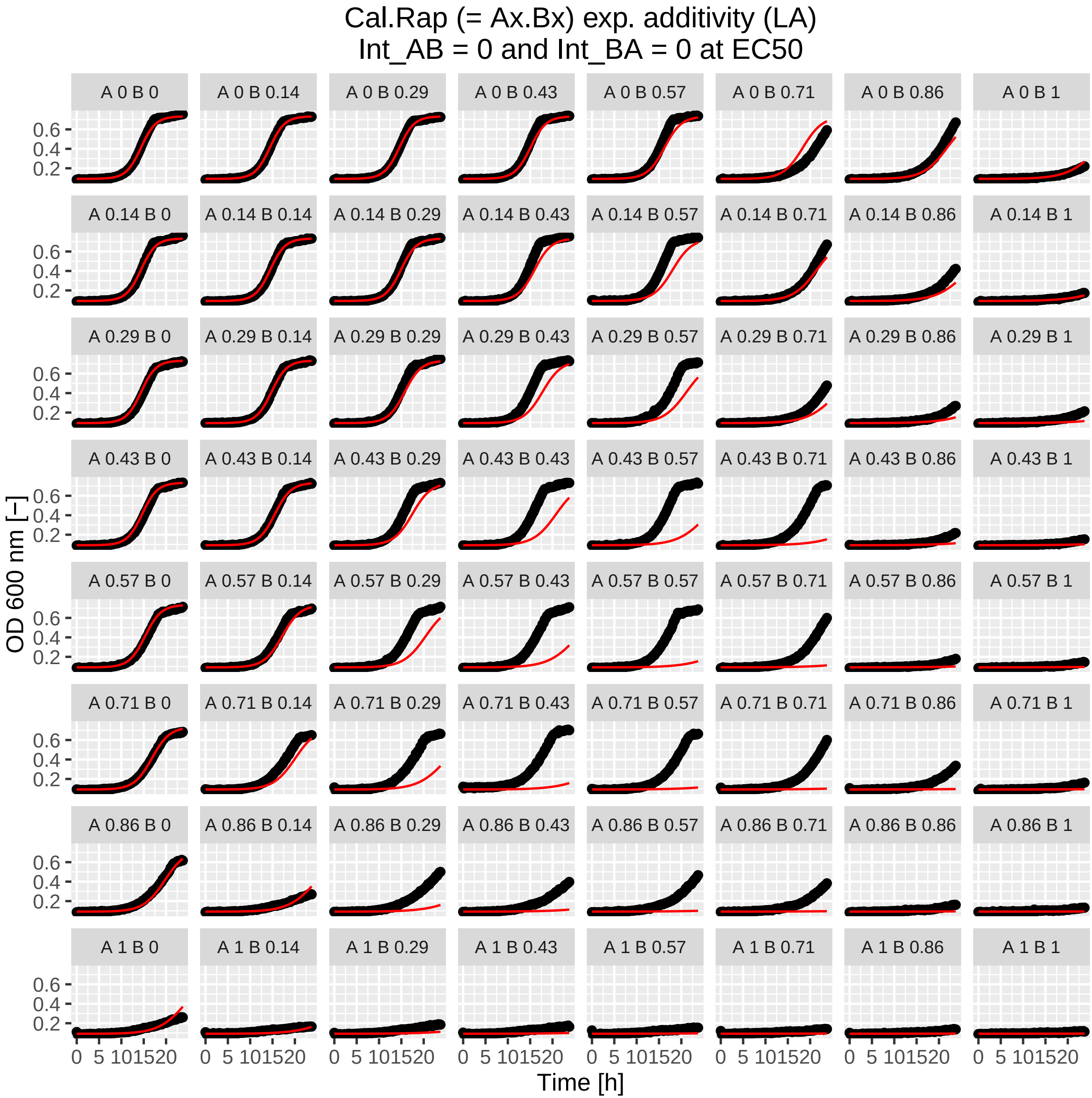

Cal.Rap (= Ax.Bx) exp. additivity (LA)
Int_AB = 0 and Int_BA = 0 at EC50
A 0 B 0
A 0 B 0.14
A 0 B 0.29
A 0 B 0.43
A 0 B 0.57
A 0 B 0.71
A 0 B 0.86
A 0 B 1
A 0.14 B 0
A 0.14 B 0.14
A 0.14 B 0.29
A 0.14 B 0.43
A 0.14 B 0.57
A 0.14 B 0.71
A 0.14 B 0.86
A 0.14 B 1
A 0.29 B 0
A 0.29 B 0.14
A 0.29 B 0.29
A 0.29 B 0.43
A 0.29 B 0.57
A 0.29 B 0.71
A 0.29 B 0.86
A 0.29 B 1
A 0.43 B 0
A 0.43 B 0.14
A 0.43 B 0.29
A 0.43 B 0.43
A 0.43 B 0.57
A 0.43 B 0.71
A 0.43 B 0.86
A 0.43 B 1
A 0.57 B 0
A 0.57 B 0.14
A 0.57 B 0.29
A 0.57 B 0.43
A 0.57 B 0.57
A 0.57 B 0.71
A 0.57 B 0.86
A 0.57 B 1
A 0.71 B 0
A 0.71 B 0.14
A 0.71 B 0.29
A 0.71 B 0.43
A 0.71 B 0.57
A 0.71 B 0.71
A 0.71 B 0.86
A 0.71 B 1
A 0.86 B 0
A 0.86 B 0.14
A 0.86 B 0.29
A 0.86 B 0.43
A 0.86 B 0.57
A 0.86 B 0.71
A 0.86 B 0.86
A 0.86 B 1
A 1 B 0
A 1 B 0.14
A 1 B 0.29
A 1 B 0.43
A 1 B 0.57
A 1 B 0.71
A 1 B 0.86
A 1 B 1
OD 600 nm [–]
0.2
0.4
0.6
0 5 101520
Time [h]

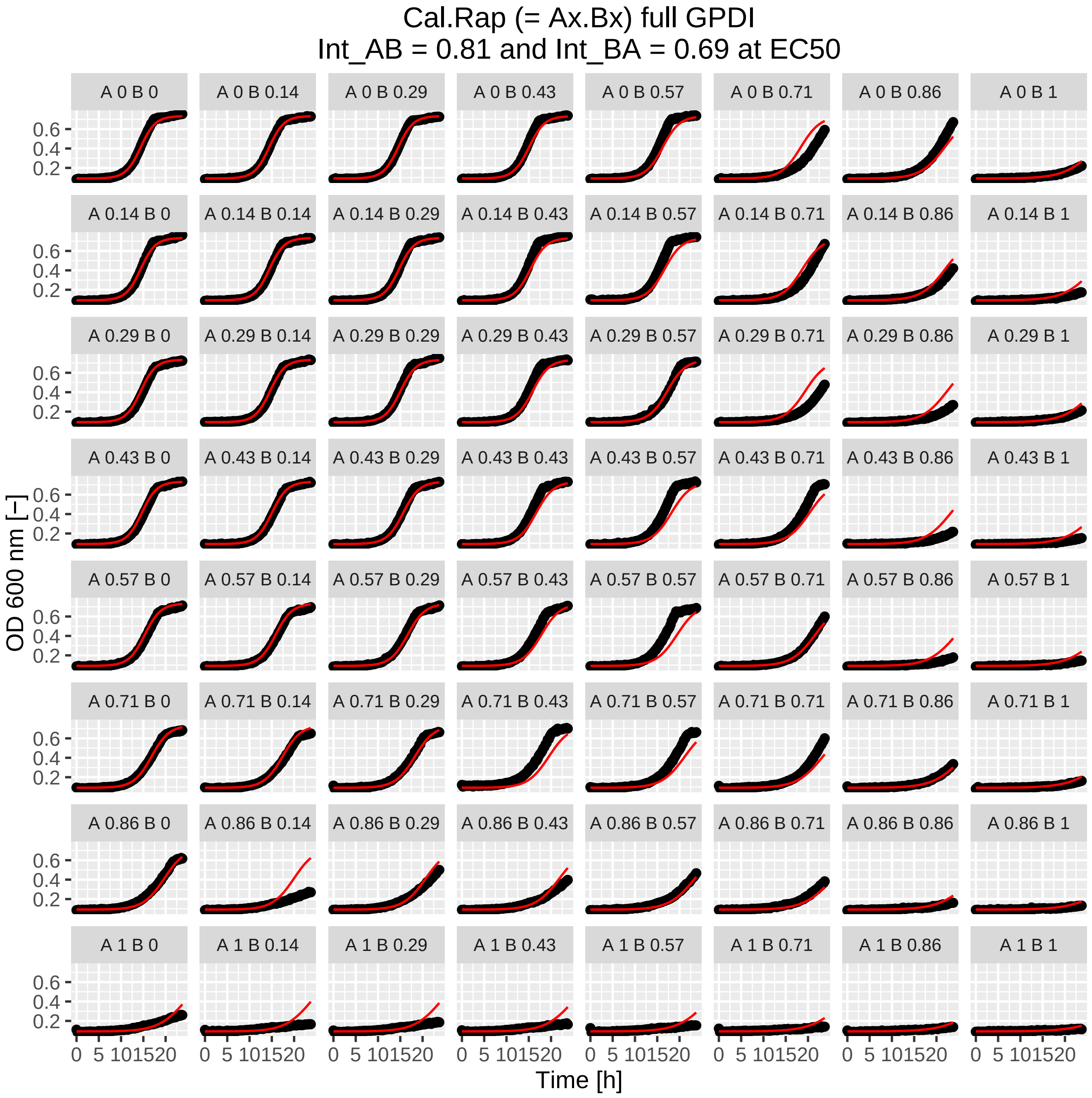
Cal.Rap (= Ax.Bx) full GPDI
Int_AB = 0.81 and Int_BA = 0.69 at EC50
A 0 B 0
A 0 B 0.14
A 0 B 0.29
A 0 B 0.43
A 0 B 0.57
A 0 B 0.71
A 0 B 0.86
A 0 B 1
A 0.14 B 0
A 0.14 B 0.14
A 0.14 B 0.29
A 0.14 B 0.43
A 0.14 B 0.57
A 0.14 B 0.71
A 0.14 B 0.86
A 0.14 B 1
A 0.29 B 0
A 0.29 B 0.14
A 0.29 B 0.29
A 0.29 B 0.43
A 0.29 B 0.57
A 0.29 B 0.71
A 0.29 B 0.86
A 0.29 B 1
A 0.43 B 0
A 0.43 B 0.14
A 0.43 B 0.29
A 0.43 B 0.43
A 0.43 B 0.57
A 0.43 B 0.71
A 0.43 B 0.86
A 0.43 B 1
A 0.57 B 0
A 0.57 B 0.14
A 0.57 B 0.29
A 0.57 B 0.43
A 0.57 B 0.57
A 0.57 B 0.71
A 0.57 B 0.86
A 0.57 B 1
A 0.71 B 0
A 0.71 B 0.14
A 0.71 B 0.29
A 0.71 B 0.43
A 0.71 B 0.57
A 0.71 B 0.71
A 0.71 B 0.86
A 0.71 B 1
A 0.86 B 0
A 0.86 B 0.14
A 0.86 B 0.29
A 0.86 B 0.43
A 0.86 B 0.57
A 0.86 B 0.71
A 0.86 B 0.86
A 0.86 B 1
A 1 B 0
A 1 B 0.14
A 1 B 0.29
A 1 B 0.43
A 1 B 0.57
A 1 B 0.71
A 1 B 0.86
A 1 B 1
OD 600 nm [–]
0.2
0.4
0.6
0 5 101520
Time [h]

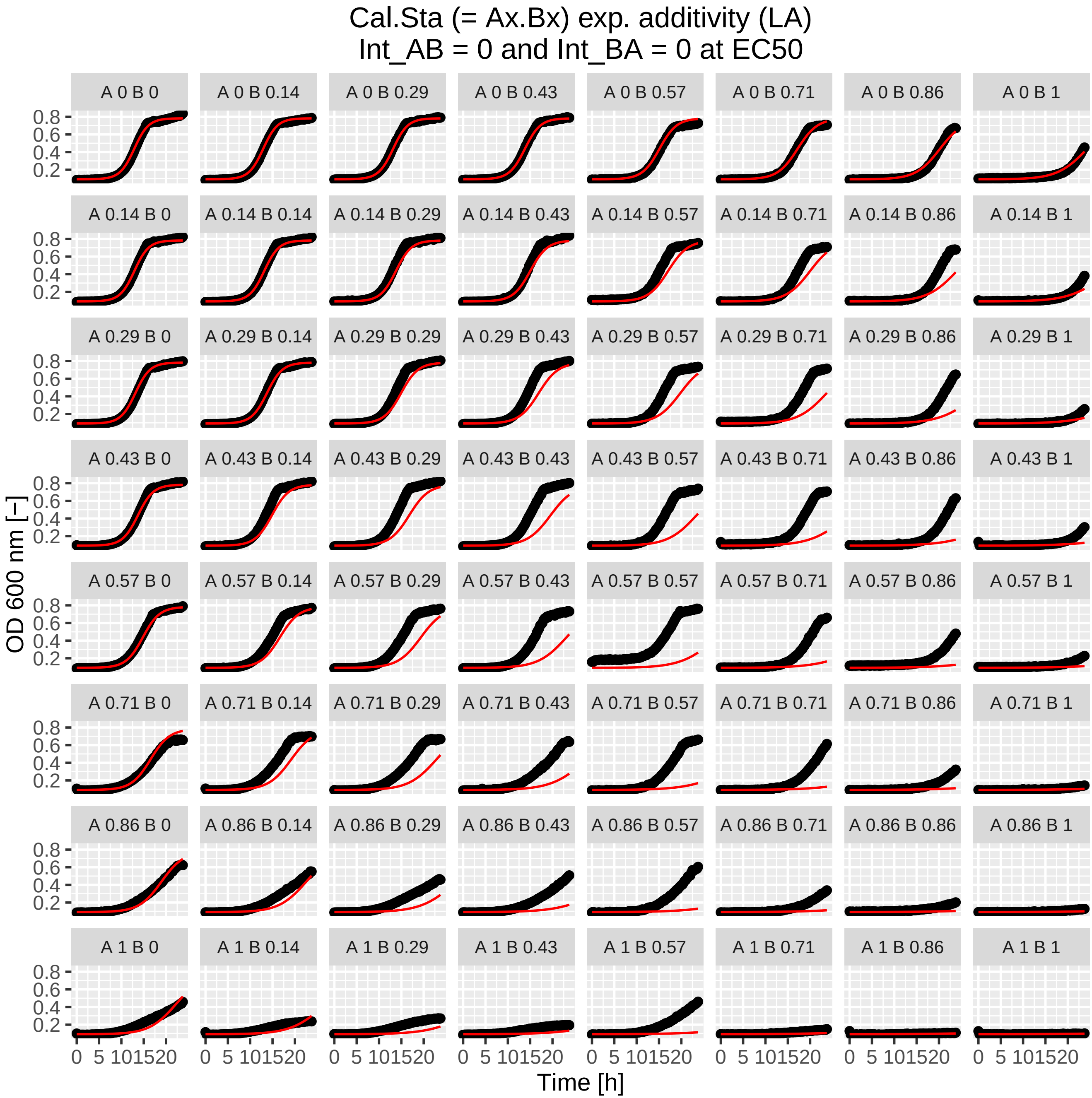

Cal.Sta (= Ax.Bx) exp. additivity (LA)
Int_AB = 0 and Int_BA = 0 at EC50
A 0 B 0
A 0 B 0.14
A 0 B 0.29
A 0 B 0.43
A 0 B 0.57
A 0 B 0.71
A 0 B 0.86
A 0 B 1
A 0.14 B 0
A 0.14 B 0.14
A 0.14 B 0.29
A 0.14 B 0.43
A 0.14 B 0.57
A 0.14 B 0.71
A 0.14 B 0.86
A 0.14 B 1
A 0.29 B 0
A 0.29 B 0.14
A 0.29 B 0.29
A 0.29 B 0.43
A 0.29 B 0.57
A 0.29 B 0.71
A 0.29 B 0.86
A 0.29 B 1
A 0.43 B 0
A 0.43 B 0.14
A 0.43 B 0.29
A 0.43 B 0.43
A 0.43 B 0.57
A 0.43 B 0.71
A 0.43 B 0.86
A 0.43 B 1
A 0.57 B 0
A 0.57 B 0.14
A 0.57 B 0.29
A 0.57 B 0.43
A 0.57 B 0.57
A 0.57 B 0.71
A 0.57 B 0.86
A 0.57 B 1
A 0.71 B 0
A 0.71 B 0.14
A 0.71 B 0.29
A 0.71 B 0.43
A 0.71 B 0.57
A 0.71 B 0.71
A 0.71 B 0.86
A 0.71 B 1
A 0.86 B 0
A 0.86 B 0.14
A 0.86 B 0.29
A 0.86 B 0.43
A 0.86 B 0.57
A 0.86 B 0.71
A 0.86 B 0.86
A 0.86 B 1
A 1 B 0
A 1 B 0.14
A 1 B 0.29
A 1 B 0.43
A 1 B 0.57
A 1 B 0.71
A 1 B 0.86
A 1 B 1
OD 600 nm [–]
0.2
0.4
0.6
0.8
0 5 101520
Time [h]

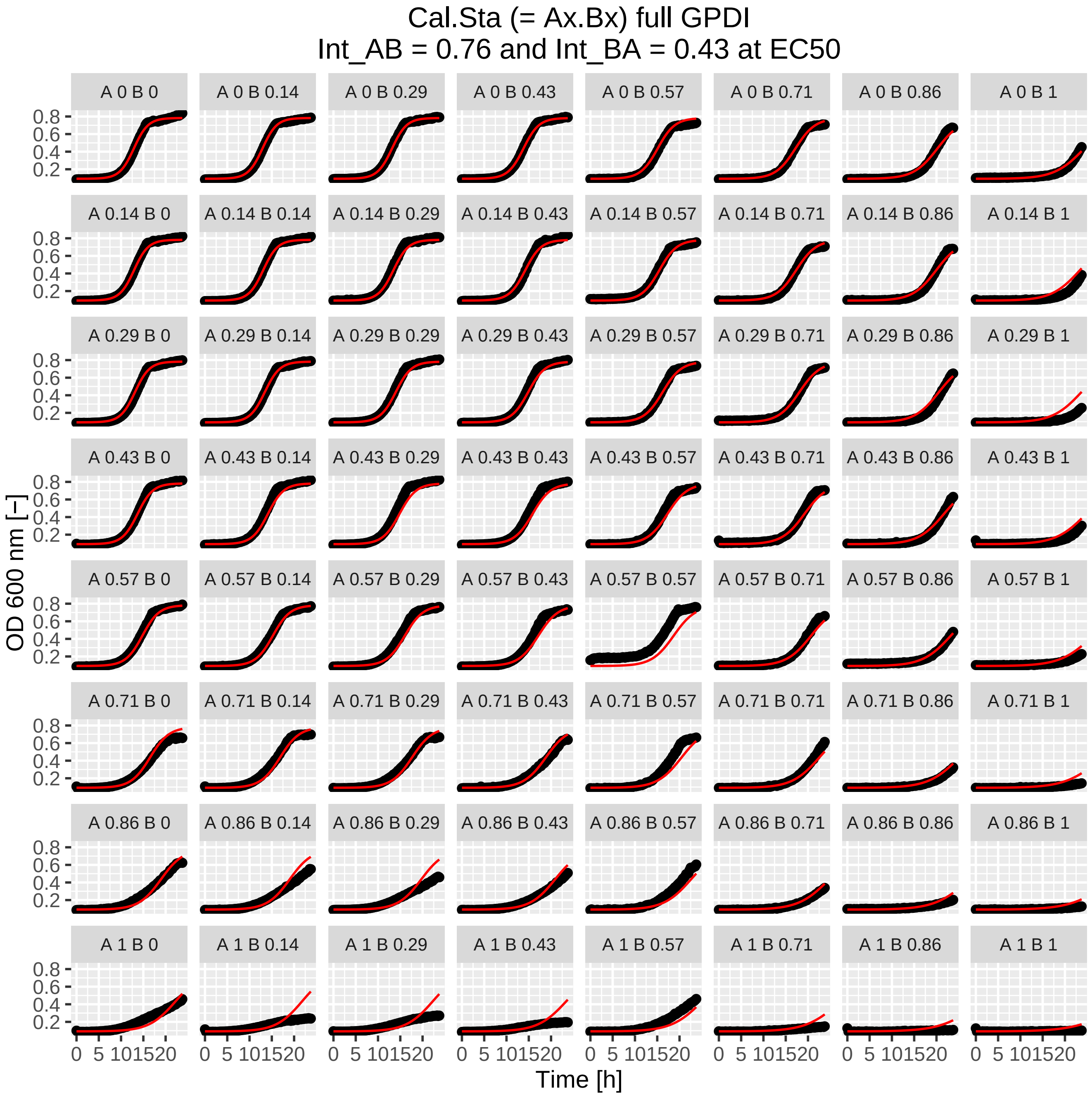
Cal.Sta (= Ax.Bx) full GPDI
Int_AB = 0.76 and Int_BA = 0.43 at EC50
A 0 B 0
A 0 B 0.14
A 0 B 0.29
A 0 B 0.43
A 0 B 0.57
A 0 B 0.71
A 0 B 0.86
A 0 B 1
A 0.14 B 0
A 0.14 B 0.14
A 0.14 B 0.29
A 0.14 B 0.43
A 0.14 B 0.57
A 0.14 B 0.71
A 0.14 B 0.86
A 0.14 B 1
A 0.29 B 0
A 0.29 B 0.14
A 0.29 B 0.29
A 0.29 B 0.43
A 0.29 B 0.57
A 0.29 B 0.71
A 0.29 B 0.86
A 0.29 B 1
A 0.43 B 0
A 0.43 B 0.14
A 0.43 B 0.29
A 0.43 B 0.43
A 0.43 B 0.57
A 0.43 B 0.71
A 0.43 B 0.86
A 0.43 B 1
A 0.57 B 0
A 0.57 B 0.14
A 0.57 B 0.29
A 0.57 B 0.43
A 0.57 B 0.57
A 0.57 B 0.71
A 0.57 B 0.86
A 0.57 B 1
A 0.71 B 0
A 0.71 B 0.14
A 0.71 B 0.29
A 0.71 B 0.43
A 0.71 B 0.57
A 0.71 B 0.71
A 0.71 B 0.86
A 0.71 B 1
A 0.86 B 0
A 0.86 B 0.14
A 0.86 B 0.29
A 0.86 B 0.43
A 0.86 B 0.57
A 0.86 B 0.71
A 0.86 B 0.86
A 0.86 B 1
A 1 B 0
A 1 B 0.14
A 1 B 0.29
A 1 B 0.43
A 1 B 0.57
A 1 B 0.71
A 1 B 0.86
A 1 B 1
OD 600 nm [−]
0.2
0.4
0.6
0.8
0
5
10
15
20
Time [h]

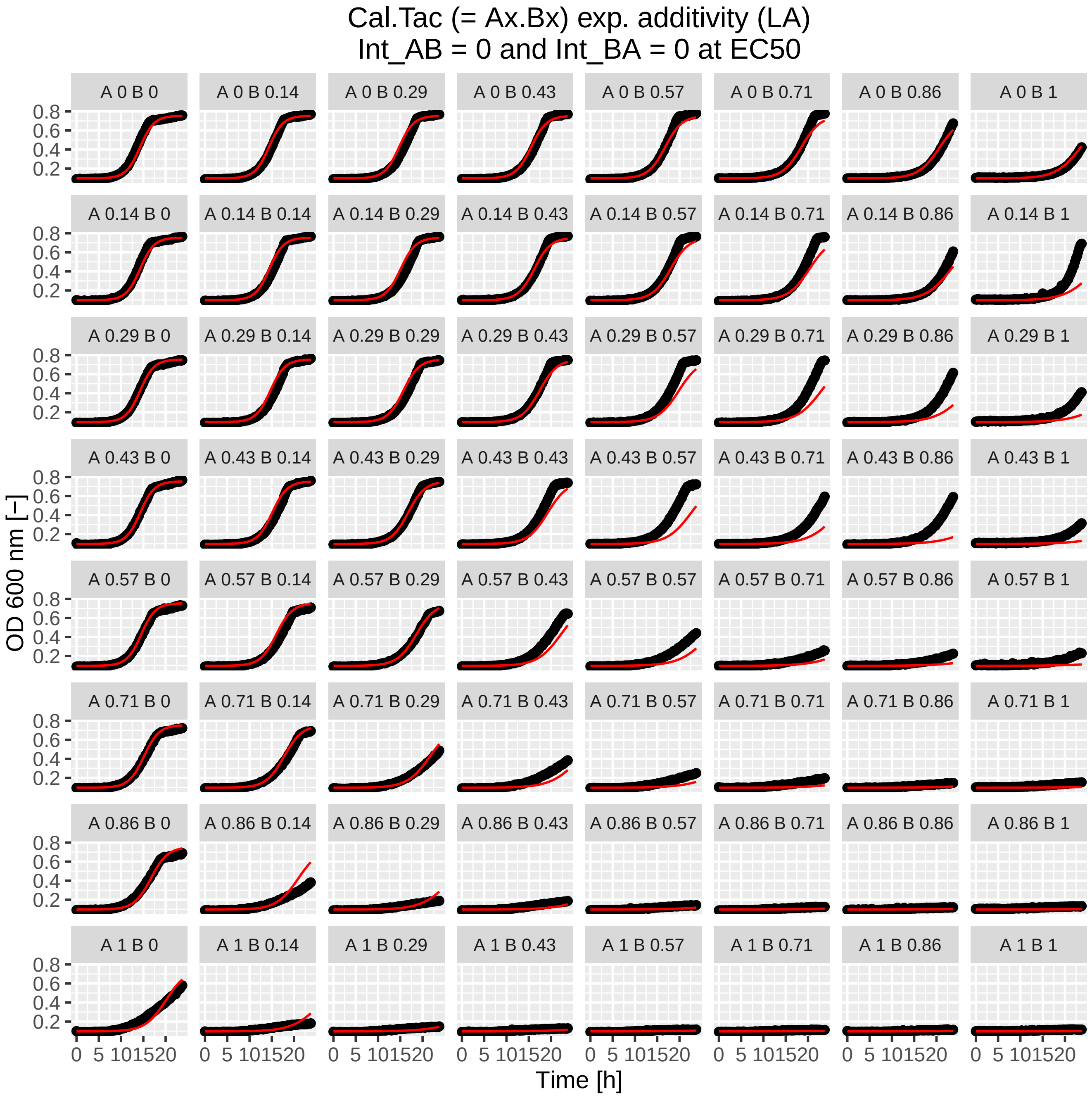
Cal.Tac (= Ax.Bx) exp. additivity (LA)
Int_AB = 0 and Int_BA = 0 at EC50
A 0 B 0
A 0 B 0.14
A 0 B 0.29
A 0 B 0.43
A 0 B 0.57
A 0 B 0.71
A 0 B 0.86
A 0 B 1
A 0.14 B 0
A 0.14 B 0.14
A 0.14 B 0.29
A 0.14 B 0.43
A 0.14 B 0.57
A 0.14 B 0.71
A 0.14 B 0.86
A 0.14 B 1
A 0.29 B 0
A 0.29 B 0.14
A 0.29 B 0.29
A 0.29 B 0.43
A 0.29 B 0.57
A 0.29 B 0.71
A 0.29 B 0.86
A 0.29 B 1
A 0.43 B 0
A 0.43 B 0.14
A 0.43 B 0.29
A 0.43 B 0.43
A 0.43 B 0.57
A 0.43 B 0.71
A 0.43 B 0.86
A 0.43 B 1
A 0.57 B 0
A 0.57 B 0.14
A 0.57 B 0.29
A 0.57 B 0.43
A 0.57 B 0.57
A 0.57 B 0.71
A 0.57 B 0.86
A 0.57 B 1
A 0.71 B 0
A 0.71 B 0.14
A 0.71 B 0.29
A 0.71 B 0.43
A 0.71 B 0.57
A 0.71 B 0.71
A 0.71 B 0.86
A 0.71 B 1
A 0.86 B 0
A 0.86 B 0.14
A 0.86 B 0.29
A 0.86 B 0.43
A 0.86 B 0.57
A 0.86 B 0.71
A 0.86 B 0.86
A 0.86 B 1
A 1 B 0
A 1 B 0.14
A 1 B 0.29
A 1 B 0.43
A 1 B 0.57
A 1 B 0.71
A 1 B 0.86
A 1 B 1
OD 600 nm [–]
0.2
0.4
0.6
0.8
0 5 101520
Time [h]

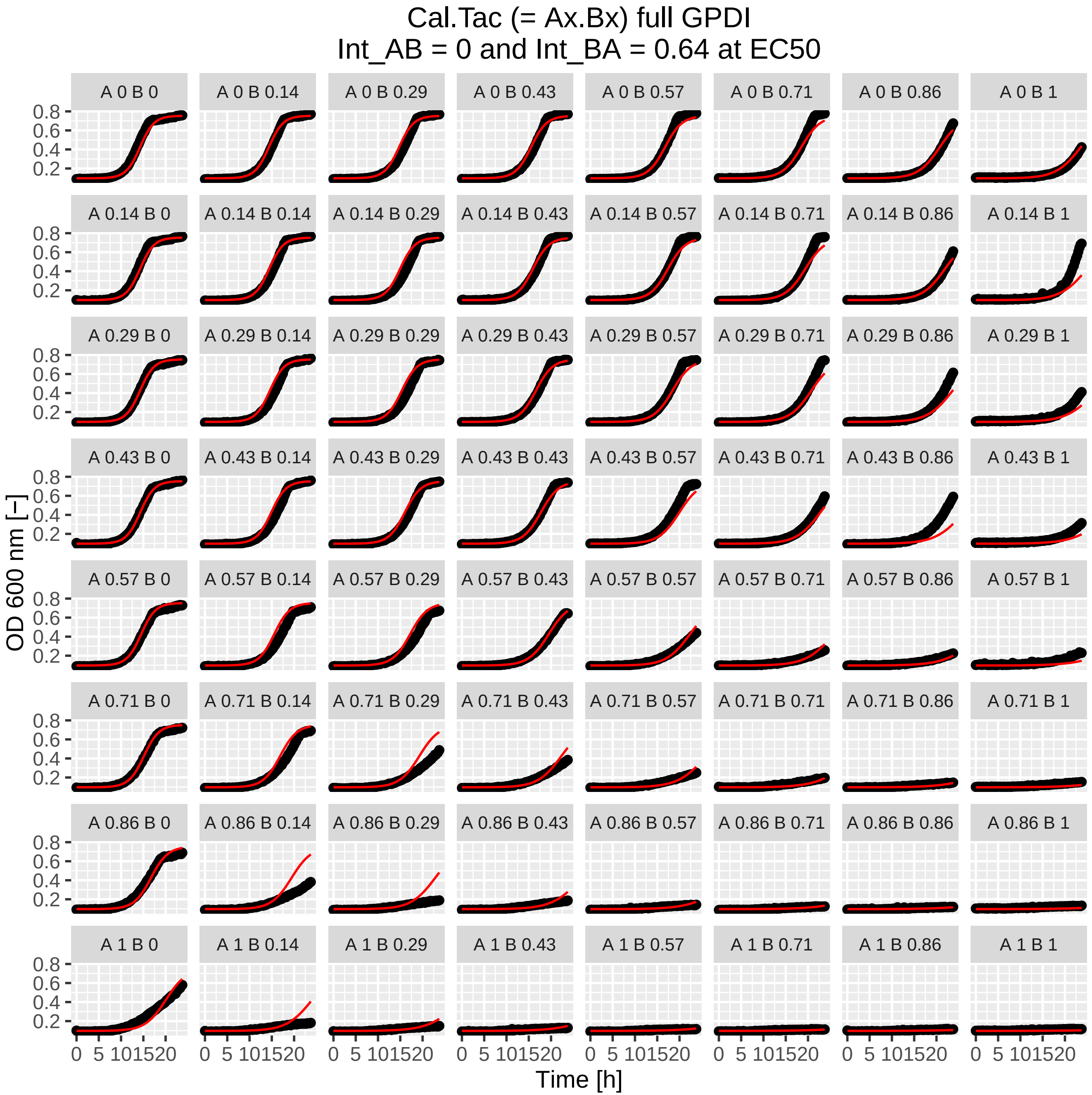

Cal.Tac (= Ax.Bx) full GPDI
Int_AB = 0 and Int_BA = 0.64 at EC50
A 0 B 0
A 0 B 0.14
A 0 B 0.29
A 0 B 0.43
A 0 B 0.57
A 0 B 0.71
A 0 B 0.86
A 0 B 1
A 0.14 B 0
A 0.14 B 0.14
A 0.14 B 0.29
A 0.14 B 0.43
A 0.14 B 0.57
A 0.14 B 0.71
A 0.14 B 0.86
A 0.14 B 1
A 0.29 B 0
A 0.29 B 0.14
A 0.29 B 0.29
A 0.29 B 0.43
A 0.29 B 0.57
A 0.29 B 0.71
A 0.29 B 0.86
A 0.29 B 1
A 0.43 B 0
A 0.43 B 0.14
A 0.43 B 0.29
A 0.43 B 0.43
A 0.43 B 0.57
A 0.43 B 0.71
A 0.43 B 0.86
A 0.43 B 1
A 0.57 B 0
A 0.57 B 0.14
A 0.57 B 0.29
A 0.57 B 0.43
A 0.57 B 0.57
A 0.57 B 0.71
A 0.57 B 0.86
A 0.57 B 1
A 0.71 B 0
A 0.71 B 0.14
A 0.71 B 0.29
A 0.71 B 0.43
A 0.71 B 0.57
A 0.71 B 0.71
A 0.71 B 0.86
A 0.71 B 1
A 0.86 B 0
A 0.86 B 0.14
A 0.86 B 0.29
A 0.86 B 0.43
A 0.86 B 0.57
A 0.86 B 0.71
A 0.86 B 0.86
A 0.86 B 1
A 1 B 0
A 1 B 0.14
A 1 B 0.29
A 1 B 0.43
A 1 B 0.57
A 1 B 0.71
A 1 B 0.86
A 1 B 1
0.8
0.6
0.4
0.2
OD 600 nm [−]
0 5 101520
Time [h]

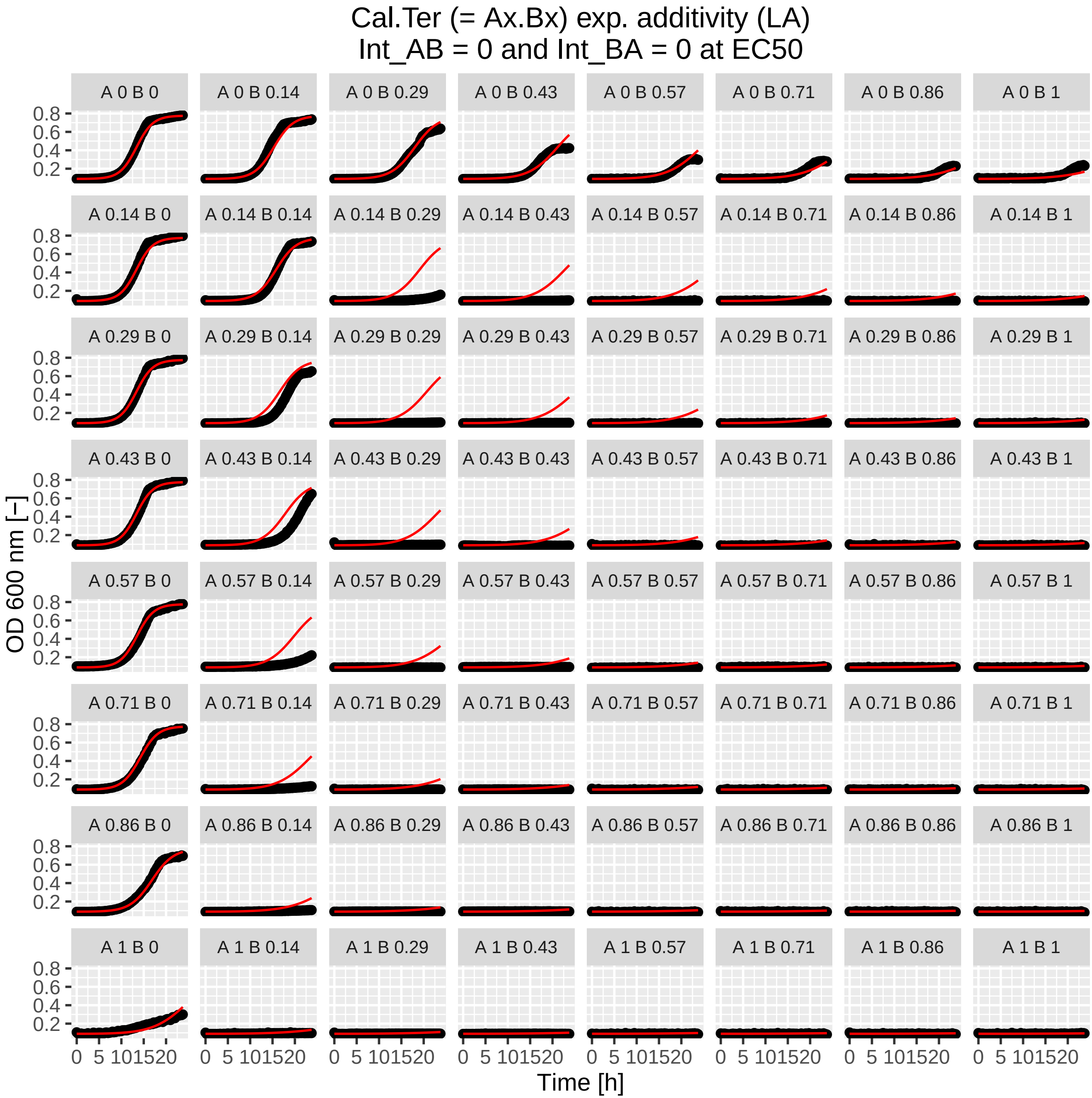
Cal.Ter (= Ax.Bx) exp. additivity (LA)
Int_AB = 0 and Int_BA = 0 at EC50
OD 600 nm [–]
Time [h]
A 0 B 0
A 0 B 0.14
A 0 B 0.29
A 0 B 0.43
A 0 B 0.57
A 0 B 0.71
A 0 B 0.86
A 0 B 1
A 1 B 0
A 1 B 0.14
A 1 B 0.29
A 1 B 0.43
A 1 B 0.57
A 1 B 0.71
A 1 B 0.86
A 1 B 1

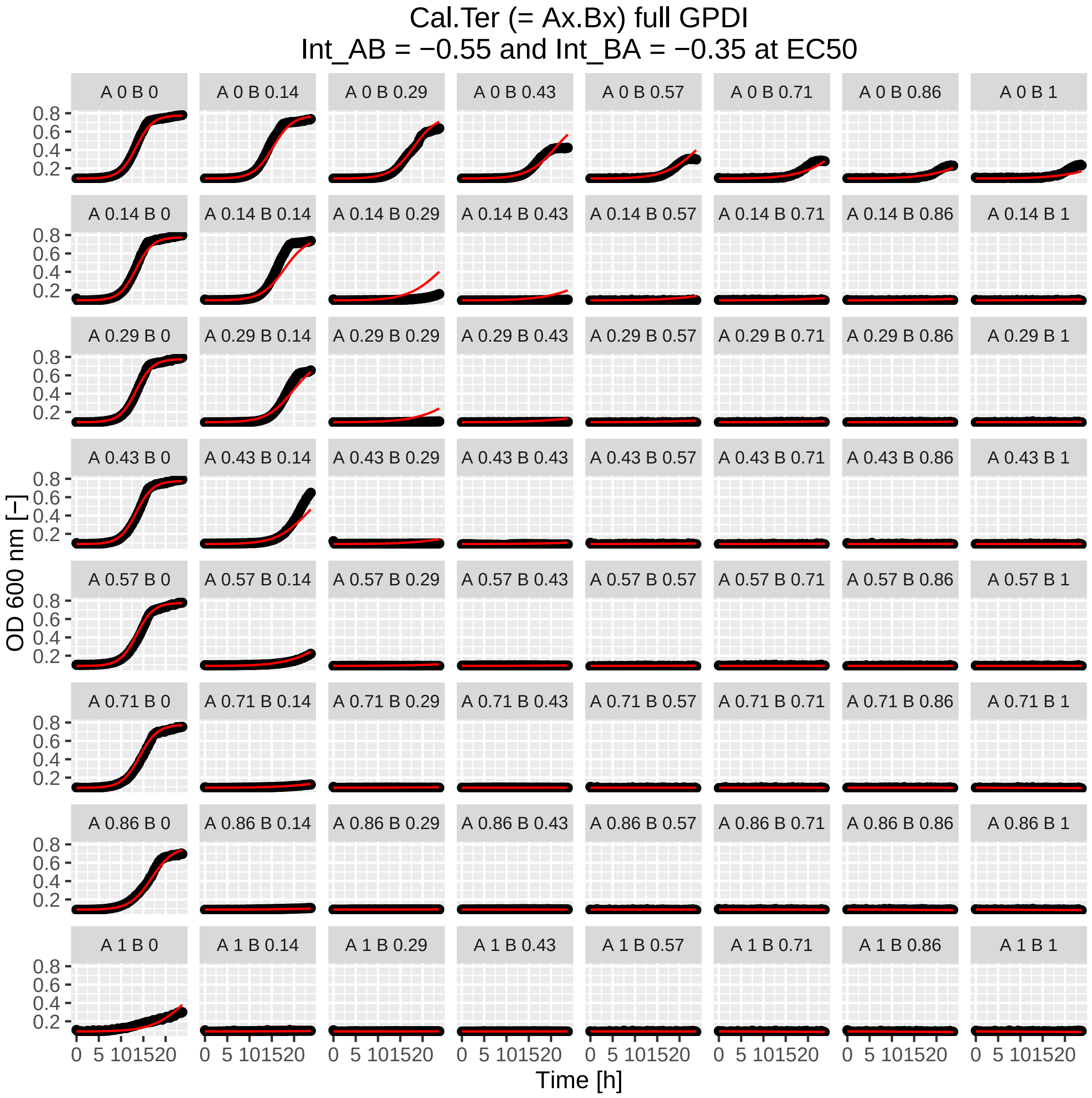

Cal.Ter (= Ax.Bx) full GPDI
Int_AB = −0.55 and Int_BA = −0.35 at EC50
A 0 B 0
A 0 B 0.14
A 0 B 0.29
A 0 B 0.43
A 0 B 0.57
A 0 B 0.71
A 0 B 0.86
A 0 B 1
A 0.14 B 0
A 0.14 B 0.14
A 0.14 B 0.29
A 0.14 B 0.43
A 0.14 B 0.57
A 0.14 B 0.71
A 0.14 B 0.86
A 0.14 B 1
A 0.29 B 0
A 0.29 B 0.14
A 0.29 B 0.29
A 0.29 B 0.43
A 0.29 B 0.57
A 0.29 B 0.71
A 0.29 B 0.86
A 0.29 B 1
A 0.43 B 0
A 0.43 B 0.14
A 0.43 B 0.29
A 0.43 B 0.43
A 0.43 B 0.57
A 0.43 B 0.71
A 0.43 B 0.86
A 0.43 B 1
A 0.57 B 0
A 0.57 B 0.14
A 0.57 B 0.29
A 0.57 B 0.43
A 0.57 B 0.57
A 0.57 B 0.71
A 0.57 B 0.86
A 0.57 B 1
A 0.71 B 0
A 0.71 B 0.14
A 0.71 B 0.29
A 0.71 B 0.43
A 0.71 B 0.57
A 0.71 B 0.71
A 0.71 B 0.86
A 0.71 B 1
A 0.86 B 0
A 0.86 B 0.14
A 0.86 B 0.29
A 0.86 B 0.43
A 0.86 B 0.57
A 0.86 B 0.71
A 0.86 B 0.86
A 0.86 B 1
A 1 B 0
A 1 B 0.14
A 1 B 0.29
A 1 B 0.43
A 1 B 0.57
A 1 B 0.71
A 1 B 0.86
A 1 B 1
0.8
0.6
0.4
0.2
OD 600 nm [−]
0 5 101520
Time [h]

Cal.Tun (= Ax.Bx) exp. additivity (LA)
Int_AB = 0 and Int_BA = 0 at EC50

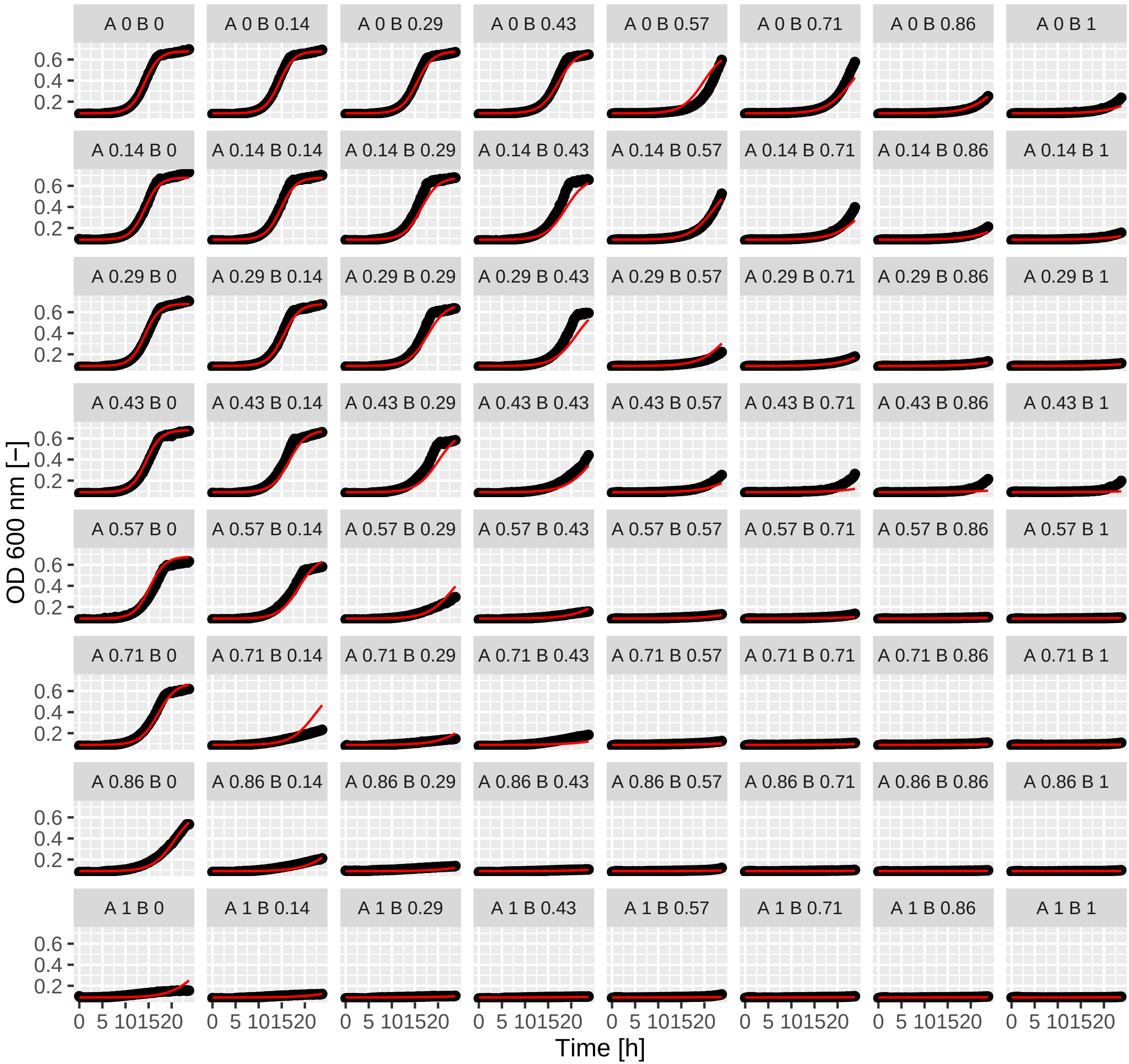

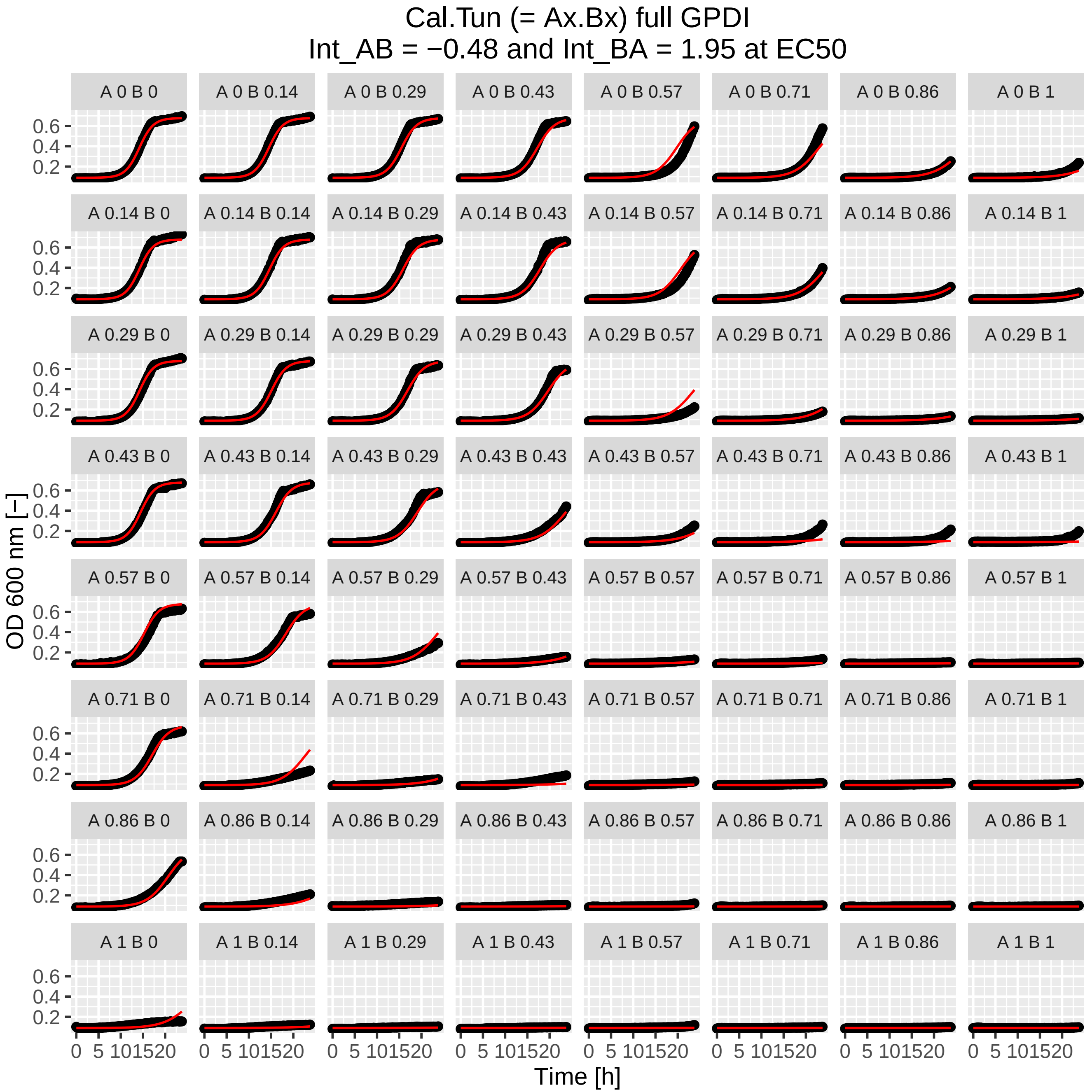
Cal.Tun (= Ax.Bx) full GPDI
Int_AB = −0.48 and Int_BA = 1.95 at EC50
A 0 B 0
A 0 B 0.14
A 0 B 0.29
A 0 B 0.43
A 0 B 0.57
A 0 B 0.71
A 0 B 0.86
A 0 B 1
A 0.14 B 0
A 0.14 B 0.14
A 0.14 B 0.29
A 0.14 B 0.43
A 0.14 B 0.57
A 0.14 B 0.71
A 0.14 B 0.86
A 0.14 B 1
A 0.29 B 0
A 0.29 B 0.14
A 0.29 B 0.29
A 0.29 B 0.43
A 0.29 B 0.57
A 0.29 B 0.71
A 0.29 B 0.86
A 0.29 B 1
A 0.43 B 0
A 0.43 B 0.14
A 0.43 B 0.29
A 0.43 B 0.43
A 0.43 B 0.57
A 0.43 B 0.71
A 0.43 B 0.86
A 0.43 B 1
A 0.57 B 0
A 0.57 B 0.14
A 0.57 B 0.29
A 0.57 B 0.43
A 0.57 B 0.57
A 0.57 B 0.71
A 0.57 B 0.86
A 0.57 B 1
A 0.71 B 0
A 0.71 B 0.14
A 0.71 B 0.29
A 0.71 B 0.43
A 0.71 B 0.57
A 0.71 B 0.71
A 0.71 B 0.86
A 0.71 B 1
A 0.86 B 0
A 0.86 B 0.14
A 0.86 B 0.29
A 0.86 B 0.43
A 0.86 B 0.57
A 0.86 B 0.71
A 0.86 B 0.86
A 0.86 B 1
A 1 B 0
A 1 B 0.14
A 1 B 0.29
A 1 B 0.43
A 1 B 0.57
A 1 B 0.71
A 1 B 0.86
A 1 B 1
OD 600 nm [−]
0.6
0.4
0.2
0 5 101520
Time [h]

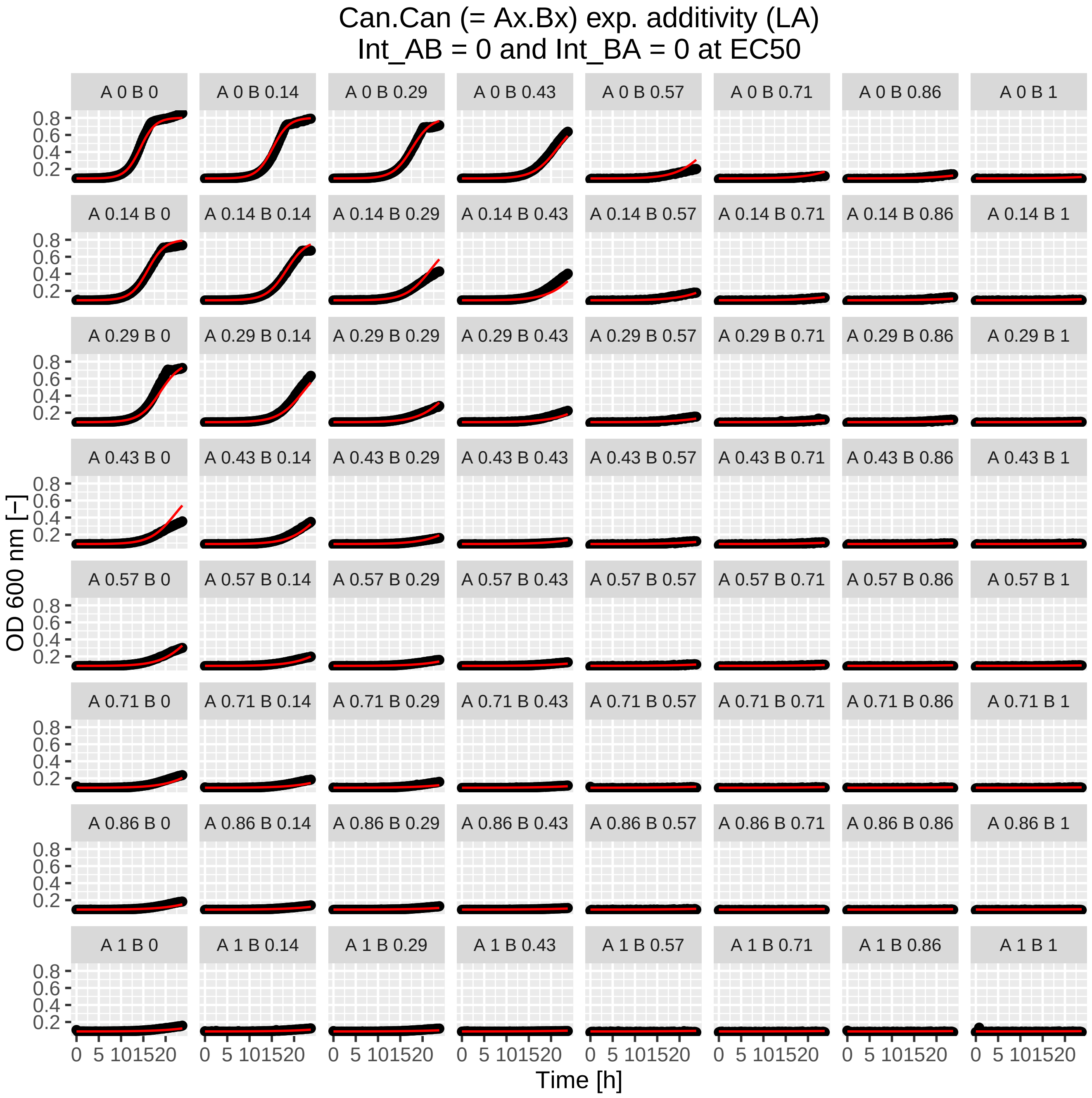

Can.Can (= Ax.Bx) exp. additivity (LA)
Int_AB = 0 and Int_BA = 0 at EC50
A 0 B 0
A 0 B 0.14
A 0 B 0.29
A 0 B 0.43
A 0 B 0.57
A 0 B 0.71
A 0 B 0.86
A 0 B 1
A 0.14 B 0
A 0.14 B 0.14
A 0.14 B 0.29
A 0.14 B 0.43
A 0.14 B 0.57
A 0.14 B 0.71
A 0.14 B 0.86
A 0.14 B 1
A 0.29 B 0
A 0.29 B 0.14
A 0.29 B 0.29
A 0.29 B 0.43
A 0.29 B 0.57
A 0.29 B 0.71
A 0.29 B 0.86
A 0.29 B 1
A 0.43 B 0
A 0.43 B 0.14
A 0.43 B 0.29
A 0.43 B 0.43
A 0.43 B 0.57
A 0.43 B 0.71
A 0.43 B 0.86
A 0.43 B 1
A 0.57 B 0
A 0.57 B 0.14
A 0.57 B 0.29
A 0.57 B 0.43
A 0.57 B 0.57
A 0.57 B 0.71
A 0.57 B 0.86
A 0.57 B 1
A 0.71 B 0
A 0.71 B 0.14
A 0.71 B 0.29
A 0.71 B 0.43
A 0.71 B 0.57
A 0.71 B 0.71
A 0.71 B 0.86
A 0.71 B 1
A 0.86 B 0
A 0.86 B 0.14
A 0.86 B 0.29
A 0.86 B 0.43
A 0.86 B 0.57
A 0.86 B 0.71
A 0.86 B 0.86
A 0.86 B 1
A 1 B 0
A 1 B 0.14
A 1 B 0.29
A 1 B 0.43
A 1 B 0.57
A 1 B 0.71
A 1 B 0.86
A 1 B 1
0.8
0.6
0.4
0.2
0 5 101520
OD 600 nm [–]
Time [h]

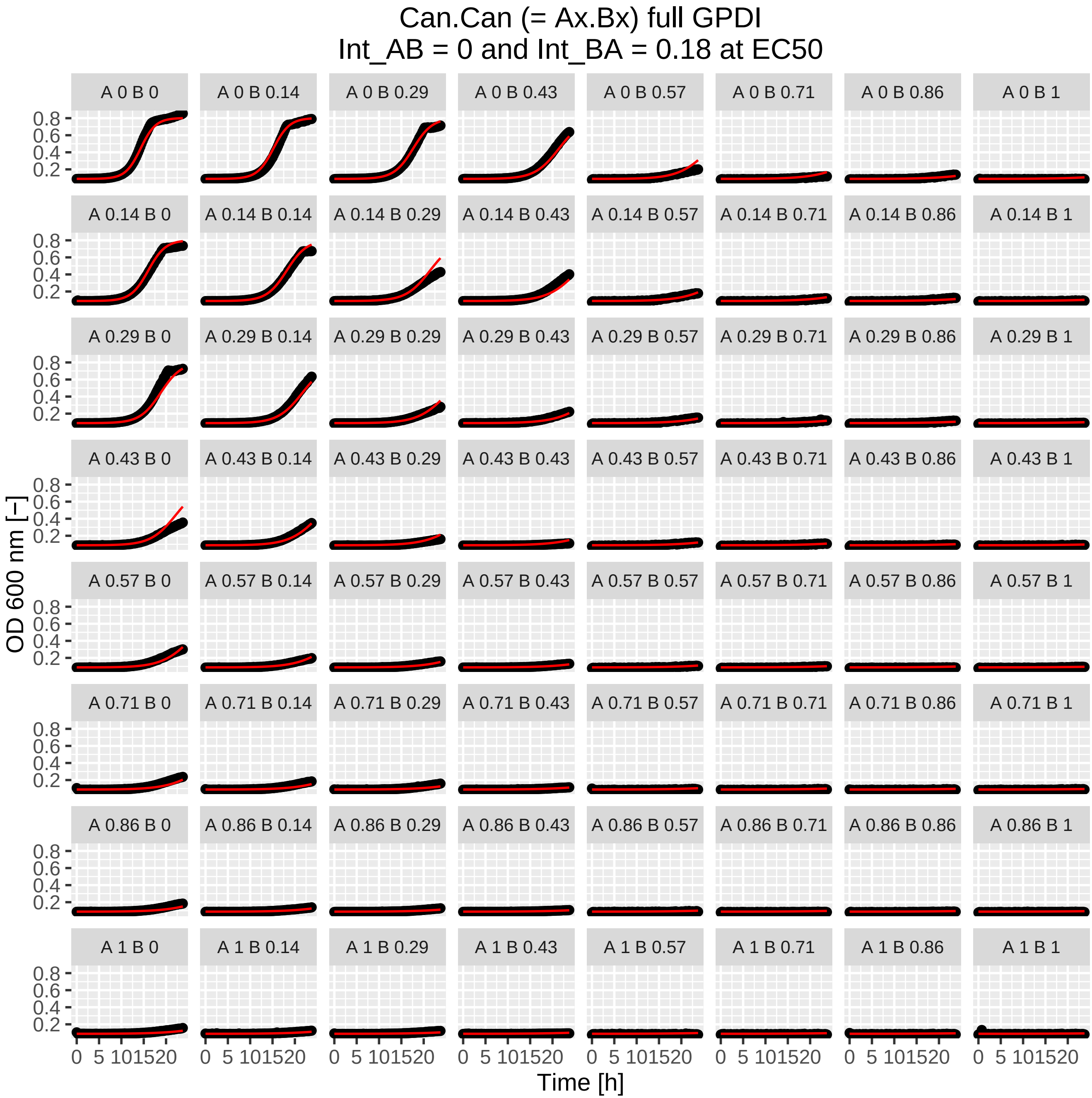
Can.Can (= Ax.Bx) full GPDI
Int_AB = 0 and Int_BA = 0.18 at EC50
A 0 B 0
A 0 B 0.14
A 0 B 0.29
A 0 B 0.43
A 0 B 0.57
A 0 B 0.71
A 0 B 0.86
A 0 B 1
A 0.14 B 0
A 0.14 B 0.14
A 0.14 B 0.29
A 0.14 B 0.43
A 0.14 B 0.57
A 0.14 B 0.71
A 0.14 B 0.86
A 0.14 B 1
A 0.29 B 0
A 0.29 B 0.14
A 0.29 B 0.29
A 0.29 B 0.43
A 0.29 B 0.57
A 0.29 B 0.71
A 0.29 B 0.86
A 0.29 B 1
A 0.43 B 0
A 0.43 B 0.14
A 0.43 B 0.29
A 0.43 B 0.43
A 0.43 B 0.57
A 0.43 B 0.71
A 0.43 B 0.86
A 0.43 B 1
A 0.57 B 0
A 0.57 B 0.14
A 0.57 B 0.29
A 0.57 B 0.43
A 0.57 B 0.57
A 0.57 B 0.71
A 0.57 B 0.86
A 0.57 B 1
A 0.71 B 0
A 0.71 B 0.14
A 0.71 B 0.29
A 0.71 B 0.43
A 0.71 B 0.57
A 0.71 B 0.71
A 0.71 B 0.86
A 0.71 B 1
A 0.86 B 0
A 0.86 B 0.14
A 0.86 B 0.29
A 0.86 B 0.43
A 0.86 B 0.57
A 0.86 B 0.71
A 0.86 B 0.86
A 0.86 B 1
A 1 B 0
A 1 B 0.14
A 1 B 0.29
A 1 B 0.43
A 1 B 0.57
A 1 B 0.71
A 1 B 0.86
A 1 B 1
0.8
0.6
0.4
0.2
OD 600 nm [–]
0 5 101520
Time [h]

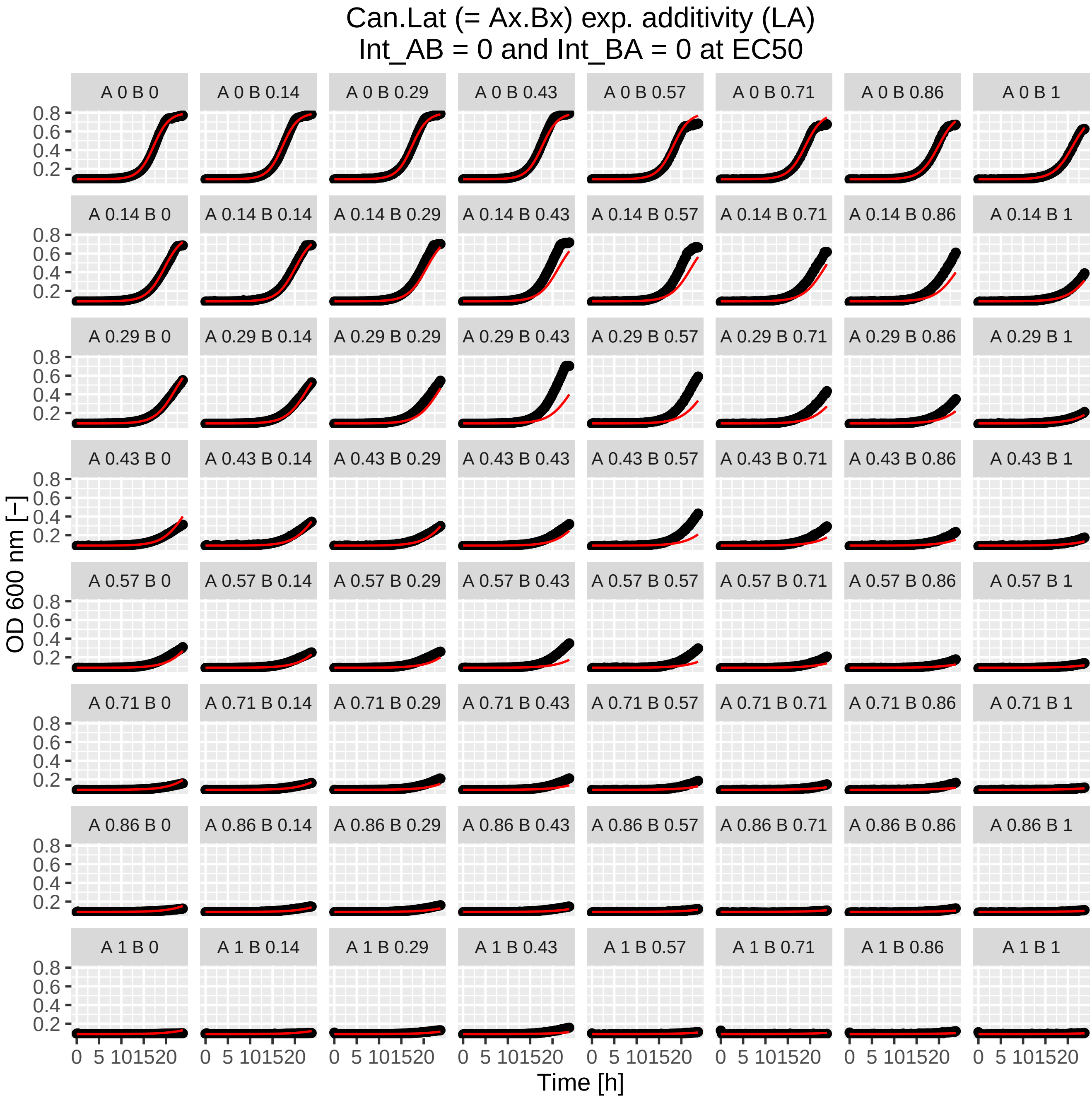
Can.Lat (= Ax.Bx) exp. additivity (LA)
Int_AB = 0 and Int_BA = 0 at EC50
OD 600 nm [–]
Time [h]
0.8
0.6
0.4
0.2
0 5 101520
A 0 B 0
A 0 B 0.14
A 0 B 0.29
A 0 B 0.43
A 0 B 0.57
A 0 B 0.71
A 0 B 0.86
A 0 B 1
A 0.14 B 0
A 0.14 B 0.14
A 0.14 B 0.29
A 0.14 B 0.43
A 0.14 B 0.57
A 0.14 B 0.71
A 0.14 B 0.86
A 0.14 B 1
A 0.29 B 0
A 0.29 B 0.14
A 0.29 B 0.29
A 0.29 B 0.43
A 0.29 B 0.57
A 0.29 B 0.71
A 0.29 B 0.86
A 0.29 B 1
A 0.43 B 0
A 0.43 B 0.14
A 0.43 B 0.29
A 0.43 B 0.43
A 0.43 B 0.57
A 0.43 B 0.71
A 0.43 B 0.86
A 0.43 B 1
A 0.57 B 0
A 0.57 B 0.14
A 0.57 B 0.29
A 0.57 B 0.43
A 0.57 B 0.57
A 0.57 B 0.71
A 0.57 B 0.86
A 0.57 B 1
A 0.71 B 0
A 0.71 B 0.14
A 0.71 B 0.29
A 0.71 B 0.43
A 0.71 B 0.57
A 0.71 B 0.71
A 0.71 B 0.86
A 0.71 B 1
A 0.86 B 0
A 0.86 B 0.14
A 0.86 B 0.29
A 0.86 B 0.43
A 0.86 B 0.57
A 0.86 B 0.71
A 0.86 B 0.86
A 0.86 B 1
A 1 B 0
A 1 B 0.14
A 1 B 0.29
A 1 B 0.43
A 1 B 0.57
A 1 B 0.71
A 1 B 0.86
A 1 B 1

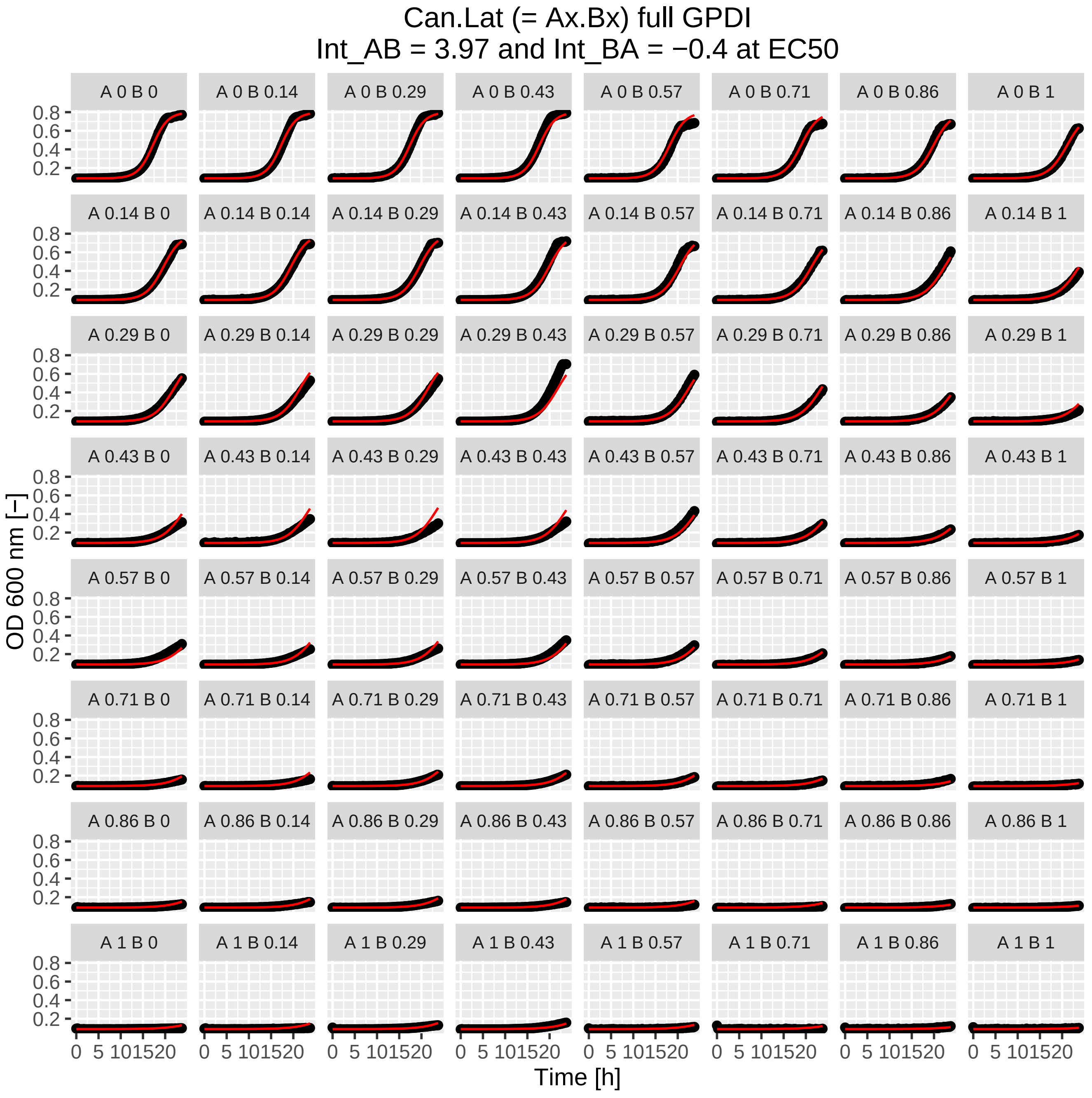
Can.Lat (= Ax.Bx) full GPDI
Int_AB = 3.97 and Int_BA = −0.4 at EC50
OD 600 nm [−]
Time [h]
A 0 B 0
A 0 B 0.14
A 0 B 0.29
A 0 B 0.43
A 0 B 0.57
A 0 B 0.71
A 0 B 0.86
A 0 B 1
A 1 B 0
A 1 B 0.14
A 1 B 0.29
A 1 B 0.43
A 1 B 0.57
A 1 B 0.71
A 1 B 0.86
A 1 B 1

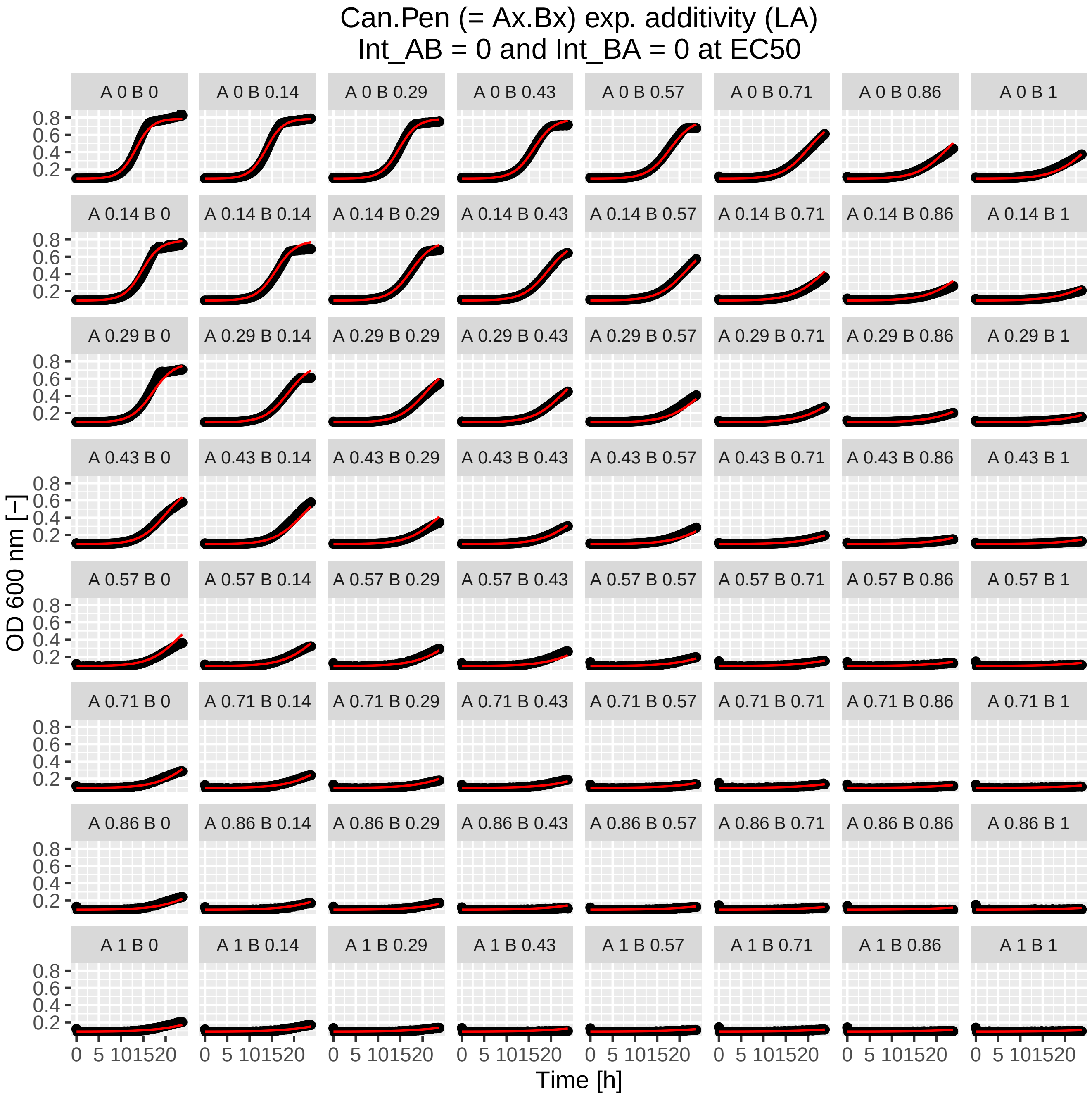
Can.Pen (= Ax.Bx) exp. additivity (LA)
Int_AB = 0 and Int_BA = 0 at EC50
OD 600 nm [–]
Time [h]
A 0 B 0
A 0 B 0.14
A 0 B 0.29
A 0 B 0.43
A 0 B 0.57
A 0 B 0.71
A 0 B 0.86
A 0 B 1
A 0.14 B 0
A 0.29 B 0
A 0.43 B 0
A 0.57 B 0
A 0.71 B 0
A 0.86 B 0
A 1 B 0
A 1 B 1

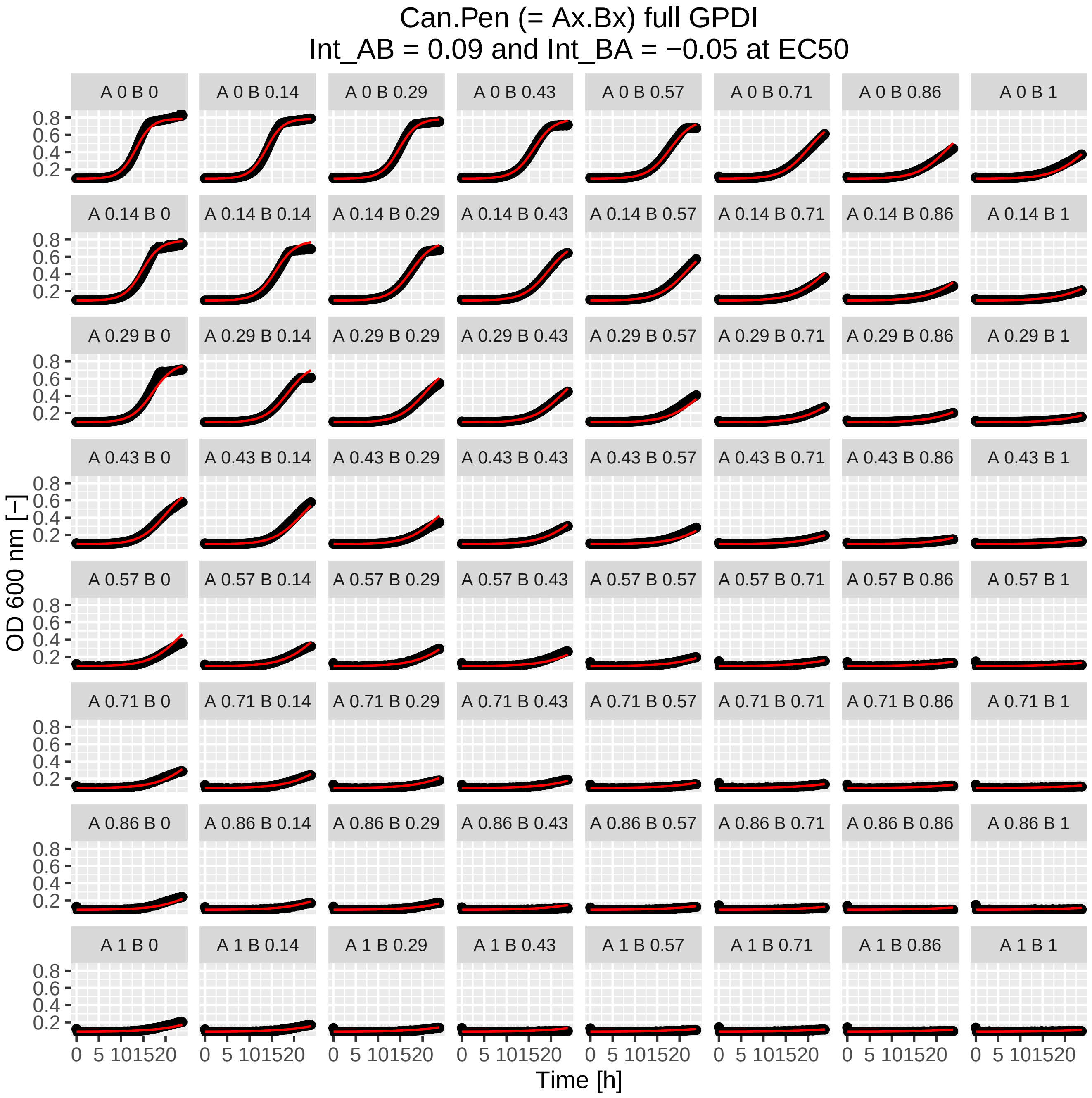
Can.Pen (= Ax.Bx) full GPDI
Int_AB = 0.09 and Int_BA = −0.05 at EC50
A 0 B 0
A 0 B 0.14
A 0 B 0.29
A 0 B 0.43
A 0 B 0.57
A 0 B 0.71
A 0 B 0.86
A 0 B 1
A 0.14 B 0
A 0.14 B 0.14
A 0.14 B 0.29
A 0.14 B 0.43
A 0.14 B 0.57
A 0.14 B 0.71
A 0.14 B 0.86
A 0.14 B 1
A 0.29 B 0
A 0.29 B 0.14
A 0.29 B 0.29
A 0.29 B 0.43
A 0.29 B 0.57
A 0.29 B 0.71
A 0.29 B 0.86
A 0.29 B 1
A 0.43 B 0
A 0.43 B 0.14
A 0.43 B 0.29
A 0.43 B 0.43
A 0.43 B 0.57
A 0.43 B 0.71
A 0.43 B 0.86
A 0.43 B 1
A 0.57 B 0
A 0.57 B 0.14
A 0.57 B 0.29
A 0.57 B 0.43
A 0.57 B 0.57
A 0.57 B 0.71
A 0.57 B 0.86
A 0.57 B 1
A 0.71 B 0
A 0.71 B 0.14
A 0.71 B 0.29
A 0.71 B 0.43
A 0.71 B 0.57
A 0.71 B 0.71
A 0.71 B 0.86
A 0.71 B 1
A 0.86 B 0
A 0.86 B 0.14
A 0.86 B 0.29
A 0.86 B 0.43
A 0.86 B 0.57
A 0.86 B 0.71
A 0.86 B 0.86
A 0.86 B 1
A 1 B 0
A 1 B 0.14
A 1 B 0.29
A 1 B 0.43
A 1 B 0.57
A 1 B 0.71
A 1 B 0.86
A 1 B 1
OD 600 nm [−]
0.2
0.4
0.6
0.8
0
5
10
15
20
Time [h]

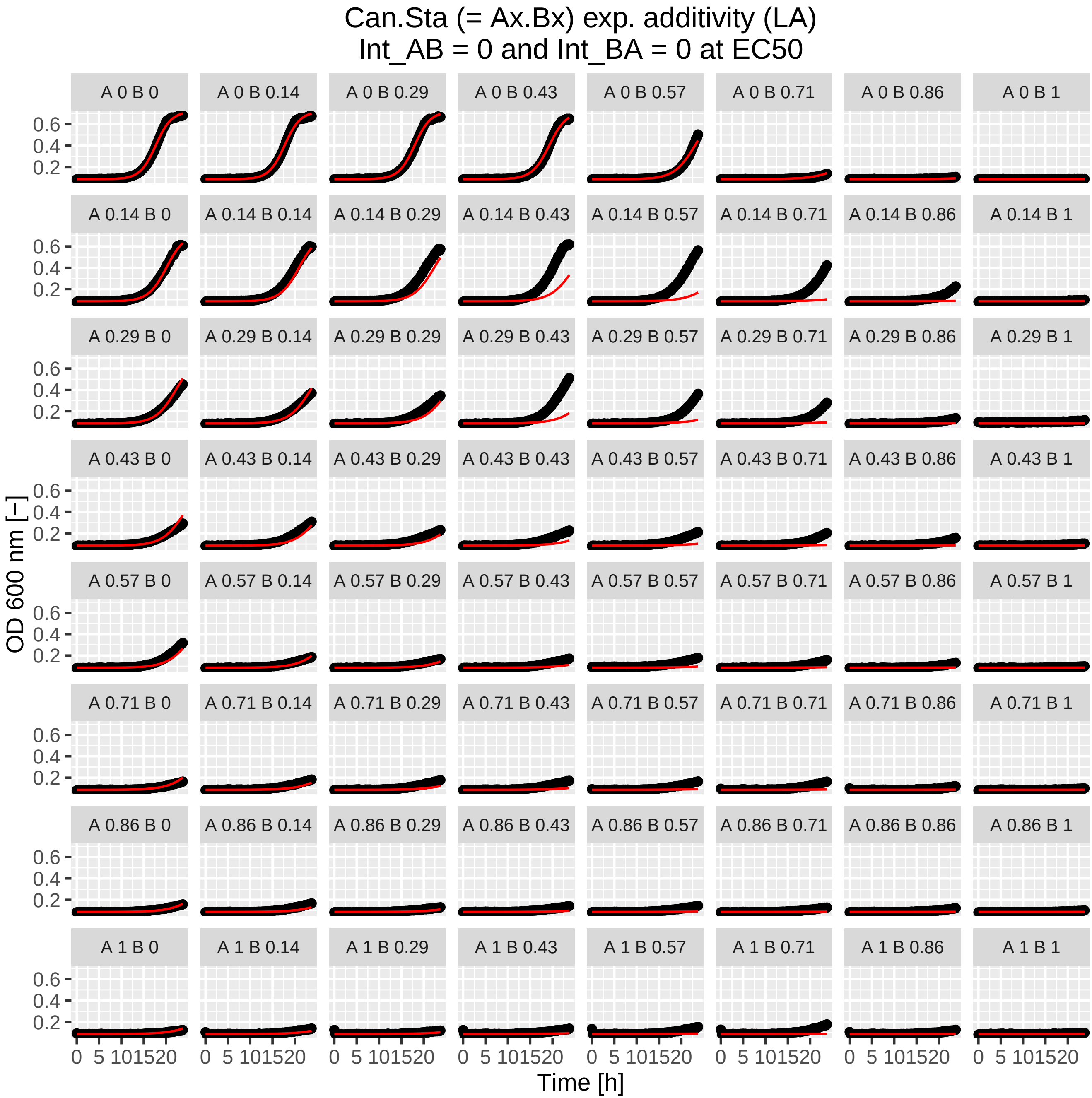
Can.Sta (= Ax.Bx) exp. additivity (LA)
Int_AB = 0 and Int_BA = 0 at EC50
OD 600 nm [–]
Time [h]
A 0 B 0
A 0 B 0.14
A 0 B 0.29
A 0 B 0.43
A 0 B 0.57
A 0 B 0.71
A 0 B 0.86
A 0 B 1
A 1 B 0
A 1 B 0.14
A 1 B 0.29
A 1 B 0.43
A 1 B 0.57
A 1 B 0.71
A 1 B 0.86
A 1 B 1
0.6
0.4
0.2
0 5 101520

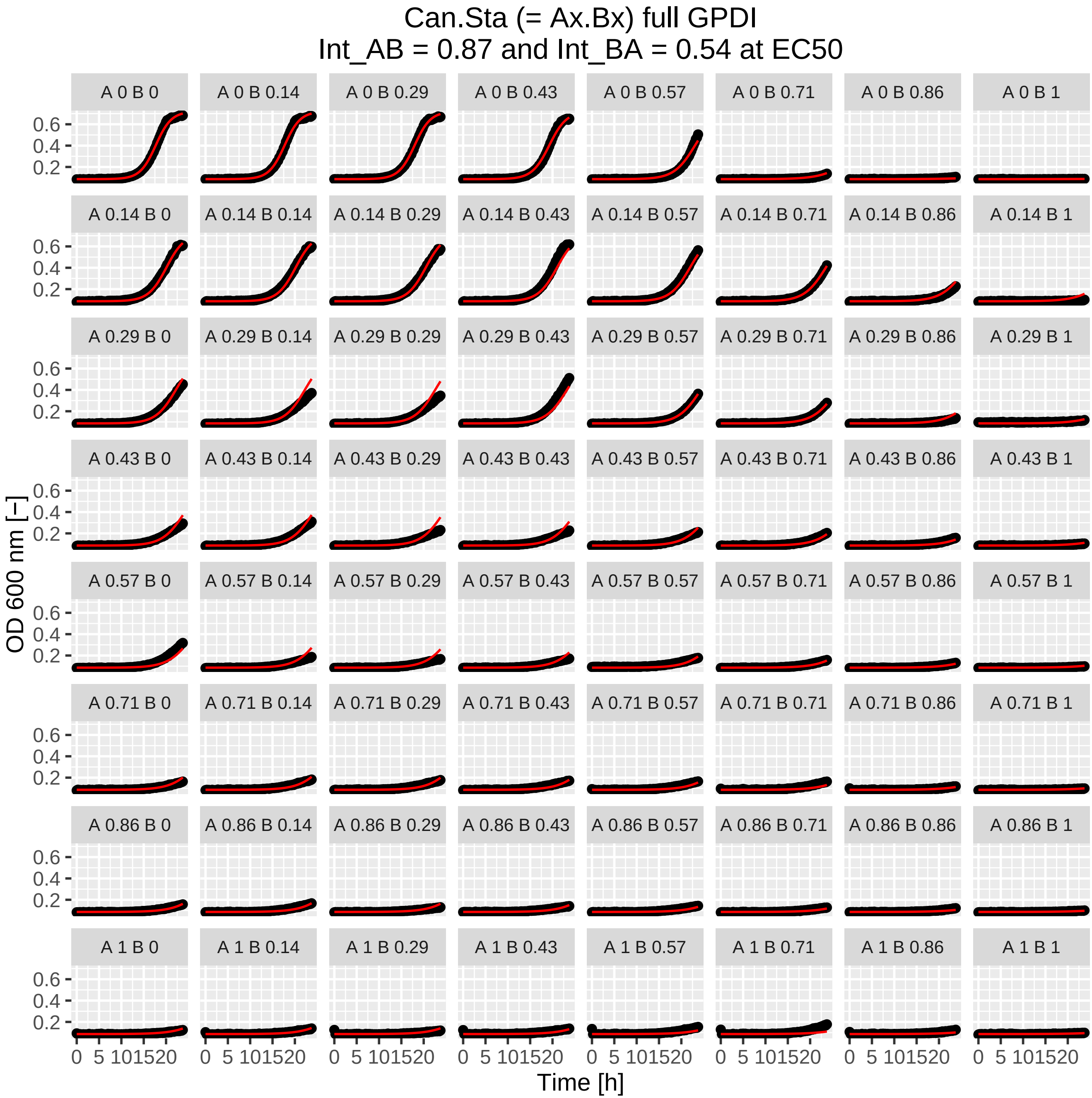
Can.Sta (= Ax.Bx) full GPDI
Int_AB = 0.87 and Int_BA = 0.54 at EC50
A 0 B 0
A 0 B 0.14
A 0 B 0.29
A 0 B 0.43
A 0 B 0.57
A 0 B 0.71
A 0 B 0.86
A 0 B 1
A 0.14 B 0
A 0.14 B 0.14
A 0.14 B 0.29
A 0.14 B 0.43
A 0.14 B 0.57
A 0.14 B 0.71
A 0.14 B 0.86
A 0.14 B 1
A 0.29 B 0
A 0.29 B 0.14
A 0.29 B 0.29
A 0.29 B 0.43
A 0.29 B 0.57
A 0.29 B 0.71
A 0.29 B 0.86
A 0.29 B 1
A 0.43 B 0
A 0.43 B 0.14
A 0.43 B 0.29
A 0.43 B 0.43
A 0.43 B 0.57
A 0.43 B 0.71
A 0.43 B 0.86
A 0.43 B 1
A 0.57 B 0
A 0.57 B 0.14
A 0.57 B 0.29
A 0.57 B 0.43
A 0.57 B 0.57
A 0.57 B 0.71
A 0.57 B 0.86
A 0.57 B 1
A 0.71 B 0
A 0.71 B 0.14
A 0.71 B 0.29
A 0.71 B 0.43
A 0.71 B 0.57
A 0.71 B 0.71
A 0.71 B 0.86
A 0.71 B 1
A 0.86 B 0
A 0.86 B 0.14
A 0.86 B 0.29
A 0.86 B 0.43
A 0.86 B 0.57
A 0.86 B 0.71
A 0.86 B 0.86
A 0.86 B 1
A 1 B 0
A 1 B 0.14
A 1 B 0.29
A 1 B 0.43
A 1 B 0.57
A 1 B 0.71
A 1 B 0.86
A 1 B 1
OD 600 nm [–]
0.6
0.4
0.2
0 5 101520
Time [h]

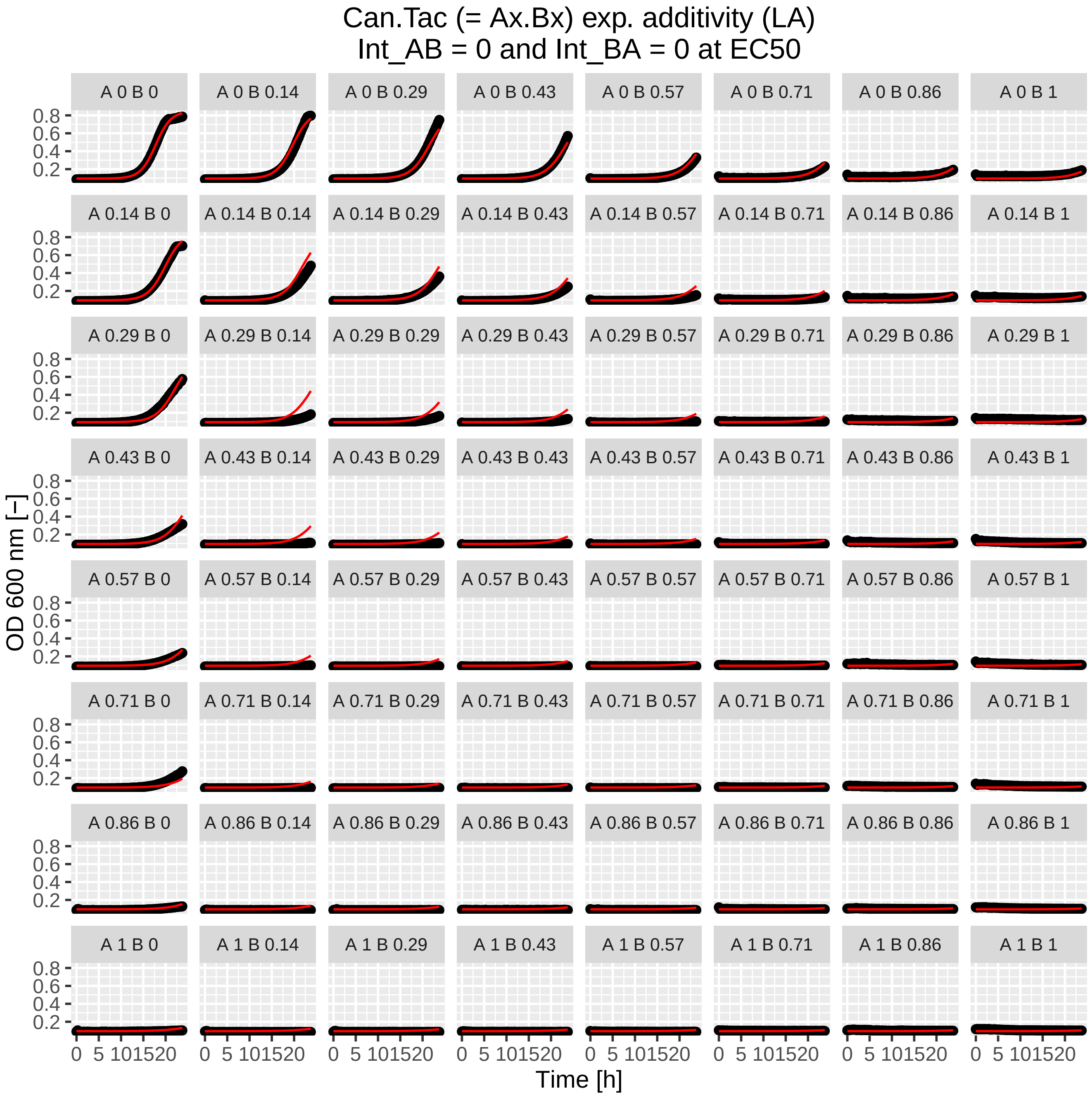

Can.Tac (= Ax.Bx) exp. additivity (LA)
Int_AB = 0 and Int_BA = 0 at EC50
A 0 B 0
A 0 B 0.14
A 0 B 0.29
A 0 B 0.43
A 0 B 0.57
A 0 B 0.71
A 0 B 0.86
A 0 B 1
A 0.14 B 0
A 0.14 B 0.14
A 0.14 B 0.29
A 0.14 B 0.43
A 0.14 B 0.57
A 0.14 B 0.71
A 0.14 B 0.86
A 0.14 B 1
A 0.29 B 0
A 0.29 B 0.14
A 0.29 B 0.29
A 0.29 B 0.43
A 0.29 B 0.57
A 0.29 B 0.71
A 0.29 B 0.86
A 0.29 B 1
A 0.43 B 0
A 0.43 B 0.14
A 0.43 B 0.29
A 0.43 B 0.43
A 0.43 B 0.57
A 0.43 B 0.71
A 0.43 B 0.86
A 0.43 B 1
A 0.57 B 0
A 0.57 B 0.14
A 0.57 B 0.29
A 0.57 B 0.43
A 0.57 B 0.57
A 0.57 B 0.71
A 0.57 B 0.86
A 0.57 B 1
A 0.71 B 0
A 0.71 B 0.14
A 0.71 B 0.29
A 0.71 B 0.43
A 0.71 B 0.57
A 0.71 B 0.71
A 0.71 B 0.86
A 0.71 B 1
A 0.86 B 0
A 0.86 B 0.14
A 0.86 B 0.29
A 0.86 B 0.43
A 0.86 B 0.57
A 0.86 B 0.71
A 0.86 B 0.86
A 0.86 B 1
A 1 B 0
A 1 B 0.14
A 1 B 0.29
A 1 B 0.43
A 1 B 0.57
A 1 B 0.71
A 1 B 0.86
A 1 B 1
OD 600 nm [–]
0.2
0.4
0.6
0.8
0
5
10
15
20
Time [h]

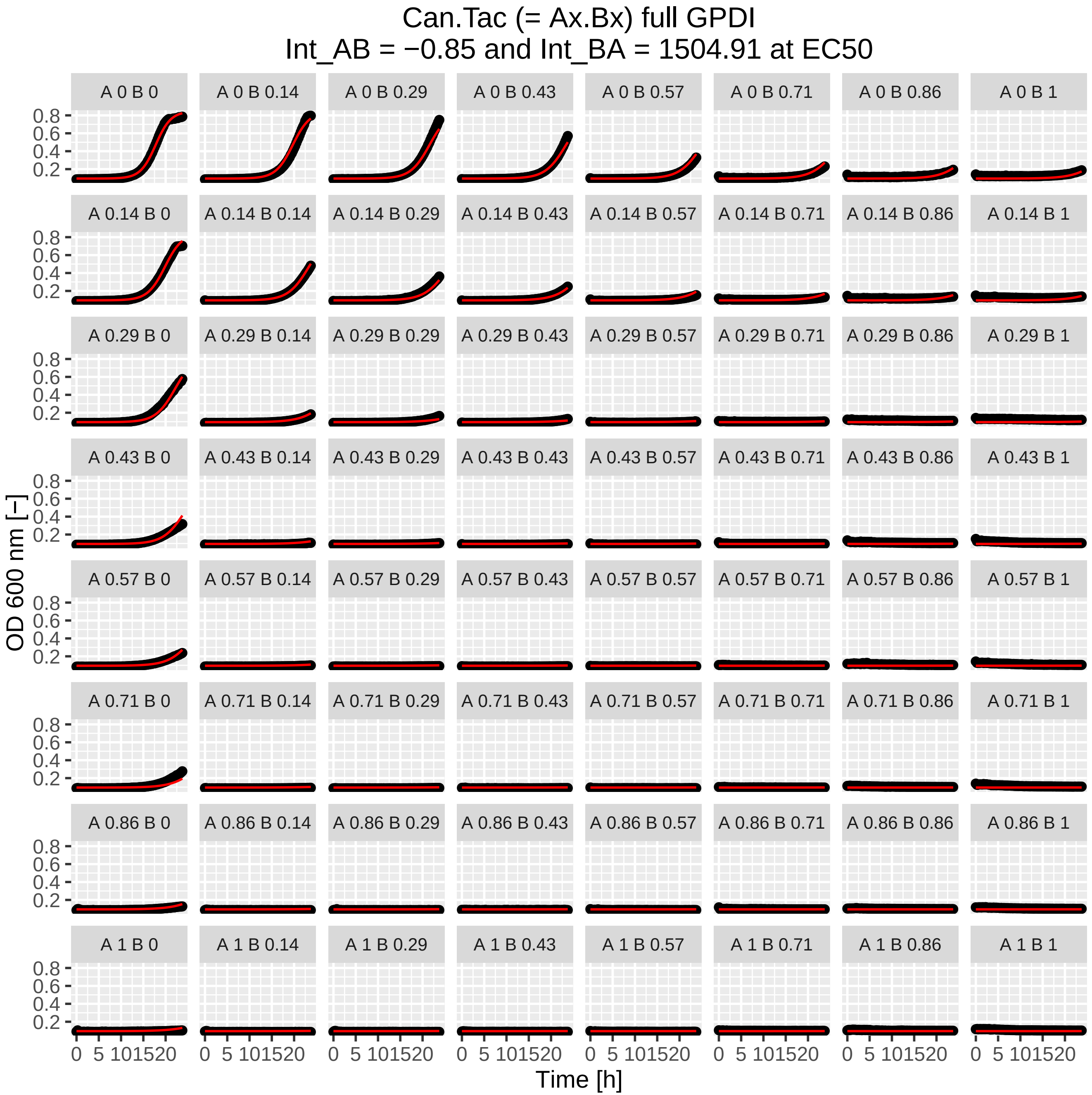
Can.Tac (= Ax.Bx) full GPDI
Int_AB = −0.85 and Int_BA = 1504.91 at EC50
OD 600 nm [−]
Time [h]
A 0 B 0
A 0 B 0.14
A 0 B 0.29
A 0 B 0.43
A 0 B 0.57
A 0 B 0.71
A 0 B 0.86
A 0 B 1
A 0.14 B 0
A 0.29 B 0
A 0.43 B 0
A 0.57 B 0
A 0.71 B 0
A 0.86 B 0
A 1 B 0
A 1 B 1

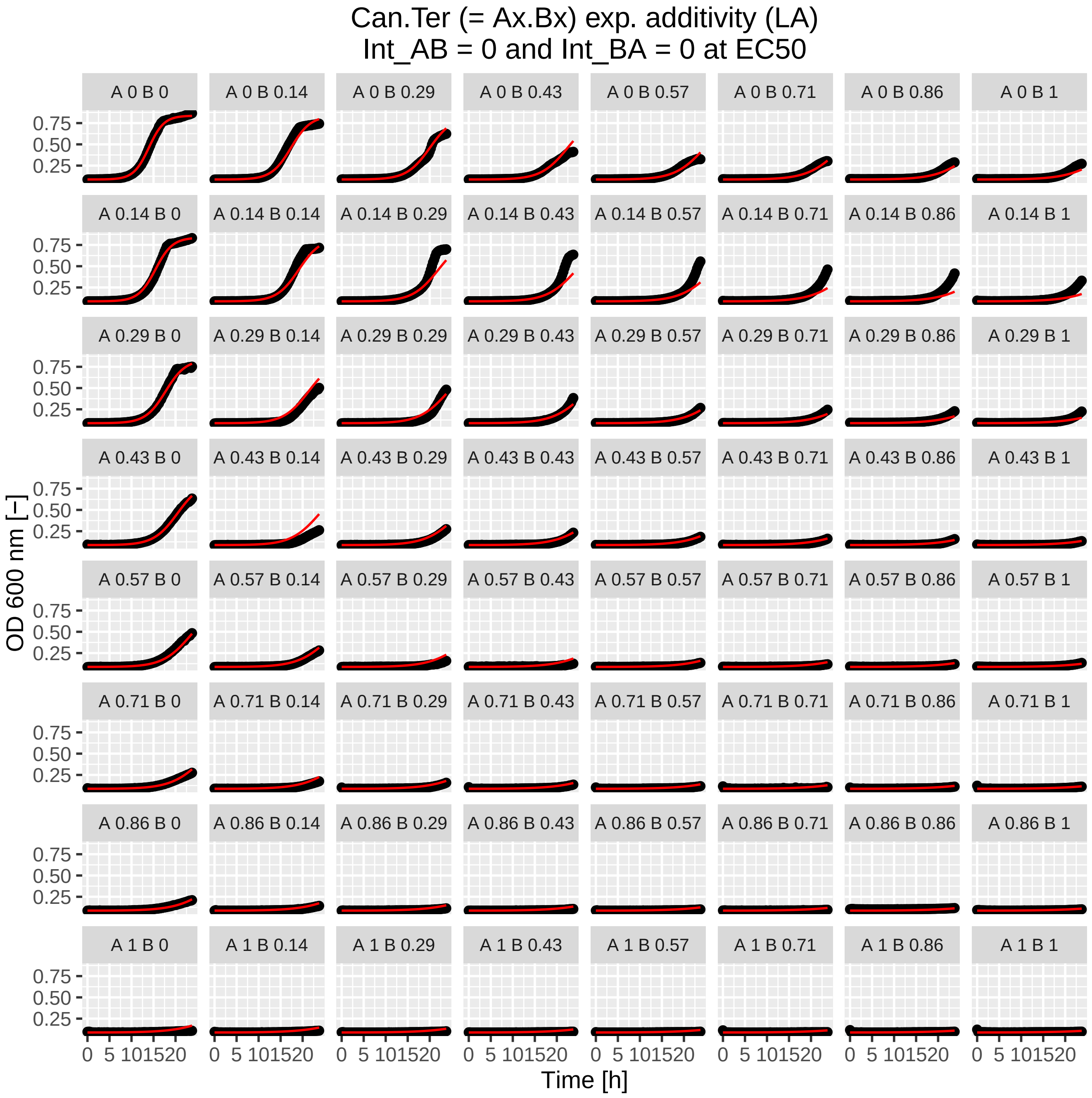

Can.Ter (= Ax.Bx) exp. additivity (LA)
Int_AB = 0 and Int_BA = 0 at EC50
OD 600 nm [–]
Time [h]
A 0 B 0
A 0 B 0.14
A 0 B 0.29
A 0 B 0.43
A 0 B 0.57
A 0 B 0.71
A 0 B 0.86
A 0 B 1
A 0.14 B 0
A 0.14 B 0.14
A 0.14 B 0.29
A 0.14 B 0.43
A 0.14 B 0.57
A 0.14 B 0.71
A 0.14 B 0.86
A 0.14 B 1
A 0.29 B 0
A 0.29 B 0.14
A 0.29 B 0.29
A 0.29 B 0.43
A 0.29 B 0.57
A 0.29 B 0.71
A 0.29 B 0.86
A 0.29 B 1
A 0.43 B 0
A 0.43 B 0.14
A 0.43 B 0.29
A 0.43 B 0.43
A 0.43 B 0.57
A 0.43 B 0.71
A 0.43 B 0.86
A 0.43 B 1
A 0.57 B 0
A 0.57 B 0.14
A 0.57 B 0.29
A 0.57 B 0.43
A 0.57 B 0.57
A 0.57 B 0.71
A 0.57 B 0.86
A 0.57 B 1
A 0.71 B 0
A 0.71 B 0.14
A 0.71 B 0.29
A 0.71 B 0.43
A 0.71 B 0.57
A 0.71 B 0.71
A 0.71 B 0.86
A 0.71 B 1
A 0.86 B 0
A 0.86 B 0.14
A 0.86 B 0.29
A 0.86 B 0.43
A 0.86 B 0.57
A 0.86 B 0.71
A 0.86 B 0.86
A 0.86 B 1
A 1 B 0
A 1 B 0.14
A 1 B 0.29
A 1 B 0.43
A 1 B 0.57
A 1 B 0.71
A 1 B 0.86
A 1 B 1
0.75
0.50
0.25
0 5 101520

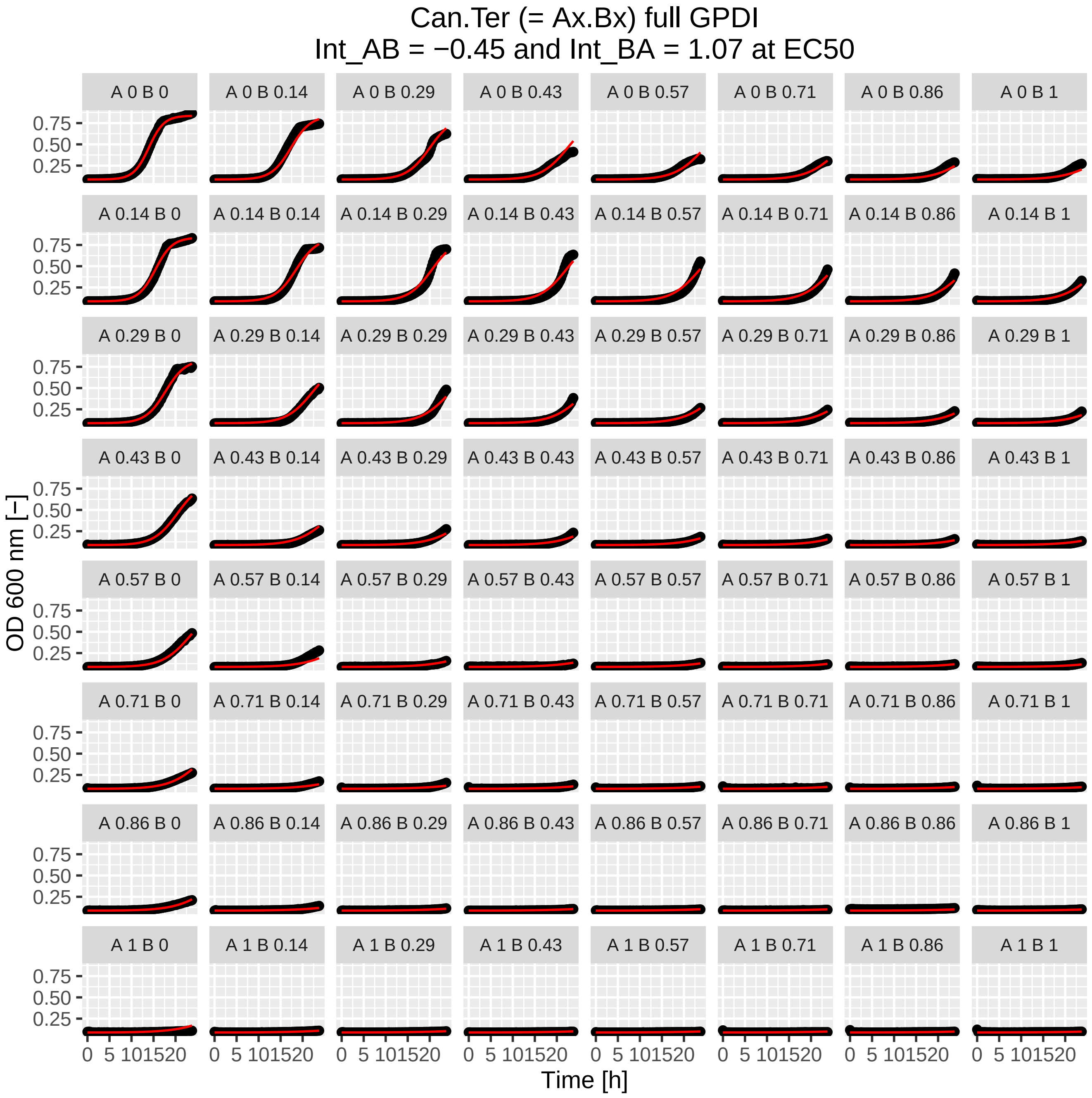
Can.Ter (= Ax.Bx) full GPDI
Int_AB = −0.45 and Int_BA = 1.07 at EC50
A 0 B 0
A 0 B 0.14
A 0 B 0.29
A 0 B 0.43
A 0 B 0.57
A 0 B 0.71
A 0 B 0.86
A 0 B 1
A 0.14 B 0
A 0.14 B 0.14
A 0.14 B 0.29
A 0.14 B 0.43
A 0.14 B 0.57
A 0.14 B 0.71
A 0.14 B 0.86
A 0.14 B 1
A 0.29 B 0
A 0.29 B 0.14
A 0.29 B 0.29
A 0.29 B 0.43
A 0.29 B 0.57
A 0.29 B 0.71
A 0.29 B 0.86
A 0.29 B 1
A 0.43 B 0
A 0.43 B 0.14
A 0.43 B 0.29
A 0.43 B 0.43
A 0.43 B 0.57
A 0.43 B 0.71
A 0.43 B 0.86
A 0.43 B 1
A 0.57 B 0
A 0.57 B 0.14
A 0.57 B 0.29
A 0.57 B 0.43
A 0.57 B 0.57
A 0.57 B 0.71
A 0.57 B 0.86
A 0.57 B 1
A 0.71 B 0
A 0.71 B 0.14
A 0.71 B 0.29
A 0.71 B 0.43
A 0.71 B 0.57
A 0.71 B 0.71
A 0.71 B 0.86
A 0.71 B 1
A 0.86 B 0
A 0.86 B 0.14
A 0.86 B 0.29
A 0.86 B 0.43
A 0.86 B 0.57
A 0.86 B 0.71
A 0.86 B 0.86
A 0.86 B 1
A 1 B 0
A 1 B 0.14
A 1 B 0.29
A 1 B 0.43
A 1 B 0.57
A 1 B 0.71
A 1 B 0.86
A 1 B 1
0.75
0.50
0.25
OD 600 nm [−]
0 5 101520
Time [h]

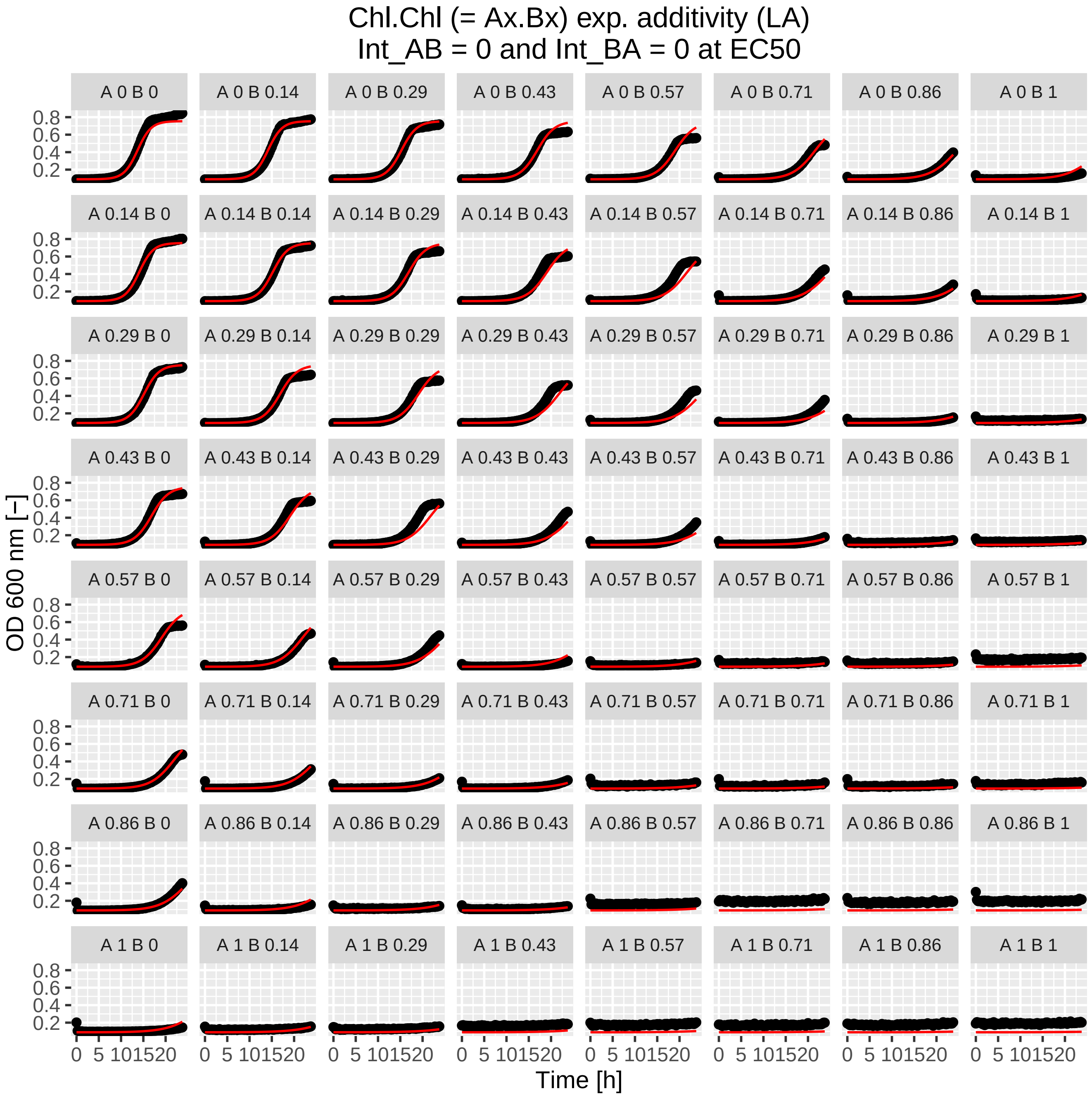
Chl.Chl (= Ax.Bx) exp. additivity (LA)
Int_AB = 0 and Int_BA = 0 at EC50
OD 600 nm [−]
Time [h]
A 0 B 0
A 0 B 0.14
A 0 B 0.29
A 0 B 0.43
A 0 B 0.57
A 0 B 0.71
A 0 B 0.86
A 0 B 1
A 0.14 B 0
A 0.14 B 0.14
A 0.14 B 0.29
A 0.14 B 0.43
A 0.14 B 0.57
A 0.14 B 0.71
A 0.14 B 0.86
A 0.14 B 1
A 0.29 B 0
A 0.29 B 0.14
A 0.29 B 0.29
A 0.29 B 0.43
A 0.29 B 0.57
A 0.29 B 0.71
A 0.29 B 0.86
A 0.29 B 1
A 0.43 B 0
A 0.43 B 0.14
A 0.43 B 0.29
A 0.43 B 0.43
A 0.43 B 0.57
A 0.43 B 0.71
A 0.43 B 0.86
A 0.43 B 1
A 0.57 B 0
A 0.57 B 0.14
A 0.57 B 0.29
A 0.57 B 0.43
A 0.57 B 0.57
A 0.57 B 0.71
A 0.57 B 0.86
A 0.57 B 1
A 0.71 B 0
A 0.71 B 0.14
A 0.71 B 0.29
A 0.71 B 0.43
A 0.71 B 0.57
A 0.71 B 0.71
A 0.71 B 0.86
A 0.71 B 1
A 0.86 B 0
A 0.86 B 0.14
A 0.86 B 0.29
A 0.86 B 0.43
A 0.86 B 0.57
A 0.86 B 0.71
A 0.86 B 0.86
A 0.86 B 1
A 1 B 0
A 1 B 0.14
A 1 B 0.29
A 1 B 0.43
A 1 B 0.57
A 1 B 0.71
A 1 B 0.86
A 1 B 1
0.8
0.6
0.4
0.2
0 5 101520

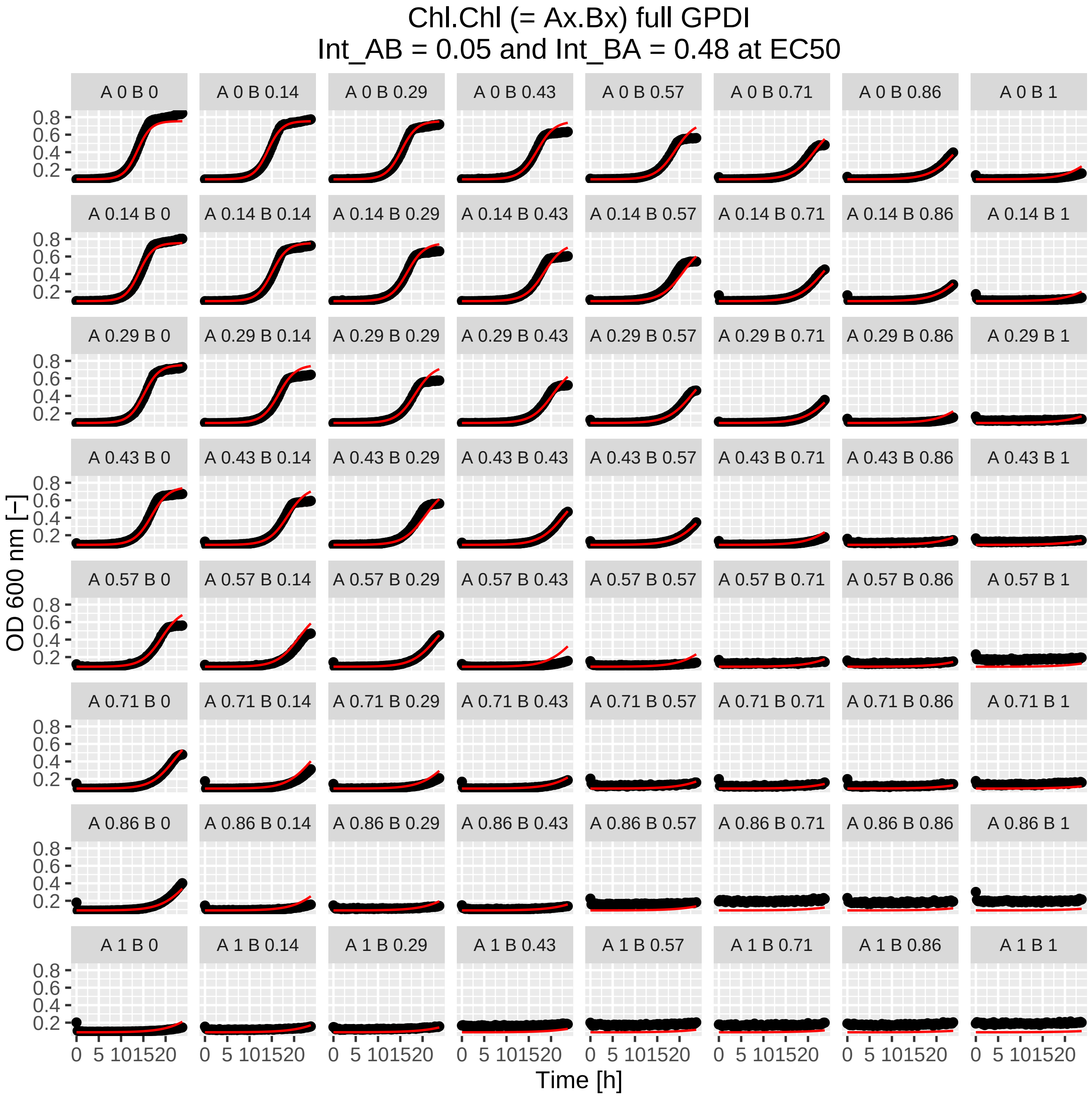
Chl.Chl (= Ax.Bx) full GPDI
Int_AB = 0.05 and Int_BA = 0.48 at EC50
OD 600 nm [−]
Time [h]
A 0 B 0
A 0 B 0.14
A 0 B 0.29
A 0 B 0.43
A 0 B 0.57
A 0 B 0.71
A 0 B 0.86
A 0 B 1
A 0.14 B 0
A 0.14 B 0.14
A 0.14 B 0.29
A 0.14 B 0.43
A 0.14 B 0.57
A 0.14 B 0.71
A 0.14 B 0.86
A 0.14 B 1
A 0.29 B 0
A 0.29 B 0.14
A 0.29 B 0.29
A 0.29 B 0.43
A 0.29 B 0.57
A 0.29 B 0.71
A 0.29 B 0.86
A 0.29 B 1
A 0.43 B 0
A 0.43 B 0.14
A 0.43 B 0.29
A 0.43 B 0.43
A 0.43 B 0.57
A 0.43 B 0.71
A 0.43 B 0.86
A 0.43 B 1
A 0.57 B 0
A 0.57 B 0.14
A 0.57 B 0.29
A 0.57 B 0.43
A 0.57 B 0.57
A 0.57 B 0.71
A 0.57 B 0.86
A 0.57 B 1
A 0.71 B 0
A 0.71 B 0.14
A 0.71 B 0.29
A 0.71 B 0.43
A 0.71 B 0.57
A 0.71 B 0.71
A 0.71 B 0.86
A 0.71 B 1
A 0.86 B 0
A 0.86 B 0.14
A 0.86 B 0.29
A 0.86 B 0.43
A 0.86 B 0.57
A 0.86 B 0.71
A 0.86 B 0.86
A 0.86 B 1
A 1 B 0
A 1 B 0.14
A 1 B 0.29
A 1 B 0.43
A 1 B 0.57
A 1 B 0.71
A 1 B 0.86
A 1 B 1
0.8
0.6
0.4
0.2
0 5 101520

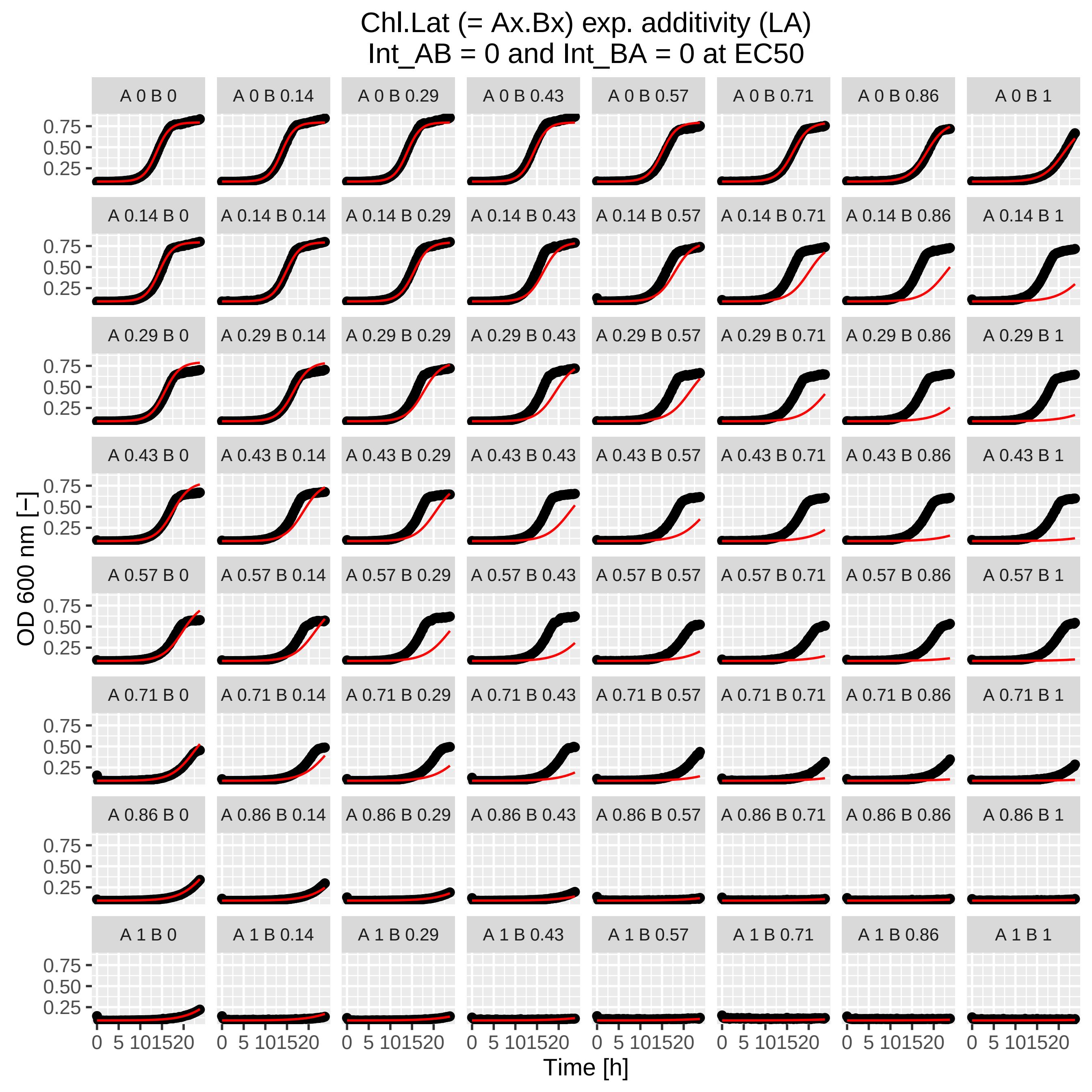

Chl.Lat (= Ax.Bx) exp. additivity (LA)
Int_AB = 0 and Int_BA = 0 at EC50
A 0 B 0
A 0 B 0.14
A 0 B 0.29
A 0 B 0.43
A 0 B 0.57
A 0 B 0.71
A 0 B 0.86
A 0 B 1
A 0.14 B 0
A 0.14 B 0.14
A 0.14 B 0.29
A 0.14 B 0.43
A 0.14 B 0.57
A 0.14 B 0.71
A 0.14 B 0.86
A 0.14 B 1
A 0.29 B 0
A 0.29 B 0.14
A 0.29 B 0.29
A 0.29 B 0.43
A 0.29 B 0.57
A 0.29 B 0.71
A 0.29 B 0.86
A 0.29 B 1
A 0.43 B 0
A 0.43 B 0.14
A 0.43 B 0.29
A 0.43 B 0.43
A 0.43 B 0.57
A 0.43 B 0.71
A 0.43 B 0.86
A 0.43 B 1
A 0.57 B 0
A 0.57 B 0.14
A 0.57 B 0.29
A 0.57 B 0.43
A 0.57 B 0.57
A 0.57 B 0.71
A 0.57 B 0.86
A 0.57 B 1
A 0.71 B 0
A 0.71 B 0.14
A 0.71 B 0.29
A 0.71 B 0.43
A 0.71 B 0.57
A 0.71 B 0.71
A 0.71 B 0.86
A 0.71 B 1
A 0.86 B 0
A 0.86 B 0.14
A 0.86 B 0.29
A 0.86 B 0.43
A 0.86 B 0.57
A 0.86 B 0.71
A 0.86 B 0.86
A 0.86 B 1
A 1 B 0
A 1 B 0.14
A 1 B 0.29
A 1 B 0.43
A 1 B 0.57
A 1 B 0.71
A 1 B 0.86
A 1 B 1
OD 600 nm [–]
0.75
0.50
0.25
0 5 101520
Time [h]

Chl.Lat (= Ax.Bx) full GPDI
Int_AB = 0.06 and Int_BA = 3.24 at EC50

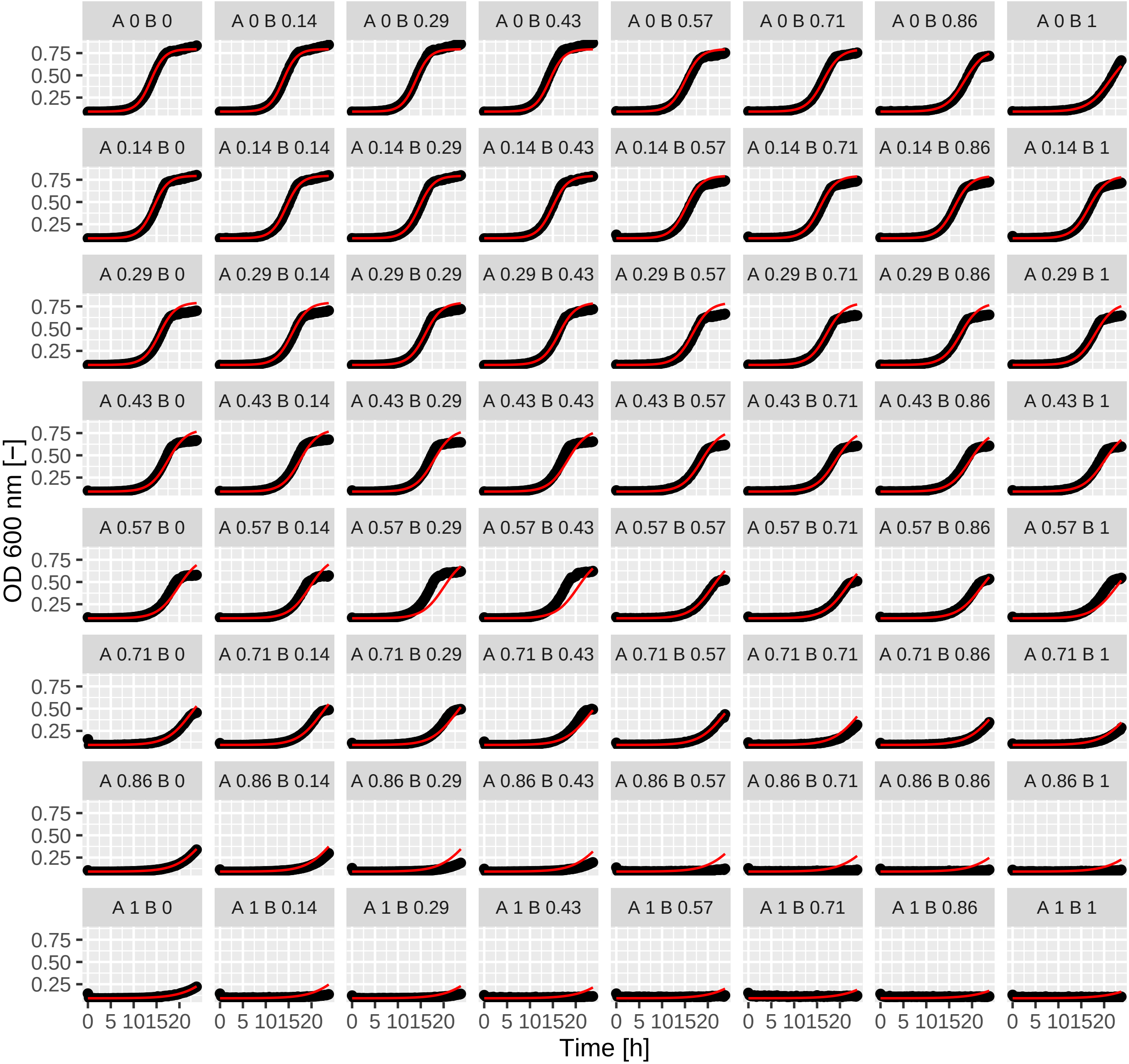

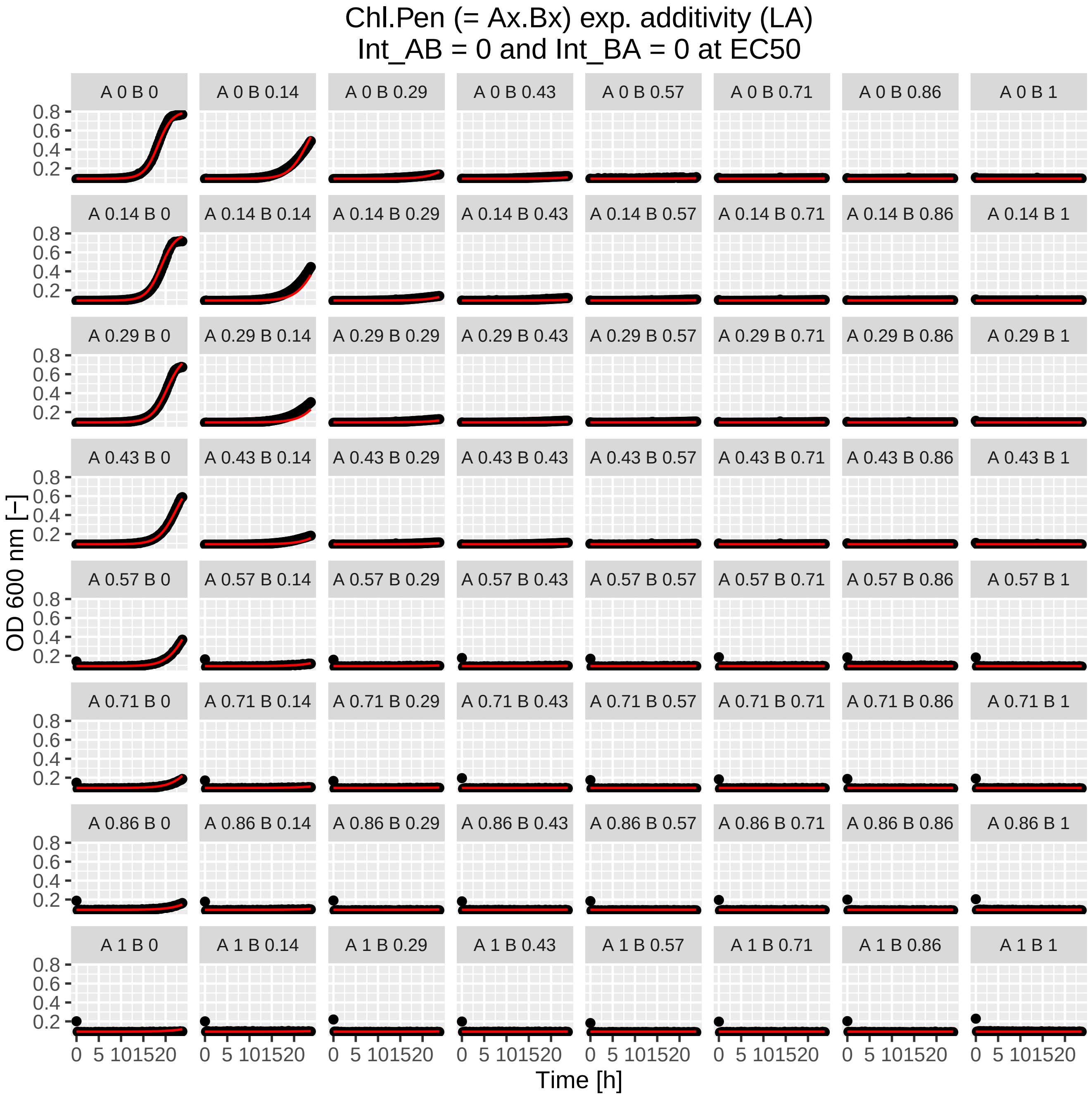

Chl.Pen (= Ax.Bx) exp. additivity (LA)
Int_AB = 0 and Int_BA = 0 at EC50
OD 600 nm [–]
Time [h]
0.2
0.4
0.6
0.8
0 5 10 15 20
A 0 B 0
A 0 B 0.14
A 0 B 0.29
A 0 B 0.43
A 0 B 0.57
A 0 B 0.71
A 0 B 0.86
A 0 B 1
A 0.14 B 0
A 0.14 B 0.14
A 0.14 B 0.29
A 0.14 B 0.43
A 0.14 B 0.57
A 0.14 B 0.71
A 0.14 B 0.86
A 0.14 B 1
A 0.29 B 0
A 0.29 B 0.14
A 0.29 B 0.29
A 0.29 B 0.43
A 0.29 B 0.57
A 0.29 B 0.71
A 0.29 B 0.86
A 0.29 B 1
A 0.43 B 0
A 0.43 B 0.14
A 0.43 B 0.29
A 0.43 B 0.43
A 0.43 B 0.57
A 0.43 B 0.71
A 0.43 B 0.86
A 0.43 B 1
A 0.57 B 0
A 0.57 B 0.14
A 0.57 B 0.29
A 0.57 B 0.43
A 0.57 B 0.57
A 0.57 B 0.71
A 0.57 B 0.86
A 0.57 B 1
A 0.71 B 0
A 0.71 B 0.14
A 0.71 B 0.29
A 0.71 B 0.43
A 0.71 B 0.57
A 0.71 B 0.71
A 0.71 B 0.86
A 0.71 B 1
A 0.86 B 0
A 0.86 B 0.14
A 0.86 B 0.29
A 0.86 B 0.43
A 0.86 B 0.57
A 0.86 B 0.71
A 0.86 B 0.86
A 0.86 B 1
A 1 B 0
A 1 B 0.14
A 1 B 0.29
A 1 B 0.43
A 1 B 0.57
A 1 B 0.71
A 1 B 0.86
A 1 B 1

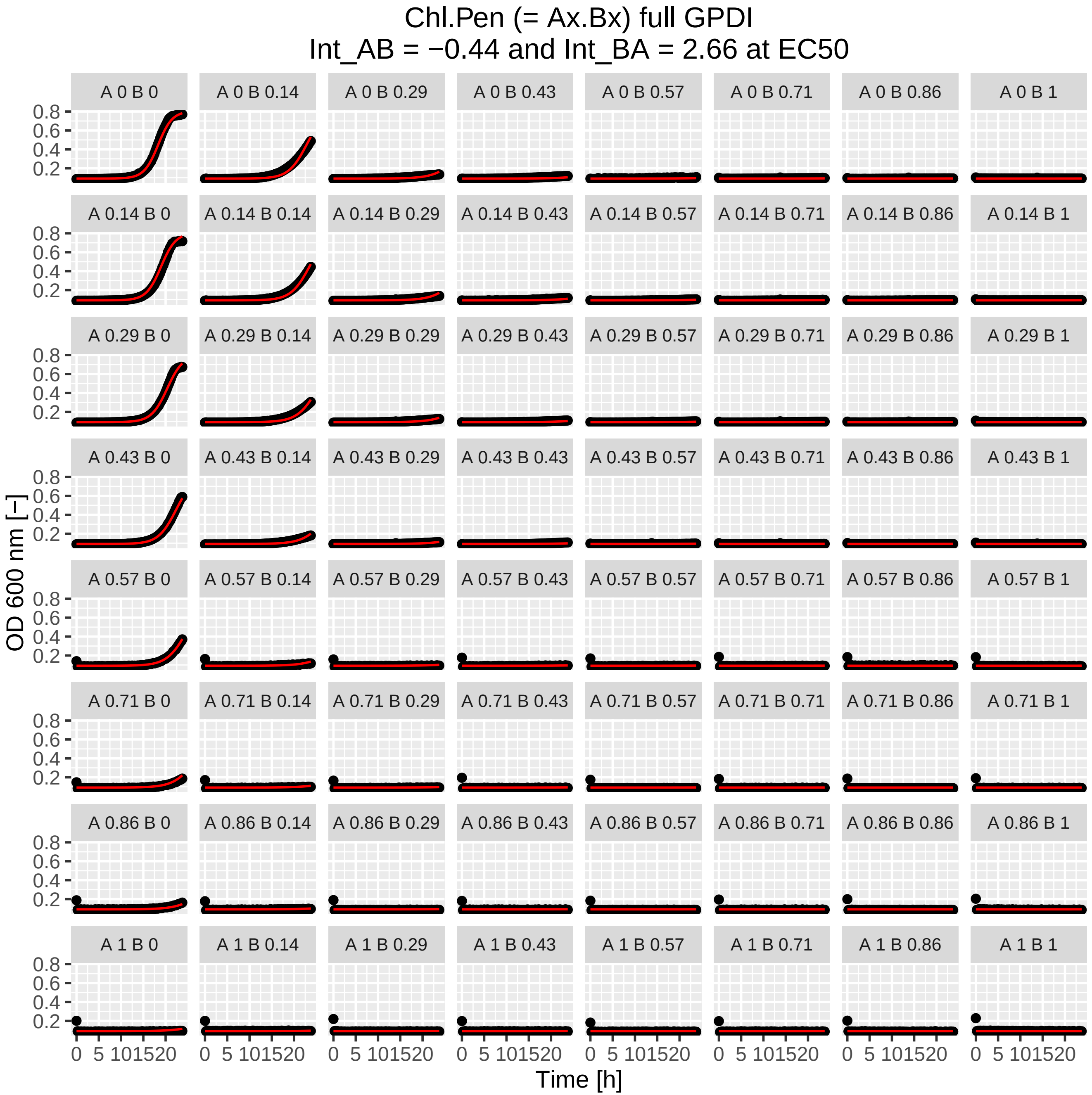
Chl.Pen (= Ax.Bx) full GPDI
Int_AB = −0.44 and Int_BA = 2.66 at EC50
OD 600 nm [−]
Time [h]
A 0 B 0
A 0 B 0.14
A 0 B 0.29
A 0 B 0.43
A 0 B 0.57
A 0 B 0.71
A 0 B 0.86
A 0 B 1
A 0.14 B 0
A 0.29 B 0
A 0.43 B 0
A 0.57 B 0
A 0.71 B 0
A 0.86 B 0
A 1 B 0
A 1 B 1

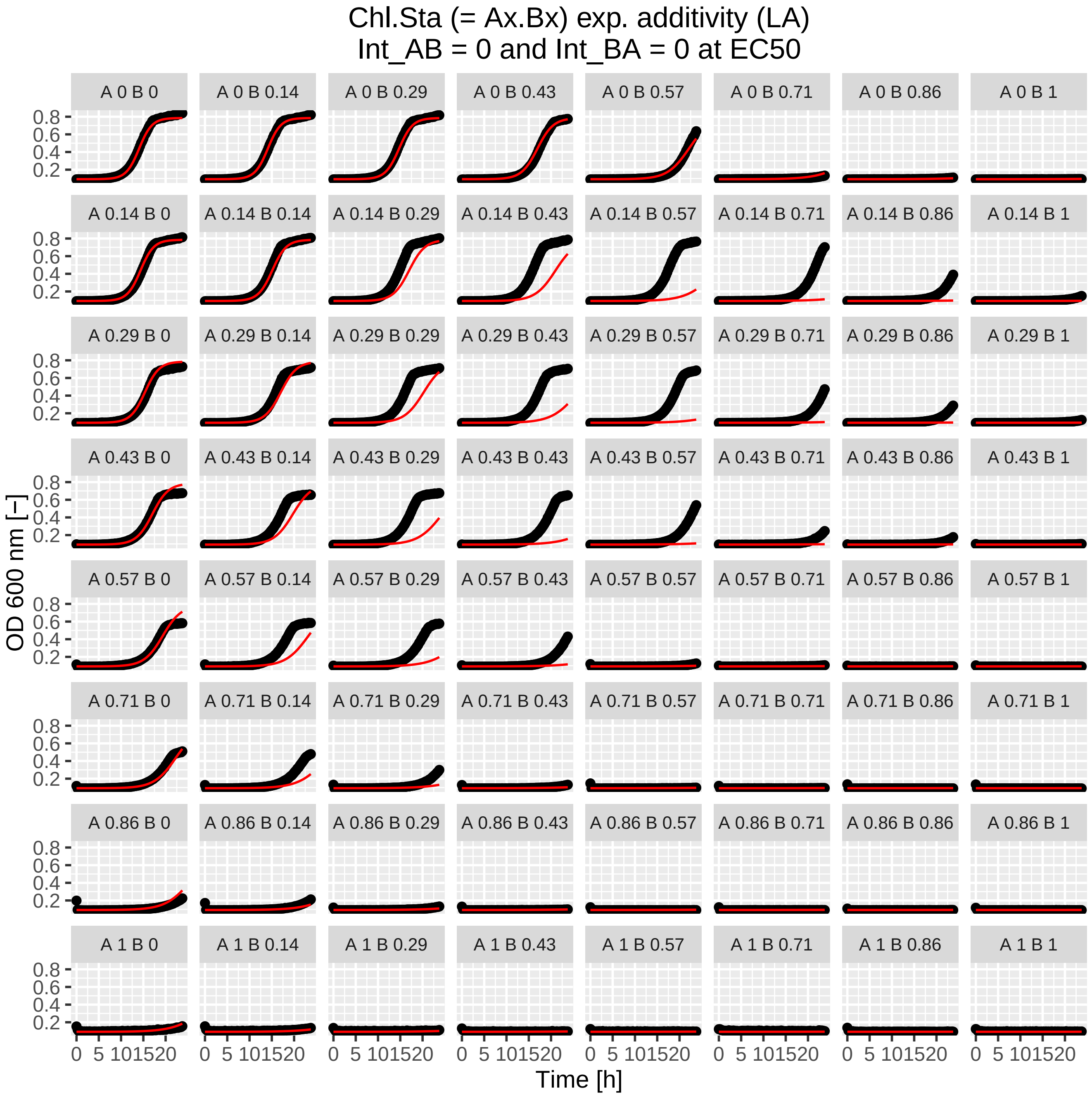

Chl.Sta (= Ax.Bx) exp. additivity (LA)
Int_AB = 0 and Int_BA = 0 at EC50
OD 600 nm [–]
Time [h]
A 0 B 0
A 0 B 0.14
A 0 B 0.29
A 0 B 0.43
A 0 B 0.57
A 0 B 0.71
A 0 B 0.86
A 0 B 1
A 0.14 B 0
A 0.14 B 0.14
A 0.14 B 0.29
A 0.14 B 0.43
A 0.14 B 0.57
A 0.14 B 0.71
A 0.14 B 0.86
A 0.14 B 1
A 0.29 B 0
A 0.29 B 0.14
A 0.29 B 0.29
A 0.29 B 0.43
A 0.29 B 0.57
A 0.29 B 0.71
A 0.29 B 0.86
A 0.29 B 1
A 0.43 B 0
A 0.43 B 0.14
A 0.43 B 0.29
A 0.43 B 0.43
A 0.43 B 0.57
A 0.43 B 0.71
A 0.43 B 0.86
A 0.43 B 1
A 0.57 B 0
A 0.57 B 0.14
A 0.57 B 0.29
A 0.57 B 0.43
A 0.57 B 0.57
A 0.57 B 0.71
A 0.57 B 0.86
A 0.57 B 1
A 0.71 B 0
A 0.71 B 0.14
A 0.71 B 0.29
A 0.71 B 0.43
A 0.71 B 0.57
A 0.71 B 0.71
A 0.71 B 0.86
A 0.71 B 1
A 0.86 B 0
A 0.86 B 0.14
A 0.86 B 0.29
A 0.86 B 0.43
A 0.86 B 0.57
A 0.86 B 0.71
A 0.86 B 0.86
A 0.86 B 1
A 1 B 0
A 1 B 0.14
A 1 B 0.29
A 1 B 0.43
A 1 B 0.57
A 1 B 0.71
A 1 B 0.86
A 1 B 1
0.8
0.6
0.4
0.2
0 5 101520

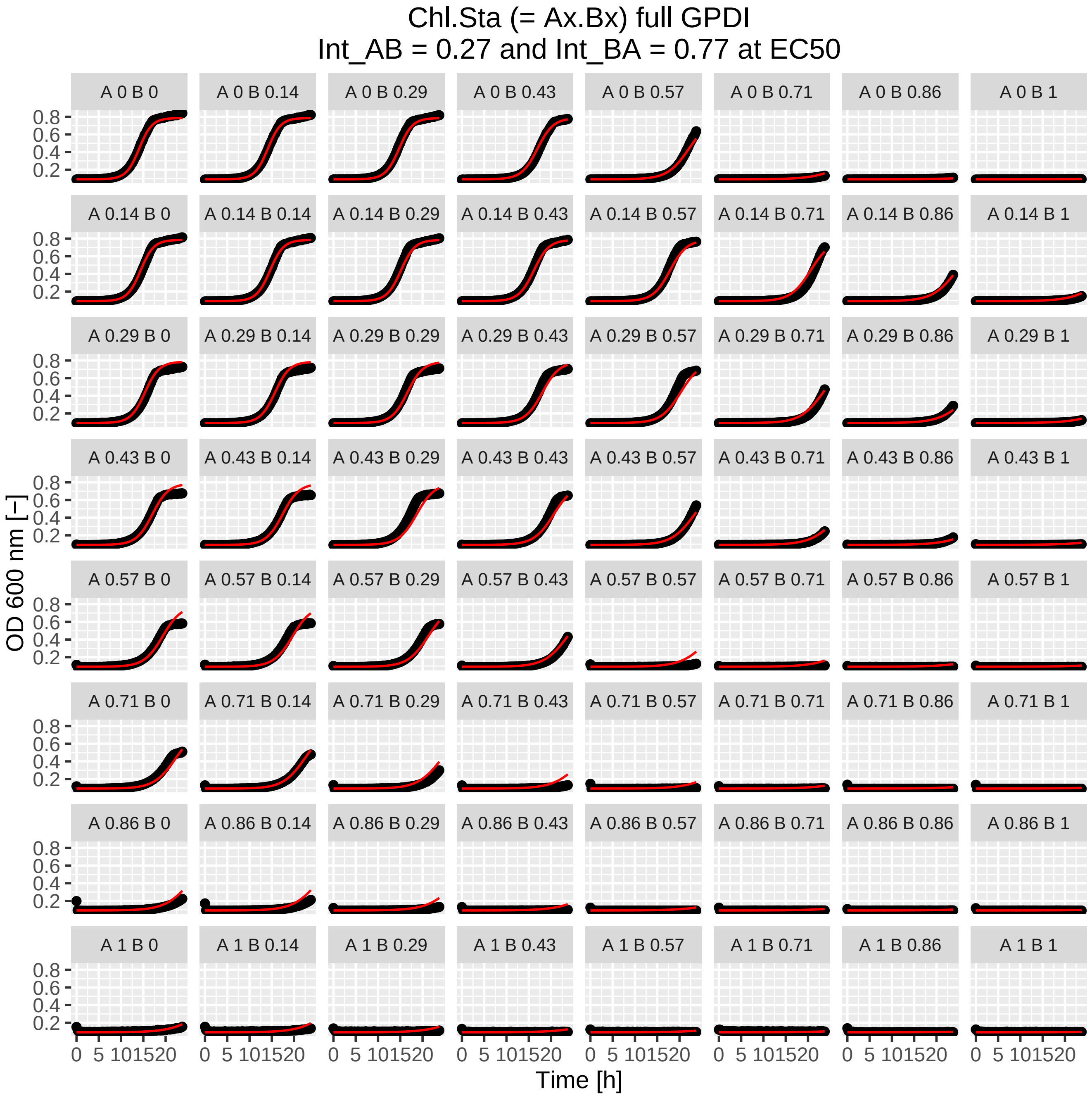

Chl.Sta (= Ax.Bx) full GPDI
Int_AB = 0.27 and Int_BA = 0.77 at EC50
OD 600 nm [–]
Time [h]

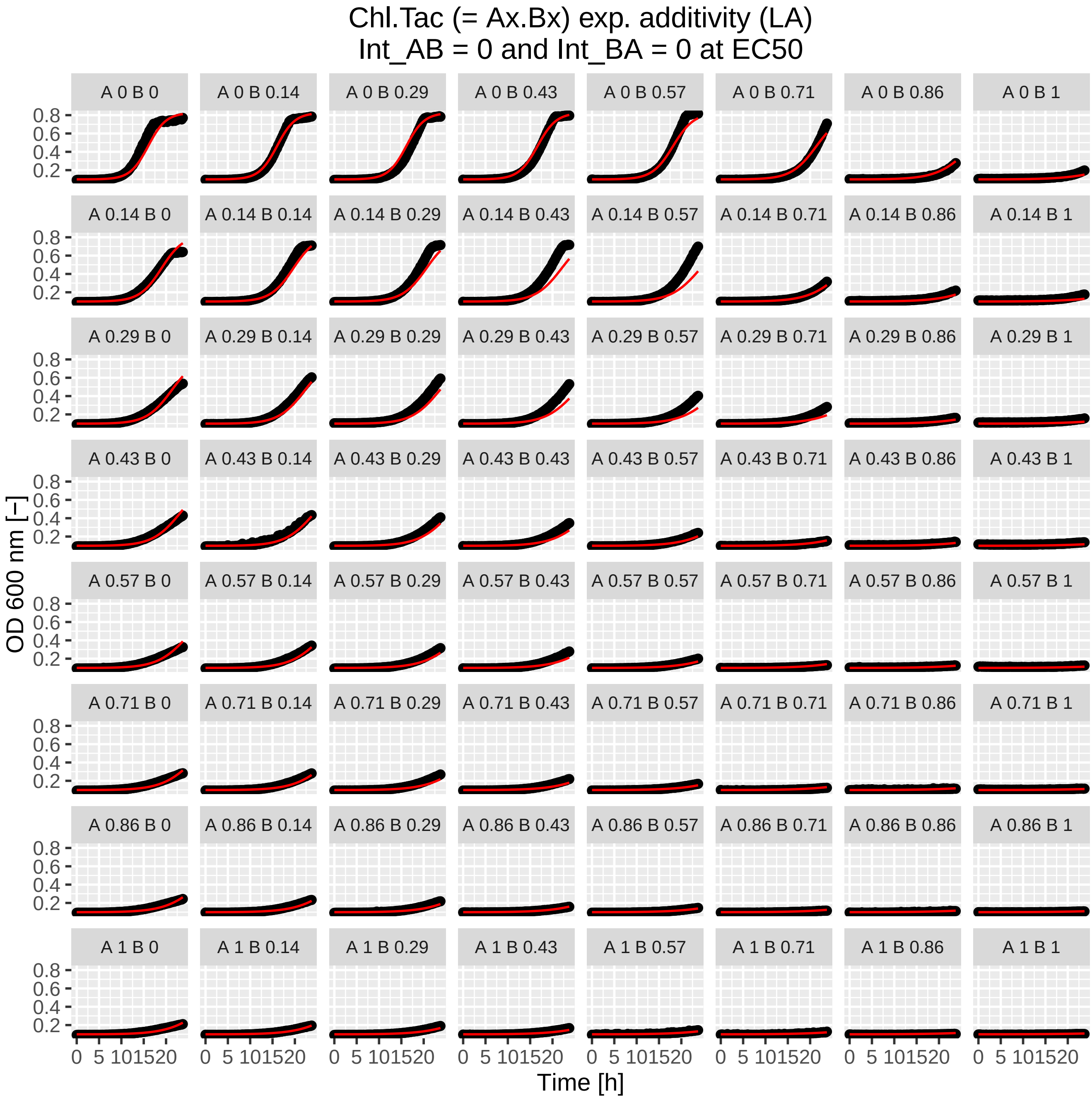

Chl.Tac (= Ax.Bx) exp. additivity (LA)
Int_AB = 0 and Int_BA = 0 at EC50
A 0 B 0
A 0 B 0.14
A 0 B 0.29
A 0 B 0.43
A 0 B 0.57
A 0 B 0.71
A 0 B 0.86
A 0 B 1
A 0.14 B 0
A 0.14 B 0.14
A 0.14 B 0.29
A 0.14 B 0.43
A 0.14 B 0.57
A 0.14 B 0.71
A 0.14 B 0.86
A 0.14 B 1
A 0.29 B 0
A 0.29 B 0.14
A 0.29 B 0.29
A 0.29 B 0.43
A 0.29 B 0.57
A 0.29 B 0.71
A 0.29 B 0.86
A 0.29 B 1
A 0.43 B 0
A 0.43 B 0.14
A 0.43 B 0.29
A 0.43 B 0.43
A 0.43 B 0.57
A 0.43 B 0.71
A 0.43 B 0.86
A 0.43 B 1
A 0.57 B 0
A 0.57 B 0.14
A 0.57 B 0.29
A 0.57 B 0.43
A 0.57 B 0.57
A 0.57 B 0.71
A 0.57 B 0.86
A 0.57 B 1
A 0.71 B 0
A 0.71 B 0.14
A 0.71 B 0.29
A 0.71 B 0.43
A 0.71 B 0.57
A 0.71 B 0.71
A 0.71 B 0.86
A 0.71 B 1
A 0.86 B 0
A 0.86 B 0.14
A 0.86 B 0.29
A 0.86 B 0.43
A 0.86 B 0.57
A 0.86 B 0.71
A 0.86 B 0.86
A 0.86 B 1
A 1 B 0
A 1 B 0.14
A 1 B 0.29
A 1 B 0.43
A 1 B 0.57
A 1 B 0.71
A 1 B 0.86
A 1 B 1
0.8
0.6
0.4
0.2
OD 600 nm [–]
0 5 101520
Time [h]

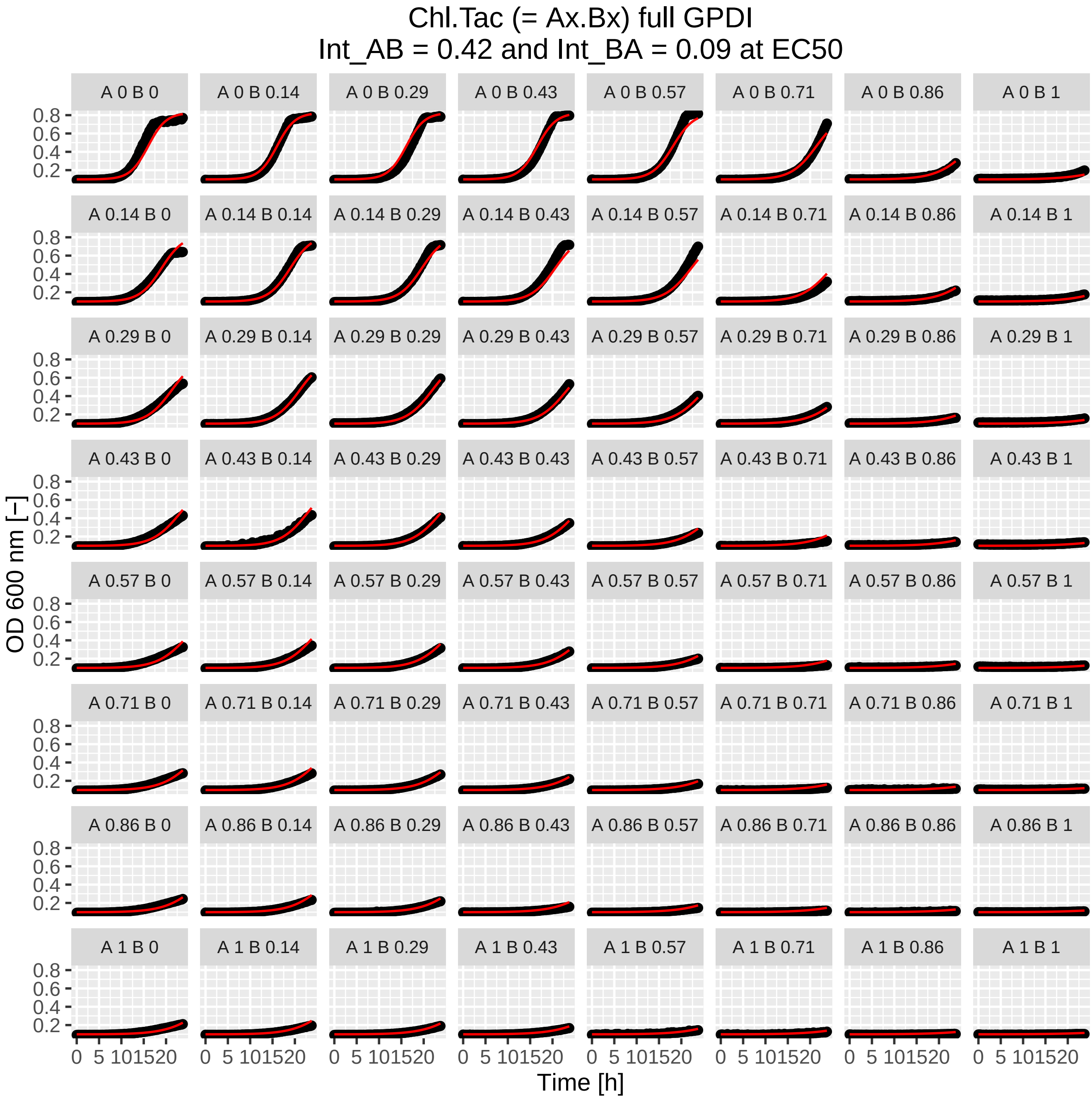

Chl.Tac (= Ax.Bx) full GPDI
Int_AB = 0.42 and Int_BA = 0.09 at EC50
A 0 B 0
A 0 B 0.14
A 0 B 0.29
A 0 B 0.43
A 0 B 0.57
A 0 B 0.71
A 0 B 0.86
A 0 B 1
A 0.14 B 0
A 0.14 B 0.14
A 0.14 B 0.29
A 0.14 B 0.43
A 0.14 B 0.57
A 0.14 B 0.71
A 0.14 B 0.86
A 0.14 B 1
A 0.29 B 0
A 0.29 B 0.14
A 0.29 B 0.29
A 0.29 B 0.43
A 0.29 B 0.57
A 0.29 B 0.71
A 0.29 B 0.86
A 0.29 B 1
A 0.43 B 0
A 0.43 B 0.14
A 0.43 B 0.29
A 0.43 B 0.43
A 0.43 B 0.57
A 0.43 B 0.71
A 0.43 B 0.86
A 0.43 B 1
A 0.57 B 0
A 0.57 B 0.14
A 0.57 B 0.29
A 0.57 B 0.43
A 0.57 B 0.57
A 0.57 B 0.71
A 0.57 B 0.86
A 0.57 B 1
A 0.71 B 0
A 0.71 B 0.14
A 0.71 B 0.29
A 0.71 B 0.43
A 0.71 B 0.57
A 0.71 B 0.71
A 0.71 B 0.86
A 0.71 B 1
A 0.86 B 0
A 0.86 B 0.14
A 0.86 B 0.29
A 0.86 B 0.43
A 0.86 B 0.57
A 0.86 B 0.71
A 0.86 B 0.86
A 0.86 B 1
A 1 B 0
A 1 B 0.14
A 1 B 0.29
A 1 B 0.43
A 1 B 0.57
A 1 B 0.71
A 1 B 0.86
A 1 B 1
OD 600 nm [–]
0.2
0.4
0.6
0.8
0 5 101520
Time [h]

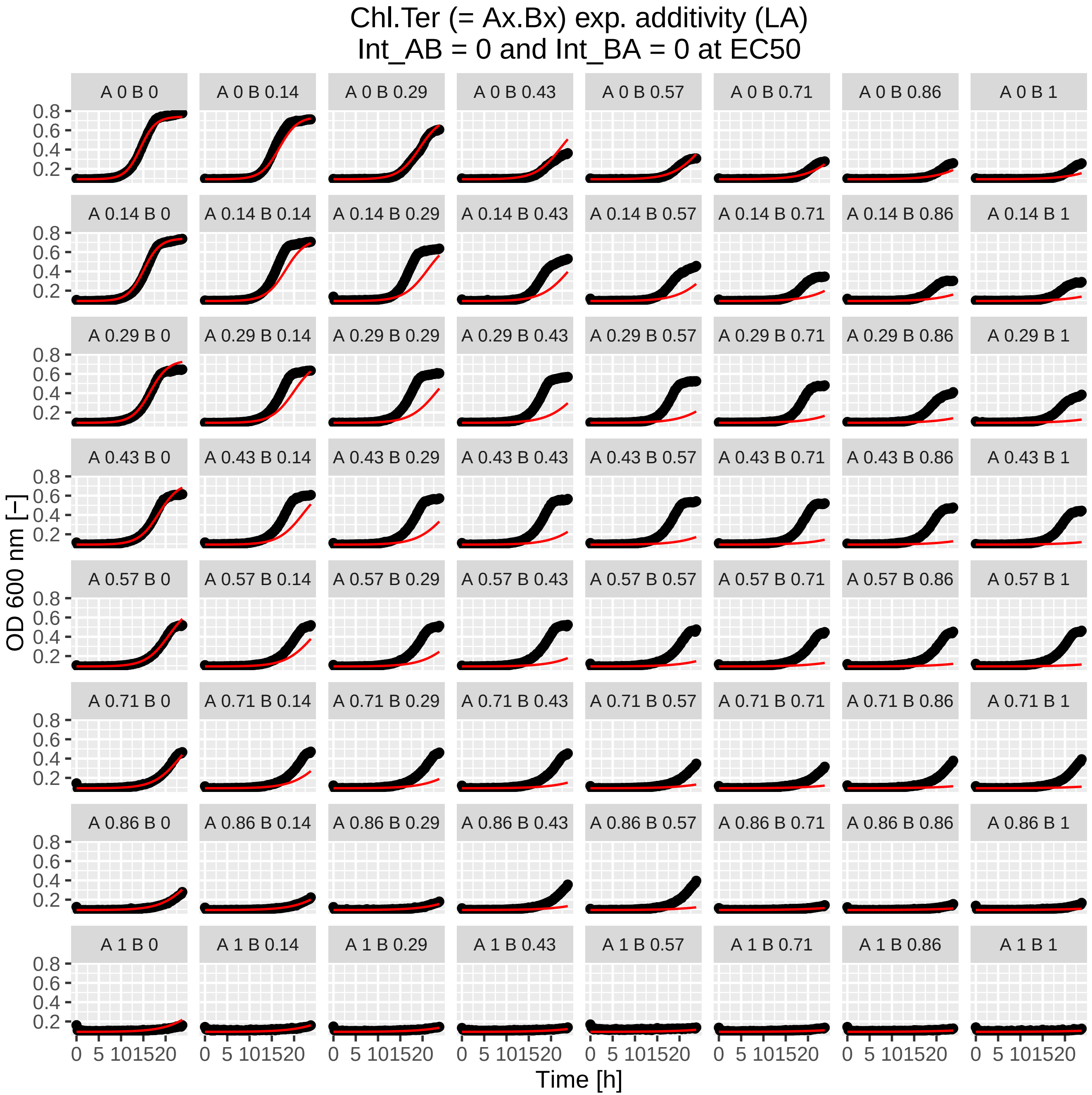

Chl.Ter (= Ax.Bx) exp. additivity (LA)
Int_AB = 0 and Int_BA = 0 at EC50
A 0 B 0
A 0 B 0.14
A 0 B 0.29
A 0 B 0.43
A 0 B 0.57
A 0 B 0.71
A 0 B 0.86
A 0 B 1
A 0.14 B 0
A 0.14 B 0.14
A 0.14 B 0.29
A 0.14 B 0.43
A 0.14 B 0.57
A 0.14 B 0.71
A 0.14 B 0.86
A 0.14 B 1
A 0.29 B 0
A 0.29 B 0.14
A 0.29 B 0.29
A 0.29 B 0.43
A 0.29 B 0.57
A 0.29 B 0.71
A 0.29 B 0.86
A 0.29 B 1
A 0.43 B 0
A 0.43 B 0.14
A 0.43 B 0.29
A 0.43 B 0.43
A 0.43 B 0.57
A 0.43 B 0.71
A 0.43 B 0.86
A 0.43 B 1
A 0.57 B 0
A 0.57 B 0.14
A 0.57 B 0.29
A 0.57 B 0.43
A 0.57 B 0.57
A 0.57 B 0.71
A 0.57 B 0.86
A 0.57 B 1
A 0.71 B 0
A 0.71 B 0.14
A 0.71 B 0.29
A 0.71 B 0.43
A 0.71 B 0.57
A 0.71 B 0.71
A 0.71 B 0.86
A 0.71 B 1
A 0.86 B 0
A 0.86 B 0.14
A 0.86 B 0.29
A 0.86 B 0.43
A 0.86 B 0.57
A 0.86 B 0.71
A 0.86 B 0.86
A 0.86 B 1
A 1 B 0
A 1 B 0.14
A 1 B 0.29
A 1 B 0.43
A 1 B 0.57
A 1 B 0.71
A 1 B 0.86
A 1 B 1
0.8
0.6
0.4
0.2
0 5 101520
OD 600 nm [–]
Time [h]

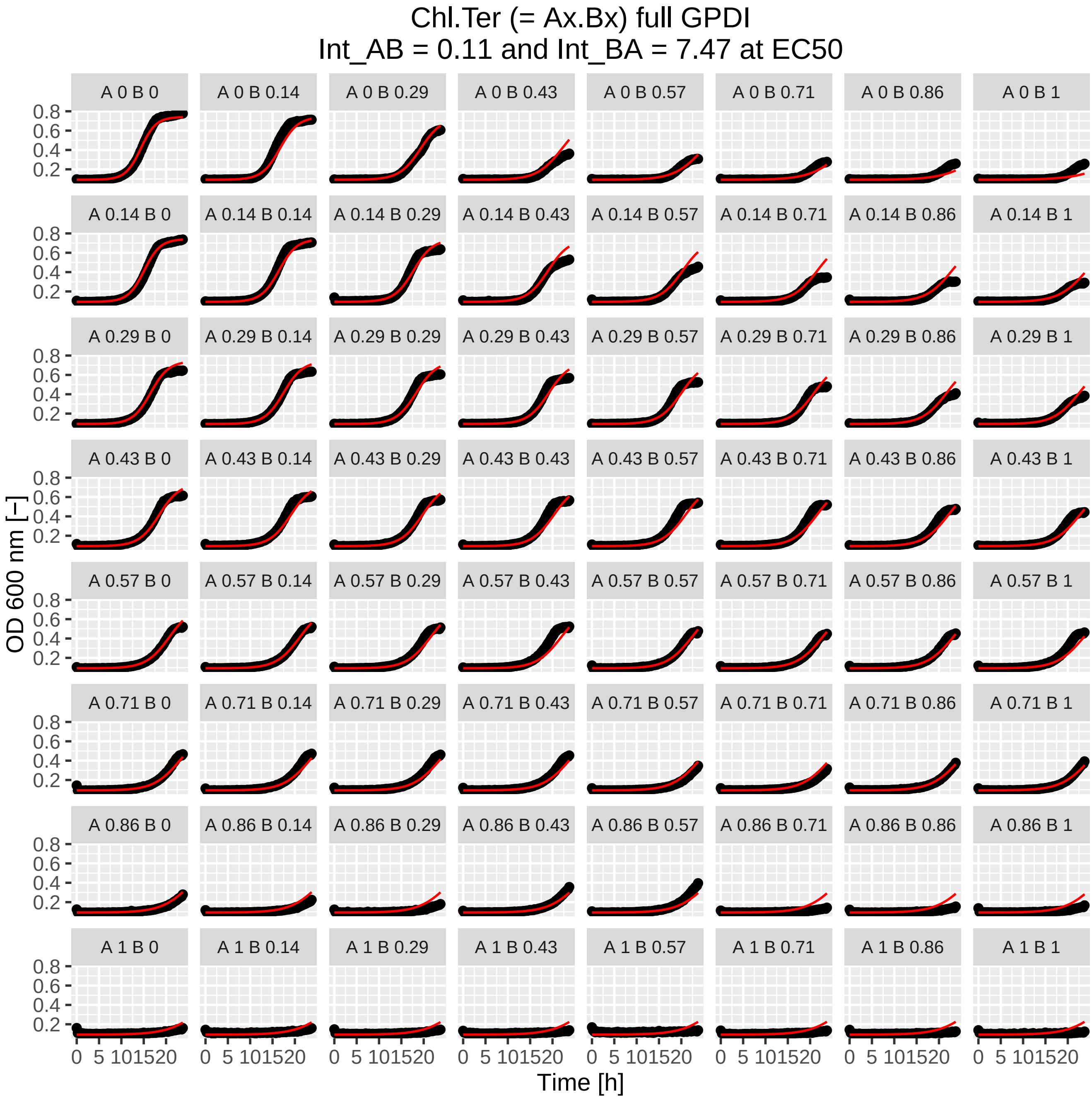
Chl.Ter (= Ax.Bx) full GPDI
Int_AB = 0.11 and Int_BA = 7.47 at EC50
A 0 B 0
A 0 B 0.14
A 0 B 0.29
A 0 B 0.43
A 0 B 0.57
A 0 B 0.71
A 0 B 0.86
A 0 B 1
A 0.14 B 0
A 0.14 B 0.14
A 0.14 B 0.29
A 0.14 B 0.43
A 0.14 B 0.57
A 0.14 B 0.71
A 0.14 B 0.86
A 0.14 B 1
A 0.29 B 0
A 0.29 B 0.14
A 0.29 B 0.29
A 0.29 B 0.43
A 0.29 B 0.57
A 0.29 B 0.71
A 0.29 B 0.86
A 0.29 B 1
A 0.43 B 0
A 0.43 B 0.14
A 0.43 B 0.29
A 0.43 B 0.43
A 0.43 B 0.57
A 0.43 B 0.71
A 0.43 B 0.86
A 0.43 B 1
A 0.57 B 0
A 0.57 B 0.14
A 0.57 B 0.29
A 0.57 B 0.43
A 0.57 B 0.57
A 0.57 B 0.71
A 0.57 B 0.86
A 0.57 B 1
A 0.71 B 0
A 0.71 B 0.14
A 0.71 B 0.29
A 0.71 B 0.43
A 0.71 B 0.57
A 0.71 B 0.71
A 0.71 B 0.86
A 0.71 B 1
A 0.86 B 0
A 0.86 B 0.14
A 0.86 B 0.29
A 0.86 B 0.43
A 0.86 B 0.57
A 0.86 B 0.71
A 0.86 B 0.86
A 0.86 B 1
A 1 B 0
A 1 B 0.14
A 1 B 0.29
A 1 B 0.43
A 1 B 0.57
A 1 B 0.71
A 1 B 0.86
A 1 B 1
OD 600 nm [−]
0.8
0.6
0.4
0.2
0 5 101520
Time [h]

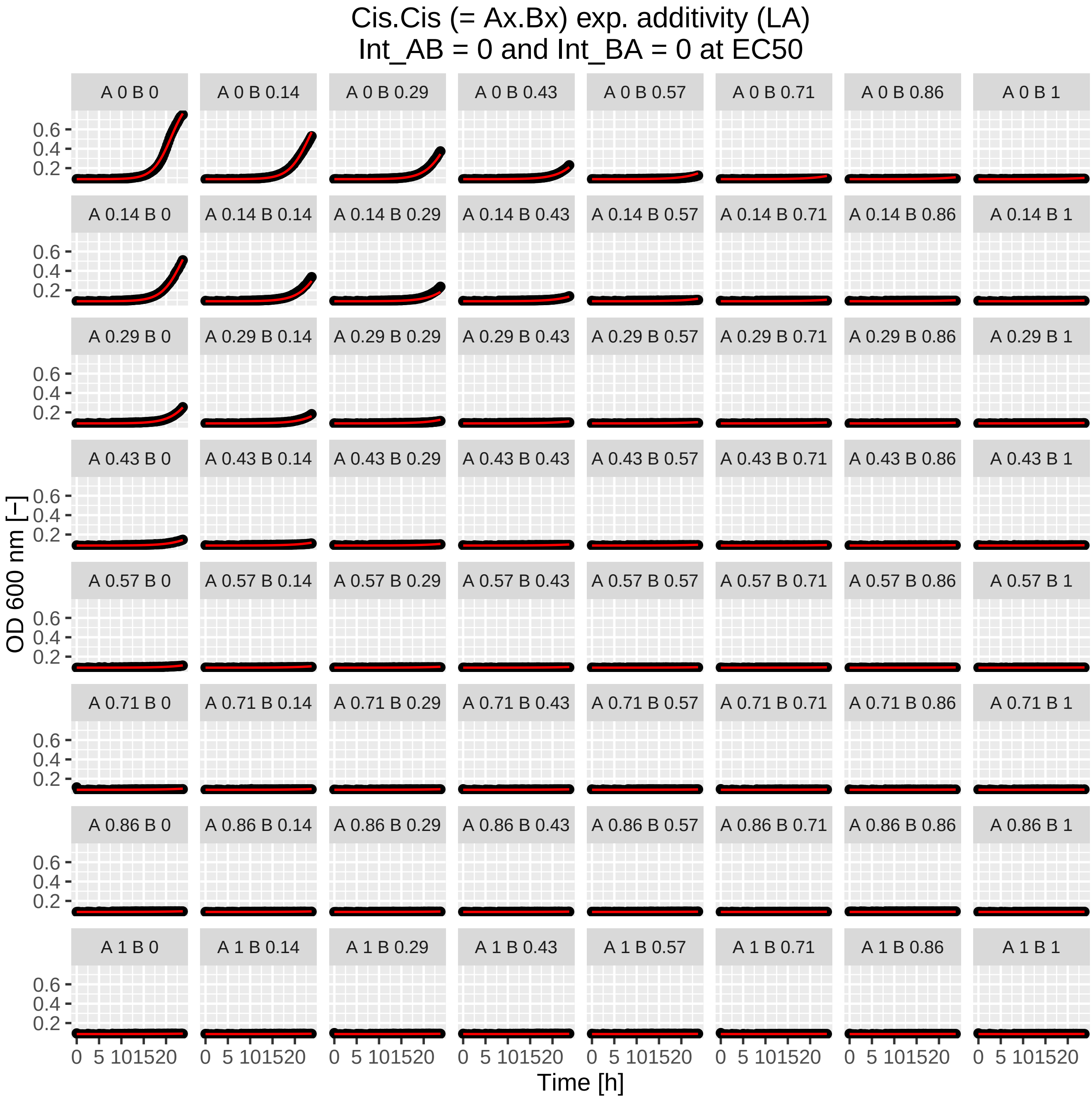

Cis.Cis (= Ax.Bx) exp. additivity (LA)
Int_AB = 0 and Int_BA = 0 at EC50
A 0 B 0
A 0 B 0.14
A 0 B 0.29
A 0 B 0.43
A 0 B 0.57
A 0 B 0.71
A 0 B 0.86
A 0 B 1
A 0.14 B 0
A 0.14 B 0.14
A 0.14 B 0.29
A 0.14 B 0.43
A 0.14 B 0.57
A 0.14 B 0.71
A 0.14 B 0.86
A 0.14 B 1
A 0.29 B 0
A 0.29 B 0.14
A 0.29 B 0.29
A 0.29 B 0.43
A 0.29 B 0.57
A 0.29 B 0.71
A 0.29 B 0.86
A 0.29 B 1
A 0.43 B 0
A 0.43 B 0.14
A 0.43 B 0.29
A 0.43 B 0.43
A 0.43 B 0.57
A 0.43 B 0.71
A 0.43 B 0.86
A 0.43 B 1
A 0.57 B 0
A 0.57 B 0.14
A 0.57 B 0.29
A 0.57 B 0.43
A 0.57 B 0.57
A 0.57 B 0.71
A 0.57 B 0.86
A 0.57 B 1
A 0.71 B 0
A 0.71 B 0.14
A 0.71 B 0.29
A 0.71 B 0.43
A 0.71 B 0.57
A 0.71 B 0.71
A 0.71 B 0.86
A 0.71 B 1
A 0.86 B 0
A 0.86 B 0.14
A 0.86 B 0.29
A 0.86 B 0.43
A 0.86 B 0.57
A 0.86 B 0.71
A 0.86 B 0.86
A 0.86 B 1
A 1 B 0
A 1 B 0.14
A 1 B 0.29
A 1 B 0.43
A 1 B 0.57
A 1 B 0.71
A 1 B 0.86
A 1 B 1
OD 600 nm [–]
0.2
0.4
0.6
0 5 101520
Time [h]

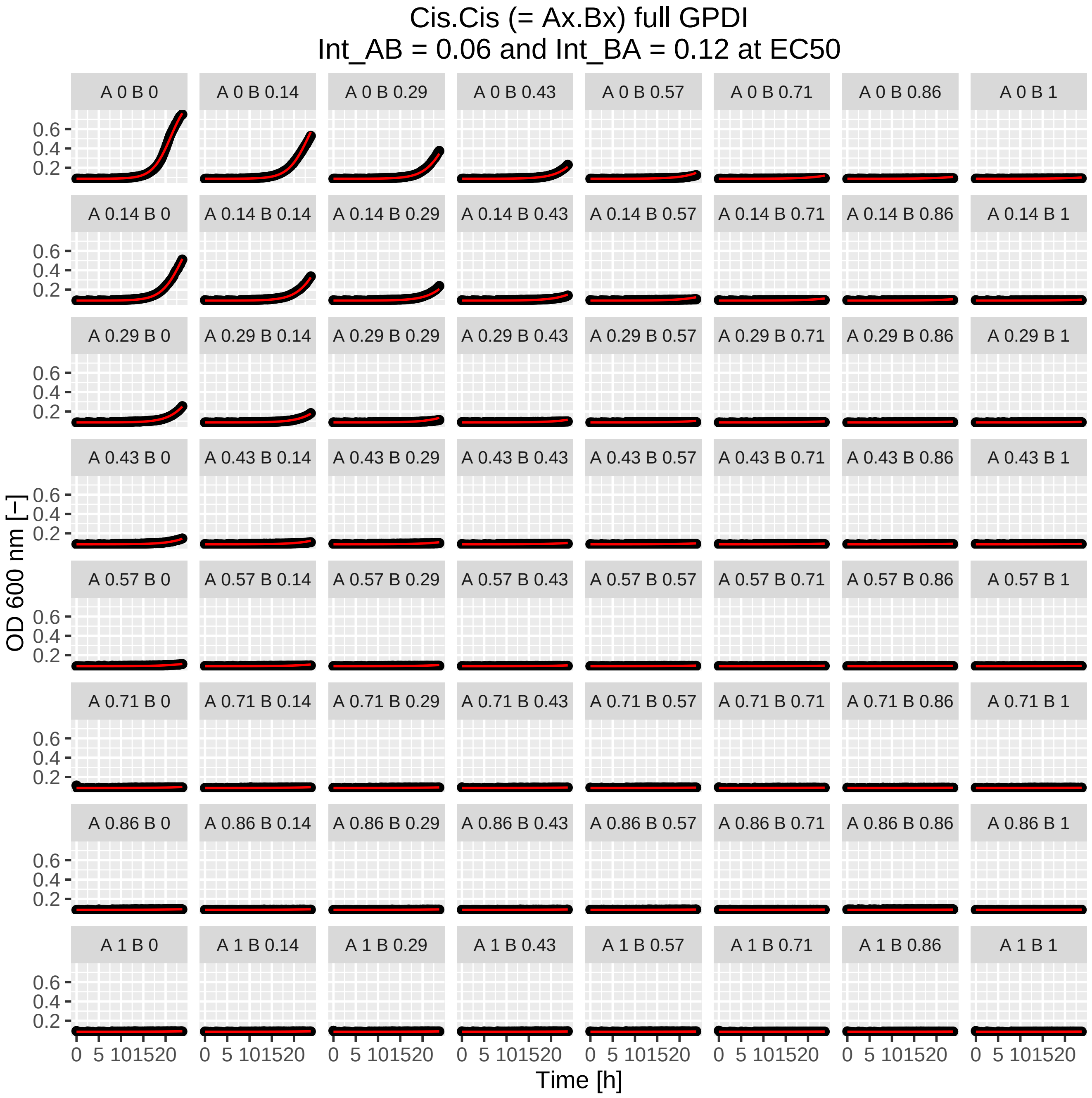
Cis.Cis (= Ax.Bx) full GPDI
Int_AB = 0.06 and Int_BA = 0.12 at EC50
A 0 B 0
A 0 B 0.14
A 0 B 0.29
A 0 B 0.43
A 0 B 0.57
A 0 B 0.71
A 0 B 0.86
A 0 B 1
A 0.14 B 0
A 0.14 B 0.14
A 0.14 B 0.29
A 0.14 B 0.43
A 0.14 B 0.57
A 0.14 B 0.71
A 0.14 B 0.86
A 0.14 B 1
A 0.29 B 0
A 0.29 B 0.14
A 0.29 B 0.29
A 0.29 B 0.43
A 0.29 B 0.57
A 0.29 B 0.71
A 0.29 B 0.86
A 0.29 B 1
A 0.43 B 0
A 0.43 B 0.14
A 0.43 B 0.29
A 0.43 B 0.43
A 0.43 B 0.57
A 0.43 B 0.71
A 0.43 B 0.86
A 0.43 B 1
A 0.57 B 0
A 0.57 B 0.14
A 0.57 B 0.29
A 0.57 B 0.43
A 0.57 B 0.57
A 0.57 B 0.71
A 0.57 B 0.86
A 0.57 B 1
A 0.71 B 0
A 0.71 B 0.14
A 0.71 B 0.29
A 0.71 B 0.43
A 0.71 B 0.57
A 0.71 B 0.71
A 0.71 B 0.86
A 0.71 B 1
A 0.86 B 0
A 0.86 B 0.14
A 0.86 B 0.29
A 0.86 B 0.43
A 0.86 B 0.57
A 0.86 B 0.71
A 0.86 B 0.86
A 0.86 B 1
A 1 B 0
A 1 B 0.14
A 1 B 0.29
A 1 B 0.43
A 1 B 0.57
A 1 B 0.71
A 1 B 0.86
A 1 B 1
OD 600 nm [–]
0.2
0.4
0.6
0 5 101520
Time [h]

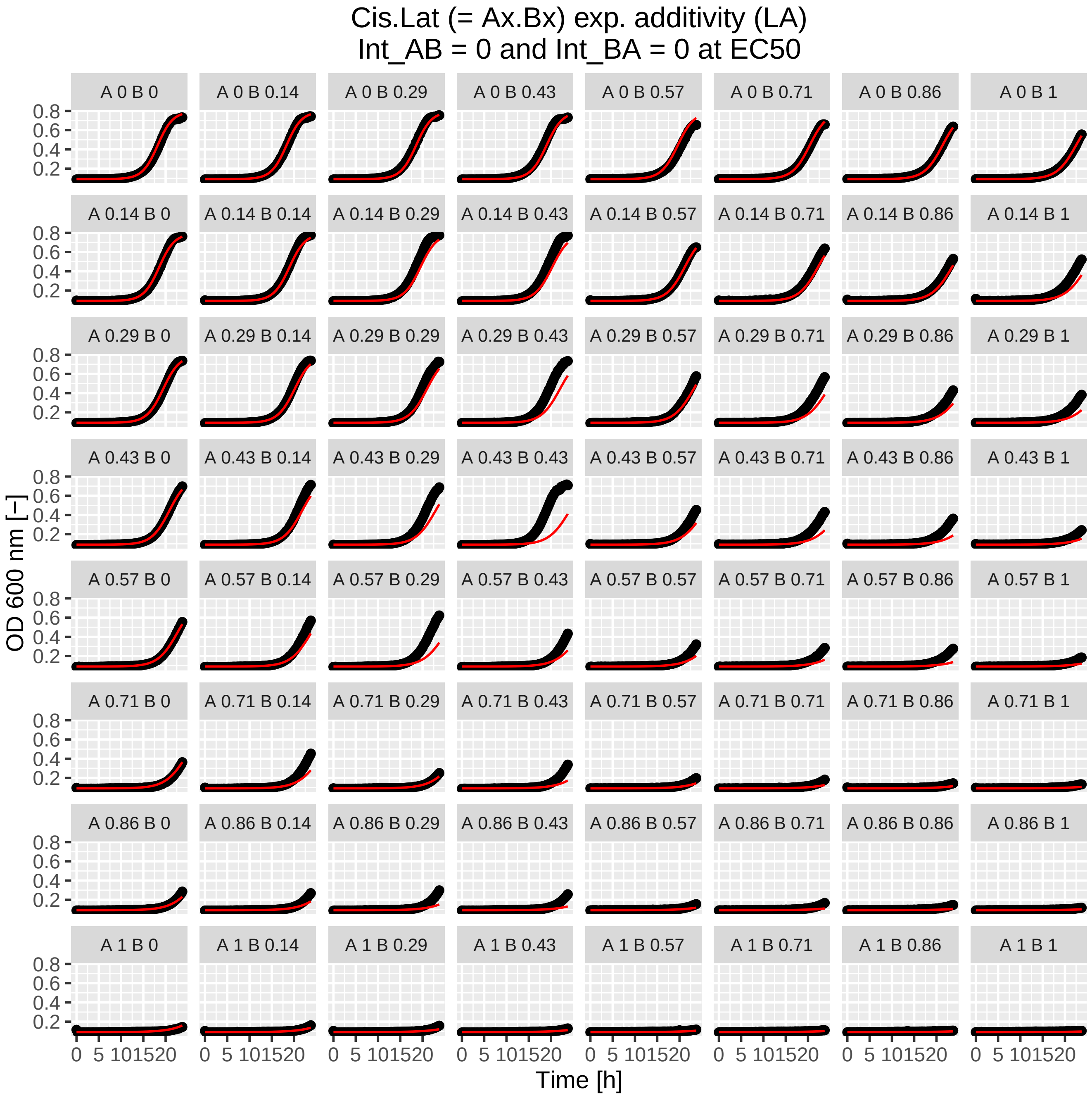
Cis.Lat (= Ax.Bx) exp. additivity (LA)
Int_AB = 0 and Int_BA = 0 at EC50
OD 600 nm [–]
Time [h]
A 0 B 0
A 0 B 0.14
A 0 B 0.29
A 0 B 0.43
A 0 B 0.57
A 0 B 0.71
A 0 B 0.86
A 0 B 1
A 0.14 B 0
A 0.14 B 0.14
A 0.14 B 0.29
A 0.14 B 0.43
A 0.14 B 0.57
A 0.14 B 0.71
A 0.14 B 0.86
A 0.14 B 1
A 0.29 B 0
A 0.29 B 0.14
A 0.29 B 0.29
A 0.29 B 0.43
A 0.29 B 0.57
A 0.29 B 0.71
A 0.29 B 0.86
A 0.29 B 1
A 0.43 B 0
A 0.43 B 0.14
A 0.43 B 0.29
A 0.43 B 0.43
A 0.43 B 0.57
A 0.43 B 0.71
A 0.43 B 0.86
A 0.43 B 1
A 0.57 B 0
A 0.57 B 0.14
A 0.57 B 0.29
A 0.57 B 0.43
A 0.57 B 0.57
A 0.57 B 0.71
A 0.57 B 0.86
A 0.57 B 1
A 0.71 B 0
A 0.71 B 0.14
A 0.71 B 0.29
A 0.71 B 0.43
A 0.71 B 0.57
A 0.71 B 0.71
A 0.71 B 0.86
A 0.71 B 1
A 0.86 B 0
A 0.86 B 0.14
A 0.86 B 0.29
A 0.86 B 0.43
A 0.86 B 0.57
A 0.86 B 0.71
A 0.86 B 0.86
A 0.86 B 1
A 1 B 0
A 1 B 0.14
A 1 B 0.29
A 1 B 0.43
A 1 B 0.57
A 1 B 0.71
A 1 B 0.86
A 1 B 1
0.8
0.6
0.4
0.2
0 5 101520

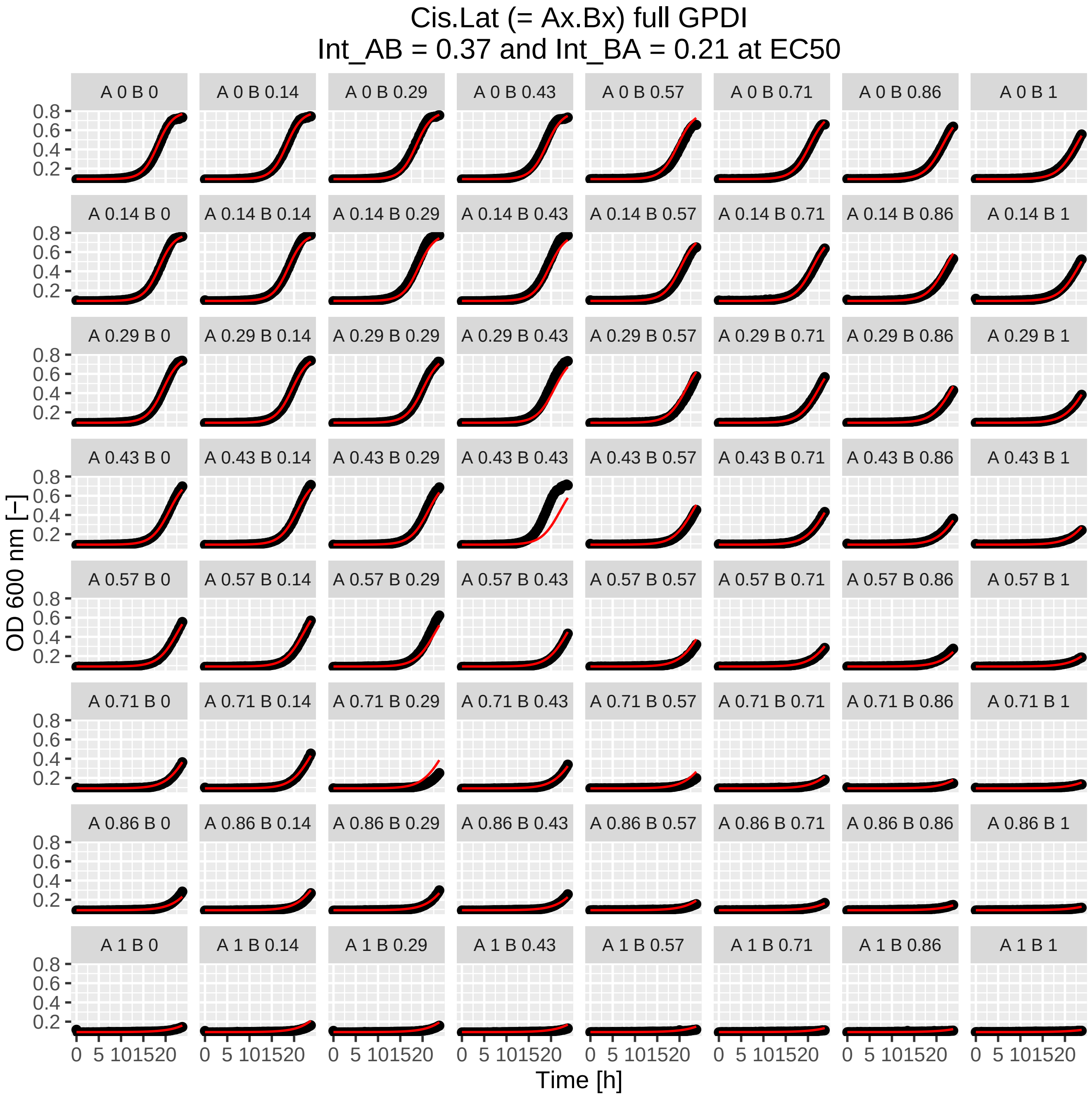
Cis.Lat (= Ax.Bx) full GPDI
Int_AB = 0.37 and Int_BA = 0.21 at EC50
A 0 B 0
A 0 B 0.14
A 0 B 0.29
A 0 B 0.43
A 0 B 0.57
A 0 B 0.71
A 0 B 0.86
A 0 B 1
A 0.14 B 0
A 0.14 B 0.14
A 0.14 B 0.29
A 0.14 B 0.43
A 0.14 B 0.57
A 0.14 B 0.71
A 0.14 B 0.86
A 0.14 B 1
A 0.29 B 0
A 0.29 B 0.14
A 0.29 B 0.29
A 0.29 B 0.43
A 0.29 B 0.57
A 0.29 B 0.71
A 0.29 B 0.86
A 0.29 B 1
A 0.43 B 0
A 0.43 B 0.14
A 0.43 B 0.29
A 0.43 B 0.43
A 0.43 B 0.57
A 0.43 B 0.71
A 0.43 B 0.86
A 0.43 B 1
A 0.57 B 0
A 0.57 B 0.14
A 0.57 B 0.29
A 0.57 B 0.43
A 0.57 B 0.57
A 0.57 B 0.71
A 0.57 B 0.86
A 0.57 B 1
A 0.71 B 0
A 0.71 B 0.14
A 0.71 B 0.29
A 0.71 B 0.43
A 0.71 B 0.57
A 0.71 B 0.71
A 0.71 B 0.86
A 0.71 B 1
A 0.86 B 0
A 0.86 B 0.14
A 0.86 B 0.29
A 0.86 B 0.43
A 0.86 B 0.57
A 0.86 B 0.71
A 0.86 B 0.86
A 0.86 B 1
A 1 B 0
A 1 B 0.14
A 1 B 0.29
A 1 B 0.43
A 1 B 0.57
A 1 B 0.71
A 1 B 0.86
A 1 B 1
OD 600 nm [–]
0.2
0.4
0.6
0.8
0
5
10
15
20
Time [h]

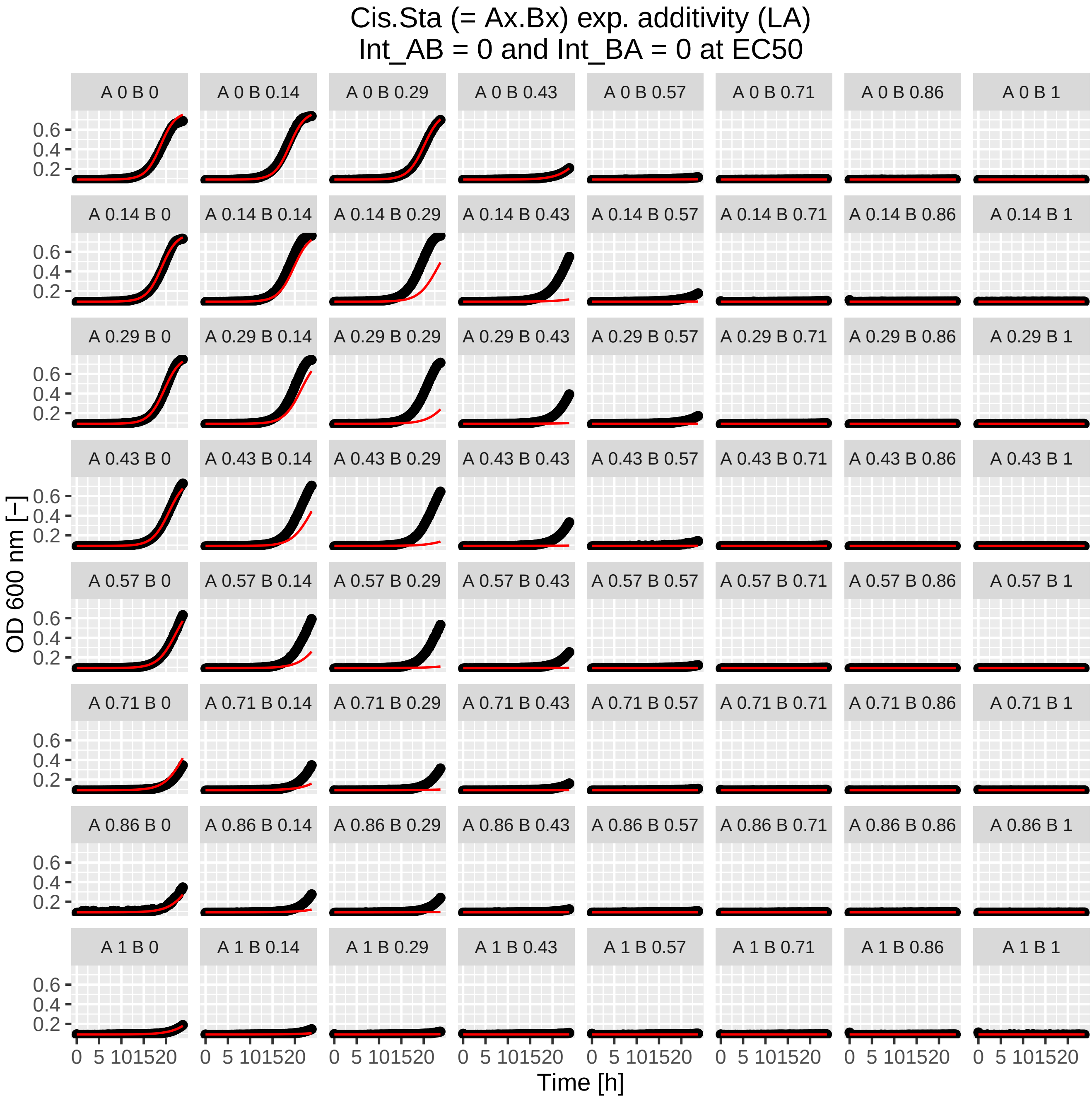

Cis.Sta (= Ax.Bx) exp. additivity (LA)
Int_AB = 0 and Int_BA = 0 at EC50
A 0 B 0
A 0 B 0.14
A 0 B 0.29
A 0 B 0.43
A 0 B 0.57
A 0 B 0.71
A 0 B 0.86
A 0 B 1
A 0.14 B 0
A 0.14 B 0.14
A 0.14 B 0.29
A 0.14 B 0.43
A 0.14 B 0.57
A 0.14 B 0.71
A 0.14 B 0.86
A 0.14 B 1
A 0.29 B 0
A 0.29 B 0.14
A 0.29 B 0.29
A 0.29 B 0.43
A 0.29 B 0.57
A 0.29 B 0.71
A 0.29 B 0.86
A 0.29 B 1
A 0.43 B 0
A 0.43 B 0.14
A 0.43 B 0.29
A 0.43 B 0.43
A 0.43 B 0.57
A 0.43 B 0.71
A 0.43 B 0.86
A 0.43 B 1
A 0.57 B 0
A 0.57 B 0.14
A 0.57 B 0.29
A 0.57 B 0.43
A 0.57 B 0.57
A 0.57 B 0.71
A 0.57 B 0.86
A 0.57 B 1
A 0.71 B 0
A 0.71 B 0.14
A 0.71 B 0.29
A 0.71 B 0.43
A 0.71 B 0.57
A 0.71 B 0.71
A 0.71 B 0.86
A 0.71 B 1
A 0.86 B 0
A 0.86 B 0.14
A 0.86 B 0.29
A 0.86 B 0.43
A 0.86 B 0.57
A 0.86 B 0.71
A 0.86 B 0.86
A 0.86 B 1
A 1 B 0
A 1 B 0.14
A 1 B 0.29
A 1 B 0.43
A 1 B 0.57
A 1 B 0.71
A 1 B 0.86
A 1 B 1
0.6
0.4
0.2
OD 600 nm [–]
0 5 101520
Time [h]

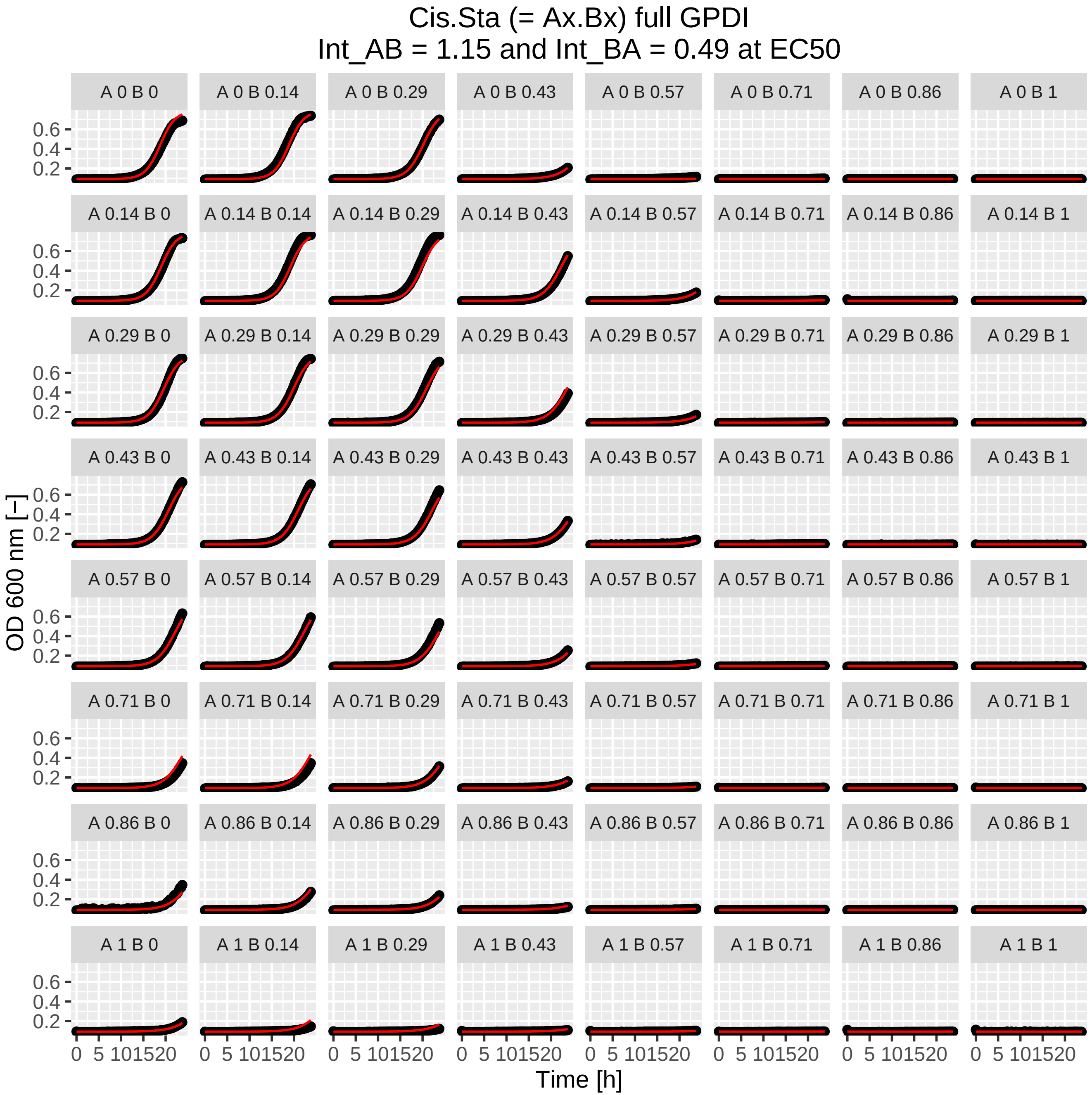

Cis.Sta (= Ax.Bx) full GPDI
Int_AB = 1.15 and Int_BA = 0.49 at EC50
A 0 B 0
A 0 B 0.14
A 0 B 0.29
A 0 B 0.43
A 0 B 0.57
A 0 B 0.71
A 0 B 0.86
A 0 B 1
A 0.14 B 0
A 0.14 B 0.14
A 0.14 B 0.29
A 0.14 B 0.43
A 0.14 B 0.57
A 0.14 B 0.71
A 0.14 B 0.86
A 0.14 B 1
A 0.29 B 0
A 0.29 B 0.14
A 0.29 B 0.29
A 0.29 B 0.43
A 0.29 B 0.57
A 0.29 B 0.71
A 0.29 B 0.86
A 0.29 B 1
A 0.43 B 0
A 0.43 B 0.14
A 0.43 B 0.29
A 0.43 B 0.43
A 0.43 B 0.57
A 0.43 B 0.71
A 0.43 B 0.86
A 0.43 B 1
A 0.57 B 0
A 0.57 B 0.14
A 0.57 B 0.29
A 0.57 B 0.43
A 0.57 B 0.57
A 0.57 B 0.71
A 0.57 B 0.86
A 0.57 B 1
A 0.71 B 0
A 0.71 B 0.14
A 0.71 B 0.29
A 0.71 B 0.43
A 0.71 B 0.57
A 0.71 B 0.71
A 0.71 B 0.86
A 0.71 B 1
A 0.86 B 0
A 0.86 B 0.14
A 0.86 B 0.29
A 0.86 B 0.43
A 0.86 B 0.57
A 0.86 B 0.71
A 0.86 B 0.86
A 0.86 B 1
A 1 B 0
A 1 B 0.14
A 1 B 0.29
A 1 B 0.43
A 1 B 0.57
A 1 B 0.71
A 1 B 0.86
A 1 B 1
OD 600 nm [–]
0.6
0.4
0.2
0 5 101520
Time [h]

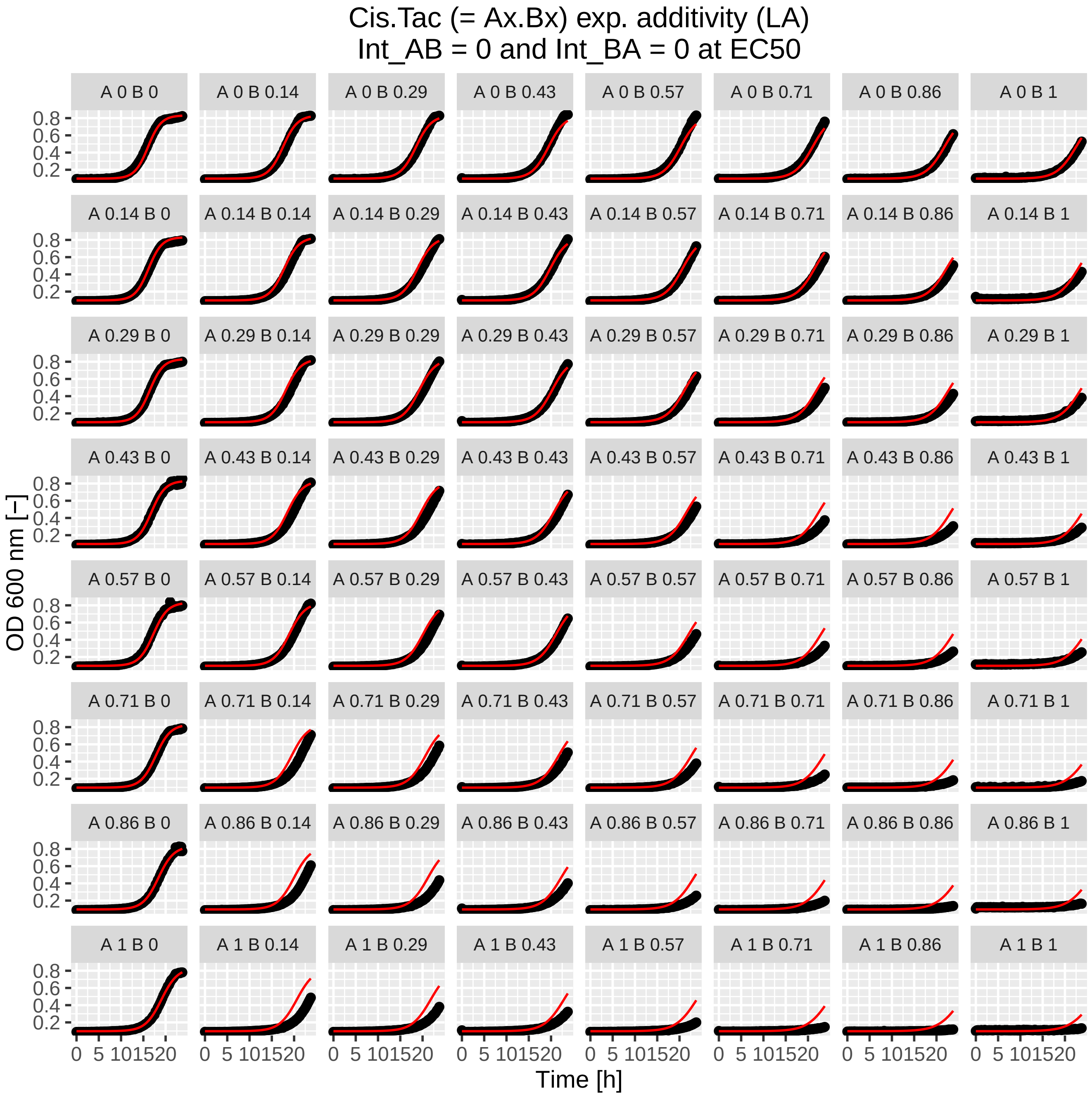

Cis.Tac (= Ax.Bx) exp. additivity (LA)
Int_AB = 0 and Int_BA = 0 at EC50
A 0 B 0
A 0 B 0.14
A 0 B 0.29
A 0 B 0.43
A 0 B 0.57
A 0 B 0.71
A 0 B 0.86
A 0 B 1
A 0.14 B 0
A 0.14 B 0.14
A 0.14 B 0.29
A 0.14 B 0.43
A 0.14 B 0.57
A 0.14 B 0.71
A 0.14 B 0.86
A 0.14 B 1
A 0.29 B 0
A 0.29 B 0.14
A 0.29 B 0.29
A 0.29 B 0.43
A 0.29 B 0.57
A 0.29 B 0.71
A 0.29 B 0.86
A 0.29 B 1
A 0.43 B 0
A 0.43 B 0.14
A 0.43 B 0.29
A 0.43 B 0.43
A 0.43 B 0.57
A 0.43 B 0.71
A 0.43 B 0.86
A 0.43 B 1
A 0.57 B 0
A 0.57 B 0.14
A 0.57 B 0.29
A 0.57 B 0.43
A 0.57 B 0.57
A 0.57 B 0.71
A 0.57 B 0.86
A 0.57 B 1
A 0.71 B 0
A 0.71 B 0.14
A 0.71 B 0.29
A 0.71 B 0.43
A 0.71 B 0.57
A 0.71 B 0.71
A 0.71 B 0.86
A 0.71 B 1
A 0.86 B 0
A 0.86 B 0.14
A 0.86 B 0.29
A 0.86 B 0.43
A 0.86 B 0.57
A 0.86 B 0.71
A 0.86 B 0.86
A 0.86 B 1
A 1 B 0
A 1 B 0.14
A 1 B 0.29
A 1 B 0.43
A 1 B 0.57
A 1 B 0.71
A 1 B 0.86
A 1 B 1
OD 600 nm [–]
0.2
0.4
0.6
0.8
0 5 101520
Time [h]

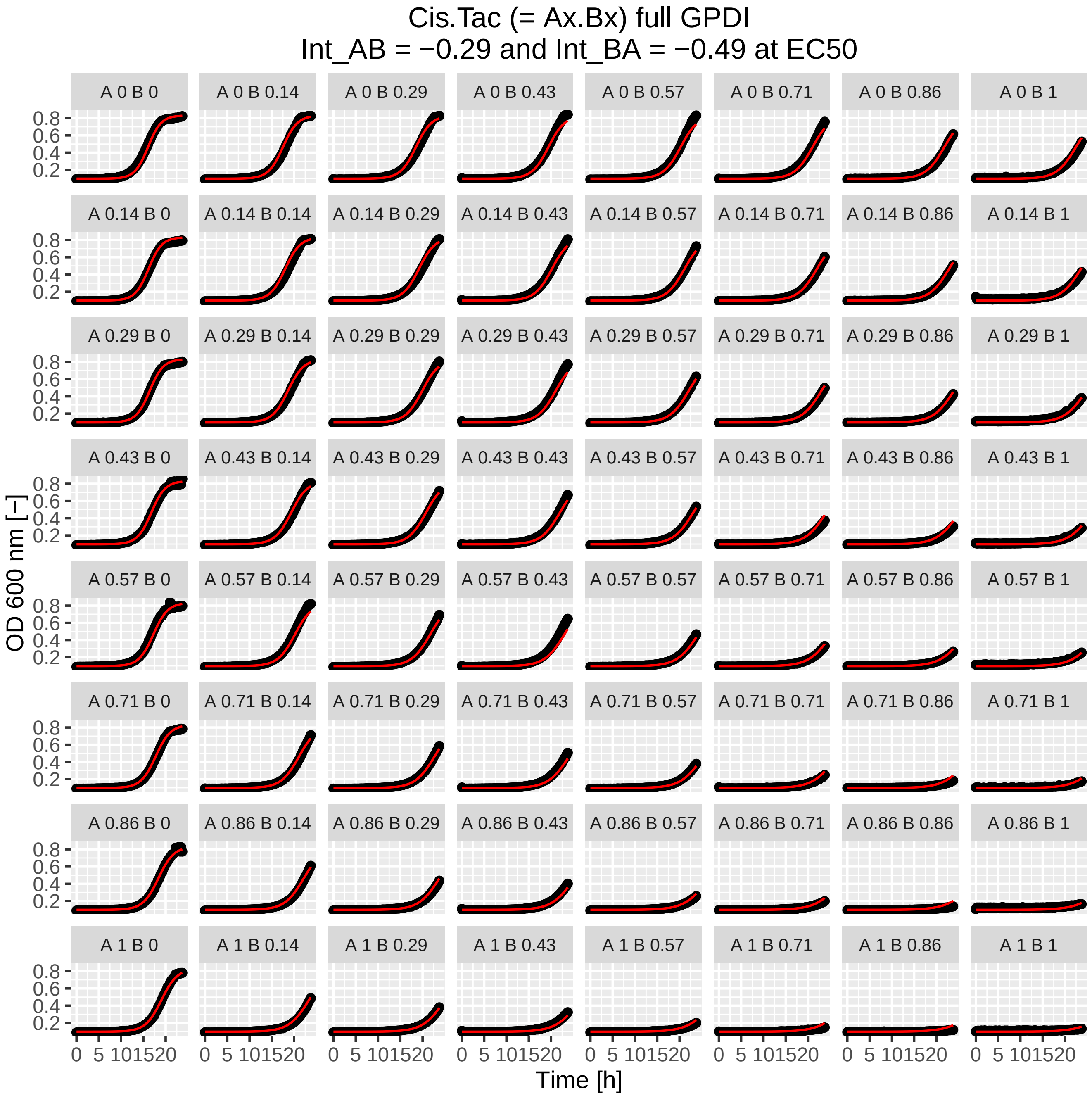

Cis.Tac (= Ax.Bx) full GPDI
Int_AB = −0.29 and Int_BA = −0.49 at EC50
A 0 B 0
A 0 B 0.14
A 0 B 0.29
A 0 B 0.43
A 0 B 0.57
A 0 B 0.71
A 0 B 0.86
A 0 B 1
A 0.14 B 0
A 0.14 B 0.14
A 0.14 B 0.29
A 0.14 B 0.43
A 0.14 B 0.57
A 0.14 B 0.71
A 0.14 B 0.86
A 0.14 B 1
A 0.29 B 0
A 0.29 B 0.14
A 0.29 B 0.29
A 0.29 B 0.43
A 0.29 B 0.57
A 0.29 B 0.71
A 0.29 B 0.86
A 0.29 B 1
A 0.43 B 0
A 0.43 B 0.14
A 0.43 B 0.29
A 0.43 B 0.43
A 0.43 B 0.57
A 0.43 B 0.71
A 0.43 B 0.86
A 0.43 B 1
A 0.57 B 0
A 0.57 B 0.14
A 0.57 B 0.29
A 0.57 B 0.43
A 0.57 B 0.57
A 0.57 B 0.71
A 0.57 B 0.86
A 0.57 B 1
A 0.71 B 0
A 0.71 B 0.14
A 0.71 B 0.29
A 0.71 B 0.43
A 0.71 B 0.57
A 0.71 B 0.71
A 0.71 B 0.86
A 0.71 B 1
A 0.86 B 0
A 0.86 B 0.14
A 0.86 B 0.29
A 0.86 B 0.43
A 0.86 B 0.57
A 0.86 B 0.71
A 0.86 B 0.86
A 0.86 B 1
A 1 B 0
A 1 B 0.14
A 1 B 0.29
A 1 B 0.43
A 1 B 0.57
A 1 B 0.71
A 1 B 0.86
A 1 B 1
OD 600 nm [−]
0.8
0.6
0.4
0.2
0 5 101520
Time [h]

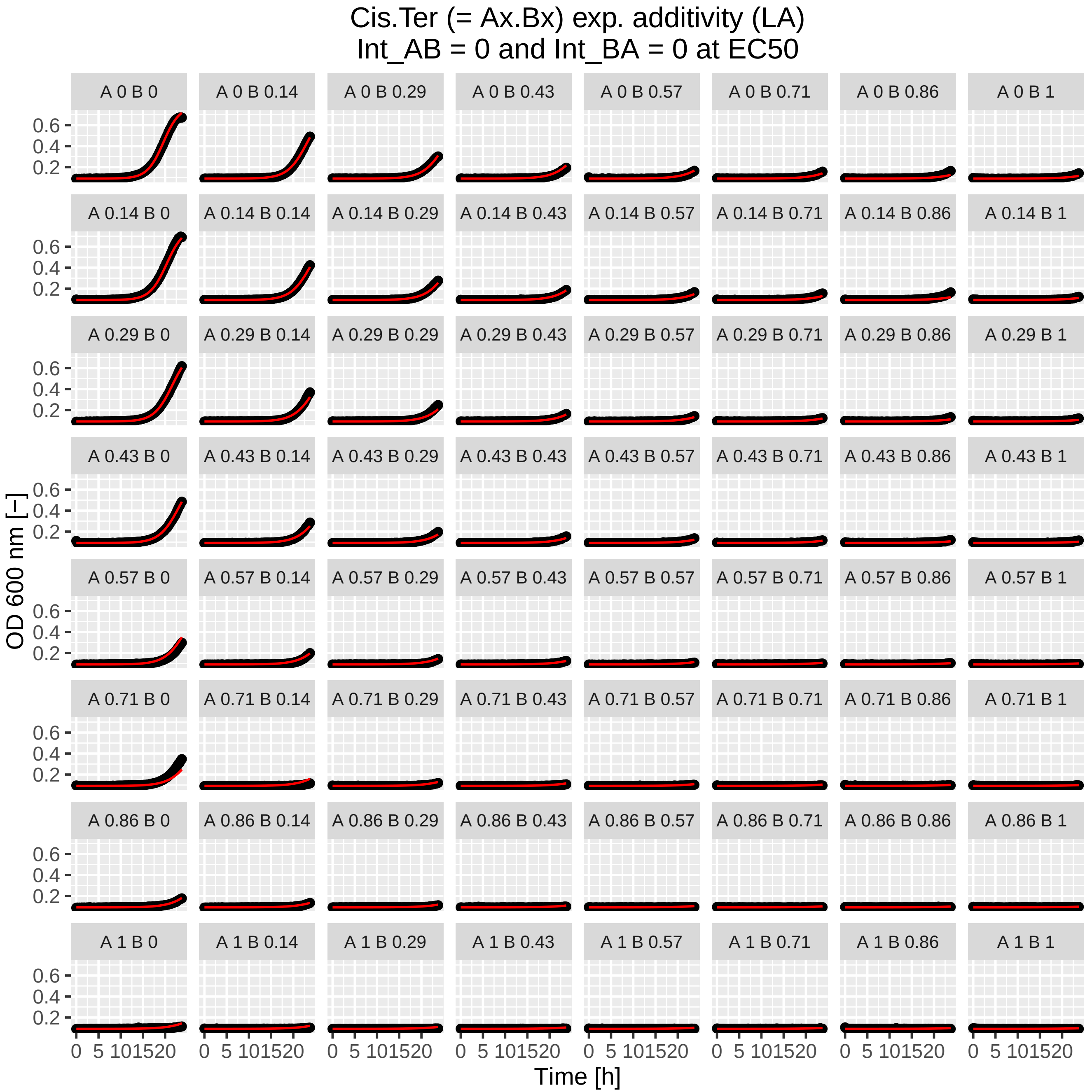

Cis.Ter (= Ax.Bx) exp. additivity (LA)
Int_AB = 0 and Int_BA = 0 at EC50
A 0 B 0
A 0 B 0.14
A 0 B 0.29
A 0 B 0.43
A 0 B 0.57
A 0 B 0.71
A 0 B 0.86
A 0 B 1
A 0.14 B 0
A 0.14 B 0.14
A 0.14 B 0.29
A 0.14 B 0.43
A 0.14 B 0.57
A 0.14 B 0.71
A 0.14 B 0.86
A 0.14 B 1
A 0.29 B 0
A 0.29 B 0.14
A 0.29 B 0.29
A 0.29 B 0.43
A 0.29 B 0.57
A 0.29 B 0.71
A 0.29 B 0.86
A 0.29 B 1
A 0.43 B 0
A 0.43 B 0.14
A 0.43 B 0.29
A 0.43 B 0.43
A 0.43 B 0.57
A 0.43 B 0.71
A 0.43 B 0.86
A 0.43 B 1
A 0.57 B 0
A 0.57 B 0.14
A 0.57 B 0.29
A 0.57 B 0.43
A 0.57 B 0.57
A 0.57 B 0.71
A 0.57 B 0.86
A 0.57 B 1
A 0.71 B 0
A 0.71 B 0.14
A 0.71 B 0.29
A 0.71 B 0.43
A 0.71 B 0.57
A 0.71 B 0.71
A 0.71 B 0.86
A 0.71 B 1
A 0.86 B 0
A 0.86 B 0.14
A 0.86 B 0.29
A 0.86 B 0.43
A 0.86 B 0.57
A 0.86 B 0.71
A 0.86 B 0.86
A 0.86 B 1
A 1 B 0
A 1 B 0.14
A 1 B 0.29
A 1 B 0.43
A 1 B 0.57
A 1 B 0.71
A 1 B 0.86
A 1 B 1
0.6
0.4
0.2
0 5 101520
OD 600 nm [–]
Time [h]

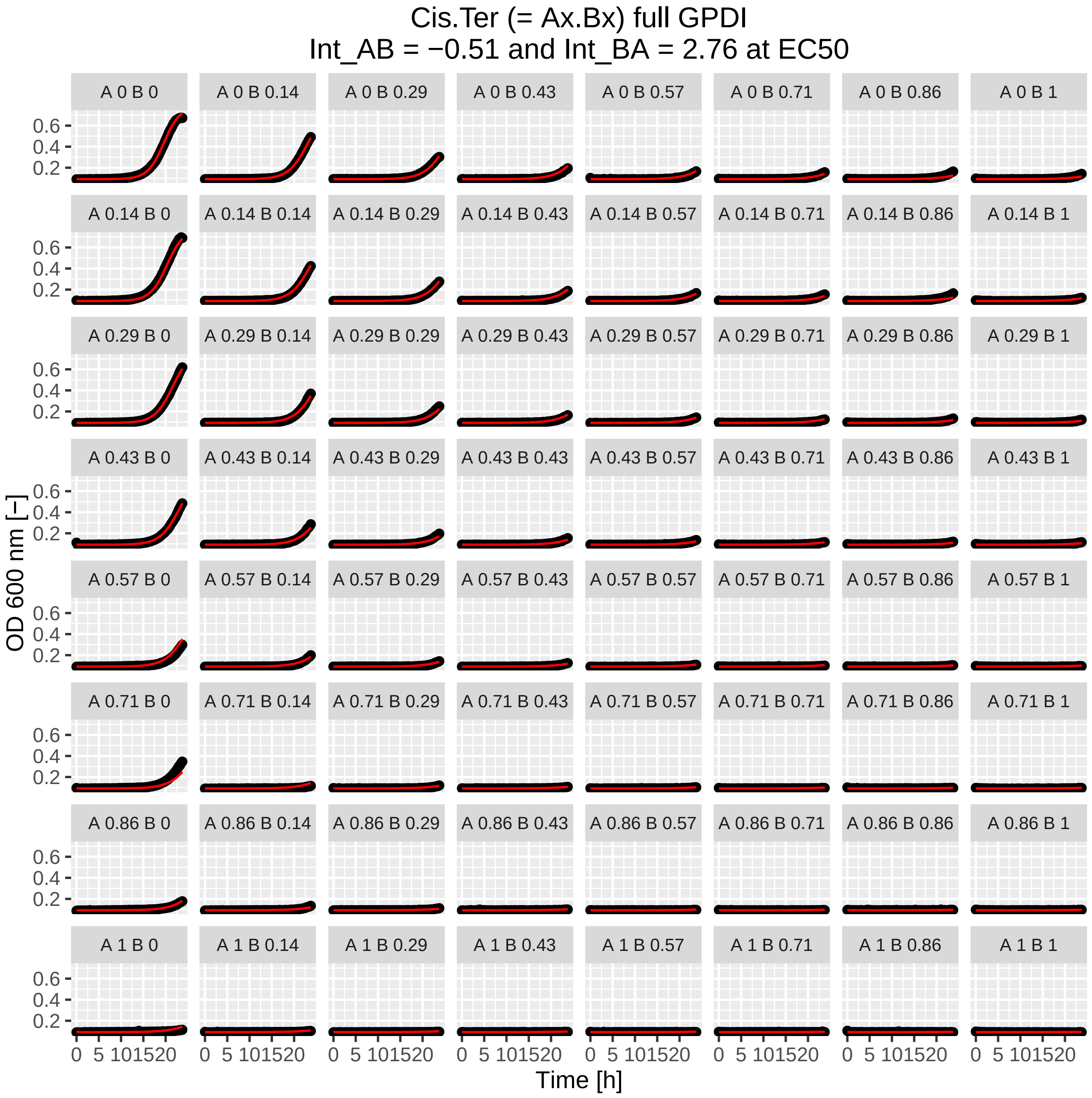
Cis.Ter (= Ax.Bx) full GPDI
Int_AB = −0.51 and Int_BA = 2.76 at EC50
A 0 B 0
A 0 B 0.14
A 0 B 0.29
A 0 B 0.43
A 0 B 0.57
A 0 B 0.71
A 0 B 0.86
A 0 B 1
A 0.14 B 0
A 0.14 B 0.14
A 0.14 B 0.29
A 0.14 B 0.43
A 0.14 B 0.57
A 0.14 B 0.71
A 0.14 B 0.86
A 0.14 B 1
A 0.29 B 0
A 0.29 B 0.14
A 0.29 B 0.29
A 0.29 B 0.43
A 0.29 B 0.57
A 0.29 B 0.71
A 0.29 B 0.86
A 0.29 B 1
A 0.43 B 0
A 0.43 B 0.14
A 0.43 B 0.29
A 0.43 B 0.43
A 0.43 B 0.57
A 0.43 B 0.71
A 0.43 B 0.86
A 0.43 B 1
A 0.57 B 0
A 0.57 B 0.14
A 0.57 B 0.29
A 0.57 B 0.43
A 0.57 B 0.57
A 0.57 B 0.71
A 0.57 B 0.86
A 0.57 B 1
A 0.71 B 0
A 0.71 B 0.14
A 0.71 B 0.29
A 0.71 B 0.43
A 0.71 B 0.57
A 0.71 B 0.71
A 0.71 B 0.86
A 0.71 B 1
A 0.86 B 0
A 0.86 B 0.14
A 0.86 B 0.29
A 0.86 B 0.43
A 0.86 B 0.57
A 0.86 B 0.71
A 0.86 B 0.86
A 0.86 B 1
A 1 B 0
A 1 B 0.14
A 1 B 0.29
A 1 B 0.43
A 1 B 0.57
A 1 B 0.71
A 1 B 0.86
A 1 B 1
OD 600 nm [−]
0.2
0.4
0.6
0 5 101520
Time [h]

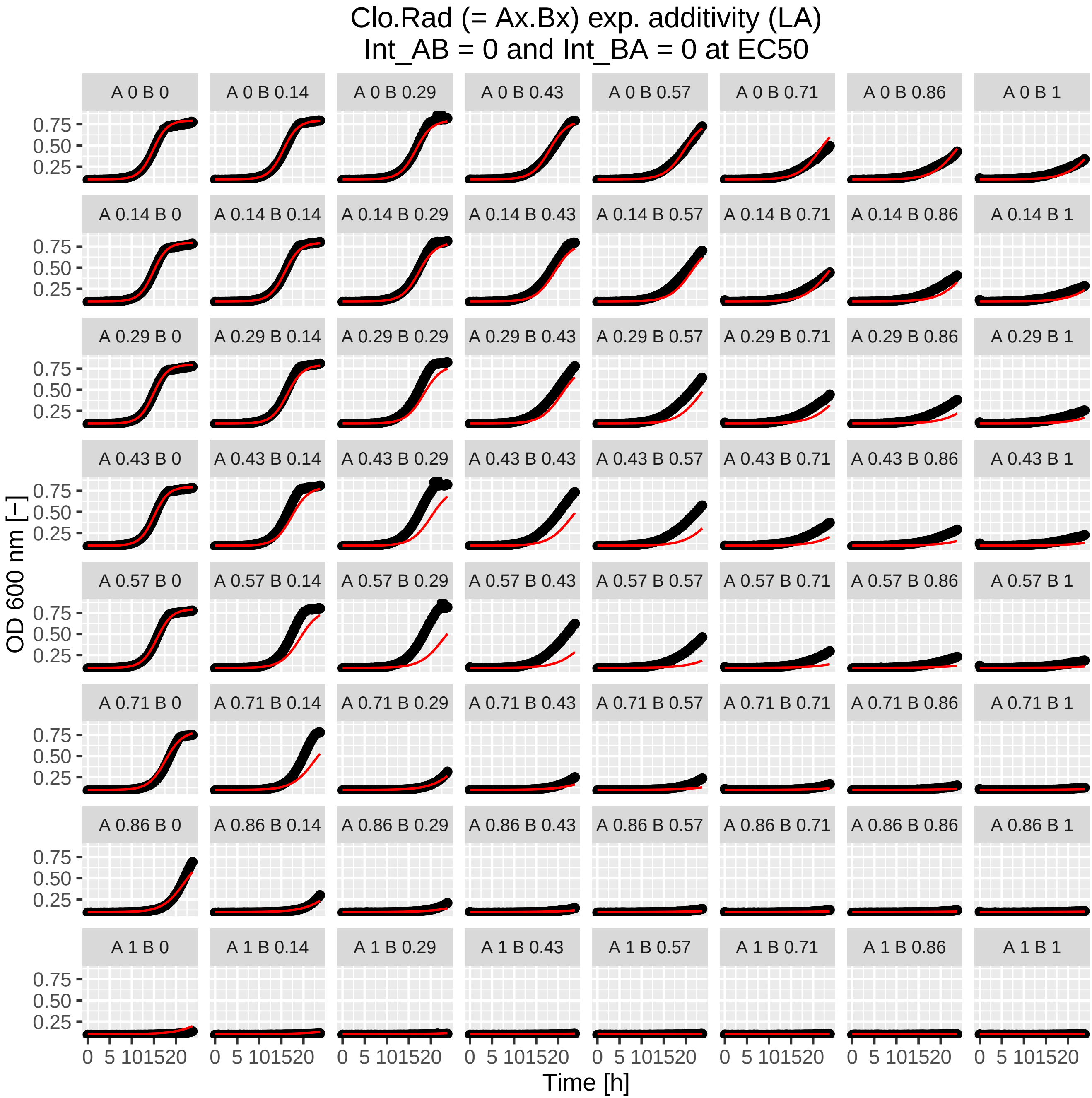

Clo.Rad (= Ax.Bx) exp. additivity (LA)
Int_AB = 0 and Int_BA = 0 at EC50
A 0 B 0
A 0 B 0.14
A 0 B 0.29
A 0 B 0.43
A 0 B 0.57
A 0 B 0.71
A 0 B 0.86
A 0 B 1
A 0.14 B 0
A 0.14 B 0.14
A 0.14 B 0.29
A 0.14 B 0.43
A 0.14 B 0.57
A 0.14 B 0.71
A 0.14 B 0.86
A 0.14 B 1
A 0.29 B 0
A 0.29 B 0.14
A 0.29 B 0.29
A 0.29 B 0.43
A 0.29 B 0.57
A 0.29 B 0.71
A 0.29 B 0.86
A 0.29 B 1
A 0.43 B 0
A 0.43 B 0.14
A 0.43 B 0.29
A 0.43 B 0.43
A 0.43 B 0.57
A 0.43 B 0.71
A 0.43 B 0.86
A 0.43 B 1
A 0.57 B 0
A 0.57 B 0.14
A 0.57 B 0.29
A 0.57 B 0.43
A 0.57 B 0.57
A 0.57 B 0.71
A 0.57 B 0.86
A 0.57 B 1
A 0.71 B 0
A 0.71 B 0.14
A 0.71 B 0.29
A 0.71 B 0.43
A 0.71 B 0.57
A 0.71 B 0.71
A 0.71 B 0.86
A 0.71 B 1
A 0.86 B 0
A 0.86 B 0.14
A 0.86 B 0.29
A 0.86 B 0.43
A 0.86 B 0.57
A 0.86 B 0.71
A 0.86 B 0.86
A 0.86 B 1
A 1 B 0
A 1 B 0.14
A 1 B 0.29
A 1 B 0.43
A 1 B 0.57
A 1 B 0.71
A 1 B 0.86
A 1 B 1
OD 600 nm [–]
0.75
0.50
0.25
0 5 101520
Time [h]

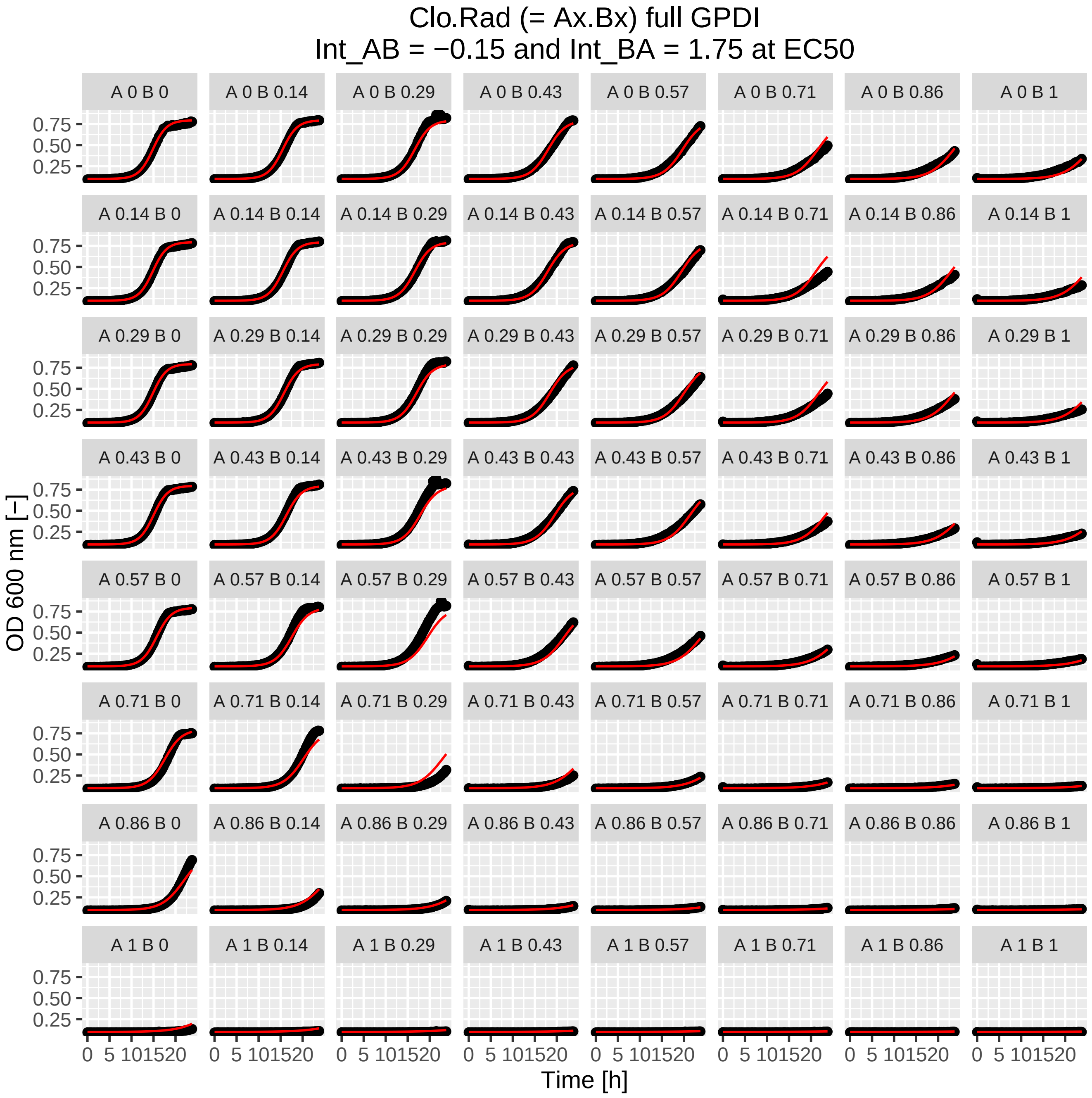
Clo.Rad (= Ax.Bx) full GPDI
Int_AB = −0.15 and Int_BA = 1.75 at EC50
A 0 B 0
A 0 B 0.14
A 0 B 0.29
A 0 B 0.43
A 0 B 0.57
A 0 B 0.71
A 0 B 0.86
A 0 B 1
A 0.14 B 0
A 0.14 B 0.14
A 0.14 B 0.29
A 0.14 B 0.43
A 0.14 B 0.57
A 0.14 B 0.71
A 0.14 B 0.86
A 0.14 B 1
A 0.29 B 0
A 0.29 B 0.14
A 0.29 B 0.29
A 0.29 B 0.43
A 0.29 B 0.57
A 0.29 B 0.71
A 0.29 B 0.86
A 0.29 B 1
A 0.43 B 0
A 0.43 B 0.14
A 0.43 B 0.29
A 0.43 B 0.43
A 0.43 B 0.57
A 0.43 B 0.71
A 0.43 B 0.86
A 0.43 B 1
A 0.57 B 0
A 0.57 B 0.14
A 0.57 B 0.29
A 0.57 B 0.43
A 0.57 B 0.57
A 0.57 B 0.71
A 0.57 B 0.86
A 0.57 B 1
A 0.71 B 0
A 0.71 B 0.14
A 0.71 B 0.29
A 0.71 B 0.43
A 0.71 B 0.57
A 0.71 B 0.71
A 0.71 B 0.86
A 0.71 B 1
A 0.86 B 0
A 0.86 B 0.14
A 0.86 B 0.29
A 0.86 B 0.43
A 0.86 B 0.57
A 0.86 B 0.71
A 0.86 B 0.86
A 0.86 B 1
A 1 B 0
A 1 B 0.14
A 1 B 0.29
A 1 B 0.43
A 1 B 0.57
A 1 B 0.71
A 1 B 0.86
A 1 B 1
0.75
0.50
0.25
OD 600 nm [−]
0 5 101520
Time [h]

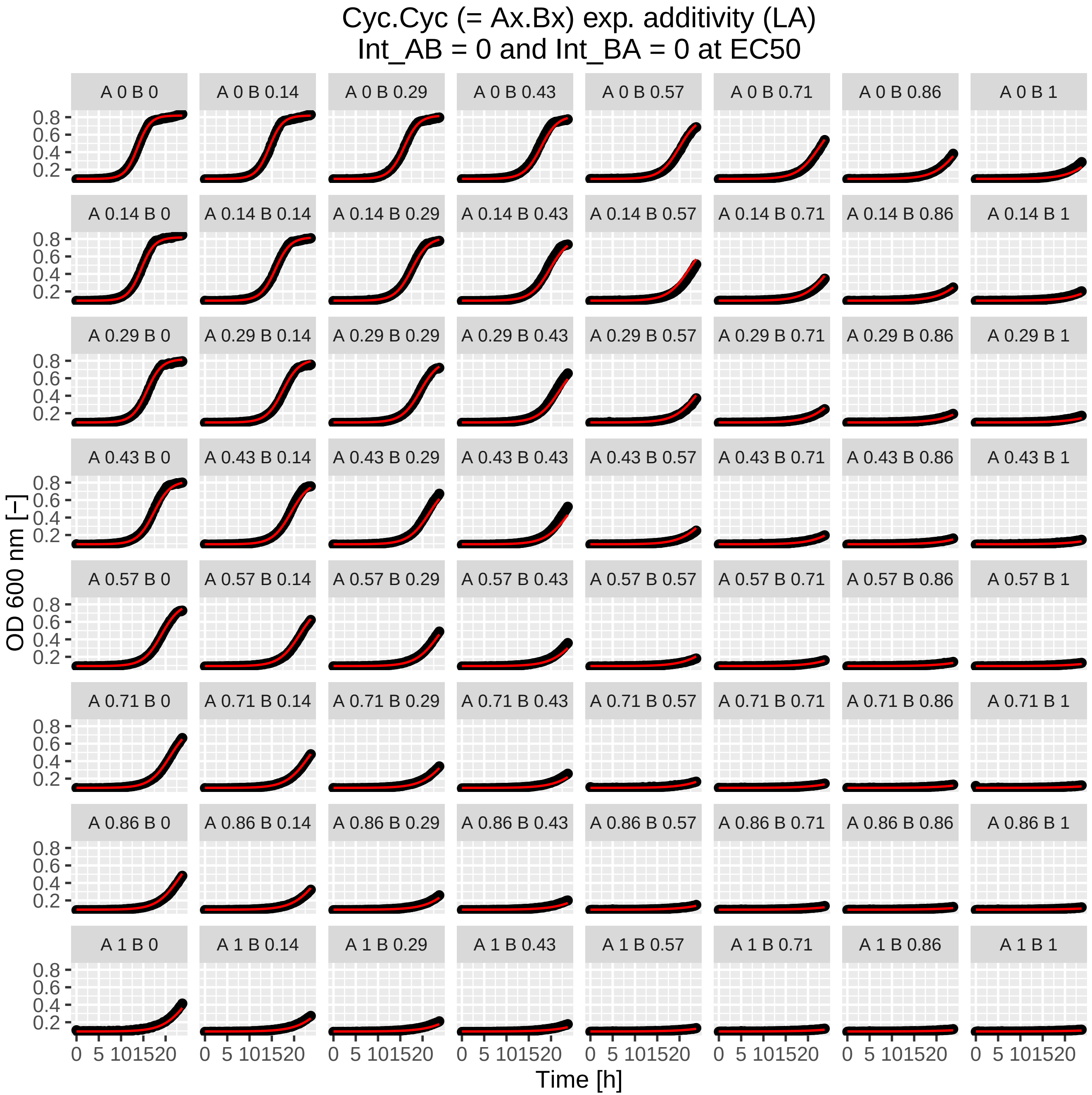
Cyc.Cyc (= Ax.Bx) exp. additivity (LA)
Int_AB = 0 and Int_BA = 0 at EC50
OD 600 nm [−]
Time [h]
0.8
0.6
0.4
0.2
0 5 101520
A 0 B 0
A 0 B 0.14
A 0 B 0.29
A 0 B 0.43
A 0 B 0.57
A 0 B 0.71
A 0 B 0.86
A 0 B 1
A 0.14 B 0
A 0.14 B 0.14
A 0.14 B 0.29
A 0.14 B 0.43
A 0.14 B 0.57
A 0.14 B 0.71
A 0.14 B 0.86
A 0.14 B 1
A 0.29 B 0
A 0.29 B 0.14
A 0.29 B 0.29
A 0.29 B 0.43
A 0.29 B 0.57
A 0.29 B 0.71
A 0.29 B 0.86
A 0.29 B 1
A 0.43 B 0
A 0.43 B 0.14
A 0.43 B 0.29
A 0.43 B 0.43
A 0.43 B 0.57
A 0.43 B 0.71
A 0.43 B 0.86
A 0.43 B 1
A 0.57 B 0
A 0.57 B 0.14
A 0.57 B 0.29
A 0.57 B 0.43
A 0.57 B 0.57
A 0.57 B 0.71
A 0.57 B 0.86
A 0.57 B 1
A 0.71 B 0
A 0.71 B 0.14
A 0.71 B 0.29
A 0.71 B 0.43
A 0.71 B 0.57
A 0.71 B 0.71
A 0.71 B 0.86
A 0.71 B 1
A 0.86 B 0
A 0.86 B 0.14
A 0.86 B 0.29
A 0.86 B 0.43
A 0.86 B 0.57
A 0.86 B 0.71
A 0.86 B 0.86
A 0.86 B 1
A 1 B 0
A 1 B 0.14
A 1 B 0.29
A 1 B 0.43
A 1 B 0.57
A 1 B 0.71
A 1 B 0.86
A 1 B 1

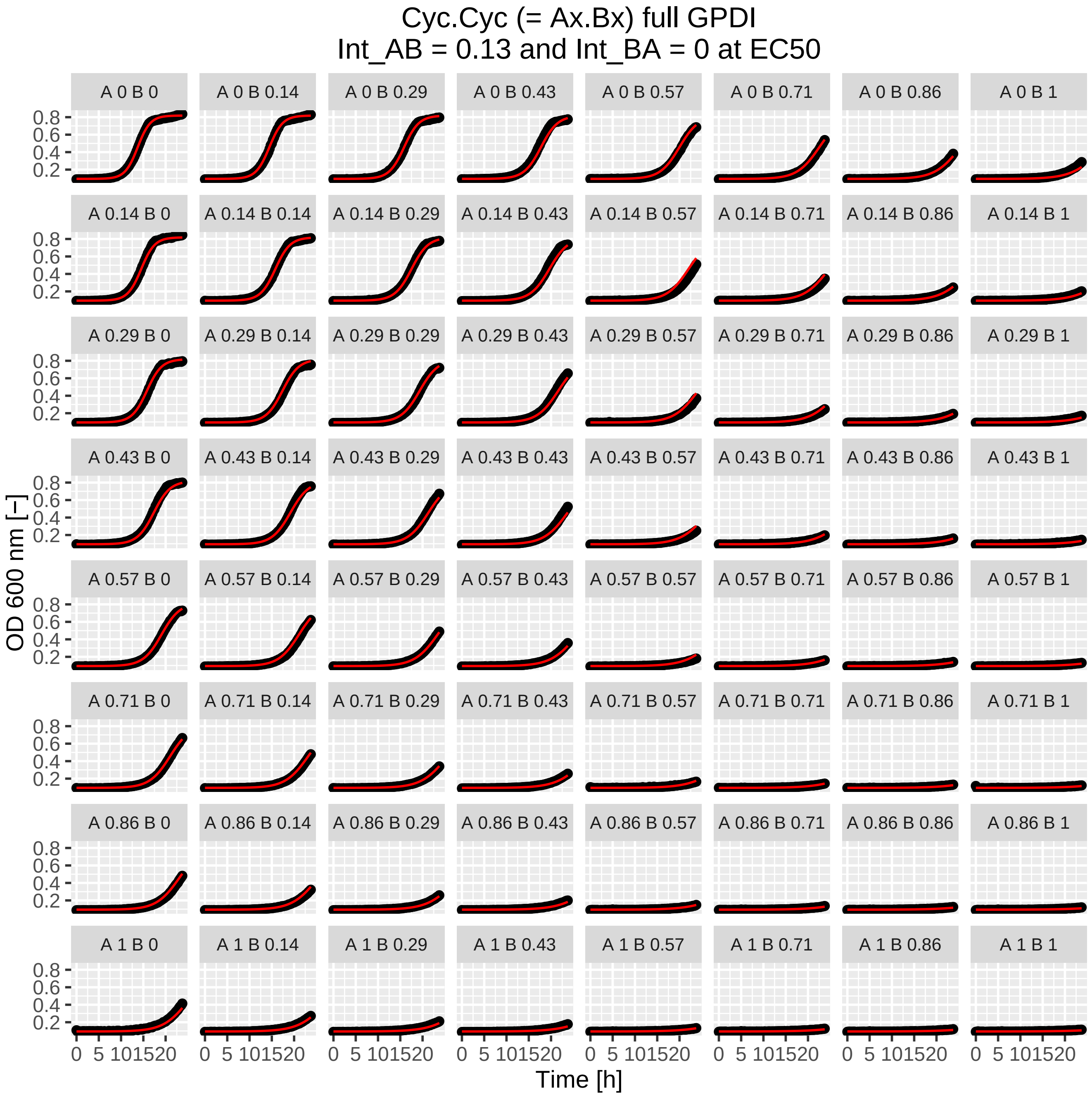
Cyc.Cyc (= Ax.Bx) full GPDI
Int_AB = 0.13 and Int_BA = 0 at EC50
A 0 B 0
A 0 B 0.14
A 0 B 0.29
A 0 B 0.43
A 0 B 0.57
A 0 B 0.71
A 0 B 0.86
A 0 B 1
A 0.14 B 0
A 0.14 B 0.14
A 0.14 B 0.29
A 0.14 B 0.43
A 0.14 B 0.57
A 0.14 B 0.71
A 0.14 B 0.86
A 0.14 B 1
A 0.29 B 0
A 0.29 B 0.14
A 0.29 B 0.29
A 0.29 B 0.43
A 0.29 B 0.57
A 0.29 B 0.71
A 0.29 B 0.86
A 0.29 B 1
A 0.43 B 0
A 0.43 B 0.14
A 0.43 B 0.29
A 0.43 B 0.43
A 0.43 B 0.57
A 0.43 B 0.71
A 0.43 B 0.86
A 0.43 B 1
A 0.57 B 0
A 0.57 B 0.14
A 0.57 B 0.29
A 0.57 B 0.43
A 0.57 B 0.57
A 0.57 B 0.71
A 0.57 B 0.86
A 0.57 B 1
A 0.71 B 0
A 0.71 B 0.14
A 0.71 B 0.29
A 0.71 B 0.43
A 0.71 B 0.57
A 0.71 B 0.71
A 0.71 B 0.86
A 0.71 B 1
A 0.86 B 0
A 0.86 B 0.14
A 0.86 B 0.29
A 0.86 B 0.43
A 0.86 B 0.57
A 0.86 B 0.71
A 0.86 B 0.86
A 0.86 B 1
A 1 B 0
A 1 B 0.14
A 1 B 0.29
A 1 B 0.43
A 1 B 0.57
A 1 B 0.71
A 1 B 0.86
A 1 B 1
0.8
0.6
0.4
0.2
0 5 101520
OD 600 nm [–]
Time [h]

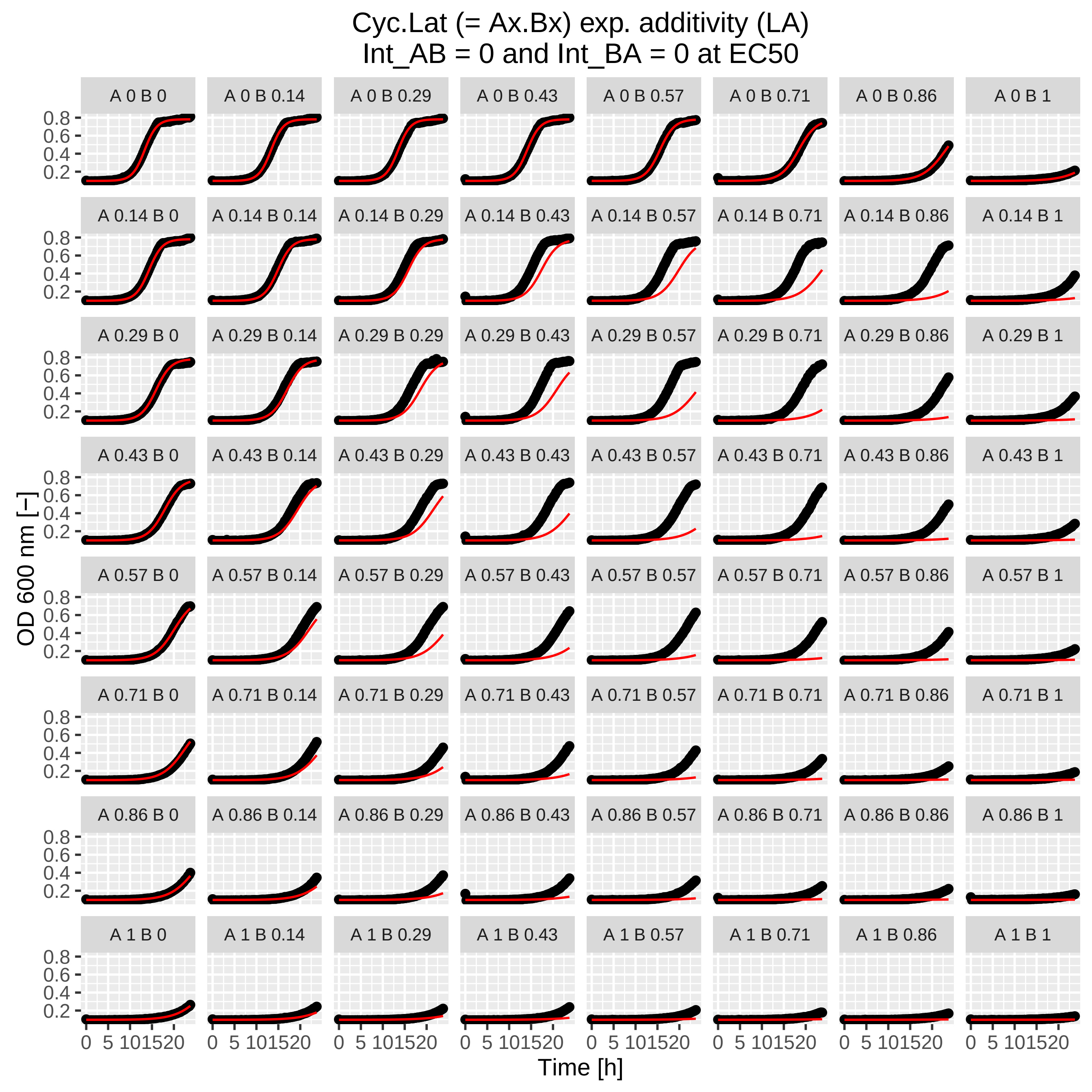

Cyc.Lat (= Ax.Bx) exp. additivity (LA)
Int_AB = 0 and Int_BA = 0 at EC50
OD 600 nm [–]
Time [h]
A 0 B 0
A 0 B 0.14
A 0 B 0.29
A 0 B 0.43
A 0 B 0.57
A 0 B 0.71
A 0 B 0.86
A 0 B 1
A 0.14 B 0
A 0.14 B 0.14
A 0.14 B 0.29
A 0.14 B 0.43
A 0.14 B 0.57
A 0.14 B 0.71
A 0.14 B 0.86
A 0.14 B 1
A 0.29 B 0
A 0.29 B 0.14
A 0.29 B 0.29
A 0.29 B 0.43
A 0.29 B 0.57
A 0.29 B 0.71
A 0.29 B 0.86
A 0.29 B 1
A 0.43 B 0
A 0.43 B 0.14
A 0.43 B 0.29
A 0.43 B 0.43
A 0.43 B 0.57
A 0.43 B 0.71
A 0.43 B 0.86
A 0.43 B 1
A 0.57 B 0
A 0.57 B 0.14
A 0.57 B 0.29
A 0.57 B 0.43
A 0.57 B 0.57
A 0.57 B 0.71
A 0.57 B 0.86
A 0.57 B 1
A 0.71 B 0
A 0.71 B 0.14
A 0.71 B 0.29
A 0.71 B 0.43
A 0.71 B 0.57
A 0.71 B 0.71
A 0.71 B 0.86
A 0.71 B 1
A 0.86 B 0
A 0.86 B 0.14
A 0.86 B 0.29
A 0.86 B 0.43
A 0.86 B 0.57
A 0.86 B 0.71
A 0.86 B 0.86
A 0.86 B 1
A 1 B 0
A 1 B 0.14
A 1 B 0.29
A 1 B 0.43
A 1 B 0.57
A 1 B 0.71
A 1 B 0.86
A 1 B 1
0.8
0.6
0.4
0.2
0 5 101520

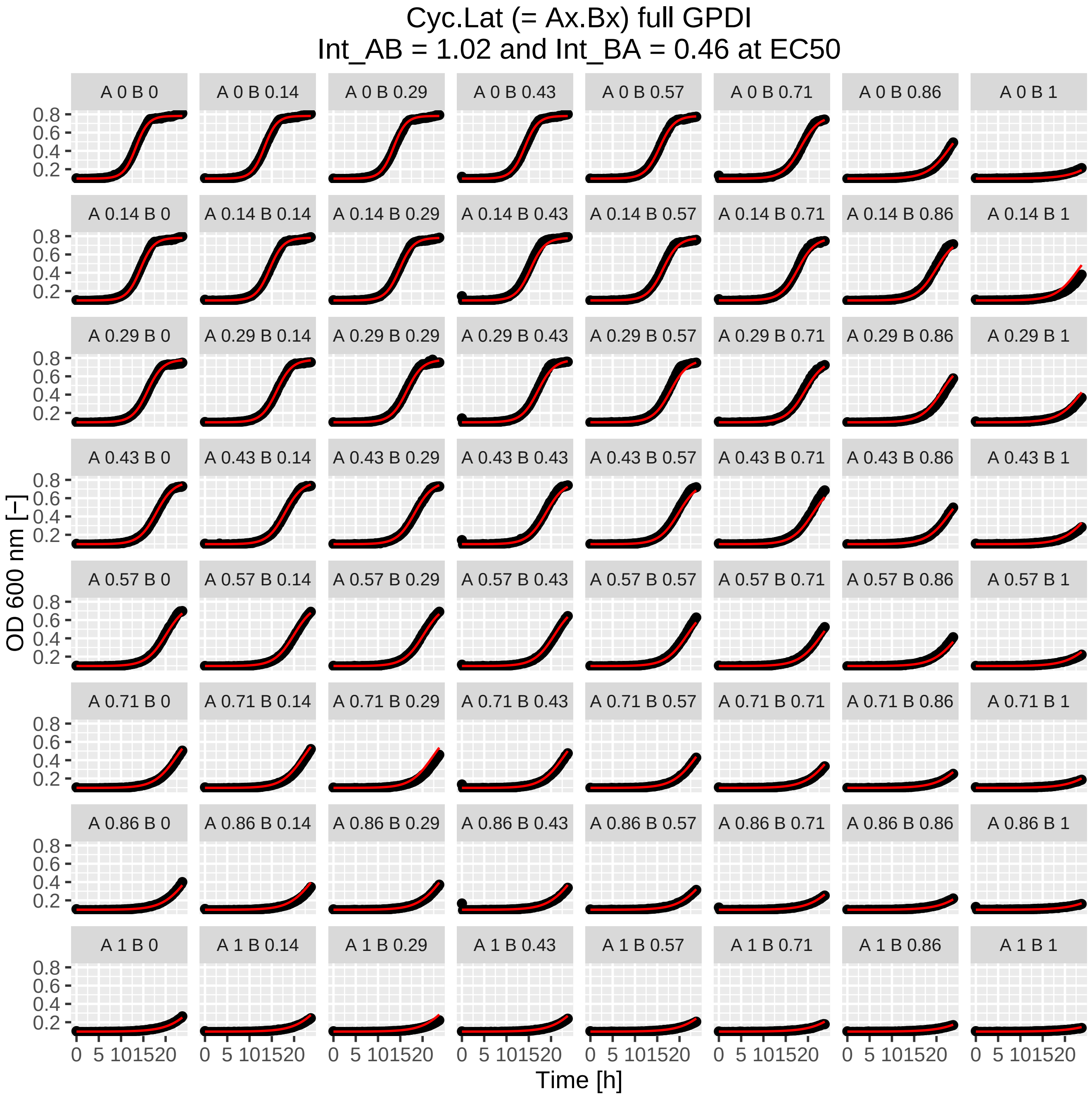

Cyc.Lat (= Ax.Bx) full GPDI
Int_AB = 1.02 and Int_BA = 0.46 at EC50
A 0 B 0
A 0 B 0.14
A 0 B 0.29
A 0 B 0.43
A 0 B 0.57
A 0 B 0.71
A 0 B 0.86
A 0 B 1
A 0.14 B 0
A 0.14 B 0.14
A 0.14 B 0.29
A 0.14 B 0.43
A 0.14 B 0.57
A 0.14 B 0.71
A 0.14 B 0.86
A 0.14 B 1
A 0.29 B 0
A 0.29 B 0.14
A 0.29 B 0.29
A 0.29 B 0.43
A 0.29 B 0.57
A 0.29 B 0.71
A 0.29 B 0.86
A 0.29 B 1
A 0.43 B 0
A 0.43 B 0.14
A 0.43 B 0.29
A 0.43 B 0.43
A 0.43 B 0.57
A 0.43 B 0.71
A 0.43 B 0.86
A 0.43 B 1
A 0.57 B 0
A 0.57 B 0.14
A 0.57 B 0.29
A 0.57 B 0.43
A 0.57 B 0.57
A 0.57 B 0.71
A 0.57 B 0.86
A 0.57 B 1
A 0.71 B 0
A 0.71 B 0.14
A 0.71 B 0.29
A 0.71 B 0.43
A 0.71 B 0.57
A 0.71 B 0.71
A 0.71 B 0.86
A 0.71 B 1
A 0.86 B 0
A 0.86 B 0.14
A 0.86 B 0.29
A 0.86 B 0.43
A 0.86 B 0.57
A 0.86 B 0.71
A 0.86 B 0.86
A 0.86 B 1
A 1 B 0
A 1 B 0.14
A 1 B 0.29
A 1 B 0.43
A 1 B 0.57
A 1 B 0.71
A 1 B 0.86
A 1 B 1
0.8
0.6
0.4
0.2
0 5 101520
OD 600 nm [−]
Time [h]

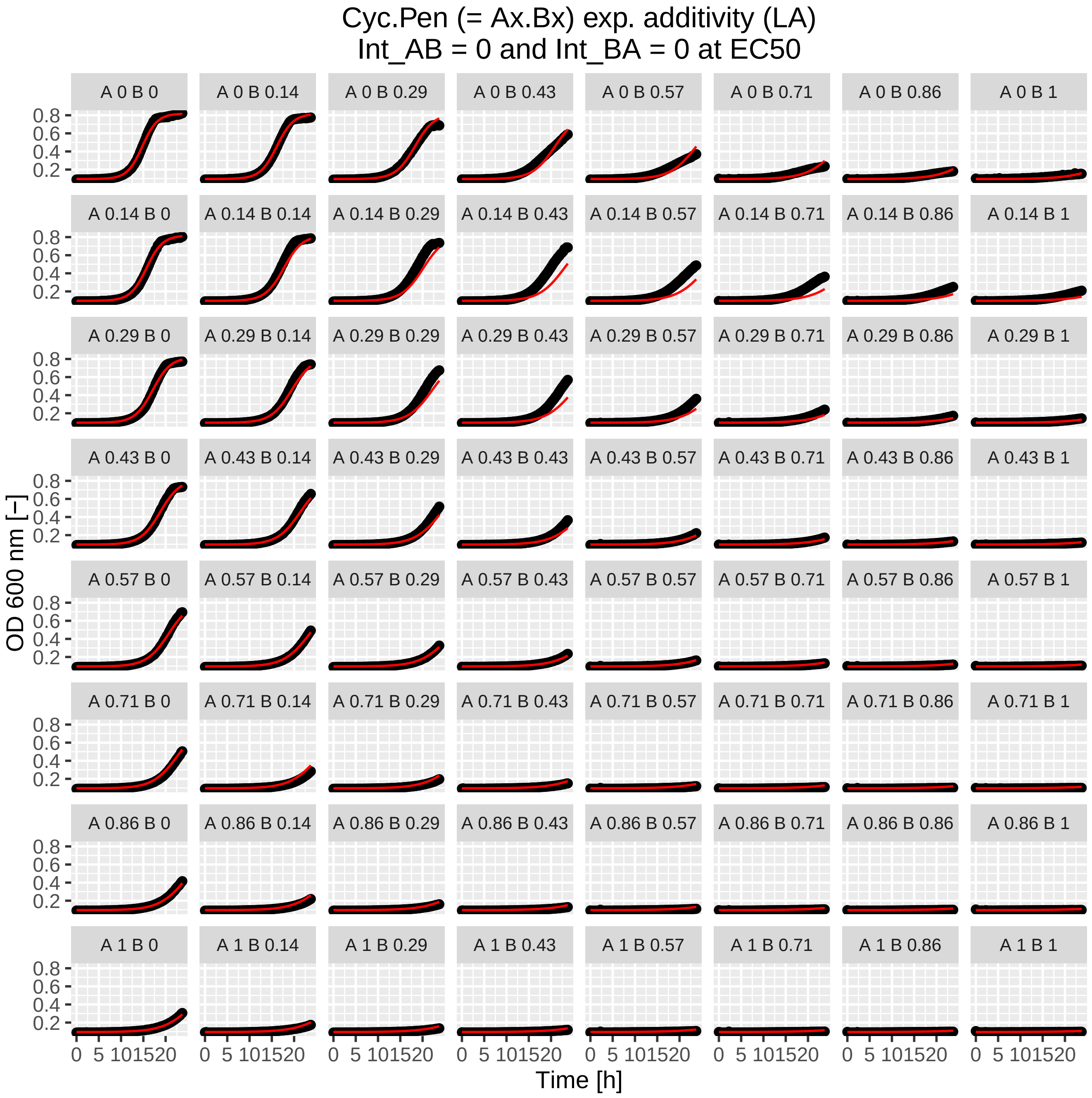

Cyc.Pen (= Ax.Bx) exp. additivity (LA)
Int_AB = 0 and Int_BA = 0 at EC50
OD 600 nm [−]
Time [h]
A 0 B 0
A 0 B 0.14
A 0 B 0.29
A 0 B 0.43
A 0 B 0.57
A 0 B 0.71
A 0 B 0.86
A 0 B 1
A 0.14 B 0
A 0.14 B 0.14
A 0.14 B 0.29
A 0.14 B 0.43
A 0.14 B 0.57
A 0.14 B 0.71
A 0.14 B 0.86
A 0.14 B 1
A 0.29 B 0
A 0.29 B 0.14
A 0.29 B 0.29
A 0.29 B 0.43
A 0.29 B 0.57
A 0.29 B 0.71
A 0.29 B 0.86
A 0.29 B 1
A 0.43 B 0
A 0.43 B 0.14
A 0.43 B 0.29
A 0.43 B 0.43
A 0.43 B 0.57
A 0.43 B 0.71
A 0.43 B 0.86
A 0.43 B 1
A 0.57 B 0
A 0.57 B 0.14
A 0.57 B 0.29
A 0.57 B 0.43
A 0.57 B 0.57
A 0.57 B 0.71
A 0.57 B 0.86
A 0.57 B 1
A 0.71 B 0
A 0.71 B 0.14
A 0.71 B 0.29
A 0.71 B 0.43
A 0.71 B 0.57
A 0.71 B 0.71
A 0.71 B 0.86
A 0.71 B 1
A 0.86 B 0
A 0.86 B 0.14
A 0.86 B 0.29
A 0.86 B 0.43
A 0.86 B 0.57
A 0.86 B 0.71
A 0.86 B 0.86
A 0.86 B 1
A 1 B 0
A 1 B 0.14
A 1 B 0.29
A 1 B 0.43
A 1 B 0.57
A 1 B 0.71
A 1 B 0.86
A 1 B 1
0.8
0.6
0.4
0.2
0
5
10
15
20

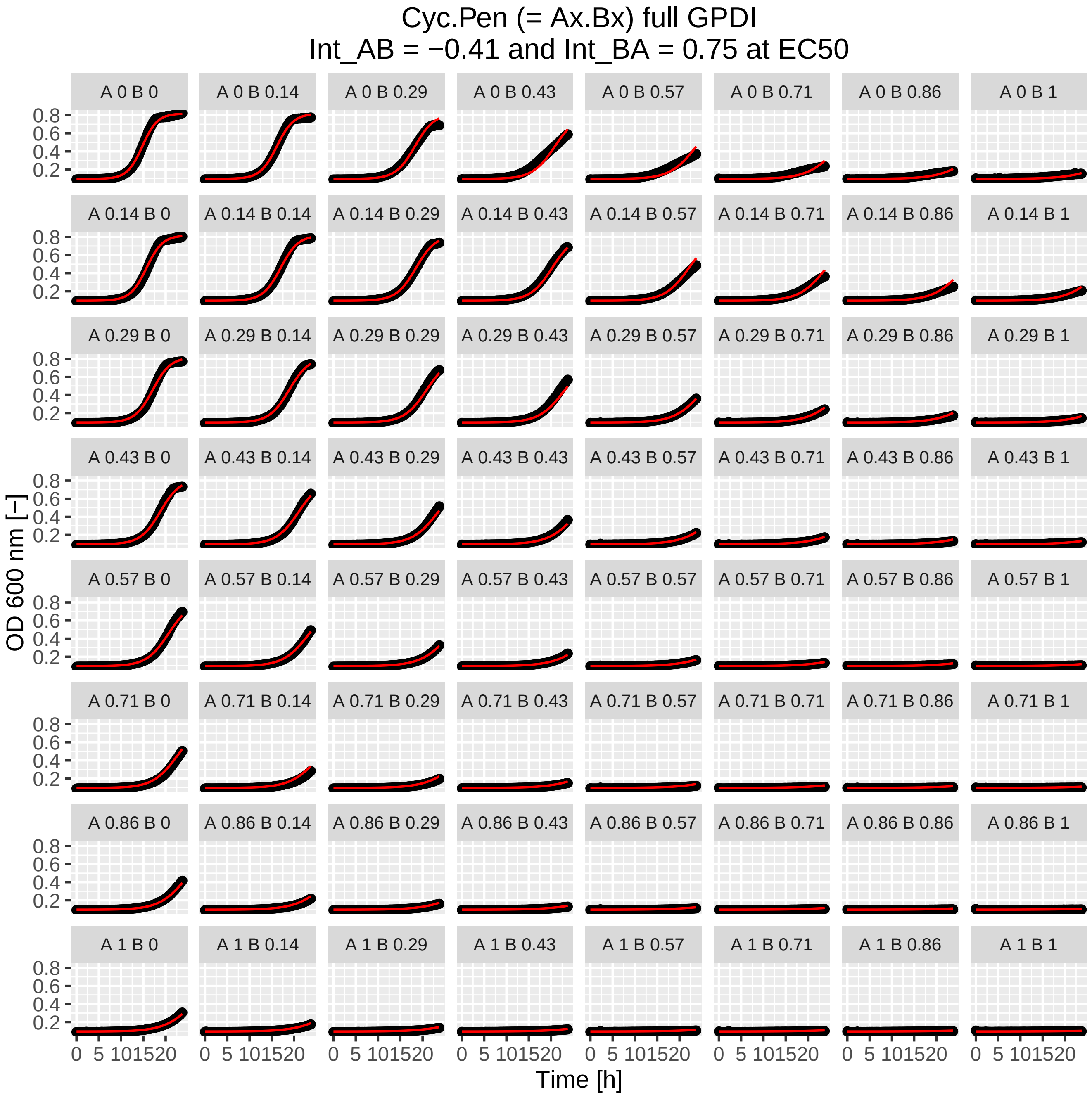

Cyc.Pen (= Ax.Bx) full GPDI
Int_AB = −0.41 and Int_BA = 0.75 at EC50
A 0 B 0
A 0 B 0.14
A 0 B 0.29
A 0 B 0.43
A 0 B 0.57
A 0 B 0.71
A 0 B 0.86
A 0 B 1
A 0.14 B 0
A 0.14 B 0.14
A 0.14 B 0.29
A 0.14 B 0.43
A 0.14 B 0.57
A 0.14 B 0.71
A 0.14 B 0.86
A 0.14 B 1
A 0.29 B 0
A 0.29 B 0.14
A 0.29 B 0.29
A 0.29 B 0.43
A 0.29 B 0.57
A 0.29 B 0.71
A 0.29 B 0.86
A 0.29 B 1
A 0.43 B 0
A 0.43 B 0.14
A 0.43 B 0.29
A 0.43 B 0.43
A 0.43 B 0.57
A 0.43 B 0.71
A 0.43 B 0.86
A 0.43 B 1
A 0.57 B 0
A 0.57 B 0.14
A 0.57 B 0.29
A 0.57 B 0.43
A 0.57 B 0.57
A 0.57 B 0.71
A 0.57 B 0.86
A 0.57 B 1
A 0.71 B 0
A 0.71 B 0.14
A 0.71 B 0.29
A 0.71 B 0.43
A 0.71 B 0.57
A 0.71 B 0.71
A 0.71 B 0.86
A 0.71 B 1
A 0.86 B 0
A 0.86 B 0.14
A 0.86 B 0.29
A 0.86 B 0.43
A 0.86 B 0.57
A 0.86 B 0.71
A 0.86 B 0.86
A 0.86 B 1
A 1 B 0
A 1 B 0.14
A 1 B 0.29
A 1 B 0.43
A 1 B 0.57
A 1 B 0.71
A 1 B 0.86
A 1 B 1
0.8
0.6
0.4
0.2
0 5 101520
OD 600 nm [−]
Time [h]

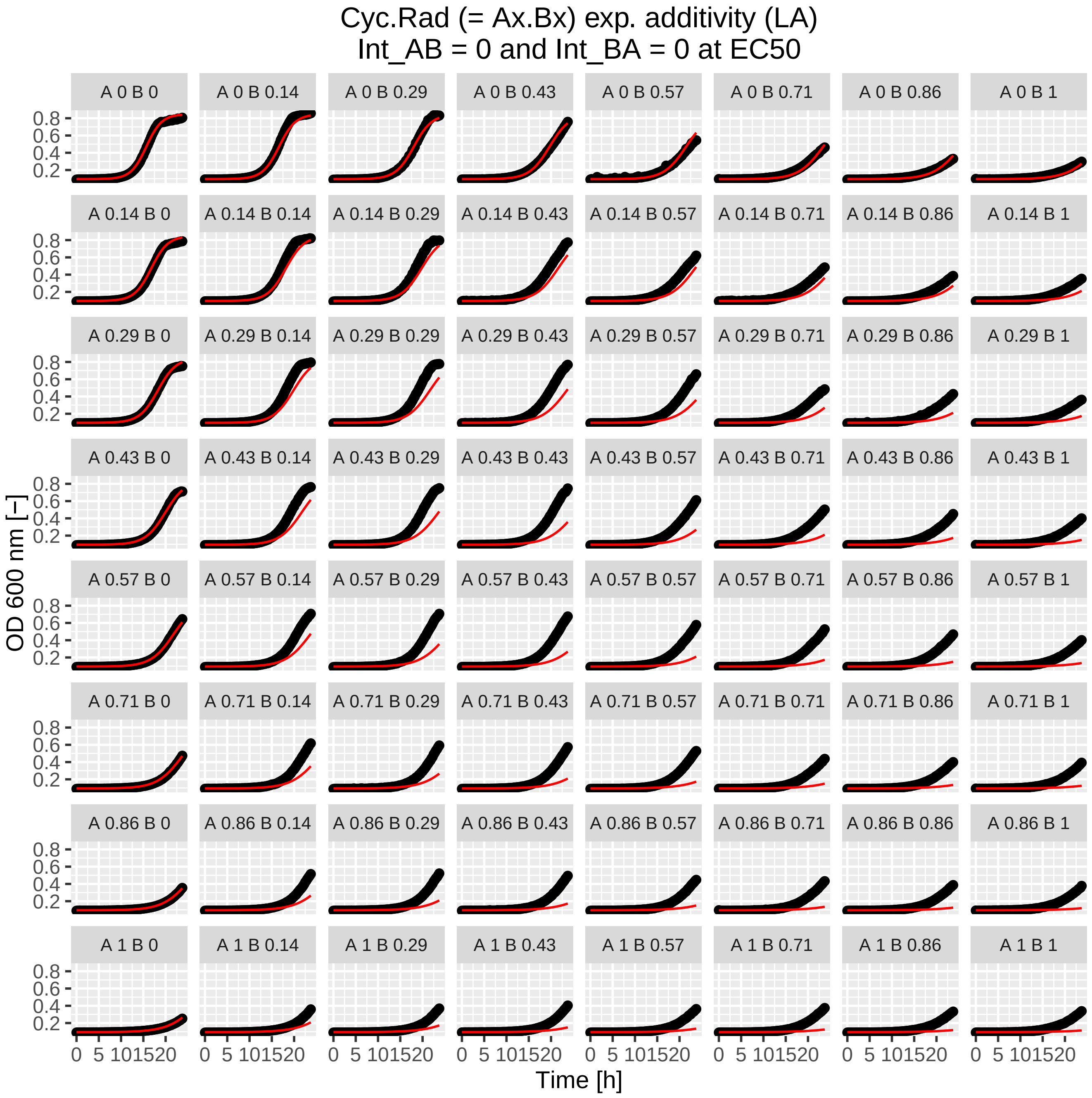

Cyc.Rad (= Ax.Bx) exp. additivity (LA)
Int_AB = 0 and Int_BA = 0 at EC50
OD 600 nm [–]
Time [h]
0.8
0.6
0.4
0.2
0 5 101520
A 0 B 0
A 0 B 0.14
A 0 B 0.29
A 0 B 0.43
A 0 B 0.57
A 0 B 0.71
A 0 B 0.86
A 0 B 1
A 0.14 B 0
A 0.14 B 0.14
A 0.14 B 0.29
A 0.14 B 0.43
A 0.14 B 0.57
A 0.14 B 0.71
A 0.14 B 0.86
A 0.14 B 1
A 0.29 B 0
A 0.29 B 0.14
A 0.29 B 0.29
A 0.29 B 0.43
A 0.29 B 0.57
A 0.29 B 0.71
A 0.29 B 0.86
A 0.29 B 1
A 0.43 B 0
A 0.43 B 0.14
A 0.43 B 0.29
A 0.43 B 0.43
A 0.43 B 0.57
A 0.43 B 0.71
A 0.43 B 0.86
A 0.43 B 1
A 0.57 B 0
A 0.57 B 0.14
A 0.57 B 0.29
A 0.57 B 0.43
A 0.57 B 0.57
A 0.57 B 0.71
A 0.57 B 0.86
A 0.57 B 1
A 0.71 B 0
A 0.71 B 0.14
A 0.71 B 0.29
A 0.71 B 0.43
A 0.71 B 0.57
A 0.71 B 0.71
A 0.71 B 0.86
A 0.71 B 1
A 0.86 B 0
A 0.86 B 0.14
A 0.86 B 0.29
A 0.86 B 0.43
A 0.86 B 0.57
A 0.86 B 0.71
A 0.86 B 0.86
A 0.86 B 1
A 1 B 0
A 1 B 0.14
A 1 B 0.29
A 1 B 0.43
A 1 B 0.57
A 1 B 0.71
A 1 B 0.86
A 1 B 1

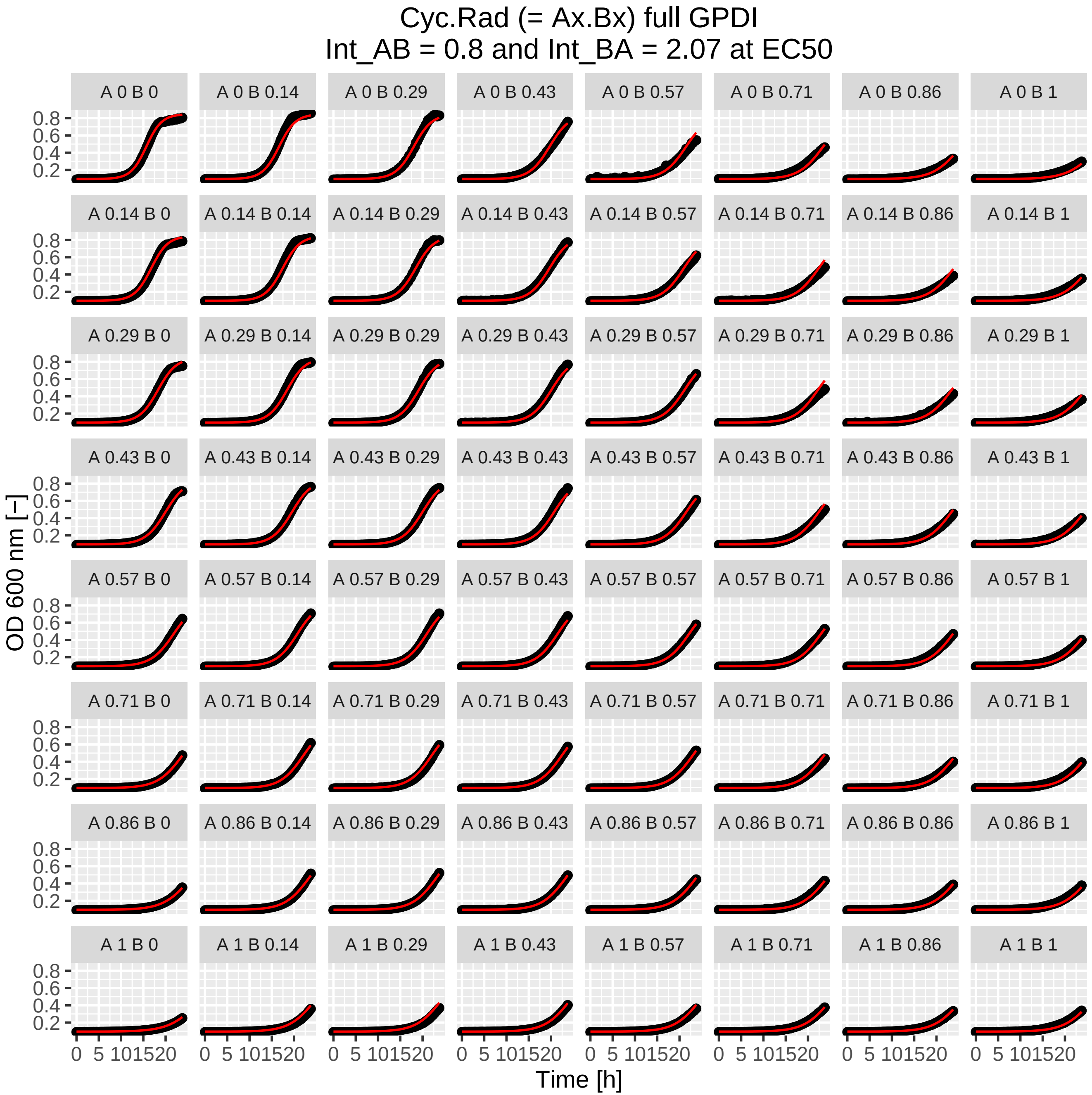
Cyc.Rad (= Ax.Bx) full GPDI
Int_AB = 0.8 and Int_BA = 2.07 at EC50
OD 600 nm [–]
Time [h]
A 0 B 0
A 0 B 0.14
A 0 B 0.29
A 0 B 0.43
A 0 B 0.57
A 0 B 0.71
A 0 B 0.86
A 0 B 1
A 1 B 0
A 1 B 0.14
A 1 B 0.29
A 1 B 0.43
A 1 B 0.57
A 1 B 0.71
A 1 B 0.86
A 1 B 1

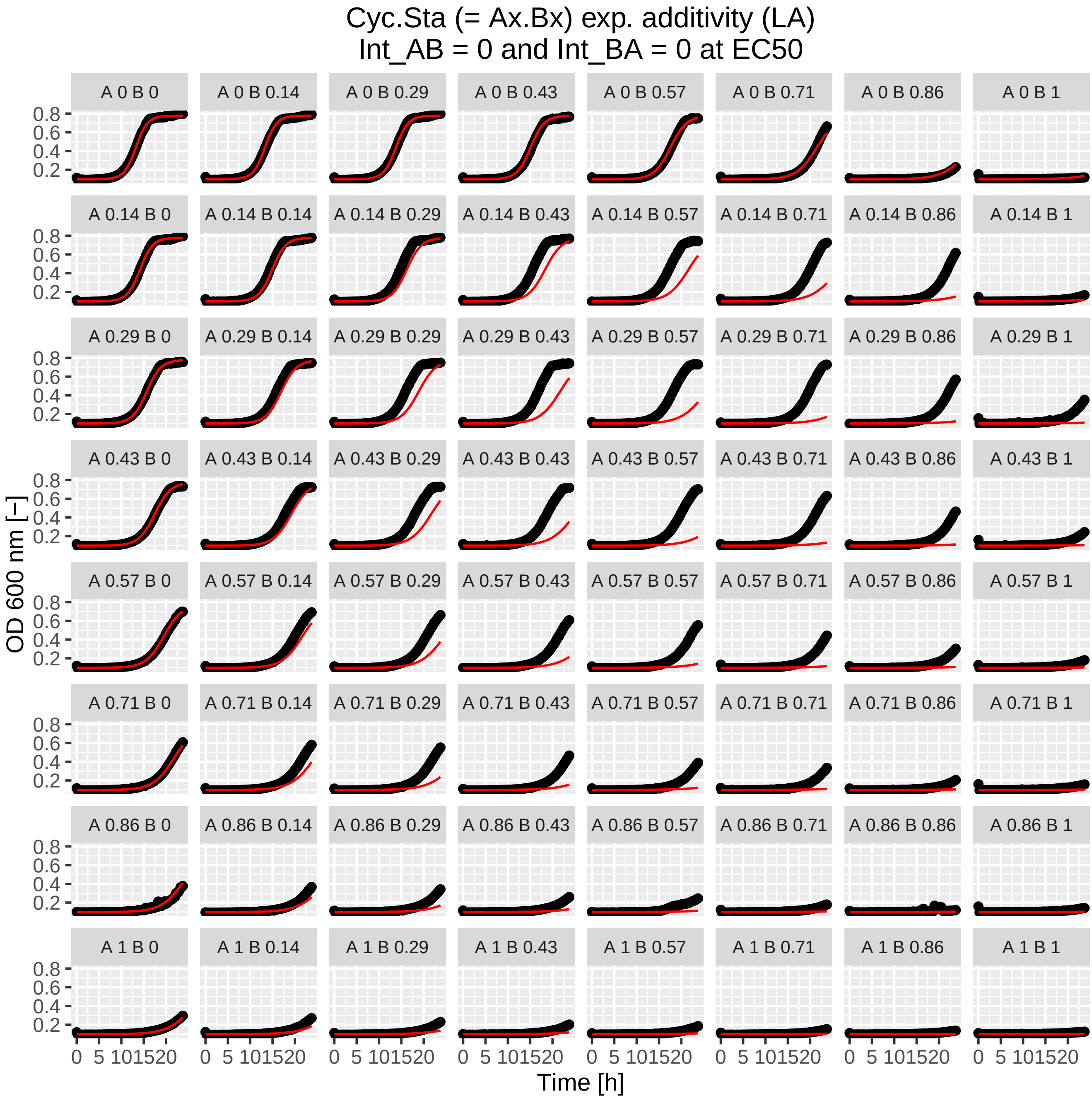
Cyc.Sta (= Ax.Bx) exp. additivity (LA)
Int_AB = 0 and Int_BA = 0 at EC50
OD 600 nm [−]
Time [h]
0 5 101520
0.2
0.4
0.6
0.8
A 0 B 0
A 0 B 0.14
A 0 B 0.29
A 0 B 0.43
A 0 B 0.57
A 0 B 0.71
A 0 B 0.86
A 0 B 1
A 0.14 B 0
A 0.14 B 0.14
A 0.14 B 0.29
A 0.14 B 0.43
A 0.14 B 0.57
A 0.14 B 0.71
A 0.14 B 0.86
A 0.14 B 1
A 0.29 B 0
A 0.29 B 0.14
A 0.29 B 0.29
A 0.29 B 0.43
A 0.29 B 0.57
A 0.29 B 0.71
A 0.29 B 0.86
A 0.29 B 1
A 0.43 B 0
A 0.43 B 0.14
A 0.43 B 0.29
A 0.43 B 0.43
A 0.43 B 0.57
A 0.43 B 0.71
A 0.43 B 0.86
A 0.43 B 1
A 0.57 B 0
A 0.57 B 0.14
A 0.57 B 0.29
A 0.57 B 0.43
A 0.57 B 0.57
A 0.57 B 0.71
A 0.57 B 0.86
A 0.57 B 1
A 0.71 B 0
A 0.71 B 0.14
A 0.71 B 0.29
A 0.71 B 0.43
A 0.71 B 0.57
A 0.71 B 0.71
A 0.71 B 0.86
A 0.71 B 1
A 0.86 B 0
A 0.86 B 0.14
A 0.86 B 0.29
A 0.86 B 0.43
A 0.86 B 0.57
A 0.86 B 0.71
A 0.86 B 0.86
A 0.86 B 1
A 1 B 0
A 1 B 0.14
A 1 B 0.29
A 1 B 0.43
A 1 B 0.57
A 1 B 0.71
A 1 B 0.86
A 1 B 1

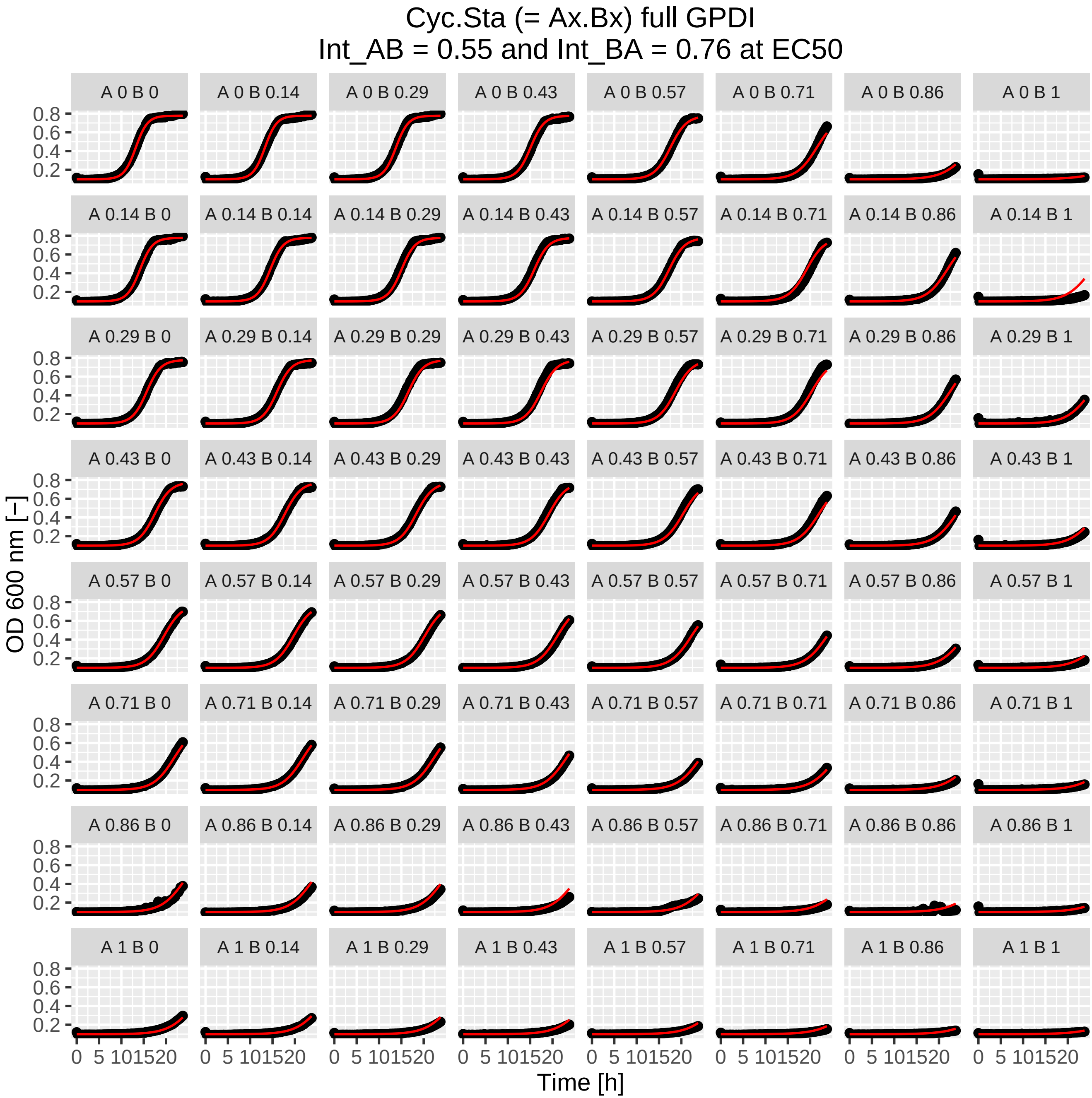

Cyc.Sta (= Ax.Bx) full GPDI
Int_AB = 0.55 and Int_BA = 0.76 at EC50
A 0 B 0
A 0 B 0.14
A 0 B 0.29
A 0 B 0.43
A 0 B 0.57
A 0 B 0.71
A 0 B 0.86
A 0 B 1
A 0.14 B 0
A 0.14 B 0.14
A 0.14 B 0.29
A 0.14 B 0.43
A 0.14 B 0.57
A 0.14 B 0.71
A 0.14 B 0.86
A 0.14 B 1
A 0.29 B 0
A 0.29 B 0.14
A 0.29 B 0.29
A 0.29 B 0.43
A 0.29 B 0.57
A 0.29 B 0.71
A 0.29 B 0.86
A 0.29 B 1
A 0.43 B 0
A 0.43 B 0.14
A 0.43 B 0.29
A 0.43 B 0.43
A 0.43 B 0.57
A 0.43 B 0.71
A 0.43 B 0.86
A 0.43 B 1
A 0.57 B 0
A 0.57 B 0.14
A 0.57 B 0.29
A 0.57 B 0.43
A 0.57 B 0.57
A 0.57 B 0.71
A 0.57 B 0.86
A 0.57 B 1
A 0.71 B 0
A 0.71 B 0.14
A 0.71 B 0.29
A 0.71 B 0.43
A 0.71 B 0.57
A 0.71 B 0.71
A 0.71 B 0.86
A 0.71 B 1
A 0.86 B 0
A 0.86 B 0.14
A 0.86 B 0.29
A 0.86 B 0.43
A 0.86 B 0.57
A 0.86 B 0.71
A 0.86 B 0.86
A 0.86 B 1
A 1 B 0
A 1 B 0.14
A 1 B 0.29
A 1 B 0.43
A 1 B 0.57
A 1 B 0.71
A 1 B 0.86
A 1 B 1
0.8
0.6
0.4
0.2
OD 600 nm [–]
0 5 101520
Time [h]

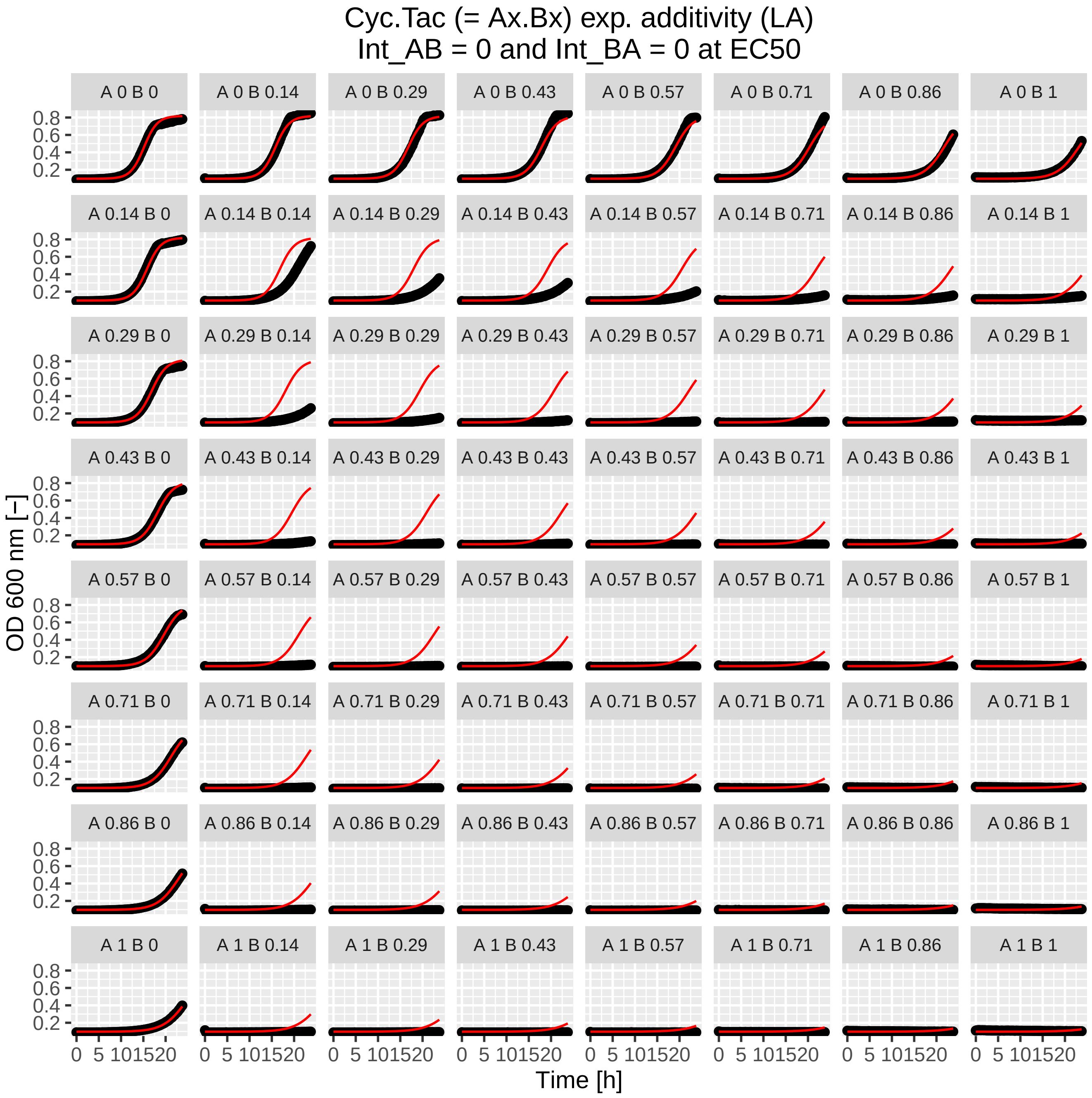

Cyc.Tac (= Ax.Bx) exp. additivity (LA)
Int_AB = 0 and Int_BA = 0 at EC50
A 0 B 0
A 0 B 0.14
A 0 B 0.29
A 0 B 0.43
A 0 B 0.57
A 0 B 0.71
A 0 B 0.86
A 0 B 1
A 0.14 B 0
A 0.14 B 0.14
A 0.14 B 0.29
A 0.14 B 0.43
A 0.14 B 0.57
A 0.14 B 0.71
A 0.14 B 0.86
A 0.14 B 1
A 0.29 B 0
A 0.29 B 0.14
A 0.29 B 0.29
A 0.29 B 0.43
A 0.29 B 0.57
A 0.29 B 0.71
A 0.29 B 0.86
A 0.29 B 1
A 0.43 B 0
A 0.43 B 0.14
A 0.43 B 0.29
A 0.43 B 0.43
A 0.43 B 0.57
A 0.43 B 0.71
A 0.43 B 0.86
A 0.43 B 1
A 0.57 B 0
A 0.57 B 0.14
A 0.57 B 0.29
A 0.57 B 0.43
A 0.57 B 0.57
A 0.57 B 0.71
A 0.57 B 0.86
A 0.57 B 1
A 0.71 B 0
A 0.71 B 0.14
A 0.71 B 0.29
A 0.71 B 0.43
A 0.71 B 0.57
A 0.71 B 0.71
A 0.71 B 0.86
A 0.71 B 1
A 0.86 B 0
A 0.86 B 0.14
A 0.86 B 0.29
A 0.86 B 0.43
A 0.86 B 0.57
A 0.86 B 0.71
A 0.86 B 0.86
A 0.86 B 1
A 1 B 0
A 1 B 0.14
A 1 B 0.29
A 1 B 0.43
A 1 B 0.57
A 1 B 0.71
A 1 B 0.86
A 1 B 1
0.8
0.6
0.4
0.2
0 5 101520
OD 600 nm [–]
Time [h]

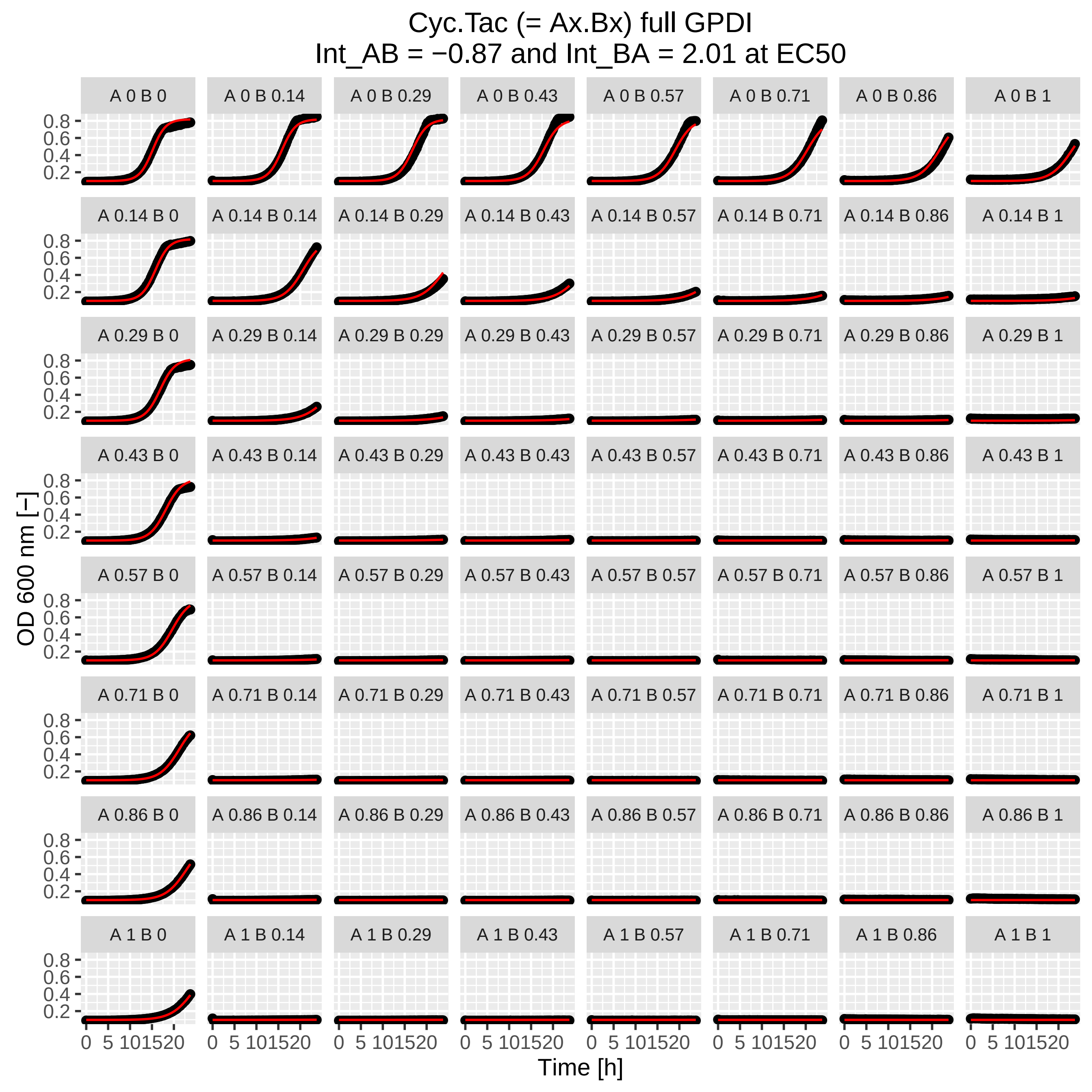

Cyc.Tac (= Ax.Bx) full GPDI
Int_AB = −0.87 and Int_BA = 2.01 at EC50
A 0 B 0
A 0 B 0.14
A 0 B 0.29
A 0 B 0.43
A 0 B 0.57
A 0 B 0.71
A 0 B 0.86
A 0 B 1
A 0.14 B 0
A 0.14 B 0.14
A 0.14 B 0.29
A 0.14 B 0.43
A 0.14 B 0.57
A 0.14 B 0.71
A 0.14 B 0.86
A 0.14 B 1
A 0.29 B 0
A 0.29 B 0.14
A 0.29 B 0.29
A 0.29 B 0.43
A 0.29 B 0.57
A 0.29 B 0.71
A 0.29 B 0.86
A 0.29 B 1
A 0.43 B 0
A 0.43 B 0.14
A 0.43 B 0.29
A 0.43 B 0.43
A 0.43 B 0.57
A 0.43 B 0.71
A 0.43 B 0.86
A 0.43 B 1
A 0.57 B 0
A 0.57 B 0.14
A 0.57 B 0.29
A 0.57 B 0.43
A 0.57 B 0.57
A 0.57 B 0.71
A 0.57 B 0.86
A 0.57 B 1
A 0.71 B 0
A 0.71 B 0.14
A 0.71 B 0.29
A 0.71 B 0.43
A 0.71 B 0.57
A 0.71 B 0.71
A 0.71 B 0.86
A 0.71 B 1
A 0.86 B 0
A 0.86 B 0.14
A 0.86 B 0.29
A 0.86 B 0.43
A 0.86 B 0.57
A 0.86 B 0.71
A 0.86 B 0.86
A 0.86 B 1
A 1 B 0
A 1 B 0.14
A 1 B 0.29
A 1 B 0.43
A 1 B 0.57
A 1 B 0.71
A 1 B 0.86
A 1 B 1
OD 600 nm [−]
0.8
0.6
0.4
0.2
0 5 101520
Time [h]

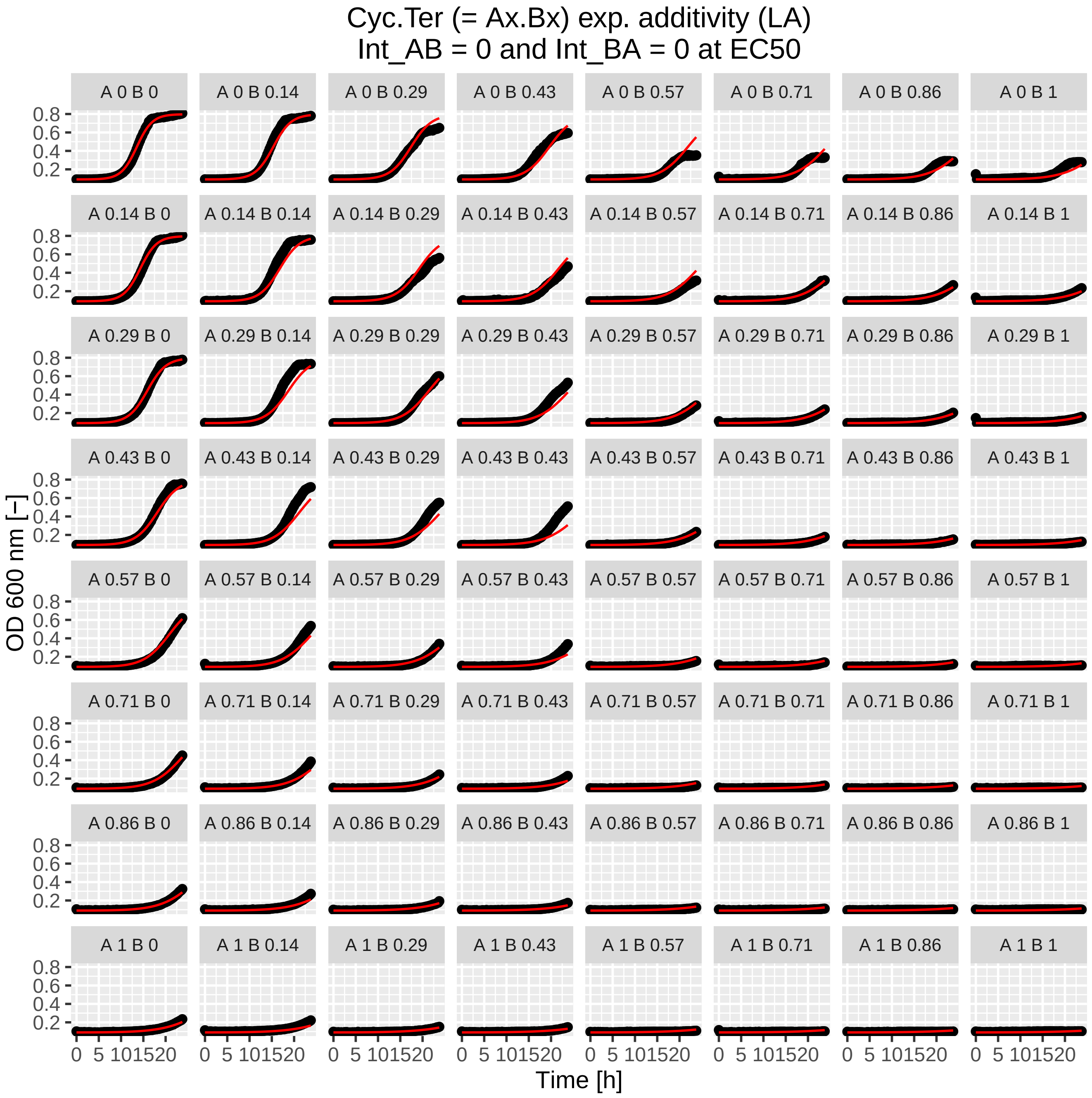

Cyc.Ter (= Ax.Bx) exp. additivity (LA)
Int_AB = 0 and Int_BA = 0 at EC50
OD 600 nm [–]
Time [h]
0.2
0.4
0.6
0.8
0
5
10
15
20
A 0 B 0
A 0 B 0.14
A 0 B 0.29
A 0 B 0.43
A 0 B 0.57
A 0 B 0.71
A 0 B 0.86
A 0 B 1
A 0.14 B 0
A 0.14 B 0.14
A 0.14 B 0.29
A 0.14 B 0.43
A 0.14 B 0.57
A 0.14 B 0.71
A 0.14 B 0.86
A 0.14 B 1
A 0.29 B 0
A 0.29 B 0.14
A 0.29 B 0.29
A 0.29 B 0.43
A 0.29 B 0.57
A 0.29 B 0.71
A 0.29 B 0.86
A 0.29 B 1
A 0.43 B 0
A 0.43 B 0.14
A 0.43 B 0.29
A 0.43 B 0.43
A 0.43 B 0.57
A 0.43 B 0.71
A 0.43 B 0.86
A 0.43 B 1
A 0.57 B 0
A 0.57 B 0.14
A 0.57 B 0.29
A 0.57 B 0.43
A 0.57 B 0.57
A 0.57 B 0.71
A 0.57 B 0.86
A 0.57 B 1
A 0.71 B 0
A 0.71 B 0.14
A 0.71 B 0.29
A 0.71 B 0.43
A 0.71 B 0.57
A 0.71 B 0.71
A 0.71 B 0.86
A 0.71 B 1
A 0.86 B 0
A 0.86 B 0.14
A 0.86 B 0.29
A 0.86 B 0.43
A 0.86 B 0.57
A 0.86 B 0.71
A 0.86 B 0.86
A 0.86 B 1
A 1 B 0
A 1 B 0.14
A 1 B 0.29
A 1 B 0.43
A 1 B 0.57
A 1 B 0.71
A 1 B 0.86
A 1 B 1

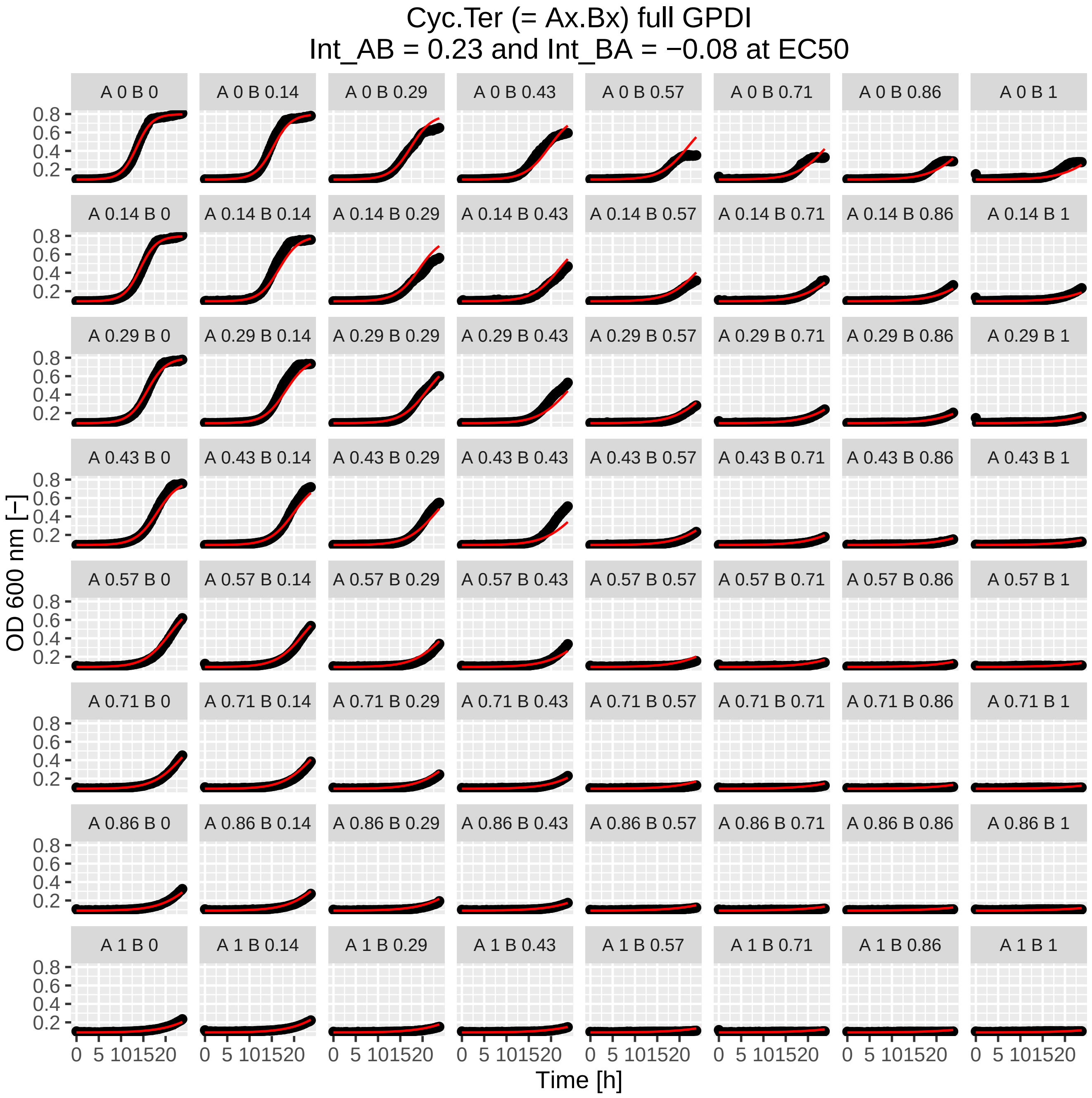

Cyc.Ter (= Ax.Bx) full GPDI
Int_AB = 0.23 and Int_BA = −0.08 at EC50
OD 600 nm [−]
Time [h]
A 0 B 0
A 0 B 0.14
A 0 B 0.29
A 0 B 0.43
A 0 B 0.57
A 0 B 0.71
A 0 B 0.86
A 0 B 1
A 0.14 B 0
A 0.14 B 0.14
A 0.14 B 0.29
A 0.14 B 0.43
A 0.14 B 0.57
A 0.14 B 0.71
A 0.14 B 0.86
A 0.14 B 1
A 0.29 B 0
A 0.29 B 0.14
A 0.29 B 0.29
A 0.29 B 0.43
A 0.29 B 0.57
A 0.29 B 0.71
A 0.29 B 0.86
A 0.29 B 1
A 0.43 B 0
A 0.43 B 0.14
A 0.43 B 0.29
A 0.43 B 0.43
A 0.43 B 0.57
A 0.43 B 0.71
A 0.43 B 0.86
A 0.43 B 1
A 0.57 B 0
A 0.57 B 0.14
A 0.57 B 0.29
A 0.57 B 0.43
A 0.57 B 0.57
A 0.57 B 0.71
A 0.57 B 0.86
A 0.57 B 1
A 0.71 B 0
A 0.71 B 0.14
A 0.71 B 0.29
A 0.71 B 0.43
A 0.71 B 0.57
A 0.71 B 0.71
A 0.71 B 0.86
A 0.71 B 1
A 0.86 B 0
A 0.86 B 0.14
A 0.86 B 0.29
A 0.86 B 0.43
A 0.86 B 0.57
A 0.86 B 0.71
A 0.86 B 0.86
A 0.86 B 1
A 1 B 0
A 1 B 0.14
A 1 B 0.29
A 1 B 0.43
A 1 B 0.57
A 1 B 0.71
A 1 B 0.86
A 1 B 1
0.2
0.4
0.6
0.8
0 5 101520

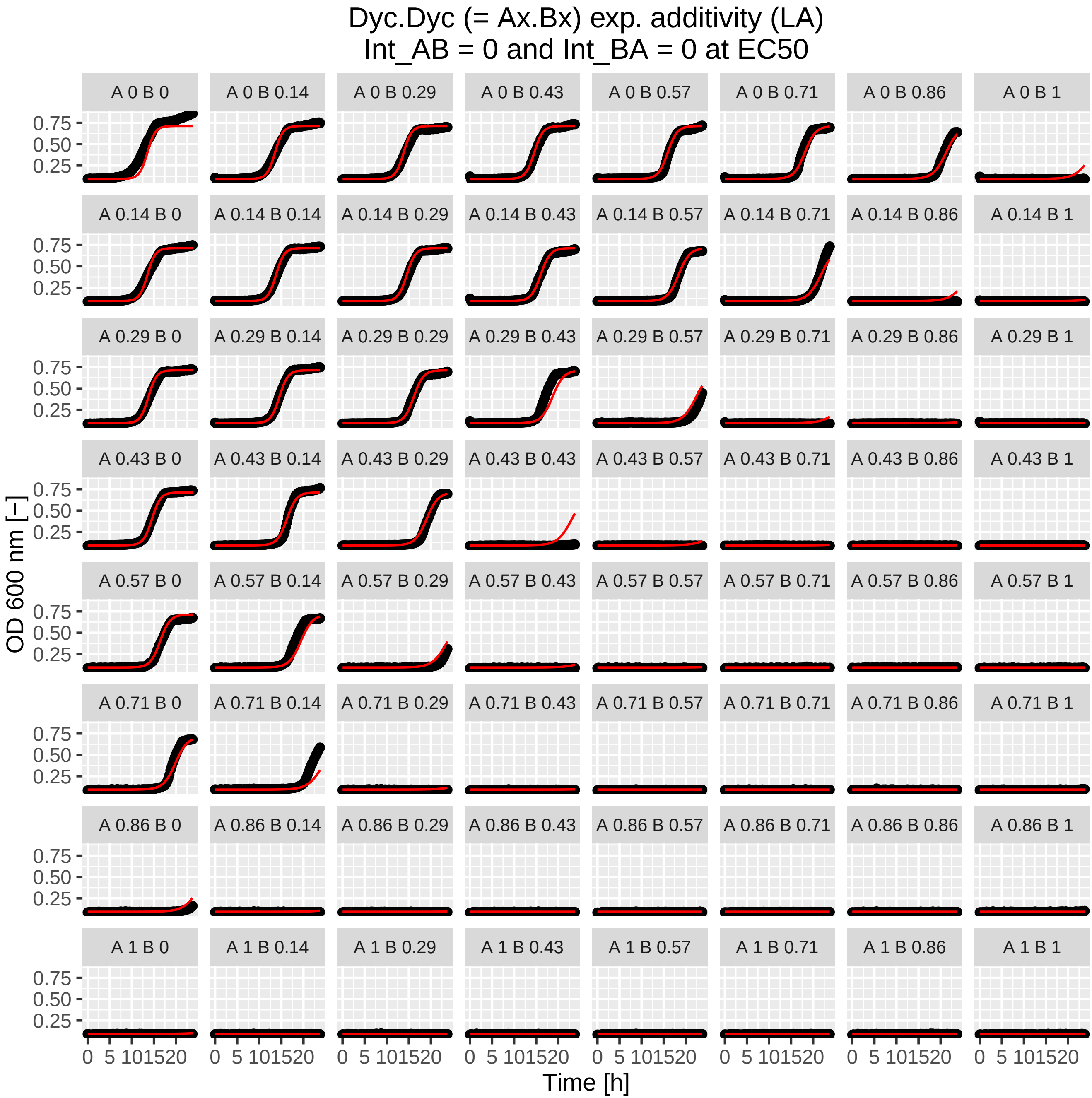

Dyc.Dyc (= Ax.Bx) exp. additivity (LA)
Int_AB = 0 and Int_BA = 0 at EC50
A 0 B 0
A 0 B 0.14
A 0 B 0.29
A 0 B 0.43
A 0 B 0.57
A 0 B 0.71
A 0 B 0.86
A 0 B 1
A 0.14 B 0
A 0.14 B 0.14
A 0.14 B 0.29
A 0.14 B 0.43
A 0.14 B 0.57
A 0.14 B 0.71
A 0.14 B 0.86
A 0.14 B 1
A 0.29 B 0
A 0.29 B 0.14
A 0.29 B 0.29
A 0.29 B 0.43
A 0.29 B 0.57
A 0.29 B 0.71
A 0.29 B 0.86
A 0.29 B 1
A 0.43 B 0
A 0.43 B 0.14
A 0.43 B 0.29
A 0.43 B 0.43
A 0.43 B 0.57
A 0.43 B 0.71
A 0.43 B 0.86
A 0.43 B 1
A 0.57 B 0
A 0.57 B 0.14
A 0.57 B 0.29
A 0.57 B 0.43
A 0.57 B 0.57
A 0.57 B 0.71
A 0.57 B 0.86
A 0.57 B 1
A 0.71 B 0
A 0.71 B 0.14
A 0.71 B 0.29
A 0.71 B 0.43
A 0.71 B 0.57
A 0.71 B 0.71
A 0.71 B 0.86
A 0.71 B 1
A 0.86 B 0
A 0.86 B 0.14
A 0.86 B 0.29
A 0.86 B 0.43
A 0.86 B 0.57
A 0.86 B 0.71
A 0.86 B 0.86
A 0.86 B 1
A 1 B 0
A 1 B 0.14
A 1 B 0.29
A 1 B 0.43
A 1 B 0.57
A 1 B 0.71
A 1 B 0.86
A 1 B 1
0.75
0.50
0.25
OD 600 nm [–]
0 5 101520
Time [h]

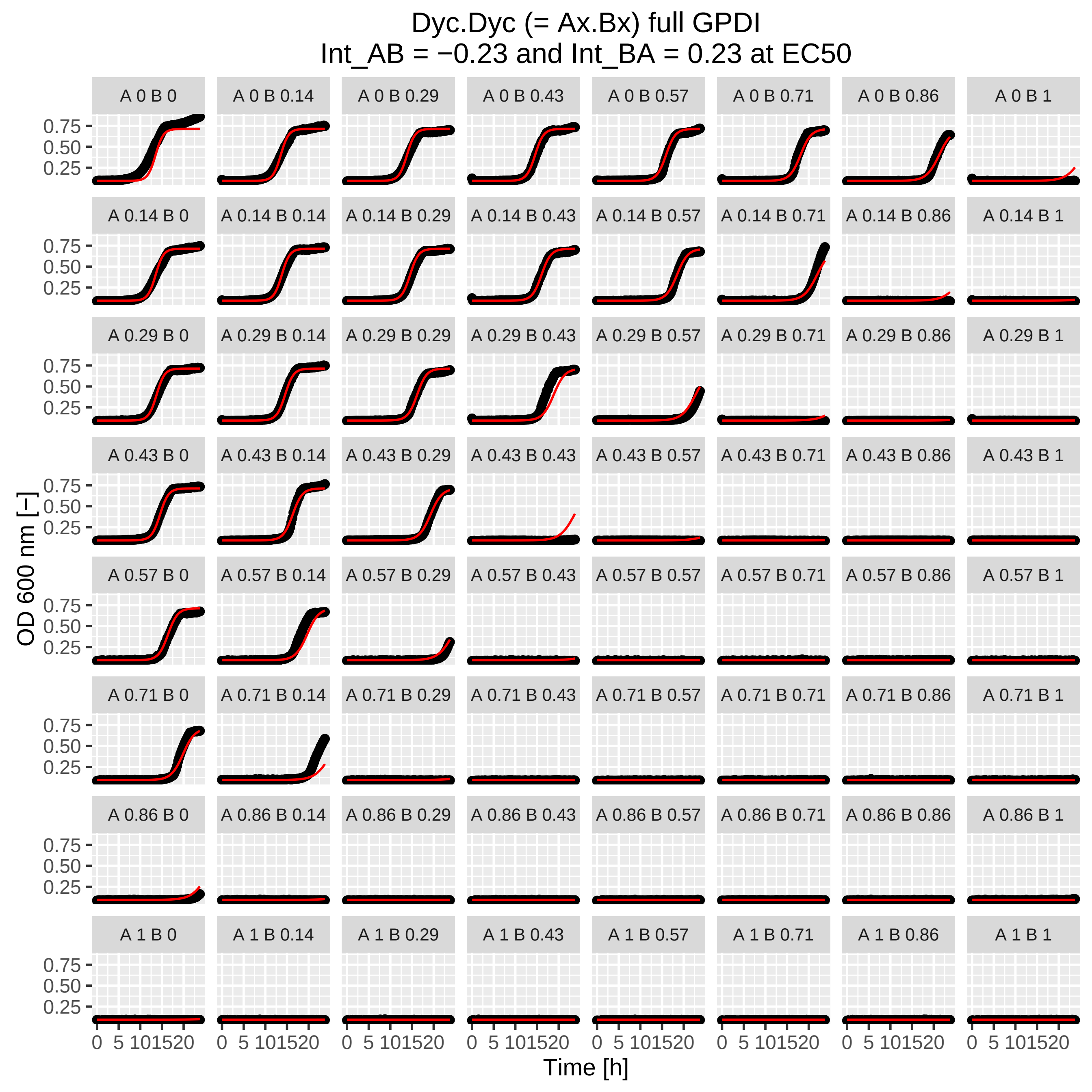

Dyc.Dyc (= Ax.Bx) full GPDI
Int_AB = −0.23 and Int_BA = 0.23 at EC50
A 0 B 0
A 0 B 0.14
A 0 B 0.29
A 0 B 0.43
A 0 B 0.57
A 0 B 0.71
A 0 B 0.86
A 0 B 1
A 0.14 B 0
A 0.14 B 0.14
A 0.14 B 0.29
A 0.14 B 0.43
A 0.14 B 0.57
A 0.14 B 0.71
A 0.14 B 0.86
A 0.14 B 1
A 0.29 B 0
A 0.29 B 0.14
A 0.29 B 0.29
A 0.29 B 0.43
A 0.29 B 0.57
A 0.29 B 0.71
A 0.29 B 0.86
A 0.29 B 1
A 0.43 B 0
A 0.43 B 0.14
A 0.43 B 0.29
A 0.43 B 0.43
A 0.43 B 0.57
A 0.43 B 0.71
A 0.43 B 0.86
A 0.43 B 1
A 0.57 B 0
A 0.57 B 0.14
A 0.57 B 0.29
A 0.57 B 0.43
A 0.57 B 0.57
A 0.57 B 0.71
A 0.57 B 0.86
A 0.57 B 1
A 0.71 B 0
A 0.71 B 0.14
A 0.71 B 0.29
A 0.71 B 0.43
A 0.71 B 0.57
A 0.71 B 0.71
A 0.71 B 0.86
A 0.71 B 1
A 0.86 B 0
A 0.86 B 0.14
A 0.86 B 0.29
A 0.86 B 0.43
A 0.86 B 0.57
A 0.86 B 0.71
A 0.86 B 0.86
A 0.86 B 1
A 1 B 0
A 1 B 0.14
A 1 B 0.29
A 1 B 0.43
A 1 B 0.57
A 1 B 0.71
A 1 B 0.86
A 1 B 1
OD 600 nm [−]
0.75
0.50
0.25
0 5 101520
Time [h]

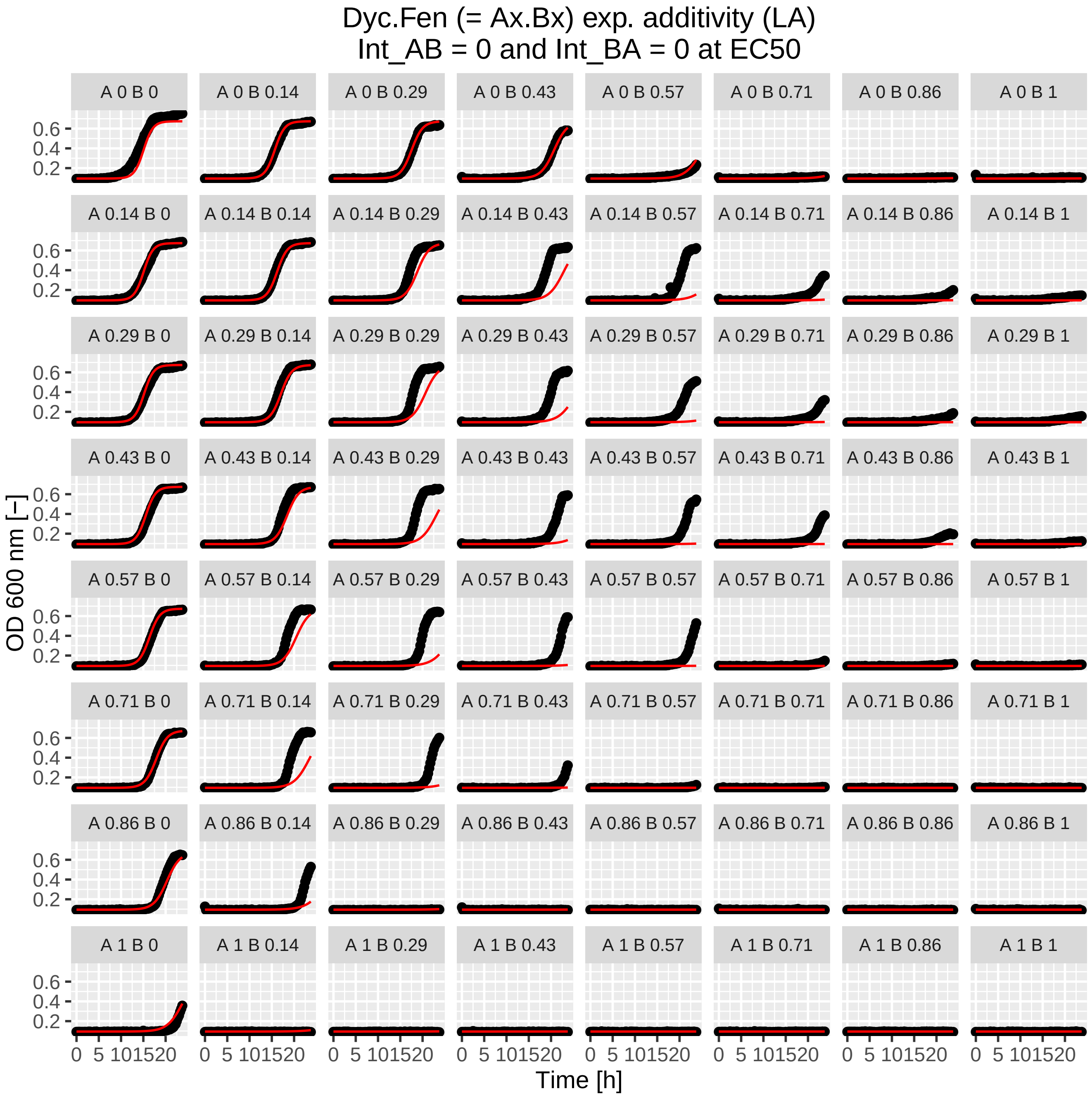
Dyc.Fen (= Ax.Bx) exp. additivity (LA)
Int_AB = 0 and Int_BA = 0 at EC50
OD 600 nm [–]
Time [h]
A 0 B 0
A 0 B 0.14
A 0 B 0.29
A 0 B 0.43
A 0 B 0.57
A 0 B 0.71
A 0 B 0.86
A 0 B 1
A 0.14 B 0
A 0.14 B 0.14
A 0.14 B 0.29
A 0.14 B 0.43
A 0.14 B 0.57
A 0.14 B 0.71
A 0.14 B 0.86
A 0.14 B 1
A 0.29 B 0
A 0.29 B 0.14
A 0.29 B 0.29
A 0.29 B 0.43
A 0.29 B 0.57
A 0.29 B 0.71
A 0.29 B 0.86
A 0.29 B 1
A 0.43 B 0
A 0.43 B 0.14
A 0.43 B 0.29
A 0.43 B 0.43
A 0.43 B 0.57
A 0.43 B 0.71
A 0.43 B 0.86
A 0.43 B 1
A 0.57 B 0
A 0.57 B 0.14
A 0.57 B 0.29
A 0.57 B 0.43
A 0.57 B 0.57
A 0.57 B 0.71
A 0.57 B 0.86
A 0.57 B 1
A 0.71 B 0
A 0.71 B 0.14
A 0.71 B 0.29
A 0.71 B 0.43
A 0.71 B 0.57
A 0.71 B 0.71
A 0.71 B 0.86
A 0.71 B 1
A 0.86 B 0
A 0.86 B 0.14
A 0.86 B 0.29
A 0.86 B 0.43
A 0.86 B 0.57
A 0.86 B 0.71
A 0.86 B 0.86
A 0.86 B 1
A 1 B 0
A 1 B 0.14
A 1 B 0.29
A 1 B 0.43
A 1 B 0.57
A 1 B 0.71
A 1 B 0.86
A 1 B 1
0.6
0.4
0.2
0 5 101520

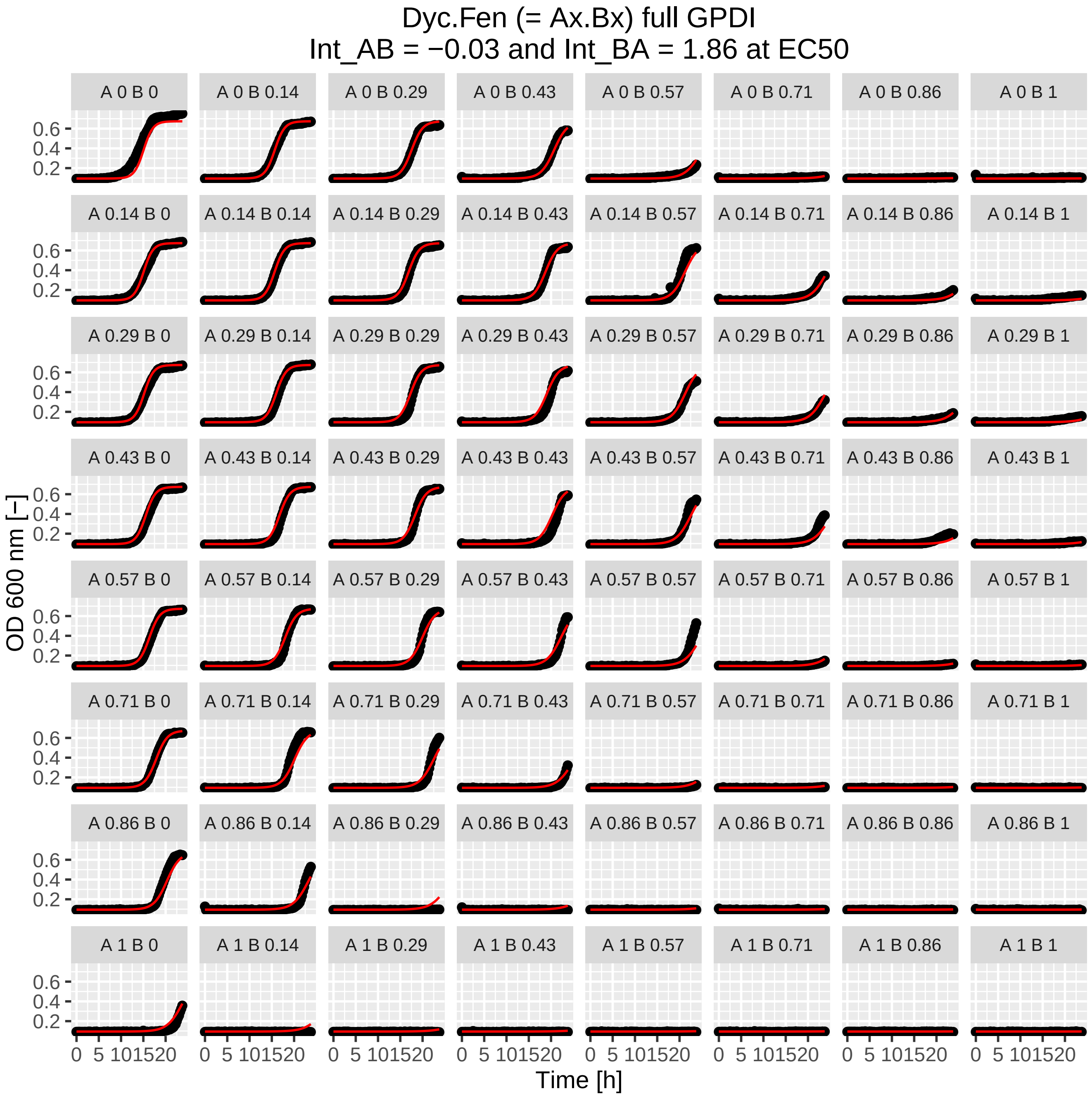

Dyc.Fen (= Ax.Bx) full GPDI
Int_AB = −0.03 and Int_BA = 1.86 at EC50
A 0 B 0
A 0 B 0.14
A 0 B 0.29
A 0 B 0.43
A 0 B 0.57
A 0 B 0.71
A 0 B 0.86
A 0 B 1
A 0.14 B 0
A 0.14 B 0.14
A 0.14 B 0.29
A 0.14 B 0.43
A 0.14 B 0.57
A 0.14 B 0.71
A 0.14 B 0.86
A 0.14 B 1
A 0.29 B 0
A 0.29 B 0.14
A 0.29 B 0.29
A 0.29 B 0.43
A 0.29 B 0.57
A 0.29 B 0.71
A 0.29 B 0.86
A 0.29 B 1
A 0.43 B 0
A 0.43 B 0.14
A 0.43 B 0.29
A 0.43 B 0.43
A 0.43 B 0.57
A 0.43 B 0.71
A 0.43 B 0.86
A 0.43 B 1
A 0.57 B 0
A 0.57 B 0.14
A 0.57 B 0.29
A 0.57 B 0.43
A 0.57 B 0.57
A 0.57 B 0.71
A 0.57 B 0.86
A 0.57 B 1
A 0.71 B 0
A 0.71 B 0.14
A 0.71 B 0.29
A 0.71 B 0.43
A 0.71 B 0.57
A 0.71 B 0.71
A 0.71 B 0.86
A 0.71 B 1
A 0.86 B 0
A 0.86 B 0.14
A 0.86 B 0.29
A 0.86 B 0.43
A 0.86 B 0.57
A 0.86 B 0.71
A 0.86 B 0.86
A 0.86 B 1
A 1 B 0
A 1 B 0.14
A 1 B 0.29
A 1 B 0.43
A 1 B 0.57
A 1 B 0.71
A 1 B 0.86
A 1 B 1
OD 600 nm [−]
0.2
0.4
0.6
0 5 101520
Time [h]

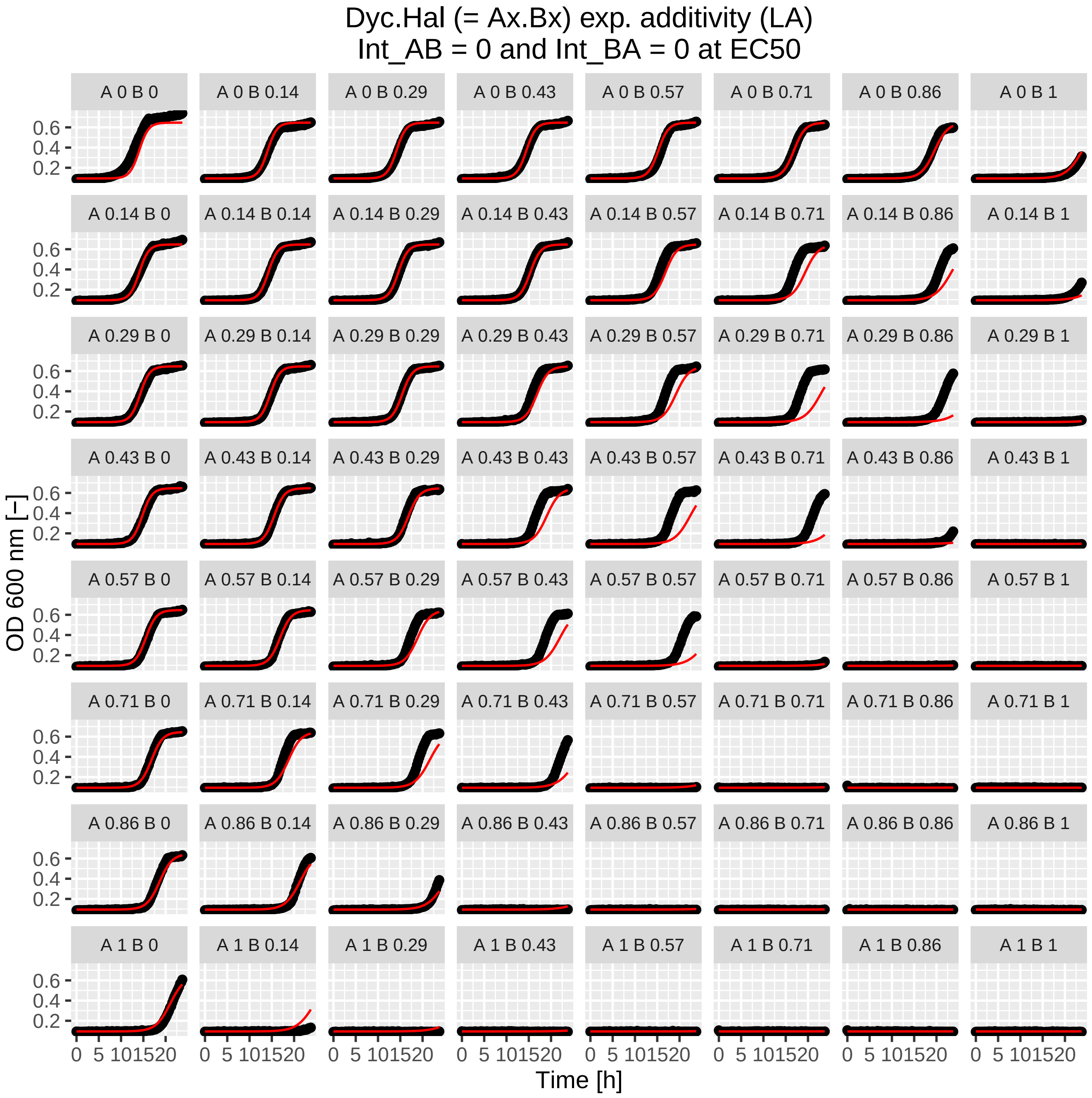
Dyc.Hal (= Ax.Bx) exp. additivity (LA)
Int_AB = 0 and Int_BA = 0 at EC50
OD 600 nm [−]
Time [h]
A 0 B 0
A 0 B 0.14
A 0 B 0.29
A 0 B 0.43
A 0 B 0.57
A 0 B 0.71
A 0 B 0.86
A 0 B 1
A 0.14 B 0
A 0.14 B 0.14
A 0.14 B 0.29
A 0.14 B 0.43
A 0.14 B 0.57
A 0.14 B 0.71
A 0.14 B 0.86
A 0.14 B 1
A 0.29 B 0
A 0.29 B 0.14
A 0.29 B 0.29
A 0.29 B 0.43
A 0.29 B 0.57
A 0.29 B 0.71
A 0.29 B 0.86
A 0.29 B 1
A 0.43 B 0
A 0.43 B 0.14
A 0.43 B 0.29
A 0.43 B 0.43
A 0.43 B 0.57
A 0.43 B 0.71
A 0.43 B 0.86
A 0.43 B 1
A 0.57 B 0
A 0.57 B 0.14
A 0.57 B 0.29
A 0.57 B 0.43
A 0.57 B 0.57
A 0.57 B 0.71
A 0.57 B 0.86
A 0.57 B 1
A 0.71 B 0
A 0.71 B 0.14
A 0.71 B 0.29
A 0.71 B 0.43
A 0.71 B 0.57
A 0.71 B 0.71
A 0.71 B 0.86
A 0.71 B 1
A 0.86 B 0
A 0.86 B 0.14
A 0.86 B 0.29
A 0.86 B 0.43
A 0.86 B 0.57
A 0.86 B 0.71
A 0.86 B 0.86
A 0.86 B 1
A 1 B 0
A 1 B 0.14
A 1 B 0.29
A 1 B 0.43
A 1 B 0.57
A 1 B 0.71
A 1 B 0.86
A 1 B 1
0.2
0.4
0.6
0 5 101520

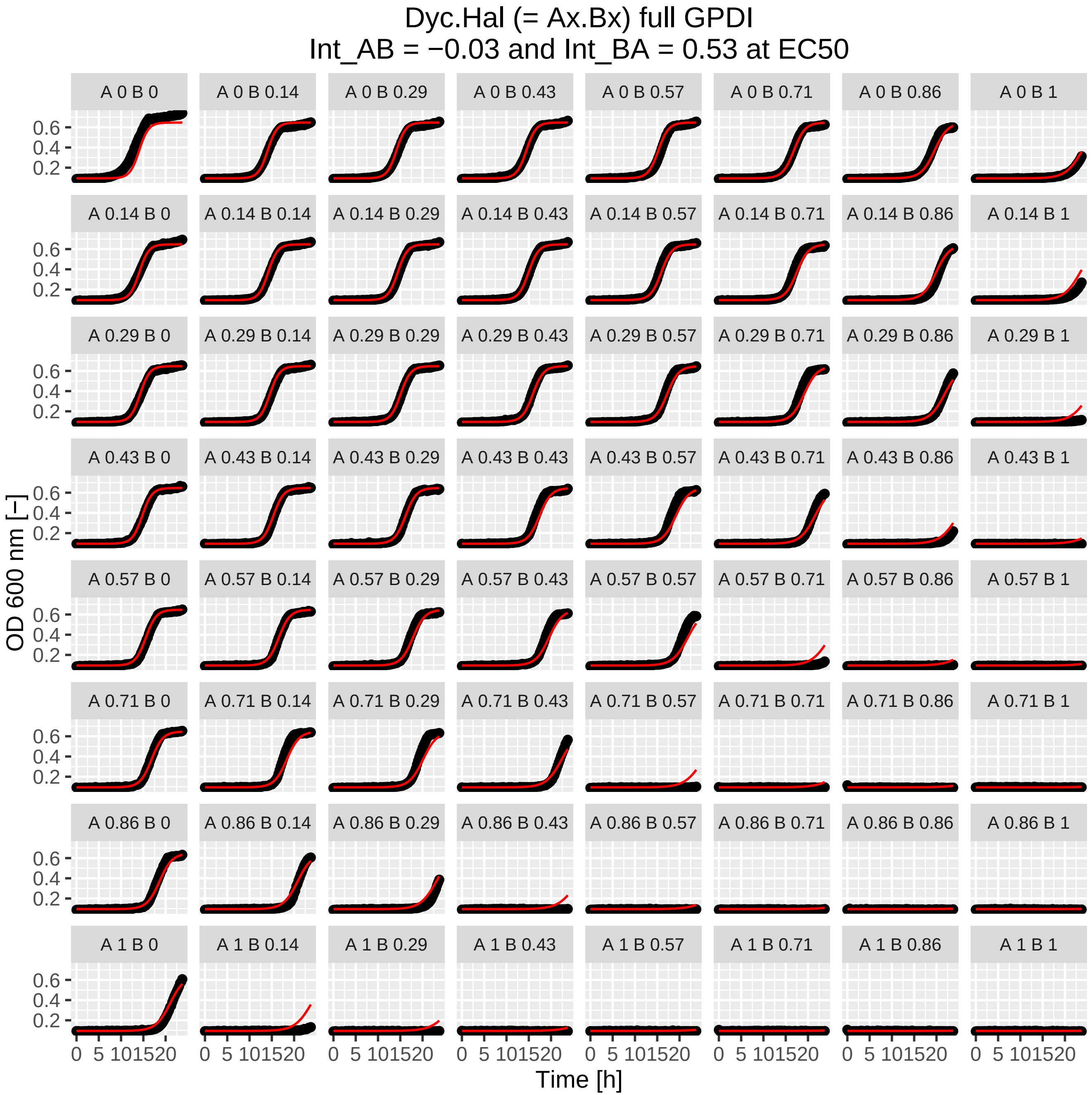

Dyc.Hal (= Ax.Bx) full GPDI
Int_AB = −0.03 and Int_BA = 0.53 at EC50
A 0 B 0
A 0 B 0.14
A 0 B 0.29
A 0 B 0.43
A 0 B 0.57
A 0 B 0.71
A 0 B 0.86
A 0 B 1
A 0.14 B 0
A 0.14 B 0.14
A 0.14 B 0.29
A 0.14 B 0.43
A 0.14 B 0.57
A 0.14 B 0.71
A 0.14 B 0.86
A 0.14 B 1
A 0.29 B 0
A 0.29 B 0.14
A 0.29 B 0.29
A 0.29 B 0.43
A 0.29 B 0.57
A 0.29 B 0.71
A 0.29 B 0.86
A 0.29 B 1
A 0.43 B 0
A 0.43 B 0.14
A 0.43 B 0.29
A 0.43 B 0.43
A 0.43 B 0.57
A 0.43 B 0.71
A 0.43 B 0.86
A 0.43 B 1
A 0.57 B 0
A 0.57 B 0.14
A 0.57 B 0.29
A 0.57 B 0.43
A 0.57 B 0.57
A 0.57 B 0.71
A 0.57 B 0.86
A 0.57 B 1
A 0.71 B 0
A 0.71 B 0.14
A 0.71 B 0.29
A 0.71 B 0.43
A 0.71 B 0.57
A 0.71 B 0.71
A 0.71 B 0.86
A 0.71 B 1
A 0.86 B 0
A 0.86 B 0.14
A 0.86 B 0.29
A 0.86 B 0.43
A 0.86 B 0.57
A 0.86 B 0.71
A 0.86 B 0.86
A 0.86 B 1
A 1 B 0
A 1 B 0.14
A 1 B 0.29
A 1 B 0.43
A 1 B 0.57
A 1 B 0.71
A 1 B 0.86
A 1 B 1
OD 600 nm [−]
0.6
0.4
0.2
0 5 101520
Time [h]

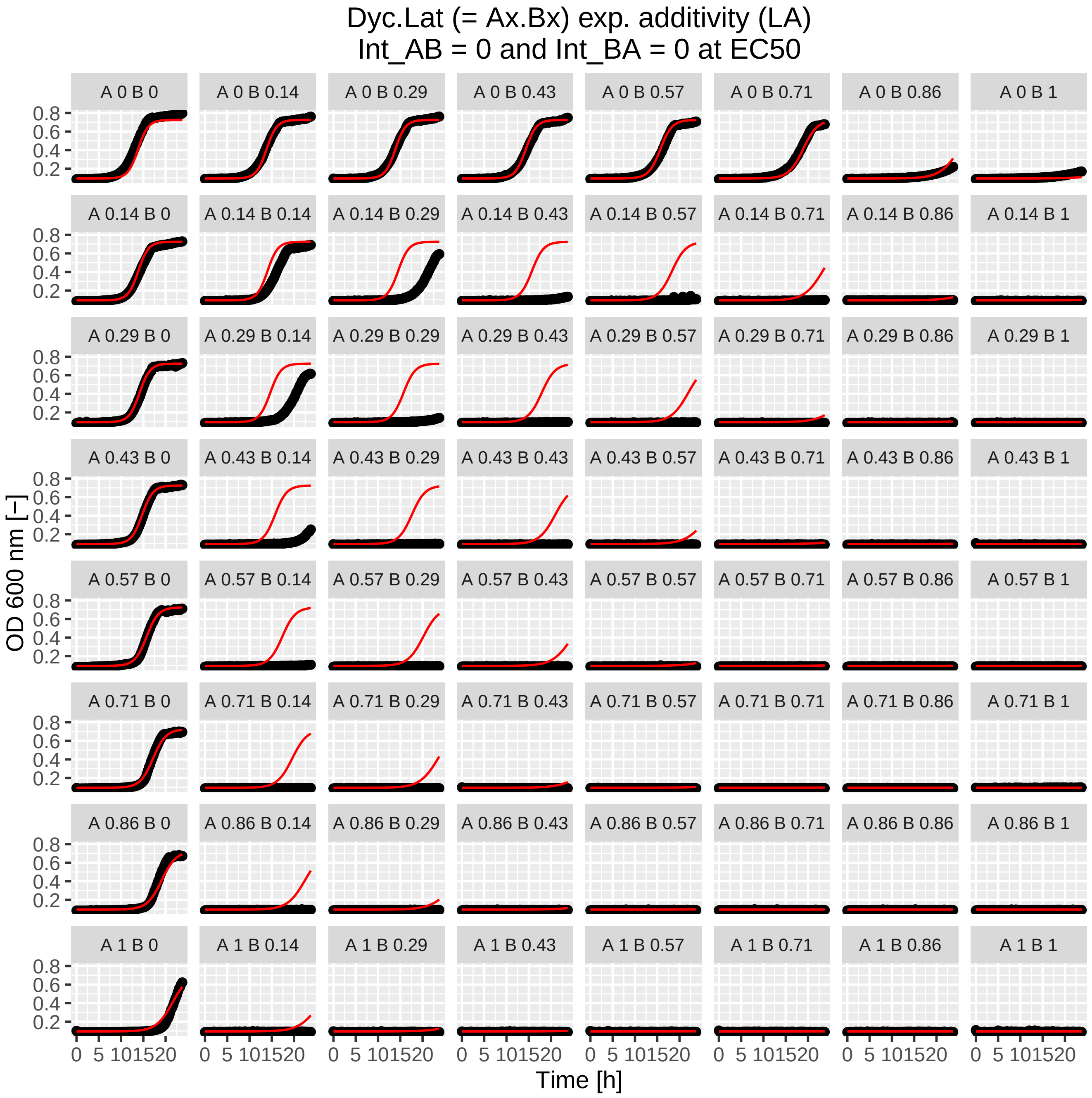

Dyc.Lat (= Ax.Bx) exp. additivity (LA)
Int_AB = 0 and Int_BA = 0 at EC50
A 0 B 0
A 0 B 0.14
A 0 B 0.29
A 0 B 0.43
A 0 B 0.57
A 0 B 0.71
A 0 B 0.86
A 0 B 1
A 0.14 B 0
A 0.14 B 0.14
A 0.14 B 0.29
A 0.14 B 0.43
A 0.14 B 0.57
A 0.14 B 0.71
A 0.14 B 0.86
A 0.14 B 1
A 0.29 B 0
A 0.29 B 0.14
A 0.29 B 0.29
A 0.29 B 0.43
A 0.29 B 0.57
A 0.29 B 0.71
A 0.29 B 0.86
A 0.29 B 1
A 0.43 B 0
A 0.43 B 0.14
A 0.43 B 0.29
A 0.43 B 0.43
A 0.43 B 0.57
A 0.43 B 0.71
A 0.43 B 0.86
A 0.43 B 1
A 0.57 B 0
A 0.57 B 0.14
A 0.57 B 0.29
A 0.57 B 0.43
A 0.57 B 0.57
A 0.57 B 0.71
A 0.57 B 0.86
A 0.57 B 1
A 0.71 B 0
A 0.71 B 0.14
A 0.71 B 0.29
A 0.71 B 0.43
A 0.71 B 0.57
A 0.71 B 0.71
A 0.71 B 0.86
A 0.71 B 1
A 0.86 B 0
A 0.86 B 0.14
A 0.86 B 0.29
A 0.86 B 0.43
A 0.86 B 0.57
A 0.86 B 0.71
A 0.86 B 0.86
A 0.86 B 1
A 1 B 0
A 1 B 0.14
A 1 B 0.29
A 1 B 0.43
A 1 B 0.57
A 1 B 0.71
A 1 B 0.86
A 1 B 1
OD 600 nm [–]
0.2
0.4
0.6
0.8
0 5 101520
Time [h]

Dyc.Lat (= Ax.Bx) full GPDI
Int_AB = −0.53 and Int_BA = −0.48 at EC50

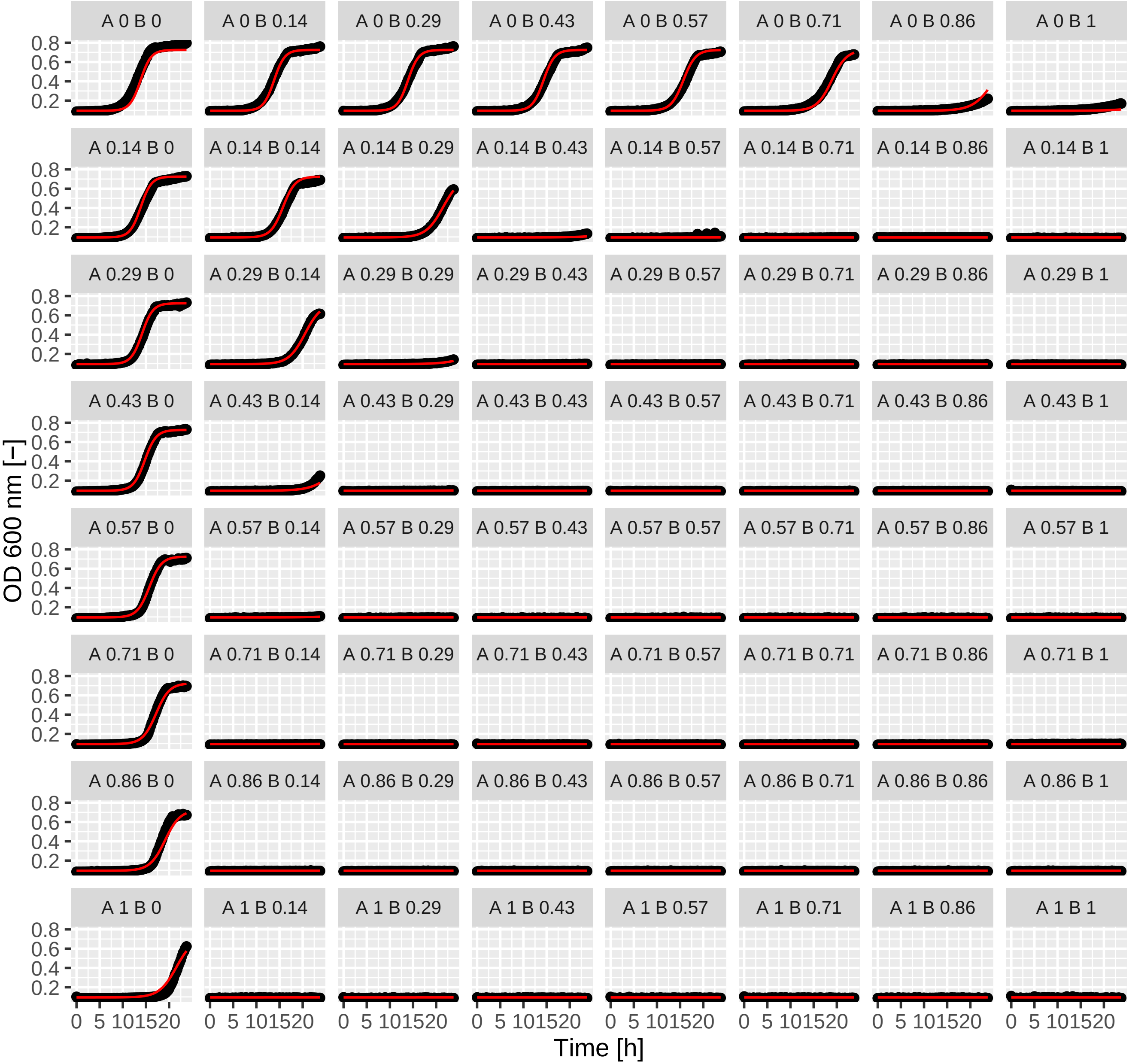

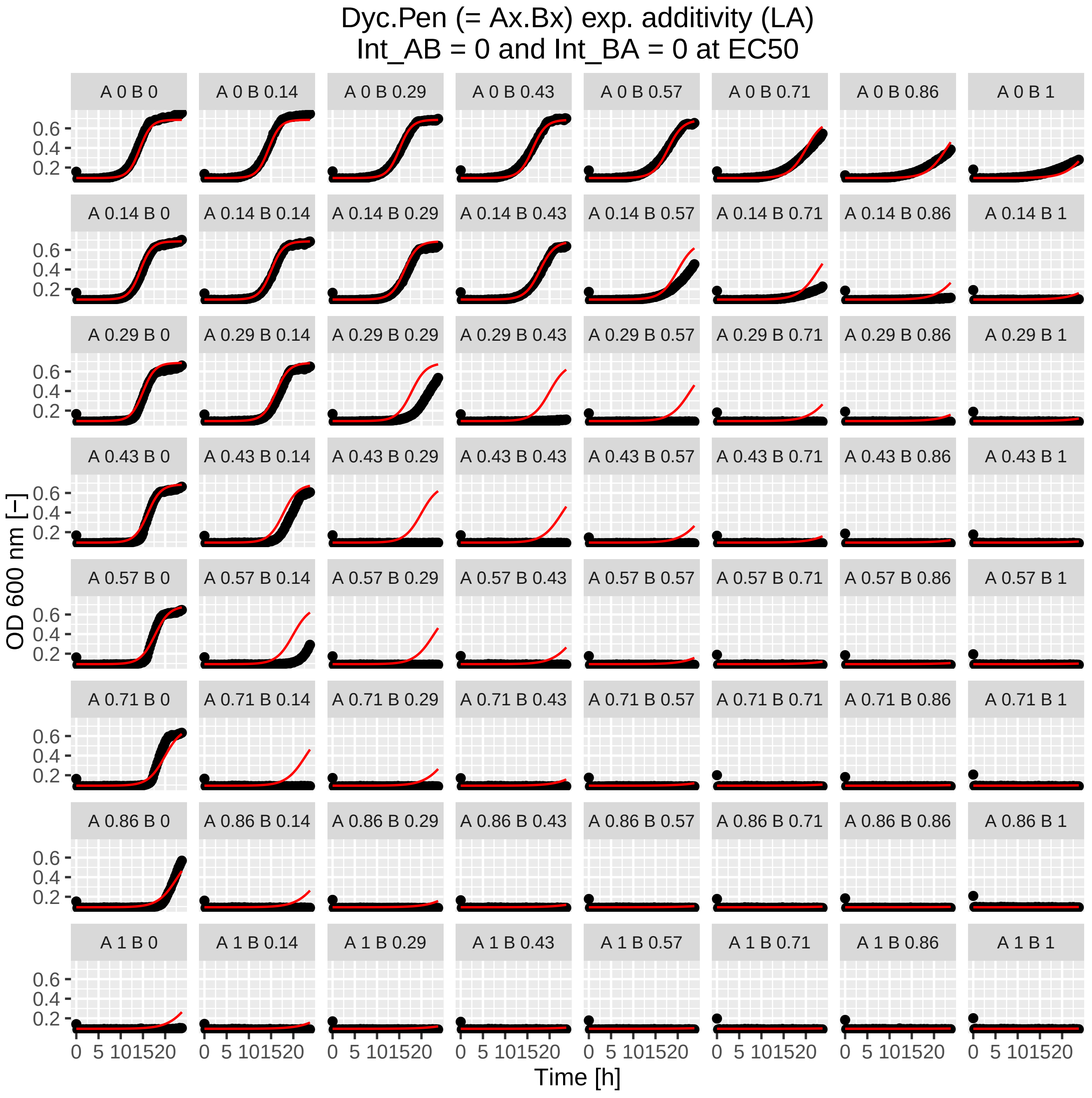

Dyc.Pen (= Ax.Bx) exp. additivity (LA)
Int_AB = 0 and Int_BA = 0 at EC50
OD 600 nm [–]
Time [h]
A 0 B 0
A 0 B 0.14
A 0 B 0.29
A 0 B 0.43
A 0 B 0.57
A 0 B 0.71
A 0 B 0.86
A 0 B 1
A 0.14 B 0
A 0.29 B 0
A 0.43 B 0
A 0.57 B 0
A 0.71 B 0
A 0.86 B 0
A 1 B 0
A 1 B 1
0.6
0.4
0.2
0 5 101520

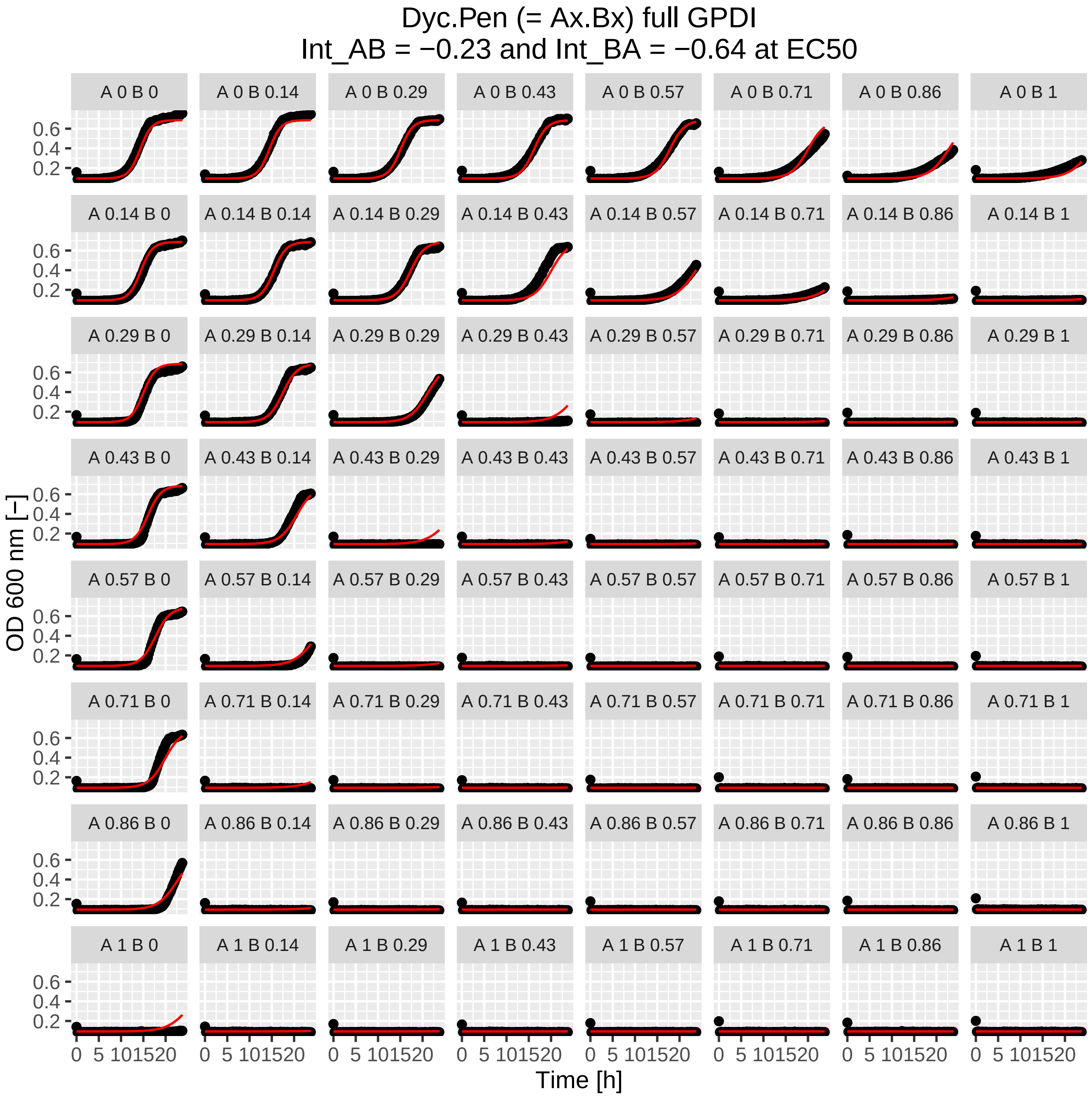

Dyc.Pen (= Ax.Bx) full GPDI
Int_AB = −0.23 and Int_BA = −0.64 at EC50
A 0 B 0
A 0 B 0.14
A 0 B 0.29
A 0 B 0.43
A 0 B 0.57
A 0 B 0.71
A 0 B 0.86
A 0 B 1
A 0.14 B 0
A 0.14 B 0.14
A 0.14 B 0.29
A 0.14 B 0.43
A 0.14 B 0.57
A 0.14 B 0.71
A 0.14 B 0.86
A 0.14 B 1
A 0.29 B 0
A 0.29 B 0.14
A 0.29 B 0.29
A 0.29 B 0.43
A 0.29 B 0.57
A 0.29 B 0.71
A 0.29 B 0.86
A 0.29 B 1
A 0.43 B 0
A 0.43 B 0.14
A 0.43 B 0.29
A 0.43 B 0.43
A 0.43 B 0.57
A 0.43 B 0.71
A 0.43 B 0.86
A 0.43 B 1
A 0.57 B 0
A 0.57 B 0.14
A 0.57 B 0.29
A 0.57 B 0.43
A 0.57 B 0.57
A 0.57 B 0.71
A 0.57 B 0.86
A 0.57 B 1
A 0.71 B 0
A 0.71 B 0.14
A 0.71 B 0.29
A 0.71 B 0.43
A 0.71 B 0.57
A 0.71 B 0.71
A 0.71 B 0.86
A 0.71 B 1
A 0.86 B 0
A 0.86 B 0.14
A 0.86 B 0.29
A 0.86 B 0.43
A 0.86 B 0.57
A 0.86 B 0.71
A 0.86 B 0.86
A 0.86 B 1
A 1 B 0
A 1 B 0.14
A 1 B 0.29
A 1 B 0.43
A 1 B 0.57
A 1 B 0.71
A 1 B 0.86
A 1 B 1
0.6
0.4
0.2
OD 600 nm [−]
0 5 101520
Time [h]

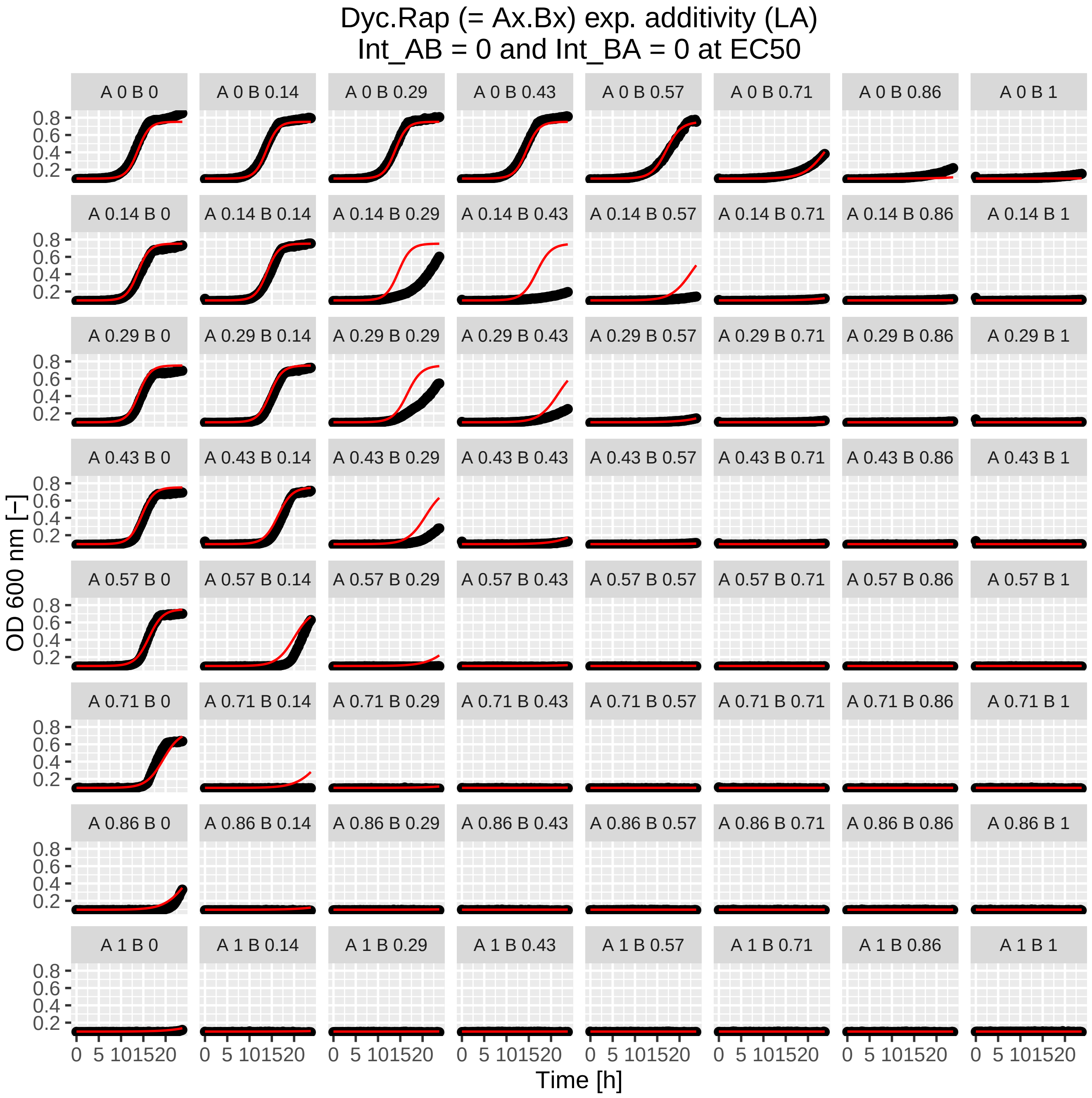

Dyc.Rap (= Ax.Bx) exp. additivity (LA)
Int_AB = 0 and Int_BA = 0 at EC50
A 0 B 0
A 0 B 0.14
A 0 B 0.29
A 0 B 0.43
A 0 B 0.57
A 0 B 0.71
A 0 B 0.86
A 0 B 1
A 0.14 B 0
A 0.14 B 0.14
A 0.14 B 0.29
A 0.14 B 0.43
A 0.14 B 0.57
A 0.14 B 0.71
A 0.14 B 0.86
A 0.14 B 1
A 0.29 B 0
A 0.29 B 0.14
A 0.29 B 0.29
A 0.29 B 0.43
A 0.29 B 0.57
A 0.29 B 0.71
A 0.29 B 0.86
A 0.29 B 1
A 0.43 B 0
A 0.43 B 0.14
A 0.43 B 0.29
A 0.43 B 0.43
A 0.43 B 0.57
A 0.43 B 0.71
A 0.43 B 0.86
A 0.43 B 1
A 0.57 B 0
A 0.57 B 0.14
A 0.57 B 0.29
A 0.57 B 0.43
A 0.57 B 0.57
A 0.57 B 0.71
A 0.57 B 0.86
A 0.57 B 1
A 0.71 B 0
A 0.71 B 0.14
A 0.71 B 0.29
A 0.71 B 0.43
A 0.71 B 0.57
A 0.71 B 0.71
A 0.71 B 0.86
A 0.71 B 1
A 0.86 B 0
A 0.86 B 0.14
A 0.86 B 0.29
A 0.86 B 0.43
A 0.86 B 0.57
A 0.86 B 0.71
A 0.86 B 0.86
A 0.86 B 1
A 1 B 0
A 1 B 0.14
A 1 B 0.29
A 1 B 0.43
A 1 B 0.57
A 1 B 0.71
A 1 B 0.86
A 1 B 1
0.8
0.6
0.4
0.2
OD 600 nm [–]
0 5 101520
Time [h]

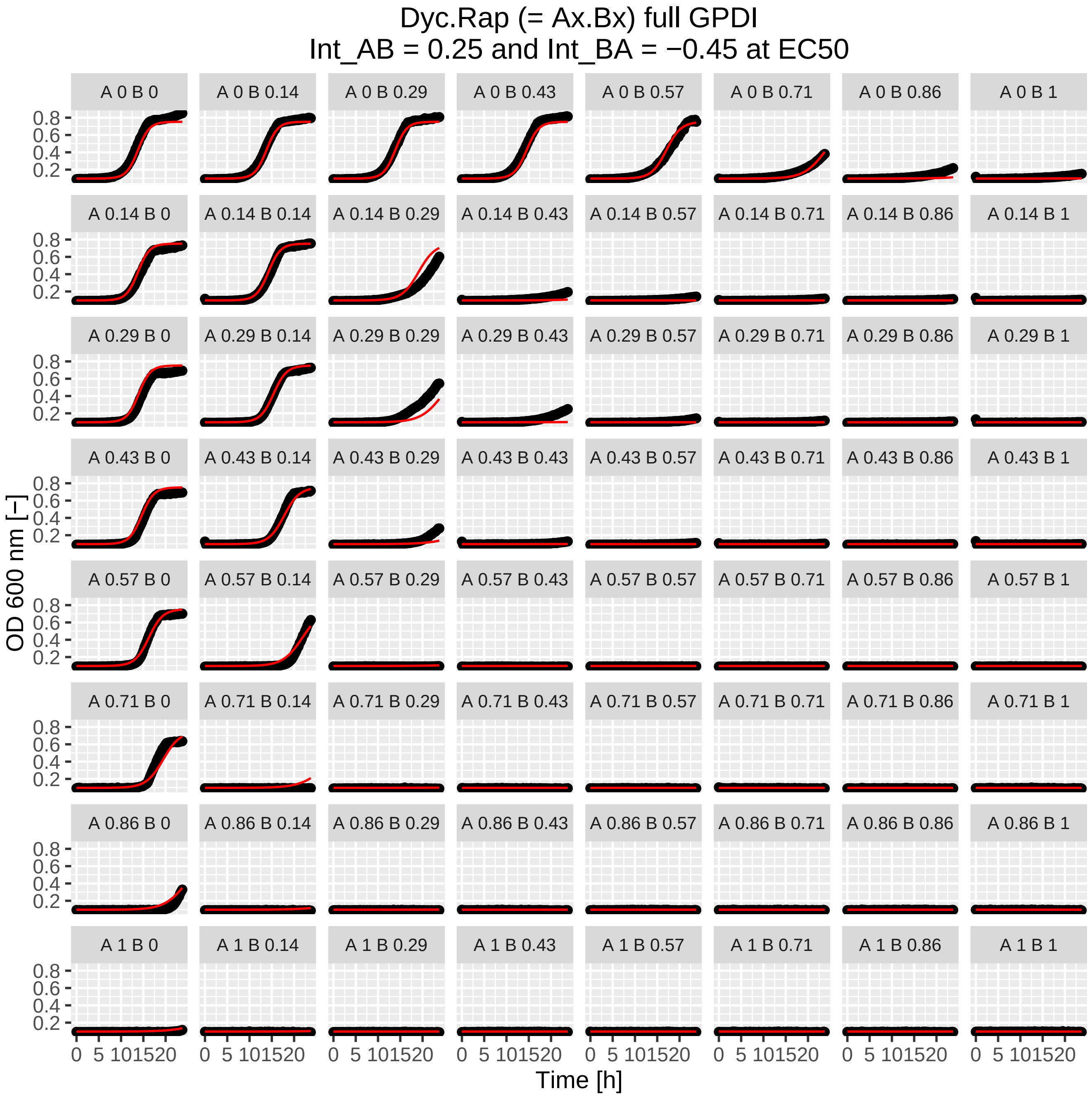

Dyc.Rap (= Ax.Bx) full GPDI
Int_AB = 0.25 and Int_BA = −0.45 at EC50
A 0 B 0
A 0 B 0.14
A 0 B 0.29
A 0 B 0.43
A 0 B 0.57
A 0 B 0.71
A 0 B 0.86
A 0 B 1
A 0.14 B 0
A 0.14 B 0.14
A 0.14 B 0.29
A 0.14 B 0.43
A 0.14 B 0.57
A 0.14 B 0.71
A 0.14 B 0.86
A 0.14 B 1
A 0.29 B 0
A 0.29 B 0.14
A 0.29 B 0.29
A 0.29 B 0.43
A 0.29 B 0.57
A 0.29 B 0.71
A 0.29 B 0.86
A 0.29 B 1
A 0.43 B 0
A 0.43 B 0.14
A 0.43 B 0.29
A 0.43 B 0.43
A 0.43 B 0.57
A 0.43 B 0.71
A 0.43 B 0.86
A 0.43 B 1
A 0.57 B 0
A 0.57 B 0.14
A 0.57 B 0.29
A 0.57 B 0.43
A 0.57 B 0.57
A 0.57 B 0.71
A 0.57 B 0.86
A 0.57 B 1
A 0.71 B 0
A 0.71 B 0.14
A 0.71 B 0.29
A 0.71 B 0.43
A 0.71 B 0.57
A 0.71 B 0.71
A 0.71 B 0.86
A 0.71 B 1
A 0.86 B 0
A 0.86 B 0.14
A 0.86 B 0.29
A 0.86 B 0.43
A 0.86 B 0.57
A 0.86 B 0.71
A 0.86 B 0.86
A 0.86 B 1
A 1 B 0
A 1 B 0.14
A 1 B 0.29
A 1 B 0.43
A 1 B 0.57
A 1 B 0.71
A 1 B 0.86
A 1 B 1
0.8
0.6
0.4
0.2
OD 600 nm [−]
0 5 101520
Time [h]

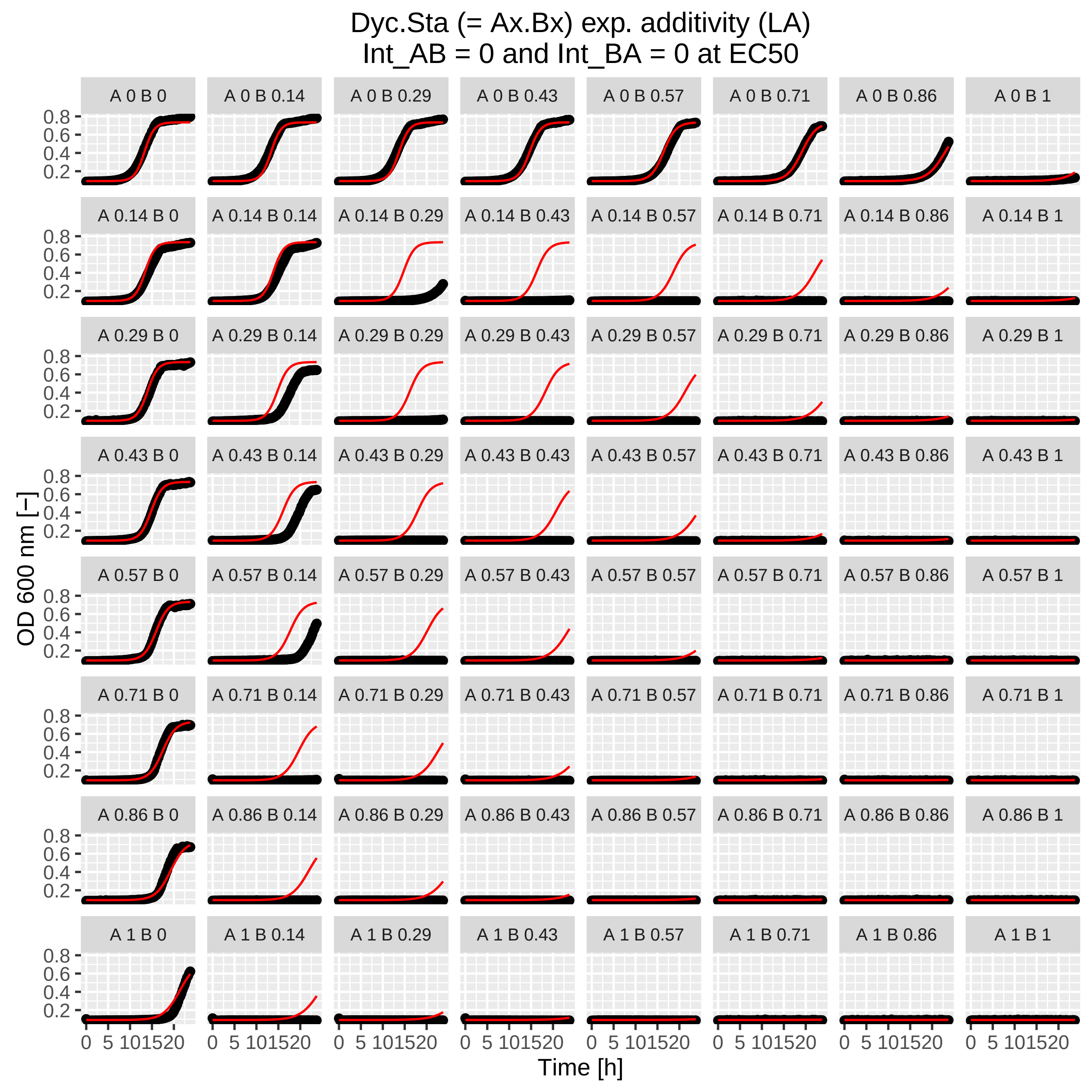
Dyc.Sta (= Ax.Bx) exp. additivity (LA)
Int_AB = 0 and Int_BA = 0 at EC50
OD 600 nm [–]
Time [h]

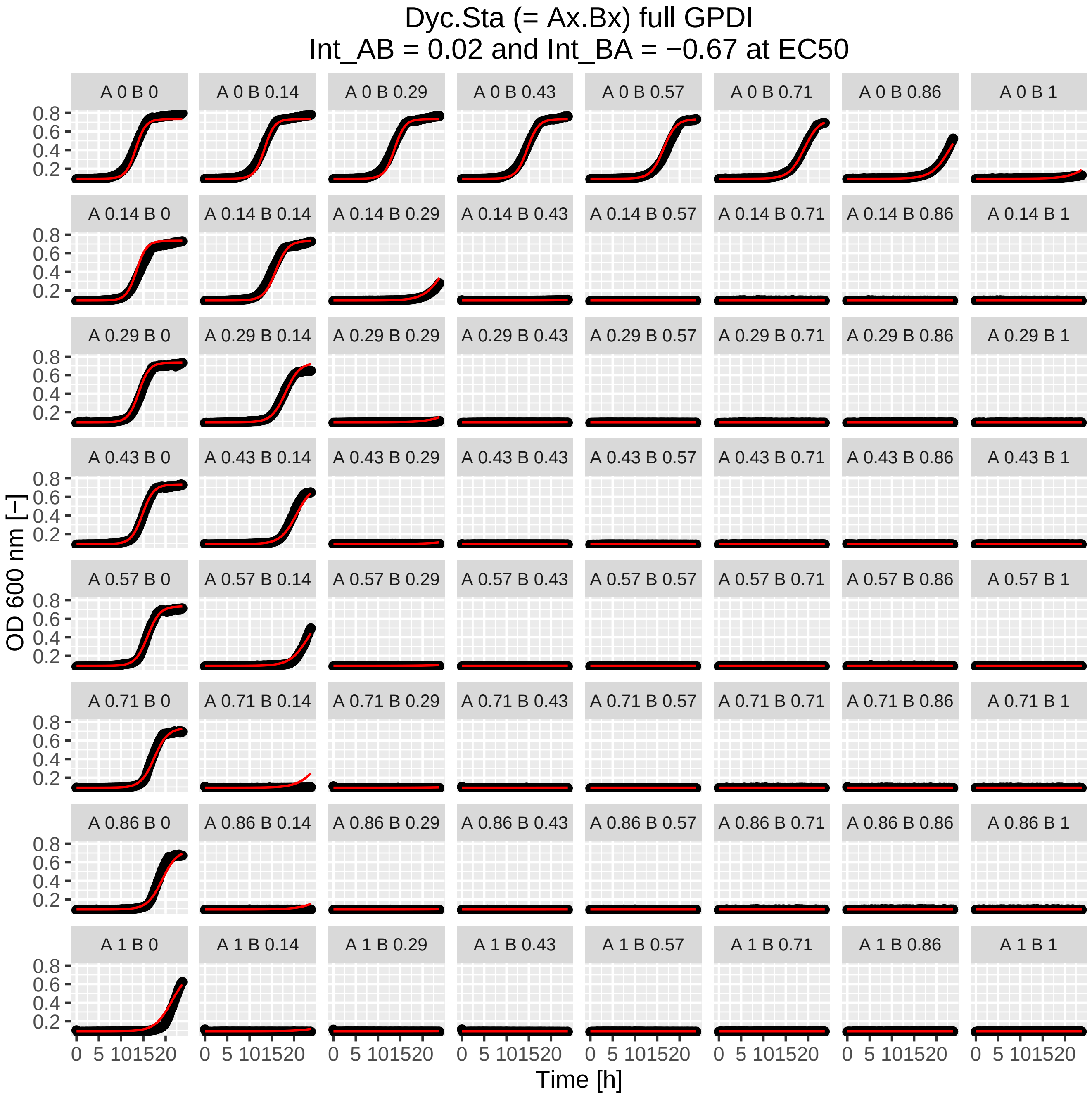
Dyc.Sta (= Ax.Bx) full GPDI
Int_AB = 0.02 and Int_BA = −0.67 at EC50
A 0 B 0
A 0 B 0.14
A 0 B 0.29
A 0 B 0.43
A 0 B 0.57
A 0 B 0.71
A 0 B 0.86
A 0 B 1
A 0.14 B 0
A 0.14 B 0.14
A 0.14 B 0.29
A 0.14 B 0.43
A 0.14 B 0.57
A 0.14 B 0.71
A 0.14 B 0.86
A 0.14 B 1
A 0.29 B 0
A 0.29 B 0.14
A 0.29 B 0.29
A 0.29 B 0.43
A 0.29 B 0.57
A 0.29 B 0.71
A 0.29 B 0.86
A 0.29 B 1
A 0.43 B 0
A 0.43 B 0.14
A 0.43 B 0.29
A 0.43 B 0.43
A 0.43 B 0.57
A 0.43 B 0.71
A 0.43 B 0.86
A 0.43 B 1
A 0.57 B 0
A 0.57 B 0.14
A 0.57 B 0.29
A 0.57 B 0.43
A 0.57 B 0.57
A 0.57 B 0.71
A 0.57 B 0.86
A 0.57 B 1
A 0.71 B 0
A 0.71 B 0.14
A 0.71 B 0.29
A 0.71 B 0.43
A 0.71 B 0.57
A 0.71 B 0.71
A 0.71 B 0.86
A 0.71 B 1
A 0.86 B 0
A 0.86 B 0.14
A 0.86 B 0.29
A 0.86 B 0.43
A 0.86 B 0.57
A 0.86 B 0.71
A 0.86 B 0.86
A 0.86 B 1
A 1 B 0
A 1 B 0.14
A 1 B 0.29
A 1 B 0.43
A 1 B 0.57
A 1 B 0.71
A 1 B 0.86
A 1 B 1
0.8
0.6
0.4
0.2
0 5 101520
OD 600 nm [−]
Time [h]

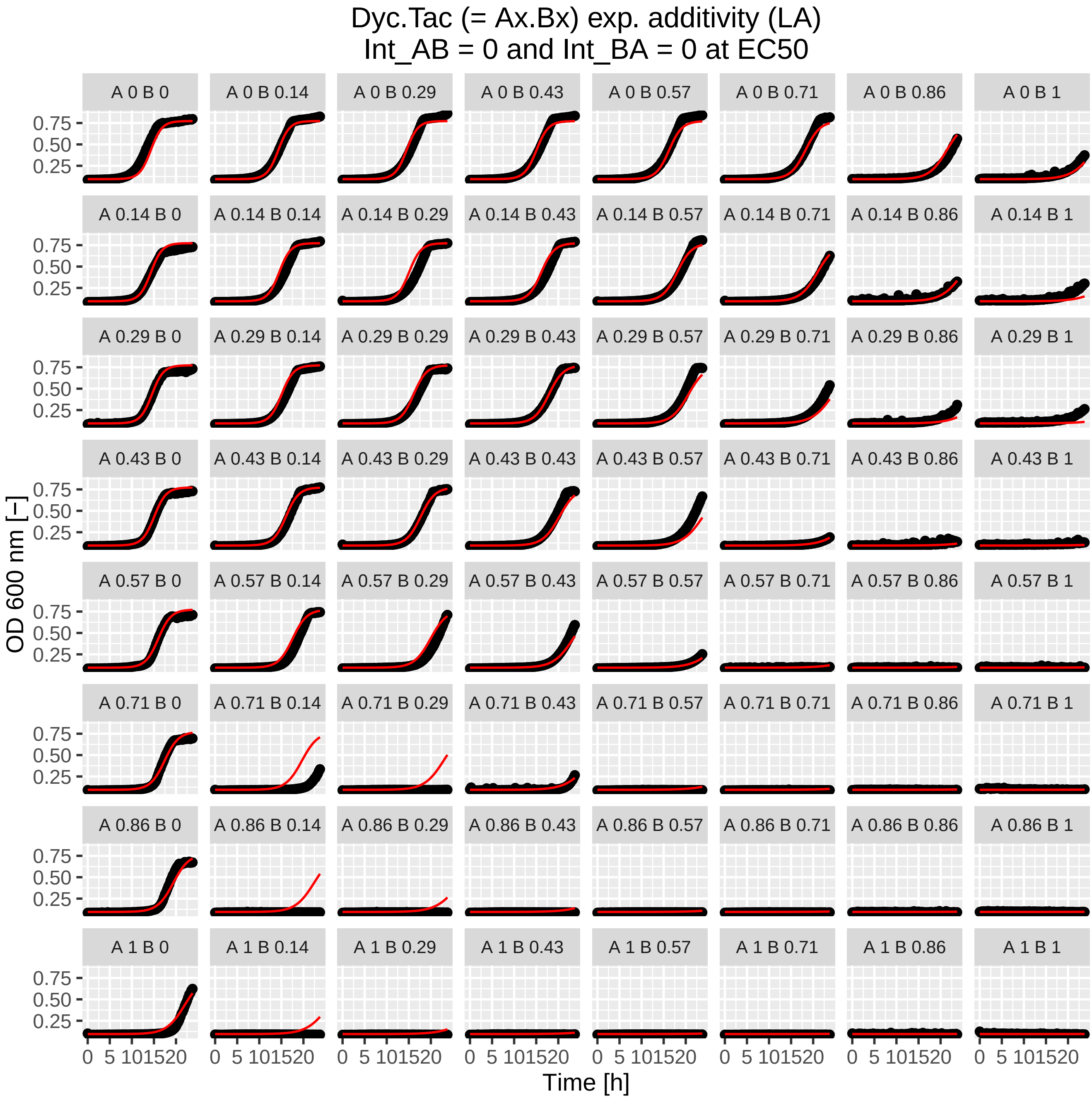
Dyc.Tac (= Ax.Bx) exp. additivity (LA)
Int_AB = 0 and Int_BA = 0 at EC50
A 0 B 0
A 0 B 0.14
A 0 B 0.29
A 0 B 0.43
A 0 B 0.57
A 0 B 0.71
A 0 B 0.86
A 0 B 1
A 0.14 B 0
A 0.14 B 0.14
A 0.14 B 0.29
A 0.14 B 0.43
A 0.14 B 0.57
A 0.14 B 0.71
A 0.14 B 0.86
A 0.14 B 1
A 0.29 B 0
A 0.29 B 0.14
A 0.29 B 0.29
A 0.29 B 0.43
A 0.29 B 0.57
A 0.29 B 0.71
A 0.29 B 0.86
A 0.29 B 1
A 0.43 B 0
A 0.43 B 0.14
A 0.43 B 0.29
A 0.43 B 0.43
A 0.43 B 0.57
A 0.43 B 0.71
A 0.43 B 0.86
A 0.43 B 1
A 0.57 B 0
A 0.57 B 0.14
A 0.57 B 0.29
A 0.57 B 0.43
A 0.57 B 0.57
A 0.57 B 0.71
A 0.57 B 0.86
A 0.57 B 1
A 0.71 B 0
A 0.71 B 0.14
A 0.71 B 0.29
A 0.71 B 0.43
A 0.71 B 0.57
A 0.71 B 0.71
A 0.71 B 0.86
A 0.71 B 1
A 0.86 B 0
A 0.86 B 0.14
A 0.86 B 0.29
A 0.86 B 0.43
A 0.86 B 0.57
A 0.86 B 0.71
A 0.86 B 0.86
A 0.86 B 1
A 1 B 0
A 1 B 0.14
A 1 B 0.29
A 1 B 0.43
A 1 B 0.57
A 1 B 0.71
A 1 B 0.86
A 1 B 1
0.75
0.50
0.25
OD 600 nm [–]
0 5 101520
Time [h]

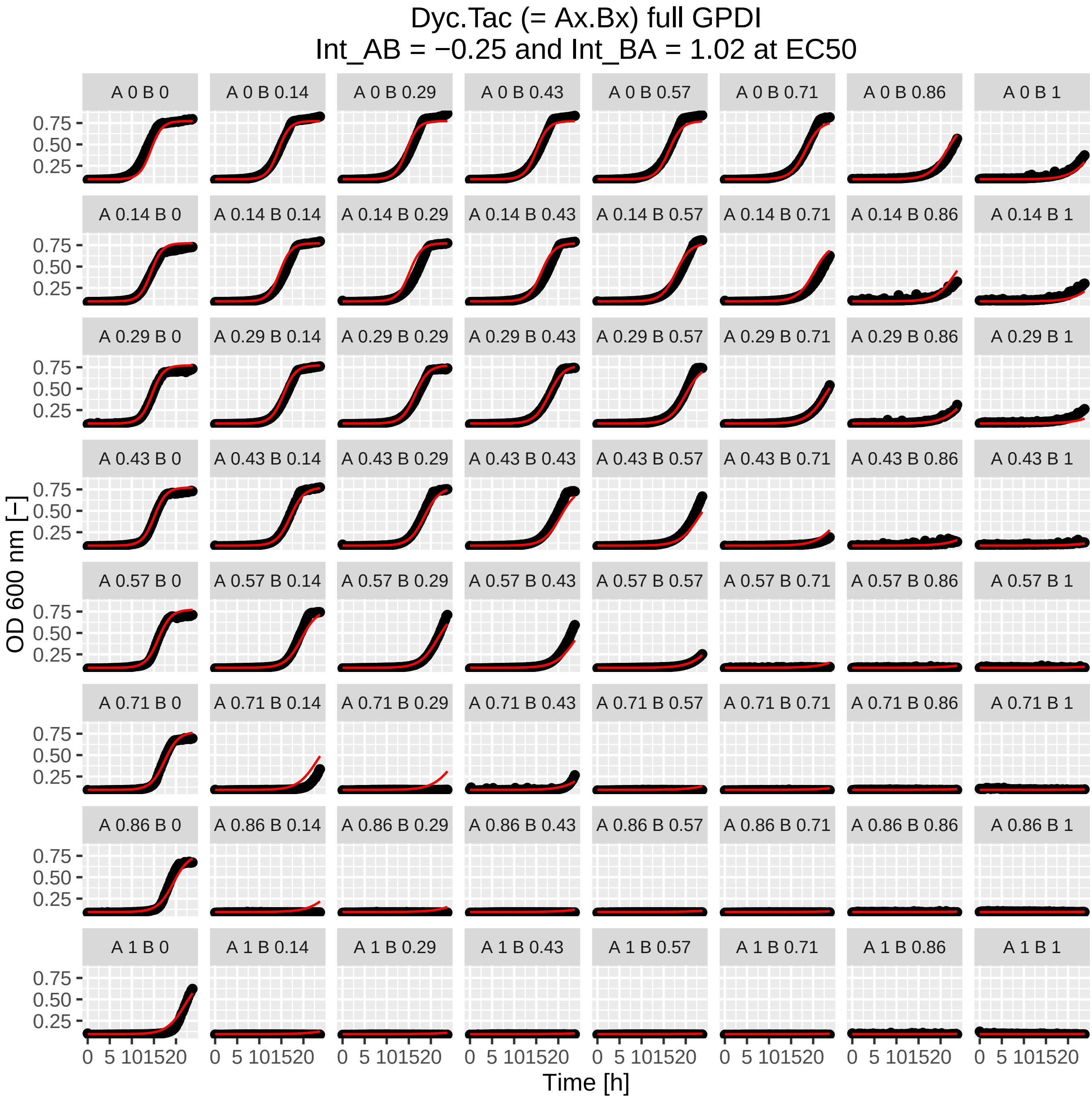

Dyc.Tac (= Ax.Bx) full GPDI
Int_AB = −0.25 and Int_BA = 1.02 at EC50
A 0 B 0
A 0 B 0.14
A 0 B 0.29
A 0 B 0.43
A 0 B 0.57
A 0 B 0.71
A 0 B 0.86
A 0 B 1
A 0.14 B 0
A 0.14 B 0.14
A 0.14 B 0.29
A 0.14 B 0.43
A 0.14 B 0.57
A 0.14 B 0.71
A 0.14 B 0.86
A 0.14 B 1
A 0.29 B 0
A 0.29 B 0.14
A 0.29 B 0.29
A 0.29 B 0.43
A 0.29 B 0.57
A 0.29 B 0.71
A 0.29 B 0.86
A 0.29 B 1
A 0.43 B 0
A 0.43 B 0.14
A 0.43 B 0.29
A 0.43 B 0.43
A 0.43 B 0.57
A 0.43 B 0.71
A 0.43 B 0.86
A 0.43 B 1
A 0.57 B 0
A 0.57 B 0.14
A 0.57 B 0.29
A 0.57 B 0.43
A 0.57 B 0.57
A 0.57 B 0.71
A 0.57 B 0.86
A 0.57 B 1
A 0.71 B 0
A 0.71 B 0.14
A 0.71 B 0.29
A 0.71 B 0.43
A 0.71 B 0.57
A 0.71 B 0.71
A 0.71 B 0.86
A 0.71 B 1
A 0.86 B 0
A 0.86 B 0.14
A 0.86 B 0.29
A 0.86 B 0.43
A 0.86 B 0.57
A 0.86 B 0.71
A 0.86 B 0.86
A 0.86 B 1
A 1 B 0
A 1 B 0.14
A 1 B 0.29
A 1 B 0.43
A 1 B 0.57
A 1 B 0.71
A 1 B 0.86
A 1 B 1
OD 600 nm [−]
0.25
0.50
0.75
0 5 101520
Time [h]

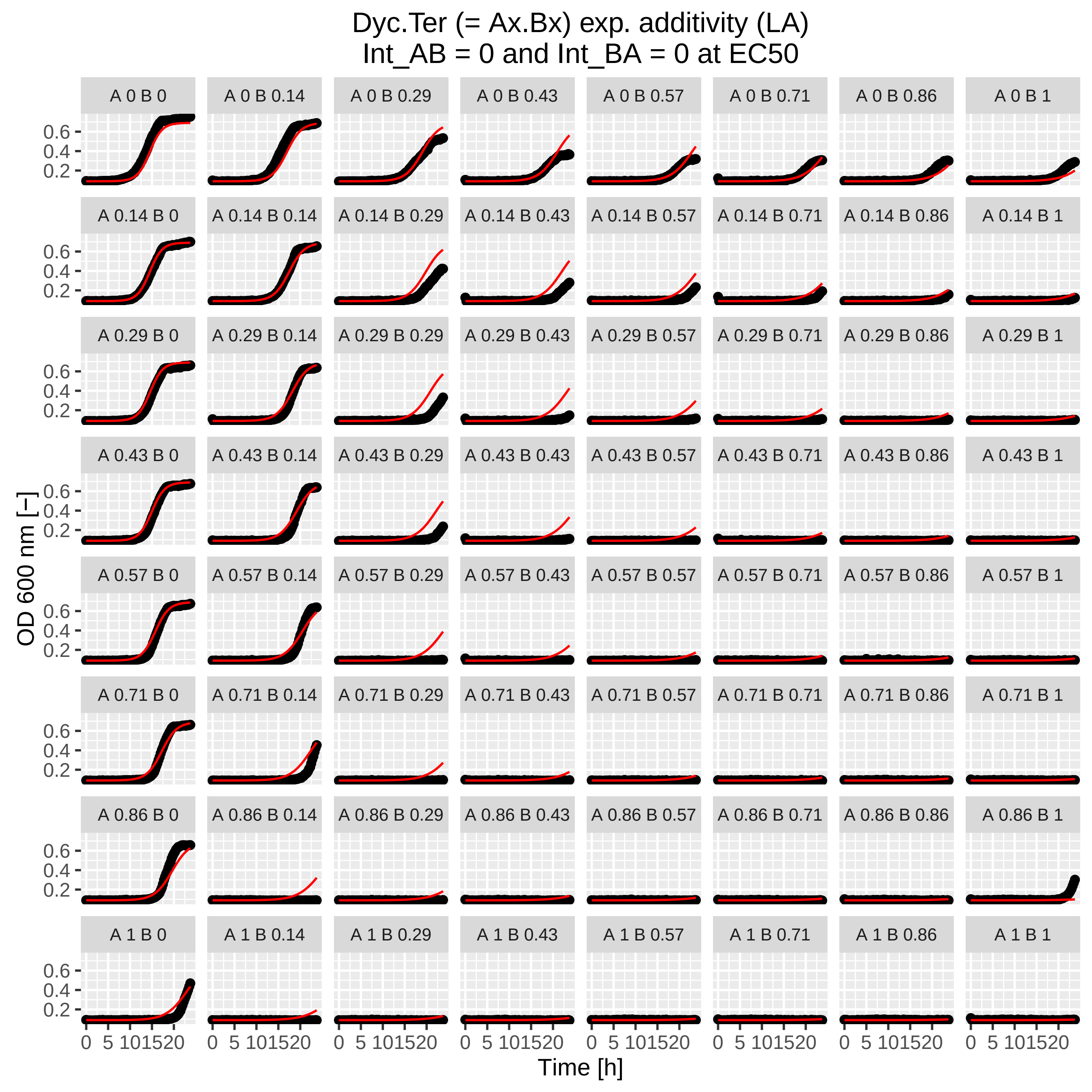
Dyc.Ter (= Ax.Bx) exp. additivity (LA)
Int_AB = 0 and Int_BA = 0 at EC50
OD 600 nm [–]
Time [h]
A 0 B 0
A 0 B 0.14
A 0 B 0.29
A 0 B 0.43
A 0 B 0.57
A 0 B 0.71
A 0 B 0.86
A 0 B 1
A 0.14 B 0
A 0.29 B 0
A 0.43 B 0
A 0.57 B 0
A 0.71 B 0
A 0.86 B 0
A 1 B 0
A 1 B 1
0.2
0.4
0.6
0
5
10
15
20

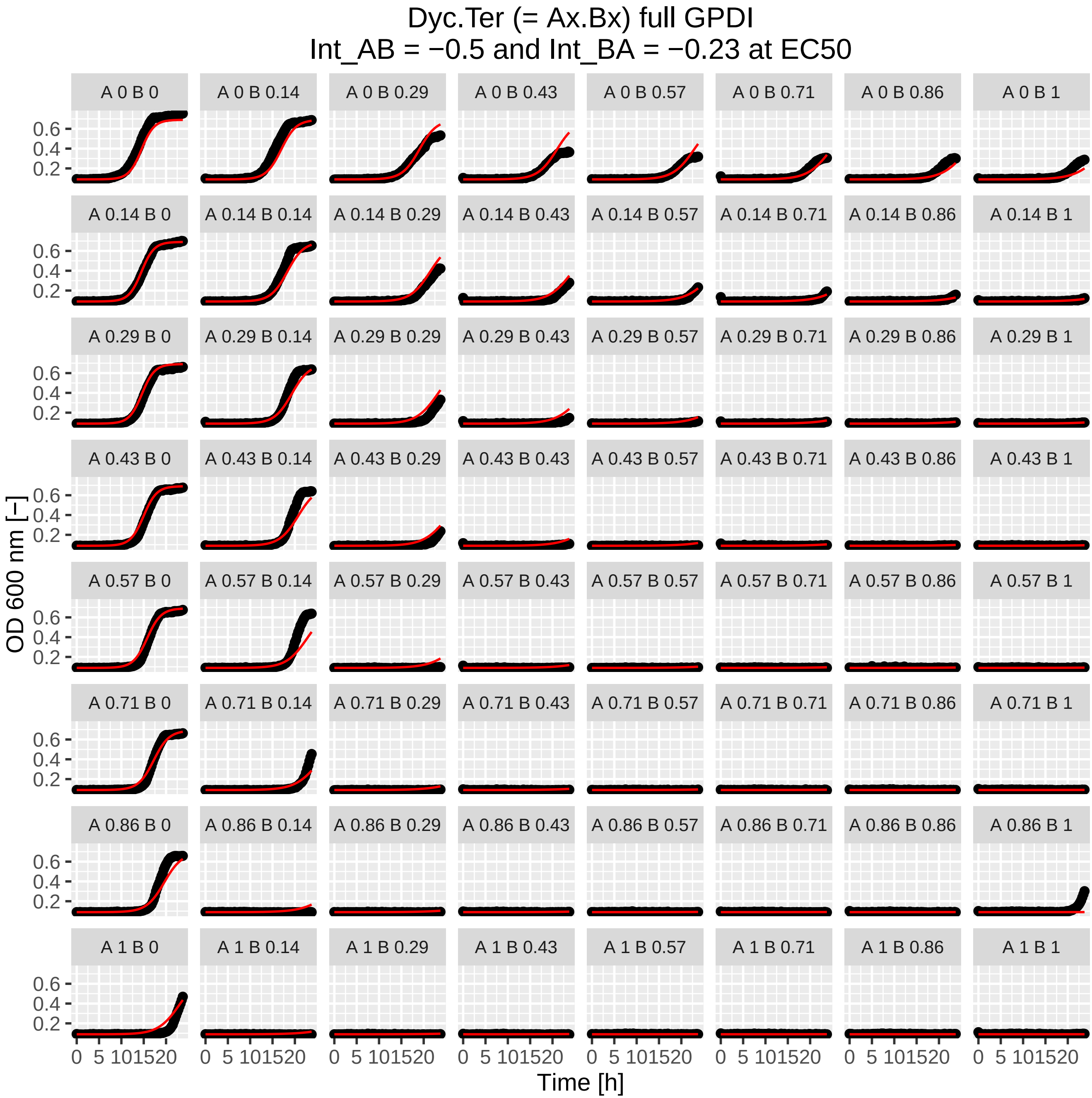

Dyc.Ter (= Ax.Bx) full GPDI
Int_AB = −0.5 and Int_BA = −0.23 at EC50
A 0 B 0
A 0 B 0.14
A 0 B 0.29
A 0 B 0.43
A 0 B 0.57
A 0 B 0.71
A 0 B 0.86
A 0 B 1
A 0.14 B 0
A 0.14 B 0.14
A 0.14 B 0.29
A 0.14 B 0.43
A 0.14 B 0.57
A 0.14 B 0.71
A 0.14 B 0.86
A 0.14 B 1
A 0.29 B 0
A 0.29 B 0.14
A 0.29 B 0.29
A 0.29 B 0.43
A 0.29 B 0.57
A 0.29 B 0.71
A 0.29 B 0.86
A 0.29 B 1
A 0.43 B 0
A 0.43 B 0.14
A 0.43 B 0.29
A 0.43 B 0.43
A 0.43 B 0.57
A 0.43 B 0.71
A 0.43 B 0.86
A 0.43 B 1
A 0.57 B 0
A 0.57 B 0.14
A 0.57 B 0.29
A 0.57 B 0.43
A 0.57 B 0.57
A 0.57 B 0.71
A 0.57 B 0.86
A 0.57 B 1
A 0.71 B 0
A 0.71 B 0.14
A 0.71 B 0.29
A 0.71 B 0.43
A 0.71 B 0.57
A 0.71 B 0.71
A 0.71 B 0.86
A 0.71 B 1
A 0.86 B 0
A 0.86 B 0.14
A 0.86 B 0.29
A 0.86 B 0.43
A 0.86 B 0.57
A 0.86 B 0.71
A 0.86 B 0.86
A 0.86 B 1
A 1 B 0
A 1 B 0.14
A 1 B 0.29
A 1 B 0.43
A 1 B 0.57
A 1 B 0.71
A 1 B 0.86
A 1 B 1
OD 600 nm [−]
0.6
0.4
0.2
0 5 101520
Time [h]

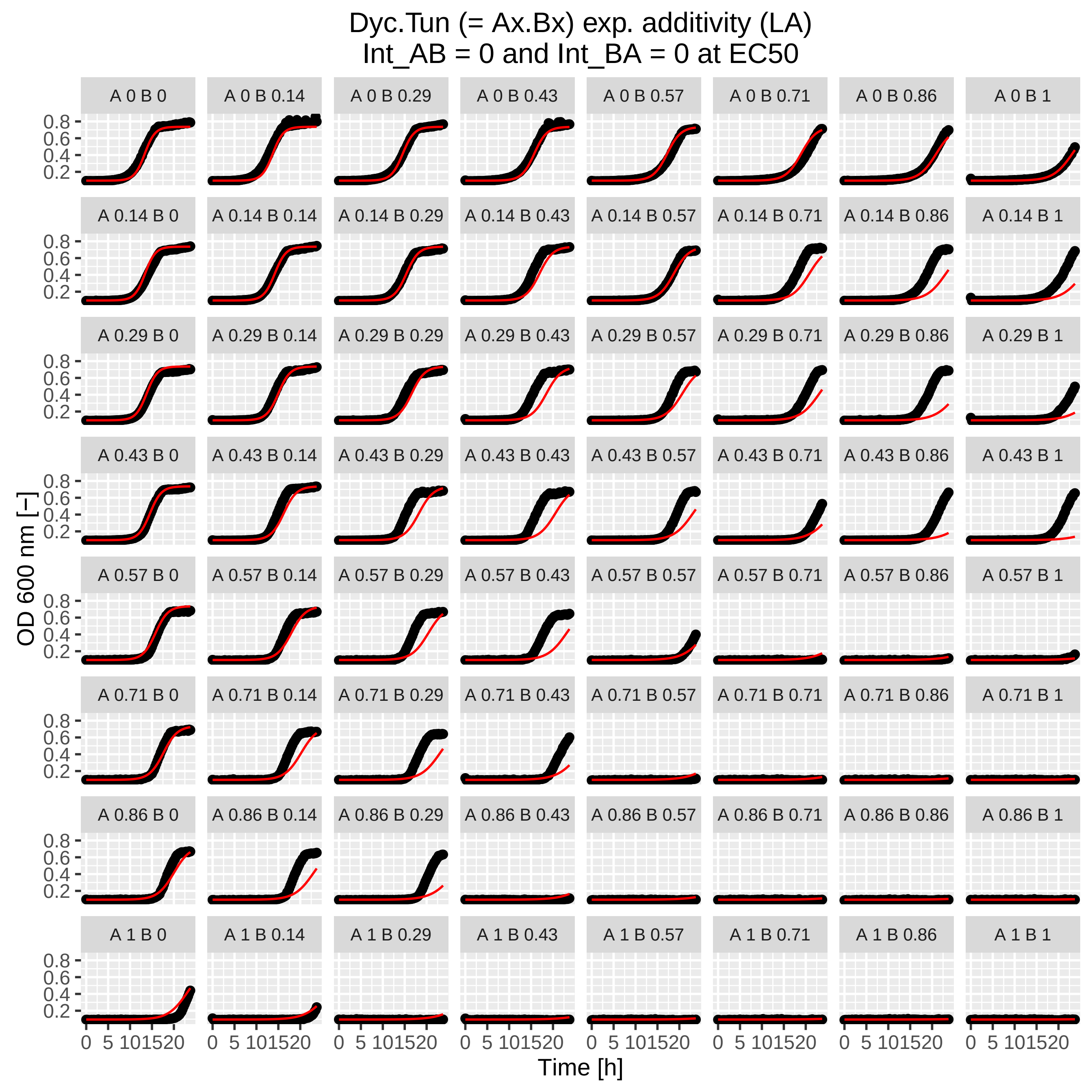

Dyc.Tun (= Ax.Bx) exp. additivity (LA)
Int_AB = 0 and Int_BA = 0 at EC50
A 0 B 0
A 0 B 0.14
A 0 B 0.29
A 0 B 0.43
A 0 B 0.57
A 0 B 0.71
A 0 B 0.86
A 0 B 1
A 0.14 B 0
A 0.14 B 0.14
A 0.14 B 0.29
A 0.14 B 0.43
A 0.14 B 0.57
A 0.14 B 0.71
A 0.14 B 0.86
A 0.14 B 1
A 0.29 B 0
A 0.29 B 0.14
A 0.29 B 0.29
A 0.29 B 0.43
A 0.29 B 0.57
A 0.29 B 0.71
A 0.29 B 0.86
A 0.29 B 1
A 0.43 B 0
A 0.43 B 0.14
A 0.43 B 0.29
A 0.43 B 0.43
A 0.43 B 0.57
A 0.43 B 0.71
A 0.43 B 0.86
A 0.43 B 1
A 0.57 B 0
A 0.57 B 0.14
A 0.57 B 0.29
A 0.57 B 0.43
A 0.57 B 0.57
A 0.57 B 0.71
A 0.57 B 0.86
A 0.57 B 1
A 0.71 B 0
A 0.71 B 0.14
A 0.71 B 0.29
A 0.71 B 0.43
A 0.71 B 0.57
A 0.71 B 0.71
A 0.71 B 0.86
A 0.71 B 1
A 0.86 B 0
A 0.86 B 0.14
A 0.86 B 0.29
A 0.86 B 0.43
A 0.86 B 0.57
A 0.86 B 0.71
A 0.86 B 0.86
A 0.86 B 1
A 1 B 0
A 1 B 0.14
A 1 B 0.29
A 1 B 0.43
A 1 B 0.57
A 1 B 0.71
A 1 B 0.86
A 1 B 1
OD 600 nm [−]
0.2
0.4
0.6
0.8
0
5
10
15
20
Time [h]

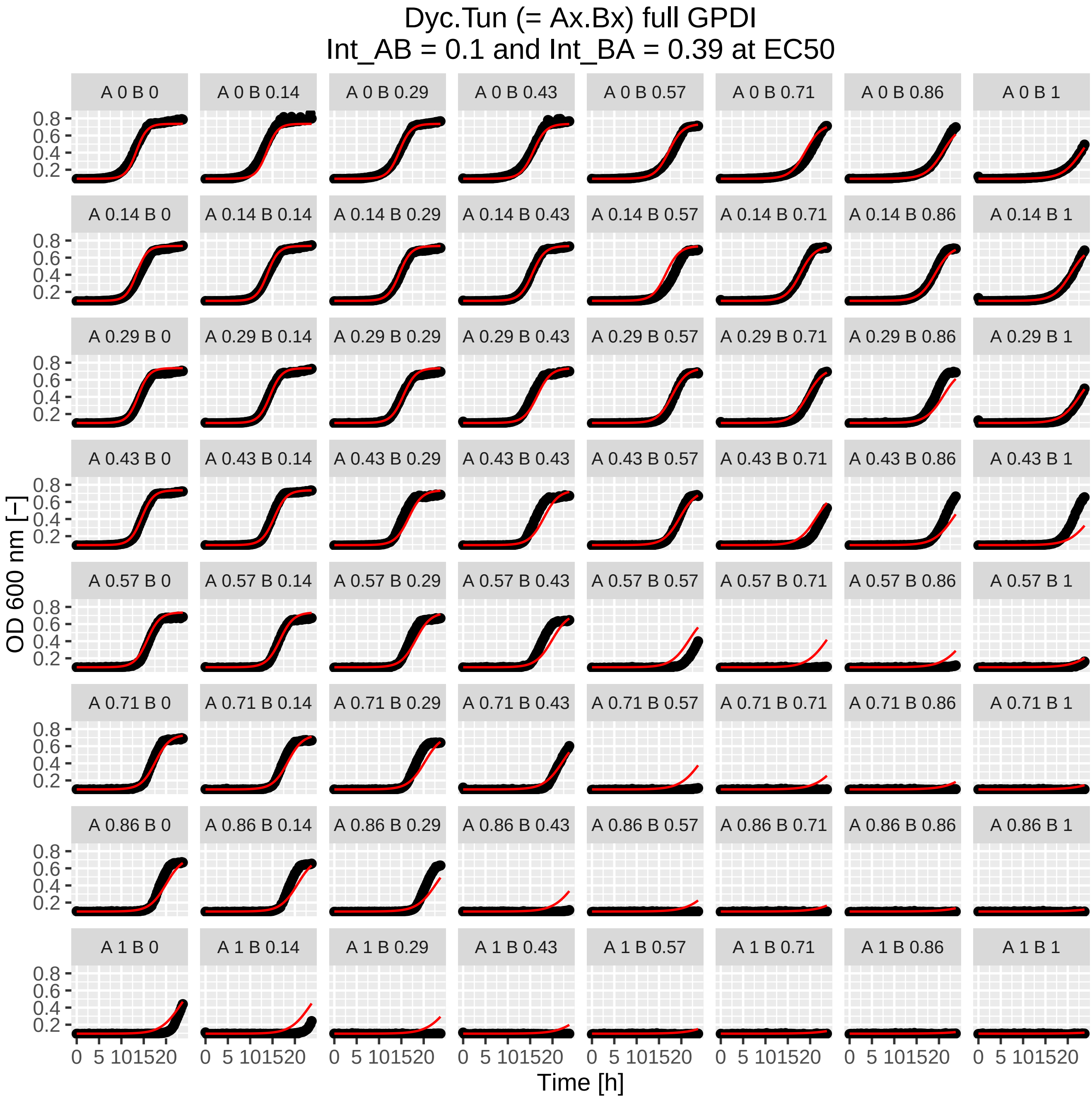
Dyc.Tun (= Ax.Bx) full GPDI
Int_AB = 0.1 and Int_BA = 0.39 at EC50
A 0 B 0
A 0 B 0.14
A 0 B 0.29
A 0 B 0.43
A 0 B 0.57
A 0 B 0.71
A 0 B 0.86
A 0 B 1
A 0.14 B 0
A 0.14 B 0.14
A 0.14 B 0.29
A 0.14 B 0.43
A 0.14 B 0.57
A 0.14 B 0.71
A 0.14 B 0.86
A 0.14 B 1
A 0.29 B 0
A 0.29 B 0.14
A 0.29 B 0.29
A 0.29 B 0.43
A 0.29 B 0.57
A 0.29 B 0.71
A 0.29 B 0.86
A 0.29 B 1
A 0.43 B 0
A 0.43 B 0.14
A 0.43 B 0.29
A 0.43 B 0.43
A 0.43 B 0.57
A 0.43 B 0.71
A 0.43 B 0.86
A 0.43 B 1
A 0.57 B 0
A 0.57 B 0.14
A 0.57 B 0.29
A 0.57 B 0.43
A 0.57 B 0.57
A 0.57 B 0.71
A 0.57 B 0.86
A 0.57 B 1
A 0.71 B 0
A 0.71 B 0.14
A 0.71 B 0.29
A 0.71 B 0.43
A 0.71 B 0.57
A 0.71 B 0.71
A 0.71 B 0.86
A 0.71 B 1
A 0.86 B 0
A 0.86 B 0.14
A 0.86 B 0.29
A 0.86 B 0.43
A 0.86 B 0.57
A 0.86 B 0.71
A 0.86 B 0.86
A 0.86 B 1
A 1 B 0
A 1 B 0.14
A 1 B 0.29
A 1 B 0.43
A 1 B 0.57
A 1 B 0.71
A 1 B 0.86
A 1 B 1
0.8
0.6
0.4
0.2
0 5 101520
OD 600 nm [–]
Time [h]

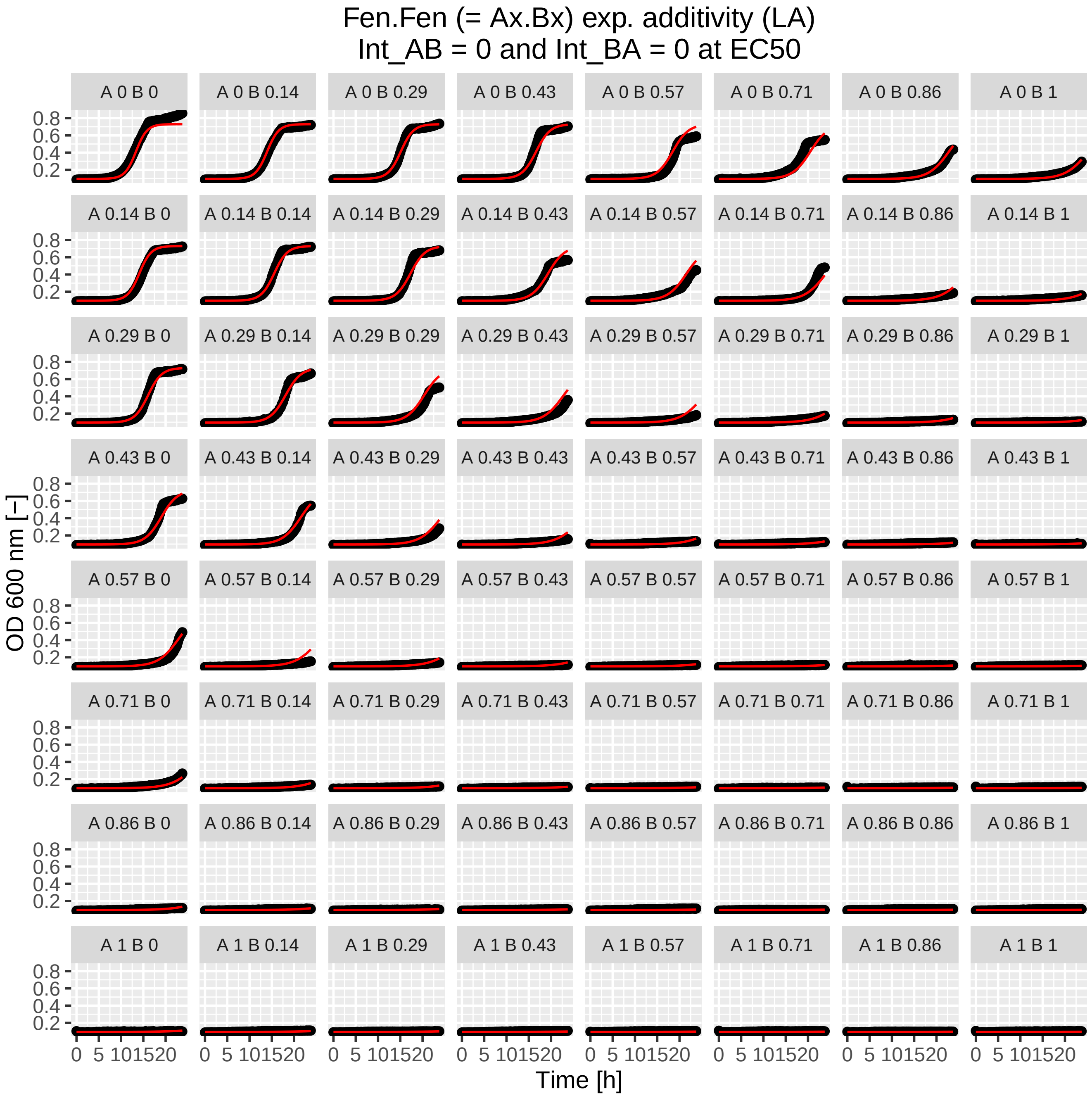
Fen.Fen (= Ax.Bx) exp. additivity (LA)
Int_AB = 0 and Int_BA = 0 at EC50
OD 600 nm [–]
Time [h]
A 0 B 0
A 0 B 0.14
A 0 B 0.29
A 0 B 0.43
A 0 B 0.57
A 0 B 0.71
A 0 B 0.86
A 0 B 1
A 1 B 0
A 1 B 1

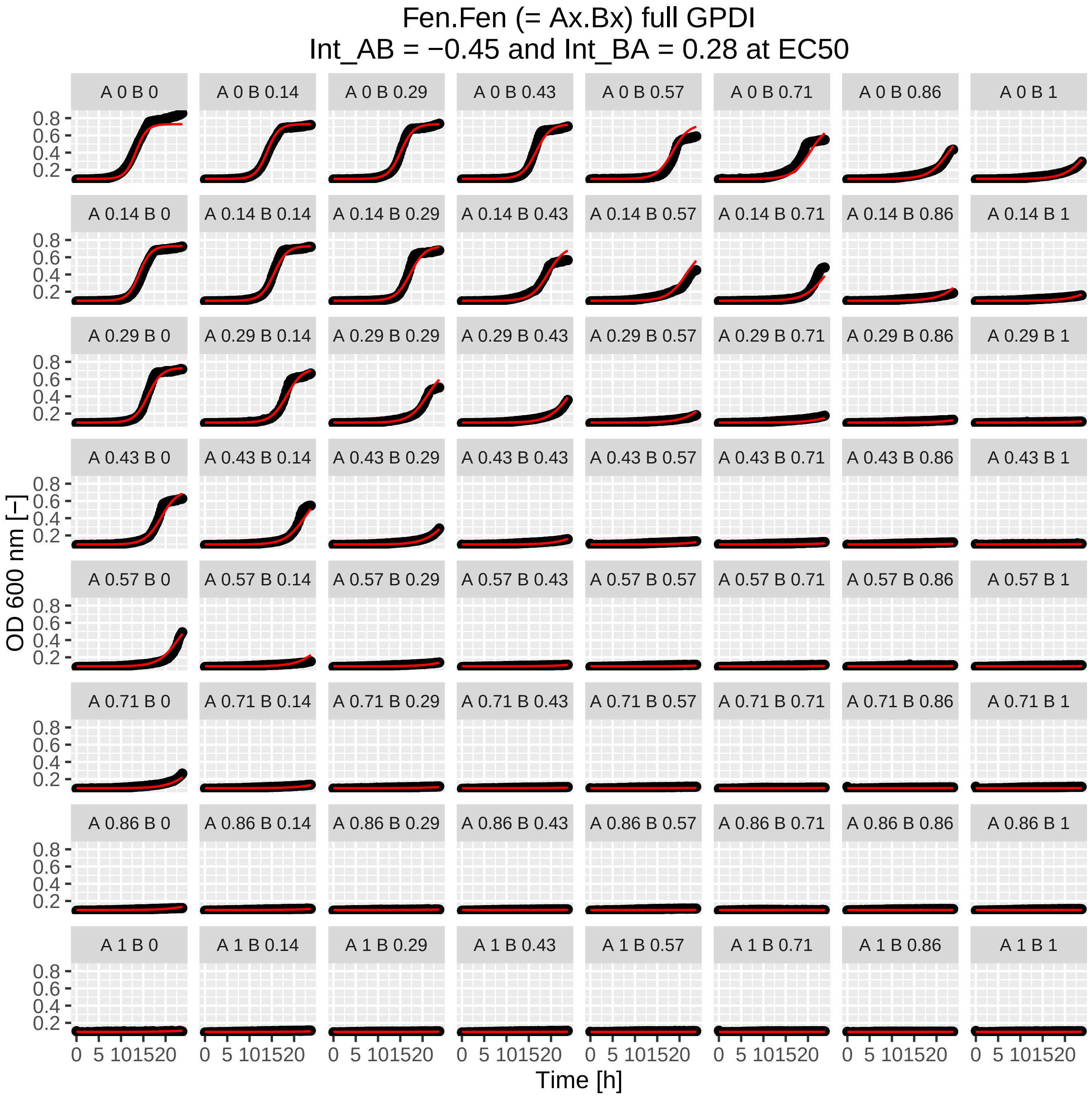

Fen.Fen (= Ax.Bx) full GPDI
Int_AB = −0.45 and Int_BA = 0.28 at EC50
A 0 B 0
A 0 B 0.14
A 0 B 0.29
A 0 B 0.43
A 0 B 0.57
A 0 B 0.71
A 0 B 0.86
A 0 B 1
A 0.14 B 0
A 0.14 B 0.14
A 0.14 B 0.29
A 0.14 B 0.43
A 0.14 B 0.57
A 0.14 B 0.71
A 0.14 B 0.86
A 0.14 B 1
A 0.29 B 0
A 0.29 B 0.14
A 0.29 B 0.29
A 0.29 B 0.43
A 0.29 B 0.57
A 0.29 B 0.71
A 0.29 B 0.86
A 0.29 B 1
A 0.43 B 0
A 0.43 B 0.14
A 0.43 B 0.29
A 0.43 B 0.43
A 0.43 B 0.57
A 0.43 B 0.71
A 0.43 B 0.86
A 0.43 B 1
A 0.57 B 0
A 0.57 B 0.14
A 0.57 B 0.29
A 0.57 B 0.43
A 0.57 B 0.57
A 0.57 B 0.71
A 0.57 B 0.86
A 0.57 B 1
A 0.71 B 0
A 0.71 B 0.14
A 0.71 B 0.29
A 0.71 B 0.43
A 0.71 B 0.57
A 0.71 B 0.71
A 0.71 B 0.86
A 0.71 B 1
A 0.86 B 0
A 0.86 B 0.14
A 0.86 B 0.29
A 0.86 B 0.43
A 0.86 B 0.57
A 0.86 B 0.71
A 0.86 B 0.86
A 0.86 B 1
A 1 B 0
A 1 B 0.14
A 1 B 0.29
A 1 B 0.43
A 1 B 0.57
A 1 B 0.71
A 1 B 0.86
A 1 B 1
0.8
0.6
0.4
0.2
OD 600 nm [−]
0 5 101520
Time [h]

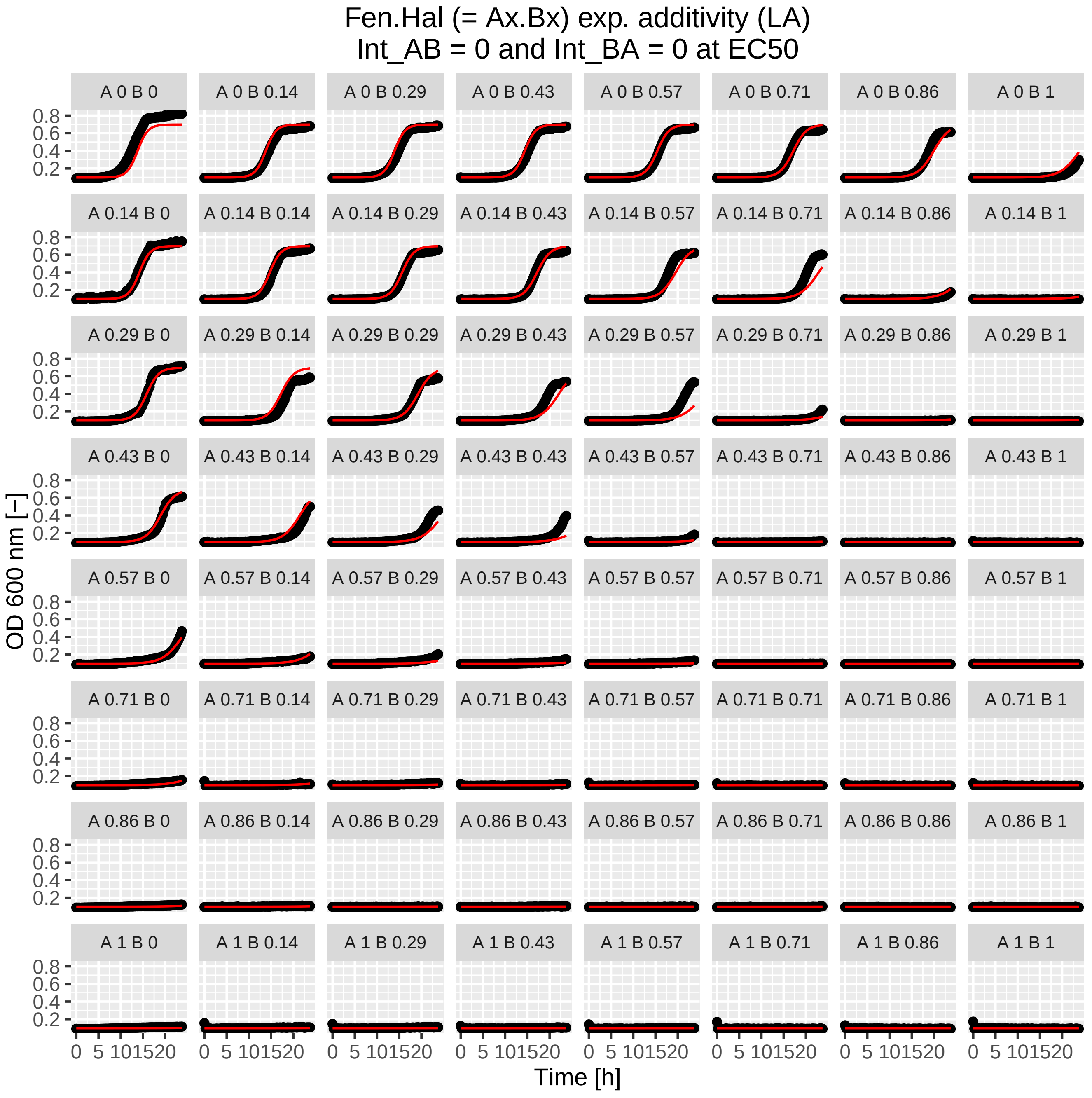
Fen.Hal (= Ax.Bx) exp. additivity (LA)
Int_AB = 0 and Int_BA = 0 at EC50
OD 600 nm [−]
Time [h]
A 0 B 0
A 0 B 0.14
A 0 B 0.29
A 0 B 0.43
A 0 B 0.57
A 0 B 0.71
A 0 B 0.86
A 0 B 1
A 0.14 B 0
A 0.29 B 0
A 0.43 B 0
A 0.57 B 0
A 0.71 B 0
A 0.86 B 0
A 1 B 0
A 1 B 1
0.2
0.4
0.6
0.8
0 5 101520

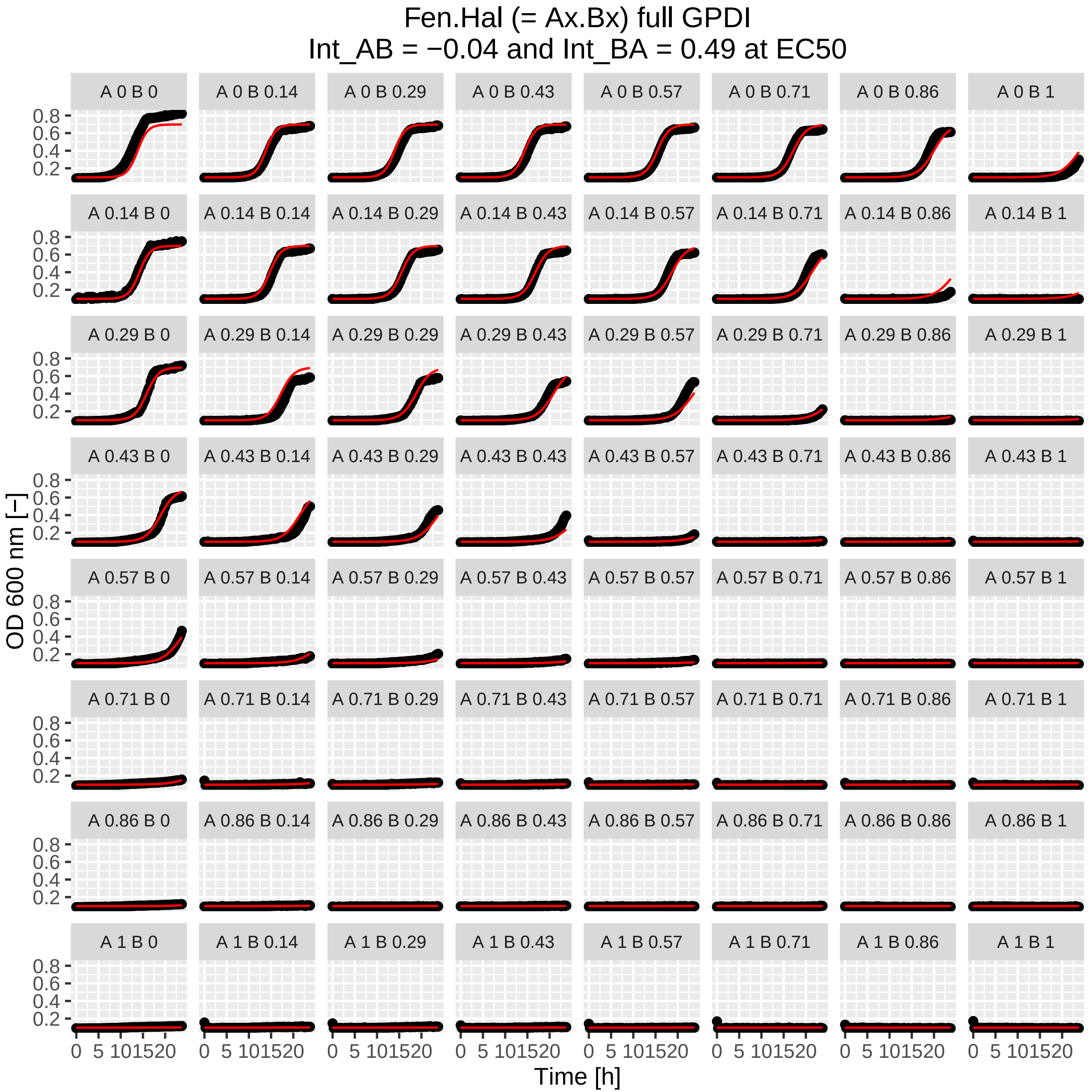
Fen.Hal (= Ax.Bx) full GPDI
Int_AB = −0.04 and Int_BA = 0.49 at EC50
OD 600 nm [−]
Time [h]
A 0 B 0
A 0 B 0.14
A 0 B 0.29
A 0 B 0.43
A 0 B 0.57
A 0 B 0.71
A 0 B 0.86
A 0 B 1
A 0.14 B 0
A 0.14 B 0.14
A 0.14 B 0.29
A 0.14 B 0.43
A 0.14 B 0.57
A 0.14 B 0.71
A 0.14 B 0.86
A 0.14 B 1
A 0.29 B 0
A 0.29 B 0.14
A 0.29 B 0.29
A 0.29 B 0.43
A 0.29 B 0.57
A 0.29 B 0.71
A 0.29 B 0.86
A 0.29 B 1
A 0.43 B 0
A 0.43 B 0.14
A 0.43 B 0.29
A 0.43 B 0.43
A 0.43 B 0.57
A 0.43 B 0.71
A 0.43 B 0.86
A 0.43 B 1
A 0.57 B 0
A 0.57 B 0.14
A 0.57 B 0.29
A 0.57 B 0.43
A 0.57 B 0.57
A 0.57 B 0.71
A 0.57 B 0.86
A 0.57 B 1
A 0.71 B 0
A 0.71 B 0.14
A 0.71 B 0.29
A 0.71 B 0.43
A 0.71 B 0.57
A 0.71 B 0.71
A 0.71 B 0.86
A 0.71 B 1
A 0.86 B 0
A 0.86 B 0.14
A 0.86 B 0.29
A 0.86 B 0.43
A 0.86 B 0.57
A 0.86 B 0.71
A 0.86 B 0.86
A 0.86 B 1
A 1 B 0
A 1 B 0.14
A 1 B 0.29
A 1 B 0.43
A 1 B 0.57
A 1 B 0.71
A 1 B 0.86
A 1 B 1
0.8
0.6
0.4
0.2
0 5 101520

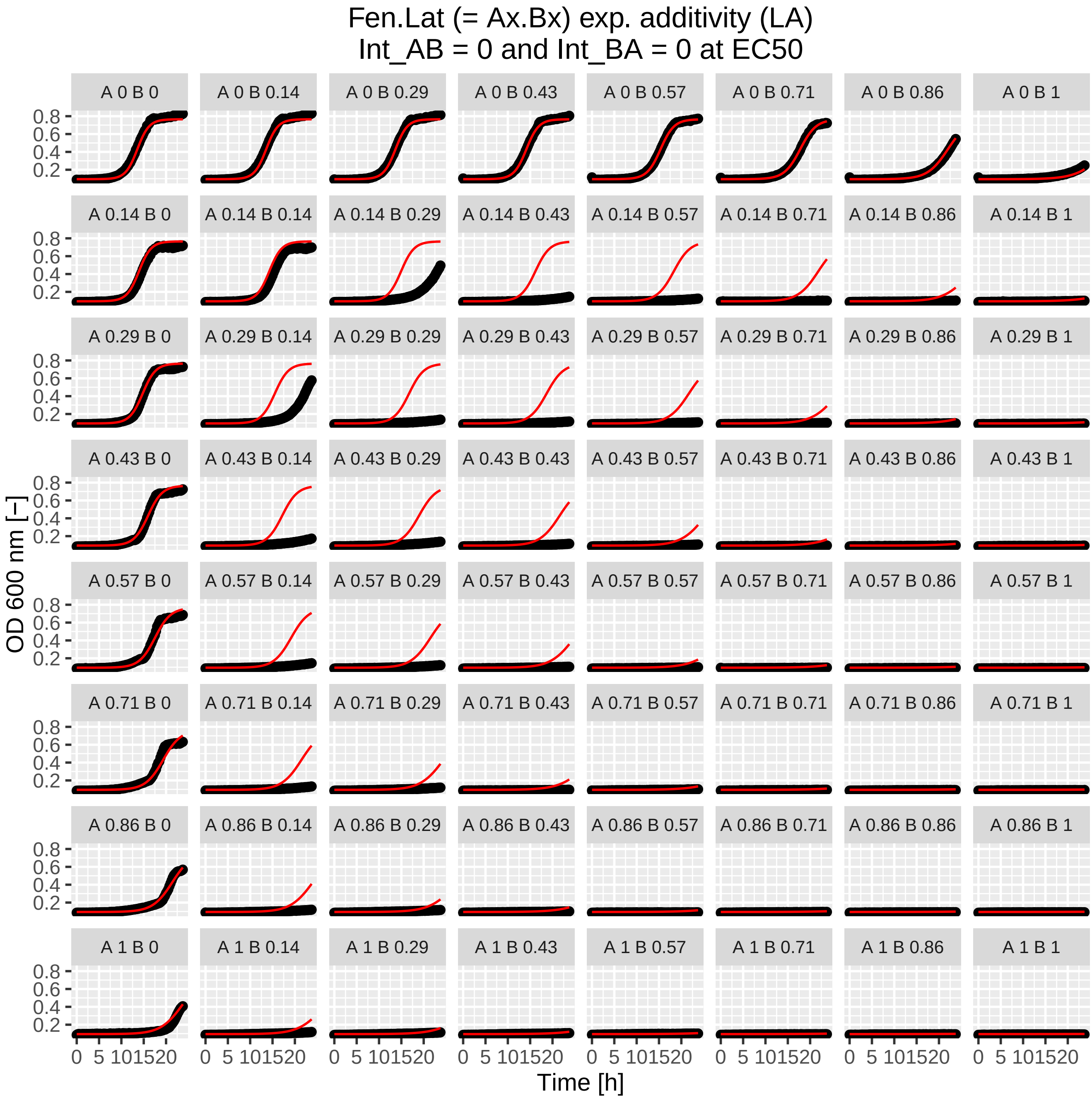

Fen.Lat (= Ax.Bx) exp. additivity (LA)
Int_AB = 0 and Int_BA = 0 at EC50
A 0 B 0
A 0 B 0.14
A 0 B 0.29
A 0 B 0.43
A 0 B 0.57
A 0 B 0.71
A 0 B 0.86
A 0 B 1
A 0.14 B 0
A 0.14 B 0.14
A 0.14 B 0.29
A 0.14 B 0.43
A 0.14 B 0.57
A 0.14 B 0.71
A 0.14 B 0.86
A 0.14 B 1
A 0.29 B 0
A 0.29 B 0.14
A 0.29 B 0.29
A 0.29 B 0.43
A 0.29 B 0.57
A 0.29 B 0.71
A 0.29 B 0.86
A 0.29 B 1
A 0.43 B 0
A 0.43 B 0.14
A 0.43 B 0.29
A 0.43 B 0.43
A 0.43 B 0.57
A 0.43 B 0.71
A 0.43 B 0.86
A 0.43 B 1
A 0.57 B 0
A 0.57 B 0.14
A 0.57 B 0.29
A 0.57 B 0.43
A 0.57 B 0.57
A 0.57 B 0.71
A 0.57 B 0.86
A 0.57 B 1
A 0.71 B 0
A 0.71 B 0.14
A 0.71 B 0.29
A 0.71 B 0.43
A 0.71 B 0.57
A 0.71 B 0.71
A 0.71 B 0.86
A 0.71 B 1
A 0.86 B 0
A 0.86 B 0.14
A 0.86 B 0.29
A 0.86 B 0.43
A 0.86 B 0.57
A 0.86 B 0.71
A 0.86 B 0.86
A 0.86 B 1
A 1 B 0
A 1 B 0.14
A 1 B 0.29
A 1 B 0.43
A 1 B 0.57
A 1 B 0.71
A 1 B 0.86
A 1 B 1
0.8
0.6
0.4
0.2
OD 600 nm [−]
0 5 101520
Time [h]

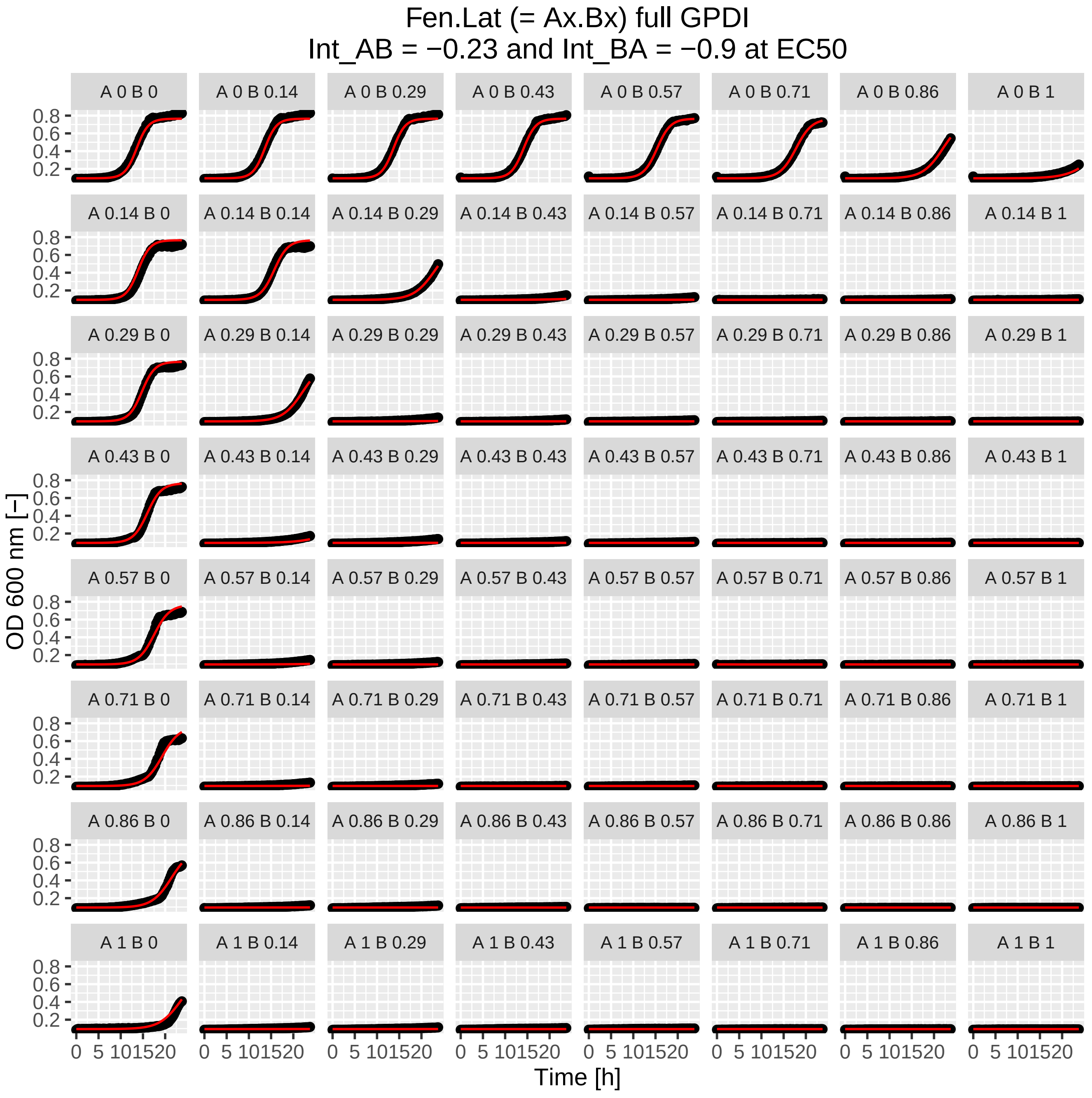
Fen.Lat (= Ax.Bx) full GPDI
Int_AB = −0.23 and Int_BA = −0.9 at EC50
A 0 B 0
A 0 B 0.14
A 0 B 0.29
A 0 B 0.43
A 0 B 0.57
A 0 B 0.71
A 0 B 0.86
A 0 B 1
A 0.14 B 0
A 0.14 B 0.14
A 0.14 B 0.29
A 0.14 B 0.43
A 0.14 B 0.57
A 0.14 B 0.71
A 0.14 B 0.86
A 0.14 B 1
A 0.29 B 0
A 0.29 B 0.14
A 0.29 B 0.29
A 0.29 B 0.43
A 0.29 B 0.57
A 0.29 B 0.71
A 0.29 B 0.86
A 0.29 B 1
A 0.43 B 0
A 0.43 B 0.14
A 0.43 B 0.29
A 0.43 B 0.43
A 0.43 B 0.57
A 0.43 B 0.71
A 0.43 B 0.86
A 0.43 B 1
A 0.57 B 0
A 0.57 B 0.14
A 0.57 B 0.29
A 0.57 B 0.43
A 0.57 B 0.57
A 0.57 B 0.71
A 0.57 B 0.86
A 0.57 B 1
A 0.71 B 0
A 0.71 B 0.14
A 0.71 B 0.29
A 0.71 B 0.43
A 0.71 B 0.57
A 0.71 B 0.71
A 0.71 B 0.86
A 0.71 B 1
A 0.86 B 0
A 0.86 B 0.14
A 0.86 B 0.29
A 0.86 B 0.43
A 0.86 B 0.57
A 0.86 B 0.71
A 0.86 B 0.86
A 0.86 B 1
A 1 B 0
A 1 B 0.14
A 1 B 0.29
A 1 B 0.43
A 1 B 0.57
A 1 B 0.71
A 1 B 0.86
A 1 B 1
OD 600 nm [−]
0.2
0.4
0.6
0.8
0 5 101520
Time [h]

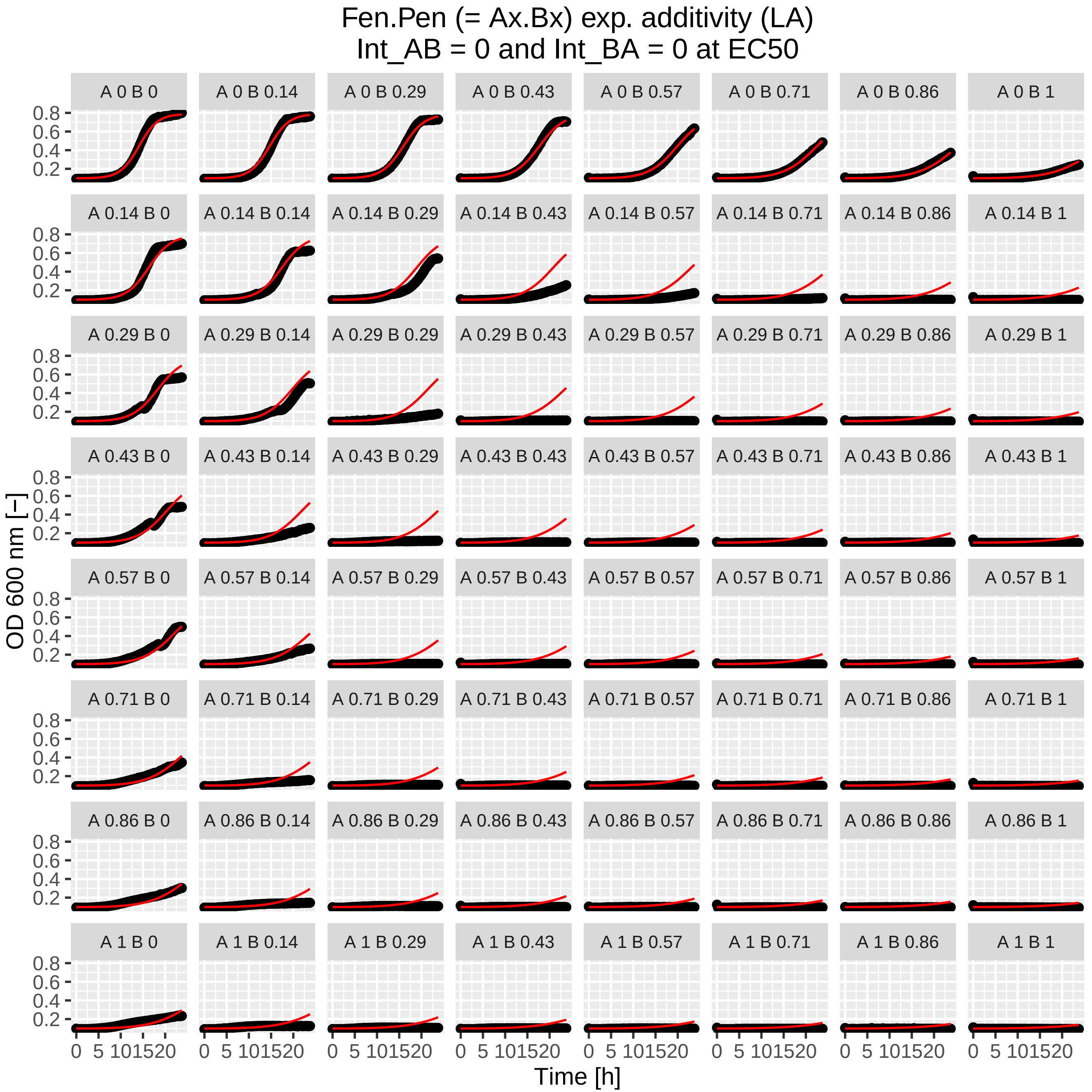
Fen.Pen (= Ax.Bx) exp. additivity (LA)
Int_AB = 0 and Int_BA = 0 at EC50
OD 600 nm [–]
Time [h]
A 0 B 0
A 0 B 0.14
A 0 B 0.29
A 0 B 0.43
A 0 B 0.57
A 0 B 0.71
A 0 B 0.86
A 0 B 1
A 1 B 0
A 1 B 0.14
A 1 B 0.29
A 1 B 0.43
A 1 B 0.57
A 1 B 0.71
A 1 B 0.86
A 1 B 1
0.8
0.6
0.4
0.2
0 5 101520

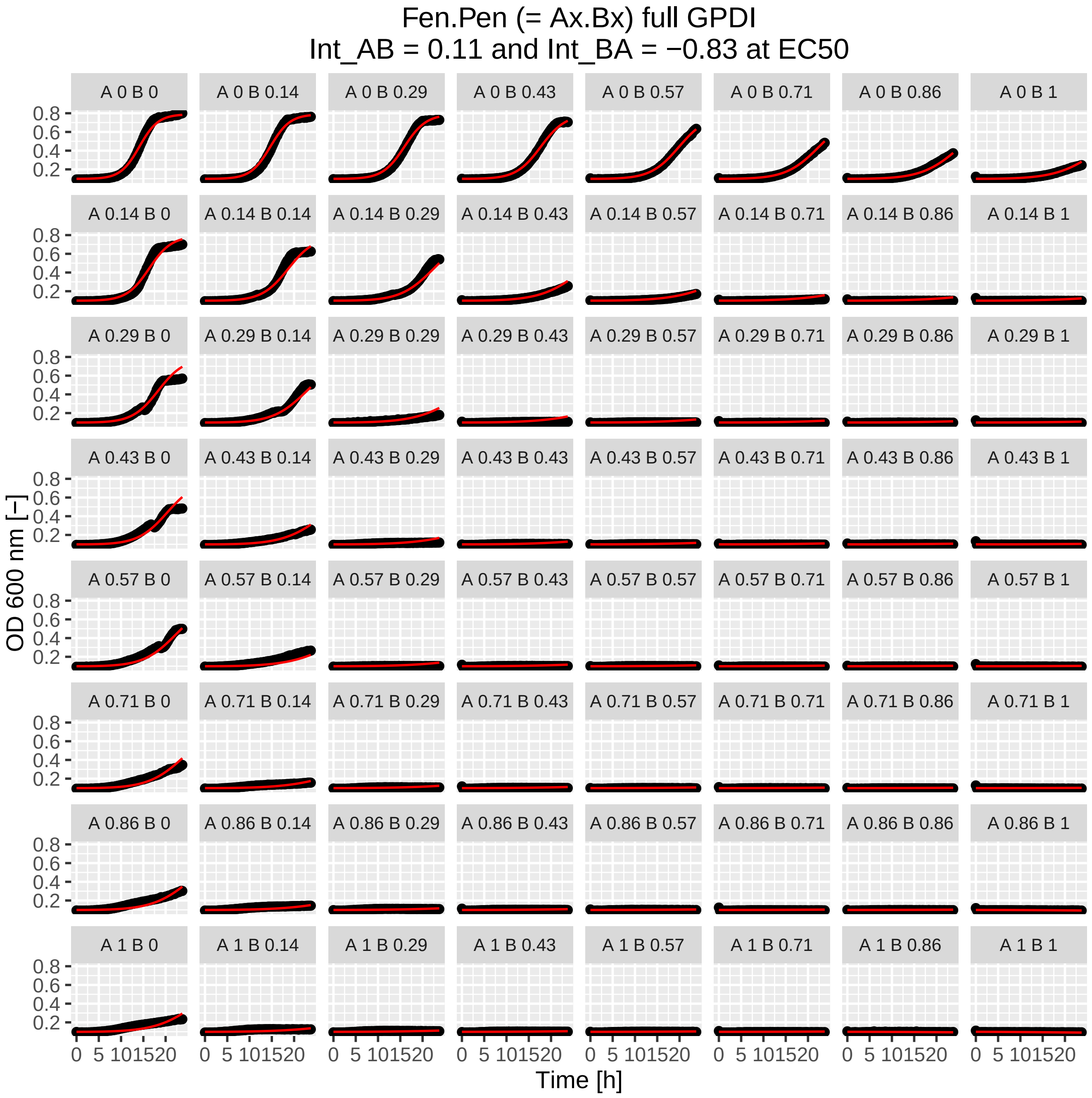

Fen.Pen (= Ax.Bx) full GPDI
Int_AB = 0.11 and Int_BA = −0.83 at EC50
OD 600 nm [−]
Time [h]

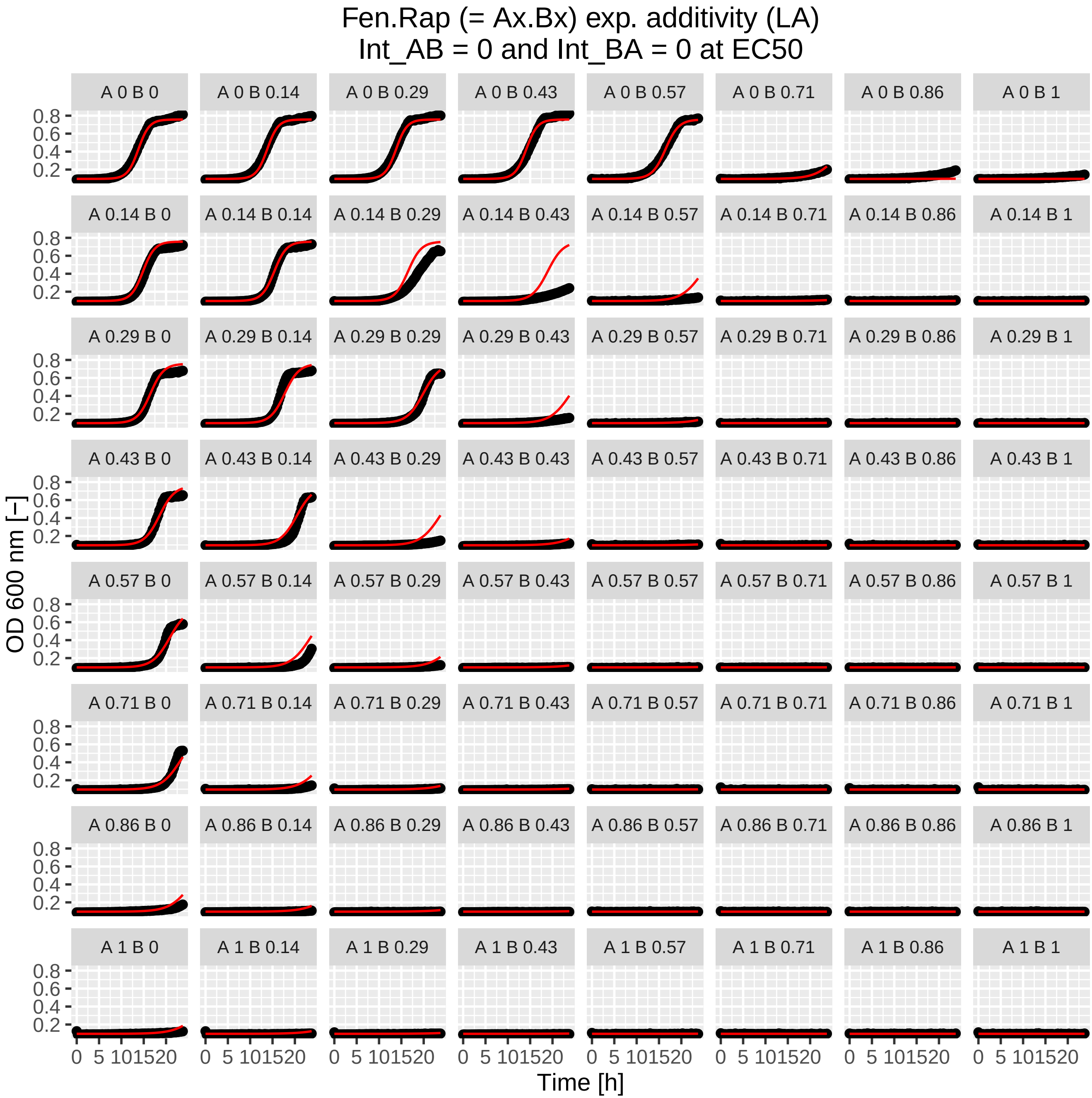
Fen.Rap (= Ax.Bx) exp. additivity (LA)
Int_AB = 0 and Int_BA = 0 at EC50
A 0 B 0
A 0 B 0.14
A 0 B 0.29
A 0 B 0.43
A 0 B 0.57
A 0 B 0.71
A 0 B 0.86
A 0 B 1
A 0.14 B 0
A 0.14 B 0.14
A 0.14 B 0.29
A 0.14 B 0.43
A 0.14 B 0.57
A 0.14 B 0.71
A 0.14 B 0.86
A 0.14 B 1
A 0.29 B 0
A 0.29 B 0.14
A 0.29 B 0.29
A 0.29 B 0.43
A 0.29 B 0.57
A 0.29 B 0.71
A 0.29 B 0.86
A 0.29 B 1
A 0.43 B 0
A 0.43 B 0.14
A 0.43 B 0.29
A 0.43 B 0.43
A 0.43 B 0.57
A 0.43 B 0.71
A 0.43 B 0.86
A 0.43 B 1
A 0.57 B 0
A 0.57 B 0.14
A 0.57 B 0.29
A 0.57 B 0.43
A 0.57 B 0.57
A 0.57 B 0.71
A 0.57 B 0.86
A 0.57 B 1
A 0.71 B 0
A 0.71 B 0.14
A 0.71 B 0.29
A 0.71 B 0.43
A 0.71 B 0.57
A 0.71 B 0.71
A 0.71 B 0.86
A 0.71 B 1
A 0.86 B 0
A 0.86 B 0.14
A 0.86 B 0.29
A 0.86 B 0.43
A 0.86 B 0.57
A 0.86 B 0.71
A 0.86 B 0.86
A 0.86 B 1
A 1 B 0
A 1 B 0.14
A 1 B 0.29
A 1 B 0.43
A 1 B 0.57
A 1 B 0.71
A 1 B 0.86
A 1 B 1
0.8
0.6
0.4
0.2
OD 600 nm [−]
0 5 101520
Time [h]

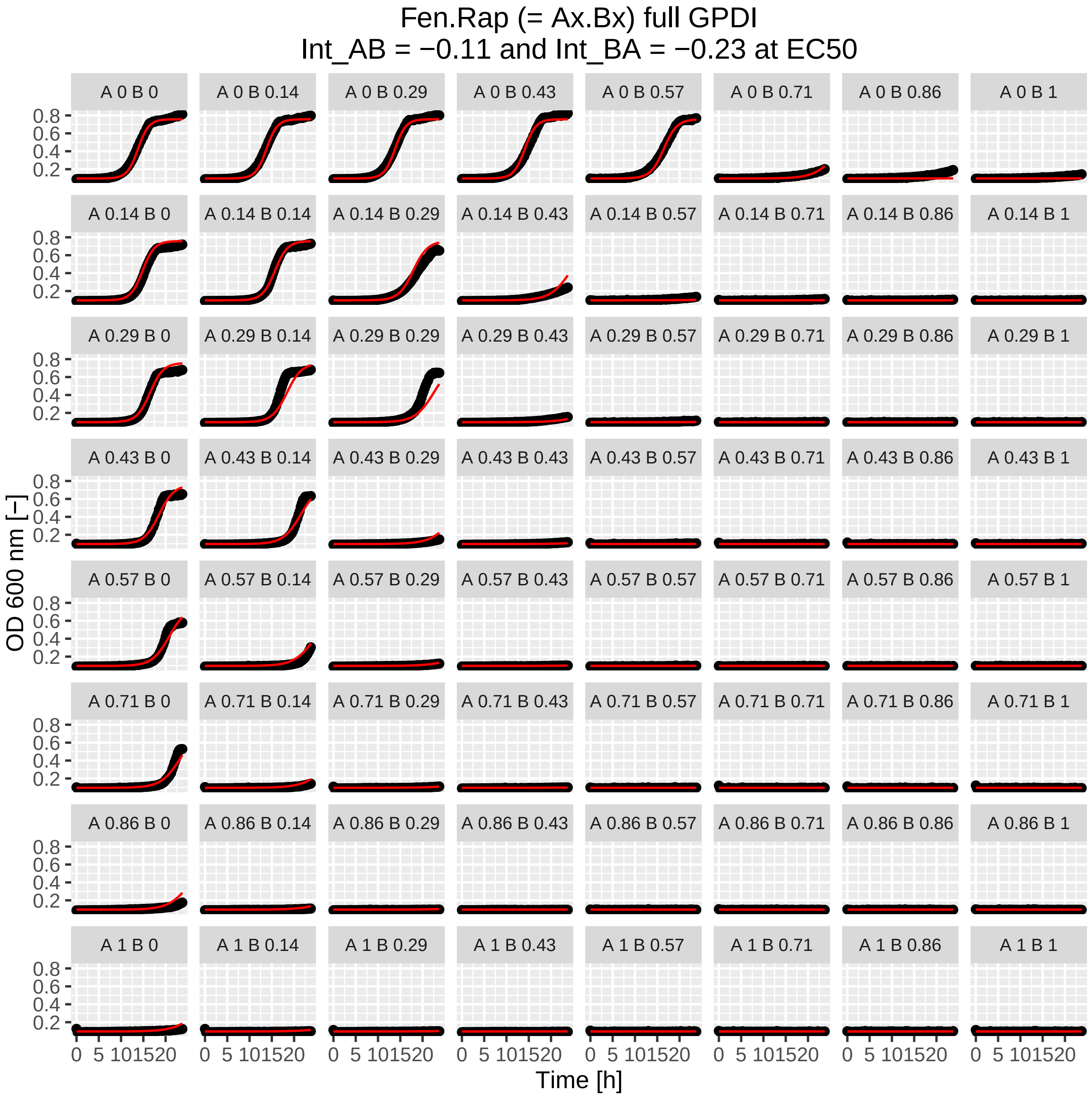
Fen.Rap (= Ax.Bx) full GPDI
Int_AB = −0.11 and Int_BA = −0.23 at EC50
OD 600 nm [−]
Time [h]
A 0 B 0
A 0 B 0.14
A 0 B 0.29
A 0 B 0.43
A 0 B 0.57
A 0 B 0.71
A 0 B 0.86
A 0 B 1
A 0.14 B 0
A 0.14 B 0.14
A 0.14 B 0.29
A 0.14 B 0.43
A 0.14 B 0.57
A 0.14 B 0.71
A 0.14 B 0.86
A 0.14 B 1
A 0.29 B 0
A 0.29 B 0.14
A 0.29 B 0.29
A 0.29 B 0.43
A 0.29 B 0.57
A 0.29 B 0.71
A 0.29 B 0.86
A 0.29 B 1
A 0.43 B 0
A 0.43 B 0.14
A 0.43 B 0.29
A 0.43 B 0.43
A 0.43 B 0.57
A 0.43 B 0.71
A 0.43 B 0.86
A 0.43 B 1
A 0.57 B 0
A 0.57 B 0.14
A 0.57 B 0.29
A 0.57 B 0.43
A 0.57 B 0.57
A 0.57 B 0.71
A 0.57 B 0.86
A 0.57 B 1
A 0.71 B 0
A 0.71 B 0.14
A 0.71 B 0.29
A 0.71 B 0.43
A 0.71 B 0.57
A 0.71 B 0.71
A 0.71 B 0.86
A 0.71 B 1
A 0.86 B 0
A 0.86 B 0.14
A 0.86 B 0.29
A 0.86 B 0.43
A 0.86 B 0.57
A 0.86 B 0.71
A 0.86 B 0.86
A 0.86 B 1
A 1 B 0
A 1 B 0.14
A 1 B 0.29
A 1 B 0.43
A 1 B 0.57
A 1 B 0.71
A 1 B 0.86
A 1 B 1
0.8
0.6
0.4
0.2
0 5 101520

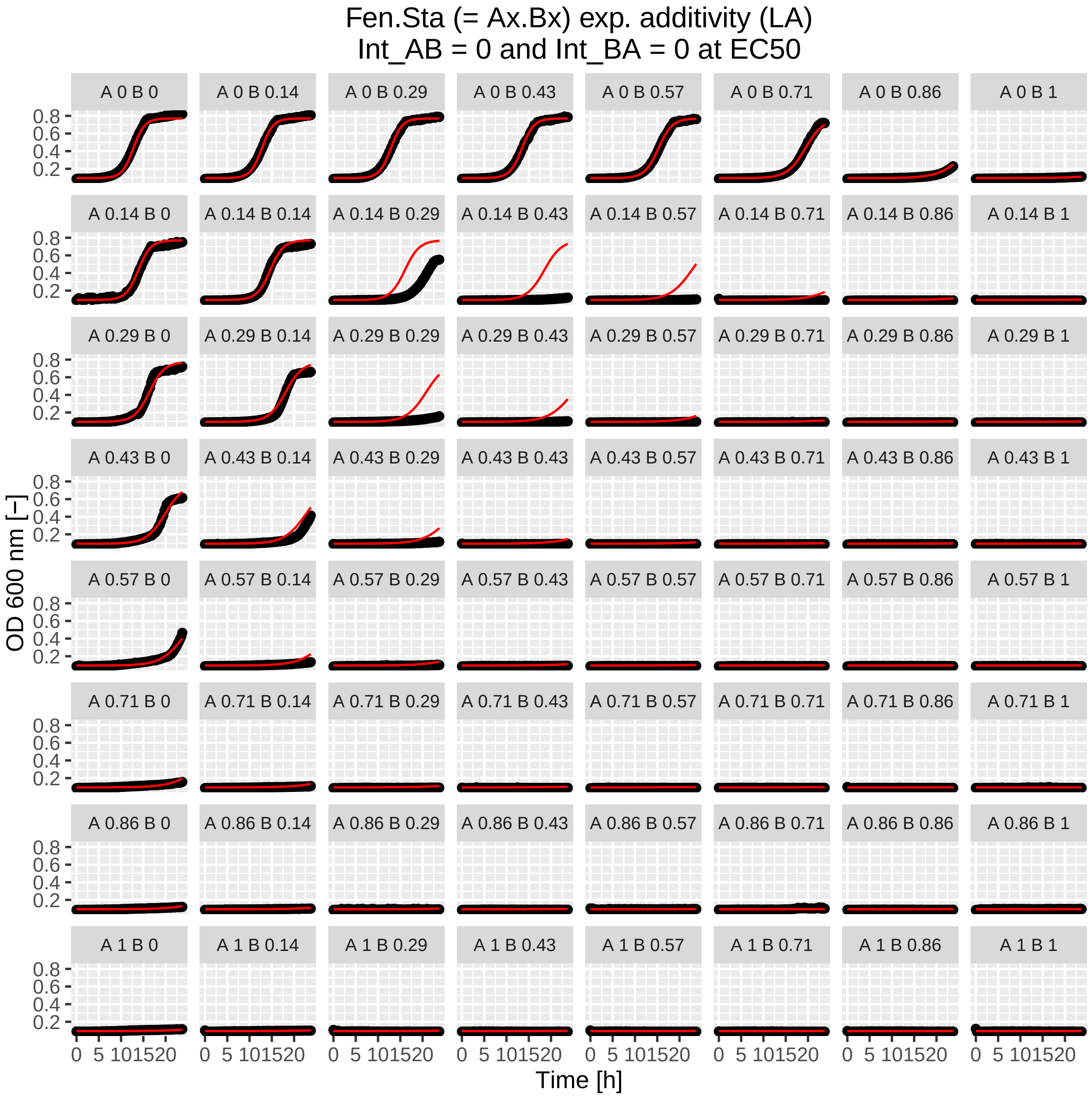
Fen.Sta (= Ax.Bx) exp. additivity (LA)
Int_AB = 0 and Int_BA = 0 at EC50
OD 600 nm [–]
Time [h]
0 5 101520
0.2
0.4
0.6
0.8
A 0 B 0
A 0 B 0.14
A 0 B 0.29
A 0 B 0.43
A 0 B 0.57
A 0 B 0.71
A 0 B 0.86
A 0 B 1
A 0.14 B 0
A 0.14 B 0.14
A 0.14 B 0.29
A 0.14 B 0.43
A 0.14 B 0.57
A 0.14 B 0.71
A 0.14 B 0.86
A 0.14 B 1
A 0.29 B 0
A 0.29 B 0.14
A 0.29 B 0.29
A 0.29 B 0.43
A 0.29 B 0.57
A 0.29 B 0.71
A 0.29 B 0.86
A 0.29 B 1
A 0.43 B 0
A 0.43 B 0.14
A 0.43 B 0.29
A 0.43 B 0.43
A 0.43 B 0.57
A 0.43 B 0.71
A 0.43 B 0.86
A 0.43 B 1
A 0.57 B 0
A 0.57 B 0.14
A 0.57 B 0.29
A 0.57 B 0.43
A 0.57 B 0.57
A 0.57 B 0.71
A 0.57 B 0.86
A 0.57 B 1
A 0.71 B 0
A 0.71 B 0.14
A 0.71 B 0.29
A 0.71 B 0.43
A 0.71 B 0.57
A 0.71 B 0.71
A 0.71 B 0.86
A 0.71 B 1
A 0.86 B 0
A 0.86 B 0.14
A 0.86 B 0.29
A 0.86 B 0.43
A 0.86 B 0.57
A 0.86 B 0.71
A 0.86 B 0.86
A 0.86 B 1
A 1 B 0
A 1 B 0.14
A 1 B 0.29
A 1 B 0.43
A 1 B 0.57
A 1 B 0.71
A 1 B 0.86
A 1 B 1

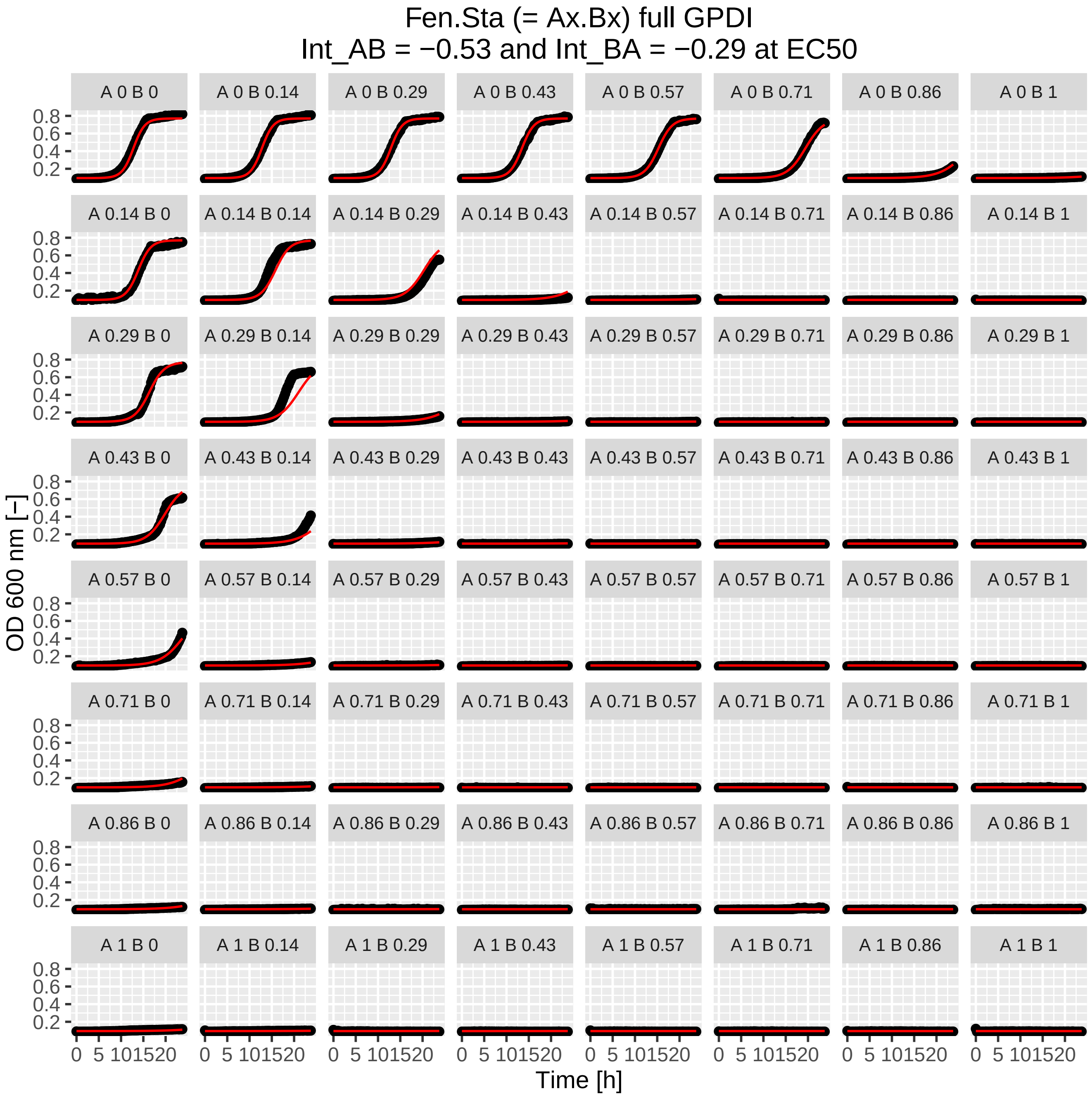

Fen.Sta (= Ax.Bx) full GPDI
Int_AB = −0.53 and Int_BA = −0.29 at EC50
A 0 B 0
A 0 B 0.14
A 0 B 0.29
A 0 B 0.43
A 0 B 0.57
A 0 B 0.71
A 0 B 0.86
A 0 B 1
A 0.14 B 0
A 0.14 B 0.14
A 0.14 B 0.29
A 0.14 B 0.43
A 0.14 B 0.57
A 0.14 B 0.71
A 0.14 B 0.86
A 0.14 B 1
A 0.29 B 0
A 0.29 B 0.14
A 0.29 B 0.29
A 0.29 B 0.43
A 0.29 B 0.57
A 0.29 B 0.71
A 0.29 B 0.86
A 0.29 B 1
A 0.43 B 0
A 0.43 B 0.14
A 0.43 B 0.29
A 0.43 B 0.43
A 0.43 B 0.57
A 0.43 B 0.71
A 0.43 B 0.86
A 0.43 B 1
A 0.57 B 0
A 0.57 B 0.14
A 0.57 B 0.29
A 0.57 B 0.43
A 0.57 B 0.57
A 0.57 B 0.71
A 0.57 B 0.86
A 0.57 B 1
A 0.71 B 0
A 0.71 B 0.14
A 0.71 B 0.29
A 0.71 B 0.43
A 0.71 B 0.57
A 0.71 B 0.71
A 0.71 B 0.86
A 0.71 B 1
A 0.86 B 0
A 0.86 B 0.14
A 0.86 B 0.29
A 0.86 B 0.43
A 0.86 B 0.57
A 0.86 B 0.71
A 0.86 B 0.86
A 0.86 B 1
A 1 B 0
A 1 B 0.14
A 1 B 0.29
A 1 B 0.43
A 1 B 0.57
A 1 B 0.71
A 1 B 0.86
A 1 B 1
0.8
0.6
0.4
0.2
OD 600 nm [−]
0 5 101520
Time [h]

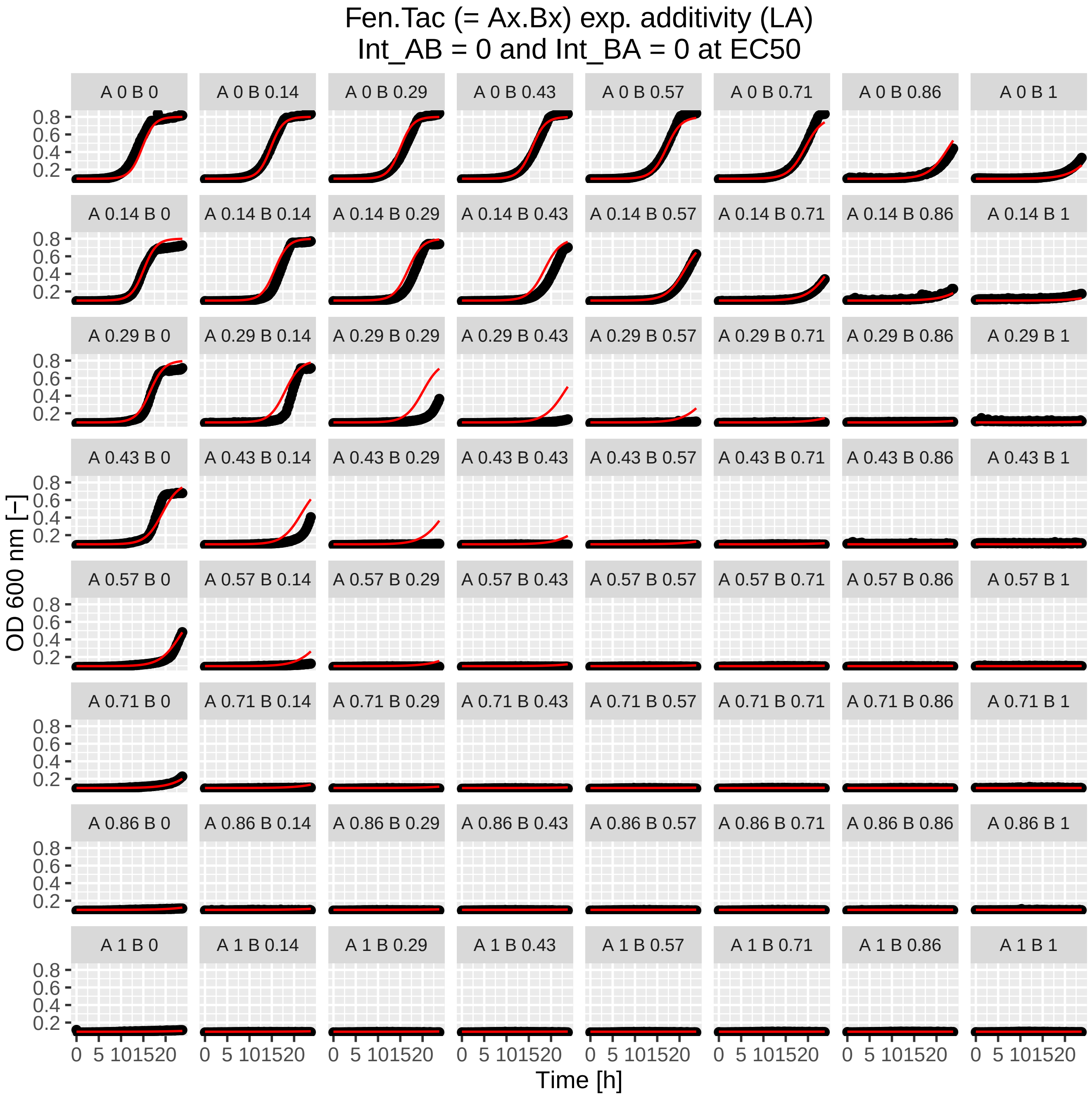

Fen.Tac (= Ax.Bx) exp. additivity (LA)
Int_AB = 0 and Int_BA = 0 at EC50
A 0 B 0
A 0 B 0.14
A 0 B 0.29
A 0 B 0.43
A 0 B 0.57
A 0 B 0.71
A 0 B 0.86
A 0 B 1
A 0.14 B 0
A 0.14 B 0.14
A 0.14 B 0.29
A 0.14 B 0.43
A 0.14 B 0.57
A 0.14 B 0.71
A 0.14 B 0.86
A 0.14 B 1
A 0.29 B 0
A 0.29 B 0.14
A 0.29 B 0.29
A 0.29 B 0.43
A 0.29 B 0.57
A 0.29 B 0.71
A 0.29 B 0.86
A 0.29 B 1
A 0.43 B 0
A 0.43 B 0.14
A 0.43 B 0.29
A 0.43 B 0.43
A 0.43 B 0.57
A 0.43 B 0.71
A 0.43 B 0.86
A 0.43 B 1
A 0.57 B 0
A 0.57 B 0.14
A 0.57 B 0.29
A 0.57 B 0.43
A 0.57 B 0.57
A 0.57 B 0.71
A 0.57 B 0.86
A 0.57 B 1
A 0.71 B 0
A 0.71 B 0.14
A 0.71 B 0.29
A 0.71 B 0.43
A 0.71 B 0.57
A 0.71 B 0.71
A 0.71 B 0.86
A 0.71 B 1
A 0.86 B 0
A 0.86 B 0.14
A 0.86 B 0.29
A 0.86 B 0.43
A 0.86 B 0.57
A 0.86 B 0.71
A 0.86 B 0.86
A 0.86 B 1
A 1 B 0
A 1 B 0.14
A 1 B 0.29
A 1 B 0.43
A 1 B 0.57
A 1 B 0.71
A 1 B 0.86
A 1 B 1
OD 600 nm [–]
0.2
0.4
0.6
0.8
0 5 101520
Time [h]

Fen.Tac (= Ax.Bx) full GPDI
Int_AB = −0.64 and Int_BA = 3.54 at EC50

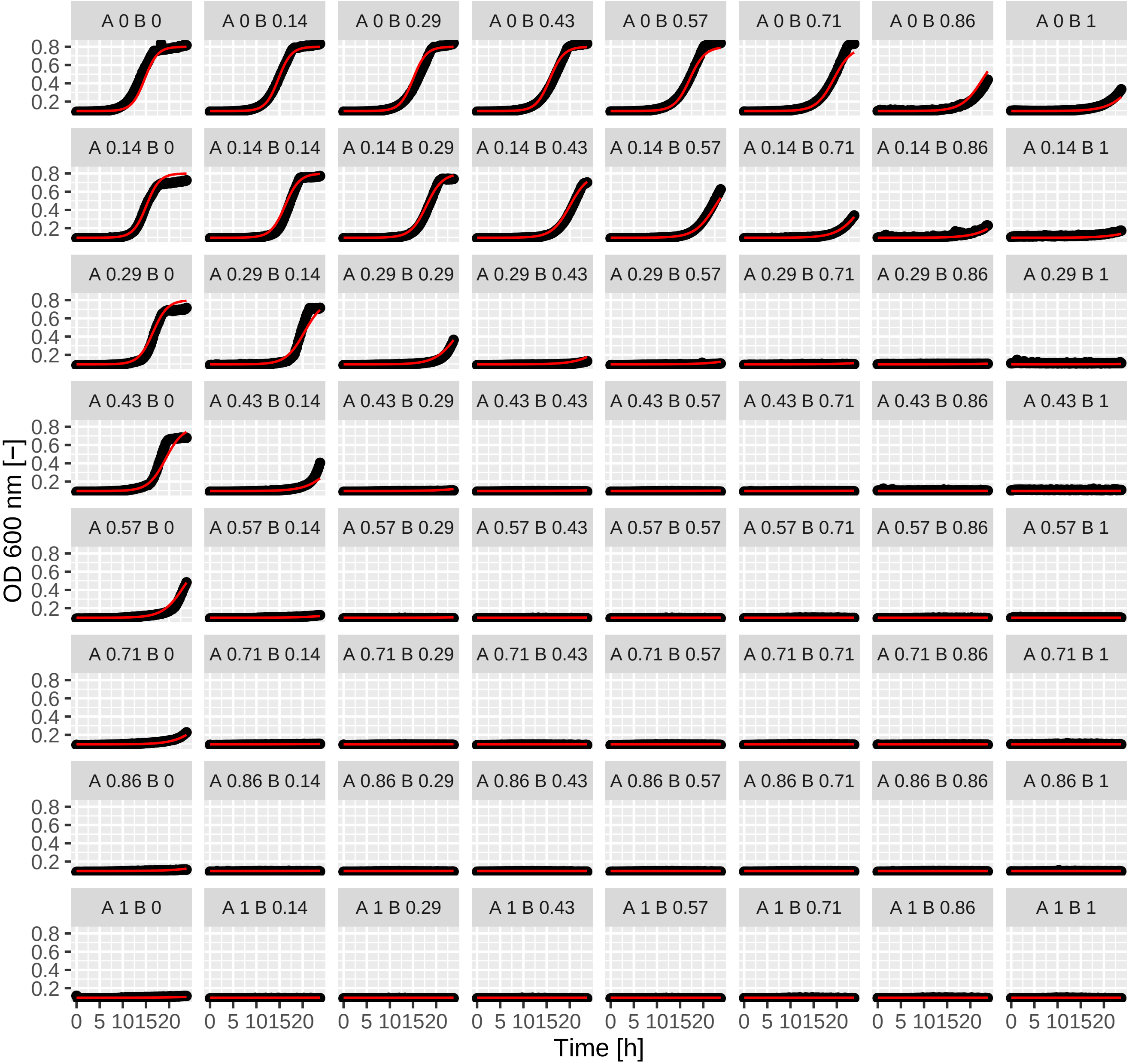

Fen.Ter (= Ax.Bx) exp. additivity (LA)
Int_AB = 0 and Int_BA = 0 at EC50

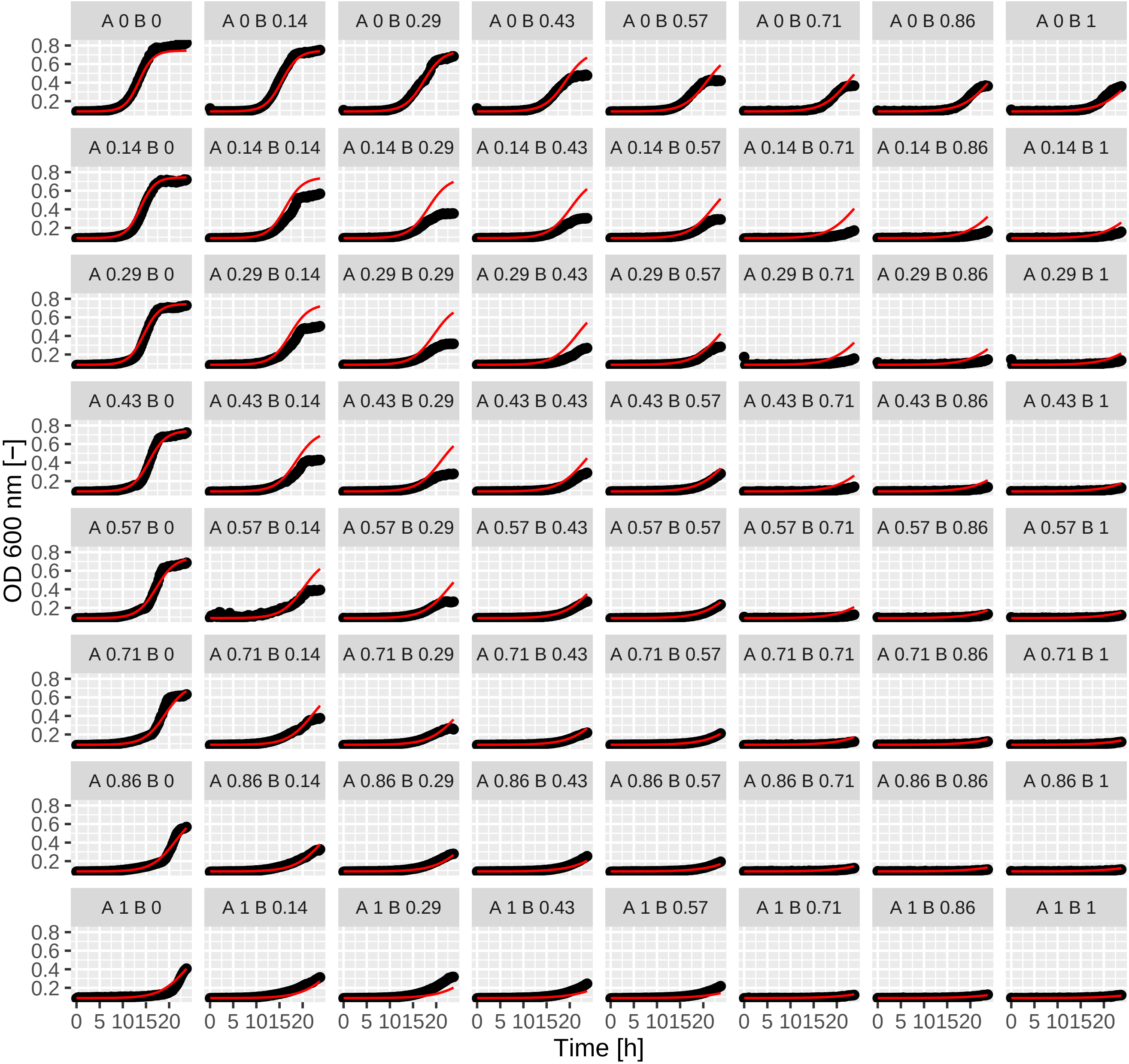

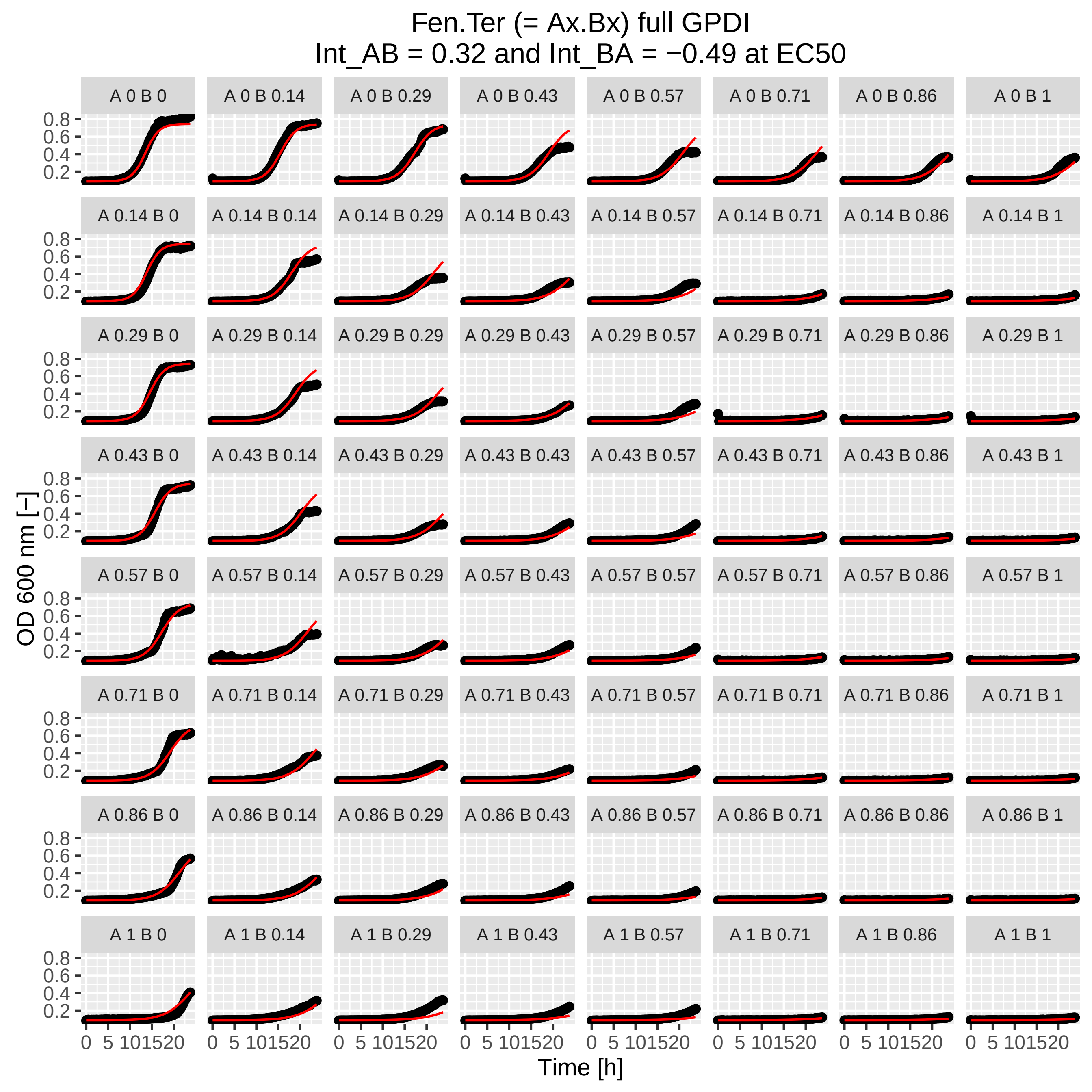

Fen.Ter (= Ax.Bx) full GPDI
Int_AB = 0.32 and Int_BA = −0.49 at EC50
OD 600 nm [−]
Time [h]
A 0 B 0
A 0 B 0.14
A 0 B 0.29
A 0 B 0.43
A 0 B 0.57
A 0 B 0.71
A 0 B 0.86
A 0 B 1
A 0.14 B 0
A 0.14 B 0.14
A 0.14 B 0.29
A 0.14 B 0.43
A 0.14 B 0.57
A 0.14 B 0.71
A 0.14 B 0.86
A 0.14 B 1
A 0.29 B 0
A 0.29 B 0.14
A 0.29 B 0.29
A 0.29 B 0.43
A 0.29 B 0.57
A 0.29 B 0.71
A 0.29 B 0.86
A 0.29 B 1
A 0.43 B 0
A 0.43 B 0.14
A 0.43 B 0.29
A 0.43 B 0.43
A 0.43 B 0.57
A 0.43 B 0.71
A 0.43 B 0.86
A 0.43 B 1
A 0.57 B 0
A 0.57 B 0.14
A 0.57 B 0.29
A 0.57 B 0.43
A 0.57 B 0.57
A 0.57 B 0.71
A 0.57 B 0.86
A 0.57 B 1
A 0.71 B 0
A 0.71 B 0.14
A 0.71 B 0.29
A 0.71 B 0.43
A 0.71 B 0.57
A 0.71 B 0.71
A 0.71 B 0.86
A 0.71 B 1
A 0.86 B 0
A 0.86 B 0.14
A 0.86 B 0.29
A 0.86 B 0.43
A 0.86 B 0.57
A 0.86 B 0.71
A 0.86 B 0.86
A 0.86 B 1
A 1 B 0
A 1 B 0.14
A 1 B 0.29
A 1 B 0.43
A 1 B 0.57
A 1 B 0.71
A 1 B 0.86
A 1 B 1
0.8
0.6
0.4
0.2
0 5 101520

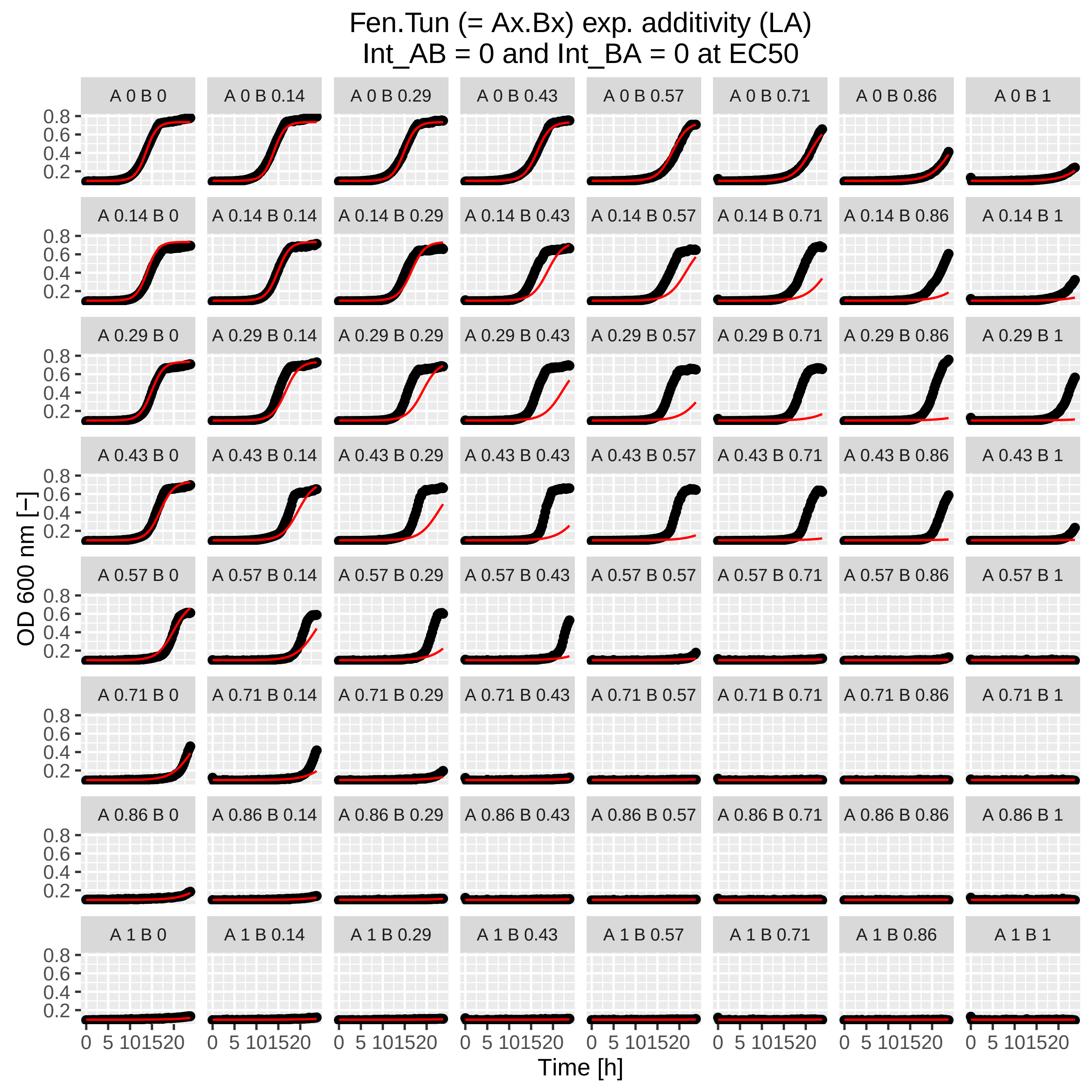

Fen.Tun (= Ax.Bx) exp. additivity (LA)
Int_AB = 0 and Int_BA = 0 at EC50
OD 600 nm [–]
Time [h]
A 0 B 0
A 0 B 0.14
A 0 B 0.29
A 0 B 0.43
A 0 B 0.57
A 0 B 0.71
A 0 B 0.86
A 0 B 1
A 0.14 B 0
A 0.14 B 0.14
A 0.14 B 0.29
A 0.14 B 0.43
A 0.14 B 0.57
A 0.14 B 0.71
A 0.14 B 0.86
A 0.14 B 1
A 0.29 B 0
A 0.29 B 0.14
A 0.29 B 0.29
A 0.29 B 0.43
A 0.29 B 0.57
A 0.29 B 0.71
A 0.29 B 0.86
A 0.29 B 1
A 0.43 B 0
A 0.43 B 0.14
A 0.43 B 0.29
A 0.43 B 0.43
A 0.43 B 0.57
A 0.43 B 0.71
A 0.43 B 0.86
A 0.43 B 1
A 0.57 B 0
A 0.57 B 0.14
A 0.57 B 0.29
A 0.57 B 0.43
A 0.57 B 0.57
A 0.57 B 0.71
A 0.57 B 0.86
A 0.57 B 1
A 0.71 B 0
A 0.71 B 0.14
A 0.71 B 0.29
A 0.71 B 0.43
A 0.71 B 0.57
A 0.71 B 0.71
A 0.71 B 0.86
A 0.71 B 1
A 0.86 B 0
A 0.86 B 0.14
A 0.86 B 0.29
A 0.86 B 0.43
A 0.86 B 0.57
A 0.86 B 0.71
A 0.86 B 0.86
A 0.86 B 1
A 1 B 0
A 1 B 0.14
A 1 B 0.29
A 1 B 0.43
A 1 B 0.57
A 1 B 0.71
A 1 B 0.86
A 1 B 1
0.2
0.4
0.6
0.8
0 5 101520

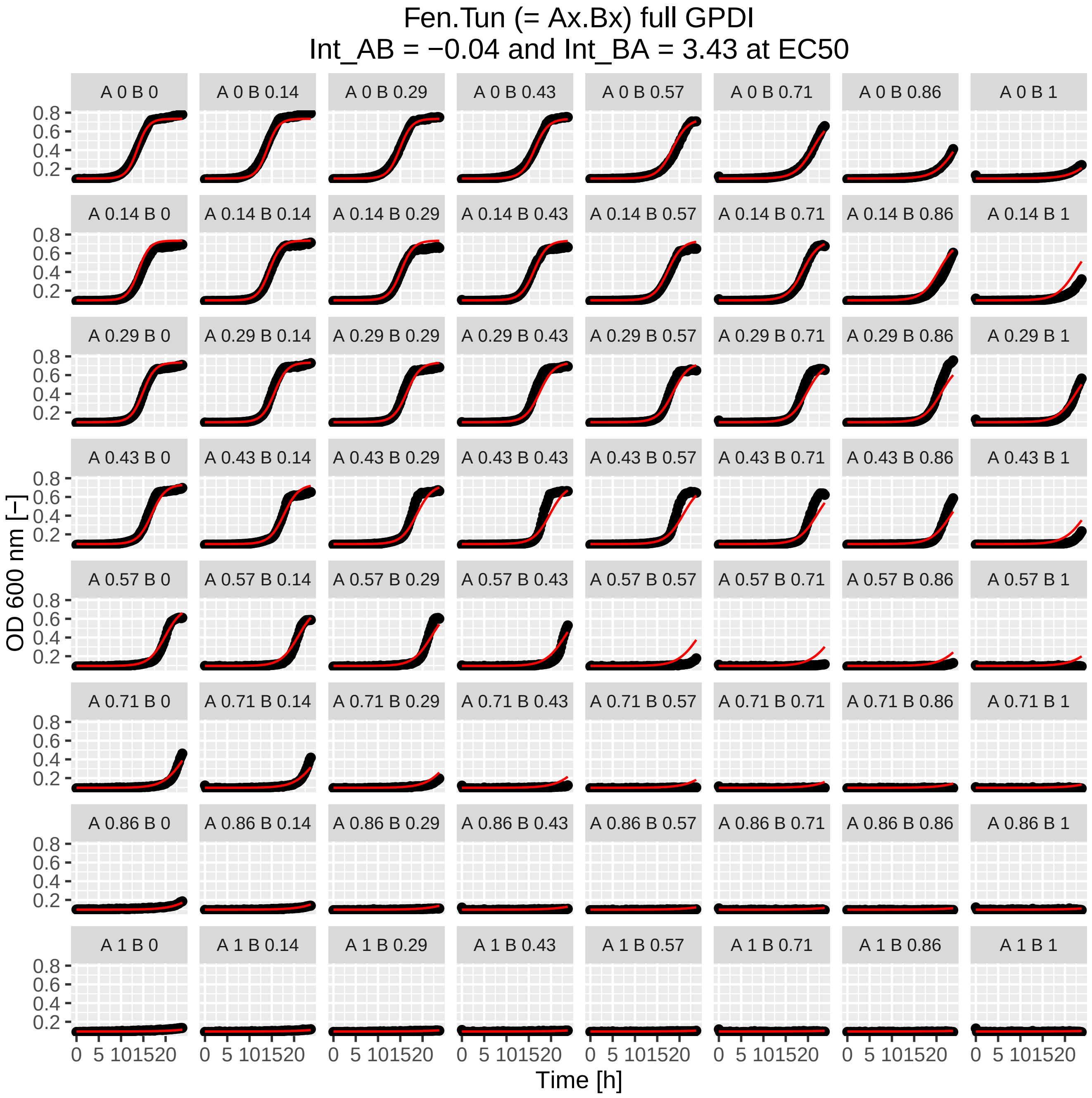
Fen.Tun (= Ax.Bx) full GPDI
Int_AB = −0.04 and Int_BA = 3.43 at EC50
A 0 B 0
A 0 B 0.14
A 0 B 0.29
A 0 B 0.43
A 0 B 0.57
A 0 B 0.71
A 0 B 0.86
A 0 B 1
A 0.14 B 0
A 0.14 B 0.14
A 0.14 B 0.29
A 0.14 B 0.43
A 0.14 B 0.57
A 0.14 B 0.71
A 0.14 B 0.86
A 0.14 B 1
A 0.29 B 0
A 0.29 B 0.14
A 0.29 B 0.29
A 0.29 B 0.43
A 0.29 B 0.57
A 0.29 B 0.71
A 0.29 B 0.86
A 0.29 B 1
A 0.43 B 0
A 0.43 B 0.14
A 0.43 B 0.29
A 0.43 B 0.43
A 0.43 B 0.57
A 0.43 B 0.71
A 0.43 B 0.86
A 0.43 B 1
A 0.57 B 0
A 0.57 B 0.14
A 0.57 B 0.29
A 0.57 B 0.43
A 0.57 B 0.57
A 0.57 B 0.71
A 0.57 B 0.86
A 0.57 B 1
A 0.71 B 0
A 0.71 B 0.14
A 0.71 B 0.29
A 0.71 B 0.43
A 0.71 B 0.57
A 0.71 B 0.71
A 0.71 B 0.86
A 0.71 B 1
A 0.86 B 0
A 0.86 B 0.14
A 0.86 B 0.29
A 0.86 B 0.43
A 0.86 B 0.57
A 0.86 B 0.71
A 0.86 B 0.86
A 0.86 B 1
A 1 B 0
A 1 B 0.14
A 1 B 0.29
A 1 B 0.43
A 1 B 0.57
A 1 B 0.71
A 1 B 0.86
A 1 B 1
OD 600 nm [−]
0.2
0.4
0.6
0.8
0
5
10
15
20
Time [h]

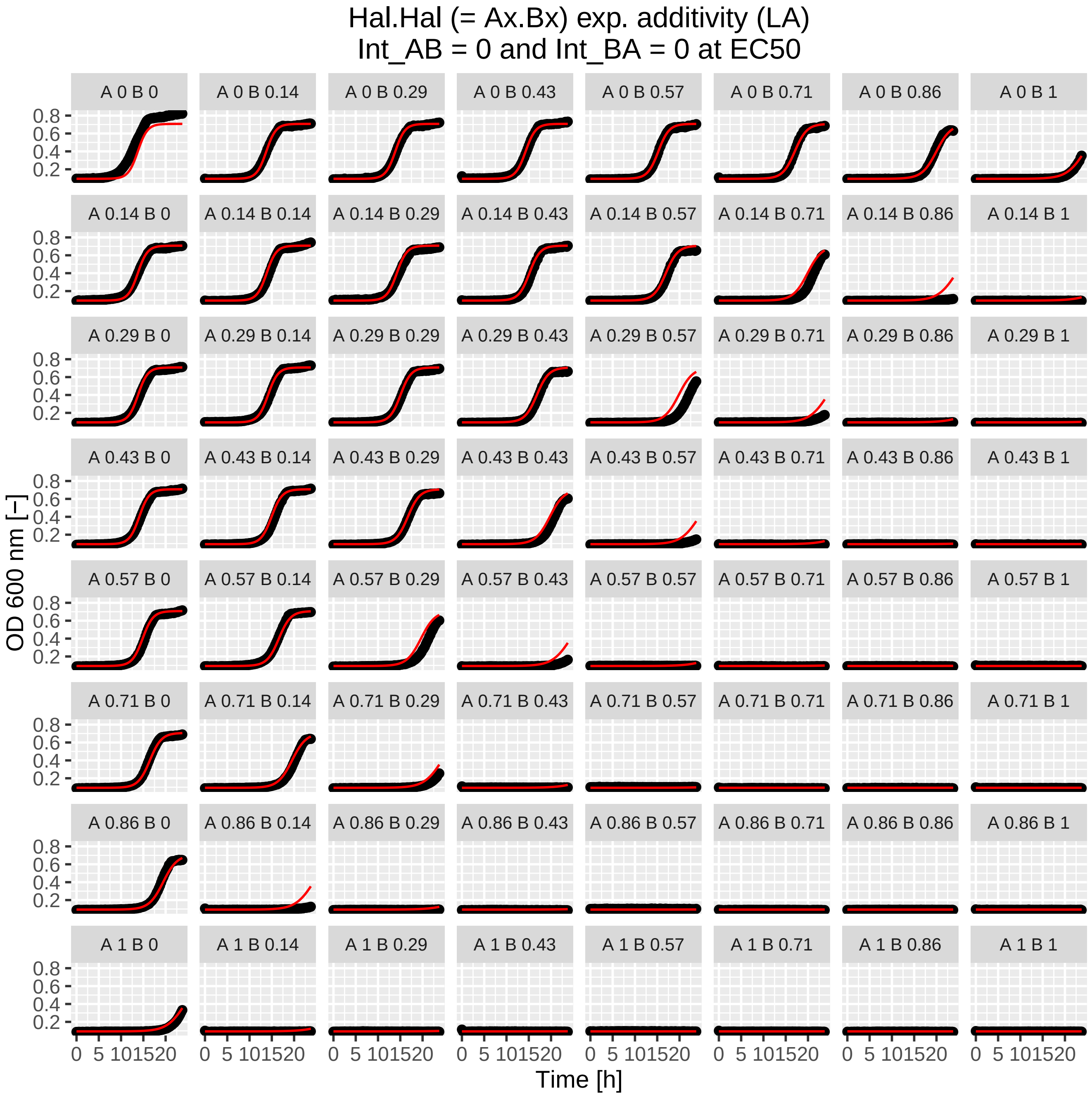
Hal.Hal (= Ax.Bx) exp. additivity (LA)
Int_AB = 0 and Int_BA = 0 at EC50
A 0 B 0
A 0 B 0.14
A 0 B 0.29
A 0 B 0.43
A 0 B 0.57
A 0 B 0.71
A 0 B 0.86
A 0 B 1
A 0.14 B 0
A 0.14 B 0.14
A 0.14 B 0.29
A 0.14 B 0.43
A 0.14 B 0.57
A 0.14 B 0.71
A 0.14 B 0.86
A 0.14 B 1
A 0.29 B 0
A 0.29 B 0.14
A 0.29 B 0.29
A 0.29 B 0.43
A 0.29 B 0.57
A 0.29 B 0.71
A 0.29 B 0.86
A 0.29 B 1
A 0.43 B 0
A 0.43 B 0.14
A 0.43 B 0.29
A 0.43 B 0.43
A 0.43 B 0.57
A 0.43 B 0.71
A 0.43 B 0.86
A 0.43 B 1
A 0.57 B 0
A 0.57 B 0.14
A 0.57 B 0.29
A 0.57 B 0.43
A 0.57 B 0.57
A 0.57 B 0.71
A 0.57 B 0.86
A 0.57 B 1
A 0.71 B 0
A 0.71 B 0.14
A 0.71 B 0.29
A 0.71 B 0.43
A 0.71 B 0.57
A 0.71 B 0.71
A 0.71 B 0.86
A 0.71 B 1
A 0.86 B 0
A 0.86 B 0.14
A 0.86 B 0.29
A 0.86 B 0.43
A 0.86 B 0.57
A 0.86 B 0.71
A 0.86 B 0.86
A 0.86 B 1
A 1 B 0
A 1 B 0.14
A 1 B 0.29
A 1 B 0.43
A 1 B 0.57
A 1 B 0.71
A 1 B 0.86
A 1 B 1
0.8
0.6
0.4
0.2
OD 600 nm [–]
0 5 101520
Time [h]

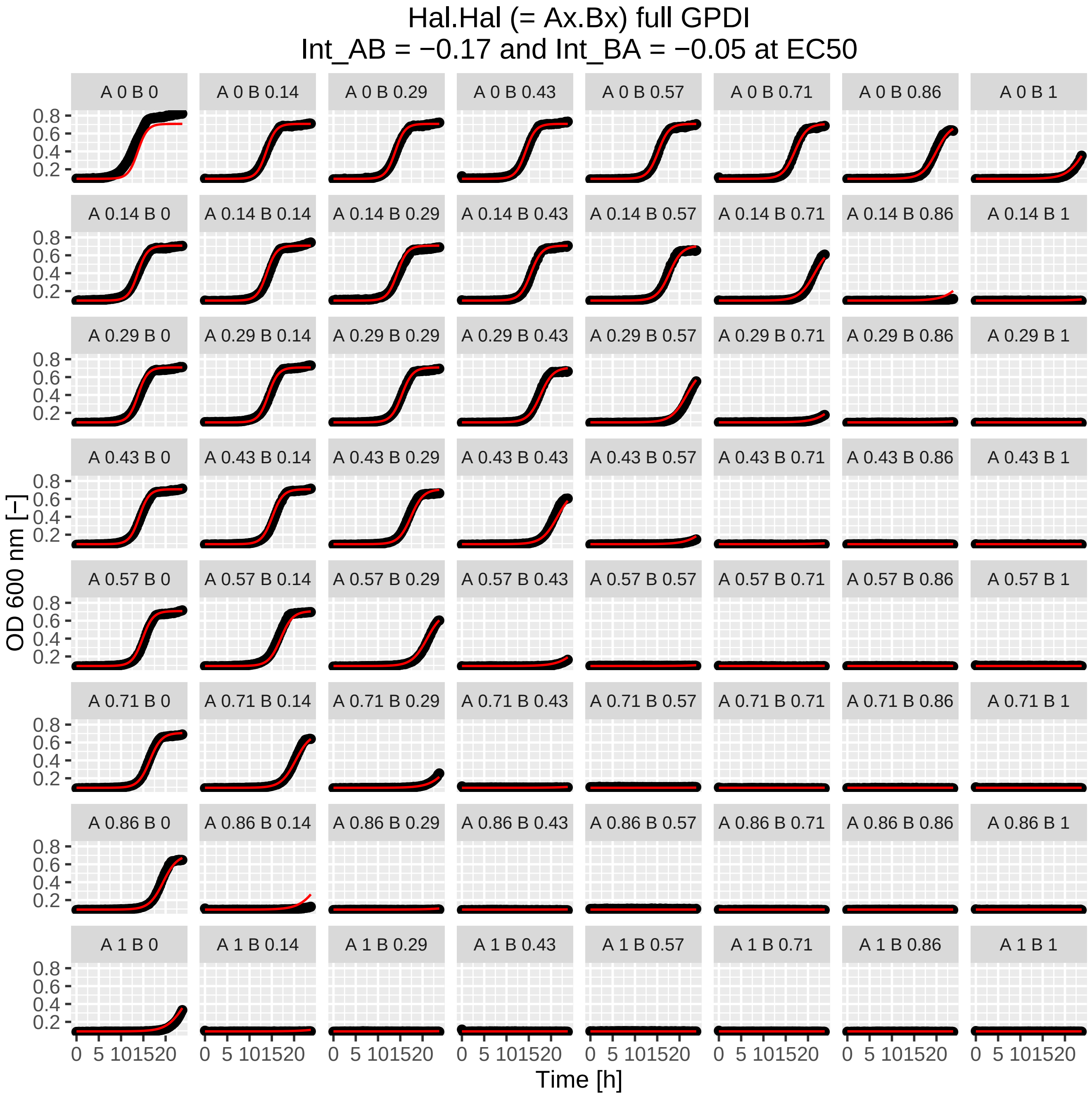

Hal.Hal (= Ax.Bx) full GPDI
Int_AB = −0.17 and Int_BA = −0.05 at EC50
OD 600 nm [−]
Time [h]
A 0 B 0
A 0 B 0.14
A 0 B 0.29
A 0 B 0.43
A 0 B 0.57
A 0 B 0.71
A 0 B 0.86
A 0 B 1
A 1 B 0
A 1 B 0.14
A 1 B 0.29
A 1 B 0.43
A 1 B 0.57
A 1 B 0.71
A 1 B 0.86
A 1 B 1

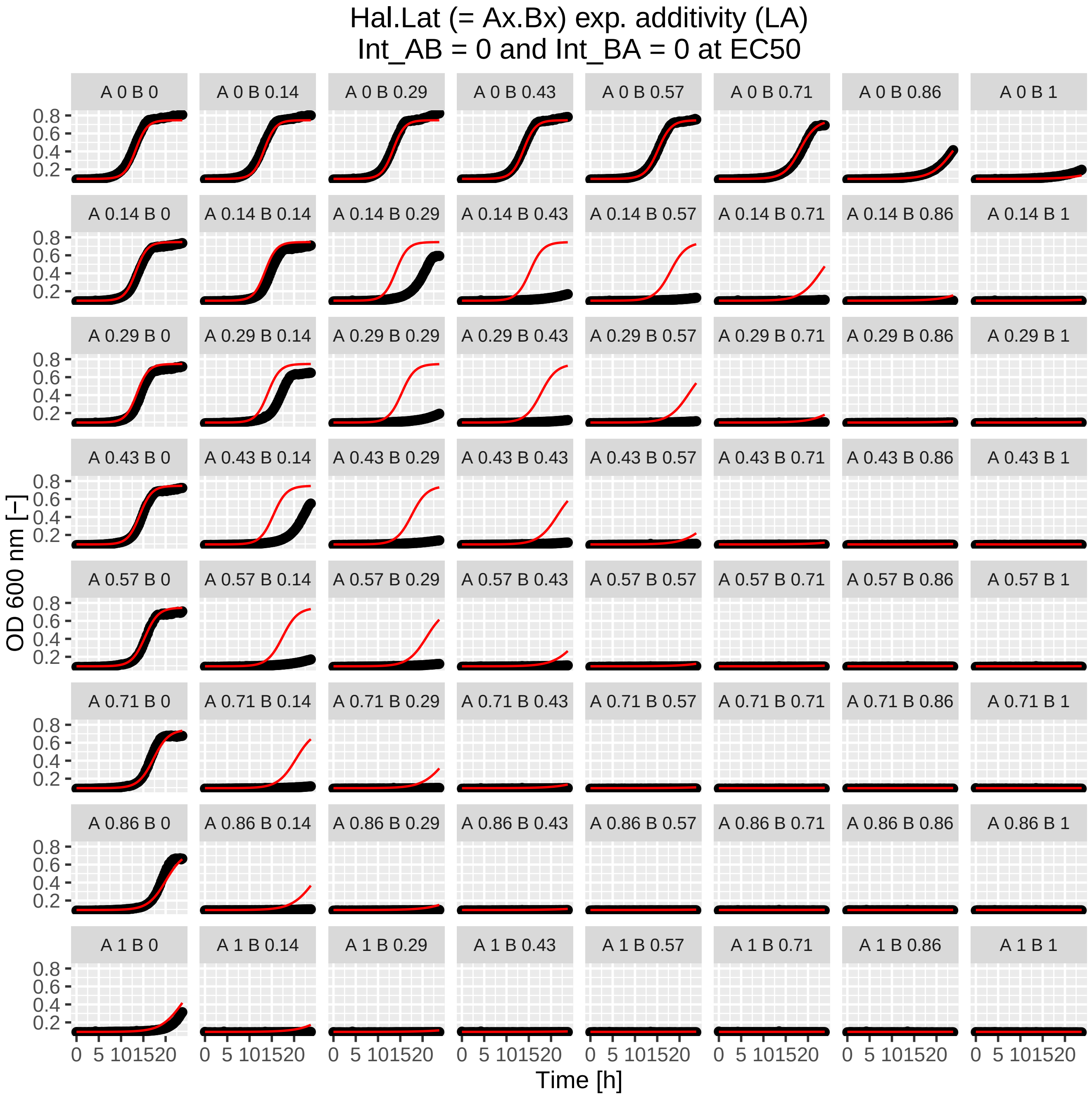

Hal.Lat (= Ax.Bx) exp. additivity (LA)
Int_AB = 0 and Int_BA = 0 at EC50
A 0 B 0
A 0 B 0.14
A 0 B 0.29
A 0 B 0.43
A 0 B 0.57
A 0 B 0.71
A 0 B 0.86
A 0 B 1
A 0.14 B 0
A 0.14 B 0.14
A 0.14 B 0.29
A 0.14 B 0.43
A 0.14 B 0.57
A 0.14 B 0.71
A 0.14 B 0.86
A 0.14 B 1
A 0.29 B 0
A 0.29 B 0.14
A 0.29 B 0.29
A 0.29 B 0.43
A 0.29 B 0.57
A 0.29 B 0.71
A 0.29 B 0.86
A 0.29 B 1
A 0.43 B 0
A 0.43 B 0.14
A 0.43 B 0.29
A 0.43 B 0.43
A 0.43 B 0.57
A 0.43 B 0.71
A 0.43 B 0.86
A 0.43 B 1
A 0.57 B 0
A 0.57 B 0.14
A 0.57 B 0.29
A 0.57 B 0.43
A 0.57 B 0.57
A 0.57 B 0.71
A 0.57 B 0.86
A 0.57 B 1
A 0.71 B 0
A 0.71 B 0.14
A 0.71 B 0.29
A 0.71 B 0.43
A 0.71 B 0.57
A 0.71 B 0.71
A 0.71 B 0.86
A 0.71 B 1
A 0.86 B 0
A 0.86 B 0.14
A 0.86 B 0.29
A 0.86 B 0.43
A 0.86 B 0.57
A 0.86 B 0.71
A 0.86 B 0.86
A 0.86 B 1
A 1 B 0
A 1 B 0.14
A 1 B 0.29
A 1 B 0.43
A 1 B 0.57
A 1 B 0.71
A 1 B 0.86
A 1 B 1
0.8
0.6
0.4
0.2
0 5 101520
OD 600 nm [–]
Time [h]

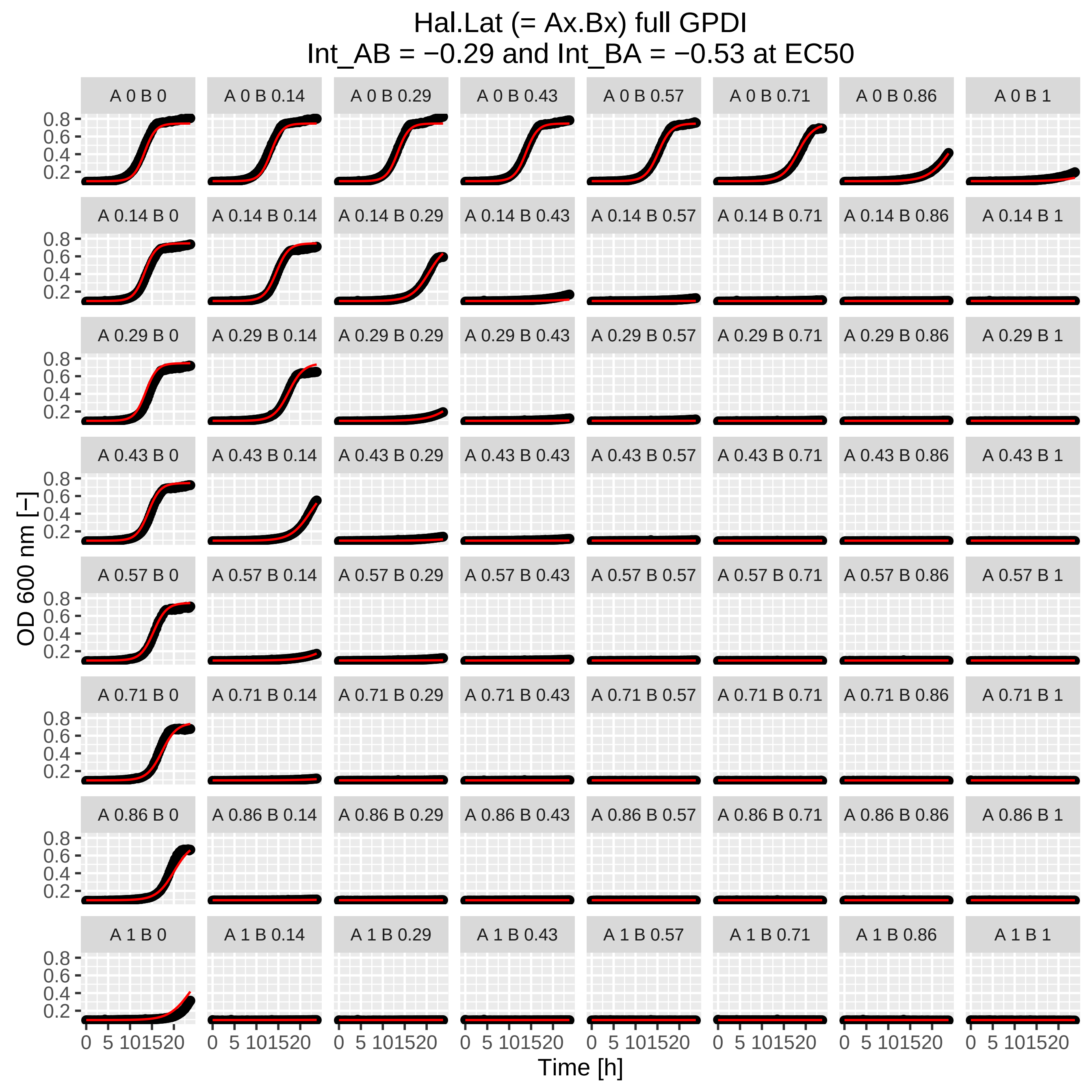
Hal.Lat (= Ax.Bx) full GPDI
Int_AB = −0.29 and Int_BA = −0.53 at EC50
OD 600 nm [−]
Time [h]

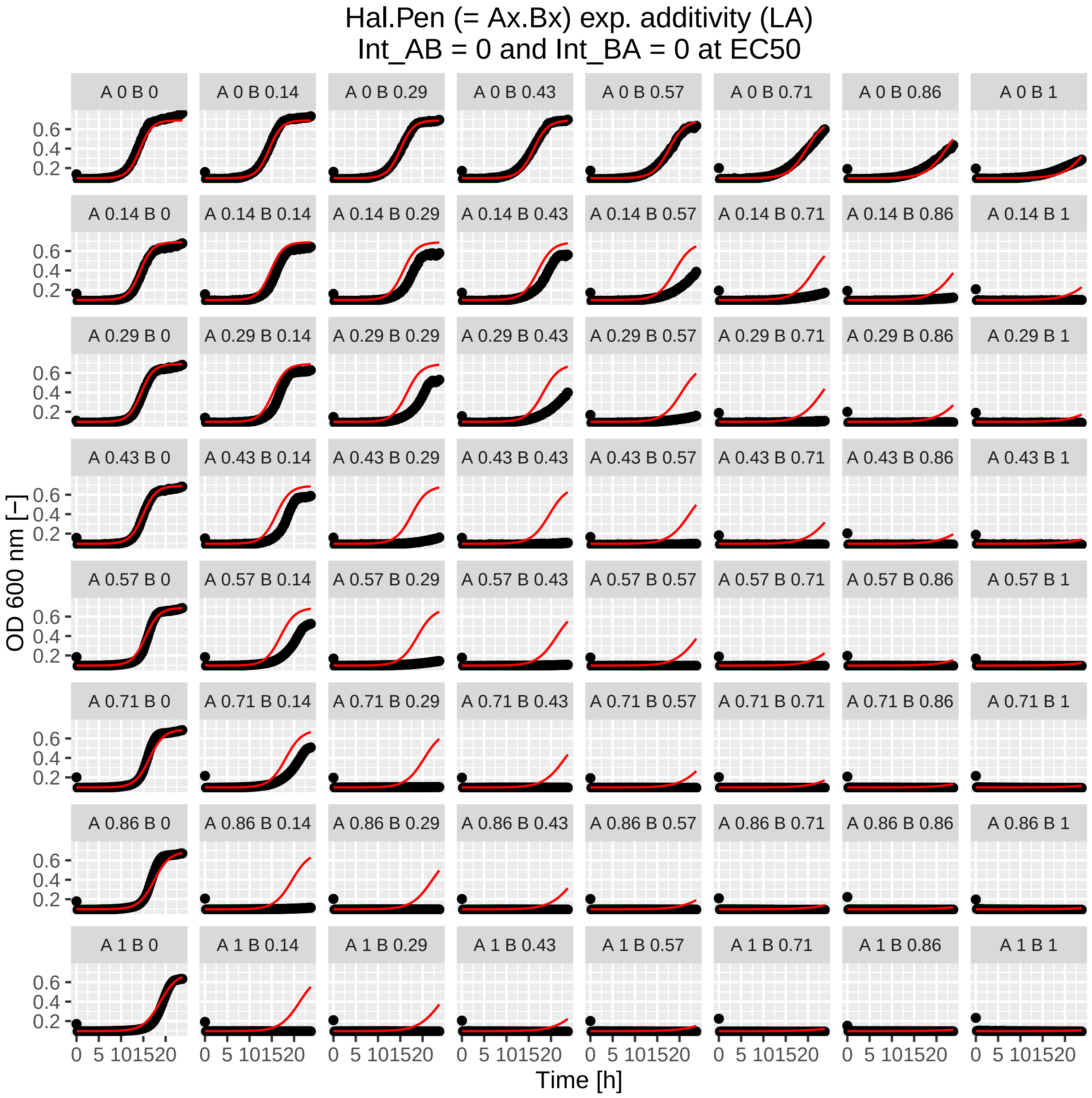
Hal.Pen (= Ax.Bx) exp. additivity (LA)
Int_AB = 0 and Int_BA = 0 at EC50
OD 600 nm [–]
Time [h]
0.6
0.4
0.2
0 5 101520
A 0 B 0
A 0 B 0.14
A 0 B 0.29
A 0 B 0.43
A 0 B 0.57
A 0 B 0.71
A 0 B 0.86
A 0 B 1
A 0.14 B 0
A 0.14 B 0.14
A 0.14 B 0.29
A 0.14 B 0.43
A 0.14 B 0.57
A 0.14 B 0.71
A 0.14 B 0.86
A 0.14 B 1
A 0.29 B 0
A 0.29 B 0.14
A 0.29 B 0.29
A 0.29 B 0.43
A 0.29 B 0.57
A 0.29 B 0.71
A 0.29 B 0.86
A 0.29 B 1
A 0.43 B 0
A 0.43 B 0.14
A 0.43 B 0.29
A 0.43 B 0.43
A 0.43 B 0.57
A 0.43 B 0.71
A 0.43 B 0.86
A 0.43 B 1
A 0.57 B 0
A 0.57 B 0.14
A 0.57 B 0.29
A 0.57 B 0.43
A 0.57 B 0.57
A 0.57 B 0.71
A 0.57 B 0.86
A 0.57 B 1
A 0.71 B 0
A 0.71 B 0.14
A 0.71 B 0.29
A 0.71 B 0.43
A 0.71 B 0.57
A 0.71 B 0.71
A 0.71 B 0.86
A 0.71 B 1
A 0.86 B 0
A 0.86 B 0.14
A 0.86 B 0.29
A 0.86 B 0.43
A 0.86 B 0.57
A 0.86 B 0.71
A 0.86 B 0.86
A 0.86 B 1
A 1 B 0
A 1 B 0.14
A 1 B 0.29
A 1 B 0.43
A 1 B 0.57
A 1 B 0.71
A 1 B 0.86
A 1 B 1

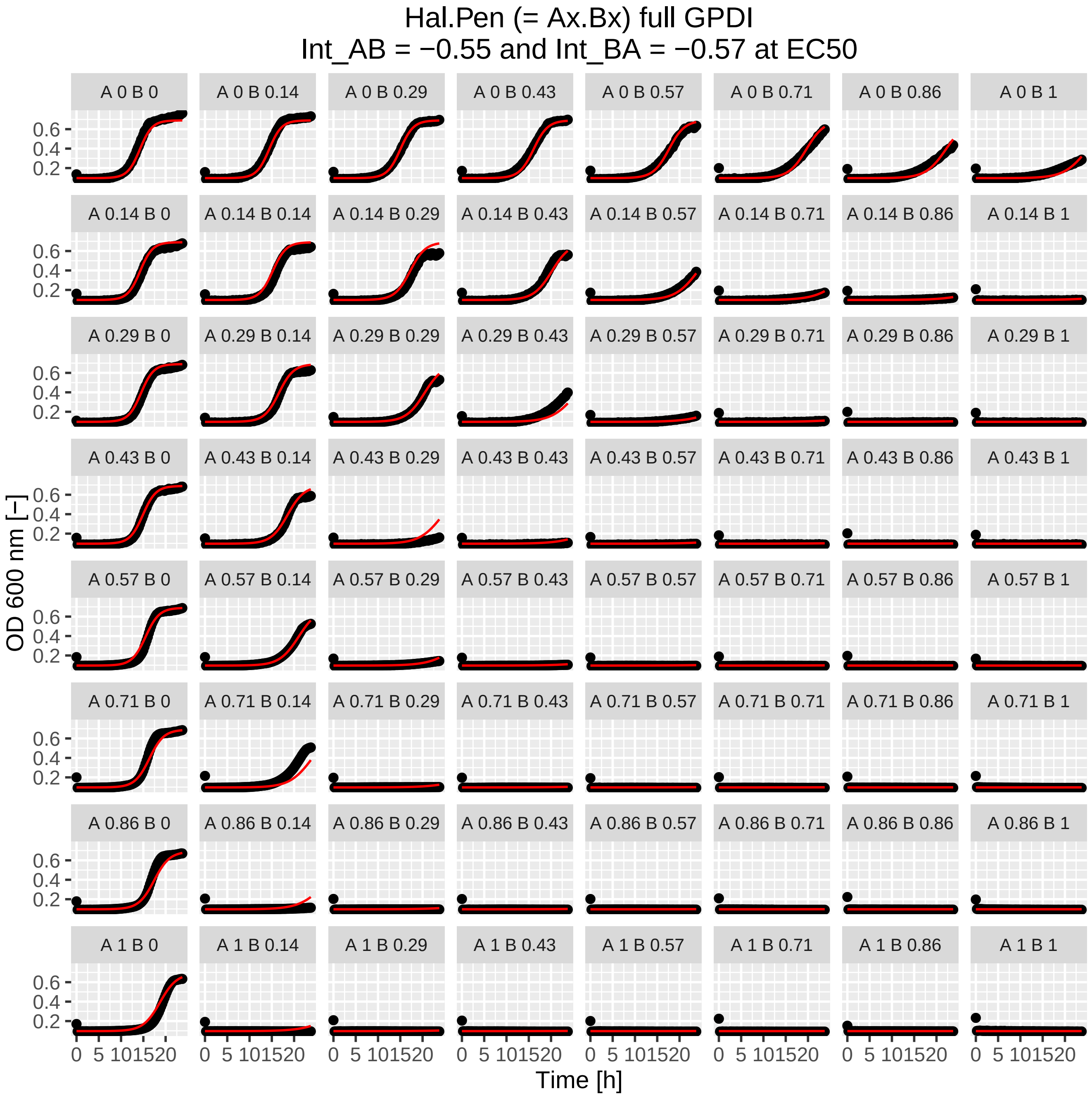

Hal.Pen (= Ax.Bx) full GPDI
Int_AB = −0.55 and Int_BA = −0.57 at EC50
A 0 B 0
A 0 B 0.14
A 0 B 0.29
A 0 B 0.43
A 0 B 0.57
A 0 B 0.71
A 0 B 0.86
A 0 B 1
A 0.14 B 0
A 0.14 B 0.14
A 0.14 B 0.29
A 0.14 B 0.43
A 0.14 B 0.57
A 0.14 B 0.71
A 0.14 B 0.86
A 0.14 B 1
A 0.29 B 0
A 0.29 B 0.14
A 0.29 B 0.29
A 0.29 B 0.43
A 0.29 B 0.57
A 0.29 B 0.71
A 0.29 B 0.86
A 0.29 B 1
A 0.43 B 0
A 0.43 B 0.14
A 0.43 B 0.29
A 0.43 B 0.43
A 0.43 B 0.57
A 0.43 B 0.71
A 0.43 B 0.86
A 0.43 B 1
A 0.57 B 0
A 0.57 B 0.14
A 0.57 B 0.29
A 0.57 B 0.43
A 0.57 B 0.57
A 0.57 B 0.71
A 0.57 B 0.86
A 0.57 B 1
A 0.71 B 0
A 0.71 B 0.14
A 0.71 B 0.29
A 0.71 B 0.43
A 0.71 B 0.57
A 0.71 B 0.71
A 0.71 B 0.86
A 0.71 B 1
A 0.86 B 0
A 0.86 B 0.14
A 0.86 B 0.29
A 0.86 B 0.43
A 0.86 B 0.57
A 0.86 B 0.71
A 0.86 B 0.86
A 0.86 B 1
A 1 B 0
A 1 B 0.14
A 1 B 0.29
A 1 B 0.43
A 1 B 0.57
A 1 B 0.71
A 1 B 0.86
A 1 B 1
0.6
0.4
0.2
OD 600 nm [–]
0 5 101520
Time [h]

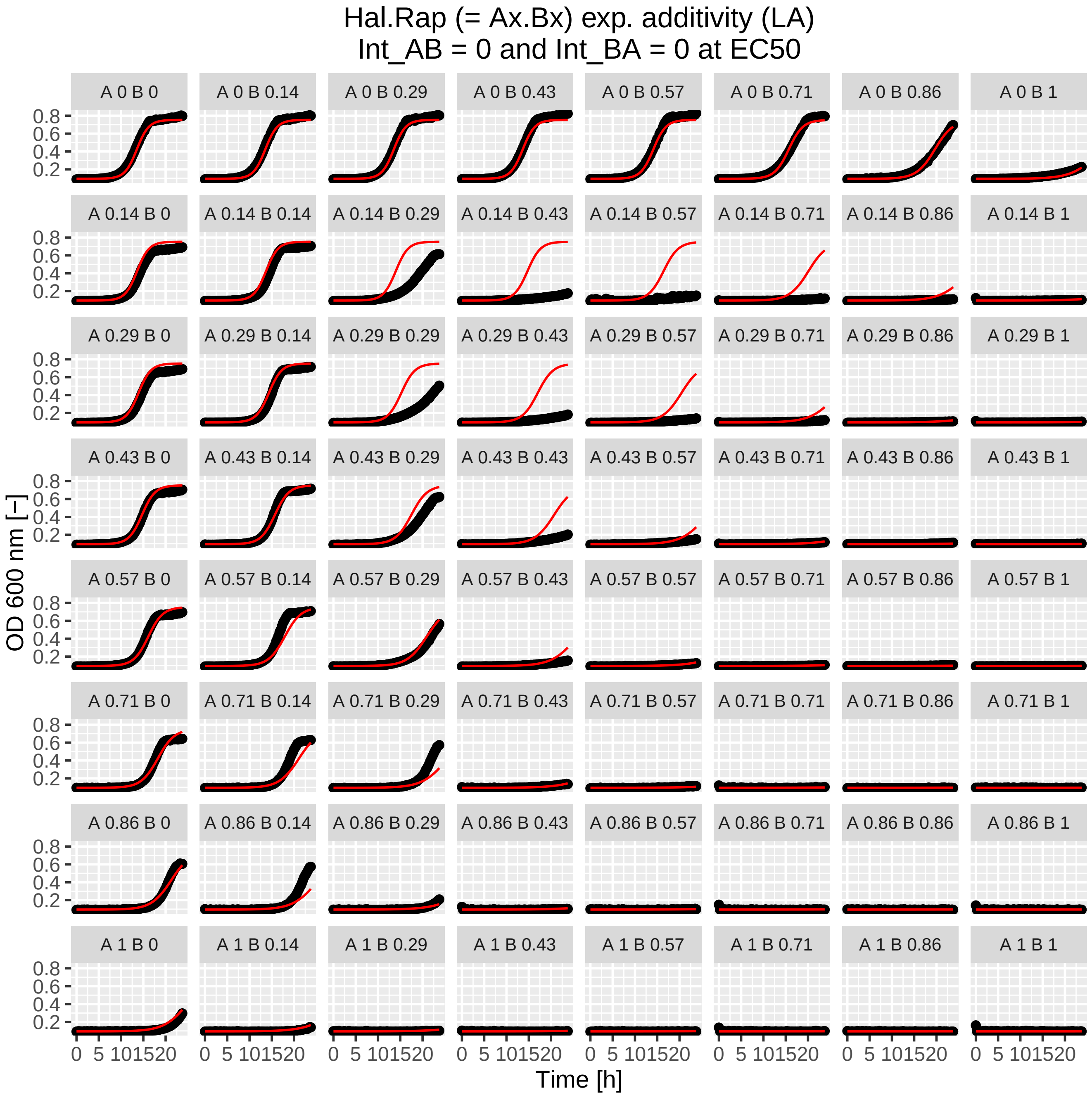

Hal.Rap (= Ax.Bx) exp. additivity (LA)
Int_AB = 0 and Int_BA = 0 at EC50
A 0 B 0
A 0 B 0.14
A 0 B 0.29
A 0 B 0.43
A 0 B 0.57
A 0 B 0.71
A 0 B 0.86
A 0 B 1
A 0.14 B 0
A 0.14 B 0.14
A 0.14 B 0.29
A 0.14 B 0.43
A 0.14 B 0.57
A 0.14 B 0.71
A 0.14 B 0.86
A 0.14 B 1
A 0.29 B 0
A 0.29 B 0.14
A 0.29 B 0.29
A 0.29 B 0.43
A 0.29 B 0.57
A 0.29 B 0.71
A 0.29 B 0.86
A 0.29 B 1
A 0.43 B 0
A 0.43 B 0.14
A 0.43 B 0.29
A 0.43 B 0.43
A 0.43 B 0.57
A 0.43 B 0.71
A 0.43 B 0.86
A 0.43 B 1
A 0.57 B 0
A 0.57 B 0.14
A 0.57 B 0.29
A 0.57 B 0.43
A 0.57 B 0.57
A 0.57 B 0.71
A 0.57 B 0.86
A 0.57 B 1
A 0.71 B 0
A 0.71 B 0.14
A 0.71 B 0.29
A 0.71 B 0.43
A 0.71 B 0.57
A 0.71 B 0.71
A 0.71 B 0.86
A 0.71 B 1
A 0.86 B 0
A 0.86 B 0.14
A 0.86 B 0.29
A 0.86 B 0.43
A 0.86 B 0.57
A 0.86 B 0.71
A 0.86 B 0.86
A 0.86 B 1
A 1 B 0
A 1 B 0.14
A 1 B 0.29
A 1 B 0.43
A 1 B 0.57
A 1 B 0.71
A 1 B 0.86
A 1 B 1
OD 600 nm [−]
0.2
0.4
0.6
0.8
0 5 101520
Time [h]

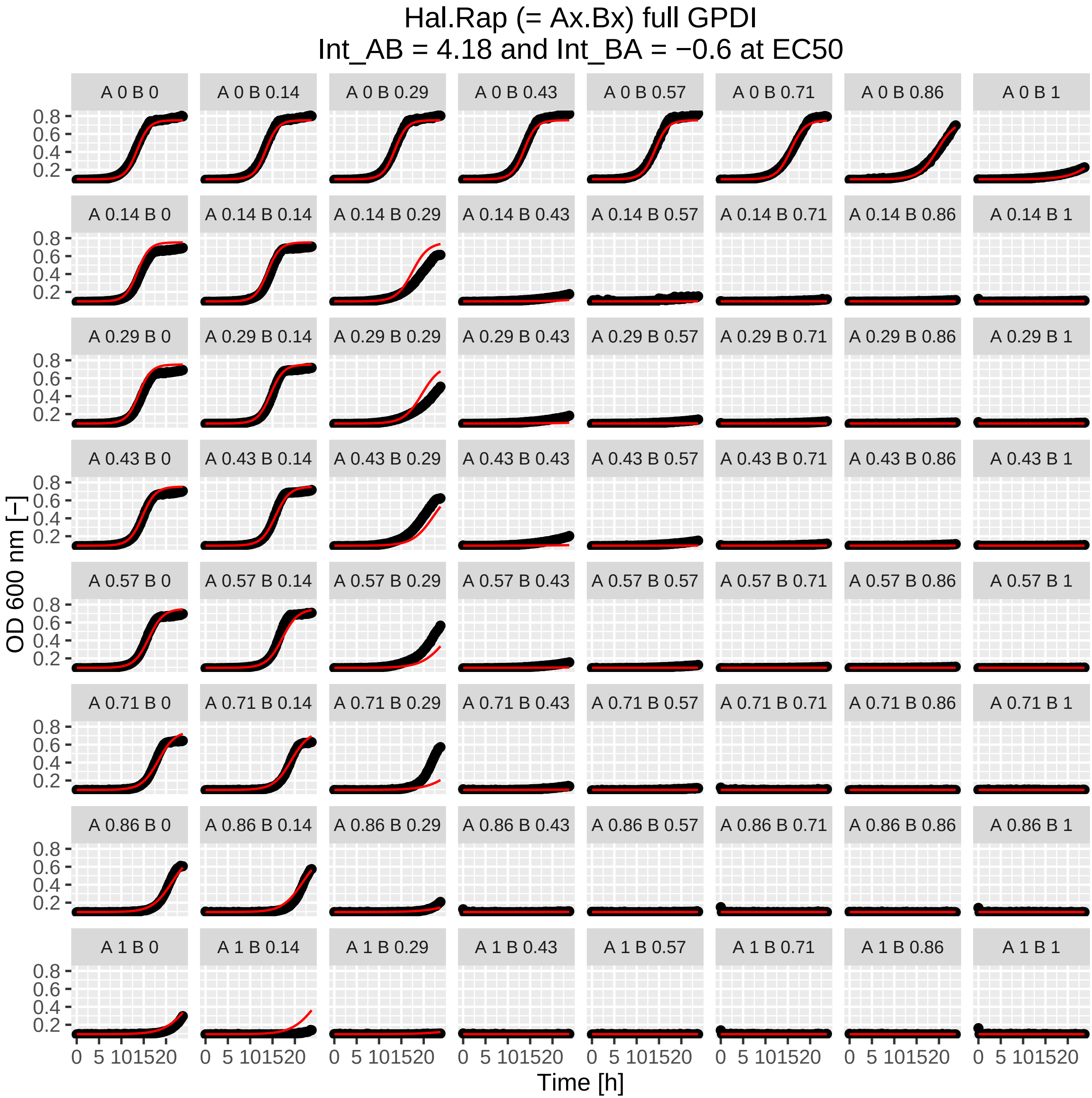
Hal.Rap (= Ax.Bx) full GPDI
Int_AB = 4.18 and Int_BA = −0.6 at EC50
OD 600 nm [−]
Time [h]
A 0 B 0
A 0 B 0.14
A 0 B 0.29
A 0 B 0.43
A 0 B 0.57
A 0 B 0.71
A 0 B 0.86
A 0 B 1
A 1 B 0
A 1 B 0.14
A 1 B 0.29
A 1 B 0.43
A 1 B 0.57
A 1 B 0.71
A 1 B 0.86
A 1 B 1

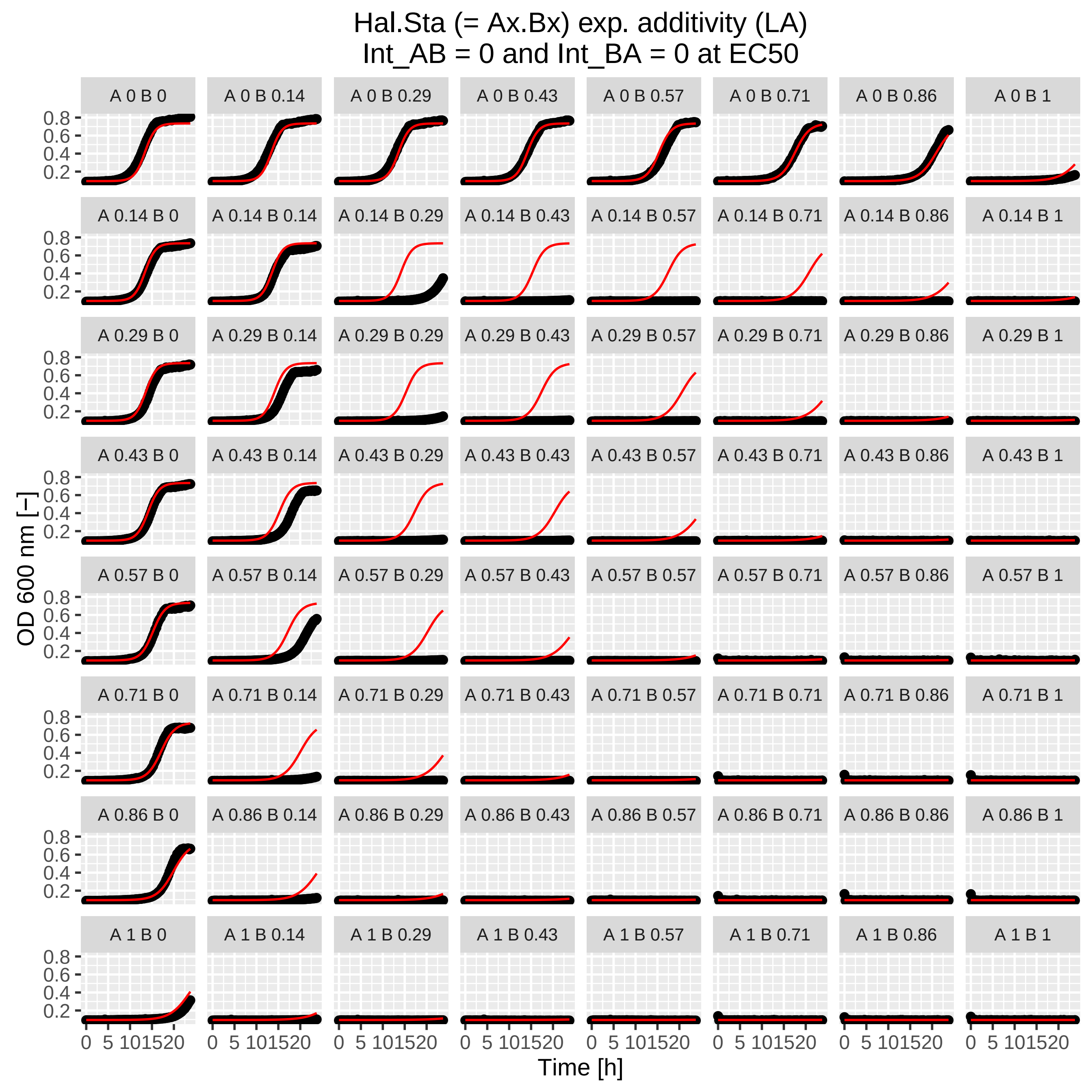

Hal.Sta (= Ax.Bx) exp. additivity (LA)
Int_AB = 0 and Int_BA = 0 at EC50
A 0 B 0
A 0 B 0.14
A 0 B 0.29
A 0 B 0.43
A 0 B 0.57
A 0 B 0.71
A 0 B 0.86
A 0 B 1
A 0.14 B 0
A 0.14 B 0.14
A 0.14 B 0.29
A 0.14 B 0.43
A 0.14 B 0.57
A 0.14 B 0.71
A 0.14 B 0.86
A 0.14 B 1
A 0.29 B 0
A 0.29 B 0.14
A 0.29 B 0.29
A 0.29 B 0.43
A 0.29 B 0.57
A 0.29 B 0.71
A 0.29 B 0.86
A 0.29 B 1
A 0.43 B 0
A 0.43 B 0.14
A 0.43 B 0.29
A 0.43 B 0.43
A 0.43 B 0.57
A 0.43 B 0.71
A 0.43 B 0.86
A 0.43 B 1
A 0.57 B 0
A 0.57 B 0.14
A 0.57 B 0.29
A 0.57 B 0.43
A 0.57 B 0.57
A 0.57 B 0.71
A 0.57 B 0.86
A 0.57 B 1
A 0.71 B 0
A 0.71 B 0.14
A 0.71 B 0.29
A 0.71 B 0.43
A 0.71 B 0.57
A 0.71 B 0.71
A 0.71 B 0.86
A 0.71 B 1
A 0.86 B 0
A 0.86 B 0.14
A 0.86 B 0.29
A 0.86 B 0.43
A 0.86 B 0.57
A 0.86 B 0.71
A 0.86 B 0.86
A 0.86 B 1
A 1 B 0
A 1 B 0.14
A 1 B 0.29
A 1 B 0.43
A 1 B 0.57
A 1 B 0.71
A 1 B 0.86
A 1 B 1
OD 600 nm [–]
0.2
0.4
0.6
0.8
0 5 101520
Time [h]

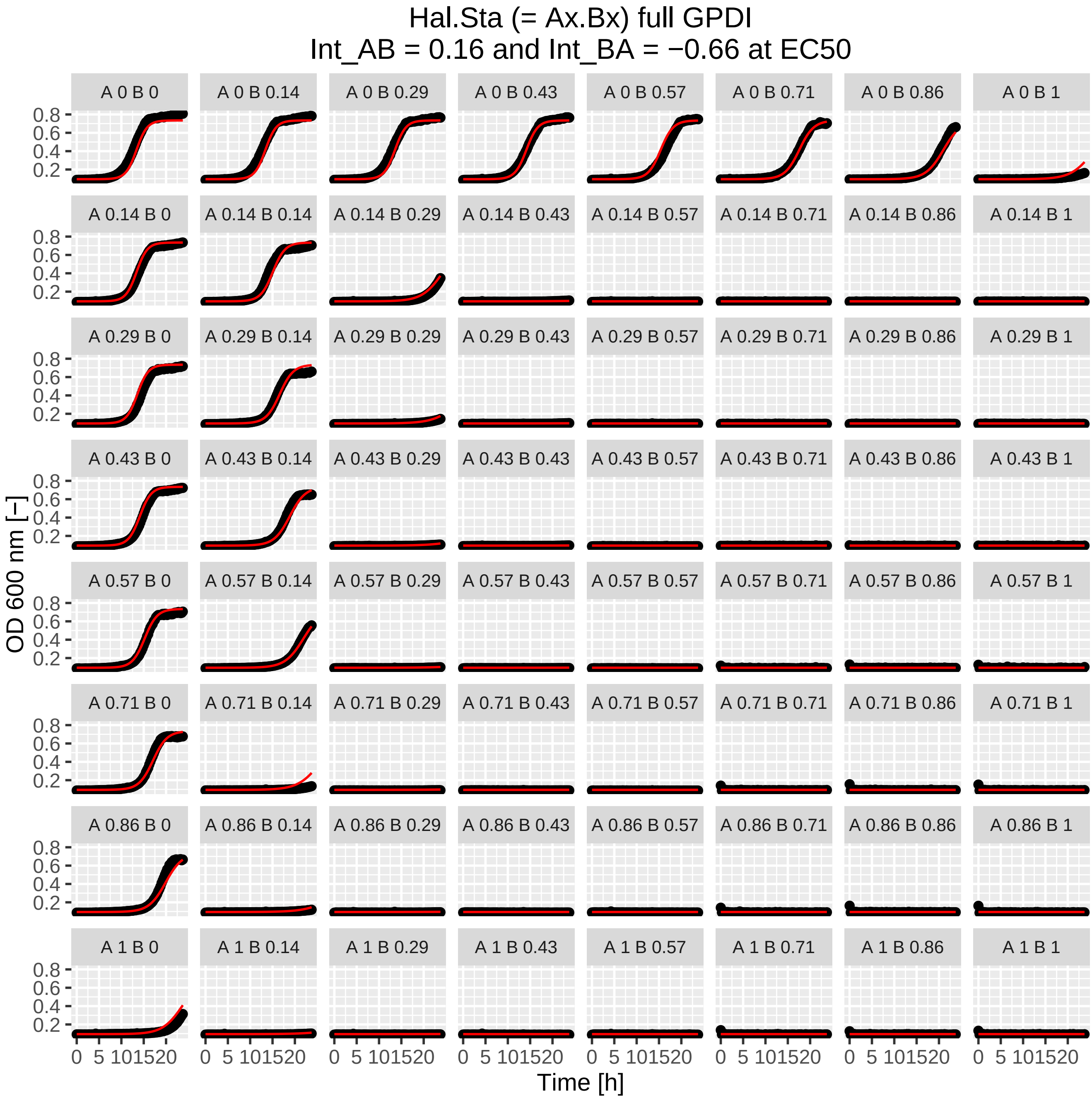
Hal.Sta (= Ax.Bx) full GPDI
Int_AB = 0.16 and Int_BA = −0.66 at EC50
A 0 B 0
A 0 B 0.14
A 0 B 0.29
A 0 B 0.43
A 0 B 0.57
A 0 B 0.71
A 0 B 0.86
A 0 B 1
A 0.14 B 0
A 0.14 B 0.14
A 0.14 B 0.29
A 0.14 B 0.43
A 0.14 B 0.57
A 0.14 B 0.71
A 0.14 B 0.86
A 0.14 B 1
A 0.29 B 0
A 0.29 B 0.14
A 0.29 B 0.29
A 0.29 B 0.43
A 0.29 B 0.57
A 0.29 B 0.71
A 0.29 B 0.86
A 0.29 B 1
A 0.43 B 0
A 0.43 B 0.14
A 0.43 B 0.29
A 0.43 B 0.43
A 0.43 B 0.57
A 0.43 B 0.71
A 0.43 B 0.86
A 0.43 B 1
A 0.57 B 0
A 0.57 B 0.14
A 0.57 B 0.29
A 0.57 B 0.43
A 0.57 B 0.57
A 0.57 B 0.71
A 0.57 B 0.86
A 0.57 B 1
A 0.71 B 0
A 0.71 B 0.14
A 0.71 B 0.29
A 0.71 B 0.43
A 0.71 B 0.57
A 0.71 B 0.71
A 0.71 B 0.86
A 0.71 B 1
A 0.86 B 0
A 0.86 B 0.14
A 0.86 B 0.29
A 0.86 B 0.43
A 0.86 B 0.57
A 0.86 B 0.71
A 0.86 B 0.86
A 0.86 B 1
A 1 B 0
A 1 B 0.14
A 1 B 0.29
A 1 B 0.43
A 1 B 0.57
A 1 B 0.71
A 1 B 0.86
A 1 B 1
OD 600 nm [−]
0.8
0.6
0.4
0.2
0
5
10
15
20
Time [h]

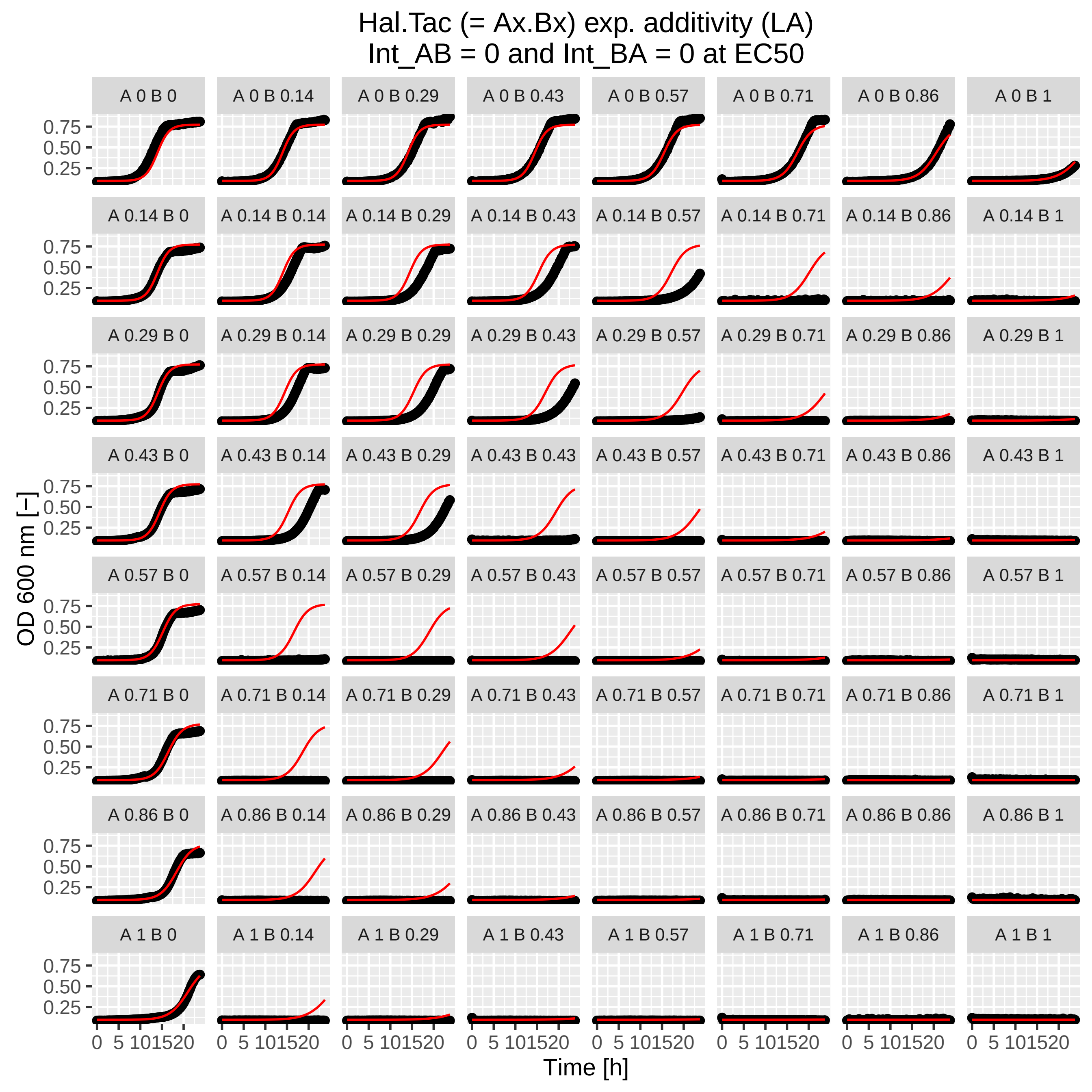
Hal.Tac (= Ax.Bx) exp. additivity (LA)
Int_AB = 0 and Int_BA = 0 at EC50
A 0 B 0
A 0 B 0.14
A 0 B 0.29
A 0 B 0.43
A 0 B 0.57
A 0 B 0.71
A 0 B 0.86
A 0 B 1
A 0.14 B 0
A 0.14 B 0.14
A 0.14 B 0.29
A 0.14 B 0.43
A 0.14 B 0.57
A 0.14 B 0.71
A 0.14 B 0.86
A 0.14 B 1
A 0.29 B 0
A 0.29 B 0.14
A 0.29 B 0.29
A 0.29 B 0.43
A 0.29 B 0.57
A 0.29 B 0.71
A 0.29 B 0.86
A 0.29 B 1
A 0.43 B 0
A 0.43 B 0.14
A 0.43 B 0.29
A 0.43 B 0.43
A 0.43 B 0.57
A 0.43 B 0.71
A 0.43 B 0.86
A 0.43 B 1
A 0.57 B 0
A 0.57 B 0.14
A 0.57 B 0.29
A 0.57 B 0.43
A 0.57 B 0.57
A 0.57 B 0.71
A 0.57 B 0.86
A 0.57 B 1
A 0.71 B 0
A 0.71 B 0.14
A 0.71 B 0.29
A 0.71 B 0.43
A 0.71 B 0.57
A 0.71 B 0.71
A 0.71 B 0.86
A 0.71 B 1
A 0.86 B 0
A 0.86 B 0.14
A 0.86 B 0.29
A 0.86 B 0.43
A 0.86 B 0.57
A 0.86 B 0.71
A 0.86 B 0.86
A 0.86 B 1
A 1 B 0
A 1 B 0.14
A 1 B 0.29
A 1 B 0.43
A 1 B 0.57
A 1 B 0.71
A 1 B 0.86
A 1 B 1
OD 600 nm [−]
0.75
0.50
0.25
0 5 101520
Time [h]

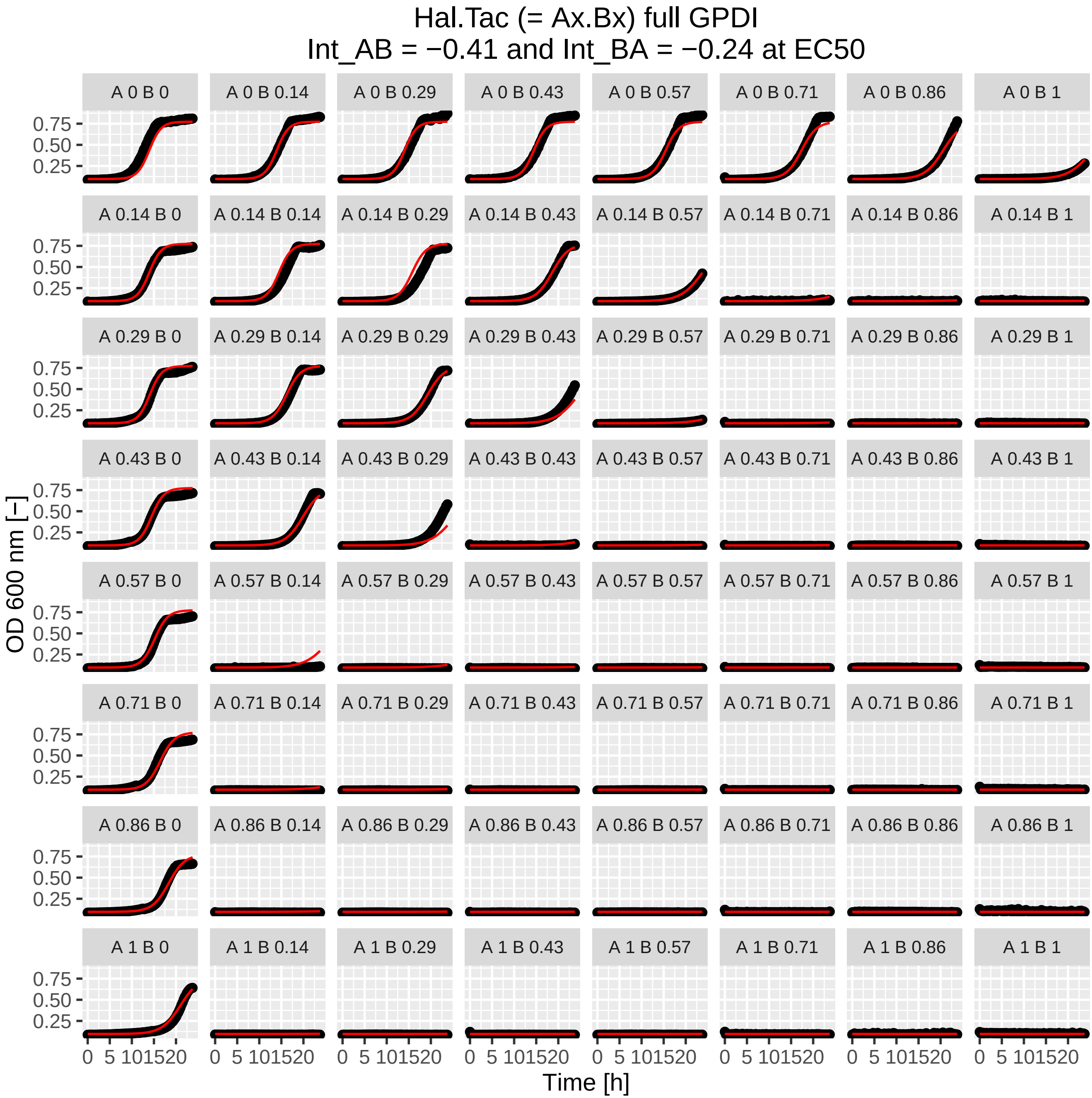
Hal.Tac (= Ax.Bx) full GPDI
Int_AB = −0.41 and Int_BA = −0.24 at EC50
A 0 B 0
A 0 B 0.14
A 0 B 0.29
A 0 B 0.43
A 0 B 0.57
A 0 B 0.71
A 0 B 0.86
A 0 B 1
A 0.14 B 0
A 0.14 B 0.14
A 0.14 B 0.29
A 0.14 B 0.43
A 0.14 B 0.57
A 0.14 B 0.71
A 0.14 B 0.86
A 0.14 B 1
A 0.29 B 0
A 0.29 B 0.14
A 0.29 B 0.29
A 0.29 B 0.43
A 0.29 B 0.57
A 0.29 B 0.71
A 0.29 B 0.86
A 0.29 B 1
A 0.43 B 0
A 0.43 B 0.14
A 0.43 B 0.29
A 0.43 B 0.43
A 0.43 B 0.57
A 0.43 B 0.71
A 0.43 B 0.86
A 0.43 B 1
A 0.57 B 0
A 0.57 B 0.14
A 0.57 B 0.29
A 0.57 B 0.43
A 0.57 B 0.57
A 0.57 B 0.71
A 0.57 B 0.86
A 0.57 B 1
A 0.71 B 0
A 0.71 B 0.14
A 0.71 B 0.29
A 0.71 B 0.43
A 0.71 B 0.57
A 0.71 B 0.71
A 0.71 B 0.86
A 0.71 B 1
A 0.86 B 0
A 0.86 B 0.14
A 0.86 B 0.29
A 0.86 B 0.43
A 0.86 B 0.57
A 0.86 B 0.71
A 0.86 B 0.86
A 0.86 B 1
A 1 B 0
A 1 B 0.14
A 1 B 0.29
A 1 B 0.43
A 1 B 0.57
A 1 B 0.71
A 1 B 0.86
A 1 B 1
0.75
0.50
0.25
OD 600 nm [−]
0 5 101520
Time [h]

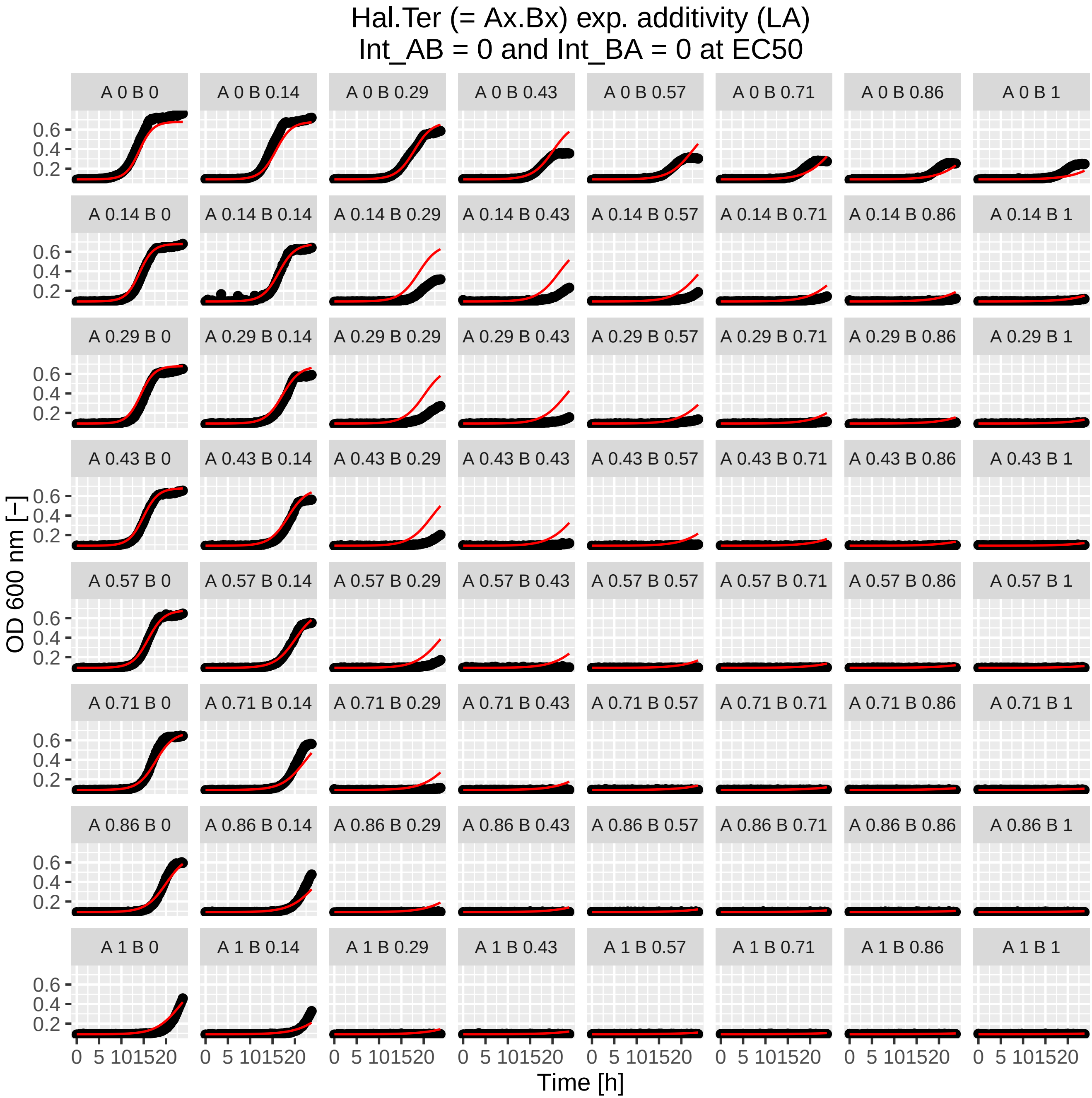

Hal.Ter (= Ax.Bx) exp. additivity (LA)
Int_AB = 0 and Int_BA = 0 at EC50
A 0 B 0
A 0 B 0.14
A 0 B 0.29
A 0 B 0.43
A 0 B 0.57
A 0 B 0.71
A 0 B 0.86
A 0 B 1
A 0.14 B 0
A 0.14 B 0.14
A 0.14 B 0.29
A 0.14 B 0.43
A 0.14 B 0.57
A 0.14 B 0.71
A 0.14 B 0.86
A 0.14 B 1
A 0.29 B 0
A 0.29 B 0.14
A 0.29 B 0.29
A 0.29 B 0.43
A 0.29 B 0.57
A 0.29 B 0.71
A 0.29 B 0.86
A 0.29 B 1
A 0.43 B 0
A 0.43 B 0.14
A 0.43 B 0.29
A 0.43 B 0.43
A 0.43 B 0.57
A 0.43 B 0.71
A 0.43 B 0.86
A 0.43 B 1
A 0.57 B 0
A 0.57 B 0.14
A 0.57 B 0.29
A 0.57 B 0.43
A 0.57 B 0.57
A 0.57 B 0.71
A 0.57 B 0.86
A 0.57 B 1
A 0.71 B 0
A 0.71 B 0.14
A 0.71 B 0.29
A 0.71 B 0.43
A 0.71 B 0.57
A 0.71 B 0.71
A 0.71 B 0.86
A 0.71 B 1
A 0.86 B 0
A 0.86 B 0.14
A 0.86 B 0.29
A 0.86 B 0.43
A 0.86 B 0.57
A 0.86 B 0.71
A 0.86 B 0.86
A 0.86 B 1
A 1 B 0
A 1 B 0.14
A 1 B 0.29
A 1 B 0.43
A 1 B 0.57
A 1 B 0.71
A 1 B 0.86
A 1 B 1
OD 600 nm [–]
0.2
0.4
0.6
0 5 101520
Time [h]

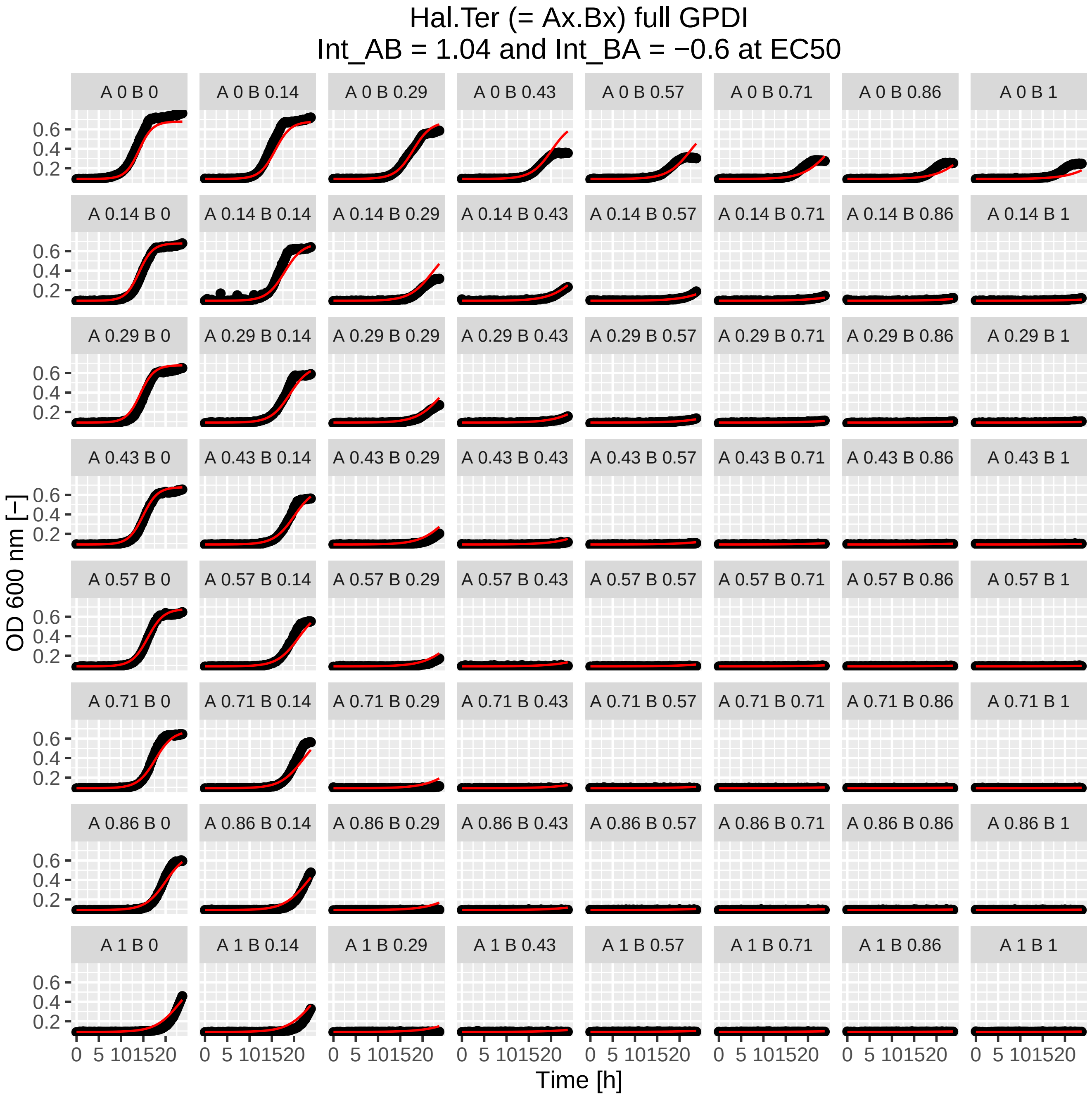
Hal.Ter (= Ax.Bx) full GPDI
Int_AB = 1.04 and Int_BA = −0.6 at EC50
OD 600 nm [−]
Time [h]
A 0 B 0
A 0 B 0.14
A 0 B 0.29
A 0 B 0.43
A 0 B 0.57
A 0 B 0.71
A 0 B 0.86
A 0 B 1
A 0.14 B 0
A 0.14 B 0.14
A 0.14 B 0.29
A 0.14 B 0.43
A 0.14 B 0.57
A 0.14 B 0.71
A 0.14 B 0.86
A 0.14 B 1
A 0.29 B 0
A 0.29 B 0.14
A 0.29 B 0.29
A 0.29 B 0.43
A 0.29 B 0.57
A 0.29 B 0.71
A 0.29 B 0.86
A 0.29 B 1
A 0.43 B 0
A 0.43 B 0.14
A 0.43 B 0.29
A 0.43 B 0.43
A 0.43 B 0.57
A 0.43 B 0.71
A 0.43 B 0.86
A 0.43 B 1
A 0.57 B 0
A 0.57 B 0.14
A 0.57 B 0.29
A 0.57 B 0.43
A 0.57 B 0.57
A 0.57 B 0.71
A 0.57 B 0.86
A 0.57 B 1
A 0.71 B 0
A 0.71 B 0.14
A 0.71 B 0.29
A 0.71 B 0.43
A 0.71 B 0.57
A 0.71 B 0.71
A 0.71 B 0.86
A 0.71 B 1
A 0.86 B 0
A 0.86 B 0.14
A 0.86 B 0.29
A 0.86 B 0.43
A 0.86 B 0.57
A 0.86 B 0.71
A 0.86 B 0.86
A 0.86 B 1
A 1 B 0
A 1 B 0.14
A 1 B 0.29
A 1 B 0.43
A 1 B 0.57
A 1 B 0.71
A 1 B 0.86
A 1 B 1
0.6
0.4
0.2
0 5 101520

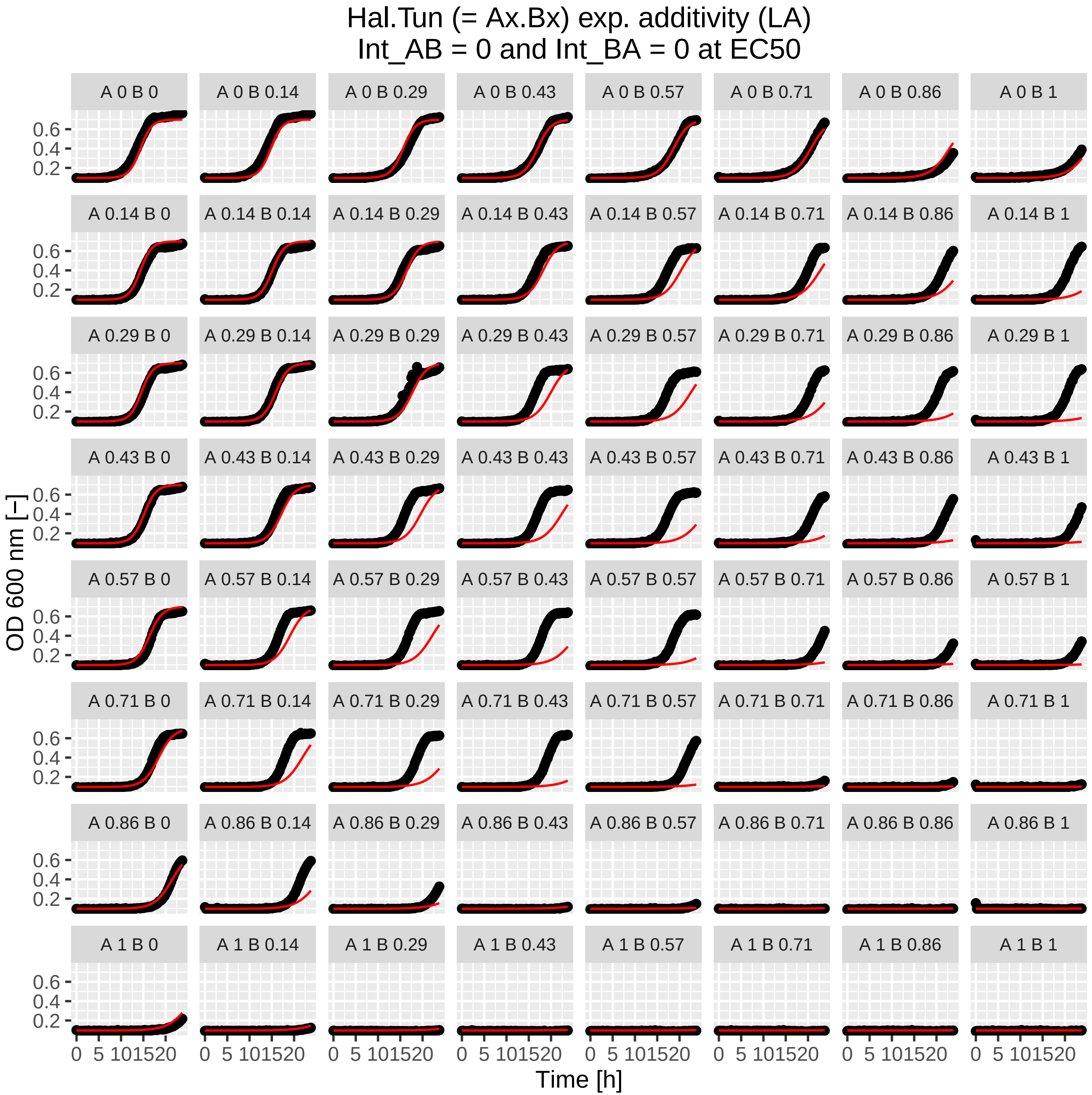

Hal.Tun (= Ax.Bx) exp. additivity (LA)
Int_AB = 0 and Int_BA = 0 at EC50
OD 600 nm [–]
Time [h]
0.6
0.4
0.2
0 5 101520
A 0 B 0
A 0 B 0.14
A 0 B 0.29
A 0 B 0.43
A 0 B 0.57
A 0 B 0.71
A 0 B 0.86
A 0 B 1
A 0.14 B 0
A 0.14 B 0.14
A 0.14 B 0.29
A 0.14 B 0.43
A 0.14 B 0.57
A 0.14 B 0.71
A 0.14 B 0.86
A 0.14 B 1
A 0.29 B 0
A 0.29 B 0.14
A 0.29 B 0.29
A 0.29 B 0.43
A 0.29 B 0.57
A 0.29 B 0.71
A 0.29 B 0.86
A 0.29 B 1
A 0.43 B 0
A 0.43 B 0.14
A 0.43 B 0.29
A 0.43 B 0.43
A 0.43 B 0.57
A 0.43 B 0.71
A 0.43 B 0.86
A 0.43 B 1
A 0.57 B 0
A 0.57 B 0.14
A 0.57 B 0.29
A 0.57 B 0.43
A 0.57 B 0.57
A 0.57 B 0.71
A 0.57 B 0.86
A 0.57 B 1
A 0.71 B 0
A 0.71 B 0.14
A 0.71 B 0.29
A 0.71 B 0.43
A 0.71 B 0.57
A 0.71 B 0.71
A 0.71 B 0.86
A 0.71 B 1
A 0.86 B 0
A 0.86 B 0.14
A 0.86 B 0.29
A 0.86 B 0.43
A 0.86 B 0.57
A 0.86 B 0.71
A 0.86 B 0.86
A 0.86 B 1
A 1 B 0
A 1 B 0.14
A 1 B 0.29
A 1 B 0.43
A 1 B 0.57
A 1 B 0.71
A 1 B 0.86
A 1 B 1

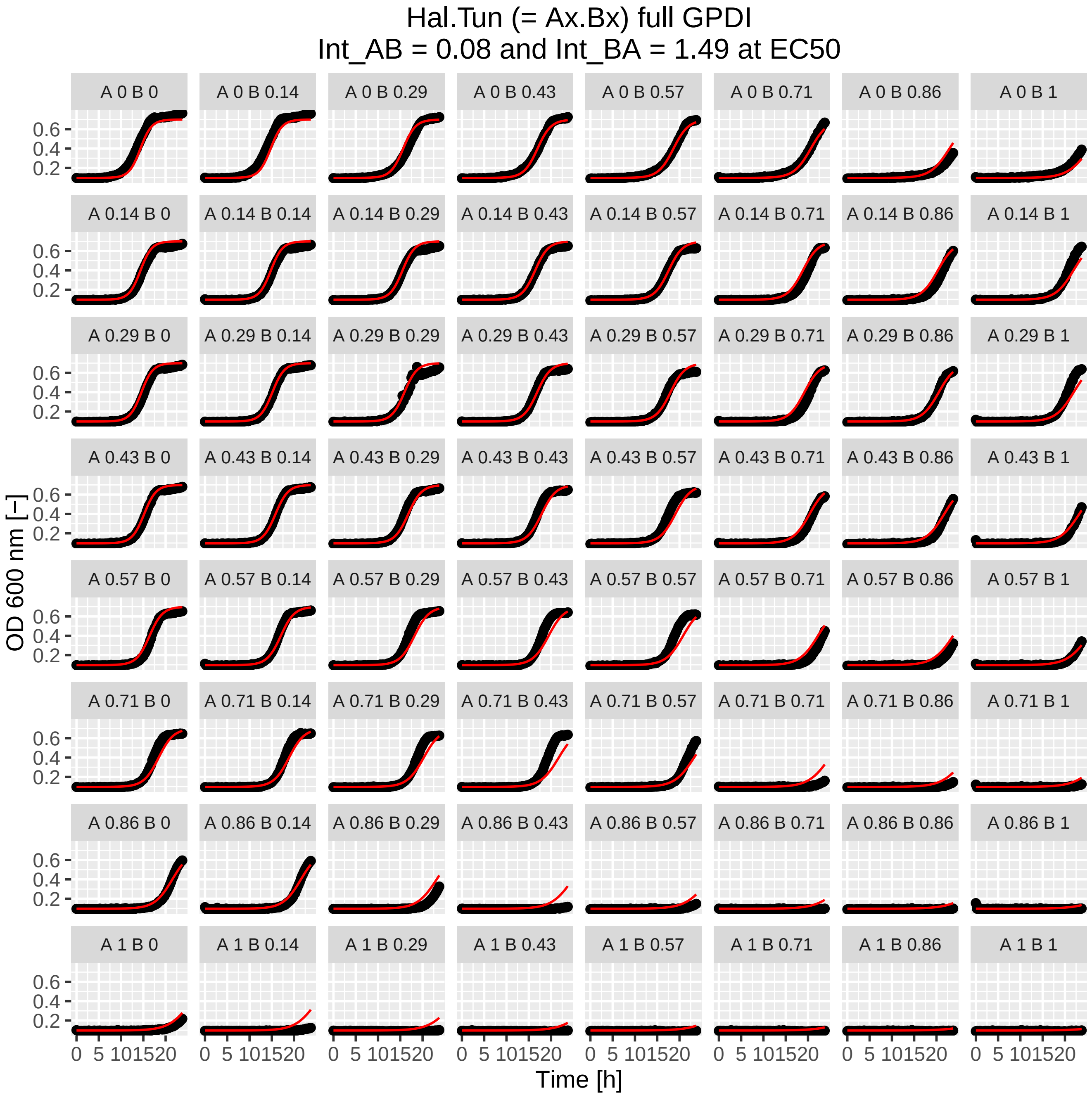
Hal.Tun (= Ax.Bx) full GPDI
Int_AB = 0.08 and Int_BA = 1.49 at EC50
A 0 B 0
A 0 B 0.14
A 0 B 0.29
A 0 B 0.43
A 0 B 0.57
A 0 B 0.71
A 0 B 0.86
A 0 B 1
A 0.14 B 0
A 0.14 B 0.14
A 0.14 B 0.29
A 0.14 B 0.43
A 0.14 B 0.57
A 0.14 B 0.71
A 0.14 B 0.86
A 0.14 B 1
A 0.29 B 0
A 0.29 B 0.14
A 0.29 B 0.29
A 0.29 B 0.43
A 0.29 B 0.57
A 0.29 B 0.71
A 0.29 B 0.86
A 0.29 B 1
A 0.43 B 0
A 0.43 B 0.14
A 0.43 B 0.29
A 0.43 B 0.43
A 0.43 B 0.57
A 0.43 B 0.71
A 0.43 B 0.86
A 0.43 B 1
A 0.57 B 0
A 0.57 B 0.14
A 0.57 B 0.29
A 0.57 B 0.43
A 0.57 B 0.57
A 0.57 B 0.71
A 0.57 B 0.86
A 0.57 B 1
A 0.71 B 0
A 0.71 B 0.14
A 0.71 B 0.29
A 0.71 B 0.43
A 0.71 B 0.57
A 0.71 B 0.71
A 0.71 B 0.86
A 0.71 B 1
A 0.86 B 0
A 0.86 B 0.14
A 0.86 B 0.29
A 0.86 B 0.43
A 0.86 B 0.57
A 0.86 B 0.71
A 0.86 B 0.86
A 0.86 B 1
A 1 B 0
A 1 B 0.14
A 1 B 0.29
A 1 B 0.43
A 1 B 0.57
A 1 B 0.71
A 1 B 0.86
A 1 B 1
OD 600 nm [–]
0.6
0.4
0.2
0 5 101520
Time [h]

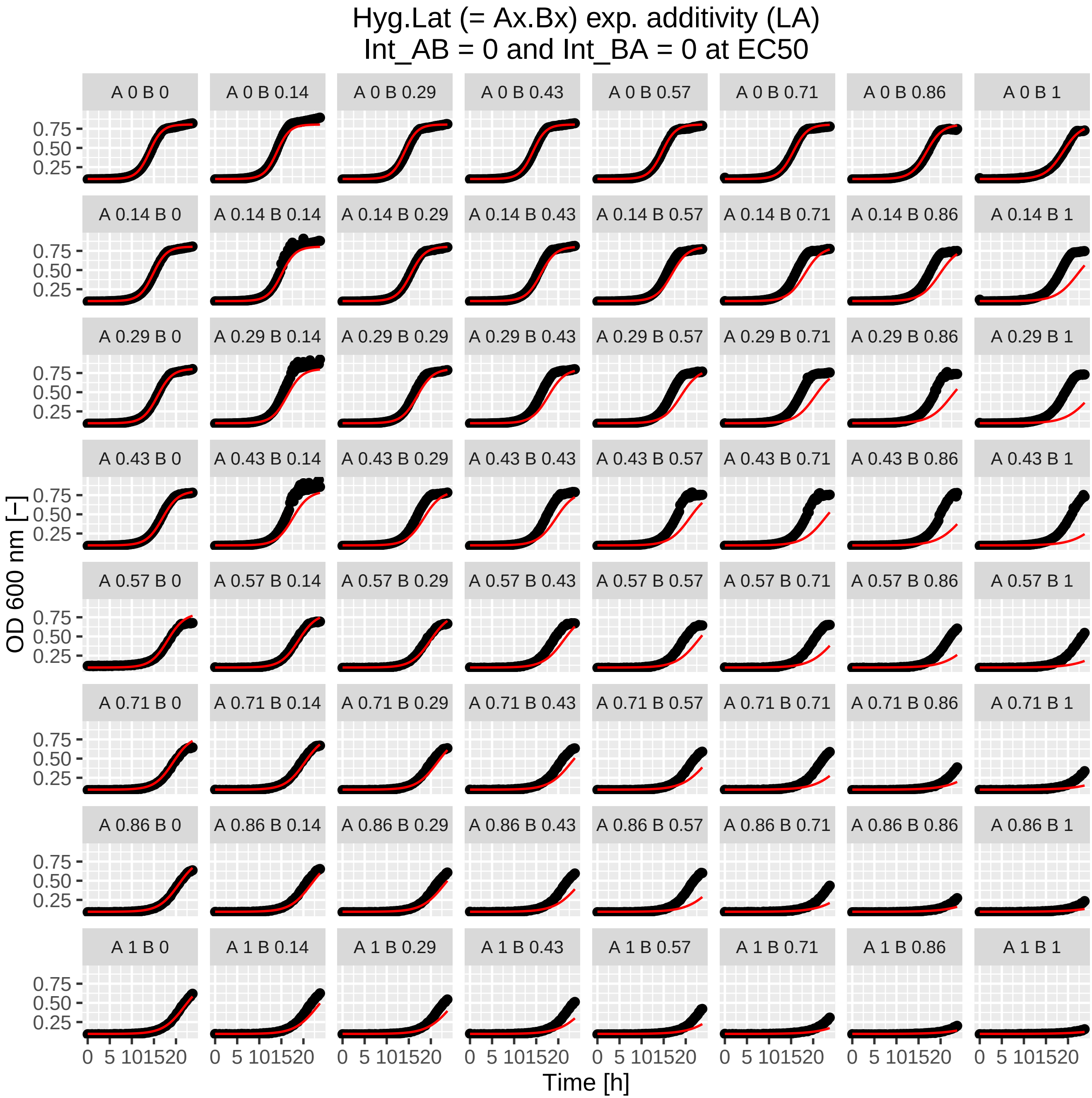
Hyg.Lat (= Ax.Bx) exp. additivity (LA)
Int_AB = 0 and Int_BA = 0 at EC50
OD 600 nm [–]
Time [h]
A 0 B 0
A 0 B 0.14
A 0 B 0.29
A 0 B 0.43
A 0 B 0.57
A 0 B 0.71
A 0 B 0.86
A 0 B 1
A 0.14 B 0
A 0.14 B 0.14
A 0.14 B 0.29
A 0.14 B 0.43
A 0.14 B 0.57
A 0.14 B 0.71
A 0.14 B 0.86
A 0.14 B 1
A 0.29 B 0
A 0.29 B 0.14
A 0.29 B 0.29
A 0.29 B 0.43
A 0.29 B 0.57
A 0.29 B 0.71
A 0.29 B 0.86
A 0.29 B 1
A 0.43 B 0
A 0.43 B 0.14
A 0.43 B 0.29
A 0.43 B 0.43
A 0.43 B 0.57
A 0.43 B 0.71
A 0.43 B 0.86
A 0.43 B 1
A 0.57 B 0
A 0.57 B 0.14
A 0.57 B 0.29
A 0.57 B 0.43
A 0.57 B 0.57
A 0.57 B 0.71
A 0.57 B 0.86
A 0.57 B 1
A 0.71 B 0
A 0.71 B 0.14
A 0.71 B 0.29
A 0.71 B 0.43
A 0.71 B 0.57
A 0.71 B 0.71
A 0.71 B 0.86
A 0.71 B 1
A 0.86 B 0
A 0.86 B 0.14
A 0.86 B 0.29
A 0.86 B 0.43
A 0.86 B 0.57
A 0.86 B 0.71
A 0.86 B 0.86
A 0.86 B 1
A 1 B 0
A 1 B 0.14
A 1 B 0.29
A 1 B 0.43
A 1 B 0.57
A 1 B 0.71
A 1 B 0.86
A 1 B 1
0.75
0.50
0.25
0 5 101520

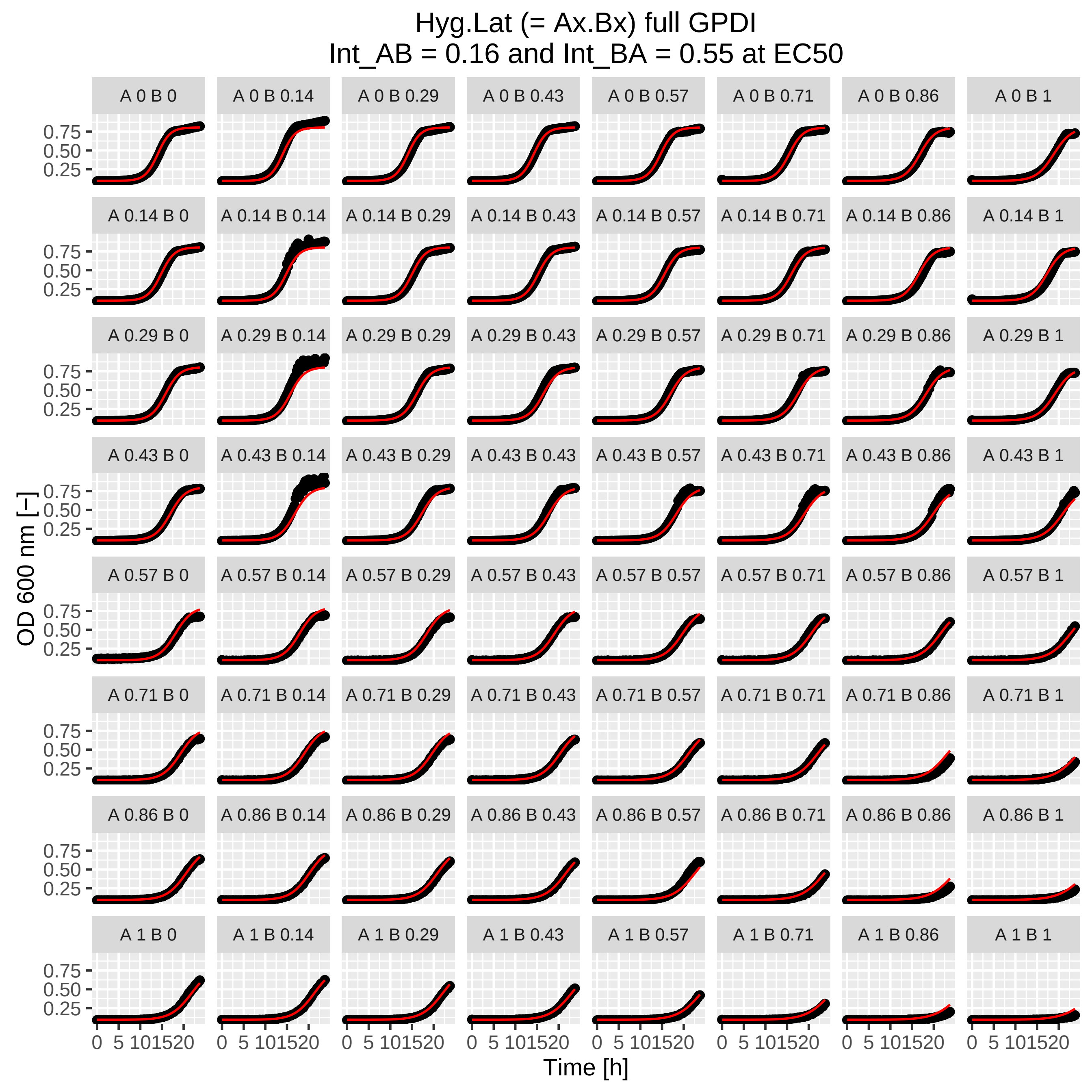

Hyg.Lat (= Ax.Bx) full GPDI
Int_AB = 0.16 and Int_BA = 0.55 at EC50
A 0 B 0
A 0 B 0.14
A 0 B 0.29
A 0 B 0.43
A 0 B 0.57
A 0 B 0.71
A 0 B 0.86
A 0 B 1
A 0.14 B 0
A 0.14 B 0.14
A 0.14 B 0.29
A 0.14 B 0.43
A 0.14 B 0.57
A 0.14 B 0.71
A 0.14 B 0.86
A 0.14 B 1
A 0.29 B 0
A 0.29 B 0.14
A 0.29 B 0.29
A 0.29 B 0.43
A 0.29 B 0.57
A 0.29 B 0.71
A 0.29 B 0.86
A 0.29 B 1
A 0.43 B 0
A 0.43 B 0.14
A 0.43 B 0.29
A 0.43 B 0.43
A 0.43 B 0.57
A 0.43 B 0.71
A 0.43 B 0.86
A 0.43 B 1
A 0.57 B 0
A 0.57 B 0.14
A 0.57 B 0.29
A 0.57 B 0.43
A 0.57 B 0.57
A 0.57 B 0.71
A 0.57 B 0.86
A 0.57 B 1
A 0.71 B 0
A 0.71 B 0.14
A 0.71 B 0.29
A 0.71 B 0.43
A 0.71 B 0.57
A 0.71 B 0.71
A 0.71 B 0.86
A 0.71 B 1
A 0.86 B 0
A 0.86 B 0.14
A 0.86 B 0.29
A 0.86 B 0.43
A 0.86 B 0.57
A 0.86 B 0.71
A 0.86 B 0.86
A 0.86 B 1
A 1 B 0
A 1 B 0.14
A 1 B 0.29
A 1 B 0.43
A 1 B 0.57
A 1 B 0.71
A 1 B 0.86
A 1 B 1
0.75
0.50
0.25
0 5 101520
OD 600 nm [–]
Time [h]

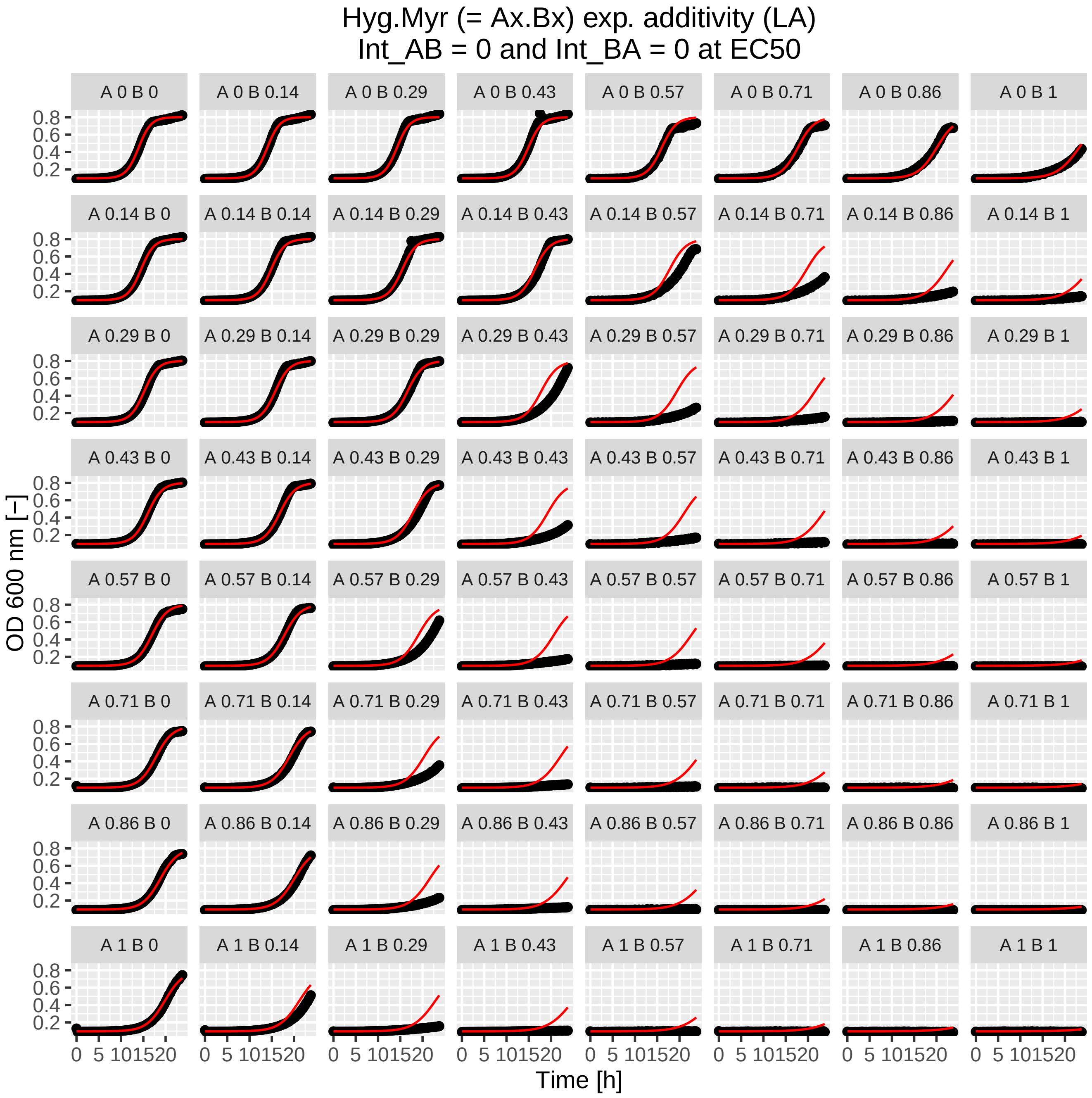

Hyg.Myr (= Ax.Bx) exp. additivity (LA)
Int_AB = 0 and Int_BA = 0 at EC50
A 0 B 0
A 0 B 0.14
A 0 B 0.29
A 0 B 0.43
A 0 B 0.57
A 0 B 0.71
A 0 B 0.86
A 0 B 1
A 0.14 B 0
A 0.14 B 0.14
A 0.14 B 0.29
A 0.14 B 0.43
A 0.14 B 0.57
A 0.14 B 0.71
A 0.14 B 0.86
A 0.14 B 1
A 0.29 B 0
A 0.29 B 0.14
A 0.29 B 0.29
A 0.29 B 0.43
A 0.29 B 0.57
A 0.29 B 0.71
A 0.29 B 0.86
A 0.29 B 1
A 0.43 B 0
A 0.43 B 0.14
A 0.43 B 0.29
A 0.43 B 0.43
A 0.43 B 0.57
A 0.43 B 0.71
A 0.43 B 0.86
A 0.43 B 1
A 0.57 B 0
A 0.57 B 0.14
A 0.57 B 0.29
A 0.57 B 0.43
A 0.57 B 0.57
A 0.57 B 0.71
A 0.57 B 0.86
A 0.57 B 1
A 0.71 B 0
A 0.71 B 0.14
A 0.71 B 0.29
A 0.71 B 0.43
A 0.71 B 0.57
A 0.71 B 0.71
A 0.71 B 0.86
A 0.71 B 1
A 0.86 B 0
A 0.86 B 0.14
A 0.86 B 0.29
A 0.86 B 0.43
A 0.86 B 0.57
A 0.86 B 0.71
A 0.86 B 0.86
A 0.86 B 1
A 1 B 0
A 1 B 0.14
A 1 B 0.29
A 1 B 0.43
A 1 B 0.57
A 1 B 0.71
A 1 B 0.86
A 1 B 1
OD 600 nm [–]
0.8
0.6
0.4
0.2
0 5 101520
Time [h]

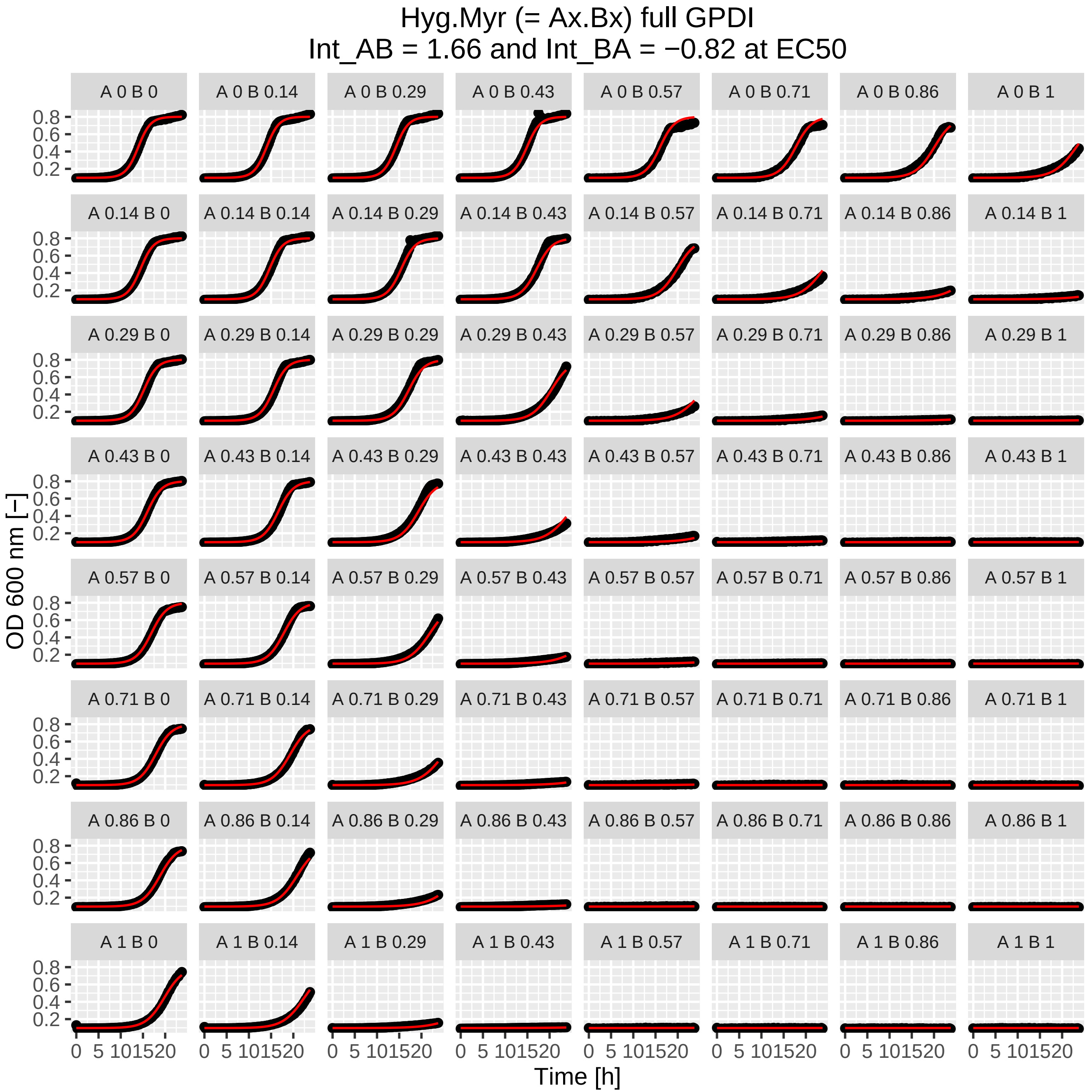
Hyg.Myr (= Ax.Bx) full GPDI
Int_AB = 1.66 and Int_BA = −0.82 at EC50
OD 600 nm [−]
Time [h]
A 0 B 0
A 0 B 0.14
A 0 B 0.29
A 0 B 0.43
A 0 B 0.57
A 0 B 0.71
A 0 B 0.86
A 0 B 1
A 0.14 B 0
A 0.29 B 0
A 0.43 B 0
A 0.57 B 0
A 0.71 B 0
A 0.86 B 0
A 1 B 0
A 1 B 1

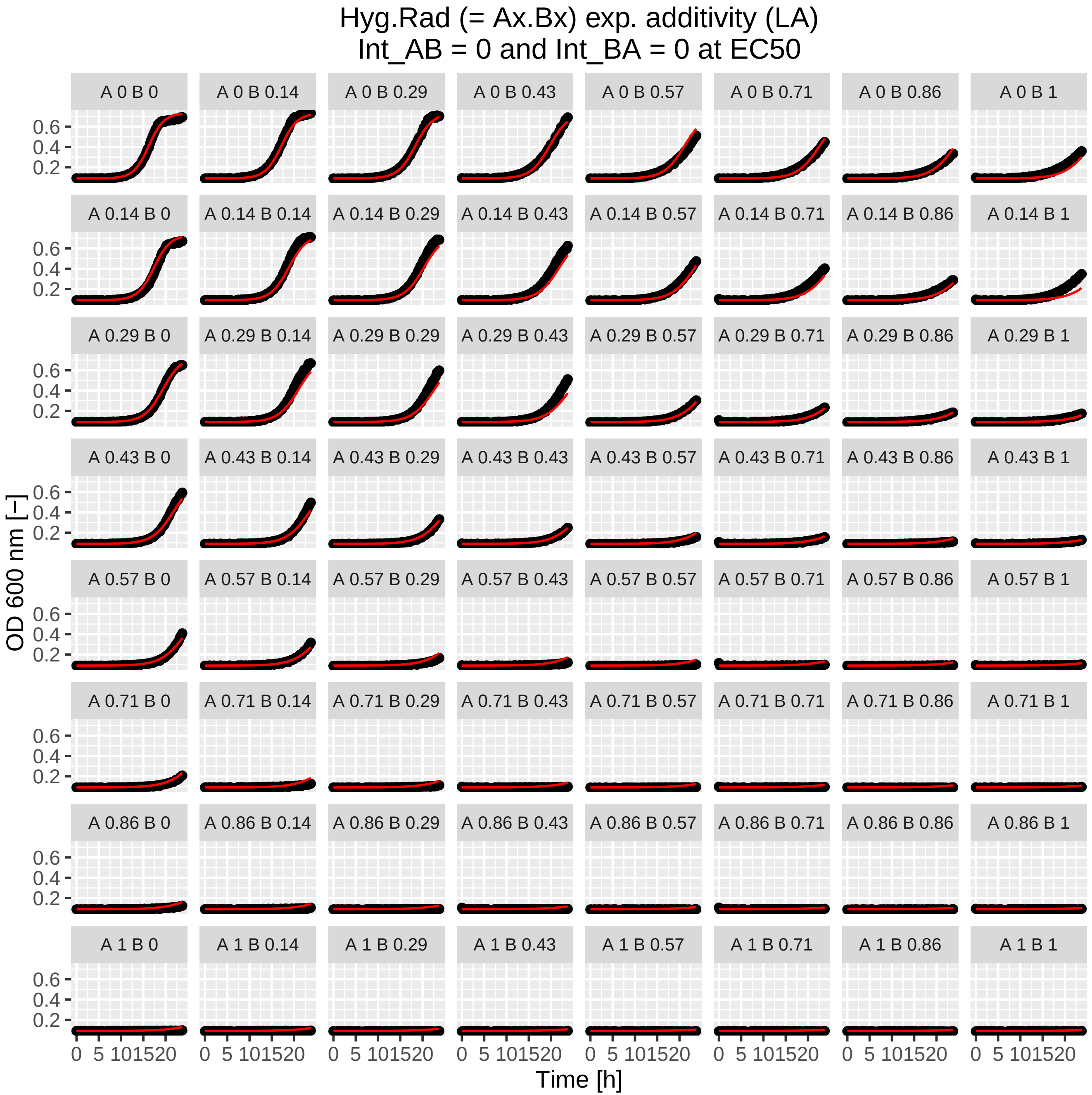
Hyg.Rad (= Ax.Bx) exp. additivity (LA)
Int_AB = 0 and Int_BA = 0 at EC50
A 0 B 0
A 0 B 0.14
A 0 B 0.29
A 0 B 0.43
A 0 B 0.57
A 0 B 0.71
A 0 B 0.86
A 0 B 1
A 0.14 B 0
A 0.14 B 0.14
A 0.14 B 0.29
A 0.14 B 0.43
A 0.14 B 0.57
A 0.14 B 0.71
A 0.14 B 0.86
A 0.14 B 1
A 0.29 B 0
A 0.29 B 0.14
A 0.29 B 0.29
A 0.29 B 0.43
A 0.29 B 0.57
A 0.29 B 0.71
A 0.29 B 0.86
A 0.29 B 1
A 0.43 B 0
A 0.43 B 0.14
A 0.43 B 0.29
A 0.43 B 0.43
A 0.43 B 0.57
A 0.43 B 0.71
A 0.43 B 0.86
A 0.43 B 1
A 0.57 B 0
A 0.57 B 0.14
A 0.57 B 0.29
A 0.57 B 0.43
A 0.57 B 0.57
A 0.57 B 0.71
A 0.57 B 0.86
A 0.57 B 1
A 0.71 B 0
A 0.71 B 0.14
A 0.71 B 0.29
A 0.71 B 0.43
A 0.71 B 0.57
A 0.71 B 0.71
A 0.71 B 0.86
A 0.71 B 1
A 0.86 B 0
A 0.86 B 0.14
A 0.86 B 0.29
A 0.86 B 0.43
A 0.86 B 0.57
A 0.86 B 0.71
A 0.86 B 0.86
A 0.86 B 1
A 1 B 0
A 1 B 0.14
A 1 B 0.29
A 1 B 0.43
A 1 B 0.57
A 1 B 0.71
A 1 B 0.86
A 1 B 1
OD 600 nm [–]
0.6
0.4
0.2
0 5 101520
Time [h]

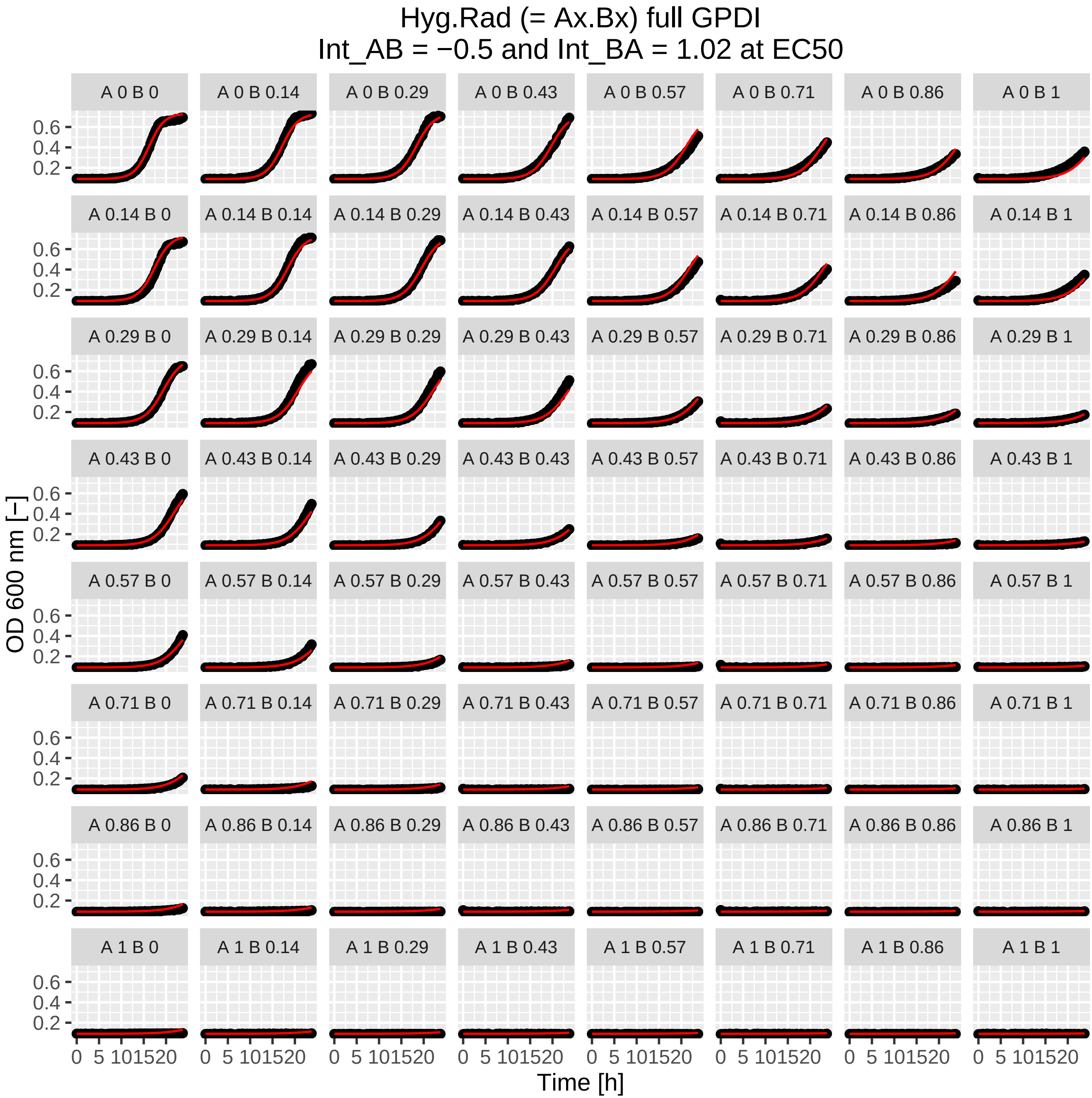
Hyg.Rad (= Ax.Bx) full GPDI
Int_AB = −0.5 and Int_BA = 1.02 at EC50
A 0 B 0
A 0 B 0.14
A 0 B 0.29
A 0 B 0.43
A 0 B 0.57
A 0 B 0.71
A 0 B 0.86
A 0 B 1
A 0.14 B 0
A 0.14 B 0.14
A 0.14 B 0.29
A 0.14 B 0.43
A 0.14 B 0.57
A 0.14 B 0.71
A 0.14 B 0.86
A 0.14 B 1
A 0.29 B 0
A 0.29 B 0.14
A 0.29 B 0.29
A 0.29 B 0.43
A 0.29 B 0.57
A 0.29 B 0.71
A 0.29 B 0.86
A 0.29 B 1
A 0.43 B 0
A 0.43 B 0.14
A 0.43 B 0.29
A 0.43 B 0.43
A 0.43 B 0.57
A 0.43 B 0.71
A 0.43 B 0.86
A 0.43 B 1
A 0.57 B 0
A 0.57 B 0.14
A 0.57 B 0.29
A 0.57 B 0.43
A 0.57 B 0.57
A 0.57 B 0.71
A 0.57 B 0.86
A 0.57 B 1
A 0.71 B 0
A 0.71 B 0.14
A 0.71 B 0.29
A 0.71 B 0.43
A 0.71 B 0.57
A 0.71 B 0.71
A 0.71 B 0.86
A 0.71 B 1
A 0.86 B 0
A 0.86 B 0.14
A 0.86 B 0.29
A 0.86 B 0.43
A 0.86 B 0.57
A 0.86 B 0.71
A 0.86 B 0.86
A 0.86 B 1
A 1 B 0
A 1 B 0.14
A 1 B 0.29
A 1 B 0.43
A 1 B 0.57
A 1 B 0.71
A 1 B 0.86
A 1 B 1
OD 600 nm [−]
0.2
0.4
0.6
0 5 101520
Time [h]

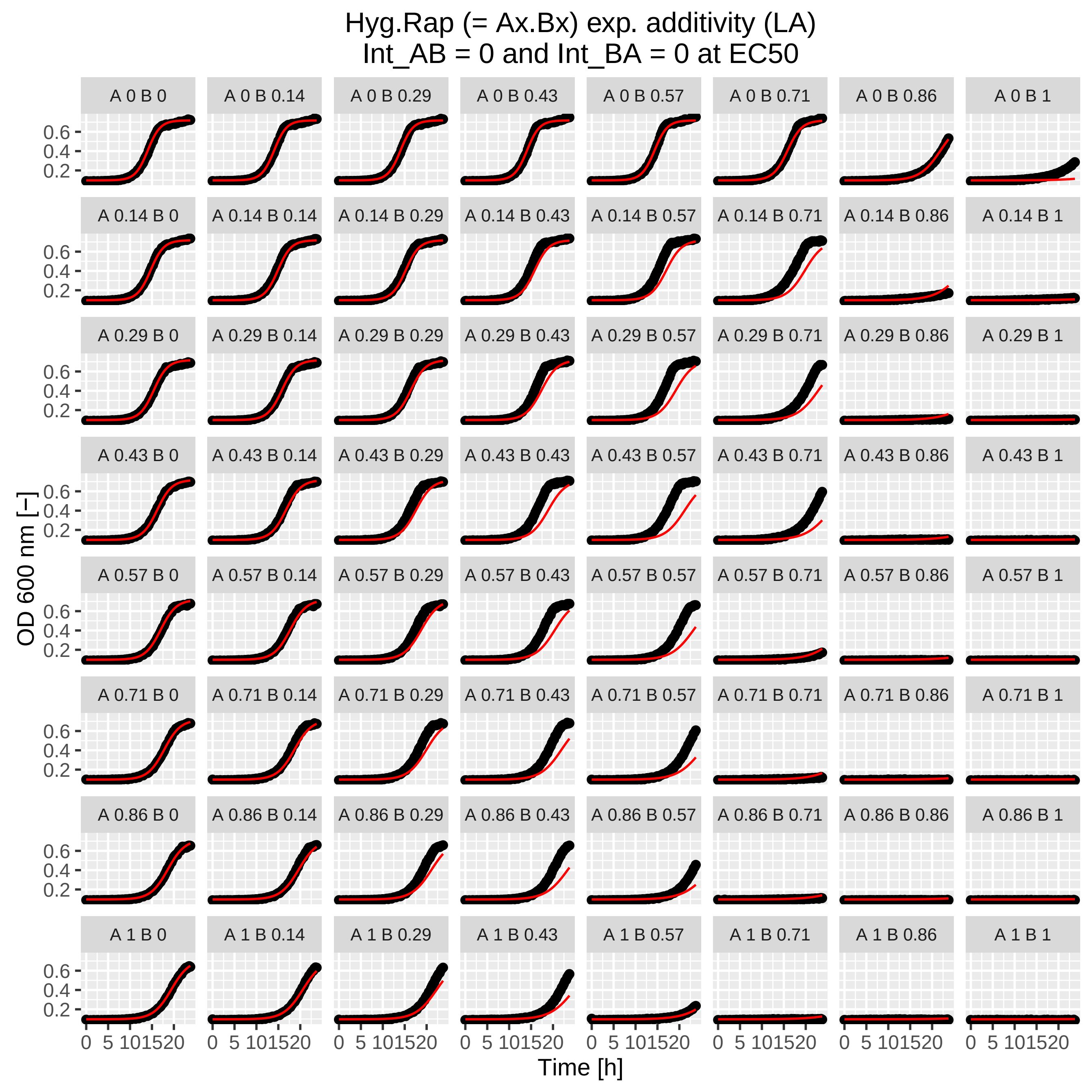

Hyg.Rap (= Ax.Bx) exp. additivity (LA)
Int_AB = 0 and Int_BA = 0 at EC50
A 0 B 0
A 0 B 0.14
A 0 B 0.29
A 0 B 0.43
A 0 B 0.57
A 0 B 0.71
A 0 B 0.86
A 0 B 1
A 0.14 B 0
A 0.14 B 0.14
A 0.14 B 0.29
A 0.14 B 0.43
A 0.14 B 0.57
A 0.14 B 0.71
A 0.14 B 0.86
A 0.14 B 1
A 0.29 B 0
A 0.29 B 0.14
A 0.29 B 0.29
A 0.29 B 0.43
A 0.29 B 0.57
A 0.29 B 0.71
A 0.29 B 0.86
A 0.29 B 1
A 0.43 B 0
A 0.43 B 0.14
A 0.43 B 0.29
A 0.43 B 0.43
A 0.43 B 0.57
A 0.43 B 0.71
A 0.43 B 0.86
A 0.43 B 1
A 0.57 B 0
A 0.57 B 0.14
A 0.57 B 0.29
A 0.57 B 0.43
A 0.57 B 0.57
A 0.57 B 0.71
A 0.57 B 0.86
A 0.57 B 1
A 0.71 B 0
A 0.71 B 0.14
A 0.71 B 0.29
A 0.71 B 0.43
A 0.71 B 0.57
A 0.71 B 0.71
A 0.71 B 0.86
A 0.71 B 1
A 0.86 B 0
A 0.86 B 0.14
A 0.86 B 0.29
A 0.86 B 0.43
A 0.86 B 0.57
A 0.86 B 0.71
A 0.86 B 0.86
A 0.86 B 1
A 1 B 0
A 1 B 0.14
A 1 B 0.29
A 1 B 0.43
A 1 B 0.57
A 1 B 0.71
A 1 B 0.86
A 1 B 1
OD 600 nm [–]
0.2
0.4
0.6
0 5 101520
Time [h]

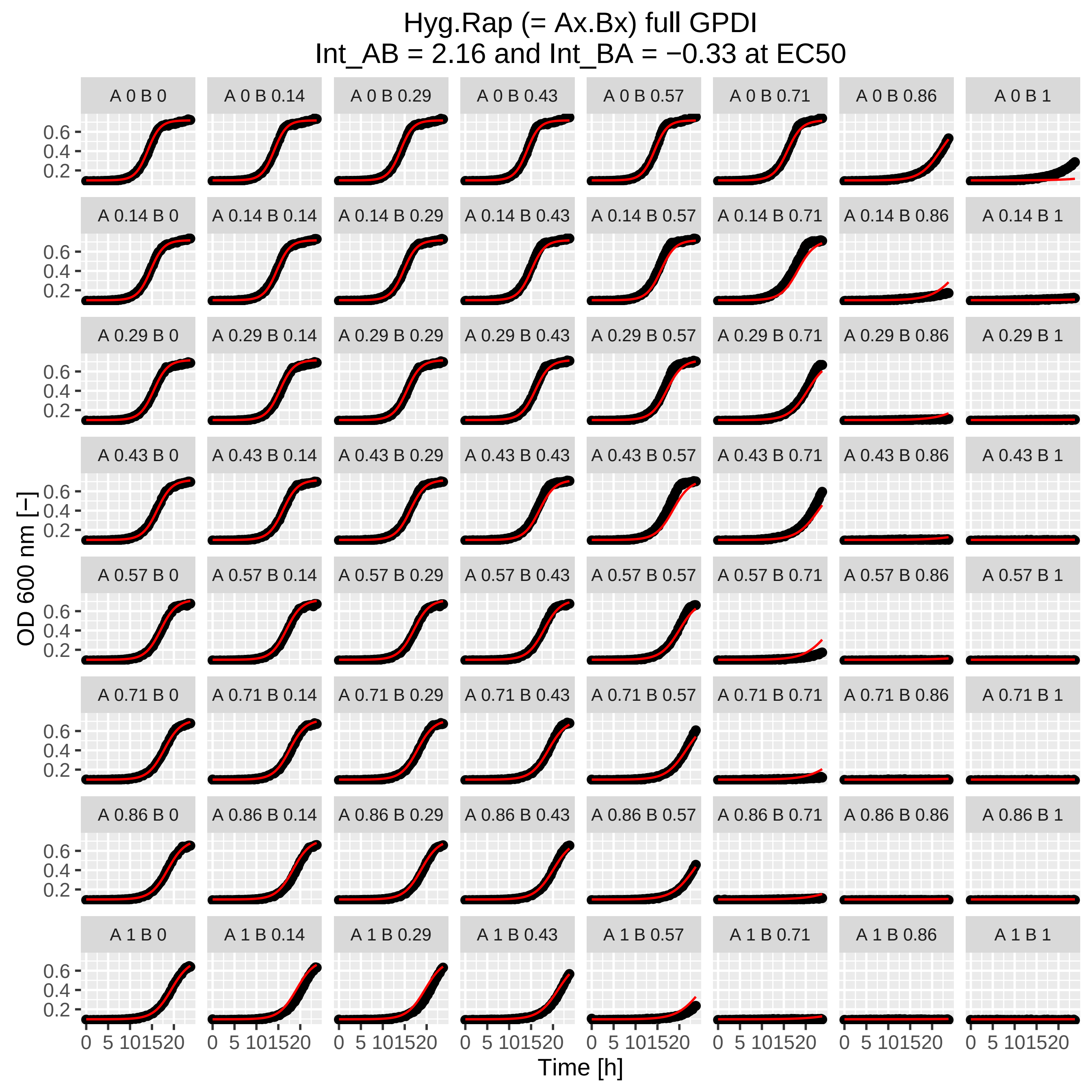
Hyg.Rap (= Ax.Bx) full GPDI
Int_AB = 2.16 and Int_BA = −0.33 at EC50
OD 600 nm [−]
Time [h]
0 5 101520
0.2 0.4 0.6
A 0 B 0
A 0 B 0.14
A 0 B 0.29
A 0 B 0.43
A 0 B 0.57
A 0 B 0.71
A 0 B 0.86
A 0 B 1
A 0.14 B 0
A 0.14 B 0.14
A 0.14 B 0.29
A 0.14 B 0.43
A 0.14 B 0.57
A 0.14 B 0.71
A 0.14 B 0.86
A 0.14 B 1
A 0.29 B 0
A 0.29 B 0.14
A 0.29 B 0.29
A 0.29 B 0.43
A 0.29 B 0.57
A 0.29 B 0.71
A 0.29 B 0.86
A 0.29 B 1
A 0.43 B 0
A 0.43 B 0.14
A 0.43 B 0.29
A 0.43 B 0.43
A 0.43 B 0.57
A 0.43 B 0.71
A 0.43 B 0.86
A 0.43 B 1
A 0.57 B 0
A 0.57 B 0.14
A 0.57 B 0.29
A 0.57 B 0.43
A 0.57 B 0.57
A 0.57 B 0.71
A 0.57 B 0.86
A 0.57 B 1
A 0.71 B 0
A 0.71 B 0.14
A 0.71 B 0.29
A 0.71 B 0.43
A 0.71 B 0.57
A 0.71 B 0.71
A 0.71 B 0.86
A 0.71 B 1
A 0.86 B 0
A 0.86 B 0.14
A 0.86 B 0.29
A 0.86 B 0.43
A 0.86 B 0.57
A 0.86 B 0.71
A 0.86 B 0.86
A 0.86 B 1
A 1 B 0
A 1 B 0.14
A 1 B 0.29
A 1 B 0.43
A 1 B 0.57
A 1 B 0.71
A 1 B 0.86
A 1 B 1

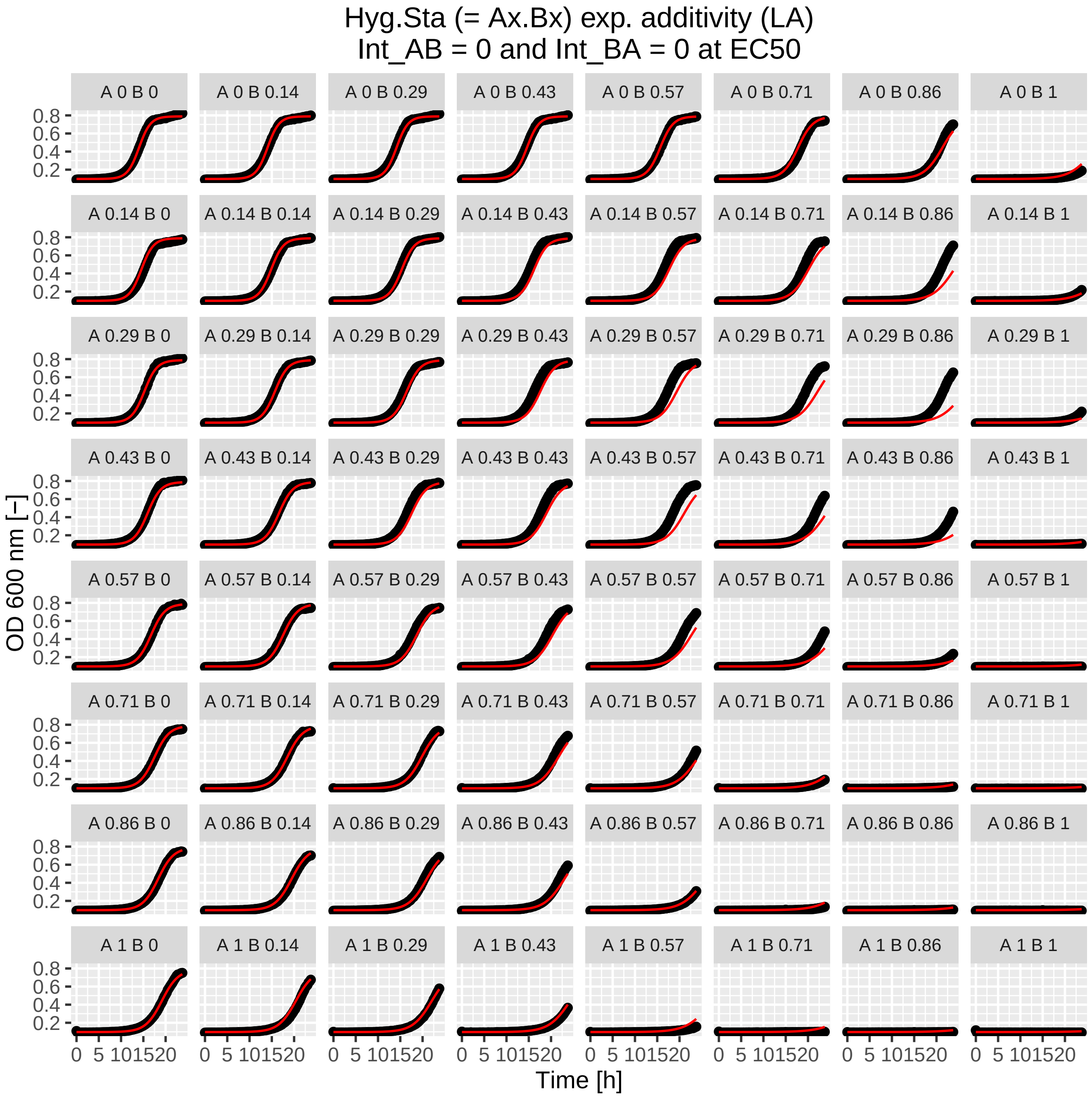

Hyg.Sta (= Ax.Bx) exp. additivity (LA)
Int_AB = 0 and Int_BA = 0 at EC50
A 0 B 0
A 0 B 0.14
A 0 B 0.29
A 0 B 0.43
A 0 B 0.57
A 0 B 0.71
A 0 B 0.86
A 0 B 1
A 0.14 B 0
A 0.14 B 0.14
A 0.14 B 0.29
A 0.14 B 0.43
A 0.14 B 0.57
A 0.14 B 0.71
A 0.14 B 0.86
A 0.14 B 1
A 0.29 B 0
A 0.29 B 0.14
A 0.29 B 0.29
A 0.29 B 0.43
A 0.29 B 0.57
A 0.29 B 0.71
A 0.29 B 0.86
A 0.29 B 1
A 0.43 B 0
A 0.43 B 0.14
A 0.43 B 0.29
A 0.43 B 0.43
A 0.43 B 0.57
A 0.43 B 0.71
A 0.43 B 0.86
A 0.43 B 1
A 0.57 B 0
A 0.57 B 0.14
A 0.57 B 0.29
A 0.57 B 0.43
A 0.57 B 0.57
A 0.57 B 0.71
A 0.57 B 0.86
A 0.57 B 1
A 0.71 B 0
A 0.71 B 0.14
A 0.71 B 0.29
A 0.71 B 0.43
A 0.71 B 0.57
A 0.71 B 0.71
A 0.71 B 0.86
A 0.71 B 1
A 0.86 B 0
A 0.86 B 0.14
A 0.86 B 0.29
A 0.86 B 0.43
A 0.86 B 0.57
A 0.86 B 0.71
A 0.86 B 0.86
A 0.86 B 1
A 1 B 0
A 1 B 0.14
A 1 B 0.29
A 1 B 0.43
A 1 B 0.57
A 1 B 0.71
A 1 B 0.86
A 1 B 1
OD 600 nm [–]
0.8
0.6
0.4
0.2
0 5 101520
Time [h]

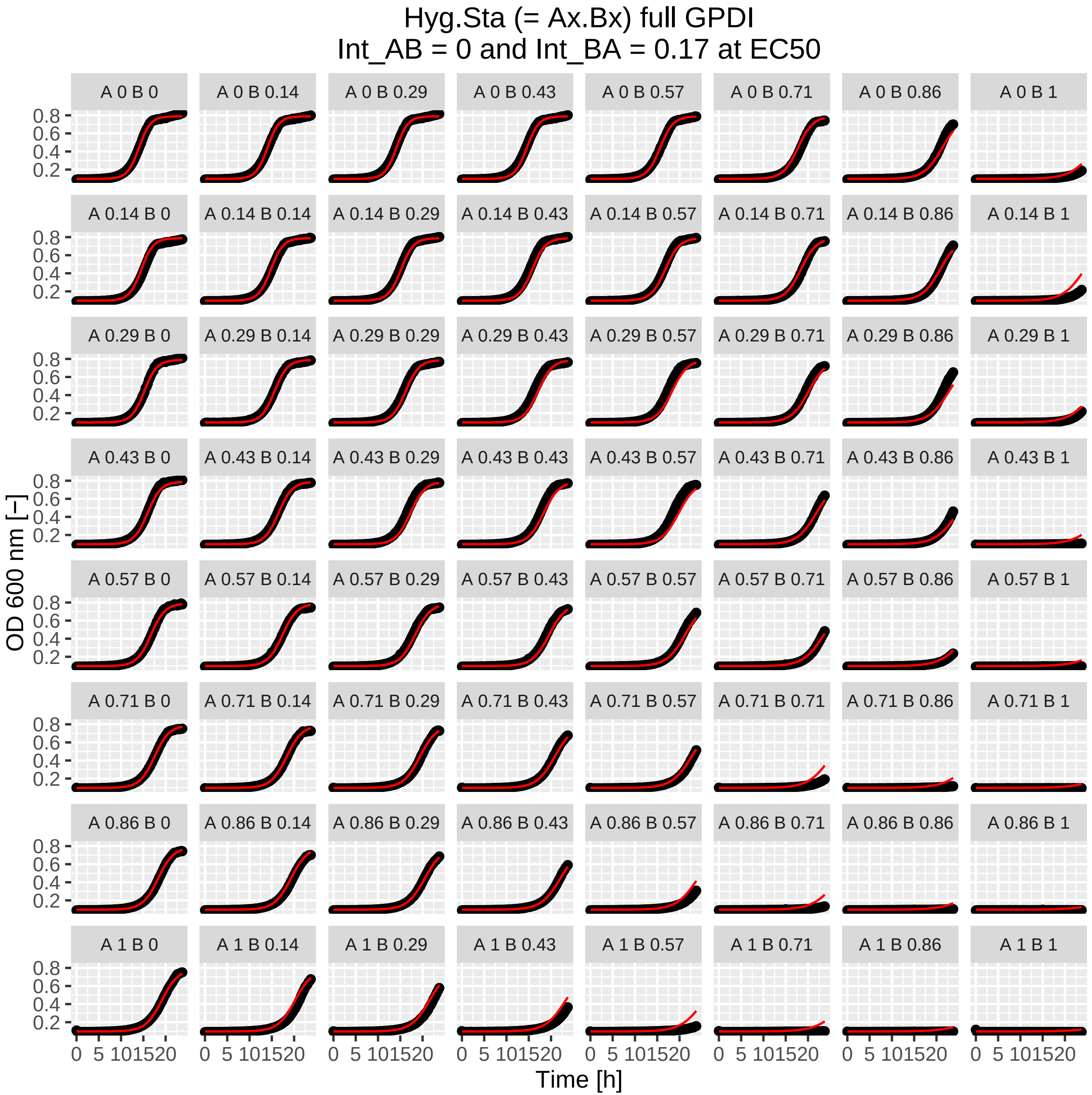
Hyg.Sta (= Ax.Bx) full GPDI
Int_AB = 0 and Int_BA = 0.17 at EC50
A 0 B 0
A 0 B 0.14
A 0 B 0.29
A 0 B 0.43
A 0 B 0.57
A 0 B 0.71
A 0 B 0.86
A 0 B 1
A 0.14 B 0
A 0.14 B 0.14
A 0.14 B 0.29
A 0.14 B 0.43
A 0.14 B 0.57
A 0.14 B 0.71
A 0.14 B 0.86
A 0.14 B 1
A 0.29 B 0
A 0.29 B 0.14
A 0.29 B 0.29
A 0.29 B 0.43
A 0.29 B 0.57
A 0.29 B 0.71
A 0.29 B 0.86
A 0.29 B 1
A 0.43 B 0
A 0.43 B 0.14
A 0.43 B 0.29
A 0.43 B 0.43
A 0.43 B 0.57
A 0.43 B 0.71
A 0.43 B 0.86
A 0.43 B 1
A 0.57 B 0
A 0.57 B 0.14
A 0.57 B 0.29
A 0.57 B 0.43
A 0.57 B 0.57
A 0.57 B 0.71
A 0.57 B 0.86
A 0.57 B 1
A 0.71 B 0
A 0.71 B 0.14
A 0.71 B 0.29
A 0.71 B 0.43
A 0.71 B 0.57
A 0.71 B 0.71
A 0.71 B 0.86
A 0.71 B 1
A 0.86 B 0
A 0.86 B 0.14
A 0.86 B 0.29
A 0.86 B 0.43
A 0.86 B 0.57
A 0.86 B 0.71
A 0.86 B 0.86
A 0.86 B 1
A 1 B 0
A 1 B 0.14
A 1 B 0.29
A 1 B 0.43
A 1 B 0.57
A 1 B 0.71
A 1 B 0.86
A 1 B 1
0.8
0.6
0.4
0.2
0 5 101520
OD 600 nm [–]
Time [h]

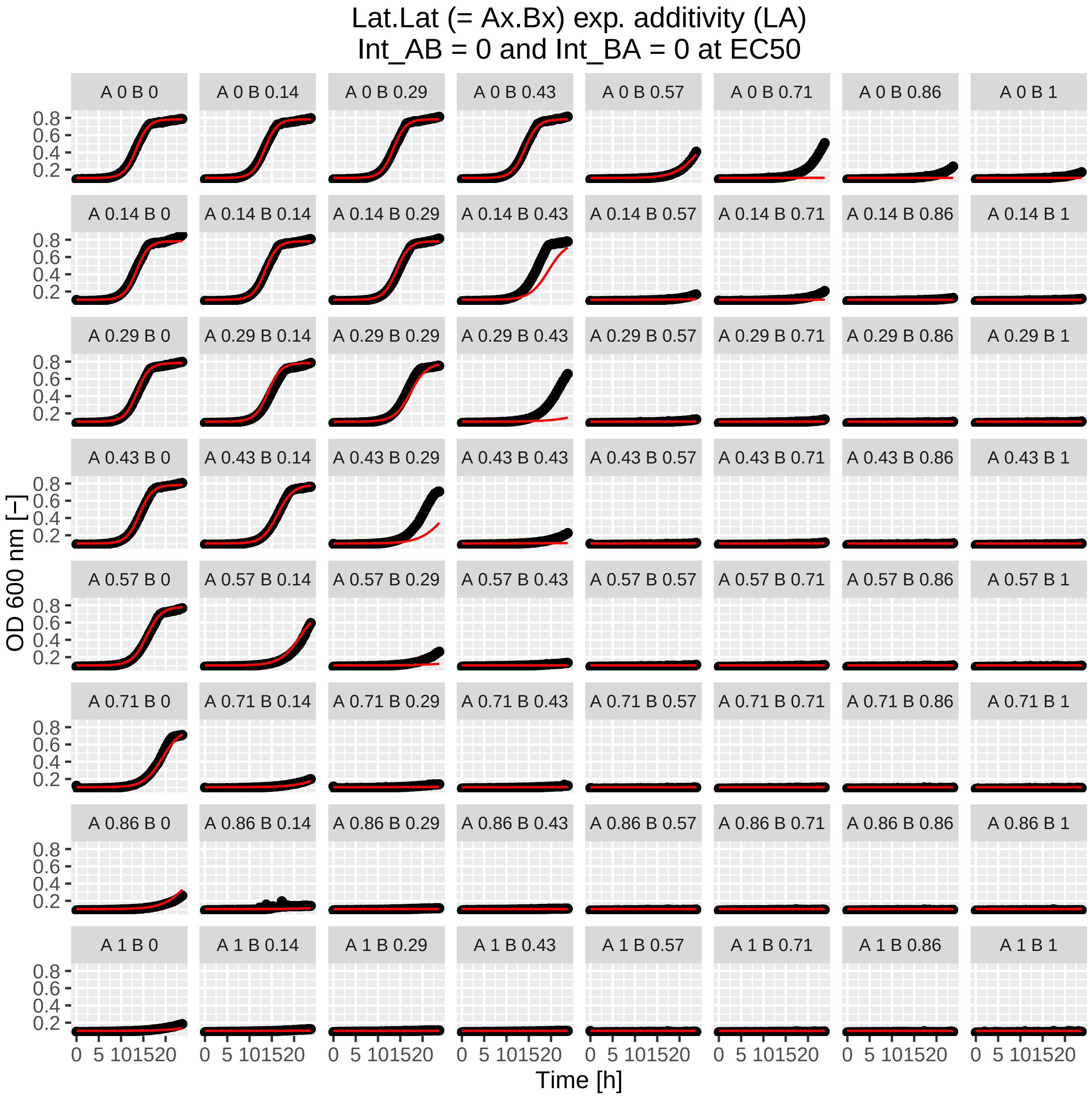
Lat.Lat (= Ax.Bx) exp. additivity (LA)
Int_AB = 0 and Int_BA = 0 at EC50
A 0 B 0
A 0 B 0.14
A 0 B 0.29
A 0 B 0.43
A 0 B 0.57
A 0 B 0.71
A 0 B 0.86
A 0 B 1
A 0.14 B 0
A 0.14 B 0.14
A 0.14 B 0.29
A 0.14 B 0.43
A 0.14 B 0.57
A 0.14 B 0.71
A 0.14 B 0.86
A 0.14 B 1
A 0.29 B 0
A 0.29 B 0.14
A 0.29 B 0.29
A 0.29 B 0.43
A 0.29 B 0.57
A 0.29 B 0.71
A 0.29 B 0.86
A 0.29 B 1
A 0.43 B 0
A 0.43 B 0.14
A 0.43 B 0.29
A 0.43 B 0.43
A 0.43 B 0.57
A 0.43 B 0.71
A 0.43 B 0.86
A 0.43 B 1
A 0.57 B 0
A 0.57 B 0.14
A 0.57 B 0.29
A 0.57 B 0.43
A 0.57 B 0.57
A 0.57 B 0.71
A 0.57 B 0.86
A 0.57 B 1
A 0.71 B 0
A 0.71 B 0.14
A 0.71 B 0.29
A 0.71 B 0.43
A 0.71 B 0.57
A 0.71 B 0.71
A 0.71 B 0.86
A 0.71 B 1
A 0.86 B 0
A 0.86 B 0.14
A 0.86 B 0.29
A 0.86 B 0.43
A 0.86 B 0.57
A 0.86 B 0.71
A 0.86 B 0.86
A 0.86 B 1
A 1 B 0
A 1 B 0.14
A 1 B 0.29
A 1 B 0.43
A 1 B 0.57
A 1 B 0.71
A 1 B 0.86
A 1 B 1
OD 600 nm [−]
0.8
0.6
0.4
0.2
0 5 101520
Time [h]

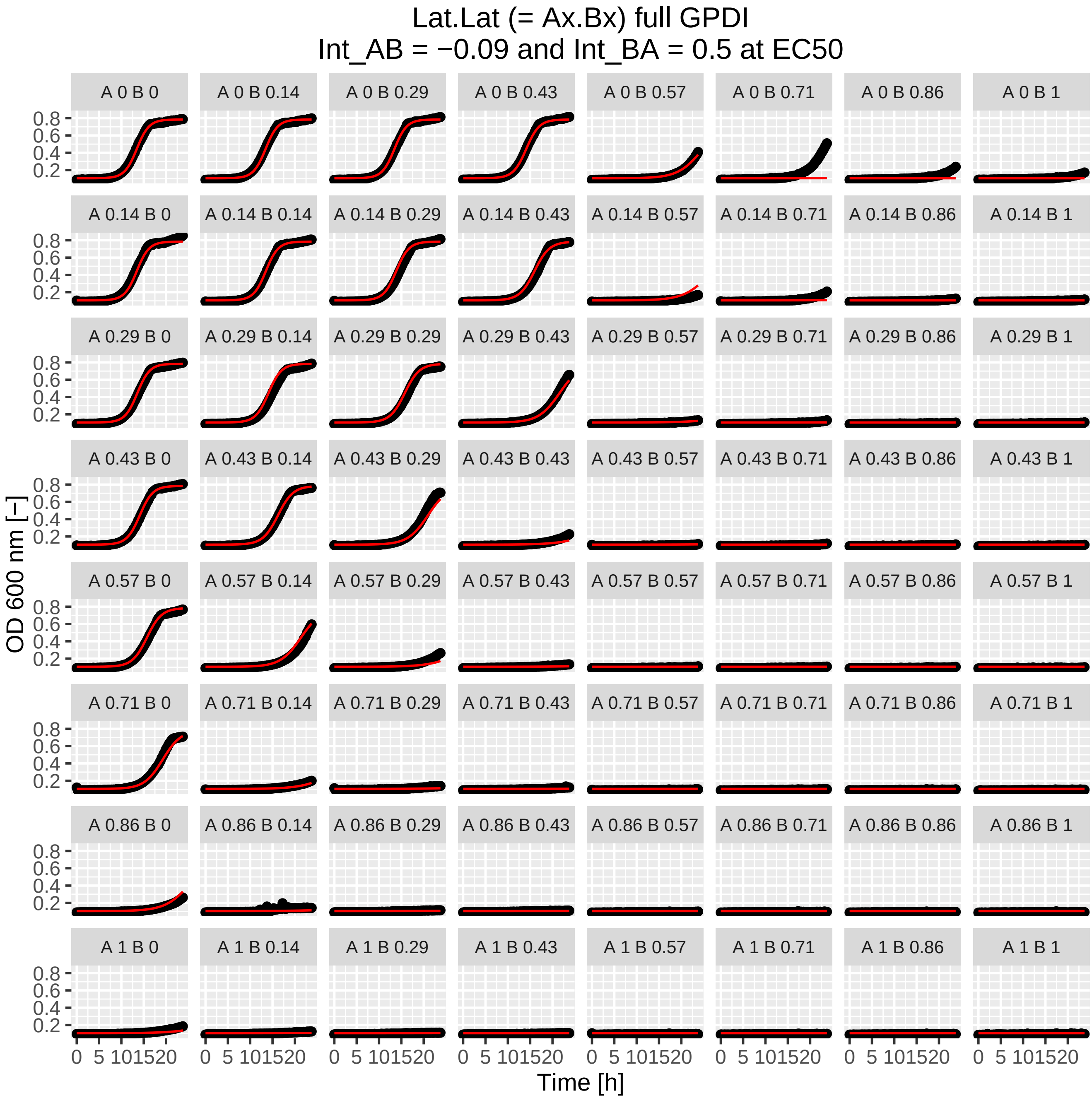

Lat.Lat (= Ax.Bx) full GPDI
Int_AB = −0.09 and Int_BA = 0.5 at EC50
OD 600 nm [−]
Time [h]
0.8
0.6
0.4
0.2
0 5 101520
A 0 B 0
A 0 B 0.14
A 0 B 0.29
A 0 B 0.43
A 0 B 0.57
A 0 B 0.71
A 0 B 0.86
A 0 B 1
A 0.14 B 0
A 0.14 B 0.14
A 0.14 B 0.29
A 0.14 B 0.43
A 0.14 B 0.57
A 0.14 B 0.71
A 0.14 B 0.86
A 0.14 B 1
A 0.29 B 0
A 0.29 B 0.14
A 0.29 B 0.29
A 0.29 B 0.43
A 0.29 B 0.57
A 0.29 B 0.71
A 0.29 B 0.86
A 0.29 B 1
A 0.43 B 0
A 0.43 B 0.14
A 0.43 B 0.29
A 0.43 B 0.43
A 0.43 B 0.57
A 0.43 B 0.71
A 0.43 B 0.86
A 0.43 B 1
A 0.57 B 0
A 0.57 B 0.14
A 0.57 B 0.29
A 0.57 B 0.43
A 0.57 B 0.57
A 0.57 B 0.71
A 0.57 B 0.86
A 0.57 B 1
A 0.71 B 0
A 0.71 B 0.14
A 0.71 B 0.29
A 0.71 B 0.43
A 0.71 B 0.57
A 0.71 B 0.71
A 0.71 B 0.86
A 0.71 B 1
A 0.86 B 0
A 0.86 B 0.14
A 0.86 B 0.29
A 0.86 B 0.43
A 0.86 B 0.57
A 0.86 B 0.71
A 0.86 B 0.86
A 0.86 B 1
A 1 B 0
A 1 B 0.14
A 1 B 0.29
A 1 B 0.43
A 1 B 0.57
A 1 B 0.71
A 1 B 0.86
A 1 B 1

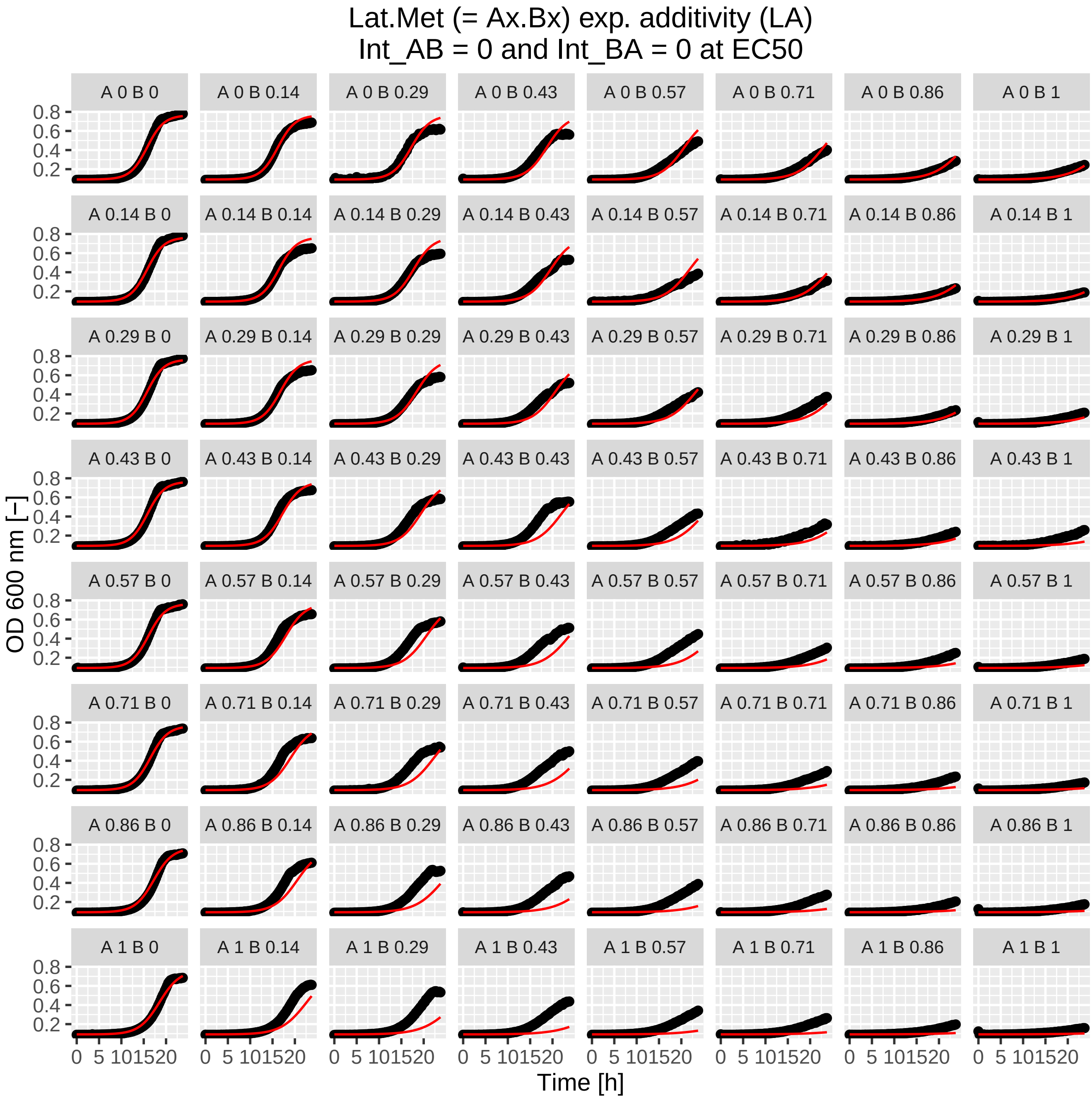

Lat.Met (= Ax.Bx) exp. additivity (LA)
Int_AB = 0 and Int_BA = 0 at EC50
A 0 B 0
A 0 B 0.14
A 0 B 0.29
A 0 B 0.43
A 0 B 0.57
A 0 B 0.71
A 0 B 0.86
A 0 B 1
A 0.14 B 0
A 0.14 B 0.14
A 0.14 B 0.29
A 0.14 B 0.43
A 0.14 B 0.57
A 0.14 B 0.71
A 0.14 B 0.86
A 0.14 B 1
A 0.29 B 0
A 0.29 B 0.14
A 0.29 B 0.29
A 0.29 B 0.43
A 0.29 B 0.57
A 0.29 B 0.71
A 0.29 B 0.86
A 0.29 B 1
A 0.43 B 0
A 0.43 B 0.14
A 0.43 B 0.29
A 0.43 B 0.43
A 0.43 B 0.57
A 0.43 B 0.71
A 0.43 B 0.86
A 0.43 B 1
A 0.57 B 0
A 0.57 B 0.14
A 0.57 B 0.29
A 0.57 B 0.43
A 0.57 B 0.57
A 0.57 B 0.71
A 0.57 B 0.86
A 0.57 B 1
A 0.71 B 0
A 0.71 B 0.14
A 0.71 B 0.29
A 0.71 B 0.43
A 0.71 B 0.57
A 0.71 B 0.71
A 0.71 B 0.86
A 0.71 B 1
A 0.86 B 0
A 0.86 B 0.14
A 0.86 B 0.29
A 0.86 B 0.43
A 0.86 B 0.57
A 0.86 B 0.71
A 0.86 B 0.86
A 0.86 B 1
A 1 B 0
A 1 B 0.14
A 1 B 0.29
A 1 B 0.43
A 1 B 0.57
A 1 B 0.71
A 1 B 0.86
A 1 B 1
OD 600 nm [–]
0.8
0.6
0.4
0.2
0 5 101520
Time [h]

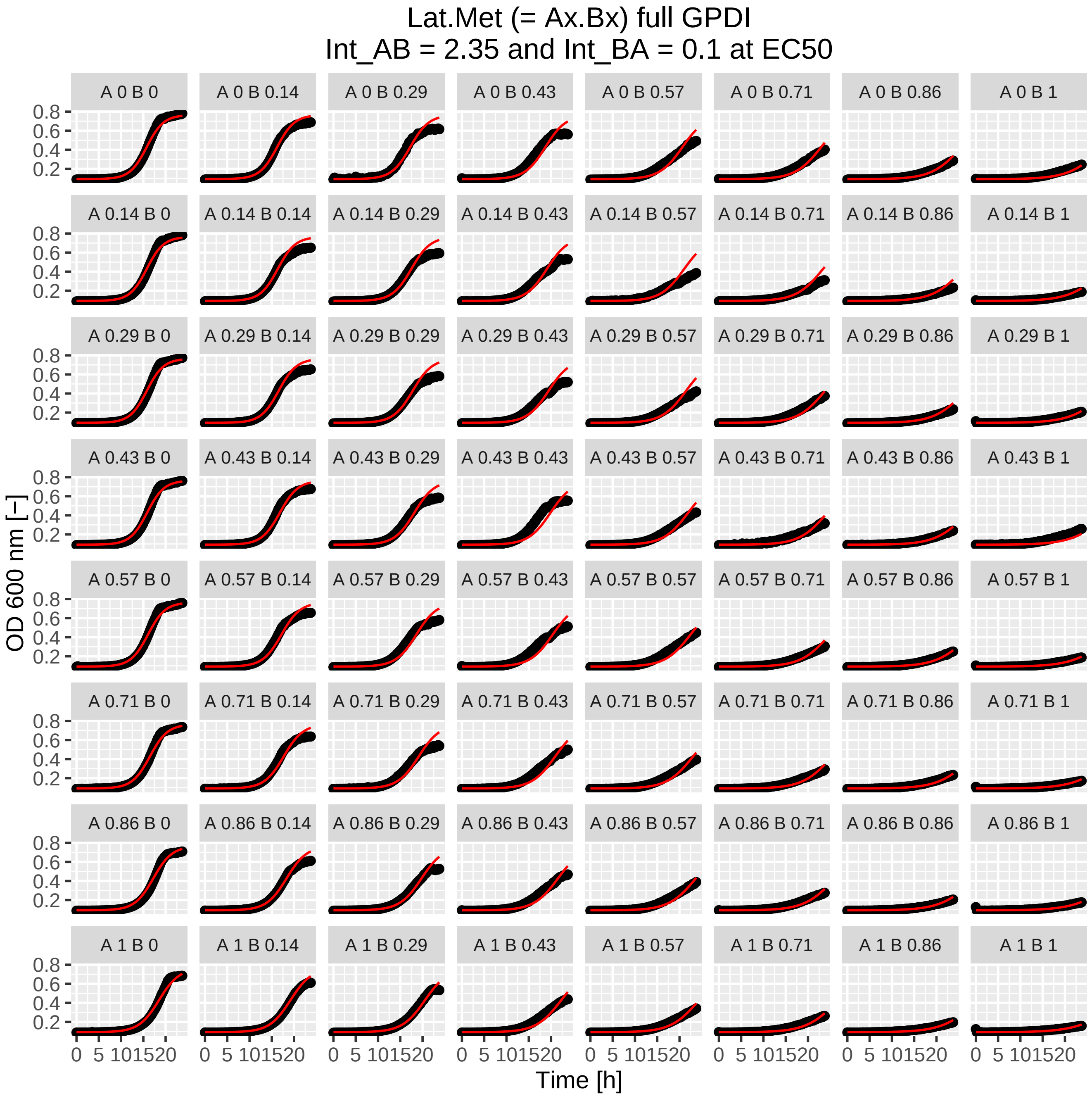
Lat.Met (= Ax.Bx) full GPDI
Int_AB = 2.35 and Int_BA = 0.1 at EC50
A 0 B 0
A 0 B 0.14
A 0 B 0.29
A 0 B 0.43
A 0 B 0.57
A 0 B 0.71
A 0 B 0.86
A 0 B 1
A 0.14 B 0
A 0.14 B 0.14
A 0.14 B 0.29
A 0.14 B 0.43
A 0.14 B 0.57
A 0.14 B 0.71
A 0.14 B 0.86
A 0.14 B 1
A 0.29 B 0
A 0.29 B 0.14
A 0.29 B 0.29
A 0.29 B 0.43
A 0.29 B 0.57
A 0.29 B 0.71
A 0.29 B 0.86
A 0.29 B 1
A 0.43 B 0
A 0.43 B 0.14
A 0.43 B 0.29
A 0.43 B 0.43
A 0.43 B 0.57
A 0.43 B 0.71
A 0.43 B 0.86
A 0.43 B 1
A 0.57 B 0
A 0.57 B 0.14
A 0.57 B 0.29
A 0.57 B 0.43
A 0.57 B 0.57
A 0.57 B 0.71
A 0.57 B 0.86
A 0.57 B 1
A 0.71 B 0
A 0.71 B 0.14
A 0.71 B 0.29
A 0.71 B 0.43
A 0.71 B 0.57
A 0.71 B 0.71
A 0.71 B 0.86
A 0.71 B 1
A 0.86 B 0
A 0.86 B 0.14
A 0.86 B 0.29
A 0.86 B 0.43
A 0.86 B 0.57
A 0.86 B 0.71
A 0.86 B 0.86
A 0.86 B 1
A 1 B 0
A 1 B 0.14
A 1 B 0.29
A 1 B 0.43
A 1 B 0.57
A 1 B 0.71
A 1 B 0.86
A 1 B 1
OD 600 nm [–]
0.2
0.4
0.6
0.8
0 5 101520
Time [h]

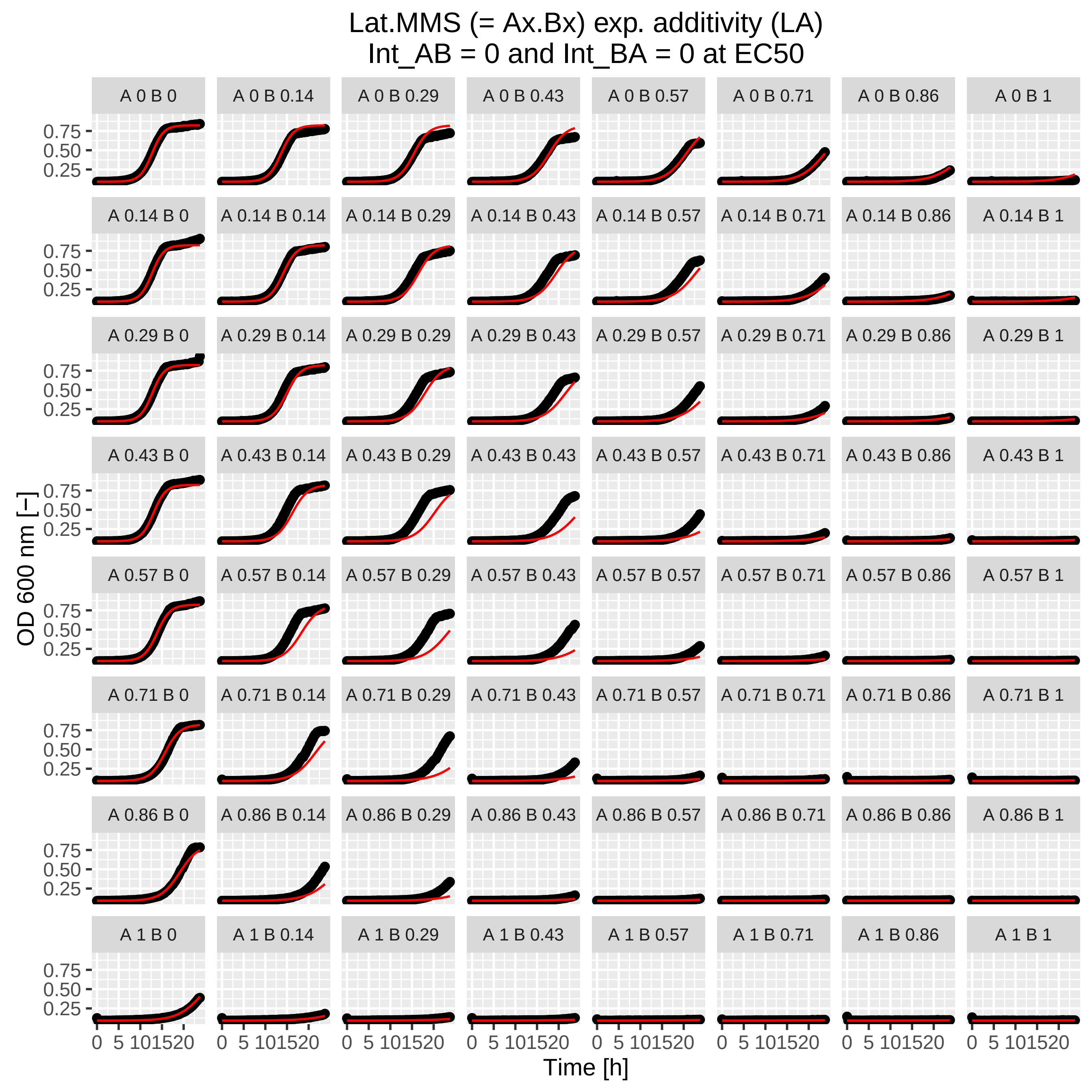
Lat.MMS (= Ax.Bx) exp. additivity (LA)
Int_AB = 0 and Int_BA = 0 at EC50
OD 600 nm [–]
Time [h]
0.75
0.50
0.25
0 5 101520
A 0 B 0
A 0 B 0.14
A 0 B 0.29
A 0 B 0.43
A 0 B 0.57
A 0 B 0.71
A 0 B 0.86
A 0 B 1
A 0.14 B 0
A 0.14 B 0.14
A 0.14 B 0.29
A 0.14 B 0.43
A 0.14 B 0.57
A 0.14 B 0.71
A 0.14 B 0.86
A 0.14 B 1
A 0.29 B 0
A 0.29 B 0.14
A 0.29 B 0.29
A 0.29 B 0.43
A 0.29 B 0.57
A 0.29 B 0.71
A 0.29 B 0.86
A 0.29 B 1
A 0.43 B 0
A 0.43 B 0.14
A 0.43 B 0.29
A 0.43 B 0.43
A 0.43 B 0.57
A 0.43 B 0.71
A 0.43 B 0.86
A 0.43 B 1
A 0.57 B 0
A 0.57 B 0.14
A 0.57 B 0.29
A 0.57 B 0.43
A 0.57 B 0.57
A 0.57 B 0.71
A 0.57 B 0.86
A 0.57 B 1
A 0.71 B 0
A 0.71 B 0.14
A 0.71 B 0.29
A 0.71 B 0.43
A 0.71 B 0.57
A 0.71 B 0.71
A 0.71 B 0.86
A 0.71 B 1
A 0.86 B 0
A 0.86 B 0.14
A 0.86 B 0.29
A 0.86 B 0.43
A 0.86 B 0.57
A 0.86 B 0.71
A 0.86 B 0.86
A 0.86 B 1
A 1 B 0
A 1 B 0.14
A 1 B 0.29
A 1 B 0.43
A 1 B 0.57
A 1 B 0.71
A 1 B 0.86
A 1 B 1

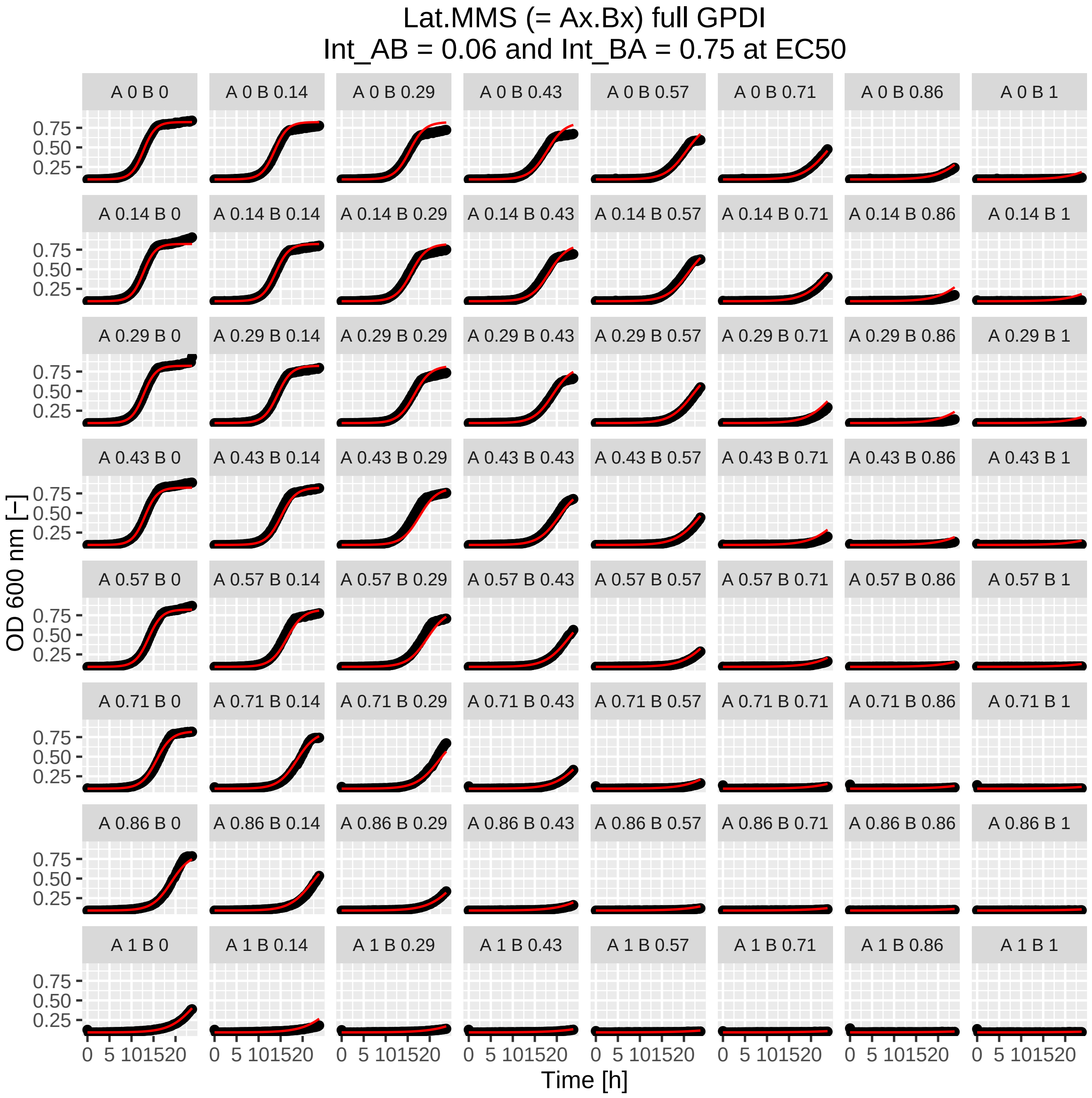
Lat.MMS (= Ax.Bx) full GPDI
Int_AB = 0.06 and Int_BA = 0.75 at EC50
OD 600 nm [–]
Time [h]
0.25
0.50
0.75
0 5 10 15 20
A 0 B 0
A 0 B 0.14
A 0 B 0.29
A 0 B 0.43
A 0 B 0.57
A 0 B 0.71
A 0 B 0.86
A 0 B 1
A 0.14 B 0
A 0.14 B 0.14
A 0.14 B 0.29
A 0.14 B 0.43
A 0.14 B 0.57
A 0.14 B 0.71
A 0.14 B 0.86
A 0.14 B 1
A 0.29 B 0
A 0.29 B 0.14
A 0.29 B 0.29
A 0.29 B 0.43
A 0.29 B 0.57
A 0.29 B 0.71
A 0.29 B 0.86
A 0.29 B 1
A 0.43 B 0
A 0.43 B 0.14
A 0.43 B 0.29
A 0.43 B 0.43
A 0.43 B 0.57
A 0.43 B 0.71
A 0.43 B 0.86
A 0.43 B 1
A 0.57 B 0
A 0.57 B 0.14
A 0.57 B 0.29
A 0.57 B 0.43
A 0.57 B 0.57
A 0.57 B 0.71
A 0.57 B 0.86
A 0.57 B 1
A 0.71 B 0
A 0.71 B 0.14
A 0.71 B 0.29
A 0.71 B 0.43
A 0.71 B 0.57
A 0.71 B 0.71
A 0.71 B 0.86
A 0.71 B 1
A 0.86 B 0
A 0.86 B 0.14
A 0.86 B 0.29
A 0.86 B 0.43
A 0.86 B 0.57
A 0.86 B 0.71
A 0.86 B 0.86
A 0.86 B 1
A 1 B 0
A 1 B 0.14
A 1 B 0.29
A 1 B 0.43
A 1 B 0.57
A 1 B 0.71
A 1 B 0.86
A 1 B 1

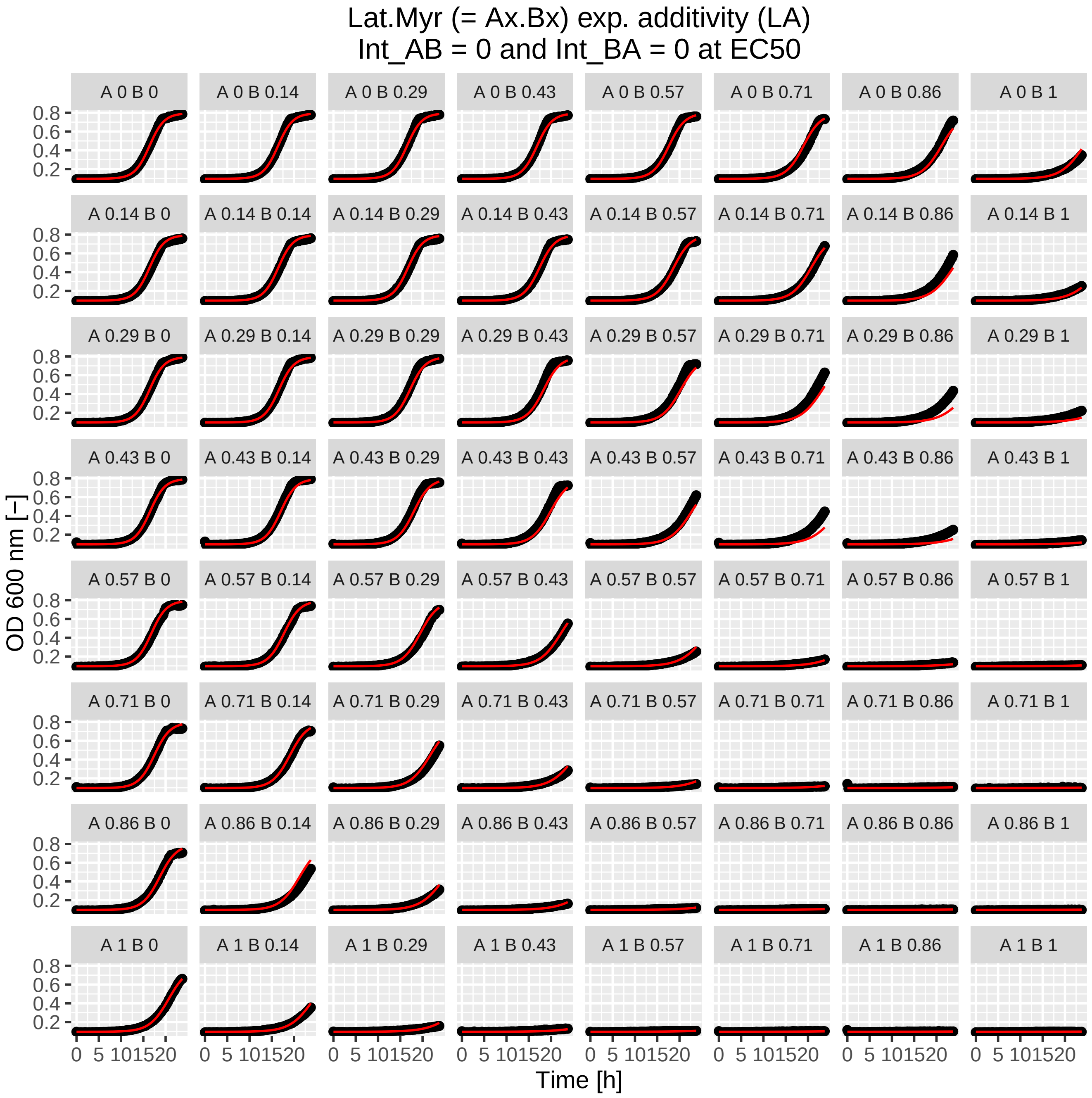
Lat.Myr (= Ax.Bx) exp. additivity (LA)
Int_AB = 0 and Int_BA = 0 at EC50
OD 600 nm [−]
Time [h]
A 0 B 0
A 0 B 0.14
A 0 B 0.29
A 0 B 0.43
A 0 B 0.57
A 0 B 0.71
A 0 B 0.86
A 0 B 1
A 1 B 0
A 1 B 0.14
A 1 B 0.29
A 1 B 0.43
A 1 B 0.57
A 1 B 0.71
A 1 B 0.86
A 1 B 1

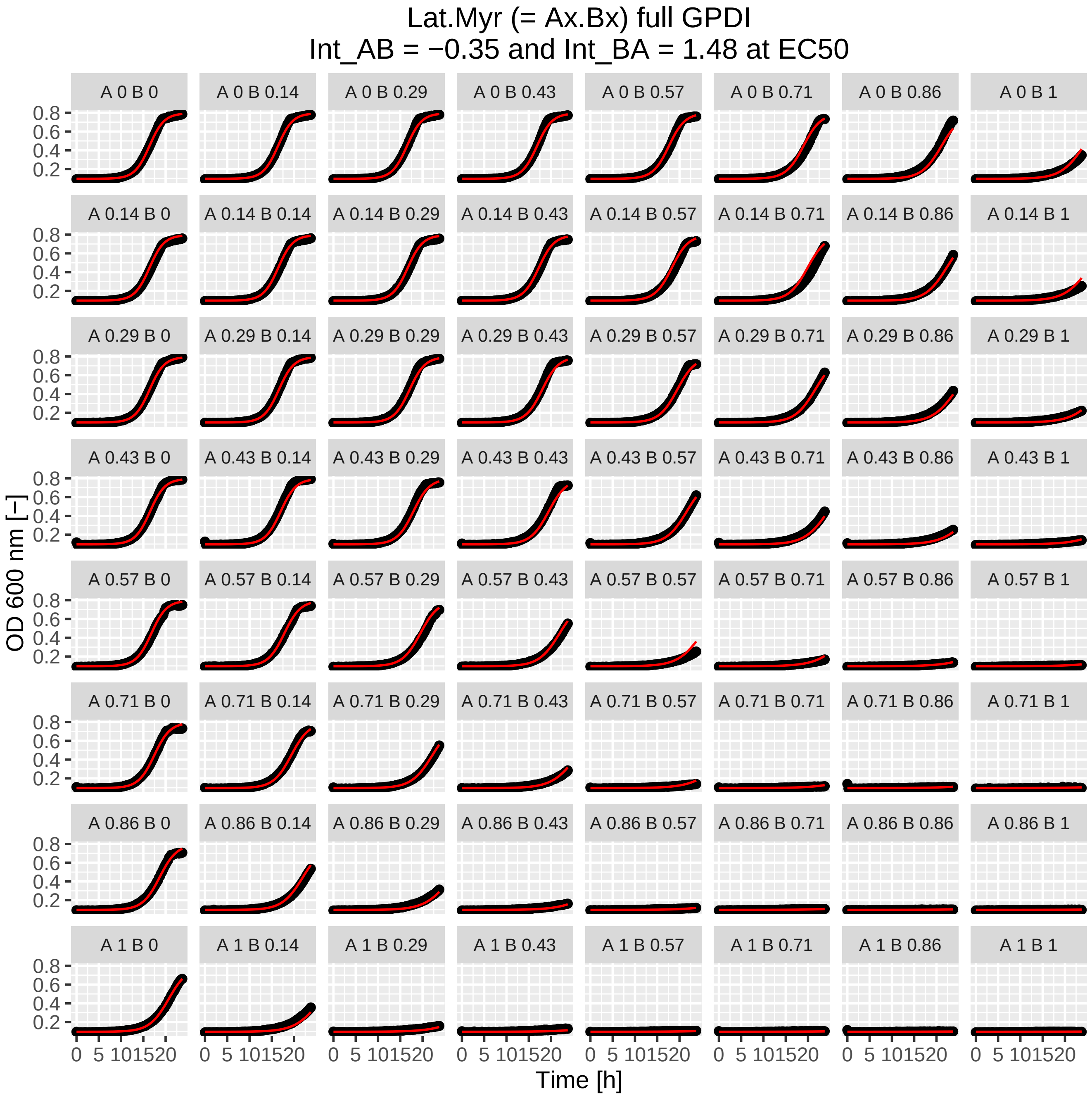

Lat.Myr (= Ax.Bx) full GPDI
Int_AB = −0.35 and Int_BA = 1.48 at EC50
A 0 B 0
A 0 B 0.14
A 0 B 0.29
A 0 B 0.43
A 0 B 0.57
A 0 B 0.71
A 0 B 0.86
A 0 B 1
A 0.14 B 0
A 0.14 B 0.14
A 0.14 B 0.29
A 0.14 B 0.43
A 0.14 B 0.57
A 0.14 B 0.71
A 0.14 B 0.86
A 0.14 B 1
A 0.29 B 0
A 0.29 B 0.14
A 0.29 B 0.29
A 0.29 B 0.43
A 0.29 B 0.57
A 0.29 B 0.71
A 0.29 B 0.86
A 0.29 B 1
A 0.43 B 0
A 0.43 B 0.14
A 0.43 B 0.29
A 0.43 B 0.43
A 0.43 B 0.57
A 0.43 B 0.71
A 0.43 B 0.86
A 0.43 B 1
A 0.57 B 0
A 0.57 B 0.14
A 0.57 B 0.29
A 0.57 B 0.43
A 0.57 B 0.57
A 0.57 B 0.71
A 0.57 B 0.86
A 0.57 B 1
A 0.71 B 0
A 0.71 B 0.14
A 0.71 B 0.29
A 0.71 B 0.43
A 0.71 B 0.57
A 0.71 B 0.71
A 0.71 B 0.86
A 0.71 B 1
A 0.86 B 0
A 0.86 B 0.14
A 0.86 B 0.29
A 0.86 B 0.43
A 0.86 B 0.57
A 0.86 B 0.71
A 0.86 B 0.86
A 0.86 B 1
A 1 B 0
A 1 B 0.14
A 1 B 0.29
A 1 B 0.43
A 1 B 0.57
A 1 B 0.71
A 1 B 0.86
A 1 B 1
OD 600 nm [−]
0.2
0.4
0.6
0.8
0 5 101520
Time [h]

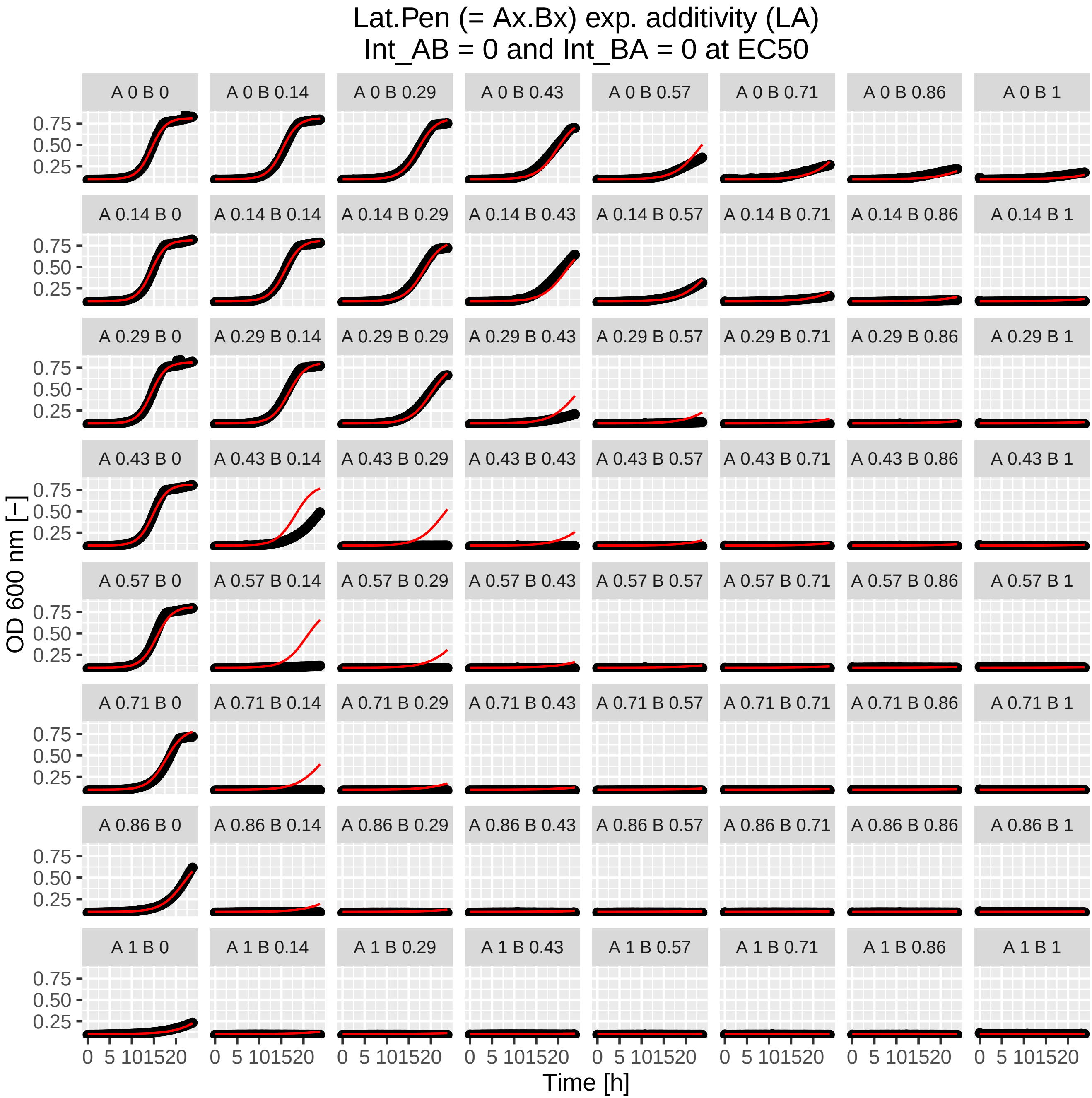

Lat.Pen (= Ax.Bx) exp. additivity (LA)
Int_AB = 0 and Int_BA = 0 at EC50
A 0 B 0
A 0 B 0.14
A 0 B 0.29
A 0 B 0.43
A 0 B 0.57
A 0 B 0.71
A 0 B 0.86
A 0 B 1
A 0.14 B 0
A 0.14 B 0.14
A 0.14 B 0.29
A 0.14 B 0.43
A 0.14 B 0.57
A 0.14 B 0.71
A 0.14 B 0.86
A 0.14 B 1
A 0.29 B 0
A 0.29 B 0.14
A 0.29 B 0.29
A 0.29 B 0.43
A 0.29 B 0.57
A 0.29 B 0.71
A 0.29 B 0.86
A 0.29 B 1
A 0.43 B 0
A 0.43 B 0.14
A 0.43 B 0.29
A 0.43 B 0.43
A 0.43 B 0.57
A 0.43 B 0.71
A 0.43 B 0.86
A 0.43 B 1
A 0.57 B 0
A 0.57 B 0.14
A 0.57 B 0.29
A 0.57 B 0.43
A 0.57 B 0.57
A 0.57 B 0.71
A 0.57 B 0.86
A 0.57 B 1
A 0.71 B 0
A 0.71 B 0.14
A 0.71 B 0.29
A 0.71 B 0.43
A 0.71 B 0.57
A 0.71 B 0.71
A 0.71 B 0.86
A 0.71 B 1
A 0.86 B 0
A 0.86 B 0.14
A 0.86 B 0.29
A 0.86 B 0.43
A 0.86 B 0.57
A 0.86 B 0.71
A 0.86 B 0.86
A 0.86 B 1
A 1 B 0
A 1 B 0.14
A 1 B 0.29
A 1 B 0.43
A 1 B 0.57
A 1 B 0.71
A 1 B 0.86
A 1 B 1
OD 600 nm [–]
0.75
0.50
0.25
0 5 101520
Time [h]

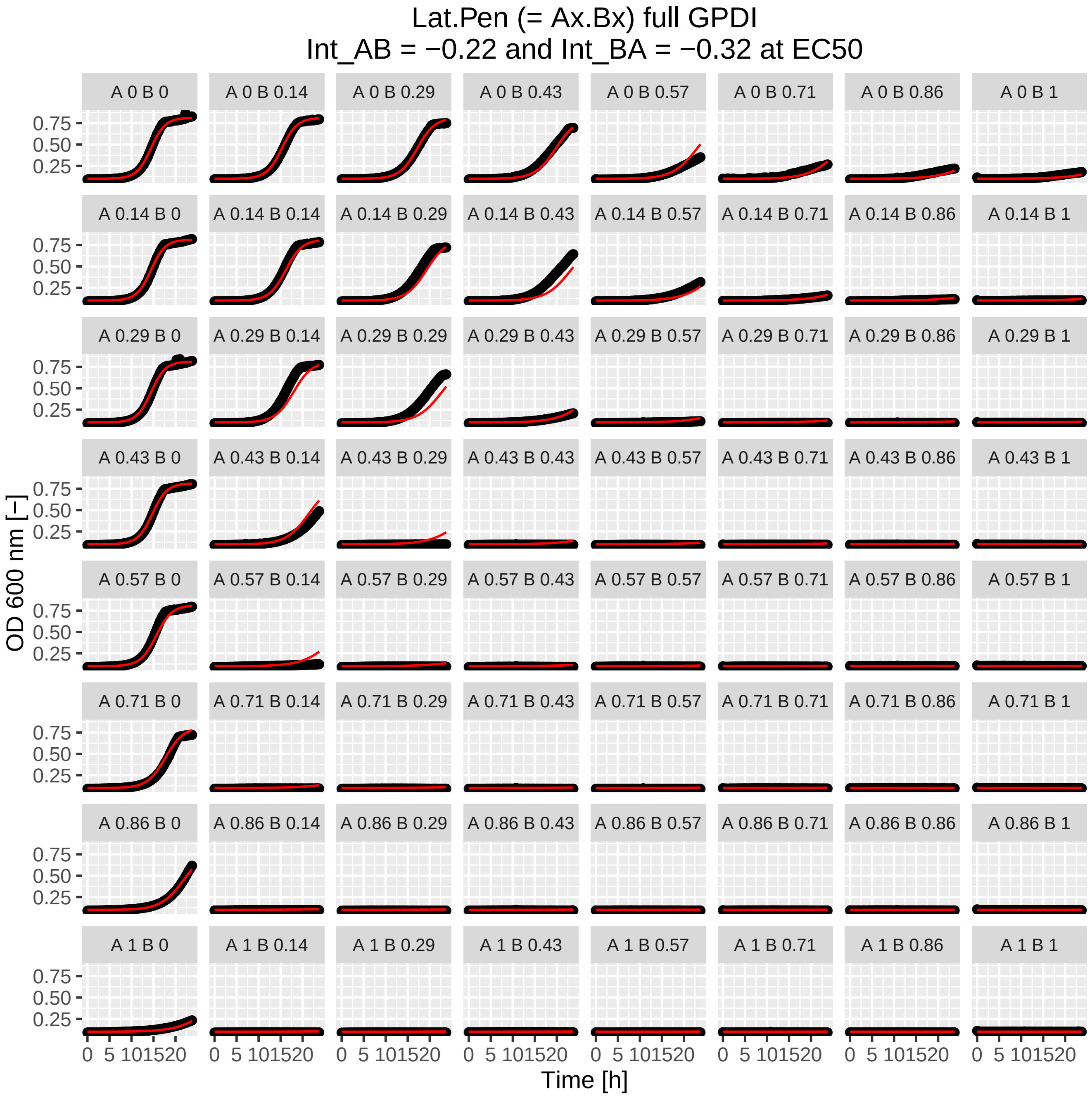
Lat.Pen (= Ax.Bx) full GPDI
Int_AB = −0.22 and Int_BA = −0.32 at EC50
OD 600 nm [−]
Time [h]
0.75
0.50
0.25
0 5 101520
A 0 B 0
A 0 B 0.14
A 0 B 0.29
A 0 B 0.43
A 0 B 0.57
A 0 B 0.71
A 0 B 0.86
A 0 B 1
A 0.14 B 0
A 0.14 B 0.14
A 0.14 B 0.29
A 0.14 B 0.43
A 0.14 B 0.57
A 0.14 B 0.71
A 0.14 B 0.86
A 0.14 B 1
A 0.29 B 0
A 0.29 B 0.14
A 0.29 B 0.29
A 0.29 B 0.43
A 0.29 B 0.57
A 0.29 B 0.71
A 0.29 B 0.86
A 0.29 B 1
A 0.43 B 0
A 0.43 B 0.14
A 0.43 B 0.29
A 0.43 B 0.43
A 0.43 B 0.57
A 0.43 B 0.71
A 0.43 B 0.86
A 0.43 B 1
A 0.57 B 0
A 0.57 B 0.14
A 0.57 B 0.29
A 0.57 B 0.43
A 0.57 B 0.57
A 0.57 B 0.71
A 0.57 B 0.86
A 0.57 B 1
A 0.71 B 0
A 0.71 B 0.14
A 0.71 B 0.29
A 0.71 B 0.43
A 0.71 B 0.57
A 0.71 B 0.71
A 0.71 B 0.86
A 0.71 B 1
A 0.86 B 0
A 0.86 B 0.14
A 0.86 B 0.29
A 0.86 B 0.43
A 0.86 B 0.57
A 0.86 B 0.71
A 0.86 B 0.86
A 0.86 B 1
A 1 B 0
A 1 B 0.14
A 1 B 0.29
A 1 B 0.43
A 1 B 0.57
A 1 B 0.71
A 1 B 0.86
A 1 B 1

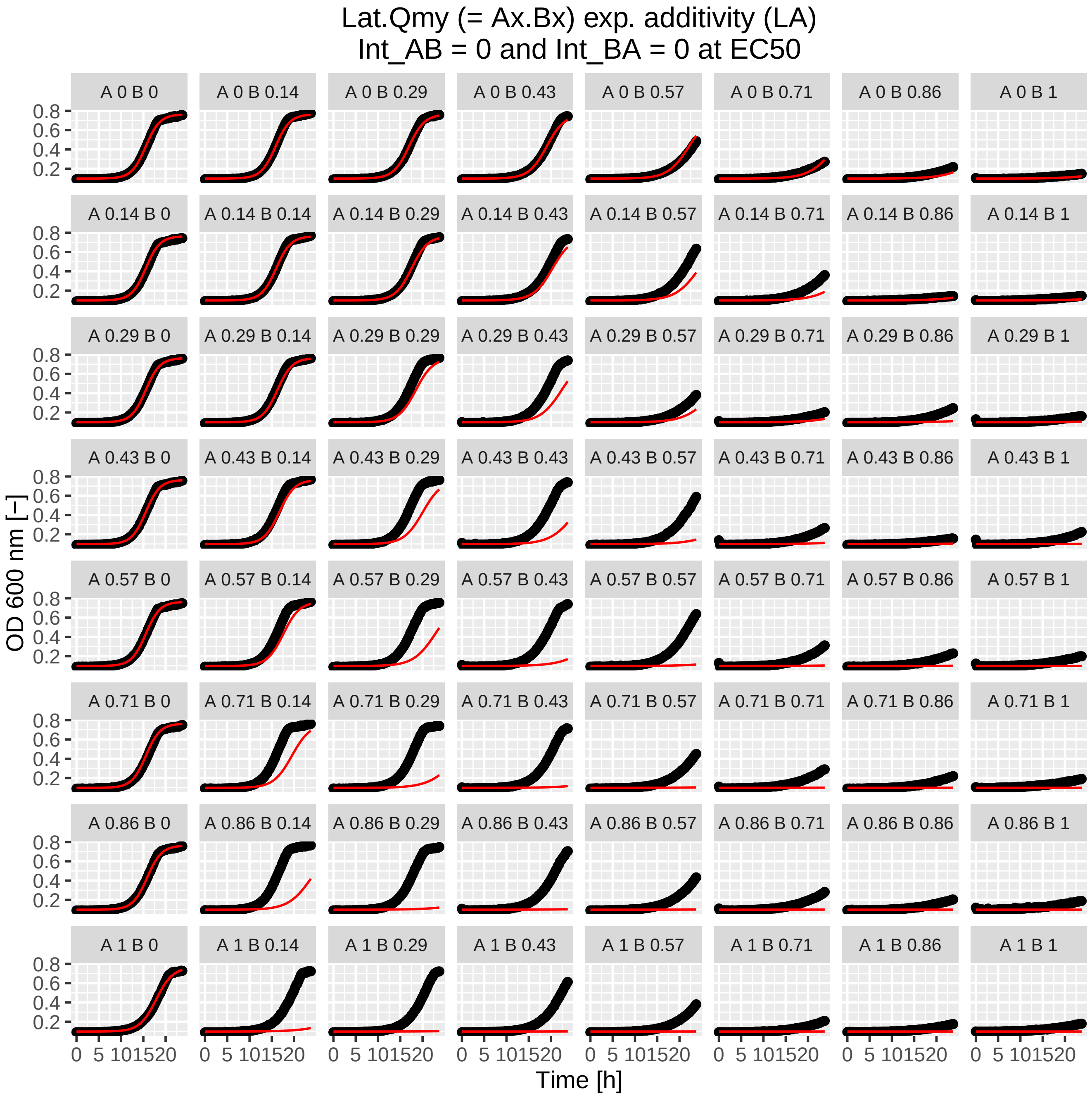

Lat.Qmy (= Ax.Bx) exp. additivity (LA)
Int_AB = 0 and Int_BA = 0 at EC50
A 0 B 0
A 0 B 0.14
A 0 B 0.29
A 0 B 0.43
A 0 B 0.57
A 0 B 0.71
A 0 B 0.86
A 0 B 1
A 0.14 B 0
A 0.14 B 0.14
A 0.14 B 0.29
A 0.14 B 0.43
A 0.14 B 0.57
A 0.14 B 0.71
A 0.14 B 0.86
A 0.14 B 1
A 0.29 B 0
A 0.29 B 0.14
A 0.29 B 0.29
A 0.29 B 0.43
A 0.29 B 0.57
A 0.29 B 0.71
A 0.29 B 0.86
A 0.29 B 1
A 0.43 B 0
A 0.43 B 0.14
A 0.43 B 0.29
A 0.43 B 0.43
A 0.43 B 0.57
A 0.43 B 0.71
A 0.43 B 0.86
A 0.43 B 1
A 0.57 B 0
A 0.57 B 0.14
A 0.57 B 0.29
A 0.57 B 0.43
A 0.57 B 0.57
A 0.57 B 0.71
A 0.57 B 0.86
A 0.57 B 1
A 0.71 B 0
A 0.71 B 0.14
A 0.71 B 0.29
A 0.71 B 0.43
A 0.71 B 0.57
A 0.71 B 0.71
A 0.71 B 0.86
A 0.71 B 1
A 0.86 B 0
A 0.86 B 0.14
A 0.86 B 0.29
A 0.86 B 0.43
A 0.86 B 0.57
A 0.86 B 0.71
A 0.86 B 0.86
A 0.86 B 1
A 1 B 0
A 1 B 0.14
A 1 B 0.29
A 1 B 0.43
A 1 B 0.57
A 1 B 0.71
A 1 B 0.86
A 1 B 1
0.8
0.6
0.4
0.2
OD 600 nm [–]
0 5 101520
Time [h]

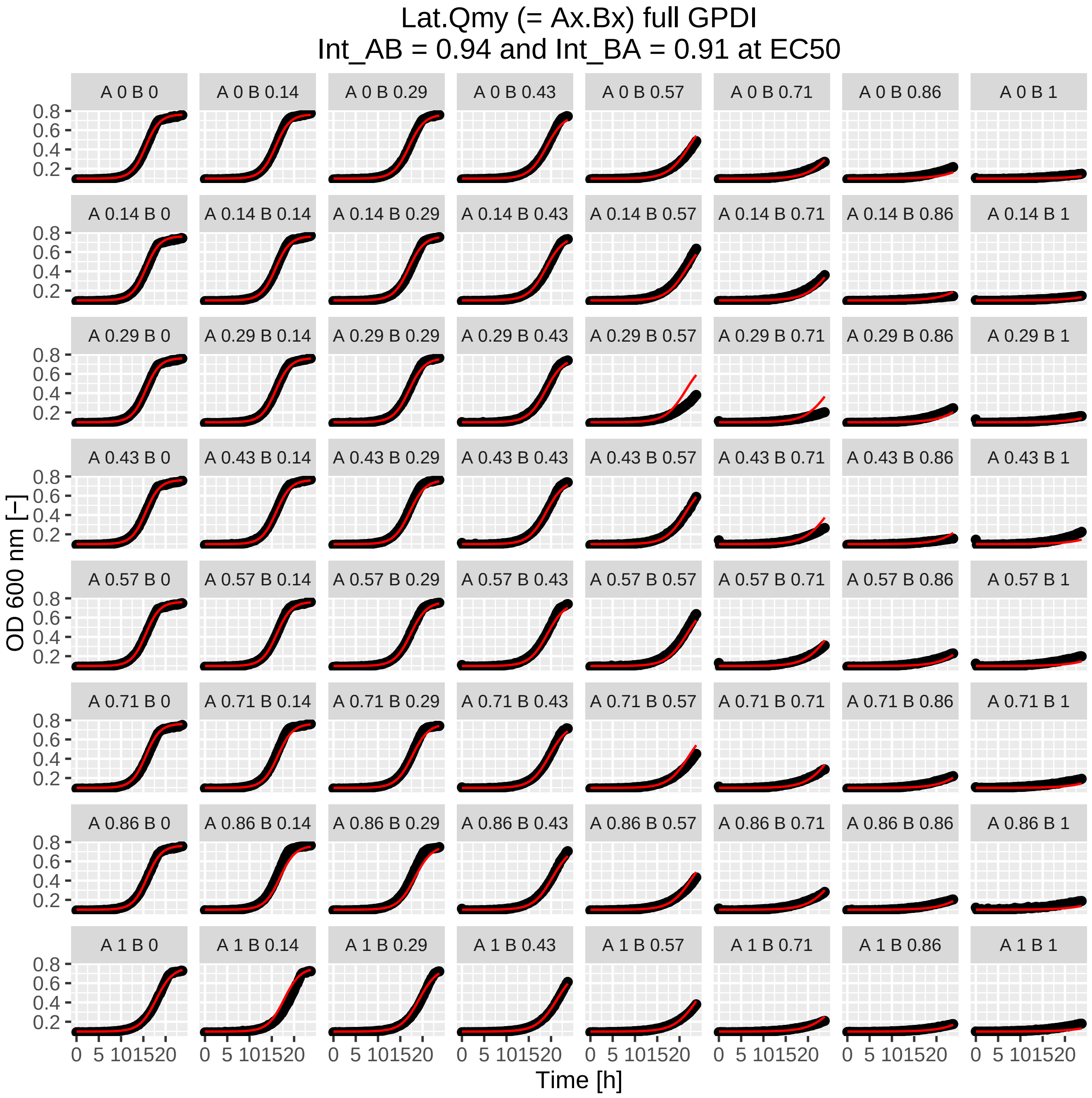

Lat.Qmy (= Ax.Bx) full GPDI
Int_AB = 0.94 and Int_BA = 0.91 at EC50
A 0 B 0
A 0 B 0.14
A 0 B 0.29
A 0 B 0.43
A 0 B 0.57
A 0 B 0.71
A 0 B 0.86
A 0 B 1
A 0.14 B 0
A 0.14 B 0.14
A 0.14 B 0.29
A 0.14 B 0.43
A 0.14 B 0.57
A 0.14 B 0.71
A 0.14 B 0.86
A 0.14 B 1
A 0.29 B 0
A 0.29 B 0.14
A 0.29 B 0.29
A 0.29 B 0.43
A 0.29 B 0.57
A 0.29 B 0.71
A 0.29 B 0.86
A 0.29 B 1
A 0.43 B 0
A 0.43 B 0.14
A 0.43 B 0.29
A 0.43 B 0.43
A 0.43 B 0.57
A 0.43 B 0.71
A 0.43 B 0.86
A 0.43 B 1
A 0.57 B 0
A 0.57 B 0.14
A 0.57 B 0.29
A 0.57 B 0.43
A 0.57 B 0.57
A 0.57 B 0.71
A 0.57 B 0.86
A 0.57 B 1
A 0.71 B 0
A 0.71 B 0.14
A 0.71 B 0.29
A 0.71 B 0.43
A 0.71 B 0.57
A 0.71 B 0.71
A 0.71 B 0.86
A 0.71 B 1
A 0.86 B 0
A 0.86 B 0.14
A 0.86 B 0.29
A 0.86 B 0.43
A 0.86 B 0.57
A 0.86 B 0.71
A 0.86 B 0.86
A 0.86 B 1
A 1 B 0
A 1 B 0.14
A 1 B 0.29
A 1 B 0.43
A 1 B 0.57
A 1 B 0.71
A 1 B 0.86
A 1 B 1
OD 600 nm [–]
0.2
0.4
0.6
0.8
0 5 101520
Time [h]

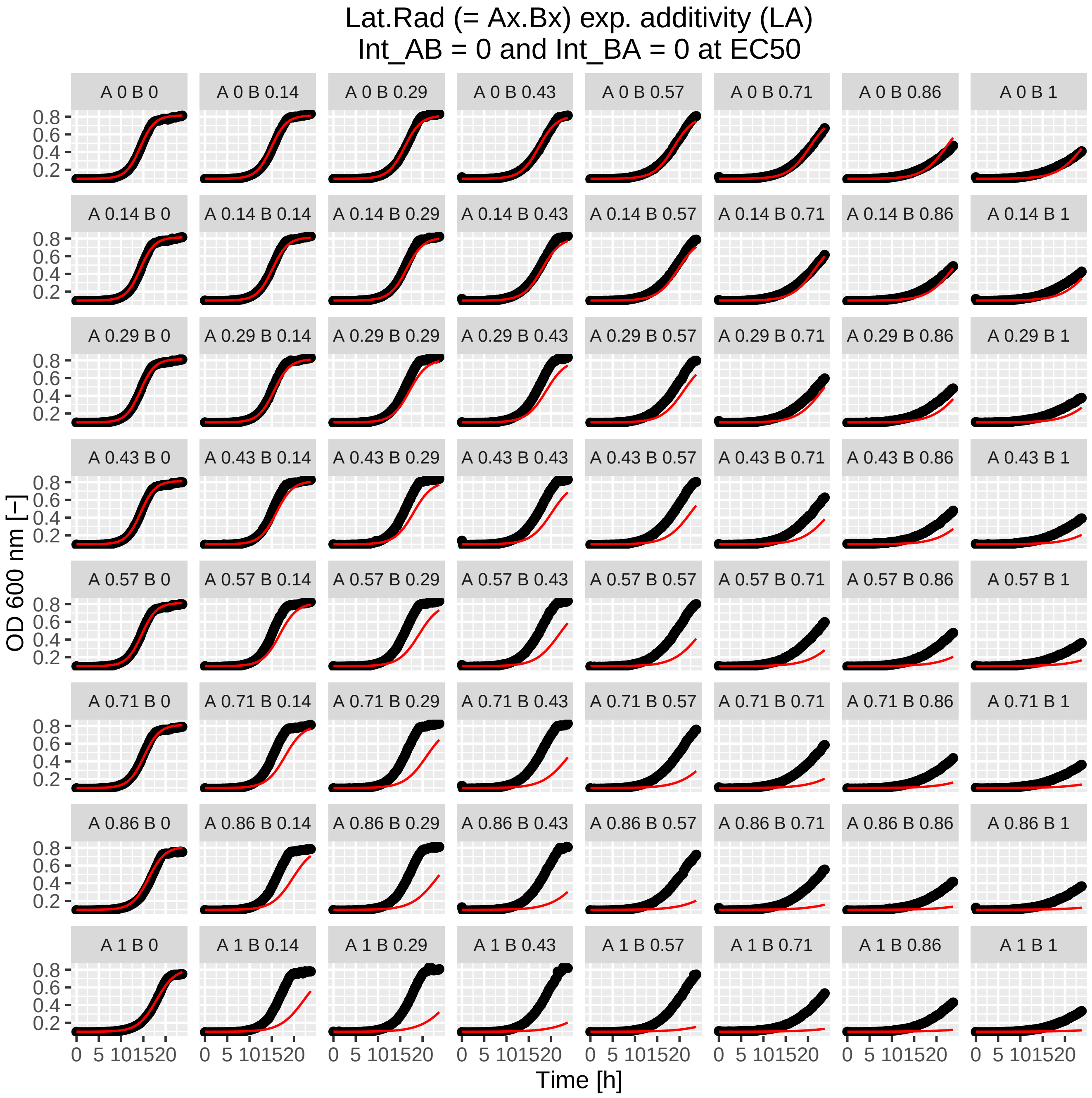
Lat.Rad (= Ax.Bx) exp. additivity (LA)
Int_AB = 0 and Int_BA = 0 at EC50
A 0 B 0
A 0 B 0.14
A 0 B 0.29
A 0 B 0.43
A 0 B 0.57
A 0 B 0.71
A 0 B 0.86
A 0 B 1
A 0.14 B 0
A 0.14 B 0.14
A 0.14 B 0.29
A 0.14 B 0.43
A 0.14 B 0.57
A 0.14 B 0.71
A 0.14 B 0.86
A 0.14 B 1
A 0.29 B 0
A 0.29 B 0.14
A 0.29 B 0.29
A 0.29 B 0.43
A 0.29 B 0.57
A 0.29 B 0.71
A 0.29 B 0.86
A 0.29 B 1
A 0.43 B 0
A 0.43 B 0.14
A 0.43 B 0.29
A 0.43 B 0.43
A 0.43 B 0.57
A 0.43 B 0.71
A 0.43 B 0.86
A 0.43 B 1
A 0.57 B 0
A 0.57 B 0.14
A 0.57 B 0.29
A 0.57 B 0.43
A 0.57 B 0.57
A 0.57 B 0.71
A 0.57 B 0.86
A 0.57 B 1
A 0.71 B 0
A 0.71 B 0.14
A 0.71 B 0.29
A 0.71 B 0.43
A 0.71 B 0.57
A 0.71 B 0.71
A 0.71 B 0.86
A 0.71 B 1
A 0.86 B 0
A 0.86 B 0.14
A 0.86 B 0.29
A 0.86 B 0.43
A 0.86 B 0.57
A 0.86 B 0.71
A 0.86 B 0.86
A 0.86 B 1
A 1 B 0
A 1 B 0.14
A 1 B 0.29
A 1 B 0.43
A 1 B 0.57
A 1 B 0.71
A 1 B 0.86
A 1 B 1
OD 600 nm [−]
0.2
0.4
0.6
0.8
0 5 101520
Time [h]

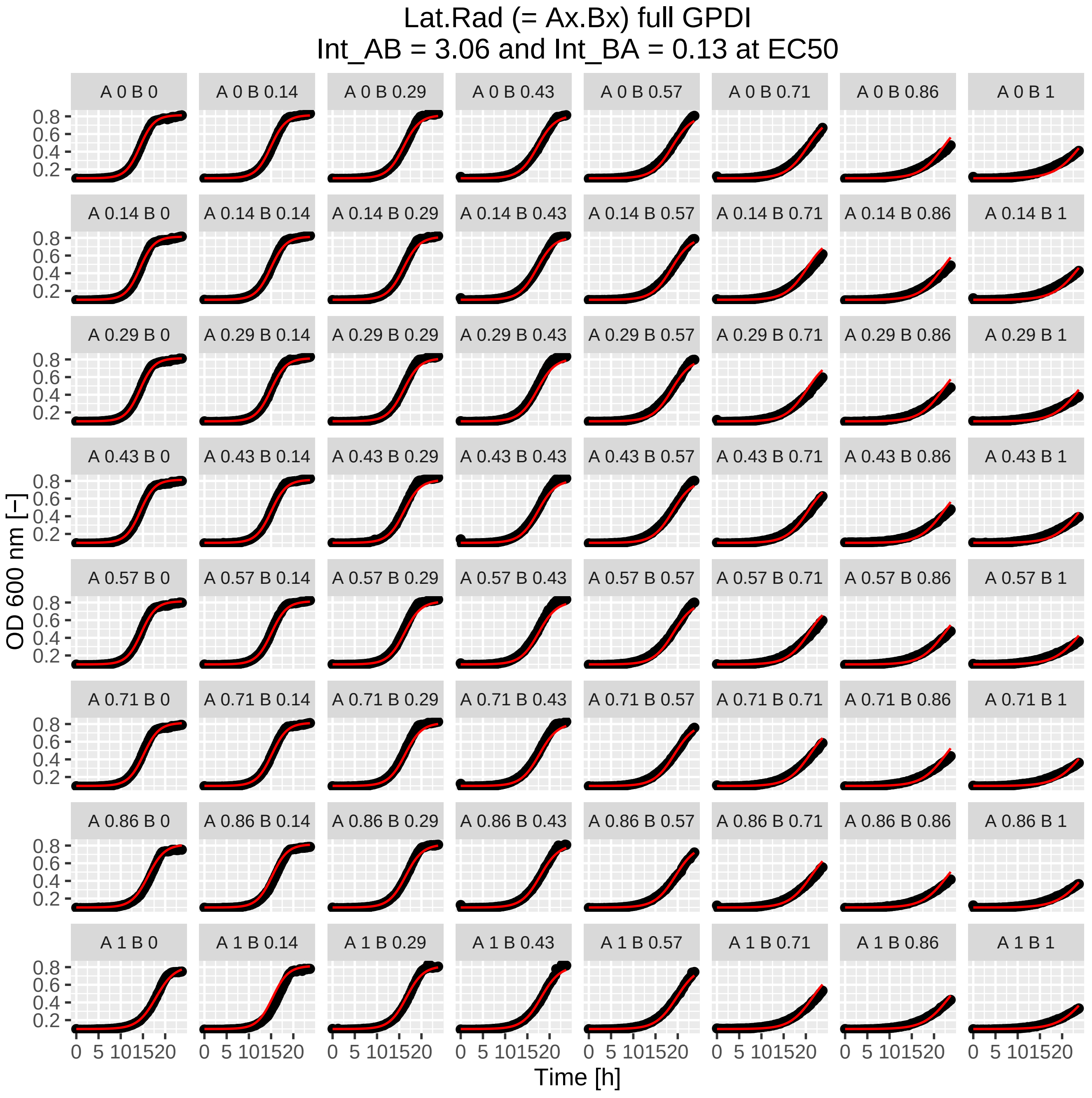

Lat.Rad (= Ax.Bx) full GPDI
Int_AB = 3.06 and Int_BA = 0.13 at EC50
A 0 B 0
A 0 B 0.14
A 0 B 0.29
A 0 B 0.43
A 0 B 0.57
A 0 B 0.71
A 0 B 0.86
A 0 B 1
A 0.14 B 0
A 0.14 B 0.14
A 0.14 B 0.29
A 0.14 B 0.43
A 0.14 B 0.57
A 0.14 B 0.71
A 0.14 B 0.86
A 0.14 B 1
A 0.29 B 0
A 0.29 B 0.14
A 0.29 B 0.29
A 0.29 B 0.43
A 0.29 B 0.57
A 0.29 B 0.71
A 0.29 B 0.86
A 0.29 B 1
A 0.43 B 0
A 0.43 B 0.14
A 0.43 B 0.29
A 0.43 B 0.43
A 0.43 B 0.57
A 0.43 B 0.71
A 0.43 B 0.86
A 0.43 B 1
A 0.57 B 0
A 0.57 B 0.14
A 0.57 B 0.29
A 0.57 B 0.43
A 0.57 B 0.57
A 0.57 B 0.71
A 0.57 B 0.86
A 0.57 B 1
A 0.71 B 0
A 0.71 B 0.14
A 0.71 B 0.29
A 0.71 B 0.43
A 0.71 B 0.57
A 0.71 B 0.71
A 0.71 B 0.86
A 0.71 B 1
A 0.86 B 0
A 0.86 B 0.14
A 0.86 B 0.29
A 0.86 B 0.43
A 0.86 B 0.57
A 0.86 B 0.71
A 0.86 B 0.86
A 0.86 B 1
A 1 B 0
A 1 B 0.14
A 1 B 0.29
A 1 B 0.43
A 1 B 0.57
A 1 B 0.71
A 1 B 0.86
A 1 B 1
OD 600 nm [–]
0.2
0.4
0.6
0.8
0
5
10
15
20
Time [h]

Lat.Rap (= Ax.Bx) exp. additivity (LA)
Int_AB = 0 and Int_BA = 0 at EC50

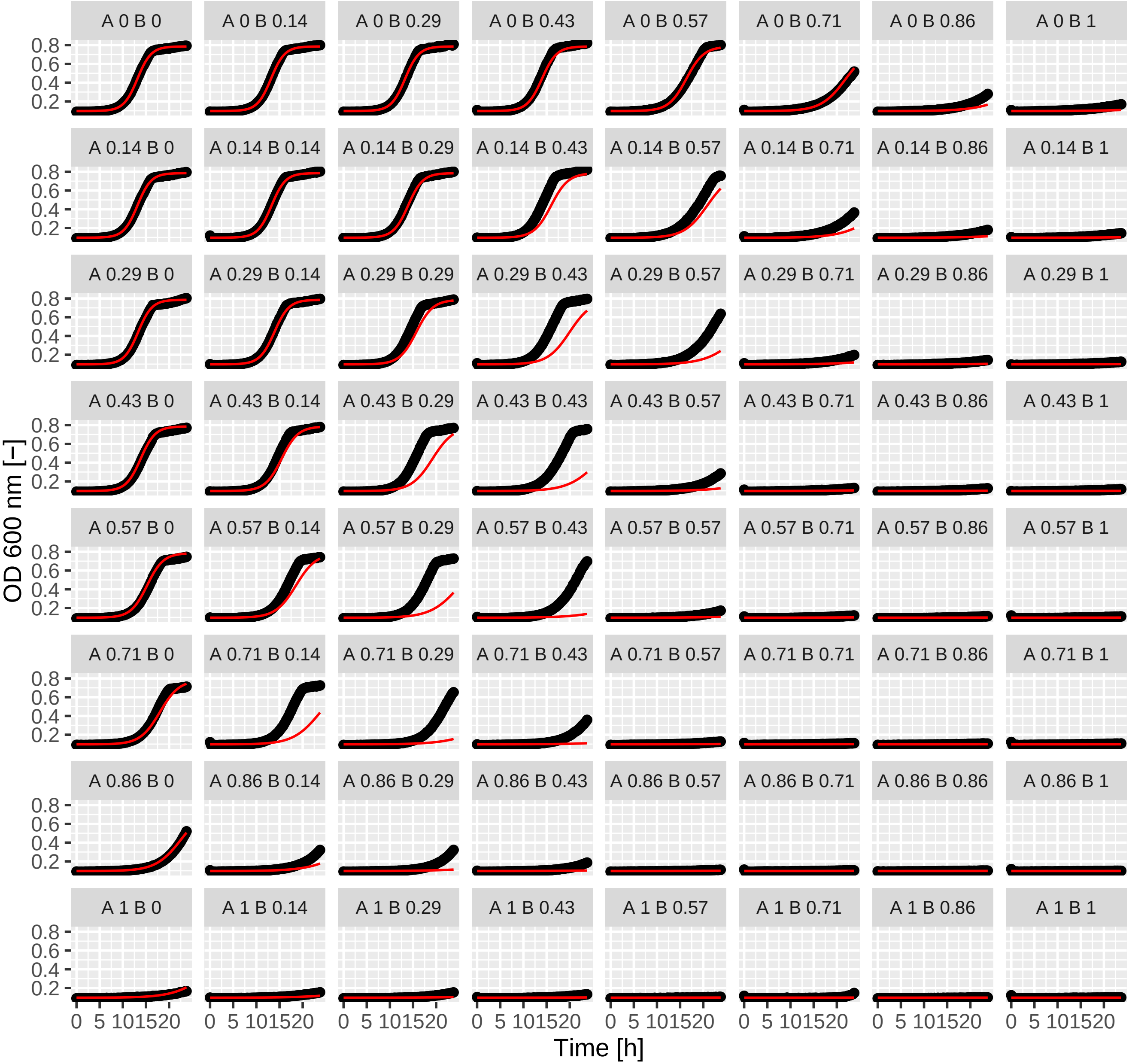

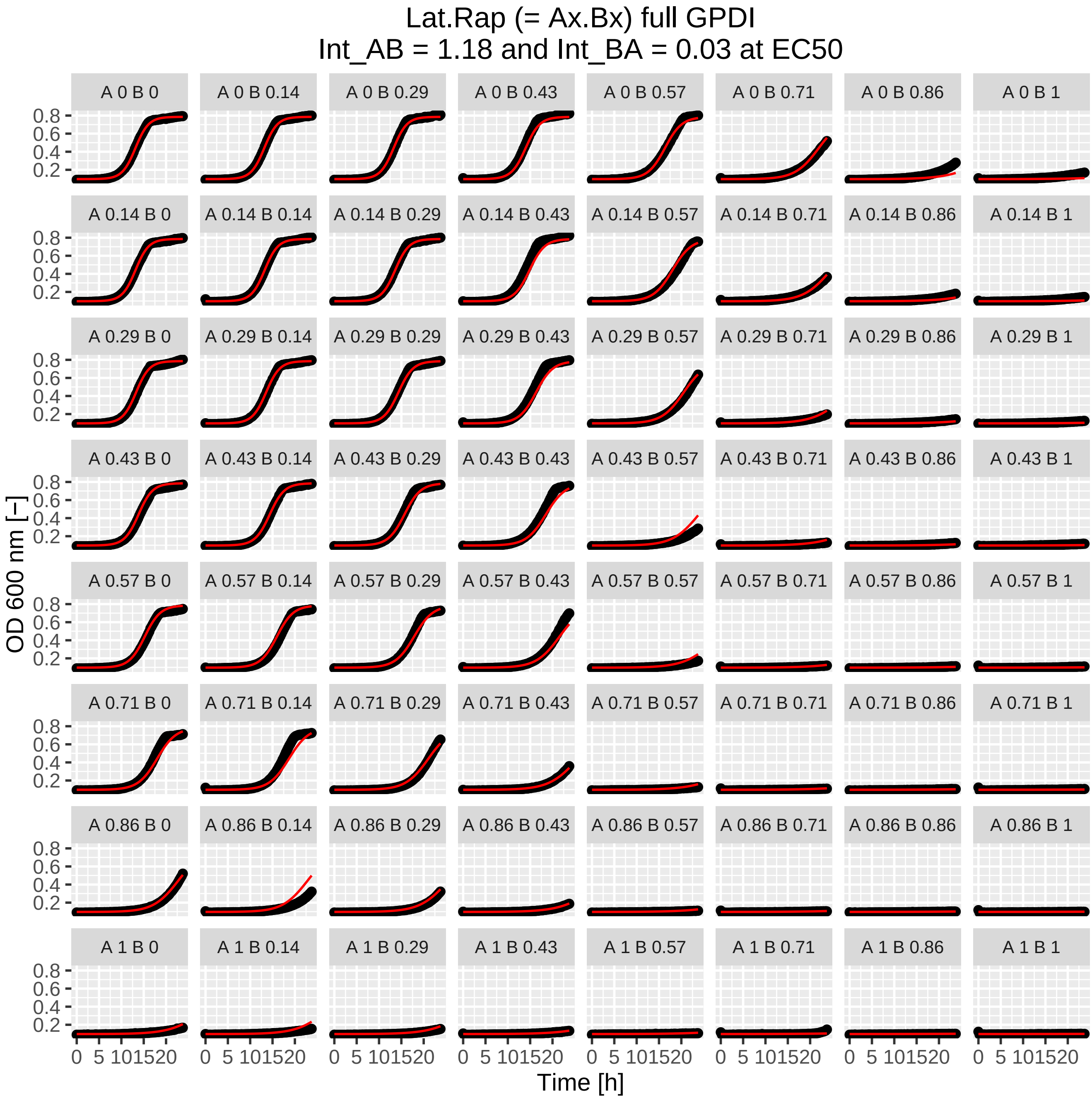
Lat.Rap (= Ax.Bx) full GPDI
Int_AB = 1.18 and Int_BA = 0.03 at EC50
A 0 B 0
A 0 B 0.14
A 0 B 0.29
A 0 B 0.43
A 0 B 0.57
A 0 B 0.71
A 0 B 0.86
A 0 B 1
A 0.14 B 0
A 0.14 B 0.14
A 0.14 B 0.29
A 0.14 B 0.43
A 0.14 B 0.57
A 0.14 B 0.71
A 0.14 B 0.86
A 0.14 B 1
A 0.29 B 0
A 0.29 B 0.14
A 0.29 B 0.29
A 0.29 B 0.43
A 0.29 B 0.57
A 0.29 B 0.71
A 0.29 B 0.86
A 0.29 B 1
A 0.43 B 0
A 0.43 B 0.14
A 0.43 B 0.29
A 0.43 B 0.43
A 0.43 B 0.57
A 0.43 B 0.71
A 0.43 B 0.86
A 0.43 B 1
A 0.57 B 0
A 0.57 B 0.14
A 0.57 B 0.29
A 0.57 B 0.43
A 0.57 B 0.57
A 0.57 B 0.71
A 0.57 B 0.86
A 0.57 B 1
A 0.71 B 0
A 0.71 B 0.14
A 0.71 B 0.29
A 0.71 B 0.43
A 0.71 B 0.57
A 0.71 B 0.71
A 0.71 B 0.86
A 0.71 B 1
A 0.86 B 0
A 0.86 B 0.14
A 0.86 B 0.29
A 0.86 B 0.43
A 0.86 B 0.57
A 0.86 B 0.71
A 0.86 B 0.86
A 0.86 B 1
A 1 B 0
A 1 B 0.14
A 1 B 0.29
A 1 B 0.43
A 1 B 0.57
A 1 B 0.71
A 1 B 0.86
A 1 B 1
OD 600 nm [–]
0.2
0.4
0.6
0.8
0 5 101520
Time [h]

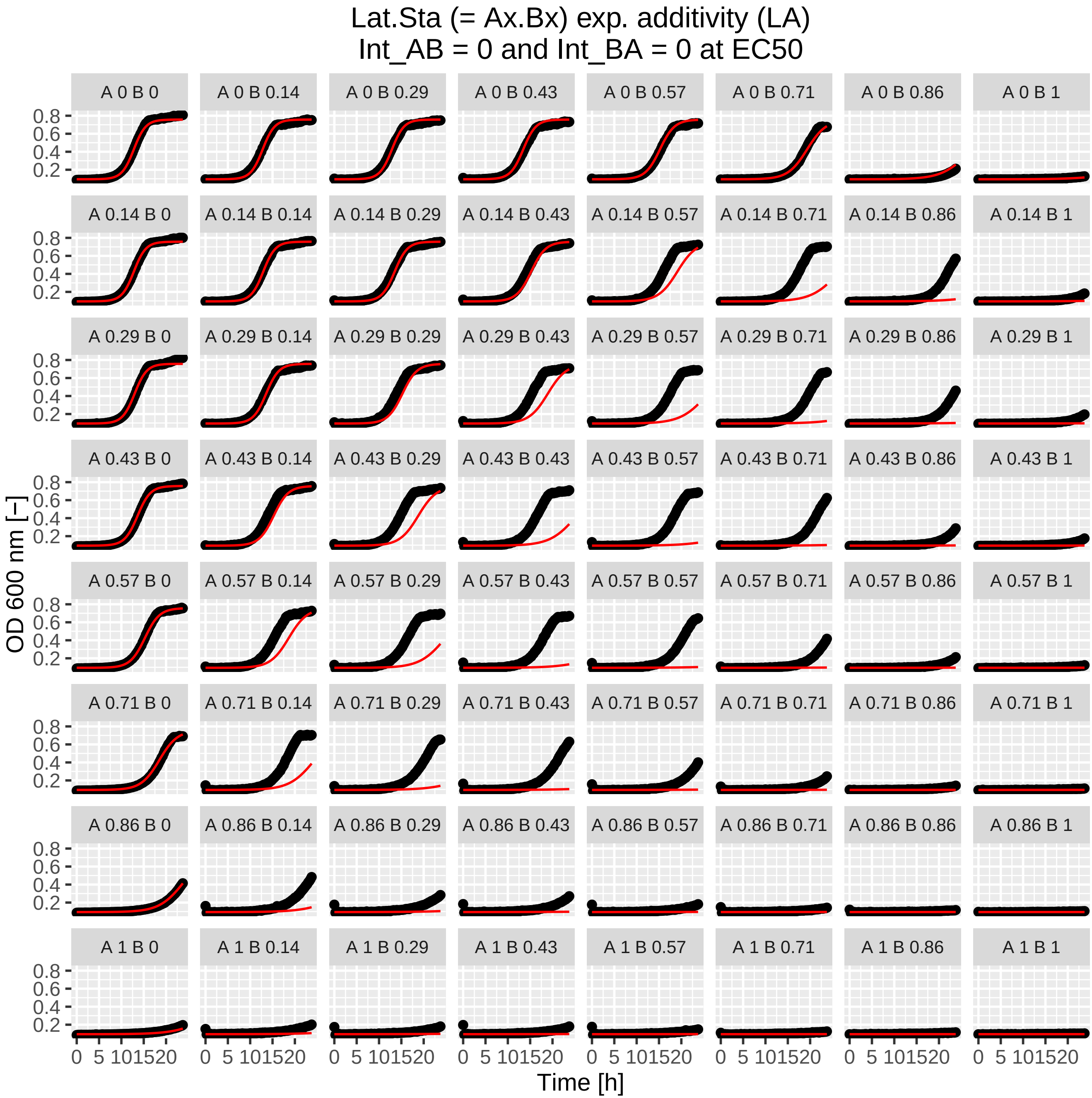
Lat.Sta (= Ax.Bx) exp. additivity (LA)
Int_AB = 0 and Int_BA = 0 at EC50
OD 600 nm [–]
Time [h]
A 0 B 0
A 0 B 0.14
A 0 B 0.29
A 0 B 0.43
A 0 B 0.57
A 0 B 0.71
A 0 B 0.86
A 0 B 1
A 1 B 0
A 1 B 0.14
A 1 B 0.29
A 1 B 0.43
A 1 B 0.57
A 1 B 0.71
A 1 B 0.86
A 1 B 1

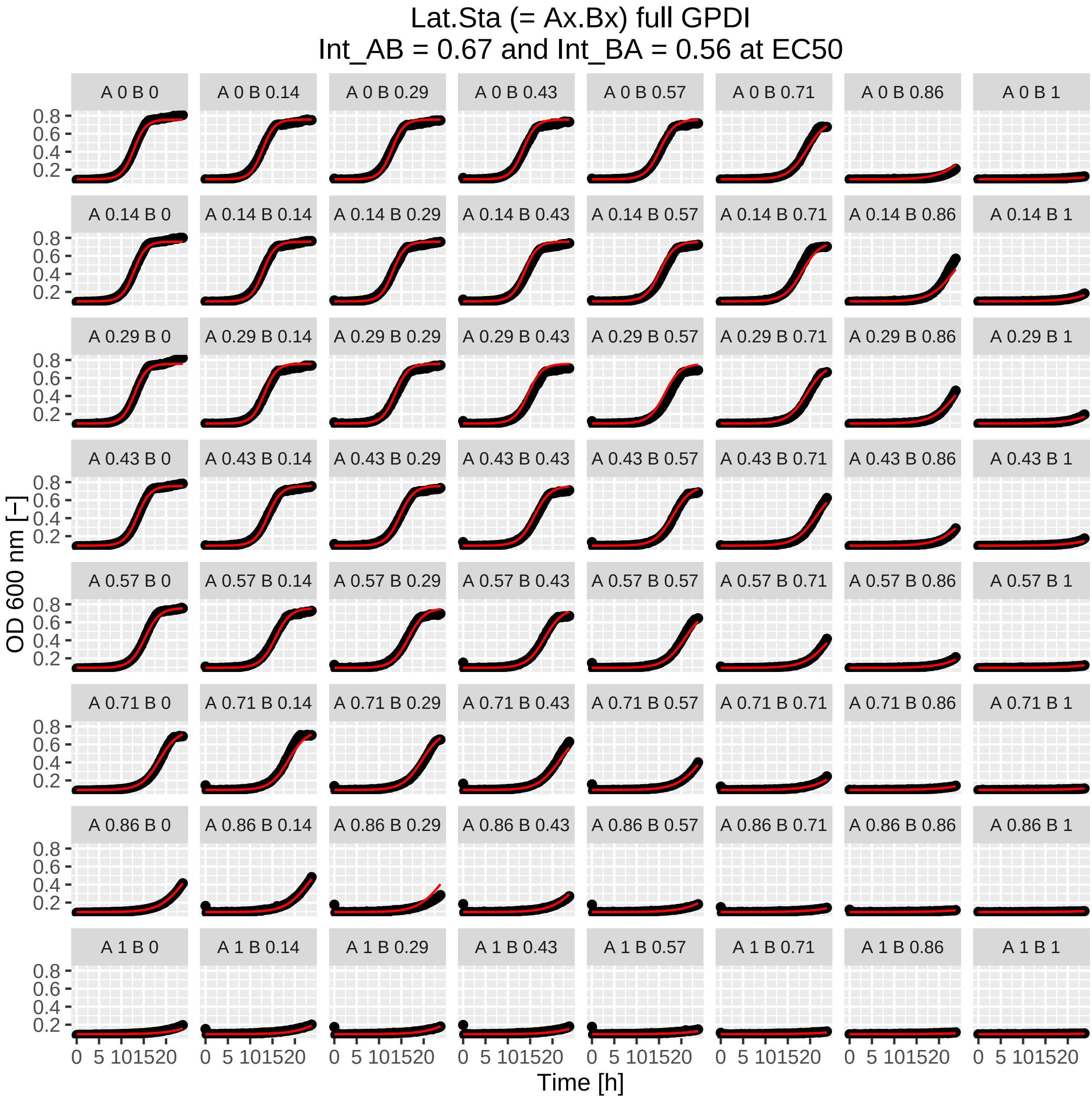
Lat.Sta (= Ax.Bx) full GPDI
Int_AB = 0.67 and Int_BA = 0.56 at EC50
A 0 B 0
A 0 B 0.14
A 0 B 0.29
A 0 B 0.43
A 0 B 0.57
A 0 B 0.71
A 0 B 0.86
A 0 B 1
A 0.14 B 0
A 0.14 B 0.14
A 0.14 B 0.29
A 0.14 B 0.43
A 0.14 B 0.57
A 0.14 B 0.71
A 0.14 B 0.86
A 0.14 B 1
A 0.29 B 0
A 0.29 B 0.14
A 0.29 B 0.29
A 0.29 B 0.43
A 0.29 B 0.57
A 0.29 B 0.71
A 0.29 B 0.86
A 0.29 B 1
A 0.43 B 0
A 0.43 B 0.14
A 0.43 B 0.29
A 0.43 B 0.43
A 0.43 B 0.57
A 0.43 B 0.71
A 0.43 B 0.86
A 0.43 B 1
A 0.57 B 0
A 0.57 B 0.14
A 0.57 B 0.29
A 0.57 B 0.43
A 0.57 B 0.57
A 0.57 B 0.71
A 0.57 B 0.86
A 0.57 B 1
A 0.71 B 0
A 0.71 B 0.14
A 0.71 B 0.29
A 0.71 B 0.43
A 0.71 B 0.57
A 0.71 B 0.71
A 0.71 B 0.86
A 0.71 B 1
A 0.86 B 0
A 0.86 B 0.14
A 0.86 B 0.29
A 0.86 B 0.43
A 0.86 B 0.57
A 0.86 B 0.71
A 0.86 B 0.86
A 0.86 B 1
A 1 B 0
A 1 B 0.14
A 1 B 0.29
A 1 B 0.43
A 1 B 0.57
A 1 B 0.71
A 1 B 0.86
A 1 B 1
OD 600 nm [–]
0.2
0.4
0.6
0.8
0
5
10
15
20
Time [h]

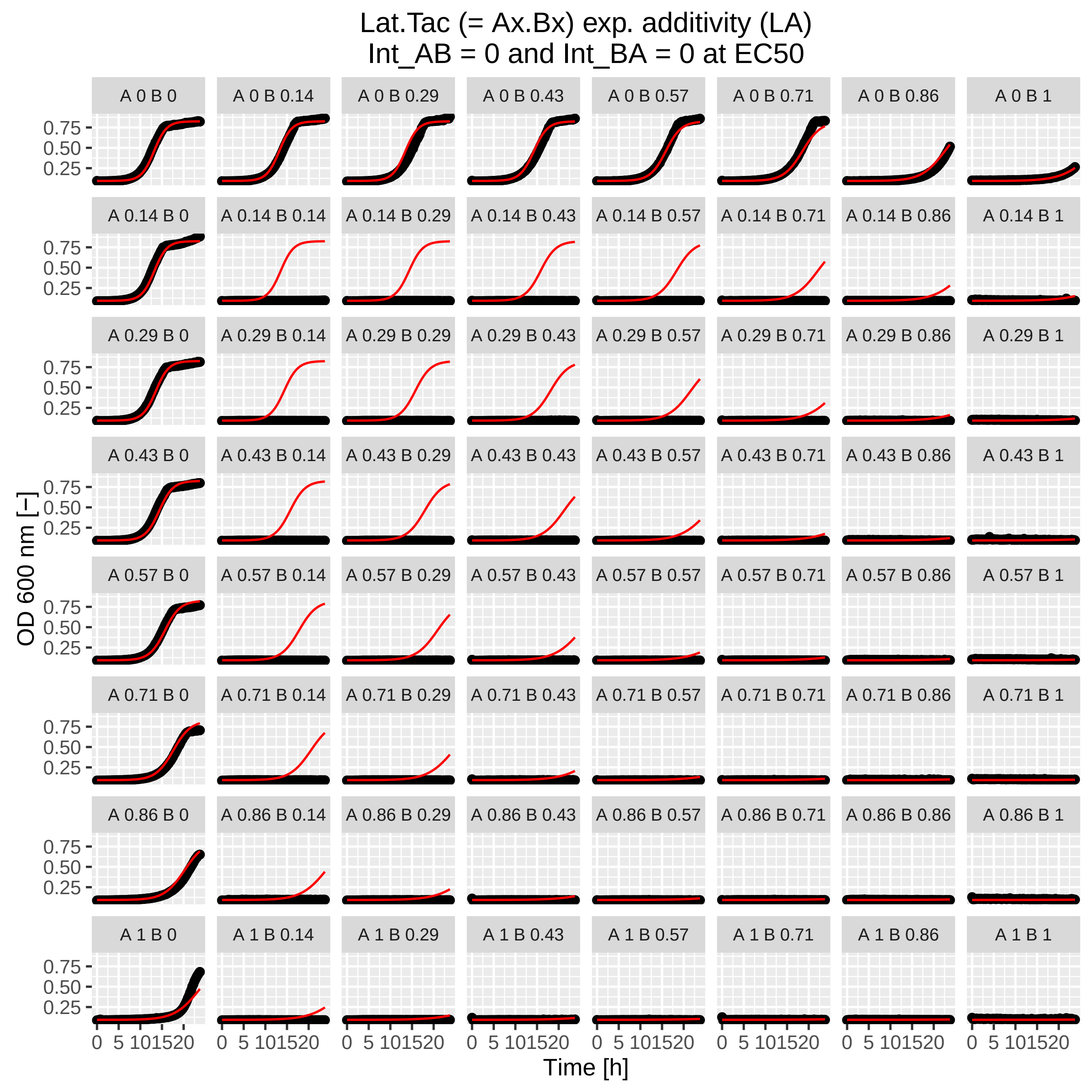

Lat.Tac (= Ax.Bx) exp. additivity (LA)
Int_AB = 0 and Int_BA = 0 at EC50
OD 600 nm [–]
Time [h]
0.75
0.50
0.25
0 5 101520
A 0 B 0
A 0 B 0.14
A 0 B 0.29
A 0 B 0.43
A 0 B 0.57
A 0 B 0.71
A 0 B 0.86
A 0 B 1
A 0.14 B 0
A 0.14 B 0.14
A 0.14 B 0.29
A 0.14 B 0.43
A 0.14 B 0.57
A 0.14 B 0.71
A 0.14 B 0.86
A 0.14 B 1
A 0.29 B 0
A 0.29 B 0.14
A 0.29 B 0.29
A 0.29 B 0.43
A 0.29 B 0.57
A 0.29 B 0.71
A 0.29 B 0.86
A 0.29 B 1
A 0.43 B 0
A 0.43 B 0.14
A 0.43 B 0.29
A 0.43 B 0.43
A 0.43 B 0.57
A 0.43 B 0.71
A 0.43 B 0.86
A 0.43 B 1
A 0.57 B 0
A 0.57 B 0.14
A 0.57 B 0.29
A 0.57 B 0.43
A 0.57 B 0.57
A 0.57 B 0.71
A 0.57 B 0.86
A 0.57 B 1
A 0.71 B 0
A 0.71 B 0.14
A 0.71 B 0.29
A 0.71 B 0.43
A 0.71 B 0.57
A 0.71 B 0.71
A 0.71 B 0.86
A 0.71 B 1
A 0.86 B 0
A 0.86 B 0.14
A 0.86 B 0.29
A 0.86 B 0.43
A 0.86 B 0.57
A 0.86 B 0.71
A 0.86 B 0.86
A 0.86 B 1
A 1 B 0
A 1 B 0.14
A 1 B 0.29
A 1 B 0.43
A 1 B 0.57
A 1 B 0.71
A 1 B 0.86
A 1 B 1

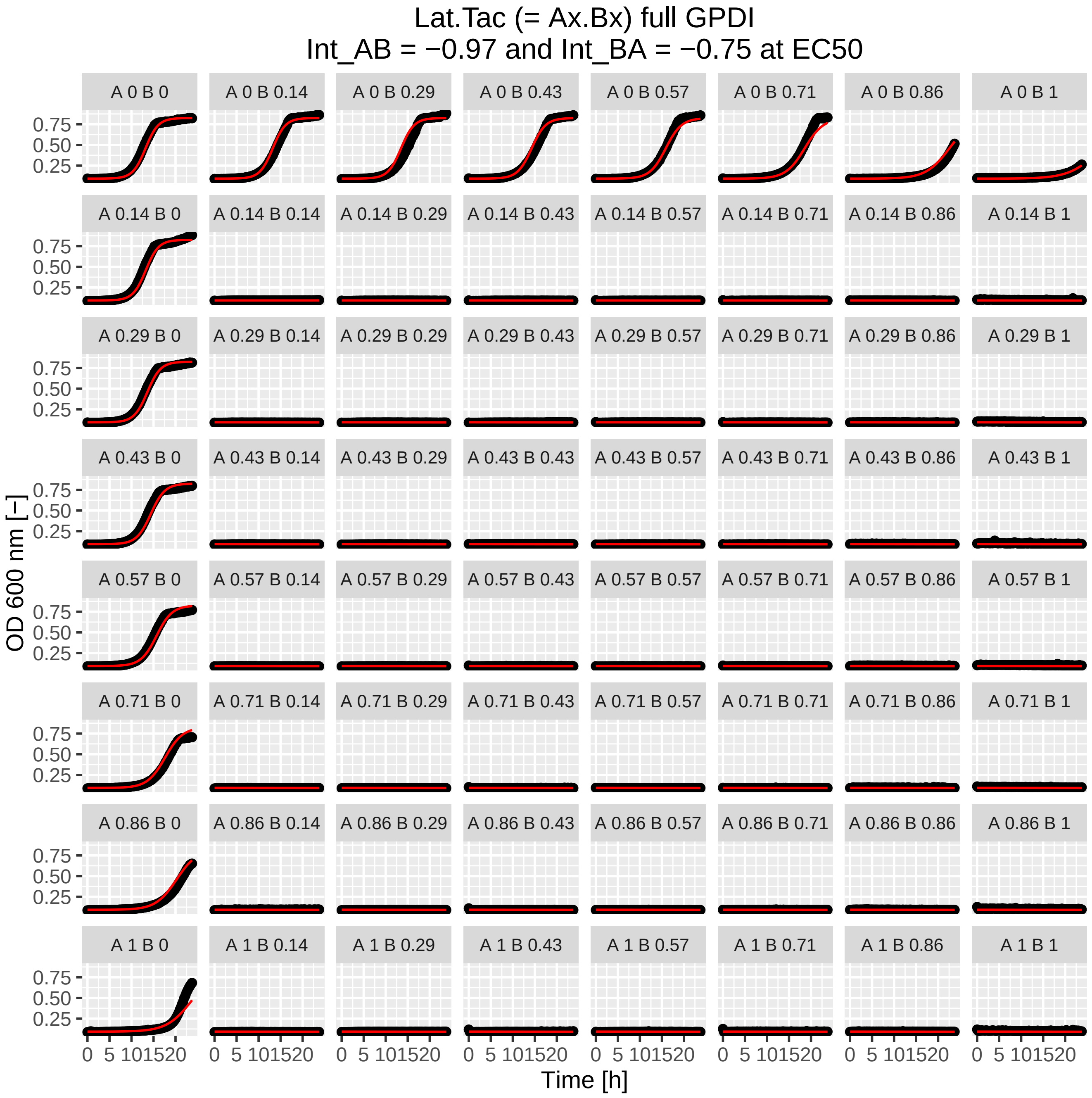

Lat.Tac (= Ax.Bx) full GPDI
Int_AB = −0.97 and Int_BA = −0.75 at EC50
OD 600 nm [−]
Time [h]

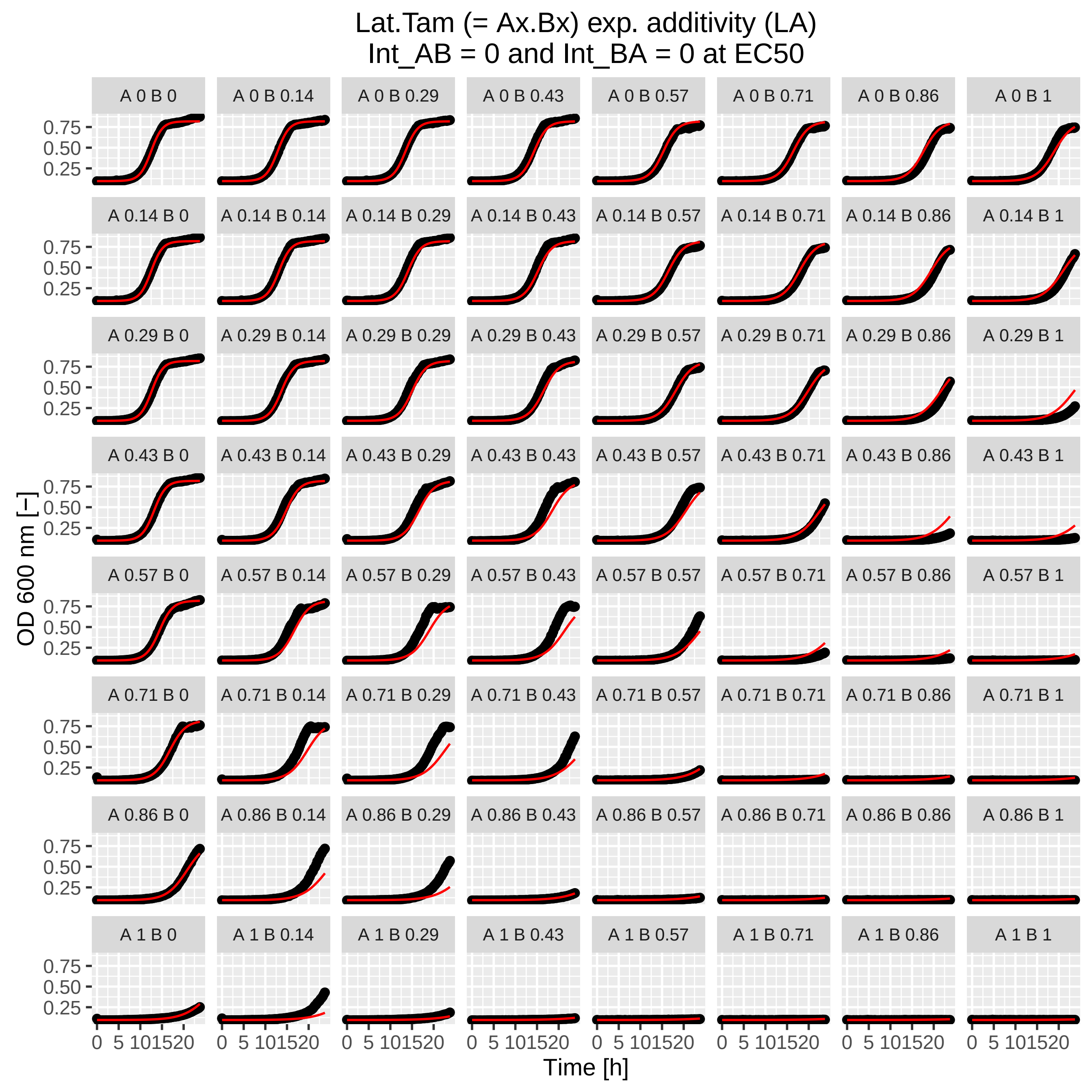
Lat.Tam (= Ax.Bx) exp. additivity (LA)
Int_AB = 0 and Int_BA = 0 at EC50
A 0 B 0
A 0 B 0.14
A 0 B 0.29
A 0 B 0.43
A 0 B 0.57
A 0 B 0.71
A 0 B 0.86
A 0 B 1
A 0.14 B 0
A 0.14 B 0.14
A 0.14 B 0.29
A 0.14 B 0.43
A 0.14 B 0.57
A 0.14 B 0.71
A 0.14 B 0.86
A 0.14 B 1
A 0.29 B 0
A 0.29 B 0.14
A 0.29 B 0.29
A 0.29 B 0.43
A 0.29 B 0.57
A 0.29 B 0.71
A 0.29 B 0.86
A 0.29 B 1
A 0.43 B 0
A 0.43 B 0.14
A 0.43 B 0.29
A 0.43 B 0.43
A 0.43 B 0.57
A 0.43 B 0.71
A 0.43 B 0.86
A 0.43 B 1
A 0.57 B 0
A 0.57 B 0.14
A 0.57 B 0.29
A 0.57 B 0.43
A 0.57 B 0.57
A 0.57 B 0.71
A 0.57 B 0.86
A 0.57 B 1
A 0.71 B 0
A 0.71 B 0.14
A 0.71 B 0.29
A 0.71 B 0.43
A 0.71 B 0.57
A 0.71 B 0.71
A 0.71 B 0.86
A 0.71 B 1
A 0.86 B 0
A 0.86 B 0.14
A 0.86 B 0.29
A 0.86 B 0.43
A 0.86 B 0.57
A 0.86 B 0.71
A 0.86 B 0.86
A 0.86 B 1
A 1 B 0
A 1 B 0.14
A 1 B 0.29
A 1 B 0.43
A 1 B 0.57
A 1 B 0.71
A 1 B 0.86
A 1 B 1
0.75
0.50
0.25
OD 600 nm [–]
0 5 101520
Time [h]

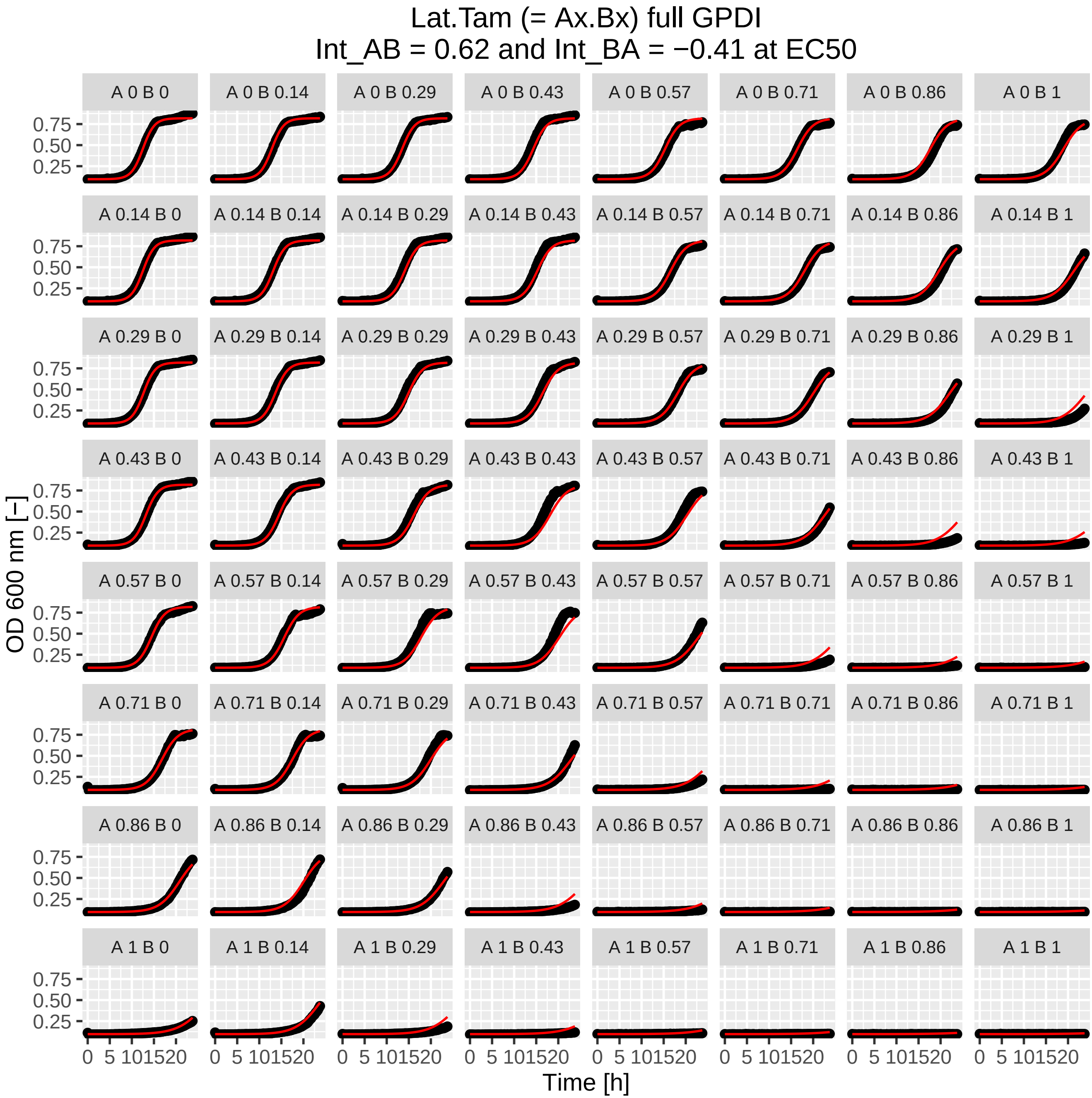

Lat.Tam (= Ax.Bx) full GPDI
Int_AB = 0.62 and Int_BA = −0.41 at EC50
A 0 B 0
A 0 B 0.14
A 0 B 0.29
A 0 B 0.43
A 0 B 0.57
A 0 B 0.71
A 0 B 0.86
A 0 B 1
A 0.14 B 0
A 0.14 B 0.14
A 0.14 B 0.29
A 0.14 B 0.43
A 0.14 B 0.57
A 0.14 B 0.71
A 0.14 B 0.86
A 0.14 B 1
A 0.29 B 0
A 0.29 B 0.14
A 0.29 B 0.29
A 0.29 B 0.43
A 0.29 B 0.57
A 0.29 B 0.71
A 0.29 B 0.86
A 0.29 B 1
A 0.43 B 0
A 0.43 B 0.14
A 0.43 B 0.29
A 0.43 B 0.43
A 0.43 B 0.57
A 0.43 B 0.71
A 0.43 B 0.86
A 0.43 B 1
A 0.57 B 0
A 0.57 B 0.14
A 0.57 B 0.29
A 0.57 B 0.43
A 0.57 B 0.57
A 0.57 B 0.71
A 0.57 B 0.86
A 0.57 B 1
A 0.71 B 0
A 0.71 B 0.14
A 0.71 B 0.29
A 0.71 B 0.43
A 0.71 B 0.57
A 0.71 B 0.71
A 0.71 B 0.86
A 0.71 B 1
A 0.86 B 0
A 0.86 B 0.14
A 0.86 B 0.29
A 0.86 B 0.43
A 0.86 B 0.57
A 0.86 B 0.71
A 0.86 B 0.86
A 0.86 B 1
A 1 B 0
A 1 B 0.14
A 1 B 0.29
A 1 B 0.43
A 1 B 0.57
A 1 B 0.71
A 1 B 0.86
A 1 B 1
0.75
0.50
0.25
OD 600 nm [−]
0 5 101520
Time [h]

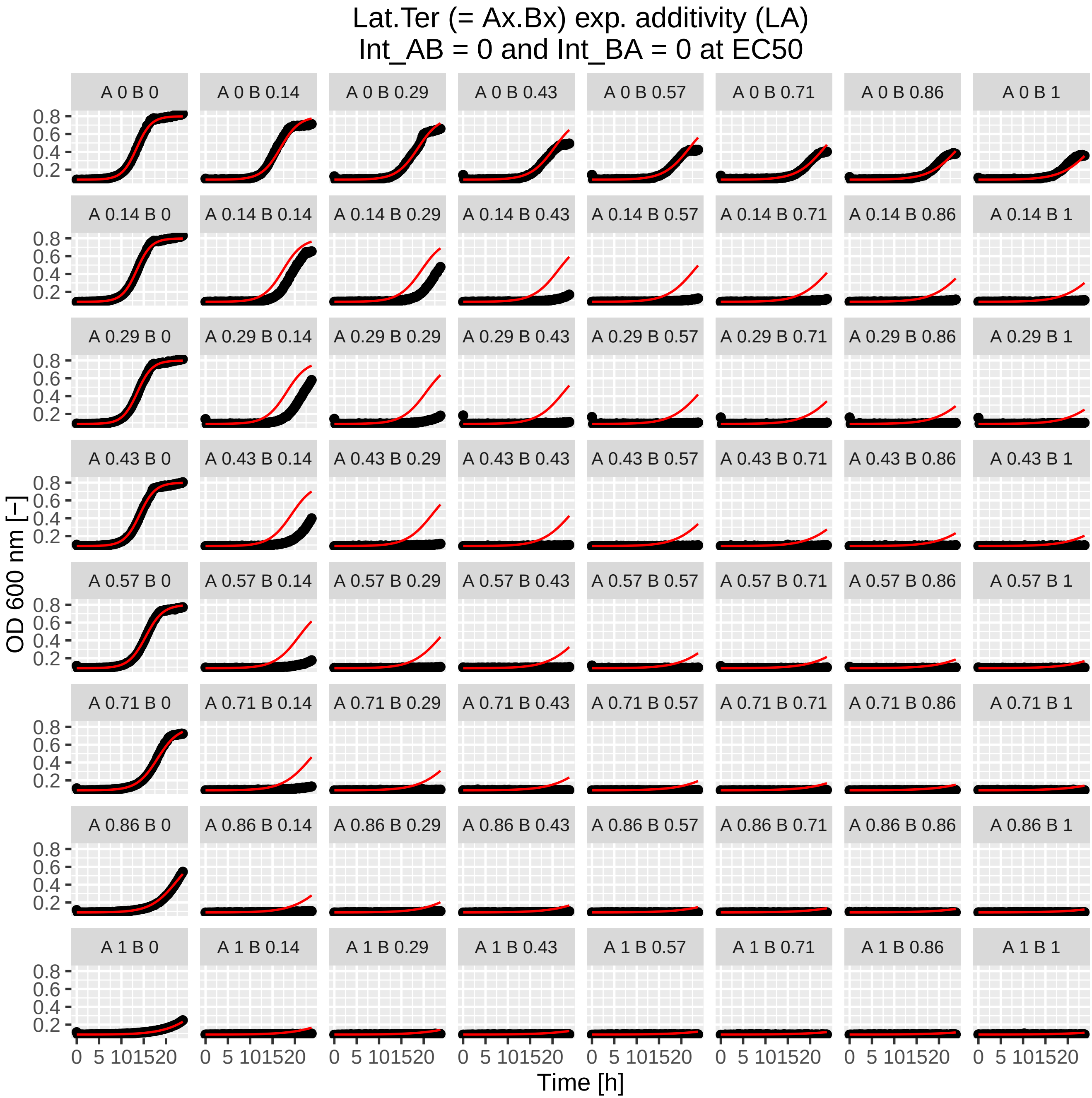

Lat.Ter (= Ax.Bx) exp. additivity (LA)
Int_AB = 0 and Int_BA = 0 at EC50
A 0 B 0
A 0 B 0.14
A 0 B 0.29
A 0 B 0.43
A 0 B 0.57
A 0 B 0.71
A 0 B 0.86
A 0 B 1
A 0.14 B 0
A 0.14 B 0.14
A 0.14 B 0.29
A 0.14 B 0.43
A 0.14 B 0.57
A 0.14 B 0.71
A 0.14 B 0.86
A 0.14 B 1
A 0.29 B 0
A 0.29 B 0.14
A 0.29 B 0.29
A 0.29 B 0.43
A 0.29 B 0.57
A 0.29 B 0.71
A 0.29 B 0.86
A 0.29 B 1
A 0.43 B 0
A 0.43 B 0.14
A 0.43 B 0.29
A 0.43 B 0.43
A 0.43 B 0.57
A 0.43 B 0.71
A 0.43 B 0.86
A 0.43 B 1
A 0.57 B 0
A 0.57 B 0.14
A 0.57 B 0.29
A 0.57 B 0.43
A 0.57 B 0.57
A 0.57 B 0.71
A 0.57 B 0.86
A 0.57 B 1
A 0.71 B 0
A 0.71 B 0.14
A 0.71 B 0.29
A 0.71 B 0.43
A 0.71 B 0.57
A 0.71 B 0.71
A 0.71 B 0.86
A 0.71 B 1
A 0.86 B 0
A 0.86 B 0.14
A 0.86 B 0.29
A 0.86 B 0.43
A 0.86 B 0.57
A 0.86 B 0.71
A 0.86 B 0.86
A 0.86 B 1
A 1 B 0
A 1 B 0.14
A 1 B 0.29
A 1 B 0.43
A 1 B 0.57
A 1 B 0.71
A 1 B 0.86
A 1 B 1
OD 600 nm [–]
0.2
0.4
0.6
0.8
0 5 101520
Time [h]

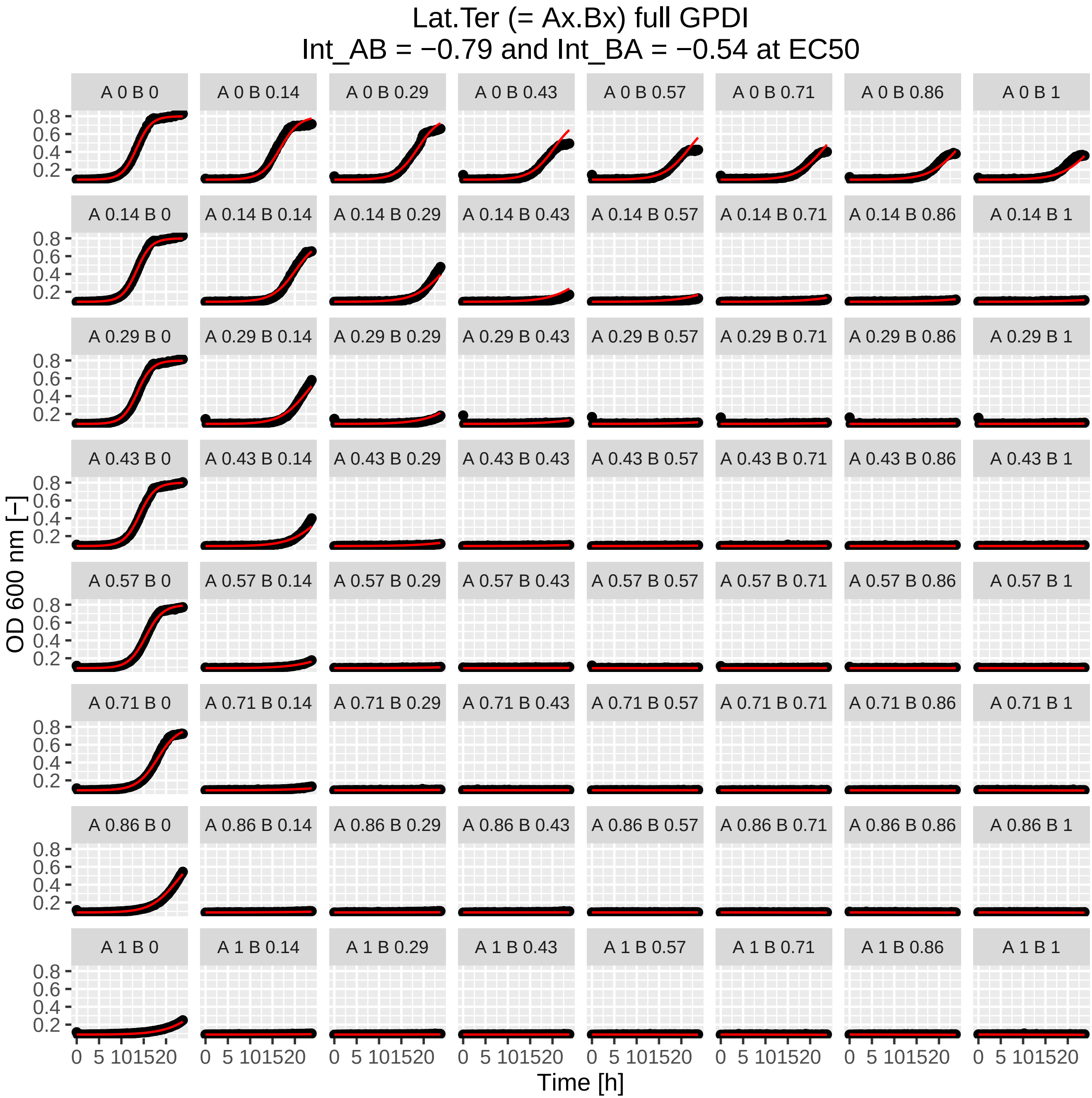

Lat.Ter (= Ax.Bx) full GPDI
Int_AB = −0.79 and Int_BA = −0.54 at EC50
A 0 B 0
A 0 B 0.14
A 0 B 0.29
A 0 B 0.43
A 0 B 0.57
A 0 B 0.71
A 0 B 0.86
A 0 B 1
A 0.14 B 0
A 0.14 B 0.14
A 0.14 B 0.29
A 0.14 B 0.43
A 0.14 B 0.57
A 0.14 B 0.71
A 0.14 B 0.86
A 0.14 B 1
A 0.29 B 0
A 0.29 B 0.14
A 0.29 B 0.29
A 0.29 B 0.43
A 0.29 B 0.57
A 0.29 B 0.71
A 0.29 B 0.86
A 0.29 B 1
A 0.43 B 0
A 0.43 B 0.14
A 0.43 B 0.29
A 0.43 B 0.43
A 0.43 B 0.57
A 0.43 B 0.71
A 0.43 B 0.86
A 0.43 B 1
A 0.57 B 0
A 0.57 B 0.14
A 0.57 B 0.29
A 0.57 B 0.43
A 0.57 B 0.57
A 0.57 B 0.71
A 0.57 B 0.86
A 0.57 B 1
A 0.71 B 0
A 0.71 B 0.14
A 0.71 B 0.29
A 0.71 B 0.43
A 0.71 B 0.57
A 0.71 B 0.71
A 0.71 B 0.86
A 0.71 B 1
A 0.86 B 0
A 0.86 B 0.14
A 0.86 B 0.29
A 0.86 B 0.43
A 0.86 B 0.57
A 0.86 B 0.71
A 0.86 B 0.86
A 0.86 B 1
A 1 B 0
A 1 B 0.14
A 1 B 0.29
A 1 B 0.43
A 1 B 0.57
A 1 B 0.71
A 1 B 0.86
A 1 B 1
OD 600 nm [−]
0.2
0.4
0.6
0.8
0 5 101520
Time [h]

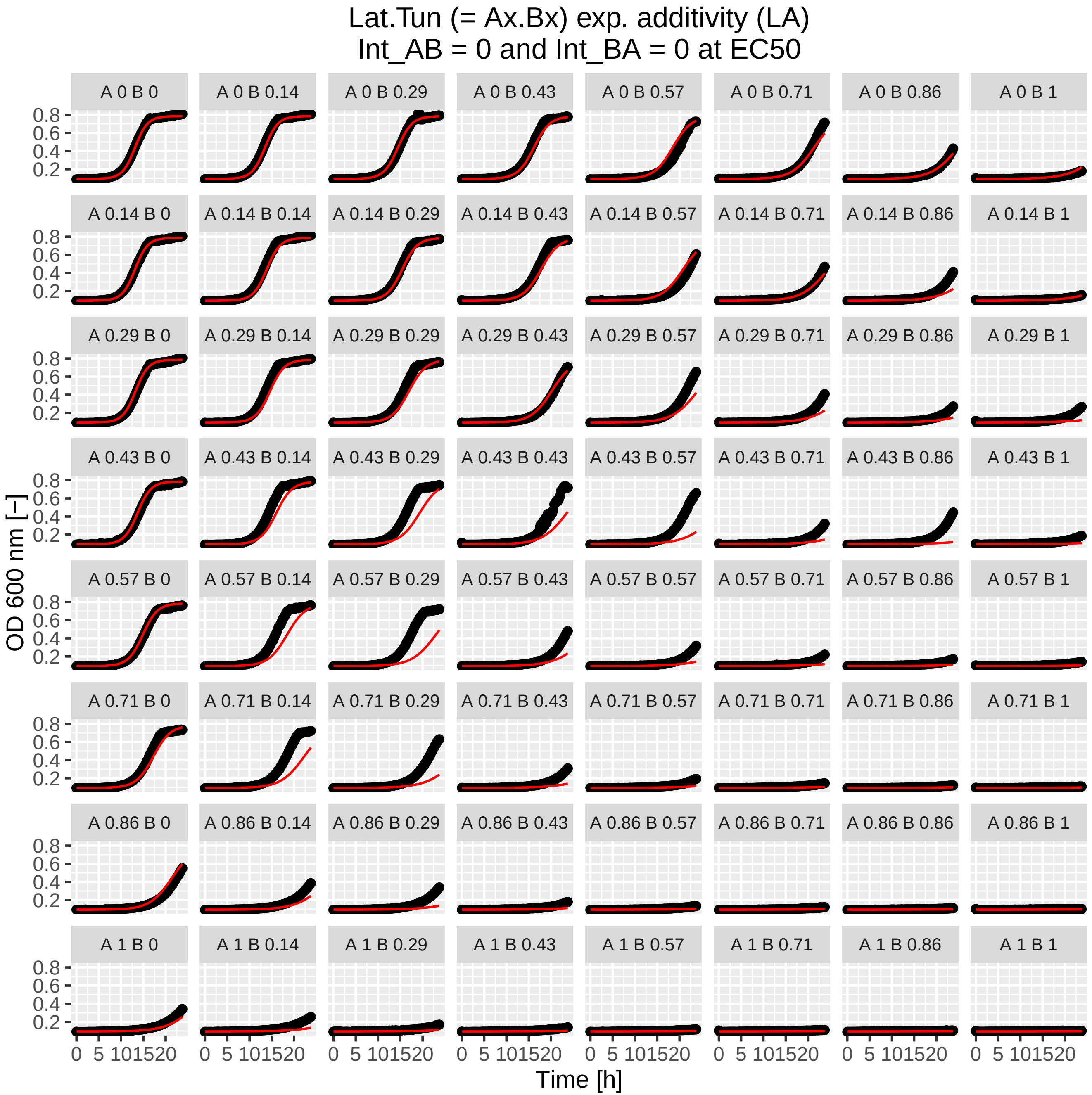

Lat.Tun (= Ax.Bx) exp. additivity (LA)
Int_AB = 0 and Int_BA = 0 at EC50
OD 600 nm [−]
Time [h]
0.2 0.4 0.6 0.8
0 5 101520
A 0 B 0
A 0 B 0.14
A 0 B 0.29
A 0 B 0.43
A 0 B 0.57
A 0 B 0.71
A 0 B 0.86
A 0 B 1
A 0.14 B 0
A 0.14 B 0.14
A 0.14 B 0.29
A 0.14 B 0.43
A 0.14 B 0.57
A 0.14 B 0.71
A 0.14 B 0.86
A 0.14 B 1
A 0.29 B 0
A 0.29 B 0.14
A 0.29 B 0.29
A 0.29 B 0.43
A 0.29 B 0.57
A 0.29 B 0.71
A 0.29 B 0.86
A 0.29 B 1
A 0.43 B 0
A 0.43 B 0.14
A 0.43 B 0.29
A 0.43 B 0.43
A 0.43 B 0.57
A 0.43 B 0.71
A 0.43 B 0.86
A 0.43 B 1
A 0.57 B 0
A 0.57 B 0.14
A 0.57 B 0.29
A 0.57 B 0.43
A 0.57 B 0.57
A 0.57 B 0.71
A 0.57 B 0.86
A 0.57 B 1
A 0.71 B 0
A 0.71 B 0.14
A 0.71 B 0.29
A 0.71 B 0.43
A 0.71 B 0.57
A 0.71 B 0.71
A 0.71 B 0.86
A 0.71 B 1
A 0.86 B 0
A 0.86 B 0.14
A 0.86 B 0.29
A 0.86 B 0.43
A 0.86 B 0.57
A 0.86 B 0.71
A 0.86 B 0.86
A 0.86 B 1
A 1 B 0
A 1 B 0.14
A 1 B 0.29
A 1 B 0.43
A 1 B 0.57
A 1 B 0.71
A 1 B 0.86
A 1 B 1

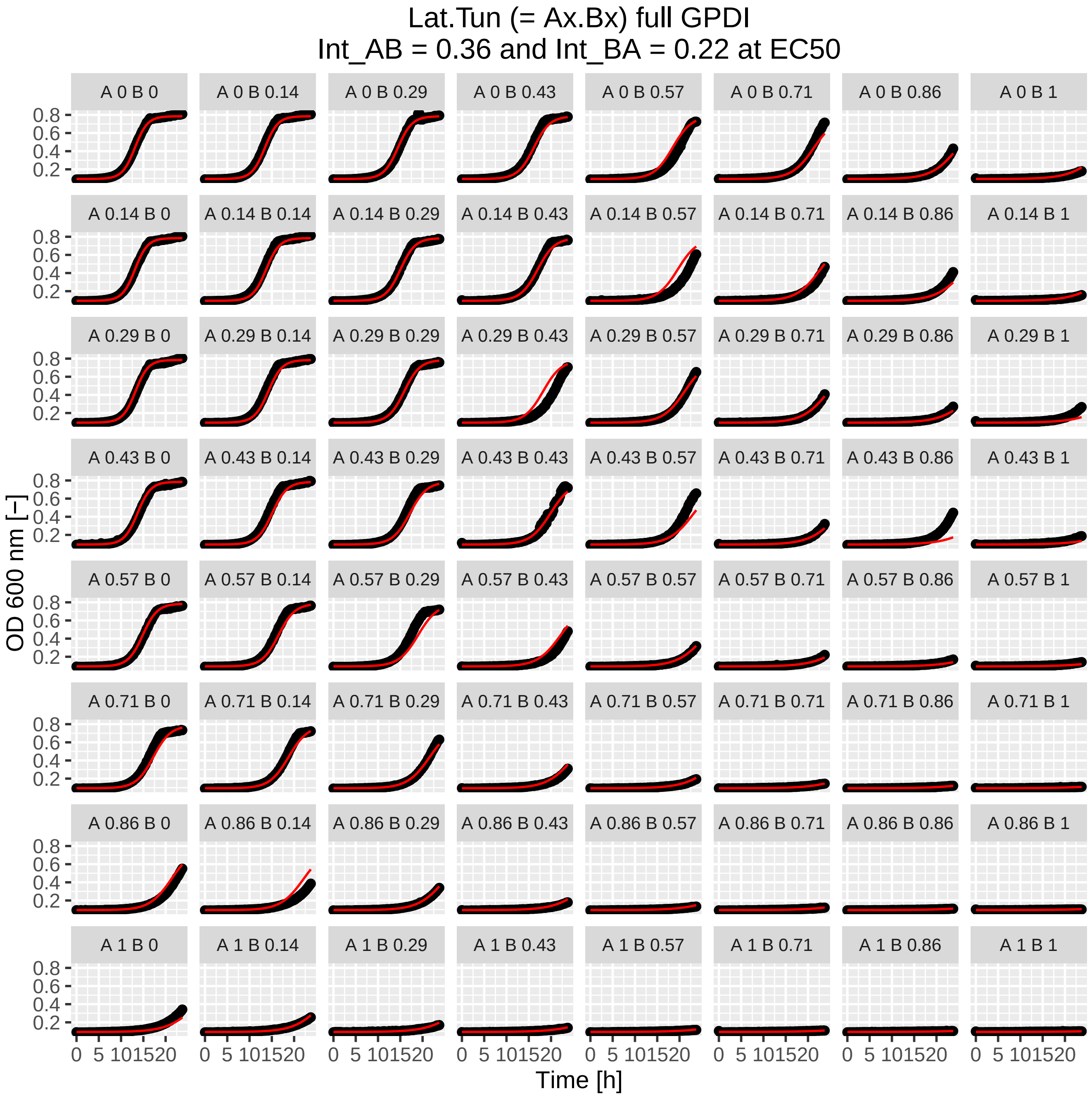
Lat.Tun (= Ax.Bx) full GPDI
Int_AB = 0.36 and Int_BA = 0.22 at EC50
A 0 B 0
A 0 B 0.14
A 0 B 0.29
A 0 B 0.43
A 0 B 0.57
A 0 B 0.71
A 0 B 0.86
A 0 B 1
A 0.14 B 0
A 0.14 B 0.14
A 0.14 B 0.29
A 0.14 B 0.43
A 0.14 B 0.57
A 0.14 B 0.71
A 0.14 B 0.86
A 0.14 B 1
A 0.29 B 0
A 0.29 B 0.14
A 0.29 B 0.29
A 0.29 B 0.43
A 0.29 B 0.57
A 0.29 B 0.71
A 0.29 B 0.86
A 0.29 B 1
A 0.43 B 0
A 0.43 B 0.14
A 0.43 B 0.29
A 0.43 B 0.43
A 0.43 B 0.57
A 0.43 B 0.71
A 0.43 B 0.86
A 0.43 B 1
A 0.57 B 0
A 0.57 B 0.14
A 0.57 B 0.29
A 0.57 B 0.43
A 0.57 B 0.57
A 0.57 B 0.71
A 0.57 B 0.86
A 0.57 B 1
A 0.71 B 0
A 0.71 B 0.14
A 0.71 B 0.29
A 0.71 B 0.43
A 0.71 B 0.57
A 0.71 B 0.71
A 0.71 B 0.86
A 0.71 B 1
A 0.86 B 0
A 0.86 B 0.14
A 0.86 B 0.29
A 0.86 B 0.43
A 0.86 B 0.57
A 0.86 B 0.71
A 0.86 B 0.86
A 0.86 B 1
A 1 B 0
A 1 B 0.14
A 1 B 0.29
A 1 B 0.43
A 1 B 0.57
A 1 B 0.71
A 1 B 0.86
A 1 B 1
OD 600 nm [–]
0.8
0.6
0.4
0.2
0 5 101520
Time [h]

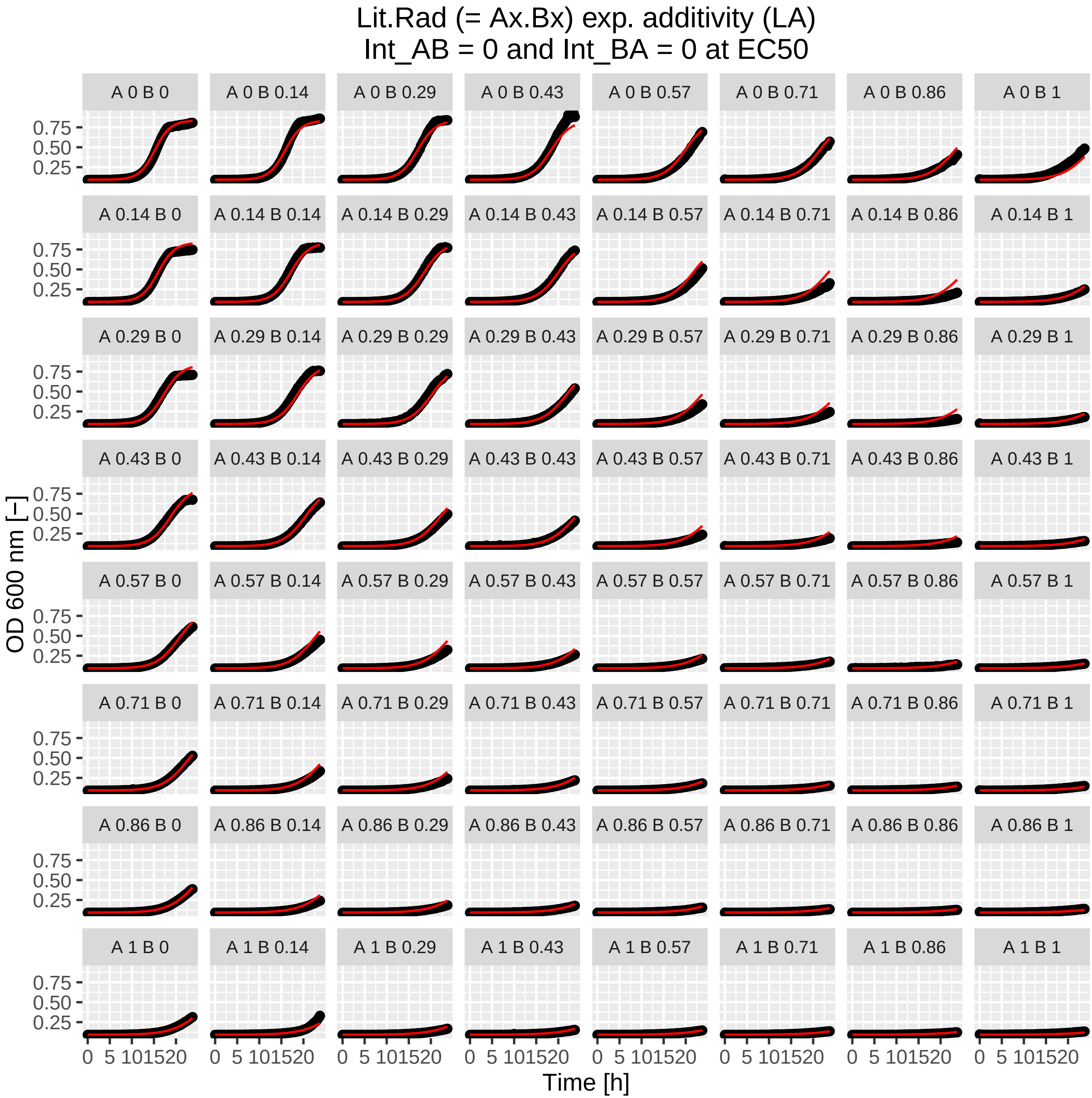

Lit.Rad (= Ax.Bx) exp. additivity (LA)
Int_AB = 0 and Int_BA = 0 at EC50
A 0 B 0
A 0 B 0.14
A 0 B 0.29
A 0 B 0.43
A 0 B 0.57
A 0 B 0.71
A 0 B 0.86
A 0 B 1
A 0.14 B 0
A 0.14 B 0.14
A 0.14 B 0.29
A 0.14 B 0.43
A 0.14 B 0.57
A 0.14 B 0.71
A 0.14 B 0.86
A 0.14 B 1
A 0.29 B 0
A 0.29 B 0.14
A 0.29 B 0.29
A 0.29 B 0.43
A 0.29 B 0.57
A 0.29 B 0.71
A 0.29 B 0.86
A 0.29 B 1
A 0.43 B 0
A 0.43 B 0.14
A 0.43 B 0.29
A 0.43 B 0.43
A 0.43 B 0.57
A 0.43 B 0.71
A 0.43 B 0.86
A 0.43 B 1
A 0.57 B 0
A 0.57 B 0.14
A 0.57 B 0.29
A 0.57 B 0.43
A 0.57 B 0.57
A 0.57 B 0.71
A 0.57 B 0.86
A 0.57 B 1
A 0.71 B 0
A 0.71 B 0.14
A 0.71 B 0.29
A 0.71 B 0.43
A 0.71 B 0.57
A 0.71 B 0.71
A 0.71 B 0.86
A 0.71 B 1
A 0.86 B 0
A 0.86 B 0.14
A 0.86 B 0.29
A 0.86 B 0.43
A 0.86 B 0.57
A 0.86 B 0.71
A 0.86 B 0.86
A 0.86 B 1
A 1 B 0
A 1 B 0.14
A 1 B 0.29
A 1 B 0.43
A 1 B 0.57
A 1 B 0.71
A 1 B 0.86
A 1 B 1
OD 600 nm [–]
0.75
0.50
0.25
0 5 101520
Time [h]

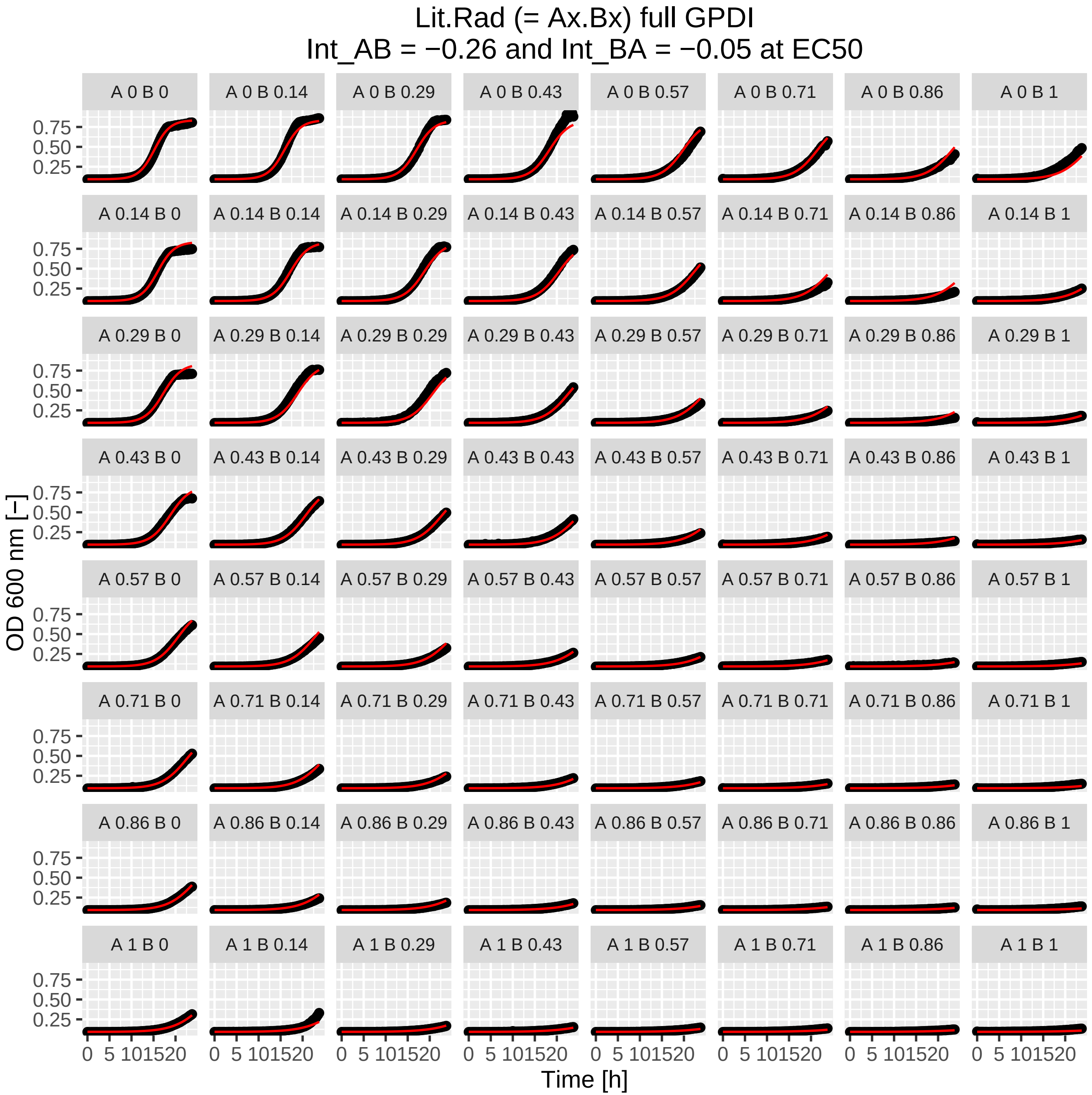
Lit.Rad (= Ax.Bx) full GPDI
Int_AB = −0.26 and Int_BA = −0.05 at EC50
A 0 B 0
A 0 B 0.14
A 0 B 0.29
A 0 B 0.43
A 0 B 0.57
A 0 B 0.71
A 0 B 0.86
A 0 B 1
A 0.14 B 0
A 0.14 B 0.14
A 0.14 B 0.29
A 0.14 B 0.43
A 0.14 B 0.57
A 0.14 B 0.71
A 0.14 B 0.86
A 0.14 B 1
A 0.29 B 0
A 0.29 B 0.14
A 0.29 B 0.29
A 0.29 B 0.43
A 0.29 B 0.57
A 0.29 B 0.71
A 0.29 B 0.86
A 0.29 B 1
A 0.43 B 0
A 0.43 B 0.14
A 0.43 B 0.29
A 0.43 B 0.43
A 0.43 B 0.57
A 0.43 B 0.71
A 0.43 B 0.86
A 0.43 B 1
A 0.57 B 0
A 0.57 B 0.14
A 0.57 B 0.29
A 0.57 B 0.43
A 0.57 B 0.57
A 0.57 B 0.71
A 0.57 B 0.86
A 0.57 B 1
A 0.71 B 0
A 0.71 B 0.14
A 0.71 B 0.29
A 0.71 B 0.43
A 0.71 B 0.57
A 0.71 B 0.71
A 0.71 B 0.86
A 0.71 B 1
A 0.86 B 0
A 0.86 B 0.14
A 0.86 B 0.29
A 0.86 B 0.43
A 0.86 B 0.57
A 0.86 B 0.71
A 0.86 B 0.86
A 0.86 B 1
A 1 B 0
A 1 B 0.14
A 1 B 0.29
A 1 B 0.43
A 1 B 0.57
A 1 B 0.71
A 1 B 0.86
A 1 B 1
0.75
0.50
0.25
OD 600 nm [−]
0 5 101520
Time [h]

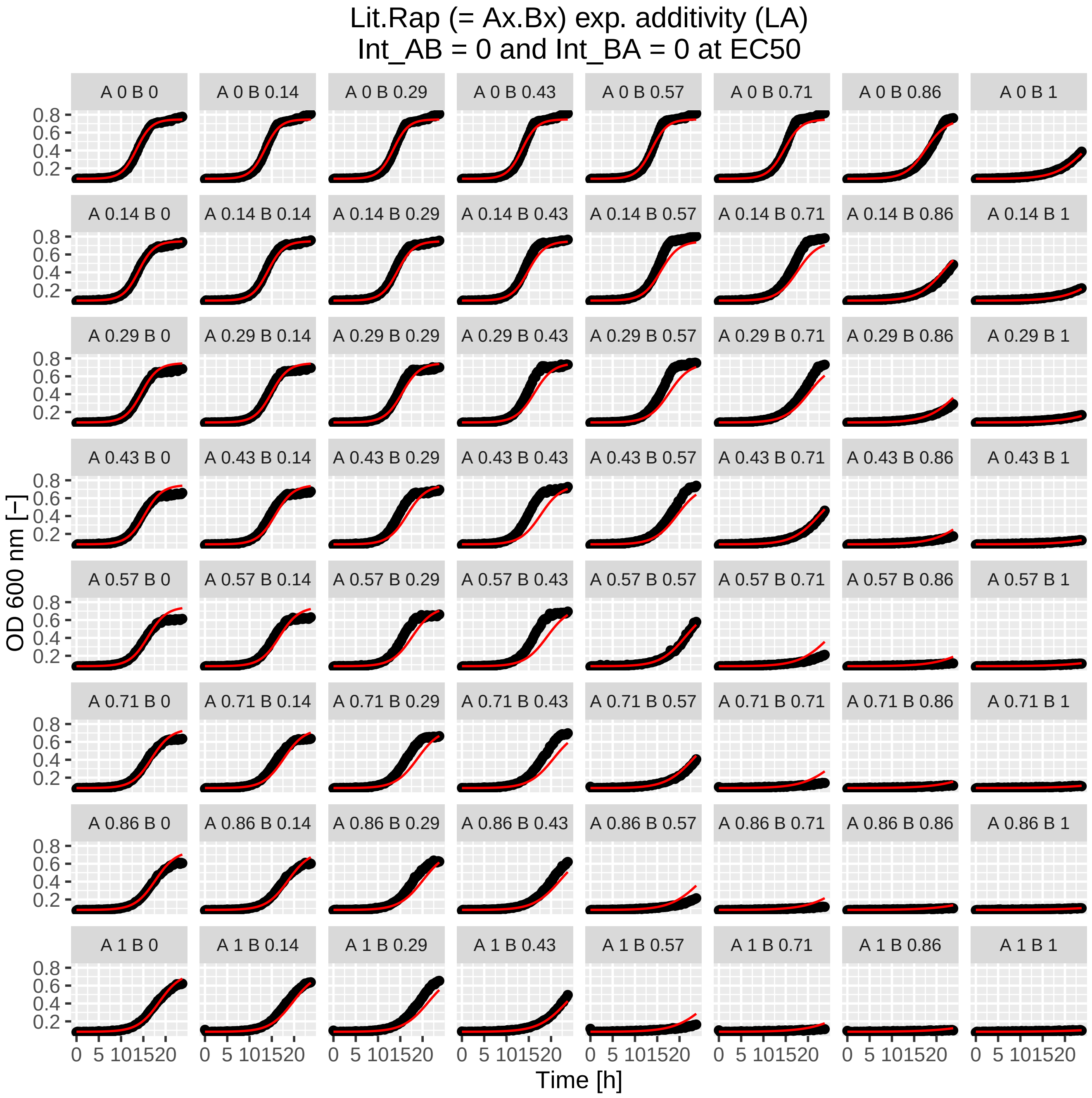

Lit.Rap (= Ax.Bx) exp. additivity (LA)
Int_AB = 0 and Int_BA = 0 at EC50
A 0 B 0
A 0 B 0.14
A 0 B 0.29
A 0 B 0.43
A 0 B 0.57
A 0 B 0.71
A 0 B 0.86
A 0 B 1
A 0.14 B 0
A 0.14 B 0.14
A 0.14 B 0.29
A 0.14 B 0.43
A 0.14 B 0.57
A 0.14 B 0.71
A 0.14 B 0.86
A 0.14 B 1
A 0.29 B 0
A 0.29 B 0.14
A 0.29 B 0.29
A 0.29 B 0.43
A 0.29 B 0.57
A 0.29 B 0.71
A 0.29 B 0.86
A 0.29 B 1
A 0.43 B 0
A 0.43 B 0.14
A 0.43 B 0.29
A 0.43 B 0.43
A 0.43 B 0.57
A 0.43 B 0.71
A 0.43 B 0.86
A 0.43 B 1
A 0.57 B 0
A 0.57 B 0.14
A 0.57 B 0.29
A 0.57 B 0.43
A 0.57 B 0.57
A 0.57 B 0.71
A 0.57 B 0.86
A 0.57 B 1
A 0.71 B 0
A 0.71 B 0.14
A 0.71 B 0.29
A 0.71 B 0.43
A 0.71 B 0.57
A 0.71 B 0.71
A 0.71 B 0.86
A 0.71 B 1
A 0.86 B 0
A 0.86 B 0.14
A 0.86 B 0.29
A 0.86 B 0.43
A 0.86 B 0.57
A 0.86 B 0.71
A 0.86 B 0.86
A 0.86 B 1
A 1 B 0
A 1 B 0.14
A 1 B 0.29
A 1 B 0.43
A 1 B 0.57
A 1 B 0.71
A 1 B 0.86
A 1 B 1
0.8
0.6
0.4
0.2
OD 600 nm [–]
0 5 101520
Time [h]

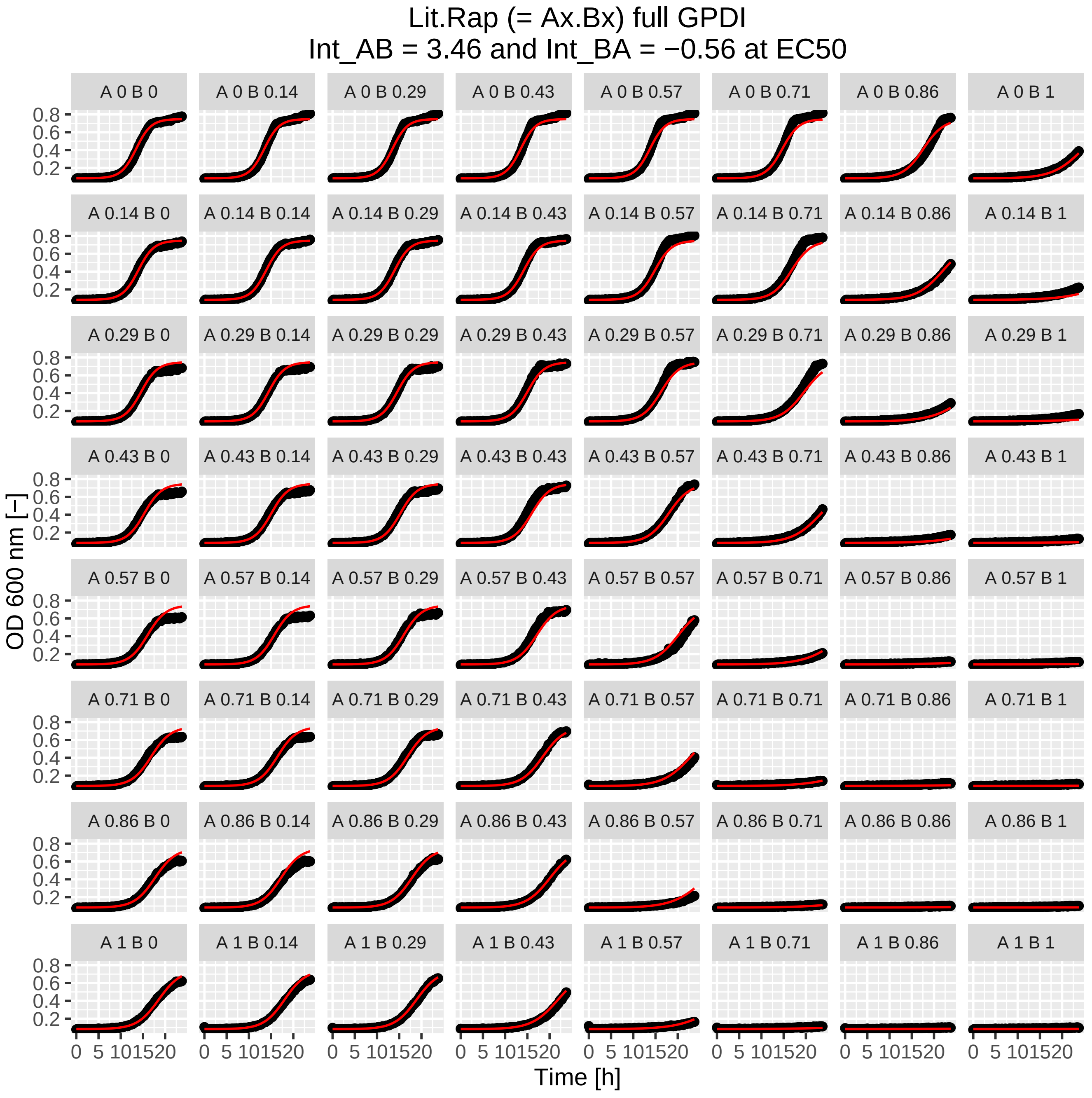

Lit.Rap (= Ax.Bx) full GPDI
Int_AB = 3.46 and Int_BA = −0.56 at EC50
A 0 B 0
A 0 B 0.14
A 0 B 0.29
A 0 B 0.43
A 0 B 0.57
A 0 B 0.71
A 0 B 0.86
A 0 B 1
A 0.14 B 0
A 0.14 B 0.14
A 0.14 B 0.29
A 0.14 B 0.43
A 0.14 B 0.57
A 0.14 B 0.71
A 0.14 B 0.86
A 0.14 B 1
A 0.29 B 0
A 0.29 B 0.14
A 0.29 B 0.29
A 0.29 B 0.43
A 0.29 B 0.57
A 0.29 B 0.71
A 0.29 B 0.86
A 0.29 B 1
A 0.43 B 0
A 0.43 B 0.14
A 0.43 B 0.29
A 0.43 B 0.43
A 0.43 B 0.57
A 0.43 B 0.71
A 0.43 B 0.86
A 0.43 B 1
A 0.57 B 0
A 0.57 B 0.14
A 0.57 B 0.29
A 0.57 B 0.43
A 0.57 B 0.57
A 0.57 B 0.71
A 0.57 B 0.86
A 0.57 B 1
A 0.71 B 0
A 0.71 B 0.14
A 0.71 B 0.29
A 0.71 B 0.43
A 0.71 B 0.57
A 0.71 B 0.71
A 0.71 B 0.86
A 0.71 B 1
A 0.86 B 0
A 0.86 B 0.14
A 0.86 B 0.29
A 0.86 B 0.43
A 0.86 B 0.57
A 0.86 B 0.71
A 0.86 B 0.86
A 0.86 B 1
A 1 B 0
A 1 B 0.14
A 1 B 0.29
A 1 B 0.43
A 1 B 0.57
A 1 B 0.71
A 1 B 0.86
A 1 B 1
0.8
0.6
0.4
0.2
OD 600 nm [−]
0 5 101520
Time [h]

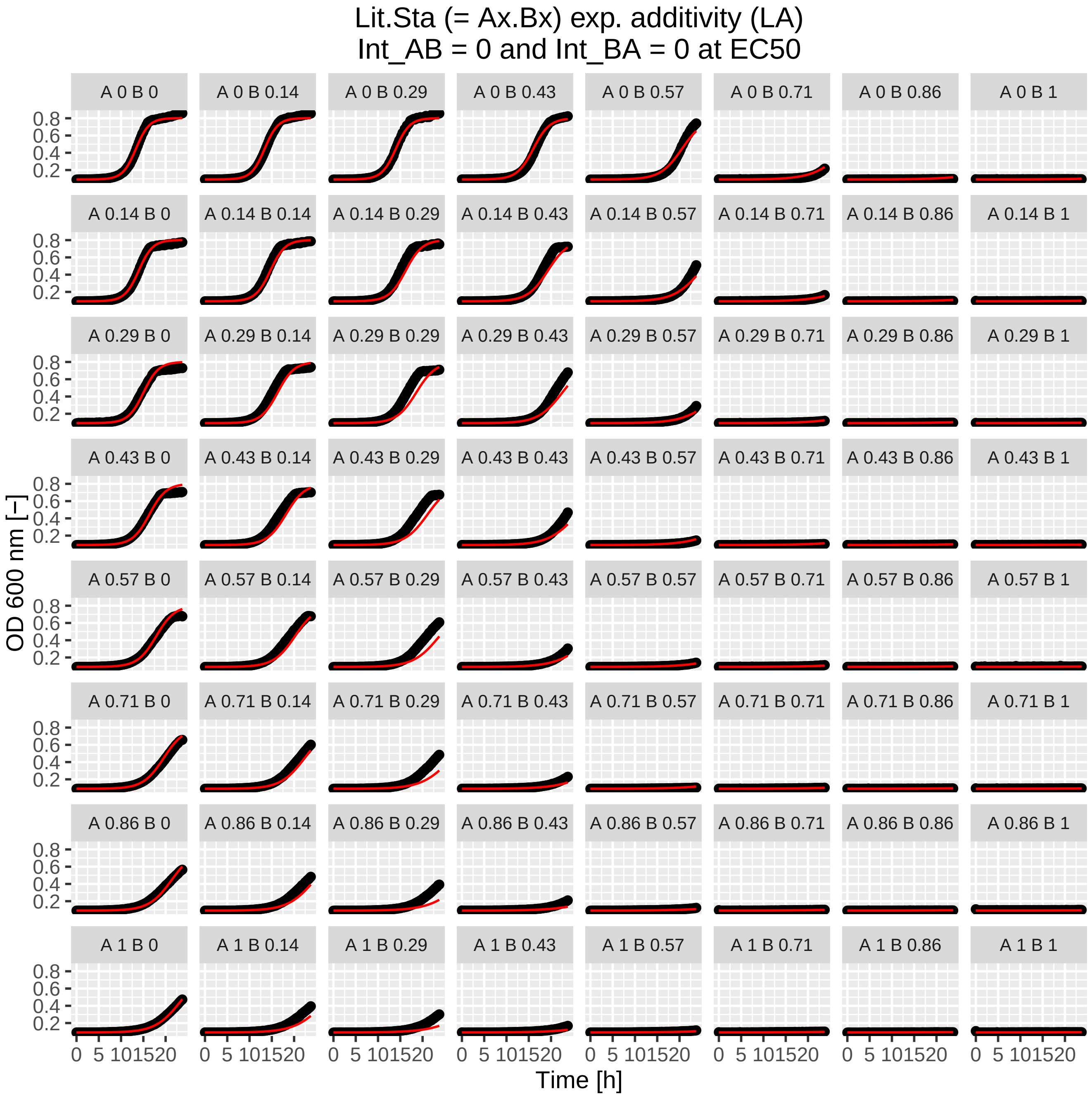
Lit.Sta (= Ax.Bx) exp. additivity (LA)
Int_AB = 0 and Int_BA = 0 at EC50
A 0 B 0
A 0 B 0.14
A 0 B 0.29
A 0 B 0.43
A 0 B 0.57
A 0 B 0.71
A 0 B 0.86
A 0 B 1
A 0.14 B 0
A 0.14 B 0.14
A 0.14 B 0.29
A 0.14 B 0.43
A 0.14 B 0.57
A 0.14 B 0.71
A 0.14 B 0.86
A 0.14 B 1
A 0.29 B 0
A 0.29 B 0.14
A 0.29 B 0.29
A 0.29 B 0.43
A 0.29 B 0.57
A 0.29 B 0.71
A 0.29 B 0.86
A 0.29 B 1
A 0.43 B 0
A 0.43 B 0.14
A 0.43 B 0.29
A 0.43 B 0.43
A 0.43 B 0.57
A 0.43 B 0.71
A 0.43 B 0.86
A 0.43 B 1
A 0.57 B 0
A 0.57 B 0.14
A 0.57 B 0.29
A 0.57 B 0.43
A 0.57 B 0.57
A 0.57 B 0.71
A 0.57 B 0.86
A 0.57 B 1
A 0.71 B 0
A 0.71 B 0.14
A 0.71 B 0.29
A 0.71 B 0.43
A 0.71 B 0.57
A 0.71 B 0.71
A 0.71 B 0.86
A 0.71 B 1
A 0.86 B 0
A 0.86 B 0.14
A 0.86 B 0.29
A 0.86 B 0.43
A 0.86 B 0.57
A 0.86 B 0.71
A 0.86 B 0.86
A 0.86 B 1
A 1 B 0
A 1 B 0.14
A 1 B 0.29
A 1 B 0.43
A 1 B 0.57
A 1 B 0.71
A 1 B 0.86
A 1 B 1
OD 600 nm [−]
0.8
0.6
0.4
0.2
0 5 101520
Time [h]

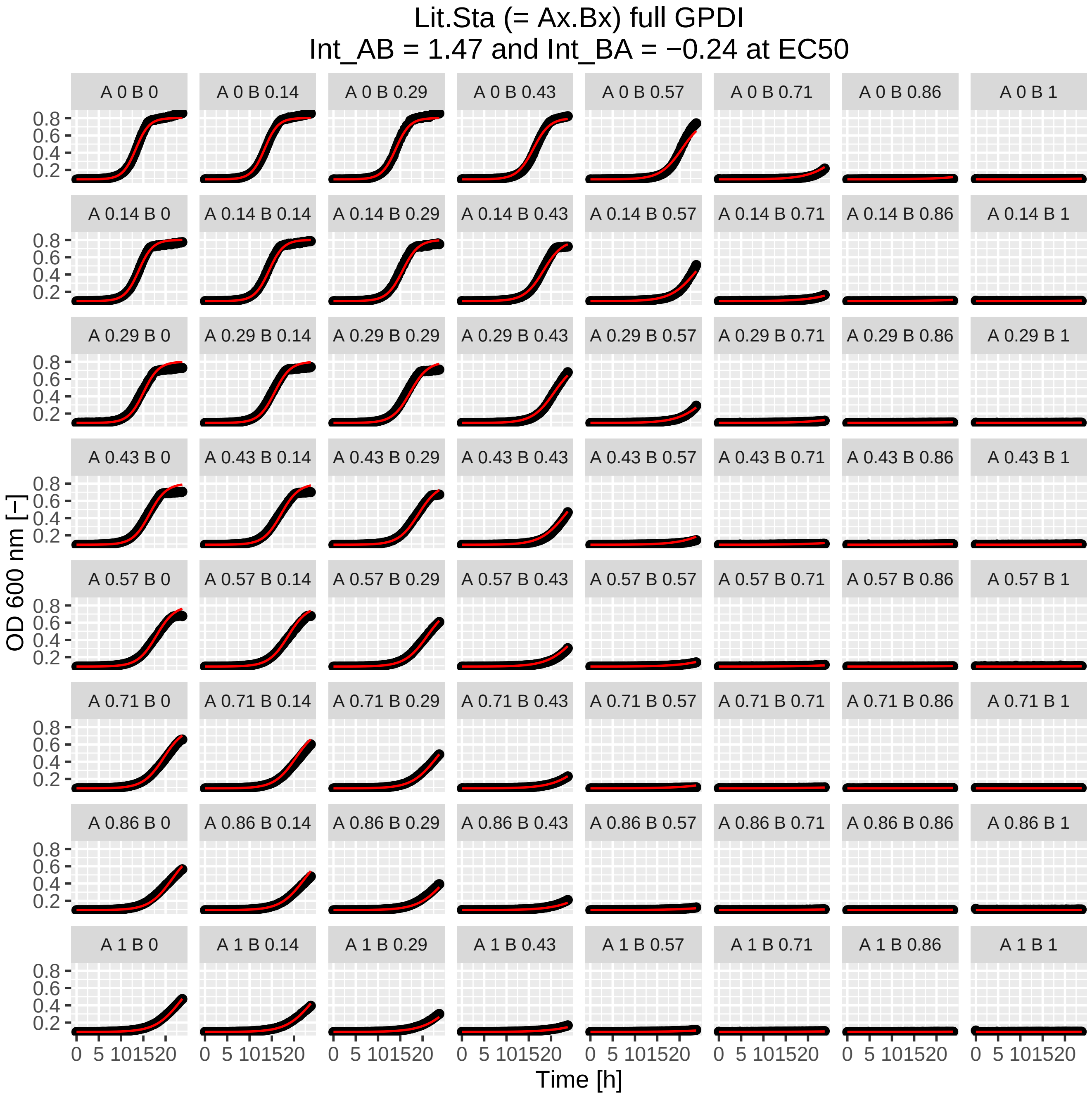

Lit.Sta (= Ax.Bx) full GPDI
Int_AB = 1.47 and Int_BA = −0.24 at EC50
A 0 B 0
A 0 B 0.14
A 0 B 0.29
A 0 B 0.43
A 0 B 0.57
A 0 B 0.71
A 0 B 0.86
A 0 B 1
A 0.14 B 0
A 0.14 B 0.14
A 0.14 B 0.29
A 0.14 B 0.43
A 0.14 B 0.57
A 0.14 B 0.71
A 0.14 B 0.86
A 0.14 B 1
A 0.29 B 0
A 0.29 B 0.14
A 0.29 B 0.29
A 0.29 B 0.43
A 0.29 B 0.57
A 0.29 B 0.71
A 0.29 B 0.86
A 0.29 B 1
A 0.43 B 0
A 0.43 B 0.14
A 0.43 B 0.29
A 0.43 B 0.43
A 0.43 B 0.57
A 0.43 B 0.71
A 0.43 B 0.86
A 0.43 B 1
A 0.57 B 0
A 0.57 B 0.14
A 0.57 B 0.29
A 0.57 B 0.43
A 0.57 B 0.57
A 0.57 B 0.71
A 0.57 B 0.86
A 0.57 B 1
A 0.71 B 0
A 0.71 B 0.14
A 0.71 B 0.29
A 0.71 B 0.43
A 0.71 B 0.57
A 0.71 B 0.71
A 0.71 B 0.86
A 0.71 B 1
A 0.86 B 0
A 0.86 B 0.14
A 0.86 B 0.29
A 0.86 B 0.43
A 0.86 B 0.57
A 0.86 B 0.71
A 0.86 B 0.86
A 0.86 B 1
A 1 B 0
A 1 B 0.14
A 1 B 0.29
A 1 B 0.43
A 1 B 0.57
A 1 B 0.71
A 1 B 0.86
A 1 B 1
OD 600 nm [−]
0.2
0.4
0.6
0.8
0
5
10
15
20
Time [h]

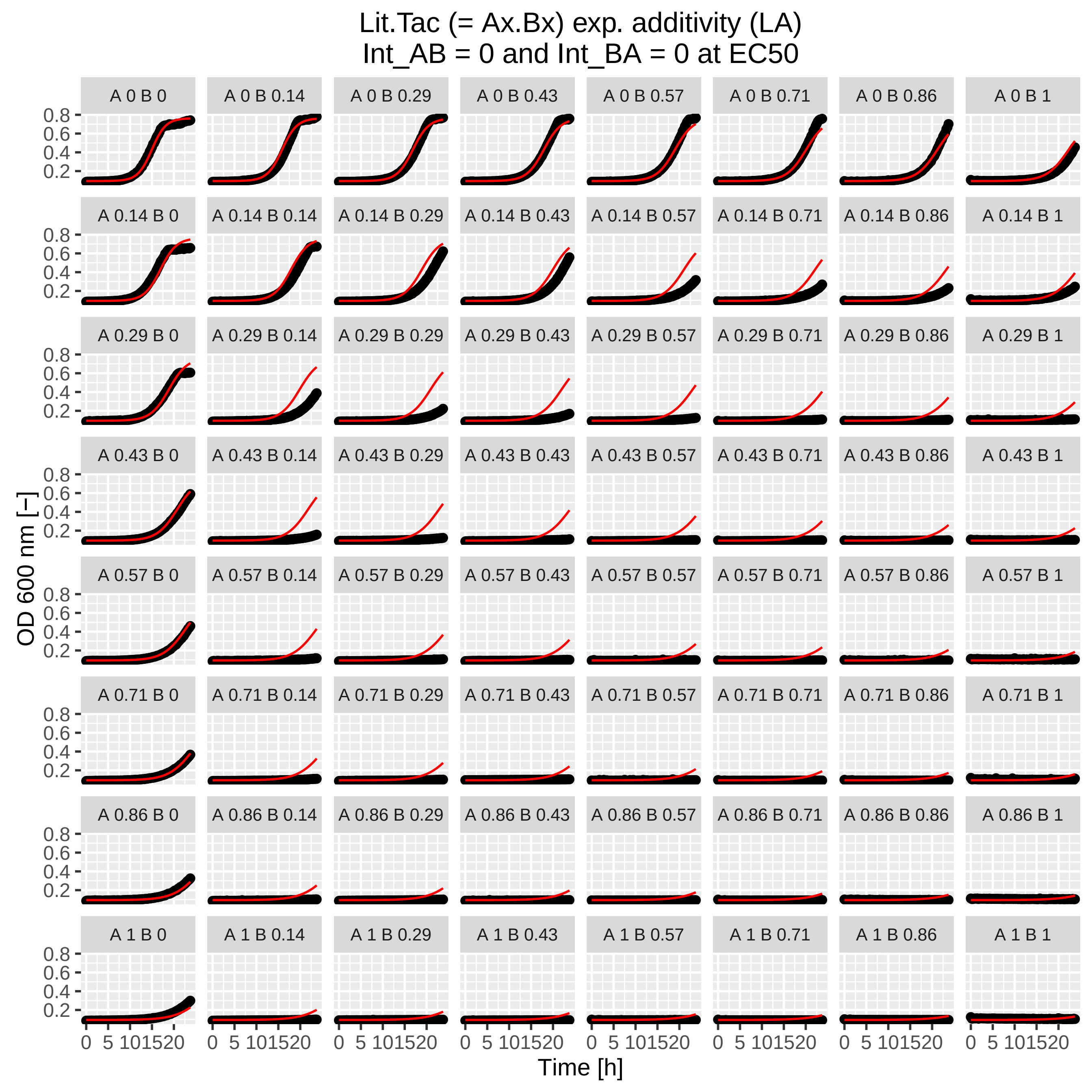
Lit.Tac (= Ax.Bx) exp. additivity (LA)
Int_AB = 0 and Int_BA = 0 at EC50
OD 600 nm [−]
Time [h]

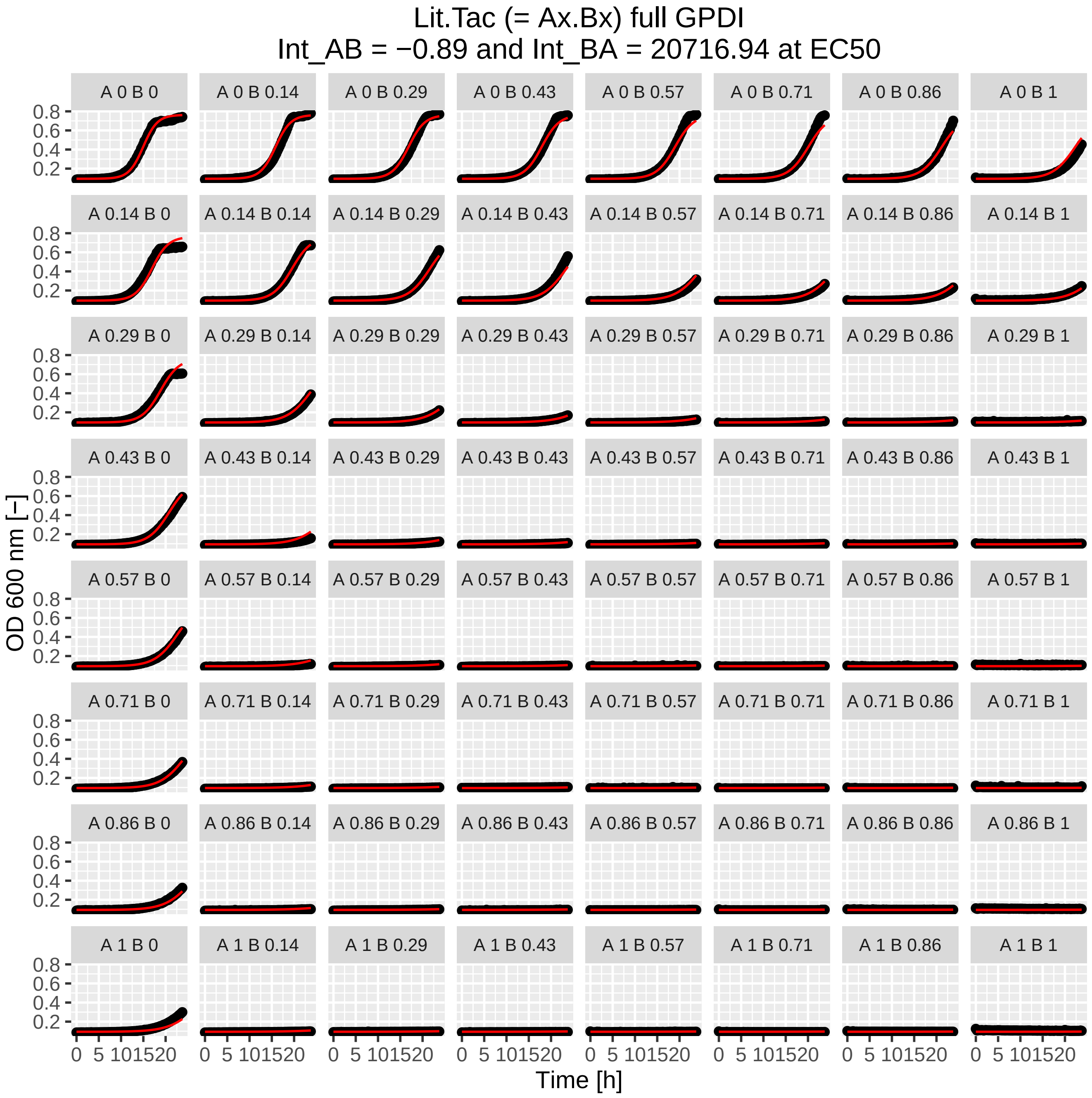

Lit.Tac (= Ax.Bx) full GPDI
Int_AB = −0.89 and Int_BA = 20716.94 at EC50
OD 600 nm [−]
Time [h]
A 0 B 0
A 0 B 0.14
A 0 B 0.29
A 0 B 0.43
A 0 B 0.57
A 0 B 0.71
A 0 B 0.86
A 0 B 1
A 1 B 0
A 1 B 1
0.2
0.4
0.6
0.8
0 5 101520

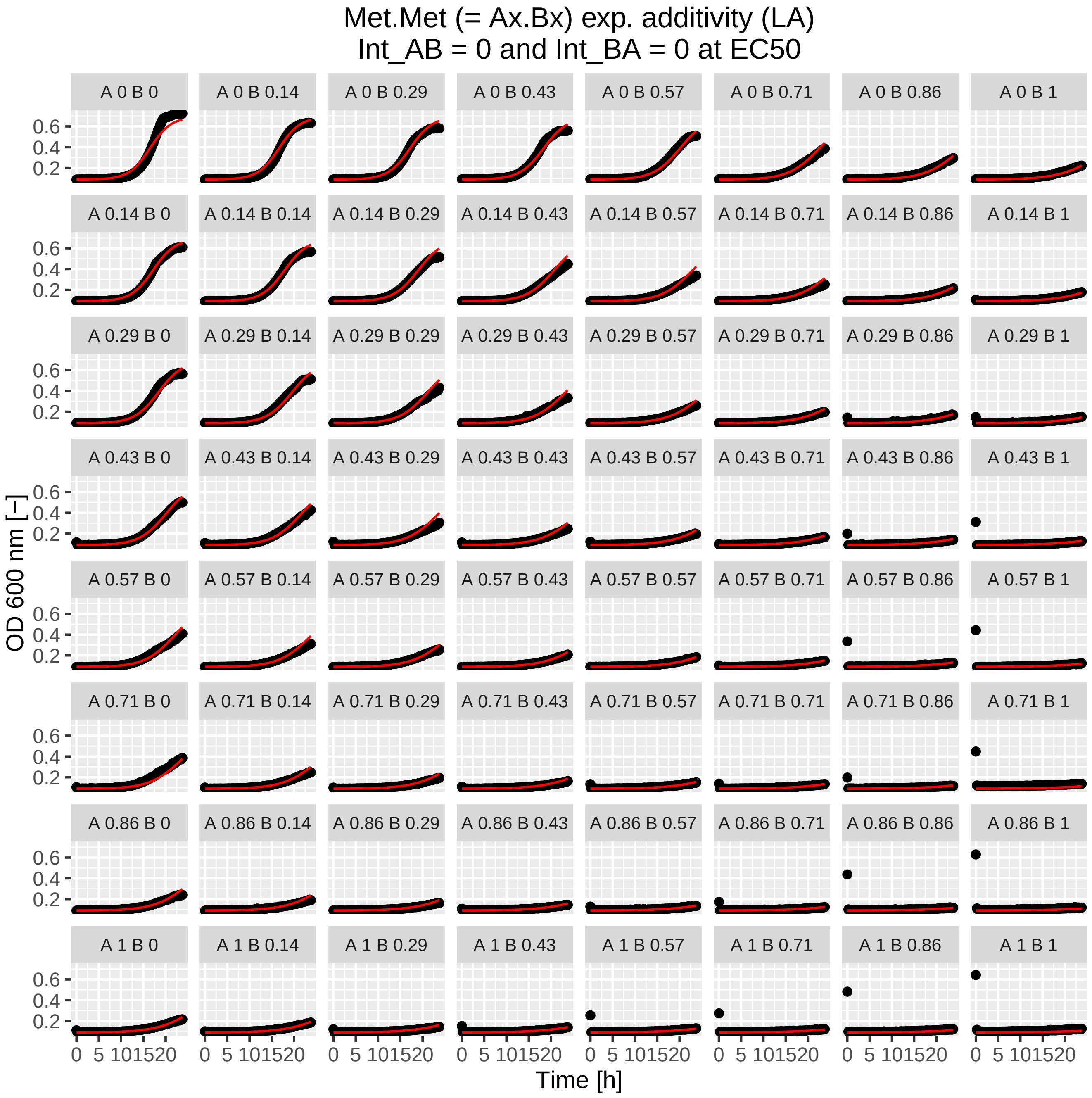

Met.Met (= Ax.Bx) exp. additivity (LA)
Int_AB = 0 and Int_BA = 0 at EC50
A 0 B 0
A 0 B 0.14
A 0 B 0.29
A 0 B 0.43
A 0 B 0.57
A 0 B 0.71
A 0 B 0.86
A 0 B 1
A 0.14 B 0
A 0.14 B 0.14
A 0.14 B 0.29
A 0.14 B 0.43
A 0.14 B 0.57
A 0.14 B 0.71
A 0.14 B 0.86
A 0.14 B 1
A 0.29 B 0
A 0.29 B 0.14
A 0.29 B 0.29
A 0.29 B 0.43
A 0.29 B 0.57
A 0.29 B 0.71
A 0.29 B 0.86
A 0.29 B 1
A 0.43 B 0
A 0.43 B 0.14
A 0.43 B 0.29
A 0.43 B 0.43
A 0.43 B 0.57
A 0.43 B 0.71
A 0.43 B 0.86
A 0.43 B 1
A 0.57 B 0
A 0.57 B 0.14
A 0.57 B 0.29
A 0.57 B 0.43
A 0.57 B 0.57
A 0.57 B 0.71
A 0.57 B 0.86
A 0.57 B 1
A 0.71 B 0
A 0.71 B 0.14
A 0.71 B 0.29
A 0.71 B 0.43
A 0.71 B 0.57
A 0.71 B 0.71
A 0.71 B 0.86
A 0.71 B 1
A 0.86 B 0
A 0.86 B 0.14
A 0.86 B 0.29
A 0.86 B 0.43
A 0.86 B 0.57
A 0.86 B 0.71
A 0.86 B 0.86
A 0.86 B 1
A 1 B 0
A 1 B 0.14
A 1 B 0.29
A 1 B 0.43
A 1 B 0.57
A 1 B 0.71
A 1 B 0.86
A 1 B 1
OD 600 nm [–]
0.6
0.4
0.2
0 5 101520
Time [h]

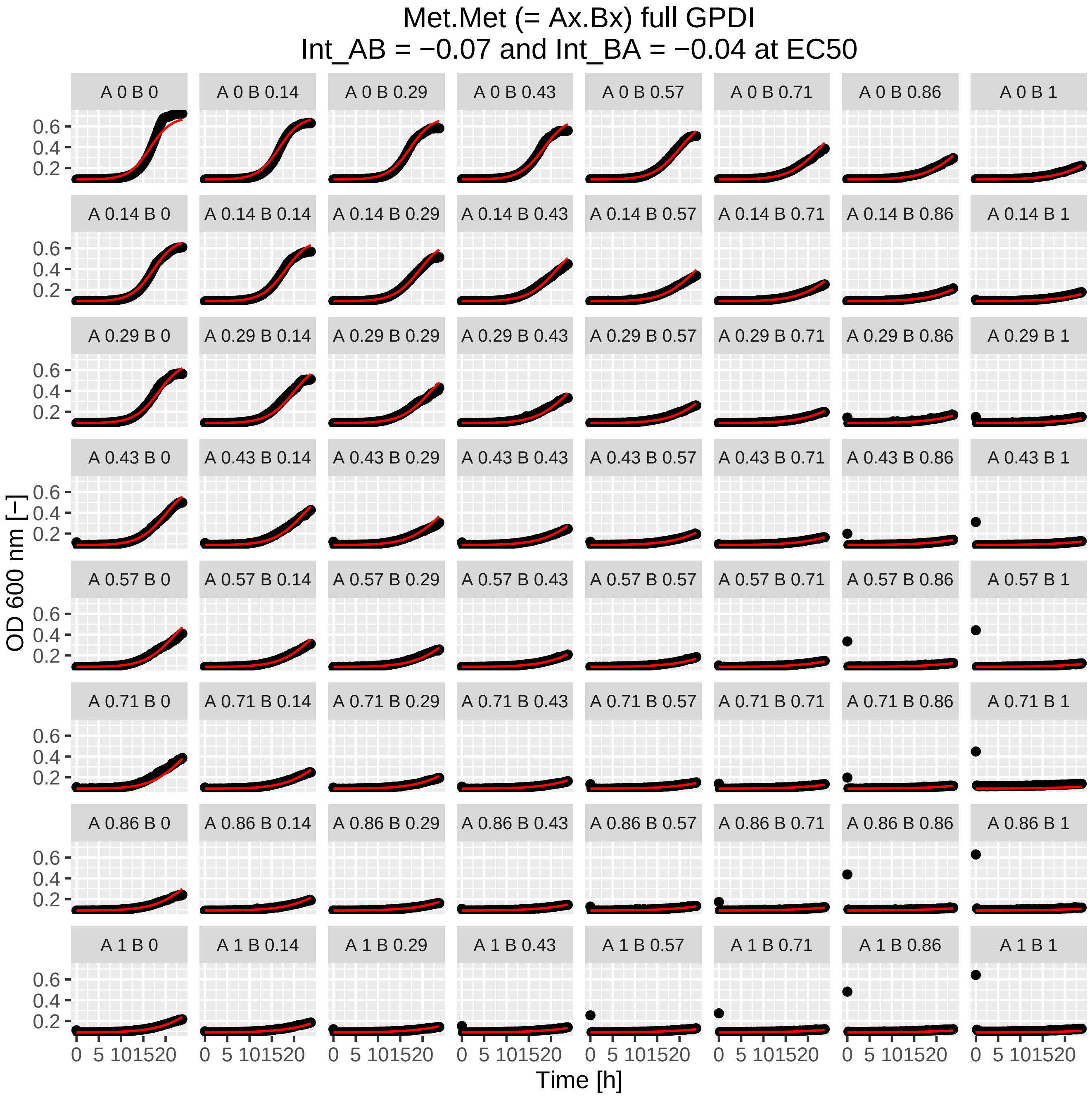
Met.Met (= Ax.Bx) full GPDI
Int_AB = −0.07 and Int_BA = −0.04 at EC50
OD 600 nm [–]
Time [h]
A 0 B 0
A 0 B 0.14
A 0 B 0.29
A 0 B 0.43
A 0 B 0.57
A 0 B 0.71
A 0 B 0.86
A 0 B 1
A 1 B 0
A 1 B 0.14
A 1 B 0.29
A 1 B 0.43
A 1 B 0.57
A 1 B 0.71
A 1 B 0.86
A 1 B 1

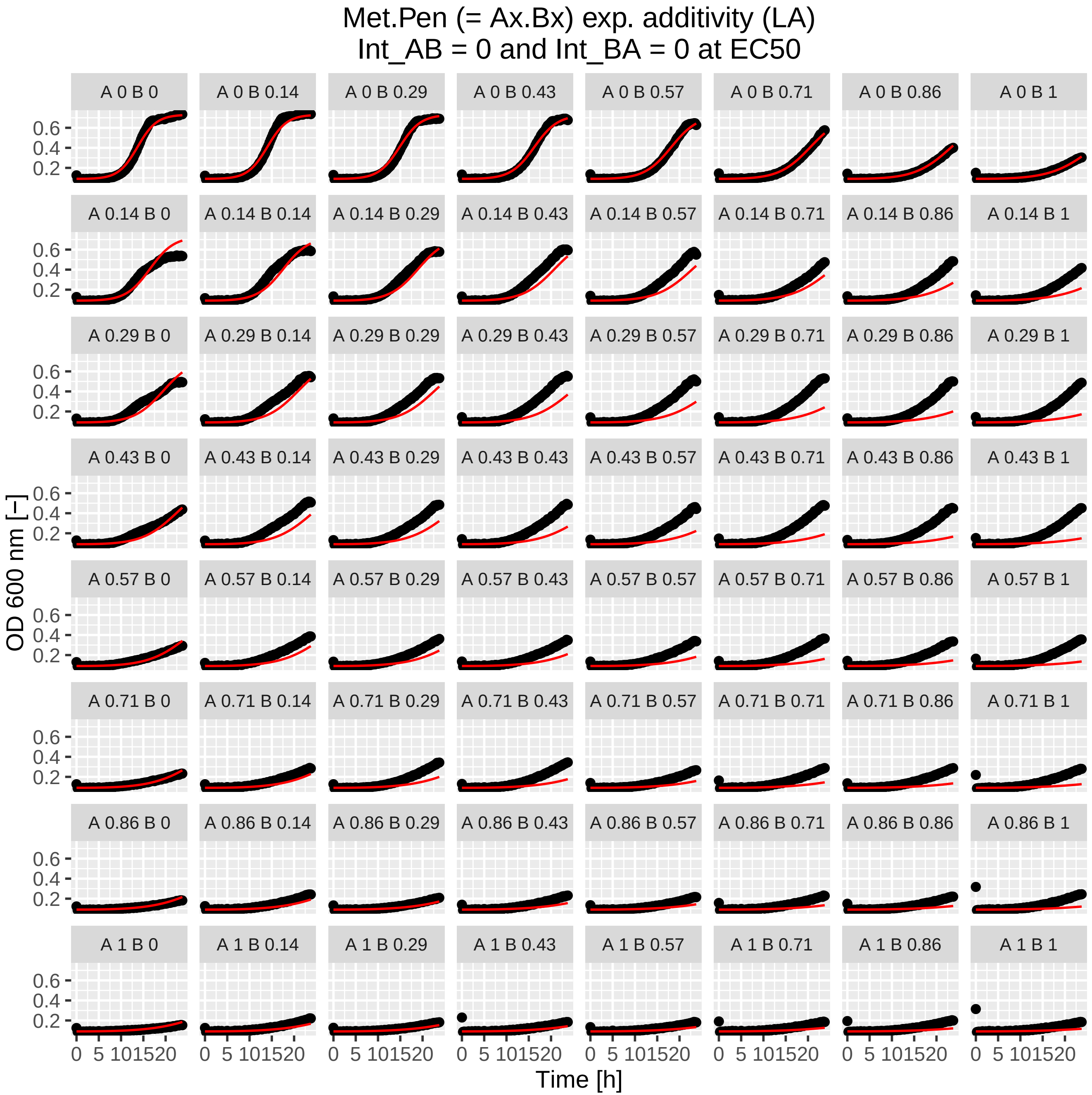

Met.Pen (= Ax.Bx) exp. additivity (LA)
Int_AB = 0 and Int_BA = 0 at EC50
A 0 B 0
A 0 B 0.14
A 0 B 0.29
A 0 B 0.43
A 0 B 0.57
A 0 B 0.71
A 0 B 0.86
A 0 B 1
A 0.14 B 0
A 0.14 B 0.14
A 0.14 B 0.29
A 0.14 B 0.43
A 0.14 B 0.57
A 0.14 B 0.71
A 0.14 B 0.86
A 0.14 B 1
A 0.29 B 0
A 0.29 B 0.14
A 0.29 B 0.29
A 0.29 B 0.43
A 0.29 B 0.57
A 0.29 B 0.71
A 0.29 B 0.86
A 0.29 B 1
A 0.43 B 0
A 0.43 B 0.14
A 0.43 B 0.29
A 0.43 B 0.43
A 0.43 B 0.57
A 0.43 B 0.71
A 0.43 B 0.86
A 0.43 B 1
A 0.57 B 0
A 0.57 B 0.14
A 0.57 B 0.29
A 0.57 B 0.43
A 0.57 B 0.57
A 0.57 B 0.71
A 0.57 B 0.86
A 0.57 B 1
A 0.71 B 0
A 0.71 B 0.14
A 0.71 B 0.29
A 0.71 B 0.43
A 0.71 B 0.57
A 0.71 B 0.71
A 0.71 B 0.86
A 0.71 B 1
A 0.86 B 0
A 0.86 B 0.14
A 0.86 B 0.29
A 0.86 B 0.43
A 0.86 B 0.57
A 0.86 B 0.71
A 0.86 B 0.86
A 0.86 B 1
A 1 B 0
A 1 B 0.14
A 1 B 0.29
A 1 B 0.43
A 1 B 0.57
A 1 B 0.71
A 1 B 0.86
A 1 B 1
OD 600 nm [−]
0.2
0.4
0.6
0 5 101520
Time [h]

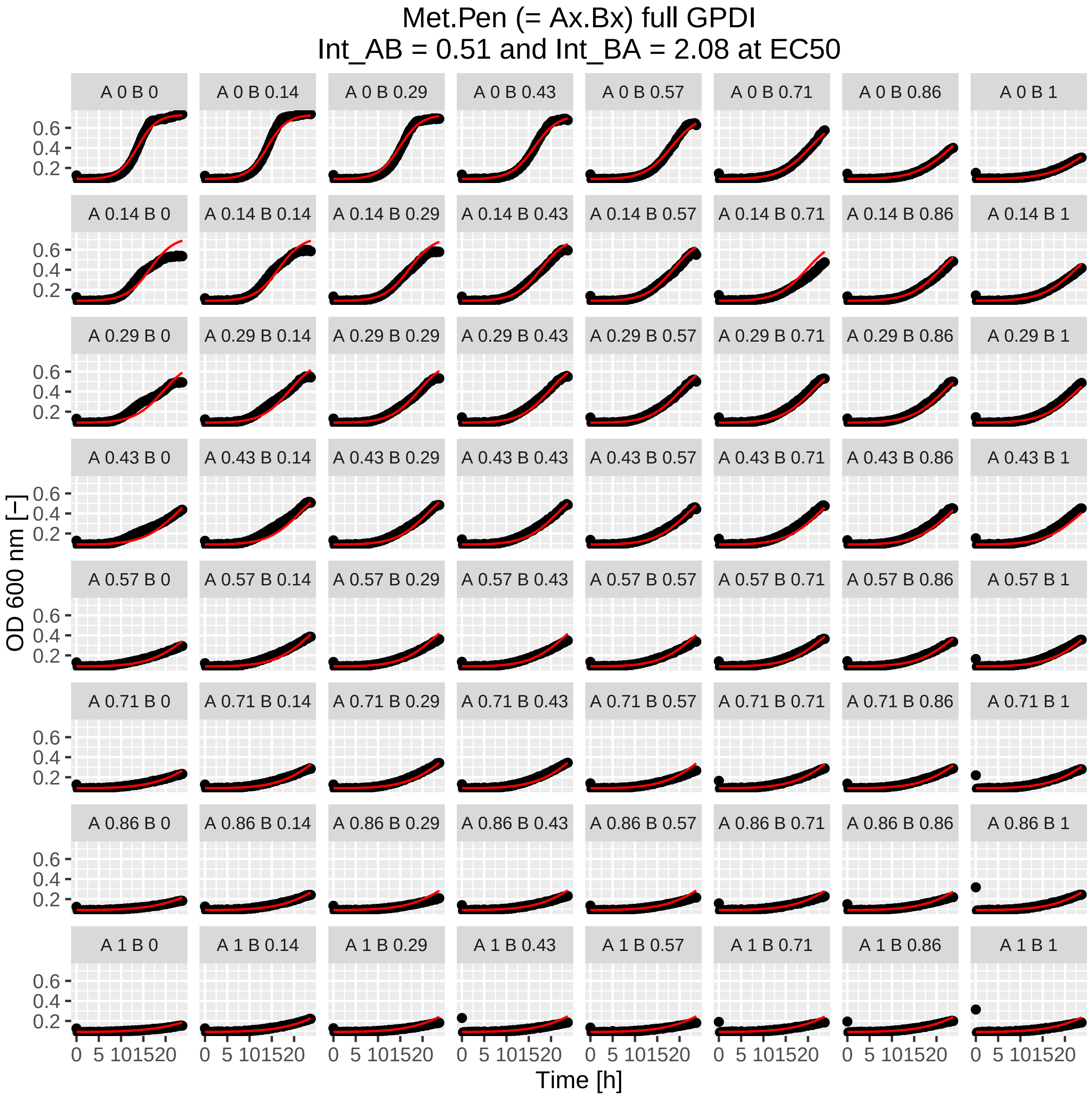
Met.Pen (= Ax.Bx) full GPDI
Int_AB = 0.51 and Int_BA = 2.08 at EC50
A 0 B 0
A 0 B 0.14
A 0 B 0.29
A 0 B 0.43
A 0 B 0.57
A 0 B 0.71
A 0 B 0.86
A 0 B 1
A 0.14 B 0
A 0.14 B 0.14
A 0.14 B 0.29
A 0.14 B 0.43
A 0.14 B 0.57
A 0.14 B 0.71
A 0.14 B 0.86
A 0.14 B 1
A 0.29 B 0
A 0.29 B 0.14
A 0.29 B 0.29
A 0.29 B 0.43
A 0.29 B 0.57
A 0.29 B 0.71
A 0.29 B 0.86
A 0.29 B 1
A 0.43 B 0
A 0.43 B 0.14
A 0.43 B 0.29
A 0.43 B 0.43
A 0.43 B 0.57
A 0.43 B 0.71
A 0.43 B 0.86
A 0.43 B 1
A 0.57 B 0
A 0.57 B 0.14
A 0.57 B 0.29
A 0.57 B 0.43
A 0.57 B 0.57
A 0.57 B 0.71
A 0.57 B 0.86
A 0.57 B 1
A 0.71 B 0
A 0.71 B 0.14
A 0.71 B 0.29
A 0.71 B 0.43
A 0.71 B 0.57
A 0.71 B 0.71
A 0.71 B 0.86
A 0.71 B 1
A 0.86 B 0
A 0.86 B 0.14
A 0.86 B 0.29
A 0.86 B 0.43
A 0.86 B 0.57
A 0.86 B 0.71
A 0.86 B 0.86
A 0.86 B 1
A 1 B 0
A 1 B 0.14
A 1 B 0.29
A 1 B 0.43
A 1 B 0.57
A 1 B 0.71
A 1 B 0.86
A 1 B 1
OD 600 nm [–]
0.6
0.4
0.2
0 5 101520
Time [h]

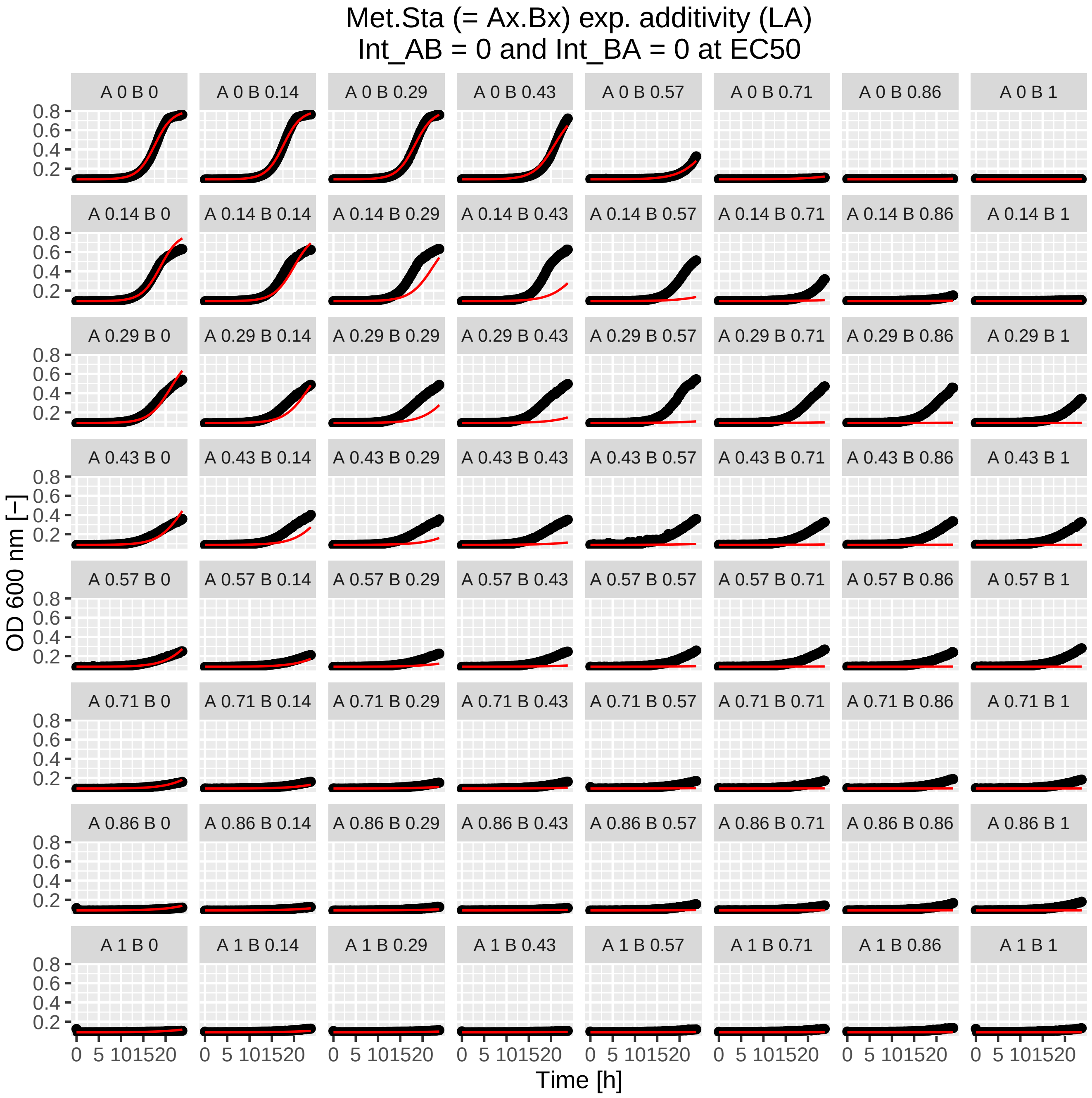
Met.Sta (= Ax.Bx) exp. additivity (LA)
Int_AB = 0 and Int_BA = 0 at EC50
A 0 B 0
A 0 B 0.14
A 0 B 0.29
A 0 B 0.43
A 0 B 0.57
A 0 B 0.71
A 0 B 0.86
A 0 B 1
A 0.14 B 0
A 0.14 B 0.14
A 0.14 B 0.29
A 0.14 B 0.43
A 0.14 B 0.57
A 0.14 B 0.71
A 0.14 B 0.86
A 0.14 B 1
A 0.29 B 0
A 0.29 B 0.14
A 0.29 B 0.29
A 0.29 B 0.43
A 0.29 B 0.57
A 0.29 B 0.71
A 0.29 B 0.86
A 0.29 B 1
A 0.43 B 0
A 0.43 B 0.14
A 0.43 B 0.29
A 0.43 B 0.43
A 0.43 B 0.57
A 0.43 B 0.71
A 0.43 B 0.86
A 0.43 B 1
A 0.57 B 0
A 0.57 B 0.14
A 0.57 B 0.29
A 0.57 B 0.43
A 0.57 B 0.57
A 0.57 B 0.71
A 0.57 B 0.86
A 0.57 B 1
A 0.71 B 0
A 0.71 B 0.14
A 0.71 B 0.29
A 0.71 B 0.43
A 0.71 B 0.57
A 0.71 B 0.71
A 0.71 B 0.86
A 0.71 B 1
A 0.86 B 0
A 0.86 B 0.14
A 0.86 B 0.29
A 0.86 B 0.43
A 0.86 B 0.57
A 0.86 B 0.71
A 0.86 B 0.86
A 0.86 B 1
A 1 B 0
A 1 B 0.14
A 1 B 0.29
A 1 B 0.43
A 1 B 0.57
A 1 B 0.71
A 1 B 0.86
A 1 B 1
OD 600 nm [–]
0.2
0.4
0.6
0.8
0 5 101520
Time [h]

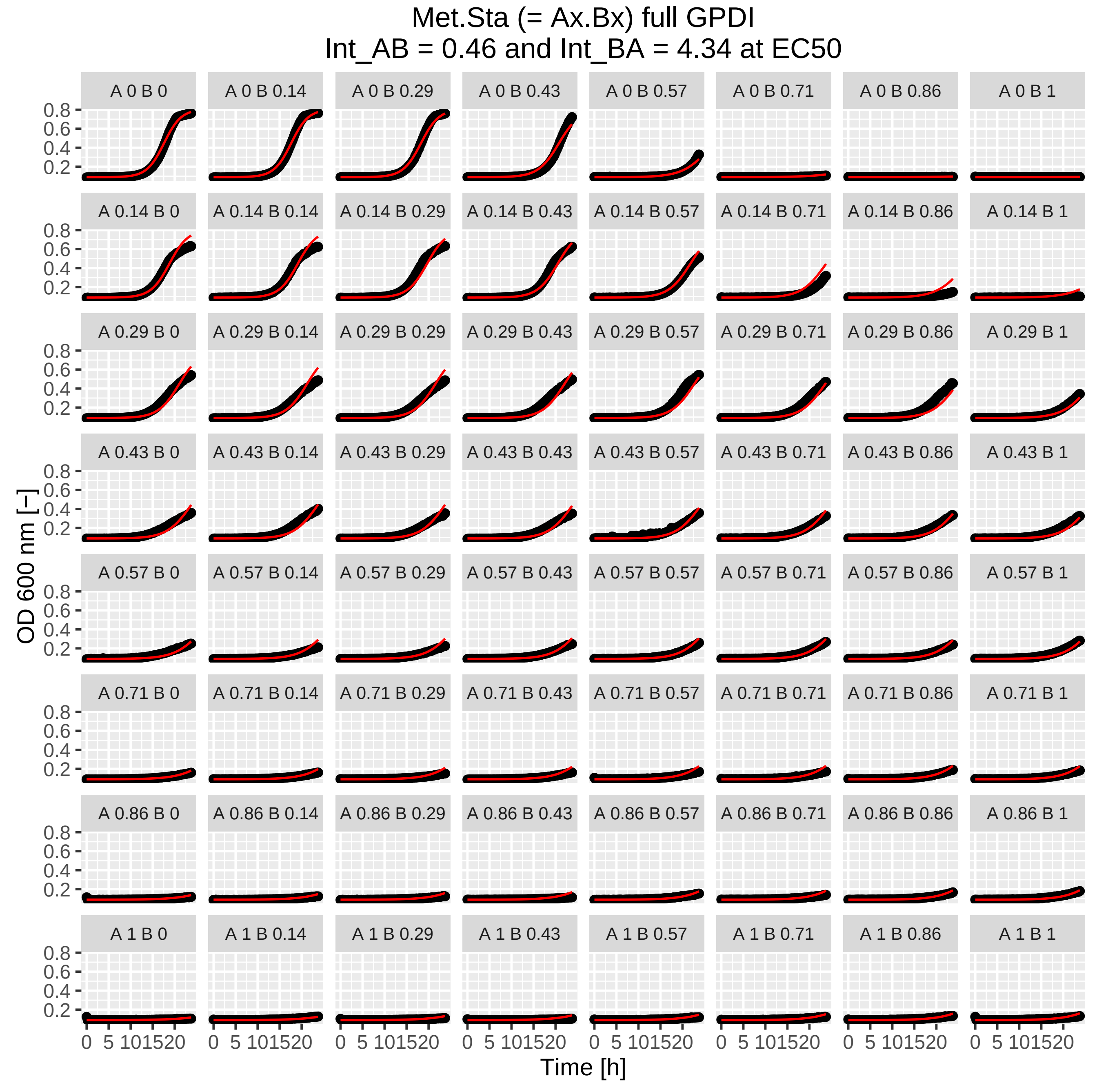

Met.Sta (= Ax.Bx) full GPDI
Int_AB = 0.46 and Int_BA = 4.34 at EC50
OD 600 nm [–]
Time [h]
0.8
0.6
0.4
0.2
0 5 101520
A 0 B 0
A 0 B 0.14
A 0 B 0.29
A 0 B 0.43
A 0 B 0.57
A 0 B 0.71
A 0 B 0.86
A 0 B 1
A 0.14 B 0
A 0.14 B 0.14
A 0.14 B 0.29
A 0.14 B 0.43
A 0.14 B 0.57
A 0.14 B 0.71
A 0.14 B 0.86
A 0.14 B 1
A 0.29 B 0
A 0.29 B 0.14
A 0.29 B 0.29
A 0.29 B 0.43
A 0.29 B 0.57
A 0.29 B 0.71
A 0.29 B 0.86
A 0.29 B 1
A 0.43 B 0
A 0.43 B 0.14
A 0.43 B 0.29
A 0.43 B 0.43
A 0.43 B 0.57
A 0.43 B 0.71
A 0.43 B 0.86
A 0.43 B 1
A 0.57 B 0
A 0.57 B 0.14
A 0.57 B 0.29
A 0.57 B 0.43
A 0.57 B 0.57
A 0.57 B 0.71
A 0.57 B 0.86
A 0.57 B 1
A 0.71 B 0
A 0.71 B 0.14
A 0.71 B 0.29
A 0.71 B 0.43
A 0.71 B 0.57
A 0.71 B 0.71
A 0.71 B 0.86
A 0.71 B 1
A 0.86 B 0
A 0.86 B 0.14
A 0.86 B 0.29
A 0.86 B 0.43
A 0.86 B 0.57
A 0.86 B 0.71
A 0.86 B 0.86
A 0.86 B 1
A 1 B 0
A 1 B 0.14
A 1 B 0.29
A 1 B 0.43
A 1 B 0.57
A 1 B 0.71
A 1 B 0.86
A 1 B 1

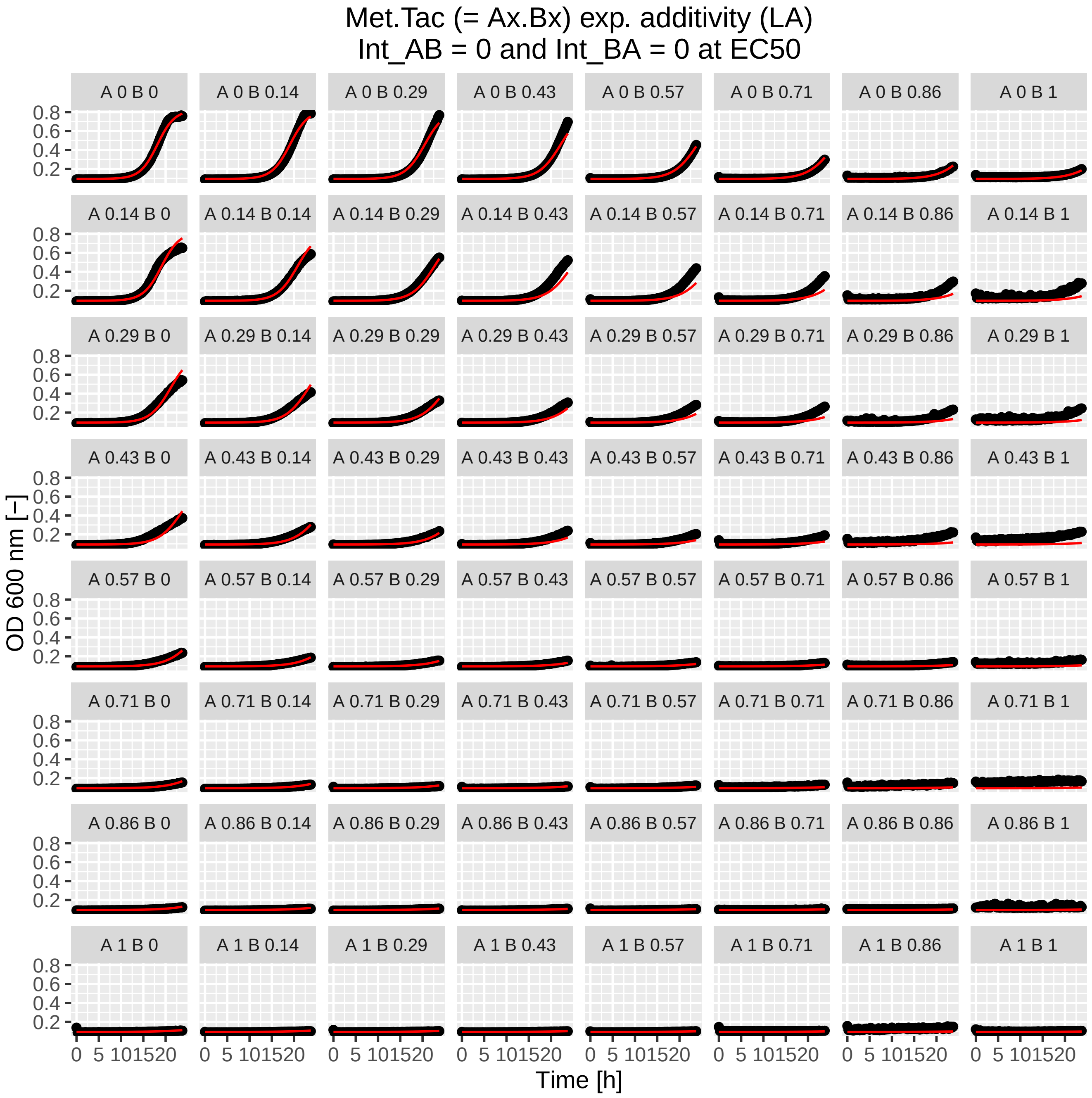
Met.Tac (= Ax.Bx) exp. additivity (LA)
Int_AB = 0 and Int_BA = 0 at EC50
A 0 B 0
A 0 B 0.14
A 0 B 0.29
A 0 B 0.43
A 0 B 0.57
A 0 B 0.71
A 0 B 0.86
A 0 B 1
A 0.14 B 0
A 0.14 B 0.14
A 0.14 B 0.29
A 0.14 B 0.43
A 0.14 B 0.57
A 0.14 B 0.71
A 0.14 B 0.86
A 0.14 B 1
A 0.29 B 0
A 0.29 B 0.14
A 0.29 B 0.29
A 0.29 B 0.43
A 0.29 B 0.57
A 0.29 B 0.71
A 0.29 B 0.86
A 0.29 B 1
A 0.43 B 0
A 0.43 B 0.14
A 0.43 B 0.29
A 0.43 B 0.43
A 0.43 B 0.57
A 0.43 B 0.71
A 0.43 B 0.86
A 0.43 B 1
A 0.57 B 0
A 0.57 B 0.14
A 0.57 B 0.29
A 0.57 B 0.43
A 0.57 B 0.57
A 0.57 B 0.71
A 0.57 B 0.86
A 0.57 B 1
A 0.71 B 0
A 0.71 B 0.14
A 0.71 B 0.29
A 0.71 B 0.43
A 0.71 B 0.57
A 0.71 B 0.71
A 0.71 B 0.86
A 0.71 B 1
A 0.86 B 0
A 0.86 B 0.14
A 0.86 B 0.29
A 0.86 B 0.43
A 0.86 B 0.57
A 0.86 B 0.71
A 0.86 B 0.86
A 0.86 B 1
A 1 B 0
A 1 B 0.14
A 1 B 0.29
A 1 B 0.43
A 1 B 0.57
A 1 B 0.71
A 1 B 0.86
A 1 B 1
0.8
0.6
0.4
0.2
0 5 101520
OD 600 nm [–]
Time [h]

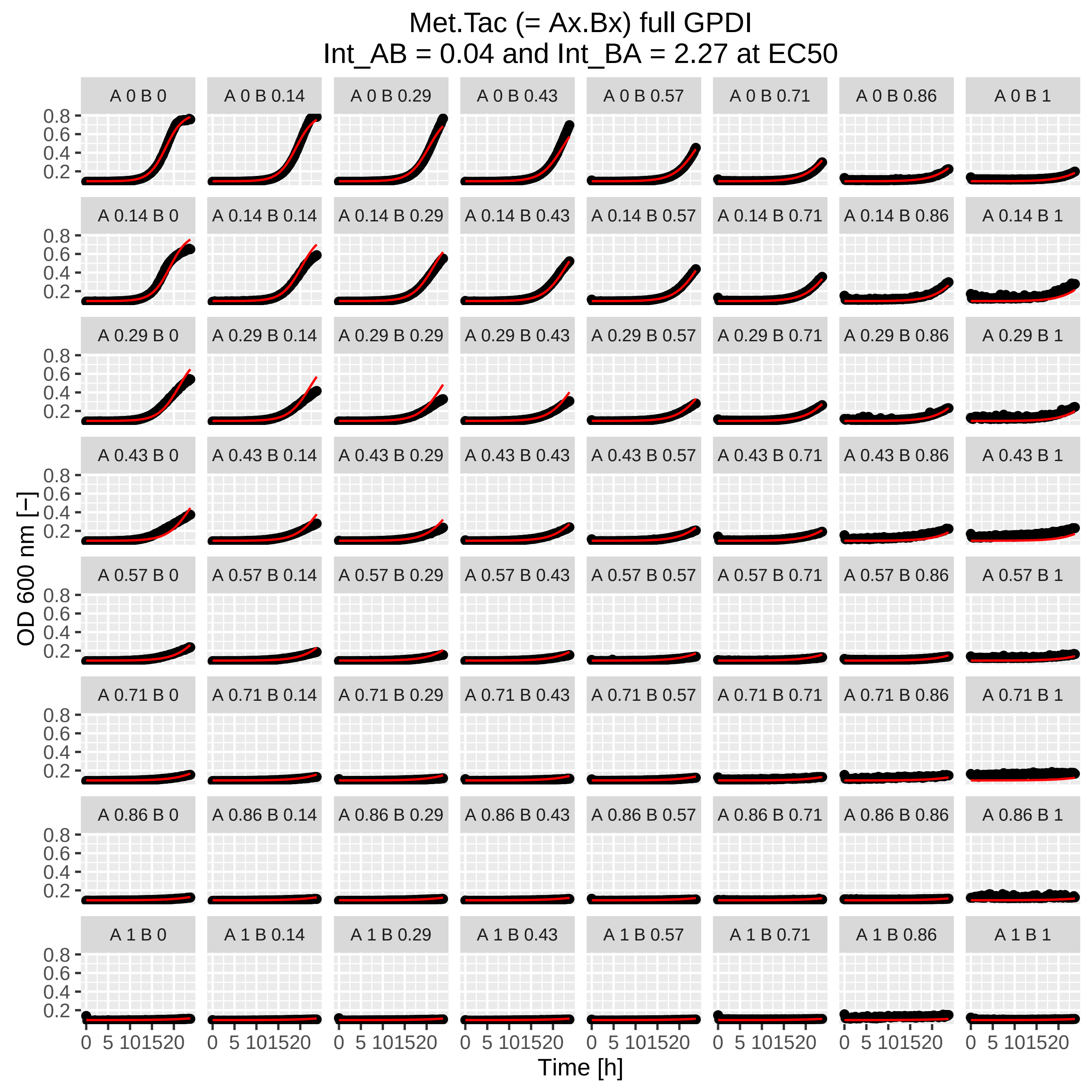

Met.Tac (= Ax.Bx) full GPDI
Int_AB = 0.04 and Int_BA = 2.27 at EC50
A 0 B 0
A 0 B 0.14
A 0 B 0.29
A 0 B 0.43
A 0 B 0.57
A 0 B 0.71
A 0 B 0.86
A 0 B 1
A 0.14 B 0
A 0.14 B 0.14
A 0.14 B 0.29
A 0.14 B 0.43
A 0.14 B 0.57
A 0.14 B 0.71
A 0.14 B 0.86
A 0.14 B 1
A 0.29 B 0
A 0.29 B 0.14
A 0.29 B 0.29
A 0.29 B 0.43
A 0.29 B 0.57
A 0.29 B 0.71
A 0.29 B 0.86
A 0.29 B 1
A 0.43 B 0
A 0.43 B 0.14
A 0.43 B 0.29
A 0.43 B 0.43
A 0.43 B 0.57
A 0.43 B 0.71
A 0.43 B 0.86
A 0.43 B 1
A 0.57 B 0
A 0.57 B 0.14
A 0.57 B 0.29
A 0.57 B 0.43
A 0.57 B 0.57
A 0.57 B 0.71
A 0.57 B 0.86
A 0.57 B 1
A 0.71 B 0
A 0.71 B 0.14
A 0.71 B 0.29
A 0.71 B 0.43
A 0.71 B 0.57
A 0.71 B 0.71
A 0.71 B 0.86
A 0.71 B 1
A 0.86 B 0
A 0.86 B 0.14
A 0.86 B 0.29
A 0.86 B 0.43
A 0.86 B 0.57
A 0.86 B 0.71
A 0.86 B 0.86
A 0.86 B 1
A 1 B 0
A 1 B 0.14
A 1 B 0.29
A 1 B 0.43
A 1 B 0.57
A 1 B 0.71
A 1 B 0.86
A 1 B 1
OD 600 nm [–]
0.2
0.4
0.6
0.8
0 5 101520
Time [h]

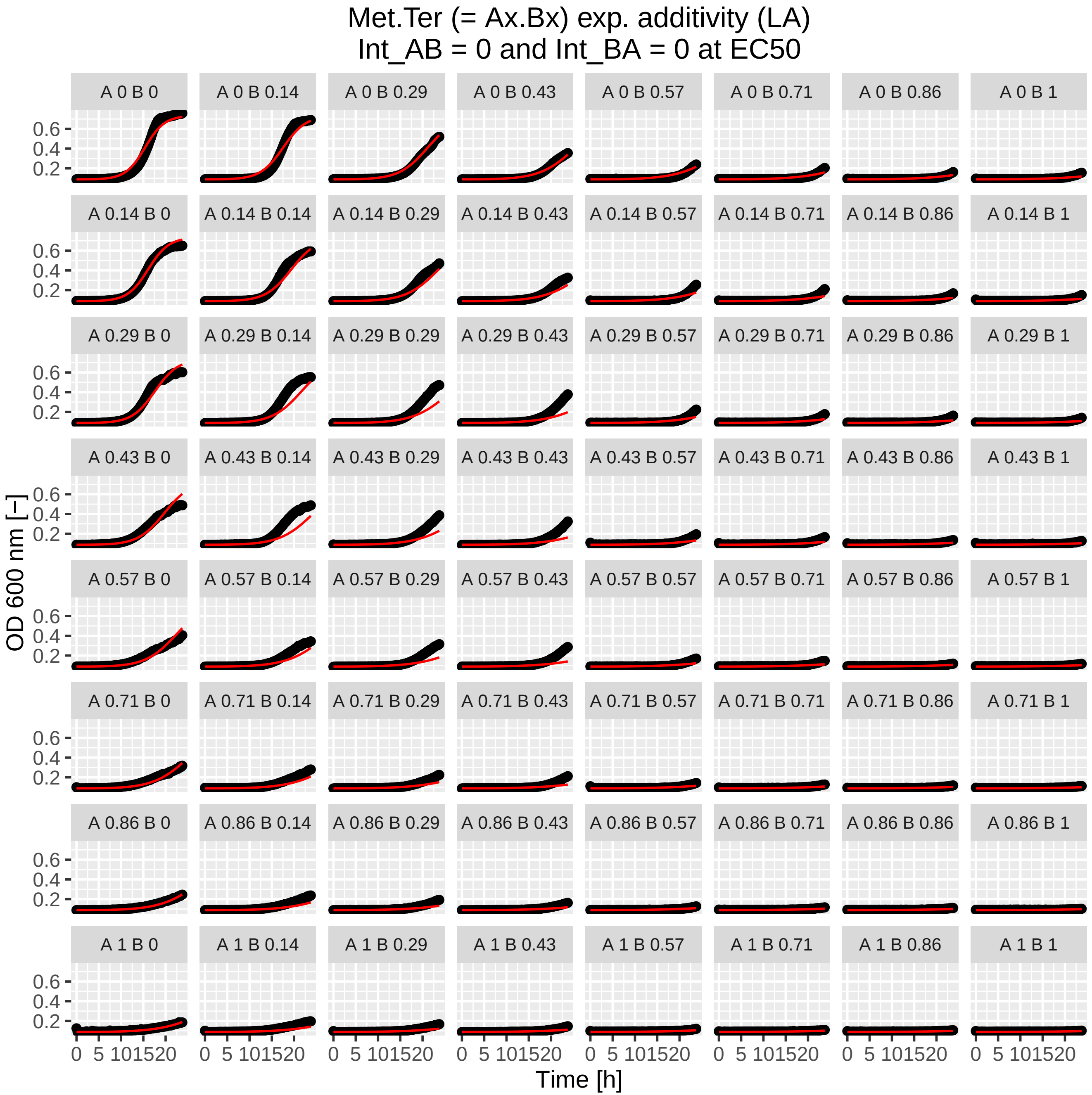

Met.Ter (= Ax.Bx) exp. additivity (LA)
Int_AB = 0 and Int_BA = 0 at EC50
A 0 B 0
A 0 B 0.14
A 0 B 0.29
A 0 B 0.43
A 0 B 0.57
A 0 B 0.71
A 0 B 0.86
A 0 B 1
A 0.14 B 0
A 0.14 B 0.14
A 0.14 B 0.29
A 0.14 B 0.43
A 0.14 B 0.57
A 0.14 B 0.71
A 0.14 B 0.86
A 0.14 B 1
A 0.29 B 0
A 0.29 B 0.14
A 0.29 B 0.29
A 0.29 B 0.43
A 0.29 B 0.57
A 0.29 B 0.71
A 0.29 B 0.86
A 0.29 B 1
A 0.43 B 0
A 0.43 B 0.14
A 0.43 B 0.29
A 0.43 B 0.43
A 0.43 B 0.57
A 0.43 B 0.71
A 0.43 B 0.86
A 0.43 B 1
A 0.57 B 0
A 0.57 B 0.14
A 0.57 B 0.29
A 0.57 B 0.43
A 0.57 B 0.57
A 0.57 B 0.71
A 0.57 B 0.86
A 0.57 B 1
A 0.71 B 0
A 0.71 B 0.14
A 0.71 B 0.29
A 0.71 B 0.43
A 0.71 B 0.57
A 0.71 B 0.71
A 0.71 B 0.86
A 0.71 B 1
A 0.86 B 0
A 0.86 B 0.14
A 0.86 B 0.29
A 0.86 B 0.43
A 0.86 B 0.57
A 0.86 B 0.71
A 0.86 B 0.86
A 0.86 B 1
A 1 B 0
A 1 B 0.14
A 1 B 0.29
A 1 B 0.43
A 1 B 0.57
A 1 B 0.71
A 1 B 0.86
A 1 B 1
OD 600 nm [–]
0.2
0.4
0.6
0 5 101520
Time [h]

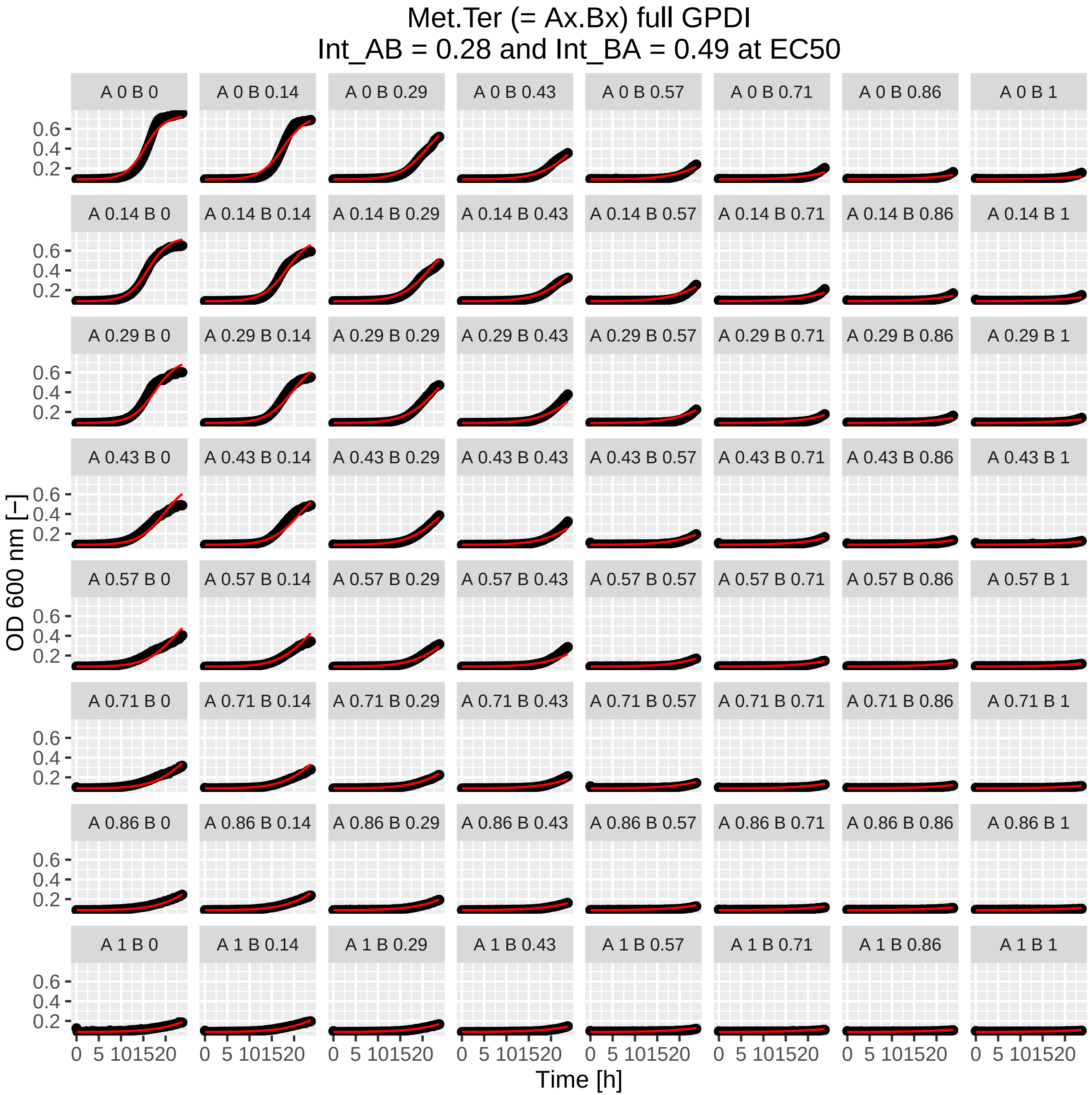
Met.Ter (= Ax.Bx) full GPDI
Int_AB = 0.28 and Int_BA = 0.49 at EC50
A 0 B 0
A 0 B 0.14
A 0 B 0.29
A 0 B 0.43
A 0 B 0.57
A 0 B 0.71
A 0 B 0.86
A 0 B 1
A 0.14 B 0
A 0.14 B 0.14
A 0.14 B 0.29
A 0.14 B 0.43
A 0.14 B 0.57
A 0.14 B 0.71
A 0.14 B 0.86
A 0.14 B 1
A 0.29 B 0
A 0.29 B 0.14
A 0.29 B 0.29
A 0.29 B 0.43
A 0.29 B 0.57
A 0.29 B 0.71
A 0.29 B 0.86
A 0.29 B 1
A 0.43 B 0
A 0.43 B 0.14
A 0.43 B 0.29
A 0.43 B 0.43
A 0.43 B 0.57
A 0.43 B 0.71
A 0.43 B 0.86
A 0.43 B 1
A 0.57 B 0
A 0.57 B 0.14
A 0.57 B 0.29
A 0.57 B 0.43
A 0.57 B 0.57
A 0.57 B 0.71
A 0.57 B 0.86
A 0.57 B 1
A 0.71 B 0
A 0.71 B 0.14
A 0.71 B 0.29
A 0.71 B 0.43
A 0.71 B 0.57
A 0.71 B 0.71
A 0.71 B 0.86
A 0.71 B 1
A 0.86 B 0
A 0.86 B 0.14
A 0.86 B 0.29
A 0.86 B 0.43
A 0.86 B 0.57
A 0.86 B 0.71
A 0.86 B 0.86
A 0.86 B 1
A 1 B 0
A 1 B 0.14
A 1 B 0.29
A 1 B 0.43
A 1 B 0.57
A 1 B 0.71
A 1 B 0.86
A 1 B 1
0.6
0.4
0.2
OD 600 nm [–]
0 5 101520
Time [h]

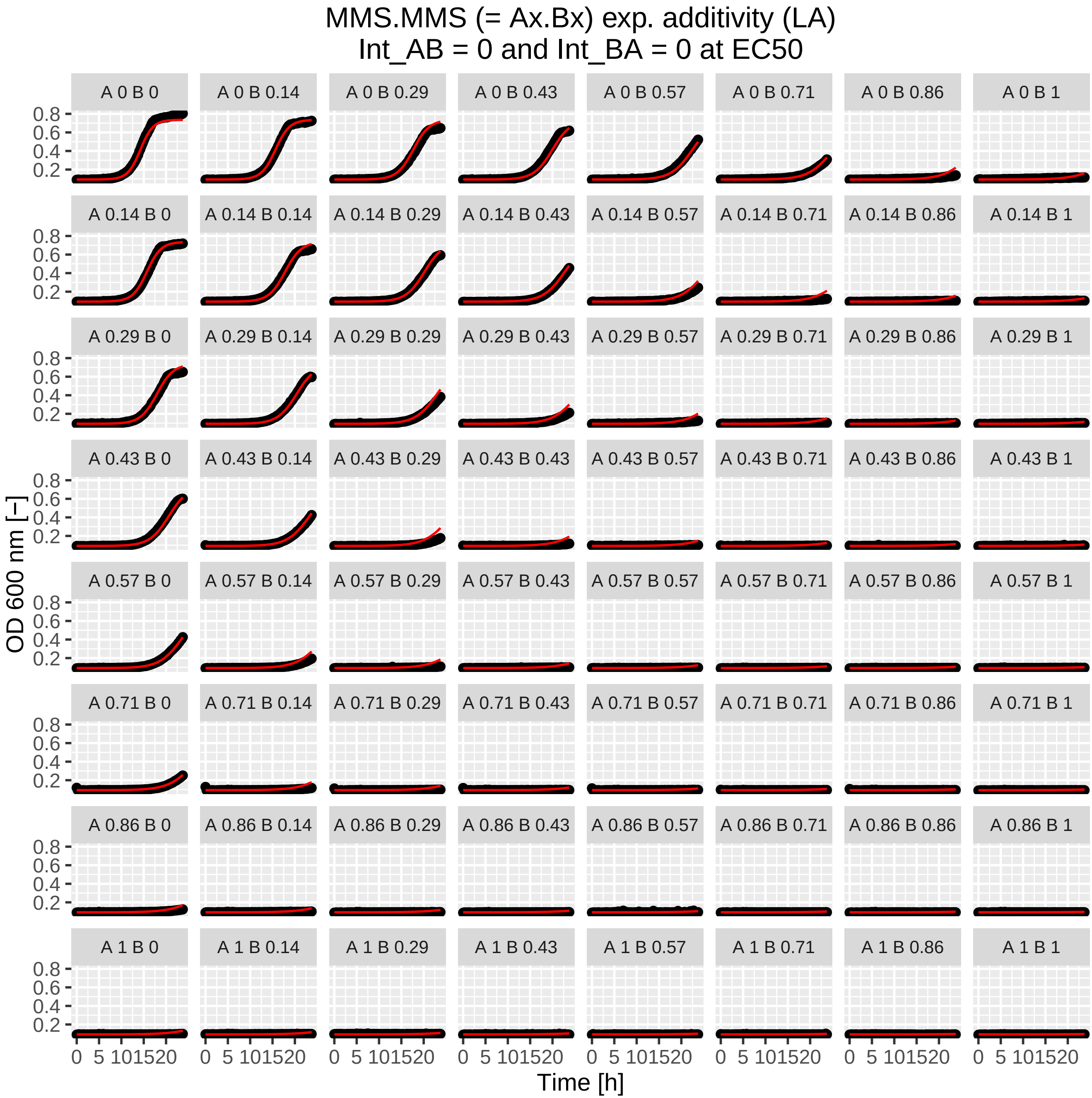

MMS.MMS (= Ax.Bx) exp. additivity (LA)
Int_AB = 0 and Int_BA = 0 at EC50
A 0 B 0
A 0 B 0.14
A 0 B 0.29
A 0 B 0.43
A 0 B 0.57
A 0 B 0.71
A 0 B 0.86
A 0 B 1
A 0.14 B 0
A 0.14 B 0.14
A 0.14 B 0.29
A 0.14 B 0.43
A 0.14 B 0.57
A 0.14 B 0.71
A 0.14 B 0.86
A 0.14 B 1
A 0.29 B 0
A 0.29 B 0.14
A 0.29 B 0.29
A 0.29 B 0.43
A 0.29 B 0.57
A 0.29 B 0.71
A 0.29 B 0.86
A 0.29 B 1
A 0.43 B 0
A 0.43 B 0.14
A 0.43 B 0.29
A 0.43 B 0.43
A 0.43 B 0.57
A 0.43 B 0.71
A 0.43 B 0.86
A 0.43 B 1
A 0.57 B 0
A 0.57 B 0.14
A 0.57 B 0.29
A 0.57 B 0.43
A 0.57 B 0.57
A 0.57 B 0.71
A 0.57 B 0.86
A 0.57 B 1
A 0.71 B 0
A 0.71 B 0.14
A 0.71 B 0.29
A 0.71 B 0.43
A 0.71 B 0.57
A 0.71 B 0.71
A 0.71 B 0.86
A 0.71 B 1
A 0.86 B 0
A 0.86 B 0.14
A 0.86 B 0.29
A 0.86 B 0.43
A 0.86 B 0.57
A 0.86 B 0.71
A 0.86 B 0.86
A 0.86 B 1
A 1 B 0
A 1 B 0.14
A 1 B 0.29
A 1 B 0.43
A 1 B 0.57
A 1 B 0.71
A 1 B 0.86
A 1 B 1
OD 600 nm [–]
0.2
0.4
0.6
0.8
0 5 101520
Time [h]

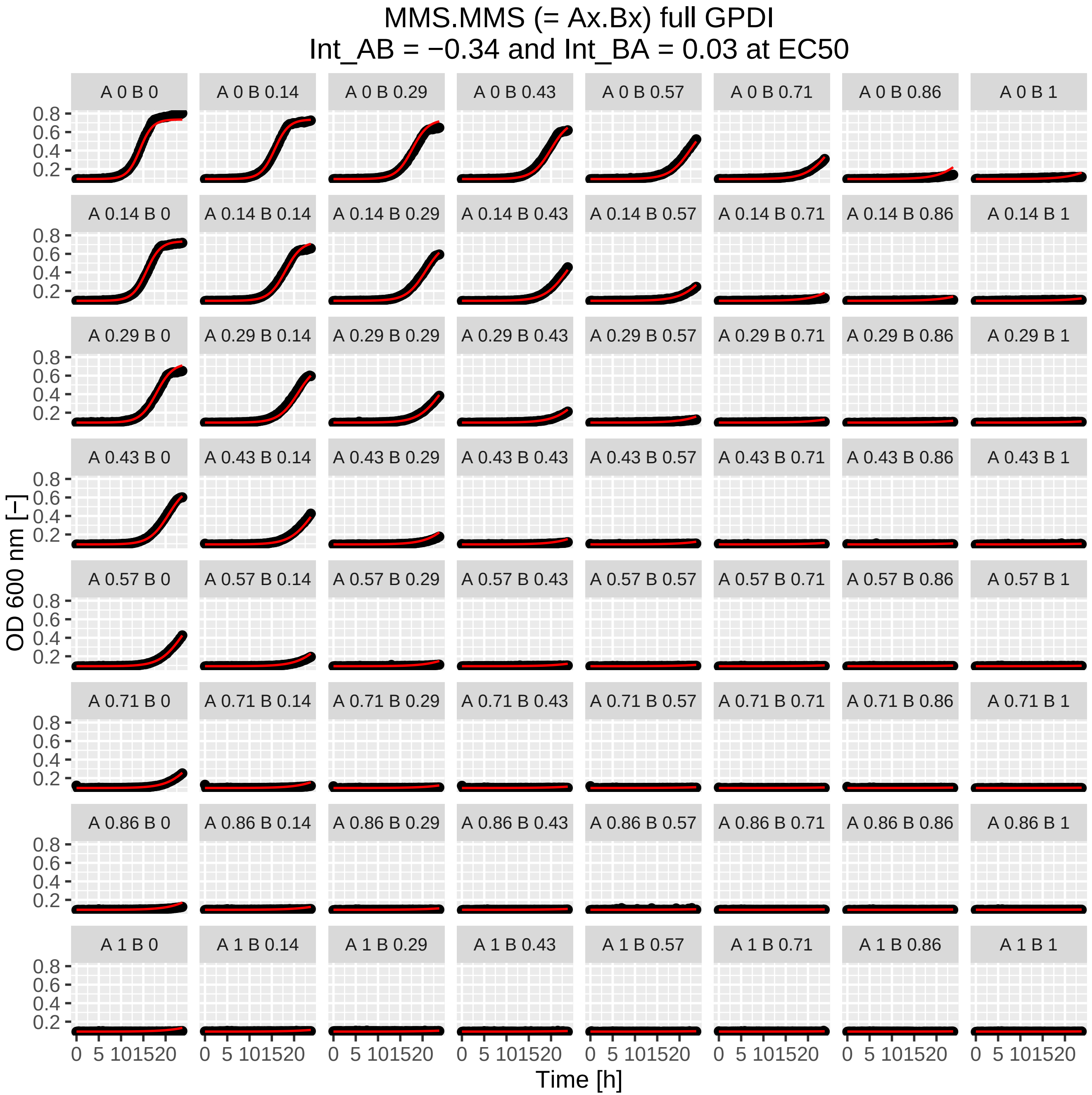
MMS.MMS (= Ax.Bx) full GPDI
Int_AB = −0.34 and Int_BA = 0.03 at EC50
OD 600 nm [−]
Time [h]
A 0 B 0
A 0 B 0.14
A 0 B 0.29
A 0 B 0.43
A 0 B 0.57
A 0 B 0.71
A 0 B 0.86
A 0 B 1
A 0.14 B 0
A 0.14 B 0.14
A 0.14 B 0.29
A 0.14 B 0.43
A 0.14 B 0.57
A 0.14 B 0.71
A 0.14 B 0.86
A 0.14 B 1
A 0.29 B 0
A 0.29 B 0.14
A 0.29 B 0.29
A 0.29 B 0.43
A 0.29 B 0.57
A 0.29 B 0.71
A 0.29 B 0.86
A 0.29 B 1
A 0.43 B 0
A 0.43 B 0.14
A 0.43 B 0.29
A 0.43 B 0.43
A 0.43 B 0.57
A 0.43 B 0.71
A 0.43 B 0.86
A 0.43 B 1
A 0.57 B 0
A 0.57 B 0.14
A 0.57 B 0.29
A 0.57 B 0.43
A 0.57 B 0.57
A 0.57 B 0.71
A 0.57 B 0.86
A 0.57 B 1
A 0.71 B 0
A 0.71 B 0.14
A 0.71 B 0.29
A 0.71 B 0.43
A 0.71 B 0.57
A 0.71 B 0.71
A 0.71 B 0.86
A 0.71 B 1
A 0.86 B 0
A 0.86 B 0.14
A 0.86 B 0.29
A 0.86 B 0.43
A 0.86 B 0.57
A 0.86 B 0.71
A 0.86 B 0.86
A 0.86 B 1
A 1 B 0
A 1 B 0.14
A 1 B 0.29
A 1 B 0.43
A 1 B 0.57
A 1 B 0.71
A 1 B 0.86
A 1 B 1
0.8
0.6
0.4
0.2
0 5 101520

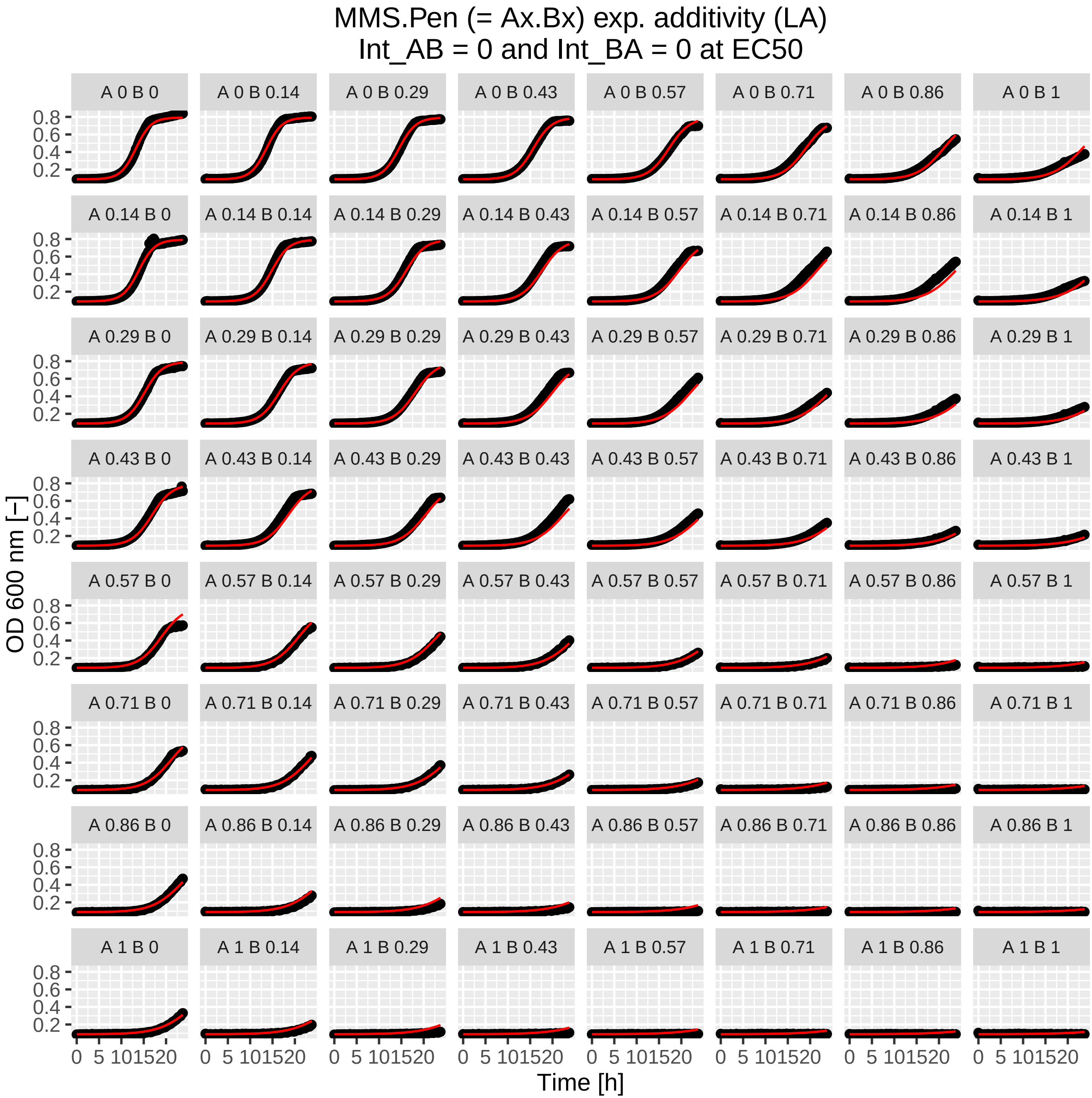

MMS.Pen (= Ax.Bx) exp. additivity (LA)
Int_AB = 0 and Int_BA = 0 at EC50
A 0 B 0
A 0 B 0.14
A 0 B 0.29
A 0 B 0.43
A 0 B 0.57
A 0 B 0.71
A 0 B 0.86
A 0 B 1
A 0.14 B 0
A 0.14 B 0.14
A 0.14 B 0.29
A 0.14 B 0.43
A 0.14 B 0.57
A 0.14 B 0.71
A 0.14 B 0.86
A 0.14 B 1
A 0.29 B 0
A 0.29 B 0.14
A 0.29 B 0.29
A 0.29 B 0.43
A 0.29 B 0.57
A 0.29 B 0.71
A 0.29 B 0.86
A 0.29 B 1
A 0.43 B 0
A 0.43 B 0.14
A 0.43 B 0.29
A 0.43 B 0.43
A 0.43 B 0.57
A 0.43 B 0.71
A 0.43 B 0.86
A 0.43 B 1
A 0.57 B 0
A 0.57 B 0.14
A 0.57 B 0.29
A 0.57 B 0.43
A 0.57 B 0.57
A 0.57 B 0.71
A 0.57 B 0.86
A 0.57 B 1
A 0.71 B 0
A 0.71 B 0.14
A 0.71 B 0.29
A 0.71 B 0.43
A 0.71 B 0.57
A 0.71 B 0.71
A 0.71 B 0.86
A 0.71 B 1
A 0.86 B 0
A 0.86 B 0.14
A 0.86 B 0.29
A 0.86 B 0.43
A 0.86 B 0.57
A 0.86 B 0.71
A 0.86 B 0.86
A 0.86 B 1
A 1 B 0
A 1 B 0.14
A 1 B 0.29
A 1 B 0.43
A 1 B 0.57
A 1 B 0.71
A 1 B 0.86
A 1 B 1
0.8
0.6
0.4
0.2
OD 600 nm [–]
0 5 101520
Time [h]

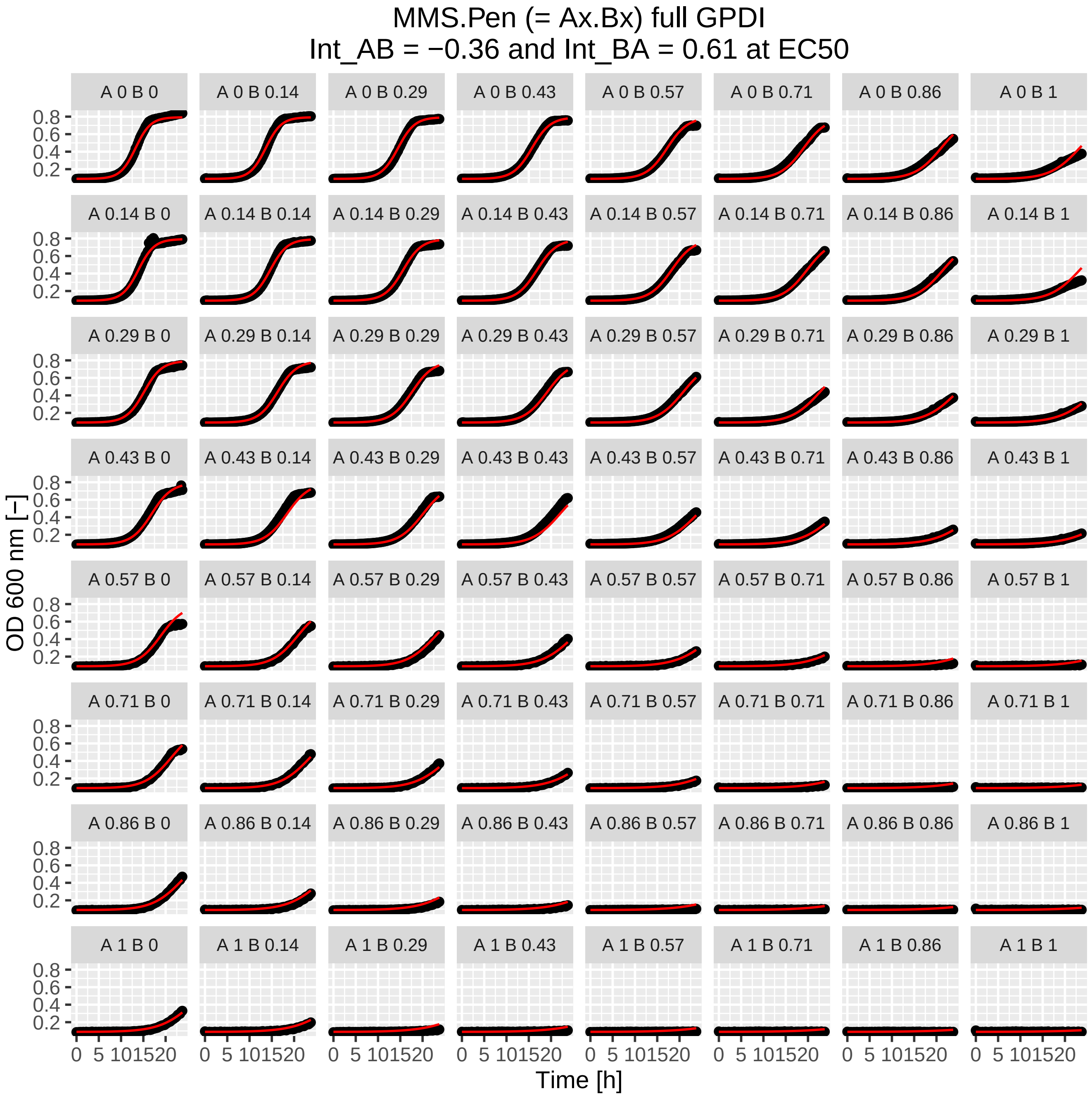

MMS.Pen (= Ax.Bx) full GPDI
Int_AB = −0.36 and Int_BA = 0.61 at EC50
A 0 B 0
A 0 B 0.14
A 0 B 0.29
A 0 B 0.43
A 0 B 0.57
A 0 B 0.71
A 0 B 0.86
A 0 B 1
A 0.14 B 0
A 0.14 B 0.14
A 0.14 B 0.29
A 0.14 B 0.43
A 0.14 B 0.57
A 0.14 B 0.71
A 0.14 B 0.86
A 0.14 B 1
A 0.29 B 0
A 0.29 B 0.14
A 0.29 B 0.29
A 0.29 B 0.43
A 0.29 B 0.57
A 0.29 B 0.71
A 0.29 B 0.86
A 0.29 B 1
A 0.43 B 0
A 0.43 B 0.14
A 0.43 B 0.29
A 0.43 B 0.43
A 0.43 B 0.57
A 0.43 B 0.71
A 0.43 B 0.86
A 0.43 B 1
A 0.57 B 0
A 0.57 B 0.14
A 0.57 B 0.29
A 0.57 B 0.43
A 0.57 B 0.57
A 0.57 B 0.71
A 0.57 B 0.86
A 0.57 B 1
A 0.71 B 0
A 0.71 B 0.14
A 0.71 B 0.29
A 0.71 B 0.43
A 0.71 B 0.57
A 0.71 B 0.71
A 0.71 B 0.86
A 0.71 B 1
A 0.86 B 0
A 0.86 B 0.14
A 0.86 B 0.29
A 0.86 B 0.43
A 0.86 B 0.57
A 0.86 B 0.71
A 0.86 B 0.86
A 0.86 B 1
A 1 B 0
A 1 B 0.14
A 1 B 0.29
A 1 B 0.43
A 1 B 0.57
A 1 B 0.71
A 1 B 0.86
A 1 B 1
OD 600 nm [−]
0.2
0.4
0.6
0.8
0
5
10
15
20
Time [h]

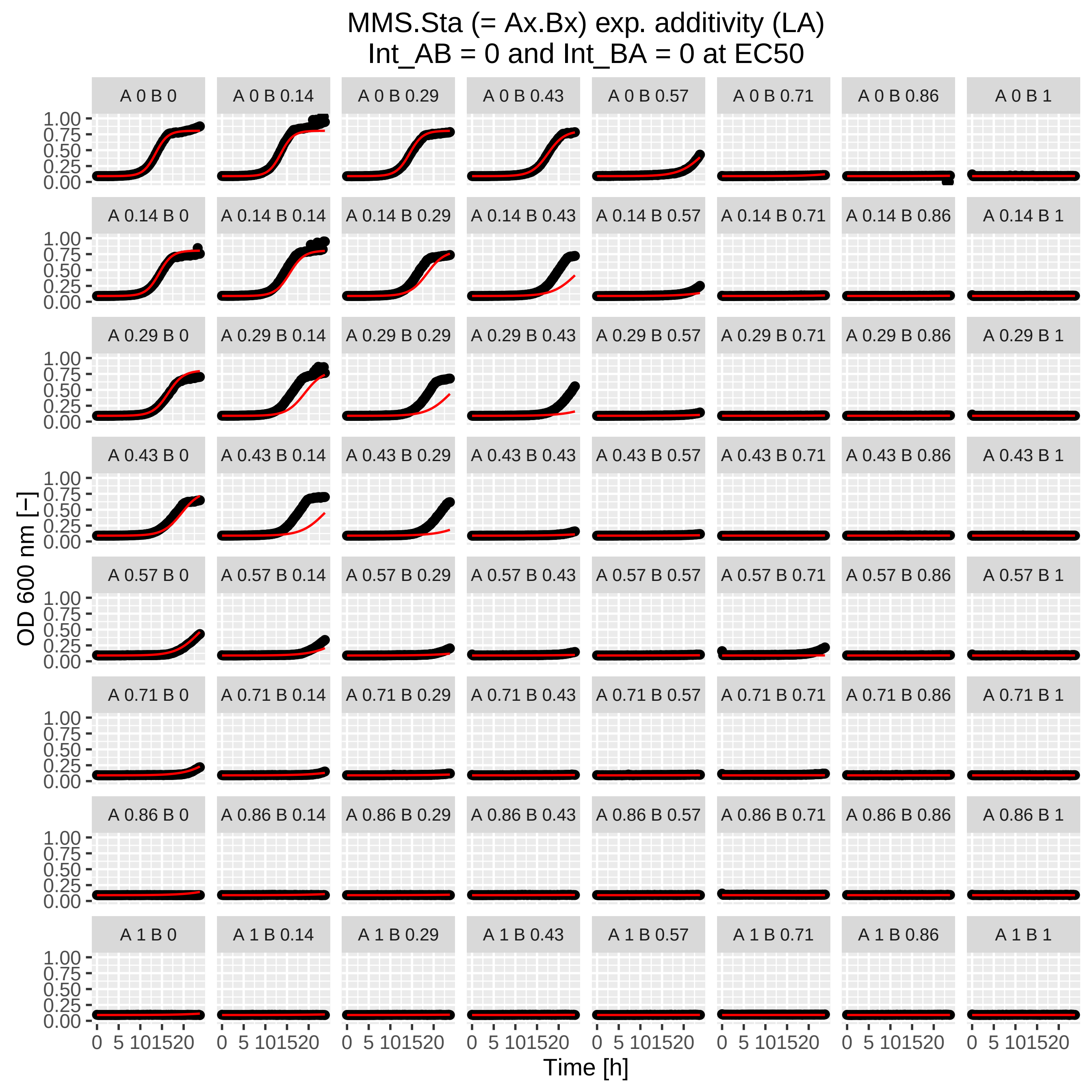
MMS.Sta (= Ax.Bx) exp. additivity (LA)
Int_AB = 0 and Int_BA = 0 at EC50
OD 600 nm [–]
Time [h]

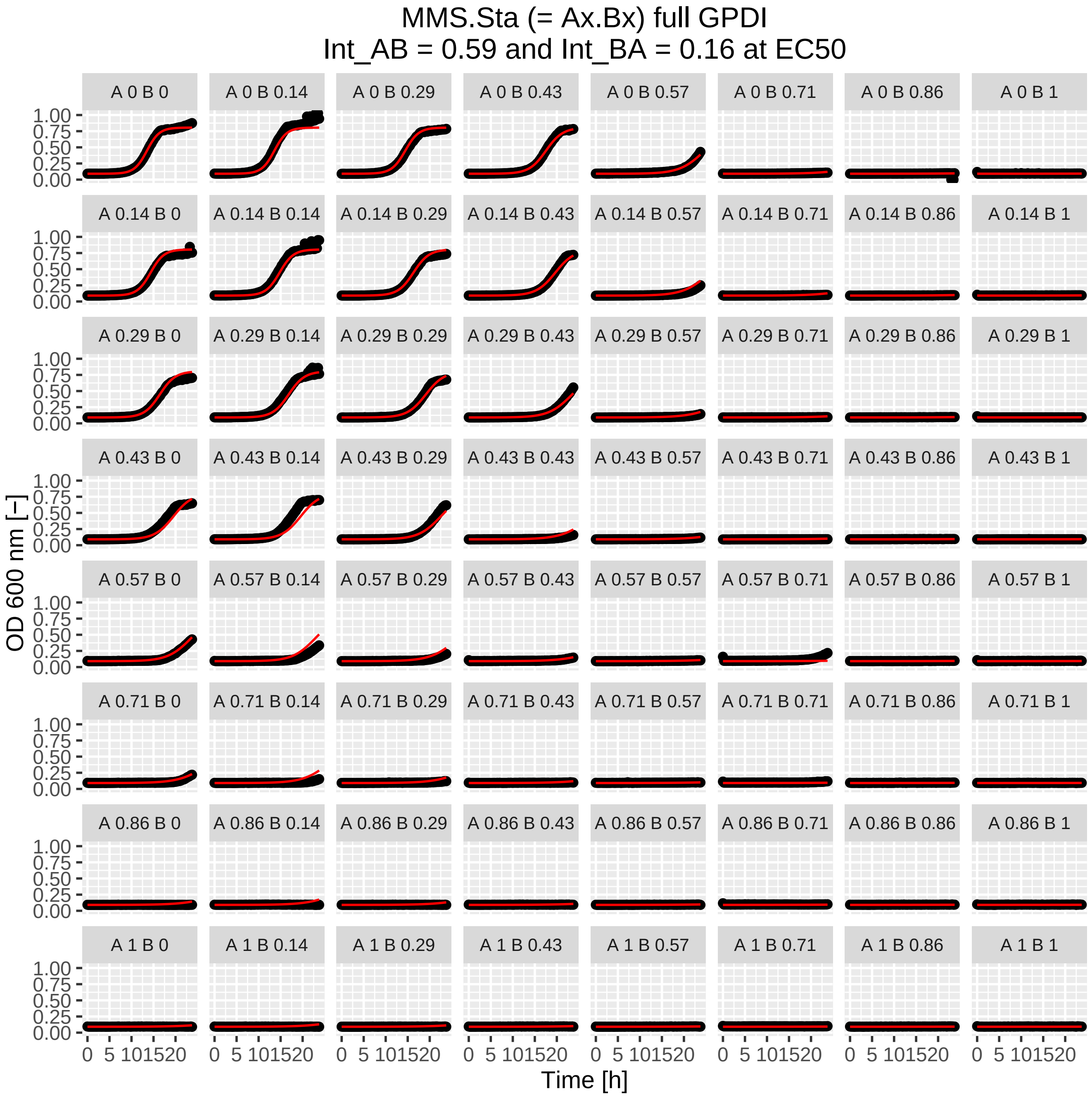

MMS.Sta (= Ax.Bx) full GPDI
Int_AB = 0.59 and Int_BA = 0.16 at EC50
OD 600 nm [–]
Time [h]
1.00
0.75
0.50
0.25
0.00
0 5 101520
A 0 B 0
A 0 B 0.14
A 0 B 0.29
A 0 B 0.43
A 0 B 0.57
A 0 B 0.71
A 0 B 0.86
A 0 B 1
A 0.14 B 0
A 0.14 B 0.14
A 0.14 B 0.29
A 0.14 B 0.43
A 0.14 B 0.57
A 0.14 B 0.71
A 0.14 B 0.86
A 0.14 B 1
A 0.29 B 0
A 0.29 B 0.14
A 0.29 B 0.29
A 0.29 B 0.43
A 0.29 B 0.57
A 0.29 B 0.71
A 0.29 B 0.86
A 0.29 B 1
A 0.43 B 0
A 0.43 B 0.14
A 0.43 B 0.29
A 0.43 B 0.43
A 0.43 B 0.57
A 0.43 B 0.71
A 0.43 B 0.86
A 0.43 B 1
A 0.57 B 0
A 0.57 B 0.14
A 0.57 B 0.29
A 0.57 B 0.43
A 0.57 B 0.57
A 0.57 B 0.71
A 0.57 B 0.86
A 0.57 B 1
A 0.71 B 0
A 0.71 B 0.14
A 0.71 B 0.29
A 0.71 B 0.43
A 0.71 B 0.57
A 0.71 B 0.71
A 0.71 B 0.86
A 0.71 B 1
A 0.86 B 0
A 0.86 B 0.14
A 0.86 B 0.29
A 0.86 B 0.43
A 0.86 B 0.57
A 0.86 B 0.71
A 0.86 B 0.86
A 0.86 B 1
A 1 B 0
A 1 B 0.14
A 1 B 0.29
A 1 B 0.43
A 1 B 0.57
A 1 B 0.71
A 1 B 0.86
A 1 B 1

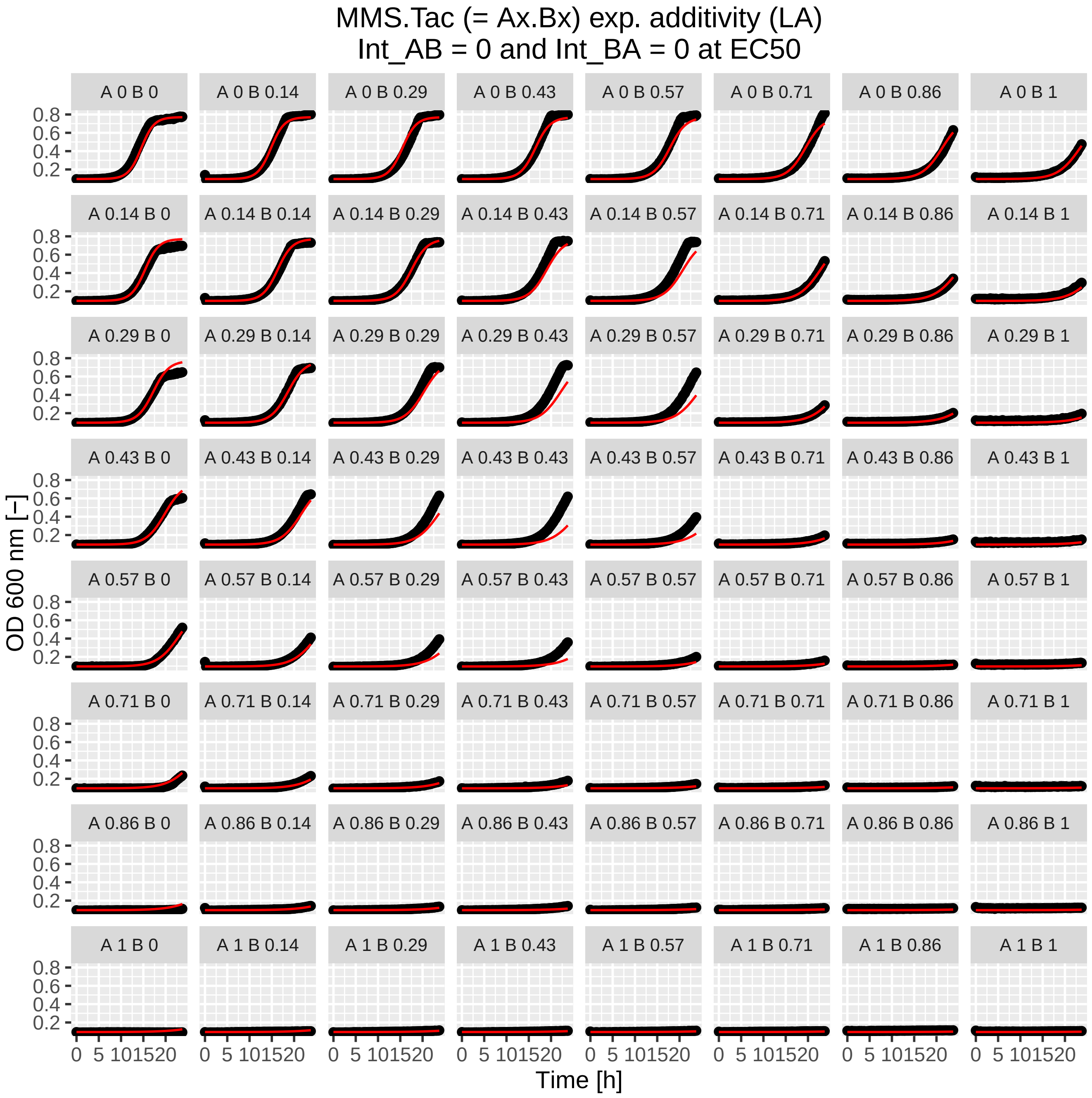
MMS.Tac (= Ax.Bx) exp. additivity (LA)
Int_AB = 0 and Int_BA = 0 at EC50
OD 600 nm [−]
Time [h]
0.8
0.6
0.4
0.2
0 5 101520
A 0 B 0
A 0 B 0.14
A 0 B 0.29
A 0 B 0.43
A 0 B 0.57
A 0 B 0.71
A 0 B 0.86
A 0 B 1
A 0.14 B 0
A 0.14 B 0.14
A 0.14 B 0.29
A 0.14 B 0.43
A 0.14 B 0.57
A 0.14 B 0.71
A 0.14 B 0.86
A 0.14 B 1
A 0.29 B 0
A 0.29 B 0.14
A 0.29 B 0.29
A 0.29 B 0.43
A 0.29 B 0.57
A 0.29 B 0.71
A 0.29 B 0.86
A 0.29 B 1
A 0.43 B 0
A 0.43 B 0.14
A 0.43 B 0.29
A 0.43 B 0.43
A 0.43 B 0.57
A 0.43 B 0.71
A 0.43 B 0.86
A 0.43 B 1
A 0.57 B 0
A 0.57 B 0.14
A 0.57 B 0.29
A 0.57 B 0.43
A 0.57 B 0.57
A 0.57 B 0.71
A 0.57 B 0.86
A 0.57 B 1
A 0.71 B 0
A 0.71 B 0.14
A 0.71 B 0.29
A 0.71 B 0.43
A 0.71 B 0.57
A 0.71 B 0.71
A 0.71 B 0.86
A 0.71 B 1
A 0.86 B 0
A 0.86 B 0.14
A 0.86 B 0.29
A 0.86 B 0.43
A 0.86 B 0.57
A 0.86 B 0.71
A 0.86 B 0.86
A 0.86 B 1
A 1 B 0
A 1 B 0.14
A 1 B 0.29
A 1 B 0.43
A 1 B 0.57
A 1 B 0.71
A 1 B 0.86
A 1 B 1

MMS.Tac (= Ax.Bx) full GPDI
Int_AB = 1.51 and Int_BA = −0.22 at EC50

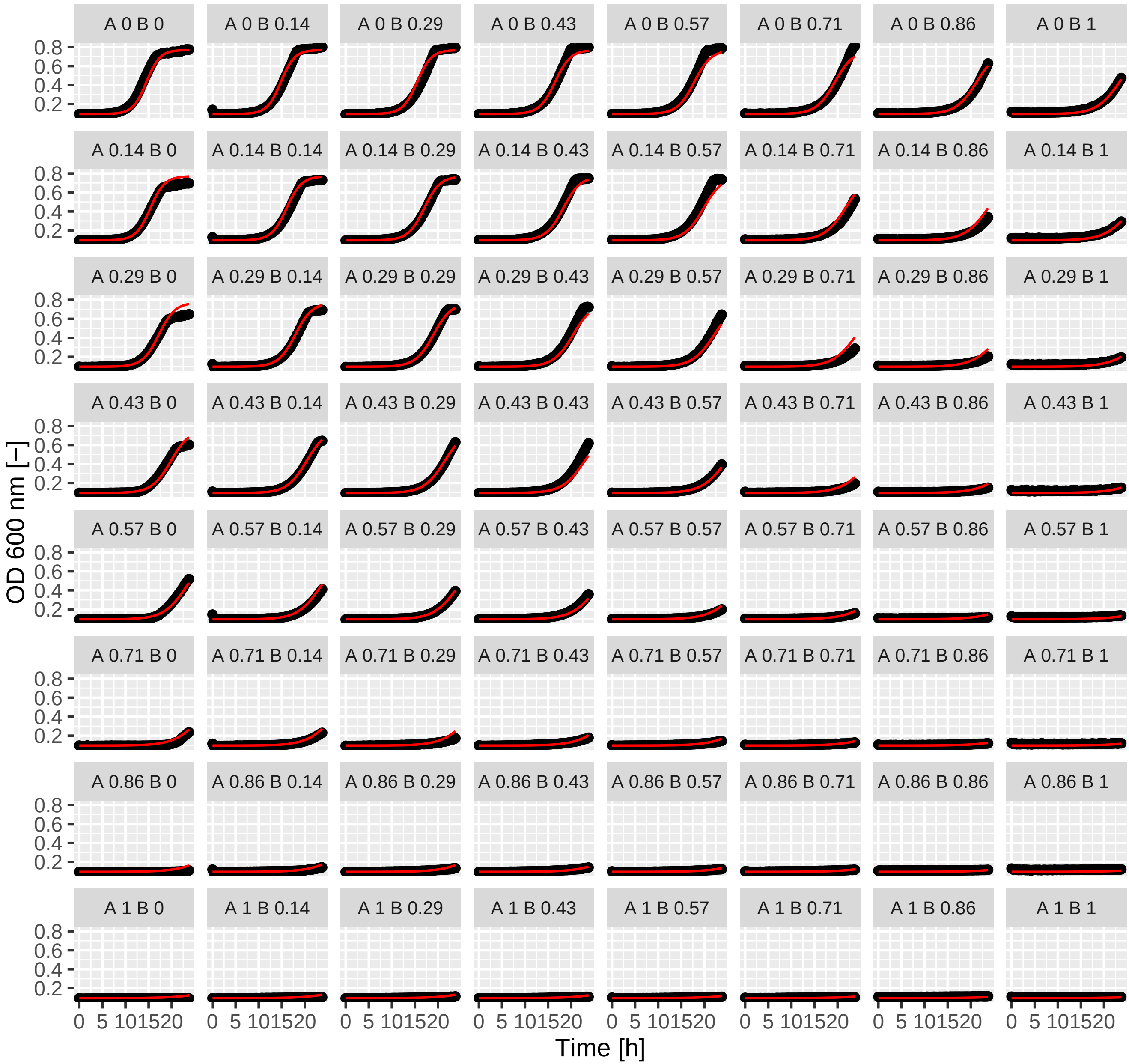

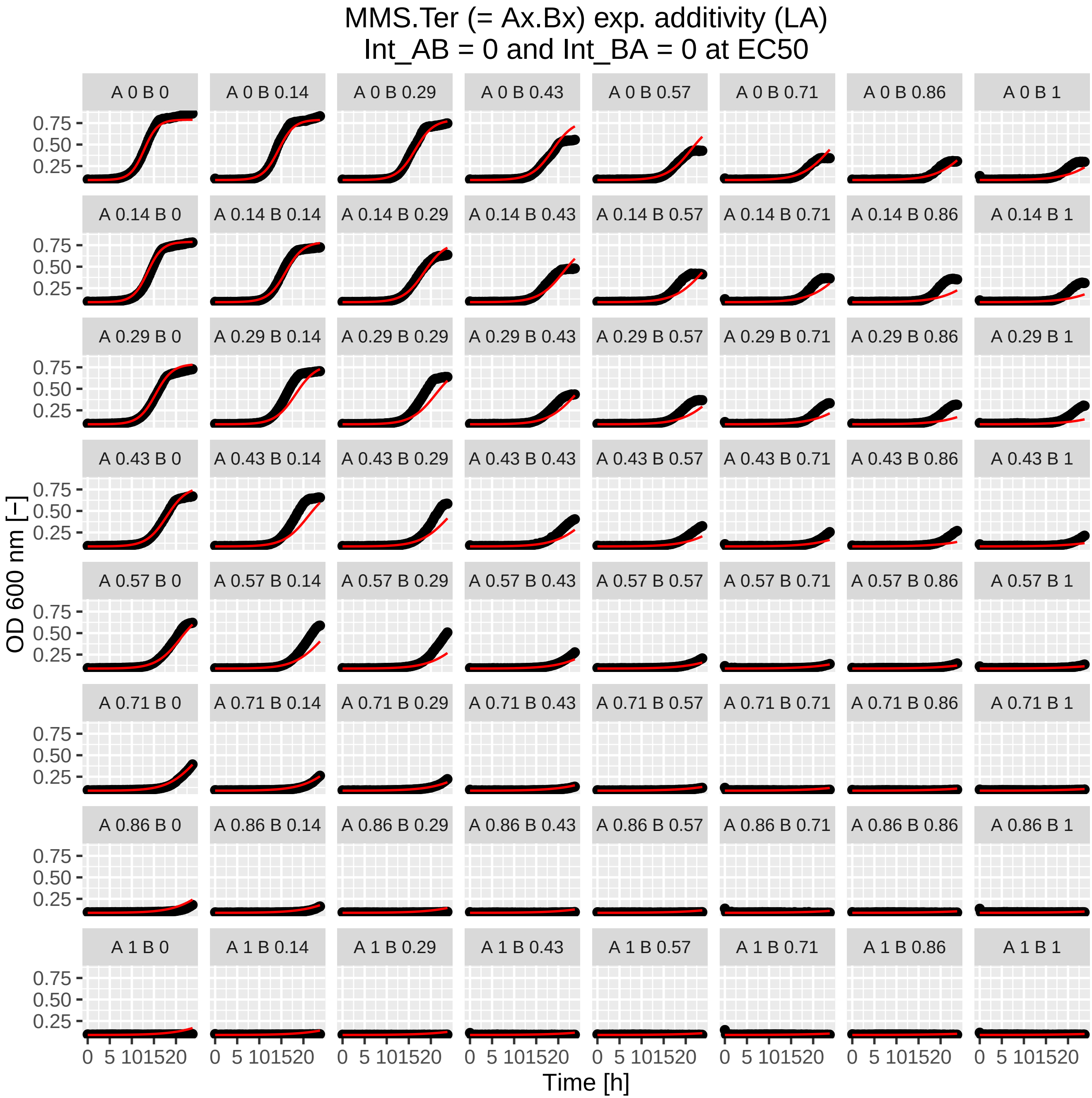
MMS.Ter (= Ax.Bx) exp. additivity (LA)
Int_AB = 0 and Int_BA = 0 at EC50
A 0 B 0
A 0 B 0.14
A 0 B 0.29
A 0 B 0.43
A 0 B 0.57
A 0 B 0.71
A 0 B 0.86
A 0 B 1
A 0.14 B 0
A 0.14 B 0.14
A 0.14 B 0.29
A 0.14 B 0.43
A 0.14 B 0.57
A 0.14 B 0.71
A 0.14 B 0.86
A 0.14 B 1
A 0.29 B 0
A 0.29 B 0.14
A 0.29 B 0.29
A 0.29 B 0.43
A 0.29 B 0.57
A 0.29 B 0.71
A 0.29 B 0.86
A 0.29 B 1
A 0.43 B 0
A 0.43 B 0.14
A 0.43 B 0.29
A 0.43 B 0.43
A 0.43 B 0.57
A 0.43 B 0.71
A 0.43 B 0.86
A 0.43 B 1
A 0.57 B 0
A 0.57 B 0.14
A 0.57 B 0.29
A 0.57 B 0.43
A 0.57 B 0.57
A 0.57 B 0.71
A 0.57 B 0.86
A 0.57 B 1
A 0.71 B 0
A 0.71 B 0.14
A 0.71 B 0.29
A 0.71 B 0.43
A 0.71 B 0.57
A 0.71 B 0.71
A 0.71 B 0.86
A 0.71 B 1
A 0.86 B 0
A 0.86 B 0.14
A 0.86 B 0.29
A 0.86 B 0.43
A 0.86 B 0.57
A 0.86 B 0.71
A 0.86 B 0.86
A 0.86 B 1
A 1 B 0
A 1 B 0.14
A 1 B 0.29
A 1 B 0.43
A 1 B 0.57
A 1 B 0.71
A 1 B 0.86
A 1 B 1
0.75
0.50
0.25
0 5 101520
OD 600 nm [–]
Time [h]

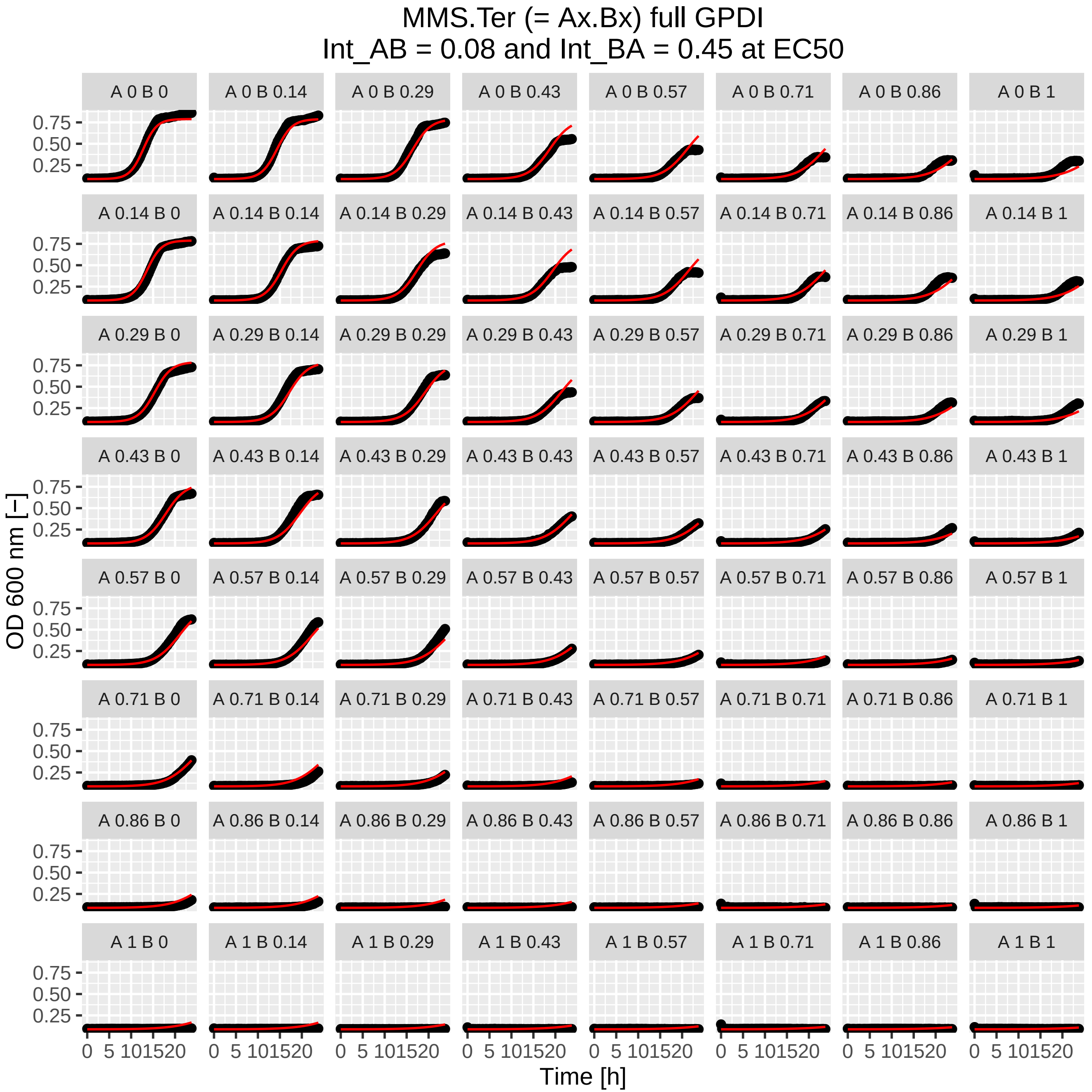

MMS.Ter (= Ax.Bx) full GPDI
Int_AB = 0.08 and Int_BA = 0.45 at EC50
A 0 B 0
A 0 B 0.14
A 0 B 0.29
A 0 B 0.43
A 0 B 0.57
A 0 B 0.71
A 0 B 0.86
A 0 B 1
A 0.14 B 0
A 0.14 B 0.14
A 0.14 B 0.29
A 0.14 B 0.43
A 0.14 B 0.57
A 0.14 B 0.71
A 0.14 B 0.86
A 0.14 B 1
A 0.29 B 0
A 0.29 B 0.14
A 0.29 B 0.29
A 0.29 B 0.43
A 0.29 B 0.57
A 0.29 B 0.71
A 0.29 B 0.86
A 0.29 B 1
A 0.43 B 0
A 0.43 B 0.14
A 0.43 B 0.29
A 0.43 B 0.43
A 0.43 B 0.57
A 0.43 B 0.71
A 0.43 B 0.86
A 0.43 B 1
A 0.57 B 0
A 0.57 B 0.14
A 0.57 B 0.29
A 0.57 B 0.43
A 0.57 B 0.57
A 0.57 B 0.71
A 0.57 B 0.86
A 0.57 B 1
A 0.71 B 0
A 0.71 B 0.14
A 0.71 B 0.29
A 0.71 B 0.43
A 0.71 B 0.57
A 0.71 B 0.71
A 0.71 B 0.86
A 0.71 B 1
A 0.86 B 0
A 0.86 B 0.14
A 0.86 B 0.29
A 0.86 B 0.43
A 0.86 B 0.57
A 0.86 B 0.71
A 0.86 B 0.86
A 0.86 B 1
A 1 B 0
A 1 B 0.14
A 1 B 0.29
A 1 B 0.43
A 1 B 0.57
A 1 B 0.71
A 1 B 0.86
A 1 B 1
0.75
0.50
0.25
0 5 101520
OD 600 nm [–]
Time [h]

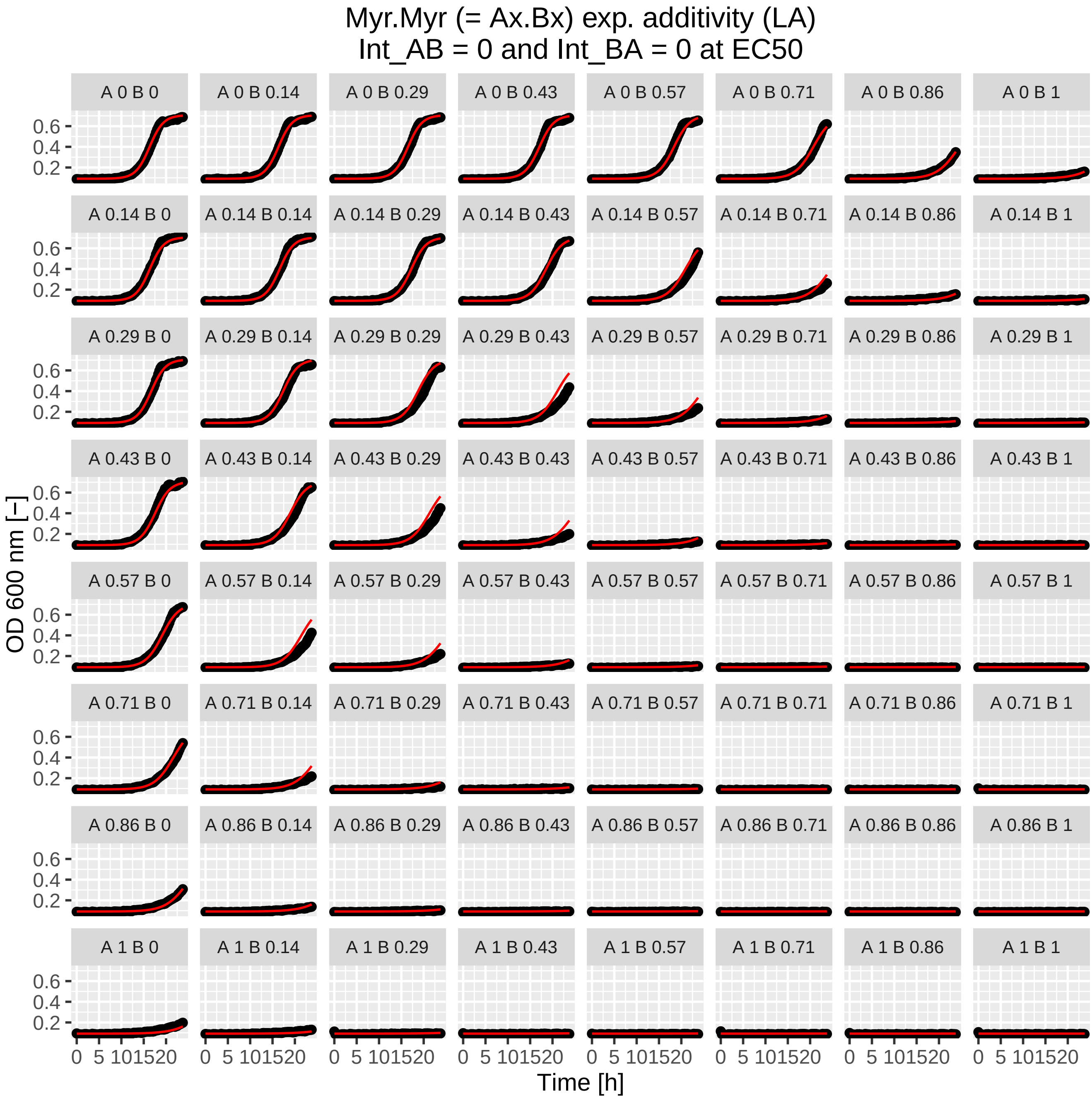
Myr.Myr (= Ax.Bx) exp. additivity (LA)
Int_AB = 0 and Int_BA = 0 at EC50
OD 600 nm [–]
Time [h]
0.6
0.4
0.2
0 5 101520
A 0 B 0
A 0 B 0.14
A 0 B 0.29
A 0 B 0.43
A 0 B 0.57
A 0 B 0.71
A 0 B 0.86
A 0 B 1
A 0.14 B 0
A 0.14 B 0.14
A 0.14 B 0.29
A 0.14 B 0.43
A 0.14 B 0.57
A 0.14 B 0.71
A 0.14 B 0.86
A 0.14 B 1
A 0.29 B 0
A 0.29 B 0.14
A 0.29 B 0.29
A 0.29 B 0.43
A 0.29 B 0.57
A 0.29 B 0.71
A 0.29 B 0.86
A 0.29 B 1
A 0.43 B 0
A 0.43 B 0.14
A 0.43 B 0.29
A 0.43 B 0.43
A 0.43 B 0.57
A 0.43 B 0.71
A 0.43 B 0.86
A 0.43 B 1
A 0.57 B 0
A 0.57 B 0.14
A 0.57 B 0.29
A 0.57 B 0.43
A 0.57 B 0.57
A 0.57 B 0.71
A 0.57 B 0.86
A 0.57 B 1
A 0.71 B 0
A 0.71 B 0.14
A 0.71 B 0.29
A 0.71 B 0.43
A 0.71 B 0.57
A 0.71 B 0.71
A 0.71 B 0.86
A 0.71 B 1
A 0.86 B 0
A 0.86 B 0.14
A 0.86 B 0.29
A 0.86 B 0.43
A 0.86 B 0.57
A 0.86 B 0.71
A 0.86 B 0.86
A 0.86 B 1
A 1 B 0
A 1 B 0.14
A 1 B 0.29
A 1 B 0.43
A 1 B 0.57
A 1 B 0.71
A 1 B 0.86
A 1 B 1

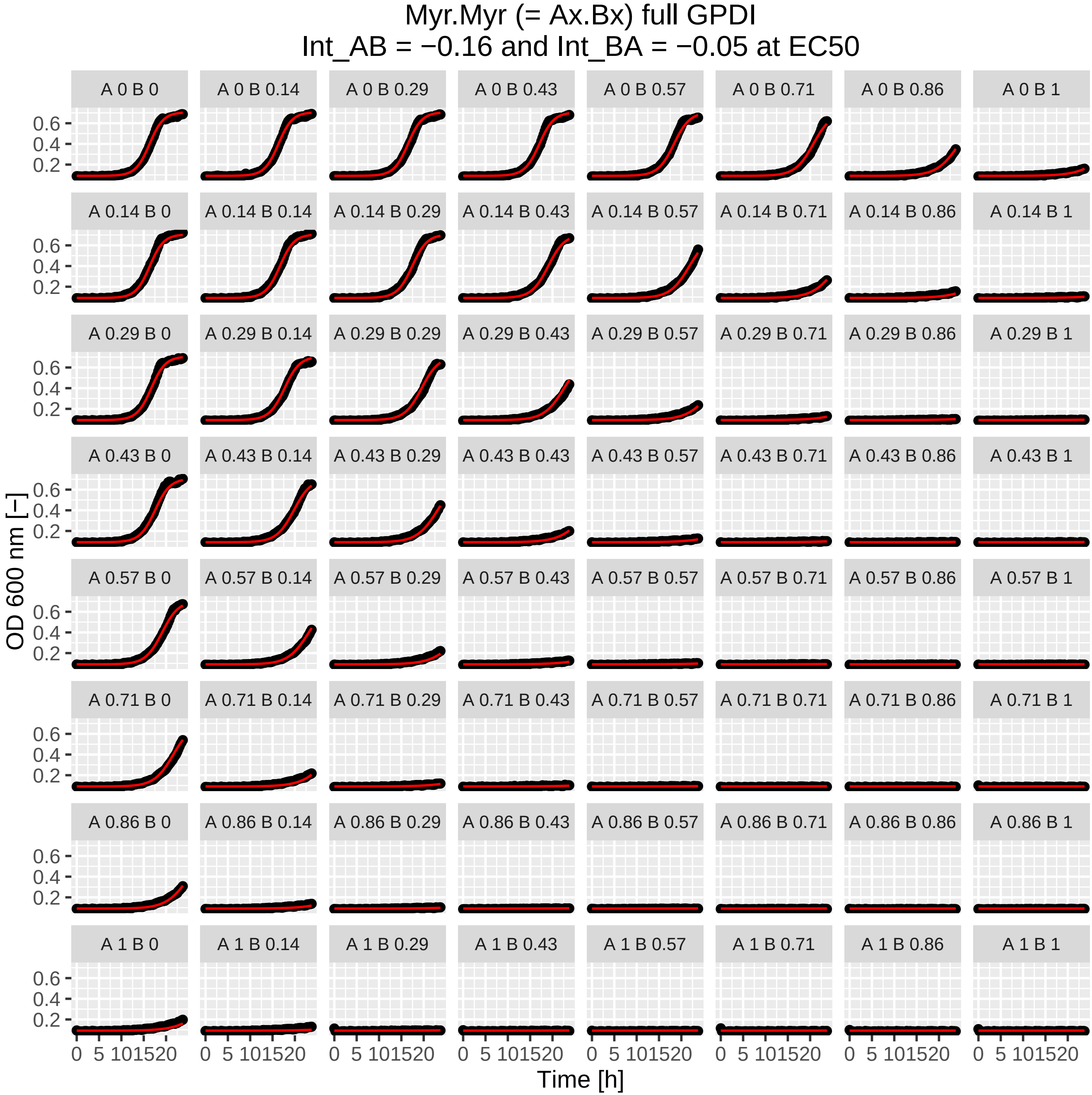
Myr.Myr (= Ax.Bx) full GPDI
Int_AB = −0.16 and Int_BA = −0.05 at EC50
OD 600 nm [−]
Time [h]
A 0 B 0
A 0 B 0.14
A 0 B 0.29
A 0 B 0.43
A 0 B 0.57
A 0 B 0.71
A 0 B 0.86
A 0 B 1
A 0.14 B 0
A 0.14 B 0.14
A 0.14 B 0.29
A 0.14 B 0.43
A 0.14 B 0.57
A 0.14 B 0.71
A 0.14 B 0.86
A 0.14 B 1
A 0.29 B 0
A 0.29 B 0.14
A 0.29 B 0.29
A 0.29 B 0.43
A 0.29 B 0.57
A 0.29 B 0.71
A 0.29 B 0.86
A 0.29 B 1
A 0.43 B 0
A 0.43 B 0.14
A 0.43 B 0.29
A 0.43 B 0.43
A 0.43 B 0.57
A 0.43 B 0.71
A 0.43 B 0.86
A 0.43 B 1
A 0.57 B 0
A 0.57 B 0.14
A 0.57 B 0.29
A 0.57 B 0.43
A 0.57 B 0.57
A 0.57 B 0.71
A 0.57 B 0.86
A 0.57 B 1
A 0.71 B 0
A 0.71 B 0.14
A 0.71 B 0.29
A 0.71 B 0.43
A 0.71 B 0.57
A 0.71 B 0.71
A 0.71 B 0.86
A 0.71 B 1
A 0.86 B 0
A 0.86 B 0.14
A 0.86 B 0.29
A 0.86 B 0.43
A 0.86 B 0.57
A 0.86 B 0.71
A 0.86 B 0.86
A 0.86 B 1
A 1 B 0
A 1 B 0.14
A 1 B 0.29
A 1 B 0.43
A 1 B 0.57
A 1 B 0.71
A 1 B 0.86
A 1 B 1
0.6
0.4
0.2
0 5 101520

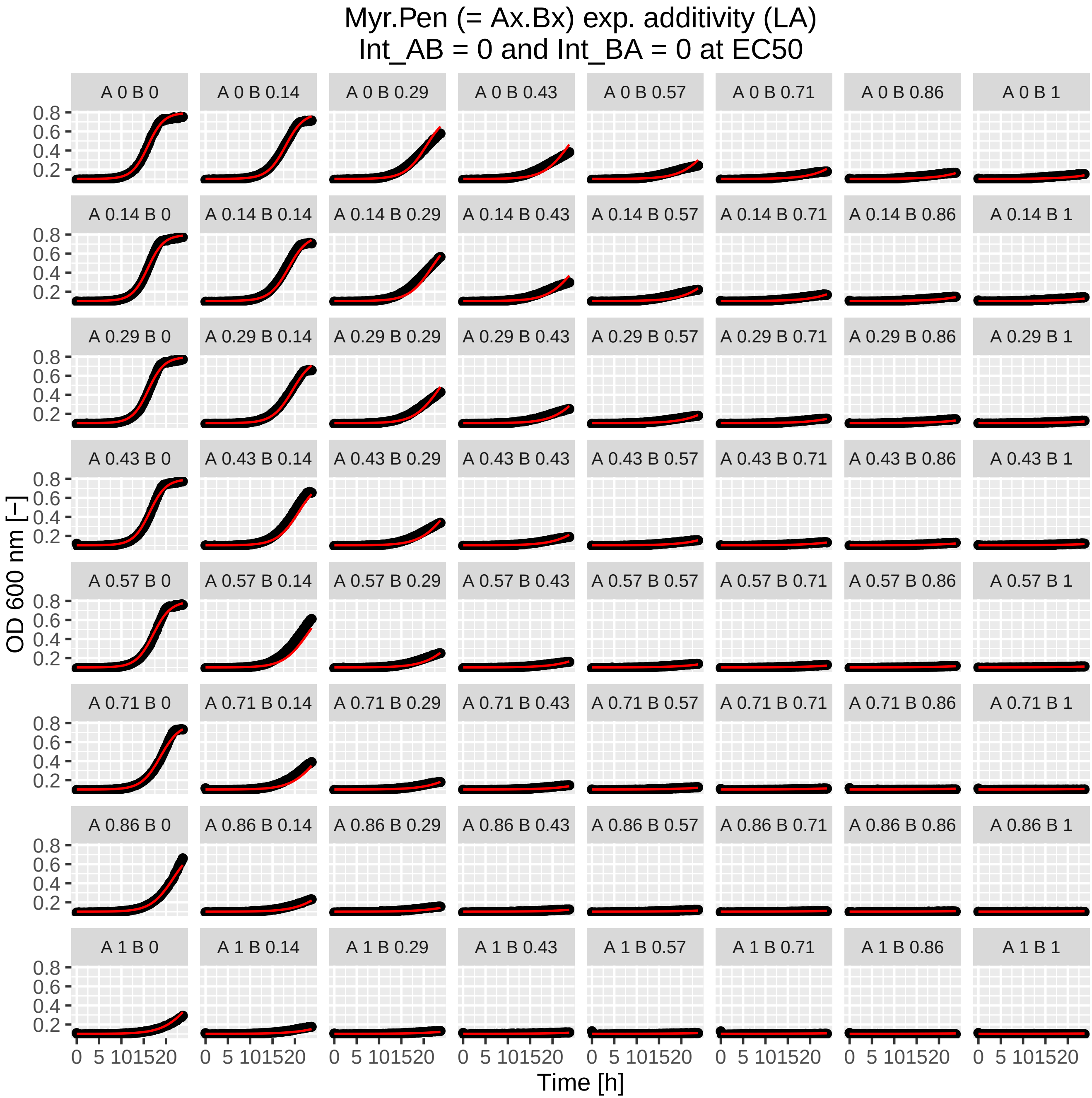

Myr.Pen (= Ax.Bx) exp. additivity (LA)
Int_AB = 0 and Int_BA = 0 at EC50
OD 600 nm [–]
Time [h]
0 5 10 15 20
0.2 0.4 0.6 0.8
A 0 B 0
A 0 B 0.14
A 0 B 0.29
A 0 B 0.43
A 0 B 0.57
A 0 B 0.71
A 0 B 0.86
A 0 B 1
A 1 B 0
A 1 B 0.14
A 1 B 0.29
A 1 B 0.43
A 1 B 0.57
A 1 B 0.71
A 1 B 0.86
A 1 B 1

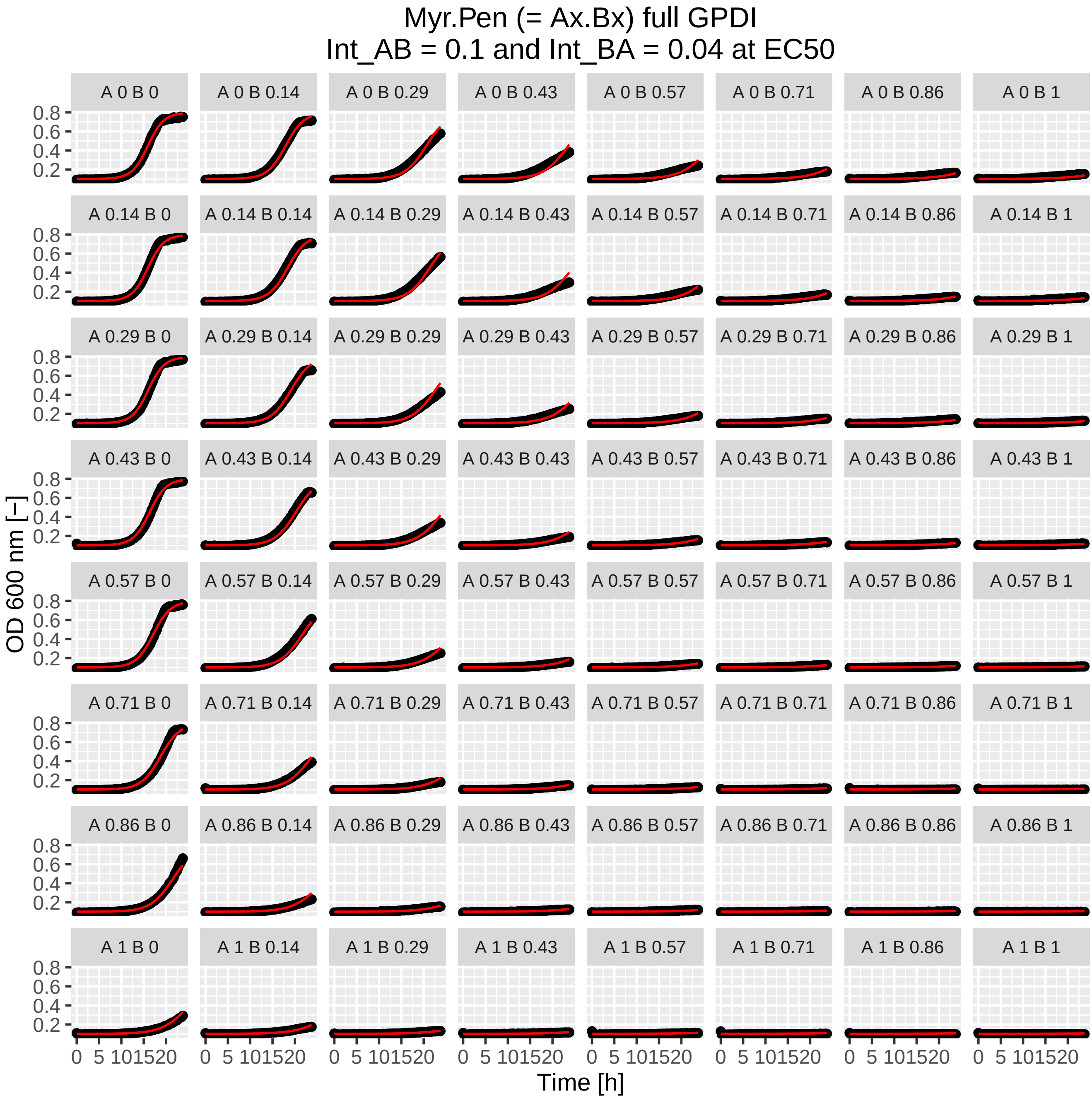

Myr.Pen (= Ax.Bx) full GPDI
Int_AB = 0.1 and Int_BA = 0.04 at EC50
A 0 B 0
A 0 B 0.14
A 0 B 0.29
A 0 B 0.43
A 0 B 0.57
A 0 B 0.71
A 0 B 0.86
A 0 B 1
A 0.14 B 0
A 0.14 B 0.14
A 0.14 B 0.29
A 0.14 B 0.43
A 0.14 B 0.57
A 0.14 B 0.71
A 0.14 B 0.86
A 0.14 B 1
A 0.29 B 0
A 0.29 B 0.14
A 0.29 B 0.29
A 0.29 B 0.43
A 0.29 B 0.57
A 0.29 B 0.71
A 0.29 B 0.86
A 0.29 B 1
A 0.43 B 0
A 0.43 B 0.14
A 0.43 B 0.29
A 0.43 B 0.43
A 0.43 B 0.57
A 0.43 B 0.71
A 0.43 B 0.86
A 0.43 B 1
A 0.57 B 0
A 0.57 B 0.14
A 0.57 B 0.29
A 0.57 B 0.43
A 0.57 B 0.57
A 0.57 B 0.71
A 0.57 B 0.86
A 0.57 B 1
A 0.71 B 0
A 0.71 B 0.14
A 0.71 B 0.29
A 0.71 B 0.43
A 0.71 B 0.57
A 0.71 B 0.71
A 0.71 B 0.86
A 0.71 B 1
A 0.86 B 0
A 0.86 B 0.14
A 0.86 B 0.29
A 0.86 B 0.43
A 0.86 B 0.57
A 0.86 B 0.71
A 0.86 B 0.86
A 0.86 B 1
A 1 B 0
A 1 B 0.14
A 1 B 0.29
A 1 B 0.43
A 1 B 0.57
A 1 B 0.71
A 1 B 0.86
A 1 B 1
OD 600 nm [–]
0.2
0.4
0.6
0.8
0 5 101520
Time [h]

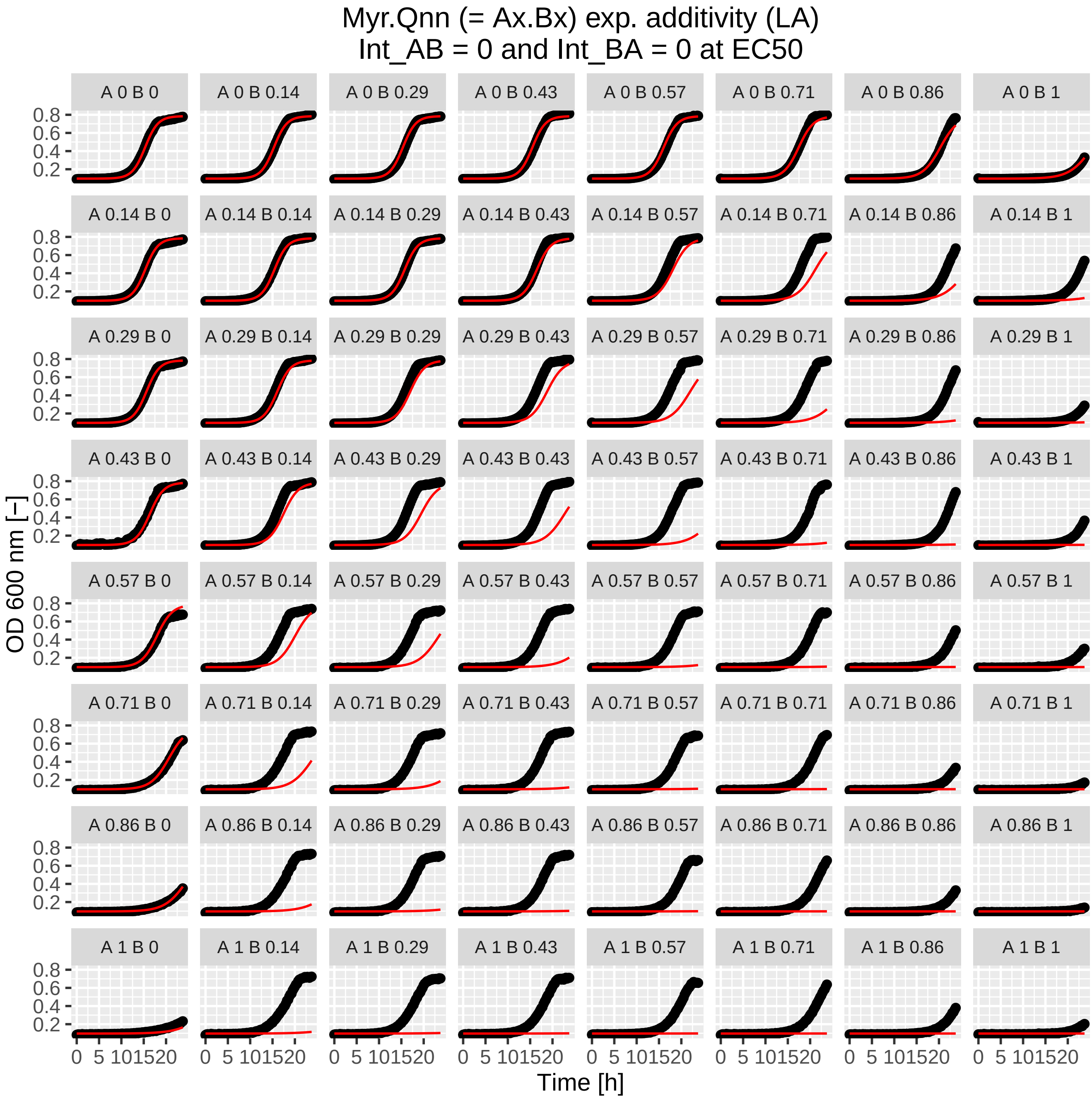

Myr.Qnn (= Ax.Bx) exp. additivity (LA)
Int_AB = 0 and Int_BA = 0 at EC50
A 0 B 0
A 0 B 0.14
A 0 B 0.29
A 0 B 0.43
A 0 B 0.57
A 0 B 0.71
A 0 B 0.86
A 0 B 1
A 0.14 B 0
A 0.14 B 0.14
A 0.14 B 0.29
A 0.14 B 0.43
A 0.14 B 0.57
A 0.14 B 0.71
A 0.14 B 0.86
A 0.14 B 1
A 0.29 B 0
A 0.29 B 0.14
A 0.29 B 0.29
A 0.29 B 0.43
A 0.29 B 0.57
A 0.29 B 0.71
A 0.29 B 0.86
A 0.29 B 1
A 0.43 B 0
A 0.43 B 0.14
A 0.43 B 0.29
A 0.43 B 0.43
A 0.43 B 0.57
A 0.43 B 0.71
A 0.43 B 0.86
A 0.43 B 1
A 0.57 B 0
A 0.57 B 0.14
A 0.57 B 0.29
A 0.57 B 0.43
A 0.57 B 0.57
A 0.57 B 0.71
A 0.57 B 0.86
A 0.57 B 1
A 0.71 B 0
A 0.71 B 0.14
A 0.71 B 0.29
A 0.71 B 0.43
A 0.71 B 0.57
A 0.71 B 0.71
A 0.71 B 0.86
A 0.71 B 1
A 0.86 B 0
A 0.86 B 0.14
A 0.86 B 0.29
A 0.86 B 0.43
A 0.86 B 0.57
A 0.86 B 0.71
A 0.86 B 0.86
A 0.86 B 1
A 1 B 0
A 1 B 0.14
A 1 B 0.29
A 1 B 0.43
A 1 B 0.57
A 1 B 0.71
A 1 B 0.86
A 1 B 1
OD 600 nm [–]
0.2
0.4
0.6
0.8
0 5 101520
Time [h]

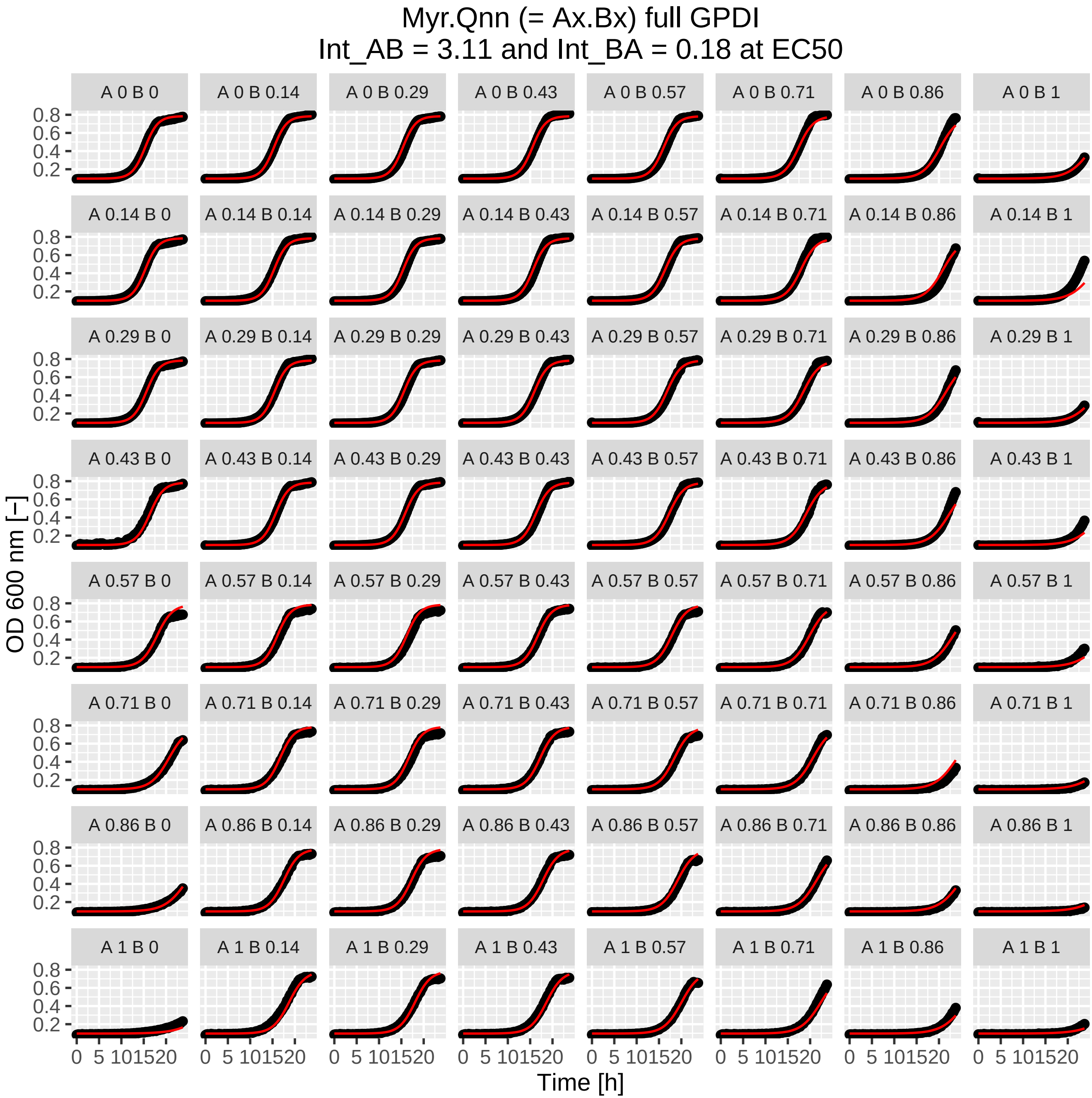

Myr.Qnn (= Ax.Bx) full GPDI
Int_AB = 3.11 and Int_BA = 0.18 at EC50
A 0 B 0
A 0 B 0.14
A 0 B 0.29
A 0 B 0.43
A 0 B 0.57
A 0 B 0.71
A 0 B 0.86
A 0 B 1
A 0.14 B 0
A 0.14 B 0.14
A 0.14 B 0.29
A 0.14 B 0.43
A 0.14 B 0.57
A 0.14 B 0.71
A 0.14 B 0.86
A 0.14 B 1
A 0.29 B 0
A 0.29 B 0.14
A 0.29 B 0.29
A 0.29 B 0.43
A 0.29 B 0.57
A 0.29 B 0.71
A 0.29 B 0.86
A 0.29 B 1
A 0.43 B 0
A 0.43 B 0.14
A 0.43 B 0.29
A 0.43 B 0.43
A 0.43 B 0.57
A 0.43 B 0.71
A 0.43 B 0.86
A 0.43 B 1
A 0.57 B 0
A 0.57 B 0.14
A 0.57 B 0.29
A 0.57 B 0.43
A 0.57 B 0.57
A 0.57 B 0.71
A 0.57 B 0.86
A 0.57 B 1
A 0.71 B 0
A 0.71 B 0.14
A 0.71 B 0.29
A 0.71 B 0.43
A 0.71 B 0.57
A 0.71 B 0.71
A 0.71 B 0.86
A 0.71 B 1
A 0.86 B 0
A 0.86 B 0.14
A 0.86 B 0.29
A 0.86 B 0.43
A 0.86 B 0.57
A 0.86 B 0.71
A 0.86 B 0.86
A 0.86 B 1
A 1 B 0
A 1 B 0.14
A 1 B 0.29
A 1 B 0.43
A 1 B 0.57
A 1 B 0.71
A 1 B 0.86
A 1 B 1
0.8
0.6
0.4
0.2
OD 600 nm [–]
0 5 101520
Time [h]

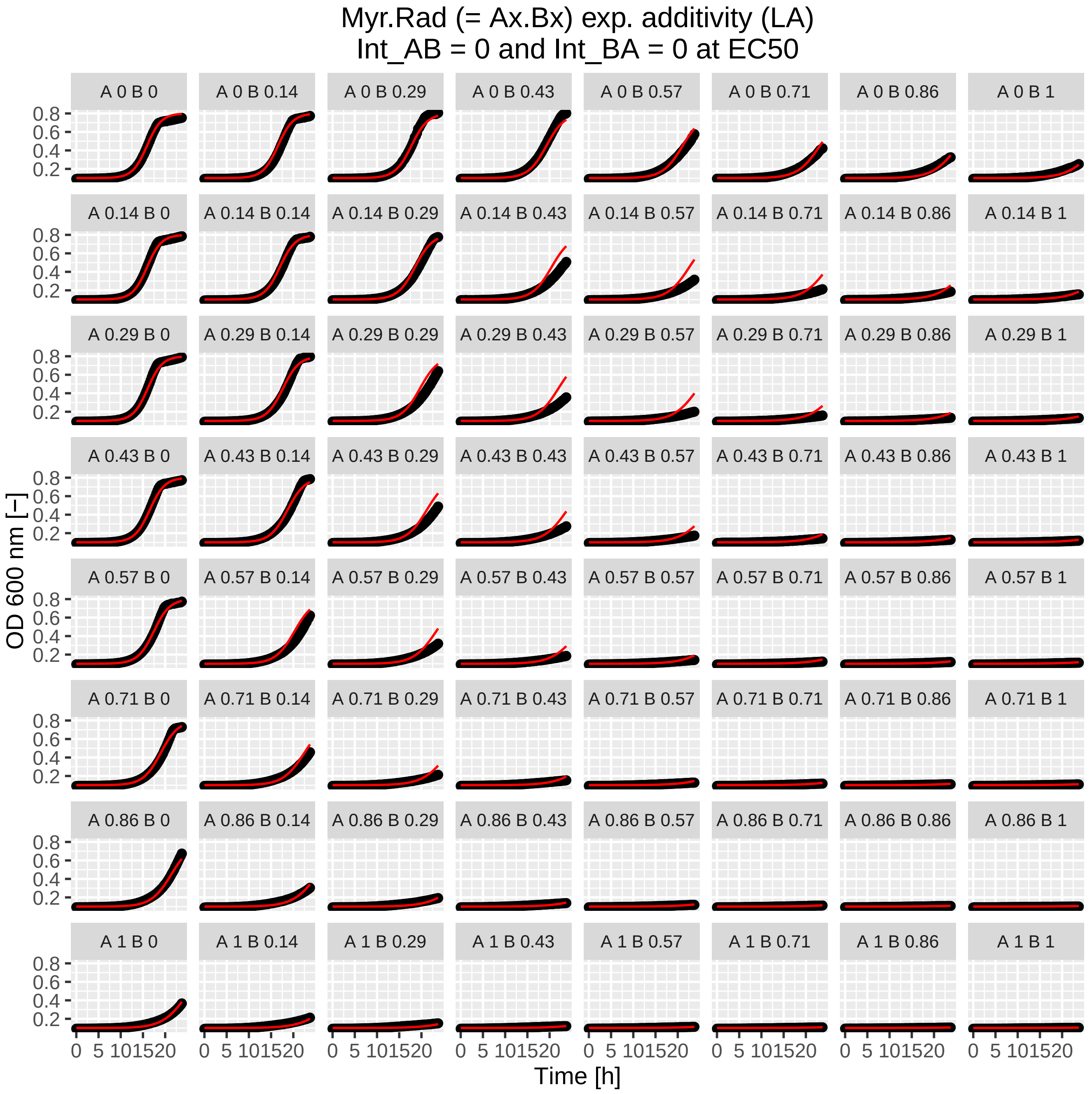
Myr.Rad (= Ax.Bx) exp. additivity (LA)
Int_AB = 0 and Int_BA = 0 at EC50
A 0 B 0
A 0 B 0.14
A 0 B 0.29
A 0 B 0.43
A 0 B 0.57
A 0 B 0.71
A 0 B 0.86
A 0 B 1
A 0.14 B 0
A 0.14 B 0.14
A 0.14 B 0.29
A 0.14 B 0.43
A 0.14 B 0.57
A 0.14 B 0.71
A 0.14 B 0.86
A 0.14 B 1
A 0.29 B 0
A 0.29 B 0.14
A 0.29 B 0.29
A 0.29 B 0.43
A 0.29 B 0.57
A 0.29 B 0.71
A 0.29 B 0.86
A 0.29 B 1
A 0.43 B 0
A 0.43 B 0.14
A 0.43 B 0.29
A 0.43 B 0.43
A 0.43 B 0.57
A 0.43 B 0.71
A 0.43 B 0.86
A 0.43 B 1
A 0.57 B 0
A 0.57 B 0.14
A 0.57 B 0.29
A 0.57 B 0.43
A 0.57 B 0.57
A 0.57 B 0.71
A 0.57 B 0.86
A 0.57 B 1
A 0.71 B 0
A 0.71 B 0.14
A 0.71 B 0.29
A 0.71 B 0.43
A 0.71 B 0.57
A 0.71 B 0.71
A 0.71 B 0.86
A 0.71 B 1
A 0.86 B 0
A 0.86 B 0.14
A 0.86 B 0.29
A 0.86 B 0.43
A 0.86 B 0.57
A 0.86 B 0.71
A 0.86 B 0.86
A 0.86 B 1
A 1 B 0
A 1 B 0.14
A 1 B 0.29
A 1 B 0.43
A 1 B 0.57
A 1 B 0.71
A 1 B 0.86
A 1 B 1
OD 600 nm [–]
0.8
0.6
0.4
0.2
0 5 101520
Time [h]

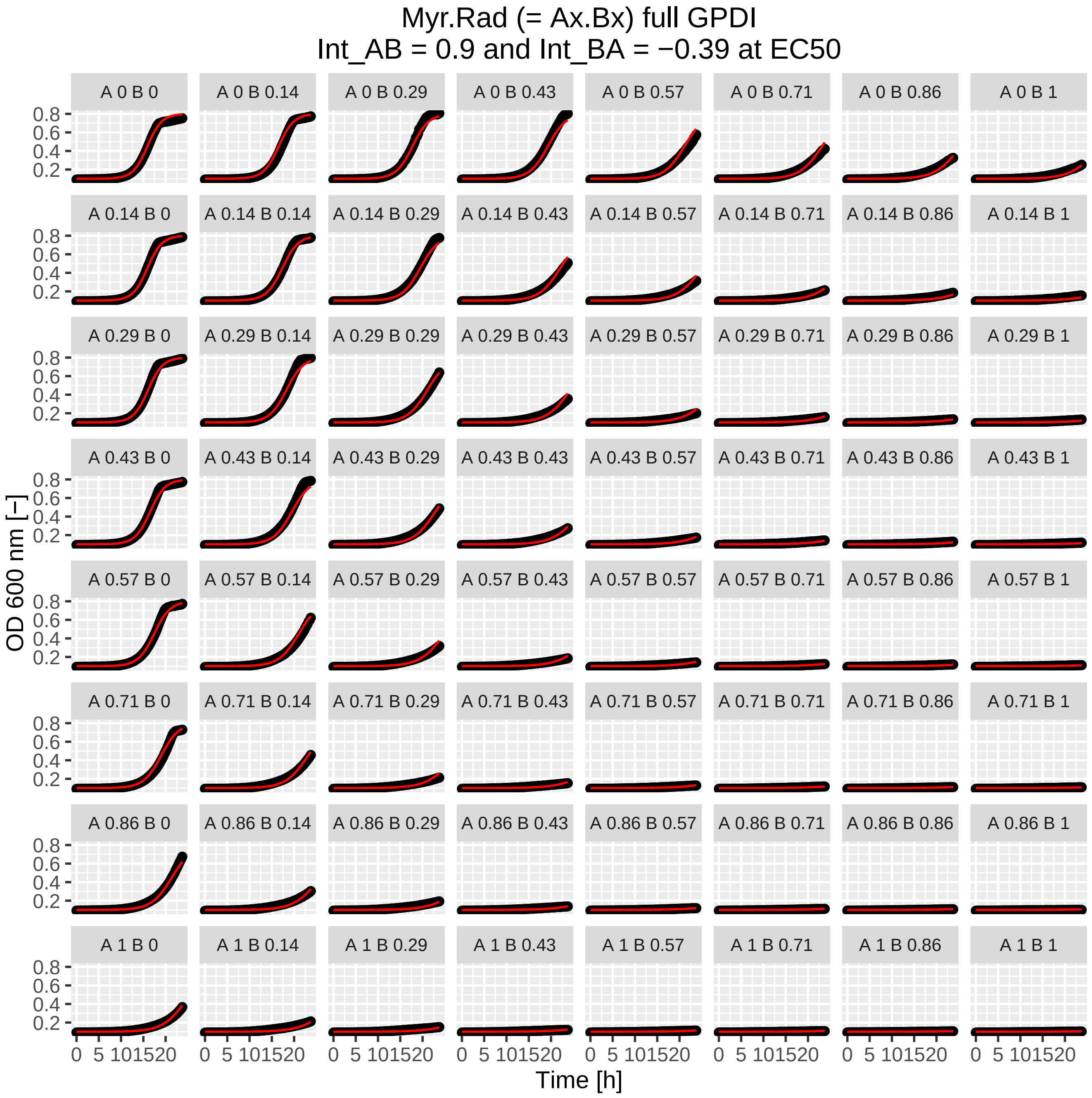
Myr.Rad (= Ax.Bx) full GPDI
Int_AB = 0.9 and Int_BA = −0.39 at EC50
OD 600 nm [−]
Time [h]
A 0 B 0
A 0 B 0.14
A 0 B 0.29
A 0 B 0.43
A 0 B 0.57
A 0 B 0.71
A 0 B 0.86
A 0 B 1
A 0.14 B 0
A 0.14 B 0.14
A 0.14 B 0.29
A 0.14 B 0.43
A 0.14 B 0.57
A 0.14 B 0.71
A 0.14 B 0.86
A 0.14 B 1
A 0.29 B 0
A 0.29 B 0.14
A 0.29 B 0.29
A 0.29 B 0.43
A 0.29 B 0.57
A 0.29 B 0.71
A 0.29 B 0.86
A 0.29 B 1
A 0.43 B 0
A 0.43 B 0.14
A 0.43 B 0.29
A 0.43 B 0.43
A 0.43 B 0.57
A 0.43 B 0.71
A 0.43 B 0.86
A 0.43 B 1
A 0.57 B 0
A 0.57 B 0.14
A 0.57 B 0.29
A 0.57 B 0.43
A 0.57 B 0.57
A 0.57 B 0.71
A 0.57 B 0.86
A 0.57 B 1
A 0.71 B 0
A 0.71 B 0.14
A 0.71 B 0.29
A 0.71 B 0.43
A 0.71 B 0.57
A 0.71 B 0.71
A 0.71 B 0.86
A 0.71 B 1
A 0.86 B 0
A 0.86 B 0.14
A 0.86 B 0.29
A 0.86 B 0.43
A 0.86 B 0.57
A 0.86 B 0.71
A 0.86 B 0.86
A 0.86 B 1
A 1 B 0
A 1 B 0.14
A 1 B 0.29
A 1 B 0.43
A 1 B 0.57
A 1 B 0.71
A 1 B 0.86
A 1 B 1
0.8
0.6
0.4
0.2
0 5 101520

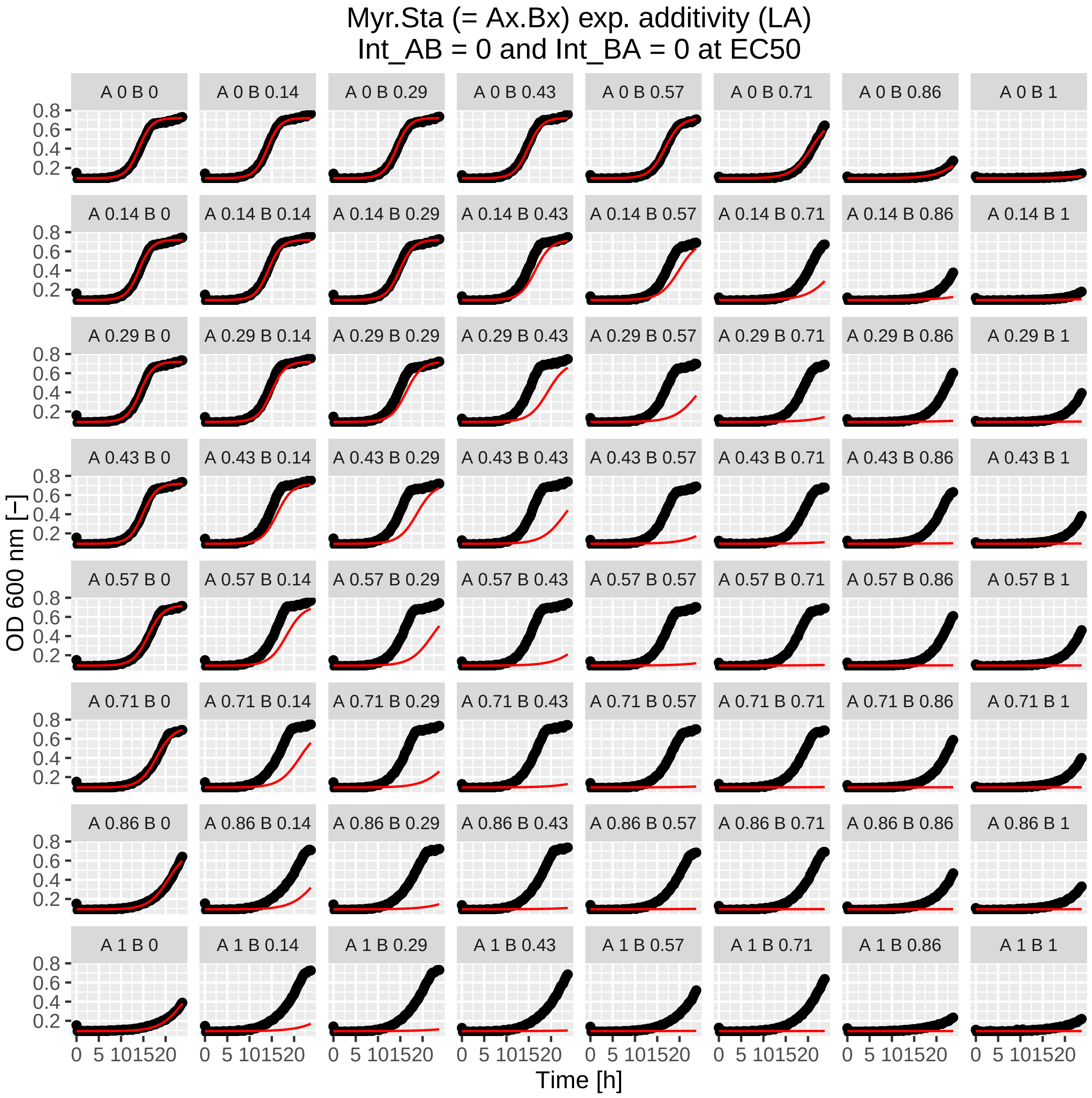
Myr.Sta (= Ax.Bx) exp. additivity (LA)
Int_AB = 0 and Int_BA = 0 at EC50
A 0 B 0
A 0 B 0.14
A 0 B 0.29
A 0 B 0.43
A 0 B 0.57
A 0 B 0.71
A 0 B 0.86
A 0 B 1
A 0.14 B 0
A 0.14 B 0.14
A 0.14 B 0.29
A 0.14 B 0.43
A 0.14 B 0.57
A 0.14 B 0.71
A 0.14 B 0.86
A 0.14 B 1
A 0.29 B 0
A 0.29 B 0.14
A 0.29 B 0.29
A 0.29 B 0.43
A 0.29 B 0.57
A 0.29 B 0.71
A 0.29 B 0.86
A 0.29 B 1
A 0.43 B 0
A 0.43 B 0.14
A 0.43 B 0.29
A 0.43 B 0.43
A 0.43 B 0.57
A 0.43 B 0.71
A 0.43 B 0.86
A 0.43 B 1
A 0.57 B 0
A 0.57 B 0.14
A 0.57 B 0.29
A 0.57 B 0.43
A 0.57 B 0.57
A 0.57 B 0.71
A 0.57 B 0.86
A 0.57 B 1
A 0.71 B 0
A 0.71 B 0.14
A 0.71 B 0.29
A 0.71 B 0.43
A 0.71 B 0.57
A 0.71 B 0.71
A 0.71 B 0.86
A 0.71 B 1
A 0.86 B 0
A 0.86 B 0.14
A 0.86 B 0.29
A 0.86 B 0.43
A 0.86 B 0.57
A 0.86 B 0.71
A 0.86 B 0.86
A 0.86 B 1
A 1 B 0
A 1 B 0.14
A 1 B 0.29
A 1 B 0.43
A 1 B 0.57
A 1 B 0.71
A 1 B 0.86
A 1 B 1
OD 600 nm [–]
0.2
0.4
0.6
0.8
0 5 101520
Time [h]

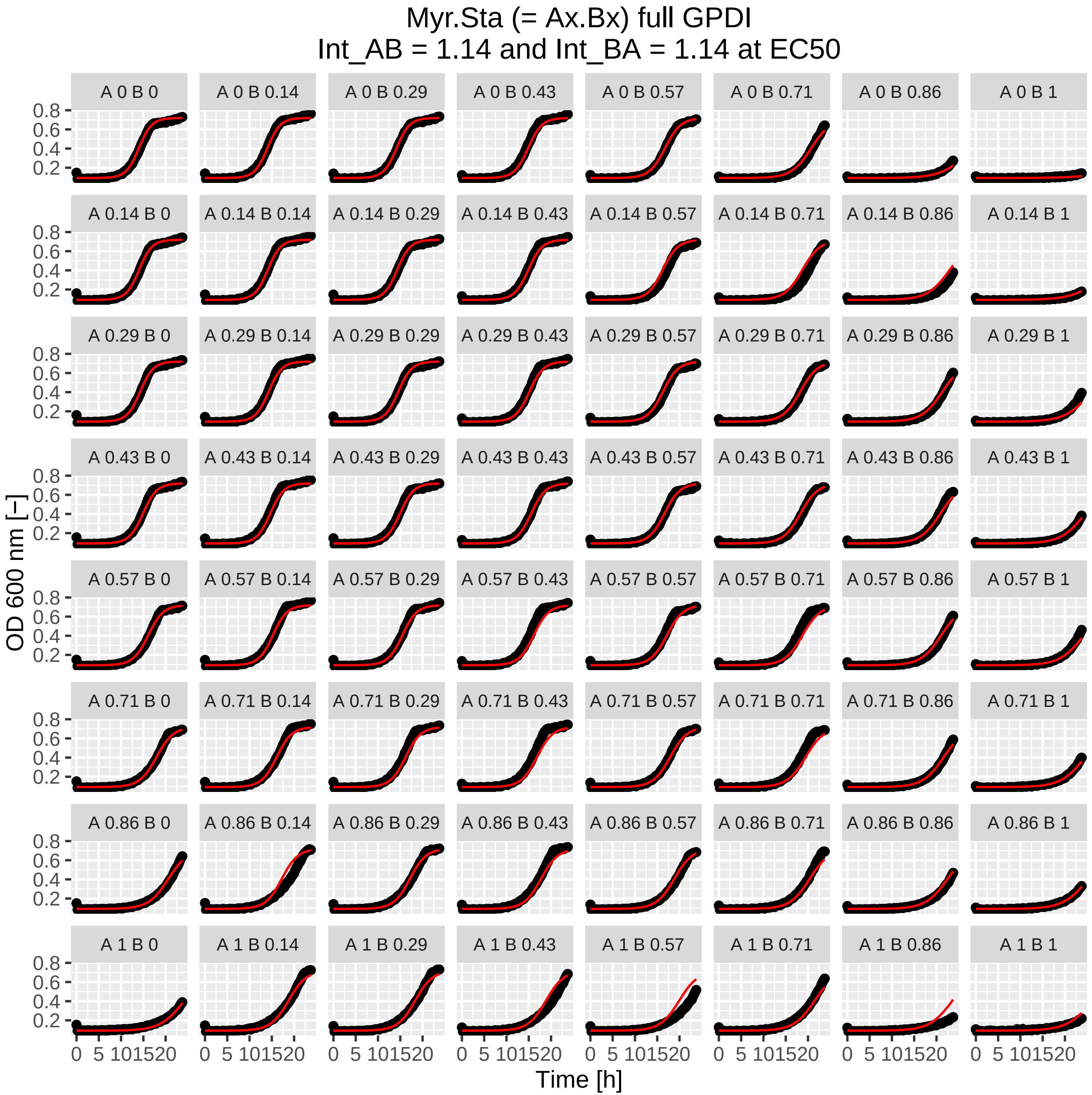

Myr.Sta (= Ax.Bx) full GPDI
Int_AB = 1.14 and Int_BA = 1.14 at EC50
A 0 B 0
A 0 B 0.14
A 0 B 0.29
A 0 B 0.43
A 0 B 0.57
A 0 B 0.71
A 0 B 0.86
A 0 B 1
A 0.14 B 0
A 0.14 B 0.14
A 0.14 B 0.29
A 0.14 B 0.43
A 0.14 B 0.57
A 0.14 B 0.71
A 0.14 B 0.86
A 0.14 B 1
A 0.29 B 0
A 0.29 B 0.14
A 0.29 B 0.29
A 0.29 B 0.43
A 0.29 B 0.57
A 0.29 B 0.71
A 0.29 B 0.86
A 0.29 B 1
A 0.43 B 0
A 0.43 B 0.14
A 0.43 B 0.29
A 0.43 B 0.43
A 0.43 B 0.57
A 0.43 B 0.71
A 0.43 B 0.86
A 0.43 B 1
A 0.57 B 0
A 0.57 B 0.14
A 0.57 B 0.29
A 0.57 B 0.43
A 0.57 B 0.57
A 0.57 B 0.71
A 0.57 B 0.86
A 0.57 B 1
A 0.71 B 0
A 0.71 B 0.14
A 0.71 B 0.29
A 0.71 B 0.43
A 0.71 B 0.57
A 0.71 B 0.71
A 0.71 B 0.86
A 0.71 B 1
A 0.86 B 0
A 0.86 B 0.14
A 0.86 B 0.29
A 0.86 B 0.43
A 0.86 B 0.57
A 0.86 B 0.71
A 0.86 B 0.86
A 0.86 B 1
A 1 B 0
A 1 B 0.14
A 1 B 0.29
A 1 B 0.43
A 1 B 0.57
A 1 B 0.71
A 1 B 0.86
A 1 B 1
OD 600 nm [–]
0.2
0.4
0.6
0.8
0
5
101520
Time [h]

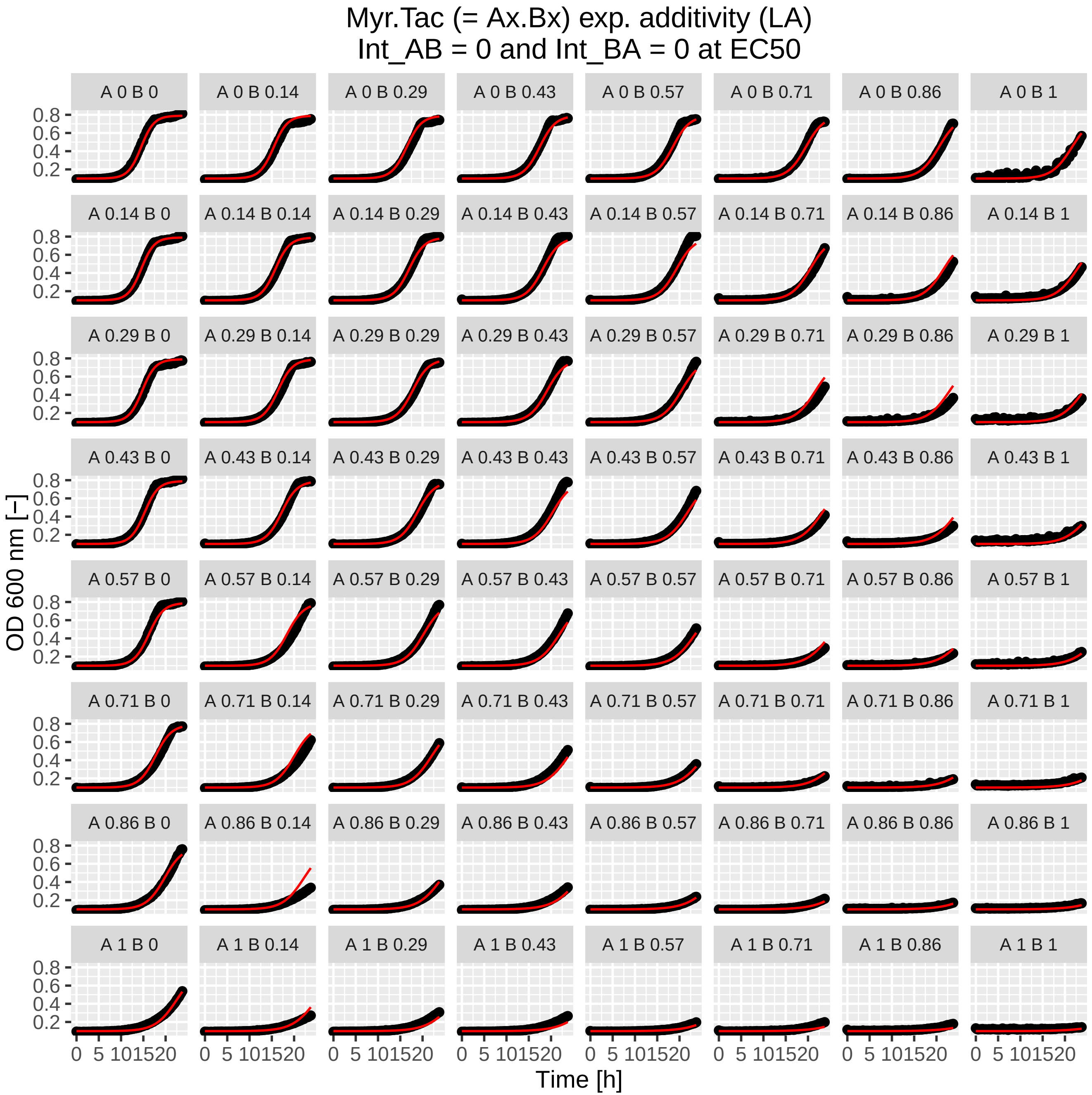
Myr.Tac (= Ax.Bx) exp. additivity (LA)
Int_AB = 0 and Int_BA = 0 at EC50
A 0 B 0
A 0 B 0.14
A 0 B 0.29
A 0 B 0.43
A 0 B 0.57
A 0 B 0.71
A 0 B 0.86
A 0 B 1
A 0.14 B 0
A 0.14 B 0.14
A 0.14 B 0.29
A 0.14 B 0.43
A 0.14 B 0.57
A 0.14 B 0.71
A 0.14 B 0.86
A 0.14 B 1
A 0.29 B 0
A 0.29 B 0.14
A 0.29 B 0.29
A 0.29 B 0.43
A 0.29 B 0.57
A 0.29 B 0.71
A 0.29 B 0.86
A 0.29 B 1
A 0.43 B 0
A 0.43 B 0.14
A 0.43 B 0.29
A 0.43 B 0.43
A 0.43 B 0.57
A 0.43 B 0.71
A 0.43 B 0.86
A 0.43 B 1
A 0.57 B 0
A 0.57 B 0.14
A 0.57 B 0.29
A 0.57 B 0.43
A 0.57 B 0.57
A 0.57 B 0.71
A 0.57 B 0.86
A 0.57 B 1
A 0.71 B 0
A 0.71 B 0.14
A 0.71 B 0.29
A 0.71 B 0.43
A 0.71 B 0.57
A 0.71 B 0.71
A 0.71 B 0.86
A 0.71 B 1
A 0.86 B 0
A 0.86 B 0.14
A 0.86 B 0.29
A 0.86 B 0.43
A 0.86 B 0.57
A 0.86 B 0.71
A 0.86 B 0.86
A 0.86 B 1
A 1 B 0
A 1 B 0.14
A 1 B 0.29
A 1 B 0.43
A 1 B 0.57
A 1 B 0.71
A 1 B 0.86
A 1 B 1
0.8
0.6
0.4
0.2
OD 600 nm [–]
0 5 101520
Time [h]

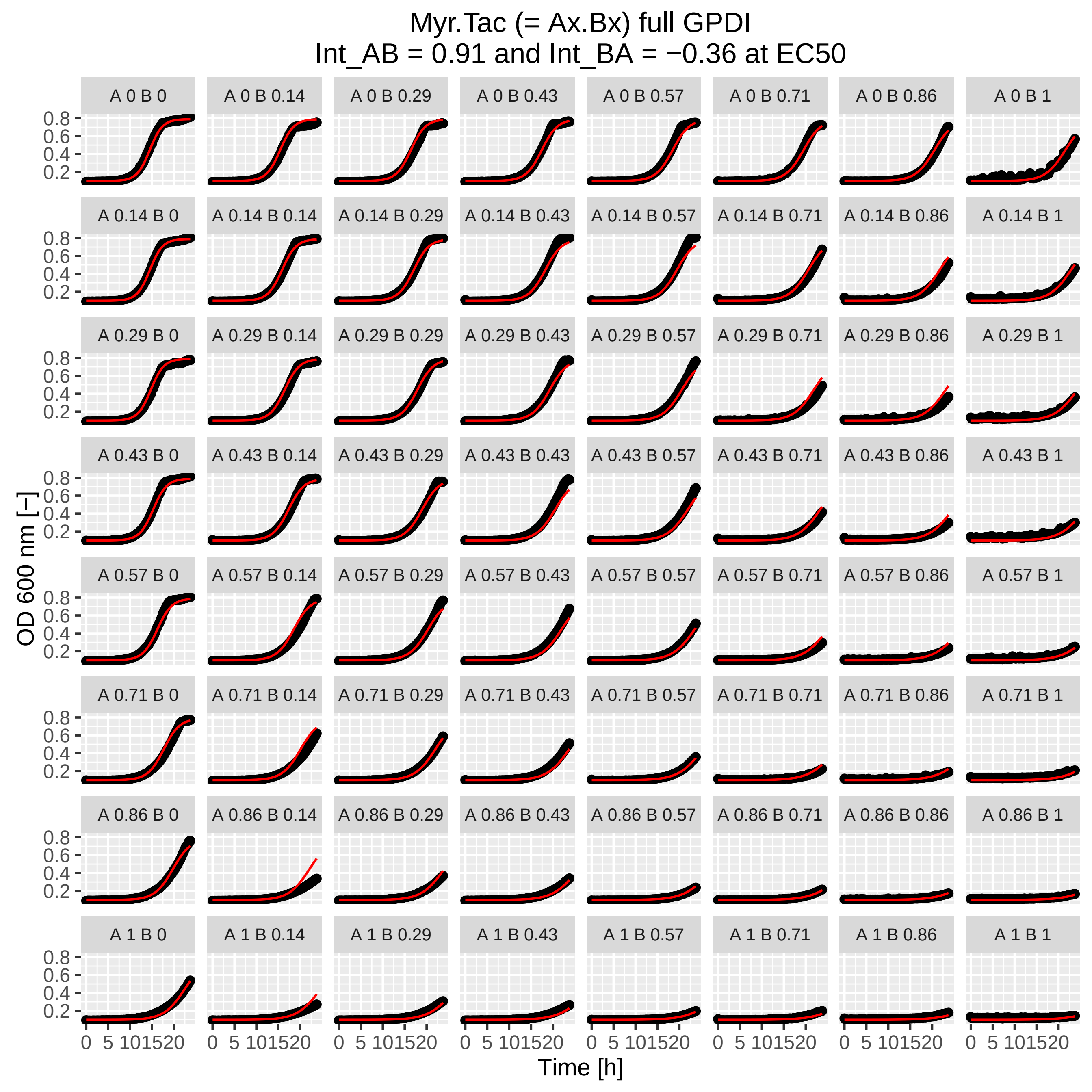
Myr.Tac (= Ax.Bx) full GPDI
Int_AB = 0.91 and Int_BA = −0.36 at EC50
OD 600 nm [−]
Time [h]

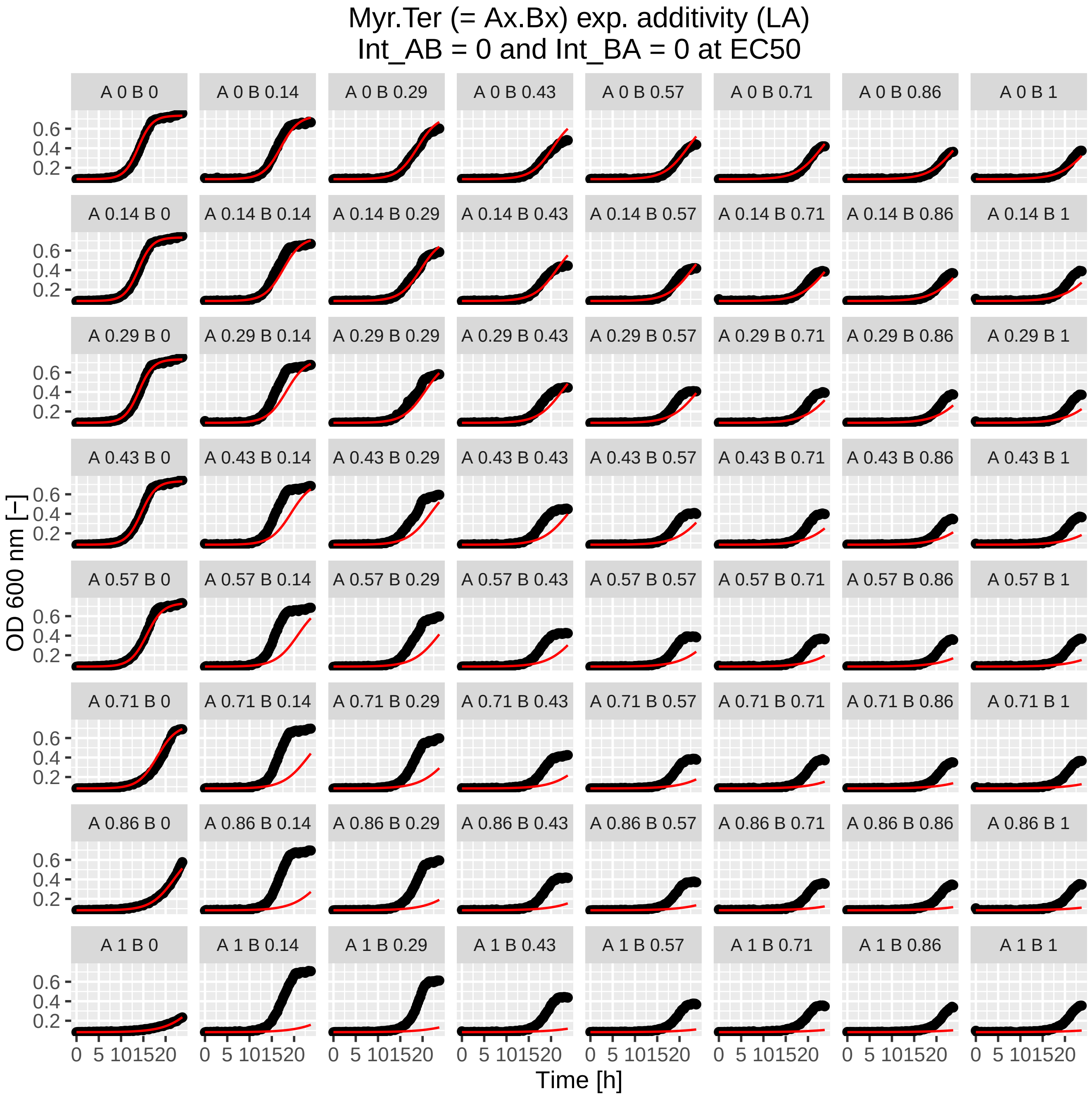

Myr.Ter (= Ax.Bx) exp. additivity (LA)
Int_AB = 0 and Int_BA = 0 at EC50
A 0 B 0
A 0 B 0.14
A 0 B 0.29
A 0 B 0.43
A 0 B 0.57
A 0 B 0.71
A 0 B 0.86
A 0 B 1
A 0.14 B 0
A 0.14 B 0.14
A 0.14 B 0.29
A 0.14 B 0.43
A 0.14 B 0.57
A 0.14 B 0.71
A 0.14 B 0.86
A 0.14 B 1
A 0.29 B 0
A 0.29 B 0.14
A 0.29 B 0.29
A 0.29 B 0.43
A 0.29 B 0.57
A 0.29 B 0.71
A 0.29 B 0.86
A 0.29 B 1
A 0.43 B 0
A 0.43 B 0.14
A 0.43 B 0.29
A 0.43 B 0.43
A 0.43 B 0.57
A 0.43 B 0.71
A 0.43 B 0.86
A 0.43 B 1
A 0.57 B 0
A 0.57 B 0.14
A 0.57 B 0.29
A 0.57 B 0.43
A 0.57 B 0.57
A 0.57 B 0.71
A 0.57 B 0.86
A 0.57 B 1
A 0.71 B 0
A 0.71 B 0.14
A 0.71 B 0.29
A 0.71 B 0.43
A 0.71 B 0.57
A 0.71 B 0.71
A 0.71 B 0.86
A 0.71 B 1
A 0.86 B 0
A 0.86 B 0.14
A 0.86 B 0.29
A 0.86 B 0.43
A 0.86 B 0.57
A 0.86 B 0.71
A 0.86 B 0.86
A 0.86 B 1
A 1 B 0
A 1 B 0.14
A 1 B 0.29
A 1 B 0.43
A 1 B 0.57
A 1 B 0.71
A 1 B 0.86
A 1 B 1
OD 600 nm [−]
0.2
0.4
0.6
0 5 101520
Time [h]

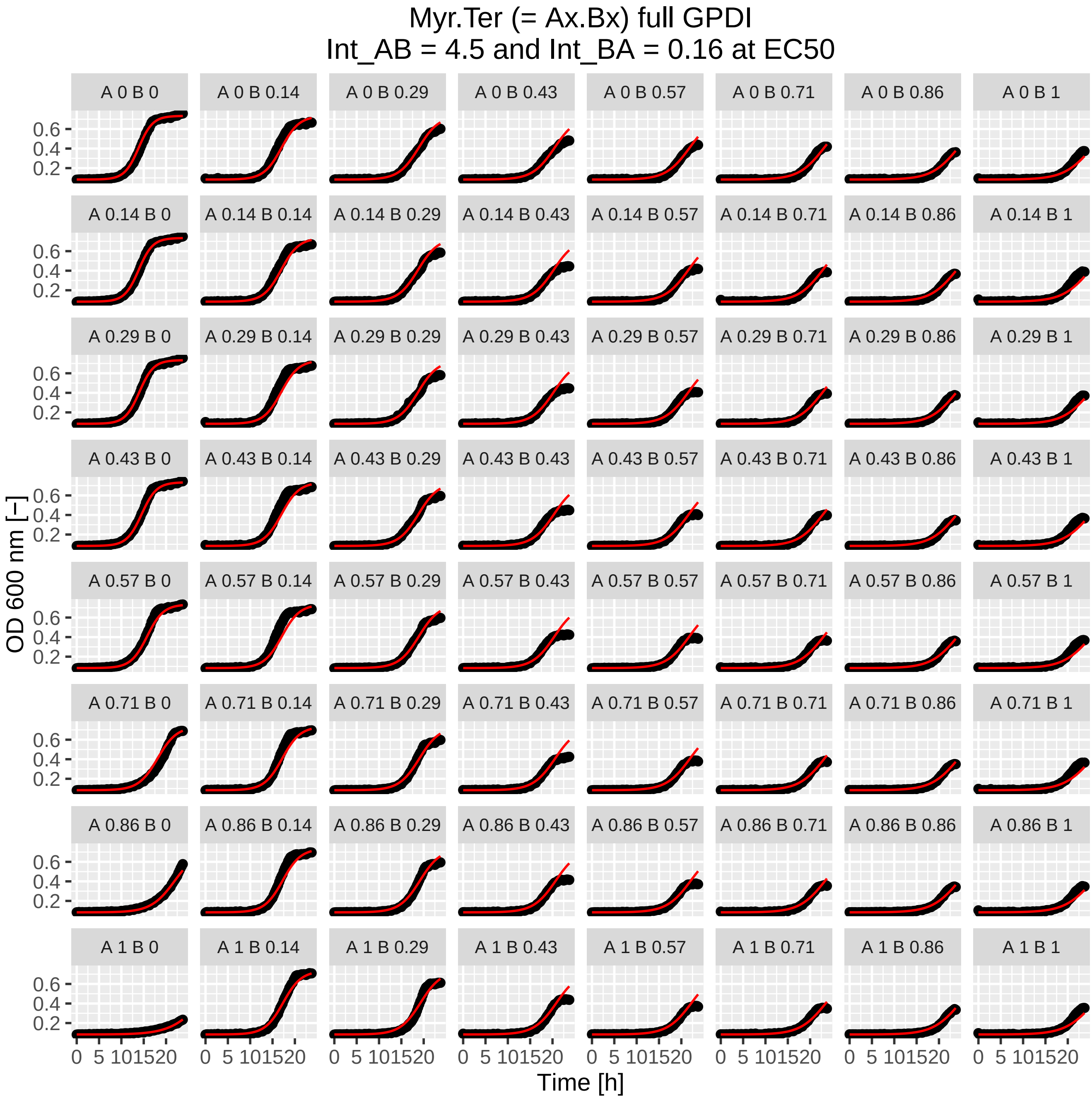

Myr.Ter (= Ax.Bx) full GPDI
Int_AB = 4.5 and Int_BA = 0.16 at EC50
OD 600 nm [−]
Time [h]
0 5 101520
0.2
0.4
0.6
A 0 B 0
A 0 B 0.14
A 0 B 0.29
A 0 B 0.43
A 0 B 0.57
A 0 B 0.71
A 0 B 0.86
A 0 B 1
A 0.14 B 0
A 0.14 B 0.14
A 0.14 B 0.29
A 0.14 B 0.43
A 0.14 B 0.57
A 0.14 B 0.71
A 0.14 B 0.86
A 0.14 B 1
A 0.29 B 0
A 0.29 B 0.14
A 0.29 B 0.29
A 0.29 B 0.43
A 0.29 B 0.57
A 0.29 B 0.71
A 0.29 B 0.86
A 0.29 B 1
A 0.43 B 0
A 0.43 B 0.14
A 0.43 B 0.29
A 0.43 B 0.43
A 0.43 B 0.57
A 0.43 B 0.71
A 0.43 B 0.86
A 0.43 B 1
A 0.57 B 0
A 0.57 B 0.14
A 0.57 B 0.29
A 0.57 B 0.43
A 0.57 B 0.57
A 0.57 B 0.71
A 0.57 B 0.86
A 0.57 B 1
A 0.71 B 0
A 0.71 B 0.14
A 0.71 B 0.29
A 0.71 B 0.43
A 0.71 B 0.57
A 0.71 B 0.71
A 0.71 B 0.86
A 0.71 B 1
A 0.86 B 0
A 0.86 B 0.14
A 0.86 B 0.29
A 0.86 B 0.43
A 0.86 B 0.57
A 0.86 B 0.71
A 0.86 B 0.86
A 0.86 B 1
A 1 B 0
A 1 B 0.14
A 1 B 0.29
A 1 B 0.43
A 1 B 0.57
A 1 B 0.71
A 1 B 0.86
A 1 B 1

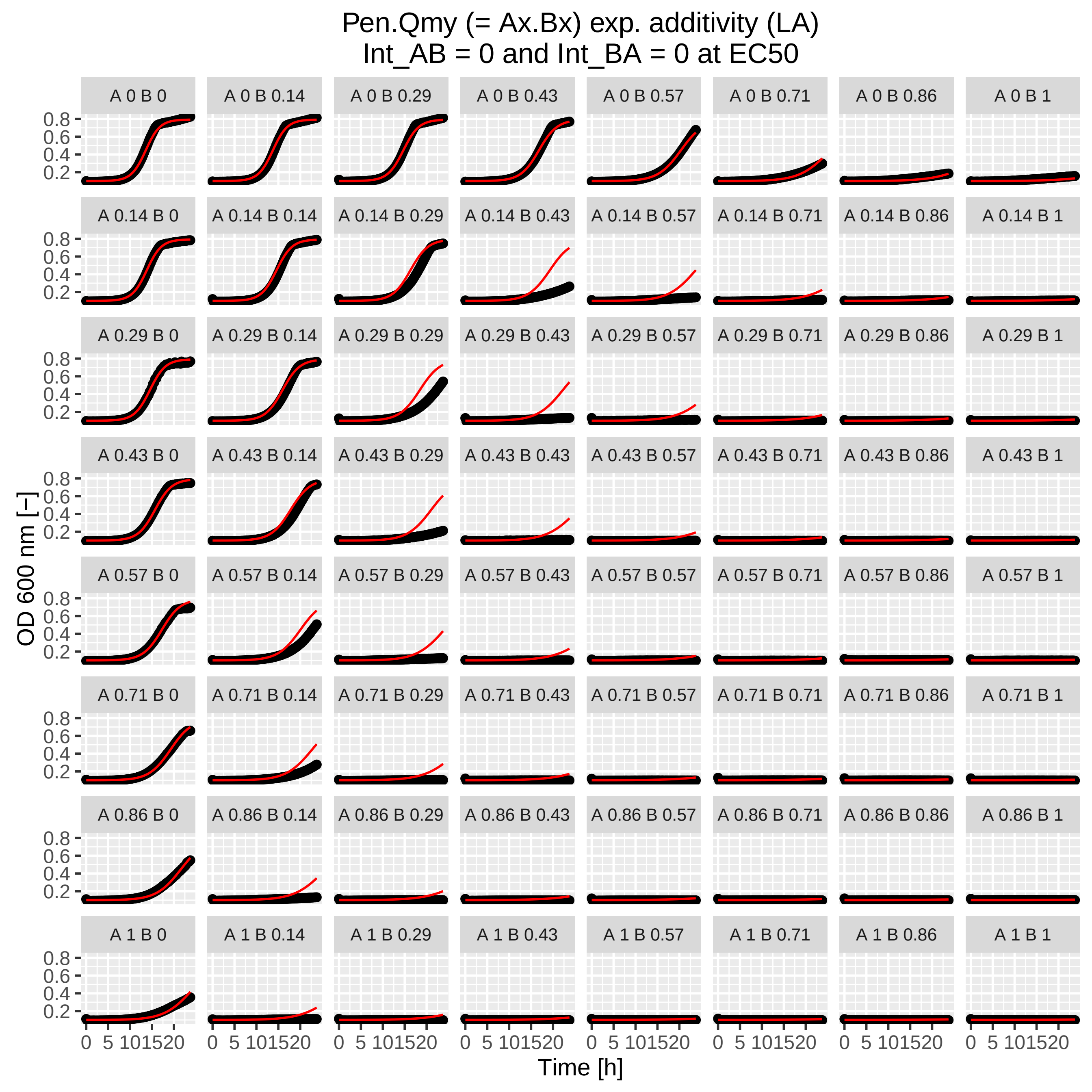
Pen.Qmy (= Ax.Bx) exp. additivity (LA)
Int_AB = 0 and Int_BA = 0 at EC50
OD 600 nm [–]
Time [h]
A 0 B 0
A 0 B 0.14
A 0 B 0.29
A 0 B 0.43
A 0 B 0.57
A 0 B 0.71
A 0 B 0.86
A 0 B 1
A 1 B 0
A 1 B 0.14
A 1 B 0.29
A 1 B 0.43
A 1 B 0.57
A 1 B 0.71
A 1 B 0.86
A 1 B 1
0.8
0.6
0.4
0.2
0 5 101520

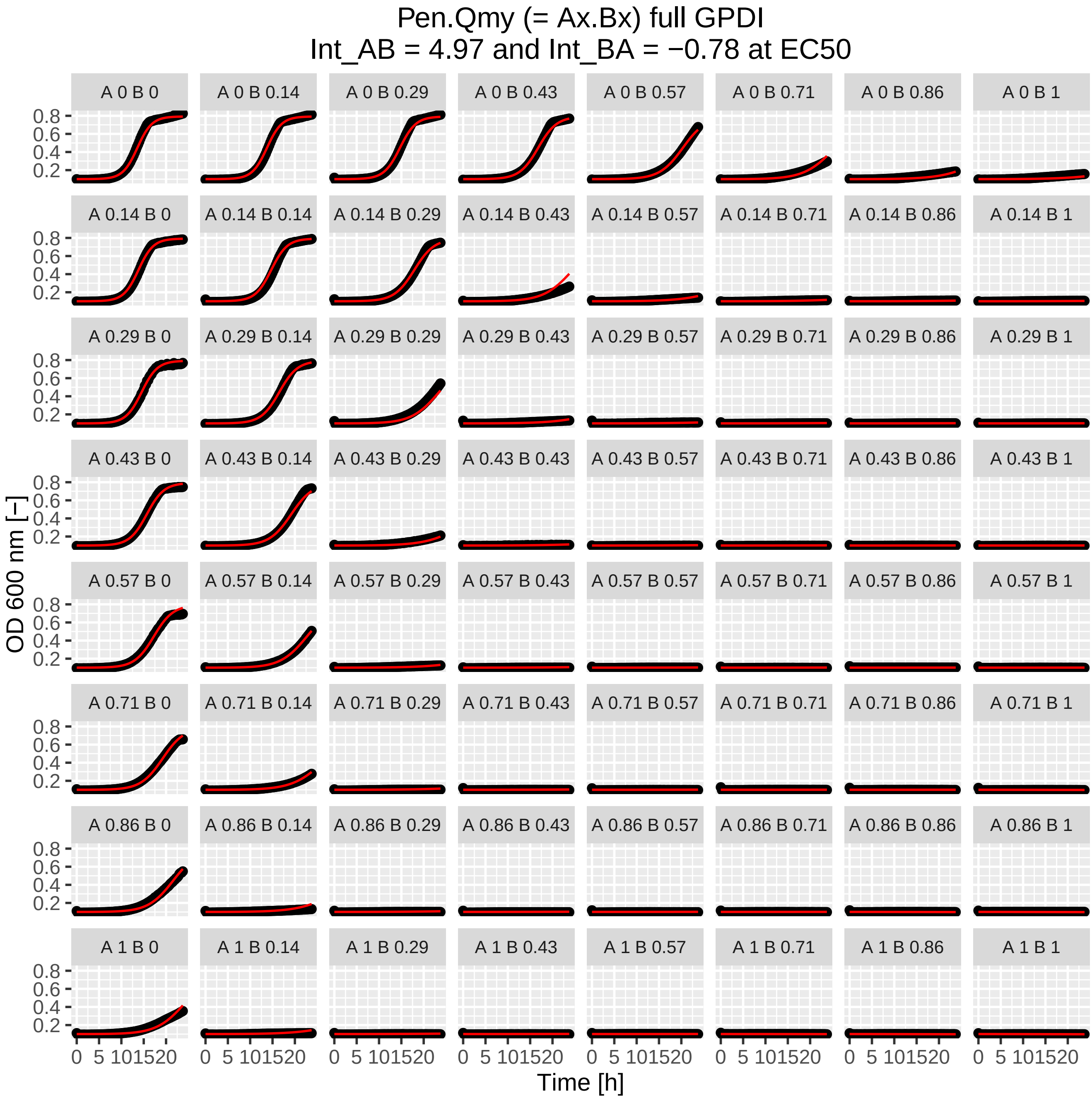
Pen.Qmy (= Ax.Bx) full GPDI
Int_AB = 4.97 and Int_BA = −0.78 at EC50
A 0 B 0
A 0 B 0.14
A 0 B 0.29
A 0 B 0.43
A 0 B 0.57
A 0 B 0.71
A 0 B 0.86
A 0 B 1
A 0.14 B 0
A 0.14 B 0.14
A 0.14 B 0.29
A 0.14 B 0.43
A 0.14 B 0.57
A 0.14 B 0.71
A 0.14 B 0.86
A 0.14 B 1
A 0.29 B 0
A 0.29 B 0.14
A 0.29 B 0.29
A 0.29 B 0.43
A 0.29 B 0.57
A 0.29 B 0.71
A 0.29 B 0.86
A 0.29 B 1
A 0.43 B 0
A 0.43 B 0.14
A 0.43 B 0.29
A 0.43 B 0.43
A 0.43 B 0.57
A 0.43 B 0.71
A 0.43 B 0.86
A 0.43 B 1
A 0.57 B 0
A 0.57 B 0.14
A 0.57 B 0.29
A 0.57 B 0.43
A 0.57 B 0.57
A 0.57 B 0.71
A 0.57 B 0.86
A 0.57 B 1
A 0.71 B 0
A 0.71 B 0.14
A 0.71 B 0.29
A 0.71 B 0.43
A 0.71 B 0.57
A 0.71 B 0.71
A 0.71 B 0.86
A 0.71 B 1
A 0.86 B 0
A 0.86 B 0.14
A 0.86 B 0.29
A 0.86 B 0.43
A 0.86 B 0.57
A 0.86 B 0.71
A 0.86 B 0.86
A 0.86 B 1
A 1 B 0
A 1 B 0.14
A 1 B 0.29
A 1 B 0.43
A 1 B 0.57
A 1 B 0.71
A 1 B 0.86
A 1 B 1
OD 600 nm [−]
0.2
0.4
0.6
0.8
0 5 101520
Time [h]

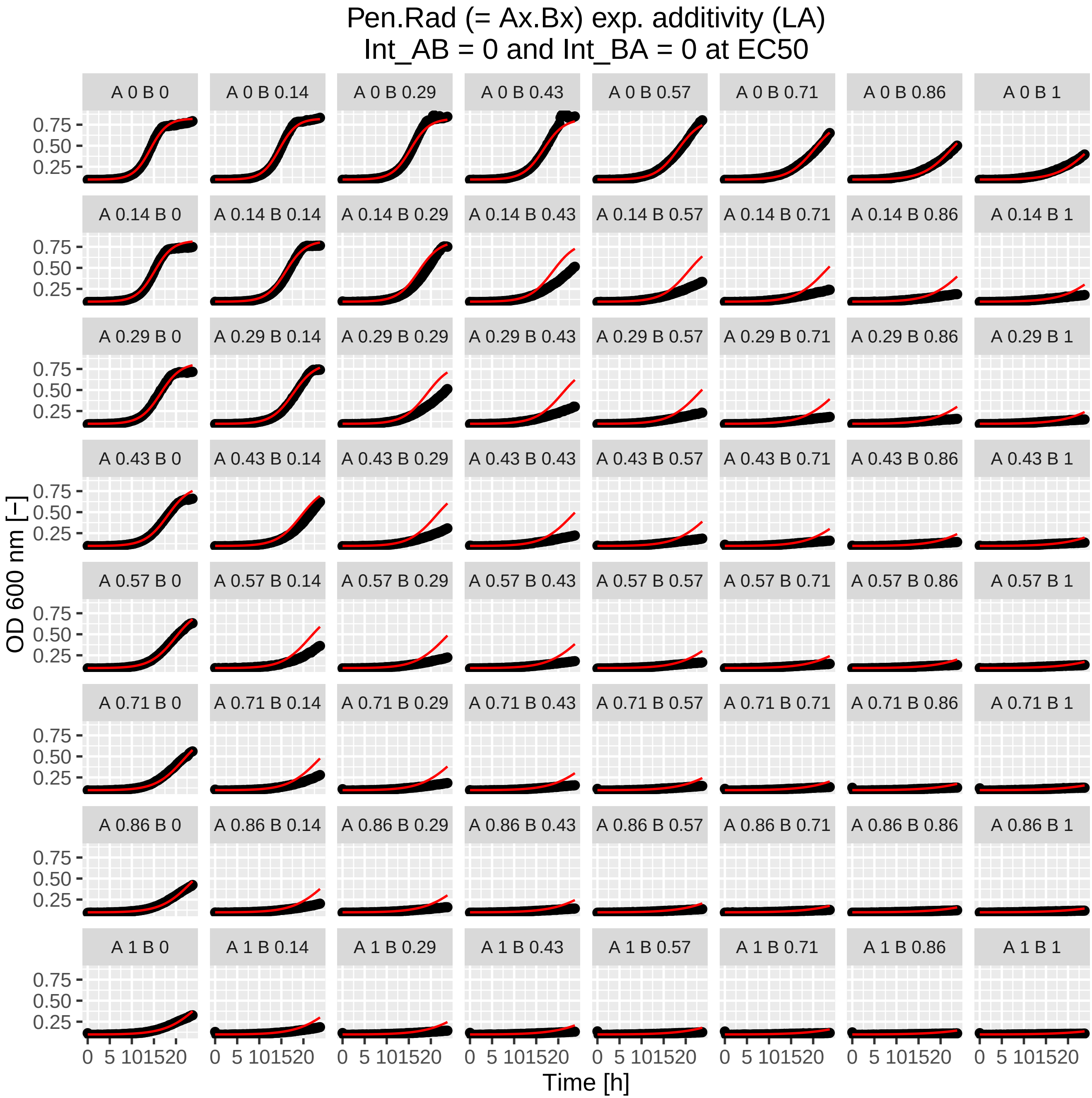

Pen.Rad (= Ax.Bx) exp. additivity (LA)
Int_AB = 0 and Int_BA = 0 at EC50
A 0 B 0
A 0 B 0.14
A 0 B 0.29
A 0 B 0.43
A 0 B 0.57
A 0 B 0.71
A 0 B 0.86
A 0 B 1
A 0.14 B 0
A 0.14 B 0.14
A 0.14 B 0.29
A 0.14 B 0.43
A 0.14 B 0.57
A 0.14 B 0.71
A 0.14 B 0.86
A 0.14 B 1
A 0.29 B 0
A 0.29 B 0.14
A 0.29 B 0.29
A 0.29 B 0.43
A 0.29 B 0.57
A 0.29 B 0.71
A 0.29 B 0.86
A 0.29 B 1
A 0.43 B 0
A 0.43 B 0.14
A 0.43 B 0.29
A 0.43 B 0.43
A 0.43 B 0.57
A 0.43 B 0.71
A 0.43 B 0.86
A 0.43 B 1
A 0.57 B 0
A 0.57 B 0.14
A 0.57 B 0.29
A 0.57 B 0.43
A 0.57 B 0.57
A 0.57 B 0.71
A 0.57 B 0.86
A 0.57 B 1
A 0.71 B 0
A 0.71 B 0.14
A 0.71 B 0.29
A 0.71 B 0.43
A 0.71 B 0.57
A 0.71 B 0.71
A 0.71 B 0.86
A 0.71 B 1
A 0.86 B 0
A 0.86 B 0.14
A 0.86 B 0.29
A 0.86 B 0.43
A 0.86 B 0.57
A 0.86 B 0.71
A 0.86 B 0.86
A 0.86 B 1
A 1 B 0
A 1 B 0.14
A 1 B 0.29
A 1 B 0.43
A 1 B 0.57
A 1 B 0.71
A 1 B 0.86
A 1 B 1
0.75
0.50
0.25
0 5 101520
OD 600 nm [–]
Time [h]

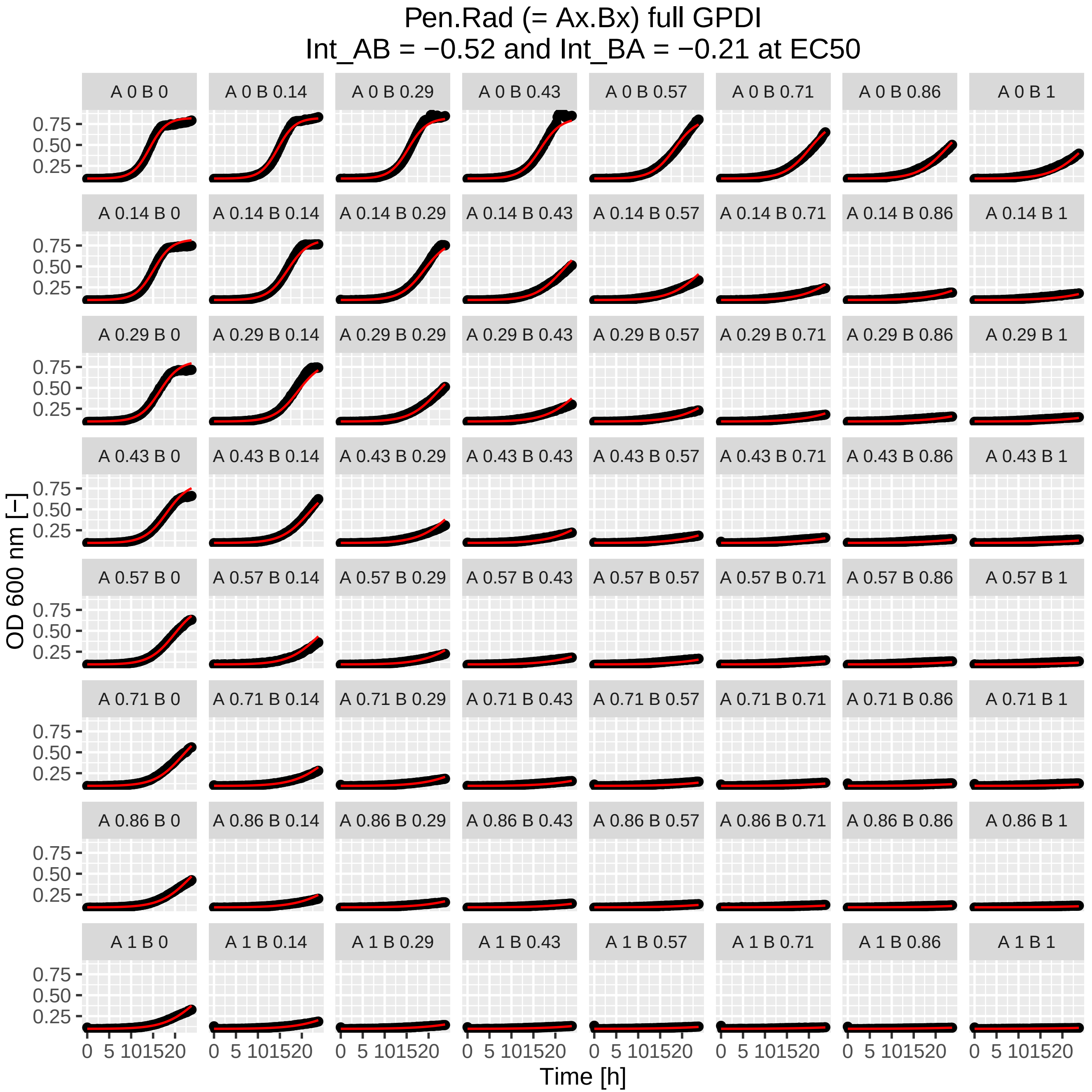
Pen.Rad (= Ax.Bx) full GPDI
Int_AB = −0.52 and Int_BA = −0.21 at EC50
A 0 B 0
A 0 B 0.14
A 0 B 0.29
A 0 B 0.43
A 0 B 0.57
A 0 B 0.71
A 0 B 0.86
A 0 B 1
A 0.14 B 0
A 0.14 B 0.14
A 0.14 B 0.29
A 0.14 B 0.43
A 0.14 B 0.57
A 0.14 B 0.71
A 0.14 B 0.86
A 0.14 B 1
A 0.29 B 0
A 0.29 B 0.14
A 0.29 B 0.29
A 0.29 B 0.43
A 0.29 B 0.57
A 0.29 B 0.71
A 0.29 B 0.86
A 0.29 B 1
A 0.43 B 0
A 0.43 B 0.14
A 0.43 B 0.29
A 0.43 B 0.43
A 0.43 B 0.57
A 0.43 B 0.71
A 0.43 B 0.86
A 0.43 B 1
A 0.57 B 0
A 0.57 B 0.14
A 0.57 B 0.29
A 0.57 B 0.43
A 0.57 B 0.57
A 0.57 B 0.71
A 0.57 B 0.86
A 0.57 B 1
A 0.71 B 0
A 0.71 B 0.14
A 0.71 B 0.29
A 0.71 B 0.43
A 0.71 B 0.57
A 0.71 B 0.71
A 0.71 B 0.86
A 0.71 B 1
A 0.86 B 0
A 0.86 B 0.14
A 0.86 B 0.29
A 0.86 B 0.43
A 0.86 B 0.57
A 0.86 B 0.71
A 0.86 B 0.86
A 0.86 B 1
A 1 B 0
A 1 B 0.14
A 1 B 0.29
A 1 B 0.43
A 1 B 0.57
A 1 B 0.71
A 1 B 0.86
A 1 B 1
OD 600 nm [−]
0.25
0.50
0.75
0 5 101520
Time [h]

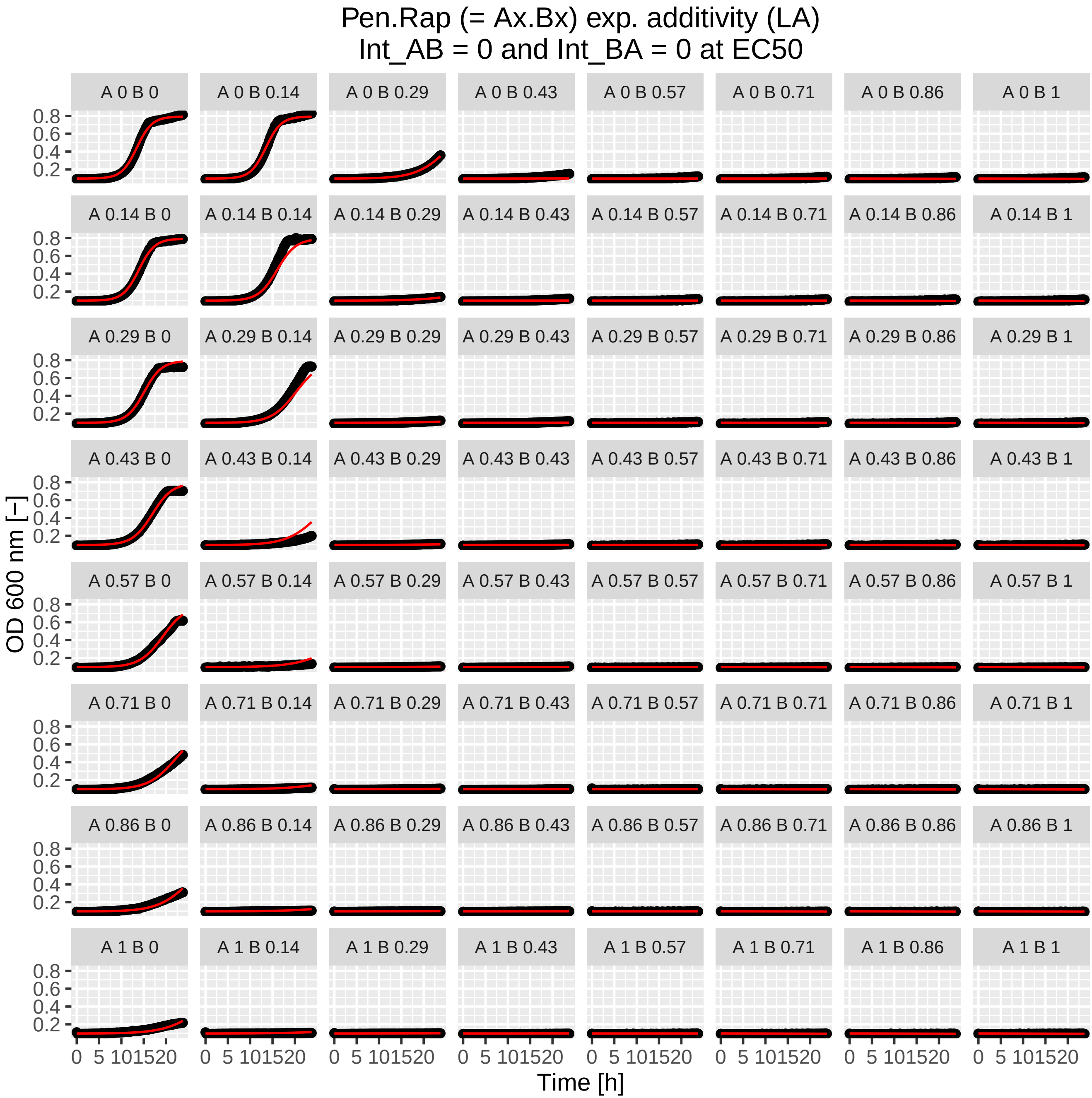

Pen.Rap (= Ax.Bx) exp. additivity (LA)
Int_AB = 0 and Int_BA = 0 at EC50
A 0 B 0
A 0 B 0.14
A 0 B 0.29
A 0 B 0.43
A 0 B 0.57
A 0 B 0.71
A 0 B 0.86
A 0 B 1
A 0.14 B 0
A 0.14 B 0.14
A 0.14 B 0.29
A 0.14 B 0.43
A 0.14 B 0.57
A 0.14 B 0.71
A 0.14 B 0.86
A 0.14 B 1
A 0.29 B 0
A 0.29 B 0.14
A 0.29 B 0.29
A 0.29 B 0.43
A 0.29 B 0.57
A 0.29 B 0.71
A 0.29 B 0.86
A 0.29 B 1
A 0.43 B 0
A 0.43 B 0.14
A 0.43 B 0.29
A 0.43 B 0.43
A 0.43 B 0.57
A 0.43 B 0.71
A 0.43 B 0.86
A 0.43 B 1
A 0.57 B 0
A 0.57 B 0.14
A 0.57 B 0.29
A 0.57 B 0.43
A 0.57 B 0.57
A 0.57 B 0.71
A 0.57 B 0.86
A 0.57 B 1
A 0.71 B 0
A 0.71 B 0.14
A 0.71 B 0.29
A 0.71 B 0.43
A 0.71 B 0.57
A 0.71 B 0.71
A 0.71 B 0.86
A 0.71 B 1
A 0.86 B 0
A 0.86 B 0.14
A 0.86 B 0.29
A 0.86 B 0.43
A 0.86 B 0.57
A 0.86 B 0.71
A 0.86 B 0.86
A 0.86 B 1
A 1 B 0
A 1 B 0.14
A 1 B 0.29
A 1 B 0.43
A 1 B 0.57
A 1 B 0.71
A 1 B 0.86
A 1 B 1
OD 600 nm [−]
0.2
0.4
0.6
0.8
0
5
10
15
20
Time [h]

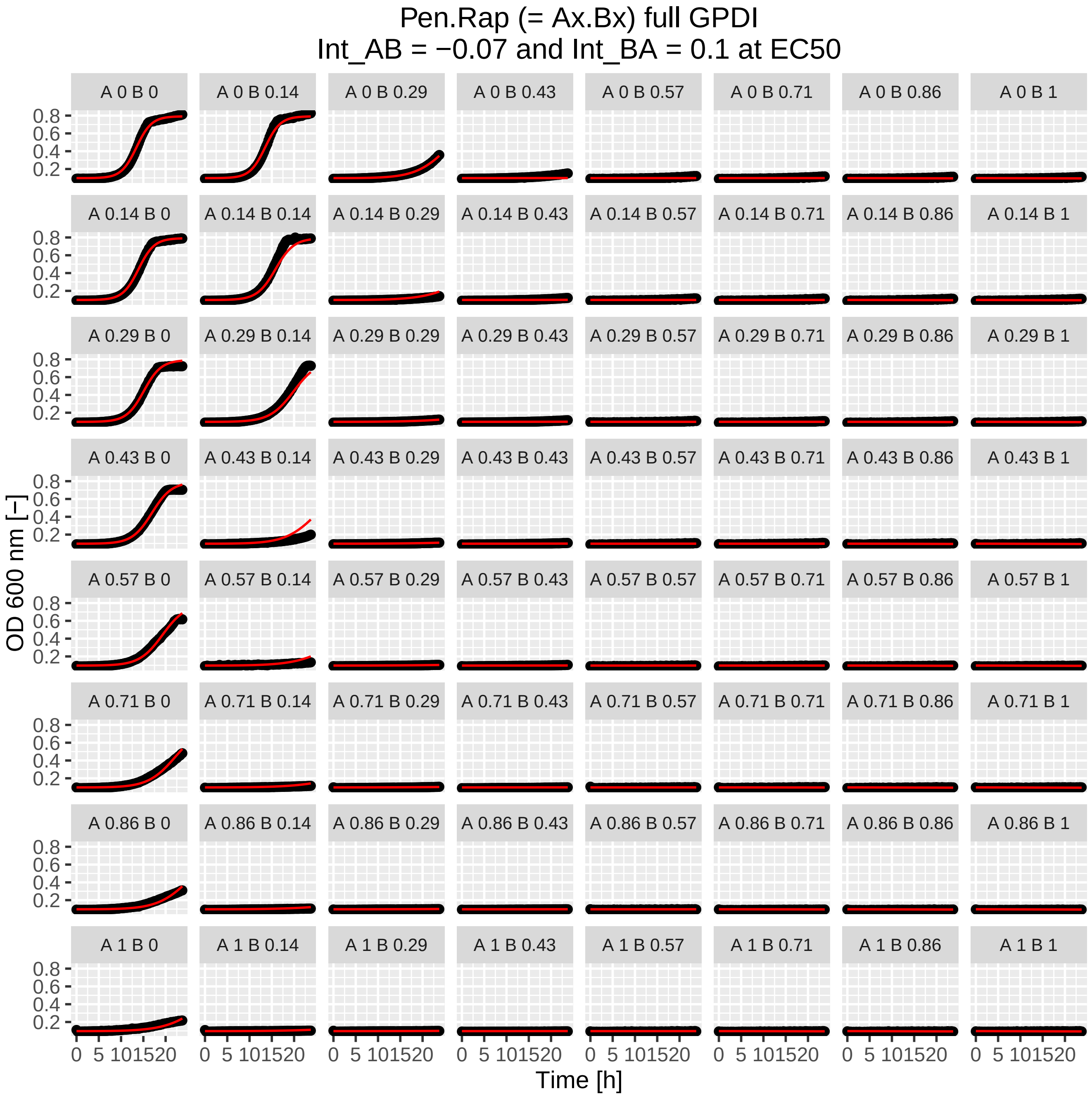

Pen.Rap (= Ax.Bx) full GPDI
Int_AB = −0.07 and Int_BA = 0.1 at EC50
A 0 B 0
A 0 B 0.14
A 0 B 0.29
A 0 B 0.43
A 0 B 0.57
A 0 B 0.71
A 0 B 0.86
A 0 B 1
A 0.14 B 0
A 0.14 B 0.14
A 0.14 B 0.29
A 0.14 B 0.43
A 0.14 B 0.57
A 0.14 B 0.71
A 0.14 B 0.86
A 0.14 B 1
A 0.29 B 0
A 0.29 B 0.14
A 0.29 B 0.29
A 0.29 B 0.43
A 0.29 B 0.57
A 0.29 B 0.71
A 0.29 B 0.86
A 0.29 B 1
A 0.43 B 0
A 0.43 B 0.14
A 0.43 B 0.29
A 0.43 B 0.43
A 0.43 B 0.57
A 0.43 B 0.71
A 0.43 B 0.86
A 0.43 B 1
A 0.57 B 0
A 0.57 B 0.14
A 0.57 B 0.29
A 0.57 B 0.43
A 0.57 B 0.57
A 0.57 B 0.71
A 0.57 B 0.86
A 0.57 B 1
A 0.71 B 0
A 0.71 B 0.14
A 0.71 B 0.29
A 0.71 B 0.43
A 0.71 B 0.57
A 0.71 B 0.71
A 0.71 B 0.86
A 0.71 B 1
A 0.86 B 0
A 0.86 B 0.14
A 0.86 B 0.29
A 0.86 B 0.43
A 0.86 B 0.57
A 0.86 B 0.71
A 0.86 B 0.86
A 0.86 B 1
A 1 B 0
A 1 B 0.14
A 1 B 0.29
A 1 B 0.43
A 1 B 0.57
A 1 B 0.71
A 1 B 0.86
A 1 B 1
OD 600 nm [−]
0.8
0.6
0.4
0.2
0 5 101520
Time [h]

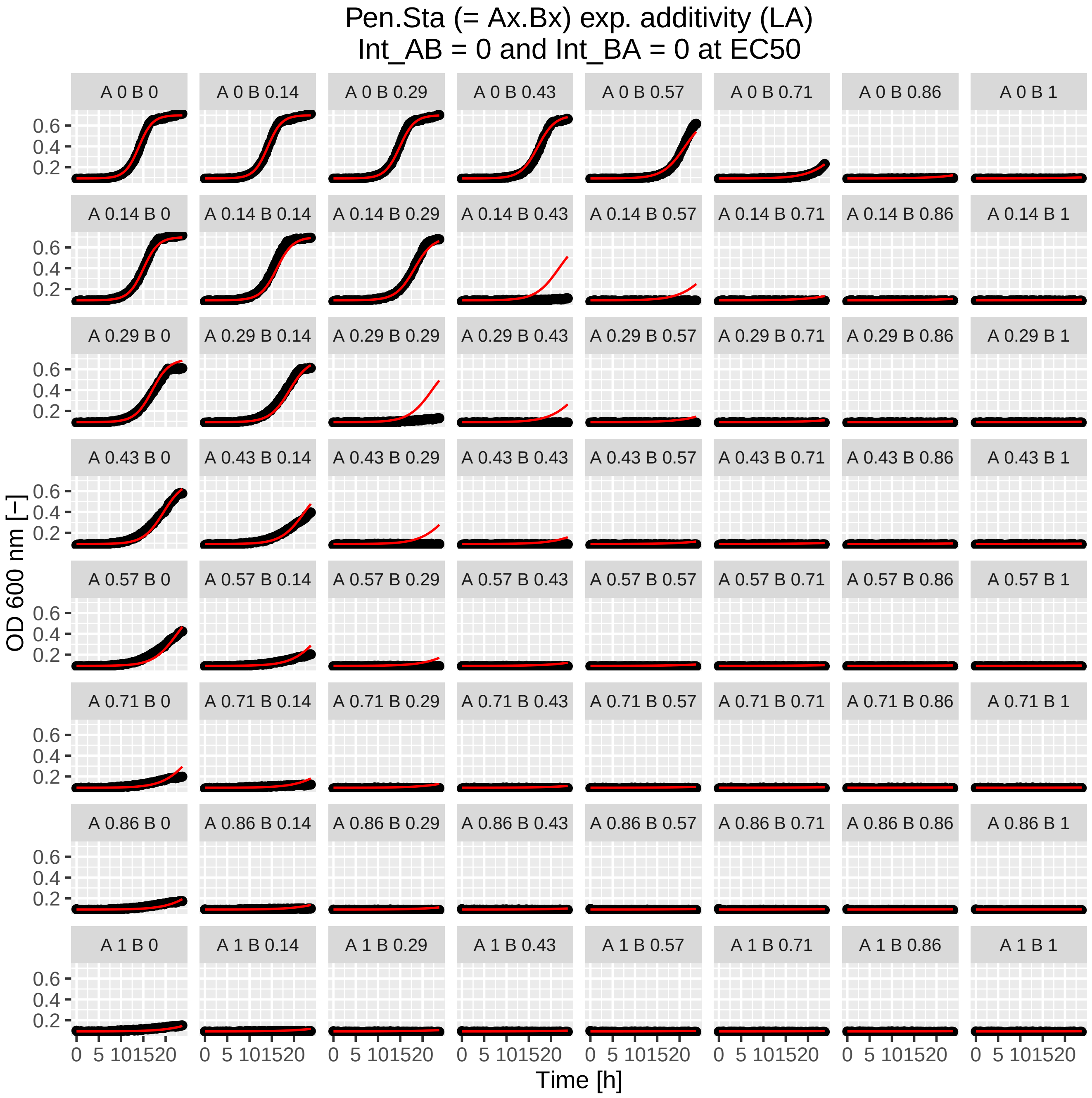

Pen.Sta (= Ax.Bx) exp. additivity (LA)
Int_AB = 0 and Int_BA = 0 at EC50
OD 600 nm [–]
Time [h]
A 0 B 0
A 0 B 0.14
A 0 B 0.29
A 0 B 0.43
A 0 B 0.57
A 0 B 0.71
A 0 B 0.86
A 0 B 1
A 0.14 B 0
A 0.14 B 0.14
A 0.14 B 0.29
A 0.14 B 0.43
A 0.14 B 0.57
A 0.14 B 0.71
A 0.14 B 0.86
A 0.14 B 1
A 0.29 B 0
A 0.29 B 0.14
A 0.29 B 0.29
A 0.29 B 0.43
A 0.29 B 0.57
A 0.29 B 0.71
A 0.29 B 0.86
A 0.29 B 1
A 0.43 B 0
A 0.43 B 0.14
A 0.43 B 0.29
A 0.43 B 0.43
A 0.43 B 0.57
A 0.43 B 0.71
A 0.43 B 0.86
A 0.43 B 1
A 0.57 B 0
A 0.57 B 0.14
A 0.57 B 0.29
A 0.57 B 0.43
A 0.57 B 0.57
A 0.57 B 0.71
A 0.57 B 0.86
A 0.57 B 1
A 0.71 B 0
A 0.71 B 0.14
A 0.71 B 0.29
A 0.71 B 0.43
A 0.71 B 0.57
A 0.71 B 0.71
A 0.71 B 0.86
A 0.71 B 1
A 0.86 B 0
A 0.86 B 0.14
A 0.86 B 0.29
A 0.86 B 0.43
A 0.86 B 0.57
A 0.86 B 0.71
A 0.86 B 0.86
A 0.86 B 1
A 1 B 0
A 1 B 0.14
A 1 B 0.29
A 1 B 0.43
A 1 B 0.57
A 1 B 0.71
A 1 B 0.86
A 1 B 1
0.6
0.4
0.2
0 5 101520

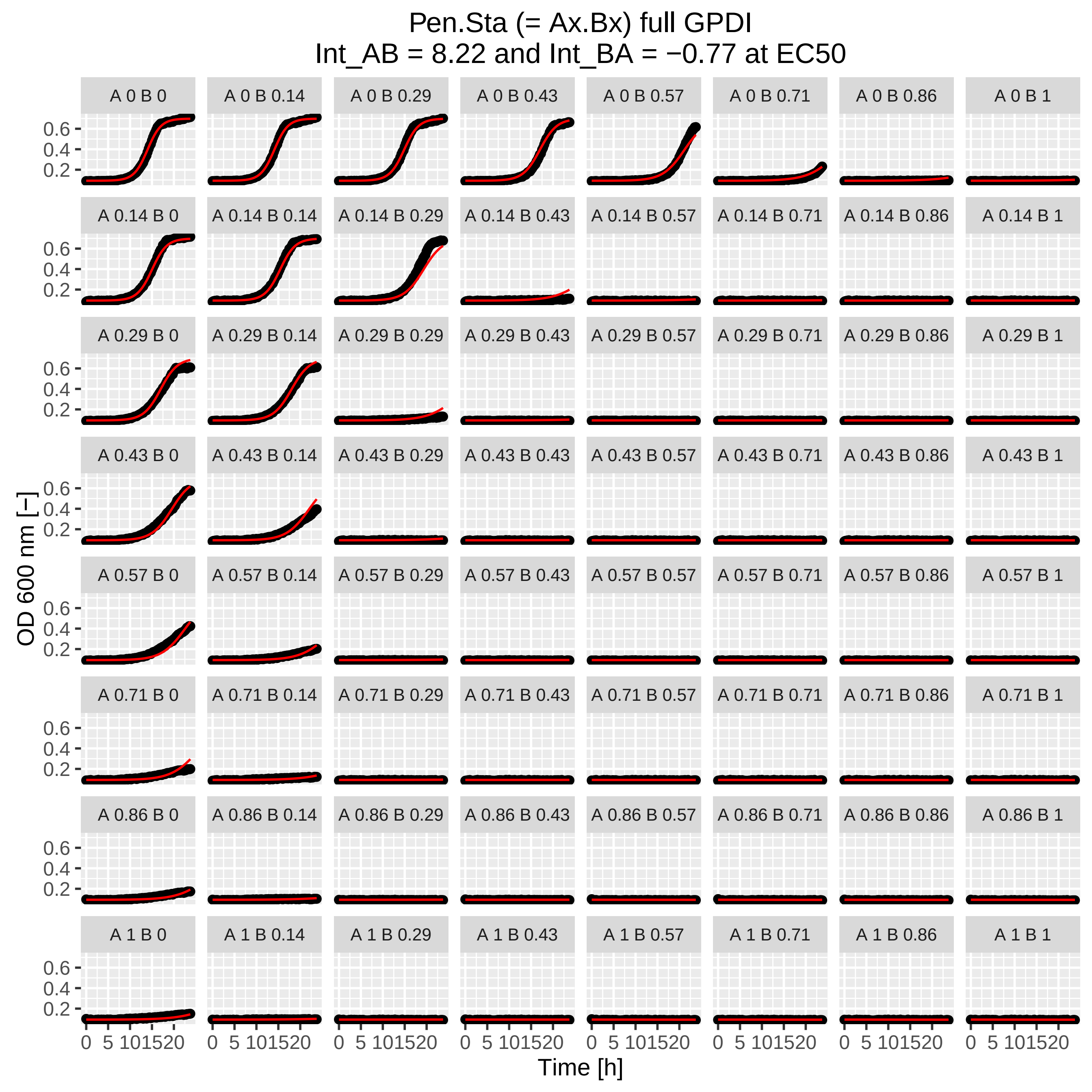
Pen.Sta (= Ax.Bx) full GPDI
Int_AB = 8.22 and Int_BA = −0.77 at EC50
OD 600 nm [–]
Time [h]
0.2
0.4
0.6
0 5 101520
A 0 B 0
A 0 B 0.14
A 0 B 0.29
A 0 B 0.43
A 0 B 0.57
A 0 B 0.71
A 0 B 0.86
A 0 B 1
A 0.14 B 0
A 0.14 B 0.14
A 0.14 B 0.29
A 0.14 B 0.43
A 0.14 B 0.57
A 0.14 B 0.71
A 0.14 B 0.86
A 0.14 B 1
A 0.29 B 0
A 0.29 B 0.14
A 0.29 B 0.29
A 0.29 B 0.43
A 0.29 B 0.57
A 0.29 B 0.71
A 0.29 B 0.86
A 0.29 B 1
A 0.43 B 0
A 0.43 B 0.14
A 0.43 B 0.29
A 0.43 B 0.43
A 0.43 B 0.57
A 0.43 B 0.71
A 0.43 B 0.86
A 0.43 B 1
A 0.57 B 0
A 0.57 B 0.14
A 0.57 B 0.29
A 0.57 B 0.43
A 0.57 B 0.57
A 0.57 B 0.71
A 0.57 B 0.86
A 0.57 B 1
A 0.71 B 0
A 0.71 B 0.14
A 0.71 B 0.29
A 0.71 B 0.43
A 0.71 B 0.57
A 0.71 B 0.71
A 0.71 B 0.86
A 0.71 B 1
A 0.86 B 0
A 0.86 B 0.14
A 0.86 B 0.29
A 0.86 B 0.43
A 0.86 B 0.57
A 0.86 B 0.71
A 0.86 B 0.86
A 0.86 B 1
A 1 B 0
A 1 B 0.14
A 1 B 0.29
A 1 B 0.43
A 1 B 0.57
A 1 B 0.71
A 1 B 0.86
A 1 B 1

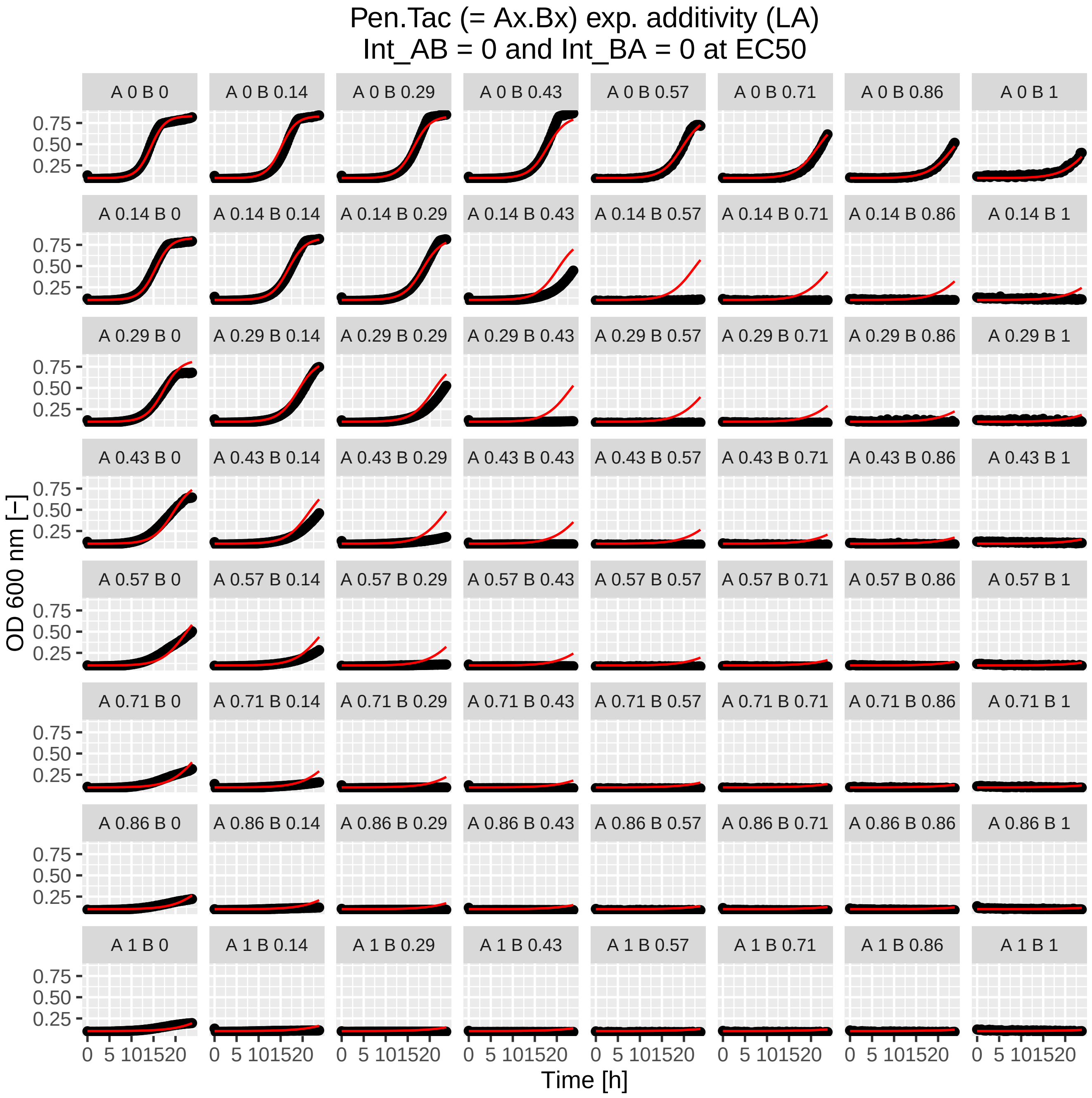

Pen.Tac (= Ax.Bx) exp. additivity (LA)
Int_AB = 0 and Int_BA = 0 at EC50
OD 600 nm [–]
Time [h]
0.75
0.50
0.25
0 5 101520
A 0 B 0
A 0 B 0.14
A 0 B 0.29
A 0 B 0.43
A 0 B 0.57
A 0 B 0.71
A 0 B 0.86
A 0 B 1
A 0.14 B 0
A 0.14 B 0.14
A 0.14 B 0.29
A 0.14 B 0.43
A 0.14 B 0.57
A 0.14 B 0.71
A 0.14 B 0.86
A 0.14 B 1
A 0.29 B 0
A 0.29 B 0.14
A 0.29 B 0.29
A 0.29 B 0.43
A 0.29 B 0.57
A 0.29 B 0.71
A 0.29 B 0.86
A 0.29 B 1
A 0.43 B 0
A 0.43 B 0.14
A 0.43 B 0.29
A 0.43 B 0.43
A 0.43 B 0.57
A 0.43 B 0.71
A 0.43 B 0.86
A 0.43 B 1
A 0.57 B 0
A 0.57 B 0.14
A 0.57 B 0.29
A 0.57 B 0.43
A 0.57 B 0.57
A 0.57 B 0.71
A 0.57 B 0.86
A 0.57 B 1
A 0.71 B 0
A 0.71 B 0.14
A 0.71 B 0.29
A 0.71 B 0.43
A 0.71 B 0.57
A 0.71 B 0.71
A 0.71 B 0.86
A 0.71 B 1
A 0.86 B 0
A 0.86 B 0.14
A 0.86 B 0.29
A 0.86 B 0.43
A 0.86 B 0.57
A 0.86 B 0.71
A 0.86 B 0.86
A 0.86 B 1
A 1 B 0
A 1 B 0.14
A 1 B 0.29
A 1 B 0.43
A 1 B 0.57
A 1 B 0.71
A 1 B 0.86
A 1 B 1

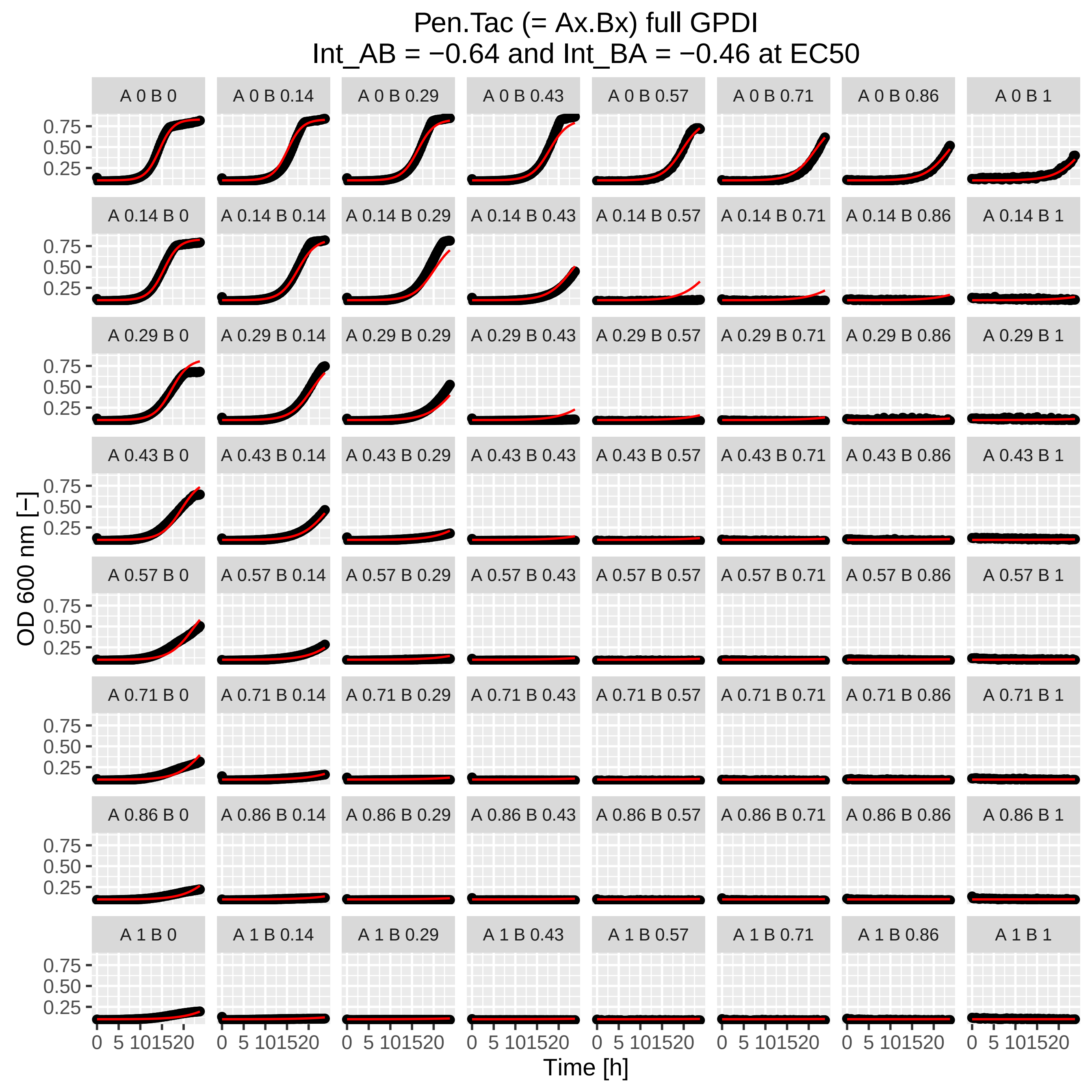

Pen.Tac (= Ax.Bx) full GPDI
Int_AB = −0.64 and Int_BA = −0.46 at EC50
A 0 B 0
A 0 B 0.14
A 0 B 0.29
A 0 B 0.43
A 0 B 0.57
A 0 B 0.71
A 0 B 0.86
A 0 B 1
A 0.14 B 0
A 0.14 B 0.14
A 0.14 B 0.29
A 0.14 B 0.43
A 0.14 B 0.57
A 0.14 B 0.71
A 0.14 B 0.86
A 0.14 B 1
A 0.29 B 0
A 0.29 B 0.14
A 0.29 B 0.29
A 0.29 B 0.43
A 0.29 B 0.57
A 0.29 B 0.71
A 0.29 B 0.86
A 0.29 B 1
A 0.43 B 0
A 0.43 B 0.14
A 0.43 B 0.29
A 0.43 B 0.43
A 0.43 B 0.57
A 0.43 B 0.71
A 0.43 B 0.86
A 0.43 B 1
A 0.57 B 0
A 0.57 B 0.14
A 0.57 B 0.29
A 0.57 B 0.43
A 0.57 B 0.57
A 0.57 B 0.71
A 0.57 B 0.86
A 0.57 B 1
A 0.71 B 0
A 0.71 B 0.14
A 0.71 B 0.29
A 0.71 B 0.43
A 0.71 B 0.57
A 0.71 B 0.71
A 0.71 B 0.86
A 0.71 B 1
A 0.86 B 0
A 0.86 B 0.14
A 0.86 B 0.29
A 0.86 B 0.43
A 0.86 B 0.57
A 0.86 B 0.71
A 0.86 B 0.86
A 0.86 B 1
A 1 B 0
A 1 B 0.14
A 1 B 0.29
A 1 B 0.43
A 1 B 0.57
A 1 B 0.71
A 1 B 0.86
A 1 B 1
0.75
0.50
0.25
OD 600 nm [–]
0 5 101520
Time [h]

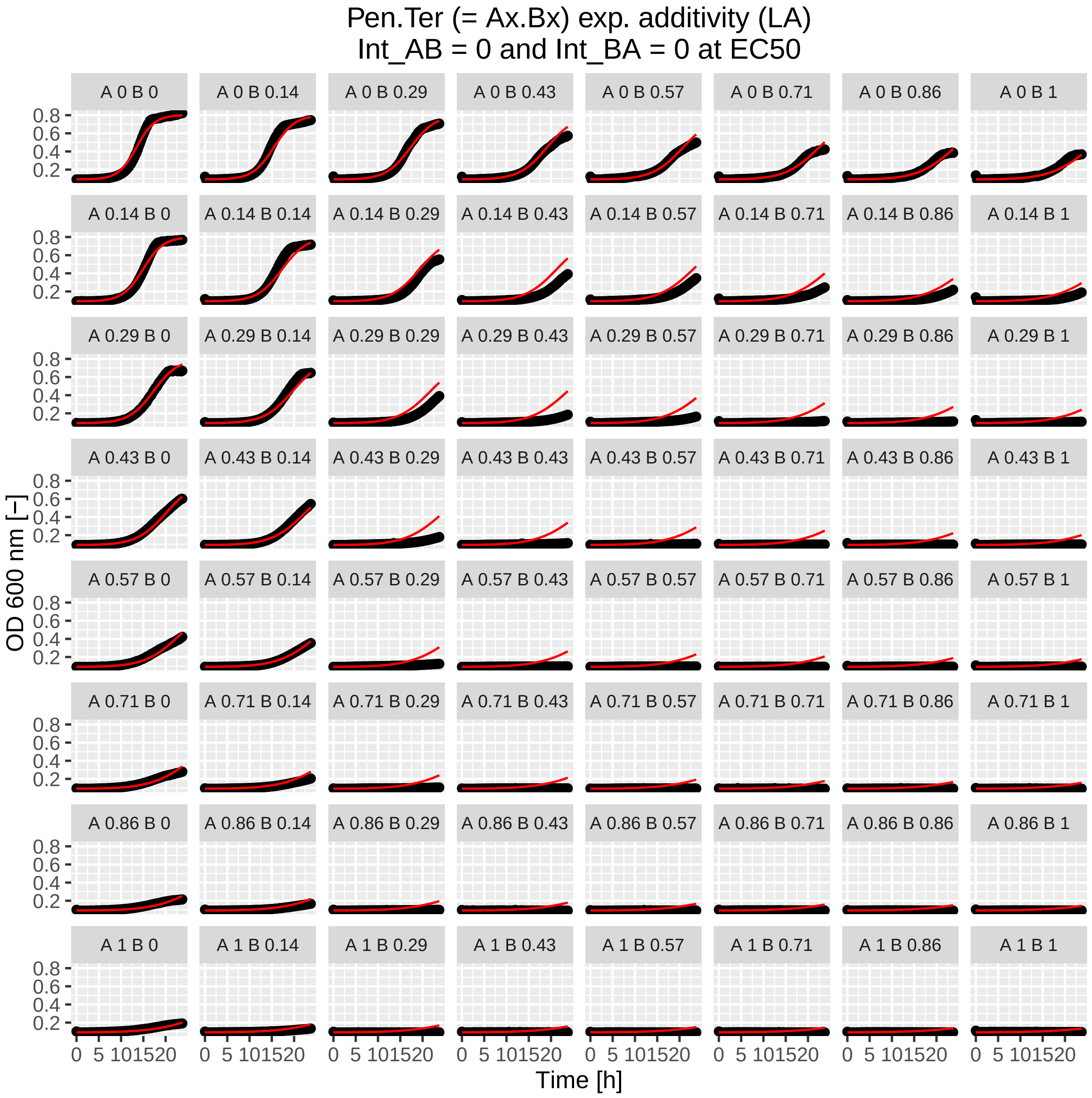

Pen.Ter (= Ax.Bx) exp. additivity (LA)
Int_AB = 0 and Int_BA = 0 at EC50
A 0 B 0
A 0 B 0.14
A 0 B 0.29
A 0 B 0.43
A 0 B 0.57
A 0 B 0.71
A 0 B 0.86
A 0 B 1
A 0.14 B 0
A 0.14 B 0.14
A 0.14 B 0.29
A 0.14 B 0.43
A 0.14 B 0.57
A 0.14 B 0.71
A 0.14 B 0.86
A 0.14 B 1
A 0.29 B 0
A 0.29 B 0.14
A 0.29 B 0.29
A 0.29 B 0.43
A 0.29 B 0.57
A 0.29 B 0.71
A 0.29 B 0.86
A 0.29 B 1
A 0.43 B 0
A 0.43 B 0.14
A 0.43 B 0.29
A 0.43 B 0.43
A 0.43 B 0.57
A 0.43 B 0.71
A 0.43 B 0.86
A 0.43 B 1
A 0.57 B 0
A 0.57 B 0.14
A 0.57 B 0.29
A 0.57 B 0.43
A 0.57 B 0.57
A 0.57 B 0.71
A 0.57 B 0.86
A 0.57 B 1
A 0.71 B 0
A 0.71 B 0.14
A 0.71 B 0.29
A 0.71 B 0.43
A 0.71 B 0.57
A 0.71 B 0.71
A 0.71 B 0.86
A 0.71 B 1
A 0.86 B 0
A 0.86 B 0.14
A 0.86 B 0.29
A 0.86 B 0.43
A 0.86 B 0.57
A 0.86 B 0.71
A 0.86 B 0.86
A 0.86 B 1
A 1 B 0
A 1 B 0.14
A 1 B 0.29
A 1 B 0.43
A 1 B 0.57
A 1 B 0.71
A 1 B 0.86
A 1 B 1
OD 600 nm [–]
0.8
0.6
0.4
0.2
0 5 101520
Time [h]

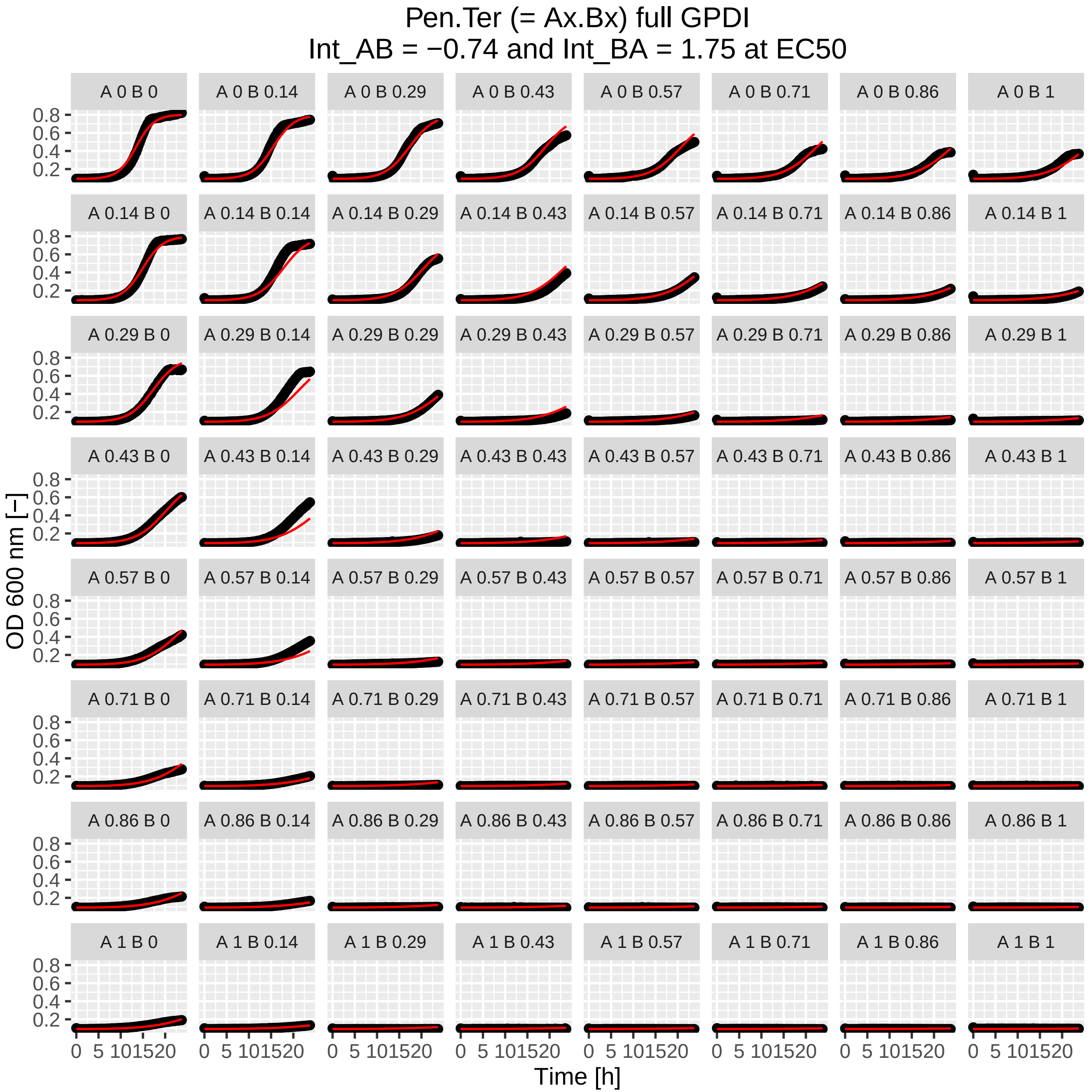
Pen.Ter (= Ax.Bx) full GPDI
Int_AB = −0.74 and Int_BA = 1.75 at EC50
A 0 B 0
A 0 B 0.14
A 0 B 0.29
A 0 B 0.43
A 0 B 0.57
A 0 B 0.71
A 0 B 0.86
A 0 B 1
A 0.14 B 0
A 0.14 B 0.14
A 0.14 B 0.29
A 0.14 B 0.43
A 0.14 B 0.57
A 0.14 B 0.71
A 0.14 B 0.86
A 0.14 B 1
A 0.29 B 0
A 0.29 B 0.14
A 0.29 B 0.29
A 0.29 B 0.43
A 0.29 B 0.57
A 0.29 B 0.71
A 0.29 B 0.86
A 0.29 B 1
A 0.43 B 0
A 0.43 B 0.14
A 0.43 B 0.29
A 0.43 B 0.43
A 0.43 B 0.57
A 0.43 B 0.71
A 0.43 B 0.86
A 0.43 B 1
A 0.57 B 0
A 0.57 B 0.14
A 0.57 B 0.29
A 0.57 B 0.43
A 0.57 B 0.57
A 0.57 B 0.71
A 0.57 B 0.86
A 0.57 B 1
A 0.71 B 0
A 0.71 B 0.14
A 0.71 B 0.29
A 0.71 B 0.43
A 0.71 B 0.57
A 0.71 B 0.71
A 0.71 B 0.86
A 0.71 B 1
A 0.86 B 0
A 0.86 B 0.14
A 0.86 B 0.29
A 0.86 B 0.43
A 0.86 B 0.57
A 0.86 B 0.71
A 0.86 B 0.86
A 0.86 B 1
A 1 B 0
A 1 B 0.14
A 1 B 0.29
A 1 B 0.43
A 1 B 0.57
A 1 B 0.71
A 1 B 0.86
A 1 B 1
0.8
0.6
0.4
0.2
OD 600 nm [−]
0 5 101520
Time [h]

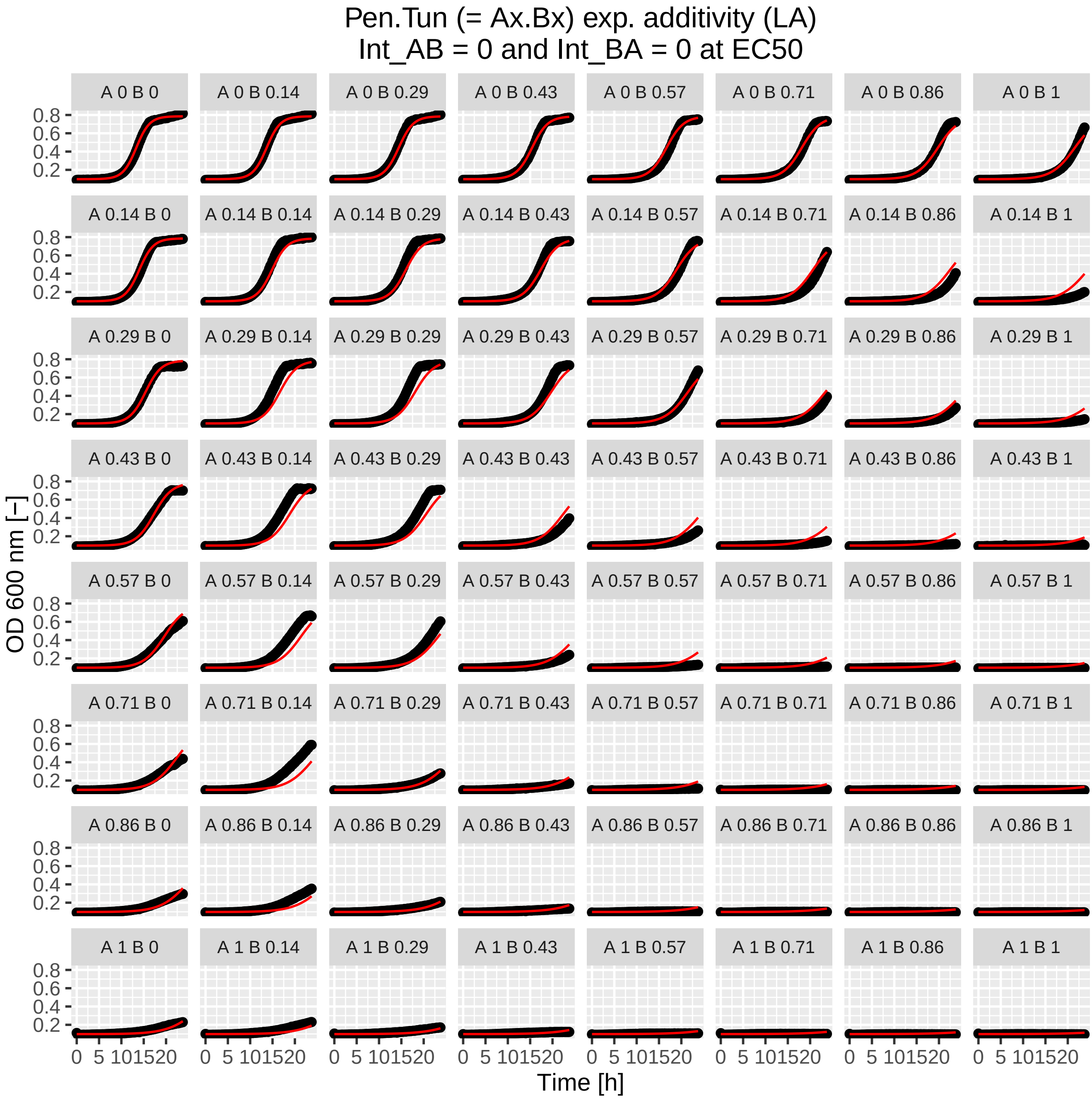
Pen.Tun (= Ax.Bx) exp. additivity (LA)
Int_AB = 0 and Int_BA = 0 at EC50
A 0 B 0
A 0 B 0.14
A 0 B 0.29
A 0 B 0.43
A 0 B 0.57
A 0 B 0.71
A 0 B 0.86
A 0 B 1
A 0.14 B 0
A 0.14 B 0.14
A 0.14 B 0.29
A 0.14 B 0.43
A 0.14 B 0.57
A 0.14 B 0.71
A 0.14 B 0.86
A 0.14 B 1
A 0.29 B 0
A 0.29 B 0.14
A 0.29 B 0.29
A 0.29 B 0.43
A 0.29 B 0.57
A 0.29 B 0.71
A 0.29 B 0.86
A 0.29 B 1
A 0.43 B 0
A 0.43 B 0.14
A 0.43 B 0.29
A 0.43 B 0.43
A 0.43 B 0.57
A 0.43 B 0.71
A 0.43 B 0.86
A 0.43 B 1
A 0.57 B 0
A 0.57 B 0.14
A 0.57 B 0.29
A 0.57 B 0.43
A 0.57 B 0.57
A 0.57 B 0.71
A 0.57 B 0.86
A 0.57 B 1
A 0.71 B 0
A 0.71 B 0.14
A 0.71 B 0.29
A 0.71 B 0.43
A 0.71 B 0.57
A 0.71 B 0.71
A 0.71 B 0.86
A 0.71 B 1
A 0.86 B 0
A 0.86 B 0.14
A 0.86 B 0.29
A 0.86 B 0.43
A 0.86 B 0.57
A 0.86 B 0.71
A 0.86 B 0.86
A 0.86 B 1
A 1 B 0
A 1 B 0.14
A 1 B 0.29
A 1 B 0.43
A 1 B 0.57
A 1 B 0.71
A 1 B 0.86
A 1 B 1
OD 600 nm [−]
0.8
0.6
0.4
0.2
0 5 101520
Time [h]

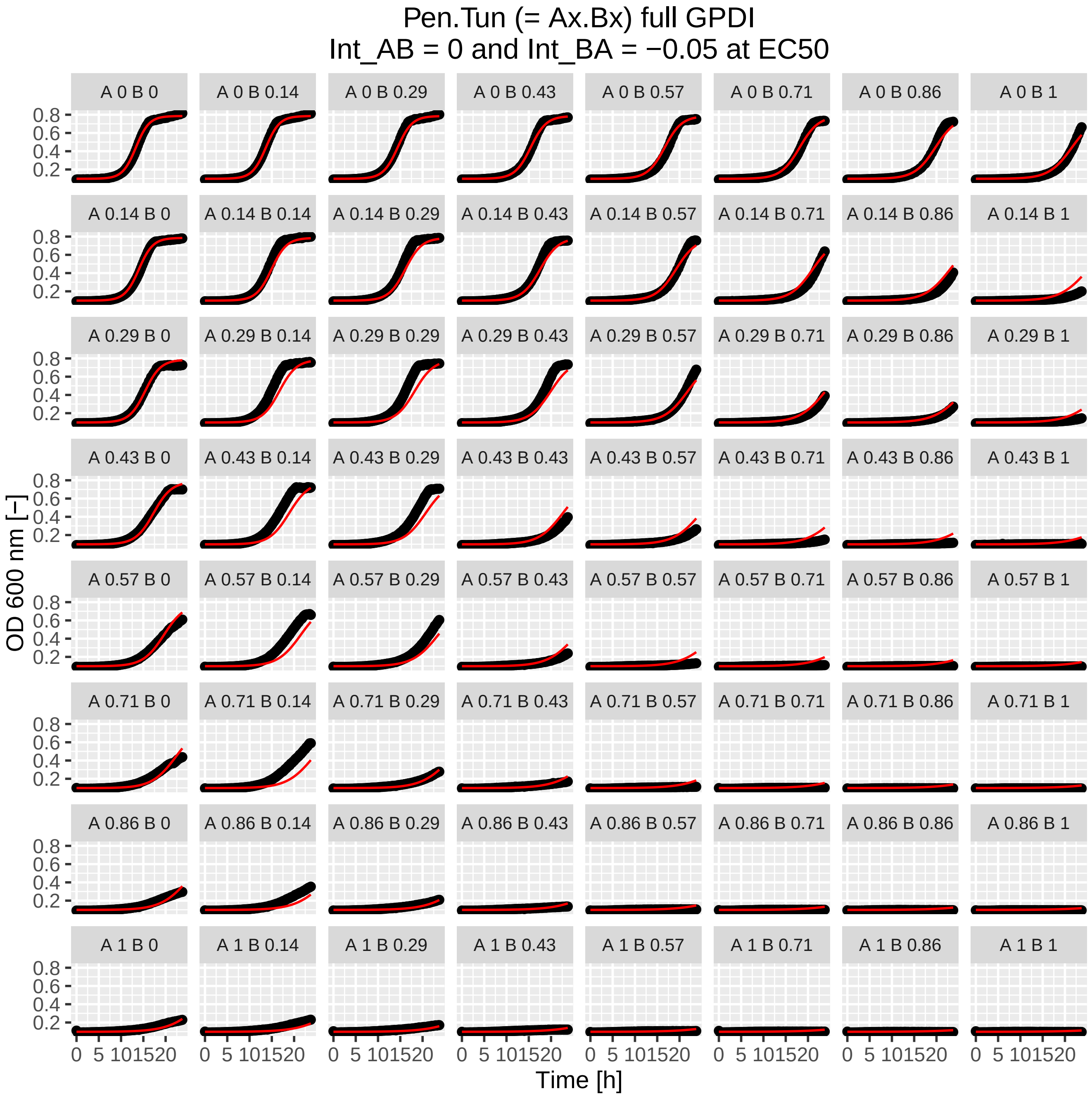
Pen.Tun (= Ax.Bx) full GPDI
Int_AB = 0 and Int_BA = −0.05 at EC50
OD 600 nm [−]
Time [h]
A 0 B 0
A 0 B 0.14
A 0 B 0.29
A 0 B 0.43
A 0 B 0.57
A 0 B 0.71
A 0 B 0.86
A 0 B 1
A 0.14 B 0
A 0.14 B 0.14
A 0.14 B 0.29
A 0.14 B 0.43
A 0.14 B 0.57
A 0.14 B 0.71
A 0.14 B 0.86
A 0.14 B 1
A 0.29 B 0
A 0.29 B 0.14
A 0.29 B 0.29
A 0.29 B 0.43
A 0.29 B 0.57
A 0.29 B 0.71
A 0.29 B 0.86
A 0.29 B 1
A 0.43 B 0
A 0.43 B 0.14
A 0.43 B 0.29
A 0.43 B 0.43
A 0.43 B 0.57
A 0.43 B 0.71
A 0.43 B 0.86
A 0.43 B 1
A 0.57 B 0
A 0.57 B 0.14
A 0.57 B 0.29
A 0.57 B 0.43
A 0.57 B 0.57
A 0.57 B 0.71
A 0.57 B 0.86
A 0.57 B 1
A 0.71 B 0
A 0.71 B 0.14
A 0.71 B 0.29
A 0.71 B 0.43
A 0.71 B 0.57
A 0.71 B 0.71
A 0.71 B 0.86
A 0.71 B 1
A 0.86 B 0
A 0.86 B 0.14
A 0.86 B 0.29
A 0.86 B 0.43
A 0.86 B 0.57
A 0.86 B 0.71
A 0.86 B 0.86
A 0.86 B 1
A 1 B 0
A 1 B 0.14
A 1 B 0.29
A 1 B 0.43
A 1 B 0.57
A 1 B 0.71
A 1 B 0.86
A 1 B 1
0.8
0.6
0.4
0.2
0 5 101520

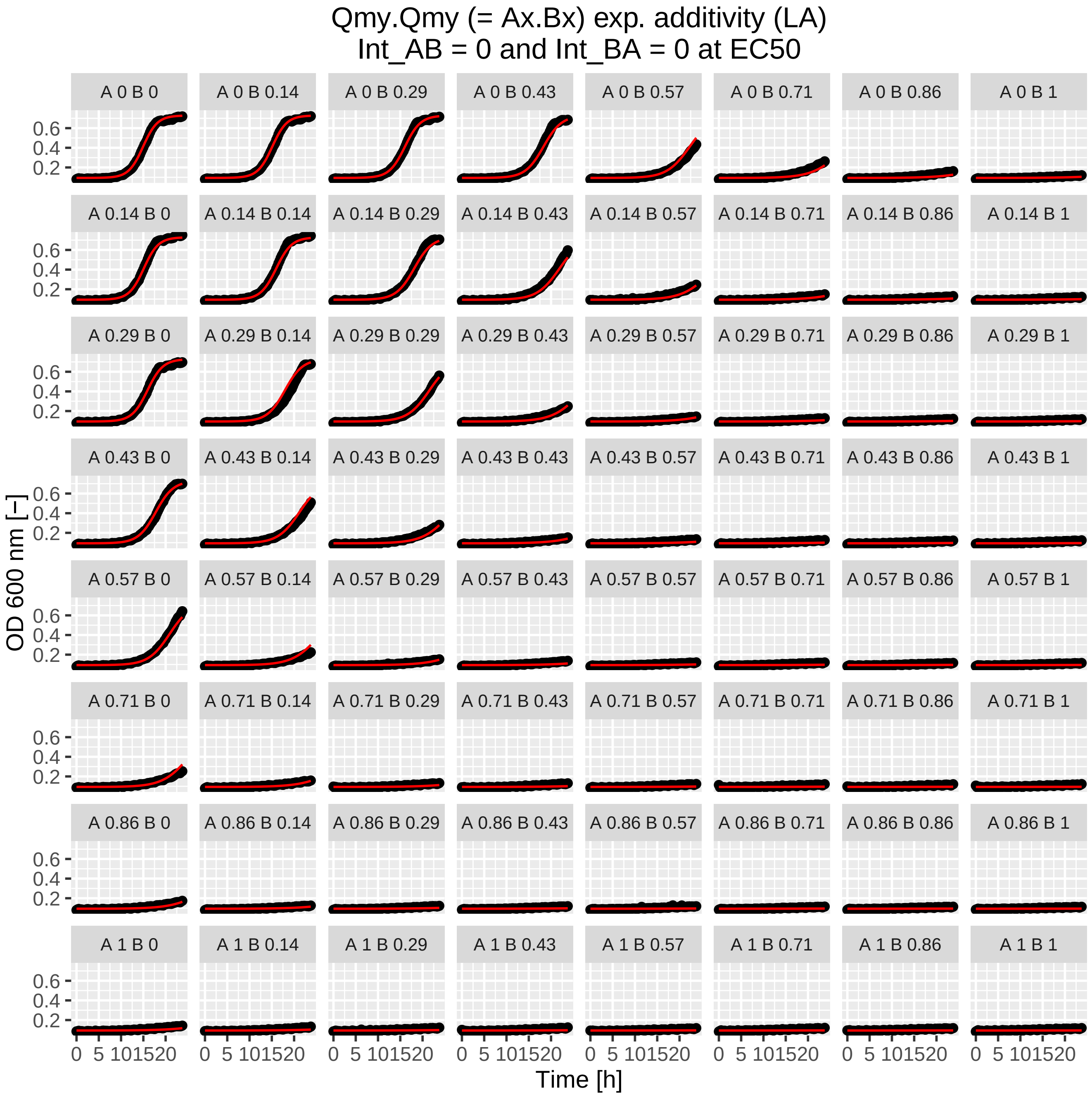

Qmy.Qmy (= Ax.Bx) exp. additivity (LA)
Int_AB = 0 and Int_BA = 0 at EC50
OD 600 nm [–]
Time [h]

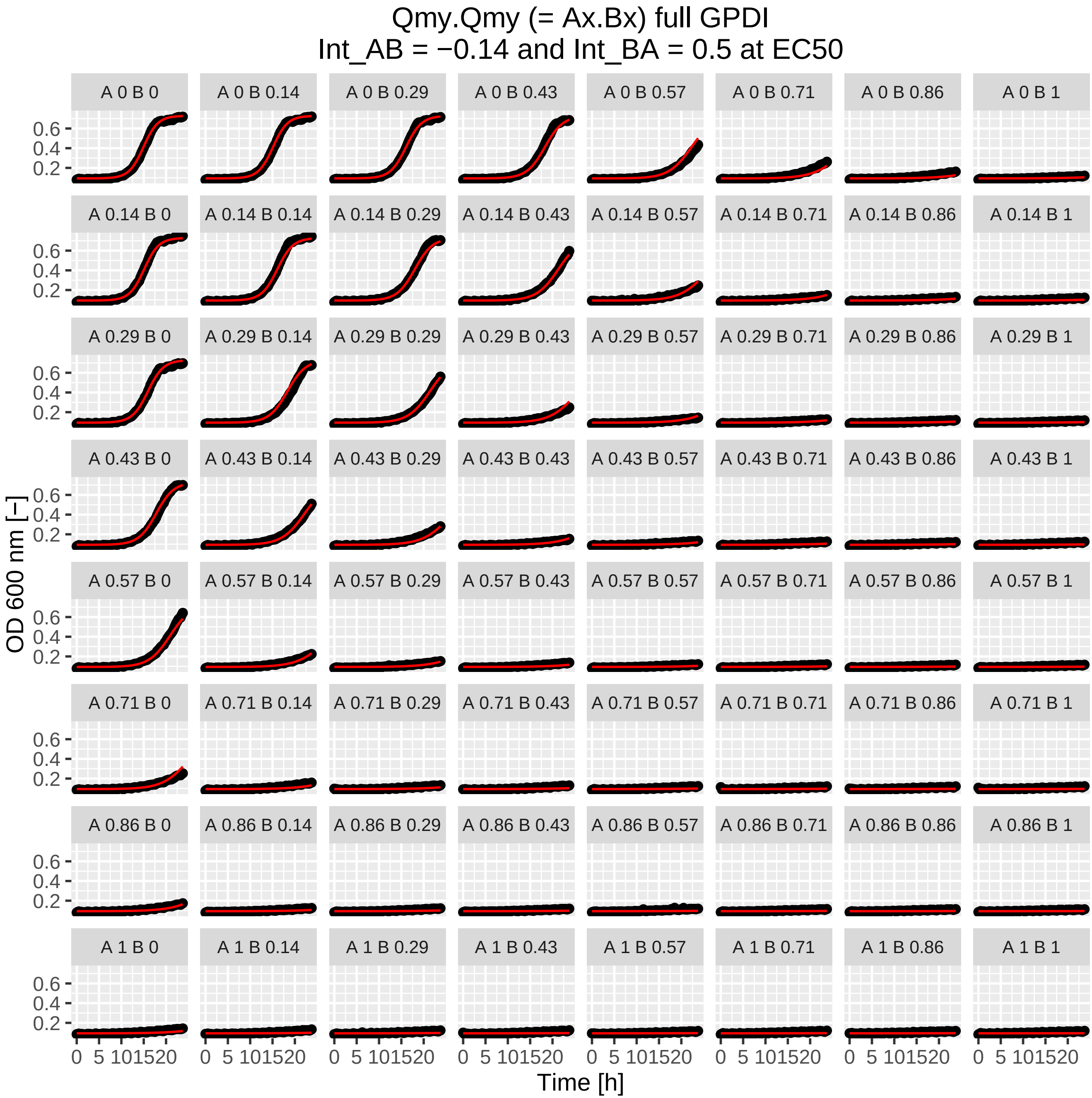
Qmy.Qmy (= Ax.Bx) full GPDI
Int_AB = −0.14 and Int_BA = 0.5 at EC50
A 0 B 0
A 0 B 0.14
A 0 B 0.29
A 0 B 0.43
A 0 B 0.57
A 0 B 0.71
A 0 B 0.86
A 0 B 1
A 0.14 B 0
A 0.14 B 0.14
A 0.14 B 0.29
A 0.14 B 0.43
A 0.14 B 0.57
A 0.14 B 0.71
A 0.14 B 0.86
A 0.14 B 1
A 0.29 B 0
A 0.29 B 0.14
A 0.29 B 0.29
A 0.29 B 0.43
A 0.29 B 0.57
A 0.29 B 0.71
A 0.29 B 0.86
A 0.29 B 1
A 0.43 B 0
A 0.43 B 0.14
A 0.43 B 0.29
A 0.43 B 0.43
A 0.43 B 0.57
A 0.43 B 0.71
A 0.43 B 0.86
A 0.43 B 1
A 0.57 B 0
A 0.57 B 0.14
A 0.57 B 0.29
A 0.57 B 0.43
A 0.57 B 0.57
A 0.57 B 0.71
A 0.57 B 0.86
A 0.57 B 1
A 0.71 B 0
A 0.71 B 0.14
A 0.71 B 0.29
A 0.71 B 0.43
A 0.71 B 0.57
A 0.71 B 0.71
A 0.71 B 0.86
A 0.71 B 1
A 0.86 B 0
A 0.86 B 0.14
A 0.86 B 0.29
A 0.86 B 0.43
A 0.86 B 0.57
A 0.86 B 0.71
A 0.86 B 0.86
A 0.86 B 1
A 1 B 0
A 1 B 0.14
A 1 B 0.29
A 1 B 0.43
A 1 B 0.57
A 1 B 0.71
A 1 B 0.86
A 1 B 1
0.6
0.4
0.2
0 5 101520
OD 600 nm [–]
Time [h]

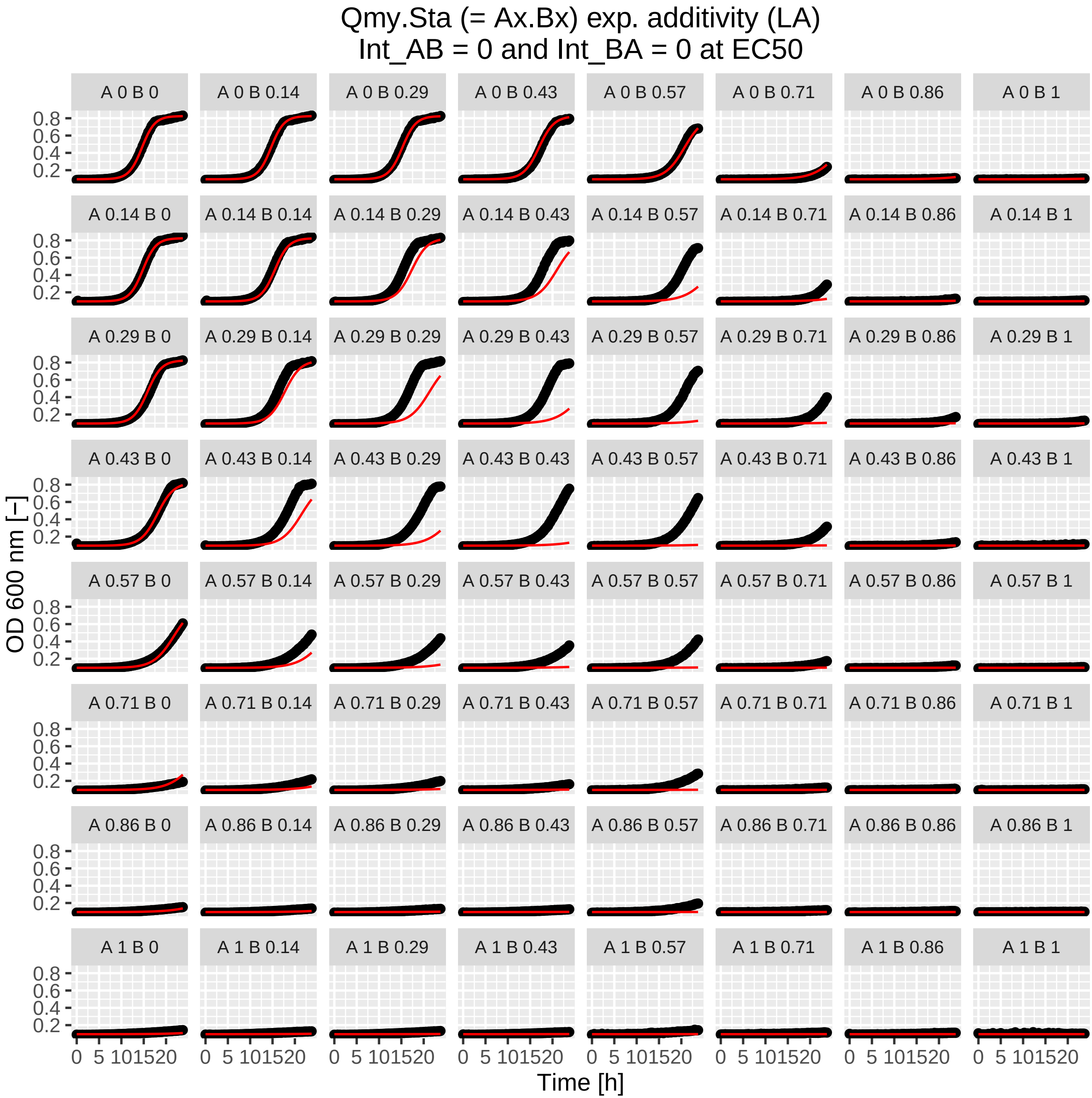

Qmy.Sta (= Ax.Bx) exp. additivity (LA)
Int_AB = 0 and Int_BA = 0 at EC50
OD 600 nm [–]
Time [h]

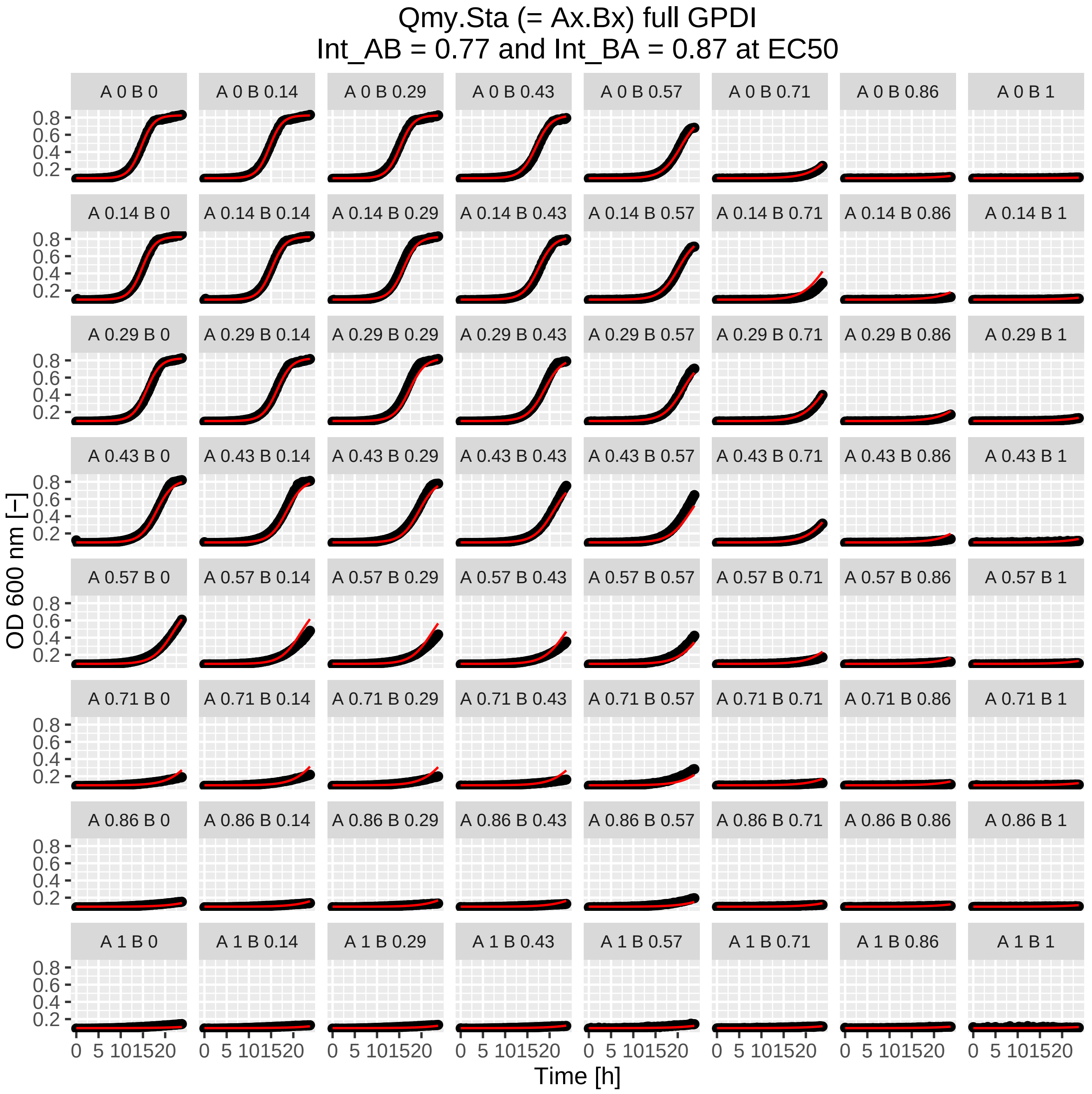

Qmy.Sta (= Ax.Bx) full GPDI
Int_AB = 0.77 and Int_BA = 0.87 at EC50
A 0 B 0
A 0 B 0.14
A 0 B 0.29
A 0 B 0.43
A 0 B 0.57
A 0 B 0.71
A 0 B 0.86
A 0 B 1
A 0.14 B 0
A 0.14 B 0.14
A 0.14 B 0.29
A 0.14 B 0.43
A 0.14 B 0.57
A 0.14 B 0.71
A 0.14 B 0.86
A 0.14 B 1
A 0.29 B 0
A 0.29 B 0.14
A 0.29 B 0.29
A 0.29 B 0.43
A 0.29 B 0.57
A 0.29 B 0.71
A 0.29 B 0.86
A 0.29 B 1
A 0.43 B 0
A 0.43 B 0.14
A 0.43 B 0.29
A 0.43 B 0.43
A 0.43 B 0.57
A 0.43 B 0.71
A 0.43 B 0.86
A 0.43 B 1
A 0.57 B 0
A 0.57 B 0.14
A 0.57 B 0.29
A 0.57 B 0.43
A 0.57 B 0.57
A 0.57 B 0.71
A 0.57 B 0.86
A 0.57 B 1
A 0.71 B 0
A 0.71 B 0.14
A 0.71 B 0.29
A 0.71 B 0.43
A 0.71 B 0.57
A 0.71 B 0.71
A 0.71 B 0.86
A 0.71 B 1
A 0.86 B 0
A 0.86 B 0.14
A 0.86 B 0.29
A 0.86 B 0.43
A 0.86 B 0.57
A 0.86 B 0.71
A 0.86 B 0.86
A 0.86 B 1
A 1 B 0
A 1 B 0.14
A 1 B 0.29
A 1 B 0.43
A 1 B 0.57
A 1 B 0.71
A 1 B 0.86
A 1 B 1
OD 600 nm [−]
0.8
0.6
0.4
0.2
0 5 101520
Time [h]

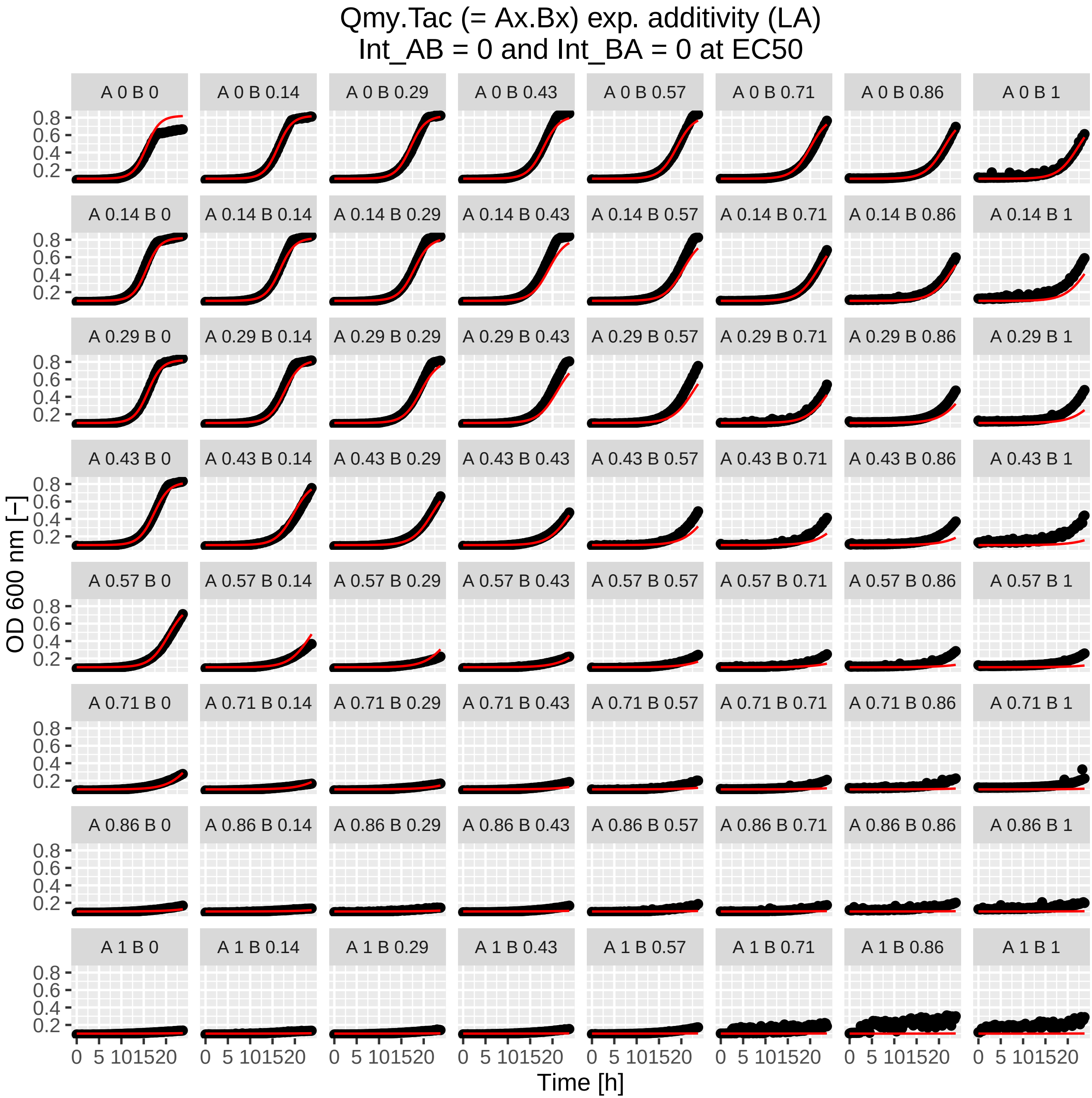

Qmy.Tac (= Ax.Bx) exp. additivity (LA)
Int_AB = 0 and Int_BA = 0 at EC50
OD 600 nm [–]
Time [h]

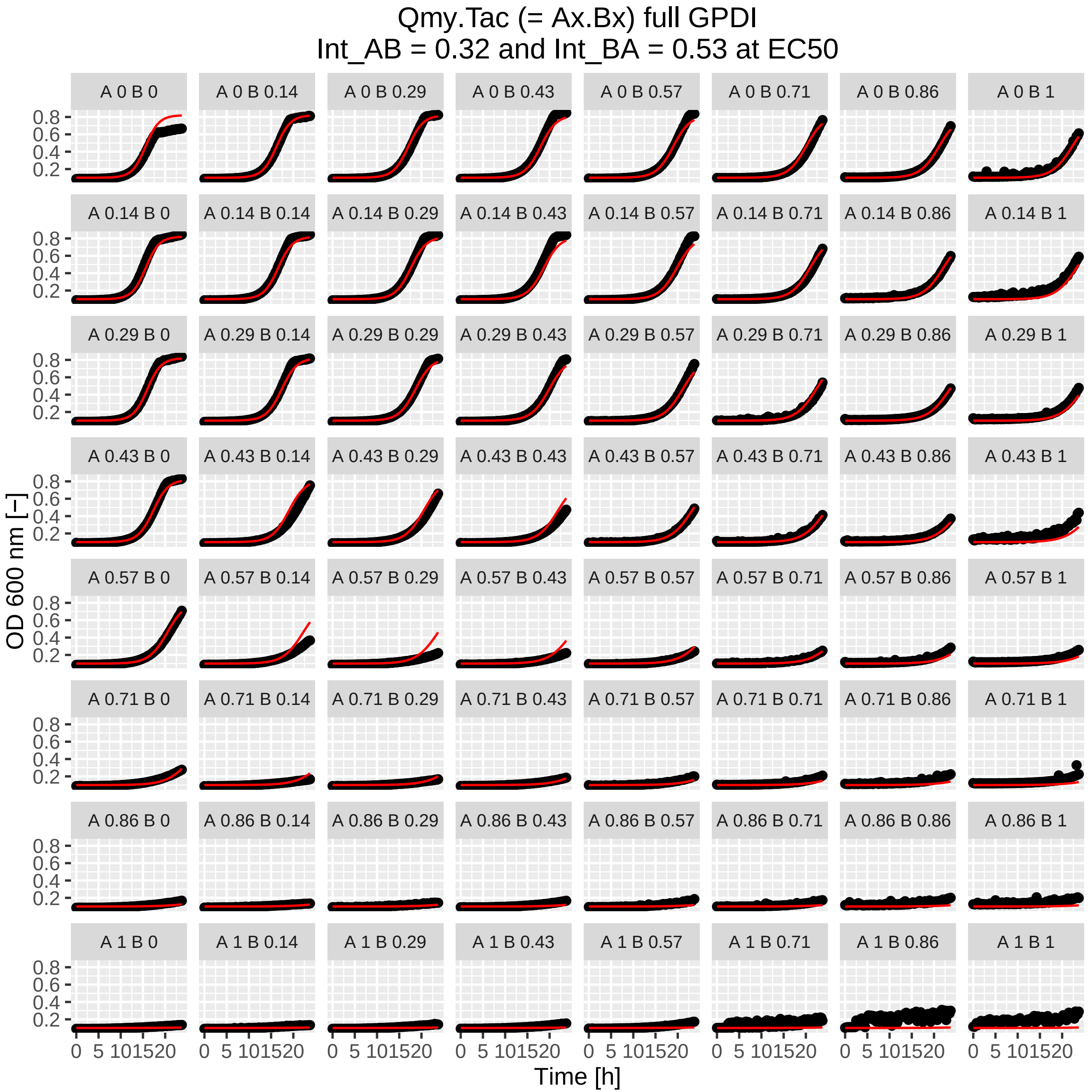

Qmy.Tac (= Ax.Bx) full GPDI
Int_AB = 0.32 and Int_BA = 0.53 at EC50
A 0 B 0
A 0 B 0.14
A 0 B 0.29
A 0 B 0.43
A 0 B 0.57
A 0 B 0.71
A 0 B 0.86
A 0 B 1
A 0.14 B 0
A 0.14 B 0.14
A 0.14 B 0.29
A 0.14 B 0.43
A 0.14 B 0.57
A 0.14 B 0.71
A 0.14 B 0.86
A 0.14 B 1
A 0.29 B 0
A 0.29 B 0.14
A 0.29 B 0.29
A 0.29 B 0.43
A 0.29 B 0.57
A 0.29 B 0.71
A 0.29 B 0.86
A 0.29 B 1
A 0.43 B 0
A 0.43 B 0.14
A 0.43 B 0.29
A 0.43 B 0.43
A 0.43 B 0.57
A 0.43 B 0.71
A 0.43 B 0.86
A 0.43 B 1
A 0.57 B 0
A 0.57 B 0.14
A 0.57 B 0.29
A 0.57 B 0.43
A 0.57 B 0.57
A 0.57 B 0.71
A 0.57 B 0.86
A 0.57 B 1
A 0.71 B 0
A 0.71 B 0.14
A 0.71 B 0.29
A 0.71 B 0.43
A 0.71 B 0.57
A 0.71 B 0.71
A 0.71 B 0.86
A 0.71 B 1
A 0.86 B 0
A 0.86 B 0.14
A 0.86 B 0.29
A 0.86 B 0.43
A 0.86 B 0.57
A 0.86 B 0.71
A 0.86 B 0.86
A 0.86 B 1
A 1 B 0
A 1 B 0.14
A 1 B 0.29
A 1 B 0.43
A 1 B 0.57
A 1 B 0.71
A 1 B 0.86
A 1 B 1
0.8
0.6
0.4
0.2
0 5 101520
OD 600 nm [–]
Time [h]

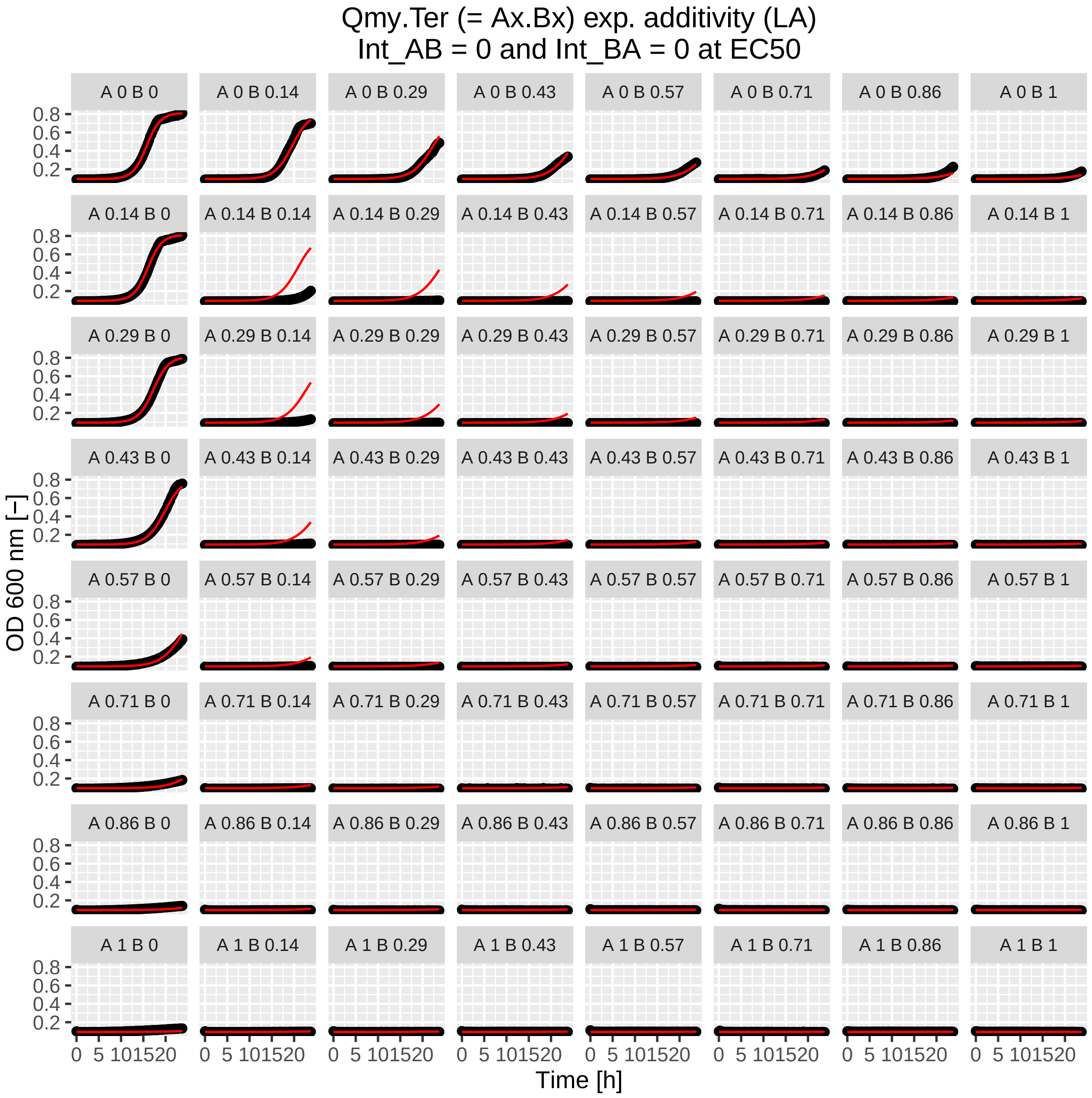
Qmy.Ter (= Ax.Bx) exp. additivity (LA)
Int_AB = 0 and Int_BA = 0 at EC50
OD 600 nm [–]
Time [h]
A 0 B 0
A 0 B 0.14
A 0 B 0.29
A 0 B 0.43
A 0 B 0.57
A 0 B 0.71
A 0 B 0.86
A 0 B 1
A 0.14 B 0
A 0.14 B 0.14
A 0.14 B 0.29
A 0.14 B 0.43
A 0.14 B 0.57
A 0.14 B 0.71
A 0.14 B 0.86
A 0.14 B 1
A 0.29 B 0
A 0.29 B 0.14
A 0.29 B 0.29
A 0.29 B 0.43
A 0.29 B 0.57
A 0.29 B 0.71
A 0.29 B 0.86
A 0.29 B 1
A 0.43 B 0
A 0.43 B 0.14
A 0.43 B 0.29
A 0.43 B 0.43
A 0.43 B 0.57
A 0.43 B 0.71
A 0.43 B 0.86
A 0.43 B 1
A 0.57 B 0
A 0.57 B 0.14
A 0.57 B 0.29
A 0.57 B 0.43
A 0.57 B 0.57
A 0.57 B 0.71
A 0.57 B 0.86
A 0.57 B 1
A 0.71 B 0
A 0.71 B 0.14
A 0.71 B 0.29
A 0.71 B 0.43
A 0.71 B 0.57
A 0.71 B 0.71
A 0.71 B 0.86
A 0.71 B 1
A 0.86 B 0
A 0.86 B 0.14
A 0.86 B 0.29
A 0.86 B 0.43
A 0.86 B 0.57
A 0.86 B 0.71
A 0.86 B 0.86
A 0.86 B 1
A 1 B 0
A 1 B 0.14
A 1 B 0.29
A 1 B 0.43
A 1 B 0.57
A 1 B 0.71
A 1 B 0.86
A 1 B 1
0.8
0.6
0.4
0.2
0 5 101520

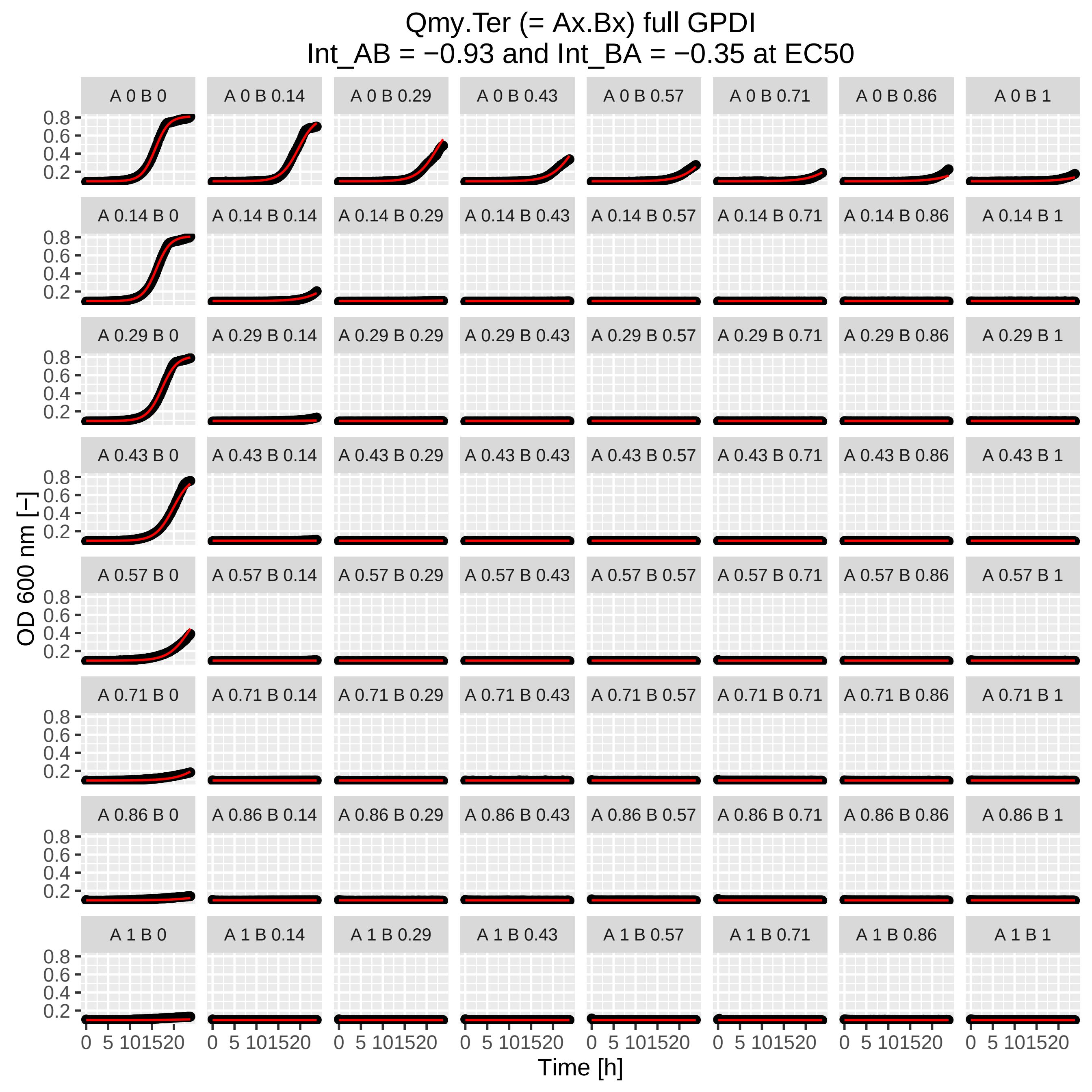
Qmy.Ter (= Ax.Bx) full GPDI
Int_AB = −0.93 and Int_BA = −0.35 at EC50
OD 600 nm [−]
Time [h]
A 0 B 0
A 0 B 0.14
A 0 B 0.29
A 0 B 0.43
A 0 B 0.57
A 0 B 0.71
A 0 B 0.86
A 0 B 1
A 0.14 B 0
A 0.29 B 0
A 0.43 B 0
A 0.57 B 0
A 0.71 B 0
A 0.86 B 0
A 1 B 0
A 1 B 1
0.2
0.4
0.6
0.8
0 5 101520

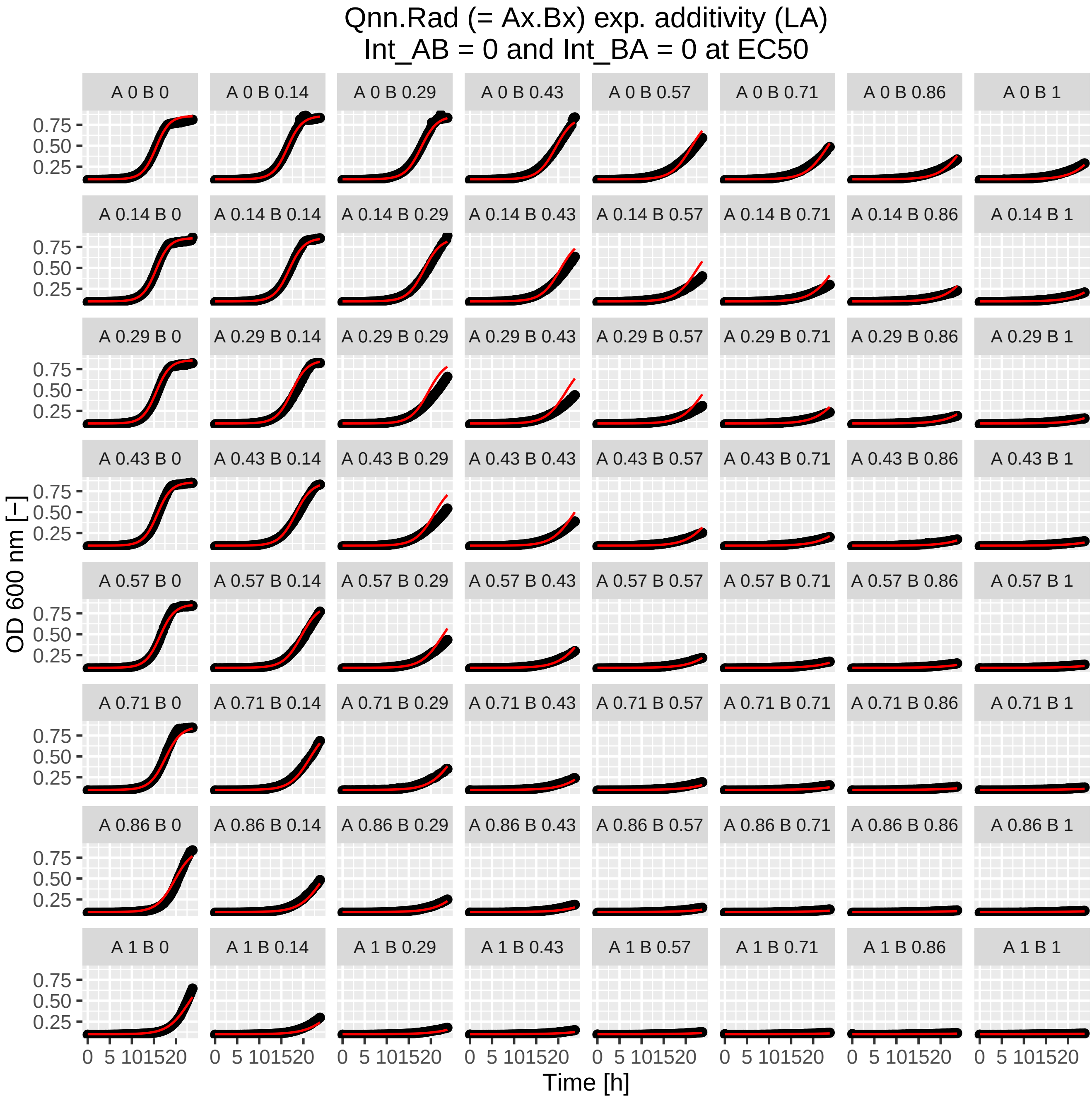

Qnn.Rad (= Ax.Bx) exp. additivity (LA)
Int_AB = 0 and Int_BA = 0 at EC50
A 0 B 0
A 0 B 0.14
A 0 B 0.29
A 0 B 0.43
A 0 B 0.57
A 0 B 0.71
A 0 B 0.86
A 0 B 1
A 0.14 B 0
A 0.14 B 0.14
A 0.14 B 0.29
A 0.14 B 0.43
A 0.14 B 0.57
A 0.14 B 0.71
A 0.14 B 0.86
A 0.14 B 1
A 0.29 B 0
A 0.29 B 0.14
A 0.29 B 0.29
A 0.29 B 0.43
A 0.29 B 0.57
A 0.29 B 0.71
A 0.29 B 0.86
A 0.29 B 1
A 0.43 B 0
A 0.43 B 0.14
A 0.43 B 0.29
A 0.43 B 0.43
A 0.43 B 0.57
A 0.43 B 0.71
A 0.43 B 0.86
A 0.43 B 1
A 0.57 B 0
A 0.57 B 0.14
A 0.57 B 0.29
A 0.57 B 0.43
A 0.57 B 0.57
A 0.57 B 0.71
A 0.57 B 0.86
A 0.57 B 1
A 0.71 B 0
A 0.71 B 0.14
A 0.71 B 0.29
A 0.71 B 0.43
A 0.71 B 0.57
A 0.71 B 0.71
A 0.71 B 0.86
A 0.71 B 1
A 0.86 B 0
A 0.86 B 0.14
A 0.86 B 0.29
A 0.86 B 0.43
A 0.86 B 0.57
A 0.86 B 0.71
A 0.86 B 0.86
A 0.86 B 1
A 1 B 0
A 1 B 0.14
A 1 B 0.29
A 1 B 0.43
A 1 B 0.57
A 1 B 0.71
A 1 B 0.86
A 1 B 1
0.75
0.50
0.25
OD 600 nm [−]
0 5 101520
Time [h]

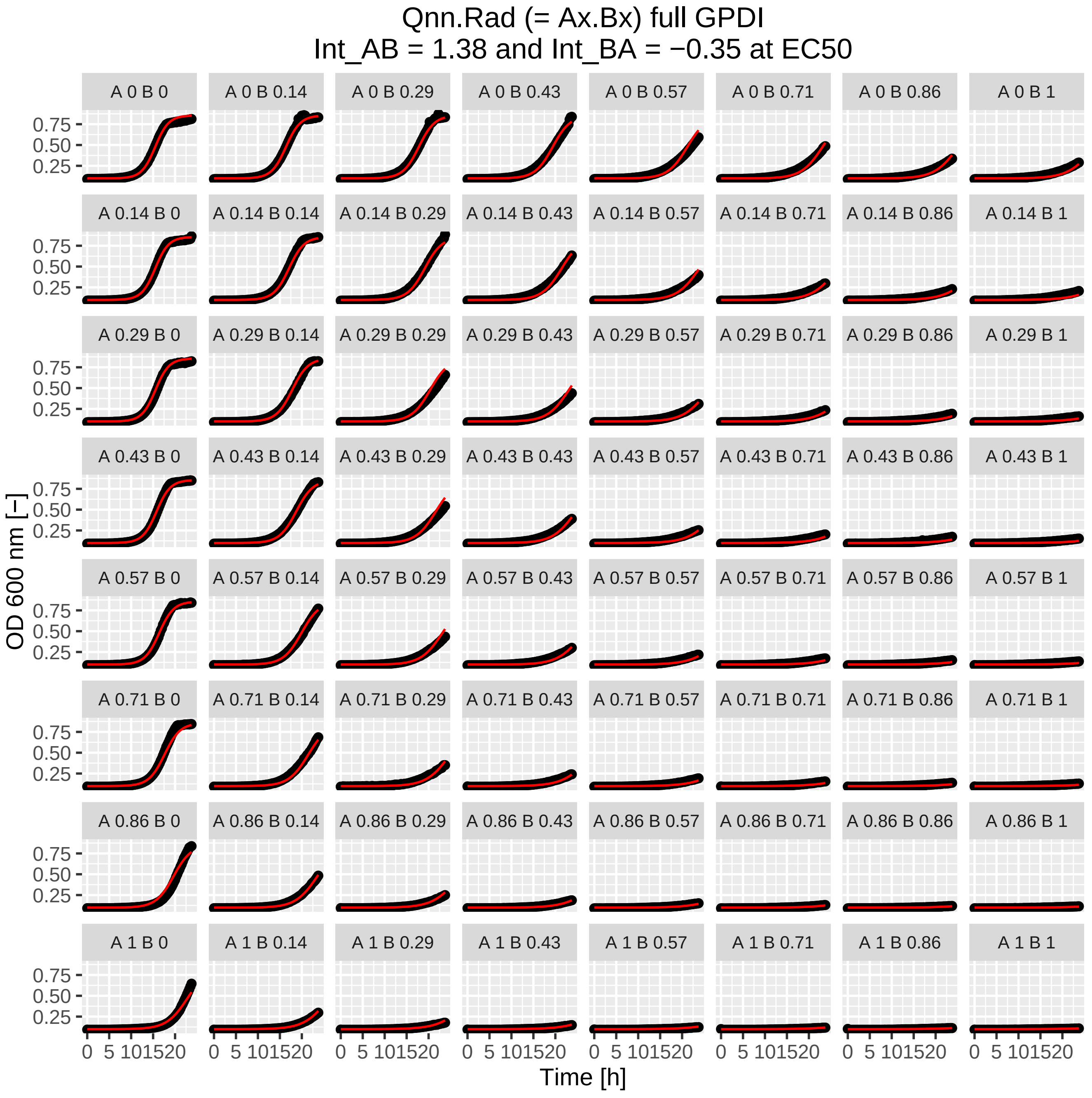
Qnn.Rad (= Ax.Bx) full GPDI
Int_AB = 1.38 and Int_BA = −0.35 at EC50
A 0 B 0
A 0 B 0.14
A 0 B 0.29
A 0 B 0.43
A 0 B 0.57
A 0 B 0.71
A 0 B 0.86
A 0 B 1
A 0.14 B 0
A 0.14 B 0.14
A 0.14 B 0.29
A 0.14 B 0.43
A 0.14 B 0.57
A 0.14 B 0.71
A 0.14 B 0.86
A 0.14 B 1
A 0.29 B 0
A 0.29 B 0.14
A 0.29 B 0.29
A 0.29 B 0.43
A 0.29 B 0.57
A 0.29 B 0.71
A 0.29 B 0.86
A 0.29 B 1
A 0.43 B 0
A 0.43 B 0.14
A 0.43 B 0.29
A 0.43 B 0.43
A 0.43 B 0.57
A 0.43 B 0.71
A 0.43 B 0.86
A 0.43 B 1
A 0.57 B 0
A 0.57 B 0.14
A 0.57 B 0.29
A 0.57 B 0.43
A 0.57 B 0.57
A 0.57 B 0.71
A 0.57 B 0.86
A 0.57 B 1
A 0.71 B 0
A 0.71 B 0.14
A 0.71 B 0.29
A 0.71 B 0.43
A 0.71 B 0.57
A 0.71 B 0.71
A 0.71 B 0.86
A 0.71 B 1
A 0.86 B 0
A 0.86 B 0.14
A 0.86 B 0.29
A 0.86 B 0.43
A 0.86 B 0.57
A 0.86 B 0.71
A 0.86 B 0.86
A 0.86 B 1
A 1 B 0
A 1 B 0.14
A 1 B 0.29
A 1 B 0.43
A 1 B 0.57
A 1 B 0.71
A 1 B 0.86
A 1 B 1
0.75
0.50
0.25
0 5 101520
OD 600 nm [−]
Time [h]

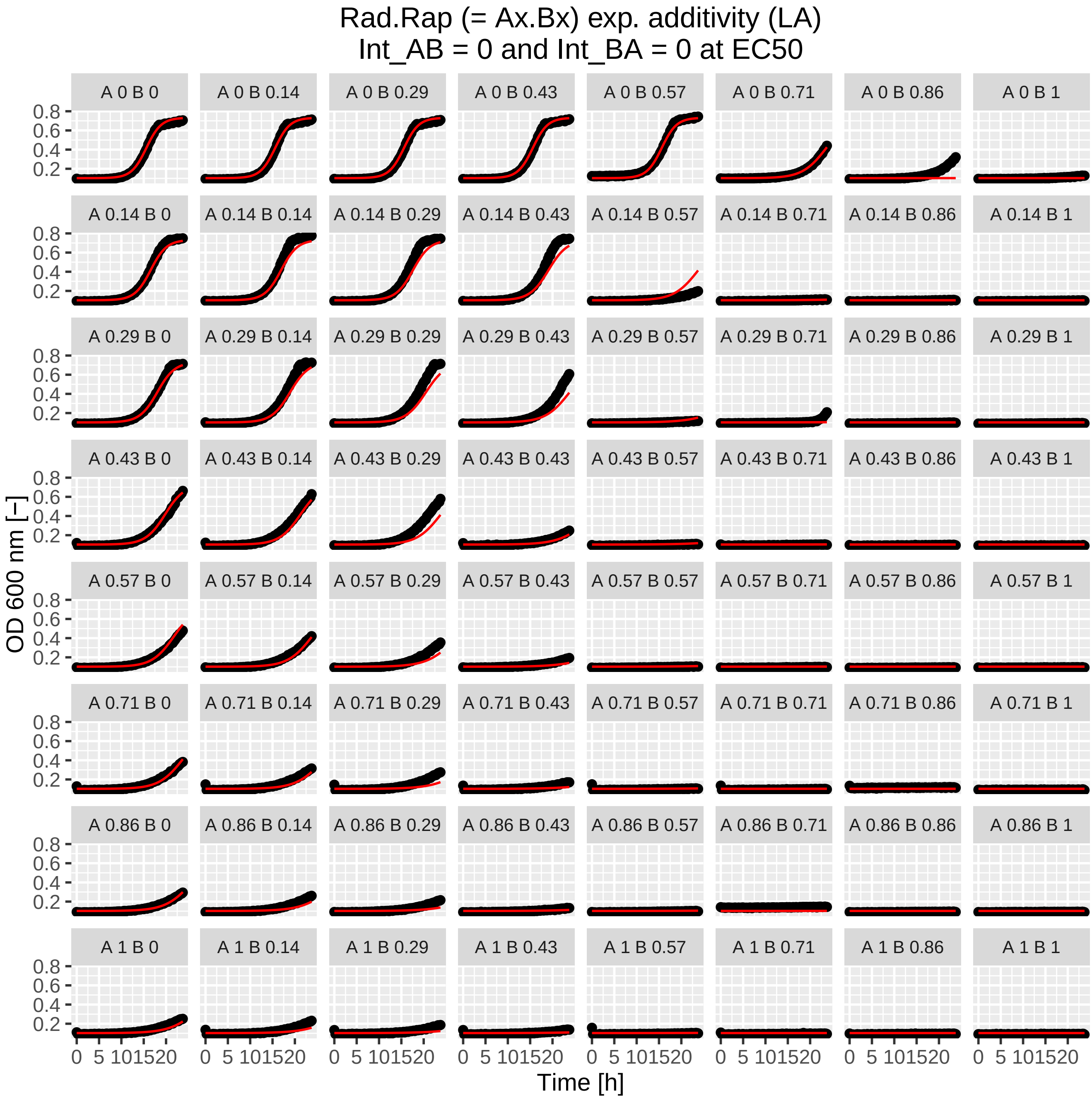

Rad.Rap (= Ax.Bx) exp. additivity (LA)
Int_AB = 0 and Int_BA = 0 at EC50
A 0 B 0
A 0 B 0.14
A 0 B 0.29
A 0 B 0.43
A 0 B 0.57
A 0 B 0.71
A 0 B 0.86
A 0 B 1
A 0.14 B 0
A 0.14 B 0.14
A 0.14 B 0.29
A 0.14 B 0.43
A 0.14 B 0.57
A 0.14 B 0.71
A 0.14 B 0.86
A 0.14 B 1
A 0.29 B 0
A 0.29 B 0.14
A 0.29 B 0.29
A 0.29 B 0.43
A 0.29 B 0.57
A 0.29 B 0.71
A 0.29 B 0.86
A 0.29 B 1
A 0.43 B 0
A 0.43 B 0.14
A 0.43 B 0.29
A 0.43 B 0.43
A 0.43 B 0.57
A 0.43 B 0.71
A 0.43 B 0.86
A 0.43 B 1
A 0.57 B 0
A 0.57 B 0.14
A 0.57 B 0.29
A 0.57 B 0.43
A 0.57 B 0.57
A 0.57 B 0.71
A 0.57 B 0.86
A 0.57 B 1
A 0.71 B 0
A 0.71 B 0.14
A 0.71 B 0.29
A 0.71 B 0.43
A 0.71 B 0.57
A 0.71 B 0.71
A 0.71 B 0.86
A 0.71 B 1
A 0.86 B 0
A 0.86 B 0.14
A 0.86 B 0.29
A 0.86 B 0.43
A 0.86 B 0.57
A 0.86 B 0.71
A 0.86 B 0.86
A 0.86 B 1
A 1 B 0
A 1 B 0.14
A 1 B 0.29
A 1 B 0.43
A 1 B 0.57
A 1 B 0.71
A 1 B 0.86
A 1 B 1
OD 600 nm [–]
0.2
0.4
0.6
0.8
0 5 101520
Time [h]

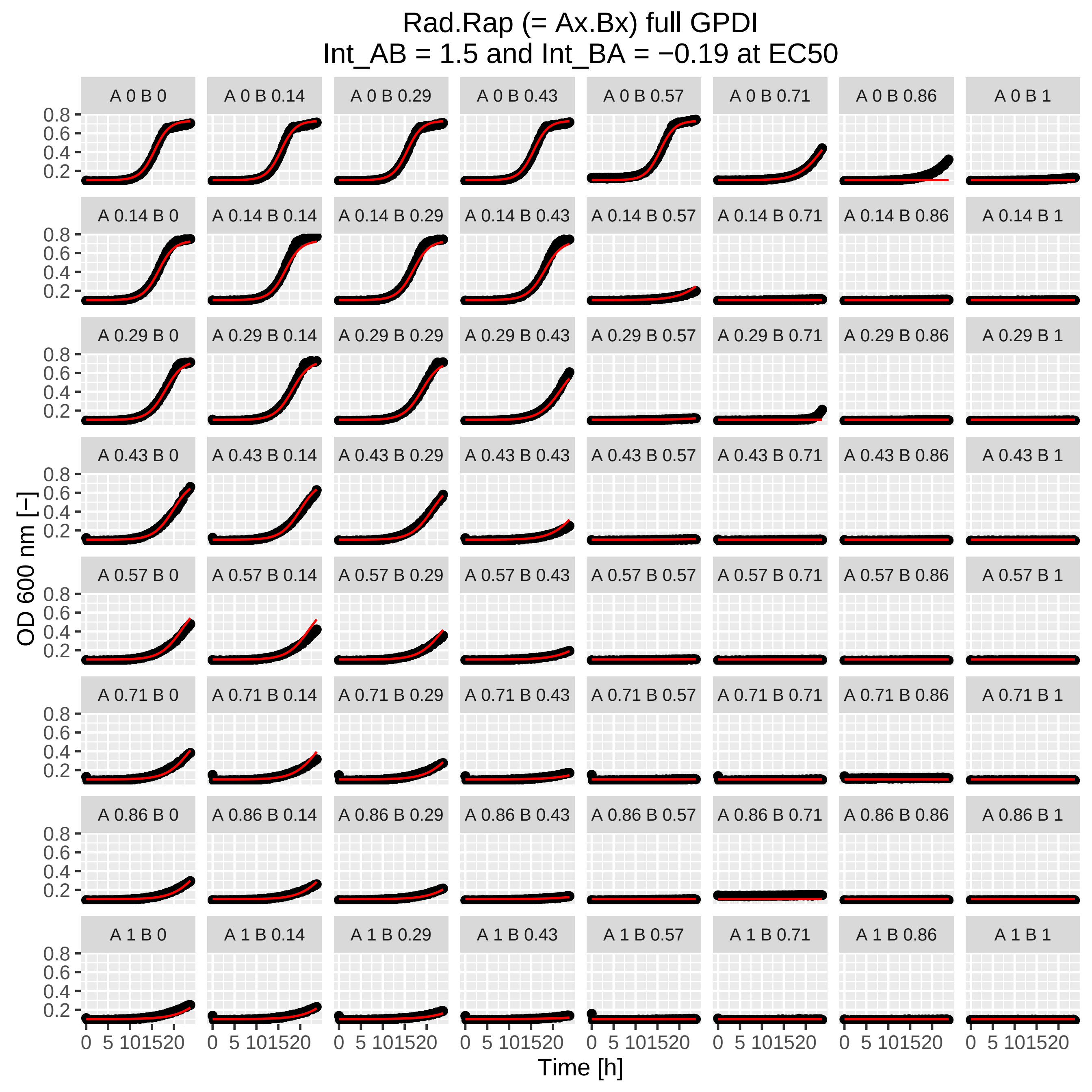

Rad.Rap (= Ax.Bx) full GPDI
Int_AB = 1.5 and Int_BA = −0.19 at EC50
A 0 B 0
A 0 B 0.14
A 0 B 0.29
A 0 B 0.43
A 0 B 0.57
A 0 B 0.71
A 0 B 0.86
A 0 B 1
A 0.14 B 0
A 0.14 B 0.14
A 0.14 B 0.29
A 0.14 B 0.43
A 0.14 B 0.57
A 0.14 B 0.71
A 0.14 B 0.86
A 0.14 B 1
A 0.29 B 0
A 0.29 B 0.14
A 0.29 B 0.29
A 0.29 B 0.43
A 0.29 B 0.57
A 0.29 B 0.71
A 0.29 B 0.86
A 0.29 B 1
A 0.43 B 0
A 0.43 B 0.14
A 0.43 B 0.29
A 0.43 B 0.43
A 0.43 B 0.57
A 0.43 B 0.71
A 0.43 B 0.86
A 0.43 B 1
A 0.57 B 0
A 0.57 B 0.14
A 0.57 B 0.29
A 0.57 B 0.43
A 0.57 B 0.57
A 0.57 B 0.71
A 0.57 B 0.86
A 0.57 B 1
A 0.71 B 0
A 0.71 B 0.14
A 0.71 B 0.29
A 0.71 B 0.43
A 0.71 B 0.57
A 0.71 B 0.71
A 0.71 B 0.86
A 0.71 B 1
A 0.86 B 0
A 0.86 B 0.14
A 0.86 B 0.29
A 0.86 B 0.43
A 0.86 B 0.57
A 0.86 B 0.71
A 0.86 B 0.86
A 0.86 B 1
A 1 B 0
A 1 B 0.14
A 1 B 0.29
A 1 B 0.43
A 1 B 0.57
A 1 B 0.71
A 1 B 0.86
A 1 B 1
OD 600 nm [−]
0.2
0.4
0.6
0.8
0 5 101520
Time [h]

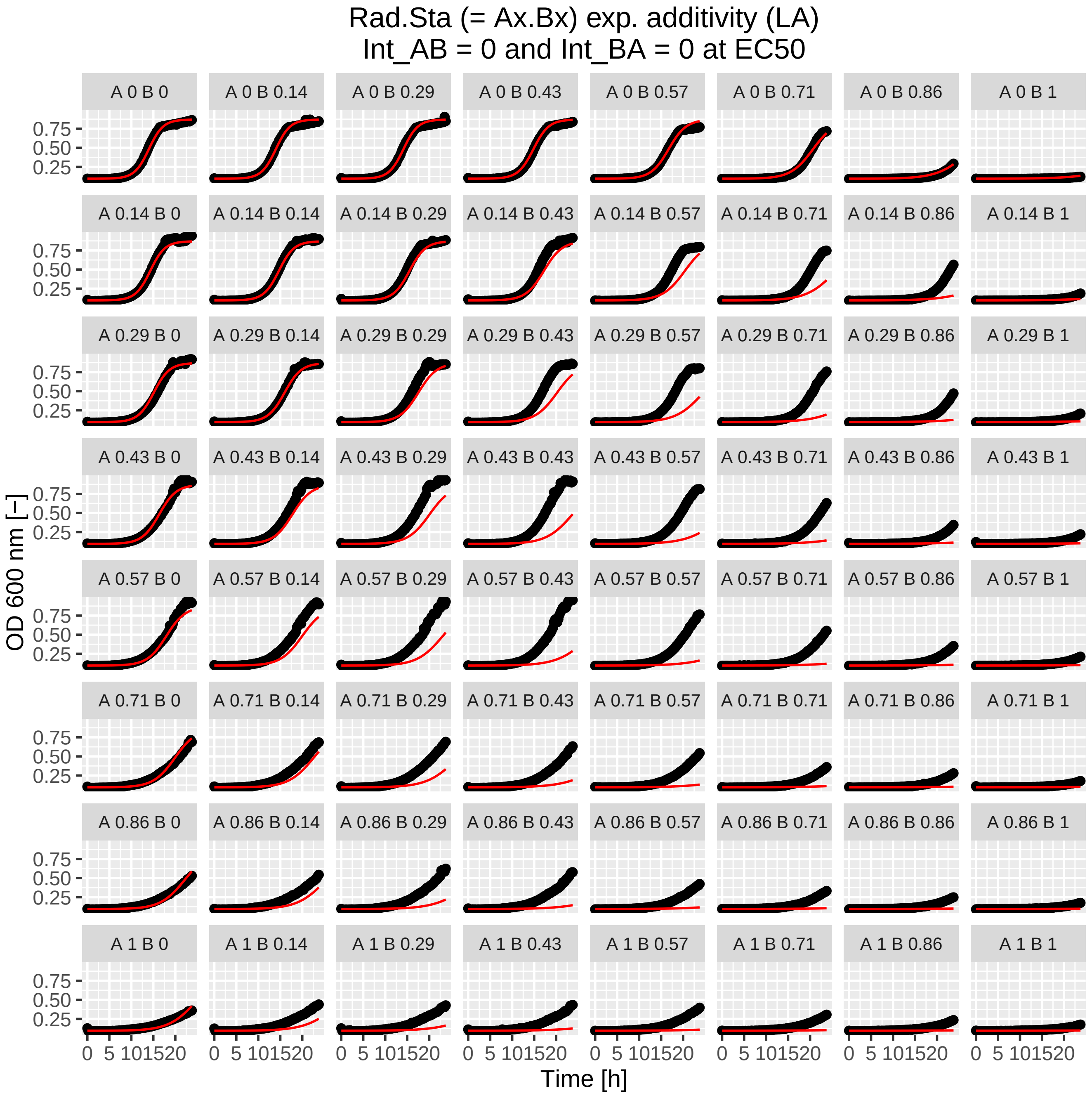

Rad.Sta (= Ax.Bx) exp. additivity (LA)
Int_AB = 0 and Int_BA = 0 at EC50
OD 600 nm [–]
Time [h]
0.75
0.50
0.25
0 5 101520
A 0 B 0
A 0 B 0.14
A 0 B 0.29
A 0 B 0.43
A 0 B 0.57
A 0 B 0.71
A 0 B 0.86
A 0 B 1
A 0.14 B 0
A 0.14 B 0.14
A 0.14 B 0.29
A 0.14 B 0.43
A 0.14 B 0.57
A 0.14 B 0.71
A 0.14 B 0.86
A 0.14 B 1
A 0.29 B 0
A 0.29 B 0.14
A 0.29 B 0.29
A 0.29 B 0.43
A 0.29 B 0.57
A 0.29 B 0.71
A 0.29 B 0.86
A 0.29 B 1
A 0.43 B 0
A 0.43 B 0.14
A 0.43 B 0.29
A 0.43 B 0.43
A 0.43 B 0.57
A 0.43 B 0.71
A 0.43 B 0.86
A 0.43 B 1
A 0.57 B 0
A 0.57 B 0.14
A 0.57 B 0.29
A 0.57 B 0.43
A 0.57 B 0.57
A 0.57 B 0.71
A 0.57 B 0.86
A 0.57 B 1
A 0.71 B 0
A 0.71 B 0.14
A 0.71 B 0.29
A 0.71 B 0.43
A 0.71 B 0.57
A 0.71 B 0.71
A 0.71 B 0.86
A 0.71 B 1
A 0.86 B 0
A 0.86 B 0.14
A 0.86 B 0.29
A 0.86 B 0.43
A 0.86 B 0.57
A 0.86 B 0.71
A 0.86 B 0.86
A 0.86 B 1
A 1 B 0
A 1 B 0.14
A 1 B 0.29
A 1 B 0.43
A 1 B 0.57
A 1 B 0.71
A 1 B 0.86
A 1 B 1

Rad.Sta (= Ax.Bx) full GPDI
Int_AB = 1.38 and Int_BA = 0.38 at EC50

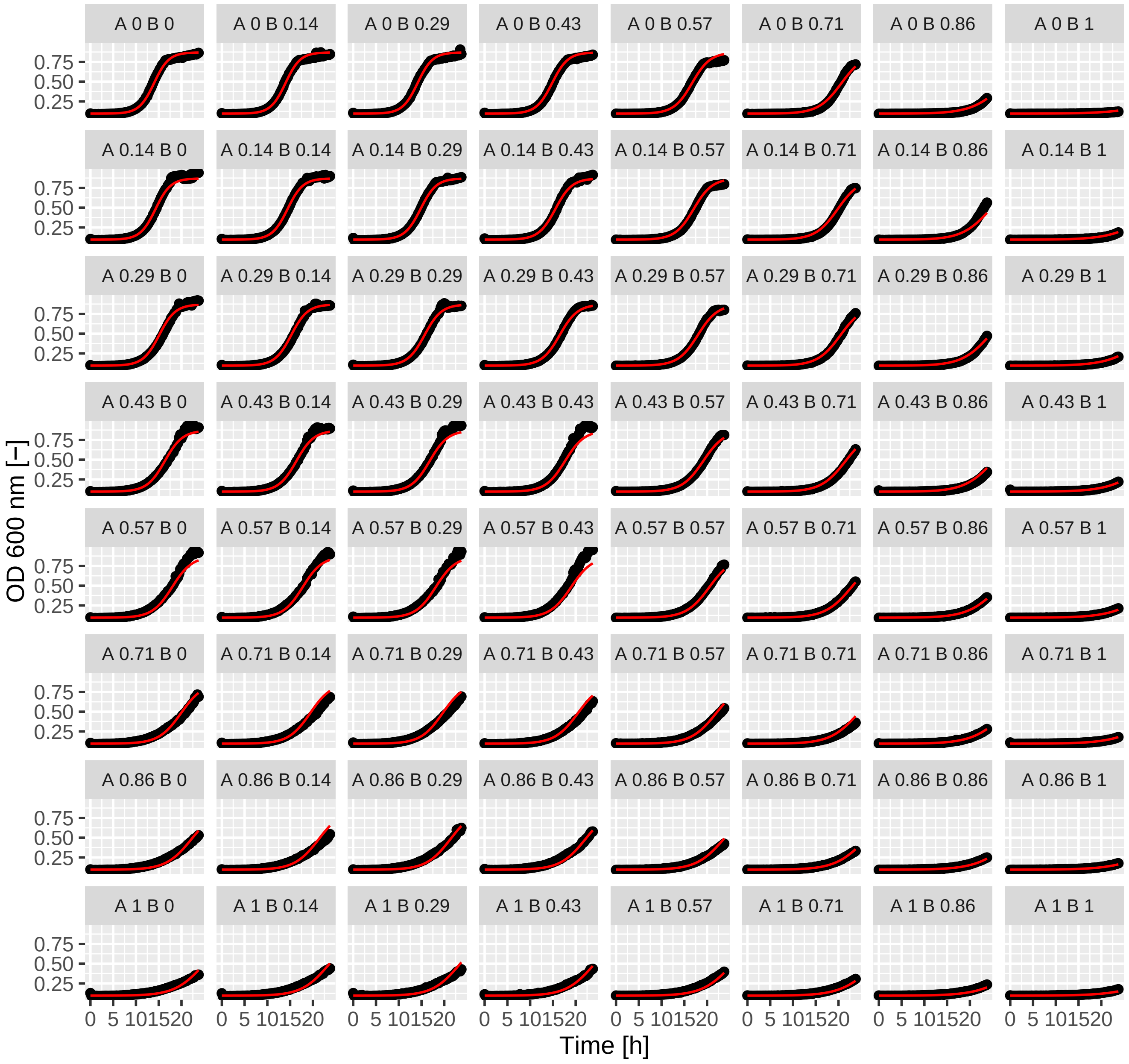

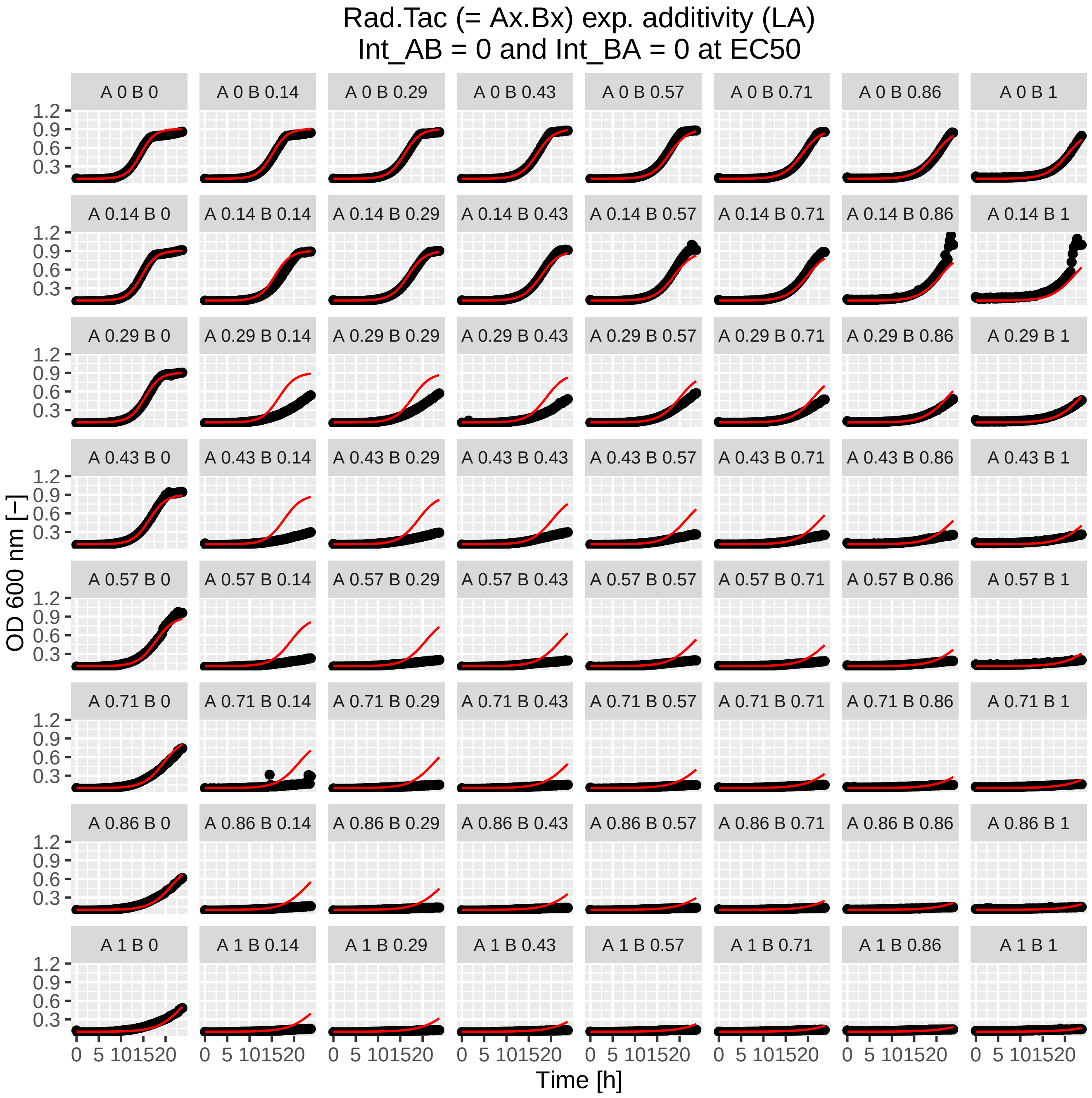

Rad.Tac (= Ax.Bx) exp. additivity (LA)
Int_AB = 0 and Int_BA = 0 at EC50
OD 600 nm [–]
Time [h]
A 0 B 0
A 0 B 0.14
A 0 B 0.29
A 0 B 0.43
A 0 B 0.57
A 0 B 0.71
A 0 B 0.86
A 0 B 1
A 0.14 B 0
A 0.14 B 0.14
A 0.14 B 0.29
A 0.14 B 0.43
A 0.14 B 0.57
A 0.14 B 0.71
A 0.14 B 0.86
A 0.14 B 1
A 0.29 B 0
A 0.29 B 0.14
A 0.29 B 0.29
A 0.29 B 0.43
A 0.29 B 0.57
A 0.29 B 0.71
A 0.29 B 0.86
A 0.29 B 1
A 0.43 B 0
A 0.43 B 0.14
A 0.43 B 0.29
A 0.43 B 0.43
A 0.43 B 0.57
A 0.43 B 0.71
A 0.43 B 0.86
A 0.43 B 1
A 0.57 B 0
A 0.57 B 0.14
A 0.57 B 0.29
A 0.57 B 0.43
A 0.57 B 0.57
A 0.57 B 0.71
A 0.57 B 0.86
A 0.57 B 1
A 0.71 B 0
A 0.71 B 0.14
A 0.71 B 0.29
A 0.71 B 0.43
A 0.71 B 0.57
A 0.71 B 0.71
A 0.71 B 0.86
A 0.71 B 1
A 0.86 B 0
A 0.86 B 0.14
A 0.86 B 0.29
A 0.86 B 0.43
A 0.86 B 0.57
A 0.86 B 0.71
A 0.86 B 0.86
A 0.86 B 1
A 1 B 0
A 1 B 0.14
A 1 B 0.29
A 1 B 0.43
A 1 B 0.57
A 1 B 0.71
A 1 B 0.86
A 1 B 1
1.2
0.9
0.6
0.3
0 5 101520

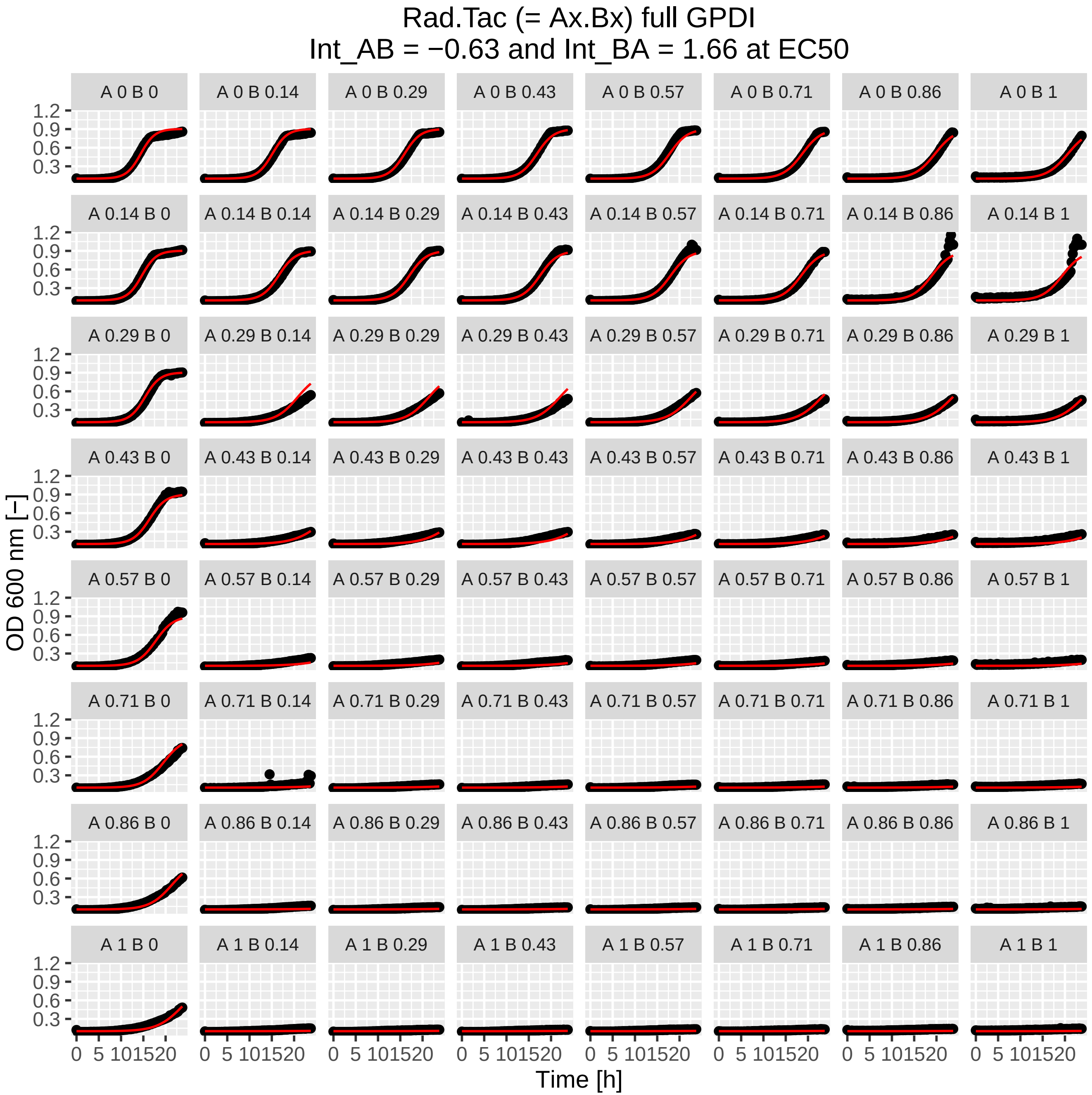
Rad.Tac (= Ax.Bx) full GPDI
Int_AB = −0.63 and Int_BA = 1.66 at EC50
A 0 B 0
A 0 B 0.14
A 0 B 0.29
A 0 B 0.43
A 0 B 0.57
A 0 B 0.71
A 0 B 0.86
A 0 B 1
A 0.14 B 0
A 0.14 B 0.14
A 0.14 B 0.29
A 0.14 B 0.43
A 0.14 B 0.57
A 0.14 B 0.71
A 0.14 B 0.86
A 0.14 B 1
A 0.29 B 0
A 0.29 B 0.14
A 0.29 B 0.29
A 0.29 B 0.43
A 0.29 B 0.57
A 0.29 B 0.71
A 0.29 B 0.86
A 0.29 B 1
A 0.43 B 0
A 0.43 B 0.14
A 0.43 B 0.29
A 0.43 B 0.43
A 0.43 B 0.57
A 0.43 B 0.71
A 0.43 B 0.86
A 0.43 B 1
A 0.57 B 0
A 0.57 B 0.14
A 0.57 B 0.29
A 0.57 B 0.43
A 0.57 B 0.57
A 0.57 B 0.71
A 0.57 B 0.86
A 0.57 B 1
A 0.71 B 0
A 0.71 B 0.14
A 0.71 B 0.29
A 0.71 B 0.43
A 0.71 B 0.57
A 0.71 B 0.71
A 0.71 B 0.86
A 0.71 B 1
A 0.86 B 0
A 0.86 B 0.14
A 0.86 B 0.29
A 0.86 B 0.43
A 0.86 B 0.57
A 0.86 B 0.71
A 0.86 B 0.86
A 0.86 B 1
A 1 B 0
A 1 B 0.14
A 1 B 0.29
A 1 B 0.43
A 1 B 0.57
A 1 B 0.71
A 1 B 0.86
A 1 B 1
1.2
0.9
0.6
0.3
OD 600 nm [−]
0 5 101520
Time [h]

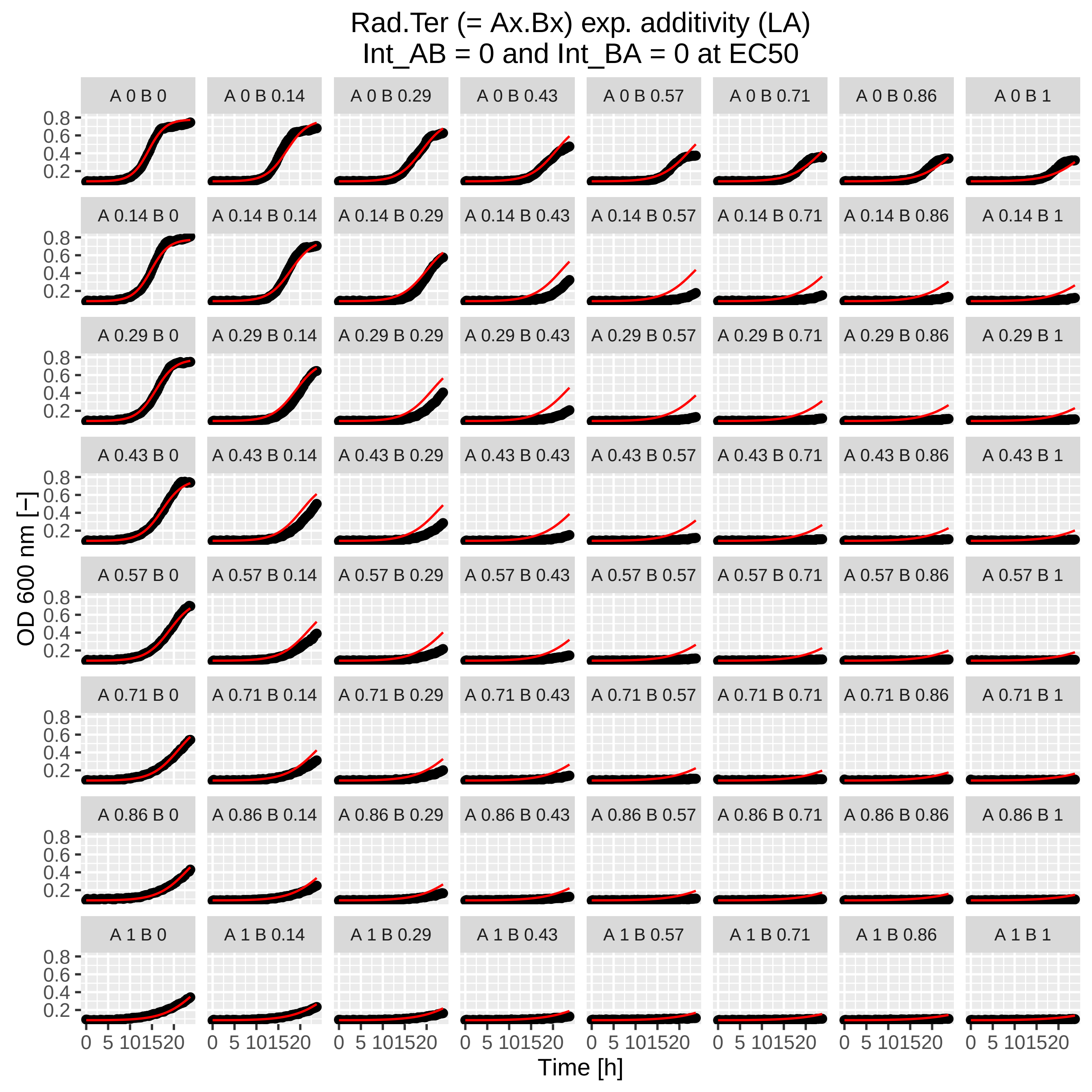
Rad.Ter (= Ax.Bx) exp. additivity (LA)
Int_AB = 0 and Int_BA = 0 at EC50
A 0 B 0
A 0 B 0.14
A 0 B 0.29
A 0 B 0.43
A 0 B 0.57
A 0 B 0.71
A 0 B 0.86
A 0 B 1
A 0.14 B 0
A 0.14 B 0.14
A 0.14 B 0.29
A 0.14 B 0.43
A 0.14 B 0.57
A 0.14 B 0.71
A 0.14 B 0.86
A 0.14 B 1
A 0.29 B 0
A 0.29 B 0.14
A 0.29 B 0.29
A 0.29 B 0.43
A 0.29 B 0.57
A 0.29 B 0.71
A 0.29 B 0.86
A 0.29 B 1
A 0.43 B 0
A 0.43 B 0.14
A 0.43 B 0.29
A 0.43 B 0.43
A 0.43 B 0.57
A 0.43 B 0.71
A 0.43 B 0.86
A 0.43 B 1
A 0.57 B 0
A 0.57 B 0.14
A 0.57 B 0.29
A 0.57 B 0.43
A 0.57 B 0.57
A 0.57 B 0.71
A 0.57 B 0.86
A 0.57 B 1
A 0.71 B 0
A 0.71 B 0.14
A 0.71 B 0.29
A 0.71 B 0.43
A 0.71 B 0.57
A 0.71 B 0.71
A 0.71 B 0.86
A 0.71 B 1
A 0.86 B 0
A 0.86 B 0.14
A 0.86 B 0.29
A 0.86 B 0.43
A 0.86 B 0.57
A 0.86 B 0.71
A 0.86 B 0.86
A 0.86 B 1
A 1 B 0
A 1 B 0.14
A 1 B 0.29
A 1 B 0.43
A 1 B 0.57
A 1 B 0.71
A 1 B 0.86
A 1 B 1
OD 600 nm [−]
0.8
0.6
0.4
0.2
0 5 101520
Time [h]

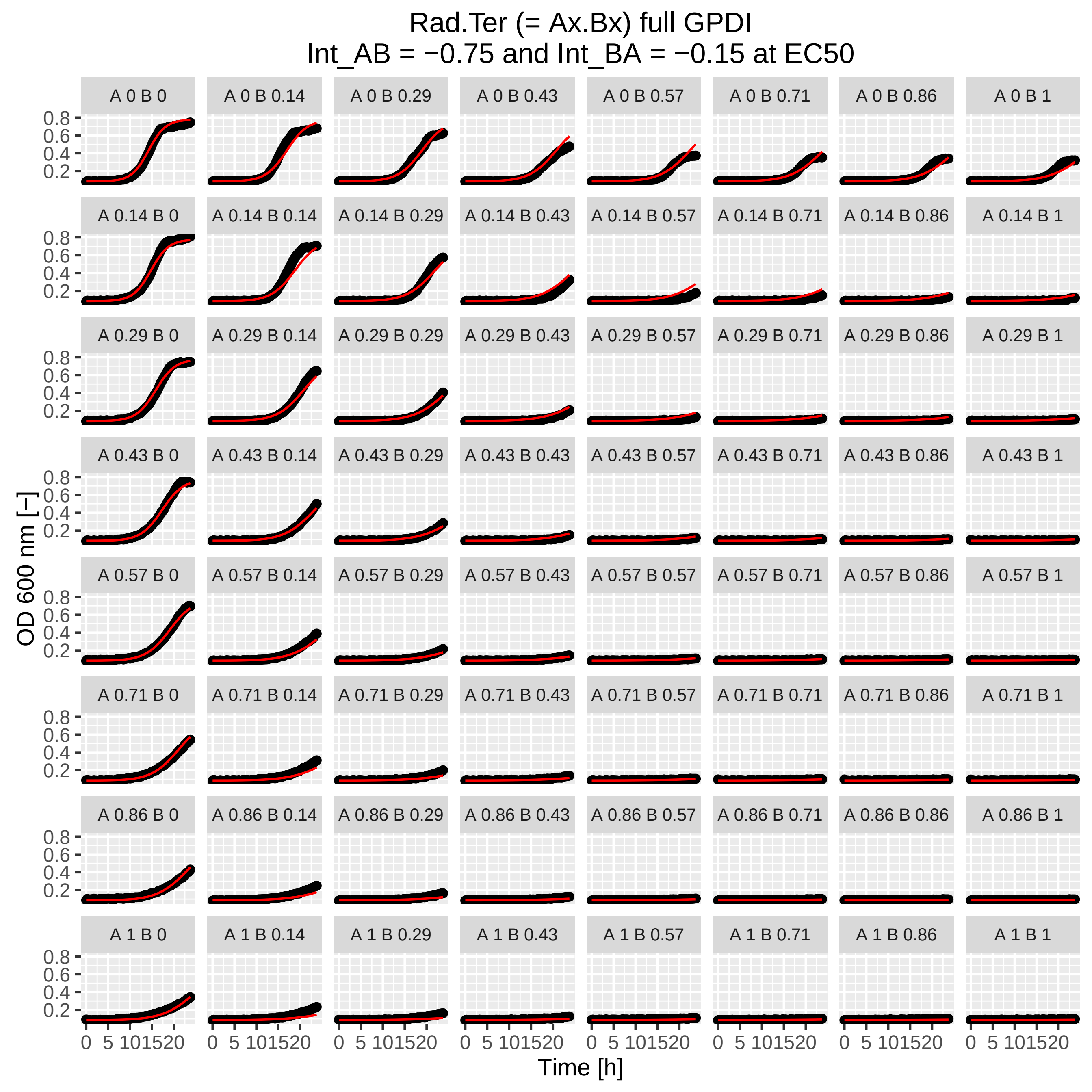
Rad.Ter (= Ax.Bx) full GPDI
Int_AB = −0.75 and Int_BA = −0.15 at EC50
OD 600 nm [−]
Time [h]
A 0 B 0
A 0 B 0.14
A 0 B 0.29
A 0 B 0.43
A 0 B 0.57
A 0 B 0.71
A 0 B 0.86
A 0 B 1
A 1 B 0
A 1 B 1
0.2
0.4
0.6
0.8
0
5
10
15
20

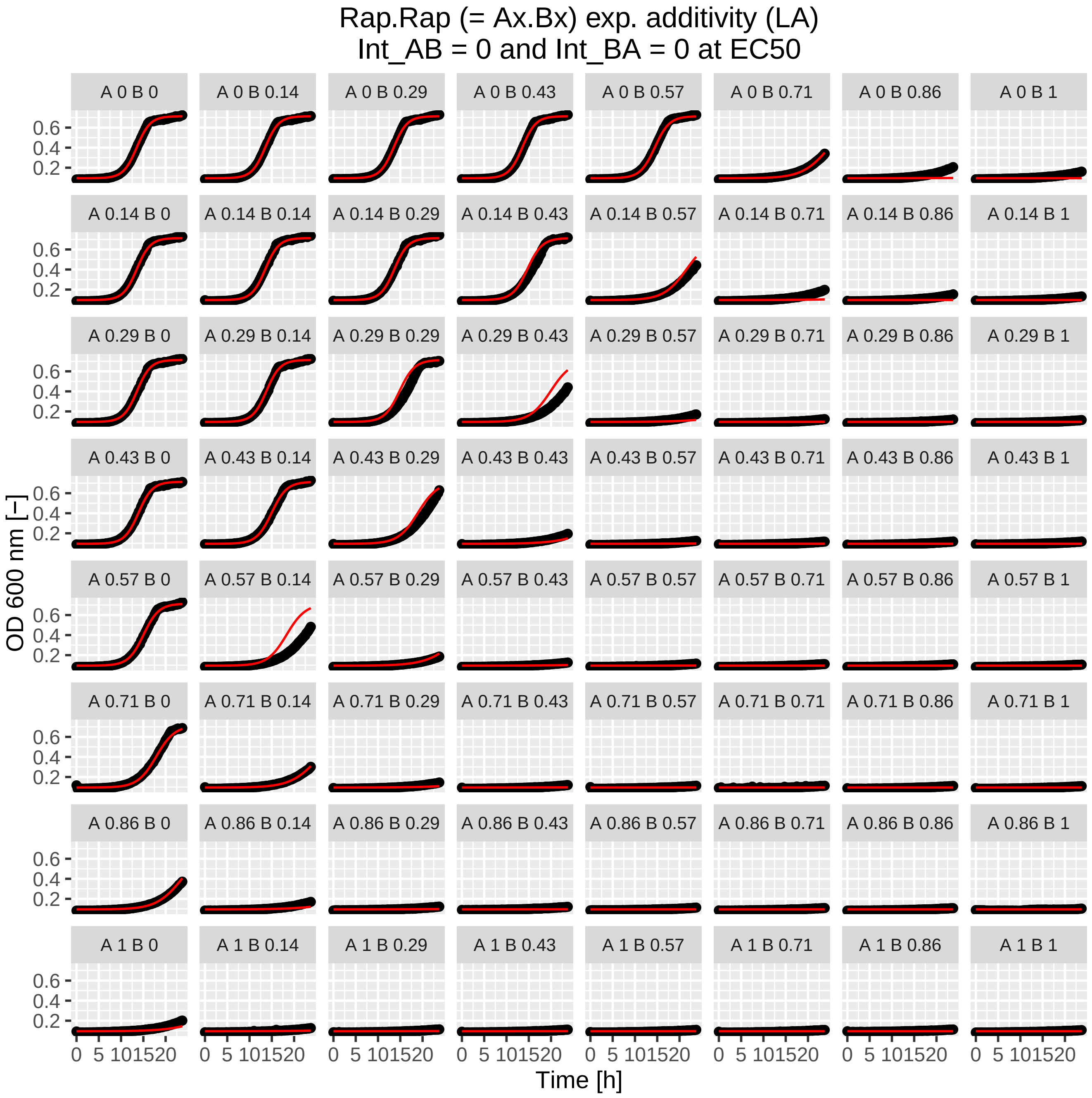
Rap.Rap (= Ax.Bx) exp. additivity (LA)
Int_AB = 0 and Int_BA = 0 at EC50
A 0 B 0
A 0 B 0.14
A 0 B 0.29
A 0 B 0.43
A 0 B 0.57
A 0 B 0.71
A 0 B 0.86
A 0 B 1
A 0.14 B 0
A 0.14 B 0.14
A 0.14 B 0.29
A 0.14 B 0.43
A 0.14 B 0.57
A 0.14 B 0.71
A 0.14 B 0.86
A 0.14 B 1
A 0.29 B 0
A 0.29 B 0.14
A 0.29 B 0.29
A 0.29 B 0.43
A 0.29 B 0.57
A 0.29 B 0.71
A 0.29 B 0.86
A 0.29 B 1
A 0.43 B 0
A 0.43 B 0.14
A 0.43 B 0.29
A 0.43 B 0.43
A 0.43 B 0.57
A 0.43 B 0.71
A 0.43 B 0.86
A 0.43 B 1
A 0.57 B 0
A 0.57 B 0.14
A 0.57 B 0.29
A 0.57 B 0.43
A 0.57 B 0.57
A 0.57 B 0.71
A 0.57 B 0.86
A 0.57 B 1
A 0.71 B 0
A 0.71 B 0.14
A 0.71 B 0.29
A 0.71 B 0.43
A 0.71 B 0.57
A 0.71 B 0.71
A 0.71 B 0.86
A 0.71 B 1
A 0.86 B 0
A 0.86 B 0.14
A 0.86 B 0.29
A 0.86 B 0.43
A 0.86 B 0.57
A 0.86 B 0.71
A 0.86 B 0.86
A 0.86 B 1
A 1 B 0
A 1 B 0.14
A 1 B 0.29
A 1 B 0.43
A 1 B 0.57
A 1 B 0.71
A 1 B 0.86
A 1 B 1
OD 600 nm [–]
0.2
0.4
0.6
0 5 101520
Time [h]

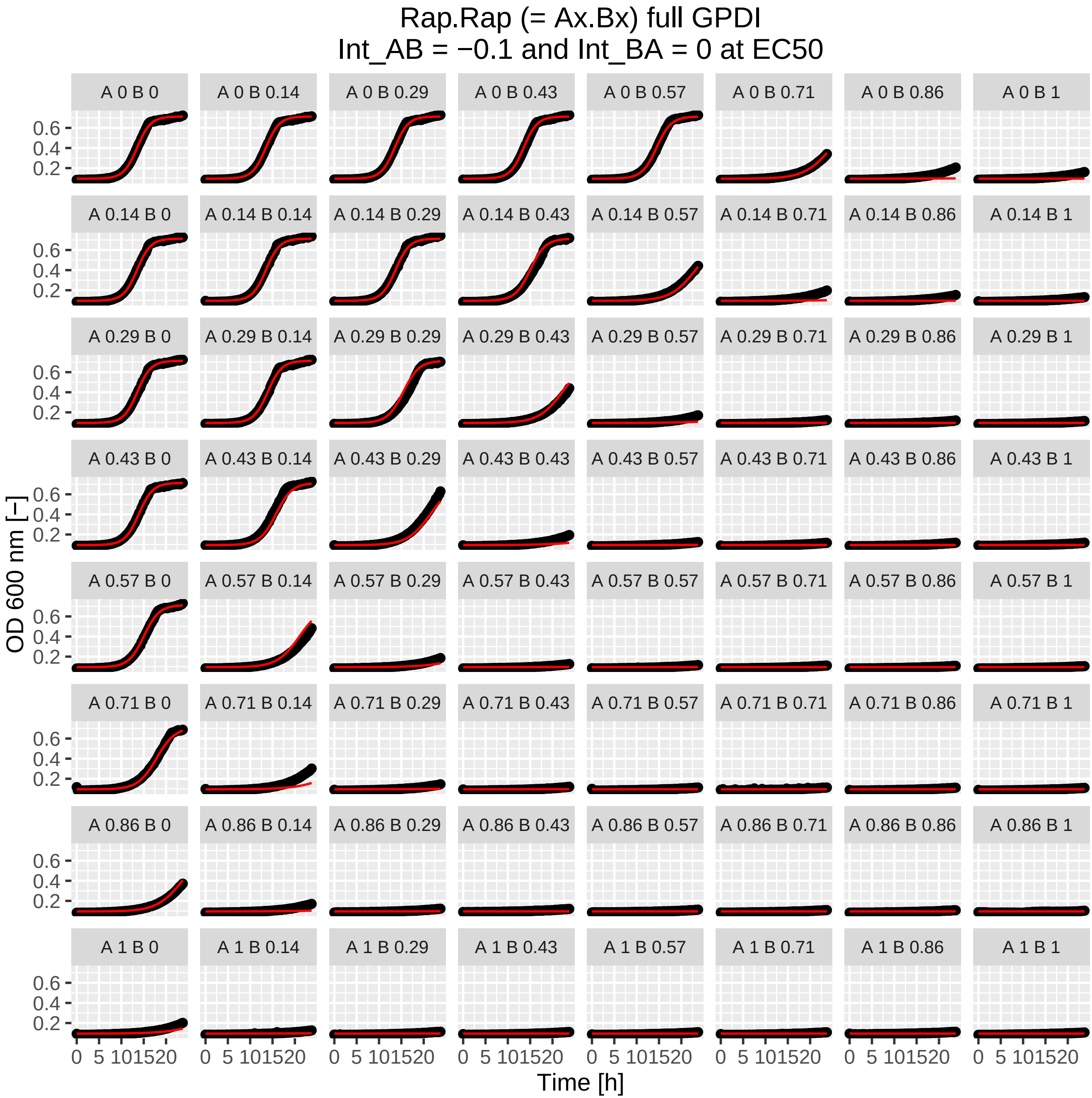

Rap.Rap (= Ax.Bx) full GPDI
Int_AB = −0.1 and Int_BA = 0 at EC50
A 0 B 0
A 0 B 0.14
A 0 B 0.29
A 0 B 0.43
A 0 B 0.57
A 0 B 0.71
A 0 B 0.86
A 0 B 1
A 0.14 B 0
A 0.14 B 0.14
A 0.14 B 0.29
A 0.14 B 0.43
A 0.14 B 0.57
A 0.14 B 0.71
A 0.14 B 0.86
A 0.14 B 1
A 0.29 B 0
A 0.29 B 0.14
A 0.29 B 0.29
A 0.29 B 0.43
A 0.29 B 0.57
A 0.29 B 0.71
A 0.29 B 0.86
A 0.29 B 1
A 0.43 B 0
A 0.43 B 0.14
A 0.43 B 0.29
A 0.43 B 0.43
A 0.43 B 0.57
A 0.43 B 0.71
A 0.43 B 0.86
A 0.43 B 1
A 0.57 B 0
A 0.57 B 0.14
A 0.57 B 0.29
A 0.57 B 0.43
A 0.57 B 0.57
A 0.57 B 0.71
A 0.57 B 0.86
A 0.57 B 1
A 0.71 B 0
A 0.71 B 0.14
A 0.71 B 0.29
A 0.71 B 0.43
A 0.71 B 0.57
A 0.71 B 0.71
A 0.71 B 0.86
A 0.71 B 1
A 0.86 B 0
A 0.86 B 0.14
A 0.86 B 0.29
A 0.86 B 0.43
A 0.86 B 0.57
A 0.86 B 0.71
A 0.86 B 0.86
A 0.86 B 1
A 1 B 0
A 1 B 0.14
A 1 B 0.29
A 1 B 0.43
A 1 B 0.57
A 1 B 0.71
A 1 B 0.86
A 1 B 1
OD 600 nm [–]
0.2
0.4
0.6
0 5 101520
Time [h]

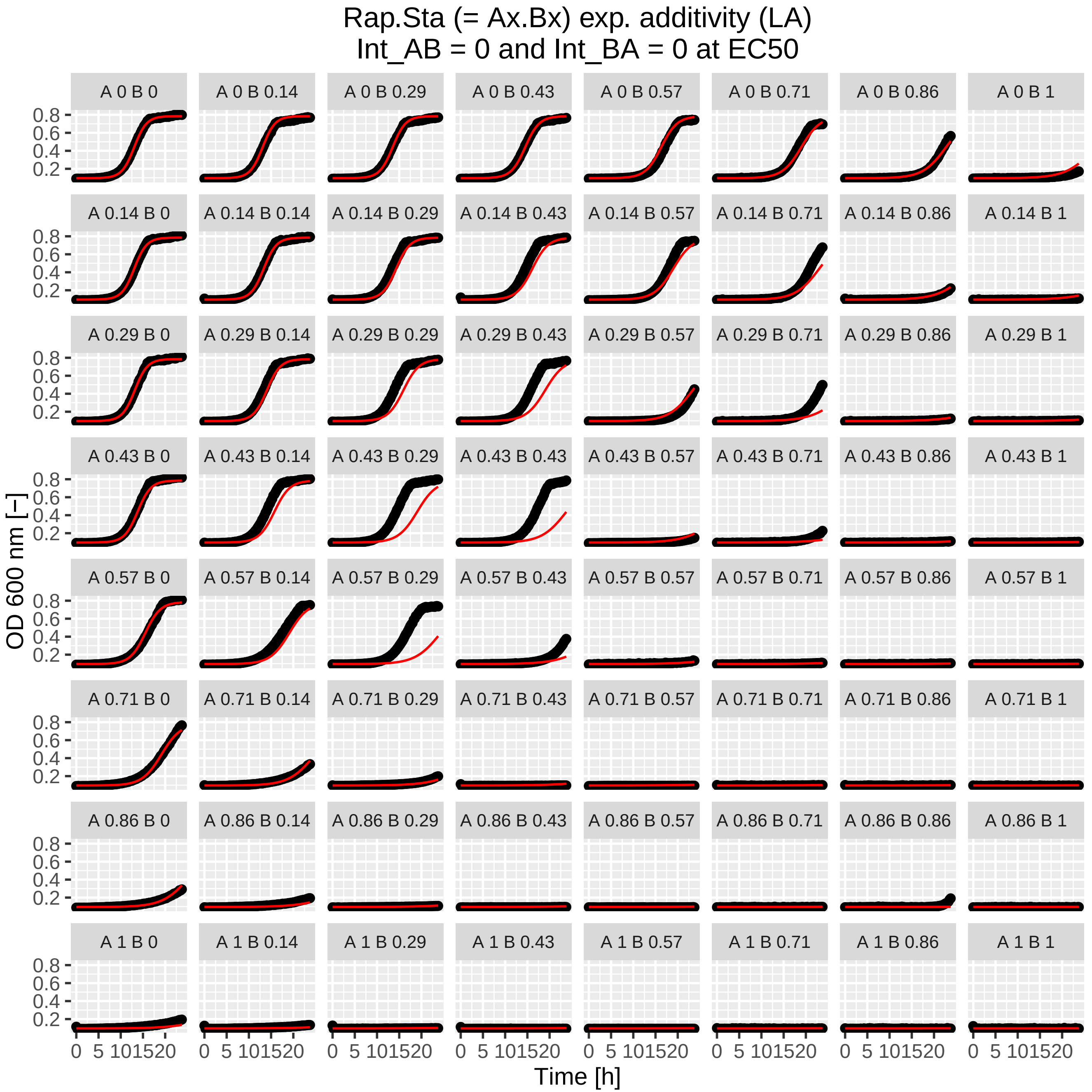

Rap.Sta (= Ax.Bx) exp. additivity (LA)
Int_AB = 0 and Int_BA = 0 at EC50
OD 600 nm [–]
Time [h]
A 0 B 0
A 0 B 0.14
A 0 B 0.29
A 0 B 0.43
A 0 B 0.57
A 0 B 0.71
A 0 B 0.86
A 0 B 1
A 0.14 B 0
A 0.14 B 0.14
A 0.14 B 0.29
A 0.14 B 0.43
A 0.14 B 0.57
A 0.14 B 0.71
A 0.14 B 0.86
A 0.14 B 1
A 0.29 B 0
A 0.29 B 0.14
A 0.29 B 0.29
A 0.29 B 0.43
A 0.29 B 0.57
A 0.29 B 0.71
A 0.29 B 0.86
A 0.29 B 1
A 0.43 B 0
A 0.43 B 0.14
A 0.43 B 0.29
A 0.43 B 0.43
A 0.43 B 0.57
A 0.43 B 0.71
A 0.43 B 0.86
A 0.43 B 1
A 0.57 B 0
A 0.57 B 0.14
A 0.57 B 0.29
A 0.57 B 0.43
A 0.57 B 0.57
A 0.57 B 0.71
A 0.57 B 0.86
A 0.57 B 1
A 0.71 B 0
A 0.71 B 0.14
A 0.71 B 0.29
A 0.71 B 0.43
A 0.71 B 0.57
A 0.71 B 0.71
A 0.71 B 0.86
A 0.71 B 1
A 0.86 B 0
A 0.86 B 0.14
A 0.86 B 0.29
A 0.86 B 0.43
A 0.86 B 0.57
A 0.86 B 0.71
A 0.86 B 0.86
A 0.86 B 1
A 1 B 0
A 1 B 0.14
A 1 B 0.29
A 1 B 0.43
A 1 B 0.57
A 1 B 0.71
A 1 B 0.86
A 1 B 1
0.8
0.6
0.4
0.2
0 5 101520

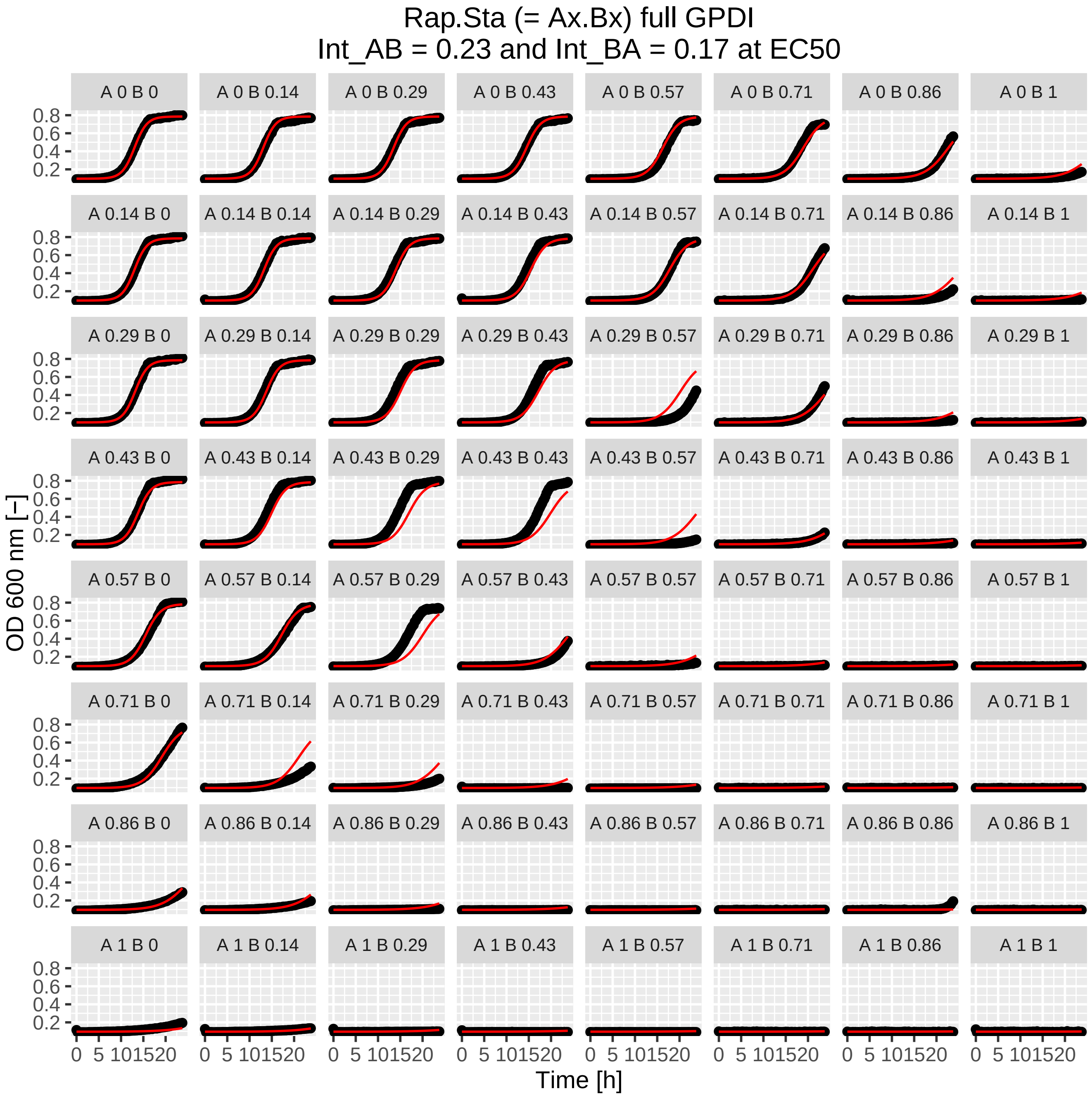
Rap.Sta (= Ax.Bx) full GPDI
Int_AB = 0.23 and Int_BA = 0.17 at EC50
OD 600 nm [–]
Time [h]
A 0 B 0
A 0 B 0.14
A 0 B 0.29
A 0 B 0.43
A 0 B 0.57
A 0 B 0.71
A 0 B 0.86
A 0 B 1
A 0.14 B 0
A 0.14 B 0.14
A 0.14 B 0.29
A 0.14 B 0.43
A 0.14 B 0.57
A 0.14 B 0.71
A 0.14 B 0.86
A 0.14 B 1
A 0.29 B 0
A 0.29 B 0.14
A 0.29 B 0.29
A 0.29 B 0.43
A 0.29 B 0.57
A 0.29 B 0.71
A 0.29 B 0.86
A 0.29 B 1
A 0.43 B 0
A 0.43 B 0.14
A 0.43 B 0.29
A 0.43 B 0.43
A 0.43 B 0.57
A 0.43 B 0.71
A 0.43 B 0.86
A 0.43 B 1
A 0.57 B 0
A 0.57 B 0.14
A 0.57 B 0.29
A 0.57 B 0.43
A 0.57 B 0.57
A 0.57 B 0.71
A 0.57 B 0.86
A 0.57 B 1
A 0.71 B 0
A 0.71 B 0.14
A 0.71 B 0.29
A 0.71 B 0.43
A 0.71 B 0.57
A 0.71 B 0.71
A 0.71 B 0.86
A 0.71 B 1
A 0.86 B 0
A 0.86 B 0.14
A 0.86 B 0.29
A 0.86 B 0.43
A 0.86 B 0.57
A 0.86 B 0.71
A 0.86 B 0.86
A 0.86 B 1
A 1 B 0
A 1 B 0.14
A 1 B 0.29
A 1 B 0.43
A 1 B 0.57
A 1 B 0.71
A 1 B 0.86
A 1 B 1
0.8
0.6
0.4
0.2
0 5 101520

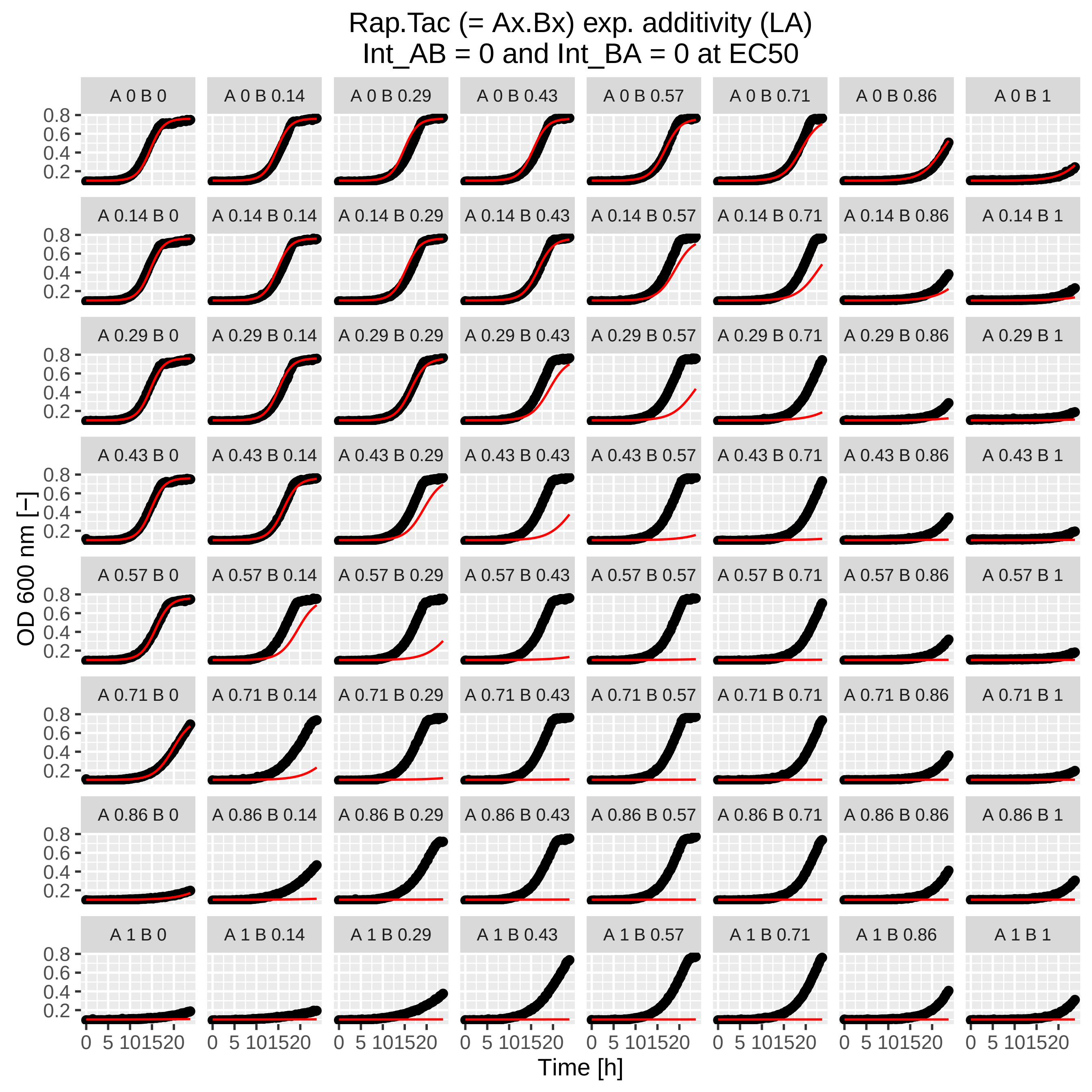
Rap.Tac (= Ax.Bx) exp. additivity (LA)
Int_AB = 0 and Int_BA = 0 at EC50
A 0 B 0
A 0 B 0.14
A 0 B 0.29
A 0 B 0.43
A 0 B 0.57
A 0 B 0.71
A 0 B 0.86
A 0 B 1
A 0.14 B 0
A 0.14 B 0.14
A 0.14 B 0.29
A 0.14 B 0.43
A 0.14 B 0.57
A 0.14 B 0.71
A 0.14 B 0.86
A 0.14 B 1
A 0.29 B 0
A 0.29 B 0.14
A 0.29 B 0.29
A 0.29 B 0.43
A 0.29 B 0.57
A 0.29 B 0.71
A 0.29 B 0.86
A 0.29 B 1
A 0.43 B 0
A 0.43 B 0.14
A 0.43 B 0.29
A 0.43 B 0.43
A 0.43 B 0.57
A 0.43 B 0.71
A 0.43 B 0.86
A 0.43 B 1
A 0.57 B 0
A 0.57 B 0.14
A 0.57 B 0.29
A 0.57 B 0.43
A 0.57 B 0.57
A 0.57 B 0.71
A 0.57 B 0.86
A 0.57 B 1
A 0.71 B 0
A 0.71 B 0.14
A 0.71 B 0.29
A 0.71 B 0.43
A 0.71 B 0.57
A 0.71 B 0.71
A 0.71 B 0.86
A 0.71 B 1
A 0.86 B 0
A 0.86 B 0.14
A 0.86 B 0.29
A 0.86 B 0.43
A 0.86 B 0.57
A 0.86 B 0.71
A 0.86 B 0.86
A 0.86 B 1
A 1 B 0
A 1 B 0.14
A 1 B 0.29
A 1 B 0.43
A 1 B 0.57
A 1 B 0.71
A 1 B 0.86
A 1 B 1
OD 600 nm [−]
0.2
0.4
0.6
0.8
0 5 101520
Time [h]

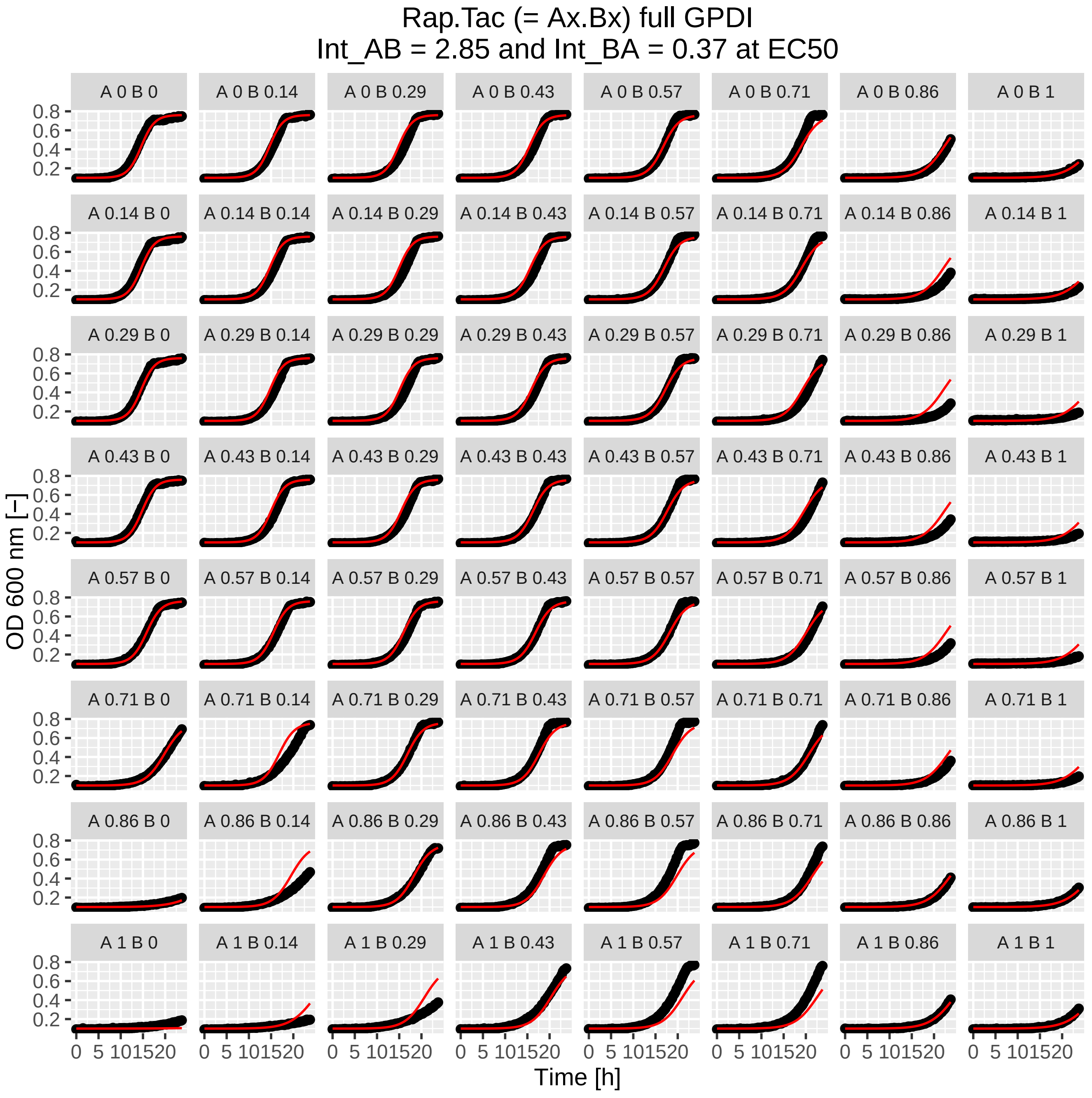

Rap.Tac (= Ax.Bx) full GPDI
Int_AB = 2.85 and Int_BA = 0.37 at EC50
A 0 B 0
A 0 B 0.14
A 0 B 0.29
A 0 B 0.43
A 0 B 0.57
A 0 B 0.71
A 0 B 0.86
A 0 B 1
A 0.14 B 0
A 0.14 B 0.14
A 0.14 B 0.29
A 0.14 B 0.43
A 0.14 B 0.57
A 0.14 B 0.71
A 0.14 B 0.86
A 0.14 B 1
A 0.29 B 0
A 0.29 B 0.14
A 0.29 B 0.29
A 0.29 B 0.43
A 0.29 B 0.57
A 0.29 B 0.71
A 0.29 B 0.86
A 0.29 B 1
A 0.43 B 0
A 0.43 B 0.14
A 0.43 B 0.29
A 0.43 B 0.43
A 0.43 B 0.57
A 0.43 B 0.71
A 0.43 B 0.86
A 0.43 B 1
A 0.57 B 0
A 0.57 B 0.14
A 0.57 B 0.29
A 0.57 B 0.43
A 0.57 B 0.57
A 0.57 B 0.71
A 0.57 B 0.86
A 0.57 B 1
A 0.71 B 0
A 0.71 B 0.14
A 0.71 B 0.29
A 0.71 B 0.43
A 0.71 B 0.57
A 0.71 B 0.71
A 0.71 B 0.86
A 0.71 B 1
A 0.86 B 0
A 0.86 B 0.14
A 0.86 B 0.29
A 0.86 B 0.43
A 0.86 B 0.57
A 0.86 B 0.71
A 0.86 B 0.86
A 0.86 B 1
A 1 B 0
A 1 B 0.14
A 1 B 0.29
A 1 B 0.43
A 1 B 0.57
A 1 B 0.71
A 1 B 0.86
A 1 B 1
OD 600 nm [–]
0.8
0.6
0.4
0.2
0 5 101520
Time [h]

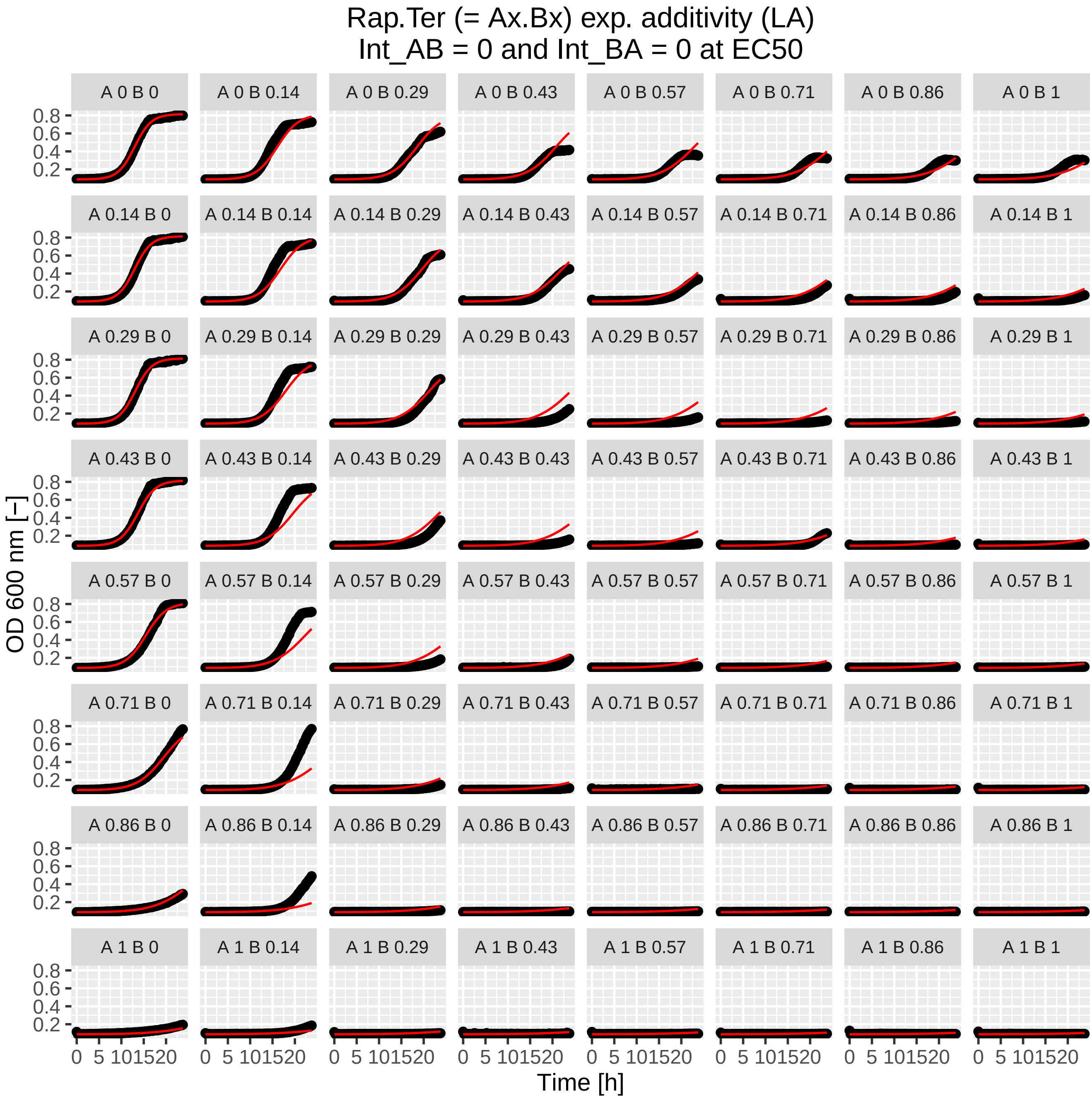

Rap.Ter (= Ax.Bx) exp. additivity (LA)
Int_AB = 0 and Int_BA = 0 at EC50
A 0 B 0
A 0 B 0.14
A 0 B 0.29
A 0 B 0.43
A 0 B 0.57
A 0 B 0.71
A 0 B 0.86
A 0 B 1
A 0.14 B 0
A 0.14 B 0.14
A 0.14 B 0.29
A 0.14 B 0.43
A 0.14 B 0.57
A 0.14 B 0.71
A 0.14 B 0.86
A 0.14 B 1
A 0.29 B 0
A 0.29 B 0.14
A 0.29 B 0.29
A 0.29 B 0.43
A 0.29 B 0.57
A 0.29 B 0.71
A 0.29 B 0.86
A 0.29 B 1
A 0.43 B 0
A 0.43 B 0.14
A 0.43 B 0.29
A 0.43 B 0.43
A 0.43 B 0.57
A 0.43 B 0.71
A 0.43 B 0.86
A 0.43 B 1
A 0.57 B 0
A 0.57 B 0.14
A 0.57 B 0.29
A 0.57 B 0.43
A 0.57 B 0.57
A 0.57 B 0.71
A 0.57 B 0.86
A 0.57 B 1
A 0.71 B 0
A 0.71 B 0.14
A 0.71 B 0.29
A 0.71 B 0.43
A 0.71 B 0.57
A 0.71 B 0.71
A 0.71 B 0.86
A 0.71 B 1
A 0.86 B 0
A 0.86 B 0.14
A 0.86 B 0.29
A 0.86 B 0.43
A 0.86 B 0.57
A 0.86 B 0.71
A 0.86 B 0.86
A 0.86 B 1
A 1 B 0
A 1 B 0.14
A 1 B 0.29
A 1 B 0.43
A 1 B 0.57
A 1 B 0.71
A 1 B 0.86
A 1 B 1
0.8
0.6
0.4
0.2
OD 600 nm [–]
0 5 101520
Time [h]

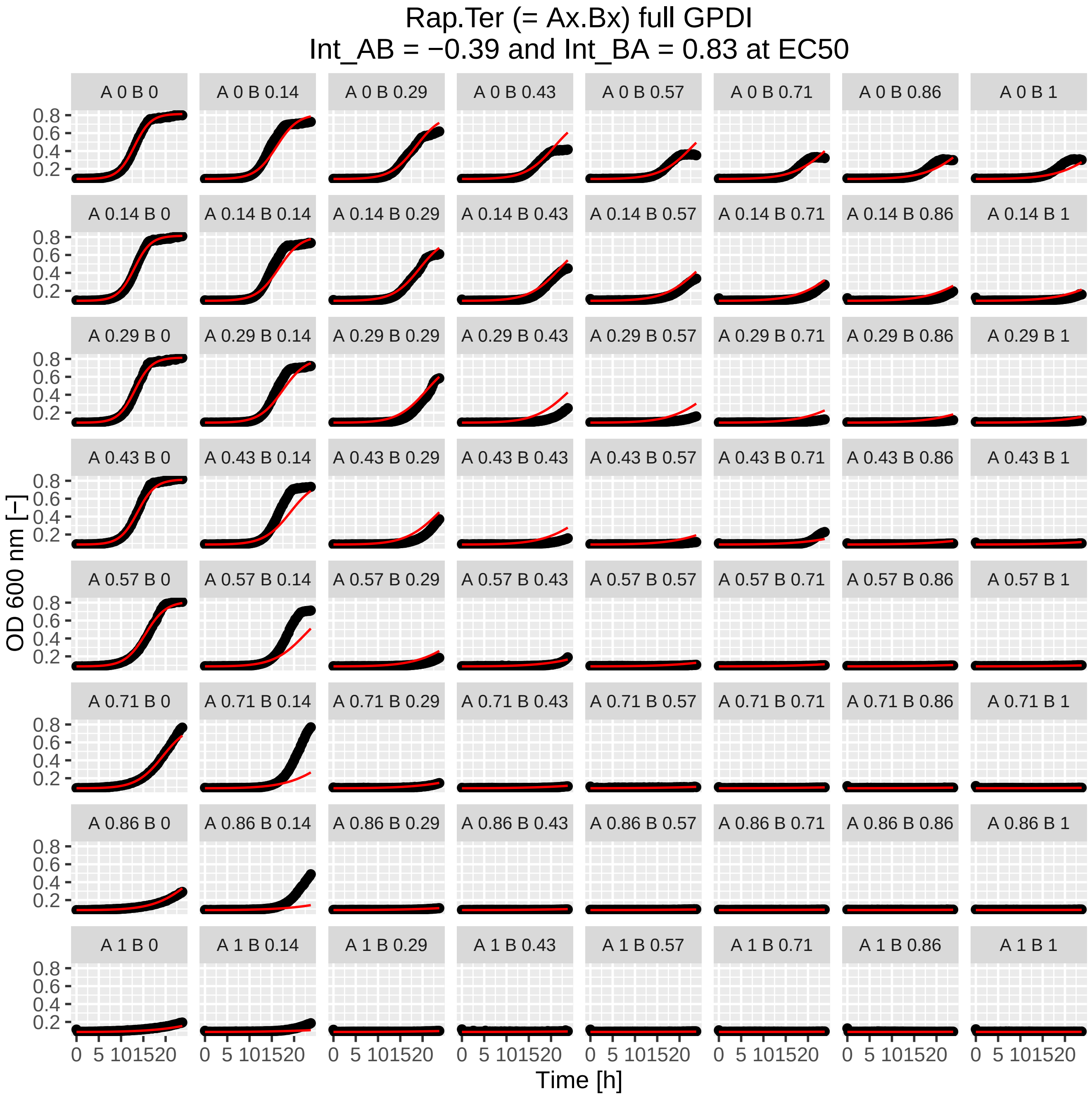
Rap.Ter (= Ax.Bx) full GPDI
Int_AB = −0.39 and Int_BA = 0.83 at EC50
OD 600 nm [−]
Time [h]
0.8
0.6
0.4
0.2
0 5 101520

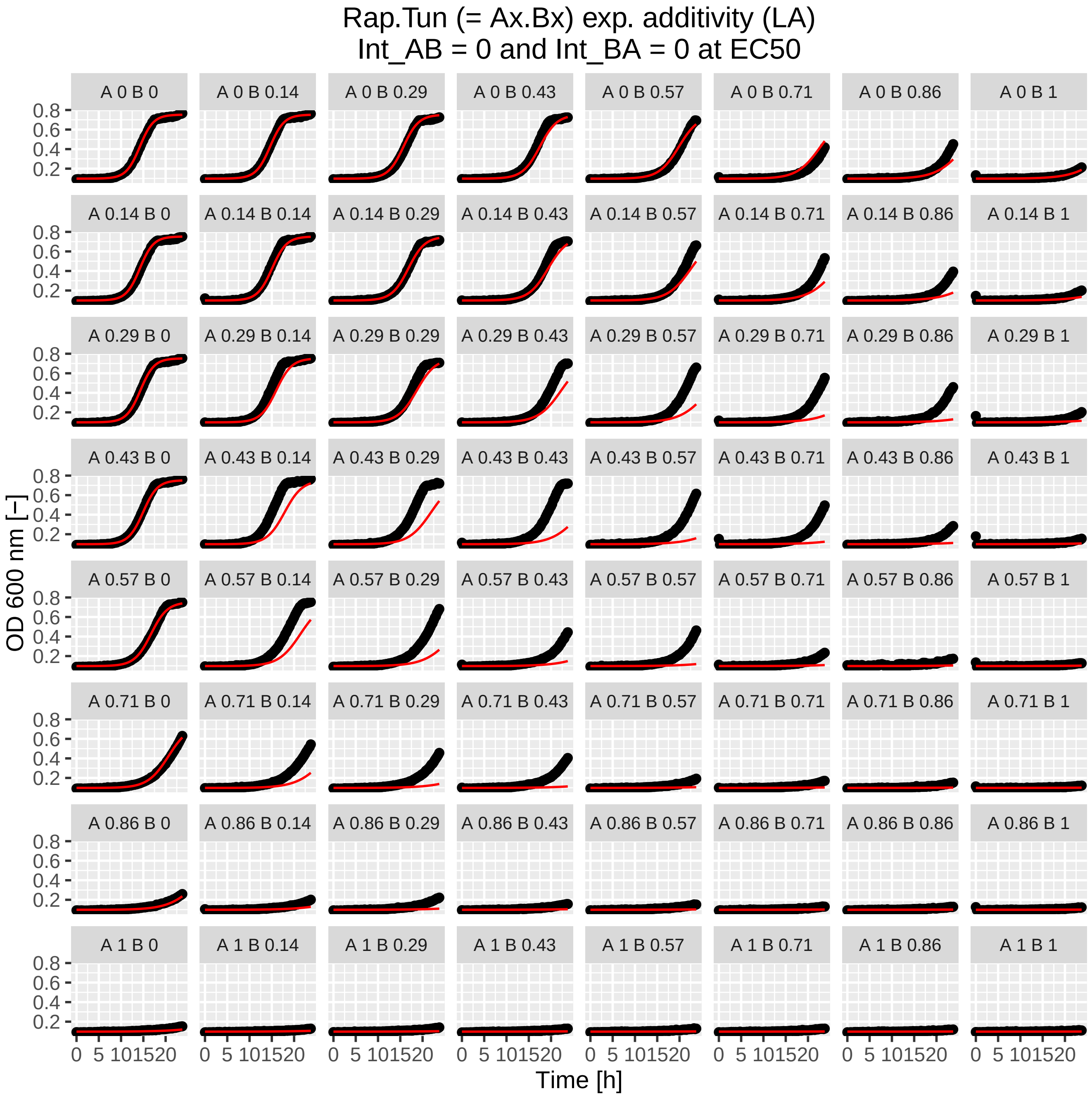
Rap.Tun (= Ax.Bx) exp. additivity (LA)
Int_AB = 0 and Int_BA = 0 at EC50
A 0 B 0
A 0 B 0.14
A 0 B 0.29
A 0 B 0.43
A 0 B 0.57
A 0 B 0.71
A 0 B 0.86
A 0 B 1
A 0.14 B 0
A 0.14 B 0.14
A 0.14 B 0.29
A 0.14 B 0.43
A 0.14 B 0.57
A 0.14 B 0.71
A 0.14 B 0.86
A 0.14 B 1
A 0.29 B 0
A 0.29 B 0.14
A 0.29 B 0.29
A 0.29 B 0.43
A 0.29 B 0.57
A 0.29 B 0.71
A 0.29 B 0.86
A 0.29 B 1
A 0.43 B 0
A 0.43 B 0.14
A 0.43 B 0.29
A 0.43 B 0.43
A 0.43 B 0.57
A 0.43 B 0.71
A 0.43 B 0.86
A 0.43 B 1
A 0.57 B 0
A 0.57 B 0.14
A 0.57 B 0.29
A 0.57 B 0.43
A 0.57 B 0.57
A 0.57 B 0.71
A 0.57 B 0.86
A 0.57 B 1
A 0.71 B 0
A 0.71 B 0.14
A 0.71 B 0.29
A 0.71 B 0.43
A 0.71 B 0.57
A 0.71 B 0.71
A 0.71 B 0.86
A 0.71 B 1
A 0.86 B 0
A 0.86 B 0.14
A 0.86 B 0.29
A 0.86 B 0.43
A 0.86 B 0.57
A 0.86 B 0.71
A 0.86 B 0.86
A 0.86 B 1
A 1 B 0
A 1 B 0.14
A 1 B 0.29
A 1 B 0.43
A 1 B 0.57
A 1 B 0.71
A 1 B 0.86
A 1 B 1
OD 600 nm [–]
0.2
0.4
0.6
0.8
0
5
10
15
20
Time [h]

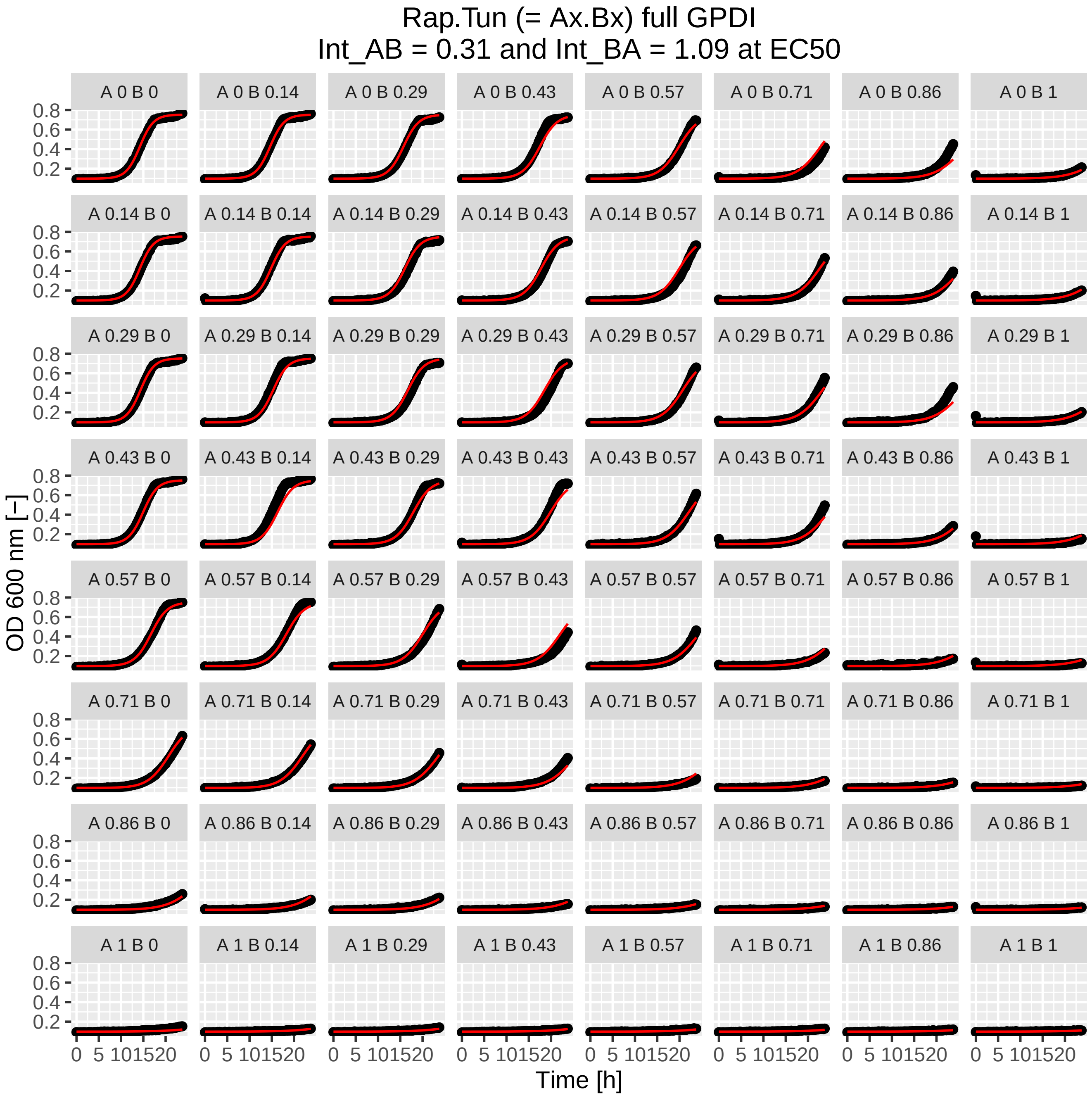

Rap.Tun (= Ax.Bx) full GPDI
Int_AB = 0.31 and Int_BA = 1.09 at EC50
OD 600 nm [–]
Time [h]
A 0 B 0
A 0 B 0.14
A 0 B 0.29
A 0 B 0.43
A 0 B 0.57
A 0 B 0.71
A 0 B 0.86
A 0 B 1
A 0.14 B 0
A 0.14 B 0.14
A 0.14 B 0.29
A 0.14 B 0.43
A 0.14 B 0.57
A 0.14 B 0.71
A 0.14 B 0.86
A 0.14 B 1
A 0.29 B 0
A 0.29 B 0.14
A 0.29 B 0.29
A 0.29 B 0.43
A 0.29 B 0.57
A 0.29 B 0.71
A 0.29 B 0.86
A 0.29 B 1
A 0.43 B 0
A 0.43 B 0.14
A 0.43 B 0.29
A 0.43 B 0.43
A 0.43 B 0.57
A 0.43 B 0.71
A 0.43 B 0.86
A 0.43 B 1
A 0.57 B 0
A 0.57 B 0.14
A 0.57 B 0.29
A 0.57 B 0.43
A 0.57 B 0.57
A 0.57 B 0.71
A 0.57 B 0.86
A 0.57 B 1
A 0.71 B 0
A 0.71 B 0.14
A 0.71 B 0.29
A 0.71 B 0.43
A 0.71 B 0.57
A 0.71 B 0.71
A 0.71 B 0.86
A 0.71 B 1
A 0.86 B 0
A 0.86 B 0.14
A 0.86 B 0.29
A 0.86 B 0.43
A 0.86 B 0.57
A 0.86 B 0.71
A 0.86 B 0.86
A 0.86 B 1
A 1 B 0
A 1 B 0.14
A 1 B 0.29
A 1 B 0.43
A 1 B 0.57
A 1 B 0.71
A 1 B 0.86
A 1 B 1
0.8
0.6
0.4
0.2
0 5 101520

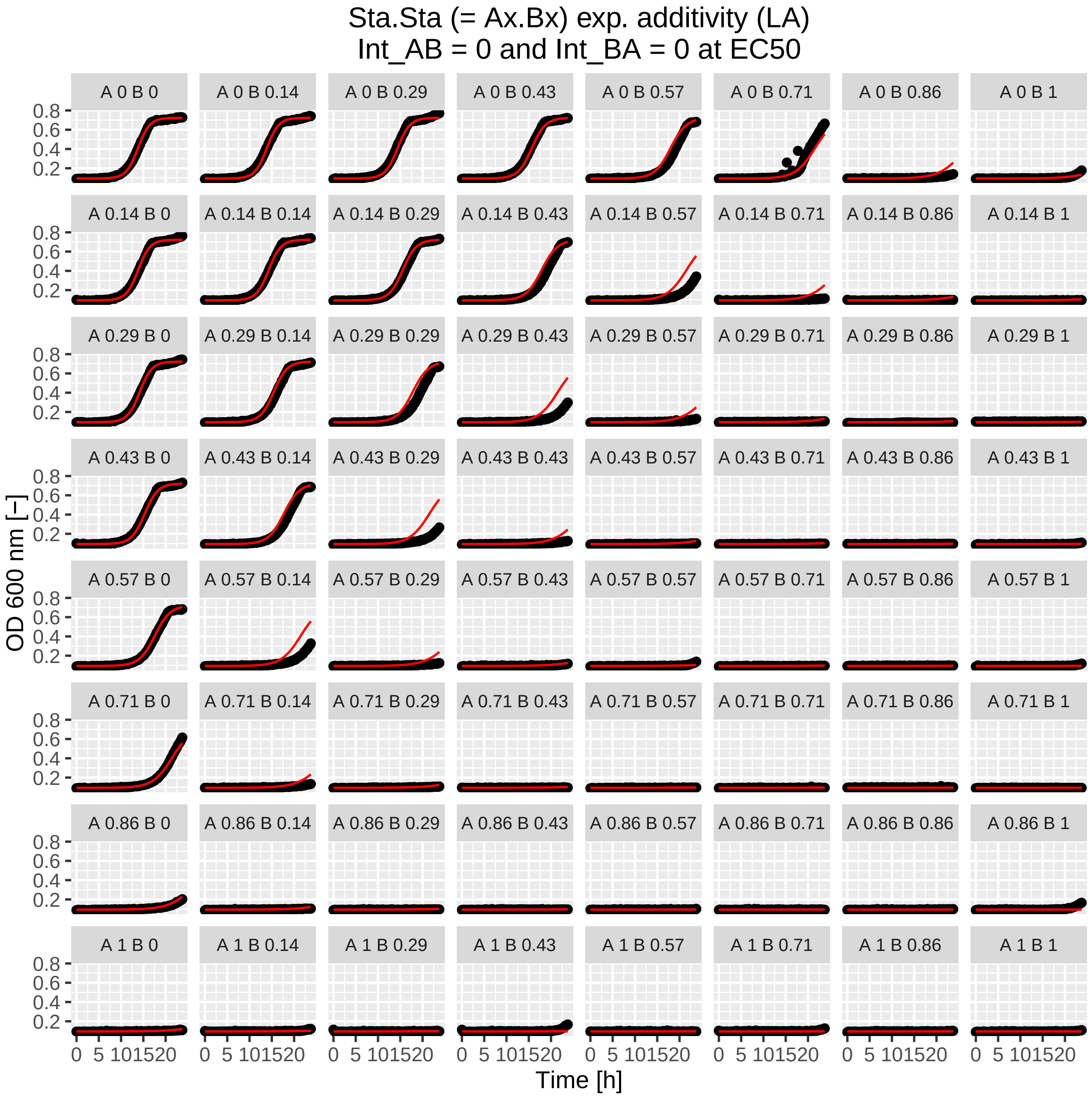

Sta.Sta (= Ax.Bx) exp. additivity (LA)
Int_AB = 0 and Int_BA = 0 at EC50
A 0 B 0
A 0 B 0.14
A 0 B 0.29
A 0 B 0.43
A 0 B 0.57
A 0 B 0.71
A 0 B 0.86
A 0 B 1
A 0.14 B 0
A 0.14 B 0.14
A 0.14 B 0.29
A 0.14 B 0.43
A 0.14 B 0.57
A 0.14 B 0.71
A 0.14 B 0.86
A 0.14 B 1
A 0.29 B 0
A 0.29 B 0.14
A 0.29 B 0.29
A 0.29 B 0.43
A 0.29 B 0.57
A 0.29 B 0.71
A 0.29 B 0.86
A 0.29 B 1
A 0.43 B 0
A 0.43 B 0.14
A 0.43 B 0.29
A 0.43 B 0.43
A 0.43 B 0.57
A 0.43 B 0.71
A 0.43 B 0.86
A 0.43 B 1
A 0.57 B 0
A 0.57 B 0.14
A 0.57 B 0.29
A 0.57 B 0.43
A 0.57 B 0.57
A 0.57 B 0.71
A 0.57 B 0.86
A 0.57 B 1
A 0.71 B 0
A 0.71 B 0.14
A 0.71 B 0.29
A 0.71 B 0.43
A 0.71 B 0.57
A 0.71 B 0.71
A 0.71 B 0.86
A 0.71 B 1
A 0.86 B 0
A 0.86 B 0.14
A 0.86 B 0.29
A 0.86 B 0.43
A 0.86 B 0.57
A 0.86 B 0.71
A 0.86 B 0.86
A 0.86 B 1
A 1 B 0
A 1 B 0.14
A 1 B 0.29
A 1 B 0.43
A 1 B 0.57
A 1 B 0.71
A 1 B 0.86
A 1 B 1
OD 600 nm [−]
0.2
0.4
0.6
0.8
0
5
10
15
20
Time [h]

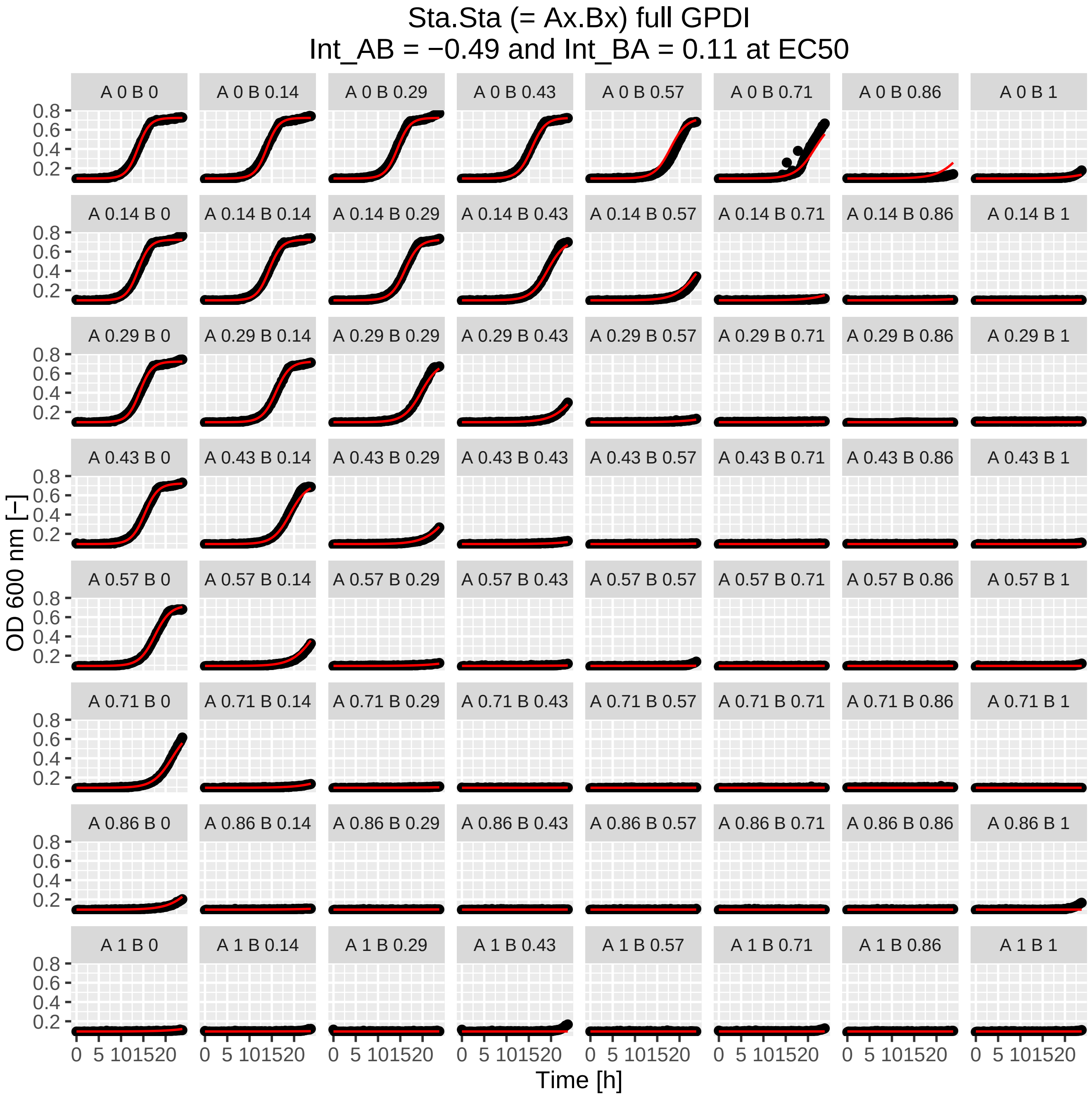

Sta.Sta (= Ax.Bx) full GPDI
Int_AB = −0.49 and Int_BA = 0.11 at EC50
OD 600 nm [−]
Time [h]
A 0 B 0
A 0 B 0.14
A 0 B 0.29
A 0 B 0.43
A 0 B 0.57
A 0 B 0.71
A 0 B 0.86
A 0 B 1
A 0.14 B 0
A 0.14 B 0.14
A 0.14 B 0.29
A 0.14 B 0.43
A 0.14 B 0.57
A 0.14 B 0.71
A 0.14 B 0.86
A 0.14 B 1
A 0.29 B 0
A 0.29 B 0.14
A 0.29 B 0.29
A 0.29 B 0.43
A 0.29 B 0.57
A 0.29 B 0.71
A 0.29 B 0.86
A 0.29 B 1
A 0.43 B 0
A 0.43 B 0.14
A 0.43 B 0.29
A 0.43 B 0.43
A 0.43 B 0.57
A 0.43 B 0.71
A 0.43 B 0.86
A 0.43 B 1
A 0.57 B 0
A 0.57 B 0.14
A 0.57 B 0.29
A 0.57 B 0.43
A 0.57 B 0.57
A 0.57 B 0.71
A 0.57 B 0.86
A 0.57 B 1
A 0.71 B 0
A 0.71 B 0.14
A 0.71 B 0.29
A 0.71 B 0.43
A 0.71 B 0.57
A 0.71 B 0.71
A 0.71 B 0.86
A 0.71 B 1
A 0.86 B 0
A 0.86 B 0.14
A 0.86 B 0.29
A 0.86 B 0.43
A 0.86 B 0.57
A 0.86 B 0.71
A 0.86 B 0.86
A 0.86 B 1
A 1 B 0
A 1 B 0.14
A 1 B 0.29
A 1 B 0.43
A 1 B 0.57
A 1 B 0.71
A 1 B 0.86
A 1 B 1
0.8
0.6
0.4
0.2
0 5 101520

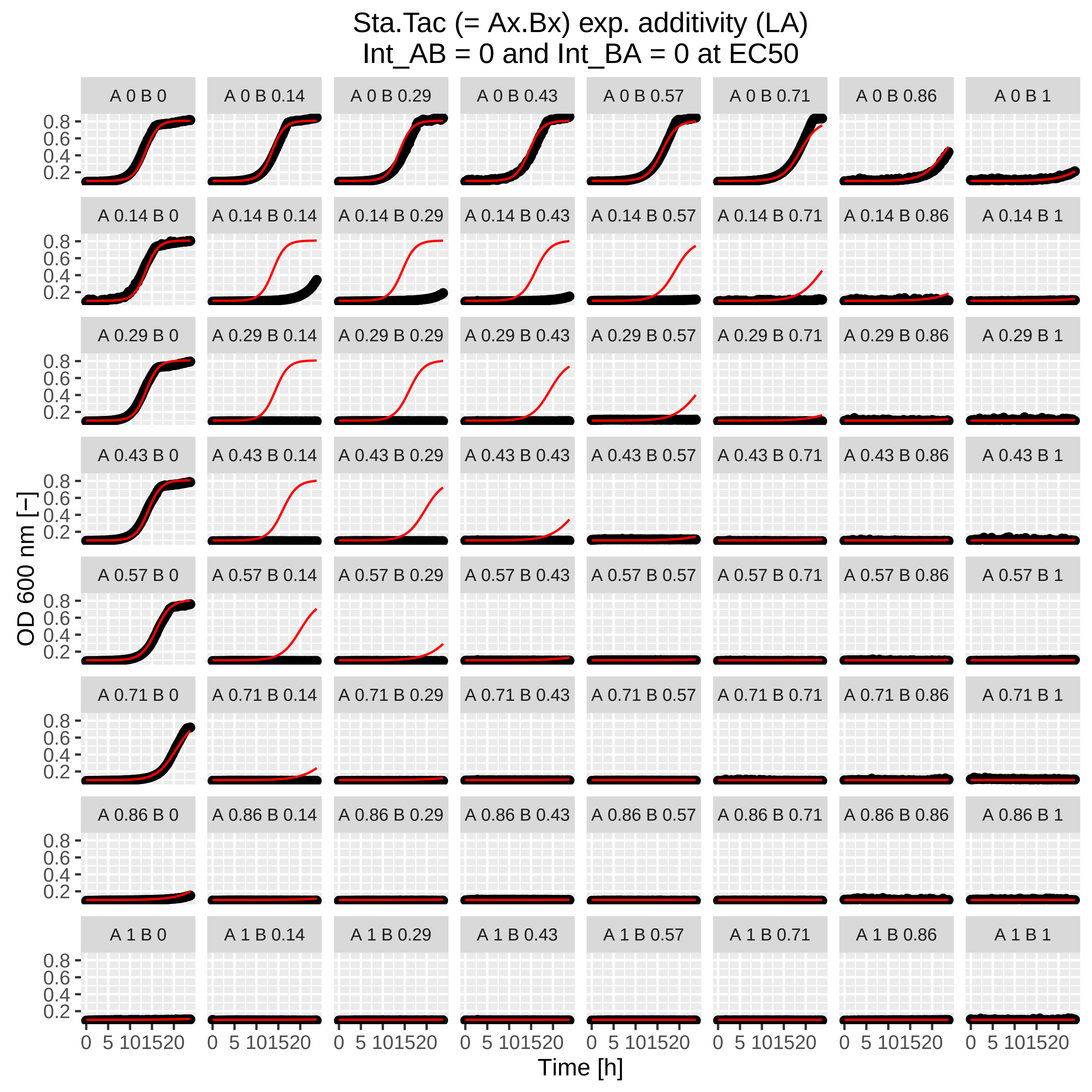
Sta.Tac (= Ax.Bx) exp. additivity (LA)
Int_AB = 0 and Int_BA = 0 at EC50
OD 600 nm [–]
Time [h]
A 0 B 0
A 0 B 0.14
A 0 B 0.29
A 0 B 0.43
A 0 B 0.57
A 0 B 0.71
A 0 B 0.86
A 0 B 1
A 0.14 B 0
A 0.29 B 0
A 0.43 B 0
A 0.57 B 0
A 0.71 B 0
A 0.86 B 0
A 1 B 0
A 1 B 1
0.8
0.6
0.4
0.2
0 5 101520

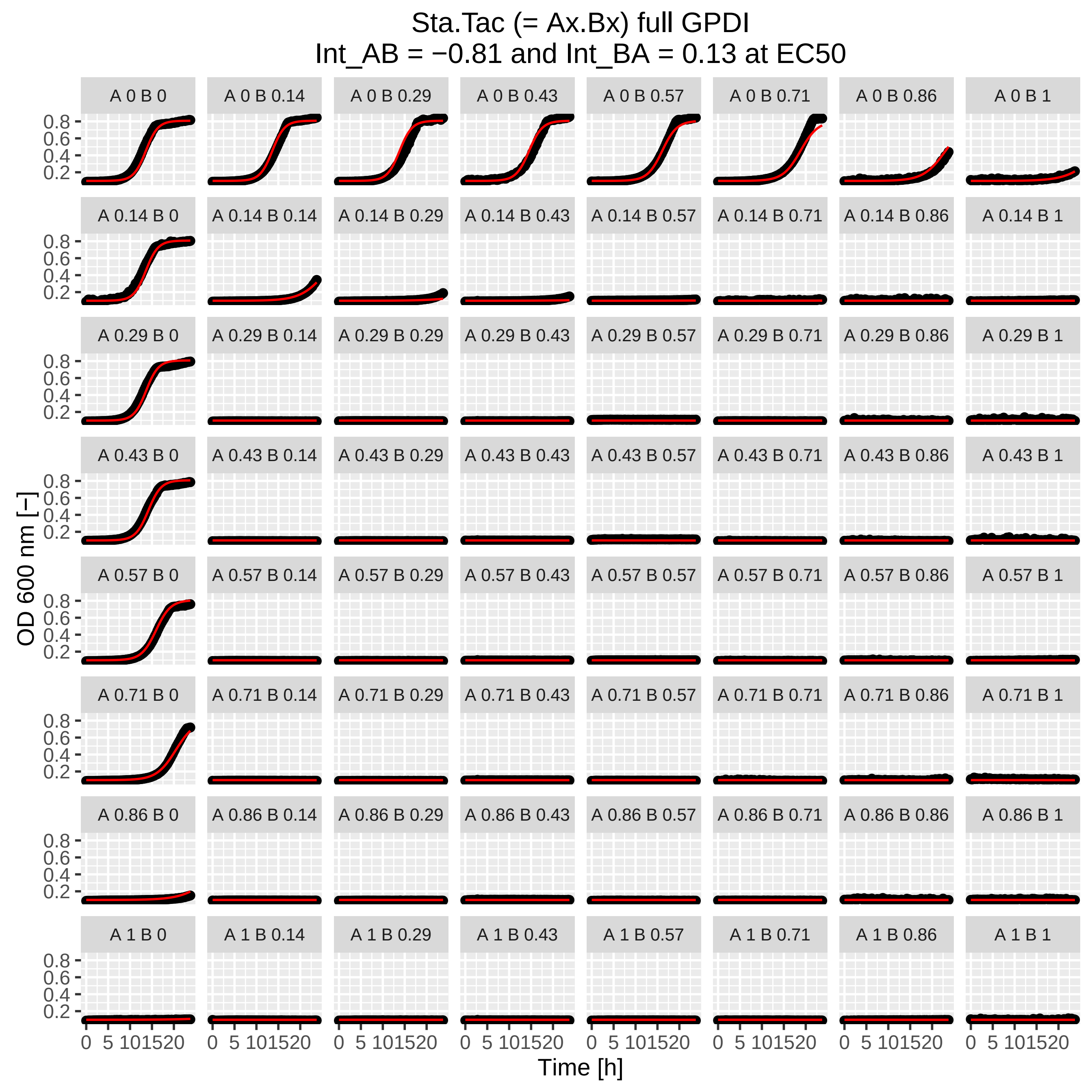

Sta.Tac (= Ax.Bx) full GPDI
Int_AB = −0.81 and Int_BA = 0.13 at EC50
A 0 B 0
A 0 B 0.14
A 0 B 0.29
A 0 B 0.43
A 0 B 0.57
A 0 B 0.71
A 0 B 0.86
A 0 B 1
A 0.14 B 0
A 0.14 B 0.14
A 0.14 B 0.29
A 0.14 B 0.43
A 0.14 B 0.57
A 0.14 B 0.71
A 0.14 B 0.86
A 0.14 B 1
A 0.29 B 0
A 0.29 B 0.14
A 0.29 B 0.29
A 0.29 B 0.43
A 0.29 B 0.57
A 0.29 B 0.71
A 0.29 B 0.86
A 0.29 B 1
A 0.43 B 0
A 0.43 B 0.14
A 0.43 B 0.29
A 0.43 B 0.43
A 0.43 B 0.57
A 0.43 B 0.71
A 0.43 B 0.86
A 0.43 B 1
A 0.57 B 0
A 0.57 B 0.14
A 0.57 B 0.29
A 0.57 B 0.43
A 0.57 B 0.57
A 0.57 B 0.71
A 0.57 B 0.86
A 0.57 B 1
A 0.71 B 0
A 0.71 B 0.14
A 0.71 B 0.29
A 0.71 B 0.43
A 0.71 B 0.57
A 0.71 B 0.71
A 0.71 B 0.86
A 0.71 B 1
A 0.86 B 0
A 0.86 B 0.14
A 0.86 B 0.29
A 0.86 B 0.43
A 0.86 B 0.57
A 0.86 B 0.71
A 0.86 B 0.86
A 0.86 B 1
A 1 B 0
A 1 B 0.14
A 1 B 0.29
A 1 B 0.43
A 1 B 0.57
A 1 B 0.71
A 1 B 0.86
A 1 B 1
0.8
0.6
0.4
0.2
0 5 101520
OD 600 nm [−]
Time [h]

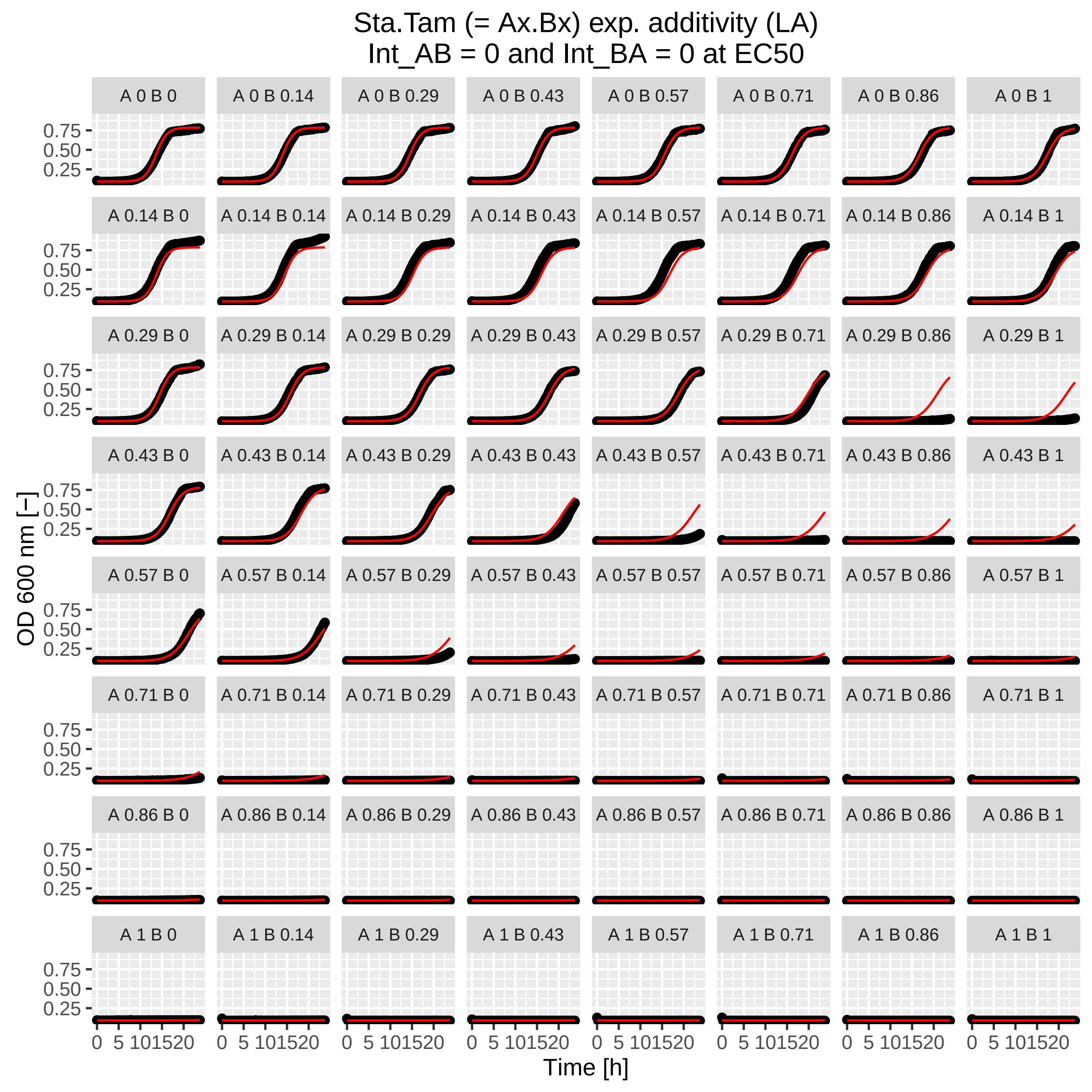

Sta.Tam (= Ax.Bx) exp. additivity (LA)
Int_AB = 0 and Int_BA = 0 at EC50
A 0 B 0
A 0 B 0.14
A 0 B 0.29
A 0 B 0.43
A 0 B 0.57
A 0 B 0.71
A 0 B 0.86
A 0 B 1
A 0.14 B 0
A 0.14 B 0.14
A 0.14 B 0.29
A 0.14 B 0.43
A 0.14 B 0.57
A 0.14 B 0.71
A 0.14 B 0.86
A 0.14 B 1
A 0.29 B 0
A 0.29 B 0.14
A 0.29 B 0.29
A 0.29 B 0.43
A 0.29 B 0.57
A 0.29 B 0.71
A 0.29 B 0.86
A 0.29 B 1
A 0.43 B 0
A 0.43 B 0.14
A 0.43 B 0.29
A 0.43 B 0.43
A 0.43 B 0.57
A 0.43 B 0.71
A 0.43 B 0.86
A 0.43 B 1
A 0.57 B 0
A 0.57 B 0.14
A 0.57 B 0.29
A 0.57 B 0.43
A 0.57 B 0.57
A 0.57 B 0.71
A 0.57 B 0.86
A 0.57 B 1
A 0.71 B 0
A 0.71 B 0.14
A 0.71 B 0.29
A 0.71 B 0.43
A 0.71 B 0.57
A 0.71 B 0.71
A 0.71 B 0.86
A 0.71 B 1
A 0.86 B 0
A 0.86 B 0.14
A 0.86 B 0.29
A 0.86 B 0.43
A 0.86 B 0.57
A 0.86 B 0.71
A 0.86 B 0.86
A 0.86 B 1
A 1 B 0
A 1 B 0.14
A 1 B 0.29
A 1 B 0.43
A 1 B 0.57
A 1 B 0.71
A 1 B 0.86
A 1 B 1
OD 600 nm [–]
0.75
0.50
0.25
0 5 101520
Time [h]

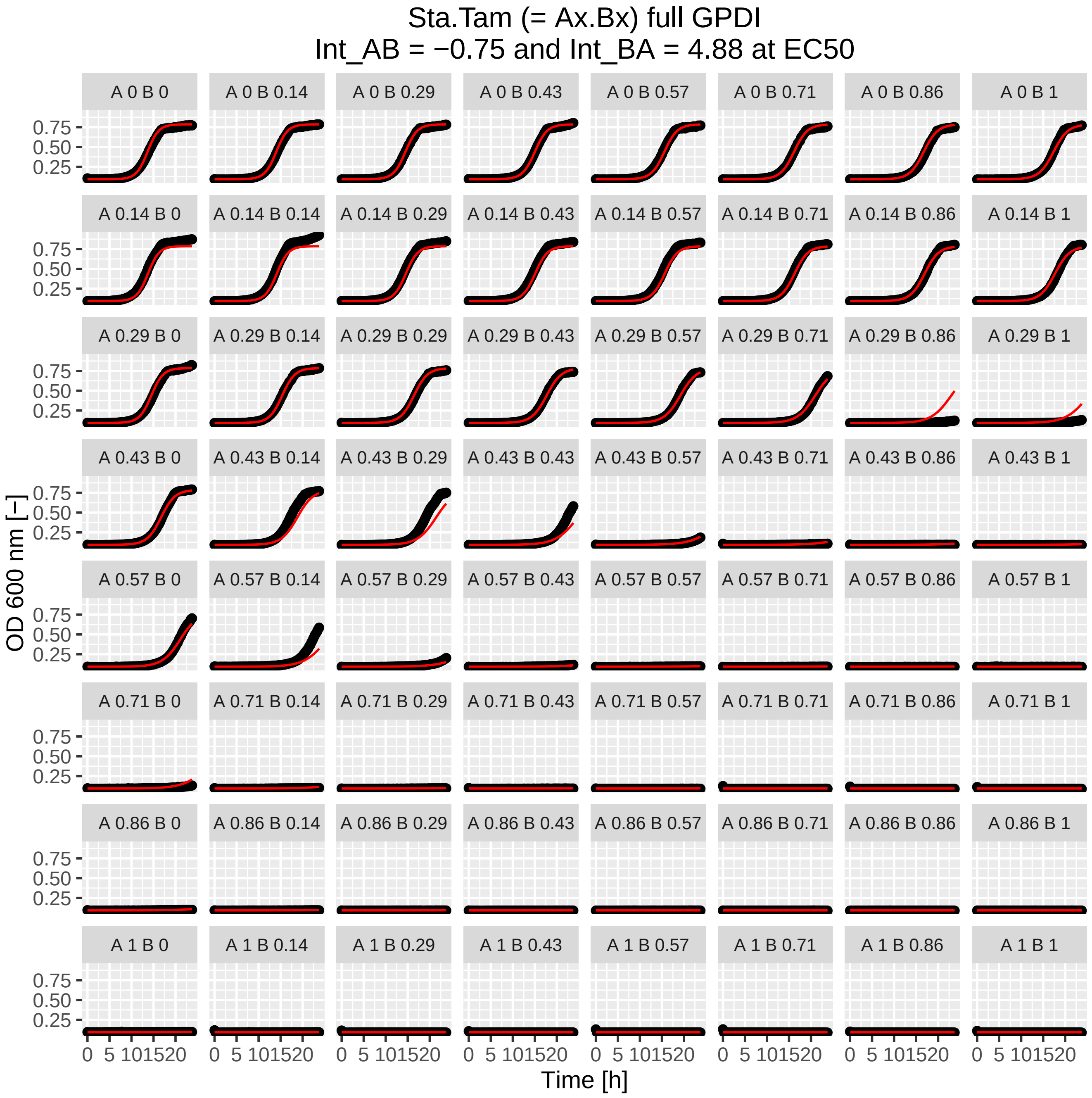

Sta.Tam (= Ax.Bx) full GPDI
Int_AB = −0.75 and Int_BA = 4.88 at EC50
OD 600 nm [−]
Time [h]
A 0 B 0
A 0 B 0.14
A 0 B 0.29
A 0 B 0.43
A 0 B 0.57
A 0 B 0.71
A 0 B 0.86
A 0 B 1
A 0.14 B 0
A 0.14 B 0.14
A 0.14 B 0.29
A 0.14 B 0.43
A 0.14 B 0.57
A 0.14 B 0.71
A 0.14 B 0.86
A 0.14 B 1
A 0.29 B 0
A 0.29 B 0.14
A 0.29 B 0.29
A 0.29 B 0.43
A 0.29 B 0.57
A 0.29 B 0.71
A 0.29 B 0.86
A 0.29 B 1
A 0.43 B 0
A 0.43 B 0.14
A 0.43 B 0.29
A 0.43 B 0.43
A 0.43 B 0.57
A 0.43 B 0.71
A 0.43 B 0.86
A 0.43 B 1
A 0.57 B 0
A 0.57 B 0.14
A 0.57 B 0.29
A 0.57 B 0.43
A 0.57 B 0.57
A 0.57 B 0.71
A 0.57 B 0.86
A 0.57 B 1
A 0.71 B 0
A 0.71 B 0.14
A 0.71 B 0.29
A 0.71 B 0.43
A 0.71 B 0.57
A 0.71 B 0.71
A 0.71 B 0.86
A 0.71 B 1
A 0.86 B 0
A 0.86 B 0.14
A 0.86 B 0.29
A 0.86 B 0.43
A 0.86 B 0.57
A 0.86 B 0.71
A 0.86 B 0.86
A 0.86 B 1
A 1 B 0
A 1 B 0.14
A 1 B 0.29
A 1 B 0.43
A 1 B 0.57
A 1 B 0.71
A 1 B 0.86
A 1 B 1
0.75
0.50
0.25
0 5 101520

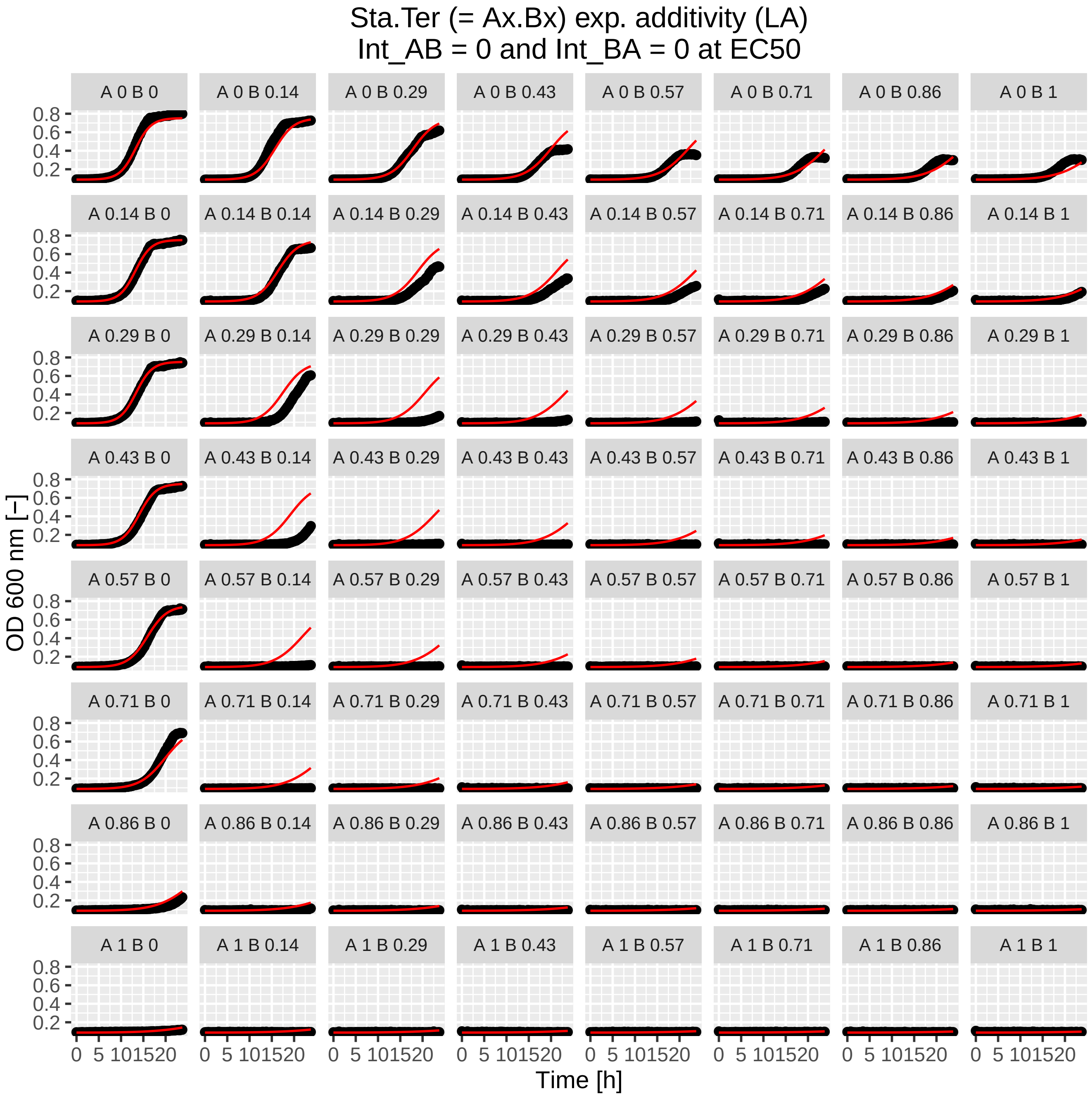

Sta.Ter (= Ax.Bx) exp. additivity (LA)
Int_AB = 0 and Int_BA = 0 at EC50
A 0 B 0
A 0 B 0.14
A 0 B 0.29
A 0 B 0.43
A 0 B 0.57
A 0 B 0.71
A 0 B 0.86
A 0 B 1
A 0.14 B 0
A 0.14 B 0.14
A 0.14 B 0.29
A 0.14 B 0.43
A 0.14 B 0.57
A 0.14 B 0.71
A 0.14 B 0.86
A 0.14 B 1
A 0.29 B 0
A 0.29 B 0.14
A 0.29 B 0.29
A 0.29 B 0.43
A 0.29 B 0.57
A 0.29 B 0.71
A 0.29 B 0.86
A 0.29 B 1
A 0.43 B 0
A 0.43 B 0.14
A 0.43 B 0.29
A 0.43 B 0.43
A 0.43 B 0.57
A 0.43 B 0.71
A 0.43 B 0.86
A 0.43 B 1
A 0.57 B 0
A 0.57 B 0.14
A 0.57 B 0.29
A 0.57 B 0.43
A 0.57 B 0.57
A 0.57 B 0.71
A 0.57 B 0.86
A 0.57 B 1
A 0.71 B 0
A 0.71 B 0.14
A 0.71 B 0.29
A 0.71 B 0.43
A 0.71 B 0.57
A 0.71 B 0.71
A 0.71 B 0.86
A 0.71 B 1
A 0.86 B 0
A 0.86 B 0.14
A 0.86 B 0.29
A 0.86 B 0.43
A 0.86 B 0.57
A 0.86 B 0.71
A 0.86 B 0.86
A 0.86 B 1
A 1 B 0
A 1 B 0.14
A 1 B 0.29
A 1 B 0.43
A 1 B 0.57
A 1 B 0.71
A 1 B 0.86
A 1 B 1
0.8
0.6
0.4
0.2
OD 600 nm [–]
0 5 101520
Time [h]

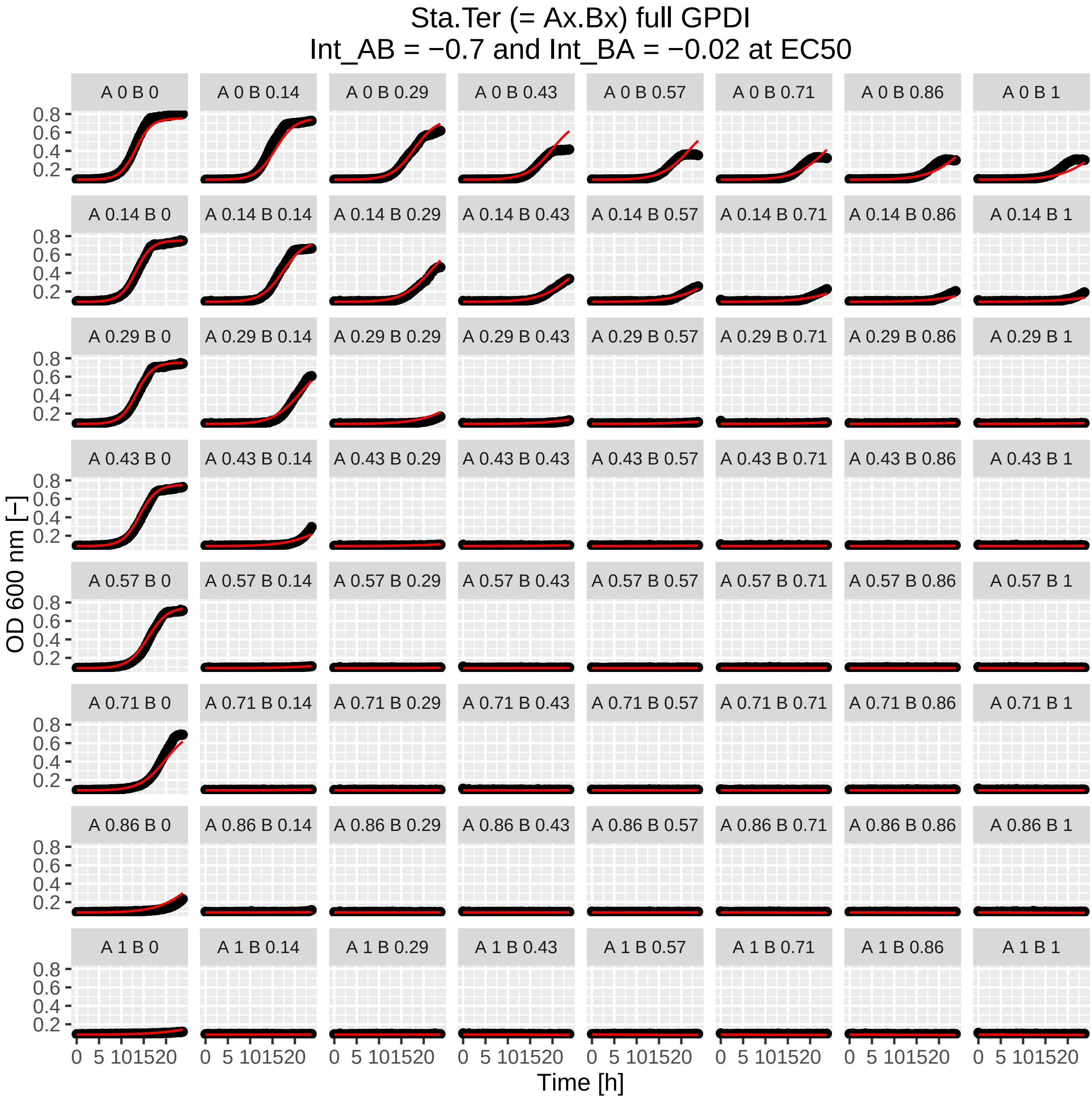
Sta.Ter (= Ax.Bx) full GPDI
Int_AB = −0.7 and Int_BA = −0.02 at EC50
A 0 B 0
A 0 B 0.14
A 0 B 0.29
A 0 B 0.43
A 0 B 0.57
A 0 B 0.71
A 0 B 0.86
A 0 B 1
A 0.14 B 0
A 0.14 B 0.14
A 0.14 B 0.29
A 0.14 B 0.43
A 0.14 B 0.57
A 0.14 B 0.71
A 0.14 B 0.86
A 0.14 B 1
A 0.29 B 0
A 0.29 B 0.14
A 0.29 B 0.29
A 0.29 B 0.43
A 0.29 B 0.57
A 0.29 B 0.71
A 0.29 B 0.86
A 0.29 B 1
A 0.43 B 0
A 0.43 B 0.14
A 0.43 B 0.29
A 0.43 B 0.43
A 0.43 B 0.57
A 0.43 B 0.71
A 0.43 B 0.86
A 0.43 B 1
A 0.57 B 0
A 0.57 B 0.14
A 0.57 B 0.29
A 0.57 B 0.43
A 0.57 B 0.57
A 0.57 B 0.71
A 0.57 B 0.86
A 0.57 B 1
A 0.71 B 0
A 0.71 B 0.14
A 0.71 B 0.29
A 0.71 B 0.43
A 0.71 B 0.57
A 0.71 B 0.71
A 0.71 B 0.86
A 0.71 B 1
A 0.86 B 0
A 0.86 B 0.14
A 0.86 B 0.29
A 0.86 B 0.43
A 0.86 B 0.57
A 0.86 B 0.71
A 0.86 B 0.86
A 0.86 B 1
A 1 B 0
A 1 B 0.14
A 1 B 0.29
A 1 B 0.43
A 1 B 0.57
A 1 B 0.71
A 1 B 0.86
A 1 B 1
OD 600 nm [−]
0.8
0.6
0.4
0.2
0 5 101520
Time [h]

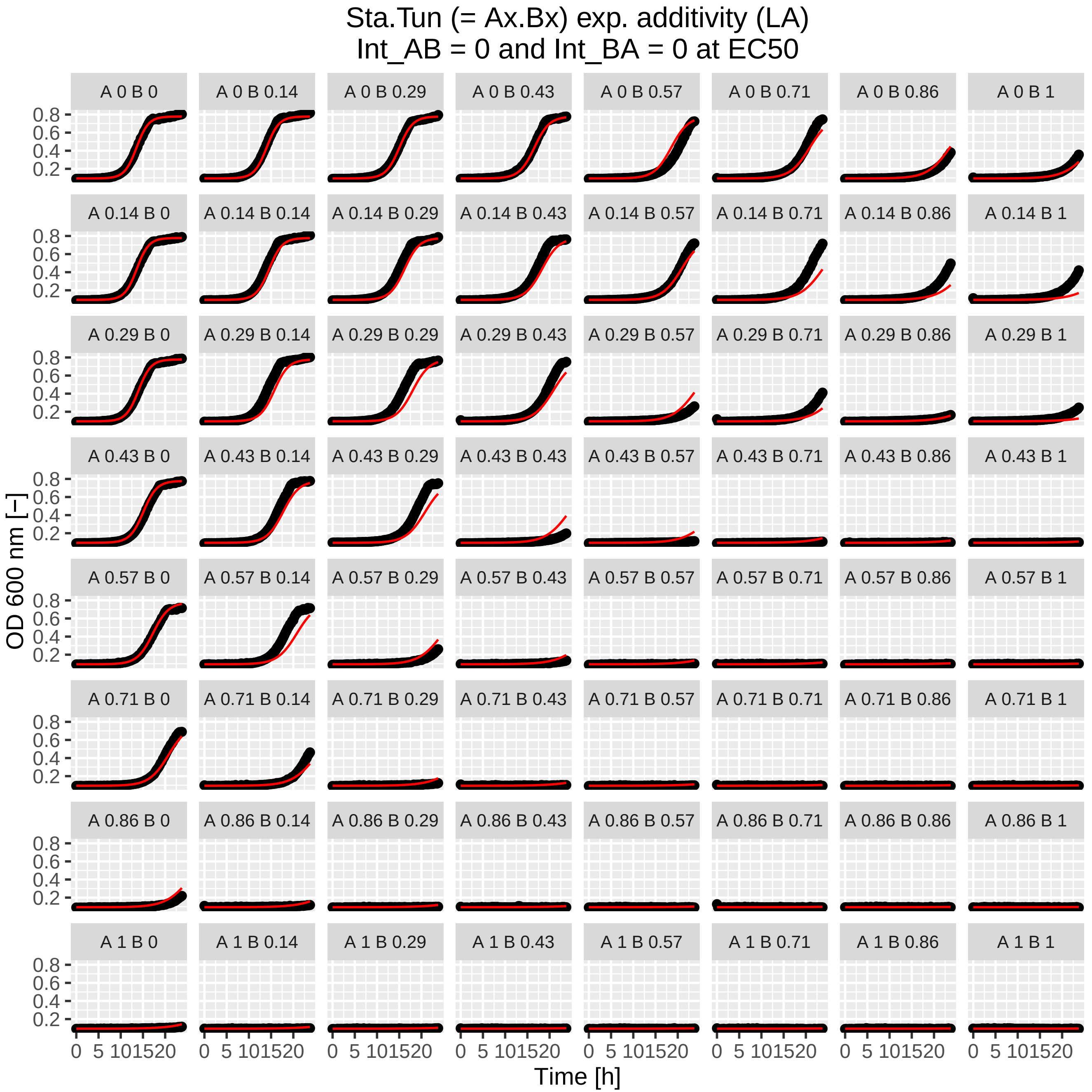

Sta.Tun (= Ax.Bx) exp. additivity (LA)
Int_AB = 0 and Int_BA = 0 at EC50
A 0 B 0
A 0 B 0.14
A 0 B 0.29
A 0 B 0.43
A 0 B 0.57
A 0 B 0.71
A 0 B 0.86
A 0 B 1
A 0.14 B 0
A 0.14 B 0.14
A 0.14 B 0.29
A 0.14 B 0.43
A 0.14 B 0.57
A 0.14 B 0.71
A 0.14 B 0.86
A 0.14 B 1
A 0.29 B 0
A 0.29 B 0.14
A 0.29 B 0.29
A 0.29 B 0.43
A 0.29 B 0.57
A 0.29 B 0.71
A 0.29 B 0.86
A 0.29 B 1
A 0.43 B 0
A 0.43 B 0.14
A 0.43 B 0.29
A 0.43 B 0.43
A 0.43 B 0.57
A 0.43 B 0.71
A 0.43 B 0.86
A 0.43 B 1
A 0.57 B 0
A 0.57 B 0.14
A 0.57 B 0.29
A 0.57 B 0.43
A 0.57 B 0.57
A 0.57 B 0.71
A 0.57 B 0.86
A 0.57 B 1
A 0.71 B 0
A 0.71 B 0.14
A 0.71 B 0.29
A 0.71 B 0.43
A 0.71 B 0.57
A 0.71 B 0.71
A 0.71 B 0.86
A 0.71 B 1
A 0.86 B 0
A 0.86 B 0.14
A 0.86 B 0.29
A 0.86 B 0.43
A 0.86 B 0.57
A 0.86 B 0.71
A 0.86 B 0.86
A 0.86 B 1
A 1 B 0
A 1 B 0.14
A 1 B 0.29
A 1 B 0.43
A 1 B 0.57
A 1 B 0.71
A 1 B 0.86
A 1 B 1
OD 600 nm [–]
0.2
0.4
0.6
0.8
0 5 101520
Time [h]

Sta.Tun (= Ax.Bx) full GPDI
Int_AB = 0.02 and Int_BA = 0.15 at EC50

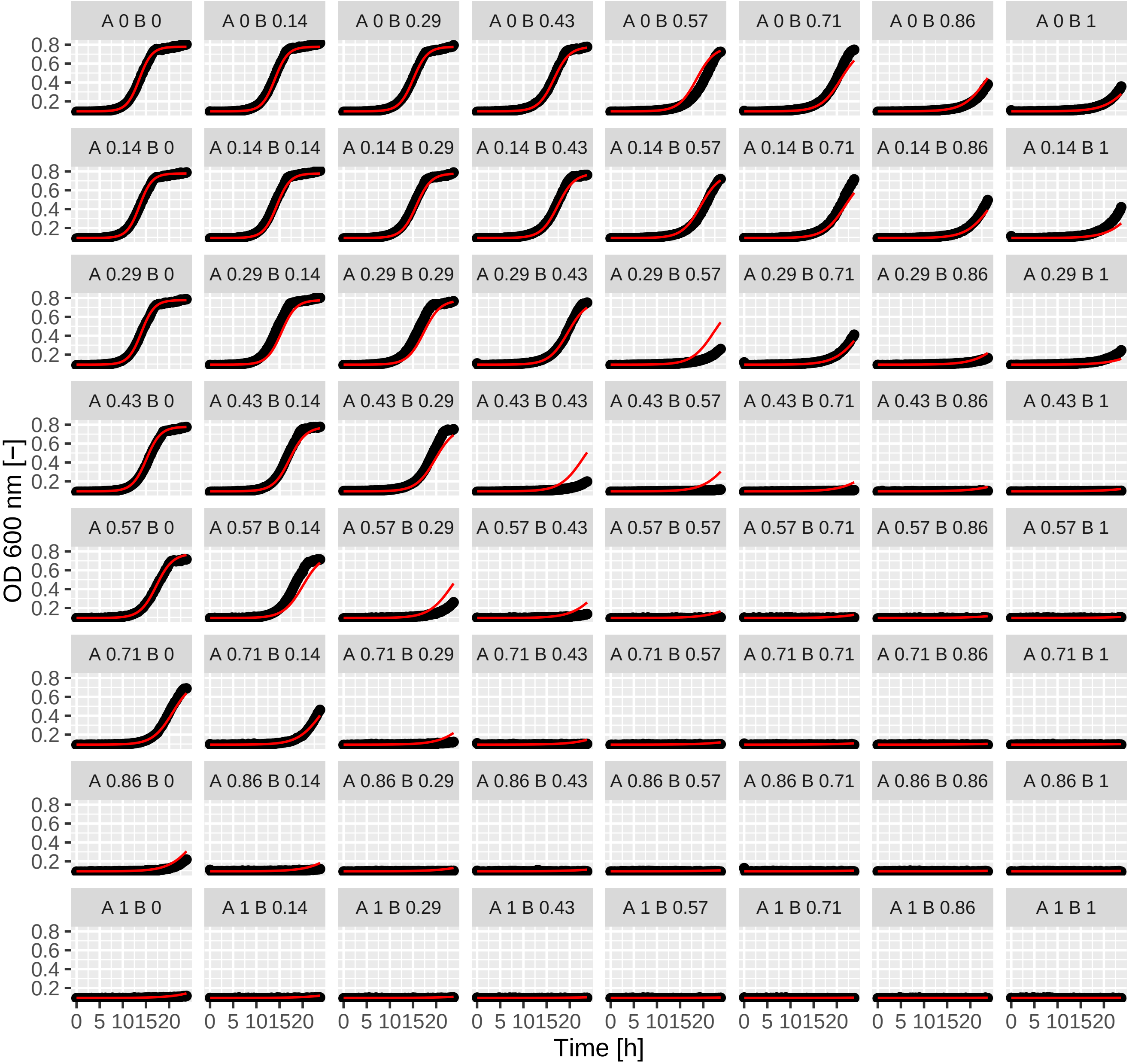

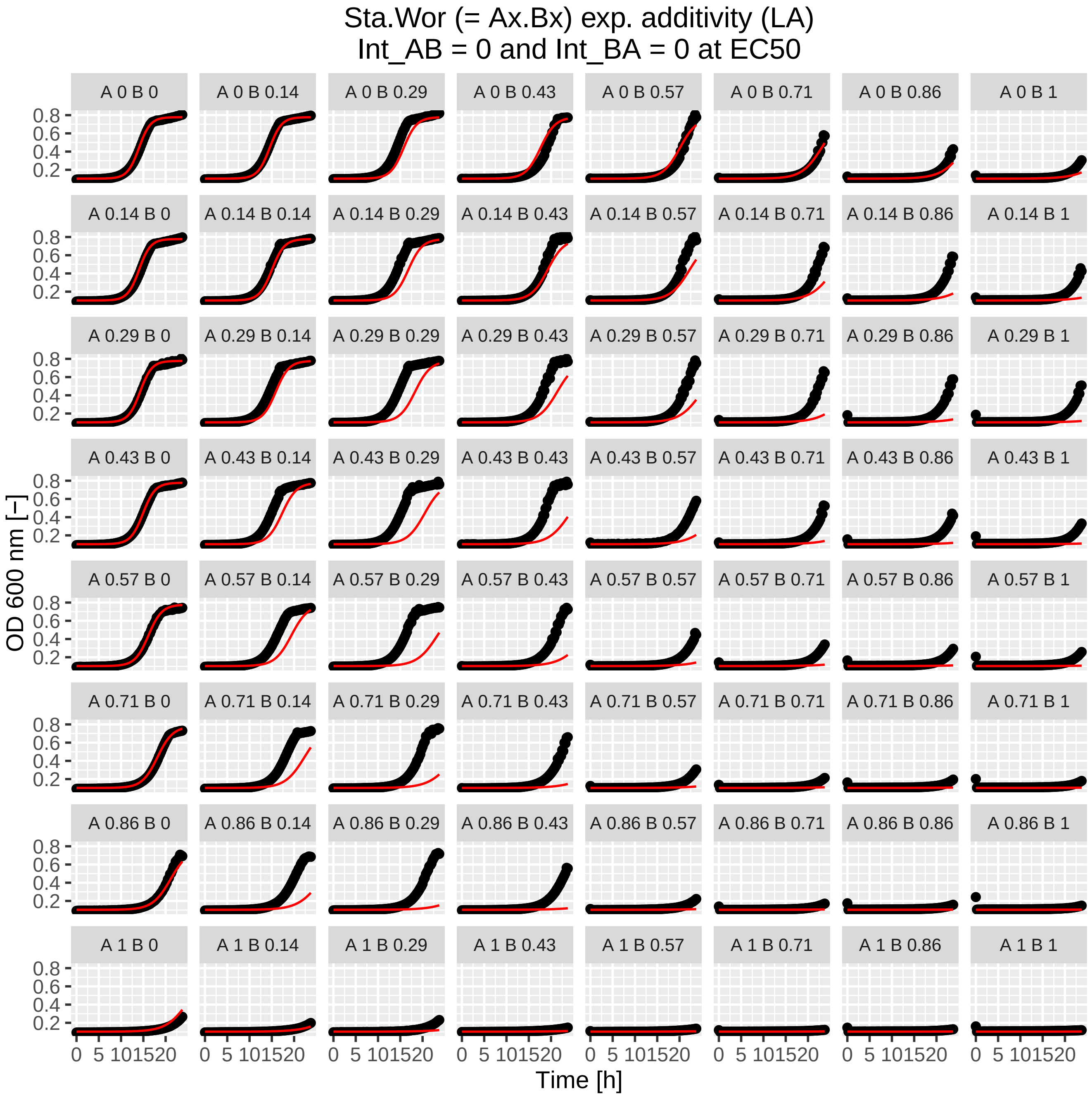
Sta.Wor (= Ax.Bx) exp. additivity (LA)
Int_AB = 0 and Int_BA = 0 at EC50
OD 600 nm [–]
Time [h]
A 0 B 0
A 0 B 0.14
A 0 B 0.29
A 0 B 0.43
A 0 B 0.57
A 0 B 0.71
A 0 B 0.86
A 0 B 1
A 1 B 0
A 1 B 0.14
A 1 B 0.29
A 1 B 0.43
A 1 B 0.57
A 1 B 0.71
A 1 B 0.86
A 1 B 1
0.2
0.4
0.6
0.8
0 5 101520

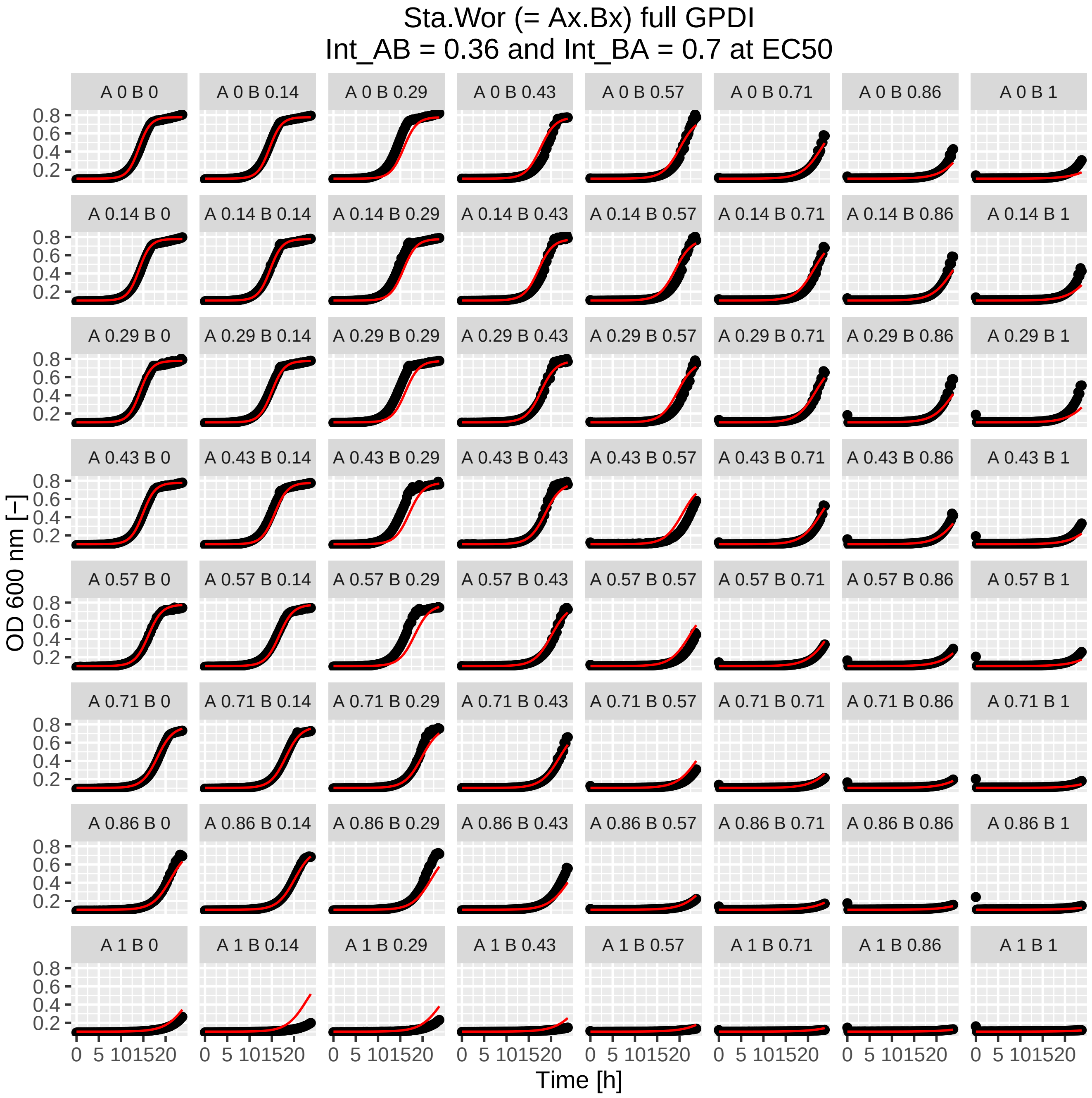

Sta.Wor (= Ax.Bx) full GPDI
Int_AB = 0.36 and Int_BA = 0.7 at EC50
A 0 B 0
A 0 B 0.14
A 0 B 0.29
A 0 B 0.43
A 0 B 0.57
A 0 B 0.71
A 0 B 0.86
A 0 B 1
A 0.14 B 0
A 0.14 B 0.14
A 0.14 B 0.29
A 0.14 B 0.43
A 0.14 B 0.57
A 0.14 B 0.71
A 0.14 B 0.86
A 0.14 B 1
A 0.29 B 0
A 0.29 B 0.14
A 0.29 B 0.29
A 0.29 B 0.43
A 0.29 B 0.57
A 0.29 B 0.71
A 0.29 B 0.86
A 0.29 B 1
A 0.43 B 0
A 0.43 B 0.14
A 0.43 B 0.29
A 0.43 B 0.43
A 0.43 B 0.57
A 0.43 B 0.71
A 0.43 B 0.86
A 0.43 B 1
A 0.57 B 0
A 0.57 B 0.14
A 0.57 B 0.29
A 0.57 B 0.43
A 0.57 B 0.57
A 0.57 B 0.71
A 0.57 B 0.86
A 0.57 B 1
A 0.71 B 0
A 0.71 B 0.14
A 0.71 B 0.29
A 0.71 B 0.43
A 0.71 B 0.57
A 0.71 B 0.71
A 0.71 B 0.86
A 0.71 B 1
A 0.86 B 0
A 0.86 B 0.14
A 0.86 B 0.29
A 0.86 B 0.43
A 0.86 B 0.57
A 0.86 B 0.71
A 0.86 B 0.86
A 0.86 B 1
A 1 B 0
A 1 B 0.14
A 1 B 0.29
A 1 B 0.43
A 1 B 0.57
A 1 B 0.71
A 1 B 0.86
A 1 B 1
OD 600 nm [–]
0.2
0.4
0.6
0.8
0
5
10
15
20
Time [h]

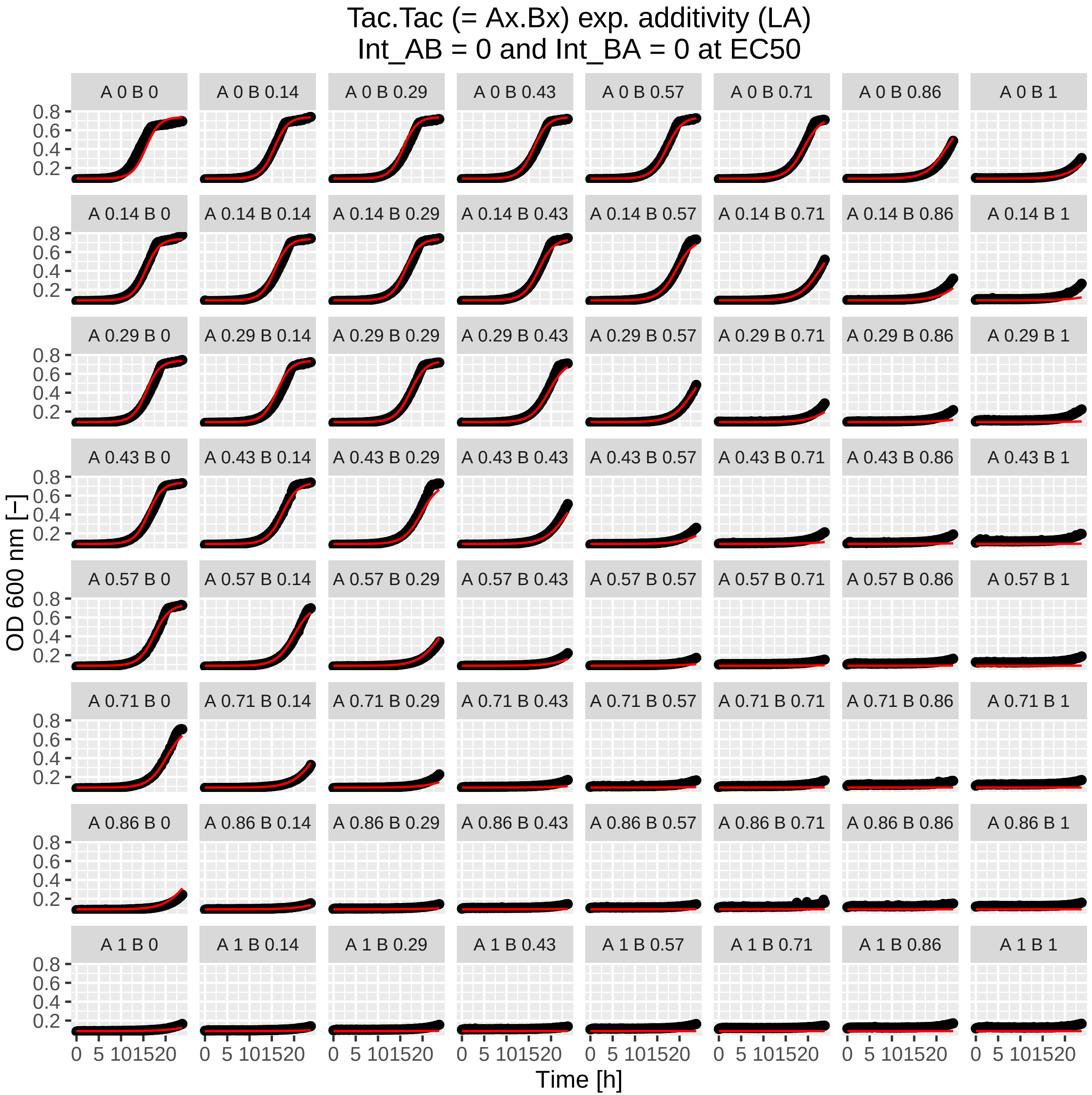
Tac.Tac (= Ax.Bx) exp. additivity (LA)
Int_AB = 0 and Int_BA = 0 at EC50
OD 600 nm [–]
Time [h]
A 0 B 0
A 0 B 0.14
A 0 B 0.29
A 0 B 0.43
A 0 B 0.57
A 0 B 0.71
A 0 B 0.86
A 0 B 1
A 0.14 B 0
A 0.14 B 0.14
A 0.14 B 0.29
A 0.14 B 0.43
A 0.14 B 0.57
A 0.14 B 0.71
A 0.14 B 0.86
A 0.14 B 1
A 0.29 B 0
A 0.29 B 0.14
A 0.29 B 0.29
A 0.29 B 0.43
A 0.29 B 0.57
A 0.29 B 0.71
A 0.29 B 0.86
A 0.29 B 1
A 0.43 B 0
A 0.43 B 0.14
A 0.43 B 0.29
A 0.43 B 0.43
A 0.43 B 0.57
A 0.43 B 0.71
A 0.43 B 0.86
A 0.43 B 1
A 0.57 B 0
A 0.57 B 0.14
A 0.57 B 0.29
A 0.57 B 0.43
A 0.57 B 0.57
A 0.57 B 0.71
A 0.57 B 0.86
A 0.57 B 1
A 0.71 B 0
A 0.71 B 0.14
A 0.71 B 0.29
A 0.71 B 0.43
A 0.71 B 0.57
A 0.71 B 0.71
A 0.71 B 0.86
A 0.71 B 1
A 0.86 B 0
A 0.86 B 0.14
A 0.86 B 0.29
A 0.86 B 0.43
A 0.86 B 0.57
A 0.86 B 0.71
A 0.86 B 0.86
A 0.86 B 1
A 1 B 0
A 1 B 0.14
A 1 B 0.29
A 1 B 0.43
A 1 B 0.57
A 1 B 0.71
A 1 B 0.86
A 1 B 1
0.8
0.6
0.4
0.2
0 5 101520

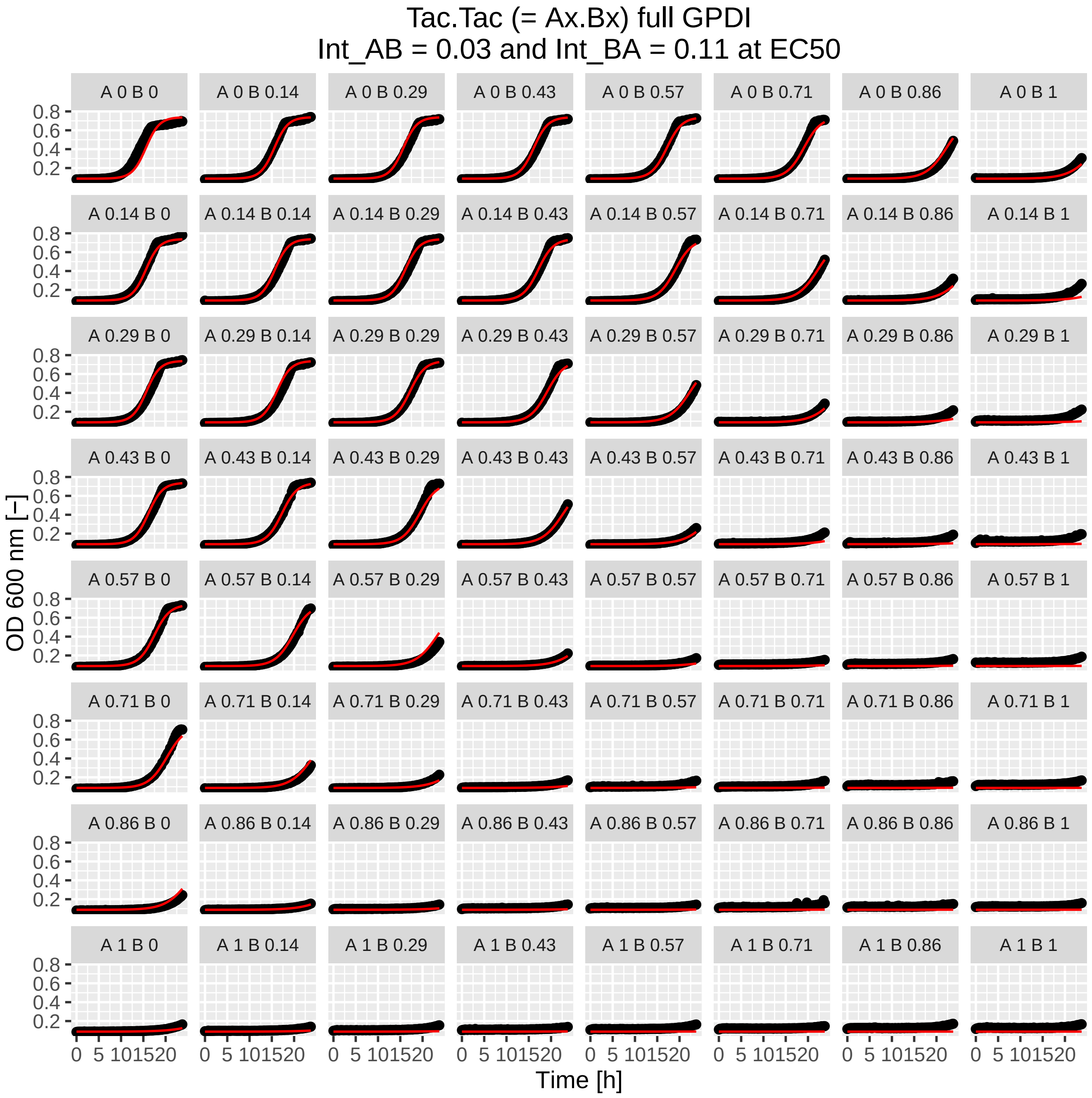

Tac.Tac (= Ax.Bx) full GPDI
Int_AB = 0.03 and Int_BA = 0.11 at EC50
A 0 B 0
A 0 B 0.14
A 0 B 0.29
A 0 B 0.43
A 0 B 0.57
A 0 B 0.71
A 0 B 0.86
A 0 B 1
A 0.14 B 0
A 0.14 B 0.14
A 0.14 B 0.29
A 0.14 B 0.43
A 0.14 B 0.57
A 0.14 B 0.71
A 0.14 B 0.86
A 0.14 B 1
A 0.29 B 0
A 0.29 B 0.14
A 0.29 B 0.29
A 0.29 B 0.43
A 0.29 B 0.57
A 0.29 B 0.71
A 0.29 B 0.86
A 0.29 B 1
A 0.43 B 0
A 0.43 B 0.14
A 0.43 B 0.29
A 0.43 B 0.43
A 0.43 B 0.57
A 0.43 B 0.71
A 0.43 B 0.86
A 0.43 B 1
A 0.57 B 0
A 0.57 B 0.14
A 0.57 B 0.29
A 0.57 B 0.43
A 0.57 B 0.57
A 0.57 B 0.71
A 0.57 B 0.86
A 0.57 B 1
A 0.71 B 0
A 0.71 B 0.14
A 0.71 B 0.29
A 0.71 B 0.43
A 0.71 B 0.57
A 0.71 B 0.71
A 0.71 B 0.86
A 0.71 B 1
A 0.86 B 0
A 0.86 B 0.14
A 0.86 B 0.29
A 0.86 B 0.43
A 0.86 B 0.57
A 0.86 B 0.71
A 0.86 B 0.86
A 0.86 B 1
A 1 B 0
A 1 B 0.14
A 1 B 0.29
A 1 B 0.43
A 1 B 0.57
A 1 B 0.71
A 1 B 0.86
A 1 B 1
OD 600 nm [–]
0.2
0.4
0.6
0.8
0
5
10
15
20
Time [h]

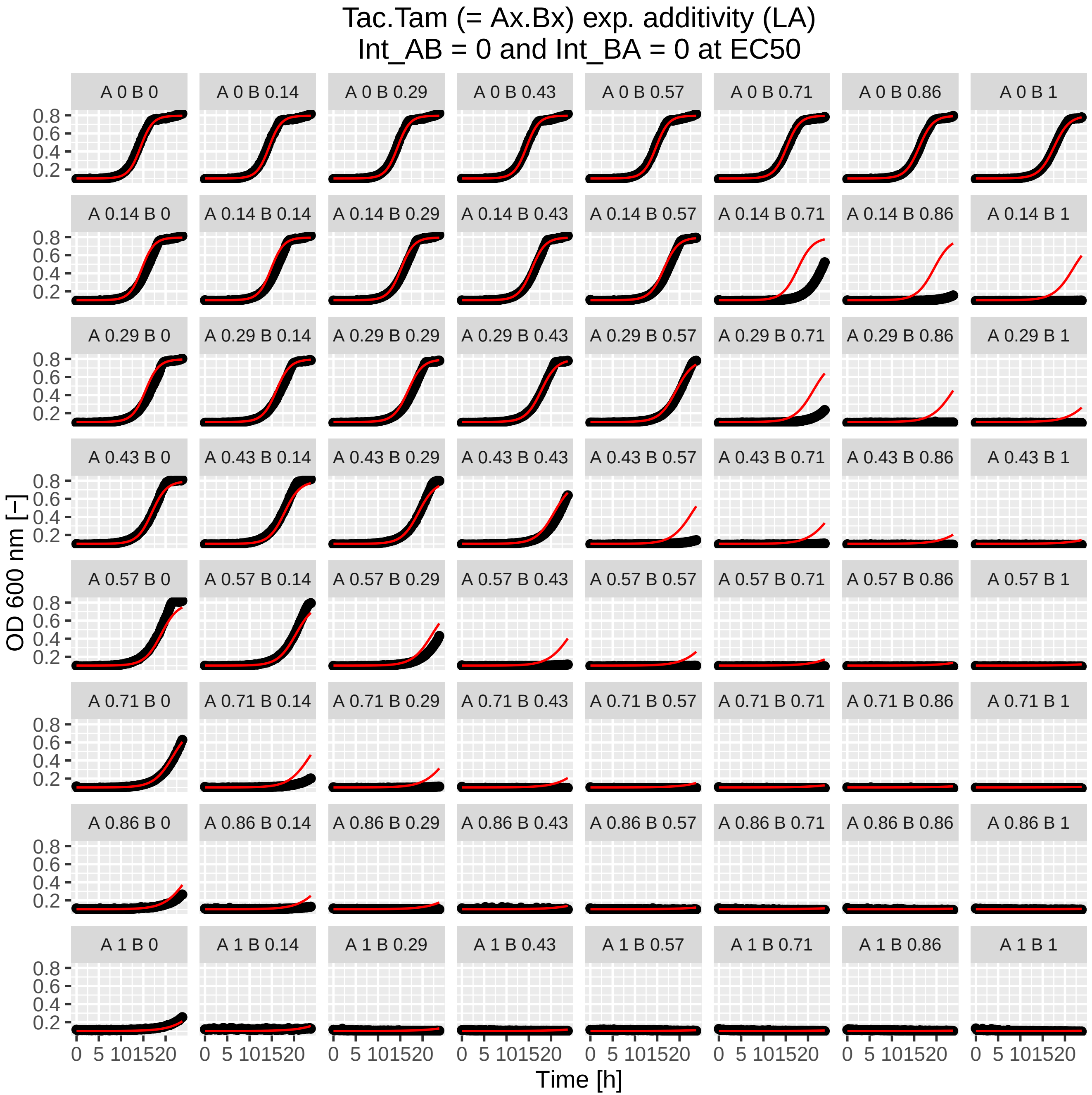

Tac.Tam (= Ax.Bx) exp. additivity (LA)
Int_AB = 0 and Int_BA = 0 at EC50
OD 600 nm [–]
Time [h]
A 0 B 0
A 0 B 0.14
A 0 B 0.29
A 0 B 0.43
A 0 B 0.57
A 0 B 0.71
A 0 B 0.86
A 0 B 1
A 0.14 B 0
A 0.14 B 0.14
A 0.14 B 0.29
A 0.14 B 0.43
A 0.14 B 0.57
A 0.14 B 0.71
A 0.14 B 0.86
A 0.14 B 1
A 0.29 B 0
A 0.29 B 0.14
A 0.29 B 0.29
A 0.29 B 0.43
A 0.29 B 0.57
A 0.29 B 0.71
A 0.29 B 0.86
A 0.29 B 1
A 0.43 B 0
A 0.43 B 0.14
A 0.43 B 0.29
A 0.43 B 0.43
A 0.43 B 0.57
A 0.43 B 0.71
A 0.43 B 0.86
A 0.43 B 1
A 0.57 B 0
A 0.57 B 0.14
A 0.57 B 0.29
A 0.57 B 0.43
A 0.57 B 0.57
A 0.57 B 0.71
A 0.57 B 0.86
A 0.57 B 1
A 0.71 B 0
A 0.71 B 0.14
A 0.71 B 0.29
A 0.71 B 0.43
A 0.71 B 0.57
A 0.71 B 0.71
A 0.71 B 0.86
A 0.71 B 1
A 0.86 B 0
A 0.86 B 0.14
A 0.86 B 0.29
A 0.86 B 0.43
A 0.86 B 0.57
A 0.86 B 0.71
A 0.86 B 0.86
A 0.86 B 1
A 1 B 0
A 1 B 0.14
A 1 B 0.29
A 1 B 0.43
A 1 B 0.57
A 1 B 0.71
A 1 B 0.86
A 1 B 1
0.8
0.6
0.4
0.2
0
5
10
15
20

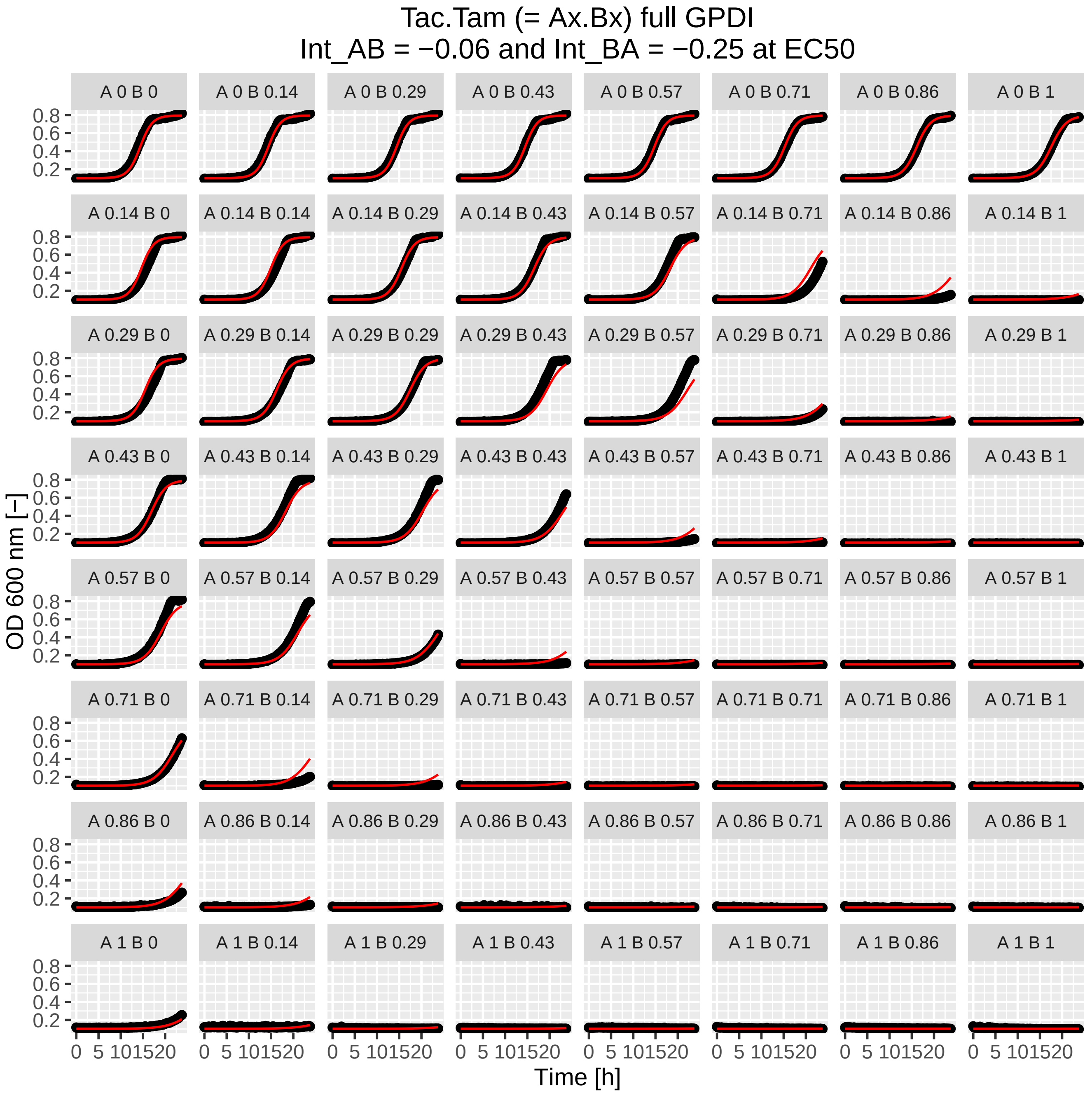
Tac.Tam (= Ax.Bx) full GPDI
Int_AB = −0.06 and Int_BA = −0.25 at EC50
OD 600 nm [−]
Time [h]
0.8
0.6
0.4
0.2
0 5 101520
A 0 B 0
A 0 B 0.14
A 0 B 0.29
A 0 B 0.43
A 0 B 0.57
A 0 B 0.71
A 0 B 0.86
A 0 B 1
A 0.14 B 0
A 0.14 B 0.14
A 0.14 B 0.29
A 0.14 B 0.43
A 0.14 B 0.57
A 0.14 B 0.71
A 0.14 B 0.86
A 0.14 B 1
A 0.29 B 0
A 0.29 B 0.14
A 0.29 B 0.29
A 0.29 B 0.43
A 0.29 B 0.57
A 0.29 B 0.71
A 0.29 B 0.86
A 0.29 B 1
A 0.43 B 0
A 0.43 B 0.14
A 0.43 B 0.29
A 0.43 B 0.43
A 0.43 B 0.57
A 0.43 B 0.71
A 0.43 B 0.86
A 0.43 B 1
A 0.57 B 0
A 0.57 B 0.14
A 0.57 B 0.29
A 0.57 B 0.43
A 0.57 B 0.57
A 0.57 B 0.71
A 0.57 B 0.86
A 0.57 B 1
A 0.71 B 0
A 0.71 B 0.14
A 0.71 B 0.29
A 0.71 B 0.43
A 0.71 B 0.57
A 0.71 B 0.71
A 0.71 B 0.86
A 0.71 B 1
A 0.86 B 0
A 0.86 B 0.14
A 0.86 B 0.29
A 0.86 B 0.43
A 0.86 B 0.57
A 0.86 B 0.71
A 0.86 B 0.86
A 0.86 B 1
A 1 B 0
A 1 B 0.14
A 1 B 0.29
A 1 B 0.43
A 1 B 0.57
A 1 B 0.71
A 1 B 0.86
A 1 B 1

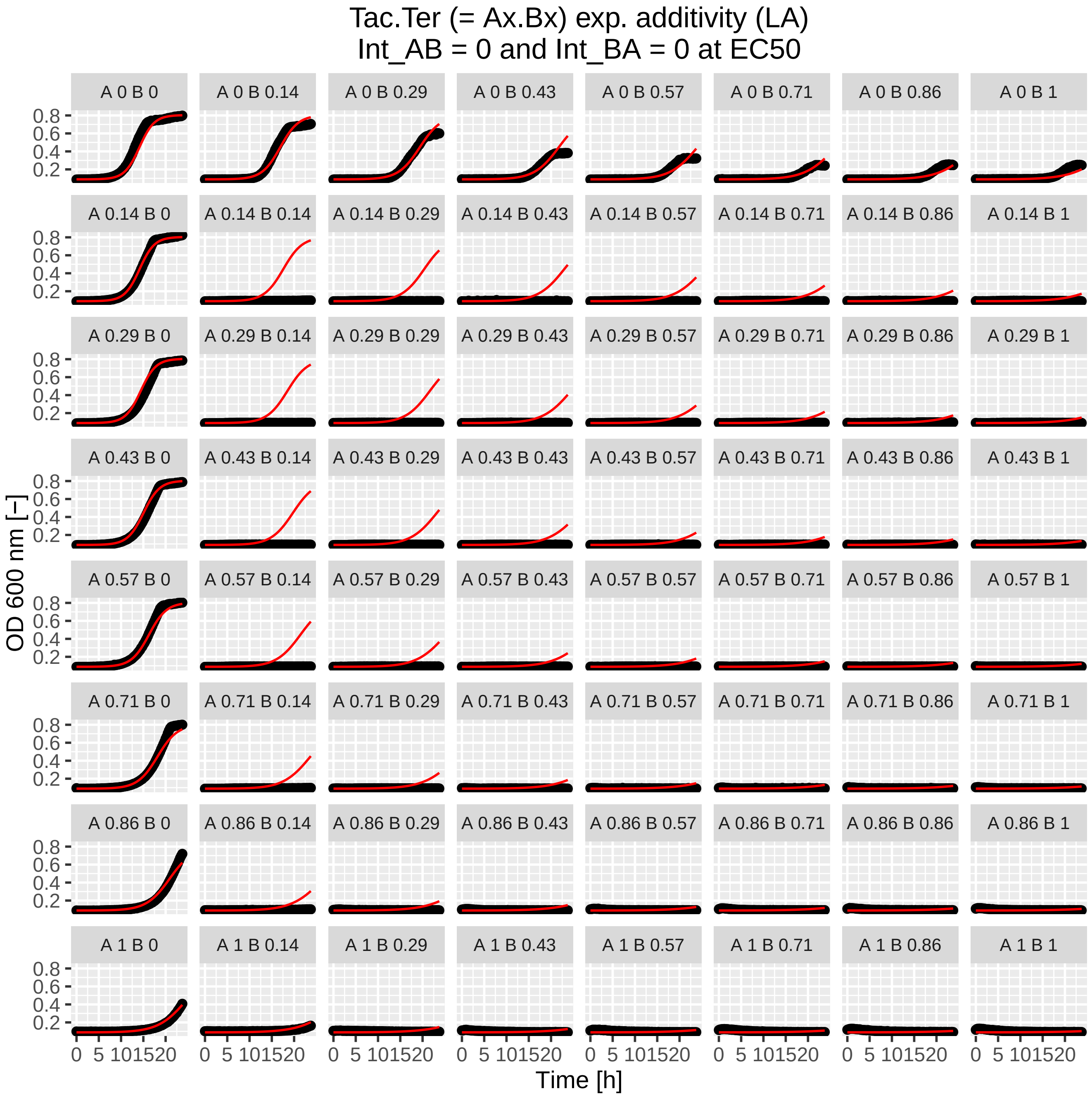
Tac.Ter (= Ax.Bx) exp. additivity (LA)
Int_AB = 0 and Int_BA = 0 at EC50
A 0 B 0
A 0 B 0.14
A 0 B 0.29
A 0 B 0.43
A 0 B 0.57
A 0 B 0.71
A 0 B 0.86
A 0 B 1
A 0.14 B 0
A 0.14 B 0.14
A 0.14 B 0.29
A 0.14 B 0.43
A 0.14 B 0.57
A 0.14 B 0.71
A 0.14 B 0.86
A 0.14 B 1
A 0.29 B 0
A 0.29 B 0.14
A 0.29 B 0.29
A 0.29 B 0.43
A 0.29 B 0.57
A 0.29 B 0.71
A 0.29 B 0.86
A 0.29 B 1
A 0.43 B 0
A 0.43 B 0.14
A 0.43 B 0.29
A 0.43 B 0.43
A 0.43 B 0.57
A 0.43 B 0.71
A 0.43 B 0.86
A 0.43 B 1
A 0.57 B 0
A 0.57 B 0.14
A 0.57 B 0.29
A 0.57 B 0.43
A 0.57 B 0.57
A 0.57 B 0.71
A 0.57 B 0.86
A 0.57 B 1
A 0.71 B 0
A 0.71 B 0.14
A 0.71 B 0.29
A 0.71 B 0.43
A 0.71 B 0.57
A 0.71 B 0.71
A 0.71 B 0.86
A 0.71 B 1
A 0.86 B 0
A 0.86 B 0.14
A 0.86 B 0.29
A 0.86 B 0.43
A 0.86 B 0.57
A 0.86 B 0.71
A 0.86 B 0.86
A 0.86 B 1
A 1 B 0
A 1 B 0.14
A 1 B 0.29
A 1 B 0.43
A 1 B 0.57
A 1 B 0.71
A 1 B 0.86
A 1 B 1
0.8
0.6
0.4
0.2
OD 600 nm [−]
0 5 101520
Time [h]

Tac.Ter (= Ax.Bx) full GPDI
Int_AB = 2.69 and Int_BA = −0.93 at EC50

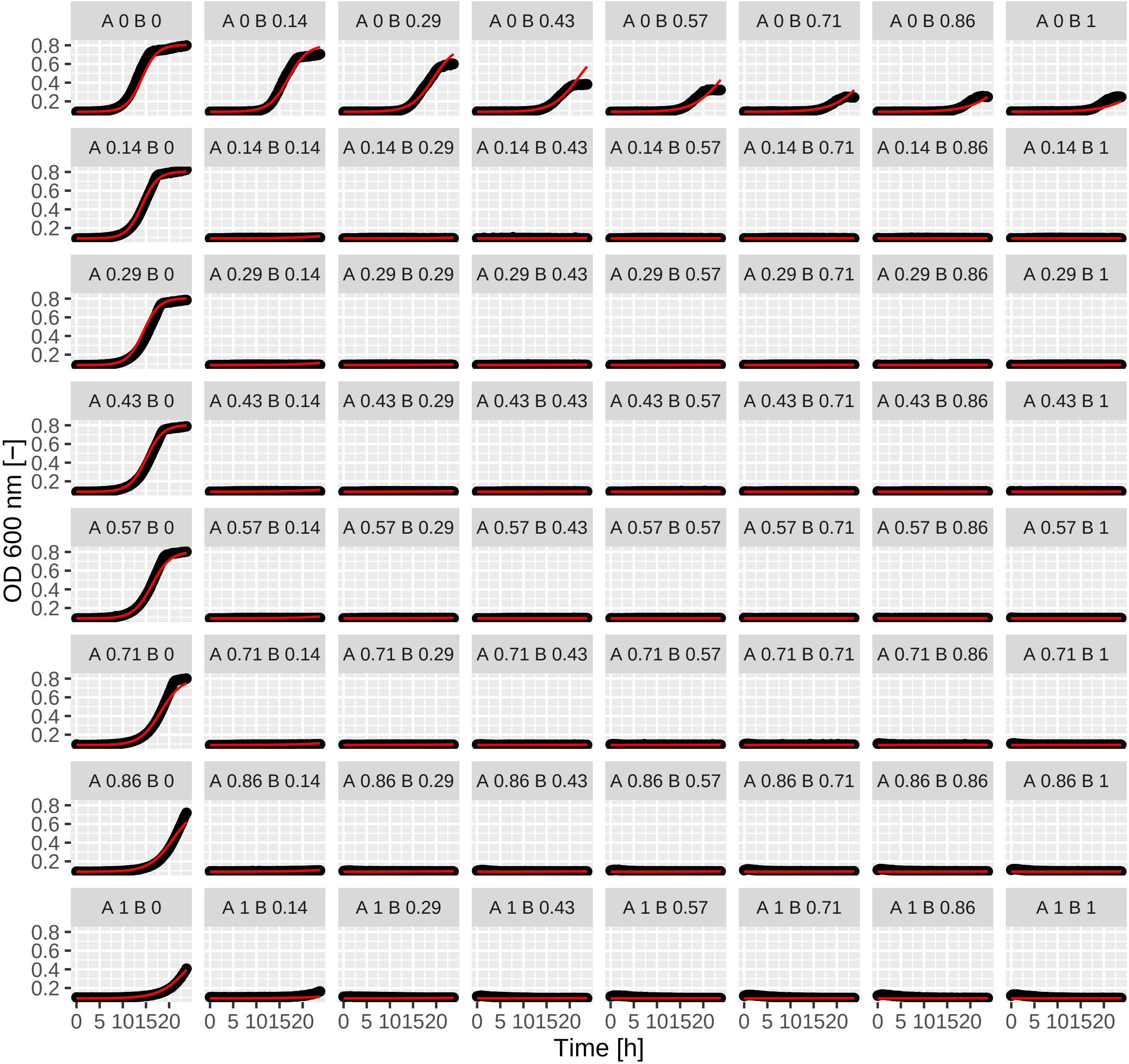

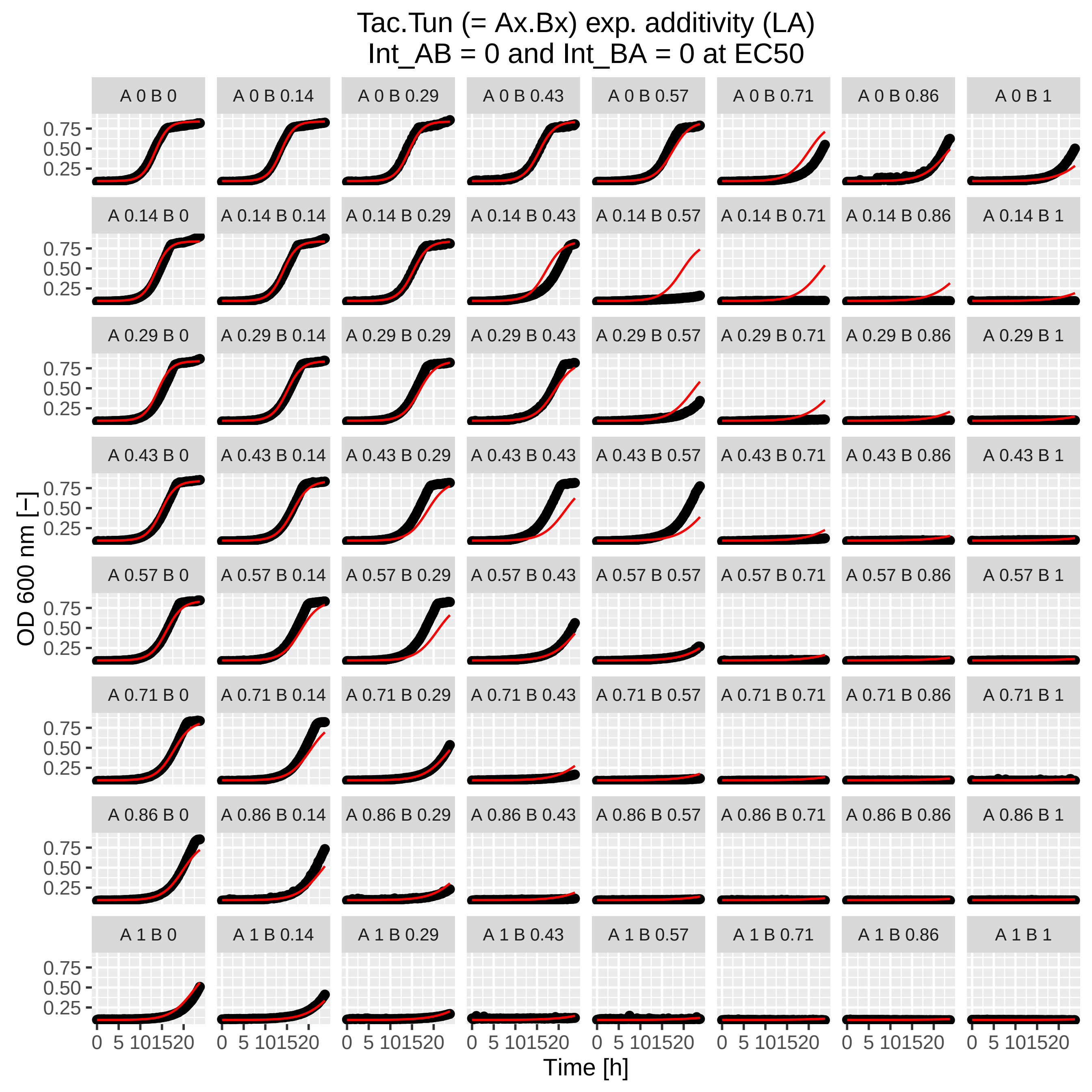
Tac.Tun (= Ax.Bx) exp. additivity (LA)
Int_AB = 0 and Int_BA = 0 at EC50
A 0 B 0
A 0 B 0.14
A 0 B 0.29
A 0 B 0.43
A 0 B 0.57
A 0 B 0.71
A 0 B 0.86
A 0 B 1
A 0.14 B 0
A 0.14 B 0.14
A 0.14 B 0.29
A 0.14 B 0.43
A 0.14 B 0.57
A 0.14 B 0.71
A 0.14 B 0.86
A 0.14 B 1
A 0.29 B 0
A 0.29 B 0.14
A 0.29 B 0.29
A 0.29 B 0.43
A 0.29 B 0.57
A 0.29 B 0.71
A 0.29 B 0.86
A 0.29 B 1
A 0.43 B 0
A 0.43 B 0.14
A 0.43 B 0.29
A 0.43 B 0.43
A 0.43 B 0.57
A 0.43 B 0.71
A 0.43 B 0.86
A 0.43 B 1
A 0.57 B 0
A 0.57 B 0.14
A 0.57 B 0.29
A 0.57 B 0.43
A 0.57 B 0.57
A 0.57 B 0.71
A 0.57 B 0.86
A 0.57 B 1
A 0.71 B 0
A 0.71 B 0.14
A 0.71 B 0.29
A 0.71 B 0.43
A 0.71 B 0.57
A 0.71 B 0.71
A 0.71 B 0.86
A 0.71 B 1
A 0.86 B 0
A 0.86 B 0.14
A 0.86 B 0.29
A 0.86 B 0.43
A 0.86 B 0.57
A 0.86 B 0.71
A 0.86 B 0.86
A 0.86 B 1
A 1 B 0
A 1 B 0.14
A 1 B 0.29
A 1 B 0.43
A 1 B 0.57
A 1 B 0.71
A 1 B 0.86
A 1 B 1
OD 600 nm [–]
0.75
0.50
0.25
0 5 101520
Time [h]

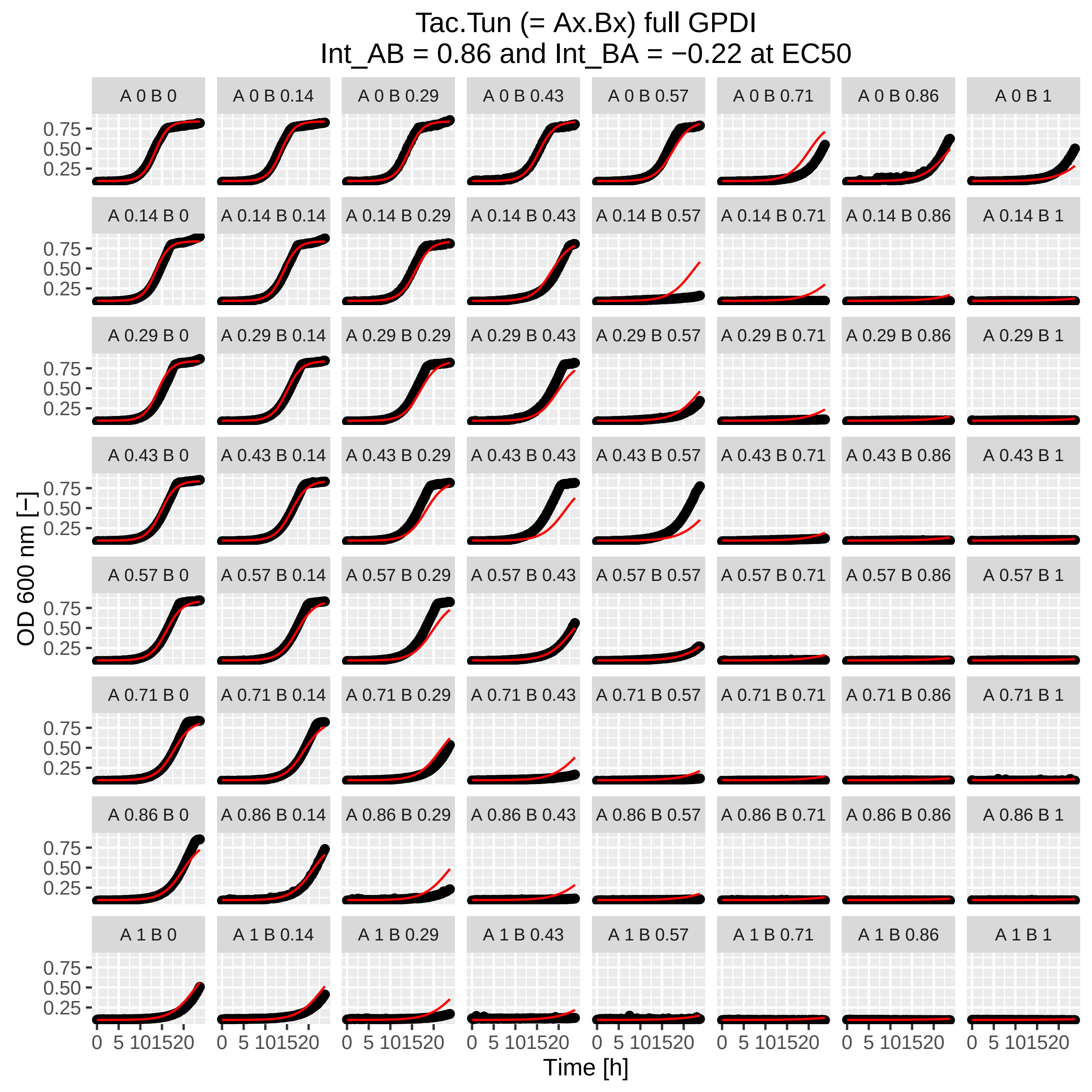

Tac.Tun (= Ax.Bx) full GPDI
Int_AB = 0.86 and Int_BA = −0.22 at EC50
OD 600 nm [−]
Time [h]
0.25
0.50
0.75
0 5 101520
A 0 B 0
A 0 B 0.14
A 0 B 0.29
A 0 B 0.43
A 0 B 0.57
A 0 B 0.71
A 0 B 0.86
A 0 B 1
A 1 B 0
A 1 B 0.14
A 1 B 0.29
A 1 B 0.43
A 1 B 0.57
A 1 B 0.71
A 1 B 0.86
A 1 B 1

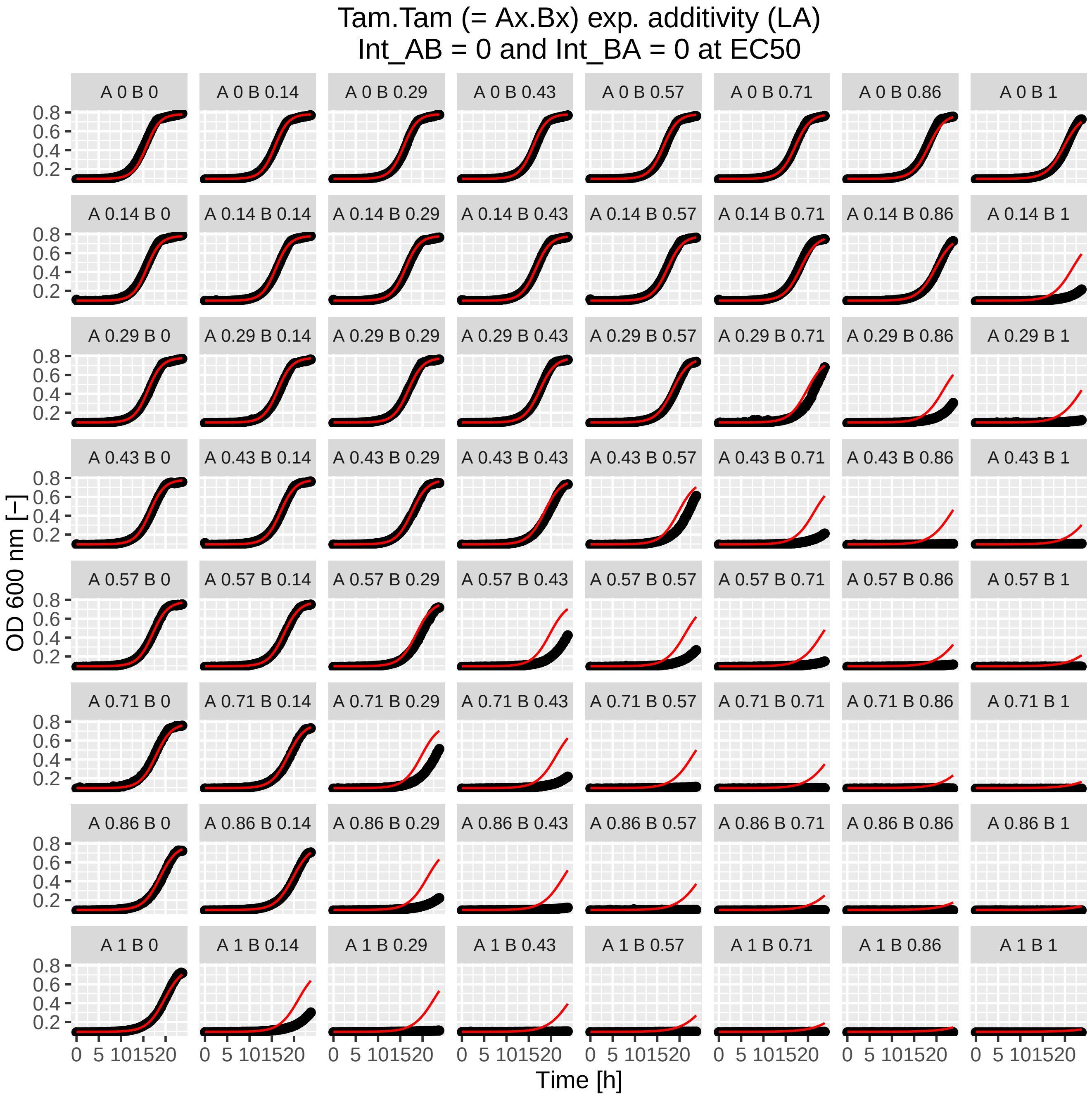

Tam.Tam (= Ax.Bx) exp. additivity (LA)
Int_AB = 0 and Int_BA = 0 at EC50
OD 600 nm [–]
Time [h]
0.8
0.6
0.4
0.2
0 5 101520
A 0 B 0
A 0 B 0.14
A 0 B 0.29
A 0 B 0.43
A 0 B 0.57
A 0 B 0.71
A 0 B 0.86
A 0 B 1
A 0.14 B 0
A 0.14 B 0.14
A 0.14 B 0.29
A 0.14 B 0.43
A 0.14 B 0.57
A 0.14 B 0.71
A 0.14 B 0.86
A 0.14 B 1
A 0.29 B 0
A 0.29 B 0.14
A 0.29 B 0.29
A 0.29 B 0.43
A 0.29 B 0.57
A 0.29 B 0.71
A 0.29 B 0.86
A 0.29 B 1
A 0.43 B 0
A 0.43 B 0.14
A 0.43 B 0.29
A 0.43 B 0.43
A 0.43 B 0.57
A 0.43 B 0.71
A 0.43 B 0.86
A 0.43 B 1
A 0.57 B 0
A 0.57 B 0.14
A 0.57 B 0.29
A 0.57 B 0.43
A 0.57 B 0.57
A 0.57 B 0.71
A 0.57 B 0.86
A 0.57 B 1
A 0.71 B 0
A 0.71 B 0.14
A 0.71 B 0.29
A 0.71 B 0.43
A 0.71 B 0.57
A 0.71 B 0.71
A 0.71 B 0.86
A 0.71 B 1
A 0.86 B 0
A 0.86 B 0.14
A 0.86 B 0.29
A 0.86 B 0.43
A 0.86 B 0.57
A 0.86 B 0.71
A 0.86 B 0.86
A 0.86 B 1
A 1 B 0
A 1 B 0.14
A 1 B 0.29
A 1 B 0.43
A 1 B 0.57
A 1 B 0.71
A 1 B 0.86
A 1 B 1

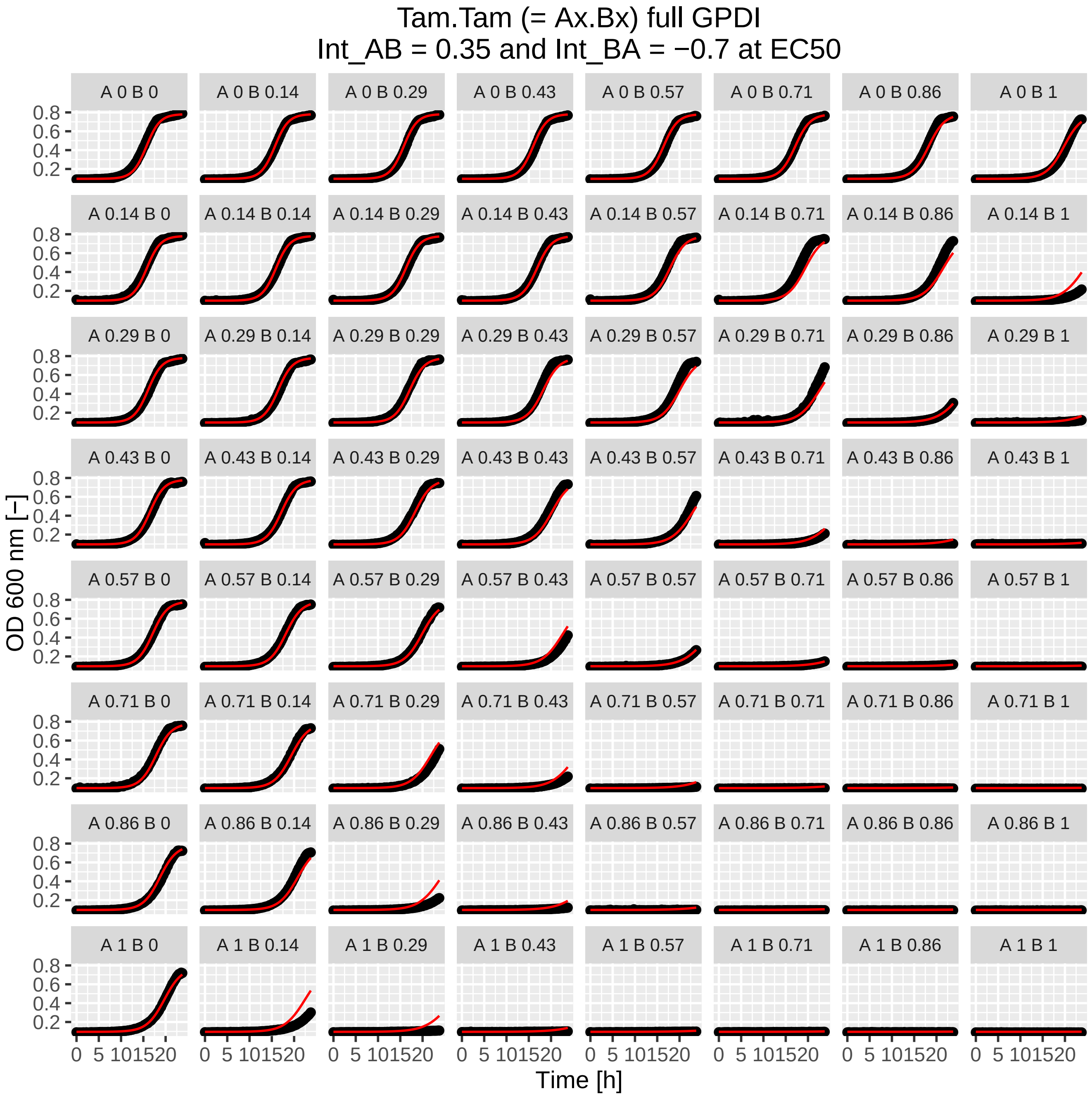

Tam.Tam (= Ax.Bx) full GPDI
Int_AB = 0.35 and Int_BA = −0.7 at EC50
A 0 B 0
A 0 B 0.14
A 0 B 0.29
A 0 B 0.43
A 0 B 0.57
A 0 B 0.71
A 0 B 0.86
A 0 B 1
A 0.14 B 0
A 0.14 B 0.14
A 0.14 B 0.29
A 0.14 B 0.43
A 0.14 B 0.57
A 0.14 B 0.71
A 0.14 B 0.86
A 0.14 B 1
A 0.29 B 0
A 0.29 B 0.14
A 0.29 B 0.29
A 0.29 B 0.43
A 0.29 B 0.57
A 0.29 B 0.71
A 0.29 B 0.86
A 0.29 B 1
A 0.43 B 0
A 0.43 B 0.14
A 0.43 B 0.29
A 0.43 B 0.43
A 0.43 B 0.57
A 0.43 B 0.71
A 0.43 B 0.86
A 0.43 B 1
A 0.57 B 0
A 0.57 B 0.14
A 0.57 B 0.29
A 0.57 B 0.43
A 0.57 B 0.57
A 0.57 B 0.71
A 0.57 B 0.86
A 0.57 B 1
A 0.71 B 0
A 0.71 B 0.14
A 0.71 B 0.29
A 0.71 B 0.43
A 0.71 B 0.57
A 0.71 B 0.71
A 0.71 B 0.86
A 0.71 B 1
A 0.86 B 0
A 0.86 B 0.14
A 0.86 B 0.29
A 0.86 B 0.43
A 0.86 B 0.57
A 0.86 B 0.71
A 0.86 B 0.86
A 0.86 B 1
A 1 B 0
A 1 B 0.14
A 1 B 0.29
A 1 B 0.43
A 1 B 0.57
A 1 B 0.71
A 1 B 0.86
A 1 B 1
0.8
0.6
0.4
0.2
OD 600 nm [−]
0 5 101520
Time [h]

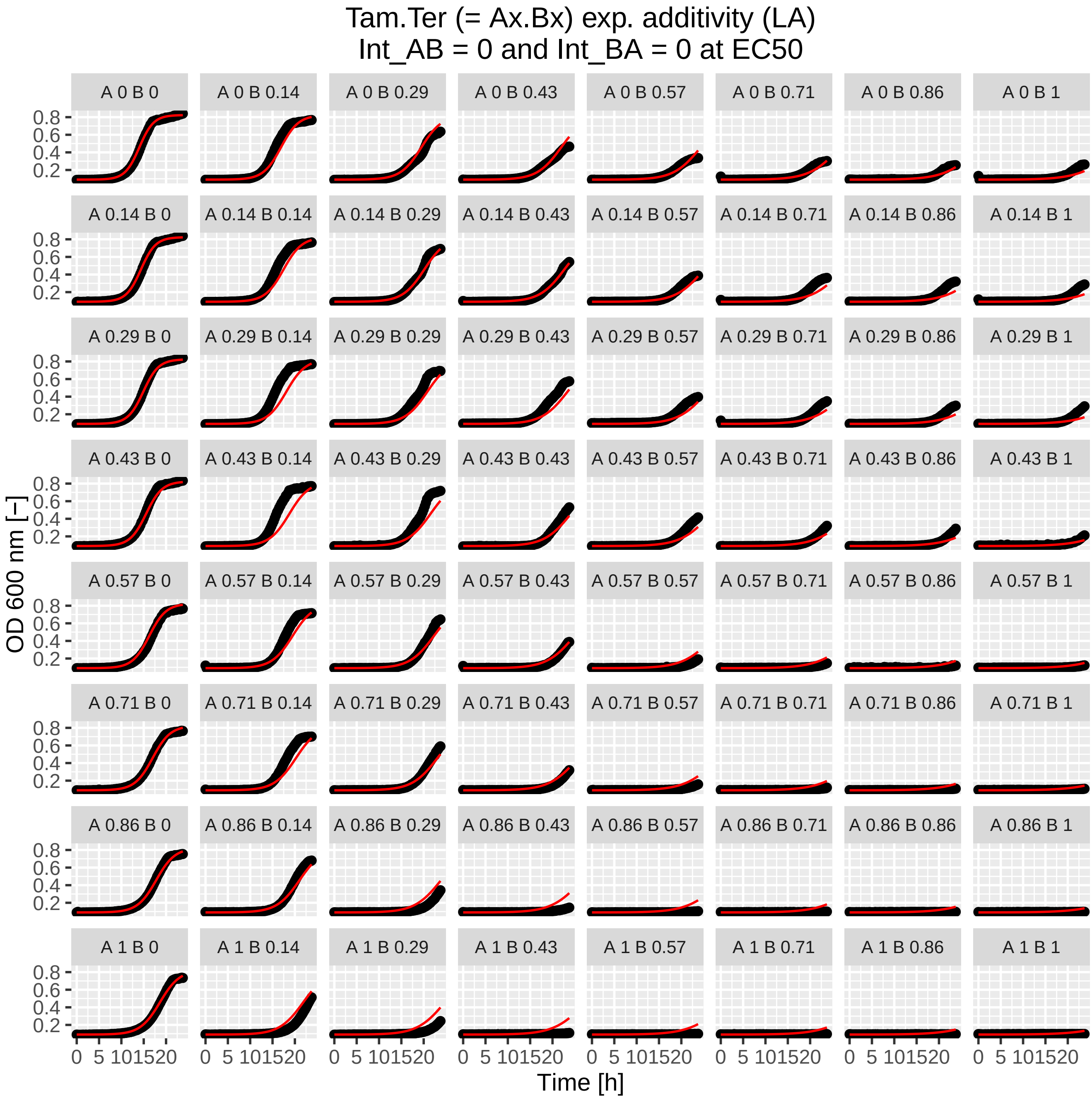

Tam.Ter (= Ax.Bx) exp. additivity (LA)
Int_AB = 0 and Int_BA = 0 at EC50
A 0 B 0
A 0 B 0.14
A 0 B 0.29
A 0 B 0.43
A 0 B 0.57
A 0 B 0.71
A 0 B 0.86
A 0 B 1
A 0.14 B 0
A 0.14 B 0.14
A 0.14 B 0.29
A 0.14 B 0.43
A 0.14 B 0.57
A 0.14 B 0.71
A 0.14 B 0.86
A 0.14 B 1
A 0.29 B 0
A 0.29 B 0.14
A 0.29 B 0.29
A 0.29 B 0.43
A 0.29 B 0.57
A 0.29 B 0.71
A 0.29 B 0.86
A 0.29 B 1
A 0.43 B 0
A 0.43 B 0.14
A 0.43 B 0.29
A 0.43 B 0.43
A 0.43 B 0.57
A 0.43 B 0.71
A 0.43 B 0.86
A 0.43 B 1
A 0.57 B 0
A 0.57 B 0.14
A 0.57 B 0.29
A 0.57 B 0.43
A 0.57 B 0.57
A 0.57 B 0.71
A 0.57 B 0.86
A 0.57 B 1
A 0.71 B 0
A 0.71 B 0.14
A 0.71 B 0.29
A 0.71 B 0.43
A 0.71 B 0.57
A 0.71 B 0.71
A 0.71 B 0.86
A 0.71 B 1
A 0.86 B 0
A 0.86 B 0.14
A 0.86 B 0.29
A 0.86 B 0.43
A 0.86 B 0.57
A 0.86 B 0.71
A 0.86 B 0.86
A 0.86 B 1
A 1 B 0
A 1 B 0.14
A 1 B 0.29
A 1 B 0.43
A 1 B 0.57
A 1 B 0.71
A 1 B 0.86
A 1 B 1
OD 600 nm [–]
0.2
0.4
0.6
0.8
0
5
10
15
20
Time [h]

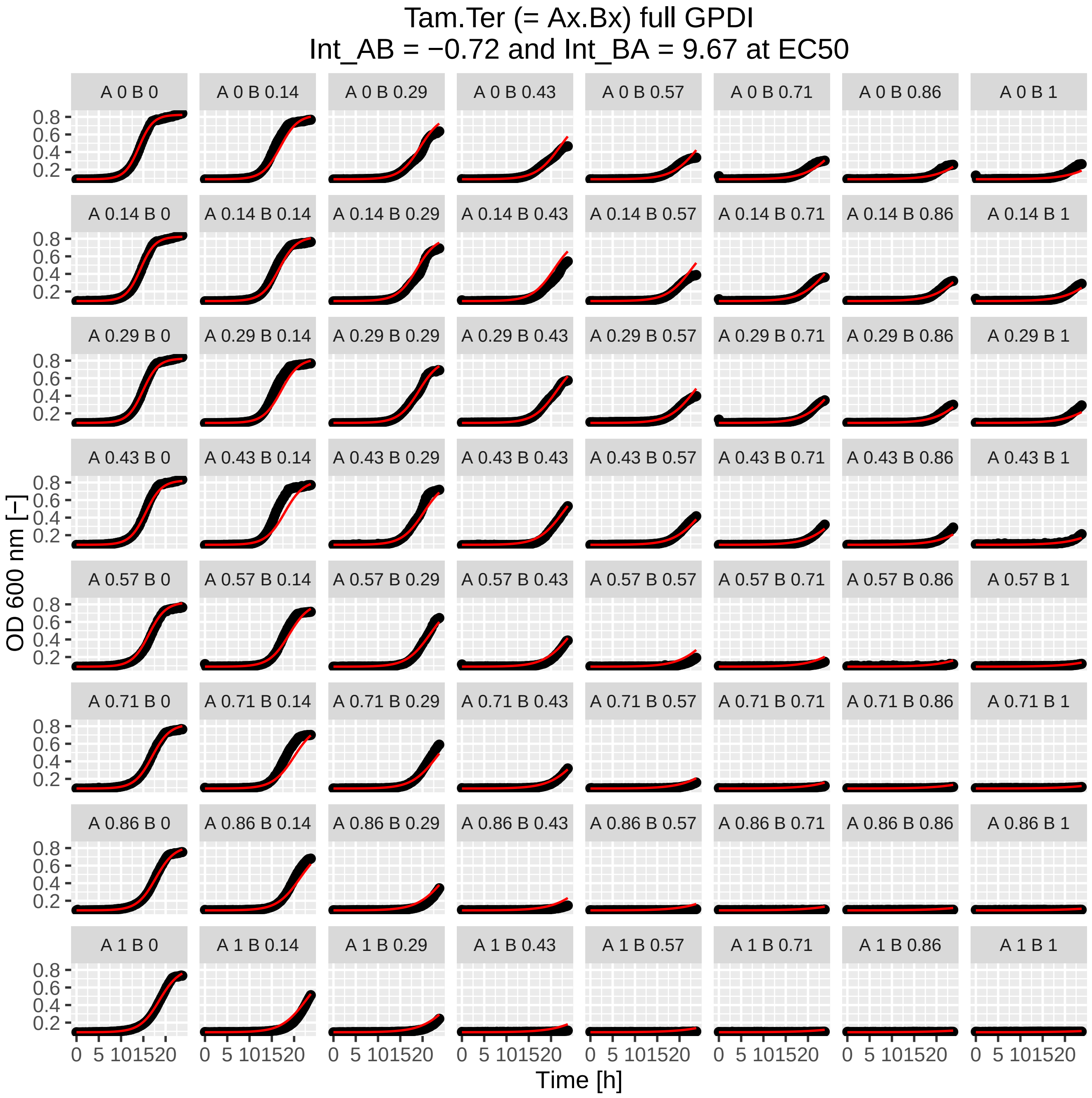
Tam.Ter (= Ax.Bx) full GPDI
Int_AB = −0.72 and Int_BA = 9.67 at EC50
A 0 B 0
A 0 B 0.14
A 0 B 0.29
A 0 B 0.43
A 0 B 0.57
A 0 B 0.71
A 0 B 0.86
A 0 B 1
A 0.14 B 0
A 0.14 B 0.14
A 0.14 B 0.29
A 0.14 B 0.43
A 0.14 B 0.57
A 0.14 B 0.71
A 0.14 B 0.86
A 0.14 B 1
A 0.29 B 0
A 0.29 B 0.14
A 0.29 B 0.29
A 0.29 B 0.43
A 0.29 B 0.57
A 0.29 B 0.71
A 0.29 B 0.86
A 0.29 B 1
A 0.43 B 0
A 0.43 B 0.14
A 0.43 B 0.29
A 0.43 B 0.43
A 0.43 B 0.57
A 0.43 B 0.71
A 0.43 B 0.86
A 0.43 B 1
A 0.57 B 0
A 0.57 B 0.14
A 0.57 B 0.29
A 0.57 B 0.43
A 0.57 B 0.57
A 0.57 B 0.71
A 0.57 B 0.86
A 0.57 B 1
A 0.71 B 0
A 0.71 B 0.14
A 0.71 B 0.29
A 0.71 B 0.43
A 0.71 B 0.57
A 0.71 B 0.71
A 0.71 B 0.86
A 0.71 B 1
A 0.86 B 0
A 0.86 B 0.14
A 0.86 B 0.29
A 0.86 B 0.43
A 0.86 B 0.57
A 0.86 B 0.71
A 0.86 B 0.86
A 0.86 B 1
A 1 B 0
A 1 B 0.14
A 1 B 0.29
A 1 B 0.43
A 1 B 0.57
A 1 B 0.71
A 1 B 0.86
A 1 B 1
0.8
0.6
0.4
0.2
0 5 101520
OD 600 nm [−]
Time [h]

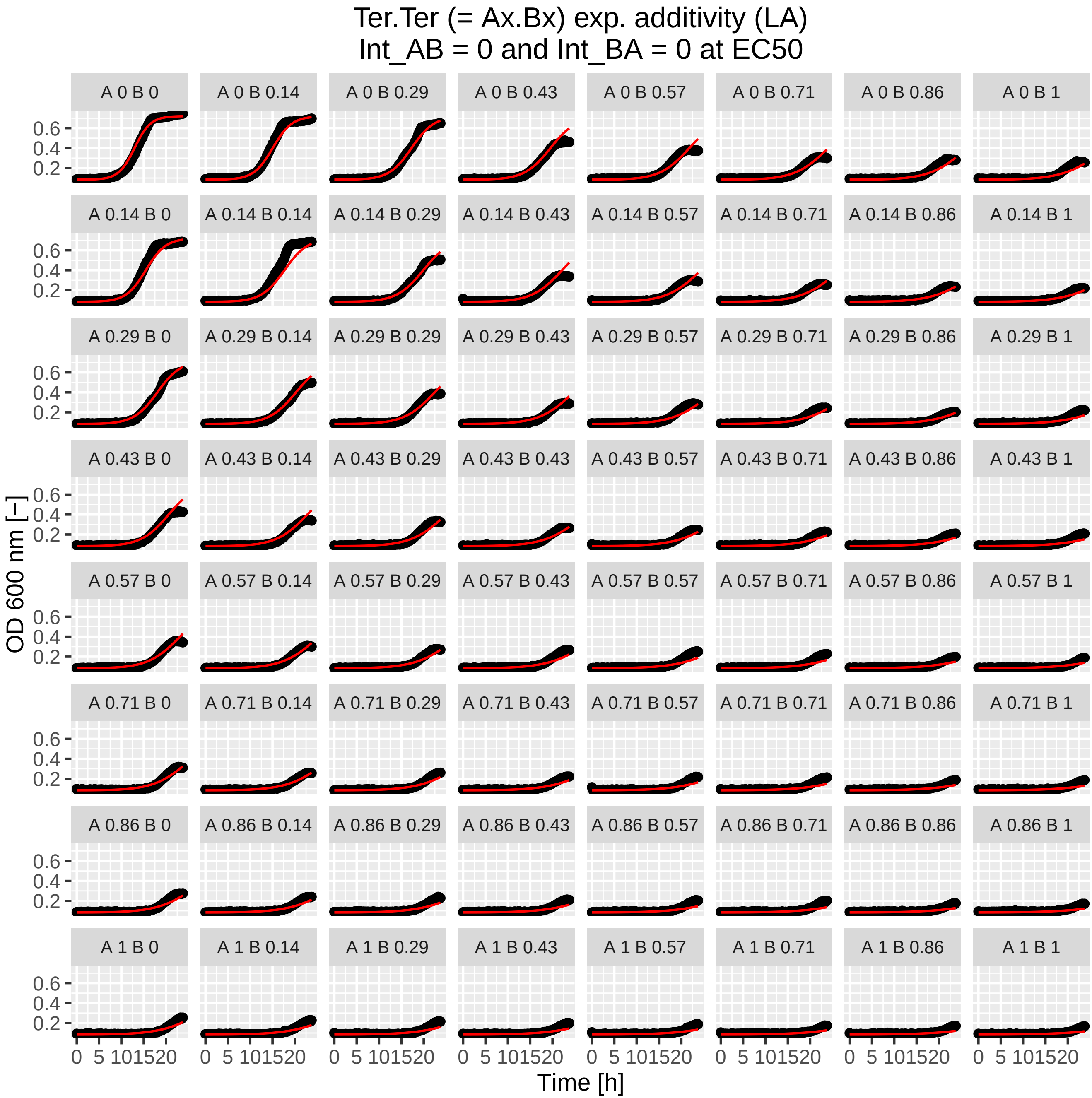
Ter.Ter (= Ax.Bx) exp. additivity (LA)
Int_AB = 0 and Int_BA = 0 at EC50
A 0 B 0
A 0 B 0.14
A 0 B 0.29
A 0 B 0.43
A 0 B 0.57
A 0 B 0.71
A 0 B 0.86
A 0 B 1
A 0.14 B 0
A 0.14 B 0.14
A 0.14 B 0.29
A 0.14 B 0.43
A 0.14 B 0.57
A 0.14 B 0.71
A 0.14 B 0.86
A 0.14 B 1
A 0.29 B 0
A 0.29 B 0.14
A 0.29 B 0.29
A 0.29 B 0.43
A 0.29 B 0.57
A 0.29 B 0.71
A 0.29 B 0.86
A 0.29 B 1
A 0.43 B 0
A 0.43 B 0.14
A 0.43 B 0.29
A 0.43 B 0.43
A 0.43 B 0.57
A 0.43 B 0.71
A 0.43 B 0.86
A 0.43 B 1
A 0.57 B 0
A 0.57 B 0.14
A 0.57 B 0.29
A 0.57 B 0.43
A 0.57 B 0.57
A 0.57 B 0.71
A 0.57 B 0.86
A 0.57 B 1
A 0.71 B 0
A 0.71 B 0.14
A 0.71 B 0.29
A 0.71 B 0.43
A 0.71 B 0.57
A 0.71 B 0.71
A 0.71 B 0.86
A 0.71 B 1
A 0.86 B 0
A 0.86 B 0.14
A 0.86 B 0.29
A 0.86 B 0.43
A 0.86 B 0.57
A 0.86 B 0.71
A 0.86 B 0.86
A 0.86 B 1
A 1 B 0
A 1 B 0.14
A 1 B 0.29
A 1 B 0.43
A 1 B 0.57
A 1 B 0.71
A 1 B 0.86
A 1 B 1
OD 600 nm [−]
0.2
0.4
0.6
0
5
10
15
20
Time [h]

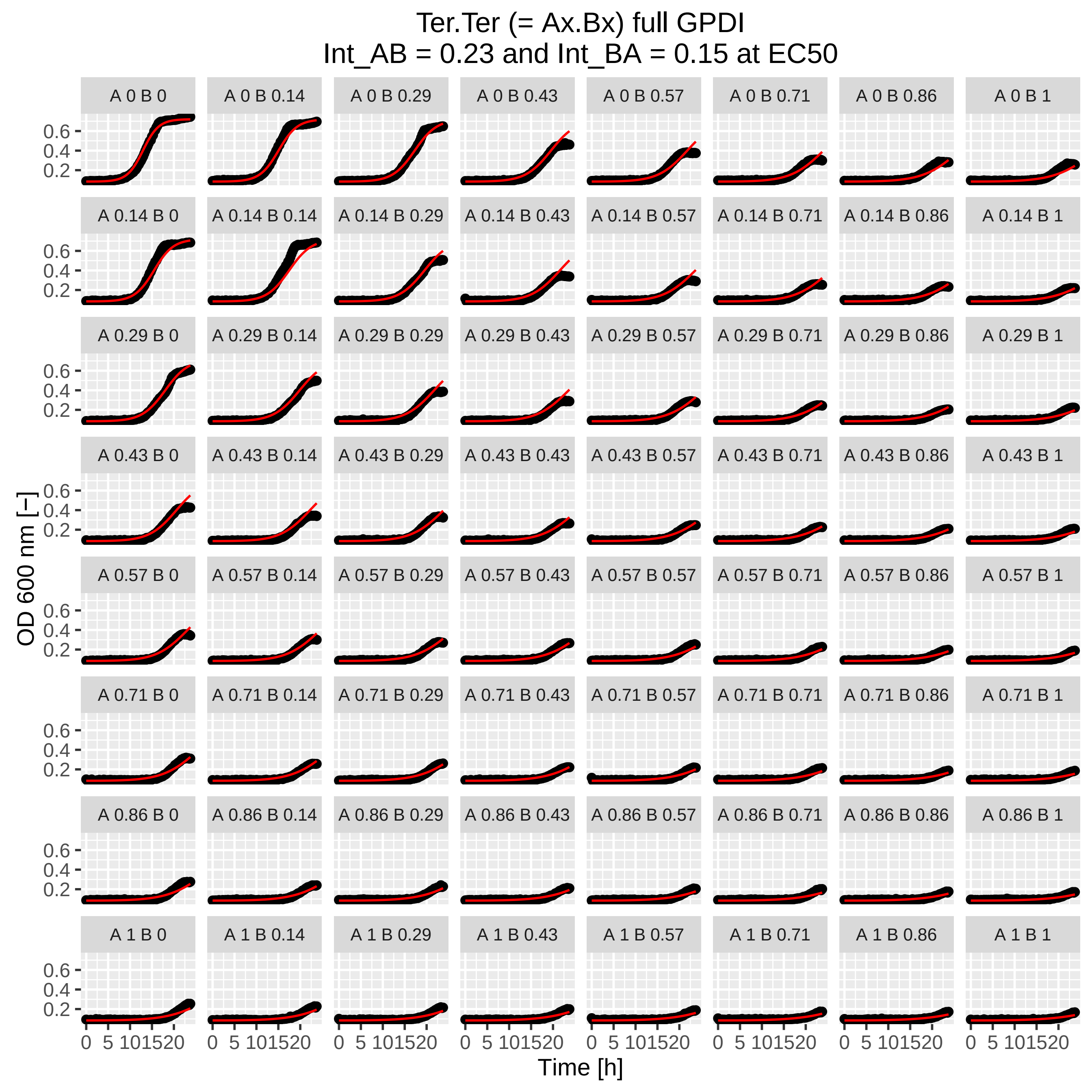
Ter.Ter (= Ax.Bx) full GPDI
Int_AB = 0.23 and Int_BA = 0.15 at EC50
A 0 B 0
A 0 B 0.14
A 0 B 0.29
A 0 B 0.43
A 0 B 0.57
A 0 B 0.71
A 0 B 0.86
A 0 B 1
A 0.14 B 0
A 0.14 B 0.14
A 0.14 B 0.29
A 0.14 B 0.43
A 0.14 B 0.57
A 0.14 B 0.71
A 0.14 B 0.86
A 0.14 B 1
A 0.29 B 0
A 0.29 B 0.14
A 0.29 B 0.29
A 0.29 B 0.43
A 0.29 B 0.57
A 0.29 B 0.71
A 0.29 B 0.86
A 0.29 B 1
A 0.43 B 0
A 0.43 B 0.14
A 0.43 B 0.29
A 0.43 B 0.43
A 0.43 B 0.57
A 0.43 B 0.71
A 0.43 B 0.86
A 0.43 B 1
A 0.57 B 0
A 0.57 B 0.14
A 0.57 B 0.29
A 0.57 B 0.43
A 0.57 B 0.57
A 0.57 B 0.71
A 0.57 B 0.86
A 0.57 B 1
A 0.71 B 0
A 0.71 B 0.14
A 0.71 B 0.29
A 0.71 B 0.43
A 0.71 B 0.57
A 0.71 B 0.71
A 0.71 B 0.86
A 0.71 B 1
A 0.86 B 0
A 0.86 B 0.14
A 0.86 B 0.29
A 0.86 B 0.43
A 0.86 B 0.57
A 0.86 B 0.71
A 0.86 B 0.86
A 0.86 B 1
A 1 B 0
A 1 B 0.14
A 1 B 0.29
A 1 B 0.43
A 1 B 0.57
A 1 B 0.71
A 1 B 0.86
A 1 B 1
OD 600 nm [–]
0.6
0.4
0.2
0 5 101520
Time [h]

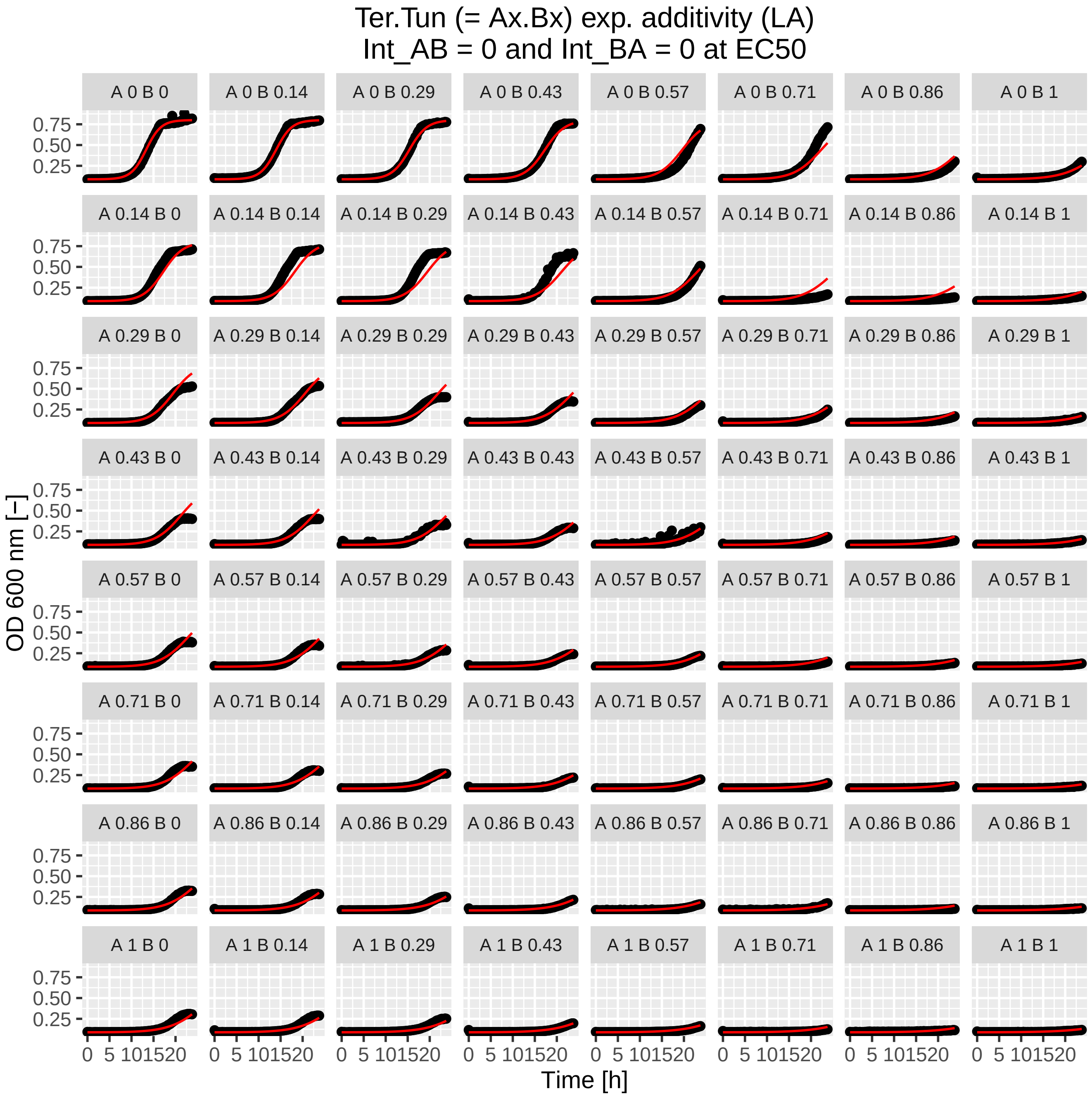

Ter.Tun (= Ax.Bx) exp. additivity (LA)
Int_AB = 0 and Int_BA = 0 at EC50
A 0 B 0
A 0 B 0.14
A 0 B 0.29
A 0 B 0.43
A 0 B 0.57
A 0 B 0.71
A 0 B 0.86
A 0 B 1
A 0.14 B 0
A 0.14 B 0.14
A 0.14 B 0.29
A 0.14 B 0.43
A 0.14 B 0.57
A 0.14 B 0.71
A 0.14 B 0.86
A 0.14 B 1
A 0.29 B 0
A 0.29 B 0.14
A 0.29 B 0.29
A 0.29 B 0.43
A 0.29 B 0.57
A 0.29 B 0.71
A 0.29 B 0.86
A 0.29 B 1
A 0.43 B 0
A 0.43 B 0.14
A 0.43 B 0.29
A 0.43 B 0.43
A 0.43 B 0.57
A 0.43 B 0.71
A 0.43 B 0.86
A 0.43 B 1
A 0.57 B 0
A 0.57 B 0.14
A 0.57 B 0.29
A 0.57 B 0.43
A 0.57 B 0.57
A 0.57 B 0.71
A 0.57 B 0.86
A 0.57 B 1
A 0.71 B 0
A 0.71 B 0.14
A 0.71 B 0.29
A 0.71 B 0.43
A 0.71 B 0.57
A 0.71 B 0.71
A 0.71 B 0.86
A 0.71 B 1
A 0.86 B 0
A 0.86 B 0.14
A 0.86 B 0.29
A 0.86 B 0.43
A 0.86 B 0.57
A 0.86 B 0.71
A 0.86 B 0.86
A 0.86 B 1
A 1 B 0
A 1 B 0.14
A 1 B 0.29
A 1 B 0.43
A 1 B 0.57
A 1 B 0.71
A 1 B 0.86
A 1 B 1
0.75
0.50
0.25
0 5 101520
OD 600 nm [–]
Time [h]

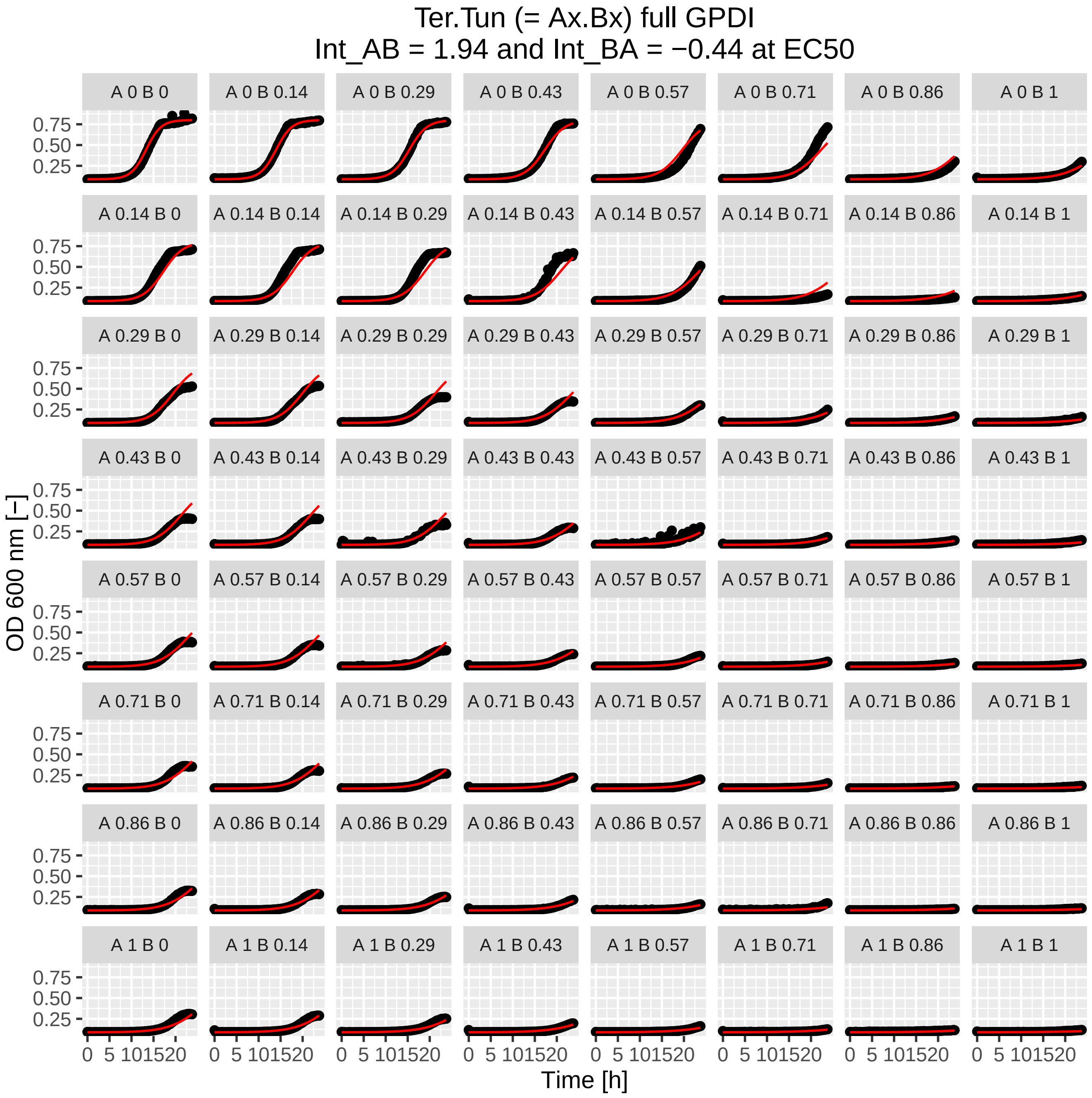
Ter.Tun (= Ax.Bx) full GPDI
Int_AB = 1.94 and Int_BA = −0.44 at EC50
A 0 B 0
A 0 B 0.14
A 0 B 0.29
A 0 B 0.43
A 0 B 0.57
A 0 B 0.71
A 0 B 0.86
A 0 B 1
A 0.14 B 0
A 0.14 B 0.14
A 0.14 B 0.29
A 0.14 B 0.43
A 0.14 B 0.57
A 0.14 B 0.71
A 0.14 B 0.86
A 0.14 B 1
A 0.29 B 0
A 0.29 B 0.14
A 0.29 B 0.29
A 0.29 B 0.43
A 0.29 B 0.57
A 0.29 B 0.71
A 0.29 B 0.86
A 0.29 B 1
A 0.43 B 0
A 0.43 B 0.14
A 0.43 B 0.29
A 0.43 B 0.43
A 0.43 B 0.57
A 0.43 B 0.71
A 0.43 B 0.86
A 0.43 B 1
A 0.57 B 0
A 0.57 B 0.14
A 0.57 B 0.29
A 0.57 B 0.43
A 0.57 B 0.57
A 0.57 B 0.71
A 0.57 B 0.86
A 0.57 B 1
A 0.71 B 0
A 0.71 B 0.14
A 0.71 B 0.29
A 0.71 B 0.43
A 0.71 B 0.57
A 0.71 B 0.71
A 0.71 B 0.86
A 0.71 B 1
A 0.86 B 0
A 0.86 B 0.14
A 0.86 B 0.29
A 0.86 B 0.43
A 0.86 B 0.57
A 0.86 B 0.71
A 0.86 B 0.86
A 0.86 B 1
A 1 B 0
A 1 B 0.14
A 1 B 0.29
A 1 B 0.43
A 1 B 0.57
A 1 B 0.71
A 1 B 0.86
A 1 B 1
0.75
0.50
0.25
0 5 101520
OD 600 nm [−]
Time [h]

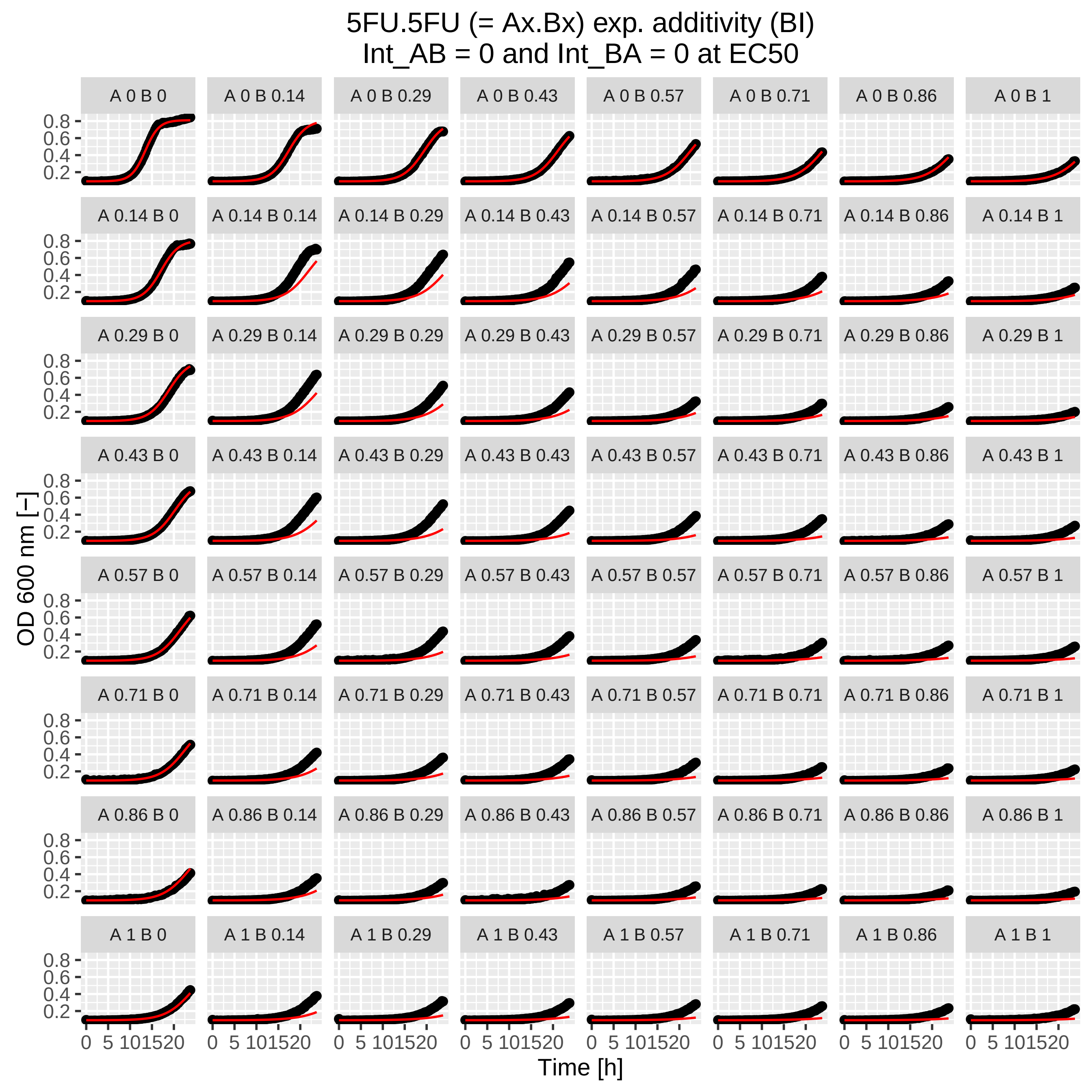
5FU.5FU (= Ax.Bx) exp. additivity (BI)
Int_AB = 0 and Int_BA = 0 at EC50
OD 600 nm [−]
Time [h]
A 0 B 0
A 0 B 0.14
A 0 B 0.29
A 0 B 0.43
A 0 B 0.57
A 0 B 0.71
A 0 B 0.86
A 0 B 1
A 0.14 B 0
A 0.14 B 0.14
A 0.14 B 0.29
A 0.14 B 0.43
A 0.14 B 0.57
A 0.14 B 0.71
A 0.14 B 0.86
A 0.14 B 1
A 0.29 B 0
A 0.29 B 0.14
A 0.29 B 0.29
A 0.29 B 0.43
A 0.29 B 0.57
A 0.29 B 0.71
A 0.29 B 0.86
A 0.29 B 1
A 0.43 B 0
A 0.43 B 0.14
A 0.43 B 0.29
A 0.43 B 0.43
A 0.43 B 0.57
A 0.43 B 0.71
A 0.43 B 0.86
A 0.43 B 1
A 0.57 B 0
A 0.57 B 0.14
A 0.57 B 0.29
A 0.57 B 0.43
A 0.57 B 0.57
A 0.57 B 0.71
A 0.57 B 0.86
A 0.57 B 1
A 0.71 B 0
A 0.71 B 0.14
A 0.71 B 0.29
A 0.71 B 0.43
A 0.71 B 0.57
A 0.71 B 0.71
A 0.71 B 0.86
A 0.71 B 1
A 0.86 B 0
A 0.86 B 0.14
A 0.86 B 0.29
A 0.86 B 0.43
A 0.86 B 0.57
A 0.86 B 0.71
A 0.86 B 0.86
A 0.86 B 1
A 1 B 0
A 1 B 0.14
A 1 B 0.29
A 1 B 0.43
A 1 B 0.57
A 1 B 0.71
A 1 B 0.86
A 1 B 1
0.8
0.6
0.4
0.2
0 5 10 15 20

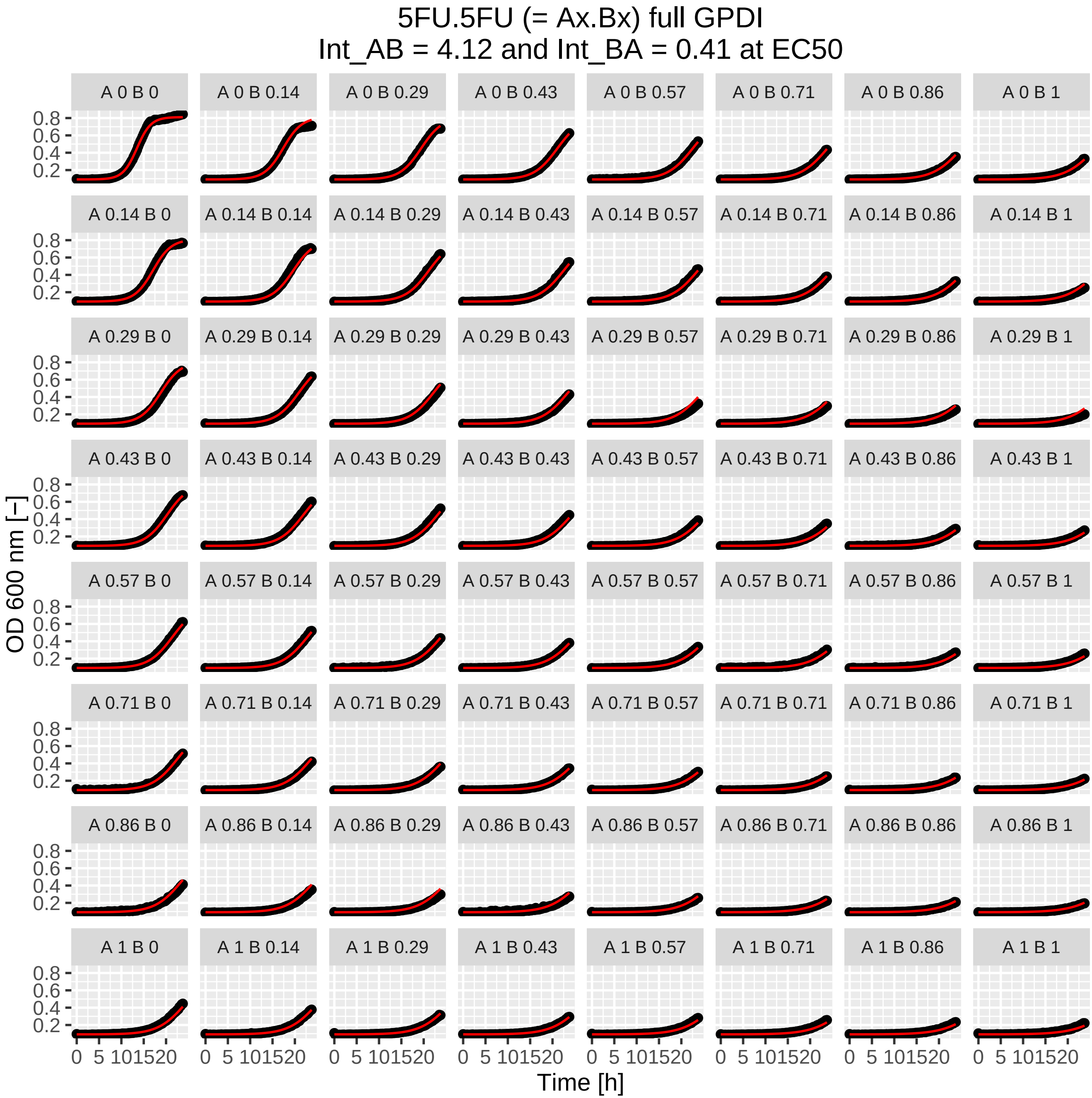
5FU.5FU (= Ax.Bx) full GPDI
Int_AB = 4.12 and Int_BA = 0.41 at EC50
A 0 B 0
A 0 B 0.14
A 0 B 0.29
A 0 B 0.43
A 0 B 0.57
A 0 B 0.71
A 0 B 0.86
A 0 B 1
A 0.14 B 0
A 0.14 B 0.14
A 0.14 B 0.29
A 0.14 B 0.43
A 0.14 B 0.57
A 0.14 B 0.71
A 0.14 B 0.86
A 0.14 B 1
A 0.29 B 0
A 0.29 B 0.14
A 0.29 B 0.29
A 0.29 B 0.43
A 0.29 B 0.57
A 0.29 B 0.71
A 0.29 B 0.86
A 0.29 B 1
A 0.43 B 0
A 0.43 B 0.14
A 0.43 B 0.29
A 0.43 B 0.43
A 0.43 B 0.57
A 0.43 B 0.71
A 0.43 B 0.86
A 0.43 B 1
A 0.57 B 0
A 0.57 B 0.14
A 0.57 B 0.29
A 0.57 B 0.43
A 0.57 B 0.57
A 0.57 B 0.71
A 0.57 B 0.86
A 0.57 B 1
A 0.71 B 0
A 0.71 B 0.14
A 0.71 B 0.29
A 0.71 B 0.43
A 0.71 B 0.57
A 0.71 B 0.71
A 0.71 B 0.86
A 0.71 B 1
A 0.86 B 0
A 0.86 B 0.14
A 0.86 B 0.29
A 0.86 B 0.43
A 0.86 B 0.57
A 0.86 B 0.71
A 0.86 B 0.86
A 0.86 B 1
A 1 B 0
A 1 B 0.14
A 1 B 0.29
A 1 B 0.43
A 1 B 0.57
A 1 B 0.71
A 1 B 0.86
A 1 B 1
OD 600 nm [–]
0.8
0.6
0.4
0.2
0 5 101520
Time [h]

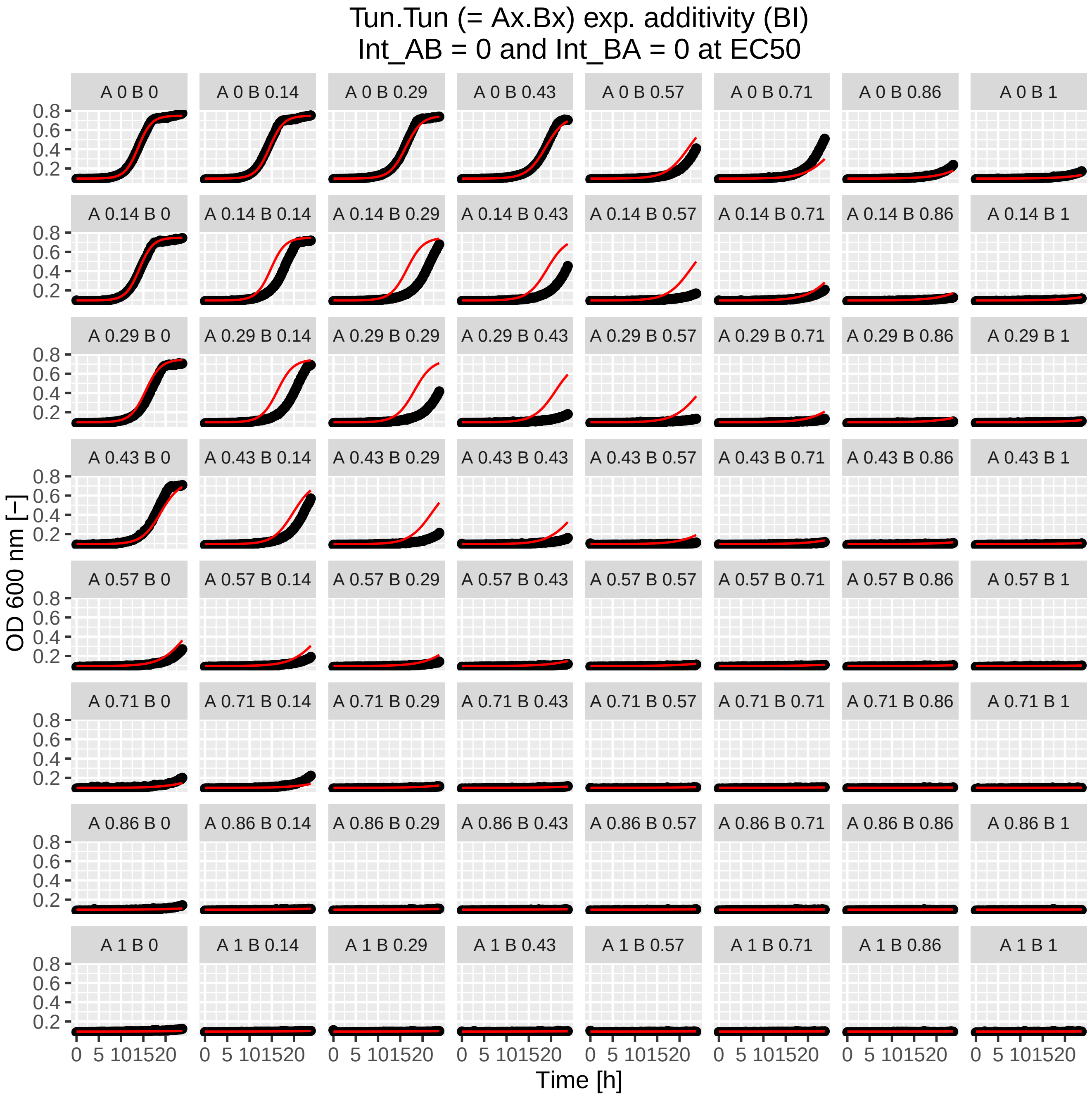
Tun.Tun (= Ax.Bx) exp. additivity (BI)
Int_AB = 0 and Int_BA = 0 at EC50
A 0 B 0
A 0 B 0.14
A 0 B 0.29
A 0 B 0.43
A 0 B 0.57
A 0 B 0.71
A 0 B 0.86
A 0 B 1
A 0.14 B 0
A 0.14 B 0.14
A 0.14 B 0.29
A 0.14 B 0.43
A 0.14 B 0.57
A 0.14 B 0.71
A 0.14 B 0.86
A 0.14 B 1
A 0.29 B 0
A 0.29 B 0.14
A 0.29 B 0.29
A 0.29 B 0.43
A 0.29 B 0.57
A 0.29 B 0.71
A 0.29 B 0.86
A 0.29 B 1
A 0.43 B 0
A 0.43 B 0.14
A 0.43 B 0.29
A 0.43 B 0.43
A 0.43 B 0.57
A 0.43 B 0.71
A 0.43 B 0.86
A 0.43 B 1
A 0.57 B 0
A 0.57 B 0.14
A 0.57 B 0.29
A 0.57 B 0.43
A 0.57 B 0.57
A 0.57 B 0.71
A 0.57 B 0.86
A 0.57 B 1
A 0.71 B 0
A 0.71 B 0.14
A 0.71 B 0.29
A 0.71 B 0.43
A 0.71 B 0.57
A 0.71 B 0.71
A 0.71 B 0.86
A 0.71 B 1
A 0.86 B 0
A 0.86 B 0.14
A 0.86 B 0.29
A 0.86 B 0.43
A 0.86 B 0.57
A 0.86 B 0.71
A 0.86 B 0.86
A 0.86 B 1
A 1 B 0
A 1 B 0.14
A 1 B 0.29
A 1 B 0.43
A 1 B 0.57
A 1 B 0.71
A 1 B 0.86
A 1 B 1
0.8
0.6
0.4
0.2
OD 600 nm [–]
0 5 101520
Time [h]

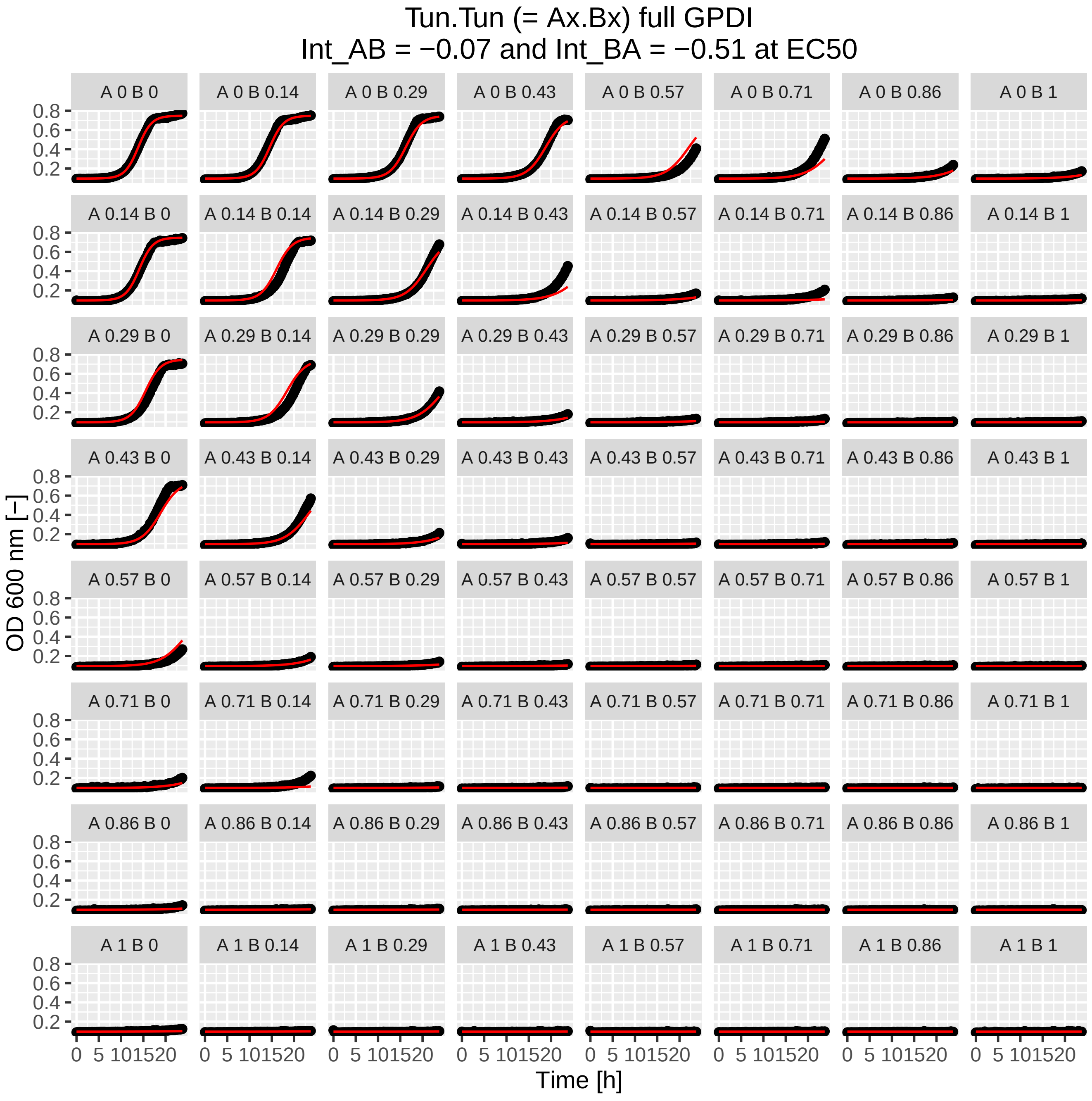
Tun.Tun (= Ax.Bx) full GPDI
Int_AB = −0.07 and Int_BA = −0.51 at EC50
A 0 B 0
A 0 B 0.14
A 0 B 0.29
A 0 B 0.43
A 0 B 0.57
A 0 B 0.71
A 0 B 0.86
A 0 B 1
A 0.14 B 0
A 0.14 B 0.14
A 0.14 B 0.29
A 0.14 B 0.43
A 0.14 B 0.57
A 0.14 B 0.71
A 0.14 B 0.86
A 0.14 B 1
A 0.29 B 0
A 0.29 B 0.14
A 0.29 B 0.29
A 0.29 B 0.43
A 0.29 B 0.57
A 0.29 B 0.71
A 0.29 B 0.86
A 0.29 B 1
A 0.43 B 0
A 0.43 B 0.14
A 0.43 B 0.29
A 0.43 B 0.43
A 0.43 B 0.57
A 0.43 B 0.71
A 0.43 B 0.86
A 0.43 B 1
A 0.57 B 0
A 0.57 B 0.14
A 0.57 B 0.29
A 0.57 B 0.43
A 0.57 B 0.57
A 0.57 B 0.71
A 0.57 B 0.86
A 0.57 B 1
A 0.71 B 0
A 0.71 B 0.14
A 0.71 B 0.29
A 0.71 B 0.43
A 0.71 B 0.57
A 0.71 B 0.71
A 0.71 B 0.86
A 0.71 B 1
A 0.86 B 0
A 0.86 B 0.14
A 0.86 B 0.29
A 0.86 B 0.43
A 0.86 B 0.57
A 0.86 B 0.71
A 0.86 B 0.86
A 0.86 B 1
A 1 B 0
A 1 B 0.14
A 1 B 0.29
A 1 B 0.43
A 1 B 0.57
A 1 B 0.71
A 1 B 0.86
A 1 B 1
0.8
0.6
0.4
0.2
OD 600 nm [−]
0 5 101520
Time [h]

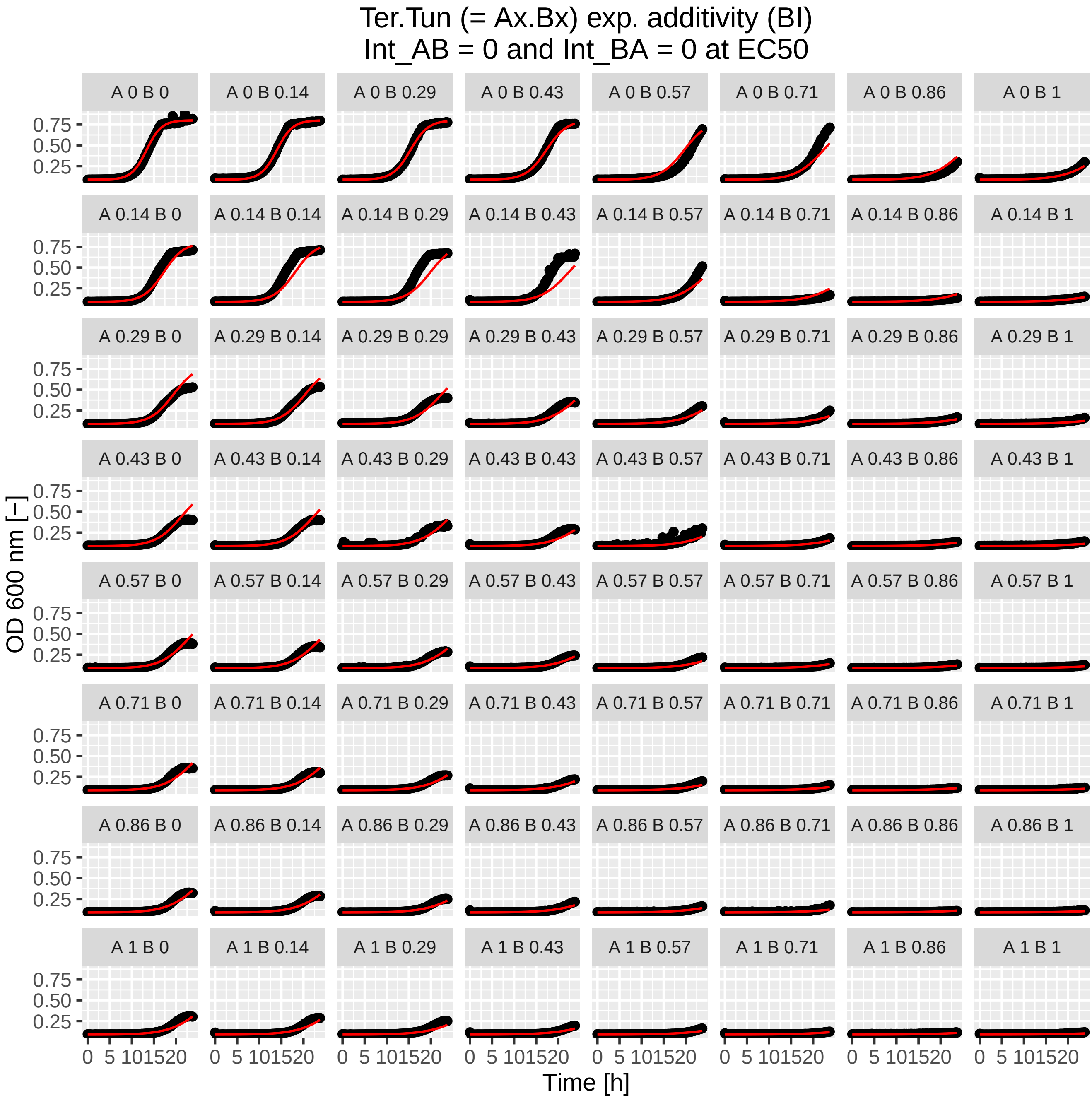

Ter.Tun (= Ax.Bx) exp. additivity (BI)
Int_AB = 0 and Int_BA = 0 at EC50
A 0 B 0
A 0 B 0.14
A 0 B 0.29
A 0 B 0.43
A 0 B 0.57
A 0 B 0.71
A 0 B 0.86
A 0 B 1
A 0.14 B 0
A 0.14 B 0.14
A 0.14 B 0.29
A 0.14 B 0.43
A 0.14 B 0.57
A 0.14 B 0.71
A 0.14 B 0.86
A 0.14 B 1
A 0.29 B 0
A 0.29 B 0.14
A 0.29 B 0.29
A 0.29 B 0.43
A 0.29 B 0.57
A 0.29 B 0.71
A 0.29 B 0.86
A 0.29 B 1
A 0.43 B 0
A 0.43 B 0.14
A 0.43 B 0.29
A 0.43 B 0.43
A 0.43 B 0.57
A 0.43 B 0.71
A 0.43 B 0.86
A 0.43 B 1
A 0.57 B 0
A 0.57 B 0.14
A 0.57 B 0.29
A 0.57 B 0.43
A 0.57 B 0.57
A 0.57 B 0.71
A 0.57 B 0.86
A 0.57 B 1
A 0.71 B 0
A 0.71 B 0.14
A 0.71 B 0.29
A 0.71 B 0.43
A 0.71 B 0.57
A 0.71 B 0.71
A 0.71 B 0.86
A 0.71 B 1
A 0.86 B 0
A 0.86 B 0.14
A 0.86 B 0.29
A 0.86 B 0.43
A 0.86 B 0.57
A 0.86 B 0.71
A 0.86 B 0.86
A 0.86 B 1
A 1 B 0
A 1 B 0.14
A 1 B 0.29
A 1 B 0.43
A 1 B 0.57
A 1 B 0.71
A 1 B 0.86
A 1 B 1
0.75
0.50
0.25
OD 600 nm [–]
0 5 101520
Time [h]

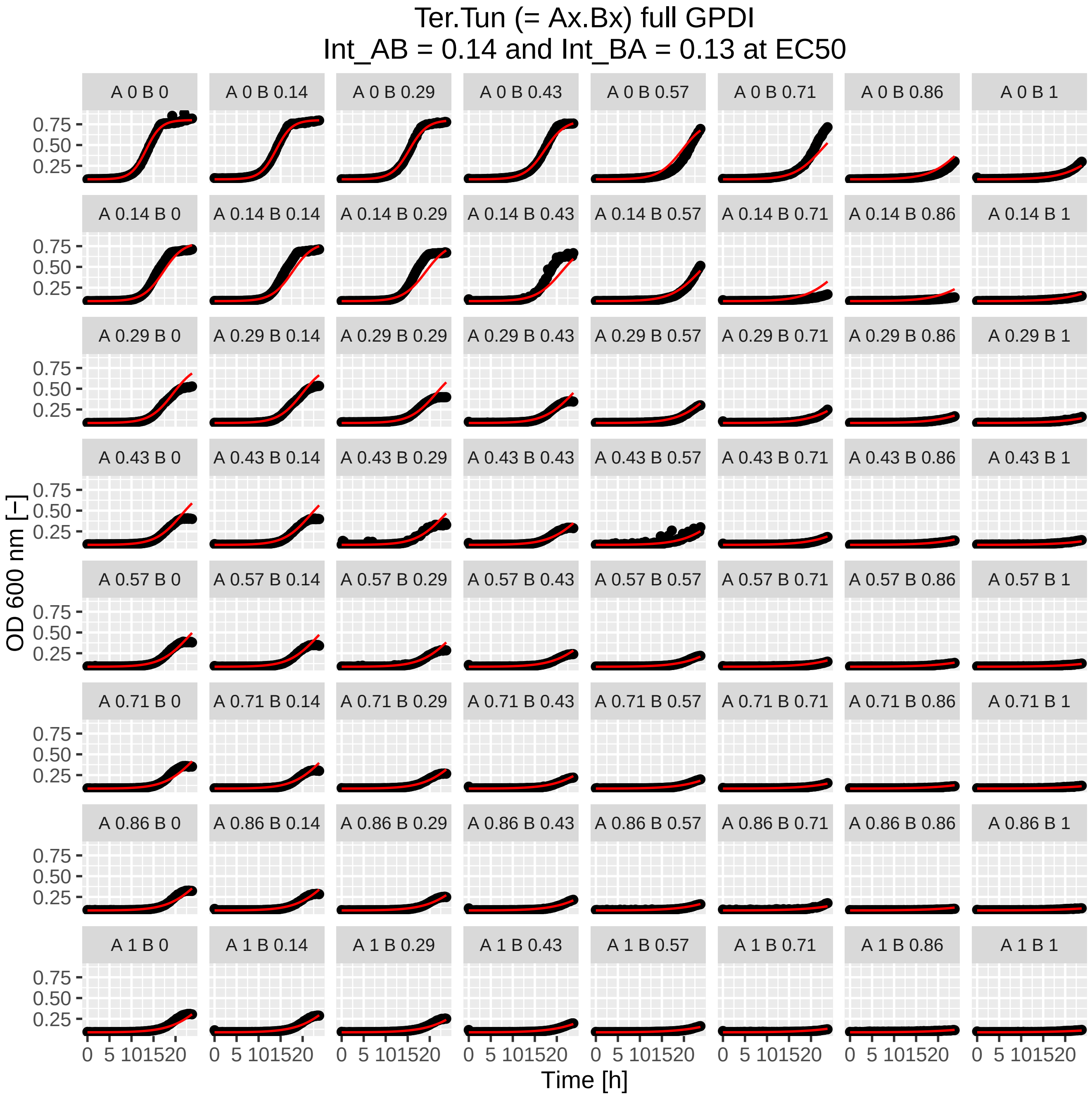
Ter.Tun (= Ax.Bx) full GPDI
Int_AB = 0.14 and Int_BA = 0.13 at EC50
A 0 B 0
A 0 B 0.14
A 0 B 0.29
A 0 B 0.43
A 0 B 0.57
A 0 B 0.71
A 0 B 0.86
A 0 B 1
A 0.14 B 0
A 0.14 B 0.14
A 0.14 B 0.29
A 0.14 B 0.43
A 0.14 B 0.57
A 0.14 B 0.71
A 0.14 B 0.86
A 0.14 B 1
A 0.29 B 0
A 0.29 B 0.14
A 0.29 B 0.29
A 0.29 B 0.43
A 0.29 B 0.57
A 0.29 B 0.71
A 0.29 B 0.86
A 0.29 B 1
A 0.43 B 0
A 0.43 B 0.14
A 0.43 B 0.29
A 0.43 B 0.43
A 0.43 B 0.57
A 0.43 B 0.71
A 0.43 B 0.86
A 0.43 B 1
A 0.57 B 0
A 0.57 B 0.14
A 0.57 B 0.29
A 0.57 B 0.43
A 0.57 B 0.57
A 0.57 B 0.71
A 0.57 B 0.86
A 0.57 B 1
A 0.71 B 0
A 0.71 B 0.14
A 0.71 B 0.29
A 0.71 B 0.43
A 0.71 B 0.57
A 0.71 B 0.71
A 0.71 B 0.86
A 0.71 B 1
A 0.86 B 0
A 0.86 B 0.14
A 0.86 B 0.29
A 0.86 B 0.43
A 0.86 B 0.57
A 0.86 B 0.71
A 0.86 B 0.86
A 0.86 B 1
A 1 B 0
A 1 B 0.14
A 1 B 0.29
A 1 B 0.43
A 1 B 0.57
A 1 B 0.71
A 1 B 0.86
A 1 B 1
0.75
0.50
0.25
0 5 101520
OD 600 nm [–]
Time [h]

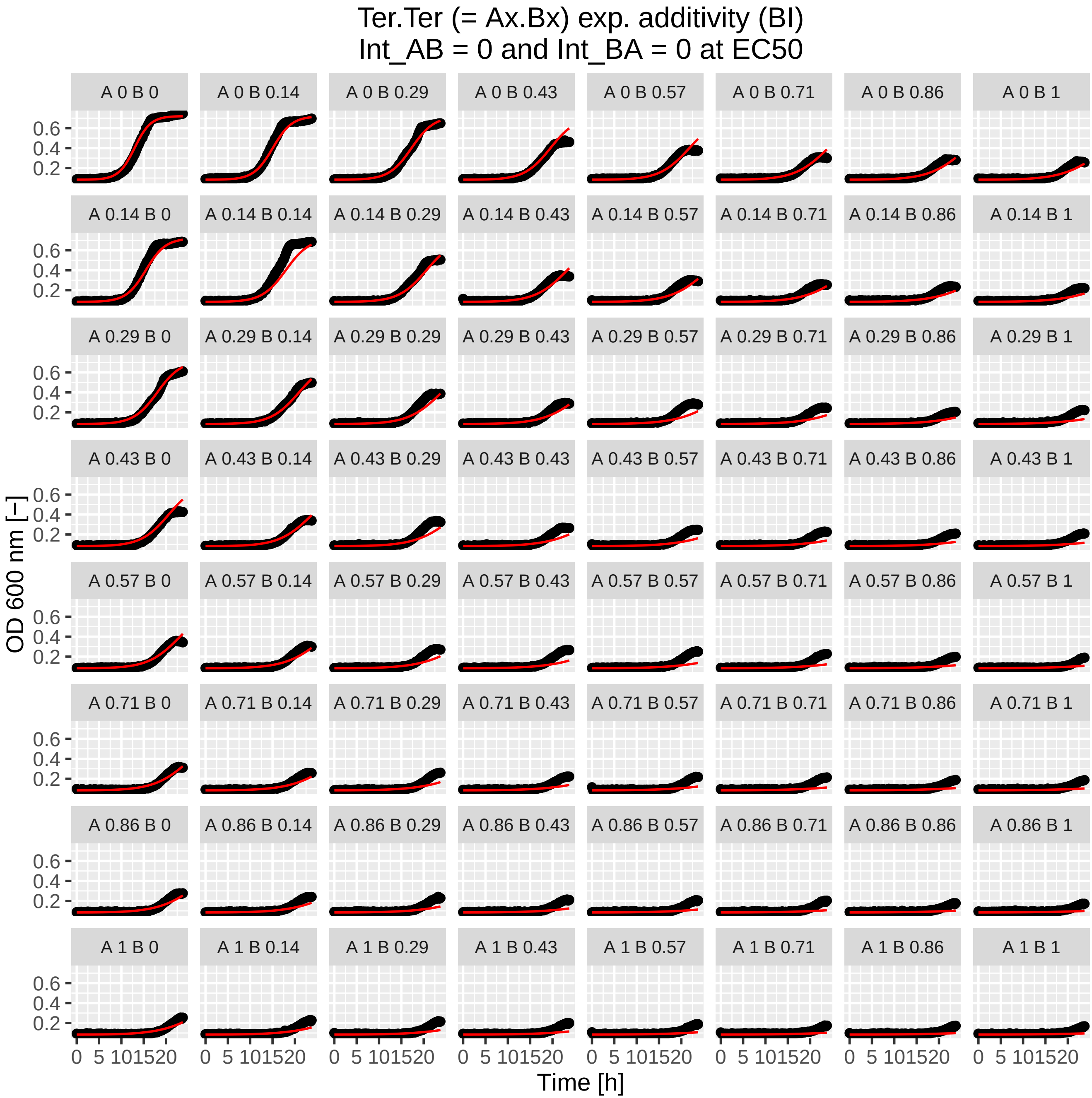
Ter.Ter (= Ax.Bx) exp. additivity (BI)
Int_AB = 0 and Int_BA = 0 at EC50
A 0 B 0
A 0 B 0.14
A 0 B 0.29
A 0 B 0.43
A 0 B 0.57
A 0 B 0.71
A 0 B 0.86
A 0 B 1
A 0.14 B 0
A 0.14 B 0.14
A 0.14 B 0.29
A 0.14 B 0.43
A 0.14 B 0.57
A 0.14 B 0.71
A 0.14 B 0.86
A 0.14 B 1
A 0.29 B 0
A 0.29 B 0.14
A 0.29 B 0.29
A 0.29 B 0.43
A 0.29 B 0.57
A 0.29 B 0.71
A 0.29 B 0.86
A 0.29 B 1
A 0.43 B 0
A 0.43 B 0.14
A 0.43 B 0.29
A 0.43 B 0.43
A 0.43 B 0.57
A 0.43 B 0.71
A 0.43 B 0.86
A 0.43 B 1
A 0.57 B 0
A 0.57 B 0.14
A 0.57 B 0.29
A 0.57 B 0.43
A 0.57 B 0.57
A 0.57 B 0.71
A 0.57 B 0.86
A 0.57 B 1
A 0.71 B 0
A 0.71 B 0.14
A 0.71 B 0.29
A 0.71 B 0.43
A 0.71 B 0.57
A 0.71 B 0.71
A 0.71 B 0.86
A 0.71 B 1
A 0.86 B 0
A 0.86 B 0.14
A 0.86 B 0.29
A 0.86 B 0.43
A 0.86 B 0.57
A 0.86 B 0.71
A 0.86 B 0.86
A 0.86 B 1
A 1 B 0
A 1 B 0.14
A 1 B 0.29
A 1 B 0.43
A 1 B 0.57
A 1 B 0.71
A 1 B 0.86
A 1 B 1
OD 600 nm [–]
0.2
0.4
0.6
0
5
10
15
20
Time [h]

Ter.Ter (= Ax.Bx) full GPDI
Int_AB = 0.52 and Int_BA = 0.46 at EC50

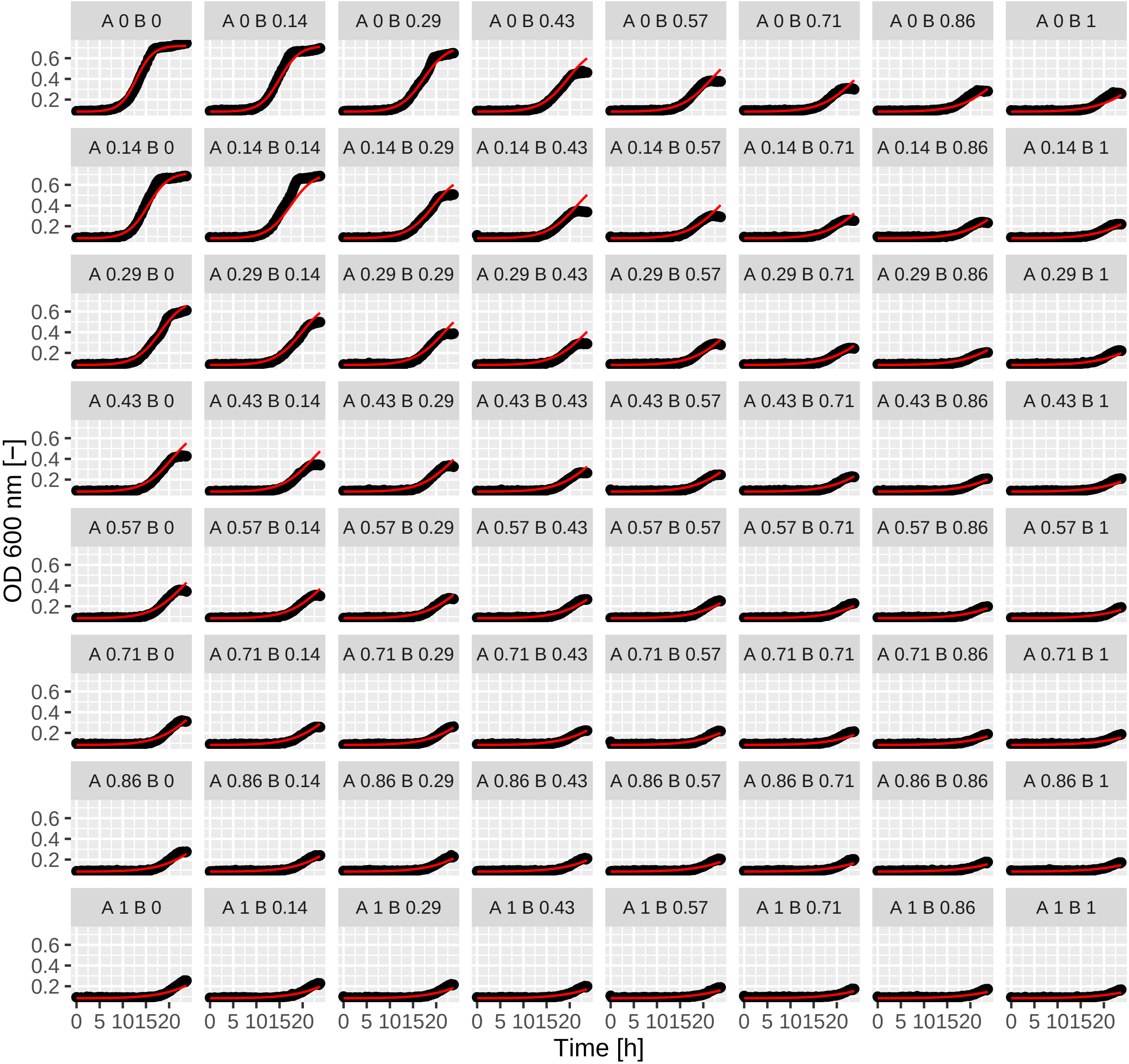

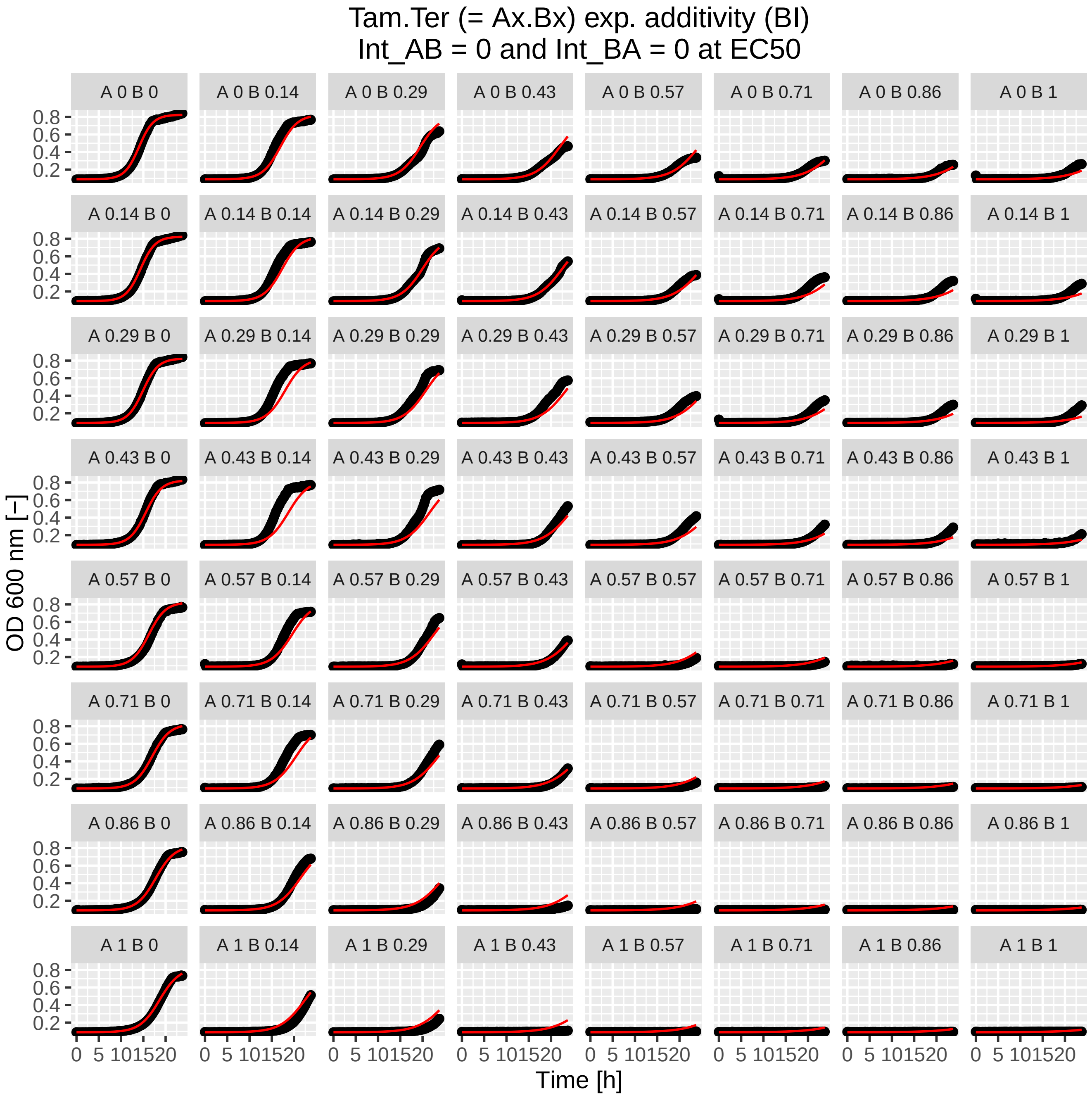
Tam.Ter (= Ax.Bx) exp. additivity (BI)
Int_AB = 0 and Int_BA = 0 at EC50
A 0 B 0
A 0 B 0.14
A 0 B 0.29
A 0 B 0.43
A 0 B 0.57
A 0 B 0.71
A 0 B 0.86
A 0 B 1
A 0.14 B 0
A 0.14 B 0.14
A 0.14 B 0.29
A 0.14 B 0.43
A 0.14 B 0.57
A 0.14 B 0.71
A 0.14 B 0.86
A 0.14 B 1
A 0.29 B 0
A 0.29 B 0.14
A 0.29 B 0.29
A 0.29 B 0.43
A 0.29 B 0.57
A 0.29 B 0.71
A 0.29 B 0.86
A 0.29 B 1
A 0.43 B 0
A 0.43 B 0.14
A 0.43 B 0.29
A 0.43 B 0.43
A 0.43 B 0.57
A 0.43 B 0.71
A 0.43 B 0.86
A 0.43 B 1
A 0.57 B 0
A 0.57 B 0.14
A 0.57 B 0.29
A 0.57 B 0.43
A 0.57 B 0.57
A 0.57 B 0.71
A 0.57 B 0.86
A 0.57 B 1
A 0.71 B 0
A 0.71 B 0.14
A 0.71 B 0.29
A 0.71 B 0.43
A 0.71 B 0.57
A 0.71 B 0.71
A 0.71 B 0.86
A 0.71 B 1
A 0.86 B 0
A 0.86 B 0.14
A 0.86 B 0.29
A 0.86 B 0.43
A 0.86 B 0.57
A 0.86 B 0.71
A 0.86 B 0.86
A 0.86 B 1
A 1 B 0
A 1 B 0.14
A 1 B 0.29
A 1 B 0.43
A 1 B 0.57
A 1 B 0.71
A 1 B 0.86
A 1 B 1
OD 600 nm [−]
0.2
0.4
0.6
0.8
0 5 101520
Time [h]

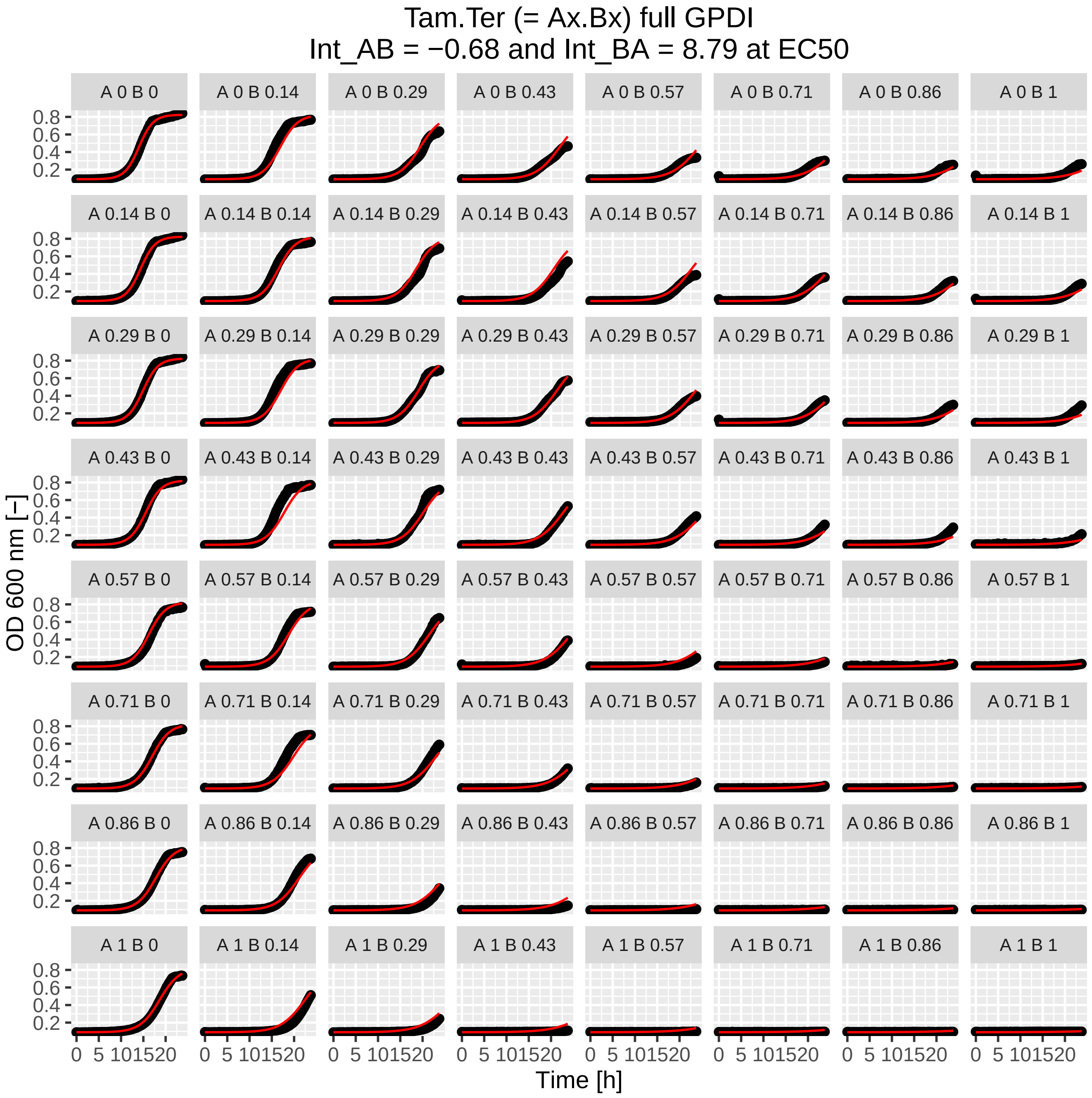
Tam.Ter (= Ax.Bx) full GPDI
Int_AB = −0.68 and Int_BA = 8.79 at EC50
OD 600 nm [−]
Time [h]
A 0 B 0
A 0 B 0.14
A 0 B 0.29
A 0 B 0.43
A 0 B 0.57
A 0 B 0.71
A 0 B 0.86
A 0 B 1
A 1 B 0
A 1 B 1

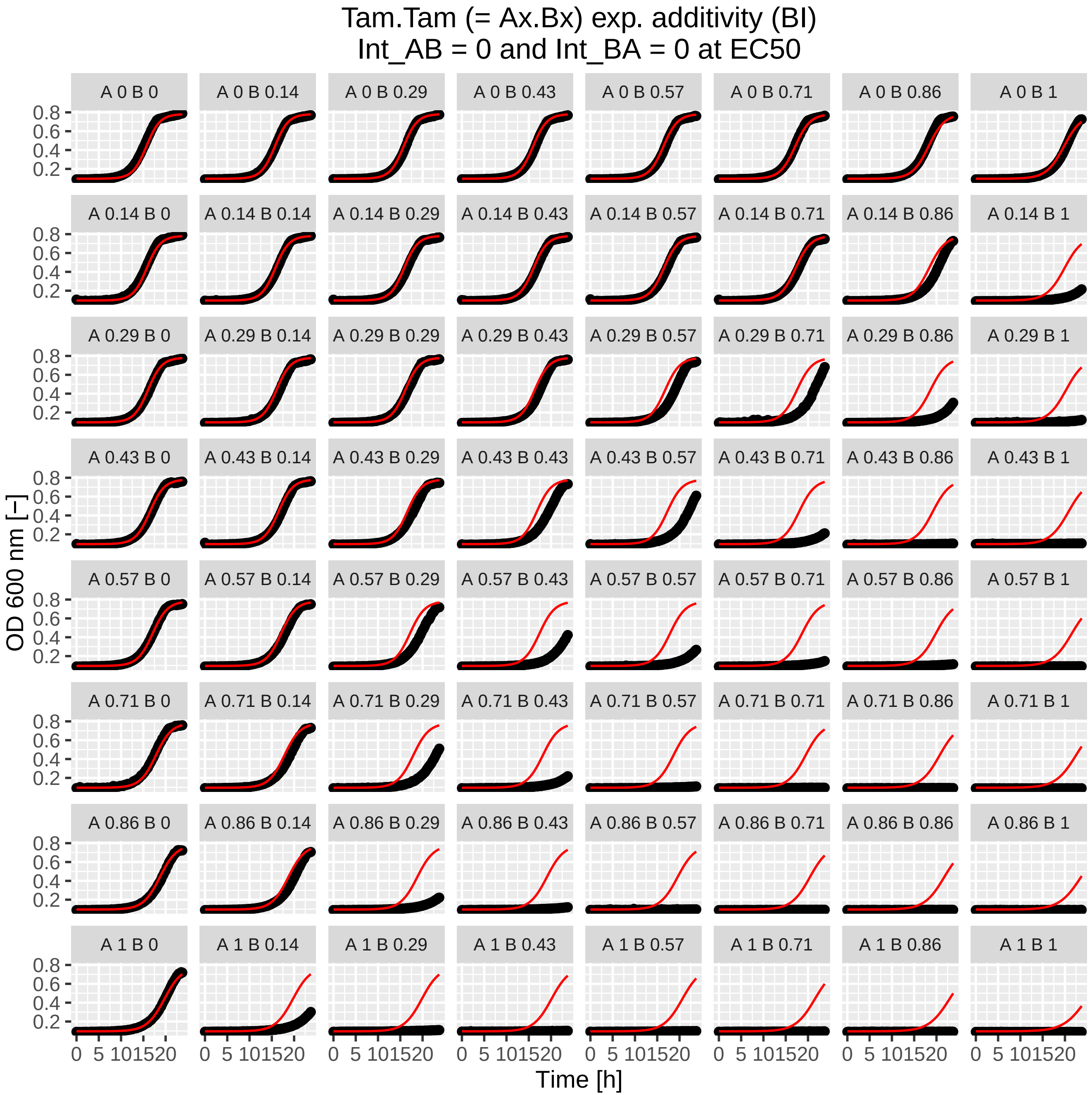
Tam.Tam (= Ax.Bx) exp. additivity (BI)
Int_AB = 0 and Int_BA = 0 at EC50
OD 600 nm [–]
Time [h]
A 0 B 0
A 0 B 0.14
A 0 B 0.29
A 0 B 0.43
A 0 B 0.57
A 0 B 0.71
A 0 B 0.86
A 0 B 1
A 1 B 0
A 1 B 0.14
A 1 B 0.29
A 1 B 0.43
A 1 B 0.57
A 1 B 0.71
A 1 B 0.86
A 1 B 1

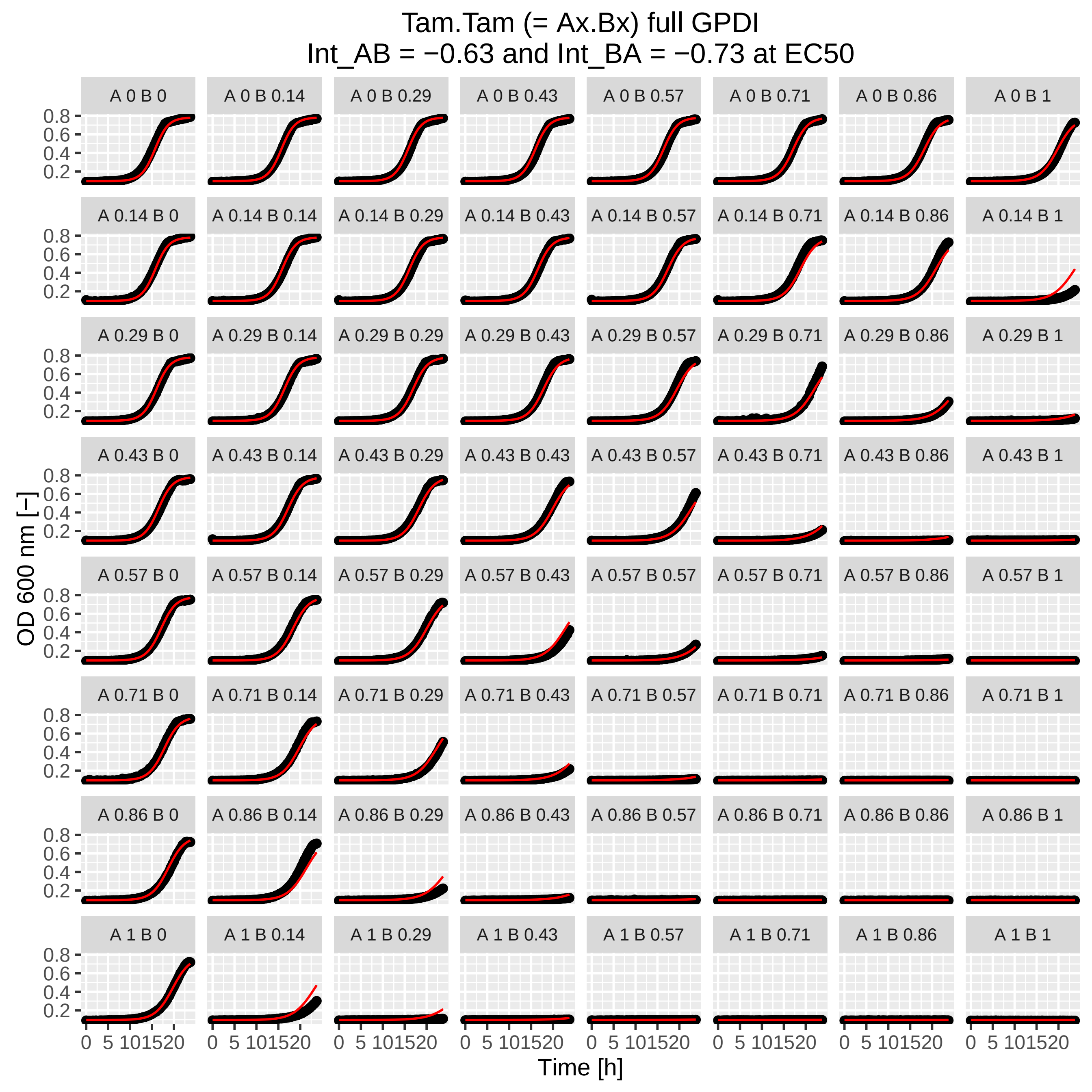
Tam.Tam (= Ax.Bx) full GPDI
Int_AB = −0.63 and Int_BA = −0.73 at EC50
OD 600 nm [−]
Time [h]
A 0 B 0
A 0 B 0.14
A 0 B 0.29
A 0 B 0.43
A 0 B 0.57
A 0 B 0.71
A 0 B 0.86
A 0 B 1
A 1 B 0
A 1 B 1
0.8
0.6
0.4
0.2
0 5 101520

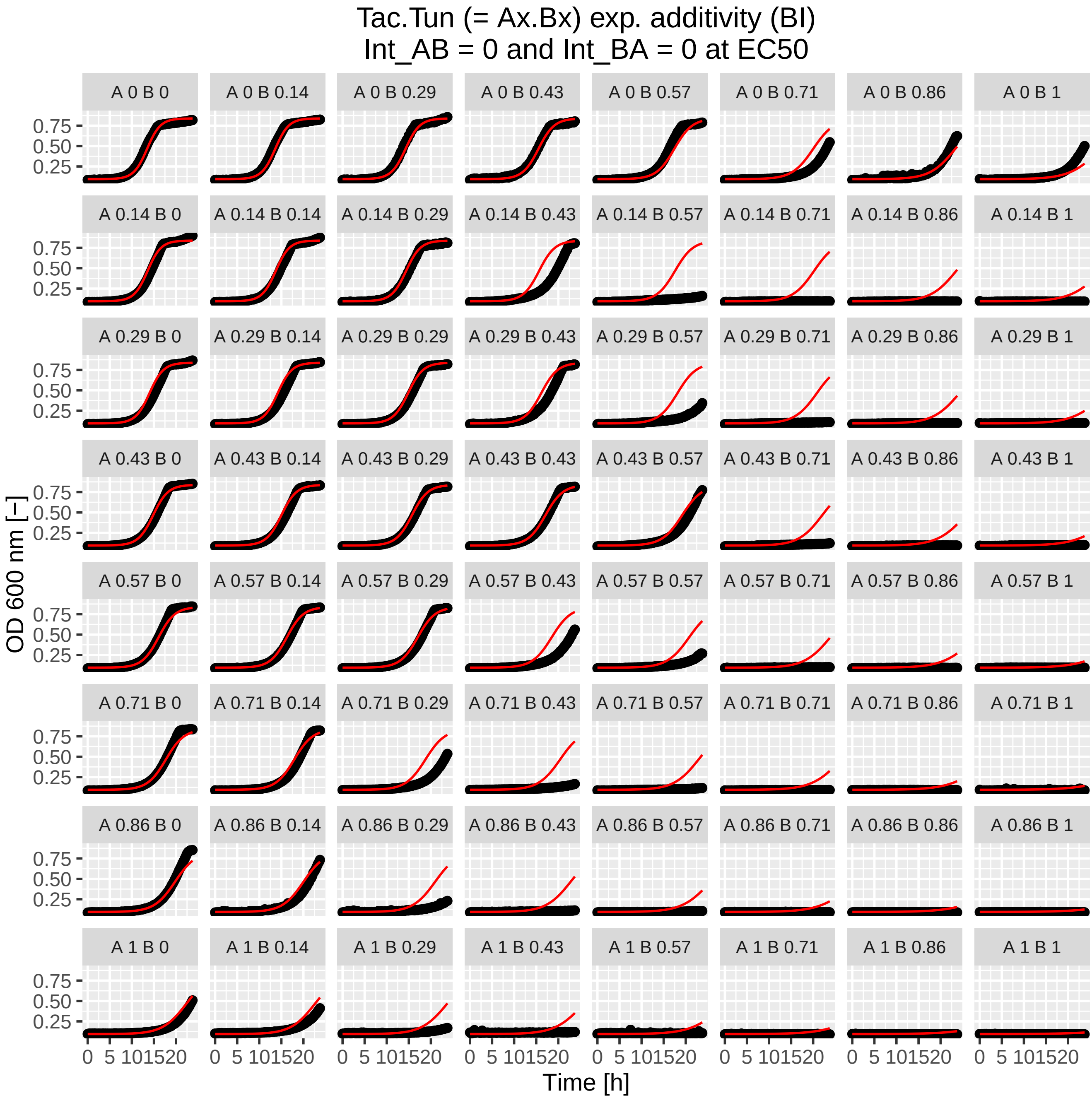

Tac.Tun (= Ax.Bx) exp. additivity (BI)
Int_AB = 0 and Int_BA = 0 at EC50
A 0 B 0
A 0 B 0.14
A 0 B 0.29
A 0 B 0.43
A 0 B 0.57
A 0 B 0.71
A 0 B 0.86
A 0 B 1
A 0.14 B 0
A 0.14 B 0.14
A 0.14 B 0.29
A 0.14 B 0.43
A 0.14 B 0.57
A 0.14 B 0.71
A 0.14 B 0.86
A 0.14 B 1
A 0.29 B 0
A 0.29 B 0.14
A 0.29 B 0.29
A 0.29 B 0.43
A 0.29 B 0.57
A 0.29 B 0.71
A 0.29 B 0.86
A 0.29 B 1
A 0.43 B 0
A 0.43 B 0.14
A 0.43 B 0.29
A 0.43 B 0.43
A 0.43 B 0.57
A 0.43 B 0.71
A 0.43 B 0.86
A 0.43 B 1
A 0.57 B 0
A 0.57 B 0.14
A 0.57 B 0.29
A 0.57 B 0.43
A 0.57 B 0.57
A 0.57 B 0.71
A 0.57 B 0.86
A 0.57 B 1
A 0.71 B 0
A 0.71 B 0.14
A 0.71 B 0.29
A 0.71 B 0.43
A 0.71 B 0.57
A 0.71 B 0.71
A 0.71 B 0.86
A 0.71 B 1
A 0.86 B 0
A 0.86 B 0.14
A 0.86 B 0.29
A 0.86 B 0.43
A 0.86 B 0.57
A 0.86 B 0.71
A 0.86 B 0.86
A 0.86 B 1
A 1 B 0
A 1 B 0.14
A 1 B 0.29
A 1 B 0.43
A 1 B 0.57
A 1 B 0.71
A 1 B 0.86
A 1 B 1
0.75
0.50
0.25
0 5 101520
OD 600 nm [–]
Time [h]

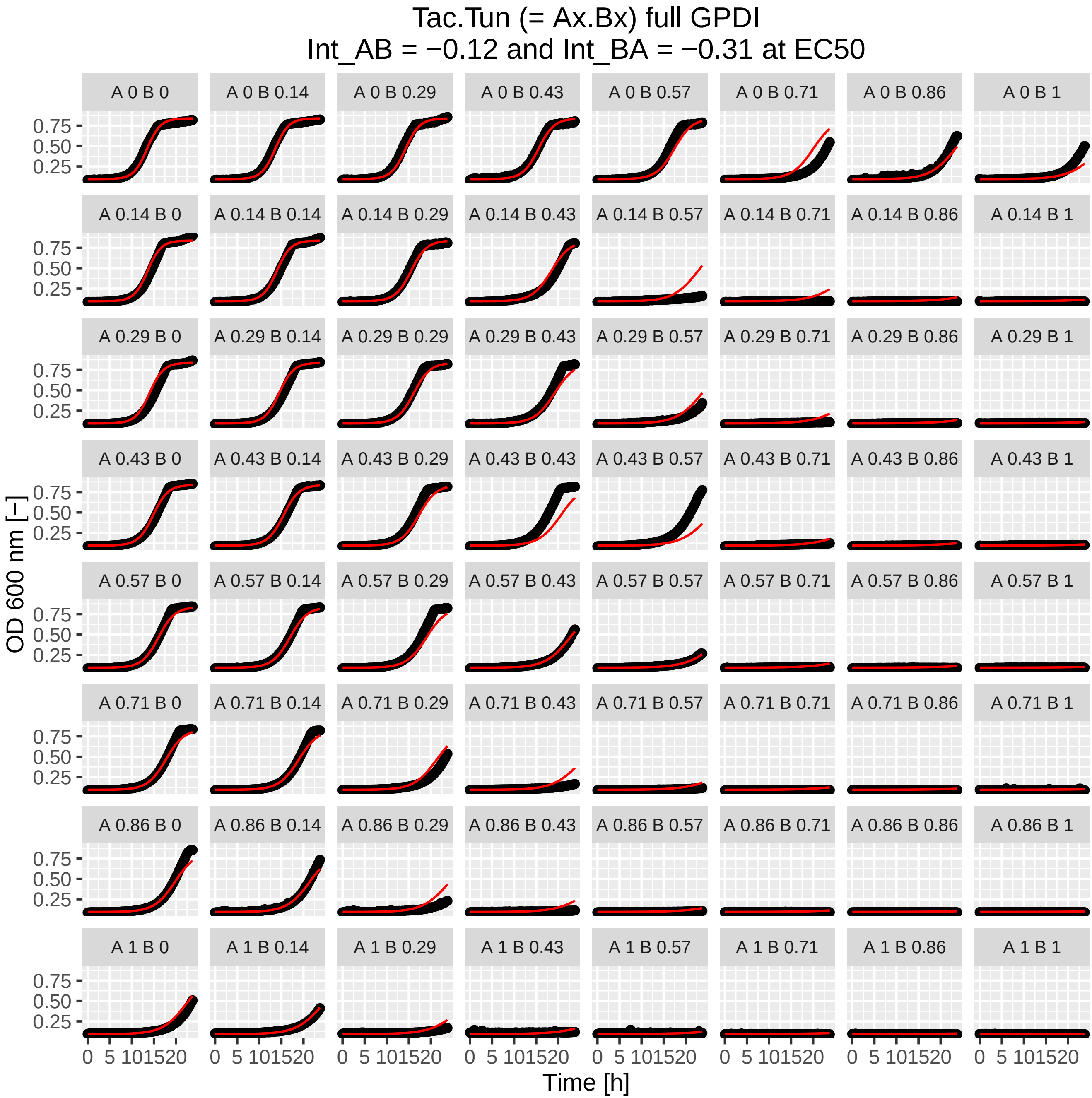
Tac.Tun (= Ax.Bx) full GPDI
Int_AB = −0.12 and Int_BA = −0.31 at EC50
A 0 B 0
A 0 B 0.14
A 0 B 0.29
A 0 B 0.43
A 0 B 0.57
A 0 B 0.71
A 0 B 0.86
A 0 B 1
A 0.14 B 0
A 0.14 B 0.14
A 0.14 B 0.29
A 0.14 B 0.43
A 0.14 B 0.57
A 0.14 B 0.71
A 0.14 B 0.86
A 0.14 B 1
A 0.29 B 0
A 0.29 B 0.14
A 0.29 B 0.29
A 0.29 B 0.43
A 0.29 B 0.57
A 0.29 B 0.71
A 0.29 B 0.86
A 0.29 B 1
A 0.43 B 0
A 0.43 B 0.14
A 0.43 B 0.29
A 0.43 B 0.43
A 0.43 B 0.57
A 0.43 B 0.71
A 0.43 B 0.86
A 0.43 B 1
A 0.57 B 0
A 0.57 B 0.14
A 0.57 B 0.29
A 0.57 B 0.43
A 0.57 B 0.57
A 0.57 B 0.71
A 0.57 B 0.86
A 0.57 B 1
A 0.71 B 0
A 0.71 B 0.14
A 0.71 B 0.29
A 0.71 B 0.43
A 0.71 B 0.57
A 0.71 B 0.71
A 0.71 B 0.86
A 0.71 B 1
A 0.86 B 0
A 0.86 B 0.14
A 0.86 B 0.29
A 0.86 B 0.43
A 0.86 B 0.57
A 0.86 B 0.71
A 0.86 B 0.86
A 0.86 B 1
A 1 B 0
A 1 B 0.14
A 1 B 0.29
A 1 B 0.43
A 1 B 0.57
A 1 B 0.71
A 1 B 0.86
A 1 B 1
OD 600 nm [−]
0.75
0.50
0.25
0 5 101520
Time [h]

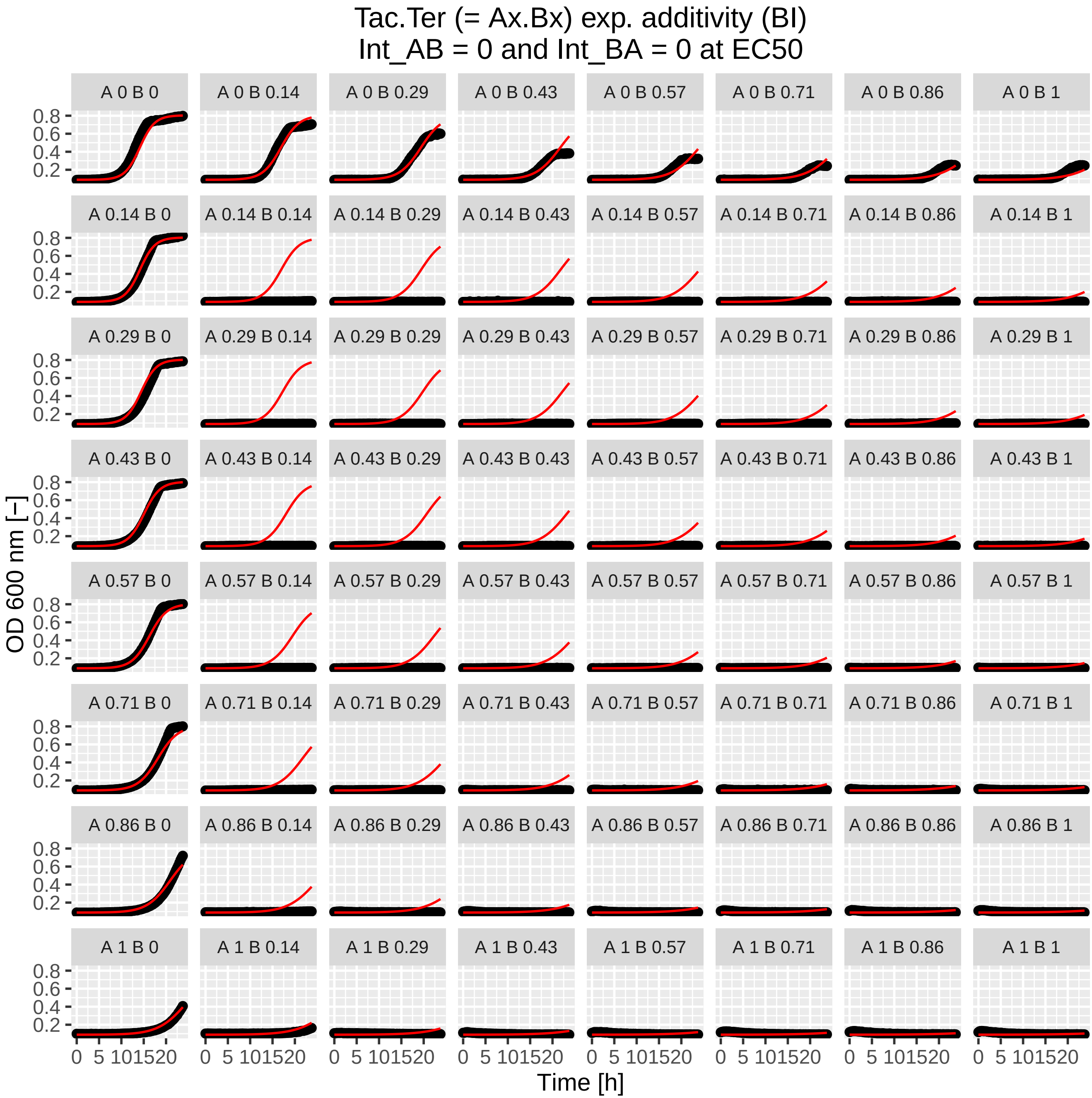

Tac.Ter (= Ax.Bx) exp. additivity (BI)
Int_AB = 0 and Int_BA = 0 at EC50
A 0 B 0
A 0 B 0.14
A 0 B 0.29
A 0 B 0.43
A 0 B 0.57
A 0 B 0.71
A 0 B 0.86
A 0 B 1
A 0.14 B 0
A 0.14 B 0.14
A 0.14 B 0.29
A 0.14 B 0.43
A 0.14 B 0.57
A 0.14 B 0.71
A 0.14 B 0.86
A 0.14 B 1
A 0.29 B 0
A 0.29 B 0.14
A 0.29 B 0.29
A 0.29 B 0.43
A 0.29 B 0.57
A 0.29 B 0.71
A 0.29 B 0.86
A 0.29 B 1
A 0.43 B 0
A 0.43 B 0.14
A 0.43 B 0.29
A 0.43 B 0.43
A 0.43 B 0.57
A 0.43 B 0.71
A 0.43 B 0.86
A 0.43 B 1
A 0.57 B 0
A 0.57 B 0.14
A 0.57 B 0.29
A 0.57 B 0.43
A 0.57 B 0.57
A 0.57 B 0.71
A 0.57 B 0.86
A 0.57 B 1
A 0.71 B 0
A 0.71 B 0.14
A 0.71 B 0.29
A 0.71 B 0.43
A 0.71 B 0.57
A 0.71 B 0.71
A 0.71 B 0.86
A 0.71 B 1
A 0.86 B 0
A 0.86 B 0.14
A 0.86 B 0.29
A 0.86 B 0.43
A 0.86 B 0.57
A 0.86 B 0.71
A 0.86 B 0.86
A 0.86 B 1
A 1 B 0
A 1 B 0.14
A 1 B 0.29
A 1 B 0.43
A 1 B 0.57
A 1 B 0.71
A 1 B 0.86
A 1 B 1
0.8
0.6
0.4
0.2
OD 600 nm [–]
0 5 101520
Time [h]

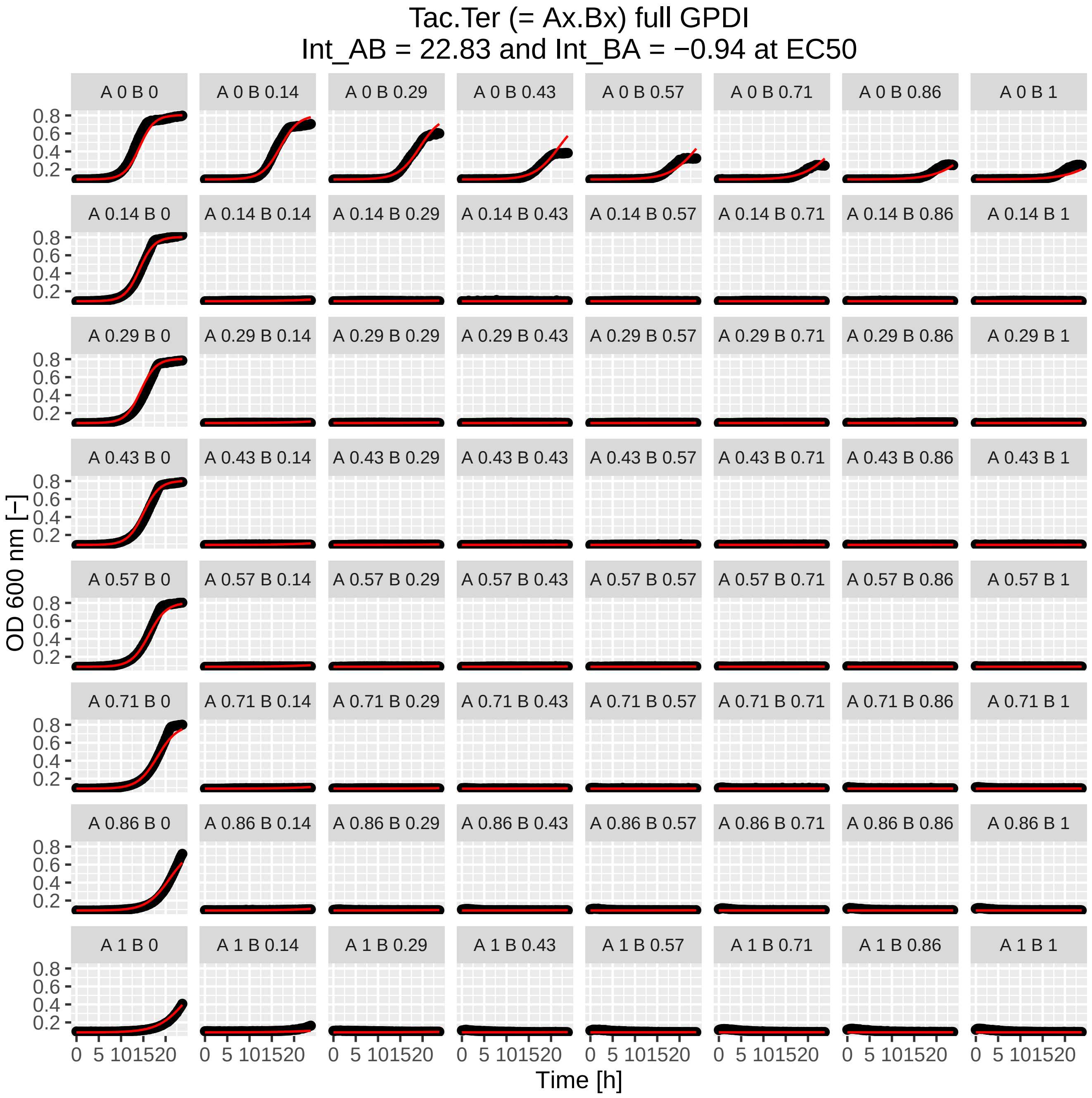
Tac.Ter (= Ax.Bx) full GPDI
Int_AB = 22.83 and Int_BA = −0.94 at EC50
OD 600 nm [−]
Time [h]
0.8
0.6
0.4
0.2
0 5 101520
A 0 B 0
A 0 B 0.14
A 0 B 0.29
A 0 B 0.43
A 0 B 0.57
A 0 B 0.71
A 0 B 0.86
A 0 B 1
A 0.14 B 0
A 0.14 B 0.14
A 0.14 B 0.29
A 0.14 B 0.43
A 0.14 B 0.57
A 0.14 B 0.71
A 0.14 B 0.86
A 0.14 B 1
A 0.29 B 0
A 0.29 B 0.14
A 0.29 B 0.29
A 0.29 B 0.43
A 0.29 B 0.57
A 0.29 B 0.71
A 0.29 B 0.86
A 0.29 B 1
A 0.43 B 0
A 0.43 B 0.14
A 0.43 B 0.29
A 0.43 B 0.43
A 0.43 B 0.57
A 0.43 B 0.71
A 0.43 B 0.86
A 0.43 B 1
A 0.57 B 0
A 0.57 B 0.14
A 0.57 B 0.29
A 0.57 B 0.43
A 0.57 B 0.57
A 0.57 B 0.71
A 0.57 B 0.86
A 0.57 B 1
A 0.71 B 0
A 0.71 B 0.14
A 0.71 B 0.29
A 0.71 B 0.43
A 0.71 B 0.57
A 0.71 B 0.71
A 0.71 B 0.86
A 0.71 B 1
A 0.86 B 0
A 0.86 B 0.14
A 0.86 B 0.29
A 0.86 B 0.43
A 0.86 B 0.57
A 0.86 B 0.71
A 0.86 B 0.86
A 0.86 B 1
A 1 B 0
A 1 B 0.14
A 1 B 0.29
A 1 B 0.43
A 1 B 0.57
A 1 B 0.71
A 1 B 0.86
A 1 B 1

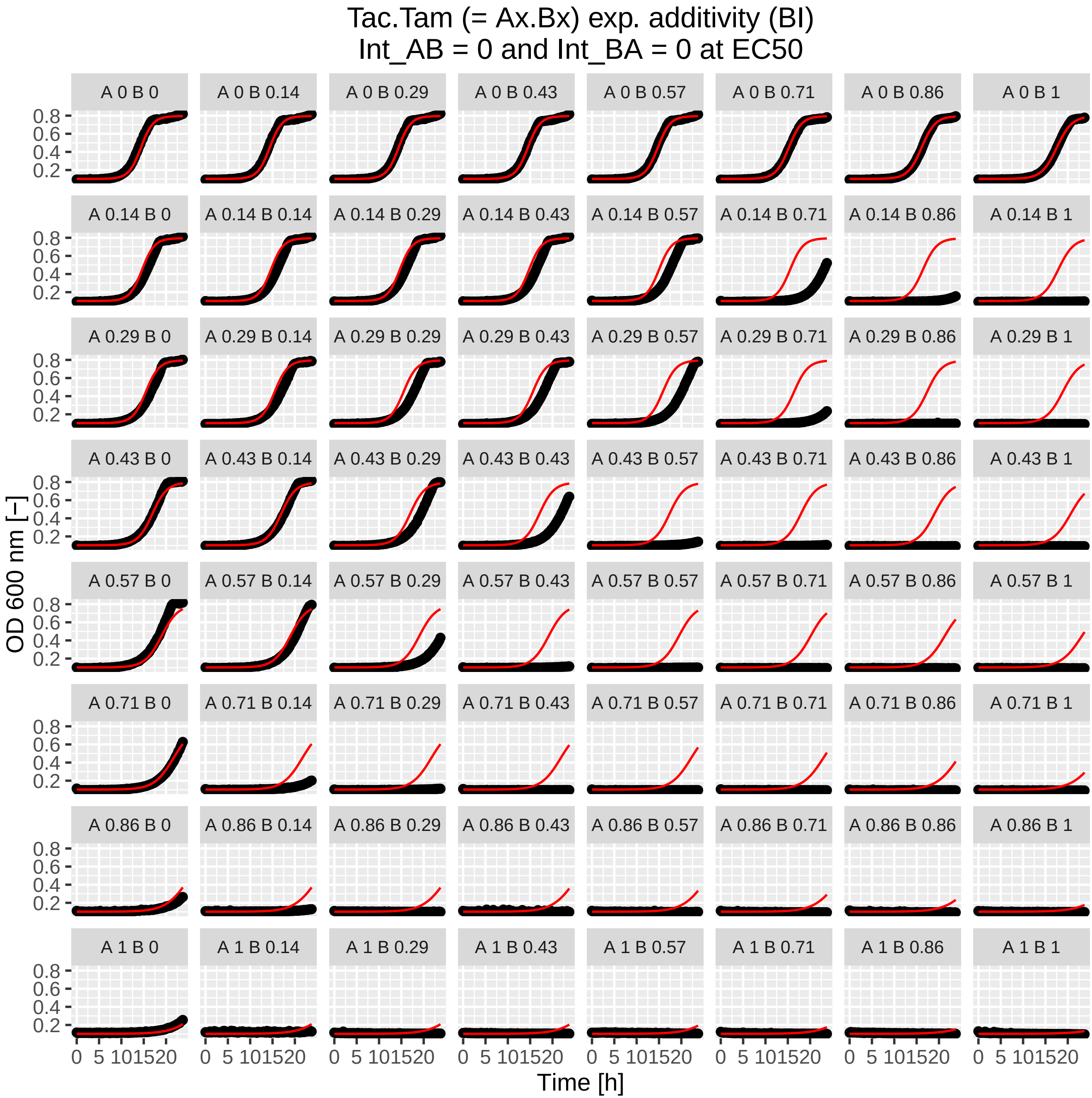

Tac.Tam (= Ax.Bx) exp. additivity (BI)
Int_AB = 0 and Int_BA = 0 at EC50
A 0 B 0
A 0 B 0.14
A 0 B 0.29
A 0 B 0.43
A 0 B 0.57
A 0 B 0.71
A 0 B 0.86
A 0 B 1
A 0.14 B 0
A 0.14 B 0.14
A 0.14 B 0.29
A 0.14 B 0.43
A 0.14 B 0.57
A 0.14 B 0.71
A 0.14 B 0.86
A 0.14 B 1
A 0.29 B 0
A 0.29 B 0.14
A 0.29 B 0.29
A 0.29 B 0.43
A 0.29 B 0.57
A 0.29 B 0.71
A 0.29 B 0.86
A 0.29 B 1
A 0.43 B 0
A 0.43 B 0.14
A 0.43 B 0.29
A 0.43 B 0.43
A 0.43 B 0.57
A 0.43 B 0.71
A 0.43 B 0.86
A 0.43 B 1
A 0.57 B 0
A 0.57 B 0.14
A 0.57 B 0.29
A 0.57 B 0.43
A 0.57 B 0.57
A 0.57 B 0.71
A 0.57 B 0.86
A 0.57 B 1
A 0.71 B 0
A 0.71 B 0.14
A 0.71 B 0.29
A 0.71 B 0.43
A 0.71 B 0.57
A 0.71 B 0.71
A 0.71 B 0.86
A 0.71 B 1
A 0.86 B 0
A 0.86 B 0.14
A 0.86 B 0.29
A 0.86 B 0.43
A 0.86 B 0.57
A 0.86 B 0.71
A 0.86 B 0.86
A 0.86 B 1
A 1 B 0
A 1 B 0.14
A 1 B 0.29
A 1 B 0.43
A 1 B 0.57
A 1 B 0.71
A 1 B 0.86
A 1 B 1
OD 600 nm [–]
0.8
0.6
0.4
0.2
0 5 101520
Time [h]

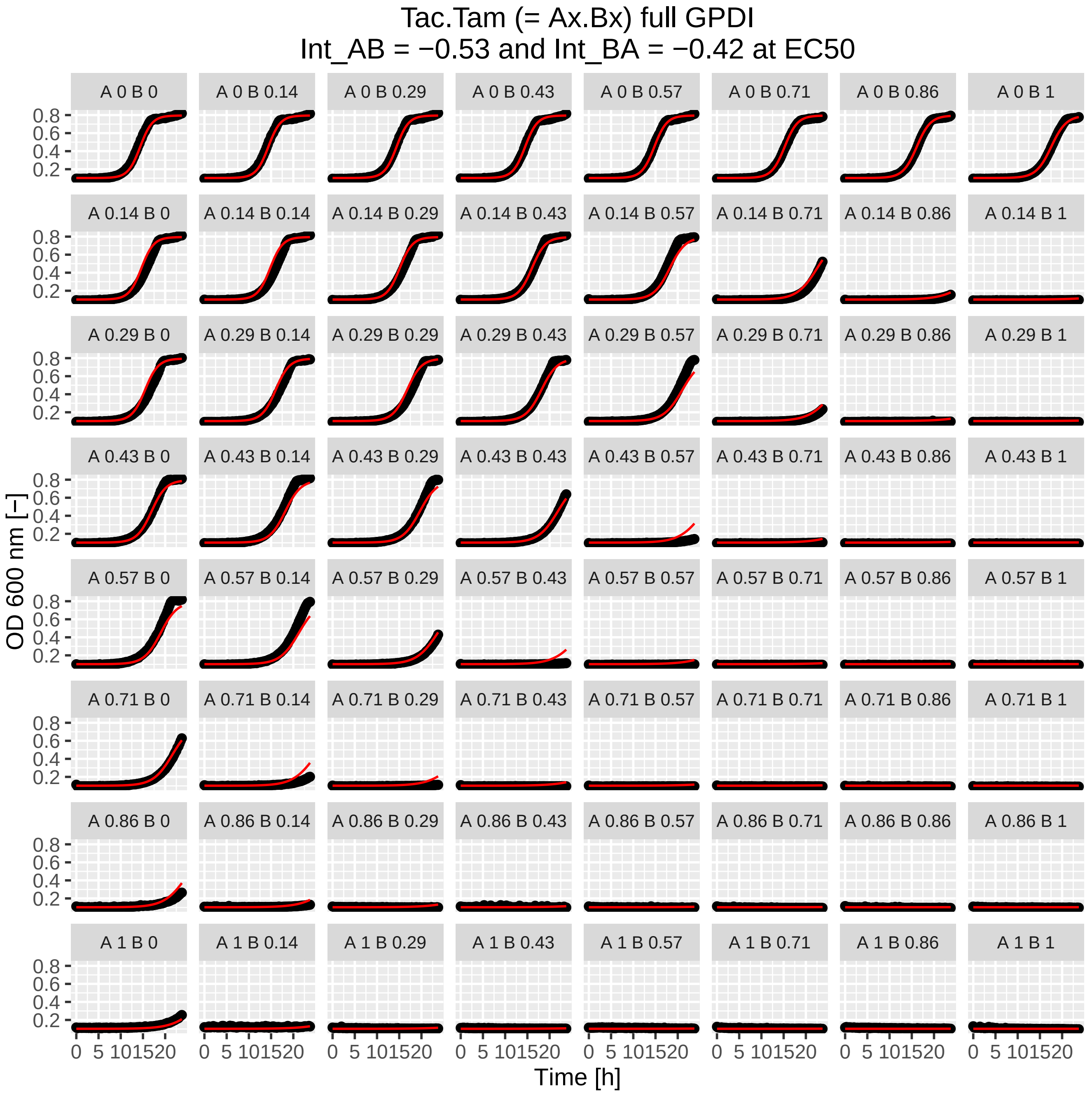
Tac.Tam (= Ax.Bx) full GPDI
Int_AB = −0.53 and Int_BA = −0.42 at EC50
OD 600 nm [−]
Time [h]
A 0 B 0
A 0 B 0.14
A 0 B 0.29
A 0 B 0.43
A 0 B 0.57
A 0 B 0.71
A 0 B 0.86
A 0 B 1
A 0.14 B 0
A 0.14 B 0.14
A 0.14 B 0.29
A 0.14 B 0.43
A 0.14 B 0.57
A 0.14 B 0.71
A 0.14 B 0.86
A 0.14 B 1
A 0.29 B 0
A 0.29 B 0.14
A 0.29 B 0.29
A 0.29 B 0.43
A 0.29 B 0.57
A 0.29 B 0.71
A 0.29 B 0.86
A 0.29 B 1
A 0.43 B 0
A 0.43 B 0.14
A 0.43 B 0.29
A 0.43 B 0.43
A 0.43 B 0.57
A 0.43 B 0.71
A 0.43 B 0.86
A 0.43 B 1
A 0.57 B 0
A 0.57 B 0.14
A 0.57 B 0.29
A 0.57 B 0.43
A 0.57 B 0.57
A 0.57 B 0.71
A 0.57 B 0.86
A 0.57 B 1
A 0.71 B 0
A 0.71 B 0.14
A 0.71 B 0.29
A 0.71 B 0.43
A 0.71 B 0.57
A 0.71 B 0.71
A 0.71 B 0.86
A 0.71 B 1
A 0.86 B 0
A 0.86 B 0.14
A 0.86 B 0.29
A 0.86 B 0.43
A 0.86 B 0.57
A 0.86 B 0.71
A 0.86 B 0.86
A 0.86 B 1
A 1 B 0
A 1 B 0.14
A 1 B 0.29
A 1 B 0.43
A 1 B 0.57
A 1 B 0.71
A 1 B 0.86
A 1 B 1
0.8
0.6
0.4
0.2
0 5 101520

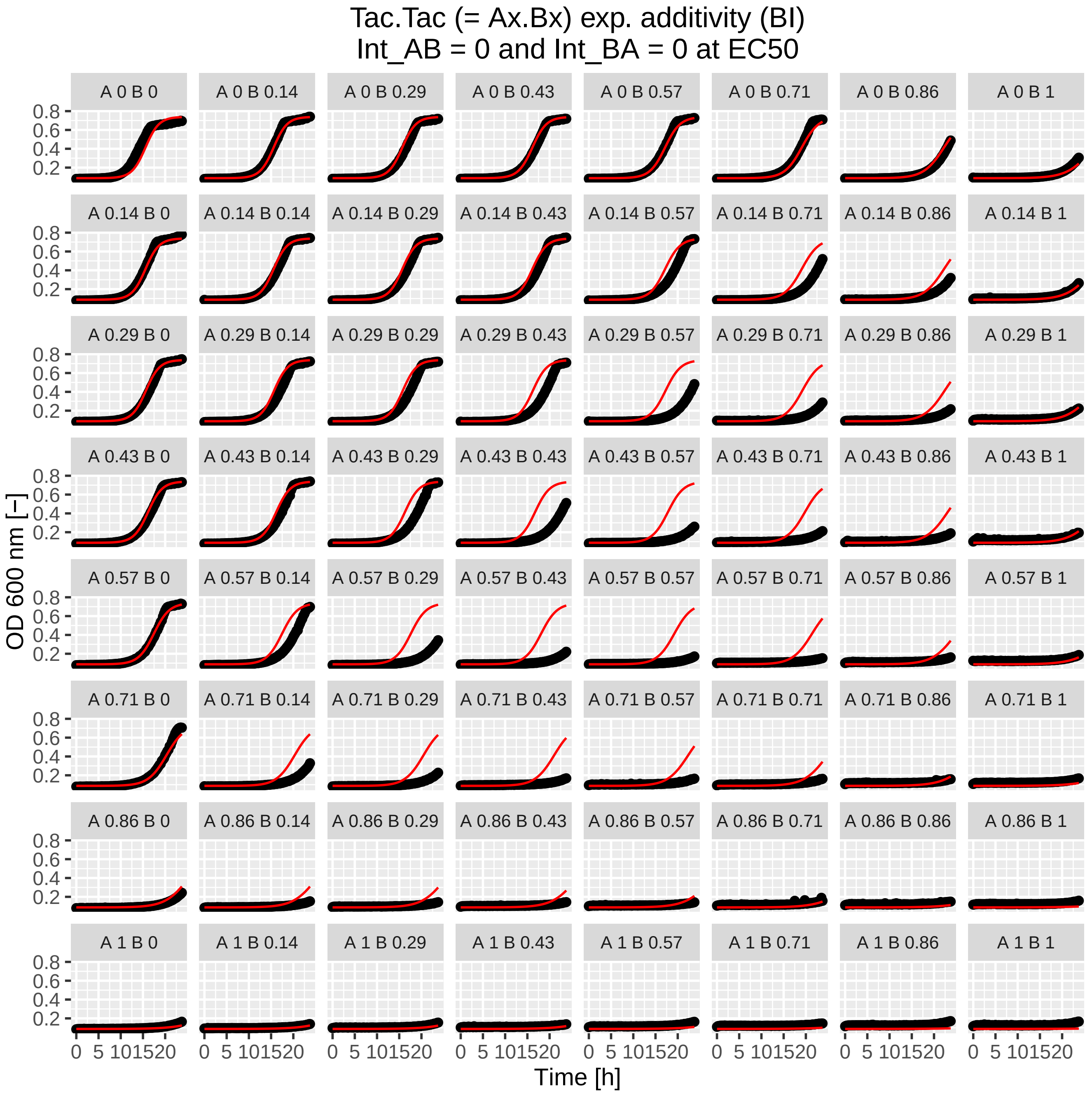

Tac.Tac (= Ax.Bx) exp. additivity (BI)
Int_AB = 0 and Int_BA = 0 at EC50
OD 600 nm [−]
Time [h]
A 0 B 0
A 0 B 0.14
A 0 B 0.29
A 0 B 0.43
A 0 B 0.57
A 0 B 0.71
A 0 B 0.86
A 0 B 1
A 0.14 B 0
A 0.14 B 0.14
A 0.14 B 0.29
A 0.14 B 0.43
A 0.14 B 0.57
A 0.14 B 0.71
A 0.14 B 0.86
A 0.14 B 1
A 0.29 B 0
A 0.29 B 0.14
A 0.29 B 0.29
A 0.29 B 0.43
A 0.29 B 0.57
A 0.29 B 0.71
A 0.29 B 0.86
A 0.29 B 1
A 0.43 B 0
A 0.43 B 0.14
A 0.43 B 0.29
A 0.43 B 0.43
A 0.43 B 0.57
A 0.43 B 0.71
A 0.43 B 0.86
A 0.43 B 1
A 0.57 B 0
A 0.57 B 0.14
A 0.57 B 0.29
A 0.57 B 0.43
A 0.57 B 0.57
A 0.57 B 0.71
A 0.57 B 0.86
A 0.57 B 1
A 0.71 B 0
A 0.71 B 0.14
A 0.71 B 0.29
A 0.71 B 0.43
A 0.71 B 0.57
A 0.71 B 0.71
A 0.71 B 0.86
A 0.71 B 1
A 0.86 B 0
A 0.86 B 0.14
A 0.86 B 0.29
A 0.86 B 0.43
A 0.86 B 0.57
A 0.86 B 0.71
A 0.86 B 0.86
A 0.86 B 1
A 1 B 0
A 1 B 0.14
A 1 B 0.29
A 1 B 0.43
A 1 B 0.57
A 1 B 0.71
A 1 B 0.86
A 1 B 1
0.2
0.4
0.6
0.8
0 5 101520

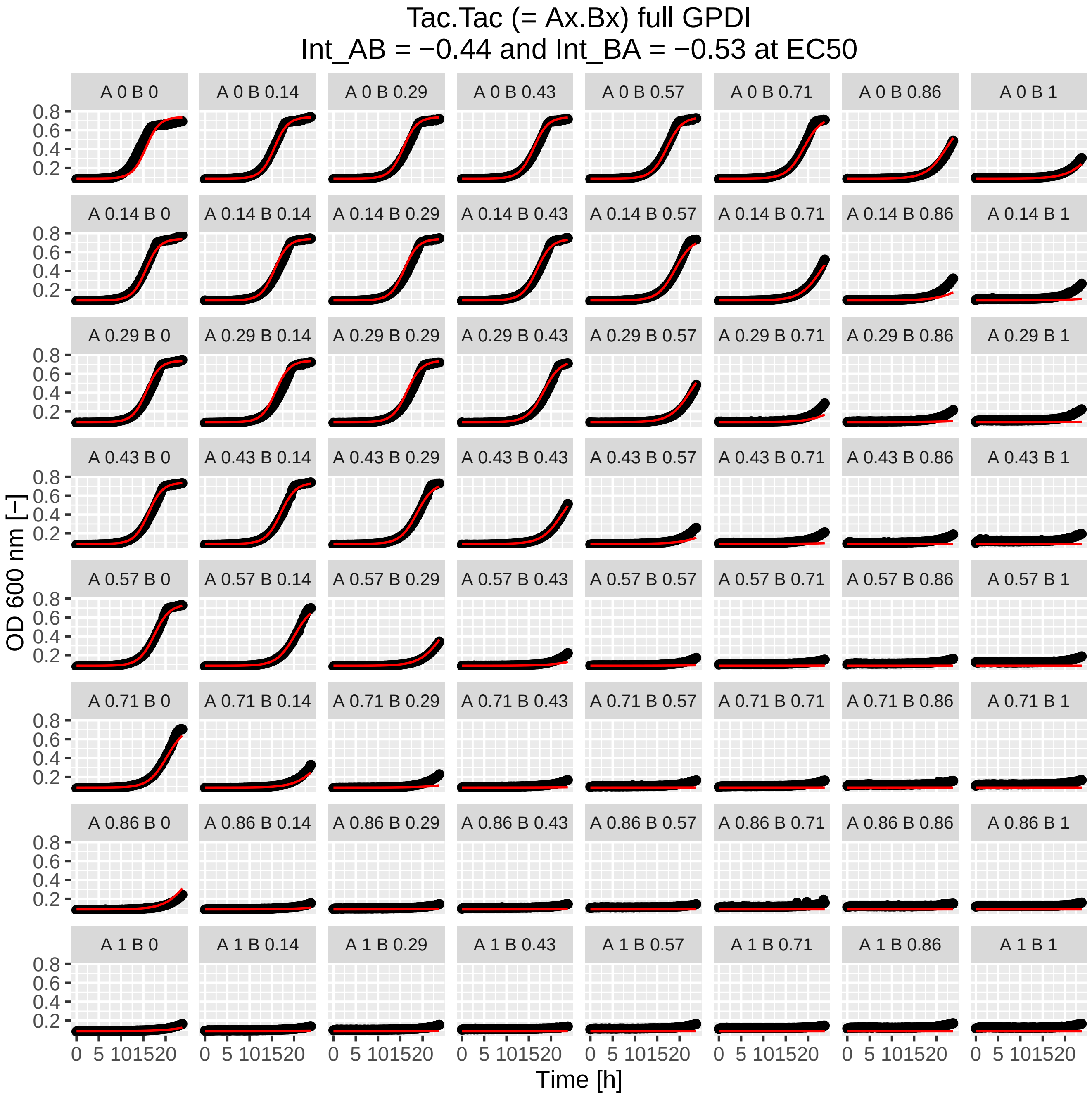

Tac.Tac (= Ax.Bx) full GPDI
Int_AB = −0.44 and Int_BA = −0.53 at EC50
A 0 B 0
A 0 B 0.14
A 0 B 0.29
A 0 B 0.43
A 0 B 0.57
A 0 B 0.71
A 0 B 0.86
A 0 B 1
A 0.14 B 0
A 0.14 B 0.14
A 0.14 B 0.29
A 0.14 B 0.43
A 0.14 B 0.57
A 0.14 B 0.71
A 0.14 B 0.86
A 0.14 B 1
A 0.29 B 0
A 0.29 B 0.14
A 0.29 B 0.29
A 0.29 B 0.43
A 0.29 B 0.57
A 0.29 B 0.71
A 0.29 B 0.86
A 0.29 B 1
A 0.43 B 0
A 0.43 B 0.14
A 0.43 B 0.29
A 0.43 B 0.43
A 0.43 B 0.57
A 0.43 B 0.71
A 0.43 B 0.86
A 0.43 B 1
A 0.57 B 0
A 0.57 B 0.14
A 0.57 B 0.29
A 0.57 B 0.43
A 0.57 B 0.57
A 0.57 B 0.71
A 0.57 B 0.86
A 0.57 B 1
A 0.71 B 0
A 0.71 B 0.14
A 0.71 B 0.29
A 0.71 B 0.43
A 0.71 B 0.57
A 0.71 B 0.71
A 0.71 B 0.86
A 0.71 B 1
A 0.86 B 0
A 0.86 B 0.14
A 0.86 B 0.29
A 0.86 B 0.43
A 0.86 B 0.57
A 0.86 B 0.71
A 0.86 B 0.86
A 0.86 B 1
A 1 B 0
A 1 B 0.14
A 1 B 0.29
A 1 B 0.43
A 1 B 0.57
A 1 B 0.71
A 1 B 0.86
A 1 B 1
0.8
0.6
0.4
0.2
OD 600 nm [−]
0 5 101520
Time [h]

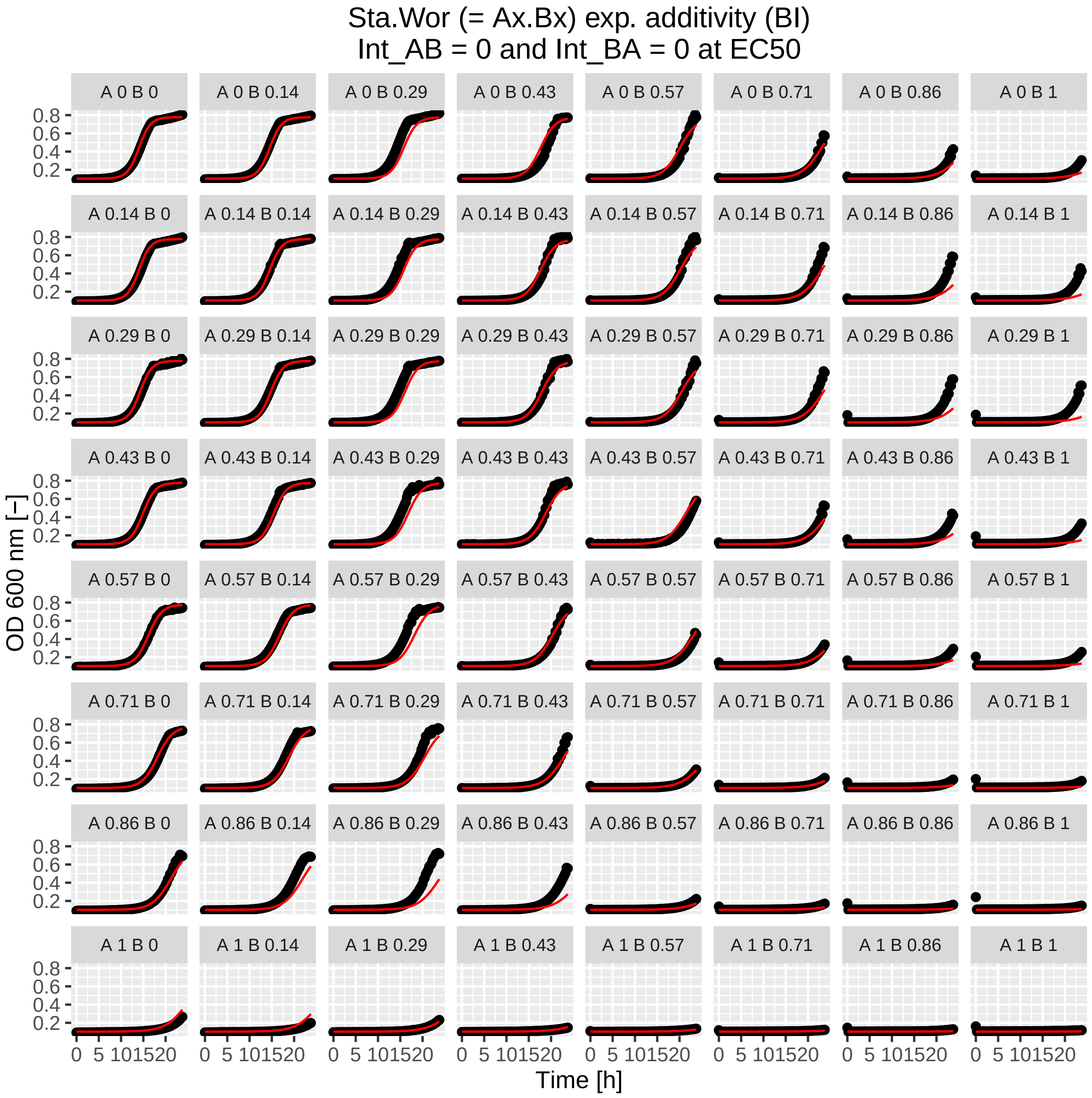
Sta.Wor (= Ax.Bx) exp. additivity (BI)
Int_AB = 0 and Int_BA = 0 at EC50
A 0 B 0
A 0 B 0.14
A 0 B 0.29
A 0 B 0.43
A 0 B 0.57
A 0 B 0.71
A 0 B 0.86
A 0 B 1
A 0.14 B 0
A 0.14 B 0.14
A 0.14 B 0.29
A 0.14 B 0.43
A 0.14 B 0.57
A 0.14 B 0.71
A 0.14 B 0.86
A 0.14 B 1
A 0.29 B 0
A 0.29 B 0.14
A 0.29 B 0.29
A 0.29 B 0.43
A 0.29 B 0.57
A 0.29 B 0.71
A 0.29 B 0.86
A 0.29 B 1
A 0.43 B 0
A 0.43 B 0.14
A 0.43 B 0.29
A 0.43 B 0.43
A 0.43 B 0.57
A 0.43 B 0.71
A 0.43 B 0.86
A 0.43 B 1
A 0.57 B 0
A 0.57 B 0.14
A 0.57 B 0.29
A 0.57 B 0.43
A 0.57 B 0.57
A 0.57 B 0.71
A 0.57 B 0.86
A 0.57 B 1
A 0.71 B 0
A 0.71 B 0.14
A 0.71 B 0.29
A 0.71 B 0.43
A 0.71 B 0.57
A 0.71 B 0.71
A 0.71 B 0.86
A 0.71 B 1
A 0.86 B 0
A 0.86 B 0.14
A 0.86 B 0.29
A 0.86 B 0.43
A 0.86 B 0.57
A 0.86 B 0.71
A 0.86 B 0.86
A 0.86 B 1
A 1 B 0
A 1 B 0.14
A 1 B 0.29
A 1 B 0.43
A 1 B 0.57
A 1 B 0.71
A 1 B 0.86
A 1 B 1
OD 600 nm [−]
0.8
0.6
0.4
0.2
0 5 101520
Time [h]

Sta.Wor (= Ax.Bx) full GPDI
Int_AB = 0.08 and Int_BA = 0.07 at EC50

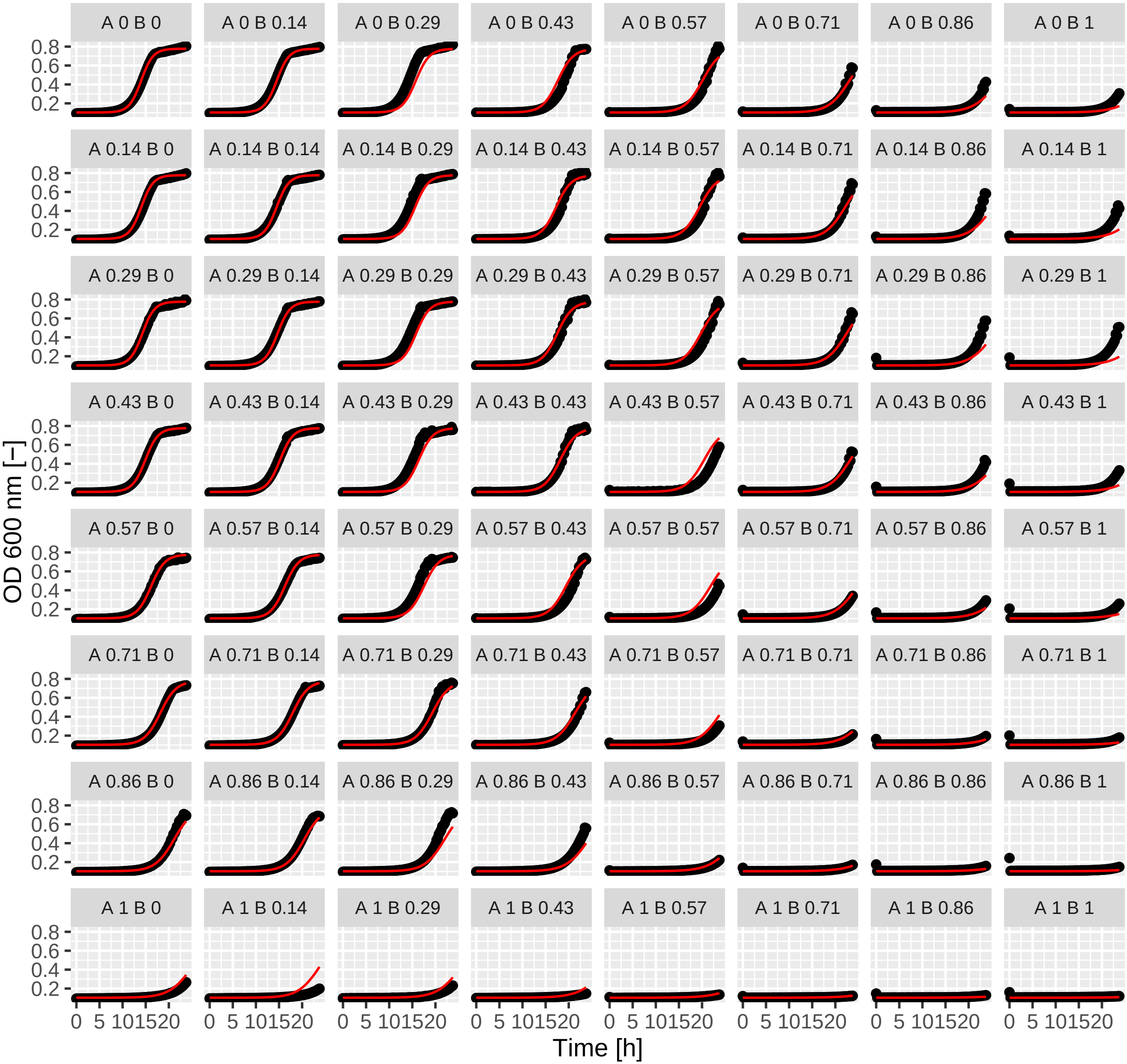

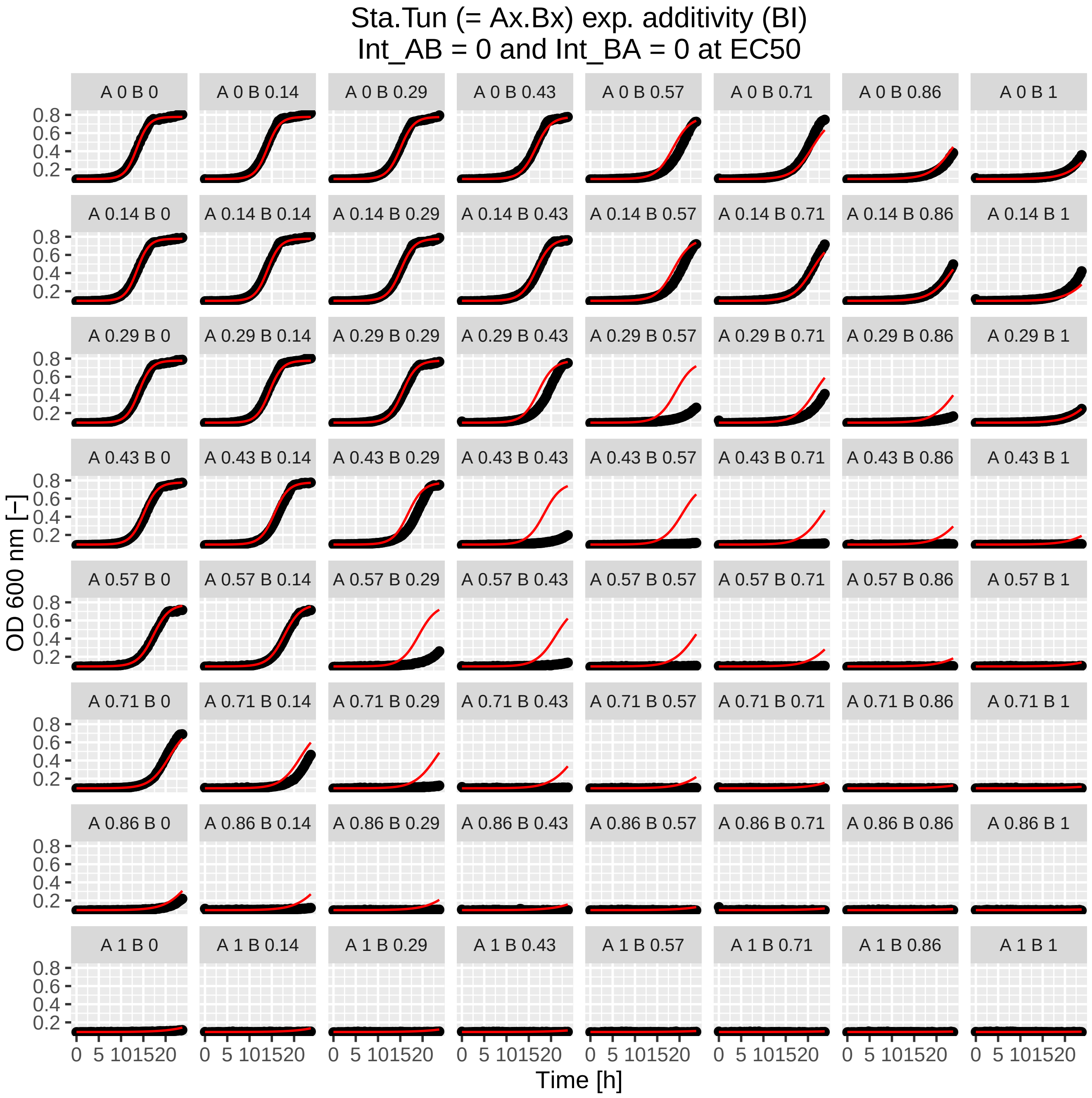

Sta.Tun (= Ax.Bx) exp. additivity (BI)
Int_AB = 0 and Int_BA = 0 at EC50
A 0 B 0
A 0 B 0.14
A 0 B 0.29
A 0 B 0.43
A 0 B 0.57
A 0 B 0.71
A 0 B 0.86
A 0 B 1
A 0.14 B 0
A 0.14 B 0.14
A 0.14 B 0.29
A 0.14 B 0.43
A 0.14 B 0.57
A 0.14 B 0.71
A 0.14 B 0.86
A 0.14 B 1
A 0.29 B 0
A 0.29 B 0.14
A 0.29 B 0.29
A 0.29 B 0.43
A 0.29 B 0.57
A 0.29 B 0.71
A 0.29 B 0.86
A 0.29 B 1
A 0.43 B 0
A 0.43 B 0.14
A 0.43 B 0.29
A 0.43 B 0.43
A 0.43 B 0.57
A 0.43 B 0.71
A 0.43 B 0.86
A 0.43 B 1
A 0.57 B 0
A 0.57 B 0.14
A 0.57 B 0.29
A 0.57 B 0.43
A 0.57 B 0.57
A 0.57 B 0.71
A 0.57 B 0.86
A 0.57 B 1
A 0.71 B 0
A 0.71 B 0.14
A 0.71 B 0.29
A 0.71 B 0.43
A 0.71 B 0.57
A 0.71 B 0.71
A 0.71 B 0.86
A 0.71 B 1
A 0.86 B 0
A 0.86 B 0.14
A 0.86 B 0.29
A 0.86 B 0.43
A 0.86 B 0.57
A 0.86 B 0.71
A 0.86 B 0.86
A 0.86 B 1
A 1 B 0
A 1 B 0.14
A 1 B 0.29
A 1 B 0.43
A 1 B 0.57
A 1 B 0.71
A 1 B 0.86
A 1 B 1
OD 600 nm [–]
0.2
0.4
0.6
0.8
0
5
10
15
20
Time [h]

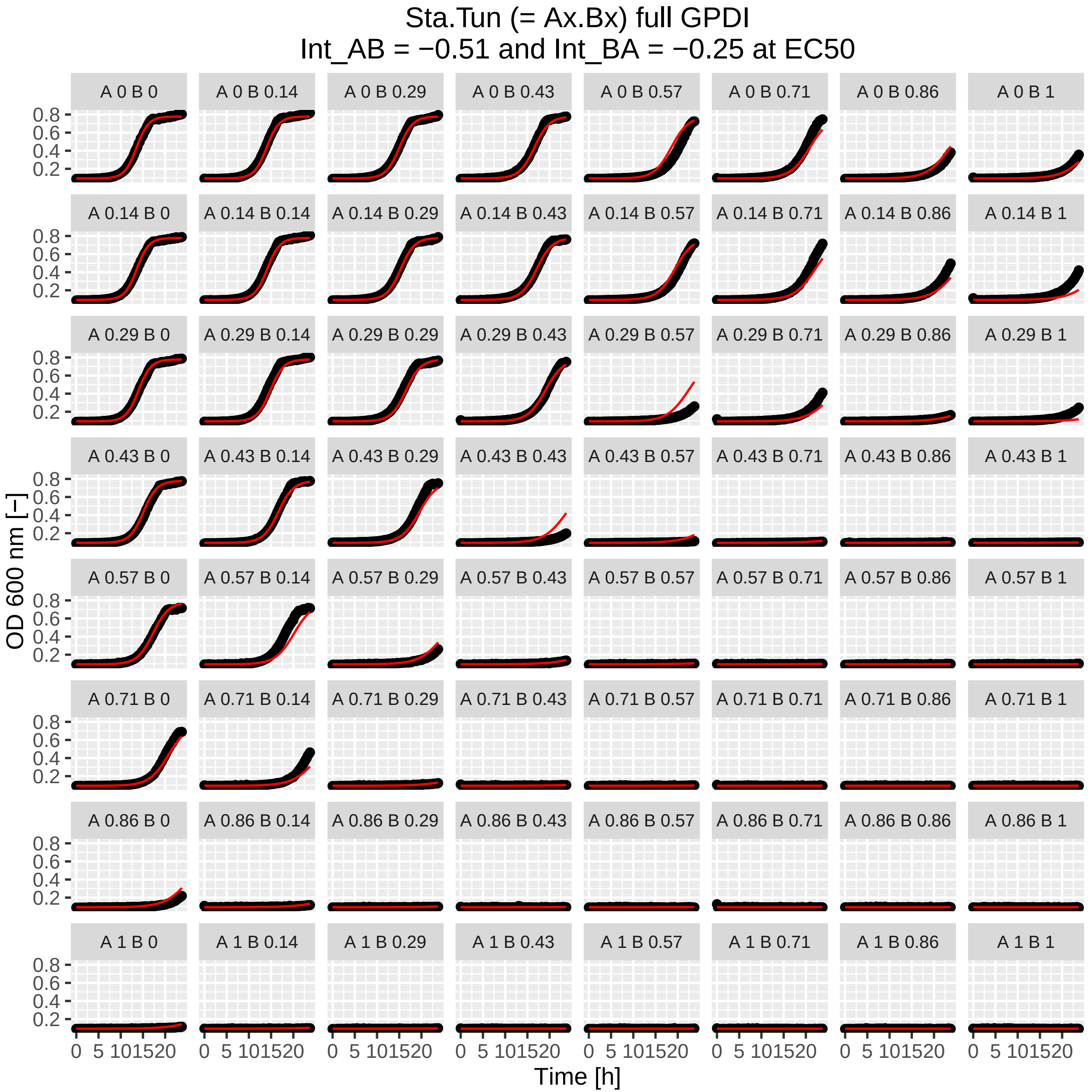
Sta.Tun (= Ax.Bx) full GPDI
Int_AB = −0.51 and Int_BA = −0.25 at EC50
OD 600 nm [−]
Time [h]
A 0 B 0
A 0 B 0.14
A 0 B 0.29
A 0 B 0.43
A 0 B 0.57
A 0 B 0.71
A 0 B 0.86
A 0 B 1
A 1 B 0
A 1 B 0.14
A 1 B 0.29
A 1 B 0.43
A 1 B 0.57
A 1 B 0.71
A 1 B 0.86
A 1 B 1

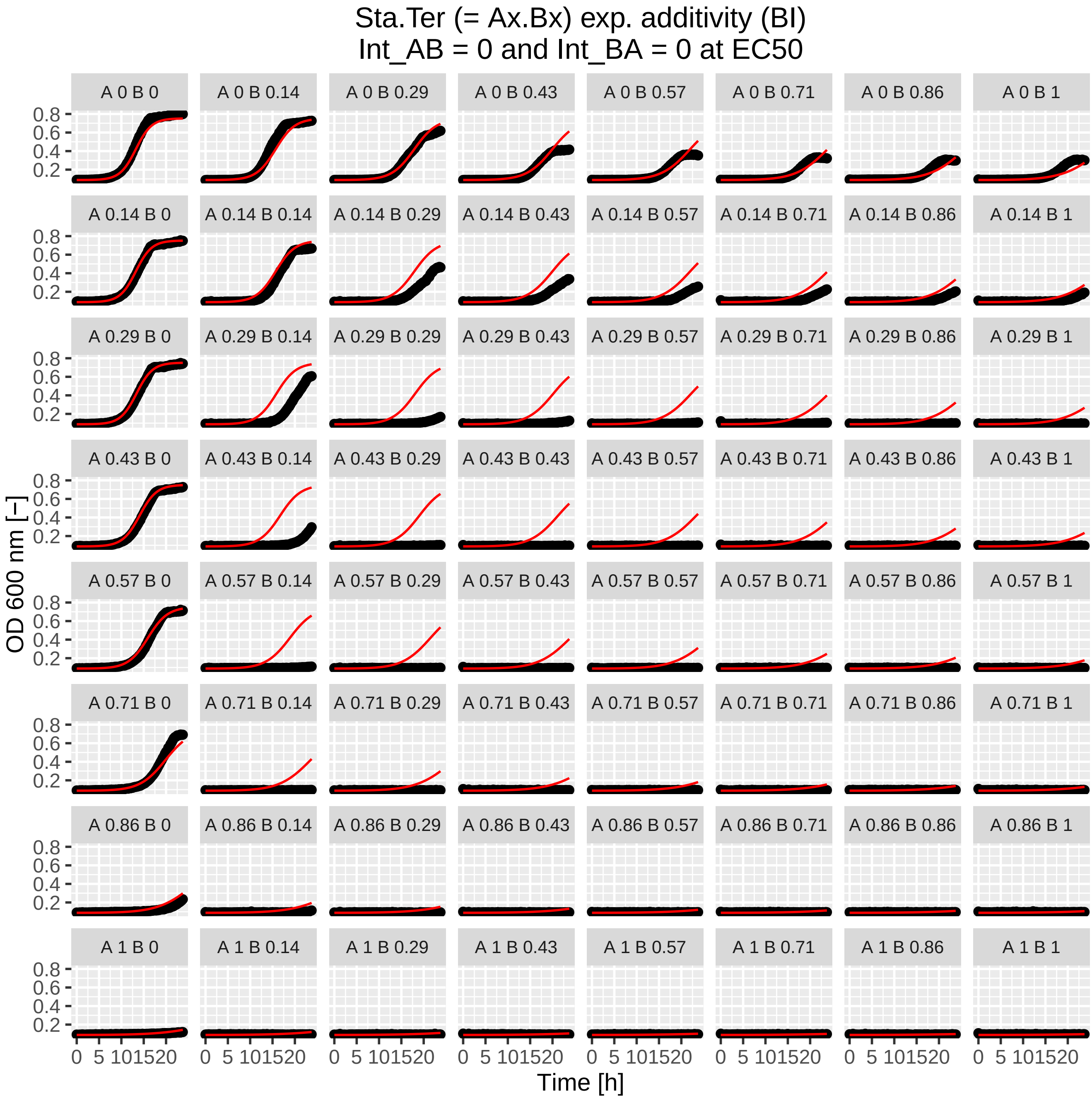

Sta.Ter (= Ax.Bx) exp. additivity (BI)
Int_AB = 0 and Int_BA = 0 at EC50
A 0 B 0
A 0 B 0.14
A 0 B 0.29
A 0 B 0.43
A 0 B 0.57
A 0 B 0.71
A 0 B 0.86
A 0 B 1
A 0.14 B 0
A 0.14 B 0.14
A 0.14 B 0.29
A 0.14 B 0.43
A 0.14 B 0.57
A 0.14 B 0.71
A 0.14 B 0.86
A 0.14 B 1
A 0.29 B 0
A 0.29 B 0.14
A 0.29 B 0.29
A 0.29 B 0.43
A 0.29 B 0.57
A 0.29 B 0.71
A 0.29 B 0.86
A 0.29 B 1
A 0.43 B 0
A 0.43 B 0.14
A 0.43 B 0.29
A 0.43 B 0.43
A 0.43 B 0.57
A 0.43 B 0.71
A 0.43 B 0.86
A 0.43 B 1
A 0.57 B 0
A 0.57 B 0.14
A 0.57 B 0.29
A 0.57 B 0.43
A 0.57 B 0.57
A 0.57 B 0.71
A 0.57 B 0.86
A 0.57 B 1
A 0.71 B 0
A 0.71 B 0.14
A 0.71 B 0.29
A 0.71 B 0.43
A 0.71 B 0.57
A 0.71 B 0.71
A 0.71 B 0.86
A 0.71 B 1
A 0.86 B 0
A 0.86 B 0.14
A 0.86 B 0.29
A 0.86 B 0.43
A 0.86 B 0.57
A 0.86 B 0.71
A 0.86 B 0.86
A 0.86 B 1
A 1 B 0
A 1 B 0.14
A 1 B 0.29
A 1 B 0.43
A 1 B 0.57
A 1 B 0.71
A 1 B 0.86
A 1 B 1
OD 600 nm [–]
0.2
0.4
0.6
0.8
0
5
10
15
20
Time [h]

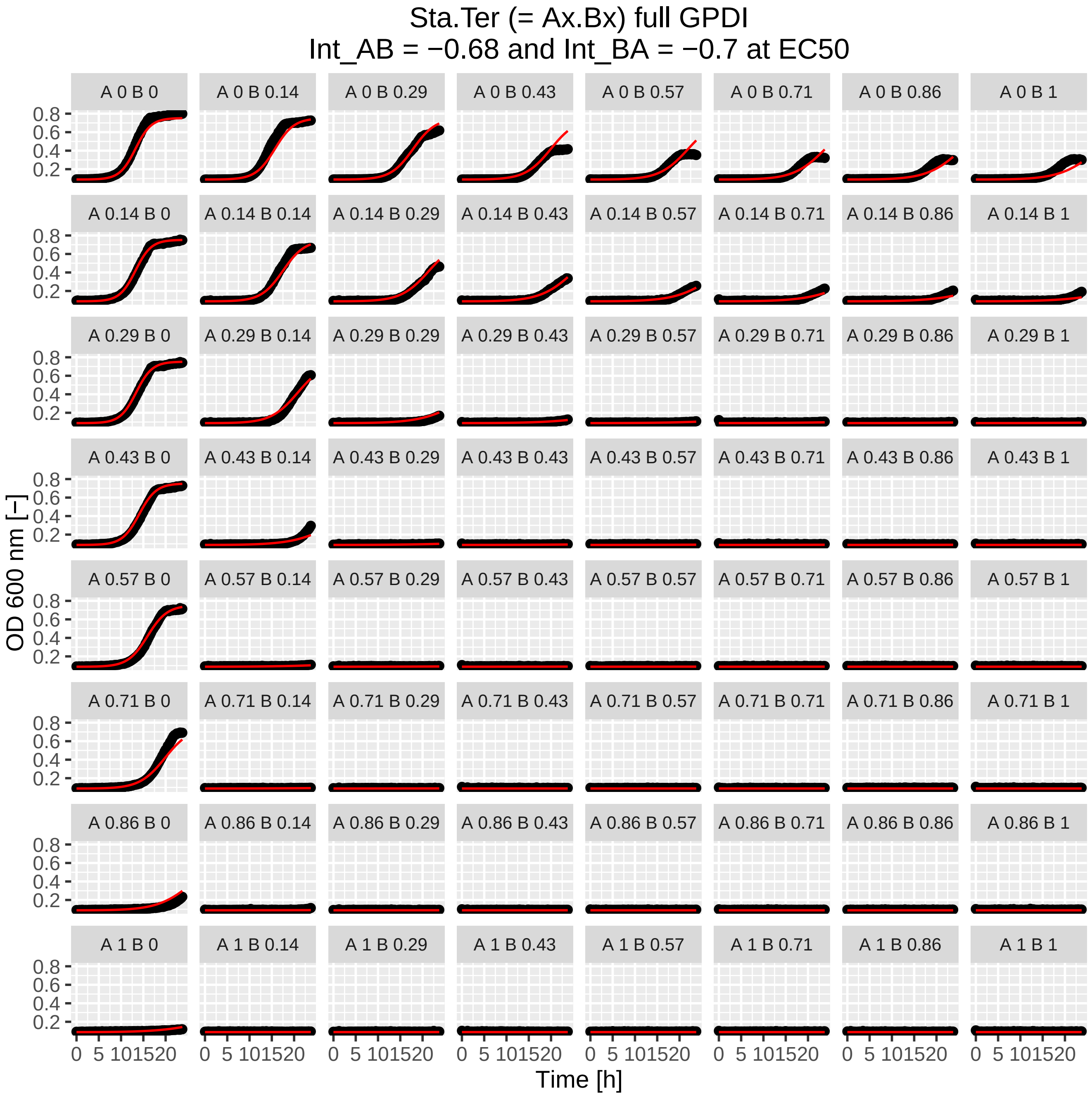
Sta.Ter (= Ax.Bx) full GPDI
Int_AB = −0.68 and Int_BA = −0.7 at EC50
A 0 B 0
A 0 B 0.14
A 0 B 0.29
A 0 B 0.43
A 0 B 0.57
A 0 B 0.71
A 0 B 0.86
A 0 B 1
A 0.14 B 0
A 0.14 B 0.14
A 0.14 B 0.29
A 0.14 B 0.43
A 0.14 B 0.57
A 0.14 B 0.71
A 0.14 B 0.86
A 0.14 B 1
A 0.29 B 0
A 0.29 B 0.14
A 0.29 B 0.29
A 0.29 B 0.43
A 0.29 B 0.57
A 0.29 B 0.71
A 0.29 B 0.86
A 0.29 B 1
A 0.43 B 0
A 0.43 B 0.14
A 0.43 B 0.29
A 0.43 B 0.43
A 0.43 B 0.57
A 0.43 B 0.71
A 0.43 B 0.86
A 0.43 B 1
A 0.57 B 0
A 0.57 B 0.14
A 0.57 B 0.29
A 0.57 B 0.43
A 0.57 B 0.57
A 0.57 B 0.71
A 0.57 B 0.86
A 0.57 B 1
A 0.71 B 0
A 0.71 B 0.14
A 0.71 B 0.29
A 0.71 B 0.43
A 0.71 B 0.57
A 0.71 B 0.71
A 0.71 B 0.86
A 0.71 B 1
A 0.86 B 0
A 0.86 B 0.14
A 0.86 B 0.29
A 0.86 B 0.43
A 0.86 B 0.57
A 0.86 B 0.71
A 0.86 B 0.86
A 0.86 B 1
A 1 B 0
A 1 B 0.14
A 1 B 0.29
A 1 B 0.43
A 1 B 0.57
A 1 B 0.71
A 1 B 0.86
A 1 B 1
OD 600 nm [−]
0.2
0.4
0.6
0.8
0 5 101520
Time [h]

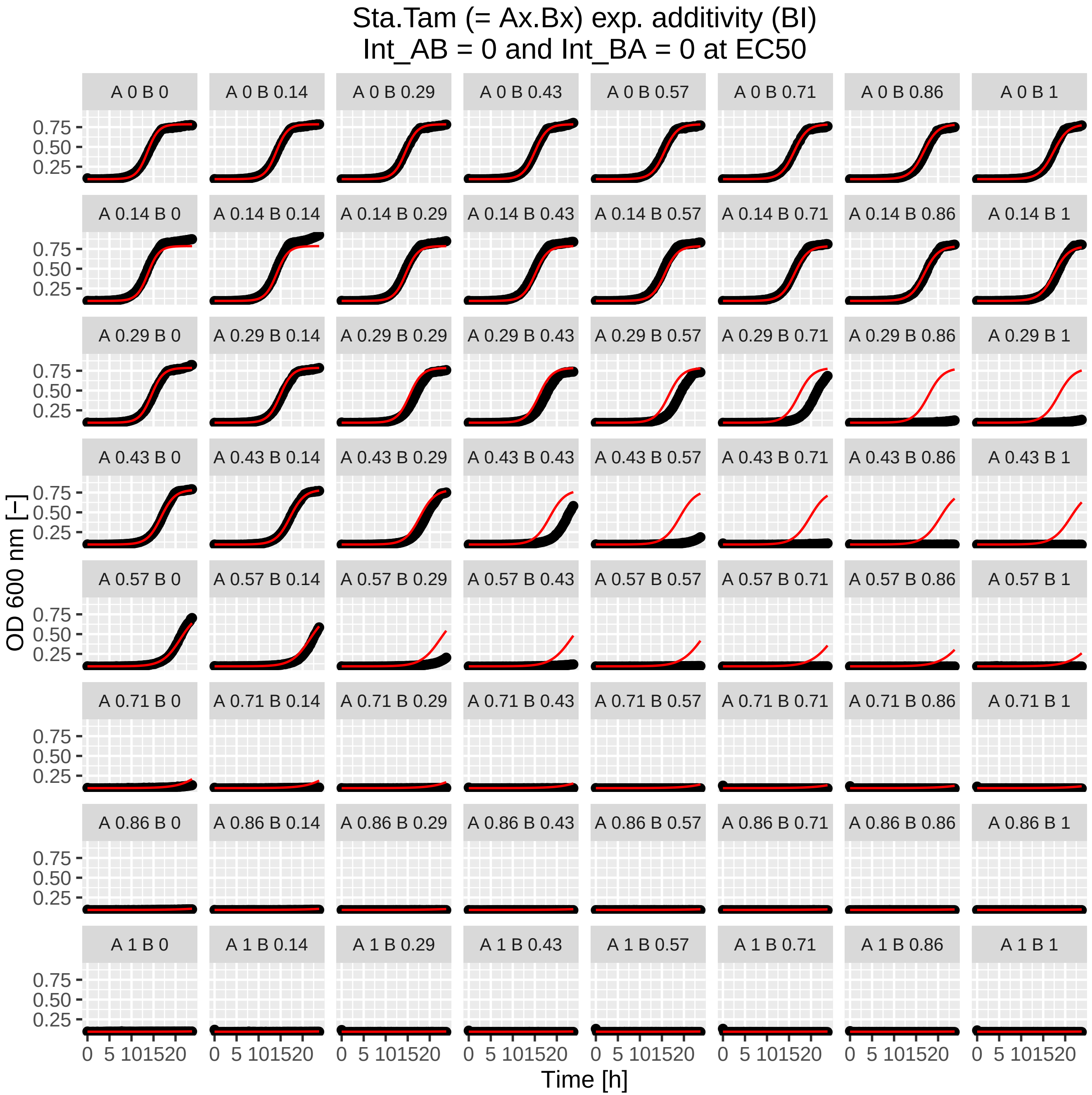

Sta.Tam (= Ax.Bx) exp. additivity (BI)
Int_AB = 0 and Int_BA = 0 at EC50
A 0 B 0
A 0 B 0.14
A 0 B 0.29
A 0 B 0.43
A 0 B 0.57
A 0 B 0.71
A 0 B 0.86
A 0 B 1
A 0.14 B 0
A 0.14 B 0.14
A 0.14 B 0.29
A 0.14 B 0.43
A 0.14 B 0.57
A 0.14 B 0.71
A 0.14 B 0.86
A 0.14 B 1
A 0.29 B 0
A 0.29 B 0.14
A 0.29 B 0.29
A 0.29 B 0.43
A 0.29 B 0.57
A 0.29 B 0.71
A 0.29 B 0.86
A 0.29 B 1
A 0.43 B 0
A 0.43 B 0.14
A 0.43 B 0.29
A 0.43 B 0.43
A 0.43 B 0.57
A 0.43 B 0.71
A 0.43 B 0.86
A 0.43 B 1
A 0.57 B 0
A 0.57 B 0.14
A 0.57 B 0.29
A 0.57 B 0.43
A 0.57 B 0.57
A 0.57 B 0.71
A 0.57 B 0.86
A 0.57 B 1
A 0.71 B 0
A 0.71 B 0.14
A 0.71 B 0.29
A 0.71 B 0.43
A 0.71 B 0.57
A 0.71 B 0.71
A 0.71 B 0.86
A 0.71 B 1
A 0.86 B 0
A 0.86 B 0.14
A 0.86 B 0.29
A 0.86 B 0.43
A 0.86 B 0.57
A 0.86 B 0.71
A 0.86 B 0.86
A 0.86 B 1
A 1 B 0
A 1 B 0.14
A 1 B 0.29
A 1 B 0.43
A 1 B 0.57
A 1 B 0.71
A 1 B 0.86
A 1 B 1
0.75
0.50
0.25
0 5 101520
OD 600 nm [–]
Time [h]

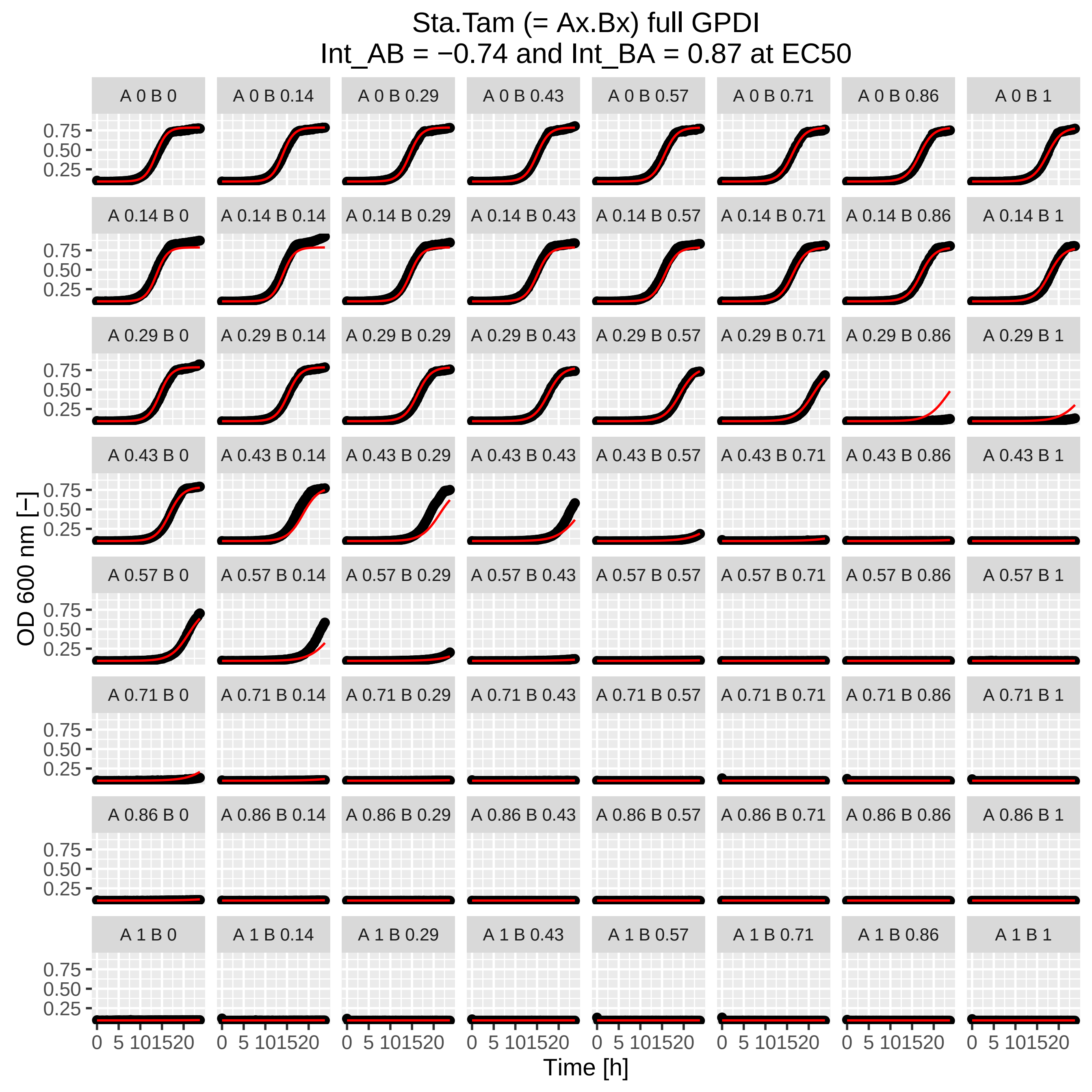

Sta.Tam (= Ax.Bx) full GPDI
Int_AB = −0.74 and Int_BA = 0.87 at EC50
A 0 B 0
A 0 B 0.14
A 0 B 0.29
A 0 B 0.43
A 0 B 0.57
A 0 B 0.71
A 0 B 0.86
A 0 B 1
A 0.14 B 0
A 0.14 B 0.14
A 0.14 B 0.29
A 0.14 B 0.43
A 0.14 B 0.57
A 0.14 B 0.71
A 0.14 B 0.86
A 0.14 B 1
A 0.29 B 0
A 0.29 B 0.14
A 0.29 B 0.29
A 0.29 B 0.43
A 0.29 B 0.57
A 0.29 B 0.71
A 0.29 B 0.86
A 0.29 B 1
A 0.43 B 0
A 0.43 B 0.14
A 0.43 B 0.29
A 0.43 B 0.43
A 0.43 B 0.57
A 0.43 B 0.71
A 0.43 B 0.86
A 0.43 B 1
A 0.57 B 0
A 0.57 B 0.14
A 0.57 B 0.29
A 0.57 B 0.43
A 0.57 B 0.57
A 0.57 B 0.71
A 0.57 B 0.86
A 0.57 B 1
A 0.71 B 0
A 0.71 B 0.14
A 0.71 B 0.29
A 0.71 B 0.43
A 0.71 B 0.57
A 0.71 B 0.71
A 0.71 B 0.86
A 0.71 B 1
A 0.86 B 0
A 0.86 B 0.14
A 0.86 B 0.29
A 0.86 B 0.43
A 0.86 B 0.57
A 0.86 B 0.71
A 0.86 B 0.86
A 0.86 B 1
A 1 B 0
A 1 B 0.14
A 1 B 0.29
A 1 B 0.43
A 1 B 0.57
A 1 B 0.71
A 1 B 0.86
A 1 B 1
0.75
0.50
0.25
0 5 101520
OD 600 nm [−]
Time [h]

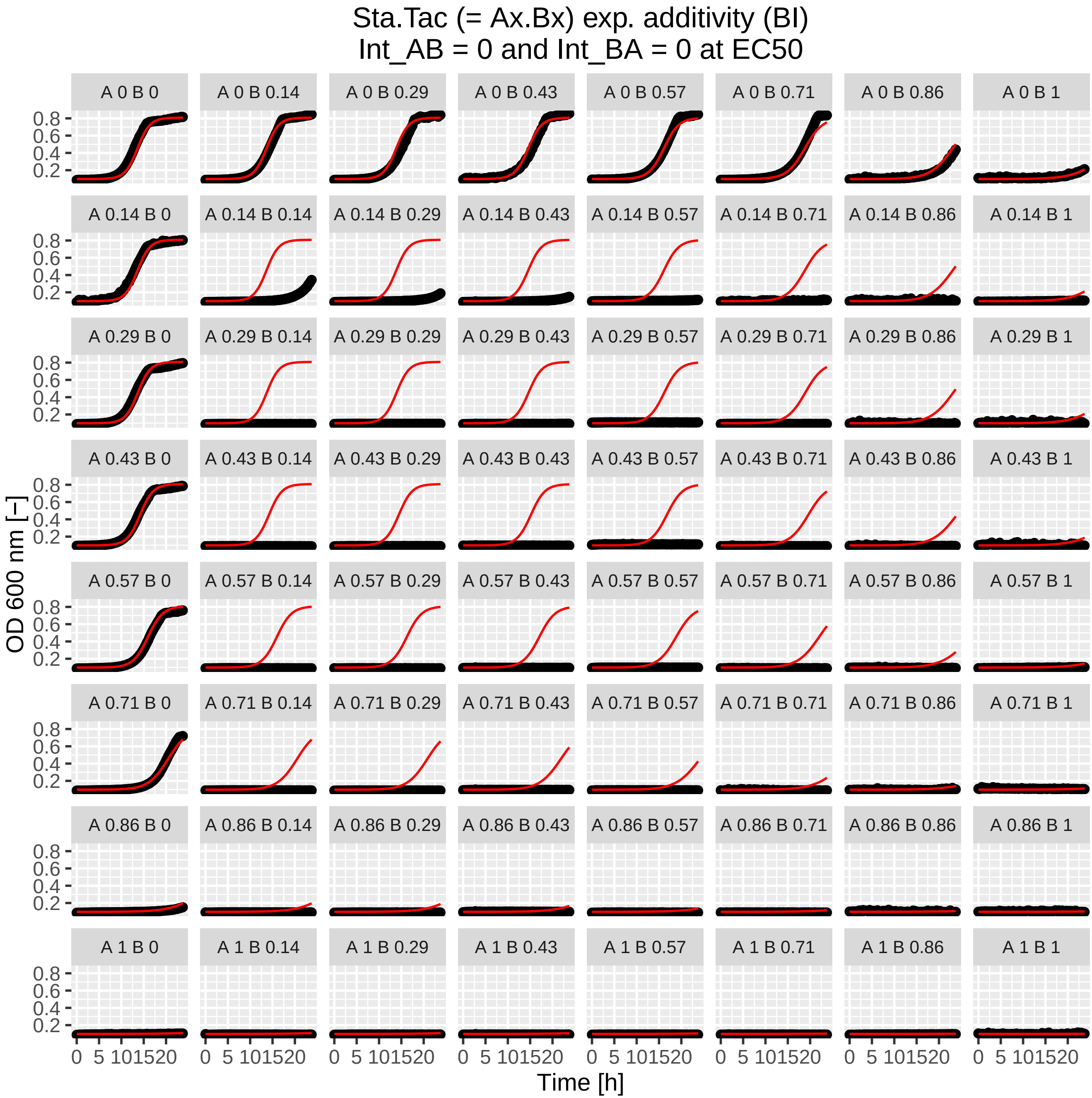
Sta.Tac (= Ax.Bx) exp. additivity (BI)
Int_AB = 0 and Int_BA = 0 at EC50
A 0 B 0
A 0 B 0.14
A 0 B 0.29
A 0 B 0.43
A 0 B 0.57
A 0 B 0.71
A 0 B 0.86
A 0 B 1
A 0.14 B 0
A 0.14 B 0.14
A 0.14 B 0.29
A 0.14 B 0.43
A 0.14 B 0.57
A 0.14 B 0.71
A 0.14 B 0.86
A 0.14 B 1
A 0.29 B 0
A 0.29 B 0.14
A 0.29 B 0.29
A 0.29 B 0.43
A 0.29 B 0.57
A 0.29 B 0.71
A 0.29 B 0.86
A 0.29 B 1
A 0.43 B 0
A 0.43 B 0.14
A 0.43 B 0.29
A 0.43 B 0.43
A 0.43 B 0.57
A 0.43 B 0.71
A 0.43 B 0.86
A 0.43 B 1
A 0.57 B 0
A 0.57 B 0.14
A 0.57 B 0.29
A 0.57 B 0.43
A 0.57 B 0.57
A 0.57 B 0.71
A 0.57 B 0.86
A 0.57 B 1
A 0.71 B 0
A 0.71 B 0.14
A 0.71 B 0.29
A 0.71 B 0.43
A 0.71 B 0.57
A 0.71 B 0.71
A 0.71 B 0.86
A 0.71 B 1
A 0.86 B 0
A 0.86 B 0.14
A 0.86 B 0.29
A 0.86 B 0.43
A 0.86 B 0.57
A 0.86 B 0.71
A 0.86 B 0.86
A 0.86 B 1
A 1 B 0
A 1 B 0.14
A 1 B 0.29
A 1 B 0.43
A 1 B 0.57
A 1 B 0.71
A 1 B 0.86
A 1 B 1
0.8
0.6
0.4
0.2
OD 600 nm [–]
0 5 101520
Time [h]

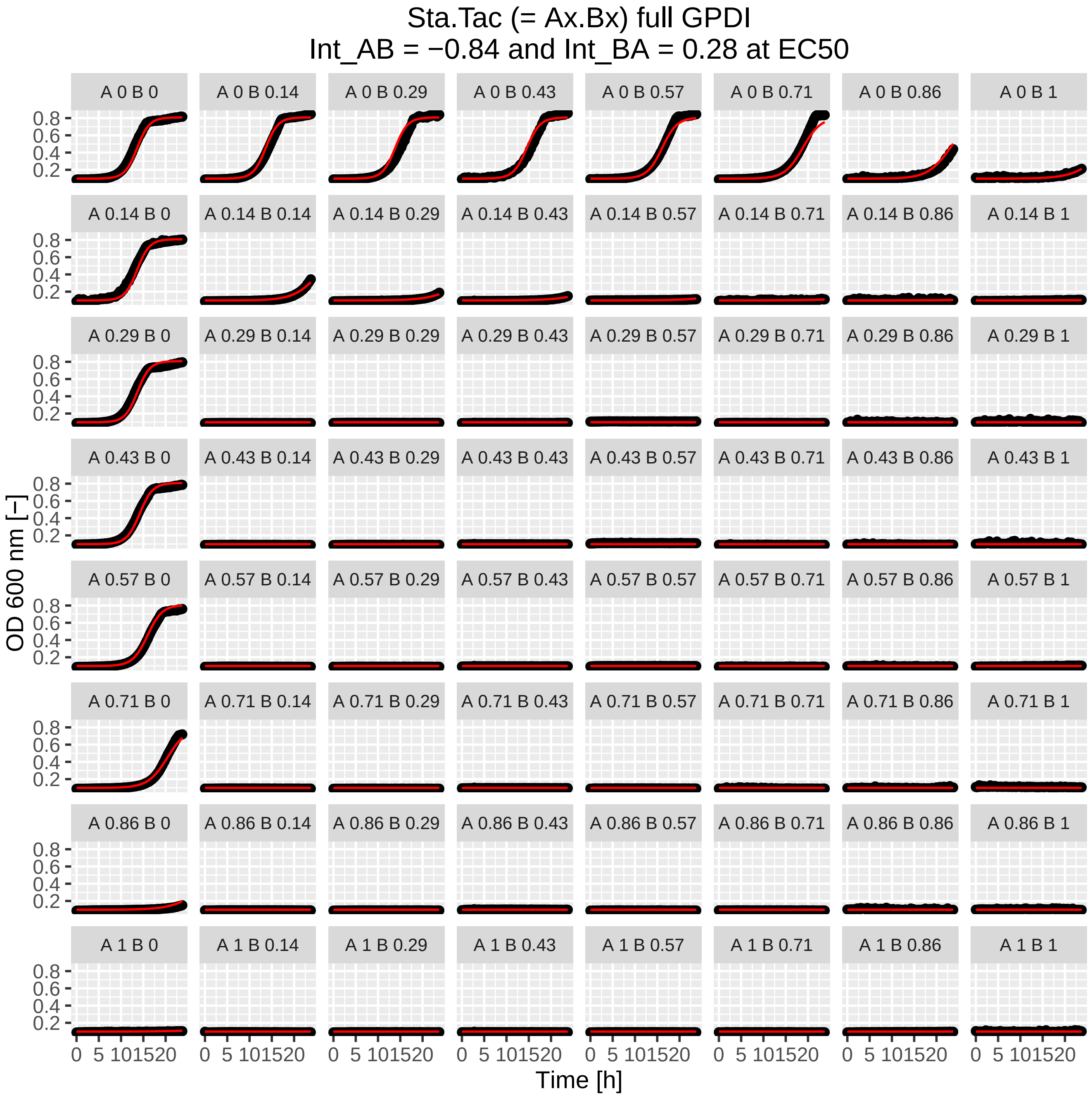

Sta.Tac (= Ax.Bx) full GPDI
Int_AB = −0.84 and Int_BA = 0.28 at EC50
A 0 B 0
A 0 B 0.14
A 0 B 0.29
A 0 B 0.43
A 0 B 0.57
A 0 B 0.71
A 0 B 0.86
A 0 B 1
A 0.14 B 0
A 0.14 B 0.14
A 0.14 B 0.29
A 0.14 B 0.43
A 0.14 B 0.57
A 0.14 B 0.71
A 0.14 B 0.86
A 0.14 B 1
A 0.29 B 0
A 0.29 B 0.14
A 0.29 B 0.29
A 0.29 B 0.43
A 0.29 B 0.57
A 0.29 B 0.71
A 0.29 B 0.86
A 0.29 B 1
A 0.43 B 0
A 0.43 B 0.14
A 0.43 B 0.29
A 0.43 B 0.43
A 0.43 B 0.57
A 0.43 B 0.71
A 0.43 B 0.86
A 0.43 B 1
A 0.57 B 0
A 0.57 B 0.14
A 0.57 B 0.29
A 0.57 B 0.43
A 0.57 B 0.57
A 0.57 B 0.71
A 0.57 B 0.86
A 0.57 B 1
A 0.71 B 0
A 0.71 B 0.14
A 0.71 B 0.29
A 0.71 B 0.43
A 0.71 B 0.57
A 0.71 B 0.71
A 0.71 B 0.86
A 0.71 B 1
A 0.86 B 0
A 0.86 B 0.14
A 0.86 B 0.29
A 0.86 B 0.43
A 0.86 B 0.57
A 0.86 B 0.71
A 0.86 B 0.86
A 0.86 B 1
A 1 B 0
A 1 B 0.14
A 1 B 0.29
A 1 B 0.43
A 1 B 0.57
A 1 B 0.71
A 1 B 0.86
A 1 B 1
OD 600 nm [−]
0.2
0.4
0.6
0.8
0 5 101520
Time [h]

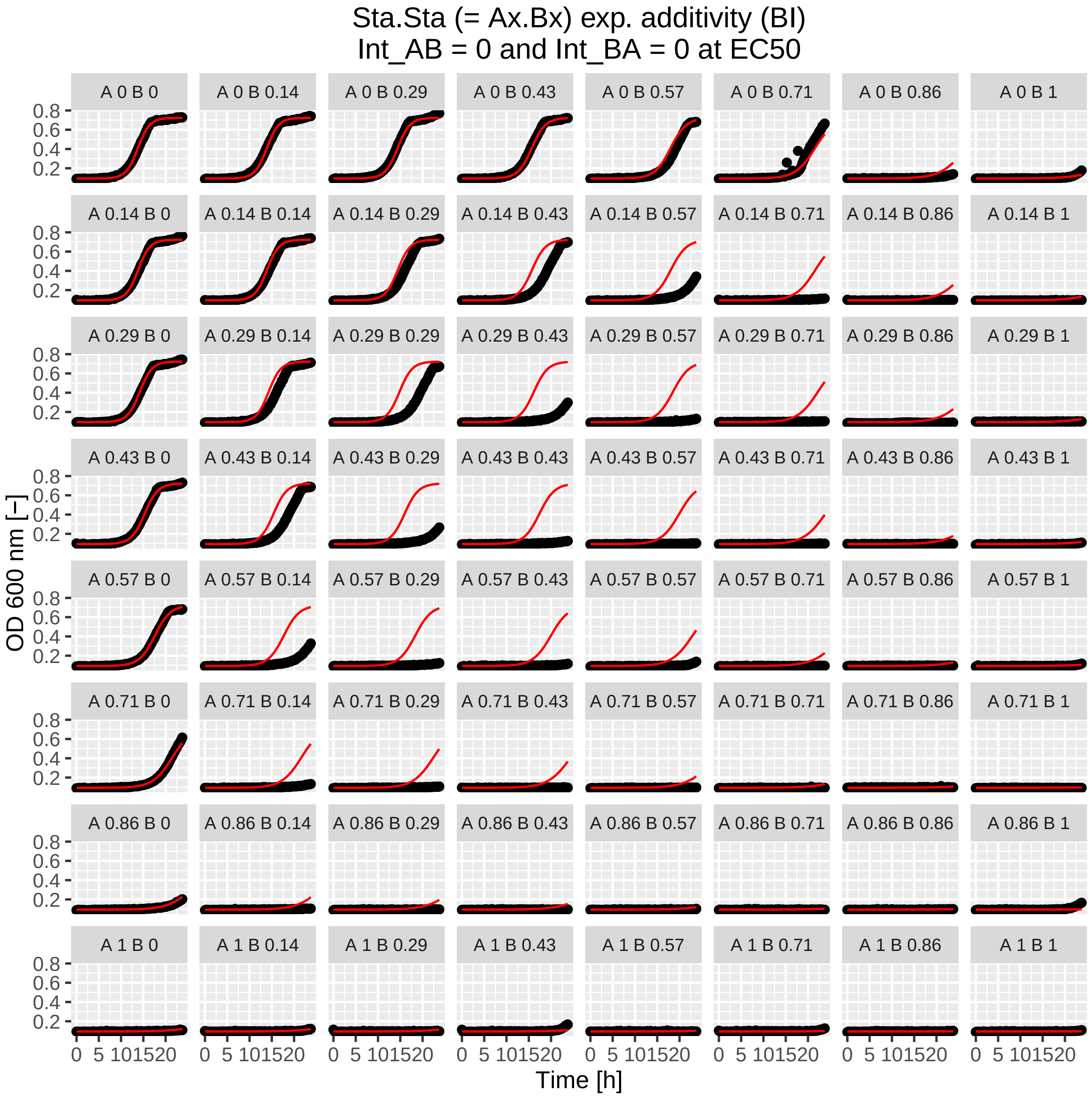

Sta.Sta (= Ax.Bx) exp. additivity (BI)
Int_AB = 0 and Int_BA = 0 at EC50
A 0 B 0
A 0 B 0.14
A 0 B 0.29
A 0 B 0.43
A 0 B 0.57
A 0 B 0.71
A 0 B 0.86
A 0 B 1
A 0.14 B 0
A 0.14 B 0.14
A 0.14 B 0.29
A 0.14 B 0.43
A 0.14 B 0.57
A 0.14 B 0.71
A 0.14 B 0.86
A 0.14 B 1
A 0.29 B 0
A 0.29 B 0.14
A 0.29 B 0.29
A 0.29 B 0.43
A 0.29 B 0.57
A 0.29 B 0.71
A 0.29 B 0.86
A 0.29 B 1
A 0.43 B 0
A 0.43 B 0.14
A 0.43 B 0.29
A 0.43 B 0.43
A 0.43 B 0.57
A 0.43 B 0.71
A 0.43 B 0.86
A 0.43 B 1
A 0.57 B 0
A 0.57 B 0.14
A 0.57 B 0.29
A 0.57 B 0.43
A 0.57 B 0.57
A 0.57 B 0.71
A 0.57 B 0.86
A 0.57 B 1
A 0.71 B 0
A 0.71 B 0.14
A 0.71 B 0.29
A 0.71 B 0.43
A 0.71 B 0.57
A 0.71 B 0.71
A 0.71 B 0.86
A 0.71 B 1
A 0.86 B 0
A 0.86 B 0.14
A 0.86 B 0.29
A 0.86 B 0.43
A 0.86 B 0.57
A 0.86 B 0.71
A 0.86 B 0.86
A 0.86 B 1
A 1 B 0
A 1 B 0.14
A 1 B 0.29
A 1 B 0.43
A 1 B 0.57
A 1 B 0.71
A 1 B 0.86
A 1 B 1
OD 600 nm [−]
0.8
0.6
0.4
0.2
0 5 101520
Time [h]

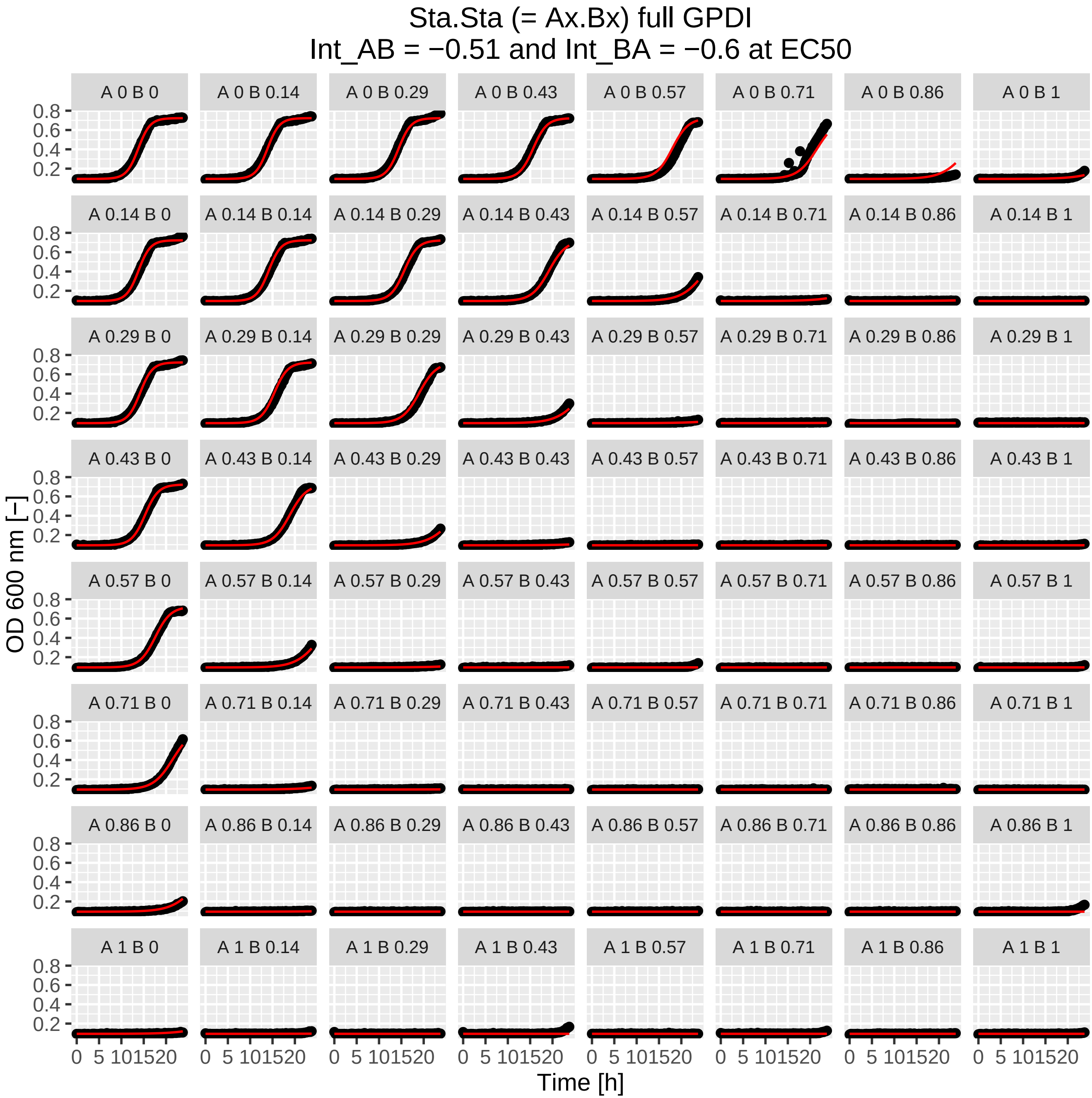

Sta.Sta (= Ax.Bx) full GPDI
Int_AB = −0.51 and Int_BA = −0.6 at EC50
A 0 B 0
A 0 B 0.14
A 0 B 0.29
A 0 B 0.43
A 0 B 0.57
A 0 B 0.71
A 0 B 0.86
A 0 B 1
A 0.14 B 0
A 0.14 B 0.14
A 0.14 B 0.29
A 0.14 B 0.43
A 0.14 B 0.57
A 0.14 B 0.71
A 0.14 B 0.86
A 0.14 B 1
A 0.29 B 0
A 0.29 B 0.14
A 0.29 B 0.29
A 0.29 B 0.43
A 0.29 B 0.57
A 0.29 B 0.71
A 0.29 B 0.86
A 0.29 B 1
A 0.43 B 0
A 0.43 B 0.14
A 0.43 B 0.29
A 0.43 B 0.43
A 0.43 B 0.57
A 0.43 B 0.71
A 0.43 B 0.86
A 0.43 B 1
A 0.57 B 0
A 0.57 B 0.14
A 0.57 B 0.29
A 0.57 B 0.43
A 0.57 B 0.57
A 0.57 B 0.71
A 0.57 B 0.86
A 0.57 B 1
A 0.71 B 0
A 0.71 B 0.14
A 0.71 B 0.29
A 0.71 B 0.43
A 0.71 B 0.57
A 0.71 B 0.71
A 0.71 B 0.86
A 0.71 B 1
A 0.86 B 0
A 0.86 B 0.14
A 0.86 B 0.29
A 0.86 B 0.43
A 0.86 B 0.57
A 0.86 B 0.71
A 0.86 B 0.86
A 0.86 B 1
A 1 B 0
A 1 B 0.14
A 1 B 0.29
A 1 B 0.43
A 1 B 0.57
A 1 B 0.71
A 1 B 0.86
A 1 B 1
0.8
0.6
0.4
0.2
OD 600 nm [−]
0 5 101520
Time [h]

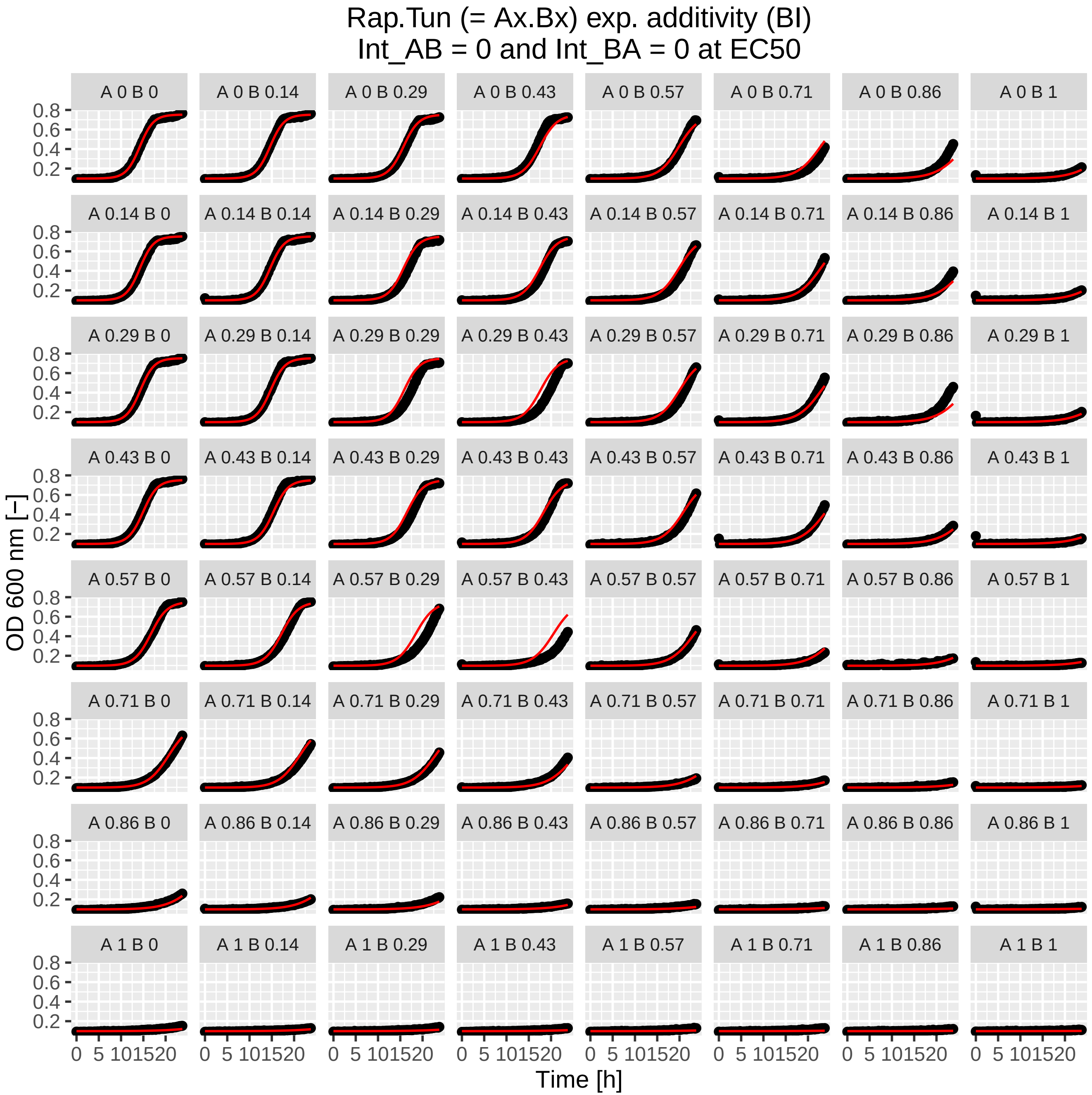

Rap.Tun (= Ax.Bx) exp. additivity (BI)
Int_AB = 0 and Int_BA = 0 at EC50
A 0 B 0
A 0 B 0.14
A 0 B 0.29
A 0 B 0.43
A 0 B 0.57
A 0 B 0.71
A 0 B 0.86
A 0 B 1
A 0.14 B 0
A 0.14 B 0.14
A 0.14 B 0.29
A 0.14 B 0.43
A 0.14 B 0.57
A 0.14 B 0.71
A 0.14 B 0.86
A 0.14 B 1
A 0.29 B 0
A 0.29 B 0.14
A 0.29 B 0.29
A 0.29 B 0.43
A 0.29 B 0.57
A 0.29 B 0.71
A 0.29 B 0.86
A 0.29 B 1
A 0.43 B 0
A 0.43 B 0.14
A 0.43 B 0.29
A 0.43 B 0.43
A 0.43 B 0.57
A 0.43 B 0.71
A 0.43 B 0.86
A 0.43 B 1
A 0.57 B 0
A 0.57 B 0.14
A 0.57 B 0.29
A 0.57 B 0.43
A 0.57 B 0.57
A 0.57 B 0.71
A 0.57 B 0.86
A 0.57 B 1
A 0.71 B 0
A 0.71 B 0.14
A 0.71 B 0.29
A 0.71 B 0.43
A 0.71 B 0.57
A 0.71 B 0.71
A 0.71 B 0.86
A 0.71 B 1
A 0.86 B 0
A 0.86 B 0.14
A 0.86 B 0.29
A 0.86 B 0.43
A 0.86 B 0.57
A 0.86 B 0.71
A 0.86 B 0.86
A 0.86 B 1
A 1 B 0
A 1 B 0.14
A 1 B 0.29
A 1 B 0.43
A 1 B 0.57
A 1 B 0.71
A 1 B 0.86
A 1 B 1
0.8
0.6
0.4
0.2
OD 600 nm [−]
0 5 101520
Time [h]

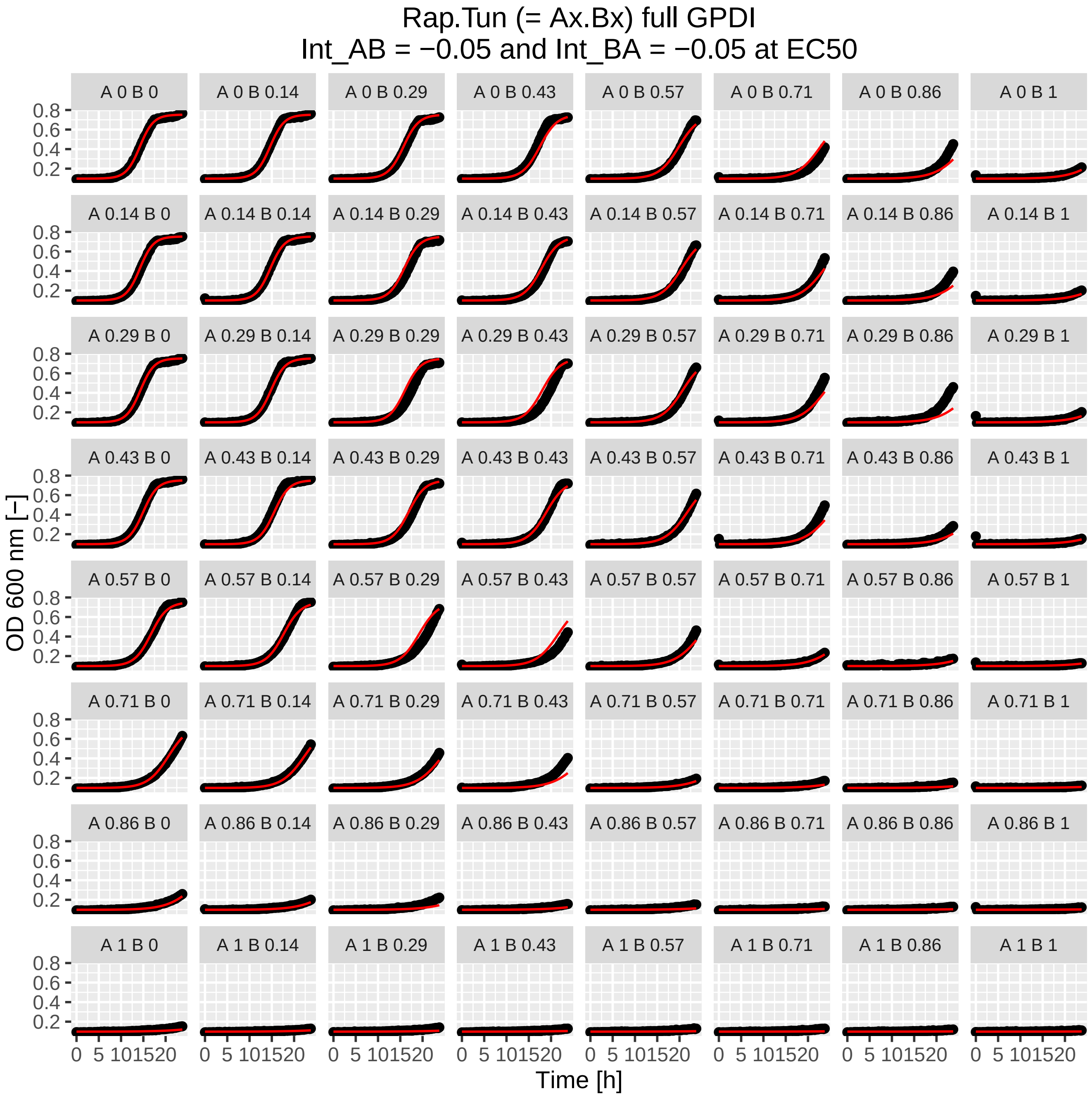
Rap.Tun (= Ax.Bx) full GPDI
Int_AB = −0.05 and Int_BA = −0.05 at EC50
OD 600 nm [−]
Time [h]
A 0 B 0
A 0 B 0.14
A 0 B 0.29
A 0 B 0.43
A 0 B 0.57
A 0 B 0.71
A 0 B 0.86
A 0 B 1
A 1 B 0
A 1 B 0.14
A 1 B 0.29
A 1 B 0.43
A 1 B 0.57
A 1 B 0.71
A 1 B 0.86
A 1 B 1

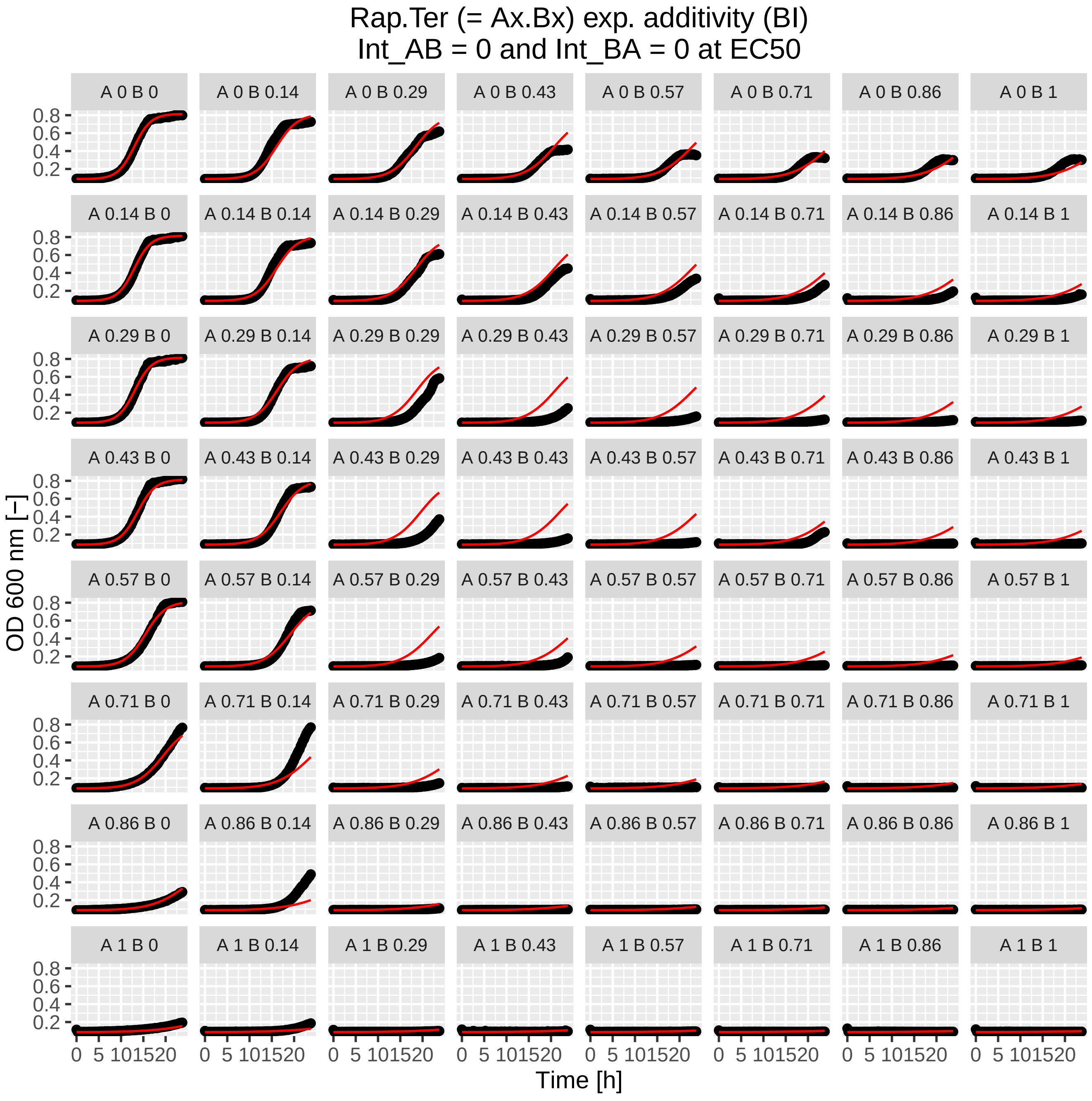

Rap.Ter (= Ax.Bx) exp. additivity (BI)
Int_AB = 0 and Int_BA = 0 at EC50
A 0 B 0
A 0 B 0.14
A 0 B 0.29
A 0 B 0.43
A 0 B 0.57
A 0 B 0.71
A 0 B 0.86
A 0 B 1
A 0.14 B 0
A 0.14 B 0.14
A 0.14 B 0.29
A 0.14 B 0.43
A 0.14 B 0.57
A 0.14 B 0.71
A 0.14 B 0.86
A 0.14 B 1
A 0.29 B 0
A 0.29 B 0.14
A 0.29 B 0.29
A 0.29 B 0.43
A 0.29 B 0.57
A 0.29 B 0.71
A 0.29 B 0.86
A 0.29 B 1
A 0.43 B 0
A 0.43 B 0.14
A 0.43 B 0.29
A 0.43 B 0.43
A 0.43 B 0.57
A 0.43 B 0.71
A 0.43 B 0.86
A 0.43 B 1
A 0.57 B 0
A 0.57 B 0.14
A 0.57 B 0.29
A 0.57 B 0.43
A 0.57 B 0.57
A 0.57 B 0.71
A 0.57 B 0.86
A 0.57 B 1
A 0.71 B 0
A 0.71 B 0.14
A 0.71 B 0.29
A 0.71 B 0.43
A 0.71 B 0.57
A 0.71 B 0.71
A 0.71 B 0.86
A 0.71 B 1
A 0.86 B 0
A 0.86 B 0.14
A 0.86 B 0.29
A 0.86 B 0.43
A 0.86 B 0.57
A 0.86 B 0.71
A 0.86 B 0.86
A 0.86 B 1
A 1 B 0
A 1 B 0.14
A 1 B 0.29
A 1 B 0.43
A 1 B 0.57
A 1 B 0.71
A 1 B 0.86
A 1 B 1
OD 600 nm [–]
0.2
0.4
0.6
0.8
0 5 101520
Time [h]

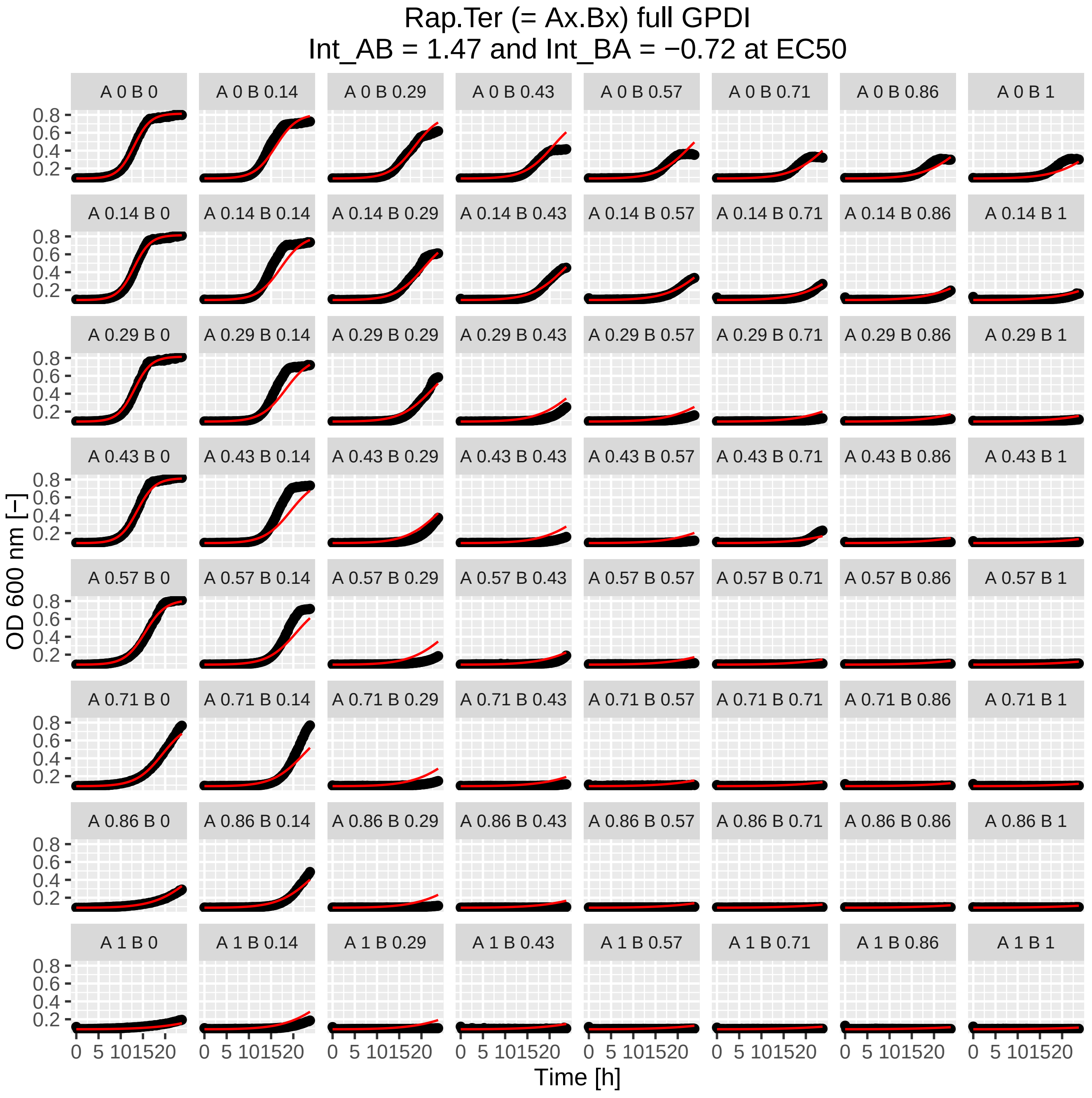
Rap.Ter (= Ax.Bx) full GPDI
Int_AB = 1.47 and Int_BA = −0.72 at EC50
A 0 B 0
A 0 B 0.14
A 0 B 0.29
A 0 B 0.43
A 0 B 0.57
A 0 B 0.71
A 0 B 0.86
A 0 B 1
A 0.14 B 0
A 0.14 B 0.14
A 0.14 B 0.29
A 0.14 B 0.43
A 0.14 B 0.57
A 0.14 B 0.71
A 0.14 B 0.86
A 0.14 B 1
A 0.29 B 0
A 0.29 B 0.14
A 0.29 B 0.29
A 0.29 B 0.43
A 0.29 B 0.57
A 0.29 B 0.71
A 0.29 B 0.86
A 0.29 B 1
A 0.43 B 0
A 0.43 B 0.14
A 0.43 B 0.29
A 0.43 B 0.43
A 0.43 B 0.57
A 0.43 B 0.71
A 0.43 B 0.86
A 0.43 B 1
A 0.57 B 0
A 0.57 B 0.14
A 0.57 B 0.29
A 0.57 B 0.43
A 0.57 B 0.57
A 0.57 B 0.71
A 0.57 B 0.86
A 0.57 B 1
A 0.71 B 0
A 0.71 B 0.14
A 0.71 B 0.29
A 0.71 B 0.43
A 0.71 B 0.57
A 0.71 B 0.71
A 0.71 B 0.86
A 0.71 B 1
A 0.86 B 0
A 0.86 B 0.14
A 0.86 B 0.29
A 0.86 B 0.43
A 0.86 B 0.57
A 0.86 B 0.71
A 0.86 B 0.86
A 0.86 B 1
A 1 B 0
A 1 B 0.14
A 1 B 0.29
A 1 B 0.43
A 1 B 0.57
A 1 B 0.71
A 1 B 0.86
A 1 B 1
OD 600 nm [−]
0.2
0.4
0.6
0.8
0 5 101520
Time [h]

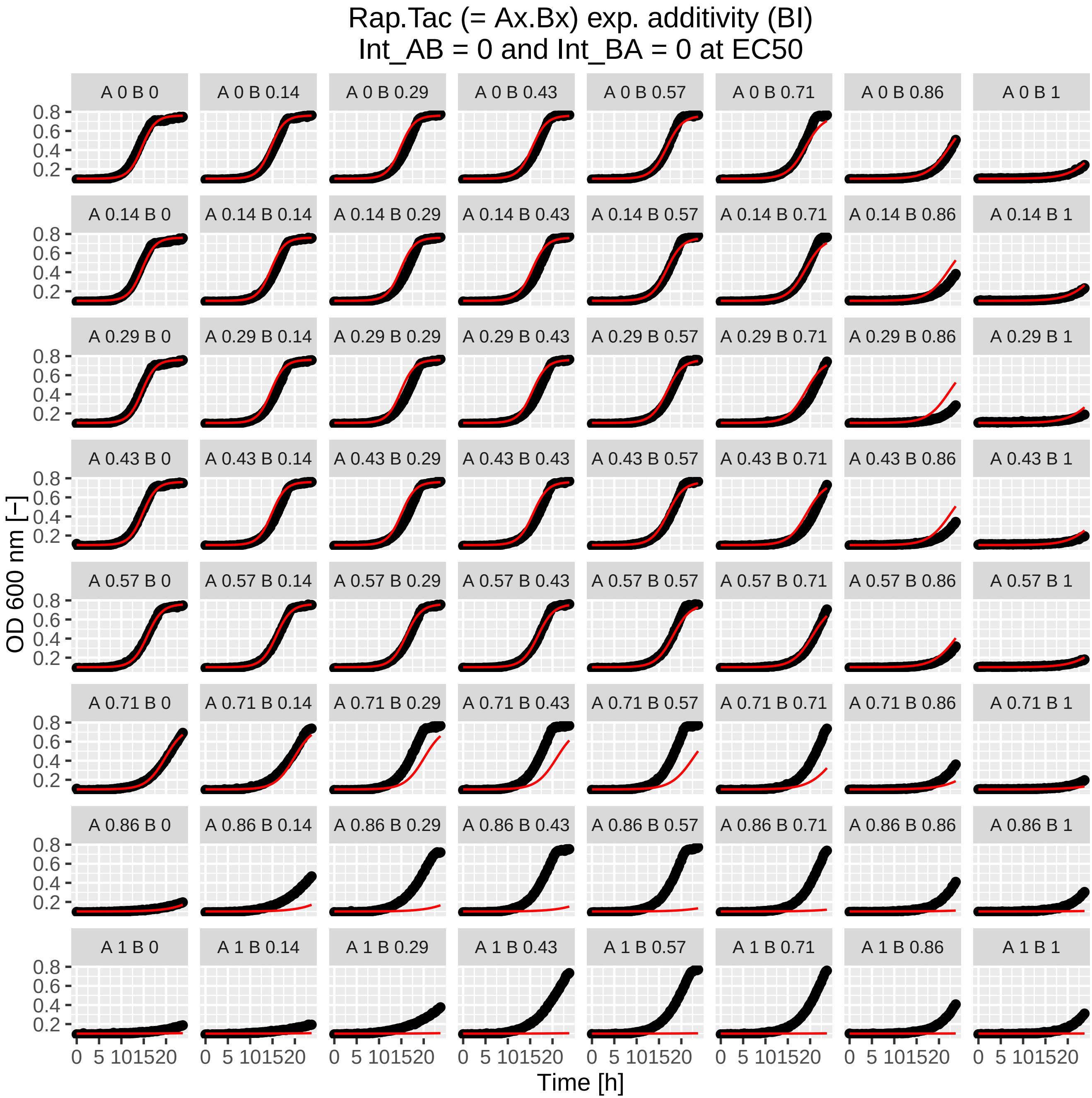

Rap.Tac (= Ax.Bx) exp. additivity (BI)
Int_AB = 0 and Int_BA = 0 at EC50
A 0 B 0
A 0 B 0.14
A 0 B 0.29
A 0 B 0.43
A 0 B 0.57
A 0 B 0.71
A 0 B 0.86
A 0 B 1
A 0.14 B 0
A 0.14 B 0.14
A 0.14 B 0.29
A 0.14 B 0.43
A 0.14 B 0.57
A 0.14 B 0.71
A 0.14 B 0.86
A 0.14 B 1
A 0.29 B 0
A 0.29 B 0.14
A 0.29 B 0.29
A 0.29 B 0.43
A 0.29 B 0.57
A 0.29 B 0.71
A 0.29 B 0.86
A 0.29 B 1
A 0.43 B 0
A 0.43 B 0.14
A 0.43 B 0.29
A 0.43 B 0.43
A 0.43 B 0.57
A 0.43 B 0.71
A 0.43 B 0.86
A 0.43 B 1
A 0.57 B 0
A 0.57 B 0.14
A 0.57 B 0.29
A 0.57 B 0.43
A 0.57 B 0.57
A 0.57 B 0.71
A 0.57 B 0.86
A 0.57 B 1
A 0.71 B 0
A 0.71 B 0.14
A 0.71 B 0.29
A 0.71 B 0.43
A 0.71 B 0.57
A 0.71 B 0.71
A 0.71 B 0.86
A 0.71 B 1
A 0.86 B 0
A 0.86 B 0.14
A 0.86 B 0.29
A 0.86 B 0.43
A 0.86 B 0.57
A 0.86 B 0.71
A 0.86 B 0.86
A 0.86 B 1
A 1 B 0
A 1 B 0.14
A 1 B 0.29
A 1 B 0.43
A 1 B 0.57
A 1 B 0.71
A 1 B 0.86
A 1 B 1
OD 600 nm [–]
0.8
0.6
0.4
0.2
0
5
10
15
20
Time [h]

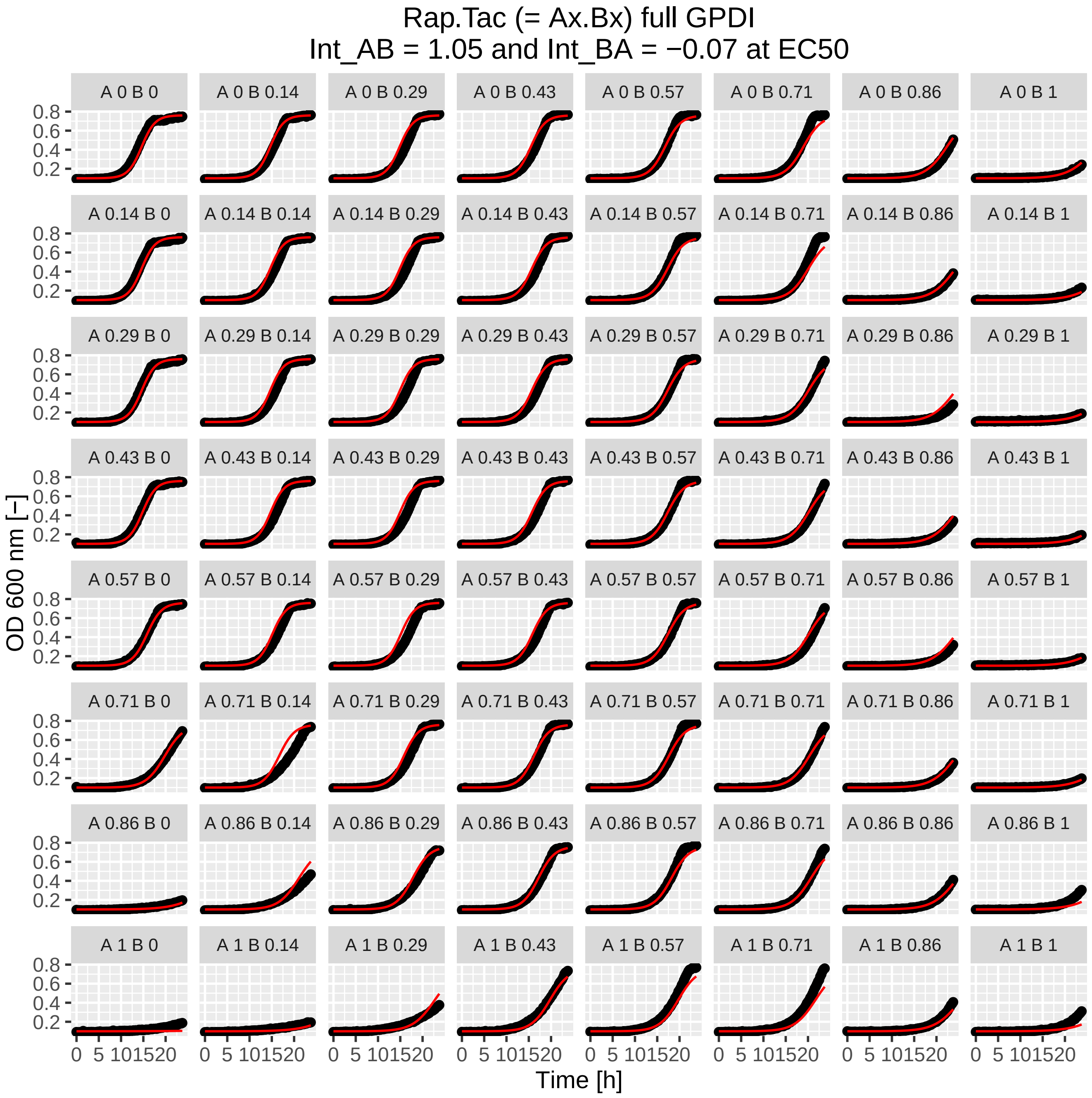

Rap.Tac (= Ax.Bx) full GPDI
Int_AB = 1.05 and Int_BA = −0.07 at EC50
A 0 B 0
A 0 B 0.14
A 0 B 0.29
A 0 B 0.43
A 0 B 0.57
A 0 B 0.71
A 0 B 0.86
A 0 B 1
A 0.14 B 0
A 0.14 B 0.14
A 0.14 B 0.29
A 0.14 B 0.43
A 0.14 B 0.57
A 0.14 B 0.71
A 0.14 B 0.86
A 0.14 B 1
A 0.29 B 0
A 0.29 B 0.14
A 0.29 B 0.29
A 0.29 B 0.43
A 0.29 B 0.57
A 0.29 B 0.71
A 0.29 B 0.86
A 0.29 B 1
A 0.43 B 0
A 0.43 B 0.14
A 0.43 B 0.29
A 0.43 B 0.43
A 0.43 B 0.57
A 0.43 B 0.71
A 0.43 B 0.86
A 0.43 B 1
A 0.57 B 0
A 0.57 B 0.14
A 0.57 B 0.29
A 0.57 B 0.43
A 0.57 B 0.57
A 0.57 B 0.71
A 0.57 B 0.86
A 0.57 B 1
A 0.71 B 0
A 0.71 B 0.14
A 0.71 B 0.29
A 0.71 B 0.43
A 0.71 B 0.57
A 0.71 B 0.71
A 0.71 B 0.86
A 0.71 B 1
A 0.86 B 0
A 0.86 B 0.14
A 0.86 B 0.29
A 0.86 B 0.43
A 0.86 B 0.57
A 0.86 B 0.71
A 0.86 B 0.86
A 0.86 B 1
A 1 B 0
A 1 B 0.14
A 1 B 0.29
A 1 B 0.43
A 1 B 0.57
A 1 B 0.71
A 1 B 0.86
A 1 B 1
0.8
0.6
0.4
0.2
OD 600 nm [−]
0 5 101520
Time [h]

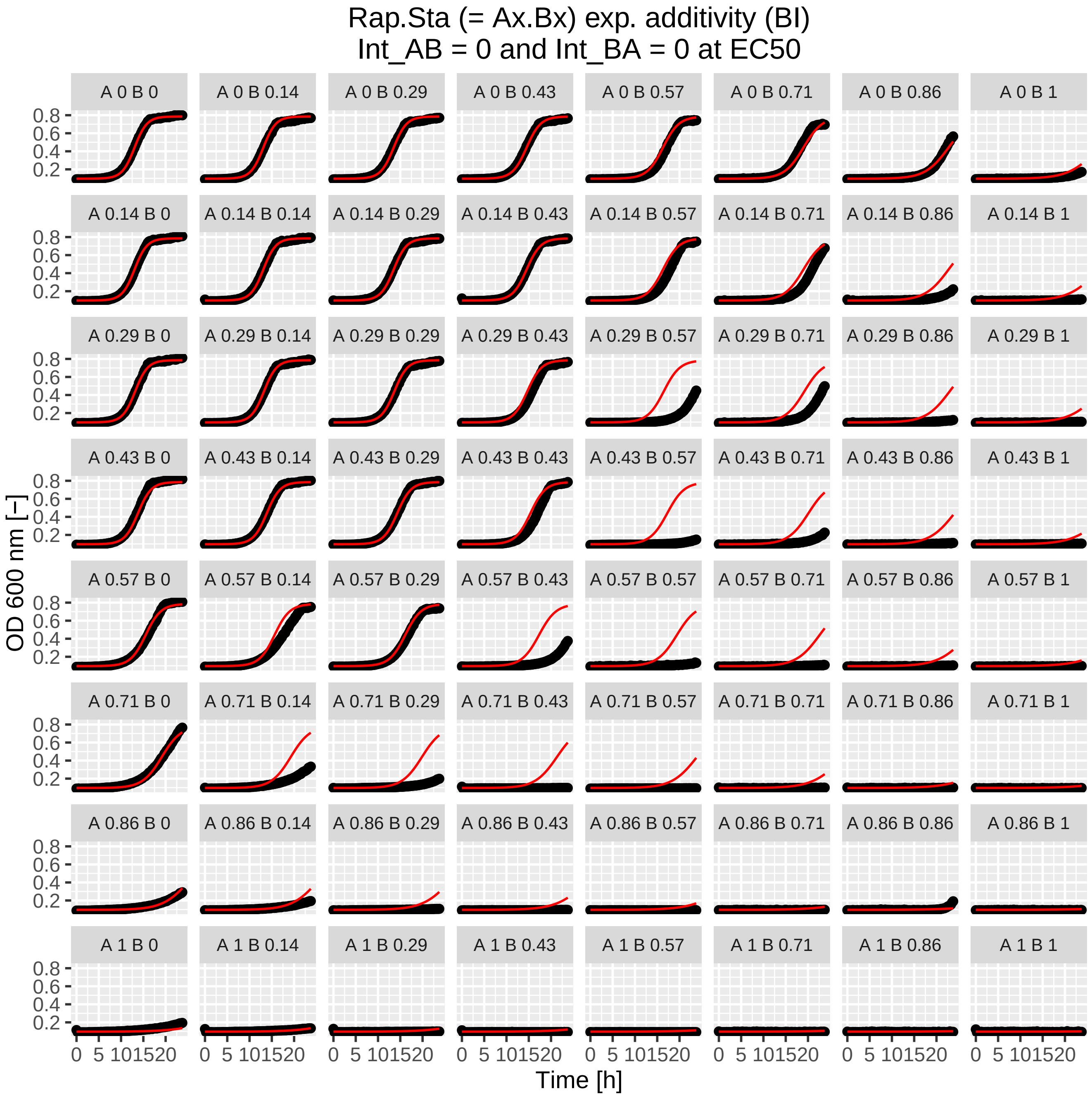
Rap.Sta (= Ax.Bx) exp. additivity (BI)
Int_AB = 0 and Int_BA = 0 at EC50
A 0 B 0
A 0 B 0.14
A 0 B 0.29
A 0 B 0.43
A 0 B 0.57
A 0 B 0.71
A 0 B 0.86
A 0 B 1
A 0.14 B 0
A 0.14 B 0.14
A 0.14 B 0.29
A 0.14 B 0.43
A 0.14 B 0.57
A 0.14 B 0.71
A 0.14 B 0.86
A 0.14 B 1
A 0.29 B 0
A 0.29 B 0.14
A 0.29 B 0.29
A 0.29 B 0.43
A 0.29 B 0.57
A 0.29 B 0.71
A 0.29 B 0.86
A 0.29 B 1
A 0.43 B 0
A 0.43 B 0.14
A 0.43 B 0.29
A 0.43 B 0.43
A 0.43 B 0.57
A 0.43 B 0.71
A 0.43 B 0.86
A 0.43 B 1
A 0.57 B 0
A 0.57 B 0.14
A 0.57 B 0.29
A 0.57 B 0.43
A 0.57 B 0.57
A 0.57 B 0.71
A 0.57 B 0.86
A 0.57 B 1
A 0.71 B 0
A 0.71 B 0.14
A 0.71 B 0.29
A 0.71 B 0.43
A 0.71 B 0.57
A 0.71 B 0.71
A 0.71 B 0.86
A 0.71 B 1
A 0.86 B 0
A 0.86 B 0.14
A 0.86 B 0.29
A 0.86 B 0.43
A 0.86 B 0.57
A 0.86 B 0.71
A 0.86 B 0.86
A 0.86 B 1
A 1 B 0
A 1 B 0.14
A 1 B 0.29
A 1 B 0.43
A 1 B 0.57
A 1 B 0.71
A 1 B 0.86
A 1 B 1
0.8
0.6
0.4
0.2
0 5 101520
OD 600 nm [–]
Time [h]

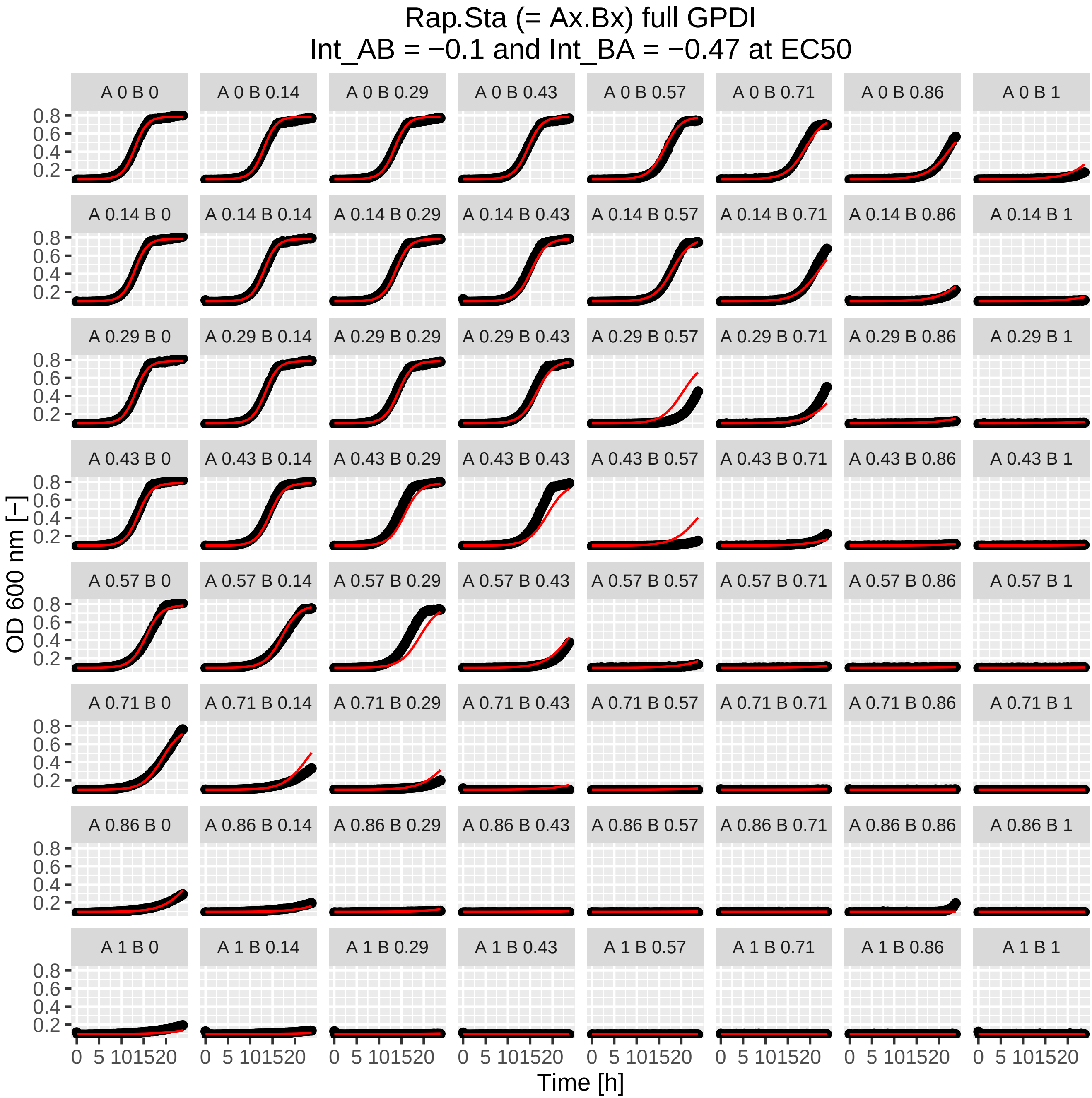
Rap.Sta (= Ax.Bx) full GPDI
Int_AB = −0.1 and Int_BA = −0.47 at EC50
OD 600 nm [−]
Time [h]
A 0 B 0
A 0 B 0.14
A 0 B 0.29
A 0 B 0.43
A 0 B 0.57
A 0 B 0.71
A 0 B 0.86
A 0 B 1
A 0.14 B 0
A 0.14 B 0.14
A 0.14 B 0.29
A 0.14 B 0.43
A 0.14 B 0.57
A 0.14 B 0.71
A 0.14 B 0.86
A 0.14 B 1
A 0.29 B 0
A 0.29 B 0.14
A 0.29 B 0.29
A 0.29 B 0.43
A 0.29 B 0.57
A 0.29 B 0.71
A 0.29 B 0.86
A 0.29 B 1
A 0.43 B 0
A 0.43 B 0.14
A 0.43 B 0.29
A 0.43 B 0.43
A 0.43 B 0.57
A 0.43 B 0.71
A 0.43 B 0.86
A 0.43 B 1
A 0.57 B 0
A 0.57 B 0.14
A 0.57 B 0.29
A 0.57 B 0.43
A 0.57 B 0.57
A 0.57 B 0.71
A 0.57 B 0.86
A 0.57 B 1
A 0.71 B 0
A 0.71 B 0.14
A 0.71 B 0.29
A 0.71 B 0.43
A 0.71 B 0.57
A 0.71 B 0.71
A 0.71 B 0.86
A 0.71 B 1
A 0.86 B 0
A 0.86 B 0.14
A 0.86 B 0.29
A 0.86 B 0.43
A 0.86 B 0.57
A 0.86 B 0.71
A 0.86 B 0.86
A 0.86 B 1
A 1 B 0
A 1 B 0.14
A 1 B 0.29
A 1 B 0.43
A 1 B 0.57
A 1 B 0.71
A 1 B 0.86
A 1 B 1
0.8
0.6
0.4
0.2
0 5 101520

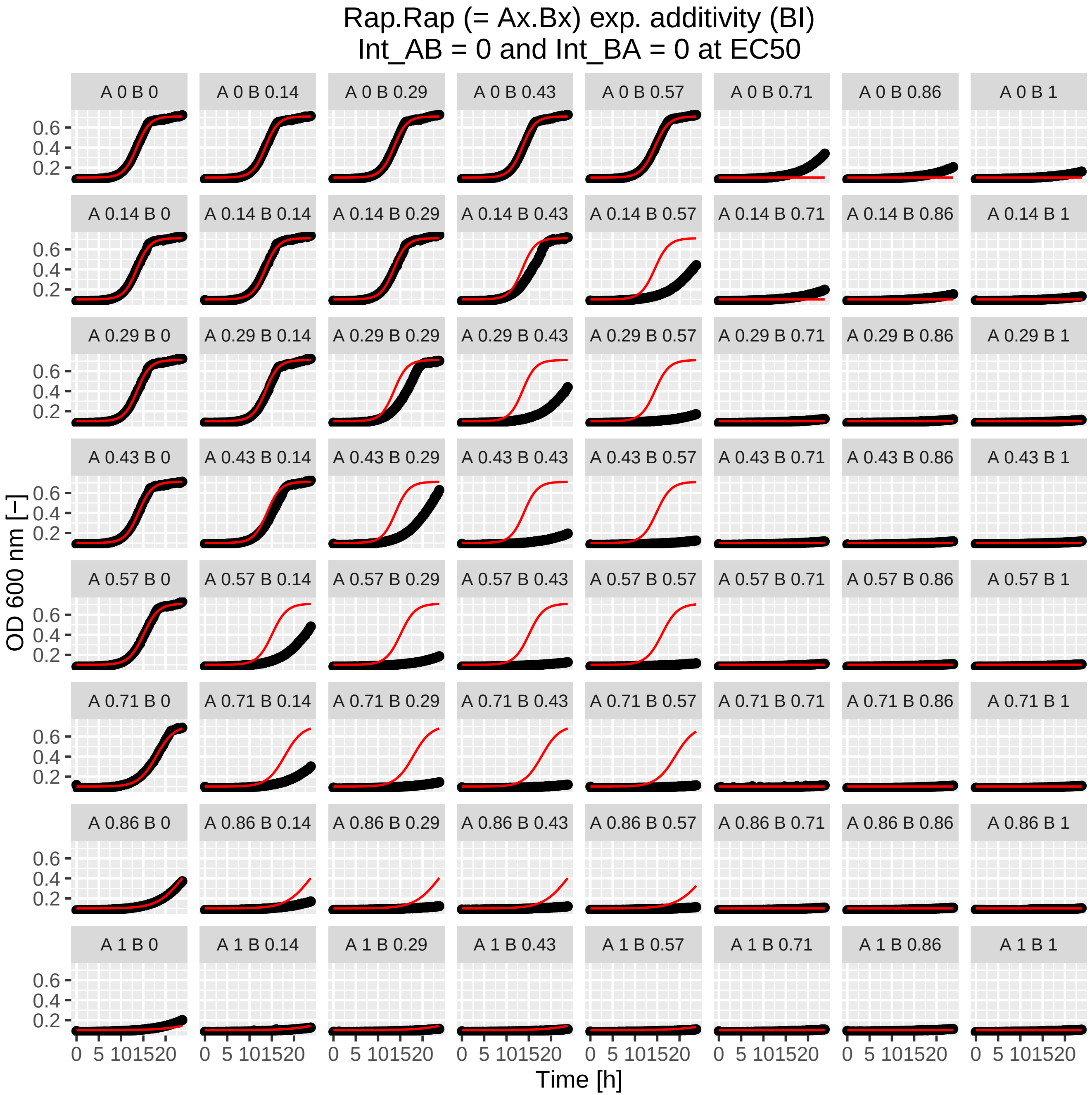

Rap.Rap (= Ax.Bx) exp. additivity (BI)
Int_AB = 0 and Int_BA = 0 at EC50
A 0 B 0
A 0 B 0.14
A 0 B 0.29
A 0 B 0.43
A 0 B 0.57
A 0 B 0.71
A 0 B 0.86
A 0 B 1
A 0.14 B 0
A 0.14 B 0.14
A 0.14 B 0.29
A 0.14 B 0.43
A 0.14 B 0.57
A 0.14 B 0.71
A 0.14 B 0.86
A 0.14 B 1
A 0.29 B 0
A 0.29 B 0.14
A 0.29 B 0.29
A 0.29 B 0.43
A 0.29 B 0.57
A 0.29 B 0.71
A 0.29 B 0.86
A 0.29 B 1
A 0.43 B 0
A 0.43 B 0.14
A 0.43 B 0.29
A 0.43 B 0.43
A 0.43 B 0.57
A 0.43 B 0.71
A 0.43 B 0.86
A 0.43 B 1
A 0.57 B 0
A 0.57 B 0.14
A 0.57 B 0.29
A 0.57 B 0.43
A 0.57 B 0.57
A 0.57 B 0.71
A 0.57 B 0.86
A 0.57 B 1
A 0.71 B 0
A 0.71 B 0.14
A 0.71 B 0.29
A 0.71 B 0.43
A 0.71 B 0.57
A 0.71 B 0.71
A 0.71 B 0.86
A 0.71 B 1
A 0.86 B 0
A 0.86 B 0.14
A 0.86 B 0.29
A 0.86 B 0.43
A 0.86 B 0.57
A 0.86 B 0.71
A 0.86 B 0.86
A 0.86 B 1
A 1 B 0
A 1 B 0.14
A 1 B 0.29
A 1 B 0.43
A 1 B 0.57
A 1 B 0.71
A 1 B 0.86
A 1 B 1
OD 600 nm [–]
0.2
0.4
0.6
0 5 101520
Time [h]

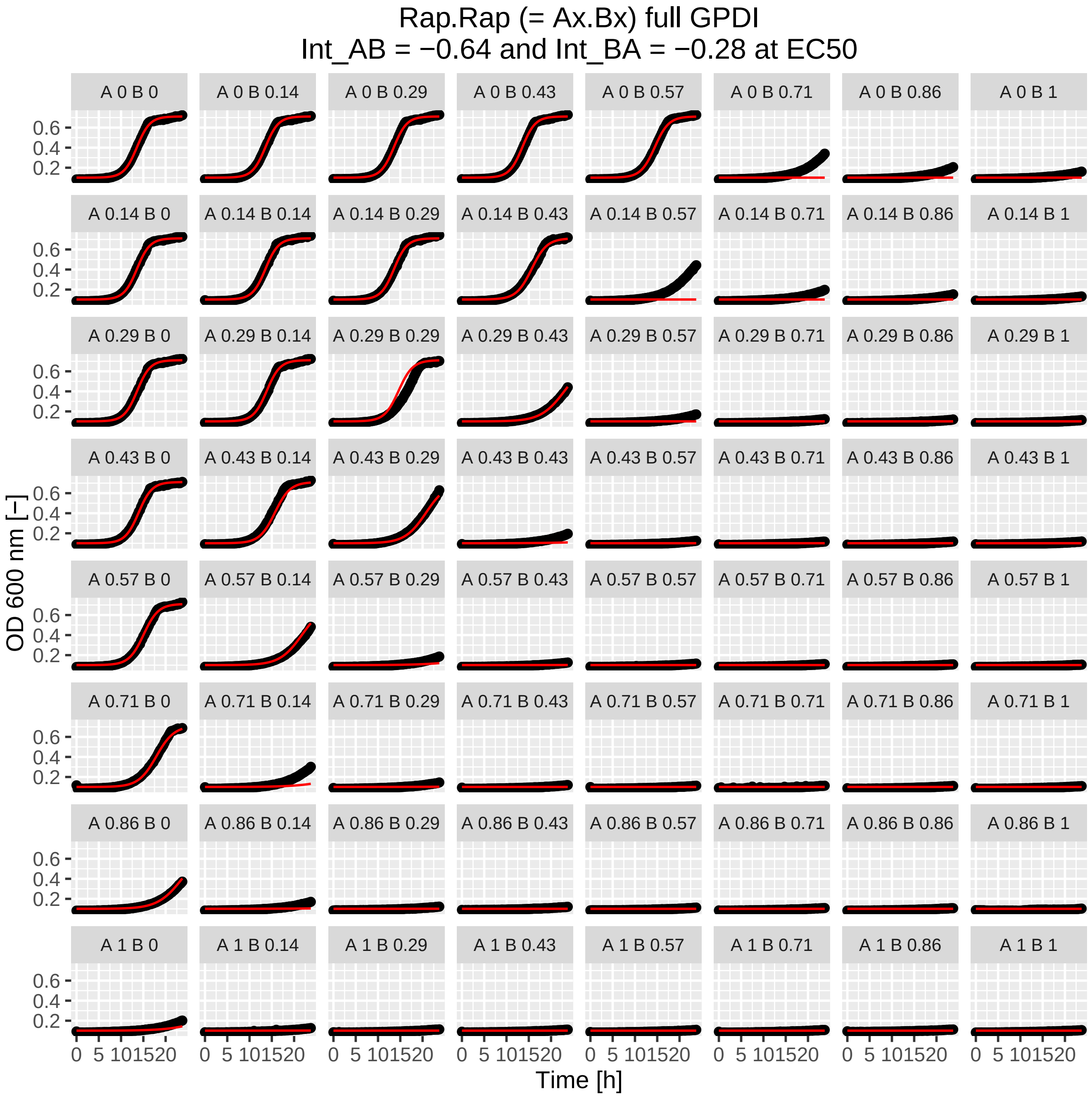

Rap.Rap (= Ax.Bx) full GPDI
Int_AB = −0.64 and Int_BA = −0.28 at EC50
A 0 B 0
A 0 B 0.14
A 0 B 0.29
A 0 B 0.43
A 0 B 0.57
A 0 B 0.71
A 0 B 0.86
A 0 B 1
A 0.14 B 0
A 0.14 B 0.14
A 0.14 B 0.29
A 0.14 B 0.43
A 0.14 B 0.57
A 0.14 B 0.71
A 0.14 B 0.86
A 0.14 B 1
A 0.29 B 0
A 0.29 B 0.14
A 0.29 B 0.29
A 0.29 B 0.43
A 0.29 B 0.57
A 0.29 B 0.71
A 0.29 B 0.86
A 0.29 B 1
A 0.43 B 0
A 0.43 B 0.14
A 0.43 B 0.29
A 0.43 B 0.43
A 0.43 B 0.57
A 0.43 B 0.71
A 0.43 B 0.86
A 0.43 B 1
A 0.57 B 0
A 0.57 B 0.14
A 0.57 B 0.29
A 0.57 B 0.43
A 0.57 B 0.57
A 0.57 B 0.71
A 0.57 B 0.86
A 0.57 B 1
A 0.71 B 0
A 0.71 B 0.14
A 0.71 B 0.29
A 0.71 B 0.43
A 0.71 B 0.57
A 0.71 B 0.71
A 0.71 B 0.86
A 0.71 B 1
A 0.86 B 0
A 0.86 B 0.14
A 0.86 B 0.29
A 0.86 B 0.43
A 0.86 B 0.57
A 0.86 B 0.71
A 0.86 B 0.86
A 0.86 B 1
A 1 B 0
A 1 B 0.14
A 1 B 0.29
A 1 B 0.43
A 1 B 0.57
A 1 B 0.71
A 1 B 0.86
A 1 B 1
0.6
0.4
0.2
0 5 101520
OD 600 nm [−]
Time [h]

Rad.Ter (= Ax.Bx) exp. additivity (BI)
Int_AB = 0 and Int_BA = 0 at EC50

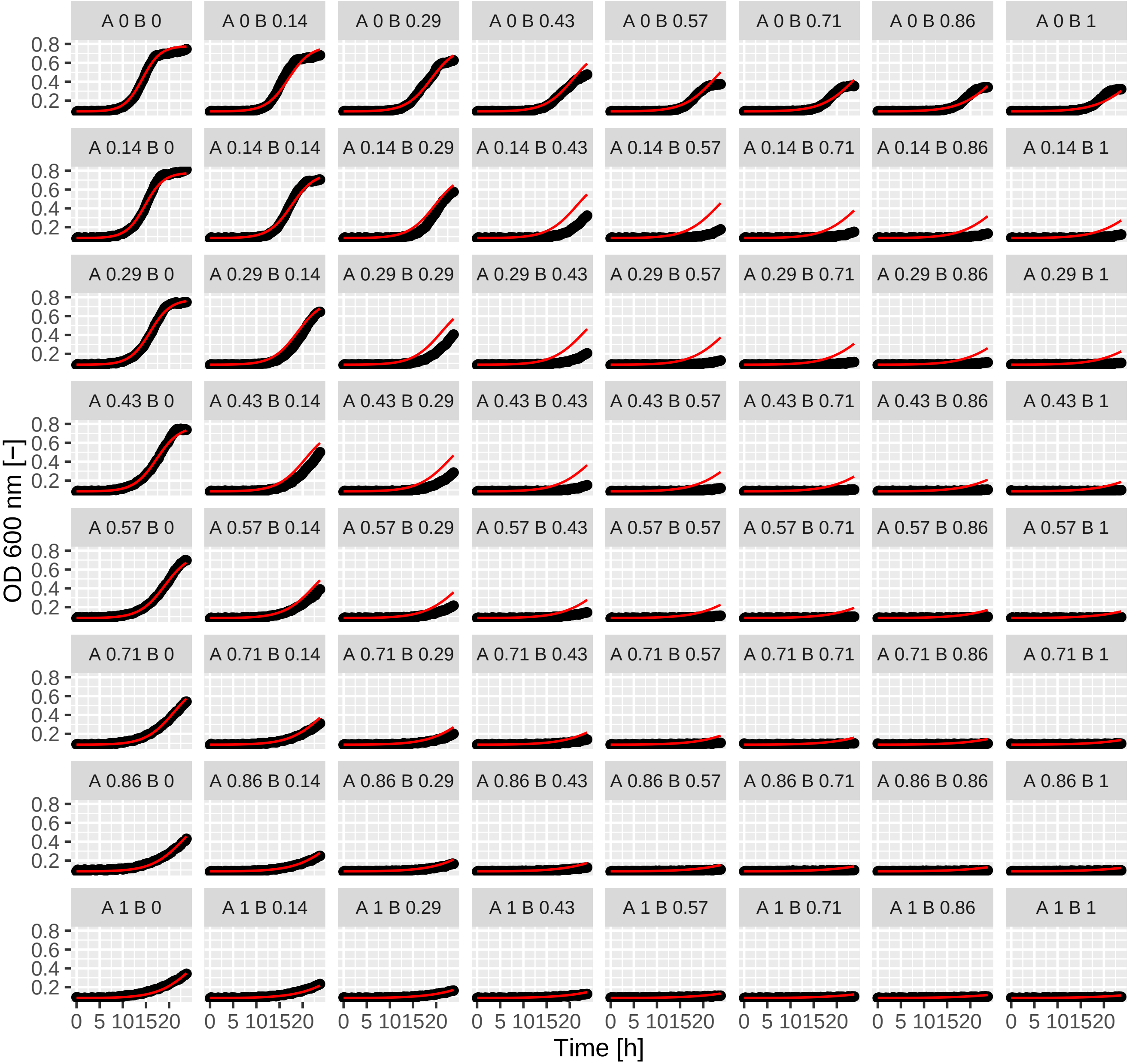

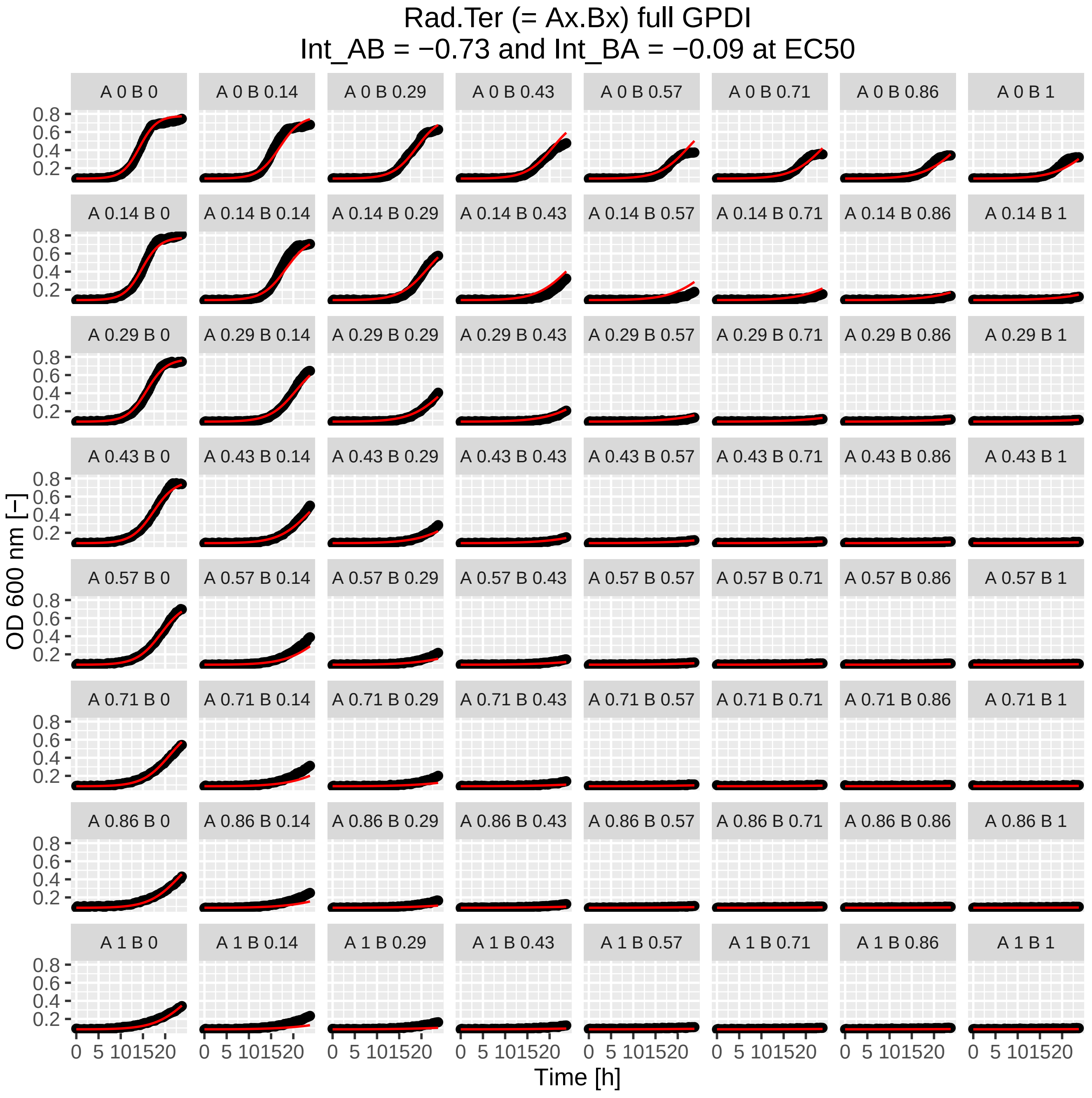
Rad.Ter (= Ax.Bx) full GPDI
Int_AB = −0.73 and Int_BA = −0.09 at EC50
A 0 B 0
A 0 B 0.14
A 0 B 0.29
A 0 B 0.43
A 0 B 0.57
A 0 B 0.71
A 0 B 0.86
A 0 B 1
A 0.14 B 0
A 0.14 B 0.14
A 0.14 B 0.29
A 0.14 B 0.43
A 0.14 B 0.57
A 0.14 B 0.71
A 0.14 B 0.86
A 0.14 B 1
A 0.29 B 0
A 0.29 B 0.14
A 0.29 B 0.29
A 0.29 B 0.43
A 0.29 B 0.57
A 0.29 B 0.71
A 0.29 B 0.86
A 0.29 B 1
A 0.43 B 0
A 0.43 B 0.14
A 0.43 B 0.29
A 0.43 B 0.43
A 0.43 B 0.57
A 0.43 B 0.71
A 0.43 B 0.86
A 0.43 B 1
A 0.57 B 0
A 0.57 B 0.14
A 0.57 B 0.29
A 0.57 B 0.43
A 0.57 B 0.57
A 0.57 B 0.71
A 0.57 B 0.86
A 0.57 B 1
A 0.71 B 0
A 0.71 B 0.14
A 0.71 B 0.29
A 0.71 B 0.43
A 0.71 B 0.57
A 0.71 B 0.71
A 0.71 B 0.86
A 0.71 B 1
A 0.86 B 0
A 0.86 B 0.14
A 0.86 B 0.29
A 0.86 B 0.43
A 0.86 B 0.57
A 0.86 B 0.71
A 0.86 B 0.86
A 0.86 B 1
A 1 B 0
A 1 B 0.14
A 1 B 0.29
A 1 B 0.43
A 1 B 0.57
A 1 B 0.71
A 1 B 0.86
A 1 B 1
OD 600 nm [−]
0.2
0.4
0.6
0.8
0 5 101520
Time [h]

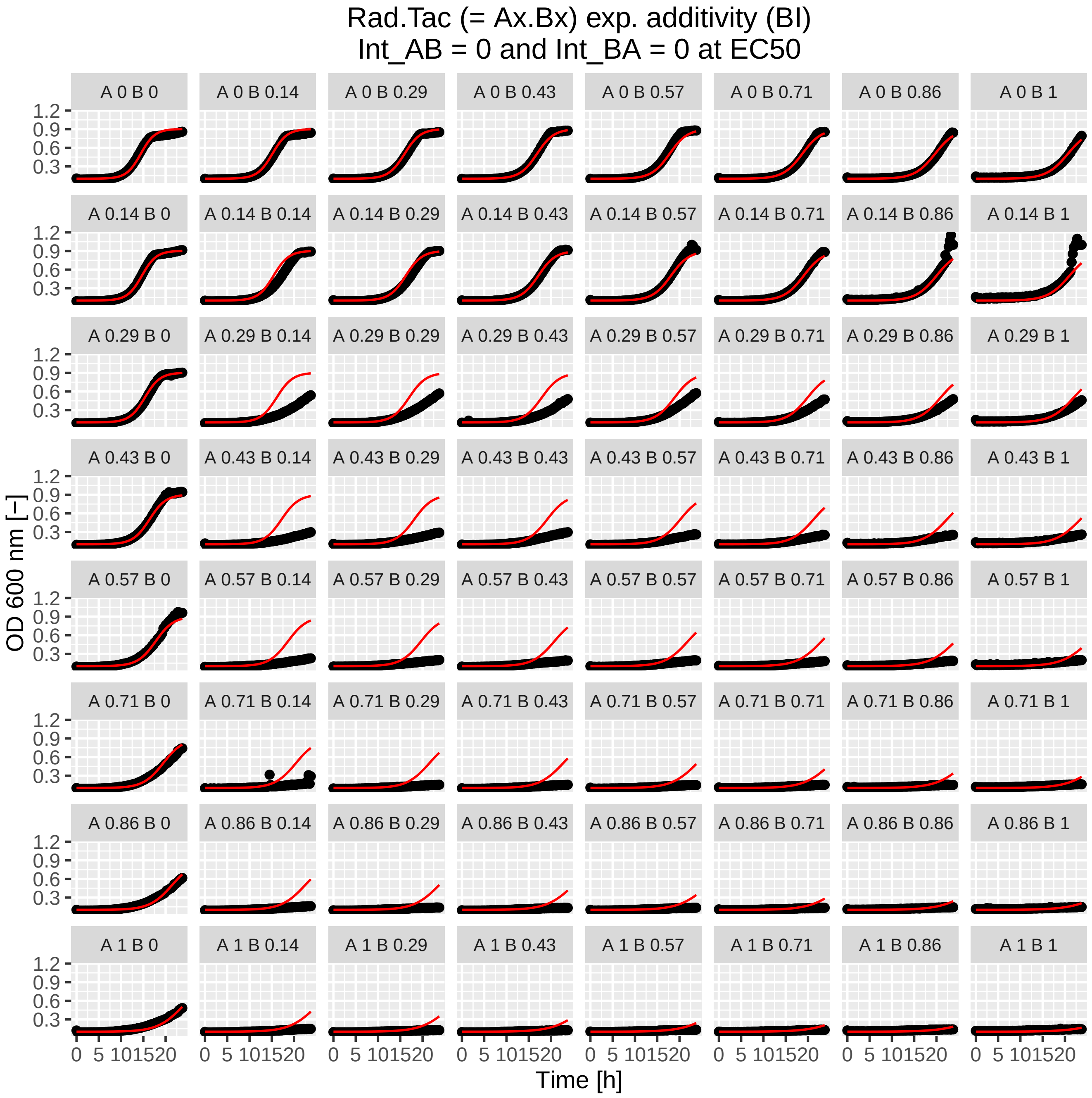

Rad.Tac (= Ax.Bx) exp. additivity (BI)
Int_AB = 0 and Int_BA = 0 at EC50
OD 600 nm [–]
Time [h]
A 0 B 0
A 0 B 0.14
A 0 B 0.29
A 0 B 0.43
A 0 B 0.57
A 0 B 0.71
A 0 B 0.86
A 0 B 1
A 1 B 0
A 1 B 1
0 5 101520
1.2
0.9
0.6
0.3

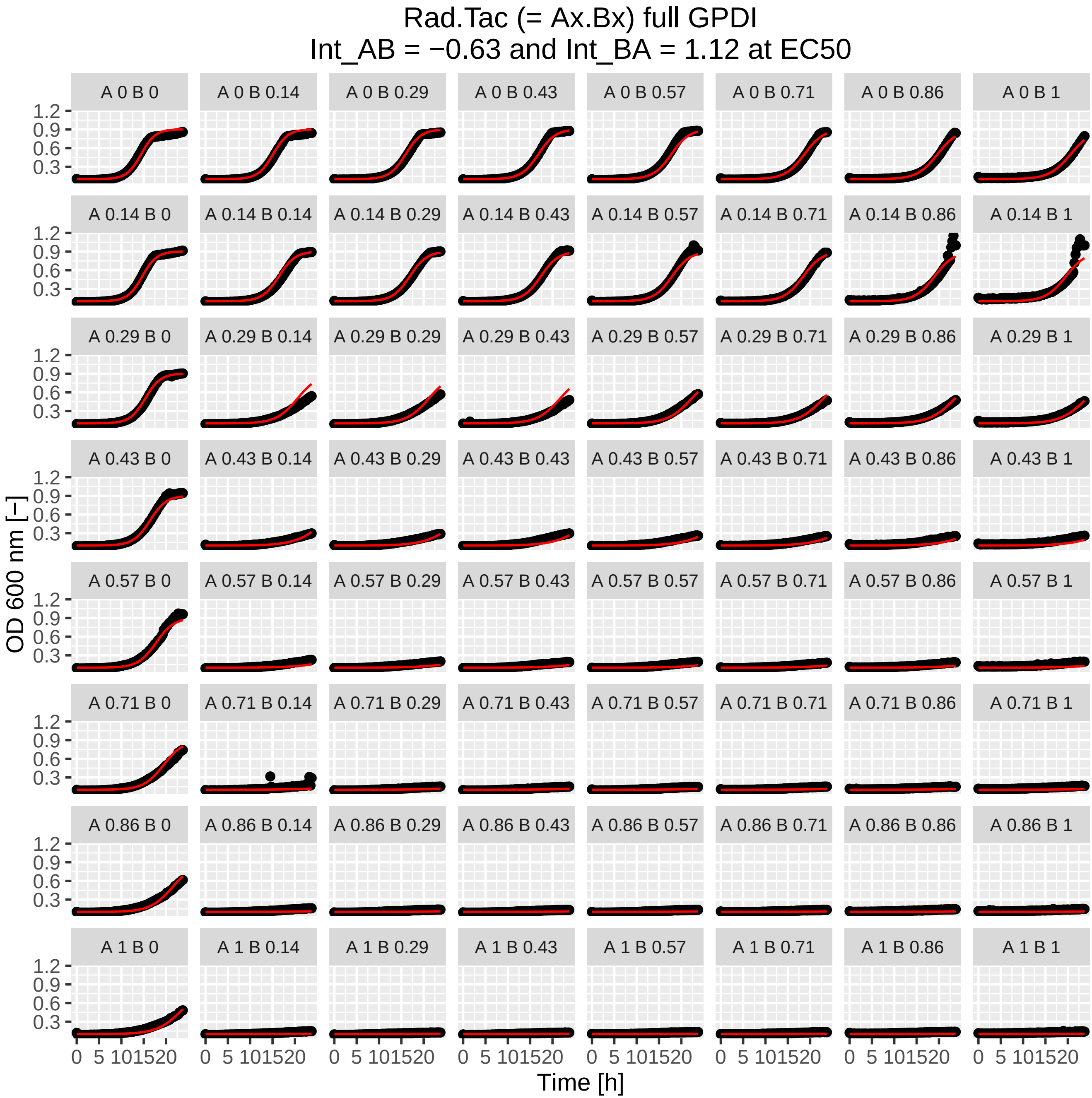

Rad.Tac (= Ax.Bx) full GPDI
Int_AB = −0.63 and Int_BA = 1.12 at EC50
A 0 B 0
A 0 B 0.14
A 0 B 0.29
A 0 B 0.43
A 0 B 0.57
A 0 B 0.71
A 0 B 0.86
A 0 B 1
A 0.14 B 0
A 0.14 B 0.14
A 0.14 B 0.29
A 0.14 B 0.43
A 0.14 B 0.57
A 0.14 B 0.71
A 0.14 B 0.86
A 0.14 B 1
A 0.29 B 0
A 0.29 B 0.14
A 0.29 B 0.29
A 0.29 B 0.43
A 0.29 B 0.57
A 0.29 B 0.71
A 0.29 B 0.86
A 0.29 B 1
A 0.43 B 0
A 0.43 B 0.14
A 0.43 B 0.29
A 0.43 B 0.43
A 0.43 B 0.57
A 0.43 B 0.71
A 0.43 B 0.86
A 0.43 B 1
A 0.57 B 0
A 0.57 B 0.14
A 0.57 B 0.29
A 0.57 B 0.43
A 0.57 B 0.57
A 0.57 B 0.71
A 0.57 B 0.86
A 0.57 B 1
A 0.71 B 0
A 0.71 B 0.14
A 0.71 B 0.29
A 0.71 B 0.43
A 0.71 B 0.57
A 0.71 B 0.71
A 0.71 B 0.86
A 0.71 B 1
A 0.86 B 0
A 0.86 B 0.14
A 0.86 B 0.29
A 0.86 B 0.43
A 0.86 B 0.57
A 0.86 B 0.71
A 0.86 B 0.86
A 0.86 B 1
A 1 B 0
A 1 B 0.14
A 1 B 0.29
A 1 B 0.43
A 1 B 0.57
A 1 B 0.71
A 1 B 0.86
A 1 B 1
1.2
0.9
0.6
0.3
OD 600 nm [−]
0 5 101520
Time [h]

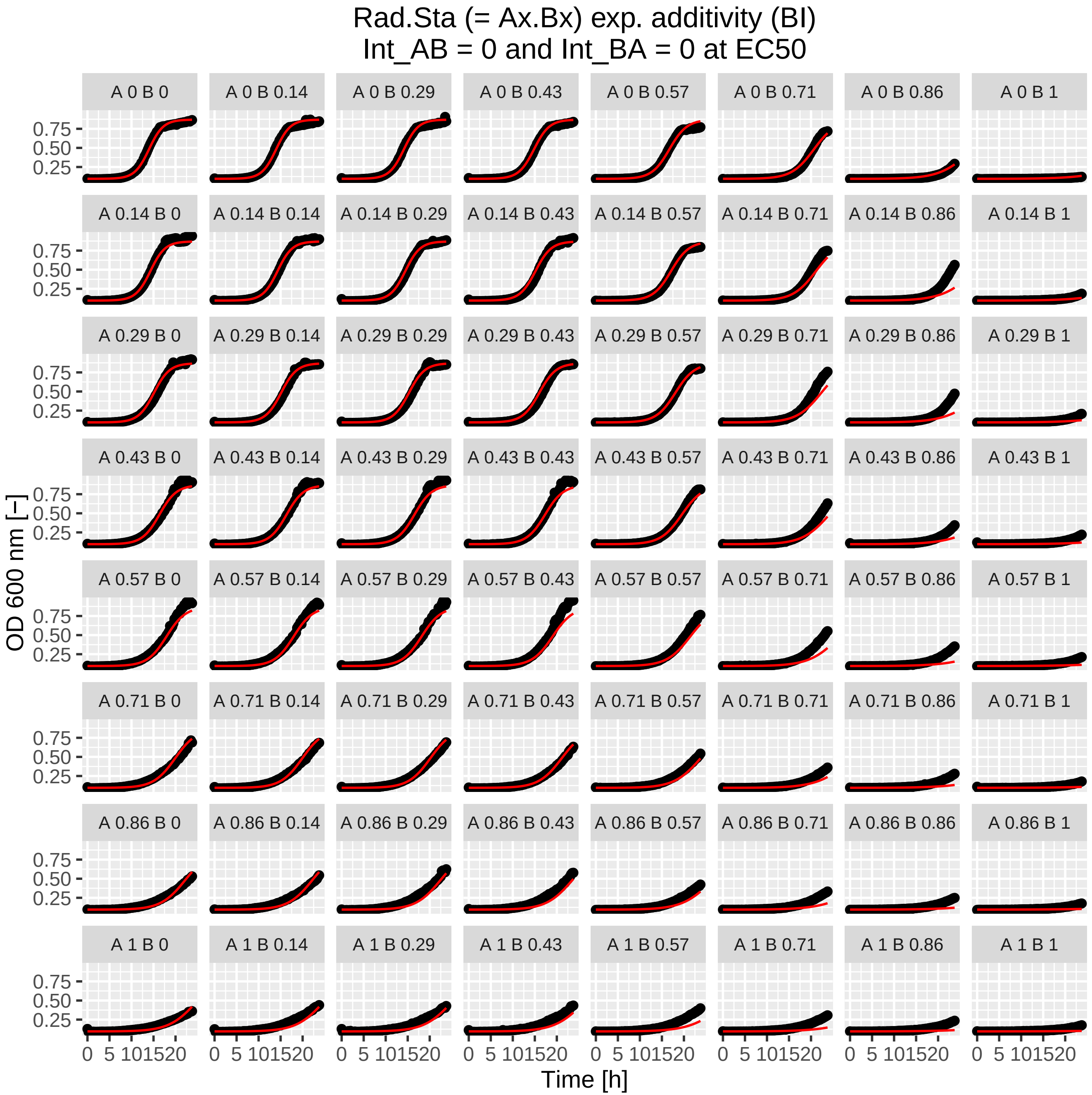

Rad.Sta (= Ax.Bx) exp. additivity (BI)
Int_AB = 0 and Int_BA = 0 at EC50
A 0 B 0
A 0 B 0.14
A 0 B 0.29
A 0 B 0.43
A 0 B 0.57
A 0 B 0.71
A 0 B 0.86
A 0 B 1
A 0.14 B 0
A 0.14 B 0.14
A 0.14 B 0.29
A 0.14 B 0.43
A 0.14 B 0.57
A 0.14 B 0.71
A 0.14 B 0.86
A 0.14 B 1
A 0.29 B 0
A 0.29 B 0.14
A 0.29 B 0.29
A 0.29 B 0.43
A 0.29 B 0.57
A 0.29 B 0.71
A 0.29 B 0.86
A 0.29 B 1
A 0.43 B 0
A 0.43 B 0.14
A 0.43 B 0.29
A 0.43 B 0.43
A 0.43 B 0.57
A 0.43 B 0.71
A 0.43 B 0.86
A 0.43 B 1
A 0.57 B 0
A 0.57 B 0.14
A 0.57 B 0.29
A 0.57 B 0.43
A 0.57 B 0.57
A 0.57 B 0.71
A 0.57 B 0.86
A 0.57 B 1
A 0.71 B 0
A 0.71 B 0.14
A 0.71 B 0.29
A 0.71 B 0.43
A 0.71 B 0.57
A 0.71 B 0.71
A 0.71 B 0.86
A 0.71 B 1
A 0.86 B 0
A 0.86 B 0.14
A 0.86 B 0.29
A 0.86 B 0.43
A 0.86 B 0.57
A 0.86 B 0.71
A 0.86 B 0.86
A 0.86 B 1
A 1 B 0
A 1 B 0.14
A 1 B 0.29
A 1 B 0.43
A 1 B 0.57
A 1 B 0.71
A 1 B 0.86
A 1 B 1
OD 600 nm [–]
0.75
0.50
0.25
0 5 101520
Time [h]

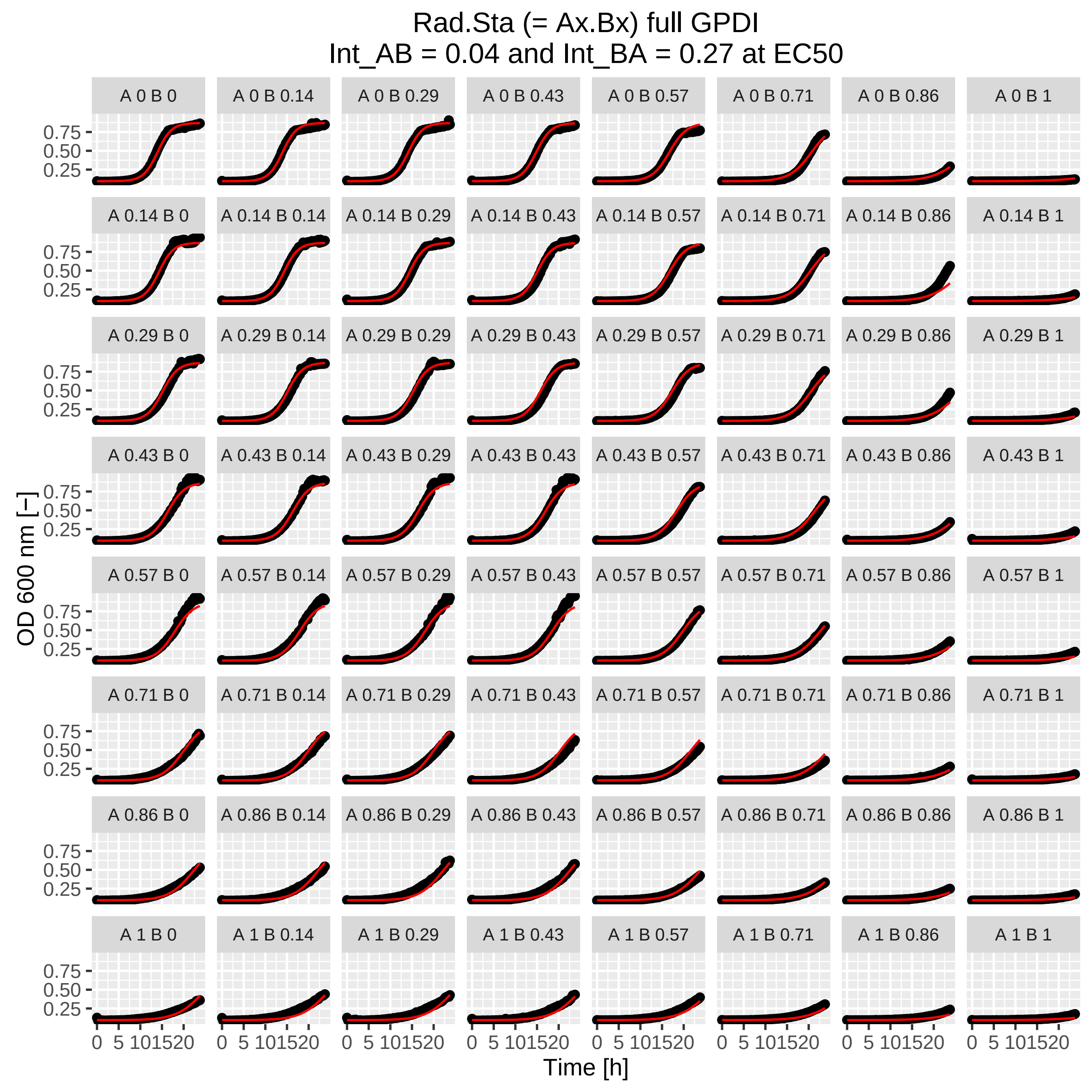
Rad.Sta (= Ax.Bx) full GPDI
Int_AB = 0.04 and Int_BA = 0.27 at EC50
A 0 B 0
A 0 B 0.14
A 0 B 0.29
A 0 B 0.43
A 0 B 0.57
A 0 B 0.71
A 0 B 0.86
A 0 B 1
A 0.14 B 0
A 0.14 B 0.14
A 0.14 B 0.29
A 0.14 B 0.43
A 0.14 B 0.57
A 0.14 B 0.71
A 0.14 B 0.86
A 0.14 B 1
A 0.29 B 0
A 0.29 B 0.14
A 0.29 B 0.29
A 0.29 B 0.43
A 0.29 B 0.57
A 0.29 B 0.71
A 0.29 B 0.86
A 0.29 B 1
A 0.43 B 0
A 0.43 B 0.14
A 0.43 B 0.29
A 0.43 B 0.43
A 0.43 B 0.57
A 0.43 B 0.71
A 0.43 B 0.86
A 0.43 B 1
A 0.57 B 0
A 0.57 B 0.14
A 0.57 B 0.29
A 0.57 B 0.43
A 0.57 B 0.57
A 0.57 B 0.71
A 0.57 B 0.86
A 0.57 B 1
A 0.71 B 0
A 0.71 B 0.14
A 0.71 B 0.29
A 0.71 B 0.43
A 0.71 B 0.57
A 0.71 B 0.71
A 0.71 B 0.86
A 0.71 B 1
A 0.86 B 0
A 0.86 B 0.14
A 0.86 B 0.29
A 0.86 B 0.43
A 0.86 B 0.57
A 0.86 B 0.71
A 0.86 B 0.86
A 0.86 B 1
A 1 B 0
A 1 B 0.14
A 1 B 0.29
A 1 B 0.43
A 1 B 0.57
A 1 B 0.71
A 1 B 0.86
A 1 B 1
OD 600 nm [–]
0.75
0.50
0.25
0 5 101520
Time [h]

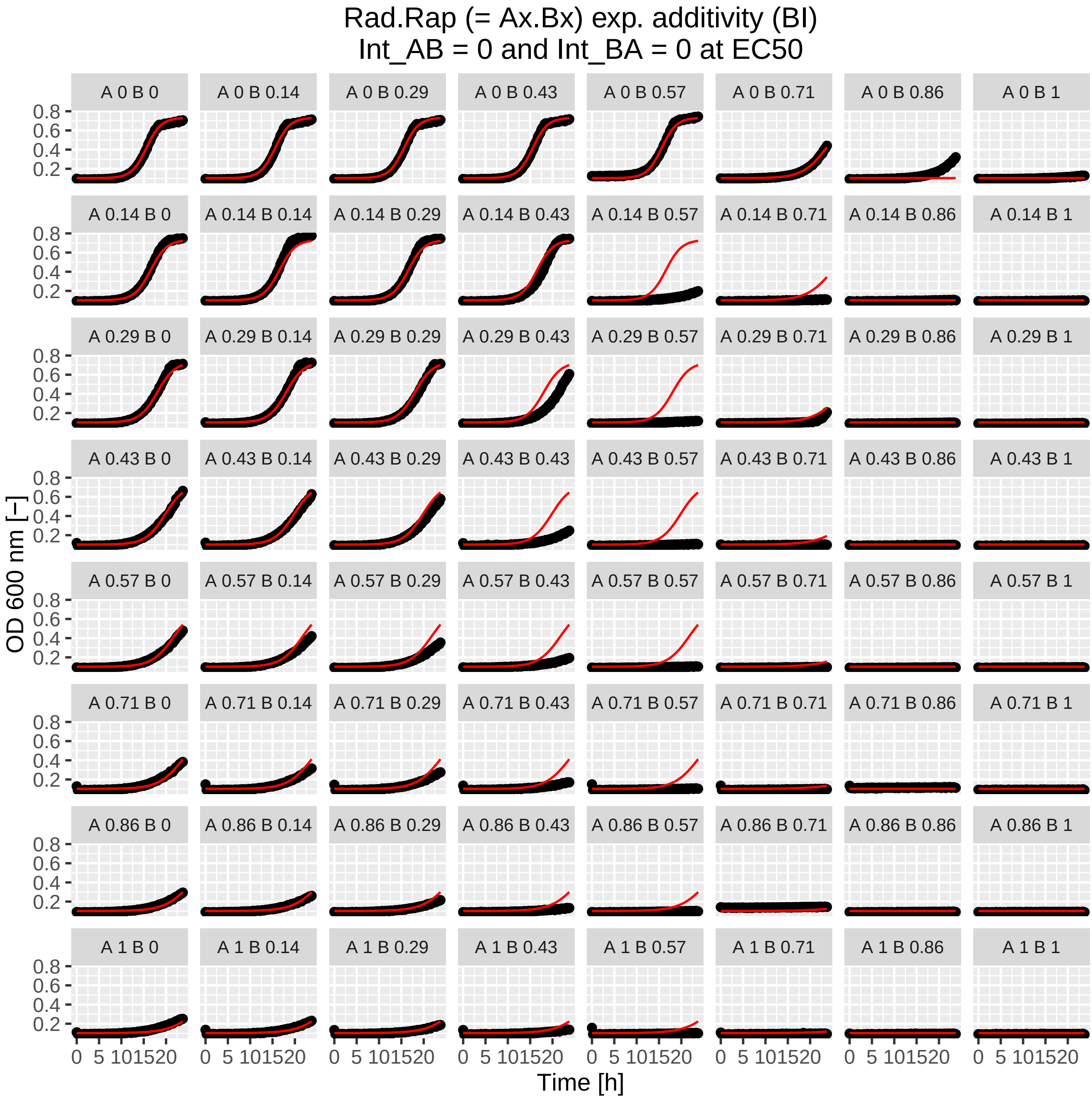

Rad.Rap (= Ax.Bx) exp. additivity (BI)
Int_AB = 0 and Int_BA = 0 at EC50
OD 600 nm [–]
Time [h]
A 0 B 0
A 0 B 0.14
A 0 B 0.29
A 0 B 0.43
A 0 B 0.57
A 0 B 0.71
A 0 B 0.86
A 0 B 1
A 0.14 B 0
A 0.14 B 0.14
A 0.14 B 0.29
A 0.14 B 0.43
A 0.14 B 0.57
A 0.14 B 0.71
A 0.14 B 0.86
A 0.14 B 1
A 0.29 B 0
A 0.29 B 0.14
A 0.29 B 0.29
A 0.29 B 0.43
A 0.29 B 0.57
A 0.29 B 0.71
A 0.29 B 0.86
A 0.29 B 1
A 0.43 B 0
A 0.43 B 0.14
A 0.43 B 0.29
A 0.43 B 0.43
A 0.43 B 0.57
A 0.43 B 0.71
A 0.43 B 0.86
A 0.43 B 1
A 0.57 B 0
A 0.57 B 0.14
A 0.57 B 0.29
A 0.57 B 0.43
A 0.57 B 0.57
A 0.57 B 0.71
A 0.57 B 0.86
A 0.57 B 1
A 0.71 B 0
A 0.71 B 0.14
A 0.71 B 0.29
A 0.71 B 0.43
A 0.71 B 0.57
A 0.71 B 0.71
A 0.71 B 0.86
A 0.71 B 1
A 0.86 B 0
A 0.86 B 0.14
A 0.86 B 0.29
A 0.86 B 0.43
A 0.86 B 0.57
A 0.86 B 0.71
A 0.86 B 0.86
A 0.86 B 1
A 1 B 0
A 1 B 0.14
A 1 B 0.29
A 1 B 0.43
A 1 B 0.57
A 1 B 0.71
A 1 B 0.86
A 1 B 1
0.2
0.4
0.6
0.8
0
5
10
15
20

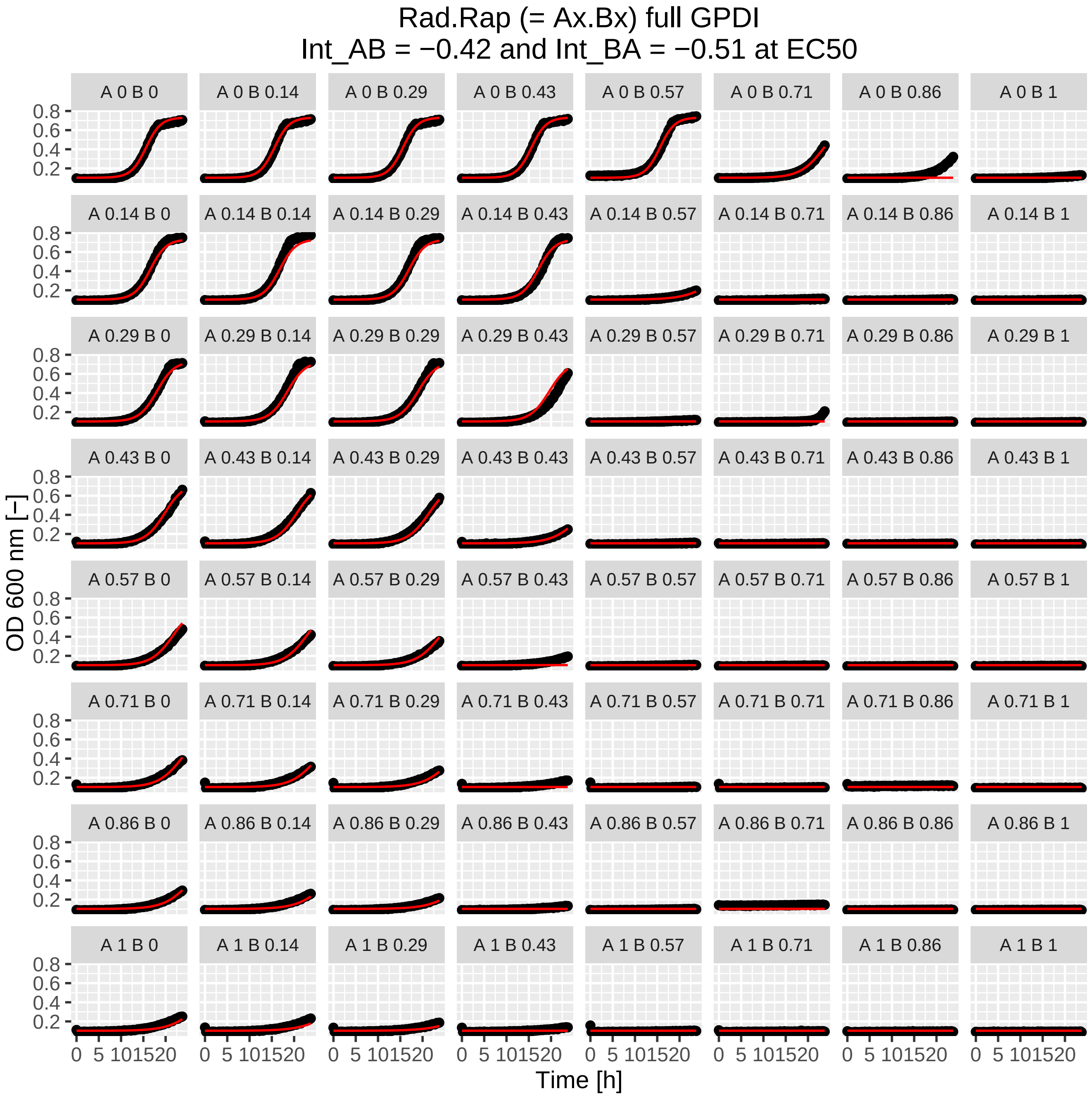
Rad.Rap (= Ax.Bx) full GPDI
Int_AB = −0.42 and Int_BA = −0.51 at EC50
OD 600 nm [−]
Time [h]
A 0 B 0
A 0 B 0.14
A 0 B 0.29
A 0 B 0.43
A 0 B 0.57
A 0 B 0.71
A 0 B 0.86
A 0 B 1
A 0.14 B 0
A 0.14 B 0.14
A 0.14 B 0.29
A 0.14 B 0.43
A 0.14 B 0.57
A 0.14 B 0.71
A 0.14 B 0.86
A 0.14 B 1
A 0.29 B 0
A 0.29 B 0.14
A 0.29 B 0.29
A 0.29 B 0.43
A 0.29 B 0.57
A 0.29 B 0.71
A 0.29 B 0.86
A 0.29 B 1
A 0.43 B 0
A 0.43 B 0.14
A 0.43 B 0.29
A 0.43 B 0.43
A 0.43 B 0.57
A 0.43 B 0.71
A 0.43 B 0.86
A 0.43 B 1
A 0.57 B 0
A 0.57 B 0.14
A 0.57 B 0.29
A 0.57 B 0.43
A 0.57 B 0.57
A 0.57 B 0.71
A 0.57 B 0.86
A 0.57 B 1
A 0.71 B 0
A 0.71 B 0.14
A 0.71 B 0.29
A 0.71 B 0.43
A 0.71 B 0.57
A 0.71 B 0.71
A 0.71 B 0.86
A 0.71 B 1
A 0.86 B 0
A 0.86 B 0.14
A 0.86 B 0.29
A 0.86 B 0.43
A 0.86 B 0.57
A 0.86 B 0.71
A 0.86 B 0.86
A 0.86 B 1
A 1 B 0
A 1 B 0.14
A 1 B 0.29
A 1 B 0.43
A 1 B 0.57
A 1 B 0.71
A 1 B 0.86
A 1 B 1
0.8
0.6
0.4
0.2
0
5
10
15
20

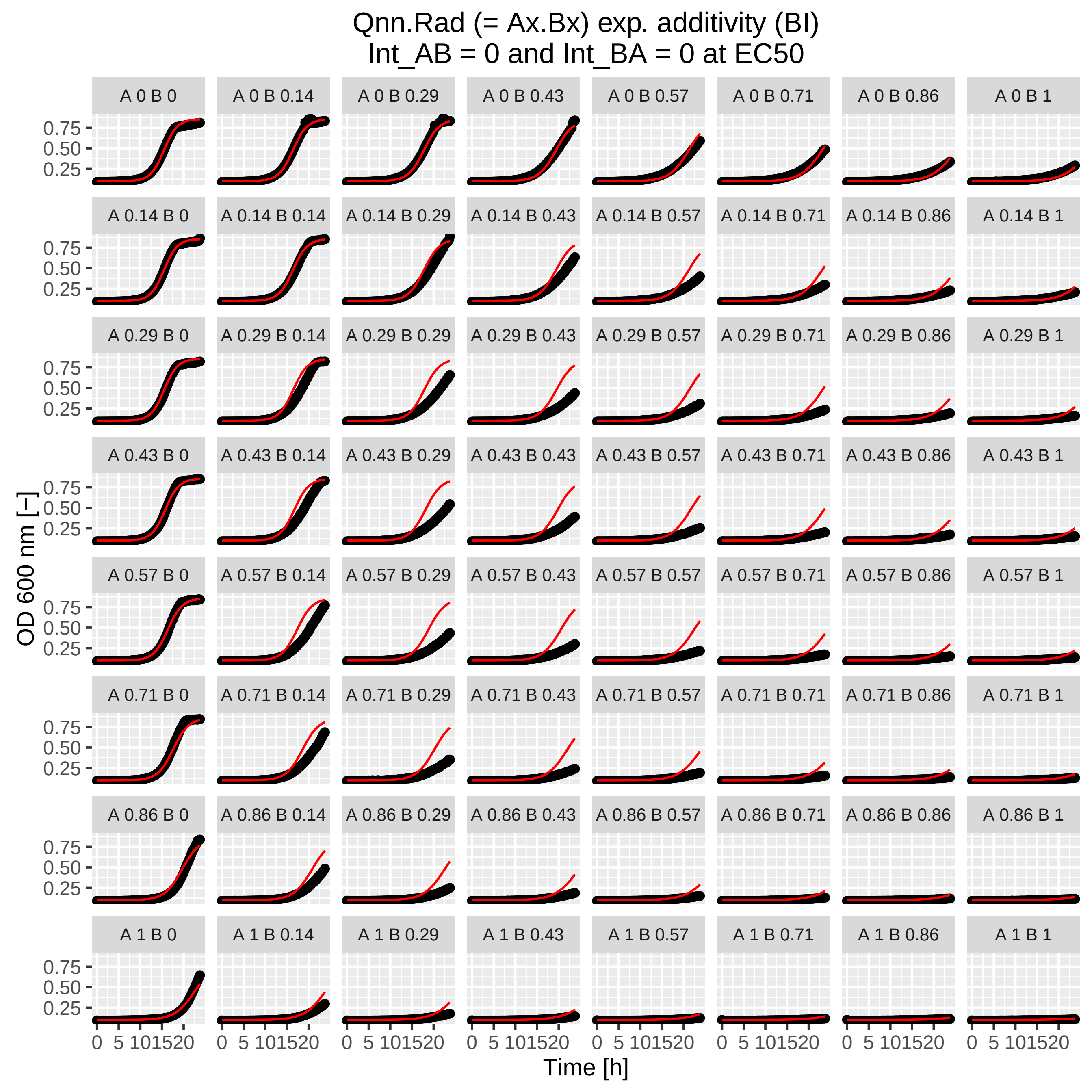
Qnn.Rad (= Ax.Bx) exp. additivity (BI)
Int_AB = 0 and Int_BA = 0 at EC50
OD 600 nm [–]
Time [h]
0.25
0.50
0.75
0 5 101520
A 0 B 0
A 0 B 0.14
A 0 B 0.29
A 0 B 0.43
A 0 B 0.57
A 0 B 0.71
A 0 B 0.86
A 0 B 1
A 0.14 B 0
A 0.14 B 0.14
A 0.14 B 0.29
A 0.14 B 0.43
A 0.14 B 0.57
A 0.14 B 0.71
A 0.14 B 0.86
A 0.14 B 1
A 0.29 B 0
A 0.29 B 0.14
A 0.29 B 0.29
A 0.29 B 0.43
A 0.29 B 0.57
A 0.29 B 0.71
A 0.29 B 0.86
A 0.29 B 1
A 0.43 B 0
A 0.43 B 0.14
A 0.43 B 0.29
A 0.43 B 0.43
A 0.43 B 0.57
A 0.43 B 0.71
A 0.43 B 0.86
A 0.43 B 1
A 0.57 B 0
A 0.57 B 0.14
A 0.57 B 0.29
A 0.57 B 0.43
A 0.57 B 0.57
A 0.57 B 0.71
A 0.57 B 0.86
A 0.57 B 1
A 0.71 B 0
A 0.71 B 0.14
A 0.71 B 0.29
A 0.71 B 0.43
A 0.71 B 0.57
A 0.71 B 0.71
A 0.71 B 0.86
A 0.71 B 1
A 0.86 B 0
A 0.86 B 0.14
A 0.86 B 0.29
A 0.86 B 0.43
A 0.86 B 0.57
A 0.86 B 0.71
A 0.86 B 0.86
A 0.86 B 1
A 1 B 0
A 1 B 0.14
A 1 B 0.29
A 1 B 0.43
A 1 B 0.57
A 1 B 0.71
A 1 B 0.86
A 1 B 1

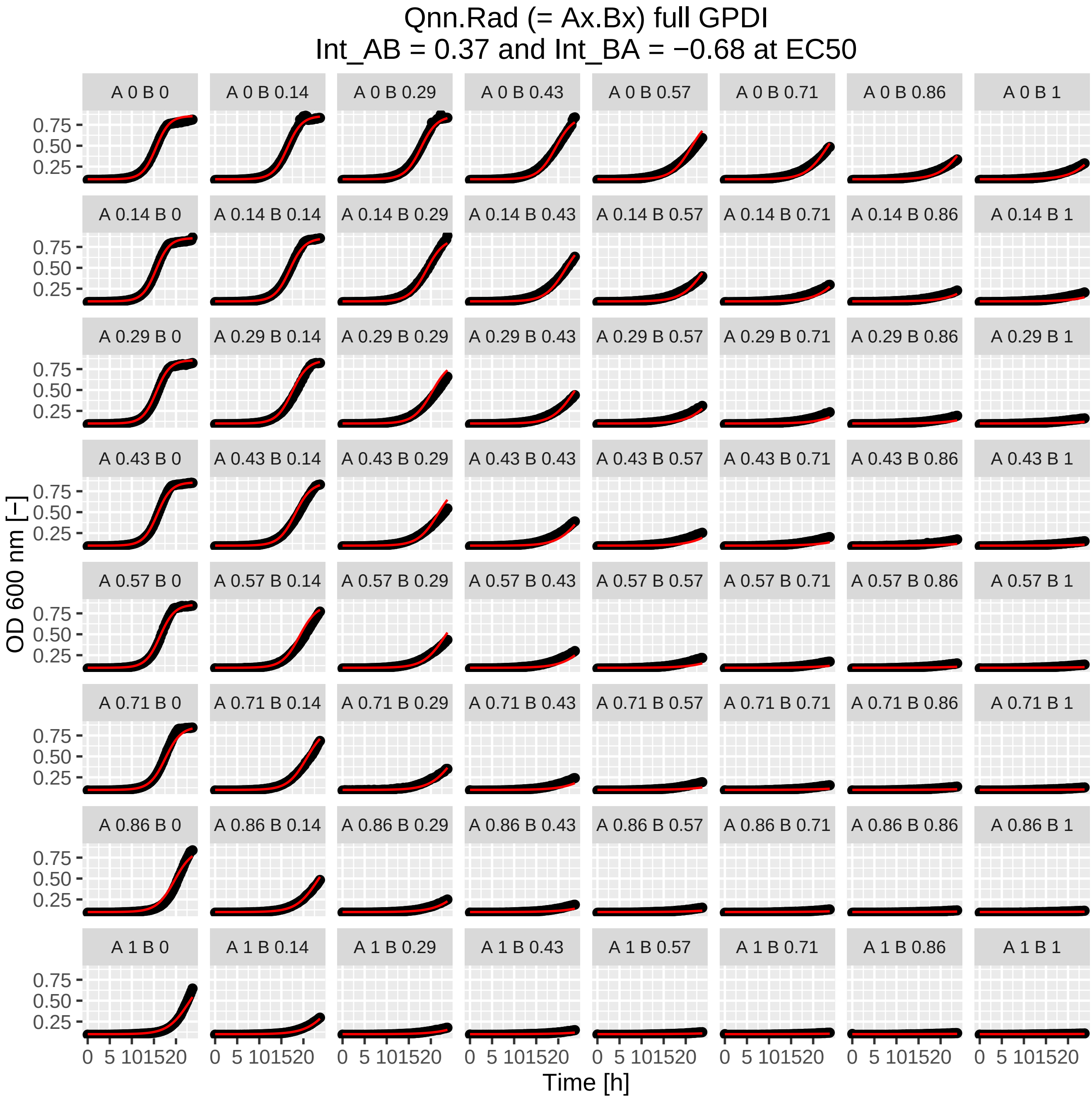
Qnn.Rad (= Ax.Bx) full GPDI
Int_AB = 0.37 and Int_BA = −0.68 at EC50
A 0 B 0
A 0 B 0.14
A 0 B 0.29
A 0 B 0.43
A 0 B 0.57
A 0 B 0.71
A 0 B 0.86
A 0 B 1
A 0.14 B 0
A 0.14 B 0.14
A 0.14 B 0.29
A 0.14 B 0.43
A 0.14 B 0.57
A 0.14 B 0.71
A 0.14 B 0.86
A 0.14 B 1
A 0.29 B 0
A 0.29 B 0.14
A 0.29 B 0.29
A 0.29 B 0.43
A 0.29 B 0.57
A 0.29 B 0.71
A 0.29 B 0.86
A 0.29 B 1
A 0.43 B 0
A 0.43 B 0.14
A 0.43 B 0.29
A 0.43 B 0.43
A 0.43 B 0.57
A 0.43 B 0.71
A 0.43 B 0.86
A 0.43 B 1
A 0.57 B 0
A 0.57 B 0.14
A 0.57 B 0.29
A 0.57 B 0.43
A 0.57 B 0.57
A 0.57 B 0.71
A 0.57 B 0.86
A 0.57 B 1
A 0.71 B 0
A 0.71 B 0.14
A 0.71 B 0.29
A 0.71 B 0.43
A 0.71 B 0.57
A 0.71 B 0.71
A 0.71 B 0.86
A 0.71 B 1
A 0.86 B 0
A 0.86 B 0.14
A 0.86 B 0.29
A 0.86 B 0.43
A 0.86 B 0.57
A 0.86 B 0.71
A 0.86 B 0.86
A 0.86 B 1
A 1 B 0
A 1 B 0.14
A 1 B 0.29
A 1 B 0.43
A 1 B 0.57
A 1 B 0.71
A 1 B 0.86
A 1 B 1
0.75
0.50
0.25
OD 600 nm [−]
0 5 101520
Time [h]

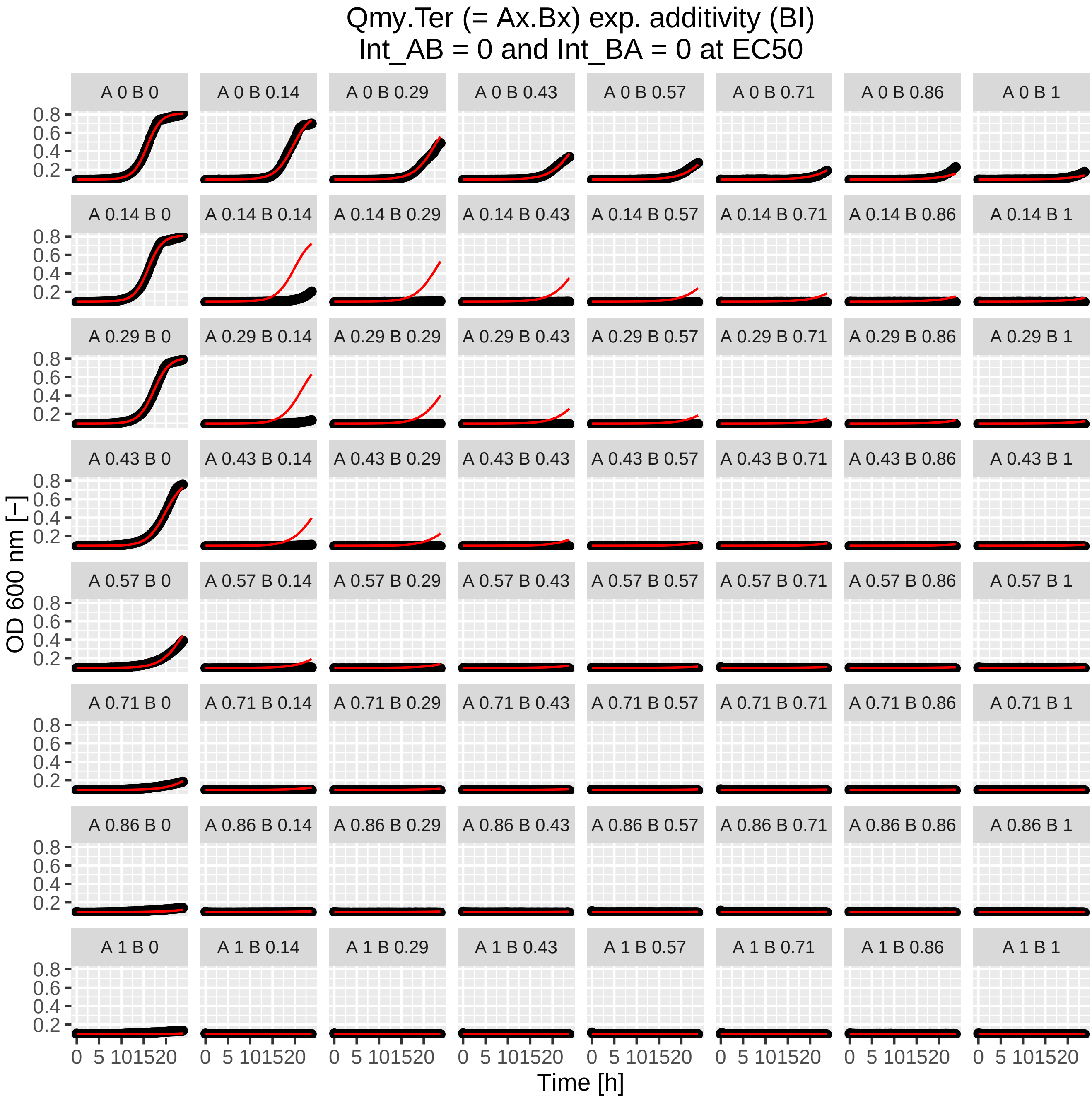
Qmy.Ter (= Ax.Bx) exp. additivity (BI)
Int_AB = 0 and Int_BA = 0 at EC50
OD 600 nm [–]
Time [h]
A 0 B 0
A 0 B 0.14
A 0 B 0.29
A 0 B 0.43
A 0 B 0.57
A 0 B 0.71
A 0 B 0.86
A 0 B 1
A 1 B 0
A 1 B 1
0.8
0.6
0.4
0.2
0 5 101520

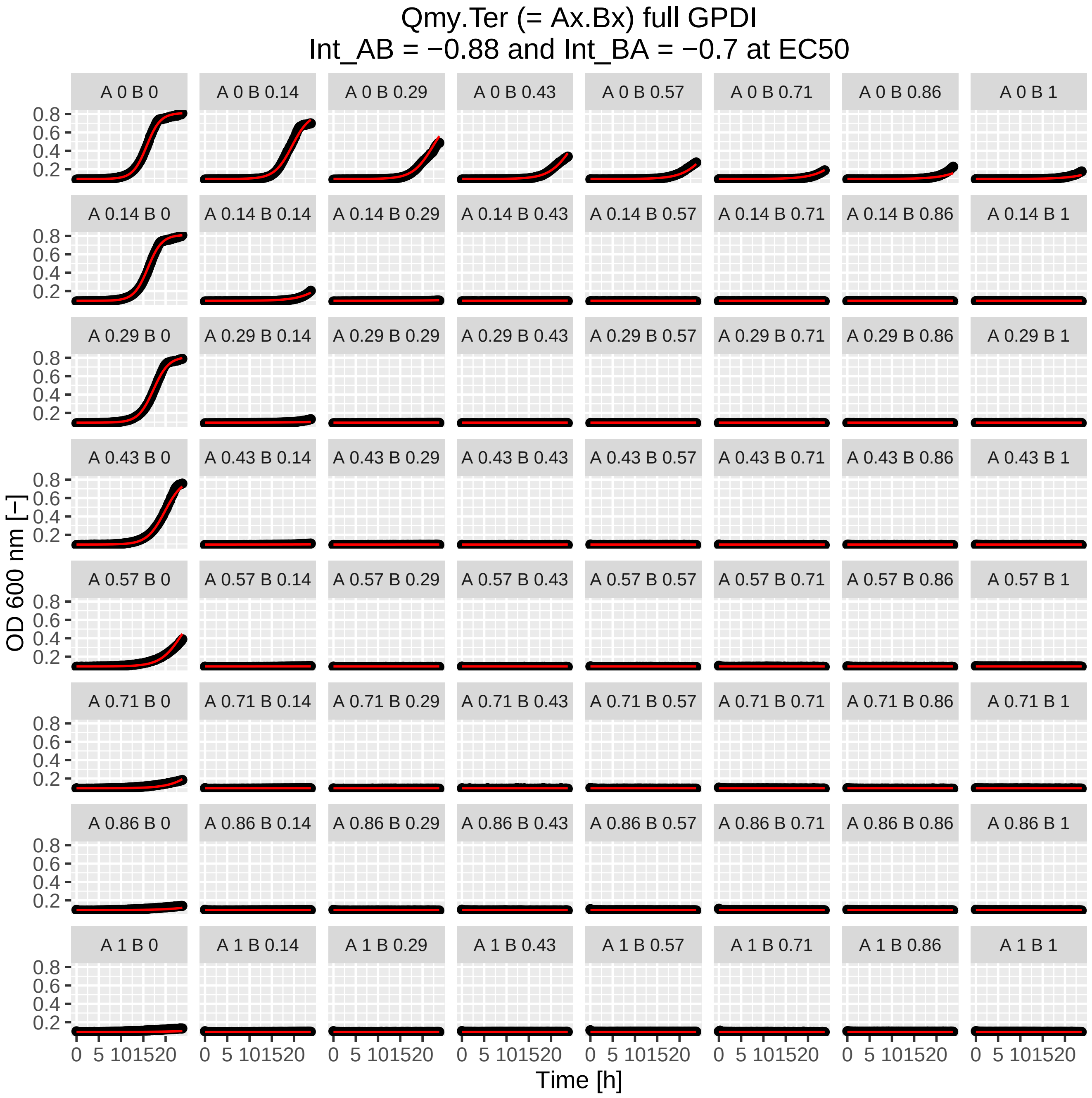

Qmy.Ter (= Ax.Bx) full GPDI
Int_AB = −0.88 and Int_BA = −0.7 at EC50
OD 600 nm [−]
Time [h]
A 0 B 0
A 0 B 0.14
A 0 B 0.29
A 0 B 0.43
A 0 B 0.57
A 0 B 0.71
A 0 B 0.86
A 0 B 1
A 1 B 0
A 1 B 1

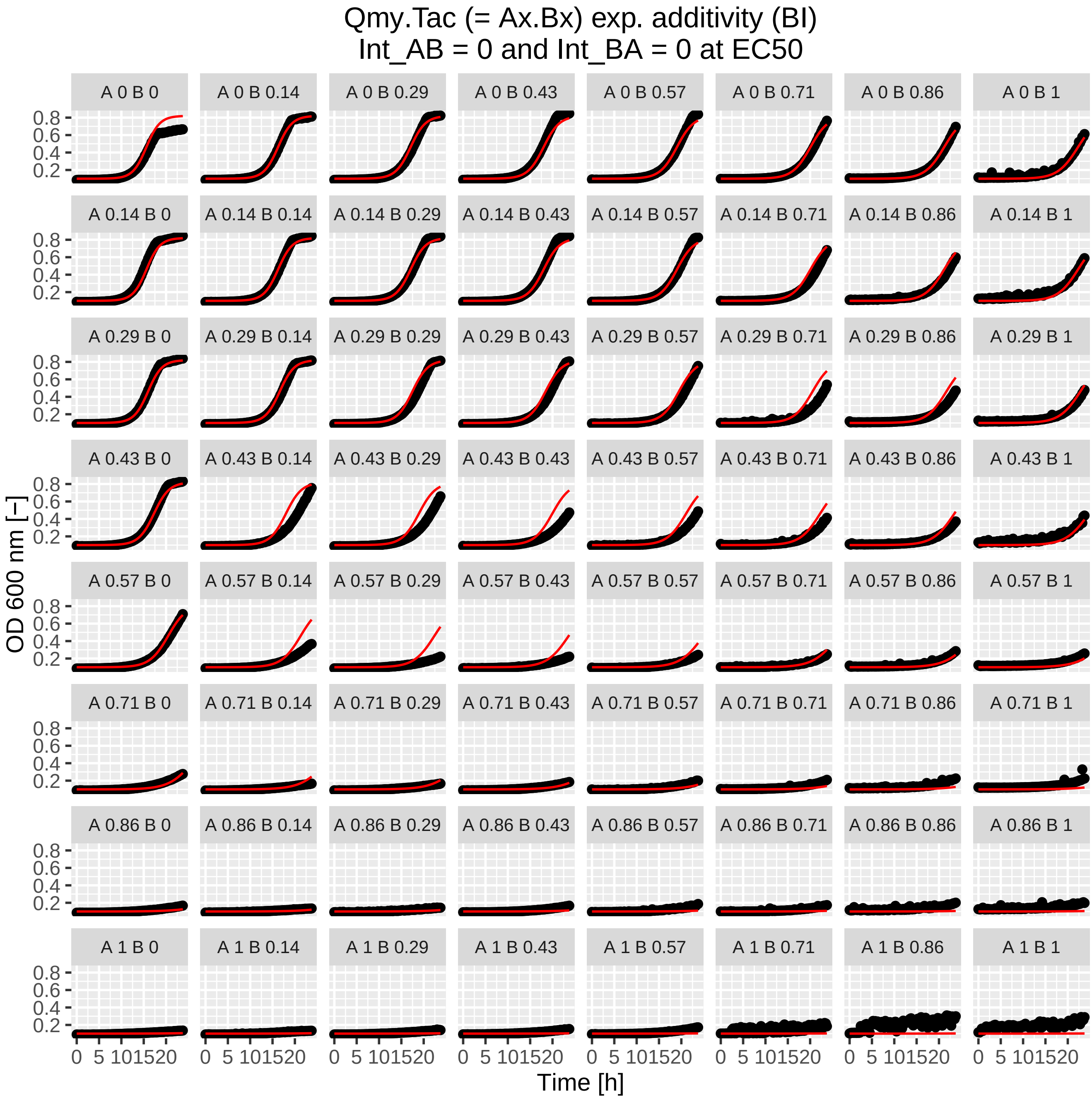

Qmy.Tac (= Ax.Bx) exp. additivity (BI)
Int_AB = 0 and Int_BA = 0 at EC50
A 0 B 0
A 0 B 0.14
A 0 B 0.29
A 0 B 0.43
A 0 B 0.57
A 0 B 0.71
A 0 B 0.86
A 0 B 1
A 0.14 B 0
A 0.14 B 0.14
A 0.14 B 0.29
A 0.14 B 0.43
A 0.14 B 0.57
A 0.14 B 0.71
A 0.14 B 0.86
A 0.14 B 1
A 0.29 B 0
A 0.29 B 0.14
A 0.29 B 0.29
A 0.29 B 0.43
A 0.29 B 0.57
A 0.29 B 0.71
A 0.29 B 0.86
A 0.29 B 1
A 0.43 B 0
A 0.43 B 0.14
A 0.43 B 0.29
A 0.43 B 0.43
A 0.43 B 0.57
A 0.43 B 0.71
A 0.43 B 0.86
A 0.43 B 1
A 0.57 B 0
A 0.57 B 0.14
A 0.57 B 0.29
A 0.57 B 0.43
A 0.57 B 0.57
A 0.57 B 0.71
A 0.57 B 0.86
A 0.57 B 1
A 0.71 B 0
A 0.71 B 0.14
A 0.71 B 0.29
A 0.71 B 0.43
A 0.71 B 0.57
A 0.71 B 0.71
A 0.71 B 0.86
A 0.71 B 1
A 0.86 B 0
A 0.86 B 0.14
A 0.86 B 0.29
A 0.86 B 0.43
A 0.86 B 0.57
A 0.86 B 0.71
A 0.86 B 0.86
A 0.86 B 1
A 1 B 0
A 1 B 0.14
A 1 B 0.29
A 1 B 0.43
A 1 B 0.57
A 1 B 0.71
A 1 B 0.86
A 1 B 1
OD 600 nm [–]
0.8
0.6
0.4
0.2
0 5 101520
Time [h]

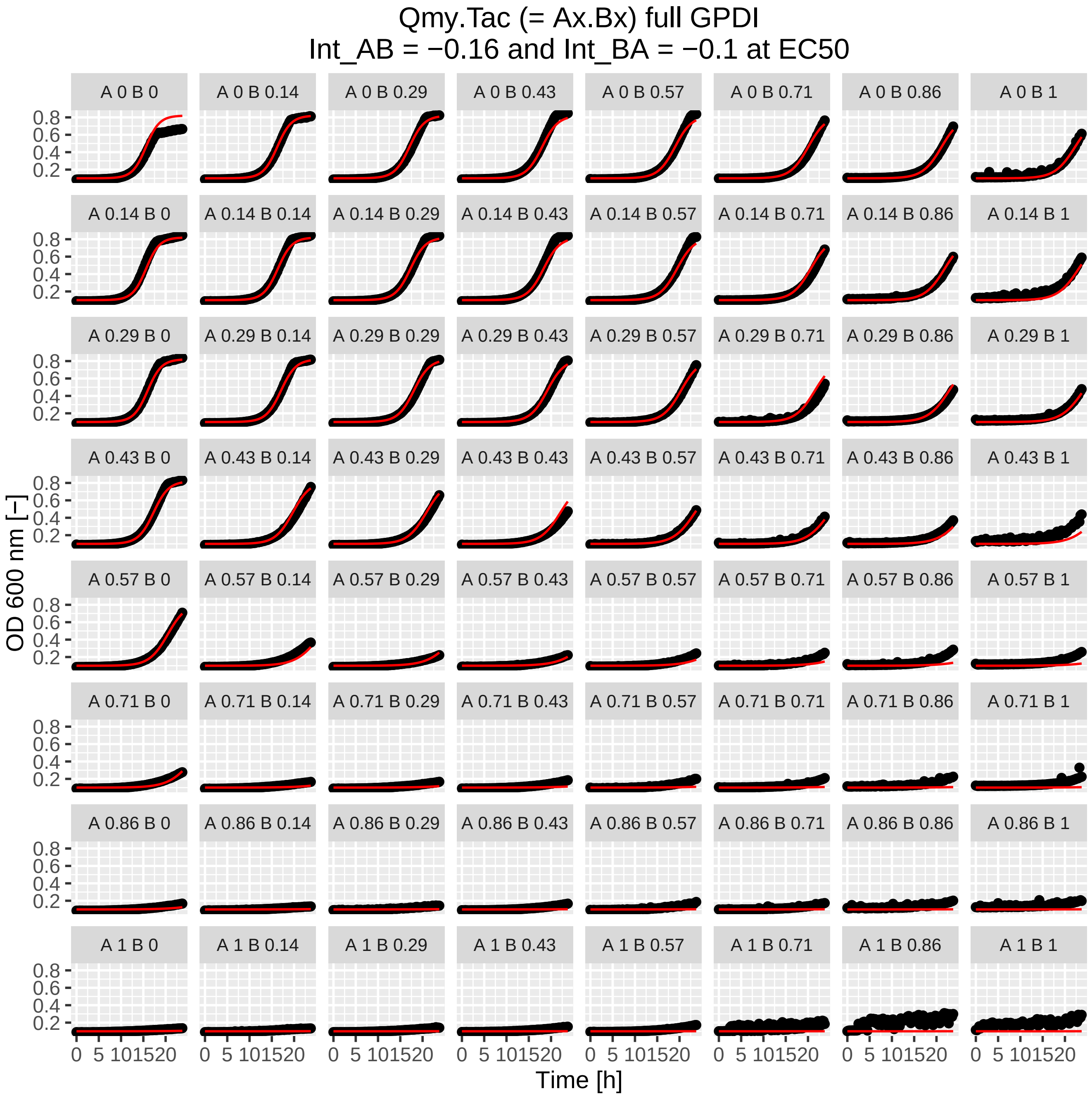

Qmy.Tac (= Ax.Bx) full GPDI
Int_AB = −0.16 and Int_BA = −0.1 at EC50
A 0 B 0
A 0 B 0.14
A 0 B 0.29
A 0 B 0.43
A 0 B 0.57
A 0 B 0.71
A 0 B 0.86
A 0 B 1
A 0.14 B 0
A 0.14 B 0.14
A 0.14 B 0.29
A 0.14 B 0.43
A 0.14 B 0.57
A 0.14 B 0.71
A 0.14 B 0.86
A 0.14 B 1
A 0.29 B 0
A 0.29 B 0.14
A 0.29 B 0.29
A 0.29 B 0.43
A 0.29 B 0.57
A 0.29 B 0.71
A 0.29 B 0.86
A 0.29 B 1
A 0.43 B 0
A 0.43 B 0.14
A 0.43 B 0.29
A 0.43 B 0.43
A 0.43 B 0.57
A 0.43 B 0.71
A 0.43 B 0.86
A 0.43 B 1
A 0.57 B 0
A 0.57 B 0.14
A 0.57 B 0.29
A 0.57 B 0.43
A 0.57 B 0.57
A 0.57 B 0.71
A 0.57 B 0.86
A 0.57 B 1
A 0.71 B 0
A 0.71 B 0.14
A 0.71 B 0.29
A 0.71 B 0.43
A 0.71 B 0.57
A 0.71 B 0.71
A 0.71 B 0.86
A 0.71 B 1
A 0.86 B 0
A 0.86 B 0.14
A 0.86 B 0.29
A 0.86 B 0.43
A 0.86 B 0.57
A 0.86 B 0.71
A 0.86 B 0.86
A 0.86 B 1
A 1 B 0
A 1 B 0.14
A 1 B 0.29
A 1 B 0.43
A 1 B 0.57
A 1 B 0.71
A 1 B 0.86
A 1 B 1
OD 600 nm [−]
0.2
0.4
0.6
0.8
0
5
10
15
20
Time [h]

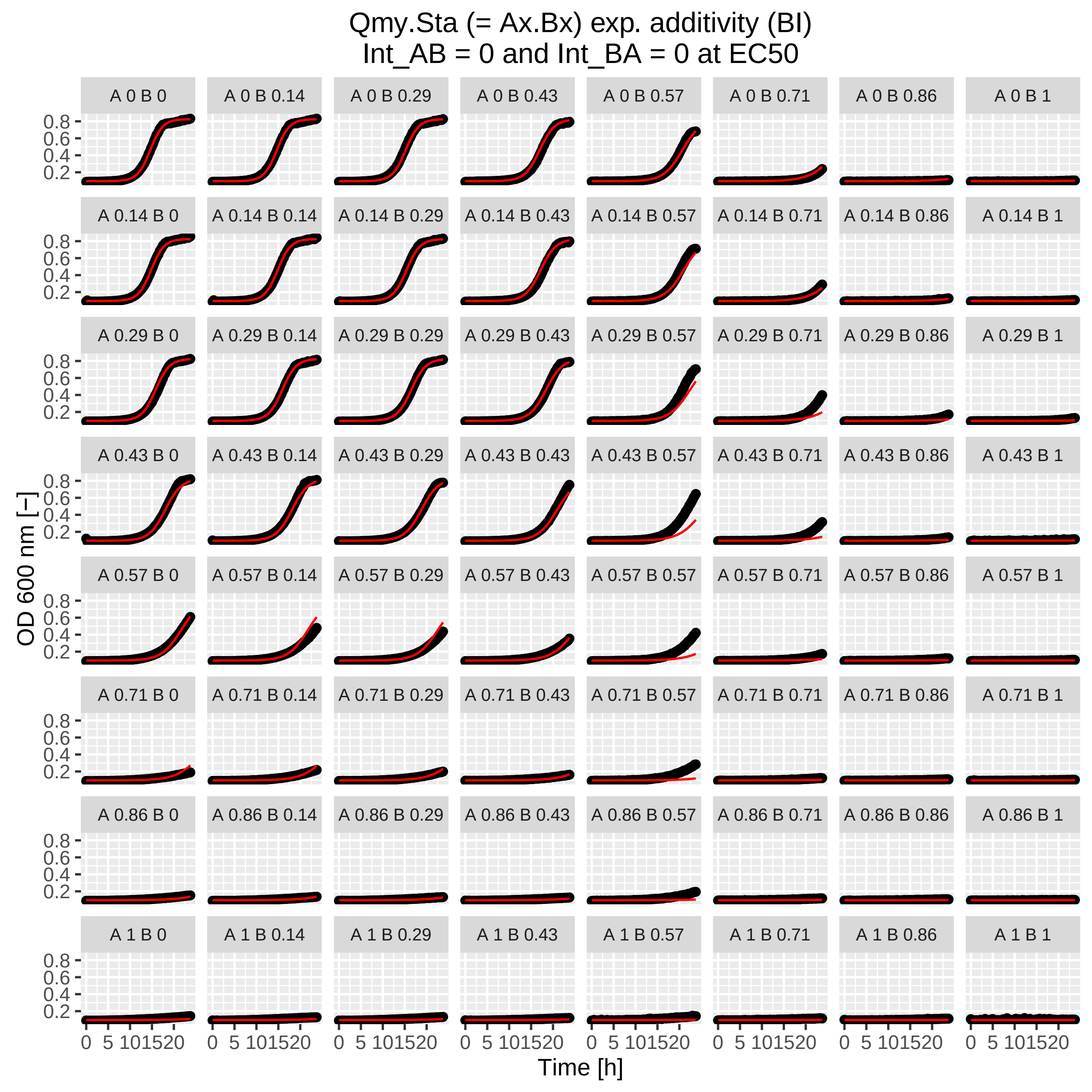
Qmy.Sta (= Ax.Bx) exp. additivity (BI)
Int_AB = 0 and Int_BA = 0 at EC50
OD 600 nm [−]
Time [h]

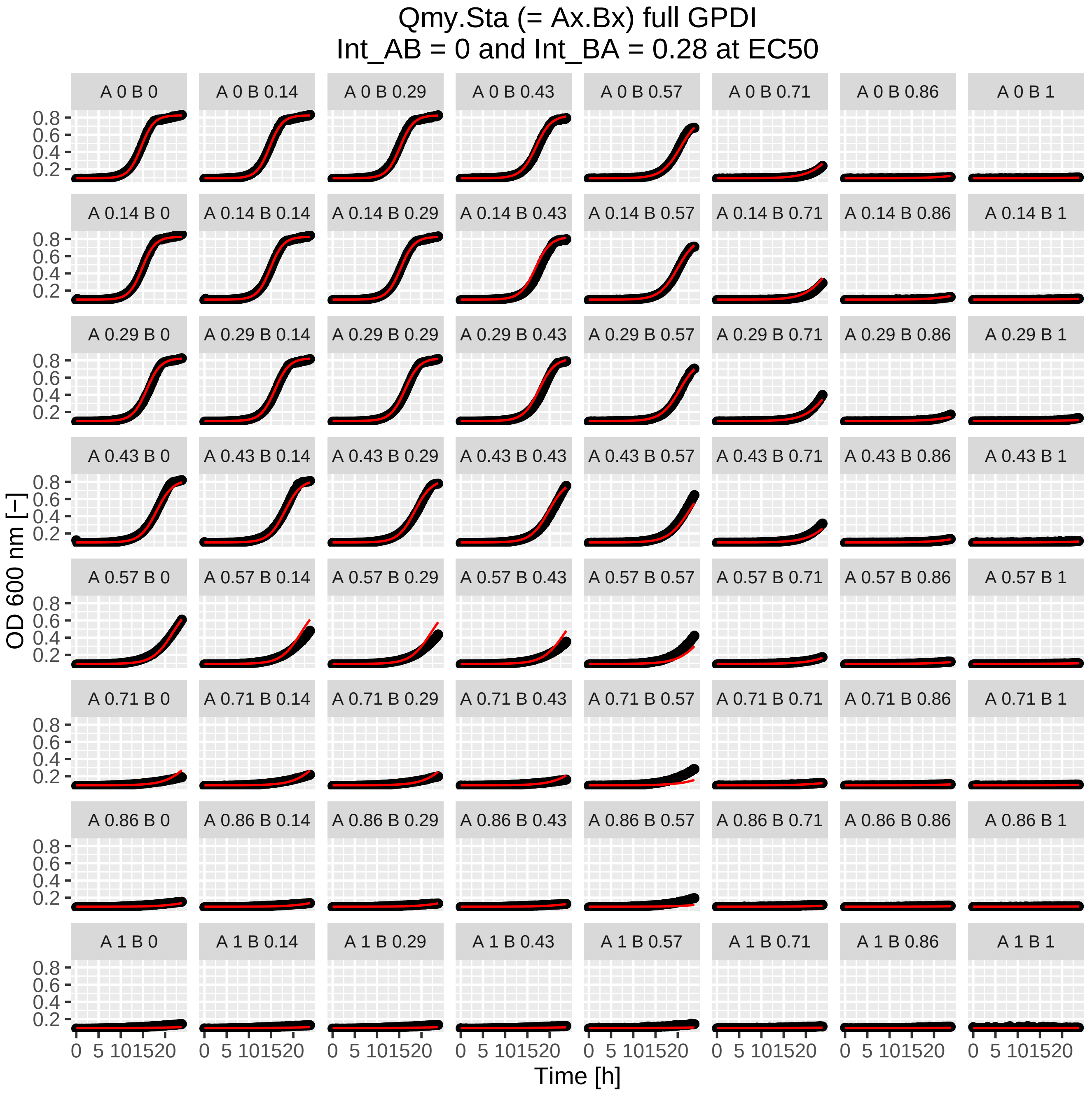

Qmy.Sta (= Ax.Bx) full GPDI
Int_AB = 0 and Int_BA = 0.28 at EC50
OD 600 nm [–]
Time [h]

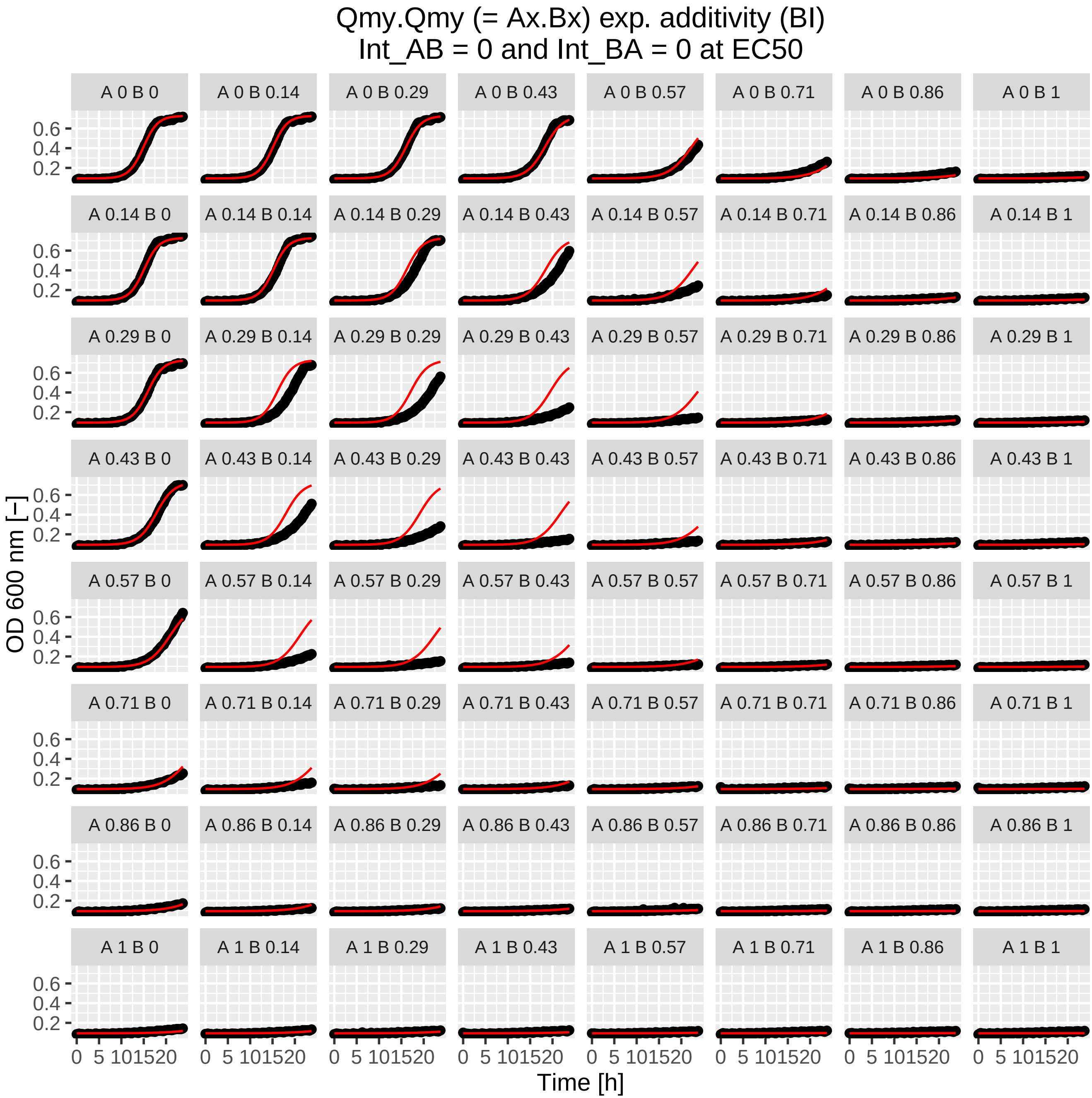

Qmy.Qmy (= Ax.Bx) exp. additivity (BI)
Int_AB = 0 and Int_BA = 0 at EC50
A 0 B 0
A 0 B 0.14
A 0 B 0.29
A 0 B 0.43
A 0 B 0.57
A 0 B 0.71
A 0 B 0.86
A 0 B 1
A 0.14 B 0
A 0.14 B 0.14
A 0.14 B 0.29
A 0.14 B 0.43
A 0.14 B 0.57
A 0.14 B 0.71
A 0.14 B 0.86
A 0.14 B 1
A 0.29 B 0
A 0.29 B 0.14
A 0.29 B 0.29
A 0.29 B 0.43
A 0.29 B 0.57
A 0.29 B 0.71
A 0.29 B 0.86
A 0.29 B 1
A 0.43 B 0
A 0.43 B 0.14
A 0.43 B 0.29
A 0.43 B 0.43
A 0.43 B 0.57
A 0.43 B 0.71
A 0.43 B 0.86
A 0.43 B 1
A 0.57 B 0
A 0.57 B 0.14
A 0.57 B 0.29
A 0.57 B 0.43
A 0.57 B 0.57
A 0.57 B 0.71
A 0.57 B 0.86
A 0.57 B 1
A 0.71 B 0
A 0.71 B 0.14
A 0.71 B 0.29
A 0.71 B 0.43
A 0.71 B 0.57
A 0.71 B 0.71
A 0.71 B 0.86
A 0.71 B 1
A 0.86 B 0
A 0.86 B 0.14
A 0.86 B 0.29
A 0.86 B 0.43
A 0.86 B 0.57
A 0.86 B 0.71
A 0.86 B 0.86
A 0.86 B 1
A 1 B 0
A 1 B 0.14
A 1 B 0.29
A 1 B 0.43
A 1 B 0.57
A 1 B 0.71
A 1 B 0.86
A 1 B 1
OD 600 nm [–]
0.2
0.4
0.6
0 5 101520
Time [h]

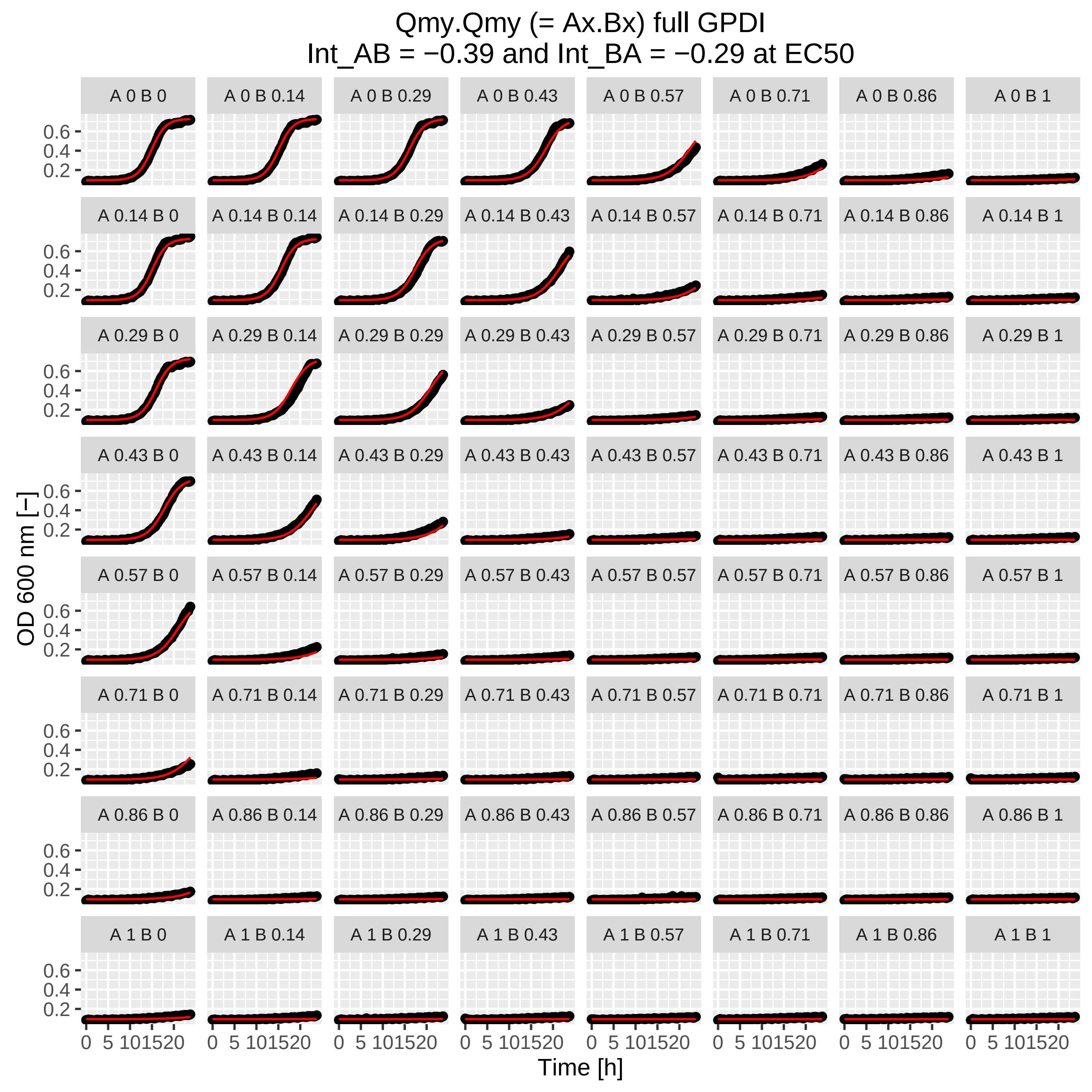

Qmy.Qmy (= Ax.Bx) full GPDI
Int_AB = −0.39 and Int_BA = −0.29 at EC50
A 0 B 0
A 0 B 0.14
A 0 B 0.29
A 0 B 0.43
A 0 B 0.57
A 0 B 0.71
A 0 B 0.86
A 0 B 1
A 0.14 B 0
A 0.14 B 0.14
A 0.14 B 0.29
A 0.14 B 0.43
A 0.14 B 0.57
A 0.14 B 0.71
A 0.14 B 0.86
A 0.14 B 1
A 0.29 B 0
A 0.29 B 0.14
A 0.29 B 0.29
A 0.29 B 0.43
A 0.29 B 0.57
A 0.29 B 0.71
A 0.29 B 0.86
A 0.29 B 1
A 0.43 B 0
A 0.43 B 0.14
A 0.43 B 0.29
A 0.43 B 0.43
A 0.43 B 0.57
A 0.43 B 0.71
A 0.43 B 0.86
A 0.43 B 1
A 0.57 B 0
A 0.57 B 0.14
A 0.57 B 0.29
A 0.57 B 0.43
A 0.57 B 0.57
A 0.57 B 0.71
A 0.57 B 0.86
A 0.57 B 1
A 0.71 B 0
A 0.71 B 0.14
A 0.71 B 0.29
A 0.71 B 0.43
A 0.71 B 0.57
A 0.71 B 0.71
A 0.71 B 0.86
A 0.71 B 1
A 0.86 B 0
A 0.86 B 0.14
A 0.86 B 0.29
A 0.86 B 0.43
A 0.86 B 0.57
A 0.86 B 0.71
A 0.86 B 0.86
A 0.86 B 1
A 1 B 0
A 1 B 0.14
A 1 B 0.29
A 1 B 0.43
A 1 B 0.57
A 1 B 0.71
A 1 B 0.86
A 1 B 1
OD 600 nm [−]
0.6
0.4
0.2
Time [h]
0 5 101520

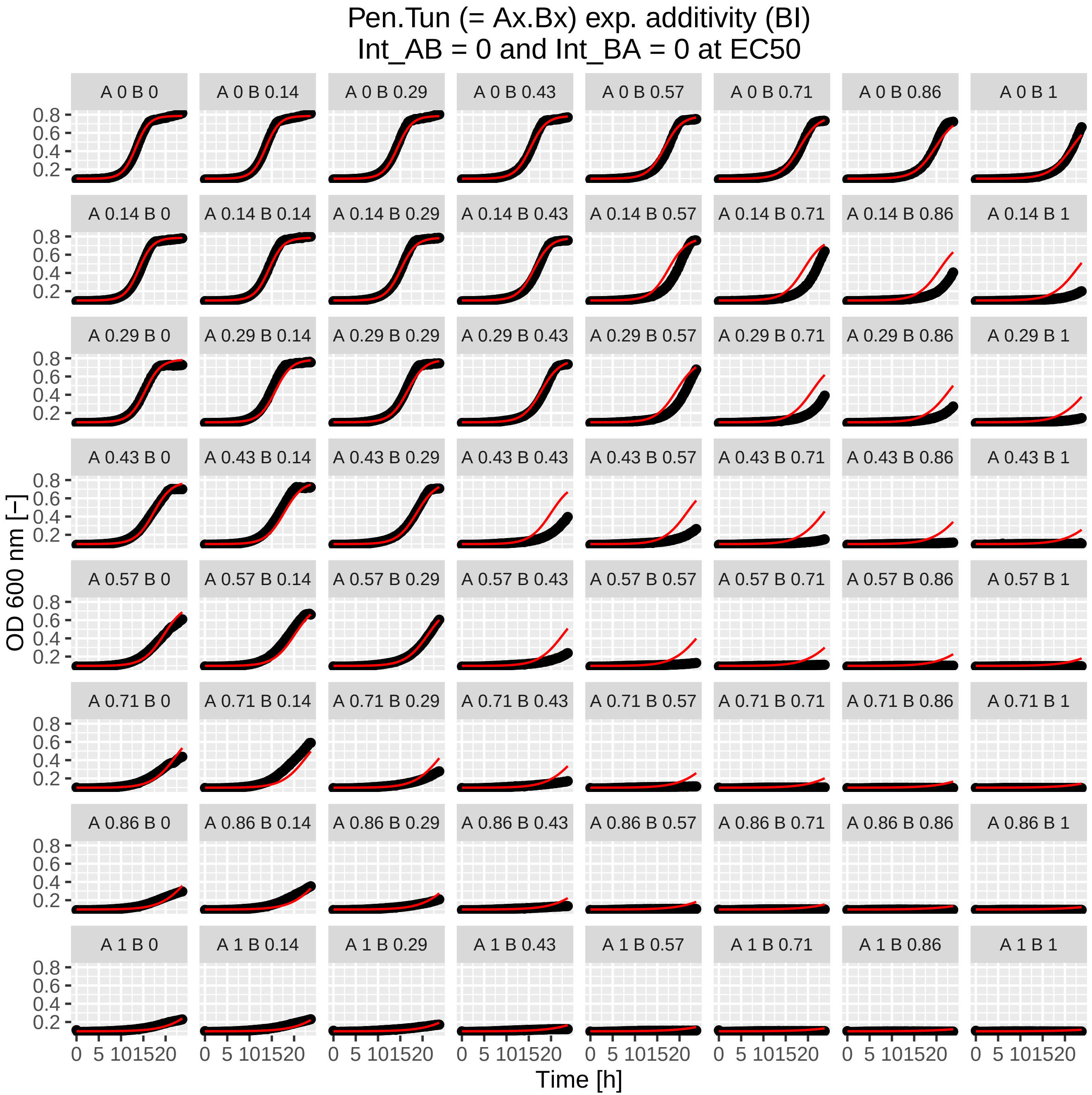
Pen.Tun (= Ax.Bx) exp. additivity (BI)
Int_AB = 0 and Int_BA = 0 at EC50
OD 600 nm [−]
Time [h]
A 0 B 0
A 0 B 0.14
A 0 B 0.29
A 0 B 0.43
A 0 B 0.57
A 0 B 0.71
A 0 B 0.86
A 0 B 1
A 0.14 B 0
A 0.14 B 0.14
A 0.14 B 0.29
A 0.14 B 0.43
A 0.14 B 0.57
A 0.14 B 0.71
A 0.14 B 0.86
A 0.14 B 1
A 0.29 B 0
A 0.29 B 0.14
A 0.29 B 0.29
A 0.29 B 0.43
A 0.29 B 0.57
A 0.29 B 0.71
A 0.29 B 0.86
A 0.29 B 1
A 0.43 B 0
A 0.43 B 0.14
A 0.43 B 0.29
A 0.43 B 0.43
A 0.43 B 0.57
A 0.43 B 0.71
A 0.43 B 0.86
A 0.43 B 1
A 0.57 B 0
A 0.57 B 0.14
A 0.57 B 0.29
A 0.57 B 0.43
A 0.57 B 0.57
A 0.57 B 0.71
A 0.57 B 0.86
A 0.57 B 1
A 0.71 B 0
A 0.71 B 0.14
A 0.71 B 0.29
A 0.71 B 0.43
A 0.71 B 0.57
A 0.71 B 0.71
A 0.71 B 0.86
A 0.71 B 1
A 0.86 B 0
A 0.86 B 0.14
A 0.86 B 0.29
A 0.86 B 0.43
A 0.86 B 0.57
A 0.86 B 0.71
A 0.86 B 0.86
A 0.86 B 1
A 1 B 0
A 1 B 0.14
A 1 B 0.29
A 1 B 0.43
A 1 B 0.57
A 1 B 0.71
A 1 B 0.86
A 1 B 1
0.8
0.6
0.4
0.2
0 5 101520

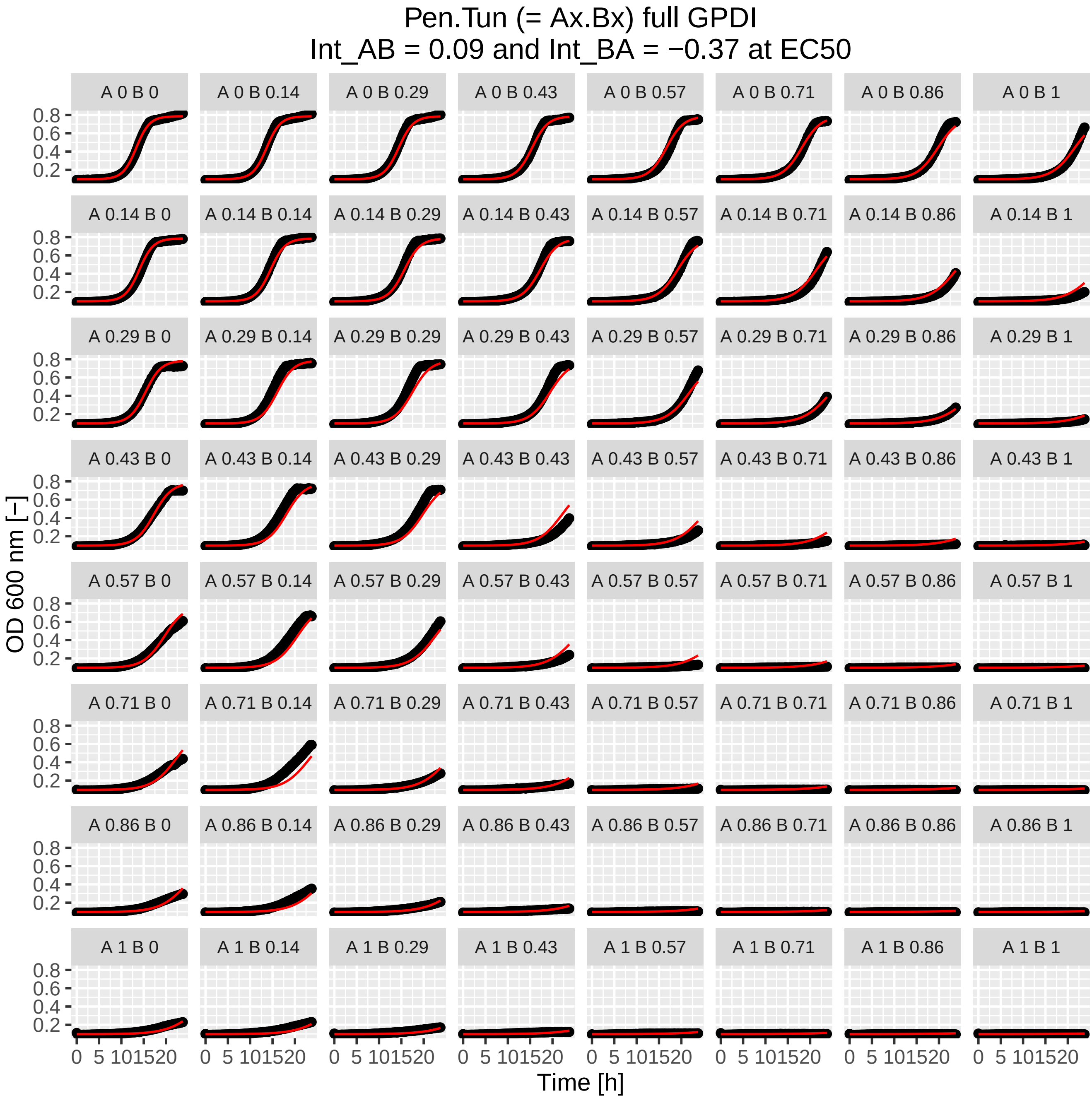

Pen.Tun (= Ax.Bx) full GPDI
Int_AB = 0.09 and Int_BA = −0.37 at EC50
A 0 B 0
A 0 B 0.14
A 0 B 0.29
A 0 B 0.43
A 0 B 0.57
A 0 B 0.71
A 0 B 0.86
A 0 B 1
A 0.14 B 0
A 0.14 B 0.14
A 0.14 B 0.29
A 0.14 B 0.43
A 0.14 B 0.57
A 0.14 B 0.71
A 0.14 B 0.86
A 0.14 B 1
A 0.29 B 0
A 0.29 B 0.14
A 0.29 B 0.29
A 0.29 B 0.43
A 0.29 B 0.57
A 0.29 B 0.71
A 0.29 B 0.86
A 0.29 B 1
A 0.43 B 0
A 0.43 B 0.14
A 0.43 B 0.29
A 0.43 B 0.43
A 0.43 B 0.57
A 0.43 B 0.71
A 0.43 B 0.86
A 0.43 B 1
A 0.57 B 0
A 0.57 B 0.14
A 0.57 B 0.29
A 0.57 B 0.43
A 0.57 B 0.57
A 0.57 B 0.71
A 0.57 B 0.86
A 0.57 B 1
A 0.71 B 0
A 0.71 B 0.14
A 0.71 B 0.29
A 0.71 B 0.43
A 0.71 B 0.57
A 0.71 B 0.71
A 0.71 B 0.86
A 0.71 B 1
A 0.86 B 0
A 0.86 B 0.14
A 0.86 B 0.29
A 0.86 B 0.43
A 0.86 B 0.57
A 0.86 B 0.71
A 0.86 B 0.86
A 0.86 B 1
A 1 B 0
A 1 B 0.14
A 1 B 0.29
A 1 B 0.43
A 1 B 0.57
A 1 B 0.71
A 1 B 0.86
A 1 B 1
OD 600 nm [−]
0.2
0.4
0.6
0.8
0
5
10
15
20
Time [h]

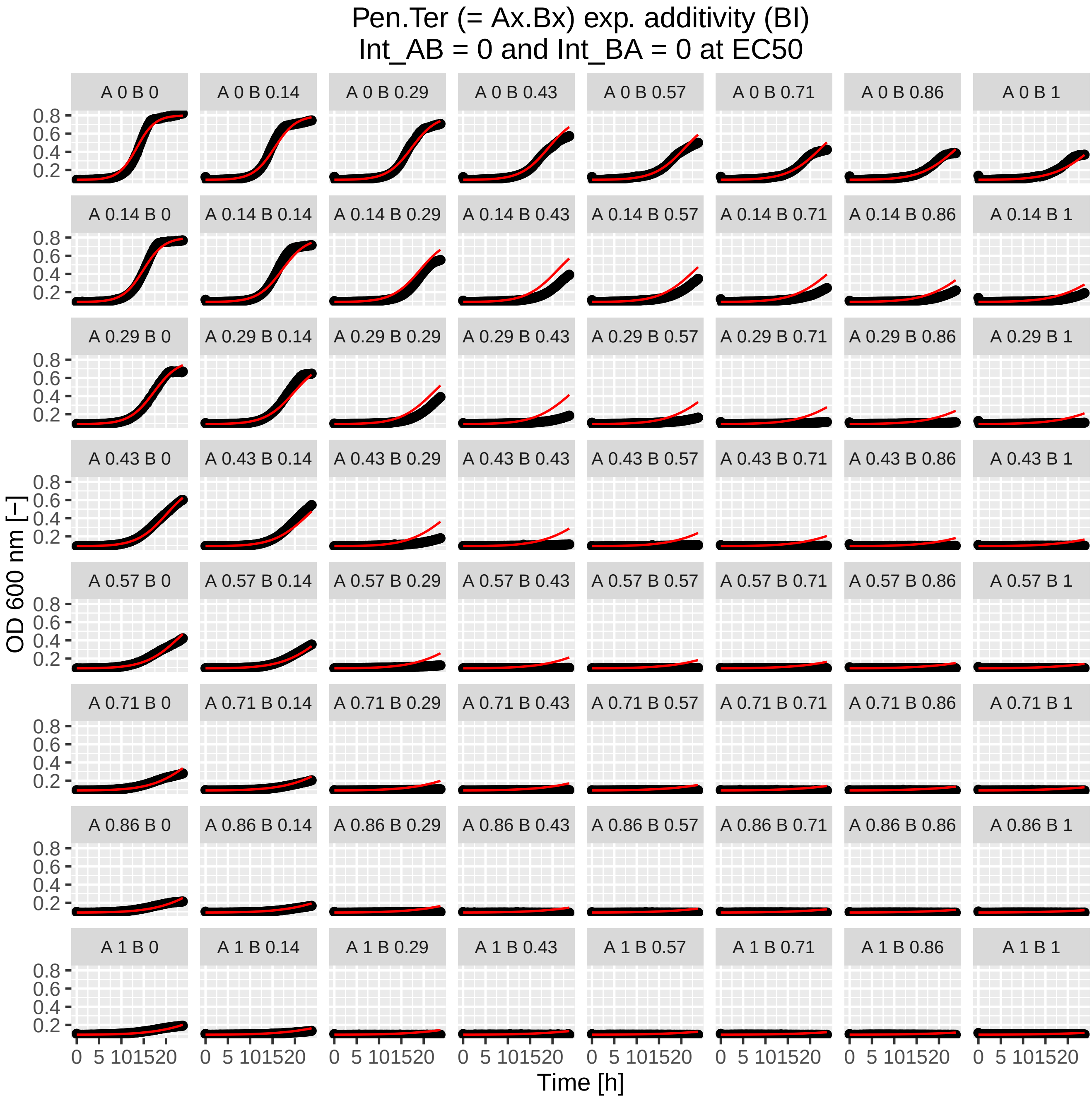
Pen.Ter (= Ax.Bx) exp. additivity (BI)
Int_AB = 0 and Int_BA = 0 at EC50
A 0 B 0
A 0 B 0.14
A 0 B 0.29
A 0 B 0.43
A 0 B 0.57
A 0 B 0.71
A 0 B 0.86
A 0 B 1
A 0.14 B 0
A 0.14 B 0.14
A 0.14 B 0.29
A 0.14 B 0.43
A 0.14 B 0.57
A 0.14 B 0.71
A 0.14 B 0.86
A 0.14 B 1
A 0.29 B 0
A 0.29 B 0.14
A 0.29 B 0.29
A 0.29 B 0.43
A 0.29 B 0.57
A 0.29 B 0.71
A 0.29 B 0.86
A 0.29 B 1
A 0.43 B 0
A 0.43 B 0.14
A 0.43 B 0.29
A 0.43 B 0.43
A 0.43 B 0.57
A 0.43 B 0.71
A 0.43 B 0.86
A 0.43 B 1
A 0.57 B 0
A 0.57 B 0.14
A 0.57 B 0.29
A 0.57 B 0.43
A 0.57 B 0.57
A 0.57 B 0.71
A 0.57 B 0.86
A 0.57 B 1
A 0.71 B 0
A 0.71 B 0.14
A 0.71 B 0.29
A 0.71 B 0.43
A 0.71 B 0.57
A 0.71 B 0.71
A 0.71 B 0.86
A 0.71 B 1
A 0.86 B 0
A 0.86 B 0.14
A 0.86 B 0.29
A 0.86 B 0.43
A 0.86 B 0.57
A 0.86 B 0.71
A 0.86 B 0.86
A 0.86 B 1
A 1 B 0
A 1 B 0.14
A 1 B 0.29
A 1 B 0.43
A 1 B 0.57
A 1 B 0.71
A 1 B 0.86
A 1 B 1
OD 600 nm [–]
0.2 0.4 0.6 0.8
0 5 101520
Time [h]

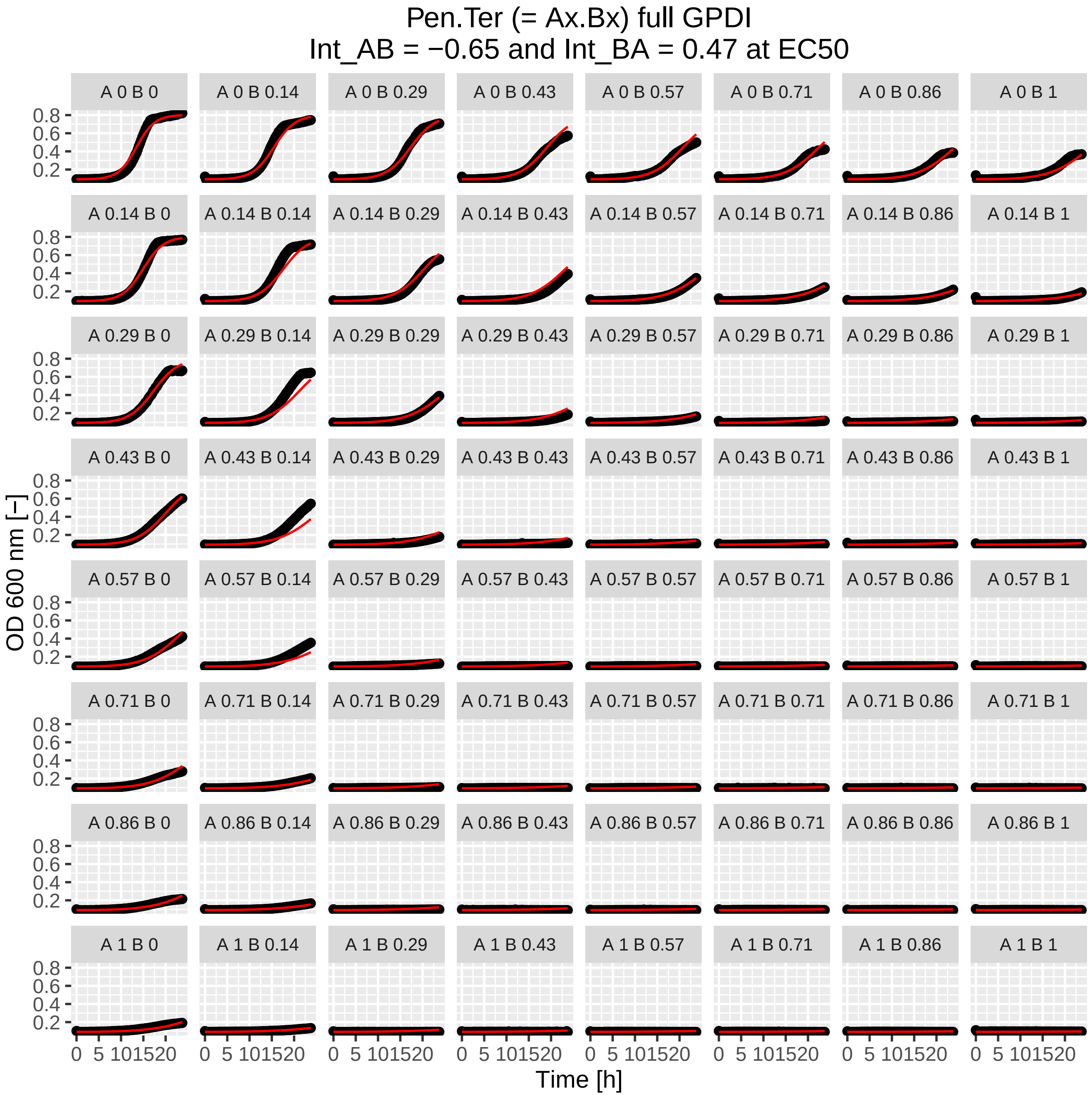

Pen.Ter (= Ax.Bx) full GPDI
Int_AB = −0.65 and Int_BA = 0.47 at EC50
OD 600 nm [−]
Time [h]
A 0 B 0
A 0 B 0.14
A 0 B 0.29
A 0 B 0.43
A 0 B 0.57
A 0 B 0.71
A 0 B 0.86
A 0 B 1
A 0.14 B 0
A 0.14 B 0.14
A 0.14 B 0.29
A 0.14 B 0.43
A 0.14 B 0.57
A 0.14 B 0.71
A 0.14 B 0.86
A 0.14 B 1
A 0.29 B 0
A 0.29 B 0.14
A 0.29 B 0.29
A 0.29 B 0.43
A 0.29 B 0.57
A 0.29 B 0.71
A 0.29 B 0.86
A 0.29 B 1
A 0.43 B 0
A 0.43 B 0.14
A 0.43 B 0.29
A 0.43 B 0.43
A 0.43 B 0.57
A 0.43 B 0.71
A 0.43 B 0.86
A 0.43 B 1
A 0.57 B 0
A 0.57 B 0.14
A 0.57 B 0.29
A 0.57 B 0.43
A 0.57 B 0.57
A 0.57 B 0.71
A 0.57 B 0.86
A 0.57 B 1
A 0.71 B 0
A 0.71 B 0.14
A 0.71 B 0.29
A 0.71 B 0.43
A 0.71 B 0.57
A 0.71 B 0.71
A 0.71 B 0.86
A 0.71 B 1
A 0.86 B 0
A 0.86 B 0.14
A 0.86 B 0.29
A 0.86 B 0.43
A 0.86 B 0.57
A 0.86 B 0.71
A 0.86 B 0.86
A 0.86 B 1
A 1 B 0
A 1 B 0.14
A 1 B 0.29
A 1 B 0.43
A 1 B 0.57
A 1 B 0.71
A 1 B 0.86
A 1 B 1
0.8
0.6
0.4
0.2
0 5 101520

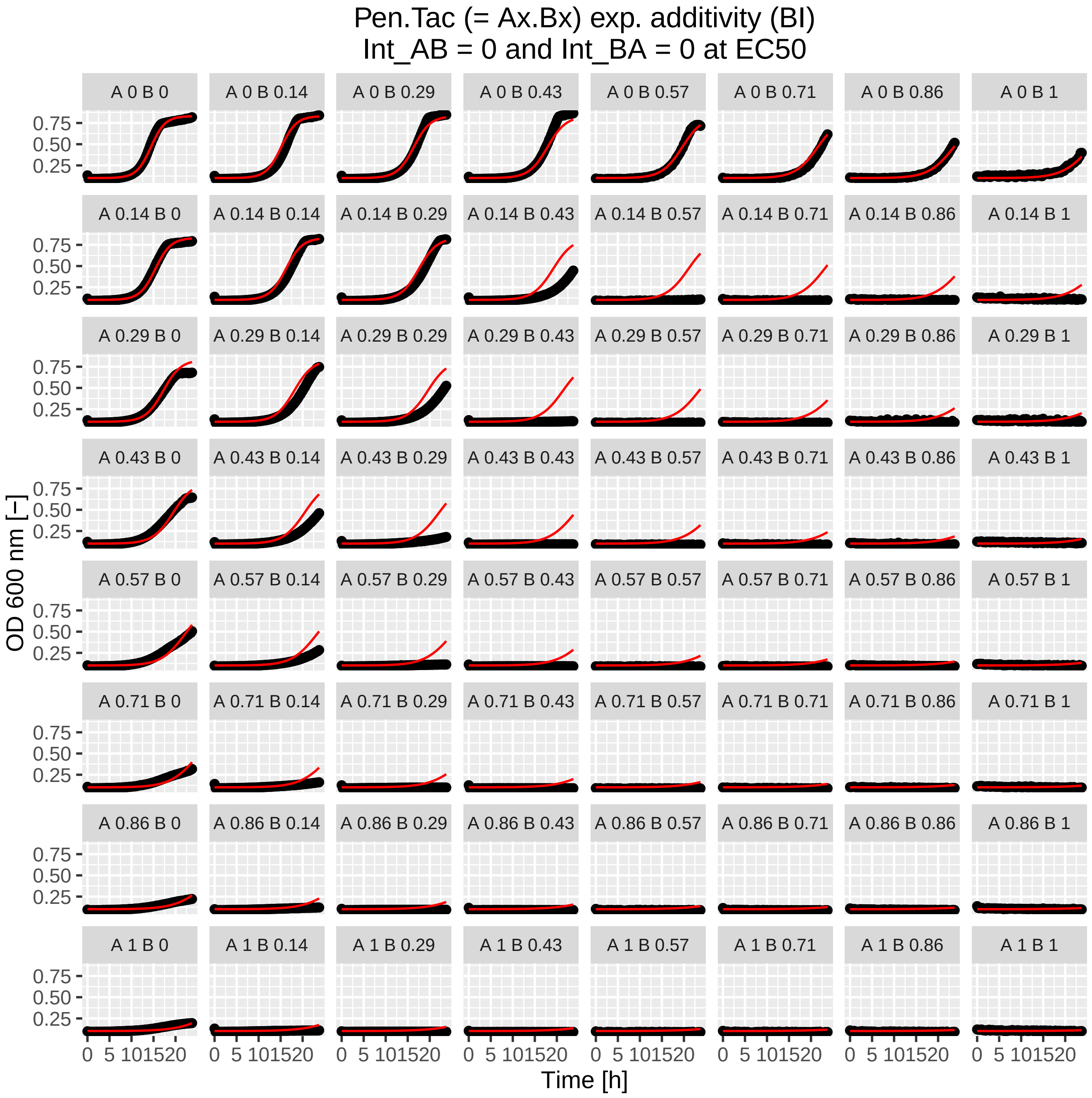

Pen.Tac (= Ax.Bx) exp. additivity (BI)
Int_AB = 0 and Int_BA = 0 at EC50
A 0 B 0
A 0 B 0.14
A 0 B 0.29
A 0 B 0.43
A 0 B 0.57
A 0 B 0.71
A 0 B 0.86
A 0 B 1
A 0.14 B 0
A 0.14 B 0.14
A 0.14 B 0.29
A 0.14 B 0.43
A 0.14 B 0.57
A 0.14 B 0.71
A 0.14 B 0.86
A 0.14 B 1
A 0.29 B 0
A 0.29 B 0.14
A 0.29 B 0.29
A 0.29 B 0.43
A 0.29 B 0.57
A 0.29 B 0.71
A 0.29 B 0.86
A 0.29 B 1
A 0.43 B 0
A 0.43 B 0.14
A 0.43 B 0.29
A 0.43 B 0.43
A 0.43 B 0.57
A 0.43 B 0.71
A 0.43 B 0.86
A 0.43 B 1
A 0.57 B 0
A 0.57 B 0.14
A 0.57 B 0.29
A 0.57 B 0.43
A 0.57 B 0.57
A 0.57 B 0.71
A 0.57 B 0.86
A 0.57 B 1
A 0.71 B 0
A 0.71 B 0.14
A 0.71 B 0.29
A 0.71 B 0.43
A 0.71 B 0.57
A 0.71 B 0.71
A 0.71 B 0.86
A 0.71 B 1
A 0.86 B 0
A 0.86 B 0.14
A 0.86 B 0.29
A 0.86 B 0.43
A 0.86 B 0.57
A 0.86 B 0.71
A 0.86 B 0.86
A 0.86 B 1
A 1 B 0
A 1 B 0.14
A 1 B 0.29
A 1 B 0.43
A 1 B 0.57
A 1 B 0.71
A 1 B 0.86
A 1 B 1
OD 600 nm [–]
0.75
0.50
0.25
0 5 101520
Time [h]

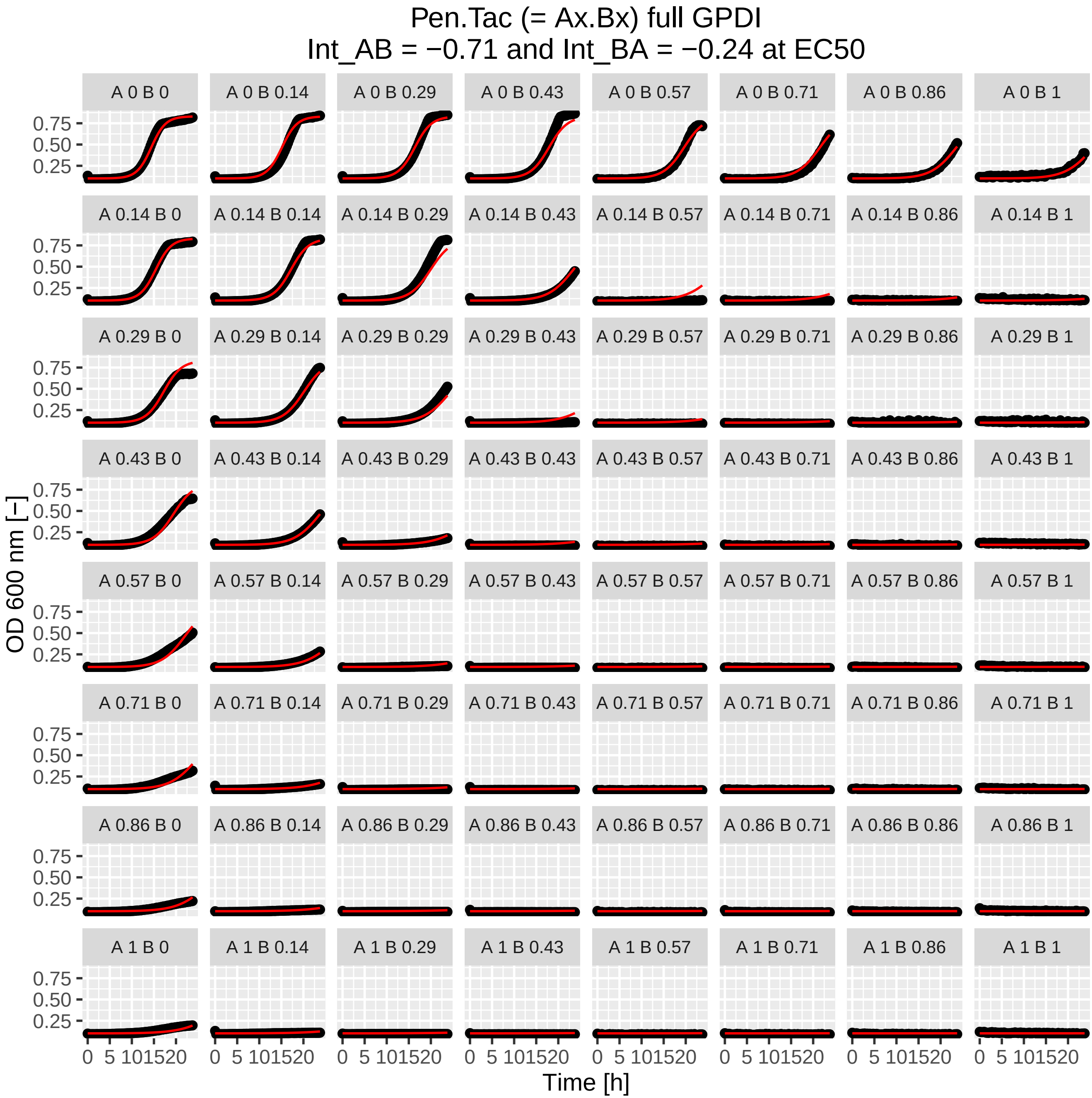

Pen.Tac (= Ax.Bx) full GPDI
Int_AB = −0.71 and Int_BA = −0.24 at EC50
OD 600 nm [−]
Time [h]
0.75
0.50
0.25
0 5 101520
A 0 B 0
A 0 B 0.14
A 0 B 0.29
A 0 B 0.43
A 0 B 0.57
A 0 B 0.71
A 0 B 0.86
A 0 B 1
A 0.14 B 0
A 0.14 B 0.14
A 0.14 B 0.29
A 0.14 B 0.43
A 0.14 B 0.57
A 0.14 B 0.71
A 0.14 B 0.86
A 0.14 B 1
A 0.29 B 0
A 0.29 B 0.14
A 0.29 B 0.29
A 0.29 B 0.43
A 0.29 B 0.57
A 0.29 B 0.71
A 0.29 B 0.86
A 0.29 B 1
A 0.43 B 0
A 0.43 B 0.14
A 0.43 B 0.29
A 0.43 B 0.43
A 0.43 B 0.57
A 0.43 B 0.71
A 0.43 B 0.86
A 0.43 B 1
A 0.57 B 0
A 0.57 B 0.14
A 0.57 B 0.29
A 0.57 B 0.43
A 0.57 B 0.57
A 0.57 B 0.71
A 0.57 B 0.86
A 0.57 B 1
A 0.71 B 0
A 0.71 B 0.14
A 0.71 B 0.29
A 0.71 B 0.43
A 0.71 B 0.57
A 0.71 B 0.71
A 0.71 B 0.86
A 0.71 B 1
A 0.86 B 0
A 0.86 B 0.14
A 0.86 B 0.29
A 0.86 B 0.43
A 0.86 B 0.57
A 0.86 B 0.71
A 0.86 B 0.86
A 0.86 B 1
A 1 B 0
A 1 B 0.14
A 1 B 0.29
A 1 B 0.43
A 1 B 0.57
A 1 B 0.71
A 1 B 0.86
A 1 B 1

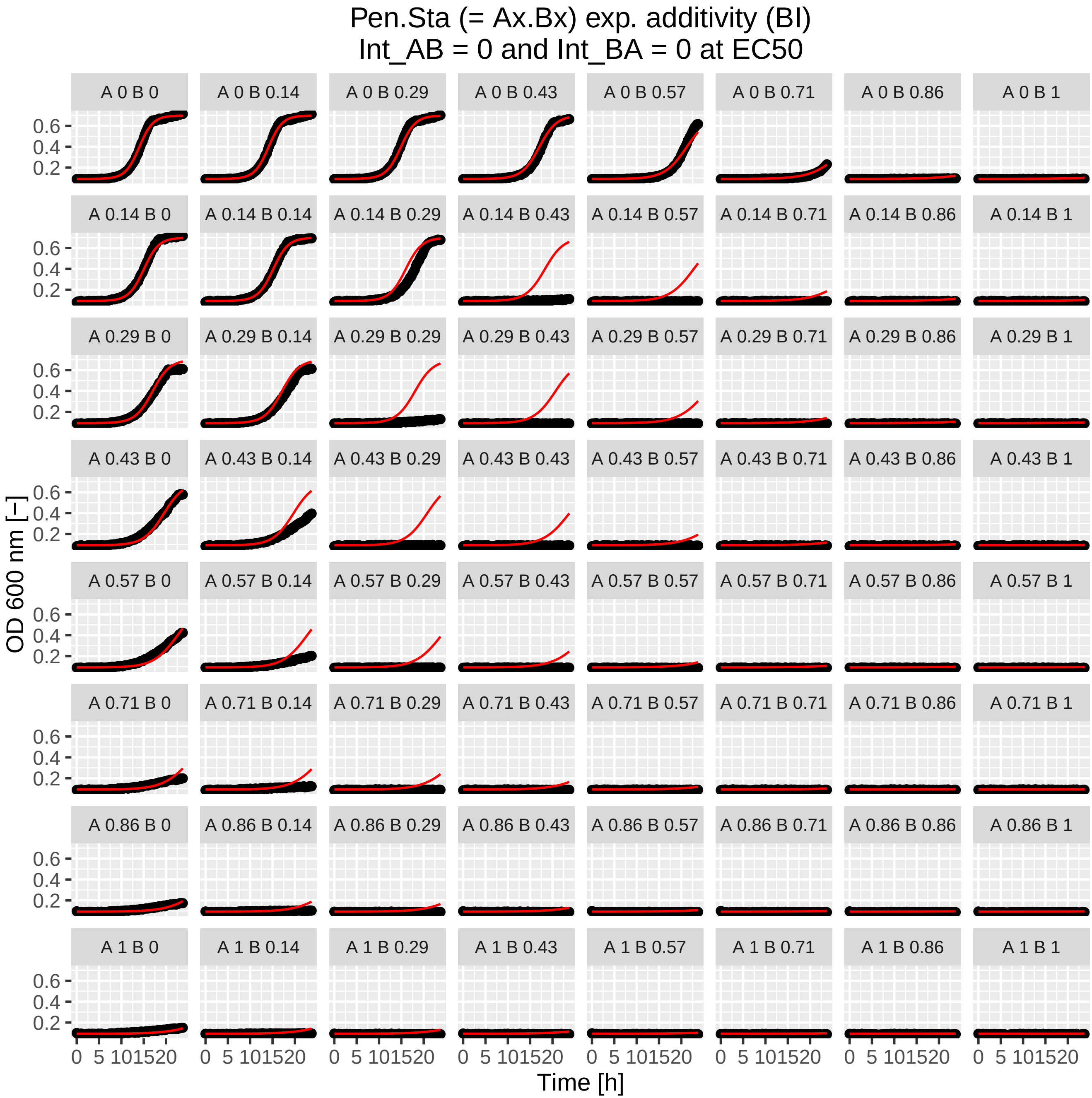
Pen.Sta (= Ax.Bx) exp. additivity (BI)
Int_AB = 0 and Int_BA = 0 at EC50
A 0 B 0
A 0 B 0.14
A 0 B 0.29
A 0 B 0.43
A 0 B 0.57
A 0 B 0.71
A 0 B 0.86
A 0 B 1
A 0.14 B 0
A 0.14 B 0.14
A 0.14 B 0.29
A 0.14 B 0.43
A 0.14 B 0.57
A 0.14 B 0.71
A 0.14 B 0.86
A 0.14 B 1
A 0.29 B 0
A 0.29 B 0.14
A 0.29 B 0.29
A 0.29 B 0.43
A 0.29 B 0.57
A 0.29 B 0.71
A 0.29 B 0.86
A 0.29 B 1
A 0.43 B 0
A 0.43 B 0.14
A 0.43 B 0.29
A 0.43 B 0.43
A 0.43 B 0.57
A 0.43 B 0.71
A 0.43 B 0.86
A 0.43 B 1
A 0.57 B 0
A 0.57 B 0.14
A 0.57 B 0.29
A 0.57 B 0.43
A 0.57 B 0.57
A 0.57 B 0.71
A 0.57 B 0.86
A 0.57 B 1
A 0.71 B 0
A 0.71 B 0.14
A 0.71 B 0.29
A 0.71 B 0.43
A 0.71 B 0.57
A 0.71 B 0.71
A 0.71 B 0.86
A 0.71 B 1
A 0.86 B 0
A 0.86 B 0.14
A 0.86 B 0.29
A 0.86 B 0.43
A 0.86 B 0.57
A 0.86 B 0.71
A 0.86 B 0.86
A 0.86 B 1
A 1 B 0
A 1 B 0.14
A 1 B 0.29
A 1 B 0.43
A 1 B 0.57
A 1 B 0.71
A 1 B 0.86
A 1 B 1
OD 600 nm [–]
0.6
0.4
0.2
0 5 101520
Time [h]

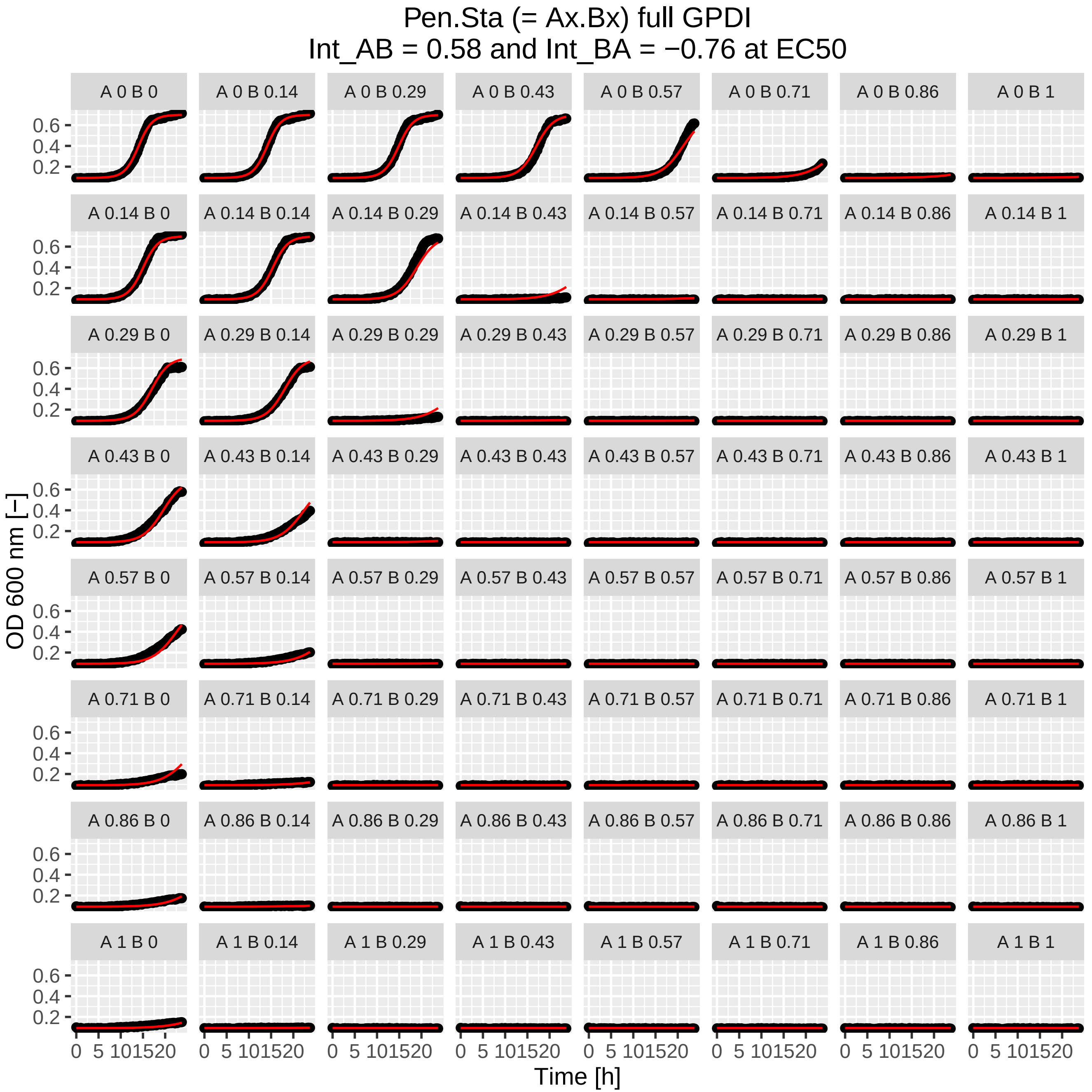
Pen.Sta (= Ax.Bx) full GPDI
Int_AB = 0.58 and Int_BA = −0.76 at EC50
OD 600 nm [−]
Time [h]

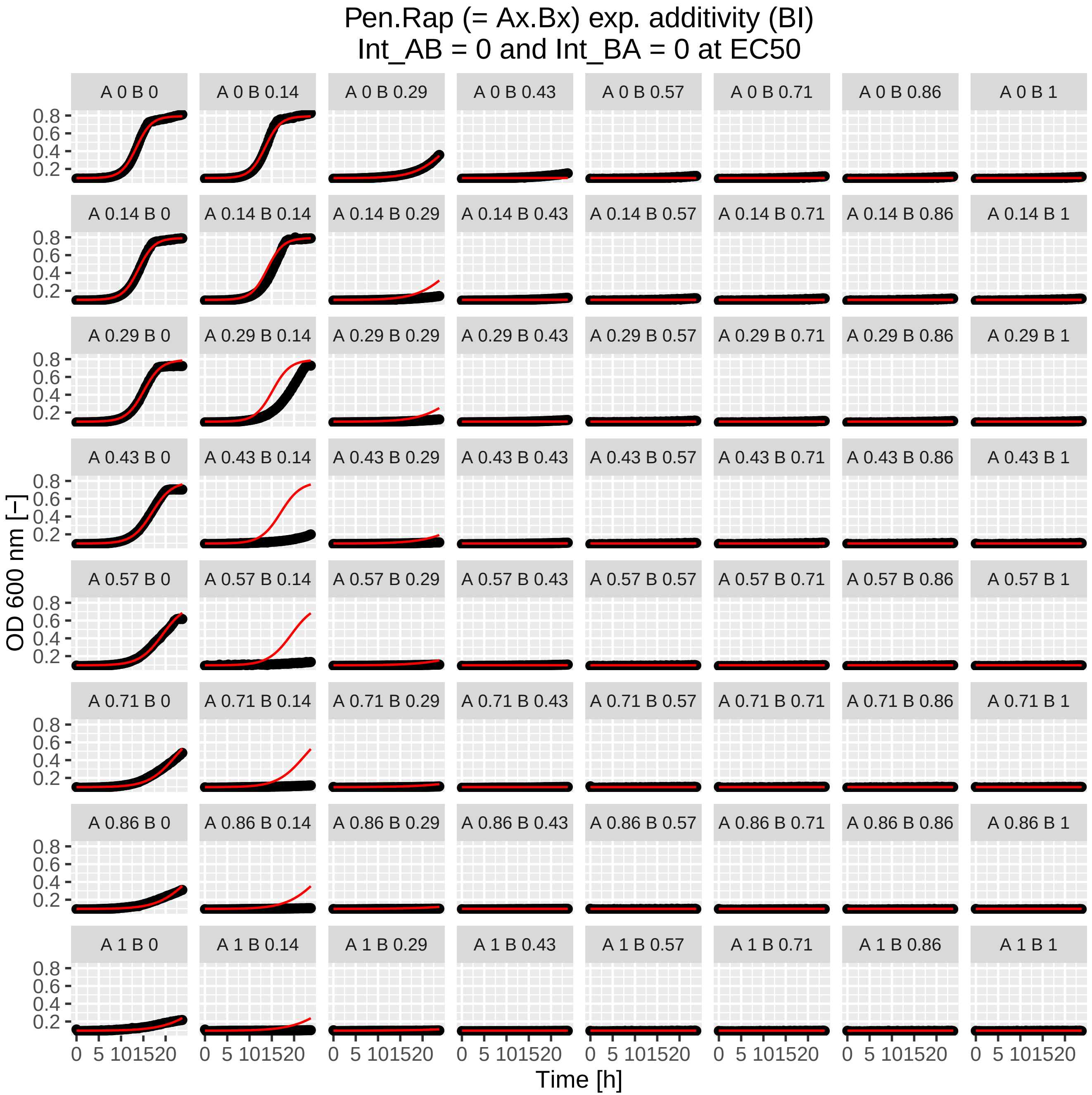

Pen.Rap (= Ax.Bx) exp. additivity (BI)
Int_AB = 0 and Int_BA = 0 at EC50
A 0 B 0
A 0 B 0.14
A 0 B 0.29
A 0 B 0.43
A 0 B 0.57
A 0 B 0.71
A 0 B 0.86
A 0 B 1
A 0.14 B 0
A 0.14 B 0.14
A 0.14 B 0.29
A 0.14 B 0.43
A 0.14 B 0.57
A 0.14 B 0.71
A 0.14 B 0.86
A 0.14 B 1
A 0.29 B 0
A 0.29 B 0.14
A 0.29 B 0.29
A 0.29 B 0.43
A 0.29 B 0.57
A 0.29 B 0.71
A 0.29 B 0.86
A 0.29 B 1
A 0.43 B 0
A 0.43 B 0.14
A 0.43 B 0.29
A 0.43 B 0.43
A 0.43 B 0.57
A 0.43 B 0.71
A 0.43 B 0.86
A 0.43 B 1
A 0.57 B 0
A 0.57 B 0.14
A 0.57 B 0.29
A 0.57 B 0.43
A 0.57 B 0.57
A 0.57 B 0.71
A 0.57 B 0.86
A 0.57 B 1
A 0.71 B 0
A 0.71 B 0.14
A 0.71 B 0.29
A 0.71 B 0.43
A 0.71 B 0.57
A 0.71 B 0.71
A 0.71 B 0.86
A 0.71 B 1
A 0.86 B 0
A 0.86 B 0.14
A 0.86 B 0.29
A 0.86 B 0.43
A 0.86 B 0.57
A 0.86 B 0.71
A 0.86 B 0.86
A 0.86 B 1
A 1 B 0
A 1 B 0.14
A 1 B 0.29
A 1 B 0.43
A 1 B 0.57
A 1 B 0.71
A 1 B 0.86
A 1 B 1
OD 600 nm [–]
0.2
0.4
0.6
0.8
0 5 101520
Time [h]

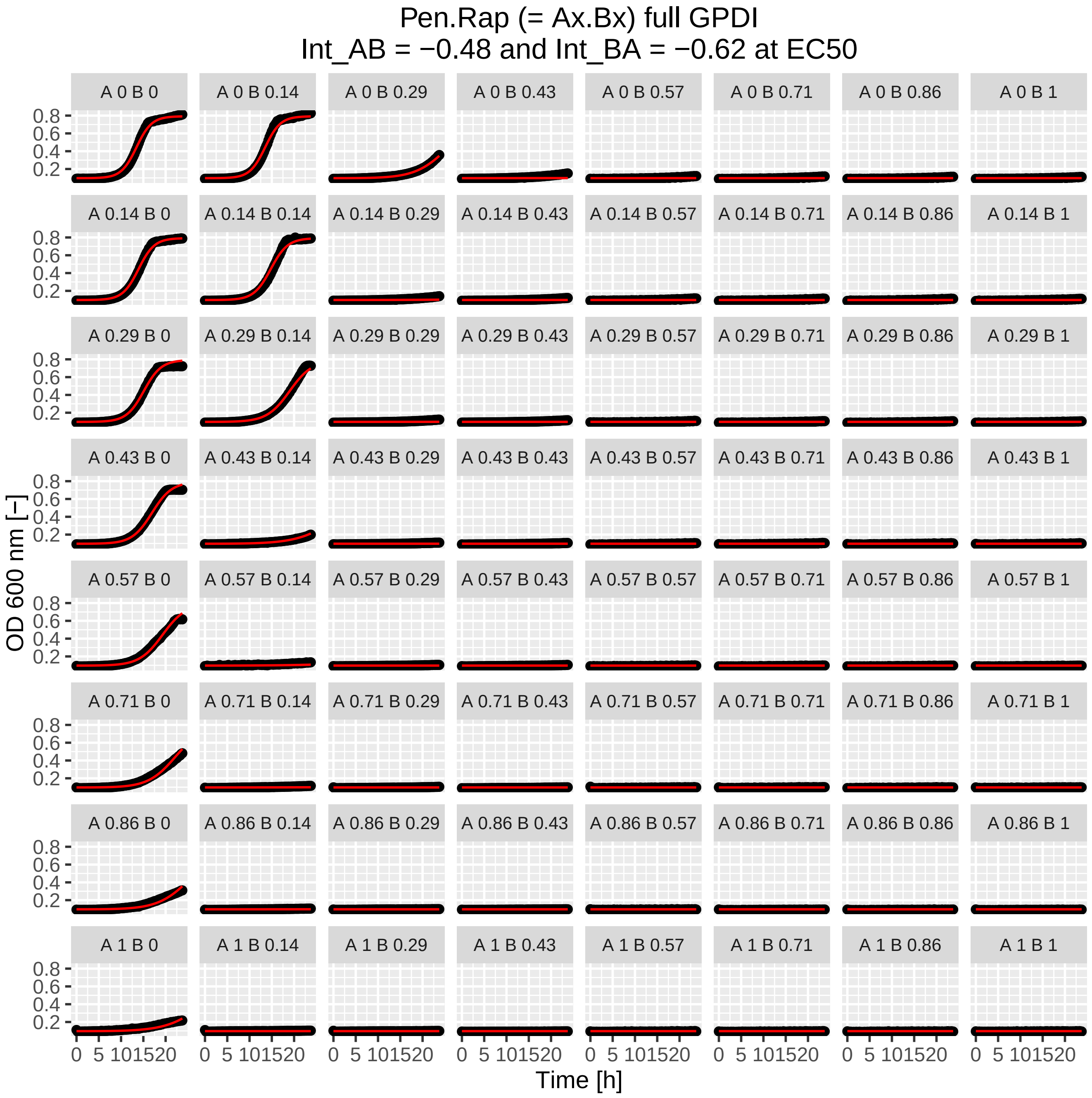

Pen.Rap (= Ax.Bx) full GPDI
Int_AB = −0.48 and Int_BA = −0.62 at EC50
A 0 B 0
A 0 B 0.14
A 0 B 0.29
A 0 B 0.43
A 0 B 0.57
A 0 B 0.71
A 0 B 0.86
A 0 B 1
A 0.14 B 0
A 0.14 B 0.14
A 0.14 B 0.29
A 0.14 B 0.43
A 0.14 B 0.57
A 0.14 B 0.71
A 0.14 B 0.86
A 0.14 B 1
A 0.29 B 0
A 0.29 B 0.14
A 0.29 B 0.29
A 0.29 B 0.43
A 0.29 B 0.57
A 0.29 B 0.71
A 0.29 B 0.86
A 0.29 B 1
A 0.43 B 0
A 0.43 B 0.14
A 0.43 B 0.29
A 0.43 B 0.43
A 0.43 B 0.57
A 0.43 B 0.71
A 0.43 B 0.86
A 0.43 B 1
A 0.57 B 0
A 0.57 B 0.14
A 0.57 B 0.29
A 0.57 B 0.43
A 0.57 B 0.57
A 0.57 B 0.71
A 0.57 B 0.86
A 0.57 B 1
A 0.71 B 0
A 0.71 B 0.14
A 0.71 B 0.29
A 0.71 B 0.43
A 0.71 B 0.57
A 0.71 B 0.71
A 0.71 B 0.86
A 0.71 B 1
A 0.86 B 0
A 0.86 B 0.14
A 0.86 B 0.29
A 0.86 B 0.43
A 0.86 B 0.57
A 0.86 B 0.71
A 0.86 B 0.86
A 0.86 B 1
A 1 B 0
A 1 B 0.14
A 1 B 0.29
A 1 B 0.43
A 1 B 0.57
A 1 B 0.71
A 1 B 0.86
A 1 B 1
OD 600 nm [−]
0.2
0.4
0.6
0.8
0 5 101520
Time [h]

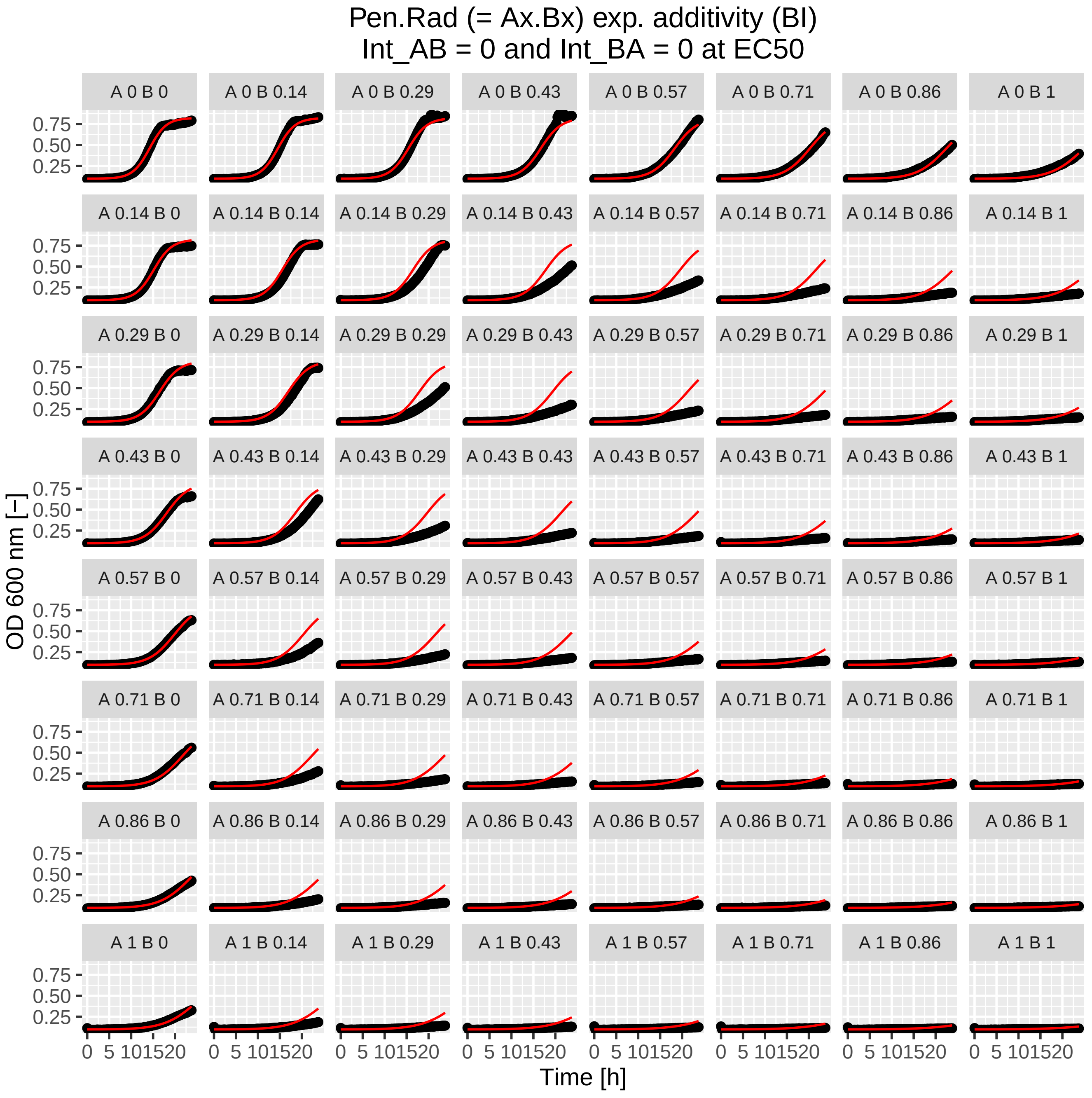
Pen.Rad (= Ax.Bx) exp. additivity (BI)
Int_AB = 0 and Int_BA = 0 at EC50
A 0 B 0
A 0 B 0.14
A 0 B 0.29
A 0 B 0.43
A 0 B 0.57
A 0 B 0.71
A 0 B 0.86
A 0 B 1
A 0.14 B 0
A 0.14 B 0.14
A 0.14 B 0.29
A 0.14 B 0.43
A 0.14 B 0.57
A 0.14 B 0.71
A 0.14 B 0.86
A 0.14 B 1
A 0.29 B 0
A 0.29 B 0.14
A 0.29 B 0.29
A 0.29 B 0.43
A 0.29 B 0.57
A 0.29 B 0.71
A 0.29 B 0.86
A 0.29 B 1
A 0.43 B 0
A 0.43 B 0.14
A 0.43 B 0.29
A 0.43 B 0.43
A 0.43 B 0.57
A 0.43 B 0.71
A 0.43 B 0.86
A 0.43 B 1
A 0.57 B 0
A 0.57 B 0.14
A 0.57 B 0.29
A 0.57 B 0.43
A 0.57 B 0.57
A 0.57 B 0.71
A 0.57 B 0.86
A 0.57 B 1
A 0.71 B 0
A 0.71 B 0.14
A 0.71 B 0.29
A 0.71 B 0.43
A 0.71 B 0.57
A 0.71 B 0.71
A 0.71 B 0.86
A 0.71 B 1
A 0.86 B 0
A 0.86 B 0.14
A 0.86 B 0.29
A 0.86 B 0.43
A 0.86 B 0.57
A 0.86 B 0.71
A 0.86 B 0.86
A 0.86 B 1
A 1 B 0
A 1 B 0.14
A 1 B 0.29
A 1 B 0.43
A 1 B 0.57
A 1 B 0.71
A 1 B 0.86
A 1 B 1
0.75
0.50
0.25
OD 600 nm [–]
0 5 101520
Time [h]

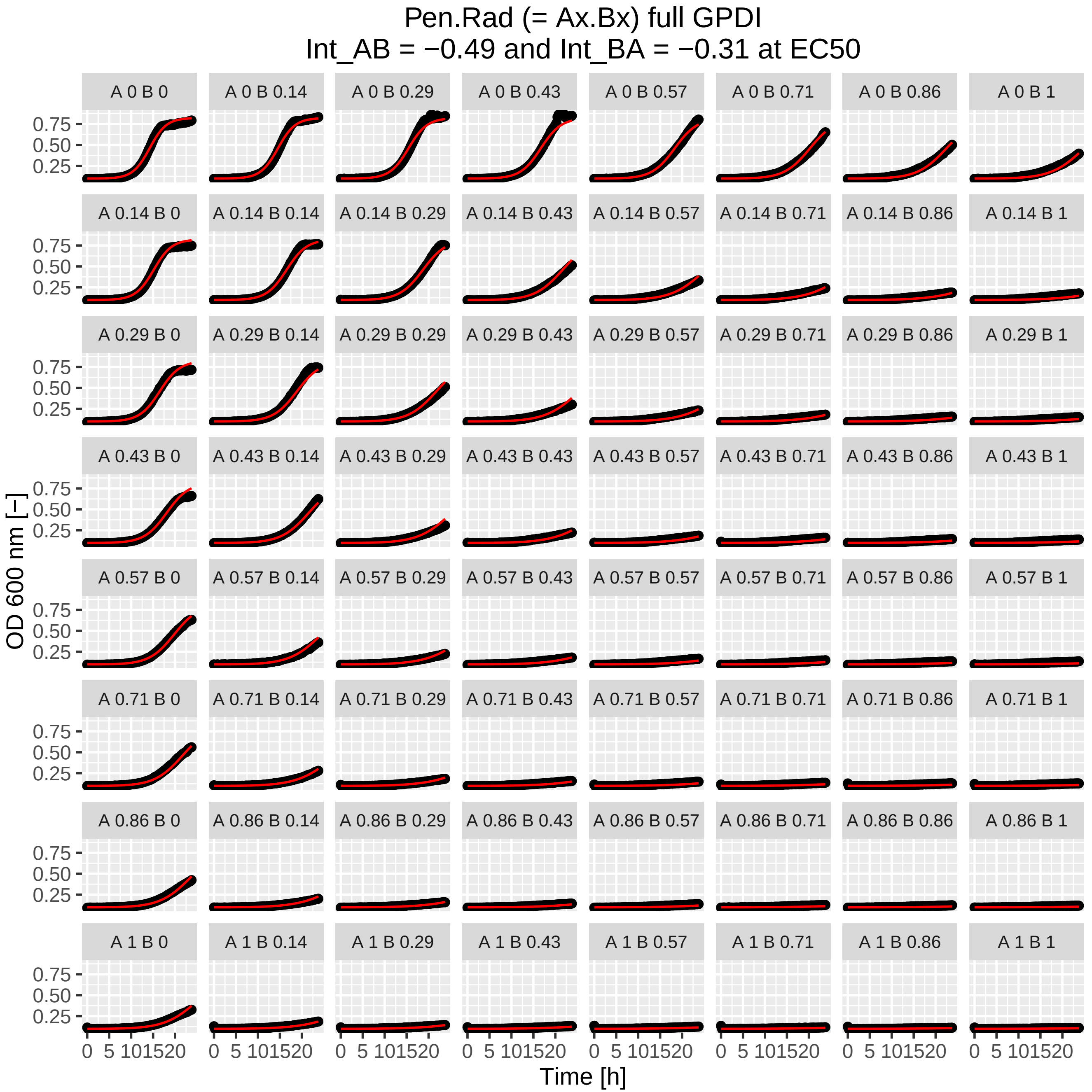

Pen.Rad (= Ax.Bx) full GPDI
Int_AB = −0.49 and Int_BA = −0.31 at EC50
OD 600 nm [–]
Time [h]
0.75
0.50
0.25
0 5 101520
A 0 B 0
A 0 B 0.14
A 0 B 0.29
A 0 B 0.43
A 0 B 0.57
A 0 B 0.71
A 0 B 0.86
A 0 B 1
A 0.14 B 0
A 0.14 B 0.14
A 0.14 B 0.29
A 0.14 B 0.43
A 0.14 B 0.57
A 0.14 B 0.71
A 0.14 B 0.86
A 0.14 B 1
A 0.29 B 0
A 0.29 B 0.14
A 0.29 B 0.29
A 0.29 B 0.43
A 0.29 B 0.57
A 0.29 B 0.71
A 0.29 B 0.86
A 0.29 B 1
A 0.43 B 0
A 0.43 B 0.14
A 0.43 B 0.29
A 0.43 B 0.43
A 0.43 B 0.57
A 0.43 B 0.71
A 0.43 B 0.86
A 0.43 B 1
A 0.57 B 0
A 0.57 B 0.14
A 0.57 B 0.29
A 0.57 B 0.43
A 0.57 B 0.57
A 0.57 B 0.71
A 0.57 B 0.86
A 0.57 B 1
A 0.71 B 0
A 0.71 B 0.14
A 0.71 B 0.29
A 0.71 B 0.43
A 0.71 B 0.57
A 0.71 B 0.71
A 0.71 B 0.86
A 0.71 B 1
A 0.86 B 0
A 0.86 B 0.14
A 0.86 B 0.29
A 0.86 B 0.43
A 0.86 B 0.57
A 0.86 B 0.71
A 0.86 B 0.86
A 0.86 B 1
A 1 B 0
A 1 B 0.14
A 1 B 0.29
A 1 B 0.43
A 1 B 0.57
A 1 B 0.71
A 1 B 0.86
A 1 B 1

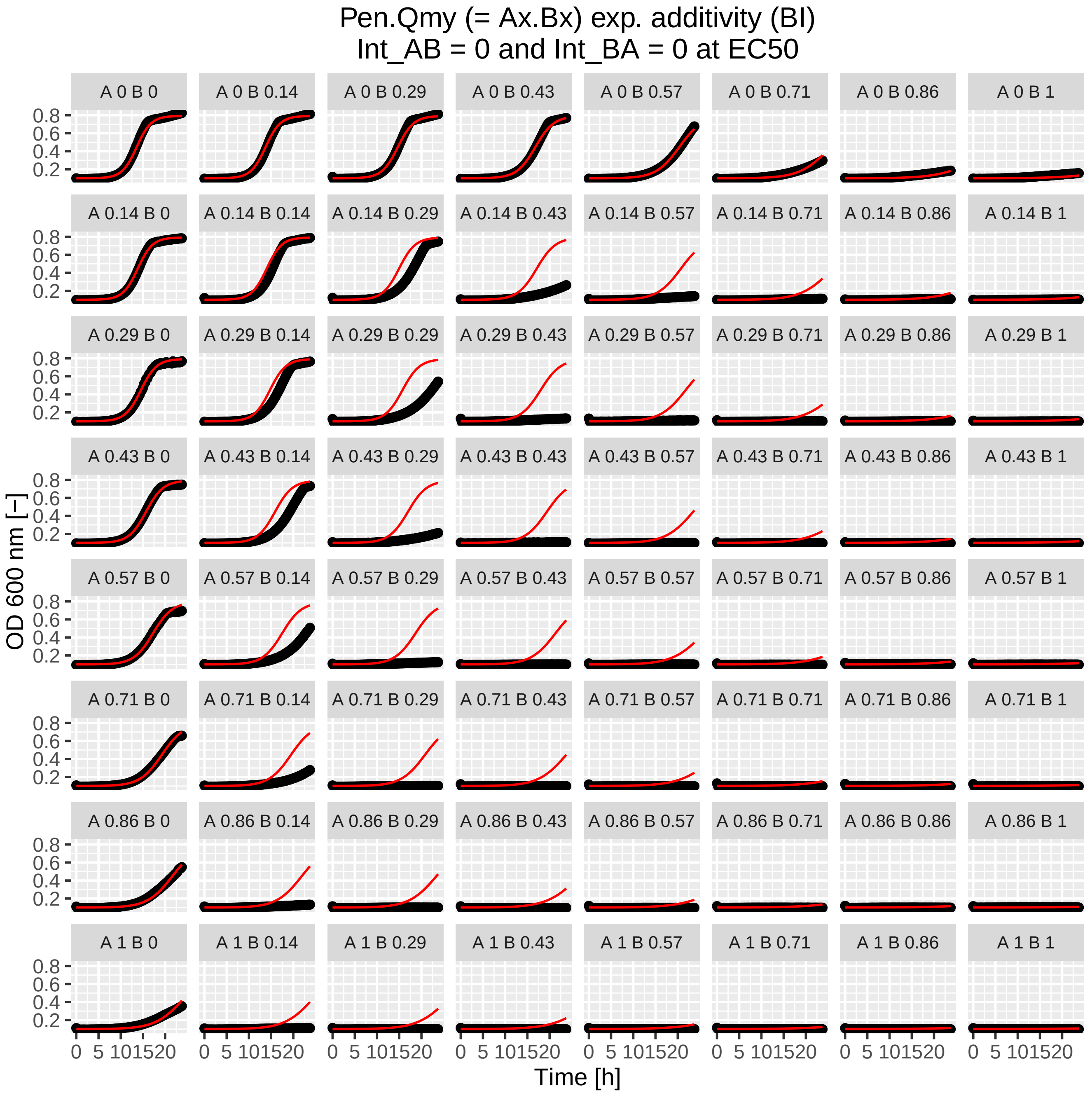
Pen.Qmy (= Ax.Bx) exp. additivity (BI)
Int_AB = 0 and Int_BA = 0 at EC50
OD 600 nm [−]
Time [h]
0.8
0.6
0.4
0.2
0 5 101520
A 0 B 0
A 0 B 0.14
A 0 B 0.29
A 0 B 0.43
A 0 B 0.57
A 0 B 0.71
A 0 B 0.86
A 0 B 1
A 0.14 B 0
A 0.14 B 0.14
A 0.14 B 0.29
A 0.14 B 0.43
A 0.14 B 0.57
A 0.14 B 0.71
A 0.14 B 0.86
A 0.14 B 1
A 0.29 B 0
A 0.29 B 0.14
A 0.29 B 0.29
A 0.29 B 0.43
A 0.29 B 0.57
A 0.29 B 0.71
A 0.29 B 0.86
A 0.29 B 1
A 0.43 B 0
A 0.43 B 0.14
A 0.43 B 0.29
A 0.43 B 0.43
A 0.43 B 0.57
A 0.43 B 0.71
A 0.43 B 0.86
A 0.43 B 1
A 0.57 B 0
A 0.57 B 0.14
A 0.57 B 0.29
A 0.57 B 0.43
A 0.57 B 0.57
A 0.57 B 0.71
A 0.57 B 0.86
A 0.57 B 1
A 0.71 B 0
A 0.71 B 0.14
A 0.71 B 0.29
A 0.71 B 0.43
A 0.71 B 0.57
A 0.71 B 0.71
A 0.71 B 0.86
A 0.71 B 1
A 0.86 B 0
A 0.86 B 0.14
A 0.86 B 0.29
A 0.86 B 0.43
A 0.86 B 0.57
A 0.86 B 0.71
A 0.86 B 0.86
A 0.86 B 1
A 1 B 0
A 1 B 0.14
A 1 B 0.29
A 1 B 0.43
A 1 B 0.57
A 1 B 0.71
A 1 B 0.86
A 1 B 1

Pen.Qmy (= Ax.Bx) full GPDI
Int_AB = −0.72 and Int_BA = −0.41 at EC50

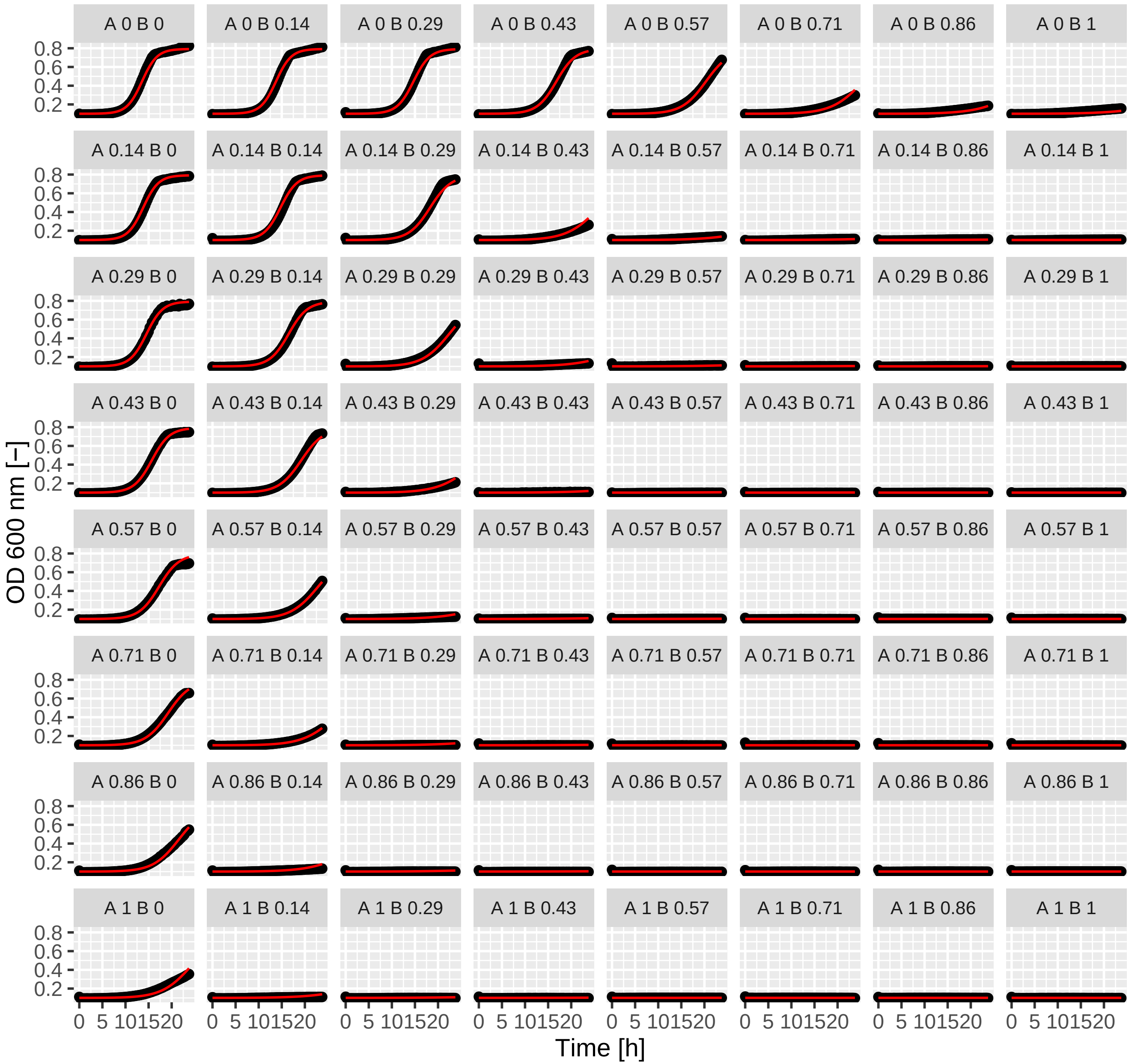

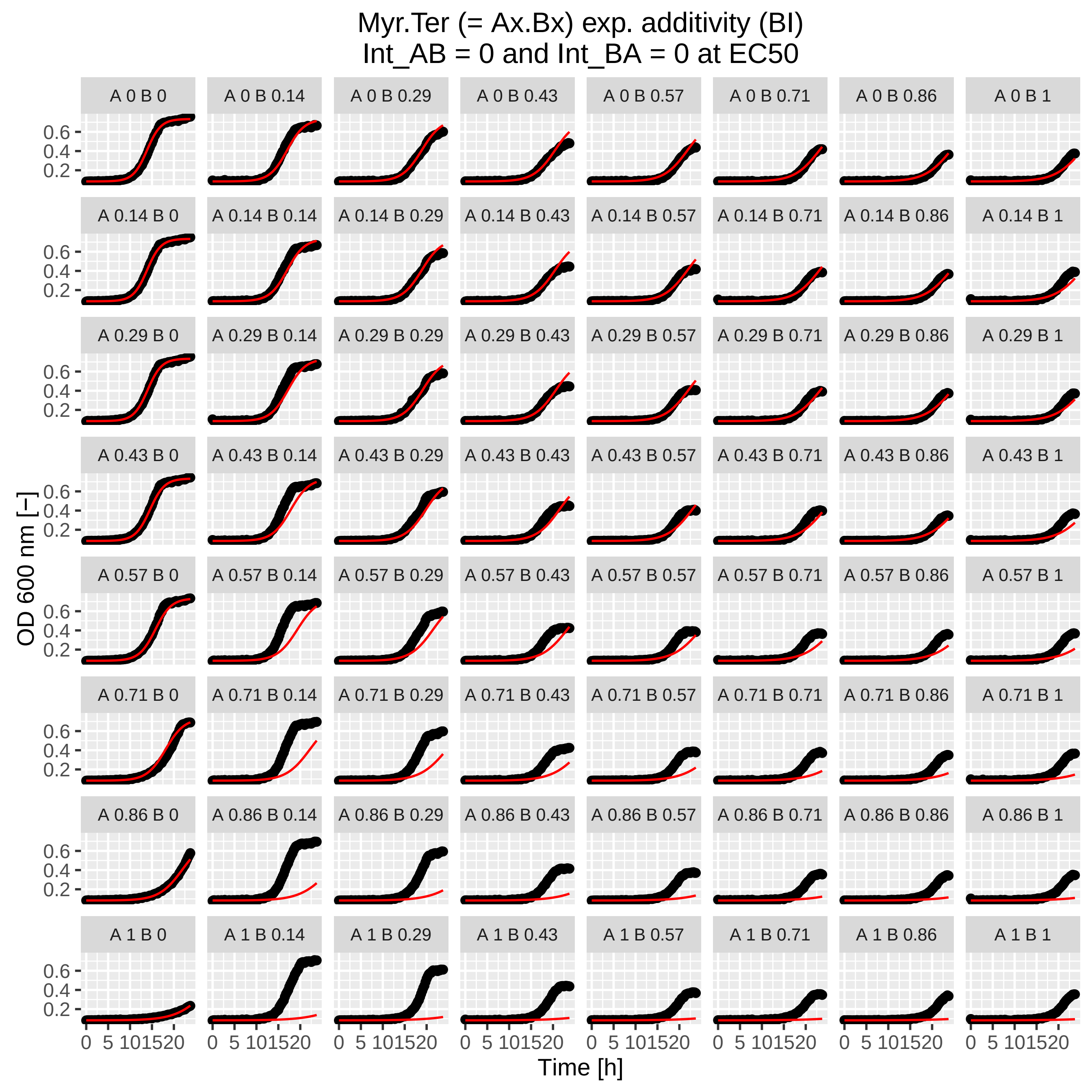

Myr.Ter (= Ax.Bx) exp. additivity (BI)
Int_AB = 0 and Int_BA = 0 at EC50
A 0 B 0
A 0 B 0.14
A 0 B 0.29
A 0 B 0.43
A 0 B 0.57
A 0 B 0.71
A 0 B 0.86
A 0 B 1
A 0.14 B 0
A 0.14 B 0.14
A 0.14 B 0.29
A 0.14 B 0.43
A 0.14 B 0.57
A 0.14 B 0.71
A 0.14 B 0.86
A 0.14 B 1
A 0.29 B 0
A 0.29 B 0.14
A 0.29 B 0.29
A 0.29 B 0.43
A 0.29 B 0.57
A 0.29 B 0.71
A 0.29 B 0.86
A 0.29 B 1
A 0.43 B 0
A 0.43 B 0.14
A 0.43 B 0.29
A 0.43 B 0.43
A 0.43 B 0.57
A 0.43 B 0.71
A 0.43 B 0.86
A 0.43 B 1
A 0.57 B 0
A 0.57 B 0.14
A 0.57 B 0.29
A 0.57 B 0.43
A 0.57 B 0.57
A 0.57 B 0.71
A 0.57 B 0.86
A 0.57 B 1
A 0.71 B 0
A 0.71 B 0.14
A 0.71 B 0.29
A 0.71 B 0.43
A 0.71 B 0.57
A 0.71 B 0.71
A 0.71 B 0.86
A 0.71 B 1
A 0.86 B 0
A 0.86 B 0.14
A 0.86 B 0.29
A 0.86 B 0.43
A 0.86 B 0.57
A 0.86 B 0.71
A 0.86 B 0.86
A 0.86 B 1
A 1 B 0
A 1 B 0.14
A 1 B 0.29
A 1 B 0.43
A 1 B 0.57
A 1 B 0.71
A 1 B 0.86
A 1 B 1
OD 600 nm [–]
0.2
0.4
0.6
0 5 101520
Time [h]

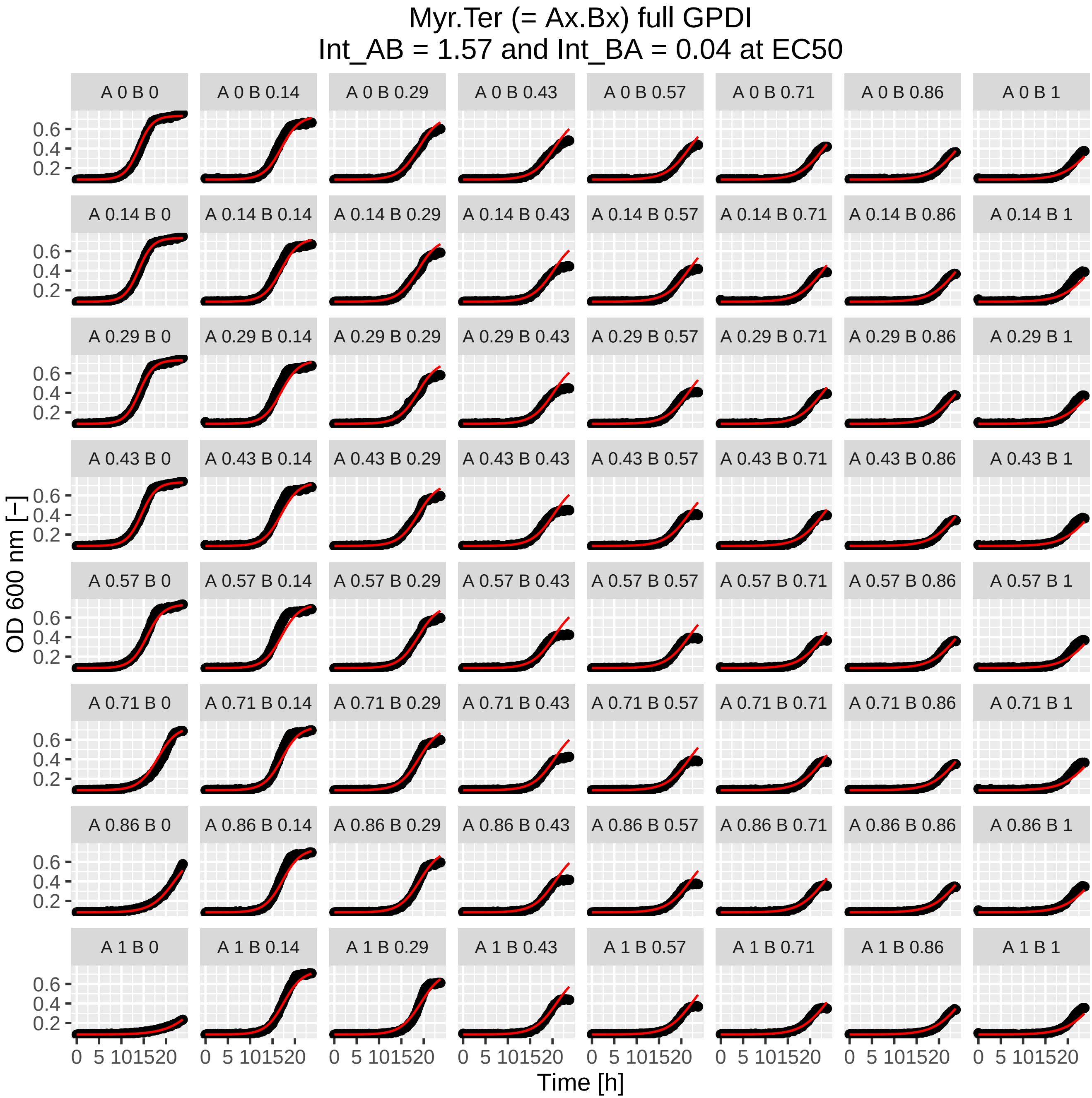
Myr.Ter (= Ax.Bx) full GPDI
Int_AB = 1.57 and Int_BA = 0.04 at EC50
A 0 B 0
A 0 B 0.14
A 0 B 0.29
A 0 B 0.43
A 0 B 0.57
A 0 B 0.71
A 0 B 0.86
A 0 B 1
A 0.14 B 0
A 0.14 B 0.14
A 0.14 B 0.29
A 0.14 B 0.43
A 0.14 B 0.57
A 0.14 B 0.71
A 0.14 B 0.86
A 0.14 B 1
A 0.29 B 0
A 0.29 B 0.14
A 0.29 B 0.29
A 0.29 B 0.43
A 0.29 B 0.57
A 0.29 B 0.71
A 0.29 B 0.86
A 0.29 B 1
A 0.43 B 0
A 0.43 B 0.14
A 0.43 B 0.29
A 0.43 B 0.43
A 0.43 B 0.57
A 0.43 B 0.71
A 0.43 B 0.86
A 0.43 B 1
A 0.57 B 0
A 0.57 B 0.14
A 0.57 B 0.29
A 0.57 B 0.43
A 0.57 B 0.57
A 0.57 B 0.71
A 0.57 B 0.86
A 0.57 B 1
A 0.71 B 0
A 0.71 B 0.14
A 0.71 B 0.29
A 0.71 B 0.43
A 0.71 B 0.57
A 0.71 B 0.71
A 0.71 B 0.86
A 0.71 B 1
A 0.86 B 0
A 0.86 B 0.14
A 0.86 B 0.29
A 0.86 B 0.43
A 0.86 B 0.57
A 0.86 B 0.71
A 0.86 B 0.86
A 0.86 B 1
A 1 B 0
A 1 B 0.14
A 1 B 0.29
A 1 B 0.43
A 1 B 0.57
A 1 B 0.71
A 1 B 0.86
A 1 B 1
OD 600 nm [–]
0.6
0.4
0.2
0 5 101520
Time [h]

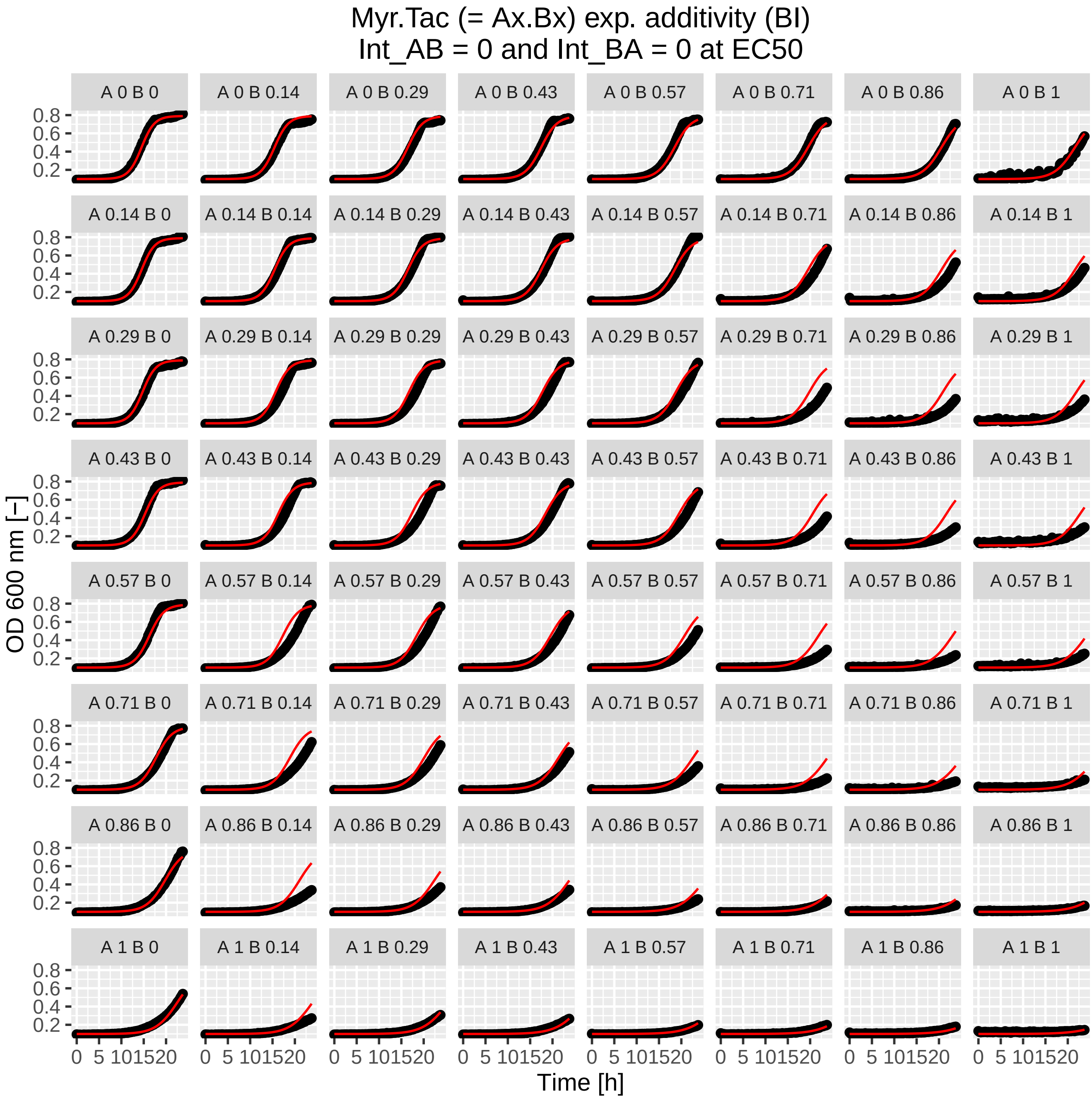

Myr.Tac (= Ax.Bx) exp. additivity (BI)
Int_AB = 0 and Int_BA = 0 at EC50
A 0 B 0
A 0 B 0.14
A 0 B 0.29
A 0 B 0.43
A 0 B 0.57
A 0 B 0.71
A 0 B 0.86
A 0 B 1
A 0.14 B 0
A 0.14 B 0.14
A 0.14 B 0.29
A 0.14 B 0.43
A 0.14 B 0.57
A 0.14 B 0.71
A 0.14 B 0.86
A 0.14 B 1
A 0.29 B 0
A 0.29 B 0.14
A 0.29 B 0.29
A 0.29 B 0.43
A 0.29 B 0.57
A 0.29 B 0.71
A 0.29 B 0.86
A 0.29 B 1
A 0.43 B 0
A 0.43 B 0.14
A 0.43 B 0.29
A 0.43 B 0.43
A 0.43 B 0.57
A 0.43 B 0.71
A 0.43 B 0.86
A 0.43 B 1
A 0.57 B 0
A 0.57 B 0.14
A 0.57 B 0.29
A 0.57 B 0.43
A 0.57 B 0.57
A 0.57 B 0.71
A 0.57 B 0.86
A 0.57 B 1
A 0.71 B 0
A 0.71 B 0.14
A 0.71 B 0.29
A 0.71 B 0.43
A 0.71 B 0.57
A 0.71 B 0.71
A 0.71 B 0.86
A 0.71 B 1
A 0.86 B 0
A 0.86 B 0.14
A 0.86 B 0.29
A 0.86 B 0.43
A 0.86 B 0.57
A 0.86 B 0.71
A 0.86 B 0.86
A 0.86 B 1
A 1 B 0
A 1 B 0.14
A 1 B 0.29
A 1 B 0.43
A 1 B 0.57
A 1 B 0.71
A 1 B 0.86
A 1 B 1
OD 600 nm [–]
0.2
0.4
0.6
0.8
0 5 101520
Time [h]

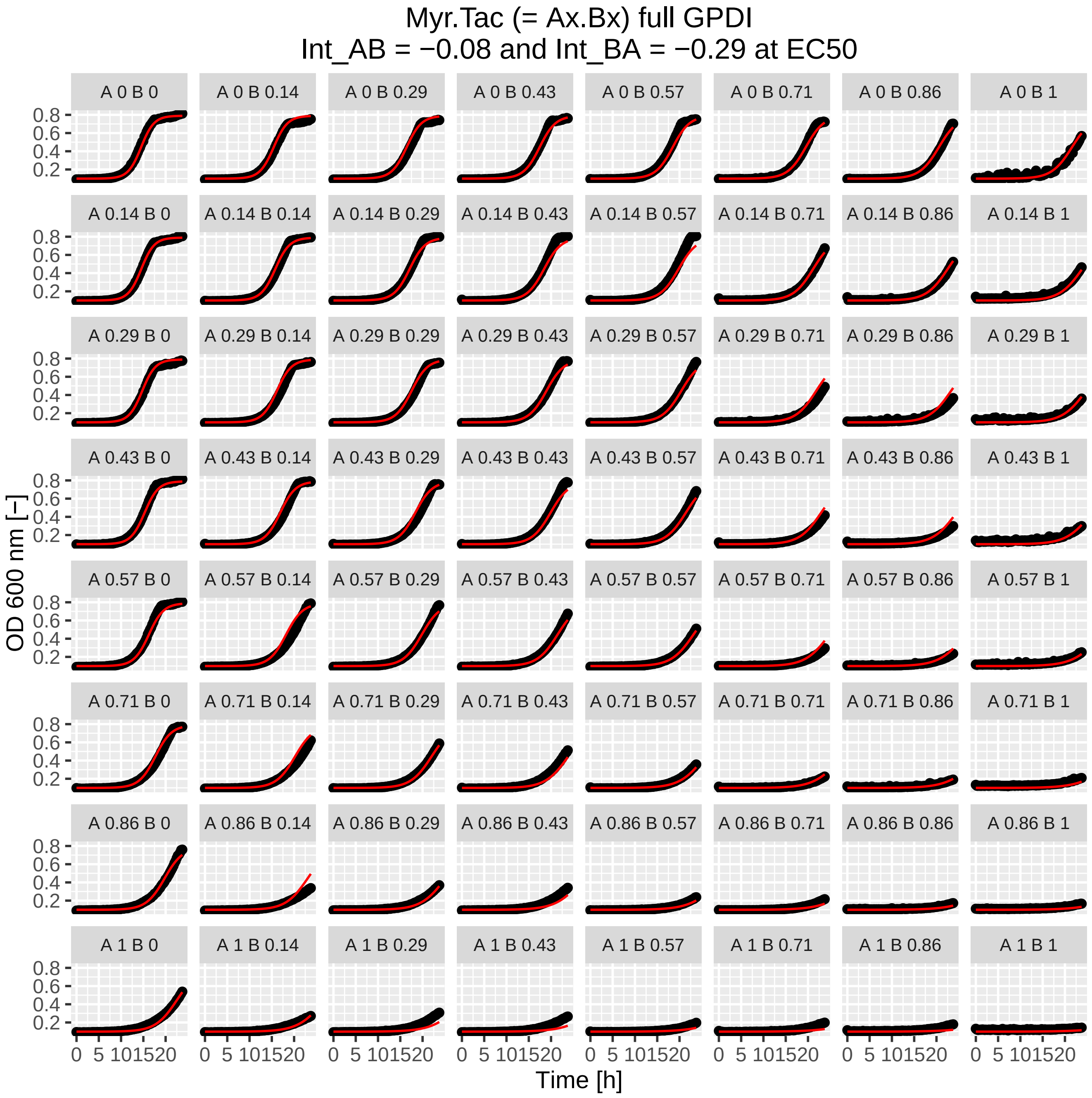

Myr.Tac (= Ax.Bx) full GPDI
Int_AB = −0.08 and Int_BA = −0.29 at EC50
A 0 B 0
A 0 B 0.14
A 0 B 0.29
A 0 B 0.43
A 0 B 0.57
A 0 B 0.71
A 0 B 0.86
A 0 B 1
A 0.14 B 0
A 0.14 B 0.14
A 0.14 B 0.29
A 0.14 B 0.43
A 0.14 B 0.57
A 0.14 B 0.71
A 0.14 B 0.86
A 0.14 B 1
A 0.29 B 0
A 0.29 B 0.14
A 0.29 B 0.29
A 0.29 B 0.43
A 0.29 B 0.57
A 0.29 B 0.71
A 0.29 B 0.86
A 0.29 B 1
A 0.43 B 0
A 0.43 B 0.14
A 0.43 B 0.29
A 0.43 B 0.43
A 0.43 B 0.57
A 0.43 B 0.71
A 0.43 B 0.86
A 0.43 B 1
A 0.57 B 0
A 0.57 B 0.14
A 0.57 B 0.29
A 0.57 B 0.43
A 0.57 B 0.57
A 0.57 B 0.71
A 0.57 B 0.86
A 0.57 B 1
A 0.71 B 0
A 0.71 B 0.14
A 0.71 B 0.29
A 0.71 B 0.43
A 0.71 B 0.57
A 0.71 B 0.71
A 0.71 B 0.86
A 0.71 B 1
A 0.86 B 0
A 0.86 B 0.14
A 0.86 B 0.29
A 0.86 B 0.43
A 0.86 B 0.57
A 0.86 B 0.71
A 0.86 B 0.86
A 0.86 B 1
A 1 B 0
A 1 B 0.14
A 1 B 0.29
A 1 B 0.43
A 1 B 0.57
A 1 B 0.71
A 1 B 0.86
A 1 B 1
OD 600 nm [−]
0.2
0.4
0.6
0.8
0
5
10
15
20
Time [h]

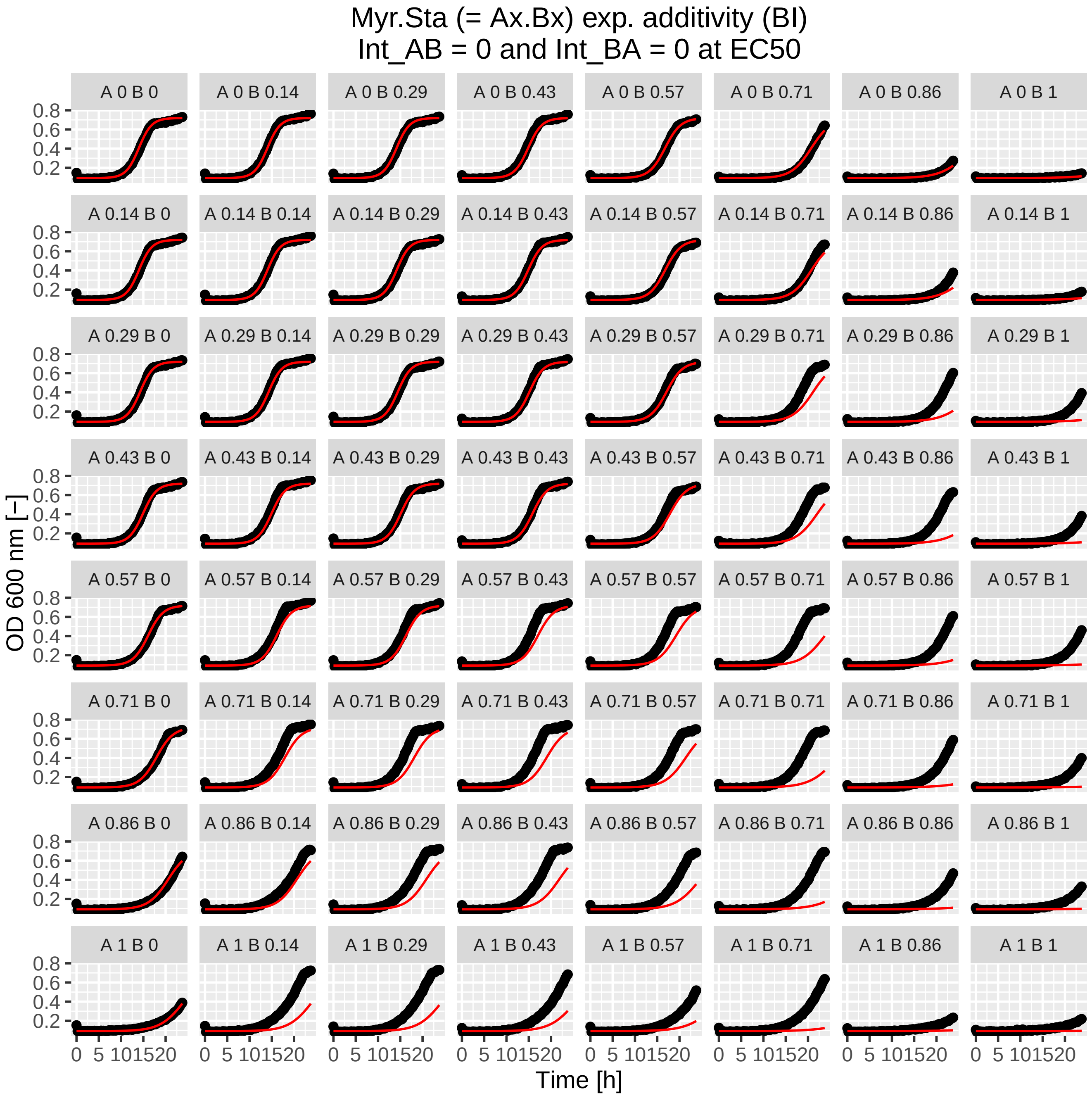
Myr.Sta (= Ax.Bx) exp. additivity (BI)
Int_AB = 0 and Int_BA = 0 at EC50
OD 600 nm [–]
Time [h]
A 0 B 0
A 0 B 0.14
A 0 B 0.29
A 0 B 0.43
A 0 B 0.57
A 0 B 0.71
A 0 B 0.86
A 0 B 1
A 1 B 0
A 1 B 0.14
A 1 B 0.29
A 1 B 0.43
A 1 B 0.57
A 1 B 0.71
A 1 B 0.86
A 1 B 1
0.8
0.6
0.4
0.2
0 5 101520

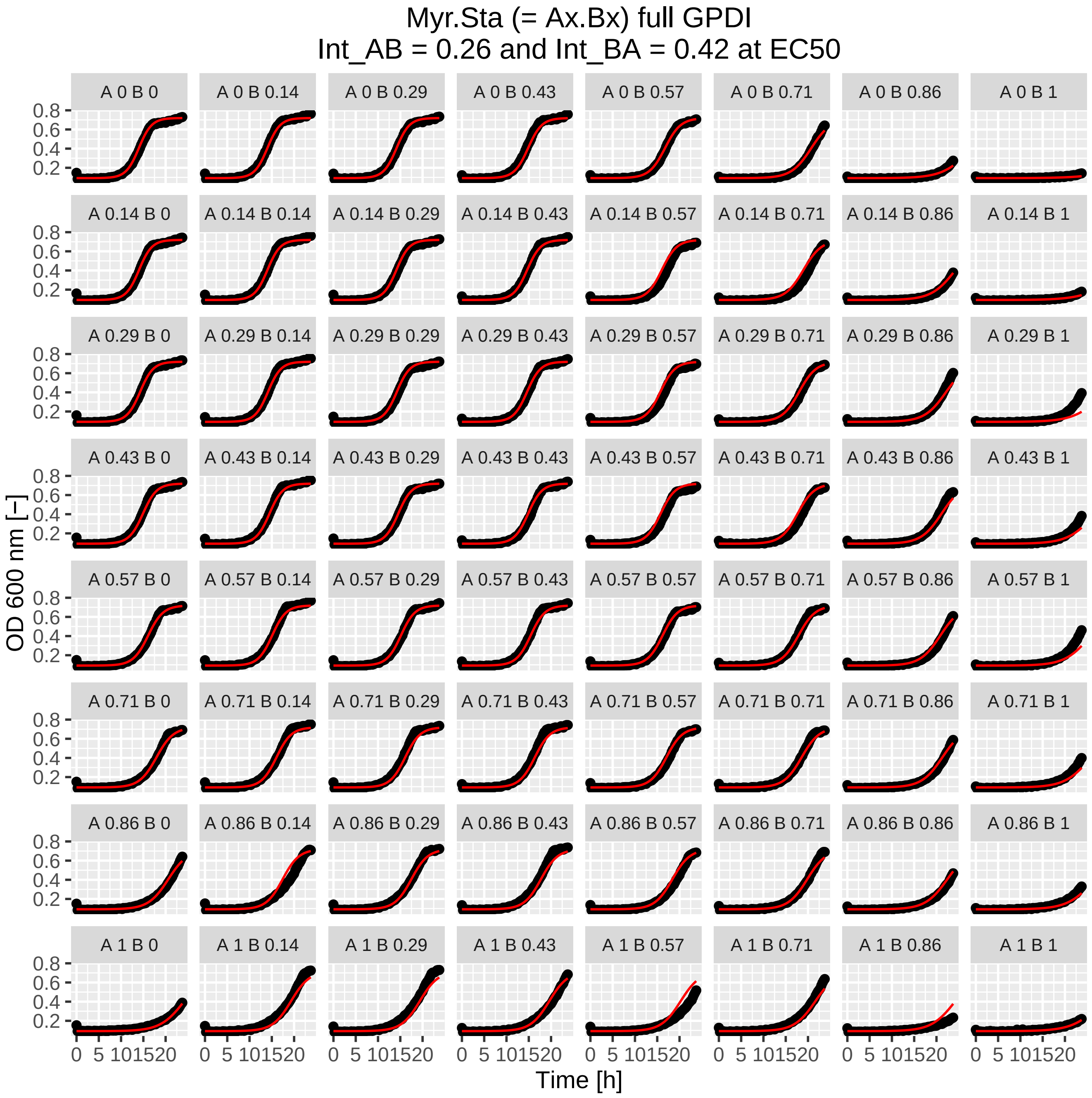
Myr.Sta (= Ax.Bx) full GPDI
Int_AB = 0.26 and Int_BA = 0.42 at EC50
A 0 B 0
A 0 B 0.14
A 0 B 0.29
A 0 B 0.43
A 0 B 0.57
A 0 B 0.71
A 0 B 0.86
A 0 B 1
A 0.14 B 0
A 0.14 B 0.14
A 0.14 B 0.29
A 0.14 B 0.43
A 0.14 B 0.57
A 0.14 B 0.71
A 0.14 B 0.86
A 0.14 B 1
A 0.29 B 0
A 0.29 B 0.14
A 0.29 B 0.29
A 0.29 B 0.43
A 0.29 B 0.57
A 0.29 B 0.71
A 0.29 B 0.86
A 0.29 B 1
A 0.43 B 0
A 0.43 B 0.14
A 0.43 B 0.29
A 0.43 B 0.43
A 0.43 B 0.57
A 0.43 B 0.71
A 0.43 B 0.86
A 0.43 B 1
A 0.57 B 0
A 0.57 B 0.14
A 0.57 B 0.29
A 0.57 B 0.43
A 0.57 B 0.57
A 0.57 B 0.71
A 0.57 B 0.86
A 0.57 B 1
A 0.71 B 0
A 0.71 B 0.14
A 0.71 B 0.29
A 0.71 B 0.43
A 0.71 B 0.57
A 0.71 B 0.71
A 0.71 B 0.86
A 0.71 B 1
A 0.86 B 0
A 0.86 B 0.14
A 0.86 B 0.29
A 0.86 B 0.43
A 0.86 B 0.57
A 0.86 B 0.71
A 0.86 B 0.86
A 0.86 B 1
A 1 B 0
A 1 B 0.14
A 1 B 0.29
A 1 B 0.43
A 1 B 0.57
A 1 B 0.71
A 1 B 0.86
A 1 B 1
OD 600 nm [–]
0.2
0.4
0.6
0.8
0
5
10
15
20
Time [h]

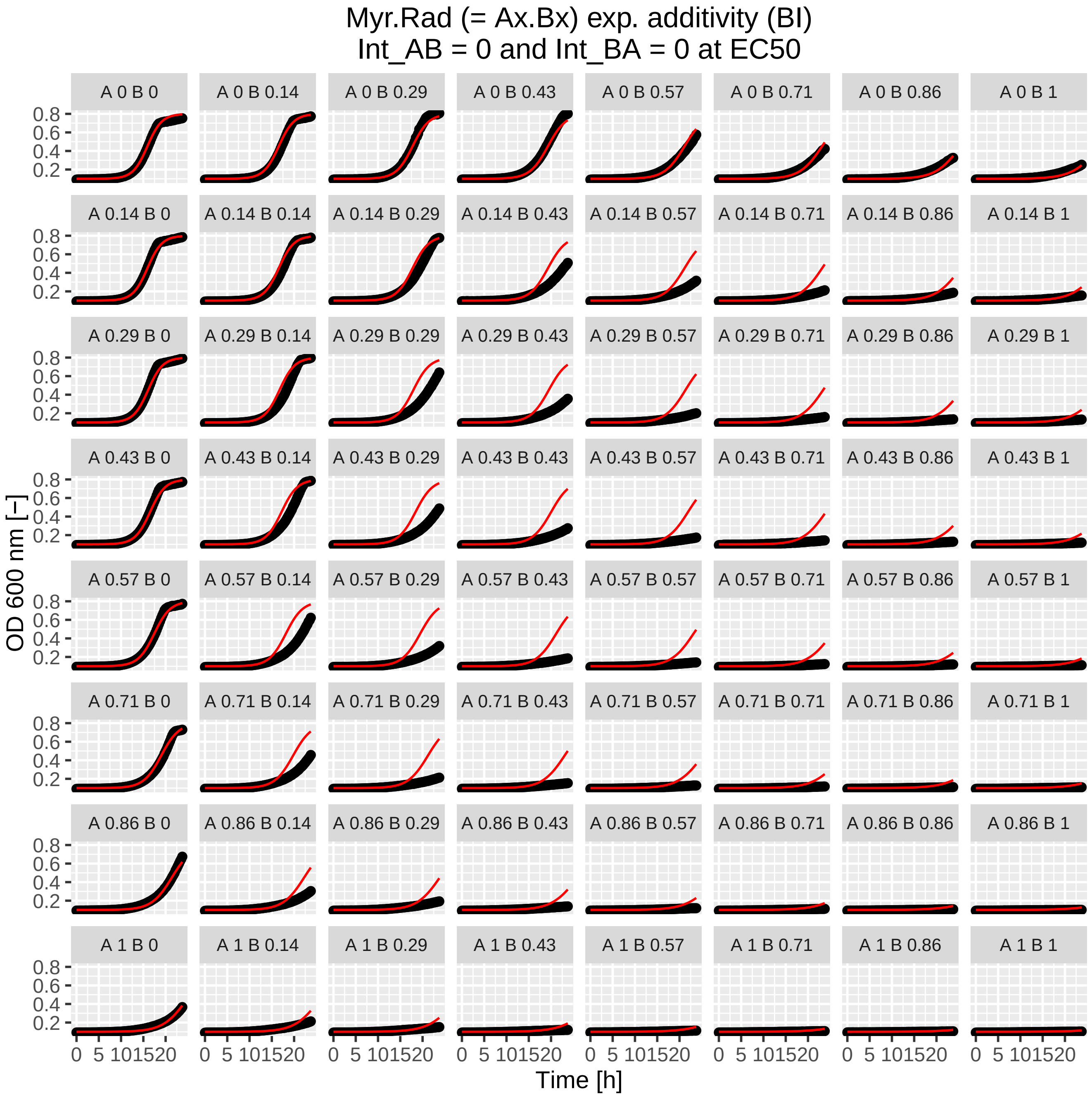
Myr.Rad (= Ax.Bx) exp. additivity (BI)
Int_AB = 0 and Int_BA = 0 at EC50
OD 600 nm [–]
Time [h]
A 0 B 0
A 0 B 0.14
A 0 B 0.29
A 0 B 0.43
A 0 B 0.57
A 0 B 0.71
A 0 B 0.86
A 0 B 1
A 0.14 B 0
A 0.14 B 0.14
A 0.14 B 0.29
A 0.14 B 0.43
A 0.14 B 0.57
A 0.14 B 0.71
A 0.14 B 0.86
A 0.14 B 1
A 0.29 B 0
A 0.29 B 0.14
A 0.29 B 0.29
A 0.29 B 0.43
A 0.29 B 0.57
A 0.29 B 0.71
A 0.29 B 0.86
A 0.29 B 1
A 0.43 B 0
A 0.43 B 0.14
A 0.43 B 0.29
A 0.43 B 0.43
A 0.43 B 0.57
A 0.43 B 0.71
A 0.43 B 0.86
A 0.43 B 1
A 0.57 B 0
A 0.57 B 0.14
A 0.57 B 0.29
A 0.57 B 0.43
A 0.57 B 0.57
A 0.57 B 0.71
A 0.57 B 0.86
A 0.57 B 1
A 0.71 B 0
A 0.71 B 0.14
A 0.71 B 0.29
A 0.71 B 0.43
A 0.71 B 0.57
A 0.71 B 0.71
A 0.71 B 0.86
A 0.71 B 1
A 0.86 B 0
A 0.86 B 0.14
A 0.86 B 0.29
A 0.86 B 0.43
A 0.86 B 0.57
A 0.86 B 0.71
A 0.86 B 0.86
A 0.86 B 1
A 1 B 0
A 1 B 0.14
A 1 B 0.29
A 1 B 0.43
A 1 B 0.57
A 1 B 0.71
A 1 B 0.86
A 1 B 1
0.2
0.4
0.6
0.8
0
5
10
15
20

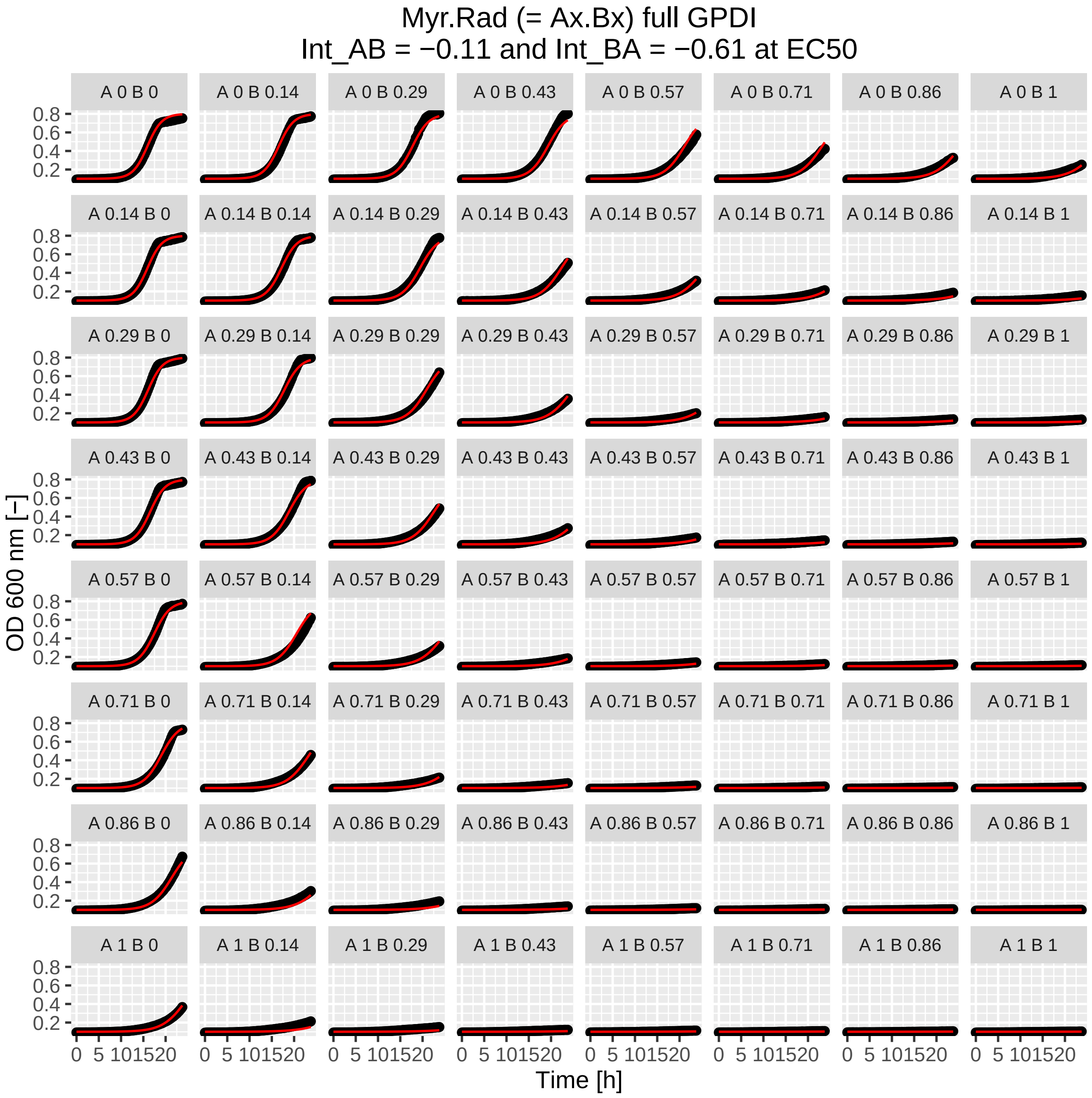
Myr.Rad (= Ax.Bx) full GPDI
Int_AB = −0.11 and Int_BA = −0.61 at EC50
A 0 B 0
A 0 B 0.14
A 0 B 0.29
A 0 B 0.43
A 0 B 0.57
A 0 B 0.71
A 0 B 0.86
A 0 B 1
A 0.14 B 0
A 0.14 B 0.14
A 0.14 B 0.29
A 0.14 B 0.43
A 0.14 B 0.57
A 0.14 B 0.71
A 0.14 B 0.86
A 0.14 B 1
A 0.29 B 0
A 0.29 B 0.14
A 0.29 B 0.29
A 0.29 B 0.43
A 0.29 B 0.57
A 0.29 B 0.71
A 0.29 B 0.86
A 0.29 B 1
A 0.43 B 0
A 0.43 B 0.14
A 0.43 B 0.29
A 0.43 B 0.43
A 0.43 B 0.57
A 0.43 B 0.71
A 0.43 B 0.86
A 0.43 B 1
A 0.57 B 0
A 0.57 B 0.14
A 0.57 B 0.29
A 0.57 B 0.43
A 0.57 B 0.57
A 0.57 B 0.71
A 0.57 B 0.86
A 0.57 B 1
A 0.71 B 0
A 0.71 B 0.14
A 0.71 B 0.29
A 0.71 B 0.43
A 0.71 B 0.57
A 0.71 B 0.71
A 0.71 B 0.86
A 0.71 B 1
A 0.86 B 0
A 0.86 B 0.14
A 0.86 B 0.29
A 0.86 B 0.43
A 0.86 B 0.57
A 0.86 B 0.71
A 0.86 B 0.86
A 0.86 B 1
A 1 B 0
A 1 B 0.14
A 1 B 0.29
A 1 B 0.43
A 1 B 0.57
A 1 B 0.71
A 1 B 0.86
A 1 B 1
OD 600 nm [−]
0.8
0.6
0.4
0.2
0 5 101520
Time [h]

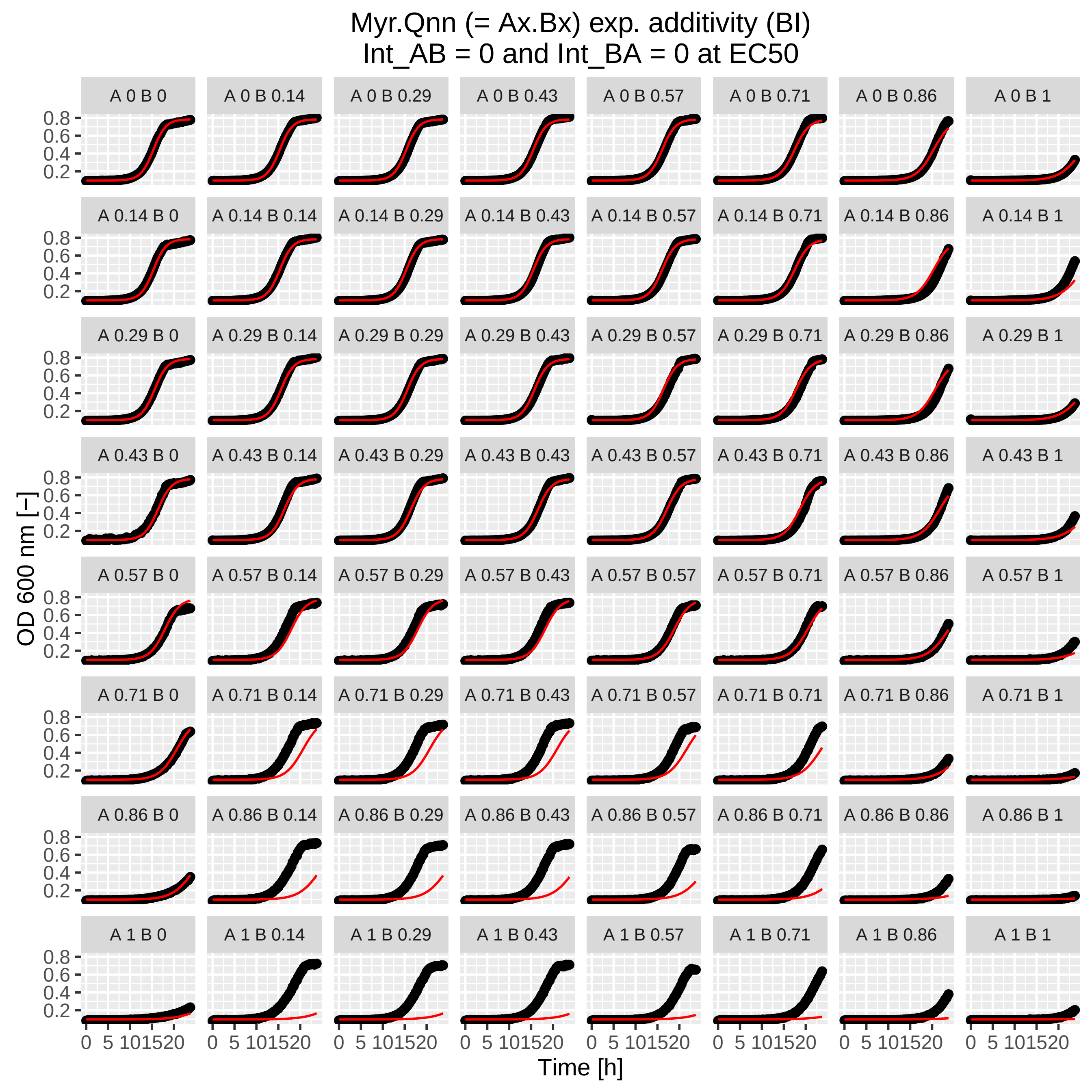
Myr.Qnn (= Ax.Bx) exp. additivity (BI)
Int_AB = 0 and Int_BA = 0 at EC50
A 0 B 0
A 0 B 0.14
A 0 B 0.29
A 0 B 0.43
A 0 B 0.57
A 0 B 0.71
A 0 B 0.86
A 0 B 1
A 0.14 B 0
A 0.14 B 0.14
A 0.14 B 0.29
A 0.14 B 0.43
A 0.14 B 0.57
A 0.14 B 0.71
A 0.14 B 0.86
A 0.14 B 1
A 0.29 B 0
A 0.29 B 0.14
A 0.29 B 0.29
A 0.29 B 0.43
A 0.29 B 0.57
A 0.29 B 0.71
A 0.29 B 0.86
A 0.29 B 1
A 0.43 B 0
A 0.43 B 0.14
A 0.43 B 0.29
A 0.43 B 0.43
A 0.43 B 0.57
A 0.43 B 0.71
A 0.43 B 0.86
A 0.43 B 1
A 0.57 B 0
A 0.57 B 0.14
A 0.57 B 0.29
A 0.57 B 0.43
A 0.57 B 0.57
A 0.57 B 0.71
A 0.57 B 0.86
A 0.57 B 1
A 0.71 B 0
A 0.71 B 0.14
A 0.71 B 0.29
A 0.71 B 0.43
A 0.71 B 0.57
A 0.71 B 0.71
A 0.71 B 0.86
A 0.71 B 1
A 0.86 B 0
A 0.86 B 0.14
A 0.86 B 0.29
A 0.86 B 0.43
A 0.86 B 0.57
A 0.86 B 0.71
A 0.86 B 0.86
A 0.86 B 1
A 1 B 0
A 1 B 0.14
A 1 B 0.29
A 1 B 0.43
A 1 B 0.57
A 1 B 0.71
A 1 B 0.86
A 1 B 1
OD 600 nm [–]
0.2
0.4
0.6
0.8
0
5
10
15
20
Time [h]

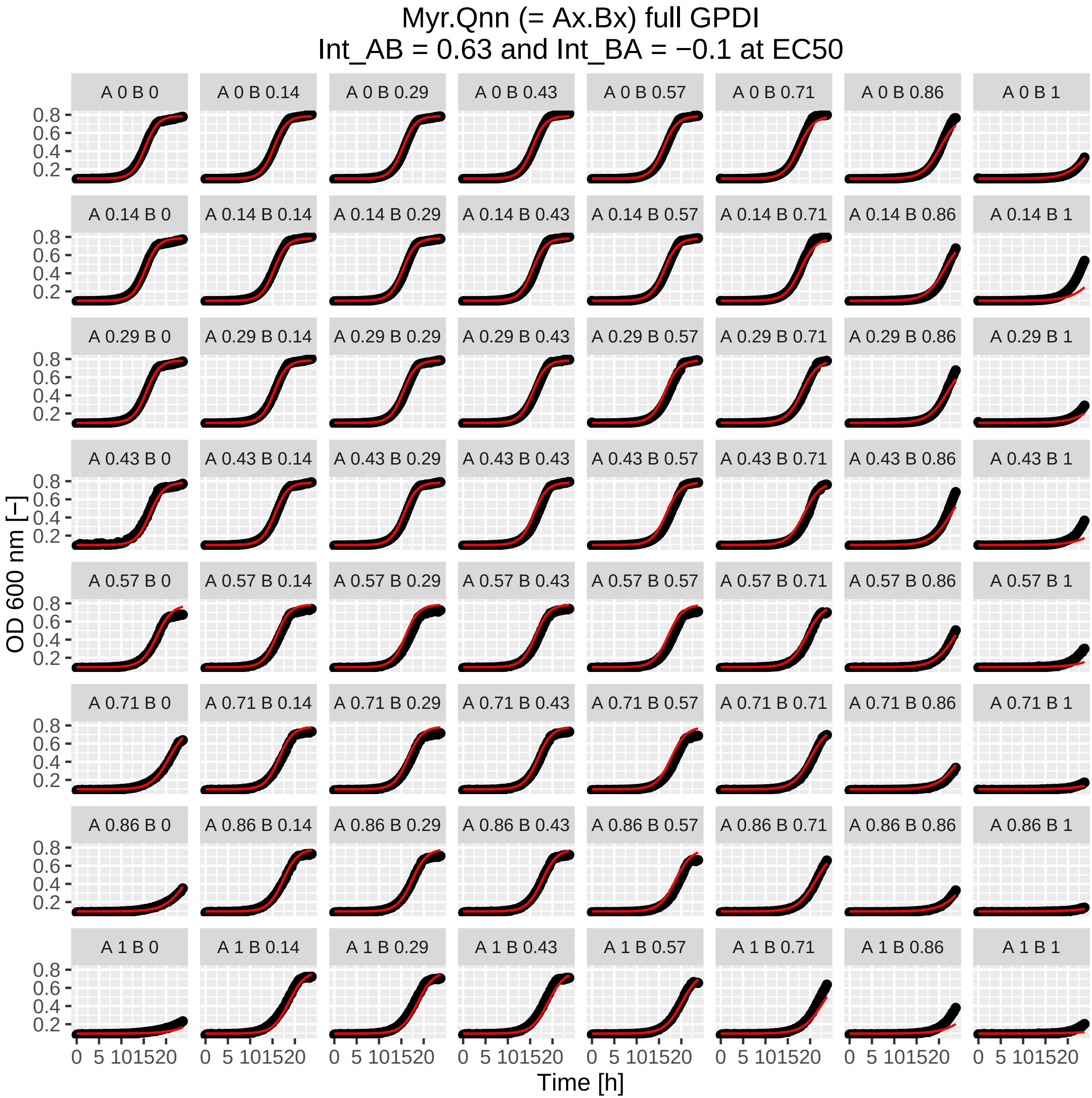

Myr.Qnn (= Ax.Bx) full GPDI
Int_AB = 0.63 and Int_BA = −0.1 at EC50
A 0 B 0
A 0 B 0.14
A 0 B 0.29
A 0 B 0.43
A 0 B 0.57
A 0 B 0.71
A 0 B 0.86
A 0 B 1
A 0.14 B 0
A 0.14 B 0.14
A 0.14 B 0.29
A 0.14 B 0.43
A 0.14 B 0.57
A 0.14 B 0.71
A 0.14 B 0.86
A 0.14 B 1
A 0.29 B 0
A 0.29 B 0.14
A 0.29 B 0.29
A 0.29 B 0.43
A 0.29 B 0.57
A 0.29 B 0.71
A 0.29 B 0.86
A 0.29 B 1
A 0.43 B 0
A 0.43 B 0.14
A 0.43 B 0.29
A 0.43 B 0.43
A 0.43 B 0.57
A 0.43 B 0.71
A 0.43 B 0.86
A 0.43 B 1
A 0.57 B 0
A 0.57 B 0.14
A 0.57 B 0.29
A 0.57 B 0.43
A 0.57 B 0.57
A 0.57 B 0.71
A 0.57 B 0.86
A 0.57 B 1
A 0.71 B 0
A 0.71 B 0.14
A 0.71 B 0.29
A 0.71 B 0.43
A 0.71 B 0.57
A 0.71 B 0.71
A 0.71 B 0.86
A 0.71 B 1
A 0.86 B 0
A 0.86 B 0.14
A 0.86 B 0.29
A 0.86 B 0.43
A 0.86 B 0.57
A 0.86 B 0.71
A 0.86 B 0.86
A 0.86 B 1
A 1 B 0
A 1 B 0.14
A 1 B 0.29
A 1 B 0.43
A 1 B 0.57
A 1 B 0.71
A 1 B 0.86
A 1 B 1
OD 600 nm [−]
0.8
0.6
0.4
0.2
0 5 101520
Time [h]

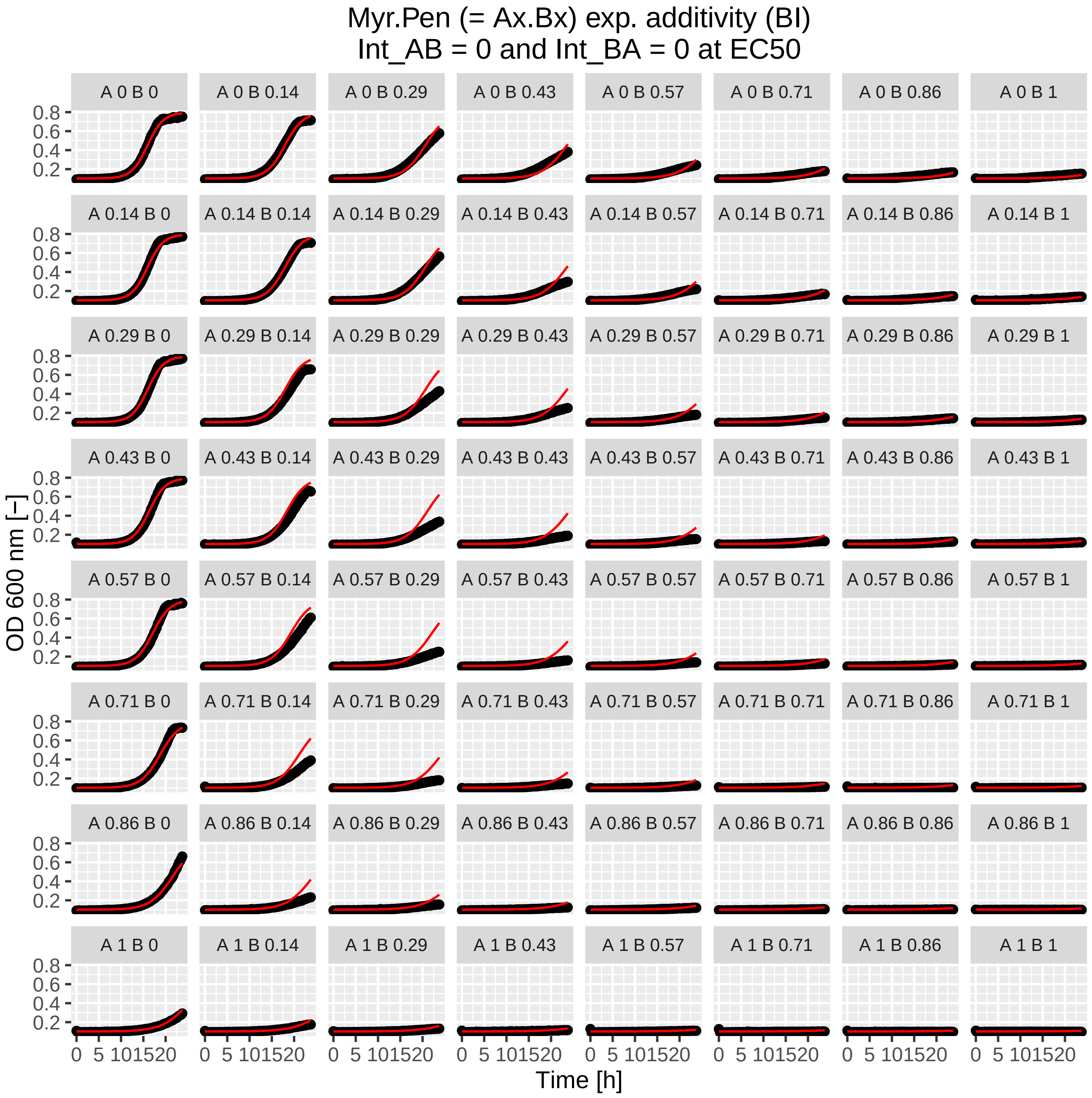
Myr.Pen (= Ax.Bx) exp. additivity (BI)
Int_AB = 0 and Int_BA = 0 at EC50
A 0 B 0
A 0 B 0.14
A 0 B 0.29
A 0 B 0.43
A 0 B 0.57
A 0 B 0.71
A 0 B 0.86
A 0 B 1
A 0.14 B 0
A 0.14 B 0.14
A 0.14 B 0.29
A 0.14 B 0.43
A 0.14 B 0.57
A 0.14 B 0.71
A 0.14 B 0.86
A 0.14 B 1
A 0.29 B 0
A 0.29 B 0.14
A 0.29 B 0.29
A 0.29 B 0.43
A 0.29 B 0.57
A 0.29 B 0.71
A 0.29 B 0.86
A 0.29 B 1
A 0.43 B 0
A 0.43 B 0.14
A 0.43 B 0.29
A 0.43 B 0.43
A 0.43 B 0.57
A 0.43 B 0.71
A 0.43 B 0.86
A 0.43 B 1
A 0.57 B 0
A 0.57 B 0.14
A 0.57 B 0.29
A 0.57 B 0.43
A 0.57 B 0.57
A 0.57 B 0.71
A 0.57 B 0.86
A 0.57 B 1
A 0.71 B 0
A 0.71 B 0.14
A 0.71 B 0.29
A 0.71 B 0.43
A 0.71 B 0.57
A 0.71 B 0.71
A 0.71 B 0.86
A 0.71 B 1
A 0.86 B 0
A 0.86 B 0.14
A 0.86 B 0.29
A 0.86 B 0.43
A 0.86 B 0.57
A 0.86 B 0.71
A 0.86 B 0.86
A 0.86 B 1
A 1 B 0
A 1 B 0.14
A 1 B 0.29
A 1 B 0.43
A 1 B 0.57
A 1 B 0.71
A 1 B 0.86
A 1 B 1
OD 600 nm [–]
0.2
0.4
0.6
0.8
0 5 101520
Time [h]

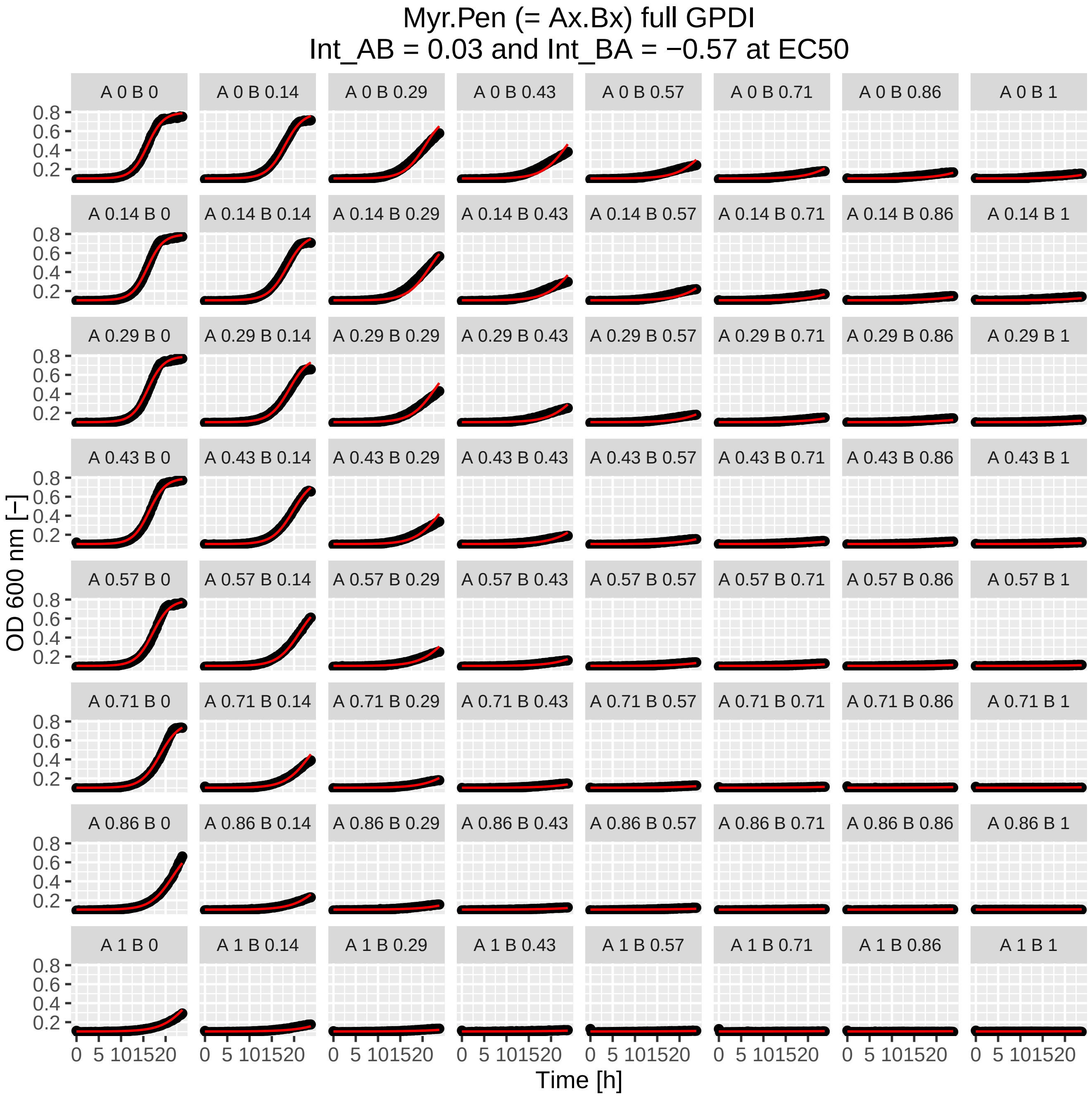

Myr.Pen (= Ax.Bx) full GPDI
Int_AB = 0.03 and Int_BA = −0.57 at EC50
A 0 B 0
A 0 B 0.14
A 0 B 0.29
A 0 B 0.43
A 0 B 0.57
A 0 B 0.71
A 0 B 0.86
A 0 B 1
A 0.14 B 0
A 0.14 B 0.14
A 0.14 B 0.29
A 0.14 B 0.43
A 0.14 B 0.57
A 0.14 B 0.71
A 0.14 B 0.86
A 0.14 B 1
A 0.29 B 0
A 0.29 B 0.14
A 0.29 B 0.29
A 0.29 B 0.43
A 0.29 B 0.57
A 0.29 B 0.71
A 0.29 B 0.86
A 0.29 B 1
A 0.43 B 0
A 0.43 B 0.14
A 0.43 B 0.29
A 0.43 B 0.43
A 0.43 B 0.57
A 0.43 B 0.71
A 0.43 B 0.86
A 0.43 B 1
A 0.57 B 0
A 0.57 B 0.14
A 0.57 B 0.29
A 0.57 B 0.43
A 0.57 B 0.57
A 0.57 B 0.71
A 0.57 B 0.86
A 0.57 B 1
A 0.71 B 0
A 0.71 B 0.14
A 0.71 B 0.29
A 0.71 B 0.43
A 0.71 B 0.57
A 0.71 B 0.71
A 0.71 B 0.86
A 0.71 B 1
A 0.86 B 0
A 0.86 B 0.14
A 0.86 B 0.29
A 0.86 B 0.43
A 0.86 B 0.57
A 0.86 B 0.71
A 0.86 B 0.86
A 0.86 B 1
A 1 B 0
A 1 B 0.14
A 1 B 0.29
A 1 B 0.43
A 1 B 0.57
A 1 B 0.71
A 1 B 0.86
A 1 B 1
OD 600 nm [−]
0.2
0.4
0.6
0.8
0 5 101520
Time [h]

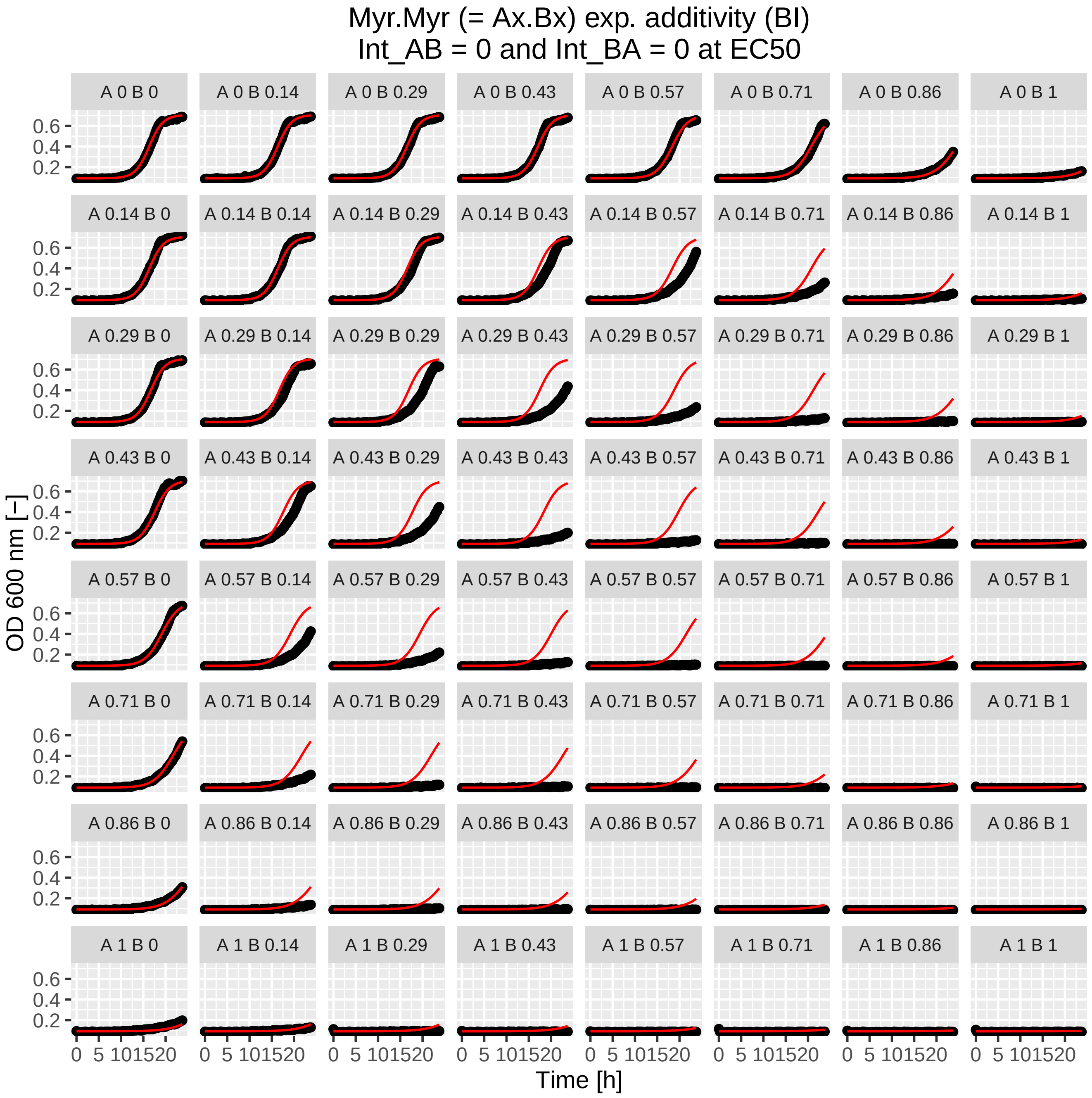
Myr.Myr (= Ax.Bx) exp. additivity (BI)
Int_AB = 0 and Int_BA = 0 at EC50
A 0 B 0
A 0 B 0.14
A 0 B 0.29
A 0 B 0.43
A 0 B 0.57
A 0 B 0.71
A 0 B 0.86
A 0 B 1
A 0.14 B 0
A 0.14 B 0.14
A 0.14 B 0.29
A 0.14 B 0.43
A 0.14 B 0.57
A 0.14 B 0.71
A 0.14 B 0.86
A 0.14 B 1
A 0.29 B 0
A 0.29 B 0.14
A 0.29 B 0.29
A 0.29 B 0.43
A 0.29 B 0.57
A 0.29 B 0.71
A 0.29 B 0.86
A 0.29 B 1
A 0.43 B 0
A 0.43 B 0.14
A 0.43 B 0.29
A 0.43 B 0.43
A 0.43 B 0.57
A 0.43 B 0.71
A 0.43 B 0.86
A 0.43 B 1
A 0.57 B 0
A 0.57 B 0.14
A 0.57 B 0.29
A 0.57 B 0.43
A 0.57 B 0.57
A 0.57 B 0.71
A 0.57 B 0.86
A 0.57 B 1
A 0.71 B 0
A 0.71 B 0.14
A 0.71 B 0.29
A 0.71 B 0.43
A 0.71 B 0.57
A 0.71 B 0.71
A 0.71 B 0.86
A 0.71 B 1
A 0.86 B 0
A 0.86 B 0.14
A 0.86 B 0.29
A 0.86 B 0.43
A 0.86 B 0.57
A 0.86 B 0.71
A 0.86 B 0.86
A 0.86 B 1
A 1 B 0
A 1 B 0.14
A 1 B 0.29
A 1 B 0.43
A 1 B 0.57
A 1 B 0.71
A 1 B 0.86
A 1 B 1
0.6
0.4
0.2
OD 600 nm [–]
0 5 101520
Time [h]

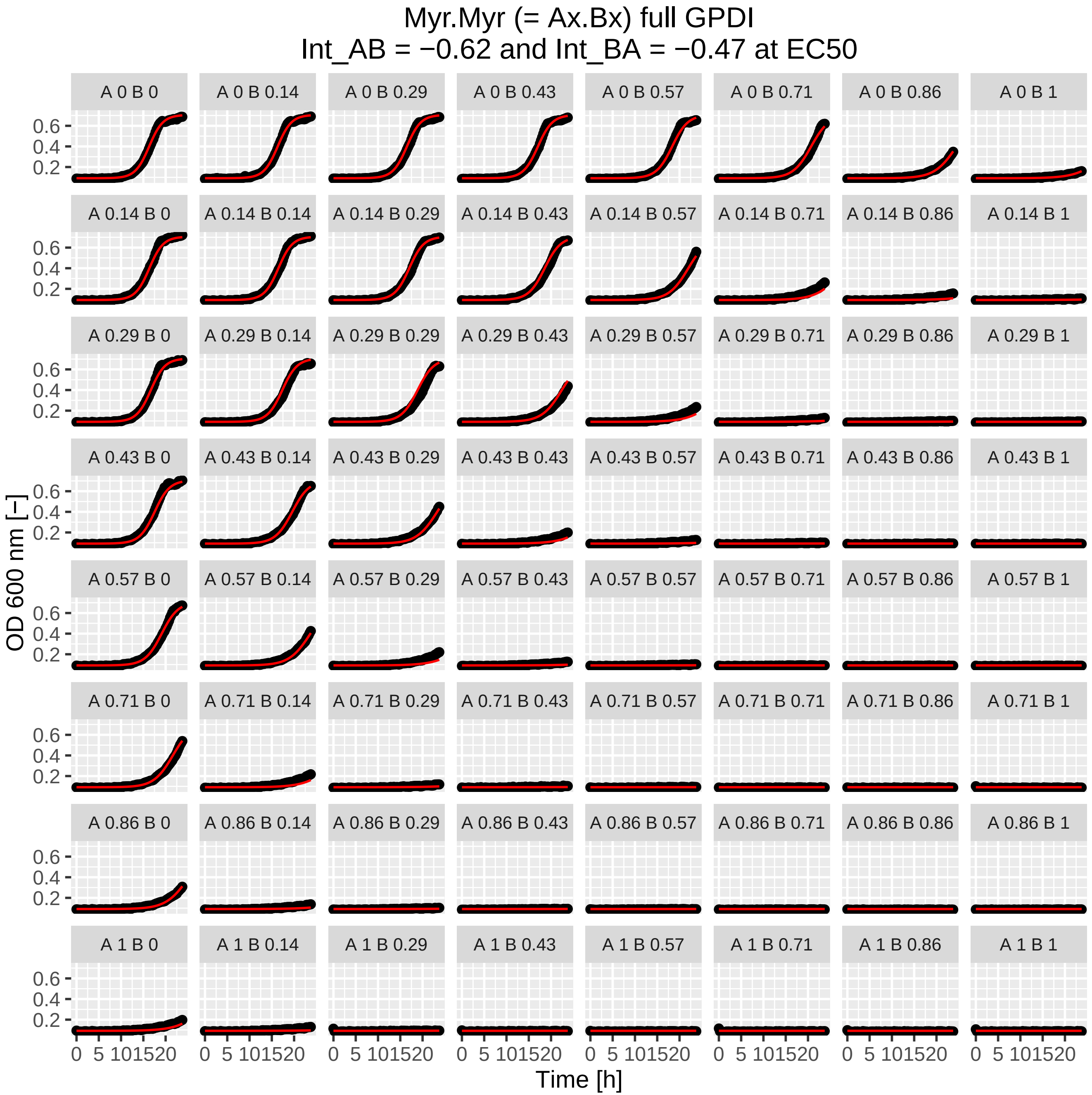

Myr.Myr (= Ax.Bx) full GPDI
Int_AB = −0.62 and Int_BA = −0.47 at EC50
A 0 B 0
A 0 B 0.14
A 0 B 0.29
A 0 B 0.43
A 0 B 0.57
A 0 B 0.71
A 0 B 0.86
A 0 B 1
A 0.14 B 0
A 0.14 B 0.14
A 0.14 B 0.29
A 0.14 B 0.43
A 0.14 B 0.57
A 0.14 B 0.71
A 0.14 B 0.86
A 0.14 B 1
A 0.29 B 0
A 0.29 B 0.14
A 0.29 B 0.29
A 0.29 B 0.43
A 0.29 B 0.57
A 0.29 B 0.71
A 0.29 B 0.86
A 0.29 B 1
A 0.43 B 0
A 0.43 B 0.14
A 0.43 B 0.29
A 0.43 B 0.43
A 0.43 B 0.57
A 0.43 B 0.71
A 0.43 B 0.86
A 0.43 B 1
A 0.57 B 0
A 0.57 B 0.14
A 0.57 B 0.29
A 0.57 B 0.43
A 0.57 B 0.57
A 0.57 B 0.71
A 0.57 B 0.86
A 0.57 B 1
A 0.71 B 0
A 0.71 B 0.14
A 0.71 B 0.29
A 0.71 B 0.43
A 0.71 B 0.57
A 0.71 B 0.71
A 0.71 B 0.86
A 0.71 B 1
A 0.86 B 0
A 0.86 B 0.14
A 0.86 B 0.29
A 0.86 B 0.43
A 0.86 B 0.57
A 0.86 B 0.71
A 0.86 B 0.86
A 0.86 B 1
A 1 B 0
A 1 B 0.14
A 1 B 0.29
A 1 B 0.43
A 1 B 0.57
A 1 B 0.71
A 1 B 0.86
A 1 B 1
OD 600 nm [−]
0.6
0.4
0.2
0 5 101520
Time [h]

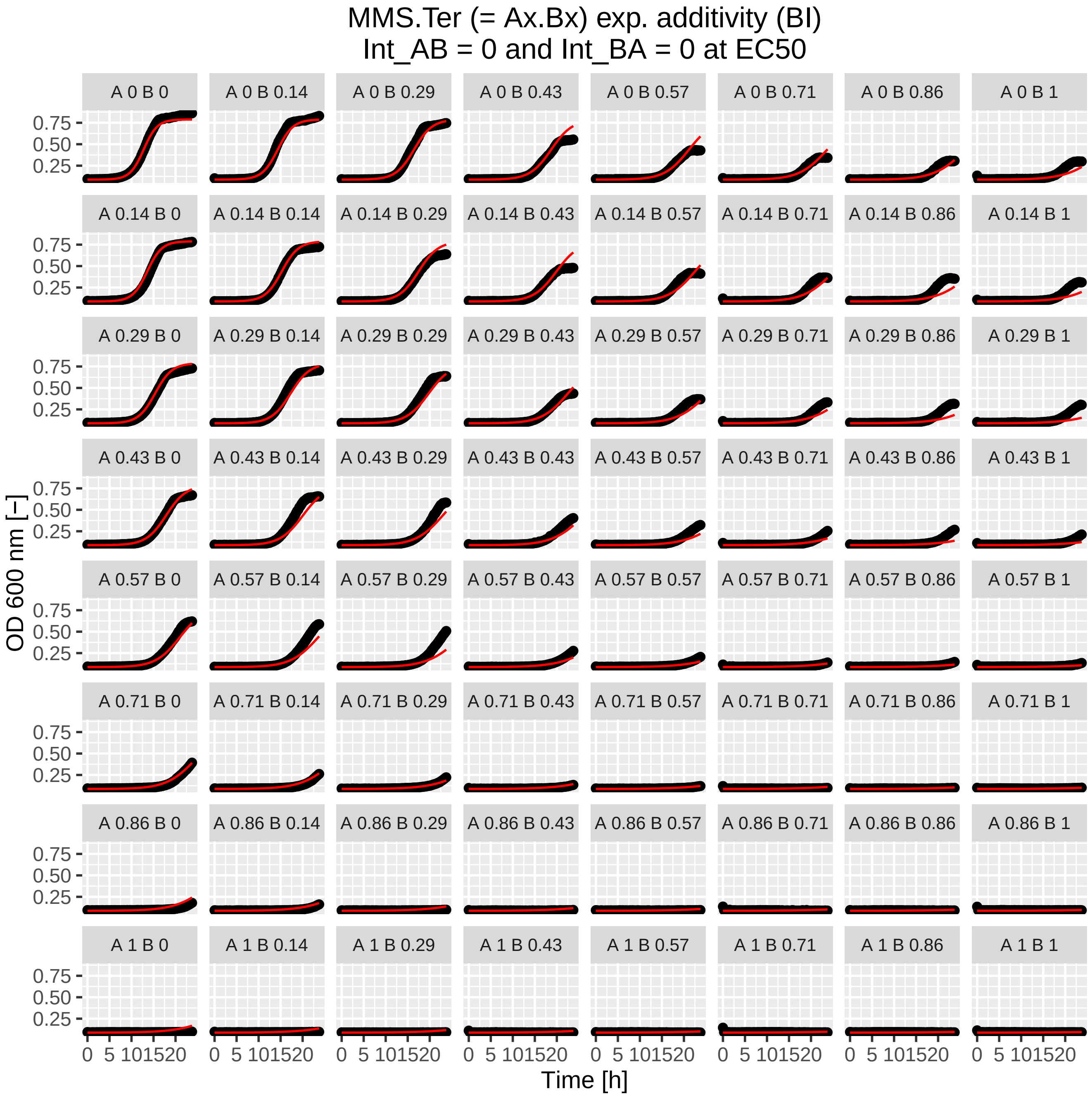

MMS.Ter (= Ax.Bx) exp. additivity (BI)
Int_AB = 0 and Int_BA = 0 at EC50
OD 600 nm [−]
Time [h]
0.75
0.50
0.25
0 5 101520
A 0 B 0
A 0 B 0.14
A 0 B 0.29
A 0 B 0.43
A 0 B 0.57
A 0 B 0.71
A 0 B 0.86
A 0 B 1
A 0.14 B 0
A 0.14 B 0.14
A 0.14 B 0.29
A 0.14 B 0.43
A 0.14 B 0.57
A 0.14 B 0.71
A 0.14 B 0.86
A 0.14 B 1
A 0.29 B 0
A 0.29 B 0.14
A 0.29 B 0.29
A 0.29 B 0.43
A 0.29 B 0.57
A 0.29 B 0.71
A 0.29 B 0.86
A 0.29 B 1
A 0.43 B 0
A 0.43 B 0.14
A 0.43 B 0.29
A 0.43 B 0.43
A 0.43 B 0.57
A 0.43 B 0.71
A 0.43 B 0.86
A 0.43 B 1
A 0.57 B 0
A 0.57 B 0.14
A 0.57 B 0.29
A 0.57 B 0.43
A 0.57 B 0.57
A 0.57 B 0.71
A 0.57 B 0.86
A 0.57 B 1
A 0.71 B 0
A 0.71 B 0.14
A 0.71 B 0.29
A 0.71 B 0.43
A 0.71 B 0.57
A 0.71 B 0.71
A 0.71 B 0.86
A 0.71 B 1
A 0.86 B 0
A 0.86 B 0.14
A 0.86 B 0.29
A 0.86 B 0.43
A 0.86 B 0.57
A 0.86 B 0.71
A 0.86 B 0.86
A 0.86 B 1
A 1 B 0
A 1 B 0.14
A 1 B 0.29
A 1 B 0.43
A 1 B 0.57
A 1 B 0.71
A 1 B 0.86
A 1 B 1

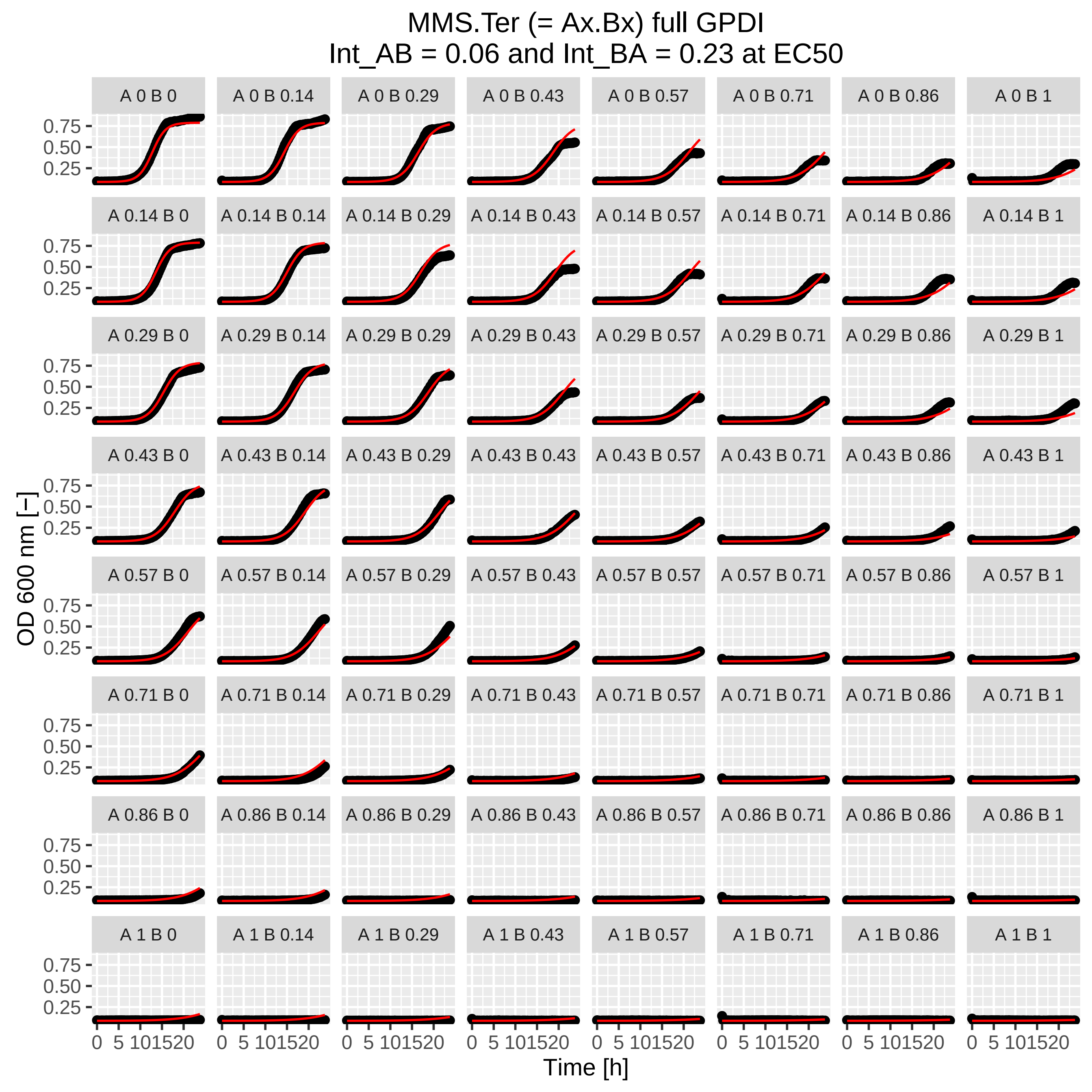

MMS.Ter (= Ax.Bx) full GPDI
Int_AB = 0.06 and Int_BA = 0.23 at EC50
A 0 B 0
A 0 B 0.14
A 0 B 0.29
A 0 B 0.43
A 0 B 0.57
A 0 B 0.71
A 0 B 0.86
A 0 B 1
A 0.14 B 0
A 0.14 B 0.14
A 0.14 B 0.29
A 0.14 B 0.43
A 0.14 B 0.57
A 0.14 B 0.71
A 0.14 B 0.86
A 0.14 B 1
A 0.29 B 0
A 0.29 B 0.14
A 0.29 B 0.29
A 0.29 B 0.43
A 0.29 B 0.57
A 0.29 B 0.71
A 0.29 B 0.86
A 0.29 B 1
A 0.43 B 0
A 0.43 B 0.14
A 0.43 B 0.29
A 0.43 B 0.43
A 0.43 B 0.57
A 0.43 B 0.71
A 0.43 B 0.86
A 0.43 B 1
A 0.57 B 0
A 0.57 B 0.14
A 0.57 B 0.29
A 0.57 B 0.43
A 0.57 B 0.57
A 0.57 B 0.71
A 0.57 B 0.86
A 0.57 B 1
A 0.71 B 0
A 0.71 B 0.14
A 0.71 B 0.29
A 0.71 B 0.43
A 0.71 B 0.57
A 0.71 B 0.71
A 0.71 B 0.86
A 0.71 B 1
A 0.86 B 0
A 0.86 B 0.14
A 0.86 B 0.29
A 0.86 B 0.43
A 0.86 B 0.57
A 0.86 B 0.71
A 0.86 B 0.86
A 0.86 B 1
A 1 B 0
A 1 B 0.14
A 1 B 0.29
A 1 B 0.43
A 1 B 0.57
A 1 B 0.71
A 1 B 0.86
A 1 B 1
0.75
0.50
0.25
0 5 101520
OD 600 nm [−]
Time [h]

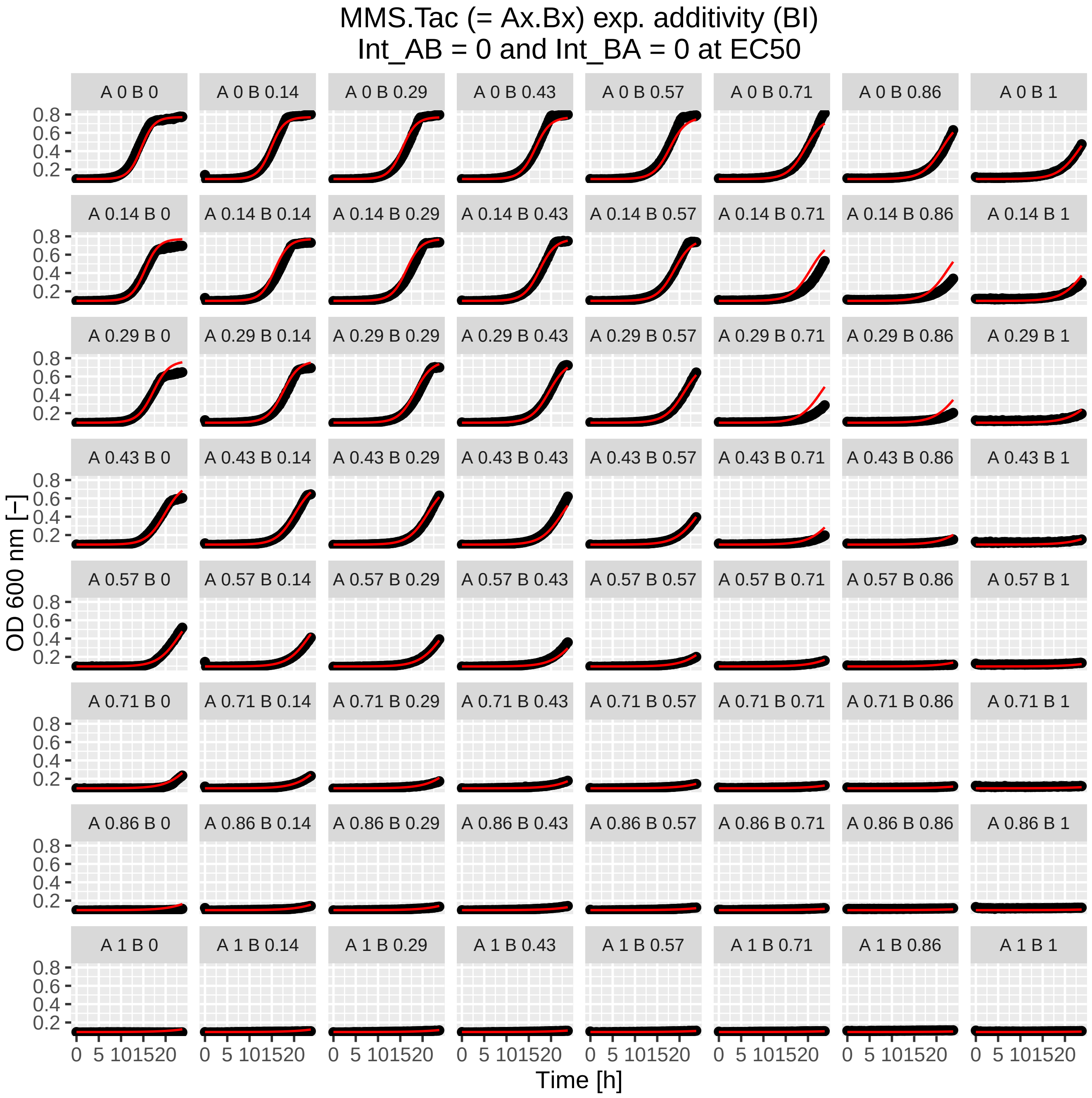
MMS.Tac (= Ax.Bx) exp. additivity (BI)
Int_AB = 0 and Int_BA = 0 at EC50
OD 600 nm [–]
Time [h]
0 5 101520
0.2
0.4
0.6
0.8
A 0 B 0
A 0 B 0.14
A 0 B 0.29
A 0 B 0.43
A 0 B 0.57
A 0 B 0.71
A 0 B 0.86
A 0 B 1
A 0.14 B 0
A 0.14 B 0.14
A 0.14 B 0.29
A 0.14 B 0.43
A 0.14 B 0.57
A 0.14 B 0.71
A 0.14 B 0.86
A 0.14 B 1
A 0.29 B 0
A 0.29 B 0.14
A 0.29 B 0.29
A 0.29 B 0.43
A 0.29 B 0.57
A 0.29 B 0.71
A 0.29 B 0.86
A 0.29 B 1
A 0.43 B 0
A 0.43 B 0.14
A 0.43 B 0.29
A 0.43 B 0.43
A 0.43 B 0.57
A 0.43 B 0.71
A 0.43 B 0.86
A 0.43 B 1
A 0.57 B 0
A 0.57 B 0.14
A 0.57 B 0.29
A 0.57 B 0.43
A 0.57 B 0.57
A 0.57 B 0.71
A 0.57 B 0.86
A 0.57 B 1
A 0.71 B 0
A 0.71 B 0.14
A 0.71 B 0.29
A 0.71 B 0.43
A 0.71 B 0.57
A 0.71 B 0.71
A 0.71 B 0.86
A 0.71 B 1
A 0.86 B 0
A 0.86 B 0.14
A 0.86 B 0.29
A 0.86 B 0.43
A 0.86 B 0.57
A 0.86 B 0.71
A 0.86 B 0.86
A 0.86 B 1
A 1 B 0
A 1 B 0.14
A 1 B 0.29
A 1 B 0.43
A 1 B 0.57
A 1 B 0.71
A 1 B 0.86
A 1 B 1

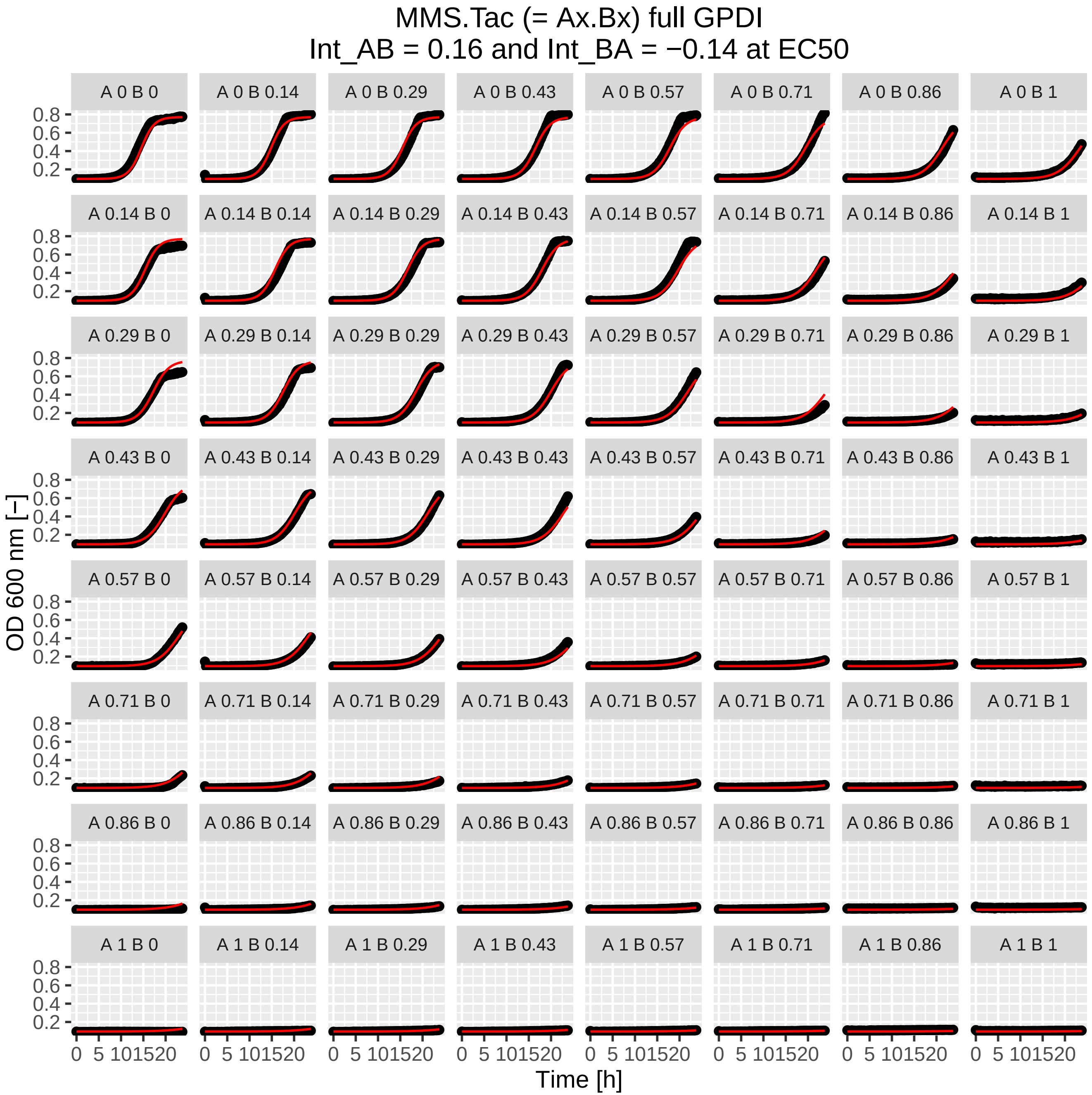
MMS.Tac (= Ax.Bx) full GPDI
Int_AB = 0.16 and Int_BA = −0.14 at EC50
A 0 B 0
A 0 B 0.14
A 0 B 0.29
A 0 B 0.43
A 0 B 0.57
A 0 B 0.71
A 0 B 0.86
A 0 B 1
A 0.14 B 0
A 0.14 B 0.14
A 0.14 B 0.29
A 0.14 B 0.43
A 0.14 B 0.57
A 0.14 B 0.71
A 0.14 B 0.86
A 0.14 B 1
A 0.29 B 0
A 0.29 B 0.14
A 0.29 B 0.29
A 0.29 B 0.43
A 0.29 B 0.57
A 0.29 B 0.71
A 0.29 B 0.86
A 0.29 B 1
A 0.43 B 0
A 0.43 B 0.14
A 0.43 B 0.29
A 0.43 B 0.43
A 0.43 B 0.57
A 0.43 B 0.71
A 0.43 B 0.86
A 0.43 B 1
A 0.57 B 0
A 0.57 B 0.14
A 0.57 B 0.29
A 0.57 B 0.43
A 0.57 B 0.57
A 0.57 B 0.71
A 0.57 B 0.86
A 0.57 B 1
A 0.71 B 0
A 0.71 B 0.14
A 0.71 B 0.29
A 0.71 B 0.43
A 0.71 B 0.57
A 0.71 B 0.71
A 0.71 B 0.86
A 0.71 B 1
A 0.86 B 0
A 0.86 B 0.14
A 0.86 B 0.29
A 0.86 B 0.43
A 0.86 B 0.57
A 0.86 B 0.71
A 0.86 B 0.86
A 0.86 B 1
A 1 B 0
A 1 B 0.14
A 1 B 0.29
A 1 B 0.43
A 1 B 0.57
A 1 B 0.71
A 1 B 0.86
A 1 B 1
OD 600 nm [–]
0.2
0.4
0.6
0.8
0 5 101520
Time [h]

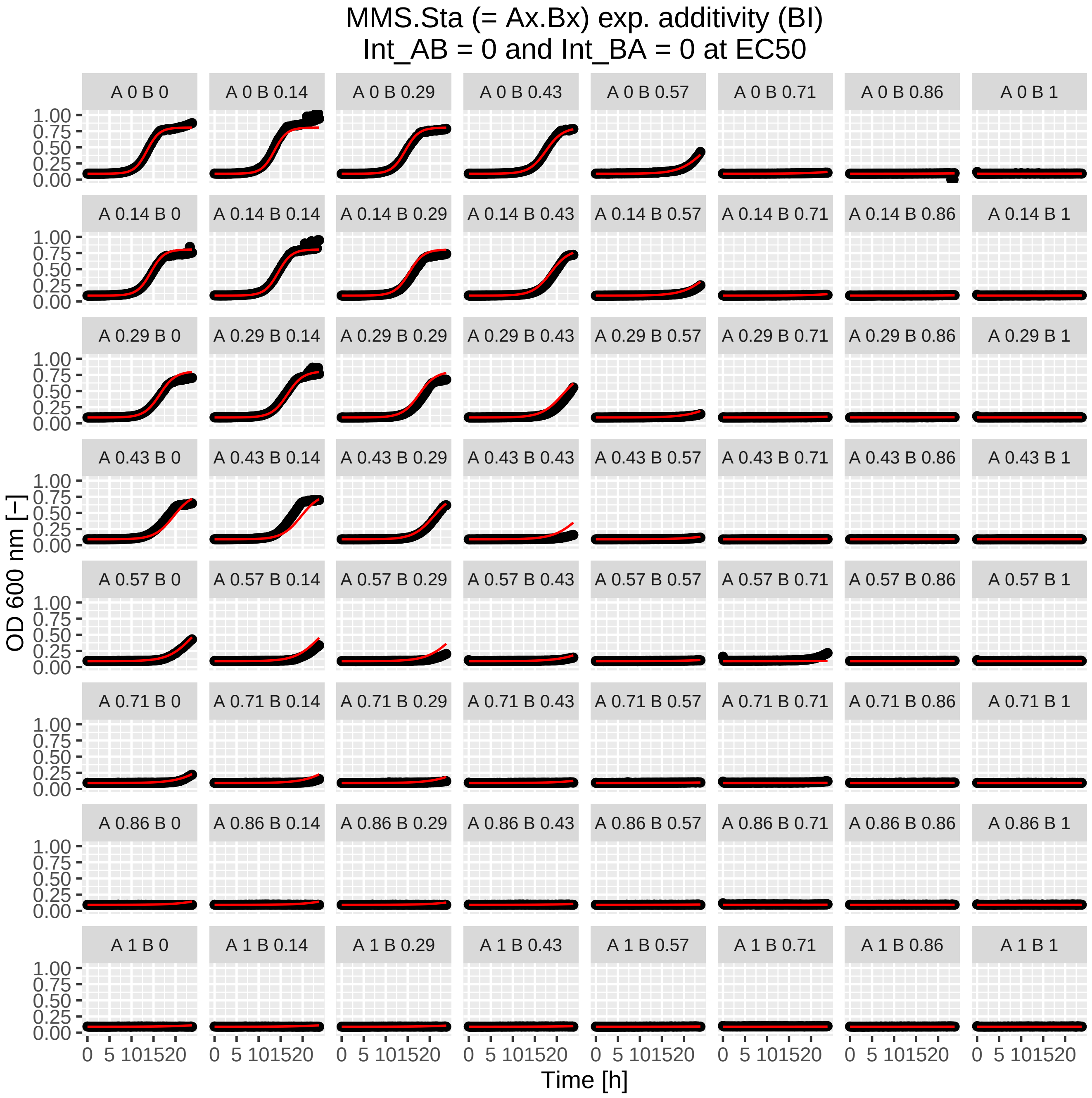
MMS.Sta (= Ax.Bx) exp. additivity (BI)
Int_AB = 0 and Int_BA = 0 at EC50
A 0 B 0
A 0 B 0.14
A 0 B 0.29
A 0 B 0.43
A 0 B 0.57
A 0 B 0.71
A 0 B 0.86
A 0 B 1
A 0.14 B 0
A 0.14 B 0.14
A 0.14 B 0.29
A 0.14 B 0.43
A 0.14 B 0.57
A 0.14 B 0.71
A 0.14 B 0.86
A 0.14 B 1
A 0.29 B 0
A 0.29 B 0.14
A 0.29 B 0.29
A 0.29 B 0.43
A 0.29 B 0.57
A 0.29 B 0.71
A 0.29 B 0.86
A 0.29 B 1
A 0.43 B 0
A 0.43 B 0.14
A 0.43 B 0.29
A 0.43 B 0.43
A 0.43 B 0.57
A 0.43 B 0.71
A 0.43 B 0.86
A 0.43 B 1
A 0.57 B 0
A 0.57 B 0.14
A 0.57 B 0.29
A 0.57 B 0.43
A 0.57 B 0.57
A 0.57 B 0.71
A 0.57 B 0.86
A 0.57 B 1
A 0.71 B 0
A 0.71 B 0.14
A 0.71 B 0.29
A 0.71 B 0.43
A 0.71 B 0.57
A 0.71 B 0.71
A 0.71 B 0.86
A 0.71 B 1
A 0.86 B 0
A 0.86 B 0.14
A 0.86 B 0.29
A 0.86 B 0.43
A 0.86 B 0.57
A 0.86 B 0.71
A 0.86 B 0.86
A 0.86 B 1
A 1 B 0
A 1 B 0.14
A 1 B 0.29
A 1 B 0.43
A 1 B 0.57
A 1 B 0.71
A 1 B 0.86
A 1 B 1
OD 600 nm [–]
1.00
0.75
0.50
0.25
0.00
0 5 101520
Time [h]

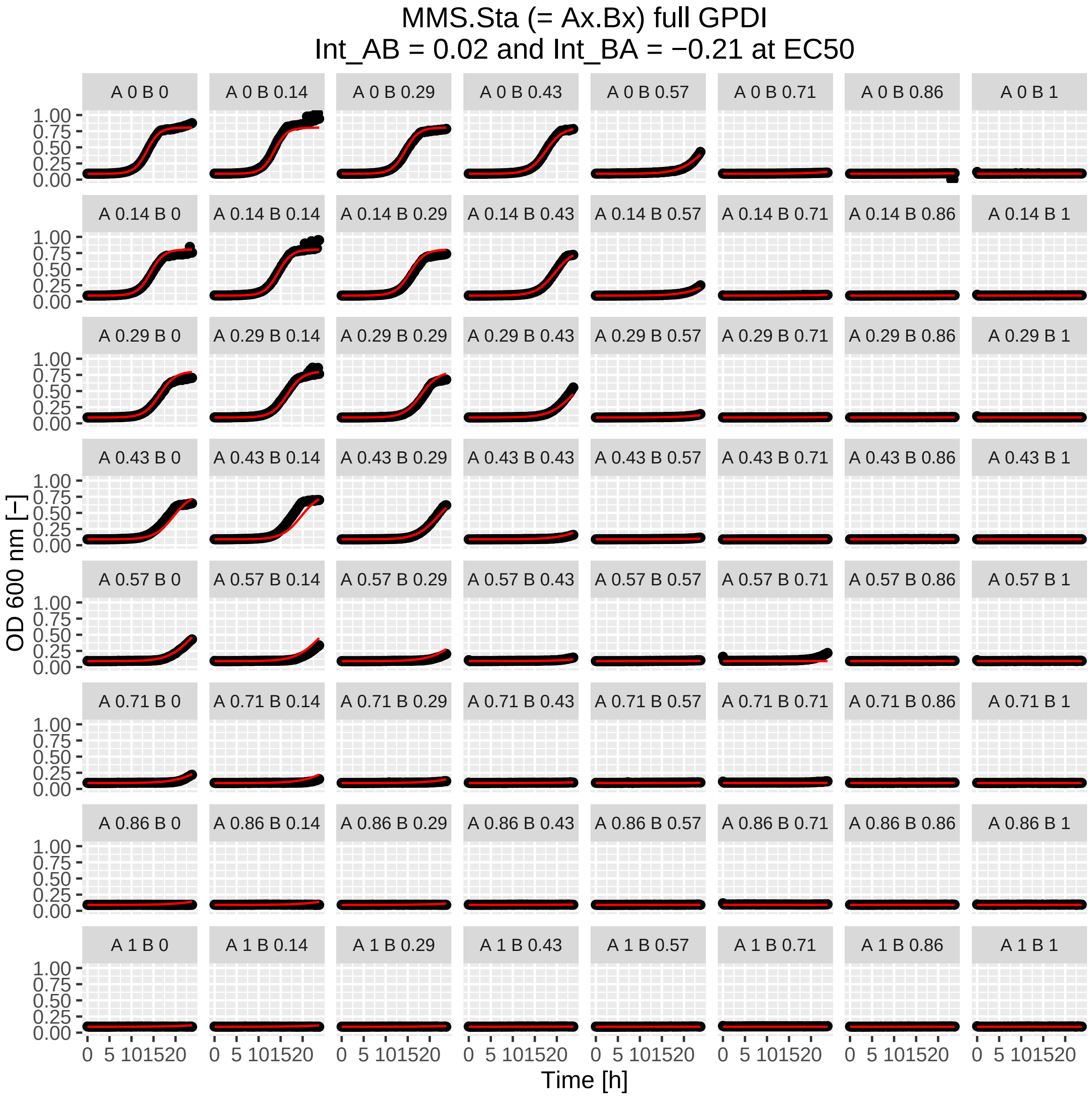
MMS.Sta (= Ax.Bx) full GPDI
Int_AB = 0.02 and Int_BA = −0.21 at EC50
A 0 B 0
A 0 B 0.14
A 0 B 0.29
A 0 B 0.43
A 0 B 0.57
A 0 B 0.71
A 0 B 0.86
A 0 B 1
A 0.14 B 0
A 0.14 B 0.14
A 0.14 B 0.29
A 0.14 B 0.43
A 0.14 B 0.57
A 0.14 B 0.71
A 0.14 B 0.86
A 0.14 B 1
A 0.29 B 0
A 0.29 B 0.14
A 0.29 B 0.29
A 0.29 B 0.43
A 0.29 B 0.57
A 0.29 B 0.71
A 0.29 B 0.86
A 0.29 B 1
A 0.43 B 0
A 0.43 B 0.14
A 0.43 B 0.29
A 0.43 B 0.43
A 0.43 B 0.57
A 0.43 B 0.71
A 0.43 B 0.86
A 0.43 B 1
A 0.57 B 0
A 0.57 B 0.14
A 0.57 B 0.29
A 0.57 B 0.43
A 0.57 B 0.57
A 0.57 B 0.71
A 0.57 B 0.86
A 0.57 B 1
A 0.71 B 0
A 0.71 B 0.14
A 0.71 B 0.29
A 0.71 B 0.43
A 0.71 B 0.57
A 0.71 B 0.71
A 0.71 B 0.86
A 0.71 B 1
A 0.86 B 0
A 0.86 B 0.14
A 0.86 B 0.29
A 0.86 B 0.43
A 0.86 B 0.57
A 0.86 B 0.71
A 0.86 B 0.86
A 0.86 B 1
A 1 B 0
A 1 B 0.14
A 1 B 0.29
A 1 B 0.43
A 1 B 0.57
A 1 B 0.71
A 1 B 0.86
A 1 B 1
OD 600 nm [−]
1.00
0.75
0.50
0.25
0.00
0 5 101520
Time [h]

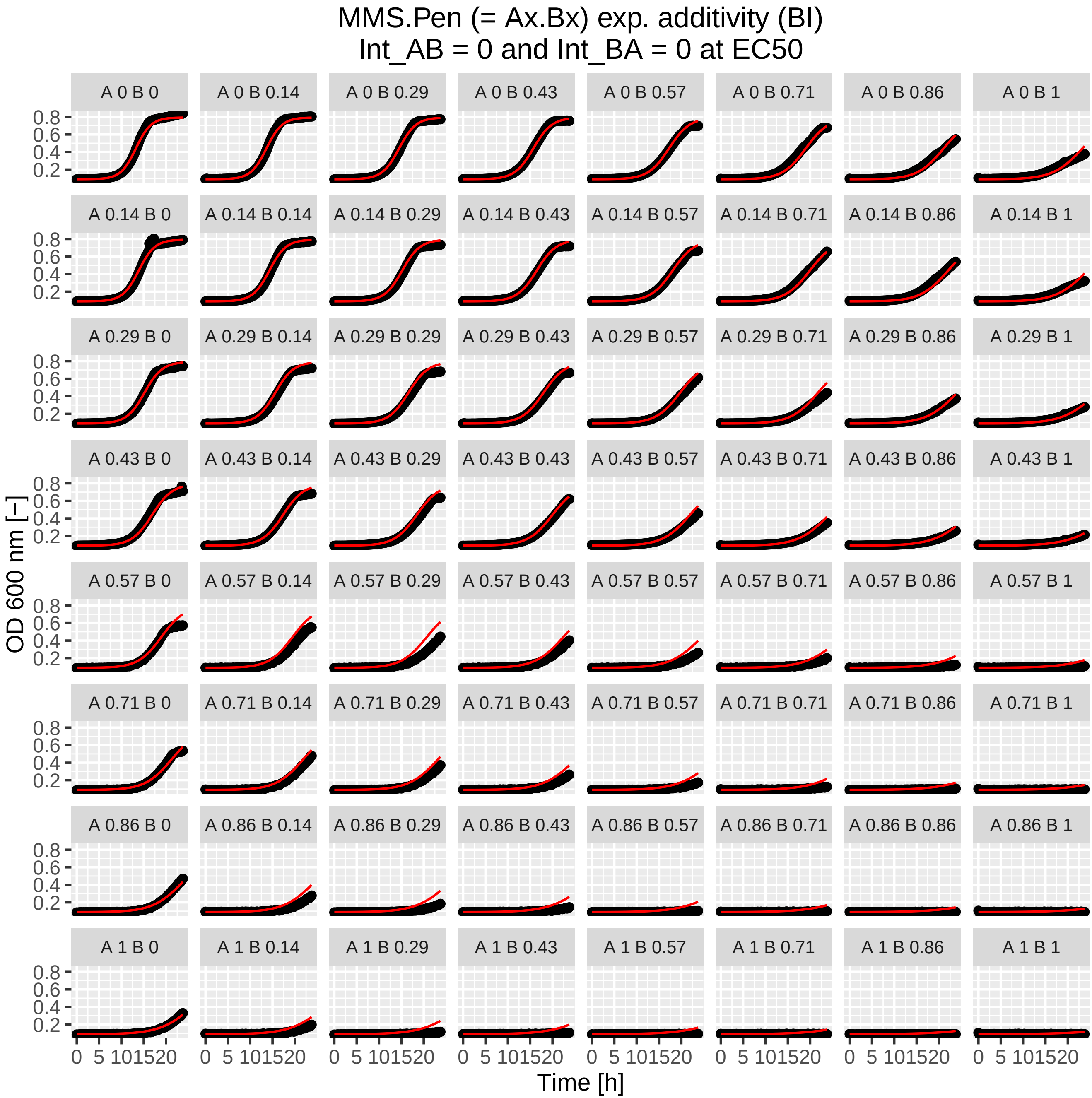
MMS.Pen (= Ax.Bx) exp. additivity (BI)
Int_AB = 0 and Int_BA = 0 at EC50
OD 600 nm [–]
Time [h]
0 5 101520
0.2 0.4 0.6 0.8
A 0 B 0
A 0 B 0.14
A 0 B 0.29
A 0 B 0.43
A 0 B 0.57
A 0 B 0.71
A 0 B 0.86
A 0 B 1
A 1 B 0
A 1 B 0.14
A 1 B 0.29
A 1 B 0.43
A 1 B 0.57
A 1 B 0.71
A 1 B 0.86
A 1 B 1

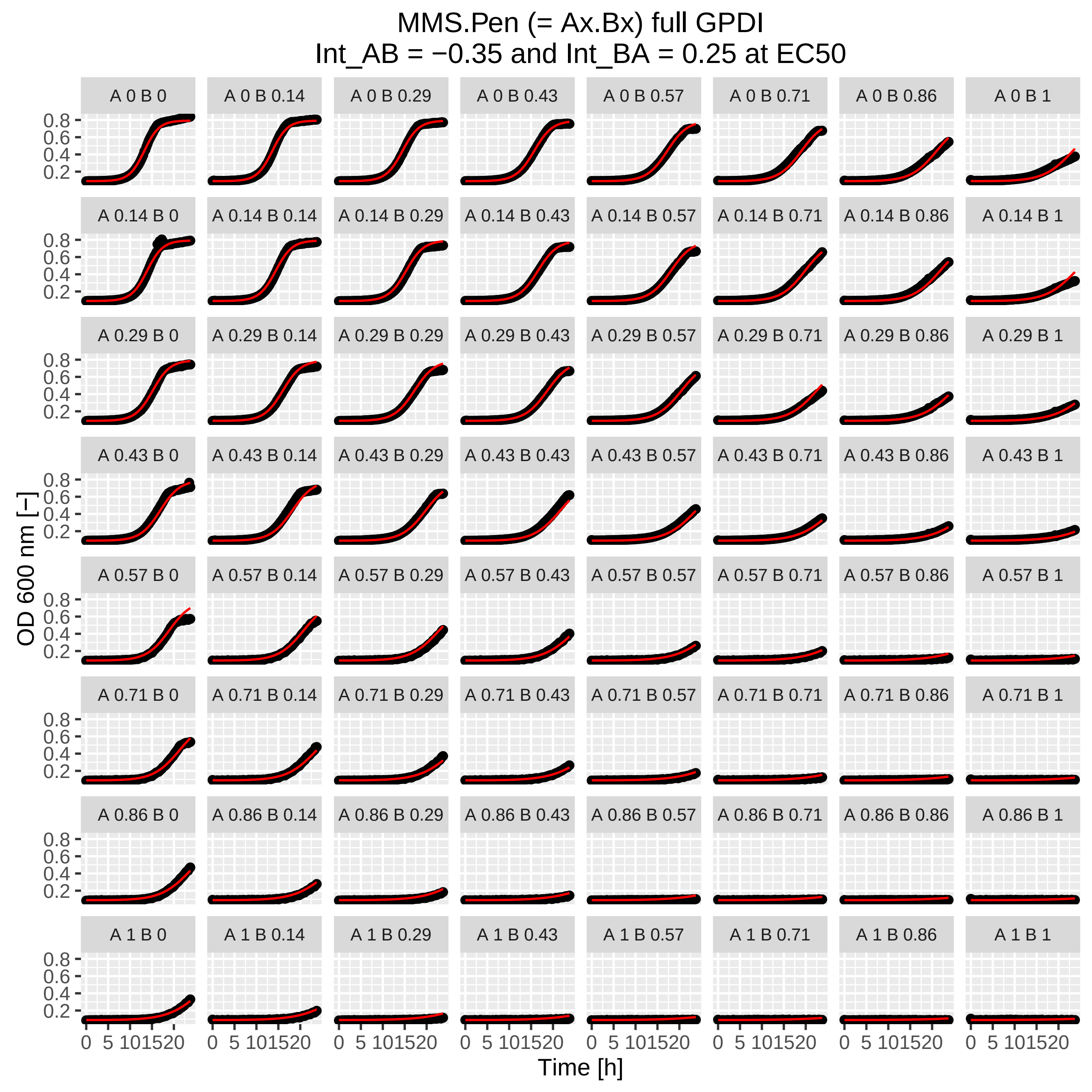
MMS.Pen (= Ax.Bx) full GPDI
Int_AB = −0.35 and Int_BA = 0.25 at EC50
A 0 B 0
A 0 B 0.14
A 0 B 0.29
A 0 B 0.43
A 0 B 0.57
A 0 B 0.71
A 0 B 0.86
A 0 B 1
A 0.14 B 0
A 0.14 B 0.14
A 0.14 B 0.29
A 0.14 B 0.43
A 0.14 B 0.57
A 0.14 B 0.71
A 0.14 B 0.86
A 0.14 B 1
A 0.29 B 0
A 0.29 B 0.14
A 0.29 B 0.29
A 0.29 B 0.43
A 0.29 B 0.57
A 0.29 B 0.71
A 0.29 B 0.86
A 0.29 B 1
A 0.43 B 0
A 0.43 B 0.14
A 0.43 B 0.29
A 0.43 B 0.43
A 0.43 B 0.57
A 0.43 B 0.71
A 0.43 B 0.86
A 0.43 B 1
A 0.57 B 0
A 0.57 B 0.14
A 0.57 B 0.29
A 0.57 B 0.43
A 0.57 B 0.57
A 0.57 B 0.71
A 0.57 B 0.86
A 0.57 B 1
A 0.71 B 0
A 0.71 B 0.14
A 0.71 B 0.29
A 0.71 B 0.43
A 0.71 B 0.57
A 0.71 B 0.71
A 0.71 B 0.86
A 0.71 B 1
A 0.86 B 0
A 0.86 B 0.14
A 0.86 B 0.29
A 0.86 B 0.43
A 0.86 B 0.57
A 0.86 B 0.71
A 0.86 B 0.86
A 0.86 B 1
A 1 B 0
A 1 B 0.14
A 1 B 0.29
A 1 B 0.43
A 1 B 0.57
A 1 B 0.71
A 1 B 0.86
A 1 B 1
OD 600 nm [−]
0.2
0.4
0.6
0.8
0 5 101520
Time [h]

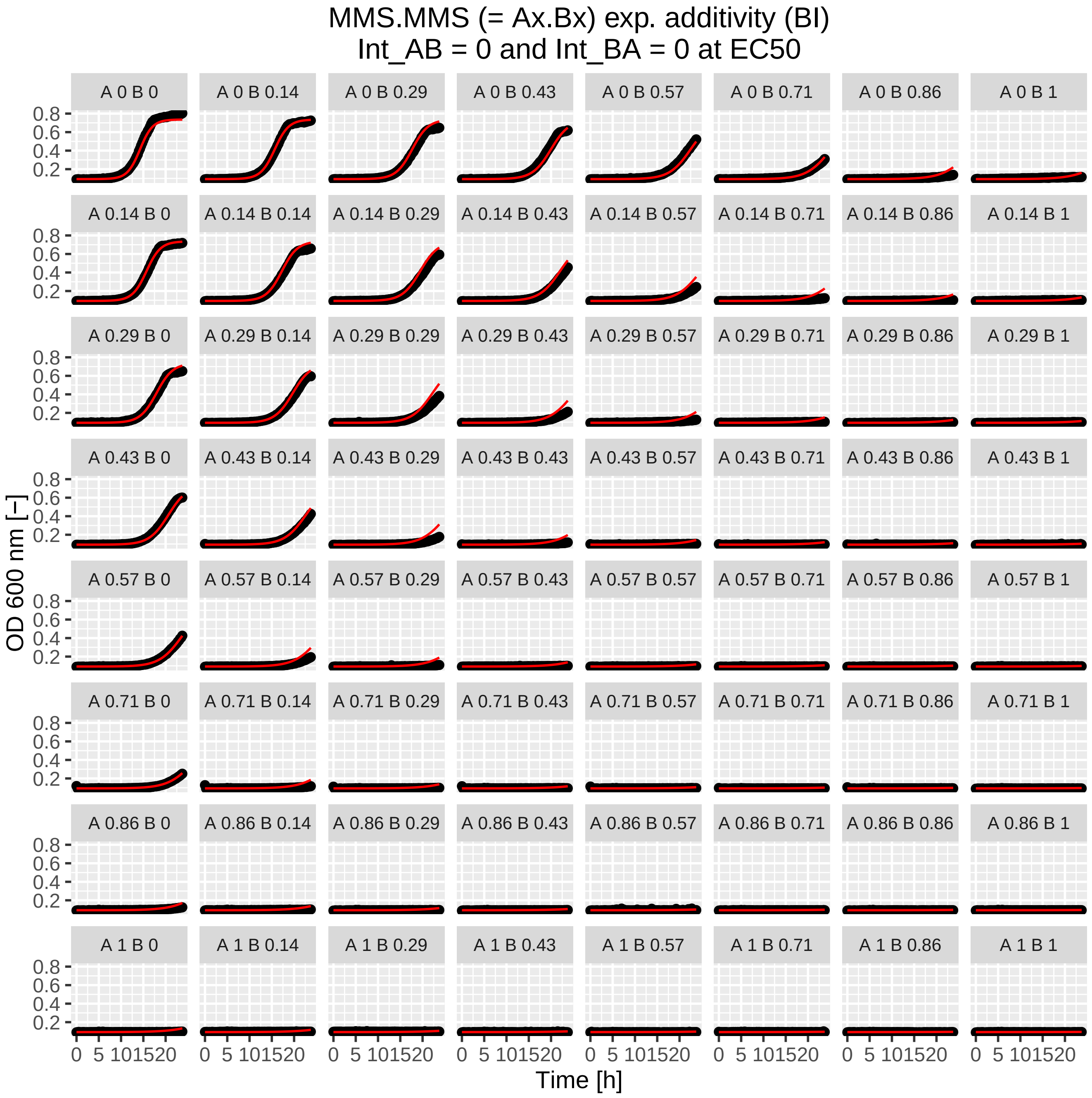
MMS.MMS (= Ax.Bx) exp. additivity (BI)
Int_AB = 0 and Int_BA = 0 at EC50
OD 600 nm [–]
Time [h]
A 0 B 0
A 0 B 0.14
A 0 B 0.29
A 0 B 0.43
A 0 B 0.57
A 0 B 0.71
A 0 B 0.86
A 0 B 1
A 1 B 0
A 1 B 0.14
A 1 B 0.29
A 1 B 0.43
A 1 B 0.57
A 1 B 0.71
A 1 B 0.86
A 1 B 1
0.2
0.4
0.6
0.8
0 5 101520

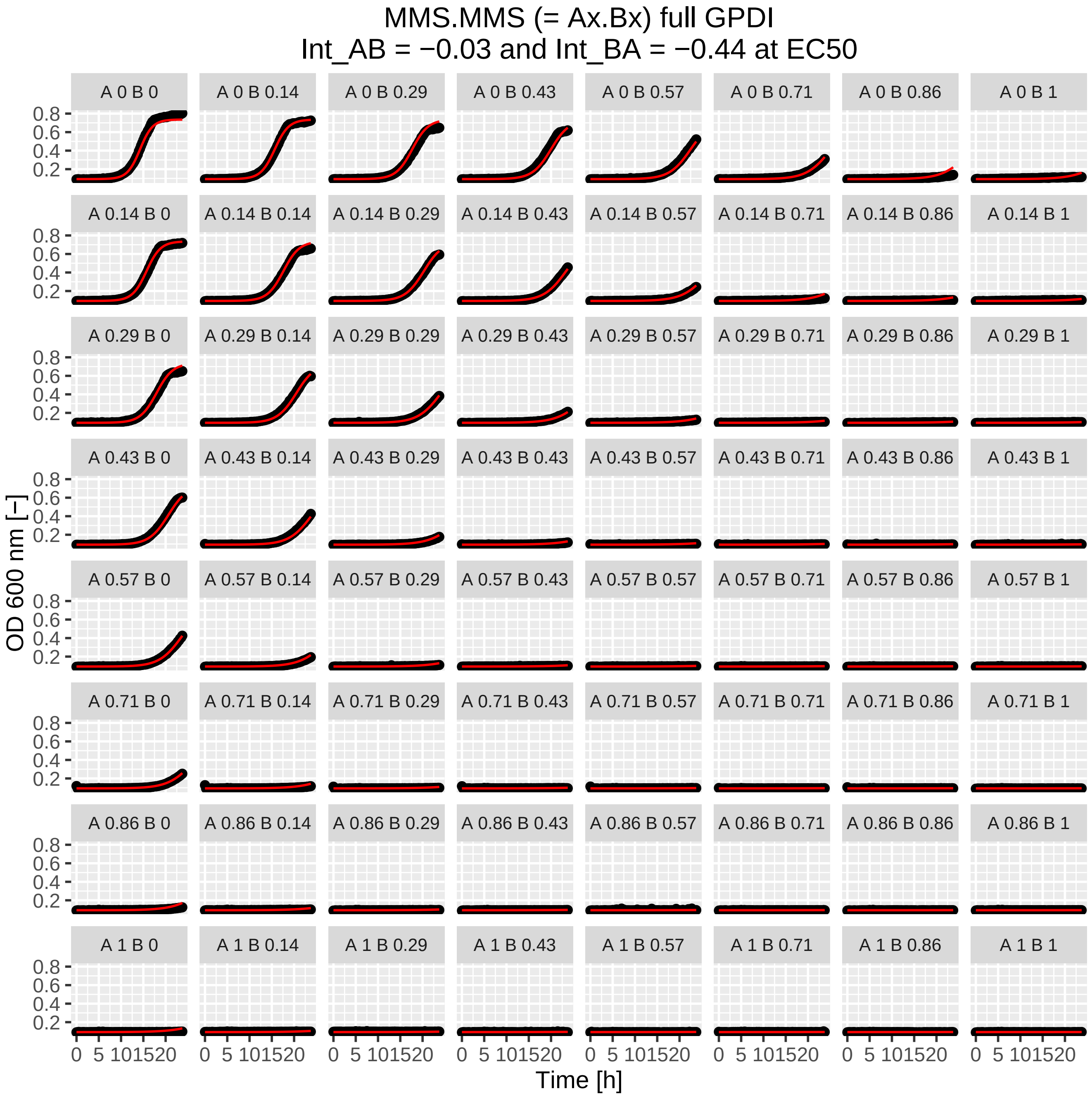
MMS.MMS (= Ax.Bx) full GPDI
Int_AB = −0.03 and Int_BA = −0.44 at EC50
OD 600 nm [−]
Time [h]
0 5 101520
0.2
0.4
0.6
0.8
A 0 B 0
A 0 B 0.14
A 0 B 0.29
A 0 B 0.43
A 0 B 0.57
A 0 B 0.71
A 0 B 0.86
A 0 B 1
A 0.14 B 0
A 0.14 B 0.14
A 0.14 B 0.29
A 0.14 B 0.43
A 0.14 B 0.57
A 0.14 B 0.71
A 0.14 B 0.86
A 0.14 B 1
A 0.29 B 0
A 0.29 B 0.14
A 0.29 B 0.29
A 0.29 B 0.43
A 0.29 B 0.57
A 0.29 B 0.71
A 0.29 B 0.86
A 0.29 B 1
A 0.43 B 0
A 0.43 B 0.14
A 0.43 B 0.29
A 0.43 B 0.43
A 0.43 B 0.57
A 0.43 B 0.71
A 0.43 B 0.86
A 0.43 B 1
A 0.57 B 0
A 0.57 B 0.14
A 0.57 B 0.29
A 0.57 B 0.43
A 0.57 B 0.57
A 0.57 B 0.71
A 0.57 B 0.86
A 0.57 B 1
A 0.71 B 0
A 0.71 B 0.14
A 0.71 B 0.29
A 0.71 B 0.43
A 0.71 B 0.57
A 0.71 B 0.71
A 0.71 B 0.86
A 0.71 B 1
A 0.86 B 0
A 0.86 B 0.14
A 0.86 B 0.29
A 0.86 B 0.43
A 0.86 B 0.57
A 0.86 B 0.71
A 0.86 B 0.86
A 0.86 B 1
A 1 B 0
A 1 B 0.14
A 1 B 0.29
A 1 B 0.43
A 1 B 0.57
A 1 B 0.71
A 1 B 0.86
A 1 B 1

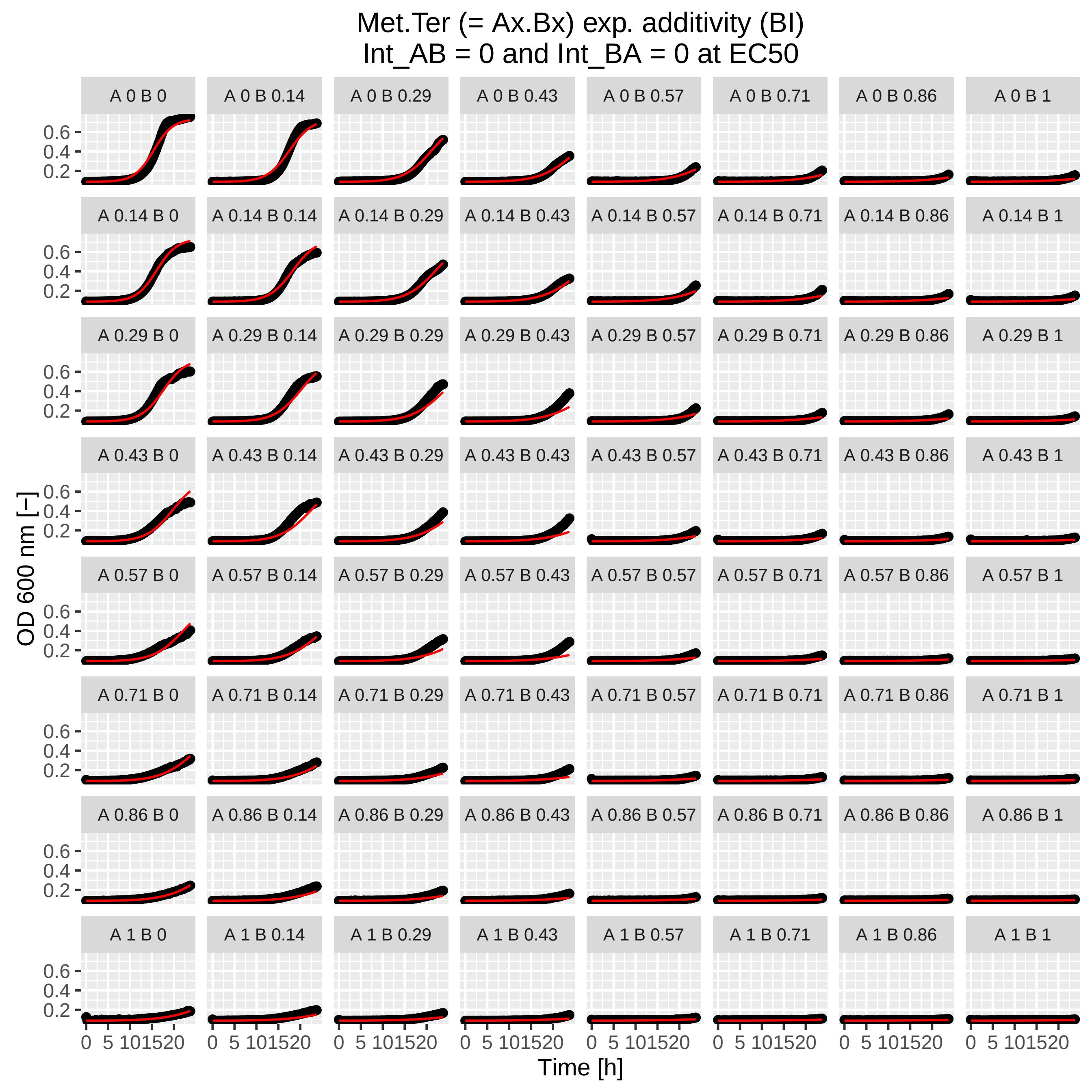

Met.Ter (= Ax.Bx) exp. additivity (BI)
Int_AB = 0 and Int_BA = 0 at EC50
A 0 B 0
A 0 B 0.14
A 0 B 0.29
A 0 B 0.43
A 0 B 0.57
A 0 B 0.71
A 0 B 0.86
A 0 B 1
A 0.14 B 0
A 0.14 B 0.14
A 0.14 B 0.29
A 0.14 B 0.43
A 0.14 B 0.57
A 0.14 B 0.71
A 0.14 B 0.86
A 0.14 B 1
A 0.29 B 0
A 0.29 B 0.14
A 0.29 B 0.29
A 0.29 B 0.43
A 0.29 B 0.57
A 0.29 B 0.71
A 0.29 B 0.86
A 0.29 B 1
A 0.43 B 0
A 0.43 B 0.14
A 0.43 B 0.29
A 0.43 B 0.43
A 0.43 B 0.57
A 0.43 B 0.71
A 0.43 B 0.86
A 0.43 B 1
A 0.57 B 0
A 0.57 B 0.14
A 0.57 B 0.29
A 0.57 B 0.43
A 0.57 B 0.57
A 0.57 B 0.71
A 0.57 B 0.86
A 0.57 B 1
A 0.71 B 0
A 0.71 B 0.14
A 0.71 B 0.29
A 0.71 B 0.43
A 0.71 B 0.57
A 0.71 B 0.71
A 0.71 B 0.86
A 0.71 B 1
A 0.86 B 0
A 0.86 B 0.14
A 0.86 B 0.29
A 0.86 B 0.43
A 0.86 B 0.57
A 0.86 B 0.71
A 0.86 B 0.86
A 0.86 B 1
A 1 B 0
A 1 B 0.14
A 1 B 0.29
A 1 B 0.43
A 1 B 0.57
A 1 B 0.71
A 1 B 0.86
A 1 B 1
OD 600 nm [–]
0.6
0.4
0.2
0 5 101520
Time [h]

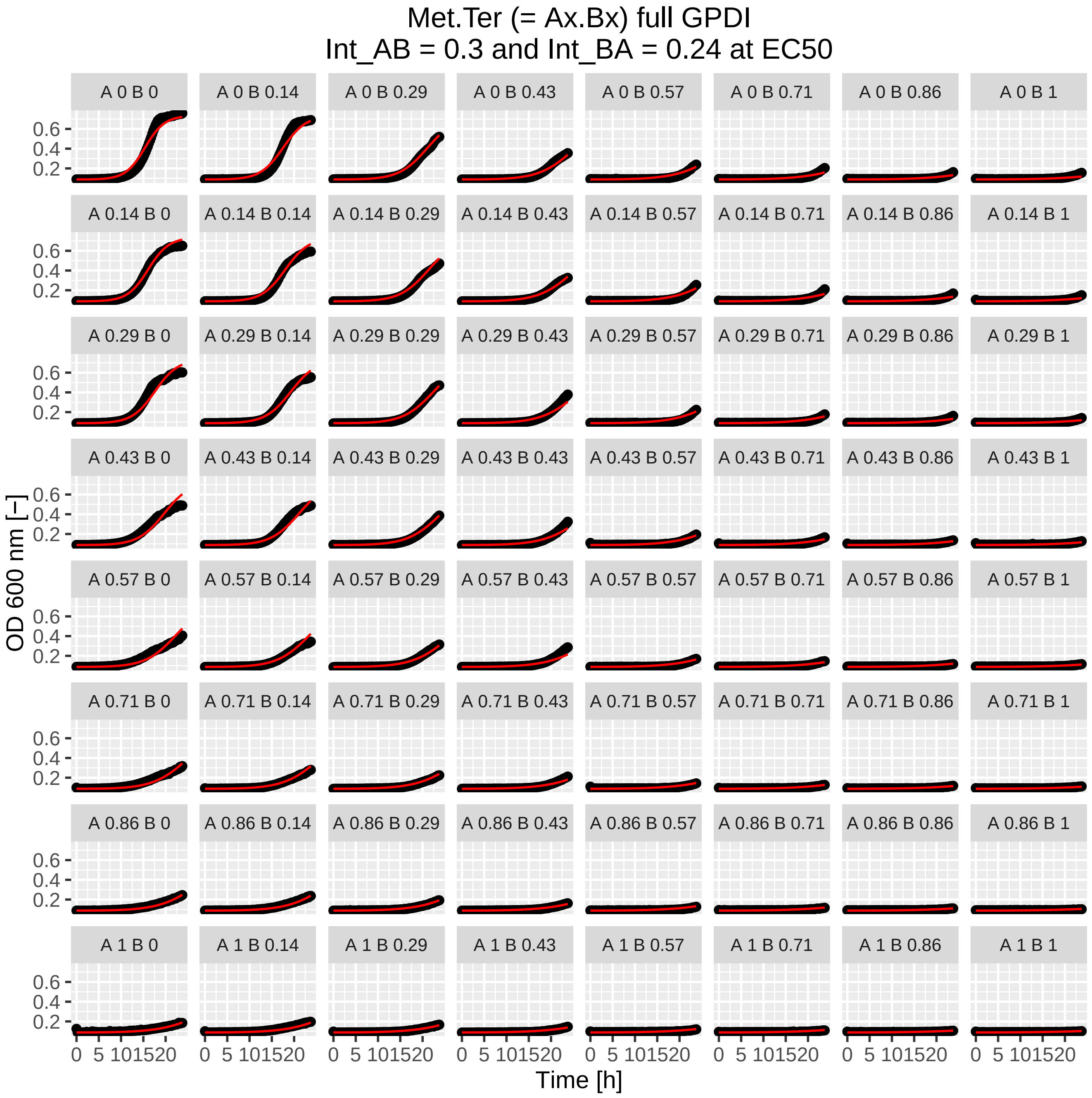
Met.Ter (= Ax.Bx) full GPDI
Int_AB = 0.3 and Int_BA = 0.24 at EC50
A 0 B 0
A 0 B 0.14
A 0 B 0.29
A 0 B 0.43
A 0 B 0.57
A 0 B 0.71
A 0 B 0.86
A 0 B 1
A 0.14 B 0
A 0.14 B 0.14
A 0.14 B 0.29
A 0.14 B 0.43
A 0.14 B 0.57
A 0.14 B 0.71
A 0.14 B 0.86
A 0.14 B 1
A 0.29 B 0
A 0.29 B 0.14
A 0.29 B 0.29
A 0.29 B 0.43
A 0.29 B 0.57
A 0.29 B 0.71
A 0.29 B 0.86
A 0.29 B 1
A 0.43 B 0
A 0.43 B 0.14
A 0.43 B 0.29
A 0.43 B 0.43
A 0.43 B 0.57
A 0.43 B 0.71
A 0.43 B 0.86
A 0.43 B 1
A 0.57 B 0
A 0.57 B 0.14
A 0.57 B 0.29
A 0.57 B 0.43
A 0.57 B 0.57
A 0.57 B 0.71
A 0.57 B 0.86
A 0.57 B 1
A 0.71 B 0
A 0.71 B 0.14
A 0.71 B 0.29
A 0.71 B 0.43
A 0.71 B 0.57
A 0.71 B 0.71
A 0.71 B 0.86
A 0.71 B 1
A 0.86 B 0
A 0.86 B 0.14
A 0.86 B 0.29
A 0.86 B 0.43
A 0.86 B 0.57
A 0.86 B 0.71
A 0.86 B 0.86
A 0.86 B 1
A 1 B 0
A 1 B 0.14
A 1 B 0.29
A 1 B 0.43
A 1 B 0.57
A 1 B 0.71
A 1 B 0.86
A 1 B 1
OD 600 nm [–]
0.2
0.4
0.6
Time [h]
0
5
10
15
20

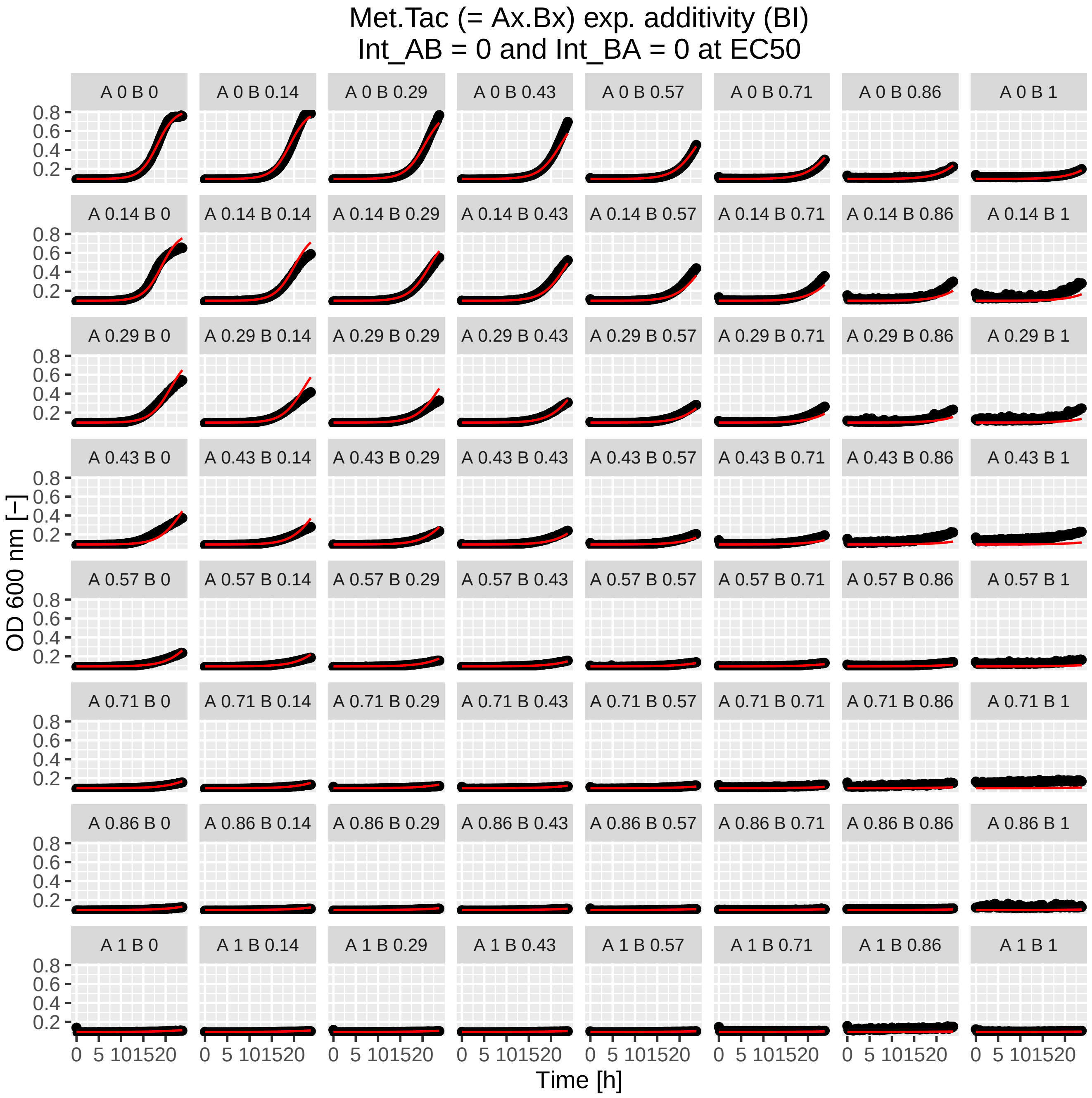

Met.Tac (= Ax.Bx) exp. additivity (BI)
Int_AB = 0 and Int_BA = 0 at EC50
A 0 B 0
A 0 B 0.14
A 0 B 0.29
A 0 B 0.43
A 0 B 0.57
A 0 B 0.71
A 0 B 0.86
A 0 B 1
A 0.14 B 0
A 0.14 B 0.14
A 0.14 B 0.29
A 0.14 B 0.43
A 0.14 B 0.57
A 0.14 B 0.71
A 0.14 B 0.86
A 0.14 B 1
A 0.29 B 0
A 0.29 B 0.14
A 0.29 B 0.29
A 0.29 B 0.43
A 0.29 B 0.57
A 0.29 B 0.71
A 0.29 B 0.86
A 0.29 B 1
A 0.43 B 0
A 0.43 B 0.14
A 0.43 B 0.29
A 0.43 B 0.43
A 0.43 B 0.57
A 0.43 B 0.71
A 0.43 B 0.86
A 0.43 B 1
A 0.57 B 0
A 0.57 B 0.14
A 0.57 B 0.29
A 0.57 B 0.43
A 0.57 B 0.57
A 0.57 B 0.71
A 0.57 B 0.86
A 0.57 B 1
A 0.71 B 0
A 0.71 B 0.14
A 0.71 B 0.29
A 0.71 B 0.43
A 0.71 B 0.57
A 0.71 B 0.71
A 0.71 B 0.86
A 0.71 B 1
A 0.86 B 0
A 0.86 B 0.14
A 0.86 B 0.29
A 0.86 B 0.43
A 0.86 B 0.57
A 0.86 B 0.71
A 0.86 B 0.86
A 0.86 B 1
A 1 B 0
A 1 B 0.14
A 1 B 0.29
A 1 B 0.43
A 1 B 0.57
A 1 B 0.71
A 1 B 0.86
A 1 B 1
0.8
0.6
0.4
0.2
OD 600 nm [−]
0 5 101520
Time [h]

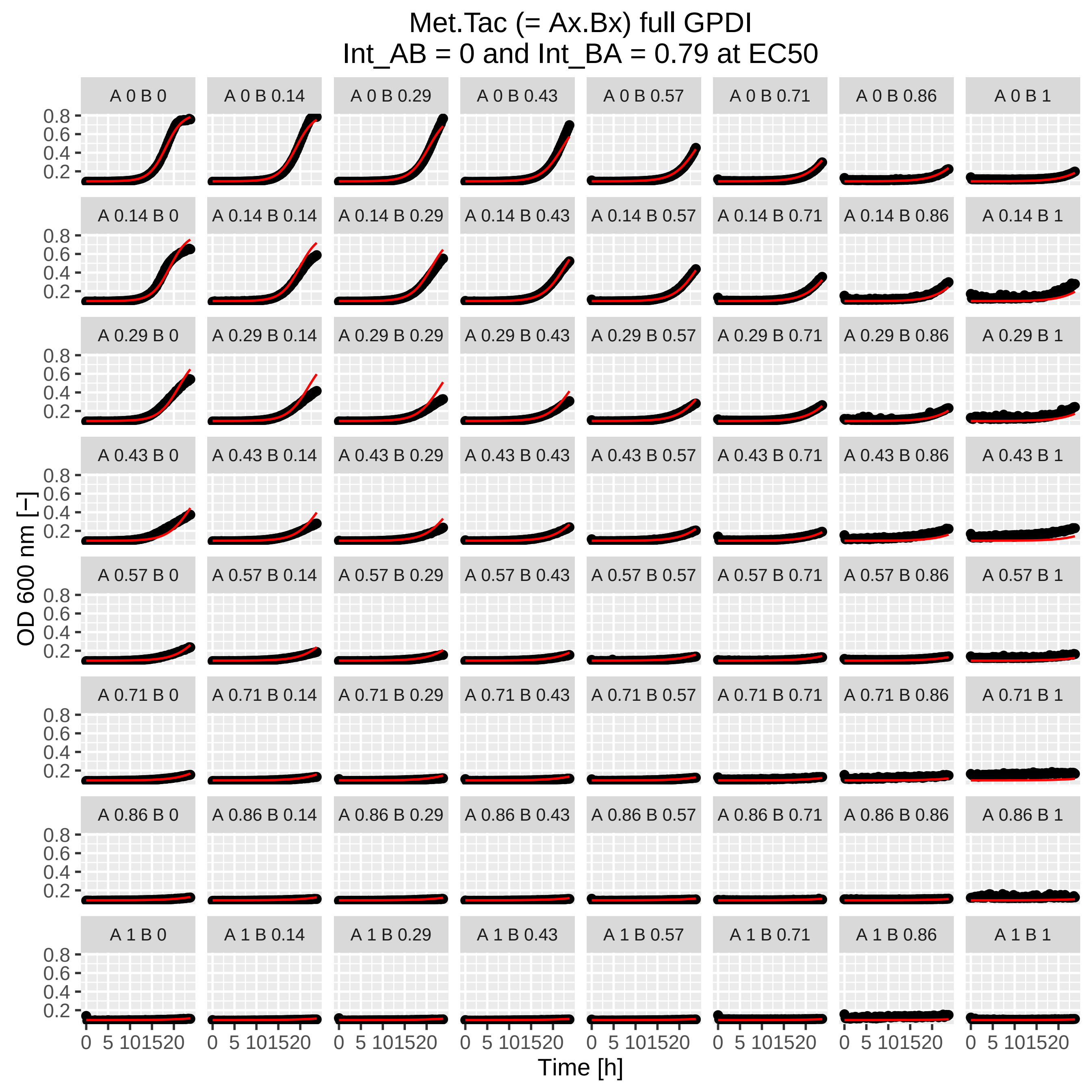
Met.Tac (= Ax.Bx) full GPDI
Int_AB = 0 and Int_BA = 0.79 at EC50
OD 600 nm [–]
Time [h]
A 0 B 0
A 0 B 0.14
A 0 B 0.29
A 0 B 0.43
A 0 B 0.57
A 0 B 0.71
A 0 B 0.86
A 0 B 1
A 1 B 0
A 1 B 0.14
A 1 B 0.29
A 1 B 0.43
A 1 B 0.57
A 1 B 0.71
A 1 B 0.86
A 1 B 1

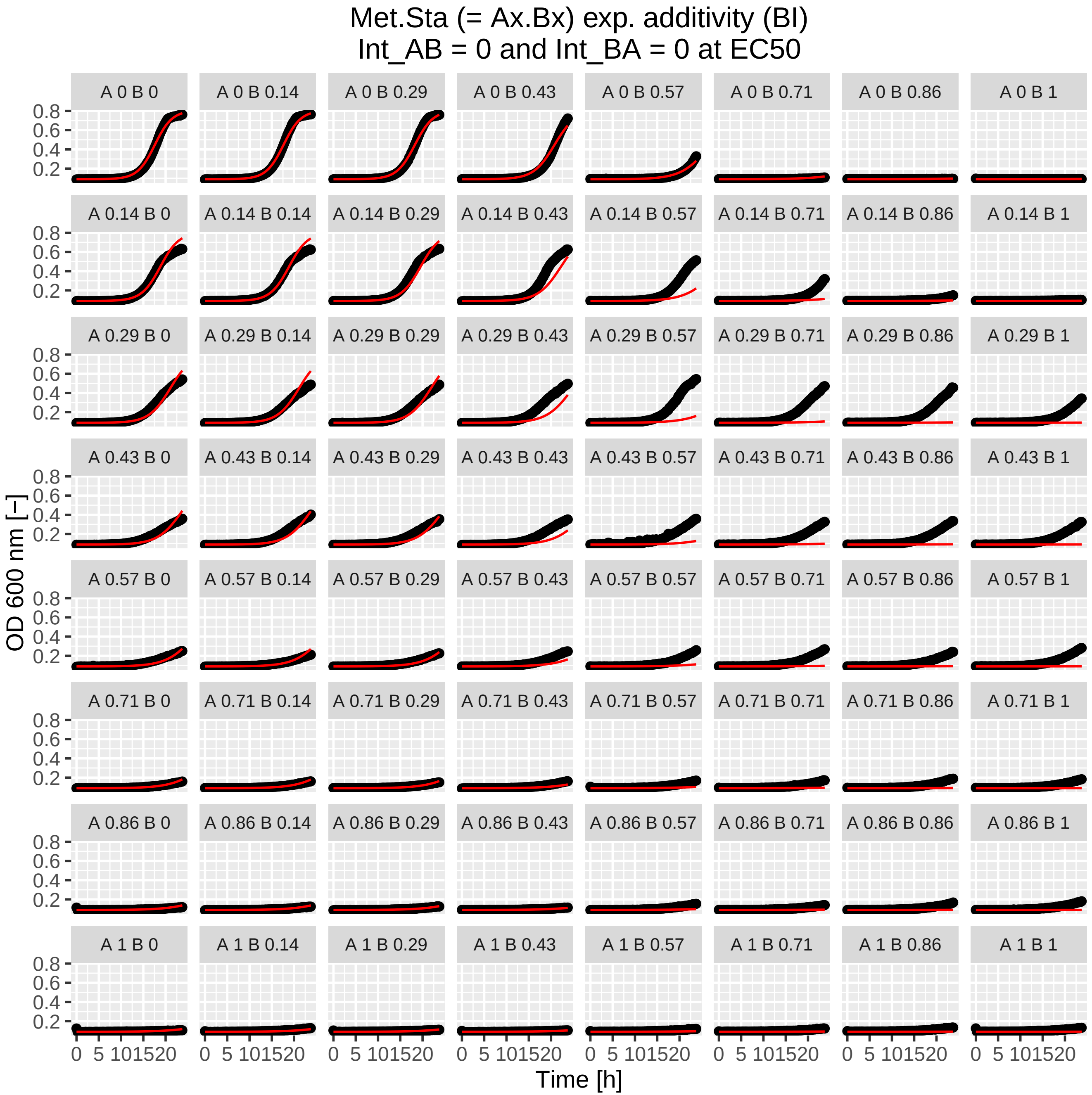
Met.Sta (= Ax.Bx) exp. additivity (BI)
Int_AB = 0 and Int_BA = 0 at EC50
OD 600 nm [–]
Time [h]
A 0 B 0
A 0 B 0.14
A 0 B 0.29
A 0 B 0.43
A 0 B 0.57
A 0 B 0.71
A 0 B 0.86
A 0 B 1
A 0.14 B 0
A 0.29 B 0
A 0.43 B 0
A 0.57 B 0
A 0.71 B 0
A 0.86 B 0
A 1 B 0
A 1 B 1
0.2
0.4
0.6
0.8
0
5
10
15
20

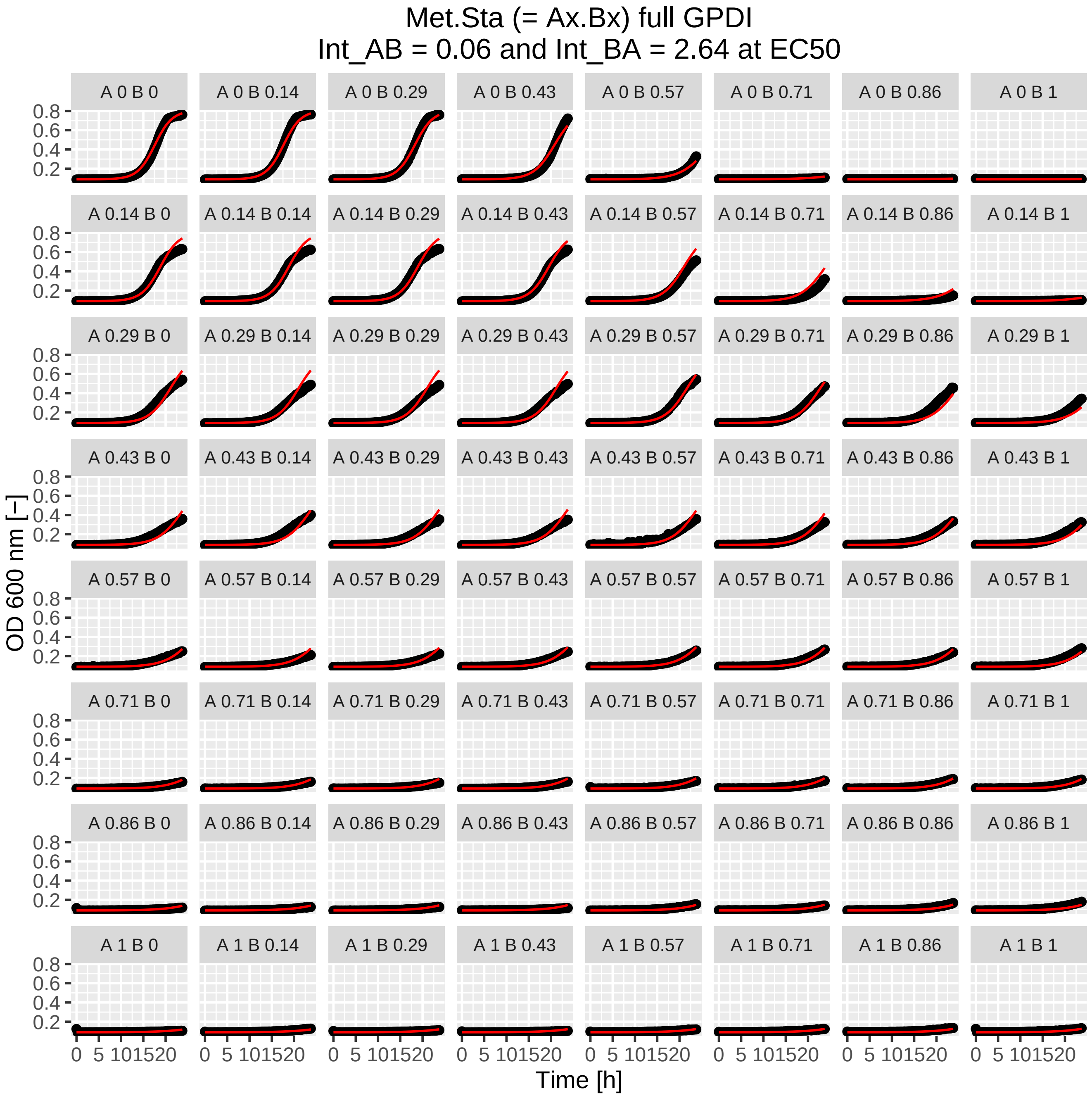
Met.Sta (= Ax.Bx) full GPDI
Int_AB = 0.06 and Int_BA = 2.64 at EC50
OD 600 nm [–]
Time [h]
A 0 B 0
A 0 B 0.14
A 0 B 0.29
A 0 B 0.43
A 0 B 0.57
A 0 B 0.71
A 0 B 0.86
A 0 B 1
A 0.14 B 0
A 0.14 B 0.14
A 0.14 B 0.29
A 0.14 B 0.43
A 0.14 B 0.57
A 0.14 B 0.71
A 0.14 B 0.86
A 0.14 B 1
A 0.29 B 0
A 0.29 B 0.14
A 0.29 B 0.29
A 0.29 B 0.43
A 0.29 B 0.57
A 0.29 B 0.71
A 0.29 B 0.86
A 0.29 B 1
A 0.43 B 0
A 0.43 B 0.14
A 0.43 B 0.29
A 0.43 B 0.43
A 0.43 B 0.57
A 0.43 B 0.71
A 0.43 B 0.86
A 0.43 B 1
A 0.57 B 0
A 0.57 B 0.14
A 0.57 B 0.29
A 0.57 B 0.43
A 0.57 B 0.57
A 0.57 B 0.71
A 0.57 B 0.86
A 0.57 B 1
A 0.71 B 0
A 0.71 B 0.14
A 0.71 B 0.29
A 0.71 B 0.43
A 0.71 B 0.57
A 0.71 B 0.71
A 0.71 B 0.86
A 0.71 B 1
A 0.86 B 0
A 0.86 B 0.14
A 0.86 B 0.29
A 0.86 B 0.43
A 0.86 B 0.57
A 0.86 B 0.71
A 0.86 B 0.86
A 0.86 B 1
A 1 B 0
A 1 B 0.14
A 1 B 0.29
A 1 B 0.43
A 1 B 0.57
A 1 B 0.71
A 1 B 0.86
A 1 B 1
0.8
0.6
0.4
0.2
0 5 101520

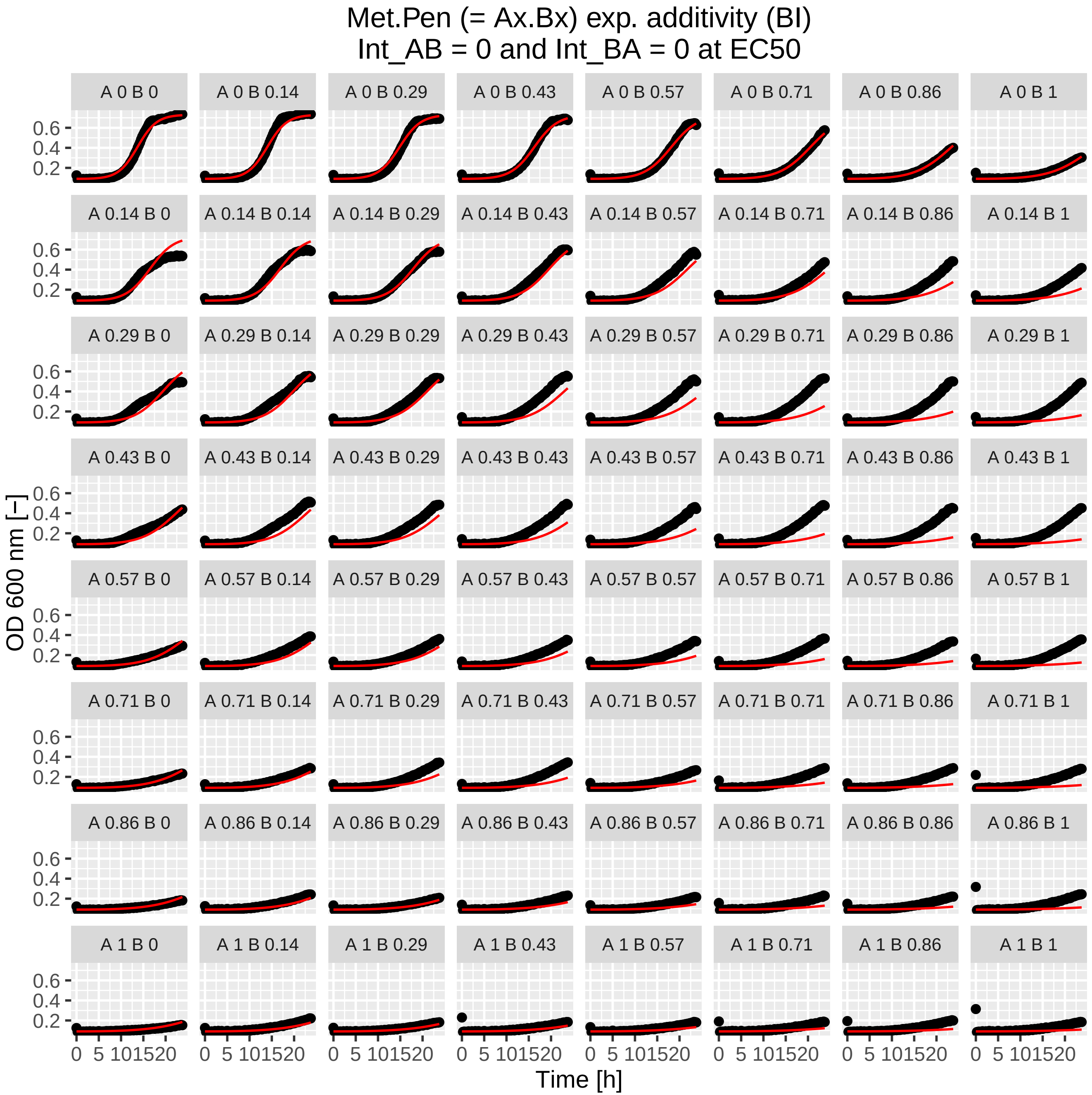
Met.Pen (= Ax.Bx) exp. additivity (BI)
Int_AB = 0 and Int_BA = 0 at EC50
A 0 B 0
A 0 B 0.14
A 0 B 0.29
A 0 B 0.43
A 0 B 0.57
A 0 B 0.71
A 0 B 0.86
A 0 B 1
A 0.14 B 0
A 0.14 B 0.14
A 0.14 B 0.29
A 0.14 B 0.43
A 0.14 B 0.57
A 0.14 B 0.71
A 0.14 B 0.86
A 0.14 B 1
A 0.29 B 0
A 0.29 B 0.14
A 0.29 B 0.29
A 0.29 B 0.43
A 0.29 B 0.57
A 0.29 B 0.71
A 0.29 B 0.86
A 0.29 B 1
A 0.43 B 0
A 0.43 B 0.14
A 0.43 B 0.29
A 0.43 B 0.43
A 0.43 B 0.57
A 0.43 B 0.71
A 0.43 B 0.86
A 0.43 B 1
A 0.57 B 0
A 0.57 B 0.14
A 0.57 B 0.29
A 0.57 B 0.43
A 0.57 B 0.57
A 0.57 B 0.71
A 0.57 B 0.86
A 0.57 B 1
A 0.71 B 0
A 0.71 B 0.14
A 0.71 B 0.29
A 0.71 B 0.43
A 0.71 B 0.57
A 0.71 B 0.71
A 0.71 B 0.86
A 0.71 B 1
A 0.86 B 0
A 0.86 B 0.14
A 0.86 B 0.29
A 0.86 B 0.43
A 0.86 B 0.57
A 0.86 B 0.71
A 0.86 B 0.86
A 0.86 B 1
A 1 B 0
A 1 B 0.14
A 1 B 0.29
A 1 B 0.43
A 1 B 0.57
A 1 B 0.71
A 1 B 0.86
A 1 B 1
OD 600 nm [–]
0.6
0.4
0.2
0 5 101520
Time [h]

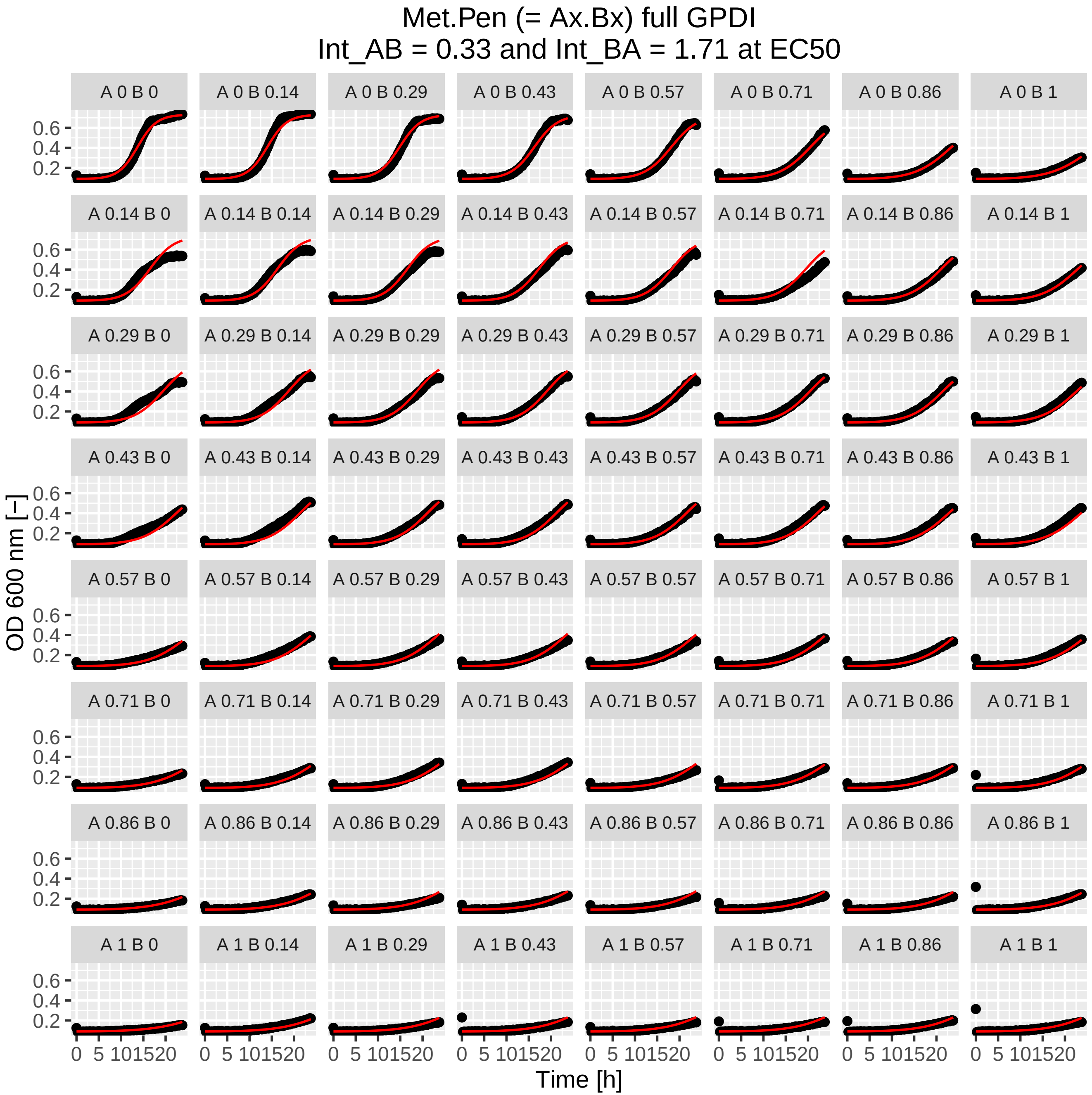

Met.Pen (= Ax.Bx) full GPDI
Int_AB = 0.33 and Int_BA = 1.71 at EC50
A 0 B 0
A 0 B 0.14
A 0 B 0.29
A 0 B 0.43
A 0 B 0.57
A 0 B 0.71
A 0 B 0.86
A 0 B 1
A 0.14 B 0
A 0.14 B 0.14
A 0.14 B 0.29
A 0.14 B 0.43
A 0.14 B 0.57
A 0.14 B 0.71
A 0.14 B 0.86
A 0.14 B 1
A 0.29 B 0
A 0.29 B 0.14
A 0.29 B 0.29
A 0.29 B 0.43
A 0.29 B 0.57
A 0.29 B 0.71
A 0.29 B 0.86
A 0.29 B 1
A 0.43 B 0
A 0.43 B 0.14
A 0.43 B 0.29
A 0.43 B 0.43
A 0.43 B 0.57
A 0.43 B 0.71
A 0.43 B 0.86
A 0.43 B 1
A 0.57 B 0
A 0.57 B 0.14
A 0.57 B 0.29
A 0.57 B 0.43
A 0.57 B 0.57
A 0.57 B 0.71
A 0.57 B 0.86
A 0.57 B 1
A 0.71 B 0
A 0.71 B 0.14
A 0.71 B 0.29
A 0.71 B 0.43
A 0.71 B 0.57
A 0.71 B 0.71
A 0.71 B 0.86
A 0.71 B 1
A 0.86 B 0
A 0.86 B 0.14
A 0.86 B 0.29
A 0.86 B 0.43
A 0.86 B 0.57
A 0.86 B 0.71
A 0.86 B 0.86
A 0.86 B 1
A 1 B 0
A 1 B 0.14
A 1 B 0.29
A 1 B 0.43
A 1 B 0.57
A 1 B 0.71
A 1 B 0.86
A 1 B 1
OD 600 nm [–]
0.2
0.4
0.6
0 5 101520
Time [h]

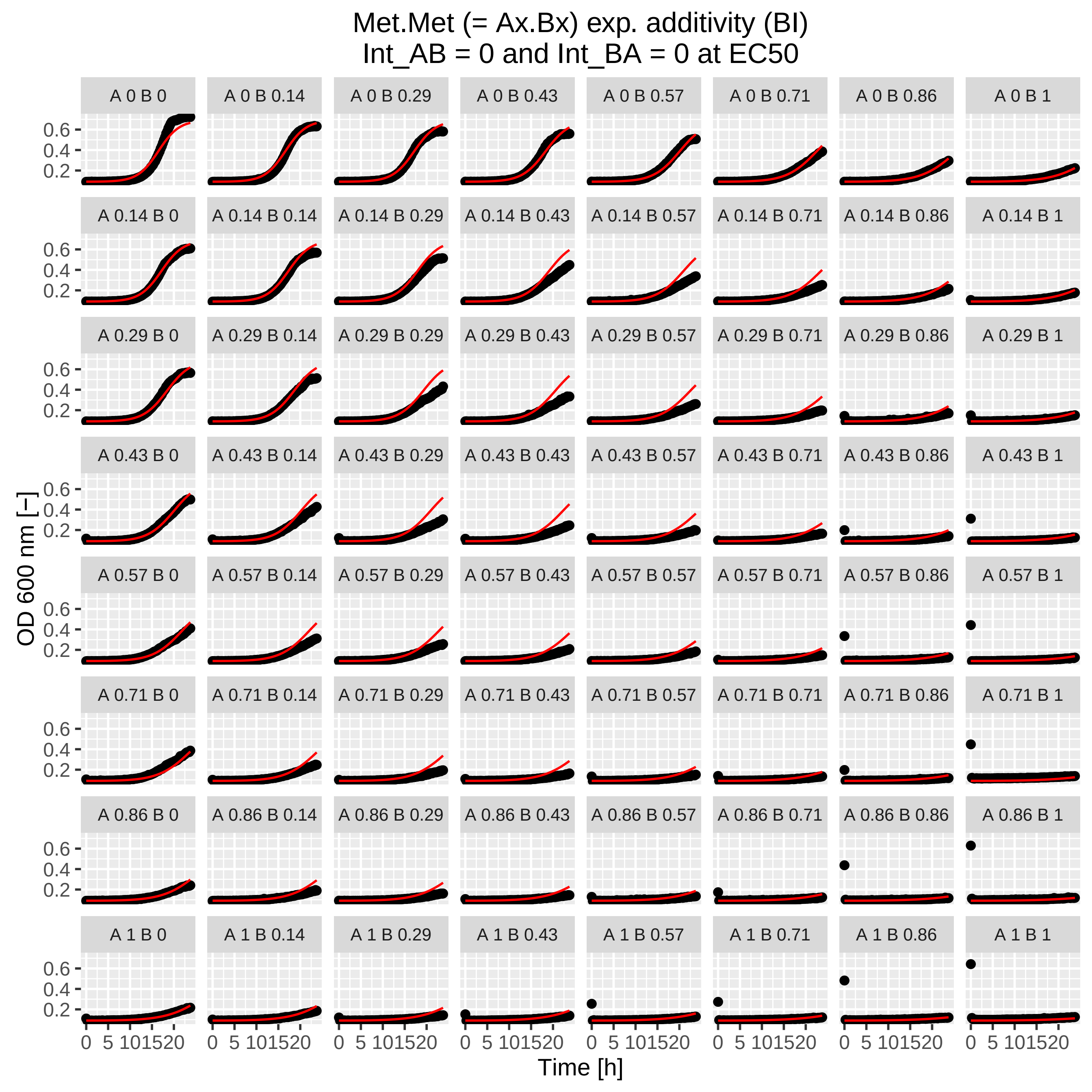

Met.Met (= Ax.Bx) exp. additivity (BI)
Int_AB = 0 and Int_BA = 0 at EC50
A 0 B 0
A 0 B 0.14
A 0 B 0.29
A 0 B 0.43
A 0 B 0.57
A 0 B 0.71
A 0 B 0.86
A 0 B 1
A 0.14 B 0
A 0.14 B 0.14
A 0.14 B 0.29
A 0.14 B 0.43
A 0.14 B 0.57
A 0.14 B 0.71
A 0.14 B 0.86
A 0.14 B 1
A 0.29 B 0
A 0.29 B 0.14
A 0.29 B 0.29
A 0.29 B 0.43
A 0.29 B 0.57
A 0.29 B 0.71
A 0.29 B 0.86
A 0.29 B 1
A 0.43 B 0
A 0.43 B 0.14
A 0.43 B 0.29
A 0.43 B 0.43
A 0.43 B 0.57
A 0.43 B 0.71
A 0.43 B 0.86
A 0.43 B 1
A 0.57 B 0
A 0.57 B 0.14
A 0.57 B 0.29
A 0.57 B 0.43
A 0.57 B 0.57
A 0.57 B 0.71
A 0.57 B 0.86
A 0.57 B 1
A 0.71 B 0
A 0.71 B 0.14
A 0.71 B 0.29
A 0.71 B 0.43
A 0.71 B 0.57
A 0.71 B 0.71
A 0.71 B 0.86
A 0.71 B 1
A 0.86 B 0
A 0.86 B 0.14
A 0.86 B 0.29
A 0.86 B 0.43
A 0.86 B 0.57
A 0.86 B 0.71
A 0.86 B 0.86
A 0.86 B 1
A 1 B 0
A 1 B 0.14
A 1 B 0.29
A 1 B 0.43
A 1 B 0.57
A 1 B 0.71
A 1 B 0.86
A 1 B 1
OD 600 nm [–]
0.2
0.4
0.6
0 5 101520
Time [h]

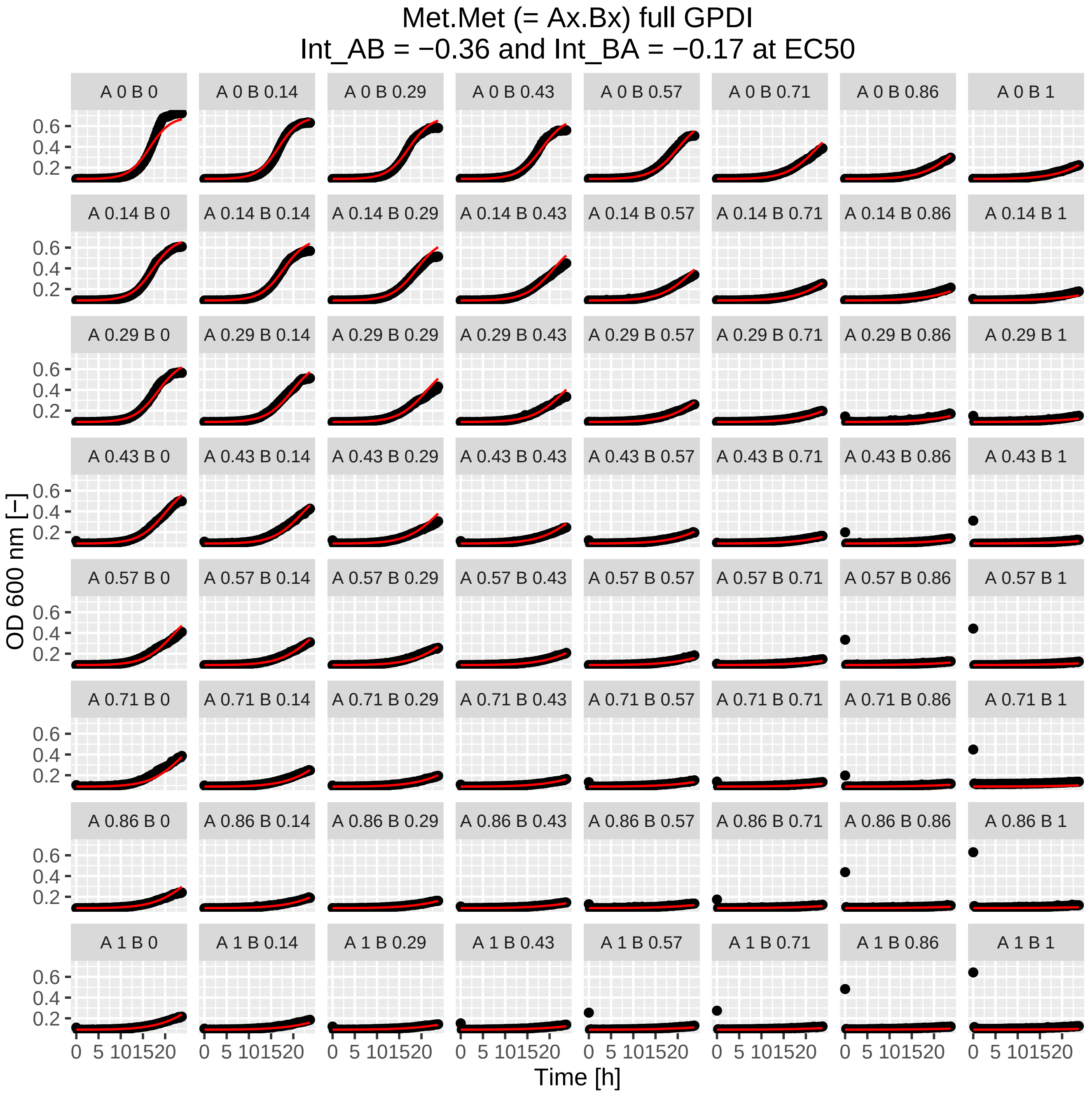
Met.Met (= Ax.Bx) full GPDI
Int_AB = −0.36 and Int_BA = −0.17 at EC50
OD 600 nm [−]
Time [h]
0.2
0.4
0.6
0 5 101520
A 0 B 0
A 0 B 0.14
A 0 B 0.29
A 0 B 0.43
A 0 B 0.57
A 0 B 0.71
A 0 B 0.86
A 0 B 1
A 0.14 B 0
A 0.14 B 0.14
A 0.14 B 0.29
A 0.14 B 0.43
A 0.14 B 0.57
A 0.14 B 0.71
A 0.14 B 0.86
A 0.14 B 1
A 0.29 B 0
A 0.29 B 0.14
A 0.29 B 0.29
A 0.29 B 0.43
A 0.29 B 0.57
A 0.29 B 0.71
A 0.29 B 0.86
A 0.29 B 1
A 0.43 B 0
A 0.43 B 0.14
A 0.43 B 0.29
A 0.43 B 0.43
A 0.43 B 0.57
A 0.43 B 0.71
A 0.43 B 0.86
A 0.43 B 1
A 0.57 B 0
A 0.57 B 0.14
A 0.57 B 0.29
A 0.57 B 0.43
A 0.57 B 0.57
A 0.57 B 0.71
A 0.57 B 0.86
A 0.57 B 1
A 0.71 B 0
A 0.71 B 0.14
A 0.71 B 0.29
A 0.71 B 0.43
A 0.71 B 0.57
A 0.71 B 0.71
A 0.71 B 0.86
A 0.71 B 1
A 0.86 B 0
A 0.86 B 0.14
A 0.86 B 0.29
A 0.86 B 0.43
A 0.86 B 0.57
A 0.86 B 0.71
A 0.86 B 0.86
A 0.86 B 1
A 1 B 0
A 1 B 0.14
A 1 B 0.29
A 1 B 0.43
A 1 B 0.57
A 1 B 0.71
A 1 B 0.86
A 1 B 1

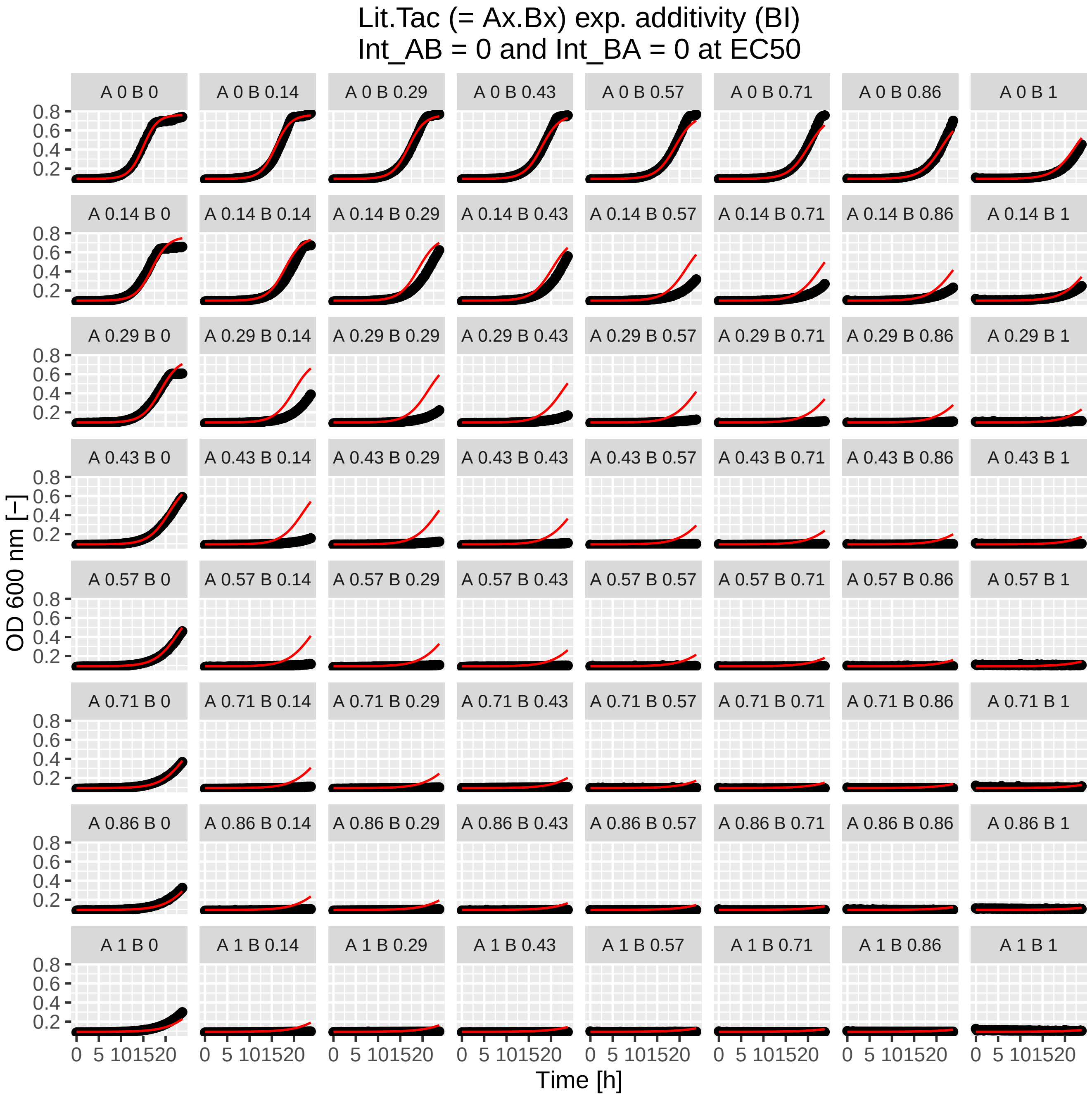
Lit.Tac (= Ax.Bx) exp. additivity (BI)
Int_AB = 0 and Int_BA = 0 at EC50
OD 600 nm [–]
Time [h]
A 0 B 0
A 0 B 0.14
A 0 B 0.29
A 0 B 0.43
A 0 B 0.57
A 0 B 0.71
A 0 B 0.86
A 0 B 1
A 0.14 B 0
A 0.14 B 0.14
A 0.14 B 0.29
A 0.14 B 0.43
A 0.14 B 0.57
A 0.14 B 0.71
A 0.14 B 0.86
A 0.14 B 1
A 0.29 B 0
A 0.29 B 0.14
A 0.29 B 0.29
A 0.29 B 0.43
A 0.29 B 0.57
A 0.29 B 0.71
A 0.29 B 0.86
A 0.29 B 1
A 0.43 B 0
A 0.43 B 0.14
A 0.43 B 0.29
A 0.43 B 0.43
A 0.43 B 0.57
A 0.43 B 0.71
A 0.43 B 0.86
A 0.43 B 1
A 0.57 B 0
A 0.57 B 0.14
A 0.57 B 0.29
A 0.57 B 0.43
A 0.57 B 0.57
A 0.57 B 0.71
A 0.57 B 0.86
A 0.57 B 1
A 0.71 B 0
A 0.71 B 0.14
A 0.71 B 0.29
A 0.71 B 0.43
A 0.71 B 0.57
A 0.71 B 0.71
A 0.71 B 0.86
A 0.71 B 1
A 0.86 B 0
A 0.86 B 0.14
A 0.86 B 0.29
A 0.86 B 0.43
A 0.86 B 0.57
A 0.86 B 0.71
A 0.86 B 0.86
A 0.86 B 1
A 1 B 0
A 1 B 0.14
A 1 B 0.29
A 1 B 0.43
A 1 B 0.57
A 1 B 0.71
A 1 B 0.86
A 1 B 1
0.8
0.6
0.4
0.2
0 5 101520

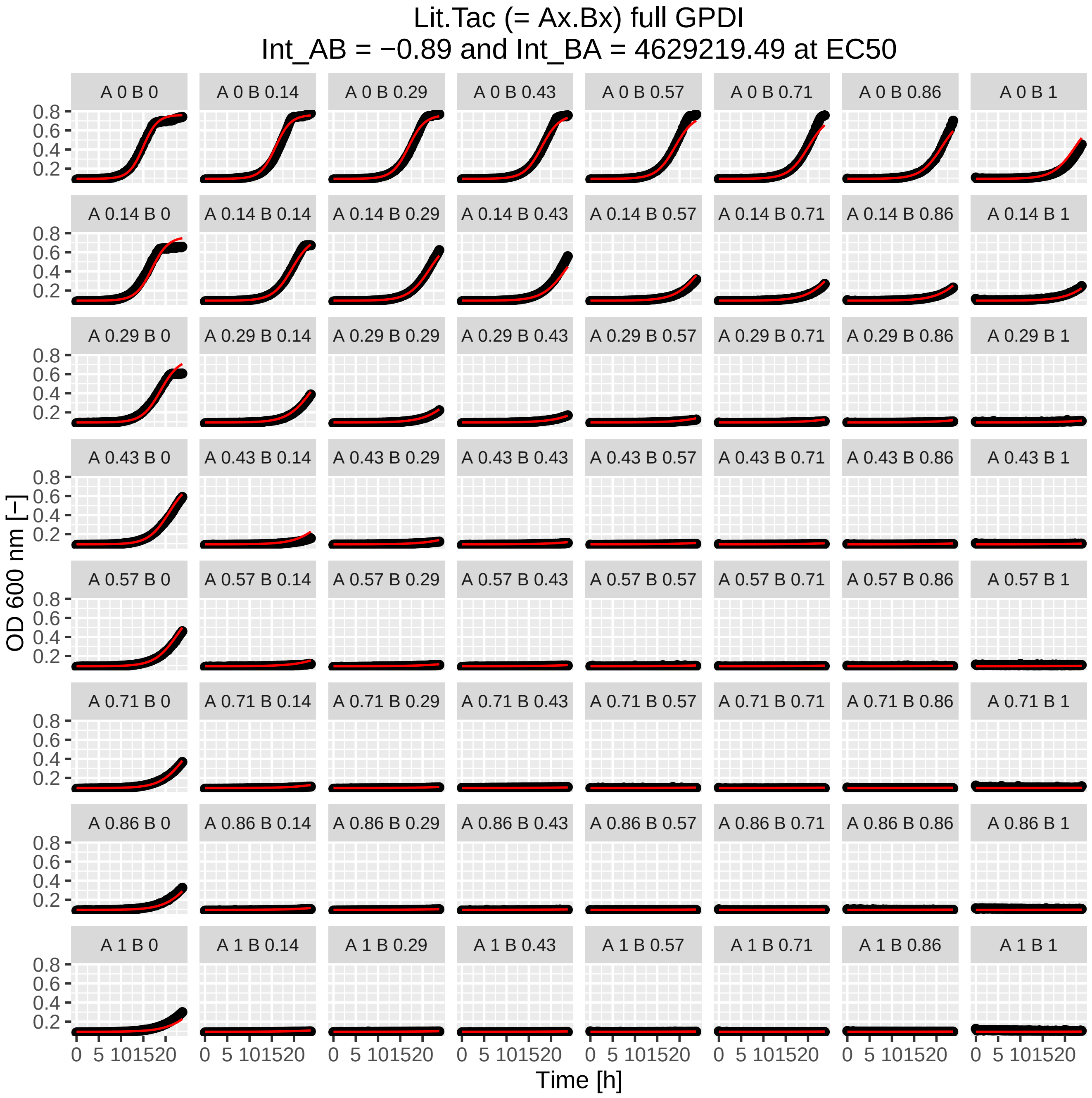

Lit.Tac (= Ax.Bx) full GPDI
Int_AB = −0.89 and Int_BA = 4629219.49 at EC50
A 0 B 0
A 0 B 0.14
A 0 B 0.29
A 0 B 0.43
A 0 B 0.57
A 0 B 0.71
A 0 B 0.86
A 0 B 1
A 0.14 B 0
A 0.14 B 0.14
A 0.14 B 0.29
A 0.14 B 0.43
A 0.14 B 0.57
A 0.14 B 0.71
A 0.14 B 0.86
A 0.14 B 1
A 0.29 B 0
A 0.29 B 0.14
A 0.29 B 0.29
A 0.29 B 0.43
A 0.29 B 0.57
A 0.29 B 0.71
A 0.29 B 0.86
A 0.29 B 1
A 0.43 B 0
A 0.43 B 0.14
A 0.43 B 0.29
A 0.43 B 0.43
A 0.43 B 0.57
A 0.43 B 0.71
A 0.43 B 0.86
A 0.43 B 1
A 0.57 B 0
A 0.57 B 0.14
A 0.57 B 0.29
A 0.57 B 0.43
A 0.57 B 0.57
A 0.57 B 0.71
A 0.57 B 0.86
A 0.57 B 1
A 0.71 B 0
A 0.71 B 0.14
A 0.71 B 0.29
A 0.71 B 0.43
A 0.71 B 0.57
A 0.71 B 0.71
A 0.71 B 0.86
A 0.71 B 1
A 0.86 B 0
A 0.86 B 0.14
A 0.86 B 0.29
A 0.86 B 0.43
A 0.86 B 0.57
A 0.86 B 0.71
A 0.86 B 0.86
A 0.86 B 1
A 1 B 0
A 1 B 0.14
A 1 B 0.29
A 1 B 0.43
A 1 B 0.57
A 1 B 0.71
A 1 B 0.86
A 1 B 1
OD 600 nm [−]
0.2
0.4
0.6
0.8
0 5 101520
Time [h]

Lit.Sta (= Ax.Bx) exp. additivity (BI)
Int_AB = 0 and Int_BA = 0 at EC50

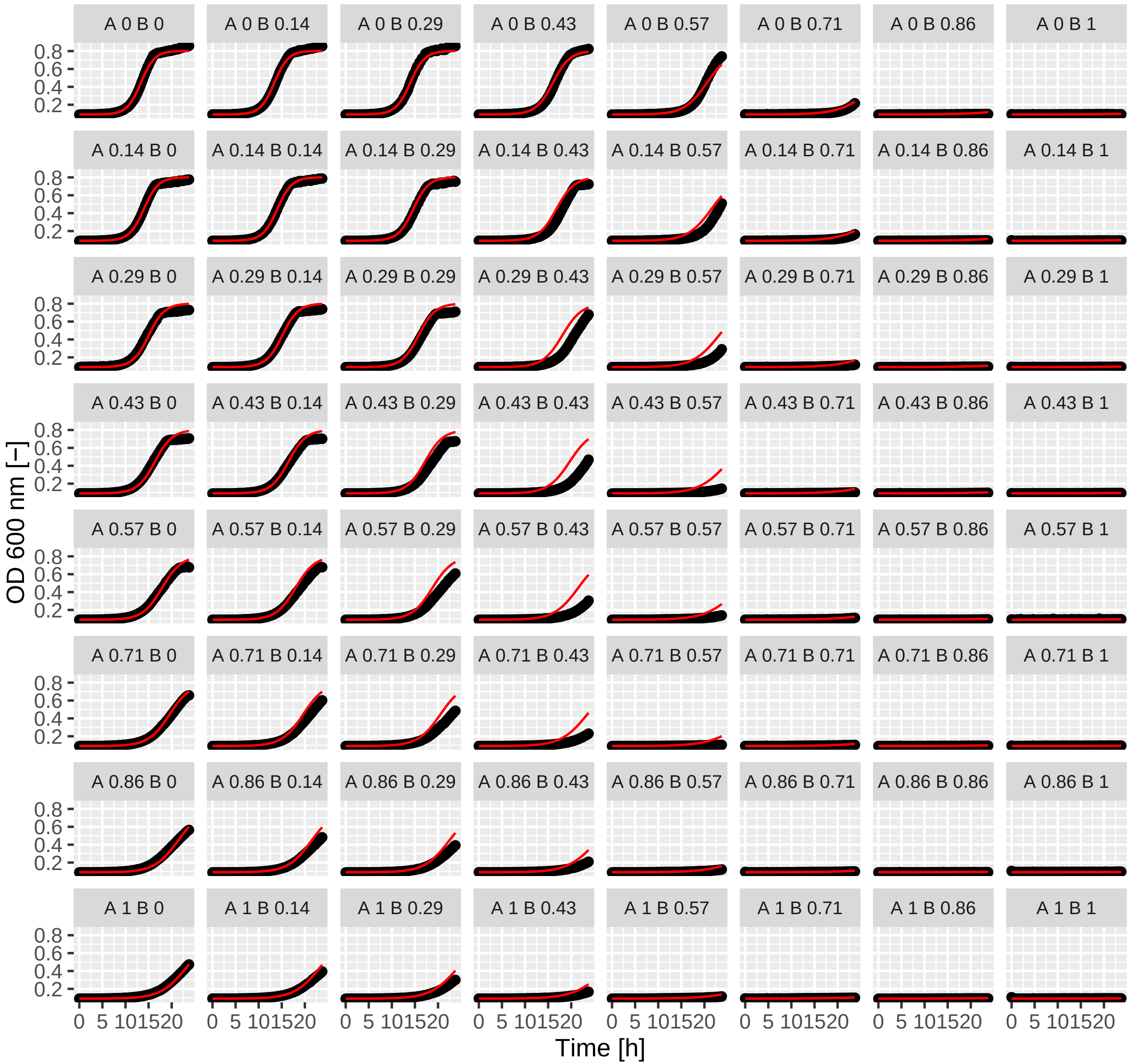

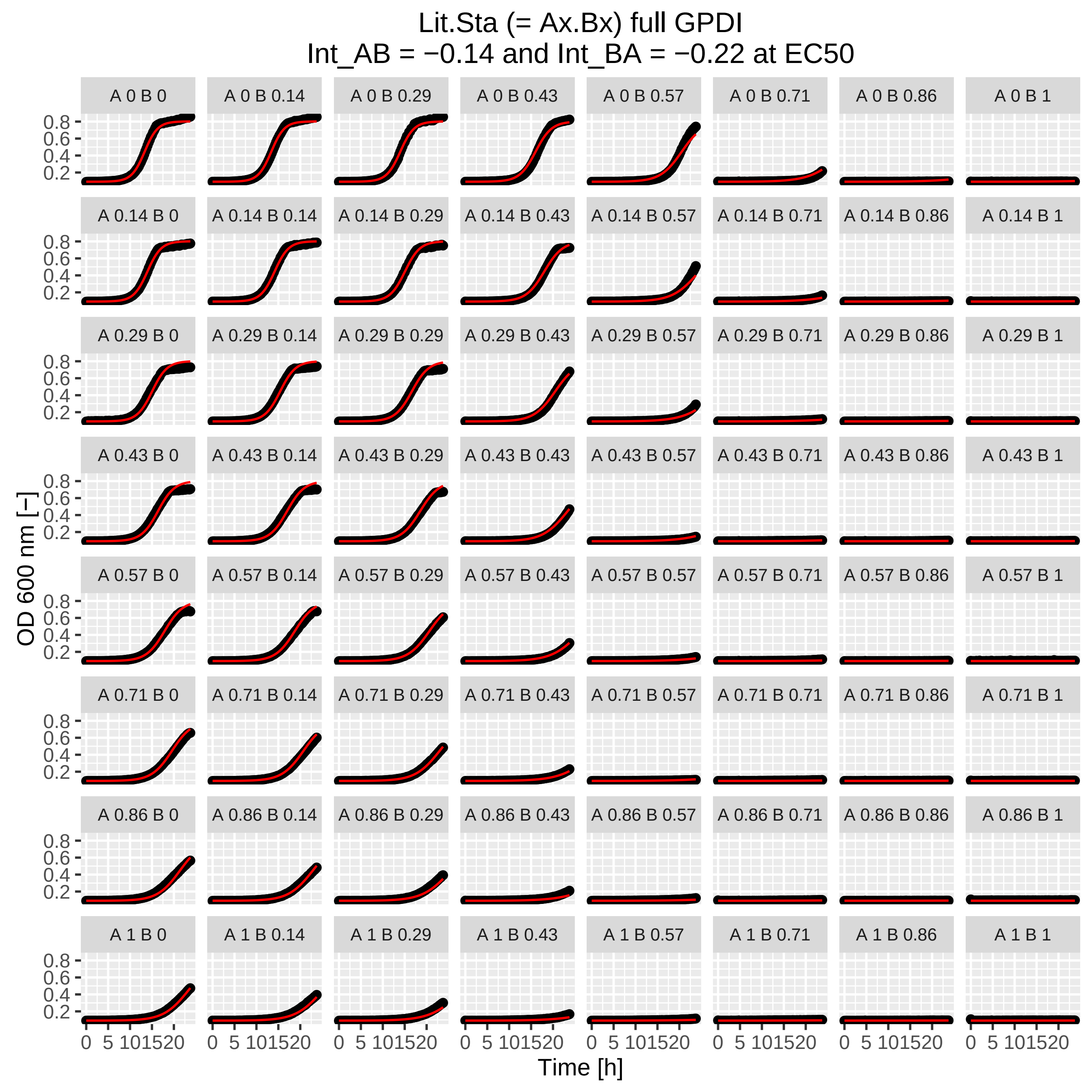

Lit.Sta (= Ax.Bx) full GPDI
Int_AB = −0.14 and Int_BA = −0.22 at EC50
A 0 B 0
A 0 B 0.14
A 0 B 0.29
A 0 B 0.43
A 0 B 0.57
A 0 B 0.71
A 0 B 0.86
A 0 B 1
A 0.14 B 0
A 0.14 B 0.14
A 0.14 B 0.29
A 0.14 B 0.43
A 0.14 B 0.57
A 0.14 B 0.71
A 0.14 B 0.86
A 0.14 B 1
A 0.29 B 0
A 0.29 B 0.14
A 0.29 B 0.29
A 0.29 B 0.43
A 0.29 B 0.57
A 0.29 B 0.71
A 0.29 B 0.86
A 0.29 B 1
A 0.43 B 0
A 0.43 B 0.14
A 0.43 B 0.29
A 0.43 B 0.43
A 0.43 B 0.57
A 0.43 B 0.71
A 0.43 B 0.86
A 0.43 B 1
A 0.57 B 0
A 0.57 B 0.14
A 0.57 B 0.29
A 0.57 B 0.43
A 0.57 B 0.57
A 0.57 B 0.71
A 0.57 B 0.86
A 0.57 B 1
A 0.71 B 0
A 0.71 B 0.14
A 0.71 B 0.29
A 0.71 B 0.43
A 0.71 B 0.57
A 0.71 B 0.71
A 0.71 B 0.86
A 0.71 B 1
A 0.86 B 0
A 0.86 B 0.14
A 0.86 B 0.29
A 0.86 B 0.43
A 0.86 B 0.57
A 0.86 B 0.71
A 0.86 B 0.86
A 0.86 B 1
A 1 B 0
A 1 B 0.14
A 1 B 0.29
A 1 B 0.43
A 1 B 0.57
A 1 B 0.71
A 1 B 0.86
A 1 B 1
OD 600 nm [−]
0.8
0.6
0.4
0.2
0 5 101520
Time [h]

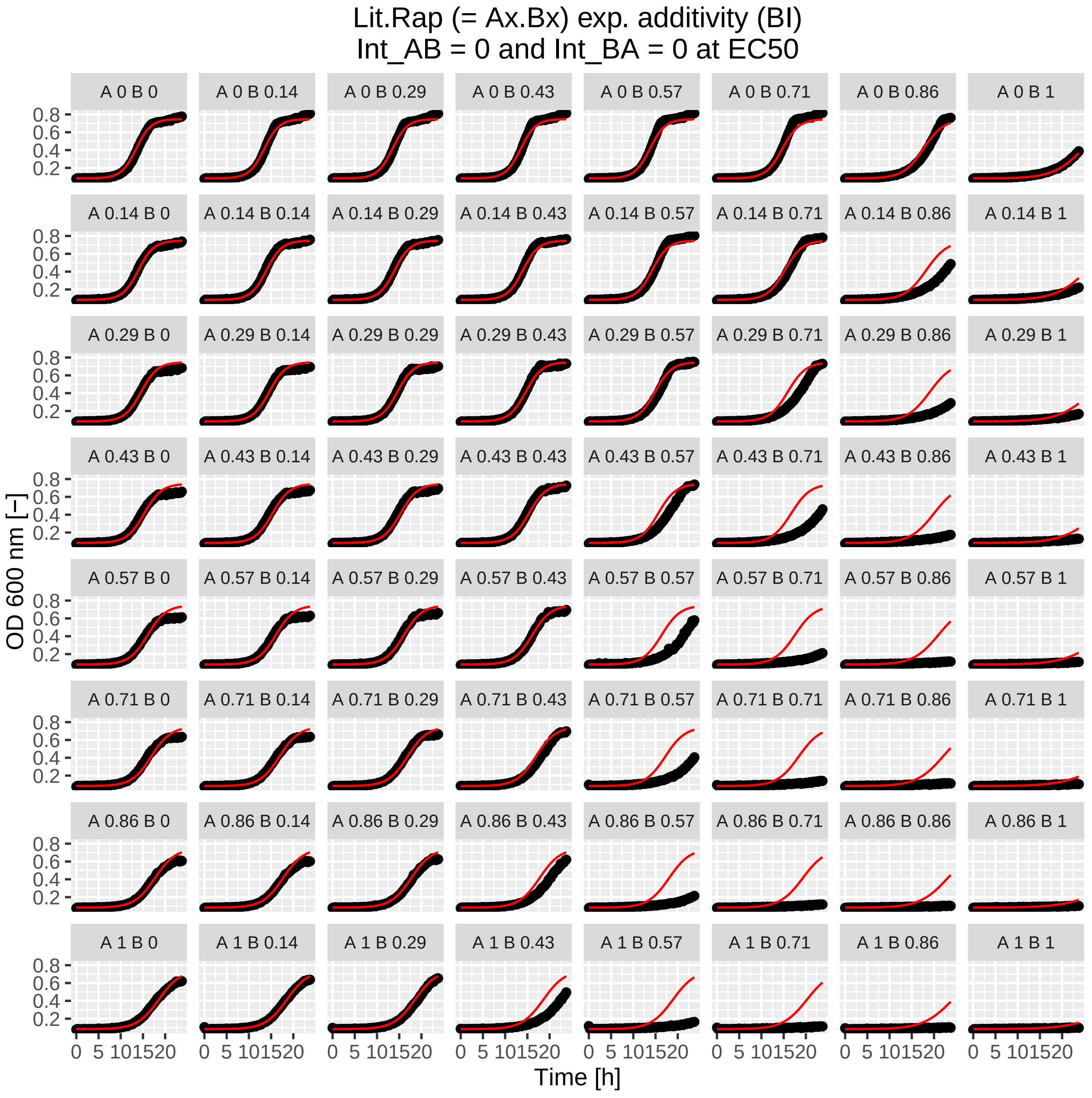

Lit.Rap (= Ax.Bx) exp. additivity (BI)
Int_AB = 0 and Int_BA = 0 at EC50
OD 600 nm [–]
Time [h]
A 0 B 0
A 0 B 0.14
A 0 B 0.29
A 0 B 0.43
A 0 B 0.57
A 0 B 0.71
A 0 B 0.86
A 0 B 1
A 0.14 B 0
A 0.29 B 0
A 0.43 B 0
A 0.57 B 0
A 0.71 B 0
A 0.86 B 0
A 1 B 0
A 1 B 1
0.2
0.4
0.6
0.8
0 5 101520

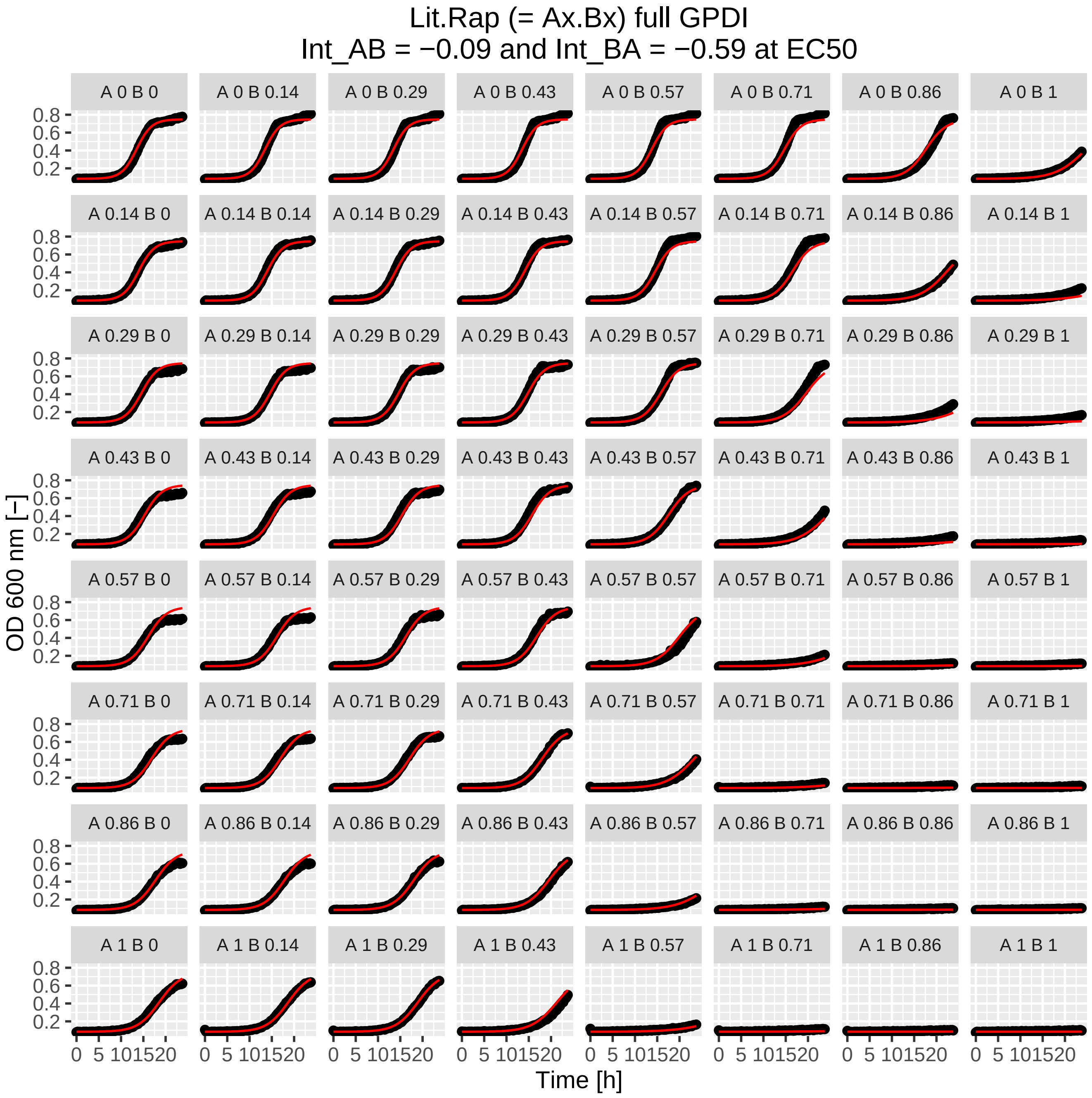

Lit.Rap (= Ax.Bx) full GPDI
Int_AB = −0.09 and Int_BA = −0.59 at EC50
A 0 B 0
A 0 B 0.14
A 0 B 0.29
A 0 B 0.43
A 0 B 0.57
A 0 B 0.71
A 0 B 0.86
A 0 B 1
A 0.14 B 0
A 0.14 B 0.14
A 0.14 B 0.29
A 0.14 B 0.43
A 0.14 B 0.57
A 0.14 B 0.71
A 0.14 B 0.86
A 0.14 B 1
A 0.29 B 0
A 0.29 B 0.14
A 0.29 B 0.29
A 0.29 B 0.43
A 0.29 B 0.57
A 0.29 B 0.71
A 0.29 B 0.86
A 0.29 B 1
A 0.43 B 0
A 0.43 B 0.14
A 0.43 B 0.29
A 0.43 B 0.43
A 0.43 B 0.57
A 0.43 B 0.71
A 0.43 B 0.86
A 0.43 B 1
A 0.57 B 0
A 0.57 B 0.14
A 0.57 B 0.29
A 0.57 B 0.43
A 0.57 B 0.57
A 0.57 B 0.71
A 0.57 B 0.86
A 0.57 B 1
A 0.71 B 0
A 0.71 B 0.14
A 0.71 B 0.29
A 0.71 B 0.43
A 0.71 B 0.57
A 0.71 B 0.71
A 0.71 B 0.86
A 0.71 B 1
A 0.86 B 0
A 0.86 B 0.14
A 0.86 B 0.29
A 0.86 B 0.43
A 0.86 B 0.57
A 0.86 B 0.71
A 0.86 B 0.86
A 0.86 B 1
A 1 B 0
A 1 B 0.14
A 1 B 0.29
A 1 B 0.43
A 1 B 0.57
A 1 B 0.71
A 1 B 0.86
A 1 B 1
0.8
0.6
0.4
0.2
0 5 101520
OD 600 nm [−]
Time [h]

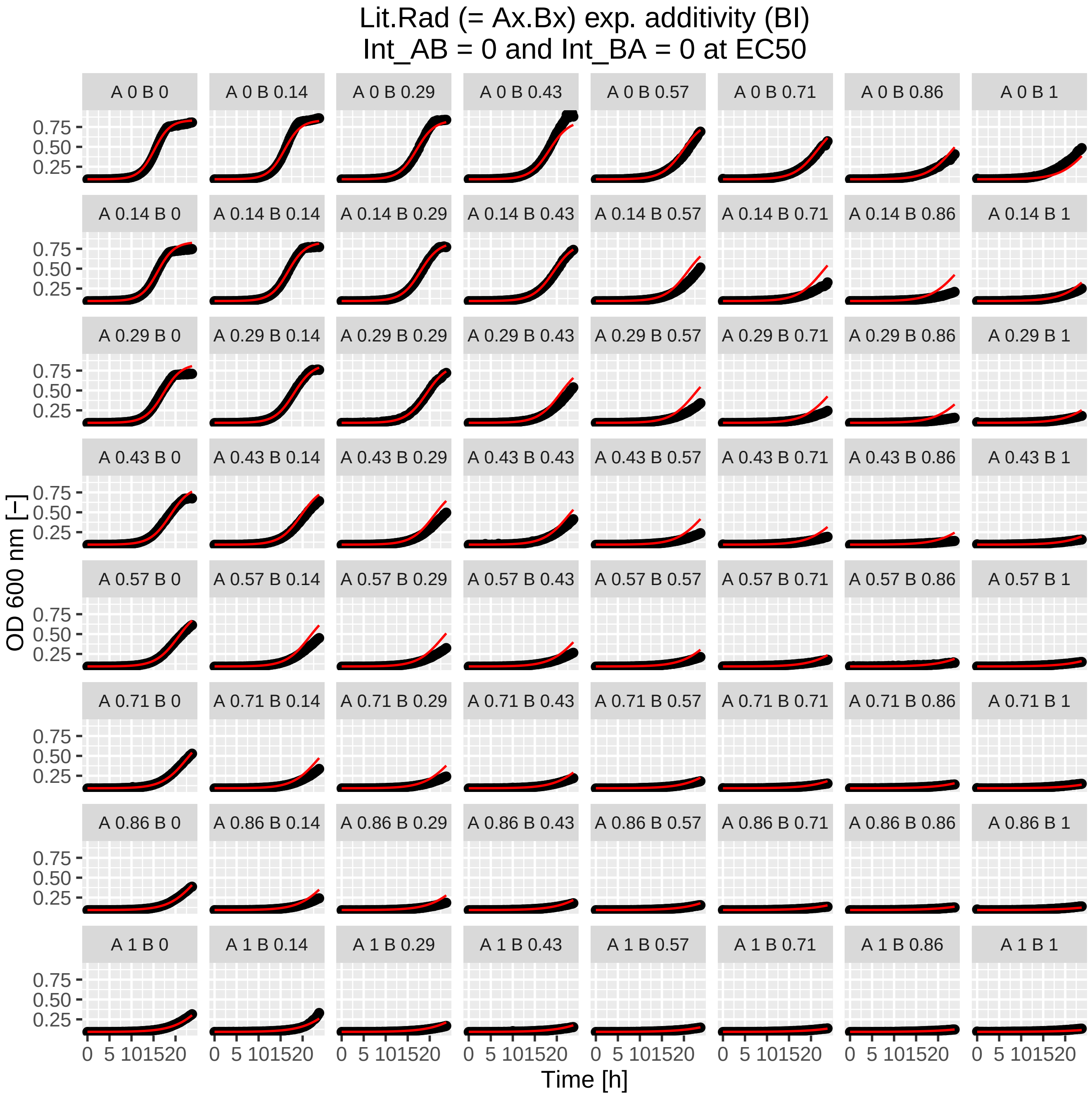

Lit.Rad (= Ax.Bx) exp. additivity (BI)
Int_AB = 0 and Int_BA = 0 at EC50
A 0 B 0
A 0 B 0.14
A 0 B 0.29
A 0 B 0.43
A 0 B 0.57
A 0 B 0.71
A 0 B 0.86
A 0 B 1
A 0.14 B 0
A 0.14 B 0.14
A 0.14 B 0.29
A 0.14 B 0.43
A 0.14 B 0.57
A 0.14 B 0.71
A 0.14 B 0.86
A 0.14 B 1
A 0.29 B 0
A 0.29 B 0.14
A 0.29 B 0.29
A 0.29 B 0.43
A 0.29 B 0.57
A 0.29 B 0.71
A 0.29 B 0.86
A 0.29 B 1
A 0.43 B 0
A 0.43 B 0.14
A 0.43 B 0.29
A 0.43 B 0.43
A 0.43 B 0.57
A 0.43 B 0.71
A 0.43 B 0.86
A 0.43 B 1
A 0.57 B 0
A 0.57 B 0.14
A 0.57 B 0.29
A 0.57 B 0.43
A 0.57 B 0.57
A 0.57 B 0.71
A 0.57 B 0.86
A 0.57 B 1
A 0.71 B 0
A 0.71 B 0.14
A 0.71 B 0.29
A 0.71 B 0.43
A 0.71 B 0.57
A 0.71 B 0.71
A 0.71 B 0.86
A 0.71 B 1
A 0.86 B 0
A 0.86 B 0.14
A 0.86 B 0.29
A 0.86 B 0.43
A 0.86 B 0.57
A 0.86 B 0.71
A 0.86 B 0.86
A 0.86 B 1
A 1 B 0
A 1 B 0.14
A 1 B 0.29
A 1 B 0.43
A 1 B 0.57
A 1 B 0.71
A 1 B 0.86
A 1 B 1
0.75
0.50
0.25
OD 600 nm [–]
0 5 101520
Time [h]

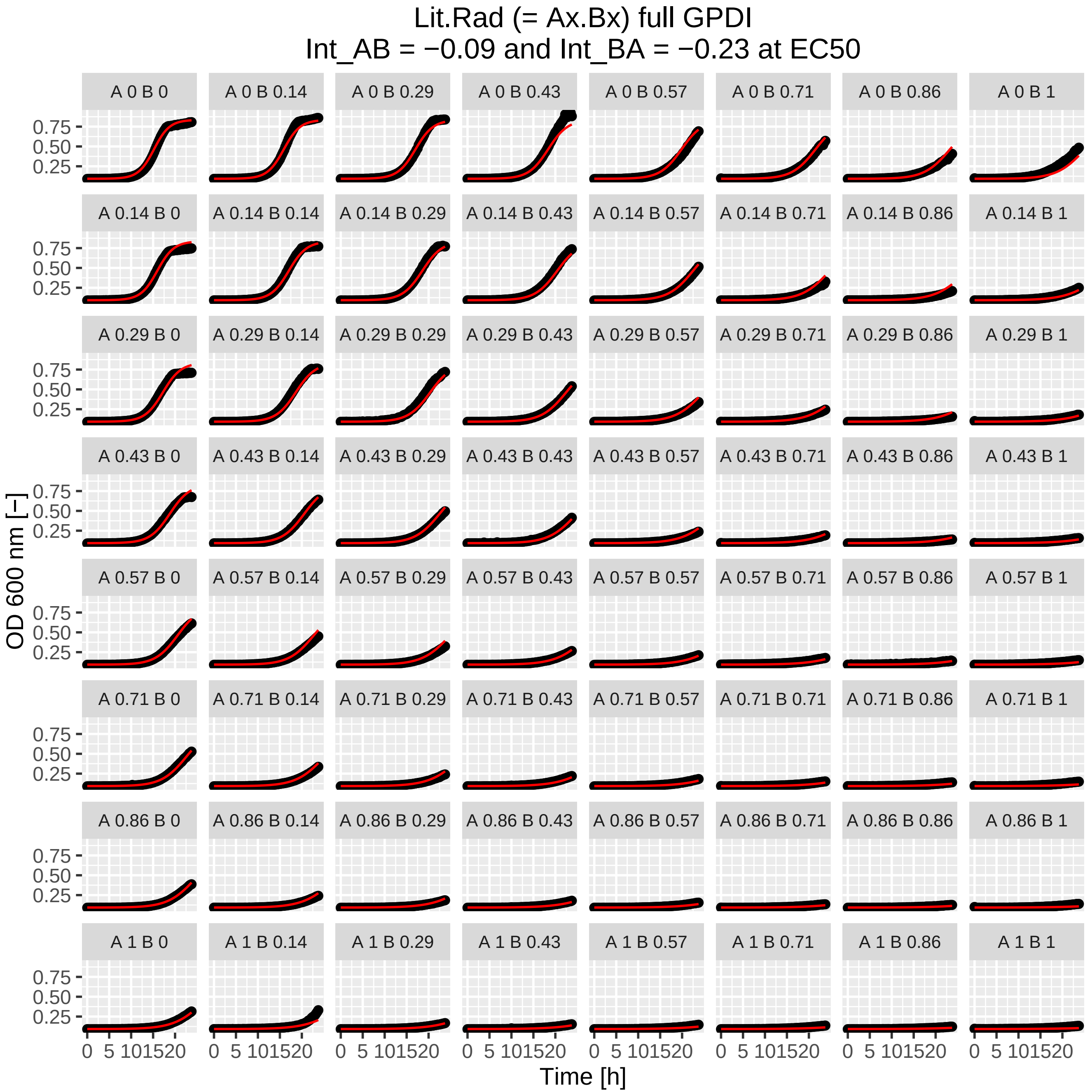

Lit.Rad (= Ax.Bx) full GPDI
Int_AB = −0.09 and Int_BA = −0.23 at EC50
A 0 B 0
A 0 B 0.14
A 0 B 0.29
A 0 B 0.43
A 0 B 0.57
A 0 B 0.71
A 0 B 0.86
A 0 B 1
A 0.14 B 0
A 0.14 B 0.14
A 0.14 B 0.29
A 0.14 B 0.43
A 0.14 B 0.57
A 0.14 B 0.71
A 0.14 B 0.86
A 0.14 B 1
A 0.29 B 0
A 0.29 B 0.14
A 0.29 B 0.29
A 0.29 B 0.43
A 0.29 B 0.57
A 0.29 B 0.71
A 0.29 B 0.86
A 0.29 B 1
A 0.43 B 0
A 0.43 B 0.14
A 0.43 B 0.29
A 0.43 B 0.43
A 0.43 B 0.57
A 0.43 B 0.71
A 0.43 B 0.86
A 0.43 B 1
A 0.57 B 0
A 0.57 B 0.14
A 0.57 B 0.29
A 0.57 B 0.43
A 0.57 B 0.57
A 0.57 B 0.71
A 0.57 B 0.86
A 0.57 B 1
A 0.71 B 0
A 0.71 B 0.14
A 0.71 B 0.29
A 0.71 B 0.43
A 0.71 B 0.57
A 0.71 B 0.71
A 0.71 B 0.86
A 0.71 B 1
A 0.86 B 0
A 0.86 B 0.14
A 0.86 B 0.29
A 0.86 B 0.43
A 0.86 B 0.57
A 0.86 B 0.71
A 0.86 B 0.86
A 0.86 B 1
A 1 B 0
A 1 B 0.14
A 1 B 0.29
A 1 B 0.43
A 1 B 0.57
A 1 B 0.71
A 1 B 0.86
A 1 B 1
0.75
0.50
0.25
OD 600 nm [−]
0 5 101520
Time [h]

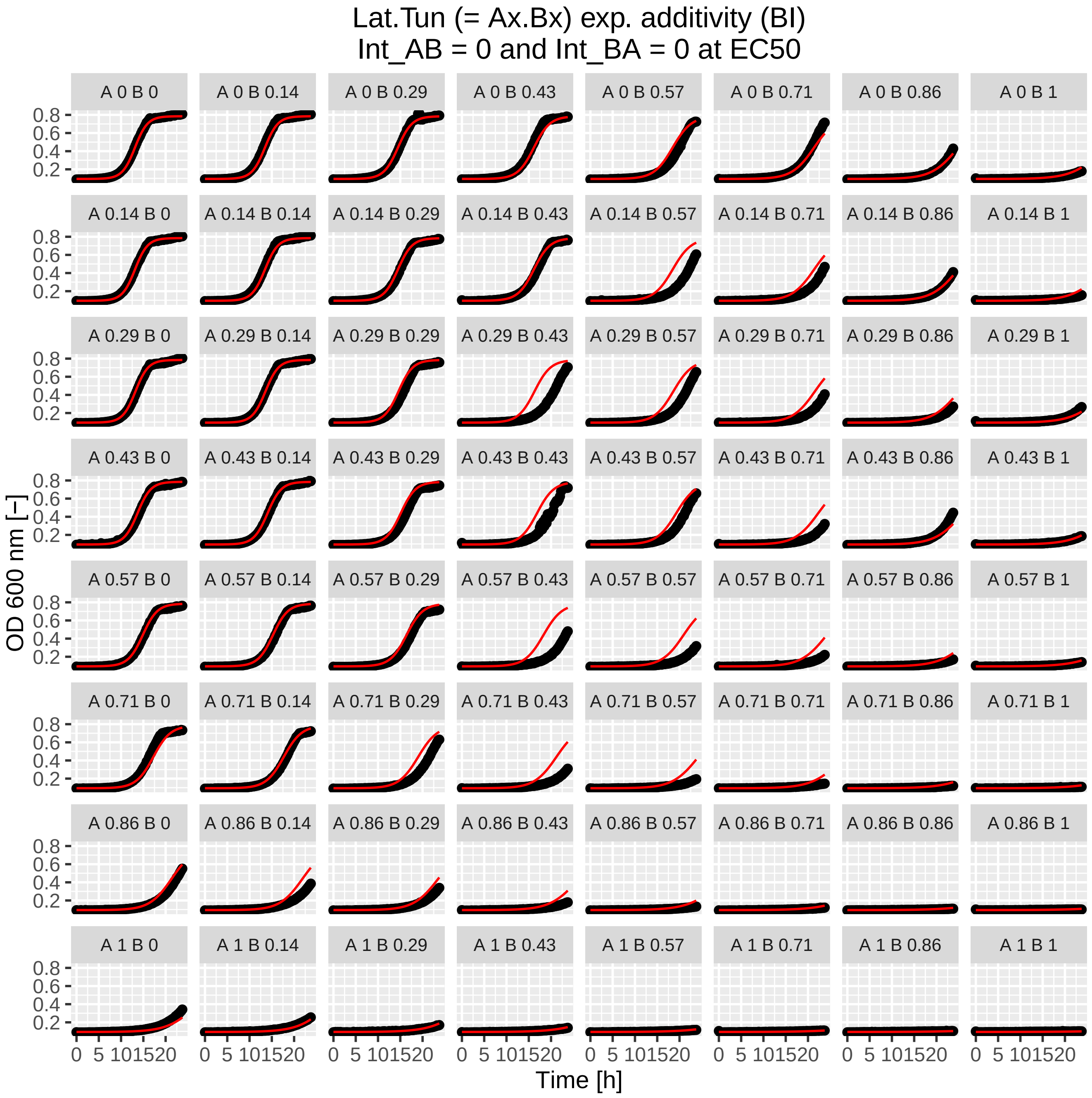
Lat.Tun (= Ax.Bx) exp. additivity (BI)
Int_AB = 0 and Int_BA = 0 at EC50
OD 600 nm [–]
Time [h]
A 0 B 0
A 0 B 0.14
A 0 B 0.29
A 0 B 0.43
A 0 B 0.57
A 0 B 0.71
A 0 B 0.86
A 0 B 1
A 0.14 B 0
A 0.14 B 0.14
A 0.14 B 0.29
A 0.14 B 0.43
A 0.14 B 0.57
A 0.14 B 0.71
A 0.14 B 0.86
A 0.14 B 1
A 0.29 B 0
A 0.29 B 0.14
A 0.29 B 0.29
A 0.29 B 0.43
A 0.29 B 0.57
A 0.29 B 0.71
A 0.29 B 0.86
A 0.29 B 1
A 0.43 B 0
A 0.43 B 0.14
A 0.43 B 0.29
A 0.43 B 0.43
A 0.43 B 0.57
A 0.43 B 0.71
A 0.43 B 0.86
A 0.43 B 1
A 0.57 B 0
A 0.57 B 0.14
A 0.57 B 0.29
A 0.57 B 0.43
A 0.57 B 0.57
A 0.57 B 0.71
A 0.57 B 0.86
A 0.57 B 1
A 0.71 B 0
A 0.71 B 0.14
A 0.71 B 0.29
A 0.71 B 0.43
A 0.71 B 0.57
A 0.71 B 0.71
A 0.71 B 0.86
A 0.71 B 1
A 0.86 B 0
A 0.86 B 0.14
A 0.86 B 0.29
A 0.86 B 0.43
A 0.86 B 0.57
A 0.86 B 0.71
A 0.86 B 0.86
A 0.86 B 1
A 1 B 0
A 1 B 0.14
A 1 B 0.29
A 1 B 0.43
A 1 B 0.57
A 1 B 0.71
A 1 B 0.86
A 1 B 1
0.8
0.6
0.4
0.2
0 5 101520

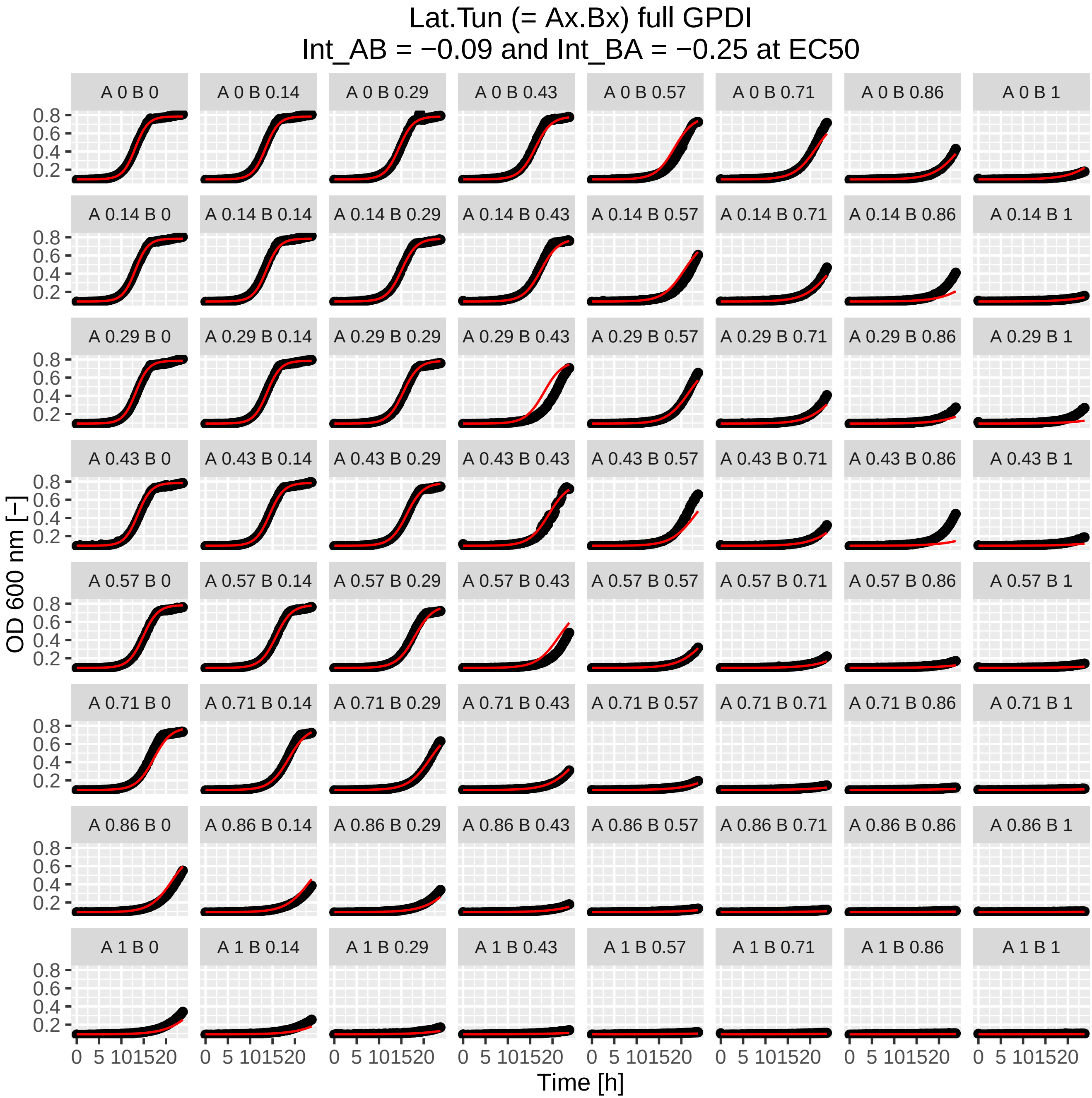
Lat.Tun (= Ax.Bx) full GPDI
Int_AB = −0.09 and Int_BA = −0.25 at EC50
OD 600 nm [−]
Time [h]
A 0 B 0
A 0 B 0.14
A 0 B 0.29
A 0 B 0.43
A 0 B 0.57
A 0 B 0.71
A 0 B 0.86
A 0 B 1
A 1 B 0
A 1 B 0.14
A 1 B 0.29
A 1 B 0.43
A 1 B 0.57
A 1 B 0.71
A 1 B 0.86
A 1 B 1

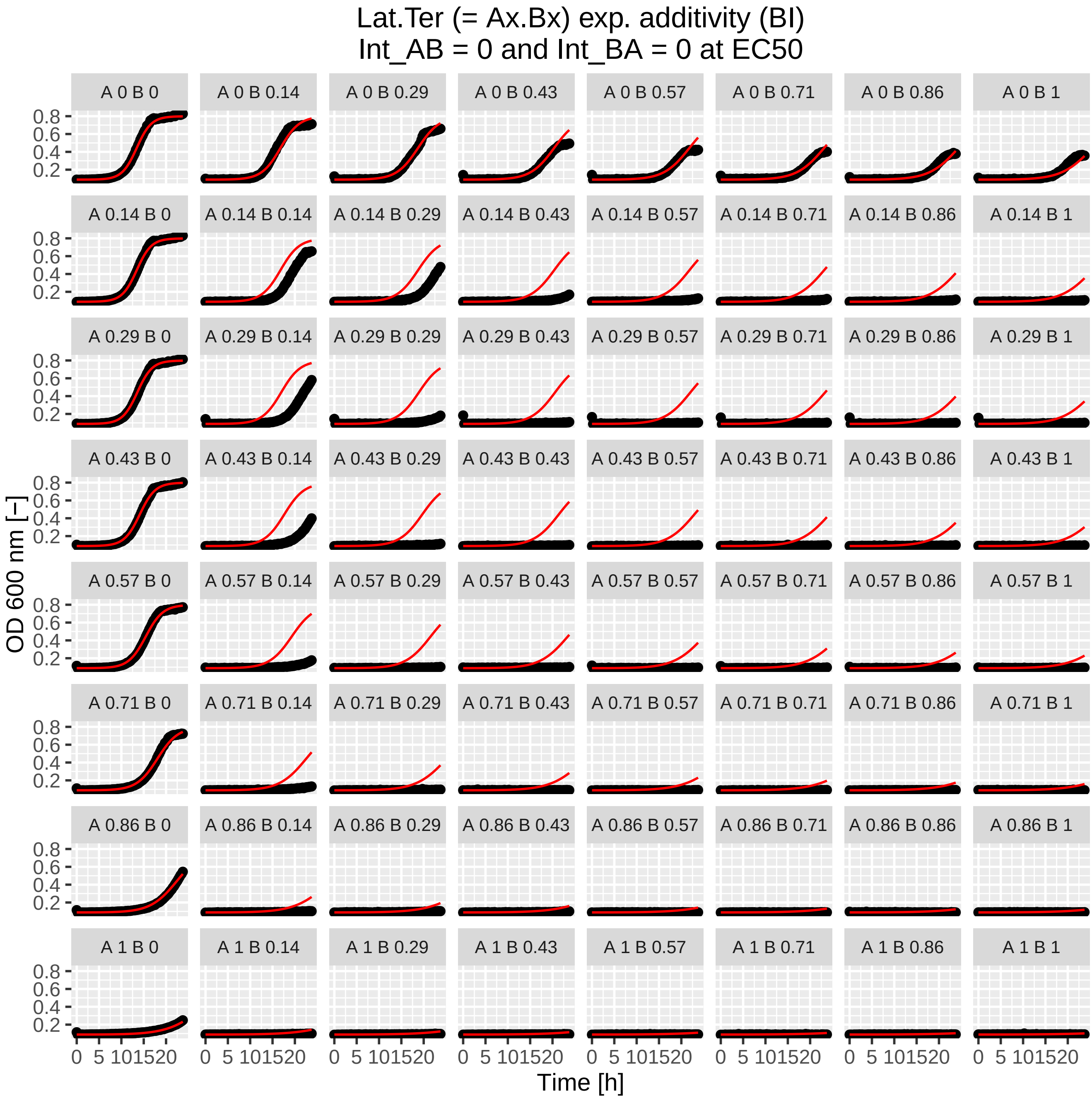
Lat.Ter (= Ax.Bx) exp. additivity (BI)
Int_AB = 0 and Int_BA = 0 at EC50
A 0 B 0
A 0 B 0.14
A 0 B 0.29
A 0 B 0.43
A 0 B 0.57
A 0 B 0.71
A 0 B 0.86
A 0 B 1
A 0.14 B 0
A 0.14 B 0.14
A 0.14 B 0.29
A 0.14 B 0.43
A 0.14 B 0.57
A 0.14 B 0.71
A 0.14 B 0.86
A 0.14 B 1
A 0.29 B 0
A 0.29 B 0.14
A 0.29 B 0.29
A 0.29 B 0.43
A 0.29 B 0.57
A 0.29 B 0.71
A 0.29 B 0.86
A 0.29 B 1
A 0.43 B 0
A 0.43 B 0.14
A 0.43 B 0.29
A 0.43 B 0.43
A 0.43 B 0.57
A 0.43 B 0.71
A 0.43 B 0.86
A 0.43 B 1
A 0.57 B 0
A 0.57 B 0.14
A 0.57 B 0.29
A 0.57 B 0.43
A 0.57 B 0.57
A 0.57 B 0.71
A 0.57 B 0.86
A 0.57 B 1
A 0.71 B 0
A 0.71 B 0.14
A 0.71 B 0.29
A 0.71 B 0.43
A 0.71 B 0.57
A 0.71 B 0.71
A 0.71 B 0.86
A 0.71 B 1
A 0.86 B 0
A 0.86 B 0.14
A 0.86 B 0.29
A 0.86 B 0.43
A 0.86 B 0.57
A 0.86 B 0.71
A 0.86 B 0.86
A 0.86 B 1
A 1 B 0
A 1 B 0.14
A 1 B 0.29
A 1 B 0.43
A 1 B 0.57
A 1 B 0.71
A 1 B 0.86
A 1 B 1
OD 600 nm [–]
0.8
0.6
0.4
0.2
0 5 101520
Time [h]

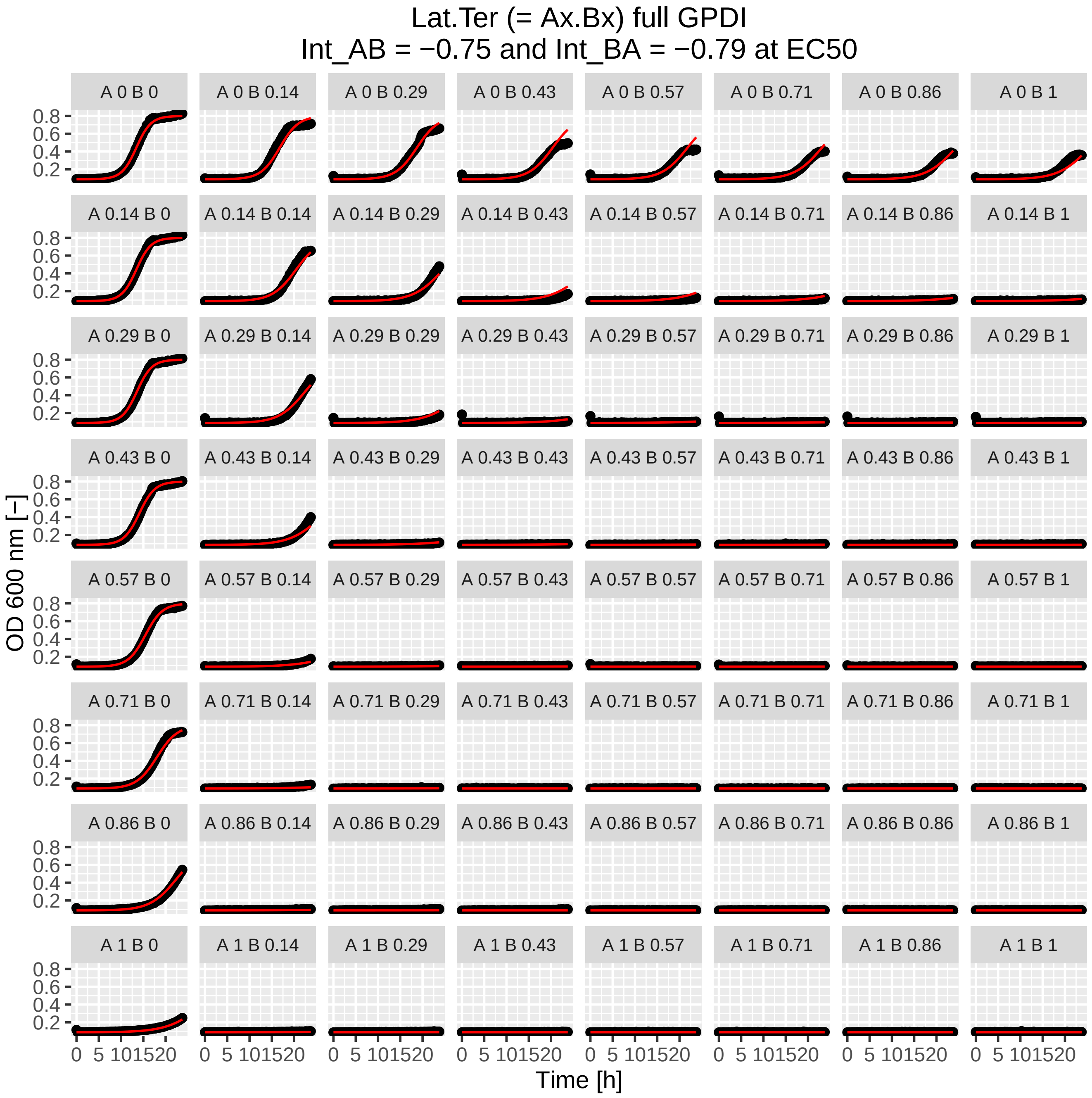

Lat.Ter (= Ax.Bx) full GPDI
Int_AB = −0.75 and Int_BA = −0.79 at EC50
OD 600 nm [−]
Time [h]
0.8
0.6
0.4
0.2
0 5 101520
A 0 B 0
A 0 B 0.14
A 0 B 0.29
A 0 B 0.43
A 0 B 0.57
A 0 B 0.71
A 0 B 0.86
A 0 B 1
A 0.14 B 0
A 0.14 B 0.14
A 0.14 B 0.29
A 0.14 B 0.43
A 0.14 B 0.57
A 0.14 B 0.71
A 0.14 B 0.86
A 0.14 B 1
A 0.29 B 0
A 0.29 B 0.14
A 0.29 B 0.29
A 0.29 B 0.43
A 0.29 B 0.57
A 0.29 B 0.71
A 0.29 B 0.86
A 0.29 B 1
A 0.43 B 0
A 0.43 B 0.14
A 0.43 B 0.29
A 0.43 B 0.43
A 0.43 B 0.57
A 0.43 B 0.71
A 0.43 B 0.86
A 0.43 B 1
A 0.57 B 0
A 0.57 B 0.14
A 0.57 B 0.29
A 0.57 B 0.43
A 0.57 B 0.57
A 0.57 B 0.71
A 0.57 B 0.86
A 0.57 B 1
A 0.71 B 0
A 0.71 B 0.14
A 0.71 B 0.29
A 0.71 B 0.43
A 0.71 B 0.57
A 0.71 B 0.71
A 0.71 B 0.86
A 0.71 B 1
A 0.86 B 0
A 0.86 B 0.14
A 0.86 B 0.29
A 0.86 B 0.43
A 0.86 B 0.57
A 0.86 B 0.71
A 0.86 B 0.86
A 0.86 B 1
A 1 B 0
A 1 B 0.14
A 1 B 0.29
A 1 B 0.43
A 1 B 0.57
A 1 B 0.71
A 1 B 0.86
A 1 B 1

Lat.Tam (= Ax.Bx) exp. additivity (BI)
Int_AB = 0 and Int_BA = 0 at EC50

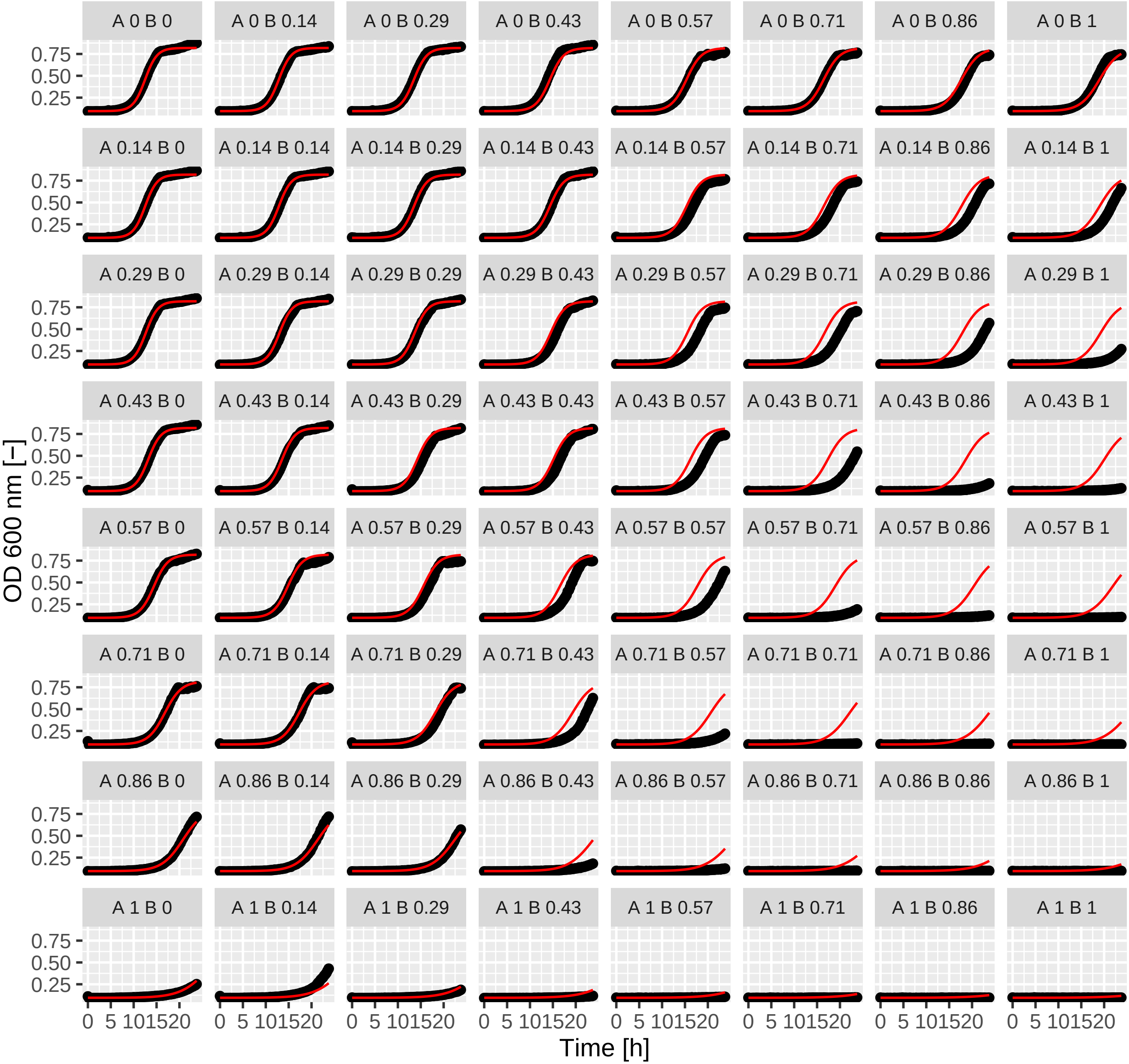

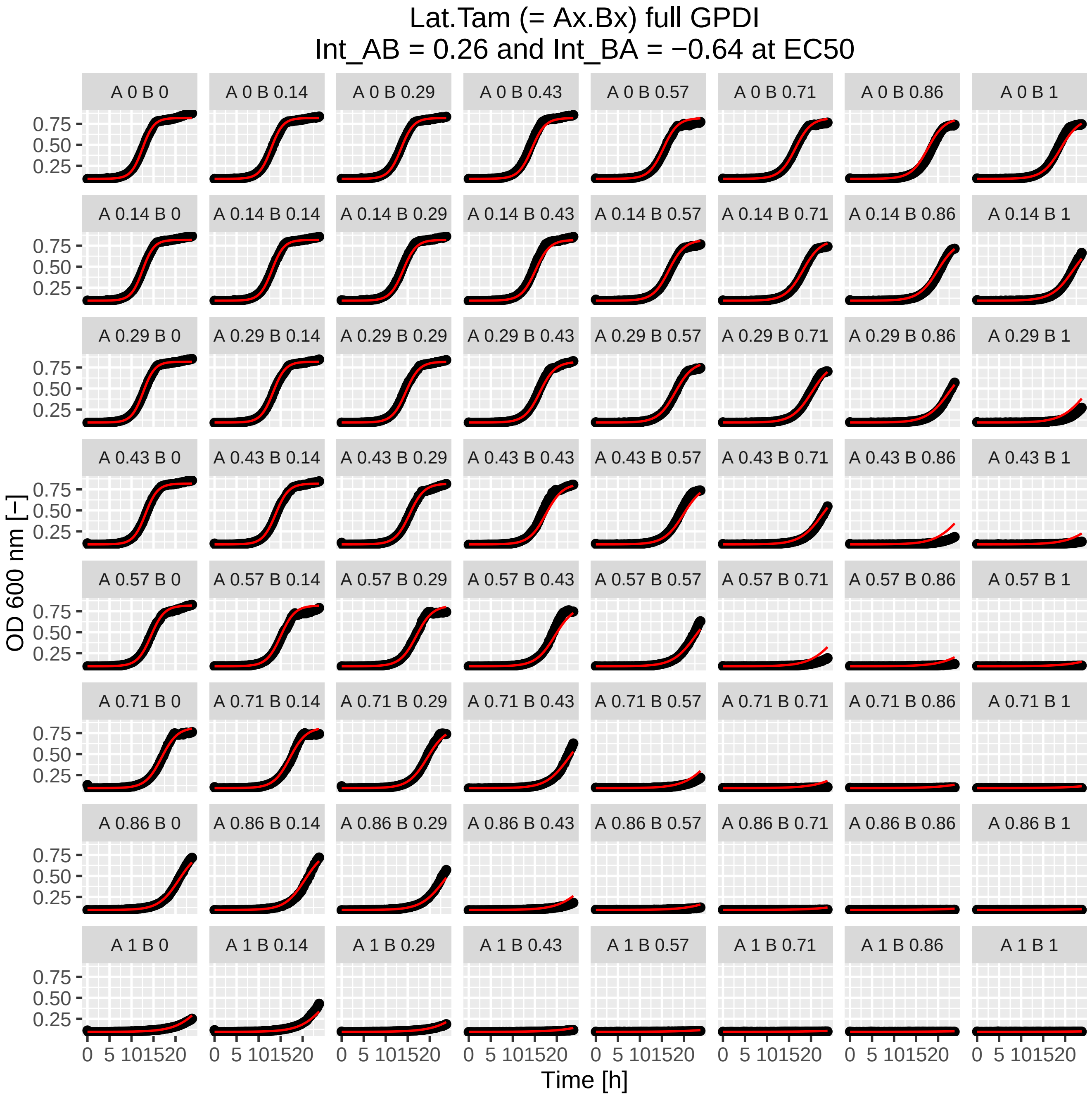

Lat.Tam (= Ax.Bx) full GPDI
Int_AB = 0.26 and Int_BA = −0.64 at EC50
OD 600 nm [−]
Time [h]
0.25
0.50
0.75
0 5 101520
A 0 B 0
A 0 B 0.14
A 0 B 0.29
A 0 B 0.43
A 0 B 0.57
A 0 B 0.71
A 0 B 0.86
A 0 B 1
A 0.14 B 0
A 0.14 B 0.14
A 0.14 B 0.29
A 0.14 B 0.43
A 0.14 B 0.57
A 0.14 B 0.71
A 0.14 B 0.86
A 0.14 B 1
A 0.29 B 0
A 0.29 B 0.14
A 0.29 B 0.29
A 0.29 B 0.43
A 0.29 B 0.57
A 0.29 B 0.71
A 0.29 B 0.86
A 0.29 B 1
A 0.43 B 0
A 0.43 B 0.14
A 0.43 B 0.29
A 0.43 B 0.43
A 0.43 B 0.57
A 0.43 B 0.71
A 0.43 B 0.86
A 0.43 B 1
A 0.57 B 0
A 0.57 B 0.14
A 0.57 B 0.29
A 0.57 B 0.43
A 0.57 B 0.57
A 0.57 B 0.71
A 0.57 B 0.86
A 0.57 B 1
A 0.71 B 0
A 0.71 B 0.14
A 0.71 B 0.29
A 0.71 B 0.43
A 0.71 B 0.57
A 0.71 B 0.71
A 0.71 B 0.86
A 0.71 B 1
A 0.86 B 0
A 0.86 B 0.14
A 0.86 B 0.29
A 0.86 B 0.43
A 0.86 B 0.57
A 0.86 B 0.71
A 0.86 B 0.86
A 0.86 B 1
A 1 B 0
A 1 B 0.14
A 1 B 0.29
A 1 B 0.43
A 1 B 0.57
A 1 B 0.71
A 1 B 0.86
A 1 B 1

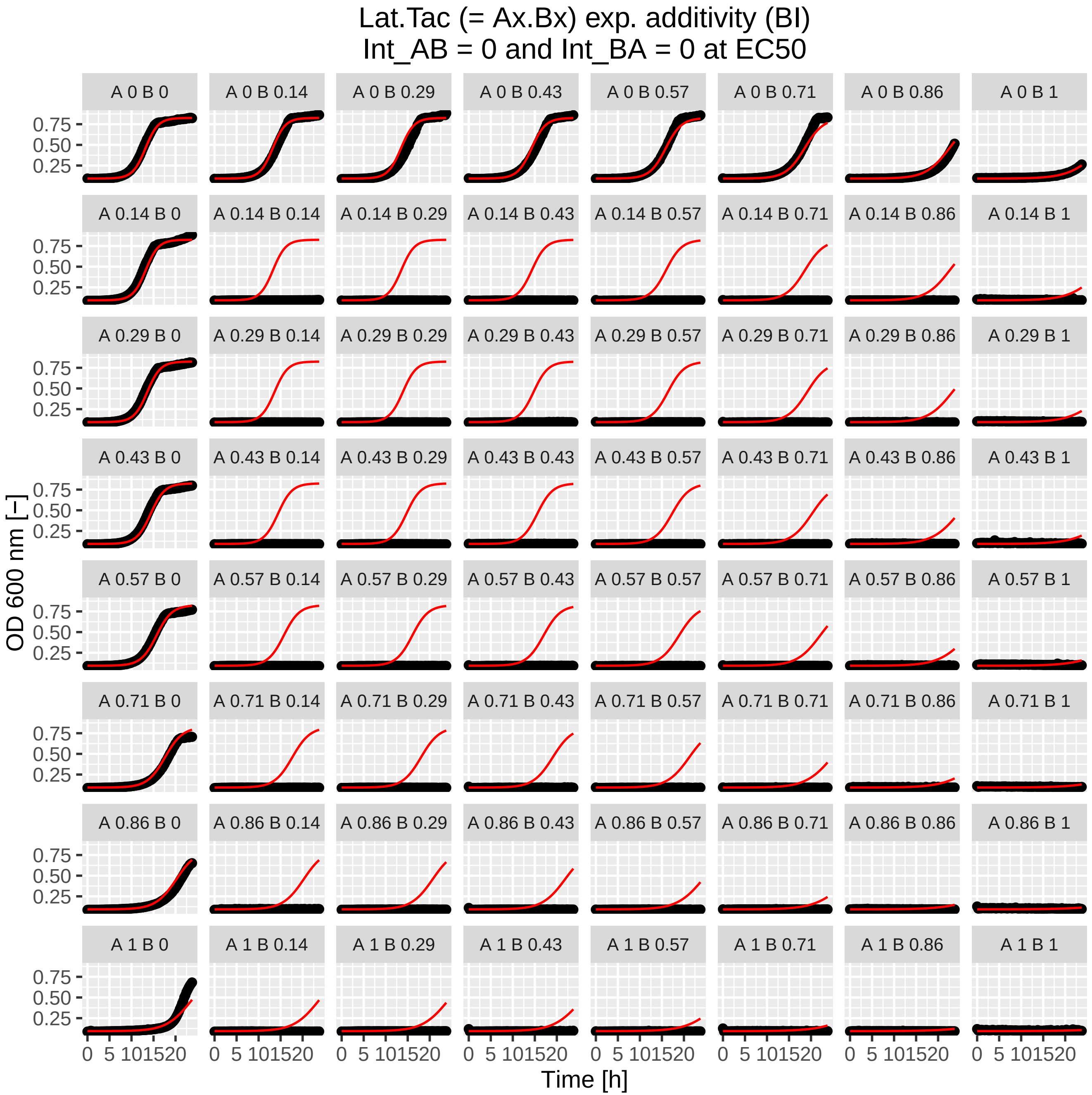

Lat.Tac (= Ax.Bx) exp. additivity (BI)
Int_AB = 0 and Int_BA = 0 at EC50
A 0 B 0
A 0 B 0.14
A 0 B 0.29
A 0 B 0.43
A 0 B 0.57
A 0 B 0.71
A 0 B 0.86
A 0 B 1
A 0.14 B 0
A 0.14 B 0.14
A 0.14 B 0.29
A 0.14 B 0.43
A 0.14 B 0.57
A 0.14 B 0.71
A 0.14 B 0.86
A 0.14 B 1
A 0.29 B 0
A 0.29 B 0.14
A 0.29 B 0.29
A 0.29 B 0.43
A 0.29 B 0.57
A 0.29 B 0.71
A 0.29 B 0.86
A 0.29 B 1
A 0.43 B 0
A 0.43 B 0.14
A 0.43 B 0.29
A 0.43 B 0.43
A 0.43 B 0.57
A 0.43 B 0.71
A 0.43 B 0.86
A 0.43 B 1
A 0.57 B 0
A 0.57 B 0.14
A 0.57 B 0.29
A 0.57 B 0.43
A 0.57 B 0.57
A 0.57 B 0.71
A 0.57 B 0.86
A 0.57 B 1
A 0.71 B 0
A 0.71 B 0.14
A 0.71 B 0.29
A 0.71 B 0.43
A 0.71 B 0.57
A 0.71 B 0.71
A 0.71 B 0.86
A 0.71 B 1
A 0.86 B 0
A 0.86 B 0.14
A 0.86 B 0.29
A 0.86 B 0.43
A 0.86 B 0.57
A 0.86 B 0.71
A 0.86 B 0.86
A 0.86 B 1
A 1 B 0
A 1 B 0.14
A 1 B 0.29
A 1 B 0.43
A 1 B 0.57
A 1 B 0.71
A 1 B 0.86
A 1 B 1
0.75
0.50
0.25
0 5 101520
OD 600 nm [–]
Time [h]

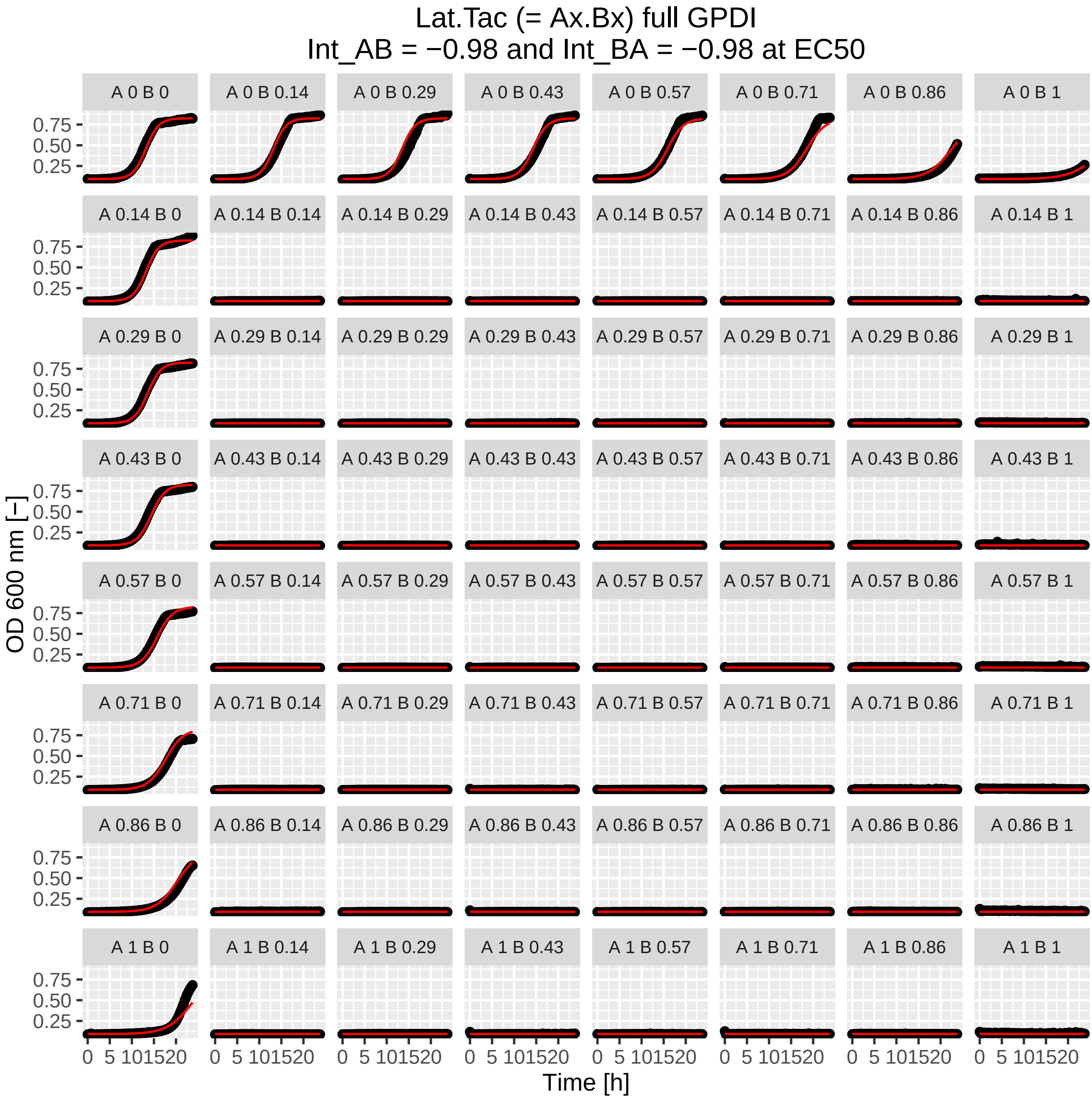
Lat.Tac (= Ax.Bx) full GPDI
Int_AB = −0.98 and Int_BA = −0.98 at EC50
A 0 B 0
A 0 B 0.14
A 0 B 0.29
A 0 B 0.43
A 0 B 0.57
A 0 B 0.71
A 0 B 0.86
A 0 B 1
A 0.14 B 0
A 0.14 B 0.14
A 0.14 B 0.29
A 0.14 B 0.43
A 0.14 B 0.57
A 0.14 B 0.71
A 0.14 B 0.86
A 0.14 B 1
A 0.29 B 0
A 0.29 B 0.14
A 0.29 B 0.29
A 0.29 B 0.43
A 0.29 B 0.57
A 0.29 B 0.71
A 0.29 B 0.86
A 0.29 B 1
A 0.43 B 0
A 0.43 B 0.14
A 0.43 B 0.29
A 0.43 B 0.43
A 0.43 B 0.57
A 0.43 B 0.71
A 0.43 B 0.86
A 0.43 B 1
A 0.57 B 0
A 0.57 B 0.14
A 0.57 B 0.29
A 0.57 B 0.43
A 0.57 B 0.57
A 0.57 B 0.71
A 0.57 B 0.86
A 0.57 B 1
A 0.71 B 0
A 0.71 B 0.14
A 0.71 B 0.29
A 0.71 B 0.43
A 0.71 B 0.57
A 0.71 B 0.71
A 0.71 B 0.86
A 0.71 B 1
A 0.86 B 0
A 0.86 B 0.14
A 0.86 B 0.29
A 0.86 B 0.43
A 0.86 B 0.57
A 0.86 B 0.71
A 0.86 B 0.86
A 0.86 B 1
A 1 B 0
A 1 B 0.14
A 1 B 0.29
A 1 B 0.43
A 1 B 0.57
A 1 B 0.71
A 1 B 0.86
A 1 B 1
0.75
0.50
0.25
0 5 101520
OD 600 nm [−]
Time [h]

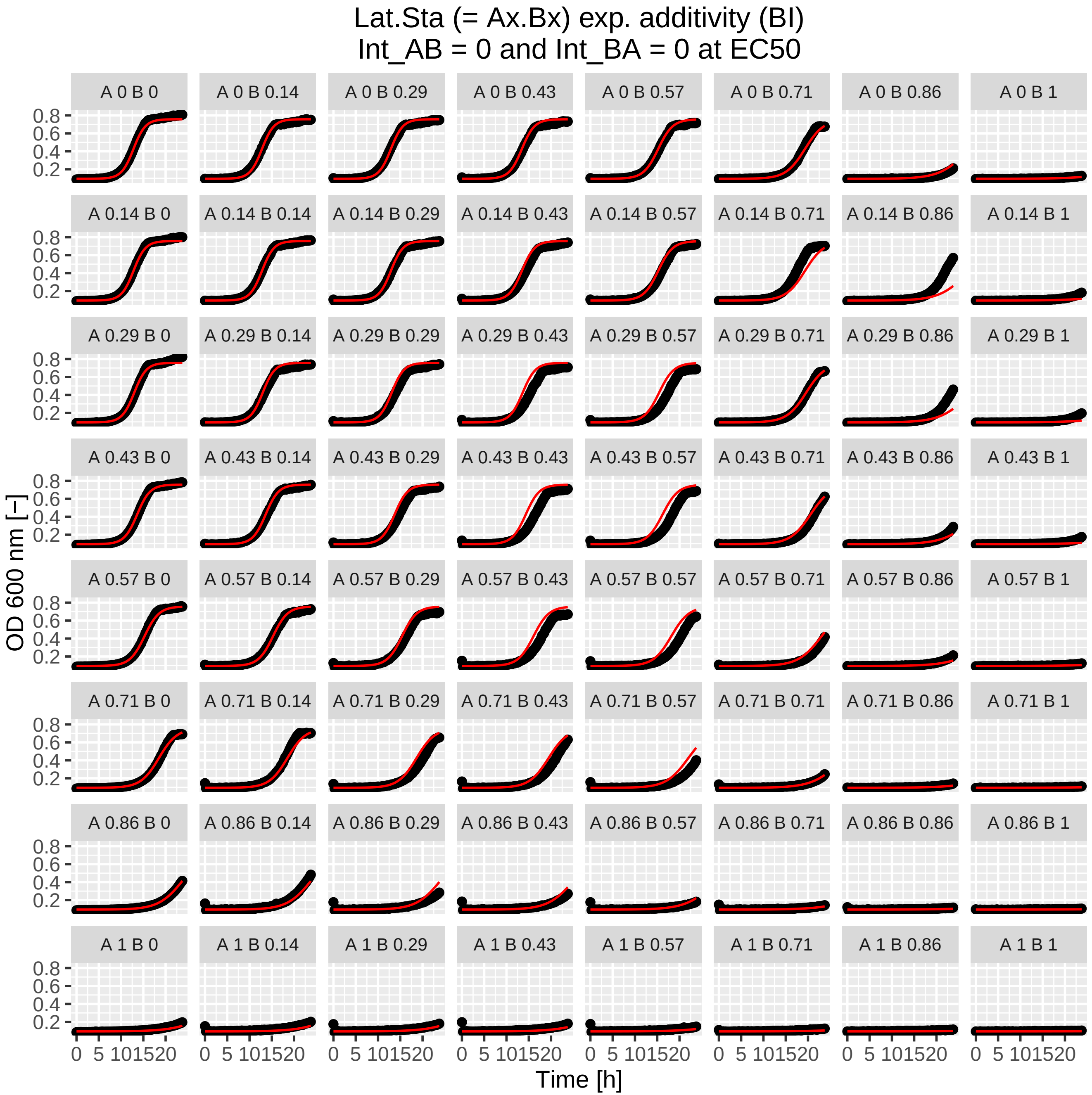

Lat.Sta (= Ax.Bx) exp. additivity (BI)
Int_AB = 0 and Int_BA = 0 at EC50
A 0 B 0
A 0 B 0.14
A 0 B 0.29
A 0 B 0.43
A 0 B 0.57
A 0 B 0.71
A 0 B 0.86
A 0 B 1
A 0.14 B 0
A 0.14 B 0.14
A 0.14 B 0.29
A 0.14 B 0.43
A 0.14 B 0.57
A 0.14 B 0.71
A 0.14 B 0.86
A 0.14 B 1
A 0.29 B 0
A 0.29 B 0.14
A 0.29 B 0.29
A 0.29 B 0.43
A 0.29 B 0.57
A 0.29 B 0.71
A 0.29 B 0.86
A 0.29 B 1
A 0.43 B 0
A 0.43 B 0.14
A 0.43 B 0.29
A 0.43 B 0.43
A 0.43 B 0.57
A 0.43 B 0.71
A 0.43 B 0.86
A 0.43 B 1
A 0.57 B 0
A 0.57 B 0.14
A 0.57 B 0.29
A 0.57 B 0.43
A 0.57 B 0.57
A 0.57 B 0.71
A 0.57 B 0.86
A 0.57 B 1
A 0.71 B 0
A 0.71 B 0.14
A 0.71 B 0.29
A 0.71 B 0.43
A 0.71 B 0.57
A 0.71 B 0.71
A 0.71 B 0.86
A 0.71 B 1
A 0.86 B 0
A 0.86 B 0.14
A 0.86 B 0.29
A 0.86 B 0.43
A 0.86 B 0.57
A 0.86 B 0.71
A 0.86 B 0.86
A 0.86 B 1
A 1 B 0
A 1 B 0.14
A 1 B 0.29
A 1 B 0.43
A 1 B 0.57
A 1 B 0.71
A 1 B 0.86
A 1 B 1
OD 600 nm [–]
0.8
0.6
0.4
0.2
0 5 101520
Time [h]

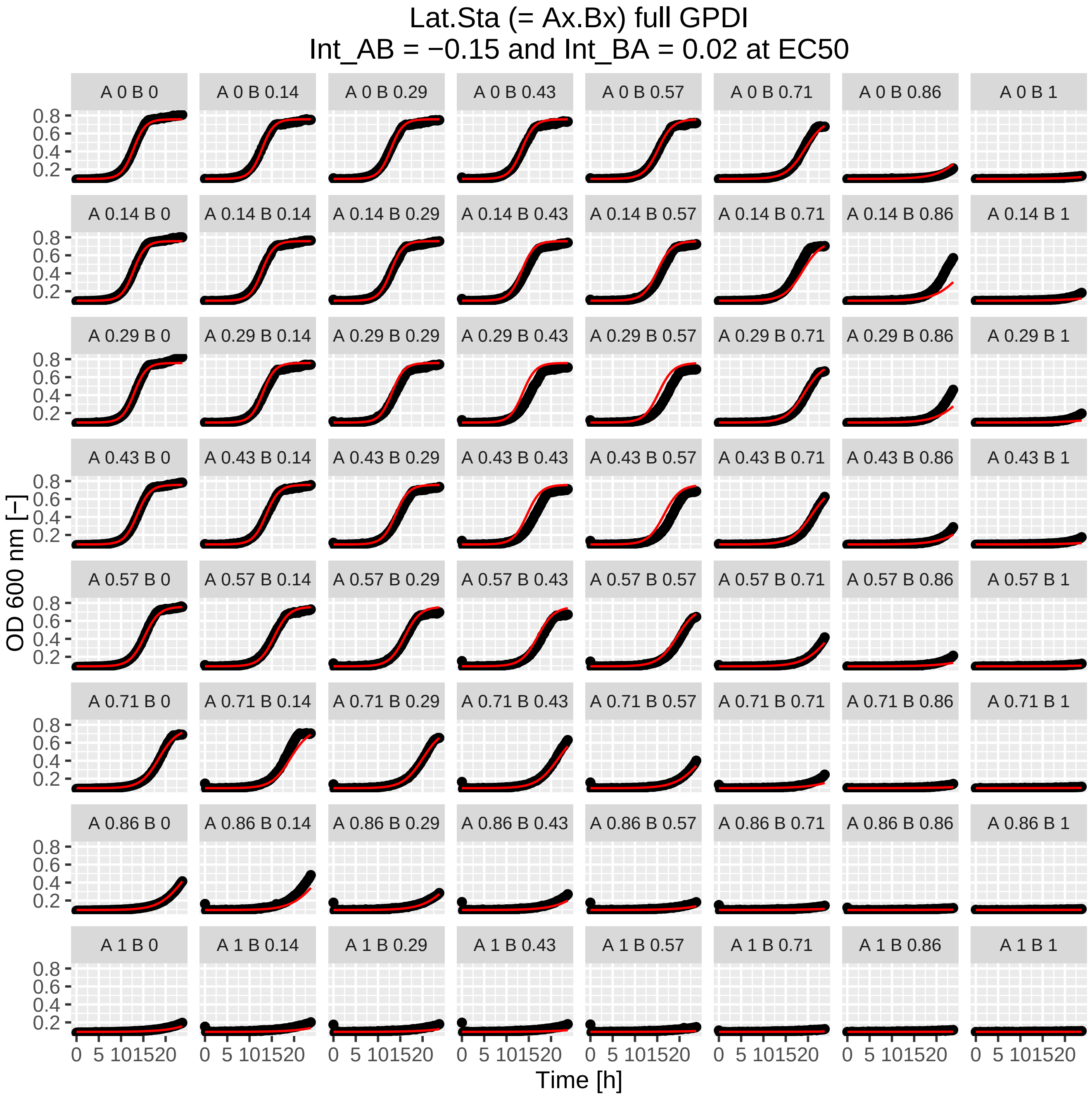

Lat.Sta (= Ax.Bx) full GPDI
Int_AB = −0.15 and Int_BA = 0.02 at EC50
A 0 B 0
A 0 B 0.14
A 0 B 0.29
A 0 B 0.43
A 0 B 0.57
A 0 B 0.71
A 0 B 0.86
A 0 B 1
A 0.14 B 0
A 0.14 B 0.14
A 0.14 B 0.29
A 0.14 B 0.43
A 0.14 B 0.57
A 0.14 B 0.71
A 0.14 B 0.86
A 0.14 B 1
A 0.29 B 0
A 0.29 B 0.14
A 0.29 B 0.29
A 0.29 B 0.43
A 0.29 B 0.57
A 0.29 B 0.71
A 0.29 B 0.86
A 0.29 B 1
A 0.43 B 0
A 0.43 B 0.14
A 0.43 B 0.29
A 0.43 B 0.43
A 0.43 B 0.57
A 0.43 B 0.71
A 0.43 B 0.86
A 0.43 B 1
A 0.57 B 0
A 0.57 B 0.14
A 0.57 B 0.29
A 0.57 B 0.43
A 0.57 B 0.57
A 0.57 B 0.71
A 0.57 B 0.86
A 0.57 B 1
A 0.71 B 0
A 0.71 B 0.14
A 0.71 B 0.29
A 0.71 B 0.43
A 0.71 B 0.57
A 0.71 B 0.71
A 0.71 B 0.86
A 0.71 B 1
A 0.86 B 0
A 0.86 B 0.14
A 0.86 B 0.29
A 0.86 B 0.43
A 0.86 B 0.57
A 0.86 B 0.71
A 0.86 B 0.86
A 0.86 B 1
A 1 B 0
A 1 B 0.14
A 1 B 0.29
A 1 B 0.43
A 1 B 0.57
A 1 B 0.71
A 1 B 0.86
A 1 B 1
OD 600 nm [−]
0.8
0.6
0.4
0.2
0 5 101520
Time [h]

Lat.Rap (= Ax.Bx) exp. additivity (BI)
Int_AB = 0 and Int_BA = 0 at EC50

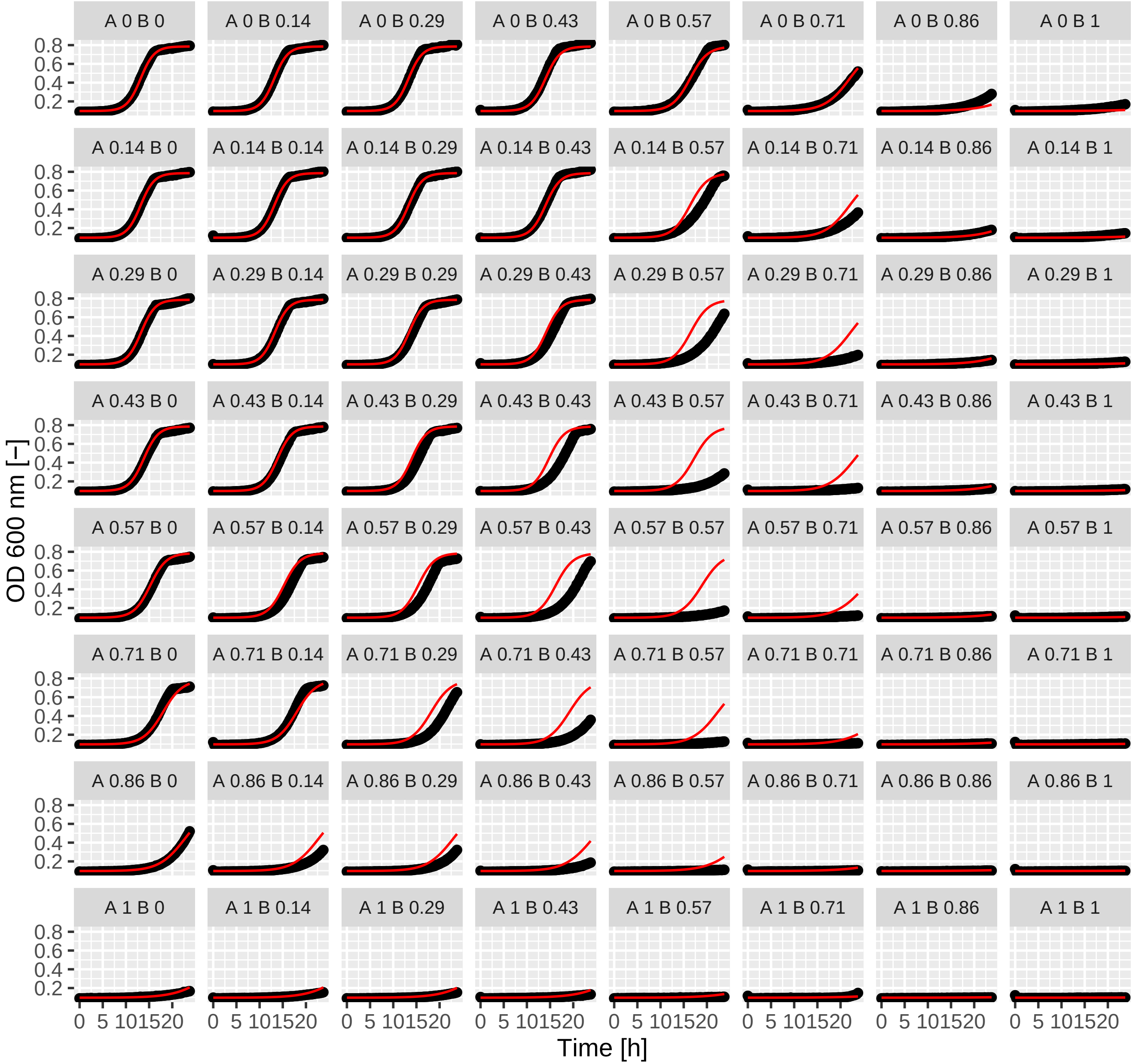

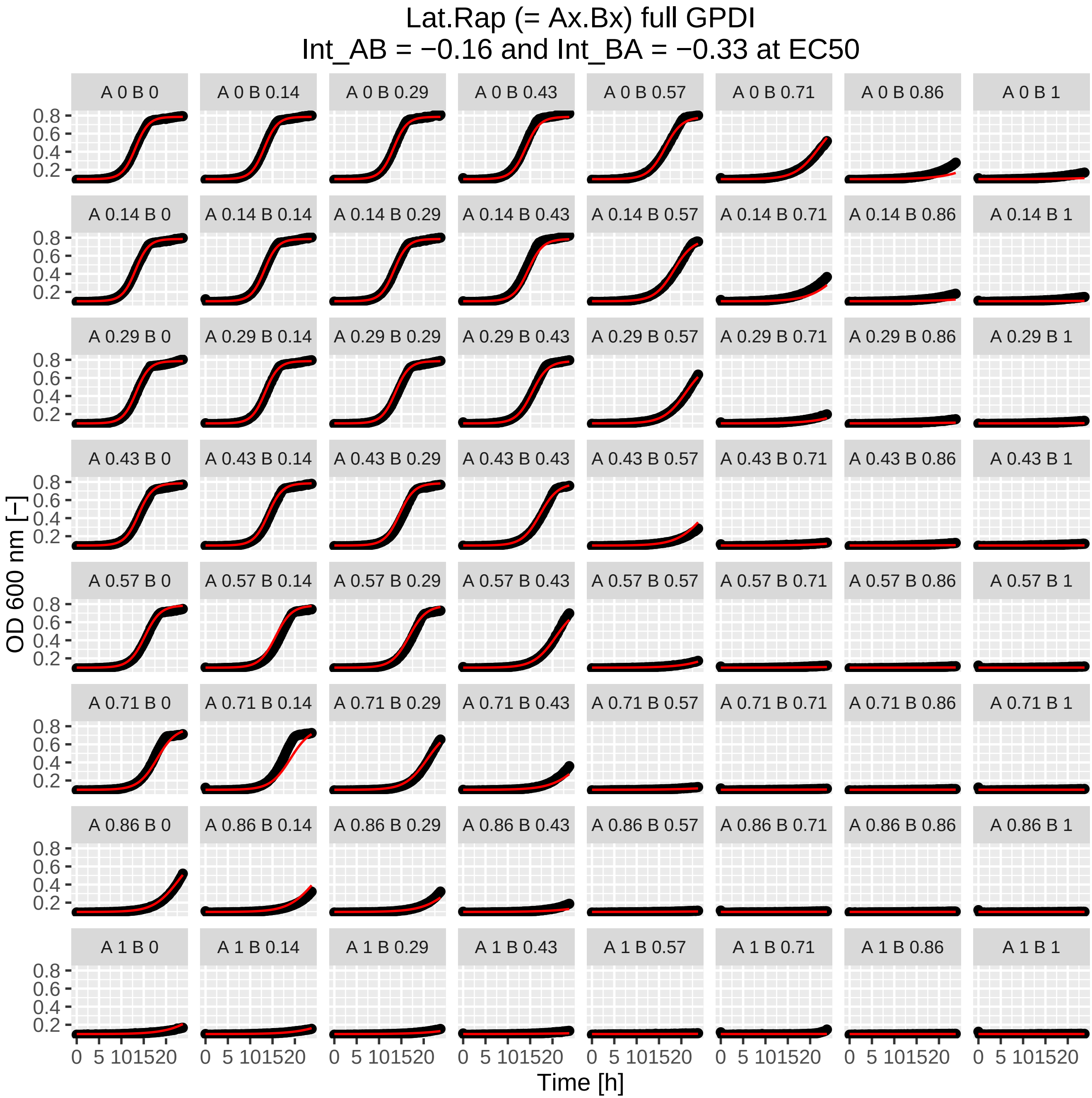
Lat.Rap (= Ax.Bx) full GPDI
Int_AB = −0.16 and Int_BA = −0.33 at EC50
A 0 B 0
A 0 B 0.14
A 0 B 0.29
A 0 B 0.43
A 0 B 0.57
A 0 B 0.71
A 0 B 0.86
A 0 B 1
A 0.14 B 0
A 0.14 B 0.14
A 0.14 B 0.29
A 0.14 B 0.43
A 0.14 B 0.57
A 0.14 B 0.71
A 0.14 B 0.86
A 0.14 B 1
A 0.29 B 0
A 0.29 B 0.14
A 0.29 B 0.29
A 0.29 B 0.43
A 0.29 B 0.57
A 0.29 B 0.71
A 0.29 B 0.86
A 0.29 B 1
A 0.43 B 0
A 0.43 B 0.14
A 0.43 B 0.29
A 0.43 B 0.43
A 0.43 B 0.57
A 0.43 B 0.71
A 0.43 B 0.86
A 0.43 B 1
A 0.57 B 0
A 0.57 B 0.14
A 0.57 B 0.29
A 0.57 B 0.43
A 0.57 B 0.57
A 0.57 B 0.71
A 0.57 B 0.86
A 0.57 B 1
A 0.71 B 0
A 0.71 B 0.14
A 0.71 B 0.29
A 0.71 B 0.43
A 0.71 B 0.57
A 0.71 B 0.71
A 0.71 B 0.86
A 0.71 B 1
A 0.86 B 0
A 0.86 B 0.14
A 0.86 B 0.29
A 0.86 B 0.43
A 0.86 B 0.57
A 0.86 B 0.71
A 0.86 B 0.86
A 0.86 B 1
A 1 B 0
A 1 B 0.14
A 1 B 0.29
A 1 B 0.43
A 1 B 0.57
A 1 B 0.71
A 1 B 0.86
A 1 B 1
0.8
0.6
0.4
0.2
OD 600 nm [−]
0 5 101520
Time [h]

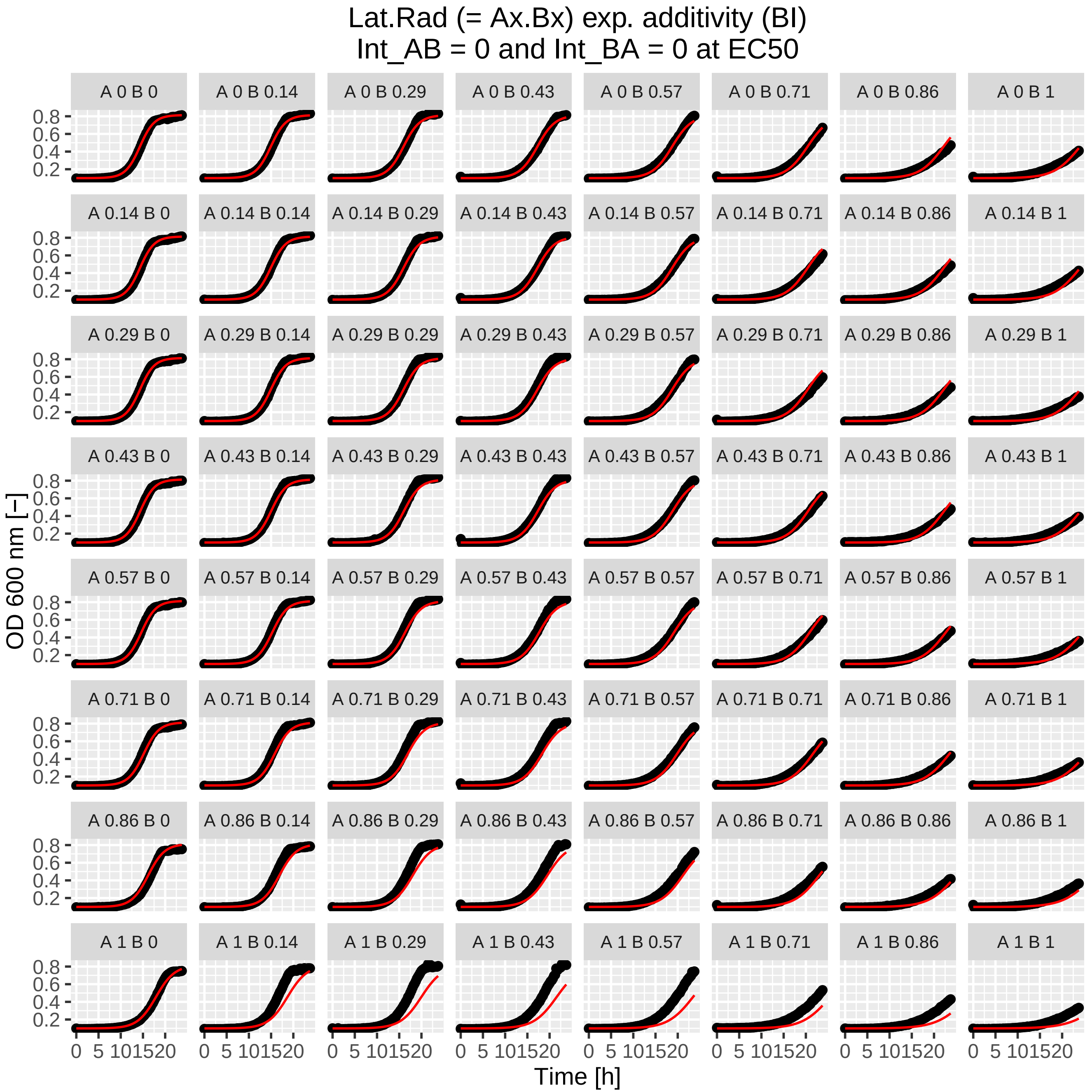
Lat.Rad (= Ax.Bx) exp. additivity (BI)
Int_AB = 0 and Int_BA = 0 at EC50
A 0 B 0
A 0 B 0.14
A 0 B 0.29
A 0 B 0.43
A 0 B 0.57
A 0 B 0.71
A 0 B 0.86
A 0 B 1
A 0.14 B 0
A 0.14 B 0.14
A 0.14 B 0.29
A 0.14 B 0.43
A 0.14 B 0.57
A 0.14 B 0.71
A 0.14 B 0.86
A 0.14 B 1
A 0.29 B 0
A 0.29 B 0.14
A 0.29 B 0.29
A 0.29 B 0.43
A 0.29 B 0.57
A 0.29 B 0.71
A 0.29 B 0.86
A 0.29 B 1
A 0.43 B 0
A 0.43 B 0.14
A 0.43 B 0.29
A 0.43 B 0.43
A 0.43 B 0.57
A 0.43 B 0.71
A 0.43 B 0.86
A 0.43 B 1
A 0.57 B 0
A 0.57 B 0.14
A 0.57 B 0.29
A 0.57 B 0.43
A 0.57 B 0.57
A 0.57 B 0.71
A 0.57 B 0.86
A 0.57 B 1
A 0.71 B 0
A 0.71 B 0.14
A 0.71 B 0.29
A 0.71 B 0.43
A 0.71 B 0.57
A 0.71 B 0.71
A 0.71 B 0.86
A 0.71 B 1
A 0.86 B 0
A 0.86 B 0.14
A 0.86 B 0.29
A 0.86 B 0.43
A 0.86 B 0.57
A 0.86 B 0.71
A 0.86 B 0.86
A 0.86 B 1
A 1 B 0
A 1 B 0.14
A 1 B 0.29
A 1 B 0.43
A 1 B 0.57
A 1 B 0.71
A 1 B 0.86
A 1 B 1
OD 600 nm [–]
0.2
0.4
0.6
0.8
0
5
10
15
20
Time [h]

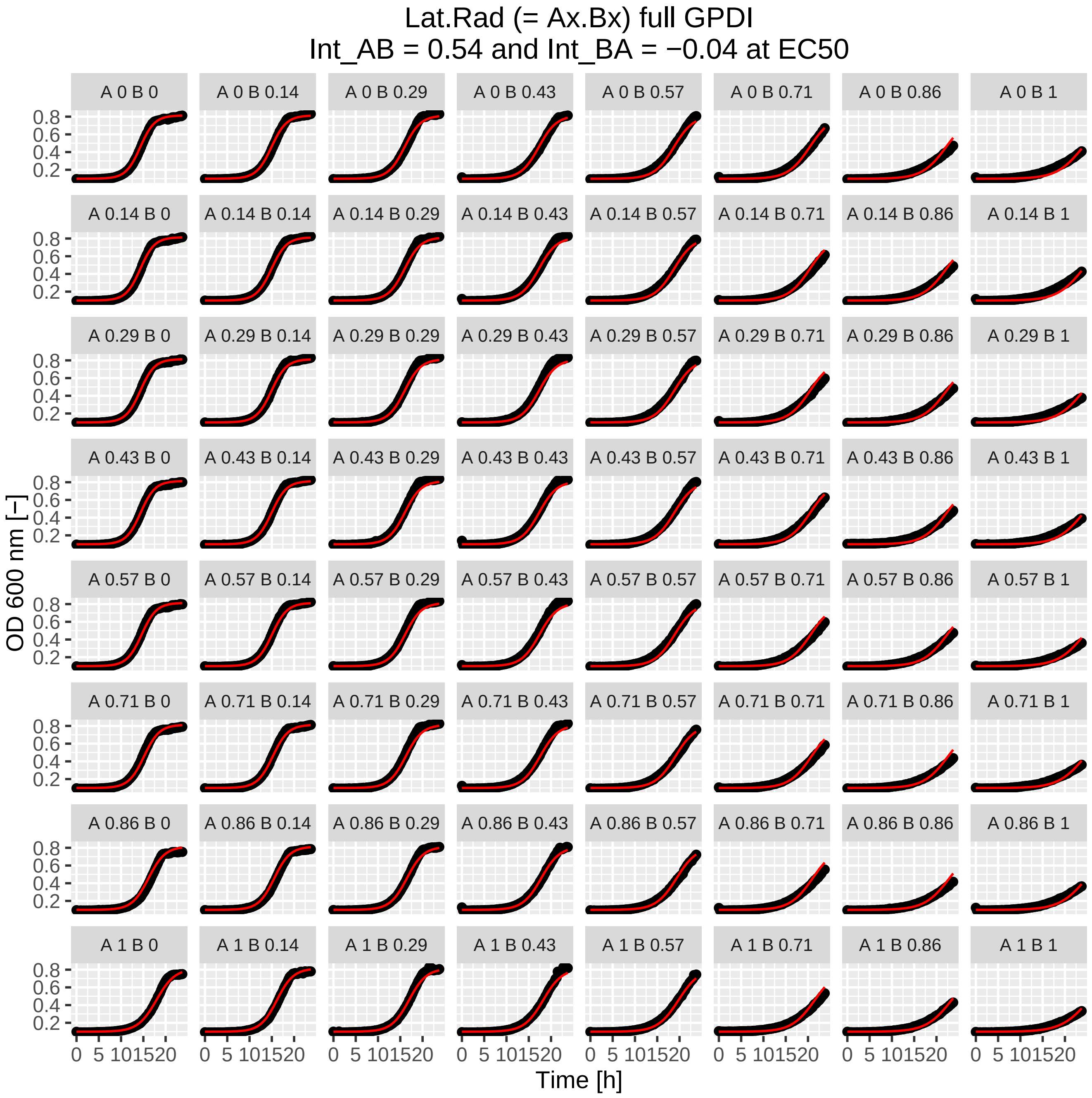

Lat.Rad (= Ax.Bx) full GPDI
Int_AB = 0.54 and Int_BA = −0.04 at EC50
A 0 B 0
A 0 B 0.14
A 0 B 0.29
A 0 B 0.43
A 0 B 0.57
A 0 B 0.71
A 0 B 0.86
A 0 B 1
A 0.14 B 0
A 0.14 B 0.14
A 0.14 B 0.29
A 0.14 B 0.43
A 0.14 B 0.57
A 0.14 B 0.71
A 0.14 B 0.86
A 0.14 B 1
A 0.29 B 0
A 0.29 B 0.14
A 0.29 B 0.29
A 0.29 B 0.43
A 0.29 B 0.57
A 0.29 B 0.71
A 0.29 B 0.86
A 0.29 B 1
A 0.43 B 0
A 0.43 B 0.14
A 0.43 B 0.29
A 0.43 B 0.43
A 0.43 B 0.57
A 0.43 B 0.71
A 0.43 B 0.86
A 0.43 B 1
A 0.57 B 0
A 0.57 B 0.14
A 0.57 B 0.29
A 0.57 B 0.43
A 0.57 B 0.57
A 0.57 B 0.71
A 0.57 B 0.86
A 0.57 B 1
A 0.71 B 0
A 0.71 B 0.14
A 0.71 B 0.29
A 0.71 B 0.43
A 0.71 B 0.57
A 0.71 B 0.71
A 0.71 B 0.86
A 0.71 B 1
A 0.86 B 0
A 0.86 B 0.14
A 0.86 B 0.29
A 0.86 B 0.43
A 0.86 B 0.57
A 0.86 B 0.71
A 0.86 B 0.86
A 0.86 B 1
A 1 B 0
A 1 B 0.14
A 1 B 0.29
A 1 B 0.43
A 1 B 0.57
A 1 B 0.71
A 1 B 0.86
A 1 B 1
OD 600 nm [−]
0.2
0.4
0.6
0.8
0
5
10
15
20
Time [h]

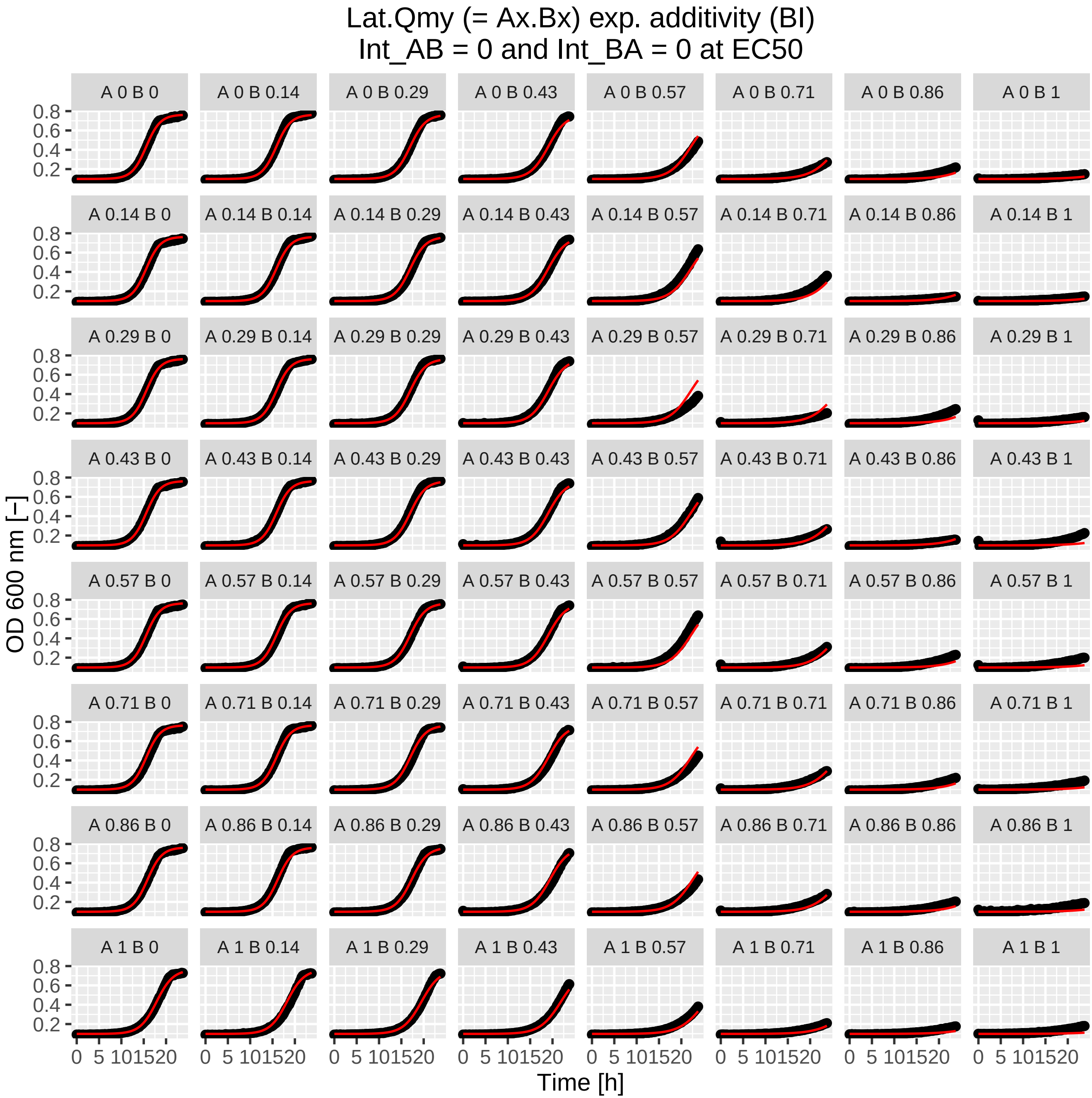

Lat.Qmy (= Ax.Bx) exp. additivity (BI)
Int_AB = 0 and Int_BA = 0 at EC50
A 0 B 0
A 0 B 0.14
A 0 B 0.29
A 0 B 0.43
A 0 B 0.57
A 0 B 0.71
A 0 B 0.86
A 0 B 1
A 0.14 B 0
A 0.14 B 0.14
A 0.14 B 0.29
A 0.14 B 0.43
A 0.14 B 0.57
A 0.14 B 0.71
A 0.14 B 0.86
A 0.14 B 1
A 0.29 B 0
A 0.29 B 0.14
A 0.29 B 0.29
A 0.29 B 0.43
A 0.29 B 0.57
A 0.29 B 0.71
A 0.29 B 0.86
A 0.29 B 1
A 0.43 B 0
A 0.43 B 0.14
A 0.43 B 0.29
A 0.43 B 0.43
A 0.43 B 0.57
A 0.43 B 0.71
A 0.43 B 0.86
A 0.43 B 1
A 0.57 B 0
A 0.57 B 0.14
A 0.57 B 0.29
A 0.57 B 0.43
A 0.57 B 0.57
A 0.57 B 0.71
A 0.57 B 0.86
A 0.57 B 1
A 0.71 B 0
A 0.71 B 0.14
A 0.71 B 0.29
A 0.71 B 0.43
A 0.71 B 0.57
A 0.71 B 0.71
A 0.71 B 0.86
A 0.71 B 1
A 0.86 B 0
A 0.86 B 0.14
A 0.86 B 0.29
A 0.86 B 0.43
A 0.86 B 0.57
A 0.86 B 0.71
A 0.86 B 0.86
A 0.86 B 1
A 1 B 0
A 1 B 0.14
A 1 B 0.29
A 1 B 0.43
A 1 B 0.57
A 1 B 0.71
A 1 B 0.86
A 1 B 1
OD 600 nm [–]
0.2
0.4
0.6
0.8
0 5 101520
Time [h]

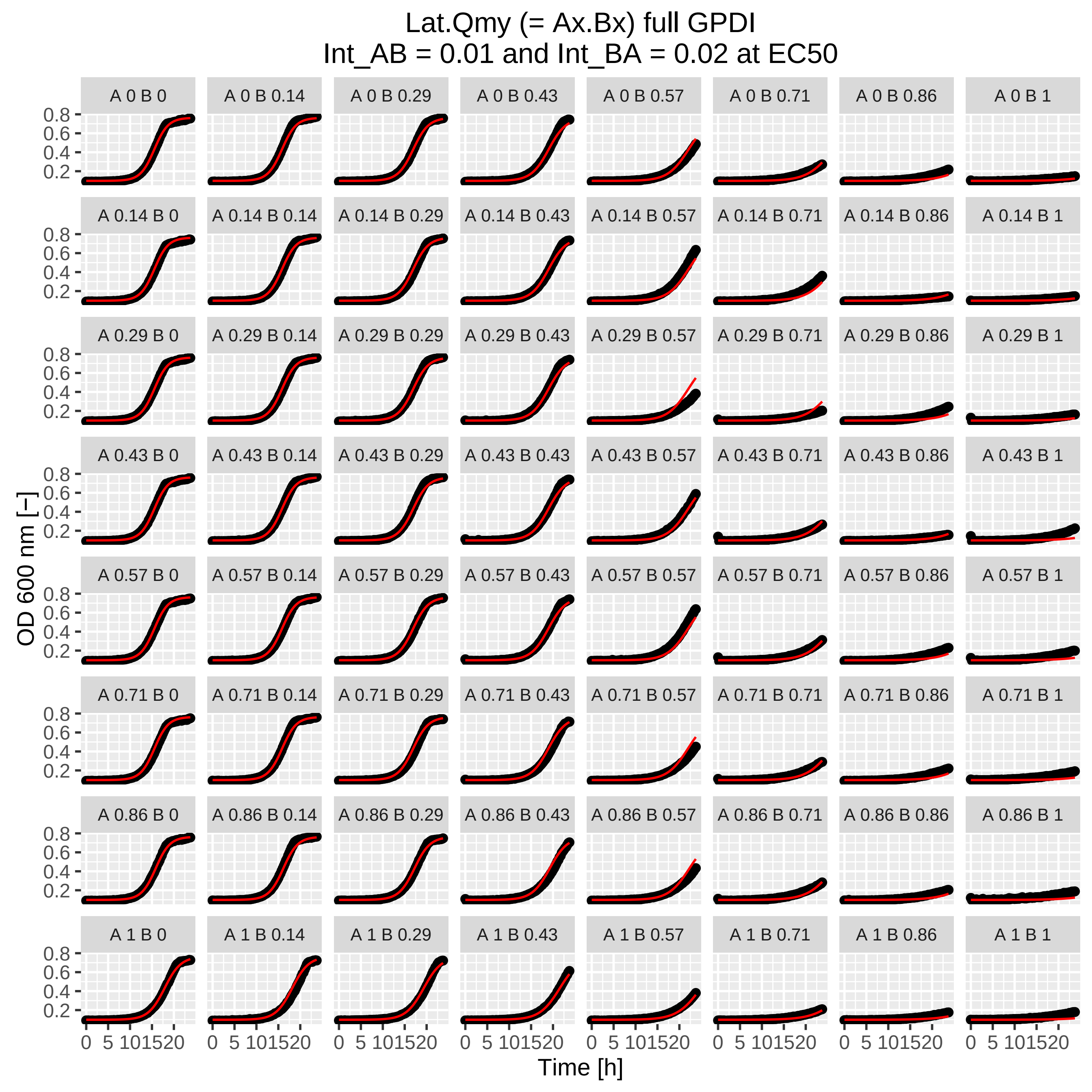

Lat.Qmy (= Ax.Bx) full GPDI
Int_AB = 0.01 and Int_BA = 0.02 at EC50
OD 600 nm [−]
Time [h]

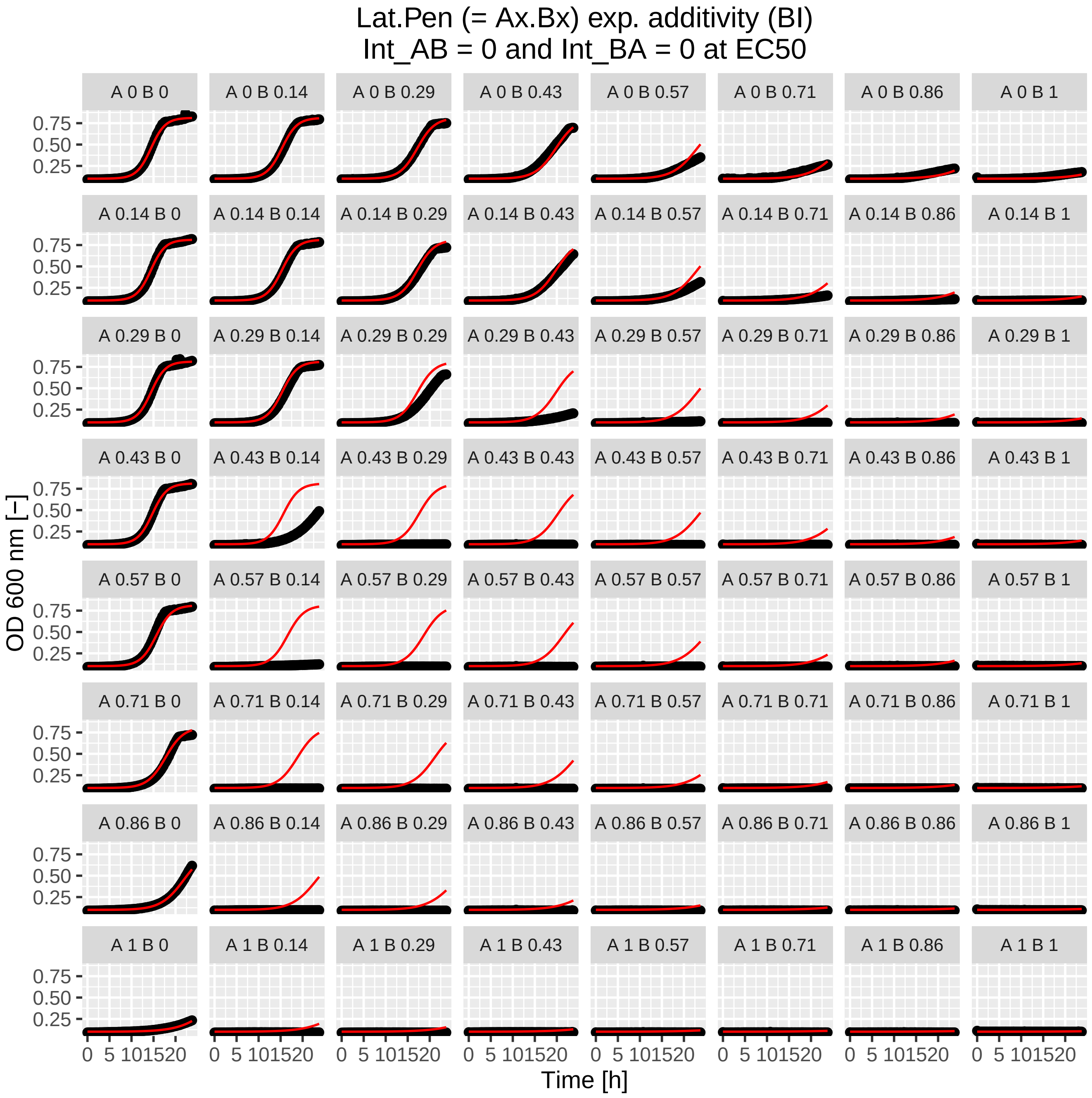
Lat.Pen (= Ax.Bx) exp. additivity (BI)
Int_AB = 0 and Int_BA = 0 at EC50
A 0 B 0
A 0 B 0.14
A 0 B 0.29
A 0 B 0.43
A 0 B 0.57
A 0 B 0.71
A 0 B 0.86
A 0 B 1
A 0.14 B 0
A 0.14 B 0.14
A 0.14 B 0.29
A 0.14 B 0.43
A 0.14 B 0.57
A 0.14 B 0.71
A 0.14 B 0.86
A 0.14 B 1
A 0.29 B 0
A 0.29 B 0.14
A 0.29 B 0.29
A 0.29 B 0.43
A 0.29 B 0.57
A 0.29 B 0.71
A 0.29 B 0.86
A 0.29 B 1
A 0.43 B 0
A 0.43 B 0.14
A 0.43 B 0.29
A 0.43 B 0.43
A 0.43 B 0.57
A 0.43 B 0.71
A 0.43 B 0.86
A 0.43 B 1
A 0.57 B 0
A 0.57 B 0.14
A 0.57 B 0.29
A 0.57 B 0.43
A 0.57 B 0.57
A 0.57 B 0.71
A 0.57 B 0.86
A 0.57 B 1
A 0.71 B 0
A 0.71 B 0.14
A 0.71 B 0.29
A 0.71 B 0.43
A 0.71 B 0.57
A 0.71 B 0.71
A 0.71 B 0.86
A 0.71 B 1
A 0.86 B 0
A 0.86 B 0.14
A 0.86 B 0.29
A 0.86 B 0.43
A 0.86 B 0.57
A 0.86 B 0.71
A 0.86 B 0.86
A 0.86 B 1
A 1 B 0
A 1 B 0.14
A 1 B 0.29
A 1 B 0.43
A 1 B 0.57
A 1 B 0.71
A 1 B 0.86
A 1 B 1
0.75
0.50
0.25
OD 600 nm [–]
0 5 101520
Time [h]

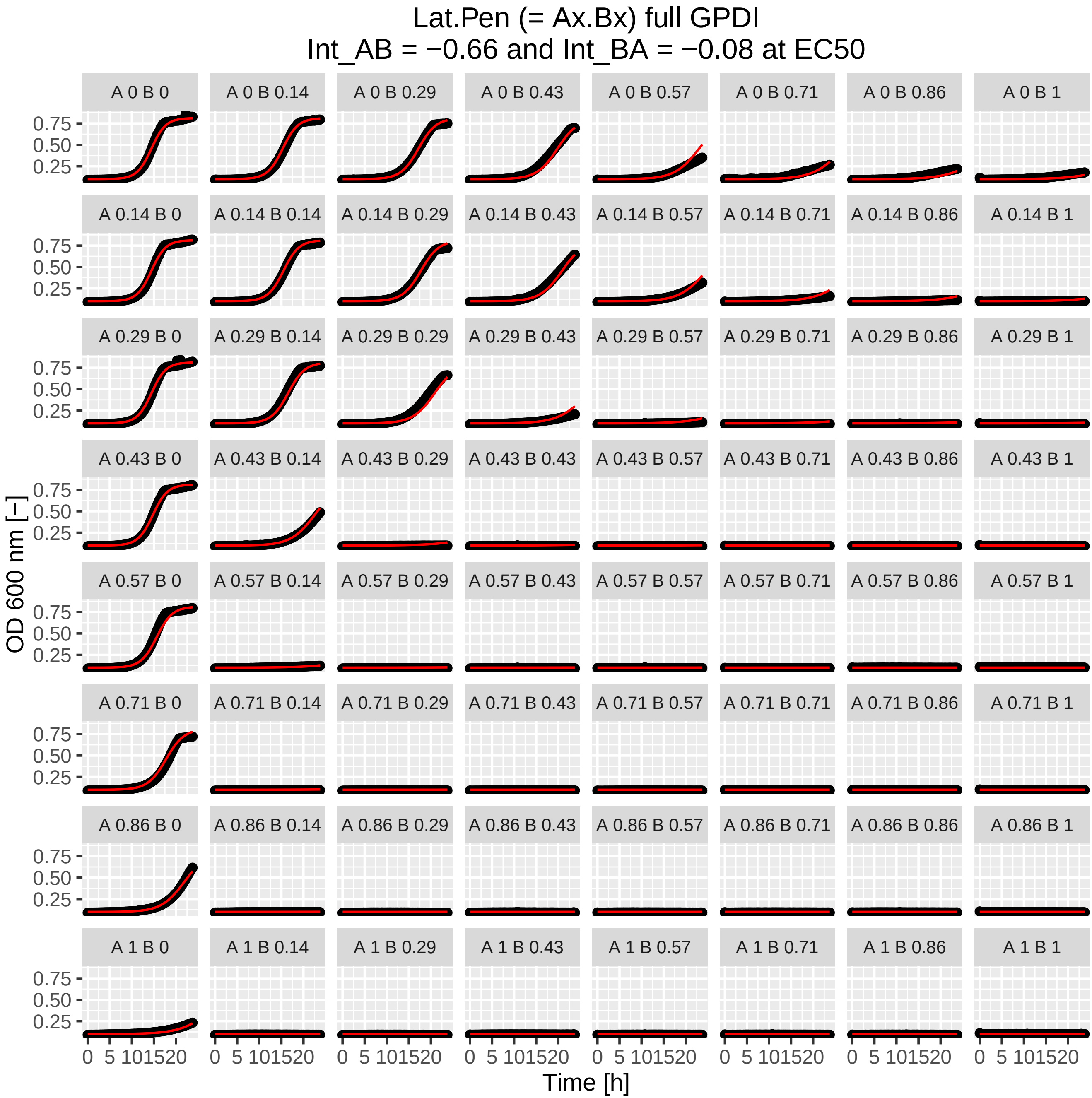

Lat.Pen (= Ax.Bx) full GPDI
Int_AB = −0.66 and Int_BA = −0.08 at EC50
A 0 B 0
A 0 B 0.14
A 0 B 0.29
A 0 B 0.43
A 0 B 0.57
A 0 B 0.71
A 0 B 0.86
A 0 B 1
A 0.14 B 0
A 0.14 B 0.14
A 0.14 B 0.29
A 0.14 B 0.43
A 0.14 B 0.57
A 0.14 B 0.71
A 0.14 B 0.86
A 0.14 B 1
A 0.29 B 0
A 0.29 B 0.14
A 0.29 B 0.29
A 0.29 B 0.43
A 0.29 B 0.57
A 0.29 B 0.71
A 0.29 B 0.86
A 0.29 B 1
A 0.43 B 0
A 0.43 B 0.14
A 0.43 B 0.29
A 0.43 B 0.43
A 0.43 B 0.57
A 0.43 B 0.71
A 0.43 B 0.86
A 0.43 B 1
A 0.57 B 0
A 0.57 B 0.14
A 0.57 B 0.29
A 0.57 B 0.43
A 0.57 B 0.57
A 0.57 B 0.71
A 0.57 B 0.86
A 0.57 B 1
A 0.71 B 0
A 0.71 B 0.14
A 0.71 B 0.29
A 0.71 B 0.43
A 0.71 B 0.57
A 0.71 B 0.71
A 0.71 B 0.86
A 0.71 B 1
A 0.86 B 0
A 0.86 B 0.14
A 0.86 B 0.29
A 0.86 B 0.43
A 0.86 B 0.57
A 0.86 B 0.71
A 0.86 B 0.86
A 0.86 B 1
A 1 B 0
A 1 B 0.14
A 1 B 0.29
A 1 B 0.43
A 1 B 0.57
A 1 B 0.71
A 1 B 0.86
A 1 B 1
0.75
0.50
0.25
OD 600 nm [−]
0 5 101520
Time [h]

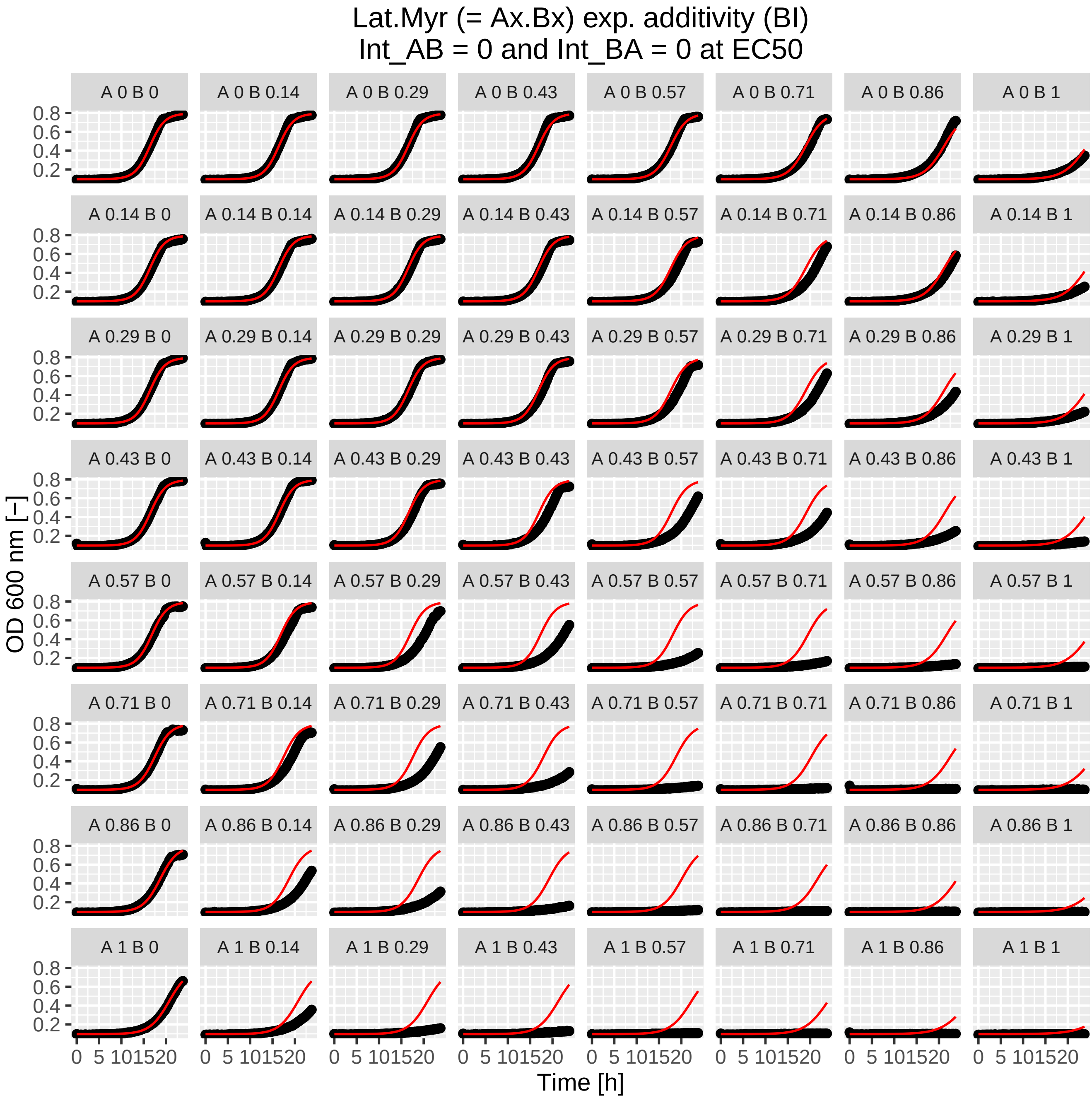

Lat.Myr (= Ax.Bx) exp. additivity (BI)
Int_AB = 0 and Int_BA = 0 at EC50
A 0 B 0
A 0 B 0.14
A 0 B 0.29
A 0 B 0.43
A 0 B 0.57
A 0 B 0.71
A 0 B 0.86
A 0 B 1
A 0.14 B 0
A 0.14 B 0.14
A 0.14 B 0.29
A 0.14 B 0.43
A 0.14 B 0.57
A 0.14 B 0.71
A 0.14 B 0.86
A 0.14 B 1
A 0.29 B 0
A 0.29 B 0.14
A 0.29 B 0.29
A 0.29 B 0.43
A 0.29 B 0.57
A 0.29 B 0.71
A 0.29 B 0.86
A 0.29 B 1
A 0.43 B 0
A 0.43 B 0.14
A 0.43 B 0.29
A 0.43 B 0.43
A 0.43 B 0.57
A 0.43 B 0.71
A 0.43 B 0.86
A 0.43 B 1
A 0.57 B 0
A 0.57 B 0.14
A 0.57 B 0.29
A 0.57 B 0.43
A 0.57 B 0.57
A 0.57 B 0.71
A 0.57 B 0.86
A 0.57 B 1
A 0.71 B 0
A 0.71 B 0.14
A 0.71 B 0.29
A 0.71 B 0.43
A 0.71 B 0.57
A 0.71 B 0.71
A 0.71 B 0.86
A 0.71 B 1
A 0.86 B 0
A 0.86 B 0.14
A 0.86 B 0.29
A 0.86 B 0.43
A 0.86 B 0.57
A 0.86 B 0.71
A 0.86 B 0.86
A 0.86 B 1
A 1 B 0
A 1 B 0.14
A 1 B 0.29
A 1 B 0.43
A 1 B 0.57
A 1 B 0.71
A 1 B 0.86
A 1 B 1
OD 600 nm [−]
0.2
0.4
0.6
0.8
0
5
10
15
20
Time [h]

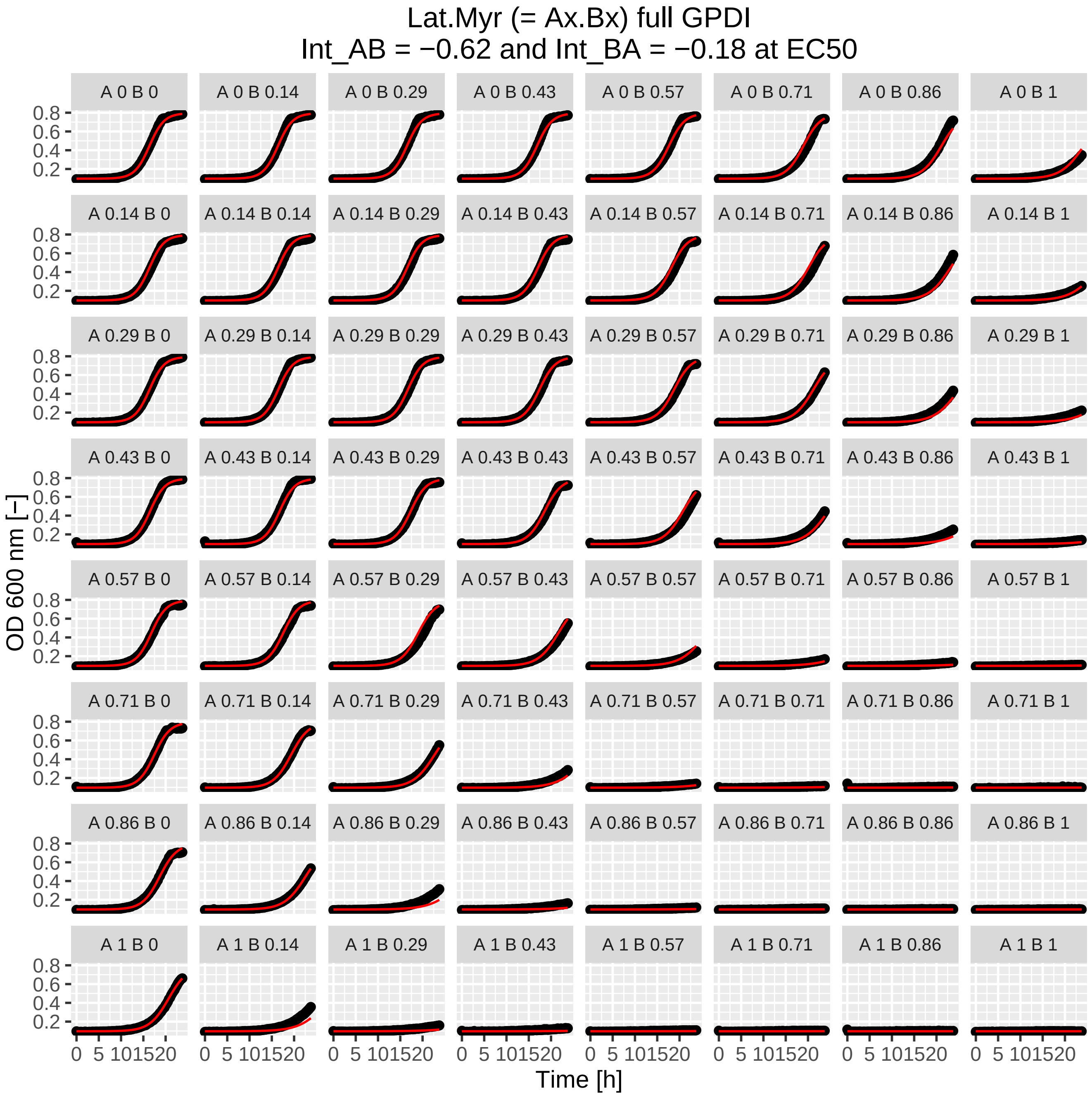
Lat.Myr (= Ax.Bx) full GPDI
Int_AB = −0.62 and Int_BA = −0.18 at EC50
A 0 B 0
A 0 B 0.14
A 0 B 0.29
A 0 B 0.43
A 0 B 0.57
A 0 B 0.71
A 0 B 0.86
A 0 B 1
A 0.14 B 0
A 0.14 B 0.14
A 0.14 B 0.29
A 0.14 B 0.43
A 0.14 B 0.57
A 0.14 B 0.71
A 0.14 B 0.86
A 0.14 B 1
A 0.29 B 0
A 0.29 B 0.14
A 0.29 B 0.29
A 0.29 B 0.43
A 0.29 B 0.57
A 0.29 B 0.71
A 0.29 B 0.86
A 0.29 B 1
A 0.43 B 0
A 0.43 B 0.14
A 0.43 B 0.29
A 0.43 B 0.43
A 0.43 B 0.57
A 0.43 B 0.71
A 0.43 B 0.86
A 0.43 B 1
A 0.57 B 0
A 0.57 B 0.14
A 0.57 B 0.29
A 0.57 B 0.43
A 0.57 B 0.57
A 0.57 B 0.71
A 0.57 B 0.86
A 0.57 B 1
A 0.71 B 0
A 0.71 B 0.14
A 0.71 B 0.29
A 0.71 B 0.43
A 0.71 B 0.57
A 0.71 B 0.71
A 0.71 B 0.86
A 0.71 B 1
A 0.86 B 0
A 0.86 B 0.14
A 0.86 B 0.29
A 0.86 B 0.43
A 0.86 B 0.57
A 0.86 B 0.71
A 0.86 B 0.86
A 0.86 B 1
A 1 B 0
A 1 B 0.14
A 1 B 0.29
A 1 B 0.43
A 1 B 0.57
A 1 B 0.71
A 1 B 0.86
A 1 B 1
0.8
0.6
0.4
0.2
OD 600 nm [−]
0 5 101520
Time [h]

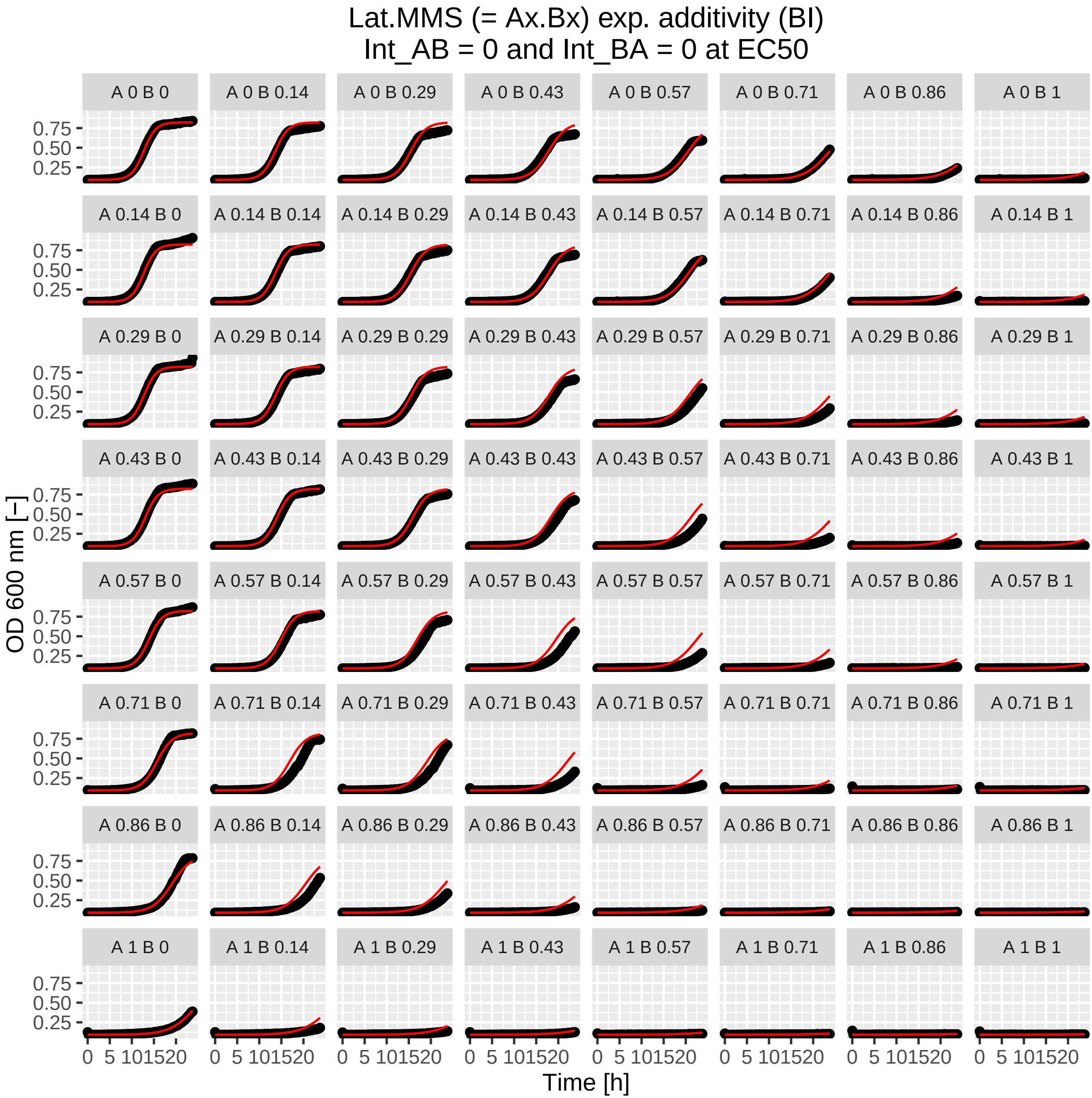
Lat.MMS (= Ax.Bx) exp. additivity (BI)
Int_AB = 0 and Int_BA = 0 at EC50
OD 600 nm [−]
Time [h]
0.75
0.50
0.25
0 5 101520
A 0 B 0
A 0 B 0.14
A 0 B 0.29
A 0 B 0.43
A 0 B 0.57
A 0 B 0.71
A 0 B 0.86
A 0 B 1
A 0.14 B 0
A 0.14 B 0.14
A 0.14 B 0.29
A 0.14 B 0.43
A 0.14 B 0.57
A 0.14 B 0.71
A 0.14 B 0.86
A 0.14 B 1
A 0.29 B 0
A 0.29 B 0.14
A 0.29 B 0.29
A 0.29 B 0.43
A 0.29 B 0.57
A 0.29 B 0.71
A 0.29 B 0.86
A 0.29 B 1
A 0.43 B 0
A 0.43 B 0.14
A 0.43 B 0.29
A 0.43 B 0.43
A 0.43 B 0.57
A 0.43 B 0.71
A 0.43 B 0.86
A 0.43 B 1
A 0.57 B 0
A 0.57 B 0.14
A 0.57 B 0.29
A 0.57 B 0.43
A 0.57 B 0.57
A 0.57 B 0.71
A 0.57 B 0.86
A 0.57 B 1
A 0.71 B 0
A 0.71 B 0.14
A 0.71 B 0.29
A 0.71 B 0.43
A 0.71 B 0.57
A 0.71 B 0.71
A 0.71 B 0.86
A 0.71 B 1
A 0.86 B 0
A 0.86 B 0.14
A 0.86 B 0.29
A 0.86 B 0.43
A 0.86 B 0.57
A 0.86 B 0.71
A 0.86 B 0.86
A 0.86 B 1
A 1 B 0
A 1 B 0.14
A 1 B 0.29
A 1 B 0.43
A 1 B 0.57
A 1 B 0.71
A 1 B 0.86
A 1 B 1

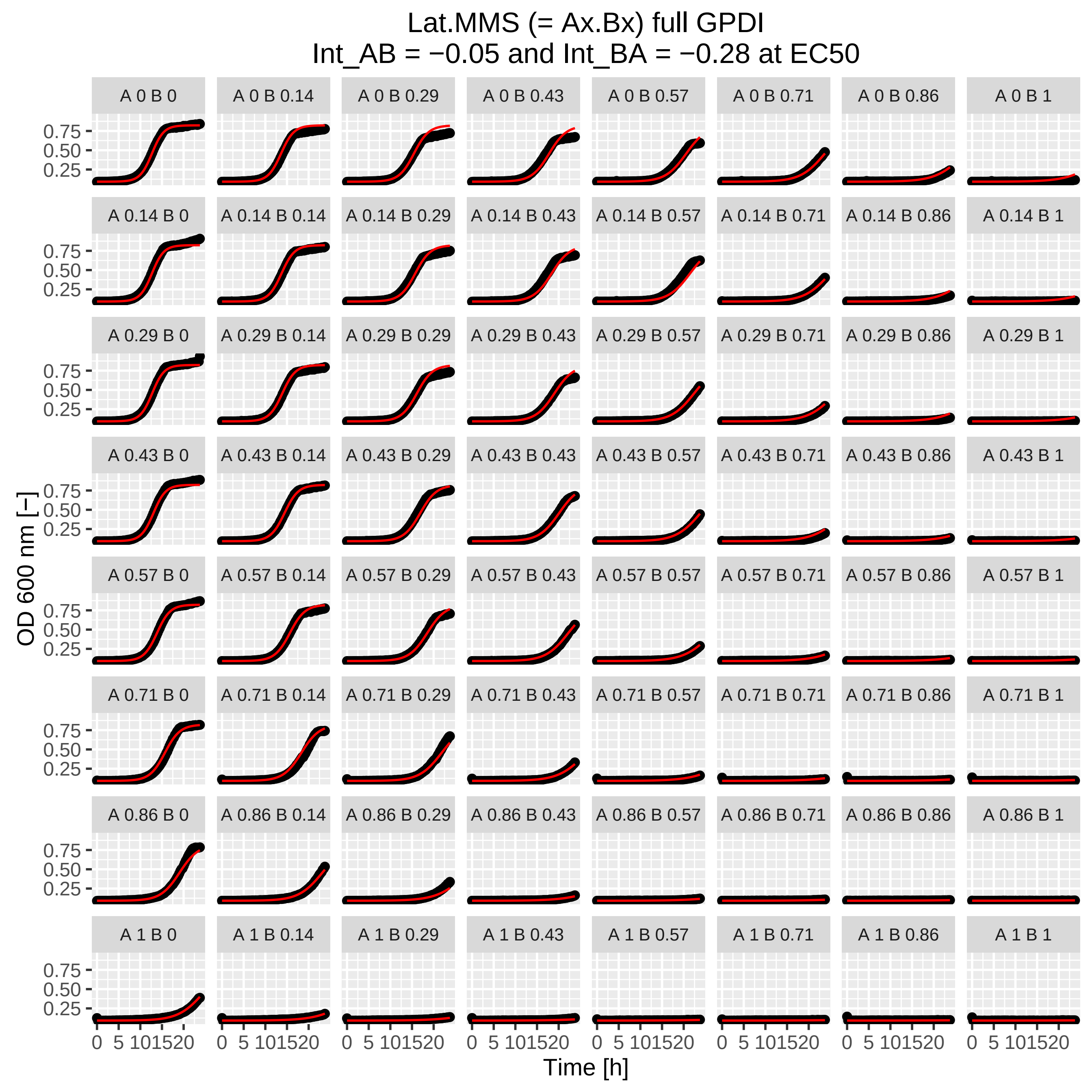
Lat.MMS (= Ax.Bx) full GPDI
Int_AB = −0.05 and Int_BA = −0.28 at EC50
A 0 B 0
A 0 B 0.14
A 0 B 0.29
A 0 B 0.43
A 0 B 0.57
A 0 B 0.71
A 0 B 0.86
A 0 B 1
A 0.14 B 0
A 0.14 B 0.14
A 0.14 B 0.29
A 0.14 B 0.43
A 0.14 B 0.57
A 0.14 B 0.71
A 0.14 B 0.86
A 0.14 B 1
A 0.29 B 0
A 0.29 B 0.14
A 0.29 B 0.29
A 0.29 B 0.43
A 0.29 B 0.57
A 0.29 B 0.71
A 0.29 B 0.86
A 0.29 B 1
A 0.43 B 0
A 0.43 B 0.14
A 0.43 B 0.29
A 0.43 B 0.43
A 0.43 B 0.57
A 0.43 B 0.71
A 0.43 B 0.86
A 0.43 B 1
A 0.57 B 0
A 0.57 B 0.14
A 0.57 B 0.29
A 0.57 B 0.43
A 0.57 B 0.57
A 0.57 B 0.71
A 0.57 B 0.86
A 0.57 B 1
A 0.71 B 0
A 0.71 B 0.14
A 0.71 B 0.29
A 0.71 B 0.43
A 0.71 B 0.57
A 0.71 B 0.71
A 0.71 B 0.86
A 0.71 B 1
A 0.86 B 0
A 0.86 B 0.14
A 0.86 B 0.29
A 0.86 B 0.43
A 0.86 B 0.57
A 0.86 B 0.71
A 0.86 B 0.86
A 0.86 B 1
A 1 B 0
A 1 B 0.14
A 1 B 0.29
A 1 B 0.43
A 1 B 0.57
A 1 B 0.71
A 1 B 0.86
A 1 B 1
0.75
0.50
0.25
0 5 101520
OD 600 nm [−]
Time [h]

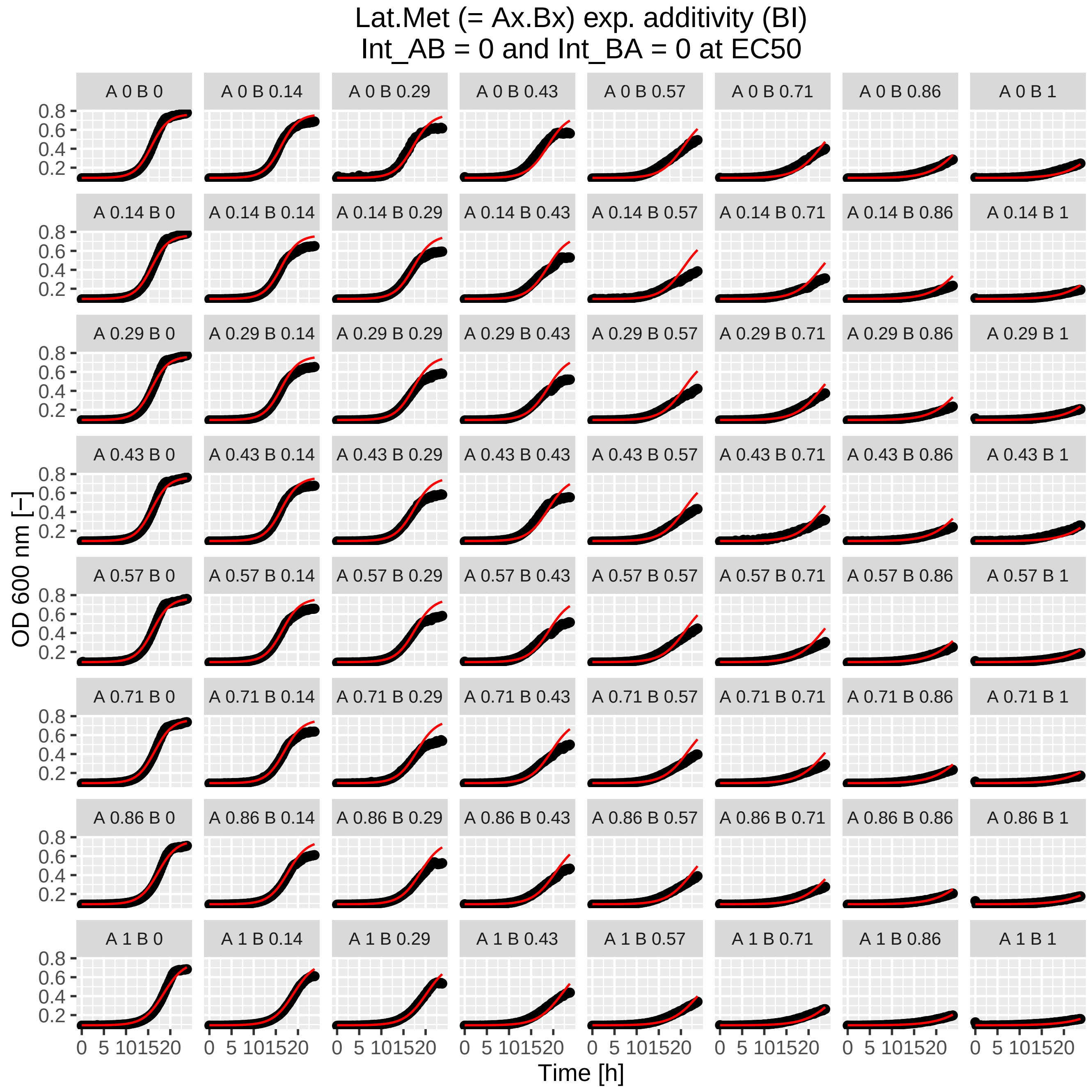

Lat.Met (= Ax.Bx) exp. additivity (BI)
Int_AB = 0 and Int_BA = 0 at EC50
A 0 B 0
A 0 B 0.14
A 0 B 0.29
A 0 B 0.43
A 0 B 0.57
A 0 B 0.71
A 0 B 0.86
A 0 B 1
A 0.14 B 0
A 0.14 B 0.14
A 0.14 B 0.29
A 0.14 B 0.43
A 0.14 B 0.57
A 0.14 B 0.71
A 0.14 B 0.86
A 0.14 B 1
A 0.29 B 0
A 0.29 B 0.14
A 0.29 B 0.29
A 0.29 B 0.43
A 0.29 B 0.57
A 0.29 B 0.71
A 0.29 B 0.86
A 0.29 B 1
A 0.43 B 0
A 0.43 B 0.14
A 0.43 B 0.29
A 0.43 B 0.43
A 0.43 B 0.57
A 0.43 B 0.71
A 0.43 B 0.86
A 0.43 B 1
A 0.57 B 0
A 0.57 B 0.14
A 0.57 B 0.29
A 0.57 B 0.43
A 0.57 B 0.57
A 0.57 B 0.71
A 0.57 B 0.86
A 0.57 B 1
A 0.71 B 0
A 0.71 B 0.14
A 0.71 B 0.29
A 0.71 B 0.43
A 0.71 B 0.57
A 0.71 B 0.71
A 0.71 B 0.86
A 0.71 B 1
A 0.86 B 0
A 0.86 B 0.14
A 0.86 B 0.29
A 0.86 B 0.43
A 0.86 B 0.57
A 0.86 B 0.71
A 0.86 B 0.86
A 0.86 B 1
A 1 B 0
A 1 B 0.14
A 1 B 0.29
A 1 B 0.43
A 1 B 0.57
A 1 B 0.71
A 1 B 0.86
A 1 B 1
OD 600 nm [–]
0.2
0.4
0.6
0.8
0 5 101520
Time [h]

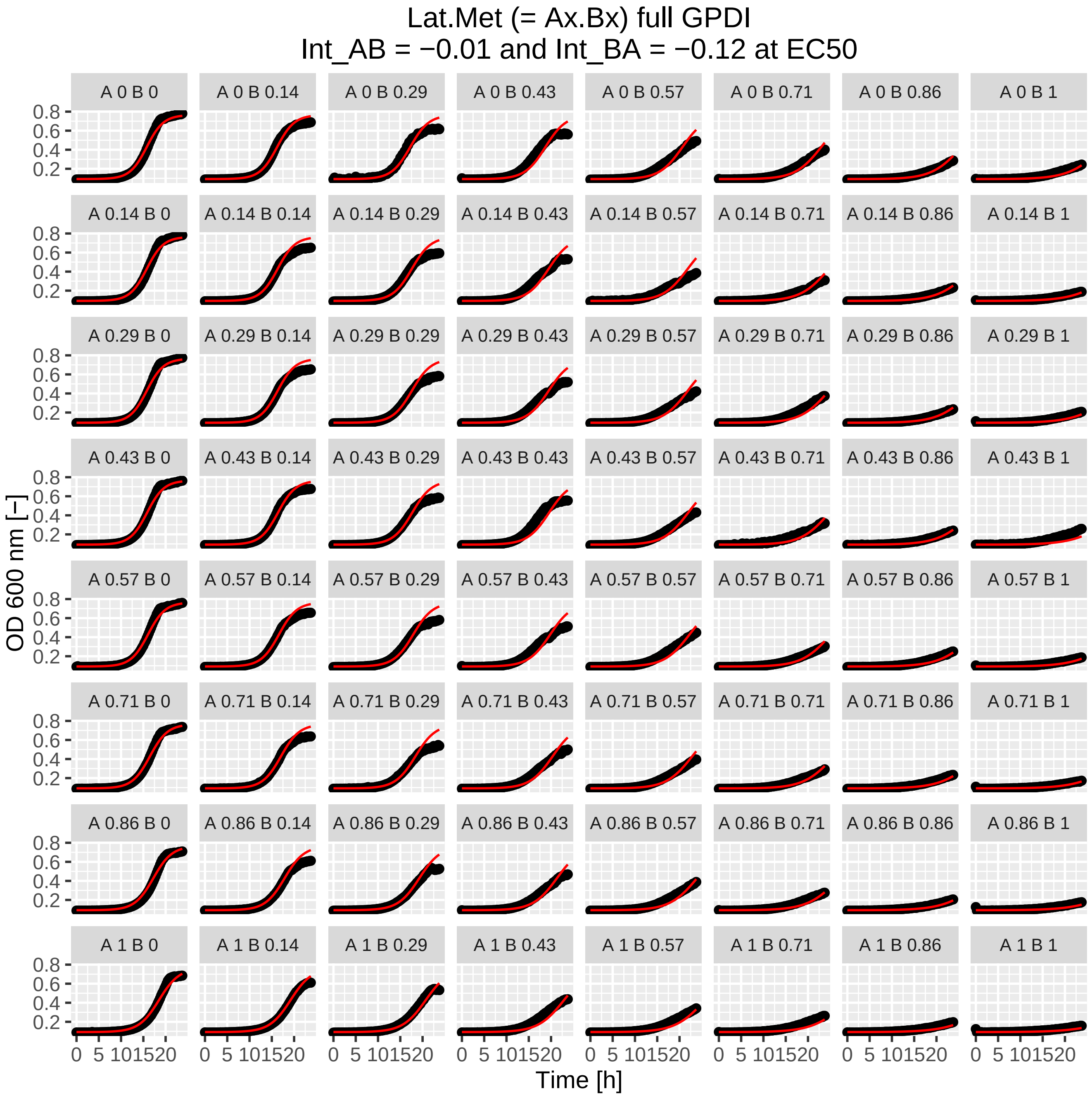
Lat.Met (= Ax.Bx) full GPDI
Int_AB = −0.01 and Int_BA = −0.12 at EC50
OD 600 nm [−]
Time [h]
A 0 B 0
A 0 B 0.14
A 0 B 0.29
A 0 B 0.43
A 0 B 0.57
A 0 B 0.71
A 0 B 0.86
A 0 B 1
A 1 B 0
A 1 B 0.14
A 1 B 0.29
A 1 B 0.43
A 1 B 0.57
A 1 B 0.71
A 1 B 0.86
A 1 B 1

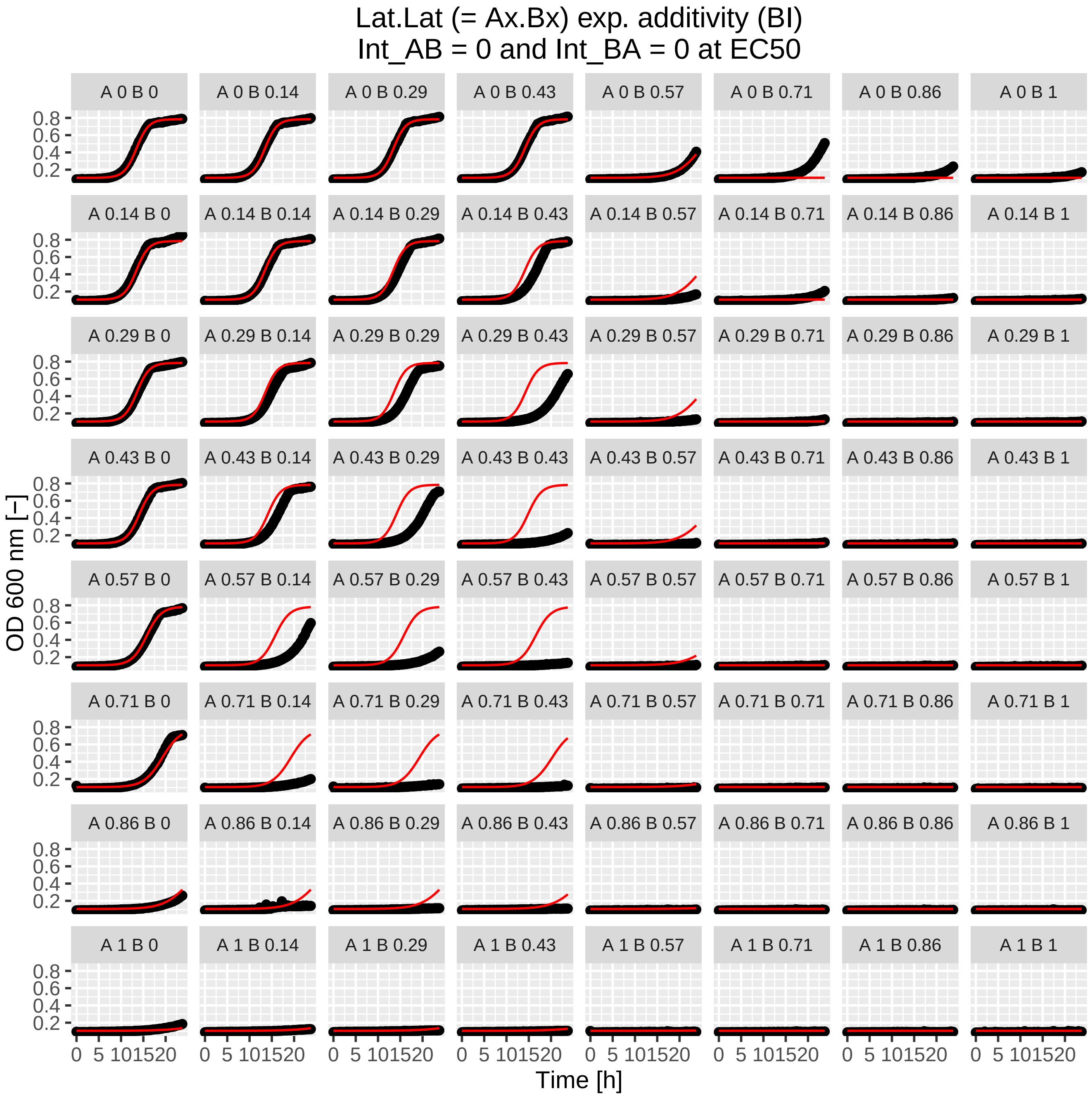
Lat.Lat (= Ax.Bx) exp. additivity (BI)
Int_AB = 0 and Int_BA = 0 at EC50
OD 600 nm [−]
Time [h]
A 0 B 0
A 0 B 0.14
A 0 B 0.29
A 0 B 0.43
A 0 B 0.57
A 0 B 0.71
A 0 B 0.86
A 0 B 1
A 0.14 B 0
A 0.14 B 0.14
A 0.14 B 0.29
A 0.14 B 0.43
A 0.14 B 0.57
A 0.14 B 0.71
A 0.14 B 0.86
A 0.14 B 1
A 0.29 B 0
A 0.29 B 0.14
A 0.29 B 0.29
A 0.29 B 0.43
A 0.29 B 0.57
A 0.29 B 0.71
A 0.29 B 0.86
A 0.29 B 1
A 0.43 B 0
A 0.43 B 0.14
A 0.43 B 0.29
A 0.43 B 0.43
A 0.43 B 0.57
A 0.43 B 0.71
A 0.43 B 0.86
A 0.43 B 1
A 0.57 B 0
A 0.57 B 0.14
A 0.57 B 0.29
A 0.57 B 0.43
A 0.57 B 0.57
A 0.57 B 0.71
A 0.57 B 0.86
A 0.57 B 1
A 0.71 B 0
A 0.71 B 0.14
A 0.71 B 0.29
A 0.71 B 0.43
A 0.71 B 0.57
A 0.71 B 0.71
A 0.71 B 0.86
A 0.71 B 1
A 0.86 B 0
A 0.86 B 0.14
A 0.86 B 0.29
A 0.86 B 0.43
A 0.86 B 0.57
A 0.86 B 0.71
A 0.86 B 0.86
A 0.86 B 1
A 1 B 0
A 1 B 0.14
A 1 B 0.29
A 1 B 0.43
A 1 B 0.57
A 1 B 0.71
A 1 B 0.86
A 1 B 1
0.8
0.6
0.4
0.2
0 5 101520

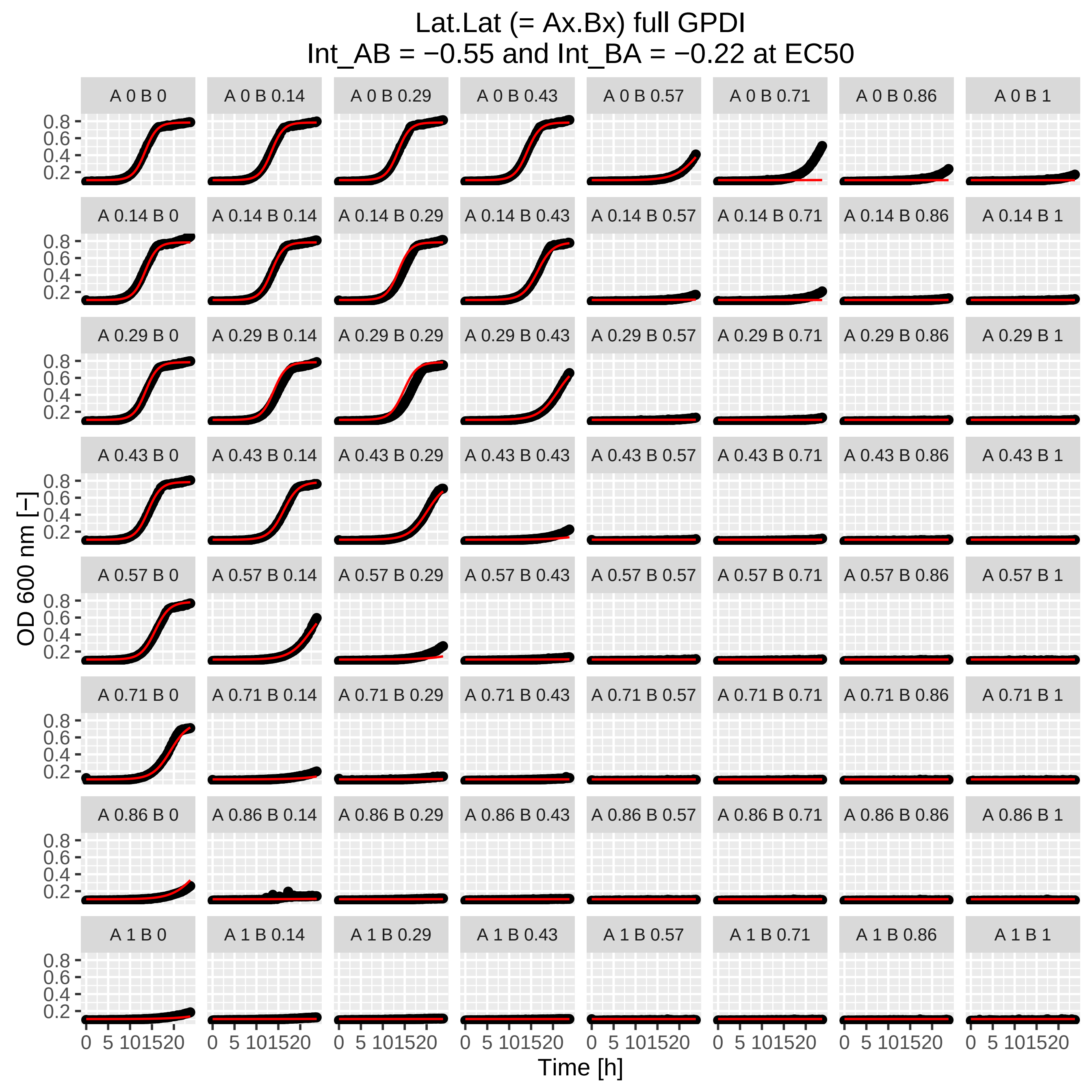

Lat.Lat (= Ax.Bx) full GPDI
Int_AB = −0.55 and Int_BA = −0.22 at EC50
A 0 B 0
A 0 B 0.14
A 0 B 0.29
A 0 B 0.43
A 0 B 0.57
A 0 B 0.71
A 0 B 0.86
A 0 B 1
A 0.14 B 0
A 0.14 B 0.14
A 0.14 B 0.29
A 0.14 B 0.43
A 0.14 B 0.57
A 0.14 B 0.71
A 0.14 B 0.86
A 0.14 B 1
A 0.29 B 0
A 0.29 B 0.14
A 0.29 B 0.29
A 0.29 B 0.43
A 0.29 B 0.57
A 0.29 B 0.71
A 0.29 B 0.86
A 0.29 B 1
A 0.43 B 0
A 0.43 B 0.14
A 0.43 B 0.29
A 0.43 B 0.43
A 0.43 B 0.57
A 0.43 B 0.71
A 0.43 B 0.86
A 0.43 B 1
A 0.57 B 0
A 0.57 B 0.14
A 0.57 B 0.29
A 0.57 B 0.43
A 0.57 B 0.57
A 0.57 B 0.71
A 0.57 B 0.86
A 0.57 B 1
A 0.71 B 0
A 0.71 B 0.14
A 0.71 B 0.29
A 0.71 B 0.43
A 0.71 B 0.57
A 0.71 B 0.71
A 0.71 B 0.86
A 0.71 B 1
A 0.86 B 0
A 0.86 B 0.14
A 0.86 B 0.29
A 0.86 B 0.43
A 0.86 B 0.57
A 0.86 B 0.71
A 0.86 B 0.86
A 0.86 B 1
A 1 B 0
A 1 B 0.14
A 1 B 0.29
A 1 B 0.43
A 1 B 0.57
A 1 B 0.71
A 1 B 0.86
A 1 B 1
OD 600 nm [−]
0.2
0.4
0.6
0.8
0 5 101520
Time [h]

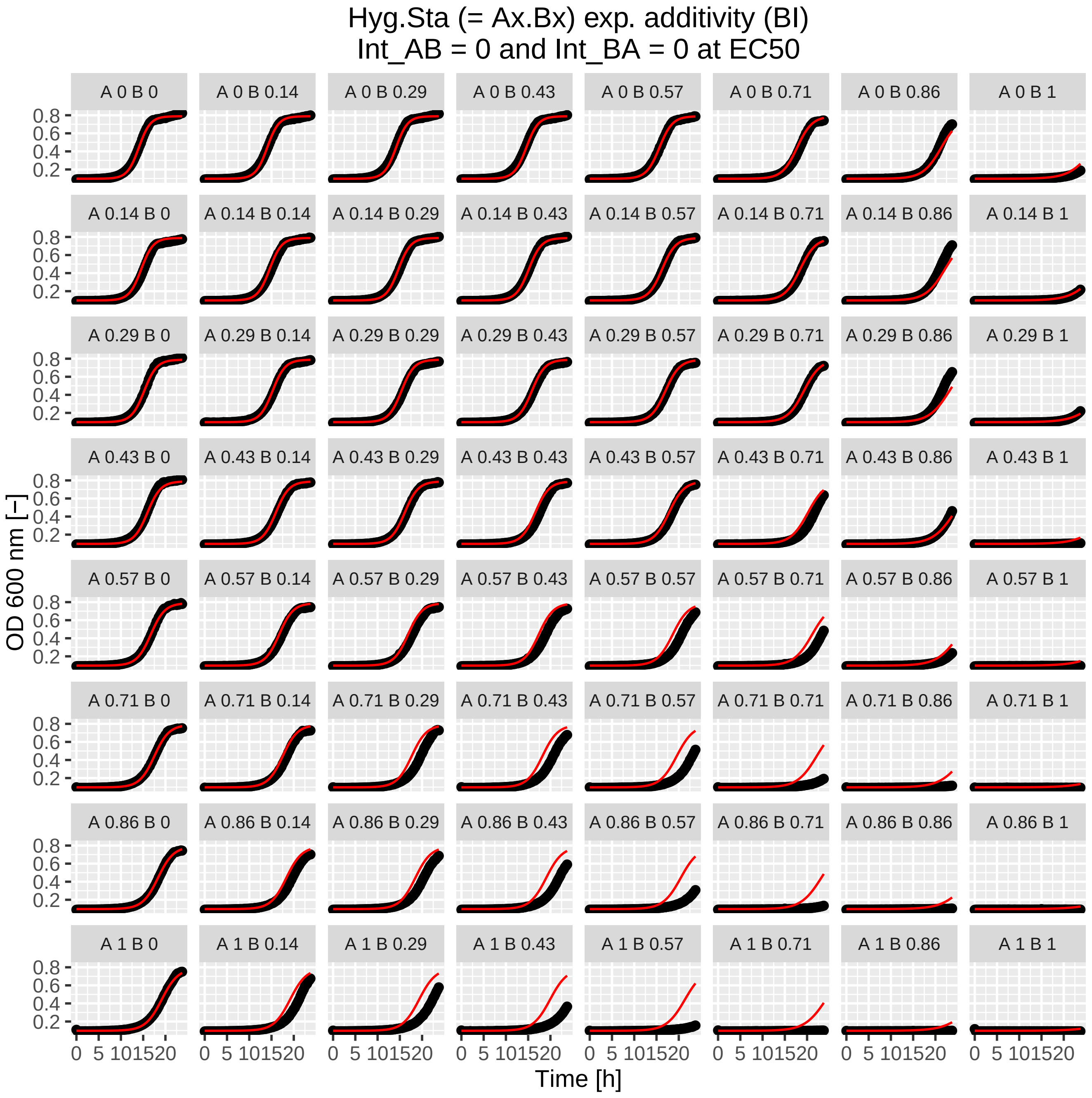

Hyg.Sta (= Ax.Bx) exp. additivity (BI)
Int_AB = 0 and Int_BA = 0 at EC50
OD 600 nm [−]
Time [h]
0.8
0.6
0.4
0.2
0
5
10
15
20
A 0 B 0
A 0 B 0.14
A 0 B 0.29
A 0 B 0.43
A 0 B 0.57
A 0 B 0.71
A 0 B 0.86
A 0 B 1
A 0.14 B 0
A 0.14 B 0.14
A 0.14 B 0.29
A 0.14 B 0.43
A 0.14 B 0.57
A 0.14 B 0.71
A 0.14 B 0.86
A 0.14 B 1
A 0.29 B 0
A 0.29 B 0.14
A 0.29 B 0.29
A 0.29 B 0.43
A 0.29 B 0.57
A 0.29 B 0.71
A 0.29 B 0.86
A 0.29 B 1
A 0.43 B 0
A 0.43 B 0.14
A 0.43 B 0.29
A 0.43 B 0.43
A 0.43 B 0.57
A 0.43 B 0.71
A 0.43 B 0.86
A 0.43 B 1
A 0.57 B 0
A 0.57 B 0.14
A 0.57 B 0.29
A 0.57 B 0.43
A 0.57 B 0.57
A 0.57 B 0.71
A 0.57 B 0.86
A 0.57 B 1
A 0.71 B 0
A 0.71 B 0.14
A 0.71 B 0.29
A 0.71 B 0.43
A 0.71 B 0.57
A 0.71 B 0.71
A 0.71 B 0.86
A 0.71 B 1
A 0.86 B 0
A 0.86 B 0.14
A 0.86 B 0.29
A 0.86 B 0.43
A 0.86 B 0.57
A 0.86 B 0.71
A 0.86 B 0.86
A 0.86 B 1
A 1 B 0
A 1 B 0.14
A 1 B 0.29
A 1 B 0.43
A 1 B 0.57
A 1 B 0.71
A 1 B 0.86
A 1 B 1

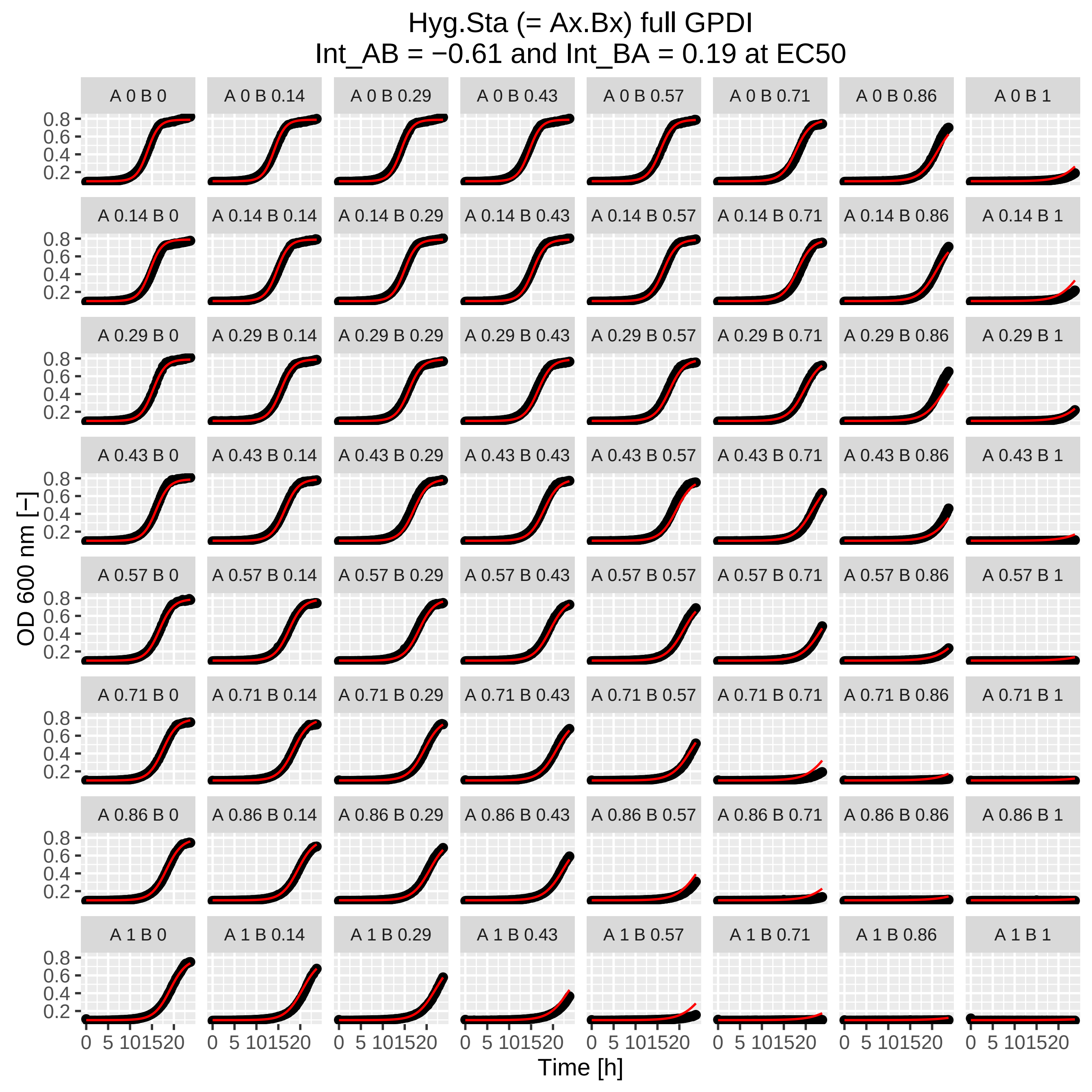

Hyg.Sta (= Ax.Bx) full GPDI
Int_AB = −0.61 and Int_BA = 0.19 at EC50
OD 600 nm [−]
Time [h]
A 0 B 0
A 0 B 0.14
A 0 B 0.29
A 0 B 0.43
A 0 B 0.57
A 0 B 0.71
A 0 B 0.86
A 0 B 1
A 0.14 B 0
A 0.14 B 0.14
A 0.14 B 0.29
A 0.14 B 0.43
A 0.14 B 0.57
A 0.14 B 0.71
A 0.14 B 0.86
A 0.14 B 1
A 0.29 B 0
A 0.29 B 0.14
A 0.29 B 0.29
A 0.29 B 0.43
A 0.29 B 0.57
A 0.29 B 0.71
A 0.29 B 0.86
A 0.29 B 1
A 0.43 B 0
A 0.43 B 0.14
A 0.43 B 0.29
A 0.43 B 0.43
A 0.43 B 0.57
A 0.43 B 0.71
A 0.43 B 0.86
A 0.43 B 1
A 0.57 B 0
A 0.57 B 0.14
A 0.57 B 0.29
A 0.57 B 0.43
A 0.57 B 0.57
A 0.57 B 0.71
A 0.57 B 0.86
A 0.57 B 1
A 0.71 B 0
A 0.71 B 0.14
A 0.71 B 0.29
A 0.71 B 0.43
A 0.71 B 0.57
A 0.71 B 0.71
A 0.71 B 0.86
A 0.71 B 1
A 0.86 B 0
A 0.86 B 0.14
A 0.86 B 0.29
A 0.86 B 0.43
A 0.86 B 0.57
A 0.86 B 0.71
A 0.86 B 0.86
A 0.86 B 1
A 1 B 0
A 1 B 0.14
A 1 B 0.29
A 1 B 0.43
A 1 B 0.57
A 1 B 0.71
A 1 B 0.86
A 1 B 1
0.8
0.6
0.4
0.2
0 5 101520

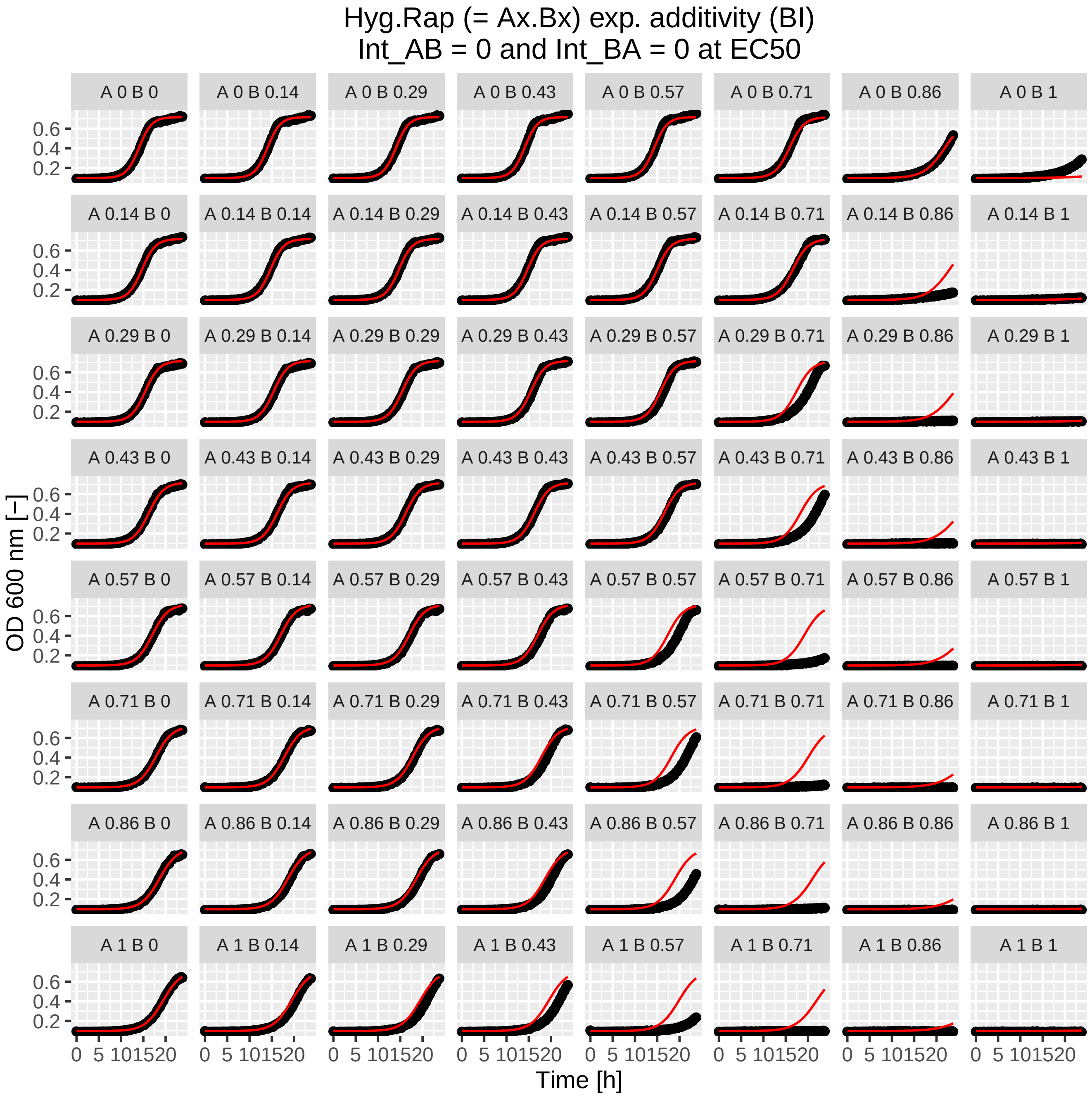

Hyg.Rap (= Ax.Bx) exp. additivity (BI)
Int_AB = 0 and Int_BA = 0 at EC50
A 0 B 0
A 0 B 0.14
A 0 B 0.29
A 0 B 0.43
A 0 B 0.57
A 0 B 0.71
A 0 B 0.86
A 0 B 1
A 0.14 B 0
A 0.14 B 0.14
A 0.14 B 0.29
A 0.14 B 0.43
A 0.14 B 0.57
A 0.14 B 0.71
A 0.14 B 0.86
A 0.14 B 1
A 0.29 B 0
A 0.29 B 0.14
A 0.29 B 0.29
A 0.29 B 0.43
A 0.29 B 0.57
A 0.29 B 0.71
A 0.29 B 0.86
A 0.29 B 1
A 0.43 B 0
A 0.43 B 0.14
A 0.43 B 0.29
A 0.43 B 0.43
A 0.43 B 0.57
A 0.43 B 0.71
A 0.43 B 0.86
A 0.43 B 1
A 0.57 B 0
A 0.57 B 0.14
A 0.57 B 0.29
A 0.57 B 0.43
A 0.57 B 0.57
A 0.57 B 0.71
A 0.57 B 0.86
A 0.57 B 1
A 0.71 B 0
A 0.71 B 0.14
A 0.71 B 0.29
A 0.71 B 0.43
A 0.71 B 0.57
A 0.71 B 0.71
A 0.71 B 0.86
A 0.71 B 1
A 0.86 B 0
A 0.86 B 0.14
A 0.86 B 0.29
A 0.86 B 0.43
A 0.86 B 0.57
A 0.86 B 0.71
A 0.86 B 0.86
A 0.86 B 1
A 1 B 0
A 1 B 0.14
A 1 B 0.29
A 1 B 0.43
A 1 B 0.57
A 1 B 0.71
A 1 B 0.86
A 1 B 1
0.6
0.4
0.2
OD 600 nm [–]
0 5 101520
Time [h]

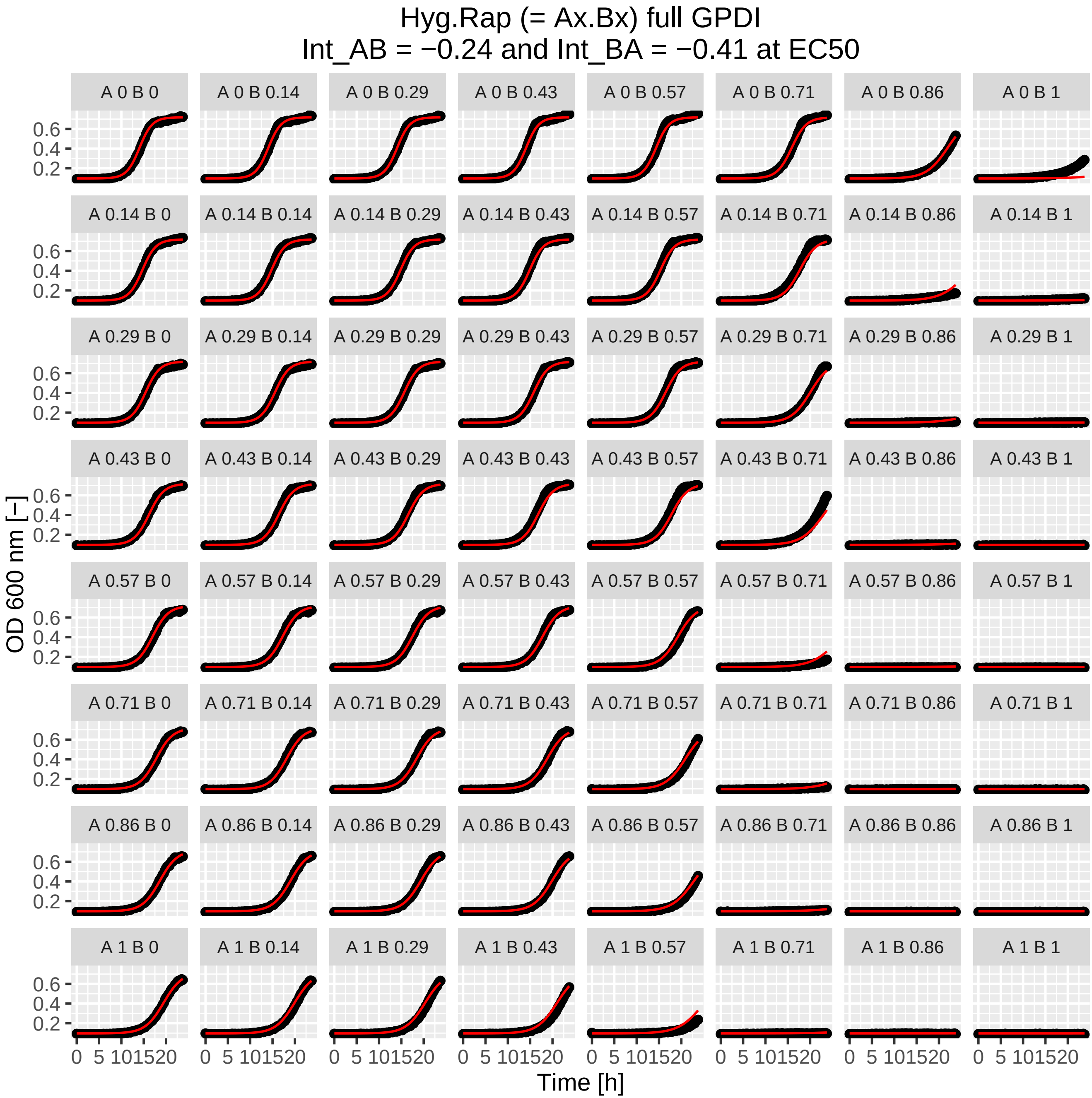

Hyg.Rap (= Ax.Bx) full GPDI
Int_AB = −0.24 and Int_BA = −0.41 at EC50
A 0 B 0
A 0 B 0.14
A 0 B 0.29
A 0 B 0.43
A 0 B 0.57
A 0 B 0.71
A 0 B 0.86
A 0 B 1
A 0.14 B 0
A 0.14 B 0.14
A 0.14 B 0.29
A 0.14 B 0.43
A 0.14 B 0.57
A 0.14 B 0.71
A 0.14 B 0.86
A 0.14 B 1
A 0.29 B 0
A 0.29 B 0.14
A 0.29 B 0.29
A 0.29 B 0.43
A 0.29 B 0.57
A 0.29 B 0.71
A 0.29 B 0.86
A 0.29 B 1
A 0.43 B 0
A 0.43 B 0.14
A 0.43 B 0.29
A 0.43 B 0.43
A 0.43 B 0.57
A 0.43 B 0.71
A 0.43 B 0.86
A 0.43 B 1
A 0.57 B 0
A 0.57 B 0.14
A 0.57 B 0.29
A 0.57 B 0.43
A 0.57 B 0.57
A 0.57 B 0.71
A 0.57 B 0.86
A 0.57 B 1
A 0.71 B 0
A 0.71 B 0.14
A 0.71 B 0.29
A 0.71 B 0.43
A 0.71 B 0.57
A 0.71 B 0.71
A 0.71 B 0.86
A 0.71 B 1
A 0.86 B 0
A 0.86 B 0.14
A 0.86 B 0.29
A 0.86 B 0.43
A 0.86 B 0.57
A 0.86 B 0.71
A 0.86 B 0.86
A 0.86 B 1
A 1 B 0
A 1 B 0.14
A 1 B 0.29
A 1 B 0.43
A 1 B 0.57
A 1 B 0.71
A 1 B 0.86
A 1 B 1
0.6
0.4
0.2
OD 600 nm [−]
0 5 101520
Time [h]

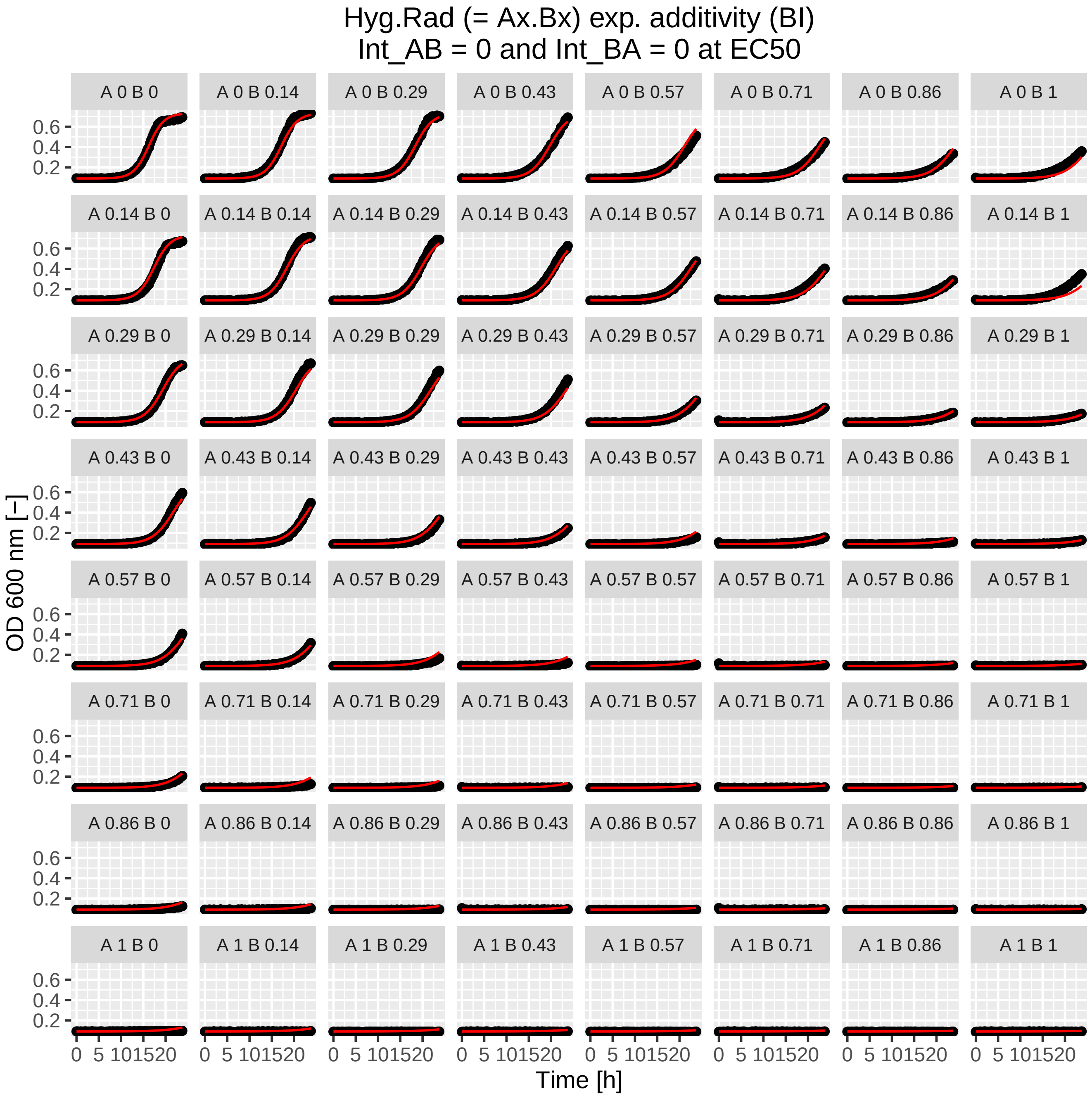

Hyg.Rad (= Ax.Bx) exp. additivity (BI)
Int_AB = 0 and Int_BA = 0 at EC50
A 0 B 0
A 0 B 0.14
A 0 B 0.29
A 0 B 0.43
A 0 B 0.57
A 0 B 0.71
A 0 B 0.86
A 0 B 1
A 0.14 B 0
A 0.14 B 0.14
A 0.14 B 0.29
A 0.14 B 0.43
A 0.14 B 0.57
A 0.14 B 0.71
A 0.14 B 0.86
A 0.14 B 1
A 0.29 B 0
A 0.29 B 0.14
A 0.29 B 0.29
A 0.29 B 0.43
A 0.29 B 0.57
A 0.29 B 0.71
A 0.29 B 0.86
A 0.29 B 1
A 0.43 B 0
A 0.43 B 0.14
A 0.43 B 0.29
A 0.43 B 0.43
A 0.43 B 0.57
A 0.43 B 0.71
A 0.43 B 0.86
A 0.43 B 1
A 0.57 B 0
A 0.57 B 0.14
A 0.57 B 0.29
A 0.57 B 0.43
A 0.57 B 0.57
A 0.57 B 0.71
A 0.57 B 0.86
A 0.57 B 1
A 0.71 B 0
A 0.71 B 0.14
A 0.71 B 0.29
A 0.71 B 0.43
A 0.71 B 0.57
A 0.71 B 0.71
A 0.71 B 0.86
A 0.71 B 1
A 0.86 B 0
A 0.86 B 0.14
A 0.86 B 0.29
A 0.86 B 0.43
A 0.86 B 0.57
A 0.86 B 0.71
A 0.86 B 0.86
A 0.86 B 1
A 1 B 0
A 1 B 0.14
A 1 B 0.29
A 1 B 0.43
A 1 B 0.57
A 1 B 0.71
A 1 B 0.86
A 1 B 1
OD 600 nm [–]
0.6
0.4
0.2
0 5 101520
Time [h]

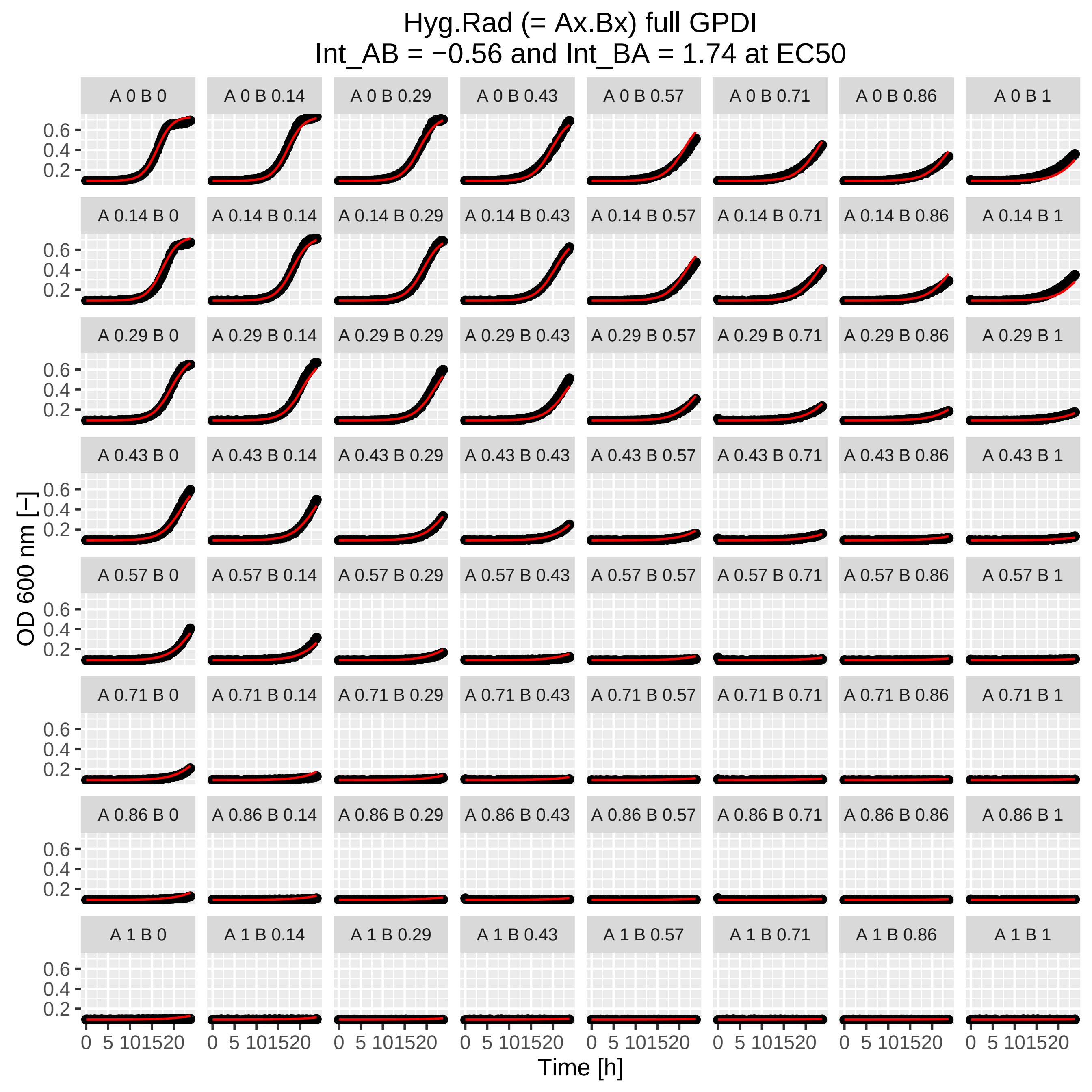

Hyg.Rad (= Ax.Bx) full GPDI
Int_AB = −0.56 and Int_BA = 1.74 at EC50
A 0 B 0
A 0 B 0.14
A 0 B 0.29
A 0 B 0.43
A 0 B 0.57
A 0 B 0.71
A 0 B 0.86
A 0 B 1
A 0.14 B 0
A 0.14 B 0.14
A 0.14 B 0.29
A 0.14 B 0.43
A 0.14 B 0.57
A 0.14 B 0.71
A 0.14 B 0.86
A 0.14 B 1
A 0.29 B 0
A 0.29 B 0.14
A 0.29 B 0.29
A 0.29 B 0.43
A 0.29 B 0.57
A 0.29 B 0.71
A 0.29 B 0.86
A 0.29 B 1
A 0.43 B 0
A 0.43 B 0.14
A 0.43 B 0.29
A 0.43 B 0.43
A 0.43 B 0.57
A 0.43 B 0.71
A 0.43 B 0.86
A 0.43 B 1
A 0.57 B 0
A 0.57 B 0.14
A 0.57 B 0.29
A 0.57 B 0.43
A 0.57 B 0.57
A 0.57 B 0.71
A 0.57 B 0.86
A 0.57 B 1
A 0.71 B 0
A 0.71 B 0.14
A 0.71 B 0.29
A 0.71 B 0.43
A 0.71 B 0.57
A 0.71 B 0.71
A 0.71 B 0.86
A 0.71 B 1
A 0.86 B 0
A 0.86 B 0.14
A 0.86 B 0.29
A 0.86 B 0.43
A 0.86 B 0.57
A 0.86 B 0.71
A 0.86 B 0.86
A 0.86 B 1
A 1 B 0
A 1 B 0.14
A 1 B 0.29
A 1 B 0.43
A 1 B 0.57
A 1 B 0.71
A 1 B 0.86
A 1 B 1
OD 600 nm [−]
0.2
0.4
0.6
0 5 101520
Time [h]

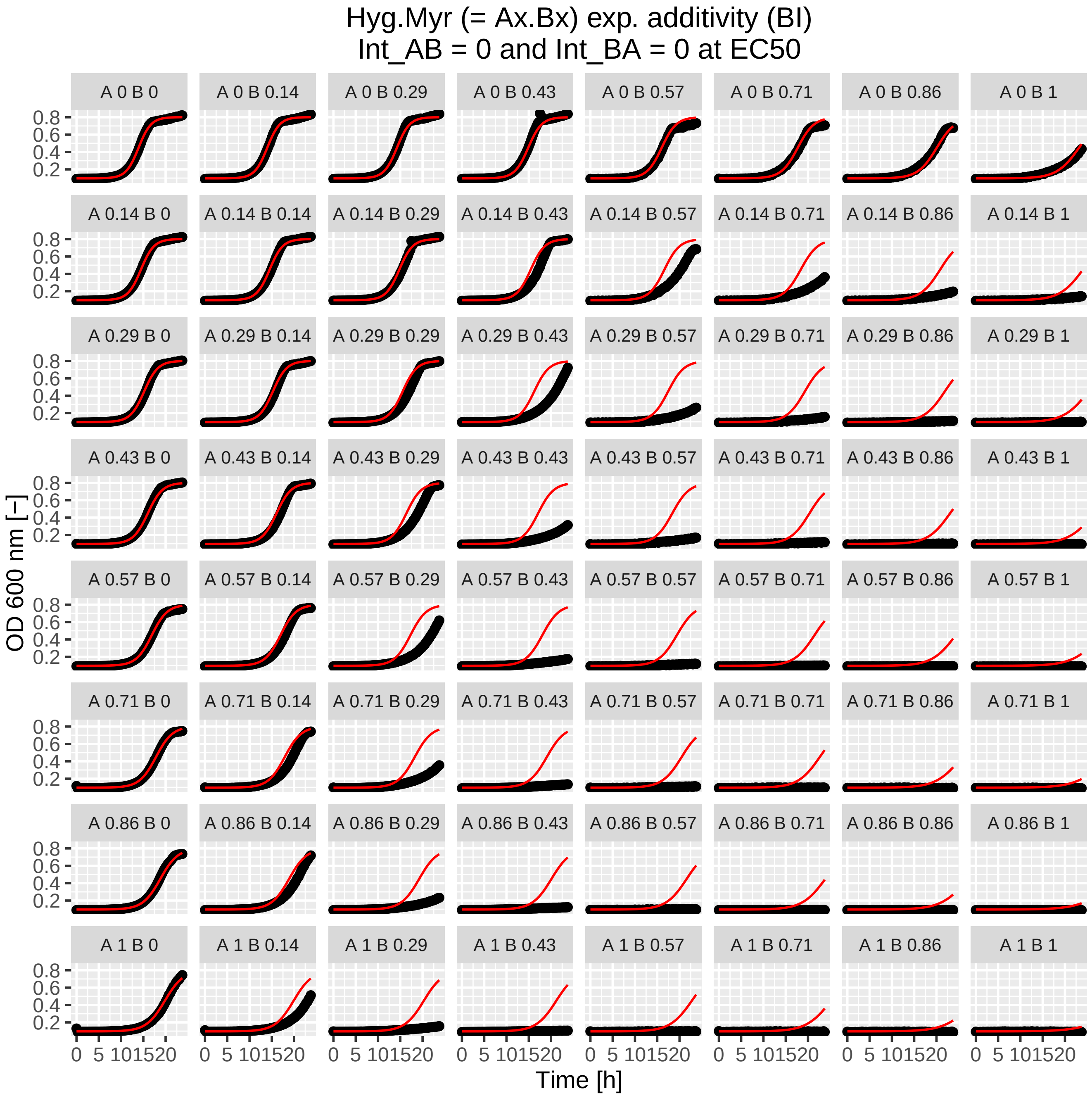

Hyg.Myr (= Ax.Bx) exp. additivity (BI)
Int_AB = 0 and Int_BA = 0 at EC50
OD 600 nm [−]
Time [h]
0.2
0.4
0.6
0.8
0 5 101520
A 0 B 0
A 0 B 0.14
A 0 B 0.29
A 0 B 0.43
A 0 B 0.57
A 0 B 0.71
A 0 B 0.86
A 0 B 1
A 0.14 B 0
A 0.14 B 0.14
A 0.14 B 0.29
A 0.14 B 0.43
A 0.14 B 0.57
A 0.14 B 0.71
A 0.14 B 0.86
A 0.14 B 1
A 0.29 B 0
A 0.29 B 0.14
A 0.29 B 0.29
A 0.29 B 0.43
A 0.29 B 0.57
A 0.29 B 0.71
A 0.29 B 0.86
A 0.29 B 1
A 0.43 B 0
A 0.43 B 0.14
A 0.43 B 0.29
A 0.43 B 0.43
A 0.43 B 0.57
A 0.43 B 0.71
A 0.43 B 0.86
A 0.43 B 1
A 0.57 B 0
A 0.57 B 0.14
A 0.57 B 0.29
A 0.57 B 0.43
A 0.57 B 0.57
A 0.57 B 0.71
A 0.57 B 0.86
A 0.57 B 1
A 0.71 B 0
A 0.71 B 0.14
A 0.71 B 0.29
A 0.71 B 0.43
A 0.71 B 0.57
A 0.71 B 0.71
A 0.71 B 0.86
A 0.71 B 1
A 0.86 B 0
A 0.86 B 0.14
A 0.86 B 0.29
A 0.86 B 0.43
A 0.86 B 0.57
A 0.86 B 0.71
A 0.86 B 0.86
A 0.86 B 1
A 1 B 0
A 1 B 0.14
A 1 B 0.29
A 1 B 0.43
A 1 B 0.57
A 1 B 0.71
A 1 B 0.86
A 1 B 1

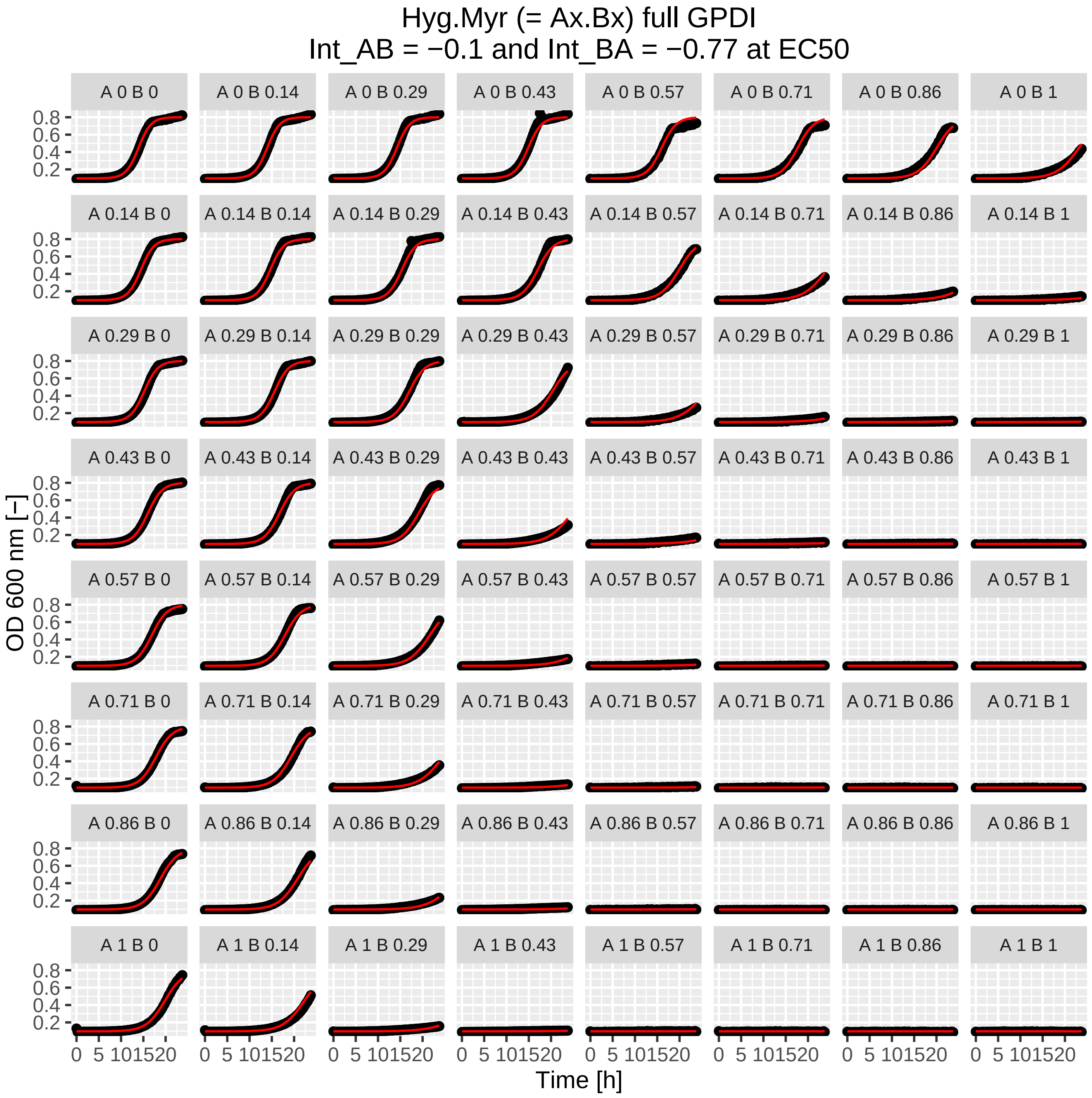

Hyg.Myr (= Ax.Bx) full GPDI
Int_AB = −0.1 and Int_BA = −0.77 at EC50
A 0 B 0
A 0 B 0.14
A 0 B 0.29
A 0 B 0.43
A 0 B 0.57
A 0 B 0.71
A 0 B 0.86
A 0 B 1
A 0.14 B 0
A 0.14 B 0.14
A 0.14 B 0.29
A 0.14 B 0.43
A 0.14 B 0.57
A 0.14 B 0.71
A 0.14 B 0.86
A 0.14 B 1
A 0.29 B 0
A 0.29 B 0.14
A 0.29 B 0.29
A 0.29 B 0.43
A 0.29 B 0.57
A 0.29 B 0.71
A 0.29 B 0.86
A 0.29 B 1
A 0.43 B 0
A 0.43 B 0.14
A 0.43 B 0.29
A 0.43 B 0.43
A 0.43 B 0.57
A 0.43 B 0.71
A 0.43 B 0.86
A 0.43 B 1
A 0.57 B 0
A 0.57 B 0.14
A 0.57 B 0.29
A 0.57 B 0.43
A 0.57 B 0.57
A 0.57 B 0.71
A 0.57 B 0.86
A 0.57 B 1
A 0.71 B 0
A 0.71 B 0.14
A 0.71 B 0.29
A 0.71 B 0.43
A 0.71 B 0.57
A 0.71 B 0.71
A 0.71 B 0.86
A 0.71 B 1
A 0.86 B 0
A 0.86 B 0.14
A 0.86 B 0.29
A 0.86 B 0.43
A 0.86 B 0.57
A 0.86 B 0.71
A 0.86 B 0.86
A 0.86 B 1
A 1 B 0
A 1 B 0.14
A 1 B 0.29
A 1 B 0.43
A 1 B 0.57
A 1 B 0.71
A 1 B 0.86
A 1 B 1
OD 600 nm [−]
0.8
0.6
0.4
0.2
0 5 101520
Time [h]

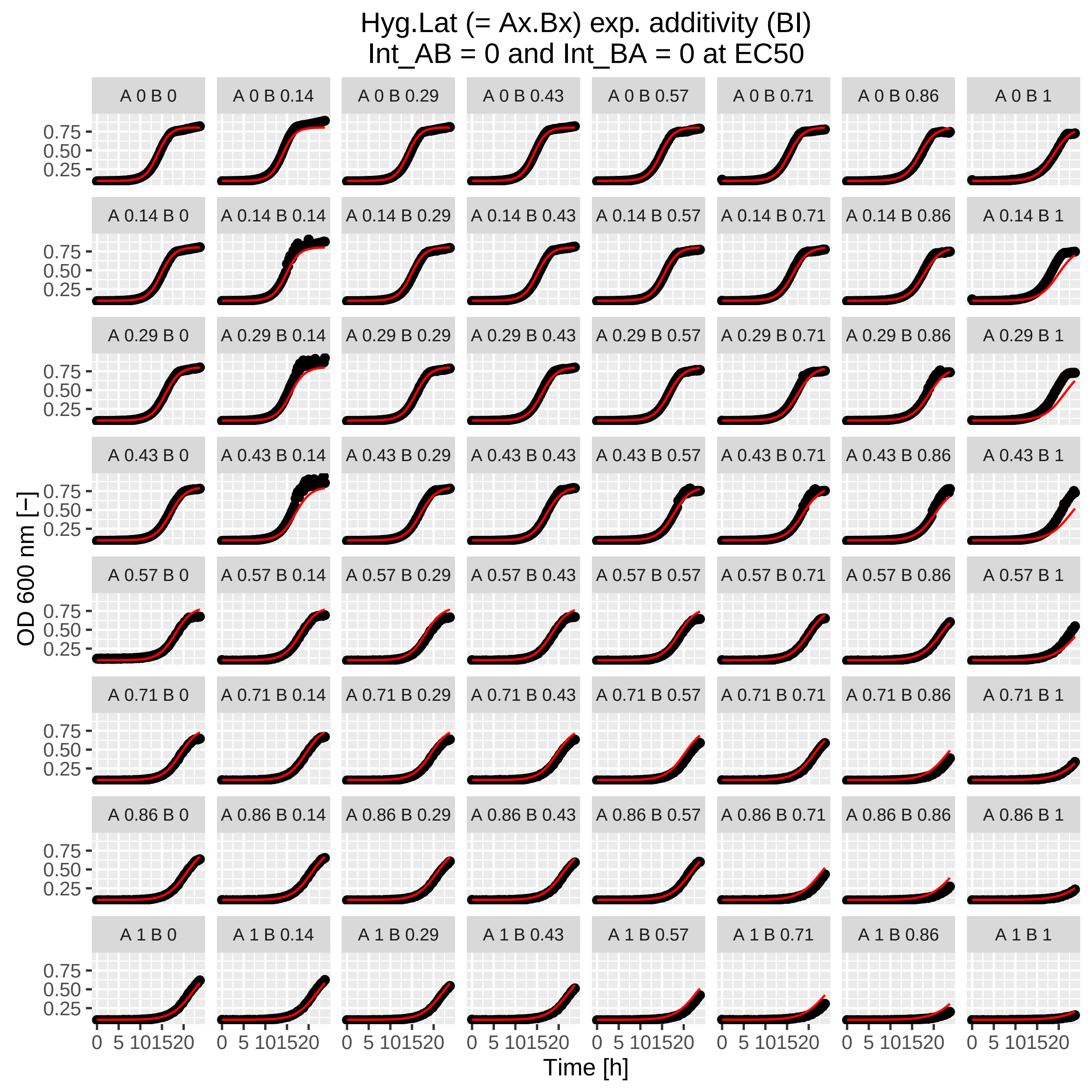

Hyg.Lat (= Ax.Bx) exp. additivity (BI)
Int_AB = 0 and Int_BA = 0 at EC50
OD 600 nm [–]
Time [h]
0.75
0.50
0.25
0 5 101520
A 0 B 0
A 0 B 0.14
A 0 B 0.29
A 0 B 0.43
A 0 B 0.57
A 0 B 0.71
A 0 B 0.86
A 0 B 1
A 0.14 B 0
A 0.14 B 0.14
A 0.14 B 0.29
A 0.14 B 0.43
A 0.14 B 0.57
A 0.14 B 0.71
A 0.14 B 0.86
A 0.14 B 1
A 0.29 B 0
A 0.29 B 0.14
A 0.29 B 0.29
A 0.29 B 0.43
A 0.29 B 0.57
A 0.29 B 0.71
A 0.29 B 0.86
A 0.29 B 1
A 0.43 B 0
A 0.43 B 0.14
A 0.43 B 0.29
A 0.43 B 0.43
A 0.43 B 0.57
A 0.43 B 0.71
A 0.43 B 0.86
A 0.43 B 1
A 0.57 B 0
A 0.57 B 0.14
A 0.57 B 0.29
A 0.57 B 0.43
A 0.57 B 0.57
A 0.57 B 0.71
A 0.57 B 0.86
A 0.57 B 1
A 0.71 B 0
A 0.71 B 0.14
A 0.71 B 0.29
A 0.71 B 0.43
A 0.71 B 0.57
A 0.71 B 0.71
A 0.71 B 0.86
A 0.71 B 1
A 0.86 B 0
A 0.86 B 0.14
A 0.86 B 0.29
A 0.86 B 0.43
A 0.86 B 0.57
A 0.86 B 0.71
A 0.86 B 0.86
A 0.86 B 1
A 1 B 0
A 1 B 0.14
A 1 B 0.29
A 1 B 0.43
A 1 B 0.57
A 1 B 0.71
A 1 B 0.86
A 1 B 1

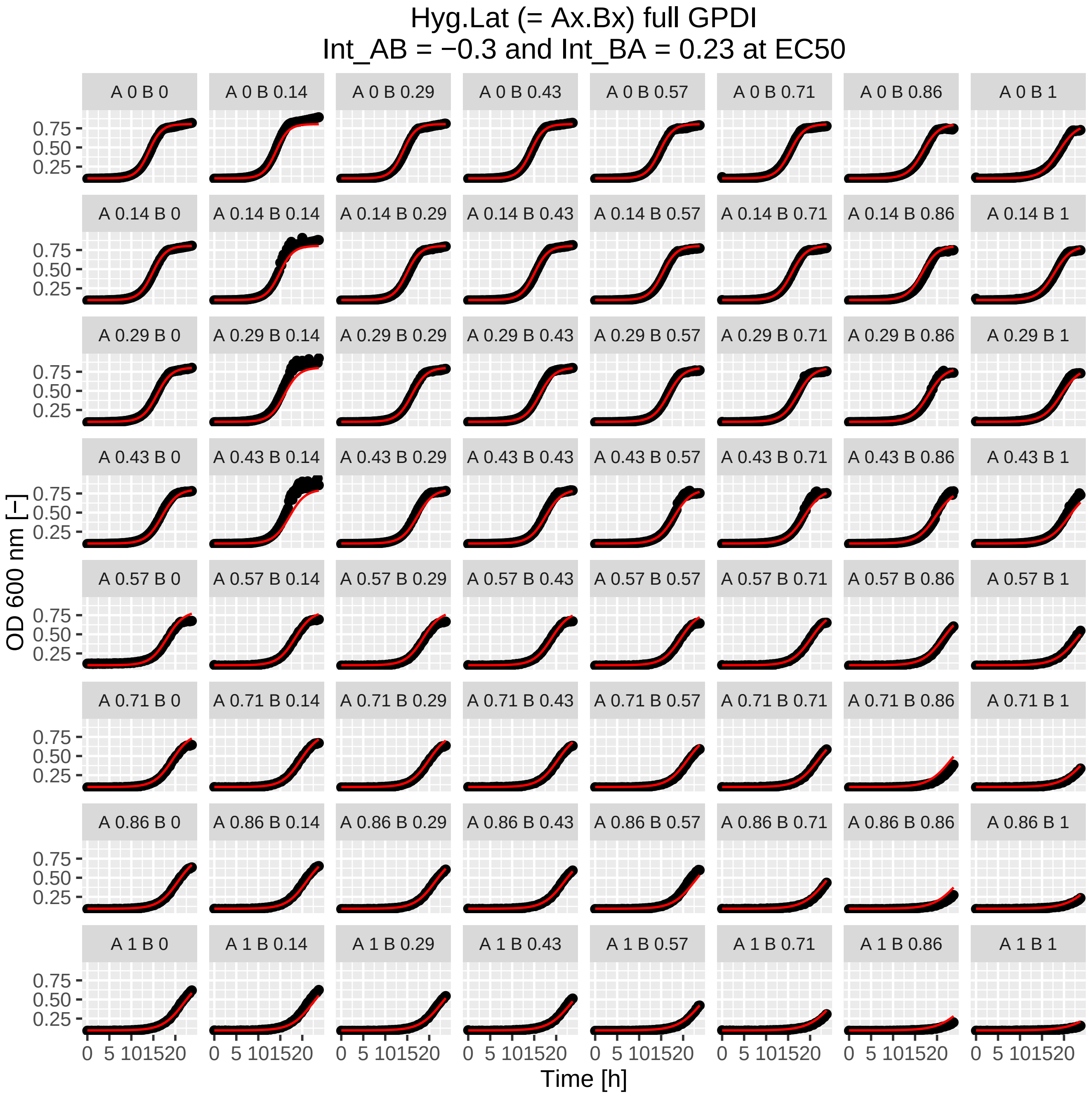

Hyg.Lat (= Ax.Bx) full GPDI
Int_AB = −0.3 and Int_BA = 0.23 at EC50
OD 600 nm [−]
Time [h]
0 5 101520
0.25
0.50
0.75
A 0 B 0
A 0 B 0.14
A 0 B 0.29
A 0 B 0.43
A 0 B 0.57
A 0 B 0.71
A 0 B 0.86
A 0 B 1
A 0.14 B 0
A 0.14 B 0.14
A 0.14 B 0.29
A 0.14 B 0.43
A 0.14 B 0.57
A 0.14 B 0.71
A 0.14 B 0.86
A 0.14 B 1
A 0.29 B 0
A 0.29 B 0.14
A 0.29 B 0.29
A 0.29 B 0.43
A 0.29 B 0.57
A 0.29 B 0.71
A 0.29 B 0.86
A 0.29 B 1
A 0.43 B 0
A 0.43 B 0.14
A 0.43 B 0.29
A 0.43 B 0.43
A 0.43 B 0.57
A 0.43 B 0.71
A 0.43 B 0.86
A 0.43 B 1
A 0.57 B 0
A 0.57 B 0.14
A 0.57 B 0.29
A 0.57 B 0.43
A 0.57 B 0.57
A 0.57 B 0.71
A 0.57 B 0.86
A 0.57 B 1
A 0.71 B 0
A 0.71 B 0.14
A 0.71 B 0.29
A 0.71 B 0.43
A 0.71 B 0.57
A 0.71 B 0.71
A 0.71 B 0.86
A 0.71 B 1
A 0.86 B 0
A 0.86 B 0.14
A 0.86 B 0.29
A 0.86 B 0.43
A 0.86 B 0.57
A 0.86 B 0.71
A 0.86 B 0.86
A 0.86 B 1
A 1 B 0
A 1 B 0.14
A 1 B 0.29
A 1 B 0.43
A 1 B 0.57
A 1 B 0.71
A 1 B 0.86
A 1 B 1

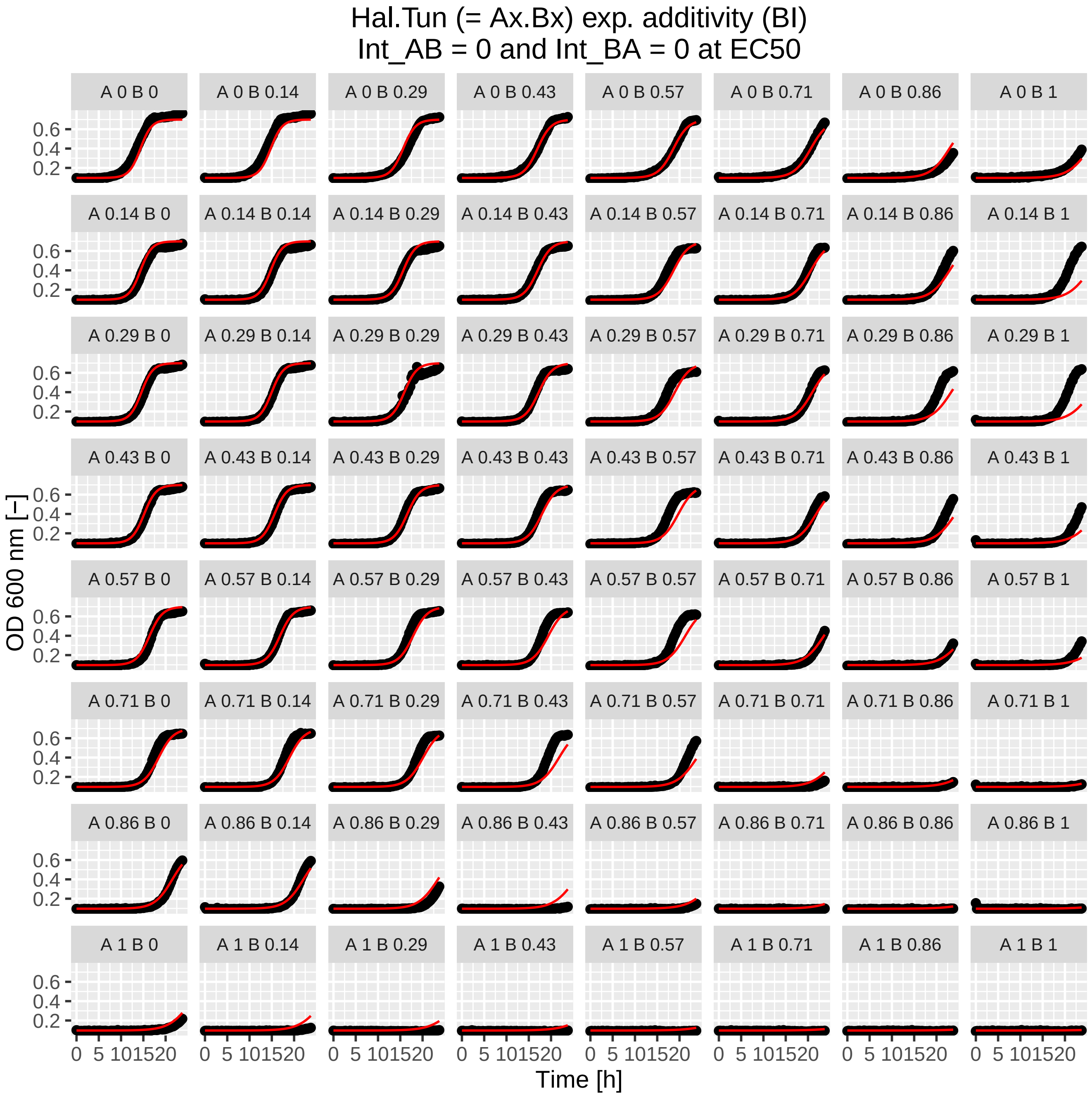
Hal.Tun (= Ax.Bx) exp. additivity (BI)
Int_AB = 0 and Int_BA = 0 at EC50
A 0 B 0
A 0 B 0.14
A 0 B 0.29
A 0 B 0.43
A 0 B 0.57
A 0 B 0.71
A 0 B 0.86
A 0 B 1
A 0.14 B 0
A 0.14 B 0.14
A 0.14 B 0.29
A 0.14 B 0.43
A 0.14 B 0.57
A 0.14 B 0.71
A 0.14 B 0.86
A 0.14 B 1
A 0.29 B 0
A 0.29 B 0.14
A 0.29 B 0.29
A 0.29 B 0.43
A 0.29 B 0.57
A 0.29 B 0.71
A 0.29 B 0.86
A 0.29 B 1
A 0.43 B 0
A 0.43 B 0.14
A 0.43 B 0.29
A 0.43 B 0.43
A 0.43 B 0.57
A 0.43 B 0.71
A 0.43 B 0.86
A 0.43 B 1
A 0.57 B 0
A 0.57 B 0.14
A 0.57 B 0.29
A 0.57 B 0.43
A 0.57 B 0.57
A 0.57 B 0.71
A 0.57 B 0.86
A 0.57 B 1
A 0.71 B 0
A 0.71 B 0.14
A 0.71 B 0.29
A 0.71 B 0.43
A 0.71 B 0.57
A 0.71 B 0.71
A 0.71 B 0.86
A 0.71 B 1
A 0.86 B 0
A 0.86 B 0.14
A 0.86 B 0.29
A 0.86 B 0.43
A 0.86 B 0.57
A 0.86 B 0.71
A 0.86 B 0.86
A 0.86 B 1
A 1 B 0
A 1 B 0.14
A 1 B 0.29
A 1 B 0.43
A 1 B 0.57
A 1 B 0.71
A 1 B 0.86
A 1 B 1
OD 600 nm [–]
0.2
0.4
0.6
0
5
10
15
20
Time [h]

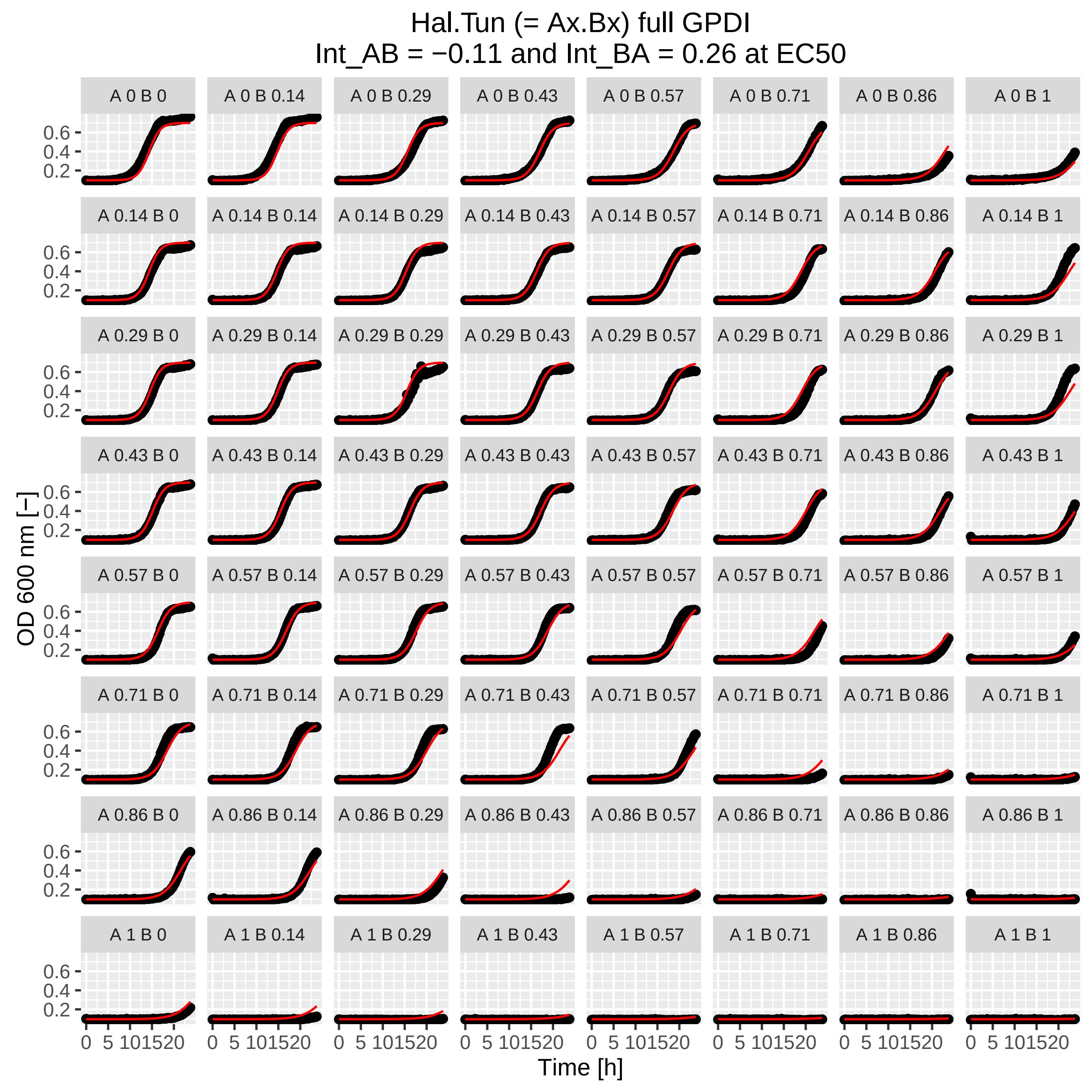

Hal.Tun (= Ax.Bx) full GPDI
Int_AB = −0.11 and Int_BA = 0.26 at EC50
OD 600 nm [−]
Time [h]
0.2
0.4
0.6
0 5 101520
A 0 B 0
A 0 B 0.14
A 0 B 0.29
A 0 B 0.43
A 0 B 0.57
A 0 B 0.71
A 0 B 0.86
A 0 B 1
A 0.14 B 0
A 0.14 B 0.14
A 0.14 B 0.29
A 0.14 B 0.43
A 0.14 B 0.57
A 0.14 B 0.71
A 0.14 B 0.86
A 0.14 B 1
A 0.29 B 0
A 0.29 B 0.14
A 0.29 B 0.29
A 0.29 B 0.43
A 0.29 B 0.57
A 0.29 B 0.71
A 0.29 B 0.86
A 0.29 B 1
A 0.43 B 0
A 0.43 B 0.14
A 0.43 B 0.29
A 0.43 B 0.43
A 0.43 B 0.57
A 0.43 B 0.71
A 0.43 B 0.86
A 0.43 B 1
A 0.57 B 0
A 0.57 B 0.14
A 0.57 B 0.29
A 0.57 B 0.43
A 0.57 B 0.57
A 0.57 B 0.71
A 0.57 B 0.86
A 0.57 B 1
A 0.71 B 0
A 0.71 B 0.14
A 0.71 B 0.29
A 0.71 B 0.43
A 0.71 B 0.57
A 0.71 B 0.71
A 0.71 B 0.86
A 0.71 B 1
A 0.86 B 0
A 0.86 B 0.14
A 0.86 B 0.29
A 0.86 B 0.43
A 0.86 B 0.57
A 0.86 B 0.71
A 0.86 B 0.86
A 0.86 B 1
A 1 B 0
A 1 B 0.14
A 1 B 0.29
A 1 B 0.43
A 1 B 0.57
A 1 B 0.71
A 1 B 0.86
A 1 B 1

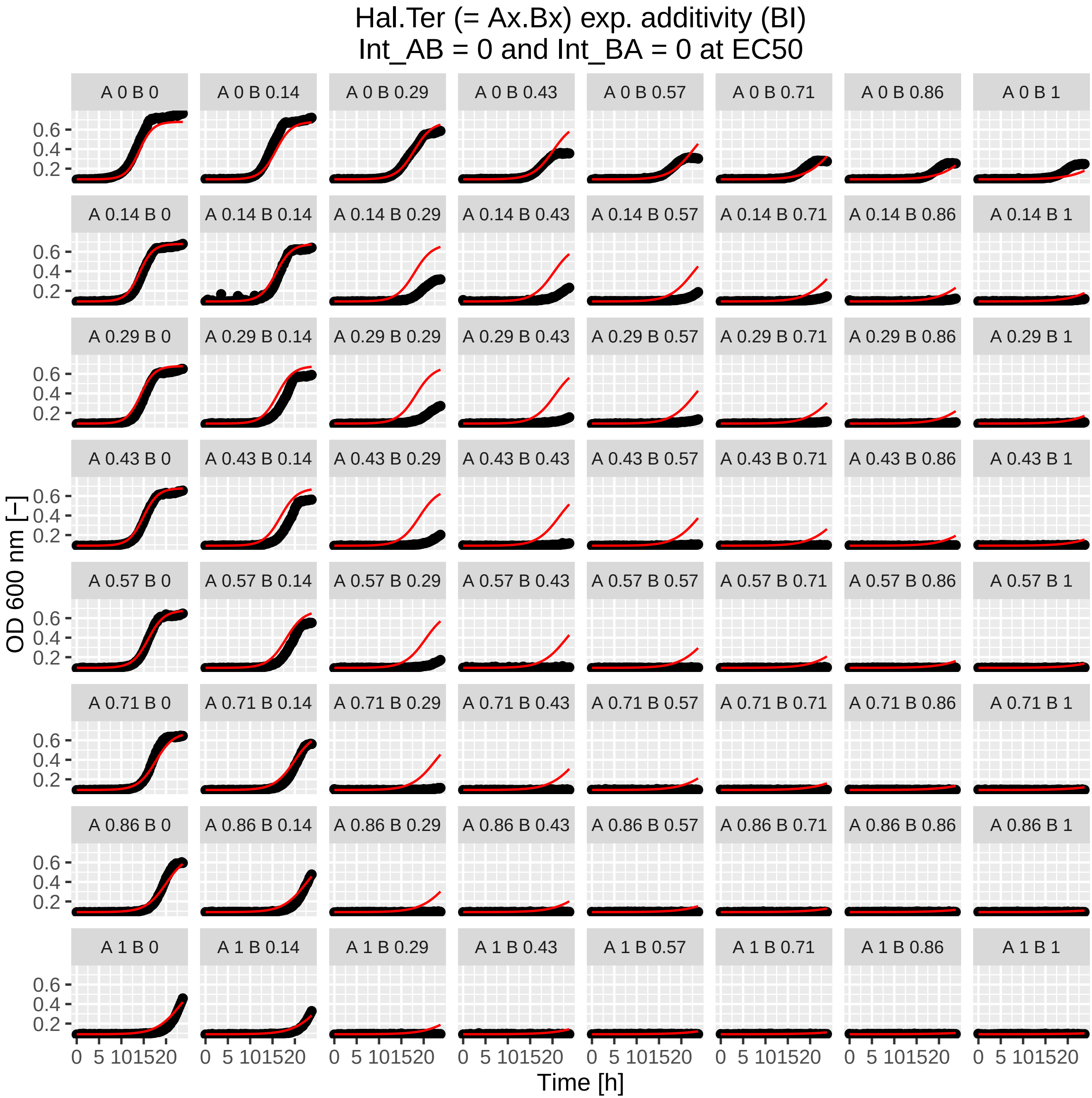

Hal.Ter (= Ax.Bx) exp. additivity (BI)
Int_AB = 0 and Int_BA = 0 at EC50
A 0 B 0
A 0 B 0.14
A 0 B 0.29
A 0 B 0.43
A 0 B 0.57
A 0 B 0.71
A 0 B 0.86
A 0 B 1
A 0.14 B 0
A 0.14 B 0.14
A 0.14 B 0.29
A 0.14 B 0.43
A 0.14 B 0.57
A 0.14 B 0.71
A 0.14 B 0.86
A 0.14 B 1
A 0.29 B 0
A 0.29 B 0.14
A 0.29 B 0.29
A 0.29 B 0.43
A 0.29 B 0.57
A 0.29 B 0.71
A 0.29 B 0.86
A 0.29 B 1
A 0.43 B 0
A 0.43 B 0.14
A 0.43 B 0.29
A 0.43 B 0.43
A 0.43 B 0.57
A 0.43 B 0.71
A 0.43 B 0.86
A 0.43 B 1
A 0.57 B 0
A 0.57 B 0.14
A 0.57 B 0.29
A 0.57 B 0.43
A 0.57 B 0.57
A 0.57 B 0.71
A 0.57 B 0.86
A 0.57 B 1
A 0.71 B 0
A 0.71 B 0.14
A 0.71 B 0.29
A 0.71 B 0.43
A 0.71 B 0.57
A 0.71 B 0.71
A 0.71 B 0.86
A 0.71 B 1
A 0.86 B 0
A 0.86 B 0.14
A 0.86 B 0.29
A 0.86 B 0.43
A 0.86 B 0.57
A 0.86 B 0.71
A 0.86 B 0.86
A 0.86 B 1
A 1 B 0
A 1 B 0.14
A 1 B 0.29
A 1 B 0.43
A 1 B 0.57
A 1 B 0.71
A 1 B 0.86
A 1 B 1
OD 600 nm [–]
0.2
0.4
0.6
0 5 101520
Time [h]

Hal.Ter (= Ax.Bx) full GPDI
Int_AB = 0.43 and Int_BA = −0.67 at EC50

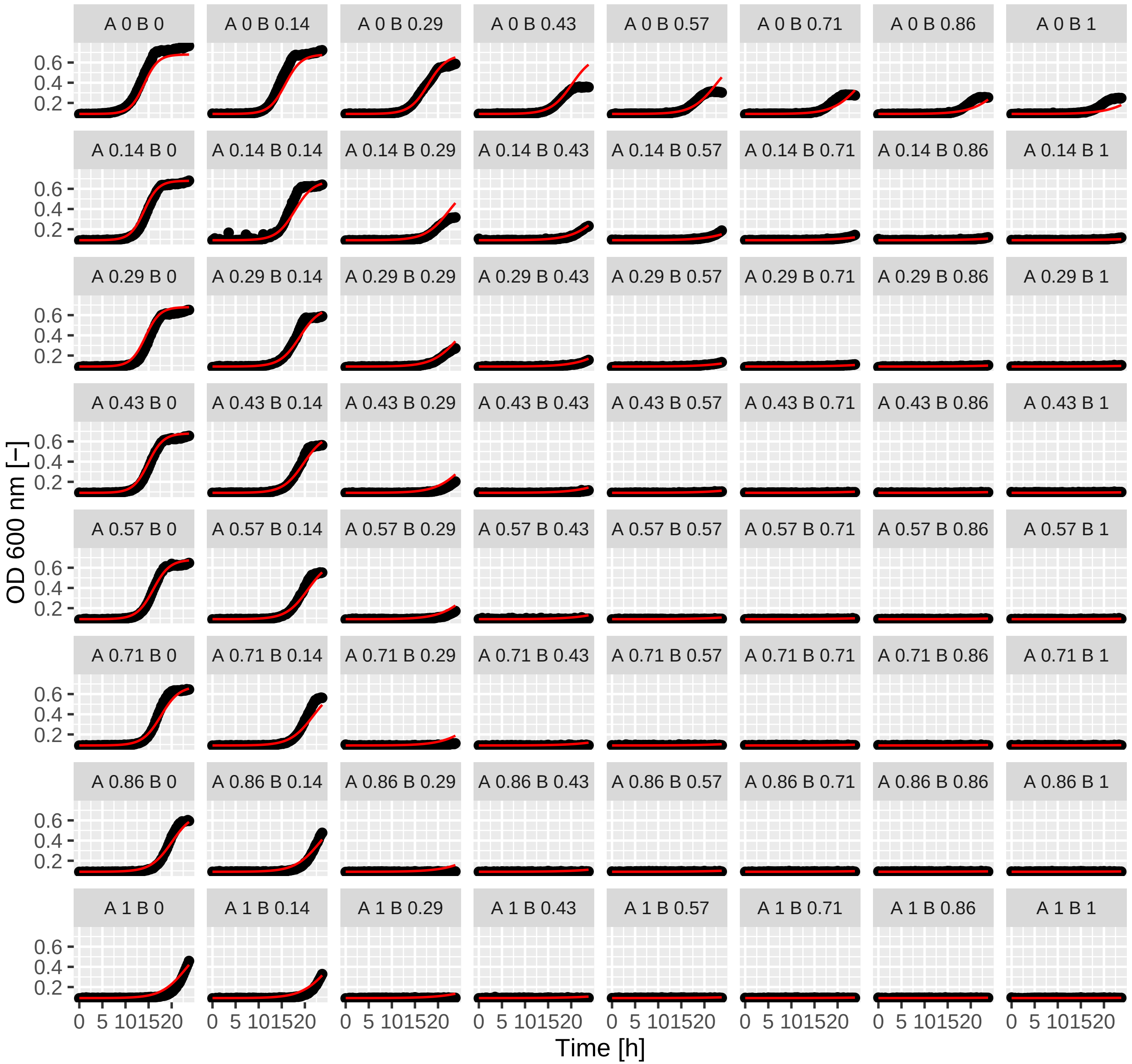

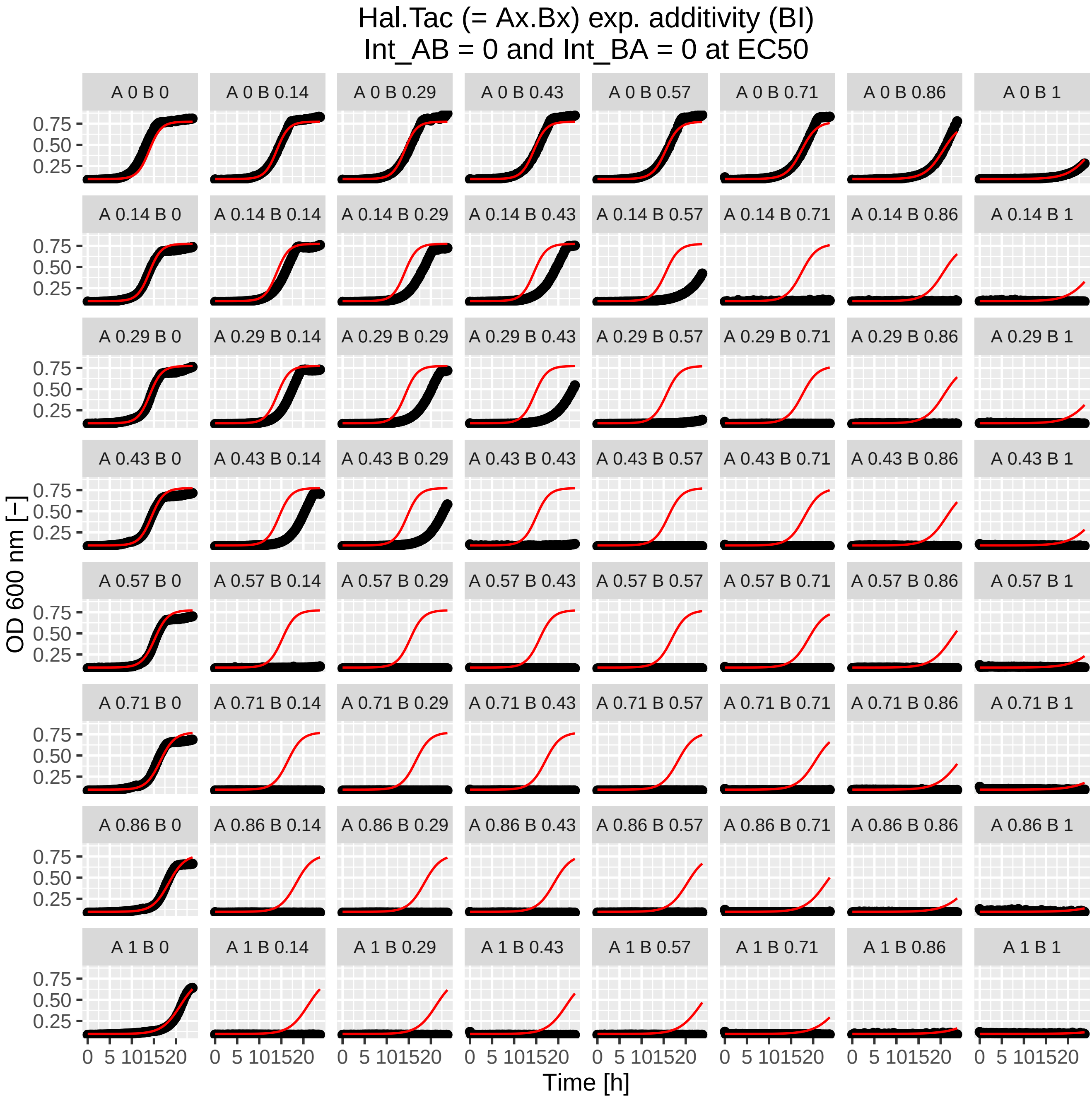
Hal.Tac (= Ax.Bx) exp. additivity (BI)
Int_AB = 0 and Int_BA = 0 at EC50
OD 600 nm [−]
Time [h]
0.75
0.50
0.25
0 5 101520
A 0 B 0
A 0 B 0.14
A 0 B 0.29
A 0 B 0.43
A 0 B 0.57
A 0 B 0.71
A 0 B 0.86
A 0 B 1
A 0.14 B 0
A 0.14 B 0.14
A 0.14 B 0.29
A 0.14 B 0.43
A 0.14 B 0.57
A 0.14 B 0.71
A 0.14 B 0.86
A 0.14 B 1
A 0.29 B 0
A 0.29 B 0.14
A 0.29 B 0.29
A 0.29 B 0.43
A 0.29 B 0.57
A 0.29 B 0.71
A 0.29 B 0.86
A 0.29 B 1
A 0.43 B 0
A 0.43 B 0.14
A 0.43 B 0.29
A 0.43 B 0.43
A 0.43 B 0.57
A 0.43 B 0.71
A 0.43 B 0.86
A 0.43 B 1
A 0.57 B 0
A 0.57 B 0.14
A 0.57 B 0.29
A 0.57 B 0.43
A 0.57 B 0.57
A 0.57 B 0.71
A 0.57 B 0.86
A 0.57 B 1
A 0.71 B 0
A 0.71 B 0.14
A 0.71 B 0.29
A 0.71 B 0.43
A 0.71 B 0.57
A 0.71 B 0.71
A 0.71 B 0.86
A 0.71 B 1
A 0.86 B 0
A 0.86 B 0.14
A 0.86 B 0.29
A 0.86 B 0.43
A 0.86 B 0.57
A 0.86 B 0.71
A 0.86 B 0.86
A 0.86 B 1
A 1 B 0
A 1 B 0.14
A 1 B 0.29
A 1 B 0.43
A 1 B 0.57
A 1 B 0.71
A 1 B 0.86
A 1 B 1

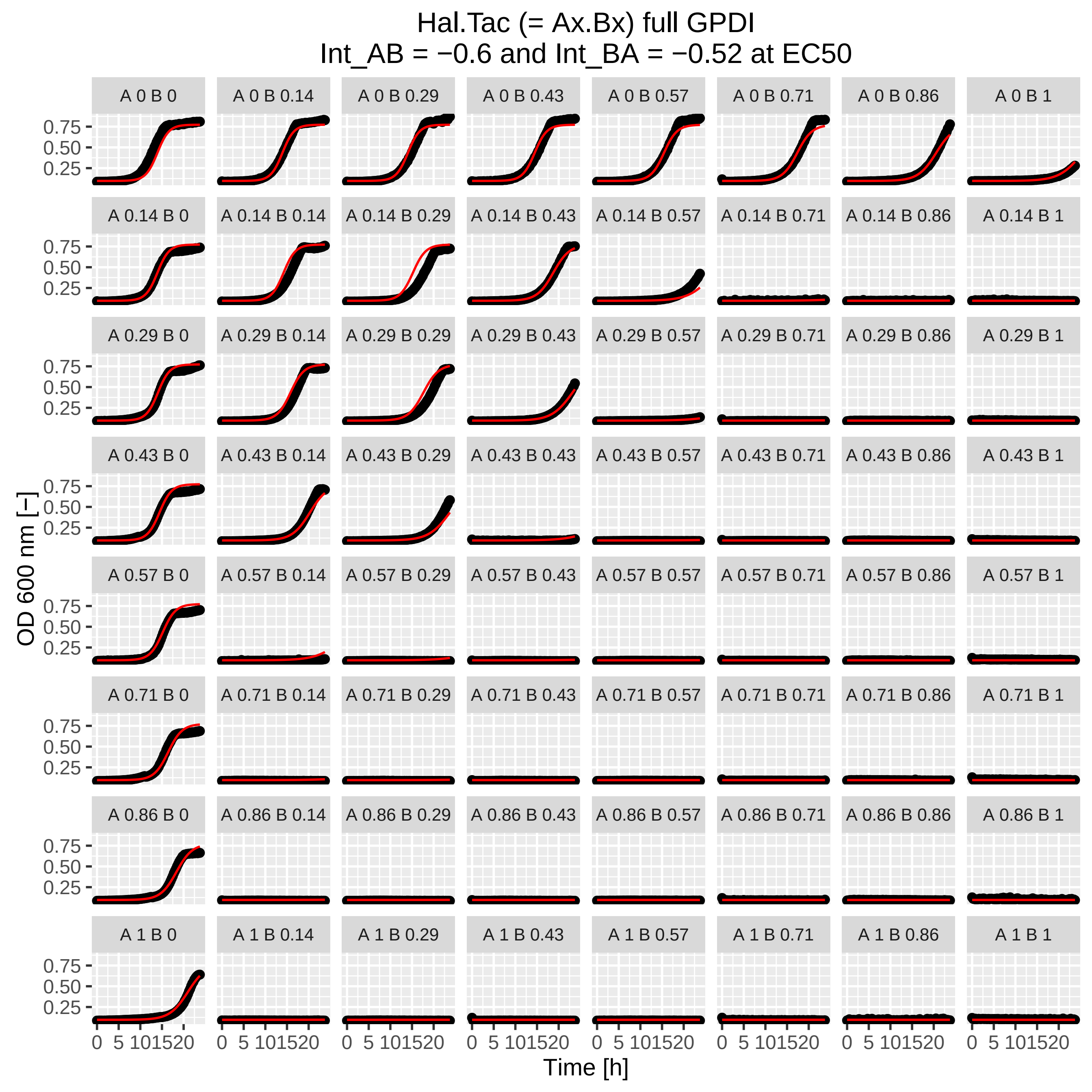
Hal.Tac (= Ax.Bx) full GPDI
Int_AB = −0.6 and Int_BA = −0.52 at EC50
OD 600 nm [−]
Time [h]

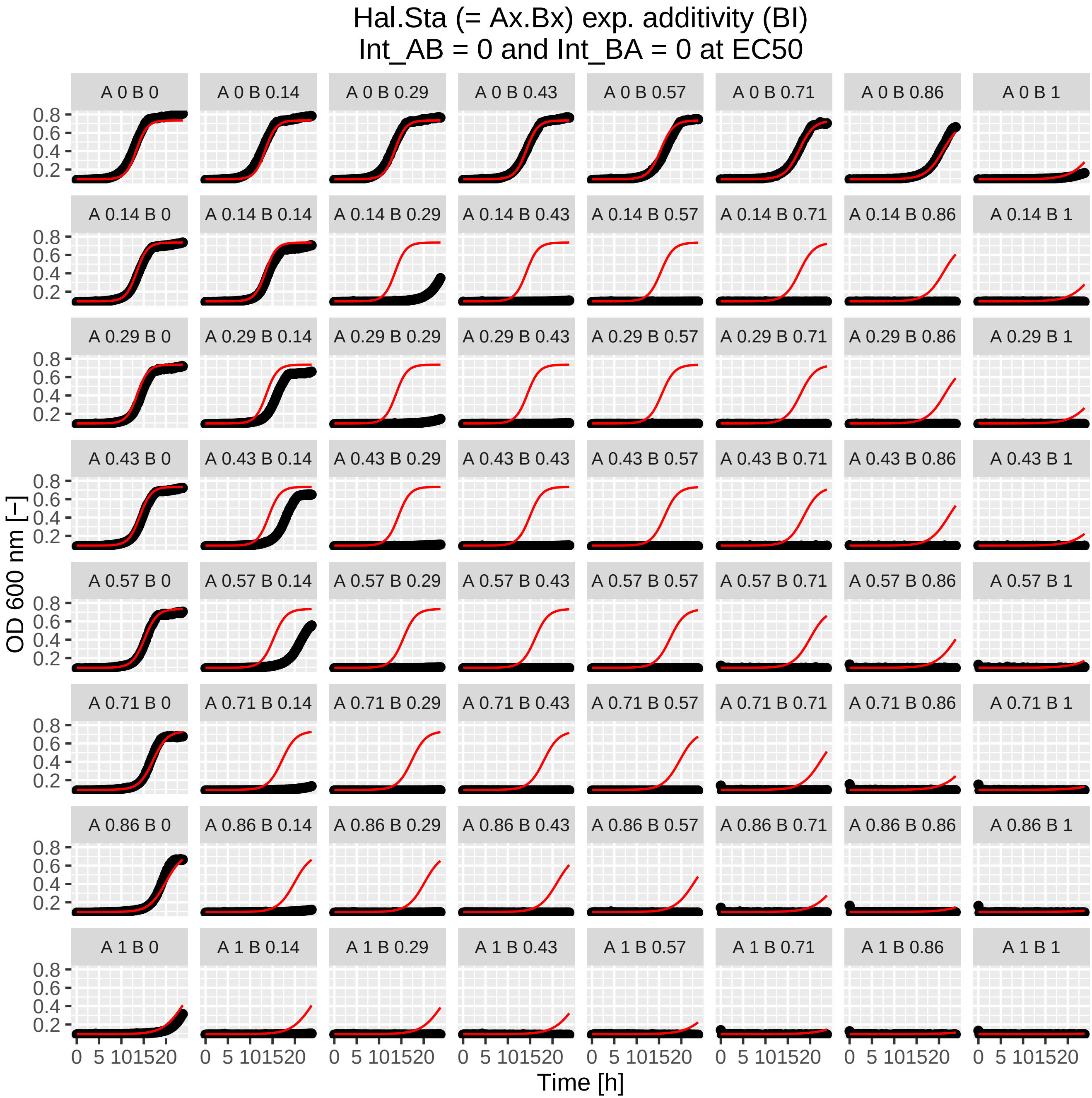
Hal.Sta (= Ax.Bx) exp. additivity (BI)
Int_AB = 0 and Int_BA = 0 at EC50
OD 600 nm [−]
Time [h]
A 0 B 0
A 0 B 0.14
A 0 B 0.29
A 0 B 0.43
A 0 B 0.57
A 0 B 0.71
A 0 B 0.86
A 0 B 1
A 0.14 B 0
A 0.14 B 0.14
A 0.14 B 0.29
A 0.14 B 0.43
A 0.14 B 0.57
A 0.14 B 0.71
A 0.14 B 0.86
A 0.14 B 1
A 0.29 B 0
A 0.29 B 0.14
A 0.29 B 0.29
A 0.29 B 0.43
A 0.29 B 0.57
A 0.29 B 0.71
A 0.29 B 0.86
A 0.29 B 1
A 0.43 B 0
A 0.43 B 0.14
A 0.43 B 0.29
A 0.43 B 0.43
A 0.43 B 0.57
A 0.43 B 0.71
A 0.43 B 0.86
A 0.43 B 1
A 0.57 B 0
A 0.57 B 0.14
A 0.57 B 0.29
A 0.57 B 0.43
A 0.57 B 0.57
A 0.57 B 0.71
A 0.57 B 0.86
A 0.57 B 1
A 0.71 B 0
A 0.71 B 0.14
A 0.71 B 0.29
A 0.71 B 0.43
A 0.71 B 0.57
A 0.71 B 0.71
A 0.71 B 0.86
A 0.71 B 1
A 0.86 B 0
A 0.86 B 0.14
A 0.86 B 0.29
A 0.86 B 0.43
A 0.86 B 0.57
A 0.86 B 0.71
A 0.86 B 0.86
A 0.86 B 1
A 1 B 0
A 1 B 0.14
A 1 B 0.29
A 1 B 0.43
A 1 B 0.57
A 1 B 0.71
A 1 B 0.86
A 1 B 1
0.8
0.6
0.4
0.2
0 5 101520

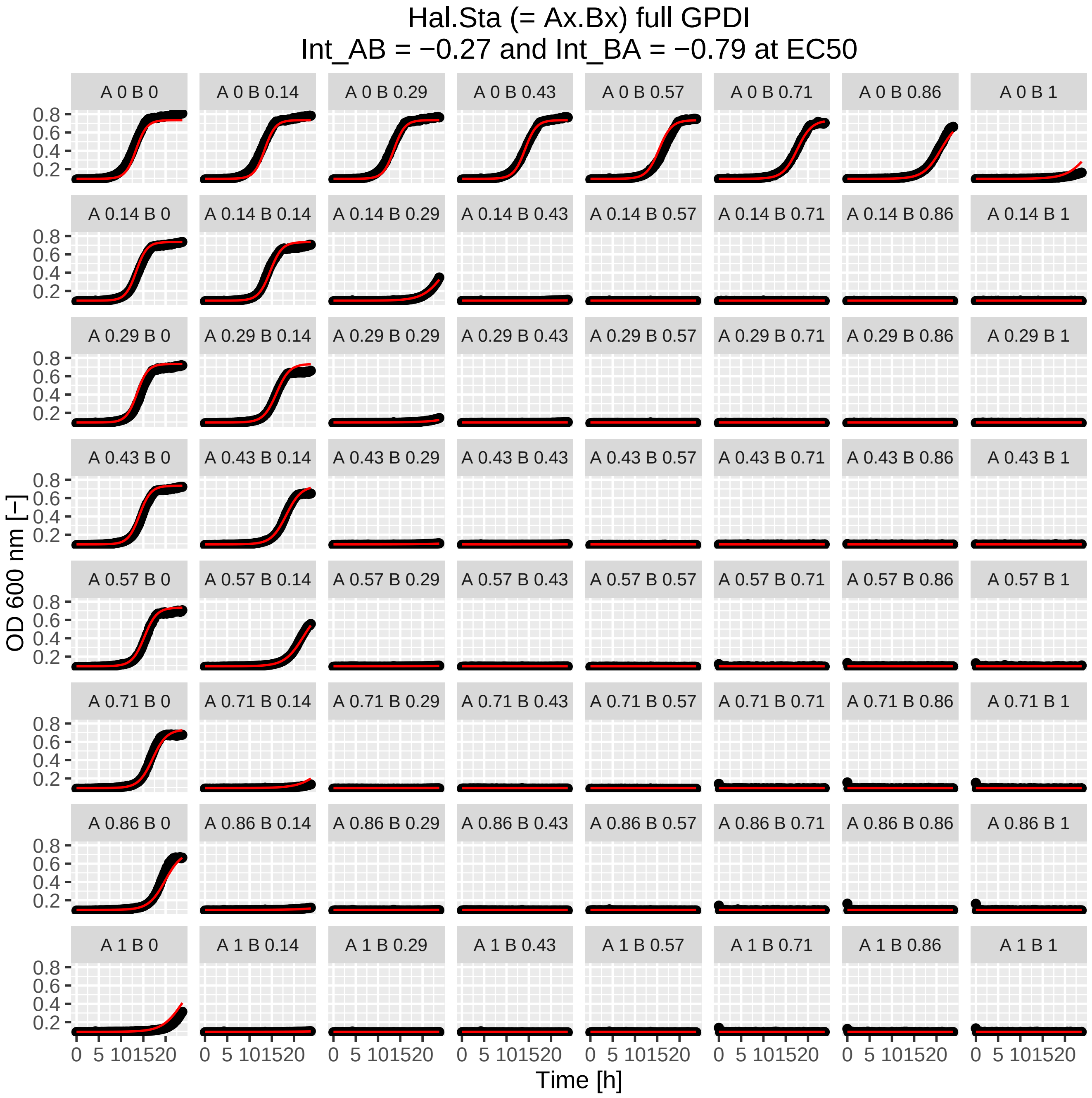

Hal.Sta (= Ax.Bx) full GPDI
Int_AB = −0.27 and Int_BA = −0.79 at EC50
A 0 B 0
A 0 B 0.14
A 0 B 0.29
A 0 B 0.43
A 0 B 0.57
A 0 B 0.71
A 0 B 0.86
A 0 B 1
A 0.14 B 0
A 0.14 B 0.14
A 0.14 B 0.29
A 0.14 B 0.43
A 0.14 B 0.57
A 0.14 B 0.71
A 0.14 B 0.86
A 0.14 B 1
A 0.29 B 0
A 0.29 B 0.14
A 0.29 B 0.29
A 0.29 B 0.43
A 0.29 B 0.57
A 0.29 B 0.71
A 0.29 B 0.86
A 0.29 B 1
A 0.43 B 0
A 0.43 B 0.14
A 0.43 B 0.29
A 0.43 B 0.43
A 0.43 B 0.57
A 0.43 B 0.71
A 0.43 B 0.86
A 0.43 B 1
A 0.57 B 0
A 0.57 B 0.14
A 0.57 B 0.29
A 0.57 B 0.43
A 0.57 B 0.57
A 0.57 B 0.71
A 0.57 B 0.86
A 0.57 B 1
A 0.71 B 0
A 0.71 B 0.14
A 0.71 B 0.29
A 0.71 B 0.43
A 0.71 B 0.57
A 0.71 B 0.71
A 0.71 B 0.86
A 0.71 B 1
A 0.86 B 0
A 0.86 B 0.14
A 0.86 B 0.29
A 0.86 B 0.43
A 0.86 B 0.57
A 0.86 B 0.71
A 0.86 B 0.86
A 0.86 B 1
A 1 B 0
A 1 B 0.14
A 1 B 0.29
A 1 B 0.43
A 1 B 0.57
A 1 B 0.71
A 1 B 0.86
A 1 B 1
0.8
0.6
0.4
0.2
OD 600 nm [−]
0 5 101520
Time [h]

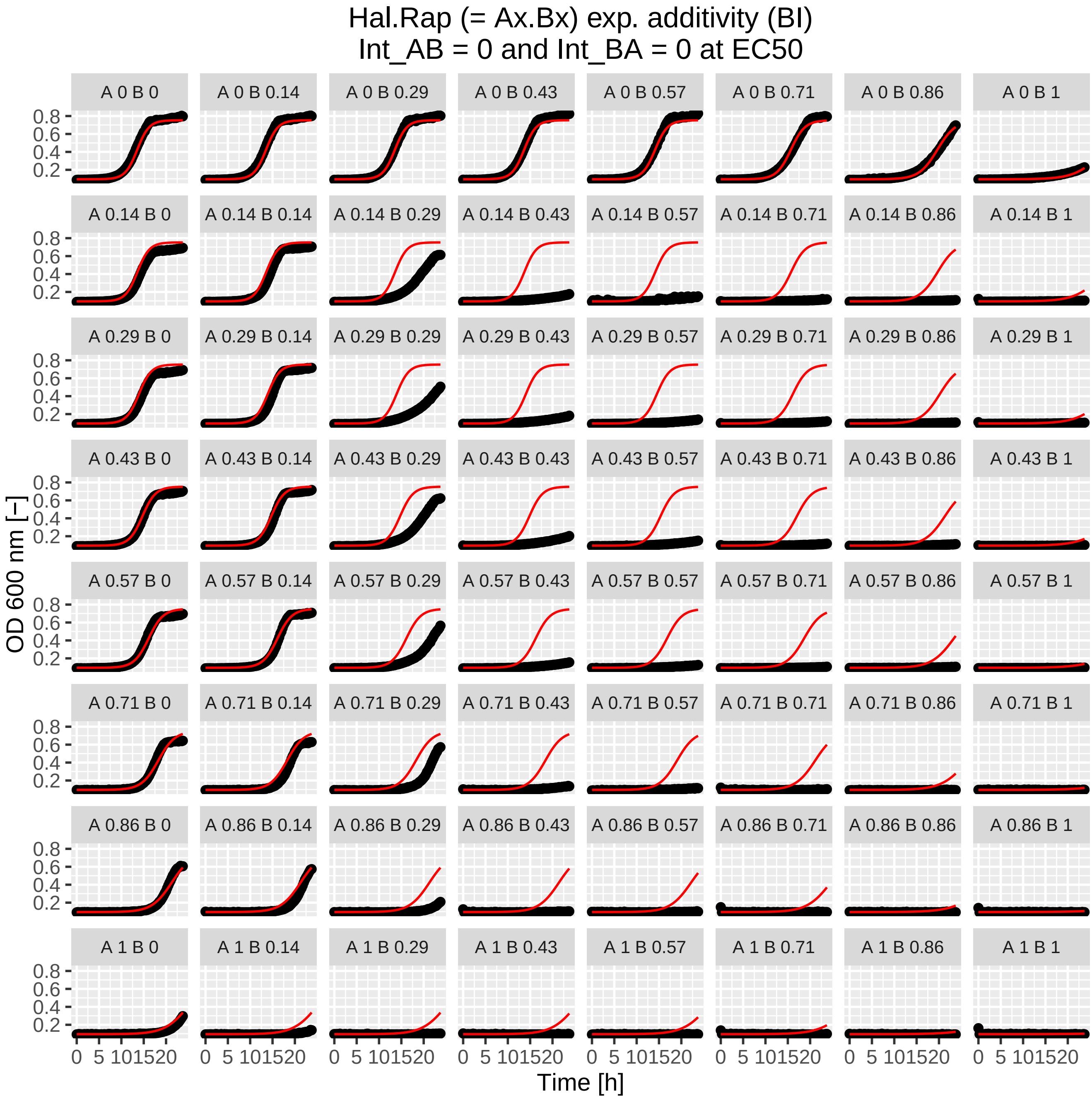
Hal.Rap (= Ax.Bx) exp. additivity (BI)
Int_AB = 0 and Int_BA = 0 at EC50
A 0 B 0
A 0 B 0.14
A 0 B 0.29
A 0 B 0.43
A 0 B 0.57
A 0 B 0.71
A 0 B 0.86
A 0 B 1
A 0.14 B 0
A 0.14 B 0.14
A 0.14 B 0.29
A 0.14 B 0.43
A 0.14 B 0.57
A 0.14 B 0.71
A 0.14 B 0.86
A 0.14 B 1
A 0.29 B 0
A 0.29 B 0.14
A 0.29 B 0.29
A 0.29 B 0.43
A 0.29 B 0.57
A 0.29 B 0.71
A 0.29 B 0.86
A 0.29 B 1
A 0.43 B 0
A 0.43 B 0.14
A 0.43 B 0.29
A 0.43 B 0.43
A 0.43 B 0.57
A 0.43 B 0.71
A 0.43 B 0.86
A 0.43 B 1
A 0.57 B 0
A 0.57 B 0.14
A 0.57 B 0.29
A 0.57 B 0.43
A 0.57 B 0.57
A 0.57 B 0.71
A 0.57 B 0.86
A 0.57 B 1
A 0.71 B 0
A 0.71 B 0.14
A 0.71 B 0.29
A 0.71 B 0.43
A 0.71 B 0.57
A 0.71 B 0.71
A 0.71 B 0.86
A 0.71 B 1
A 0.86 B 0
A 0.86 B 0.14
A 0.86 B 0.29
A 0.86 B 0.43
A 0.86 B 0.57
A 0.86 B 0.71
A 0.86 B 0.86
A 0.86 B 1
A 1 B 0
A 1 B 0.14
A 1 B 0.29
A 1 B 0.43
A 1 B 0.57
A 1 B 0.71
A 1 B 0.86
A 1 B 1
0.2
0.4
0.6
0.8
0 5 101520
OD 600 nm [–]
Time [h]

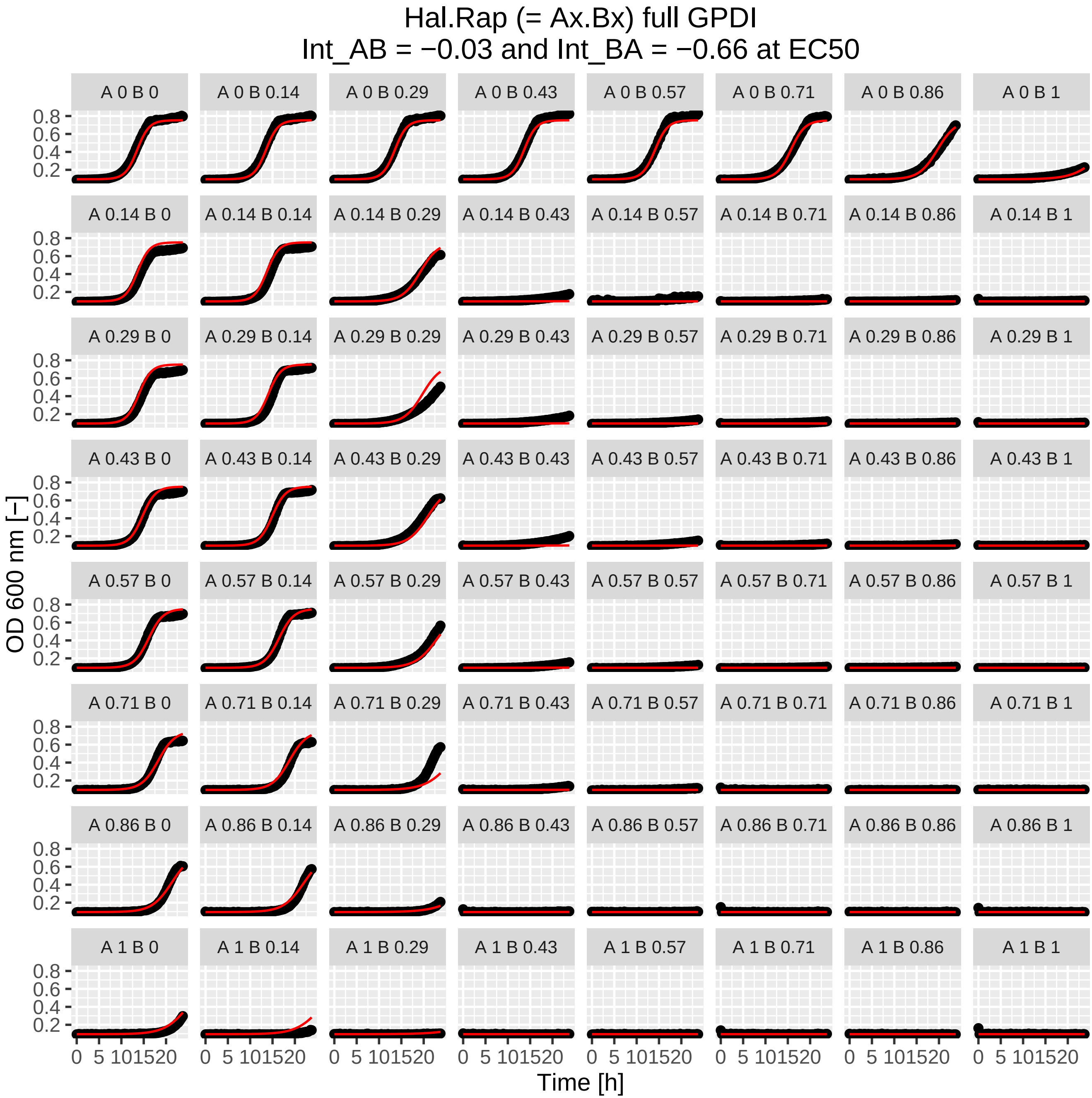

Hal.Rap (= Ax.Bx) full GPDI
Int_AB = −0.03 and Int_BA = −0.66 at EC50
A 0 B 0
A 0 B 0.14
A 0 B 0.29
A 0 B 0.43
A 0 B 0.57
A 0 B 0.71
A 0 B 0.86
A 0 B 1
A 0.14 B 0
A 0.14 B 0.14
A 0.14 B 0.29
A 0.14 B 0.43
A 0.14 B 0.57
A 0.14 B 0.71
A 0.14 B 0.86
A 0.14 B 1
A 0.29 B 0
A 0.29 B 0.14
A 0.29 B 0.29
A 0.29 B 0.43
A 0.29 B 0.57
A 0.29 B 0.71
A 0.29 B 0.86
A 0.29 B 1
A 0.43 B 0
A 0.43 B 0.14
A 0.43 B 0.29
A 0.43 B 0.43
A 0.43 B 0.57
A 0.43 B 0.71
A 0.43 B 0.86
A 0.43 B 1
A 0.57 B 0
A 0.57 B 0.14
A 0.57 B 0.29
A 0.57 B 0.43
A 0.57 B 0.57
A 0.57 B 0.71
A 0.57 B 0.86
A 0.57 B 1
A 0.71 B 0
A 0.71 B 0.14
A 0.71 B 0.29
A 0.71 B 0.43
A 0.71 B 0.57
A 0.71 B 0.71
A 0.71 B 0.86
A 0.71 B 1
A 0.86 B 0
A 0.86 B 0.14
A 0.86 B 0.29
A 0.86 B 0.43
A 0.86 B 0.57
A 0.86 B 0.71
A 0.86 B 0.86
A 0.86 B 1
A 1 B 0
A 1 B 0.14
A 1 B 0.29
A 1 B 0.43
A 1 B 0.57
A 1 B 0.71
A 1 B 0.86
A 1 B 1
OD 600 nm [−]
0.8
0.6
0.4
0.2
0 5 101520
Time [h]

Hal.Pen (= Ax.Bx) exp. additivity (BI)
Int_AB = 0 and Int_BA = 0 at EC50

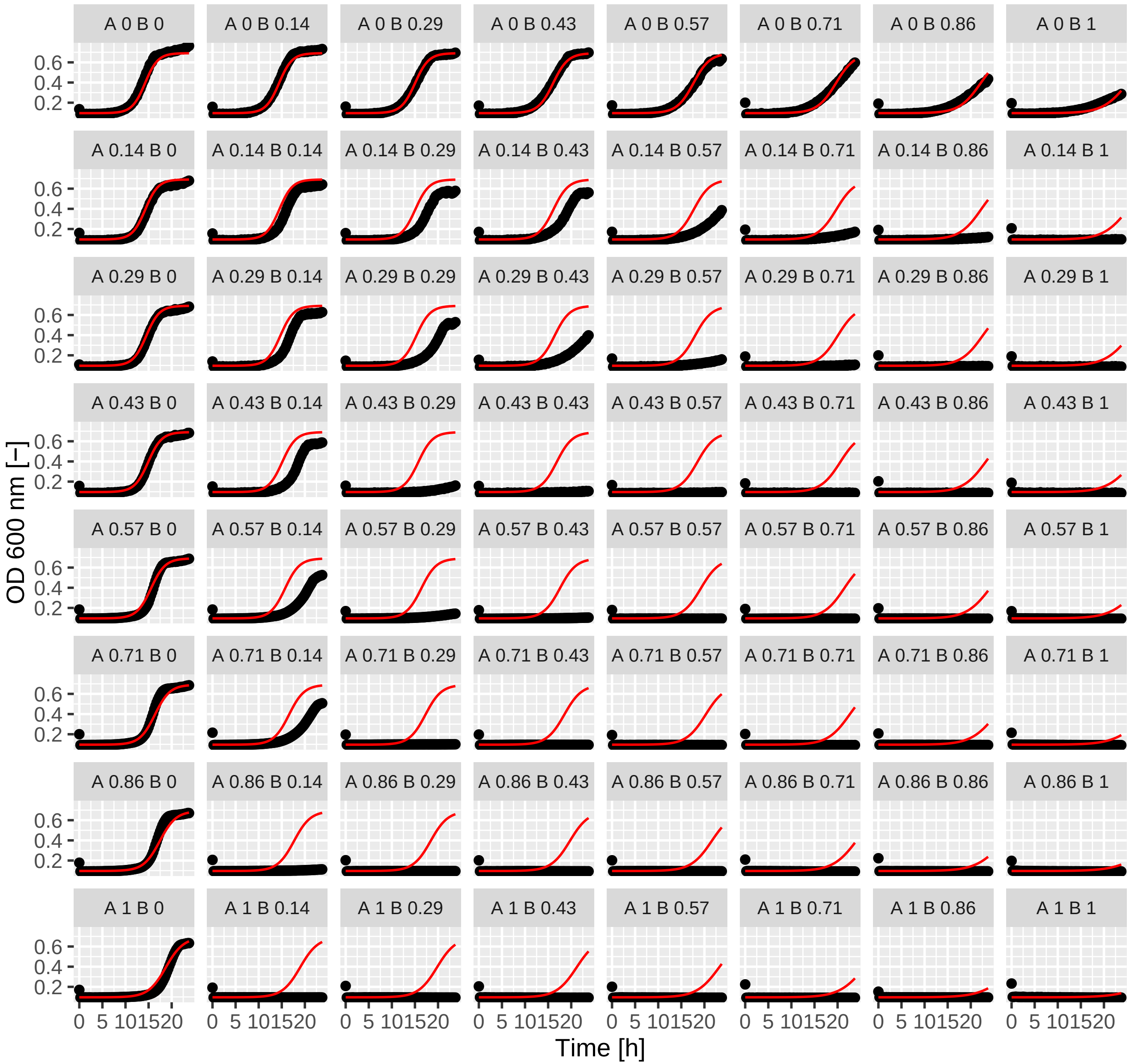

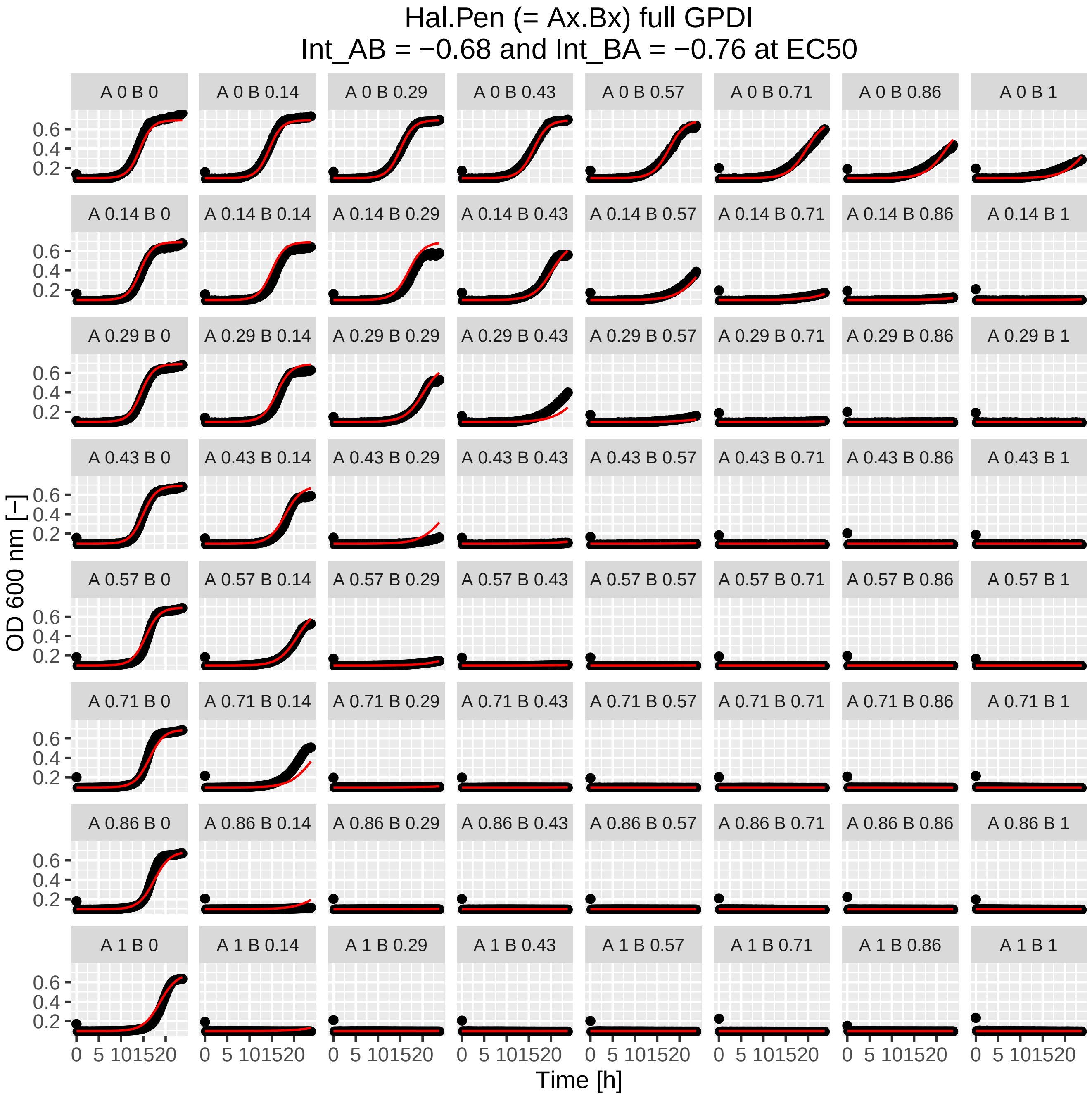
Hal.Pen (= Ax.Bx) full GPDI
Int_AB = −0.68 and Int_BA = −0.76 at EC50
A 0 B 0
A 0 B 0.14
A 0 B 0.29
A 0 B 0.43
A 0 B 0.57
A 0 B 0.71
A 0 B 0.86
A 0 B 1
A 0.14 B 0
A 0.14 B 0.14
A 0.14 B 0.29
A 0.14 B 0.43
A 0.14 B 0.57
A 0.14 B 0.71
A 0.14 B 0.86
A 0.14 B 1
A 0.29 B 0
A 0.29 B 0.14
A 0.29 B 0.29
A 0.29 B 0.43
A 0.29 B 0.57
A 0.29 B 0.71
A 0.29 B 0.86
A 0.29 B 1
A 0.43 B 0
A 0.43 B 0.14
A 0.43 B 0.29
A 0.43 B 0.43
A 0.43 B 0.57
A 0.43 B 0.71
A 0.43 B 0.86
A 0.43 B 1
A 0.57 B 0
A 0.57 B 0.14
A 0.57 B 0.29
A 0.57 B 0.43
A 0.57 B 0.57
A 0.57 B 0.71
A 0.57 B 0.86
A 0.57 B 1
A 0.71 B 0
A 0.71 B 0.14
A 0.71 B 0.29
A 0.71 B 0.43
A 0.71 B 0.57
A 0.71 B 0.71
A 0.71 B 0.86
A 0.71 B 1
A 0.86 B 0
A 0.86 B 0.14
A 0.86 B 0.29
A 0.86 B 0.43
A 0.86 B 0.57
A 0.86 B 0.71
A 0.86 B 0.86
A 0.86 B 1
A 1 B 0
A 1 B 0.14
A 1 B 0.29
A 1 B 0.43
A 1 B 0.57
A 1 B 0.71
A 1 B 0.86
A 1 B 1
0.6
0.4
0.2
0 5 101520
OD 600 nm [−]
Time [h]

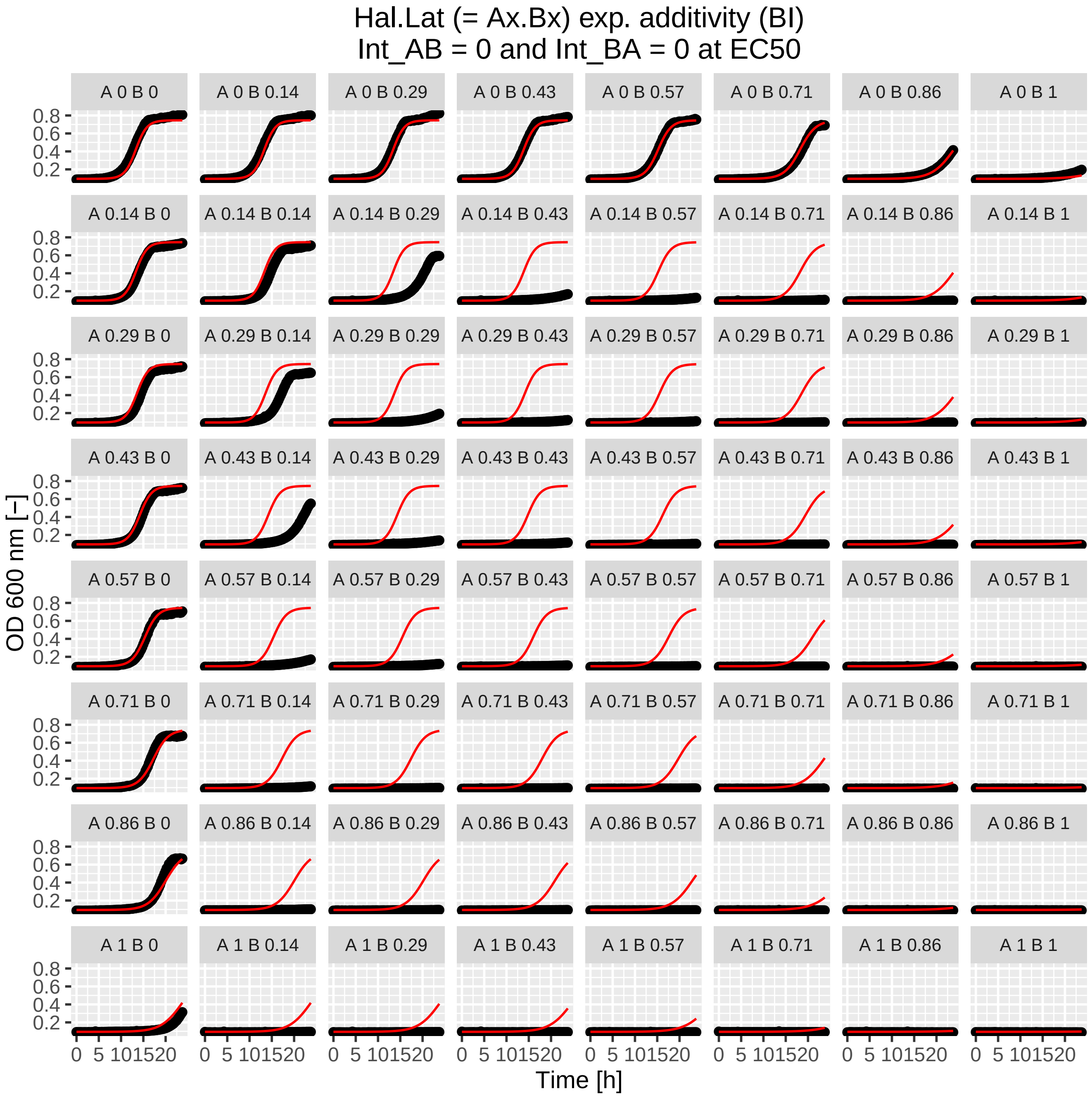

Hal.Lat (= Ax.Bx) exp. additivity (BI)
Int_AB = 0 and Int_BA = 0 at EC50
A 0 B 0
A 0 B 0.14
A 0 B 0.29
A 0 B 0.43
A 0 B 0.57
A 0 B 0.71
A 0 B 0.86
A 0 B 1
A 0.14 B 0
A 0.14 B 0.14
A 0.14 B 0.29
A 0.14 B 0.43
A 0.14 B 0.57
A 0.14 B 0.71
A 0.14 B 0.86
A 0.14 B 1
A 0.29 B 0
A 0.29 B 0.14
A 0.29 B 0.29
A 0.29 B 0.43
A 0.29 B 0.57
A 0.29 B 0.71
A 0.29 B 0.86
A 0.29 B 1
A 0.43 B 0
A 0.43 B 0.14
A 0.43 B 0.29
A 0.43 B 0.43
A 0.43 B 0.57
A 0.43 B 0.71
A 0.43 B 0.86
A 0.43 B 1
A 0.57 B 0
A 0.57 B 0.14
A 0.57 B 0.29
A 0.57 B 0.43
A 0.57 B 0.57
A 0.57 B 0.71
A 0.57 B 0.86
A 0.57 B 1
A 0.71 B 0
A 0.71 B 0.14
A 0.71 B 0.29
A 0.71 B 0.43
A 0.71 B 0.57
A 0.71 B 0.71
A 0.71 B 0.86
A 0.71 B 1
A 0.86 B 0
A 0.86 B 0.14
A 0.86 B 0.29
A 0.86 B 0.43
A 0.86 B 0.57
A 0.86 B 0.71
A 0.86 B 0.86
A 0.86 B 1
A 1 B 0
A 1 B 0.14
A 1 B 0.29
A 1 B 0.43
A 1 B 0.57
A 1 B 0.71
A 1 B 0.86
A 1 B 1
0.8
0.6
0.4
0.2
OD 600 nm [–]
0 5 101520
Time [h]

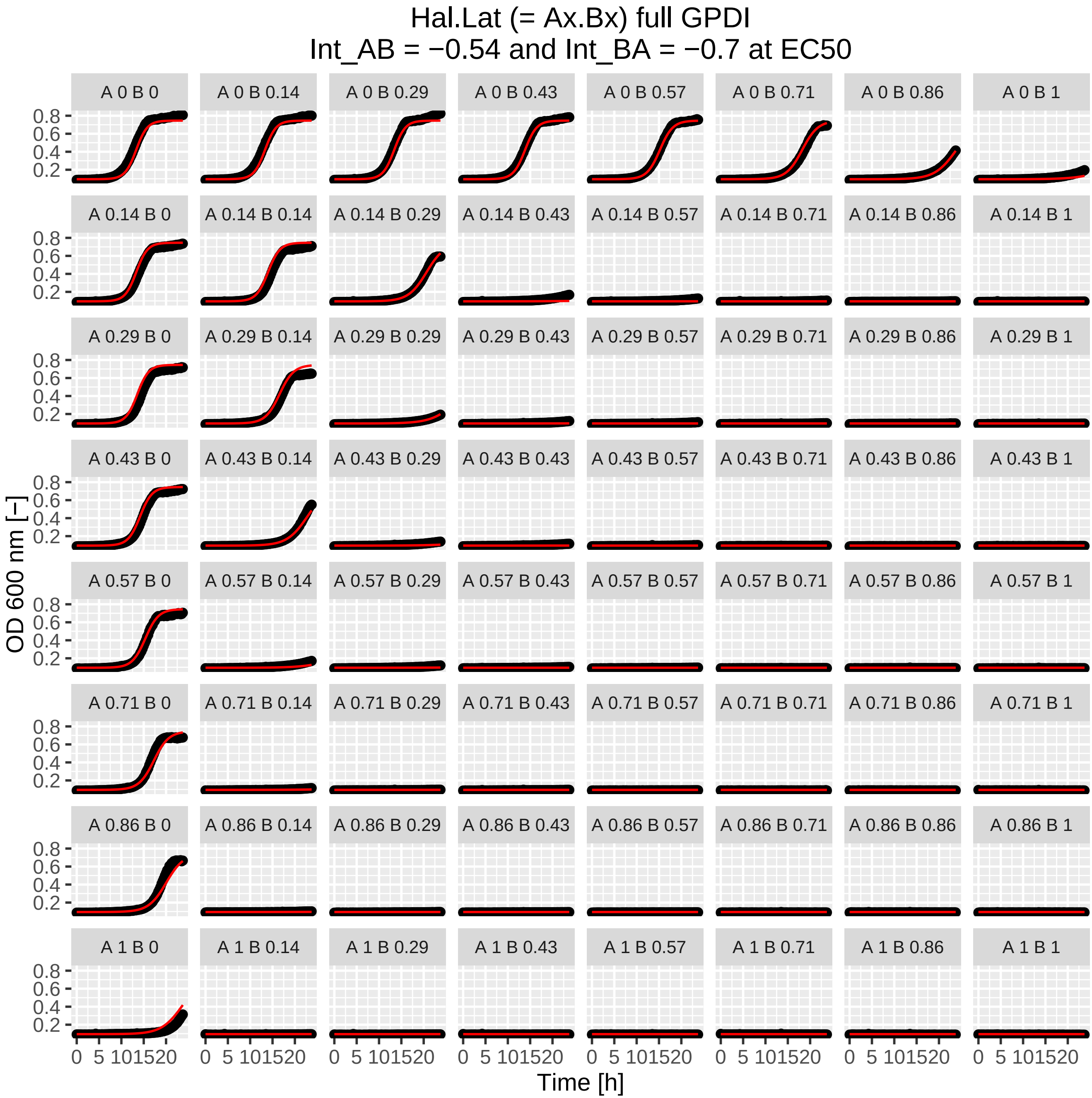

Hal.Lat (= Ax.Bx) full GPDI
Int_AB = −0.54 and Int_BA = −0.7 at EC50
A 0 B 0
A 0 B 0.14
A 0 B 0.29
A 0 B 0.43
A 0 B 0.57
A 0 B 0.71
A 0 B 0.86
A 0 B 1
A 0.14 B 0
A 0.14 B 0.14
A 0.14 B 0.29
A 0.14 B 0.43
A 0.14 B 0.57
A 0.14 B 0.71
A 0.14 B 0.86
A 0.14 B 1
A 0.29 B 0
A 0.29 B 0.14
A 0.29 B 0.29
A 0.29 B 0.43
A 0.29 B 0.57
A 0.29 B 0.71
A 0.29 B 0.86
A 0.29 B 1
A 0.43 B 0
A 0.43 B 0.14
A 0.43 B 0.29
A 0.43 B 0.43
A 0.43 B 0.57
A 0.43 B 0.71
A 0.43 B 0.86
A 0.43 B 1
A 0.57 B 0
A 0.57 B 0.14
A 0.57 B 0.29
A 0.57 B 0.43
A 0.57 B 0.57
A 0.57 B 0.71
A 0.57 B 0.86
A 0.57 B 1
A 0.71 B 0
A 0.71 B 0.14
A 0.71 B 0.29
A 0.71 B 0.43
A 0.71 B 0.57
A 0.71 B 0.71
A 0.71 B 0.86
A 0.71 B 1
A 0.86 B 0
A 0.86 B 0.14
A 0.86 B 0.29
A 0.86 B 0.43
A 0.86 B 0.57
A 0.86 B 0.71
A 0.86 B 0.86
A 0.86 B 1
A 1 B 0
A 1 B 0.14
A 1 B 0.29
A 1 B 0.43
A 1 B 0.57
A 1 B 0.71
A 1 B 0.86
A 1 B 1
0.8
0.6
0.4
0.2
0 5 101520
OD 600 nm [−]
Time [h]

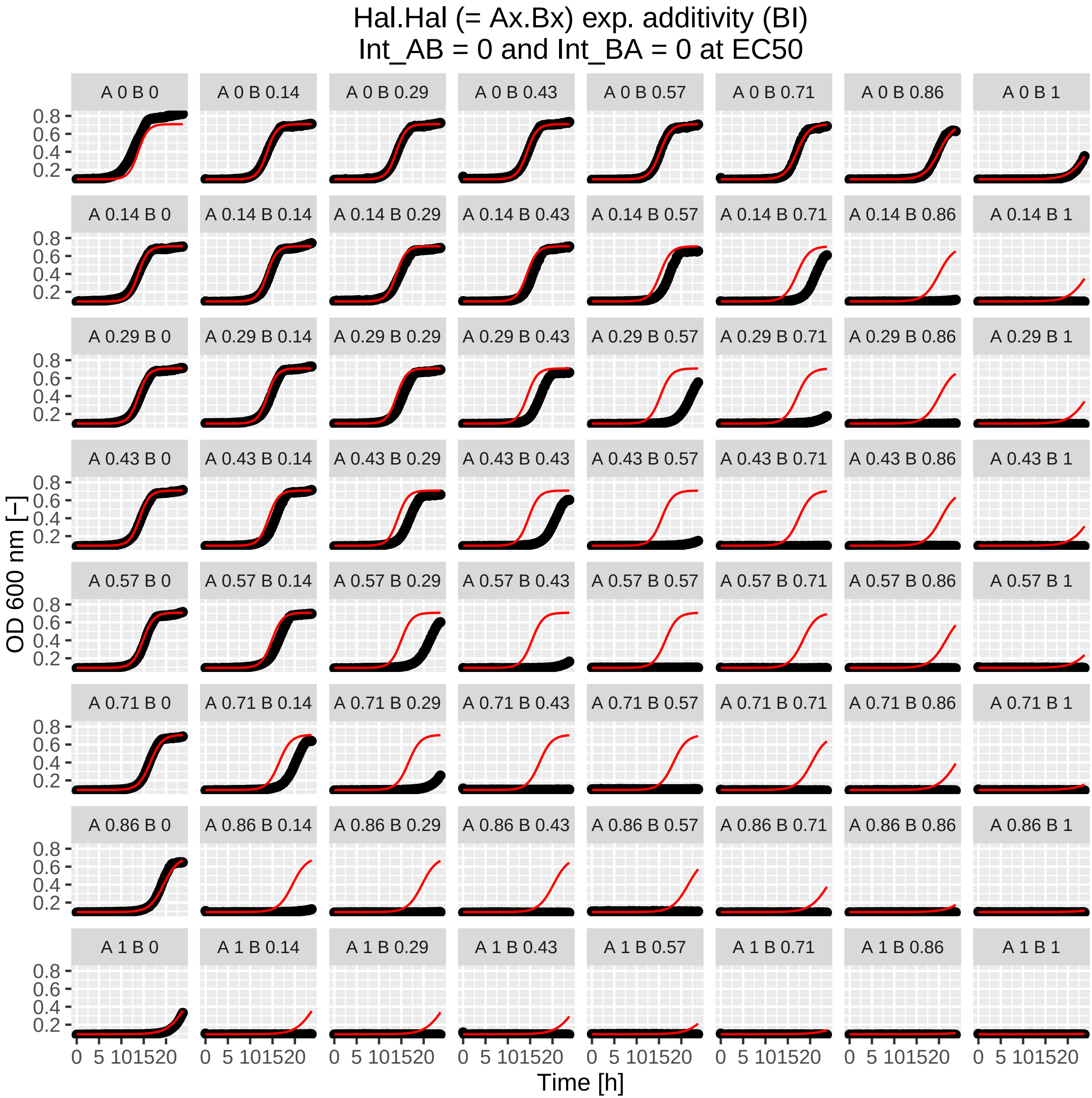

Hal.Hal (= Ax.Bx) exp. additivity (BI)
Int_AB = 0 and Int_BA = 0 at EC50
A 0 B 0
A 0 B 0.14
A 0 B 0.29
A 0 B 0.43
A 0 B 0.57
A 0 B 0.71
A 0 B 0.86
A 0 B 1
A 0.14 B 0
A 0.14 B 0.14
A 0.14 B 0.29
A 0.14 B 0.43
A 0.14 B 0.57
A 0.14 B 0.71
A 0.14 B 0.86
A 0.14 B 1
A 0.29 B 0
A 0.29 B 0.14
A 0.29 B 0.29
A 0.29 B 0.43
A 0.29 B 0.57
A 0.29 B 0.71
A 0.29 B 0.86
A 0.29 B 1
A 0.43 B 0
A 0.43 B 0.14
A 0.43 B 0.29
A 0.43 B 0.43
A 0.43 B 0.57
A 0.43 B 0.71
A 0.43 B 0.86
A 0.43 B 1
A 0.57 B 0
A 0.57 B 0.14
A 0.57 B 0.29
A 0.57 B 0.43
A 0.57 B 0.57
A 0.57 B 0.71
A 0.57 B 0.86
A 0.57 B 1
A 0.71 B 0
A 0.71 B 0.14
A 0.71 B 0.29
A 0.71 B 0.43
A 0.71 B 0.57
A 0.71 B 0.71
A 0.71 B 0.86
A 0.71 B 1
A 0.86 B 0
A 0.86 B 0.14
A 0.86 B 0.29
A 0.86 B 0.43
A 0.86 B 0.57
A 0.86 B 0.71
A 0.86 B 0.86
A 0.86 B 1
A 1 B 0
A 1 B 0.14
A 1 B 0.29
A 1 B 0.43
A 1 B 0.57
A 1 B 0.71
A 1 B 0.86
A 1 B 1
OD 600 nm [−]
0.8
0.6
0.4
0.2
0 5 101520
Time [h]

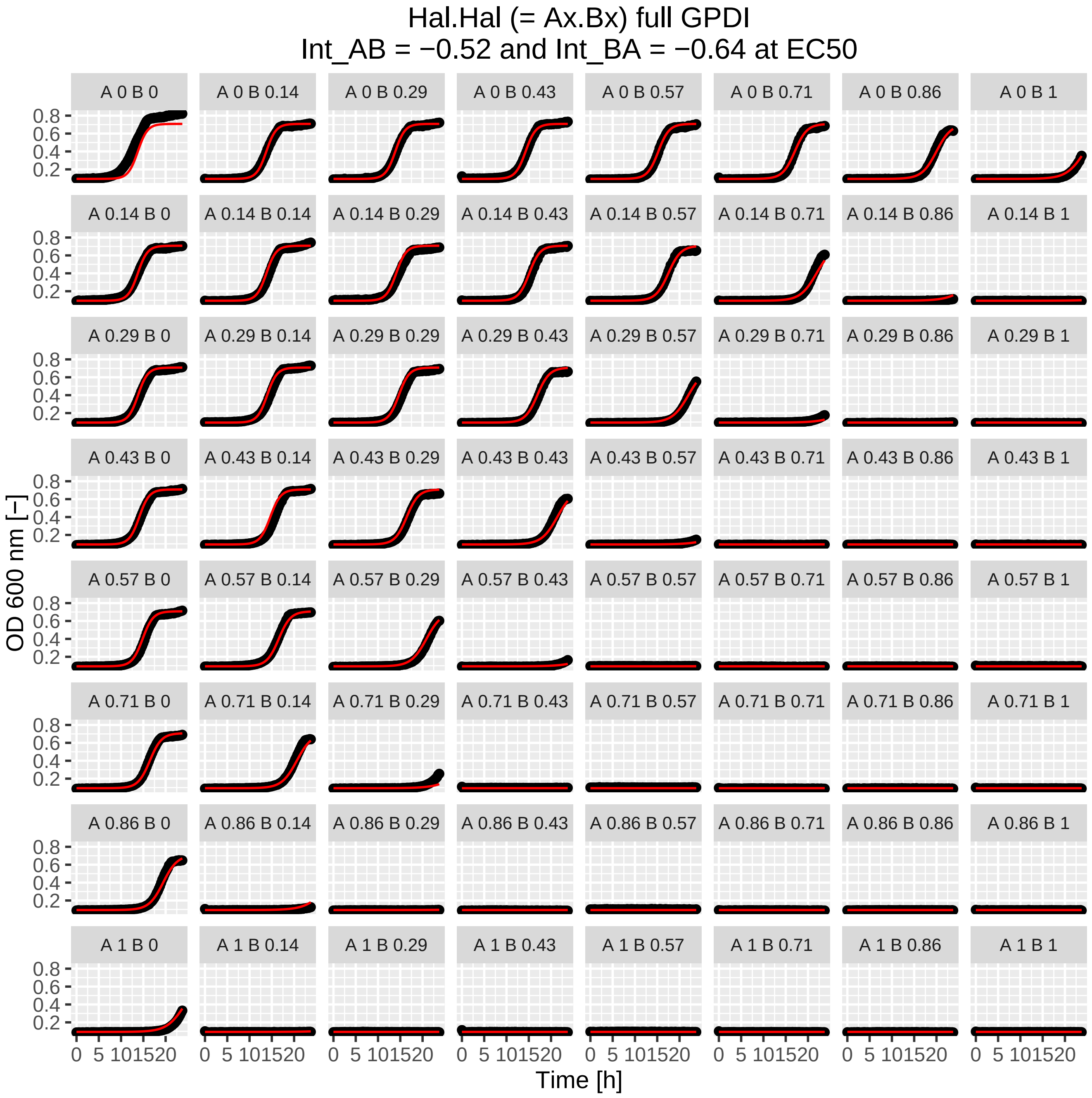

Hal.Hal (= Ax.Bx) full GPDI
Int_AB = −0.52 and Int_BA = −0.64 at EC50
A 0 B 0
A 0 B 0.14
A 0 B 0.29
A 0 B 0.43
A 0 B 0.57
A 0 B 0.71
A 0 B 0.86
A 0 B 1
A 0.14 B 0
A 0.14 B 0.14
A 0.14 B 0.29
A 0.14 B 0.43
A 0.14 B 0.57
A 0.14 B 0.71
A 0.14 B 0.86
A 0.14 B 1
A 0.29 B 0
A 0.29 B 0.14
A 0.29 B 0.29
A 0.29 B 0.43
A 0.29 B 0.57
A 0.29 B 0.71
A 0.29 B 0.86
A 0.29 B 1
A 0.43 B 0
A 0.43 B 0.14
A 0.43 B 0.29
A 0.43 B 0.43
A 0.43 B 0.57
A 0.43 B 0.71
A 0.43 B 0.86
A 0.43 B 1
A 0.57 B 0
A 0.57 B 0.14
A 0.57 B 0.29
A 0.57 B 0.43
A 0.57 B 0.57
A 0.57 B 0.71
A 0.57 B 0.86
A 0.57 B 1
A 0.71 B 0
A 0.71 B 0.14
A 0.71 B 0.29
A 0.71 B 0.43
A 0.71 B 0.57
A 0.71 B 0.71
A 0.71 B 0.86
A 0.71 B 1
A 0.86 B 0
A 0.86 B 0.14
A 0.86 B 0.29
A 0.86 B 0.43
A 0.86 B 0.57
A 0.86 B 0.71
A 0.86 B 0.86
A 0.86 B 1
A 1 B 0
A 1 B 0.14
A 1 B 0.29
A 1 B 0.43
A 1 B 0.57
A 1 B 0.71
A 1 B 0.86
A 1 B 1
0.8
0.6
0.4
0.2
OD 600 nm [−]
0 5 101520
Time [h]

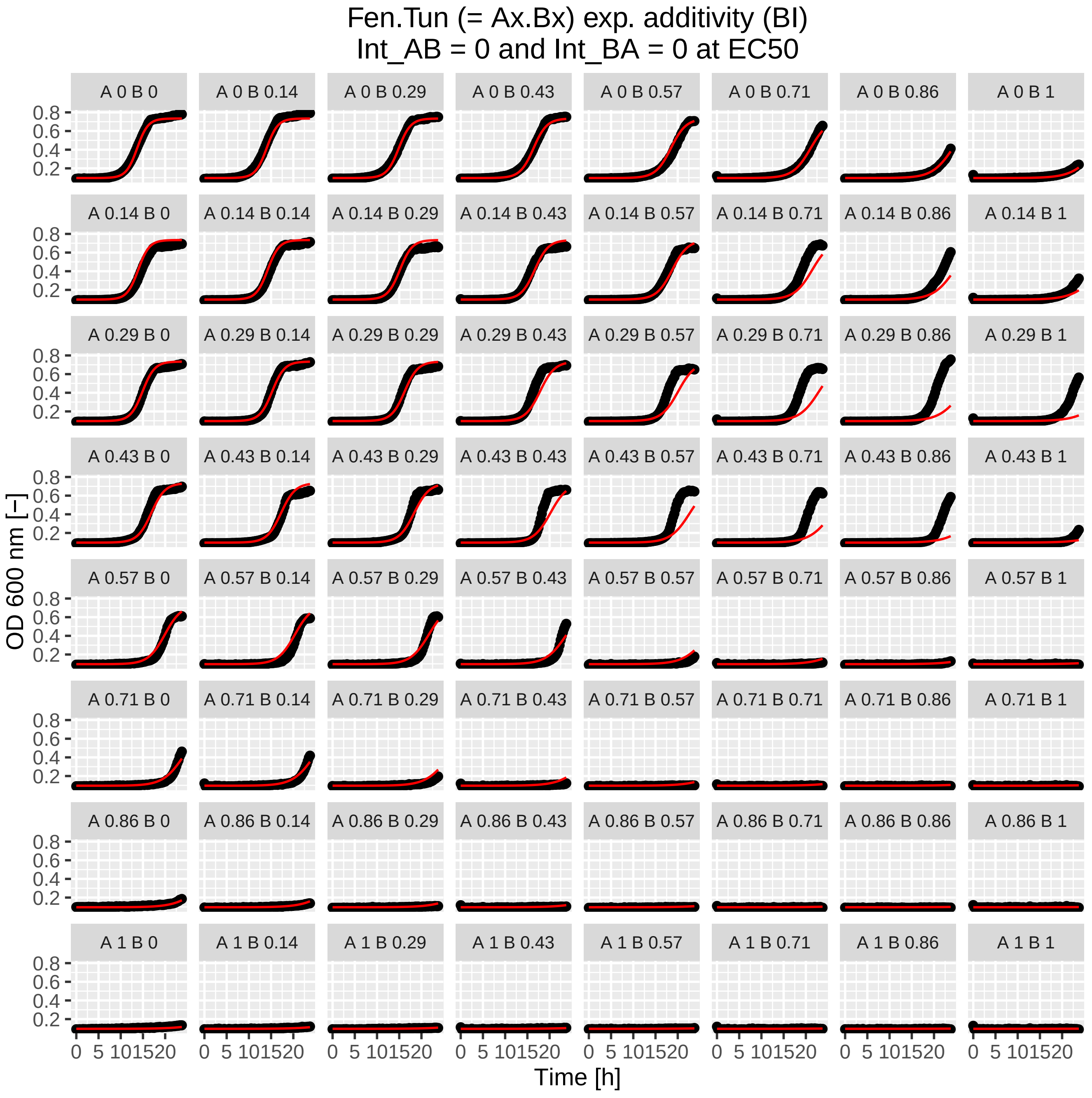

Fen.Tun (= Ax.Bx) exp. additivity (BI)
Int_AB = 0 and Int_BA = 0 at EC50
OD 600 nm [−]
Time [h]

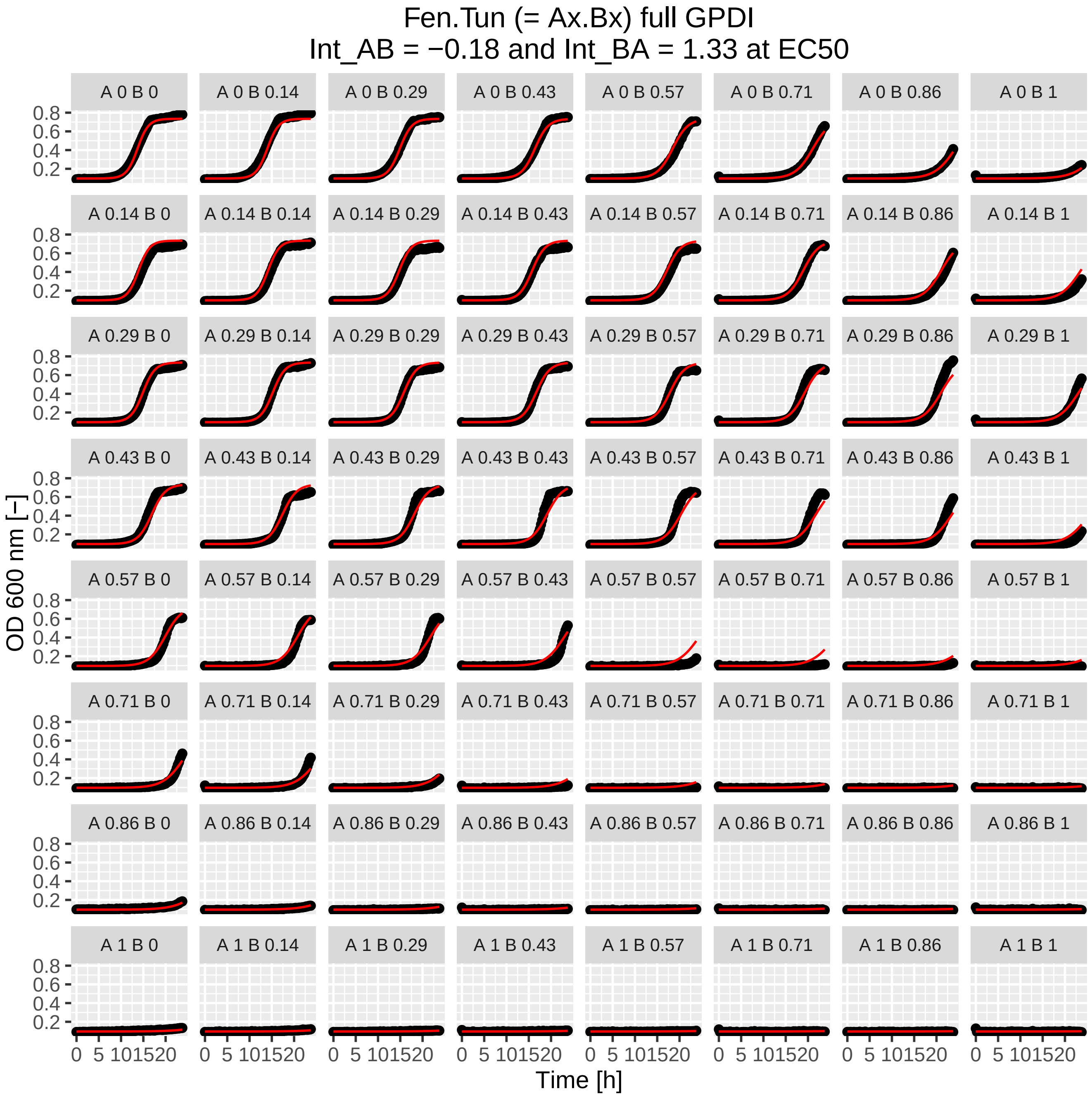
Fen.Tun (= Ax.Bx) full GPDI
Int_AB = −0.18 and Int_BA = 1.33 at EC50
A 0 B 0
A 0 B 0.14
A 0 B 0.29
A 0 B 0.43
A 0 B 0.57
A 0 B 0.71
A 0 B 0.86
A 0 B 1
A 0.14 B 0
A 0.14 B 0.14
A 0.14 B 0.29
A 0.14 B 0.43
A 0.14 B 0.57
A 0.14 B 0.71
A 0.14 B 0.86
A 0.14 B 1
A 0.29 B 0
A 0.29 B 0.14
A 0.29 B 0.29
A 0.29 B 0.43
A 0.29 B 0.57
A 0.29 B 0.71
A 0.29 B 0.86
A 0.29 B 1
A 0.43 B 0
A 0.43 B 0.14
A 0.43 B 0.29
A 0.43 B 0.43
A 0.43 B 0.57
A 0.43 B 0.71
A 0.43 B 0.86
A 0.43 B 1
A 0.57 B 0
A 0.57 B 0.14
A 0.57 B 0.29
A 0.57 B 0.43
A 0.57 B 0.57
A 0.57 B 0.71
A 0.57 B 0.86
A 0.57 B 1
A 0.71 B 0
A 0.71 B 0.14
A 0.71 B 0.29
A 0.71 B 0.43
A 0.71 B 0.57
A 0.71 B 0.71
A 0.71 B 0.86
A 0.71 B 1
A 0.86 B 0
A 0.86 B 0.14
A 0.86 B 0.29
A 0.86 B 0.43
A 0.86 B 0.57
A 0.86 B 0.71
A 0.86 B 0.86
A 0.86 B 1
A 1 B 0
A 1 B 0.14
A 1 B 0.29
A 1 B 0.43
A 1 B 0.57
A 1 B 0.71
A 1 B 0.86
A 1 B 1
OD 600 nm [−]
0.2
0.4
0.6
0.8
0
5
10
15
20
Time [h]

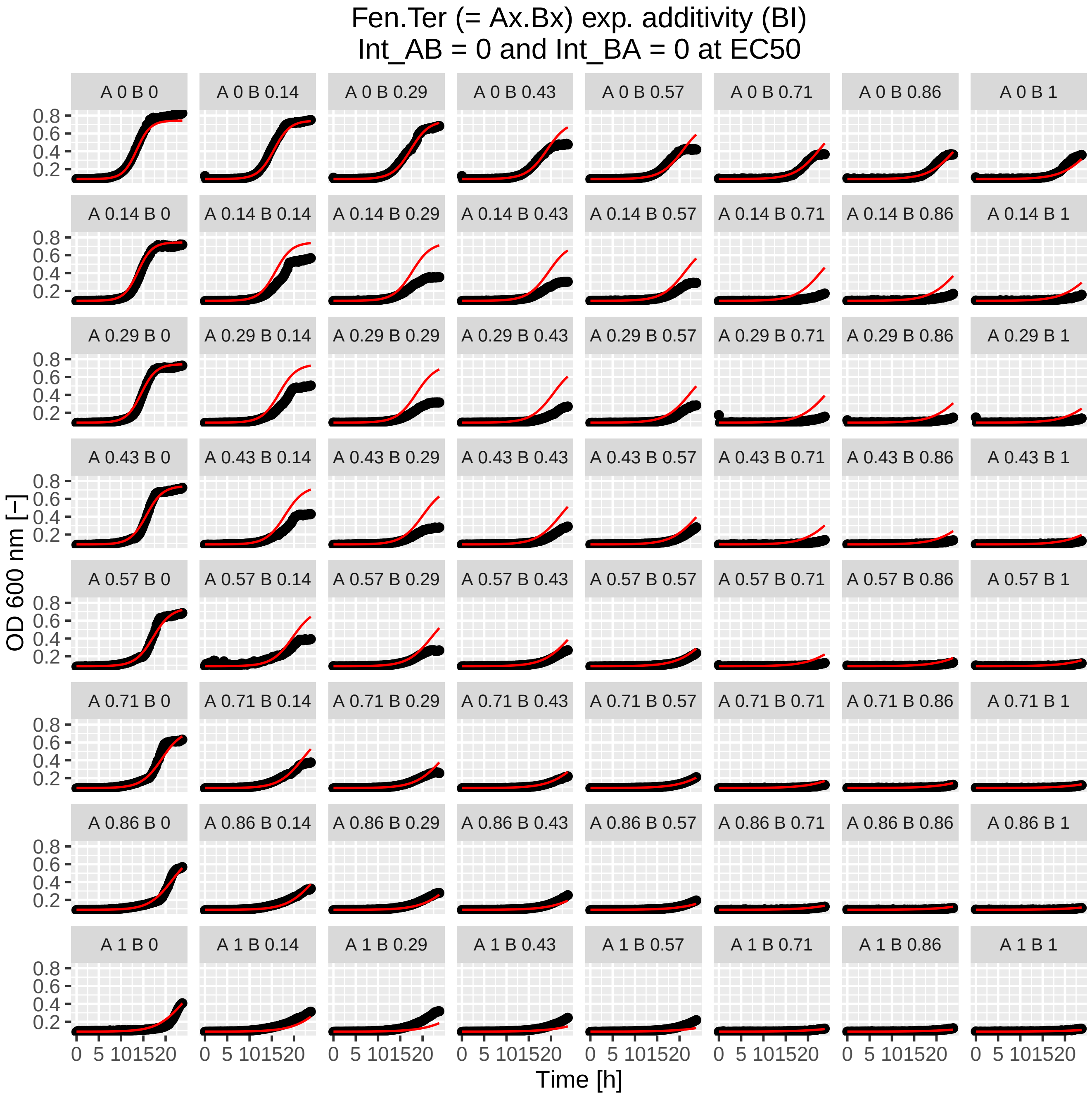
Fen.Ter (= Ax.Bx) exp. additivity (BI)
Int_AB = 0 and Int_BA = 0 at EC50
OD 600 nm [–]
Time [h]
A 0 B 0
A 0 B 0.14
A 0 B 0.29
A 0 B 0.43
A 0 B 0.57
A 0 B 0.71
A 0 B 0.86
A 0 B 1
A 0.14 B 0
A 0.14 B 0.14
A 0.14 B 0.29
A 0.14 B 0.43
A 0.14 B 0.57
A 0.14 B 0.71
A 0.14 B 0.86
A 0.14 B 1
A 0.29 B 0
A 0.29 B 0.14
A 0.29 B 0.29
A 0.29 B 0.43
A 0.29 B 0.57
A 0.29 B 0.71
A 0.29 B 0.86
A 0.29 B 1
A 0.43 B 0
A 0.43 B 0.14
A 0.43 B 0.29
A 0.43 B 0.43
A 0.43 B 0.57
A 0.43 B 0.71
A 0.43 B 0.86
A 0.43 B 1
A 0.57 B 0
A 0.57 B 0.14
A 0.57 B 0.29
A 0.57 B 0.43
A 0.57 B 0.57
A 0.57 B 0.71
A 0.57 B 0.86
A 0.57 B 1
A 0.71 B 0
A 0.71 B 0.14
A 0.71 B 0.29
A 0.71 B 0.43
A 0.71 B 0.57
A 0.71 B 0.71
A 0.71 B 0.86
A 0.71 B 1
A 0.86 B 0
A 0.86 B 0.14
A 0.86 B 0.29
A 0.86 B 0.43
A 0.86 B 0.57
A 0.86 B 0.71
A 0.86 B 0.86
A 0.86 B 1
A 1 B 0
A 1 B 0.14
A 1 B 0.29
A 1 B 0.43
A 1 B 0.57
A 1 B 0.71
A 1 B 0.86
A 1 B 1
0.8
0.6
0.4
0.2
0 5 101520

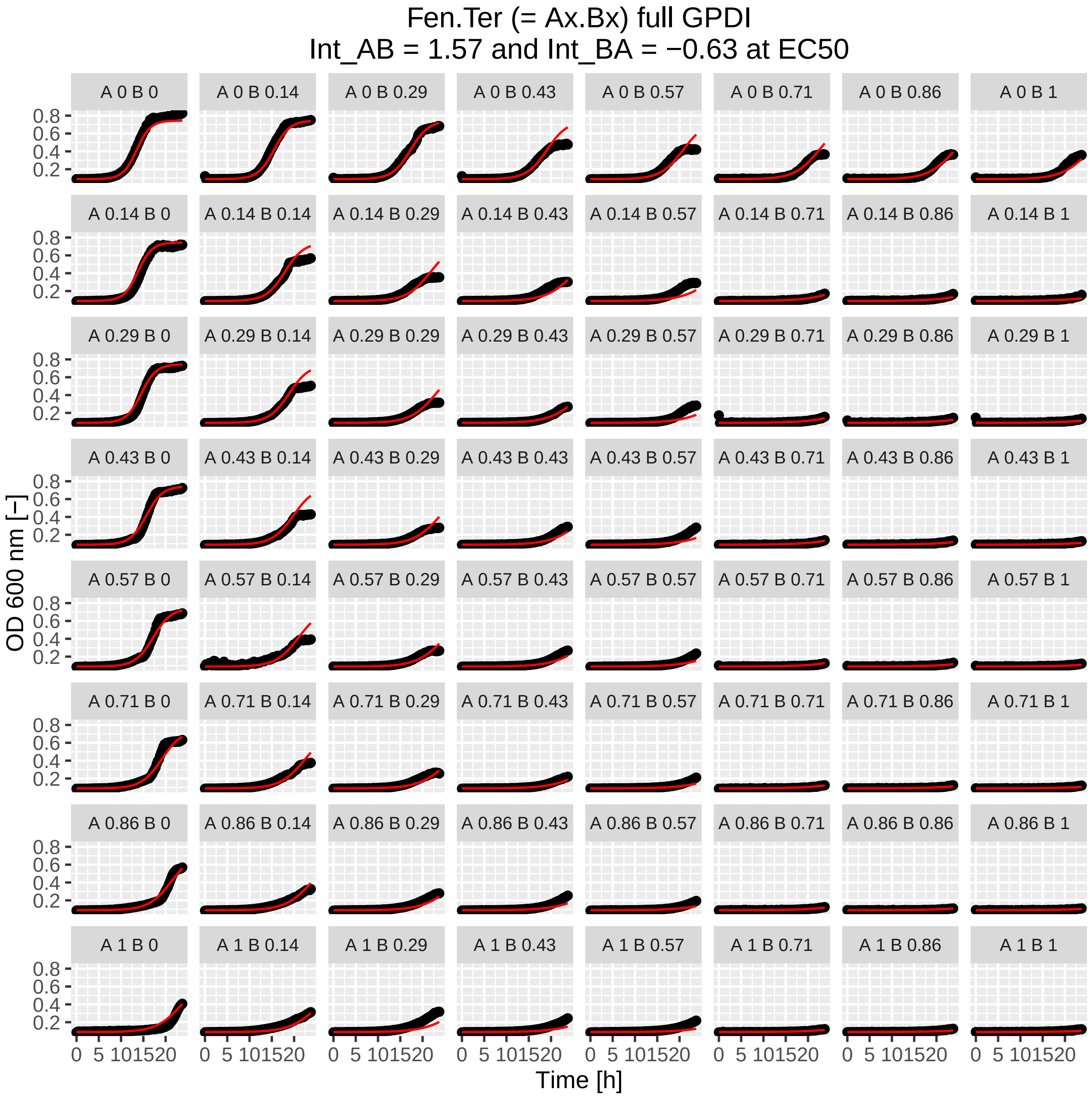

Fen.Ter (= Ax.Bx) full GPDI
Int_AB = 1.57 and Int_BA = −0.63 at EC50
A 0 B 0
A 0 B 0.14
A 0 B 0.29
A 0 B 0.43
A 0 B 0.57
A 0 B 0.71
A 0 B 0.86
A 0 B 1
A 0.14 B 0
A 0.14 B 0.14
A 0.14 B 0.29
A 0.14 B 0.43
A 0.14 B 0.57
A 0.14 B 0.71
A 0.14 B 0.86
A 0.14 B 1
A 0.29 B 0
A 0.29 B 0.14
A 0.29 B 0.29
A 0.29 B 0.43
A 0.29 B 0.57
A 0.29 B 0.71
A 0.29 B 0.86
A 0.29 B 1
A 0.43 B 0
A 0.43 B 0.14
A 0.43 B 0.29
A 0.43 B 0.43
A 0.43 B 0.57
A 0.43 B 0.71
A 0.43 B 0.86
A 0.43 B 1
A 0.57 B 0
A 0.57 B 0.14
A 0.57 B 0.29
A 0.57 B 0.43
A 0.57 B 0.57
A 0.57 B 0.71
A 0.57 B 0.86
A 0.57 B 1
A 0.71 B 0
A 0.71 B 0.14
A 0.71 B 0.29
A 0.71 B 0.43
A 0.71 B 0.57
A 0.71 B 0.71
A 0.71 B 0.86
A 0.71 B 1
A 0.86 B 0
A 0.86 B 0.14
A 0.86 B 0.29
A 0.86 B 0.43
A 0.86 B 0.57
A 0.86 B 0.71
A 0.86 B 0.86
A 0.86 B 1
A 1 B 0
A 1 B 0.14
A 1 B 0.29
A 1 B 0.43
A 1 B 0.57
A 1 B 0.71
A 1 B 0.86
A 1 B 1
OD 600 nm [−]
0.2
0.4
0.6
0.8
0
5
10
15
20
Time [h]

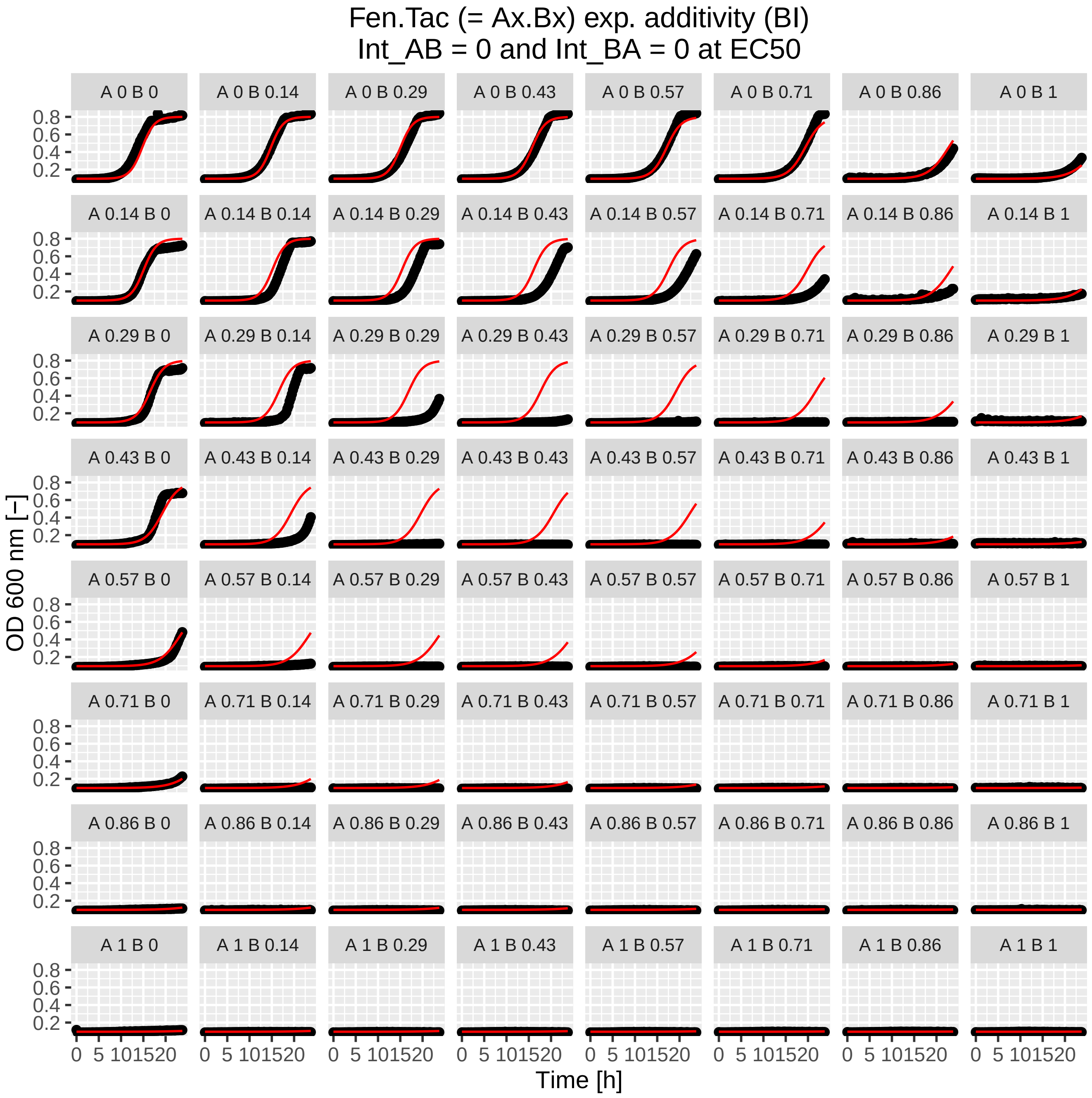
Fen.Tac (= Ax.Bx) exp. additivity (BI)
Int_AB = 0 and Int_BA = 0 at EC50
A 0 B 0
A 0 B 0.14
A 0 B 0.29
A 0 B 0.43
A 0 B 0.57
A 0 B 0.71
A 0 B 0.86
A 0 B 1
A 0.14 B 0
A 0.14 B 0.14
A 0.14 B 0.29
A 0.14 B 0.43
A 0.14 B 0.57
A 0.14 B 0.71
A 0.14 B 0.86
A 0.14 B 1
A 0.29 B 0
A 0.29 B 0.14
A 0.29 B 0.29
A 0.29 B 0.43
A 0.29 B 0.57
A 0.29 B 0.71
A 0.29 B 0.86
A 0.29 B 1
A 0.43 B 0
A 0.43 B 0.14
A 0.43 B 0.29
A 0.43 B 0.43
A 0.43 B 0.57
A 0.43 B 0.71
A 0.43 B 0.86
A 0.43 B 1
A 0.57 B 0
A 0.57 B 0.14
A 0.57 B 0.29
A 0.57 B 0.43
A 0.57 B 0.57
A 0.57 B 0.71
A 0.57 B 0.86
A 0.57 B 1
A 0.71 B 0
A 0.71 B 0.14
A 0.71 B 0.29
A 0.71 B 0.43
A 0.71 B 0.57
A 0.71 B 0.71
A 0.71 B 0.86
A 0.71 B 1
A 0.86 B 0
A 0.86 B 0.14
A 0.86 B 0.29
A 0.86 B 0.43
A 0.86 B 0.57
A 0.86 B 0.71
A 0.86 B 0.86
A 0.86 B 1
A 1 B 0
A 1 B 0.14
A 1 B 0.29
A 1 B 0.43
A 1 B 0.57
A 1 B 0.71
A 1 B 0.86
A 1 B 1
OD 600 nm [–]
0.8
0.6
0.4
0.2
0 5 101520
Time [h]

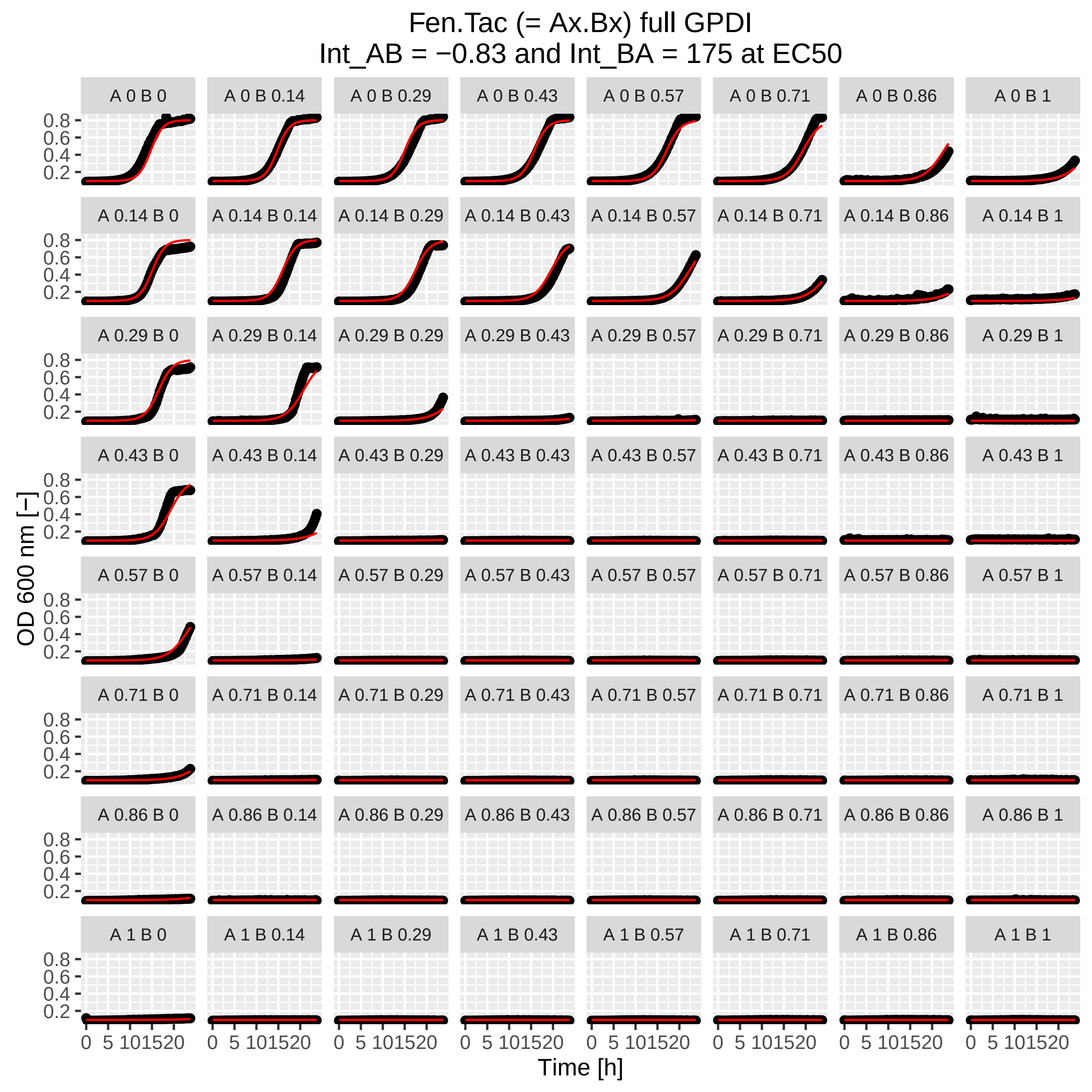

Fen.Tac (= Ax.Bx) full GPDI
Int_AB = −0.83 and Int_BA = 175 at EC50
A 0 B 0
A 0 B 0.14
A 0 B 0.29
A 0 B 0.43
A 0 B 0.57
A 0 B 0.71
A 0 B 0.86
A 0 B 1
A 0.14 B 0
A 0.14 B 0.14
A 0.14 B 0.29
A 0.14 B 0.43
A 0.14 B 0.57
A 0.14 B 0.71
A 0.14 B 0.86
A 0.14 B 1
A 0.29 B 0
A 0.29 B 0.14
A 0.29 B 0.29
A 0.29 B 0.43
A 0.29 B 0.57
A 0.29 B 0.71
A 0.29 B 0.86
A 0.29 B 1
A 0.43 B 0
A 0.43 B 0.14
A 0.43 B 0.29
A 0.43 B 0.43
A 0.43 B 0.57
A 0.43 B 0.71
A 0.43 B 0.86
A 0.43 B 1
A 0.57 B 0
A 0.57 B 0.14
A 0.57 B 0.29
A 0.57 B 0.43
A 0.57 B 0.57
A 0.57 B 0.71
A 0.57 B 0.86
A 0.57 B 1
A 0.71 B 0
A 0.71 B 0.14
A 0.71 B 0.29
A 0.71 B 0.43
A 0.71 B 0.57
A 0.71 B 0.71
A 0.71 B 0.86
A 0.71 B 1
A 0.86 B 0
A 0.86 B 0.14
A 0.86 B 0.29
A 0.86 B 0.43
A 0.86 B 0.57
A 0.86 B 0.71
A 0.86 B 0.86
A 0.86 B 1
A 1 B 0
A 1 B 0.14
A 1 B 0.29
A 1 B 0.43
A 1 B 0.57
A 1 B 0.71
A 1 B 0.86
A 1 B 1
0.8
0.6
0.4
0.2
OD 600 nm [−]
0 5 101520
Time [h]

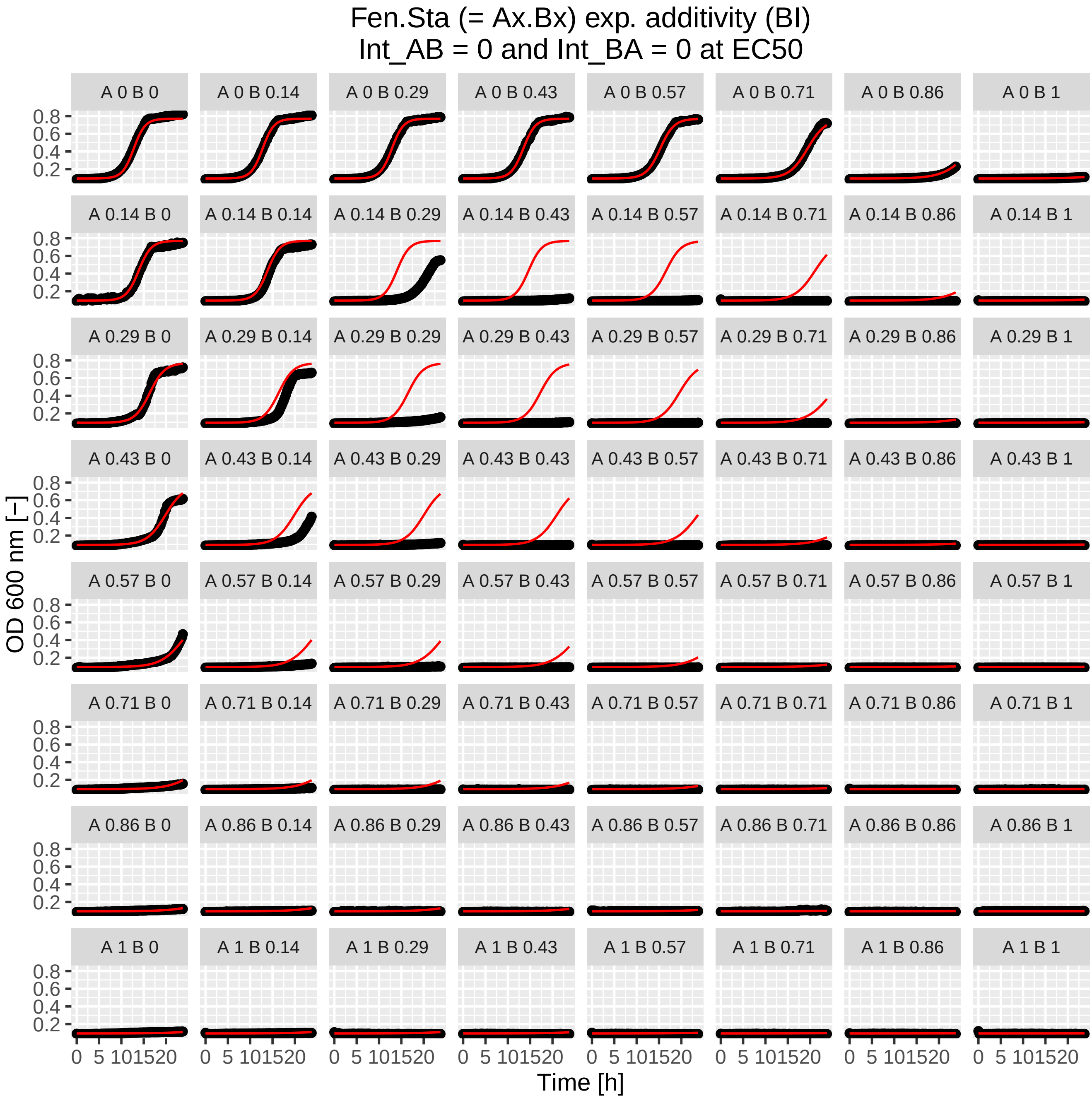

Fen.Sta (= Ax.Bx) exp. additivity (BI)
Int_AB = 0 and Int_BA = 0 at EC50
A 0 B 0
A 0 B 0.14
A 0 B 0.29
A 0 B 0.43
A 0 B 0.57
A 0 B 0.71
A 0 B 0.86
A 0 B 1
A 0.14 B 0
A 0.14 B 0.14
A 0.14 B 0.29
A 0.14 B 0.43
A 0.14 B 0.57
A 0.14 B 0.71
A 0.14 B 0.86
A 0.14 B 1
A 0.29 B 0
A 0.29 B 0.14
A 0.29 B 0.29
A 0.29 B 0.43
A 0.29 B 0.57
A 0.29 B 0.71
A 0.29 B 0.86
A 0.29 B 1
A 0.43 B 0
A 0.43 B 0.14
A 0.43 B 0.29
A 0.43 B 0.43
A 0.43 B 0.57
A 0.43 B 0.71
A 0.43 B 0.86
A 0.43 B 1
A 0.57 B 0
A 0.57 B 0.14
A 0.57 B 0.29
A 0.57 B 0.43
A 0.57 B 0.57
A 0.57 B 0.71
A 0.57 B 0.86
A 0.57 B 1
A 0.71 B 0
A 0.71 B 0.14
A 0.71 B 0.29
A 0.71 B 0.43
A 0.71 B 0.57
A 0.71 B 0.71
A 0.71 B 0.86
A 0.71 B 1
A 0.86 B 0
A 0.86 B 0.14
A 0.86 B 0.29
A 0.86 B 0.43
A 0.86 B 0.57
A 0.86 B 0.71
A 0.86 B 0.86
A 0.86 B 1
A 1 B 0
A 1 B 0.14
A 1 B 0.29
A 1 B 0.43
A 1 B 0.57
A 1 B 0.71
A 1 B 0.86
A 1 B 1
0.8
0.6
0.4
0.2
OD 600 nm [−]
0 5 101520
Time [h]

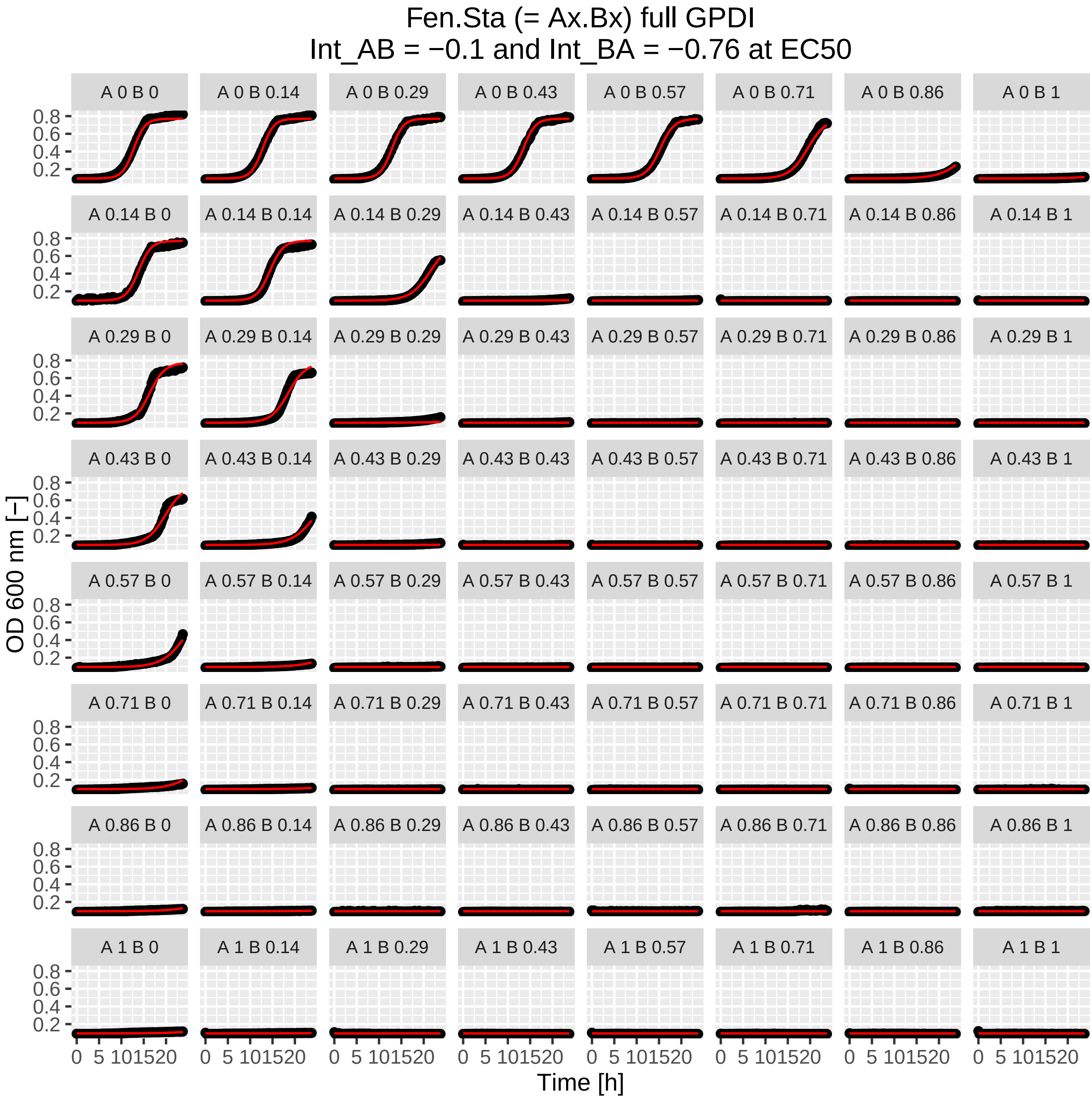

Fen.Sta (= Ax.Bx) full GPDI
Int_AB = −0.1 and Int_BA = −0.76 at EC50
A 0 B 0
A 0 B 0.14
A 0 B 0.29
A 0 B 0.43
A 0 B 0.57
A 0 B 0.71
A 0 B 0.86
A 0 B 1
A 0.14 B 0
A 0.14 B 0.14
A 0.14 B 0.29
A 0.14 B 0.43
A 0.14 B 0.57
A 0.14 B 0.71
A 0.14 B 0.86
A 0.14 B 1
A 0.29 B 0
A 0.29 B 0.14
A 0.29 B 0.29
A 0.29 B 0.43
A 0.29 B 0.57
A 0.29 B 0.71
A 0.29 B 0.86
A 0.29 B 1
A 0.43 B 0
A 0.43 B 0.14
A 0.43 B 0.29
A 0.43 B 0.43
A 0.43 B 0.57
A 0.43 B 0.71
A 0.43 B 0.86
A 0.43 B 1
A 0.57 B 0
A 0.57 B 0.14
A 0.57 B 0.29
A 0.57 B 0.43
A 0.57 B 0.57
A 0.57 B 0.71
A 0.57 B 0.86
A 0.57 B 1
A 0.71 B 0
A 0.71 B 0.14
A 0.71 B 0.29
A 0.71 B 0.43
A 0.71 B 0.57
A 0.71 B 0.71
A 0.71 B 0.86
A 0.71 B 1
A 0.86 B 0
A 0.86 B 0.14
A 0.86 B 0.29
A 0.86 B 0.43
A 0.86 B 0.57
A 0.86 B 0.71
A 0.86 B 0.86
A 0.86 B 1
A 1 B 0
A 1 B 0.14
A 1 B 0.29
A 1 B 0.43
A 1 B 0.57
A 1 B 0.71
A 1 B 0.86
A 1 B 1
0.8
0.6
0.4
0.2
0 5 101520
OD 600 nm [−]
Time [h]

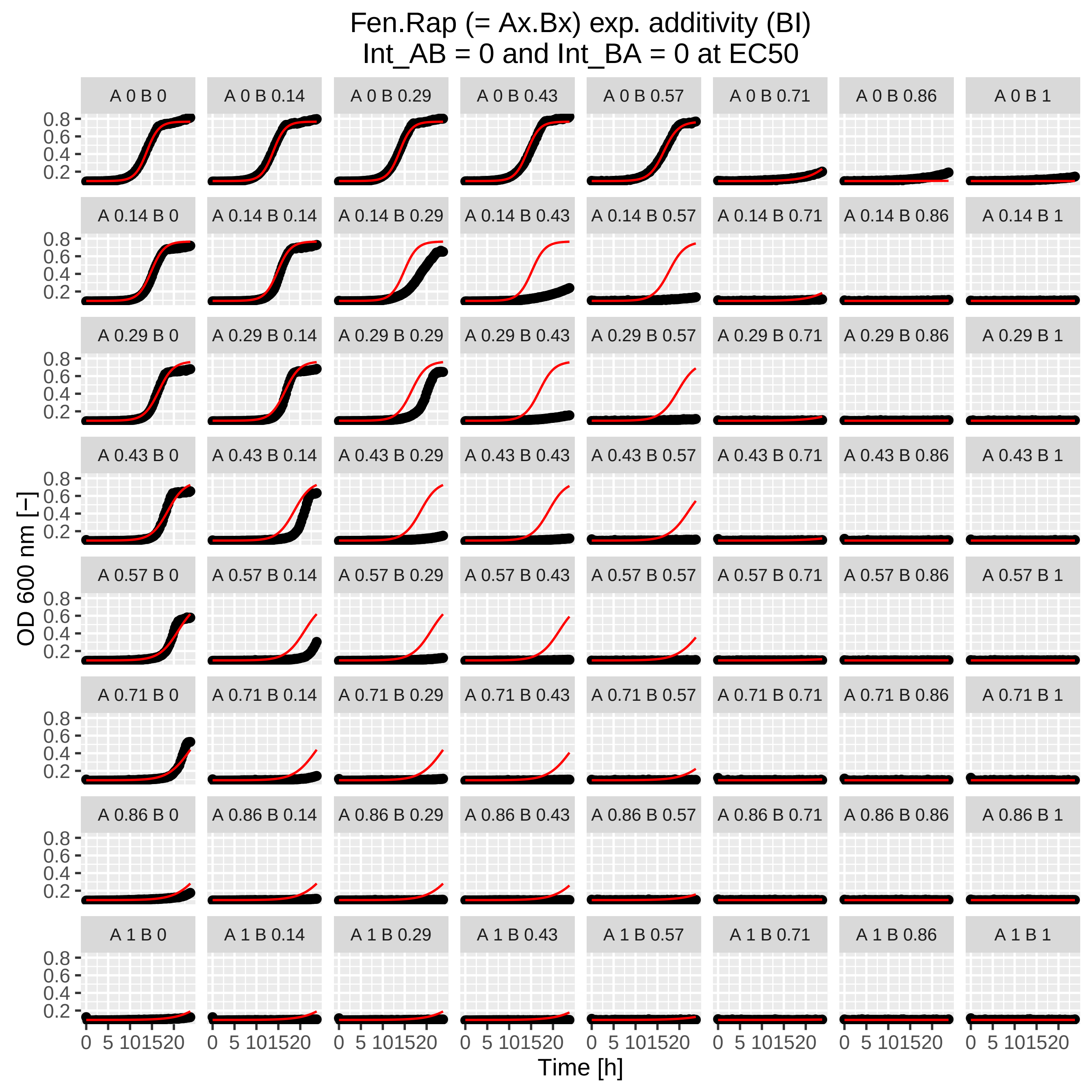

Fen.Rap (= Ax.Bx) exp. additivity (BI)
Int_AB = 0 and Int_BA = 0 at EC50
A 0 B 0
A 0 B 0.14
A 0 B 0.29
A 0 B 0.43
A 0 B 0.57
A 0 B 0.71
A 0 B 0.86
A 0 B 1
A 0.14 B 0
A 0.14 B 0.14
A 0.14 B 0.29
A 0.14 B 0.43
A 0.14 B 0.57
A 0.14 B 0.71
A 0.14 B 0.86
A 0.14 B 1
A 0.29 B 0
A 0.29 B 0.14
A 0.29 B 0.29
A 0.29 B 0.43
A 0.29 B 0.57
A 0.29 B 0.71
A 0.29 B 0.86
A 0.29 B 1
A 0.43 B 0
A 0.43 B 0.14
A 0.43 B 0.29
A 0.43 B 0.43
A 0.43 B 0.57
A 0.43 B 0.71
A 0.43 B 0.86
A 0.43 B 1
A 0.57 B 0
A 0.57 B 0.14
A 0.57 B 0.29
A 0.57 B 0.43
A 0.57 B 0.57
A 0.57 B 0.71
A 0.57 B 0.86
A 0.57 B 1
A 0.71 B 0
A 0.71 B 0.14
A 0.71 B 0.29
A 0.71 B 0.43
A 0.71 B 0.57
A 0.71 B 0.71
A 0.71 B 0.86
A 0.71 B 1
A 0.86 B 0
A 0.86 B 0.14
A 0.86 B 0.29
A 0.86 B 0.43
A 0.86 B 0.57
A 0.86 B 0.71
A 0.86 B 0.86
A 0.86 B 1
A 1 B 0
A 1 B 0.14
A 1 B 0.29
A 1 B 0.43
A 1 B 0.57
A 1 B 0.71
A 1 B 0.86
A 1 B 1
OD 600 nm [–]
0.2
0.4
0.6
0.8
0 5 101520
Time [h]

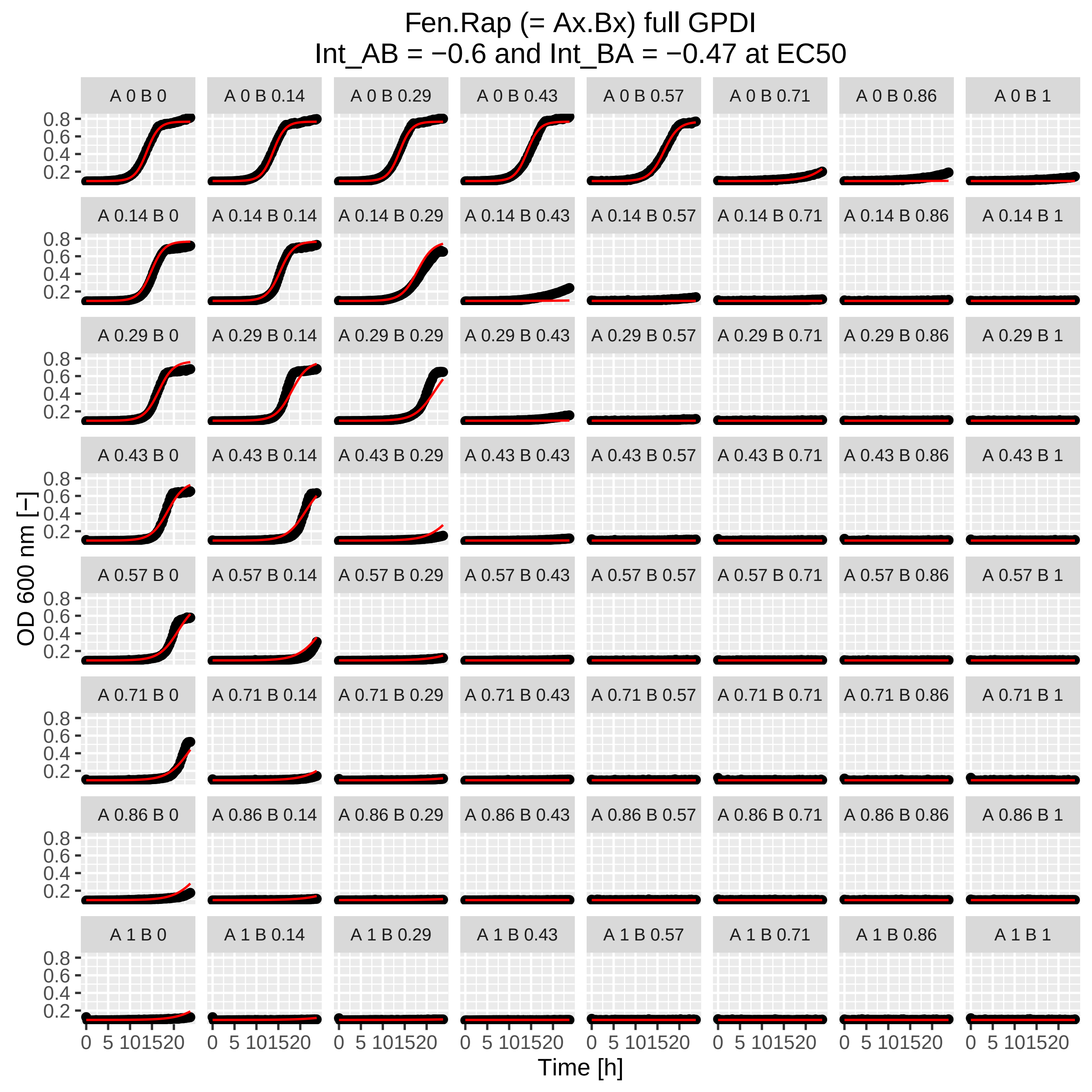
Fen.Rap (= Ax.Bx) full GPDI
Int_AB = −0.6 and Int_BA = −0.47 at EC50
OD 600 nm [−]
Time [h]
A 0 B 0
A 0 B 0.14
A 0 B 0.29
A 0 B 0.43
A 0 B 0.57
A 0 B 0.71
A 0 B 0.86
A 0 B 1
A 1 B 0
A 1 B 1

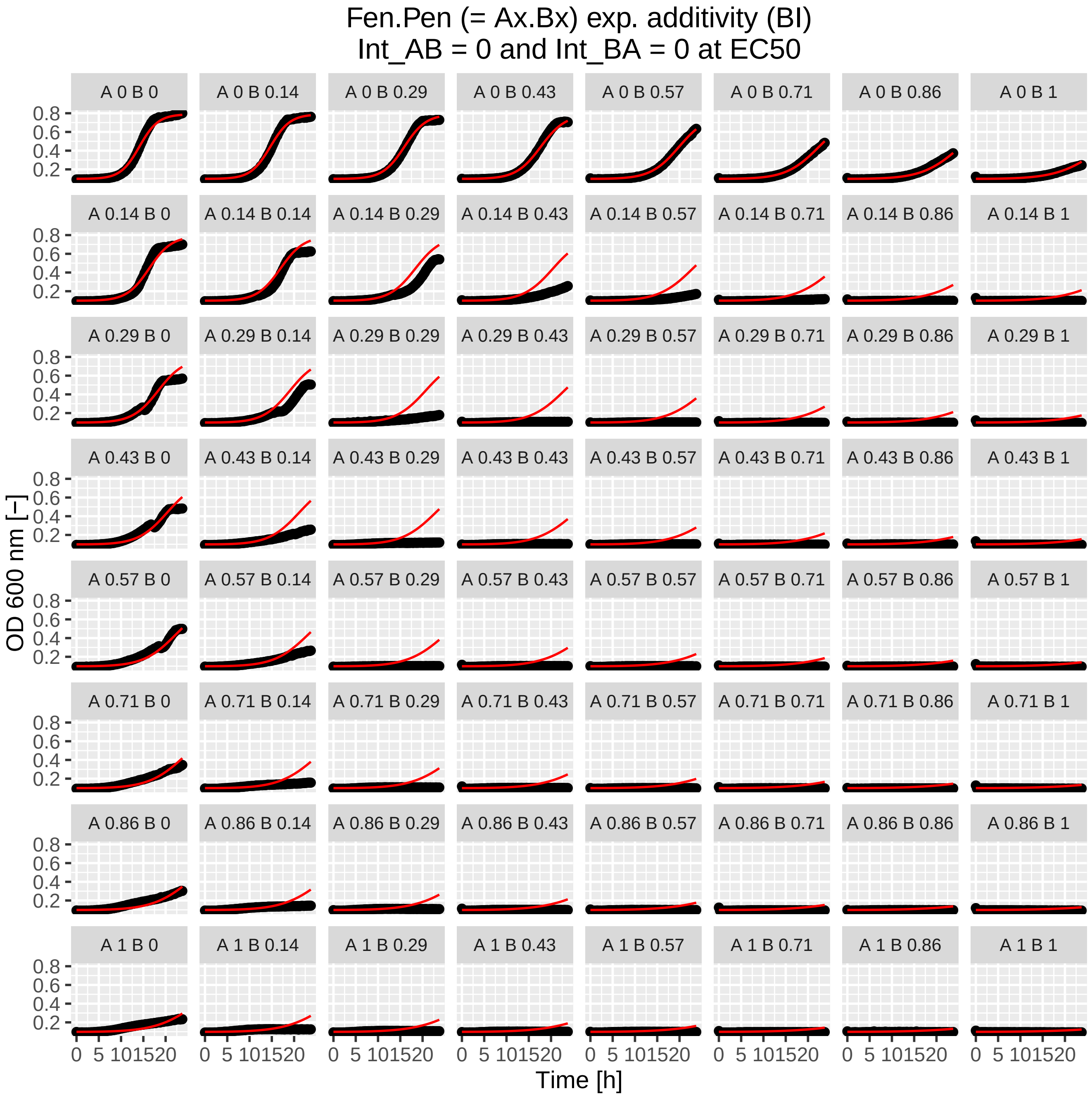

Fen.Pen (= Ax.Bx) exp. additivity (BI)
Int_AB = 0 and Int_BA = 0 at EC50
A 0 B 0
A 0 B 0.14
A 0 B 0.29
A 0 B 0.43
A 0 B 0.57
A 0 B 0.71
A 0 B 0.86
A 0 B 1
A 0.14 B 0
A 0.14 B 0.14
A 0.14 B 0.29
A 0.14 B 0.43
A 0.14 B 0.57
A 0.14 B 0.71
A 0.14 B 0.86
A 0.14 B 1
A 0.29 B 0
A 0.29 B 0.14
A 0.29 B 0.29
A 0.29 B 0.43
A 0.29 B 0.57
A 0.29 B 0.71
A 0.29 B 0.86
A 0.29 B 1
A 0.43 B 0
A 0.43 B 0.14
A 0.43 B 0.29
A 0.43 B 0.43
A 0.43 B 0.57
A 0.43 B 0.71
A 0.43 B 0.86
A 0.43 B 1
A 0.57 B 0
A 0.57 B 0.14
A 0.57 B 0.29
A 0.57 B 0.43
A 0.57 B 0.57
A 0.57 B 0.71
A 0.57 B 0.86
A 0.57 B 1
A 0.71 B 0
A 0.71 B 0.14
A 0.71 B 0.29
A 0.71 B 0.43
A 0.71 B 0.57
A 0.71 B 0.71
A 0.71 B 0.86
A 0.71 B 1
A 0.86 B 0
A 0.86 B 0.14
A 0.86 B 0.29
A 0.86 B 0.43
A 0.86 B 0.57
A 0.86 B 0.71
A 0.86 B 0.86
A 0.86 B 1
A 1 B 0
A 1 B 0.14
A 1 B 0.29
A 1 B 0.43
A 1 B 0.57
A 1 B 0.71
A 1 B 0.86
A 1 B 1
OD 600 nm [–]
0.8
0.6
0.4
0.2
0 5 101520
Time [h]

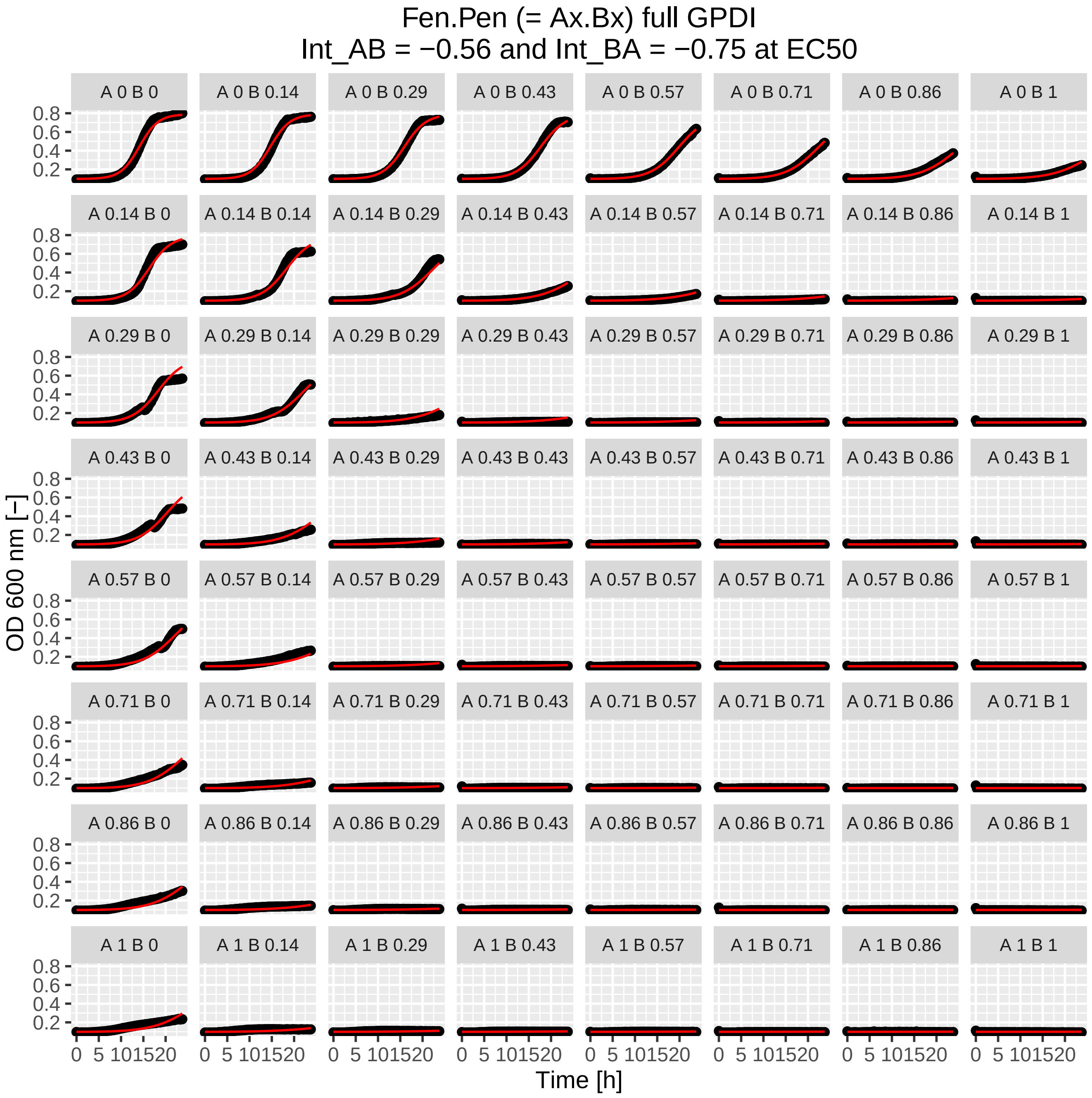

Fen.Pen (= Ax.Bx) full GPDI
Int_AB = −0.56 and Int_BA = −0.75 at EC50
A 0 B 0
A 0 B 0.14
A 0 B 0.29
A 0 B 0.43
A 0 B 0.57
A 0 B 0.71
A 0 B 0.86
A 0 B 1
A 0.14 B 0
A 0.14 B 0.14
A 0.14 B 0.29
A 0.14 B 0.43
A 0.14 B 0.57
A 0.14 B 0.71
A 0.14 B 0.86
A 0.14 B 1
A 0.29 B 0
A 0.29 B 0.14
A 0.29 B 0.29
A 0.29 B 0.43
A 0.29 B 0.57
A 0.29 B 0.71
A 0.29 B 0.86
A 0.29 B 1
A 0.43 B 0
A 0.43 B 0.14
A 0.43 B 0.29
A 0.43 B 0.43
A 0.43 B 0.57
A 0.43 B 0.71
A 0.43 B 0.86
A 0.43 B 1
A 0.57 B 0
A 0.57 B 0.14
A 0.57 B 0.29
A 0.57 B 0.43
A 0.57 B 0.57
A 0.57 B 0.71
A 0.57 B 0.86
A 0.57 B 1
A 0.71 B 0
A 0.71 B 0.14
A 0.71 B 0.29
A 0.71 B 0.43
A 0.71 B 0.57
A 0.71 B 0.71
A 0.71 B 0.86
A 0.71 B 1
A 0.86 B 0
A 0.86 B 0.14
A 0.86 B 0.29
A 0.86 B 0.43
A 0.86 B 0.57
A 0.86 B 0.71
A 0.86 B 0.86
A 0.86 B 1
A 1 B 0
A 1 B 0.14
A 1 B 0.29
A 1 B 0.43
A 1 B 0.57
A 1 B 0.71
A 1 B 0.86
A 1 B 1
OD 600 nm [−]
0.2
0.4
0.6
0.8
0 5 101520
Time [h]

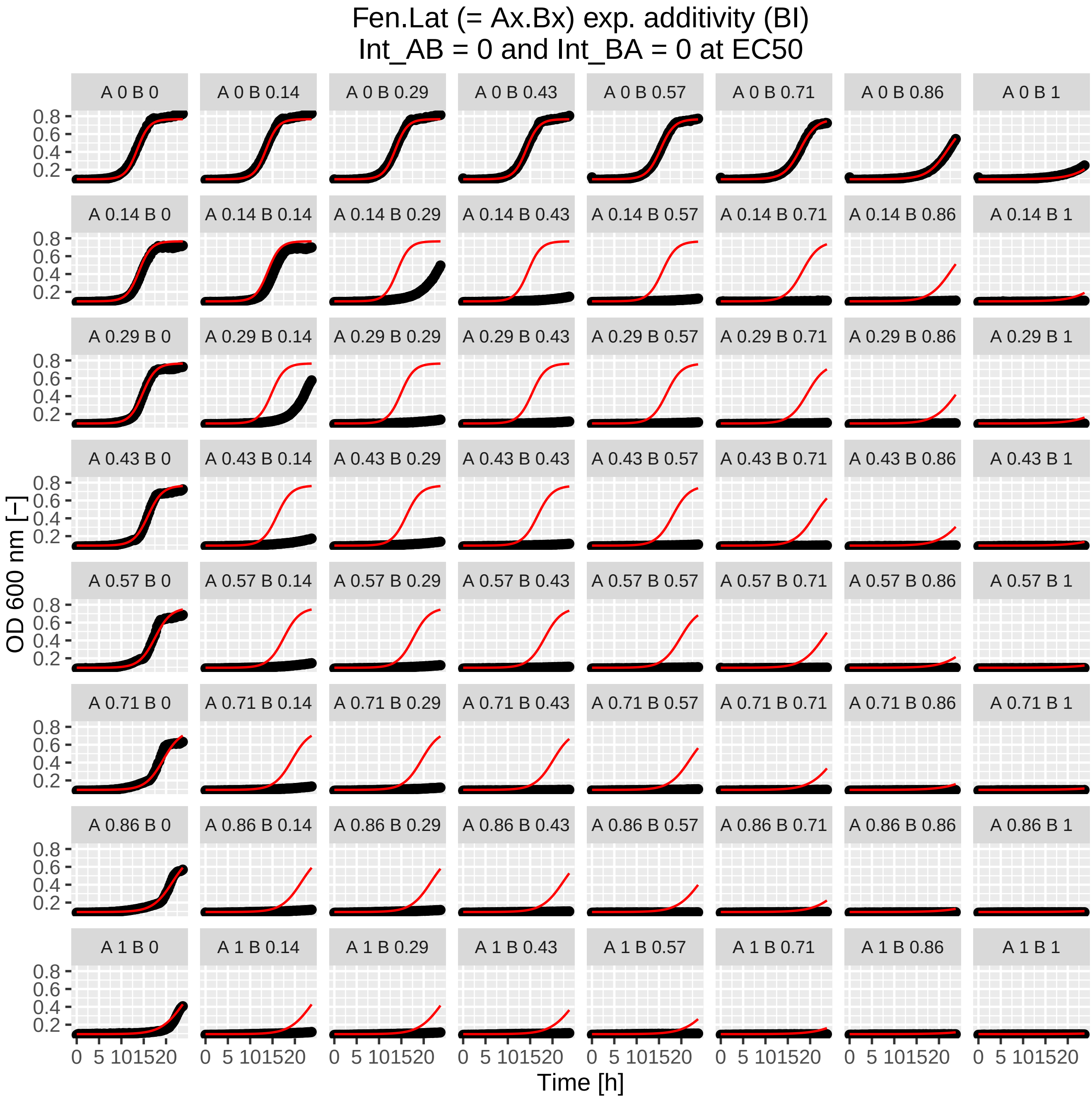
Fen.Lat (= Ax.Bx) exp. additivity (BI)
Int_AB = 0 and Int_BA = 0 at EC50
A 0 B 0
A 0 B 0.14
A 0 B 0.29
A 0 B 0.43
A 0 B 0.57
A 0 B 0.71
A 0 B 0.86
A 0 B 1
A 0.14 B 0
A 0.14 B 0.14
A 0.14 B 0.29
A 0.14 B 0.43
A 0.14 B 0.57
A 0.14 B 0.71
A 0.14 B 0.86
A 0.14 B 1
A 0.29 B 0
A 0.29 B 0.14
A 0.29 B 0.29
A 0.29 B 0.43
A 0.29 B 0.57
A 0.29 B 0.71
A 0.29 B 0.86
A 0.29 B 1
A 0.43 B 0
A 0.43 B 0.14
A 0.43 B 0.29
A 0.43 B 0.43
A 0.43 B 0.57
A 0.43 B 0.71
A 0.43 B 0.86
A 0.43 B 1
A 0.57 B 0
A 0.57 B 0.14
A 0.57 B 0.29
A 0.57 B 0.43
A 0.57 B 0.57
A 0.57 B 0.71
A 0.57 B 0.86
A 0.57 B 1
A 0.71 B 0
A 0.71 B 0.14
A 0.71 B 0.29
A 0.71 B 0.43
A 0.71 B 0.57
A 0.71 B 0.71
A 0.71 B 0.86
A 0.71 B 1
A 0.86 B 0
A 0.86 B 0.14
A 0.86 B 0.29
A 0.86 B 0.43
A 0.86 B 0.57
A 0.86 B 0.71
A 0.86 B 0.86
A 0.86 B 1
A 1 B 0
A 1 B 0.14
A 1 B 0.29
A 1 B 0.43
A 1 B 0.57
A 1 B 0.71
A 1 B 0.86
A 1 B 1
OD 600 nm [–]
0.2
0.4
0.6
0.8
0 5 101520
Time [h]

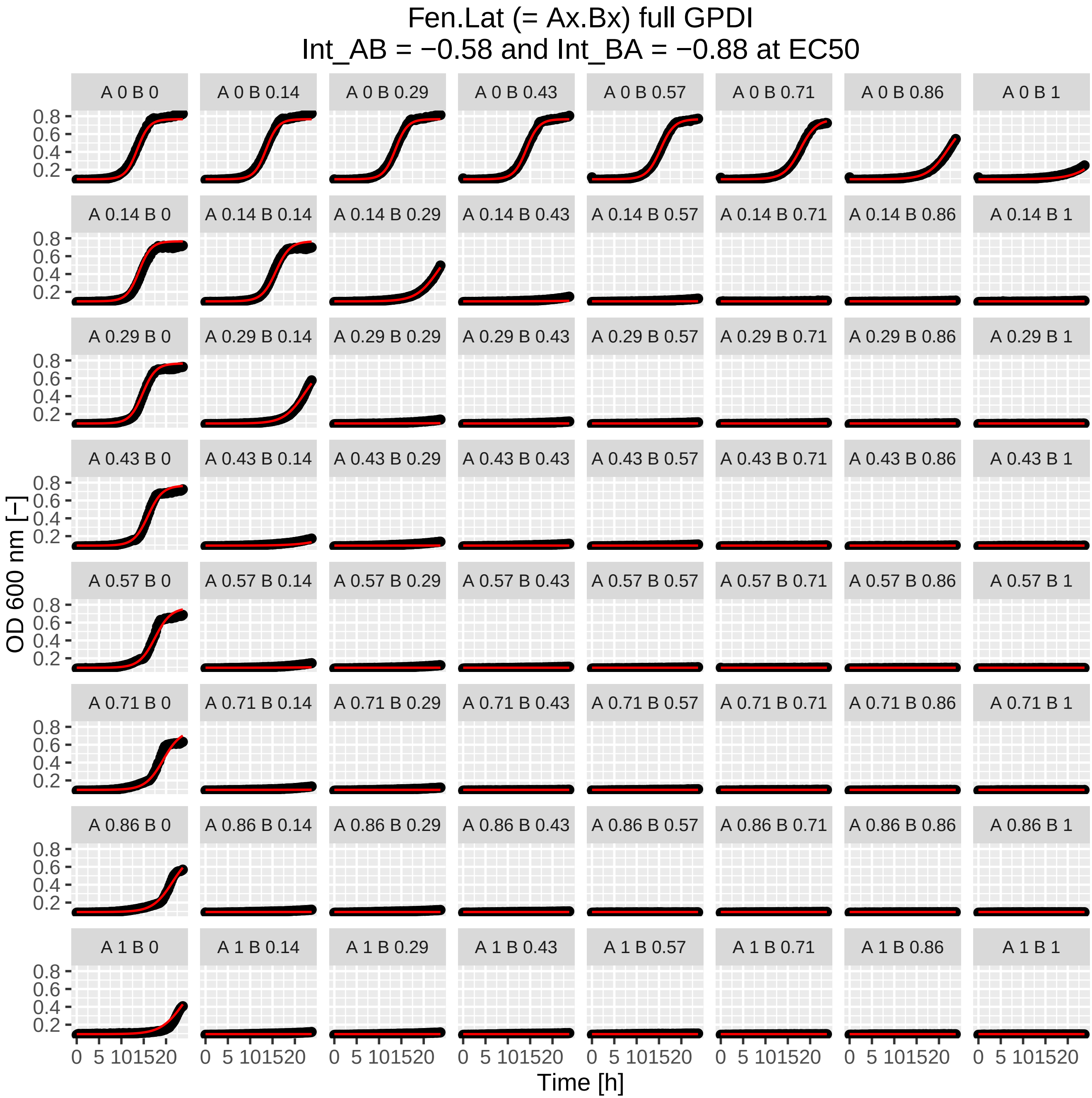

Fen.Lat (= Ax.Bx) full GPDI
Int_AB = −0.58 and Int_BA = −0.88 at EC50
A 0 B 0
A 0 B 0.14
A 0 B 0.29
A 0 B 0.43
A 0 B 0.57
A 0 B 0.71
A 0 B 0.86
A 0 B 1
A 0.14 B 0
A 0.14 B 0.14
A 0.14 B 0.29
A 0.14 B 0.43
A 0.14 B 0.57
A 0.14 B 0.71
A 0.14 B 0.86
A 0.14 B 1
A 0.29 B 0
A 0.29 B 0.14
A 0.29 B 0.29
A 0.29 B 0.43
A 0.29 B 0.57
A 0.29 B 0.71
A 0.29 B 0.86
A 0.29 B 1
A 0.43 B 0
A 0.43 B 0.14
A 0.43 B 0.29
A 0.43 B 0.43
A 0.43 B 0.57
A 0.43 B 0.71
A 0.43 B 0.86
A 0.43 B 1
A 0.57 B 0
A 0.57 B 0.14
A 0.57 B 0.29
A 0.57 B 0.43
A 0.57 B 0.57
A 0.57 B 0.71
A 0.57 B 0.86
A 0.57 B 1
A 0.71 B 0
A 0.71 B 0.14
A 0.71 B 0.29
A 0.71 B 0.43
A 0.71 B 0.57
A 0.71 B 0.71
A 0.71 B 0.86
A 0.71 B 1
A 0.86 B 0
A 0.86 B 0.14
A 0.86 B 0.29
A 0.86 B 0.43
A 0.86 B 0.57
A 0.86 B 0.71
A 0.86 B 0.86
A 0.86 B 1
A 1 B 0
A 1 B 0.14
A 1 B 0.29
A 1 B 0.43
A 1 B 0.57
A 1 B 0.71
A 1 B 0.86
A 1 B 1
OD 600 nm [−]
0.2
0.4
0.6
0.8
0
5
10
15
20
Time [h]

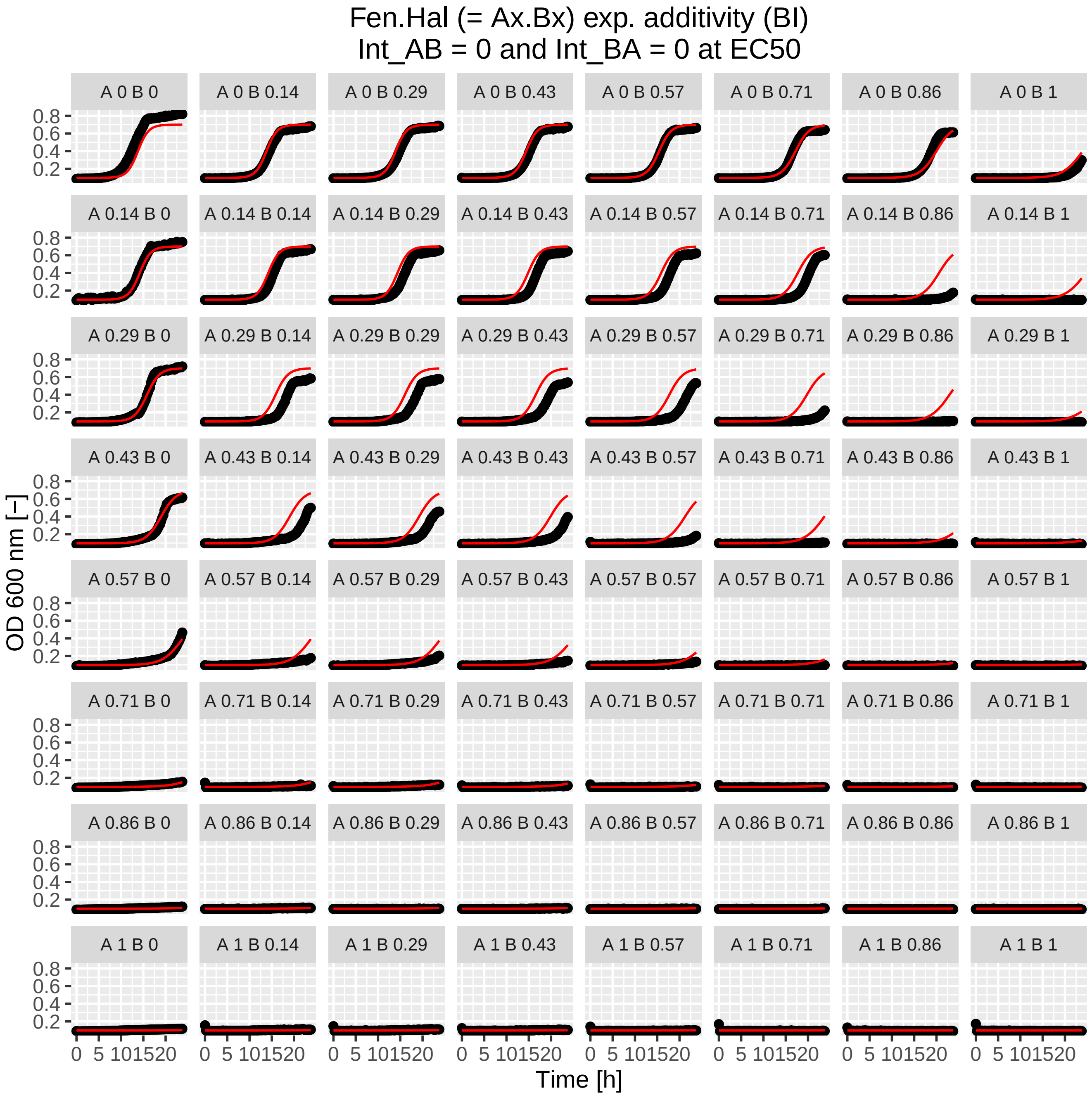

Fen.Hal (= Ax.Bx) exp. additivity (BI)
Int_AB = 0 and Int_BA = 0 at EC50
A 0 B 0
A 0 B 0.14
A 0 B 0.29
A 0 B 0.43
A 0 B 0.57
A 0 B 0.71
A 0 B 0.86
A 0 B 1
A 0.14 B 0
A 0.14 B 0.14
A 0.14 B 0.29
A 0.14 B 0.43
A 0.14 B 0.57
A 0.14 B 0.71
A 0.14 B 0.86
A 0.14 B 1
A 0.29 B 0
A 0.29 B 0.14
A 0.29 B 0.29
A 0.29 B 0.43
A 0.29 B 0.57
A 0.29 B 0.71
A 0.29 B 0.86
A 0.29 B 1
A 0.43 B 0
A 0.43 B 0.14
A 0.43 B 0.29
A 0.43 B 0.43
A 0.43 B 0.57
A 0.43 B 0.71
A 0.43 B 0.86
A 0.43 B 1
A 0.57 B 0
A 0.57 B 0.14
A 0.57 B 0.29
A 0.57 B 0.43
A 0.57 B 0.57
A 0.57 B 0.71
A 0.57 B 0.86
A 0.57 B 1
A 0.71 B 0
A 0.71 B 0.14
A 0.71 B 0.29
A 0.71 B 0.43
A 0.71 B 0.57
A 0.71 B 0.71
A 0.71 B 0.86
A 0.71 B 1
A 0.86 B 0
A 0.86 B 0.14
A 0.86 B 0.29
A 0.86 B 0.43
A 0.86 B 0.57
A 0.86 B 0.71
A 0.86 B 0.86
A 0.86 B 1
A 1 B 0
A 1 B 0.14
A 1 B 0.29
A 1 B 0.43
A 1 B 0.57
A 1 B 0.71
A 1 B 0.86
A 1 B 1
OD 600 nm [−]
0.2
0.4
0.6
0.8
0 5 101520
Time [h]

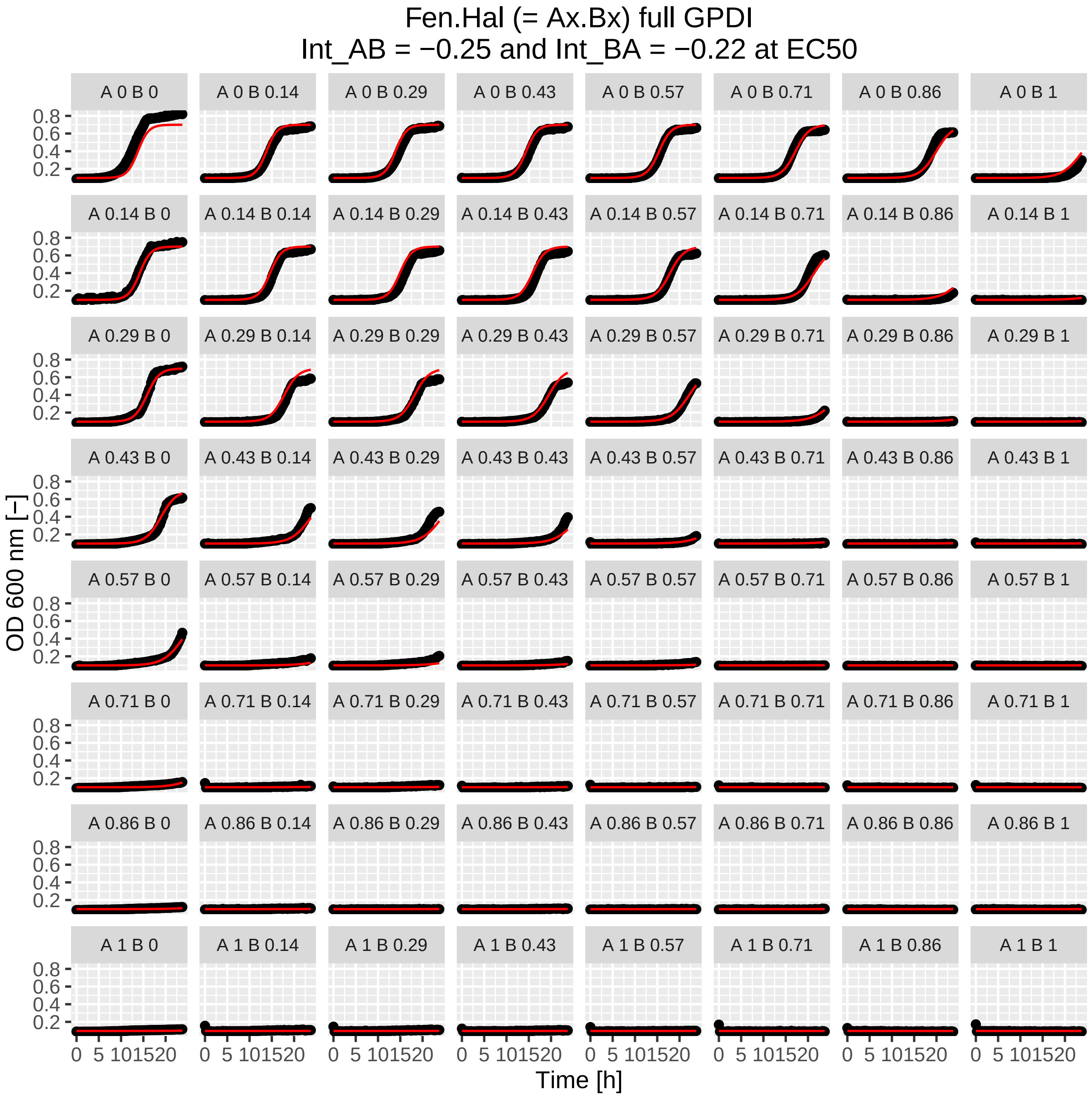

Fen.Hal (= Ax.Bx) full GPDI
Int_AB = −0.25 and Int_BA = −0.22 at EC50
A 0 B 0
A 0 B 0.14
A 0 B 0.29
A 0 B 0.43
A 0 B 0.57
A 0 B 0.71
A 0 B 0.86
A 0 B 1
A 0.14 B 0
A 0.14 B 0.14
A 0.14 B 0.29
A 0.14 B 0.43
A 0.14 B 0.57
A 0.14 B 0.71
A 0.14 B 0.86
A 0.14 B 1
A 0.29 B 0
A 0.29 B 0.14
A 0.29 B 0.29
A 0.29 B 0.43
A 0.29 B 0.57
A 0.29 B 0.71
A 0.29 B 0.86
A 0.29 B 1
A 0.43 B 0
A 0.43 B 0.14
A 0.43 B 0.29
A 0.43 B 0.43
A 0.43 B 0.57
A 0.43 B 0.71
A 0.43 B 0.86
A 0.43 B 1
A 0.57 B 0
A 0.57 B 0.14
A 0.57 B 0.29
A 0.57 B 0.43
A 0.57 B 0.57
A 0.57 B 0.71
A 0.57 B 0.86
A 0.57 B 1
A 0.71 B 0
A 0.71 B 0.14
A 0.71 B 0.29
A 0.71 B 0.43
A 0.71 B 0.57
A 0.71 B 0.71
A 0.71 B 0.86
A 0.71 B 1
A 0.86 B 0
A 0.86 B 0.14
A 0.86 B 0.29
A 0.86 B 0.43
A 0.86 B 0.57
A 0.86 B 0.71
A 0.86 B 0.86
A 0.86 B 1
A 1 B 0
A 1 B 0.14
A 1 B 0.29
A 1 B 0.43
A 1 B 0.57
A 1 B 0.71
A 1 B 0.86
A 1 B 1
0.8
0.6
0.4
0.2
OD 600 nm [−]
0 5 101520
Time [h]

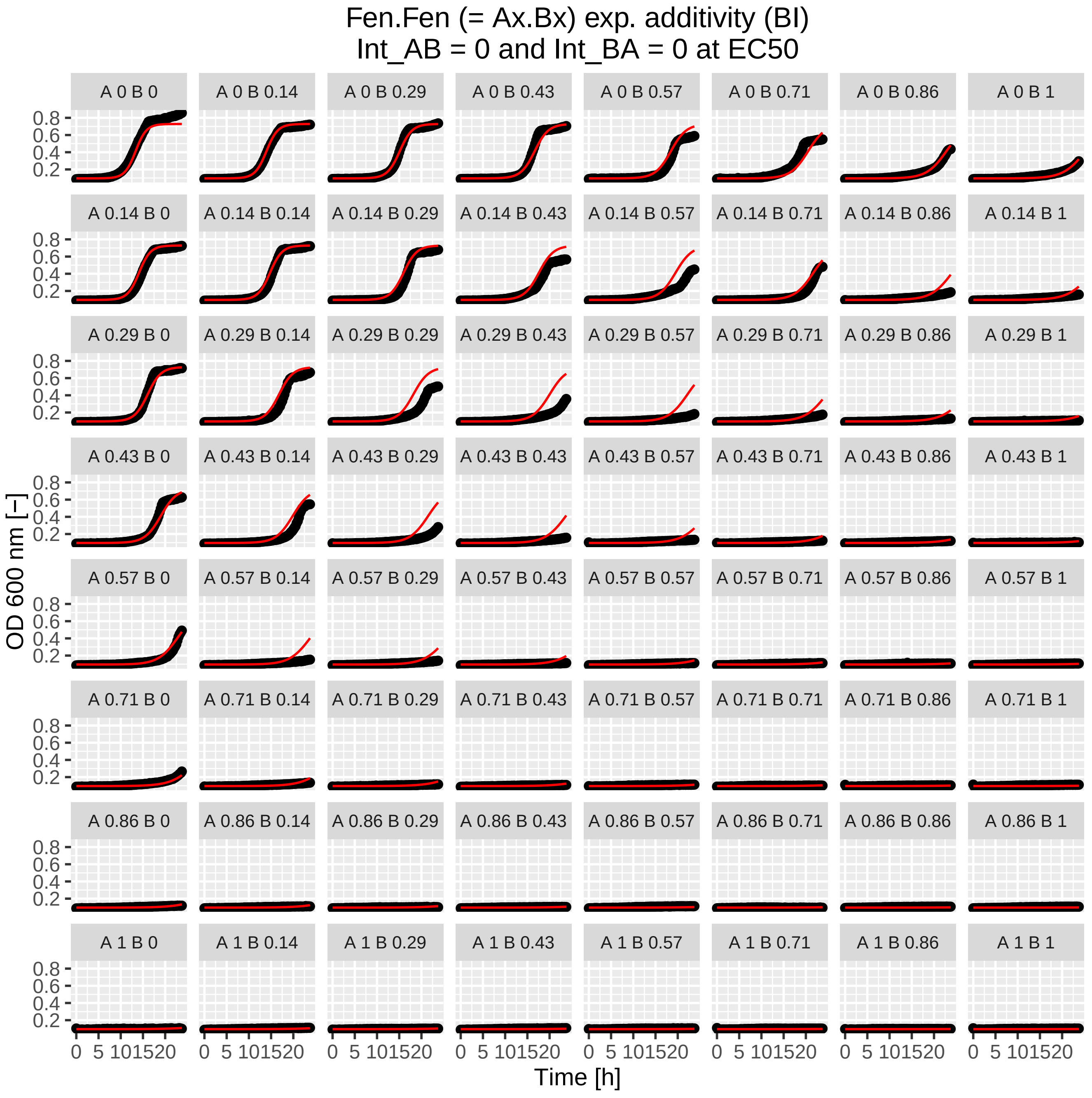

Fen.Fen (= Ax.Bx) exp. additivity (BI)
Int_AB = 0 and Int_BA = 0 at EC50
A 0 B 0
A 0 B 0.14
A 0 B 0.29
A 0 B 0.43
A 0 B 0.57
A 0 B 0.71
A 0 B 0.86
A 0 B 1
A 0.14 B 0
A 0.14 B 0.14
A 0.14 B 0.29
A 0.14 B 0.43
A 0.14 B 0.57
A 0.14 B 0.71
A 0.14 B 0.86
A 0.14 B 1
A 0.29 B 0
A 0.29 B 0.14
A 0.29 B 0.29
A 0.29 B 0.43
A 0.29 B 0.57
A 0.29 B 0.71
A 0.29 B 0.86
A 0.29 B 1
A 0.43 B 0
A 0.43 B 0.14
A 0.43 B 0.29
A 0.43 B 0.43
A 0.43 B 0.57
A 0.43 B 0.71
A 0.43 B 0.86
A 0.43 B 1
A 0.57 B 0
A 0.57 B 0.14
A 0.57 B 0.29
A 0.57 B 0.43
A 0.57 B 0.57
A 0.57 B 0.71
A 0.57 B 0.86
A 0.57 B 1
A 0.71 B 0
A 0.71 B 0.14
A 0.71 B 0.29
A 0.71 B 0.43
A 0.71 B 0.57
A 0.71 B 0.71
A 0.71 B 0.86
A 0.71 B 1
A 0.86 B 0
A 0.86 B 0.14
A 0.86 B 0.29
A 0.86 B 0.43
A 0.86 B 0.57
A 0.86 B 0.71
A 0.86 B 0.86
A 0.86 B 1
A 1 B 0
A 1 B 0.14
A 1 B 0.29
A 1 B 0.43
A 1 B 0.57
A 1 B 0.71
A 1 B 0.86
A 1 B 1
OD 600 nm [−]
0.2
0.4
0.6
0.8
0
5
10
15
20
Time [h]

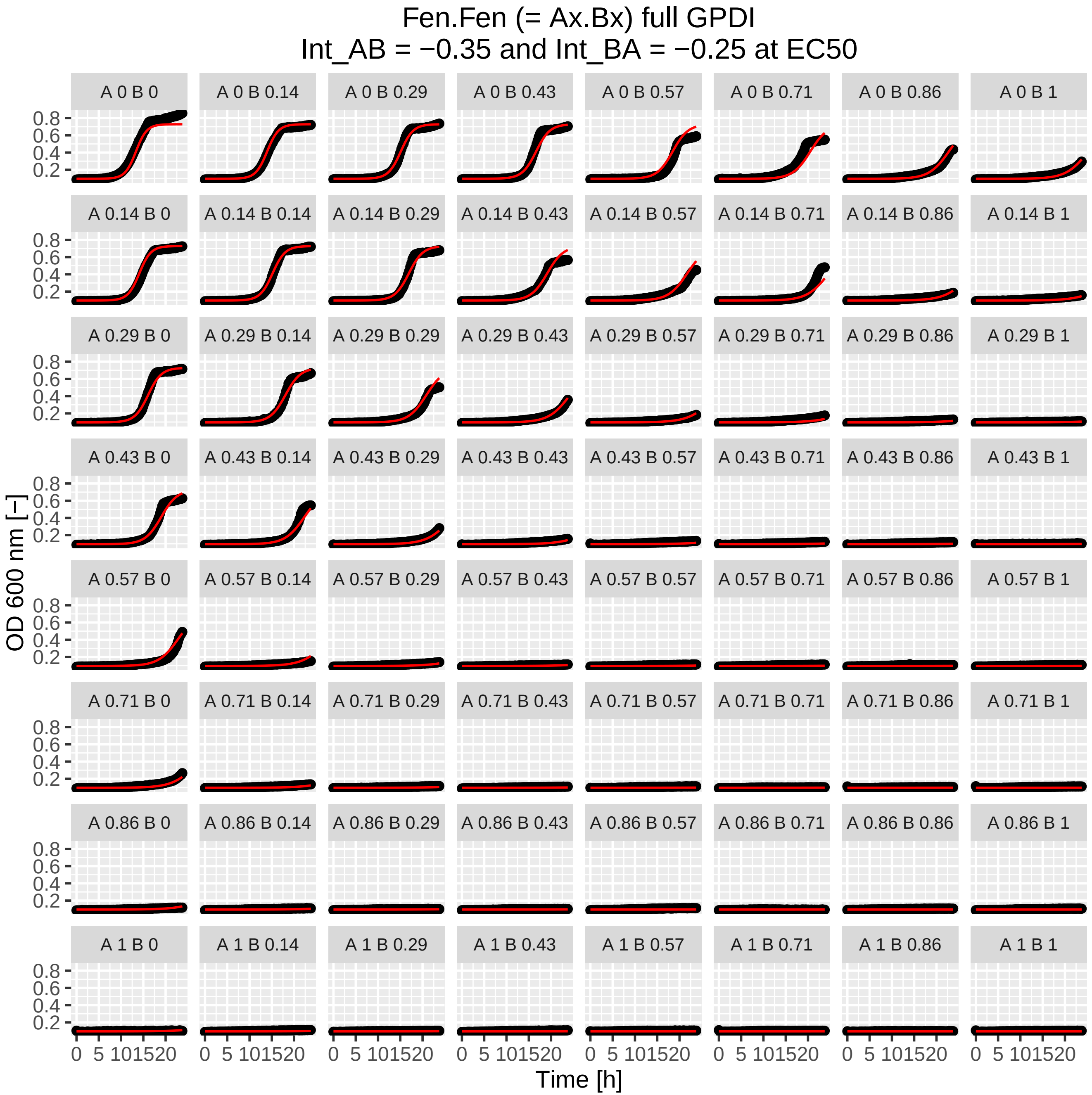

Fen.Fen (= Ax.Bx) full GPDI
Int_AB = −0.35 and Int_BA = −0.25 at EC50
OD 600 nm [−]
Time [h]
A 0 B 0
A 0 B 0.14
A 0 B 0.29
A 0 B 0.43
A 0 B 0.57
A 0 B 0.71
A 0 B 0.86
A 0 B 1
A 1 B 0
A 1 B 0.14
A 1 B 0.29
A 1 B 0.43
A 1 B 0.57
A 1 B 0.71
A 1 B 0.86
A 1 B 1

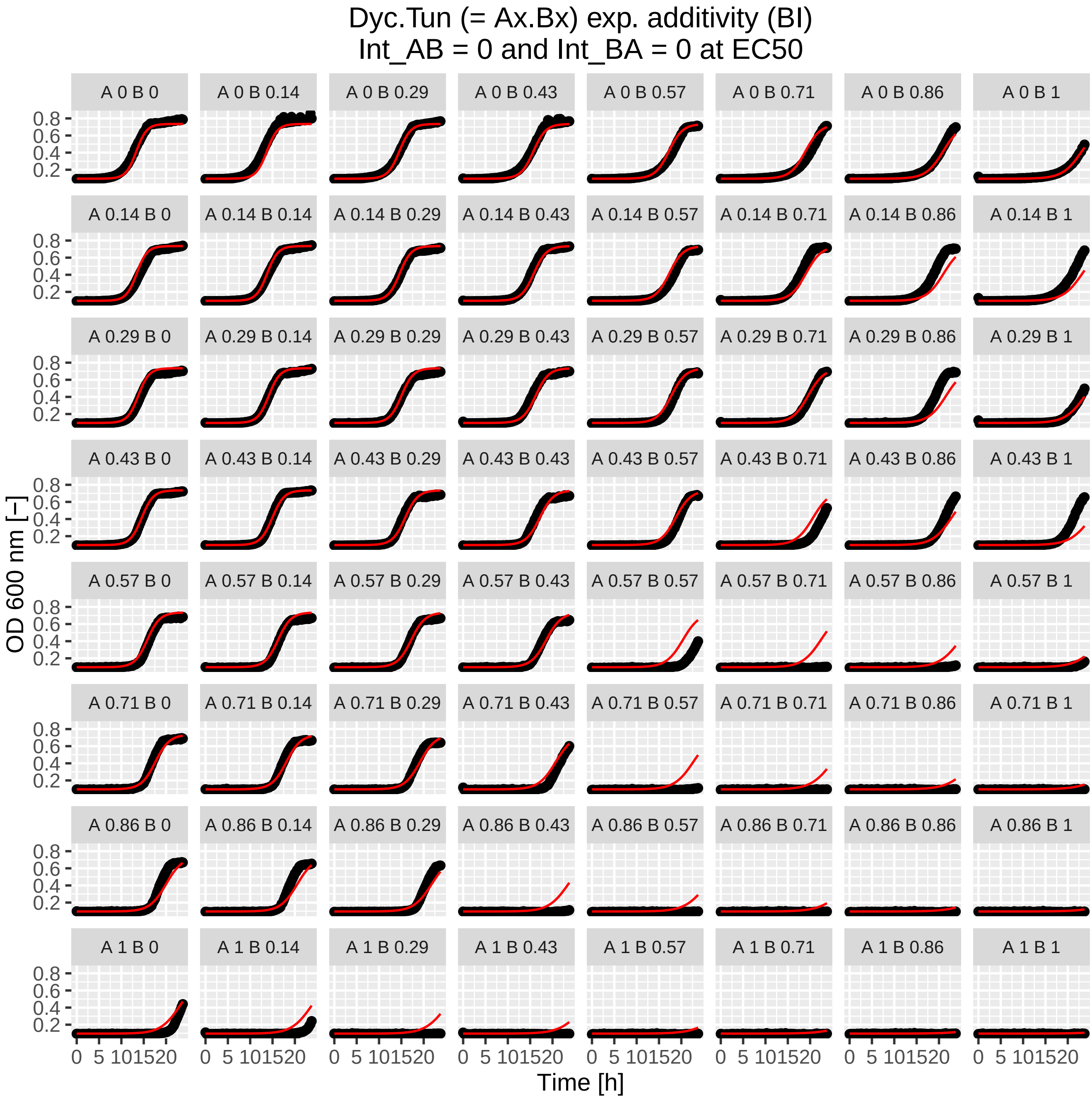

Dyc.Tun (= Ax.Bx) exp. additivity (BI)
Int_AB = 0 and Int_BA = 0 at EC50
OD 600 nm [−]
Time [h]
0.8
0.6
0.4
0.2
0 5 101520
A 0 B 0
A 0 B 0.14
A 0 B 0.29
A 0 B 0.43
A 0 B 0.57
A 0 B 0.71
A 0 B 0.86
A 0 B 1
A 0.14 B 0
A 0.14 B 0.14
A 0.14 B 0.29
A 0.14 B 0.43
A 0.14 B 0.57
A 0.14 B 0.71
A 0.14 B 0.86
A 0.14 B 1
A 0.29 B 0
A 0.29 B 0.14
A 0.29 B 0.29
A 0.29 B 0.43
A 0.29 B 0.57
A 0.29 B 0.71
A 0.29 B 0.86
A 0.29 B 1
A 0.43 B 0
A 0.43 B 0.14
A 0.43 B 0.29
A 0.43 B 0.43
A 0.43 B 0.57
A 0.43 B 0.71
A 0.43 B 0.86
A 0.43 B 1
A 0.57 B 0
A 0.57 B 0.14
A 0.57 B 0.29
A 0.57 B 0.43
A 0.57 B 0.57
A 0.57 B 0.71
A 0.57 B 0.86
A 0.57 B 1
A 0.71 B 0
A 0.71 B 0.14
A 0.71 B 0.29
A 0.71 B 0.43
A 0.71 B 0.57
A 0.71 B 0.71
A 0.71 B 0.86
A 0.71 B 1
A 0.86 B 0
A 0.86 B 0.14
A 0.86 B 0.29
A 0.86 B 0.43
A 0.86 B 0.57
A 0.86 B 0.71
A 0.86 B 0.86
A 0.86 B 1
A 1 B 0
A 1 B 0.14
A 1 B 0.29
A 1 B 0.43
A 1 B 0.57
A 1 B 0.71
A 1 B 0.86
A 1 B 1

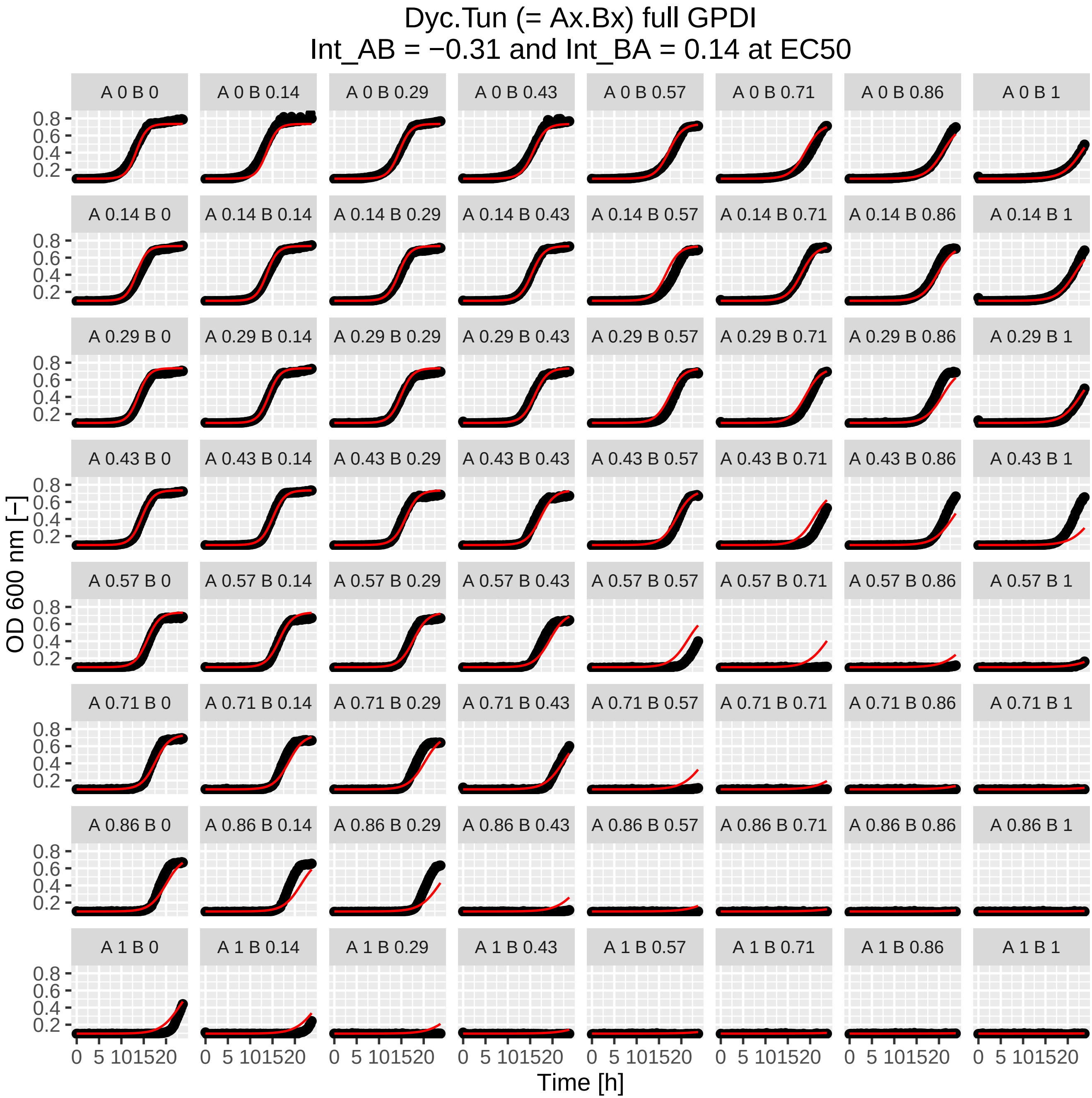
Dyc.Tun (= Ax.Bx) full GPDI
Int_AB = −0.31 and Int_BA = 0.14 at EC50
OD 600 nm [−]
Time [h]
A 0 B 0
A 0 B 0.14
A 0 B 0.29
A 0 B 0.43
A 0 B 0.57
A 0 B 0.71
A 0 B 0.86
A 0 B 1
A 0.14 B 0
A 0.14 B 0.14
A 0.14 B 0.29
A 0.14 B 0.43
A 0.14 B 0.57
A 0.14 B 0.71
A 0.14 B 0.86
A 0.14 B 1
A 0.29 B 0
A 0.29 B 0.14
A 0.29 B 0.29
A 0.29 B 0.43
A 0.29 B 0.57
A 0.29 B 0.71
A 0.29 B 0.86
A 0.29 B 1
A 0.43 B 0
A 0.43 B 0.14
A 0.43 B 0.29
A 0.43 B 0.43
A 0.43 B 0.57
A 0.43 B 0.71
A 0.43 B 0.86
A 0.43 B 1
A 0.57 B 0
A 0.57 B 0.14
A 0.57 B 0.29
A 0.57 B 0.43
A 0.57 B 0.57
A 0.57 B 0.71
A 0.57 B 0.86
A 0.57 B 1
A 0.71 B 0
A 0.71 B 0.14
A 0.71 B 0.29
A 0.71 B 0.43
A 0.71 B 0.57
A 0.71 B 0.71
A 0.71 B 0.86
A 0.71 B 1
A 0.86 B 0
A 0.86 B 0.14
A 0.86 B 0.29
A 0.86 B 0.43
A 0.86 B 0.57
A 0.86 B 0.71
A 0.86 B 0.86
A 0.86 B 1
A 1 B 0
A 1 B 0.14
A 1 B 0.29
A 1 B 0.43
A 1 B 0.57
A 1 B 0.71
A 1 B 0.86
A 1 B 1
0.8
0.6
0.4
0.2
0
5
10
15
20

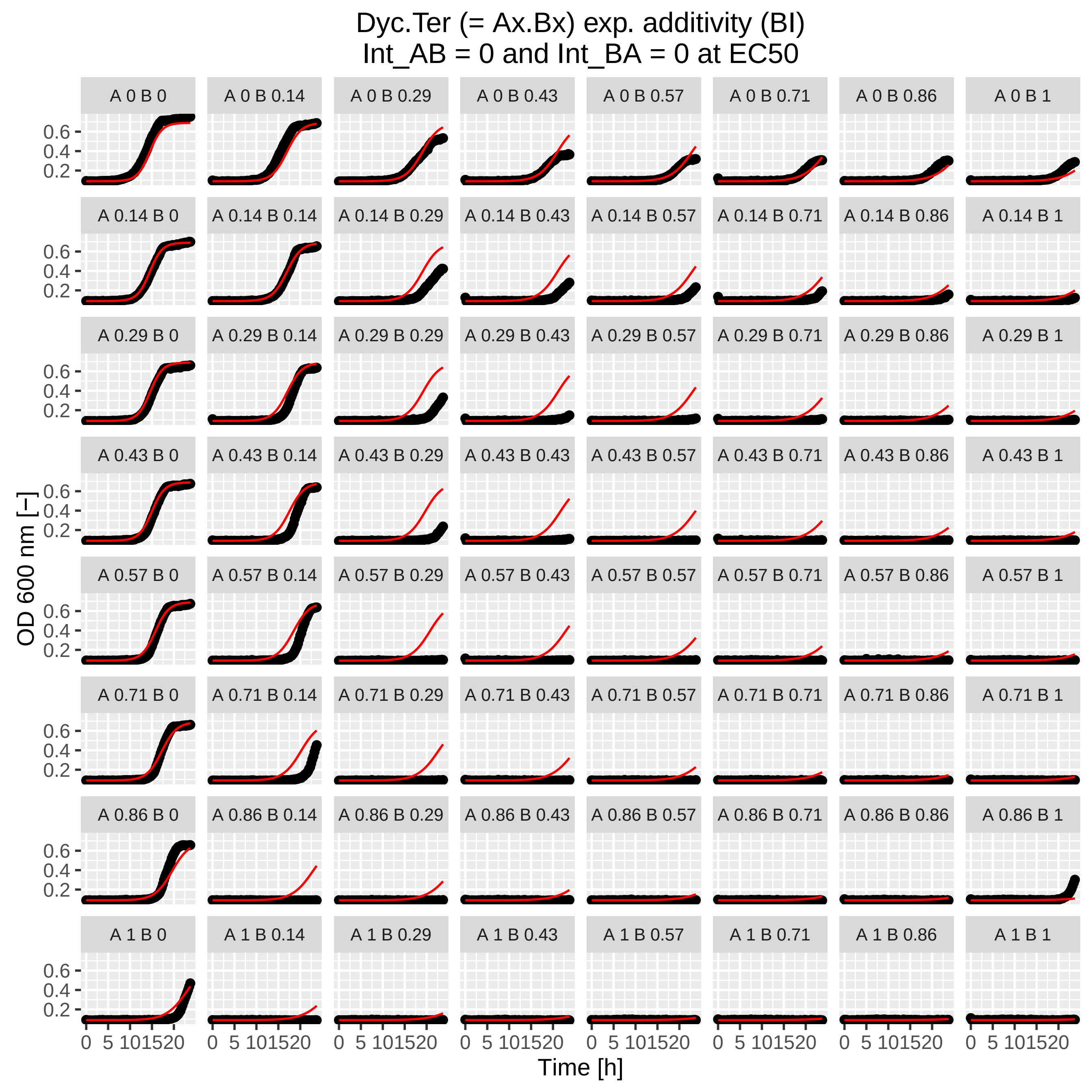

Dyc.Ter (= Ax.Bx) exp. additivity (BI)
Int_AB = 0 and Int_BA = 0 at EC50
A 0 B 0
A 0 B 0.14
A 0 B 0.29
A 0 B 0.43
A 0 B 0.57
A 0 B 0.71
A 0 B 0.86
A 0 B 1
A 0.14 B 0
A 0.14 B 0.14
A 0.14 B 0.29
A 0.14 B 0.43
A 0.14 B 0.57
A 0.14 B 0.71
A 0.14 B 0.86
A 0.14 B 1
A 0.29 B 0
A 0.29 B 0.14
A 0.29 B 0.29
A 0.29 B 0.43
A 0.29 B 0.57
A 0.29 B 0.71
A 0.29 B 0.86
A 0.29 B 1
A 0.43 B 0
A 0.43 B 0.14
A 0.43 B 0.29
A 0.43 B 0.43
A 0.43 B 0.57
A 0.43 B 0.71
A 0.43 B 0.86
A 0.43 B 1
A 0.57 B 0
A 0.57 B 0.14
A 0.57 B 0.29
A 0.57 B 0.43
A 0.57 B 0.57
A 0.57 B 0.71
A 0.57 B 0.86
A 0.57 B 1
A 0.71 B 0
A 0.71 B 0.14
A 0.71 B 0.29
A 0.71 B 0.43
A 0.71 B 0.57
A 0.71 B 0.71
A 0.71 B 0.86
A 0.71 B 1
A 0.86 B 0
A 0.86 B 0.14
A 0.86 B 0.29
A 0.86 B 0.43
A 0.86 B 0.57
A 0.86 B 0.71
A 0.86 B 0.86
A 0.86 B 1
A 1 B 0
A 1 B 0.14
A 1 B 0.29
A 1 B 0.43
A 1 B 0.57
A 1 B 0.71
A 1 B 0.86
A 1 B 1
OD 600 nm [–]
0.2
0.4
0.6
0
5
10
15
20
Time [h]

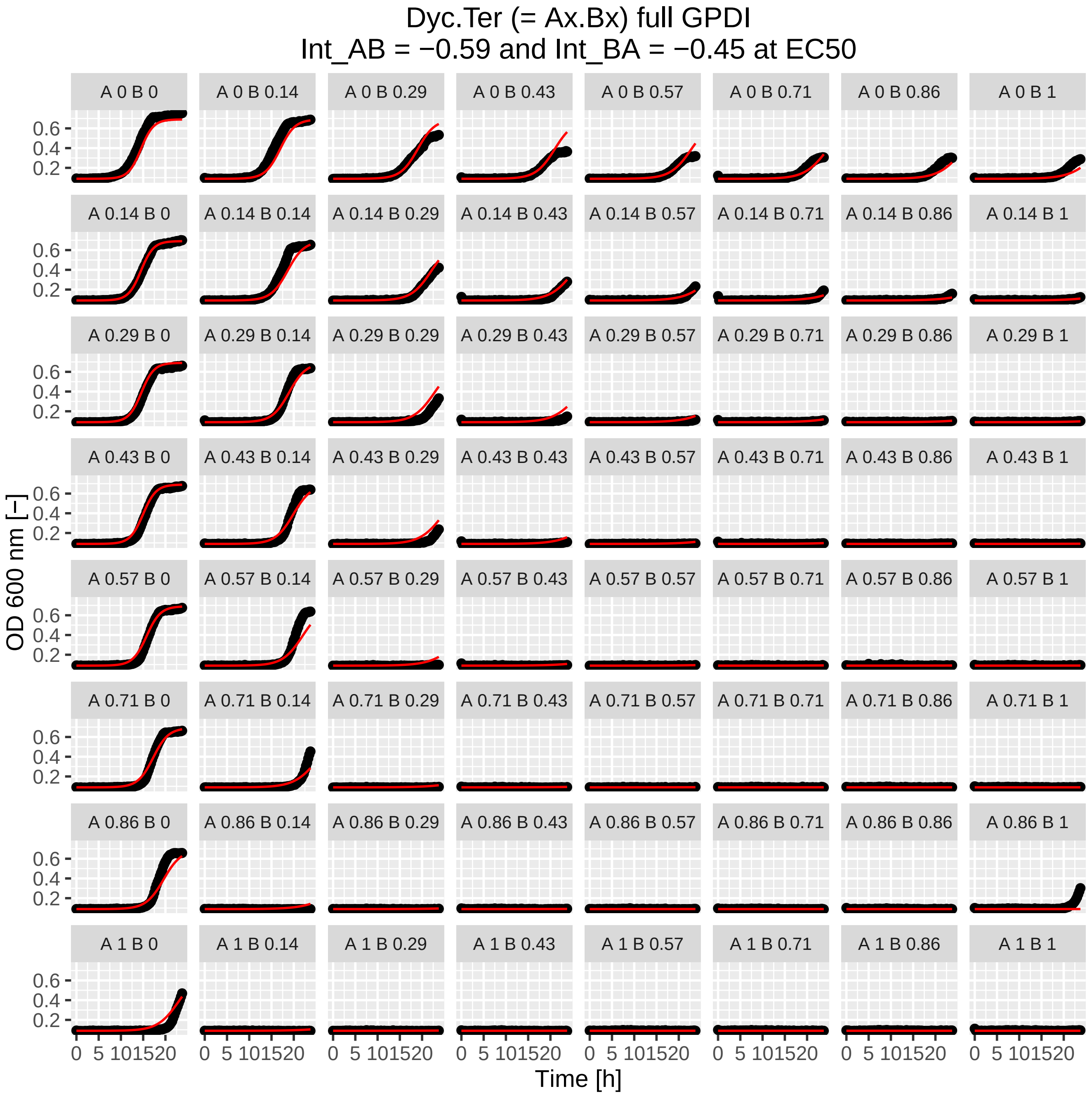

Dyc.Ter (= Ax.Bx) full GPDI
Int_AB = −0.59 and Int_BA = −0.45 at EC50
OD 600 nm [−]
Time [h]
A 0 B 0
A 0 B 0.14
A 0 B 0.29
A 0 B 0.43
A 0 B 0.57
A 0 B 0.71
A 0 B 0.86
A 0 B 1
A 0.14 B 0
A 0.14 B 0.14
A 0.14 B 0.29
A 0.14 B 0.43
A 0.14 B 0.57
A 0.14 B 0.71
A 0.14 B 0.86
A 0.14 B 1
A 0.29 B 0
A 0.29 B 0.14
A 0.29 B 0.29
A 0.29 B 0.43
A 0.29 B 0.57
A 0.29 B 0.71
A 0.29 B 0.86
A 0.29 B 1
A 0.43 B 0
A 0.43 B 0.14
A 0.43 B 0.29
A 0.43 B 0.43
A 0.43 B 0.57
A 0.43 B 0.71
A 0.43 B 0.86
A 0.43 B 1
A 0.57 B 0
A 0.57 B 0.14
A 0.57 B 0.29
A 0.57 B 0.43
A 0.57 B 0.57
A 0.57 B 0.71
A 0.57 B 0.86
A 0.57 B 1
A 0.71 B 0
A 0.71 B 0.14
A 0.71 B 0.29
A 0.71 B 0.43
A 0.71 B 0.57
A 0.71 B 0.71
A 0.71 B 0.86
A 0.71 B 1
A 0.86 B 0
A 0.86 B 0.14
A 0.86 B 0.29
A 0.86 B 0.43
A 0.86 B 0.57
A 0.86 B 0.71
A 0.86 B 0.86
A 0.86 B 1
A 1 B 0
A 1 B 0.14
A 1 B 0.29
A 1 B 0.43
A 1 B 0.57
A 1 B 0.71
A 1 B 0.86
A 1 B 1
0.2
0.4
0.6
0 5 101520

Dyc.Tac (= Ax.Bx) exp. additivity (BI)
Int_AB = 0 and Int_BA = 0 at EC50

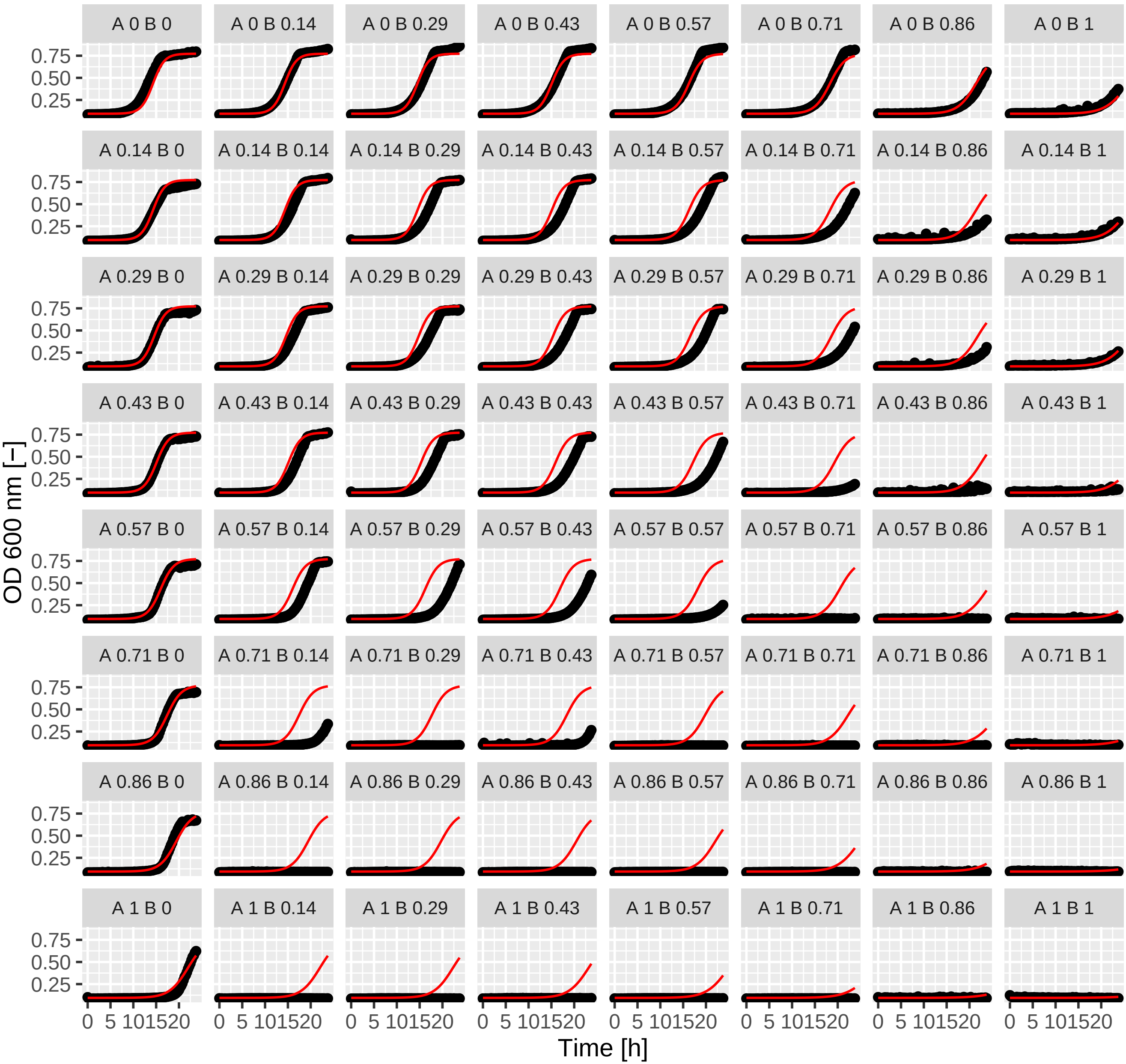

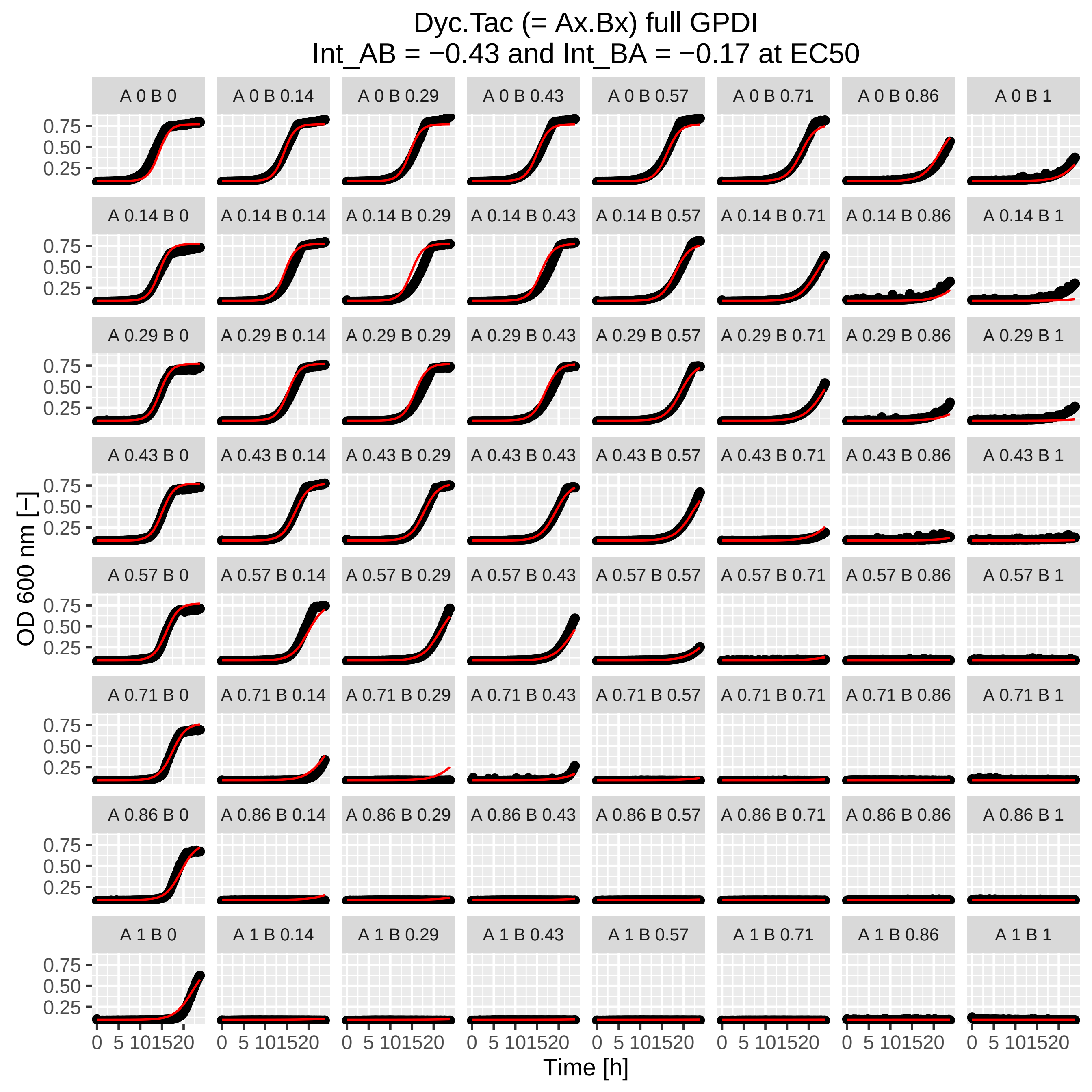

Dyc.Tac (= Ax.Bx) full GPDI
Int_AB = −0.43 and Int_BA = −0.17 at EC50
OD 600 nm [−]
Time [h]

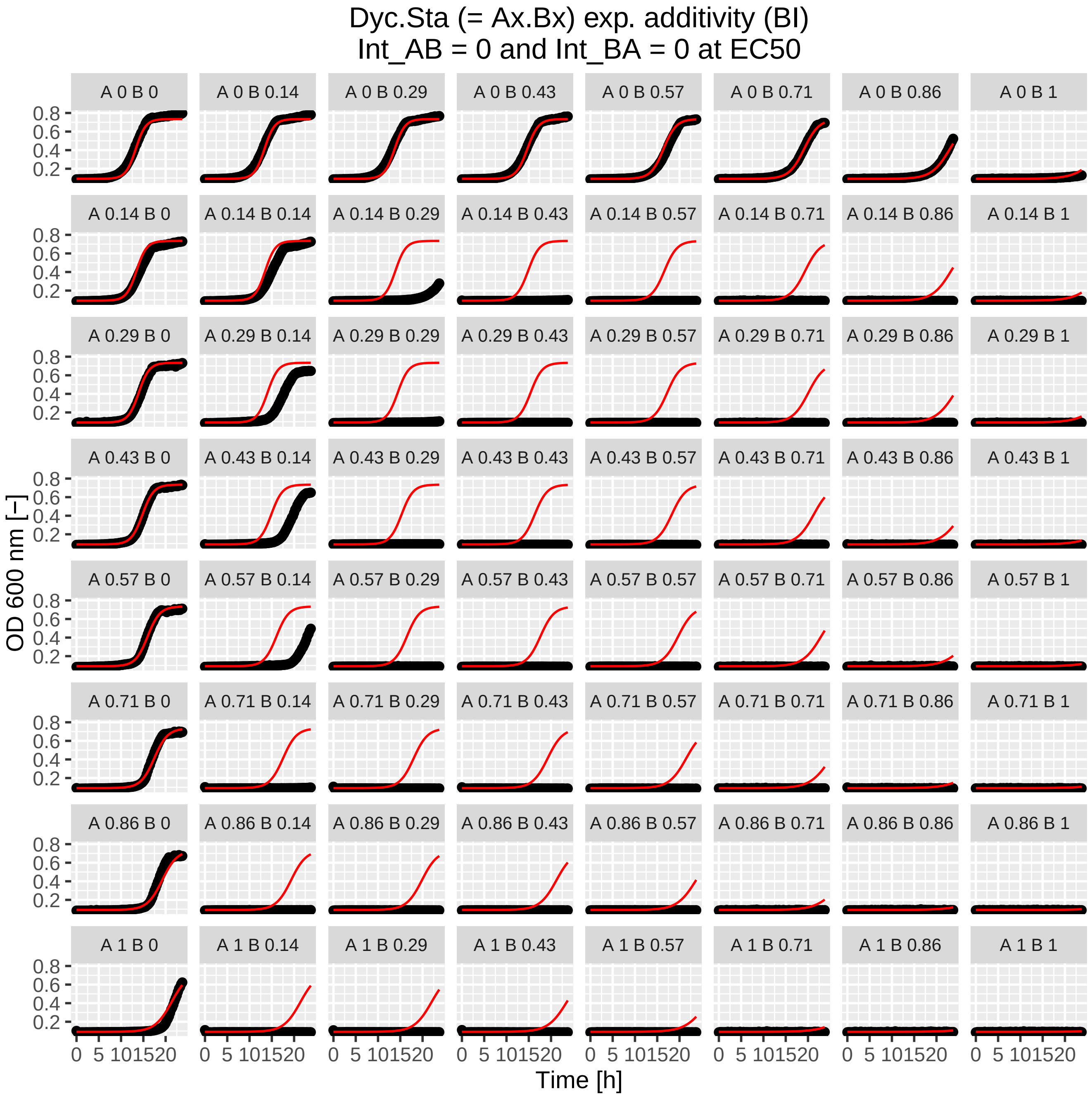
Dyc.Sta (= Ax.Bx) exp. additivity (BI)
Int_AB = 0 and Int_BA = 0 at EC50
A 0 B 0
A 0 B 0.14
A 0 B 0.29
A 0 B 0.43
A 0 B 0.57
A 0 B 0.71
A 0 B 0.86
A 0 B 1
A 0.14 B 0
A 0.14 B 0.14
A 0.14 B 0.29
A 0.14 B 0.43
A 0.14 B 0.57
A 0.14 B 0.71
A 0.14 B 0.86
A 0.14 B 1
A 0.29 B 0
A 0.29 B 0.14
A 0.29 B 0.29
A 0.29 B 0.43
A 0.29 B 0.57
A 0.29 B 0.71
A 0.29 B 0.86
A 0.29 B 1
A 0.43 B 0
A 0.43 B 0.14
A 0.43 B 0.29
A 0.43 B 0.43
A 0.43 B 0.57
A 0.43 B 0.71
A 0.43 B 0.86
A 0.43 B 1
A 0.57 B 0
A 0.57 B 0.14
A 0.57 B 0.29
A 0.57 B 0.43
A 0.57 B 0.57
A 0.57 B 0.71
A 0.57 B 0.86
A 0.57 B 1
A 0.71 B 0
A 0.71 B 0.14
A 0.71 B 0.29
A 0.71 B 0.43
A 0.71 B 0.57
A 0.71 B 0.71
A 0.71 B 0.86
A 0.71 B 1
A 0.86 B 0
A 0.86 B 0.14
A 0.86 B 0.29
A 0.86 B 0.43
A 0.86 B 0.57
A 0.86 B 0.71
A 0.86 B 0.86
A 0.86 B 1
A 1 B 0
A 1 B 0.14
A 1 B 0.29
A 1 B 0.43
A 1 B 0.57
A 1 B 0.71
A 1 B 0.86
A 1 B 1
0.8
0.6
0.4
0.2
OD 600 nm [−]
0 5 101520
Time [h]

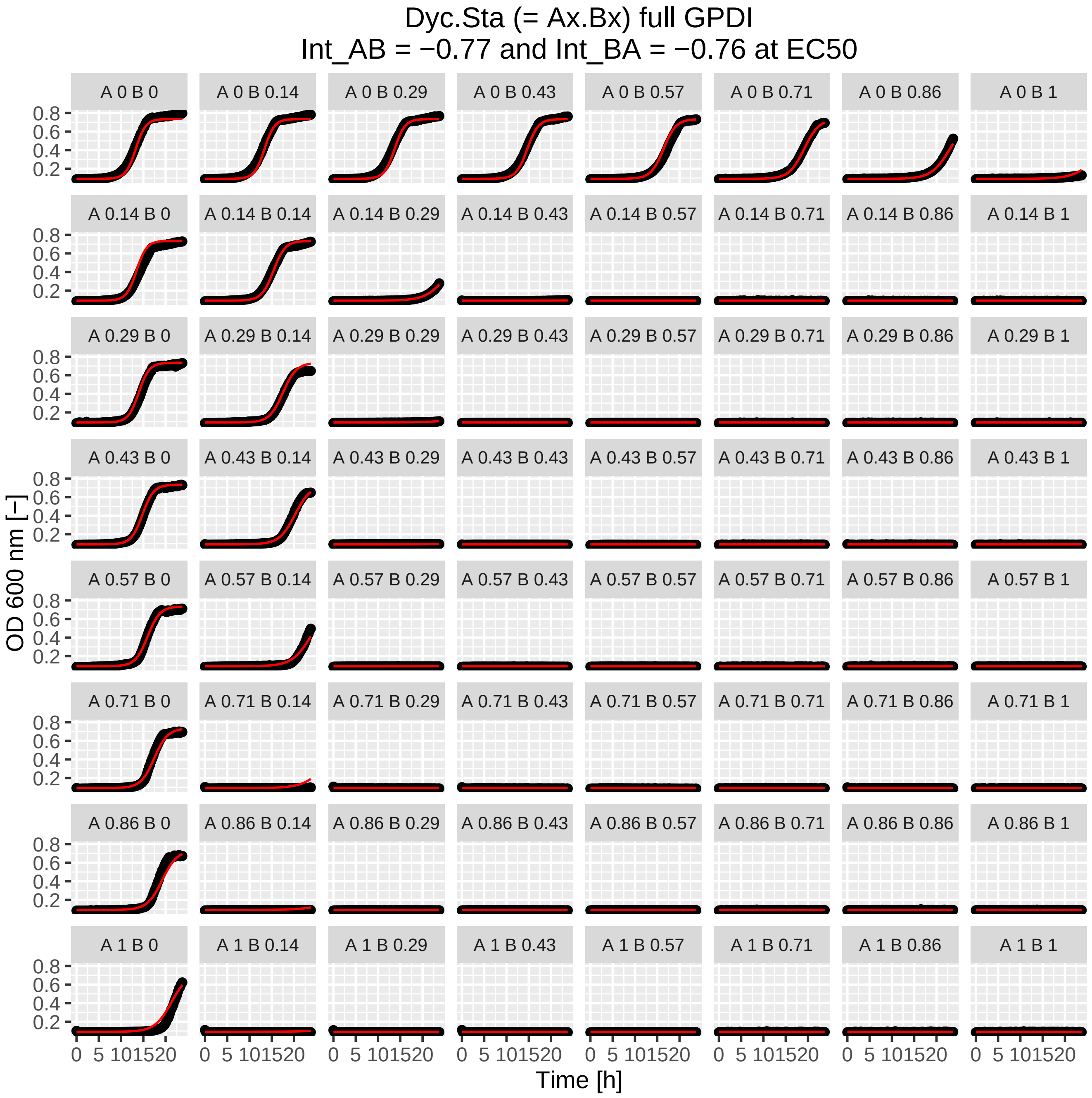

Dyc.Sta (= Ax.Bx) full GPDI
Int_AB = −0.77 and Int_BA = −0.76 at EC50
A 0 B 0
A 0 B 0.14
A 0 B 0.29
A 0 B 0.43
A 0 B 0.57
A 0 B 0.71
A 0 B 0.86
A 0 B 1
A 0.14 B 0
A 0.14 B 0.14
A 0.14 B 0.29
A 0.14 B 0.43
A 0.14 B 0.57
A 0.14 B 0.71
A 0.14 B 0.86
A 0.14 B 1
A 0.29 B 0
A 0.29 B 0.14
A 0.29 B 0.29
A 0.29 B 0.43
A 0.29 B 0.57
A 0.29 B 0.71
A 0.29 B 0.86
A 0.29 B 1
A 0.43 B 0
A 0.43 B 0.14
A 0.43 B 0.29
A 0.43 B 0.43
A 0.43 B 0.57
A 0.43 B 0.71
A 0.43 B 0.86
A 0.43 B 1
A 0.57 B 0
A 0.57 B 0.14
A 0.57 B 0.29
A 0.57 B 0.43
A 0.57 B 0.57
A 0.57 B 0.71
A 0.57 B 0.86
A 0.57 B 1
A 0.71 B 0
A 0.71 B 0.14
A 0.71 B 0.29
A 0.71 B 0.43
A 0.71 B 0.57
A 0.71 B 0.71
A 0.71 B 0.86
A 0.71 B 1
A 0.86 B 0
A 0.86 B 0.14
A 0.86 B 0.29
A 0.86 B 0.43
A 0.86 B 0.57
A 0.86 B 0.71
A 0.86 B 0.86
A 0.86 B 1
A 1 B 0
A 1 B 0.14
A 1 B 0.29
A 1 B 0.43
A 1 B 0.57
A 1 B 0.71
A 1 B 0.86
A 1 B 1
0.2
0.4
0.6
0.8
0 5 101520
OD 600 nm [−]
Time [h]

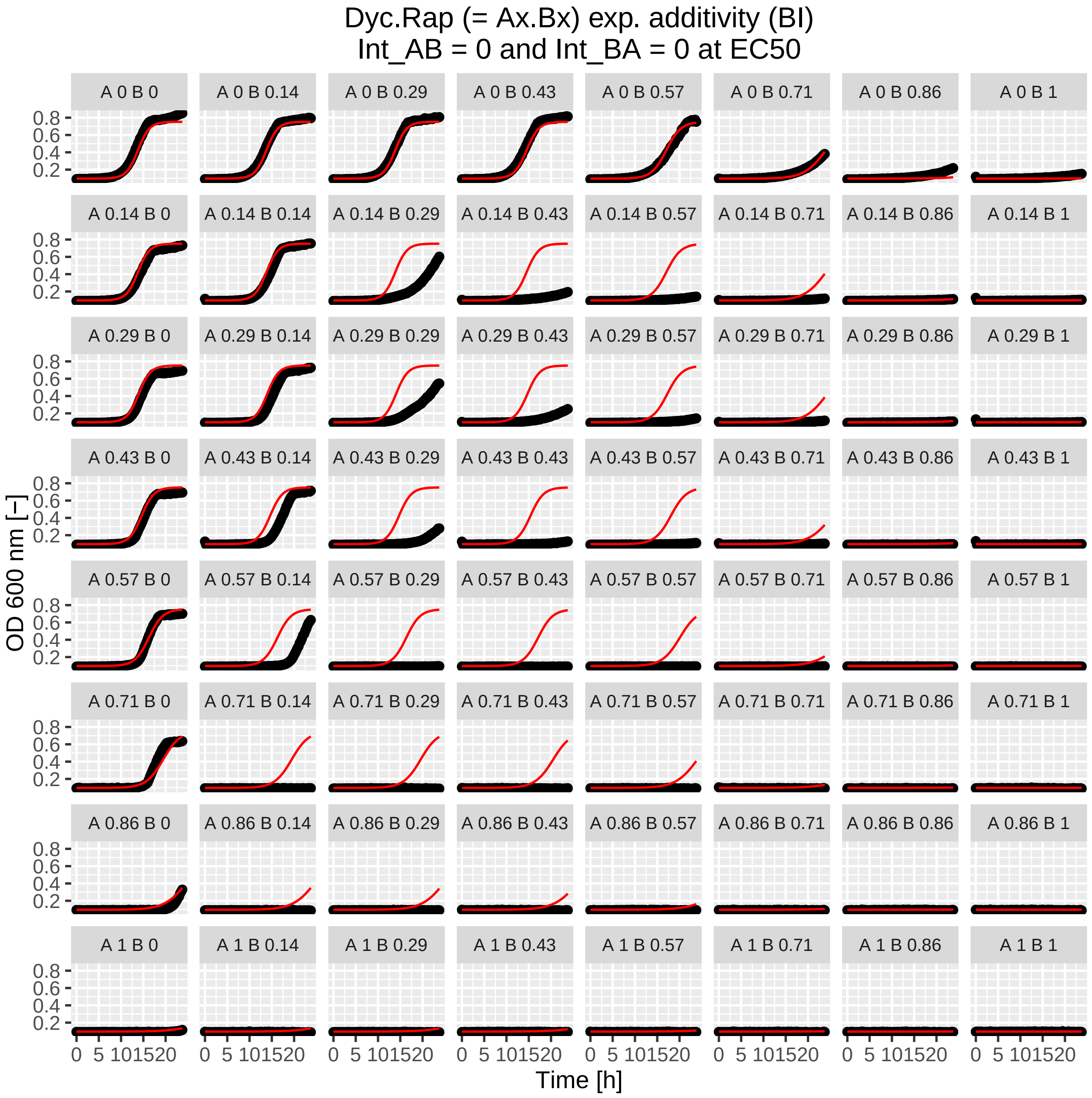

Dyc.Rap (= Ax.Bx) exp. additivity (BI)
Int_AB = 0 and Int_BA = 0 at EC50
OD 600 nm [–]
Time [h]
0.8
0.6
0.4
0.2
0 5 10 15 20
A 0 B 0
A 0 B 0.14
A 0 B 0.29
A 0 B 0.43
A 0 B 0.57
A 0 B 0.71
A 0 B 0.86
A 0 B 1
A 0.14 B 0
A 0.14 B 0.14
A 0.14 B 0.29
A 0.14 B 0.43
A 0.14 B 0.57
A 0.14 B 0.71
A 0.14 B 0.86
A 0.14 B 1
A 0.29 B 0
A 0.29 B 0.14
A 0.29 B 0.29
A 0.29 B 0.43
A 0.29 B 0.57
A 0.29 B 0.71
A 0.29 B 0.86
A 0.29 B 1
A 0.43 B 0
A 0.43 B 0.14
A 0.43 B 0.29
A 0.43 B 0.43
A 0.43 B 0.57
A 0.43 B 0.71
A 0.43 B 0.86
A 0.43 B 1
A 0.57 B 0
A 0.57 B 0.14
A 0.57 B 0.29
A 0.57 B 0.43
A 0.57 B 0.57
A 0.57 B 0.71
A 0.57 B 0.86
A 0.57 B 1
A 0.71 B 0
A 0.71 B 0.14
A 0.71 B 0.29
A 0.71 B 0.43
A 0.71 B 0.57
A 0.71 B 0.71
A 0.71 B 0.86
A 0.71 B 1
A 0.86 B 0
A 0.86 B 0.14
A 0.86 B 0.29
A 0.86 B 0.43
A 0.86 B 0.57
A 0.86 B 0.71
A 0.86 B 0.86
A 0.86 B 1
A 1 B 0
A 1 B 0.14
A 1 B 0.29
A 1 B 0.43
A 1 B 0.57
A 1 B 0.71
A 1 B 0.86
A 1 B 1

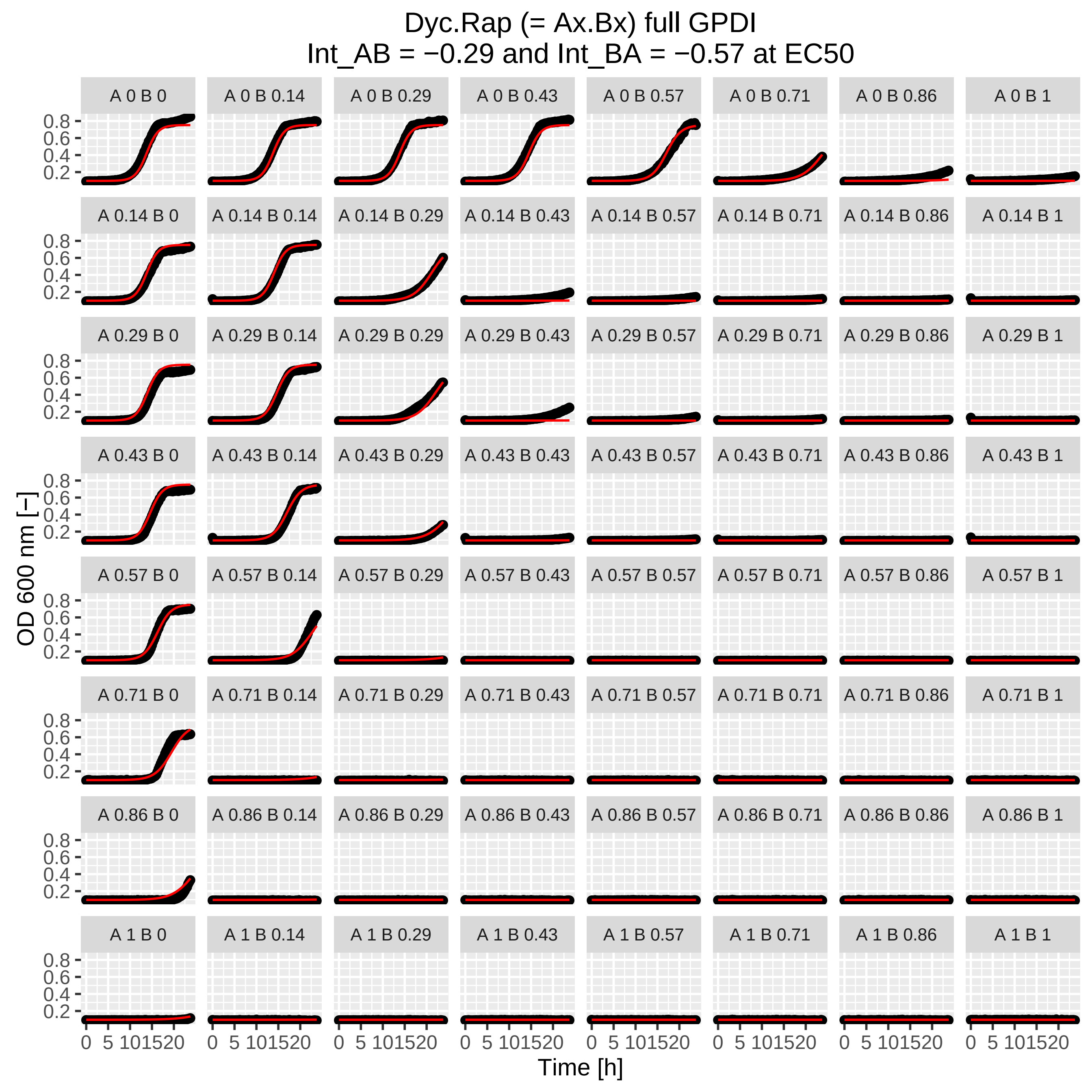
Dyc.Rap (= Ax.Bx) full GPDI
Int_AB = −0.29 and Int_BA = −0.57 at EC50
OD 600 nm [−]
Time [h]
0.8
0.6
0.4
0.2
0 5 101520
A 0 B 0
A 0 B 0.14
A 0 B 0.29
A 0 B 0.43
A 0 B 0.57
A 0 B 0.71
A 0 B 0.86
A 0 B 1
A 0.14 B 0
A 0.14 B 0.14
A 0.14 B 0.29
A 0.14 B 0.43
A 0.14 B 0.57
A 0.14 B 0.71
A 0.14 B 0.86
A 0.14 B 1
A 0.29 B 0
A 0.29 B 0.14
A 0.29 B 0.29
A 0.29 B 0.43
A 0.29 B 0.57
A 0.29 B 0.71
A 0.29 B 0.86
A 0.29 B 1
A 0.43 B 0
A 0.43 B 0.14
A 0.43 B 0.29
A 0.43 B 0.43
A 0.43 B 0.57
A 0.43 B 0.71
A 0.43 B 0.86
A 0.43 B 1
A 0.57 B 0
A 0.57 B 0.14
A 0.57 B 0.29
A 0.57 B 0.43
A 0.57 B 0.57
A 0.57 B 0.71
A 0.57 B 0.86
A 0.57 B 1
A 0.71 B 0
A 0.71 B 0.14
A 0.71 B 0.29
A 0.71 B 0.43
A 0.71 B 0.57
A 0.71 B 0.71
A 0.71 B 0.86
A 0.71 B 1
A 0.86 B 0
A 0.86 B 0.14
A 0.86 B 0.29
A 0.86 B 0.43
A 0.86 B 0.57
A 0.86 B 0.71
A 0.86 B 0.86
A 0.86 B 1
A 1 B 0
A 1 B 0.14
A 1 B 0.29
A 1 B 0.43
A 1 B 0.57
A 1 B 0.71
A 1 B 0.86
A 1 B 1

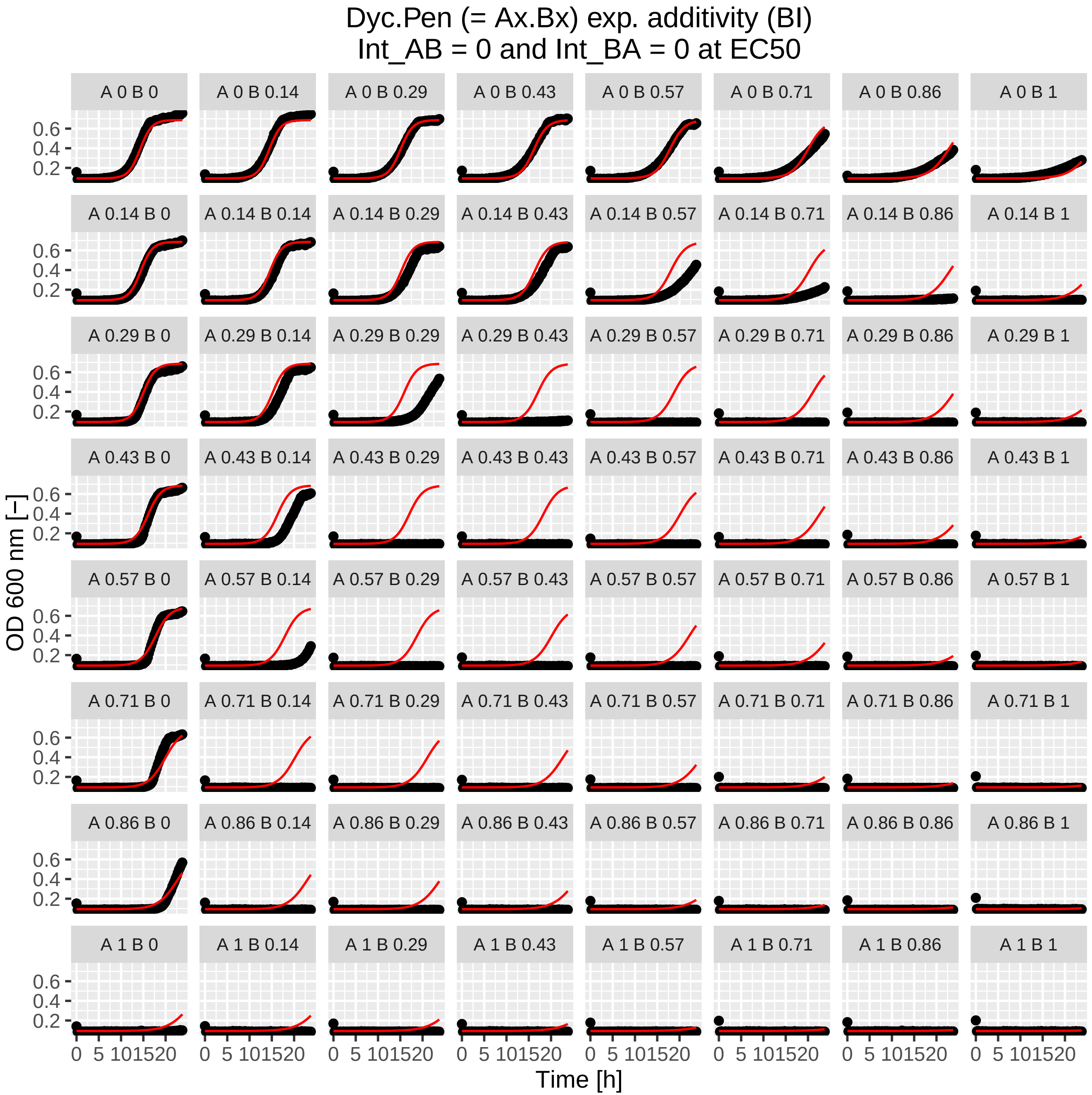

Dyc.Pen (= Ax.Bx) exp. additivity (BI)
Int_AB = 0 and Int_BA = 0 at EC50
A 0 B 0
A 0 B 0.14
A 0 B 0.29
A 0 B 0.43
A 0 B 0.57
A 0 B 0.71
A 0 B 0.86
A 0 B 1
A 0.14 B 0
A 0.14 B 0.14
A 0.14 B 0.29
A 0.14 B 0.43
A 0.14 B 0.57
A 0.14 B 0.71
A 0.14 B 0.86
A 0.14 B 1
A 0.29 B 0
A 0.29 B 0.14
A 0.29 B 0.29
A 0.29 B 0.43
A 0.29 B 0.57
A 0.29 B 0.71
A 0.29 B 0.86
A 0.29 B 1
A 0.43 B 0
A 0.43 B 0.14
A 0.43 B 0.29
A 0.43 B 0.43
A 0.43 B 0.57
A 0.43 B 0.71
A 0.43 B 0.86
A 0.43 B 1
A 0.57 B 0
A 0.57 B 0.14
A 0.57 B 0.29
A 0.57 B 0.43
A 0.57 B 0.57
A 0.57 B 0.71
A 0.57 B 0.86
A 0.57 B 1
A 0.71 B 0
A 0.71 B 0.14
A 0.71 B 0.29
A 0.71 B 0.43
A 0.71 B 0.57
A 0.71 B 0.71
A 0.71 B 0.86
A 0.71 B 1
A 0.86 B 0
A 0.86 B 0.14
A 0.86 B 0.29
A 0.86 B 0.43
A 0.86 B 0.57
A 0.86 B 0.71
A 0.86 B 0.86
A 0.86 B 1
A 1 B 0
A 1 B 0.14
A 1 B 0.29
A 1 B 0.43
A 1 B 0.57
A 1 B 0.71
A 1 B 0.86
A 1 B 1
OD 600 nm [–]
0.2
0.4
0.6
0 5 101520
Time [h]

# Dyc.Pen (= Ax.Bx) full GPDI
Int_AB = −0.84 and Int_BA = −0.33 at EC50

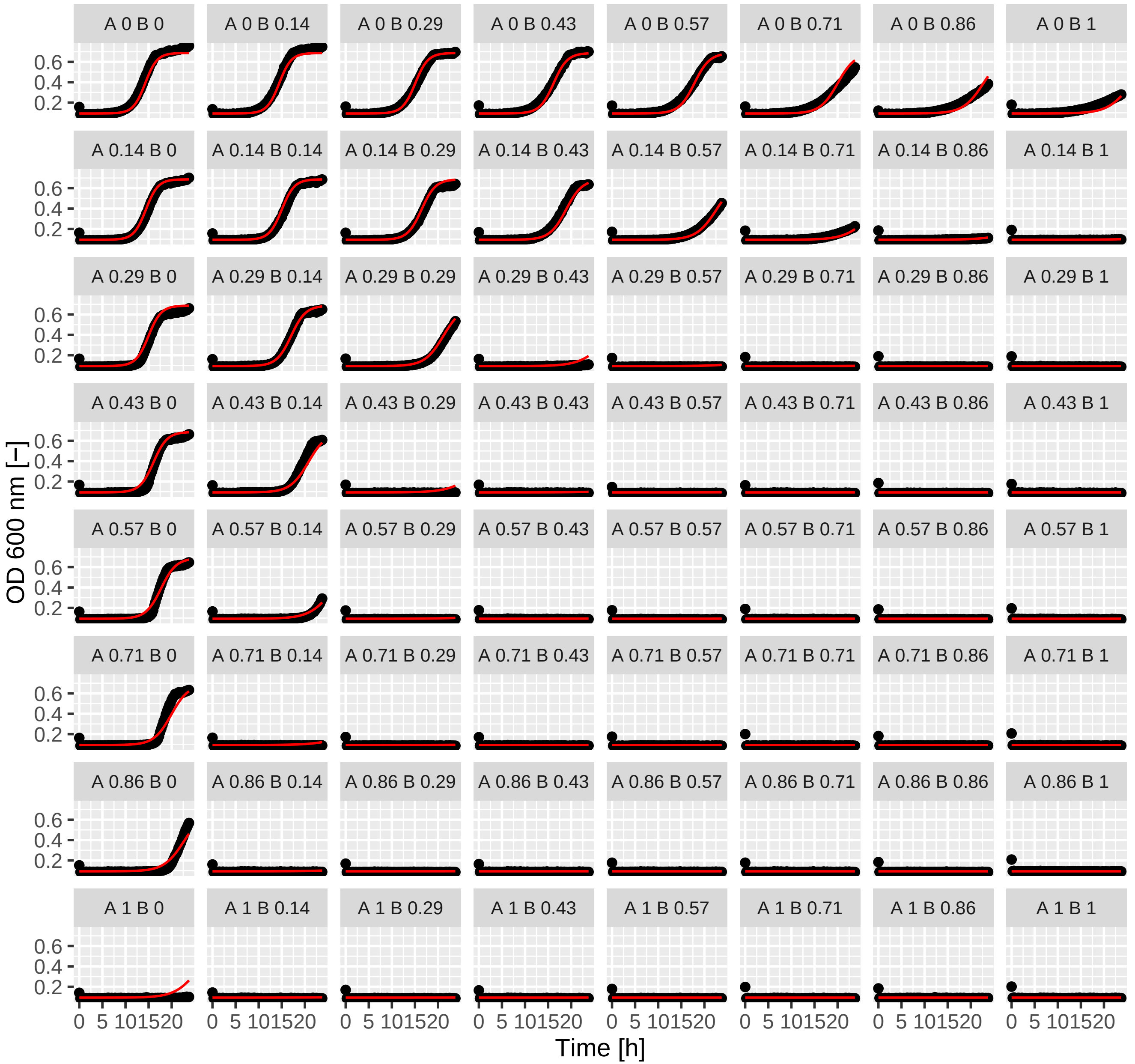

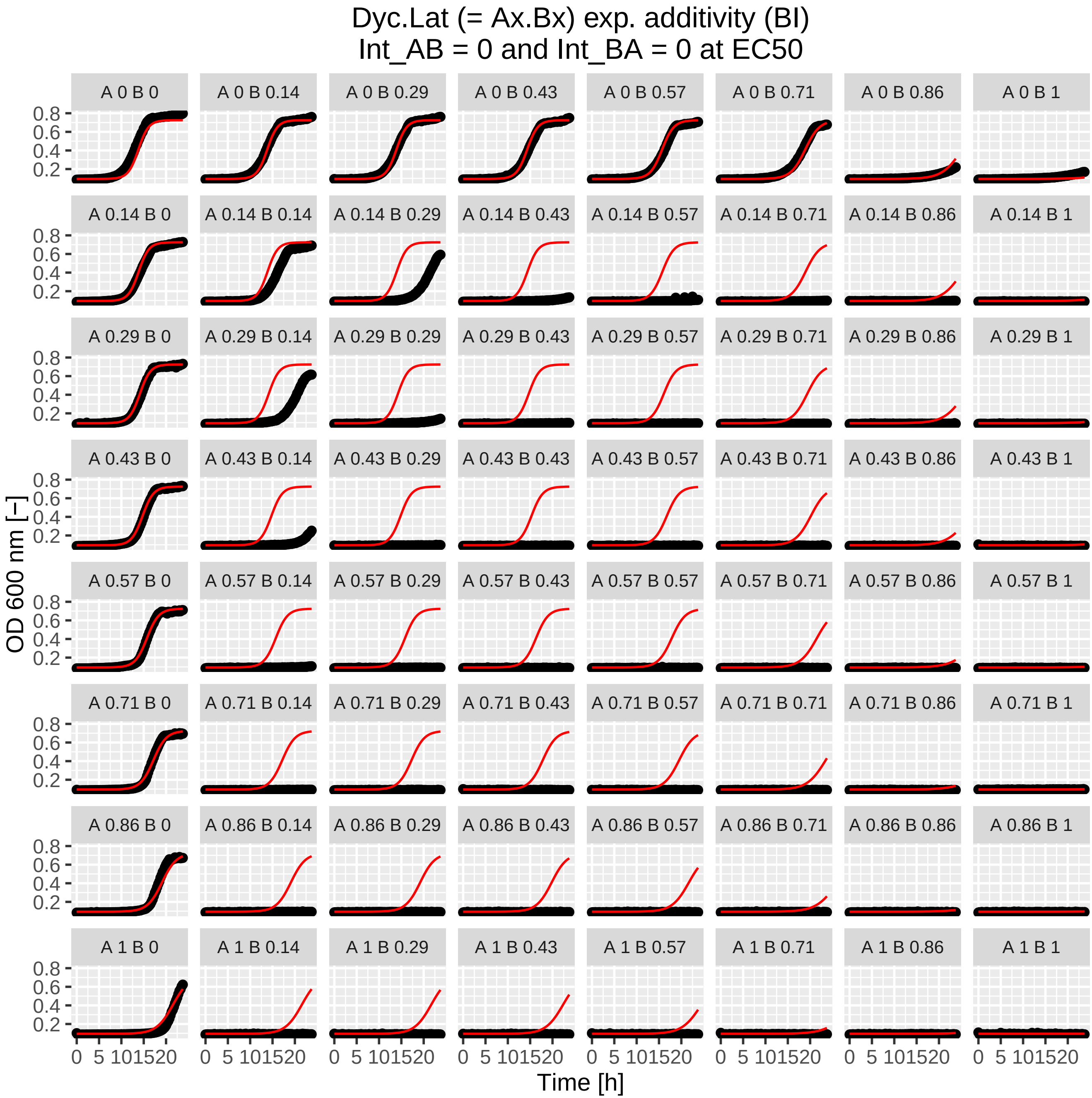
Dyc.Lat (= Ax.Bx) exp. additivity (BI)
Int_AB = 0 and Int_BA = 0 at EC50
A 0 B 0
A 0 B 0.14
A 0 B 0.29
A 0 B 0.43
A 0 B 0.57
A 0 B 0.71
A 0 B 0.86
A 0 B 1
A 0.14 B 0
A 0.14 B 0.14
A 0.14 B 0.29
A 0.14 B 0.43
A 0.14 B 0.57
A 0.14 B 0.71
A 0.14 B 0.86
A 0.14 B 1
A 0.29 B 0
A 0.29 B 0.14
A 0.29 B 0.29
A 0.29 B 0.43
A 0.29 B 0.57
A 0.29 B 0.71
A 0.29 B 0.86
A 0.29 B 1
A 0.43 B 0
A 0.43 B 0.14
A 0.43 B 0.29
A 0.43 B 0.43
A 0.43 B 0.57
A 0.43 B 0.71
A 0.43 B 0.86
A 0.43 B 1
A 0.57 B 0
A 0.57 B 0.14
A 0.57 B 0.29
A 0.57 B 0.43
A 0.57 B 0.57
A 0.57 B 0.71
A 0.57 B 0.86
A 0.57 B 1
A 0.71 B 0
A 0.71 B 0.14
A 0.71 B 0.29
A 0.71 B 0.43
A 0.71 B 0.57
A 0.71 B 0.71
A 0.71 B 0.86
A 0.71 B 1
A 0.86 B 0
A 0.86 B 0.14
A 0.86 B 0.29
A 0.86 B 0.43
A 0.86 B 0.57
A 0.86 B 0.71
A 0.86 B 0.86
A 0.86 B 1
A 1 B 0
A 1 B 0.14
A 1 B 0.29
A 1 B 0.43
A 1 B 0.57
A 1 B 0.71
A 1 B 0.86
A 1 B 1
0.8
0.6
0.4
0.2
OD 600 nm [−]
0 5 101520
Time [h]

Dyc.Lat (= Ax.Bx) full GPDI
Int_AB = −0.9 and Int_BA = −0.56 at EC50

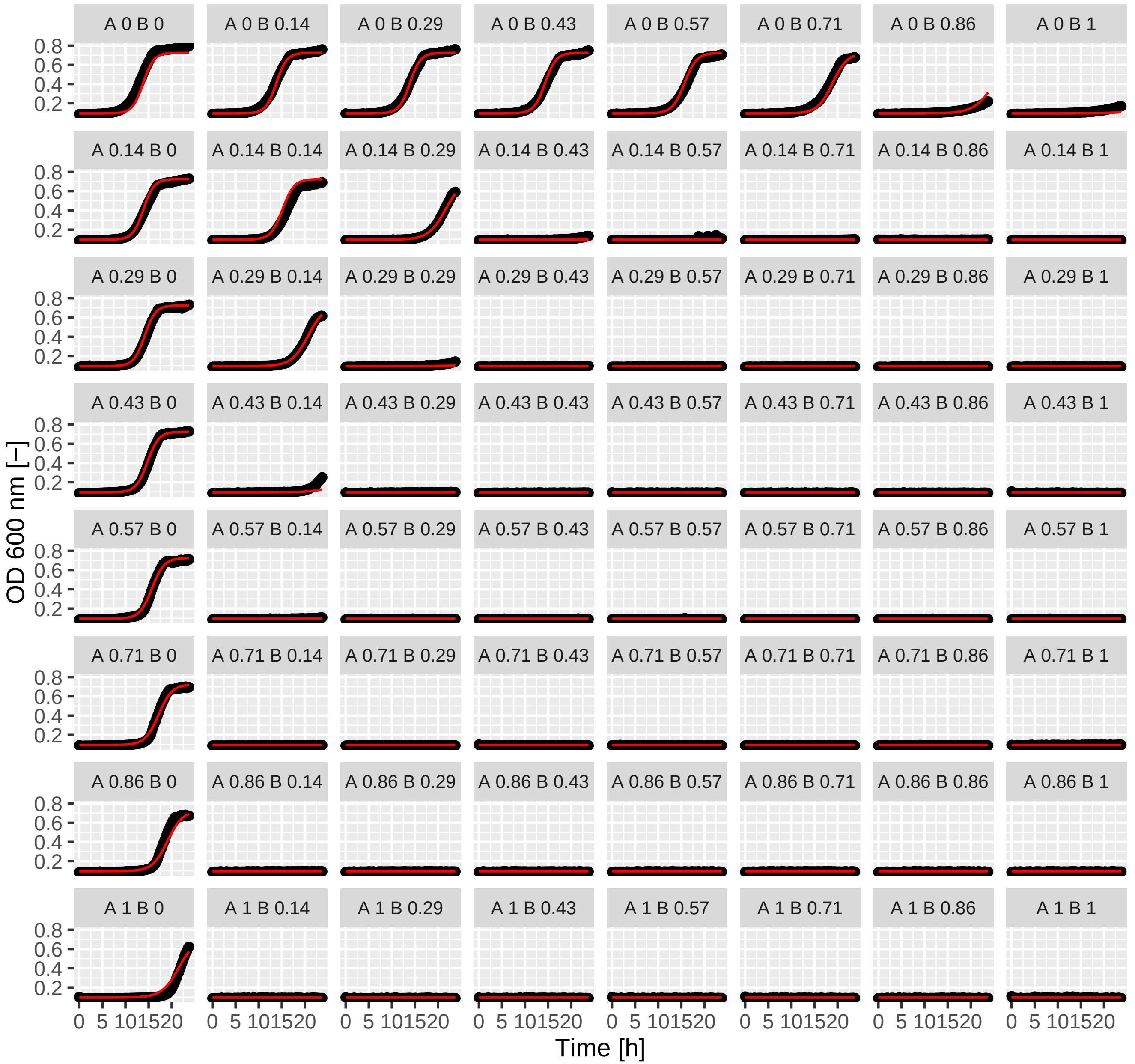

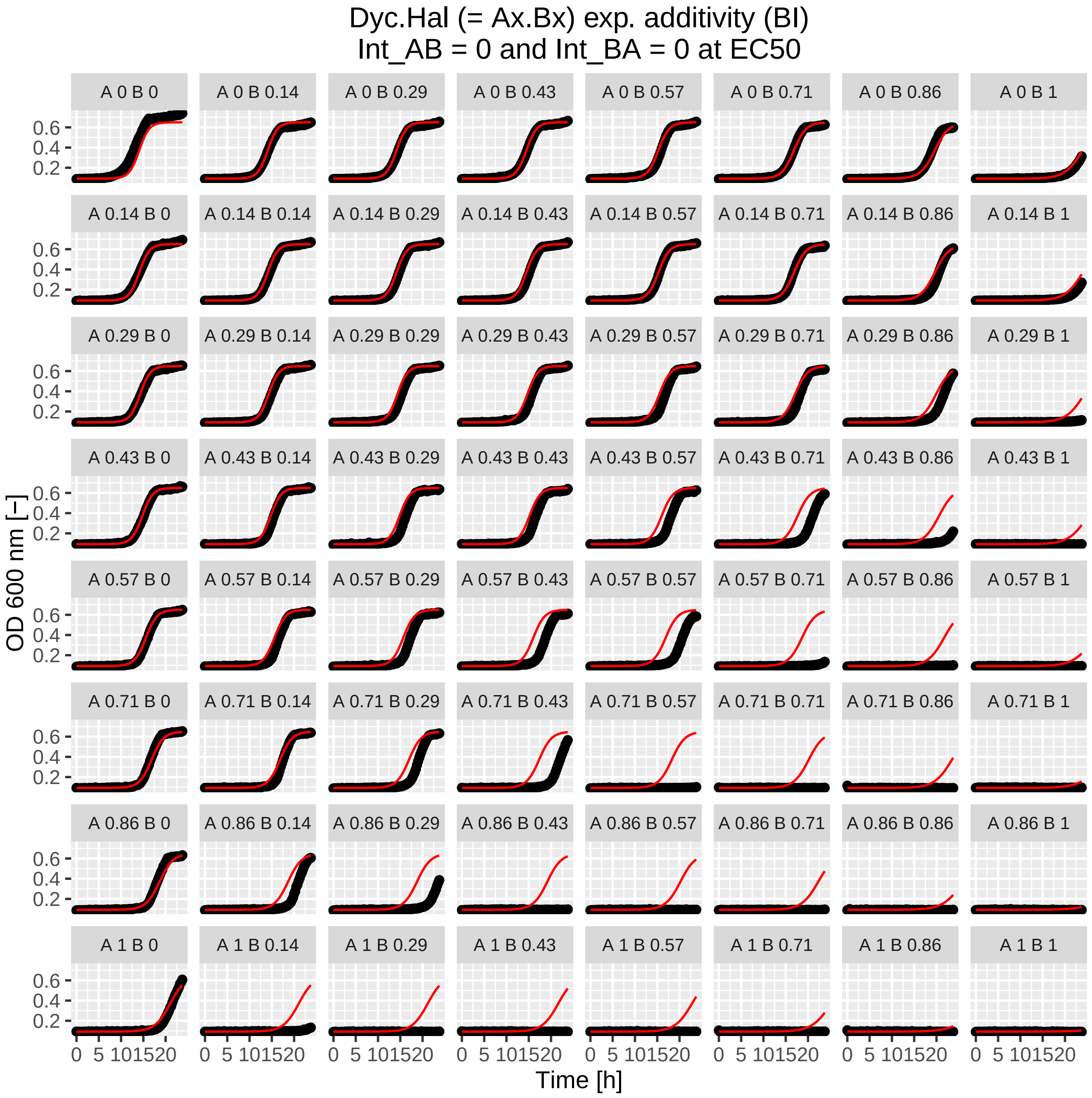
Dyc.Hal (= Ax.Bx) exp. additivity (BI)
Int_AB = 0 and Int_BA = 0 at EC50
OD 600 nm [–]
Time [h]
0.2
0.4
0.6
0 5 101520
A 0 B 0
A 0 B 0.14
A 0 B 0.29
A 0 B 0.43
A 0 B 0.57
A 0 B 0.71
A 0 B 0.86
A 0 B 1
A 0.14 B 0
A 0.14 B 0.14
A 0.14 B 0.29
A 0.14 B 0.43
A 0.14 B 0.57
A 0.14 B 0.71
A 0.14 B 0.86
A 0.14 B 1
A 0.29 B 0
A 0.29 B 0.14
A 0.29 B 0.29
A 0.29 B 0.43
A 0.29 B 0.57
A 0.29 B 0.71
A 0.29 B 0.86
A 0.29 B 1
A 0.43 B 0
A 0.43 B 0.14
A 0.43 B 0.29
A 0.43 B 0.43
A 0.43 B 0.57
A 0.43 B 0.71
A 0.43 B 0.86
A 0.43 B 1
A 0.57 B 0
A 0.57 B 0.14
A 0.57 B 0.29
A 0.57 B 0.43
A 0.57 B 0.57
A 0.57 B 0.71
A 0.57 B 0.86
A 0.57 B 1
A 0.71 B 0
A 0.71 B 0.14
A 0.71 B 0.29
A 0.71 B 0.43
A 0.71 B 0.57
A 0.71 B 0.71
A 0.71 B 0.86
A 0.71 B 1
A 0.86 B 0
A 0.86 B 0.14
A 0.86 B 0.29
A 0.86 B 0.43
A 0.86 B 0.57
A 0.86 B 0.71
A 0.86 B 0.86
A 0.86 B 1
A 1 B 0
A 1 B 0.14
A 1 B 0.29
A 1 B 0.43
A 1 B 0.57
A 1 B 0.71
A 1 B 0.86
A 1 B 1

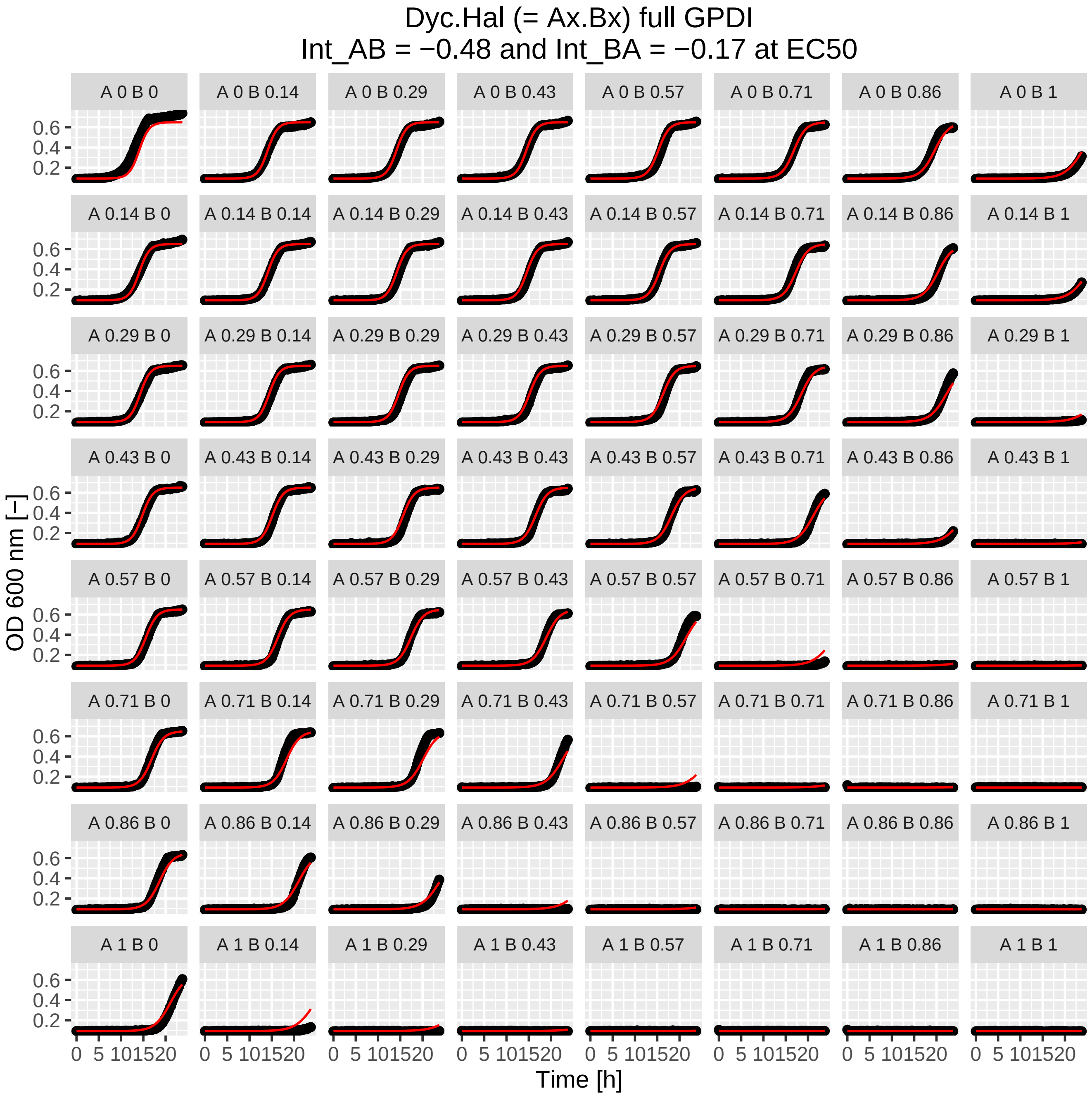
Dyc.Hal (= Ax.Bx) full GPDI
Int_AB = −0.48 and Int_BA = −0.17 at EC50
A 0 B 0
A 0 B 0.14
A 0 B 0.29
A 0 B 0.43
A 0 B 0.57
A 0 B 0.71
A 0 B 0.86
A 0 B 1
A 0.14 B 0
A 0.14 B 0.14
A 0.14 B 0.29
A 0.14 B 0.43
A 0.14 B 0.57
A 0.14 B 0.71
A 0.14 B 0.86
A 0.14 B 1
A 0.29 B 0
A 0.29 B 0.14
A 0.29 B 0.29
A 0.29 B 0.43
A 0.29 B 0.57
A 0.29 B 0.71
A 0.29 B 0.86
A 0.29 B 1
A 0.43 B 0
A 0.43 B 0.14
A 0.43 B 0.29
A 0.43 B 0.43
A 0.43 B 0.57
A 0.43 B 0.71
A 0.43 B 0.86
A 0.43 B 1
A 0.57 B 0
A 0.57 B 0.14
A 0.57 B 0.29
A 0.57 B 0.43
A 0.57 B 0.57
A 0.57 B 0.71
A 0.57 B 0.86
A 0.57 B 1
A 0.71 B 0
A 0.71 B 0.14
A 0.71 B 0.29
A 0.71 B 0.43
A 0.71 B 0.57
A 0.71 B 0.71
A 0.71 B 0.86
A 0.71 B 1
A 0.86 B 0
A 0.86 B 0.14
A 0.86 B 0.29
A 0.86 B 0.43
A 0.86 B 0.57
A 0.86 B 0.71
A 0.86 B 0.86
A 0.86 B 1
A 1 B 0
A 1 B 0.14
A 1 B 0.29
A 1 B 0.43
A 1 B 0.57
A 1 B 0.71
A 1 B 0.86
A 1 B 1
OD 600 nm [−]
0.6
0.4
0.2
0 5 101520
Time [h]

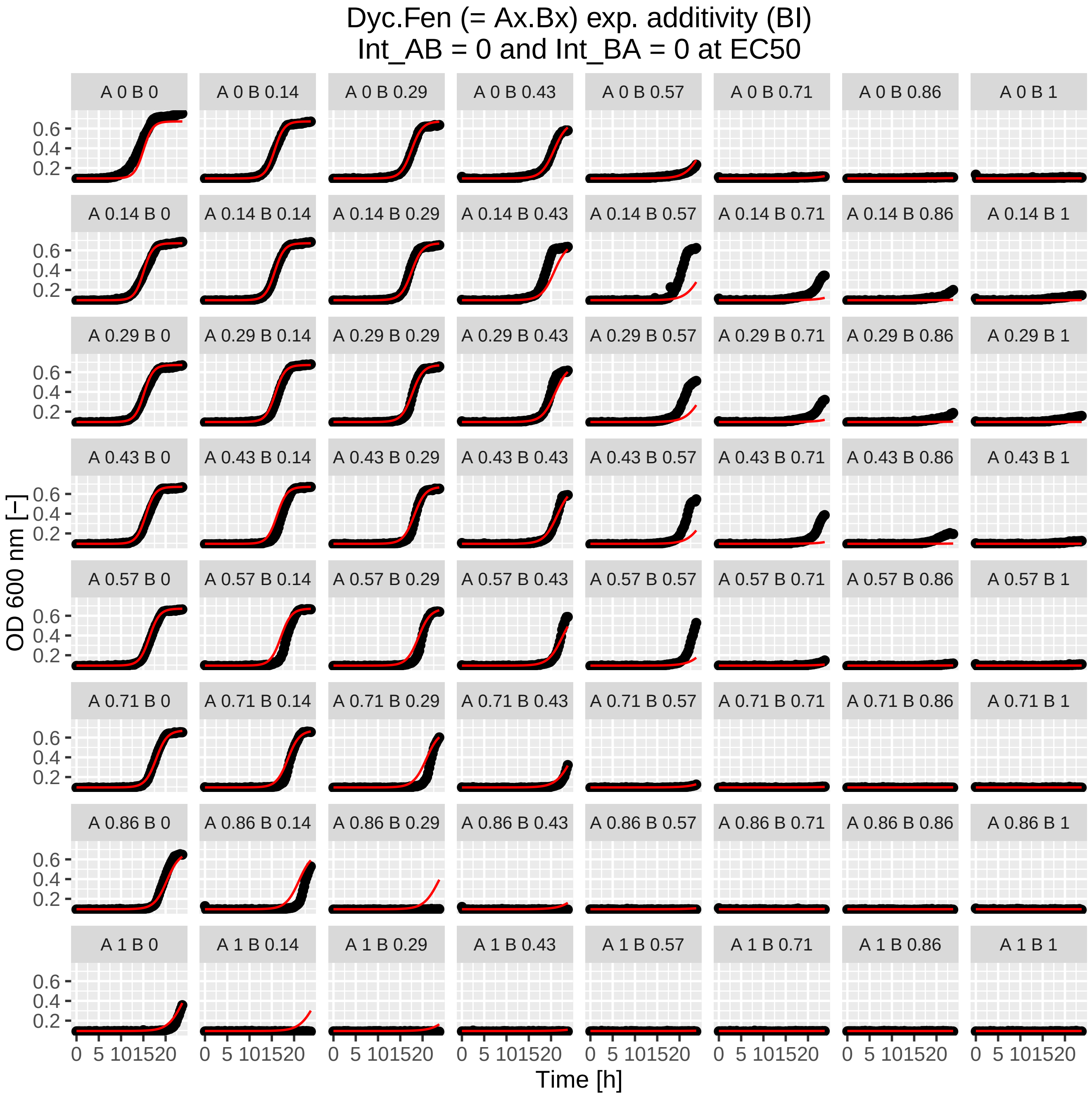

Dyc.Fen (= Ax.Bx) exp. additivity (BI)
Int_AB = 0 and Int_BA = 0 at EC50
OD 600 nm [–]
Time [h]
0.6
0.4
0.2
0 5 101520
A 0 B 0
A 0 B 0.14
A 0 B 0.29
A 0 B 0.43
A 0 B 0.57
A 0 B 0.71
A 0 B 0.86
A 0 B 1
A 0.14 B 0
A 0.14 B 0.14
A 0.14 B 0.29
A 0.14 B 0.43
A 0.14 B 0.57
A 0.14 B 0.71
A 0.14 B 0.86
A 0.14 B 1
A 0.29 B 0
A 0.29 B 0.14
A 0.29 B 0.29
A 0.29 B 0.43
A 0.29 B 0.57
A 0.29 B 0.71
A 0.29 B 0.86
A 0.29 B 1
A 0.43 B 0
A 0.43 B 0.14
A 0.43 B 0.29
A 0.43 B 0.43
A 0.43 B 0.57
A 0.43 B 0.71
A 0.43 B 0.86
A 0.43 B 1
A 0.57 B 0
A 0.57 B 0.14
A 0.57 B 0.29
A 0.57 B 0.43
A 0.57 B 0.57
A 0.57 B 0.71
A 0.57 B 0.86
A 0.57 B 1
A 0.71 B 0
A 0.71 B 0.14
A 0.71 B 0.29
A 0.71 B 0.43
A 0.71 B 0.57
A 0.71 B 0.71
A 0.71 B 0.86
A 0.71 B 1
A 0.86 B 0
A 0.86 B 0.14
A 0.86 B 0.29
A 0.86 B 0.43
A 0.86 B 0.57
A 0.86 B 0.71
A 0.86 B 0.86
A 0.86 B 1
A 1 B 0
A 1 B 0.14
A 1 B 0.29
A 1 B 0.43
A 1 B 0.57
A 1 B 0.71
A 1 B 0.86
A 1 B 1

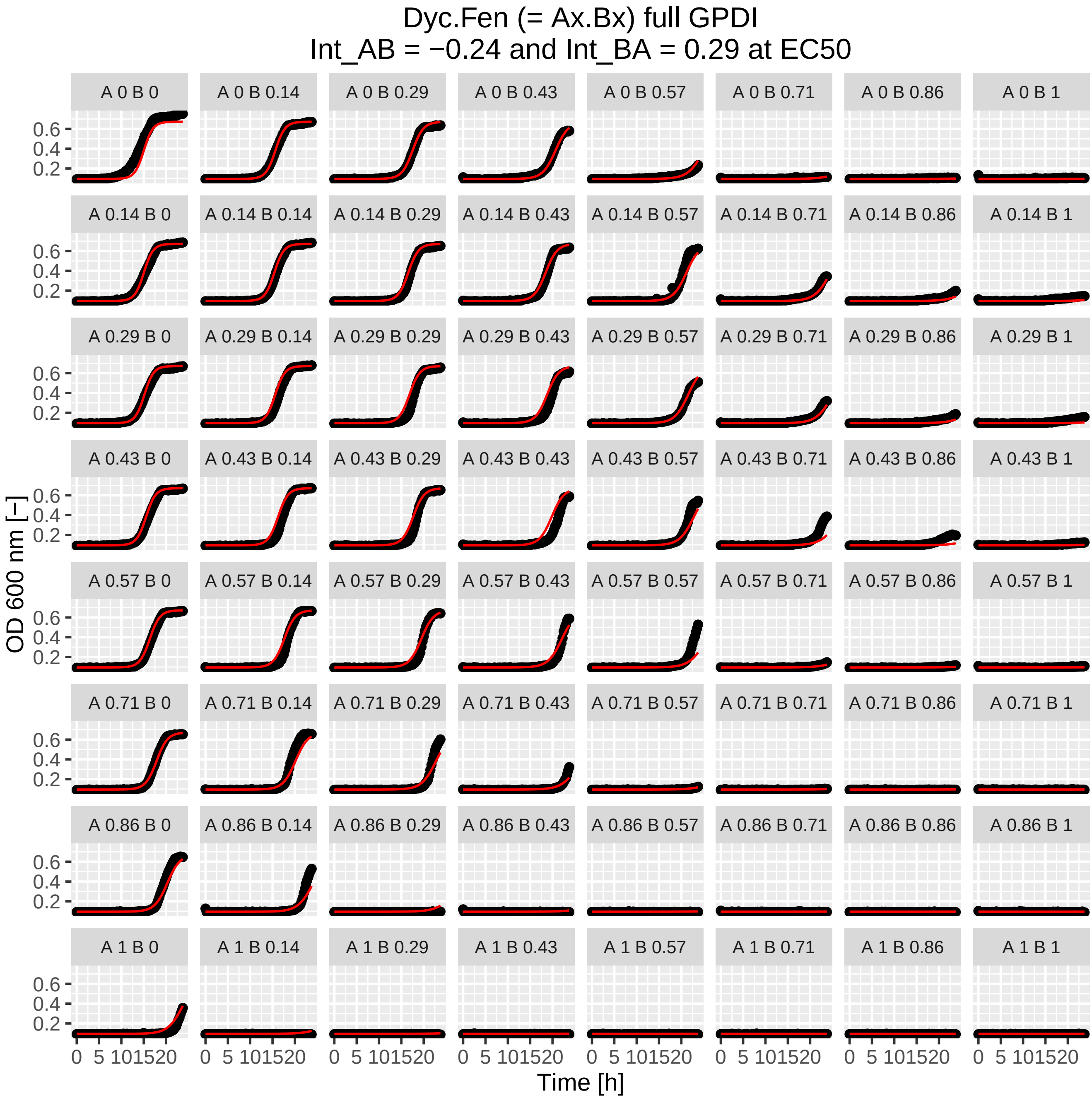

Dyc.Fen (= Ax.Bx) full GPDI
Int_AB = −0.24 and Int_BA = 0.29 at EC50
A 0 B 0
A 0 B 0.14
A 0 B 0.29
A 0 B 0.43
A 0 B 0.57
A 0 B 0.71
A 0 B 0.86
A 0 B 1
A 0.14 B 0
A 0.14 B 0.14
A 0.14 B 0.29
A 0.14 B 0.43
A 0.14 B 0.57
A 0.14 B 0.71
A 0.14 B 0.86
A 0.14 B 1
A 0.29 B 0
A 0.29 B 0.14
A 0.29 B 0.29
A 0.29 B 0.43
A 0.29 B 0.57
A 0.29 B 0.71
A 0.29 B 0.86
A 0.29 B 1
A 0.43 B 0
A 0.43 B 0.14
A 0.43 B 0.29
A 0.43 B 0.43
A 0.43 B 0.57
A 0.43 B 0.71
A 0.43 B 0.86
A 0.43 B 1
A 0.57 B 0
A 0.57 B 0.14
A 0.57 B 0.29
A 0.57 B 0.43
A 0.57 B 0.57
A 0.57 B 0.71
A 0.57 B 0.86
A 0.57 B 1
A 0.71 B 0
A 0.71 B 0.14
A 0.71 B 0.29
A 0.71 B 0.43
A 0.71 B 0.57
A 0.71 B 0.71
A 0.71 B 0.86
A 0.71 B 1
A 0.86 B 0
A 0.86 B 0.14
A 0.86 B 0.29
A 0.86 B 0.43
A 0.86 B 0.57
A 0.86 B 0.71
A 0.86 B 0.86
A 0.86 B 1
A 1 B 0
A 1 B 0.14
A 1 B 0.29
A 1 B 0.43
A 1 B 0.57
A 1 B 0.71
A 1 B 0.86
A 1 B 1
OD 600 nm [−]
0.6
0.4
0.2
0 5 101520
Time [h]

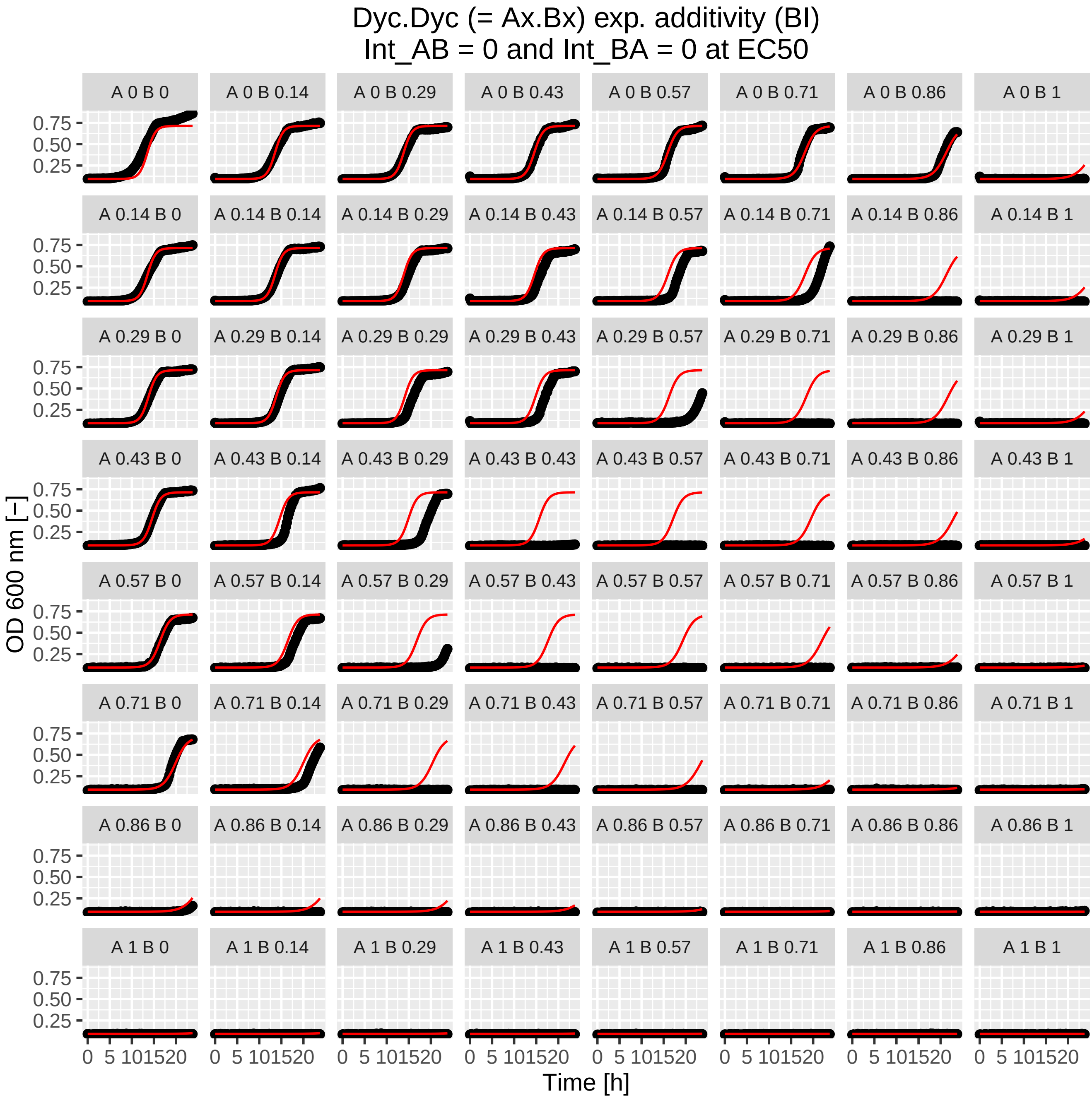
Dyc.Dyc (= Ax.Bx) exp. additivity (BI)
Int_AB = 0 and Int_BA = 0 at EC50
OD 600 nm [–]
Time [h]
A 0 B 0
A 0 B 0.14
A 0 B 0.29
A 0 B 0.43
A 0 B 0.57
A 0 B 0.71
A 0 B 0.86
A 0 B 1
A 0.14 B 0
A 0.14 B 0.14
A 0.14 B 0.29
A 0.14 B 0.43
A 0.14 B 0.57
A 0.14 B 0.71
A 0.14 B 0.86
A 0.14 B 1
A 0.29 B 0
A 0.29 B 0.14
A 0.29 B 0.29
A 0.29 B 0.43
A 0.29 B 0.57
A 0.29 B 0.71
A 0.29 B 0.86
A 0.29 B 1
A 0.43 B 0
A 0.43 B 0.14
A 0.43 B 0.29
A 0.43 B 0.43
A 0.43 B 0.57
A 0.43 B 0.71
A 0.43 B 0.86
A 0.43 B 1
A 0.57 B 0
A 0.57 B 0.14
A 0.57 B 0.29
A 0.57 B 0.43
A 0.57 B 0.57
A 0.57 B 0.71
A 0.57 B 0.86
A 0.57 B 1
A 0.71 B 0
A 0.71 B 0.14
A 0.71 B 0.29
A 0.71 B 0.43
A 0.71 B 0.57
A 0.71 B 0.71
A 0.71 B 0.86
A 0.71 B 1
A 0.86 B 0
A 0.86 B 0.14
A 0.86 B 0.29
A 0.86 B 0.43
A 0.86 B 0.57
A 0.86 B 0.71
A 0.86 B 0.86
A 0.86 B 1
A 1 B 0
A 1 B 0.14
A 1 B 0.29
A 1 B 0.43
A 1 B 0.57
A 1 B 0.71
A 1 B 0.86
A 1 B 1
0.75
0.50
0.25
0 5 101520

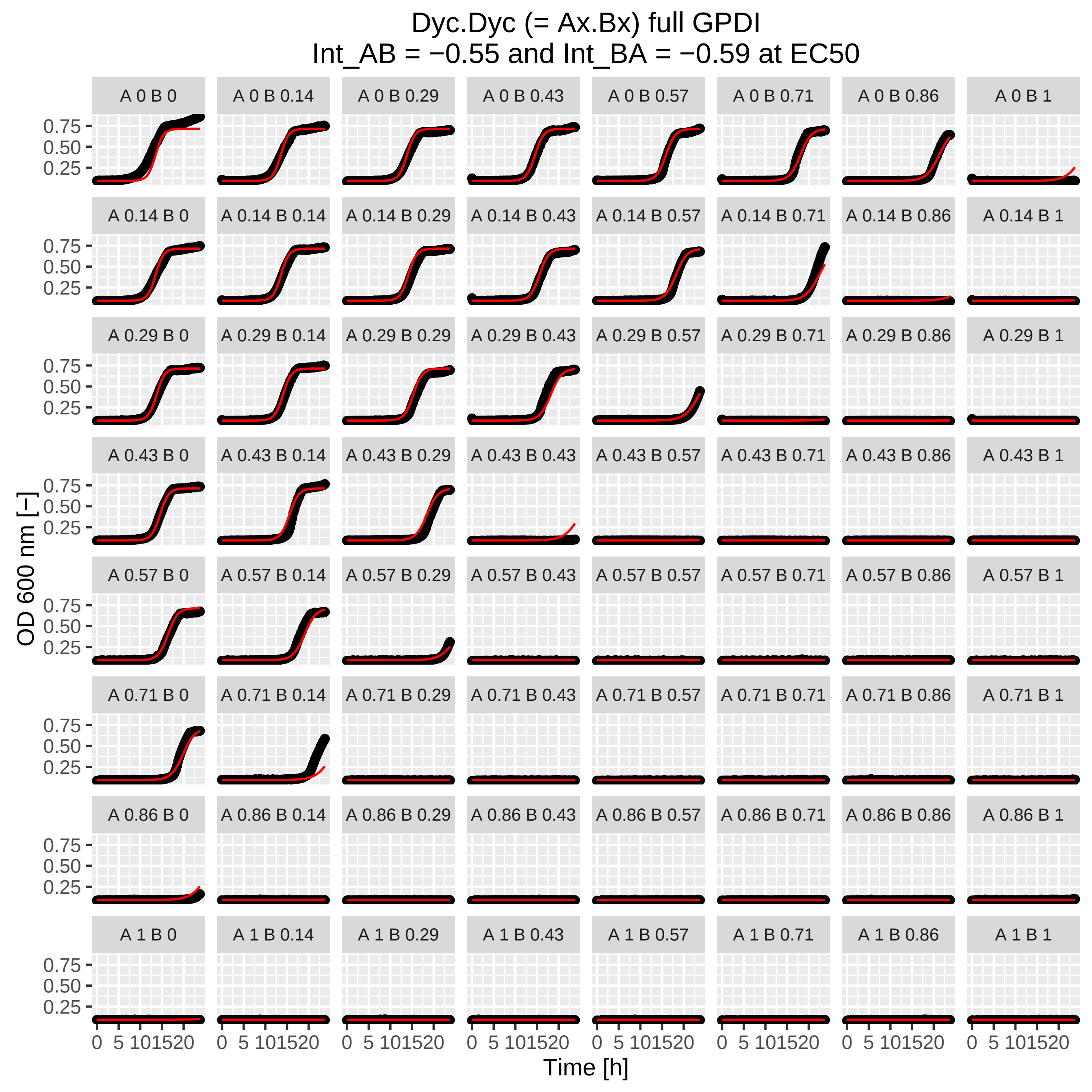
Dyc.Dyc (= Ax.Bx) full GPDI
Int_AB = −0.55 and Int_BA = −0.59 at EC50
OD 600 nm [−]
Time [h]
0.75
0.50
0.25
0 5 101520
A 0 B 0
A 0 B 0.14
A 0 B 0.29
A 0 B 0.43
A 0 B 0.57
A 0 B 0.71
A 0 B 0.86
A 0 B 1
A 0.14 B 0
A 0.14 B 0.14
A 0.14 B 0.29
A 0.14 B 0.43
A 0.14 B 0.57
A 0.14 B 0.71
A 0.14 B 0.86
A 0.14 B 1
A 0.29 B 0
A 0.29 B 0.14
A 0.29 B 0.29
A 0.29 B 0.43
A 0.29 B 0.57
A 0.29 B 0.71
A 0.29 B 0.86
A 0.29 B 1
A 0.43 B 0
A 0.43 B 0.14
A 0.43 B 0.29
A 0.43 B 0.43
A 0.43 B 0.57
A 0.43 B 0.71
A 0.43 B 0.86
A 0.43 B 1
A 0.57 B 0
A 0.57 B 0.14
A 0.57 B 0.29
A 0.57 B 0.43
A 0.57 B 0.57
A 0.57 B 0.71
A 0.57 B 0.86
A 0.57 B 1
A 0.71 B 0
A 0.71 B 0.14
A 0.71 B 0.29
A 0.71 B 0.43
A 0.71 B 0.57
A 0.71 B 0.71
A 0.71 B 0.86
A 0.71 B 1
A 0.86 B 0
A 0.86 B 0.14
A 0.86 B 0.29
A 0.86 B 0.43
A 0.86 B 0.57
A 0.86 B 0.71
A 0.86 B 0.86
A 0.86 B 1
A 1 B 0
A 1 B 0.14
A 1 B 0.29
A 1 B 0.43
A 1 B 0.57
A 1 B 0.71
A 1 B 0.86
A 1 B 1

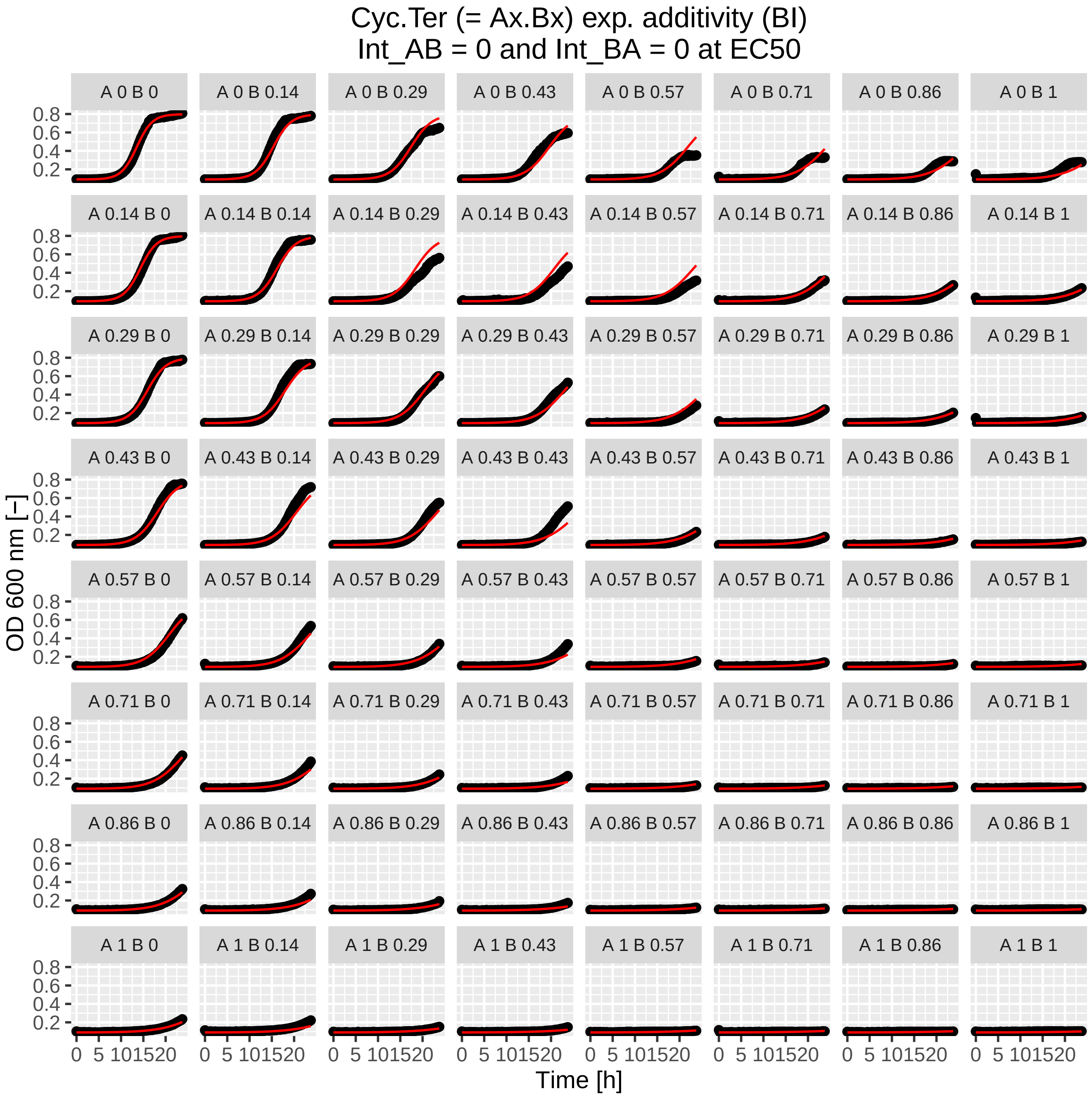

Cyc.Ter (= Ax.Bx) exp. additivity (BI)
Int_AB = 0 and Int_BA = 0 at EC50
A 0 B 0
A 0 B 0.14
A 0 B 0.29
A 0 B 0.43
A 0 B 0.57
A 0 B 0.71
A 0 B 0.86
A 0 B 1
A 0.14 B 0
A 0.14 B 0.14
A 0.14 B 0.29
A 0.14 B 0.43
A 0.14 B 0.57
A 0.14 B 0.71
A 0.14 B 0.86
A 0.14 B 1
A 0.29 B 0
A 0.29 B 0.14
A 0.29 B 0.29
A 0.29 B 0.43
A 0.29 B 0.57
A 0.29 B 0.71
A 0.29 B 0.86
A 0.29 B 1
A 0.43 B 0
A 0.43 B 0.14
A 0.43 B 0.29
A 0.43 B 0.43
A 0.43 B 0.57
A 0.43 B 0.71
A 0.43 B 0.86
A 0.43 B 1
A 0.57 B 0
A 0.57 B 0.14
A 0.57 B 0.29
A 0.57 B 0.43
A 0.57 B 0.57
A 0.57 B 0.71
A 0.57 B 0.86
A 0.57 B 1
A 0.71 B 0
A 0.71 B 0.14
A 0.71 B 0.29
A 0.71 B 0.43
A 0.71 B 0.57
A 0.71 B 0.71
A 0.71 B 0.86
A 0.71 B 1
A 0.86 B 0
A 0.86 B 0.14
A 0.86 B 0.29
A 0.86 B 0.43
A 0.86 B 0.57
A 0.86 B 0.71
A 0.86 B 0.86
A 0.86 B 1
A 1 B 0
A 1 B 0.14
A 1 B 0.29
A 1 B 0.43
A 1 B 0.57
A 1 B 0.71
A 1 B 0.86
A 1 B 1
0.8
0.6
0.4
0.2
OD 600 nm [–]
0 5 101520
Time [h]

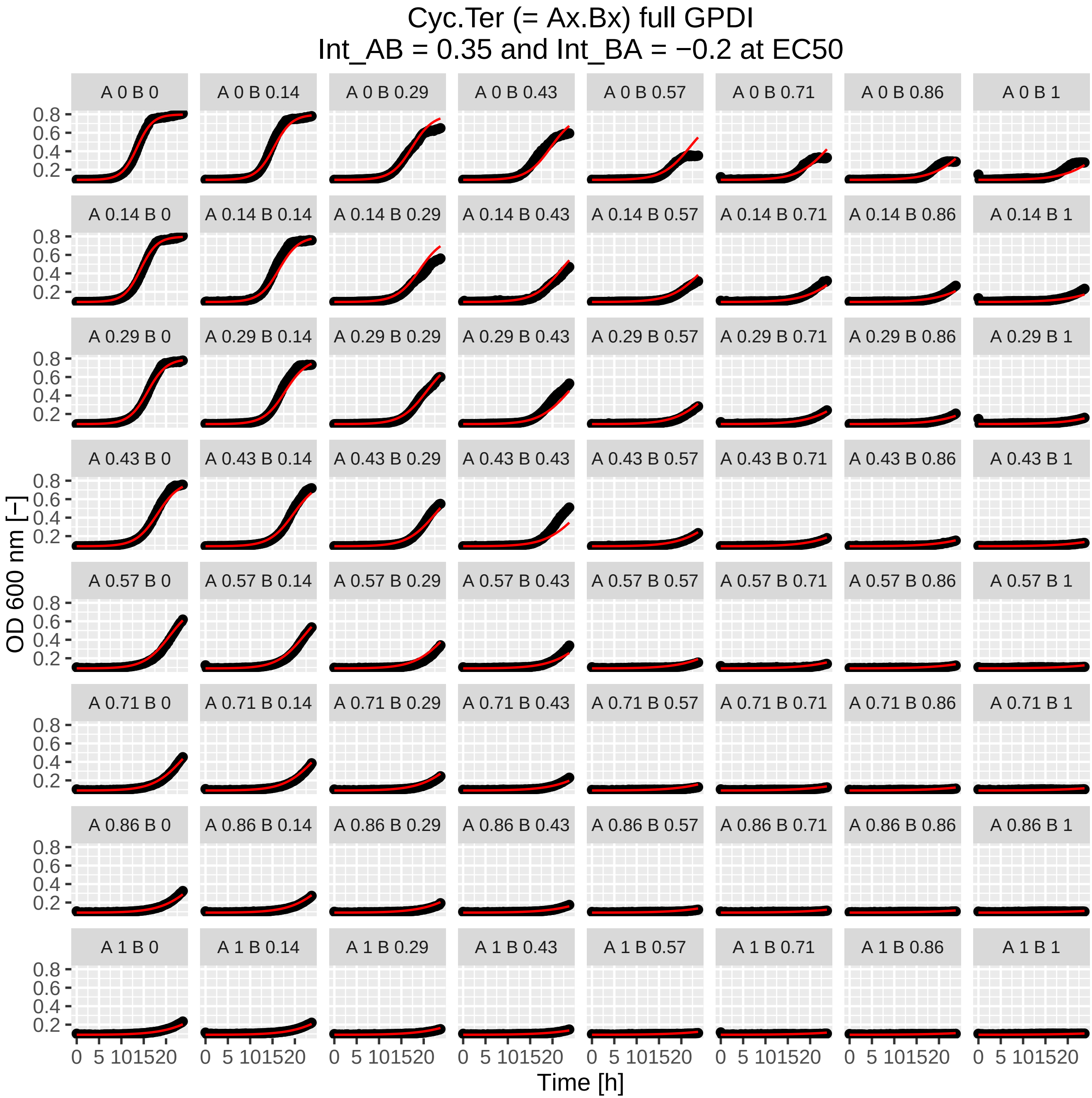

Cyc.Ter (= Ax.Bx) full GPDI
Int_AB = 0.35 and Int_BA = −0.2 at EC50
A 0 B 0
A 0 B 0.14
A 0 B 0.29
A 0 B 0.43
A 0 B 0.57
A 0 B 0.71
A 0 B 0.86
A 0 B 1
A 0.14 B 0
A 0.14 B 0.14
A 0.14 B 0.29
A 0.14 B 0.43
A 0.14 B 0.57
A 0.14 B 0.71
A 0.14 B 0.86
A 0.14 B 1
A 0.29 B 0
A 0.29 B 0.14
A 0.29 B 0.29
A 0.29 B 0.43
A 0.29 B 0.57
A 0.29 B 0.71
A 0.29 B 0.86
A 0.29 B 1
A 0.43 B 0
A 0.43 B 0.14
A 0.43 B 0.29
A 0.43 B 0.43
A 0.43 B 0.57
A 0.43 B 0.71
A 0.43 B 0.86
A 0.43 B 1
A 0.57 B 0
A 0.57 B 0.14
A 0.57 B 0.29
A 0.57 B 0.43
A 0.57 B 0.57
A 0.57 B 0.71
A 0.57 B 0.86
A 0.57 B 1
A 0.71 B 0
A 0.71 B 0.14
A 0.71 B 0.29
A 0.71 B 0.43
A 0.71 B 0.57
A 0.71 B 0.71
A 0.71 B 0.86
A 0.71 B 1
A 0.86 B 0
A 0.86 B 0.14
A 0.86 B 0.29
A 0.86 B 0.43
A 0.86 B 0.57
A 0.86 B 0.71
A 0.86 B 0.86
A 0.86 B 1
A 1 B 0
A 1 B 0.14
A 1 B 0.29
A 1 B 0.43
A 1 B 0.57
A 1 B 0.71
A 1 B 0.86
A 1 B 1
OD 600 nm [−]
0.2
0.4
0.6
0.8
0
5
10
15
20
Time [h]

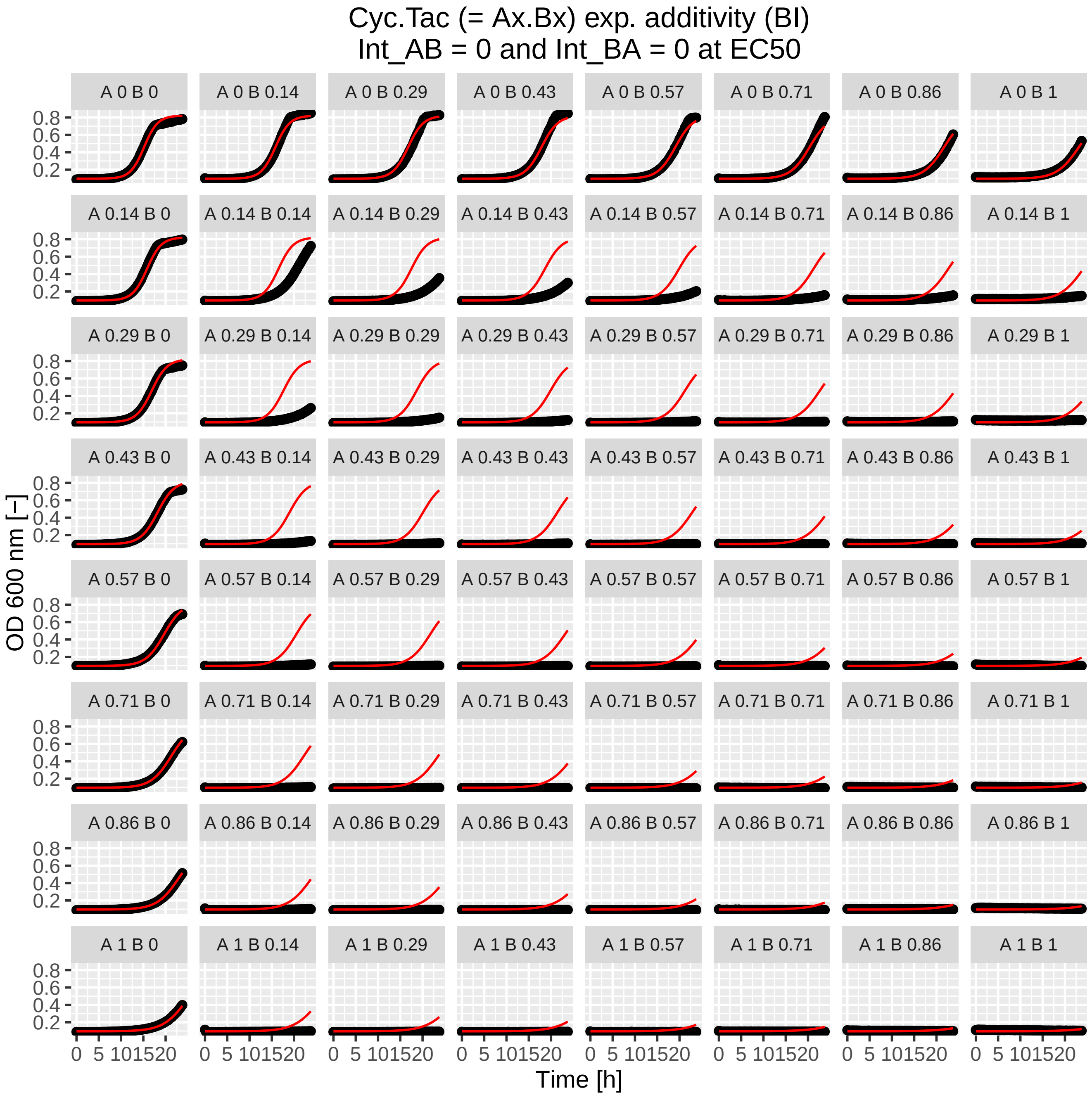

Cyc.Tac (= Ax.Bx) exp. additivity (BI)
Int_AB = 0 and Int_BA = 0 at EC50
A 0 B 0
A 0 B 0.14
A 0 B 0.29
A 0 B 0.43
A 0 B 0.57
A 0 B 0.71
A 0 B 0.86
A 0 B 1
A 0.14 B 0
A 0.14 B 0.14
A 0.14 B 0.29
A 0.14 B 0.43
A 0.14 B 0.57
A 0.14 B 0.71
A 0.14 B 0.86
A 0.14 B 1
A 0.29 B 0
A 0.29 B 0.14
A 0.29 B 0.29
A 0.29 B 0.43
A 0.29 B 0.57
A 0.29 B 0.71
A 0.29 B 0.86
A 0.29 B 1
A 0.43 B 0
A 0.43 B 0.14
A 0.43 B 0.29
A 0.43 B 0.43
A 0.43 B 0.57
A 0.43 B 0.71
A 0.43 B 0.86
A 0.43 B 1
A 0.57 B 0
A 0.57 B 0.14
A 0.57 B 0.29
A 0.57 B 0.43
A 0.57 B 0.57
A 0.57 B 0.71
A 0.57 B 0.86
A 0.57 B 1
A 0.71 B 0
A 0.71 B 0.14
A 0.71 B 0.29
A 0.71 B 0.43
A 0.71 B 0.57
A 0.71 B 0.71
A 0.71 B 0.86
A 0.71 B 1
A 0.86 B 0
A 0.86 B 0.14
A 0.86 B 0.29
A 0.86 B 0.43
A 0.86 B 0.57
A 0.86 B 0.71
A 0.86 B 0.86
A 0.86 B 1
A 1 B 0
A 1 B 0.14
A 1 B 0.29
A 1 B 0.43
A 1 B 0.57
A 1 B 0.71
A 1 B 0.86
A 1 B 1
OD 600 nm [−]
0.8
0.6
0.4
0.2
0 5 101520
Time [h]

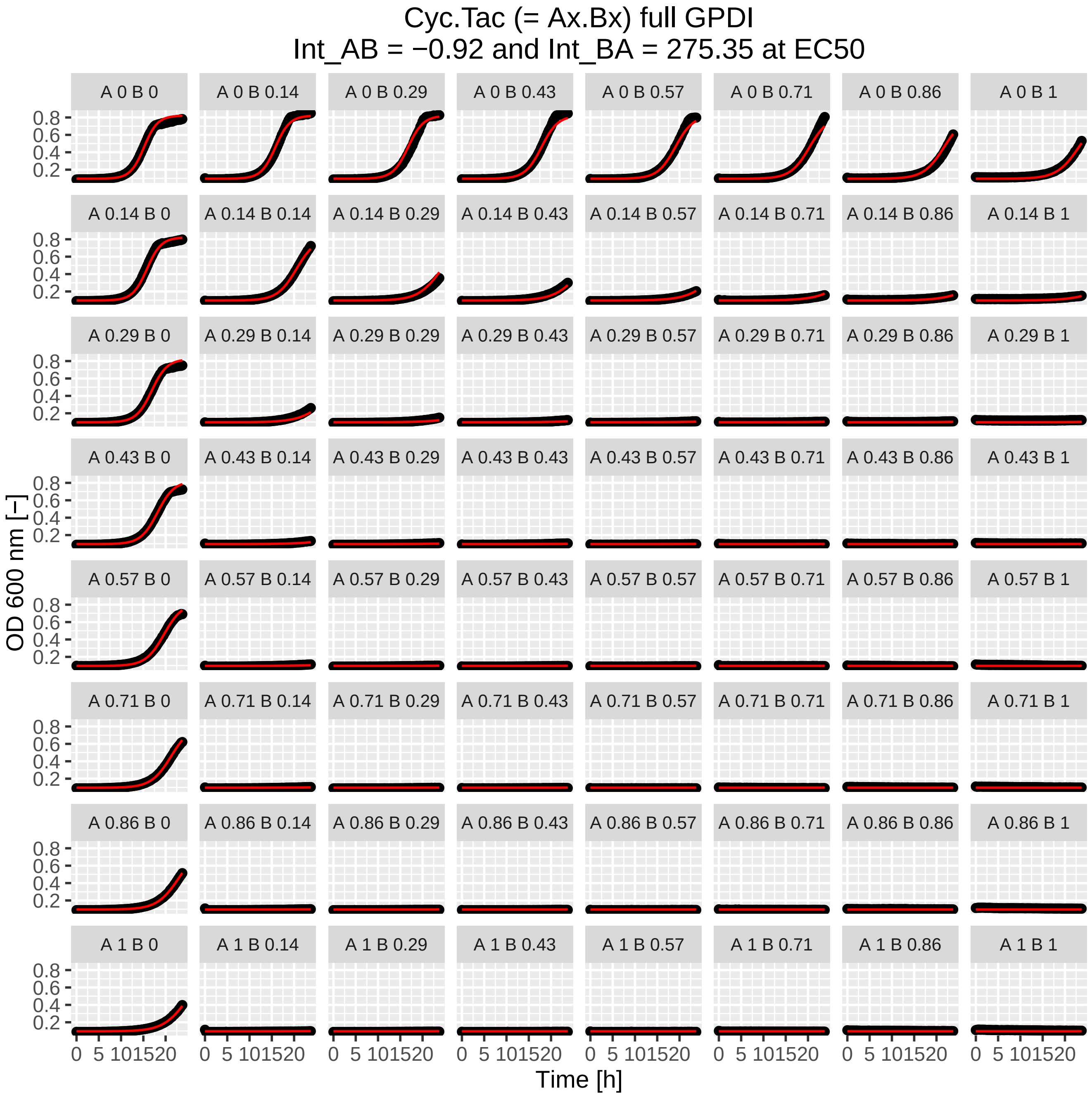

Cyc.Tac (= Ax.Bx) full GPDI
Int_AB = −0.92 and Int_BA = 275.35 at EC50
A 0 B 0
A 0 B 0.14
A 0 B 0.29
A 0 B 0.43
A 0 B 0.57
A 0 B 0.71
A 0 B 0.86
A 0 B 1
A 0.14 B 0
A 0.14 B 0.14
A 0.14 B 0.29
A 0.14 B 0.43
A 0.14 B 0.57
A 0.14 B 0.71
A 0.14 B 0.86
A 0.14 B 1
A 0.29 B 0
A 0.29 B 0.14
A 0.29 B 0.29
A 0.29 B 0.43
A 0.29 B 0.57
A 0.29 B 0.71
A 0.29 B 0.86
A 0.29 B 1
A 0.43 B 0
A 0.43 B 0.14
A 0.43 B 0.29
A 0.43 B 0.43
A 0.43 B 0.57
A 0.43 B 0.71
A 0.43 B 0.86
A 0.43 B 1
A 0.57 B 0
A 0.57 B 0.14
A 0.57 B 0.29
A 0.57 B 0.43
A 0.57 B 0.57
A 0.57 B 0.71
A 0.57 B 0.86
A 0.57 B 1
A 0.71 B 0
A 0.71 B 0.14
A 0.71 B 0.29
A 0.71 B 0.43
A 0.71 B 0.57
A 0.71 B 0.71
A 0.71 B 0.86
A 0.71 B 1
A 0.86 B 0
A 0.86 B 0.14
A 0.86 B 0.29
A 0.86 B 0.43
A 0.86 B 0.57
A 0.86 B 0.71
A 0.86 B 0.86
A 0.86 B 1
A 1 B 0
A 1 B 0.14
A 1 B 0.29
A 1 B 0.43
A 1 B 0.57
A 1 B 0.71
A 1 B 0.86
A 1 B 1
OD 600 nm [−]
0.8
0.6
0.4
0.2
0 5 101520
Time [h]

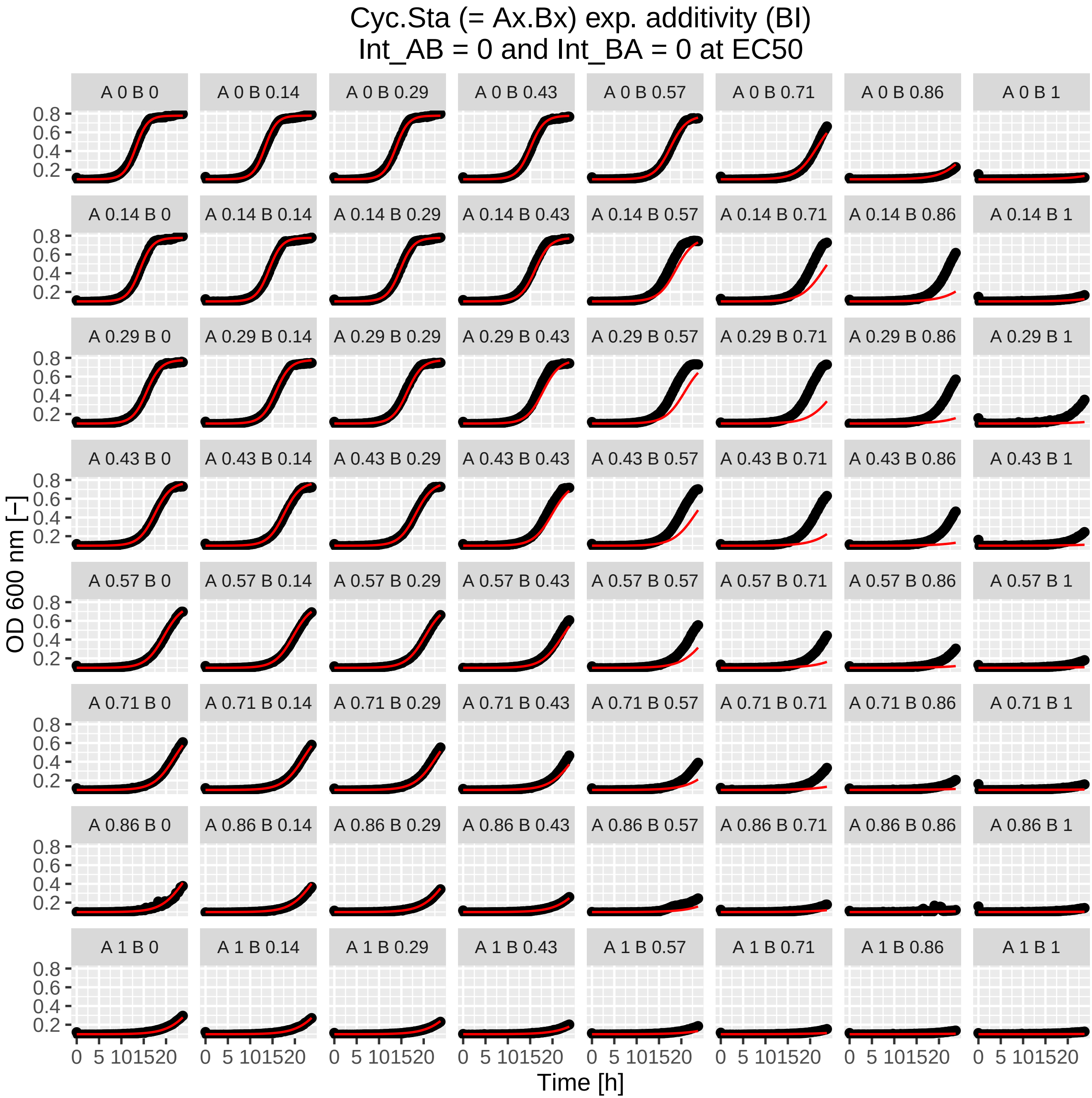
Cyc.Sta (= Ax.Bx) exp. additivity (BI)
Int_AB = 0 and Int_BA = 0 at EC50
OD 600 nm [–]
Time [h]
0.8
0.6
0.4
0.2
0 5 101520
A 0 B 0
A 0 B 0.14
A 0 B 0.29
A 0 B 0.43
A 0 B 0.57
A 0 B 0.71
A 0 B 0.86
A 0 B 1
A 0.14 B 0
A 0.14 B 0.14
A 0.14 B 0.29
A 0.14 B 0.43
A 0.14 B 0.57
A 0.14 B 0.71
A 0.14 B 0.86
A 0.14 B 1
A 0.29 B 0
A 0.29 B 0.14
A 0.29 B 0.29
A 0.29 B 0.43
A 0.29 B 0.57
A 0.29 B 0.71
A 0.29 B 0.86
A 0.29 B 1
A 0.43 B 0
A 0.43 B 0.14
A 0.43 B 0.29
A 0.43 B 0.43
A 0.43 B 0.57
A 0.43 B 0.71
A 0.43 B 0.86
A 0.43 B 1
A 0.57 B 0
A 0.57 B 0.14
A 0.57 B 0.29
A 0.57 B 0.43
A 0.57 B 0.57
A 0.57 B 0.71
A 0.57 B 0.86
A 0.57 B 1
A 0.71 B 0
A 0.71 B 0.14
A 0.71 B 0.29
A 0.71 B 0.43
A 0.71 B 0.57
A 0.71 B 0.71
A 0.71 B 0.86
A 0.71 B 1
A 0.86 B 0
A 0.86 B 0.14
A 0.86 B 0.29
A 0.86 B 0.43
A 0.86 B 0.57
A 0.86 B 0.71
A 0.86 B 0.86
A 0.86 B 1
A 1 B 0
A 1 B 0.14
A 1 B 0.29
A 1 B 0.43
A 1 B 0.57
A 1 B 0.71
A 1 B 0.86
A 1 B 1

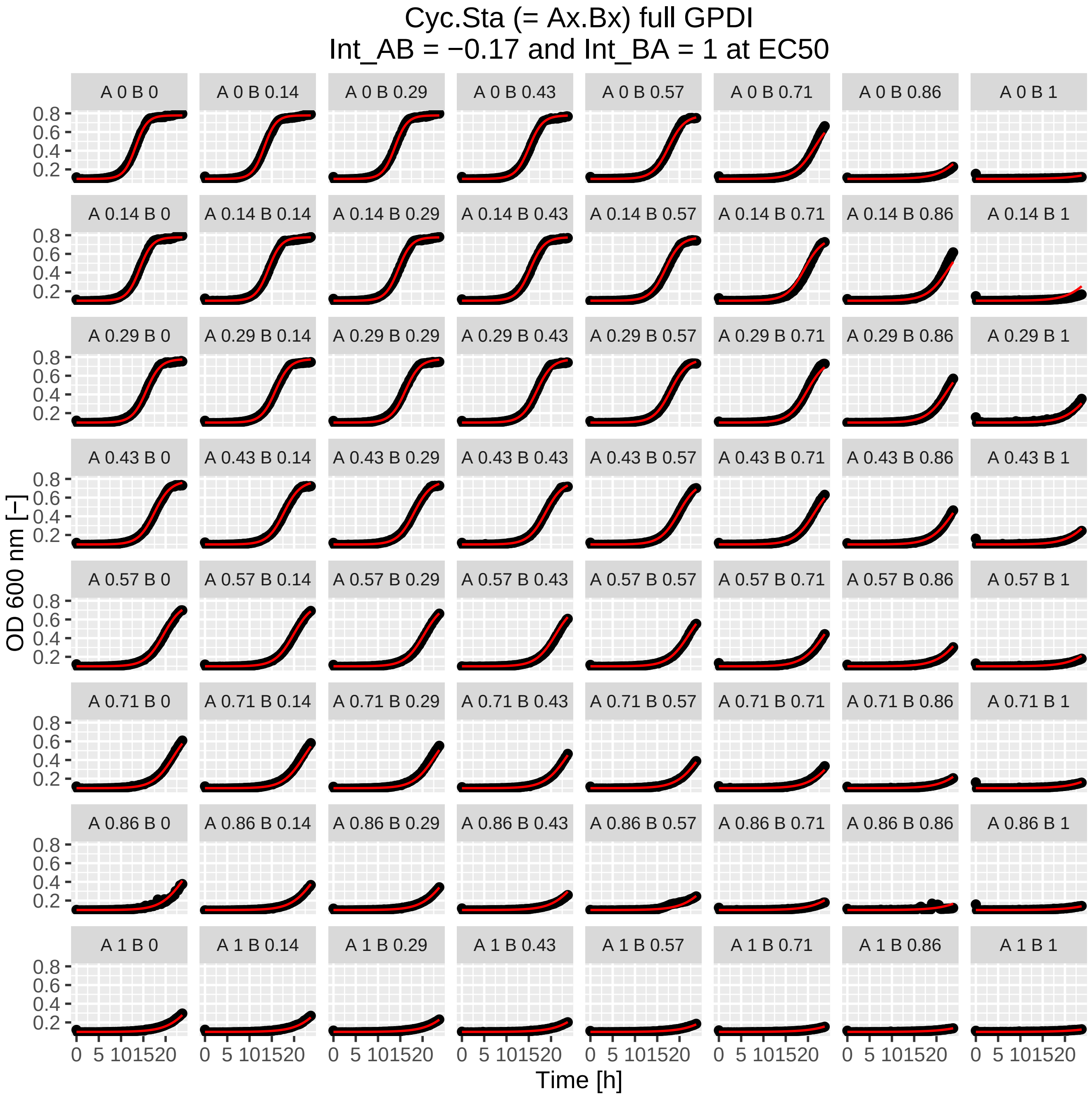
Cyc.Sta (= Ax.Bx) full GPDI
Int_AB = −0.17 and Int_BA = 1 at EC50
OD 600 nm [−]
Time [h]
A 0 B 0
A 0 B 0.14
A 0 B 0.29
A 0 B 0.43
A 0 B 0.57
A 0 B 0.71
A 0 B 0.86
A 0 B 1
A 1 B 0
A 1 B 0.14
A 1 B 0.29
A 1 B 0.43
A 1 B 0.57
A 1 B 0.71
A 1 B 0.86
A 1 B 1

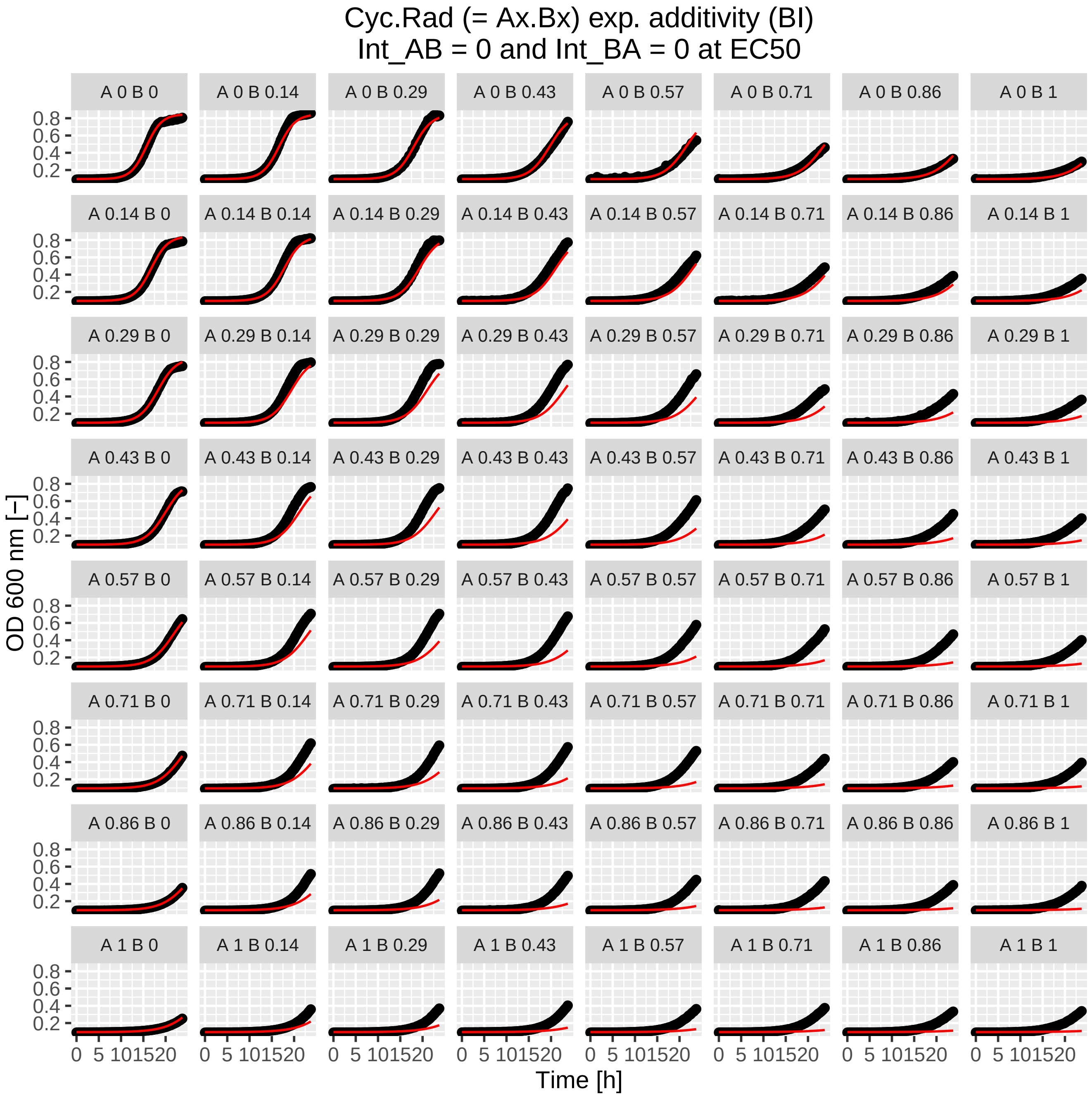

Cyc.Rad (= Ax.Bx) exp. additivity (BI)
Int_AB = 0 and Int_BA = 0 at EC50
OD 600 nm [–]
Time [h]
A 0 B 0
A 0 B 0.14
A 0 B 0.29
A 0 B 0.43
A 0 B 0.57
A 0 B 0.71
A 0 B 0.86
A 0 B 1
A 0.14 B 0
A 0.14 B 0.14
A 0.14 B 0.29
A 0.14 B 0.43
A 0.14 B 0.57
A 0.14 B 0.71
A 0.14 B 0.86
A 0.14 B 1
A 0.29 B 0
A 0.29 B 0.14
A 0.29 B 0.29
A 0.29 B 0.43
A 0.29 B 0.57
A 0.29 B 0.71
A 0.29 B 0.86
A 0.29 B 1
A 0.43 B 0
A 0.43 B 0.14
A 0.43 B 0.29
A 0.43 B 0.43
A 0.43 B 0.57
A 0.43 B 0.71
A 0.43 B 0.86
A 0.43 B 1
A 0.57 B 0
A 0.57 B 0.14
A 0.57 B 0.29
A 0.57 B 0.43
A 0.57 B 0.57
A 0.57 B 0.71
A 0.57 B 0.86
A 0.57 B 1
A 0.71 B 0
A 0.71 B 0.14
A 0.71 B 0.29
A 0.71 B 0.43
A 0.71 B 0.57
A 0.71 B 0.71
A 0.71 B 0.86
A 0.71 B 1
A 0.86 B 0
A 0.86 B 0.14
A 0.86 B 0.29
A 0.86 B 0.43
A 0.86 B 0.57
A 0.86 B 0.71
A 0.86 B 0.86
A 0.86 B 1
A 1 B 0
A 1 B 0.14
A 1 B 0.29
A 1 B 0.43
A 1 B 0.57
A 1 B 0.71
A 1 B 0.86
A 1 B 1
0.2
0.4
0.6
0.8
0 5 101520

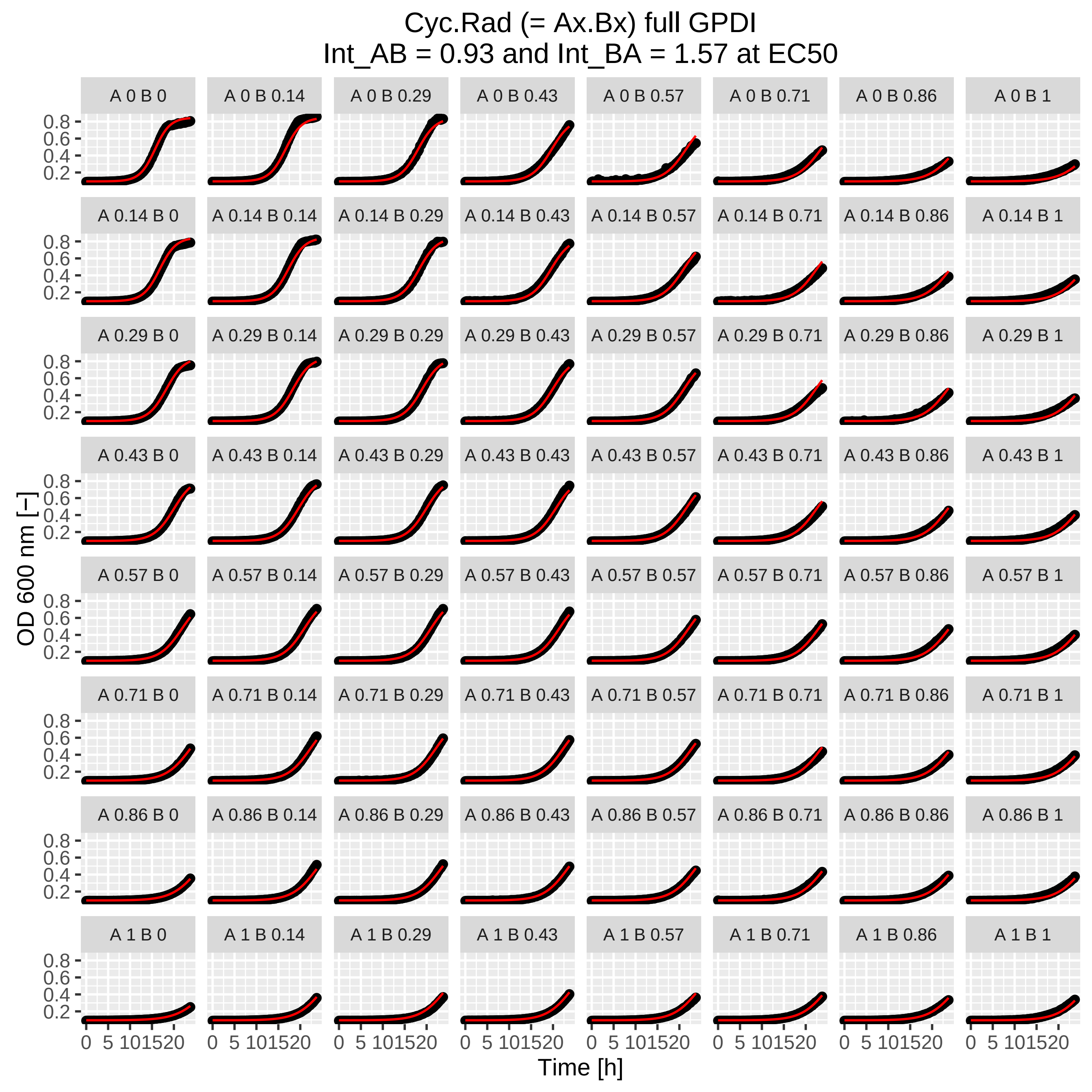

Cyc.Rad (= Ax.Bx) full GPDI
Int_AB = 0.93 and Int_BA = 1.57 at EC50
A 0 B 0
A 0 B 0.14
A 0 B 0.29
A 0 B 0.43
A 0 B 0.57
A 0 B 0.71
A 0 B 0.86
A 0 B 1
A 0.14 B 0
A 0.14 B 0.14
A 0.14 B 0.29
A 0.14 B 0.43
A 0.14 B 0.57
A 0.14 B 0.71
A 0.14 B 0.86
A 0.14 B 1
A 0.29 B 0
A 0.29 B 0.14
A 0.29 B 0.29
A 0.29 B 0.43
A 0.29 B 0.57
A 0.29 B 0.71
A 0.29 B 0.86
A 0.29 B 1
A 0.43 B 0
A 0.43 B 0.14
A 0.43 B 0.29
A 0.43 B 0.43
A 0.43 B 0.57
A 0.43 B 0.71
A 0.43 B 0.86
A 0.43 B 1
A 0.57 B 0
A 0.57 B 0.14
A 0.57 B 0.29
A 0.57 B 0.43
A 0.57 B 0.57
A 0.57 B 0.71
A 0.57 B 0.86
A 0.57 B 1
A 0.71 B 0
A 0.71 B 0.14
A 0.71 B 0.29
A 0.71 B 0.43
A 0.71 B 0.57
A 0.71 B 0.71
A 0.71 B 0.86
A 0.71 B 1
A 0.86 B 0
A 0.86 B 0.14
A 0.86 B 0.29
A 0.86 B 0.43
A 0.86 B 0.57
A 0.86 B 0.71
A 0.86 B 0.86
A 0.86 B 1
A 1 B 0
A 1 B 0.14
A 1 B 0.29
A 1 B 0.43
A 1 B 0.57
A 1 B 0.71
A 1 B 0.86
A 1 B 1
OD 600 nm [–]
0.2
0.4
0.6
0.8
0
5
10
15
20
Time [h]

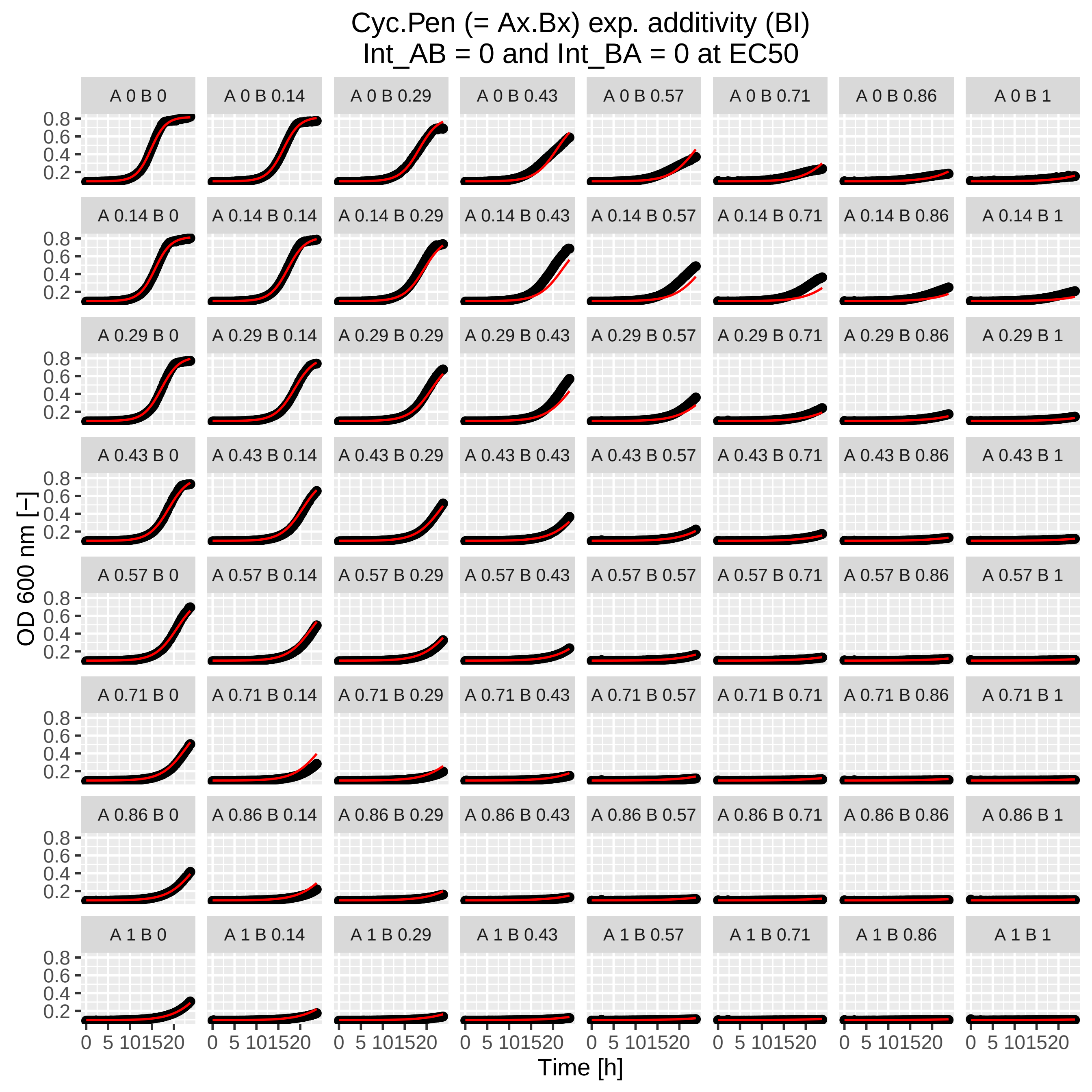
Cyc.Pen (= Ax.Bx) exp. additivity (BI)
Int_AB = 0 and Int_BA = 0 at EC50
A 0 B 0
A 0 B 0.14
A 0 B 0.29
A 0 B 0.43
A 0 B 0.57
A 0 B 0.71
A 0 B 0.86
A 0 B 1
A 0.14 B 0
A 0.14 B 0.14
A 0.14 B 0.29
A 0.14 B 0.43
A 0.14 B 0.57
A 0.14 B 0.71
A 0.14 B 0.86
A 0.14 B 1
A 0.29 B 0
A 0.29 B 0.14
A 0.29 B 0.29
A 0.29 B 0.43
A 0.29 B 0.57
A 0.29 B 0.71
A 0.29 B 0.86
A 0.29 B 1
A 0.43 B 0
A 0.43 B 0.14
A 0.43 B 0.29
A 0.43 B 0.43
A 0.43 B 0.57
A 0.43 B 0.71
A 0.43 B 0.86
A 0.43 B 1
A 0.57 B 0
A 0.57 B 0.14
A 0.57 B 0.29
A 0.57 B 0.43
A 0.57 B 0.57
A 0.57 B 0.71
A 0.57 B 0.86
A 0.57 B 1
A 0.71 B 0
A 0.71 B 0.14
A 0.71 B 0.29
A 0.71 B 0.43
A 0.71 B 0.57
A 0.71 B 0.71
A 0.71 B 0.86
A 0.71 B 1
A 0.86 B 0
A 0.86 B 0.14
A 0.86 B 0.29
A 0.86 B 0.43
A 0.86 B 0.57
A 0.86 B 0.71
A 0.86 B 0.86
A 0.86 B 1
A 1 B 0
A 1 B 0.14
A 1 B 0.29
A 1 B 0.43
A 1 B 0.57
A 1 B 0.71
A 1 B 0.86
A 1 B 1
0.8
0.6
0.4
0.2
0 5 101520
OD 600 nm [−]
Time [h]

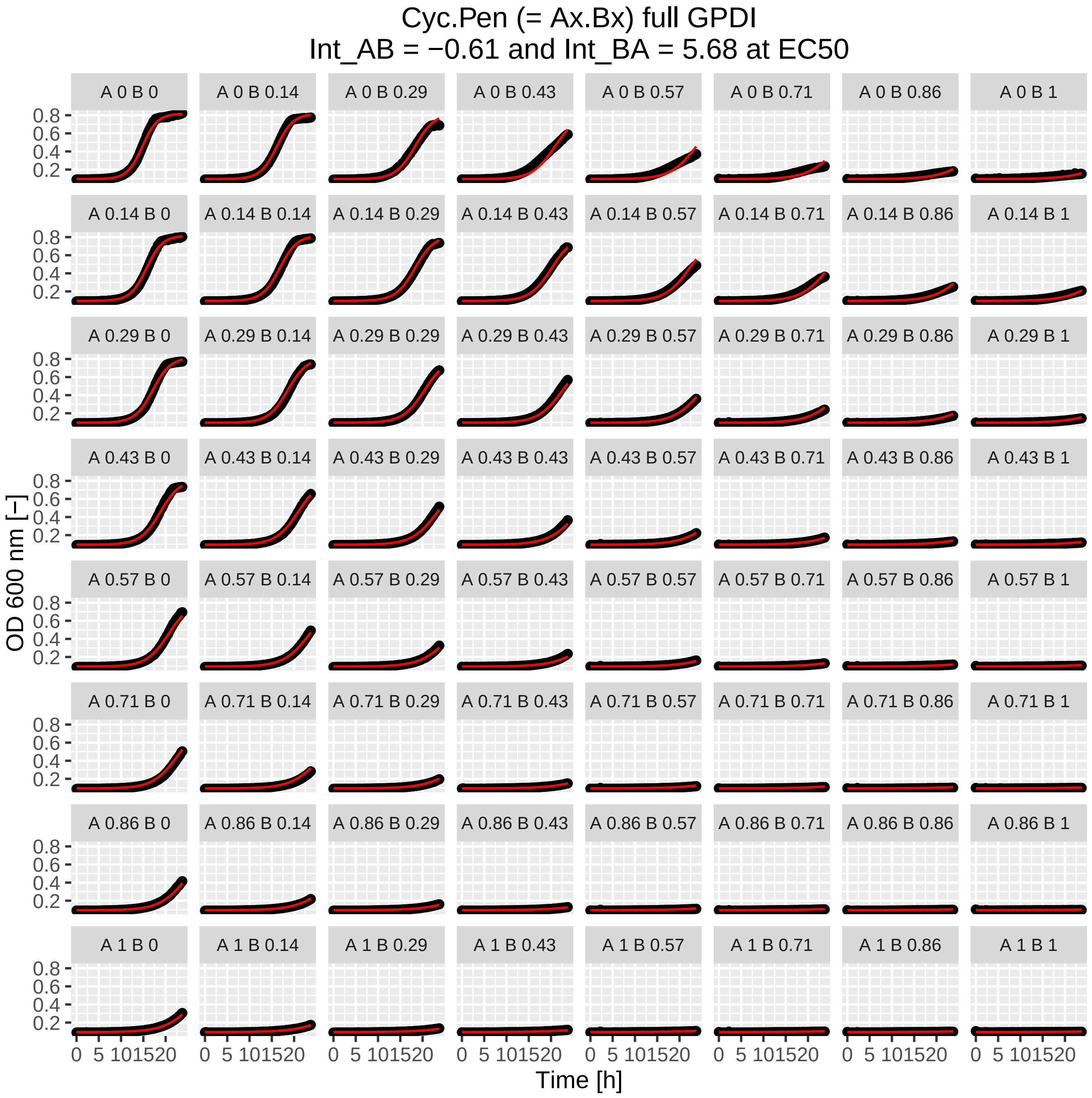
Cyc.Pen (= Ax.Bx) full GPDI
Int_AB = −0.61 and Int_BA = 5.68 at EC50
A 0 B 0
A 0 B 0.14
A 0 B 0.29
A 0 B 0.43
A 0 B 0.57
A 0 B 0.71
A 0 B 0.86
A 0 B 1
A 0.14 B 0
A 0.14 B 0.14
A 0.14 B 0.29
A 0.14 B 0.43
A 0.14 B 0.57
A 0.14 B 0.71
A 0.14 B 0.86
A 0.14 B 1
A 0.29 B 0
A 0.29 B 0.14
A 0.29 B 0.29
A 0.29 B 0.43
A 0.29 B 0.57
A 0.29 B 0.71
A 0.29 B 0.86
A 0.29 B 1
A 0.43 B 0
A 0.43 B 0.14
A 0.43 B 0.29
A 0.43 B 0.43
A 0.43 B 0.57
A 0.43 B 0.71
A 0.43 B 0.86
A 0.43 B 1
A 0.57 B 0
A 0.57 B 0.14
A 0.57 B 0.29
A 0.57 B 0.43
A 0.57 B 0.57
A 0.57 B 0.71
A 0.57 B 0.86
A 0.57 B 1
A 0.71 B 0
A 0.71 B 0.14
A 0.71 B 0.29
A 0.71 B 0.43
A 0.71 B 0.57
A 0.71 B 0.71
A 0.71 B 0.86
A 0.71 B 1
A 0.86 B 0
A 0.86 B 0.14
A 0.86 B 0.29
A 0.86 B 0.43
A 0.86 B 0.57
A 0.86 B 0.71
A 0.86 B 0.86
A 0.86 B 1
A 1 B 0
A 1 B 0.14
A 1 B 0.29
A 1 B 0.43
A 1 B 0.57
A 1 B 0.71
A 1 B 0.86
A 1 B 1
0.8
0.6
0.4
0.2
0 5 101520
OD 600 nm [−]
Time [h]

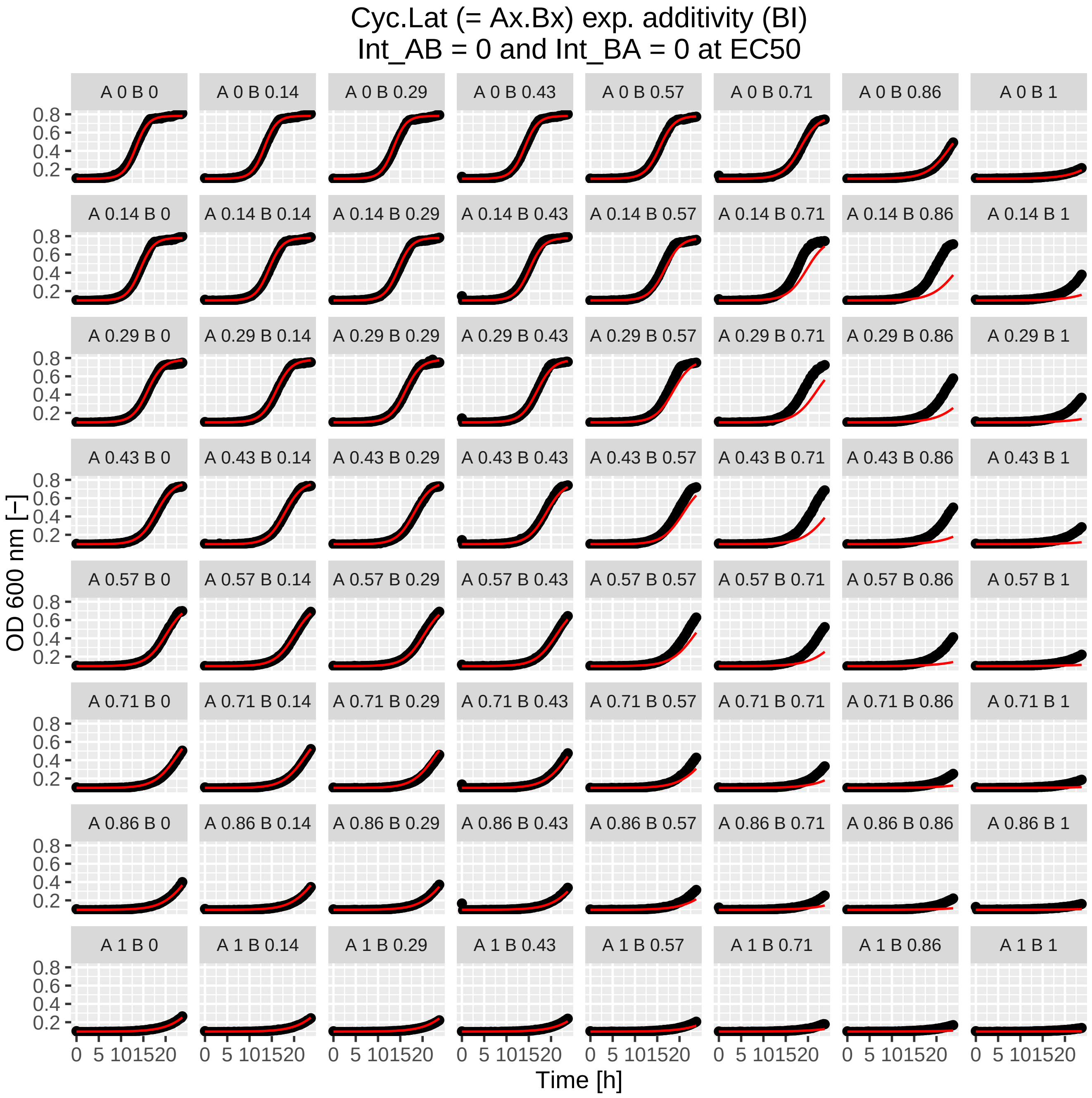

Cyc.Lat (= Ax.Bx) exp. additivity (BI)
Int_AB = 0 and Int_BA = 0 at EC50
A 0 B 0
A 0 B 0.14
A 0 B 0.29
A 0 B 0.43
A 0 B 0.57
A 0 B 0.71
A 0 B 0.86
A 0 B 1
A 0.14 B 0
A 0.14 B 0.14
A 0.14 B 0.29
A 0.14 B 0.43
A 0.14 B 0.57
A 0.14 B 0.71
A 0.14 B 0.86
A 0.14 B 1
A 0.29 B 0
A 0.29 B 0.14
A 0.29 B 0.29
A 0.29 B 0.43
A 0.29 B 0.57
A 0.29 B 0.71
A 0.29 B 0.86
A 0.29 B 1
A 0.43 B 0
A 0.43 B 0.14
A 0.43 B 0.29
A 0.43 B 0.43
A 0.43 B 0.57
A 0.43 B 0.71
A 0.43 B 0.86
A 0.43 B 1
A 0.57 B 0
A 0.57 B 0.14
A 0.57 B 0.29
A 0.57 B 0.43
A 0.57 B 0.57
A 0.57 B 0.71
A 0.57 B 0.86
A 0.57 B 1
A 0.71 B 0
A 0.71 B 0.14
A 0.71 B 0.29
A 0.71 B 0.43
A 0.71 B 0.57
A 0.71 B 0.71
A 0.71 B 0.86
A 0.71 B 1
A 0.86 B 0
A 0.86 B 0.14
A 0.86 B 0.29
A 0.86 B 0.43
A 0.86 B 0.57
A 0.86 B 0.71
A 0.86 B 0.86
A 0.86 B 1
A 1 B 0
A 1 B 0.14
A 1 B 0.29
A 1 B 0.43
A 1 B 0.57
A 1 B 0.71
A 1 B 0.86
A 1 B 1
OD 600 nm [−]
0.2
0.4
0.6
0.8
0 5 101520
Time [h]

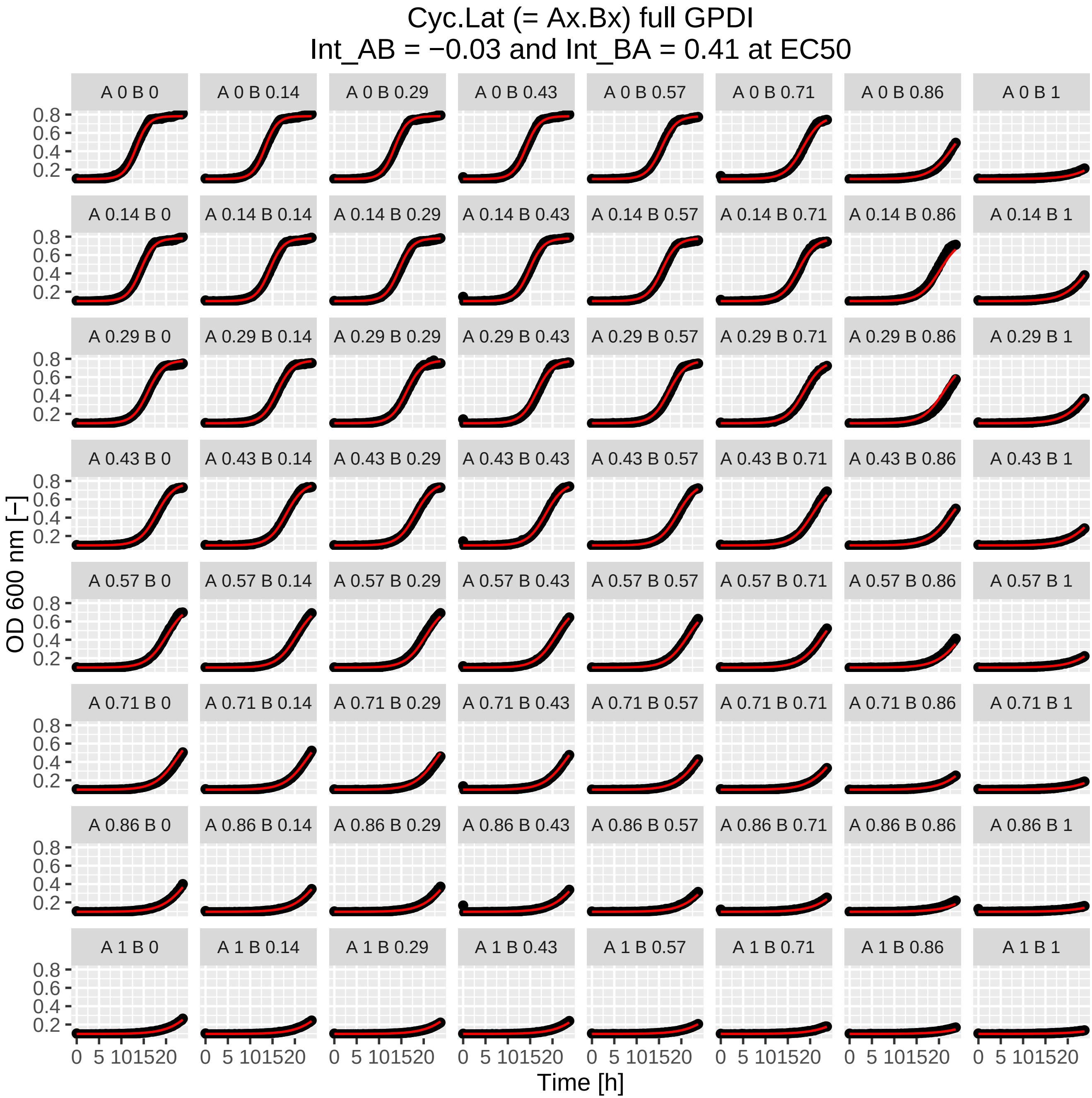

Cyc.Lat (= Ax.Bx) full GPDI
Int_AB = −0.03 and Int_BA = 0.41 at EC50
A 0 B 0
A 0 B 0.14
A 0 B 0.29
A 0 B 0.43
A 0 B 0.57
A 0 B 0.71
A 0 B 0.86
A 0 B 1
A 0.14 B 0
A 0.14 B 0.14
A 0.14 B 0.29
A 0.14 B 0.43
A 0.14 B 0.57
A 0.14 B 0.71
A 0.14 B 0.86
A 0.14 B 1
A 0.29 B 0
A 0.29 B 0.14
A 0.29 B 0.29
A 0.29 B 0.43
A 0.29 B 0.57
A 0.29 B 0.71
A 0.29 B 0.86
A 0.29 B 1
A 0.43 B 0
A 0.43 B 0.14
A 0.43 B 0.29
A 0.43 B 0.43
A 0.43 B 0.57
A 0.43 B 0.71
A 0.43 B 0.86
A 0.43 B 1
A 0.57 B 0
A 0.57 B 0.14
A 0.57 B 0.29
A 0.57 B 0.43
A 0.57 B 0.57
A 0.57 B 0.71
A 0.57 B 0.86
A 0.57 B 1
A 0.71 B 0
A 0.71 B 0.14
A 0.71 B 0.29
A 0.71 B 0.43
A 0.71 B 0.57
A 0.71 B 0.71
A 0.71 B 0.86
A 0.71 B 1
A 0.86 B 0
A 0.86 B 0.14
A 0.86 B 0.29
A 0.86 B 0.43
A 0.86 B 0.57
A 0.86 B 0.71
A 0.86 B 0.86
A 0.86 B 1
A 1 B 0
A 1 B 0.14
A 1 B 0.29
A 1 B 0.43
A 1 B 0.57
A 1 B 0.71
A 1 B 0.86
A 1 B 1
OD 600 nm [−]
0.2
0.4
0.6
0.8
0
5
10
15
20
Time [h]

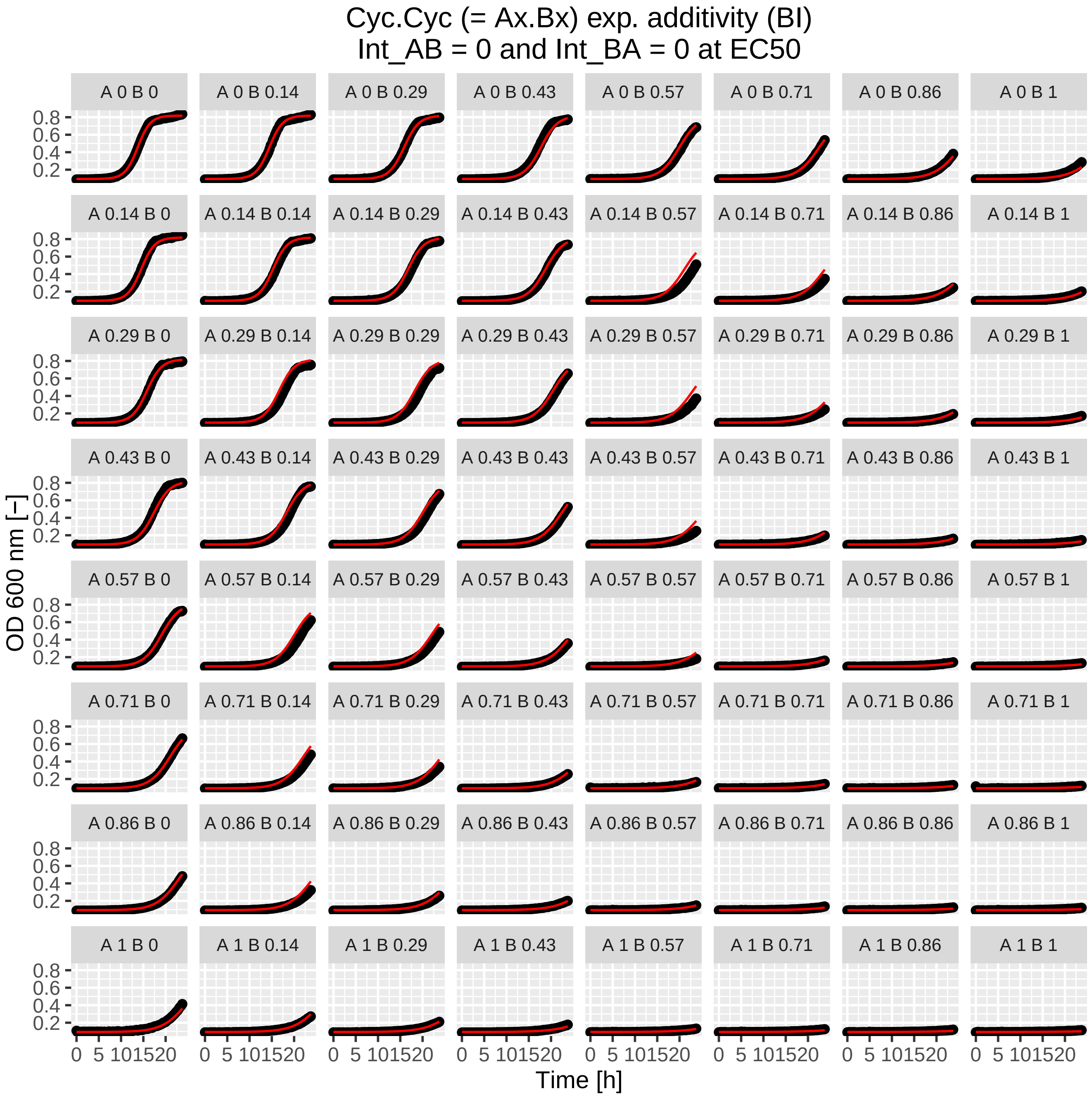

Cyc.Cyc (= Ax.Bx) exp. additivity (BI)
Int_AB = 0 and Int_BA = 0 at EC50
OD 600 nm [–]
Time [h]
0.8
0.6
0.4
0.2
0 5 101520
A 0 B 0
A 0 B 0.14
A 0 B 0.29
A 0 B 0.43
A 0 B 0.57
A 0 B 0.71
A 0 B 0.86
A 0 B 1
A 0.14 B 0
A 0.14 B 0.14
A 0.14 B 0.29
A 0.14 B 0.43
A 0.14 B 0.57
A 0.14 B 0.71
A 0.14 B 0.86
A 0.14 B 1
A 0.29 B 0
A 0.29 B 0.14
A 0.29 B 0.29
A 0.29 B 0.43
A 0.29 B 0.57
A 0.29 B 0.71
A 0.29 B 0.86
A 0.29 B 1
A 0.43 B 0
A 0.43 B 0.14
A 0.43 B 0.29
A 0.43 B 0.43
A 0.43 B 0.57
A 0.43 B 0.71
A 0.43 B 0.86
A 0.43 B 1
A 0.57 B 0
A 0.57 B 0.14
A 0.57 B 0.29
A 0.57 B 0.43
A 0.57 B 0.57
A 0.57 B 0.71
A 0.57 B 0.86
A 0.57 B 1
A 0.71 B 0
A 0.71 B 0.14
A 0.71 B 0.29
A 0.71 B 0.43
A 0.71 B 0.57
A 0.71 B 0.71
A 0.71 B 0.86
A 0.71 B 1
A 0.86 B 0
A 0.86 B 0.14
A 0.86 B 0.29
A 0.86 B 0.43
A 0.86 B 0.57
A 0.86 B 0.71
A 0.86 B 0.86
A 0.86 B 1
A 1 B 0
A 1 B 0.14
A 1 B 0.29
A 1 B 0.43
A 1 B 0.57
A 1 B 0.71
A 1 B 0.86
A 1 B 1

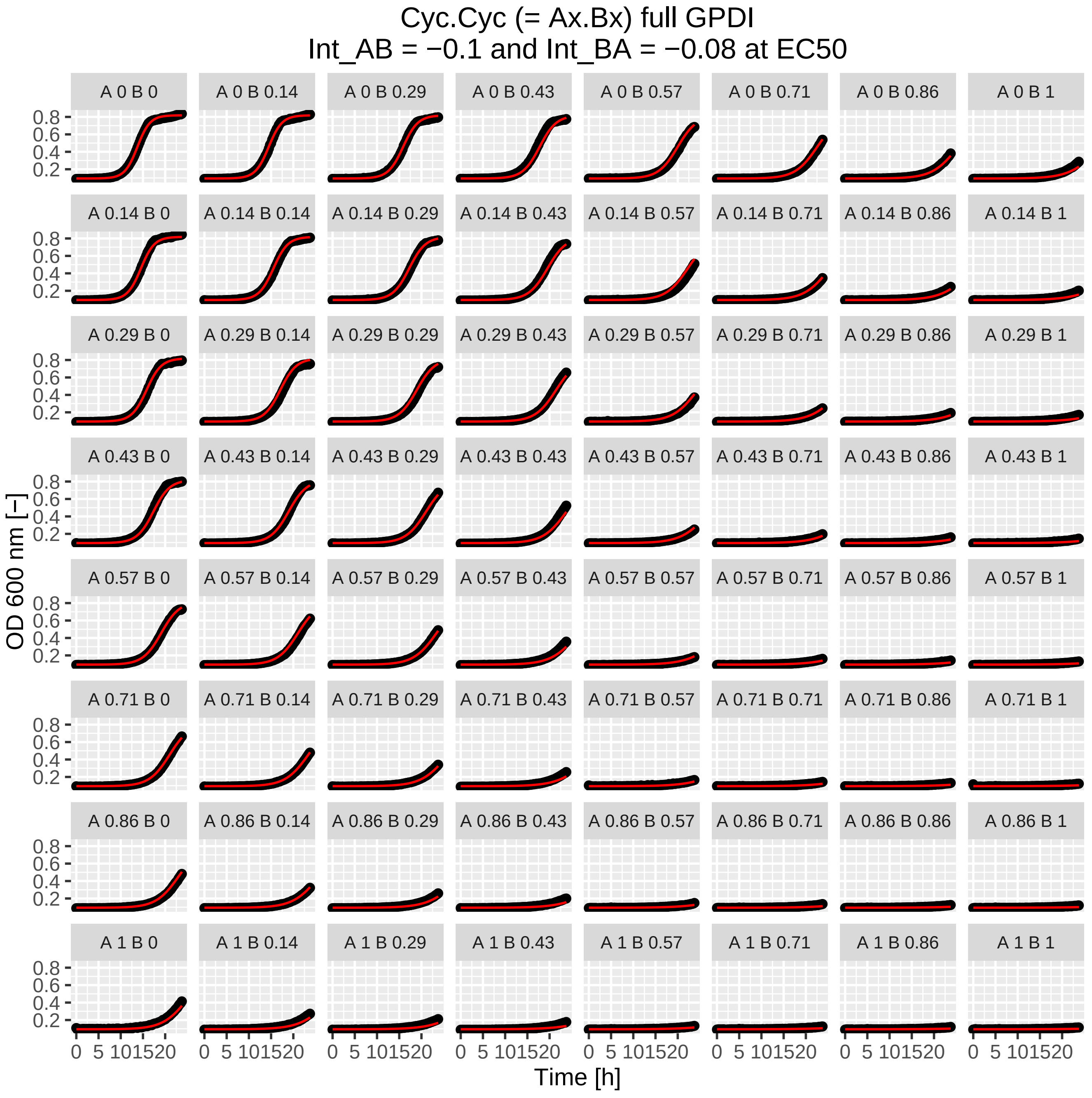
Cyc.Cyc (= Ax.Bx) full GPDI
Int_AB = −0.1 and Int_BA = −0.08 at EC50
A 0 B 0
A 0 B 0.14
A 0 B 0.29
A 0 B 0.43
A 0 B 0.57
A 0 B 0.71
A 0 B 0.86
A 0 B 1
A 0.14 B 0
A 0.14 B 0.14
A 0.14 B 0.29
A 0.14 B 0.43
A 0.14 B 0.57
A 0.14 B 0.71
A 0.14 B 0.86
A 0.14 B 1
A 0.29 B 0
A 0.29 B 0.14
A 0.29 B 0.29
A 0.29 B 0.43
A 0.29 B 0.57
A 0.29 B 0.71
A 0.29 B 0.86
A 0.29 B 1
A 0.43 B 0
A 0.43 B 0.14
A 0.43 B 0.29
A 0.43 B 0.43
A 0.43 B 0.57
A 0.43 B 0.71
A 0.43 B 0.86
A 0.43 B 1
A 0.57 B 0
A 0.57 B 0.14
A 0.57 B 0.29
A 0.57 B 0.43
A 0.57 B 0.57
A 0.57 B 0.71
A 0.57 B 0.86
A 0.57 B 1
A 0.71 B 0
A 0.71 B 0.14
A 0.71 B 0.29
A 0.71 B 0.43
A 0.71 B 0.57
A 0.71 B 0.71
A 0.71 B 0.86
A 0.71 B 1
A 0.86 B 0
A 0.86 B 0.14
A 0.86 B 0.29
A 0.86 B 0.43
A 0.86 B 0.57
A 0.86 B 0.71
A 0.86 B 0.86
A 0.86 B 1
A 1 B 0
A 1 B 0.14
A 1 B 0.29
A 1 B 0.43
A 1 B 0.57
A 1 B 0.71
A 1 B 0.86
A 1 B 1
OD 600 nm [−]
0.8
0.6
0.4
0.2
0 5 101520
Time [h]

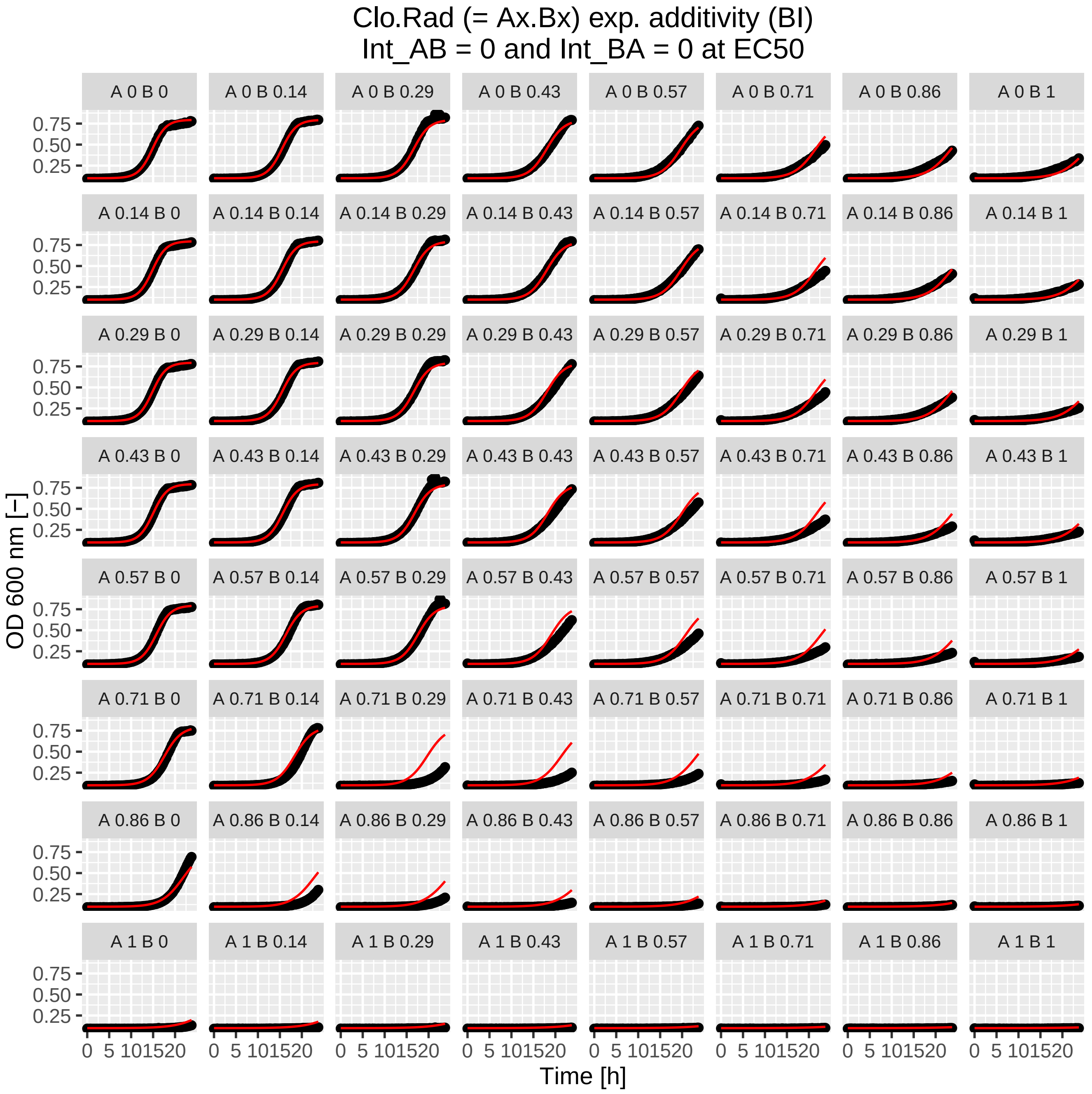

Clo.Rad (= Ax.Bx) exp. additivity (BI)
Int_AB = 0 and Int_BA = 0 at EC50
A 0 B 0
A 0 B 0.14
A 0 B 0.29
A 0 B 0.43
A 0 B 0.57
A 0 B 0.71
A 0 B 0.86
A 0 B 1
A 0.14 B 0
A 0.14 B 0.14
A 0.14 B 0.29
A 0.14 B 0.43
A 0.14 B 0.57
A 0.14 B 0.71
A 0.14 B 0.86
A 0.14 B 1
A 0.29 B 0
A 0.29 B 0.14
A 0.29 B 0.29
A 0.29 B 0.43
A 0.29 B 0.57
A 0.29 B 0.71
A 0.29 B 0.86
A 0.29 B 1
A 0.43 B 0
A 0.43 B 0.14
A 0.43 B 0.29
A 0.43 B 0.43
A 0.43 B 0.57
A 0.43 B 0.71
A 0.43 B 0.86
A 0.43 B 1
A 0.57 B 0
A 0.57 B 0.14
A 0.57 B 0.29
A 0.57 B 0.43
A 0.57 B 0.57
A 0.57 B 0.71
A 0.57 B 0.86
A 0.57 B 1
A 0.71 B 0
A 0.71 B 0.14
A 0.71 B 0.29
A 0.71 B 0.43
A 0.71 B 0.57
A 0.71 B 0.71
A 0.71 B 0.86
A 0.71 B 1
A 0.86 B 0
A 0.86 B 0.14
A 0.86 B 0.29
A 0.86 B 0.43
A 0.86 B 0.57
A 0.86 B 0.71
A 0.86 B 0.86
A 0.86 B 1
A 1 B 0
A 1 B 0.14
A 1 B 0.29
A 1 B 0.43
A 1 B 0.57
A 1 B 0.71
A 1 B 0.86
A 1 B 1
OD 600 nm [–]
0.75
0.50
0.25
0 5 101520
Time [h]

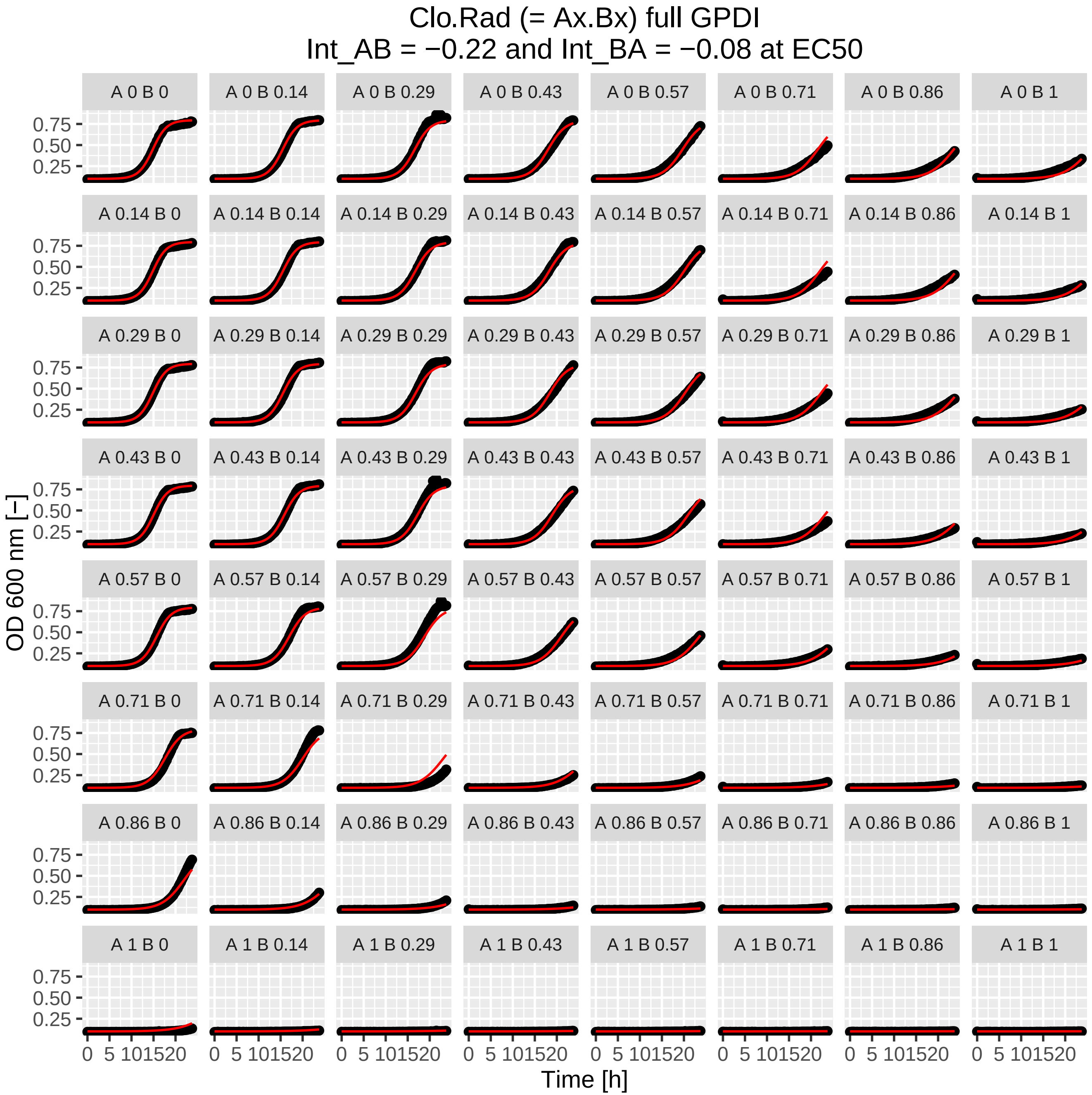
Clo.Rad (= Ax.Bx) full GPDI
Int_AB = −0.22 and Int_BA = −0.08 at EC50
A 0 B 0
A 0 B 0.14
A 0 B 0.29
A 0 B 0.43
A 0 B 0.57
A 0 B 0.71
A 0 B 0.86
A 0 B 1
A 0.14 B 0
A 0.14 B 0.14
A 0.14 B 0.29
A 0.14 B 0.43
A 0.14 B 0.57
A 0.14 B 0.71
A 0.14 B 0.86
A 0.14 B 1
A 0.29 B 0
A 0.29 B 0.14
A 0.29 B 0.29
A 0.29 B 0.43
A 0.29 B 0.57
A 0.29 B 0.71
A 0.29 B 0.86
A 0.29 B 1
A 0.43 B 0
A 0.43 B 0.14
A 0.43 B 0.29
A 0.43 B 0.43
A 0.43 B 0.57
A 0.43 B 0.71
A 0.43 B 0.86
A 0.43 B 1
A 0.57 B 0
A 0.57 B 0.14
A 0.57 B 0.29
A 0.57 B 0.43
A 0.57 B 0.57
A 0.57 B 0.71
A 0.57 B 0.86
A 0.57 B 1
A 0.71 B 0
A 0.71 B 0.14
A 0.71 B 0.29
A 0.71 B 0.43
A 0.71 B 0.57
A 0.71 B 0.71
A 0.71 B 0.86
A 0.71 B 1
A 0.86 B 0
A 0.86 B 0.14
A 0.86 B 0.29
A 0.86 B 0.43
A 0.86 B 0.57
A 0.86 B 0.71
A 0.86 B 0.86
A 0.86 B 1
A 1 B 0
A 1 B 0.14
A 1 B 0.29
A 1 B 0.43
A 1 B 0.57
A 1 B 0.71
A 1 B 0.86
A 1 B 1
OD 600 nm [−]
0.75
0.50
0.25
0 5 101520
Time [h]

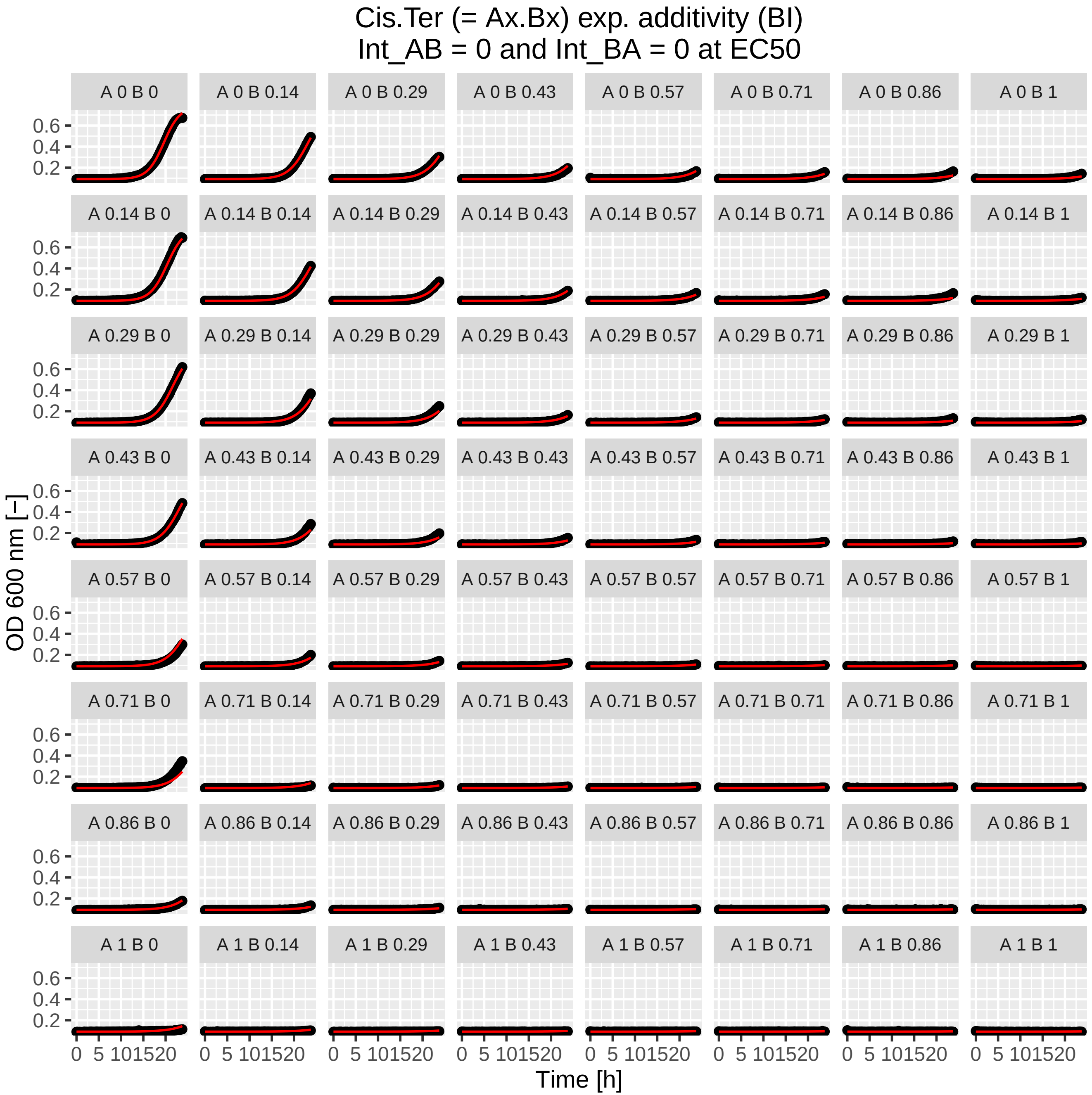

Cis.Ter (= Ax.Bx) exp. additivity (BI)
Int_AB = 0 and Int_BA = 0 at EC50
A 0 B 0
A 0 B 0.14
A 0 B 0.29
A 0 B 0.43
A 0 B 0.57
A 0 B 0.71
A 0 B 0.86
A 0 B 1
A 0.14 B 0
A 0.14 B 0.14
A 0.14 B 0.29
A 0.14 B 0.43
A 0.14 B 0.57
A 0.14 B 0.71
A 0.14 B 0.86
A 0.14 B 1
A 0.29 B 0
A 0.29 B 0.14
A 0.29 B 0.29
A 0.29 B 0.43
A 0.29 B 0.57
A 0.29 B 0.71
A 0.29 B 0.86
A 0.29 B 1
A 0.43 B 0
A 0.43 B 0.14
A 0.43 B 0.29
A 0.43 B 0.43
A 0.43 B 0.57
A 0.43 B 0.71
A 0.43 B 0.86
A 0.43 B 1
A 0.57 B 0
A 0.57 B 0.14
A 0.57 B 0.29
A 0.57 B 0.43
A 0.57 B 0.57
A 0.57 B 0.71
A 0.57 B 0.86
A 0.57 B 1
A 0.71 B 0
A 0.71 B 0.14
A 0.71 B 0.29
A 0.71 B 0.43
A 0.71 B 0.57
A 0.71 B 0.71
A 0.71 B 0.86
A 0.71 B 1
A 0.86 B 0
A 0.86 B 0.14
A 0.86 B 0.29
A 0.86 B 0.43
A 0.86 B 0.57
A 0.86 B 0.71
A 0.86 B 0.86
A 0.86 B 1
A 1 B 0
A 1 B 0.14
A 1 B 0.29
A 1 B 0.43
A 1 B 0.57
A 1 B 0.71
A 1 B 0.86
A 1 B 1
OD 600 nm [–]
0.6
0.4
0.2
0 5 101520
Time [h]

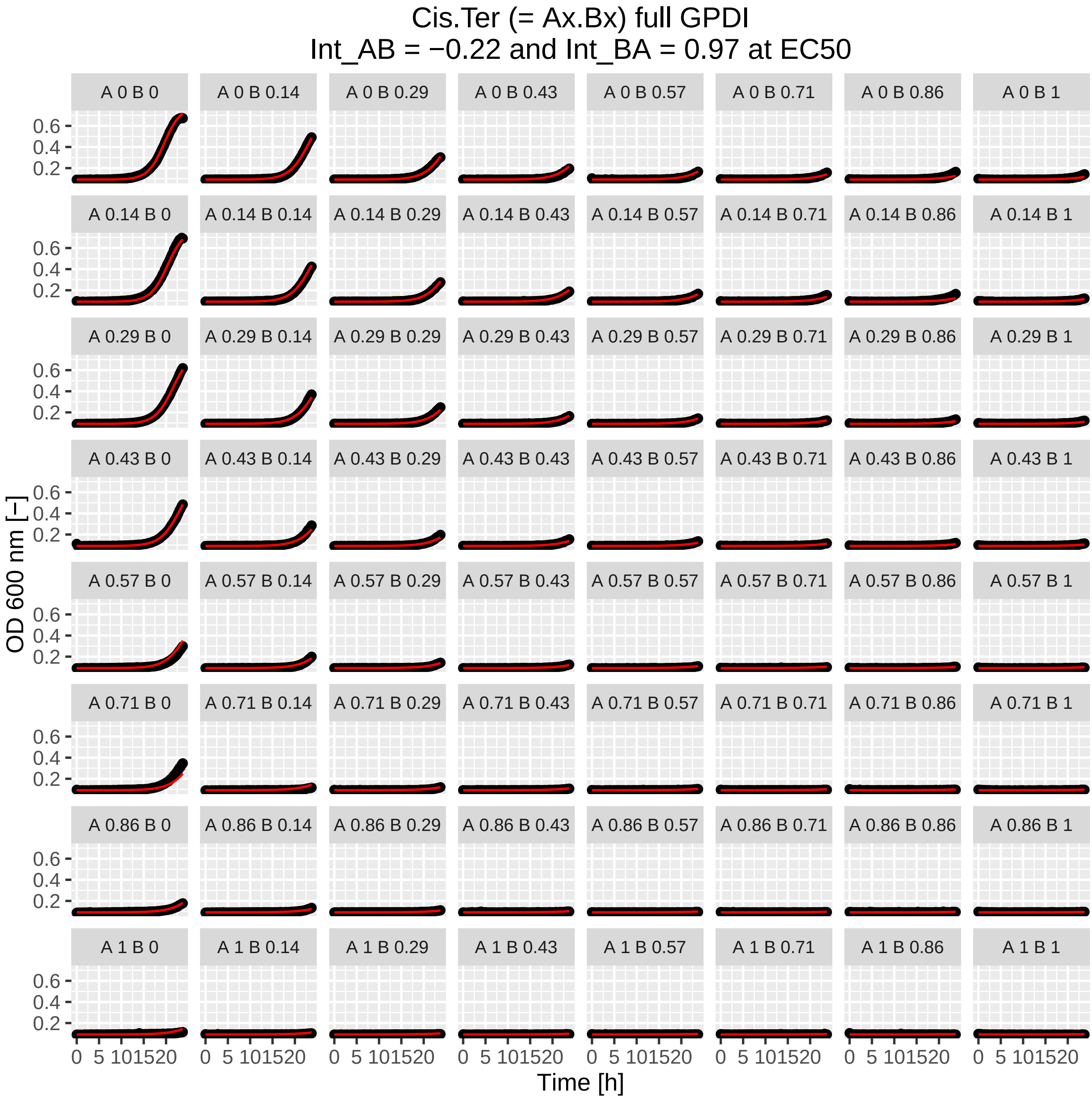
Cis.Ter (= Ax.Bx) full GPDI
Int_AB = −0.22 and Int_BA = 0.97 at EC50
OD 600 nm [−]
Time [h]
A 0 B 0
A 0 B 0.14
A 0 B 0.29
A 0 B 0.43
A 0 B 0.57
A 0 B 0.71
A 0 B 0.86
A 0 B 1
A 0.14 B 0
A 0.14 B 0.14
A 0.14 B 0.29
A 0.14 B 0.43
A 0.14 B 0.57
A 0.14 B 0.71
A 0.14 B 0.86
A 0.14 B 1
A 0.29 B 0
A 0.29 B 0.14
A 0.29 B 0.29
A 0.29 B 0.43
A 0.29 B 0.57
A 0.29 B 0.71
A 0.29 B 0.86
A 0.29 B 1
A 0.43 B 0
A 0.43 B 0.14
A 0.43 B 0.29
A 0.43 B 0.43
A 0.43 B 0.57
A 0.43 B 0.71
A 0.43 B 0.86
A 0.43 B 1
A 0.57 B 0
A 0.57 B 0.14
A 0.57 B 0.29
A 0.57 B 0.43
A 0.57 B 0.57
A 0.57 B 0.71
A 0.57 B 0.86
A 0.57 B 1
A 0.71 B 0
A 0.71 B 0.14
A 0.71 B 0.29
A 0.71 B 0.43
A 0.71 B 0.57
A 0.71 B 0.71
A 0.71 B 0.86
A 0.71 B 1
A 0.86 B 0
A 0.86 B 0.14
A 0.86 B 0.29
A 0.86 B 0.43
A 0.86 B 0.57
A 0.86 B 0.71
A 0.86 B 0.86
A 0.86 B 1
A 1 B 0
A 1 B 0.14
A 1 B 0.29
A 1 B 0.43
A 1 B 0.57
A 1 B 0.71
A 1 B 0.86
A 1 B 1
0.6
0.4
0.2
0 5 101520

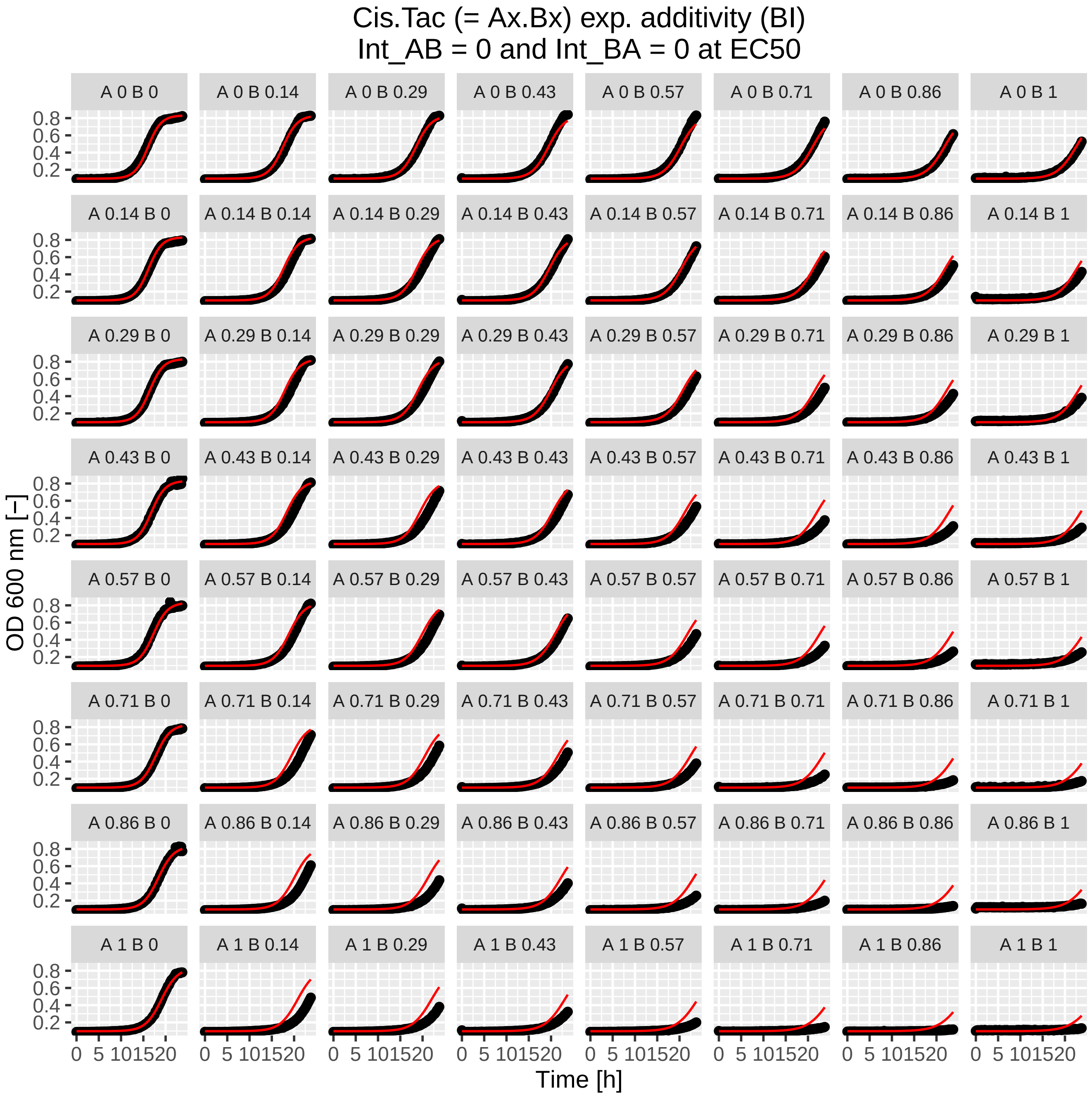

Cis.Tac (= Ax.Bx) exp. additivity (BI)
Int_AB = 0 and Int_BA = 0 at EC50
A 0 B 0
A 0 B 0.14
A 0 B 0.29
A 0 B 0.43
A 0 B 0.57
A 0 B 0.71
A 0 B 0.86
A 0 B 1
A 0.14 B 0
A 0.14 B 0.14
A 0.14 B 0.29
A 0.14 B 0.43
A 0.14 B 0.57
A 0.14 B 0.71
A 0.14 B 0.86
A 0.14 B 1
A 0.29 B 0
A 0.29 B 0.14
A 0.29 B 0.29
A 0.29 B 0.43
A 0.29 B 0.57
A 0.29 B 0.71
A 0.29 B 0.86
A 0.29 B 1
A 0.43 B 0
A 0.43 B 0.14
A 0.43 B 0.29
A 0.43 B 0.43
A 0.43 B 0.57
A 0.43 B 0.71
A 0.43 B 0.86
A 0.43 B 1
A 0.57 B 0
A 0.57 B 0.14
A 0.57 B 0.29
A 0.57 B 0.43
A 0.57 B 0.57
A 0.57 B 0.71
A 0.57 B 0.86
A 0.57 B 1
A 0.71 B 0
A 0.71 B 0.14
A 0.71 B 0.29
A 0.71 B 0.43
A 0.71 B 0.57
A 0.71 B 0.71
A 0.71 B 0.86
A 0.71 B 1
A 0.86 B 0
A 0.86 B 0.14
A 0.86 B 0.29
A 0.86 B 0.43
A 0.86 B 0.57
A 0.86 B 0.71
A 0.86 B 0.86
A 0.86 B 1
A 1 B 0
A 1 B 0.14
A 1 B 0.29
A 1 B 0.43
A 1 B 0.57
A 1 B 0.71
A 1 B 0.86
A 1 B 1
OD 600 nm [–]
0.2
0.4
0.6
0.8
0
5
10
15
20
Time [h]

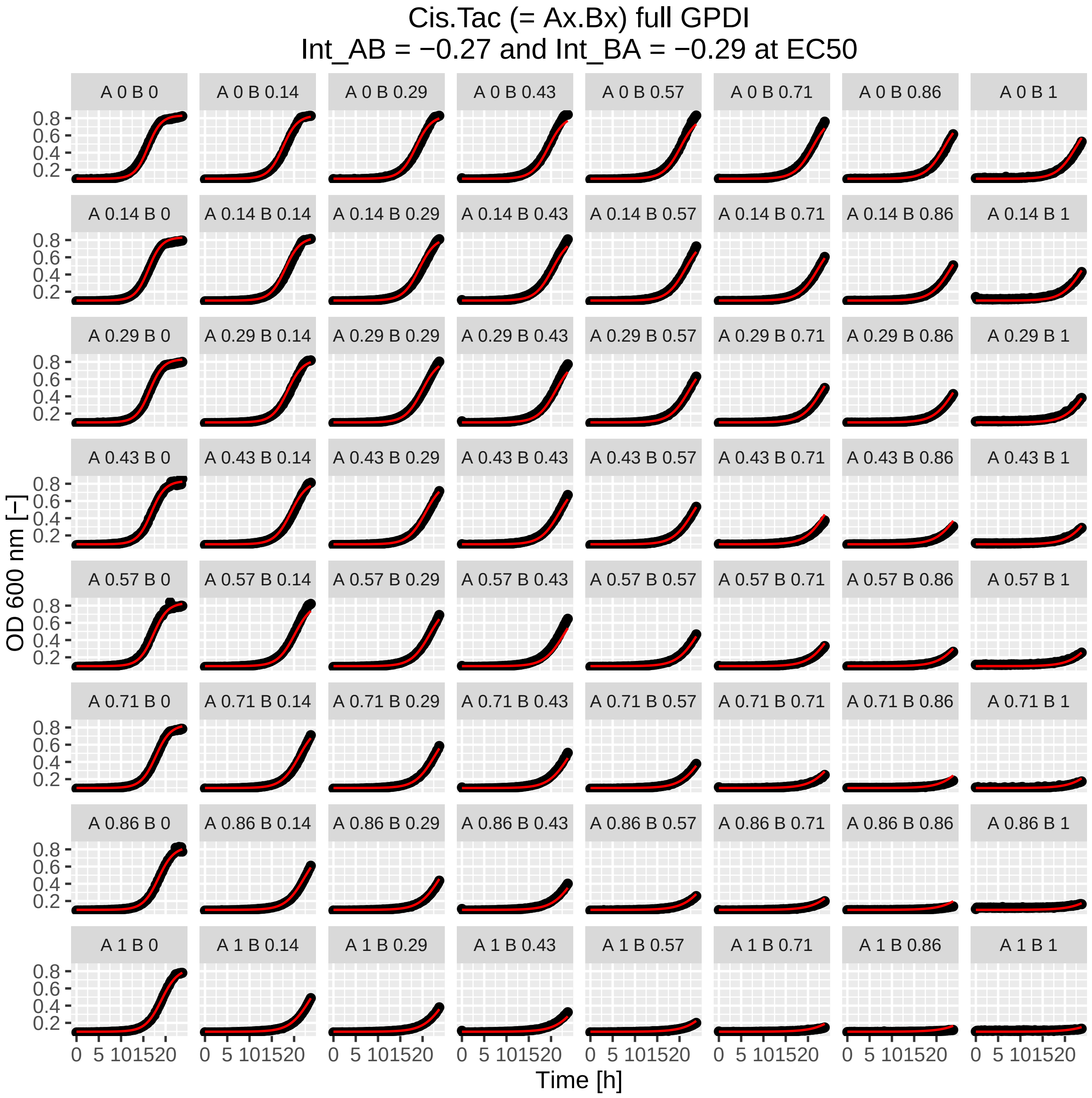

Cis.Tac (= Ax.Bx) full GPDI
Int_AB = −0.27 and Int_BA = −0.29 at EC50
A 0 B 0
A 0 B 0.14
A 0 B 0.29
A 0 B 0.43
A 0 B 0.57
A 0 B 0.71
A 0 B 0.86
A 0 B 1
A 0.14 B 0
A 0.14 B 0.14
A 0.14 B 0.29
A 0.14 B 0.43
A 0.14 B 0.57
A 0.14 B 0.71
A 0.14 B 0.86
A 0.14 B 1
A 0.29 B 0
A 0.29 B 0.14
A 0.29 B 0.29
A 0.29 B 0.43
A 0.29 B 0.57
A 0.29 B 0.71
A 0.29 B 0.86
A 0.29 B 1
A 0.43 B 0
A 0.43 B 0.14
A 0.43 B 0.29
A 0.43 B 0.43
A 0.43 B 0.57
A 0.43 B 0.71
A 0.43 B 0.86
A 0.43 B 1
A 0.57 B 0
A 0.57 B 0.14
A 0.57 B 0.29
A 0.57 B 0.43
A 0.57 B 0.57
A 0.57 B 0.71
A 0.57 B 0.86
A 0.57 B 1
A 0.71 B 0
A 0.71 B 0.14
A 0.71 B 0.29
A 0.71 B 0.43
A 0.71 B 0.57
A 0.71 B 0.71
A 0.71 B 0.86
A 0.71 B 1
A 0.86 B 0
A 0.86 B 0.14
A 0.86 B 0.29
A 0.86 B 0.43
A 0.86 B 0.57
A 0.86 B 0.71
A 0.86 B 0.86
A 0.86 B 1
A 1 B 0
A 1 B 0.14
A 1 B 0.29
A 1 B 0.43
A 1 B 0.57
A 1 B 0.71
A 1 B 0.86
A 1 B 1
OD 600 nm [−]
0.2
0.4
0.6
0.8
0 5 101520
Time [h]

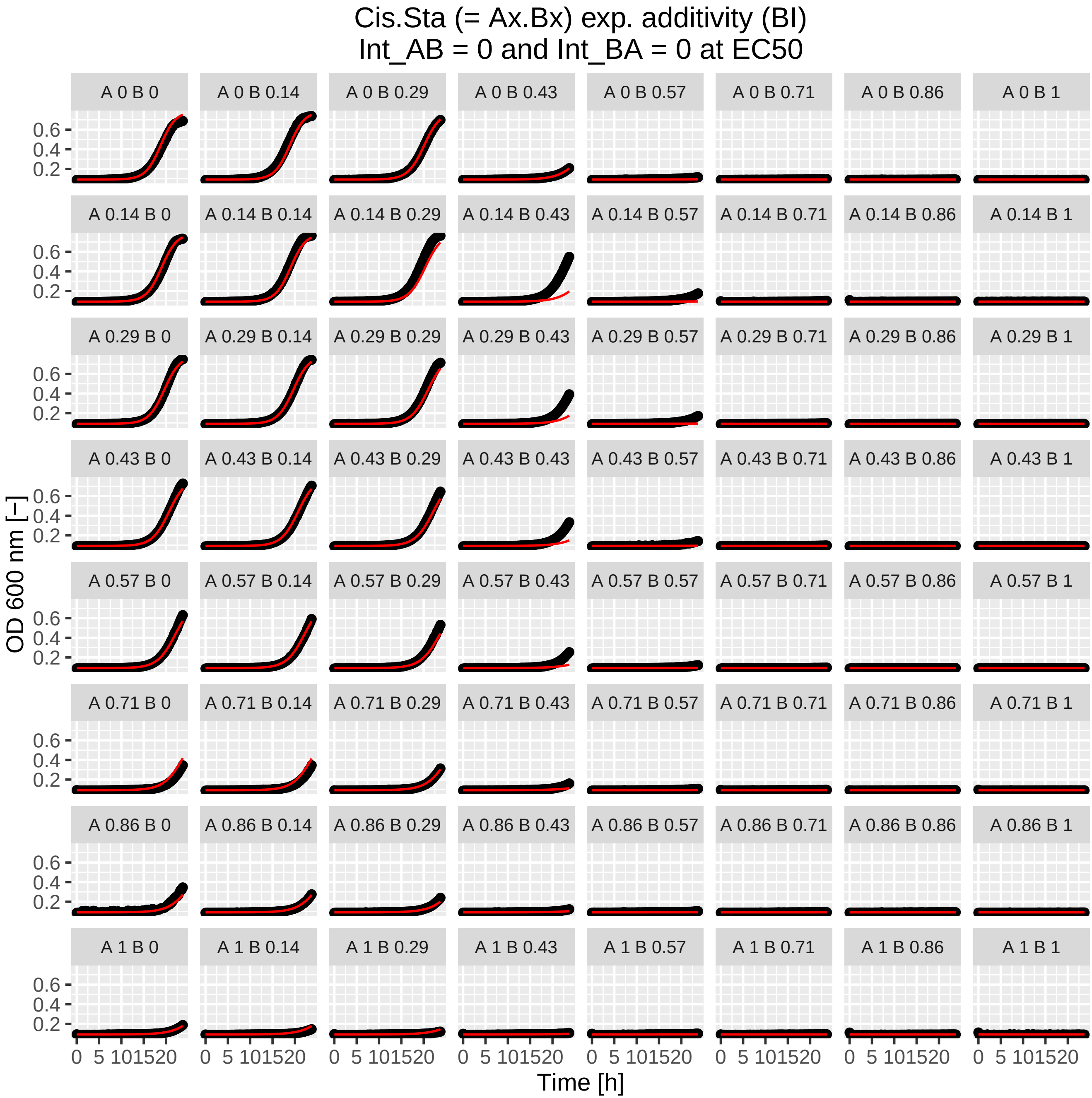

Cis.Sta (= Ax.Bx) exp. additivity (BI)
Int_AB = 0 and Int_BA = 0 at EC50
A 0 B 0
A 0 B 0.14
A 0 B 0.29
A 0 B 0.43
A 0 B 0.57
A 0 B 0.71
A 0 B 0.86
A 0 B 1
A 0.14 B 0
A 0.14 B 0.14
A 0.14 B 0.29
A 0.14 B 0.43
A 0.14 B 0.57
A 0.14 B 0.71
A 0.14 B 0.86
A 0.14 B 1
A 0.29 B 0
A 0.29 B 0.14
A 0.29 B 0.29
A 0.29 B 0.43
A 0.29 B 0.57
A 0.29 B 0.71
A 0.29 B 0.86
A 0.29 B 1
A 0.43 B 0
A 0.43 B 0.14
A 0.43 B 0.29
A 0.43 B 0.43
A 0.43 B 0.57
A 0.43 B 0.71
A 0.43 B 0.86
A 0.43 B 1
A 0.57 B 0
A 0.57 B 0.14
A 0.57 B 0.29
A 0.57 B 0.43
A 0.57 B 0.57
A 0.57 B 0.71
A 0.57 B 0.86
A 0.57 B 1
A 0.71 B 0
A 0.71 B 0.14
A 0.71 B 0.29
A 0.71 B 0.43
A 0.71 B 0.57
A 0.71 B 0.71
A 0.71 B 0.86
A 0.71 B 1
A 0.86 B 0
A 0.86 B 0.14
A 0.86 B 0.29
A 0.86 B 0.43
A 0.86 B 0.57
A 0.86 B 0.71
A 0.86 B 0.86
A 0.86 B 1
A 1 B 0
A 1 B 0.14
A 1 B 0.29
A 1 B 0.43
A 1 B 0.57
A 1 B 0.71
A 1 B 0.86
A 1 B 1
0.6
0.4
0.2
0 5 101520
OD 600 nm [–]
Time [h]

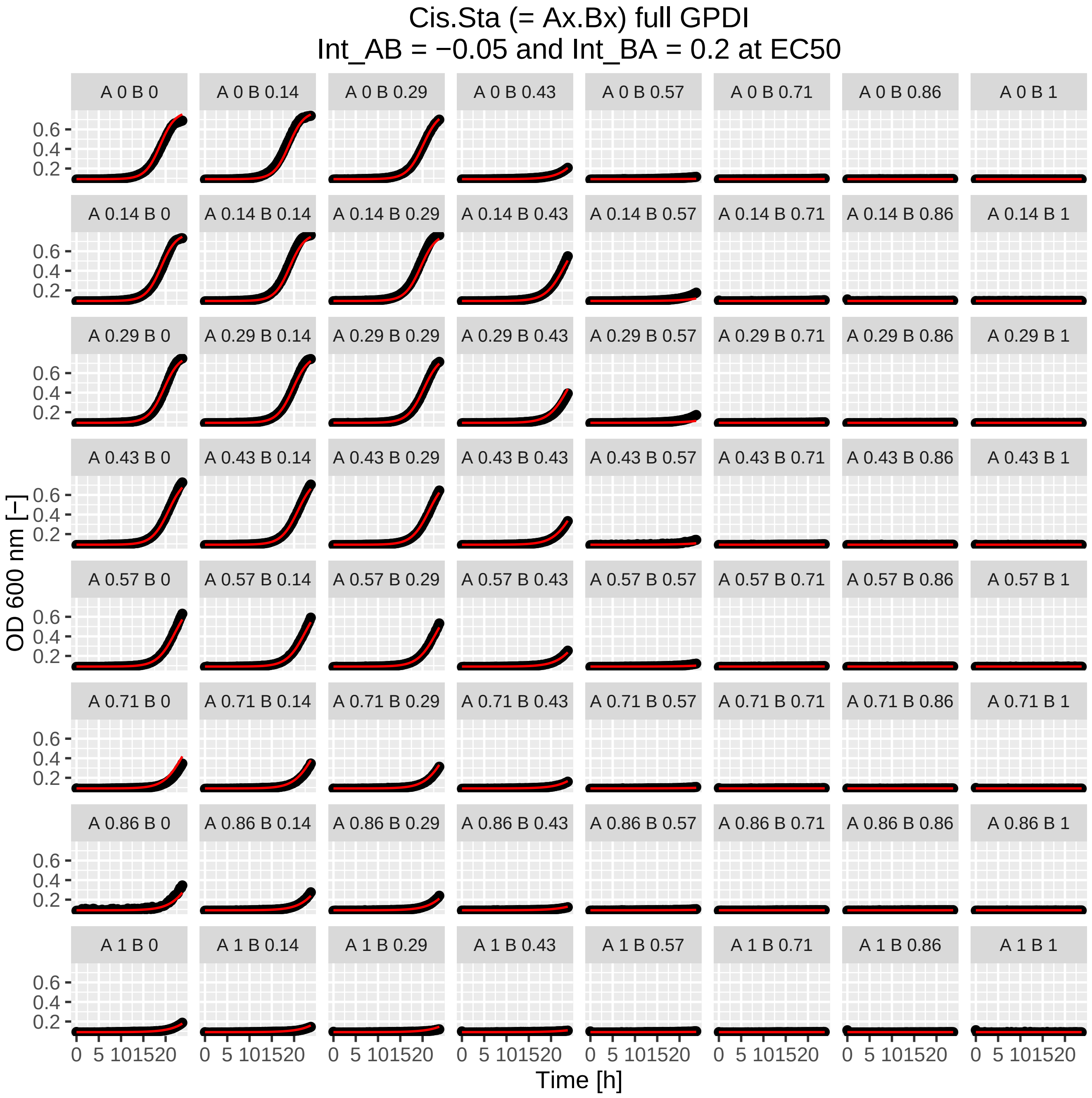

Cis.Sta (= Ax.Bx) full GPDI
Int_AB = −0.05 and Int_BA = 0.2 at EC50
A 0 B 0
A 0 B 0.14
A 0 B 0.29
A 0 B 0.43
A 0 B 0.57
A 0 B 0.71
A 0 B 0.86
A 0 B 1
A 0.14 B 0
A 0.14 B 0.14
A 0.14 B 0.29
A 0.14 B 0.43
A 0.14 B 0.57
A 0.14 B 0.71
A 0.14 B 0.86
A 0.14 B 1
A 0.29 B 0
A 0.29 B 0.14
A 0.29 B 0.29
A 0.29 B 0.43
A 0.29 B 0.57
A 0.29 B 0.71
A 0.29 B 0.86
A 0.29 B 1
A 0.43 B 0
A 0.43 B 0.14
A 0.43 B 0.29
A 0.43 B 0.43
A 0.43 B 0.57
A 0.43 B 0.71
A 0.43 B 0.86
A 0.43 B 1
A 0.57 B 0
A 0.57 B 0.14
A 0.57 B 0.29
A 0.57 B 0.43
A 0.57 B 0.57
A 0.57 B 0.71
A 0.57 B 0.86
A 0.57 B 1
A 0.71 B 0
A 0.71 B 0.14
A 0.71 B 0.29
A 0.71 B 0.43
A 0.71 B 0.57
A 0.71 B 0.71
A 0.71 B 0.86
A 0.71 B 1
A 0.86 B 0
A 0.86 B 0.14
A 0.86 B 0.29
A 0.86 B 0.43
A 0.86 B 0.57
A 0.86 B 0.71
A 0.86 B 0.86
A 0.86 B 1
A 1 B 0
A 1 B 0.14
A 1 B 0.29
A 1 B 0.43
A 1 B 0.57
A 1 B 0.71
A 1 B 0.86
A 1 B 1
0.6
0.4
0.2
OD 600 nm [−]
0 5 101520
Time [h]

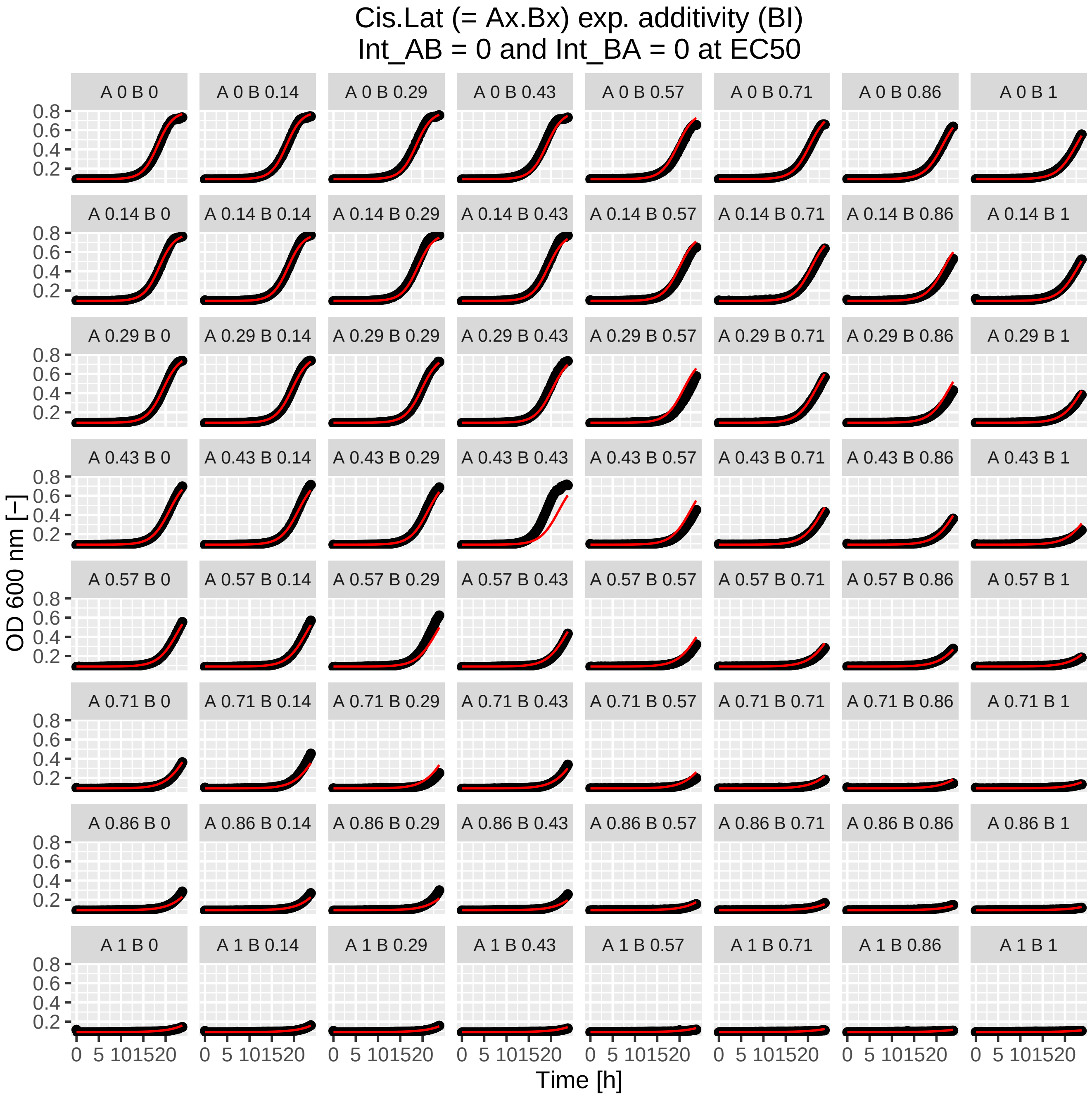

Cis.Lat (= Ax.Bx) exp. additivity (BI)
Int_AB = 0 and Int_BA = 0 at EC50
OD 600 nm [−]
Time [h]
A 0 B 0
A 0 B 0.14
A 0 B 0.29
A 0 B 0.43
A 0 B 0.57
A 0 B 0.71
A 0 B 0.86
A 0 B 1
A 0.14 B 0
A 0.14 B 0.14
A 0.14 B 0.29
A 0.14 B 0.43
A 0.14 B 0.57
A 0.14 B 0.71
A 0.14 B 0.86
A 0.14 B 1
A 0.29 B 0
A 0.29 B 0.14
A 0.29 B 0.29
A 0.29 B 0.43
A 0.29 B 0.57
A 0.29 B 0.71
A 0.29 B 0.86
A 0.29 B 1
A 0.43 B 0
A 0.43 B 0.14
A 0.43 B 0.29
A 0.43 B 0.43
A 0.43 B 0.57
A 0.43 B 0.71
A 0.43 B 0.86
A 0.43 B 1
A 0.57 B 0
A 0.57 B 0.14
A 0.57 B 0.29
A 0.57 B 0.43
A 0.57 B 0.57
A 0.57 B 0.71
A 0.57 B 0.86
A 0.57 B 1
A 0.71 B 0
A 0.71 B 0.14
A 0.71 B 0.29
A 0.71 B 0.43
A 0.71 B 0.57
A 0.71 B 0.71
A 0.71 B 0.86
A 0.71 B 1
A 0.86 B 0
A 0.86 B 0.14
A 0.86 B 0.29
A 0.86 B 0.43
A 0.86 B 0.57
A 0.86 B 0.71
A 0.86 B 0.86
A 0.86 B 1
A 1 B 0
A 1 B 0.14
A 1 B 0.29
A 1 B 0.43
A 1 B 0.57
A 1 B 0.71
A 1 B 0.86
A 1 B 1
0.8
0.6
0.4
0.2
0 5 101520

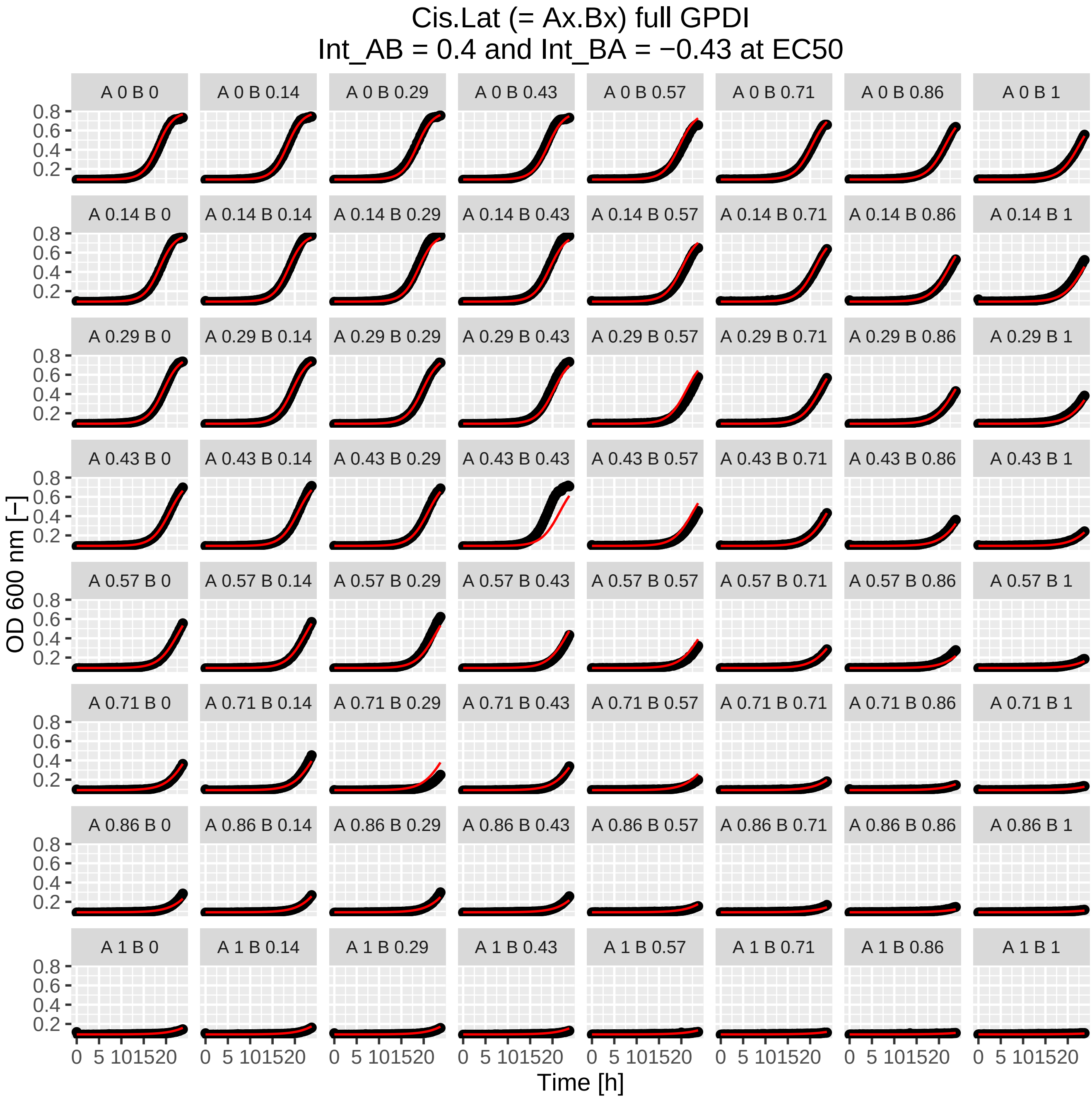

Cis.Lat (= Ax.Bx) full GPDI
Int_AB = 0.4 and Int_BA = −0.43 at EC50
OD 600 nm [−]
Time [h]
A 0 B 0
A 0 B 0.14
A 0 B 0.29
A 0 B 0.43
A 0 B 0.57
A 0 B 0.71
A 0 B 0.86
A 0 B 1
A 1 B 0
A 1 B 0.14
A 1 B 0.29
A 1 B 0.43
A 1 B 0.57
A 1 B 0.71
A 1 B 0.86
A 1 B 1

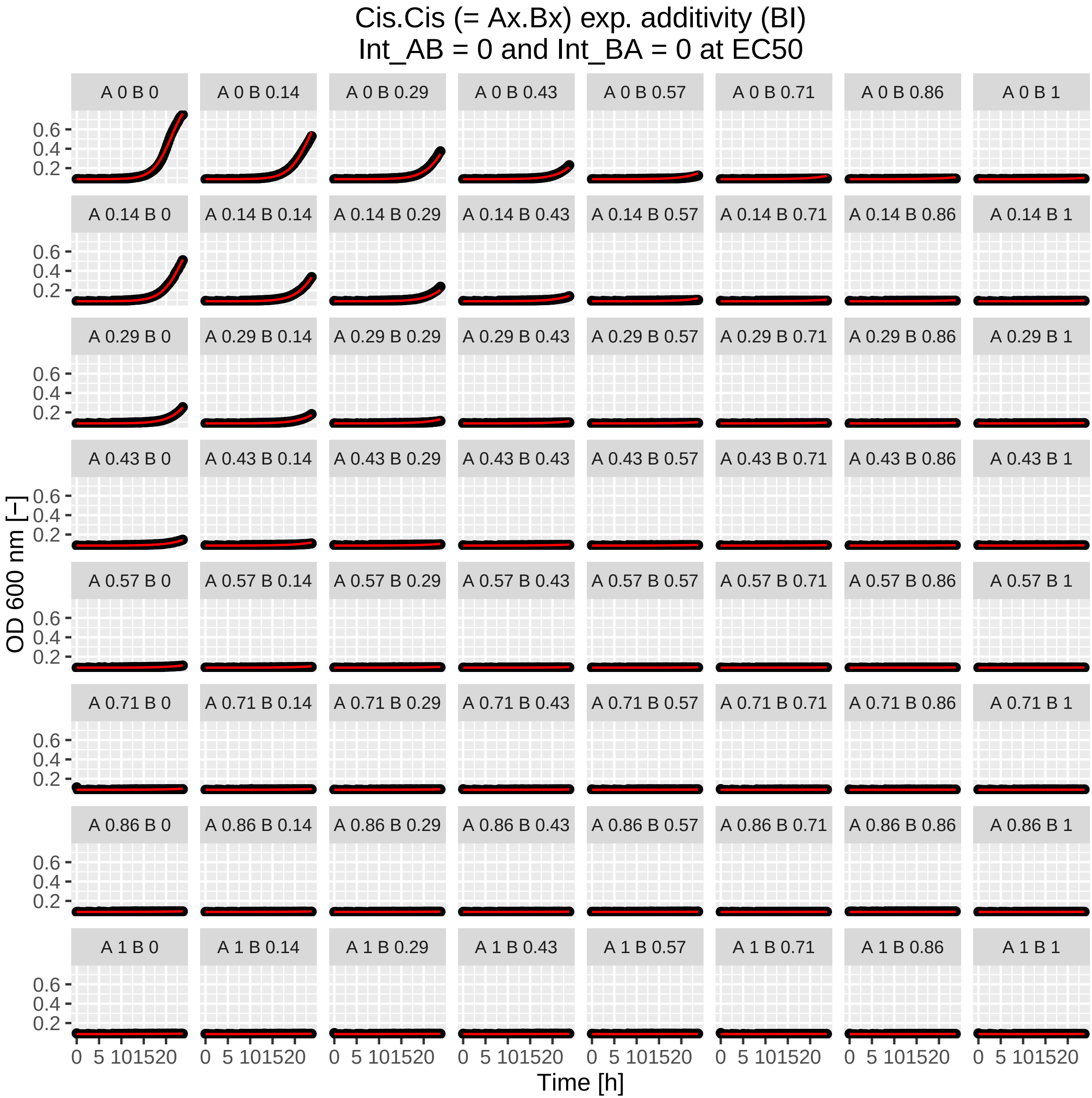

Cis.Cis (= Ax.Bx) exp. additivity (BI)
Int_AB = 0 and Int_BA = 0 at EC50
A 0 B 0
A 0 B 0.14
A 0 B 0.29
A 0 B 0.43
A 0 B 0.57
A 0 B 0.71
A 0 B 0.86
A 0 B 1
A 0.14 B 0
A 0.14 B 0.14
A 0.14 B 0.29
A 0.14 B 0.43
A 0.14 B 0.57
A 0.14 B 0.71
A 0.14 B 0.86
A 0.14 B 1
A 0.29 B 0
A 0.29 B 0.14
A 0.29 B 0.29
A 0.29 B 0.43
A 0.29 B 0.57
A 0.29 B 0.71
A 0.29 B 0.86
A 0.29 B 1
A 0.43 B 0
A 0.43 B 0.14
A 0.43 B 0.29
A 0.43 B 0.43
A 0.43 B 0.57
A 0.43 B 0.71
A 0.43 B 0.86
A 0.43 B 1
A 0.57 B 0
A 0.57 B 0.14
A 0.57 B 0.29
A 0.57 B 0.43
A 0.57 B 0.57
A 0.57 B 0.71
A 0.57 B 0.86
A 0.57 B 1
A 0.71 B 0
A 0.71 B 0.14
A 0.71 B 0.29
A 0.71 B 0.43
A 0.71 B 0.57
A 0.71 B 0.71
A 0.71 B 0.86
A 0.71 B 1
A 0.86 B 0
A 0.86 B 0.14
A 0.86 B 0.29
A 0.86 B 0.43
A 0.86 B 0.57
A 0.86 B 0.71
A 0.86 B 0.86
A 0.86 B 1
A 1 B 0
A 1 B 0.14
A 1 B 0.29
A 1 B 0.43
A 1 B 0.57
A 1 B 0.71
A 1 B 0.86
A 1 B 1
OD 600 nm [–]
0.6
0.4
0.2
0 5 101520
Time [h]

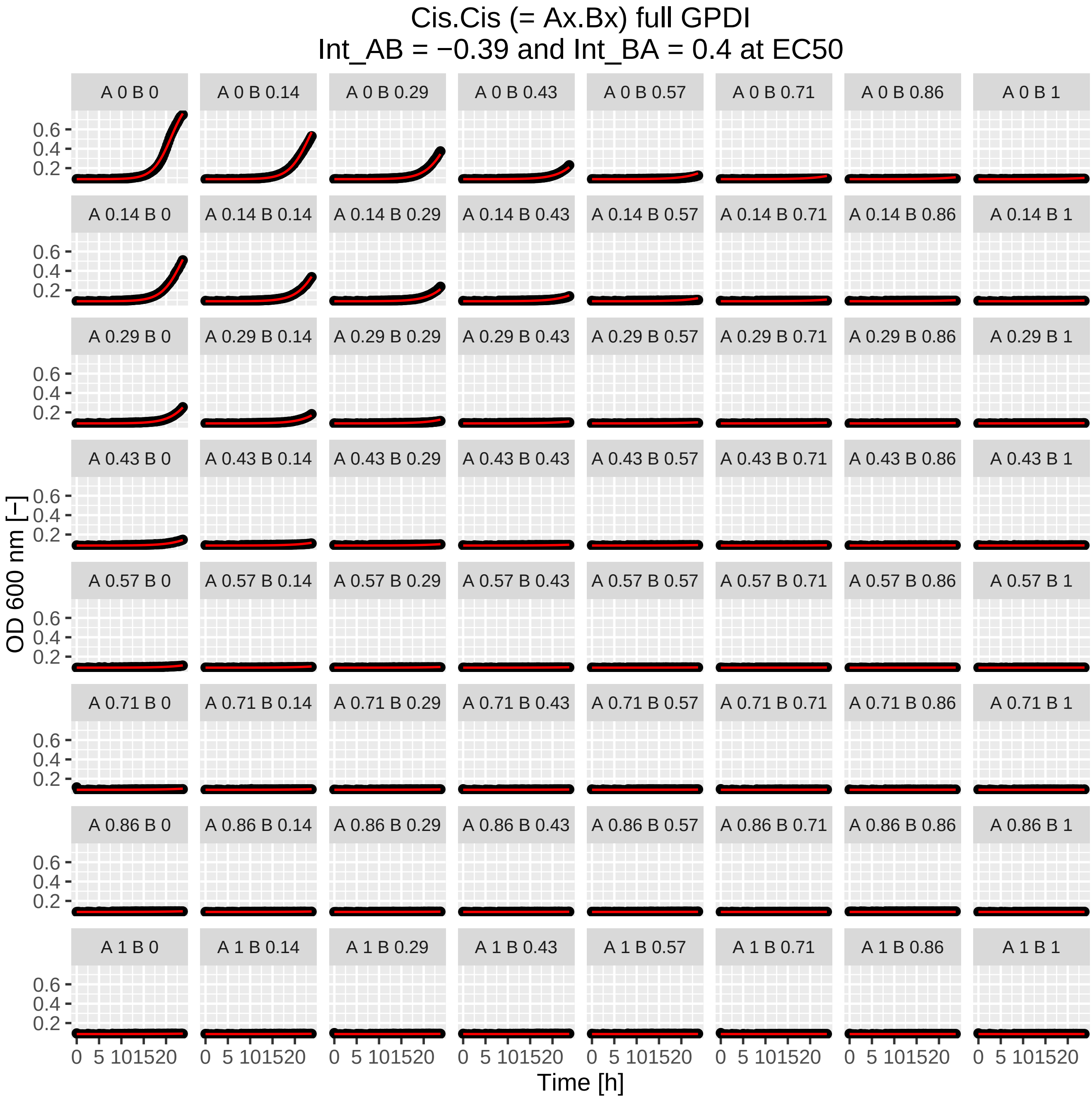

Cis.Cis (= Ax.Bx) full GPDI
Int_AB = −0.39 and Int_BA = 0.4 at EC50
A 0 B 0
A 0 B 0.14
A 0 B 0.29
A 0 B 0.43
A 0 B 0.57
A 0 B 0.71
A 0 B 0.86
A 0 B 1
A 0.14 B 0
A 0.14 B 0.14
A 0.14 B 0.29
A 0.14 B 0.43
A 0.14 B 0.57
A 0.14 B 0.71
A 0.14 B 0.86
A 0.14 B 1
A 0.29 B 0
A 0.29 B 0.14
A 0.29 B 0.29
A 0.29 B 0.43
A 0.29 B 0.57
A 0.29 B 0.71
A 0.29 B 0.86
A 0.29 B 1
A 0.43 B 0
A 0.43 B 0.14
A 0.43 B 0.29
A 0.43 B 0.43
A 0.43 B 0.57
A 0.43 B 0.71
A 0.43 B 0.86
A 0.43 B 1
A 0.57 B 0
A 0.57 B 0.14
A 0.57 B 0.29
A 0.57 B 0.43
A 0.57 B 0.57
A 0.57 B 0.71
A 0.57 B 0.86
A 0.57 B 1
A 0.71 B 0
A 0.71 B 0.14
A 0.71 B 0.29
A 0.71 B 0.43
A 0.71 B 0.57
A 0.71 B 0.71
A 0.71 B 0.86
A 0.71 B 1
A 0.86 B 0
A 0.86 B 0.14
A 0.86 B 0.29
A 0.86 B 0.43
A 0.86 B 0.57
A 0.86 B 0.71
A 0.86 B 0.86
A 0.86 B 1
A 1 B 0
A 1 B 0.14
A 1 B 0.29
A 1 B 0.43
A 1 B 0.57
A 1 B 0.71
A 1 B 0.86
A 1 B 1
0.6
0.4
0.2
OD 600 nm [−]
0 5 101520
Time [h]

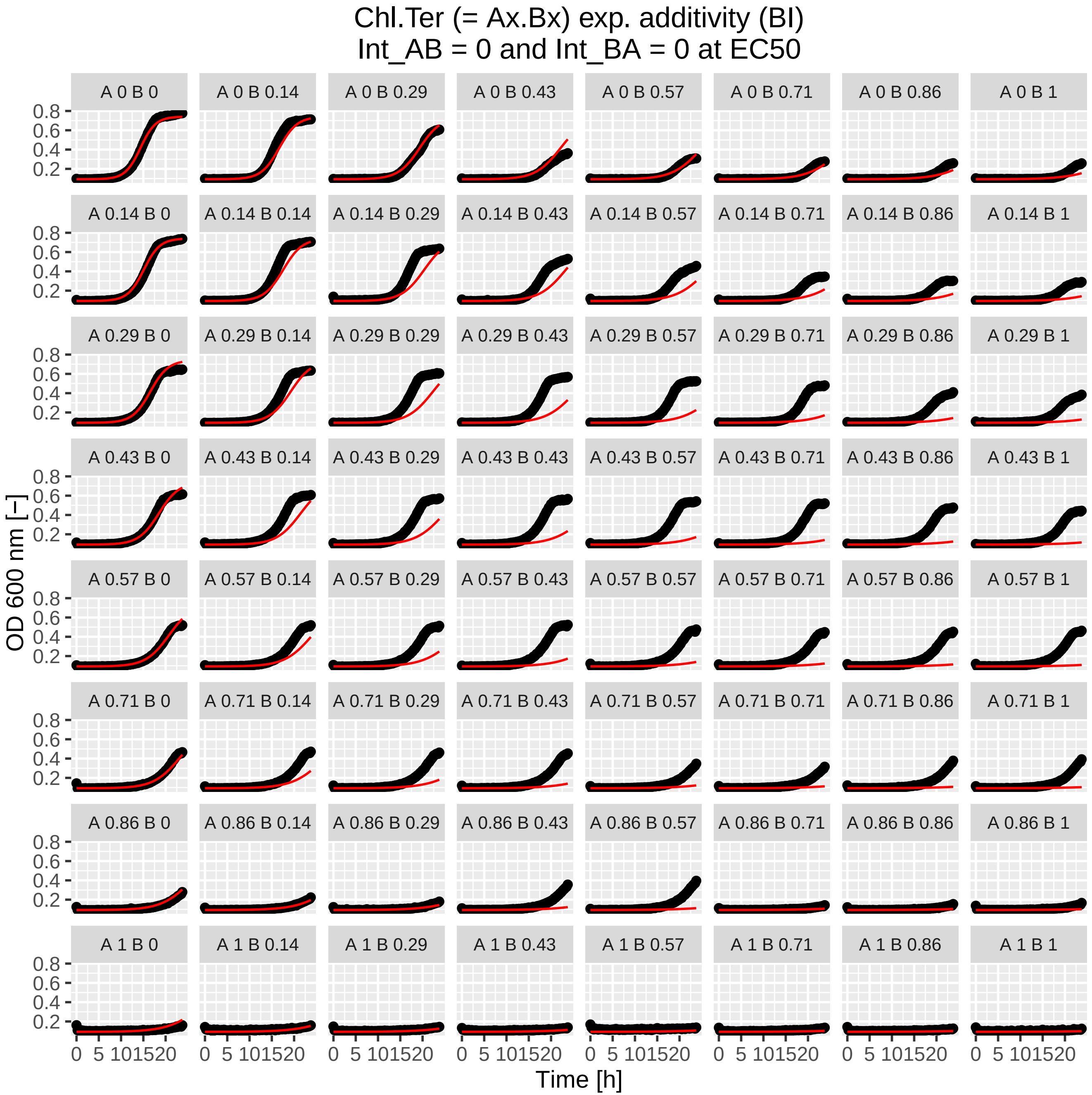
Chl.Ter (= Ax.Bx) exp. additivity (BI)
Int_AB = 0 and Int_BA = 0 at EC50
A 0 B 0
A 0 B 0.14
A 0 B 0.29
A 0 B 0.43
A 0 B 0.57
A 0 B 0.71
A 0 B 0.86
A 0 B 1
A 0.14 B 0
A 0.14 B 0.14
A 0.14 B 0.29
A 0.14 B 0.43
A 0.14 B 0.57
A 0.14 B 0.71
A 0.14 B 0.86
A 0.14 B 1
A 0.29 B 0
A 0.29 B 0.14
A 0.29 B 0.29
A 0.29 B 0.43
A 0.29 B 0.57
A 0.29 B 0.71
A 0.29 B 0.86
A 0.29 B 1
A 0.43 B 0
A 0.43 B 0.14
A 0.43 B 0.29
A 0.43 B 0.43
A 0.43 B 0.57
A 0.43 B 0.71
A 0.43 B 0.86
A 0.43 B 1
A 0.57 B 0
A 0.57 B 0.14
A 0.57 B 0.29
A 0.57 B 0.43
A 0.57 B 0.57
A 0.57 B 0.71
A 0.57 B 0.86
A 0.57 B 1
A 0.71 B 0
A 0.71 B 0.14
A 0.71 B 0.29
A 0.71 B 0.43
A 0.71 B 0.57
A 0.71 B 0.71
A 0.71 B 0.86
A 0.71 B 1
A 0.86 B 0
A 0.86 B 0.14
A 0.86 B 0.29
A 0.86 B 0.43
A 0.86 B 0.57
A 0.86 B 0.71
A 0.86 B 0.86
A 0.86 B 1
A 1 B 0
A 1 B 0.14
A 1 B 0.29
A 1 B 0.43
A 1 B 0.57
A 1 B 0.71
A 1 B 0.86
A 1 B 1
OD 600 nm [−]
0.2
0.4
0.6
0.8
0 5 101520
Time [h]

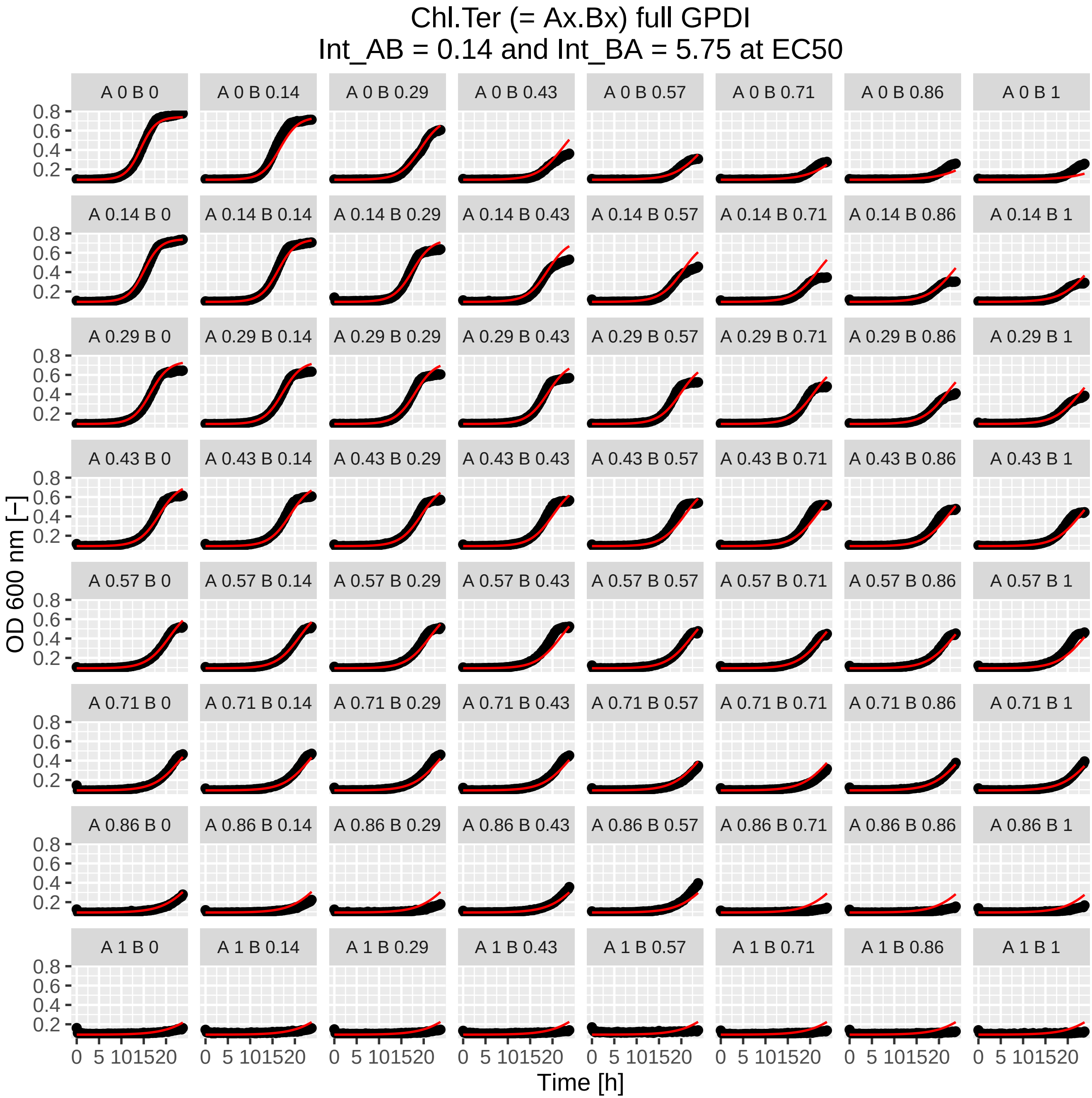

Chl.Ter (= Ax.Bx) full GPDI
Int_AB = 0.14 and Int_BA = 5.75 at EC50
A 0 B 0
A 0 B 0.14
A 0 B 0.29
A 0 B 0.43
A 0 B 0.57
A 0 B 0.71
A 0 B 0.86
A 0 B 1
A 0.14 B 0
A 0.14 B 0.14
A 0.14 B 0.29
A 0.14 B 0.43
A 0.14 B 0.57
A 0.14 B 0.71
A 0.14 B 0.86
A 0.14 B 1
A 0.29 B 0
A 0.29 B 0.14
A 0.29 B 0.29
A 0.29 B 0.43
A 0.29 B 0.57
A 0.29 B 0.71
A 0.29 B 0.86
A 0.29 B 1
A 0.43 B 0
A 0.43 B 0.14
A 0.43 B 0.29
A 0.43 B 0.43
A 0.43 B 0.57
A 0.43 B 0.71
A 0.43 B 0.86
A 0.43 B 1
A 0.57 B 0
A 0.57 B 0.14
A 0.57 B 0.29
A 0.57 B 0.43
A 0.57 B 0.57
A 0.57 B 0.71
A 0.57 B 0.86
A 0.57 B 1
A 0.71 B 0
A 0.71 B 0.14
A 0.71 B 0.29
A 0.71 B 0.43
A 0.71 B 0.57
A 0.71 B 0.71
A 0.71 B 0.86
A 0.71 B 1
A 0.86 B 0
A 0.86 B 0.14
A 0.86 B 0.29
A 0.86 B 0.43
A 0.86 B 0.57
A 0.86 B 0.71
A 0.86 B 0.86
A 0.86 B 1
A 1 B 0
A 1 B 0.14
A 1 B 0.29
A 1 B 0.43
A 1 B 0.57
A 1 B 0.71
A 1 B 0.86
A 1 B 1
0.8
0.6
0.4
0.2
0 5 101520
OD 600 nm [–]
Time [h]

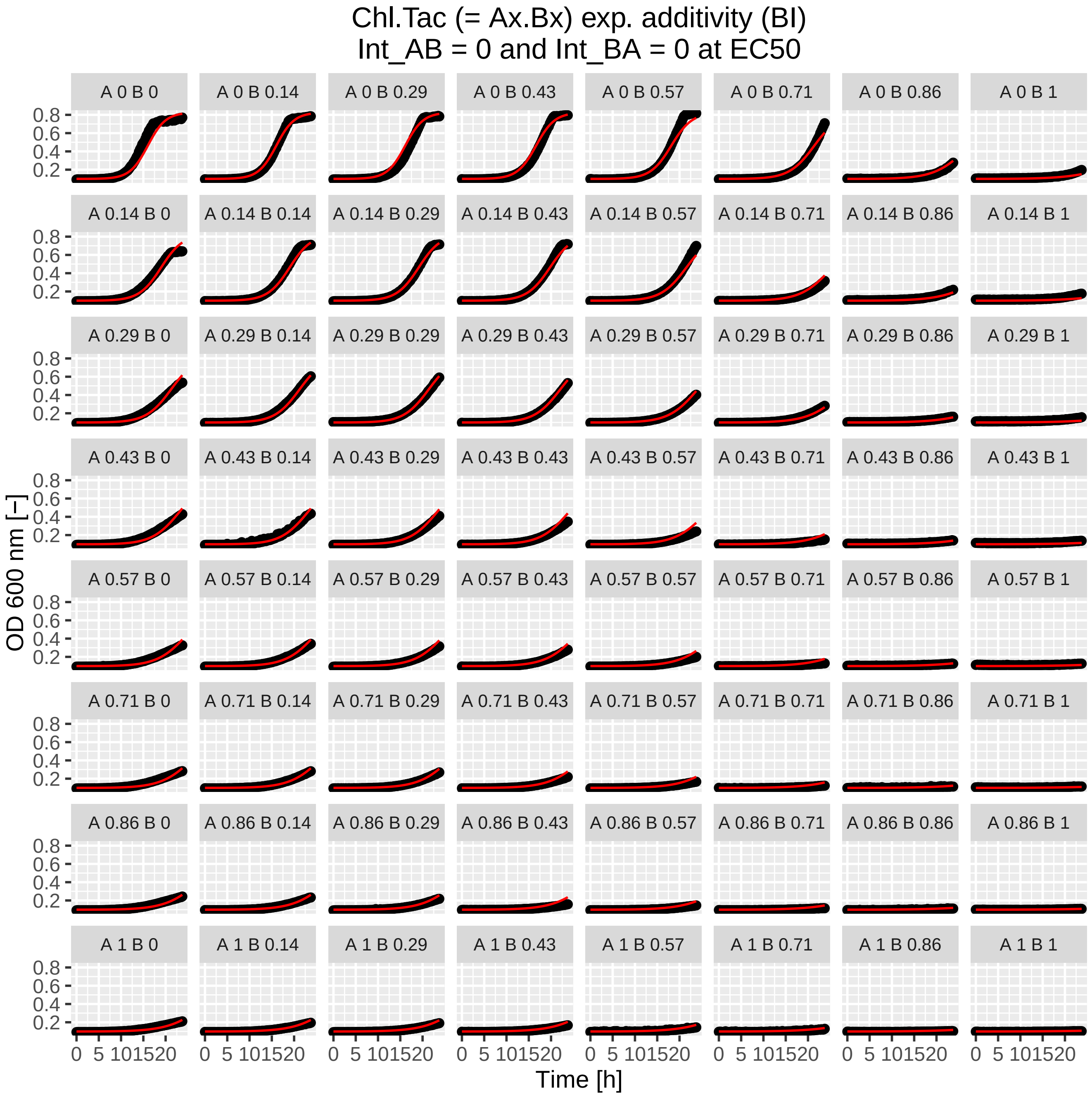
Chl.Tac (= Ax.Bx) exp. additivity (BI)
Int_AB = 0 and Int_BA = 0 at EC50
OD 600 nm [–]
Time [h]
0.8
0.6
0.4
0.2
0 5 101520
A 0 B 0
A 0 B 0.14
A 0 B 0.29
A 0 B 0.43
A 0 B 0.57
A 0 B 0.71
A 0 B 0.86
A 0 B 1
A 0.14 B 0
A 0.14 B 0.14
A 0.14 B 0.29
A 0.14 B 0.43
A 0.14 B 0.57
A 0.14 B 0.71
A 0.14 B 0.86
A 0.14 B 1
A 0.29 B 0
A 0.29 B 0.14
A 0.29 B 0.29
A 0.29 B 0.43
A 0.29 B 0.57
A 0.29 B 0.71
A 0.29 B 0.86
A 0.29 B 1
A 0.43 B 0
A 0.43 B 0.14
A 0.43 B 0.29
A 0.43 B 0.43
A 0.43 B 0.57
A 0.43 B 0.71
A 0.43 B 0.86
A 0.43 B 1
A 0.57 B 0
A 0.57 B 0.14
A 0.57 B 0.29
A 0.57 B 0.43
A 0.57 B 0.57
A 0.57 B 0.71
A 0.57 B 0.86
A 0.57 B 1
A 0.71 B 0
A 0.71 B 0.14
A 0.71 B 0.29
A 0.71 B 0.43
A 0.71 B 0.57
A 0.71 B 0.71
A 0.71 B 0.86
A 0.71 B 1
A 0.86 B 0
A 0.86 B 0.14
A 0.86 B 0.29
A 0.86 B 0.43
A 0.86 B 0.57
A 0.86 B 0.71
A 0.86 B 0.86
A 0.86 B 1
A 1 B 0
A 1 B 0.14
A 1 B 0.29
A 1 B 0.43
A 1 B 0.57
A 1 B 0.71
A 1 B 0.86
A 1 B 1

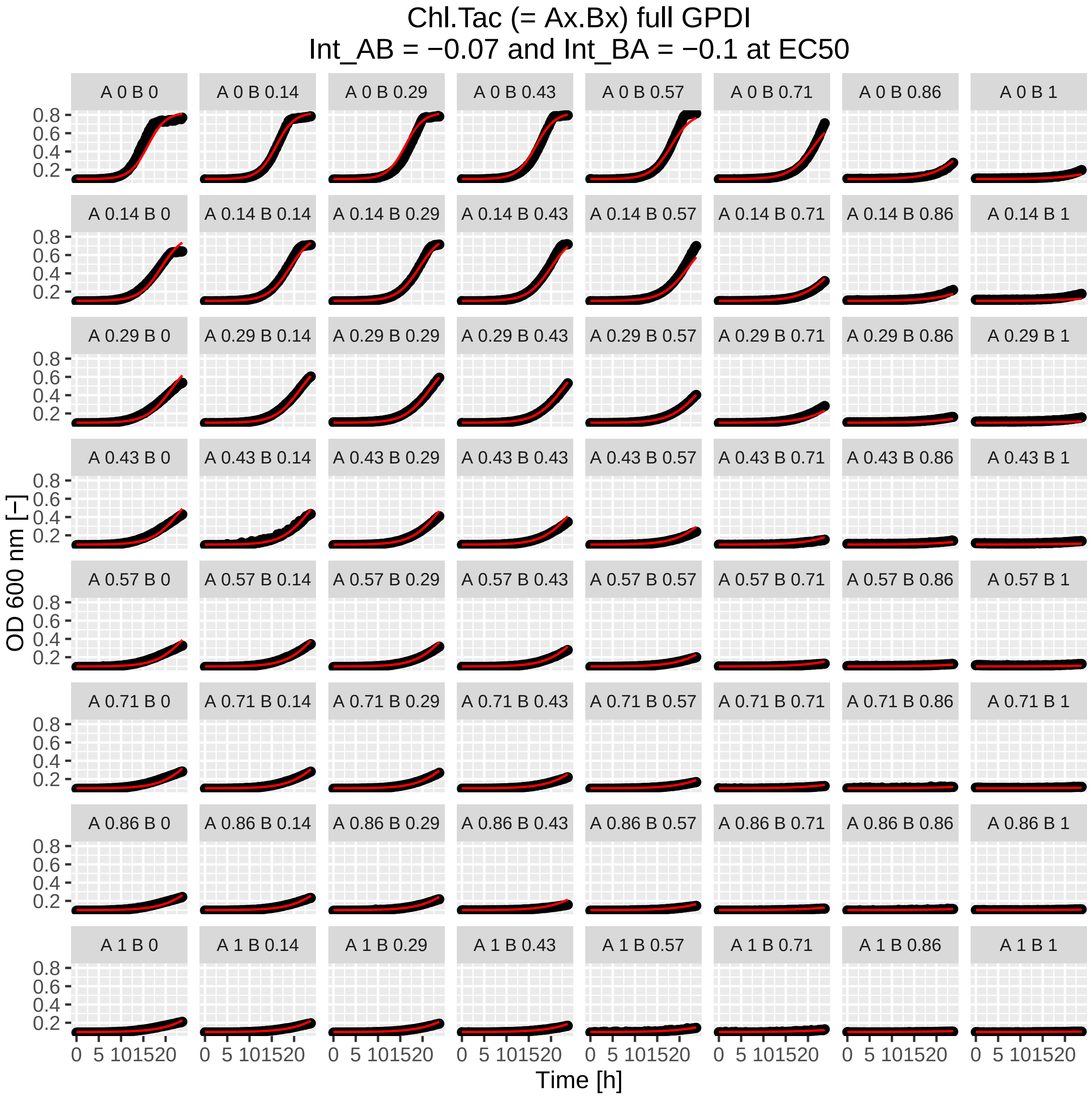
Chl.Tac (= Ax.Bx) full GPDI
Int_AB = −0.07 and Int_BA = −0.1 at EC50
A 0 B 0
A 0 B 0.14
A 0 B 0.29
A 0 B 0.43
A 0 B 0.57
A 0 B 0.71
A 0 B 0.86
A 0 B 1
A 0.14 B 0
A 0.14 B 0.14
A 0.14 B 0.29
A 0.14 B 0.43
A 0.14 B 0.57
A 0.14 B 0.71
A 0.14 B 0.86
A 0.14 B 1
A 0.29 B 0
A 0.29 B 0.14
A 0.29 B 0.29
A 0.29 B 0.43
A 0.29 B 0.57
A 0.29 B 0.71
A 0.29 B 0.86
A 0.29 B 1
A 0.43 B 0
A 0.43 B 0.14
A 0.43 B 0.29
A 0.43 B 0.43
A 0.43 B 0.57
A 0.43 B 0.71
A 0.43 B 0.86
A 0.43 B 1
A 0.57 B 0
A 0.57 B 0.14
A 0.57 B 0.29
A 0.57 B 0.43
A 0.57 B 0.57
A 0.57 B 0.71
A 0.57 B 0.86
A 0.57 B 1
A 0.71 B 0
A 0.71 B 0.14
A 0.71 B 0.29
A 0.71 B 0.43
A 0.71 B 0.57
A 0.71 B 0.71
A 0.71 B 0.86
A 0.71 B 1
A 0.86 B 0
A 0.86 B 0.14
A 0.86 B 0.29
A 0.86 B 0.43
A 0.86 B 0.57
A 0.86 B 0.71
A 0.86 B 0.86
A 0.86 B 1
A 1 B 0
A 1 B 0.14
A 1 B 0.29
A 1 B 0.43
A 1 B 0.57
A 1 B 0.71
A 1 B 0.86
A 1 B 1
OD 600 nm [−]
0.2
0.4
0.6
0.8
0 5 101520
Time [h]

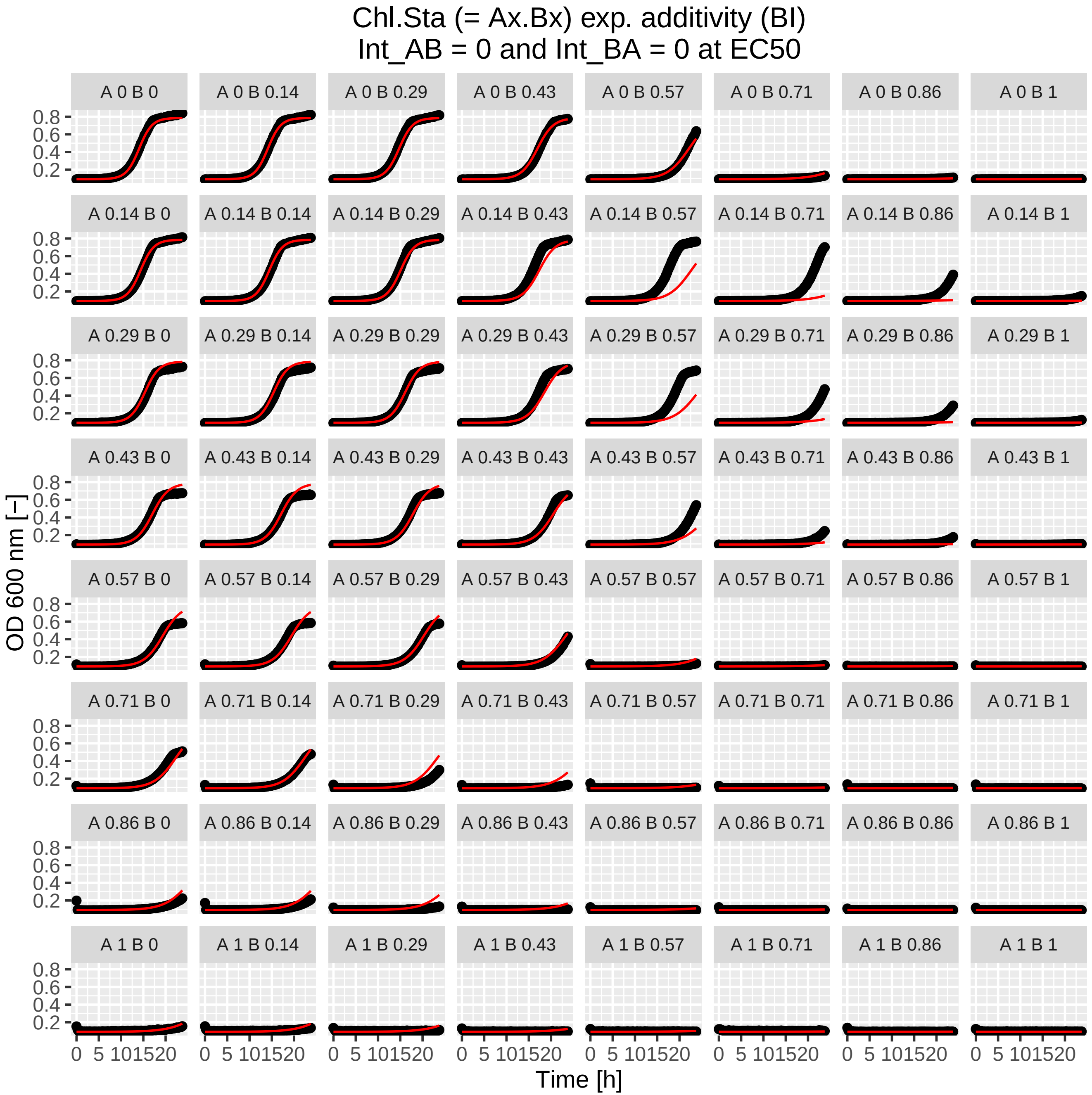
Chl.Sta (= Ax.Bx) exp. additivity (BI)
Int_AB = 0 and Int_BA = 0 at EC50
A 0 B 0
A 0 B 0.14
A 0 B 0.29
A 0 B 0.43
A 0 B 0.57
A 0 B 0.71
A 0 B 0.86
A 0 B 1
A 0.14 B 0
A 0.14 B 0.14
A 0.14 B 0.29
A 0.14 B 0.43
A 0.14 B 0.57
A 0.14 B 0.71
A 0.14 B 0.86
A 0.14 B 1
A 0.29 B 0
A 0.29 B 0.14
A 0.29 B 0.29
A 0.29 B 0.43
A 0.29 B 0.57
A 0.29 B 0.71
A 0.29 B 0.86
A 0.29 B 1
A 0.43 B 0
A 0.43 B 0.14
A 0.43 B 0.29
A 0.43 B 0.43
A 0.43 B 0.57
A 0.43 B 0.71
A 0.43 B 0.86
A 0.43 B 1
A 0.57 B 0
A 0.57 B 0.14
A 0.57 B 0.29
A 0.57 B 0.43
A 0.57 B 0.57
A 0.57 B 0.71
A 0.57 B 0.86
A 0.57 B 1
A 0.71 B 0
A 0.71 B 0.14
A 0.71 B 0.29
A 0.71 B 0.43
A 0.71 B 0.57
A 0.71 B 0.71
A 0.71 B 0.86
A 0.71 B 1
A 0.86 B 0
A 0.86 B 0.14
A 0.86 B 0.29
A 0.86 B 0.43
A 0.86 B 0.57
A 0.86 B 0.71
A 0.86 B 0.86
A 0.86 B 1
A 1 B 0
A 1 B 0.14
A 1 B 0.29
A 1 B 0.43
A 1 B 0.57
A 1 B 0.71
A 1 B 0.86
A 1 B 1
0.8
0.6
0.4
0.2
0 5 101520
OD 600 nm [–]
Time [h]

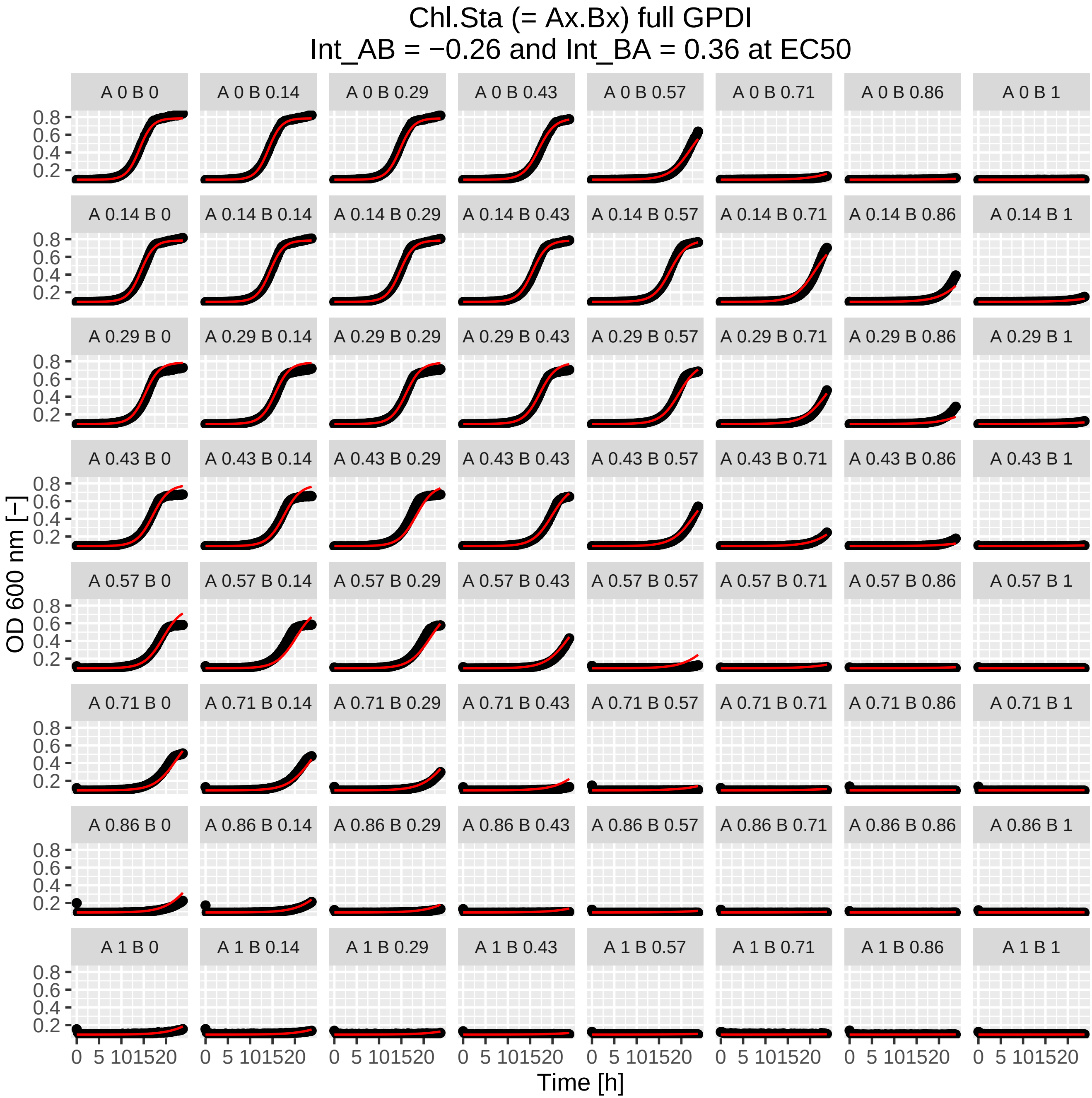
Chl.Sta (= Ax.Bx) full GPDI
Int_AB = −0.26 and Int_BA = 0.36 at EC50
OD 600 nm [−]
Time [h]
A 0 B 0
A 0 B 0.14
A 0 B 0.29
A 0 B 0.43
A 0 B 0.57
A 0 B 0.71
A 0 B 0.86
A 0 B 1
A 0.14 B 0
A 0.14 B 0.14
A 0.14 B 0.29
A 0.14 B 0.43
A 0.14 B 0.57
A 0.14 B 0.71
A 0.14 B 0.86
A 0.14 B 1
A 0.29 B 0
A 0.29 B 0.14
A 0.29 B 0.29
A 0.29 B 0.43
A 0.29 B 0.57
A 0.29 B 0.71
A 0.29 B 0.86
A 0.29 B 1
A 0.43 B 0
A 0.43 B 0.14
A 0.43 B 0.29
A 0.43 B 0.43
A 0.43 B 0.57
A 0.43 B 0.71
A 0.43 B 0.86
A 0.43 B 1
A 0.57 B 0
A 0.57 B 0.14
A 0.57 B 0.29
A 0.57 B 0.43
A 0.57 B 0.57
A 0.57 B 0.71
A 0.57 B 0.86
A 0.57 B 1
A 0.71 B 0
A 0.71 B 0.14
A 0.71 B 0.29
A 0.71 B 0.43
A 0.71 B 0.57
A 0.71 B 0.71
A 0.71 B 0.86
A 0.71 B 1
A 0.86 B 0
A 0.86 B 0.14
A 0.86 B 0.29
A 0.86 B 0.43
A 0.86 B 0.57
A 0.86 B 0.71
A 0.86 B 0.86
A 0.86 B 1
A 1 B 0
A 1 B 0.14
A 1 B 0.29
A 1 B 0.43
A 1 B 0.57
A 1 B 0.71
A 1 B 0.86
A 1 B 1
0.8
0.6
0.4
0.2
0 5 101520

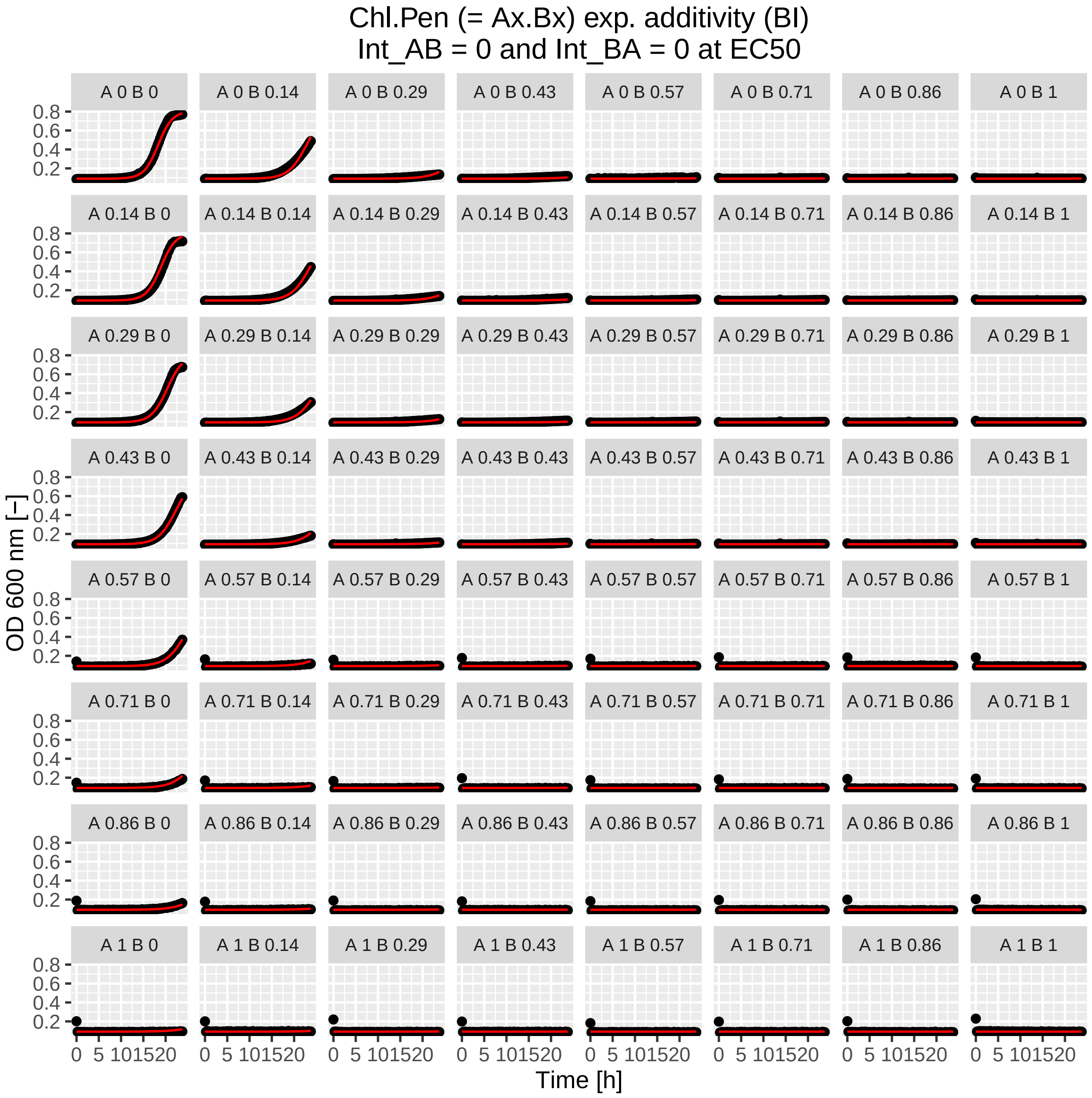
Chl.Pen (= Ax.Bx) exp. additivity (BI)
Int_AB = 0 and Int_BA = 0 at EC50
OD 600 nm [–]
Time [h]
A 0 B 0
A 0 B 0.14
A 0 B 0.29
A 0 B 0.43
A 0 B 0.57
A 0 B 0.71
A 0 B 0.86
A 0 B 1
A 0.14 B 0
A 0.14 B 0.14
A 0.14 B 0.29
A 0.14 B 0.43
A 0.14 B 0.57
A 0.14 B 0.71
A 0.14 B 0.86
A 0.14 B 1
A 0.29 B 0
A 0.29 B 0.14
A 0.29 B 0.29
A 0.29 B 0.43
A 0.29 B 0.57
A 0.29 B 0.71
A 0.29 B 0.86
A 0.29 B 1
A 0.43 B 0
A 0.43 B 0.14
A 0.43 B 0.29
A 0.43 B 0.43
A 0.43 B 0.57
A 0.43 B 0.71
A 0.43 B 0.86
A 0.43 B 1
A 0.57 B 0
A 0.57 B 0.14
A 0.57 B 0.29
A 0.57 B 0.43
A 0.57 B 0.57
A 0.57 B 0.71
A 0.57 B 0.86
A 0.57 B 1
A 0.71 B 0
A 0.71 B 0.14
A 0.71 B 0.29
A 0.71 B 0.43
A 0.71 B 0.57
A 0.71 B 0.71
A 0.71 B 0.86
A 0.71 B 1
A 0.86 B 0
A 0.86 B 0.14
A 0.86 B 0.29
A 0.86 B 0.43
A 0.86 B 0.57
A 0.86 B 0.71
A 0.86 B 0.86
A 0.86 B 1
A 1 B 0
A 1 B 0.14
A 1 B 0.29
A 1 B 0.43
A 1 B 0.57
A 1 B 0.71
A 1 B 0.86
A 1 B 1
0.8
0.6
0.4
0.2
0 5 101520

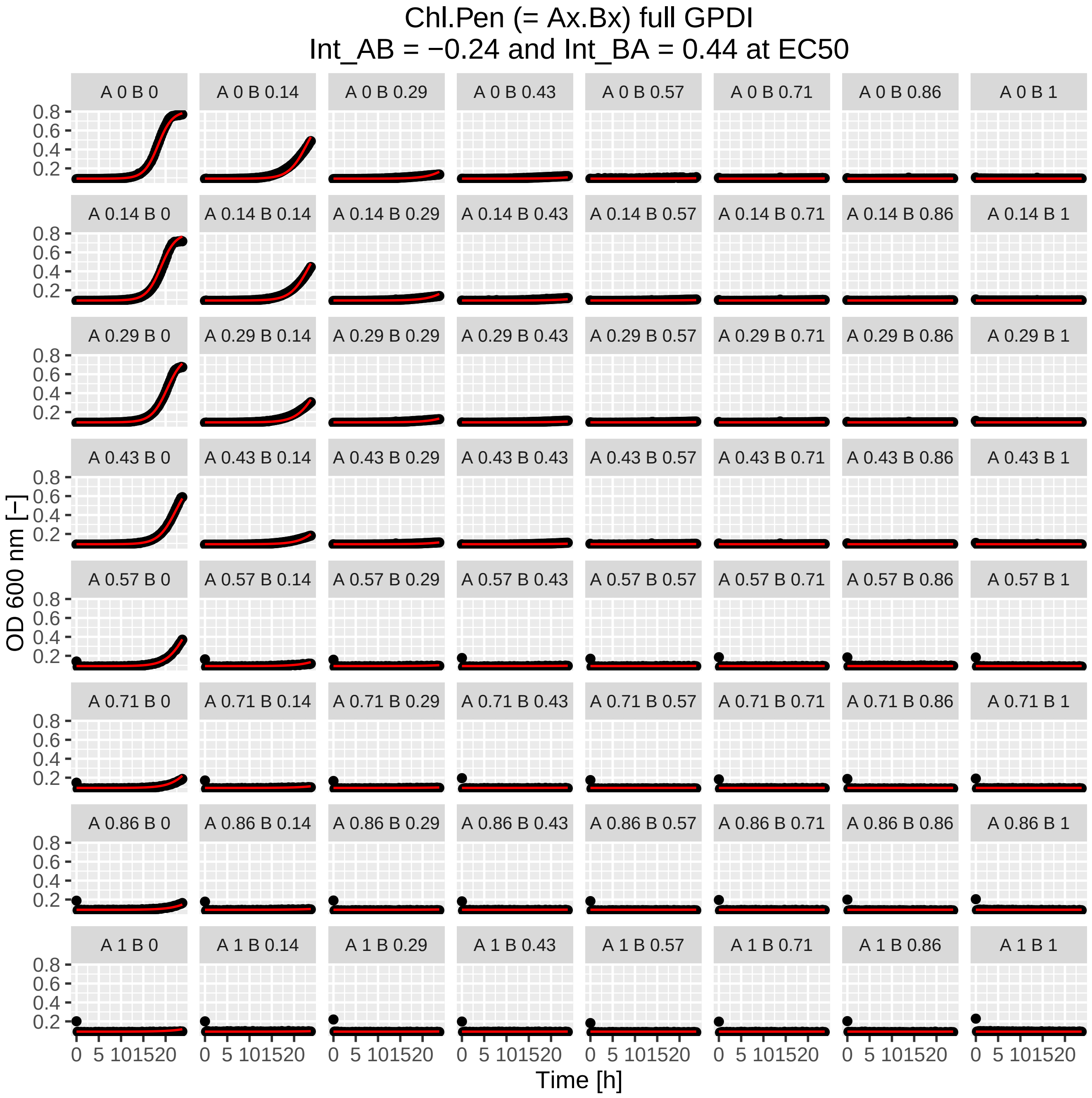

Chl.Pen (= Ax.Bx) full GPDI
Int_AB = −0.24 and Int_BA = 0.44 at EC50
A 0 B 0
A 0 B 0.14
A 0 B 0.29
A 0 B 0.43
A 0 B 0.57
A 0 B 0.71
A 0 B 0.86
A 0 B 1
A 0.14 B 0
A 0.14 B 0.14
A 0.14 B 0.29
A 0.14 B 0.43
A 0.14 B 0.57
A 0.14 B 0.71
A 0.14 B 0.86
A 0.14 B 1
A 0.29 B 0
A 0.29 B 0.14
A 0.29 B 0.29
A 0.29 B 0.43
A 0.29 B 0.57
A 0.29 B 0.71
A 0.29 B 0.86
A 0.29 B 1
A 0.43 B 0
A 0.43 B 0.14
A 0.43 B 0.29
A 0.43 B 0.43
A 0.43 B 0.57
A 0.43 B 0.71
A 0.43 B 0.86
A 0.43 B 1
A 0.57 B 0
A 0.57 B 0.14
A 0.57 B 0.29
A 0.57 B 0.43
A 0.57 B 0.57
A 0.57 B 0.71
A 0.57 B 0.86
A 0.57 B 1
A 0.71 B 0
A 0.71 B 0.14
A 0.71 B 0.29
A 0.71 B 0.43
A 0.71 B 0.57
A 0.71 B 0.71
A 0.71 B 0.86
A 0.71 B 1
A 0.86 B 0
A 0.86 B 0.14
A 0.86 B 0.29
A 0.86 B 0.43
A 0.86 B 0.57
A 0.86 B 0.71
A 0.86 B 0.86
A 0.86 B 1
A 1 B 0
A 1 B 0.14
A 1 B 0.29
A 1 B 0.43
A 1 B 0.57
A 1 B 0.71
A 1 B 0.86
A 1 B 1
0.8
0.6
0.4
0.2
OD 600 nm [−]
0 5 101520
Time [h]

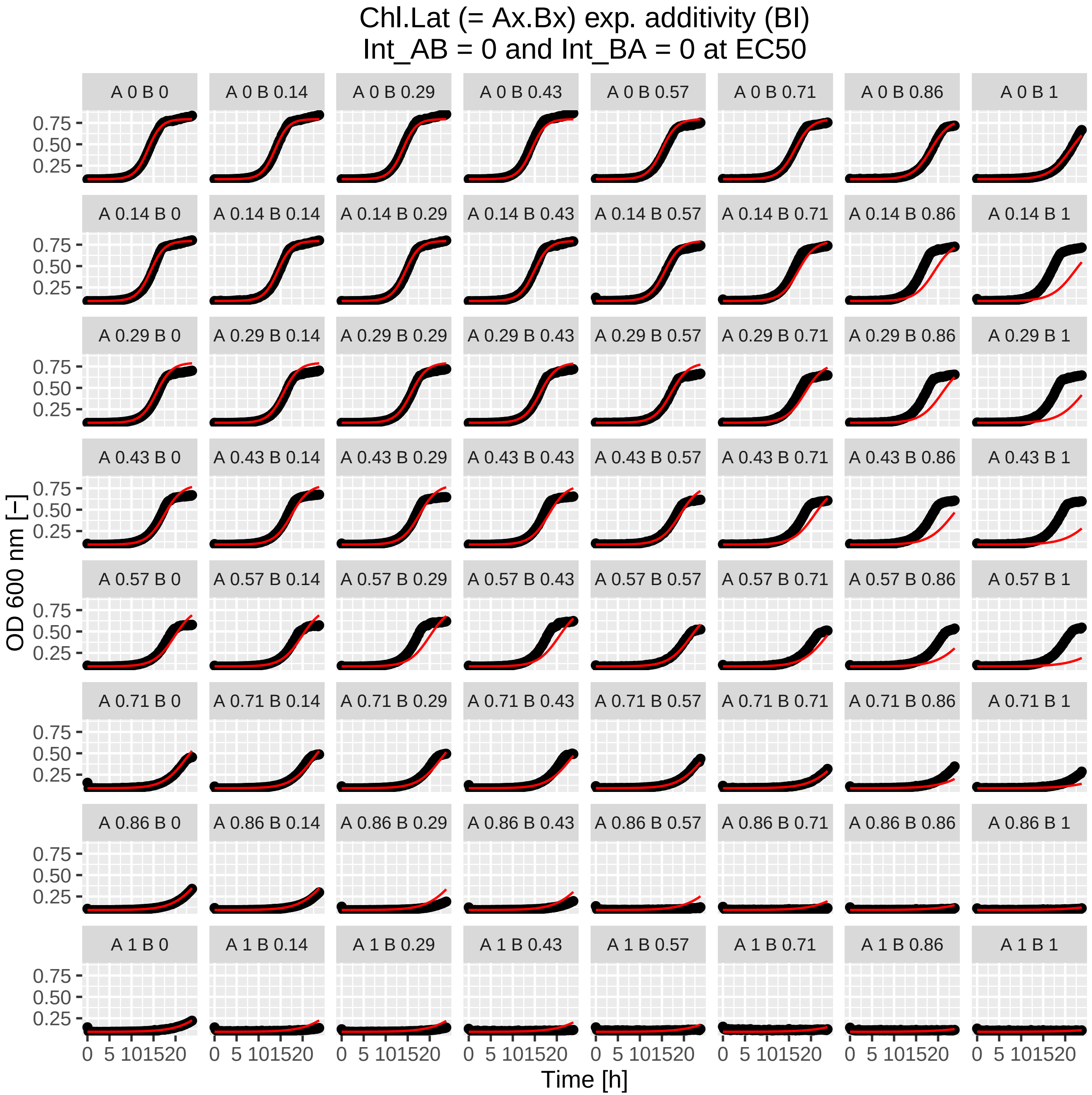

Chl.Lat (= Ax.Bx) exp. additivity (BI)
Int_AB = 0 and Int_BA = 0 at EC50
A 0 B 0
A 0 B 0.14
A 0 B 0.29
A 0 B 0.43
A 0 B 0.57
A 0 B 0.71
A 0 B 0.86
A 0 B 1
A 0.14 B 0
A 0.14 B 0.14
A 0.14 B 0.29
A 0.14 B 0.43
A 0.14 B 0.57
A 0.14 B 0.71
A 0.14 B 0.86
A 0.14 B 1
A 0.29 B 0
A 0.29 B 0.14
A 0.29 B 0.29
A 0.29 B 0.43
A 0.29 B 0.57
A 0.29 B 0.71
A 0.29 B 0.86
A 0.29 B 1
A 0.43 B 0
A 0.43 B 0.14
A 0.43 B 0.29
A 0.43 B 0.43
A 0.43 B 0.57
A 0.43 B 0.71
A 0.43 B 0.86
A 0.43 B 1
A 0.57 B 0
A 0.57 B 0.14
A 0.57 B 0.29
A 0.57 B 0.43
A 0.57 B 0.57
A 0.57 B 0.71
A 0.57 B 0.86
A 0.57 B 1
A 0.71 B 0
A 0.71 B 0.14
A 0.71 B 0.29
A 0.71 B 0.43
A 0.71 B 0.57
A 0.71 B 0.71
A 0.71 B 0.86
A 0.71 B 1
A 0.86 B 0
A 0.86 B 0.14
A 0.86 B 0.29
A 0.86 B 0.43
A 0.86 B 0.57
A 0.86 B 0.71
A 0.86 B 0.86
A 0.86 B 1
A 1 B 0
A 1 B 0.14
A 1 B 0.29
A 1 B 0.43
A 1 B 0.57
A 1 B 0.71
A 1 B 0.86
A 1 B 1
OD 600 nm [–]
0.75
0.50
0.25
0 5 101520
Time [h]

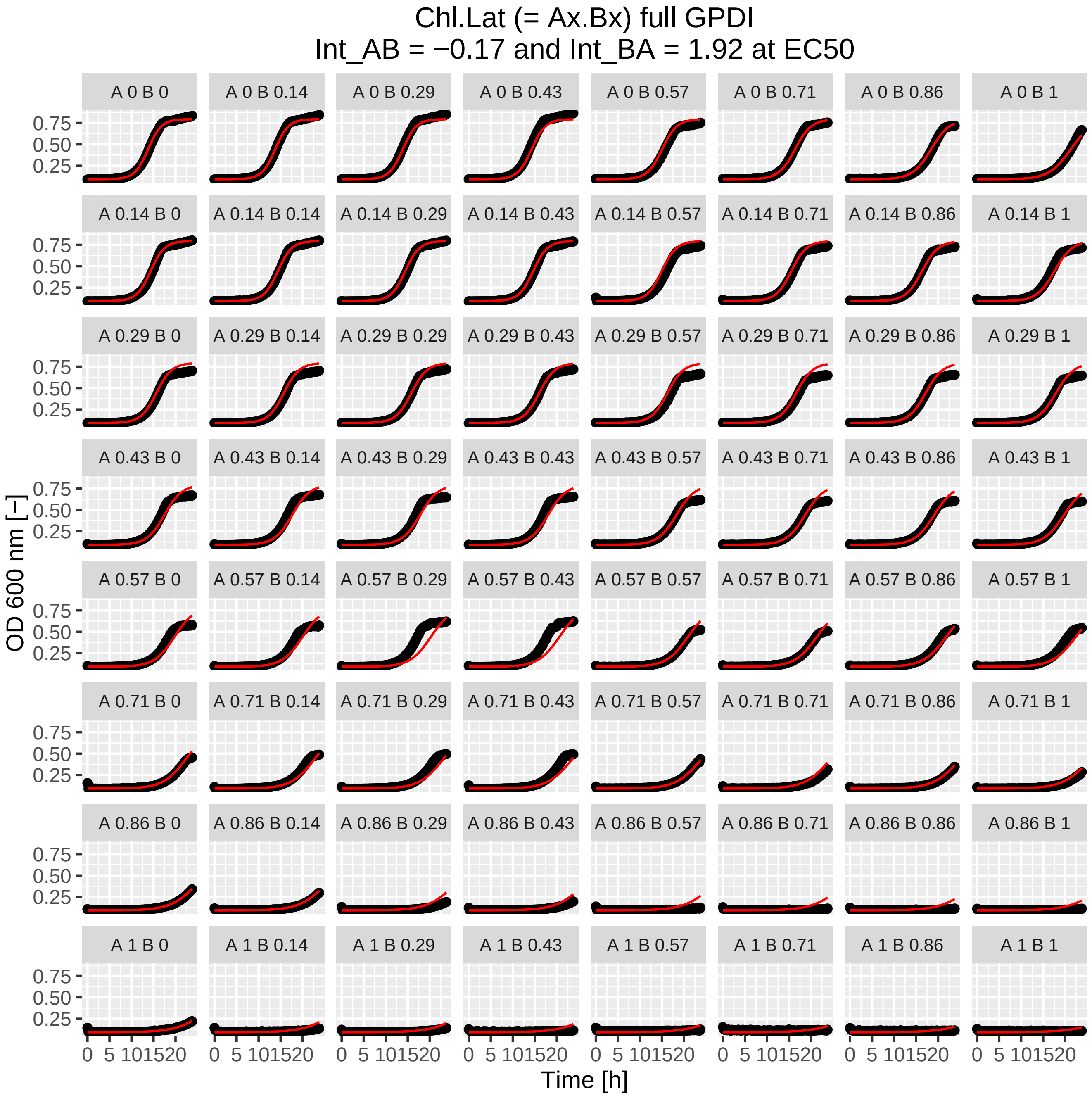
Chl.Lat (= Ax.Bx) full GPDI
Int_AB = −0.17 and Int_BA = 1.92 at EC50
OD 600 nm [−]
Time [h]
0.75
0.50
0.25
0 5 101520
A 0 B 0
A 0 B 0.14
A 0 B 0.29
A 0 B 0.43
A 0 B 0.57
A 0 B 0.71
A 0 B 0.86
A 0 B 1
A 0.14 B 0
A 0.14 B 0.14
A 0.14 B 0.29
A 0.14 B 0.43
A 0.14 B 0.57
A 0.14 B 0.71
A 0.14 B 0.86
A 0.14 B 1
A 0.29 B 0
A 0.29 B 0.14
A 0.29 B 0.29
A 0.29 B 0.43
A 0.29 B 0.57
A 0.29 B 0.71
A 0.29 B 0.86
A 0.29 B 1
A 0.43 B 0
A 0.43 B 0.14
A 0.43 B 0.29
A 0.43 B 0.43
A 0.43 B 0.57
A 0.43 B 0.71
A 0.43 B 0.86
A 0.43 B 1
A 0.57 B 0
A 0.57 B 0.14
A 0.57 B 0.29
A 0.57 B 0.43
A 0.57 B 0.57
A 0.57 B 0.71
A 0.57 B 0.86
A 0.57 B 1
A 0.71 B 0
A 0.71 B 0.14
A 0.71 B 0.29
A 0.71 B 0.43
A 0.71 B 0.57
A 0.71 B 0.71
A 0.71 B 0.86
A 0.71 B 1
A 0.86 B 0
A 0.86 B 0.14
A 0.86 B 0.29
A 0.86 B 0.43
A 0.86 B 0.57
A 0.86 B 0.71
A 0.86 B 0.86
A 0.86 B 1
A 1 B 0
A 1 B 0.14
A 1 B 0.29
A 1 B 0.43
A 1 B 0.57
A 1 B 0.71
A 1 B 0.86
A 1 B 1

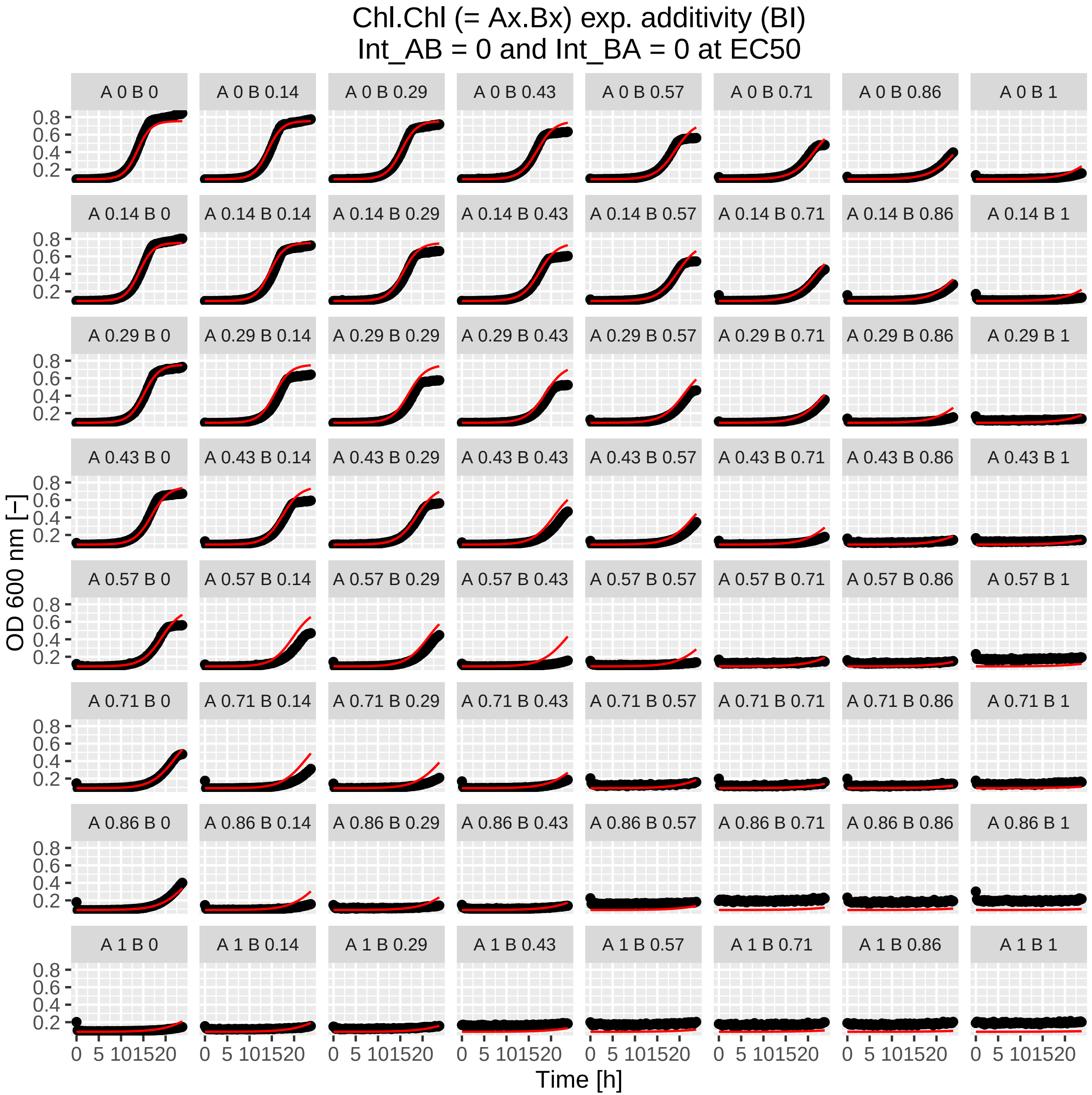

Chl.Chl (= Ax.Bx) exp. additivity (BI)
Int_AB = 0 and Int_BA = 0 at EC50
A 0 B 0
A 0 B 0.14
A 0 B 0.29
A 0 B 0.43
A 0 B 0.57
A 0 B 0.71
A 0 B 0.86
A 0 B 1
A 0.14 B 0
A 0.14 B 0.14
A 0.14 B 0.29
A 0.14 B 0.43
A 0.14 B 0.57
A 0.14 B 0.71
A 0.14 B 0.86
A 0.14 B 1
A 0.29 B 0
A 0.29 B 0.14
A 0.29 B 0.29
A 0.29 B 0.43
A 0.29 B 0.57
A 0.29 B 0.71
A 0.29 B 0.86
A 0.29 B 1
A 0.43 B 0
A 0.43 B 0.14
A 0.43 B 0.29
A 0.43 B 0.43
A 0.43 B 0.57
A 0.43 B 0.71
A 0.43 B 0.86
A 0.43 B 1
A 0.57 B 0
A 0.57 B 0.14
A 0.57 B 0.29
A 0.57 B 0.43
A 0.57 B 0.57
A 0.57 B 0.71
A 0.57 B 0.86
A 0.57 B 1
A 0.71 B 0
A 0.71 B 0.14
A 0.71 B 0.29
A 0.71 B 0.43
A 0.71 B 0.57
A 0.71 B 0.71
A 0.71 B 0.86
A 0.71 B 1
A 0.86 B 0
A 0.86 B 0.14
A 0.86 B 0.29
A 0.86 B 0.43
A 0.86 B 0.57
A 0.86 B 0.71
A 0.86 B 0.86
A 0.86 B 1
A 1 B 0
A 1 B 0.14
A 1 B 0.29
A 1 B 0.43
A 1 B 0.57
A 1 B 0.71
A 1 B 0.86
A 1 B 1
OD 600 nm [–]
0.2
0.4
0.6
0.8
0 5 101520
Time [h]

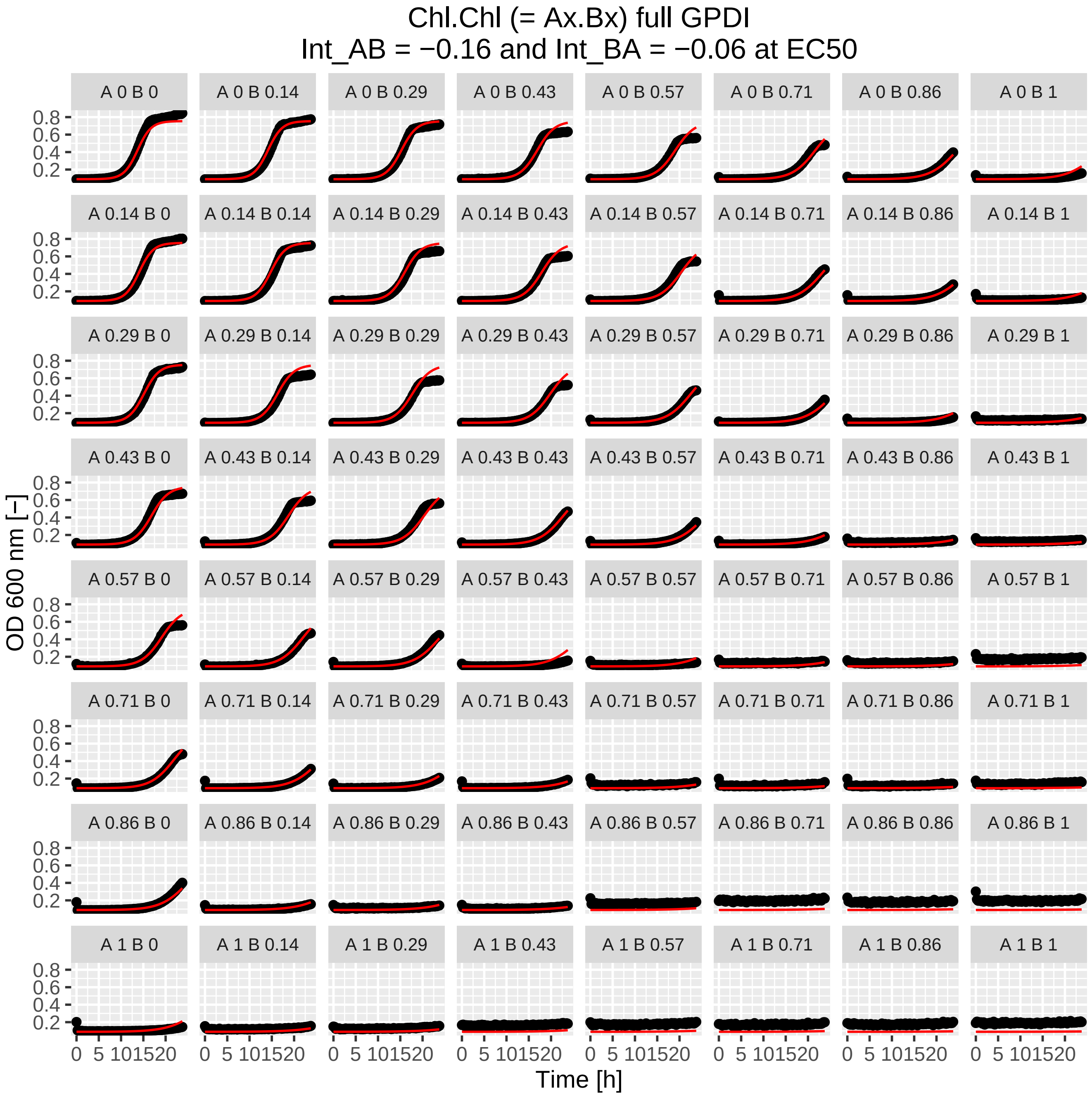
Chl.Chl (= Ax.Bx) full GPDI
Int_AB = −0.16 and Int_BA = −0.06 at EC50
A 0 B 0
A 0 B 0.14
A 0 B 0.29
A 0 B 0.43
A 0 B 0.57
A 0 B 0.71
A 0 B 0.86
A 0 B 1
A 0.14 B 0
A 0.14 B 0.14
A 0.14 B 0.29
A 0.14 B 0.43
A 0.14 B 0.57
A 0.14 B 0.71
A 0.14 B 0.86
A 0.14 B 1
A 0.29 B 0
A 0.29 B 0.14
A 0.29 B 0.29
A 0.29 B 0.43
A 0.29 B 0.57
A 0.29 B 0.71
A 0.29 B 0.86
A 0.29 B 1
A 0.43 B 0
A 0.43 B 0.14
A 0.43 B 0.29
A 0.43 B 0.43
A 0.43 B 0.57
A 0.43 B 0.71
A 0.43 B 0.86
A 0.43 B 1
A 0.57 B 0
A 0.57 B 0.14
A 0.57 B 0.29
A 0.57 B 0.43
A 0.57 B 0.57
A 0.57 B 0.71
A 0.57 B 0.86
A 0.57 B 1
A 0.71 B 0
A 0.71 B 0.14
A 0.71 B 0.29
A 0.71 B 0.43
A 0.71 B 0.57
A 0.71 B 0.71
A 0.71 B 0.86
A 0.71 B 1
A 0.86 B 0
A 0.86 B 0.14
A 0.86 B 0.29
A 0.86 B 0.43
A 0.86 B 0.57
A 0.86 B 0.71
A 0.86 B 0.86
A 0.86 B 1
A 1 B 0
A 1 B 0.14
A 1 B 0.29
A 1 B 0.43
A 1 B 0.57
A 1 B 0.71
A 1 B 0.86
A 1 B 1
OD 600 nm [−]
0.8
0.6
0.4
0.2
0 5 101520
Time [h]

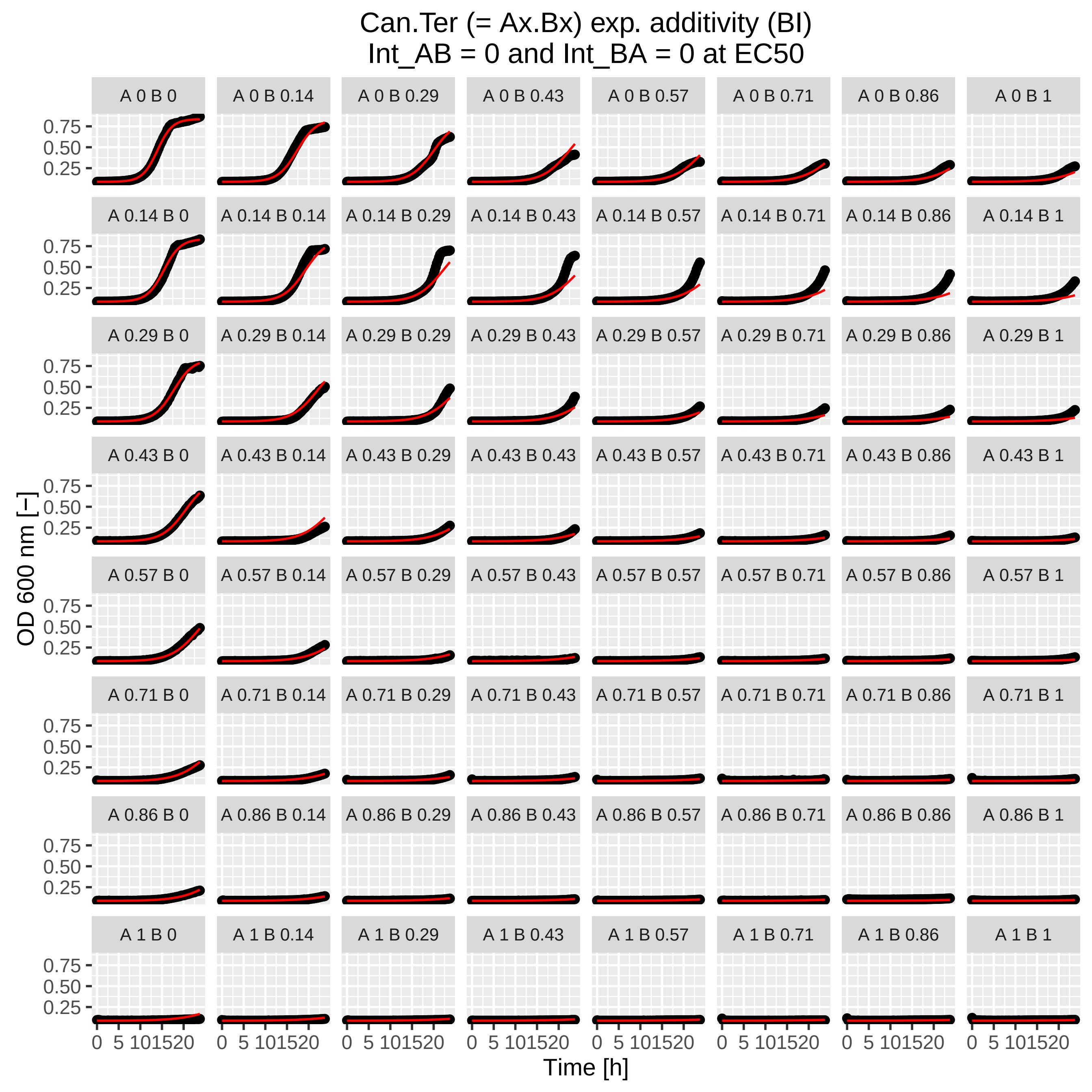

Can.Ter (= Ax.Bx) exp. additivity (BI)
Int_AB = 0 and Int_BA = 0 at EC50
A 0 B 0
A 0 B 0.14
A 0 B 0.29
A 0 B 0.43
A 0 B 0.57
A 0 B 0.71
A 0 B 0.86
A 0 B 1
A 0.14 B 0
A 0.14 B 0.14
A 0.14 B 0.29
A 0.14 B 0.43
A 0.14 B 0.57
A 0.14 B 0.71
A 0.14 B 0.86
A 0.14 B 1
A 0.29 B 0
A 0.29 B 0.14
A 0.29 B 0.29
A 0.29 B 0.43
A 0.29 B 0.57
A 0.29 B 0.71
A 0.29 B 0.86
A 0.29 B 1
A 0.43 B 0
A 0.43 B 0.14
A 0.43 B 0.29
A 0.43 B 0.43
A 0.43 B 0.57
A 0.43 B 0.71
A 0.43 B 0.86
A 0.43 B 1
A 0.57 B 0
A 0.57 B 0.14
A 0.57 B 0.29
A 0.57 B 0.43
A 0.57 B 0.57
A 0.57 B 0.71
A 0.57 B 0.86
A 0.57 B 1
A 0.71 B 0
A 0.71 B 0.14
A 0.71 B 0.29
A 0.71 B 0.43
A 0.71 B 0.57
A 0.71 B 0.71
A 0.71 B 0.86
A 0.71 B 1
A 0.86 B 0
A 0.86 B 0.14
A 0.86 B 0.29
A 0.86 B 0.43
A 0.86 B 0.57
A 0.86 B 0.71
A 0.86 B 0.86
A 0.86 B 1
A 1 B 0
A 1 B 0.14
A 1 B 0.29
A 1 B 0.43
A 1 B 0.57
A 1 B 0.71
A 1 B 0.86
A 1 B 1
0.75
0.50
0.25
OD 600 nm [–]
0 5 101520
Time [h]

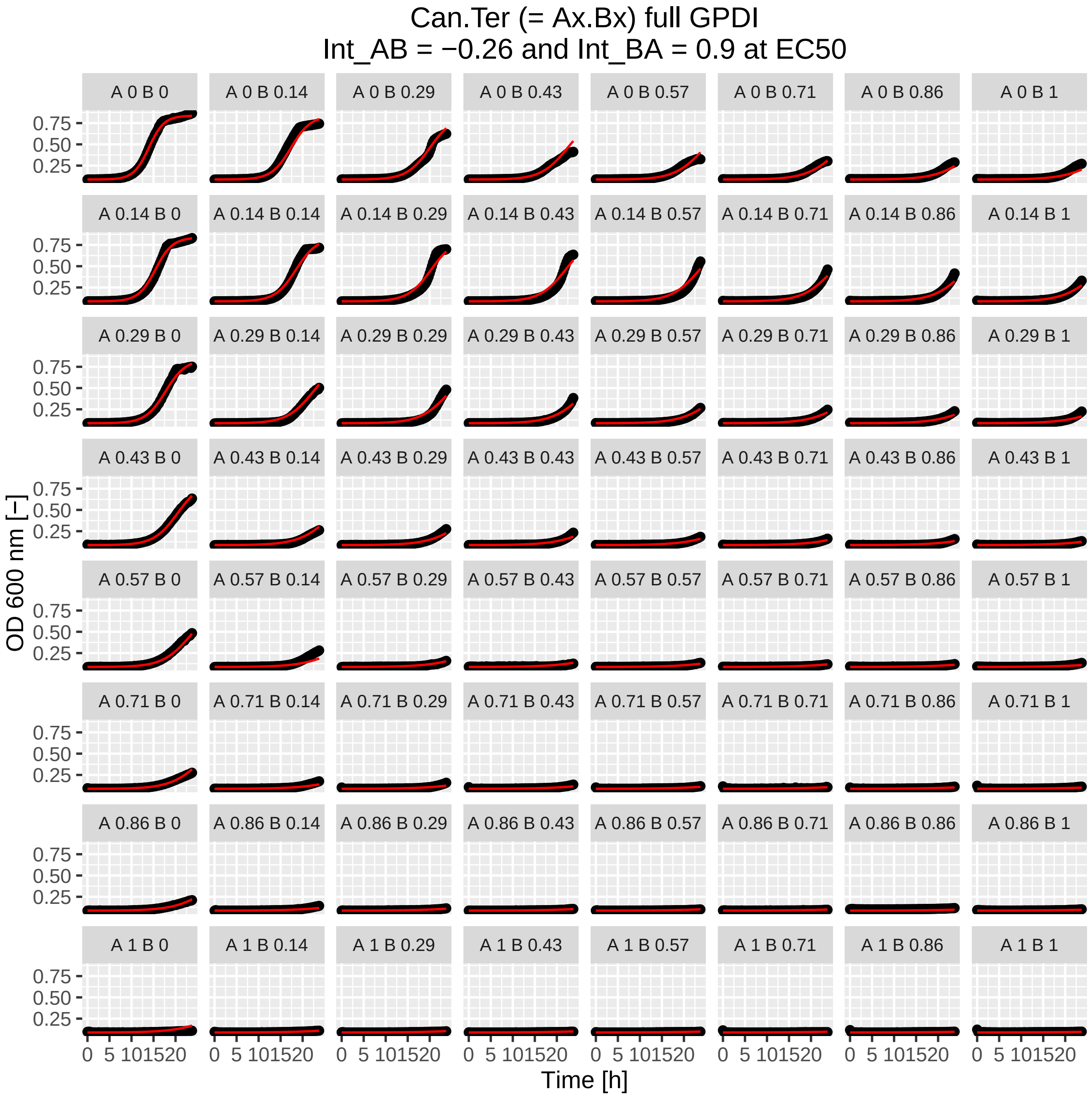
Can.Ter (= Ax.Bx) full GPDI
Int_AB = −0.26 and Int_BA = 0.9 at EC50
A 0 B 0
A 0 B 0.14
A 0 B 0.29
A 0 B 0.43
A 0 B 0.57
A 0 B 0.71
A 0 B 0.86
A 0 B 1
A 0.14 B 0
A 0.14 B 0.14
A 0.14 B 0.29
A 0.14 B 0.43
A 0.14 B 0.57
A 0.14 B 0.71
A 0.14 B 0.86
A 0.14 B 1
A 0.29 B 0
A 0.29 B 0.14
A 0.29 B 0.29
A 0.29 B 0.43
A 0.29 B 0.57
A 0.29 B 0.71
A 0.29 B 0.86
A 0.29 B 1
A 0.43 B 0
A 0.43 B 0.14
A 0.43 B 0.29
A 0.43 B 0.43
A 0.43 B 0.57
A 0.43 B 0.71
A 0.43 B 0.86
A 0.43 B 1
A 0.57 B 0
A 0.57 B 0.14
A 0.57 B 0.29
A 0.57 B 0.43
A 0.57 B 0.57
A 0.57 B 0.71
A 0.57 B 0.86
A 0.57 B 1
A 0.71 B 0
A 0.71 B 0.14
A 0.71 B 0.29
A 0.71 B 0.43
A 0.71 B 0.57
A 0.71 B 0.71
A 0.71 B 0.86
A 0.71 B 1
A 0.86 B 0
A 0.86 B 0.14
A 0.86 B 0.29
A 0.86 B 0.43
A 0.86 B 0.57
A 0.86 B 0.71
A 0.86 B 0.86
A 0.86 B 1
A 1 B 0
A 1 B 0.14
A 1 B 0.29
A 1 B 0.43
A 1 B 0.57
A 1 B 0.71
A 1 B 0.86
A 1 B 1
0.75
0.50
0.25
0 5 101520
OD 600 nm [−]
Time [h]

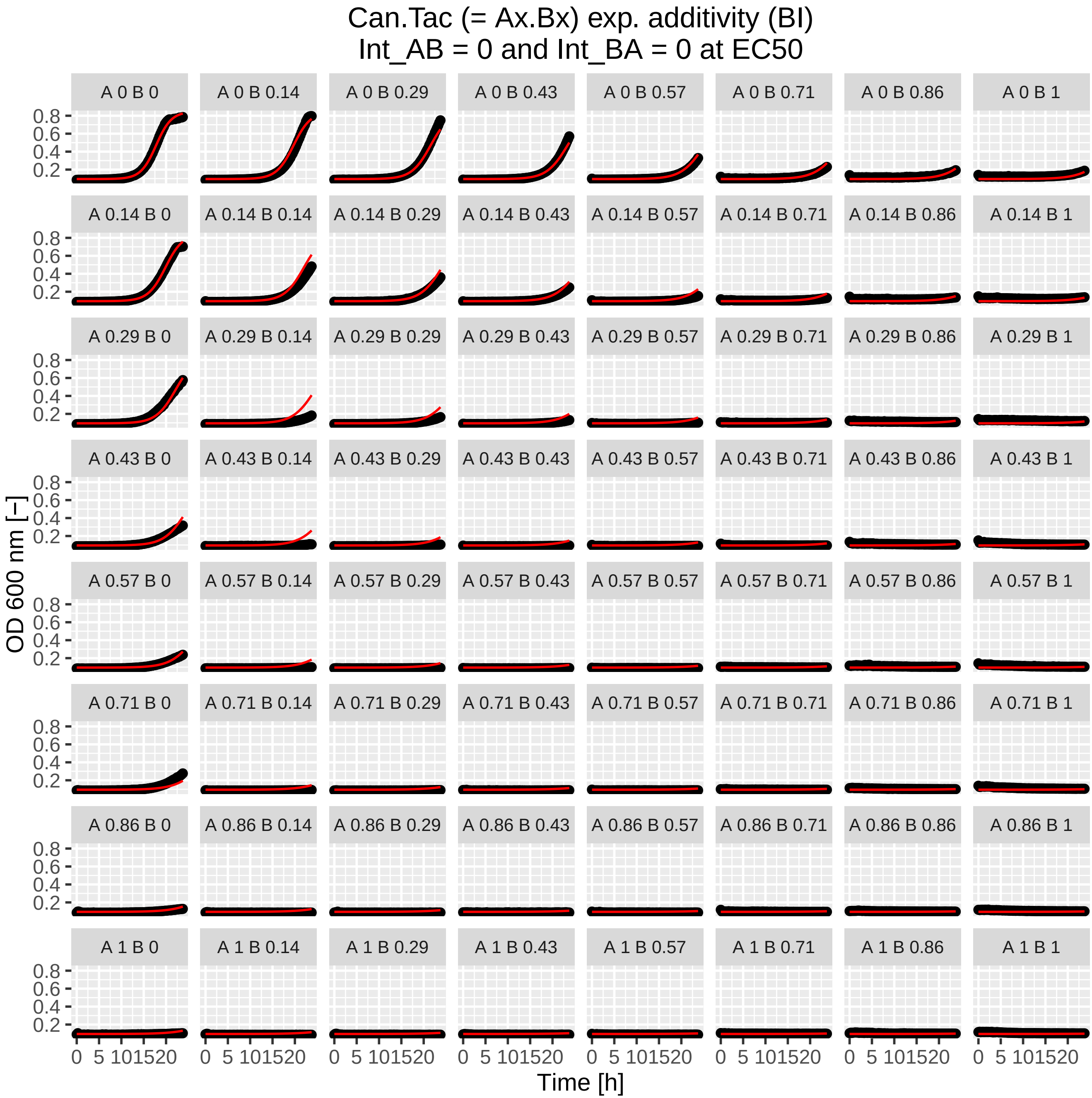

Can.Tac (= Ax.Bx) exp. additivity (BI)
Int_AB = 0 and Int_BA = 0 at EC50
OD 600 nm [–]
Time [h]
0.8
0.6
0.4
0.2
0
5
10
15
20
A 0 B 0
A 0 B 0.14
A 0 B 0.29
A 0 B 0.43
A 0 B 0.57
A 0 B 0.71
A 0 B 0.86
A 0 B 1
A 0.14 B 0
A 0.14 B 0.14
A 0.14 B 0.29
A 0.14 B 0.43
A 0.14 B 0.57
A 0.14 B 0.71
A 0.14 B 0.86
A 0.14 B 1
A 0.29 B 0
A 0.29 B 0.14
A 0.29 B 0.29
A 0.29 B 0.43
A 0.29 B 0.57
A 0.29 B 0.71
A 0.29 B 0.86
A 0.29 B 1
A 0.43 B 0
A 0.43 B 0.14
A 0.43 B 0.29
A 0.43 B 0.43
A 0.43 B 0.57
A 0.43 B 0.71
A 0.43 B 0.86
A 0.43 B 1
A 0.57 B 0
A 0.57 B 0.14
A 0.57 B 0.29
A 0.57 B 0.43
A 0.57 B 0.57
A 0.57 B 0.71
A 0.57 B 0.86
A 0.57 B 1
A 0.71 B 0
A 0.71 B 0.14
A 0.71 B 0.29
A 0.71 B 0.43
A 0.71 B 0.57
A 0.71 B 0.71
A 0.71 B 0.86
A 0.71 B 1
A 0.86 B 0
A 0.86 B 0.14
A 0.86 B 0.29
A 0.86 B 0.43
A 0.86 B 0.57
A 0.86 B 0.71
A 0.86 B 0.86
A 0.86 B 1
A 1 B 0
A 1 B 0.14
A 1 B 0.29
A 1 B 0.43
A 1 B 0.57
A 1 B 0.71
A 1 B 0.86
A 1 B 1

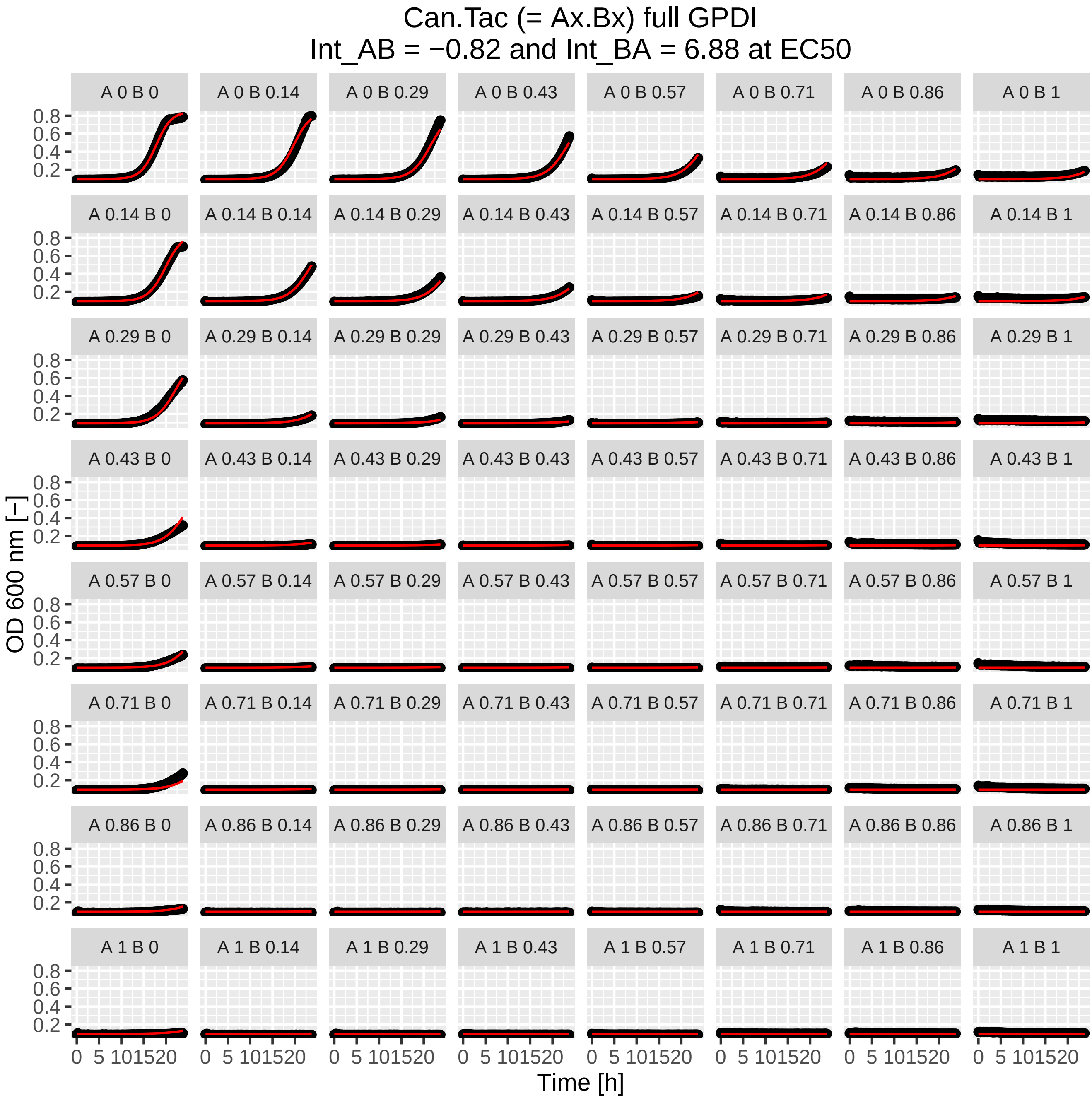

Can.Tac (= Ax.Bx) full GPDI
Int_AB = −0.82 and Int_BA = 6.88 at EC50
A 0 B 0
A 0 B 0.14
A 0 B 0.29
A 0 B 0.43
A 0 B 0.57
A 0 B 0.71
A 0 B 0.86
A 0 B 1
A 0.14 B 0
A 0.14 B 0.14
A 0.14 B 0.29
A 0.14 B 0.43
A 0.14 B 0.57
A 0.14 B 0.71
A 0.14 B 0.86
A 0.14 B 1
A 0.29 B 0
A 0.29 B 0.14
A 0.29 B 0.29
A 0.29 B 0.43
A 0.29 B 0.57
A 0.29 B 0.71
A 0.29 B 0.86
A 0.29 B 1
A 0.43 B 0
A 0.43 B 0.14
A 0.43 B 0.29
A 0.43 B 0.43
A 0.43 B 0.57
A 0.43 B 0.71
A 0.43 B 0.86
A 0.43 B 1
A 0.57 B 0
A 0.57 B 0.14
A 0.57 B 0.29
A 0.57 B 0.43
A 0.57 B 0.57
A 0.57 B 0.71
A 0.57 B 0.86
A 0.57 B 1
A 0.71 B 0
A 0.71 B 0.14
A 0.71 B 0.29
A 0.71 B 0.43
A 0.71 B 0.57
A 0.71 B 0.71
A 0.71 B 0.86
A 0.71 B 1
A 0.86 B 0
A 0.86 B 0.14
A 0.86 B 0.29
A 0.86 B 0.43
A 0.86 B 0.57
A 0.86 B 0.71
A 0.86 B 0.86
A 0.86 B 1
A 1 B 0
A 1 B 0.14
A 1 B 0.29
A 1 B 0.43
A 1 B 0.57
A 1 B 0.71
A 1 B 0.86
A 1 B 1
0.8
0.6
0.4
0.2
0 5 101520
OD 600 nm [−]
Time [h]

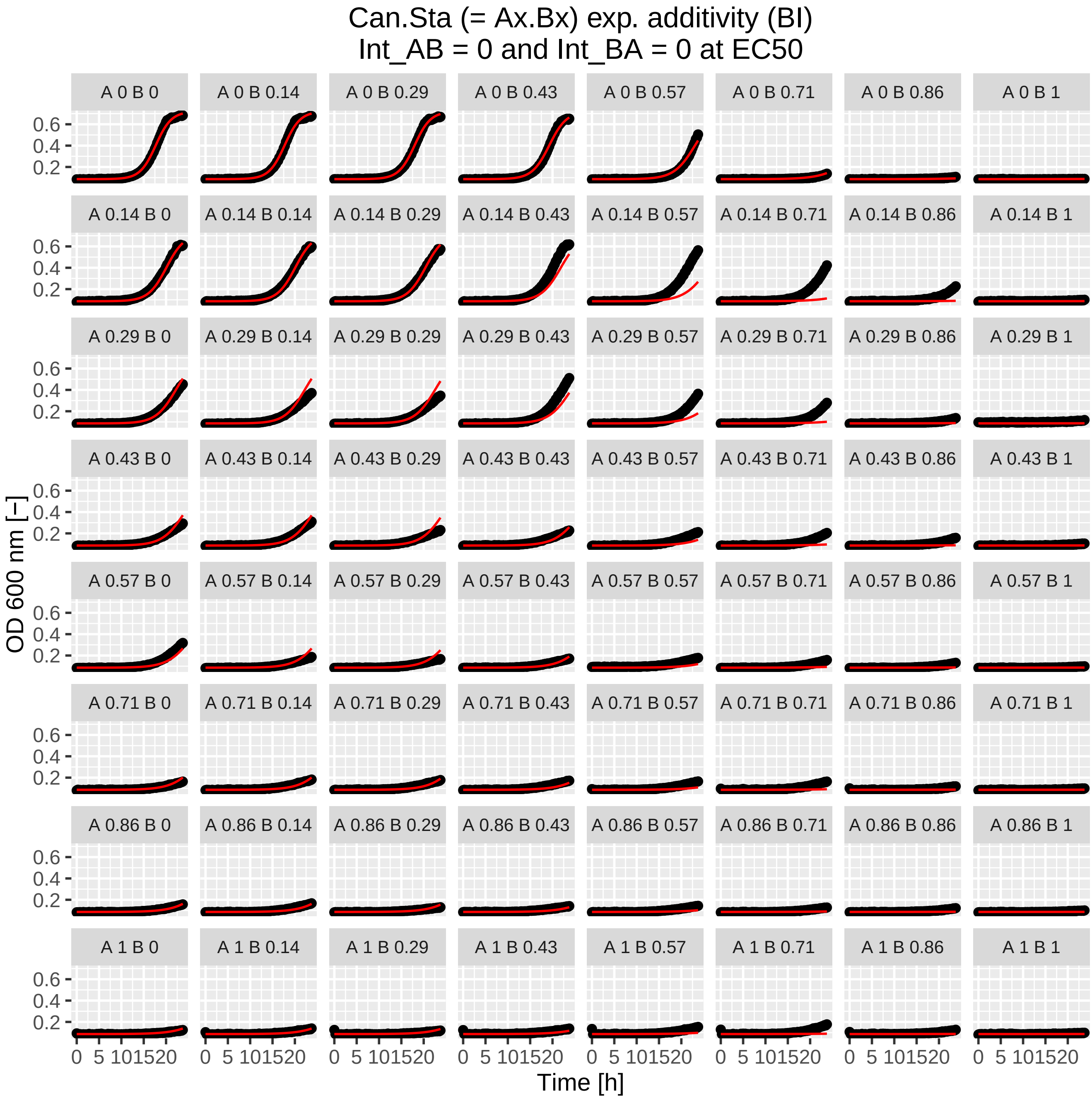

Can.Sta (= Ax.Bx) exp. additivity (BI)
Int_AB = 0 and Int_BA = 0 at EC50
A 0 B 0
A 0 B 0.14
A 0 B 0.29
A 0 B 0.43
A 0 B 0.57
A 0 B 0.71
A 0 B 0.86
A 0 B 1
A 0.14 B 0
A 0.14 B 0.14
A 0.14 B 0.29
A 0.14 B 0.43
A 0.14 B 0.57
A 0.14 B 0.71
A 0.14 B 0.86
A 0.14 B 1
A 0.29 B 0
A 0.29 B 0.14
A 0.29 B 0.29
A 0.29 B 0.43
A 0.29 B 0.57
A 0.29 B 0.71
A 0.29 B 0.86
A 0.29 B 1
A 0.43 B 0
A 0.43 B 0.14
A 0.43 B 0.29
A 0.43 B 0.43
A 0.43 B 0.57
A 0.43 B 0.71
A 0.43 B 0.86
A 0.43 B 1
A 0.57 B 0
A 0.57 B 0.14
A 0.57 B 0.29
A 0.57 B 0.43
A 0.57 B 0.57
A 0.57 B 0.71
A 0.57 B 0.86
A 0.57 B 1
A 0.71 B 0
A 0.71 B 0.14
A 0.71 B 0.29
A 0.71 B 0.43
A 0.71 B 0.57
A 0.71 B 0.71
A 0.71 B 0.86
A 0.71 B 1
A 0.86 B 0
A 0.86 B 0.14
A 0.86 B 0.29
A 0.86 B 0.43
A 0.86 B 0.57
A 0.86 B 0.71
A 0.86 B 0.86
A 0.86 B 1
A 1 B 0
A 1 B 0.14
A 1 B 0.29
A 1 B 0.43
A 1 B 0.57
A 1 B 0.71
A 1 B 0.86
A 1 B 1
OD 600 nm [−]
0.6
0.4
0.2
0 5 101520
Time [h]

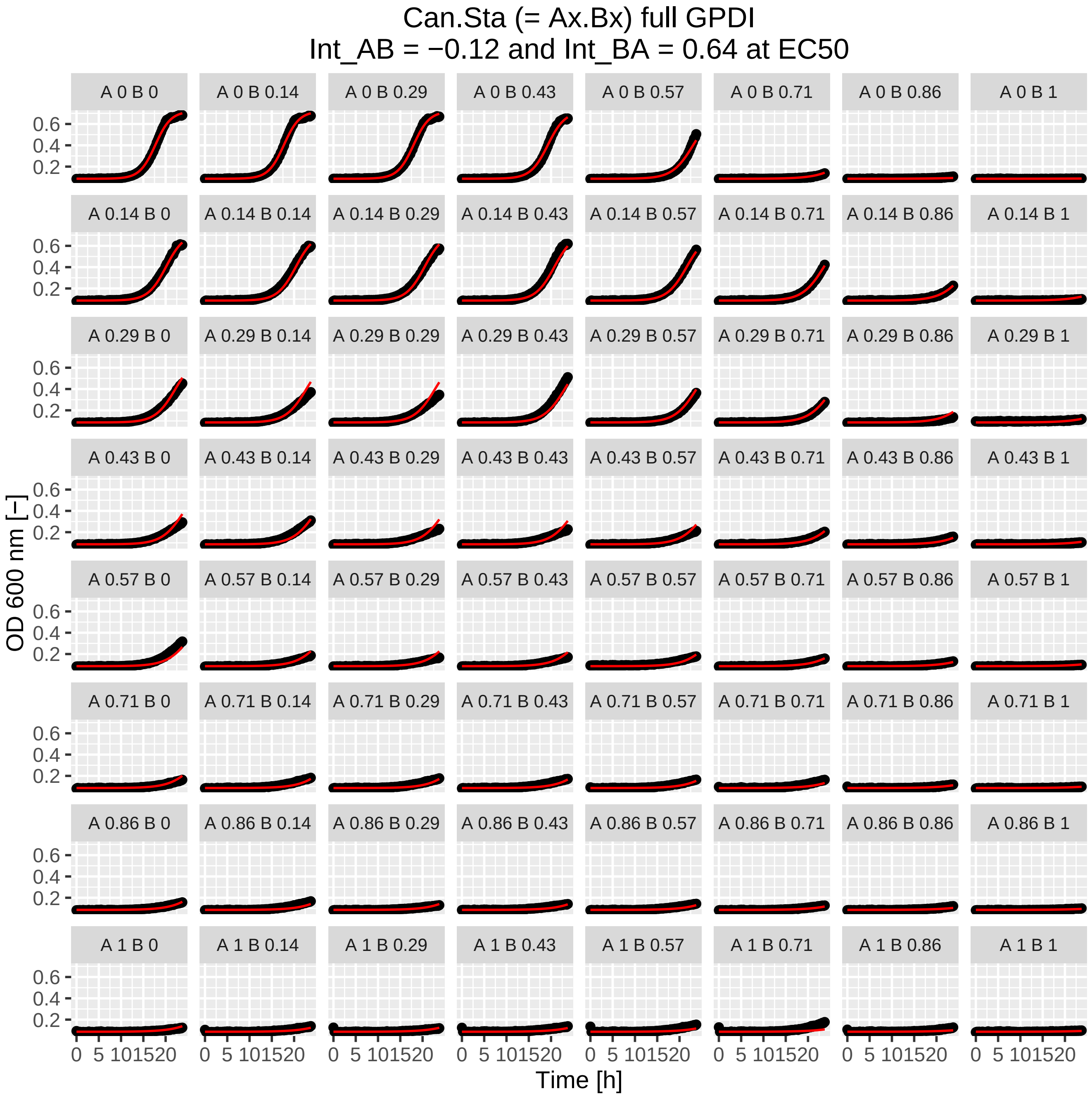

Can.Sta (= Ax.Bx) full GPDI
Int_AB = −0.12 and Int_BA = 0.64 at EC50
A 0 B 0
A 0 B 0.14
A 0 B 0.29
A 0 B 0.43
A 0 B 0.57
A 0 B 0.71
A 0 B 0.86
A 0 B 1
A 0.14 B 0
A 0.14 B 0.14
A 0.14 B 0.29
A 0.14 B 0.43
A 0.14 B 0.57
A 0.14 B 0.71
A 0.14 B 0.86
A 0.14 B 1
A 0.29 B 0
A 0.29 B 0.14
A 0.29 B 0.29
A 0.29 B 0.43
A 0.29 B 0.57
A 0.29 B 0.71
A 0.29 B 0.86
A 0.29 B 1
A 0.43 B 0
A 0.43 B 0.14
A 0.43 B 0.29
A 0.43 B 0.43
A 0.43 B 0.57
A 0.43 B 0.71
A 0.43 B 0.86
A 0.43 B 1
A 0.57 B 0
A 0.57 B 0.14
A 0.57 B 0.29
A 0.57 B 0.43
A 0.57 B 0.57
A 0.57 B 0.71
A 0.57 B 0.86
A 0.57 B 1
A 0.71 B 0
A 0.71 B 0.14
A 0.71 B 0.29
A 0.71 B 0.43
A 0.71 B 0.57
A 0.71 B 0.71
A 0.71 B 0.86
A 0.71 B 1
A 0.86 B 0
A 0.86 B 0.14
A 0.86 B 0.29
A 0.86 B 0.43
A 0.86 B 0.57
A 0.86 B 0.71
A 0.86 B 0.86
A 0.86 B 1
A 1 B 0
A 1 B 0.14
A 1 B 0.29
A 1 B 0.43
A 1 B 0.57
A 1 B 0.71
A 1 B 0.86
A 1 B 1
OD 600 nm [−]
0.6
0.4
0.2
0 5 101520
Time [h]

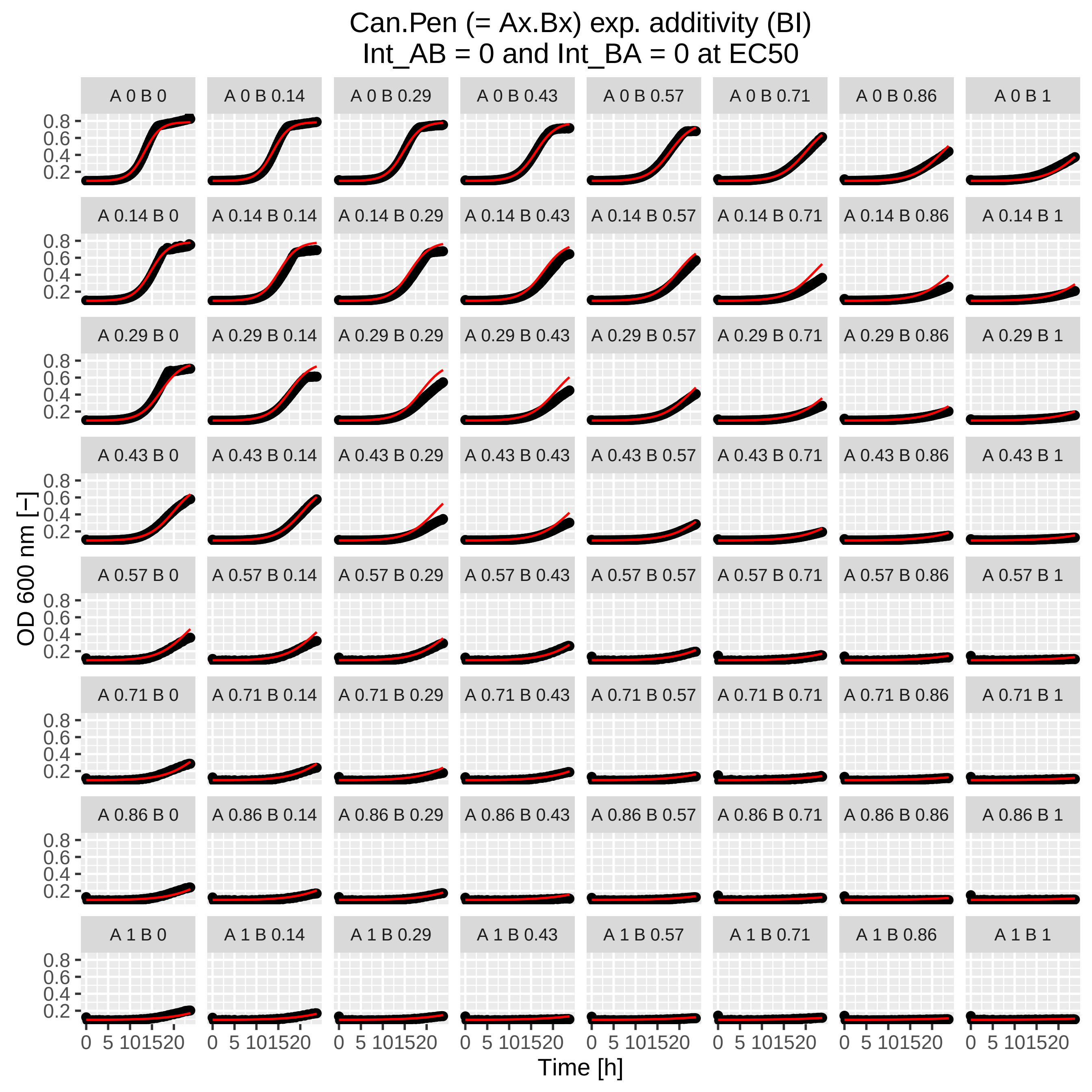

Can.Pen (= Ax.Bx) exp. additivity (BI)
Int_AB = 0 and Int_BA = 0 at EC50
A 0 B 0
A 0 B 0.14
A 0 B 0.29
A 0 B 0.43
A 0 B 0.57
A 0 B 0.71
A 0 B 0.86
A 0 B 1
A 0.14 B 0
A 0.14 B 0.14
A 0.14 B 0.29
A 0.14 B 0.43
A 0.14 B 0.57
A 0.14 B 0.71
A 0.14 B 0.86
A 0.14 B 1
A 0.29 B 0
A 0.29 B 0.14
A 0.29 B 0.29
A 0.29 B 0.43
A 0.29 B 0.57
A 0.29 B 0.71
A 0.29 B 0.86
A 0.29 B 1
A 0.43 B 0
A 0.43 B 0.14
A 0.43 B 0.29
A 0.43 B 0.43
A 0.43 B 0.57
A 0.43 B 0.71
A 0.43 B 0.86
A 0.43 B 1
A 0.57 B 0
A 0.57 B 0.14
A 0.57 B 0.29
A 0.57 B 0.43
A 0.57 B 0.57
A 0.57 B 0.71
A 0.57 B 0.86
A 0.57 B 1
A 0.71 B 0
A 0.71 B 0.14
A 0.71 B 0.29
A 0.71 B 0.43
A 0.71 B 0.57
A 0.71 B 0.71
A 0.71 B 0.86
A 0.71 B 1
A 0.86 B 0
A 0.86 B 0.14
A 0.86 B 0.29
A 0.86 B 0.43
A 0.86 B 0.57
A 0.86 B 0.71
A 0.86 B 0.86
A 0.86 B 1
A 1 B 0
A 1 B 0.14
A 1 B 0.29
A 1 B 0.43
A 1 B 0.57
A 1 B 0.71
A 1 B 0.86
A 1 B 1
OD 600 nm [–]
0.8
0.6
0.4
0.2
0 5 101520
Time [h]

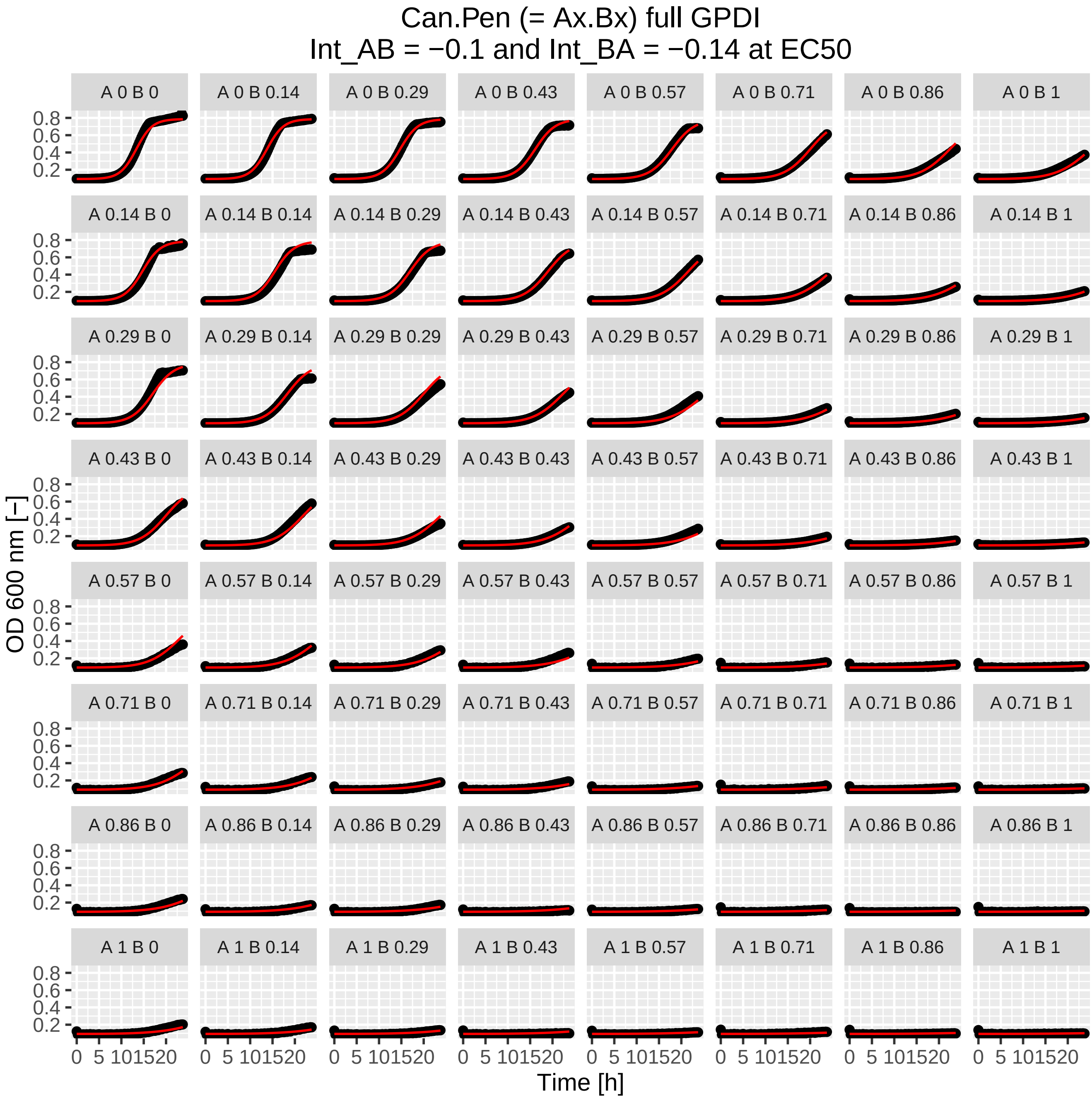

Can.Pen (= Ax.Bx) full GPDI
Int_AB = −0.1 and Int_BA = −0.14 at EC50
OD 600 nm [−]
Time [h]
A 0 B 0
A 0 B 0.14
A 0 B 0.29
A 0 B 0.43
A 0 B 0.57
A 0 B 0.71
A 0 B 0.86
A 0 B 1
A 1 B 0
A 1 B 0.14
A 1 B 0.29
A 1 B 0.43
A 1 B 0.57
A 1 B 0.71
A 1 B 0.86
A 1 B 1

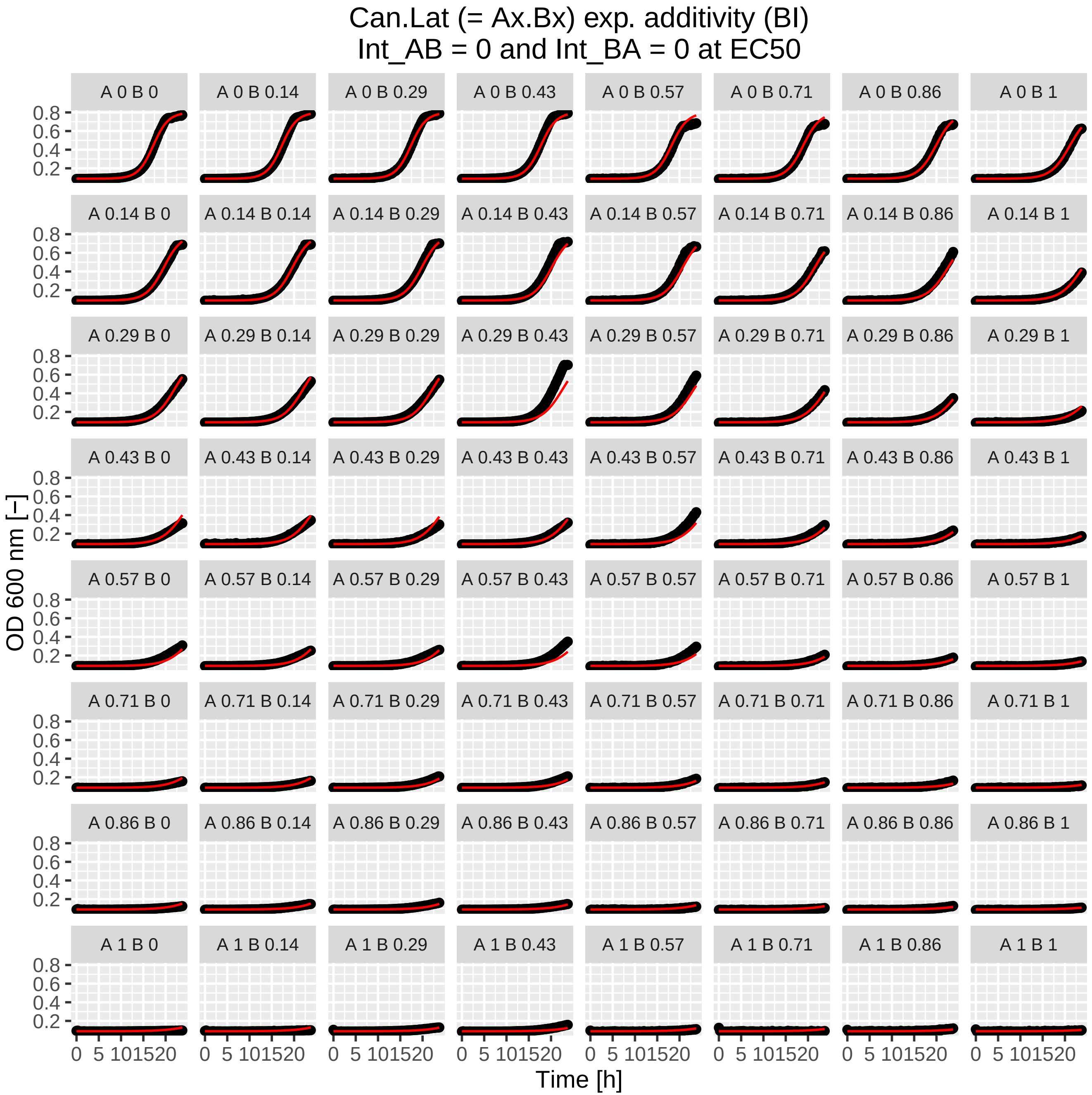
Can.Lat (= Ax.Bx) exp. additivity (BI)
Int_AB = 0 and Int_BA = 0 at EC50
OD 600 nm [–]
Time [h]
A 0 B 0
A 0 B 0.14
A 0 B 0.29
A 0 B 0.43
A 0 B 0.57
A 0 B 0.71
A 0 B 0.86
A 0 B 1
A 0.14 B 0
A 0.14 B 0.14
A 0.14 B 0.29
A 0.14 B 0.43
A 0.14 B 0.57
A 0.14 B 0.71
A 0.14 B 0.86
A 0.14 B 1
A 0.29 B 0
A 0.29 B 0.14
A 0.29 B 0.29
A 0.29 B 0.43
A 0.29 B 0.57
A 0.29 B 0.71
A 0.29 B 0.86
A 0.29 B 1
A 0.43 B 0
A 0.43 B 0.14
A 0.43 B 0.29
A 0.43 B 0.43
A 0.43 B 0.57
A 0.43 B 0.71
A 0.43 B 0.86
A 0.43 B 1
A 0.57 B 0
A 0.57 B 0.14
A 0.57 B 0.29
A 0.57 B 0.43
A 0.57 B 0.57
A 0.57 B 0.71
A 0.57 B 0.86
A 0.57 B 1
A 0.71 B 0
A 0.71 B 0.14
A 0.71 B 0.29
A 0.71 B 0.43
A 0.71 B 0.57
A 0.71 B 0.71
A 0.71 B 0.86
A 0.71 B 1
A 0.86 B 0
A 0.86 B 0.14
A 0.86 B 0.29
A 0.86 B 0.43
A 0.86 B 0.57
A 0.86 B 0.71
A 0.86 B 0.86
A 0.86 B 1
A 1 B 0
A 1 B 0.14
A 1 B 0.29
A 1 B 0.43
A 1 B 0.57
A 1 B 0.71
A 1 B 0.86
A 1 B 1
0.2
0.4
0.6
0.8
0 5 101520

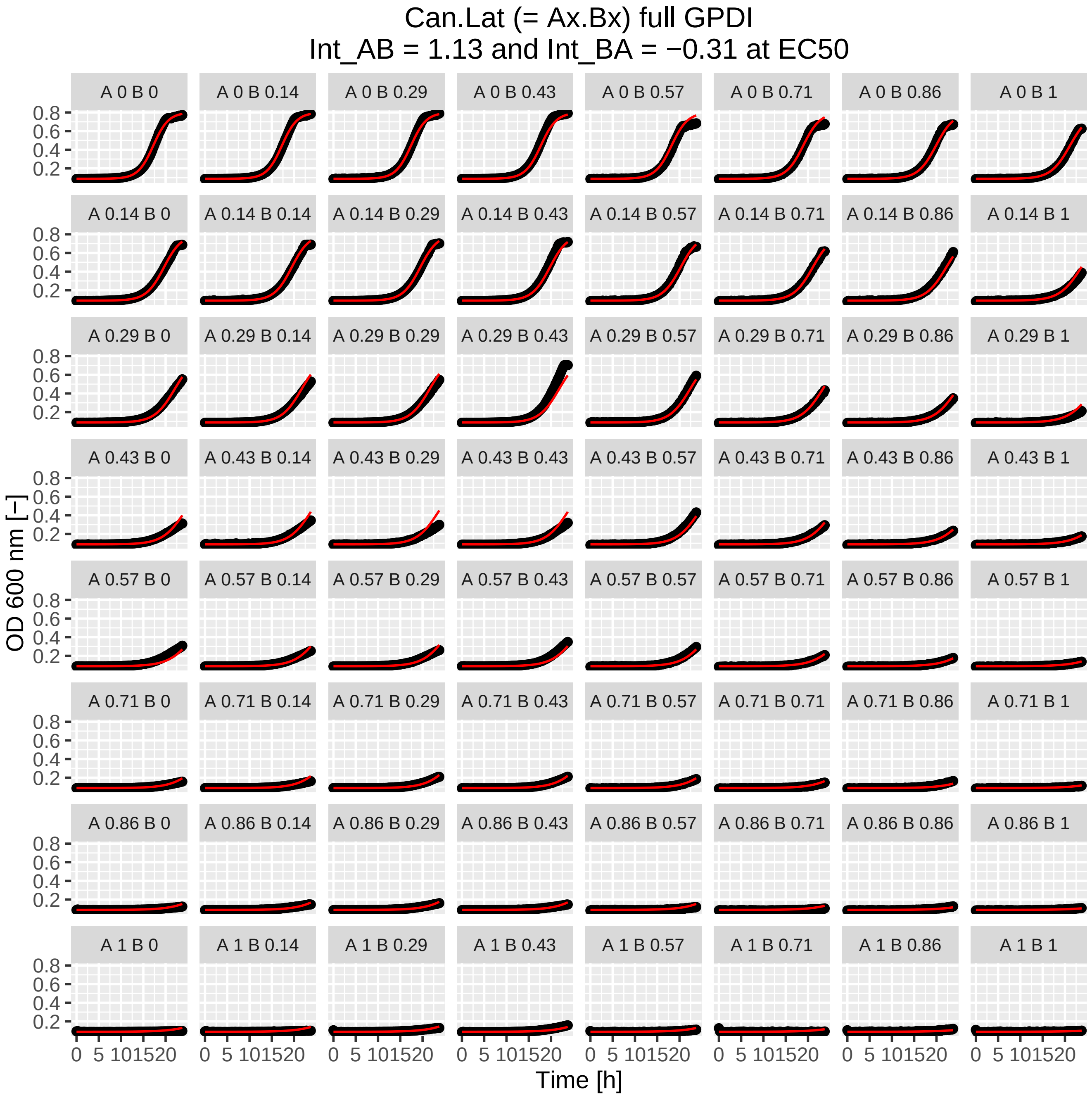

Can.Lat (= Ax.Bx) full GPDI
Int_AB = 1.13 and Int_BA = −0.31 at EC50
OD 600 nm [−]
Time [h]
A 0 B 0
A 0 B 0.14
A 0 B 0.29
A 0 B 0.43
A 0 B 0.57
A 0 B 0.71
A 0 B 0.86
A 0 B 1
A 0.14 B 0
A 0.14 B 0.14
A 0.14 B 0.29
A 0.14 B 0.43
A 0.14 B 0.57
A 0.14 B 0.71
A 0.14 B 0.86
A 0.14 B 1
A 0.29 B 0
A 0.29 B 0.14
A 0.29 B 0.29
A 0.29 B 0.43
A 0.29 B 0.57
A 0.29 B 0.71
A 0.29 B 0.86
A 0.29 B 1
A 0.43 B 0
A 0.43 B 0.14
A 0.43 B 0.29
A 0.43 B 0.43
A 0.43 B 0.57
A 0.43 B 0.71
A 0.43 B 0.86
A 0.43 B 1
A 0.57 B 0
A 0.57 B 0.14
A 0.57 B 0.29
A 0.57 B 0.43
A 0.57 B 0.57
A 0.57 B 0.71
A 0.57 B 0.86
A 0.57 B 1
A 0.71 B 0
A 0.71 B 0.14
A 0.71 B 0.29
A 0.71 B 0.43
A 0.71 B 0.57
A 0.71 B 0.71
A 0.71 B 0.86
A 0.71 B 1
A 0.86 B 0
A 0.86 B 0.14
A 0.86 B 0.29
A 0.86 B 0.43
A 0.86 B 0.57
A 0.86 B 0.71
A 0.86 B 0.86
A 0.86 B 1
A 1 B 0
A 1 B 0.14
A 1 B 0.29
A 1 B 0.43
A 1 B 0.57
A 1 B 0.71
A 1 B 0.86
A 1 B 1
0.2
0.4
0.6
0.8
0 5 101520

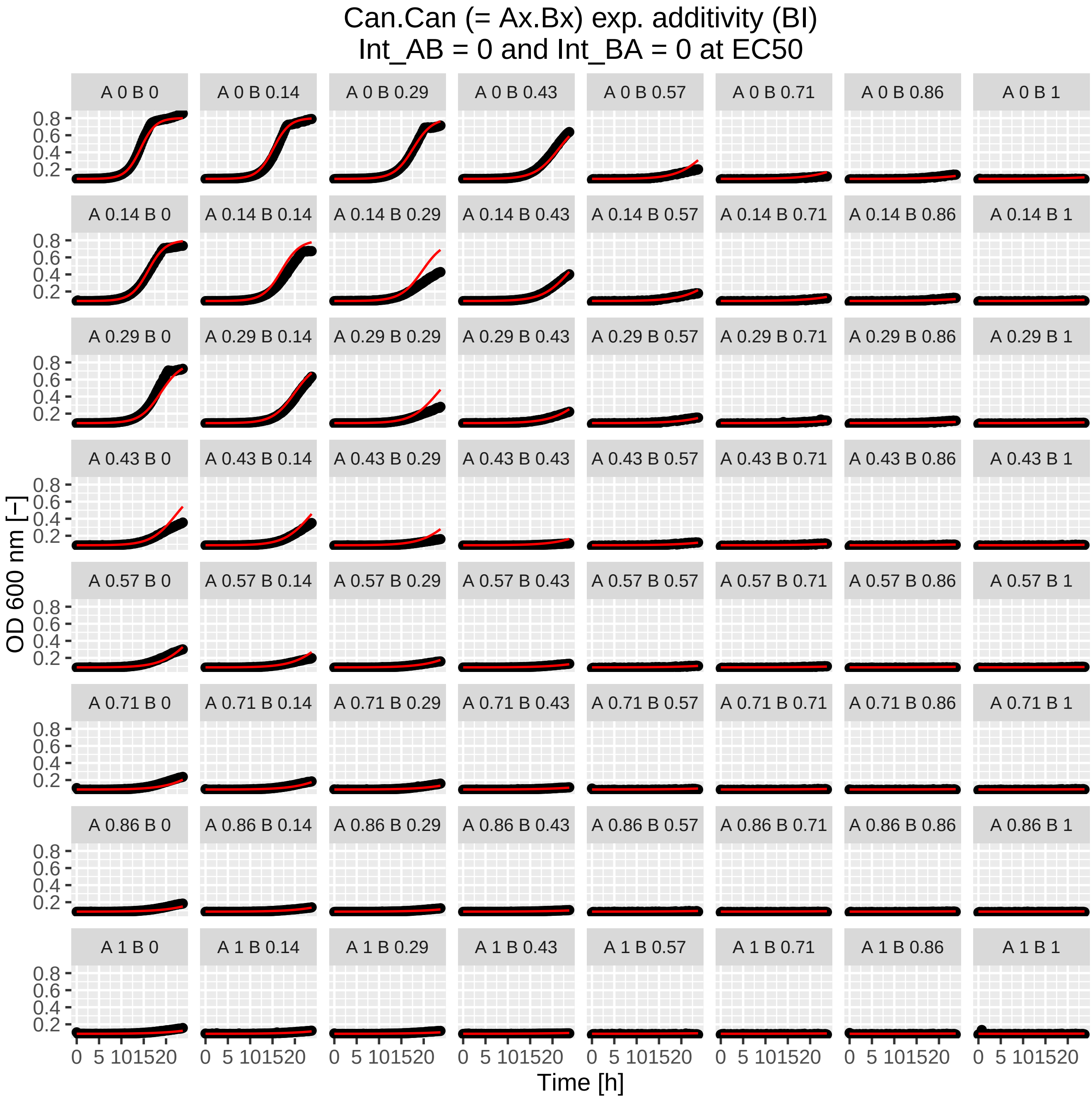
Can.Can (= Ax.Bx) exp. additivity (BI)
Int_AB = 0 and Int_BA = 0 at EC50
A 0 B 0
A 0 B 0.14
A 0 B 0.29
A 0 B 0.43
A 0 B 0.57
A 0 B 0.71
A 0 B 0.86
A 0 B 1
A 0.14 B 0
A 0.14 B 0.14
A 0.14 B 0.29
A 0.14 B 0.43
A 0.14 B 0.57
A 0.14 B 0.71
A 0.14 B 0.86
A 0.14 B 1
A 0.29 B 0
A 0.29 B 0.14
A 0.29 B 0.29
A 0.29 B 0.43
A 0.29 B 0.57
A 0.29 B 0.71
A 0.29 B 0.86
A 0.29 B 1
A 0.43 B 0
A 0.43 B 0.14
A 0.43 B 0.29
A 0.43 B 0.43
A 0.43 B 0.57
A 0.43 B 0.71
A 0.43 B 0.86
A 0.43 B 1
A 0.57 B 0
A 0.57 B 0.14
A 0.57 B 0.29
A 0.57 B 0.43
A 0.57 B 0.57
A 0.57 B 0.71
A 0.57 B 0.86
A 0.57 B 1
A 0.71 B 0
A 0.71 B 0.14
A 0.71 B 0.29
A 0.71 B 0.43
A 0.71 B 0.57
A 0.71 B 0.71
A 0.71 B 0.86
A 0.71 B 1
A 0.86 B 0
A 0.86 B 0.14
A 0.86 B 0.29
A 0.86 B 0.43
A 0.86 B 0.57
A 0.86 B 0.71
A 0.86 B 0.86
A 0.86 B 1
A 1 B 0
A 1 B 0.14
A 1 B 0.29
A 1 B 0.43
A 1 B 0.57
A 1 B 0.71
A 1 B 0.86
A 1 B 1
0.8
0.6
0.4
0.2
OD 600 nm [–]
0 5 101520
Time [h]

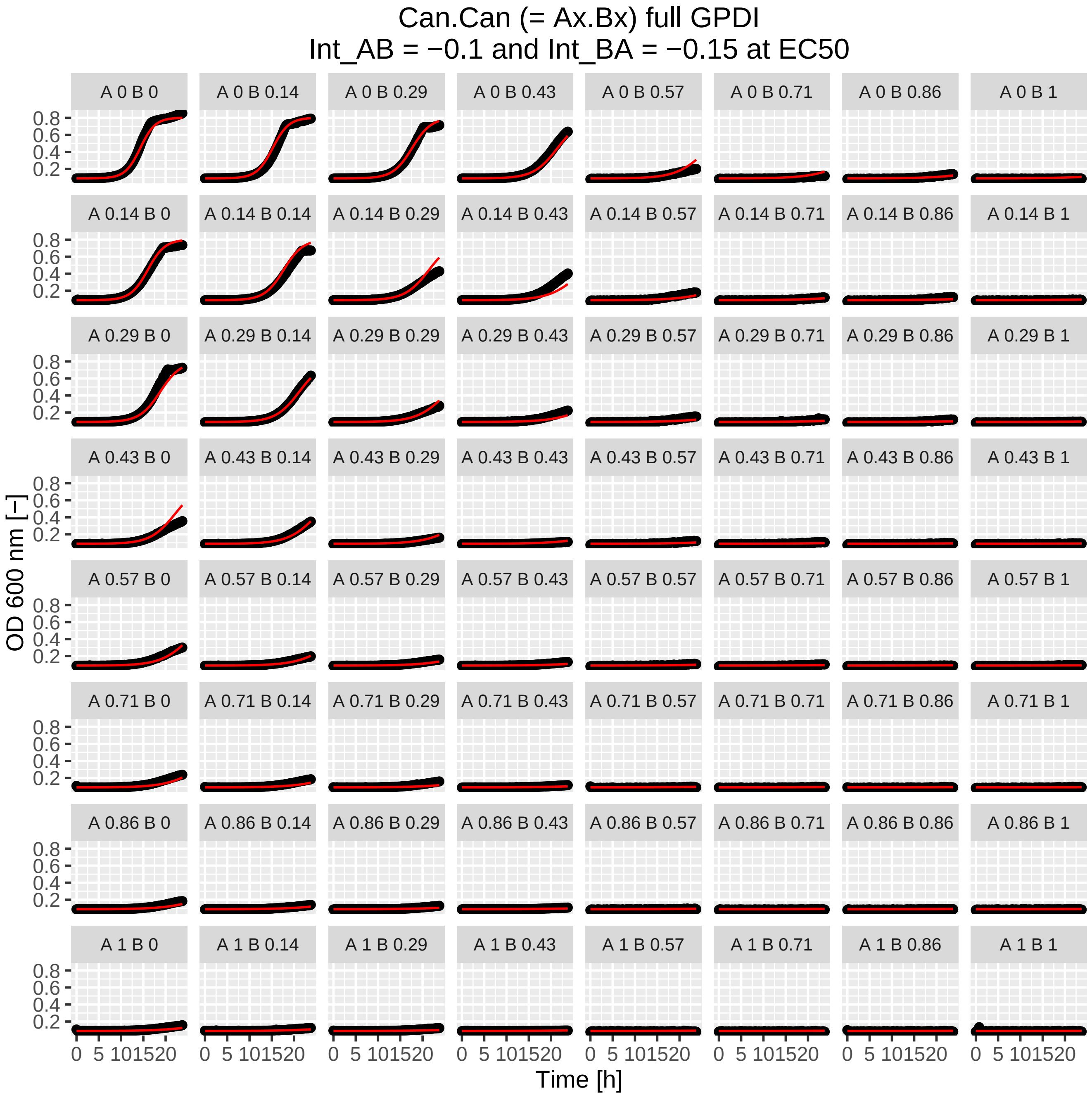
Can.Can (= Ax.Bx) full GPDI
Int_AB = −0.1 and Int_BA = −0.15 at EC50
OD 600 nm [−]
Time [h]
A 0 B 0
A 0 B 0.14
A 0 B 0.29
A 0 B 0.43
A 0 B 0.57
A 0 B 0.71
A 0 B 0.86
A 0 B 1
A 0.14 B 0
A 0.14 B 0.14
A 0.14 B 0.29
A 0.14 B 0.43
A 0.14 B 0.57
A 0.14 B 0.71
A 0.14 B 0.86
A 0.14 B 1
A 0.29 B 0
A 0.29 B 0.14
A 0.29 B 0.29
A 0.29 B 0.43
A 0.29 B 0.57
A 0.29 B 0.71
A 0.29 B 0.86
A 0.29 B 1
A 0.43 B 0
A 0.43 B 0.14
A 0.43 B 0.29
A 0.43 B 0.43
A 0.43 B 0.57
A 0.43 B 0.71
A 0.43 B 0.86
A 0.43 B 1
A 0.57 B 0
A 0.57 B 0.14
A 0.57 B 0.29
A 0.57 B 0.43
A 0.57 B 0.57
A 0.57 B 0.71
A 0.57 B 0.86
A 0.57 B 1
A 0.71 B 0
A 0.71 B 0.14
A 0.71 B 0.29
A 0.71 B 0.43
A 0.71 B 0.57
A 0.71 B 0.71
A 0.71 B 0.86
A 0.71 B 1
A 0.86 B 0
A 0.86 B 0.14
A 0.86 B 0.29
A 0.86 B 0.43
A 0.86 B 0.57
A 0.86 B 0.71
A 0.86 B 0.86
A 0.86 B 1
A 1 B 0
A 1 B 0.14
A 1 B 0.29
A 1 B 0.43
A 1 B 0.57
A 1 B 0.71
A 1 B 0.86
A 1 B 1
0.2
0.4
0.6
0.8
0
5
10
15
20

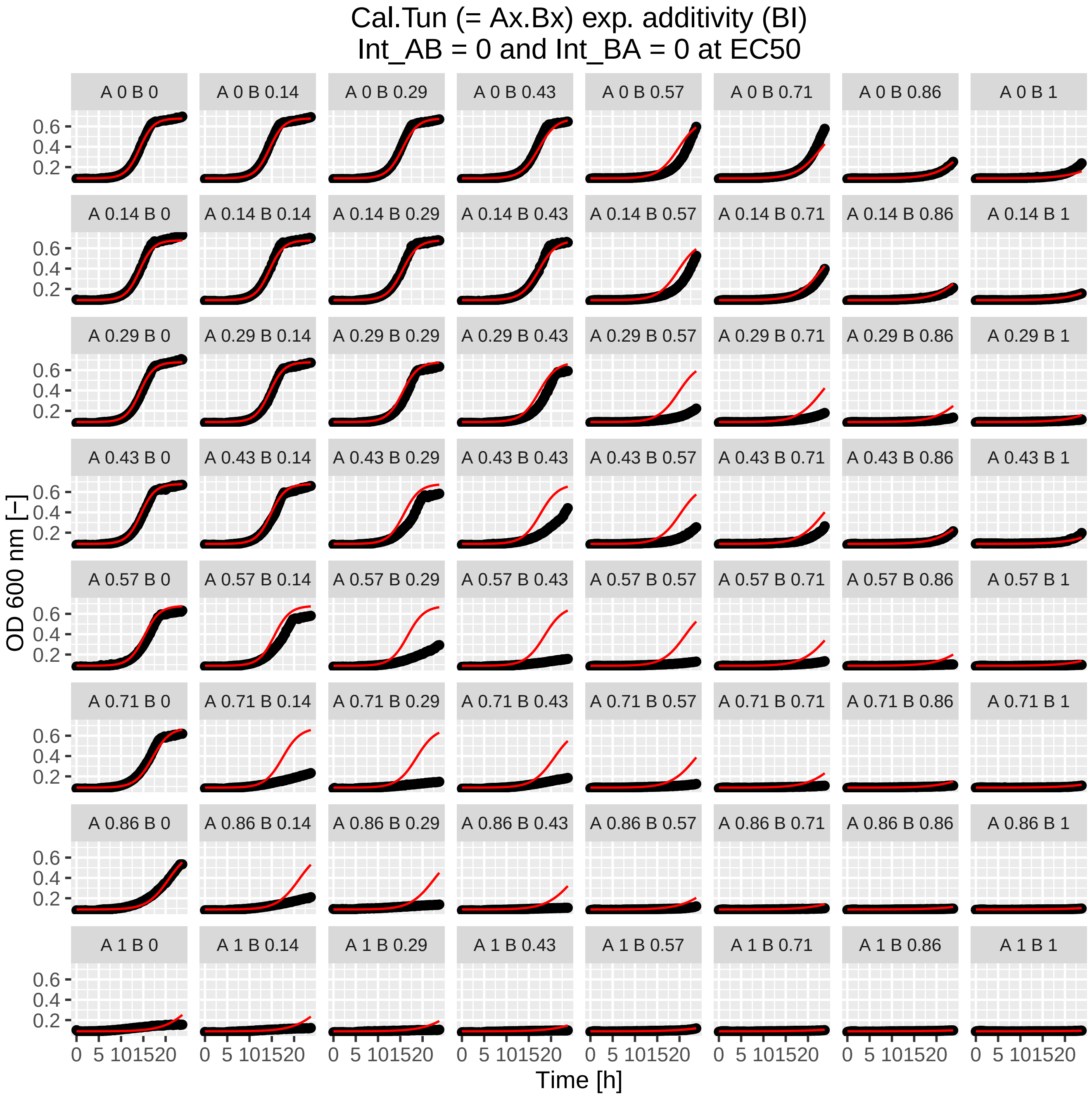
Cal.Tun (= Ax.Bx) exp. additivity (BI)
Int_AB = 0 and Int_BA = 0 at EC50
OD 600 nm [–]
Time [h]
A 0 B 0
A 0 B 0.14
A 0 B 0.29
A 0 B 0.43
A 0 B 0.57
A 0 B 0.71
A 0 B 0.86
A 0 B 1
A 0.14 B 0
A 0.14 B 0.14
A 0.14 B 0.29
A 0.14 B 0.43
A 0.14 B 0.57
A 0.14 B 0.71
A 0.14 B 0.86
A 0.14 B 1
A 0.29 B 0
A 0.29 B 0.14
A 0.29 B 0.29
A 0.29 B 0.43
A 0.29 B 0.57
A 0.29 B 0.71
A 0.29 B 0.86
A 0.29 B 1
A 0.43 B 0
A 0.43 B 0.14
A 0.43 B 0.29
A 0.43 B 0.43
A 0.43 B 0.57
A 0.43 B 0.71
A 0.43 B 0.86
A 0.43 B 1
A 0.57 B 0
A 0.57 B 0.14
A 0.57 B 0.29
A 0.57 B 0.43
A 0.57 B 0.57
A 0.57 B 0.71
A 0.57 B 0.86
A 0.57 B 1
A 0.71 B 0
A 0.71 B 0.14
A 0.71 B 0.29
A 0.71 B 0.43
A 0.71 B 0.57
A 0.71 B 0.71
A 0.71 B 0.86
A 0.71 B 1
A 0.86 B 0
A 0.86 B 0.14
A 0.86 B 0.29
A 0.86 B 0.43
A 0.86 B 0.57
A 0.86 B 0.71
A 0.86 B 0.86
A 0.86 B 1
A 1 B 0
A 1 B 0.14
A 1 B 0.29
A 1 B 0.43
A 1 B 0.57
A 1 B 0.71
A 1 B 0.86
A 1 B 1
0.6
0.4
0.2
0 5 101520

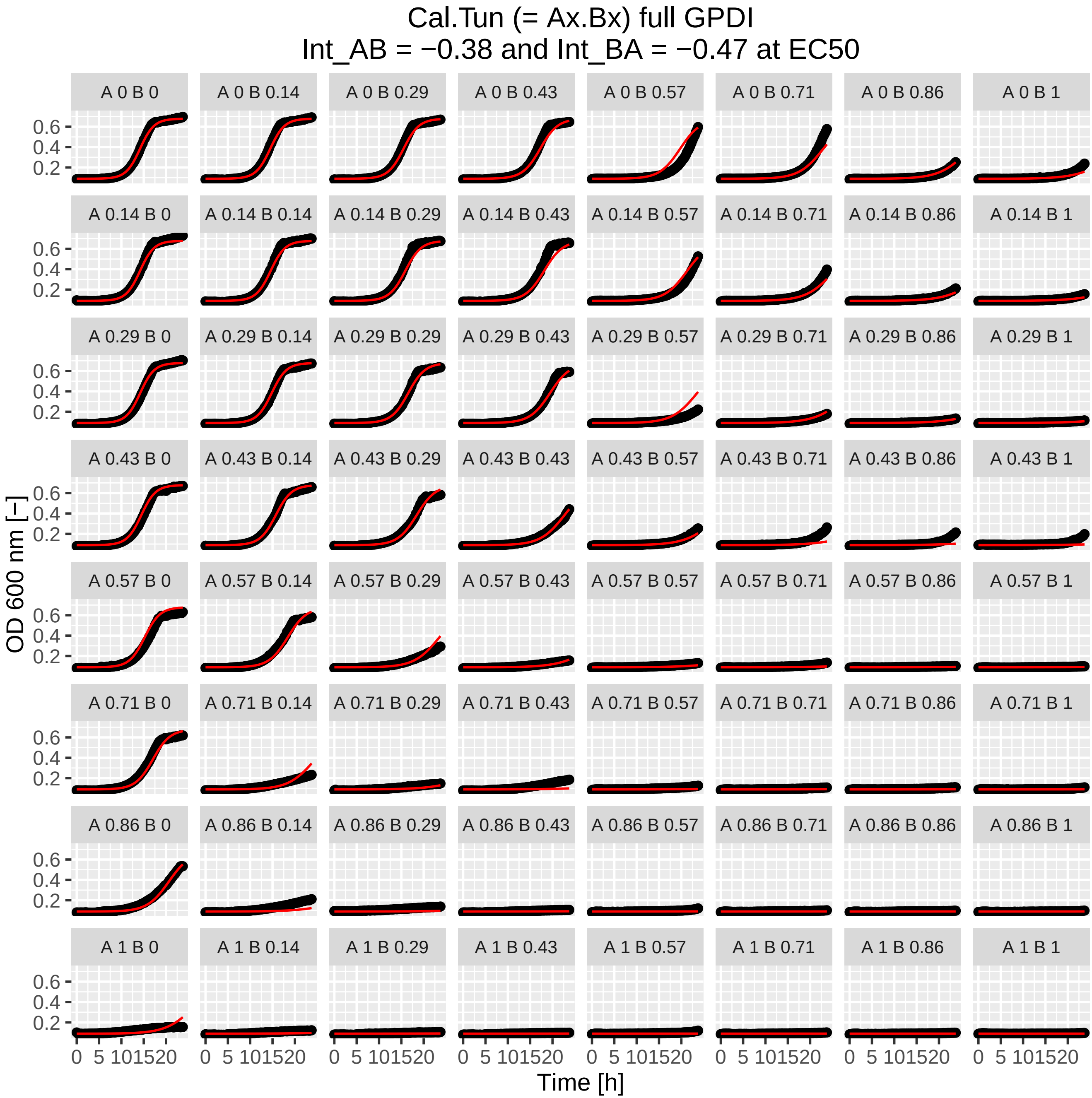

Cal.Tun (= Ax.Bx) full GPDI
Int_AB = −0.38 and Int_BA = −0.47 at EC50
OD 600 nm [−]
Time [h]
0.2
0.4
0.6
0 5 101520
A 0 B 0
A 0 B 0.14
A 0 B 0.29
A 0 B 0.43
A 0 B 0.57
A 0 B 0.71
A 0 B 0.86
A 0 B 1
A 0.14 B 0
A 0.14 B 0.14
A 0.14 B 0.29
A 0.14 B 0.43
A 0.14 B 0.57
A 0.14 B 0.71
A 0.14 B 0.86
A 0.14 B 1
A 0.29 B 0
A 0.29 B 0.14
A 0.29 B 0.29
A 0.29 B 0.43
A 0.29 B 0.57
A 0.29 B 0.71
A 0.29 B 0.86
A 0.29 B 1
A 0.43 B 0
A 0.43 B 0.14
A 0.43 B 0.29
A 0.43 B 0.43
A 0.43 B 0.57
A 0.43 B 0.71
A 0.43 B 0.86
A 0.43 B 1
A 0.57 B 0
A 0.57 B 0.14
A 0.57 B 0.29
A 0.57 B 0.43
A 0.57 B 0.57
A 0.57 B 0.71
A 0.57 B 0.86
A 0.57 B 1
A 0.71 B 0
A 0.71 B 0.14
A 0.71 B 0.29
A 0.71 B 0.43
A 0.71 B 0.57
A 0.71 B 0.71
A 0.71 B 0.86
A 0.71 B 1
A 0.86 B 0
A 0.86 B 0.14
A 0.86 B 0.29
A 0.86 B 0.43
A 0.86 B 0.57
A 0.86 B 0.71
A 0.86 B 0.86
A 0.86 B 1
A 1 B 0
A 1 B 0.14
A 1 B 0.29
A 1 B 0.43
A 1 B 0.57
A 1 B 0.71
A 1 B 0.86
A 1 B 1

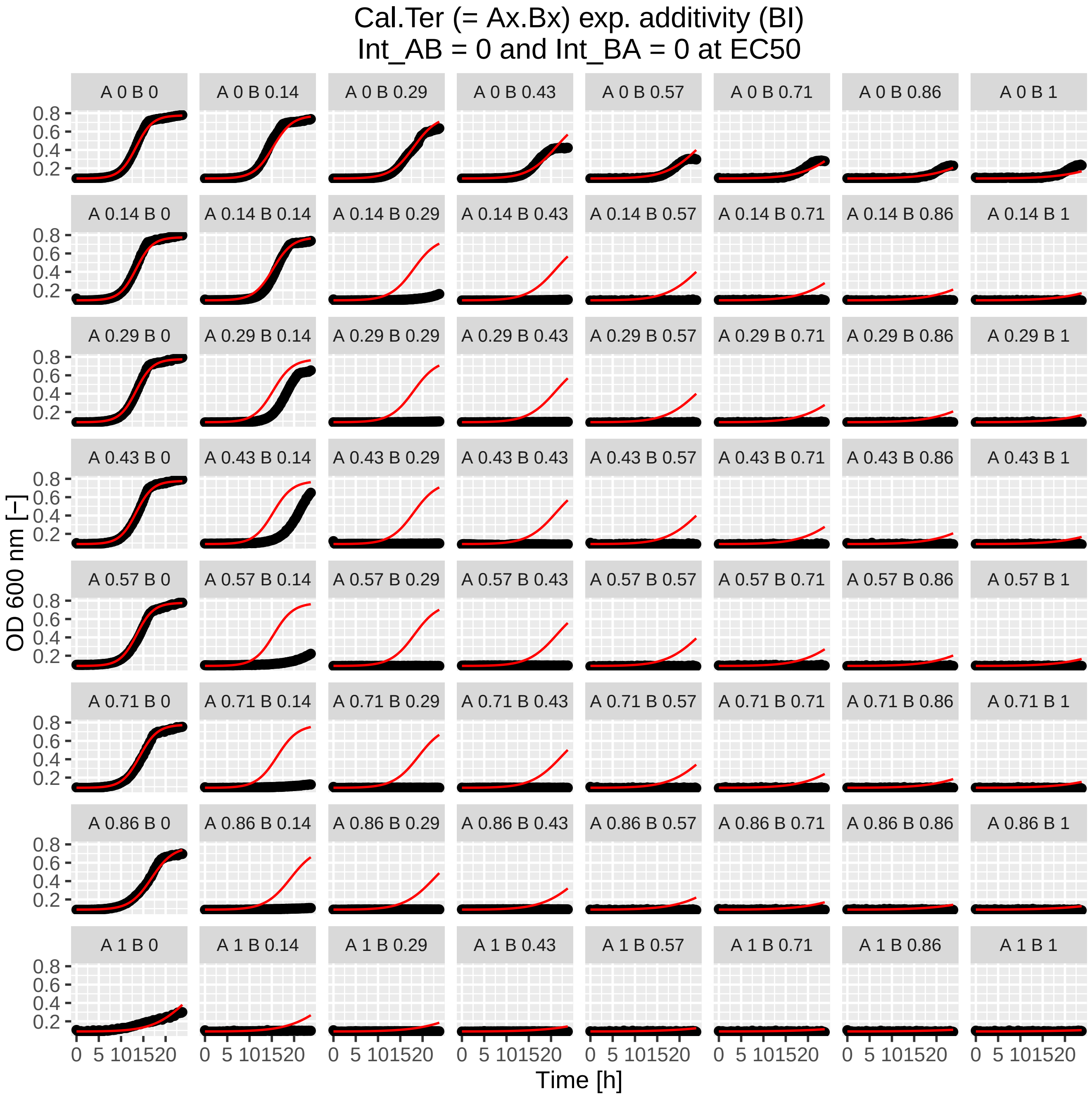

Cal.Ter (= Ax.Bx) exp. additivity (BI)
Int_AB = 0 and Int_BA = 0 at EC50
A 0 B 0
A 0 B 0.14
A 0 B 0.29
A 0 B 0.43
A 0 B 0.57
A 0 B 0.71
A 0 B 0.86
A 0 B 1
A 0.14 B 0
A 0.14 B 0.14
A 0.14 B 0.29
A 0.14 B 0.43
A 0.14 B 0.57
A 0.14 B 0.71
A 0.14 B 0.86
A 0.14 B 1
A 0.29 B 0
A 0.29 B 0.14
A 0.29 B 0.29
A 0.29 B 0.43
A 0.29 B 0.57
A 0.29 B 0.71
A 0.29 B 0.86
A 0.29 B 1
A 0.43 B 0
A 0.43 B 0.14
A 0.43 B 0.29
A 0.43 B 0.43
A 0.43 B 0.57
A 0.43 B 0.71
A 0.43 B 0.86
A 0.43 B 1
A 0.57 B 0
A 0.57 B 0.14
A 0.57 B 0.29
A 0.57 B 0.43
A 0.57 B 0.57
A 0.57 B 0.71
A 0.57 B 0.86
A 0.57 B 1
A 0.71 B 0
A 0.71 B 0.14
A 0.71 B 0.29
A 0.71 B 0.43
A 0.71 B 0.57
A 0.71 B 0.71
A 0.71 B 0.86
A 0.71 B 1
A 0.86 B 0
A 0.86 B 0.14
A 0.86 B 0.29
A 0.86 B 0.43
A 0.86 B 0.57
A 0.86 B 0.71
A 0.86 B 0.86
A 0.86 B 1
A 1 B 0
A 1 B 0.14
A 1 B 0.29
A 1 B 0.43
A 1 B 0.57
A 1 B 0.71
A 1 B 0.86
A 1 B 1
OD 600 nm [–]
0.8
0.6
0.4
0.2
0 5 101520
Time [h]

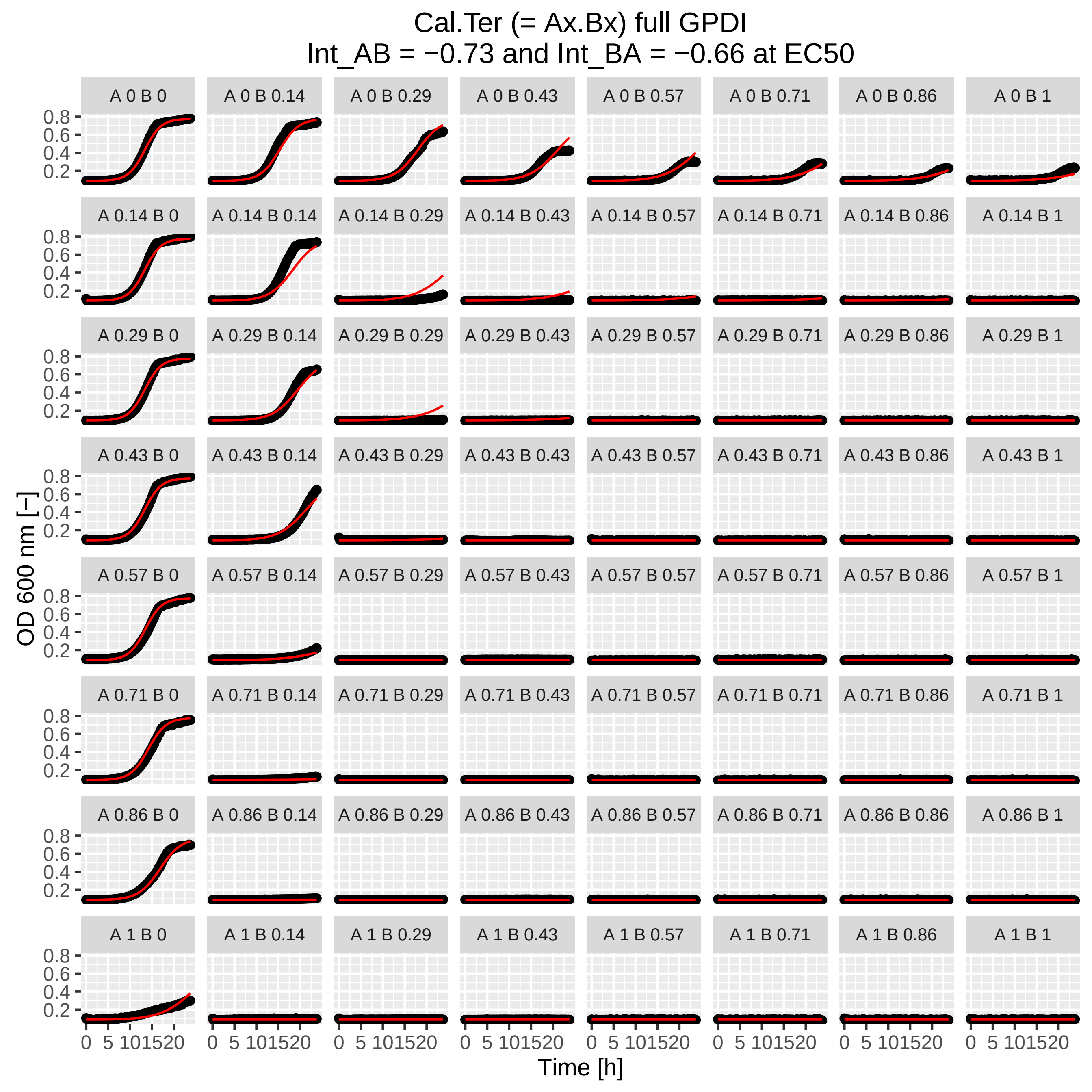

Cal.Ter (= Ax.Bx) full GPDI
Int_AB = −0.73 and Int_BA = −0.66 at EC50
A 0 B 0
A 0 B 0.14
A 0 B 0.29
A 0 B 0.43
A 0 B 0.57
A 0 B 0.71
A 0 B 0.86
A 0 B 1
A 0.14 B 0
A 0.14 B 0.14
A 0.14 B 0.29
A 0.14 B 0.43
A 0.14 B 0.57
A 0.14 B 0.71
A 0.14 B 0.86
A 0.14 B 1
A 0.29 B 0
A 0.29 B 0.14
A 0.29 B 0.29
A 0.29 B 0.43
A 0.29 B 0.57
A 0.29 B 0.71
A 0.29 B 0.86
A 0.29 B 1
A 0.43 B 0
A 0.43 B 0.14
A 0.43 B 0.29
A 0.43 B 0.43
A 0.43 B 0.57
A 0.43 B 0.71
A 0.43 B 0.86
A 0.43 B 1
A 0.57 B 0
A 0.57 B 0.14
A 0.57 B 0.29
A 0.57 B 0.43
A 0.57 B 0.57
A 0.57 B 0.71
A 0.57 B 0.86
A 0.57 B 1
A 0.71 B 0
A 0.71 B 0.14
A 0.71 B 0.29
A 0.71 B 0.43
A 0.71 B 0.57
A 0.71 B 0.71
A 0.71 B 0.86
A 0.71 B 1
A 0.86 B 0
A 0.86 B 0.14
A 0.86 B 0.29
A 0.86 B 0.43
A 0.86 B 0.57
A 0.86 B 0.71
A 0.86 B 0.86
A 0.86 B 1
A 1 B 0
A 1 B 0.14
A 1 B 0.29
A 1 B 0.43
A 1 B 0.57
A 1 B 0.71
A 1 B 0.86
A 1 B 1
0.8
0.6
0.4
0.2
OD 600 nm [−]
0 5 101520
Time [h]

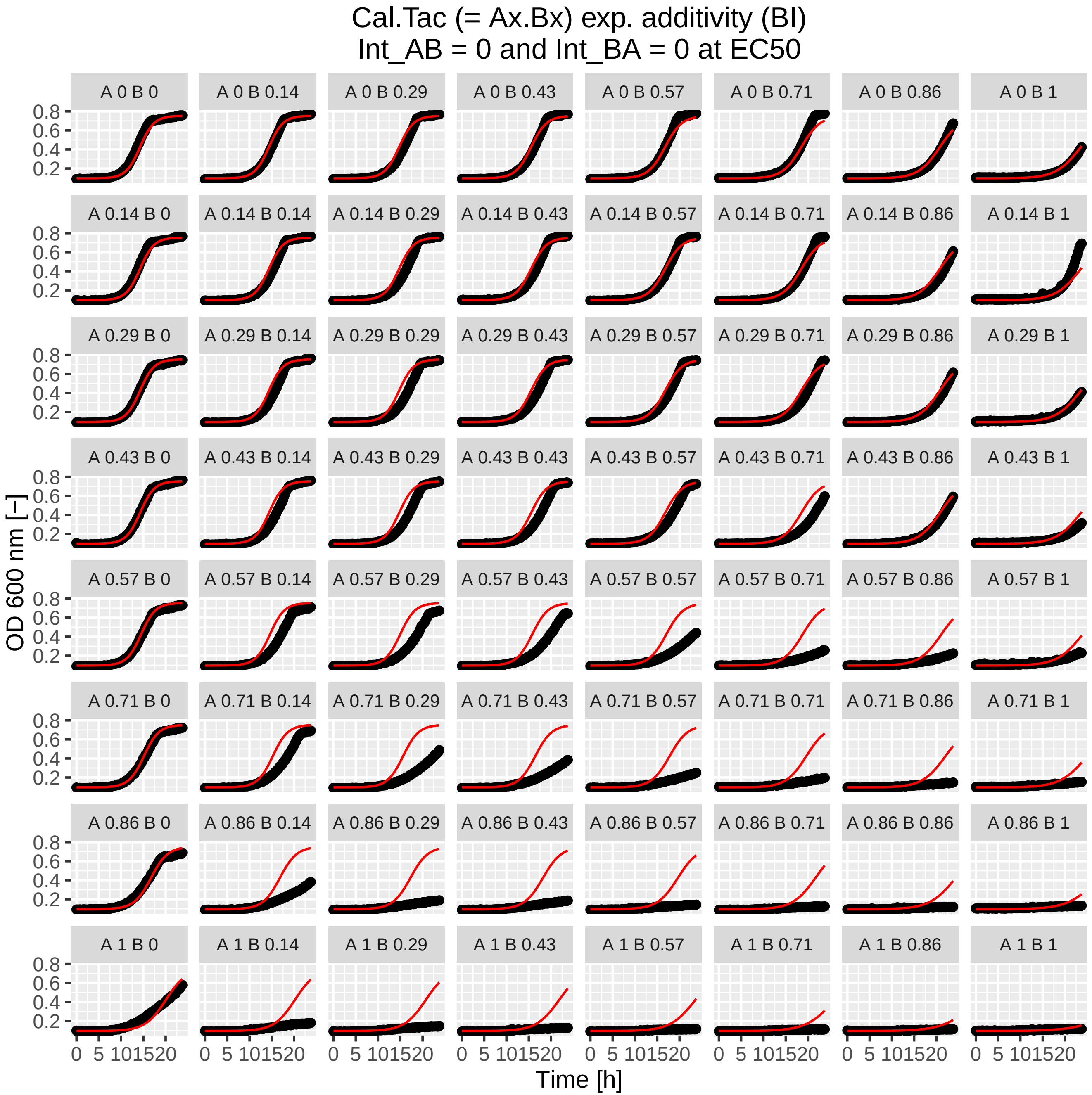
Cal.Tac (= Ax.Bx) exp. additivity (BI)
Int_AB = 0 and Int_BA = 0 at EC50
A 0 B 0
A 0 B 0.14
A 0 B 0.29
A 0 B 0.43
A 0 B 0.57
A 0 B 0.71
A 0 B 0.86
A 0 B 1
A 0.14 B 0
A 0.14 B 0.14
A 0.14 B 0.29
A 0.14 B 0.43
A 0.14 B 0.57
A 0.14 B 0.71
A 0.14 B 0.86
A 0.14 B 1
A 0.29 B 0
A 0.29 B 0.14
A 0.29 B 0.29
A 0.29 B 0.43
A 0.29 B 0.57
A 0.29 B 0.71
A 0.29 B 0.86
A 0.29 B 1
A 0.43 B 0
A 0.43 B 0.14
A 0.43 B 0.29
A 0.43 B 0.43
A 0.43 B 0.57
A 0.43 B 0.71
A 0.43 B 0.86
A 0.43 B 1
A 0.57 B 0
A 0.57 B 0.14
A 0.57 B 0.29
A 0.57 B 0.43
A 0.57 B 0.57
A 0.57 B 0.71
A 0.57 B 0.86
A 0.57 B 1
A 0.71 B 0
A 0.71 B 0.14
A 0.71 B 0.29
A 0.71 B 0.43
A 0.71 B 0.57
A 0.71 B 0.71
A 0.71 B 0.86
A 0.71 B 1
A 0.86 B 0
A 0.86 B 0.14
A 0.86 B 0.29
A 0.86 B 0.43
A 0.86 B 0.57
A 0.86 B 0.71
A 0.86 B 0.86
A 0.86 B 1
A 1 B 0
A 1 B 0.14
A 1 B 0.29
A 1 B 0.43
A 1 B 0.57
A 1 B 0.71
A 1 B 0.86
A 1 B 1
OD 600 nm [−]
0.8
0.6
0.4
0.2
0 5 101520
Time [h]

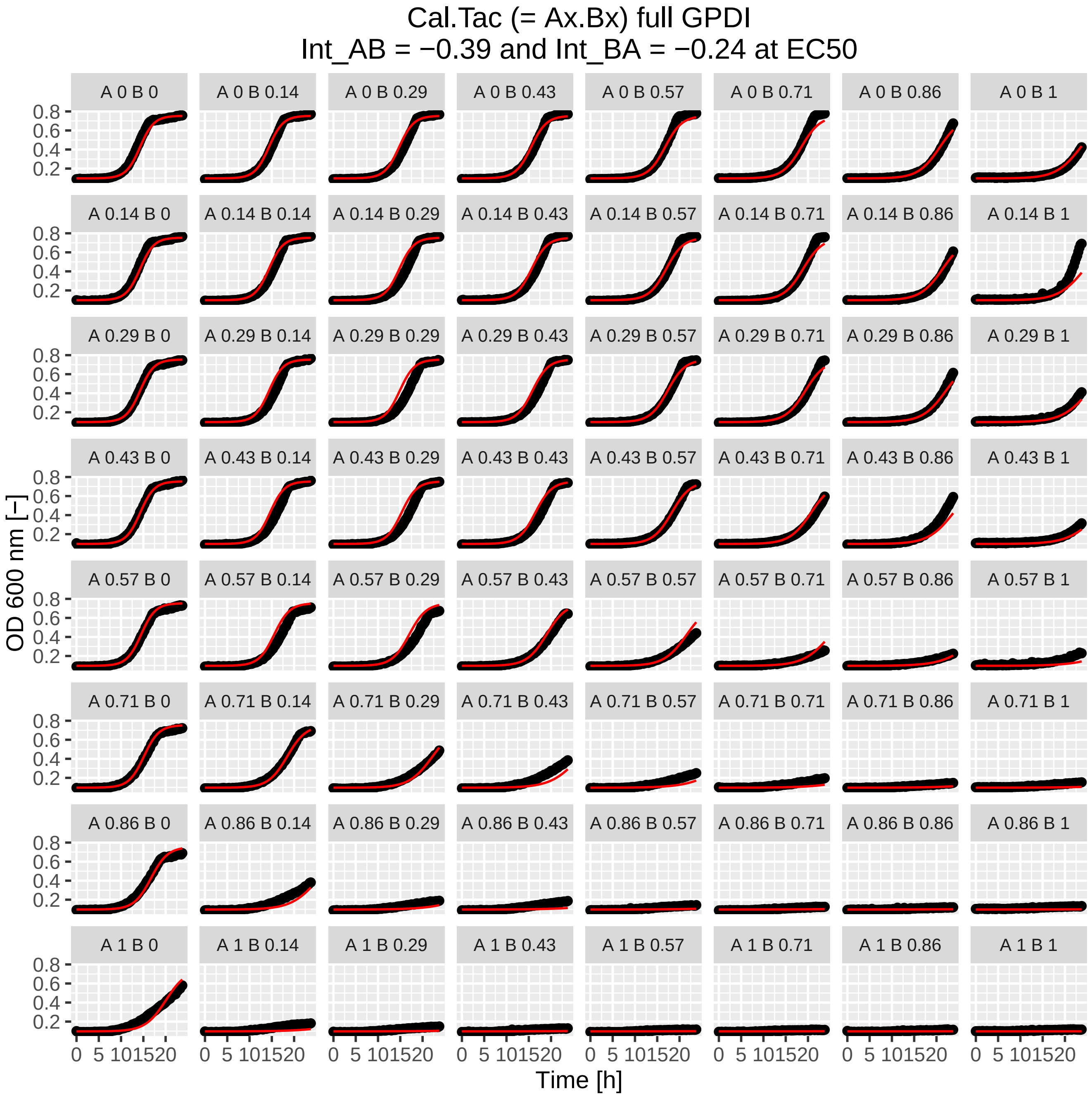
Cal.Tac (= Ax.Bx) full GPDI
Int_AB = −0.39 and Int_BA = −0.24 at EC50
OD 600 nm [−]
Time [h]
0 5 101520
0.2
0.4
0.6
0.8
A 0 B 0
A 0 B 0.14
A 0 B 0.29
A 0 B 0.43
A 0 B 0.57
A 0 B 0.71
A 0 B 0.86
A 0 B 1
A 0.14 B 0
A 0.14 B 0.14
A 0.14 B 0.29
A 0.14 B 0.43
A 0.14 B 0.57
A 0.14 B 0.71
A 0.14 B 0.86
A 0.14 B 1
A 0.29 B 0
A 0.29 B 0.14
A 0.29 B 0.29
A 0.29 B 0.43
A 0.29 B 0.57
A 0.29 B 0.71
A 0.29 B 0.86
A 0.29 B 1
A 0.43 B 0
A 0.43 B 0.14
A 0.43 B 0.29
A 0.43 B 0.43
A 0.43 B 0.57
A 0.43 B 0.71
A 0.43 B 0.86
A 0.43 B 1
A 0.57 B 0
A 0.57 B 0.14
A 0.57 B 0.29
A 0.57 B 0.43
A 0.57 B 0.57
A 0.57 B 0.71
A 0.57 B 0.86
A 0.57 B 1
A 0.71 B 0
A 0.71 B 0.14
A 0.71 B 0.29
A 0.71 B 0.43
A 0.71 B 0.57
A 0.71 B 0.71
A 0.71 B 0.86
A 0.71 B 1
A 0.86 B 0
A 0.86 B 0.14
A 0.86 B 0.29
A 0.86 B 0.43
A 0.86 B 0.57
A 0.86 B 0.71
A 0.86 B 0.86
A 0.86 B 1
A 1 B 0
A 1 B 0.14
A 1 B 0.29
A 1 B 0.43
A 1 B 0.57
A 1 B 0.71
A 1 B 0.86
A 1 B 1

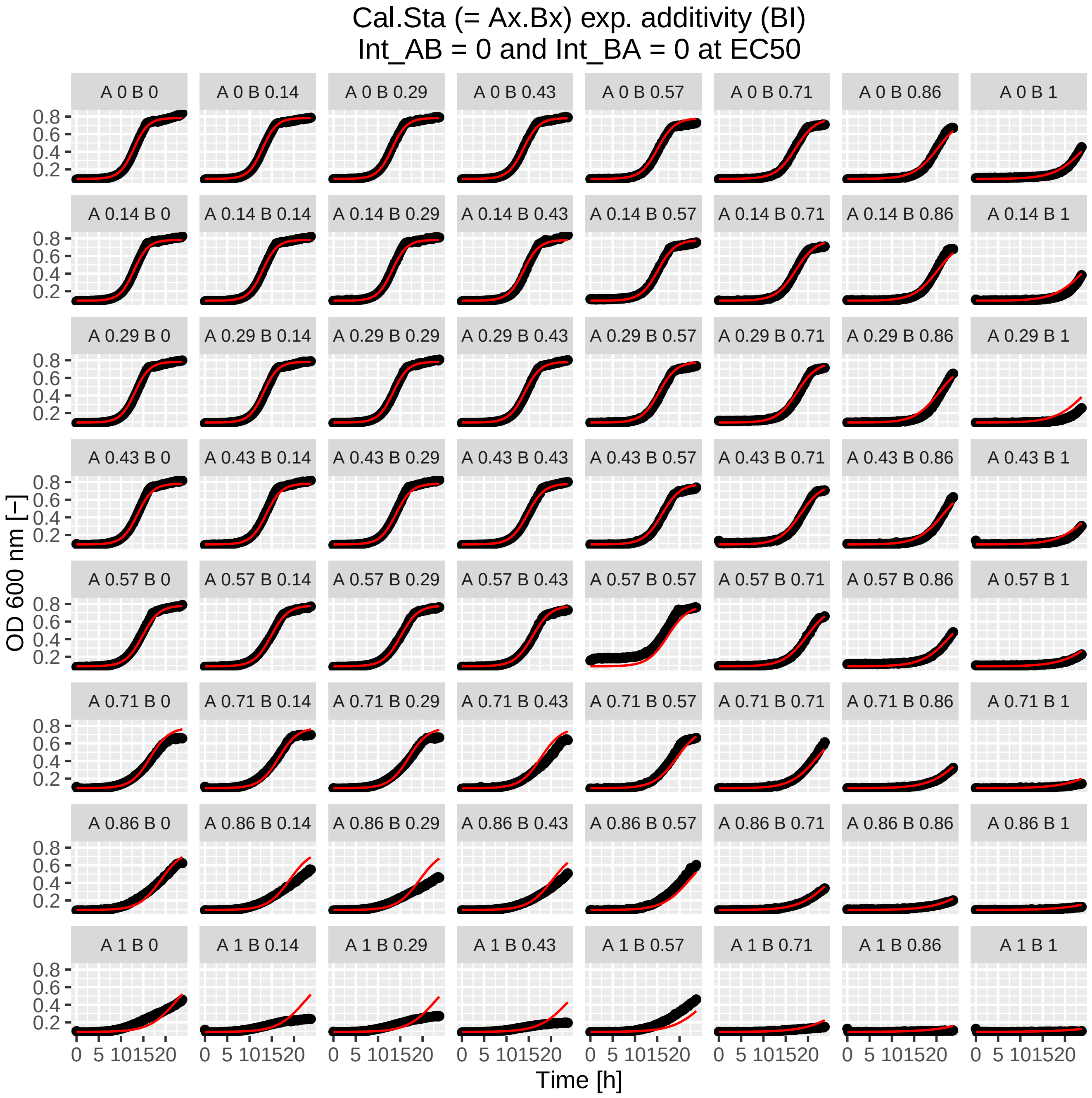
Cal.Sta (= Ax.Bx) exp. additivity (BI)
Int_AB = 0 and Int_BA = 0 at EC50
OD 600 nm [–]
Time [h]
A 0 B 0
A 0 B 0.14
A 0 B 0.29
A 0 B 0.43
A 0 B 0.57
A 0 B 0.71
A 0 B 0.86
A 0 B 1
A 0.14 B 0
A 0.14 B 0.14
A 0.14 B 0.29
A 0.14 B 0.43
A 0.14 B 0.57
A 0.14 B 0.71
A 0.14 B 0.86
A 0.14 B 1
A 0.29 B 0
A 0.29 B 0.14
A 0.29 B 0.29
A 0.29 B 0.43
A 0.29 B 0.57
A 0.29 B 0.71
A 0.29 B 0.86
A 0.29 B 1
A 0.43 B 0
A 0.43 B 0.14
A 0.43 B 0.29
A 0.43 B 0.43
A 0.43 B 0.57
A 0.43 B 0.71
A 0.43 B 0.86
A 0.43 B 1
A 0.57 B 0
A 0.57 B 0.14
A 0.57 B 0.29
A 0.57 B 0.43
A 0.57 B 0.57
A 0.57 B 0.71
A 0.57 B 0.86
A 0.57 B 1
A 0.71 B 0
A 0.71 B 0.14
A 0.71 B 0.29
A 0.71 B 0.43
A 0.71 B 0.57
A 0.71 B 0.71
A 0.71 B 0.86
A 0.71 B 1
A 0.86 B 0
A 0.86 B 0.14
A 0.86 B 0.29
A 0.86 B 0.43
A 0.86 B 0.57
A 0.86 B 0.71
A 0.86 B 0.86
A 0.86 B 1
A 1 B 0
A 1 B 0.14
A 1 B 0.29
A 1 B 0.43
A 1 B 0.57
A 1 B 0.71
A 1 B 0.86
A 1 B 1
0.8
0.6
0.4
0.2
0 5 101520

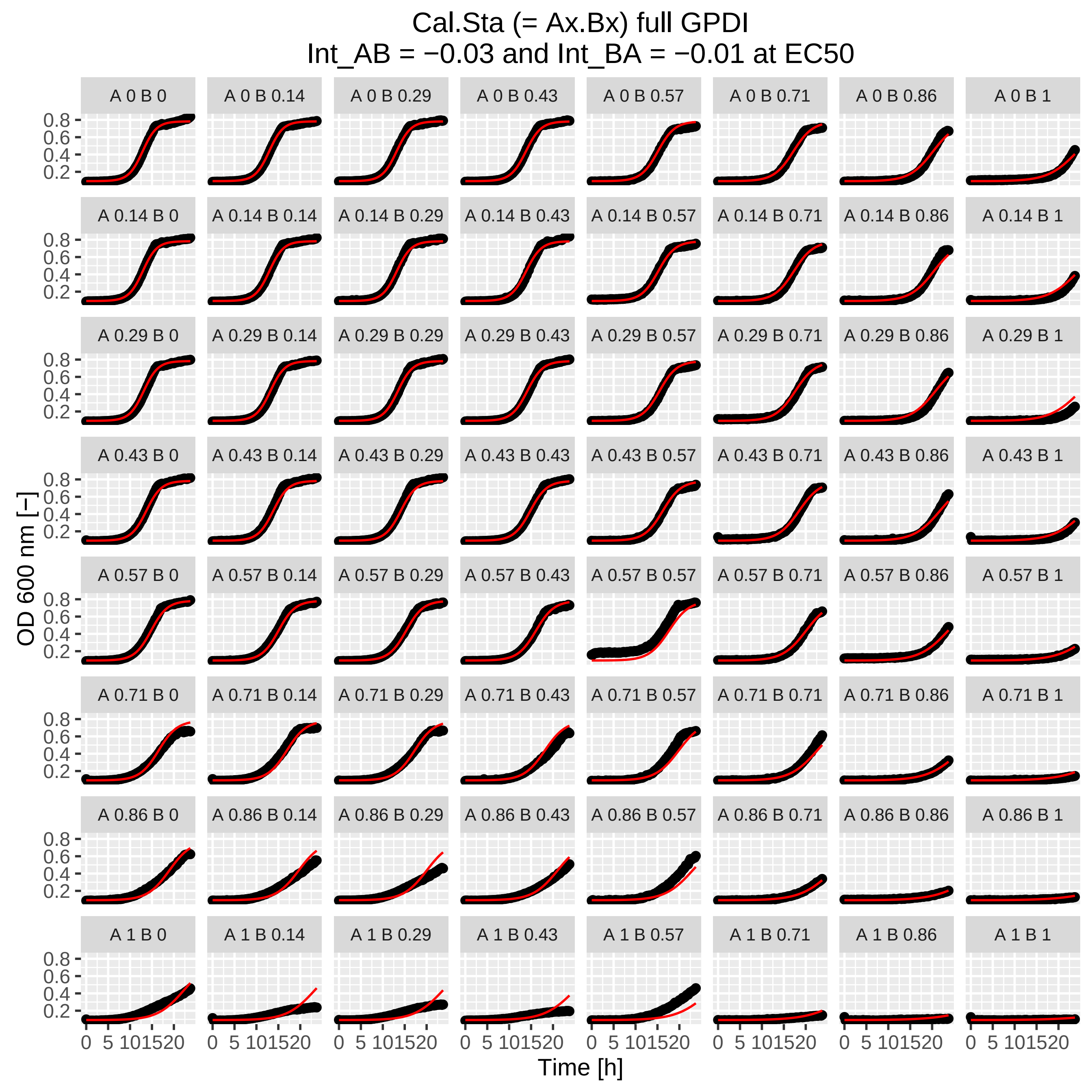
Cal.Sta (= Ax.Bx) full GPDI
Int_AB = −0.03 and Int_BA = −0.01 at EC50
A 0 B 0
A 0 B 0.14
A 0 B 0.29
A 0 B 0.43
A 0 B 0.57
A 0 B 0.71
A 0 B 0.86
A 0 B 1
A 0.14 B 0
A 0.14 B 0.14
A 0.14 B 0.29
A 0.14 B 0.43
A 0.14 B 0.57
A 0.14 B 0.71
A 0.14 B 0.86
A 0.14 B 1
A 0.29 B 0
A 0.29 B 0.14
A 0.29 B 0.29
A 0.29 B 0.43
A 0.29 B 0.57
A 0.29 B 0.71
A 0.29 B 0.86
A 0.29 B 1
A 0.43 B 0
A 0.43 B 0.14
A 0.43 B 0.29
A 0.43 B 0.43
A 0.43 B 0.57
A 0.43 B 0.71
A 0.43 B 0.86
A 0.43 B 1
A 0.57 B 0
A 0.57 B 0.14
A 0.57 B 0.29
A 0.57 B 0.43
A 0.57 B 0.57
A 0.57 B 0.71
A 0.57 B 0.86
A 0.57 B 1
A 0.71 B 0
A 0.71 B 0.14
A 0.71 B 0.29
A 0.71 B 0.43
A 0.71 B 0.57
A 0.71 B 0.71
A 0.71 B 0.86
A 0.71 B 1
A 0.86 B 0
A 0.86 B 0.14
A 0.86 B 0.29
A 0.86 B 0.43
A 0.86 B 0.57
A 0.86 B 0.71
A 0.86 B 0.86
A 0.86 B 1
A 1 B 0
A 1 B 0.14
A 1 B 0.29
A 1 B 0.43
A 1 B 0.57
A 1 B 0.71
A 1 B 0.86
A 1 B 1
OD 600 nm [−]
0.8
0.6
0.4
0.2
0 5 101520
Time [h]

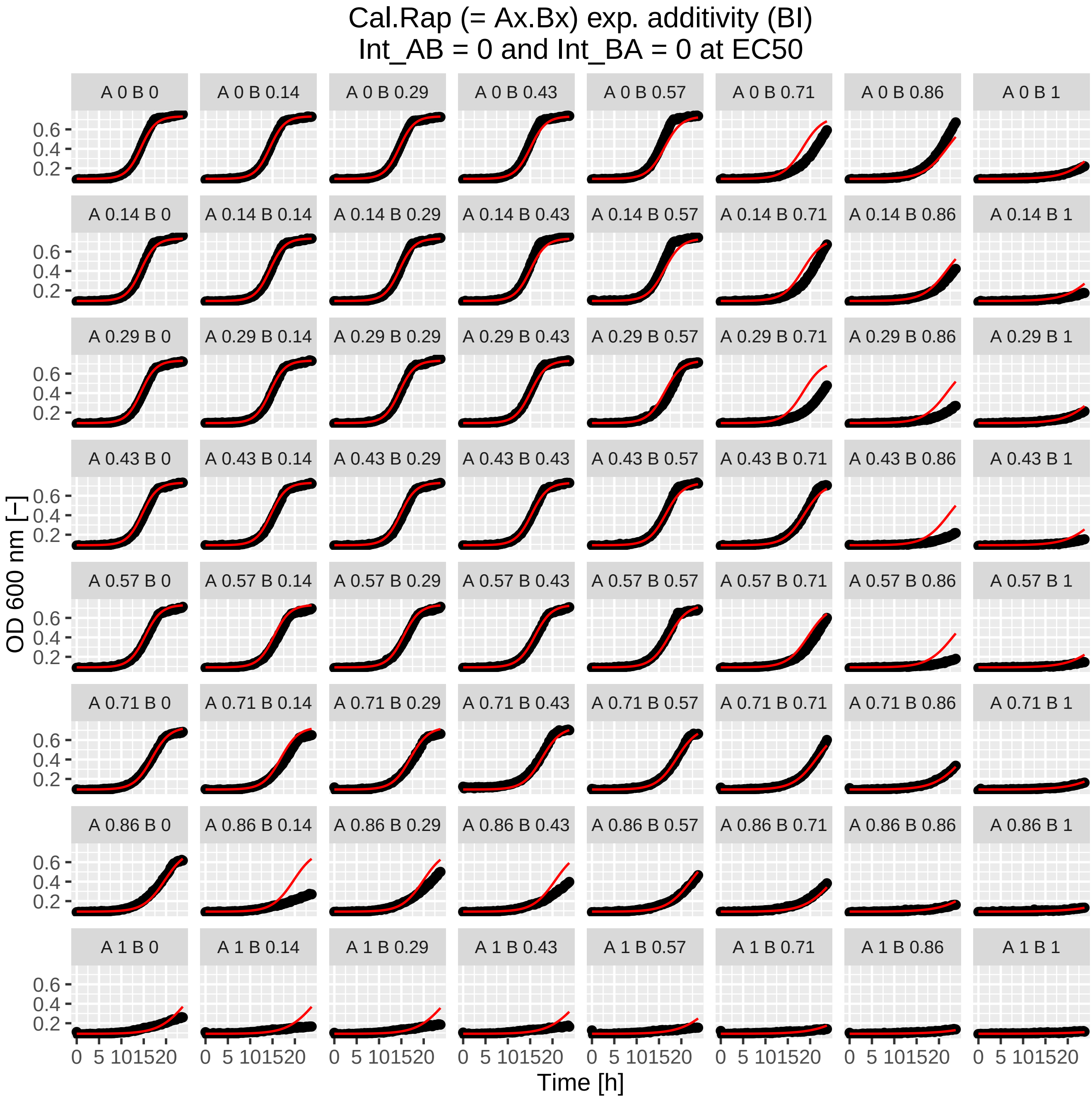

Cal.Rap (= Ax.Bx) exp. additivity (BI)
Int_AB = 0 and Int_BA = 0 at EC50
A 0 B 0
A 0 B 0.14
A 0 B 0.29
A 0 B 0.43
A 0 B 0.57
A 0 B 0.71
A 0 B 0.86
A 0 B 1
A 0.14 B 0
A 0.14 B 0.14
A 0.14 B 0.29
A 0.14 B 0.43
A 0.14 B 0.57
A 0.14 B 0.71
A 0.14 B 0.86
A 0.14 B 1
A 0.29 B 0
A 0.29 B 0.14
A 0.29 B 0.29
A 0.29 B 0.43
A 0.29 B 0.57
A 0.29 B 0.71
A 0.29 B 0.86
A 0.29 B 1
A 0.43 B 0
A 0.43 B 0.14
A 0.43 B 0.29
A 0.43 B 0.43
A 0.43 B 0.57
A 0.43 B 0.71
A 0.43 B 0.86
A 0.43 B 1
A 0.57 B 0
A 0.57 B 0.14
A 0.57 B 0.29
A 0.57 B 0.43
A 0.57 B 0.57
A 0.57 B 0.71
A 0.57 B 0.86
A 0.57 B 1
A 0.71 B 0
A 0.71 B 0.14
A 0.71 B 0.29
A 0.71 B 0.43
A 0.71 B 0.57
A 0.71 B 0.71
A 0.71 B 0.86
A 0.71 B 1
A 0.86 B 0
A 0.86 B 0.14
A 0.86 B 0.29
A 0.86 B 0.43
A 0.86 B 0.57
A 0.86 B 0.71
A 0.86 B 0.86
A 0.86 B 1
A 1 B 0
A 1 B 0.14
A 1 B 0.29
A 1 B 0.43
A 1 B 0.57
A 1 B 0.71
A 1 B 0.86
A 1 B 1
OD 600 nm [–]
0.2
0.4
0.6
0 5 101520
Time [h]

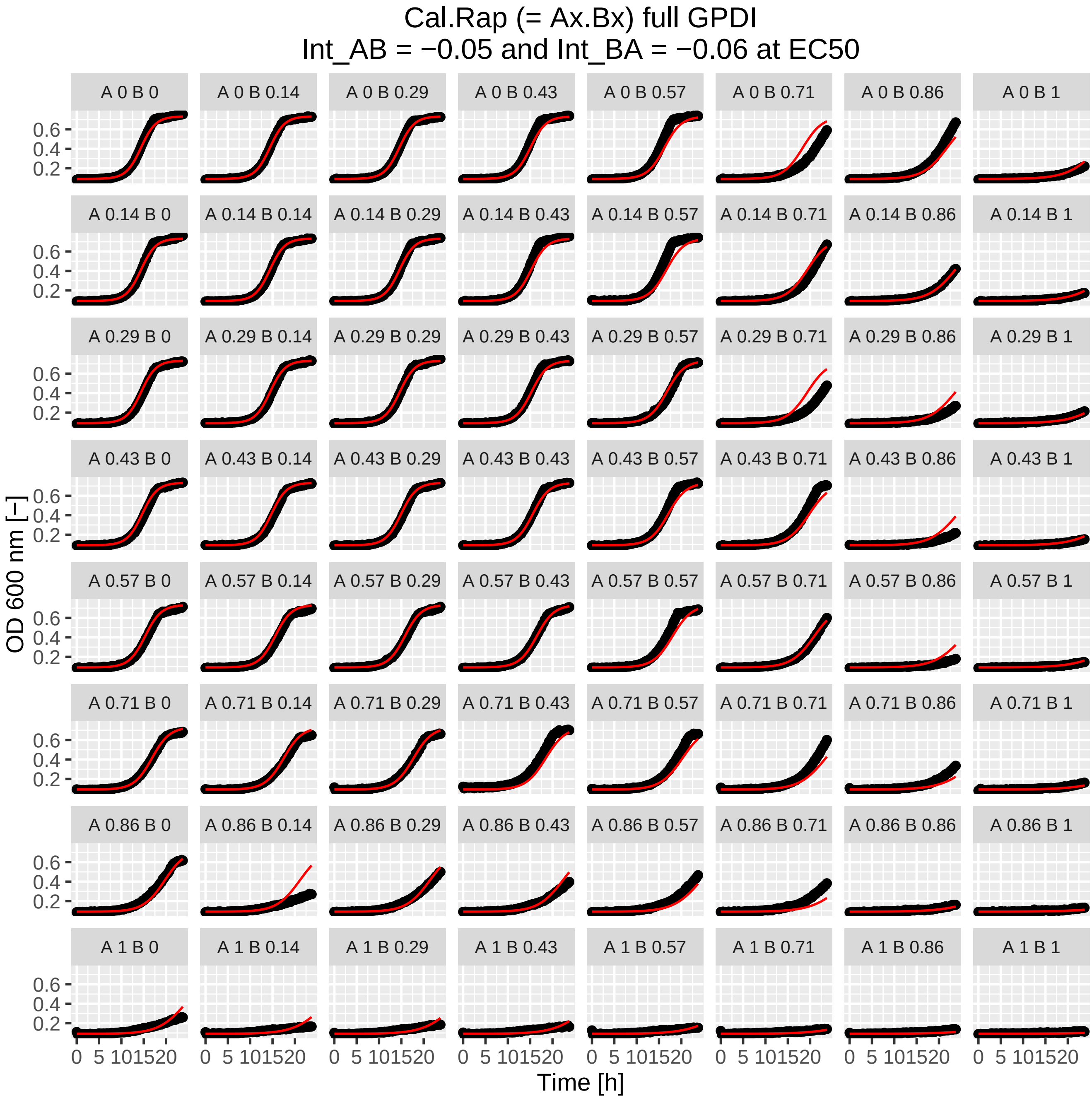

Cal.Rap (= Ax.Bx) full GPDI
Int_AB = −0.05 and Int_BA = −0.06 at EC50
OD 600 nm [−]
Time [h]
0.2
0.4
0.6
0 5 101520
A 0 B 0
A 0 B 0.14
A 0 B 0.29
A 0 B 0.43
A 0 B 0.57
A 0 B 0.71
A 0 B 0.86
A 0 B 1
A 0.14 B 0
A 0.14 B 0.14
A 0.14 B 0.29
A 0.14 B 0.43
A 0.14 B 0.57
A 0.14 B 0.71
A 0.14 B 0.86
A 0.14 B 1
A 0.29 B 0
A 0.29 B 0.14
A 0.29 B 0.29
A 0.29 B 0.43
A 0.29 B 0.57
A 0.29 B 0.71
A 0.29 B 0.86
A 0.29 B 1
A 0.43 B 0
A 0.43 B 0.14
A 0.43 B 0.29
A 0.43 B 0.43
A 0.43 B 0.57
A 0.43 B 0.71
A 0.43 B 0.86
A 0.43 B 1
A 0.57 B 0
A 0.57 B 0.14
A 0.57 B 0.29
A 0.57 B 0.43
A 0.57 B 0.57
A 0.57 B 0.71
A 0.57 B 0.86
A 0.57 B 1
A 0.71 B 0
A 0.71 B 0.14
A 0.71 B 0.29
A 0.71 B 0.43
A 0.71 B 0.57
A 0.71 B 0.71
A 0.71 B 0.86
A 0.71 B 1
A 0.86 B 0
A 0.86 B 0.14
A 0.86 B 0.29
A 0.86 B 0.43
A 0.86 B 0.57
A 0.86 B 0.71
A 0.86 B 0.86
A 0.86 B 1
A 1 B 0
A 1 B 0.14
A 1 B 0.29
A 1 B 0.43
A 1 B 0.57
A 1 B 0.71
A 1 B 0.86
A 1 B 1

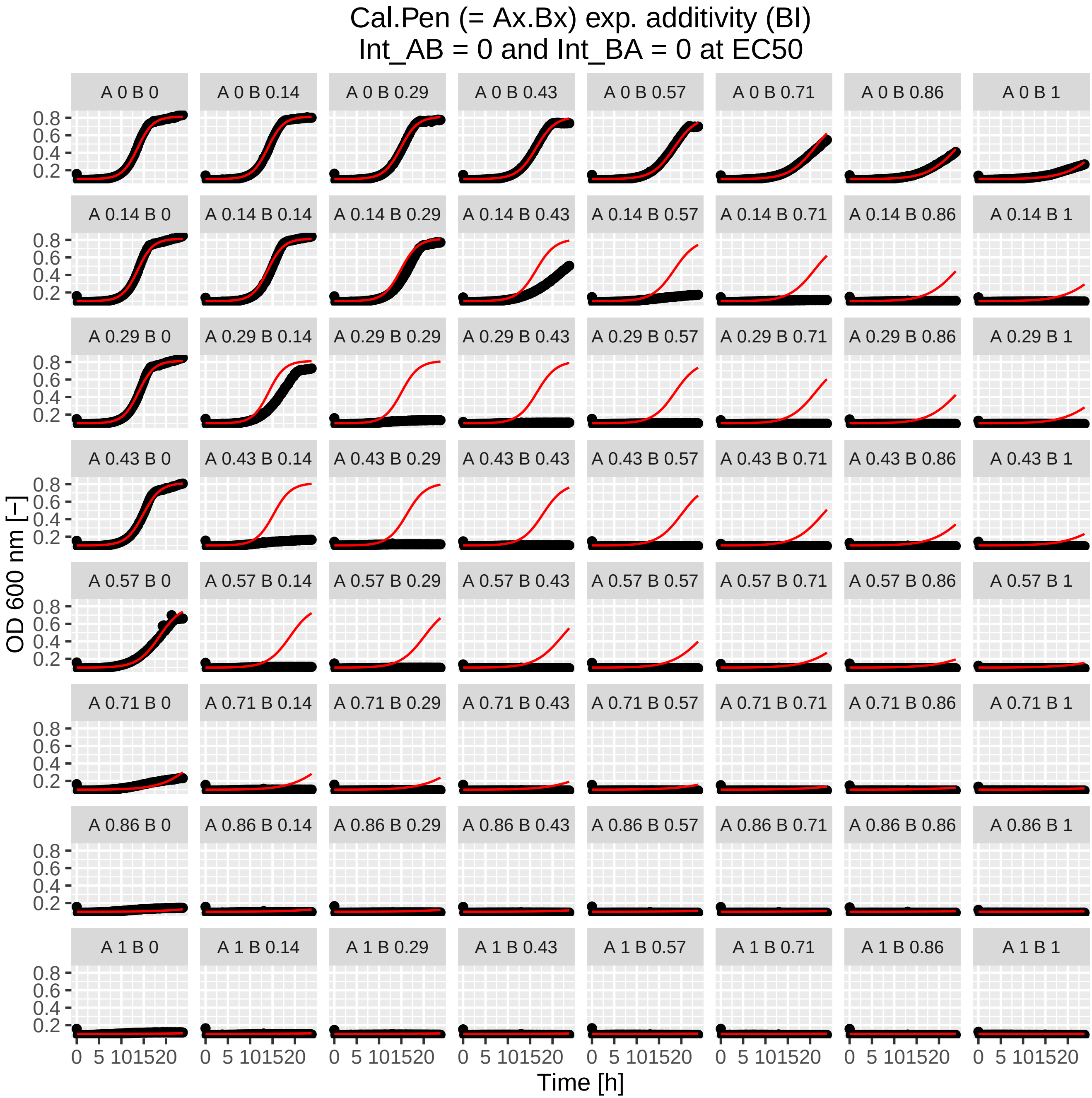

Cal.Pen (= Ax.Bx) exp. additivity (BI)
Int_AB = 0 and Int_BA = 0 at EC50
A 0 B 0
A 0 B 0.14
A 0 B 0.29
A 0 B 0.43
A 0 B 0.57
A 0 B 0.71
A 0 B 0.86
A 0 B 1
A 0.14 B 0
A 0.14 B 0.14
A 0.14 B 0.29
A 0.14 B 0.43
A 0.14 B 0.57
A 0.14 B 0.71
A 0.14 B 0.86
A 0.14 B 1
A 0.29 B 0
A 0.29 B 0.14
A 0.29 B 0.29
A 0.29 B 0.43
A 0.29 B 0.57
A 0.29 B 0.71
A 0.29 B 0.86
A 0.29 B 1
A 0.43 B 0
A 0.43 B 0.14
A 0.43 B 0.29
A 0.43 B 0.43
A 0.43 B 0.57
A 0.43 B 0.71
A 0.43 B 0.86
A 0.43 B 1
A 0.57 B 0
A 0.57 B 0.14
A 0.57 B 0.29
A 0.57 B 0.43
A 0.57 B 0.57
A 0.57 B 0.71
A 0.57 B 0.86
A 0.57 B 1
A 0.71 B 0
A 0.71 B 0.14
A 0.71 B 0.29
A 0.71 B 0.43
A 0.71 B 0.57
A 0.71 B 0.71
A 0.71 B 0.86
A 0.71 B 1
A 0.86 B 0
A 0.86 B 0.14
A 0.86 B 0.29
A 0.86 B 0.43
A 0.86 B 0.57
A 0.86 B 0.71
A 0.86 B 0.86
A 0.86 B 1
A 1 B 0
A 1 B 0.14
A 1 B 0.29
A 1 B 0.43
A 1 B 0.57
A 1 B 0.71
A 1 B 0.86
A 1 B 1
OD 600 nm [−]
0.2
0.4
0.6
0.8
0
5
10
15
20
Time [h]

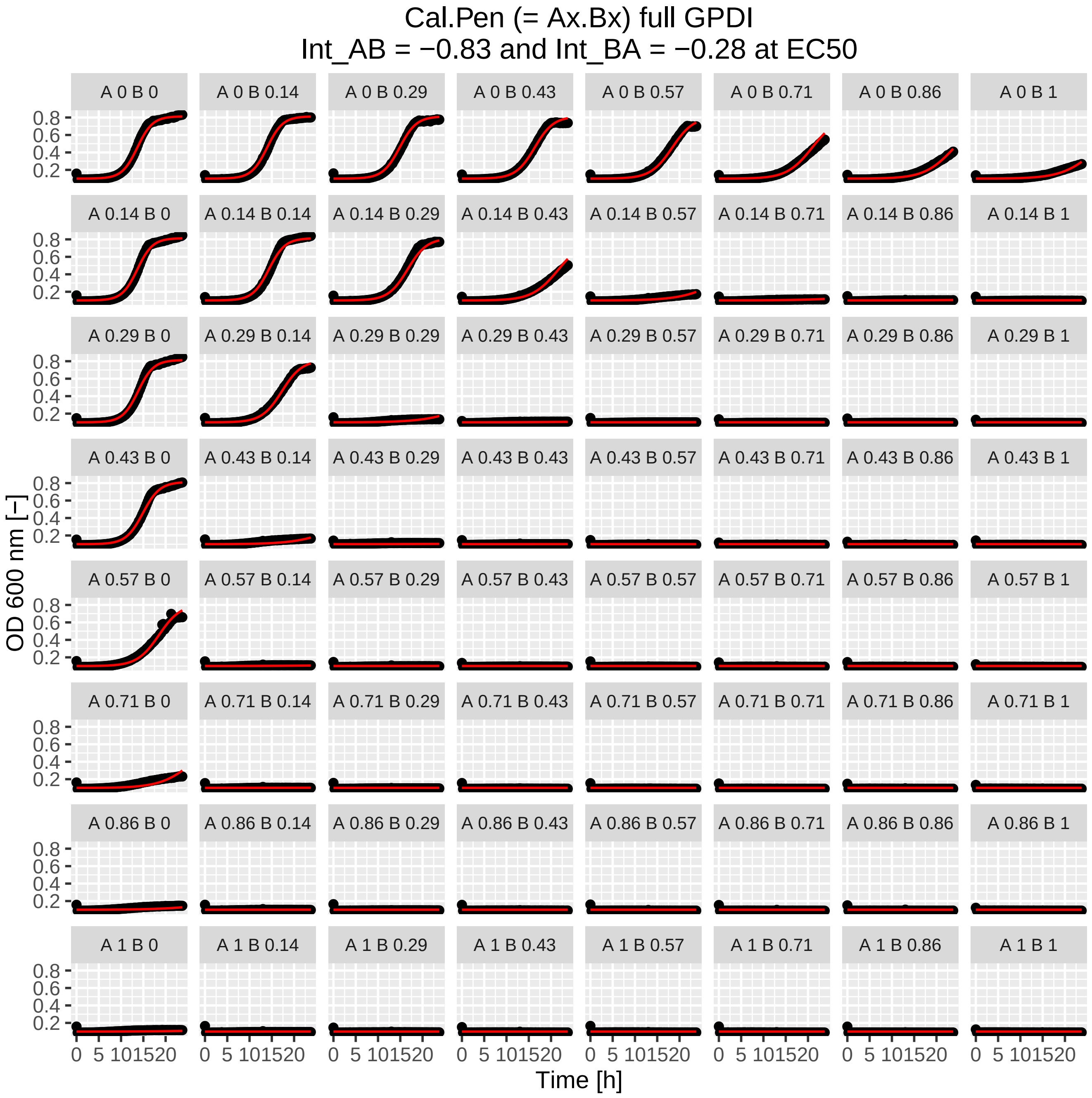

Cal.Pen (= Ax.Bx) full GPDI
Int_AB = −0.83 and Int_BA = −0.28 at EC50
A 0 B 0
A 0 B 0.14
A 0 B 0.29
A 0 B 0.43
A 0 B 0.57
A 0 B 0.71
A 0 B 0.86
A 0 B 1
A 0.14 B 0
A 0.14 B 0.14
A 0.14 B 0.29
A 0.14 B 0.43
A 0.14 B 0.57
A 0.14 B 0.71
A 0.14 B 0.86
A 0.14 B 1
A 0.29 B 0
A 0.29 B 0.14
A 0.29 B 0.29
A 0.29 B 0.43
A 0.29 B 0.57
A 0.29 B 0.71
A 0.29 B 0.86
A 0.29 B 1
A 0.43 B 0
A 0.43 B 0.14
A 0.43 B 0.29
A 0.43 B 0.43
A 0.43 B 0.57
A 0.43 B 0.71
A 0.43 B 0.86
A 0.43 B 1
A 0.57 B 0
A 0.57 B 0.14
A 0.57 B 0.29
A 0.57 B 0.43
A 0.57 B 0.57
A 0.57 B 0.71
A 0.57 B 0.86
A 0.57 B 1
A 0.71 B 0
A 0.71 B 0.14
A 0.71 B 0.29
A 0.71 B 0.43
A 0.71 B 0.57
A 0.71 B 0.71
A 0.71 B 0.86
A 0.71 B 1
A 0.86 B 0
A 0.86 B 0.14
A 0.86 B 0.29
A 0.86 B 0.43
A 0.86 B 0.57
A 0.86 B 0.71
A 0.86 B 0.86
A 0.86 B 1
A 1 B 0
A 1 B 0.14
A 1 B 0.29
A 1 B 0.43
A 1 B 0.57
A 1 B 0.71
A 1 B 0.86
A 1 B 1
OD 600 nm [−]
0.2
0.4
0.6
0.8
0 5 101520
Time [h]

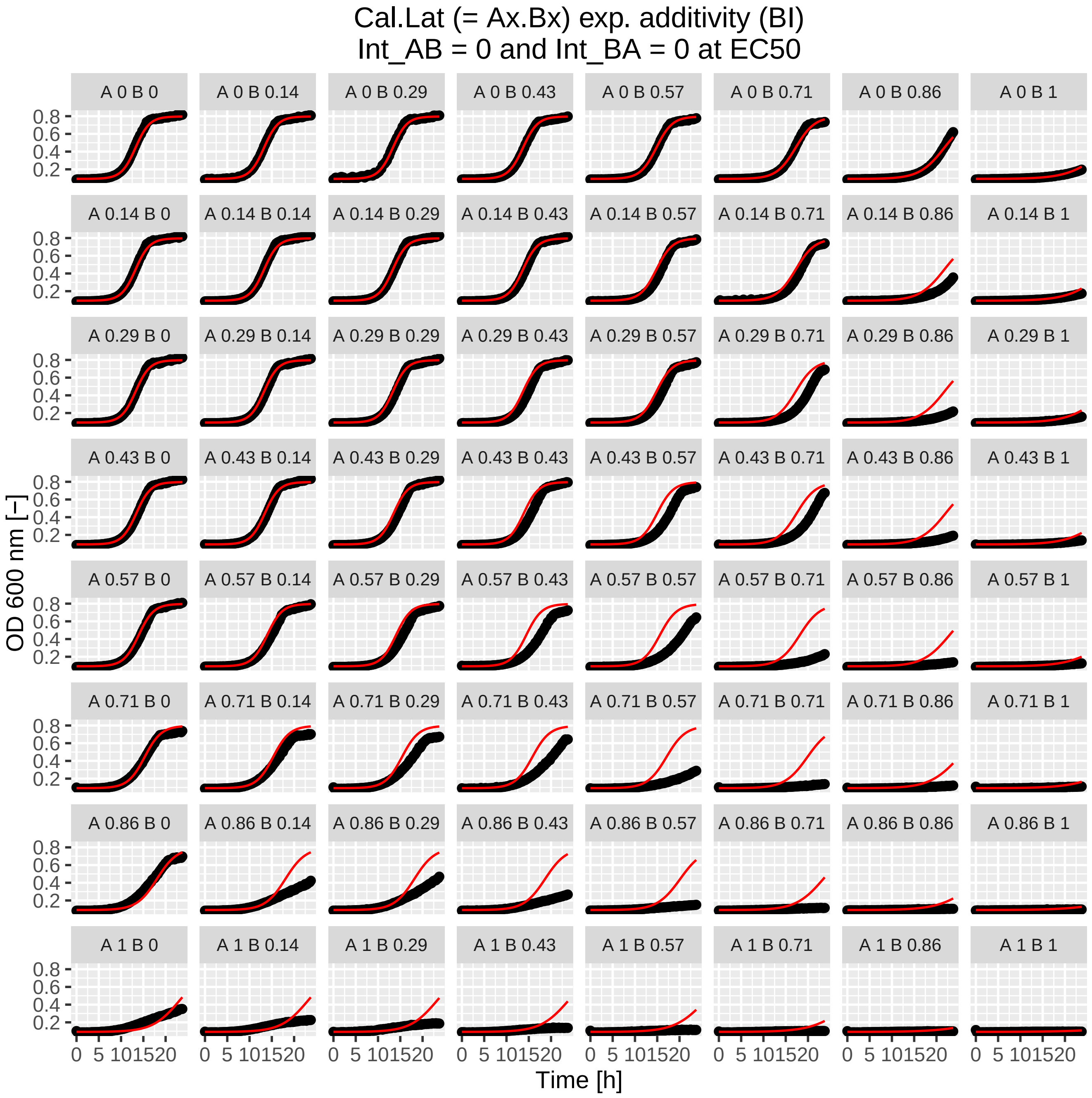

Cal.Lat (= Ax.Bx) exp. additivity (BI)
Int_AB = 0 and Int_BA = 0 at EC50
A 0 B 0
A 0 B 0.14
A 0 B 0.29
A 0 B 0.43
A 0 B 0.57
A 0 B 0.71
A 0 B 0.86
A 0 B 1
A 0.14 B 0
A 0.14 B 0.14
A 0.14 B 0.29
A 0.14 B 0.43
A 0.14 B 0.57
A 0.14 B 0.71
A 0.14 B 0.86
A 0.14 B 1
A 0.29 B 0
A 0.29 B 0.14
A 0.29 B 0.29
A 0.29 B 0.43
A 0.29 B 0.57
A 0.29 B 0.71
A 0.29 B 0.86
A 0.29 B 1
A 0.43 B 0
A 0.43 B 0.14
A 0.43 B 0.29
A 0.43 B 0.43
A 0.43 B 0.57
A 0.43 B 0.71
A 0.43 B 0.86
A 0.43 B 1
A 0.57 B 0
A 0.57 B 0.14
A 0.57 B 0.29
A 0.57 B 0.43
A 0.57 B 0.57
A 0.57 B 0.71
A 0.57 B 0.86
A 0.57 B 1
A 0.71 B 0
A 0.71 B 0.14
A 0.71 B 0.29
A 0.71 B 0.43
A 0.71 B 0.57
A 0.71 B 0.71
A 0.71 B 0.86
A 0.71 B 1
A 0.86 B 0
A 0.86 B 0.14
A 0.86 B 0.29
A 0.86 B 0.43
A 0.86 B 0.57
A 0.86 B 0.71
A 0.86 B 0.86
A 0.86 B 1
A 1 B 0
A 1 B 0.14
A 1 B 0.29
A 1 B 0.43
A 1 B 0.57
A 1 B 0.71
A 1 B 0.86
A 1 B 1
OD 600 nm [−]
0.8
0.6
0.4
0.2
0 5 101520
Time [h]

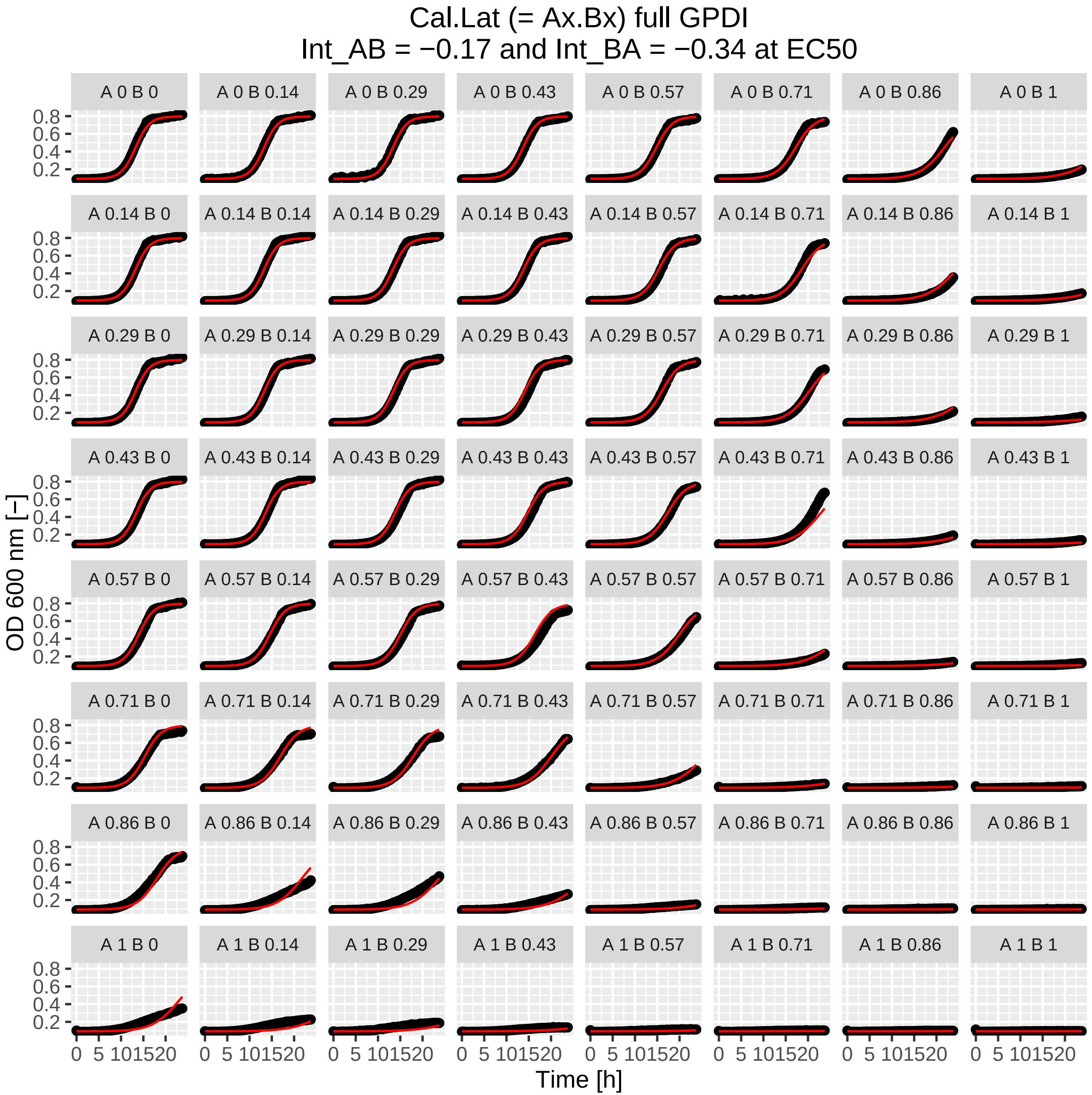

Cal.Lat (= Ax.Bx) full GPDI
Int_AB = −0.17 and Int_BA = −0.34 at EC50
A 0 B 0
A 0 B 0.14
A 0 B 0.29
A 0 B 0.43
A 0 B 0.57
A 0 B 0.71
A 0 B 0.86
A 0 B 1
A 0.14 B 0
A 0.14 B 0.14
A 0.14 B 0.29
A 0.14 B 0.43
A 0.14 B 0.57
A 0.14 B 0.71
A 0.14 B 0.86
A 0.14 B 1
A 0.29 B 0
A 0.29 B 0.14
A 0.29 B 0.29
A 0.29 B 0.43
A 0.29 B 0.57
A 0.29 B 0.71
A 0.29 B 0.86
A 0.29 B 1
A 0.43 B 0
A 0.43 B 0.14
A 0.43 B 0.29
A 0.43 B 0.43
A 0.43 B 0.57
A 0.43 B 0.71
A 0.43 B 0.86
A 0.43 B 1
A 0.57 B 0
A 0.57 B 0.14
A 0.57 B 0.29
A 0.57 B 0.43
A 0.57 B 0.57
A 0.57 B 0.71
A 0.57 B 0.86
A 0.57 B 1
A 0.71 B 0
A 0.71 B 0.14
A 0.71 B 0.29
A 0.71 B 0.43
A 0.71 B 0.57
A 0.71 B 0.71
A 0.71 B 0.86
A 0.71 B 1
A 0.86 B 0
A 0.86 B 0.14
A 0.86 B 0.29
A 0.86 B 0.43
A 0.86 B 0.57
A 0.86 B 0.71
A 0.86 B 0.86
A 0.86 B 1
A 1 B 0
A 1 B 0.14
A 1 B 0.29
A 1 B 0.43
A 1 B 0.57
A 1 B 0.71
A 1 B 0.86
A 1 B 1
0.8
0.6
0.4
0.2
0 5 101520
OD 600 nm [−]
Time [h]

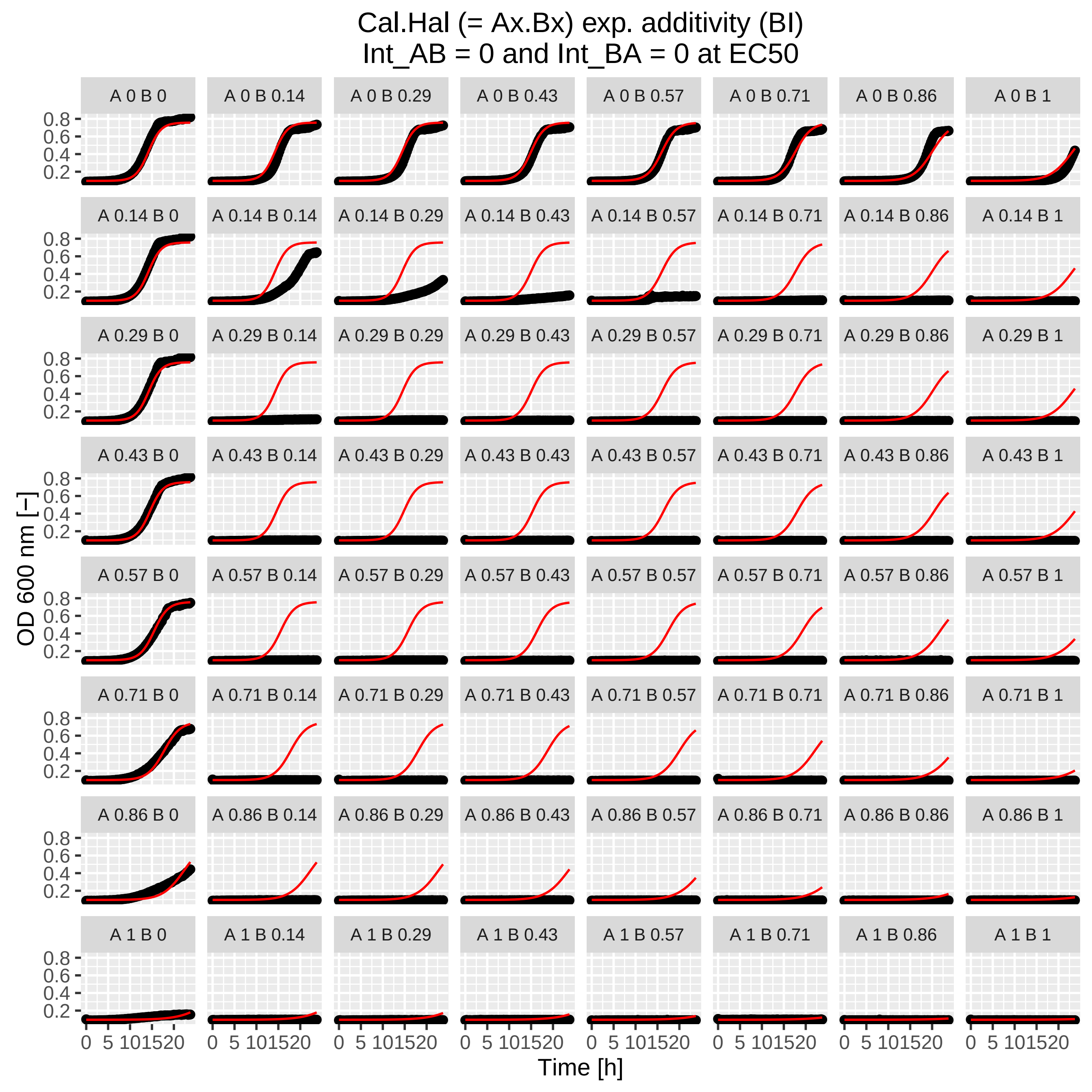

Cal.Hal (= Ax.Bx) exp. additivity (BI)
Int_AB = 0 and Int_BA = 0 at EC50
A 0 B 0
A 0 B 0.14
A 0 B 0.29
A 0 B 0.43
A 0 B 0.57
A 0 B 0.71
A 0 B 0.86
A 0 B 1
A 0.14 B 0
A 0.14 B 0.14
A 0.14 B 0.29
A 0.14 B 0.43
A 0.14 B 0.57
A 0.14 B 0.71
A 0.14 B 0.86
A 0.14 B 1
A 0.29 B 0
A 0.29 B 0.14
A 0.29 B 0.29
A 0.29 B 0.43
A 0.29 B 0.57
A 0.29 B 0.71
A 0.29 B 0.86
A 0.29 B 1
A 0.43 B 0
A 0.43 B 0.14
A 0.43 B 0.29
A 0.43 B 0.43
A 0.43 B 0.57
A 0.43 B 0.71
A 0.43 B 0.86
A 0.43 B 1
A 0.57 B 0
A 0.57 B 0.14
A 0.57 B 0.29
A 0.57 B 0.43
A 0.57 B 0.57
A 0.57 B 0.71
A 0.57 B 0.86
A 0.57 B 1
A 0.71 B 0
A 0.71 B 0.14
A 0.71 B 0.29
A 0.71 B 0.43
A 0.71 B 0.57
A 0.71 B 0.71
A 0.71 B 0.86
A 0.71 B 1
A 0.86 B 0
A 0.86 B 0.14
A 0.86 B 0.29
A 0.86 B 0.43
A 0.86 B 0.57
A 0.86 B 0.71
A 0.86 B 0.86
A 0.86 B 1
A 1 B 0
A 1 B 0.14
A 1 B 0.29
A 1 B 0.43
A 1 B 0.57
A 1 B 0.71
A 1 B 0.86
A 1 B 1
0.8
0.6
0.4
0.2
0 5 101520
OD 600 nm [–]
Time [h]

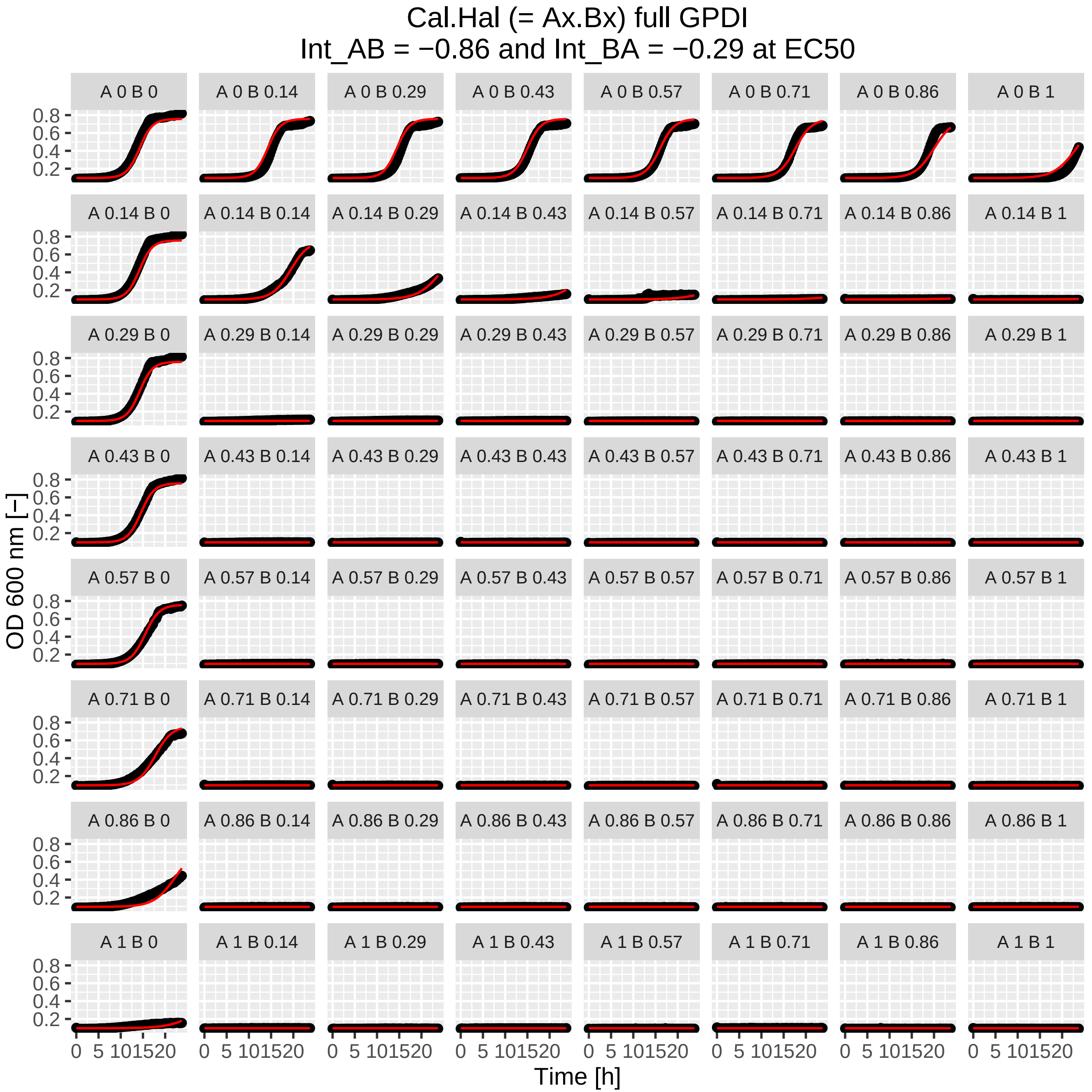
Cal.Hal (= Ax.Bx) full GPDI
Int_AB = −0.86 and Int_BA = −0.29 at EC50
A 0 B 0
A 0 B 0.14
A 0 B 0.29
A 0 B 0.43
A 0 B 0.57
A 0 B 0.71
A 0 B 0.86
A 0 B 1
A 0.14 B 0
A 0.14 B 0.14
A 0.14 B 0.29
A 0.14 B 0.43
A 0.14 B 0.57
A 0.14 B 0.71
A 0.14 B 0.86
A 0.14 B 1
A 0.29 B 0
A 0.29 B 0.14
A 0.29 B 0.29
A 0.29 B 0.43
A 0.29 B 0.57
A 0.29 B 0.71
A 0.29 B 0.86
A 0.29 B 1
A 0.43 B 0
A 0.43 B 0.14
A 0.43 B 0.29
A 0.43 B 0.43
A 0.43 B 0.57
A 0.43 B 0.71
A 0.43 B 0.86
A 0.43 B 1
A 0.57 B 0
A 0.57 B 0.14
A 0.57 B 0.29
A 0.57 B 0.43
A 0.57 B 0.57
A 0.57 B 0.71
A 0.57 B 0.86
A 0.57 B 1
A 0.71 B 0
A 0.71 B 0.14
A 0.71 B 0.29
A 0.71 B 0.43
A 0.71 B 0.57
A 0.71 B 0.71
A 0.71 B 0.86
A 0.71 B 1
A 0.86 B 0
A 0.86 B 0.14
A 0.86 B 0.29
A 0.86 B 0.43
A 0.86 B 0.57
A 0.86 B 0.71
A 0.86 B 0.86
A 0.86 B 1
A 1 B 0
A 1 B 0.14
A 1 B 0.29
A 1 B 0.43
A 1 B 0.57
A 1 B 0.71
A 1 B 0.86
A 1 B 1
OD 600 nm [−]
0.8
0.6
0.4
0.2
0 5 101520
Time [h]

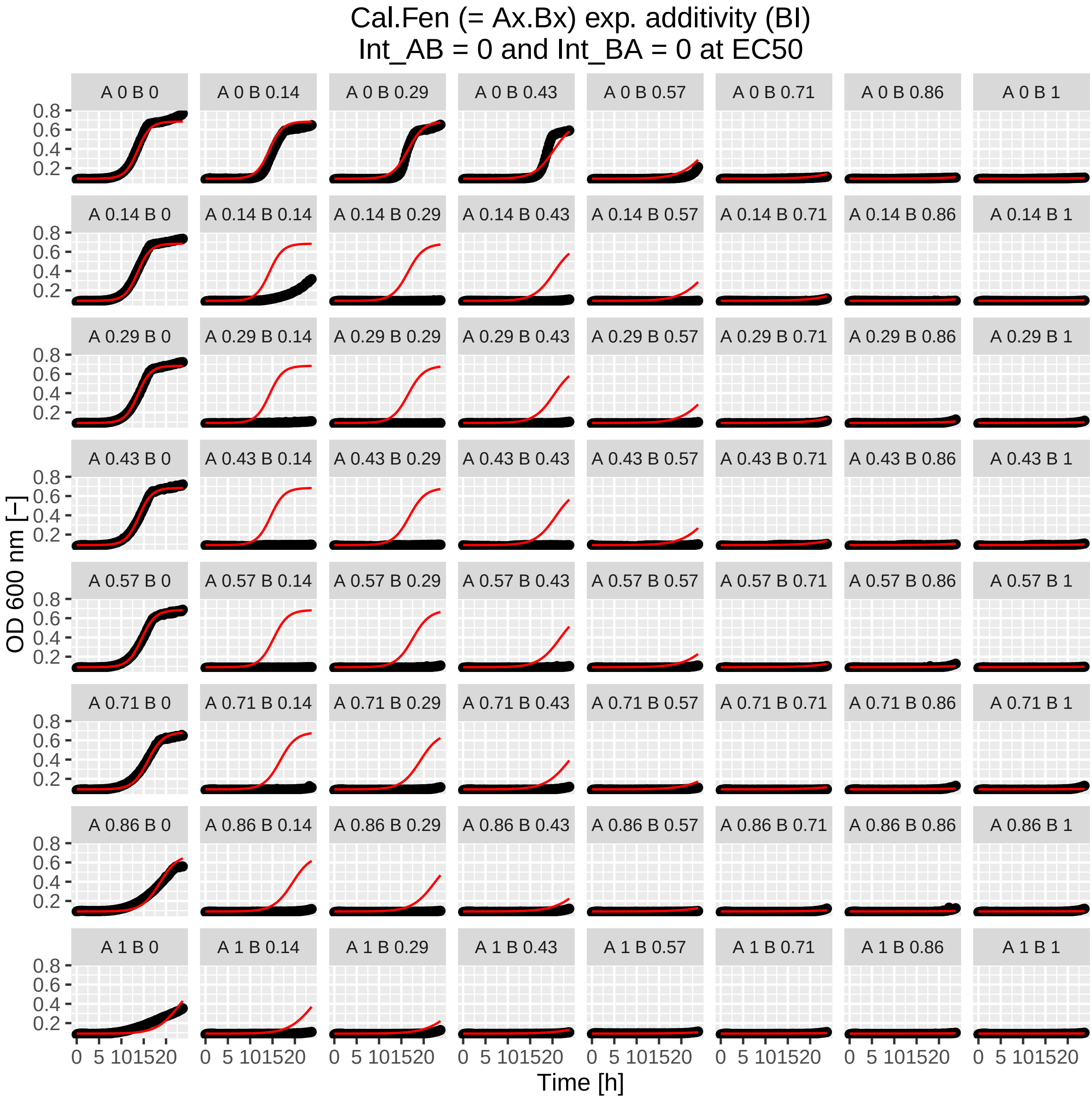

Cal.Fen (= Ax.Bx) exp. additivity (BI)
Int_AB = 0 and Int_BA = 0 at EC50
A 0 B 0
A 0 B 0.14
A 0 B 0.29
A 0 B 0.43
A 0 B 0.57
A 0 B 0.71
A 0 B 0.86
A 0 B 1
A 0.14 B 0
A 0.14 B 0.14
A 0.14 B 0.29
A 0.14 B 0.43
A 0.14 B 0.57
A 0.14 B 0.71
A 0.14 B 0.86
A 0.14 B 1
A 0.29 B 0
A 0.29 B 0.14
A 0.29 B 0.29
A 0.29 B 0.43
A 0.29 B 0.57
A 0.29 B 0.71
A 0.29 B 0.86
A 0.29 B 1
A 0.43 B 0
A 0.43 B 0.14
A 0.43 B 0.29
A 0.43 B 0.43
A 0.43 B 0.57
A 0.43 B 0.71
A 0.43 B 0.86
A 0.43 B 1
A 0.57 B 0
A 0.57 B 0.14
A 0.57 B 0.29
A 0.57 B 0.43
A 0.57 B 0.57
A 0.57 B 0.71
A 0.57 B 0.86
A 0.57 B 1
A 0.71 B 0
A 0.71 B 0.14
A 0.71 B 0.29
A 0.71 B 0.43
A 0.71 B 0.57
A 0.71 B 0.71
A 0.71 B 0.86
A 0.71 B 1
A 0.86 B 0
A 0.86 B 0.14
A 0.86 B 0.29
A 0.86 B 0.43
A 0.86 B 0.57
A 0.86 B 0.71
A 0.86 B 0.86
A 0.86 B 1
A 1 B 0
A 1 B 0.14
A 1 B 0.29
A 1 B 0.43
A 1 B 0.57
A 1 B 0.71
A 1 B 0.86
A 1 B 1
0.8
0.6
0.4
0.2
OD 600 nm [–]
0 5 101520
Time [h]

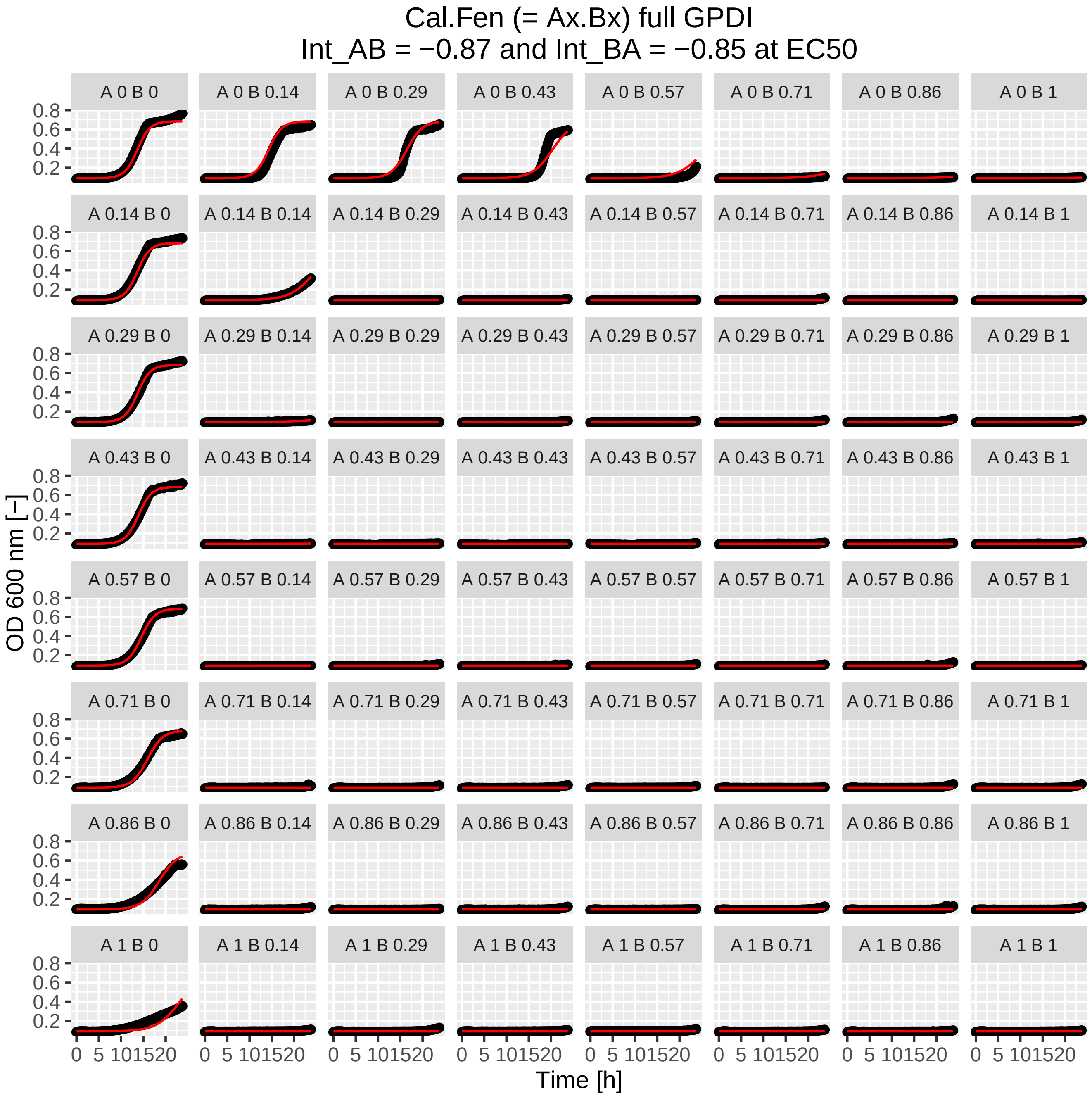
Cal.Fen (= Ax.Bx) full GPDI
Int_AB = −0.87 and Int_BA = −0.85 at EC50
OD 600 nm [−]
Time [h]
A 0 B 0
A 0 B 0.14
A 0 B 0.29
A 0 B 0.43
A 0 B 0.57
A 0 B 0.71
A 0 B 0.86
A 0 B 1
A 0.14 B 0
A 0.29 B 0
A 0.43 B 0
A 0.57 B 0
A 0.71 B 0
A 0.86 B 0
A 1 B 0
A 1 B 1

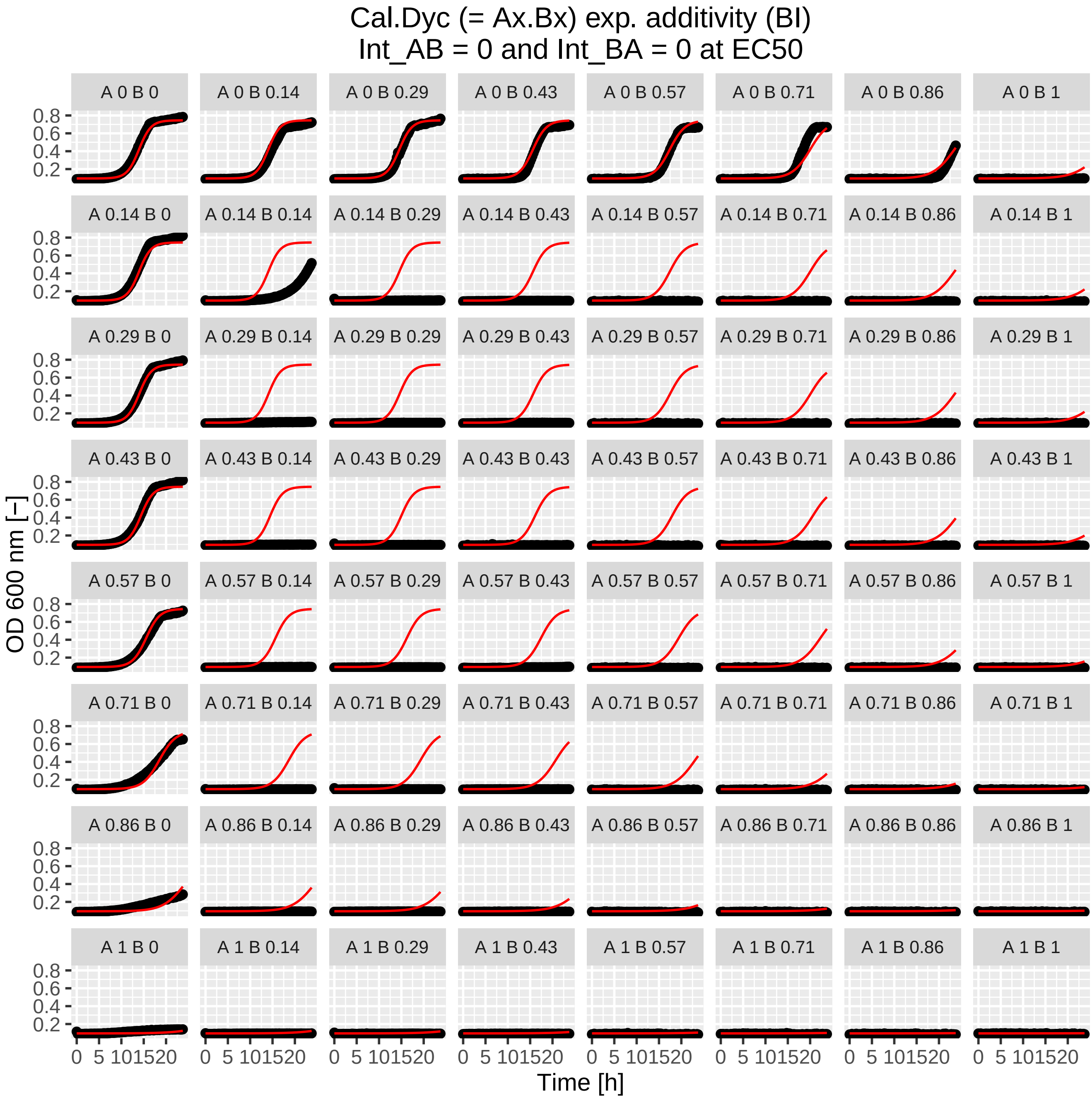

Cal.Dyc (= Ax.Bx) exp. additivity (BI)
Int_AB = 0 and Int_BA = 0 at EC50
A 0 B 0
A 0 B 0.14
A 0 B 0.29
A 0 B 0.43
A 0 B 0.57
A 0 B 0.71
A 0 B 0.86
A 0 B 1
A 0.14 B 0
A 0.14 B 0.14
A 0.14 B 0.29
A 0.14 B 0.43
A 0.14 B 0.57
A 0.14 B 0.71
A 0.14 B 0.86
A 0.14 B 1
A 0.29 B 0
A 0.29 B 0.14
A 0.29 B 0.29
A 0.29 B 0.43
A 0.29 B 0.57
A 0.29 B 0.71
A 0.29 B 0.86
A 0.29 B 1
A 0.43 B 0
A 0.43 B 0.14
A 0.43 B 0.29
A 0.43 B 0.43
A 0.43 B 0.57
A 0.43 B 0.71
A 0.43 B 0.86
A 0.43 B 1
A 0.57 B 0
A 0.57 B 0.14
A 0.57 B 0.29
A 0.57 B 0.43
A 0.57 B 0.57
A 0.57 B 0.71
A 0.57 B 0.86
A 0.57 B 1
A 0.71 B 0
A 0.71 B 0.14
A 0.71 B 0.29
A 0.71 B 0.43
A 0.71 B 0.57
A 0.71 B 0.71
A 0.71 B 0.86
A 0.71 B 1
A 0.86 B 0
A 0.86 B 0.14
A 0.86 B 0.29
A 0.86 B 0.43
A 0.86 B 0.57
A 0.86 B 0.71
A 0.86 B 0.86
A 0.86 B 1
A 1 B 0
A 1 B 0.14
A 1 B 0.29
A 1 B 0.43
A 1 B 0.57
A 1 B 0.71
A 1 B 0.86
A 1 B 1
OD 600 nm [–]
0.8
0.6
0.4
0.2
0 5 101520
Time [h]

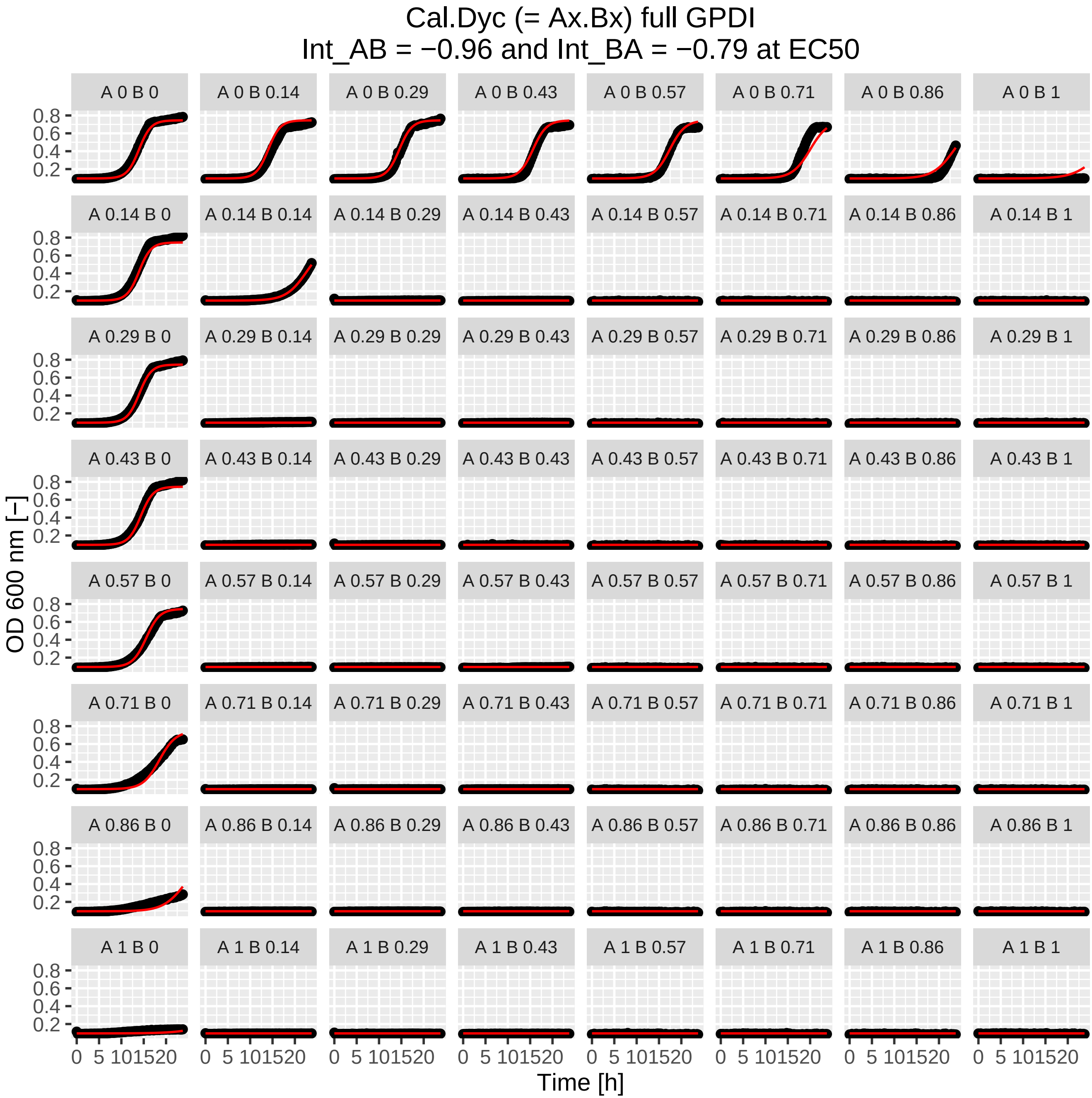
Cal.Dyc (= Ax.Bx) full GPDI
Int_AB = −0.96 and Int_BA = −0.79 at EC50
A 0 B 0
A 0 B 0.14
A 0 B 0.29
A 0 B 0.43
A 0 B 0.57
A 0 B 0.71
A 0 B 0.86
A 0 B 1
A 0.14 B 0
A 0.14 B 0.14
A 0.14 B 0.29
A 0.14 B 0.43
A 0.14 B 0.57
A 0.14 B 0.71
A 0.14 B 0.86
A 0.14 B 1
A 0.29 B 0
A 0.29 B 0.14
A 0.29 B 0.29
A 0.29 B 0.43
A 0.29 B 0.57
A 0.29 B 0.71
A 0.29 B 0.86
A 0.29 B 1
A 0.43 B 0
A 0.43 B 0.14
A 0.43 B 0.29
A 0.43 B 0.43
A 0.43 B 0.57
A 0.43 B 0.71
A 0.43 B 0.86
A 0.43 B 1
A 0.57 B 0
A 0.57 B 0.14
A 0.57 B 0.29
A 0.57 B 0.43
A 0.57 B 0.57
A 0.57 B 0.71
A 0.57 B 0.86
A 0.57 B 1
A 0.71 B 0
A 0.71 B 0.14
A 0.71 B 0.29
A 0.71 B 0.43
A 0.71 B 0.57
A 0.71 B 0.71
A 0.71 B 0.86
A 0.71 B 1
A 0.86 B 0
A 0.86 B 0.14
A 0.86 B 0.29
A 0.86 B 0.43
A 0.86 B 0.57
A 0.86 B 0.71
A 0.86 B 0.86
A 0.86 B 1
A 1 B 0
A 1 B 0.14
A 1 B 0.29
A 1 B 0.43
A 1 B 0.57
A 1 B 0.71
A 1 B 0.86
A 1 B 1
0.8
0.6
0.4
0.2
0 5 101520
OD 600 nm [−]
Time [h]

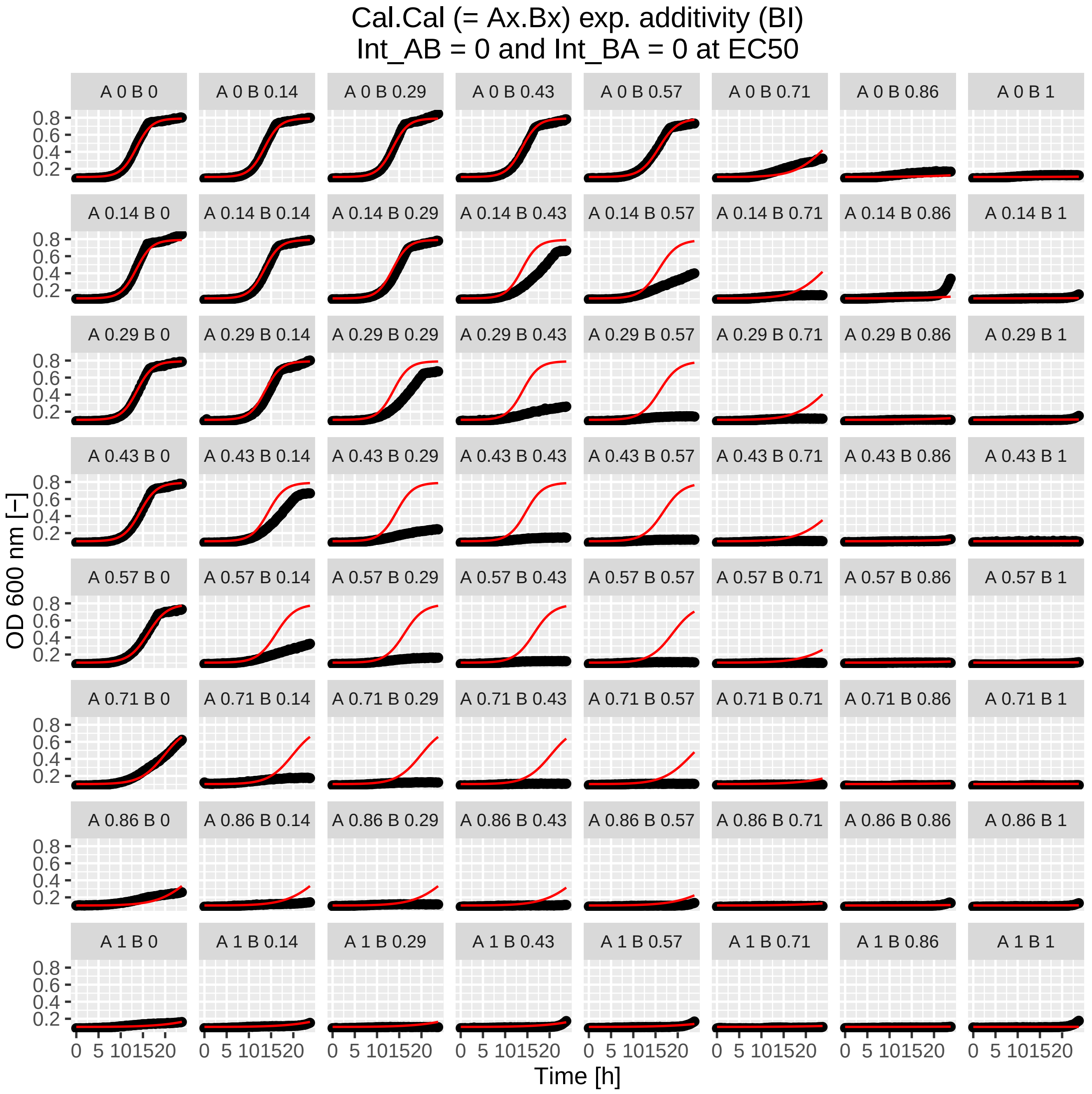

Cal.Cal (= Ax.Bx) exp. additivity (BI)
Int_AB = 0 and Int_BA = 0 at EC50
OD 600 nm [−]
Time [h]

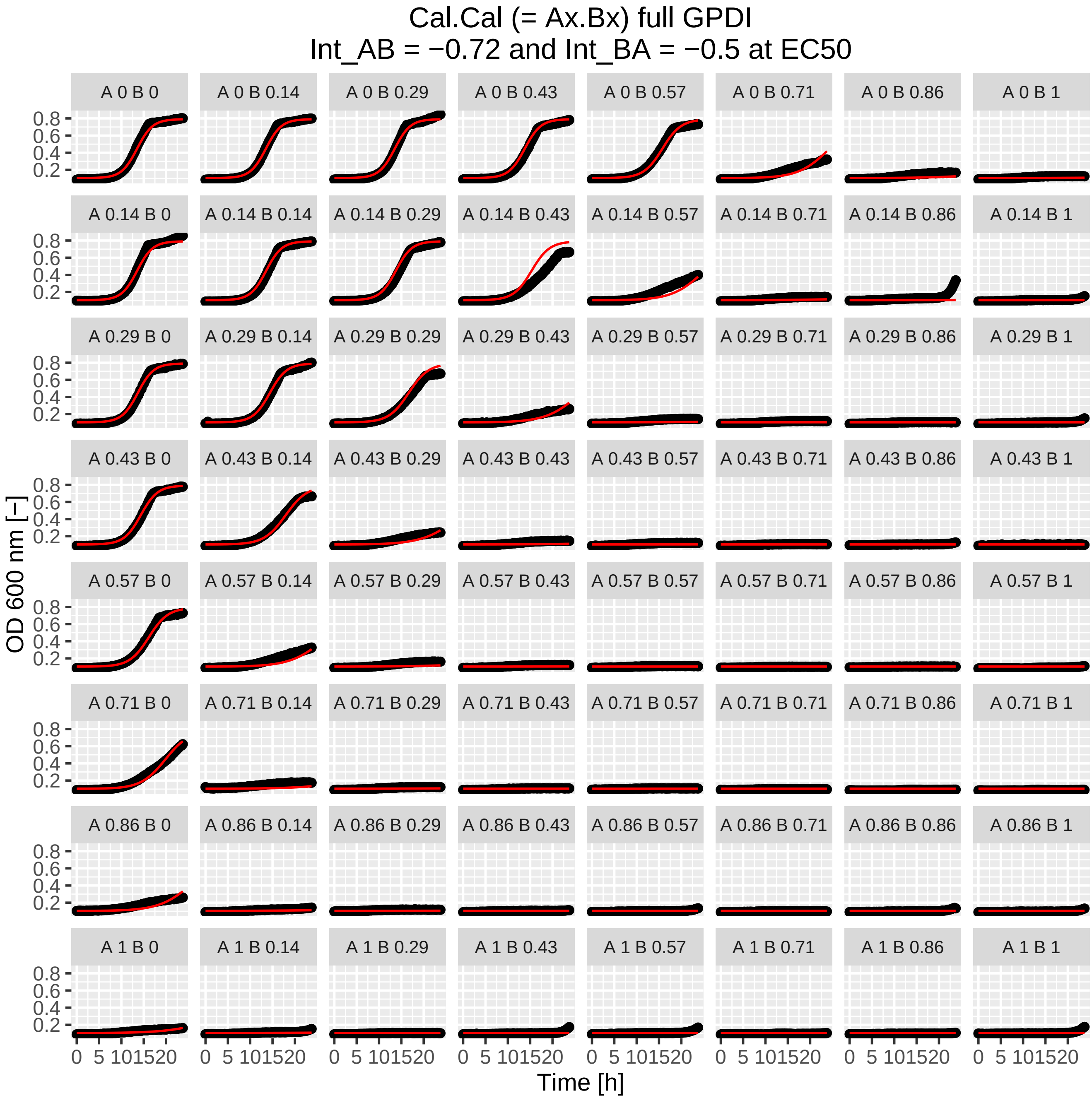
Cal.Cal (= Ax.Bx) full GPDI
Int_AB = −0.72 and Int_BA = −0.5 at EC50
OD 600 nm [−]
Time [h]
A 0 B 0
A 0 B 0.14
A 0 B 0.29
A 0 B 0.43
A 0 B 0.57
A 0 B 0.71
A 0 B 0.86
A 0 B 1
A 0.14 B 0
A 0.14 B 0.14
A 0.14 B 0.29
A 0.14 B 0.43
A 0.14 B 0.57
A 0.14 B 0.71
A 0.14 B 0.86
A 0.14 B 1
A 0.29 B 0
A 0.29 B 0.14
A 0.29 B 0.29
A 0.29 B 0.43
A 0.29 B 0.57
A 0.29 B 0.71
A 0.29 B 0.86
A 0.29 B 1
A 0.43 B 0
A 0.43 B 0.14
A 0.43 B 0.29
A 0.43 B 0.43
A 0.43 B 0.57
A 0.43 B 0.71
A 0.43 B 0.86
A 0.43 B 1
A 0.57 B 0
A 0.57 B 0.14
A 0.57 B 0.29
A 0.57 B 0.43
A 0.57 B 0.57
A 0.57 B 0.71
A 0.57 B 0.86
A 0.57 B 1
A 0.71 B 0
A 0.71 B 0.14
A 0.71 B 0.29
A 0.71 B 0.43
A 0.71 B 0.57
A 0.71 B 0.71
A 0.71 B 0.86
A 0.71 B 1
A 0.86 B 0
A 0.86 B 0.14
A 0.86 B 0.29
A 0.86 B 0.43
A 0.86 B 0.57
A 0.86 B 0.71
A 0.86 B 0.86
A 0.86 B 1
A 1 B 0
A 1 B 0.14
A 1 B 0.29
A 1 B 0.43
A 1 B 0.57
A 1 B 0.71
A 1 B 0.86
A 1 B 1
0.8
0.6
0.4
0.2
0 5 101520

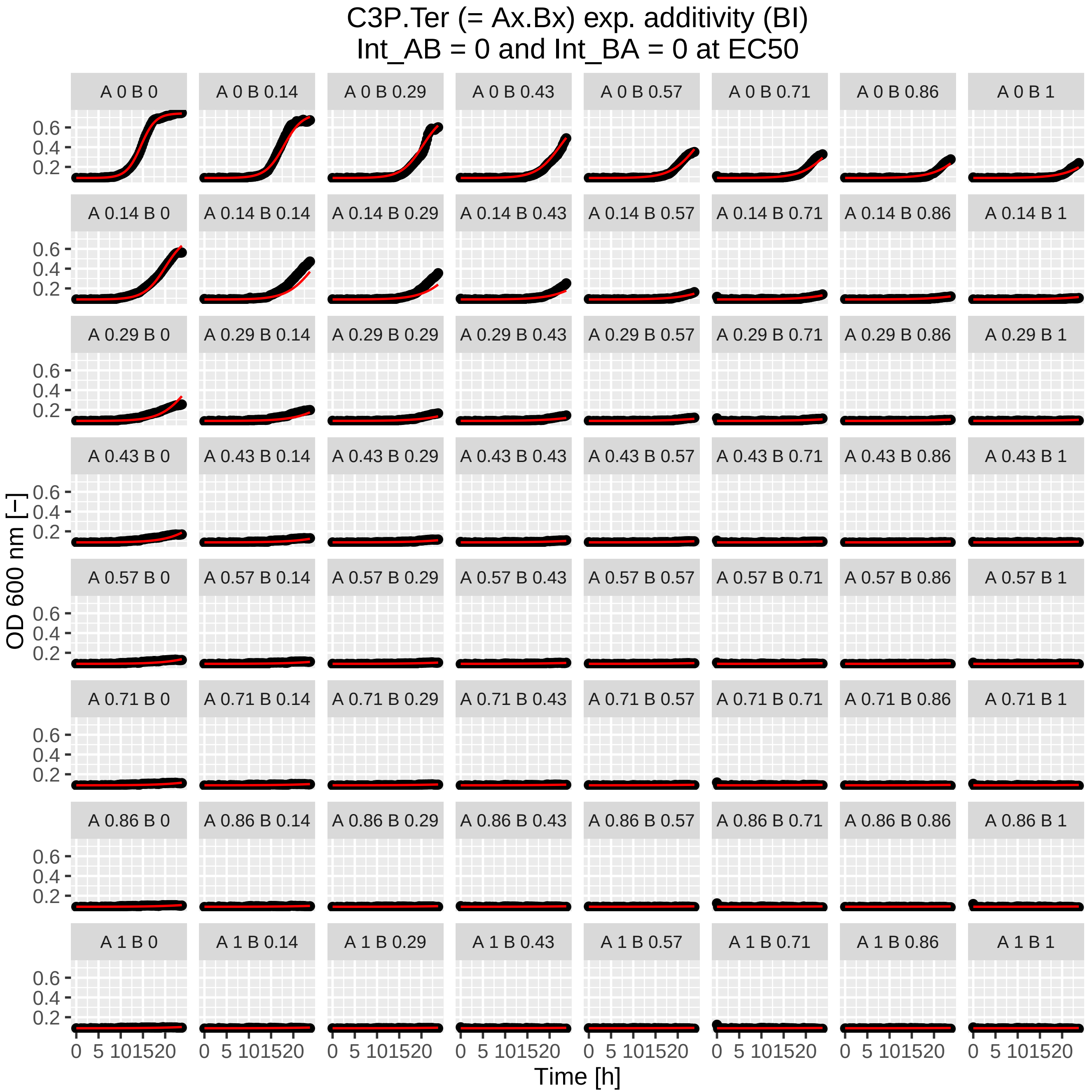
C3P.Ter (= Ax.Bx) exp. additivity (BI)
Int_AB = 0 and Int_BA = 0 at EC50
OD 600 nm [–]
Time [h]
A 0 B 0
A 0 B 0.14
A 0 B 0.29
A 0 B 0.43
A 0 B 0.57
A 0 B 0.71
A 0 B 0.86
A 0 B 1
A 0.14 B 0
A 0.14 B 0.14
A 0.14 B 0.29
A 0.14 B 0.43
A 0.14 B 0.57
A 0.14 B 0.71
A 0.14 B 0.86
A 0.14 B 1
A 0.29 B 0
A 0.29 B 0.14
A 0.29 B 0.29
A 0.29 B 0.43
A 0.29 B 0.57
A 0.29 B 0.71
A 0.29 B 0.86
A 0.29 B 1
A 0.43 B 0
A 0.43 B 0.14
A 0.43 B 0.29
A 0.43 B 0.43
A 0.43 B 0.57
A 0.43 B 0.71
A 0.43 B 0.86
A 0.43 B 1
A 0.57 B 0
A 0.57 B 0.14
A 0.57 B 0.29
A 0.57 B 0.43
A 0.57 B 0.57
A 0.57 B 0.71
A 0.57 B 0.86
A 0.57 B 1
A 0.71 B 0
A 0.71 B 0.14
A 0.71 B 0.29
A 0.71 B 0.43
A 0.71 B 0.57
A 0.71 B 0.71
A 0.71 B 0.86
A 0.71 B 1
A 0.86 B 0
A 0.86 B 0.14
A 0.86 B 0.29
A 0.86 B 0.43
A 0.86 B 0.57
A 0.86 B 0.71
A 0.86 B 0.86
A 0.86 B 1
A 1 B 0
A 1 B 0.14
A 1 B 0.29
A 1 B 0.43
A 1 B 0.57
A 1 B 0.71
A 1 B 0.86
A 1 B 1
0.6
0.4
0.2
0 5 101520

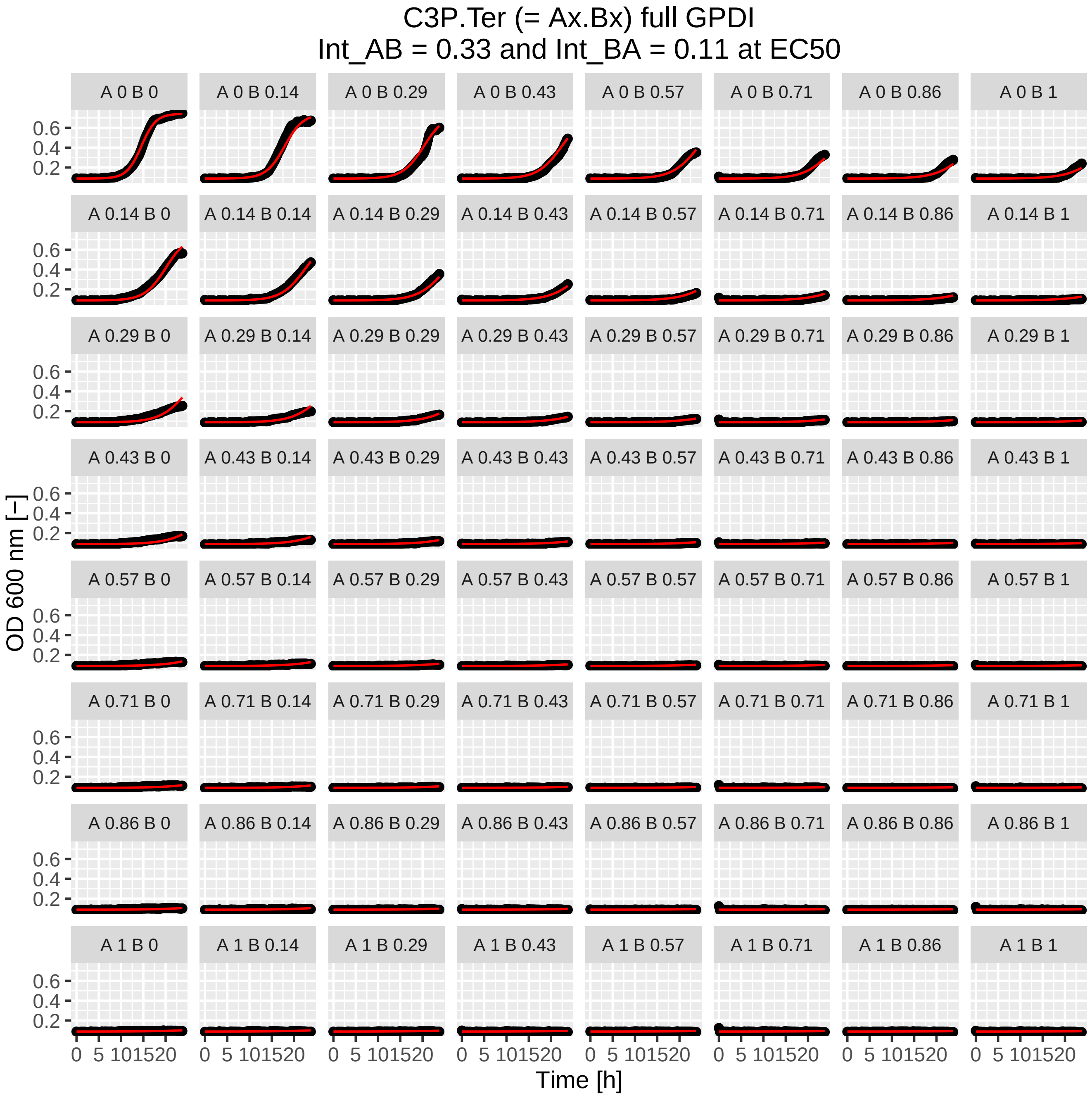
C3P.Ter (= Ax.Bx) full GPDI
Int_AB = 0.33 and Int_BA = 0.11 at EC50
OD 600 nm [–]
Time [h]
A 0 B 0
A 0 B 0.14
A 0 B 0.29
A 0 B 0.43
A 0 B 0.57
A 0 B 0.71
A 0 B 0.86
A 0 B 1
A 0.14 B 0
A 0.14 B 0.14
A 0.14 B 0.29
A 0.14 B 0.43
A 0.14 B 0.57
A 0.14 B 0.71
A 0.14 B 0.86
A 0.14 B 1
A 0.29 B 0
A 0.29 B 0.14
A 0.29 B 0.29
A 0.29 B 0.43
A 0.29 B 0.57
A 0.29 B 0.71
A 0.29 B 0.86
A 0.29 B 1
A 0.43 B 0
A 0.43 B 0.14
A 0.43 B 0.29
A 0.43 B 0.43
A 0.43 B 0.57
A 0.43 B 0.71
A 0.43 B 0.86
A 0.43 B 1
A 0.57 B 0
A 0.57 B 0.14
A 0.57 B 0.29
A 0.57 B 0.43
A 0.57 B 0.57
A 0.57 B 0.71
A 0.57 B 0.86
A 0.57 B 1
A 0.71 B 0
A 0.71 B 0.14
A 0.71 B 0.29
A 0.71 B 0.43
A 0.71 B 0.57
A 0.71 B 0.71
A 0.71 B 0.86
A 0.71 B 1
A 0.86 B 0
A 0.86 B 0.14
A 0.86 B 0.29
A 0.86 B 0.43
A 0.86 B 0.57
A 0.86 B 0.71
A 0.86 B 0.86
A 0.86 B 1
A 1 B 0
A 1 B 0.14
A 1 B 0.29
A 1 B 0.43
A 1 B 0.57
A 1 B 0.71
A 1 B 0.86
A 1 B 1
0.6
0.4
0.2
0 5 101520

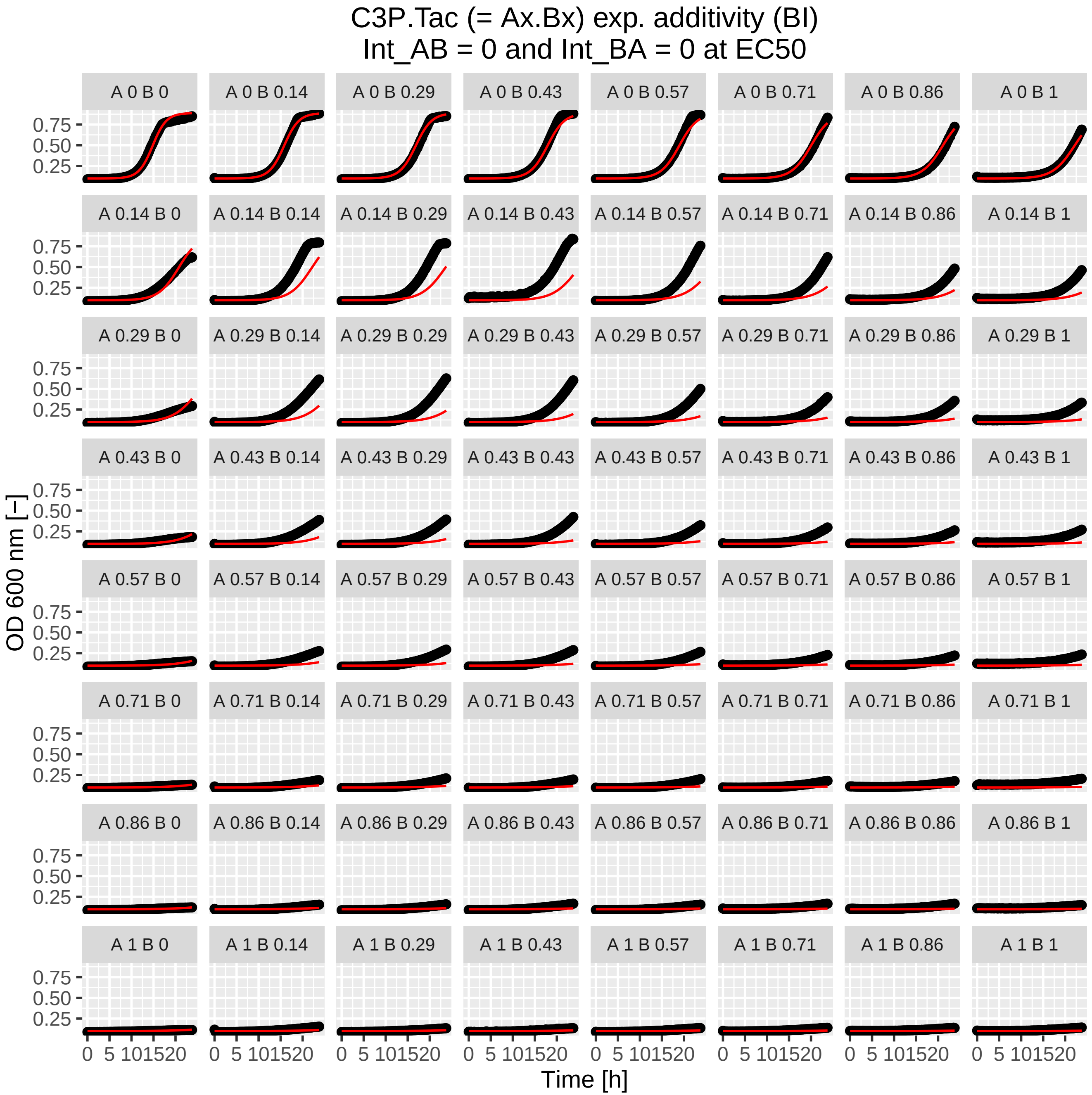

C3P.Tac (= Ax.Bx) exp. additivity (BI)
Int_AB = 0 and Int_BA = 0 at EC50
A 0 B 0
A 0 B 0.14
A 0 B 0.29
A 0 B 0.43
A 0 B 0.57
A 0 B 0.71
A 0 B 0.86
A 0 B 1
A 0.14 B 0
A 0.14 B 0.14
A 0.14 B 0.29
A 0.14 B 0.43
A 0.14 B 0.57
A 0.14 B 0.71
A 0.14 B 0.86
A 0.14 B 1
A 0.29 B 0
A 0.29 B 0.14
A 0.29 B 0.29
A 0.29 B 0.43
A 0.29 B 0.57
A 0.29 B 0.71
A 0.29 B 0.86
A 0.29 B 1
A 0.43 B 0
A 0.43 B 0.14
A 0.43 B 0.29
A 0.43 B 0.43
A 0.43 B 0.57
A 0.43 B 0.71
A 0.43 B 0.86
A 0.43 B 1
A 0.57 B 0
A 0.57 B 0.14
A 0.57 B 0.29
A 0.57 B 0.43
A 0.57 B 0.57
A 0.57 B 0.71
A 0.57 B 0.86
A 0.57 B 1
A 0.71 B 0
A 0.71 B 0.14
A 0.71 B 0.29
A 0.71 B 0.43
A 0.71 B 0.57
A 0.71 B 0.71
A 0.71 B 0.86
A 0.71 B 1
A 0.86 B 0
A 0.86 B 0.14
A 0.86 B 0.29
A 0.86 B 0.43
A 0.86 B 0.57
A 0.86 B 0.71
A 0.86 B 0.86
A 0.86 B 1
A 1 B 0
A 1 B 0.14
A 1 B 0.29
A 1 B 0.43
A 1 B 0.57
A 1 B 0.71
A 1 B 0.86
A 1 B 1
OD 600 nm [−]
0.75
0.50
0.25
0 5 101520
Time [h]

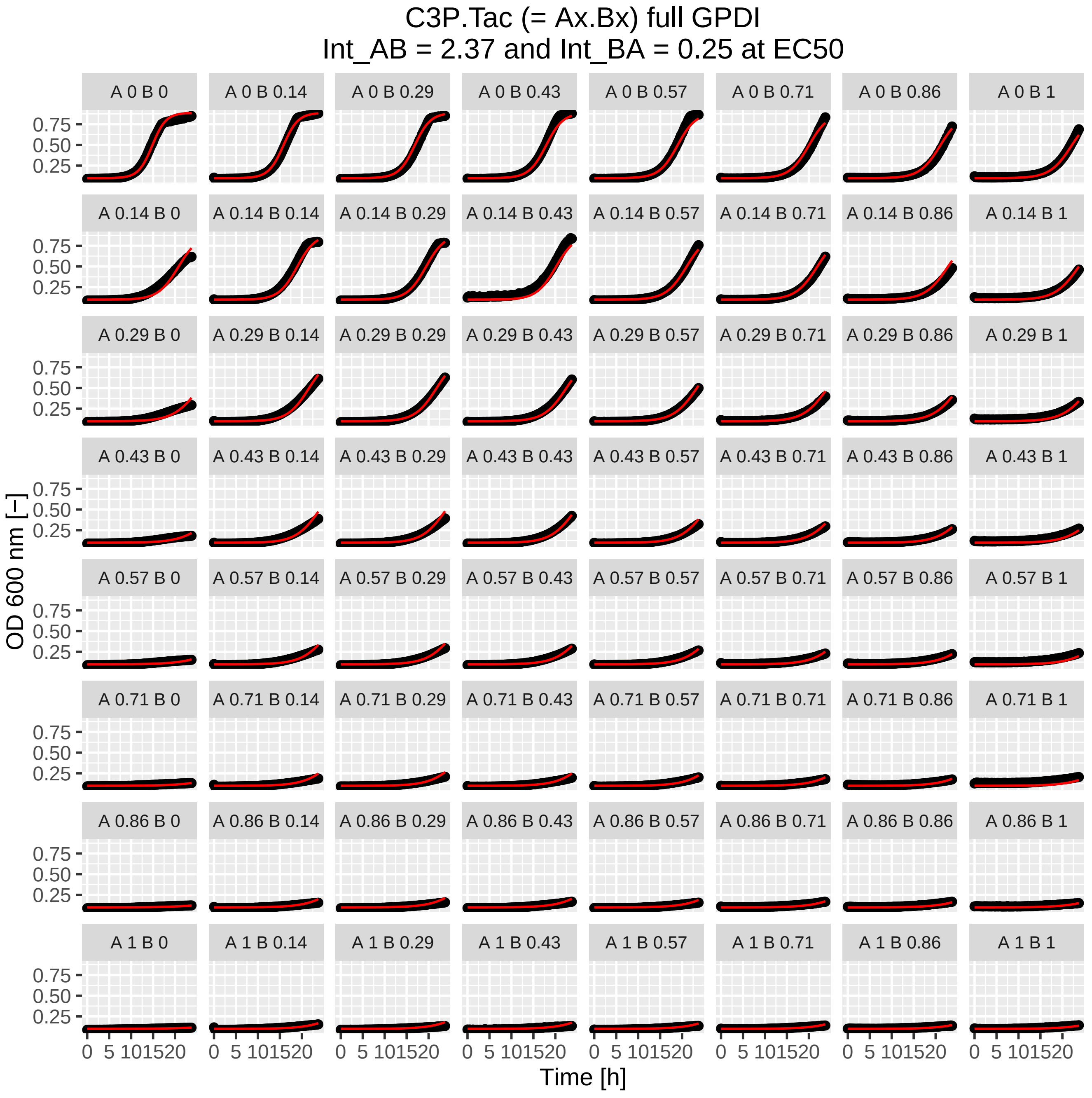
C3P.Tac (= Ax.Bx) full GPDI
Int_AB = 2.37 and Int_BA = 0.25 at EC50
OD 600 nm [−]
Time [h]
0.75
0.50
0.25
0 5 101520
A 0 B 0
A 0 B 0.14
A 0 B 0.29
A 0 B 0.43
A 0 B 0.57
A 0 B 0.71
A 0 B 0.86
A 0 B 1
A 0.14 B 0
A 0.14 B 0.14
A 0.14 B 0.29
A 0.14 B 0.43
A 0.14 B 0.57
A 0.14 B 0.71
A 0.14 B 0.86
A 0.14 B 1
A 0.29 B 0
A 0.29 B 0.14
A 0.29 B 0.29
A 0.29 B 0.43
A 0.29 B 0.57
A 0.29 B 0.71
A 0.29 B 0.86
A 0.29 B 1
A 0.43 B 0
A 0.43 B 0.14
A 0.43 B 0.29
A 0.43 B 0.43
A 0.43 B 0.57
A 0.43 B 0.71
A 0.43 B 0.86
A 0.43 B 1
A 0.57 B 0
A 0.57 B 0.14
A 0.57 B 0.29
A 0.57 B 0.43
A 0.57 B 0.57
A 0.57 B 0.71
A 0.57 B 0.86
A 0.57 B 1
A 0.71 B 0
A 0.71 B 0.14
A 0.71 B 0.29
A 0.71 B 0.43
A 0.71 B 0.57
A 0.71 B 0.71
A 0.71 B 0.86
A 0.71 B 1
A 0.86 B 0
A 0.86 B 0.14
A 0.86 B 0.29
A 0.86 B 0.43
A 0.86 B 0.57
A 0.86 B 0.71
A 0.86 B 0.86
A 0.86 B 1
A 1 B 0
A 1 B 0.14
A 1 B 0.29
A 1 B 0.43
A 1 B 0.57
A 1 B 0.71
A 1 B 0.86
A 1 B 1

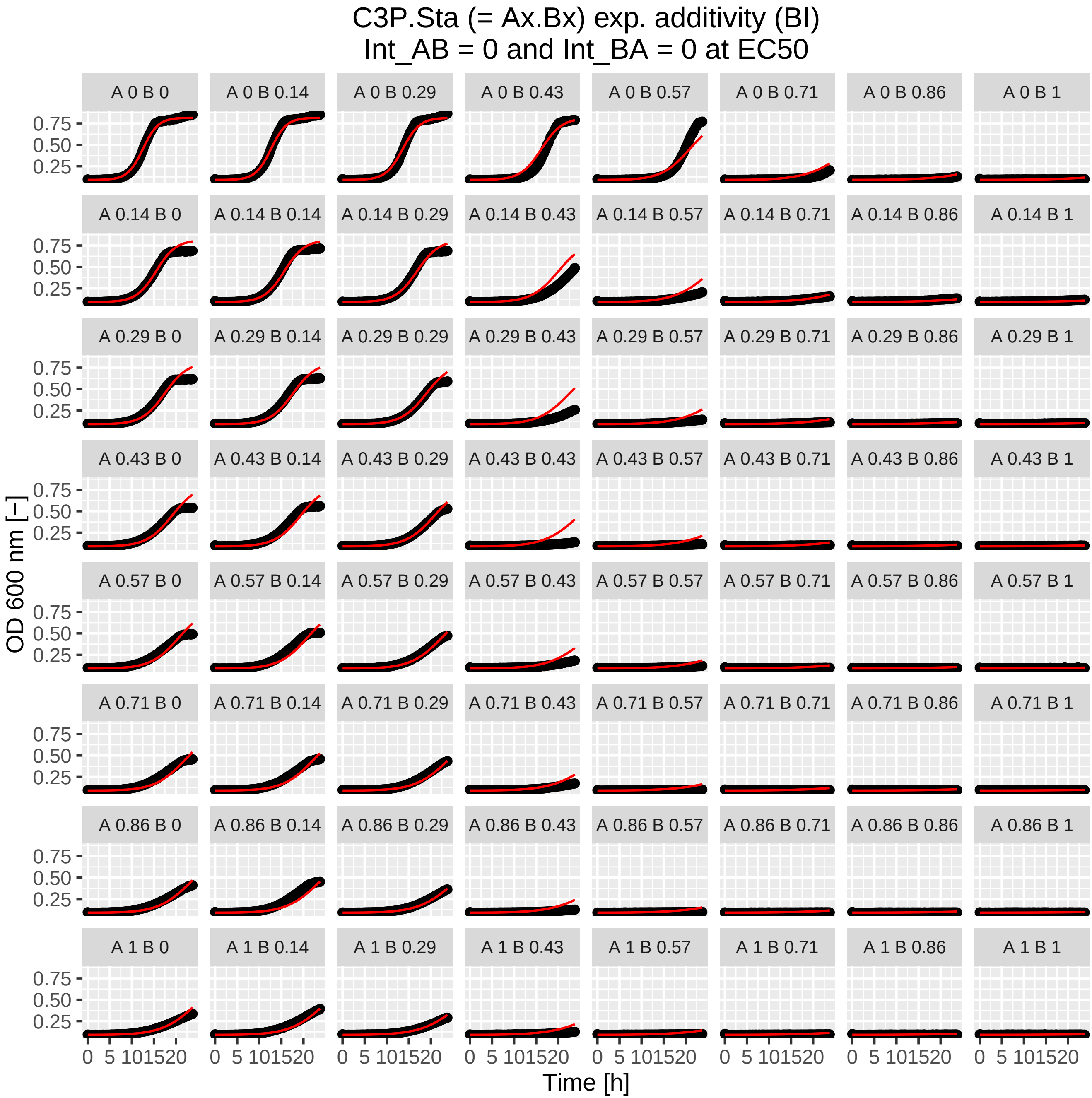

C3P.Sta (= Ax.Bx) exp. additivity (BI)
Int_AB = 0 and Int_BA = 0 at EC50
A 0 B 0
A 0 B 0.14
A 0 B 0.29
A 0 B 0.43
A 0 B 0.57
A 0 B 0.71
A 0 B 0.86
A 0 B 1
A 0.14 B 0
A 0.14 B 0.14
A 0.14 B 0.29
A 0.14 B 0.43
A 0.14 B 0.57
A 0.14 B 0.71
A 0.14 B 0.86
A 0.14 B 1
A 0.29 B 0
A 0.29 B 0.14
A 0.29 B 0.29
A 0.29 B 0.43
A 0.29 B 0.57
A 0.29 B 0.71
A 0.29 B 0.86
A 0.29 B 1
A 0.43 B 0
A 0.43 B 0.14
A 0.43 B 0.29
A 0.43 B 0.43
A 0.43 B 0.57
A 0.43 B 0.71
A 0.43 B 0.86
A 0.43 B 1
A 0.57 B 0
A 0.57 B 0.14
A 0.57 B 0.29
A 0.57 B 0.43
A 0.57 B 0.57
A 0.57 B 0.71
A 0.57 B 0.86
A 0.57 B 1
A 0.71 B 0
A 0.71 B 0.14
A 0.71 B 0.29
A 0.71 B 0.43
A 0.71 B 0.57
A 0.71 B 0.71
A 0.71 B 0.86
A 0.71 B 1
A 0.86 B 0
A 0.86 B 0.14
A 0.86 B 0.29
A 0.86 B 0.43
A 0.86 B 0.57
A 0.86 B 0.71
A 0.86 B 0.86
A 0.86 B 1
A 1 B 0
A 1 B 0.14
A 1 B 0.29
A 1 B 0.43
A 1 B 0.57
A 1 B 0.71
A 1 B 0.86
A 1 B 1
0.75
0.50
0.25
OD 600 nm [−]
0 5 101520
Time [h]

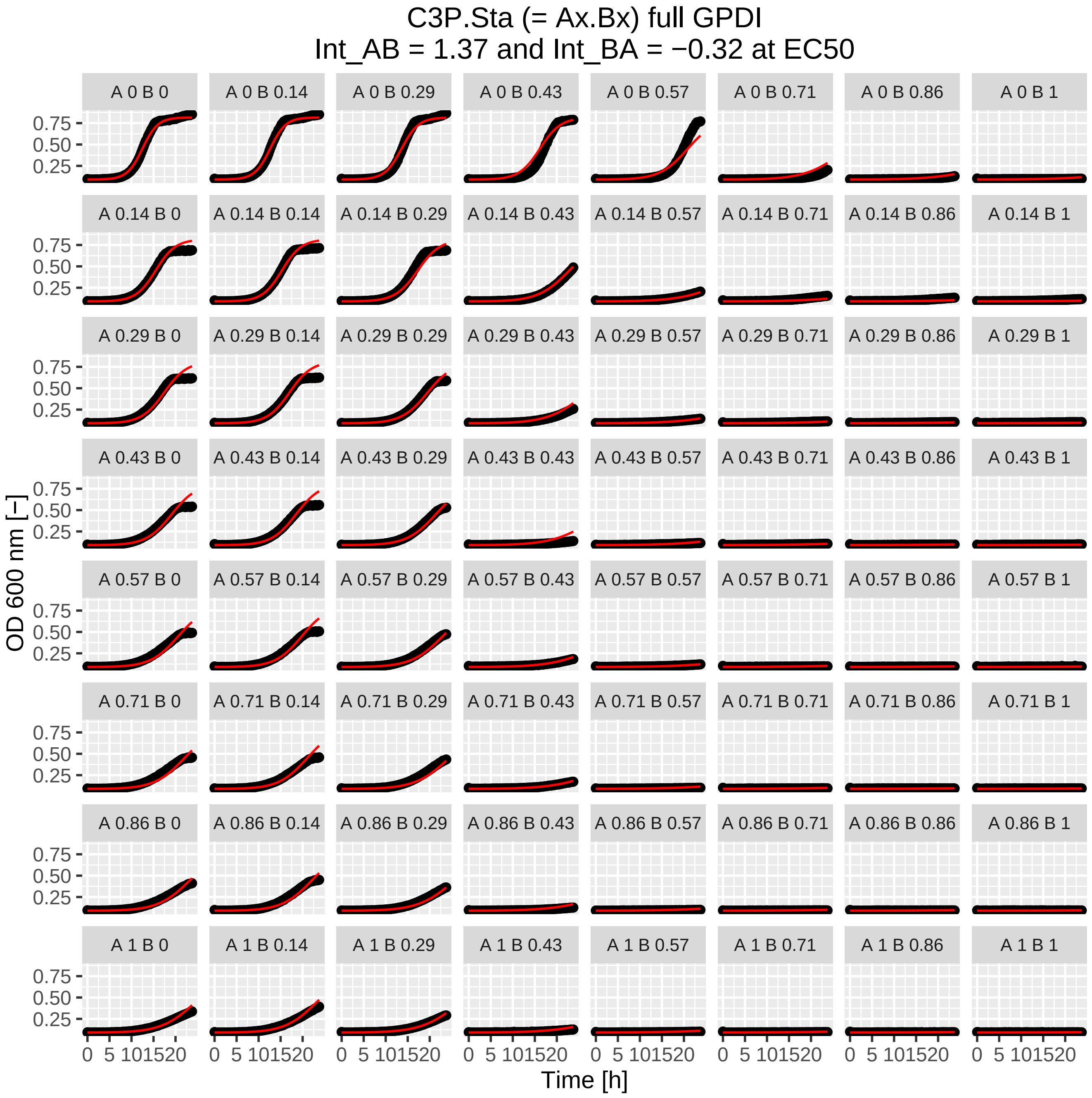
C3P.Sta (= Ax.Bx) full GPDI
Int_AB = 1.37 and Int_BA = −0.32 at EC50
OD 600 nm [−]
Time [h]
A 0 B 0
A 0 B 0.14
A 0 B 0.29
A 0 B 0.43
A 0 B 0.57
A 0 B 0.71
A 0 B 0.86
A 0 B 1
A 0.14 B 0
A 0.14 B 0.14
A 0.14 B 0.29
A 0.14 B 0.43
A 0.14 B 0.57
A 0.14 B 0.71
A 0.14 B 0.86
A 0.14 B 1
A 0.29 B 0
A 0.29 B 0.14
A 0.29 B 0.29
A 0.29 B 0.43
A 0.29 B 0.57
A 0.29 B 0.71
A 0.29 B 0.86
A 0.29 B 1
A 0.43 B 0
A 0.43 B 0.14
A 0.43 B 0.29
A 0.43 B 0.43
A 0.43 B 0.57
A 0.43 B 0.71
A 0.43 B 0.86
A 0.43 B 1
A 0.57 B 0
A 0.57 B 0.14
A 0.57 B 0.29
A 0.57 B 0.43
A 0.57 B 0.57
A 0.57 B 0.71
A 0.57 B 0.86
A 0.57 B 1
A 0.71 B 0
A 0.71 B 0.14
A 0.71 B 0.29
A 0.71 B 0.43
A 0.71 B 0.57
A 0.71 B 0.71
A 0.71 B 0.86
A 0.71 B 1
A 0.86 B 0
A 0.86 B 0.14
A 0.86 B 0.29
A 0.86 B 0.43
A 0.86 B 0.57
A 0.86 B 0.71
A 0.86 B 0.86
A 0.86 B 1
A 1 B 0
A 1 B 0.14
A 1 B 0.29
A 1 B 0.43
A 1 B 0.57
A 1 B 0.71
A 1 B 0.86
A 1 B 1
0.75
0.50
0.25
0 5 101520

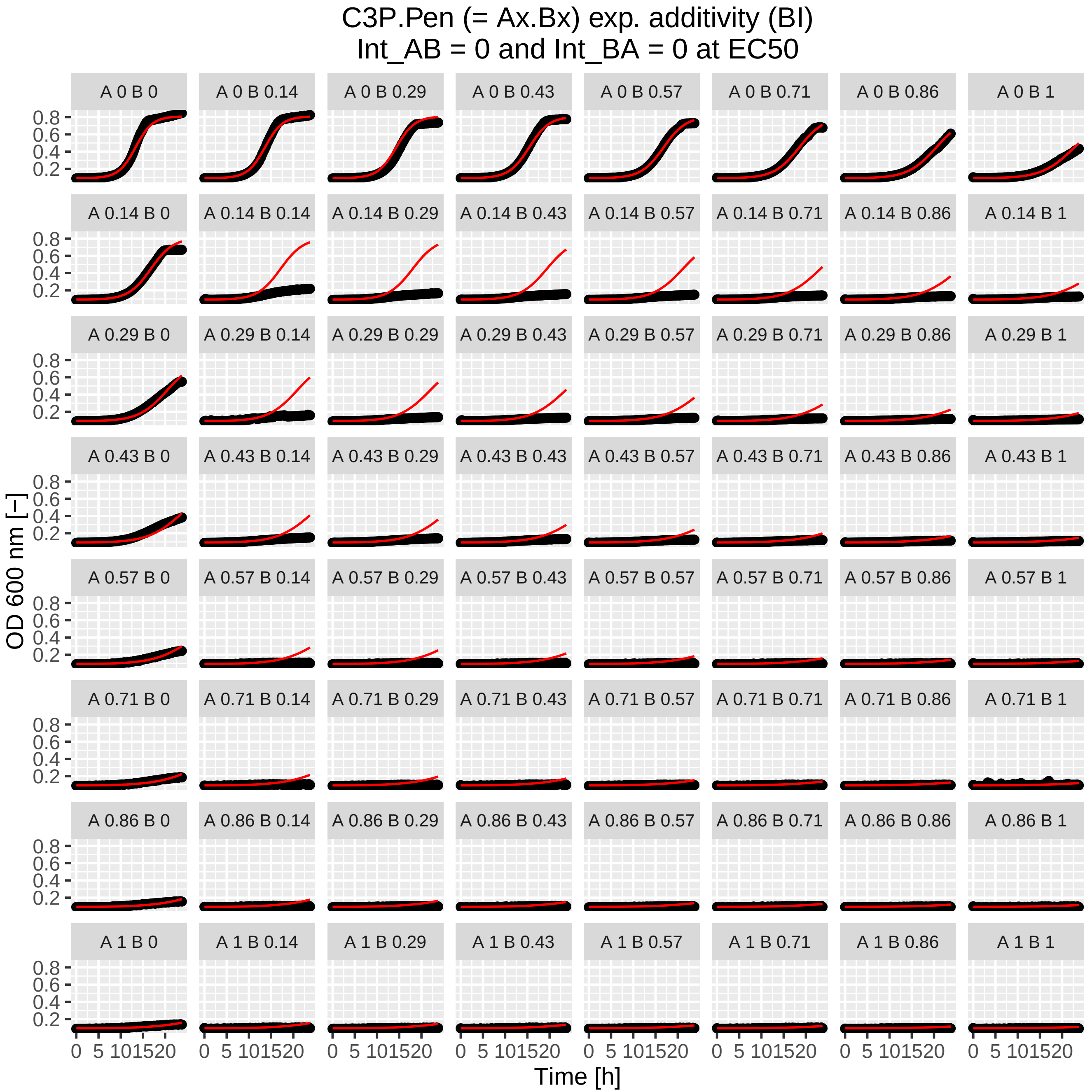
C3P.Pen (= Ax.Bx) exp. additivity (BI)
Int_AB = 0 and Int_BA = 0 at EC50
OD 600 nm [−]
Time [h]
A 0 B 0
A 0 B 0.14
A 0 B 0.29
A 0 B 0.43
A 0 B 0.57
A 0 B 0.71
A 0 B 0.86
A 0 B 1
A 0.14 B 0
A 0.29 B 0
A 0.43 B 0
A 0.57 B 0
A 0.71 B 0
A 0.86 B 0
A 1 B 0
A 1 B 1

C3P.Pen (= Ax.Bx) full GPDI
Int_AB = −0.81 and Int_BA = 1.37 at EC50

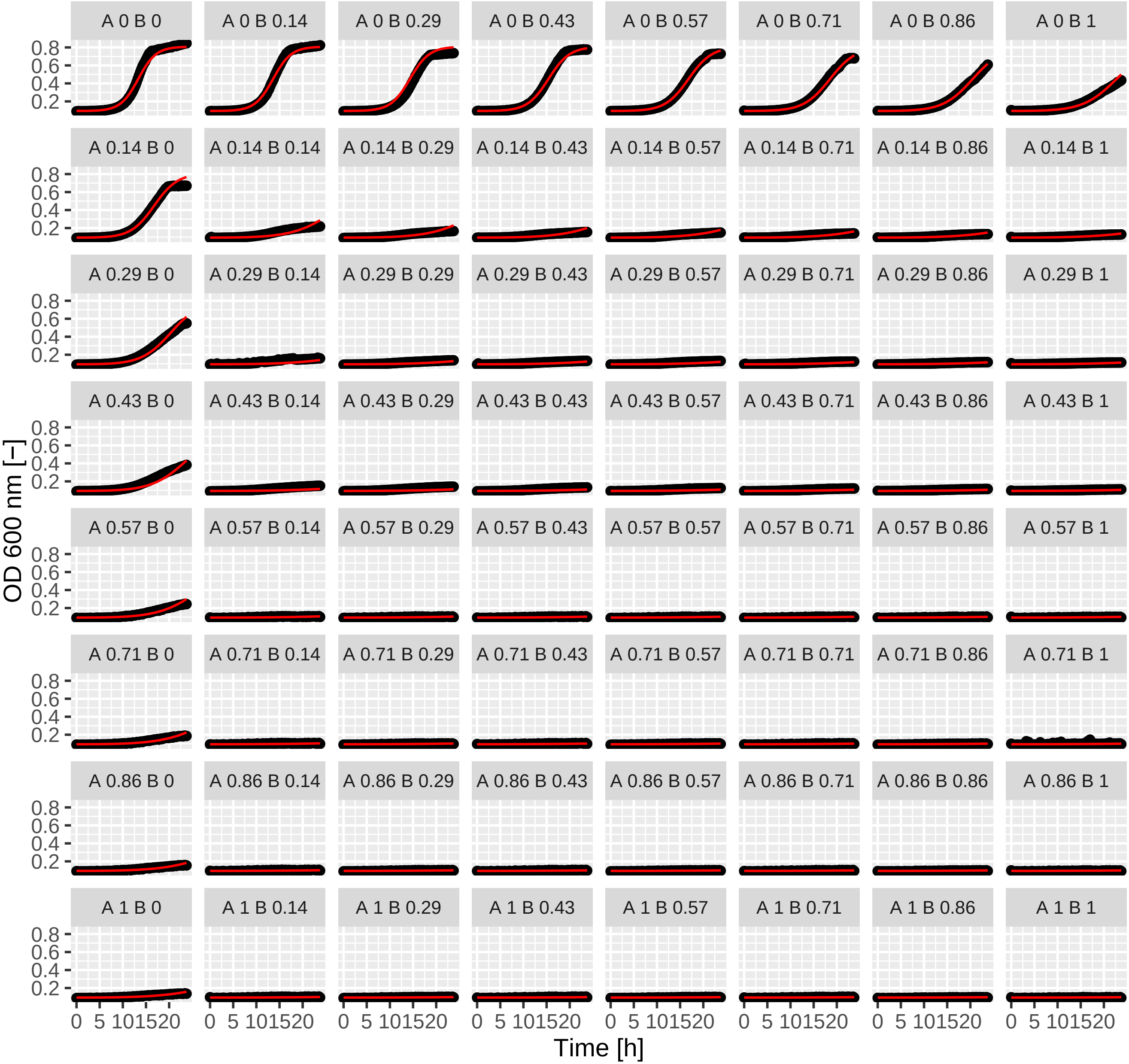

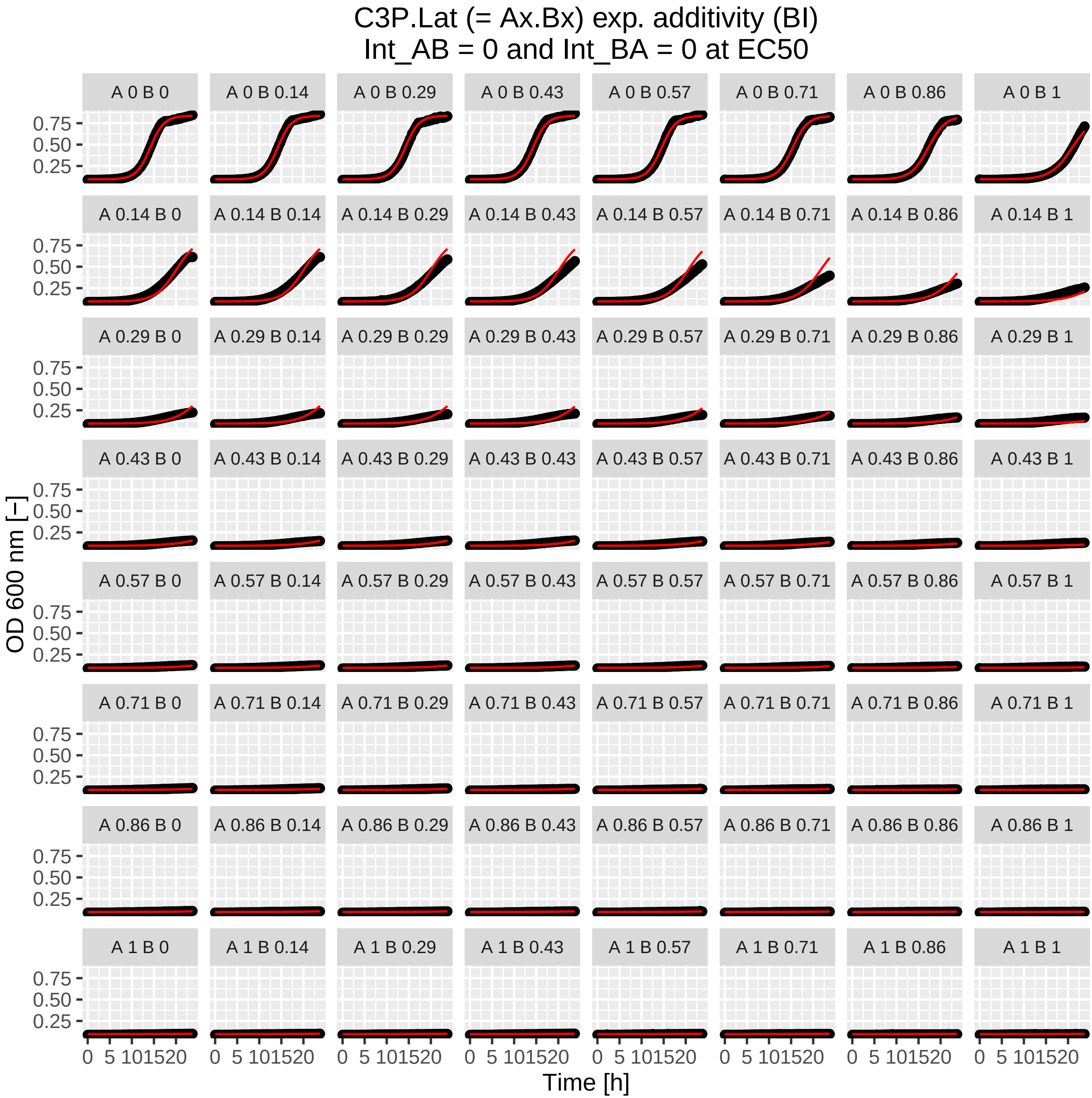
C3P.Lat (= Ax.Bx) exp. additivity (BI)
Int_AB = 0 and Int_BA = 0 at EC50
A 0 B 0
A 0 B 0.14
A 0 B 0.29
A 0 B 0.43
A 0 B 0.57
A 0 B 0.71
A 0 B 0.86
A 0 B 1
A 0.14 B 0
A 0.14 B 0.14
A 0.14 B 0.29
A 0.14 B 0.43
A 0.14 B 0.57
A 0.14 B 0.71
A 0.14 B 0.86
A 0.14 B 1
A 0.29 B 0
A 0.29 B 0.14
A 0.29 B 0.29
A 0.29 B 0.43
A 0.29 B 0.57
A 0.29 B 0.71
A 0.29 B 0.86
A 0.29 B 1
A 0.43 B 0
A 0.43 B 0.14
A 0.43 B 0.29
A 0.43 B 0.43
A 0.43 B 0.57
A 0.43 B 0.71
A 0.43 B 0.86
A 0.43 B 1
A 0.57 B 0
A 0.57 B 0.14
A 0.57 B 0.29
A 0.57 B 0.43
A 0.57 B 0.57
A 0.57 B 0.71
A 0.57 B 0.86
A 0.57 B 1
A 0.71 B 0
A 0.71 B 0.14
A 0.71 B 0.29
A 0.71 B 0.43
A 0.71 B 0.57
A 0.71 B 0.71
A 0.71 B 0.86
A 0.71 B 1
A 0.86 B 0
A 0.86 B 0.14
A 0.86 B 0.29
A 0.86 B 0.43
A 0.86 B 0.57
A 0.86 B 0.71
A 0.86 B 0.86
A 0.86 B 1
A 1 B 0
A 1 B 0.14
A 1 B 0.29
A 1 B 0.43
A 1 B 0.57
A 1 B 0.71
A 1 B 0.86
A 1 B 1
0.75
0.50
0.25
OD 600 nm [–]
0 5 101520
Time [h]

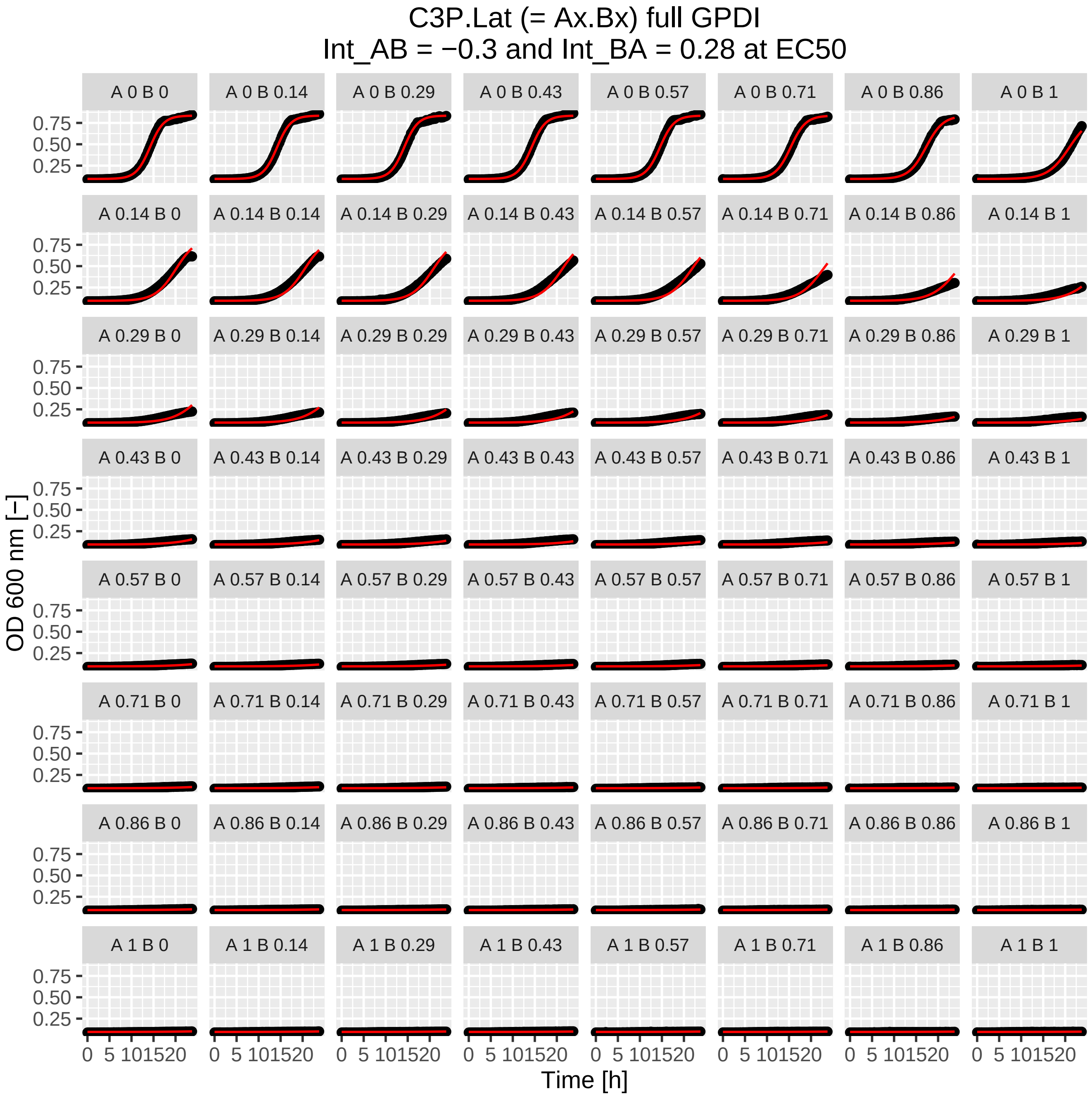
C3P.Lat (= Ax.Bx) full GPDI
Int_AB = −0.3 and Int_BA = 0.28 at EC50
A 0 B 0
A 0 B 0.14
A 0 B 0.29
A 0 B 0.43
A 0 B 0.57
A 0 B 0.71
A 0 B 0.86
A 0 B 1
A 0.14 B 0
A 0.14 B 0.14
A 0.14 B 0.29
A 0.14 B 0.43
A 0.14 B 0.57
A 0.14 B 0.71
A 0.14 B 0.86
A 0.14 B 1
A 0.29 B 0
A 0.29 B 0.14
A 0.29 B 0.29
A 0.29 B 0.43
A 0.29 B 0.57
A 0.29 B 0.71
A 0.29 B 0.86
A 0.29 B 1
A 0.43 B 0
A 0.43 B 0.14
A 0.43 B 0.29
A 0.43 B 0.43
A 0.43 B 0.57
A 0.43 B 0.71
A 0.43 B 0.86
A 0.43 B 1
A 0.57 B 0
A 0.57 B 0.14
A 0.57 B 0.29
A 0.57 B 0.43
A 0.57 B 0.57
A 0.57 B 0.71
A 0.57 B 0.86
A 0.57 B 1
A 0.71 B 0
A 0.71 B 0.14
A 0.71 B 0.29
A 0.71 B 0.43
A 0.71 B 0.57
A 0.71 B 0.71
A 0.71 B 0.86
A 0.71 B 1
A 0.86 B 0
A 0.86 B 0.14
A 0.86 B 0.29
A 0.86 B 0.43
A 0.86 B 0.57
A 0.86 B 0.71
A 0.86 B 0.86
A 0.86 B 1
A 1 B 0
A 1 B 0.14
A 1 B 0.29
A 1 B 0.43
A 1 B 0.57
A 1 B 0.71
A 1 B 0.86
A 1 B 1
0.75
0.50
0.25
OD 600 nm [−]
0 5 101520
Time [h]

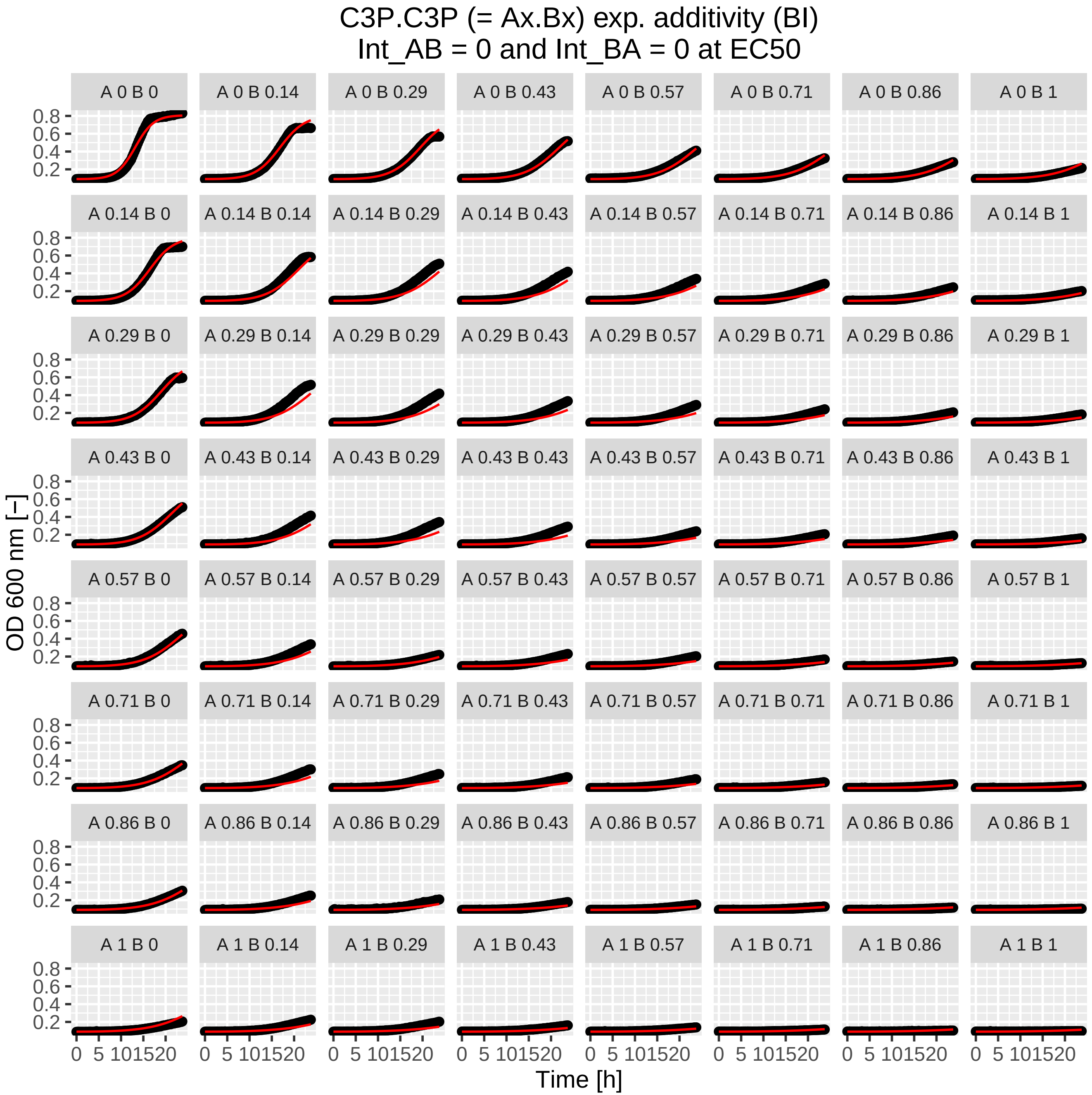

C3P.C3P (= Ax.Bx) exp. additivity (BI)
Int_AB = 0 and Int_BA = 0 at EC50
OD 600 nm [–]
Time [h]
A 0 B 0
A 0 B 0.14
A 0 B 0.29
A 0 B 0.43
A 0 B 0.57
A 0 B 0.71
A 0 B 0.86
A 0 B 1
A 0.14 B 0
A 0.14 B 0.14
A 0.14 B 0.29
A 0.14 B 0.43
A 0.14 B 0.57
A 0.14 B 0.71
A 0.14 B 0.86
A 0.14 B 1
A 0.29 B 0
A 0.29 B 0.14
A 0.29 B 0.29
A 0.29 B 0.43
A 0.29 B 0.57
A 0.29 B 0.71
A 0.29 B 0.86
A 0.29 B 1
A 0.43 B 0
A 0.43 B 0.14
A 0.43 B 0.29
A 0.43 B 0.43
A 0.43 B 0.57
A 0.43 B 0.71
A 0.43 B 0.86
A 0.43 B 1
A 0.57 B 0
A 0.57 B 0.14
A 0.57 B 0.29
A 0.57 B 0.43
A 0.57 B 0.57
A 0.57 B 0.71
A 0.57 B 0.86
A 0.57 B 1
A 0.71 B 0
A 0.71 B 0.14
A 0.71 B 0.29
A 0.71 B 0.43
A 0.71 B 0.57
A 0.71 B 0.71
A 0.71 B 0.86
A 0.71 B 1
A 0.86 B 0
A 0.86 B 0.14
A 0.86 B 0.29
A 0.86 B 0.43
A 0.86 B 0.57
A 0.86 B 0.71
A 0.86 B 0.86
A 0.86 B 1
A 1 B 0
A 1 B 0.14
A 1 B 0.29
A 1 B 0.43
A 1 B 0.57
A 1 B 0.71
A 1 B 0.86
A 1 B 1
0.8
0.6
0.4
0.2
0 5 101520

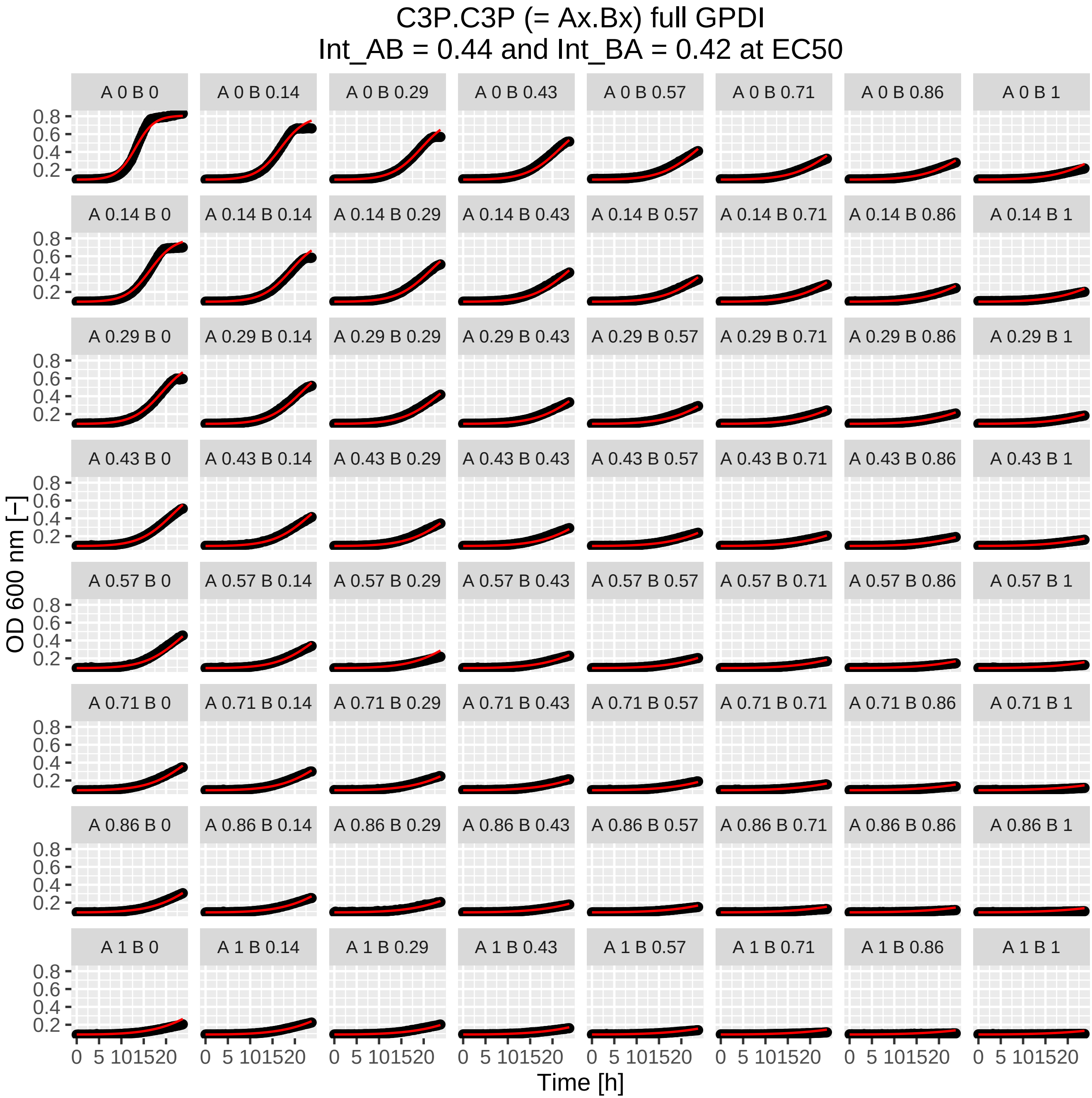

C3P.C3P (= Ax.Bx) full GPDI
Int_AB = 0.44 and Int_BA = 0.42 at EC50
OD 600 nm [−]
Time [h]
0.8
0.6
0.4
0.2
0 5 101520
A 0 B 0
A 0 B 0.14
A 0 B 0.29
A 0 B 0.43
A 0 B 0.57
A 0 B 0.71
A 0 B 0.86
A 0 B 1
A 0.14 B 0
A 0.14 B 0.14
A 0.14 B 0.29
A 0.14 B 0.43
A 0.14 B 0.57
A 0.14 B 0.71
A 0.14 B 0.86
A 0.14 B 1
A 0.29 B 0
A 0.29 B 0.14
A 0.29 B 0.29
A 0.29 B 0.43
A 0.29 B 0.57
A 0.29 B 0.71
A 0.29 B 0.86
A 0.29 B 1
A 0.43 B 0
A 0.43 B 0.14
A 0.43 B 0.29
A 0.43 B 0.43
A 0.43 B 0.57
A 0.43 B 0.71
A 0.43 B 0.86
A 0.43 B 1
A 0.57 B 0
A 0.57 B 0.14
A 0.57 B 0.29
A 0.57 B 0.43
A 0.57 B 0.57
A 0.57 B 0.71
A 0.57 B 0.86
A 0.57 B 1
A 0.71 B 0
A 0.71 B 0.14
A 0.71 B 0.29
A 0.71 B 0.43
A 0.71 B 0.57
A 0.71 B 0.71
A 0.71 B 0.86
A 0.71 B 1
A 0.86 B 0
A 0.86 B 0.14
A 0.86 B 0.29
A 0.86 B 0.43
A 0.86 B 0.57
A 0.86 B 0.71
A 0.86 B 0.86
A 0.86 B 1
A 1 B 0
A 1 B 0.14
A 1 B 0.29
A 1 B 0.43
A 1 B 0.57
A 1 B 0.71
A 1 B 0.86
A 1 B 1

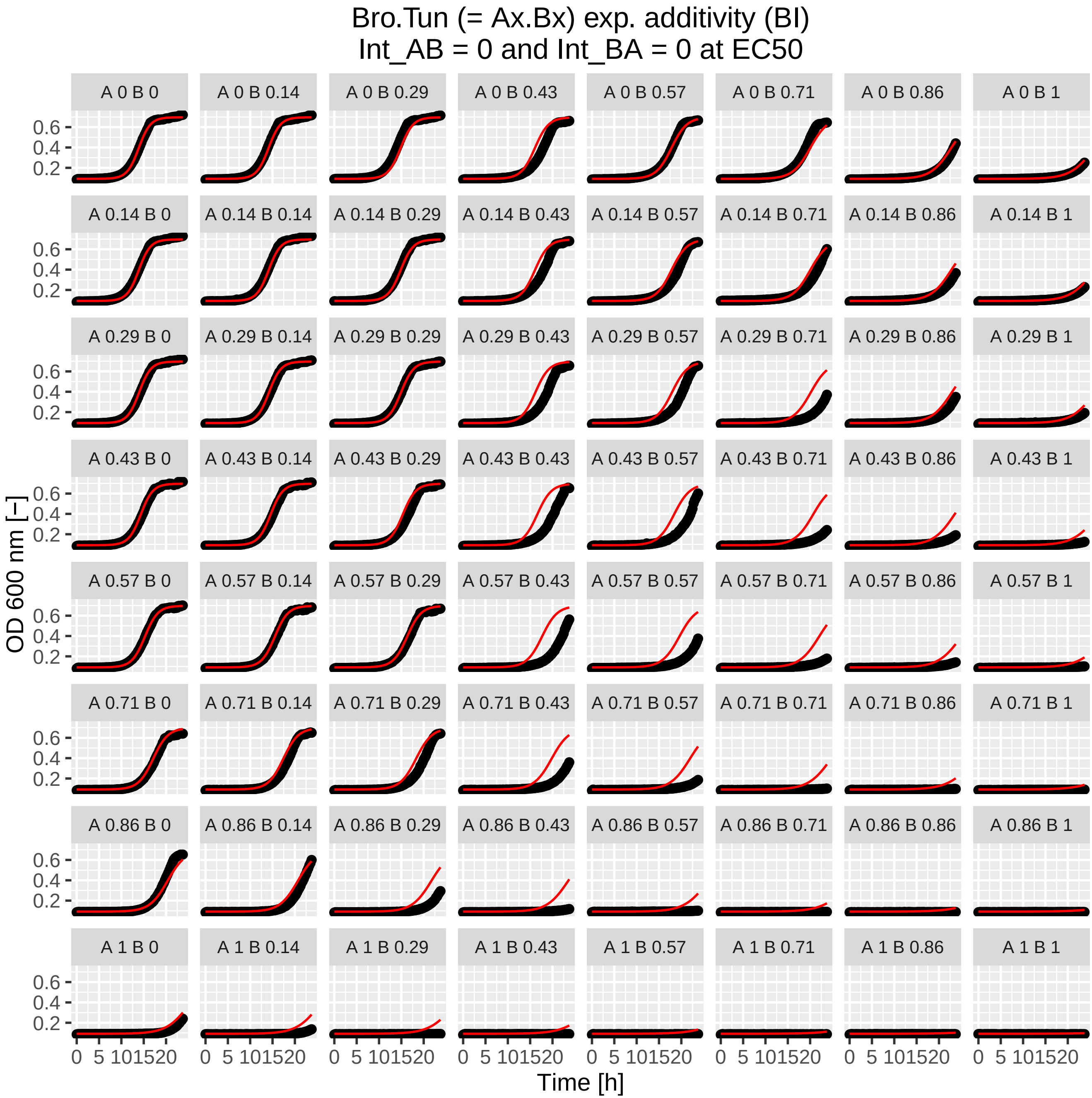

Bro.Tun (= Ax.Bx) exp. additivity (BI)
Int_AB = 0 and Int_BA = 0 at EC50
A 0 B 0
A 0 B 0.14
A 0 B 0.29
A 0 B 0.43
A 0 B 0.57
A 0 B 0.71
A 0 B 0.86
A 0 B 1
A 0.14 B 0
A 0.14 B 0.14
A 0.14 B 0.29
A 0.14 B 0.43
A 0.14 B 0.57
A 0.14 B 0.71
A 0.14 B 0.86
A 0.14 B 1
A 0.29 B 0
A 0.29 B 0.14
A 0.29 B 0.29
A 0.29 B 0.43
A 0.29 B 0.57
A 0.29 B 0.71
A 0.29 B 0.86
A 0.29 B 1
A 0.43 B 0
A 0.43 B 0.14
A 0.43 B 0.29
A 0.43 B 0.43
A 0.43 B 0.57
A 0.43 B 0.71
A 0.43 B 0.86
A 0.43 B 1
A 0.57 B 0
A 0.57 B 0.14
A 0.57 B 0.29
A 0.57 B 0.43
A 0.57 B 0.57
A 0.57 B 0.71
A 0.57 B 0.86
A 0.57 B 1
A 0.71 B 0
A 0.71 B 0.14
A 0.71 B 0.29
A 0.71 B 0.43
A 0.71 B 0.57
A 0.71 B 0.71
A 0.71 B 0.86
A 0.71 B 1
A 0.86 B 0
A 0.86 B 0.14
A 0.86 B 0.29
A 0.86 B 0.43
A 0.86 B 0.57
A 0.86 B 0.71
A 0.86 B 0.86
A 0.86 B 1
A 1 B 0
A 1 B 0.14
A 1 B 0.29
A 1 B 0.43
A 1 B 0.57
A 1 B 0.71
A 1 B 0.86
A 1 B 1
OD 600 nm [−]
0.2
0.4
0.6
0 5 101520
Time [h]

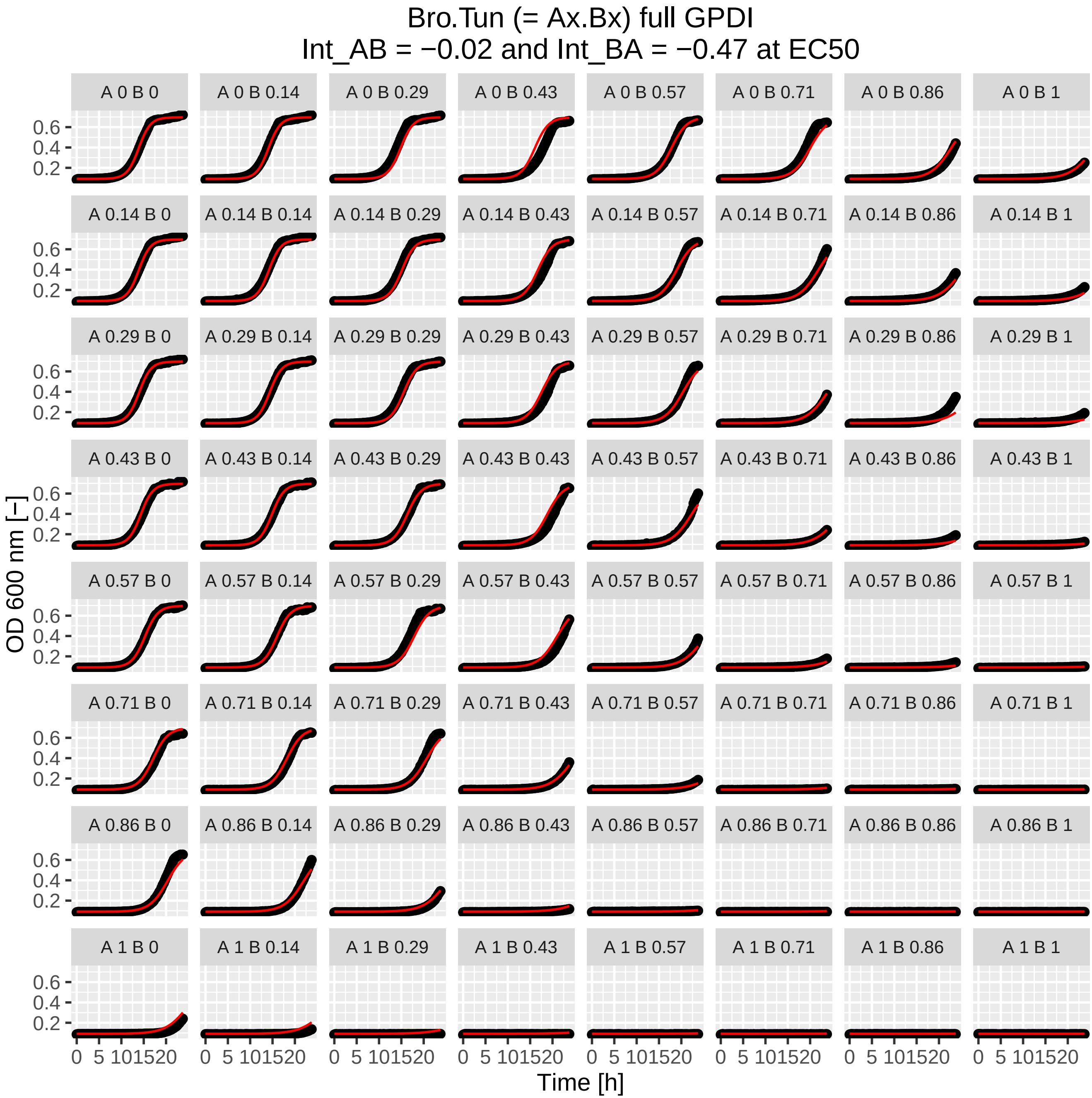
Bro.Tun (= Ax.Bx) full GPDI
Int_AB = −0.02 and Int_BA = −0.47 at EC50
OD 600 nm [−]
Time [h]
A 0 B 0
A 0 B 0.14
A 0 B 0.29
A 0 B 0.43
A 0 B 0.57
A 0 B 0.71
A 0 B 0.86
A 0 B 1
A 1 B 0
A 1 B 0.14
A 1 B 0.29
A 1 B 0.43
A 1 B 0.57
A 1 B 0.71
A 1 B 0.86
A 1 B 1

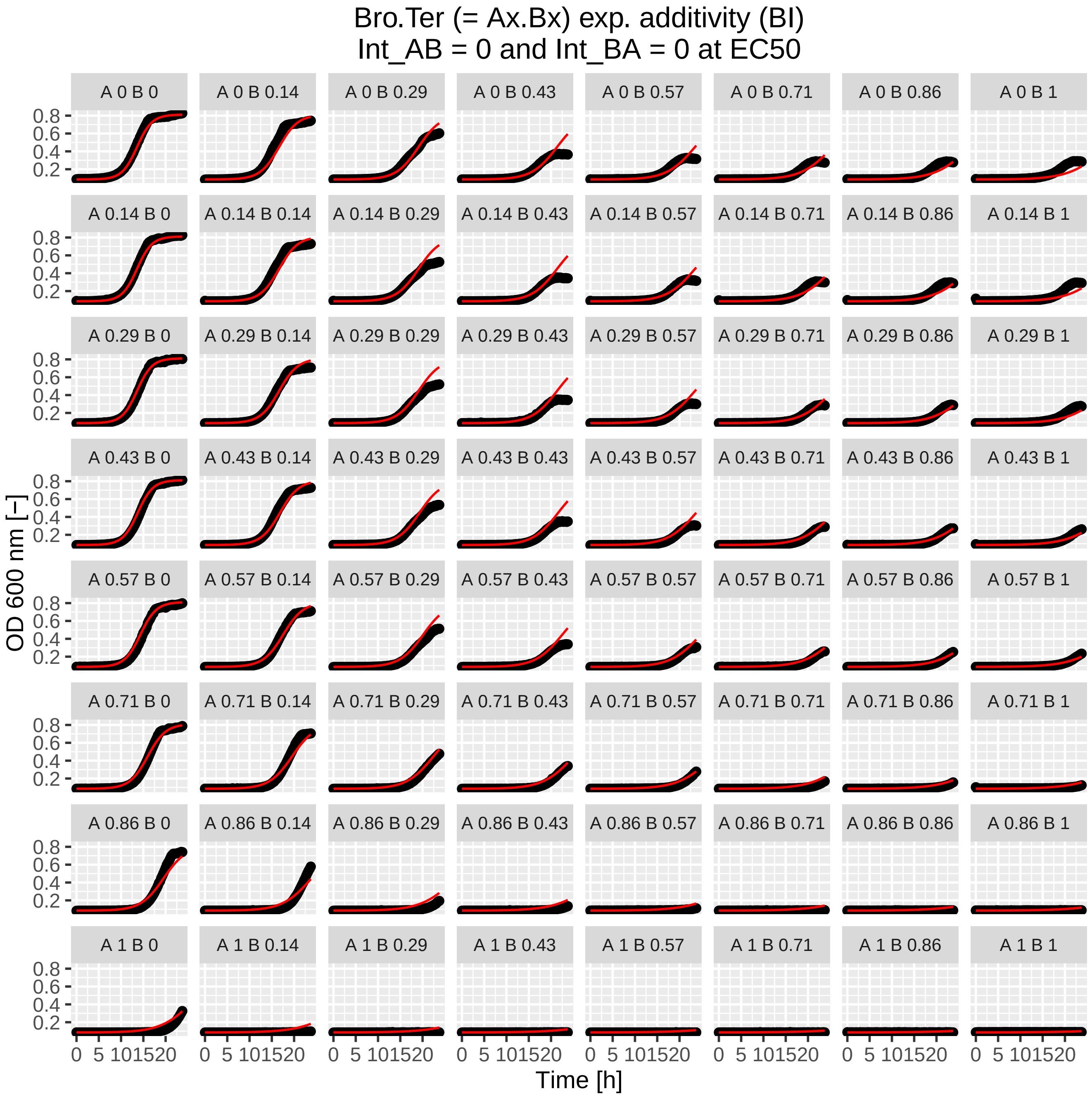

Bro.Ter (= Ax.Bx) exp. additivity (BI)
Int_AB = 0 and Int_BA = 0 at EC50
A 0 B 0
A 0 B 0.14
A 0 B 0.29
A 0 B 0.43
A 0 B 0.57
A 0 B 0.71
A 0 B 0.86
A 0 B 1
A 0.14 B 0
A 0.14 B 0.14
A 0.14 B 0.29
A 0.14 B 0.43
A 0.14 B 0.57
A 0.14 B 0.71
A 0.14 B 0.86
A 0.14 B 1
A 0.29 B 0
A 0.29 B 0.14
A 0.29 B 0.29
A 0.29 B 0.43
A 0.29 B 0.57
A 0.29 B 0.71
A 0.29 B 0.86
A 0.29 B 1
A 0.43 B 0
A 0.43 B 0.14
A 0.43 B 0.29
A 0.43 B 0.43
A 0.43 B 0.57
A 0.43 B 0.71
A 0.43 B 0.86
A 0.43 B 1
A 0.57 B 0
A 0.57 B 0.14
A 0.57 B 0.29
A 0.57 B 0.43
A 0.57 B 0.57
A 0.57 B 0.71
A 0.57 B 0.86
A 0.57 B 1
A 0.71 B 0
A 0.71 B 0.14
A 0.71 B 0.29
A 0.71 B 0.43
A 0.71 B 0.57
A 0.71 B 0.71
A 0.71 B 0.86
A 0.71 B 1
A 0.86 B 0
A 0.86 B 0.14
A 0.86 B 0.29
A 0.86 B 0.43
A 0.86 B 0.57
A 0.86 B 0.71
A 0.86 B 0.86
A 0.86 B 1
A 1 B 0
A 1 B 0.14
A 1 B 0.29
A 1 B 0.43
A 1 B 0.57
A 1 B 0.71
A 1 B 0.86
A 1 B 1
0.8
0.6
0.4
0.2
0 5 101520
OD 600 nm [–]
Time [h]

Bro.Ter (= Ax.Bx) full GPDI
Int_AB = 0.02 and Int_BA = −0.2 at EC50

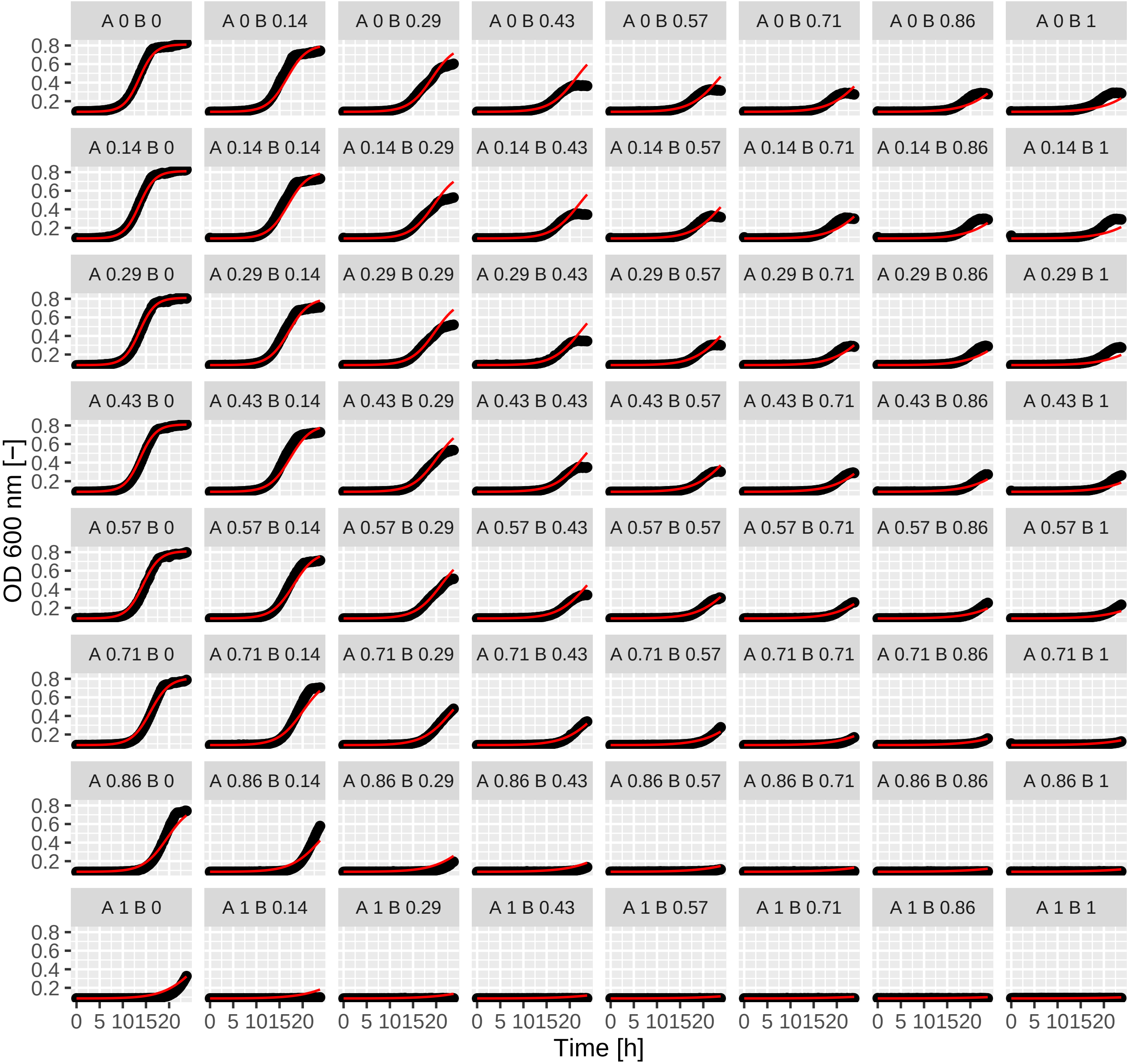

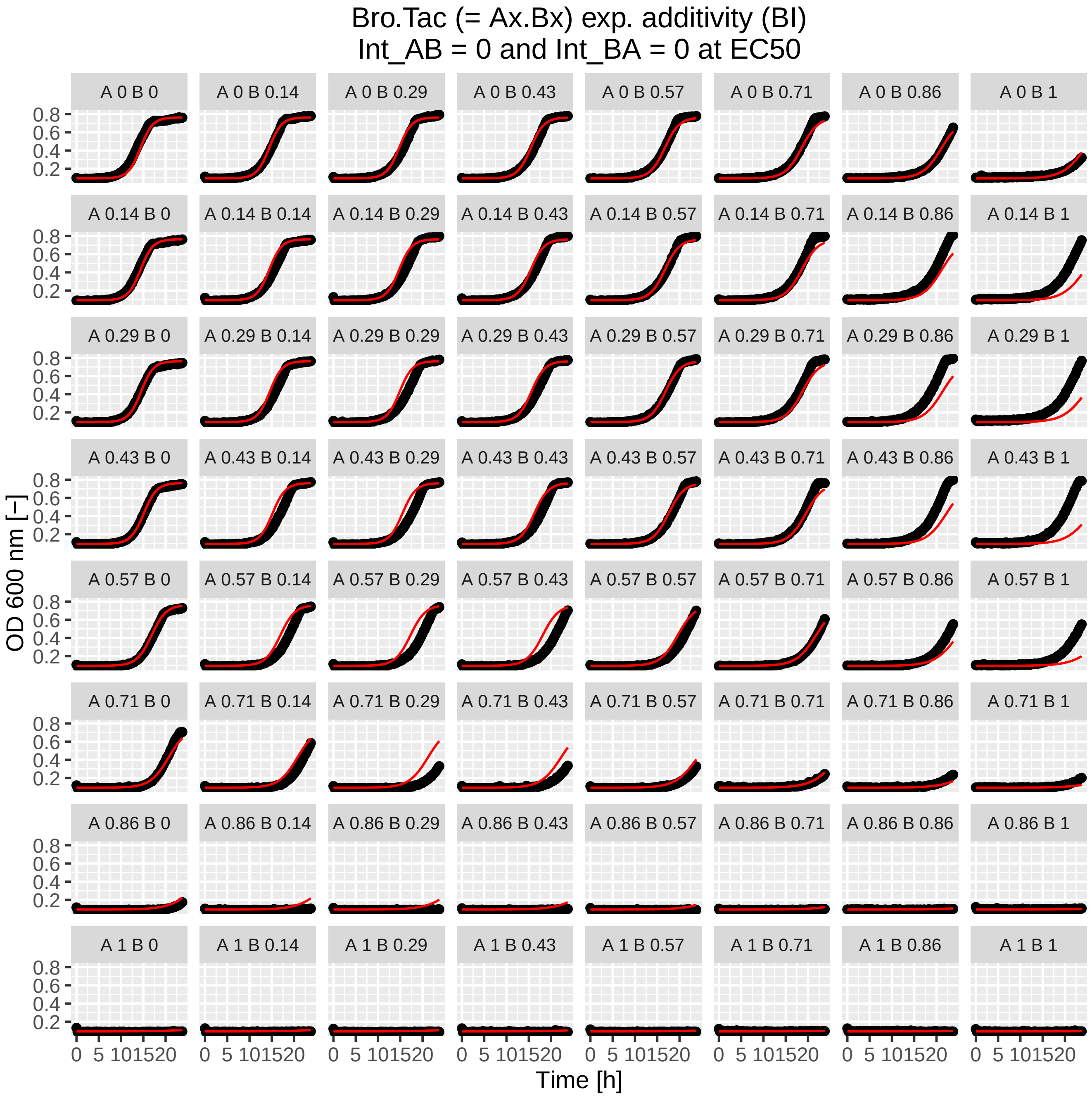
Bro.Tac (= Ax.Bx) exp. additivity (BI)
Int_AB = 0 and Int_BA = 0 at EC50
A 0 B 0
A 0 B 0.14
A 0 B 0.29
A 0 B 0.43
A 0 B 0.57
A 0 B 0.71
A 0 B 0.86
A 0 B 1
A 0.14 B 0
A 0.14 B 0.14
A 0.14 B 0.29
A 0.14 B 0.43
A 0.14 B 0.57
A 0.14 B 0.71
A 0.14 B 0.86
A 0.14 B 1
A 0.29 B 0
A 0.29 B 0.14
A 0.29 B 0.29
A 0.29 B 0.43
A 0.29 B 0.57
A 0.29 B 0.71
A 0.29 B 0.86
A 0.29 B 1
A 0.43 B 0
A 0.43 B 0.14
A 0.43 B 0.29
A 0.43 B 0.43
A 0.43 B 0.57
A 0.43 B 0.71
A 0.43 B 0.86
A 0.43 B 1
A 0.57 B 0
A 0.57 B 0.14
A 0.57 B 0.29
A 0.57 B 0.43
A 0.57 B 0.57
A 0.57 B 0.71
A 0.57 B 0.86
A 0.57 B 1
A 0.71 B 0
A 0.71 B 0.14
A 0.71 B 0.29
A 0.71 B 0.43
A 0.71 B 0.57
A 0.71 B 0.71
A 0.71 B 0.86
A 0.71 B 1
A 0.86 B 0
A 0.86 B 0.14
A 0.86 B 0.29
A 0.86 B 0.43
A 0.86 B 0.57
A 0.86 B 0.71
A 0.86 B 0.86
A 0.86 B 1
A 1 B 0
A 1 B 0.14
A 1 B 0.29
A 1 B 0.43
A 1 B 0.57
A 1 B 0.71
A 1 B 0.86
A 1 B 1
OD 600 nm [–]
0.8
0.6
0.4
0.2
0 5 101520
Time [h]

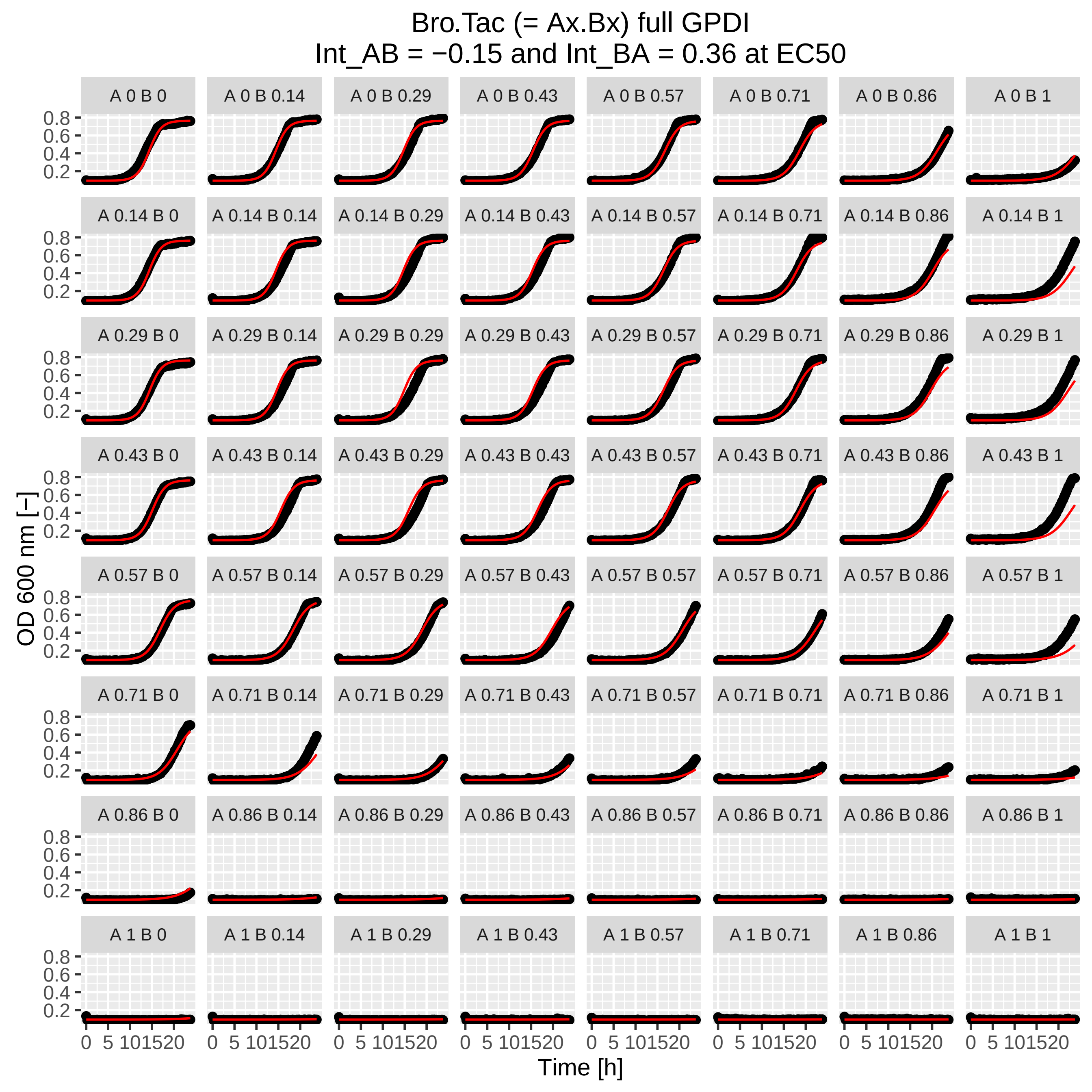
Bro.Tac (= Ax.Bx) full GPDI
Int_AB = −0.15 and Int_BA = 0.36 at EC50
A 0 B 0
A 0 B 0.14
A 0 B 0.29
A 0 B 0.43
A 0 B 0.57
A 0 B 0.71
A 0 B 0.86
A 0 B 1
A 0.14 B 0
A 0.14 B 0.14
A 0.14 B 0.29
A 0.14 B 0.43
A 0.14 B 0.57
A 0.14 B 0.71
A 0.14 B 0.86
A 0.14 B 1
A 0.29 B 0
A 0.29 B 0.14
A 0.29 B 0.29
A 0.29 B 0.43
A 0.29 B 0.57
A 0.29 B 0.71
A 0.29 B 0.86
A 0.29 B 1
A 0.43 B 0
A 0.43 B 0.14
A 0.43 B 0.29
A 0.43 B 0.43
A 0.43 B 0.57
A 0.43 B 0.71
A 0.43 B 0.86
A 0.43 B 1
A 0.57 B 0
A 0.57 B 0.14
A 0.57 B 0.29
A 0.57 B 0.43
A 0.57 B 0.57
A 0.57 B 0.71
A 0.57 B 0.86
A 0.57 B 1
A 0.71 B 0
A 0.71 B 0.14
A 0.71 B 0.29
A 0.71 B 0.43
A 0.71 B 0.57
A 0.71 B 0.71
A 0.71 B 0.86
A 0.71 B 1
A 0.86 B 0
A 0.86 B 0.14
A 0.86 B 0.29
A 0.86 B 0.43
A 0.86 B 0.57
A 0.86 B 0.71
A 0.86 B 0.86
A 0.86 B 1
A 1 B 0
A 1 B 0.14
A 1 B 0.29
A 1 B 0.43
A 1 B 0.57
A 1 B 0.71
A 1 B 0.86
A 1 B 1
0.8
0.6
0.4
0.2
0 5 101520
OD 600 nm [−]
Time [h]

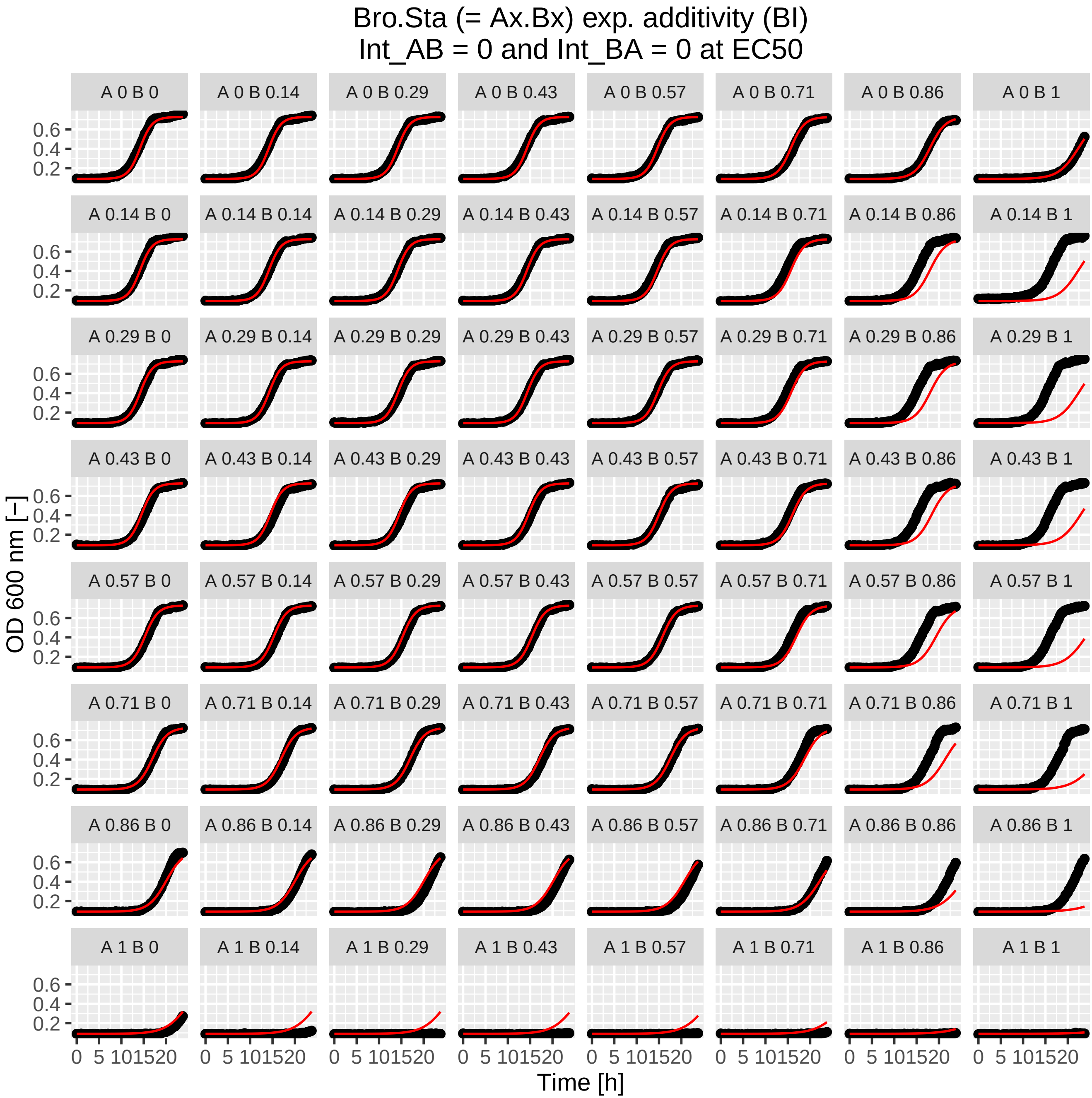

Bro.Sta (= Ax.Bx) exp. additivity (BI)
Int_AB = 0 and Int_BA = 0 at EC50
OD 600 nm [–]
Time [h]
0.2
0.4
0.6
0 5 101520
A 0 B 0
A 0 B 0.14
A 0 B 0.29
A 0 B 0.43
A 0 B 0.57
A 0 B 0.71
A 0 B 0.86
A 0 B 1
A 0.14 B 0
A 0.14 B 0.14
A 0.14 B 0.29
A 0.14 B 0.43
A 0.14 B 0.57
A 0.14 B 0.71
A 0.14 B 0.86
A 0.14 B 1
A 0.29 B 0
A 0.29 B 0.14
A 0.29 B 0.29
A 0.29 B 0.43
A 0.29 B 0.57
A 0.29 B 0.71
A 0.29 B 0.86
A 0.29 B 1
A 0.43 B 0
A 0.43 B 0.14
A 0.43 B 0.29
A 0.43 B 0.43
A 0.43 B 0.57
A 0.43 B 0.71
A 0.43 B 0.86
A 0.43 B 1
A 0.57 B 0
A 0.57 B 0.14
A 0.57 B 0.29
A 0.57 B 0.43
A 0.57 B 0.57
A 0.57 B 0.71
A 0.57 B 0.86
A 0.57 B 1
A 0.71 B 0
A 0.71 B 0.14
A 0.71 B 0.29
A 0.71 B 0.43
A 0.71 B 0.57
A 0.71 B 0.71
A 0.71 B 0.86
A 0.71 B 1
A 0.86 B 0
A 0.86 B 0.14
A 0.86 B 0.29
A 0.86 B 0.43
A 0.86 B 0.57
A 0.86 B 0.71
A 0.86 B 0.86
A 0.86 B 1
A 1 B 0
A 1 B 0.14
A 1 B 0.29
A 1 B 0.43
A 1 B 0.57
A 1 B 0.71
A 1 B 0.86
A 1 B 1

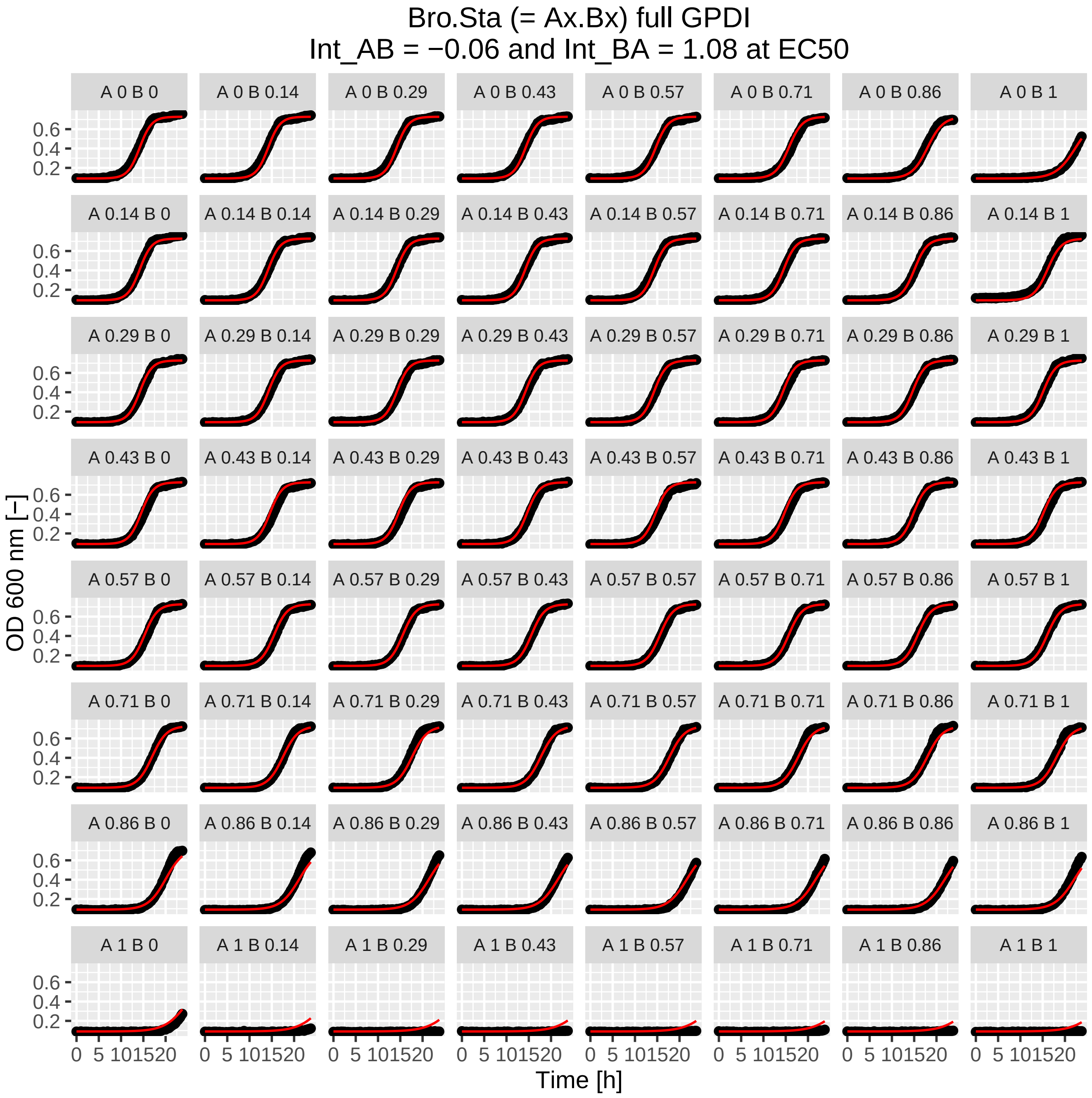

Bro.Sta (= Ax.Bx) full GPDI
Int_AB = −0.06 and Int_BA = 1.08 at EC50
A 0 B 0
A 0 B 0.14
A 0 B 0.29
A 0 B 0.43
A 0 B 0.57
A 0 B 0.71
A 0 B 0.86
A 0 B 1
A 0.14 B 0
A 0.14 B 0.14
A 0.14 B 0.29
A 0.14 B 0.43
A 0.14 B 0.57
A 0.14 B 0.71
A 0.14 B 0.86
A 0.14 B 1
A 0.29 B 0
A 0.29 B 0.14
A 0.29 B 0.29
A 0.29 B 0.43
A 0.29 B 0.57
A 0.29 B 0.71
A 0.29 B 0.86
A 0.29 B 1
A 0.43 B 0
A 0.43 B 0.14
A 0.43 B 0.29
A 0.43 B 0.43
A 0.43 B 0.57
A 0.43 B 0.71
A 0.43 B 0.86
A 0.43 B 1
A 0.57 B 0
A 0.57 B 0.14
A 0.57 B 0.29
A 0.57 B 0.43
A 0.57 B 0.57
A 0.57 B 0.71
A 0.57 B 0.86
A 0.57 B 1
A 0.71 B 0
A 0.71 B 0.14
A 0.71 B 0.29
A 0.71 B 0.43
A 0.71 B 0.57
A 0.71 B 0.71
A 0.71 B 0.86
A 0.71 B 1
A 0.86 B 0
A 0.86 B 0.14
A 0.86 B 0.29
A 0.86 B 0.43
A 0.86 B 0.57
A 0.86 B 0.71
A 0.86 B 0.86
A 0.86 B 1
A 1 B 0
A 1 B 0.14
A 1 B 0.29
A 1 B 0.43
A 1 B 0.57
A 1 B 0.71
A 1 B 0.86
A 1 B 1
0.6
0.4
0.2
OD 600 nm [−]
0 5 101520
Time [h]

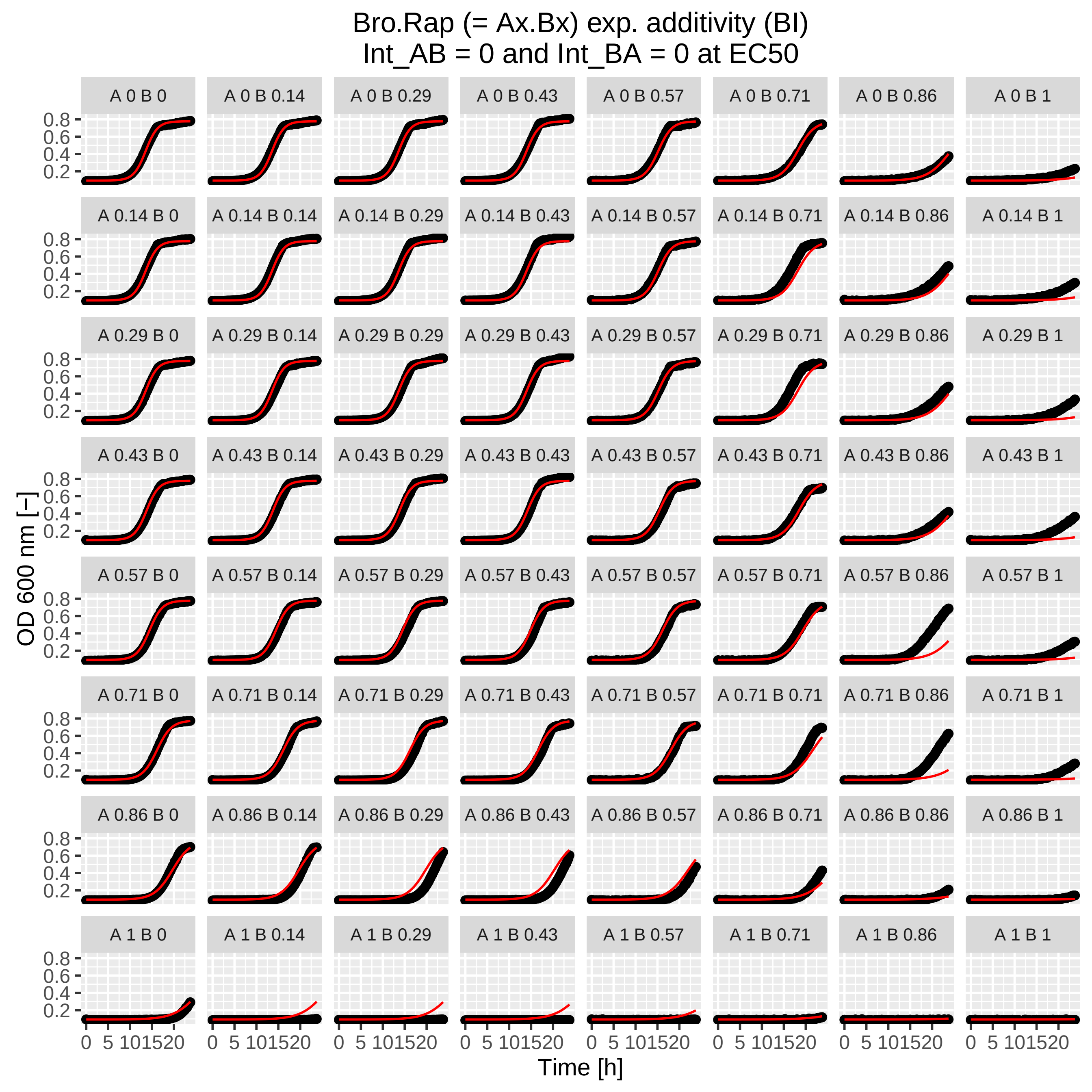

Bro.Rap (= Ax.Bx) exp. additivity (BI)
Int_AB = 0 and Int_BA = 0 at EC50
A 0 B 0
A 0 B 0.14
A 0 B 0.29
A 0 B 0.43
A 0 B 0.57
A 0 B 0.71
A 0 B 0.86
A 0 B 1
A 0.14 B 0
A 0.14 B 0.14
A 0.14 B 0.29
A 0.14 B 0.43
A 0.14 B 0.57
A 0.14 B 0.71
A 0.14 B 0.86
A 0.14 B 1
A 0.29 B 0
A 0.29 B 0.14
A 0.29 B 0.29
A 0.29 B 0.43
A 0.29 B 0.57
A 0.29 B 0.71
A 0.29 B 0.86
A 0.29 B 1
A 0.43 B 0
A 0.43 B 0.14
A 0.43 B 0.29
A 0.43 B 0.43
A 0.43 B 0.57
A 0.43 B 0.71
A 0.43 B 0.86
A 0.43 B 1
A 0.57 B 0
A 0.57 B 0.14
A 0.57 B 0.29
A 0.57 B 0.43
A 0.57 B 0.57
A 0.57 B 0.71
A 0.57 B 0.86
A 0.57 B 1
A 0.71 B 0
A 0.71 B 0.14
A 0.71 B 0.29
A 0.71 B 0.43
A 0.71 B 0.57
A 0.71 B 0.71
A 0.71 B 0.86
A 0.71 B 1
A 0.86 B 0
A 0.86 B 0.14
A 0.86 B 0.29
A 0.86 B 0.43
A 0.86 B 0.57
A 0.86 B 0.71
A 0.86 B 0.86
A 0.86 B 1
A 1 B 0
A 1 B 0.14
A 1 B 0.29
A 1 B 0.43
A 1 B 0.57
A 1 B 0.71
A 1 B 0.86
A 1 B 1
0.8
0.6
0.4
0.2
OD 600 nm [–]
0 5 101520
Time [h]

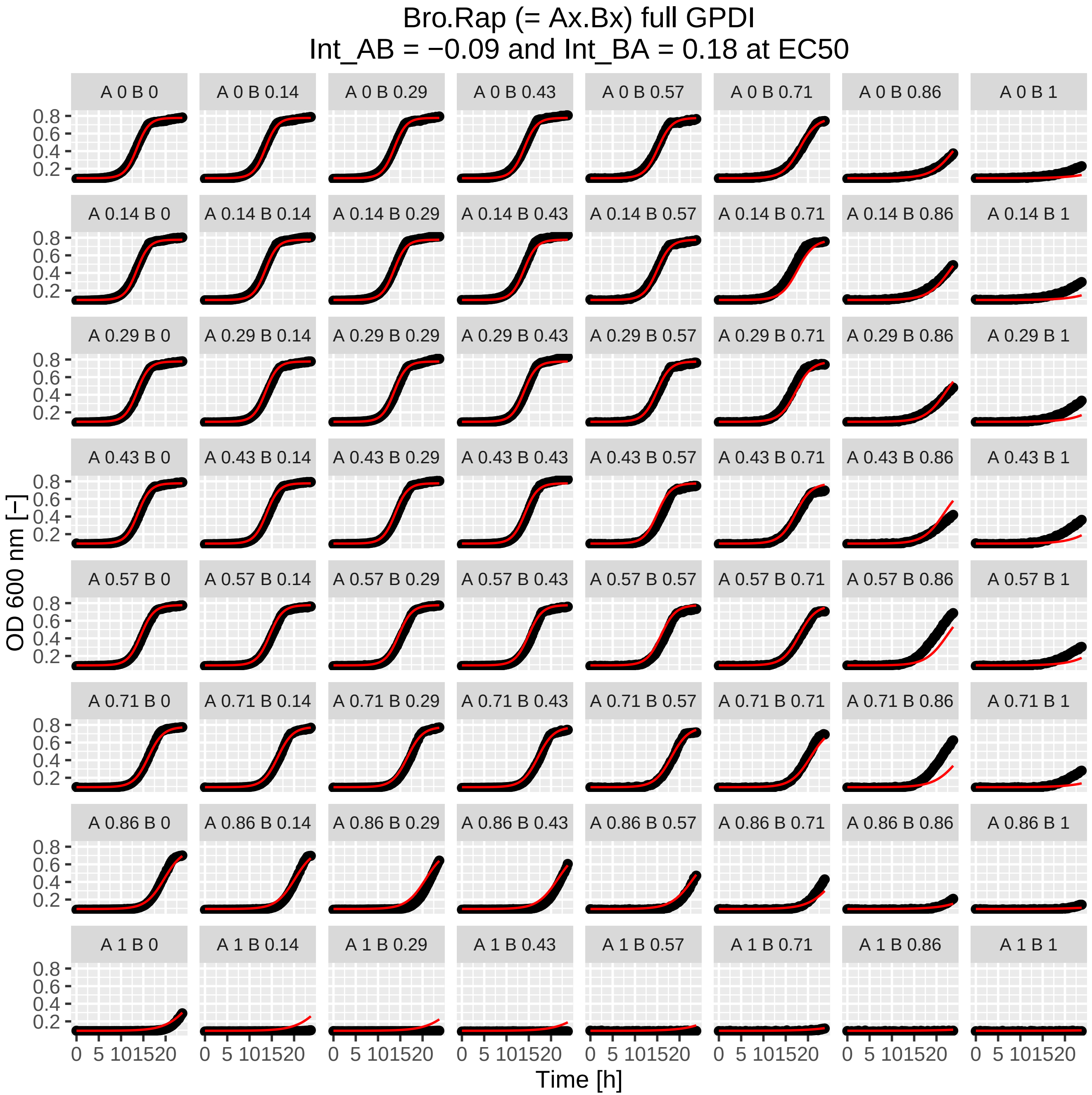
Bro.Rap (= Ax.Bx) full GPDI
Int_AB = −0.09 and Int_BA = 0.18 at EC50
OD 600 nm [−]
Time [h]
A 0 B 0
A 0 B 0.14
A 0 B 0.29
A 0 B 0.43
A 0 B 0.57
A 0 B 0.71
A 0 B 0.86
A 0 B 1
A 1 B 0
A 1 B 1

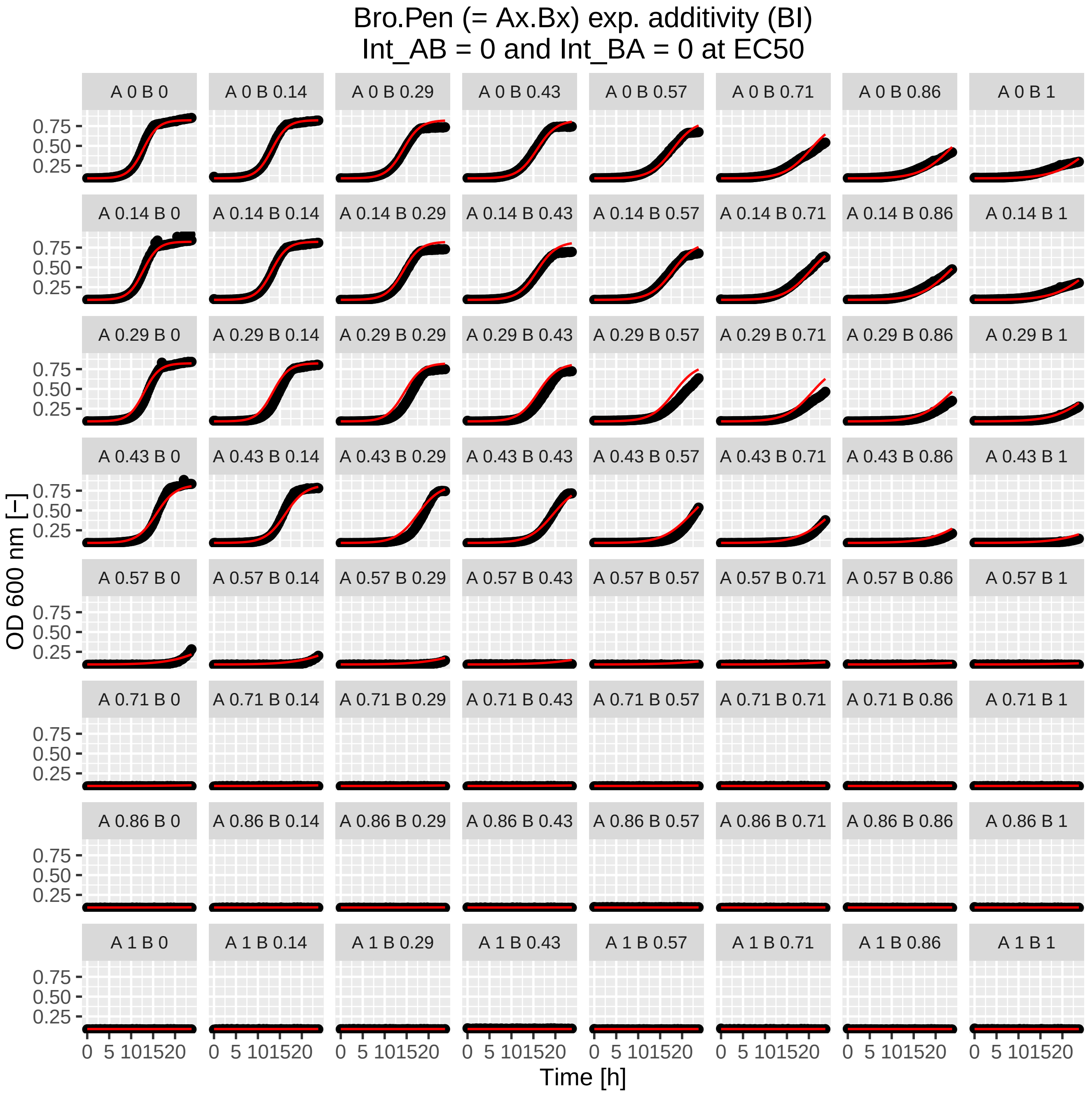
Bro.Pen (= Ax.Bx) exp. additivity (BI)
Int_AB = 0 and Int_BA = 0 at EC50
OD 600 nm [–]
Time [h]
0.75
0.50
0.25
0 5 101520
A 0 B 0
A 0 B 0.14
A 0 B 0.29
A 0 B 0.43
A 0 B 0.57
A 0 B 0.71
A 0 B 0.86
A 0 B 1
A 0.14 B 0
A 0.14 B 0.14
A 0.14 B 0.29
A 0.14 B 0.43
A 0.14 B 0.57
A 0.14 B 0.71
A 0.14 B 0.86
A 0.14 B 1
A 0.29 B 0
A 0.29 B 0.14
A 0.29 B 0.29
A 0.29 B 0.43
A 0.29 B 0.57
A 0.29 B 0.71
A 0.29 B 0.86
A 0.29 B 1
A 0.43 B 0
A 0.43 B 0.14
A 0.43 B 0.29
A 0.43 B 0.43
A 0.43 B 0.57
A 0.43 B 0.71
A 0.43 B 0.86
A 0.43 B 1
A 0.57 B 0
A 0.57 B 0.14
A 0.57 B 0.29
A 0.57 B 0.43
A 0.57 B 0.57
A 0.57 B 0.71
A 0.57 B 0.86
A 0.57 B 1
A 0.71 B 0
A 0.71 B 0.14
A 0.71 B 0.29
A 0.71 B 0.43
A 0.71 B 0.57
A 0.71 B 0.71
A 0.71 B 0.86
A 0.71 B 1
A 0.86 B 0
A 0.86 B 0.14
A 0.86 B 0.29
A 0.86 B 0.43
A 0.86 B 0.57
A 0.86 B 0.71
A 0.86 B 0.86
A 0.86 B 1
A 1 B 0
A 1 B 0.14
A 1 B 0.29
A 1 B 0.43
A 1 B 0.57
A 1 B 0.71
A 1 B 0.86
A 1 B 1

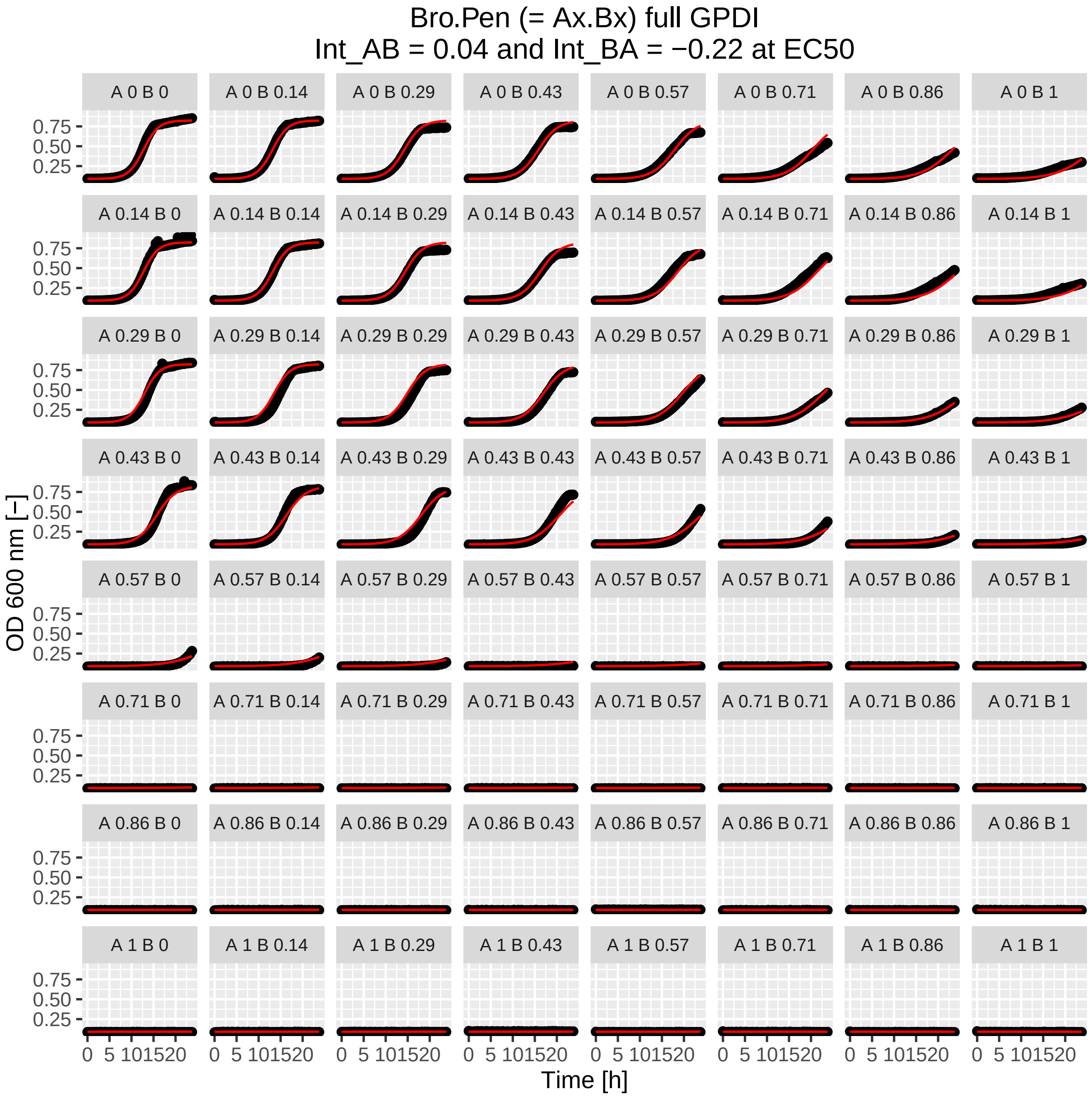

Bro.Pen (= Ax.Bx) full GPDI
Int_AB = 0.04 and Int_BA = −0.22 at EC50
A 0 B 0
A 0 B 0.14
A 0 B 0.29
A 0 B 0.43
A 0 B 0.57
A 0 B 0.71
A 0 B 0.86
A 0 B 1
A 0.14 B 0
A 0.14 B 0.14
A 0.14 B 0.29
A 0.14 B 0.43
A 0.14 B 0.57
A 0.14 B 0.71
A 0.14 B 0.86
A 0.14 B 1
A 0.29 B 0
A 0.29 B 0.14
A 0.29 B 0.29
A 0.29 B 0.43
A 0.29 B 0.57
A 0.29 B 0.71
A 0.29 B 0.86
A 0.29 B 1
A 0.43 B 0
A 0.43 B 0.14
A 0.43 B 0.29
A 0.43 B 0.43
A 0.43 B 0.57
A 0.43 B 0.71
A 0.43 B 0.86
A 0.43 B 1
A 0.57 B 0
A 0.57 B 0.14
A 0.57 B 0.29
A 0.57 B 0.43
A 0.57 B 0.57
A 0.57 B 0.71
A 0.57 B 0.86
A 0.57 B 1
A 0.71 B 0
A 0.71 B 0.14
A 0.71 B 0.29
A 0.71 B 0.43
A 0.71 B 0.57
A 0.71 B 0.71
A 0.71 B 0.86
A 0.71 B 1
A 0.86 B 0
A 0.86 B 0.14
A 0.86 B 0.29
A 0.86 B 0.43
A 0.86 B 0.57
A 0.86 B 0.71
A 0.86 B 0.86
A 0.86 B 1
A 1 B 0
A 1 B 0.14
A 1 B 0.29
A 1 B 0.43
A 1 B 0.57
A 1 B 0.71
A 1 B 0.86
A 1 B 1
0.75
0.50
0.25
0 5 101520
OD 600 nm [−]
Time [h]

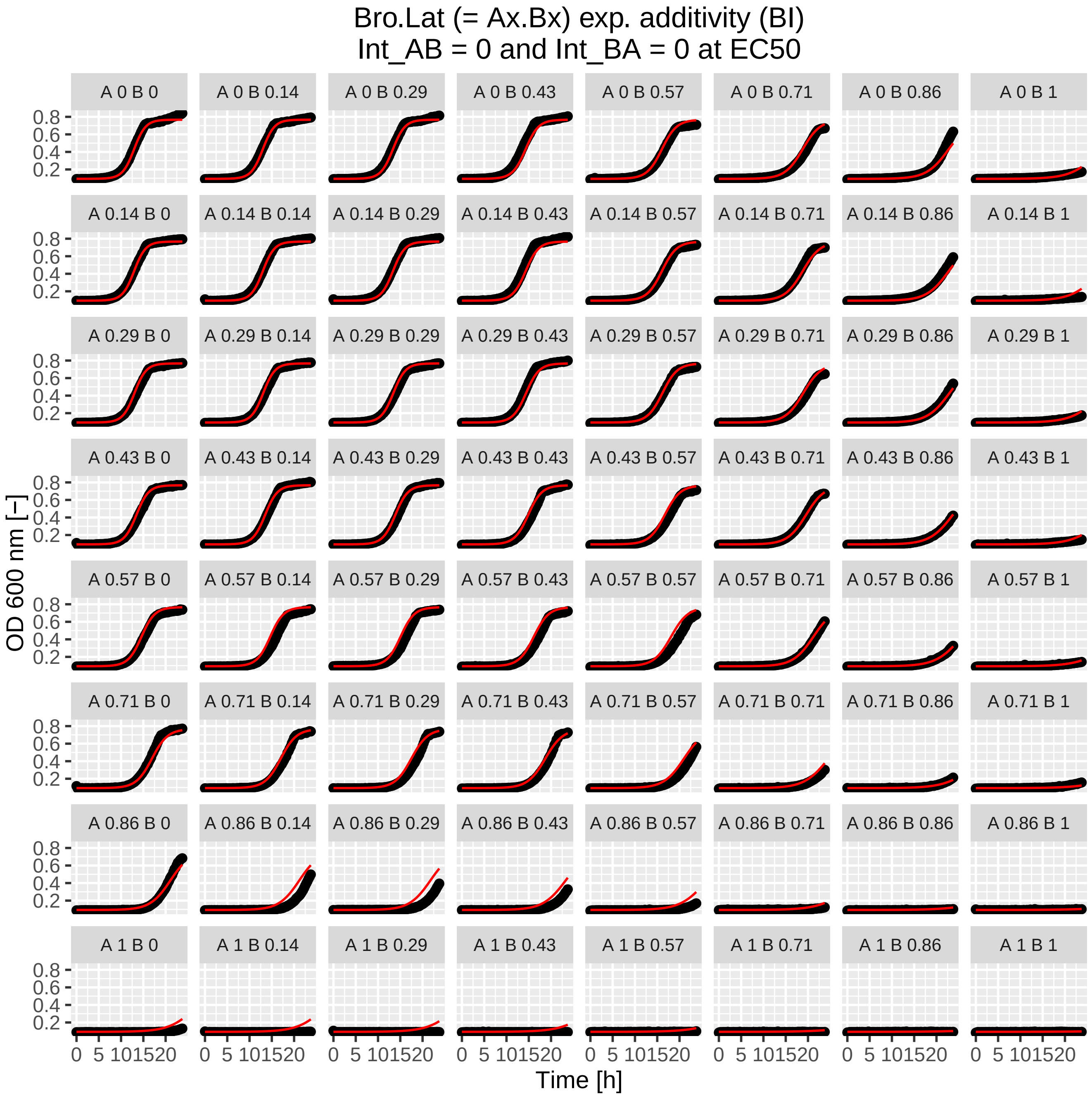
Bro.Lat (= Ax.Bx) exp. additivity (BI)
Int_AB = 0 and Int_BA = 0 at EC50
A 0 B 0
A 0 B 0.14
A 0 B 0.29
A 0 B 0.43
A 0 B 0.57
A 0 B 0.71
A 0 B 0.86
A 0 B 1
A 0.14 B 0
A 0.14 B 0.14
A 0.14 B 0.29
A 0.14 B 0.43
A 0.14 B 0.57
A 0.14 B 0.71
A 0.14 B 0.86
A 0.14 B 1
A 0.29 B 0
A 0.29 B 0.14
A 0.29 B 0.29
A 0.29 B 0.43
A 0.29 B 0.57
A 0.29 B 0.71
A 0.29 B 0.86
A 0.29 B 1
A 0.43 B 0
A 0.43 B 0.14
A 0.43 B 0.29
A 0.43 B 0.43
A 0.43 B 0.57
A 0.43 B 0.71
A 0.43 B 0.86
A 0.43 B 1
A 0.57 B 0
A 0.57 B 0.14
A 0.57 B 0.29
A 0.57 B 0.43
A 0.57 B 0.57
A 0.57 B 0.71
A 0.57 B 0.86
A 0.57 B 1
A 0.71 B 0
A 0.71 B 0.14
A 0.71 B 0.29
A 0.71 B 0.43
A 0.71 B 0.57
A 0.71 B 0.71
A 0.71 B 0.86
A 0.71 B 1
A 0.86 B 0
A 0.86 B 0.14
A 0.86 B 0.29
A 0.86 B 0.43
A 0.86 B 0.57
A 0.86 B 0.71
A 0.86 B 0.86
A 0.86 B 1
A 1 B 0
A 1 B 0.14
A 1 B 0.29
A 1 B 0.43
A 1 B 0.57
A 1 B 0.71
A 1 B 0.86
A 1 B 1
OD 600 nm [−]
0.2
0.4
0.6
0.8
0
5
10
15
20
Time [h]

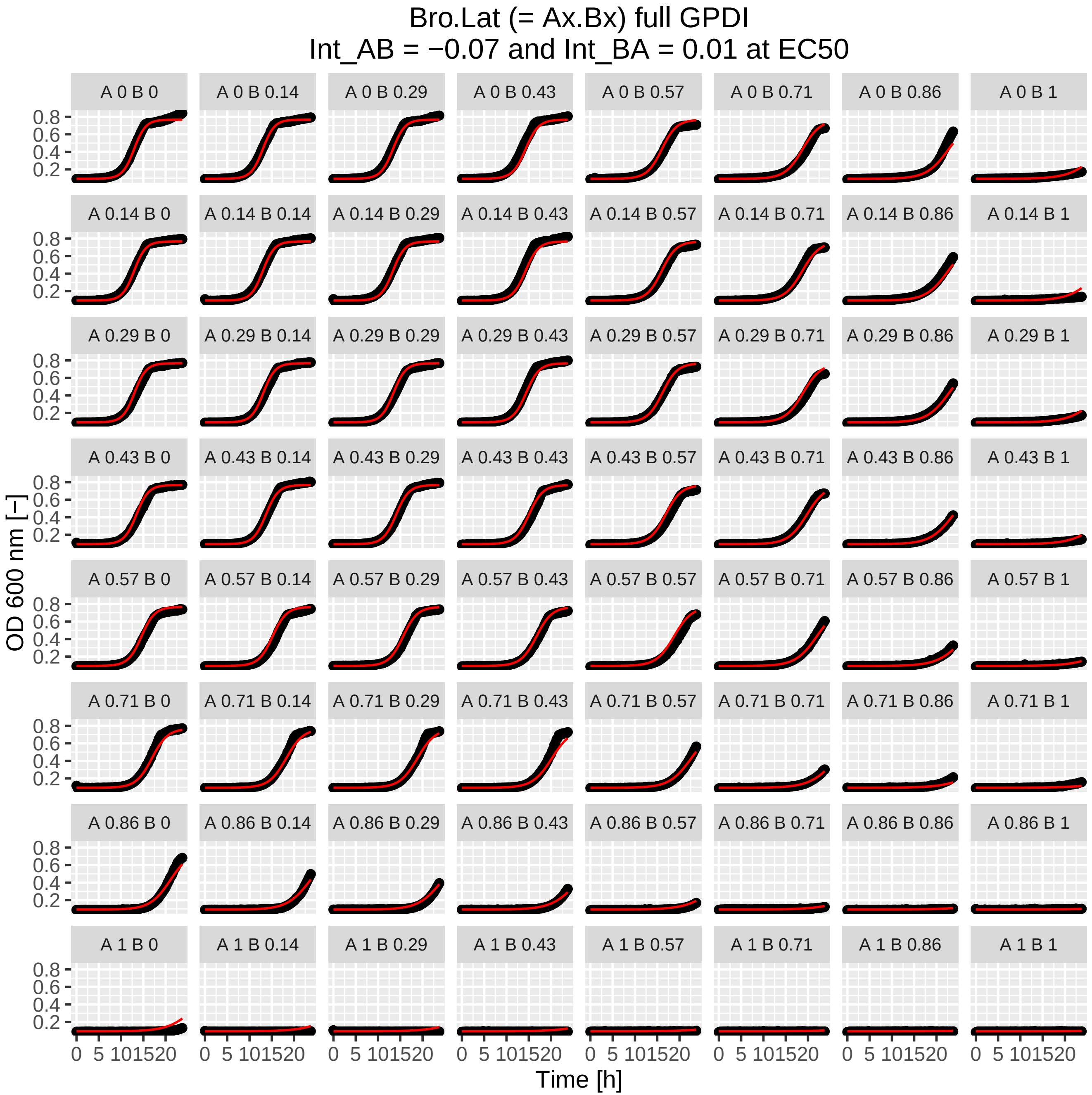

Bro.Lat (= Ax.Bx) full GPDI
Int_AB = −0.07 and Int_BA = 0.01 at EC50
A 0 B 0
A 0 B 0.14
A 0 B 0.29
A 0 B 0.43
A 0 B 0.57
A 0 B 0.71
A 0 B 0.86
A 0 B 1
A 0.14 B 0
A 0.14 B 0.14
A 0.14 B 0.29
A 0.14 B 0.43
A 0.14 B 0.57
A 0.14 B 0.71
A 0.14 B 0.86
A 0.14 B 1
A 0.29 B 0
A 0.29 B 0.14
A 0.29 B 0.29
A 0.29 B 0.43
A 0.29 B 0.57
A 0.29 B 0.71
A 0.29 B 0.86
A 0.29 B 1
A 0.43 B 0
A 0.43 B 0.14
A 0.43 B 0.29
A 0.43 B 0.43
A 0.43 B 0.57
A 0.43 B 0.71
A 0.43 B 0.86
A 0.43 B 1
A 0.57 B 0
A 0.57 B 0.14
A 0.57 B 0.29
A 0.57 B 0.43
A 0.57 B 0.57
A 0.57 B 0.71
A 0.57 B 0.86
A 0.57 B 1
A 0.71 B 0
A 0.71 B 0.14
A 0.71 B 0.29
A 0.71 B 0.43
A 0.71 B 0.57
A 0.71 B 0.71
A 0.71 B 0.86
A 0.71 B 1
A 0.86 B 0
A 0.86 B 0.14
A 0.86 B 0.29
A 0.86 B 0.43
A 0.86 B 0.57
A 0.86 B 0.71
A 0.86 B 0.86
A 0.86 B 1
A 1 B 0
A 1 B 0.14
A 1 B 0.29
A 1 B 0.43
A 1 B 0.57
A 1 B 0.71
A 1 B 0.86
A 1 B 1
OD 600 nm [−]
0.8
0.6
0.4
0.2
0 5 101520
Time [h]

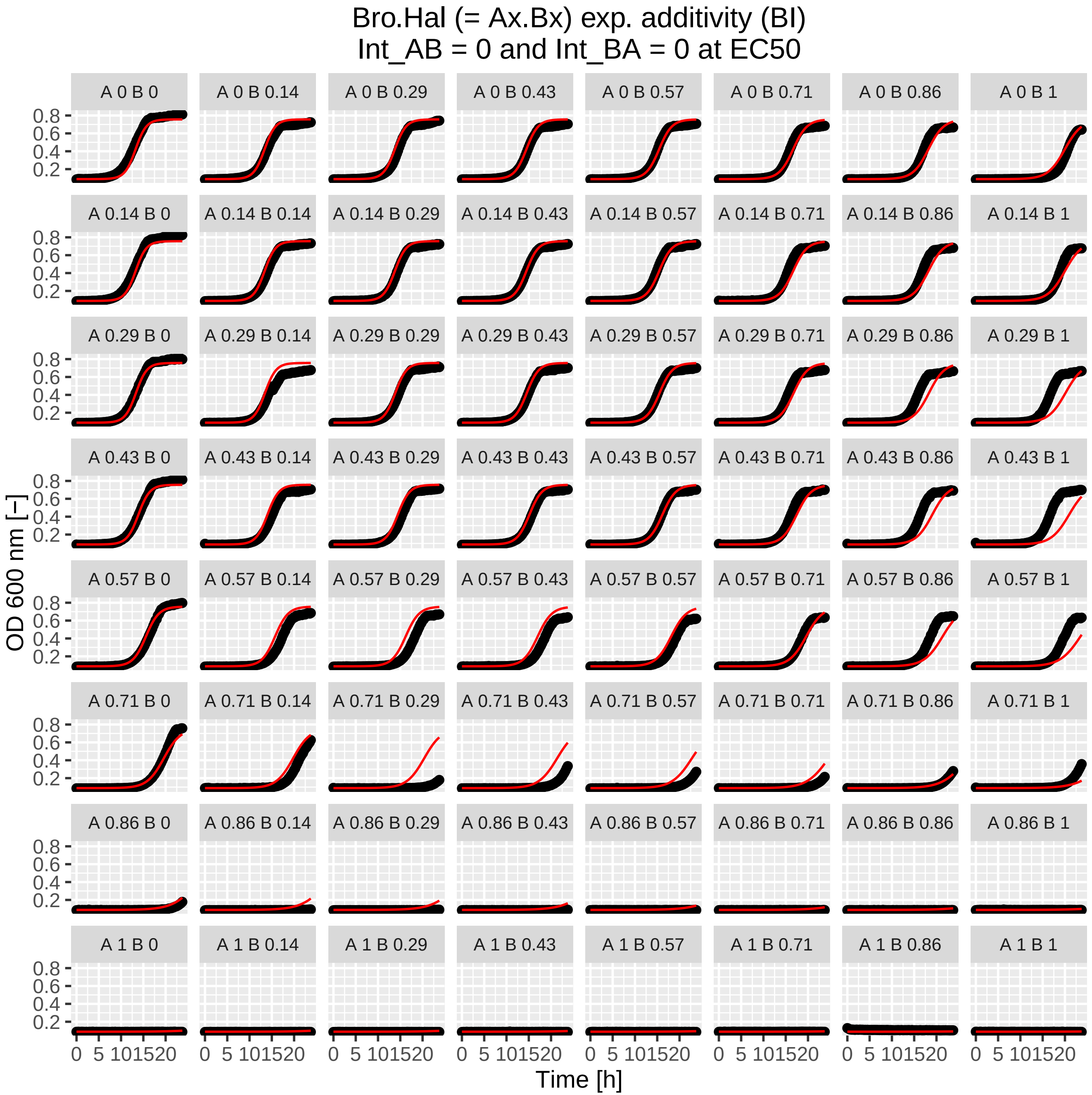

Bro.Hal (= Ax.Bx) exp. additivity (BI)
Int_AB = 0 and Int_BA = 0 at EC50
A 0 B 0
A 0 B 0.14
A 0 B 0.29
A 0 B 0.43
A 0 B 0.57
A 0 B 0.71
A 0 B 0.86
A 0 B 1
A 0.14 B 0
A 0.14 B 0.14
A 0.14 B 0.29
A 0.14 B 0.43
A 0.14 B 0.57
A 0.14 B 0.71
A 0.14 B 0.86
A 0.14 B 1
A 0.29 B 0
A 0.29 B 0.14
A 0.29 B 0.29
A 0.29 B 0.43
A 0.29 B 0.57
A 0.29 B 0.71
A 0.29 B 0.86
A 0.29 B 1
A 0.43 B 0
A 0.43 B 0.14
A 0.43 B 0.29
A 0.43 B 0.43
A 0.43 B 0.57
A 0.43 B 0.71
A 0.43 B 0.86
A 0.43 B 1
A 0.57 B 0
A 0.57 B 0.14
A 0.57 B 0.29
A 0.57 B 0.43
A 0.57 B 0.57
A 0.57 B 0.71
A 0.57 B 0.86
A 0.57 B 1
A 0.71 B 0
A 0.71 B 0.14
A 0.71 B 0.29
A 0.71 B 0.43
A 0.71 B 0.57
A 0.71 B 0.71
A 0.71 B 0.86
A 0.71 B 1
A 0.86 B 0
A 0.86 B 0.14
A 0.86 B 0.29
A 0.86 B 0.43
A 0.86 B 0.57
A 0.86 B 0.71
A 0.86 B 0.86
A 0.86 B 1
A 1 B 0
A 1 B 0.14
A 1 B 0.29
A 1 B 0.43
A 1 B 0.57
A 1 B 0.71
A 1 B 0.86
A 1 B 1
0.8
0.6
0.4
0.2
OD 600 nm [–]
0 5 101520
Time [h]

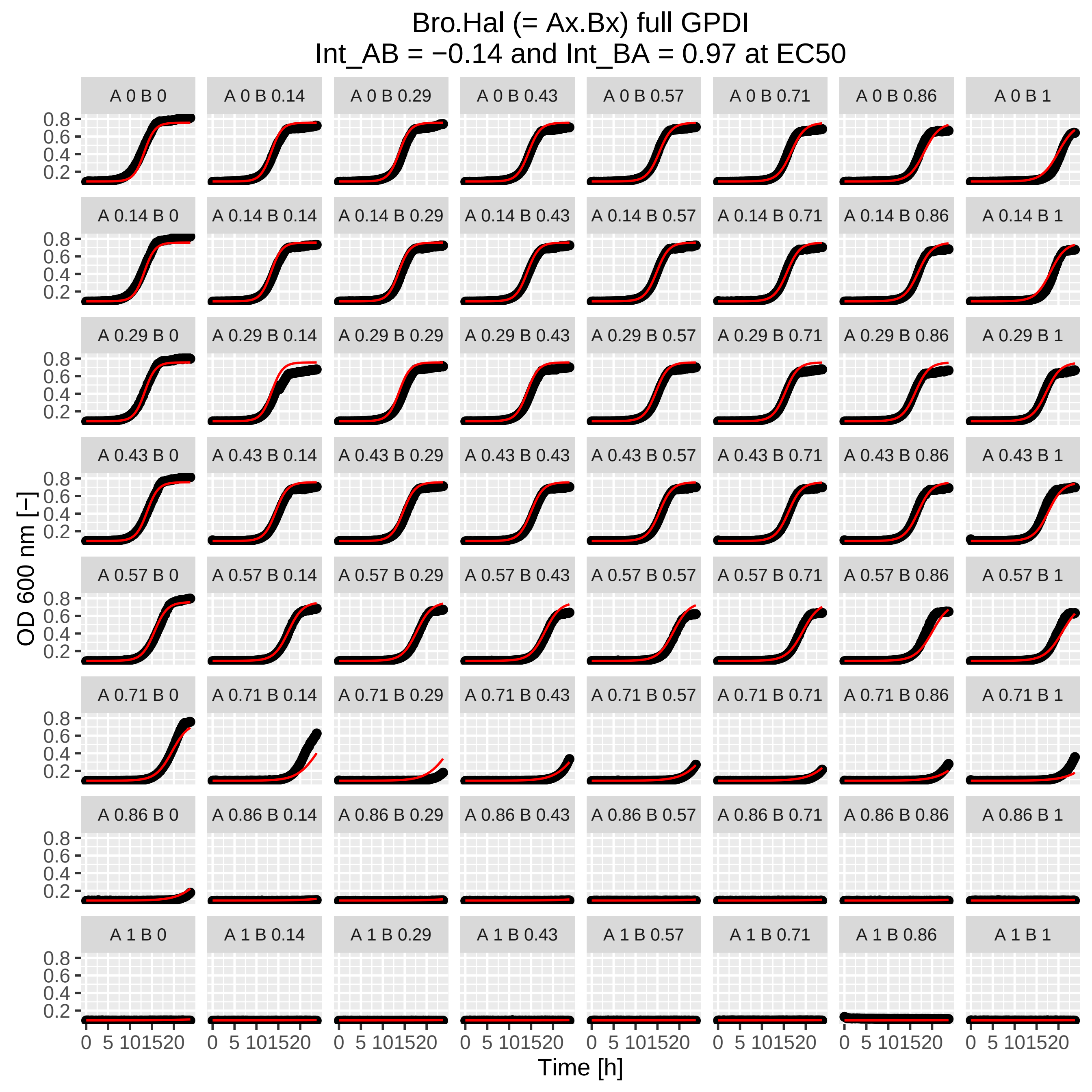

Bro.Hal (= Ax.Bx) full GPDI
Int_AB = −0.14 and Int_BA = 0.97 at EC50
OD 600 nm [−]
Time [h]
A 0 B 0
A 0 B 0.14
A 0 B 0.29
A 0 B 0.43
A 0 B 0.57
A 0 B 0.71
A 0 B 0.86
A 0 B 1
A 0.14 B 0
A 0.14 B 0.14
A 0.14 B 0.29
A 0.14 B 0.43
A 0.14 B 0.57
A 0.14 B 0.71
A 0.14 B 0.86
A 0.14 B 1
A 0.29 B 0
A 0.29 B 0.14
A 0.29 B 0.29
A 0.29 B 0.43
A 0.29 B 0.57
A 0.29 B 0.71
A 0.29 B 0.86
A 0.29 B 1
A 0.43 B 0
A 0.43 B 0.14
A 0.43 B 0.29
A 0.43 B 0.43
A 0.43 B 0.57
A 0.43 B 0.71
A 0.43 B 0.86
A 0.43 B 1
A 0.57 B 0
A 0.57 B 0.14
A 0.57 B 0.29
A 0.57 B 0.43
A 0.57 B 0.57
A 0.57 B 0.71
A 0.57 B 0.86
A 0.57 B 1
A 0.71 B 0
A 0.71 B 0.14
A 0.71 B 0.29
A 0.71 B 0.43
A 0.71 B 0.57
A 0.71 B 0.71
A 0.71 B 0.86
A 0.71 B 1
A 0.86 B 0
A 0.86 B 0.14
A 0.86 B 0.29
A 0.86 B 0.43
A 0.86 B 0.57
A 0.86 B 0.71
A 0.86 B 0.86
A 0.86 B 1
A 1 B 0
A 1 B 0.14
A 1 B 0.29
A 1 B 0.43
A 1 B 0.57
A 1 B 0.71
A 1 B 0.86
A 1 B 1
0.2
0.4
0.6
0.8
0
5
101520

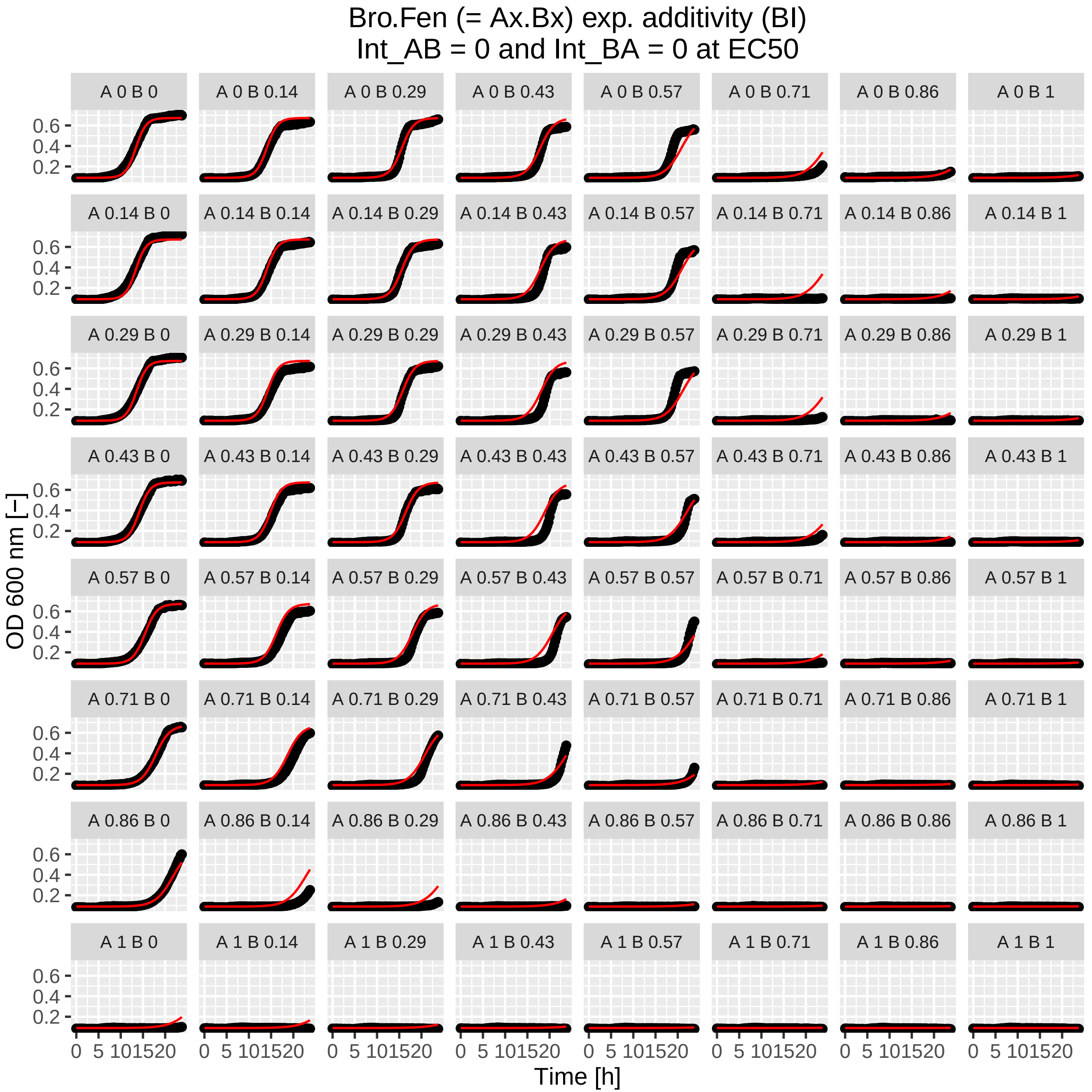

Bro.Fen (= Ax.Bx) exp. additivity (BI)
Int_AB = 0 and Int_BA = 0 at EC50
OD 600 nm [–]
Time [h]
A 0 B 0
A 0 B 0.14
A 0 B 0.29
A 0 B 0.43
A 0 B 0.57
A 0 B 0.71
A 0 B 0.86
A 0 B 1
A 0.14 B 0
A 0.14 B 0.14
A 0.14 B 0.29
A 0.14 B 0.43
A 0.14 B 0.57
A 0.14 B 0.71
A 0.14 B 0.86
A 0.14 B 1
A 0.29 B 0
A 0.29 B 0.14
A 0.29 B 0.29
A 0.29 B 0.43
A 0.29 B 0.57
A 0.29 B 0.71
A 0.29 B 0.86
A 0.29 B 1
A 0.43 B 0
A 0.43 B 0.14
A 0.43 B 0.29
A 0.43 B 0.43
A 0.43 B 0.57
A 0.43 B 0.71
A 0.43 B 0.86
A 0.43 B 1
A 0.57 B 0
A 0.57 B 0.14
A 0.57 B 0.29
A 0.57 B 0.43
A 0.57 B 0.57
A 0.57 B 0.71
A 0.57 B 0.86
A 0.57 B 1
A 0.71 B 0
A 0.71 B 0.14
A 0.71 B 0.29
A 0.71 B 0.43
A 0.71 B 0.57
A 0.71 B 0.71
A 0.71 B 0.86
A 0.71 B 1
A 0.86 B 0
A 0.86 B 0.14
A 0.86 B 0.29
A 0.86 B 0.43
A 0.86 B 0.57
A 0.86 B 0.71
A 0.86 B 0.86
A 0.86 B 1
A 1 B 0
A 1 B 0.14
A 1 B 0.29
A 1 B 0.43
A 1 B 0.57
A 1 B 0.71
A 1 B 0.86
A 1 B 1
0.2
0.4
0.6
0 5 101520

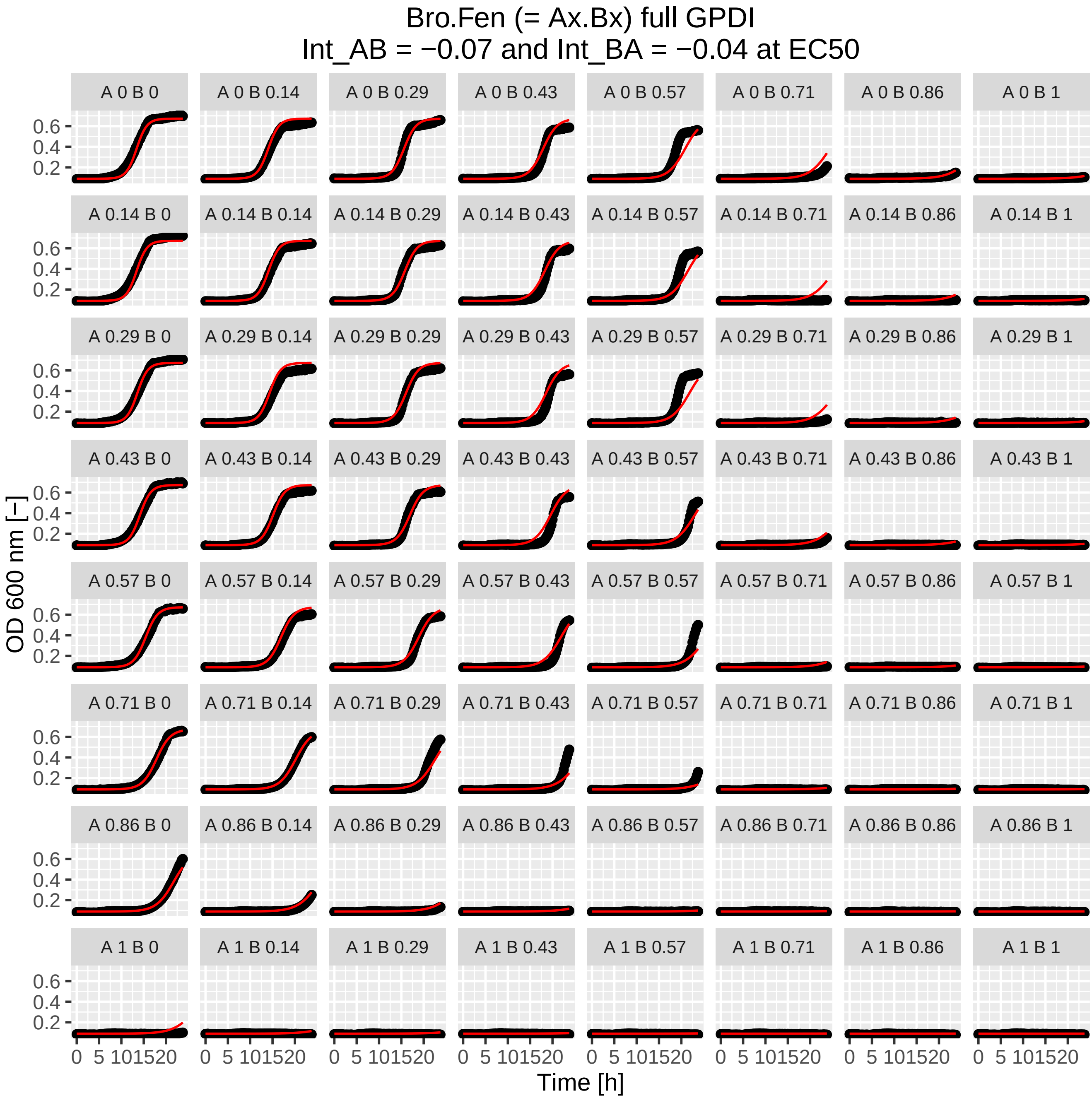
Bro.Fen (= Ax.Bx) full GPDI
Int_AB = −0.07 and Int_BA = −0.04 at EC50
A 0 B 0
A 0 B 0.14
A 0 B 0.29
A 0 B 0.43
A 0 B 0.57
A 0 B 0.71
A 0 B 0.86
A 0 B 1
A 0.14 B 0
A 0.14 B 0.14
A 0.14 B 0.29
A 0.14 B 0.43
A 0.14 B 0.57
A 0.14 B 0.71
A 0.14 B 0.86
A 0.14 B 1
A 0.29 B 0
A 0.29 B 0.14
A 0.29 B 0.29
A 0.29 B 0.43
A 0.29 B 0.57
A 0.29 B 0.71
A 0.29 B 0.86
A 0.29 B 1
A 0.43 B 0
A 0.43 B 0.14
A 0.43 B 0.29
A 0.43 B 0.43
A 0.43 B 0.57
A 0.43 B 0.71
A 0.43 B 0.86
A 0.43 B 1
A 0.57 B 0
A 0.57 B 0.14
A 0.57 B 0.29
A 0.57 B 0.43
A 0.57 B 0.57
A 0.57 B 0.71
A 0.57 B 0.86
A 0.57 B 1
A 0.71 B 0
A 0.71 B 0.14
A 0.71 B 0.29
A 0.71 B 0.43
A 0.71 B 0.57
A 0.71 B 0.71
A 0.71 B 0.86
A 0.71 B 1
A 0.86 B 0
A 0.86 B 0.14
A 0.86 B 0.29
A 0.86 B 0.43
A 0.86 B 0.57
A 0.86 B 0.71
A 0.86 B 0.86
A 0.86 B 1
A 1 B 0
A 1 B 0.14
A 1 B 0.29
A 1 B 0.43
A 1 B 0.57
A 1 B 0.71
A 1 B 0.86
A 1 B 1
OD 600 nm [−]
0.6
0.4
0.2
0 5 101520
Time [h]

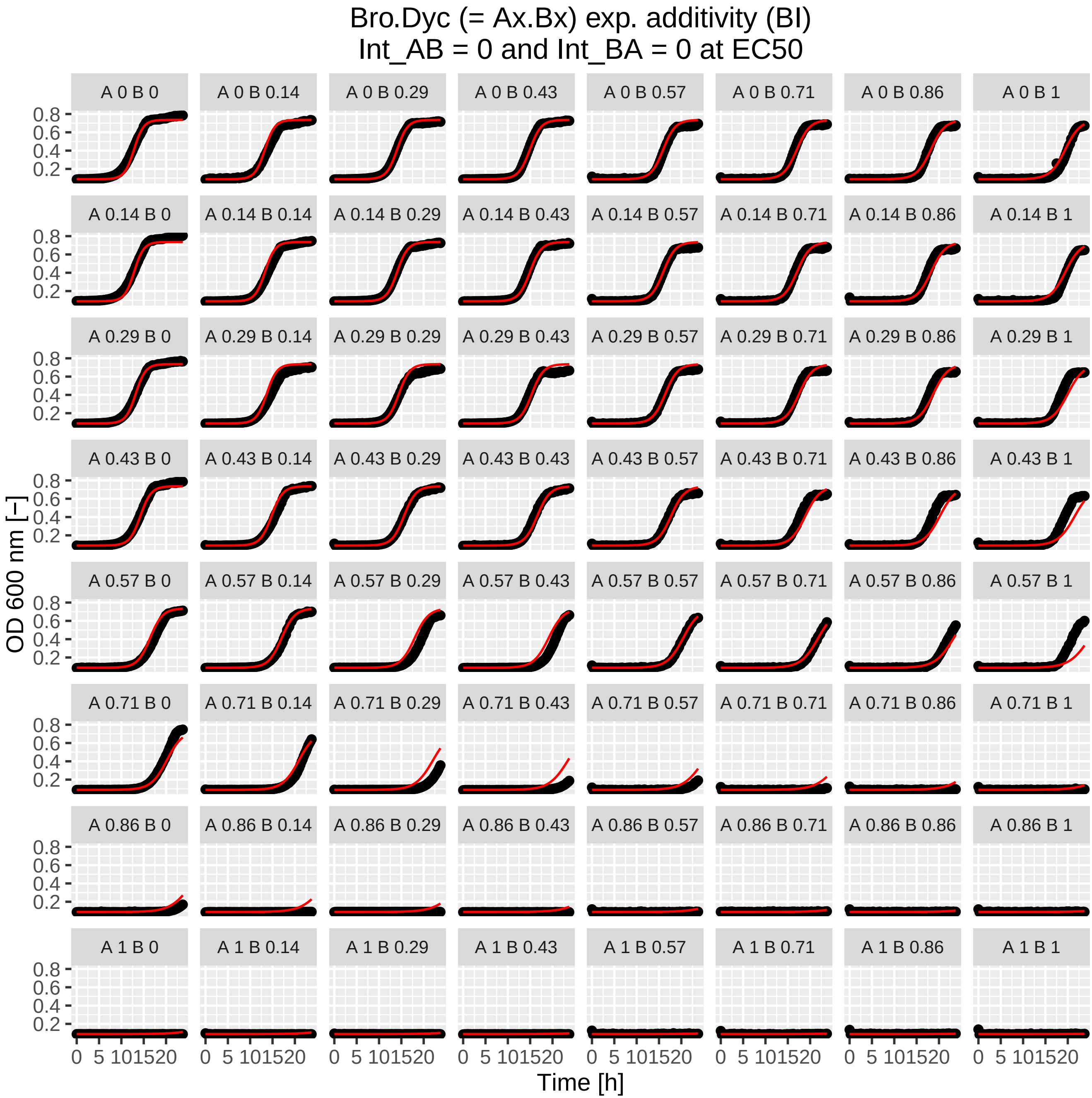

Bro.Dyc (= Ax.Bx) exp. additivity (BI)
Int_AB = 0 and Int_BA = 0 at EC50
A 0 B 0
A 0 B 0.14
A 0 B 0.29
A 0 B 0.43
A 0 B 0.57
A 0 B 0.71
A 0 B 0.86
A 0 B 1
A 0.14 B 0
A 0.14 B 0.14
A 0.14 B 0.29
A 0.14 B 0.43
A 0.14 B 0.57
A 0.14 B 0.71
A 0.14 B 0.86
A 0.14 B 1
A 0.29 B 0
A 0.29 B 0.14
A 0.29 B 0.29
A 0.29 B 0.43
A 0.29 B 0.57
A 0.29 B 0.71
A 0.29 B 0.86
A 0.29 B 1
A 0.43 B 0
A 0.43 B 0.14
A 0.43 B 0.29
A 0.43 B 0.43
A 0.43 B 0.57
A 0.43 B 0.71
A 0.43 B 0.86
A 0.43 B 1
A 0.57 B 0
A 0.57 B 0.14
A 0.57 B 0.29
A 0.57 B 0.43
A 0.57 B 0.57
A 0.57 B 0.71
A 0.57 B 0.86
A 0.57 B 1
A 0.71 B 0
A 0.71 B 0.14
A 0.71 B 0.29
A 0.71 B 0.43
A 0.71 B 0.57
A 0.71 B 0.71
A 0.71 B 0.86
A 0.71 B 1
A 0.86 B 0
A 0.86 B 0.14
A 0.86 B 0.29
A 0.86 B 0.43
A 0.86 B 0.57
A 0.86 B 0.71
A 0.86 B 0.86
A 0.86 B 1
A 1 B 0
A 1 B 0.14
A 1 B 0.29
A 1 B 0.43
A 1 B 0.57
A 1 B 0.71
A 1 B 0.86
A 1 B 1
0.8
0.6
0.4
0.2
OD 600 nm [–]
0 5 101520
Time [h]

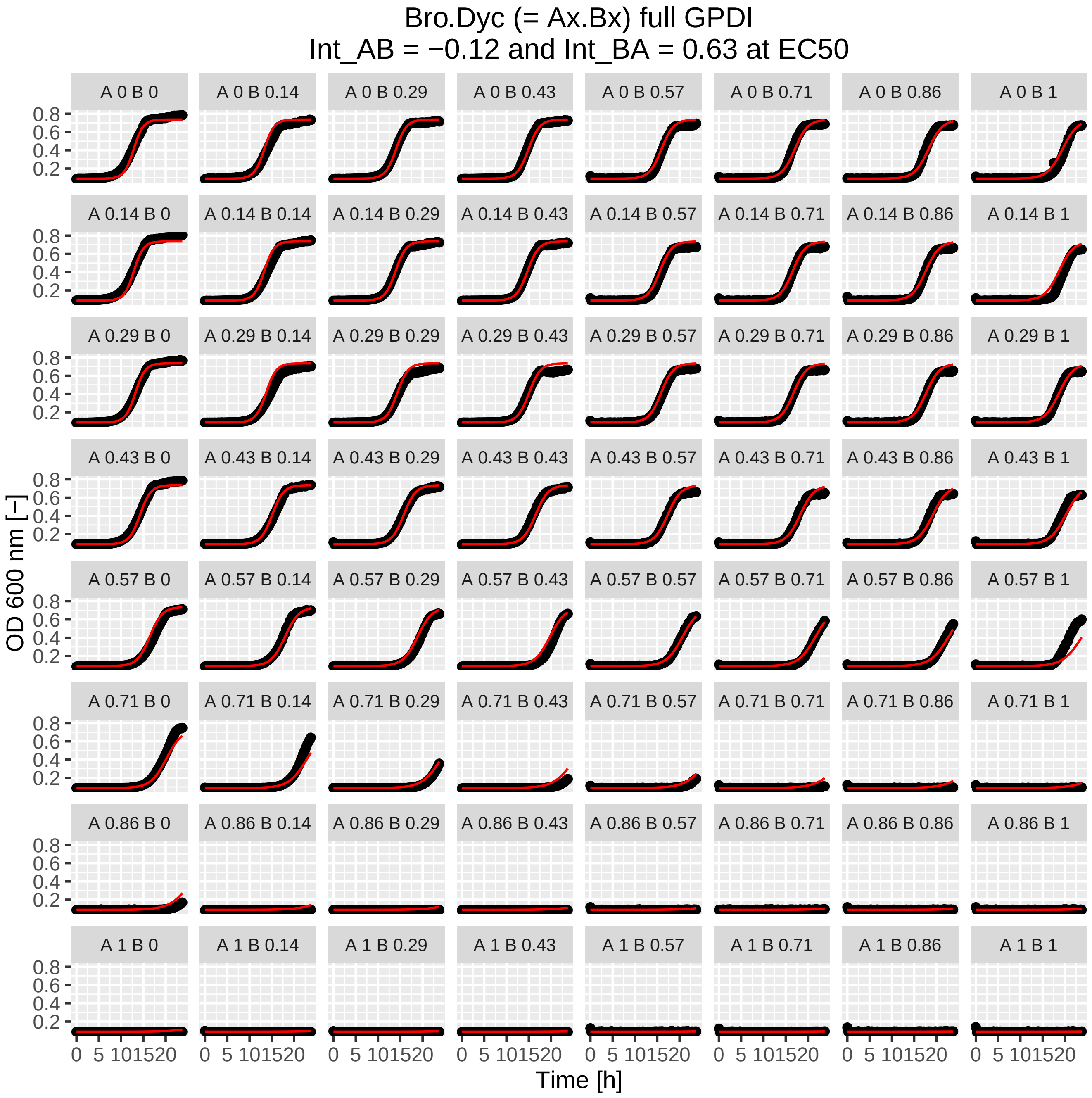
Bro.Dyc (= Ax.Bx) full GPDI
Int_AB = −0.12 and Int_BA = 0.63 at EC50
A 0 B 0
A 0 B 0.14
A 0 B 0.29
A 0 B 0.43
A 0 B 0.57
A 0 B 0.71
A 0 B 0.86
A 0 B 1
A 0.14 B 0
A 0.14 B 0.14
A 0.14 B 0.29
A 0.14 B 0.43
A 0.14 B 0.57
A 0.14 B 0.71
A 0.14 B 0.86
A 0.14 B 1
A 0.29 B 0
A 0.29 B 0.14
A 0.29 B 0.29
A 0.29 B 0.43
A 0.29 B 0.57
A 0.29 B 0.71
A 0.29 B 0.86
A 0.29 B 1
A 0.43 B 0
A 0.43 B 0.14
A 0.43 B 0.29
A 0.43 B 0.43
A 0.43 B 0.57
A 0.43 B 0.71
A 0.43 B 0.86
A 0.43 B 1
A 0.57 B 0
A 0.57 B 0.14
A 0.57 B 0.29
A 0.57 B 0.43
A 0.57 B 0.57
A 0.57 B 0.71
A 0.57 B 0.86
A 0.57 B 1
A 0.71 B 0
A 0.71 B 0.14
A 0.71 B 0.29
A 0.71 B 0.43
A 0.71 B 0.57
A 0.71 B 0.71
A 0.71 B 0.86
A 0.71 B 1
A 0.86 B 0
A 0.86 B 0.14
A 0.86 B 0.29
A 0.86 B 0.43
A 0.86 B 0.57
A 0.86 B 0.71
A 0.86 B 0.86
A 0.86 B 1
A 1 B 0
A 1 B 0.14
A 1 B 0.29
A 1 B 0.43
A 1 B 0.57
A 1 B 0.71
A 1 B 0.86
A 1 B 1
OD 600 nm [−]
0.8
0.6
0.4
0.2
0 5 101520
Time [h]

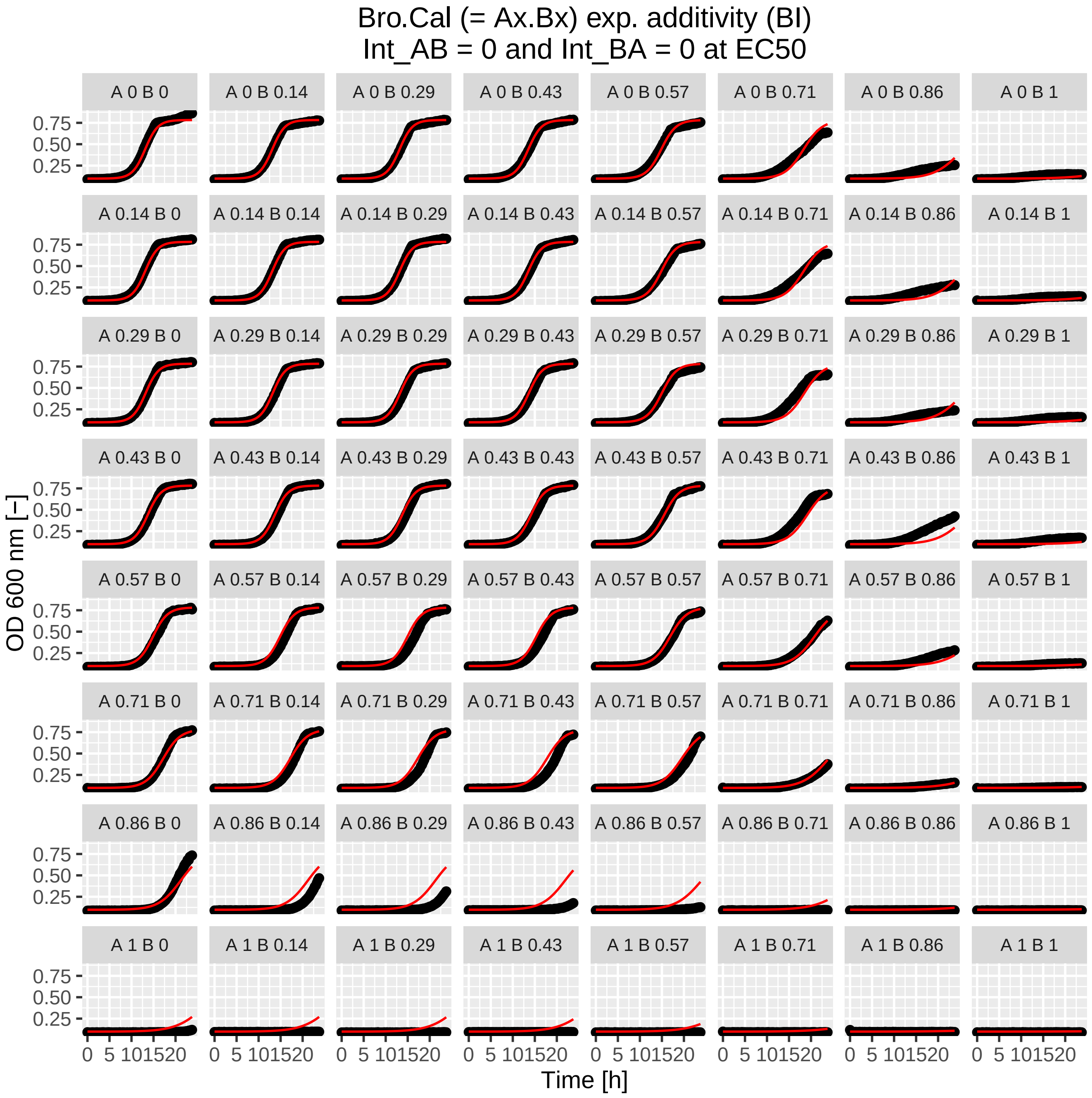
Bro.Cal (= Ax.Bx) exp. additivity (BI)
Int_AB = 0 and Int_BA = 0 at EC50
OD 600 nm [–]
Time [h]
A 0 B 0
A 0 B 0.14
A 0 B 0.29
A 0 B 0.43
A 0 B 0.57
A 0 B 0.71
A 0 B 0.86
A 0 B 1
A 0.14 B 0
A 0.14 B 0.14
A 0.14 B 0.29
A 0.14 B 0.43
A 0.14 B 0.57
A 0.14 B 0.71
A 0.14 B 0.86
A 0.14 B 1
A 0.29 B 0
A 0.29 B 0.14
A 0.29 B 0.29
A 0.29 B 0.43
A 0.29 B 0.57
A 0.29 B 0.71
A 0.29 B 0.86
A 0.29 B 1
A 0.43 B 0
A 0.43 B 0.14
A 0.43 B 0.29
A 0.43 B 0.43
A 0.43 B 0.57
A 0.43 B 0.71
A 0.43 B 0.86
A 0.43 B 1
A 0.57 B 0
A 0.57 B 0.14
A 0.57 B 0.29
A 0.57 B 0.43
A 0.57 B 0.57
A 0.57 B 0.71
A 0.57 B 0.86
A 0.57 B 1
A 0.71 B 0
A 0.71 B 0.14
A 0.71 B 0.29
A 0.71 B 0.43
A 0.71 B 0.57
A 0.71 B 0.71
A 0.71 B 0.86
A 0.71 B 1
A 0.86 B 0
A 0.86 B 0.14
A 0.86 B 0.29
A 0.86 B 0.43
A 0.86 B 0.57
A 0.86 B 0.71
A 0.86 B 0.86
A 0.86 B 1
A 1 B 0
A 1 B 0.14
A 1 B 0.29
A 1 B 0.43
A 1 B 0.57
A 1 B 0.71
A 1 B 0.86
A 1 B 1
0.25
0.50
0.75
0 5 101520

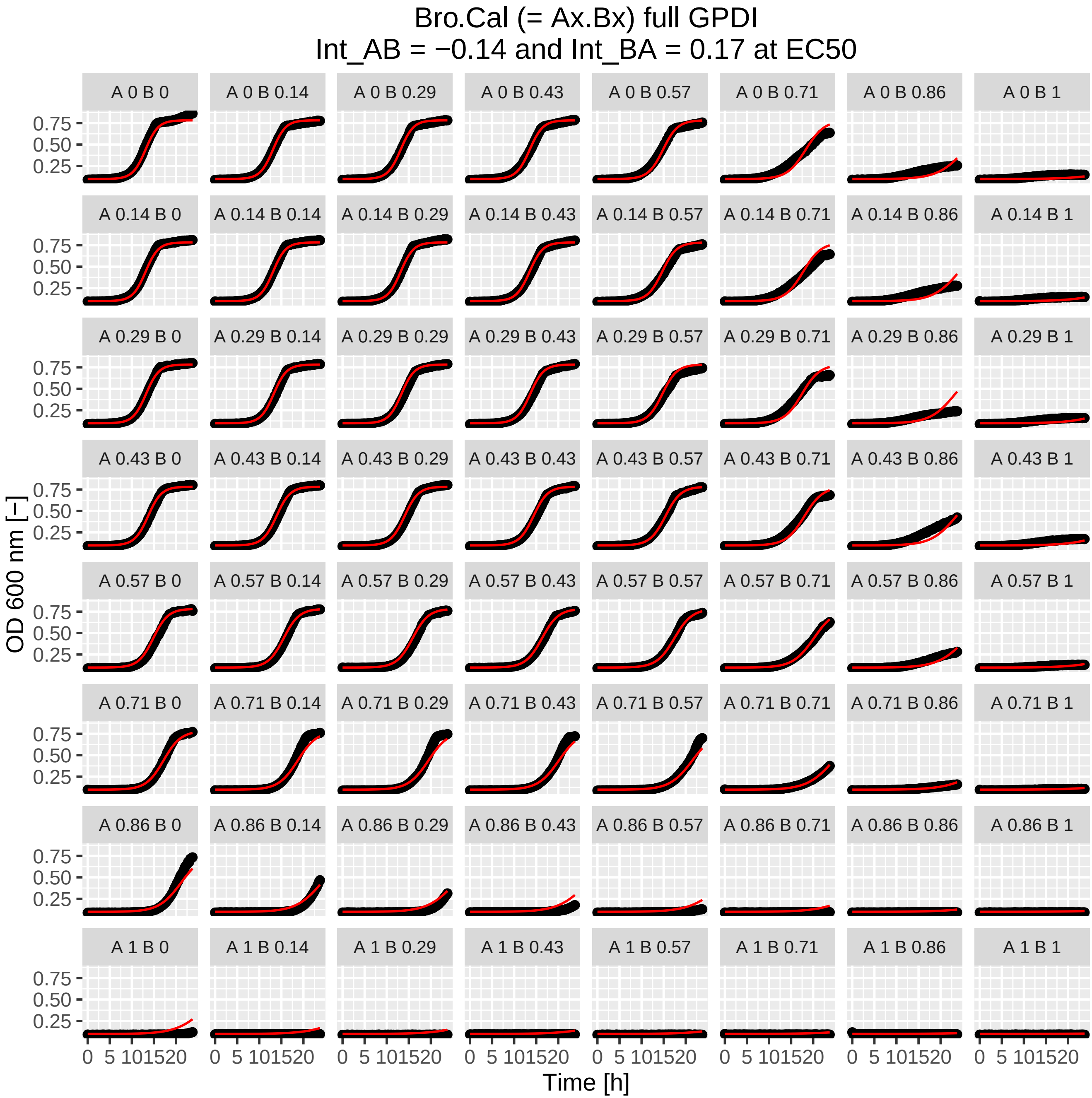

Bro.Cal (= Ax.Bx) full GPDI
Int_AB = −0.14 and Int_BA = 0.17 at EC50
A 0 B 0
A 0 B 0.14
A 0 B 0.29
A 0 B 0.43
A 0 B 0.57
A 0 B 0.71
A 0 B 0.86
A 0 B 1
A 0.14 B 0
A 0.14 B 0.14
A 0.14 B 0.29
A 0.14 B 0.43
A 0.14 B 0.57
A 0.14 B 0.71
A 0.14 B 0.86
A 0.14 B 1
A 0.29 B 0
A 0.29 B 0.14
A 0.29 B 0.29
A 0.29 B 0.43
A 0.29 B 0.57
A 0.29 B 0.71
A 0.29 B 0.86
A 0.29 B 1
A 0.43 B 0
A 0.43 B 0.14
A 0.43 B 0.29
A 0.43 B 0.43
A 0.43 B 0.57
A 0.43 B 0.71
A 0.43 B 0.86
A 0.43 B 1
A 0.57 B 0
A 0.57 B 0.14
A 0.57 B 0.29
A 0.57 B 0.43
A 0.57 B 0.57
A 0.57 B 0.71
A 0.57 B 0.86
A 0.57 B 1
A 0.71 B 0
A 0.71 B 0.14
A 0.71 B 0.29
A 0.71 B 0.43
A 0.71 B 0.57
A 0.71 B 0.71
A 0.71 B 0.86
A 0.71 B 1
A 0.86 B 0
A 0.86 B 0.14
A 0.86 B 0.29
A 0.86 B 0.43
A 0.86 B 0.57
A 0.86 B 0.71
A 0.86 B 0.86
A 0.86 B 1
A 1 B 0
A 1 B 0.14
A 1 B 0.29
A 1 B 0.43
A 1 B 0.57
A 1 B 0.71
A 1 B 0.86
A 1 B 1
0.75
0.50
0.25
OD 600 nm [−]
0 5 101520
Time [h]

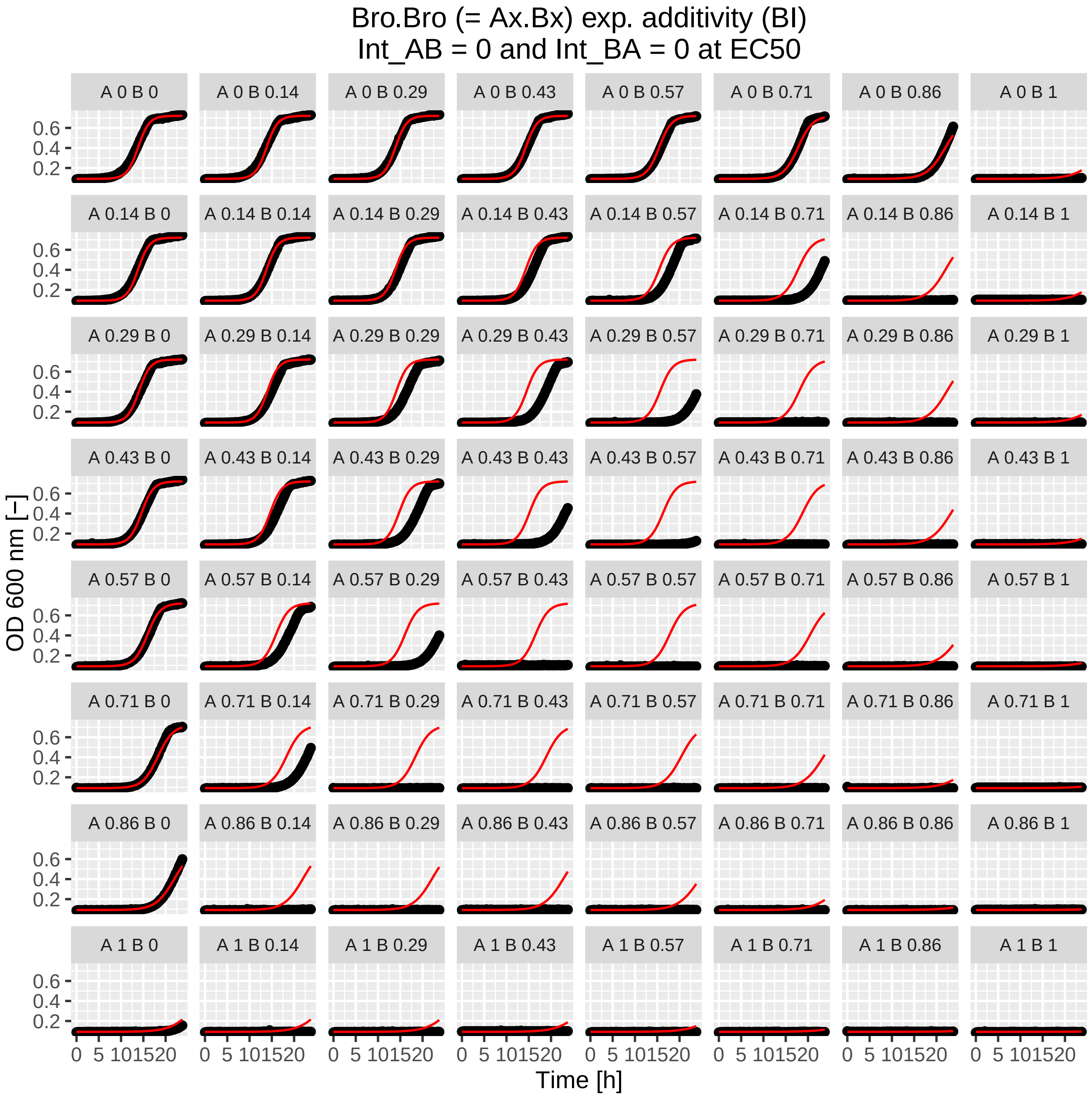
Bro.Bro (= Ax.Bx) exp. additivity (BI)
Int_AB = 0 and Int_BA = 0 at EC50
OD 600 nm [–]
Time [h]
0.6
0.4
0.2
0 5 101520
A 0 B 0
A 0 B 0.14
A 0 B 0.29
A 0 B 0.43
A 0 B 0.57
A 0 B 0.71
A 0 B 0.86
A 0 B 1
A 0.14 B 0
A 0.14 B 0.14
A 0.14 B 0.29
A 0.14 B 0.43
A 0.14 B 0.57
A 0.14 B 0.71
A 0.14 B 0.86
A 0.14 B 1
A 0.29 B 0
A 0.29 B 0.14
A 0.29 B 0.29
A 0.29 B 0.43
A 0.29 B 0.57
A 0.29 B 0.71
A 0.29 B 0.86
A 0.29 B 1
A 0.43 B 0
A 0.43 B 0.14
A 0.43 B 0.29
A 0.43 B 0.43
A 0.43 B 0.57
A 0.43 B 0.71
A 0.43 B 0.86
A 0.43 B 1
A 0.57 B 0
A 0.57 B 0.14
A 0.57 B 0.29
A 0.57 B 0.43
A 0.57 B 0.57
A 0.57 B 0.71
A 0.57 B 0.86
A 0.57 B 1
A 0.71 B 0
A 0.71 B 0.14
A 0.71 B 0.29
A 0.71 B 0.43
A 0.71 B 0.57
A 0.71 B 0.71
A 0.71 B 0.86
A 0.71 B 1
A 0.86 B 0
A 0.86 B 0.14
A 0.86 B 0.29
A 0.86 B 0.43
A 0.86 B 0.57
A 0.86 B 0.71
A 0.86 B 0.86
A 0.86 B 1
A 1 B 0
A 1 B 0.14
A 1 B 0.29
A 1 B 0.43
A 1 B 0.57
A 1 B 0.71
A 1 B 0.86
A 1 B 1

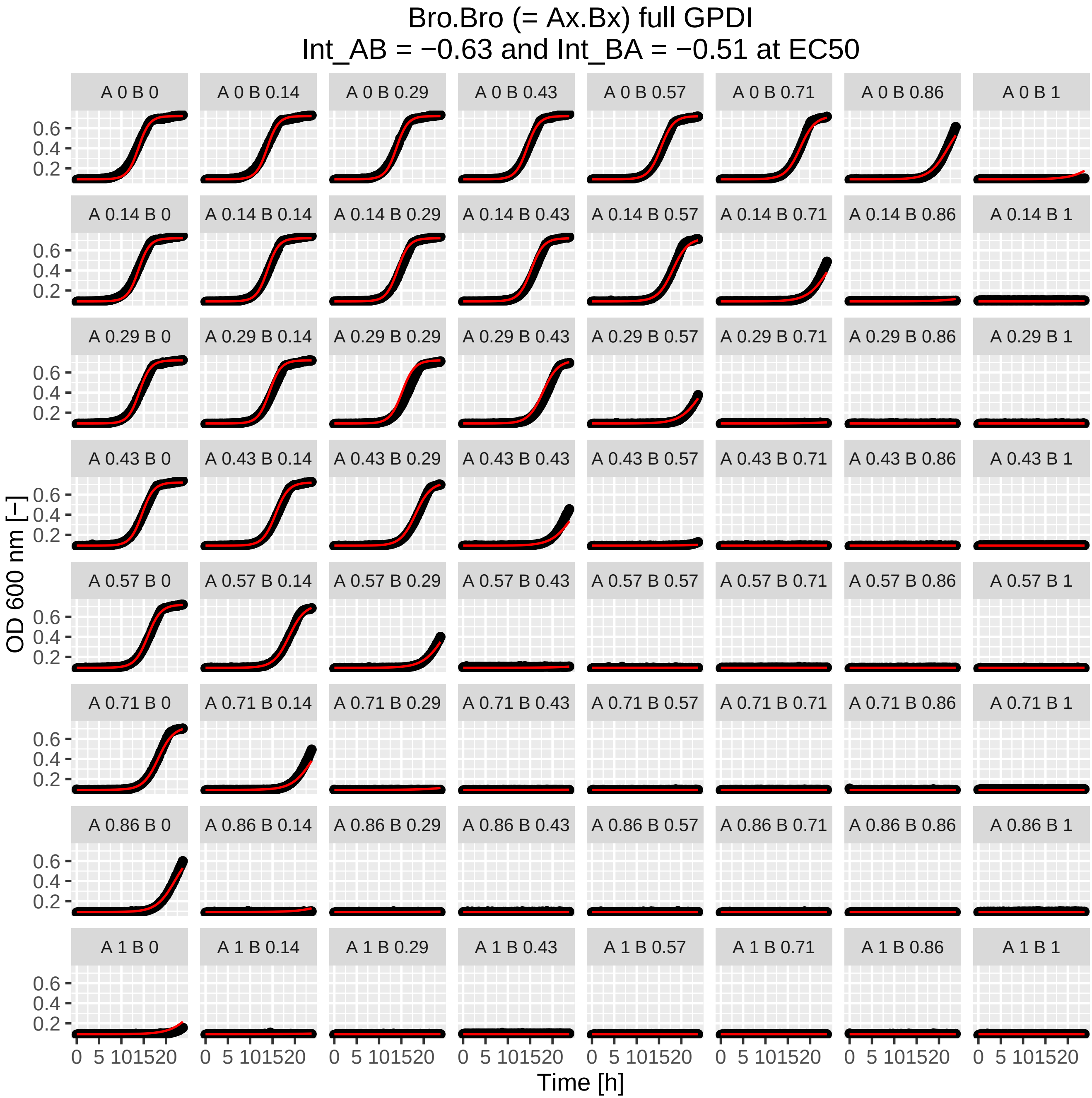
Bro.Bro (= Ax.Bx) full GPDI
Int_AB = −0.63 and Int_BA = −0.51 at EC50
A 0 B 0
A 0 B 0.14
A 0 B 0.29
A 0 B 0.43
A 0 B 0.57
A 0 B 0.71
A 0 B 0.86
A 0 B 1
A 0.14 B 0
A 0.14 B 0.14
A 0.14 B 0.29
A 0.14 B 0.43
A 0.14 B 0.57
A 0.14 B 0.71
A 0.14 B 0.86
A 0.14 B 1
A 0.29 B 0
A 0.29 B 0.14
A 0.29 B 0.29
A 0.29 B 0.43
A 0.29 B 0.57
A 0.29 B 0.71
A 0.29 B 0.86
A 0.29 B 1
A 0.43 B 0
A 0.43 B 0.14
A 0.43 B 0.29
A 0.43 B 0.43
A 0.43 B 0.57
A 0.43 B 0.71
A 0.43 B 0.86
A 0.43 B 1
A 0.57 B 0
A 0.57 B 0.14
A 0.57 B 0.29
A 0.57 B 0.43
A 0.57 B 0.57
A 0.57 B 0.71
A 0.57 B 0.86
A 0.57 B 1
A 0.71 B 0
A 0.71 B 0.14
A 0.71 B 0.29
A 0.71 B 0.43
A 0.71 B 0.57
A 0.71 B 0.71
A 0.71 B 0.86
A 0.71 B 1
A 0.86 B 0
A 0.86 B 0.14
A 0.86 B 0.29
A 0.86 B 0.43
A 0.86 B 0.57
A 0.86 B 0.71
A 0.86 B 0.86
A 0.86 B 1
A 1 B 0
A 1 B 0.14
A 1 B 0.29
A 1 B 0.43
A 1 B 0.57
A 1 B 0.71
A 1 B 0.86
A 1 B 1
0.6
0.4
0.2
0 5 101520
OD 600 nm [–]
Time [h]

Ben.Tun (= Ax.Bx) exp. additivity (BI)
Int_AB = 0 and Int_BA = 0 at EC50

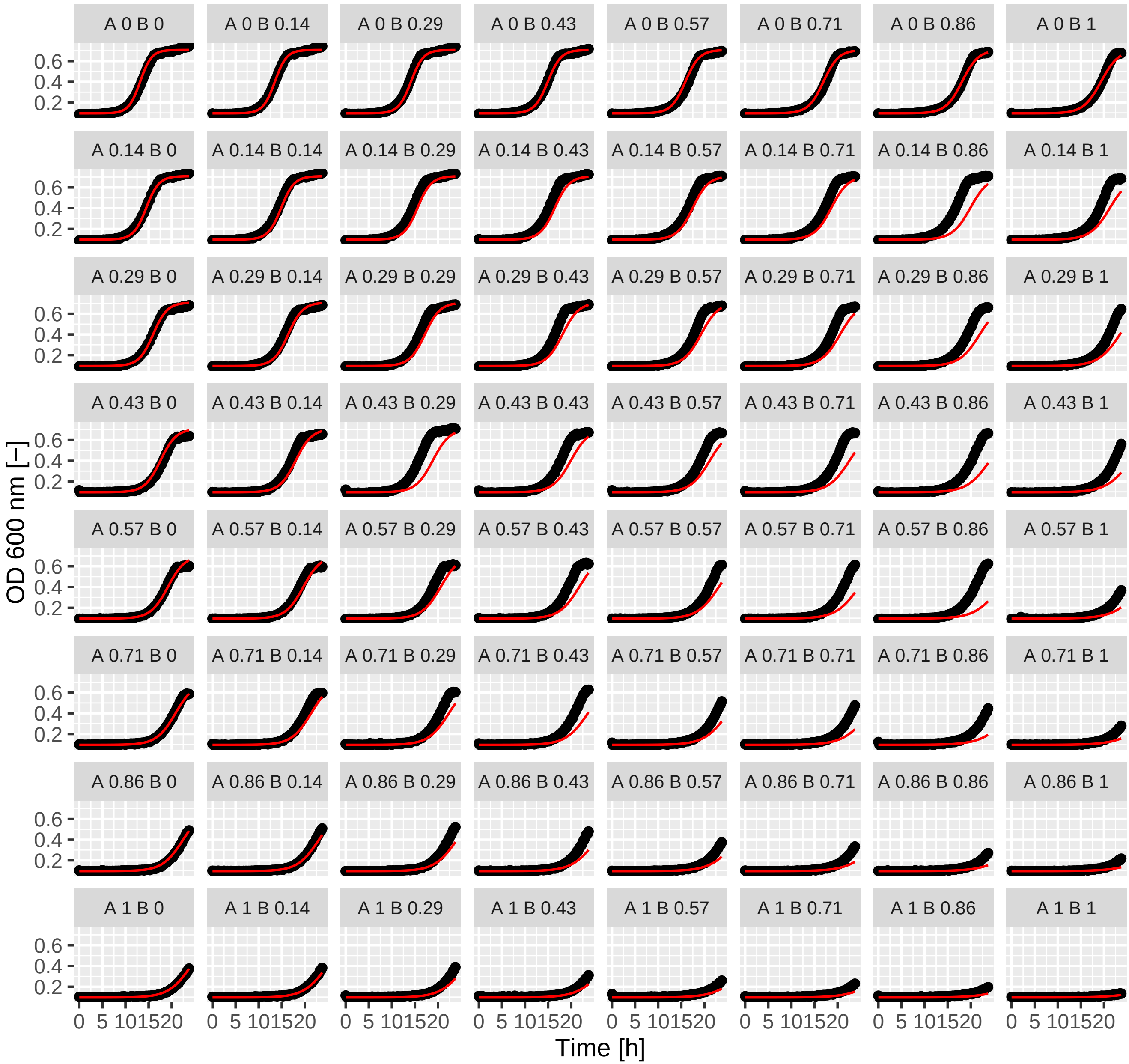

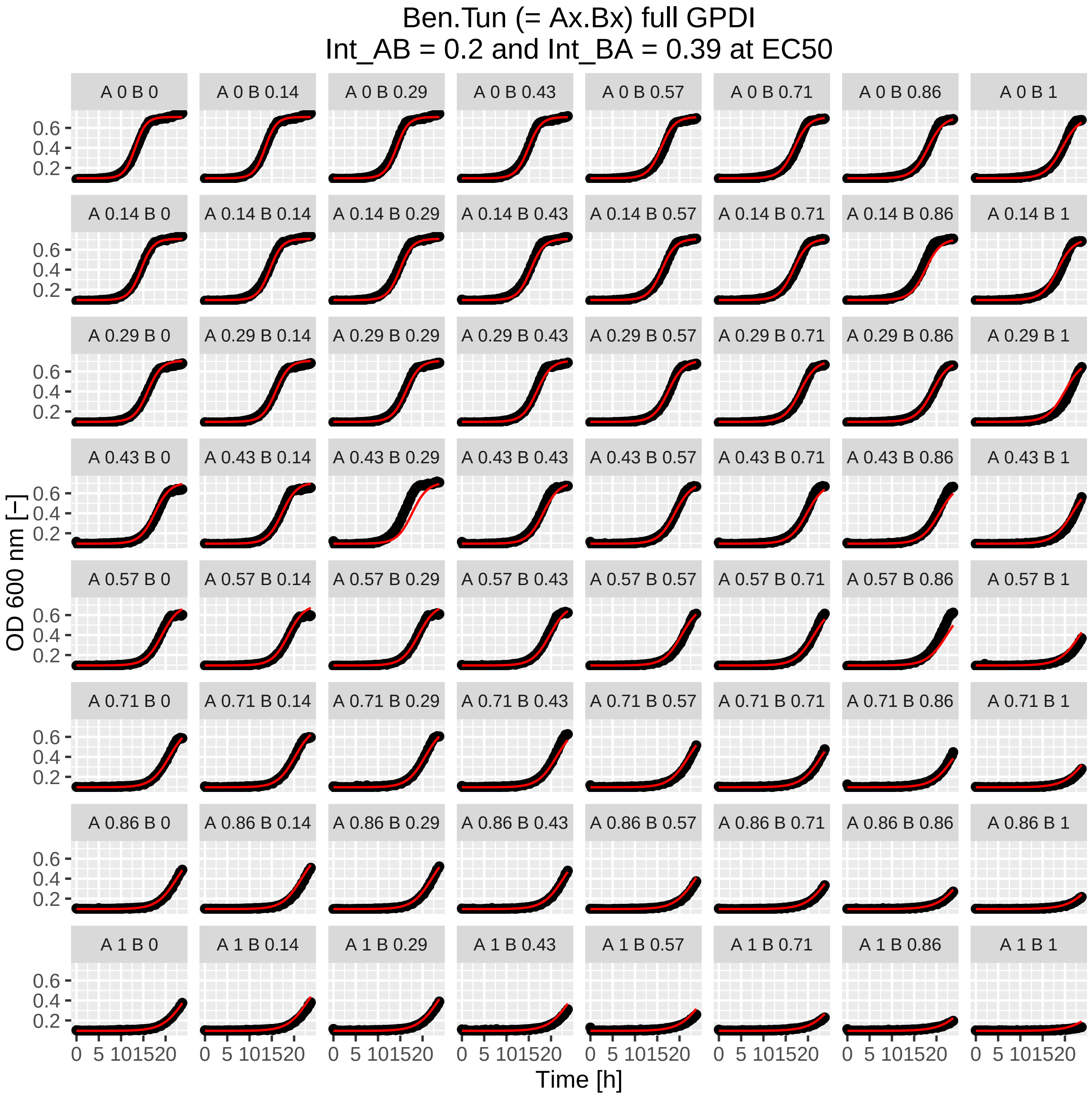
Ben.Tun (= Ax.Bx) full GPDI
Int_AB = 0.2 and Int_BA = 0.39 at EC50
A 0 B 0
A 0 B 0.14
A 0 B 0.29
A 0 B 0.43
A 0 B 0.57
A 0 B 0.71
A 0 B 0.86
A 0 B 1
A 0.14 B 0
A 0.14 B 0.14
A 0.14 B 0.29
A 0.14 B 0.43
A 0.14 B 0.57
A 0.14 B 0.71
A 0.14 B 0.86
A 0.14 B 1
A 0.29 B 0
A 0.29 B 0.14
A 0.29 B 0.29
A 0.29 B 0.43
A 0.29 B 0.57
A 0.29 B 0.71
A 0.29 B 0.86
A 0.29 B 1
A 0.43 B 0
A 0.43 B 0.14
A 0.43 B 0.29
A 0.43 B 0.43
A 0.43 B 0.57
A 0.43 B 0.71
A 0.43 B 0.86
A 0.43 B 1
A 0.57 B 0
A 0.57 B 0.14
A 0.57 B 0.29
A 0.57 B 0.43
A 0.57 B 0.57
A 0.57 B 0.71
A 0.57 B 0.86
A 0.57 B 1
A 0.71 B 0
A 0.71 B 0.14
A 0.71 B 0.29
A 0.71 B 0.43
A 0.71 B 0.57
A 0.71 B 0.71
A 0.71 B 0.86
A 0.71 B 1
A 0.86 B 0
A 0.86 B 0.14
A 0.86 B 0.29
A 0.86 B 0.43
A 0.86 B 0.57
A 0.86 B 0.71
A 0.86 B 0.86
A 0.86 B 1
A 1 B 0
A 1 B 0.14
A 1 B 0.29
A 1 B 0.43
A 1 B 0.57
A 1 B 0.71
A 1 B 0.86
A 1 B 1
OD 600 nm [–]
0.2
0.4
0.6
0 5 101520
Time [h]

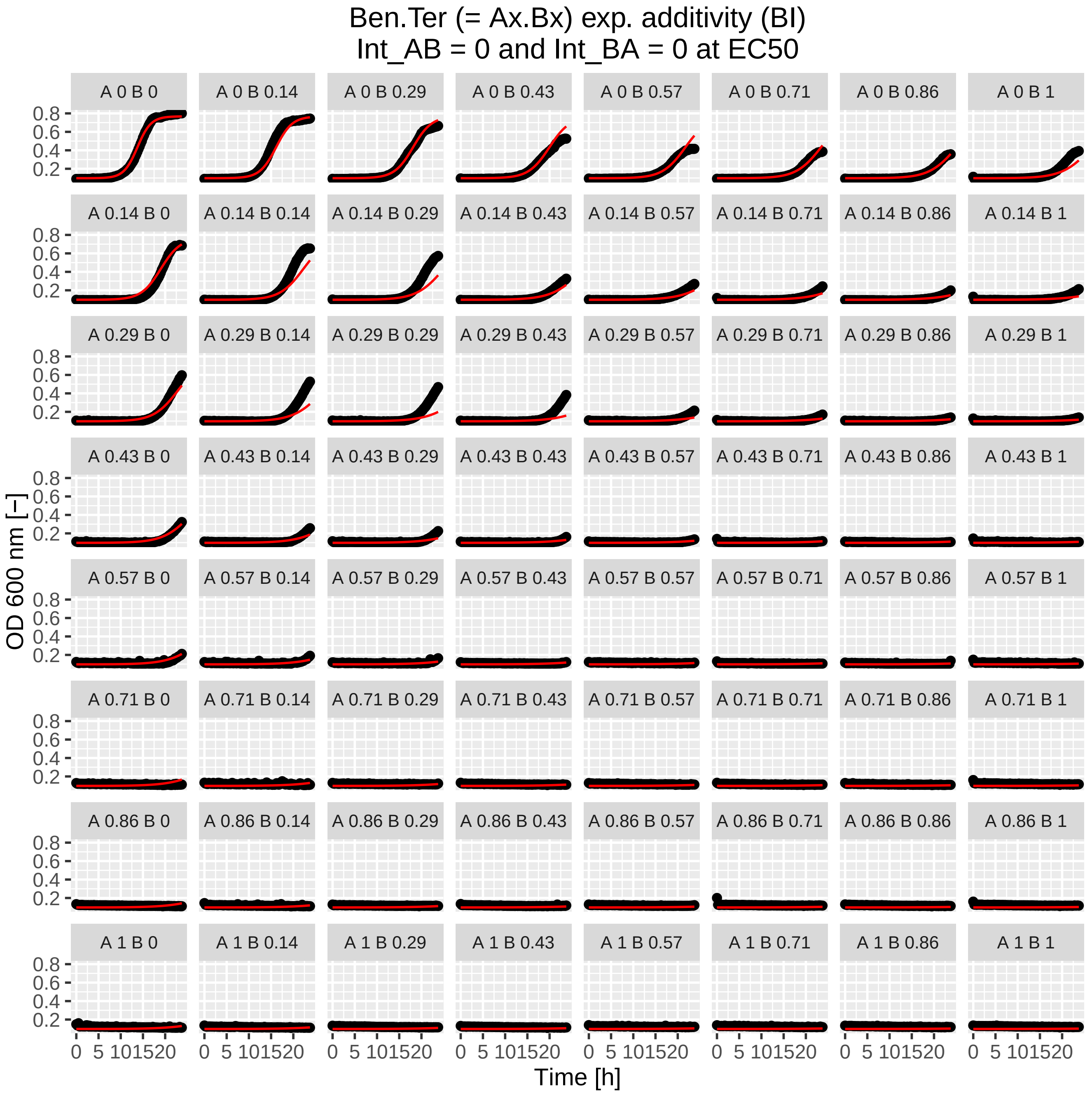

Ben.Ter (= Ax.Bx) exp. additivity (BI)
Int_AB = 0 and Int_BA = 0 at EC50
OD 600 nm [–]
Time [h]
A 0 B 0
A 0 B 0.14
A 0 B 0.29
A 0 B 0.43
A 0 B 0.57
A 0 B 0.71
A 0 B 0.86
A 0 B 1
A 0.14 B 0
A 0.14 B 0.14
A 0.14 B 0.29
A 0.14 B 0.43
A 0.14 B 0.57
A 0.14 B 0.71
A 0.14 B 0.86
A 0.14 B 1
A 0.29 B 0
A 0.29 B 0.14
A 0.29 B 0.29
A 0.29 B 0.43
A 0.29 B 0.57
A 0.29 B 0.71
A 0.29 B 0.86
A 0.29 B 1
A 0.43 B 0
A 0.43 B 0.14
A 0.43 B 0.29
A 0.43 B 0.43
A 0.43 B 0.57
A 0.43 B 0.71
A 0.43 B 0.86
A 0.43 B 1
A 0.57 B 0
A 0.57 B 0.14
A 0.57 B 0.29
A 0.57 B 0.43
A 0.57 B 0.57
A 0.57 B 0.71
A 0.57 B 0.86
A 0.57 B 1
A 0.71 B 0
A 0.71 B 0.14
A 0.71 B 0.29
A 0.71 B 0.43
A 0.71 B 0.57
A 0.71 B 0.71
A 0.71 B 0.86
A 0.71 B 1
A 0.86 B 0
A 0.86 B 0.14
A 0.86 B 0.29
A 0.86 B 0.43
A 0.86 B 0.57
A 0.86 B 0.71
A 0.86 B 0.86
A 0.86 B 1
A 1 B 0
A 1 B 0.14
A 1 B 0.29
A 1 B 0.43
A 1 B 0.57
A 1 B 0.71
A 1 B 0.86
A 1 B 1
0.8
0.6
0.4
0.2
0 5 10 15 20

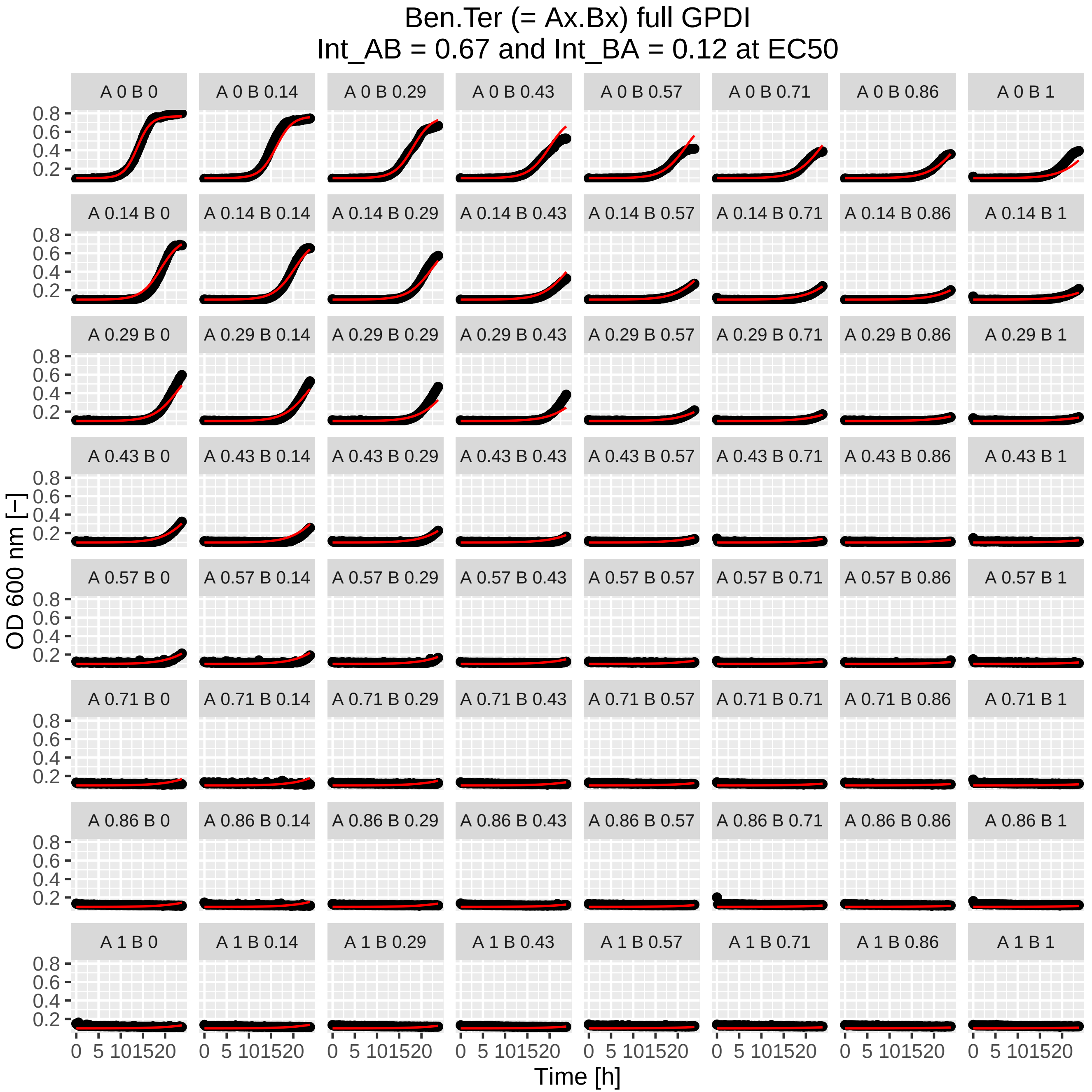

Ben.Ter (= Ax.Bx) full GPDI
Int_AB = 0.67 and Int_BA = 0.12 at EC50
A 0 B 0
A 0 B 0.14
A 0 B 0.29
A 0 B 0.43
A 0 B 0.57
A 0 B 0.71
A 0 B 0.86
A 0 B 1
A 0.14 B 0
A 0.14 B 0.14
A 0.14 B 0.29
A 0.14 B 0.43
A 0.14 B 0.57
A 0.14 B 0.71
A 0.14 B 0.86
A 0.14 B 1
A 0.29 B 0
A 0.29 B 0.14
A 0.29 B 0.29
A 0.29 B 0.43
A 0.29 B 0.57
A 0.29 B 0.71
A 0.29 B 0.86
A 0.29 B 1
A 0.43 B 0
A 0.43 B 0.14
A 0.43 B 0.29
A 0.43 B 0.43
A 0.43 B 0.57
A 0.43 B 0.71
A 0.43 B 0.86
A 0.43 B 1
A 0.57 B 0
A 0.57 B 0.14
A 0.57 B 0.29
A 0.57 B 0.43
A 0.57 B 0.57
A 0.57 B 0.71
A 0.57 B 0.86
A 0.57 B 1
A 0.71 B 0
A 0.71 B 0.14
A 0.71 B 0.29
A 0.71 B 0.43
A 0.71 B 0.57
A 0.71 B 0.71
A 0.71 B 0.86
A 0.71 B 1
A 0.86 B 0
A 0.86 B 0.14
A 0.86 B 0.29
A 0.86 B 0.43
A 0.86 B 0.57
A 0.86 B 0.71
A 0.86 B 0.86
A 0.86 B 1
A 1 B 0
A 1 B 0.14
A 1 B 0.29
A 1 B 0.43
A 1 B 0.57
A 1 B 0.71
A 1 B 0.86
A 1 B 1
0.8
0.6
0.4
0.2
OD 600 nm [–]
0 5 101520
Time [h]

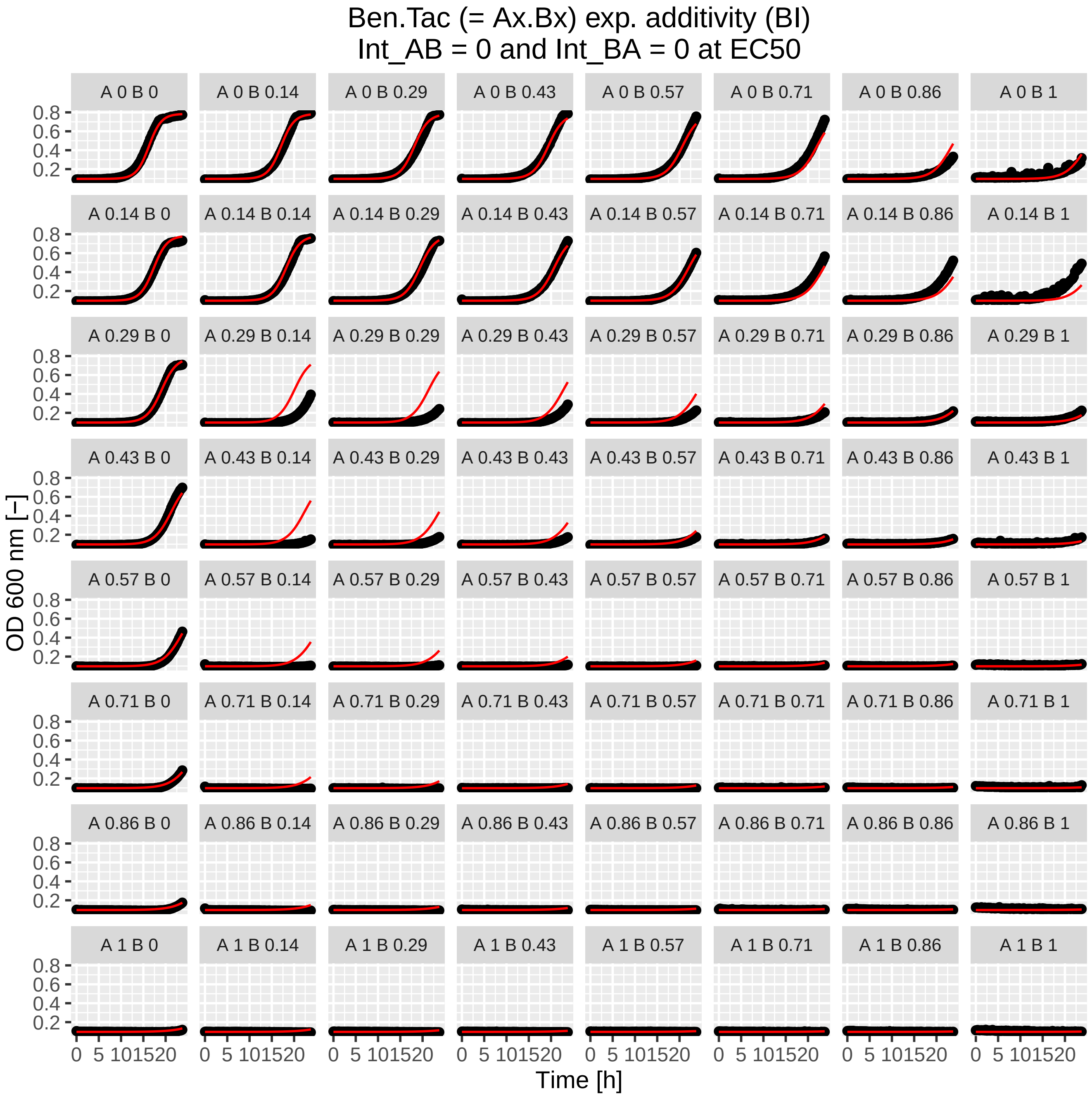

Ben.Tac (= Ax.Bx) exp. additivity (BI)
Int_AB = 0 and Int_BA = 0 at EC50
A 0 B 0
A 0 B 0.14
A 0 B 0.29
A 0 B 0.43
A 0 B 0.57
A 0 B 0.71
A 0 B 0.86
A 0 B 1
A 0.14 B 0
A 0.14 B 0.14
A 0.14 B 0.29
A 0.14 B 0.43
A 0.14 B 0.57
A 0.14 B 0.71
A 0.14 B 0.86
A 0.14 B 1
A 0.29 B 0
A 0.29 B 0.14
A 0.29 B 0.29
A 0.29 B 0.43
A 0.29 B 0.57
A 0.29 B 0.71
A 0.29 B 0.86
A 0.29 B 1
A 0.43 B 0
A 0.43 B 0.14
A 0.43 B 0.29
A 0.43 B 0.43
A 0.43 B 0.57
A 0.43 B 0.71
A 0.43 B 0.86
A 0.43 B 1
A 0.57 B 0
A 0.57 B 0.14
A 0.57 B 0.29
A 0.57 B 0.43
A 0.57 B 0.57
A 0.57 B 0.71
A 0.57 B 0.86
A 0.57 B 1
A 0.71 B 0
A 0.71 B 0.14
A 0.71 B 0.29
A 0.71 B 0.43
A 0.71 B 0.57
A 0.71 B 0.71
A 0.71 B 0.86
A 0.71 B 1
A 0.86 B 0
A 0.86 B 0.14
A 0.86 B 0.29
A 0.86 B 0.43
A 0.86 B 0.57
A 0.86 B 0.71
A 0.86 B 0.86
A 0.86 B 1
A 1 B 0
A 1 B 0.14
A 1 B 0.29
A 1 B 0.43
A 1 B 0.57
A 1 B 0.71
A 1 B 0.86
A 1 B 1
0.8
0.6
0.4
0.2
OD 600 nm [–]
0 5 101520
Time [h]

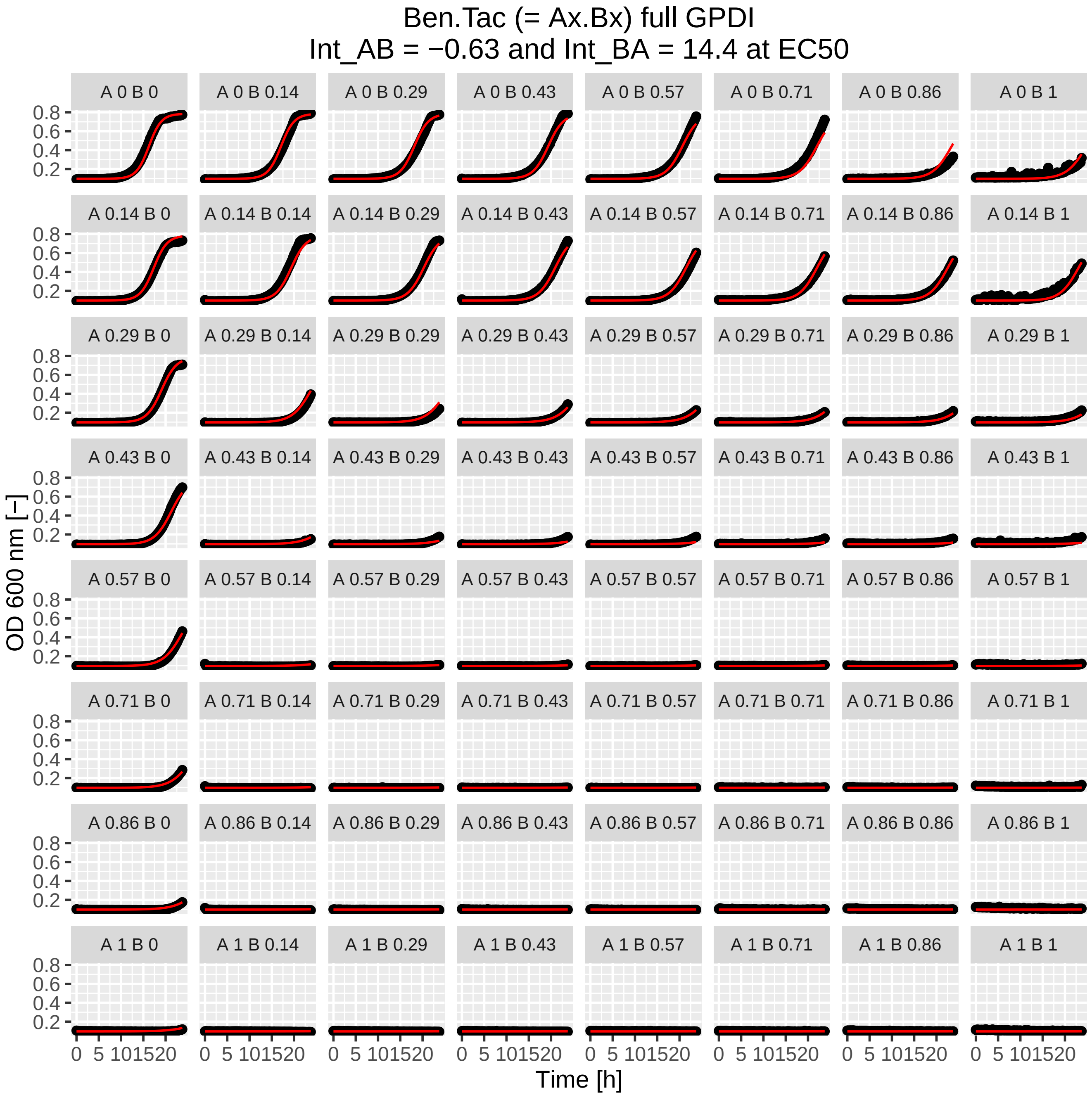

Ben.Tac (= Ax.Bx) full GPDI
Int_AB = −0.63 and Int_BA = 14.4 at EC50
A 0 B 0
A 0 B 0.14
A 0 B 0.29
A 0 B 0.43
A 0 B 0.57
A 0 B 0.71
A 0 B 0.86
A 0 B 1
A 0.14 B 0
A 0.14 B 0.14
A 0.14 B 0.29
A 0.14 B 0.43
A 0.14 B 0.57
A 0.14 B 0.71
A 0.14 B 0.86
A 0.14 B 1
A 0.29 B 0
A 0.29 B 0.14
A 0.29 B 0.29
A 0.29 B 0.43
A 0.29 B 0.57
A 0.29 B 0.71
A 0.29 B 0.86
A 0.29 B 1
A 0.43 B 0
A 0.43 B 0.14
A 0.43 B 0.29
A 0.43 B 0.43
A 0.43 B 0.57
A 0.43 B 0.71
A 0.43 B 0.86
A 0.43 B 1
A 0.57 B 0
A 0.57 B 0.14
A 0.57 B 0.29
A 0.57 B 0.43
A 0.57 B 0.57
A 0.57 B 0.71
A 0.57 B 0.86
A 0.57 B 1
A 0.71 B 0
A 0.71 B 0.14
A 0.71 B 0.29
A 0.71 B 0.43
A 0.71 B 0.57
A 0.71 B 0.71
A 0.71 B 0.86
A 0.71 B 1
A 0.86 B 0
A 0.86 B 0.14
A 0.86 B 0.29
A 0.86 B 0.43
A 0.86 B 0.57
A 0.86 B 0.71
A 0.86 B 0.86
A 0.86 B 1
A 1 B 0
A 1 B 0.14
A 1 B 0.29
A 1 B 0.43
A 1 B 0.57
A 1 B 0.71
A 1 B 0.86
A 1 B 1
0.8
0.6
0.4
0.2
OD 600 nm [−]
0 5 101520
Time [h]

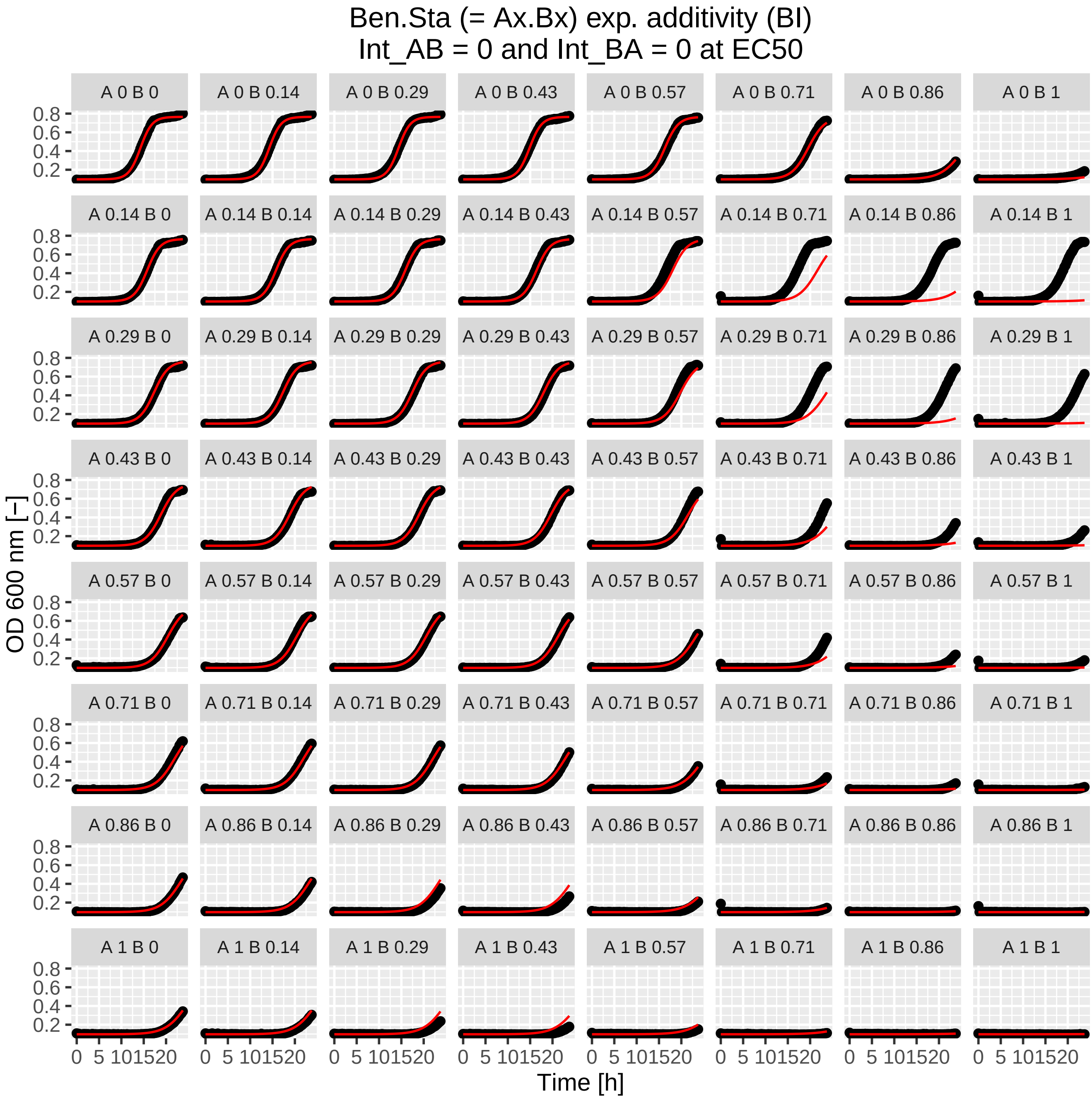

Ben.Sta (= Ax.Bx) exp. additivity (BI)
Int_AB = 0 and Int_BA = 0 at EC50
A 0 B 0
A 0 B 0.14
A 0 B 0.29
A 0 B 0.43
A 0 B 0.57
A 0 B 0.71
A 0 B 0.86
A 0 B 1
A 0.14 B 0
A 0.14 B 0.14
A 0.14 B 0.29
A 0.14 B 0.43
A 0.14 B 0.57
A 0.14 B 0.71
A 0.14 B 0.86
A 0.14 B 1
A 0.29 B 0
A 0.29 B 0.14
A 0.29 B 0.29
A 0.29 B 0.43
A 0.29 B 0.57
A 0.29 B 0.71
A 0.29 B 0.86
A 0.29 B 1
A 0.43 B 0
A 0.43 B 0.14
A 0.43 B 0.29
A 0.43 B 0.43
A 0.43 B 0.57
A 0.43 B 0.71
A 0.43 B 0.86
A 0.43 B 1
A 0.57 B 0
A 0.57 B 0.14
A 0.57 B 0.29
A 0.57 B 0.43
A 0.57 B 0.57
A 0.57 B 0.71
A 0.57 B 0.86
A 0.57 B 1
A 0.71 B 0
A 0.71 B 0.14
A 0.71 B 0.29
A 0.71 B 0.43
A 0.71 B 0.57
A 0.71 B 0.71
A 0.71 B 0.86
A 0.71 B 1
A 0.86 B 0
A 0.86 B 0.14
A 0.86 B 0.29
A 0.86 B 0.43
A 0.86 B 0.57
A 0.86 B 0.71
A 0.86 B 0.86
A 0.86 B 1
A 1 B 0
A 1 B 0.14
A 1 B 0.29
A 1 B 0.43
A 1 B 0.57
A 1 B 0.71
A 1 B 0.86
A 1 B 1
OD 600 nm [–]
0.8
0.6
0.4
0.2
0 5 101520
Time [h]

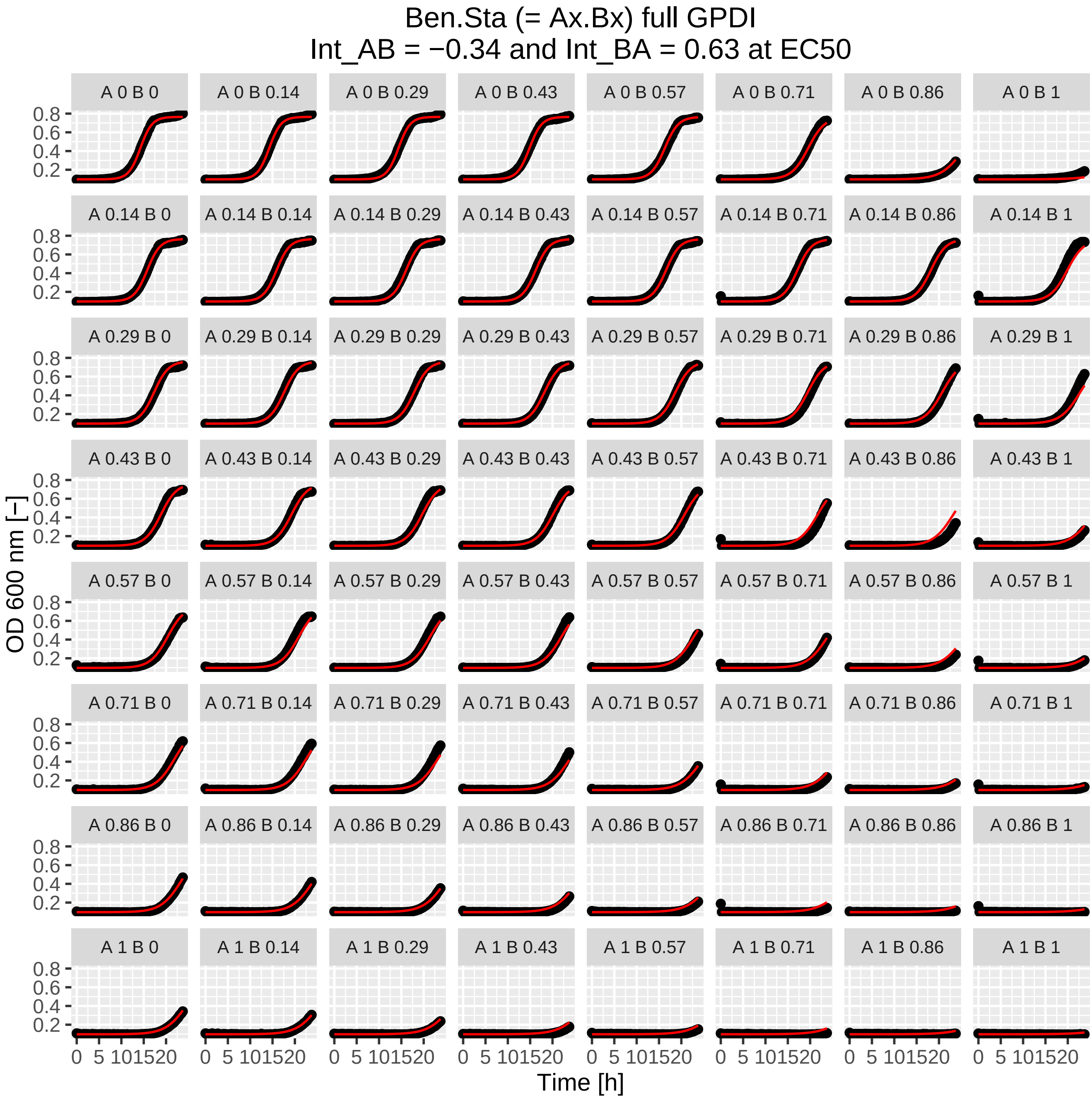

Ben.Sta (= Ax.Bx) full GPDI
Int_AB = −0.34 and Int_BA = 0.63 at EC50
A 0 B 0
A 0 B 0.14
A 0 B 0.29
A 0 B 0.43
A 0 B 0.57
A 0 B 0.71
A 0 B 0.86
A 0 B 1
A 0.14 B 0
A 0.14 B 0.14
A 0.14 B 0.29
A 0.14 B 0.43
A 0.14 B 0.57
A 0.14 B 0.71
A 0.14 B 0.86
A 0.14 B 1
A 0.29 B 0
A 0.29 B 0.14
A 0.29 B 0.29
A 0.29 B 0.43
A 0.29 B 0.57
A 0.29 B 0.71
A 0.29 B 0.86
A 0.29 B 1
A 0.43 B 0
A 0.43 B 0.14
A 0.43 B 0.29
A 0.43 B 0.43
A 0.43 B 0.57
A 0.43 B 0.71
A 0.43 B 0.86
A 0.43 B 1
A 0.57 B 0
A 0.57 B 0.14
A 0.57 B 0.29
A 0.57 B 0.43
A 0.57 B 0.57
A 0.57 B 0.71
A 0.57 B 0.86
A 0.57 B 1
A 0.71 B 0
A 0.71 B 0.14
A 0.71 B 0.29
A 0.71 B 0.43
A 0.71 B 0.57
A 0.71 B 0.71
A 0.71 B 0.86
A 0.71 B 1
A 0.86 B 0
A 0.86 B 0.14
A 0.86 B 0.29
A 0.86 B 0.43
A 0.86 B 0.57
A 0.86 B 0.71
A 0.86 B 0.86
A 0.86 B 1
A 1 B 0
A 1 B 0.14
A 1 B 0.29
A 1 B 0.43
A 1 B 0.57
A 1 B 0.71
A 1 B 0.86
A 1 B 1
OD 600 nm [−]
0.8
0.6
0.4
0.2
0 5 101520
Time [h]

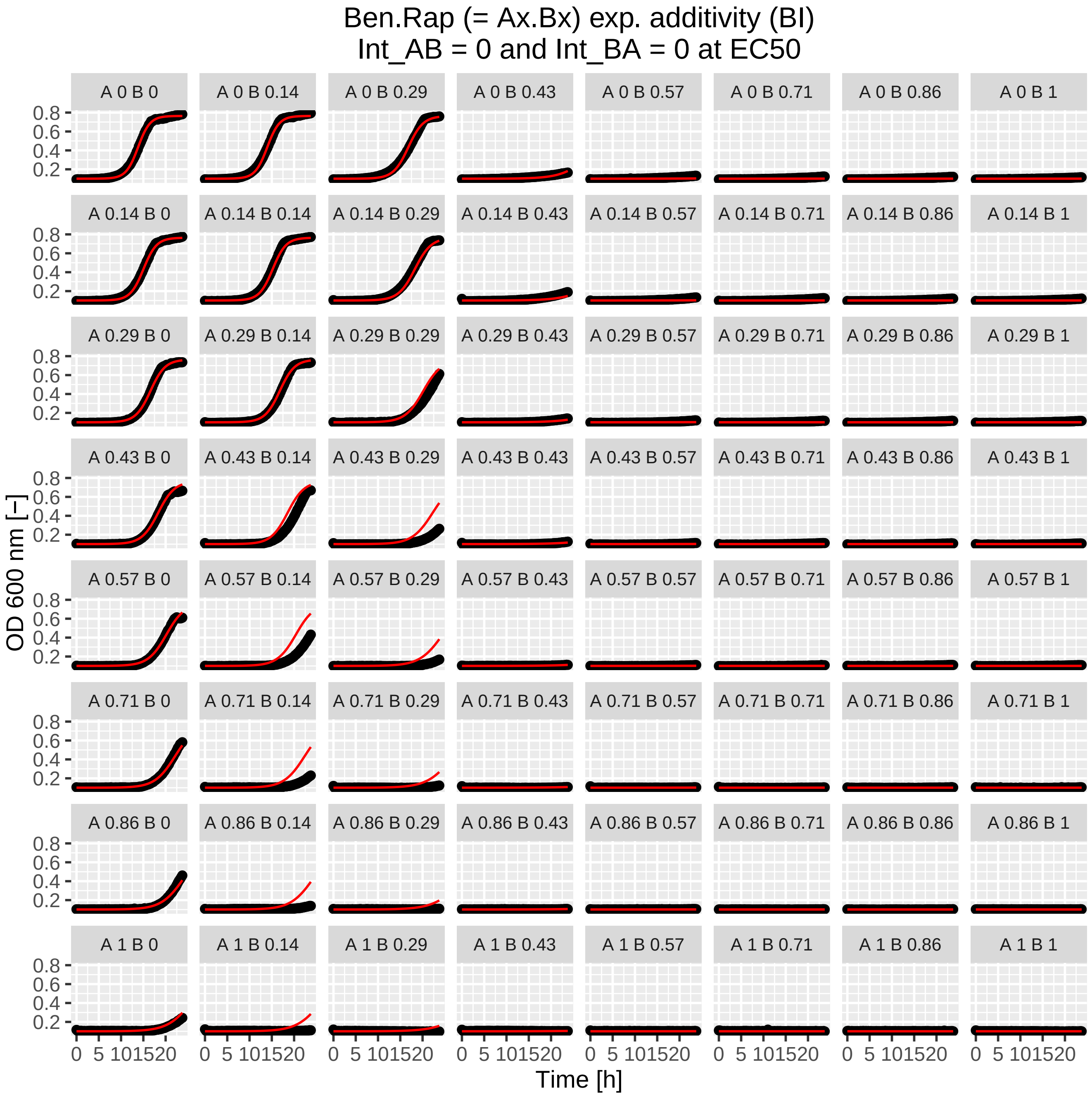

Ben.Rap (= Ax.Bx) exp. additivity (BI)
Int_AB = 0 and Int_BA = 0 at EC50
A 0 B 0
A 0 B 0.14
A 0 B 0.29
A 0 B 0.43
A 0 B 0.57
A 0 B 0.71
A 0 B 0.86
A 0 B 1
A 0.14 B 0
A 0.14 B 0.14
A 0.14 B 0.29
A 0.14 B 0.43
A 0.14 B 0.57
A 0.14 B 0.71
A 0.14 B 0.86
A 0.14 B 1
A 0.29 B 0
A 0.29 B 0.14
A 0.29 B 0.29
A 0.29 B 0.43
A 0.29 B 0.57
A 0.29 B 0.71
A 0.29 B 0.86
A 0.29 B 1
A 0.43 B 0
A 0.43 B 0.14
A 0.43 B 0.29
A 0.43 B 0.43
A 0.43 B 0.57
A 0.43 B 0.71
A 0.43 B 0.86
A 0.43 B 1
A 0.57 B 0
A 0.57 B 0.14
A 0.57 B 0.29
A 0.57 B 0.43
A 0.57 B 0.57
A 0.57 B 0.71
A 0.57 B 0.86
A 0.57 B 1
A 0.71 B 0
A 0.71 B 0.14
A 0.71 B 0.29
A 0.71 B 0.43
A 0.71 B 0.57
A 0.71 B 0.71
A 0.71 B 0.86
A 0.71 B 1
A 0.86 B 0
A 0.86 B 0.14
A 0.86 B 0.29
A 0.86 B 0.43
A 0.86 B 0.57
A 0.86 B 0.71
A 0.86 B 0.86
A 0.86 B 1
A 1 B 0
A 1 B 0.14
A 1 B 0.29
A 1 B 0.43
A 1 B 0.57
A 1 B 0.71
A 1 B 0.86
A 1 B 1
OD 600 nm [–]
0.8
0.6
0.4
0.2
0 5 101520
Time [h]

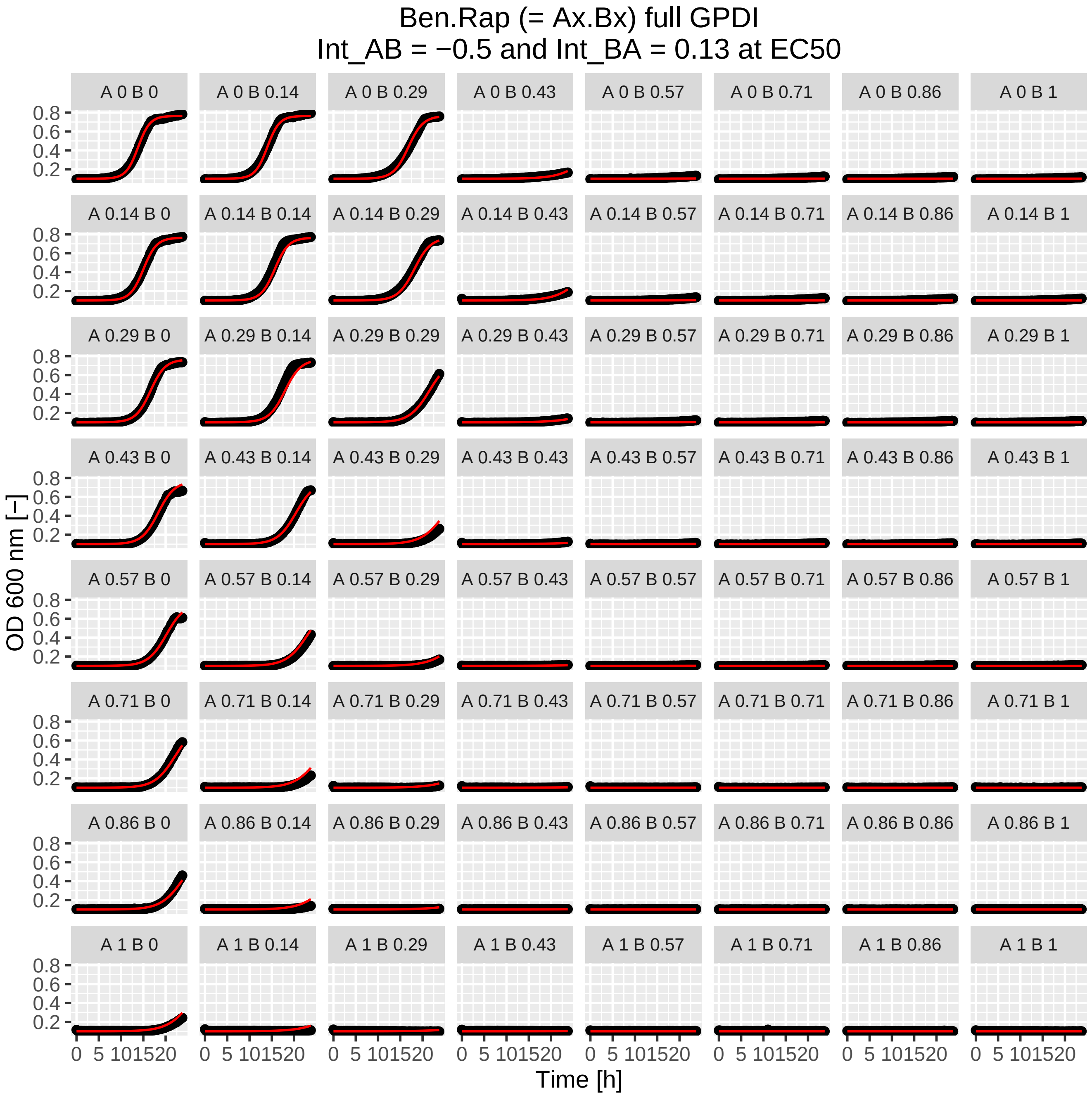

Ben.Rap (= Ax.Bx) full GPDI
Int_AB = −0.5 and Int_BA = 0.13 at EC50
A 0 B 0
A 0 B 0.14
A 0 B 0.29
A 0 B 0.43
A 0 B 0.57
A 0 B 0.71
A 0 B 0.86
A 0 B 1
A 0.14 B 0
A 0.14 B 0.14
A 0.14 B 0.29
A 0.14 B 0.43
A 0.14 B 0.57
A 0.14 B 0.71
A 0.14 B 0.86
A 0.14 B 1
A 0.29 B 0
A 0.29 B 0.14
A 0.29 B 0.29
A 0.29 B 0.43
A 0.29 B 0.57
A 0.29 B 0.71
A 0.29 B 0.86
A 0.29 B 1
A 0.43 B 0
A 0.43 B 0.14
A 0.43 B 0.29
A 0.43 B 0.43
A 0.43 B 0.57
A 0.43 B 0.71
A 0.43 B 0.86
A 0.43 B 1
A 0.57 B 0
A 0.57 B 0.14
A 0.57 B 0.29
A 0.57 B 0.43
A 0.57 B 0.57
A 0.57 B 0.71
A 0.57 B 0.86
A 0.57 B 1
A 0.71 B 0
A 0.71 B 0.14
A 0.71 B 0.29
A 0.71 B 0.43
A 0.71 B 0.57
A 0.71 B 0.71
A 0.71 B 0.86
A 0.71 B 1
A 0.86 B 0
A 0.86 B 0.14
A 0.86 B 0.29
A 0.86 B 0.43
A 0.86 B 0.57
A 0.86 B 0.71
A 0.86 B 0.86
A 0.86 B 1
A 1 B 0
A 1 B 0.14
A 1 B 0.29
A 1 B 0.43
A 1 B 0.57
A 1 B 0.71
A 1 B 0.86
A 1 B 1
OD 600 nm [−]
0.2
0.4
0.6
0.8
0 5 101520
Time [h]

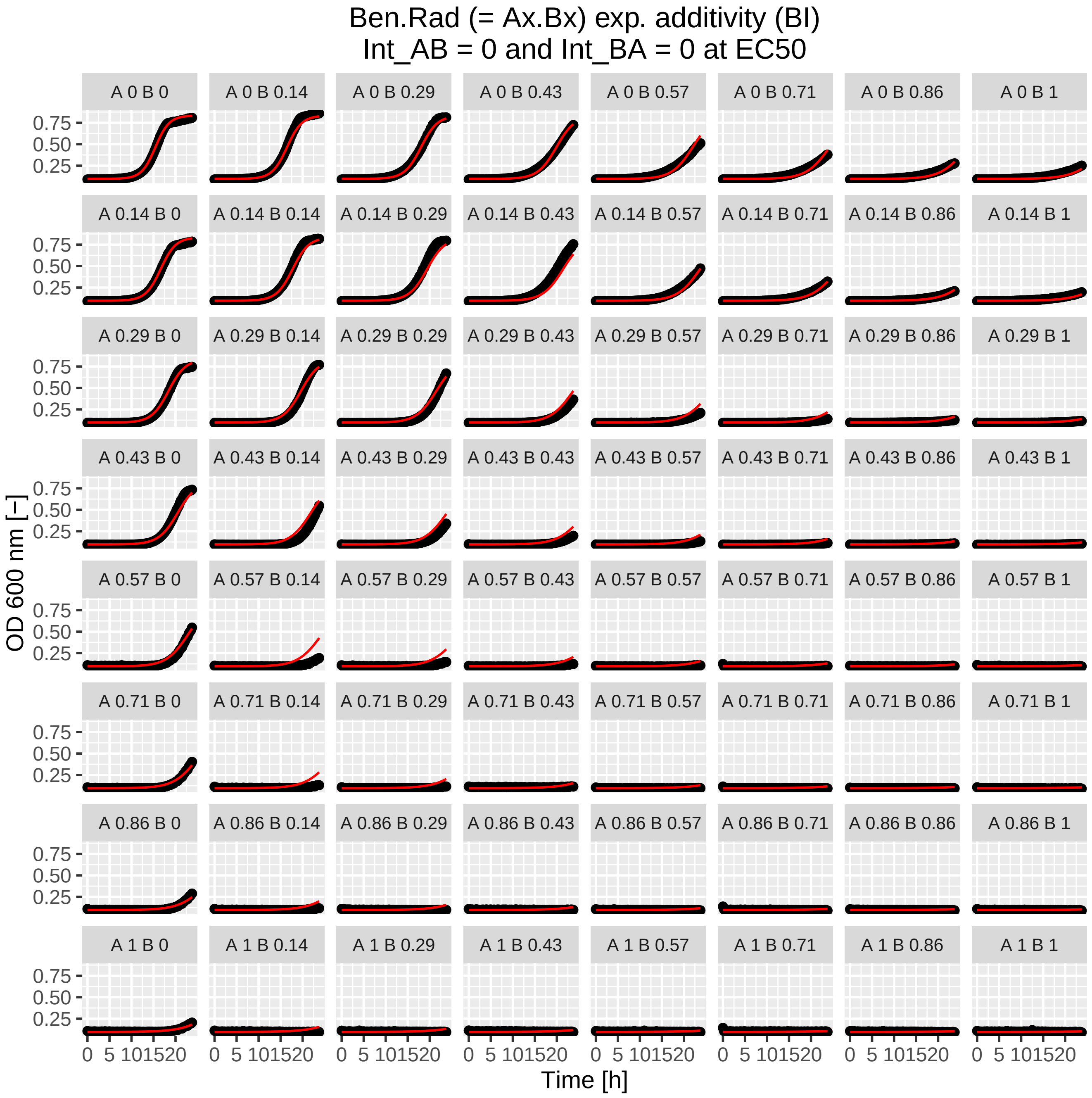

Ben.Rad (= Ax.Bx) exp. additivity (BI)
Int_AB = 0 and Int_BA = 0 at EC50
OD 600 nm [−]
Time [h]
0.75
0.50
0.25
0 5 101520
A 0 B 0
A 0 B 0.14
A 0 B 0.29
A 0 B 0.43
A 0 B 0.57
A 0 B 0.71
A 0 B 0.86
A 0 B 1
A 0.14 B 0
A 0.14 B 0.14
A 0.14 B 0.29
A 0.14 B 0.43
A 0.14 B 0.57
A 0.14 B 0.71
A 0.14 B 0.86
A 0.14 B 1
A 0.29 B 0
A 0.29 B 0.14
A 0.29 B 0.29
A 0.29 B 0.43
A 0.29 B 0.57
A 0.29 B 0.71
A 0.29 B 0.86
A 0.29 B 1
A 0.43 B 0
A 0.43 B 0.14
A 0.43 B 0.29
A 0.43 B 0.43
A 0.43 B 0.57
A 0.43 B 0.71
A 0.43 B 0.86
A 0.43 B 1
A 0.57 B 0
A 0.57 B 0.14
A 0.57 B 0.29
A 0.57 B 0.43
A 0.57 B 0.57
A 0.57 B 0.71
A 0.57 B 0.86
A 0.57 B 1
A 0.71 B 0
A 0.71 B 0.14
A 0.71 B 0.29
A 0.71 B 0.43
A 0.71 B 0.57
A 0.71 B 0.71
A 0.71 B 0.86
A 0.71 B 1
A 0.86 B 0
A 0.86 B 0.14
A 0.86 B 0.29
A 0.86 B 0.43
A 0.86 B 0.57
A 0.86 B 0.71
A 0.86 B 0.86
A 0.86 B 1
A 1 B 0
A 1 B 0.14
A 1 B 0.29
A 1 B 0.43
A 1 B 0.57
A 1 B 0.71
A 1 B 0.86
A 1 B 1

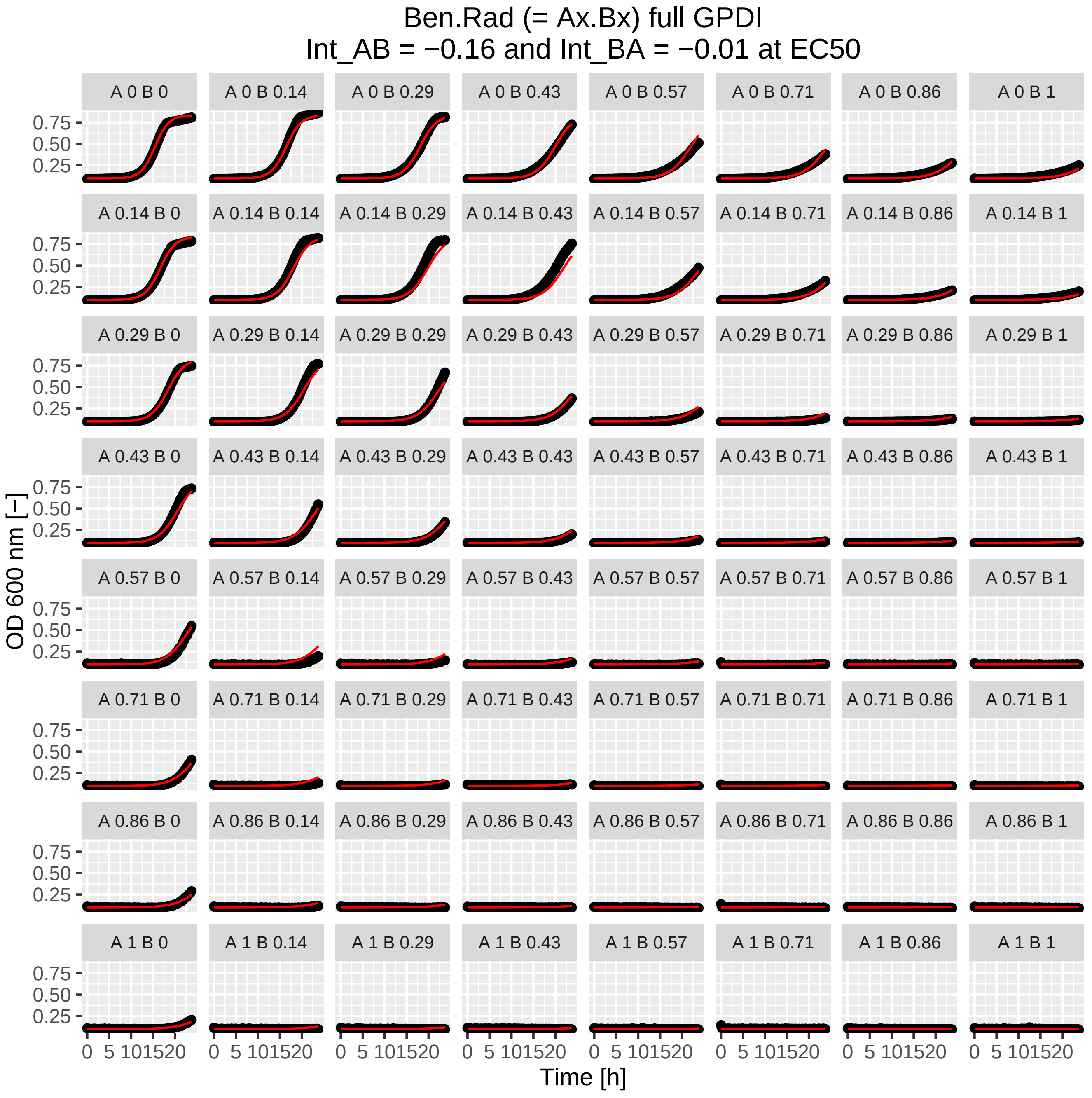

Ben.Rad (= Ax.Bx) full GPDI
Int_AB = −0.16 and Int_BA = −0.01 at EC50
A 0 B 0
A 0 B 0.14
A 0 B 0.29
A 0 B 0.43
A 0 B 0.57
A 0 B 0.71
A 0 B 0.86
A 0 B 1
A 0.14 B 0
A 0.14 B 0.14
A 0.14 B 0.29
A 0.14 B 0.43
A 0.14 B 0.57
A 0.14 B 0.71
A 0.14 B 0.86
A 0.14 B 1
A 0.29 B 0
A 0.29 B 0.14
A 0.29 B 0.29
A 0.29 B 0.43
A 0.29 B 0.57
A 0.29 B 0.71
A 0.29 B 0.86
A 0.29 B 1
A 0.43 B 0
A 0.43 B 0.14
A 0.43 B 0.29
A 0.43 B 0.43
A 0.43 B 0.57
A 0.43 B 0.71
A 0.43 B 0.86
A 0.43 B 1
A 0.57 B 0
A 0.57 B 0.14
A 0.57 B 0.29
A 0.57 B 0.43
A 0.57 B 0.57
A 0.57 B 0.71
A 0.57 B 0.86
A 0.57 B 1
A 0.71 B 0
A 0.71 B 0.14
A 0.71 B 0.29
A 0.71 B 0.43
A 0.71 B 0.57
A 0.71 B 0.71
A 0.71 B 0.86
A 0.71 B 1
A 0.86 B 0
A 0.86 B 0.14
A 0.86 B 0.29
A 0.86 B 0.43
A 0.86 B 0.57
A 0.86 B 0.71
A 0.86 B 0.86
A 0.86 B 1
A 1 B 0
A 1 B 0.14
A 1 B 0.29
A 1 B 0.43
A 1 B 0.57
A 1 B 0.71
A 1 B 0.86
A 1 B 1
0.75
0.50
0.25
OD 600 nm [−]
0 5 101520
Time [h]

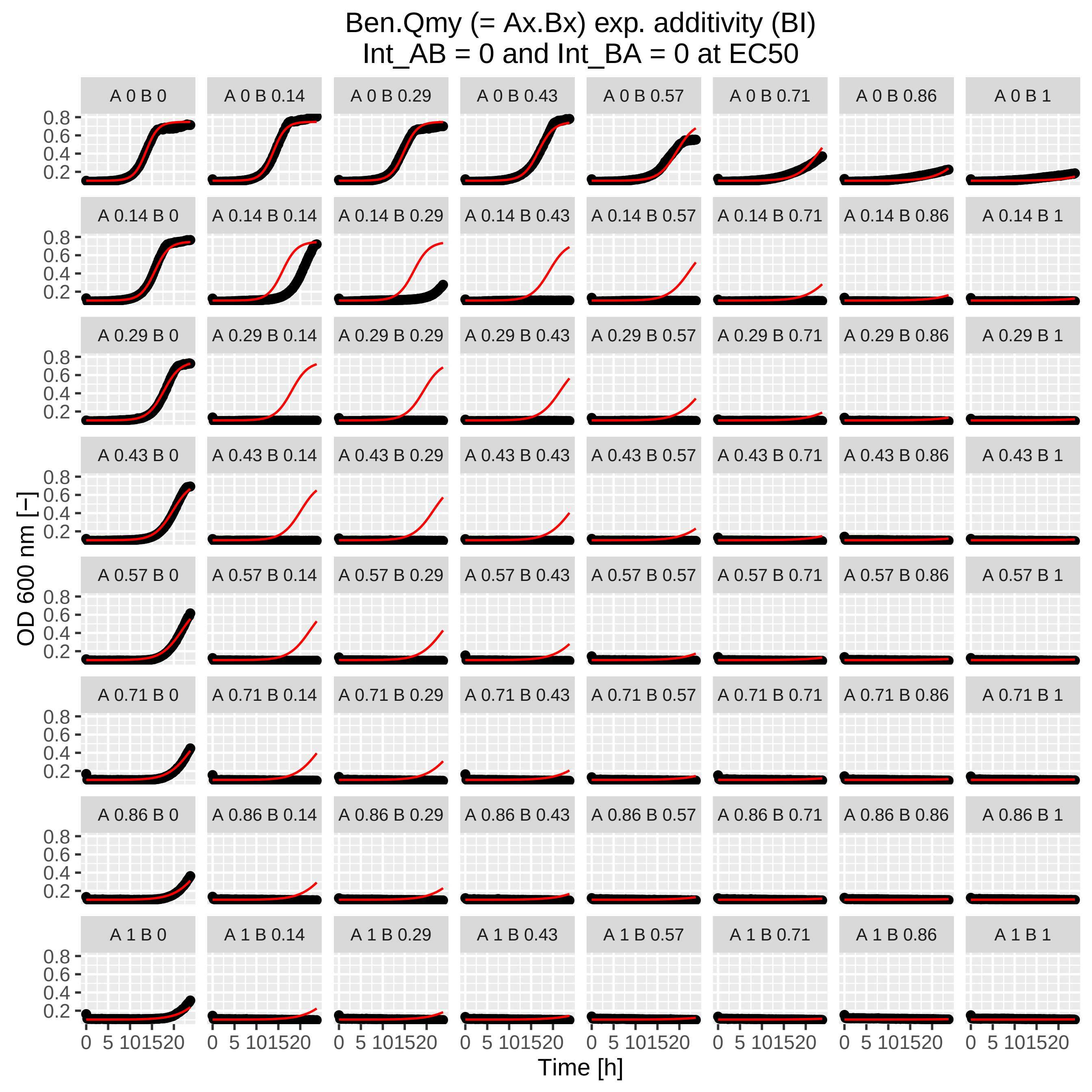
Ben.Qmy (= Ax.Bx) exp. additivity (BI)
Int_AB = 0 and Int_BA = 0 at EC50
OD 600 nm [−]
Time [h]
A 0 B 0
A 0 B 0.14
A 0 B 0.29
A 0 B 0.43
A 0 B 0.57
A 0 B 0.71
A 0 B 0.86
A 0 B 1
A 0.14 B 0
A 0.29 B 0
A 0.43 B 0
A 0.57 B 0
A 0.71 B 0
A 0.86 B 0
A 1 B 0
A 1 B 1

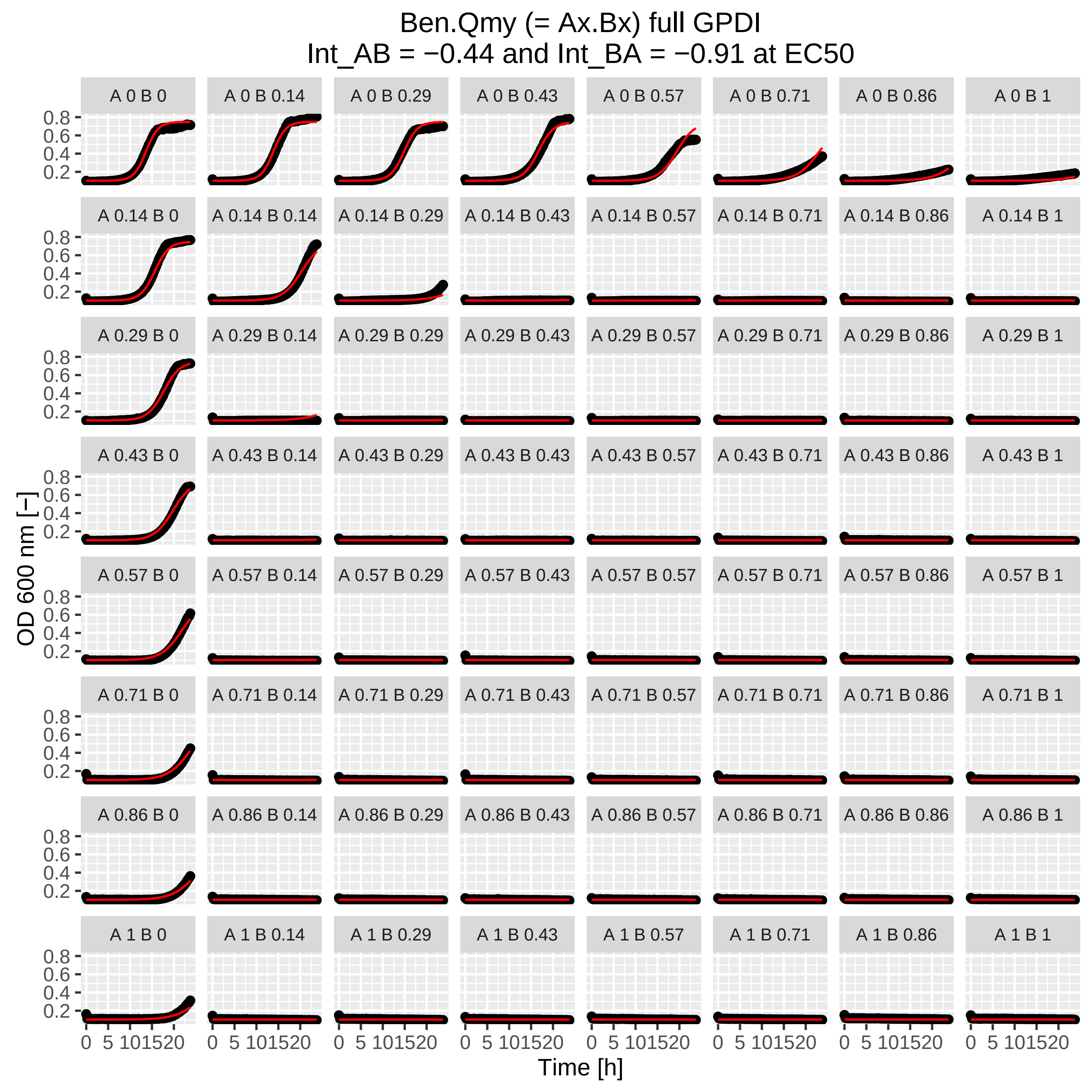
Ben.Qmy (= Ax.Bx) full GPDI
Int_AB = −0.44 and Int_BA = −0.91 at EC50
OD 600 nm [−]
Time [h]
A 0 B 0
A 0 B 0.14
A 0 B 0.29
A 0 B 0.43
A 0 B 0.57
A 0 B 0.71
A 0 B 0.86
A 0 B 1
A 0.14 B 0
A 0.14 B 0.14
A 0.14 B 0.29
A 0.14 B 0.43
A 0.14 B 0.57
A 0.14 B 0.71
A 0.14 B 0.86
A 0.14 B 1
A 0.29 B 0
A 0.29 B 0.14
A 0.29 B 0.29
A 0.29 B 0.43
A 0.29 B 0.57
A 0.29 B 0.71
A 0.29 B 0.86
A 0.29 B 1
A 0.43 B 0
A 0.43 B 0.14
A 0.43 B 0.29
A 0.43 B 0.43
A 0.43 B 0.57
A 0.43 B 0.71
A 0.43 B 0.86
A 0.43 B 1
A 0.57 B 0
A 0.57 B 0.14
A 0.57 B 0.29
A 0.57 B 0.43
A 0.57 B 0.57
A 0.57 B 0.71
A 0.57 B 0.86
A 0.57 B 1
A 0.71 B 0
A 0.71 B 0.14
A 0.71 B 0.29
A 0.71 B 0.43
A 0.71 B 0.57
A 0.71 B 0.71
A 0.71 B 0.86
A 0.71 B 1
A 0.86 B 0
A 0.86 B 0.14
A 0.86 B 0.29
A 0.86 B 0.43
A 0.86 B 0.57
A 0.86 B 0.71
A 0.86 B 0.86
A 0.86 B 1
A 1 B 0
A 1 B 0.14
A 1 B 0.29
A 1 B 0.43
A 1 B 0.57
A 1 B 0.71
A 1 B 0.86
A 1 B 1
0.2
0.4
0.6
0.8
0
5
10
15
20

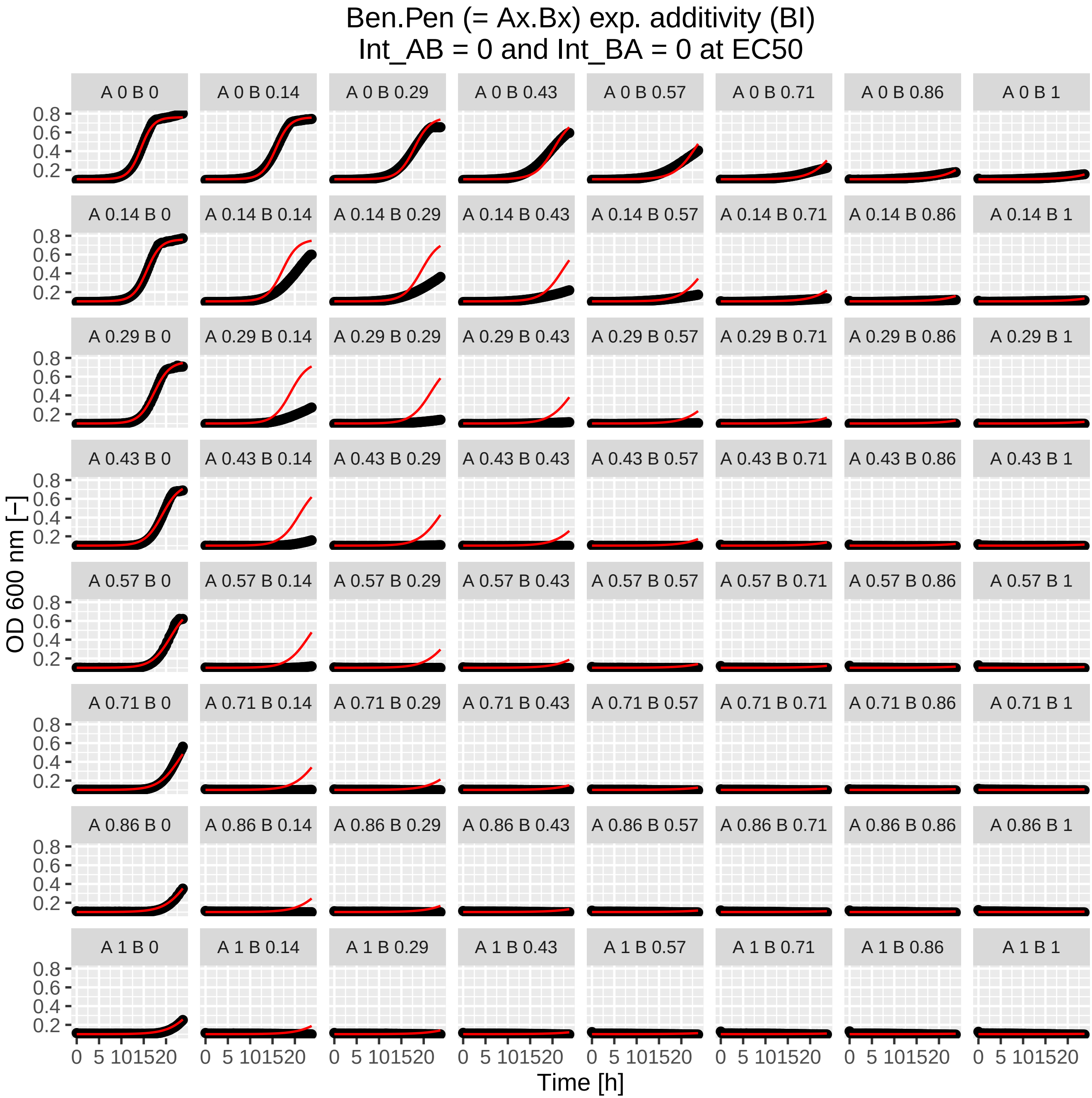
Ben.Pen (= Ax.Bx) exp. additivity (BI)
Int_AB = 0 and Int_BA = 0 at EC50
OD 600 nm [–]
Time [h]

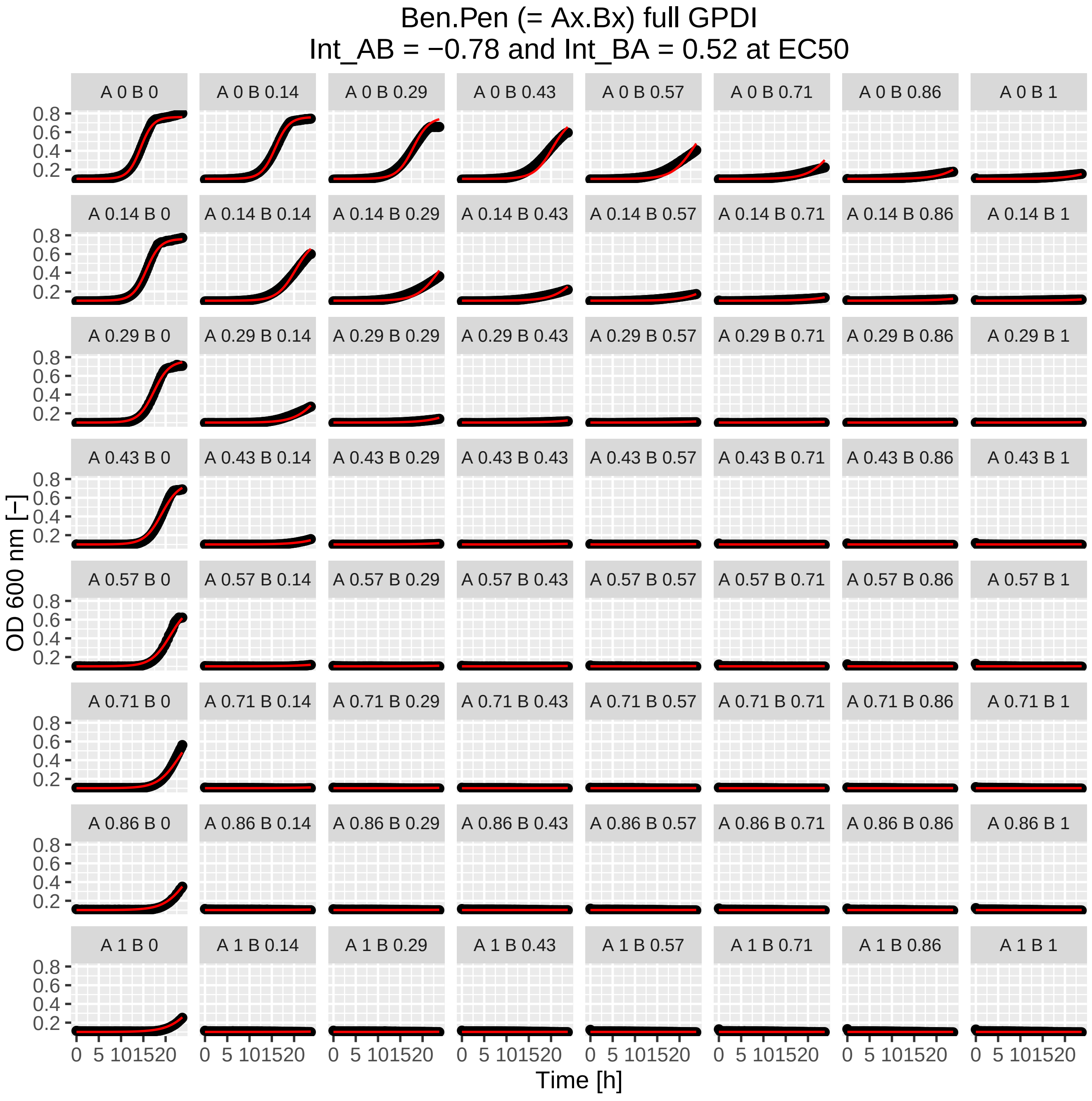

Ben.Pen (= Ax.Bx) full GPDI
Int_AB = −0.78 and Int_BA = 0.52 at EC50
OD 600 nm [–]
Time [h]

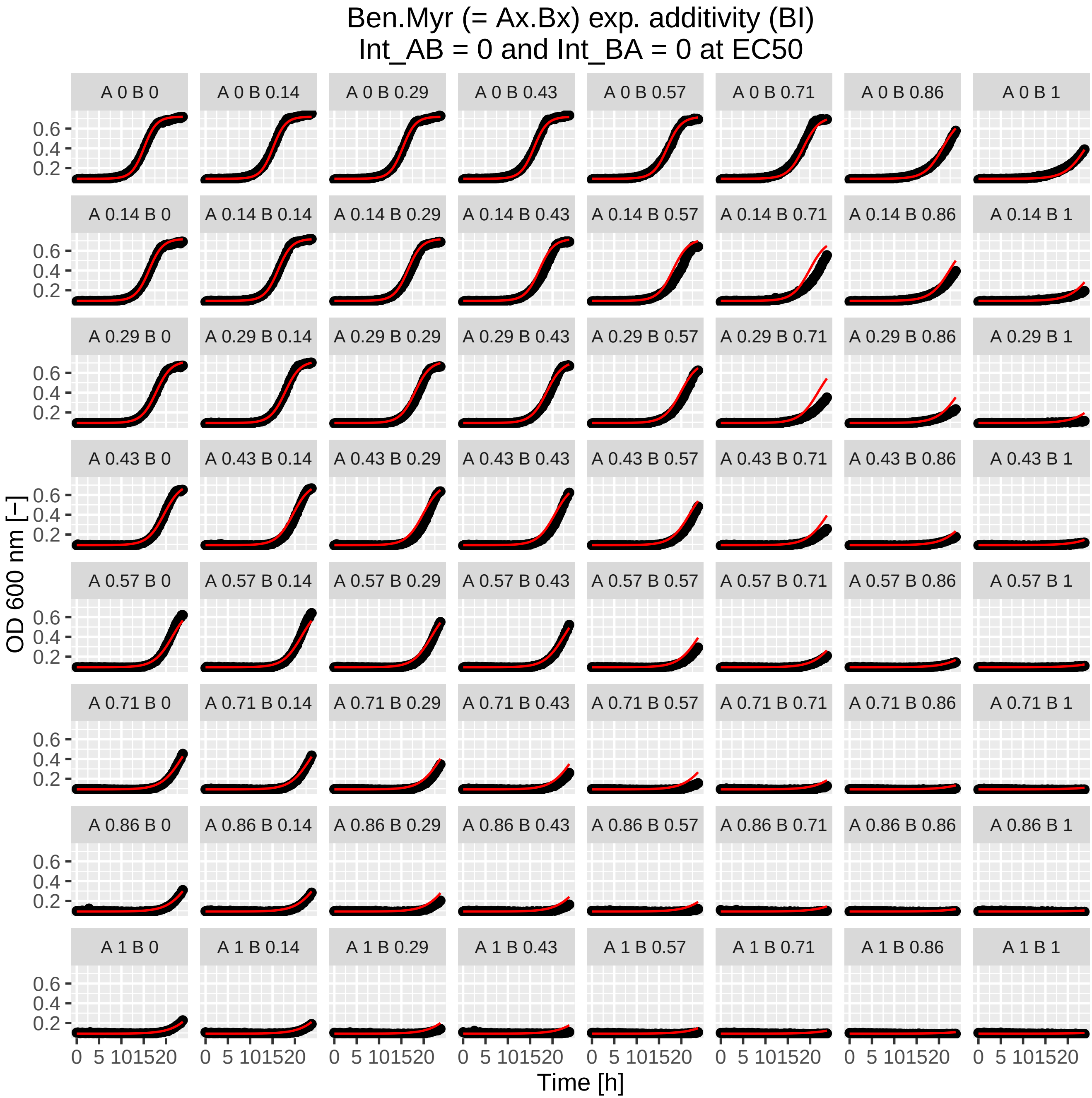
Ben.Myr (= Ax.Bx) exp. additivity (BI)
Int_AB = 0 and Int_BA = 0 at EC50
OD 600 nm [–]
Time [h]
A 0 B 0
A 0 B 0.14
A 0 B 0.29
A 0 B 0.43
A 0 B 0.57
A 0 B 0.71
A 0 B 0.86
A 0 B 1
A 0.14 B 0
A 0.29 B 0
A 0.43 B 0
A 0.57 B 0
A 0.71 B 0
A 0.86 B 0
A 1 B 0
A 1 B 1
0.2
0.4
0.6
0 5 101520

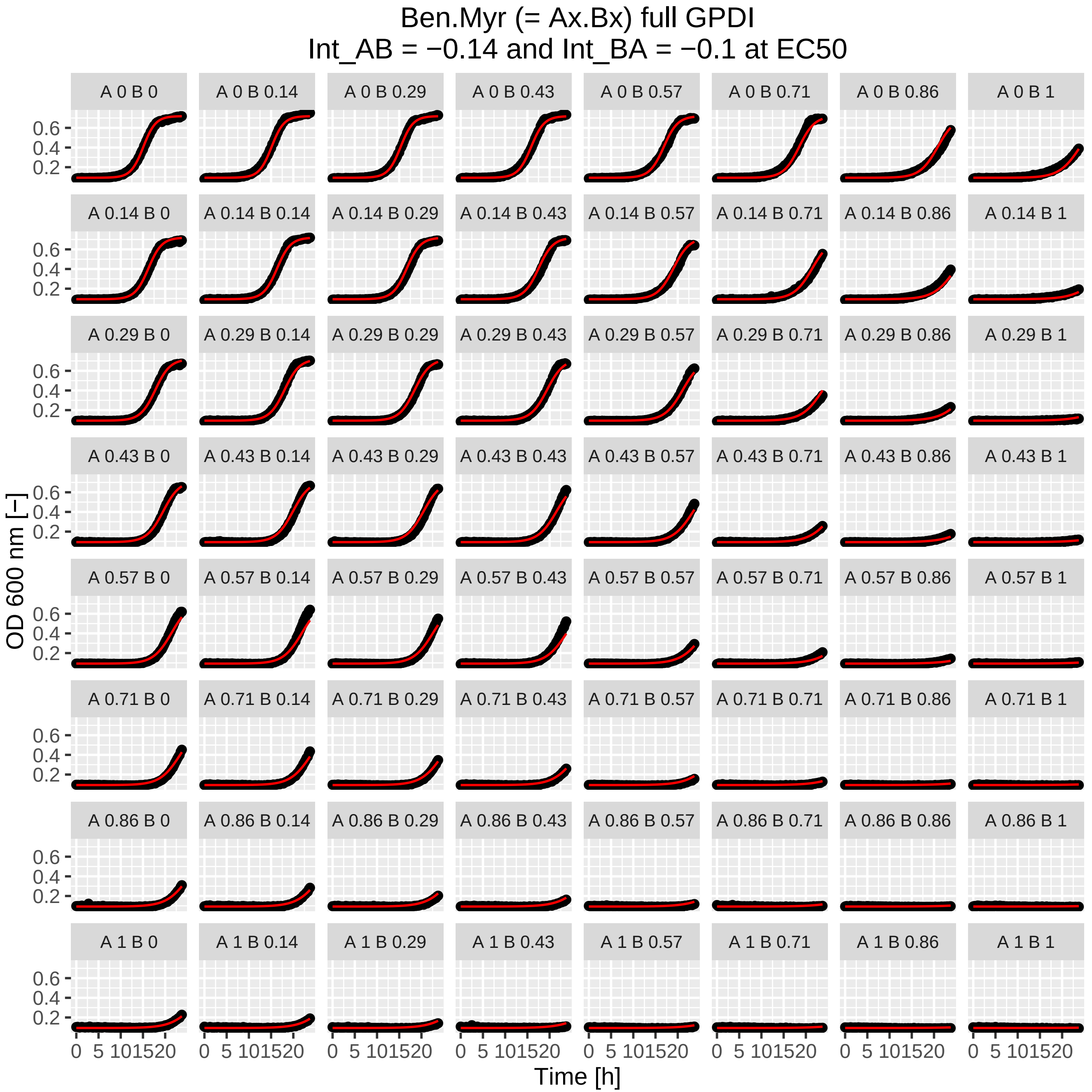

Ben.Myr (= Ax.Bx) full GPDI
Int_AB = −0.14 and Int_BA = −0.1 at EC50
OD 600 nm [−]
Time [h]

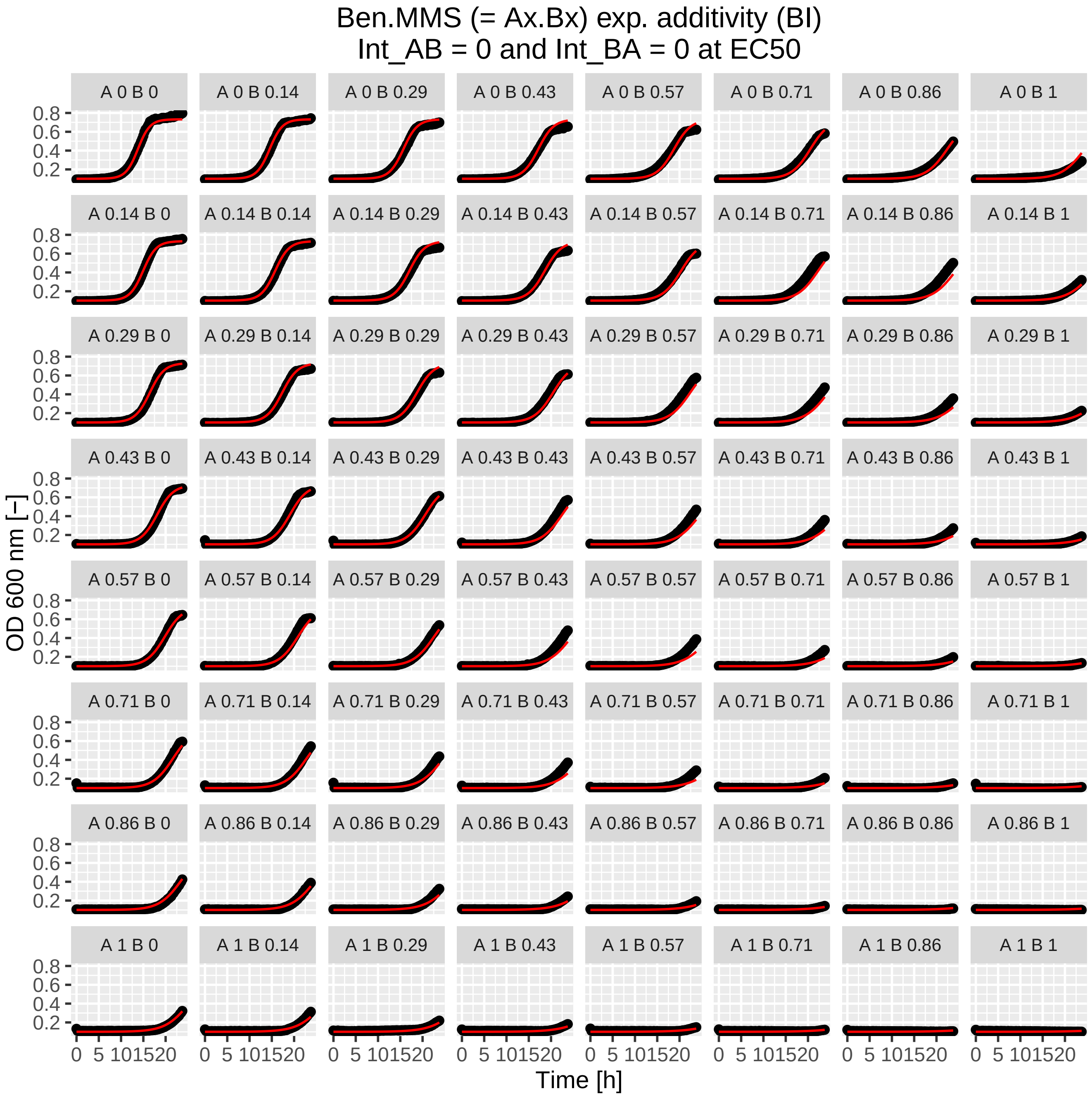
Ben.MMS (= Ax.Bx) exp. additivity (BI)
Int_AB = 0 and Int_BA = 0 at EC50
OD 600 nm [–]
Time [h]
A 0 B 0
A 0 B 0.14
A 0 B 0.29
A 0 B 0.43
A 0 B 0.57
A 0 B 0.71
A 0 B 0.86
A 0 B 1
A 0.14 B 0
A 0.14 B 0.14
A 0.14 B 0.29
A 0.14 B 0.43
A 0.14 B 0.57
A 0.14 B 0.71
A 0.14 B 0.86
A 0.14 B 1
A 0.29 B 0
A 0.29 B 0.14
A 0.29 B 0.29
A 0.29 B 0.43
A 0.29 B 0.57
A 0.29 B 0.71
A 0.29 B 0.86
A 0.29 B 1
A 0.43 B 0
A 0.43 B 0.14
A 0.43 B 0.29
A 0.43 B 0.43
A 0.43 B 0.57
A 0.43 B 0.71
A 0.43 B 0.86
A 0.43 B 1
A 0.57 B 0
A 0.57 B 0.14
A 0.57 B 0.29
A 0.57 B 0.43
A 0.57 B 0.57
A 0.57 B 0.71
A 0.57 B 0.86
A 0.57 B 1
A 0.71 B 0
A 0.71 B 0.14
A 0.71 B 0.29
A 0.71 B 0.43
A 0.71 B 0.57
A 0.71 B 0.71
A 0.71 B 0.86
A 0.71 B 1
A 0.86 B 0
A 0.86 B 0.14
A 0.86 B 0.29
A 0.86 B 0.43
A 0.86 B 0.57
A 0.86 B 0.71
A 0.86 B 0.86
A 0.86 B 1
A 1 B 0
A 1 B 0.14
A 1 B 0.29
A 1 B 0.43
A 1 B 0.57
A 1 B 0.71
A 1 B 0.86
A 1 B 1
0.8
0.6
0.4
0.2
0 5 101520

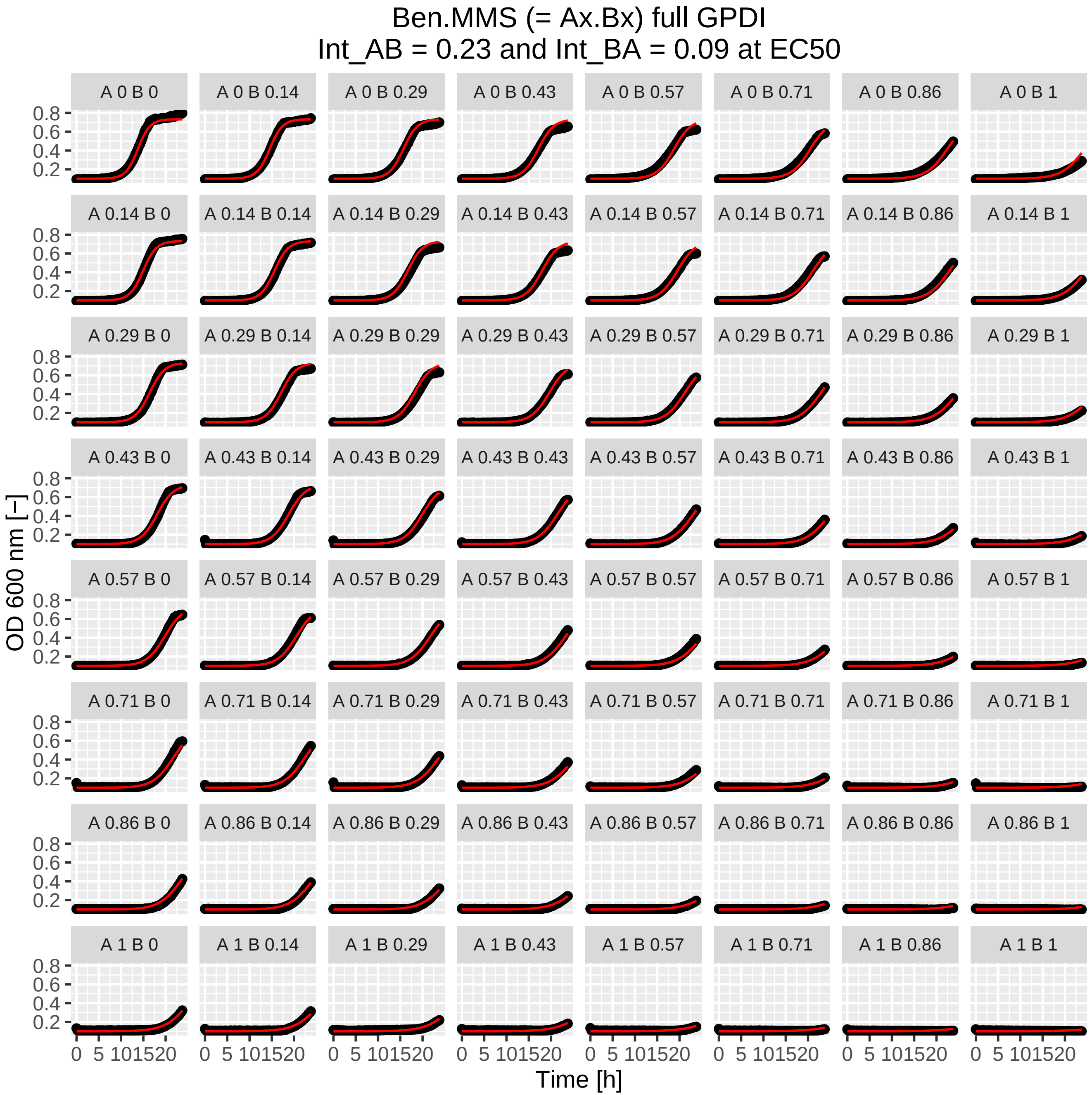

Ben.MMS (= Ax.Bx) full GPDI
Int_AB = 0.23 and Int_BA = 0.09 at EC50
OD 600 nm [–]
Time [h]
A 0 B 0
A 0 B 0.14
A 0 B 0.29
A 0 B 0.43
A 0 B 0.57
A 0 B 0.71
A 0 B 0.86
A 0 B 1
A 1 B 0
A 1 B 0.14
A 1 B 0.29
A 1 B 0.43
A 1 B 0.57
A 1 B 0.71
A 1 B 0.86
A 1 B 1

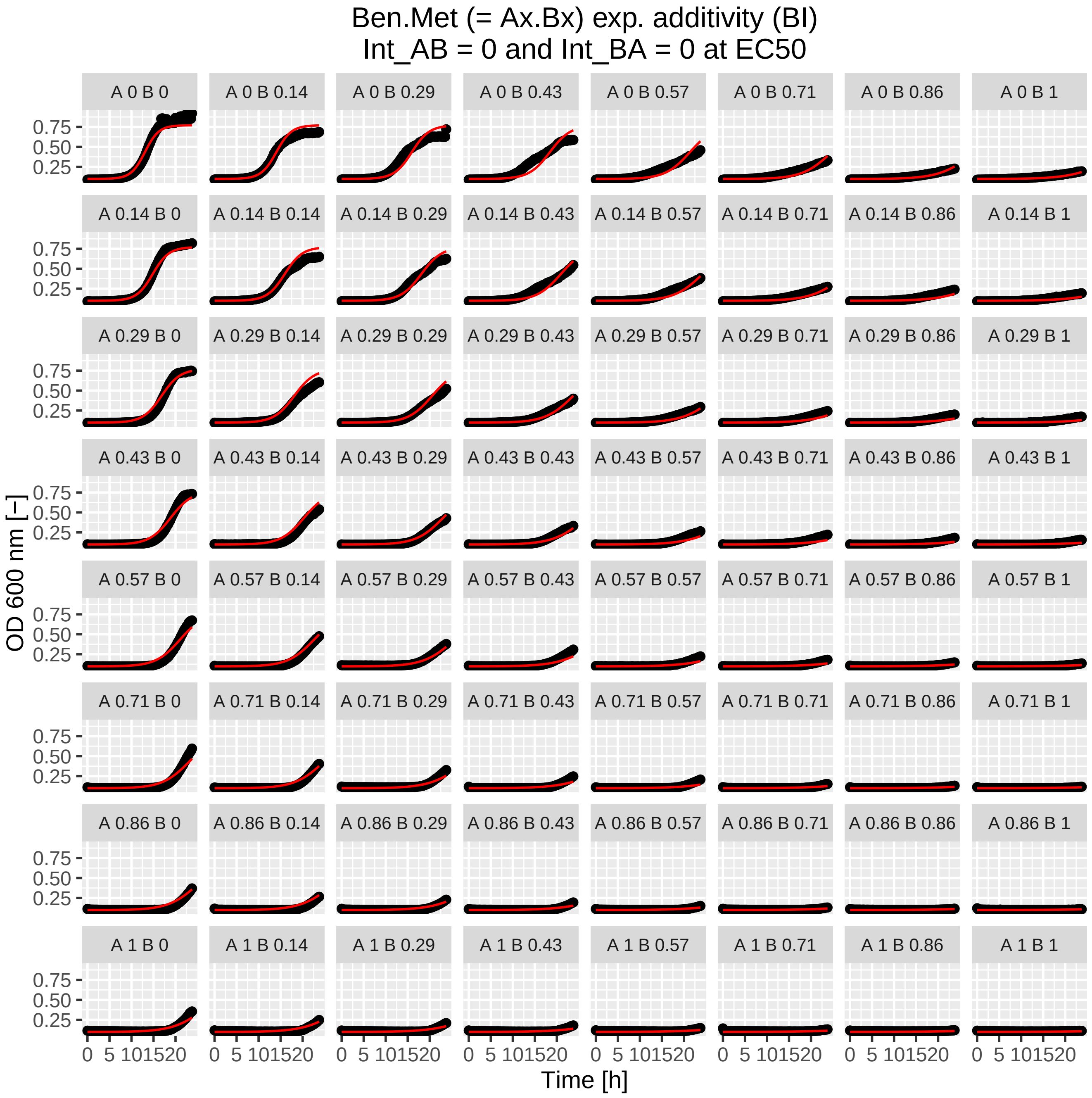

Ben.Met (= Ax.Bx) exp. additivity (BI)
Int_AB = 0 and Int_BA = 0 at EC50
A 0 B 0
A 0 B 0.14
A 0 B 0.29
A 0 B 0.43
A 0 B 0.57
A 0 B 0.71
A 0 B 0.86
A 0 B 1
A 0.14 B 0
A 0.14 B 0.14
A 0.14 B 0.29
A 0.14 B 0.43
A 0.14 B 0.57
A 0.14 B 0.71
A 0.14 B 0.86
A 0.14 B 1
A 0.29 B 0
A 0.29 B 0.14
A 0.29 B 0.29
A 0.29 B 0.43
A 0.29 B 0.57
A 0.29 B 0.71
A 0.29 B 0.86
A 0.29 B 1
A 0.43 B 0
A 0.43 B 0.14
A 0.43 B 0.29
A 0.43 B 0.43
A 0.43 B 0.57
A 0.43 B 0.71
A 0.43 B 0.86
A 0.43 B 1
A 0.57 B 0
A 0.57 B 0.14
A 0.57 B 0.29
A 0.57 B 0.43
A 0.57 B 0.57
A 0.57 B 0.71
A 0.57 B 0.86
A 0.57 B 1
A 0.71 B 0
A 0.71 B 0.14
A 0.71 B 0.29
A 0.71 B 0.43
A 0.71 B 0.57
A 0.71 B 0.71
A 0.71 B 0.86
A 0.71 B 1
A 0.86 B 0
A 0.86 B 0.14
A 0.86 B 0.29
A 0.86 B 0.43
A 0.86 B 0.57
A 0.86 B 0.71
A 0.86 B 0.86
A 0.86 B 1
A 1 B 0
A 1 B 0.14
A 1 B 0.29
A 1 B 0.43
A 1 B 0.57
A 1 B 0.71
A 1 B 0.86
A 1 B 1
0.75
0.50
0.25
OD 600 nm [–]
0 5 101520
Time [h]

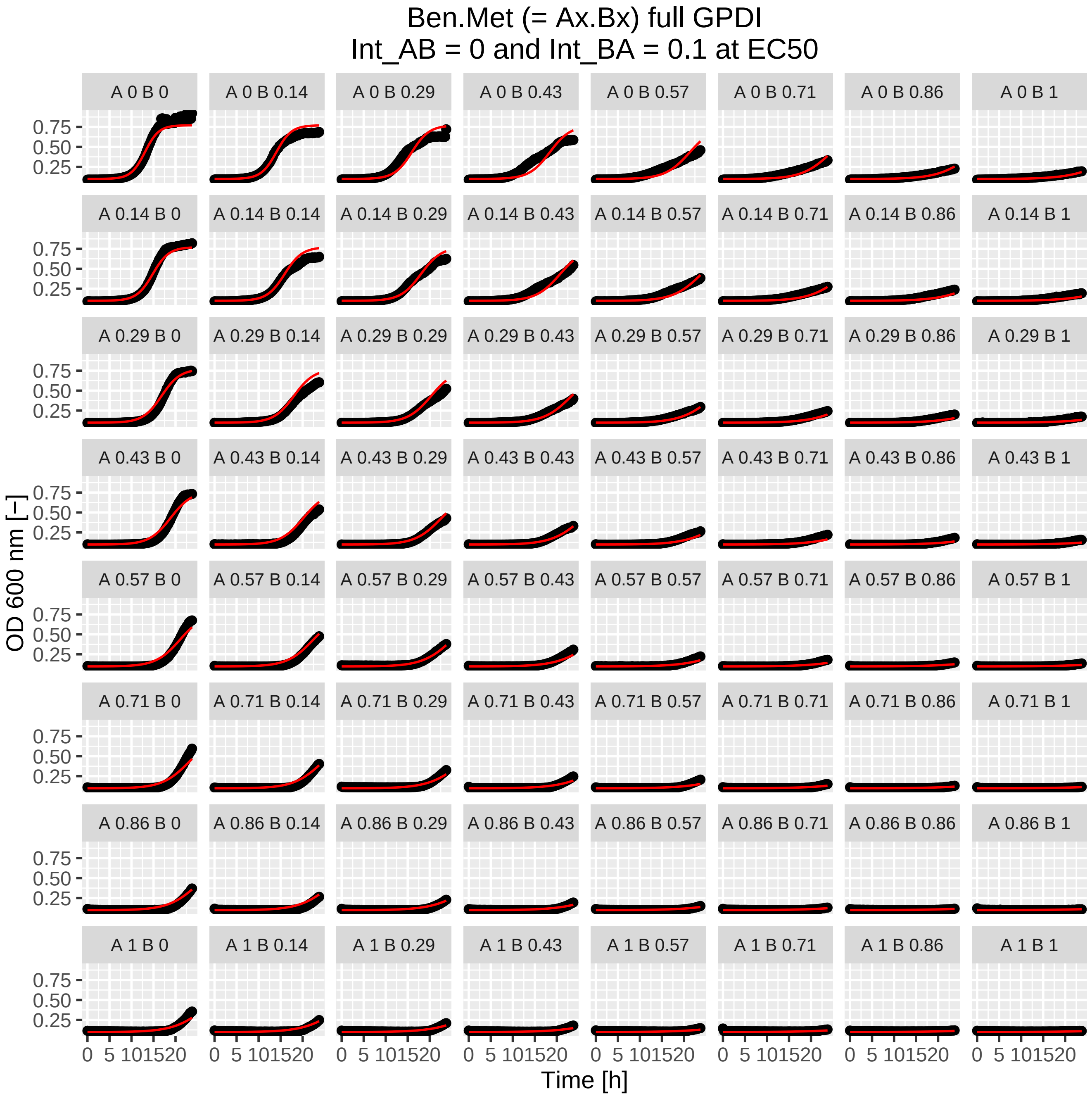

Ben.Met (= Ax.Bx) full GPDI
Int_AB = 0 and Int_BA = 0.1 at EC50
A 0 B 0
A 0 B 0.14
A 0 B 0.29
A 0 B 0.43
A 0 B 0.57
A 0 B 0.71
A 0 B 0.86
A 0 B 1
A 0.14 B 0
A 0.14 B 0.14
A 0.14 B 0.29
A 0.14 B 0.43
A 0.14 B 0.57
A 0.14 B 0.71
A 0.14 B 0.86
A 0.14 B 1
A 0.29 B 0
A 0.29 B 0.14
A 0.29 B 0.29
A 0.29 B 0.43
A 0.29 B 0.57
A 0.29 B 0.71
A 0.29 B 0.86
A 0.29 B 1
A 0.43 B 0
A 0.43 B 0.14
A 0.43 B 0.29
A 0.43 B 0.43
A 0.43 B 0.57
A 0.43 B 0.71
A 0.43 B 0.86
A 0.43 B 1
A 0.57 B 0
A 0.57 B 0.14
A 0.57 B 0.29
A 0.57 B 0.43
A 0.57 B 0.57
A 0.57 B 0.71
A 0.57 B 0.86
A 0.57 B 1
A 0.71 B 0
A 0.71 B 0.14
A 0.71 B 0.29
A 0.71 B 0.43
A 0.71 B 0.57
A 0.71 B 0.71
A 0.71 B 0.86
A 0.71 B 1
A 0.86 B 0
A 0.86 B 0.14
A 0.86 B 0.29
A 0.86 B 0.43
A 0.86 B 0.57
A 0.86 B 0.71
A 0.86 B 0.86
A 0.86 B 1
A 1 B 0
A 1 B 0.14
A 1 B 0.29
A 1 B 0.43
A 1 B 0.57
A 1 B 0.71
A 1 B 0.86
A 1 B 1
0.75
0.50
0.25
0 5 101520
OD 600 nm [–]
Time [h]

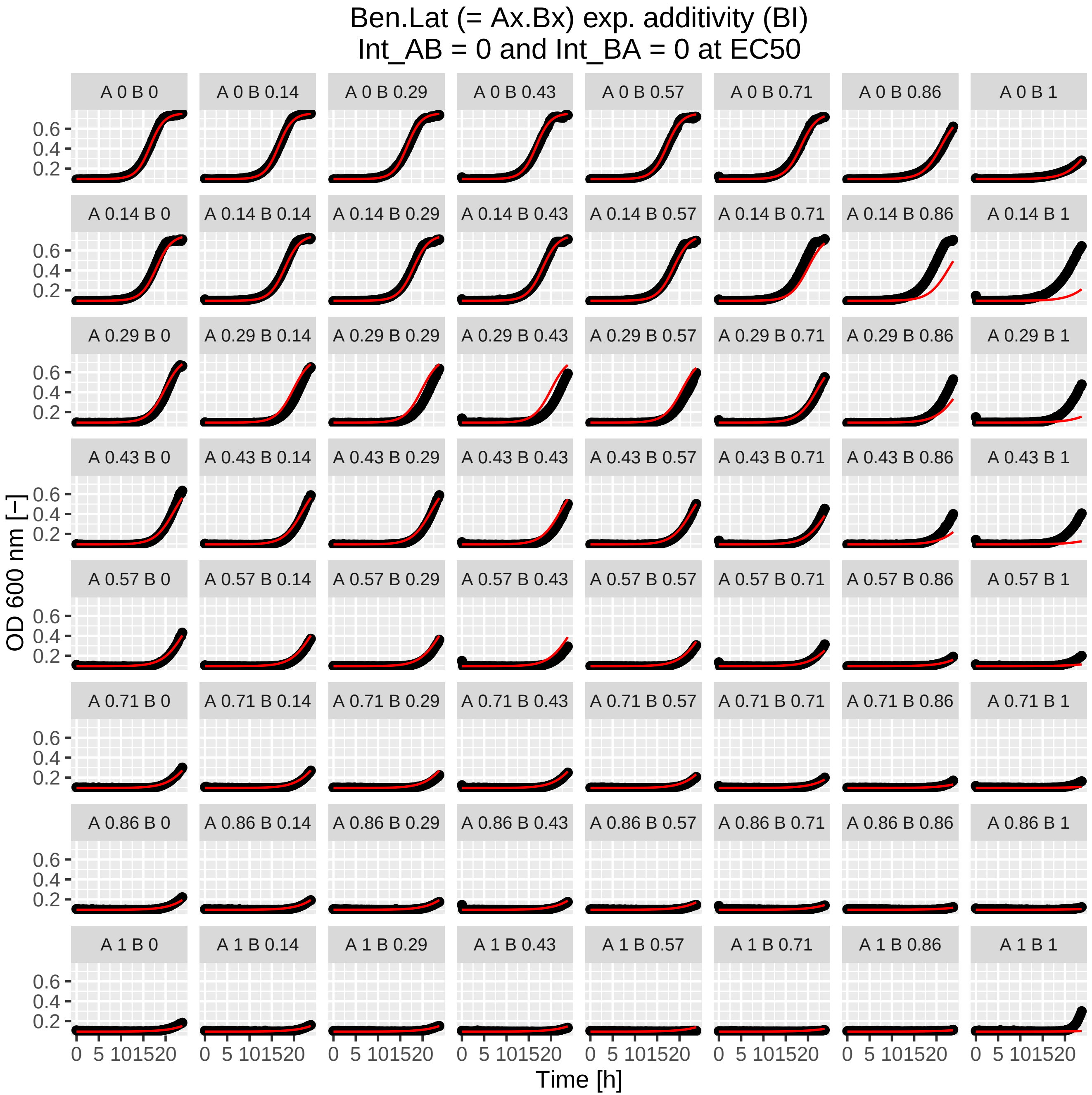
Ben.Lat (= Ax.Bx) exp. additivity (BI)
Int_AB = 0 and Int_BA = 0 at EC50
A 0 B 0
A 0 B 0.14
A 0 B 0.29
A 0 B 0.43
A 0 B 0.57
A 0 B 0.71
A 0 B 0.86
A 0 B 1
A 0.14 B 0
A 0.14 B 0.14
A 0.14 B 0.29
A 0.14 B 0.43
A 0.14 B 0.57
A 0.14 B 0.71
A 0.14 B 0.86
A 0.14 B 1
A 0.29 B 0
A 0.29 B 0.14
A 0.29 B 0.29
A 0.29 B 0.43
A 0.29 B 0.57
A 0.29 B 0.71
A 0.29 B 0.86
A 0.29 B 1
A 0.43 B 0
A 0.43 B 0.14
A 0.43 B 0.29
A 0.43 B 0.43
A 0.43 B 0.57
A 0.43 B 0.71
A 0.43 B 0.86
A 0.43 B 1
A 0.57 B 0
A 0.57 B 0.14
A 0.57 B 0.29
A 0.57 B 0.43
A 0.57 B 0.57
A 0.57 B 0.71
A 0.57 B 0.86
A 0.57 B 1
A 0.71 B 0
A 0.71 B 0.14
A 0.71 B 0.29
A 0.71 B 0.43
A 0.71 B 0.57
A 0.71 B 0.71
A 0.71 B 0.86
A 0.71 B 1
A 0.86 B 0
A 0.86 B 0.14
A 0.86 B 0.29
A 0.86 B 0.43
A 0.86 B 0.57
A 0.86 B 0.71
A 0.86 B 0.86
A 0.86 B 1
A 1 B 0
A 1 B 0.14
A 1 B 0.29
A 1 B 0.43
A 1 B 0.57
A 1 B 0.71
A 1 B 0.86
A 1 B 1
OD 600 nm [–]
0.2
0.4
0.6
0 5 101520
Time [h]

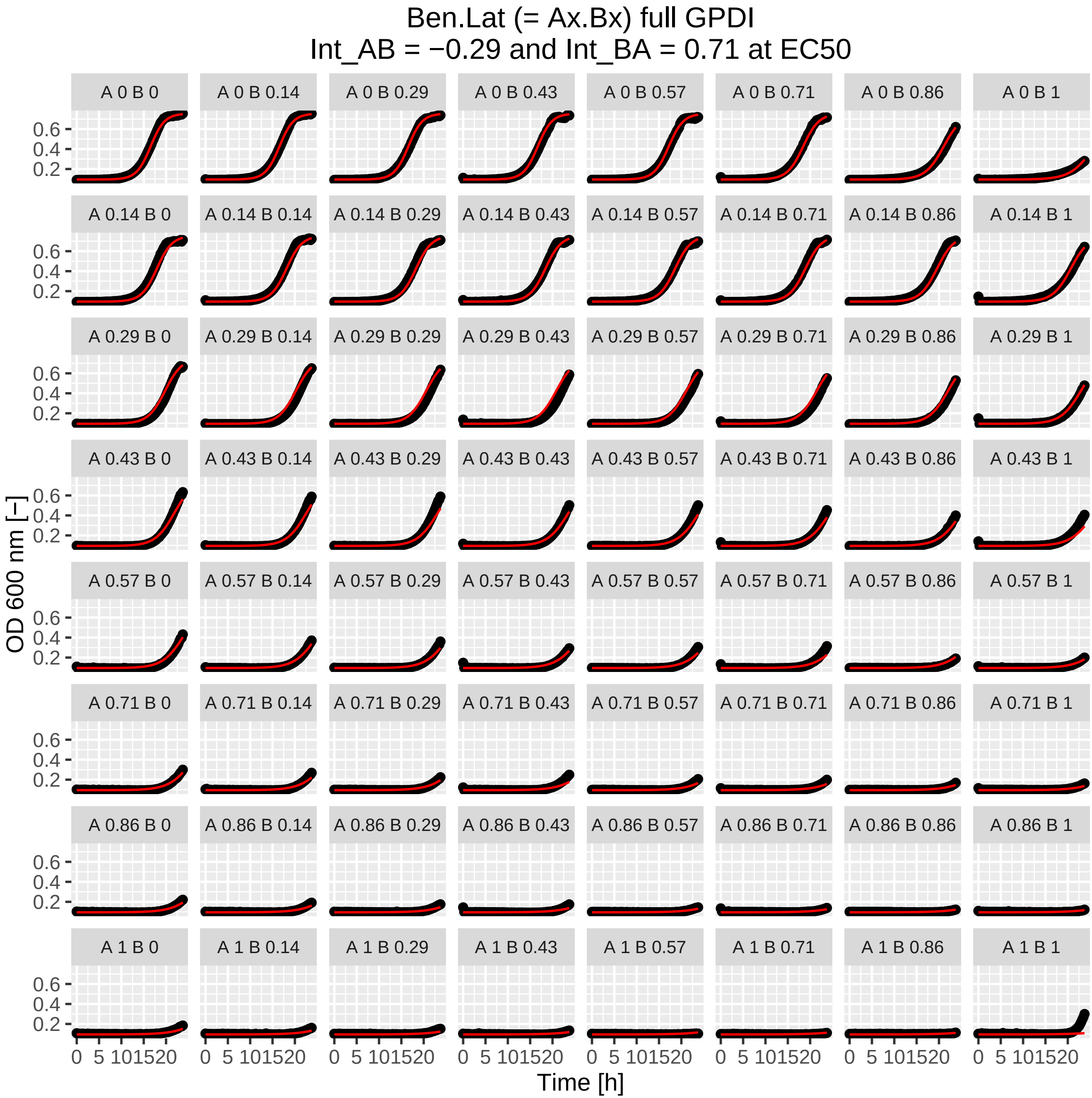

Ben.Lat (= Ax.Bx) full GPDI
Int_AB = −0.29 and Int_BA = 0.71 at EC50
A 0 B 0
A 0 B 0.14
A 0 B 0.29
A 0 B 0.43
A 0 B 0.57
A 0 B 0.71
A 0 B 0.86
A 0 B 1
A 0.14 B 0
A 0.14 B 0.14
A 0.14 B 0.29
A 0.14 B 0.43
A 0.14 B 0.57
A 0.14 B 0.71
A 0.14 B 0.86
A 0.14 B 1
A 0.29 B 0
A 0.29 B 0.14
A 0.29 B 0.29
A 0.29 B 0.43
A 0.29 B 0.57
A 0.29 B 0.71
A 0.29 B 0.86
A 0.29 B 1
A 0.43 B 0
A 0.43 B 0.14
A 0.43 B 0.29
A 0.43 B 0.43
A 0.43 B 0.57
A 0.43 B 0.71
A 0.43 B 0.86
A 0.43 B 1
A 0.57 B 0
A 0.57 B 0.14
A 0.57 B 0.29
A 0.57 B 0.43
A 0.57 B 0.57
A 0.57 B 0.71
A 0.57 B 0.86
A 0.57 B 1
A 0.71 B 0
A 0.71 B 0.14
A 0.71 B 0.29
A 0.71 B 0.43
A 0.71 B 0.57
A 0.71 B 0.71
A 0.71 B 0.86
A 0.71 B 1
A 0.86 B 0
A 0.86 B 0.14
A 0.86 B 0.29
A 0.86 B 0.43
A 0.86 B 0.57
A 0.86 B 0.71
A 0.86 B 0.86
A 0.86 B 1
A 1 B 0
A 1 B 0.14
A 1 B 0.29
A 1 B 0.43
A 1 B 0.57
A 1 B 0.71
A 1 B 0.86
A 1 B 1
OD 600 nm [−]
0.2
0.4
0.6
0 5 101520
Time [h]

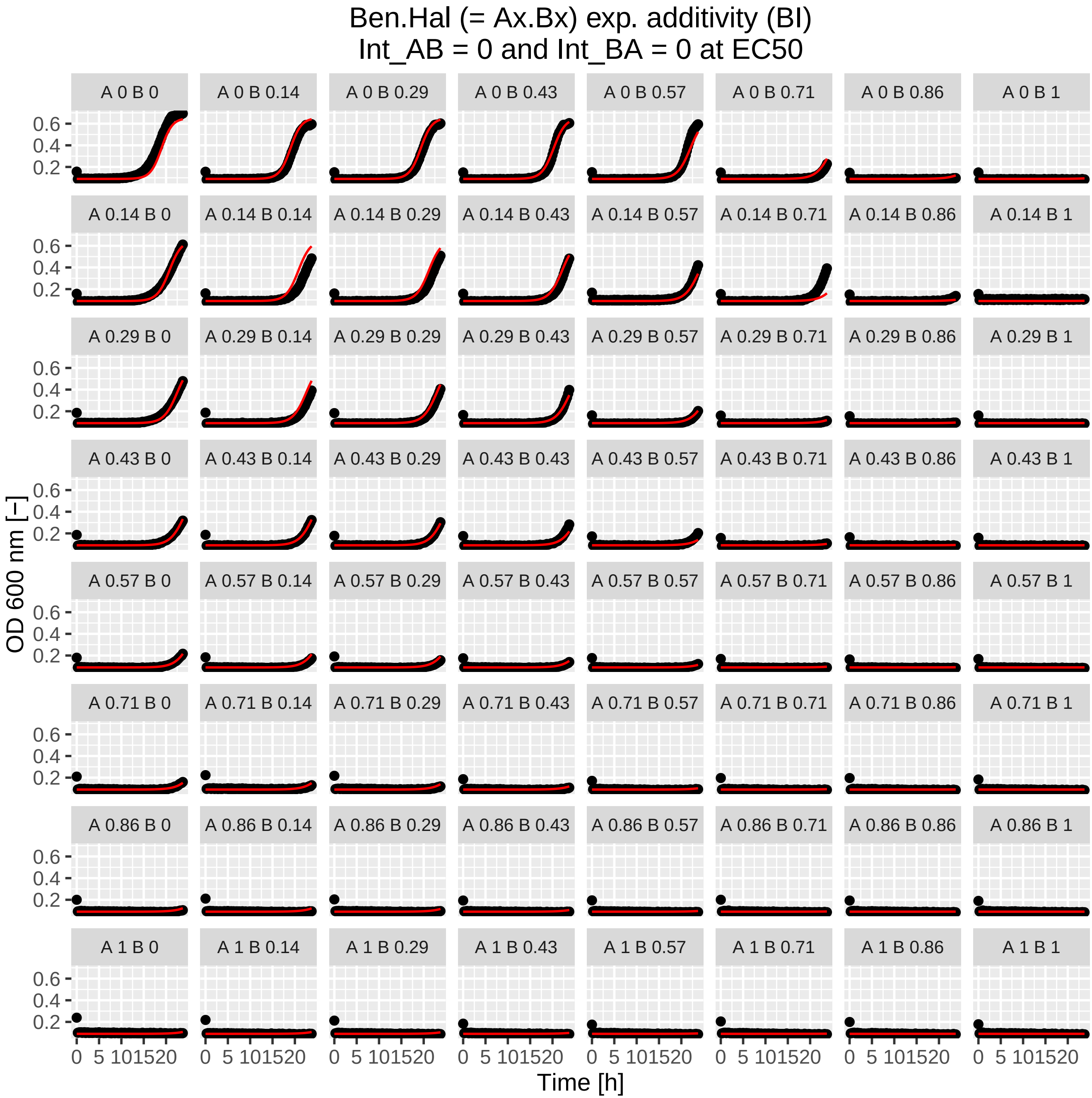
Ben.Hal (= Ax.Bx) exp. additivity (BI)
Int_AB = 0 and Int_BA = 0 at EC50
OD 600 nm [–]
Time [h]
A 0 B 0
A 0 B 0.14
A 0 B 0.29
A 0 B 0.43
A 0 B 0.57
A 0 B 0.71
A 0 B 0.86
A 0 B 1
A 0.14 B 0
A 0.14 B 0.14
A 0.14 B 0.29
A 0.14 B 0.43
A 0.14 B 0.57
A 0.14 B 0.71
A 0.14 B 0.86
A 0.14 B 1
A 0.29 B 0
A 0.29 B 0.14
A 0.29 B 0.29
A 0.29 B 0.43
A 0.29 B 0.57
A 0.29 B 0.71
A 0.29 B 0.86
A 0.29 B 1
A 0.43 B 0
A 0.43 B 0.14
A 0.43 B 0.29
A 0.43 B 0.43
A 0.43 B 0.57
A 0.43 B 0.71
A 0.43 B 0.86
A 0.43 B 1
A 0.57 B 0
A 0.57 B 0.14
A 0.57 B 0.29
A 0.57 B 0.43
A 0.57 B 0.57
A 0.57 B 0.71
A 0.57 B 0.86
A 0.57 B 1
A 0.71 B 0
A 0.71 B 0.14
A 0.71 B 0.29
A 0.71 B 0.43
A 0.71 B 0.57
A 0.71 B 0.71
A 0.71 B 0.86
A 0.71 B 1
A 0.86 B 0
A 0.86 B 0.14
A 0.86 B 0.29
A 0.86 B 0.43
A 0.86 B 0.57
A 0.86 B 0.71
A 0.86 B 0.86
A 0.86 B 1
A 1 B 0
A 1 B 0.14
A 1 B 0.29
A 1 B 0.43
A 1 B 0.57
A 1 B 0.71
A 1 B 0.86
A 1 B 1
0.6
0.4
0.2
0 5 101520

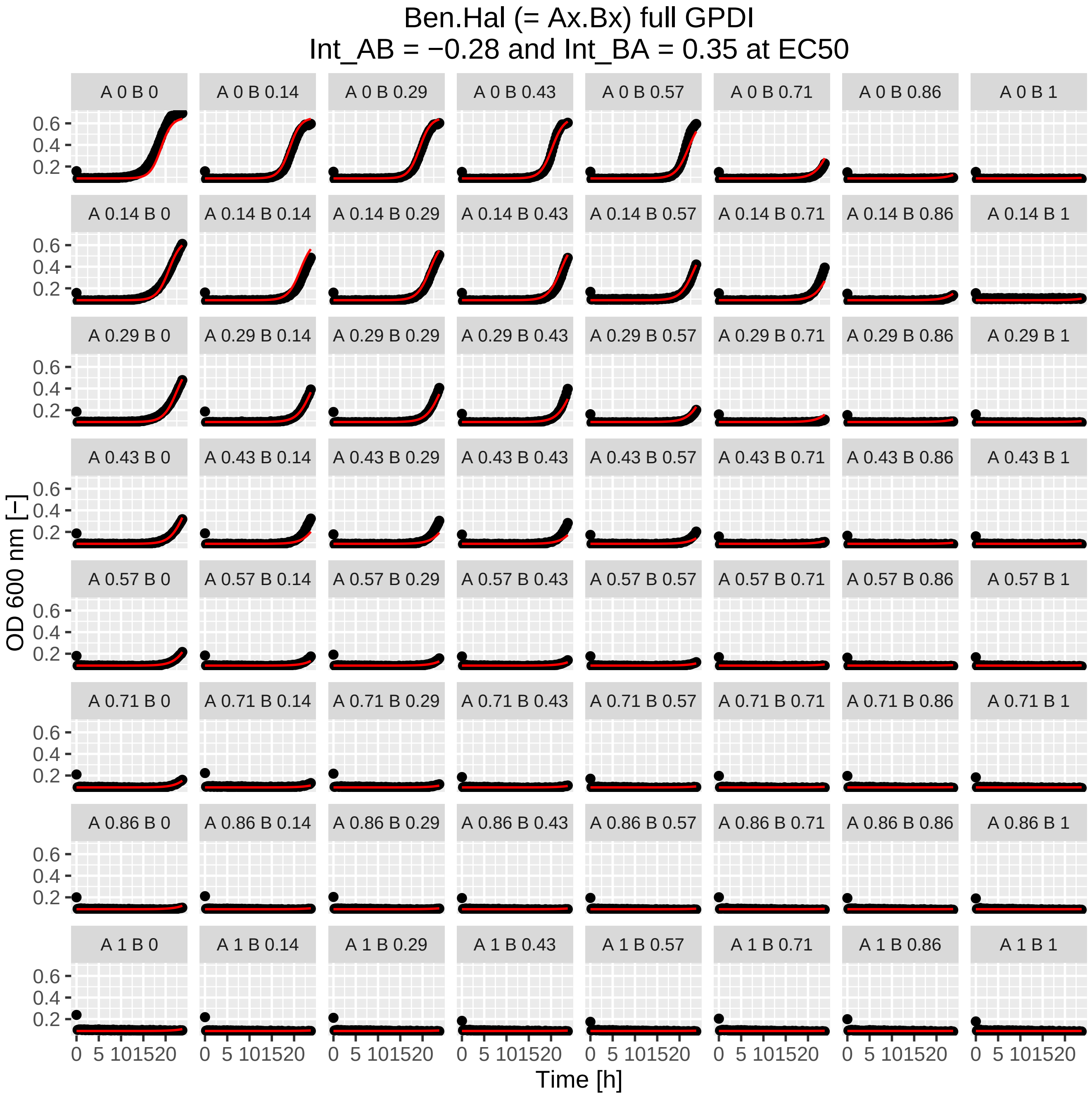

Ben.Hal (= Ax.Bx) full GPDI
Int_AB = −0.28 and Int_BA = 0.35 at EC50
A 0 B 0
A 0 B 0.14
A 0 B 0.29
A 0 B 0.43
A 0 B 0.57
A 0 B 0.71
A 0 B 0.86
A 0 B 1
A 0.14 B 0
A 0.14 B 0.14
A 0.14 B 0.29
A 0.14 B 0.43
A 0.14 B 0.57
A 0.14 B 0.71
A 0.14 B 0.86
A 0.14 B 1
A 0.29 B 0
A 0.29 B 0.14
A 0.29 B 0.29
A 0.29 B 0.43
A 0.29 B 0.57
A 0.29 B 0.71
A 0.29 B 0.86
A 0.29 B 1
A 0.43 B 0
A 0.43 B 0.14
A 0.43 B 0.29
A 0.43 B 0.43
A 0.43 B 0.57
A 0.43 B 0.71
A 0.43 B 0.86
A 0.43 B 1
A 0.57 B 0
A 0.57 B 0.14
A 0.57 B 0.29
A 0.57 B 0.43
A 0.57 B 0.57
A 0.57 B 0.71
A 0.57 B 0.86
A 0.57 B 1
A 0.71 B 0
A 0.71 B 0.14
A 0.71 B 0.29
A 0.71 B 0.43
A 0.71 B 0.57
A 0.71 B 0.71
A 0.71 B 0.86
A 0.71 B 1
A 0.86 B 0
A 0.86 B 0.14
A 0.86 B 0.29
A 0.86 B 0.43
A 0.86 B 0.57
A 0.86 B 0.71
A 0.86 B 0.86
A 0.86 B 1
A 1 B 0
A 1 B 0.14
A 1 B 0.29
A 1 B 0.43
A 1 B 0.57
A 1 B 0.71
A 1 B 0.86
A 1 B 1
OD 600 nm [−]
0.2
0.4
0.6
0 5 101520
Time [h]

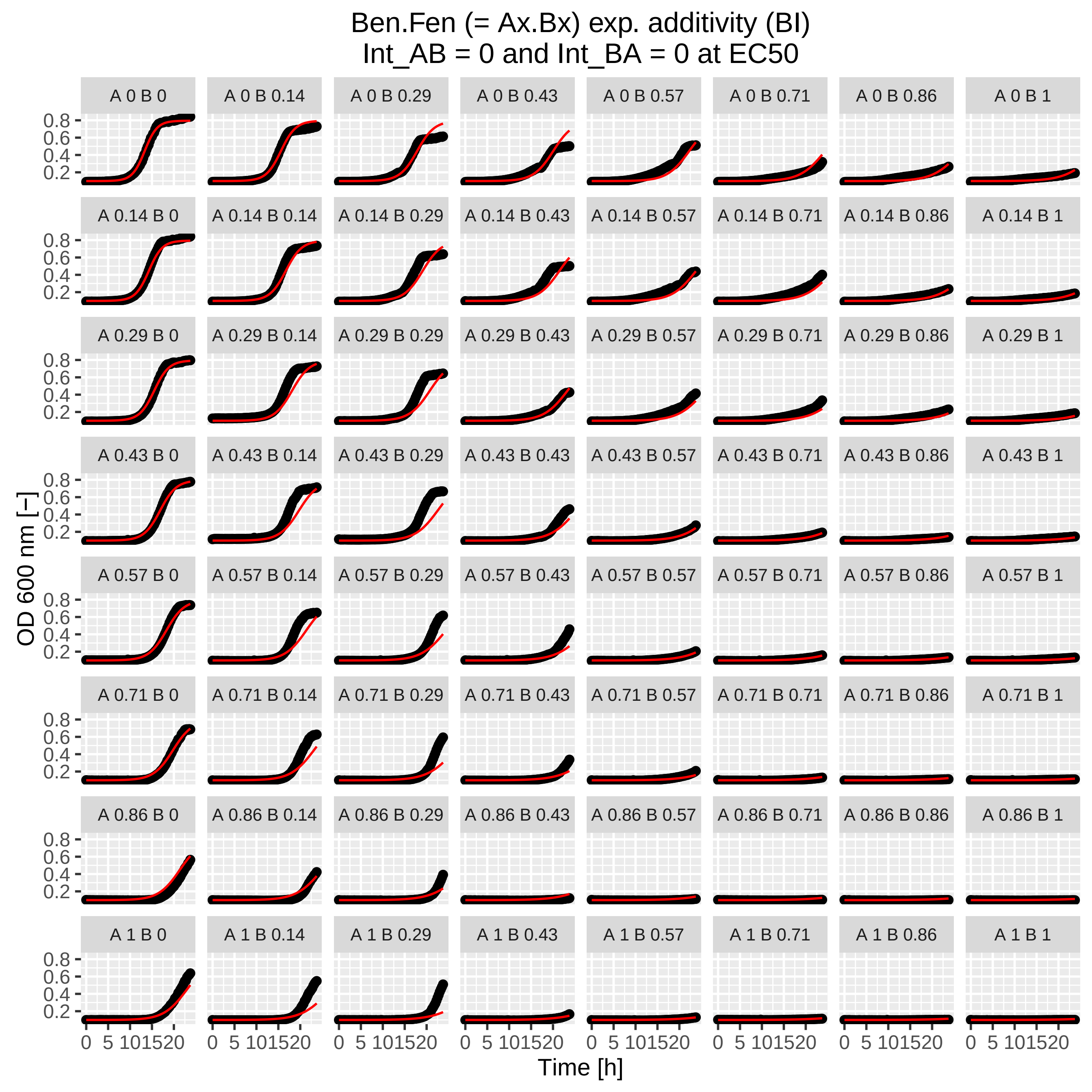
Ben.Fen (= Ax.Bx) exp. additivity (BI)
Int_AB = 0 and Int_BA = 0 at EC50
A 0 B 0
A 0 B 0.14
A 0 B 0.29
A 0 B 0.43
A 0 B 0.57
A 0 B 0.71
A 0 B 0.86
A 0 B 1
A 0.14 B 0
A 0.14 B 0.14
A 0.14 B 0.29
A 0.14 B 0.43
A 0.14 B 0.57
A 0.14 B 0.71
A 0.14 B 0.86
A 0.14 B 1
A 0.29 B 0
A 0.29 B 0.14
A 0.29 B 0.29
A 0.29 B 0.43
A 0.29 B 0.57
A 0.29 B 0.71
A 0.29 B 0.86
A 0.29 B 1
A 0.43 B 0
A 0.43 B 0.14
A 0.43 B 0.29
A 0.43 B 0.43
A 0.43 B 0.57
A 0.43 B 0.71
A 0.43 B 0.86
A 0.43 B 1
A 0.57 B 0
A 0.57 B 0.14
A 0.57 B 0.29
A 0.57 B 0.43
A 0.57 B 0.57
A 0.57 B 0.71
A 0.57 B 0.86
A 0.57 B 1
A 0.71 B 0
A 0.71 B 0.14
A 0.71 B 0.29
A 0.71 B 0.43
A 0.71 B 0.57
A 0.71 B 0.71
A 0.71 B 0.86
A 0.71 B 1
A 0.86 B 0
A 0.86 B 0.14
A 0.86 B 0.29
A 0.86 B 0.43
A 0.86 B 0.57
A 0.86 B 0.71
A 0.86 B 0.86
A 0.86 B 1
A 1 B 0
A 1 B 0.14
A 1 B 0.29
A 1 B 0.43
A 1 B 0.57
A 1 B 0.71
A 1 B 0.86
A 1 B 1
OD 600 nm [–]
0.2
0.4
0.6
0.8
0 5 101520
Time [h]

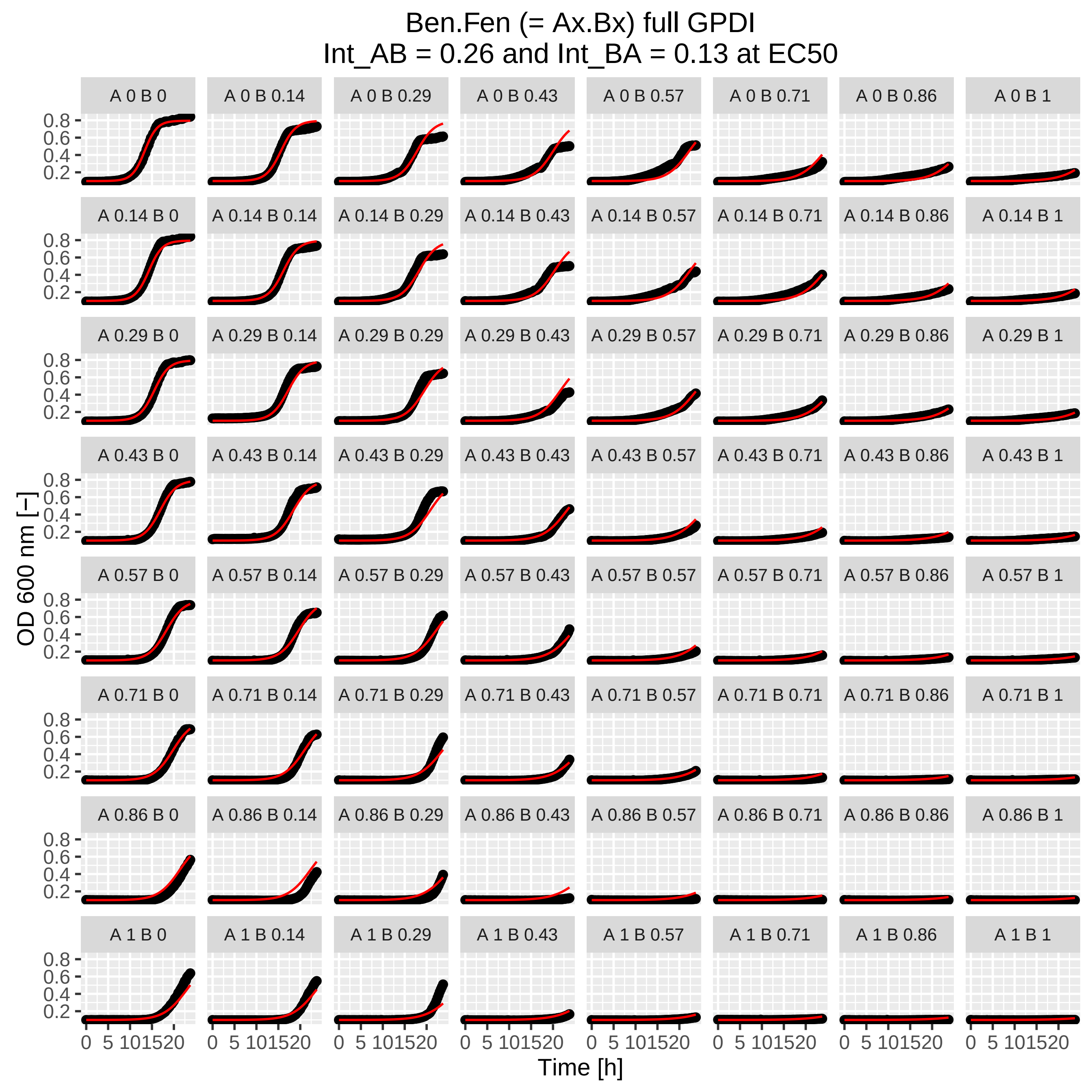

Ben.Fen (= Ax.Bx) full GPDI
Int_AB = 0.26 and Int_BA = 0.13 at EC50
A 0 B 0
A 0 B 0.14
A 0 B 0.29
A 0 B 0.43
A 0 B 0.57
A 0 B 0.71
A 0 B 0.86
A 0 B 1
A 0.14 B 0
A 0.14 B 0.14
A 0.14 B 0.29
A 0.14 B 0.43
A 0.14 B 0.57
A 0.14 B 0.71
A 0.14 B 0.86
A 0.14 B 1
A 0.29 B 0
A 0.29 B 0.14
A 0.29 B 0.29
A 0.29 B 0.43
A 0.29 B 0.57
A 0.29 B 0.71
A 0.29 B 0.86
A 0.29 B 1
A 0.43 B 0
A 0.43 B 0.14
A 0.43 B 0.29
A 0.43 B 0.43
A 0.43 B 0.57
A 0.43 B 0.71
A 0.43 B 0.86
A 0.43 B 1
A 0.57 B 0
A 0.57 B 0.14
A 0.57 B 0.29
A 0.57 B 0.43
A 0.57 B 0.57
A 0.57 B 0.71
A 0.57 B 0.86
A 0.57 B 1
A 0.71 B 0
A 0.71 B 0.14
A 0.71 B 0.29
A 0.71 B 0.43
A 0.71 B 0.57
A 0.71 B 0.71
A 0.71 B 0.86
A 0.71 B 1
A 0.86 B 0
A 0.86 B 0.14
A 0.86 B 0.29
A 0.86 B 0.43
A 0.86 B 0.57
A 0.86 B 0.71
A 0.86 B 0.86
A 0.86 B 1
A 1 B 0
A 1 B 0.14
A 1 B 0.29
A 1 B 0.43
A 1 B 0.57
A 1 B 0.71
A 1 B 0.86
A 1 B 1
OD 600 nm [–]
0.2
0.4
0.6
0.8
0
5
10
15
20
Time [h]

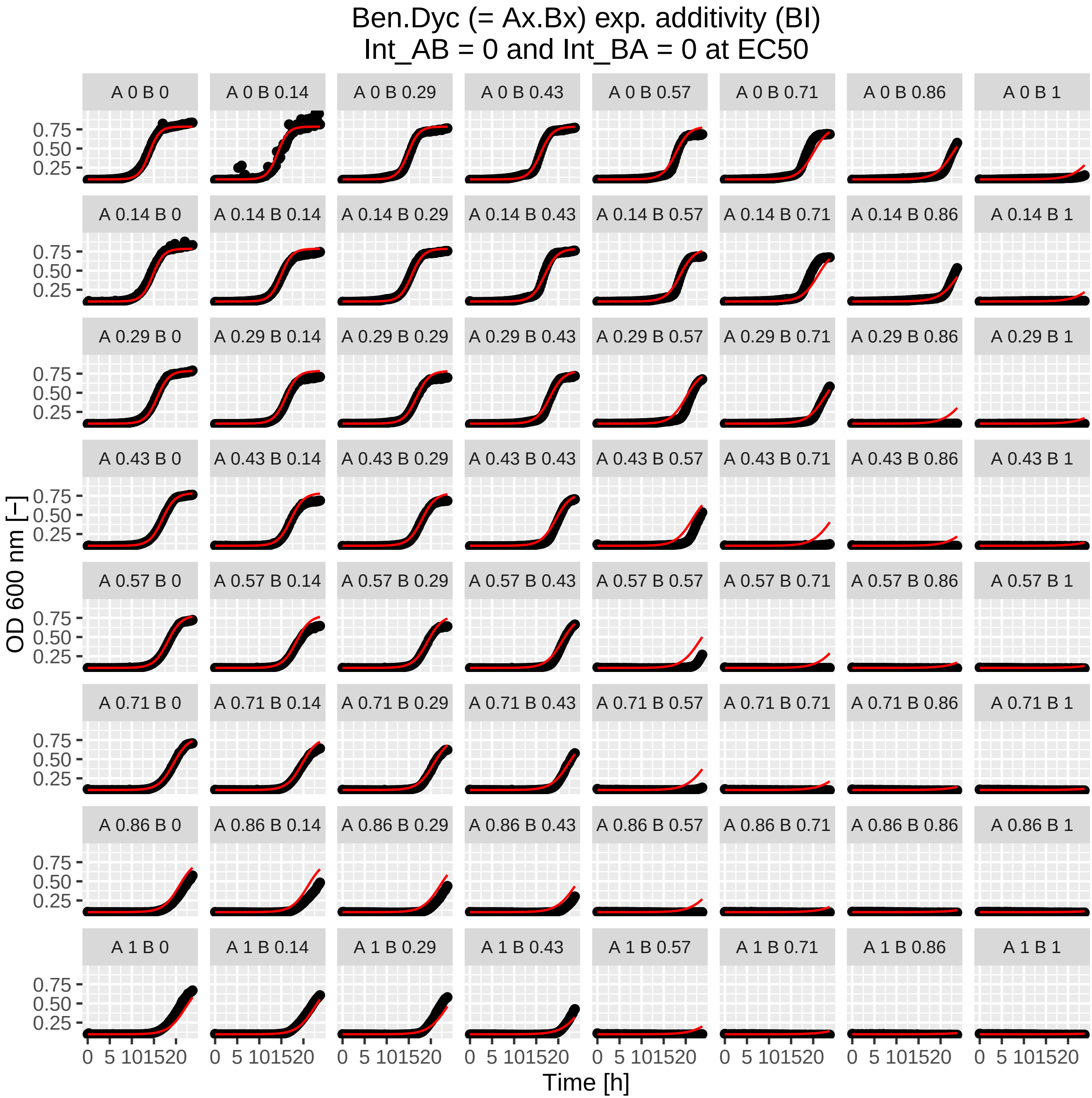

Ben.Dyc (= Ax.Bx) exp. additivity (BI)
Int_AB = 0 and Int_BA = 0 at EC50
A 0 B 0
A 0 B 0.14
A 0 B 0.29
A 0 B 0.43
A 0 B 0.57
A 0 B 0.71
A 0 B 0.86
A 0 B 1
A 0.14 B 0
A 0.14 B 0.14
A 0.14 B 0.29
A 0.14 B 0.43
A 0.14 B 0.57
A 0.14 B 0.71
A 0.14 B 0.86
A 0.14 B 1
A 0.29 B 0
A 0.29 B 0.14
A 0.29 B 0.29
A 0.29 B 0.43
A 0.29 B 0.57
A 0.29 B 0.71
A 0.29 B 0.86
A 0.29 B 1
A 0.43 B 0
A 0.43 B 0.14
A 0.43 B 0.29
A 0.43 B 0.43
A 0.43 B 0.57
A 0.43 B 0.71
A 0.43 B 0.86
A 0.43 B 1
A 0.57 B 0
A 0.57 B 0.14
A 0.57 B 0.29
A 0.57 B 0.43
A 0.57 B 0.57
A 0.57 B 0.71
A 0.57 B 0.86
A 0.57 B 1
A 0.71 B 0
A 0.71 B 0.14
A 0.71 B 0.29
A 0.71 B 0.43
A 0.71 B 0.57
A 0.71 B 0.71
A 0.71 B 0.86
A 0.71 B 1
A 0.86 B 0
A 0.86 B 0.14
A 0.86 B 0.29
A 0.86 B 0.43
A 0.86 B 0.57
A 0.86 B 0.71
A 0.86 B 0.86
A 0.86 B 1
A 1 B 0
A 1 B 0.14
A 1 B 0.29
A 1 B 0.43
A 1 B 0.57
A 1 B 0.71
A 1 B 0.86
A 1 B 1
OD 600 nm [–]
0.75
0.50
0.25
0 5 101520
Time [h]

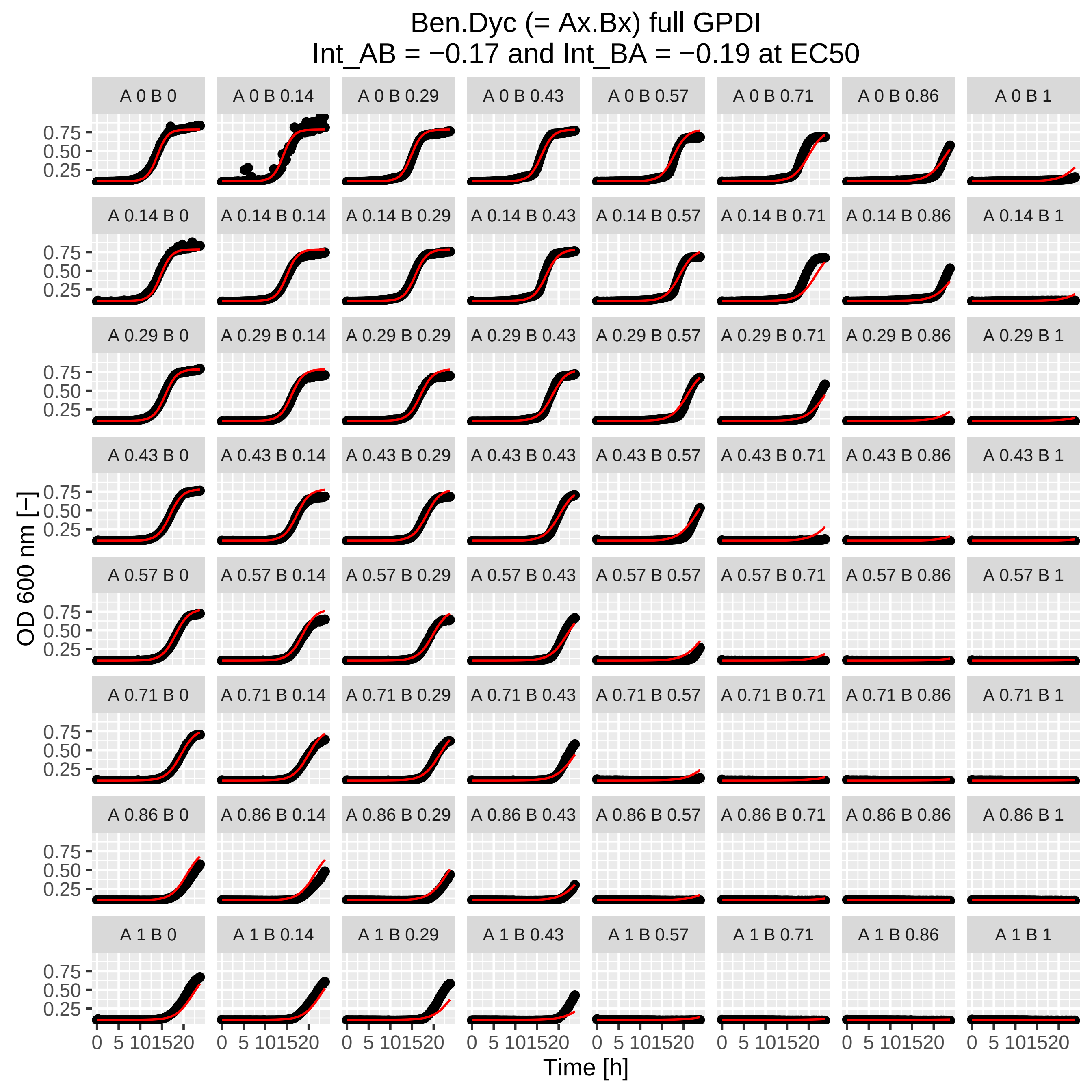
Ben.Dyc (= Ax.Bx) full GPDI
Int_AB = −0.17 and Int_BA = −0.19 at EC50
A 0 B 0
A 0 B 0.14
A 0 B 0.29
A 0 B 0.43
A 0 B 0.57
A 0 B 0.71
A 0 B 0.86
A 0 B 1
A 0.14 B 0
A 0.14 B 0.14
A 0.14 B 0.29
A 0.14 B 0.43
A 0.14 B 0.57
A 0.14 B 0.71
A 0.14 B 0.86
A 0.14 B 1
A 0.29 B 0
A 0.29 B 0.14
A 0.29 B 0.29
A 0.29 B 0.43
A 0.29 B 0.57
A 0.29 B 0.71
A 0.29 B 0.86
A 0.29 B 1
A 0.43 B 0
A 0.43 B 0.14
A 0.43 B 0.29
A 0.43 B 0.43
A 0.43 B 0.57
A 0.43 B 0.71
A 0.43 B 0.86
A 0.43 B 1
A 0.57 B 0
A 0.57 B 0.14
A 0.57 B 0.29
A 0.57 B 0.43
A 0.57 B 0.57
A 0.57 B 0.71
A 0.57 B 0.86
A 0.57 B 1
A 0.71 B 0
A 0.71 B 0.14
A 0.71 B 0.29
A 0.71 B 0.43
A 0.71 B 0.57
A 0.71 B 0.71
A 0.71 B 0.86
A 0.71 B 1
A 0.86 B 0
A 0.86 B 0.14
A 0.86 B 0.29
A 0.86 B 0.43
A 0.86 B 0.57
A 0.86 B 0.71
A 0.86 B 0.86
A 0.86 B 1
A 1 B 0
A 1 B 0.14
A 1 B 0.29
A 1 B 0.43
A 1 B 0.57
A 1 B 0.71
A 1 B 0.86
A 1 B 1
OD 600 nm [−]
0.75
0.50
0.25
0 5 101520
Time [h]

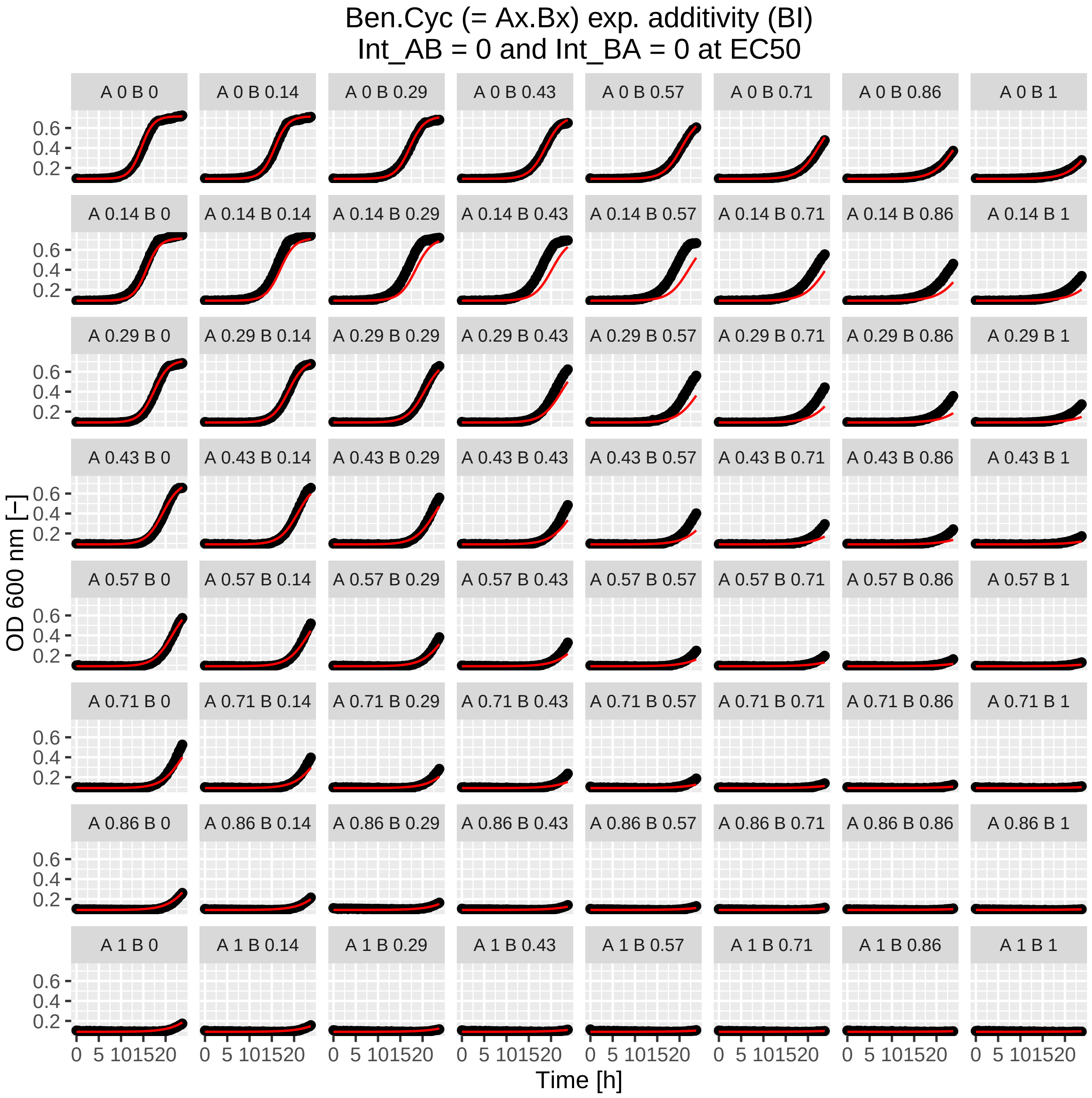
Ben.Cyc (= Ax.Bx) exp. additivity (BI)
Int_AB = 0 and Int_BA = 0 at EC50
A 0 B 0
A 0 B 0.14
A 0 B 0.29
A 0 B 0.43
A 0 B 0.57
A 0 B 0.71
A 0 B 0.86
A 0 B 1
A 0.14 B 0
A 0.14 B 0.14
A 0.14 B 0.29
A 0.14 B 0.43
A 0.14 B 0.57
A 0.14 B 0.71
A 0.14 B 0.86
A 0.14 B 1
A 0.29 B 0
A 0.29 B 0.14
A 0.29 B 0.29
A 0.29 B 0.43
A 0.29 B 0.57
A 0.29 B 0.71
A 0.29 B 0.86
A 0.29 B 1
A 0.43 B 0
A 0.43 B 0.14
A 0.43 B 0.29
A 0.43 B 0.43
A 0.43 B 0.57
A 0.43 B 0.71
A 0.43 B 0.86
A 0.43 B 1
A 0.57 B 0
A 0.57 B 0.14
A 0.57 B 0.29
A 0.57 B 0.43
A 0.57 B 0.57
A 0.57 B 0.71
A 0.57 B 0.86
A 0.57 B 1
A 0.71 B 0
A 0.71 B 0.14
A 0.71 B 0.29
A 0.71 B 0.43
A 0.71 B 0.57
A 0.71 B 0.71
A 0.71 B 0.86
A 0.71 B 1
A 0.86 B 0
A 0.86 B 0.14
A 0.86 B 0.29
A 0.86 B 0.43
A 0.86 B 0.57
A 0.86 B 0.71
A 0.86 B 0.86
A 0.86 B 1
A 1 B 0
A 1 B 0.14
A 1 B 0.29
A 1 B 0.43
A 1 B 0.57
A 1 B 0.71
A 1 B 0.86
A 1 B 1
OD 600 nm [–]
0.6
0.4
0.2
0 5 101520
Time [h]

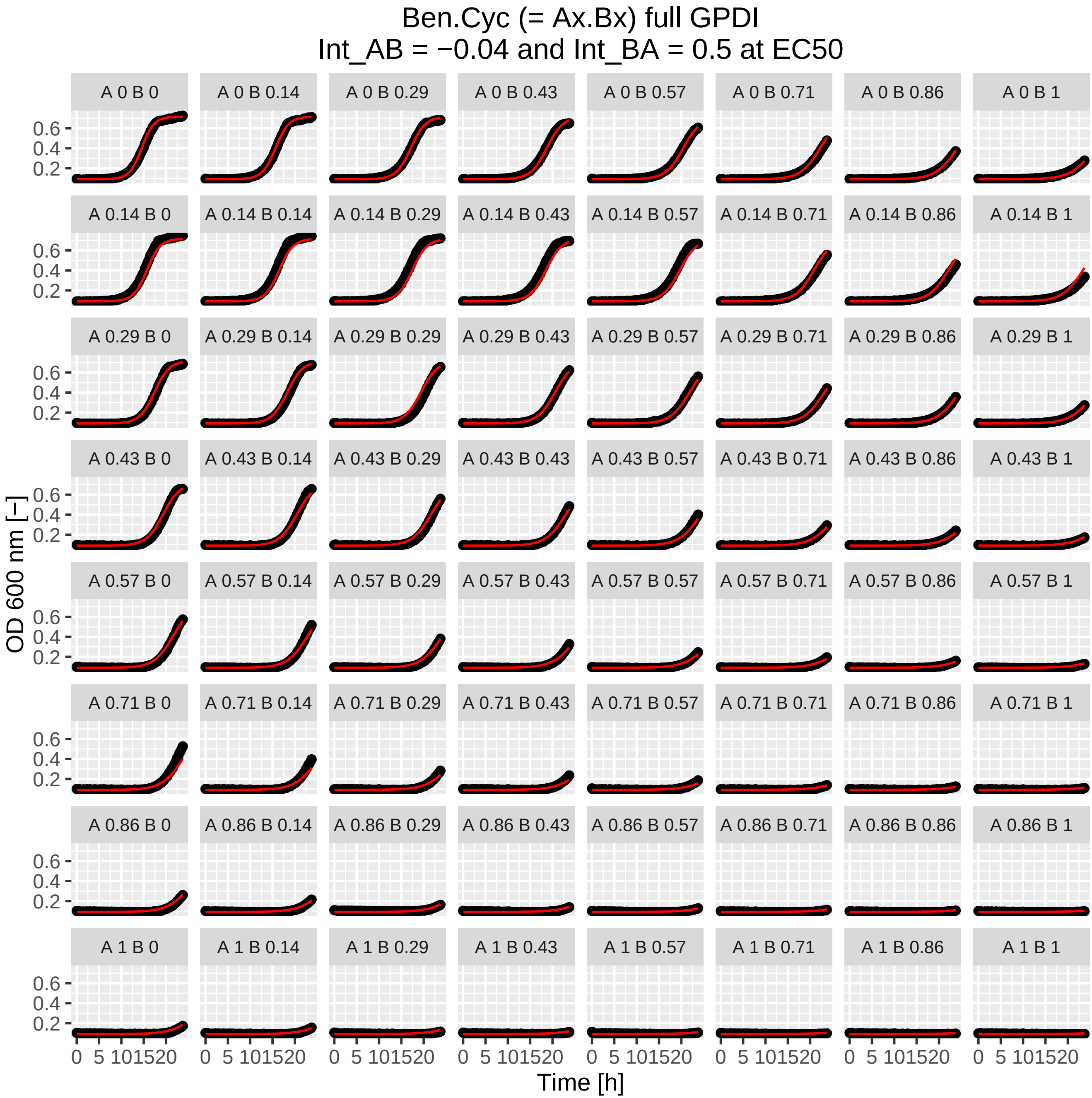

Ben.Cyc (= Ax.Bx) full GPDI
Int_AB = −0.04 and Int_BA = 0.5 at EC50
A 0 B 0
A 0 B 0.14
A 0 B 0.29
A 0 B 0.43
A 0 B 0.57
A 0 B 0.71
A 0 B 0.86
A 0 B 1
A 0.14 B 0
A 0.14 B 0.14
A 0.14 B 0.29
A 0.14 B 0.43
A 0.14 B 0.57
A 0.14 B 0.71
A 0.14 B 0.86
A 0.14 B 1
A 0.29 B 0
A 0.29 B 0.14
A 0.29 B 0.29
A 0.29 B 0.43
A 0.29 B 0.57
A 0.29 B 0.71
A 0.29 B 0.86
A 0.29 B 1
A 0.43 B 0
A 0.43 B 0.14
A 0.43 B 0.29
A 0.43 B 0.43
A 0.43 B 0.57
A 0.43 B 0.71
A 0.43 B 0.86
A 0.43 B 1
A 0.57 B 0
A 0.57 B 0.14
A 0.57 B 0.29
A 0.57 B 0.43
A 0.57 B 0.57
A 0.57 B 0.71
A 0.57 B 0.86
A 0.57 B 1
A 0.71 B 0
A 0.71 B 0.14
A 0.71 B 0.29
A 0.71 B 0.43
A 0.71 B 0.57
A 0.71 B 0.71
A 0.71 B 0.86
A 0.71 B 1
A 0.86 B 0
A 0.86 B 0.14
A 0.86 B 0.29
A 0.86 B 0.43
A 0.86 B 0.57
A 0.86 B 0.71
A 0.86 B 0.86
A 0.86 B 1
A 1 B 0
A 1 B 0.14
A 1 B 0.29
A 1 B 0.43
A 1 B 0.57
A 1 B 0.71
A 1 B 0.86
A 1 B 1
0.6
0.4
0.2
OD 600 nm [−]
0 5 101520
Time [h]

Ben.Cis (= Ax.Bx) exp. additivity (BI)
Int_AB = 0 and Int_BA = 0 at EC50

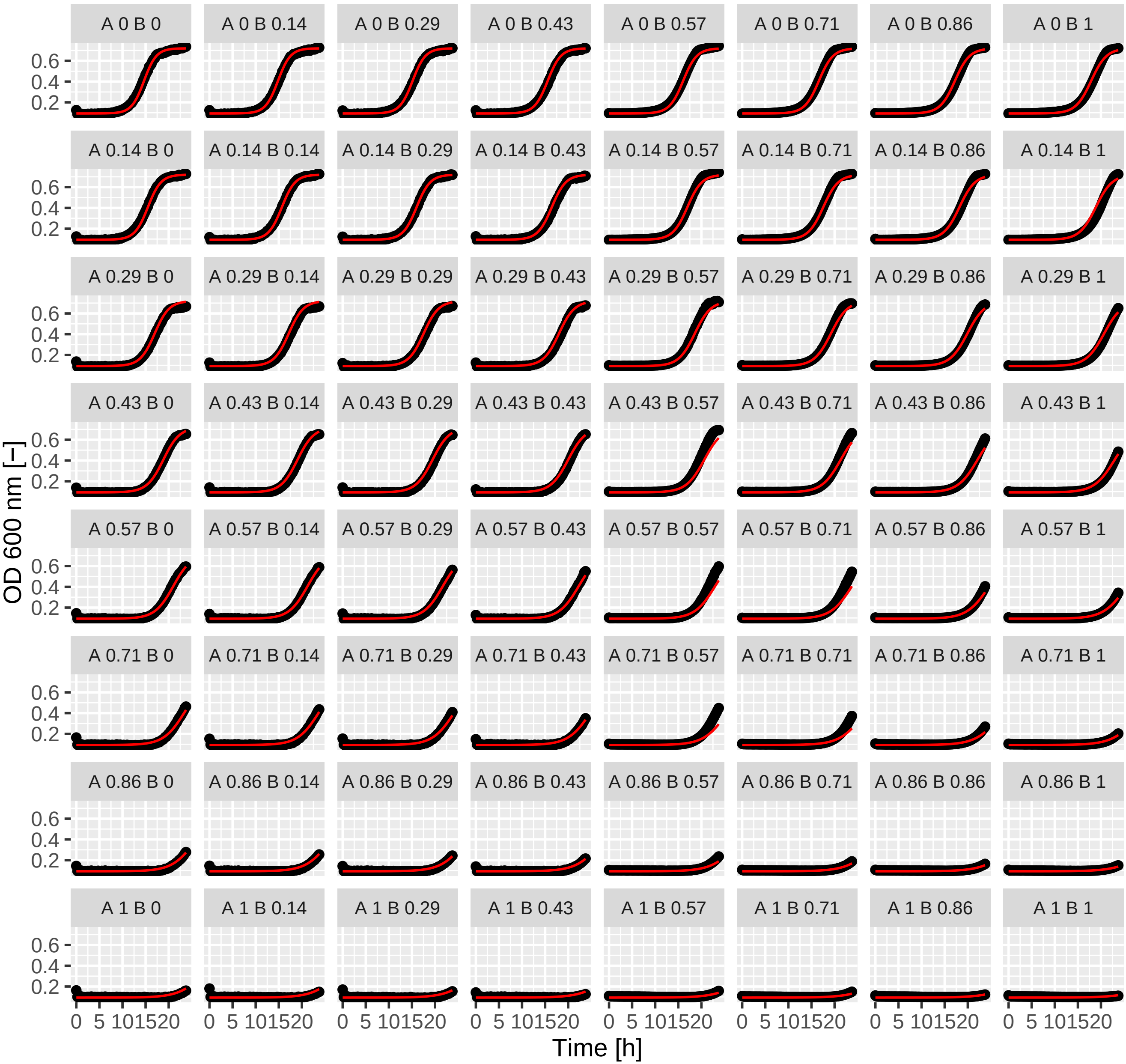

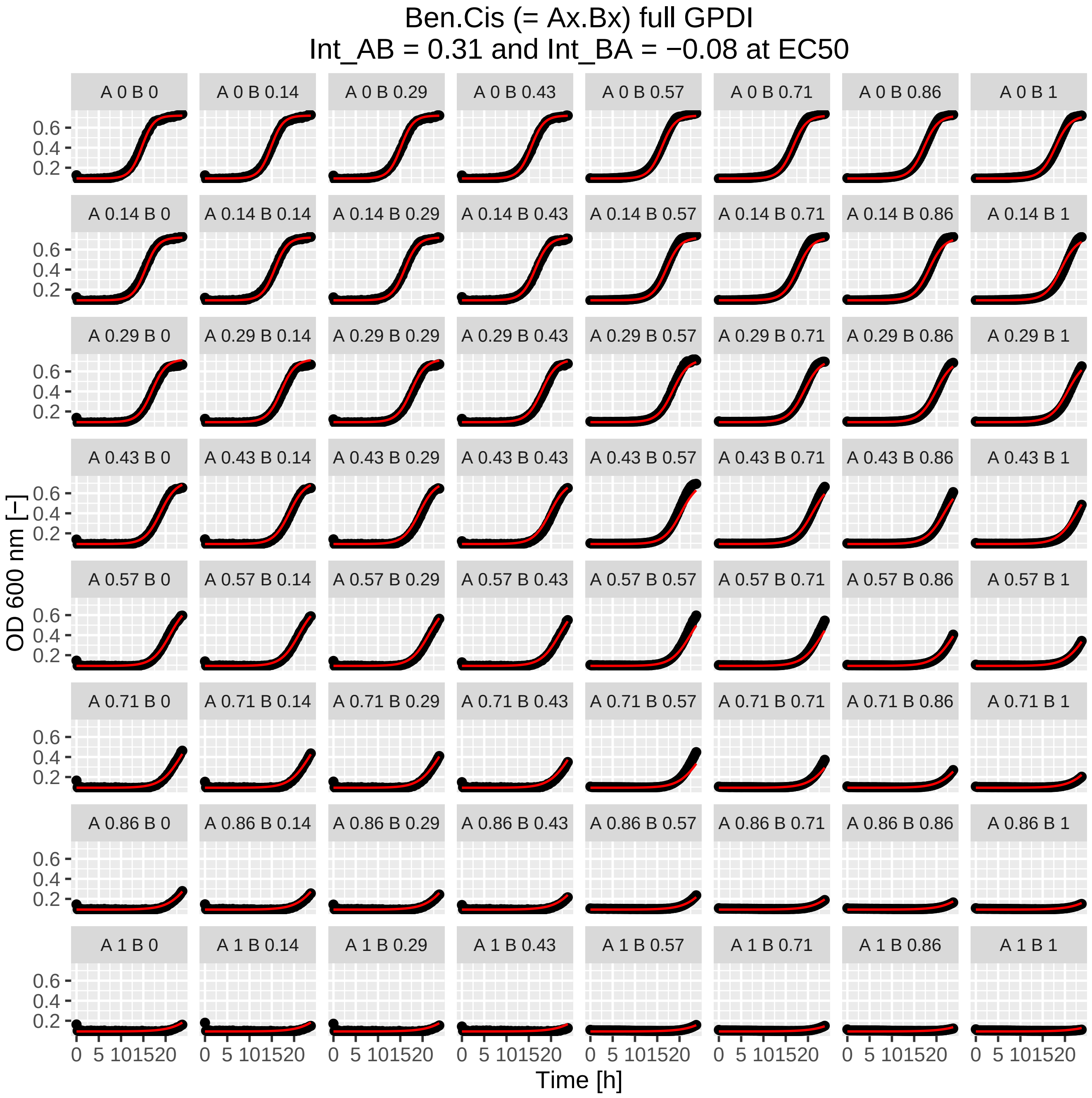

Ben.Cis (= Ax.Bx) full GPDI
Int_AB = 0.31 and Int_BA = −0.08 at EC50
A 0 B 0
A 0 B 0.14
A 0 B 0.29
A 0 B 0.43
A 0 B 0.57
A 0 B 0.71
A 0 B 0.86
A 0 B 1
A 0.14 B 0
A 0.14 B 0.14
A 0.14 B 0.29
A 0.14 B 0.43
A 0.14 B 0.57
A 0.14 B 0.71
A 0.14 B 0.86
A 0.14 B 1
A 0.29 B 0
A 0.29 B 0.14
A 0.29 B 0.29
A 0.29 B 0.43
A 0.29 B 0.57
A 0.29 B 0.71
A 0.29 B 0.86
A 0.29 B 1
A 0.43 B 0
A 0.43 B 0.14
A 0.43 B 0.29
A 0.43 B 0.43
A 0.43 B 0.57
A 0.43 B 0.71
A 0.43 B 0.86
A 0.43 B 1
A 0.57 B 0
A 0.57 B 0.14
A 0.57 B 0.29
A 0.57 B 0.43
A 0.57 B 0.57
A 0.57 B 0.71
A 0.57 B 0.86
A 0.57 B 1
A 0.71 B 0
A 0.71 B 0.14
A 0.71 B 0.29
A 0.71 B 0.43
A 0.71 B 0.57
A 0.71 B 0.71
A 0.71 B 0.86
A 0.71 B 1
A 0.86 B 0
A 0.86 B 0.14
A 0.86 B 0.29
A 0.86 B 0.43
A 0.86 B 0.57
A 0.86 B 0.71
A 0.86 B 0.86
A 0.86 B 1
A 1 B 0
A 1 B 0.14
A 1 B 0.29
A 1 B 0.43
A 1 B 0.57
A 1 B 0.71
A 1 B 0.86
A 1 B 1
OD 600 nm [−]
0.2
0.4
0.6
0 5 101520
Time [h]

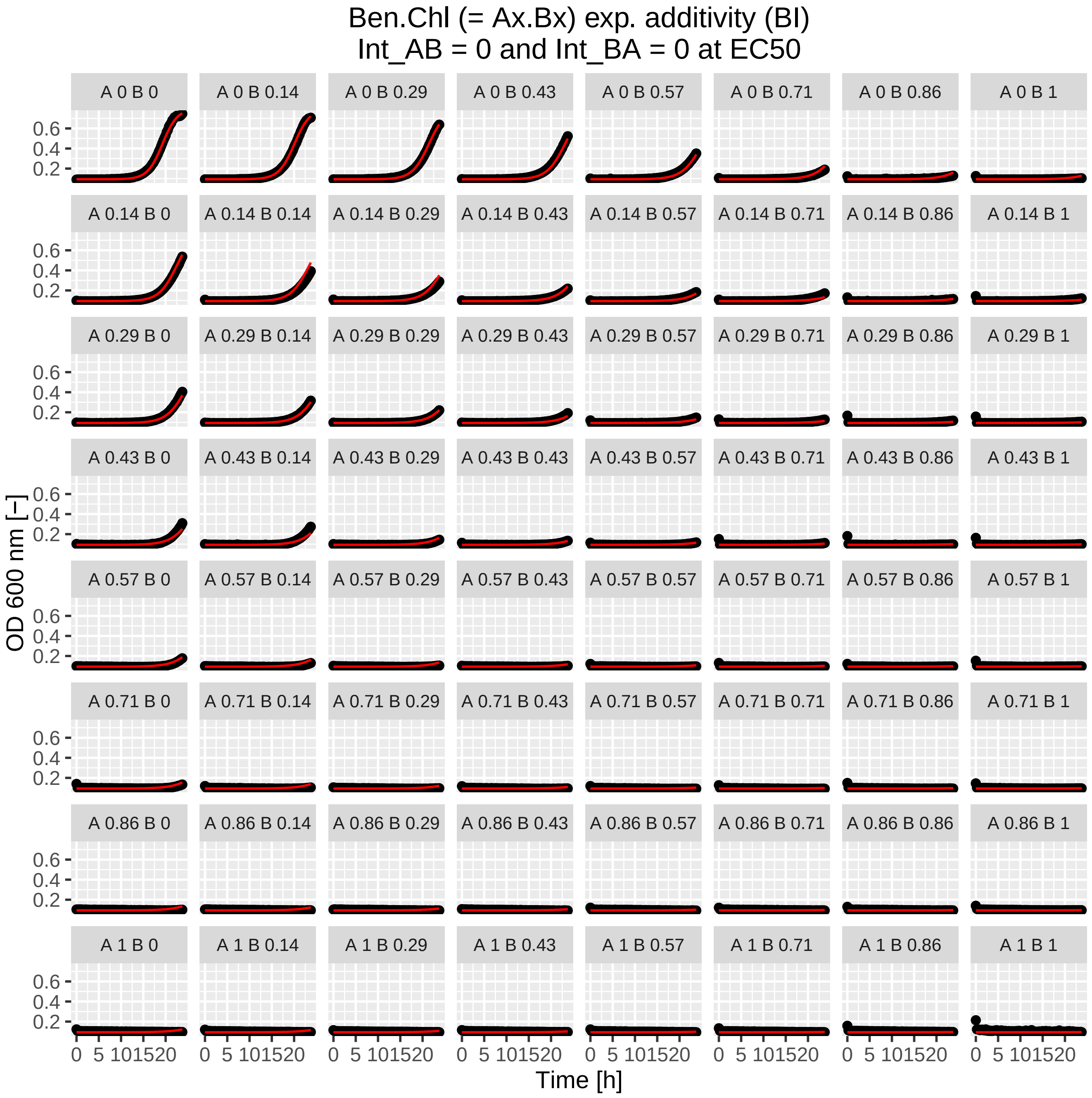
Ben.Chl (= Ax.Bx) exp. additivity (BI)
Int_AB = 0 and Int_BA = 0 at EC50
A 0 B 0
A 0 B 0.14
A 0 B 0.29
A 0 B 0.43
A 0 B 0.57
A 0 B 0.71
A 0 B 0.86
A 0 B 1
A 0.14 B 0
A 0.14 B 0.14
A 0.14 B 0.29
A 0.14 B 0.43
A 0.14 B 0.57
A 0.14 B 0.71
A 0.14 B 0.86
A 0.14 B 1
A 0.29 B 0
A 0.29 B 0.14
A 0.29 B 0.29
A 0.29 B 0.43
A 0.29 B 0.57
A 0.29 B 0.71
A 0.29 B 0.86
A 0.29 B 1
A 0.43 B 0
A 0.43 B 0.14
A 0.43 B 0.29
A 0.43 B 0.43
A 0.43 B 0.57
A 0.43 B 0.71
A 0.43 B 0.86
A 0.43 B 1
A 0.57 B 0
A 0.57 B 0.14
A 0.57 B 0.29
A 0.57 B 0.43
A 0.57 B 0.57
A 0.57 B 0.71
A 0.57 B 0.86
A 0.57 B 1
A 0.71 B 0
A 0.71 B 0.14
A 0.71 B 0.29
A 0.71 B 0.43
A 0.71 B 0.57
A 0.71 B 0.71
A 0.71 B 0.86
A 0.71 B 1
A 0.86 B 0
A 0.86 B 0.14
A 0.86 B 0.29
A 0.86 B 0.43
A 0.86 B 0.57
A 0.86 B 0.71
A 0.86 B 0.86
A 0.86 B 1
A 1 B 0
A 1 B 0.14
A 1 B 0.29
A 1 B 0.43
A 1 B 0.57
A 1 B 0.71
A 1 B 0.86
A 1 B 1
OD 600 nm [–]
0.6
0.4
0.2
0 5 101520
Time [h]

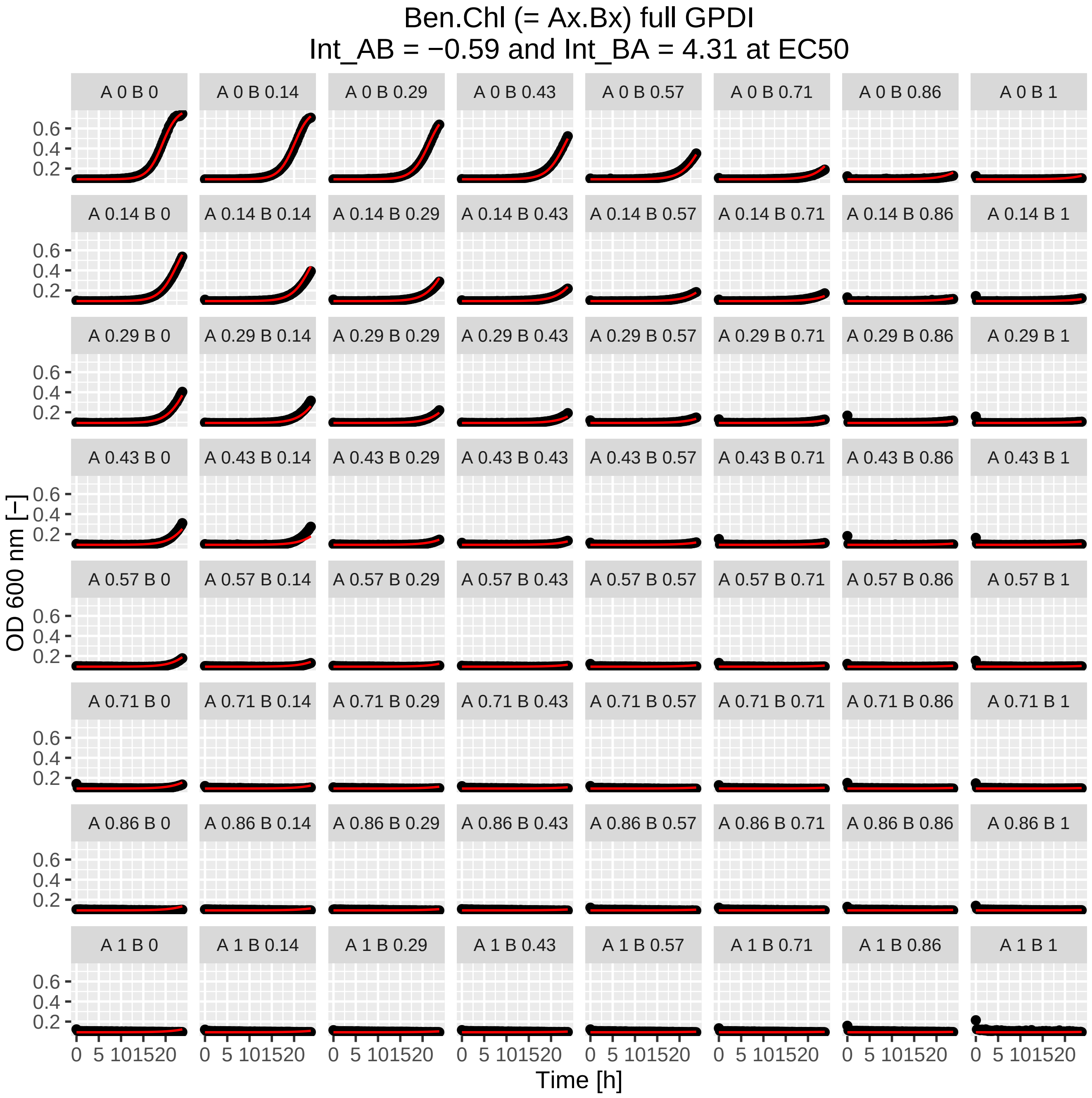

Ben.Chl (= Ax.Bx) full GPDI
Int_AB = −0.59 and Int_BA = 4.31 at EC50
A 0 B 0
A 0 B 0.14
A 0 B 0.29
A 0 B 0.43
A 0 B 0.57
A 0 B 0.71
A 0 B 0.86
A 0 B 1
A 0.14 B 0
A 0.14 B 0.14
A 0.14 B 0.29
A 0.14 B 0.43
A 0.14 B 0.57
A 0.14 B 0.71
A 0.14 B 0.86
A 0.14 B 1
A 0.29 B 0
A 0.29 B 0.14
A 0.29 B 0.29
A 0.29 B 0.43
A 0.29 B 0.57
A 0.29 B 0.71
A 0.29 B 0.86
A 0.29 B 1
A 0.43 B 0
A 0.43 B 0.14
A 0.43 B 0.29
A 0.43 B 0.43
A 0.43 B 0.57
A 0.43 B 0.71
A 0.43 B 0.86
A 0.43 B 1
A 0.57 B 0
A 0.57 B 0.14
A 0.57 B 0.29
A 0.57 B 0.43
A 0.57 B 0.57
A 0.57 B 0.71
A 0.57 B 0.86
A 0.57 B 1
A 0.71 B 0
A 0.71 B 0.14
A 0.71 B 0.29
A 0.71 B 0.43
A 0.71 B 0.57
A 0.71 B 0.71
A 0.71 B 0.86
A 0.71 B 1
A 0.86 B 0
A 0.86 B 0.14
A 0.86 B 0.29
A 0.86 B 0.43
A 0.86 B 0.57
A 0.86 B 0.71
A 0.86 B 0.86
A 0.86 B 1
A 1 B 0
A 1 B 0.14
A 1 B 0.29
A 1 B 0.43
A 1 B 0.57
A 1 B 0.71
A 1 B 0.86
A 1 B 1
OD 600 nm [−]
0.6
0.4
0.2
0 5 101520
Time [h]

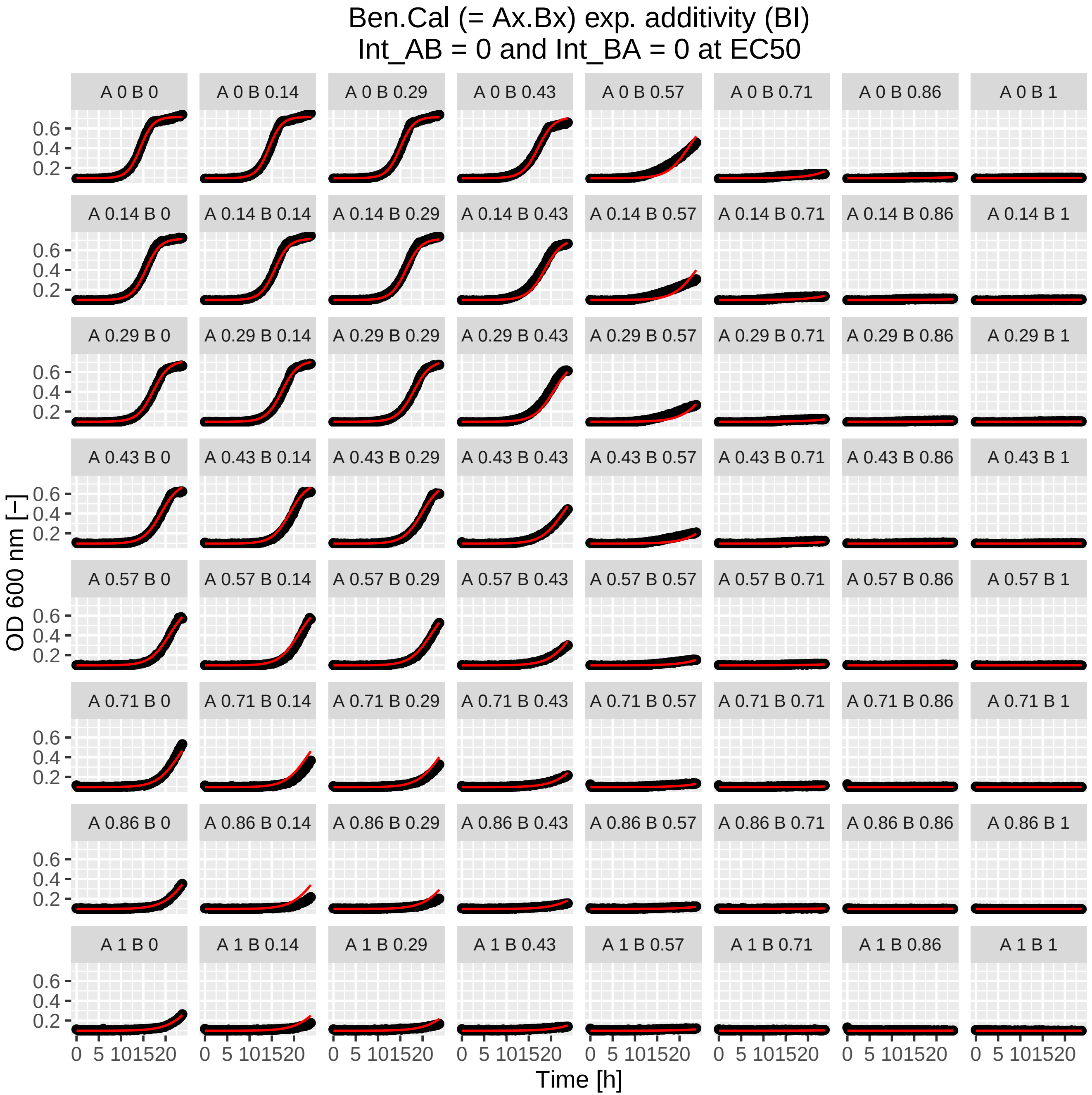
Ben.Cal (= Ax.Bx) exp. additivity (BI)
Int_AB = 0 and Int_BA = 0 at EC50
A 0 B 0
A 0 B 0.14
A 0 B 0.29
A 0 B 0.43
A 0 B 0.57
A 0 B 0.71
A 0 B 0.86
A 0 B 1
A 0.14 B 0
A 0.14 B 0.14
A 0.14 B 0.29
A 0.14 B 0.43
A 0.14 B 0.57
A 0.14 B 0.71
A 0.14 B 0.86
A 0.14 B 1
A 0.29 B 0
A 0.29 B 0.14
A 0.29 B 0.29
A 0.29 B 0.43
A 0.29 B 0.57
A 0.29 B 0.71
A 0.29 B 0.86
A 0.29 B 1
A 0.43 B 0
A 0.43 B 0.14
A 0.43 B 0.29
A 0.43 B 0.43
A 0.43 B 0.57
A 0.43 B 0.71
A 0.43 B 0.86
A 0.43 B 1
A 0.57 B 0
A 0.57 B 0.14
A 0.57 B 0.29
A 0.57 B 0.43
A 0.57 B 0.57
A 0.57 B 0.71
A 0.57 B 0.86
A 0.57 B 1
A 0.71 B 0
A 0.71 B 0.14
A 0.71 B 0.29
A 0.71 B 0.43
A 0.71 B 0.57
A 0.71 B 0.71
A 0.71 B 0.86
A 0.71 B 1
A 0.86 B 0
A 0.86 B 0.14
A 0.86 B 0.29
A 0.86 B 0.43
A 0.86 B 0.57
A 0.86 B 0.71
A 0.86 B 0.86
A 0.86 B 1
A 1 B 0
A 1 B 0.14
A 1 B 0.29
A 1 B 0.43
A 1 B 0.57
A 1 B 0.71
A 1 B 0.86
A 1 B 1
OD 600 nm [–]
0.6
0.4
0.2
0 5 101520
Time [h]

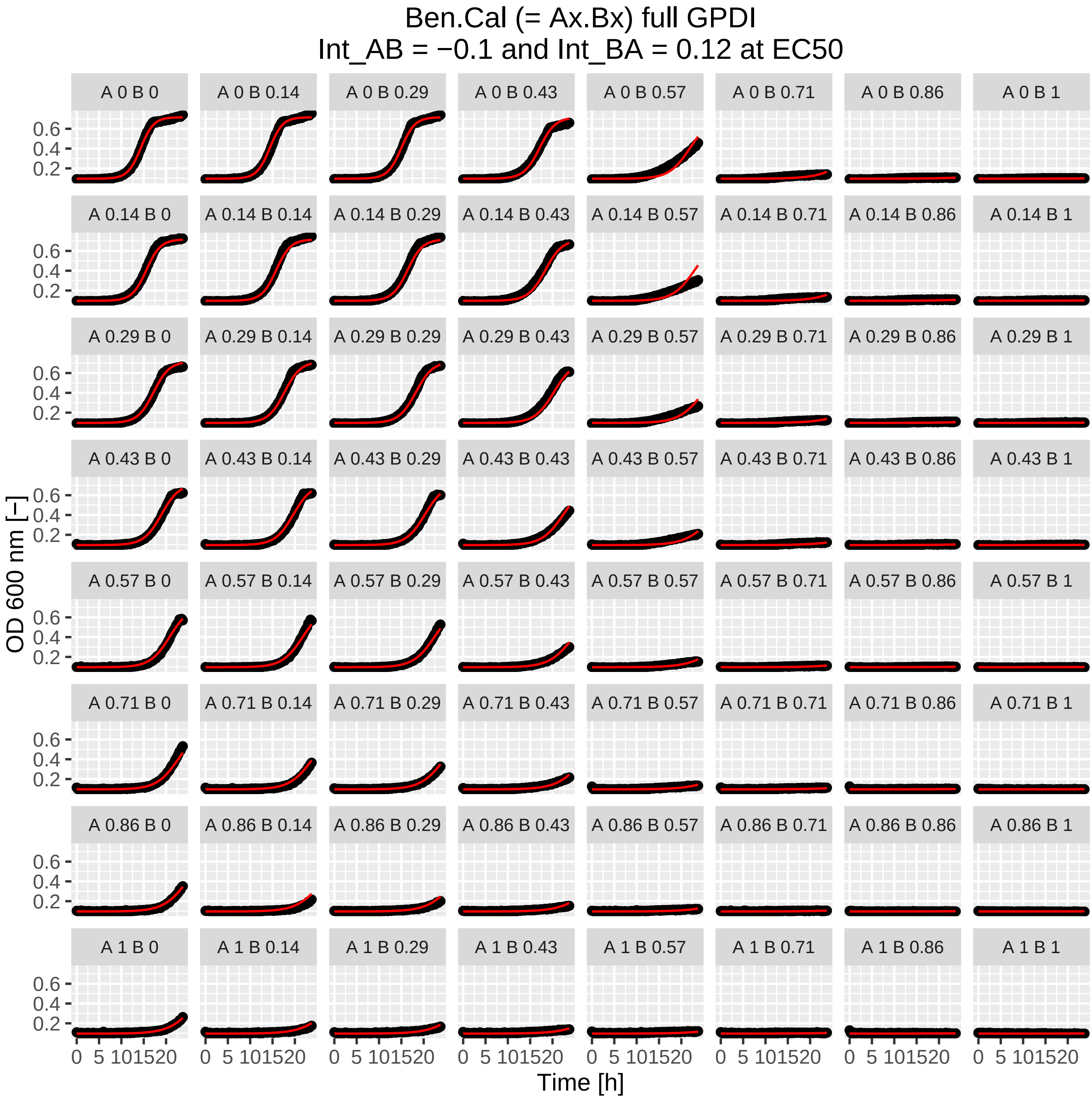
Ben.Cal (= Ax.Bx) full GPDI
Int_AB = −0.1 and Int_BA = 0.12 at EC50
A 0 B 0
A 0 B 0.14
A 0 B 0.29
A 0 B 0.43
A 0 B 0.57
A 0 B 0.71
A 0 B 0.86
A 0 B 1
A 0.14 B 0
A 0.14 B 0.14
A 0.14 B 0.29
A 0.14 B 0.43
A 0.14 B 0.57
A 0.14 B 0.71
A 0.14 B 0.86
A 0.14 B 1
A 0.29 B 0
A 0.29 B 0.14
A 0.29 B 0.29
A 0.29 B 0.43
A 0.29 B 0.57
A 0.29 B 0.71
A 0.29 B 0.86
A 0.29 B 1
A 0.43 B 0
A 0.43 B 0.14
A 0.43 B 0.29
A 0.43 B 0.43
A 0.43 B 0.57
A 0.43 B 0.71
A 0.43 B 0.86
A 0.43 B 1
A 0.57 B 0
A 0.57 B 0.14
A 0.57 B 0.29
A 0.57 B 0.43
A 0.57 B 0.57
A 0.57 B 0.71
A 0.57 B 0.86
A 0.57 B 1
A 0.71 B 0
A 0.71 B 0.14
A 0.71 B 0.29
A 0.71 B 0.43
A 0.71 B 0.57
A 0.71 B 0.71
A 0.71 B 0.86
A 0.71 B 1
A 0.86 B 0
A 0.86 B 0.14
A 0.86 B 0.29
A 0.86 B 0.43
A 0.86 B 0.57
A 0.86 B 0.71
A 0.86 B 0.86
A 0.86 B 1
A 1 B 0
A 1 B 0.14
A 1 B 0.29
A 1 B 0.43
A 1 B 0.57
A 1 B 0.71
A 1 B 0.86
A 1 B 1
OD 600 nm [−]
0.2
0.4
0.6
0 5 101520
Time [h]

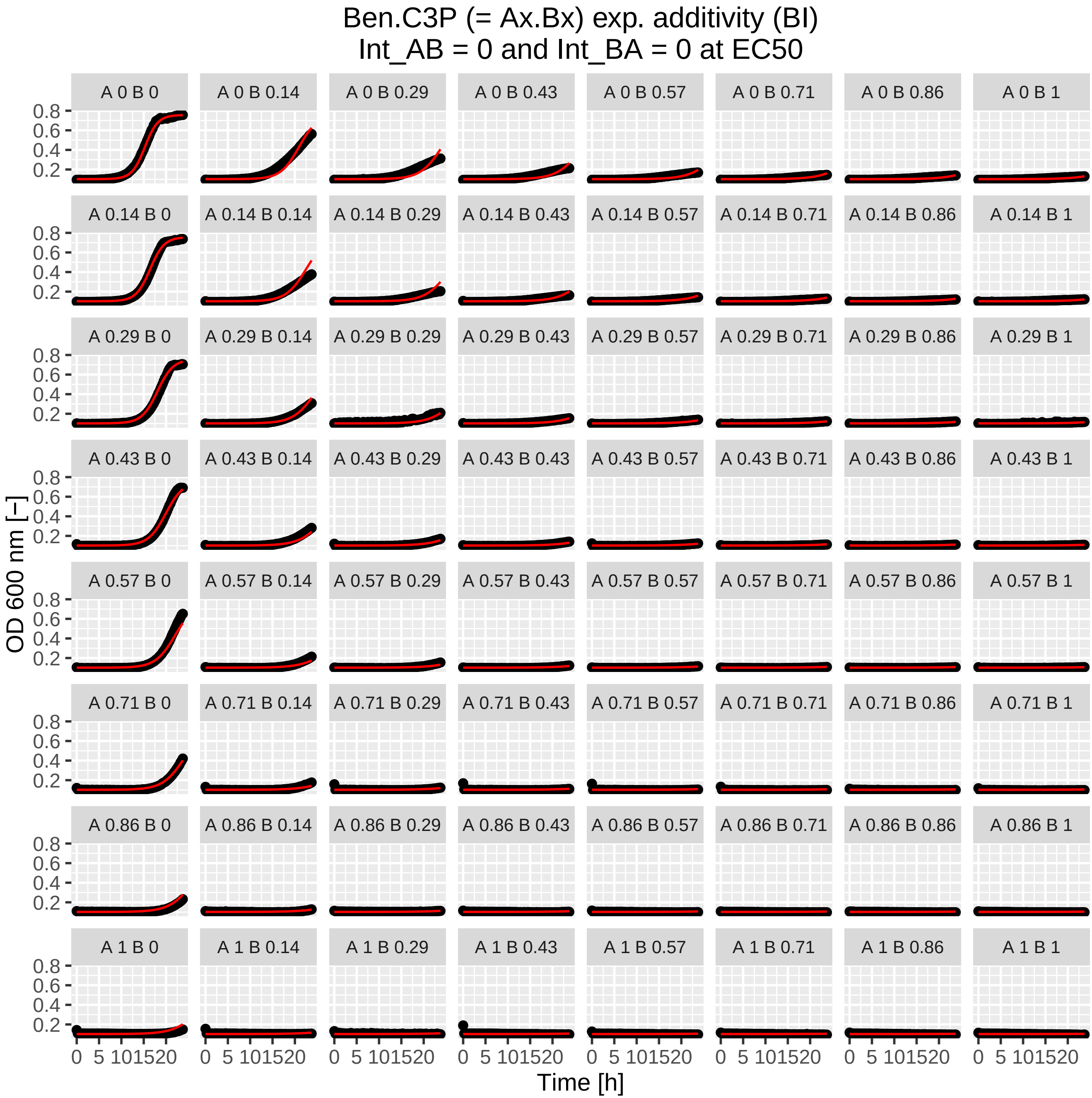
Ben.C3P (= Ax.Bx) exp. additivity (BI)
Int_AB = 0 and Int_BA = 0 at EC50
A 0 B 0
A 0 B 0.14
A 0 B 0.29
A 0 B 0.43
A 0 B 0.57
A 0 B 0.71
A 0 B 0.86
A 0 B 1
A 0.14 B 0
A 0.14 B 0.14
A 0.14 B 0.29
A 0.14 B 0.43
A 0.14 B 0.57
A 0.14 B 0.71
A 0.14 B 0.86
A 0.14 B 1
A 0.29 B 0
A 0.29 B 0.14
A 0.29 B 0.29
A 0.29 B 0.43
A 0.29 B 0.57
A 0.29 B 0.71
A 0.29 B 0.86
A 0.29 B 1
A 0.43 B 0
A 0.43 B 0.14
A 0.43 B 0.29
A 0.43 B 0.43
A 0.43 B 0.57
A 0.43 B 0.71
A 0.43 B 0.86
A 0.43 B 1
A 0.57 B 0
A 0.57 B 0.14
A 0.57 B 0.29
A 0.57 B 0.43
A 0.57 B 0.57
A 0.57 B 0.71
A 0.57 B 0.86
A 0.57 B 1
A 0.71 B 0
A 0.71 B 0.14
A 0.71 B 0.29
A 0.71 B 0.43
A 0.71 B 0.57
A 0.71 B 0.71
A 0.71 B 0.86
A 0.71 B 1
A 0.86 B 0
A 0.86 B 0.14
A 0.86 B 0.29
A 0.86 B 0.43
A 0.86 B 0.57
A 0.86 B 0.71
A 0.86 B 0.86
A 0.86 B 1
A 1 B 0
A 1 B 0.14
A 1 B 0.29
A 1 B 0.43
A 1 B 0.57
A 1 B 0.71
A 1 B 0.86
A 1 B 1
OD 600 nm [–]
0.8
0.6
0.4
0.2
0 5 101520
Time [h]

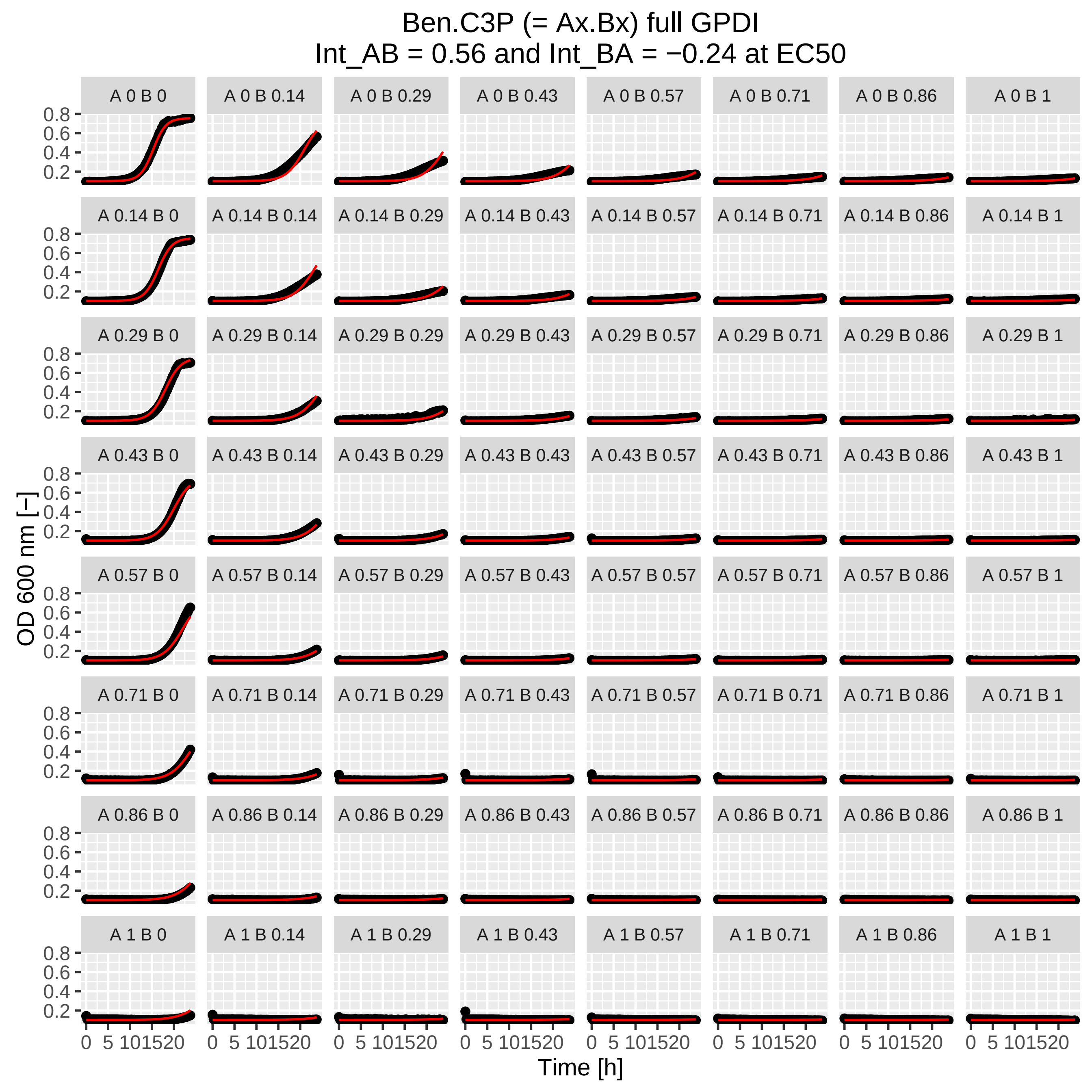

Ben.C3P (= Ax.Bx) full GPDI
Int_AB = 0.56 and Int_BA = −0.24 at EC50
OD 600 nm [−]
Time [h]
A 0 B 0
A 0 B 0.14
A 0 B 0.29
A 0 B 0.43
A 0 B 0.57
A 0 B 0.71
A 0 B 0.86
A 0 B 1
A 0.14 B 0
A 0.14 B 0.14
A 0.14 B 0.29
A 0.14 B 0.43
A 0.14 B 0.57
A 0.14 B 0.71
A 0.14 B 0.86
A 0.14 B 1
A 0.29 B 0
A 0.29 B 0.14
A 0.29 B 0.29
A 0.29 B 0.43
A 0.29 B 0.57
A 0.29 B 0.71
A 0.29 B 0.86
A 0.29 B 1
A 0.43 B 0
A 0.43 B 0.14
A 0.43 B 0.29
A 0.43 B 0.43
A 0.43 B 0.57
A 0.43 B 0.71
A 0.43 B 0.86
A 0.43 B 1
A 0.57 B 0
A 0.57 B 0.14
A 0.57 B 0.29
A 0.57 B 0.43
A 0.57 B 0.57
A 0.57 B 0.71
A 0.57 B 0.86
A 0.57 B 1
A 0.71 B 0
A 0.71 B 0.14
A 0.71 B 0.29
A 0.71 B 0.43
A 0.71 B 0.57
A 0.71 B 0.71
A 0.71 B 0.86
A 0.71 B 1
A 0.86 B 0
A 0.86 B 0.14
A 0.86 B 0.29
A 0.86 B 0.43
A 0.86 B 0.57
A 0.86 B 0.71
A 0.86 B 0.86
A 0.86 B 1
A 1 B 0
A 1 B 0.14
A 1 B 0.29
A 1 B 0.43
A 1 B 0.57
A 1 B 0.71
A 1 B 0.86
A 1 B 1
0.8
0.6
0.4
0.2
0 5 101520

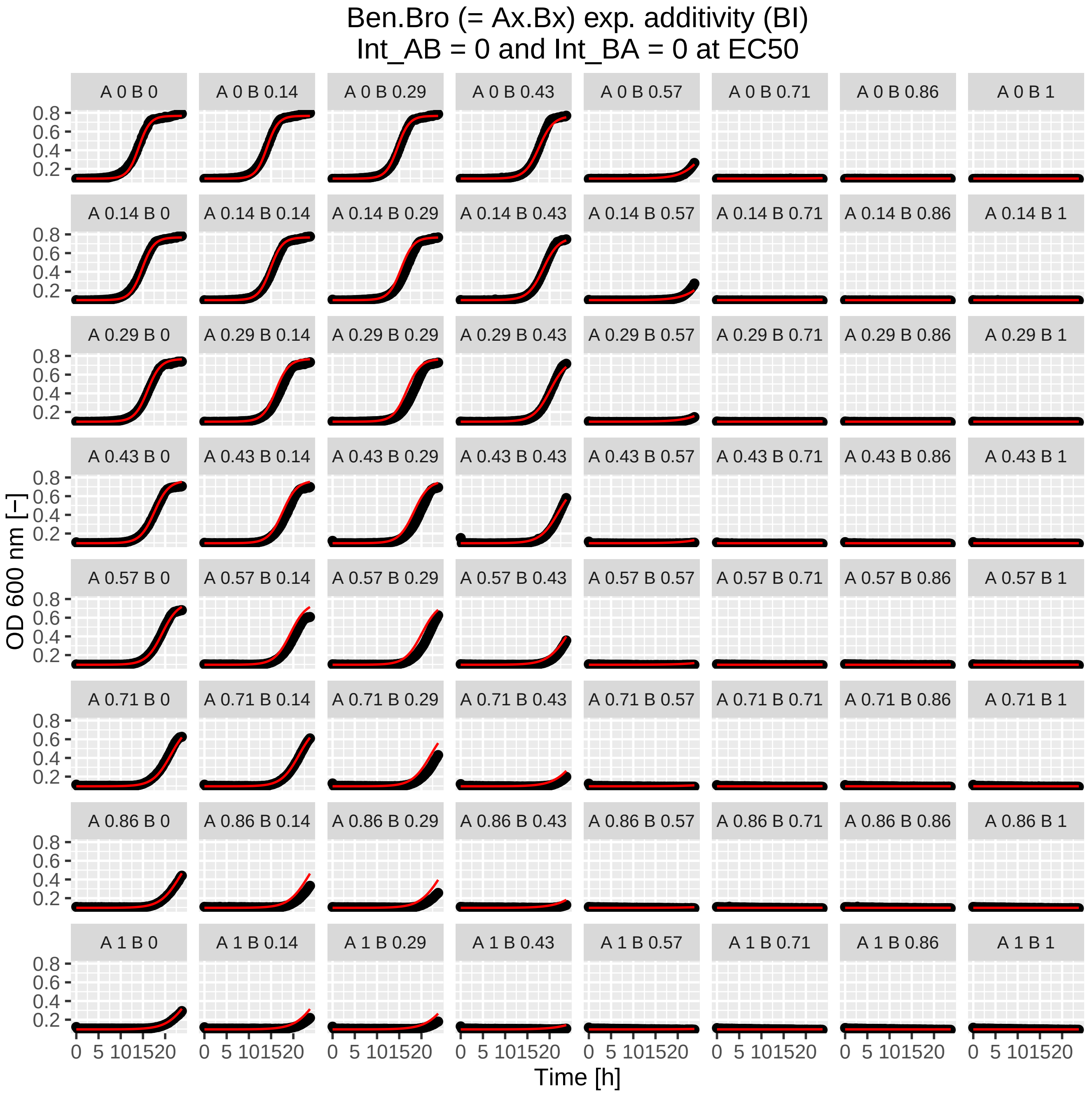
Ben.Bro (= Ax.Bx) exp. additivity (BI)
Int_AB = 0 and Int_BA = 0 at EC50
OD 600 nm [–]
Time [h]
A 0 B 0
A 0 B 0.14
A 0 B 0.29
A 0 B 0.43
A 0 B 0.57
A 0 B 0.71
A 0 B 0.86
A 0 B 1
A 0.14 B 0
A 0.14 B 0.14
A 0.14 B 0.29
A 0.14 B 0.43
A 0.14 B 0.57
A 0.14 B 0.71
A 0.14 B 0.86
A 0.14 B 1
A 0.29 B 0
A 0.29 B 0.14
A 0.29 B 0.29
A 0.29 B 0.43
A 0.29 B 0.57
A 0.29 B 0.71
A 0.29 B 0.86
A 0.29 B 1
A 0.43 B 0
A 0.43 B 0.14
A 0.43 B 0.29
A 0.43 B 0.43
A 0.43 B 0.57
A 0.43 B 0.71
A 0.43 B 0.86
A 0.43 B 1
A 0.57 B 0
A 0.57 B 0.14
A 0.57 B 0.29
A 0.57 B 0.43
A 0.57 B 0.57
A 0.57 B 0.71
A 0.57 B 0.86
A 0.57 B 1
A 0.71 B 0
A 0.71 B 0.14
A 0.71 B 0.29
A 0.71 B 0.43
A 0.71 B 0.57
A 0.71 B 0.71
A 0.71 B 0.86
A 0.71 B 1
A 0.86 B 0
A 0.86 B 0.14
A 0.86 B 0.29
A 0.86 B 0.43
A 0.86 B 0.57
A 0.86 B 0.71
A 0.86 B 0.86
A 0.86 B 1
A 1 B 0
A 1 B 0.14
A 1 B 0.29
A 1 B 0.43
A 1 B 0.57
A 1 B 0.71
A 1 B 0.86
A 1 B 1
0.2
0.4
0.6
0.8
0
5
10
15
20

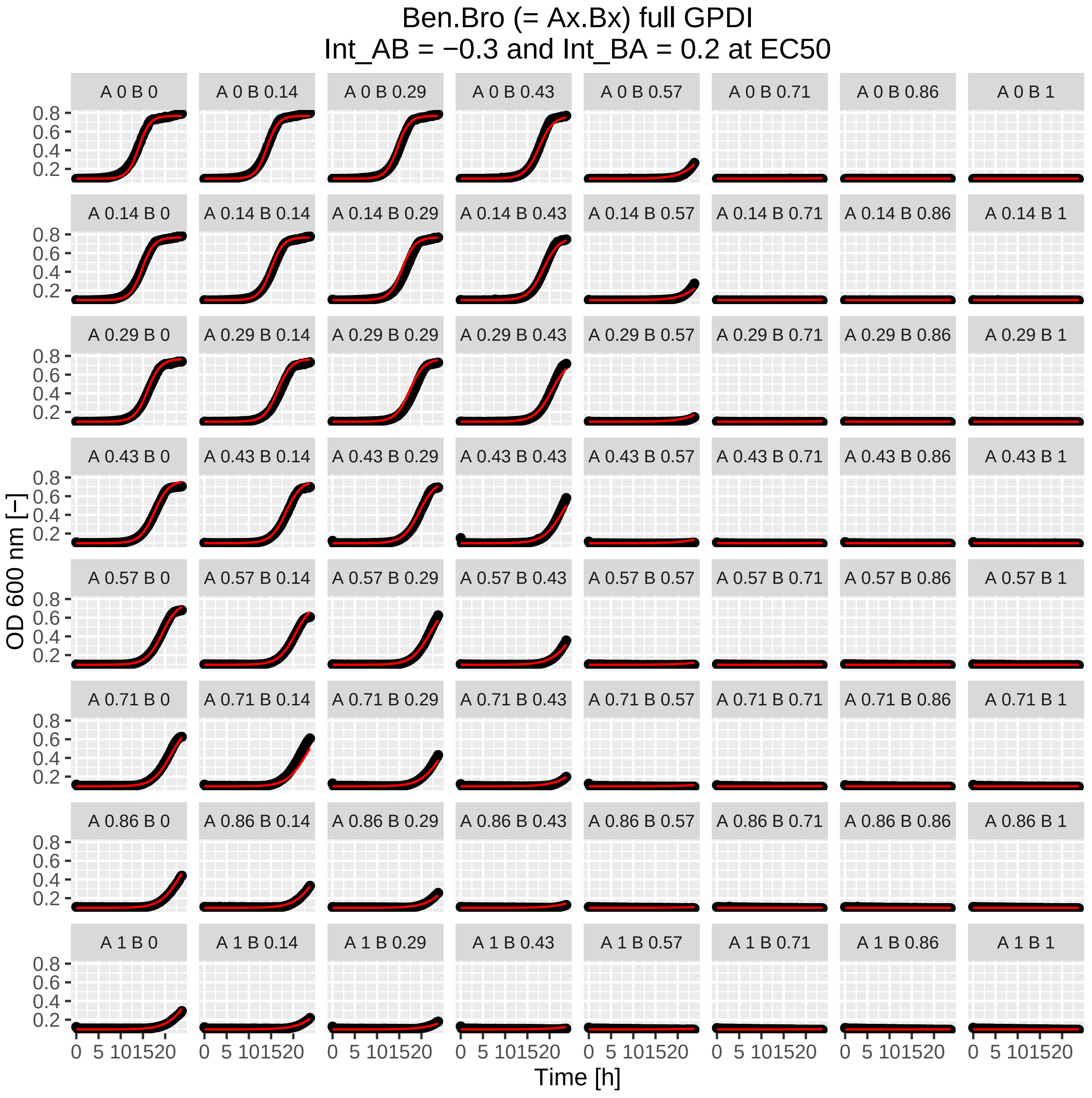

Ben.Bro (= Ax.Bx) full GPDI
Int_AB = −0.3 and Int_BA = 0.2 at EC50
A 0 B 0
A 0 B 0.14
A 0 B 0.29
A 0 B 0.43
A 0 B 0.57
A 0 B 0.71
A 0 B 0.86
A 0 B 1
A 0.14 B 0
A 0.14 B 0.14
A 0.14 B 0.29
A 0.14 B 0.43
A 0.14 B 0.57
A 0.14 B 0.71
A 0.14 B 0.86
A 0.14 B 1
A 0.29 B 0
A 0.29 B 0.14
A 0.29 B 0.29
A 0.29 B 0.43
A 0.29 B 0.57
A 0.29 B 0.71
A 0.29 B 0.86
A 0.29 B 1
A 0.43 B 0
A 0.43 B 0.14
A 0.43 B 0.29
A 0.43 B 0.43
A 0.43 B 0.57
A 0.43 B 0.71
A 0.43 B 0.86
A 0.43 B 1
A 0.57 B 0
A 0.57 B 0.14
A 0.57 B 0.29
A 0.57 B 0.43
A 0.57 B 0.57
A 0.57 B 0.71
A 0.57 B 0.86
A 0.57 B 1
A 0.71 B 0
A 0.71 B 0.14
A 0.71 B 0.29
A 0.71 B 0.43
A 0.71 B 0.57
A 0.71 B 0.71
A 0.71 B 0.86
A 0.71 B 1
A 0.86 B 0
A 0.86 B 0.14
A 0.86 B 0.29
A 0.86 B 0.43
A 0.86 B 0.57
A 0.86 B 0.71
A 0.86 B 0.86
A 0.86 B 1
A 1 B 0
A 1 B 0.14
A 1 B 0.29
A 1 B 0.43
A 1 B 0.57
A 1 B 0.71
A 1 B 0.86
A 1 B 1
0.8
0.6
0.4
0.2
OD 600 nm [–]
0 5 101520
Time [h]

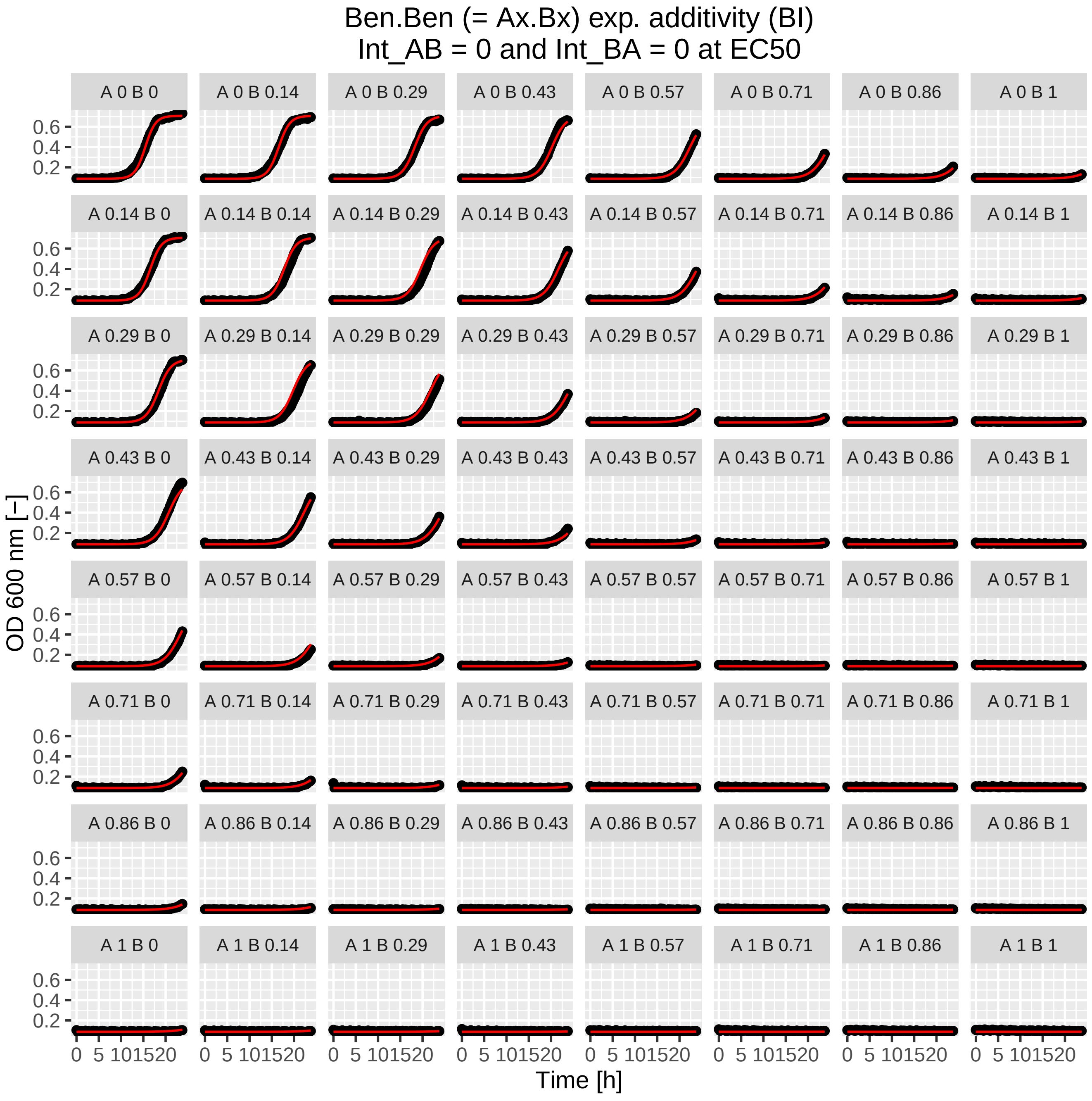

Ben.Ben (= Ax.Bx) exp. additivity (BI)
Int_AB = 0 and Int_BA = 0 at EC50
A 0 B 0
A 0 B 0.14
A 0 B 0.29
A 0 B 0.43
A 0 B 0.57
A 0 B 0.71
A 0 B 0.86
A 0 B 1
A 0.14 B 0
A 0.14 B 0.14
A 0.14 B 0.29
A 0.14 B 0.43
A 0.14 B 0.57
A 0.14 B 0.71
A 0.14 B 0.86
A 0.14 B 1
A 0.29 B 0
A 0.29 B 0.14
A 0.29 B 0.29
A 0.29 B 0.43
A 0.29 B 0.57
A 0.29 B 0.71
A 0.29 B 0.86
A 0.29 B 1
A 0.43 B 0
A 0.43 B 0.14
A 0.43 B 0.29
A 0.43 B 0.43
A 0.43 B 0.57
A 0.43 B 0.71
A 0.43 B 0.86
A 0.43 B 1
A 0.57 B 0
A 0.57 B 0.14
A 0.57 B 0.29
A 0.57 B 0.43
A 0.57 B 0.57
A 0.57 B 0.71
A 0.57 B 0.86
A 0.57 B 1
A 0.71 B 0
A 0.71 B 0.14
A 0.71 B 0.29
A 0.71 B 0.43
A 0.71 B 0.57
A 0.71 B 0.71
A 0.71 B 0.86
A 0.71 B 1
A 0.86 B 0
A 0.86 B 0.14
A 0.86 B 0.29
A 0.86 B 0.43
A 0.86 B 0.57
A 0.86 B 0.71
A 0.86 B 0.86
A 0.86 B 1
A 1 B 0
A 1 B 0.14
A 1 B 0.29
A 1 B 0.43
A 1 B 0.57
A 1 B 0.71
A 1 B 0.86
A 1 B 1
OD 600 nm [–]
0.6
0.4
0.2
0 5 101520
Time [h]

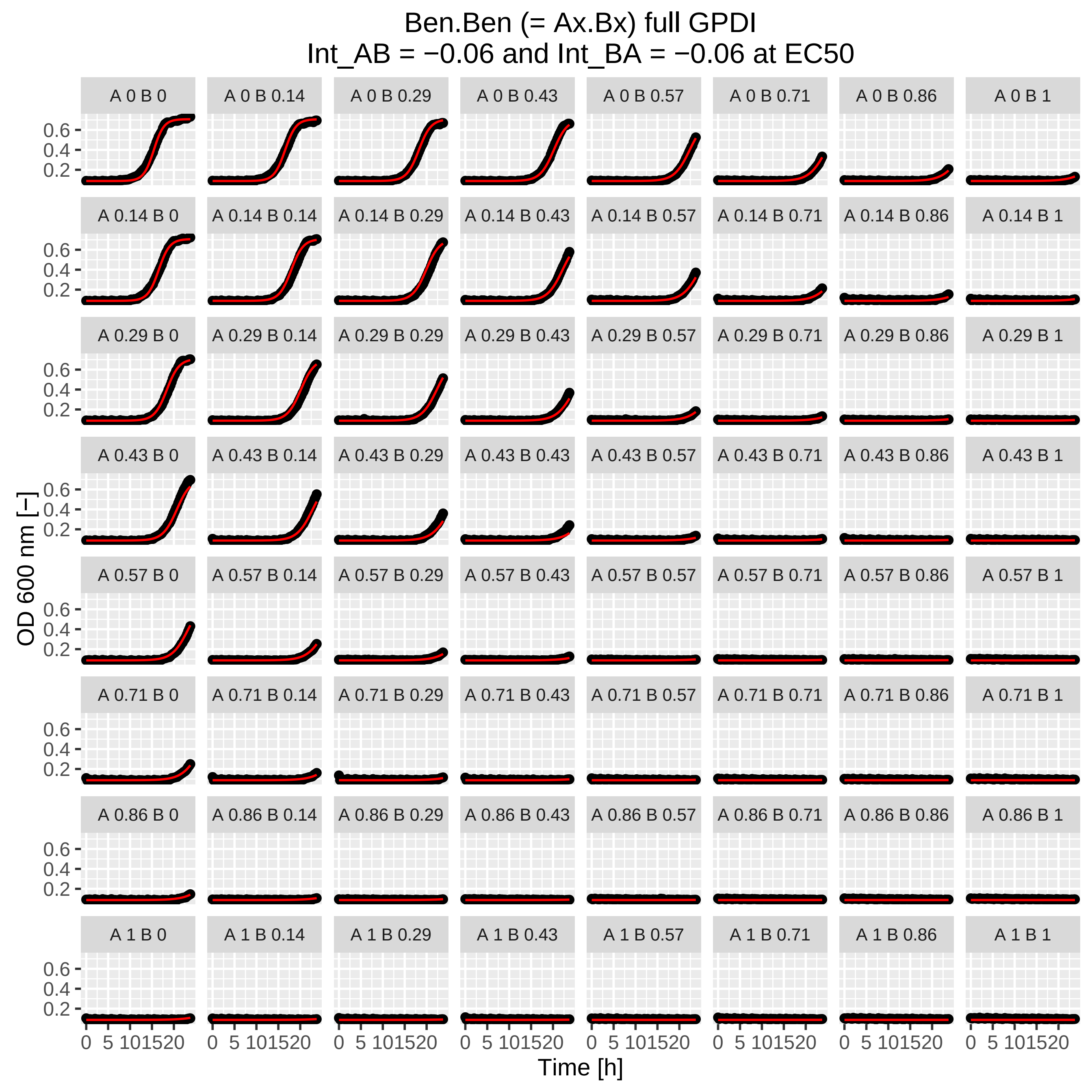
Ben.Ben (= Ax.Bx) full GPDI
Int_AB = −0.06 and Int_BA = −0.06 at EC50
A 0 B 0
A 0 B 0.14
A 0 B 0.29
A 0 B 0.43
A 0 B 0.57
A 0 B 0.71
A 0 B 0.86
A 0 B 1
A 0.14 B 0
A 0.14 B 0.14
A 0.14 B 0.29
A 0.14 B 0.43
A 0.14 B 0.57
A 0.14 B 0.71
A 0.14 B 0.86
A 0.14 B 1
A 0.29 B 0
A 0.29 B 0.14
A 0.29 B 0.29
A 0.29 B 0.43
A 0.29 B 0.57
A 0.29 B 0.71
A 0.29 B 0.86
A 0.29 B 1
A 0.43 B 0
A 0.43 B 0.14
A 0.43 B 0.29
A 0.43 B 0.43
A 0.43 B 0.57
A 0.43 B 0.71
A 0.43 B 0.86
A 0.43 B 1
A 0.57 B 0
A 0.57 B 0.14
A 0.57 B 0.29
A 0.57 B 0.43
A 0.57 B 0.57
A 0.57 B 0.71
A 0.57 B 0.86
A 0.57 B 1
A 0.71 B 0
A 0.71 B 0.14
A 0.71 B 0.29
A 0.71 B 0.43
A 0.71 B 0.57
A 0.71 B 0.71
A 0.71 B 0.86
A 0.71 B 1
A 0.86 B 0
A 0.86 B 0.14
A 0.86 B 0.29
A 0.86 B 0.43
A 0.86 B 0.57
A 0.86 B 0.71
A 0.86 B 0.86
A 0.86 B 1
A 1 B 0
A 1 B 0.14
A 1 B 0.29
A 1 B 0.43
A 1 B 0.57
A 1 B 0.71
A 1 B 0.86
A 1 B 1
OD 600 nm [−]
0.6
0.4
0.2
0 5 101520
Time [h]

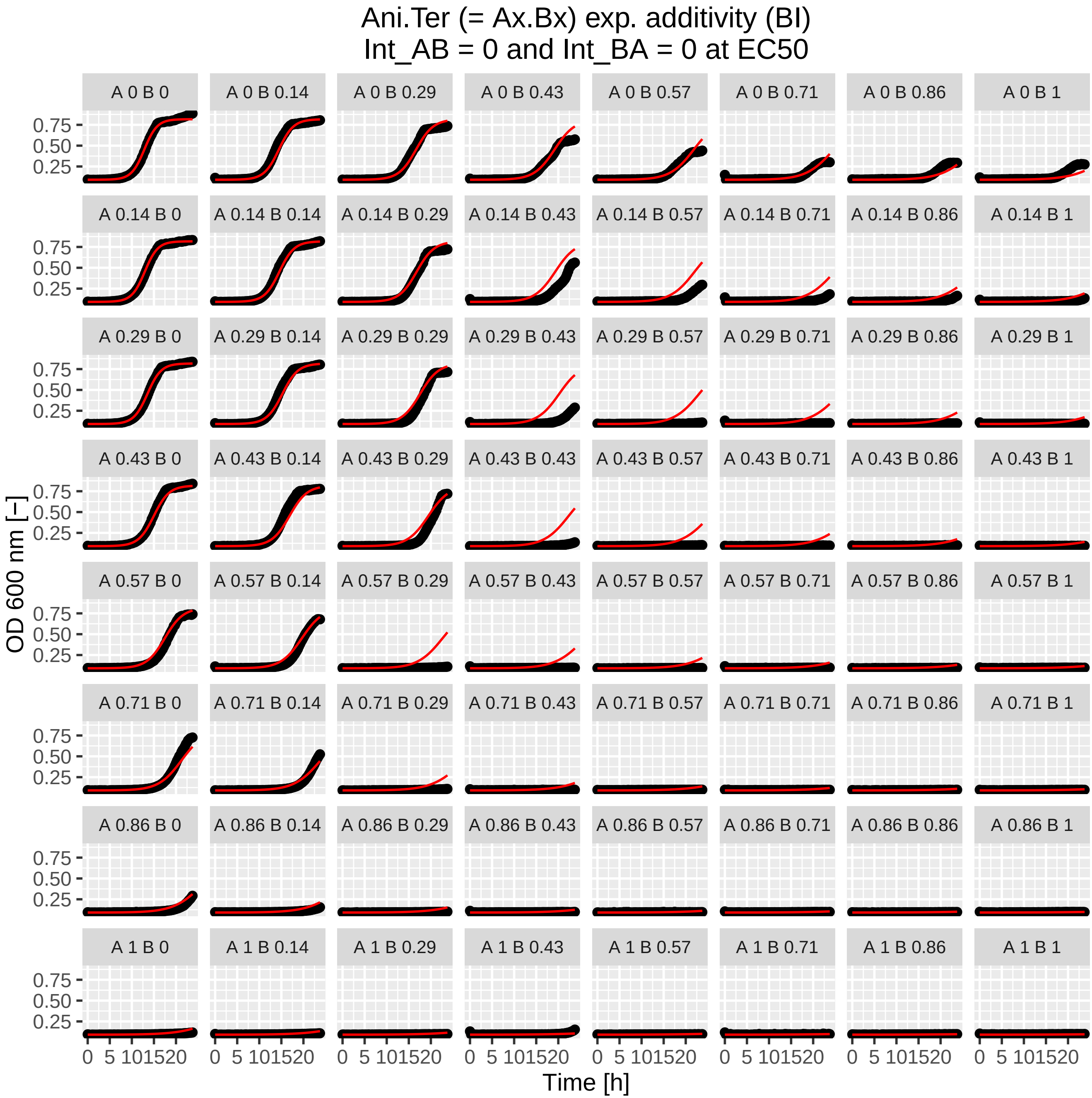

Ani.Ter (= Ax.Bx) exp. additivity (BI)
Int_AB = 0 and Int_BA = 0 at EC50
A 0 B 0
A 0 B 0.14
A 0 B 0.29
A 0 B 0.43
A 0 B 0.57
A 0 B 0.71
A 0 B 0.86
A 0 B 1
A 0.14 B 0
A 0.14 B 0.14
A 0.14 B 0.29
A 0.14 B 0.43
A 0.14 B 0.57
A 0.14 B 0.71
A 0.14 B 0.86
A 0.14 B 1
A 0.29 B 0
A 0.29 B 0.14
A 0.29 B 0.29
A 0.29 B 0.43
A 0.29 B 0.57
A 0.29 B 0.71
A 0.29 B 0.86
A 0.29 B 1
A 0.43 B 0
A 0.43 B 0.14
A 0.43 B 0.29
A 0.43 B 0.43
A 0.43 B 0.57
A 0.43 B 0.71
A 0.43 B 0.86
A 0.43 B 1
A 0.57 B 0
A 0.57 B 0.14
A 0.57 B 0.29
A 0.57 B 0.43
A 0.57 B 0.57
A 0.57 B 0.71
A 0.57 B 0.86
A 0.57 B 1
A 0.71 B 0
A 0.71 B 0.14
A 0.71 B 0.29
A 0.71 B 0.43
A 0.71 B 0.57
A 0.71 B 0.71
A 0.71 B 0.86
A 0.71 B 1
A 0.86 B 0
A 0.86 B 0.14
A 0.86 B 0.29
A 0.86 B 0.43
A 0.86 B 0.57
A 0.86 B 0.71
A 0.86 B 0.86
A 0.86 B 1
A 1 B 0
A 1 B 0.14
A 1 B 0.29
A 1 B 0.43
A 1 B 0.57
A 1 B 0.71
A 1 B 0.86
A 1 B 1
OD 600 nm [−]
0.75
0.50
0.25
0 5 101520
Time [h]

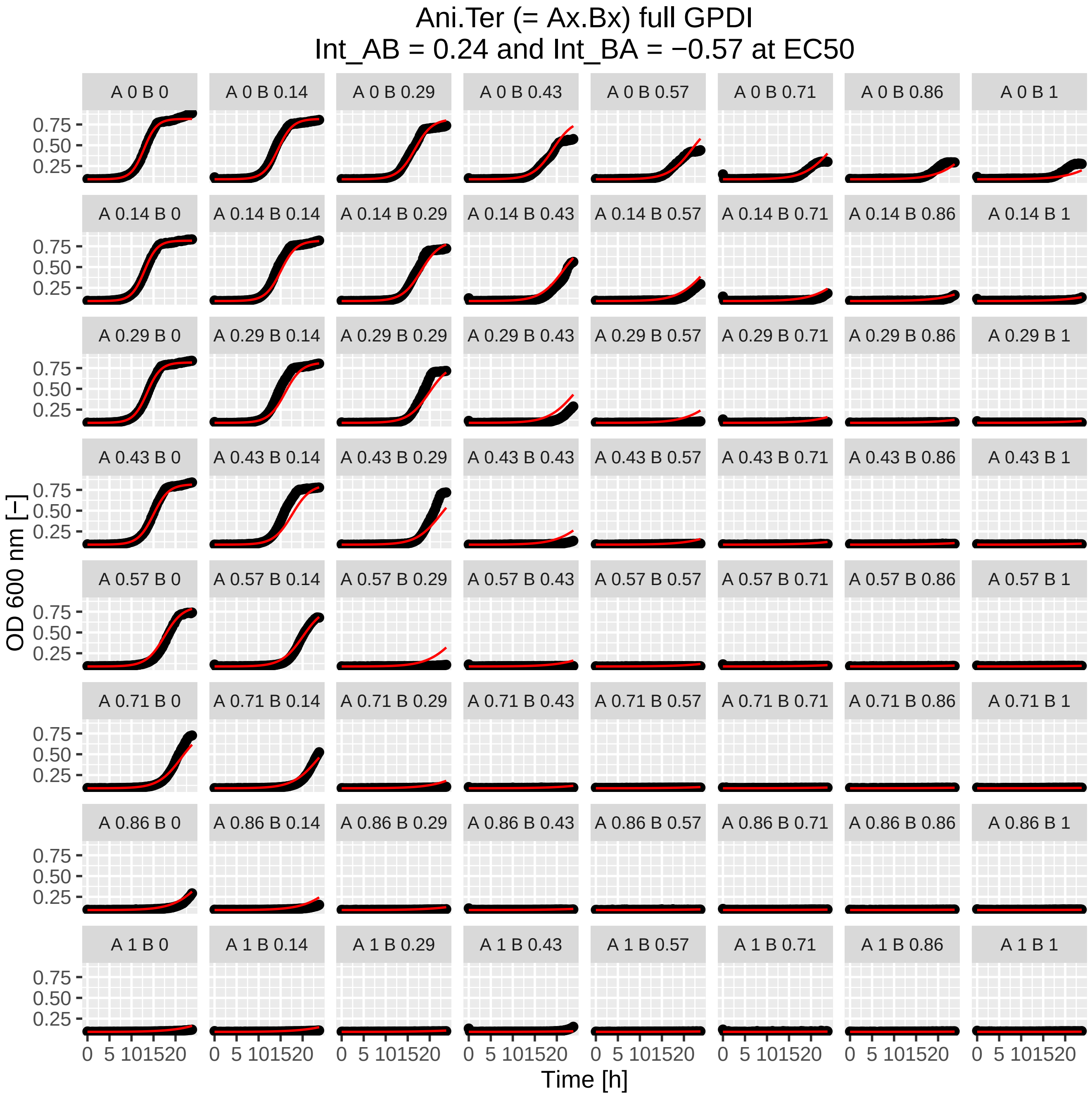

Ani.Ter (= Ax.Bx) full GPDI
Int_AB = 0.24 and Int_BA = −0.57 at EC50
A 0 B 0
A 0 B 0.14
A 0 B 0.29
A 0 B 0.43
A 0 B 0.57
A 0 B 0.71
A 0 B 0.86
A 0 B 1
A 0.14 B 0
A 0.14 B 0.14
A 0.14 B 0.29
A 0.14 B 0.43
A 0.14 B 0.57
A 0.14 B 0.71
A 0.14 B 0.86
A 0.14 B 1
A 0.29 B 0
A 0.29 B 0.14
A 0.29 B 0.29
A 0.29 B 0.43
A 0.29 B 0.57
A 0.29 B 0.71
A 0.29 B 0.86
A 0.29 B 1
A 0.43 B 0
A 0.43 B 0.14
A 0.43 B 0.29
A 0.43 B 0.43
A 0.43 B 0.57
A 0.43 B 0.71
A 0.43 B 0.86
A 0.43 B 1
A 0.57 B 0
A 0.57 B 0.14
A 0.57 B 0.29
A 0.57 B 0.43
A 0.57 B 0.57
A 0.57 B 0.71
A 0.57 B 0.86
A 0.57 B 1
A 0.71 B 0
A 0.71 B 0.14
A 0.71 B 0.29
A 0.71 B 0.43
A 0.71 B 0.57
A 0.71 B 0.71
A 0.71 B 0.86
A 0.71 B 1
A 0.86 B 0
A 0.86 B 0.14
A 0.86 B 0.29
A 0.86 B 0.43
A 0.86 B 0.57
A 0.86 B 0.71
A 0.86 B 0.86
A 0.86 B 1
A 1 B 0
A 1 B 0.14
A 1 B 0.29
A 1 B 0.43
A 1 B 0.57
A 1 B 0.71
A 1 B 0.86
A 1 B 1
OD 600 nm [−]
0.75
0.50
0.25
0 5 101520
Time [h]

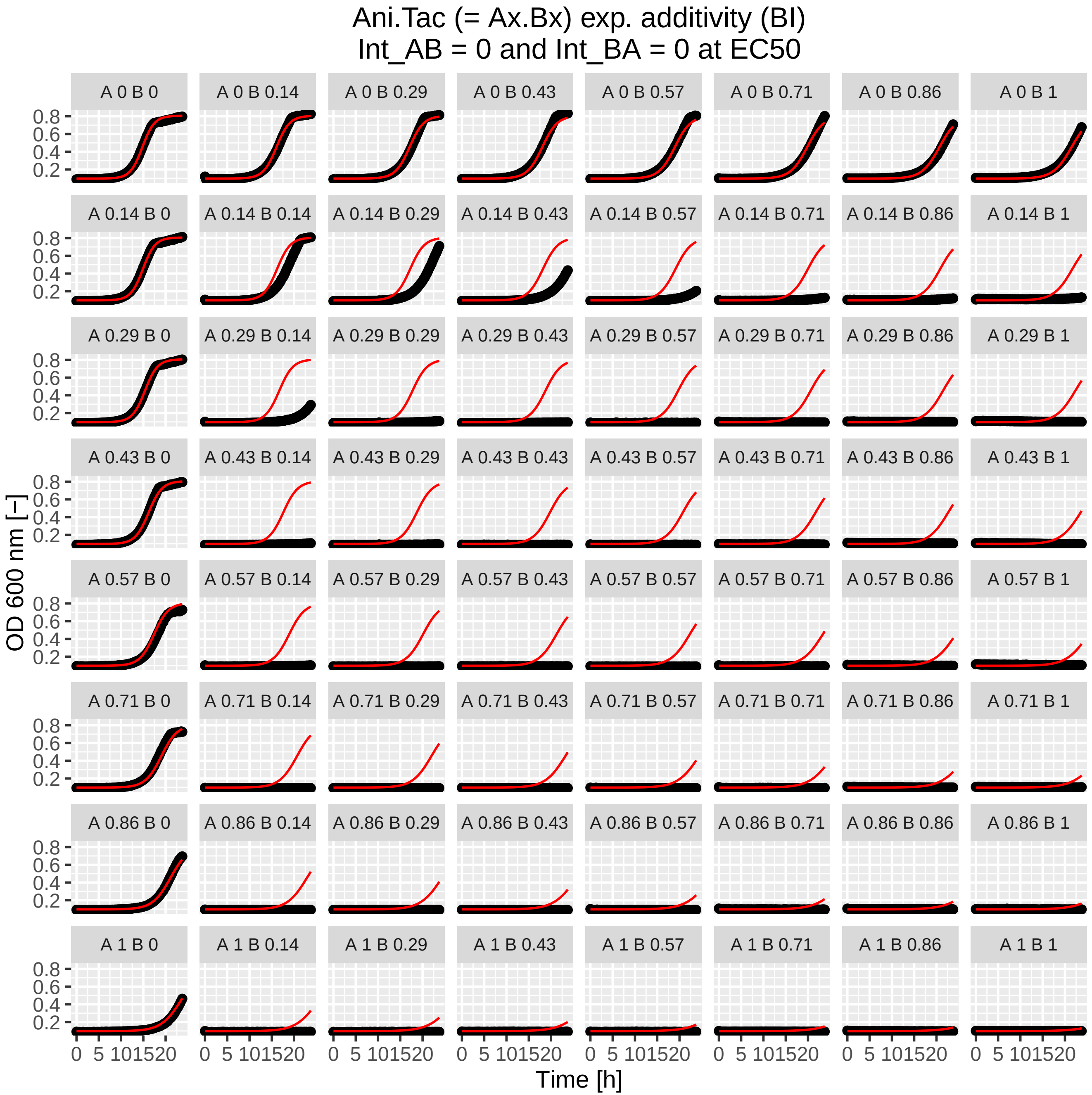

Ani.Tac (= Ax.Bx) exp. additivity (BI)
Int_AB = 0 and Int_BA = 0 at EC50
A 0 B 0
A 0 B 0.14
A 0 B 0.29
A 0 B 0.43
A 0 B 0.57
A 0 B 0.71
A 0 B 0.86
A 0 B 1
A 0.14 B 0
A 0.14 B 0.14
A 0.14 B 0.29
A 0.14 B 0.43
A 0.14 B 0.57
A 0.14 B 0.71
A 0.14 B 0.86
A 0.14 B 1
A 0.29 B 0
A 0.29 B 0.14
A 0.29 B 0.29
A 0.29 B 0.43
A 0.29 B 0.57
A 0.29 B 0.71
A 0.29 B 0.86
A 0.29 B 1
A 0.43 B 0
A 0.43 B 0.14
A 0.43 B 0.29
A 0.43 B 0.43
A 0.43 B 0.57
A 0.43 B 0.71
A 0.43 B 0.86
A 0.43 B 1
A 0.57 B 0
A 0.57 B 0.14
A 0.57 B 0.29
A 0.57 B 0.43
A 0.57 B 0.57
A 0.57 B 0.71
A 0.57 B 0.86
A 0.57 B 1
A 0.71 B 0
A 0.71 B 0.14
A 0.71 B 0.29
A 0.71 B 0.43
A 0.71 B 0.57
A 0.71 B 0.71
A 0.71 B 0.86
A 0.71 B 1
A 0.86 B 0
A 0.86 B 0.14
A 0.86 B 0.29
A 0.86 B 0.43
A 0.86 B 0.57
A 0.86 B 0.71
A 0.86 B 0.86
A 0.86 B 1
A 1 B 0
A 1 B 0.14
A 1 B 0.29
A 1 B 0.43
A 1 B 0.57
A 1 B 0.71
A 1 B 0.86
A 1 B 1
0.8
0.6
0.4
0.2
OD 600 nm [–]
0 5 101520
Time [h]

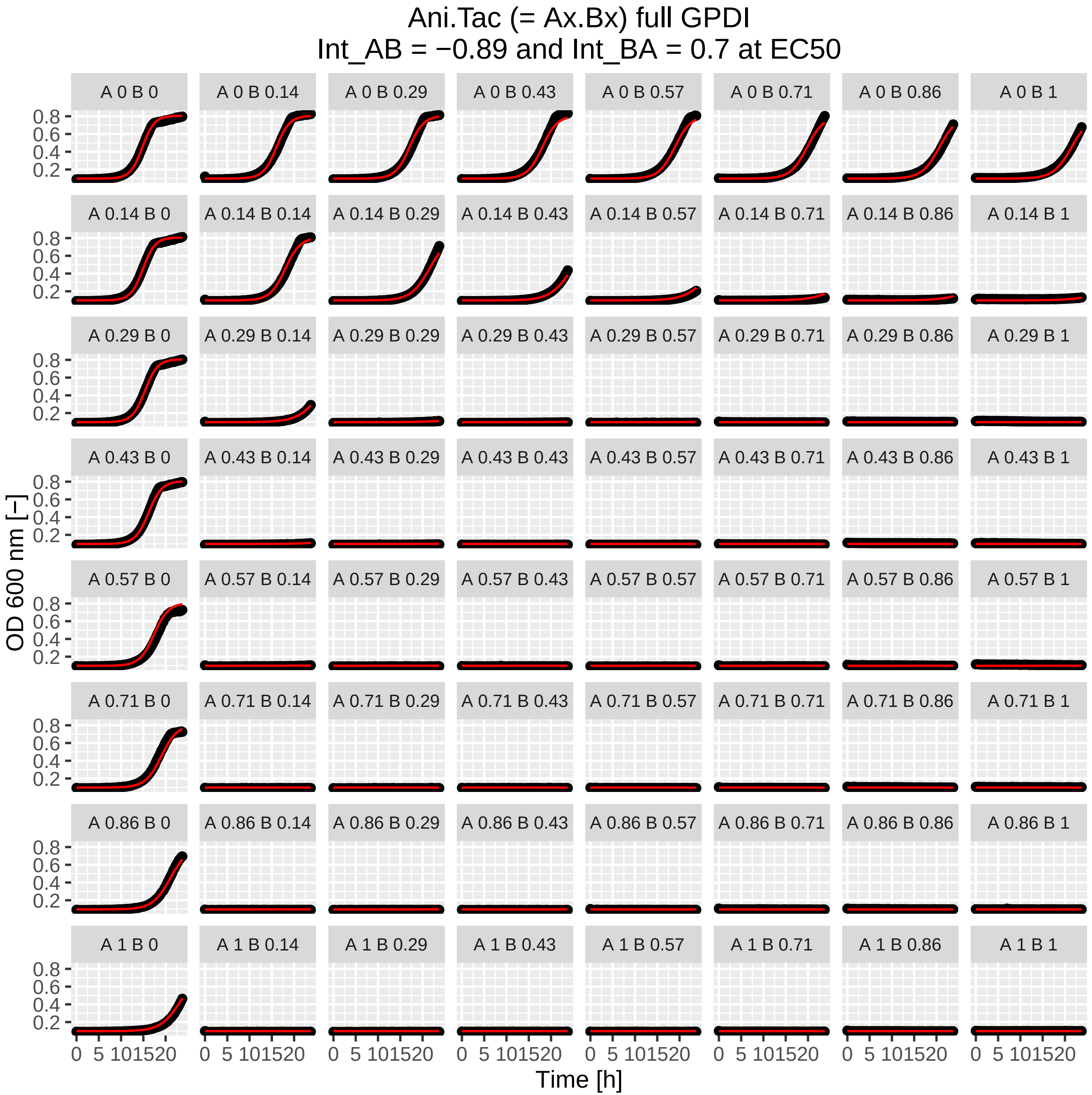

Ani.Tac (= Ax.Bx) full GPDI
Int_AB = −0.89 and Int_BA = 0.7 at EC50
OD 600 nm [−]
Time [h]
A 0 B 0
A 0 B 0.14
A 0 B 0.29
A 0 B 0.43
A 0 B 0.57
A 0 B 0.71
A 0 B 0.86
A 0 B 1
A 0.14 B 0
A 0.14 B 0.14
A 0.14 B 0.29
A 0.14 B 0.43
A 0.14 B 0.57
A 0.14 B 0.71
A 0.14 B 0.86
A 0.14 B 1
A 0.29 B 0
A 0.29 B 0.14
A 0.29 B 0.29
A 0.29 B 0.43
A 0.29 B 0.57
A 0.29 B 0.71
A 0.29 B 0.86
A 0.29 B 1
A 0.43 B 0
A 0.43 B 0.14
A 0.43 B 0.29
A 0.43 B 0.43
A 0.43 B 0.57
A 0.43 B 0.71
A 0.43 B 0.86
A 0.43 B 1
A 0.57 B 0
A 0.57 B 0.14
A 0.57 B 0.29
A 0.57 B 0.43
A 0.57 B 0.57
A 0.57 B 0.71
A 0.57 B 0.86
A 0.57 B 1
A 0.71 B 0
A 0.71 B 0.14
A 0.71 B 0.29
A 0.71 B 0.43
A 0.71 B 0.57
A 0.71 B 0.71
A 0.71 B 0.86
A 0.71 B 1
A 0.86 B 0
A 0.86 B 0.14
A 0.86 B 0.29
A 0.86 B 0.43
A 0.86 B 0.57
A 0.86 B 0.71
A 0.86 B 0.86
A 0.86 B 1
A 1 B 0
A 1 B 0.14
A 1 B 0.29
A 1 B 0.43
A 1 B 0.57
A 1 B 0.71
A 1 B 0.86
A 1 B 1
0.8
0.6
0.4
0.2
0 5 101520

Ani.Sta (= Ax.Bx) exp. additivity (BI)
Int_AB = 0 and Int_BA = 0 at EC50

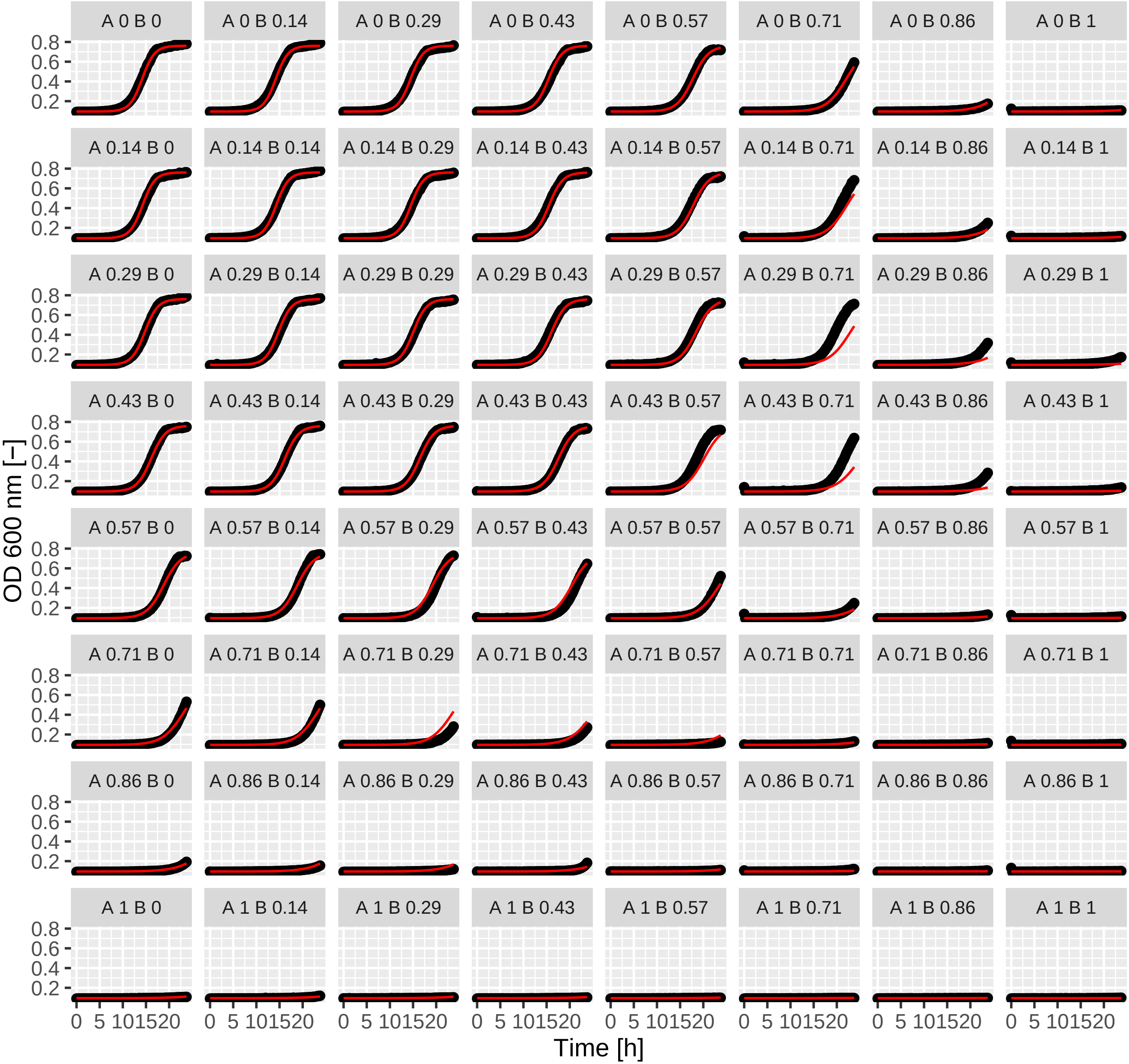

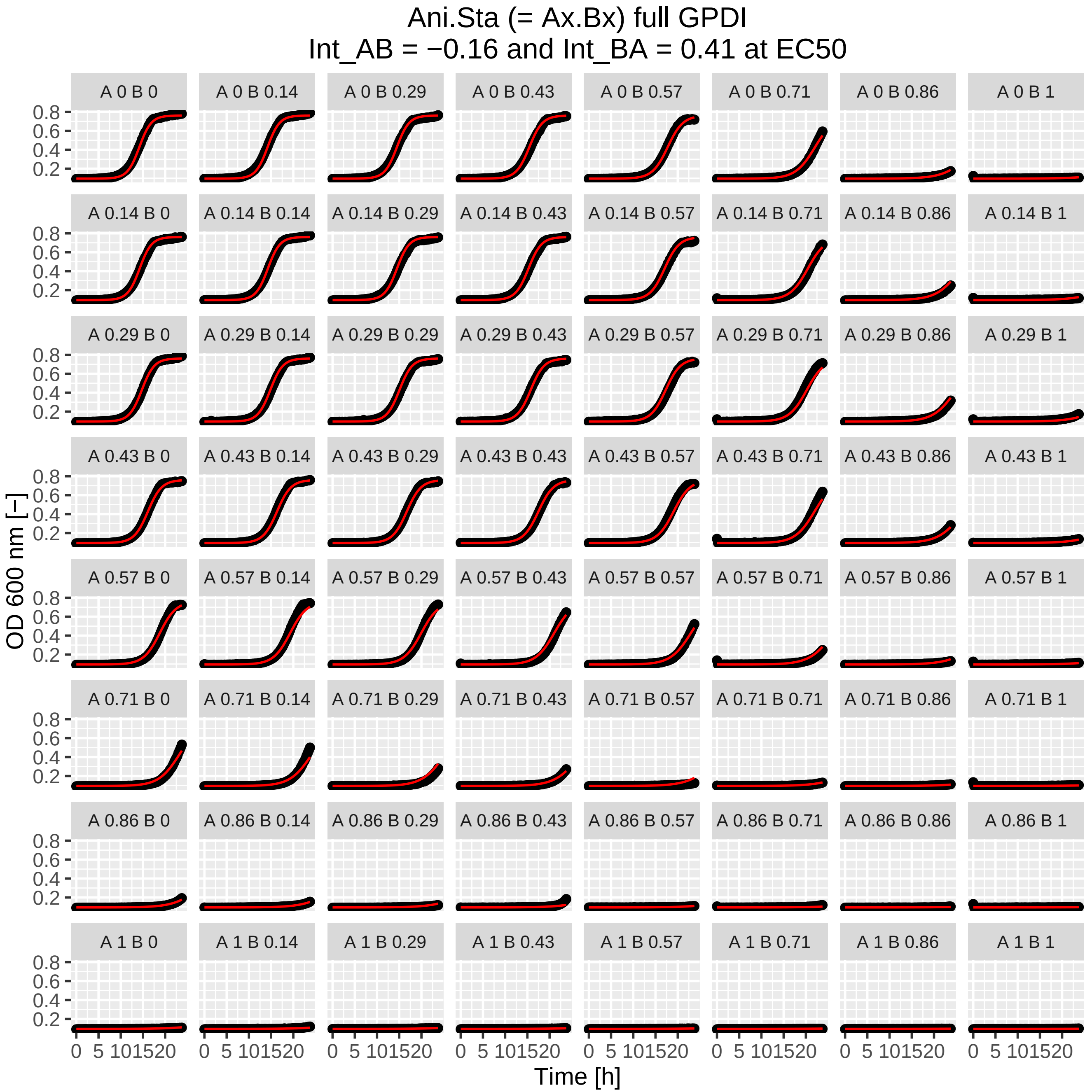

Ani.Sta (= Ax.Bx) full GPDI
Int_AB = −0.16 and Int_BA = 0.41 at EC50
OD 600 nm [−]
Time [h]
0.8
0.6
0.4
0.2
0 5 101520
A 0 B 0
A 0 B 0.14
A 0 B 0.29
A 0 B 0.43
A 0 B 0.57
A 0 B 0.71
A 0 B 0.86
A 0 B 1
A 0.14 B 0
A 0.14 B 0.14
A 0.14 B 0.29
A 0.14 B 0.43
A 0.14 B 0.57
A 0.14 B 0.71
A 0.14 B 0.86
A 0.14 B 1
A 0.29 B 0
A 0.29 B 0.14
A 0.29 B 0.29
A 0.29 B 0.43
A 0.29 B 0.57
A 0.29 B 0.71
A 0.29 B 0.86
A 0.29 B 1
A 0.43 B 0
A 0.43 B 0.14
A 0.43 B 0.29
A 0.43 B 0.43
A 0.43 B 0.57
A 0.43 B 0.71
A 0.43 B 0.86
A 0.43 B 1
A 0.57 B 0
A 0.57 B 0.14
A 0.57 B 0.29
A 0.57 B 0.43
A 0.57 B 0.57
A 0.57 B 0.71
A 0.57 B 0.86
A 0.57 B 1
A 0.71 B 0
A 0.71 B 0.14
A 0.71 B 0.29
A 0.71 B 0.43
A 0.71 B 0.57
A 0.71 B 0.71
A 0.71 B 0.86
A 0.71 B 1
A 0.86 B 0
A 0.86 B 0.14
A 0.86 B 0.29
A 0.86 B 0.43
A 0.86 B 0.57
A 0.86 B 0.71
A 0.86 B 0.86
A 0.86 B 1
A 1 B 0
A 1 B 0.14
A 1 B 0.29
A 1 B 0.43
A 1 B 0.57
A 1 B 0.71
A 1 B 0.86
A 1 B 1

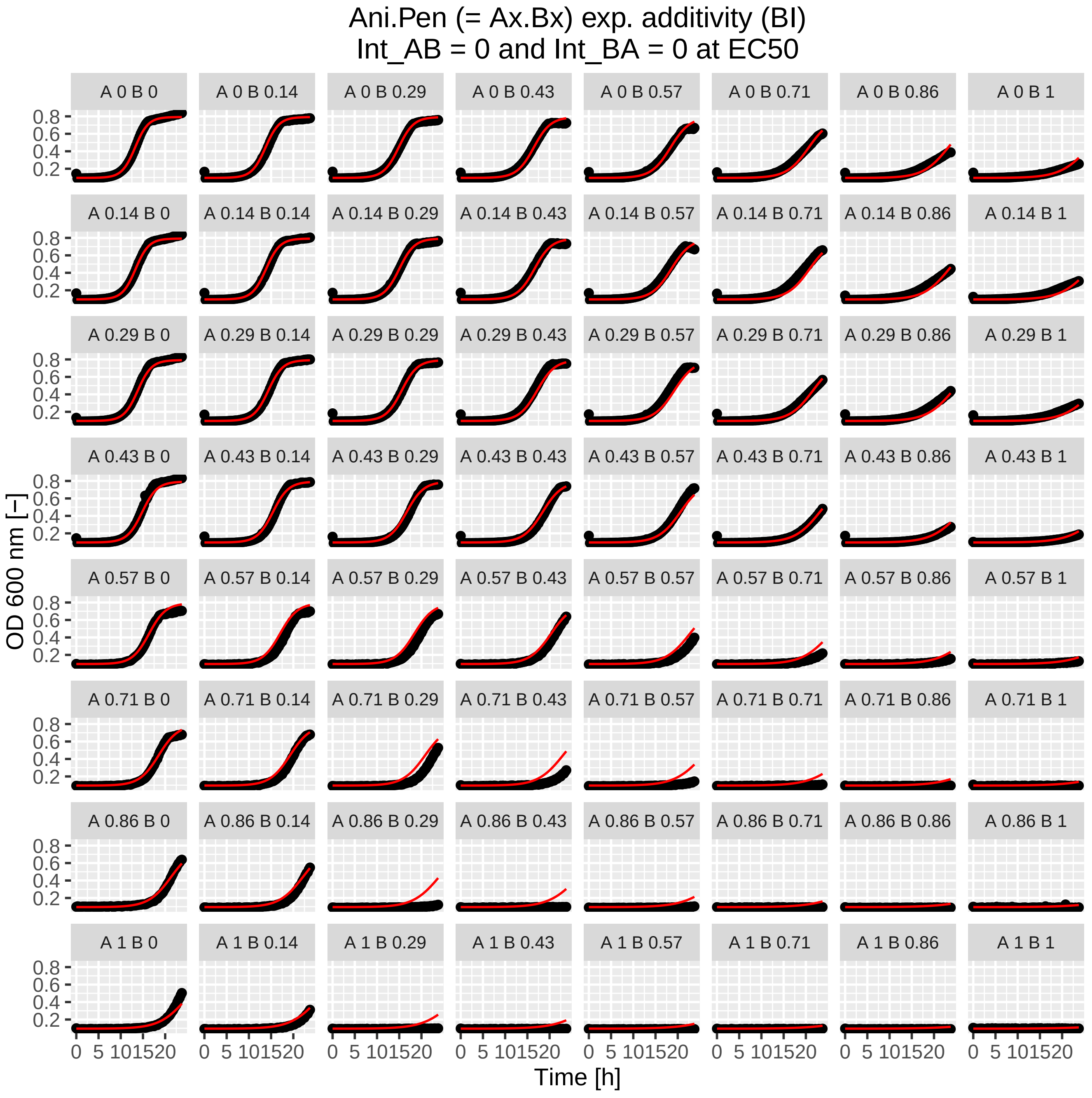

Ani.Pen (= Ax.Bx) exp. additivity (BI)
Int_AB = 0 and Int_BA = 0 at EC50
A 0 B 0
A 0 B 0.14
A 0 B 0.29
A 0 B 0.43
A 0 B 0.57
A 0 B 0.71
A 0 B 0.86
A 0 B 1
A 0.14 B 0
A 0.14 B 0.14
A 0.14 B 0.29
A 0.14 B 0.43
A 0.14 B 0.57
A 0.14 B 0.71
A 0.14 B 0.86
A 0.14 B 1
A 0.29 B 0
A 0.29 B 0.14
A 0.29 B 0.29
A 0.29 B 0.43
A 0.29 B 0.57
A 0.29 B 0.71
A 0.29 B 0.86
A 0.29 B 1
A 0.43 B 0
A 0.43 B 0.14
A 0.43 B 0.29
A 0.43 B 0.43
A 0.43 B 0.57
A 0.43 B 0.71
A 0.43 B 0.86
A 0.43 B 1
A 0.57 B 0
A 0.57 B 0.14
A 0.57 B 0.29
A 0.57 B 0.43
A 0.57 B 0.57
A 0.57 B 0.71
A 0.57 B 0.86
A 0.57 B 1
A 0.71 B 0
A 0.71 B 0.14
A 0.71 B 0.29
A 0.71 B 0.43
A 0.71 B 0.57
A 0.71 B 0.71
A 0.71 B 0.86
A 0.71 B 1
A 0.86 B 0
A 0.86 B 0.14
A 0.86 B 0.29
A 0.86 B 0.43
A 0.86 B 0.57
A 0.86 B 0.71
A 0.86 B 0.86
A 0.86 B 1
A 1 B 0
A 1 B 0.14
A 1 B 0.29
A 1 B 0.43
A 1 B 0.57
A 1 B 0.71
A 1 B 0.86
A 1 B 1
OD 600 nm [–]
0.8
0.6
0.4
0.2
0 5 101520
Time [h]

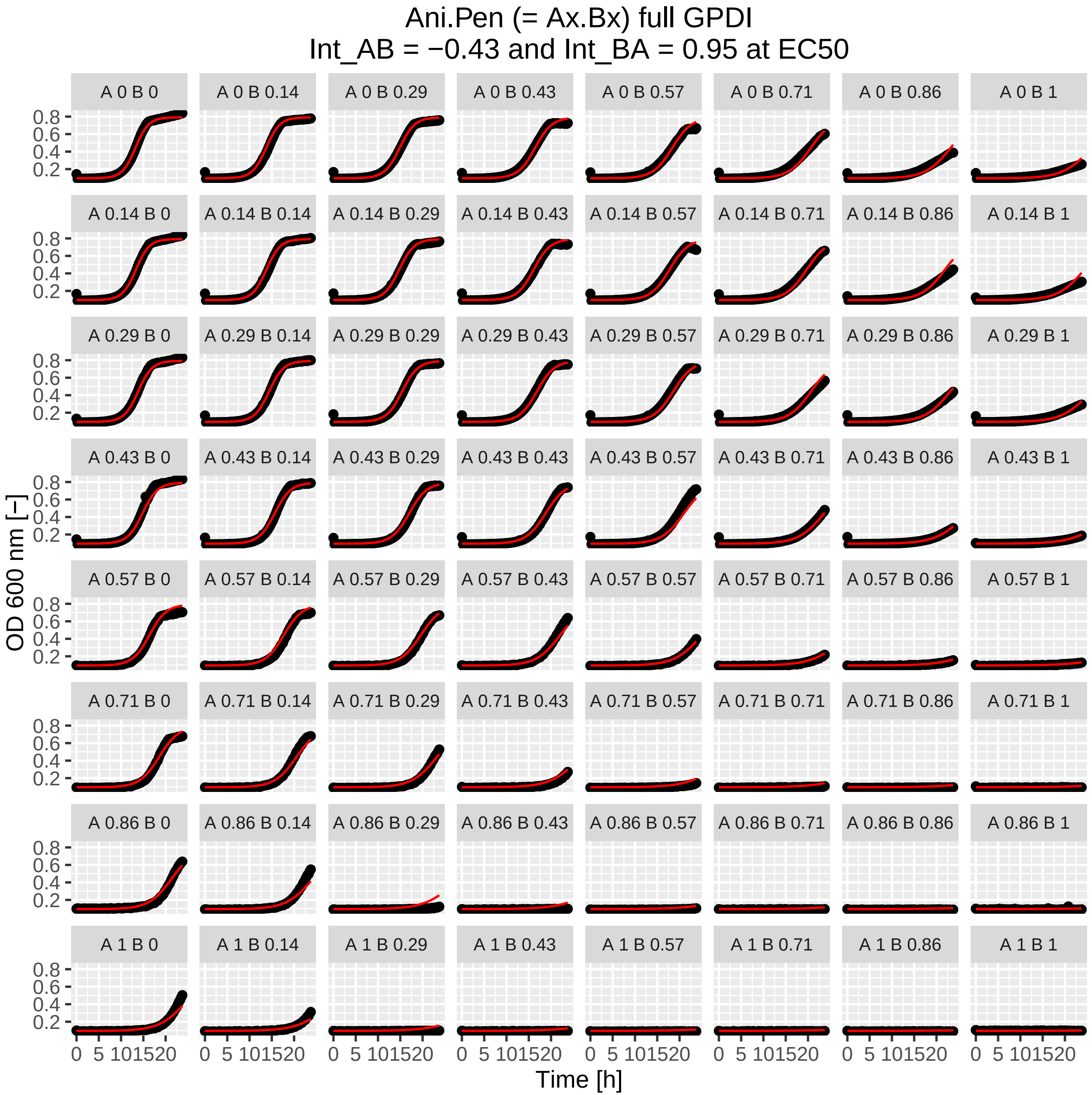
Ani.Pen (= Ax.Bx) full GPDI
Int_AB = −0.43 and Int_BA = 0.95 at EC50
OD 600 nm [−]
Time [h]
A 0 B 0
A 0 B 0.14
A 0 B 0.29
A 0 B 0.43
A 0 B 0.57
A 0 B 0.71
A 0 B 0.86
A 0 B 1
A 0.14 B 0
A 0.14 B 0.14
A 0.14 B 0.29
A 0.14 B 0.43
A 0.14 B 0.57
A 0.14 B 0.71
A 0.14 B 0.86
A 0.14 B 1
A 0.29 B 0
A 0.29 B 0.14
A 0.29 B 0.29
A 0.29 B 0.43
A 0.29 B 0.57
A 0.29 B 0.71
A 0.29 B 0.86
A 0.29 B 1
A 0.43 B 0
A 0.43 B 0.14
A 0.43 B 0.29
A 0.43 B 0.43
A 0.43 B 0.57
A 0.43 B 0.71
A 0.43 B 0.86
A 0.43 B 1
A 0.57 B 0
A 0.57 B 0.14
A 0.57 B 0.29
A 0.57 B 0.43
A 0.57 B 0.57
A 0.57 B 0.71
A 0.57 B 0.86
A 0.57 B 1
A 0.71 B 0
A 0.71 B 0.14
A 0.71 B 0.29
A 0.71 B 0.43
A 0.71 B 0.57
A 0.71 B 0.71
A 0.71 B 0.86
A 0.71 B 1
A 0.86 B 0
A 0.86 B 0.14
A 0.86 B 0.29
A 0.86 B 0.43
A 0.86 B 0.57
A 0.86 B 0.71
A 0.86 B 0.86
A 0.86 B 1
A 1 B 0
A 1 B 0.14
A 1 B 0.29
A 1 B 0.43
A 1 B 0.57
A 1 B 0.71
A 1 B 0.86
A 1 B 1
0.8
0.6
0.4
0.2
0 5 101520

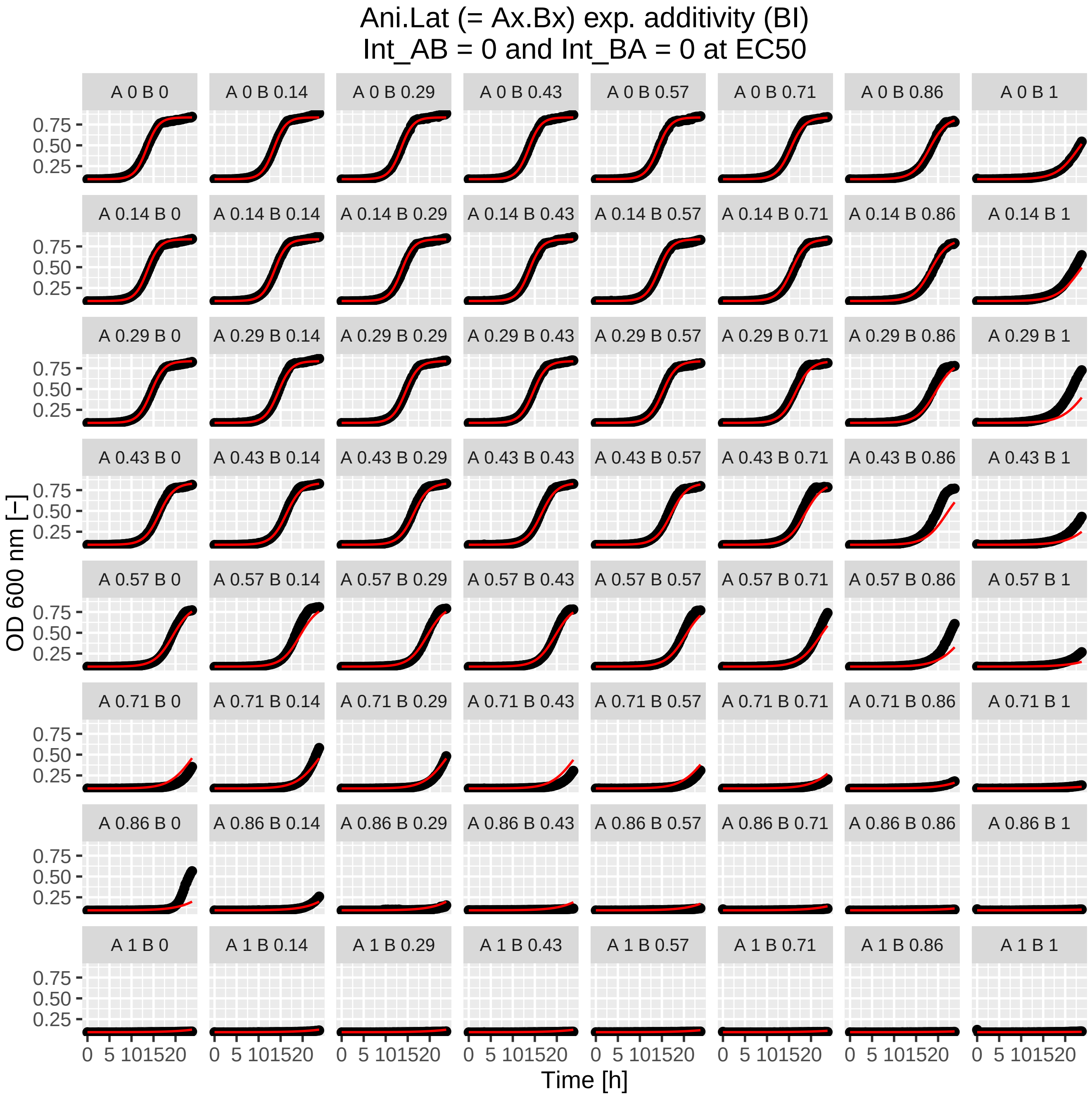

Ani.Lat (= Ax.Bx) exp. additivity (BI)
Int_AB = 0 and Int_BA = 0 at EC50
A 0 B 0
A 0 B 0.14
A 0 B 0.29
A 0 B 0.43
A 0 B 0.57
A 0 B 0.71
A 0 B 0.86
A 0 B 1
A 0.14 B 0
A 0.14 B 0.14
A 0.14 B 0.29
A 0.14 B 0.43
A 0.14 B 0.57
A 0.14 B 0.71
A 0.14 B 0.86
A 0.14 B 1
A 0.29 B 0
A 0.29 B 0.14
A 0.29 B 0.29
A 0.29 B 0.43
A 0.29 B 0.57
A 0.29 B 0.71
A 0.29 B 0.86
A 0.29 B 1
A 0.43 B 0
A 0.43 B 0.14
A 0.43 B 0.29
A 0.43 B 0.43
A 0.43 B 0.57
A 0.43 B 0.71
A 0.43 B 0.86
A 0.43 B 1
A 0.57 B 0
A 0.57 B 0.14
A 0.57 B 0.29
A 0.57 B 0.43
A 0.57 B 0.57
A 0.57 B 0.71
A 0.57 B 0.86
A 0.57 B 1
A 0.71 B 0
A 0.71 B 0.14
A 0.71 B 0.29
A 0.71 B 0.43
A 0.71 B 0.57
A 0.71 B 0.71
A 0.71 B 0.86
A 0.71 B 1
A 0.86 B 0
A 0.86 B 0.14
A 0.86 B 0.29
A 0.86 B 0.43
A 0.86 B 0.57
A 0.86 B 0.71
A 0.86 B 0.86
A 0.86 B 1
A 1 B 0
A 1 B 0.14
A 1 B 0.29
A 1 B 0.43
A 1 B 0.57
A 1 B 0.71
A 1 B 0.86
A 1 B 1
0.75
0.50
0.25
OD 600 nm [–]
0 5 101520
Time [h]

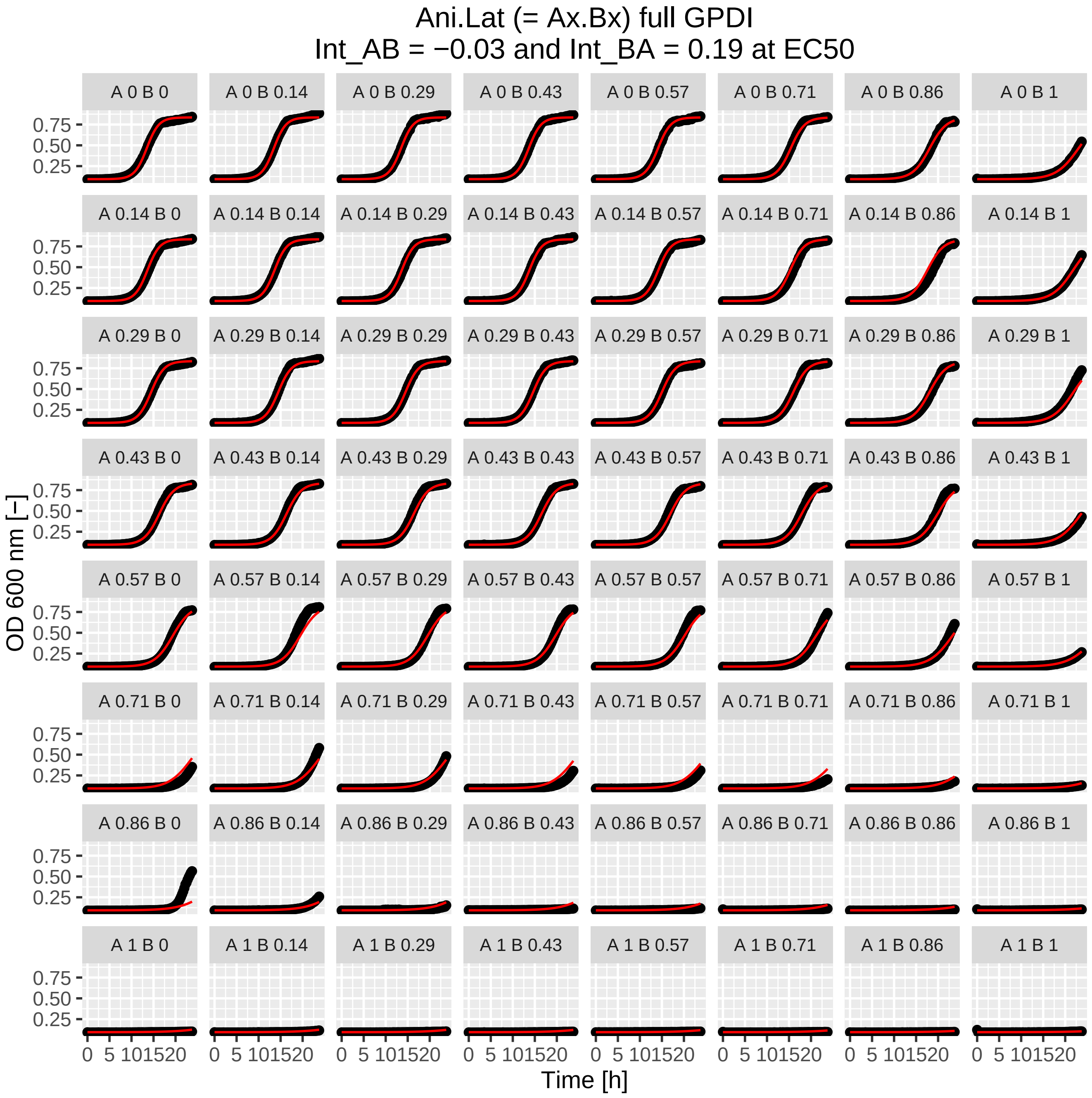

Ani.Lat (= Ax.Bx) full GPDI
Int_AB = −0.03 and Int_BA = 0.19 at EC50
A 0 B 0
A 0 B 0.14
A 0 B 0.29
A 0 B 0.43
A 0 B 0.57
A 0 B 0.71
A 0 B 0.86
A 0 B 1
A 0.14 B 0
A 0.14 B 0.14
A 0.14 B 0.29
A 0.14 B 0.43
A 0.14 B 0.57
A 0.14 B 0.71
A 0.14 B 0.86
A 0.14 B 1
A 0.29 B 0
A 0.29 B 0.14
A 0.29 B 0.29
A 0.29 B 0.43
A 0.29 B 0.57
A 0.29 B 0.71
A 0.29 B 0.86
A 0.29 B 1
A 0.43 B 0
A 0.43 B 0.14
A 0.43 B 0.29
A 0.43 B 0.43
A 0.43 B 0.57
A 0.43 B 0.71
A 0.43 B 0.86
A 0.43 B 1
A 0.57 B 0
A 0.57 B 0.14
A 0.57 B 0.29
A 0.57 B 0.43
A 0.57 B 0.57
A 0.57 B 0.71
A 0.57 B 0.86
A 0.57 B 1
A 0.71 B 0
A 0.71 B 0.14
A 0.71 B 0.29
A 0.71 B 0.43
A 0.71 B 0.57
A 0.71 B 0.71
A 0.71 B 0.86
A 0.71 B 1
A 0.86 B 0
A 0.86 B 0.14
A 0.86 B 0.29
A 0.86 B 0.43
A 0.86 B 0.57
A 0.86 B 0.71
A 0.86 B 0.86
A 0.86 B 1
A 1 B 0
A 1 B 0.14
A 1 B 0.29
A 1 B 0.43
A 1 B 0.57
A 1 B 0.71
A 1 B 0.86
A 1 B 1
0.75
0.50
0.25
OD 600 nm [−]
0 5 101520
Time [h]

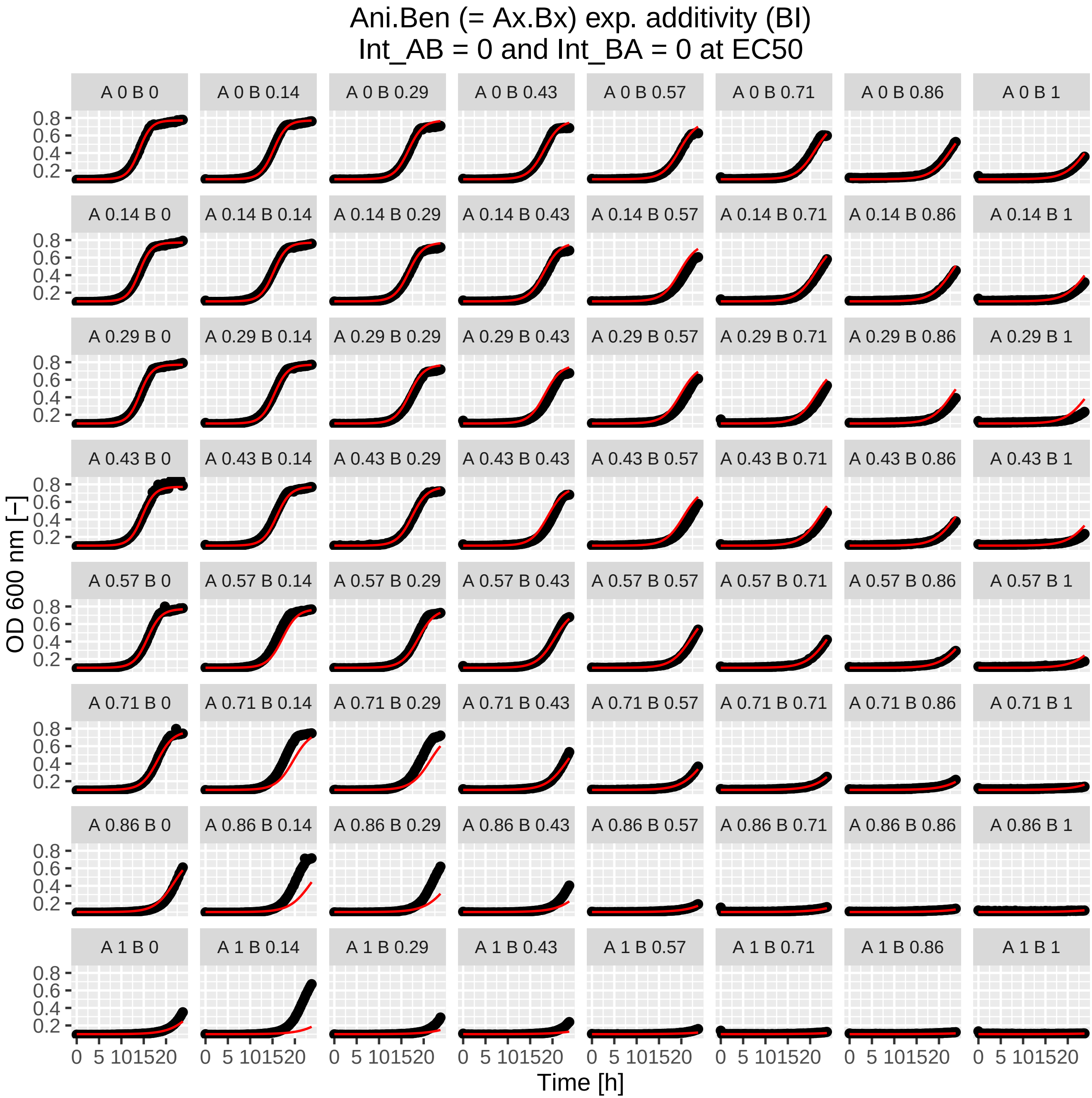
Ani.Ben (= Ax.Bx) exp. additivity (BI)
Int_AB = 0 and Int_BA = 0 at EC50
OD 600 nm [–]
Time [h]
A 0 B 0
A 0 B 0.14
A 0 B 0.29
A 0 B 0.43
A 0 B 0.57
A 0 B 0.71
A 0 B 0.86
A 0 B 1
A 1 B 0
A 1 B 0.14
A 1 B 0.29
A 1 B 0.43
A 1 B 0.57
A 1 B 0.71
A 1 B 0.86
A 1 B 1
0.2
0.4
0.6
0.8
0 5 101520

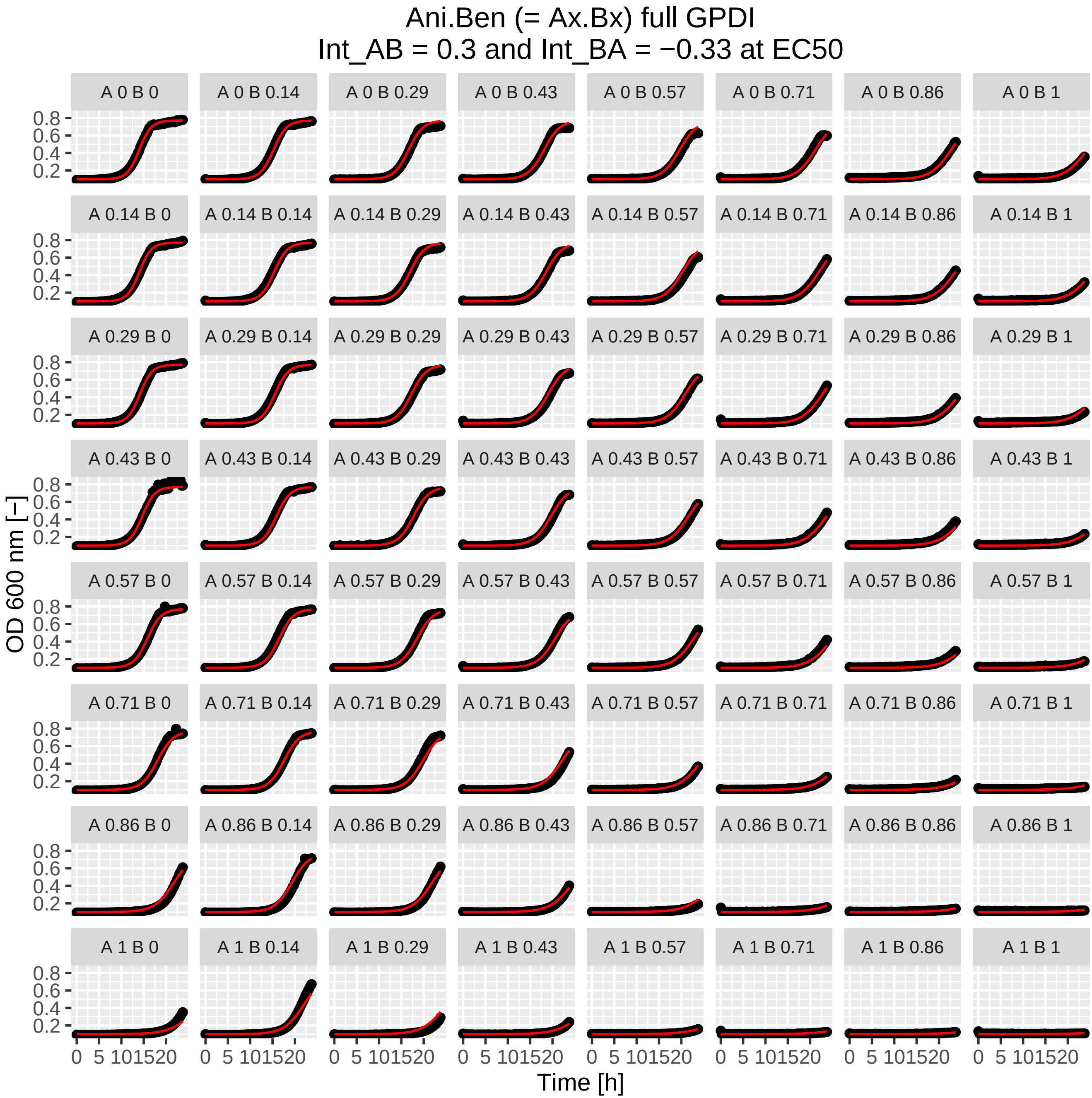
Ani.Ben (= Ax.Bx) full GPDI
Int_AB = 0.3 and Int_BA = −0.33 at EC50
A 0 B 0
A 0 B 0.14
A 0 B 0.29
A 0 B 0.43
A 0 B 0.57
A 0 B 0.71
A 0 B 0.86
A 0 B 1
A 0.14 B 0
A 0.14 B 0.14
A 0.14 B 0.29
A 0.14 B 0.43
A 0.14 B 0.57
A 0.14 B 0.71
A 0.14 B 0.86
A 0.14 B 1
A 0.29 B 0
A 0.29 B 0.14
A 0.29 B 0.29
A 0.29 B 0.43
A 0.29 B 0.57
A 0.29 B 0.71
A 0.29 B 0.86
A 0.29 B 1
A 0.43 B 0
A 0.43 B 0.14
A 0.43 B 0.29
A 0.43 B 0.43
A 0.43 B 0.57
A 0.43 B 0.71
A 0.43 B 0.86
A 0.43 B 1
A 0.57 B 0
A 0.57 B 0.14
A 0.57 B 0.29
A 0.57 B 0.43
A 0.57 B 0.57
A 0.57 B 0.71
A 0.57 B 0.86
A 0.57 B 1
A 0.71 B 0
A 0.71 B 0.14
A 0.71 B 0.29
A 0.71 B 0.43
A 0.71 B 0.57
A 0.71 B 0.71
A 0.71 B 0.86
A 0.71 B 1
A 0.86 B 0
A 0.86 B 0.14
A 0.86 B 0.29
A 0.86 B 0.43
A 0.86 B 0.57
A 0.86 B 0.71
A 0.86 B 0.86
A 0.86 B 1
A 1 B 0
A 1 B 0.14
A 1 B 0.29
A 1 B 0.43
A 1 B 0.57
A 1 B 0.71
A 1 B 0.86
A 1 B 1
0.8
0.6
0.4
0.2
0 5 101520
OD 600 nm [−]
Time [h]

Ani.Ani (= Ax.Bx) exp. additivity (BI)
Int_AB = 0 and Int_BA = 0 at EC50

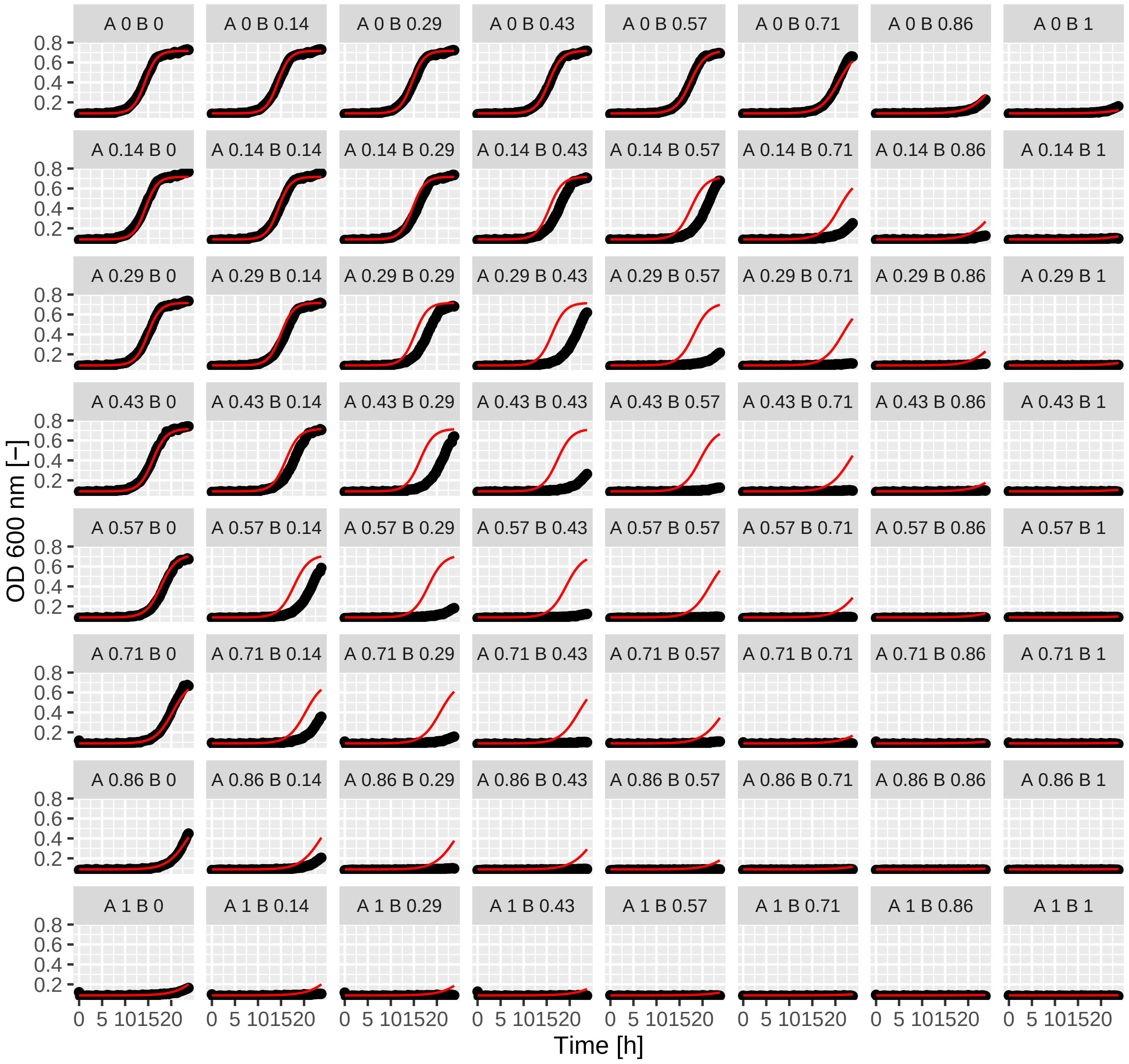

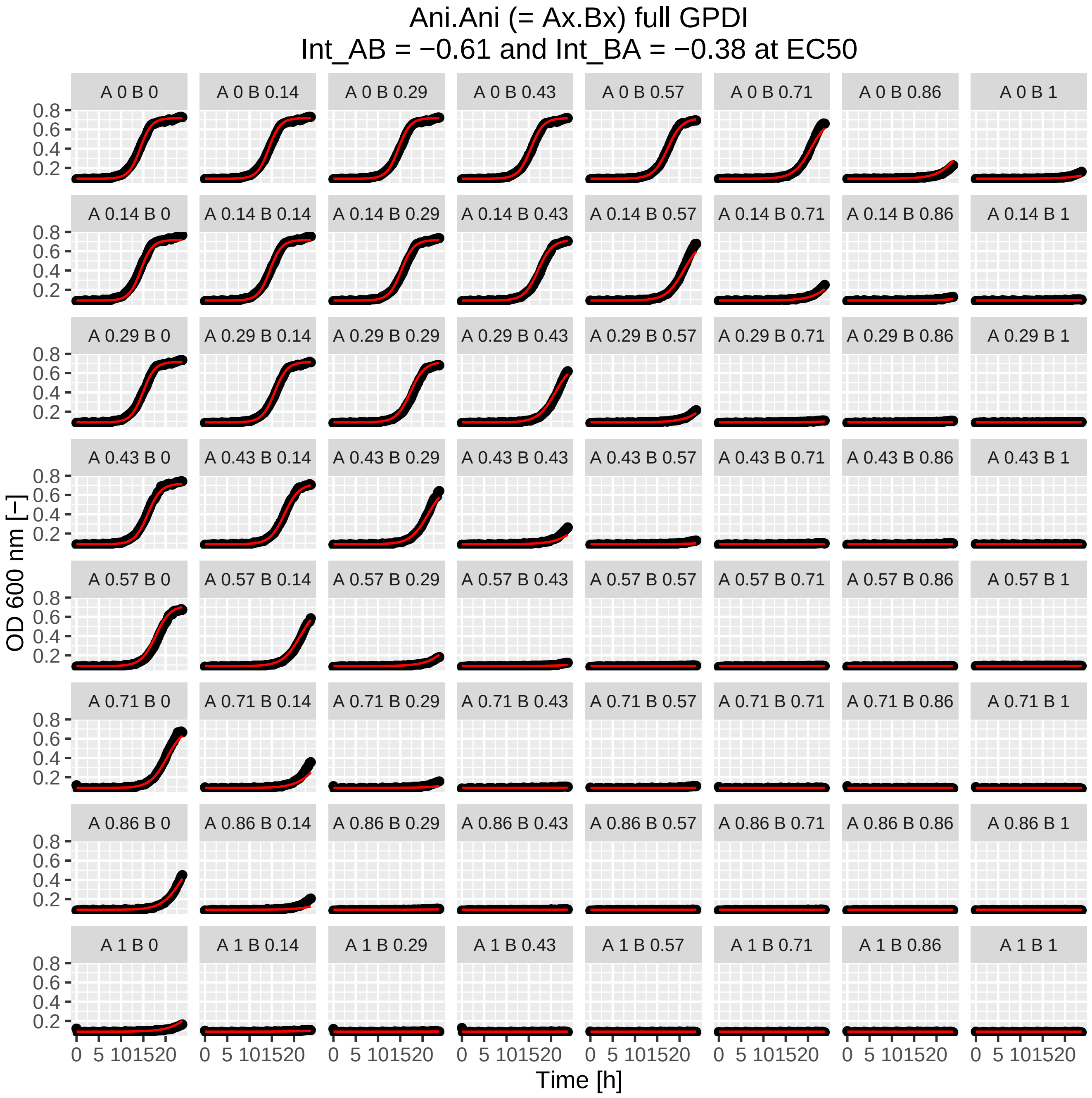
Ani.Ani (= Ax.Bx) full GPDI
Int_AB = −0.61 and Int_BA = −0.38 at EC50
A 0 B 0
A 0 B 0.14
A 0 B 0.29
A 0 B 0.43
A 0 B 0.57
A 0 B 0.71
A 0 B 0.86
A 0 B 1
A 0.14 B 0
A 0.14 B 0.14
A 0.14 B 0.29
A 0.14 B 0.43
A 0.14 B 0.57
A 0.14 B 0.71
A 0.14 B 0.86
A 0.14 B 1
A 0.29 B 0
A 0.29 B 0.14
A 0.29 B 0.29
A 0.29 B 0.43
A 0.29 B 0.57
A 0.29 B 0.71
A 0.29 B 0.86
A 0.29 B 1
A 0.43 B 0
A 0.43 B 0.14
A 0.43 B 0.29
A 0.43 B 0.43
A 0.43 B 0.57
A 0.43 B 0.71
A 0.43 B 0.86
A 0.43 B 1
A 0.57 B 0
A 0.57 B 0.14
A 0.57 B 0.29
A 0.57 B 0.43
A 0.57 B 0.57
A 0.57 B 0.71
A 0.57 B 0.86
A 0.57 B 1
A 0.71 B 0
A 0.71 B 0.14
A 0.71 B 0.29
A 0.71 B 0.43
A 0.71 B 0.57
A 0.71 B 0.71
A 0.71 B 0.86
A 0.71 B 1
A 0.86 B 0
A 0.86 B 0.14
A 0.86 B 0.29
A 0.86 B 0.43
A 0.86 B 0.57
A 0.86 B 0.71
A 0.86 B 0.86
A 0.86 B 1
A 1 B 0
A 1 B 0.14
A 1 B 0.29
A 1 B 0.43
A 1 B 0.57
A 1 B 0.71
A 1 B 0.86
A 1 B 1
OD 600 nm [−]
0.2
0.4
0.6
0.8
0
5
10
15
20
Time [h]

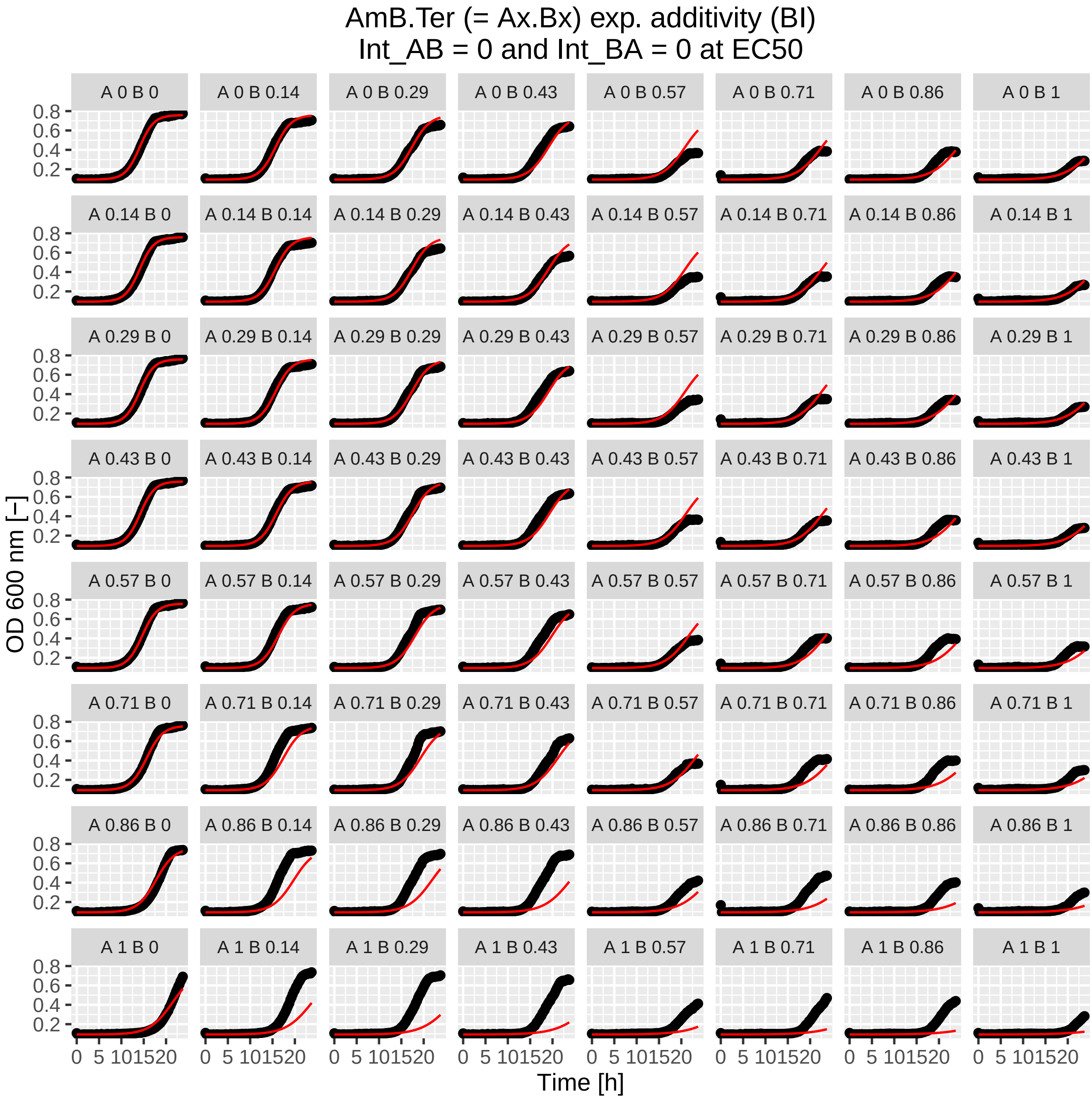
AmB.Ter (= Ax.Bx) exp. additivity (BI)
Int_AB = 0 and Int_BA = 0 at EC50
A 0 B 0
A 0 B 0.14
A 0 B 0.29
A 0 B 0.43
A 0 B 0.57
A 0 B 0.71
A 0 B 0.86
A 0 B 1
A 0.14 B 0
A 0.14 B 0.14
A 0.14 B 0.29
A 0.14 B 0.43
A 0.14 B 0.57
A 0.14 B 0.71
A 0.14 B 0.86
A 0.14 B 1
A 0.29 B 0
A 0.29 B 0.14
A 0.29 B 0.29
A 0.29 B 0.43
A 0.29 B 0.57
A 0.29 B 0.71
A 0.29 B 0.86
A 0.29 B 1
A 0.43 B 0
A 0.43 B 0.14
A 0.43 B 0.29
A 0.43 B 0.43
A 0.43 B 0.57
A 0.43 B 0.71
A 0.43 B 0.86
A 0.43 B 1
A 0.57 B 0
A 0.57 B 0.14
A 0.57 B 0.29
A 0.57 B 0.43
A 0.57 B 0.57
A 0.57 B 0.71
A 0.57 B 0.86
A 0.57 B 1
A 0.71 B 0
A 0.71 B 0.14
A 0.71 B 0.29
A 0.71 B 0.43
A 0.71 B 0.57
A 0.71 B 0.71
A 0.71 B 0.86
A 0.71 B 1
A 0.86 B 0
A 0.86 B 0.14
A 0.86 B 0.29
A 0.86 B 0.43
A 0.86 B 0.57
A 0.86 B 0.71
A 0.86 B 0.86
A 0.86 B 1
A 1 B 0
A 1 B 0.14
A 1 B 0.29
A 1 B 0.43
A 1 B 0.57
A 1 B 0.71
A 1 B 0.86
A 1 B 1
OD 600 nm [–]
0.2
0.4
0.6
0.8
0
5
10
15
20
Time [h]

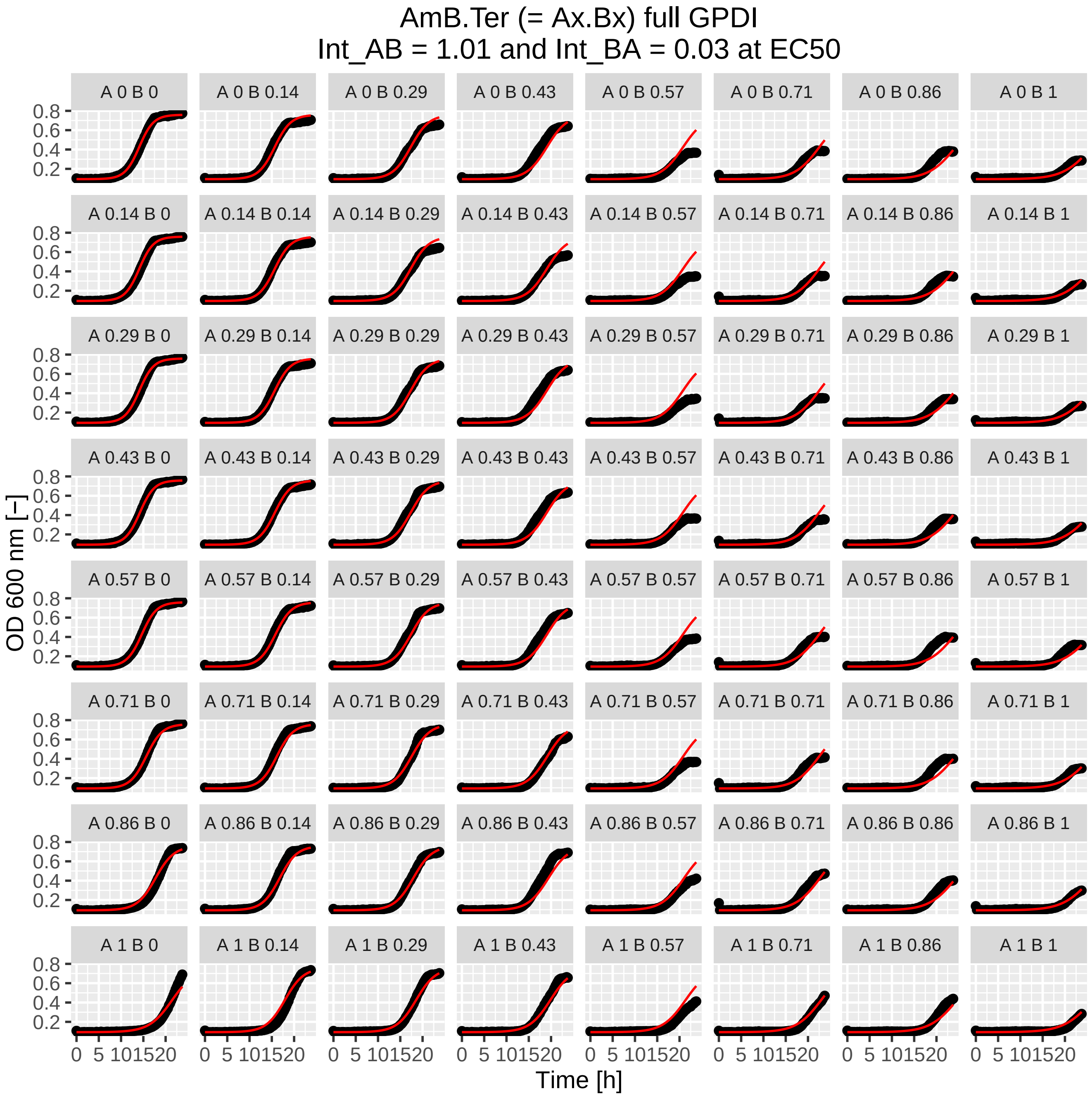
AmB.Ter (= Ax.Bx) full GPDI
Int_AB = 1.01 and Int_BA = 0.03 at EC50
A 0 B 0
A 0 B 0.14
A 0 B 0.29
A 0 B 0.43
A 0 B 0.57
A 0 B 0.71
A 0 B 0.86
A 0 B 1
A 0.14 B 0
A 0.14 B 0.14
A 0.14 B 0.29
A 0.14 B 0.43
A 0.14 B 0.57
A 0.14 B 0.71
A 0.14 B 0.86
A 0.14 B 1
A 0.29 B 0
A 0.29 B 0.14
A 0.29 B 0.29
A 0.29 B 0.43
A 0.29 B 0.57
A 0.29 B 0.71
A 0.29 B 0.86
A 0.29 B 1
A 0.43 B 0
A 0.43 B 0.14
A 0.43 B 0.29
A 0.43 B 0.43
A 0.43 B 0.57
A 0.43 B 0.71
A 0.43 B 0.86
A 0.43 B 1
A 0.57 B 0
A 0.57 B 0.14
A 0.57 B 0.29
A 0.57 B 0.43
A 0.57 B 0.57
A 0.57 B 0.71
A 0.57 B 0.86
A 0.57 B 1
A 0.71 B 0
A 0.71 B 0.14
A 0.71 B 0.29
A 0.71 B 0.43
A 0.71 B 0.57
A 0.71 B 0.71
A 0.71 B 0.86
A 0.71 B 1
A 0.86 B 0
A 0.86 B 0.14
A 0.86 B 0.29
A 0.86 B 0.43
A 0.86 B 0.57
A 0.86 B 0.71
A 0.86 B 0.86
A 0.86 B 1
A 1 B 0
A 1 B 0.14
A 1 B 0.29
A 1 B 0.43
A 1 B 0.57
A 1 B 0.71
A 1 B 0.86
A 1 B 1
OD 600 nm [−]
0.2 0.4 0.6 0.8
0 5 101520
Time [h]

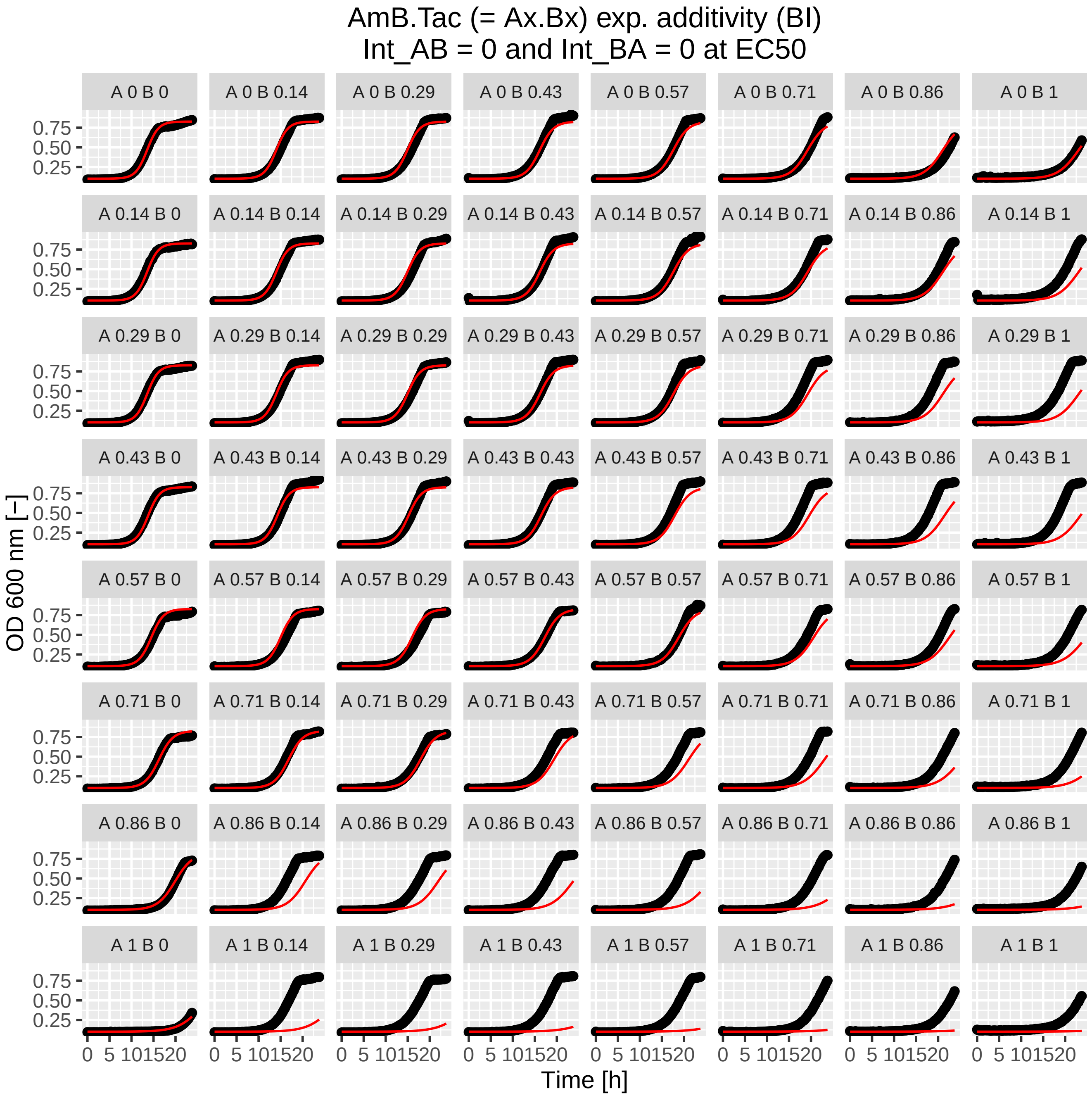

AmB.Tac (= Ax.Bx) exp. additivity (BI)
Int_AB = 0 and Int_BA = 0 at EC50
A 0 B 0
A 0 B 0.14
A 0 B 0.29
A 0 B 0.43
A 0 B 0.57
A 0 B 0.71
A 0 B 0.86
A 0 B 1
A 0.14 B 0
A 0.14 B 0.14
A 0.14 B 0.29
A 0.14 B 0.43
A 0.14 B 0.57
A 0.14 B 0.71
A 0.14 B 0.86
A 0.14 B 1
A 0.29 B 0
A 0.29 B 0.14
A 0.29 B 0.29
A 0.29 B 0.43
A 0.29 B 0.57
A 0.29 B 0.71
A 0.29 B 0.86
A 0.29 B 1
A 0.43 B 0
A 0.43 B 0.14
A 0.43 B 0.29
A 0.43 B 0.43
A 0.43 B 0.57
A 0.43 B 0.71
A 0.43 B 0.86
A 0.43 B 1
A 0.57 B 0
A 0.57 B 0.14
A 0.57 B 0.29
A 0.57 B 0.43
A 0.57 B 0.57
A 0.57 B 0.71
A 0.57 B 0.86
A 0.57 B 1
A 0.71 B 0
A 0.71 B 0.14
A 0.71 B 0.29
A 0.71 B 0.43
A 0.71 B 0.57
A 0.71 B 0.71
A 0.71 B 0.86
A 0.71 B 1
A 0.86 B 0
A 0.86 B 0.14
A 0.86 B 0.29
A 0.86 B 0.43
A 0.86 B 0.57
A 0.86 B 0.71
A 0.86 B 0.86
A 0.86 B 1
A 1 B 0
A 1 B 0.14
A 1 B 0.29
A 1 B 0.43
A 1 B 0.57
A 1 B 0.71
A 1 B 0.86
A 1 B 1
0.75
0.50
0.25
0 5 101520
OD 600 nm [–]
Time [h]

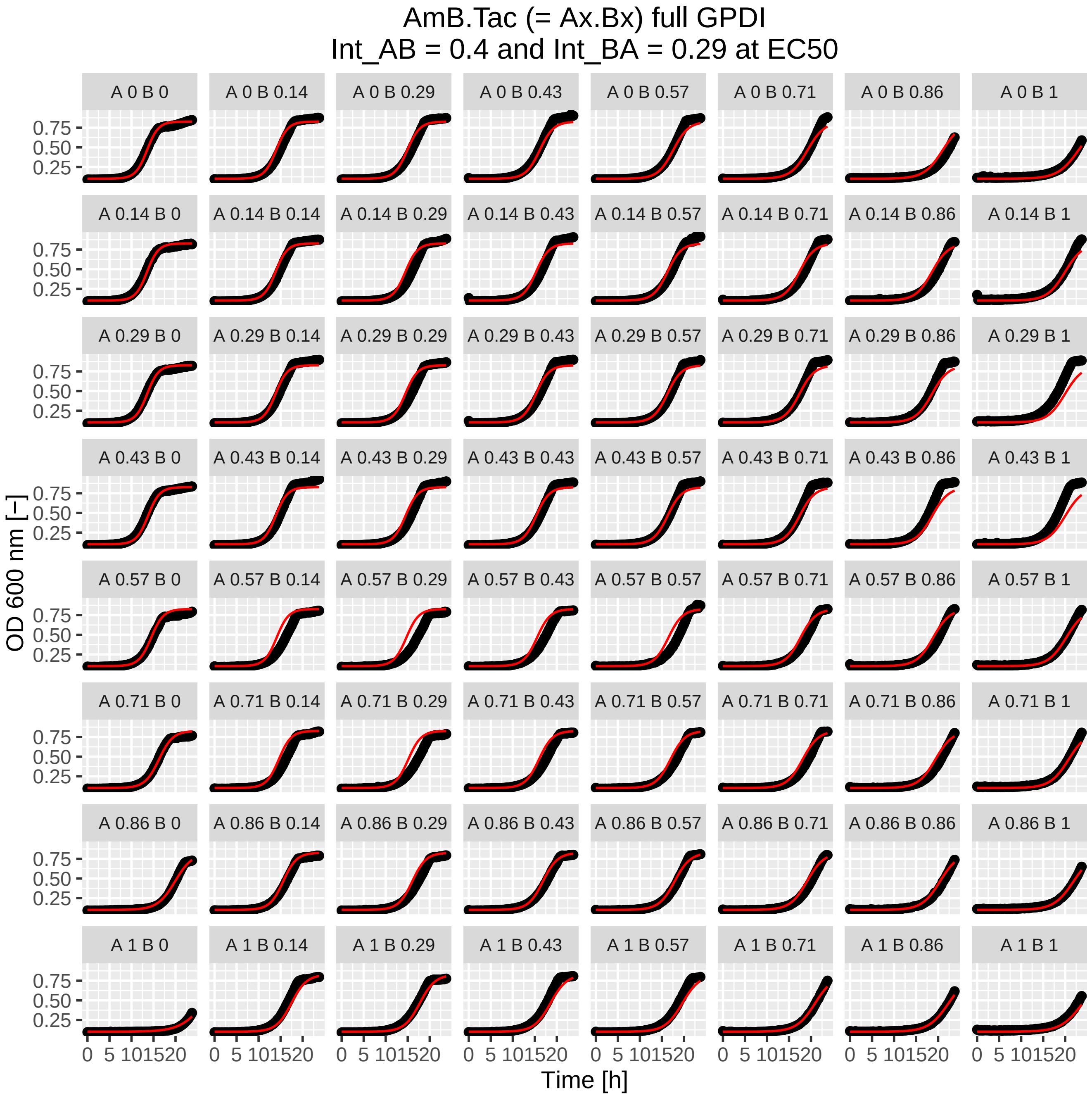
AmB.Tac (= Ax.Bx) full GPDI
Int_AB = 0.4 and Int_BA = 0.29 at EC50
OD 600 nm [–]
Time [h]
0.25
0.50
0.75
0 5 10 15 20
A 0 B 0
A 0 B 0.14
A 0 B 0.29
A 0 B 0.43
A 0 B 0.57
A 0 B 0.71
A 0 B 0.86
A 0 B 1
A 0.14 B 0
A 0.14 B 0.14
A 0.14 B 0.29
A 0.14 B 0.43
A 0.14 B 0.57
A 0.14 B 0.71
A 0.14 B 0.86
A 0.14 B 1
A 0.29 B 0
A 0.29 B 0.14
A 0.29 B 0.29
A 0.29 B 0.43
A 0.29 B 0.57
A 0.29 B 0.71
A 0.29 B 0.86
A 0.29 B 1
A 0.43 B 0
A 0.43 B 0.14
A 0.43 B 0.29
A 0.43 B 0.43
A 0.43 B 0.57
A 0.43 B 0.71
A 0.43 B 0.86
A 0.43 B 1
A 0.57 B 0
A 0.57 B 0.14
A 0.57 B 0.29
A 0.57 B 0.43
A 0.57 B 0.57
A 0.57 B 0.71
A 0.57 B 0.86
A 0.57 B 1
A 0.71 B 0
A 0.71 B 0.14
A 0.71 B 0.29
A 0.71 B 0.43
A 0.71 B 0.57
A 0.71 B 0.71
A 0.71 B 0.86
A 0.71 B 1
A 0.86 B 0
A 0.86 B 0.14
A 0.86 B 0.29
A 0.86 B 0.43
A 0.86 B 0.57
A 0.86 B 0.71
A 0.86 B 0.86
A 0.86 B 1
A 1 B 0
A 1 B 0.14
A 1 B 0.29
A 1 B 0.43
A 1 B 0.57
A 1 B 0.71
A 1 B 0.86
A 1 B 1

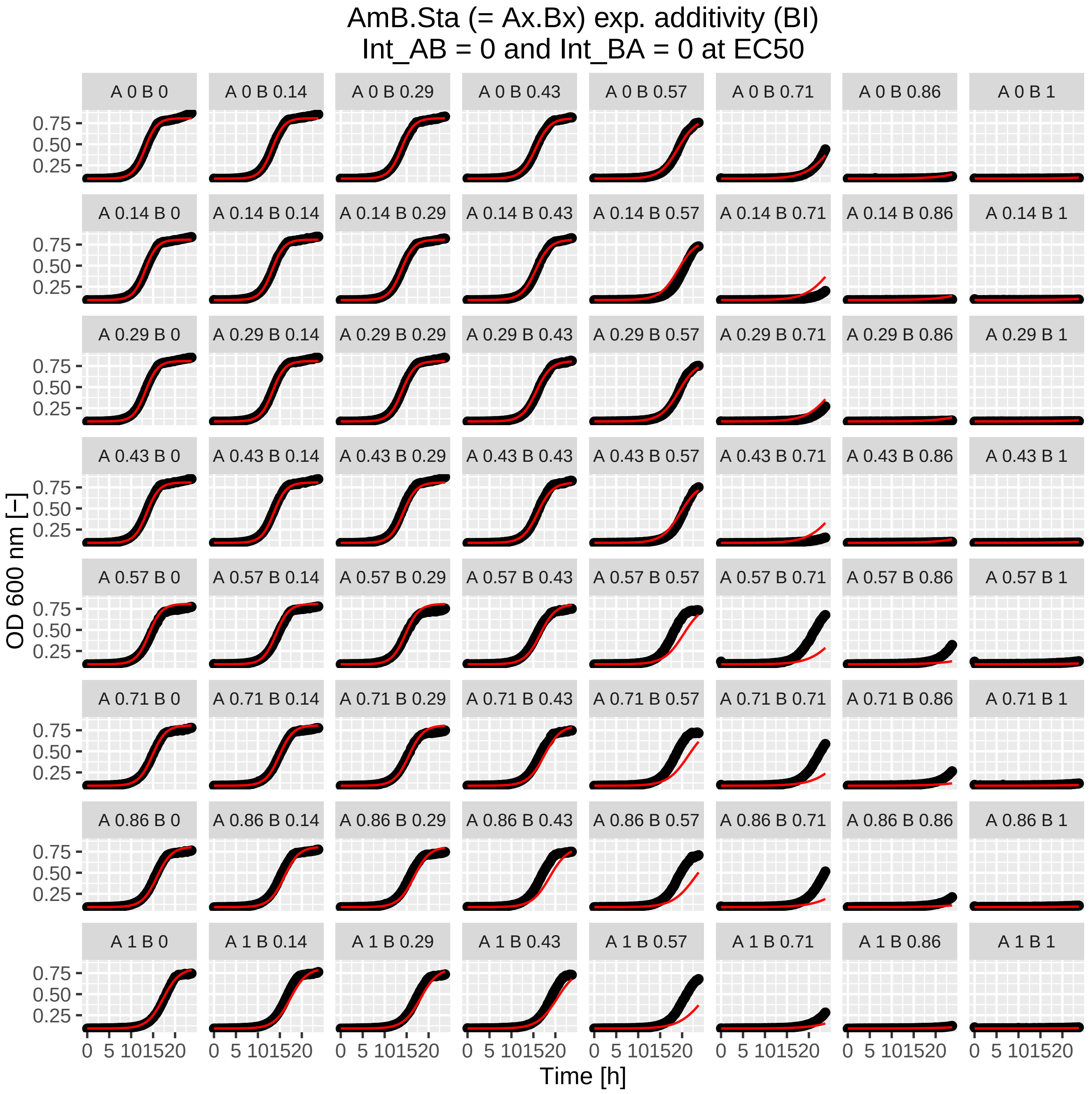
AmB.Sta (= Ax.Bx) exp. additivity (BI)
Int_AB = 0 and Int_BA = 0 at EC50
A 0 B 0
A 0 B 0.14
A 0 B 0.29
A 0 B 0.43
A 0 B 0.57
A 0 B 0.71
A 0 B 0.86
A 0 B 1
A 0.14 B 0
A 0.14 B 0.14
A 0.14 B 0.29
A 0.14 B 0.43
A 0.14 B 0.57
A 0.14 B 0.71
A 0.14 B 0.86
A 0.14 B 1
A 0.29 B 0
A 0.29 B 0.14
A 0.29 B 0.29
A 0.29 B 0.43
A 0.29 B 0.57
A 0.29 B 0.71
A 0.29 B 0.86
A 0.29 B 1
A 0.43 B 0
A 0.43 B 0.14
A 0.43 B 0.29
A 0.43 B 0.43
A 0.43 B 0.57
A 0.43 B 0.71
A 0.43 B 0.86
A 0.43 B 1
A 0.57 B 0
A 0.57 B 0.14
A 0.57 B 0.29
A 0.57 B 0.43
A 0.57 B 0.57
A 0.57 B 0.71
A 0.57 B 0.86
A 0.57 B 1
A 0.71 B 0
A 0.71 B 0.14
A 0.71 B 0.29
A 0.71 B 0.43
A 0.71 B 0.57
A 0.71 B 0.71
A 0.71 B 0.86
A 0.71 B 1
A 0.86 B 0
A 0.86 B 0.14
A 0.86 B 0.29
A 0.86 B 0.43
A 0.86 B 0.57
A 0.86 B 0.71
A 0.86 B 0.86
A 0.86 B 1
A 1 B 0
A 1 B 0.14
A 1 B 0.29
A 1 B 0.43
A 1 B 0.57
A 1 B 0.71
A 1 B 0.86
A 1 B 1
OD 600 nm [–]
0.75
0.50
0.25
0 5 101520
Time [h]

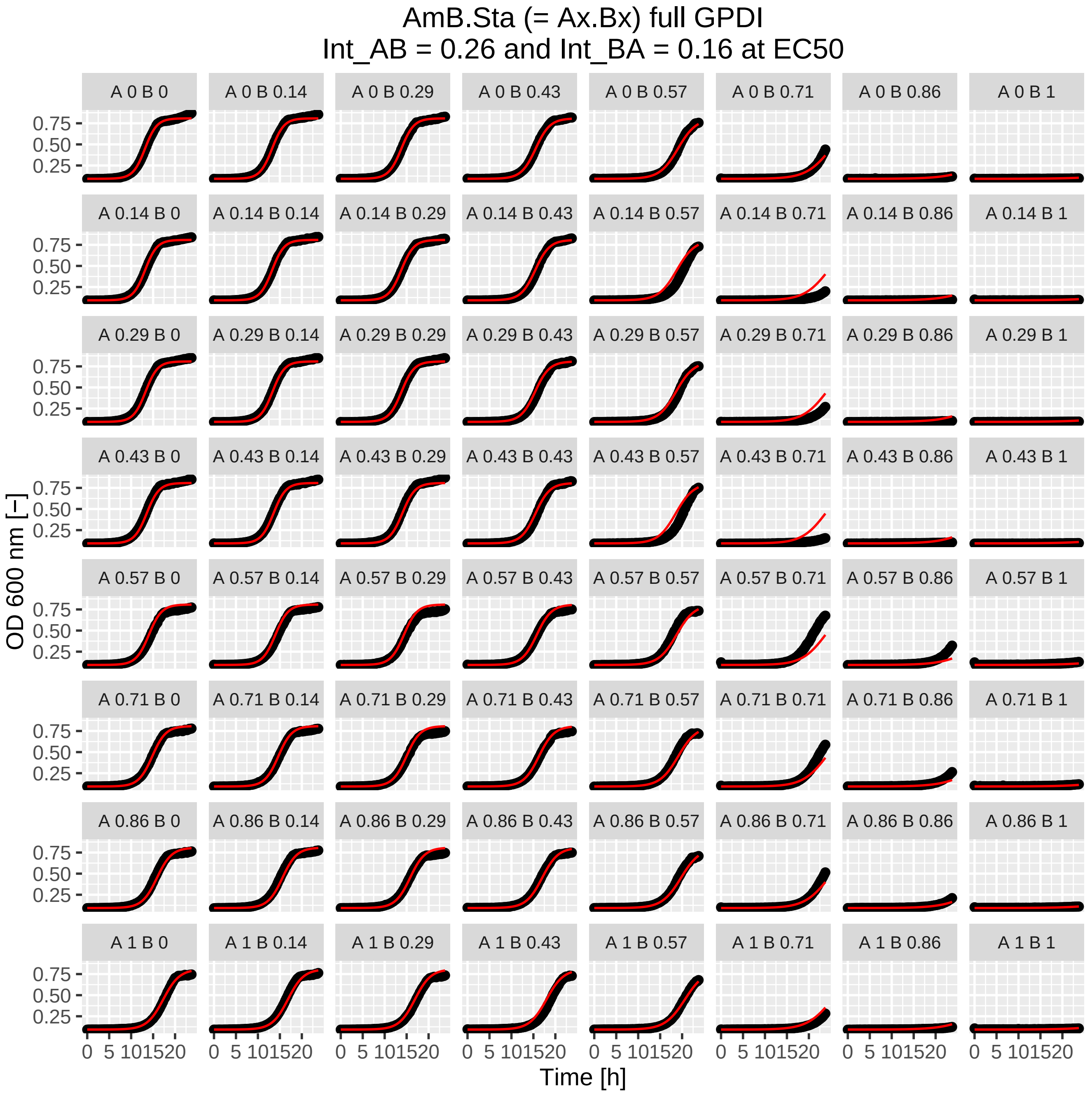

AmB.Sta (= Ax.Bx) full GPDI
Int_AB = 0.26 and Int_BA = 0.16 at EC50
A 0 B 0
A 0 B 0.14
A 0 B 0.29
A 0 B 0.43
A 0 B 0.57
A 0 B 0.71
A 0 B 0.86
A 0 B 1
A 0.14 B 0
A 0.14 B 0.14
A 0.14 B 0.29
A 0.14 B 0.43
A 0.14 B 0.57
A 0.14 B 0.71
A 0.14 B 0.86
A 0.14 B 1
A 0.29 B 0
A 0.29 B 0.14
A 0.29 B 0.29
A 0.29 B 0.43
A 0.29 B 0.57
A 0.29 B 0.71
A 0.29 B 0.86
A 0.29 B 1
A 0.43 B 0
A 0.43 B 0.14
A 0.43 B 0.29
A 0.43 B 0.43
A 0.43 B 0.57
A 0.43 B 0.71
A 0.43 B 0.86
A 0.43 B 1
A 0.57 B 0
A 0.57 B 0.14
A 0.57 B 0.29
A 0.57 B 0.43
A 0.57 B 0.57
A 0.57 B 0.71
A 0.57 B 0.86
A 0.57 B 1
A 0.71 B 0
A 0.71 B 0.14
A 0.71 B 0.29
A 0.71 B 0.43
A 0.71 B 0.57
A 0.71 B 0.71
A 0.71 B 0.86
A 0.71 B 1
A 0.86 B 0
A 0.86 B 0.14
A 0.86 B 0.29
A 0.86 B 0.43
A 0.86 B 0.57
A 0.86 B 0.71
A 0.86 B 0.86
A 0.86 B 1
A 1 B 0
A 1 B 0.14
A 1 B 0.29
A 1 B 0.43
A 1 B 0.57
A 1 B 0.71
A 1 B 0.86
A 1 B 1
OD 600 nm [−]
0.75
0.50
0.25
0 5 101520
Time [h]

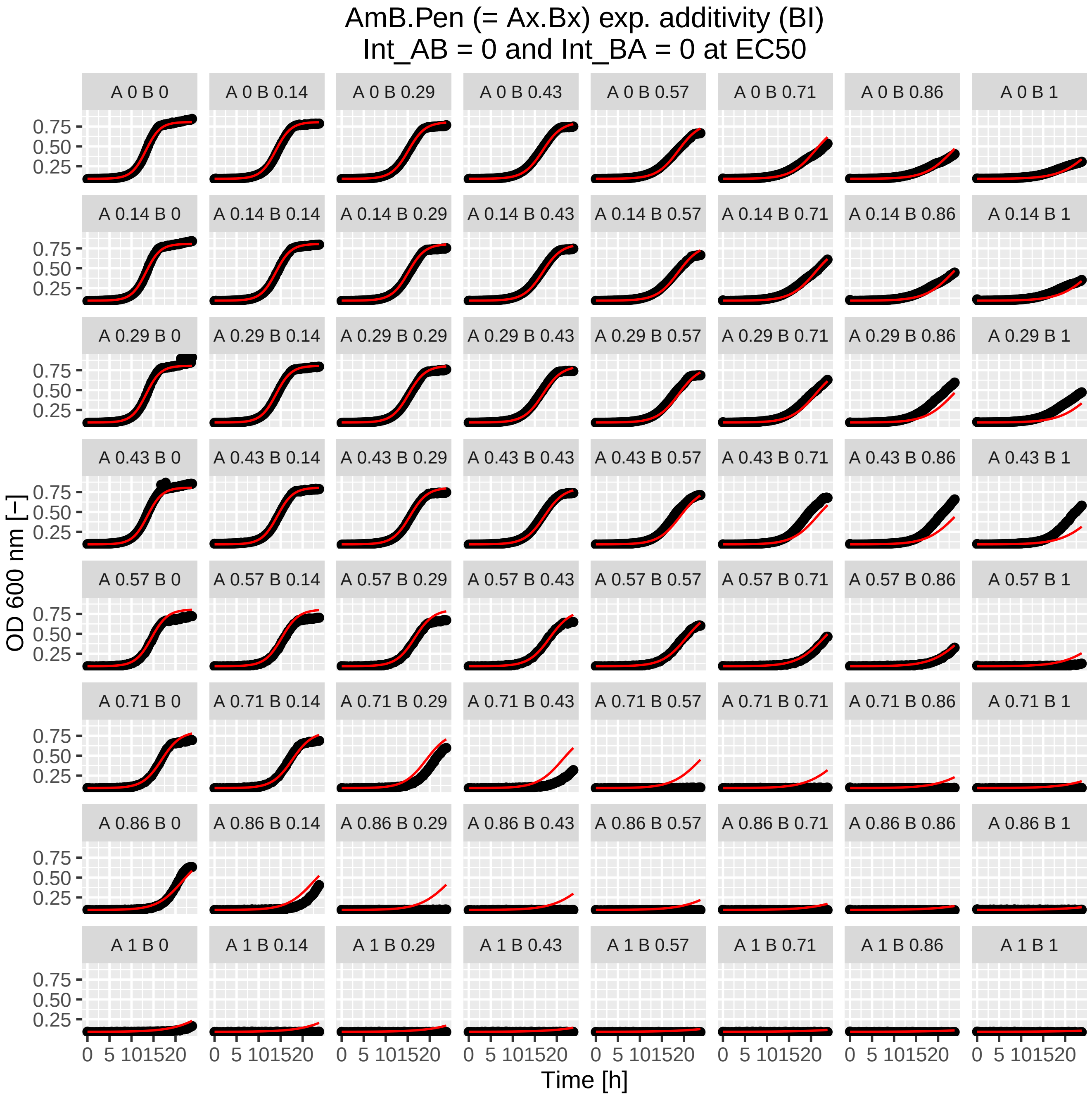
AmB.Pen (= Ax.Bx) exp. additivity (BI)
Int_AB = 0 and Int_BA = 0 at EC50
OD 600 nm [–]
Time [h]
0.75
0.50
0.25
0 5 101520
A 0 B 0
A 0 B 0.14
A 0 B 0.29
A 0 B 0.43
A 0 B 0.57
A 0 B 0.71
A 0 B 0.86
A 0 B 1
A 0.14 B 0
A 0.14 B 0.14
A 0.14 B 0.29
A 0.14 B 0.43
A 0.14 B 0.57
A 0.14 B 0.71
A 0.14 B 0.86
A 0.14 B 1
A 0.29 B 0
A 0.29 B 0.14
A 0.29 B 0.29
A 0.29 B 0.43
A 0.29 B 0.57
A 0.29 B 0.71
A 0.29 B 0.86
A 0.29 B 1
A 0.43 B 0
A 0.43 B 0.14
A 0.43 B 0.29
A 0.43 B 0.43
A 0.43 B 0.57
A 0.43 B 0.71
A 0.43 B 0.86
A 0.43 B 1
A 0.57 B 0
A 0.57 B 0.14
A 0.57 B 0.29
A 0.57 B 0.43
A 0.57 B 0.57
A 0.57 B 0.71
A 0.57 B 0.86
A 0.57 B 1
A 0.71 B 0
A 0.71 B 0.14
A 0.71 B 0.29
A 0.71 B 0.43
A 0.71 B 0.57
A 0.71 B 0.71
A 0.71 B 0.86
A 0.71 B 1
A 0.86 B 0
A 0.86 B 0.14
A 0.86 B 0.29
A 0.86 B 0.43
A 0.86 B 0.57
A 0.86 B 0.71
A 0.86 B 0.86
A 0.86 B 1
A 1 B 0
A 1 B 0.14
A 1 B 0.29
A 1 B 0.43
A 1 B 0.57
A 1 B 0.71
A 1 B 0.86
A 1 B 1

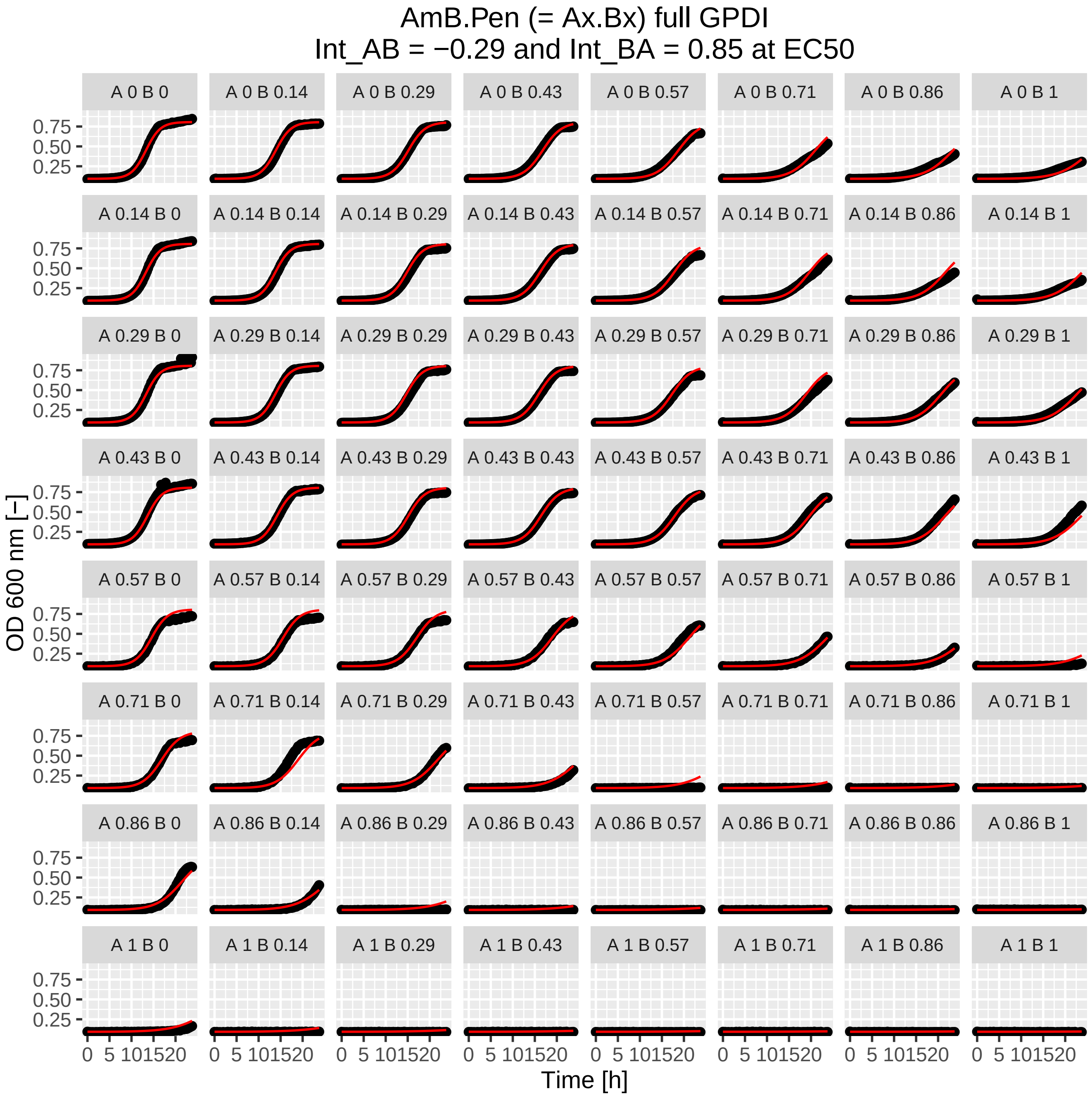
AmB.Pen (= Ax.Bx) full GPDI
Int_AB = −0.29 and Int_BA = 0.85 at EC50
A 0 B 0
A 0 B 0.14
A 0 B 0.29
A 0 B 0.43
A 0 B 0.57
A 0 B 0.71
A 0 B 0.86
A 0 B 1
A 0.14 B 0
A 0.14 B 0.14
A 0.14 B 0.29
A 0.14 B 0.43
A 0.14 B 0.57
A 0.14 B 0.71
A 0.14 B 0.86
A 0.14 B 1
A 0.29 B 0
A 0.29 B 0.14
A 0.29 B 0.29
A 0.29 B 0.43
A 0.29 B 0.57
A 0.29 B 0.71
A 0.29 B 0.86
A 0.29 B 1
A 0.43 B 0
A 0.43 B 0.14
A 0.43 B 0.29
A 0.43 B 0.43
A 0.43 B 0.57
A 0.43 B 0.71
A 0.43 B 0.86
A 0.43 B 1
A 0.57 B 0
A 0.57 B 0.14
A 0.57 B 0.29
A 0.57 B 0.43
A 0.57 B 0.57
A 0.57 B 0.71
A 0.57 B 0.86
A 0.57 B 1
A 0.71 B 0
A 0.71 B 0.14
A 0.71 B 0.29
A 0.71 B 0.43
A 0.71 B 0.57
A 0.71 B 0.71
A 0.71 B 0.86
A 0.71 B 1
A 0.86 B 0
A 0.86 B 0.14
A 0.86 B 0.29
A 0.86 B 0.43
A 0.86 B 0.57
A 0.86 B 0.71
A 0.86 B 0.86
A 0.86 B 1
A 1 B 0
A 1 B 0.14
A 1 B 0.29
A 1 B 0.43
A 1 B 0.57
A 1 B 0.71
A 1 B 0.86
A 1 B 1
OD 600 nm [−]
0.75
0.50
0.25
0 5 101520
Time [h]

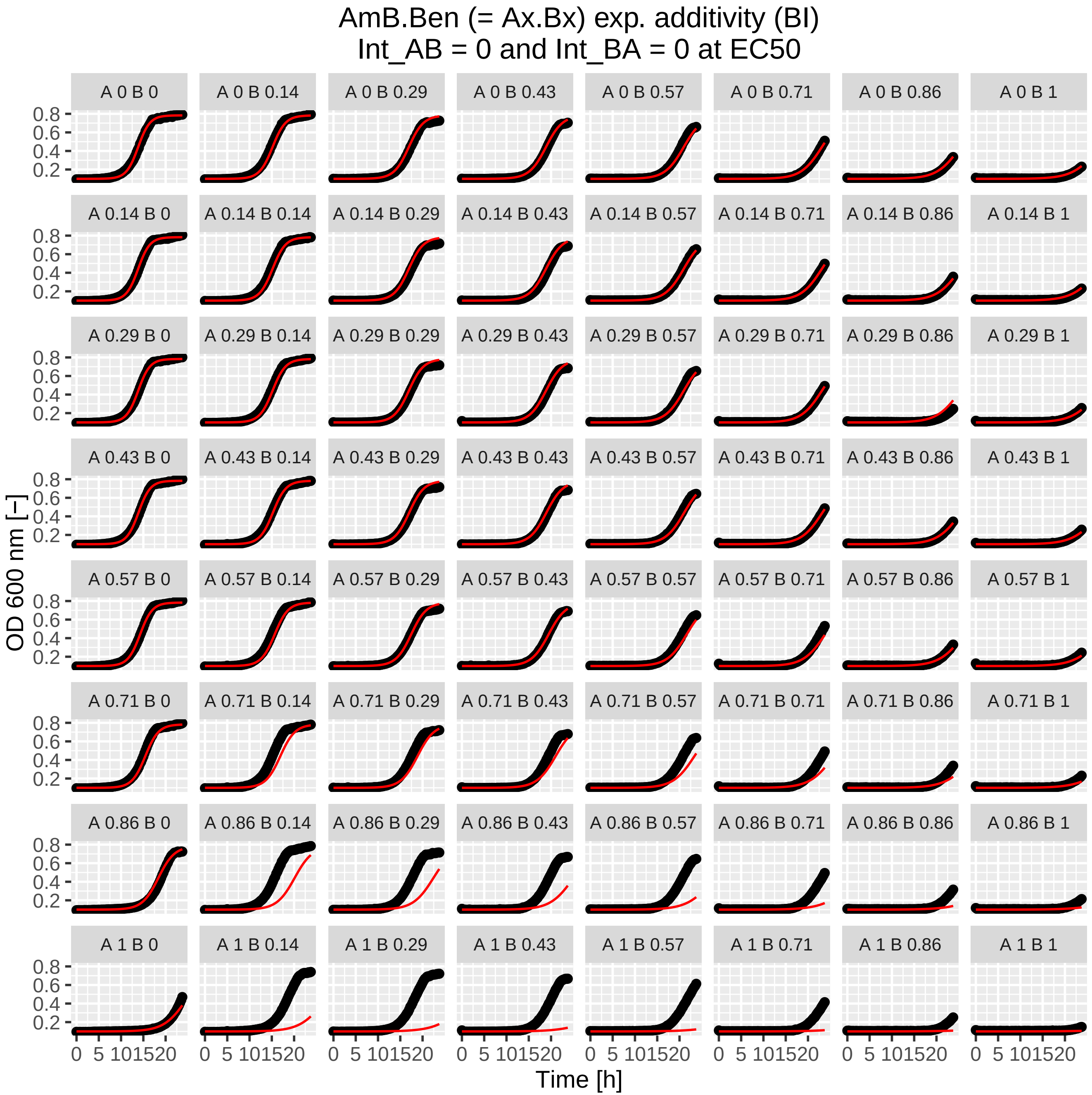
AmB.Ben (= Ax.Bx) exp. additivity (BI)
Int_AB = 0 and Int_BA = 0 at EC50
OD 600 nm [–]
Time [h]
A 0 B 0
A 0 B 0.14
A 0 B 0.29
A 0 B 0.43
A 0 B 0.57
A 0 B 0.71
A 0 B 0.86
A 0 B 1
A 1 B 0
A 1 B 1

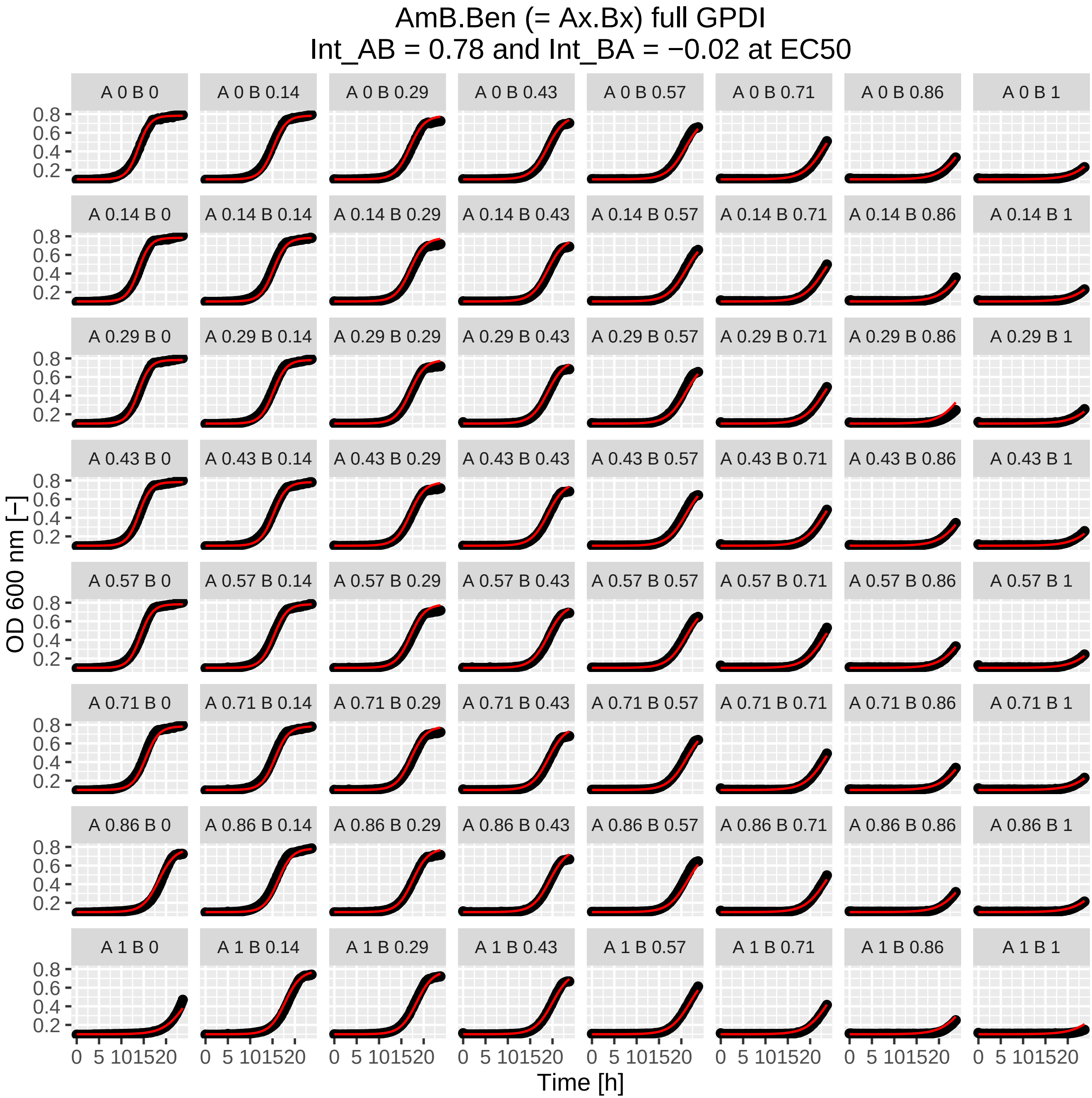
AmB.Ben (= Ax.Bx) full GPDI
Int_AB = 0.78 and Int_BA = −0.02 at EC50
A 0 B 0
A 0 B 0.14
A 0 B 0.29
A 0 B 0.43
A 0 B 0.57
A 0 B 0.71
A 0 B 0.86
A 0 B 1
A 0.14 B 0
A 0.14 B 0.14
A 0.14 B 0.29
A 0.14 B 0.43
A 0.14 B 0.57
A 0.14 B 0.71
A 0.14 B 0.86
A 0.14 B 1
A 0.29 B 0
A 0.29 B 0.14
A 0.29 B 0.29
A 0.29 B 0.43
A 0.29 B 0.57
A 0.29 B 0.71
A 0.29 B 0.86
A 0.29 B 1
A 0.43 B 0
A 0.43 B 0.14
A 0.43 B 0.29
A 0.43 B 0.43
A 0.43 B 0.57
A 0.43 B 0.71
A 0.43 B 0.86
A 0.43 B 1
A 0.57 B 0
A 0.57 B 0.14
A 0.57 B 0.29
A 0.57 B 0.43
A 0.57 B 0.57
A 0.57 B 0.71
A 0.57 B 0.86
A 0.57 B 1
A 0.71 B 0
A 0.71 B 0.14
A 0.71 B 0.29
A 0.71 B 0.43
A 0.71 B 0.57
A 0.71 B 0.71
A 0.71 B 0.86
A 0.71 B 1
A 0.86 B 0
A 0.86 B 0.14
A 0.86 B 0.29
A 0.86 B 0.43
A 0.86 B 0.57
A 0.86 B 0.71
A 0.86 B 0.86
A 0.86 B 1
A 1 B 0
A 1 B 0.14
A 1 B 0.29
A 1 B 0.43
A 1 B 0.57
A 1 B 0.71
A 1 B 0.86
A 1 B 1
OD 600 nm [−]
0.8
0.6
0.4
0.2
0 5 101520
Time [h]

AmB.AmB (= Ax.Bx) exp. additivity (BI)
Int_AB = 0 and Int_BA = 0 at EC50

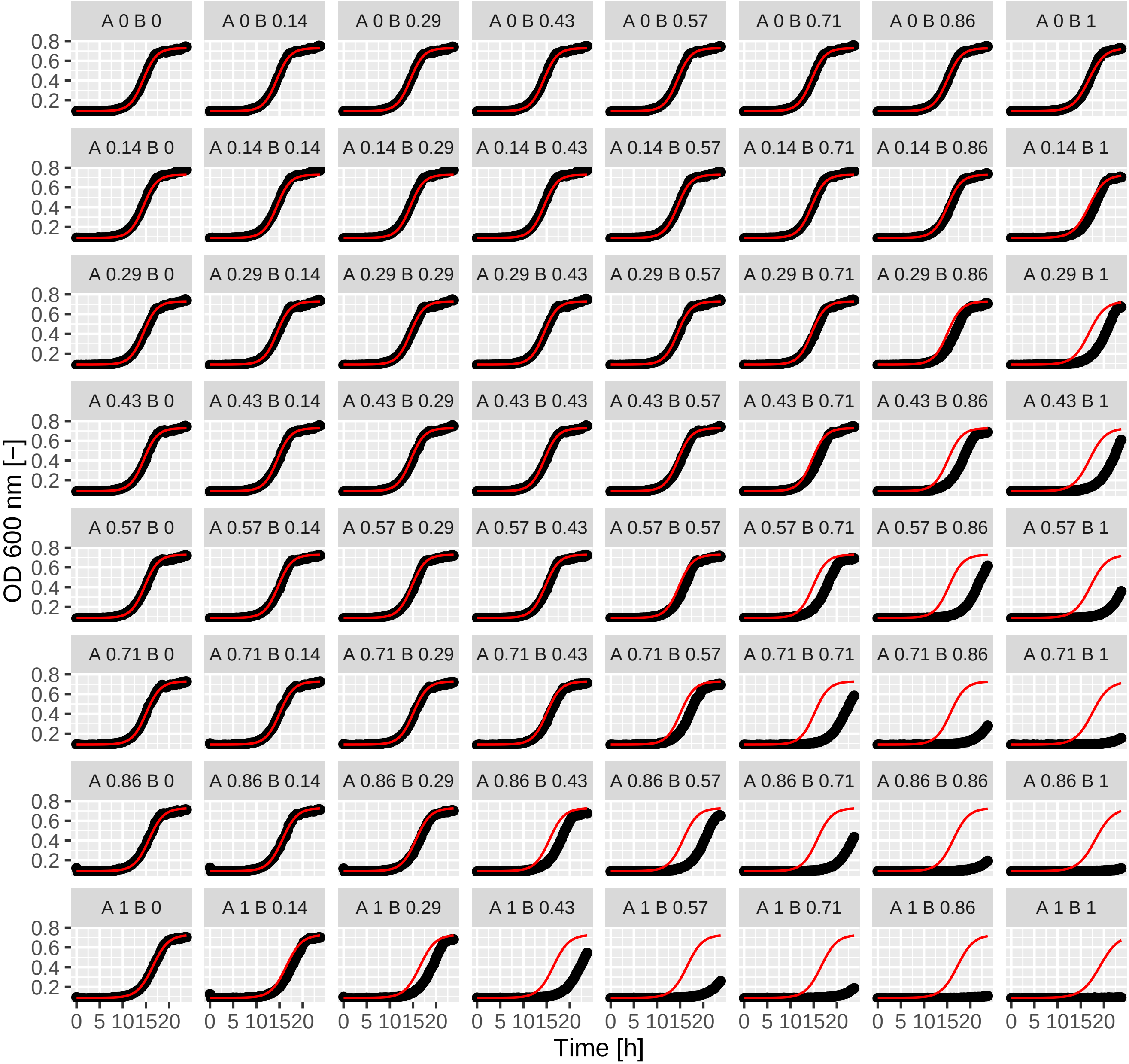

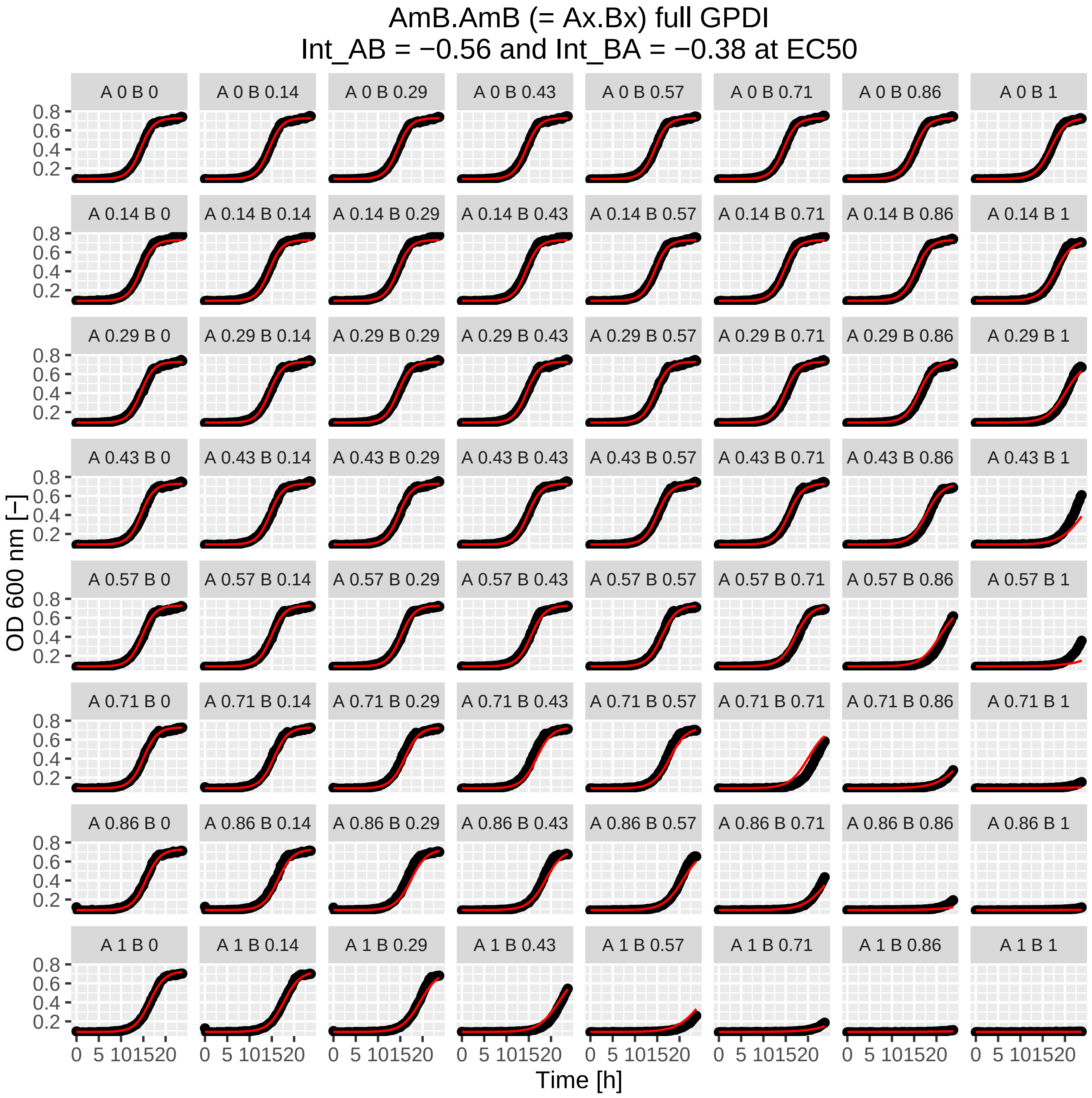
AmB.AmB (= Ax.Bx) full GPDI
Int_AB = −0.56 and Int_BA = −0.38 at EC50
OD 600 nm [−]
Time [h]
A 0 B 0
A 0 B 0.14
A 0 B 0.29
A 0 B 0.43
A 0 B 0.57
A 0 B 0.71
A 0 B 0.86
A 0 B 1
A 0.14 B 0
A 0.14 B 0.14
A 0.14 B 0.29
A 0.14 B 0.43
A 0.14 B 0.57
A 0.14 B 0.71
A 0.14 B 0.86
A 0.14 B 1
A 0.29 B 0
A 0.29 B 0.14
A 0.29 B 0.29
A 0.29 B 0.43
A 0.29 B 0.57
A 0.29 B 0.71
A 0.29 B 0.86
A 0.29 B 1
A 0.43 B 0
A 0.43 B 0.14
A 0.43 B 0.29
A 0.43 B 0.43
A 0.43 B 0.57
A 0.43 B 0.71
A 0.43 B 0.86
A 0.43 B 1
A 0.57 B 0
A 0.57 B 0.14
A 0.57 B 0.29
A 0.57 B 0.43
A 0.57 B 0.57
A 0.57 B 0.71
A 0.57 B 0.86
A 0.57 B 1
A 0.71 B 0
A 0.71 B 0.14
A 0.71 B 0.29
A 0.71 B 0.43
A 0.71 B 0.57
A 0.71 B 0.71
A 0.71 B 0.86
A 0.71 B 1
A 0.86 B 0
A 0.86 B 0.14
A 0.86 B 0.29
A 0.86 B 0.43
A 0.86 B 0.57
A 0.86 B 0.71
A 0.86 B 0.86
A 0.86 B 1
A 1 B 0
A 1 B 0.14
A 1 B 0.29
A 1 B 0.43
A 1 B 0.57
A 1 B 0.71
A 1 B 0.86
A 1 B 1
0.8
0.6
0.4
0.2
0 5 101520

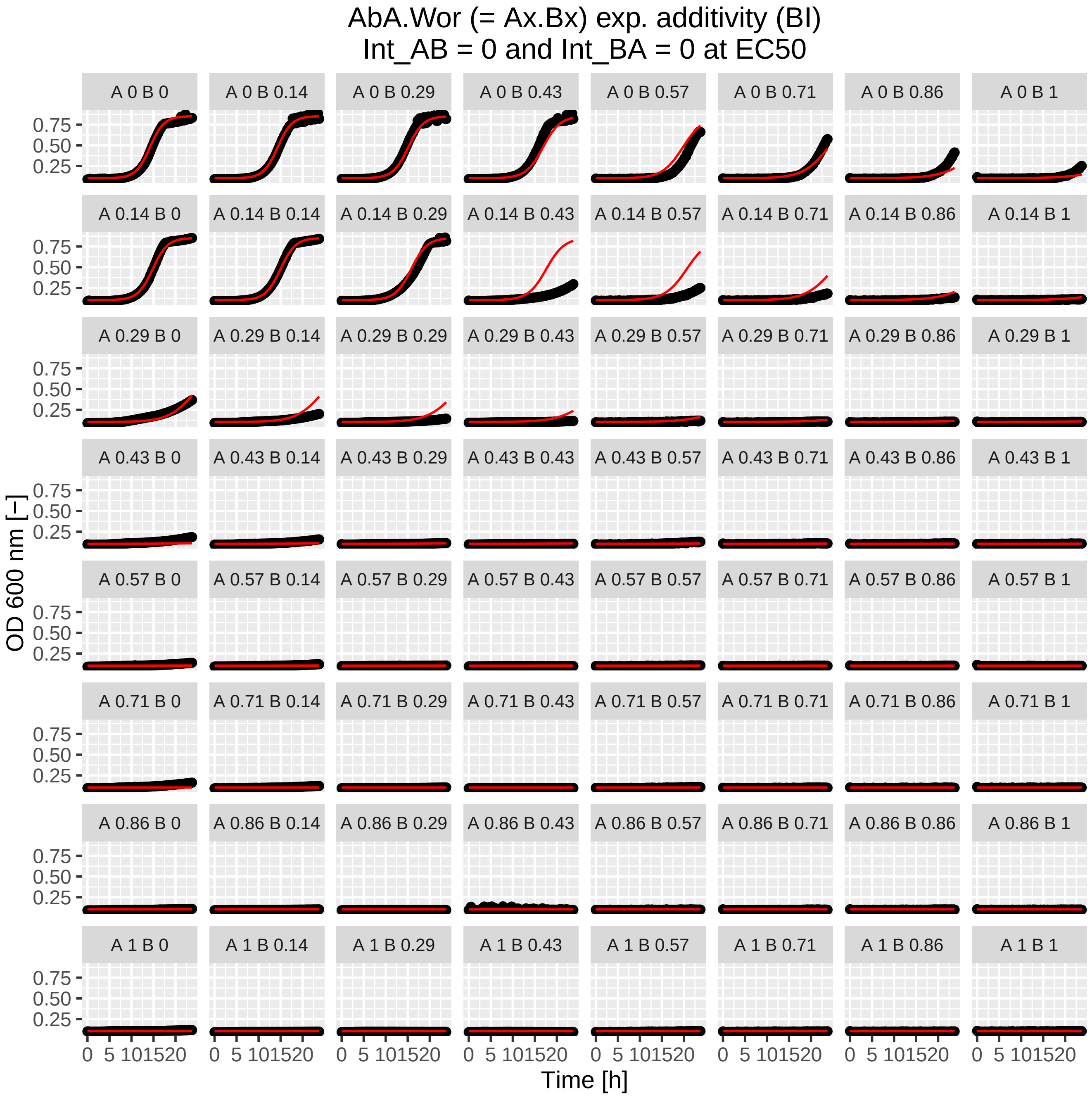
AbA.Wor (= Ax.Bx) exp. additivity (BI)
Int_AB = 0 and Int_BA = 0 at EC50
OD 600 nm [–]
Time [h]
0.75
0.50
0.25
0 5 101520
A 0 B 0
A 0 B 0.14
A 0 B 0.29
A 0 B 0.43
A 0 B 0.57
A 0 B 0.71
A 0 B 0.86
A 0 B 1
A 0.14 B 0
A 0.14 B 0.14
A 0.14 B 0.29
A 0.14 B 0.43
A 0.14 B 0.57
A 0.14 B 0.71
A 0.14 B 0.86
A 0.14 B 1
A 0.29 B 0
A 0.29 B 0.14
A 0.29 B 0.29
A 0.29 B 0.43
A 0.29 B 0.57
A 0.29 B 0.71
A 0.29 B 0.86
A 0.29 B 1
A 0.43 B 0
A 0.43 B 0.14
A 0.43 B 0.29
A 0.43 B 0.43
A 0.43 B 0.57
A 0.43 B 0.71
A 0.43 B 0.86
A 0.43 B 1
A 0.57 B 0
A 0.57 B 0.14
A 0.57 B 0.29
A 0.57 B 0.43
A 0.57 B 0.57
A 0.57 B 0.71
A 0.57 B 0.86
A 0.57 B 1
A 0.71 B 0
A 0.71 B 0.14
A 0.71 B 0.29
A 0.71 B 0.43
A 0.71 B 0.57
A 0.71 B 0.71
A 0.71 B 0.86
A 0.71 B 1
A 0.86 B 0
A 0.86 B 0.14
A 0.86 B 0.29
A 0.86 B 0.43
A 0.86 B 0.57
A 0.86 B 0.71
A 0.86 B 0.86
A 0.86 B 1
A 1 B 0
A 1 B 0.14
A 1 B 0.29
A 1 B 0.43
A 1 B 0.57
A 1 B 0.71
A 1 B 0.86
A 1 B 1

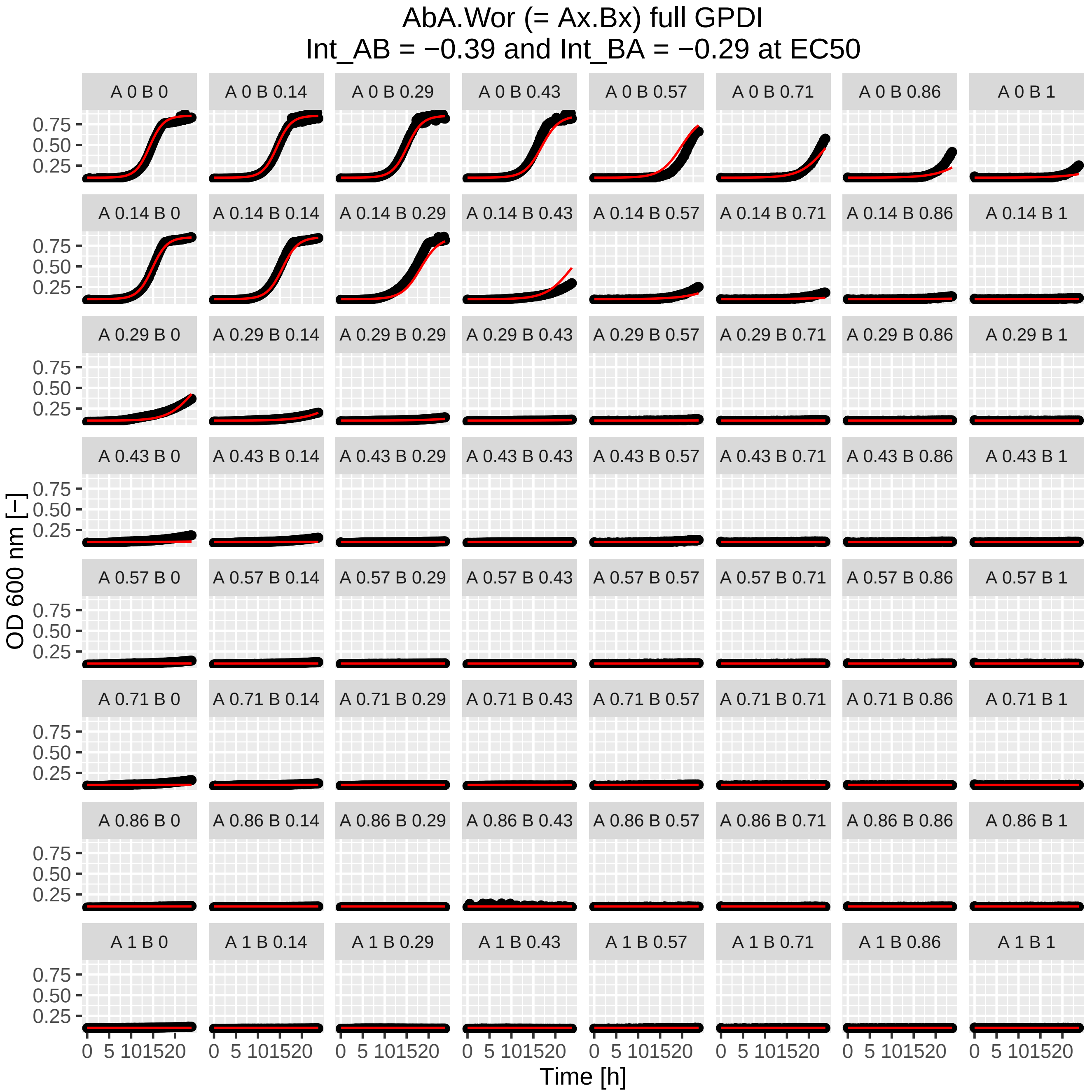
AbA.Wor (= Ax.Bx) full GPDI
Int_AB = −0.39 and Int_BA = −0.29 at EC50
A 0 B 0
A 0 B 0.14
A 0 B 0.29
A 0 B 0.43
A 0 B 0.57
A 0 B 0.71
A 0 B 0.86
A 0 B 1
A 0.14 B 0
A 0.14 B 0.14
A 0.14 B 0.29
A 0.14 B 0.43
A 0.14 B 0.57
A 0.14 B 0.71
A 0.14 B 0.86
A 0.14 B 1
A 0.29 B 0
A 0.29 B 0.14
A 0.29 B 0.29
A 0.29 B 0.43
A 0.29 B 0.57
A 0.29 B 0.71
A 0.29 B 0.86
A 0.29 B 1
A 0.43 B 0
A 0.43 B 0.14
A 0.43 B 0.29
A 0.43 B 0.43
A 0.43 B 0.57
A 0.43 B 0.71
A 0.43 B 0.86
A 0.43 B 1
A 0.57 B 0
A 0.57 B 0.14
A 0.57 B 0.29
A 0.57 B 0.43
A 0.57 B 0.57
A 0.57 B 0.71
A 0.57 B 0.86
A 0.57 B 1
A 0.71 B 0
A 0.71 B 0.14
A 0.71 B 0.29
A 0.71 B 0.43
A 0.71 B 0.57
A 0.71 B 0.71
A 0.71 B 0.86
A 0.71 B 1
A 0.86 B 0
A 0.86 B 0.14
A 0.86 B 0.29
A 0.86 B 0.43
A 0.86 B 0.57
A 0.86 B 0.71
A 0.86 B 0.86
A 0.86 B 1
A 1 B 0
A 1 B 0.14
A 1 B 0.29
A 1 B 0.43
A 1 B 0.57
A 1 B 0.71
A 1 B 0.86
A 1 B 1
OD 600 nm [−]
0.75
0.50
0.25
0 5 101520
Time [h]

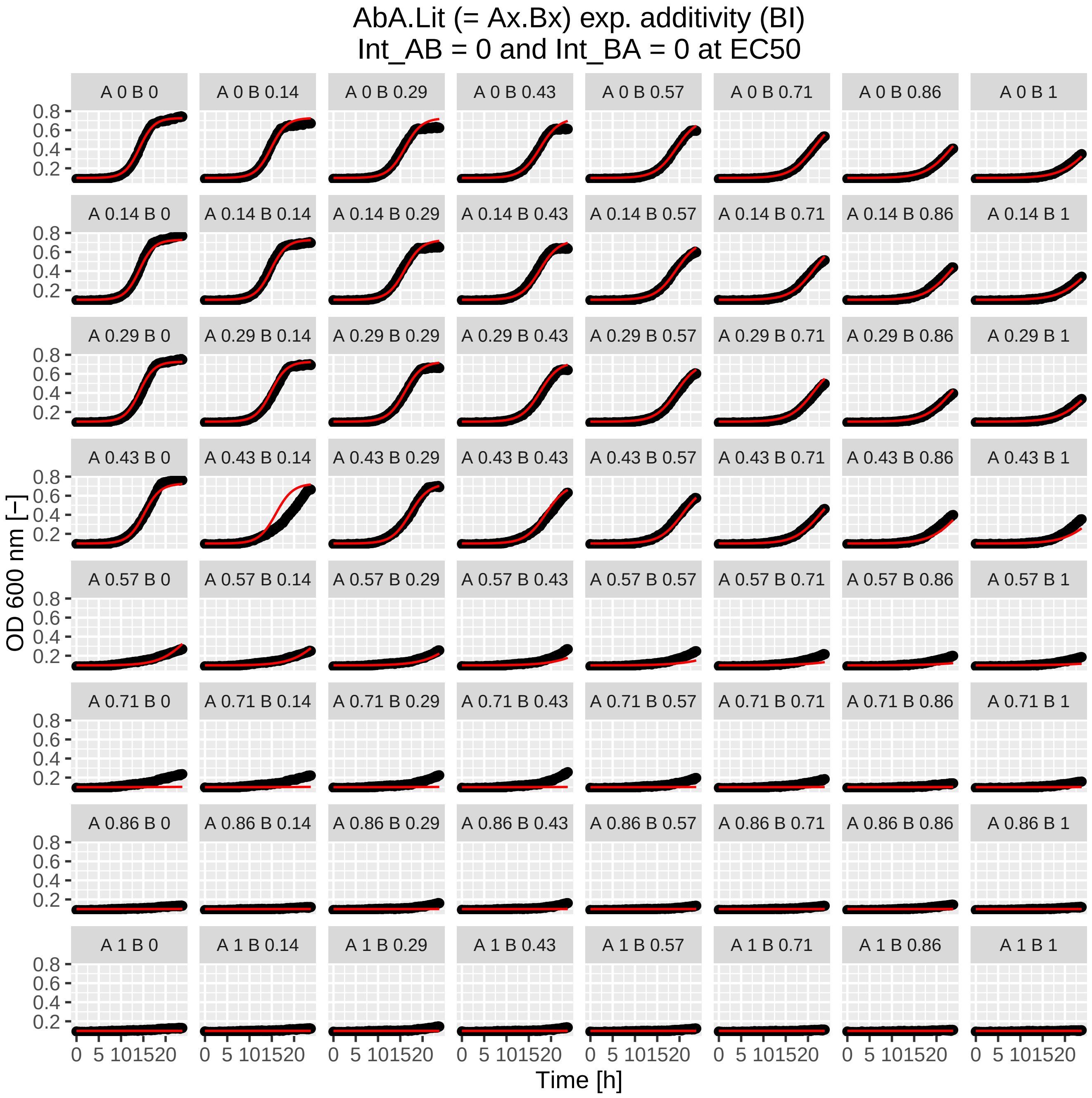

AbA.Lit (= Ax.Bx) exp. additivity (BI)
Int_AB = 0 and Int_BA = 0 at EC50
OD 600 nm [−]
Time [h]
A 0 B 0
A 0 B 0.14
A 0 B 0.29
A 0 B 0.43
A 0 B 0.57
A 0 B 0.71
A 0 B 0.86
A 0 B 1
A 0.14 B 0
A 0.14 B 0.14
A 0.14 B 0.29
A 0.14 B 0.43
A 0.14 B 0.57
A 0.14 B 0.71
A 0.14 B 0.86
A 0.14 B 1
A 0.29 B 0
A 0.29 B 0.14
A 0.29 B 0.29
A 0.29 B 0.43
A 0.29 B 0.57
A 0.29 B 0.71
A 0.29 B 0.86
A 0.29 B 1
A 0.43 B 0
A 0.43 B 0.14
A 0.43 B 0.29
A 0.43 B 0.43
A 0.43 B 0.57
A 0.43 B 0.71
A 0.43 B 0.86
A 0.43 B 1
A 0.57 B 0
A 0.57 B 0.14
A 0.57 B 0.29
A 0.57 B 0.43
A 0.57 B 0.57
A 0.57 B 0.71
A 0.57 B 0.86
A 0.57 B 1
A 0.71 B 0
A 0.71 B 0.14
A 0.71 B 0.29
A 0.71 B 0.43
A 0.71 B 0.57
A 0.71 B 0.71
A 0.71 B 0.86
A 0.71 B 1
A 0.86 B 0
A 0.86 B 0.14
A 0.86 B 0.29
A 0.86 B 0.43
A 0.86 B 0.57
A 0.86 B 0.71
A 0.86 B 0.86
A 0.86 B 1
A 1 B 0
A 1 B 0.14
A 1 B 0.29
A 1 B 0.43
A 1 B 0.57
A 1 B 0.71
A 1 B 0.86
A 1 B 1
0.8
0.6
0.4
0.2
0 5 101520

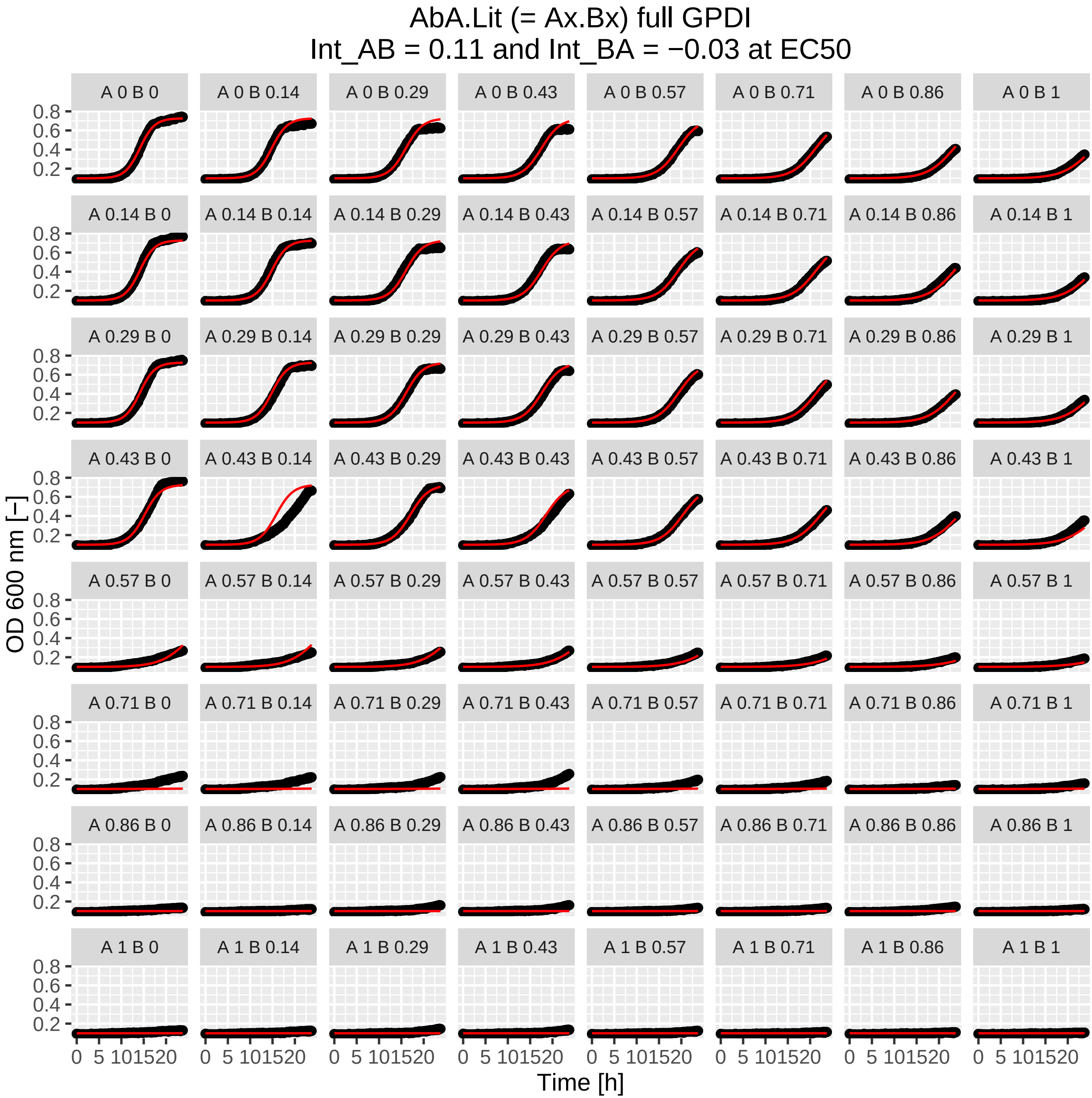
AbA.Lit (= Ax.Bx) full GPDI
Int_AB = 0.11 and Int_BA = −0.03 at EC50
A 0 B 0
A 0 B 0.14
A 0 B 0.29
A 0 B 0.43
A 0 B 0.57
A 0 B 0.71
A 0 B 0.86
A 0 B 1
A 0.14 B 0
A 0.14 B 0.14
A 0.14 B 0.29
A 0.14 B 0.43
A 0.14 B 0.57
A 0.14 B 0.71
A 0.14 B 0.86
A 0.14 B 1
A 0.29 B 0
A 0.29 B 0.14
A 0.29 B 0.29
A 0.29 B 0.43
A 0.29 B 0.57
A 0.29 B 0.71
A 0.29 B 0.86
A 0.29 B 1
A 0.43 B 0
A 0.43 B 0.14
A 0.43 B 0.29
A 0.43 B 0.43
A 0.43 B 0.57
A 0.43 B 0.71
A 0.43 B 0.86
A 0.43 B 1
A 0.57 B 0
A 0.57 B 0.14
A 0.57 B 0.29
A 0.57 B 0.43
A 0.57 B 0.57
A 0.57 B 0.71
A 0.57 B 0.86
A 0.57 B 1
A 0.71 B 0
A 0.71 B 0.14
A 0.71 B 0.29
A 0.71 B 0.43
A 0.71 B 0.57
A 0.71 B 0.71
A 0.71 B 0.86
A 0.71 B 1
A 0.86 B 0
A 0.86 B 0.14
A 0.86 B 0.29
A 0.86 B 0.43
A 0.86 B 0.57
A 0.86 B 0.71
A 0.86 B 0.86
A 0.86 B 1
A 1 B 0
A 1 B 0.14
A 1 B 0.29
A 1 B 0.43
A 1 B 0.57
A 1 B 0.71
A 1 B 0.86
A 1 B 1
OD 600 nm [−]
0.8
0.6
0.4
0.2
0 5 101520
Time [h]

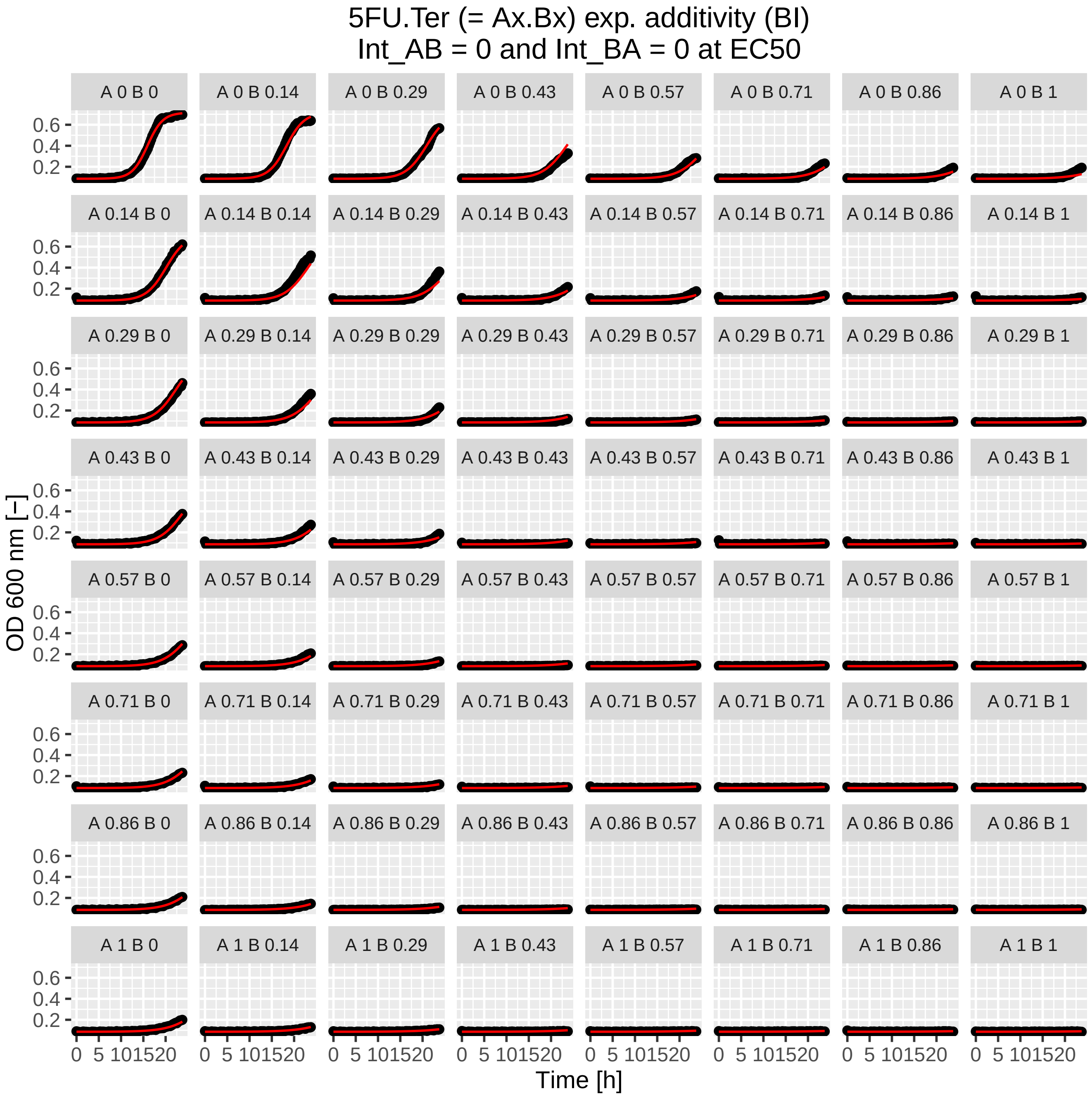

5FU.Ter (= Ax.Bx) exp. additivity (BI)
Int_AB = 0 and Int_BA = 0 at EC50
A 0 B 0
A 0 B 0.14
A 0 B 0.29
A 0 B 0.43
A 0 B 0.57
A 0 B 0.71
A 0 B 0.86
A 0 B 1
A 0.14 B 0
A 0.14 B 0.14
A 0.14 B 0.29
A 0.14 B 0.43
A 0.14 B 0.57
A 0.14 B 0.71
A 0.14 B 0.86
A 0.14 B 1
A 0.29 B 0
A 0.29 B 0.14
A 0.29 B 0.29
A 0.29 B 0.43
A 0.29 B 0.57
A 0.29 B 0.71
A 0.29 B 0.86
A 0.29 B 1
A 0.43 B 0
A 0.43 B 0.14
A 0.43 B 0.29
A 0.43 B 0.43
A 0.43 B 0.57
A 0.43 B 0.71
A 0.43 B 0.86
A 0.43 B 1
A 0.57 B 0
A 0.57 B 0.14
A 0.57 B 0.29
A 0.57 B 0.43
A 0.57 B 0.57
A 0.57 B 0.71
A 0.57 B 0.86
A 0.57 B 1
A 0.71 B 0
A 0.71 B 0.14
A 0.71 B 0.29
A 0.71 B 0.43
A 0.71 B 0.57
A 0.71 B 0.71
A 0.71 B 0.86
A 0.71 B 1
A 0.86 B 0
A 0.86 B 0.14
A 0.86 B 0.29
A 0.86 B 0.43
A 0.86 B 0.57
A 0.86 B 0.71
A 0.86 B 0.86
A 0.86 B 1
A 1 B 0
A 1 B 0.14
A 1 B 0.29
A 1 B 0.43
A 1 B 0.57
A 1 B 0.71
A 1 B 0.86
A 1 B 1
OD 600 nm [–]
0.6
0.4
0.2
0 5 101520
Time [h]

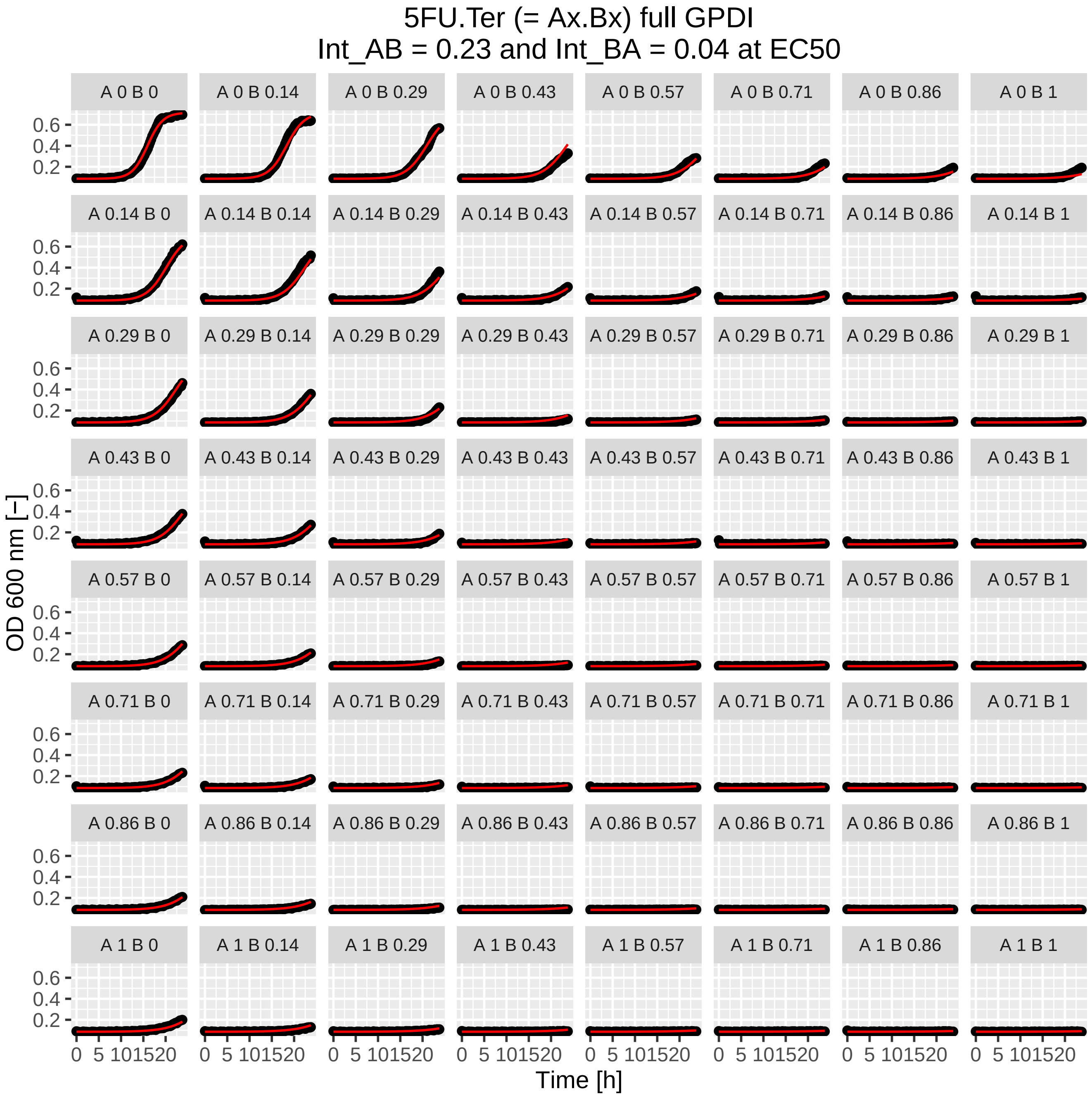
5FU.Ter (= Ax.Bx) full GPDI
Int_AB = 0.23 and Int_BA = 0.04 at EC50
A 0 B 0
A 0 B 0.14
A 0 B 0.29
A 0 B 0.43
A 0 B 0.57
A 0 B 0.71
A 0 B 0.86
A 0 B 1
A 0.14 B 0
A 0.14 B 0.14
A 0.14 B 0.29
A 0.14 B 0.43
A 0.14 B 0.57
A 0.14 B 0.71
A 0.14 B 0.86
A 0.14 B 1
A 0.29 B 0
A 0.29 B 0.14
A 0.29 B 0.29
A 0.29 B 0.43
A 0.29 B 0.57
A 0.29 B 0.71
A 0.29 B 0.86
A 0.29 B 1
A 0.43 B 0
A 0.43 B 0.14
A 0.43 B 0.29
A 0.43 B 0.43
A 0.43 B 0.57
A 0.43 B 0.71
A 0.43 B 0.86
A 0.43 B 1
A 0.57 B 0
A 0.57 B 0.14
A 0.57 B 0.29
A 0.57 B 0.43
A 0.57 B 0.57
A 0.57 B 0.71
A 0.57 B 0.86
A 0.57 B 1
A 0.71 B 0
A 0.71 B 0.14
A 0.71 B 0.29
A 0.71 B 0.43
A 0.71 B 0.57
A 0.71 B 0.71
A 0.71 B 0.86
A 0.71 B 1
A 0.86 B 0
A 0.86 B 0.14
A 0.86 B 0.29
A 0.86 B 0.43
A 0.86 B 0.57
A 0.86 B 0.71
A 0.86 B 0.86
A 0.86 B 1
A 1 B 0
A 1 B 0.14
A 1 B 0.29
A 1 B 0.43
A 1 B 0.57
A 1 B 0.71
A 1 B 0.86
A 1 B 1
0.6
0.4
0.2
OD 600 nm [–]
0 5 101520
Time [h]

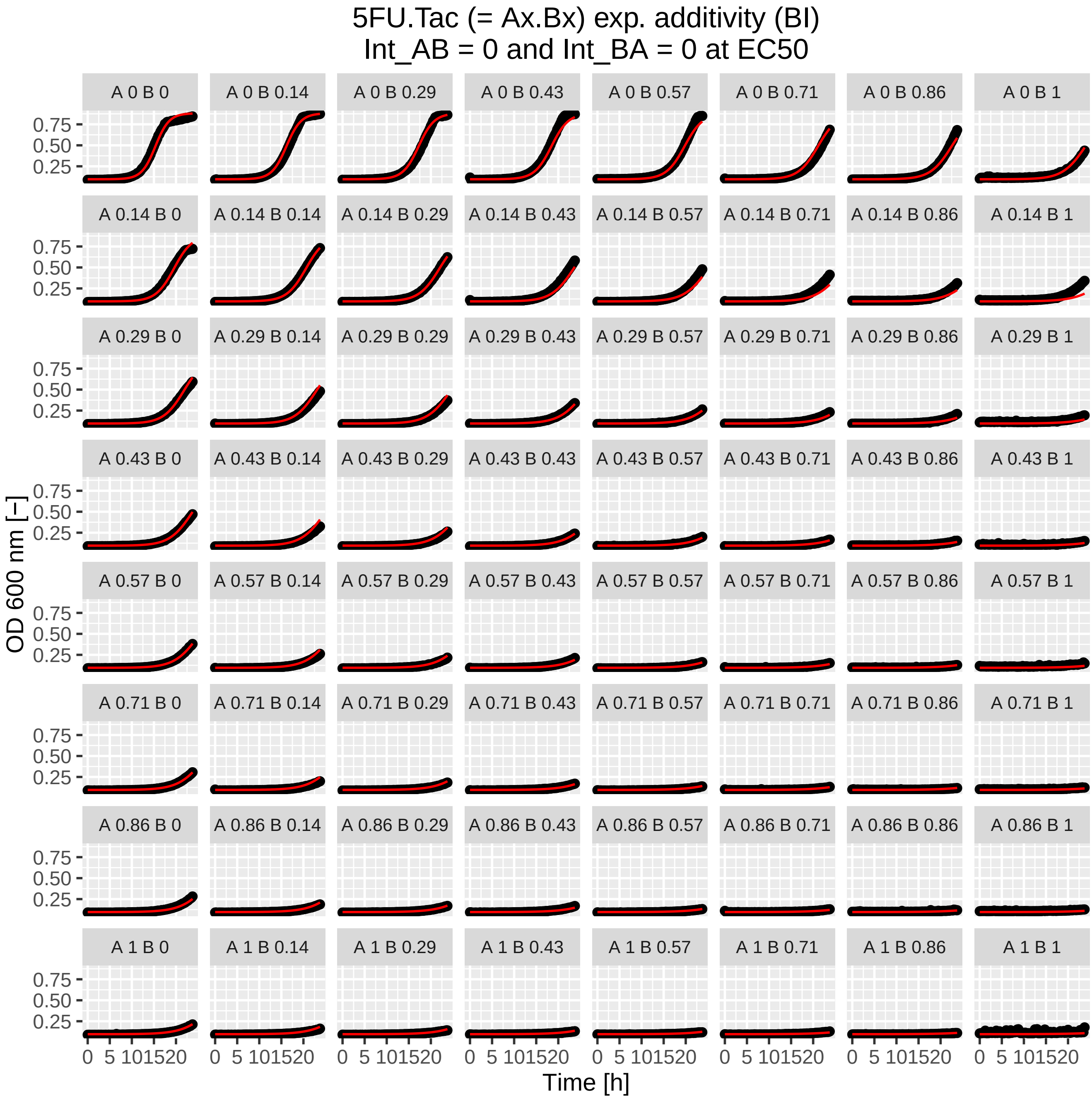

5FU.Tac (= Ax.Bx) exp. additivity (BI)
Int_AB = 0 and Int_BA = 0 at EC50
OD 600 nm [–]
Time [h]
A 0 B 0
A 0 B 0.14
A 0 B 0.29
A 0 B 0.43
A 0 B 0.57
A 0 B 0.71
A 0 B 0.86
A 0 B 1
A 0.14 B 0
A 0.29 B 0
A 0.43 B 0
A 0.57 B 0
A 0.71 B 0
A 0.86 B 0
A 1 B 0
A 1 B 1
0.75
0.50
0.25
0 5 101520

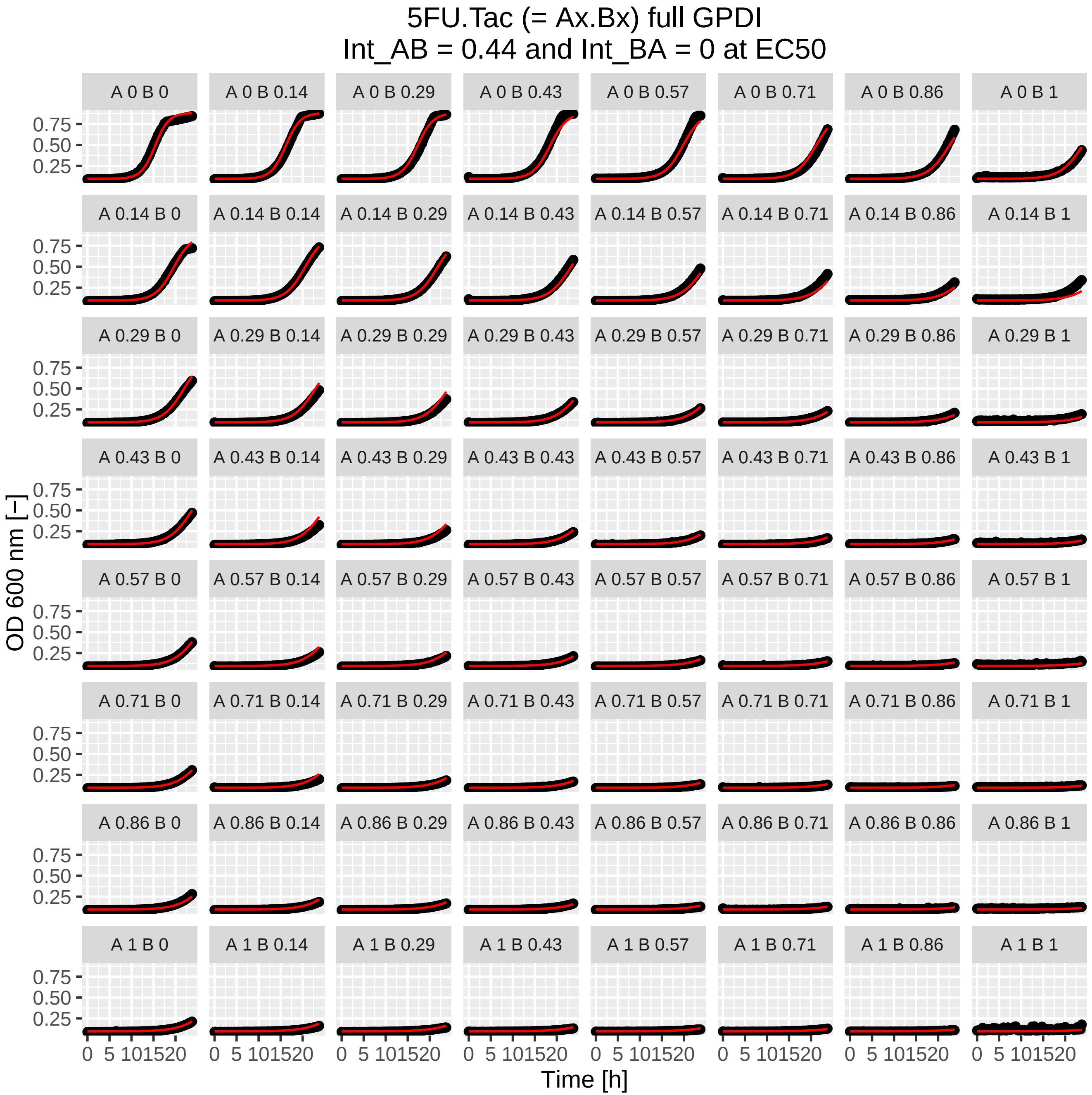

5FU.Tac (= Ax.Bx) full GPDI
Int_AB = 0.44 and Int_BA = 0 at EC50
A 0 B 0
A 0 B 0.14
A 0 B 0.29
A 0 B 0.43
A 0 B 0.57
A 0 B 0.71
A 0 B 0.86
A 0 B 1
A 0.14 B 0
A 0.14 B 0.14
A 0.14 B 0.29
A 0.14 B 0.43
A 0.14 B 0.57
A 0.14 B 0.71
A 0.14 B 0.86
A 0.14 B 1
A 0.29 B 0
A 0.29 B 0.14
A 0.29 B 0.29
A 0.29 B 0.43
A 0.29 B 0.57
A 0.29 B 0.71
A 0.29 B 0.86
A 0.29 B 1
A 0.43 B 0
A 0.43 B 0.14
A 0.43 B 0.29
A 0.43 B 0.43
A 0.43 B 0.57
A 0.43 B 0.71
A 0.43 B 0.86
A 0.43 B 1
A 0.57 B 0
A 0.57 B 0.14
A 0.57 B 0.29
A 0.57 B 0.43
A 0.57 B 0.57
A 0.57 B 0.71
A 0.57 B 0.86
A 0.57 B 1
A 0.71 B 0
A 0.71 B 0.14
A 0.71 B 0.29
A 0.71 B 0.43
A 0.71 B 0.57
A 0.71 B 0.71
A 0.71 B 0.86
A 0.71 B 1
A 0.86 B 0
A 0.86 B 0.14
A 0.86 B 0.29
A 0.86 B 0.43
A 0.86 B 0.57
A 0.86 B 0.71
A 0.86 B 0.86
A 0.86 B 1
A 1 B 0
A 1 B 0.14
A 1 B 0.29
A 1 B 0.43
A 1 B 0.57
A 1 B 0.71
A 1 B 0.86
A 1 B 1
0.75
0.50
0.25
OD 600 nm [–]
0 5 101520
Time [h]

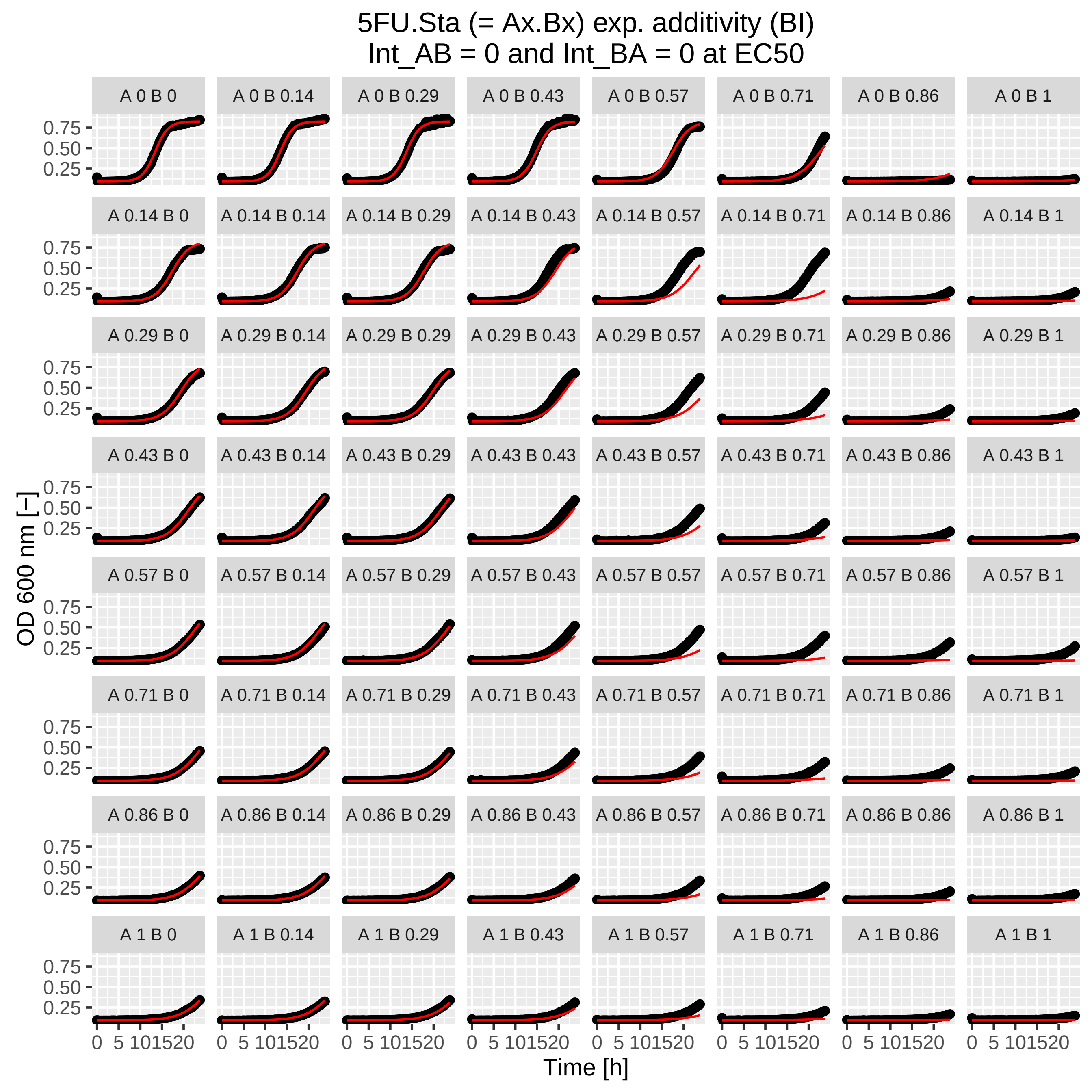
5FU.Sta (= Ax.Bx) exp. additivity (BI)
Int_AB = 0 and Int_BA = 0 at EC50
OD 600 nm [–]
Time [h]
A 0 B 0
A 0 B 0.14
A 0 B 0.29
A 0 B 0.43
A 0 B 0.57
A 0 B 0.71
A 0 B 0.86
A 0 B 1
A 0.14 B 0
A 0.14 B 0.14
A 0.14 B 0.29
A 0.14 B 0.43
A 0.14 B 0.57
A 0.14 B 0.71
A 0.14 B 0.86
A 0.14 B 1
A 0.29 B 0
A 0.29 B 0.14
A 0.29 B 0.29
A 0.29 B 0.43
A 0.29 B 0.57
A 0.29 B 0.71
A 0.29 B 0.86
A 0.29 B 1
A 0.43 B 0
A 0.43 B 0.14
A 0.43 B 0.29
A 0.43 B 0.43
A 0.43 B 0.57
A 0.43 B 0.71
A 0.43 B 0.86
A 0.43 B 1
A 0.57 B 0
A 0.57 B 0.14
A 0.57 B 0.29
A 0.57 B 0.43
A 0.57 B 0.57
A 0.57 B 0.71
A 0.57 B 0.86
A 0.57 B 1
A 0.71 B 0
A 0.71 B 0.14
A 0.71 B 0.29
A 0.71 B 0.43
A 0.71 B 0.57
A 0.71 B 0.71
A 0.71 B 0.86
A 0.71 B 1
A 0.86 B 0
A 0.86 B 0.14
A 0.86 B 0.29
A 0.86 B 0.43
A 0.86 B 0.57
A 0.86 B 0.71
A 0.86 B 0.86
A 0.86 B 1
A 1 B 0
A 1 B 0.14
A 1 B 0.29
A 1 B 0.43
A 1 B 0.57
A 1 B 0.71
A 1 B 0.86
A 1 B 1
0.75
0.50
0.25
0 5 101520

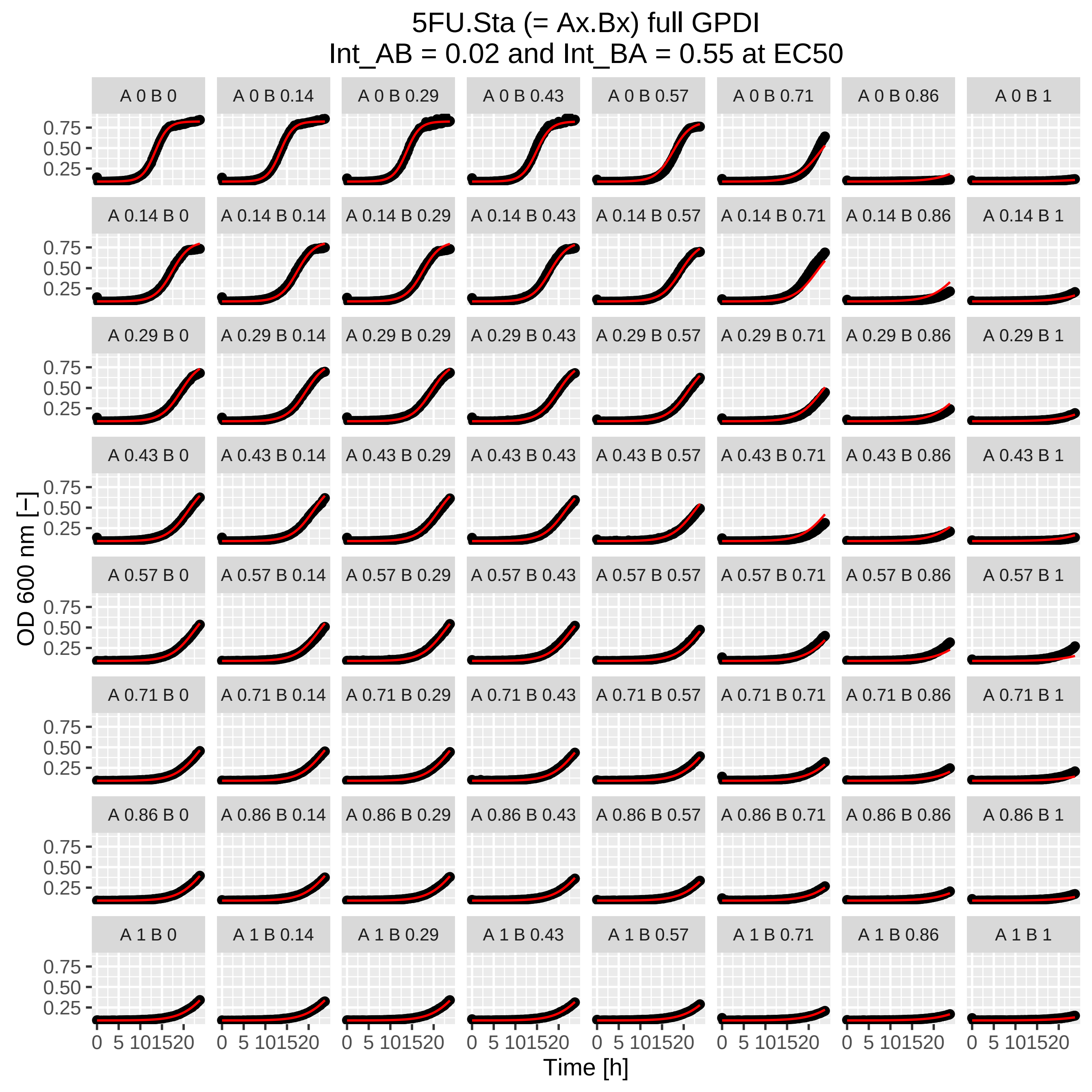
5FU.Sta (= Ax.Bx) full GPDI
Int_AB = 0.02 and Int_BA = 0.55 at EC50
OD 600 nm [–]
Time [h]
0.75
0.50
0.25
0 5 101520
A 0 B 0
A 0 B 0.14
A 0 B 0.29
A 0 B 0.43
A 0 B 0.57
A 0 B 0.71
A 0 B 0.86
A 0 B 1
A 0.14 B 0
A 0.14 B 0.14
A 0.14 B 0.29
A 0.14 B 0.43
A 0.14 B 0.57
A 0.14 B 0.71
A 0.14 B 0.86
A 0.14 B 1
A 0.29 B 0
A 0.29 B 0.14
A 0.29 B 0.29
A 0.29 B 0.43
A 0.29 B 0.57
A 0.29 B 0.71
A 0.29 B 0.86
A 0.29 B 1
A 0.43 B 0
A 0.43 B 0.14
A 0.43 B 0.29
A 0.43 B 0.43
A 0.43 B 0.57
A 0.43 B 0.71
A 0.43 B 0.86
A 0.43 B 1
A 0.57 B 0
A 0.57 B 0.14
A 0.57 B 0.29
A 0.57 B 0.43
A 0.57 B 0.57
A 0.57 B 0.71
A 0.57 B 0.86
A 0.57 B 1
A 0.71 B 0
A 0.71 B 0.14
A 0.71 B 0.29
A 0.71 B 0.43
A 0.71 B 0.57
A 0.71 B 0.71
A 0.71 B 0.86
A 0.71 B 1
A 0.86 B 0
A 0.86 B 0.14
A 0.86 B 0.29
A 0.86 B 0.43
A 0.86 B 0.57
A 0.86 B 0.71
A 0.86 B 0.86
A 0.86 B 1
A 1 B 0
A 1 B 0.14
A 1 B 0.29
A 1 B 0.43
A 1 B 0.57
A 1 B 0.71
A 1 B 0.86
A 1 B 1

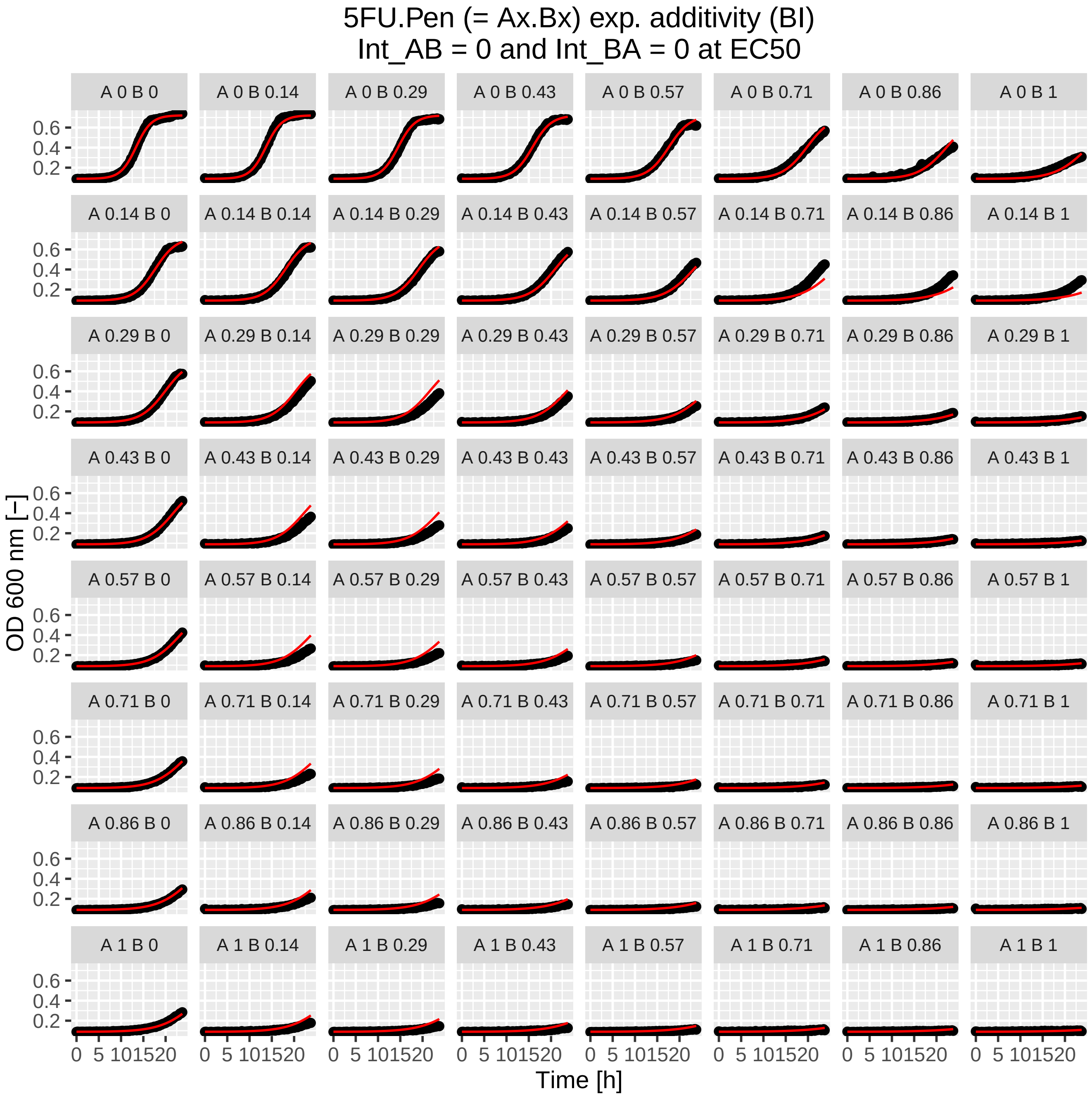
5FU.Pen (= Ax.Bx) exp. additivity (BI)
Int_AB = 0 and Int_BA = 0 at EC50
OD 600 nm [–]
Time [h]
A 0 B 0
A 0 B 0.14
A 0 B 0.29
A 0 B 0.43
A 0 B 0.57
A 0 B 0.71
A 0 B 0.86
A 0 B 1
A 1 B 0
A 1 B 0.14
A 1 B 0.29
A 1 B 0.43
A 1 B 0.57
A 1 B 0.71
A 1 B 0.86
A 1 B 1
0.6
0.4
0.2
0 5 101520

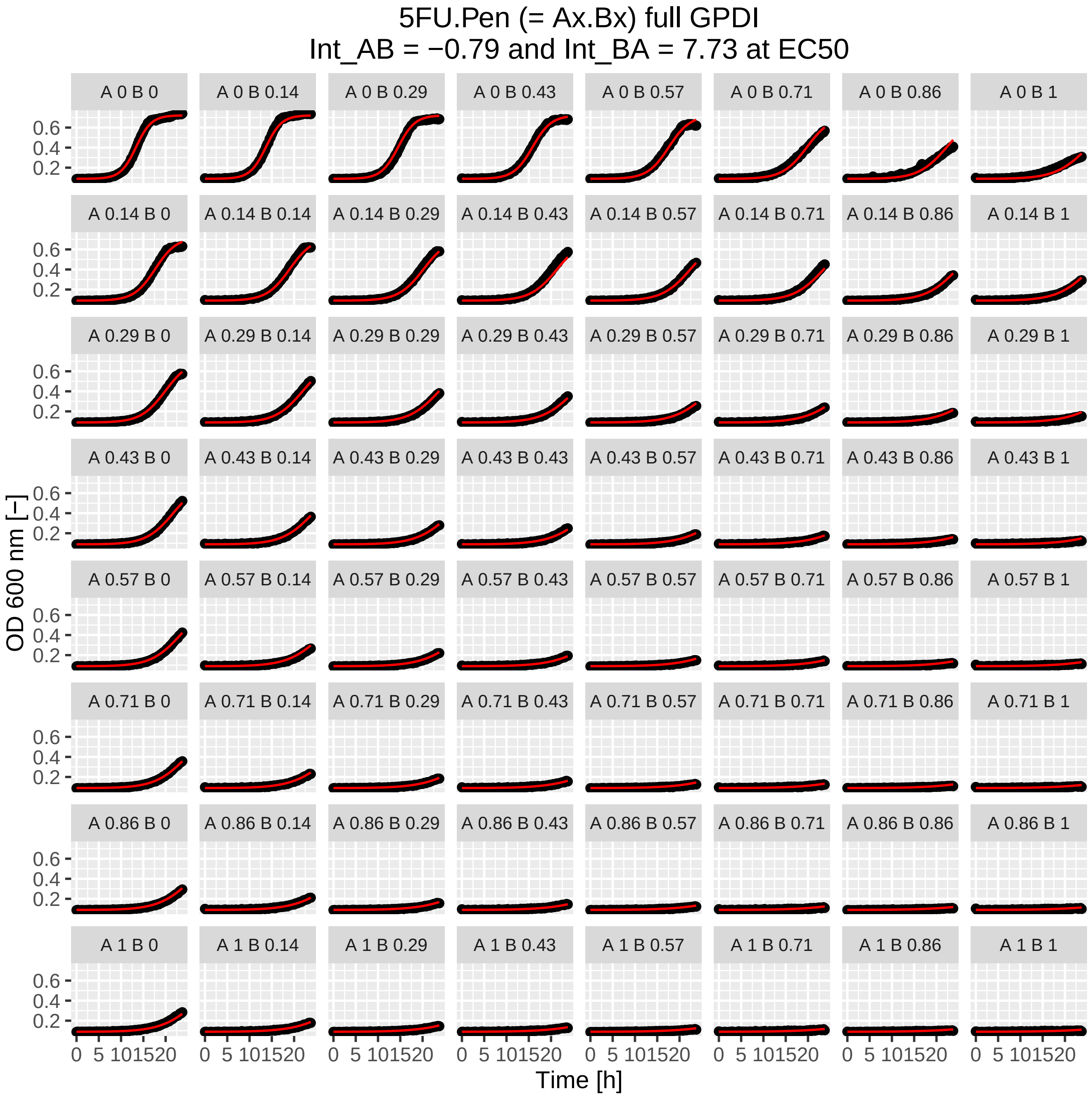
5FU.Pen (= Ax.Bx) full GPDI
Int_AB = −0.79 and Int_BA = 7.73 at EC50
OD 600 nm [–]
Time [h]
0.6
0.4
0.2
0 5 101520
A 0 B 0
A 0 B 0.14
A 0 B 0.29
A 0 B 0.43
A 0 B 0.57
A 0 B 0.71
A 0 B 0.86
A 0 B 1
A 0.14 B 0
A 0.14 B 0.14
A 0.14 B 0.29
A 0.14 B 0.43
A 0.14 B 0.57
A 0.14 B 0.71
A 0.14 B 0.86
A 0.14 B 1
A 0.29 B 0
A 0.29 B 0.14
A 0.29 B 0.29
A 0.29 B 0.43
A 0.29 B 0.57
A 0.29 B 0.71
A 0.29 B 0.86
A 0.29 B 1
A 0.43 B 0
A 0.43 B 0.14
A 0.43 B 0.29
A 0.43 B 0.43
A 0.43 B 0.57
A 0.43 B 0.71
A 0.43 B 0.86
A 0.43 B 1
A 0.57 B 0
A 0.57 B 0.14
A 0.57 B 0.29
A 0.57 B 0.43
A 0.57 B 0.57
A 0.57 B 0.71
A 0.57 B 0.86
A 0.57 B 1
A 0.71 B 0
A 0.71 B 0.14
A 0.71 B 0.29
A 0.71 B 0.43
A 0.71 B 0.57
A 0.71 B 0.71
A 0.71 B 0.86
A 0.71 B 1
A 0.86 B 0
A 0.86 B 0.14
A 0.86 B 0.29
A 0.86 B 0.43
A 0.86 B 0.57
A 0.86 B 0.71
A 0.86 B 0.86
A 0.86 B 1
A 1 B 0
A 1 B 0.14
A 1 B 0.29
A 1 B 0.43
A 1 B 0.57
A 1 B 0.71
A 1 B 0.86
A 1 B 1

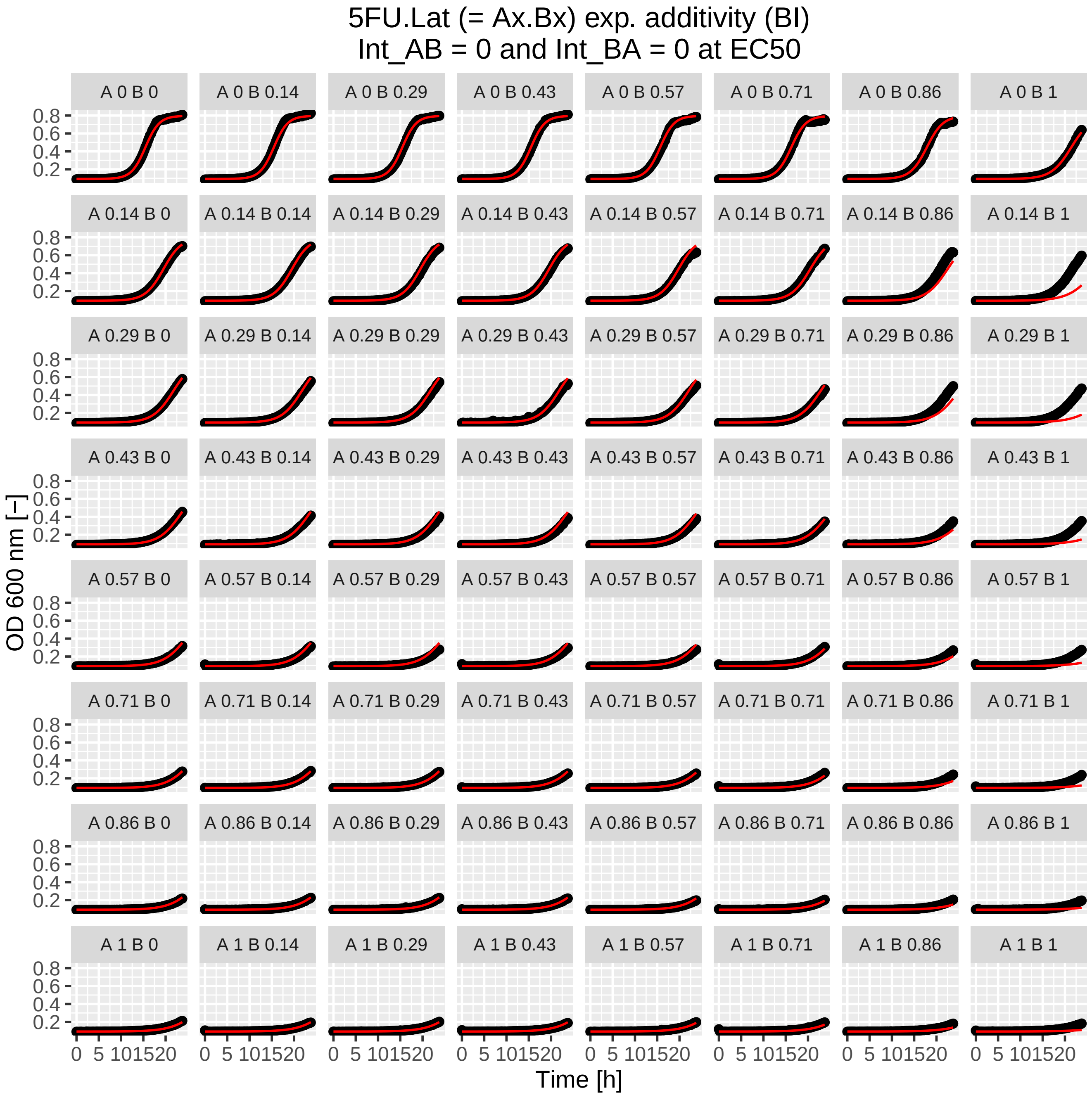

5FU.Lat (= Ax.Bx) exp. additivity (BI)
Int_AB = 0 and Int_BA = 0 at EC50
A 0 B 0
A 0 B 0.14
A 0 B 0.29
A 0 B 0.43
A 0 B 0.57
A 0 B 0.71
A 0 B 0.86
A 0 B 1
A 0.14 B 0
A 0.14 B 0.14
A 0.14 B 0.29
A 0.14 B 0.43
A 0.14 B 0.57
A 0.14 B 0.71
A 0.14 B 0.86
A 0.14 B 1
A 0.29 B 0
A 0.29 B 0.14
A 0.29 B 0.29
A 0.29 B 0.43
A 0.29 B 0.57
A 0.29 B 0.71
A 0.29 B 0.86
A 0.29 B 1
A 0.43 B 0
A 0.43 B 0.14
A 0.43 B 0.29
A 0.43 B 0.43
A 0.43 B 0.57
A 0.43 B 0.71
A 0.43 B 0.86
A 0.43 B 1
A 0.57 B 0
A 0.57 B 0.14
A 0.57 B 0.29
A 0.57 B 0.43
A 0.57 B 0.57
A 0.57 B 0.71
A 0.57 B 0.86
A 0.57 B 1
A 0.71 B 0
A 0.71 B 0.14
A 0.71 B 0.29
A 0.71 B 0.43
A 0.71 B 0.57
A 0.71 B 0.71
A 0.71 B 0.86
A 0.71 B 1
A 0.86 B 0
A 0.86 B 0.14
A 0.86 B 0.29
A 0.86 B 0.43
A 0.86 B 0.57
A 0.86 B 0.71
A 0.86 B 0.86
A 0.86 B 1
A 1 B 0
A 1 B 0.14
A 1 B 0.29
A 1 B 0.43
A 1 B 0.57
A 1 B 0.71
A 1 B 0.86
A 1 B 1
0.8
0.6
0.4
0.2
OD 600 nm [–]
0 5 101520
Time [h]

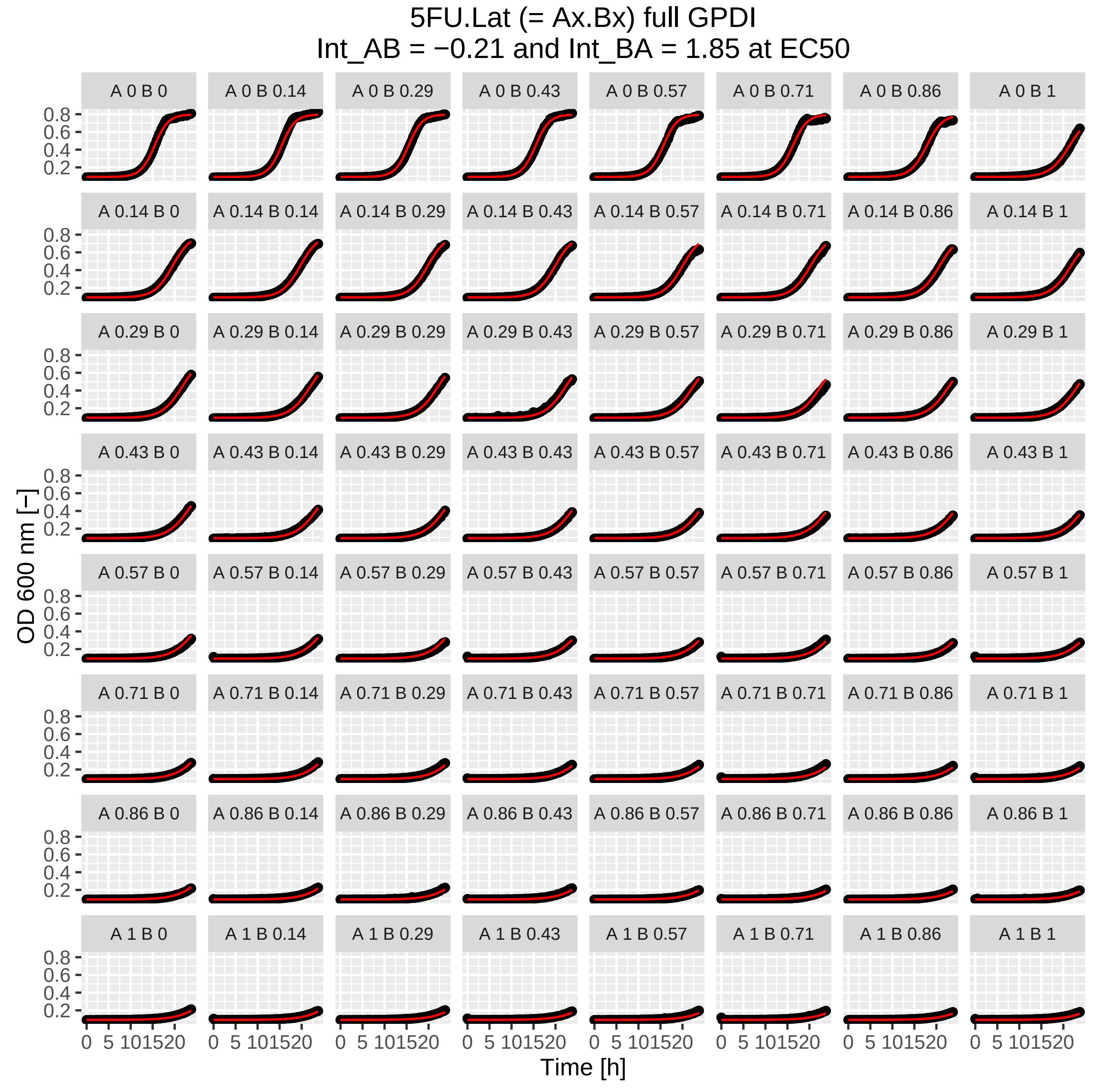
5FU.Lat (= Ax.Bx) full GPDI
Int_AB = −0.21 and Int_BA = 1.85 at EC50
OD 600 nm [−]
Time [h]
A 0 B 0
A 0 B 0.14
A 0 B 0.29
A 0 B 0.43
A 0 B 0.57
A 0 B 0.71
A 0 B 0.86
A 0 B 1
A 1 B 0
A 1 B 0.14
A 1 B 0.29
A 1 B 0.43
A 1 B 0.57
A 1 B 0.71
A 1 B 0.86
A 1 B 1

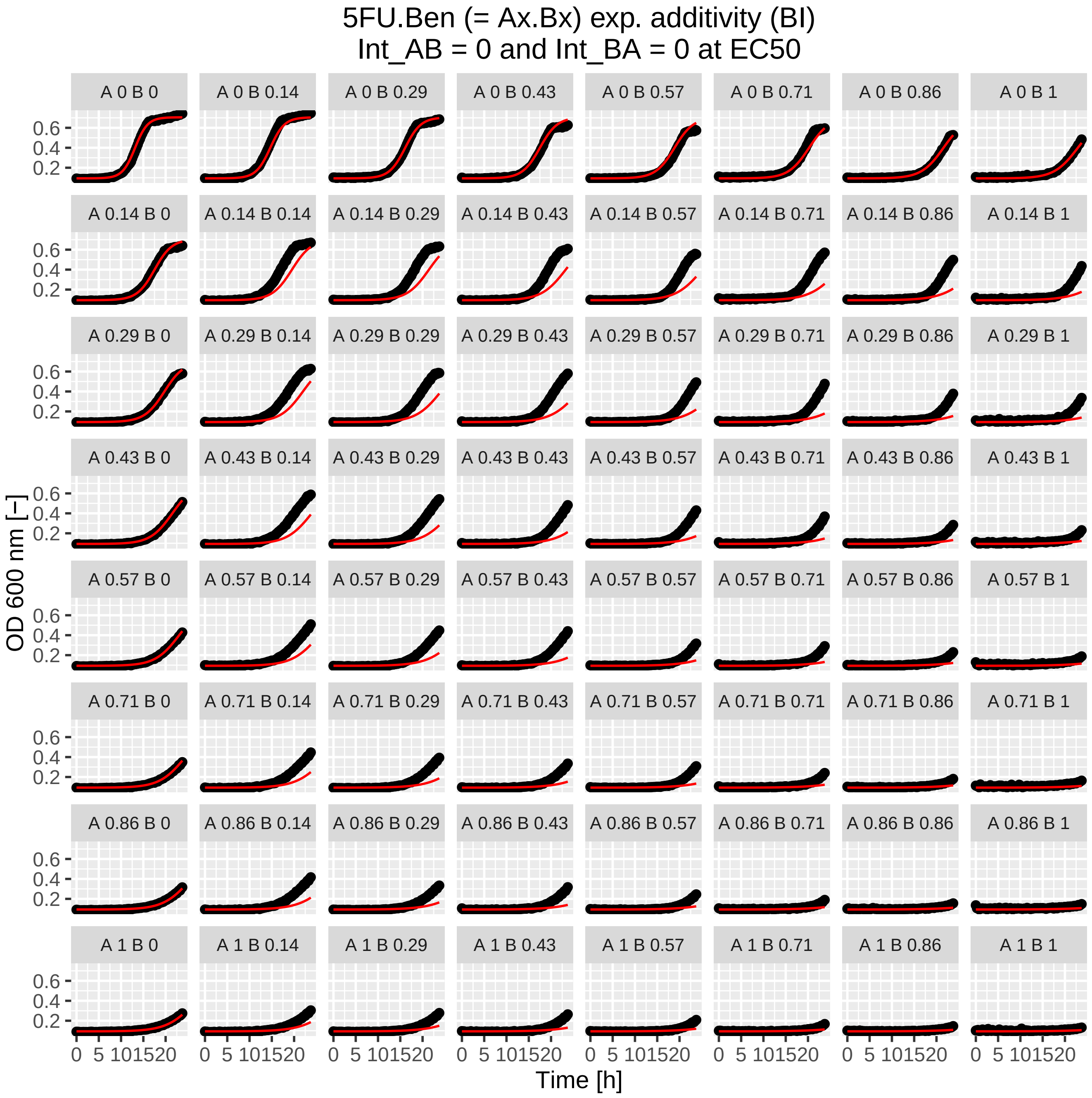

5FU.Ben (= Ax.Bx) exp. additivity (BI)
Int_AB = 0 and Int_BA = 0 at EC50
A 0 B 0
A 0 B 0.14
A 0 B 0.29
A 0 B 0.43
A 0 B 0.57
A 0 B 0.71
A 0 B 0.86
A 0 B 1
A 0.14 B 0
A 0.14 B 0.14
A 0.14 B 0.29
A 0.14 B 0.43
A 0.14 B 0.57
A 0.14 B 0.71
A 0.14 B 0.86
A 0.14 B 1
A 0.29 B 0
A 0.29 B 0.14
A 0.29 B 0.29
A 0.29 B 0.43
A 0.29 B 0.57
A 0.29 B 0.71
A 0.29 B 0.86
A 0.29 B 1
A 0.43 B 0
A 0.43 B 0.14
A 0.43 B 0.29
A 0.43 B 0.43
A 0.43 B 0.57
A 0.43 B 0.71
A 0.43 B 0.86
A 0.43 B 1
A 0.57 B 0
A 0.57 B 0.14
A 0.57 B 0.29
A 0.57 B 0.43
A 0.57 B 0.57
A 0.57 B 0.71
A 0.57 B 0.86
A 0.57 B 1
A 0.71 B 0
A 0.71 B 0.14
A 0.71 B 0.29
A 0.71 B 0.43
A 0.71 B 0.57
A 0.71 B 0.71
A 0.71 B 0.86
A 0.71 B 1
A 0.86 B 0
A 0.86 B 0.14
A 0.86 B 0.29
A 0.86 B 0.43
A 0.86 B 0.57
A 0.86 B 0.71
A 0.86 B 0.86
A 0.86 B 1
A 1 B 0
A 1 B 0.14
A 1 B 0.29
A 1 B 0.43
A 1 B 0.57
A 1 B 0.71
A 1 B 0.86
A 1 B 1
OD 600 nm [−]
0.2
0.4
0.6
0 5 101520
Time [h]

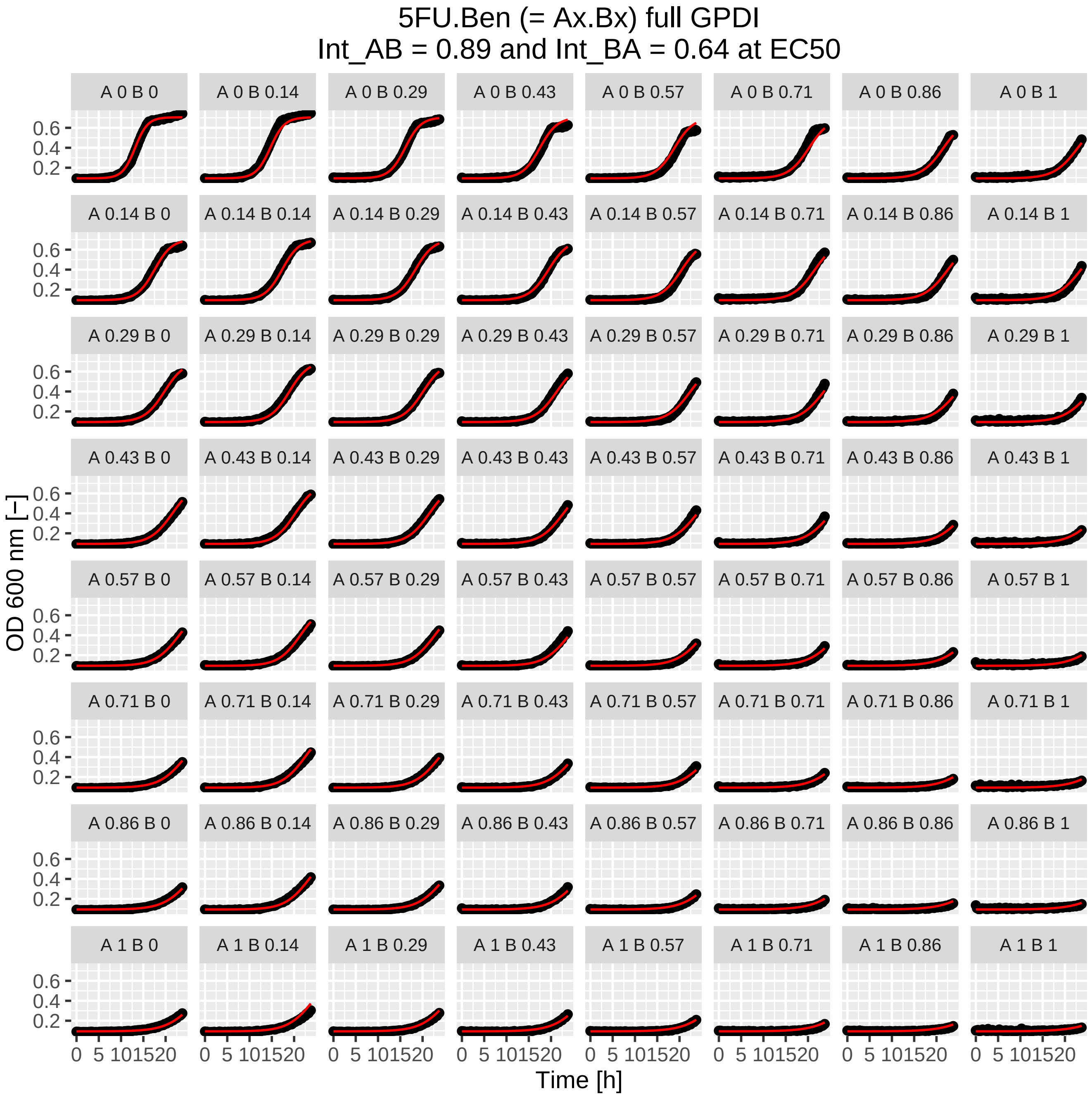
5FU.Ben (= Ax.Bx) full GPDI
Int_AB = 0.89 and Int_BA = 0.64 at EC50
A 0 B 0
A 0 B 0.14
A 0 B 0.29
A 0 B 0.43
A 0 B 0.57
A 0 B 0.71
A 0 B 0.86
A 0 B 1
A 0.14 B 0
A 0.14 B 0.14
A 0.14 B 0.29
A 0.14 B 0.43
A 0.14 B 0.57
A 0.14 B 0.71
A 0.14 B 0.86
A 0.14 B 1
A 0.29 B 0
A 0.29 B 0.14
A 0.29 B 0.29
A 0.29 B 0.43
A 0.29 B 0.57
A 0.29 B 0.71
A 0.29 B 0.86
A 0.29 B 1
A 0.43 B 0
A 0.43 B 0.14
A 0.43 B 0.29
A 0.43 B 0.43
A 0.43 B 0.57
A 0.43 B 0.71
A 0.43 B 0.86
A 0.43 B 1
A 0.57 B 0
A 0.57 B 0.14
A 0.57 B 0.29
A 0.57 B 0.43
A 0.57 B 0.57
A 0.57 B 0.71
A 0.57 B 0.86
A 0.57 B 1
A 0.71 B 0
A 0.71 B 0.14
A 0.71 B 0.29
A 0.71 B 0.43
A 0.71 B 0.57
A 0.71 B 0.71
A 0.71 B 0.86
A 0.71 B 1
A 0.86 B 0
A 0.86 B 0.14
A 0.86 B 0.29
A 0.86 B 0.43
A 0.86 B 0.57
A 0.86 B 0.71
A 0.86 B 0.86
A 0.86 B 1
A 1 B 0
A 1 B 0.14
A 1 B 0.29
A 1 B 0.43
A 1 B 0.57
A 1 B 0.71
A 1 B 0.86
A 1 B 1
OD 600 nm [–]
0.6
0.4
0.2
0 5 101520
Time [h]

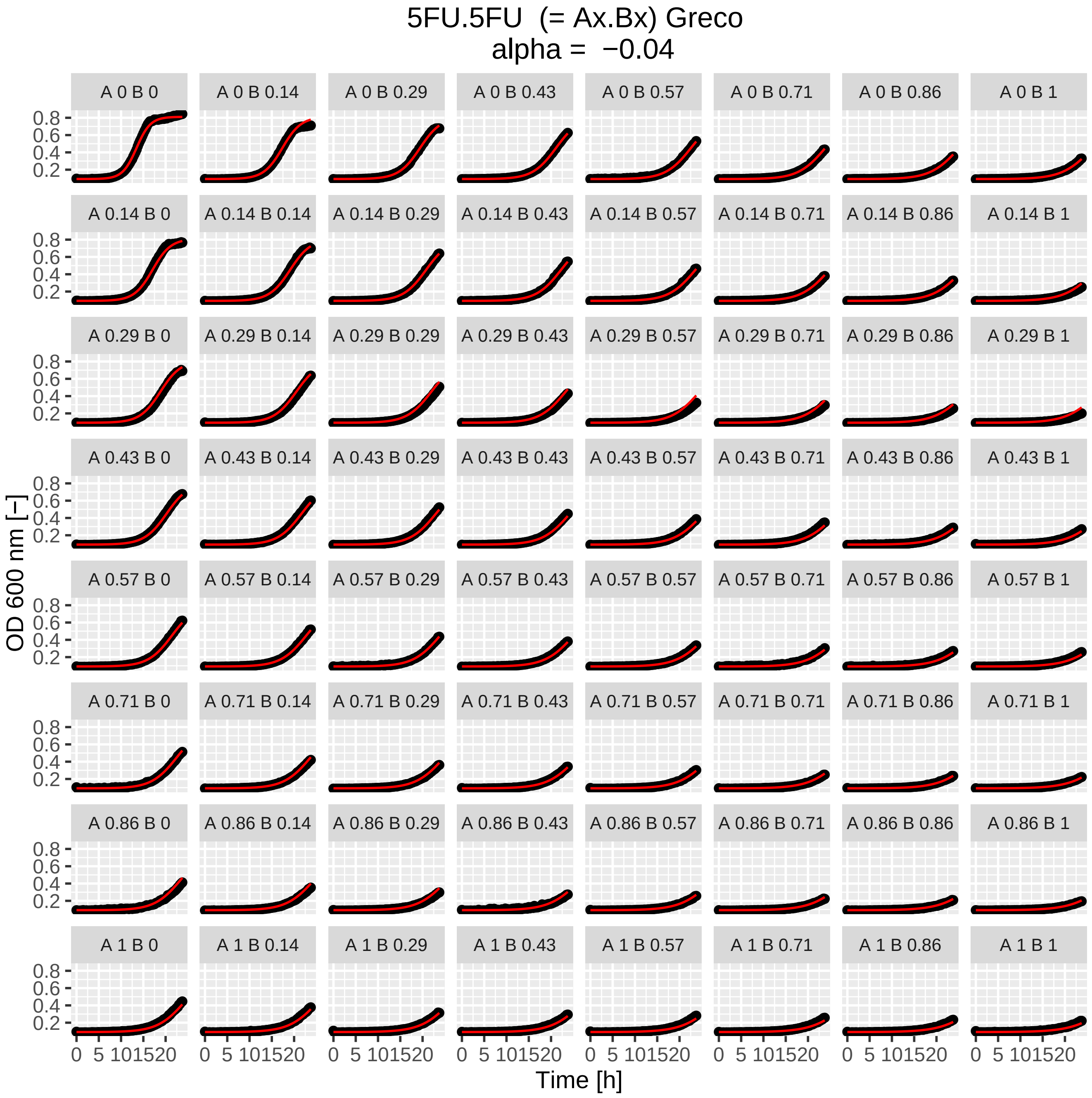
5FU.5FU (= Ax.Bx) Greco
alpha = −0.04
A 0 B 0
A 0 B 0.14
A 0 B 0.29
A 0 B 0.43
A 0 B 0.57
A 0 B 0.71
A 0 B 0.86
A 0 B 1
A 0.14 B 0
A 0.14 B 0.14
A 0.14 B 0.29
A 0.14 B 0.43
A 0.14 B 0.57
A 0.14 B 0.71
A 0.14 B 0.86
A 0.14 B 1
A 0.29 B 0
A 0.29 B 0.14
A 0.29 B 0.29
A 0.29 B 0.43
A 0.29 B 0.57
A 0.29 B 0.71
A 0.29 B 0.86
A 0.29 B 1
A 0.43 B 0
A 0.43 B 0.14
A 0.43 B 0.29
A 0.43 B 0.43
A 0.43 B 0.57
A 0.43 B 0.71
A 0.43 B 0.86
A 0.43 B 1
A 0.57 B 0
A 0.57 B 0.14
A 0.57 B 0.29
A 0.57 B 0.43
A 0.57 B 0.57
A 0.57 B 0.71
A 0.57 B 0.86
A 0.57 B 1
A 0.71 B 0
A 0.71 B 0.14
A 0.71 B 0.29
A 0.71 B 0.43
A 0.71 B 0.57
A 0.71 B 0.71
A 0.71 B 0.86
A 0.71 B 1
A 0.86 B 0
A 0.86 B 0.14
A 0.86 B 0.29
A 0.86 B 0.43
A 0.86 B 0.57
A 0.86 B 0.71
A 0.86 B 0.86
A 0.86 B 1
A 1 B 0
A 1 B 0.14
A 1 B 0.29
A 1 B 0.43
A 1 B 0.57
A 1 B 0.71
A 1 B 0.86
A 1 B 1
OD 600 nm [−]
0.2
0.4
0.6
0.8
0 5 101520
Time [h]

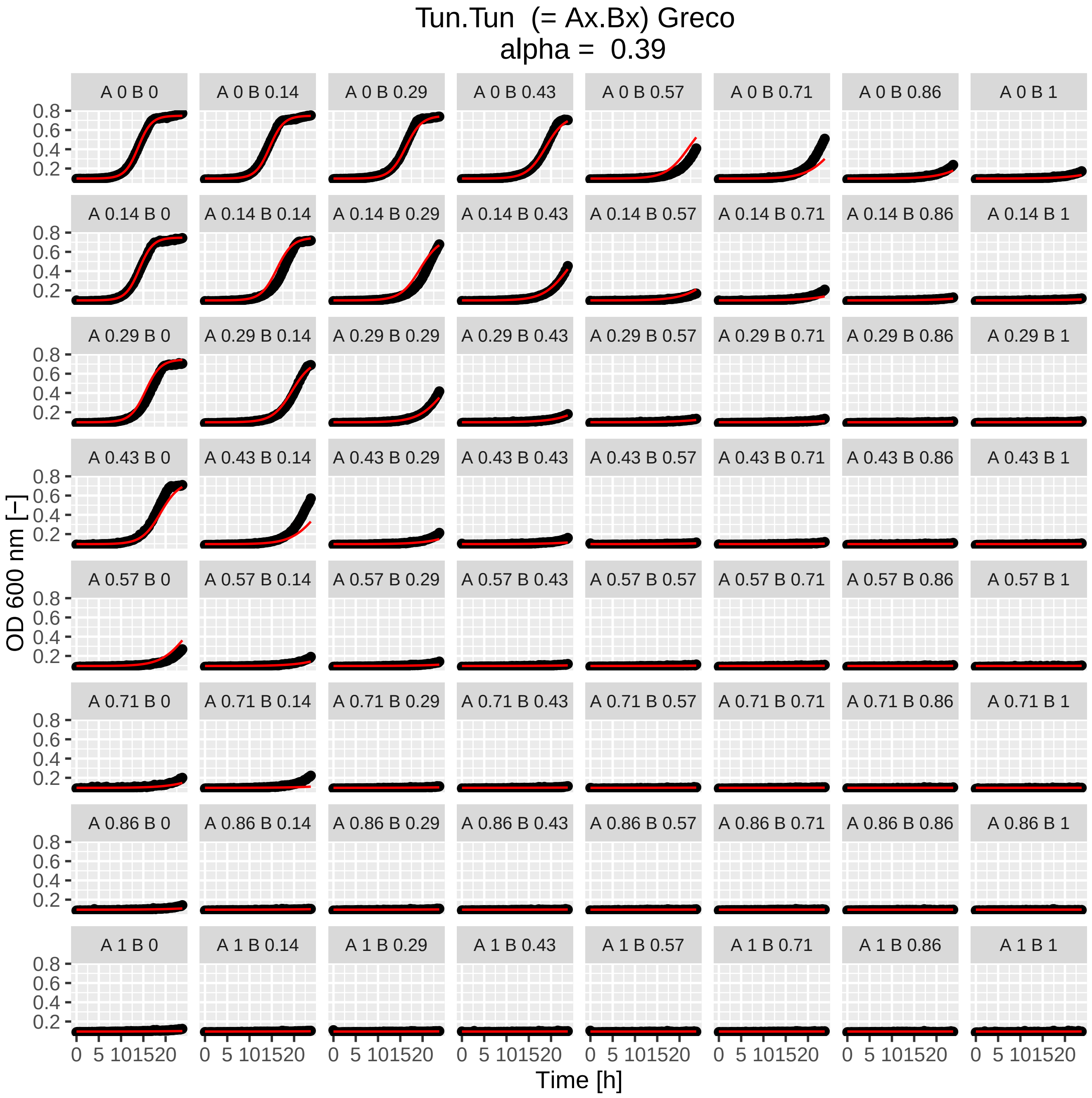
Tun.Tun (= Ax.Bx) Greco
alpha = 0.39
A 0 B 0
A 0 B 0.14
A 0 B 0.29
A 0 B 0.43
A 0 B 0.57
A 0 B 0.71
A 0 B 0.86
A 0 B 1
A 0.14 B 0
A 0.14 B 0.14
A 0.14 B 0.29
A 0.14 B 0.43
A 0.14 B 0.57
A 0.14 B 0.71
A 0.14 B 0.86
A 0.14 B 1
A 0.29 B 0
A 0.29 B 0.14
A 0.29 B 0.29
A 0.29 B 0.43
A 0.29 B 0.57
A 0.29 B 0.71
A 0.29 B 0.86
A 0.29 B 1
A 0.43 B 0
A 0.43 B 0.14
A 0.43 B 0.29
A 0.43 B 0.43
A 0.43 B 0.57
A 0.43 B 0.71
A 0.43 B 0.86
A 0.43 B 1
A 0.57 B 0
A 0.57 B 0.14
A 0.57 B 0.29
A 0.57 B 0.43
A 0.57 B 0.57
A 0.57 B 0.71
A 0.57 B 0.86
A 0.57 B 1
A 0.71 B 0
A 0.71 B 0.14
A 0.71 B 0.29
A 0.71 B 0.43
A 0.71 B 0.57
A 0.71 B 0.71
A 0.71 B 0.86
A 0.71 B 1
A 0.86 B 0
A 0.86 B 0.14
A 0.86 B 0.29
A 0.86 B 0.43
A 0.86 B 0.57
A 0.86 B 0.71
A 0.86 B 0.86
A 0.86 B 1
A 1 B 0
A 1 B 0.14
A 1 B 0.29
A 1 B 0.43
A 1 B 0.57
A 1 B 0.71
A 1 B 0.86
A 1 B 1
OD 600 nm [–]
0.8
0.6
0.4
0.2
0 5 101520
Time [h]

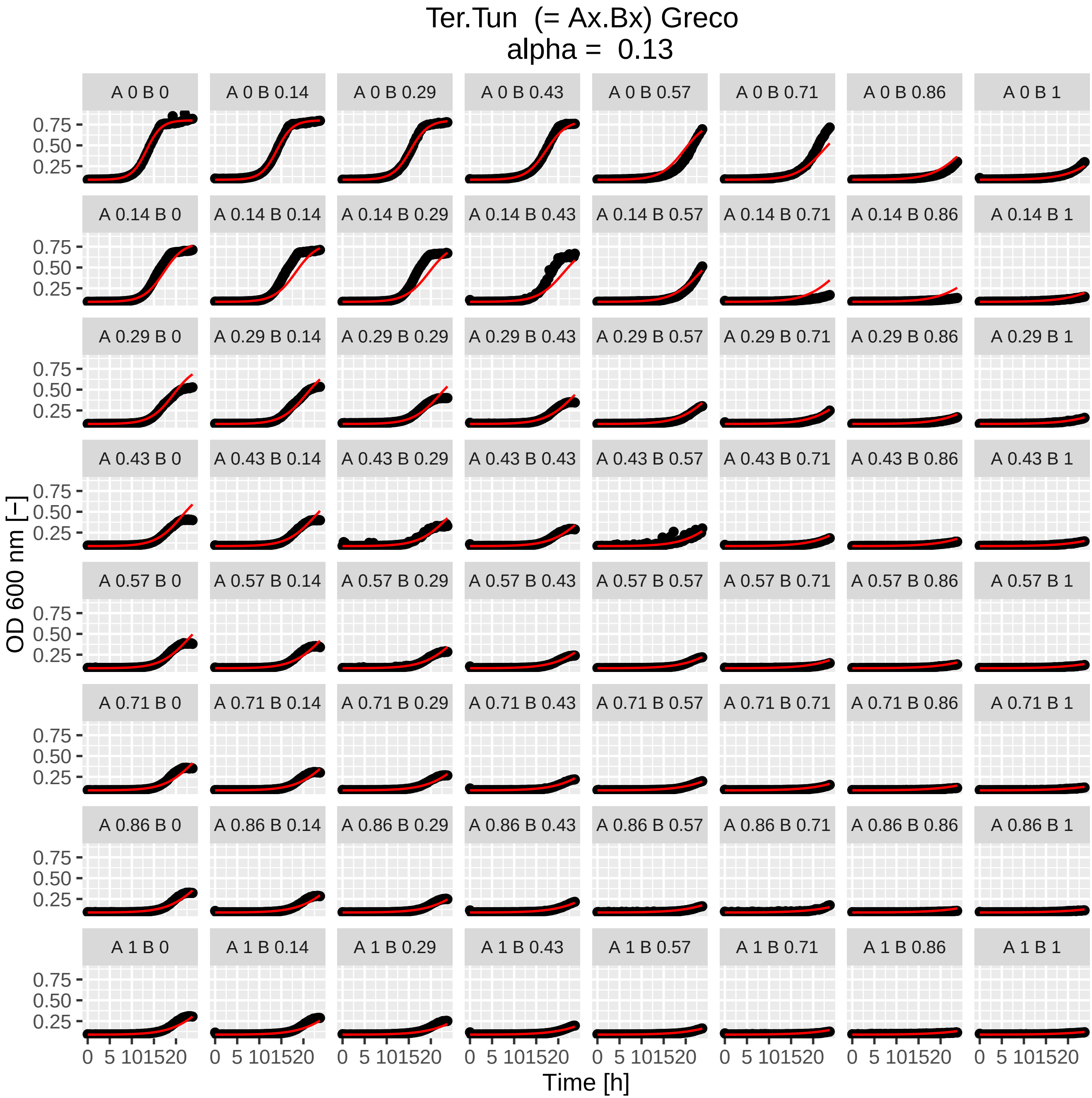
Ter.Tun (= Ax.Bx) Greco
alpha = 0.13
A 0 B 0
A 0 B 0.14
A 0 B 0.29
A 0 B 0.43
A 0 B 0.57
A 0 B 0.71
A 0 B 0.86
A 0 B 1
A 0.14 B 0
A 0.14 B 0.14
A 0.14 B 0.29
A 0.14 B 0.43
A 0.14 B 0.57
A 0.14 B 0.71
A 0.14 B 0.86
A 0.14 B 1
A 0.29 B 0
A 0.29 B 0.14
A 0.29 B 0.29
A 0.29 B 0.43
A 0.29 B 0.57
A 0.29 B 0.71
A 0.29 B 0.86
A 0.29 B 1
A 0.43 B 0
A 0.43 B 0.14
A 0.43 B 0.29
A 0.43 B 0.43
A 0.43 B 0.57
A 0.43 B 0.71
A 0.43 B 0.86
A 0.43 B 1
A 0.57 B 0
A 0.57 B 0.14
A 0.57 B 0.29
A 0.57 B 0.43
A 0.57 B 0.57
A 0.57 B 0.71
A 0.57 B 0.86
A 0.57 B 1
A 0.71 B 0
A 0.71 B 0.14
A 0.71 B 0.29
A 0.71 B 0.43
A 0.71 B 0.57
A 0.71 B 0.71
A 0.71 B 0.86
A 0.71 B 1
A 0.86 B 0
A 0.86 B 0.14
A 0.86 B 0.29
A 0.86 B 0.43
A 0.86 B 0.57
A 0.86 B 0.71
A 0.86 B 0.86
A 0.86 B 1
A 1 B 0
A 1 B 0.14
A 1 B 0.29
A 1 B 0.43
A 1 B 0.57
A 1 B 0.71
A 1 B 0.86
A 1 B 1
0.75
0.50
0.25
OD 600 nm [–]
0 5 101520
Time [h]

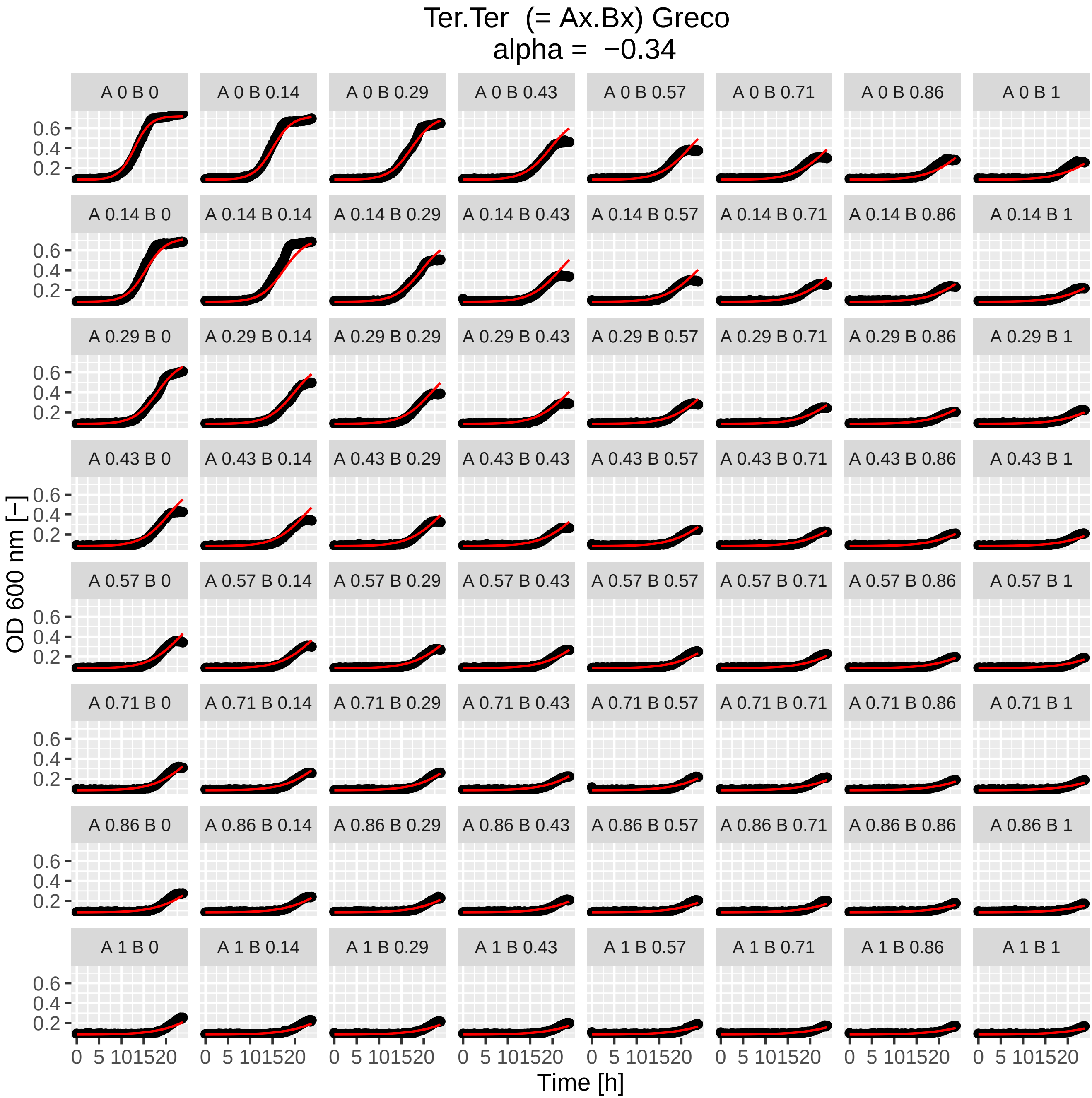

Ter.Ter (= Ax.Bx) Greco
alpha = −0.34
OD 600 nm [−]
Time [h]
A 0 B 0
A 0 B 0.14
A 0 B 0.29
A 0 B 0.43
A 0 B 0.57
A 0 B 0.71
A 0 B 0.86
A 0 B 1
A 0.14 B 0
A 0.29 B 0
A 0.43 B 0
A 0.57 B 0
A 0.71 B 0
A 0.86 B 0
A 1 B 0
A 1 B 1
0.2
0.4
0.6
0 5 101520

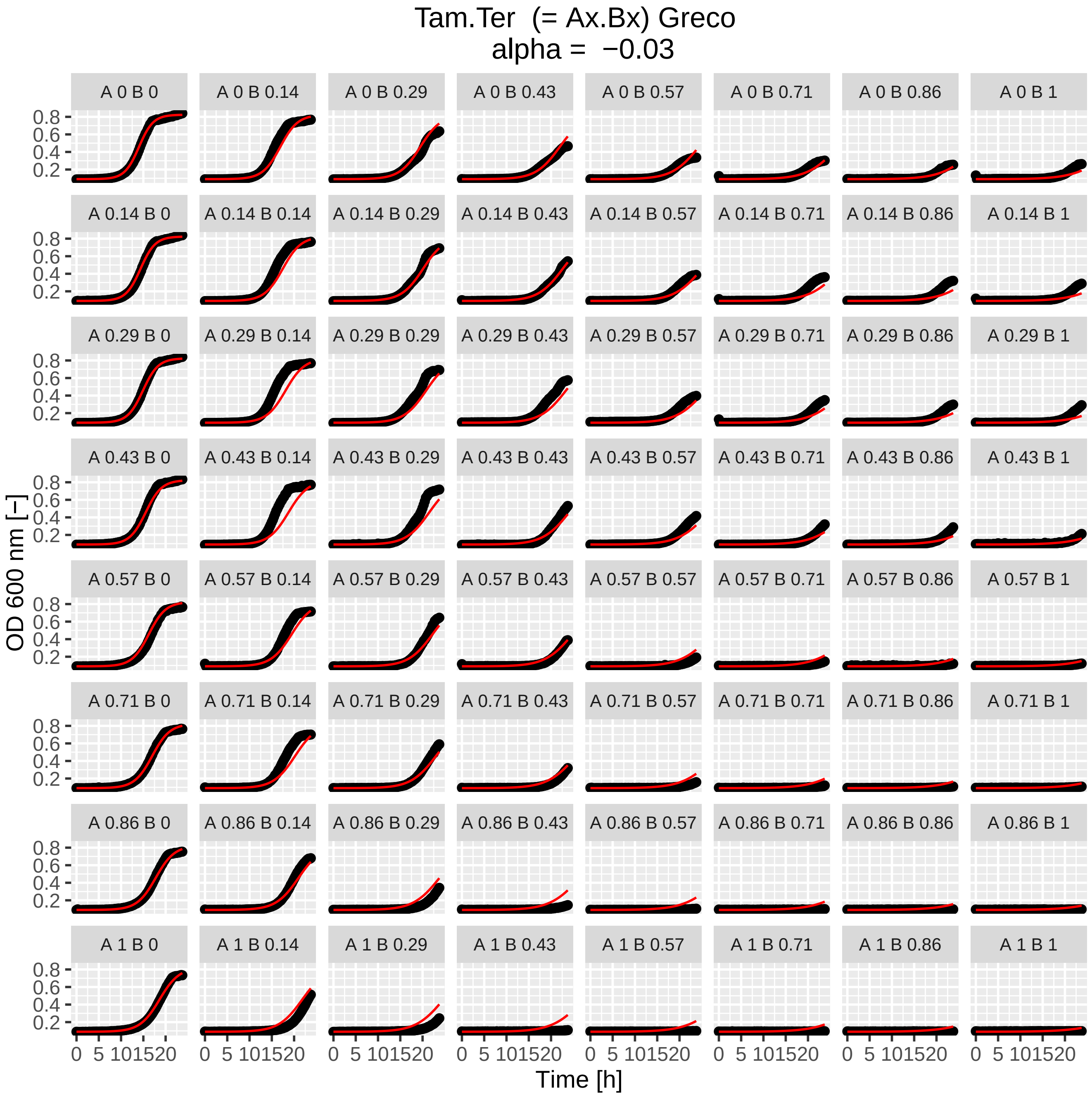

Tam.Ter (= Ax.Bx) Greco
alpha = −0.03
A 0 B 0
A 0 B 0.14
A 0 B 0.29
A 0 B 0.43
A 0 B 0.57
A 0 B 0.71
A 0 B 0.86
A 0 B 1
A 0.14 B 0
A 0.14 B 0.14
A 0.14 B 0.29
A 0.14 B 0.43
A 0.14 B 0.57
A 0.14 B 0.71
A 0.14 B 0.86
A 0.14 B 1
A 0.29 B 0
A 0.29 B 0.14
A 0.29 B 0.29
A 0.29 B 0.43
A 0.29 B 0.57
A 0.29 B 0.71
A 0.29 B 0.86
A 0.29 B 1
A 0.43 B 0
A 0.43 B 0.14
A 0.43 B 0.29
A 0.43 B 0.43
A 0.43 B 0.57
A 0.43 B 0.71
A 0.43 B 0.86
A 0.43 B 1
A 0.57 B 0
A 0.57 B 0.14
A 0.57 B 0.29
A 0.57 B 0.43
A 0.57 B 0.57
A 0.57 B 0.71
A 0.57 B 0.86
A 0.57 B 1
A 0.71 B 0
A 0.71 B 0.14
A 0.71 B 0.29
A 0.71 B 0.43
A 0.71 B 0.57
A 0.71 B 0.71
A 0.71 B 0.86
A 0.71 B 1
A 0.86 B 0
A 0.86 B 0.14
A 0.86 B 0.29
A 0.86 B 0.43
A 0.86 B 0.57
A 0.86 B 0.71
A 0.86 B 0.86
A 0.86 B 1
A 1 B 0
A 1 B 0.14
A 1 B 0.29
A 1 B 0.43
A 1 B 0.57
A 1 B 0.71
A 1 B 0.86
A 1 B 1
OD 600 nm [−]
0.8
0.6
0.4
0.2
0 5 101520
Time [h]

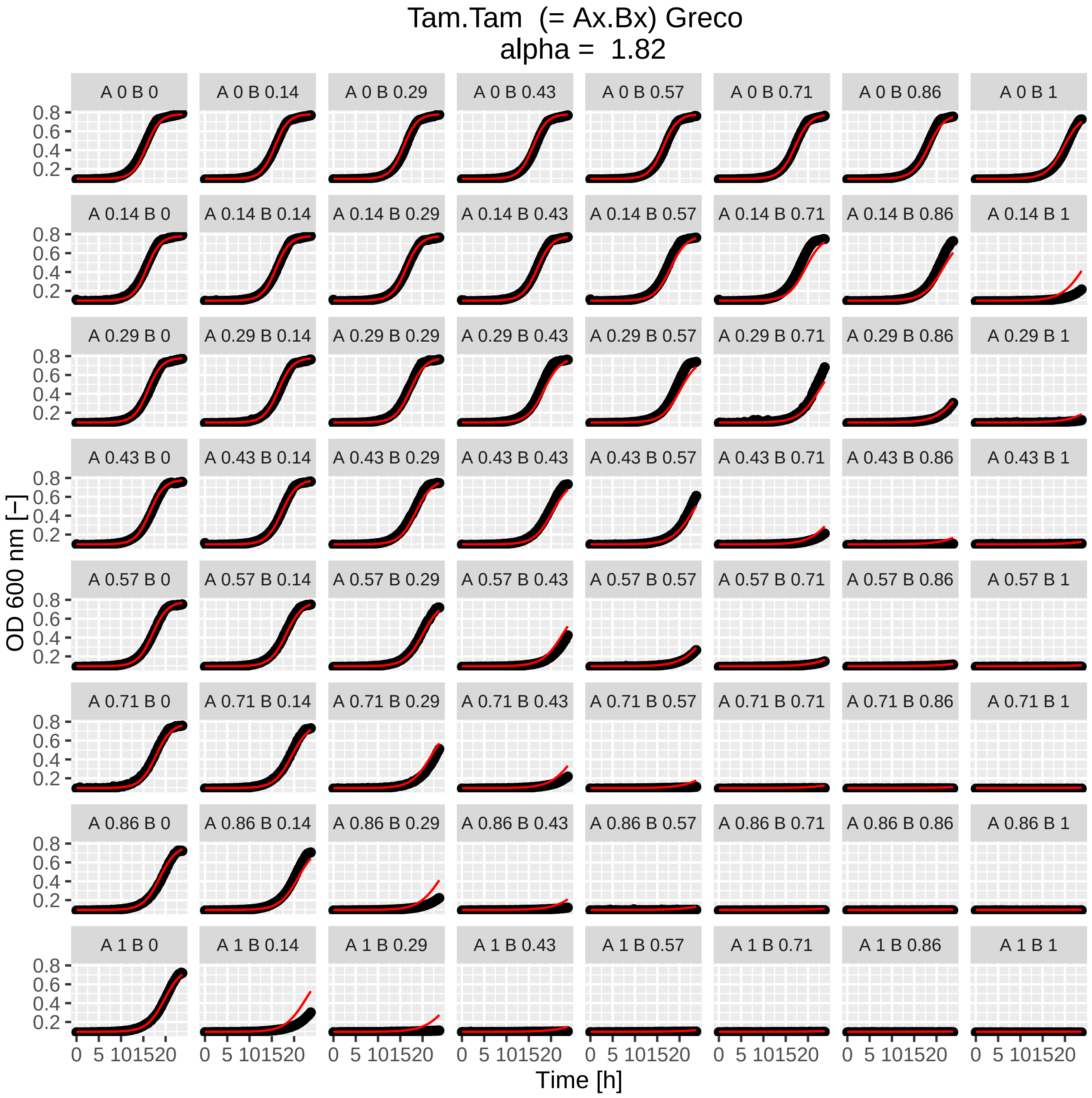

Tam.Tam (= Ax.Bx) Greco
alpha = 1.82
A 0 B 0
A 0 B 0.14
A 0 B 0.29
A 0 B 0.43
A 0 B 0.57
A 0 B 0.71
A 0 B 0.86
A 0 B 1
A 0.14 B 0
A 0.14 B 0.14
A 0.14 B 0.29
A 0.14 B 0.43
A 0.14 B 0.57
A 0.14 B 0.71
A 0.14 B 0.86
A 0.14 B 1
A 0.29 B 0
A 0.29 B 0.14
A 0.29 B 0.29
A 0.29 B 0.43
A 0.29 B 0.57
A 0.29 B 0.71
A 0.29 B 0.86
A 0.29 B 1
A 0.43 B 0
A 0.43 B 0.14
A 0.43 B 0.29
A 0.43 B 0.43
A 0.43 B 0.57
A 0.43 B 0.71
A 0.43 B 0.86
A 0.43 B 1
A 0.57 B 0
A 0.57 B 0.14
A 0.57 B 0.29
A 0.57 B 0.43
A 0.57 B 0.57
A 0.57 B 0.71
A 0.57 B 0.86
A 0.57 B 1
A 0.71 B 0
A 0.71 B 0.14
A 0.71 B 0.29
A 0.71 B 0.43
A 0.71 B 0.57
A 0.71 B 0.71
A 0.71 B 0.86
A 0.71 B 1
A 0.86 B 0
A 0.86 B 0.14
A 0.86 B 0.29
A 0.86 B 0.43
A 0.86 B 0.57
A 0.86 B 0.71
A 0.86 B 0.86
A 0.86 B 1
A 1 B 0
A 1 B 0.14
A 1 B 0.29
A 1 B 0.43
A 1 B 0.57
A 1 B 0.71
A 1 B 0.86
A 1 B 1
OD 600 nm [–]
0.2
0.4
0.6
0.8
0 5 101520
Time [h]

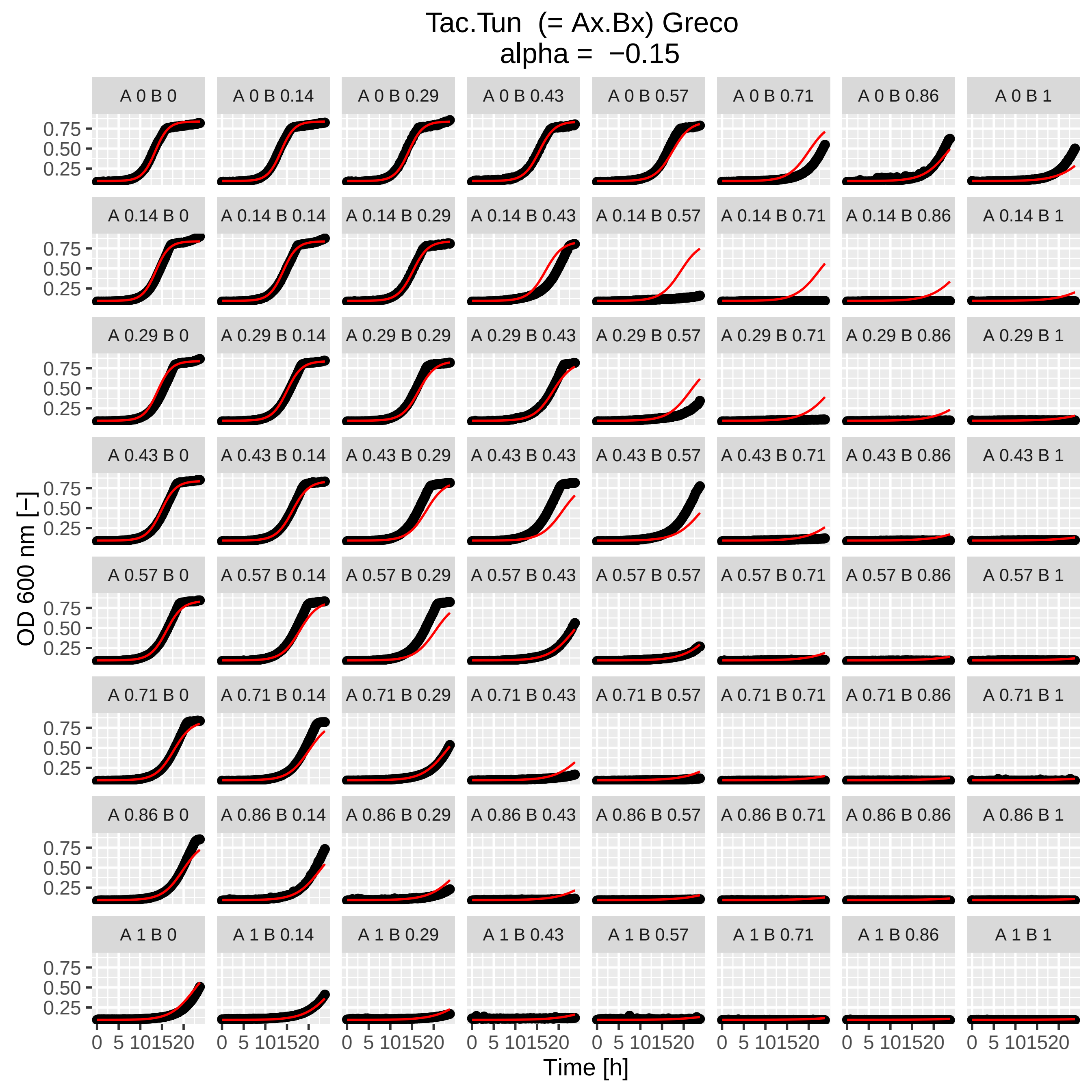
Tac.Tun (= Ax.Bx) Greco
alpha = −0.15
OD 600 nm [−]
Time [h]
0.75
0.50
0.25
0 5 101520
A 0 B 0
A 0 B 0.14
A 0 B 0.29
A 0 B 0.43
A 0 B 0.57
A 0 B 0.71
A 0 B 0.86
A 0 B 1
A 0.14 B 0
A 0.14 B 0.14
A 0.14 B 0.29
A 0.14 B 0.43
A 0.14 B 0.57
A 0.14 B 0.71
A 0.14 B 0.86
A 0.14 B 1
A 0.29 B 0
A 0.29 B 0.14
A 0.29 B 0.29
A 0.29 B 0.43
A 0.29 B 0.57
A 0.29 B 0.71
A 0.29 B 0.86
A 0.29 B 1
A 0.43 B 0
A 0.43 B 0.14
A 0.43 B 0.29
A 0.43 B 0.43
A 0.43 B 0.57
A 0.43 B 0.71
A 0.43 B 0.86
A 0.43 B 1
A 0.57 B 0
A 0.57 B 0.14
A 0.57 B 0.29
A 0.57 B 0.43
A 0.57 B 0.57
A 0.57 B 0.71
A 0.57 B 0.86
A 0.57 B 1
A 0.71 B 0
A 0.71 B 0.14
A 0.71 B 0.29
A 0.71 B 0.43
A 0.71 B 0.57
A 0.71 B 0.71
A 0.71 B 0.86
A 0.71 B 1
A 0.86 B 0
A 0.86 B 0.14
A 0.86 B 0.29
A 0.86 B 0.43
A 0.86 B 0.57
A 0.86 B 0.71
A 0.86 B 0.86
A 0.86 B 1
A 1 B 0
A 1 B 0.14
A 1 B 0.29
A 1 B 0.43
A 1 B 0.57
A 1 B 0.71
A 1 B 0.86
A 1 B 1

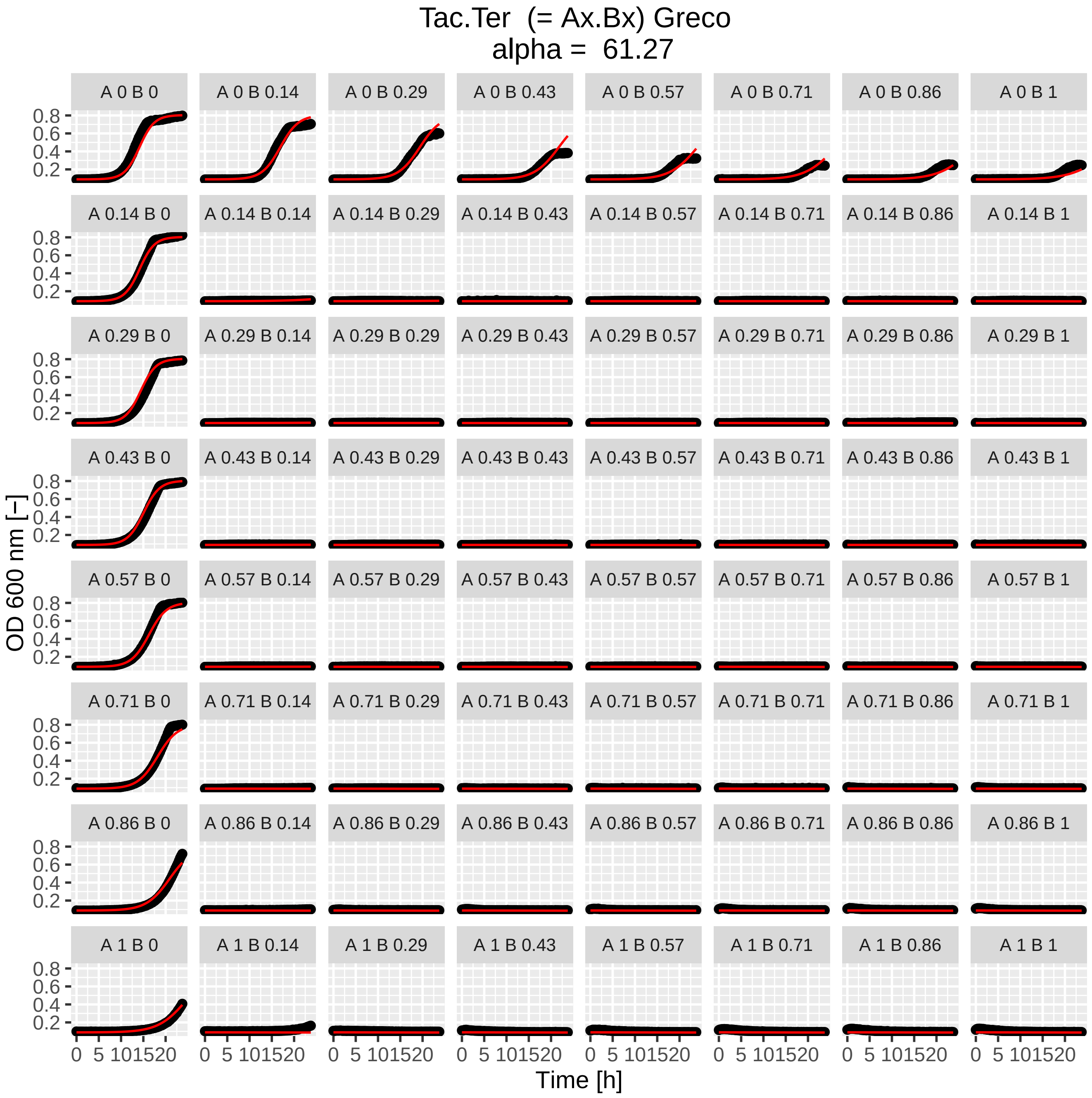
Tac.Ter (= Ax.Bx) Greco
alpha = 61.27
A 0 B 0
A 0 B 0.14
A 0 B 0.29
A 0 B 0.43
A 0 B 0.57
A 0 B 0.71
A 0 B 0.86
A 0 B 1
A 0.14 B 0
A 0.14 B 0.14
A 0.14 B 0.29
A 0.14 B 0.43
A 0.14 B 0.57
A 0.14 B 0.71
A 0.14 B 0.86
A 0.14 B 1
A 0.29 B 0
A 0.29 B 0.14
A 0.29 B 0.29
A 0.29 B 0.43
A 0.29 B 0.57
A 0.29 B 0.71
A 0.29 B 0.86
A 0.29 B 1
A 0.43 B 0
A 0.43 B 0.14
A 0.43 B 0.29
A 0.43 B 0.43
A 0.43 B 0.57
A 0.43 B 0.71
A 0.43 B 0.86
A 0.43 B 1
A 0.57 B 0
A 0.57 B 0.14
A 0.57 B 0.29
A 0.57 B 0.43
A 0.57 B 0.57
A 0.57 B 0.71
A 0.57 B 0.86
A 0.57 B 1
A 0.71 B 0
A 0.71 B 0.14
A 0.71 B 0.29
A 0.71 B 0.43
A 0.71 B 0.57
A 0.71 B 0.71
A 0.71 B 0.86
A 0.71 B 1
A 0.86 B 0
A 0.86 B 0.14
A 0.86 B 0.29
A 0.86 B 0.43
A 0.86 B 0.57
A 0.86 B 0.71
A 0.86 B 0.86
A 0.86 B 1
A 1 B 0
A 1 B 0.14
A 1 B 0.29
A 1 B 0.43
A 1 B 0.57
A 1 B 0.71
A 1 B 0.86
A 1 B 1
0.8
0.6
0.4
0.2
OD 600 nm [–]
0 5 101520
Time [h]

Tac.Tam (= Ax.Bx) Greco
alpha = 1.1

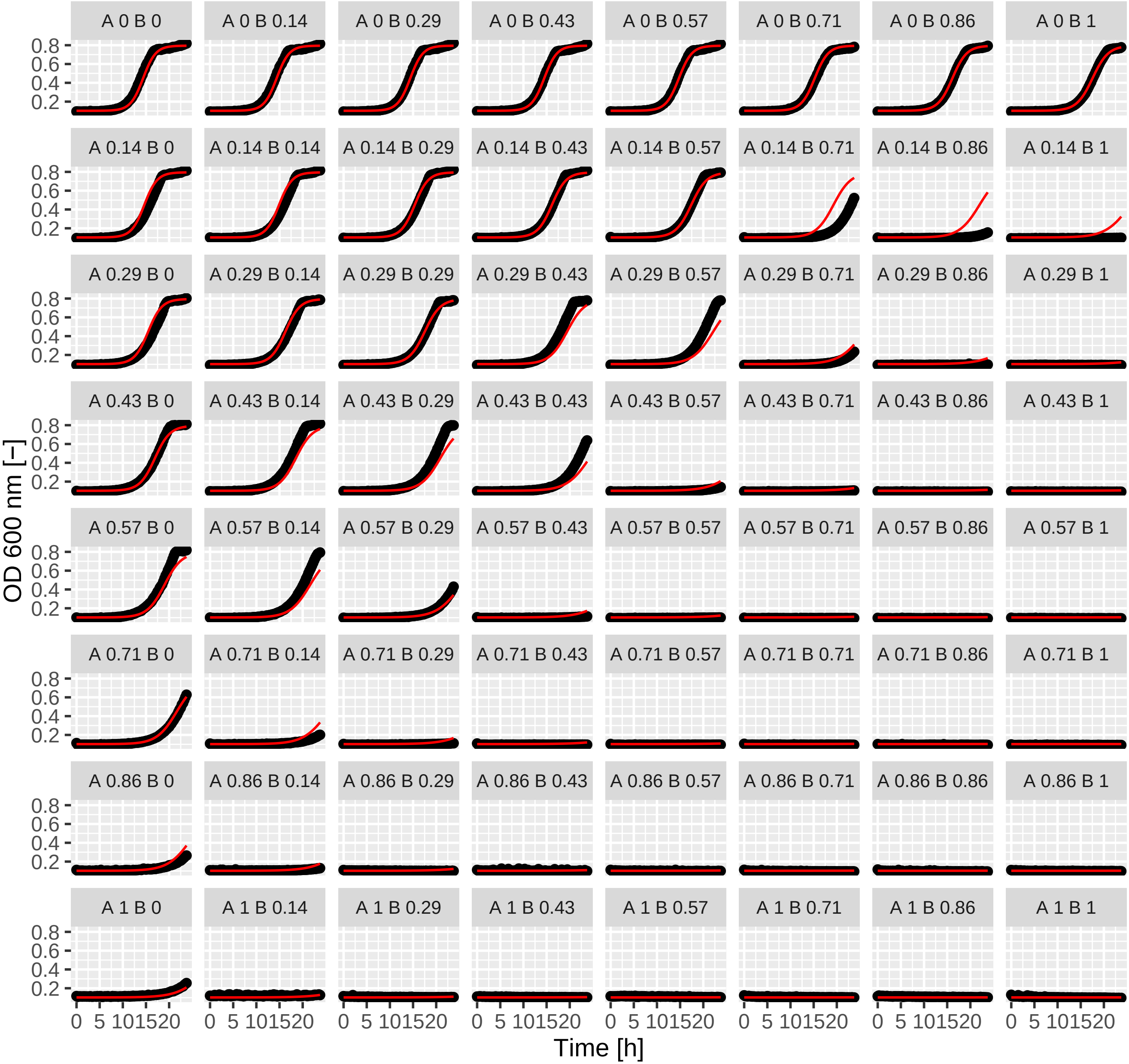

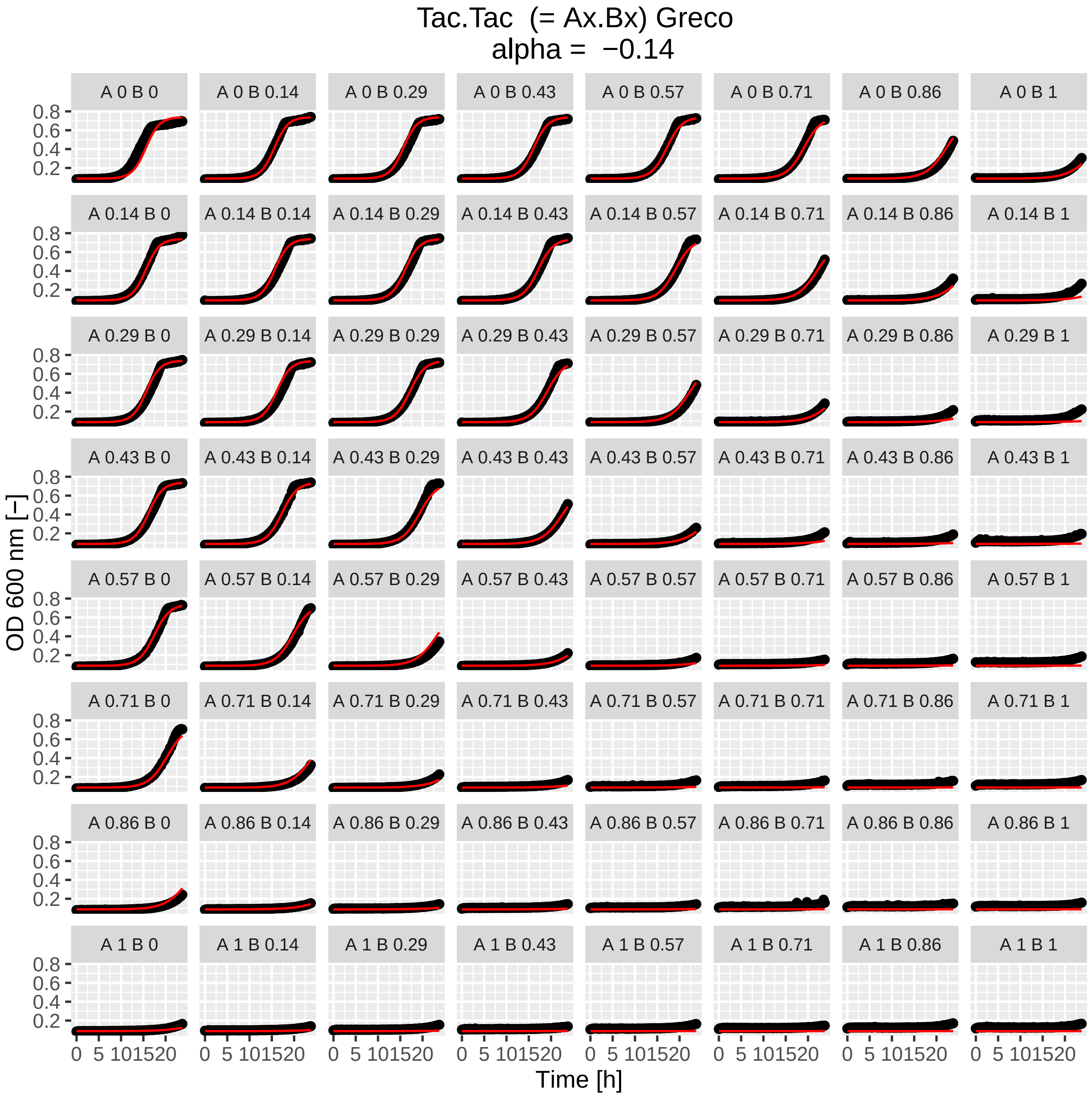
Tac.Tac (= Ax.Bx) Greco
alpha = −0.14
A 0 B 0
A 0 B 0.14
A 0 B 0.29
A 0 B 0.43
A 0 B 0.57
A 0 B 0.71
A 0 B 0.86
A 0 B 1
A 0.14 B 0
A 0.14 B 0.14
A 0.14 B 0.29
A 0.14 B 0.43
A 0.14 B 0.57
A 0.14 B 0.71
A 0.14 B 0.86
A 0.14 B 1
A 0.29 B 0
A 0.29 B 0.14
A 0.29 B 0.29
A 0.29 B 0.43
A 0.29 B 0.57
A 0.29 B 0.71
A 0.29 B 0.86
A 0.29 B 1
A 0.43 B 0
A 0.43 B 0.14
A 0.43 B 0.29
A 0.43 B 0.43
A 0.43 B 0.57
A 0.43 B 0.71
A 0.43 B 0.86
A 0.43 B 1
A 0.57 B 0
A 0.57 B 0.14
A 0.57 B 0.29
A 0.57 B 0.43
A 0.57 B 0.57
A 0.57 B 0.71
A 0.57 B 0.86
A 0.57 B 1
A 0.71 B 0
A 0.71 B 0.14
A 0.71 B 0.29
A 0.71 B 0.43
A 0.71 B 0.57
A 0.71 B 0.71
A 0.71 B 0.86
A 0.71 B 1
A 0.86 B 0
A 0.86 B 0.14
A 0.86 B 0.29
A 0.86 B 0.43
A 0.86 B 0.57
A 0.86 B 0.71
A 0.86 B 0.86
A 0.86 B 1
A 1 B 0
A 1 B 0.14
A 1 B 0.29
A 1 B 0.43
A 1 B 0.57
A 1 B 0.71
A 1 B 0.86
A 1 B 1
OD 600 nm [−]
0.2
0.4
0.6
0.8
0
5
10
15
20
Time [h]

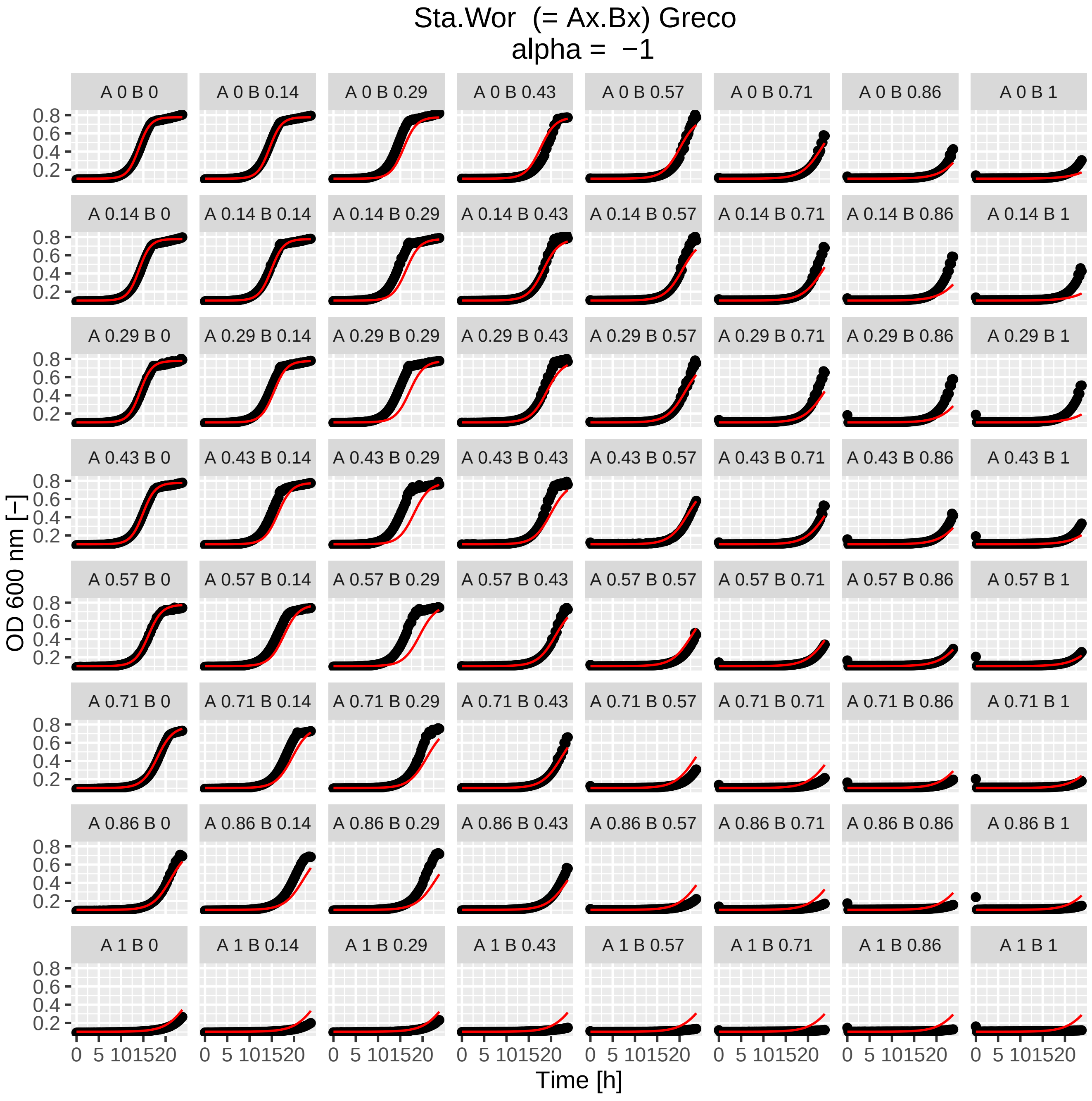
Sta.Wor (= Ax.Bx) Greco
alpha = −1
OD 600 nm [−]
Time [h]
0.8
0.6
0.4
0.2
0 5 101520
A 0 B 0
A 0 B 0.14
A 0 B 0.29
A 0 B 0.43
A 0 B 0.57
A 0 B 0.71
A 0 B 0.86
A 0 B 1
A 0.14 B 0
A 0.14 B 0.14
A 0.14 B 0.29
A 0.14 B 0.43
A 0.14 B 0.57
A 0.14 B 0.71
A 0.14 B 0.86
A 0.14 B 1
A 0.29 B 0
A 0.29 B 0.14
A 0.29 B 0.29
A 0.29 B 0.43
A 0.29 B 0.57
A 0.29 B 0.71
A 0.29 B 0.86
A 0.29 B 1
A 0.43 B 0
A 0.43 B 0.14
A 0.43 B 0.29
A 0.43 B 0.43
A 0.43 B 0.57
A 0.43 B 0.71
A 0.43 B 0.86
A 0.43 B 1
A 0.57 B 0
A 0.57 B 0.14
A 0.57 B 0.29
A 0.57 B 0.43
A 0.57 B 0.57
A 0.57 B 0.71
A 0.57 B 0.86
A 0.57 B 1
A 0.71 B 0
A 0.71 B 0.14
A 0.71 B 0.29
A 0.71 B 0.43
A 0.71 B 0.57
A 0.71 B 0.71
A 0.71 B 0.86
A 0.71 B 1
A 0.86 B 0
A 0.86 B 0.14
A 0.86 B 0.29
A 0.86 B 0.43
A 0.86 B 0.57
A 0.86 B 0.71
A 0.86 B 0.86
A 0.86 B 1
A 1 B 0
A 1 B 0.14
A 1 B 0.29
A 1 B 0.43
A 1 B 0.57
A 1 B 0.71
A 1 B 0.86
A 1 B 1

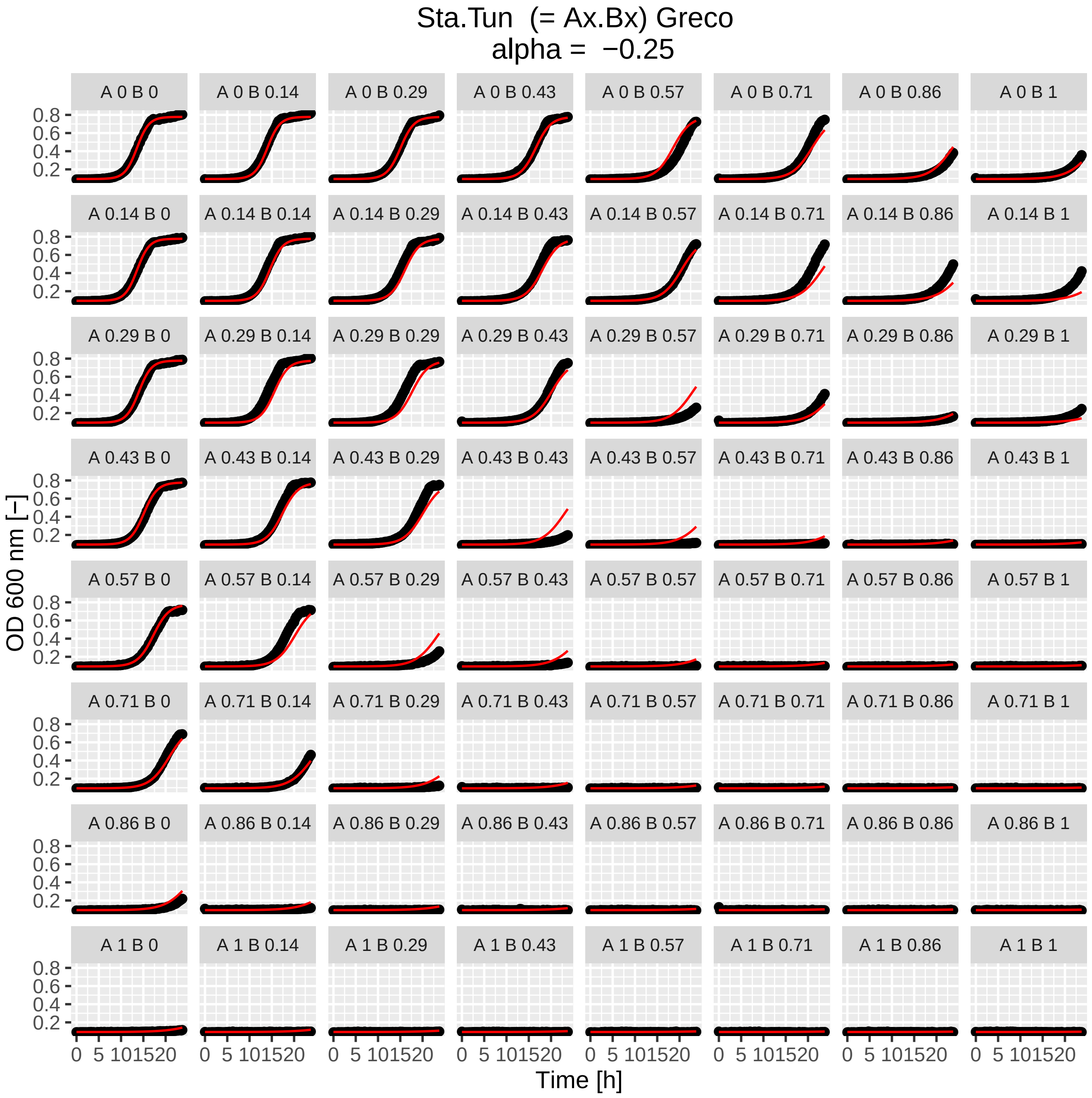
Sta.Tun (= Ax.Bx) Greco
alpha = −0.25
OD 600 nm [−]
Time [h]
A 0 B 0
A 0 B 0.14
A 0 B 0.29
A 0 B 0.43
A 0 B 0.57
A 0 B 0.71
A 0 B 0.86
A 0 B 1
A 1 B 0
A 1 B 1
0.8
0.6
0.4
0.2
0 5 101520

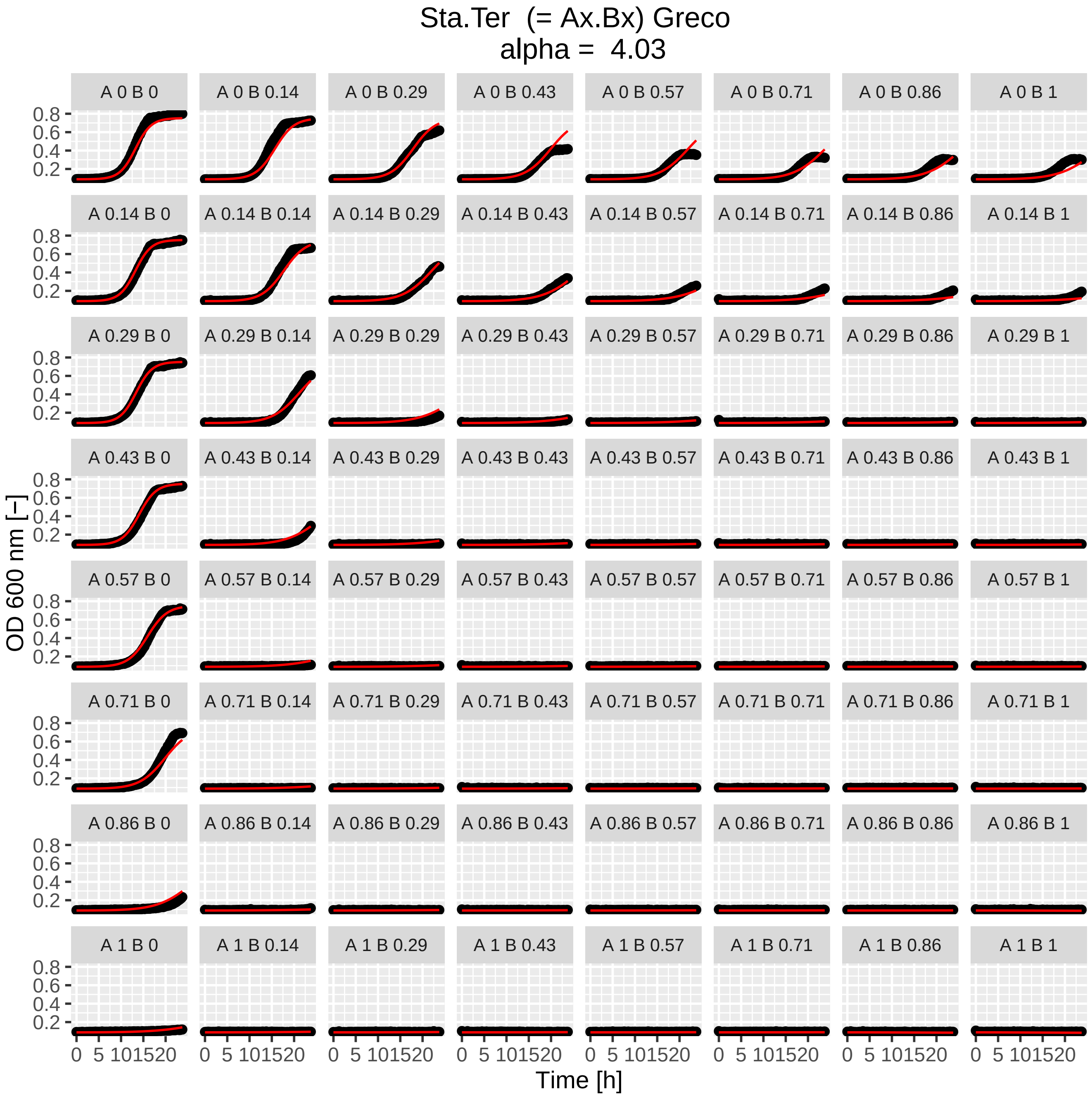
Sta.Ter (= Ax.Bx) Greco
alpha = 4.03
OD 600 nm [–]
Time [h]

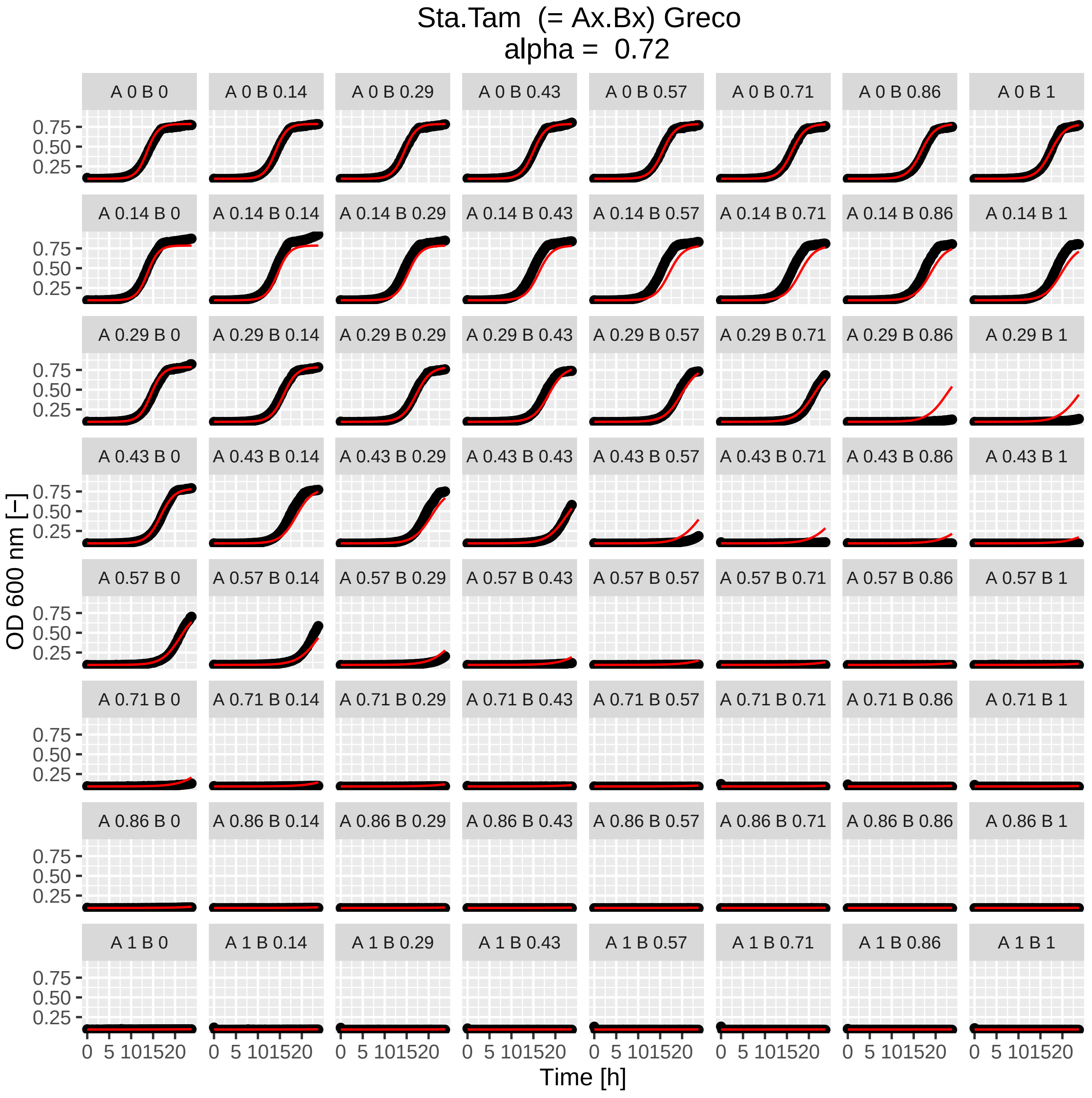
Sta.Tam (= Ax.Bx) Greco
alpha = 0.72
A 0 B 0
A 0 B 0.14
A 0 B 0.29
A 0 B 0.43
A 0 B 0.57
A 0 B 0.71
A 0 B 0.86
A 0 B 1
A 0.14 B 0
A 0.14 B 0.14
A 0.14 B 0.29
A 0.14 B 0.43
A 0.14 B 0.57
A 0.14 B 0.71
A 0.14 B 0.86
A 0.14 B 1
A 0.29 B 0
A 0.29 B 0.14
A 0.29 B 0.29
A 0.29 B 0.43
A 0.29 B 0.57
A 0.29 B 0.71
A 0.29 B 0.86
A 0.29 B 1
A 0.43 B 0
A 0.43 B 0.14
A 0.43 B 0.29
A 0.43 B 0.43
A 0.43 B 0.57
A 0.43 B 0.71
A 0.43 B 0.86
A 0.43 B 1
A 0.57 B 0
A 0.57 B 0.14
A 0.57 B 0.29
A 0.57 B 0.43
A 0.57 B 0.57
A 0.57 B 0.71
A 0.57 B 0.86
A 0.57 B 1
A 0.71 B 0
A 0.71 B 0.14
A 0.71 B 0.29
A 0.71 B 0.43
A 0.71 B 0.57
A 0.71 B 0.71
A 0.71 B 0.86
A 0.71 B 1
A 0.86 B 0
A 0.86 B 0.14
A 0.86 B 0.29
A 0.86 B 0.43
A 0.86 B 0.57
A 0.86 B 0.71
A 0.86 B 0.86
A 0.86 B 1
A 1 B 0
A 1 B 0.14
A 1 B 0.29
A 1 B 0.43
A 1 B 0.57
A 1 B 0.71
A 1 B 0.86
A 1 B 1
OD 600 nm [–]
0.75
0.50
0.25
0 5 101520
Time [h]

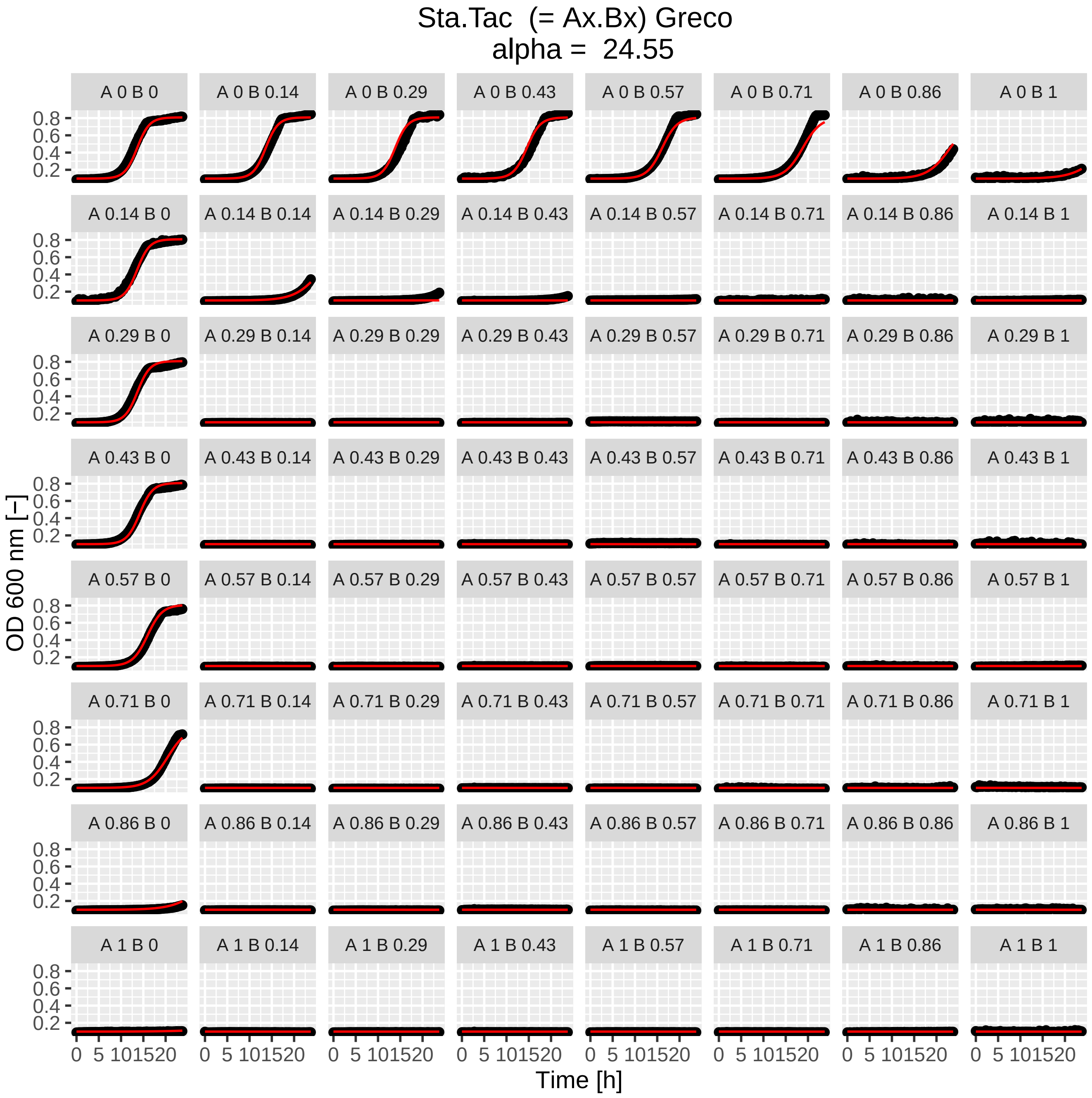
Sta.Tac (= Ax.Bx) Greco
alpha = 24.55
A 0 B 0
A 0 B 0.14
A 0 B 0.29
A 0 B 0.43
A 0 B 0.57
A 0 B 0.71
A 0 B 0.86
A 0 B 1
A 0.14 B 0
A 0.14 B 0.14
A 0.14 B 0.29
A 0.14 B 0.43
A 0.14 B 0.57
A 0.14 B 0.71
A 0.14 B 0.86
A 0.14 B 1
A 0.29 B 0
A 0.29 B 0.14
A 0.29 B 0.29
A 0.29 B 0.43
A 0.29 B 0.57
A 0.29 B 0.71
A 0.29 B 0.86
A 0.29 B 1
A 0.43 B 0
A 0.43 B 0.14
A 0.43 B 0.29
A 0.43 B 0.43
A 0.43 B 0.57
A 0.43 B 0.71
A 0.43 B 0.86
A 0.43 B 1
A 0.57 B 0
A 0.57 B 0.14
A 0.57 B 0.29
A 0.57 B 0.43
A 0.57 B 0.57
A 0.57 B 0.71
A 0.57 B 0.86
A 0.57 B 1
A 0.71 B 0
A 0.71 B 0.14
A 0.71 B 0.29
A 0.71 B 0.43
A 0.71 B 0.57
A 0.71 B 0.71
A 0.71 B 0.86
A 0.71 B 1
A 0.86 B 0
A 0.86 B 0.14
A 0.86 B 0.29
A 0.86 B 0.43
A 0.86 B 0.57
A 0.86 B 0.71
A 0.86 B 0.86
A 0.86 B 1
A 1 B 0
A 1 B 0.14
A 1 B 0.29
A 1 B 0.43
A 1 B 0.57
A 1 B 0.71
A 1 B 0.86
A 1 B 1
OD 600 nm [−]
0.2
0.4
0.6
0.8
0 5 101520
Time [h]

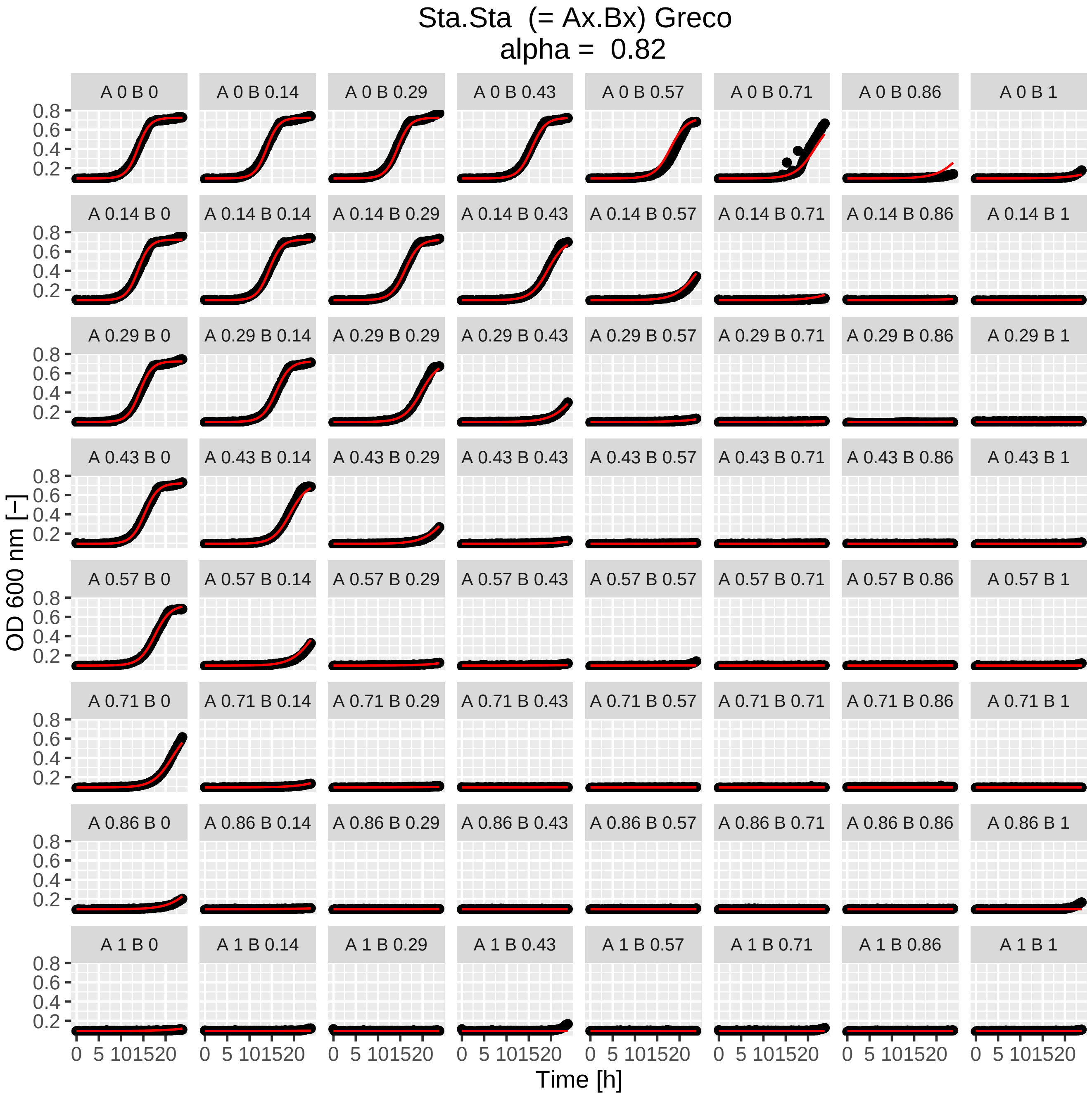

Sta.Sta (= Ax.Bx) Greco
alpha = 0.82
A 0 B 0
A 0 B 0.14
A 0 B 0.29
A 0 B 0.43
A 0 B 0.57
A 0 B 0.71
A 0 B 0.86
A 0 B 1
A 0.14 B 0
A 0.14 B 0.14
A 0.14 B 0.29
A 0.14 B 0.43
A 0.14 B 0.57
A 0.14 B 0.71
A 0.14 B 0.86
A 0.14 B 1
A 0.29 B 0
A 0.29 B 0.14
A 0.29 B 0.29
A 0.29 B 0.43
A 0.29 B 0.57
A 0.29 B 0.71
A 0.29 B 0.86
A 0.29 B 1
A 0.43 B 0
A 0.43 B 0.14
A 0.43 B 0.29
A 0.43 B 0.43
A 0.43 B 0.57
A 0.43 B 0.71
A 0.43 B 0.86
A 0.43 B 1
A 0.57 B 0
A 0.57 B 0.14
A 0.57 B 0.29
A 0.57 B 0.43
A 0.57 B 0.57
A 0.57 B 0.71
A 0.57 B 0.86
A 0.57 B 1
A 0.71 B 0
A 0.71 B 0.14
A 0.71 B 0.29
A 0.71 B 0.43
A 0.71 B 0.57
A 0.71 B 0.71
A 0.71 B 0.86
A 0.71 B 1
A 0.86 B 0
A 0.86 B 0.14
A 0.86 B 0.29
A 0.86 B 0.43
A 0.86 B 0.57
A 0.86 B 0.71
A 0.86 B 0.86
A 0.86 B 1
A 1 B 0
A 1 B 0.14
A 1 B 0.29
A 1 B 0.43
A 1 B 0.57
A 1 B 0.71
A 1 B 0.86
A 1 B 1
OD 600 nm [–]
0.8
0.6
0.4
0.2
0 5 101520
Time [h]

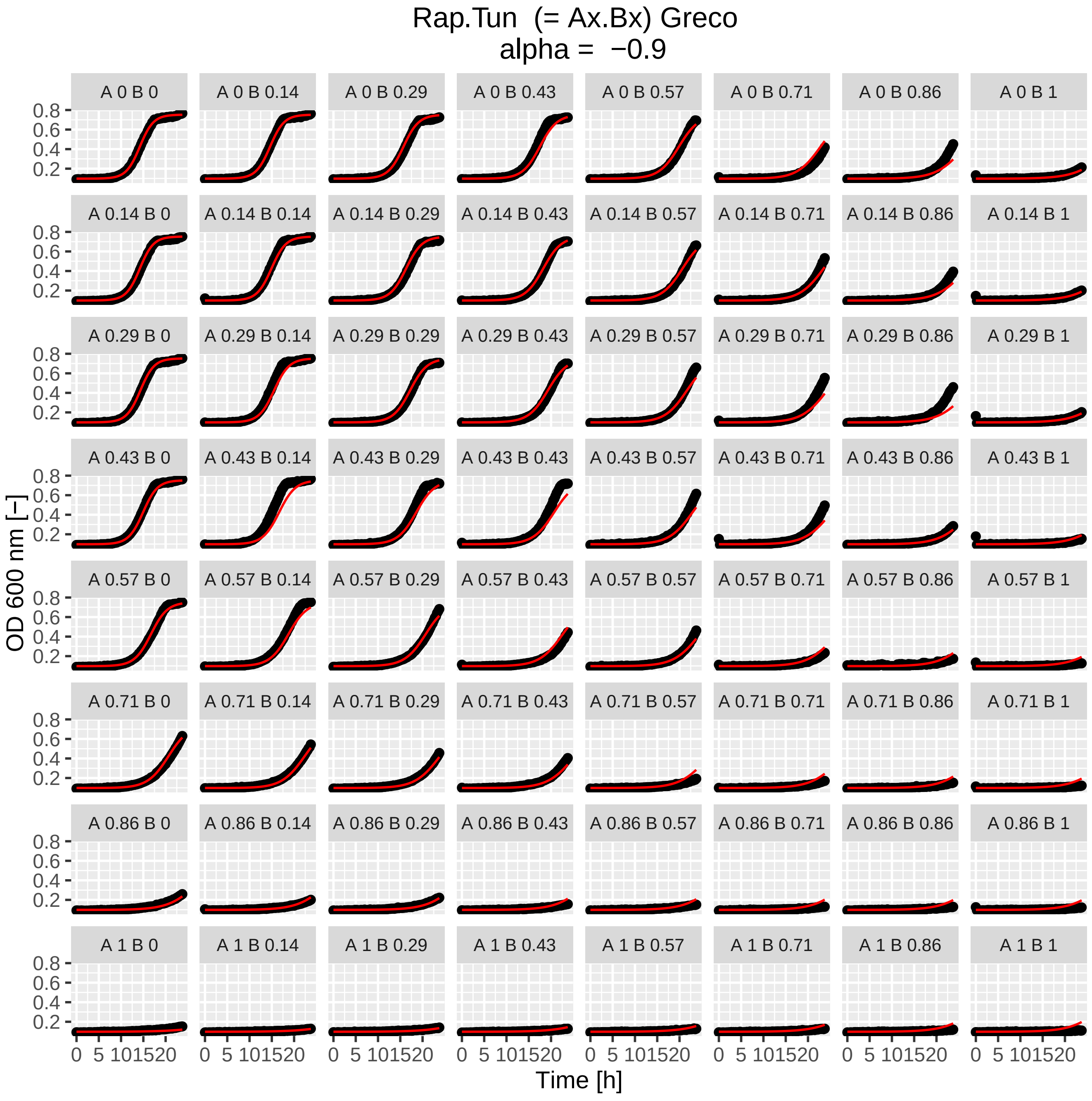

Rap.Tun (= Ax.Bx) Greco
alpha = −0.9
A 0 B 0
A 0 B 0.14
A 0 B 0.29
A 0 B 0.43
A 0 B 0.57
A 0 B 0.71
A 0 B 0.86
A 0 B 1
A 0.14 B 0
A 0.14 B 0.14
A 0.14 B 0.29
A 0.14 B 0.43
A 0.14 B 0.57
A 0.14 B 0.71
A 0.14 B 0.86
A 0.14 B 1
A 0.29 B 0
A 0.29 B 0.14
A 0.29 B 0.29
A 0.29 B 0.43
A 0.29 B 0.57
A 0.29 B 0.71
A 0.29 B 0.86
A 0.29 B 1
A 0.43 B 0
A 0.43 B 0.14
A 0.43 B 0.29
A 0.43 B 0.43
A 0.43 B 0.57
A 0.43 B 0.71
A 0.43 B 0.86
A 0.43 B 1
A 0.57 B 0
A 0.57 B 0.14
A 0.57 B 0.29
A 0.57 B 0.43
A 0.57 B 0.57
A 0.57 B 0.71
A 0.57 B 0.86
A 0.57 B 1
A 0.71 B 0
A 0.71 B 0.14
A 0.71 B 0.29
A 0.71 B 0.43
A 0.71 B 0.57
A 0.71 B 0.71
A 0.71 B 0.86
A 0.71 B 1
A 0.86 B 0
A 0.86 B 0.14
A 0.86 B 0.29
A 0.86 B 0.43
A 0.86 B 0.57
A 0.86 B 0.71
A 0.86 B 0.86
A 0.86 B 1
A 1 B 0
A 1 B 0.14
A 1 B 0.29
A 1 B 0.43
A 1 B 0.57
A 1 B 0.71
A 1 B 0.86
A 1 B 1
0.8
0.6
0.4
0.2
0 5 101520
OD 600 nm [−]
Time [h]

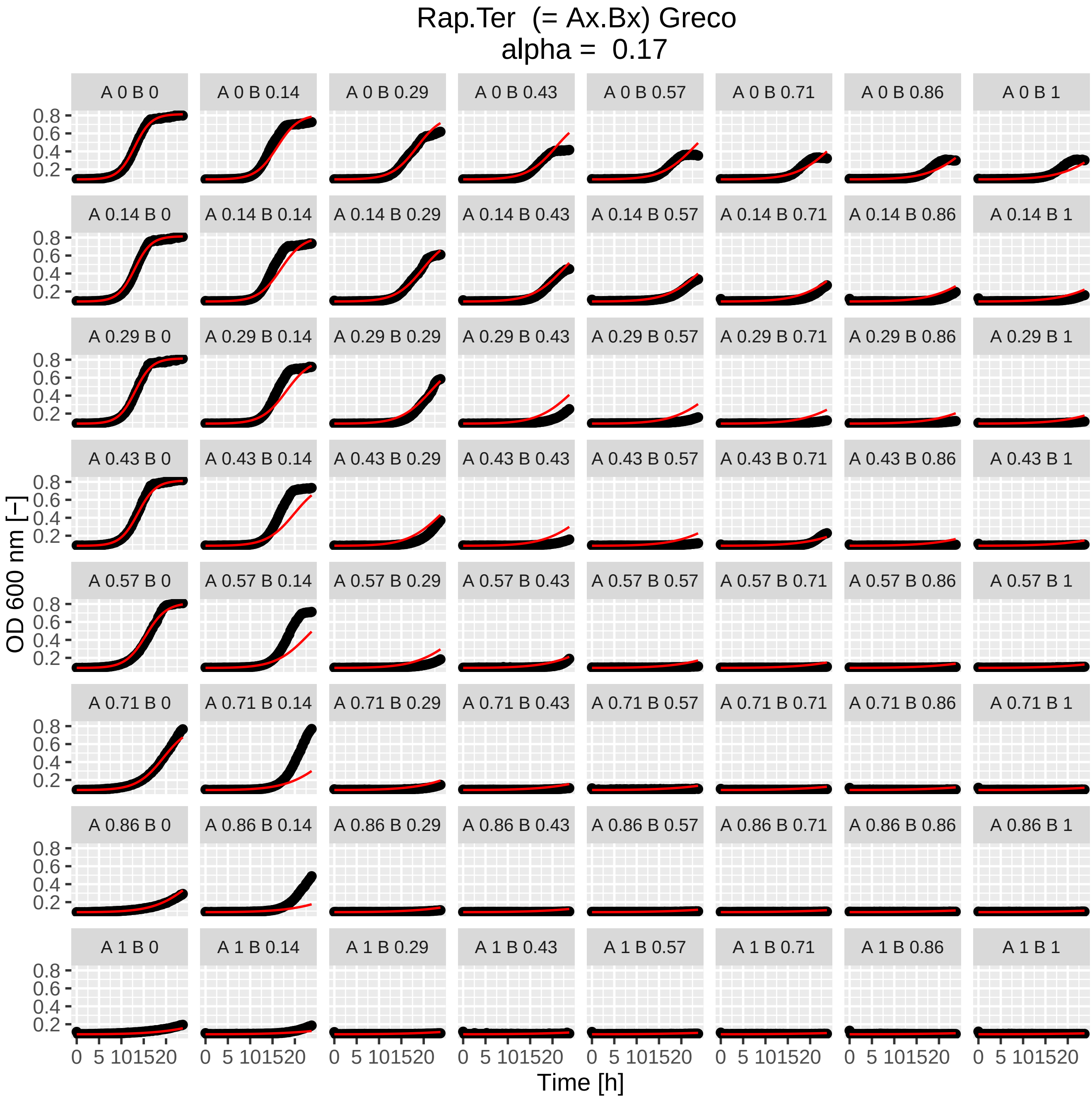
Rap.Ter (= Ax.Bx) Greco
alpha = 0.17
A 0 B 0
A 0 B 0.14
A 0 B 0.29
A 0 B 0.43
A 0 B 0.57
A 0 B 0.71
A 0 B 0.86
A 0 B 1
A 0.14 B 0
A 0.14 B 0.14
A 0.14 B 0.29
A 0.14 B 0.43
A 0.14 B 0.57
A 0.14 B 0.71
A 0.14 B 0.86
A 0.14 B 1
A 0.29 B 0
A 0.29 B 0.14
A 0.29 B 0.29
A 0.29 B 0.43
A 0.29 B 0.57
A 0.29 B 0.71
A 0.29 B 0.86
A 0.29 B 1
A 0.43 B 0
A 0.43 B 0.14
A 0.43 B 0.29
A 0.43 B 0.43
A 0.43 B 0.57
A 0.43 B 0.71
A 0.43 B 0.86
A 0.43 B 1
A 0.57 B 0
A 0.57 B 0.14
A 0.57 B 0.29
A 0.57 B 0.43
A 0.57 B 0.57
A 0.57 B 0.71
A 0.57 B 0.86
A 0.57 B 1
A 0.71 B 0
A 0.71 B 0.14
A 0.71 B 0.29
A 0.71 B 0.43
A 0.71 B 0.57
A 0.71 B 0.71
A 0.71 B 0.86
A 0.71 B 1
A 0.86 B 0
A 0.86 B 0.14
A 0.86 B 0.29
A 0.86 B 0.43
A 0.86 B 0.57
A 0.86 B 0.71
A 0.86 B 0.86
A 0.86 B 1
A 1 B 0
A 1 B 0.14
A 1 B 0.29
A 1 B 0.43
A 1 B 0.57
A 1 B 0.71
A 1 B 0.86
A 1 B 1
OD 600 nm [–]
0.8
0.6
0.4
0.2
0 5 101520
Time [h]

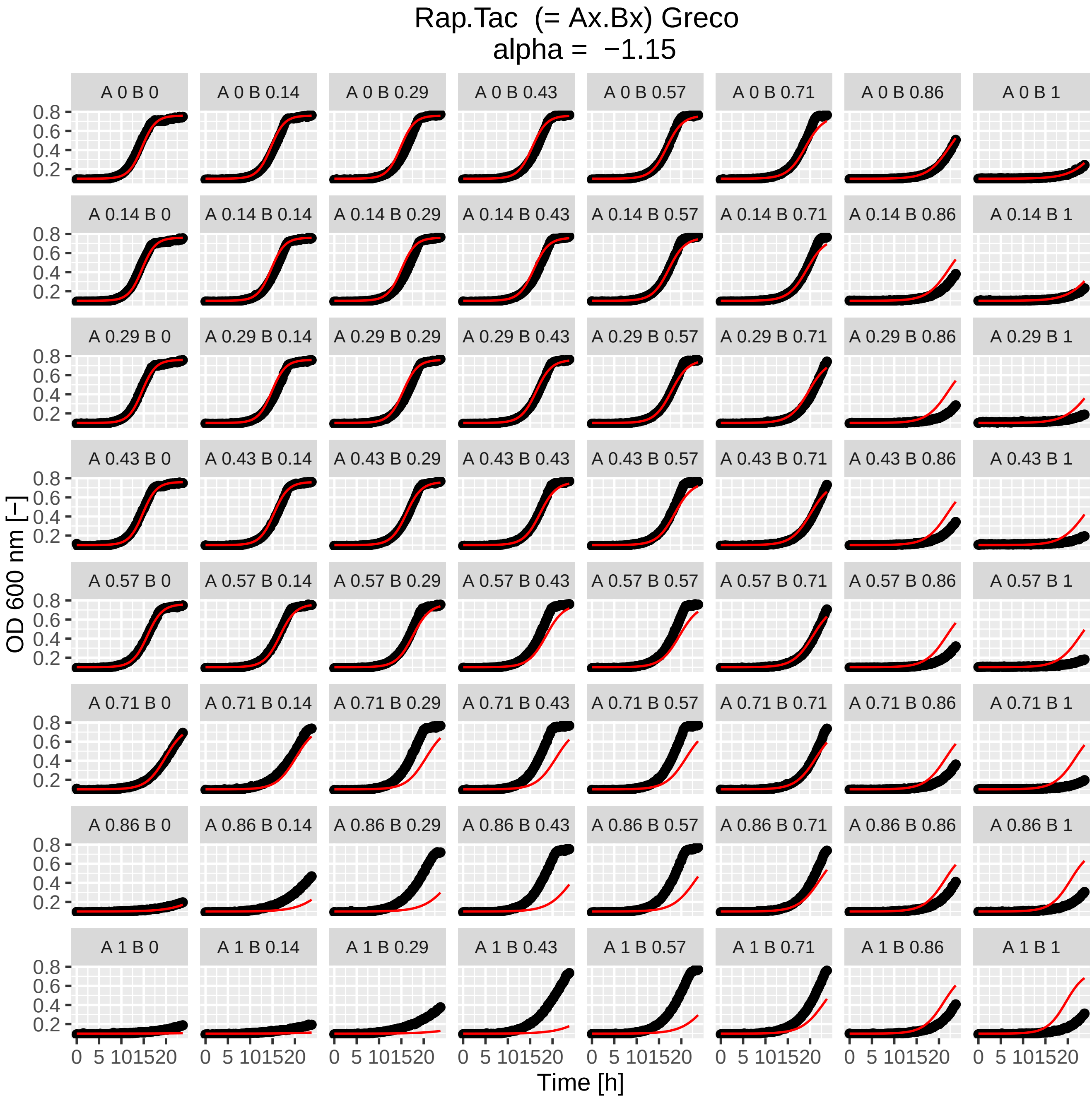

Rap.Tac (= Ax.Bx) Greco
alpha = −1.15
OD 600 nm [−]
Time [h]
A 0 B 0
A 0 B 0.14
A 0 B 0.29
A 0 B 0.43
A 0 B 0.57
A 0 B 0.71
A 0 B 0.86
A 0 B 1
A 0.14 B 0
A 0.14 B 0.14
A 0.14 B 0.29
A 0.14 B 0.43
A 0.14 B 0.57
A 0.14 B 0.71
A 0.14 B 0.86
A 0.14 B 1
A 0.29 B 0
A 0.29 B 0.14
A 0.29 B 0.29
A 0.29 B 0.43
A 0.29 B 0.57
A 0.29 B 0.71
A 0.29 B 0.86
A 0.29 B 1
A 0.43 B 0
A 0.43 B 0.14
A 0.43 B 0.29
A 0.43 B 0.43
A 0.43 B 0.57
A 0.43 B 0.71
A 0.43 B 0.86
A 0.43 B 1
A 0.57 B 0
A 0.57 B 0.14
A 0.57 B 0.29
A 0.57 B 0.43
A 0.57 B 0.57
A 0.57 B 0.71
A 0.57 B 0.86
A 0.57 B 1
A 0.71 B 0
A 0.71 B 0.14
A 0.71 B 0.29
A 0.71 B 0.43
A 0.71 B 0.57
A 0.71 B 0.71
A 0.71 B 0.86
A 0.71 B 1
A 0.86 B 0
A 0.86 B 0.14
A 0.86 B 0.29
A 0.86 B 0.43
A 0.86 B 0.57
A 0.86 B 0.71
A 0.86 B 0.86
A 0.86 B 1
A 1 B 0
A 1 B 0.14
A 1 B 0.29
A 1 B 0.43
A 1 B 0.57
A 1 B 0.71
A 1 B 0.86
A 1 B 1
0.8
0.6
0.4
0.2
0 5 101520

Rap.Sta (= Ax.Bx) Greco
alpha = −0.52

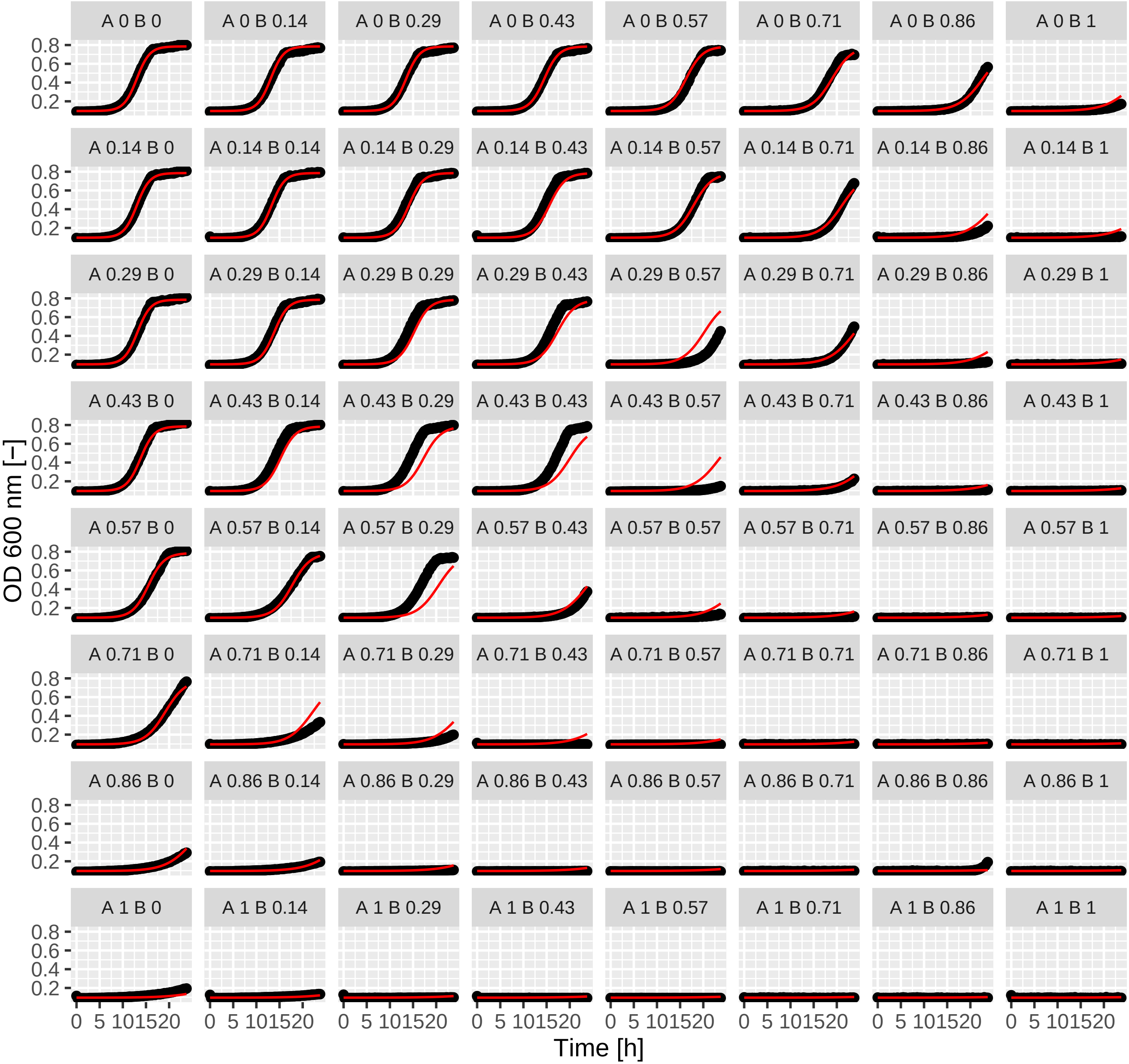

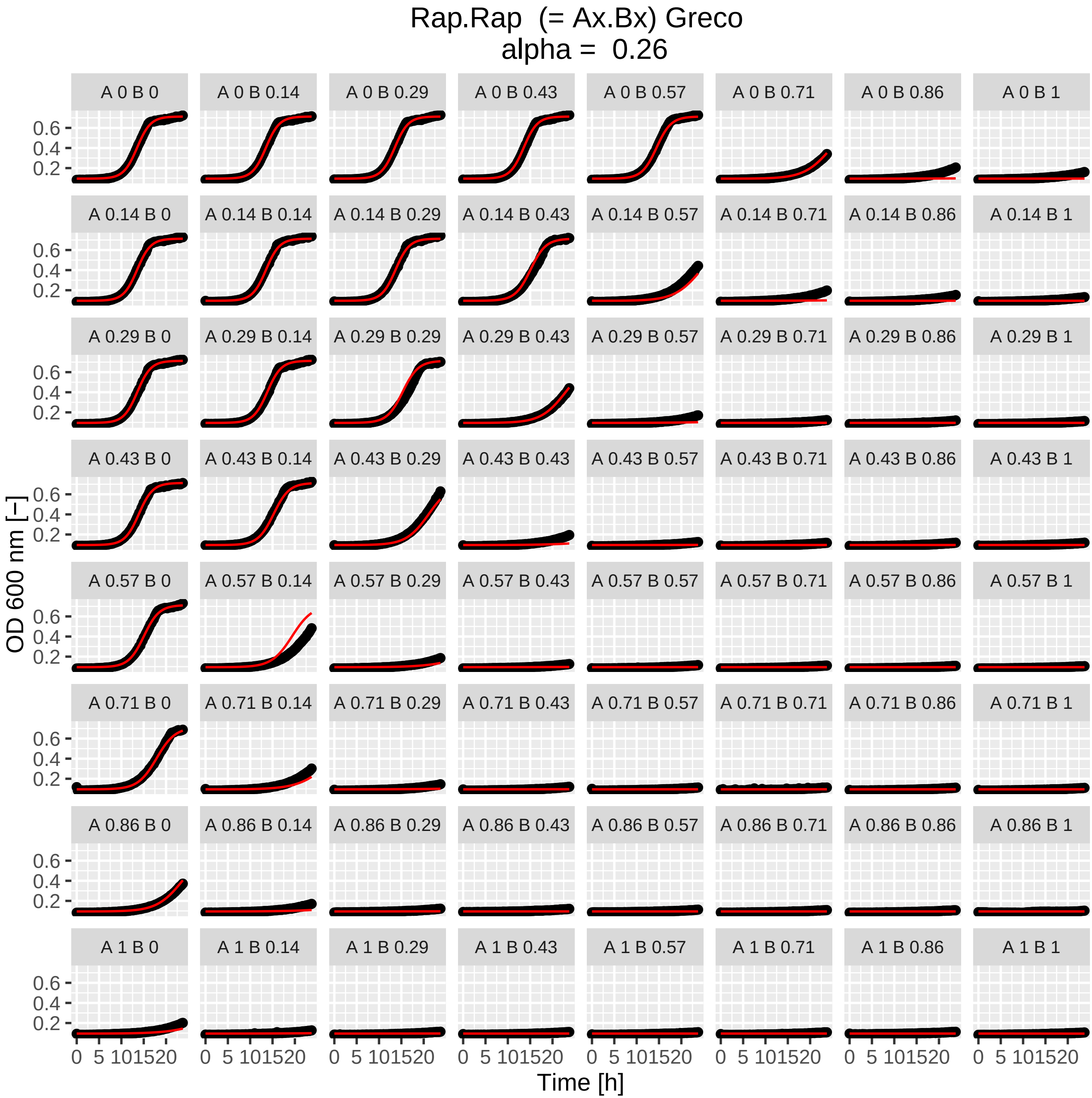
Rap.Rap (= Ax.Bx) Greco
alpha = 0.26
A 0 B 0
A 0 B 0.14
A 0 B 0.29
A 0 B 0.43
A 0 B 0.57
A 0 B 0.71
A 0 B 0.86
A 0 B 1
A 0.14 B 0
A 0.14 B 0.14
A 0.14 B 0.29
A 0.14 B 0.43
A 0.14 B 0.57
A 0.14 B 0.71
A 0.14 B 0.86
A 0.14 B 1
A 0.29 B 0
A 0.29 B 0.14
A 0.29 B 0.29
A 0.29 B 0.43
A 0.29 B 0.57
A 0.29 B 0.71
A 0.29 B 0.86
A 0.29 B 1
A 0.43 B 0
A 0.43 B 0.14
A 0.43 B 0.29
A 0.43 B 0.43
A 0.43 B 0.57
A 0.43 B 0.71
A 0.43 B 0.86
A 0.43 B 1
A 0.57 B 0
A 0.57 B 0.14
A 0.57 B 0.29
A 0.57 B 0.43
A 0.57 B 0.57
A 0.57 B 0.71
A 0.57 B 0.86
A 0.57 B 1
A 0.71 B 0
A 0.71 B 0.14
A 0.71 B 0.29
A 0.71 B 0.43
A 0.71 B 0.57
A 0.71 B 0.71
A 0.71 B 0.86
A 0.71 B 1
A 0.86 B 0
A 0.86 B 0.14
A 0.86 B 0.29
A 0.86 B 0.43
A 0.86 B 0.57
A 0.86 B 0.71
A 0.86 B 0.86
A 0.86 B 1
A 1 B 0
A 1 B 0.14
A 1 B 0.29
A 1 B 0.43
A 1 B 0.57
A 1 B 0.71
A 1 B 0.86
A 1 B 1
OD 600 nm [–]
0.2
0.4
0.6
0 5 101520
Time [h]

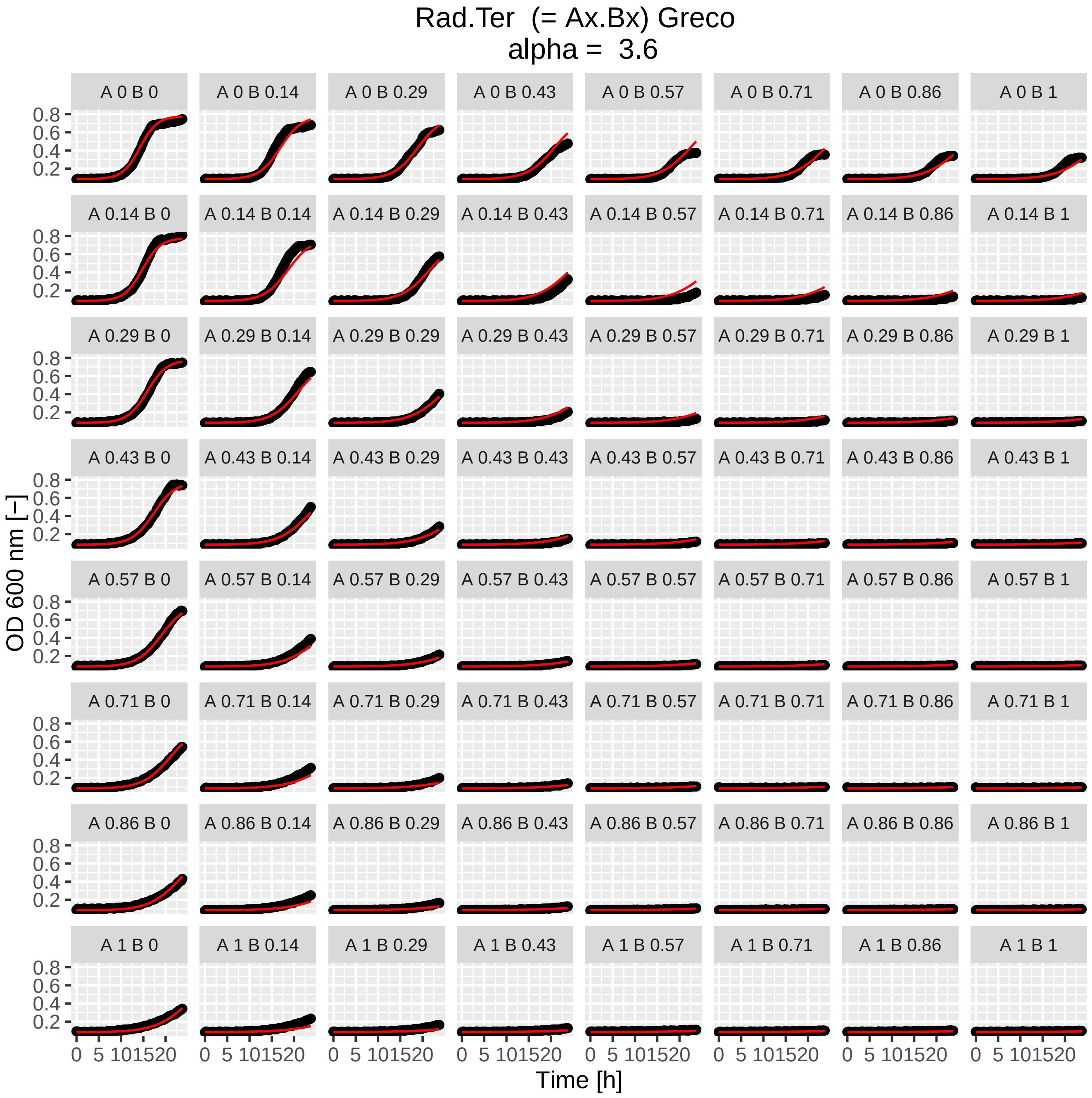

Rad.Ter (= Ax.Bx) Greco
alpha = 3.6
A 0 B 0
A 0 B 0.14
A 0 B 0.29
A 0 B 0.43
A 0 B 0.57
A 0 B 0.71
A 0 B 0.86
A 0 B 1
A 0.14 B 0
A 0.14 B 0.14
A 0.14 B 0.29
A 0.14 B 0.43
A 0.14 B 0.57
A 0.14 B 0.71
A 0.14 B 0.86
A 0.14 B 1
A 0.29 B 0
A 0.29 B 0.14
A 0.29 B 0.29
A 0.29 B 0.43
A 0.29 B 0.57
A 0.29 B 0.71
A 0.29 B 0.86
A 0.29 B 1
A 0.43 B 0
A 0.43 B 0.14
A 0.43 B 0.29
A 0.43 B 0.43
A 0.43 B 0.57
A 0.43 B 0.71
A 0.43 B 0.86
A 0.43 B 1
A 0.57 B 0
A 0.57 B 0.14
A 0.57 B 0.29
A 0.57 B 0.43
A 0.57 B 0.57
A 0.57 B 0.71
A 0.57 B 0.86
A 0.57 B 1
A 0.71 B 0
A 0.71 B 0.14
A 0.71 B 0.29
A 0.71 B 0.43
A 0.71 B 0.57
A 0.71 B 0.71
A 0.71 B 0.86
A 0.71 B 1
A 0.86 B 0
A 0.86 B 0.14
A 0.86 B 0.29
A 0.86 B 0.43
A 0.86 B 0.57
A 0.86 B 0.71
A 0.86 B 0.86
A 0.86 B 1
A 1 B 0
A 1 B 0.14
A 1 B 0.29
A 1 B 0.43
A 1 B 0.57
A 1 B 0.71
A 1 B 0.86
A 1 B 1
0.8
0.6
0.4
0.2
OD 600 nm [–]
0 5 101520
Time [h]

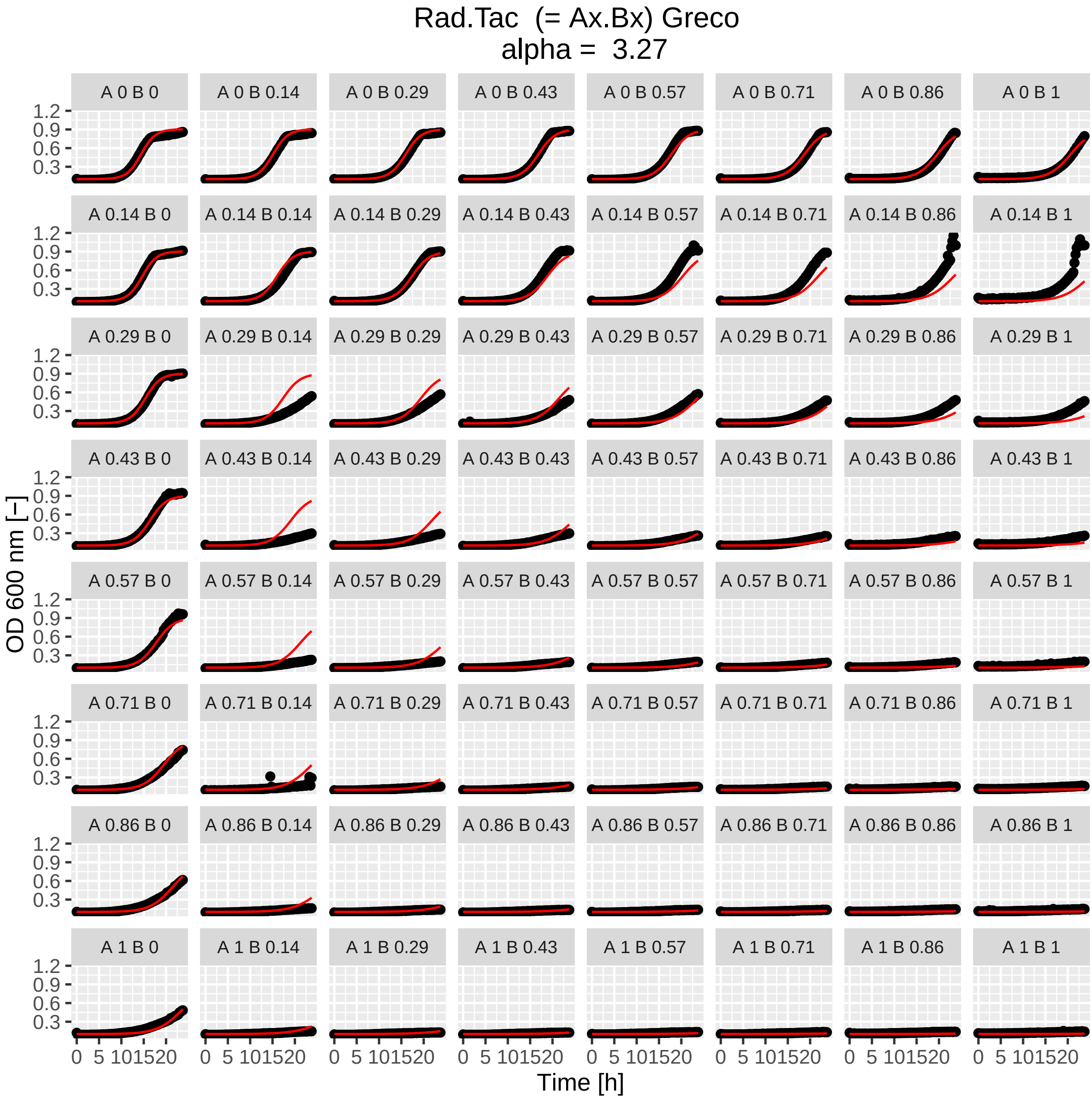

Rad.Tac (= Ax.Bx) Greco
alpha = 3.27
A 0 B 0
A 0 B 0.14
A 0 B 0.29
A 0 B 0.43
A 0 B 0.57
A 0 B 0.71
A 0 B 0.86
A 0 B 1
A 0.14 B 0
A 0.14 B 0.14
A 0.14 B 0.29
A 0.14 B 0.43
A 0.14 B 0.57
A 0.14 B 0.71
A 0.14 B 0.86
A 0.14 B 1
A 0.29 B 0
A 0.29 B 0.14
A 0.29 B 0.29
A 0.29 B 0.43
A 0.29 B 0.57
A 0.29 B 0.71
A 0.29 B 0.86
A 0.29 B 1
A 0.43 B 0
A 0.43 B 0.14
A 0.43 B 0.29
A 0.43 B 0.43
A 0.43 B 0.57
A 0.43 B 0.71
A 0.43 B 0.86
A 0.43 B 1
A 0.57 B 0
A 0.57 B 0.14
A 0.57 B 0.29
A 0.57 B 0.43
A 0.57 B 0.57
A 0.57 B 0.71
A 0.57 B 0.86
A 0.57 B 1
A 0.71 B 0
A 0.71 B 0.14
A 0.71 B 0.29
A 0.71 B 0.43
A 0.71 B 0.57
A 0.71 B 0.71
A 0.71 B 0.86
A 0.71 B 1
A 0.86 B 0
A 0.86 B 0.14
A 0.86 B 0.29
A 0.86 B 0.43
A 0.86 B 0.57
A 0.86 B 0.71
A 0.86 B 0.86
A 0.86 B 1
A 1 B 0
A 1 B 0.14
A 1 B 0.29
A 1 B 0.43
A 1 B 0.57
A 1 B 0.71
A 1 B 0.86
A 1 B 1
OD 600 nm [-]
1.2
0.9
0.6
0.3
0 5 101520
Time [h]

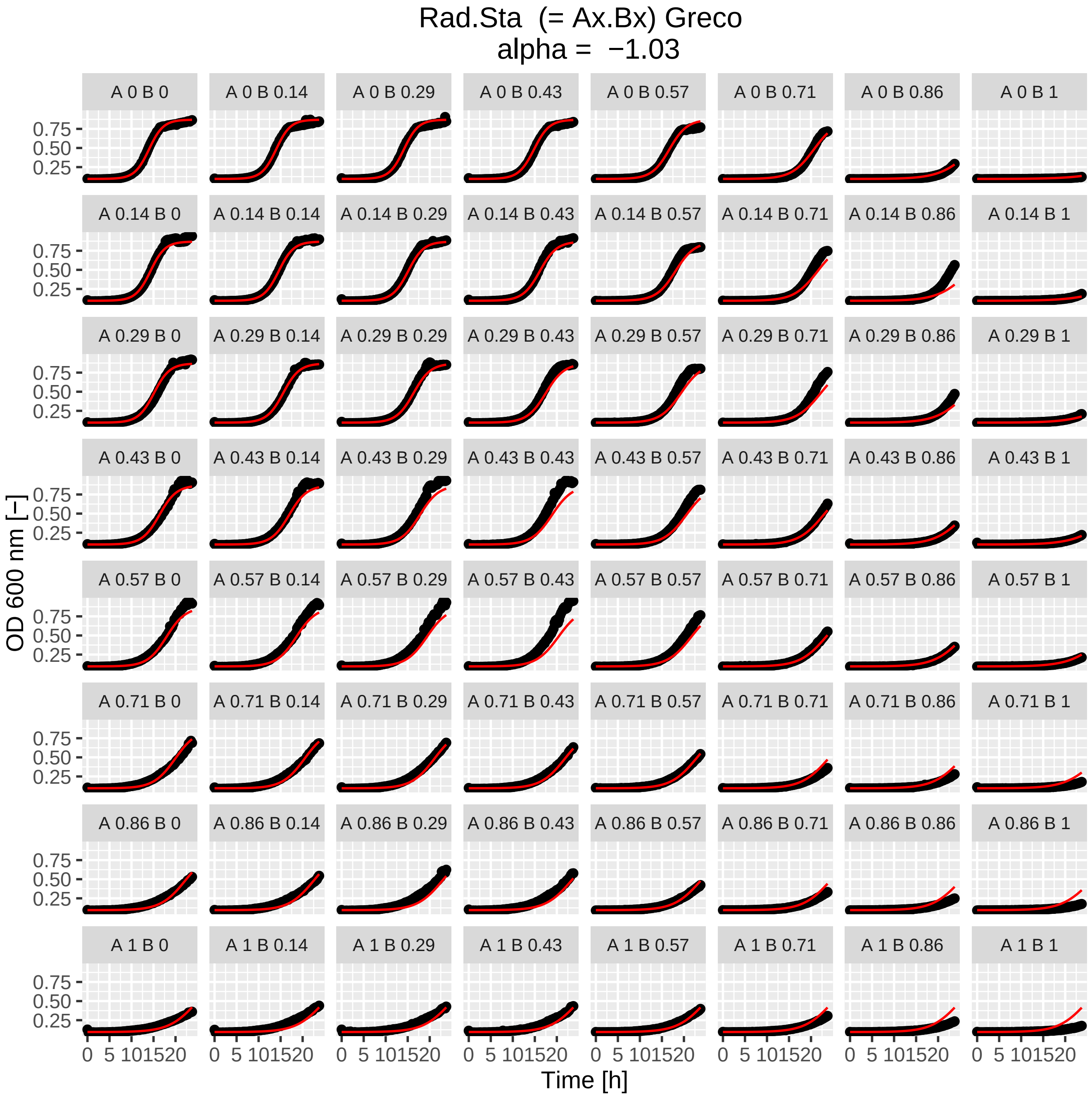
Rad.Sta (= Ax.Bx) Greco
alpha = −1.03
A 0 B 0
A 0 B 0.14
A 0 B 0.29
A 0 B 0.43
A 0 B 0.57
A 0 B 0.71
A 0 B 0.86
A 0 B 1
A 0.14 B 0
A 0.14 B 0.14
A 0.14 B 0.29
A 0.14 B 0.43
A 0.14 B 0.57
A 0.14 B 0.71
A 0.14 B 0.86
A 0.14 B 1
A 0.29 B 0
A 0.29 B 0.14
A 0.29 B 0.29
A 0.29 B 0.43
A 0.29 B 0.57
A 0.29 B 0.71
A 0.29 B 0.86
A 0.29 B 1
A 0.43 B 0
A 0.43 B 0.14
A 0.43 B 0.29
A 0.43 B 0.43
A 0.43 B 0.57
A 0.43 B 0.71
A 0.43 B 0.86
A 0.43 B 1
A 0.57 B 0
A 0.57 B 0.14
A 0.57 B 0.29
A 0.57 B 0.43
A 0.57 B 0.57
A 0.57 B 0.71
A 0.57 B 0.86
A 0.57 B 1
A 0.71 B 0
A 0.71 B 0.14
A 0.71 B 0.29
A 0.71 B 0.43
A 0.71 B 0.57
A 0.71 B 0.71
A 0.71 B 0.86
A 0.71 B 1
A 0.86 B 0
A 0.86 B 0.14
A 0.86 B 0.29
A 0.86 B 0.43
A 0.86 B 0.57
A 0.86 B 0.71
A 0.86 B 0.86
A 0.86 B 1
A 1 B 0
A 1 B 0.14
A 1 B 0.29
A 1 B 0.43
A 1 B 0.57
A 1 B 0.71
A 1 B 0.86
A 1 B 1
0.75
0.50
0.25
0 5 101520
OD 600 nm [−]
Time [h]

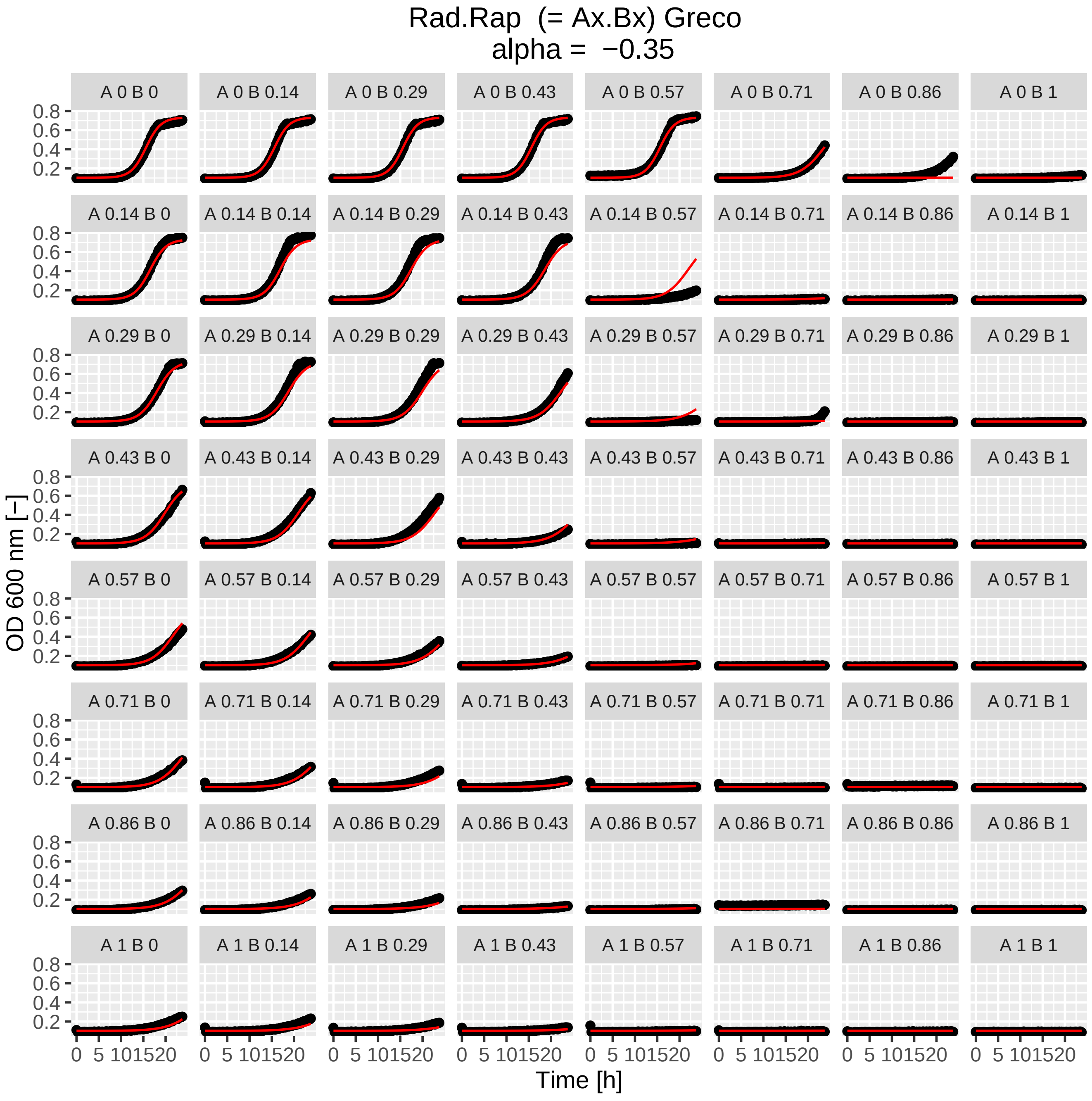
Rad.Rap (= Ax.Bx) Greco
alpha = −0.35
OD 600 nm [−]
Time [h]
A 0 B 0
A 0 B 0.14
A 0 B 0.29
A 0 B 0.43
A 0 B 0.57
A 0 B 0.71
A 0 B 0.86
A 0 B 1
A 0.14 B 0
A 0.14 B 0.14
A 0.14 B 0.29
A 0.14 B 0.43
A 0.14 B 0.57
A 0.14 B 0.71
A 0.14 B 0.86
A 0.14 B 1
A 0.29 B 0
A 0.29 B 0.14
A 0.29 B 0.29
A 0.29 B 0.43
A 0.29 B 0.57
A 0.29 B 0.71
A 0.29 B 0.86
A 0.29 B 1
A 0.43 B 0
A 0.43 B 0.14
A 0.43 B 0.29
A 0.43 B 0.43
A 0.43 B 0.57
A 0.43 B 0.71
A 0.43 B 0.86
A 0.43 B 1
A 0.57 B 0
A 0.57 B 0.14
A 0.57 B 0.29
A 0.57 B 0.43
A 0.57 B 0.57
A 0.57 B 0.71
A 0.57 B 0.86
A 0.57 B 1
A 0.71 B 0
A 0.71 B 0.14
A 0.71 B 0.29
A 0.71 B 0.43
A 0.71 B 0.57
A 0.71 B 0.71
A 0.71 B 0.86
A 0.71 B 1
A 0.86 B 0
A 0.86 B 0.14
A 0.86 B 0.29
A 0.86 B 0.43
A 0.86 B 0.57
A 0.86 B 0.71
A 0.86 B 0.86
A 0.86 B 1
A 1 B 0
A 1 B 0.14
A 1 B 0.29
A 1 B 0.43
A 1 B 0.57
A 1 B 0.71
A 1 B 0.86
A 1 B 1
0.8
0.6
0.4
0.2
0 5 101520

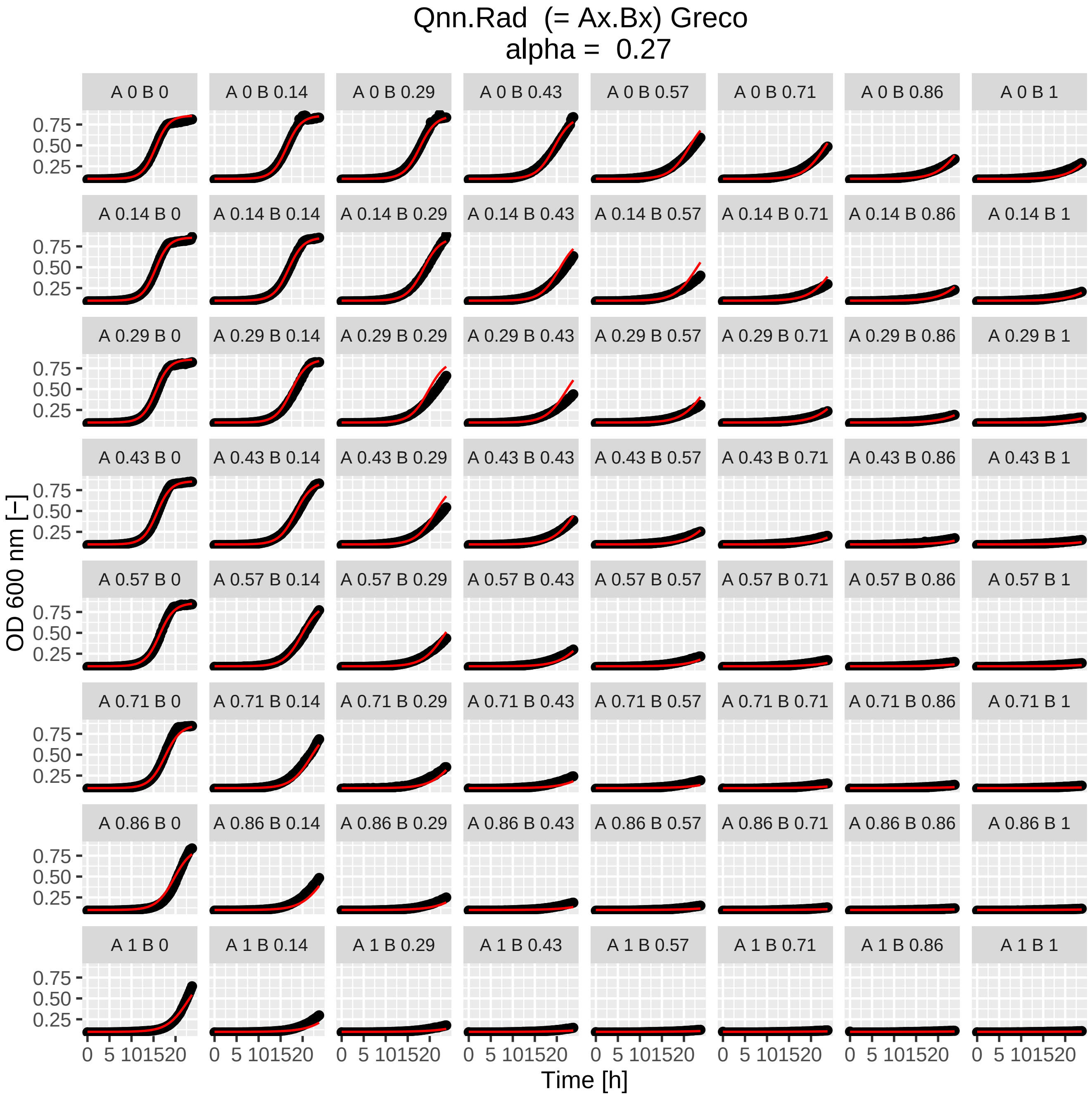

Qnn.Rad (= Ax.Bx) Greco
alpha = 0.27
A 0 B 0
A 0 B 0.14
A 0 B 0.29
A 0 B 0.43
A 0 B 0.57
A 0 B 0.71
A 0 B 0.86
A 0 B 1
A 0.14 B 0
A 0.14 B 0.14
A 0.14 B 0.29
A 0.14 B 0.43
A 0.14 B 0.57
A 0.14 B 0.71
A 0.14 B 0.86
A 0.14 B 1
A 0.29 B 0
A 0.29 B 0.14
A 0.29 B 0.29
A 0.29 B 0.43
A 0.29 B 0.57
A 0.29 B 0.71
A 0.29 B 0.86
A 0.29 B 1
A 0.43 B 0
A 0.43 B 0.14
A 0.43 B 0.29
A 0.43 B 0.43
A 0.43 B 0.57
A 0.43 B 0.71
A 0.43 B 0.86
A 0.43 B 1
A 0.57 B 0
A 0.57 B 0.14
A 0.57 B 0.29
A 0.57 B 0.43
A 0.57 B 0.57
A 0.57 B 0.71
A 0.57 B 0.86
A 0.57 B 1
A 0.71 B 0
A 0.71 B 0.14
A 0.71 B 0.29
A 0.71 B 0.43
A 0.71 B 0.57
A 0.71 B 0.71
A 0.71 B 0.86
A 0.71 B 1
A 0.86 B 0
A 0.86 B 0.14
A 0.86 B 0.29
A 0.86 B 0.43
A 0.86 B 0.57
A 0.86 B 0.71
A 0.86 B 0.86
A 0.86 B 1
A 1 B 0
A 1 B 0.14
A 1 B 0.29
A 1 B 0.43
A 1 B 0.57
A 1 B 0.71
A 1 B 0.86
A 1 B 1
OD 600 nm [–]
0.75
0.50
0.25
0 5 101520
Time [h]

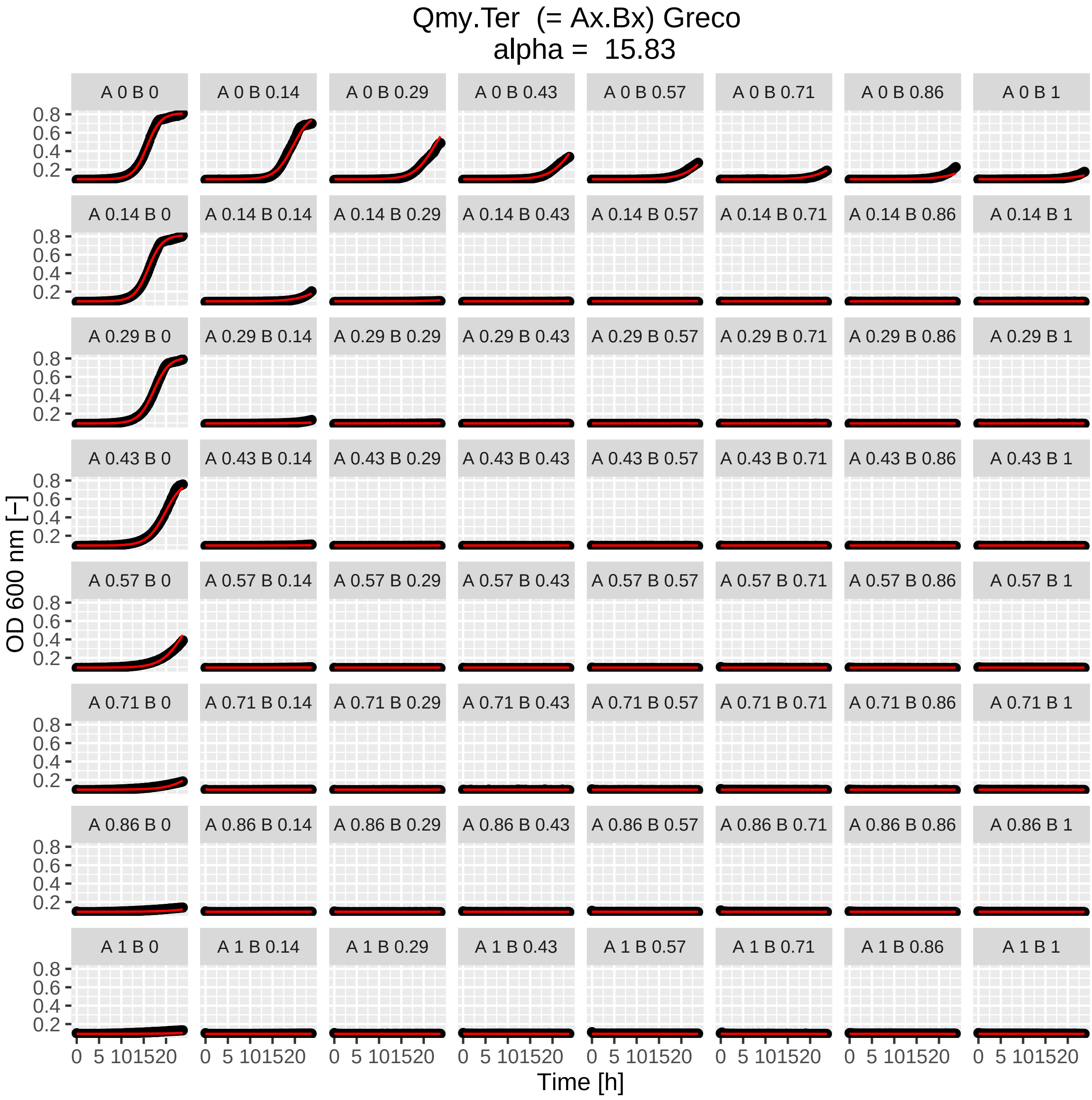
Qmy.Ter (= Ax.Bx) Greco
alpha = 15.83
A 0 B 0
A 0 B 0.14
A 0 B 0.29
A 0 B 0.43
A 0 B 0.57
A 0 B 0.71
A 0 B 0.86
A 0 B 1
A 0.14 B 0
A 0.14 B 0.14
A 0.14 B 0.29
A 0.14 B 0.43
A 0.14 B 0.57
A 0.14 B 0.71
A 0.14 B 0.86
A 0.14 B 1
A 0.29 B 0
A 0.29 B 0.14
A 0.29 B 0.29
A 0.29 B 0.43
A 0.29 B 0.57
A 0.29 B 0.71
A 0.29 B 0.86
A 0.29 B 1
A 0.43 B 0
A 0.43 B 0.14
A 0.43 B 0.29
A 0.43 B 0.43
A 0.43 B 0.57
A 0.43 B 0.71
A 0.43 B 0.86
A 0.43 B 1
A 0.57 B 0
A 0.57 B 0.14
A 0.57 B 0.29
A 0.57 B 0.43
A 0.57 B 0.57
A 0.57 B 0.71
A 0.57 B 0.86
A 0.57 B 1
A 0.71 B 0
A 0.71 B 0.14
A 0.71 B 0.29
A 0.71 B 0.43
A 0.71 B 0.57
A 0.71 B 0.71
A 0.71 B 0.86
A 0.71 B 1
A 0.86 B 0
A 0.86 B 0.14
A 0.86 B 0.29
A 0.86 B 0.43
A 0.86 B 0.57
A 0.86 B 0.71
A 0.86 B 0.86
A 0.86 B 1
A 1 B 0
A 1 B 0.14
A 1 B 0.29
A 1 B 0.43
A 1 B 0.57
A 1 B 0.71
A 1 B 0.86
A 1 B 1
OD 600 nm [–]
0.8
0.6
0.4
0.2
0 5 101520
Time [h]

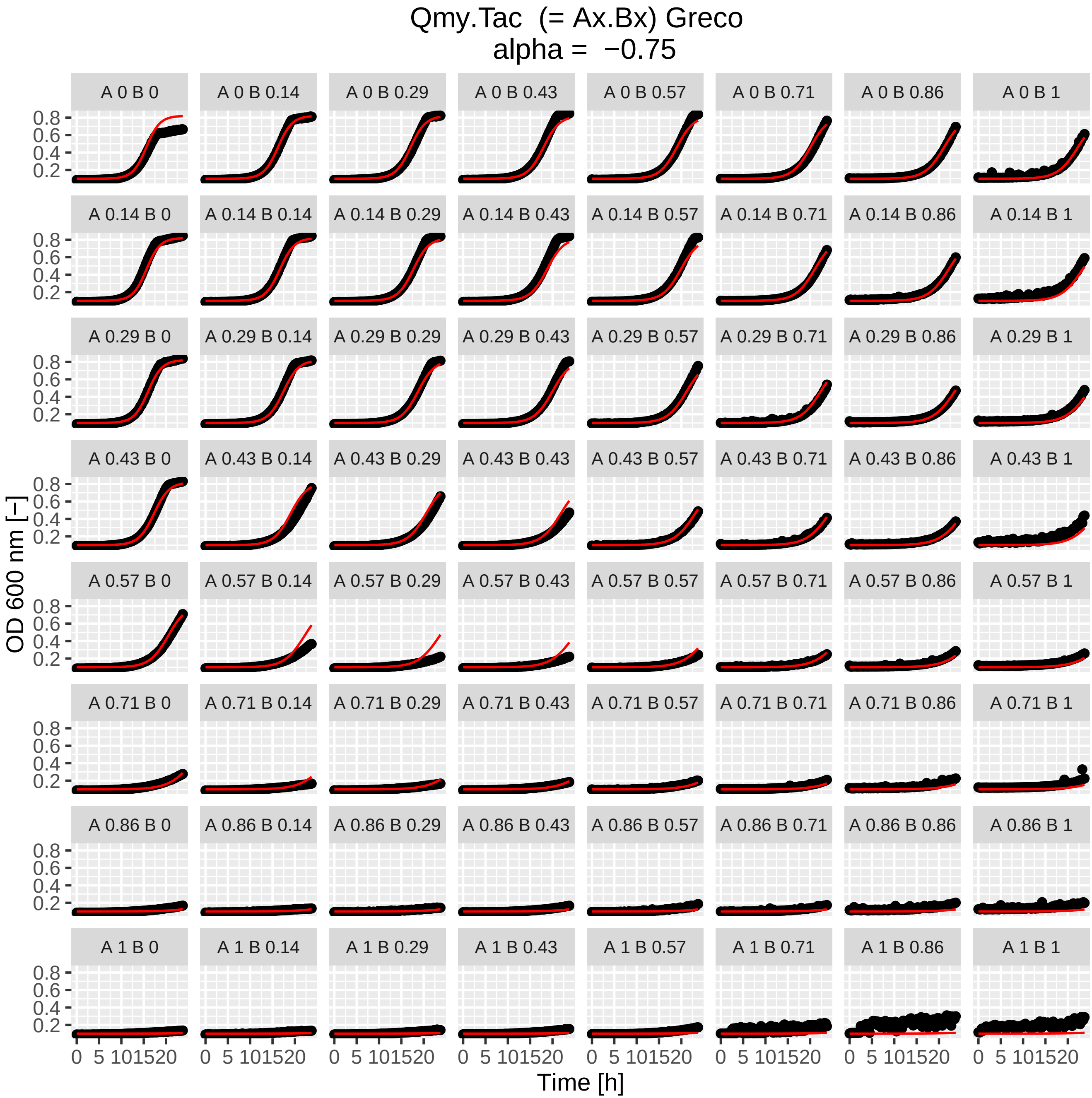

Qmy.Tac (= Ax.Bx) Greco
alpha = −0.75
OD 600 nm [−]
Time [h]
A 0 B 0
A 0 B 0.14
A 0 B 0.29
A 0 B 0.43
A 0 B 0.57
A 0 B 0.71
A 0 B 0.86
A 0 B 1
A 0.14 B 0
A 0.14 B 0.14
A 0.14 B 0.29
A 0.14 B 0.43
A 0.14 B 0.57
A 0.14 B 0.71
A 0.14 B 0.86
A 0.14 B 1
A 0.29 B 0
A 0.29 B 0.14
A 0.29 B 0.29
A 0.29 B 0.43
A 0.29 B 0.57
A 0.29 B 0.71
A 0.29 B 0.86
A 0.29 B 1
A 0.43 B 0
A 0.43 B 0.14
A 0.43 B 0.29
A 0.43 B 0.43
A 0.43 B 0.57
A 0.43 B 0.71
A 0.43 B 0.86
A 0.43 B 1
A 0.57 B 0
A 0.57 B 0.14
A 0.57 B 0.29
A 0.57 B 0.43
A 0.57 B 0.57
A 0.57 B 0.71
A 0.57 B 0.86
A 0.57 B 1
A 0.71 B 0
A 0.71 B 0.14
A 0.71 B 0.29
A 0.71 B 0.43
A 0.71 B 0.57
A 0.71 B 0.71
A 0.71 B 0.86
A 0.71 B 1
A 0.86 B 0
A 0.86 B 0.14
A 0.86 B 0.29
A 0.86 B 0.43
A 0.86 B 0.57
A 0.86 B 0.71
A 0.86 B 0.86
A 0.86 B 1
A 1 B 0
A 1 B 0.14
A 1 B 0.29
A 1 B 0.43
A 1 B 0.57
A 1 B 0.71
A 1 B 0.86
A 1 B 1
0.8
0.6
0.4
0.2
0 5 101520

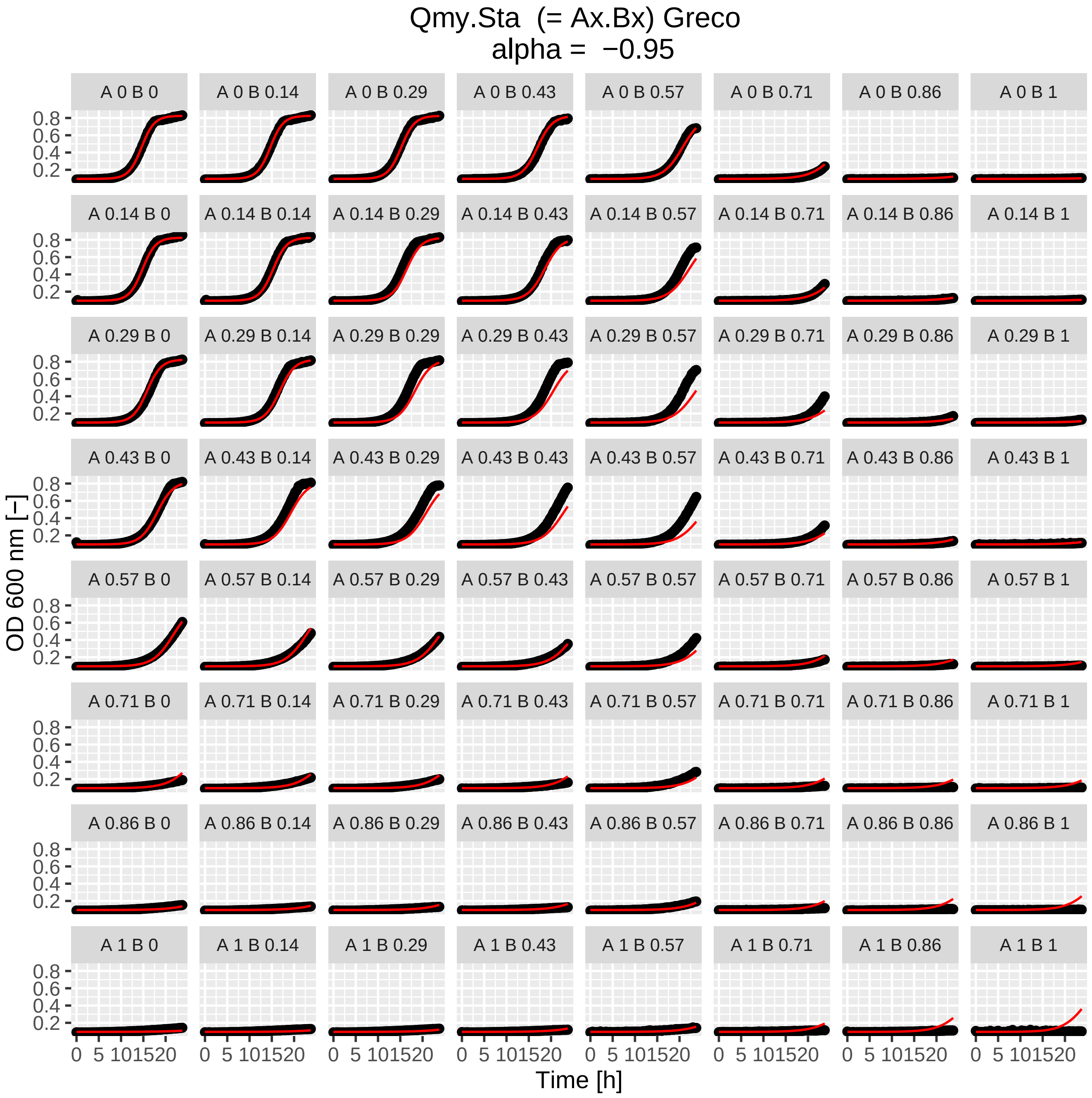
Qmy.Sta (= Ax.Bx) Greco
alpha = −0.95
OD 600 nm [−]
Time [h]
A 0 B 0
A 0 B 0.14
A 0 B 0.29
A 0 B 0.43
A 0 B 0.57
A 0 B 0.71
A 0 B 0.86
A 0 B 1
A 0.14 B 0
A 0.14 B 0.14
A 0.14 B 0.29
A 0.14 B 0.43
A 0.14 B 0.57
A 0.14 B 0.71
A 0.14 B 0.86
A 0.14 B 1
A 0.29 B 0
A 0.29 B 0.14
A 0.29 B 0.29
A 0.29 B 0.43
A 0.29 B 0.57
A 0.29 B 0.71
A 0.29 B 0.86
A 0.29 B 1
A 0.43 B 0
A 0.43 B 0.14
A 0.43 B 0.29
A 0.43 B 0.43
A 0.43 B 0.57
A 0.43 B 0.71
A 0.43 B 0.86
A 0.43 B 1
A 0.57 B 0
A 0.57 B 0.14
A 0.57 B 0.29
A 0.57 B 0.43
A 0.57 B 0.57
A 0.57 B 0.71
A 0.57 B 0.86
A 0.57 B 1
A 0.71 B 0
A 0.71 B 0.14
A 0.71 B 0.29
A 0.71 B 0.43
A 0.71 B 0.57
A 0.71 B 0.71
A 0.71 B 0.86
A 0.71 B 1
A 0.86 B 0
A 0.86 B 0.14
A 0.86 B 0.29
A 0.86 B 0.43
A 0.86 B 0.57
A 0.86 B 0.71
A 0.86 B 0.86
A 0.86 B 1
A 1 B 0
A 1 B 0.14
A 1 B 0.29
A 1 B 0.43
A 1 B 0.57
A 1 B 0.71
A 1 B 0.86
A 1 B 1
0.2
0.4
0.6
0.8
0 5 101520

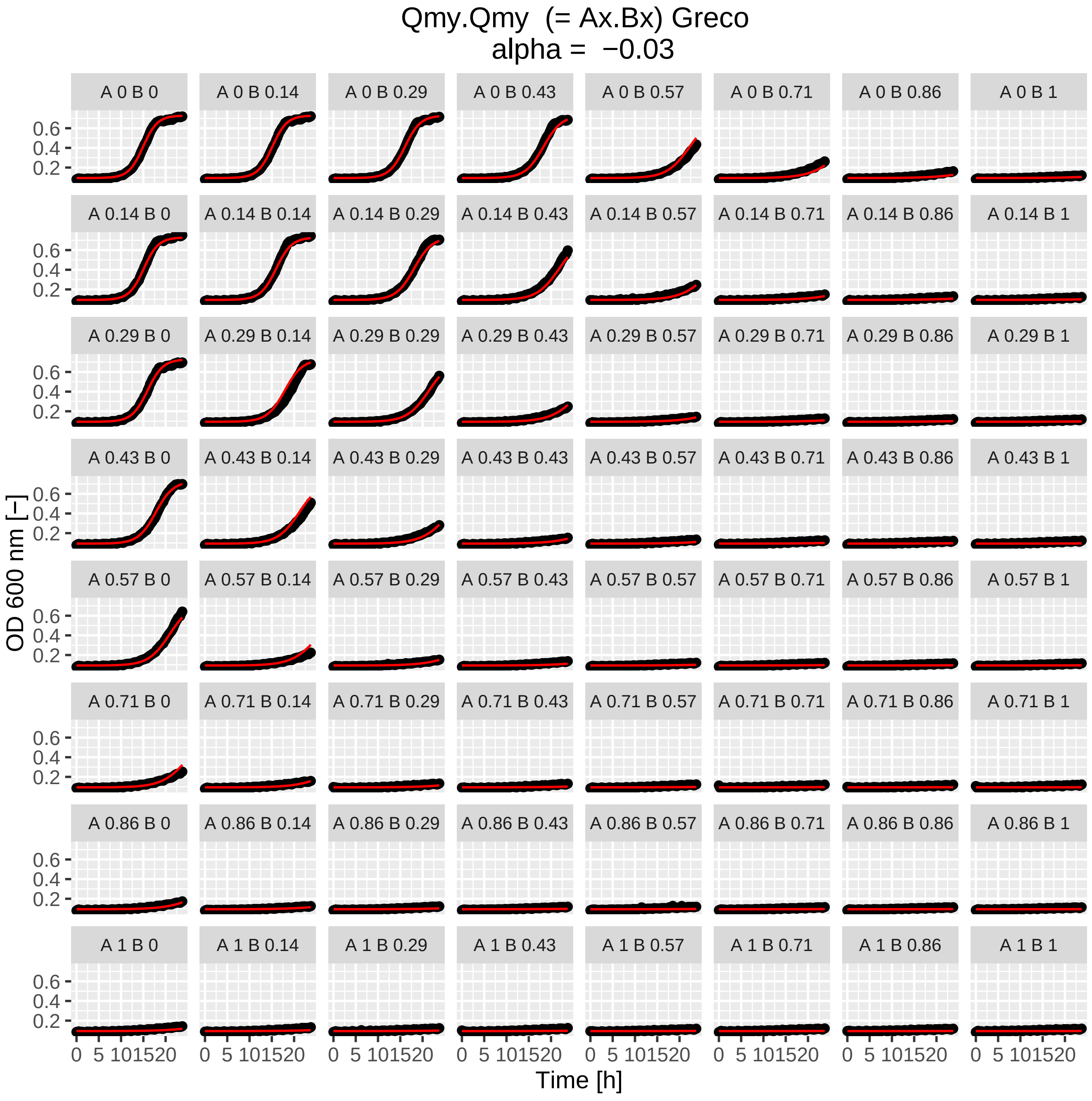
Qmy.Qmy (= Ax.Bx) Greco
alpha = −0.03
OD 600 nm [−]
Time [h]
0.6
0.4
0.2
0 5 101520
A 0 B 0
A 0 B 0.14
A 0 B 0.29
A 0 B 0.43
A 0 B 0.57
A 0 B 0.71
A 0 B 0.86
A 0 B 1
A 0.14 B 0
A 0.14 B 0.14
A 0.14 B 0.29
A 0.14 B 0.43
A 0.14 B 0.57
A 0.14 B 0.71
A 0.14 B 0.86
A 0.14 B 1
A 0.29 B 0
A 0.29 B 0.14
A 0.29 B 0.29
A 0.29 B 0.43
A 0.29 B 0.57
A 0.29 B 0.71
A 0.29 B 0.86
A 0.29 B 1
A 0.43 B 0
A 0.43 B 0.14
A 0.43 B 0.29
A 0.43 B 0.43
A 0.43 B 0.57
A 0.43 B 0.71
A 0.43 B 0.86
A 0.43 B 1
A 0.57 B 0
A 0.57 B 0.14
A 0.57 B 0.29
A 0.57 B 0.43
A 0.57 B 0.57
A 0.57 B 0.71
A 0.57 B 0.86
A 0.57 B 1
A 0.71 B 0
A 0.71 B 0.14
A 0.71 B 0.29
A 0.71 B 0.43
A 0.71 B 0.57
A 0.71 B 0.71
A 0.71 B 0.86
A 0.71 B 1
A 0.86 B 0
A 0.86 B 0.14
A 0.86 B 0.29
A 0.86 B 0.43
A 0.86 B 0.57
A 0.86 B 0.71
A 0.86 B 0.86
A 0.86 B 1
A 1 B 0
A 1 B 0.14
A 1 B 0.29
A 1 B 0.43
A 1 B 0.57
A 1 B 0.71
A 1 B 0.86
A 1 B 1

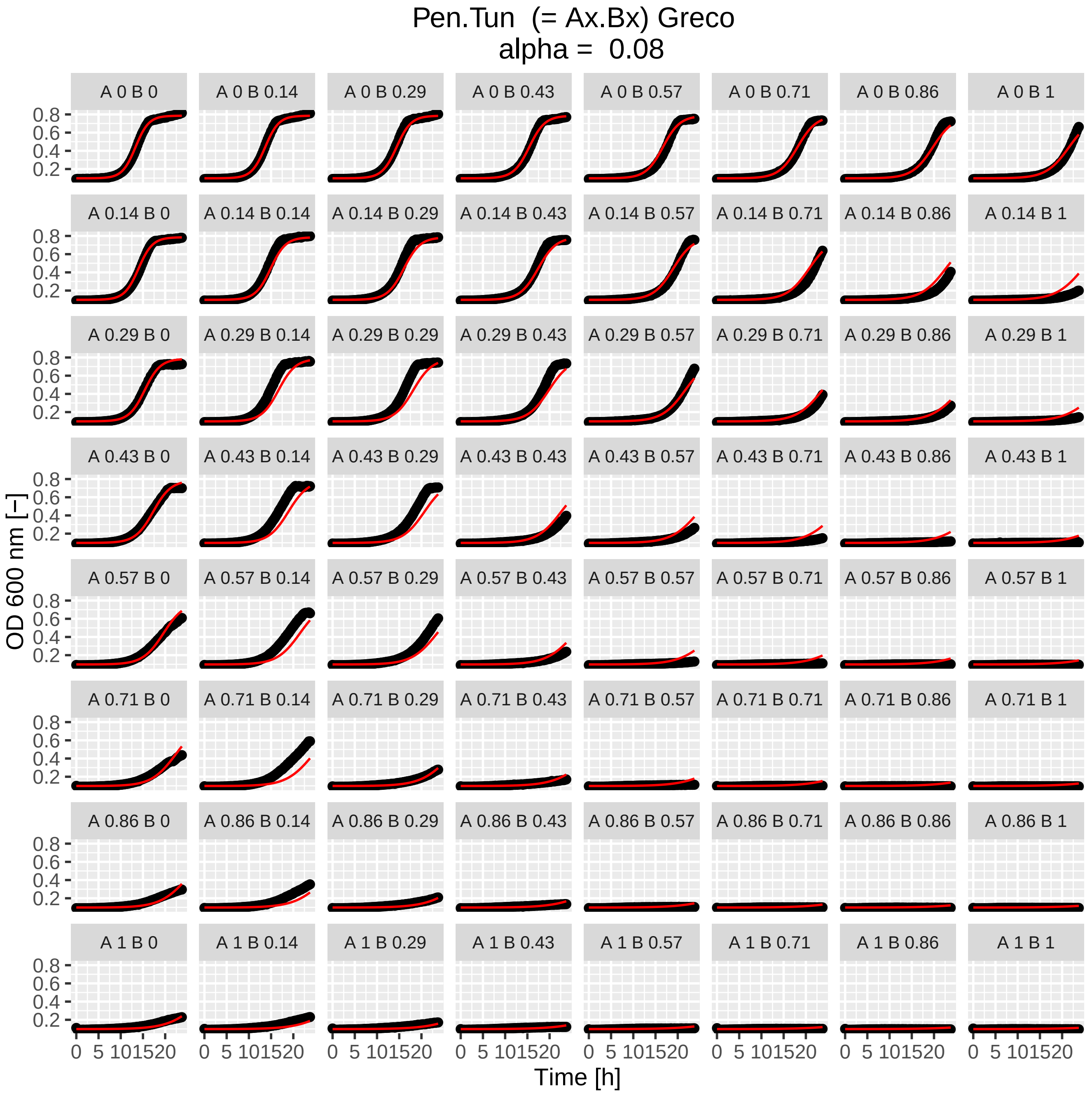

Pen.Tun (= Ax.Bx) Greco
alpha = 0.08
A 0 B 0
A 0 B 0.14
A 0 B 0.29
A 0 B 0.43
A 0 B 0.57
A 0 B 0.71
A 0 B 0.86
A 0 B 1
A 0.14 B 0
A 0.14 B 0.14
A 0.14 B 0.29
A 0.14 B 0.43
A 0.14 B 0.57
A 0.14 B 0.71
A 0.14 B 0.86
A 0.14 B 1
A 0.29 B 0
A 0.29 B 0.14
A 0.29 B 0.29
A 0.29 B 0.43
A 0.29 B 0.57
A 0.29 B 0.71
A 0.29 B 0.86
A 0.29 B 1
A 0.43 B 0
A 0.43 B 0.14
A 0.43 B 0.29
A 0.43 B 0.43
A 0.43 B 0.57
A 0.43 B 0.71
A 0.43 B 0.86
A 0.43 B 1
A 0.57 B 0
A 0.57 B 0.14
A 0.57 B 0.29
A 0.57 B 0.43
A 0.57 B 0.57
A 0.57 B 0.71
A 0.57 B 0.86
A 0.57 B 1
A 0.71 B 0
A 0.71 B 0.14
A 0.71 B 0.29
A 0.71 B 0.43
A 0.71 B 0.57
A 0.71 B 0.71
A 0.71 B 0.86
A 0.71 B 1
A 0.86 B 0
A 0.86 B 0.14
A 0.86 B 0.29
A 0.86 B 0.43
A 0.86 B 0.57
A 0.86 B 0.71
A 0.86 B 0.86
A 0.86 B 1
A 1 B 0
A 1 B 0.14
A 1 B 0.29
A 1 B 0.43
A 1 B 0.57
A 1 B 0.71
A 1 B 0.86
A 1 B 1
OD 600 nm [−]
0.2
0.4
0.6
0.8
0 5 101520
Time [h]

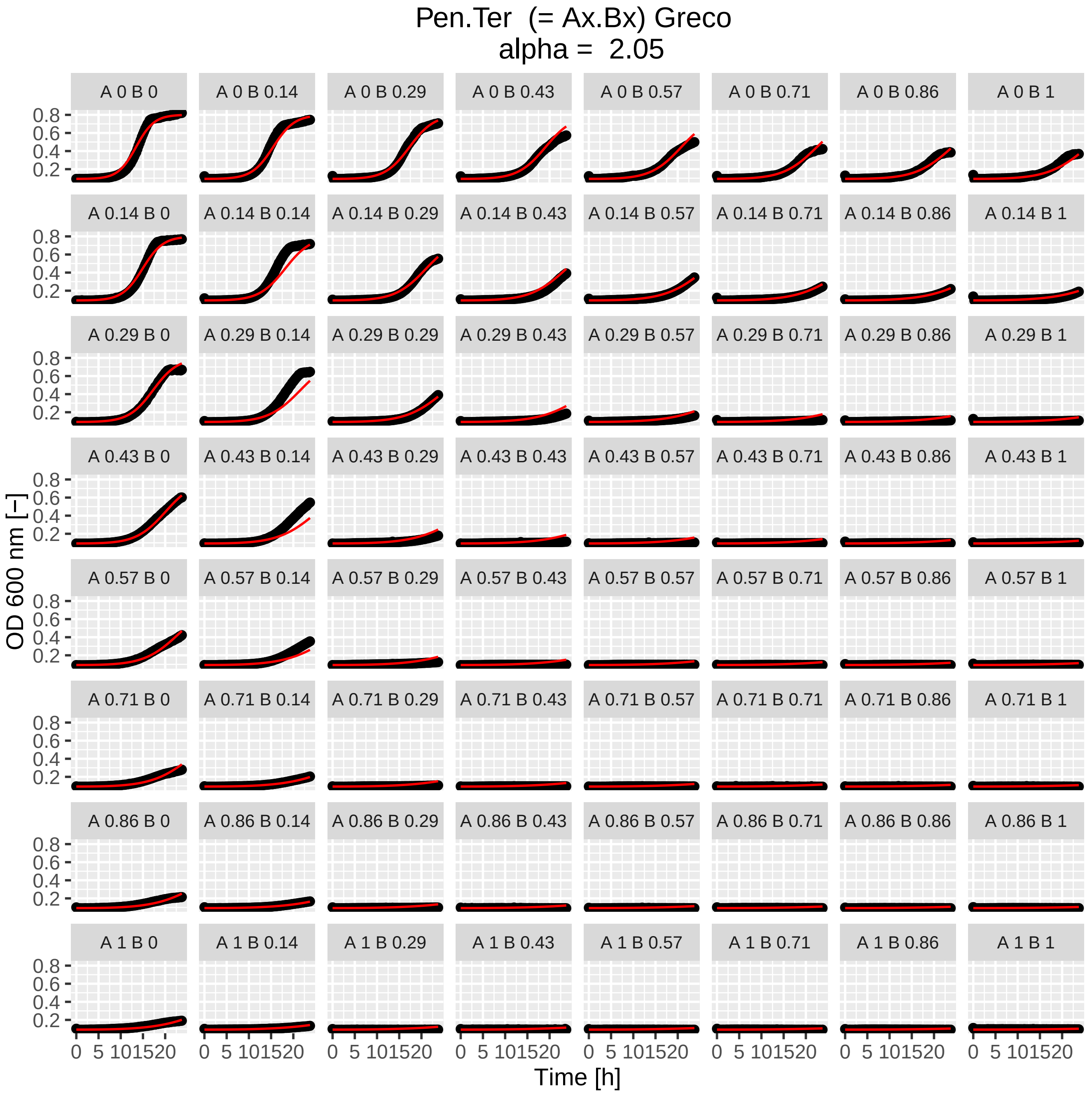
Pen.Ter (= Ax.Bx) Greco
alpha = 2.05
OD 600 nm [–]
Time [h]
0.8
0.6
0.4
0.2
0 5 101520
A 0 B 0
A 0 B 0.14
A 0 B 0.29
A 0 B 0.43
A 0 B 0.57
A 0 B 0.71
A 0 B 0.86
A 0 B 1
A 0.14 B 0
A 0.14 B 0.14
A 0.14 B 0.29
A 0.14 B 0.43
A 0.14 B 0.57
A 0.14 B 0.71
A 0.14 B 0.86
A 0.14 B 1
A 0.29 B 0
A 0.29 B 0.14
A 0.29 B 0.29
A 0.29 B 0.43
A 0.29 B 0.57
A 0.29 B 0.71
A 0.29 B 0.86
A 0.29 B 1
A 0.43 B 0
A 0.43 B 0.14
A 0.43 B 0.29
A 0.43 B 0.43
A 0.43 B 0.57
A 0.43 B 0.71
A 0.43 B 0.86
A 0.43 B 1
A 0.57 B 0
A 0.57 B 0.14
A 0.57 B 0.29
A 0.57 B 0.43
A 0.57 B 0.57
A 0.57 B 0.71
A 0.57 B 0.86
A 0.57 B 1
A 0.71 B 0
A 0.71 B 0.14
A 0.71 B 0.29
A 0.71 B 0.43
A 0.71 B 0.57
A 0.71 B 0.71
A 0.71 B 0.86
A 0.71 B 1
A 0.86 B 0
A 0.86 B 0.14
A 0.86 B 0.29
A 0.86 B 0.43
A 0.86 B 0.57
A 0.86 B 0.71
A 0.86 B 0.86
A 0.86 B 1
A 1 B 0
A 1 B 0.14
A 1 B 0.29
A 1 B 0.43
A 1 B 0.57
A 1 B 0.71
A 1 B 0.86
A 1 B 1

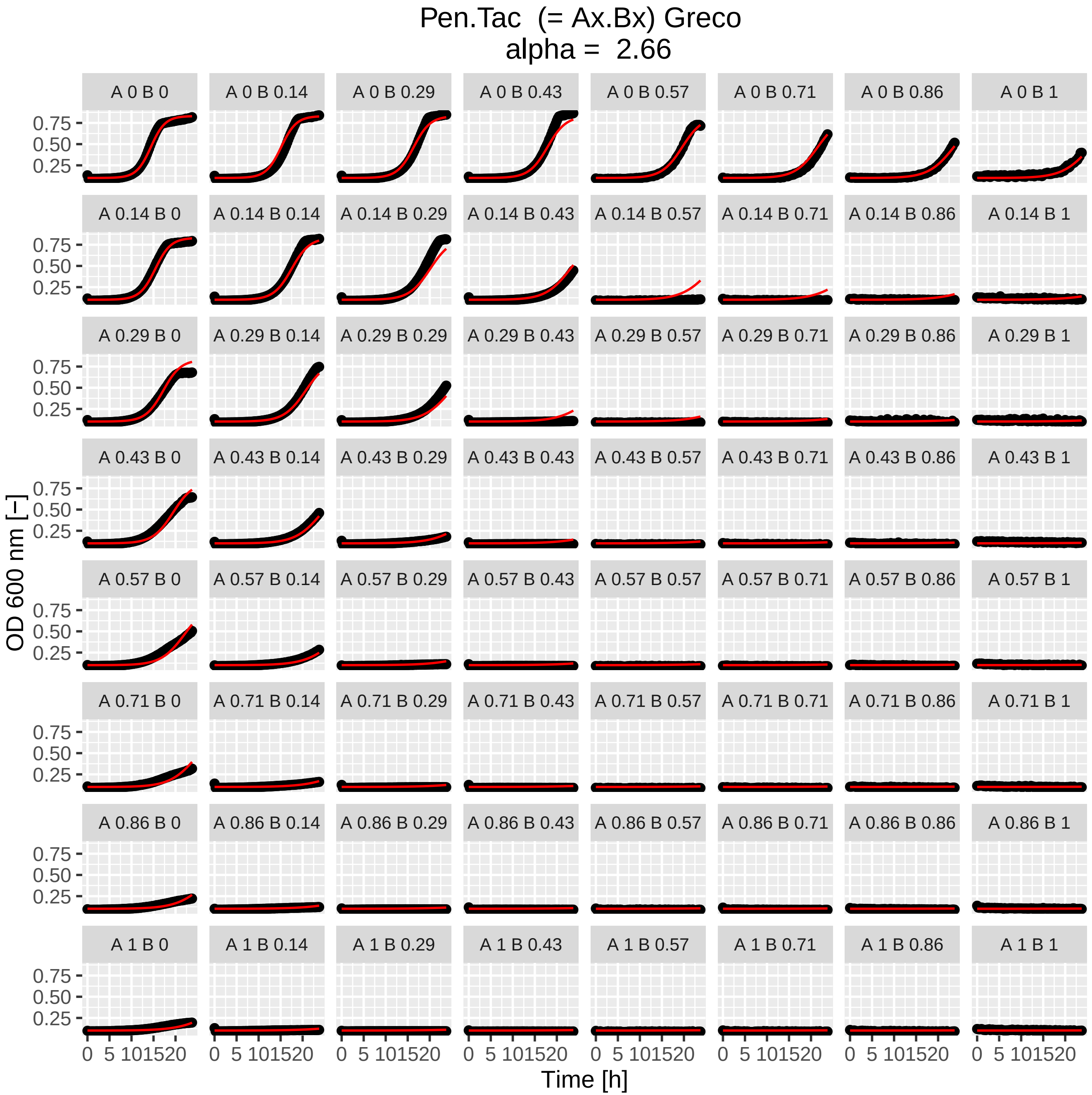

Pen.Tac (= Ax.Bx) Greco
alpha = 2.66
A 0 B 0
A 0 B 0.14
A 0 B 0.29
A 0 B 0.43
A 0 B 0.57
A 0 B 0.71
A 0 B 0.86
A 0 B 1
A 0.14 B 0
A 0.14 B 0.14
A 0.14 B 0.29
A 0.14 B 0.43
A 0.14 B 0.57
A 0.14 B 0.71
A 0.14 B 0.86
A 0.14 B 1
A 0.29 B 0
A 0.29 B 0.14
A 0.29 B 0.29
A 0.29 B 0.43
A 0.29 B 0.57
A 0.29 B 0.71
A 0.29 B 0.86
A 0.29 B 1
A 0.43 B 0
A 0.43 B 0.14
A 0.43 B 0.29
A 0.43 B 0.43
A 0.43 B 0.57
A 0.43 B 0.71
A 0.43 B 0.86
A 0.43 B 1
A 0.57 B 0
A 0.57 B 0.14
A 0.57 B 0.29
A 0.57 B 0.43
A 0.57 B 0.57
A 0.57 B 0.71
A 0.57 B 0.86
A 0.57 B 1
A 0.71 B 0
A 0.71 B 0.14
A 0.71 B 0.29
A 0.71 B 0.43
A 0.71 B 0.57
A 0.71 B 0.71
A 0.71 B 0.86
A 0.71 B 1
A 0.86 B 0
A 0.86 B 0.14
A 0.86 B 0.29
A 0.86 B 0.43
A 0.86 B 0.57
A 0.86 B 0.71
A 0.86 B 0.86
A 0.86 B 1
A 1 B 0
A 1 B 0.14
A 1 B 0.29
A 1 B 0.43
A 1 B 0.57
A 1 B 0.71
A 1 B 0.86
A 1 B 1
OD 600 nm [–]
0.75
0.50
0.25
0 5 101520
Time [h]

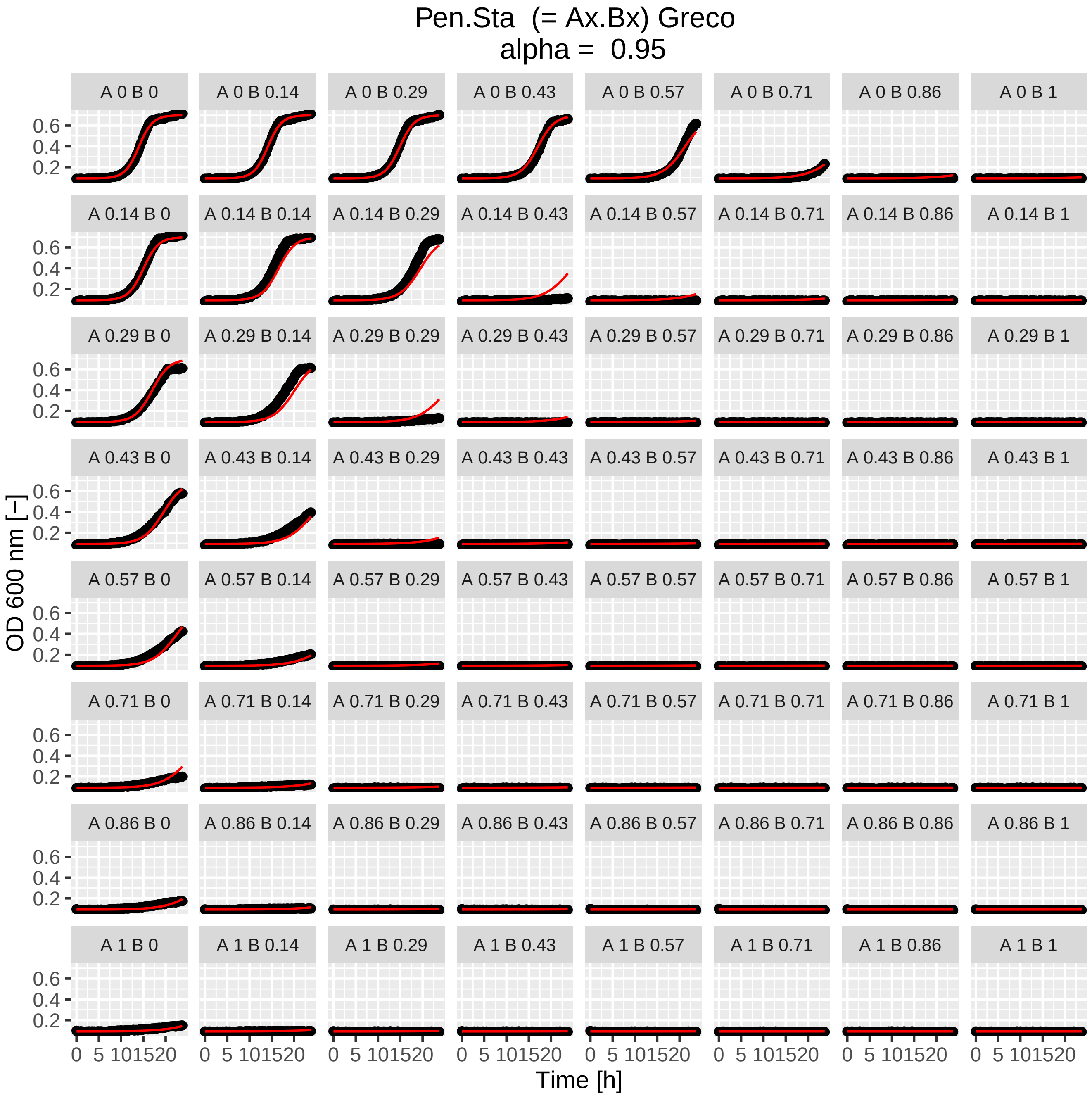

Pen.Sta (= Ax.Bx) Greco
alpha = 0.95
A 0 B 0
A 0 B 0.14
A 0 B 0.29
A 0 B 0.43
A 0 B 0.57
A 0 B 0.71
A 0 B 0.86
A 0 B 1
A 0.14 B 0
A 0.14 B 0.14
A 0.14 B 0.29
A 0.14 B 0.43
A 0.14 B 0.57
A 0.14 B 0.71
A 0.14 B 0.86
A 0.14 B 1
A 0.29 B 0
A 0.29 B 0.14
A 0.29 B 0.29
A 0.29 B 0.43
A 0.29 B 0.57
A 0.29 B 0.71
A 0.29 B 0.86
A 0.29 B 1
A 0.43 B 0
A 0.43 B 0.14
A 0.43 B 0.29
A 0.43 B 0.43
A 0.43 B 0.57
A 0.43 B 0.71
A 0.43 B 0.86
A 0.43 B 1
A 0.57 B 0
A 0.57 B 0.14
A 0.57 B 0.29
A 0.57 B 0.43
A 0.57 B 0.57
A 0.57 B 0.71
A 0.57 B 0.86
A 0.57 B 1
A 0.71 B 0
A 0.71 B 0.14
A 0.71 B 0.29
A 0.71 B 0.43
A 0.71 B 0.57
A 0.71 B 0.71
A 0.71 B 0.86
A 0.71 B 1
A 0.86 B 0
A 0.86 B 0.14
A 0.86 B 0.29
A 0.86 B 0.43
A 0.86 B 0.57
A 0.86 B 0.71
A 0.86 B 0.86
A 0.86 B 1
A 1 B 0
A 1 B 0.14
A 1 B 0.29
A 1 B 0.43
A 1 B 0.57
A 1 B 0.71
A 1 B 0.86
A 1 B 1
OD 600 nm [–]
0.2
0.4
0.6
0 5 101520
Time [h]

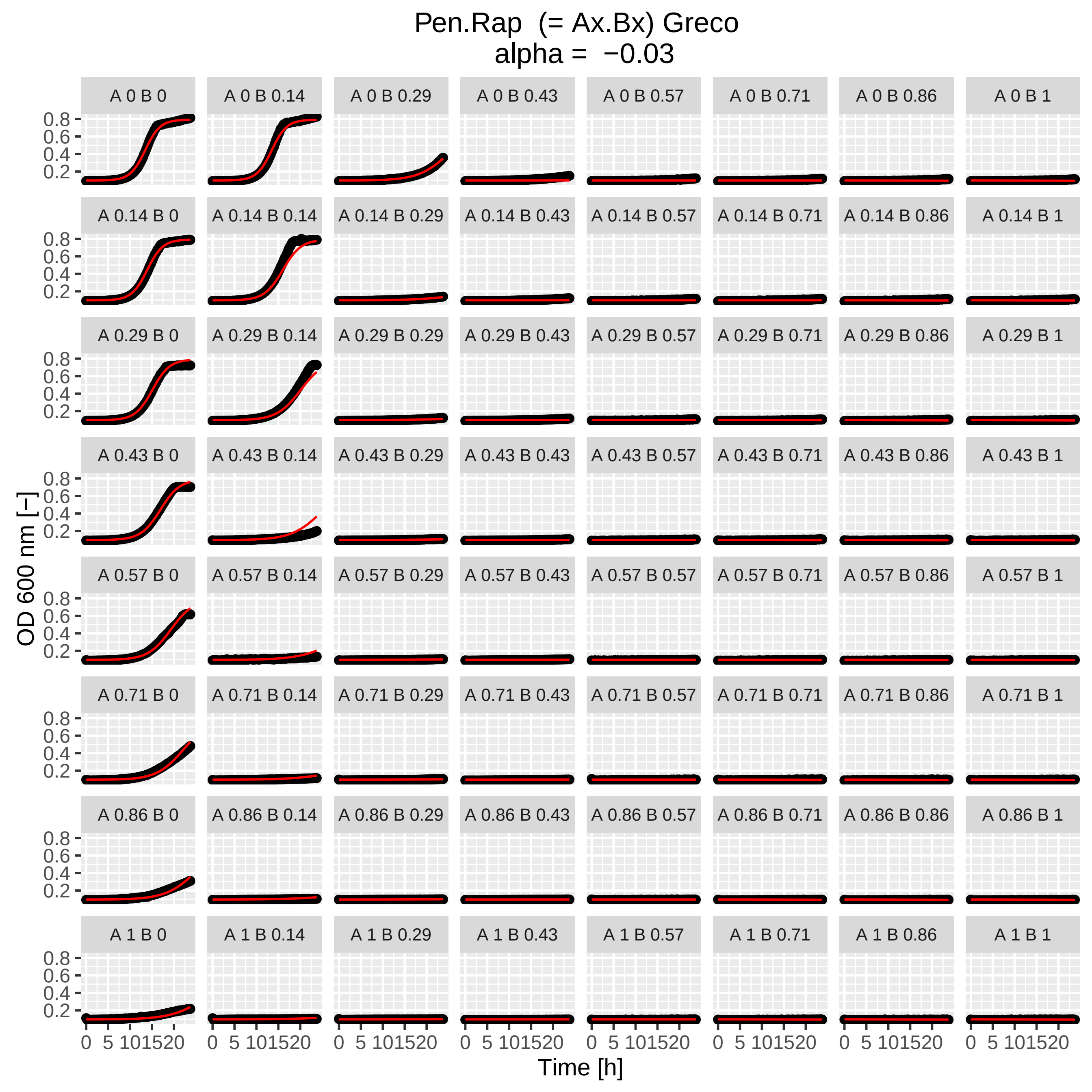
Pen.Rap (= Ax.Bx) Greco
alpha = −0.03
OD 600 nm [−]
Time [h]
A 0 B 0
A 0 B 0.14
A 0 B 0.29
A 0 B 0.43
A 0 B 0.57
A 0 B 0.71
A 0 B 0.86
A 0 B 1
A 1 B 0
A 1 B 1
0.2
0.4
0.6
0.8
0 5 101520

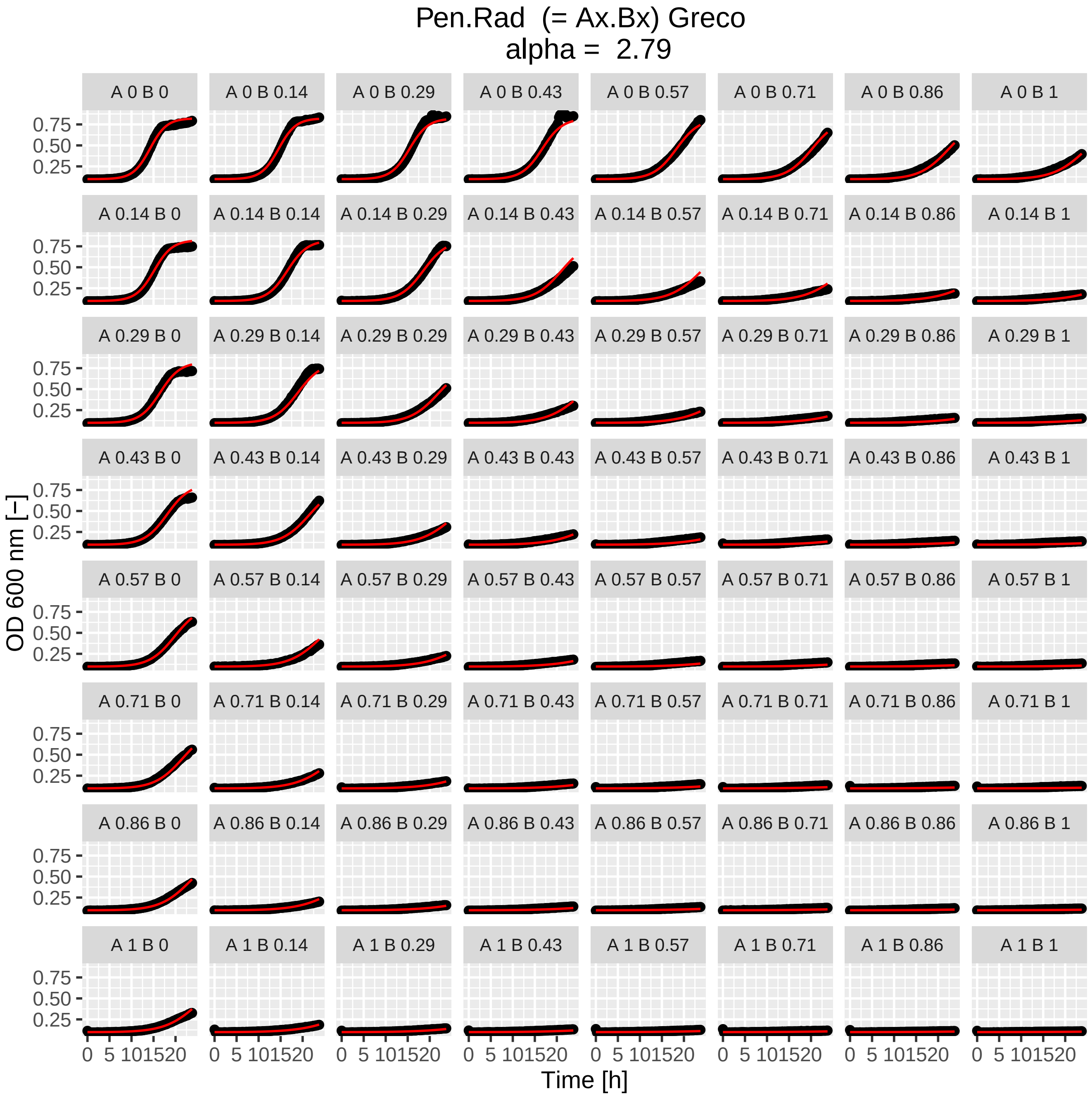

Pen.Rad (= Ax.Bx) Greco
alpha = 2.79
OD 600 nm [−]
Time [h]

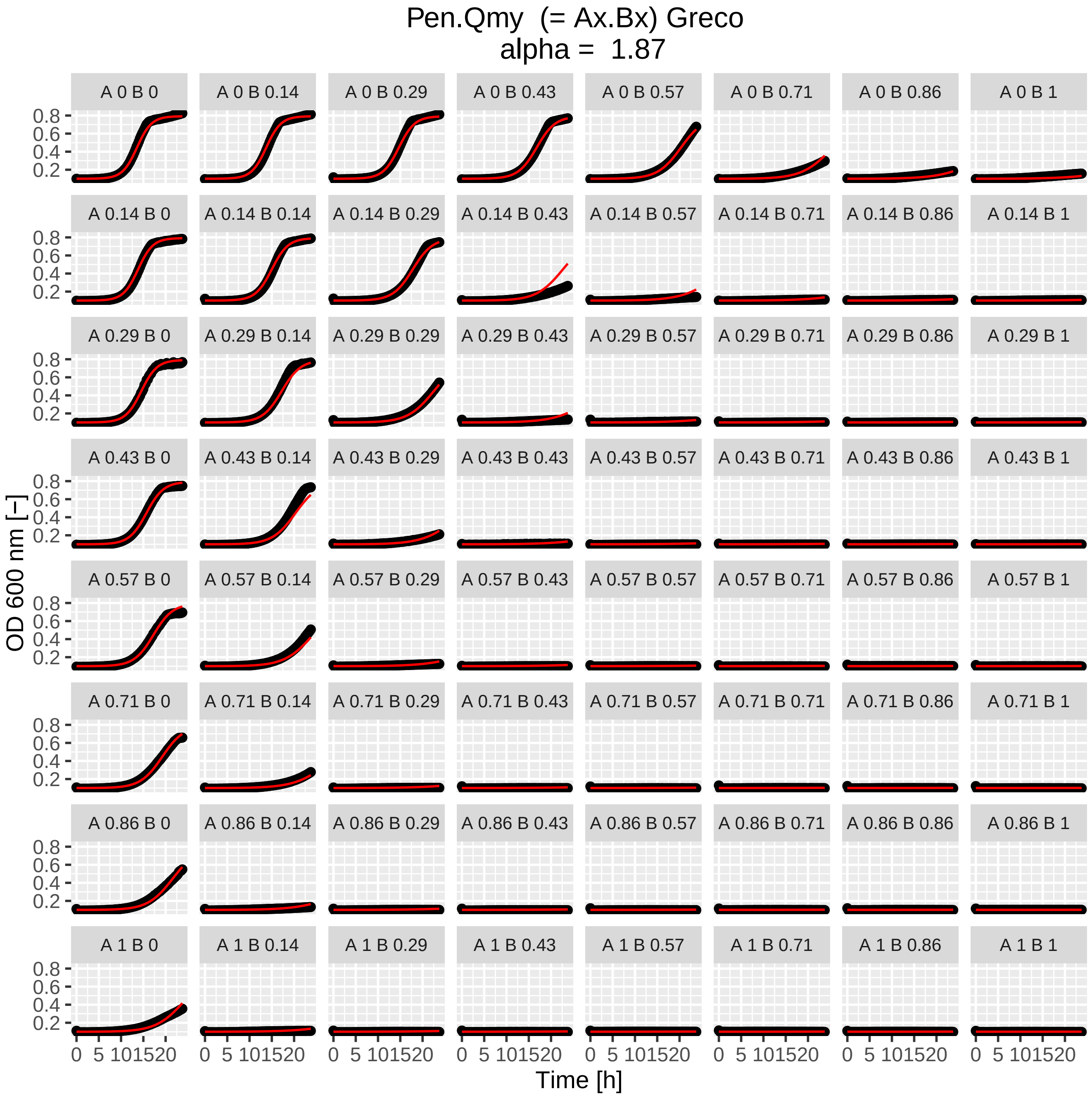

Pen.Qmy (= Ax.Bx) Greco
alpha = 1.87
A 0 B 0
A 0 B 0.14
A 0 B 0.29
A 0 B 0.43
A 0 B 0.57
A 0 B 0.71
A 0 B 0.86
A 0 B 1
A 0.14 B 0
A 0.14 B 0.14
A 0.14 B 0.29
A 0.14 B 0.43
A 0.14 B 0.57
A 0.14 B 0.71
A 0.14 B 0.86
A 0.14 B 1
A 0.29 B 0
A 0.29 B 0.14
A 0.29 B 0.29
A 0.29 B 0.43
A 0.29 B 0.57
A 0.29 B 0.71
A 0.29 B 0.86
A 0.29 B 1
A 0.43 B 0
A 0.43 B 0.14
A 0.43 B 0.29
A 0.43 B 0.43
A 0.43 B 0.57
A 0.43 B 0.71
A 0.43 B 0.86
A 0.43 B 1
A 0.57 B 0
A 0.57 B 0.14
A 0.57 B 0.29
A 0.57 B 0.43
A 0.57 B 0.57
A 0.57 B 0.71
A 0.57 B 0.86
A 0.57 B 1
A 0.71 B 0
A 0.71 B 0.14
A 0.71 B 0.29
A 0.71 B 0.43
A 0.71 B 0.57
A 0.71 B 0.71
A 0.71 B 0.86
A 0.71 B 1
A 0.86 B 0
A 0.86 B 0.14
A 0.86 B 0.29
A 0.86 B 0.43
A 0.86 B 0.57
A 0.86 B 0.71
A 0.86 B 0.86
A 0.86 B 1
A 1 B 0
A 1 B 0.14
A 1 B 0.29
A 1 B 0.43
A 1 B 0.57
A 1 B 0.71
A 1 B 0.86
A 1 B 1
OD 600 nm [−]
0.2
0.4
0.6
0.8
0 5 101520
Time [h]

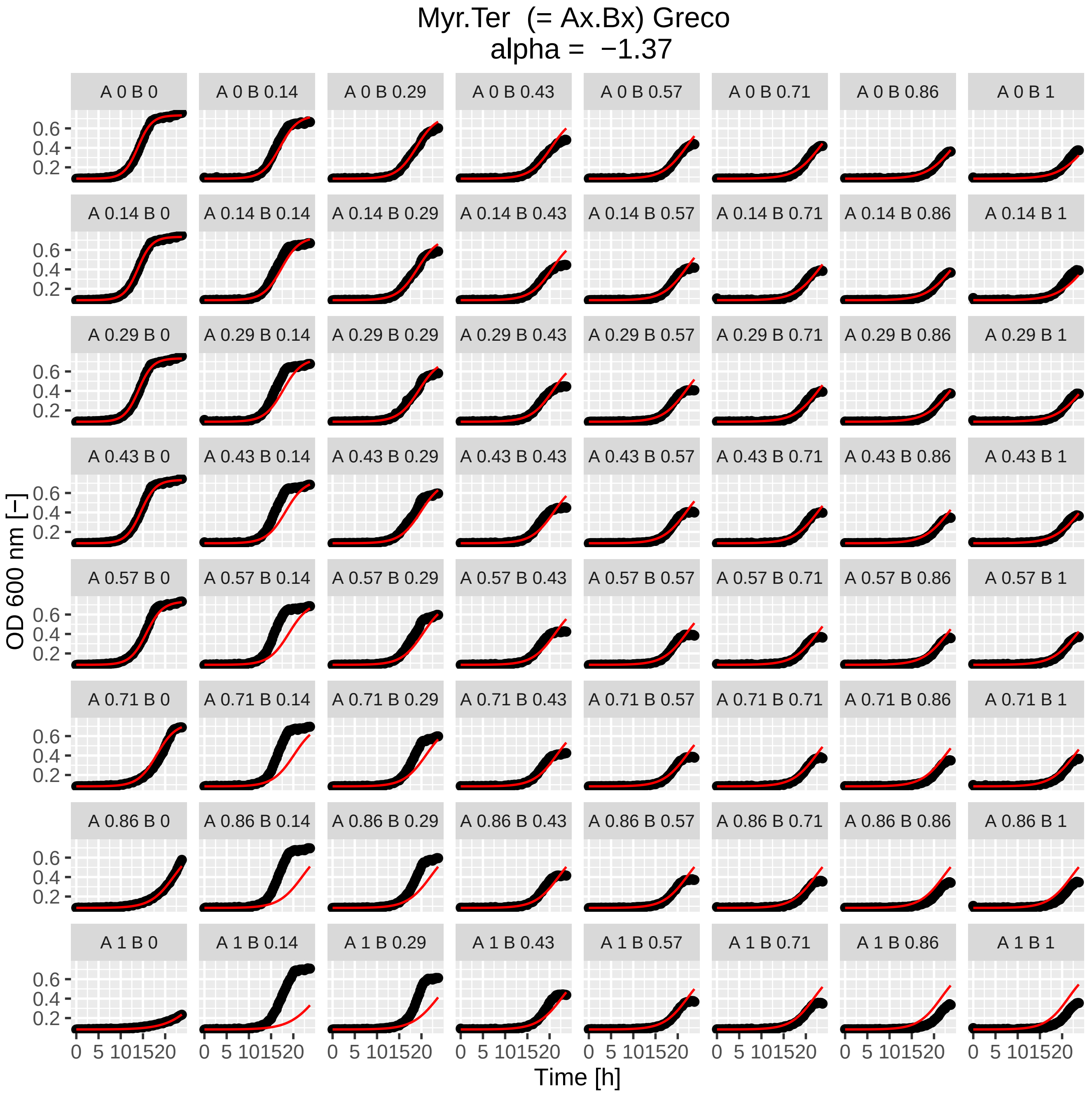

Myr.Ter (= Ax.Bx) Greco
alpha = −1.37
OD 600 nm [−]
Time [h]
0.2
0.4
0.6
0 5 101520
A 0 B 0
A 0 B 0.14
A 0 B 0.29
A 0 B 0.43
A 0 B 0.57
A 0 B 0.71
A 0 B 0.86
A 0 B 1
A 0.14 B 0
A 0.14 B 0.14
A 0.14 B 0.29
A 0.14 B 0.43
A 0.14 B 0.57
A 0.14 B 0.71
A 0.14 B 0.86
A 0.14 B 1
A 0.29 B 0
A 0.29 B 0.14
A 0.29 B 0.29
A 0.29 B 0.43
A 0.29 B 0.57
A 0.29 B 0.71
A 0.29 B 0.86
A 0.29 B 1
A 0.43 B 0
A 0.43 B 0.14
A 0.43 B 0.29
A 0.43 B 0.43
A 0.43 B 0.57
A 0.43 B 0.71
A 0.43 B 0.86
A 0.43 B 1
A 0.57 B 0
A 0.57 B 0.14
A 0.57 B 0.29
A 0.57 B 0.43
A 0.57 B 0.57
A 0.57 B 0.71
A 0.57 B 0.86
A 0.57 B 1
A 0.71 B 0
A 0.71 B 0.14
A 0.71 B 0.29
A 0.71 B 0.43
A 0.71 B 0.57
A 0.71 B 0.71
A 0.71 B 0.86
A 0.71 B 1
A 0.86 B 0
A 0.86 B 0.14
A 0.86 B 0.29
A 0.86 B 0.43
A 0.86 B 0.57
A 0.86 B 0.71
A 0.86 B 0.86
A 0.86 B 1
A 1 B 0
A 1 B 0.14
A 1 B 0.29
A 1 B 0.43
A 1 B 0.57
A 1 B 0.71
A 1 B 0.86
A 1 B 1

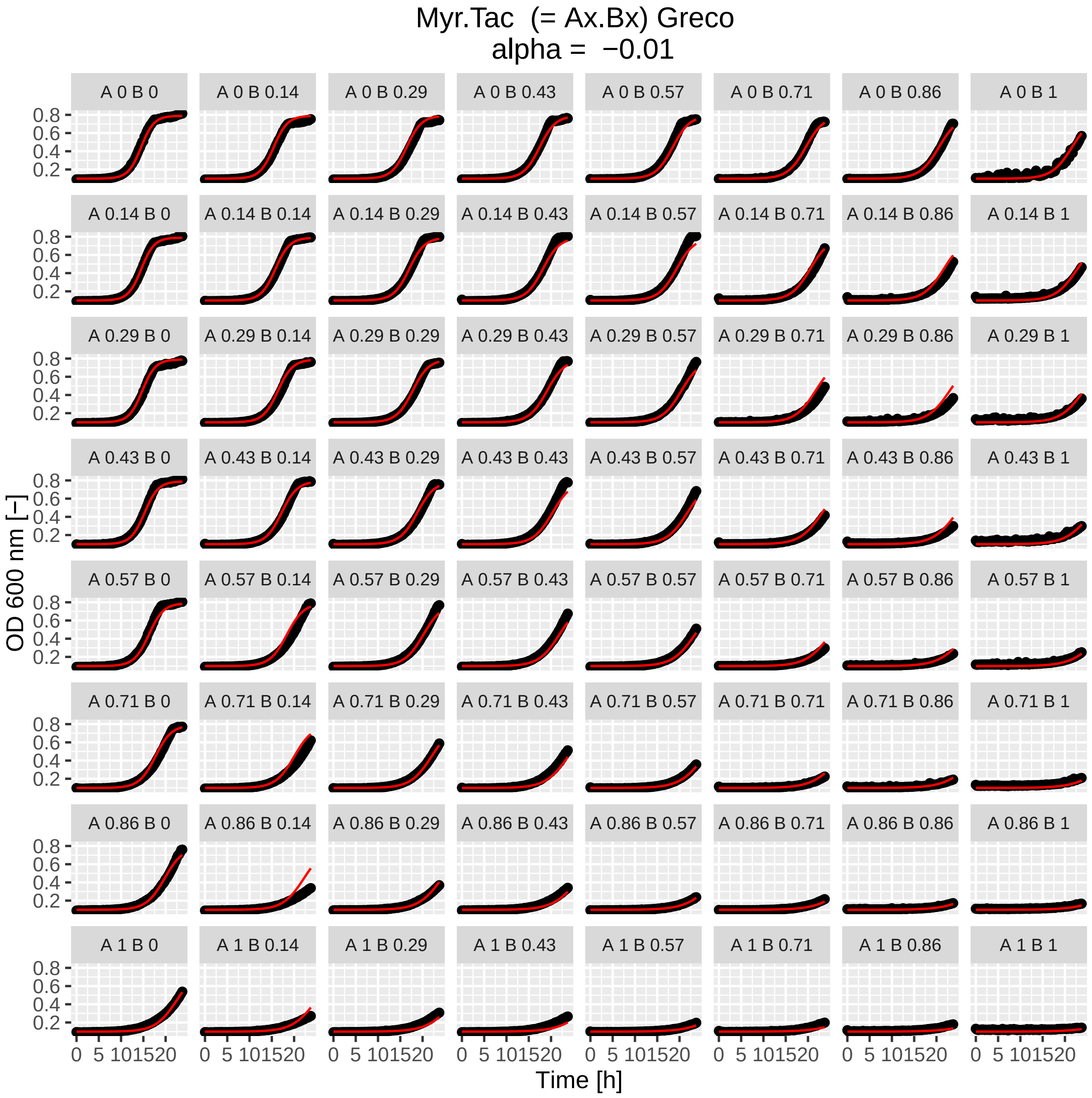

Myr.Tac (= Ax.Bx) Greco
alpha = −0.01
A 0 B 0
A 0 B 0.14
A 0 B 0.29
A 0 B 0.43
A 0 B 0.57
A 0 B 0.71
A 0 B 0.86
A 0 B 1
A 0.14 B 0
A 0.14 B 0.14
A 0.14 B 0.29
A 0.14 B 0.43
A 0.14 B 0.57
A 0.14 B 0.71
A 0.14 B 0.86
A 0.14 B 1
A 0.29 B 0
A 0.29 B 0.14
A 0.29 B 0.29
A 0.29 B 0.43
A 0.29 B 0.57
A 0.29 B 0.71
A 0.29 B 0.86
A 0.29 B 1
A 0.43 B 0
A 0.43 B 0.14
A 0.43 B 0.29
A 0.43 B 0.43
A 0.43 B 0.57
A 0.43 B 0.71
A 0.43 B 0.86
A 0.43 B 1
A 0.57 B 0
A 0.57 B 0.14
A 0.57 B 0.29
A 0.57 B 0.43
A 0.57 B 0.57
A 0.57 B 0.71
A 0.57 B 0.86
A 0.57 B 1
A 0.71 B 0
A 0.71 B 0.14
A 0.71 B 0.29
A 0.71 B 0.43
A 0.71 B 0.57
A 0.71 B 0.71
A 0.71 B 0.86
A 0.71 B 1
A 0.86 B 0
A 0.86 B 0.14
A 0.86 B 0.29
A 0.86 B 0.43
A 0.86 B 0.57
A 0.86 B 0.71
A 0.86 B 0.86
A 0.86 B 1
A 1 B 0
A 1 B 0.14
A 1 B 0.29
A 1 B 0.43
A 1 B 0.57
A 1 B 0.71
A 1 B 0.86
A 1 B 1
0.8
0.6
0.4
0.2
OD 600 nm [−]
0 5 101520
Time [h]

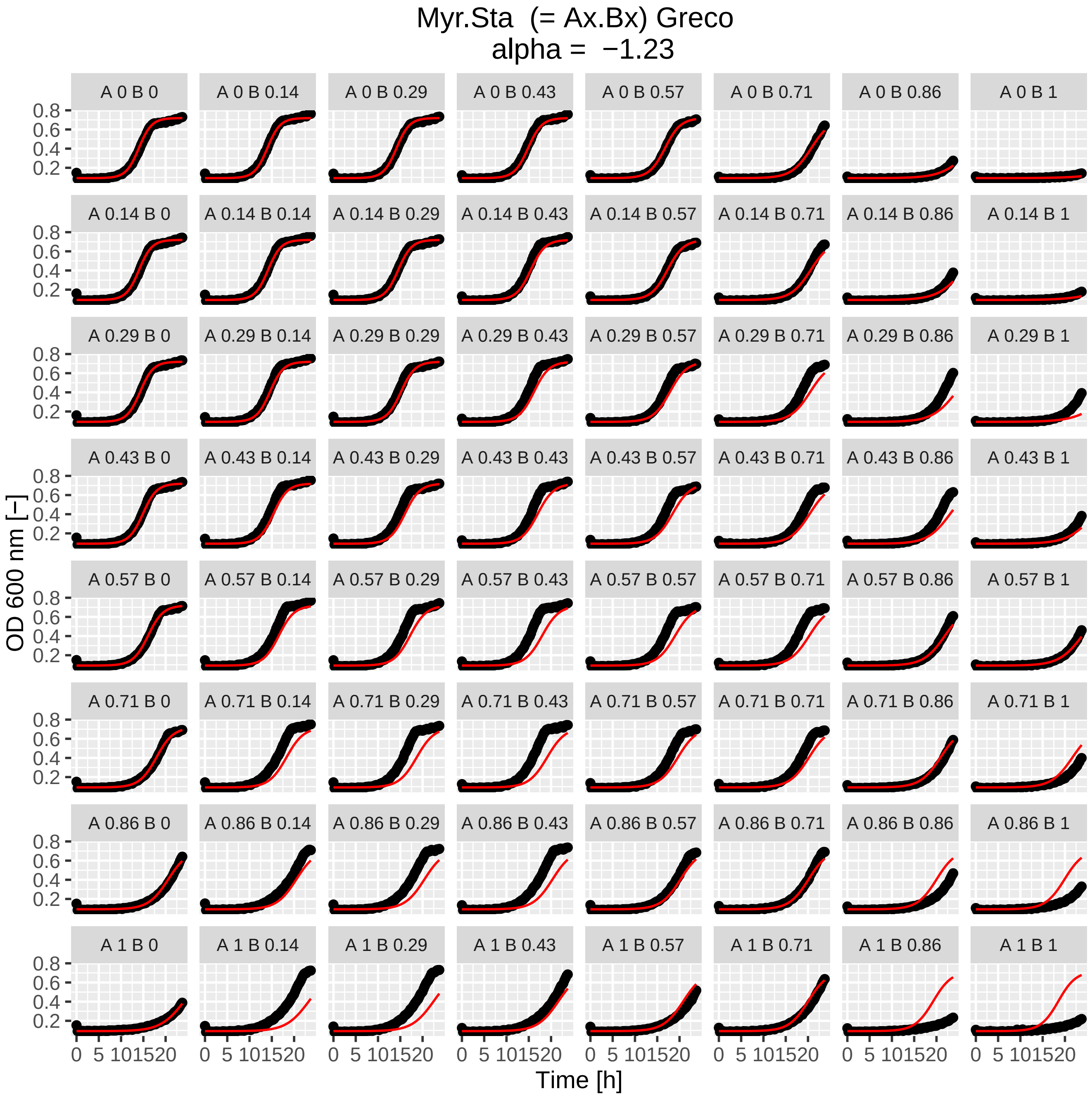

Myr.Sta (= Ax.Bx) Greco
alpha = −1.23
OD 600 nm [−]
Time [h]
A 0 B 0
A 0 B 0.14
A 0 B 0.29
A 0 B 0.43
A 0 B 0.57
A 0 B 0.71
A 0 B 0.86
A 0 B 1
A 0.14 B 0
A 0.14 B 0.14
A 0.14 B 0.29
A 0.14 B 0.43
A 0.14 B 0.57
A 0.14 B 0.71
A 0.14 B 0.86
A 0.14 B 1
A 0.29 B 0
A 0.29 B 0.14
A 0.29 B 0.29
A 0.29 B 0.43
A 0.29 B 0.57
A 0.29 B 0.71
A 0.29 B 0.86
A 0.29 B 1
A 0.43 B 0
A 0.43 B 0.14
A 0.43 B 0.29
A 0.43 B 0.43
A 0.43 B 0.57
A 0.43 B 0.71
A 0.43 B 0.86
A 0.43 B 1
A 0.57 B 0
A 0.57 B 0.14
A 0.57 B 0.29
A 0.57 B 0.43
A 0.57 B 0.57
A 0.57 B 0.71
A 0.57 B 0.86
A 0.57 B 1
A 0.71 B 0
A 0.71 B 0.14
A 0.71 B 0.29
A 0.71 B 0.43
A 0.71 B 0.57
A 0.71 B 0.71
A 0.71 B 0.86
A 0.71 B 1
A 0.86 B 0
A 0.86 B 0.14
A 0.86 B 0.29
A 0.86 B 0.43
A 0.86 B 0.57
A 0.86 B 0.71
A 0.86 B 0.86
A 0.86 B 1
A 1 B 0
A 1 B 0.14
A 1 B 0.29
A 1 B 0.43
A 1 B 0.57
A 1 B 0.71
A 1 B 0.86
A 1 B 1
0.2
0.4
0.6
0.8
0 5 101520

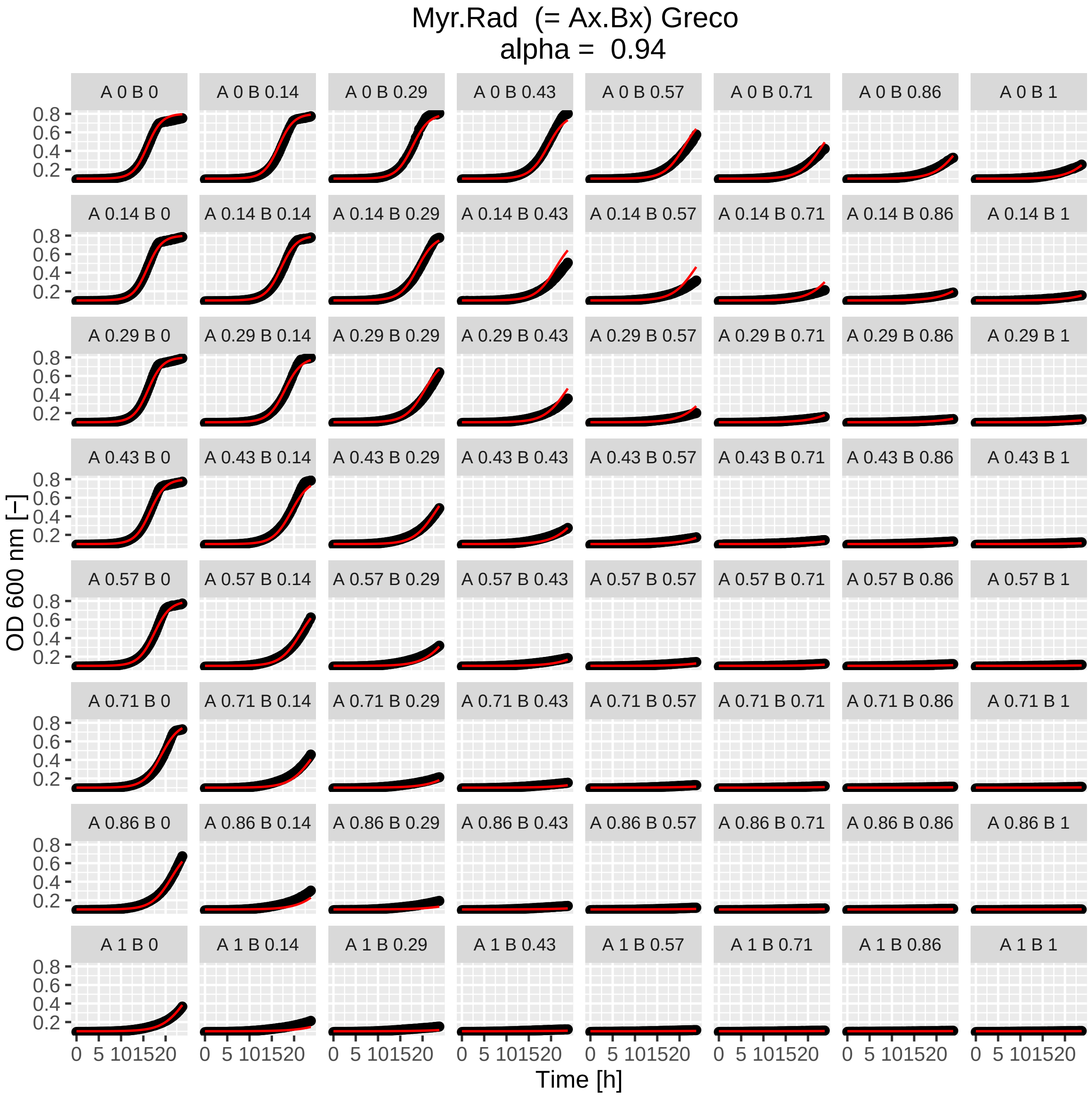

Myr.Rad (= Ax.Bx) Greco
alpha = 0.94
A 0 B 0
A 0 B 0.14
A 0 B 0.29
A 0 B 0.43
A 0 B 0.57
A 0 B 0.71
A 0 B 0.86
A 0 B 1
A 0.14 B 0
A 0.14 B 0.14
A 0.14 B 0.29
A 0.14 B 0.43
A 0.14 B 0.57
A 0.14 B 0.71
A 0.14 B 0.86
A 0.14 B 1
A 0.29 B 0
A 0.29 B 0.14
A 0.29 B 0.29
A 0.29 B 0.43
A 0.29 B 0.57
A 0.29 B 0.71
A 0.29 B 0.86
A 0.29 B 1
A 0.43 B 0
A 0.43 B 0.14
A 0.43 B 0.29
A 0.43 B 0.43
A 0.43 B 0.57
A 0.43 B 0.71
A 0.43 B 0.86
A 0.43 B 1
A 0.57 B 0
A 0.57 B 0.14
A 0.57 B 0.29
A 0.57 B 0.43
A 0.57 B 0.57
A 0.57 B 0.71
A 0.57 B 0.86
A 0.57 B 1
A 0.71 B 0
A 0.71 B 0.14
A 0.71 B 0.29
A 0.71 B 0.43
A 0.71 B 0.57
A 0.71 B 0.71
A 0.71 B 0.86
A 0.71 B 1
A 0.86 B 0
A 0.86 B 0.14
A 0.86 B 0.29
A 0.86 B 0.43
A 0.86 B 0.57
A 0.86 B 0.71
A 0.86 B 0.86
A 0.86 B 1
A 1 B 0
A 1 B 0.14
A 1 B 0.29
A 1 B 0.43
A 1 B 0.57
A 1 B 0.71
A 1 B 0.86
A 1 B 1
0.8
0.6
0.4
0.2
0 5 101520
OD 600 nm [–]
Time [h]

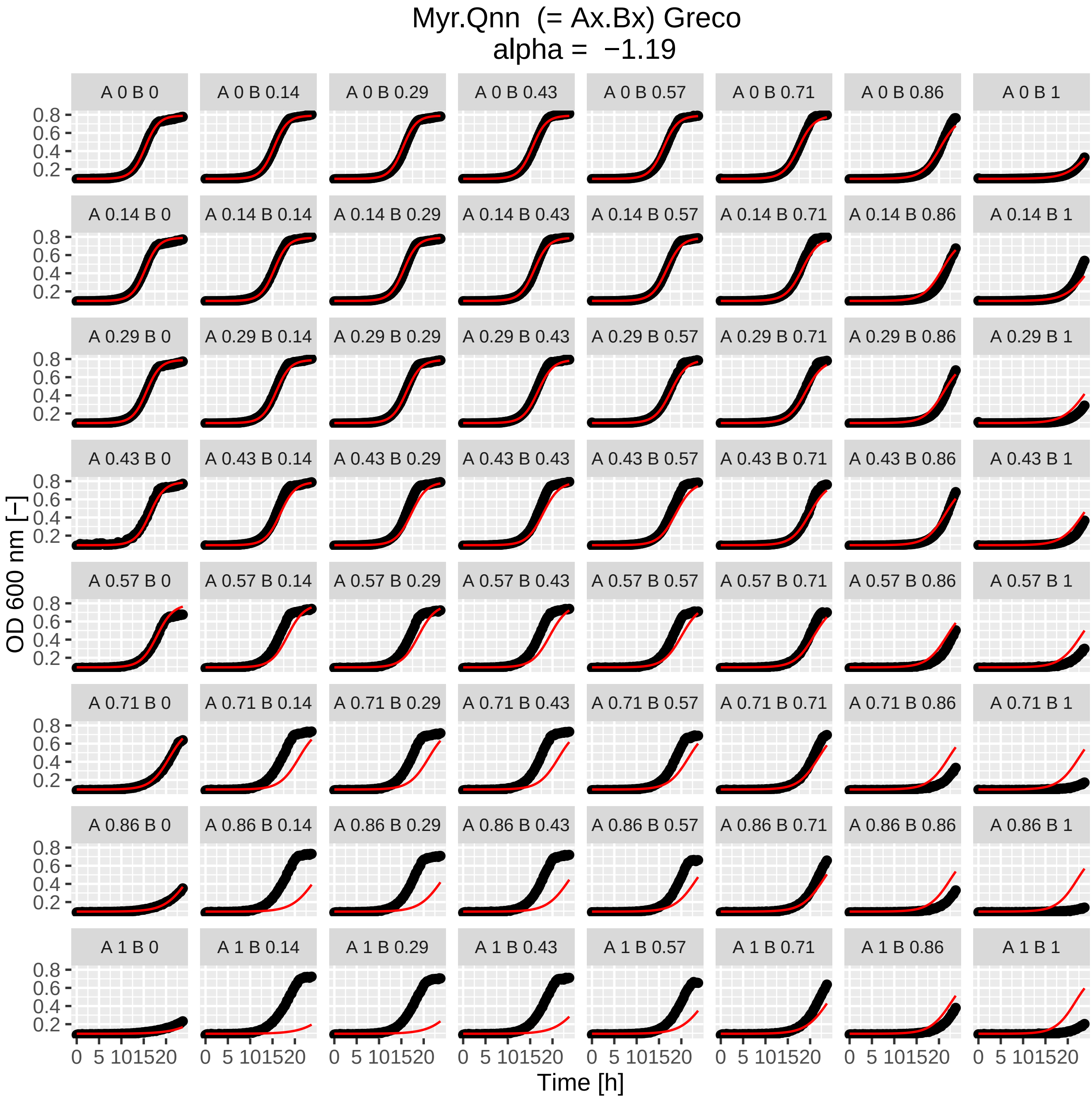
Myr.Qnn (= Ax.Bx) Greco
alpha = −1.19
A 0 B 0
A 0 B 0.14
A 0 B 0.29
A 0 B 0.43
A 0 B 0.57
A 0 B 0.71
A 0 B 0.86
A 0 B 1
A 0.14 B 0
A 0.14 B 0.14
A 0.14 B 0.29
A 0.14 B 0.43
A 0.14 B 0.57
A 0.14 B 0.71
A 0.14 B 0.86
A 0.14 B 1
A 0.29 B 0
A 0.29 B 0.14
A 0.29 B 0.29
A 0.29 B 0.43
A 0.29 B 0.57
A 0.29 B 0.71
A 0.29 B 0.86
A 0.29 B 1
A 0.43 B 0
A 0.43 B 0.14
A 0.43 B 0.29
A 0.43 B 0.43
A 0.43 B 0.57
A 0.43 B 0.71
A 0.43 B 0.86
A 0.43 B 1
A 0.57 B 0
A 0.57 B 0.14
A 0.57 B 0.29
A 0.57 B 0.43
A 0.57 B 0.57
A 0.57 B 0.71
A 0.57 B 0.86
A 0.57 B 1
A 0.71 B 0
A 0.71 B 0.14
A 0.71 B 0.29
A 0.71 B 0.43
A 0.71 B 0.57
A 0.71 B 0.71
A 0.71 B 0.86
A 0.71 B 1
A 0.86 B 0
A 0.86 B 0.14
A 0.86 B 0.29
A 0.86 B 0.43
A 0.86 B 0.57
A 0.86 B 0.71
A 0.86 B 0.86
A 0.86 B 1
A 1 B 0
A 1 B 0.14
A 1 B 0.29
A 1 B 0.43
A 1 B 0.57
A 1 B 0.71
A 1 B 0.86
A 1 B 1
OD 600 nm [−]
0.2
0.4
0.6
0.8
0 5 101520
Time [h]

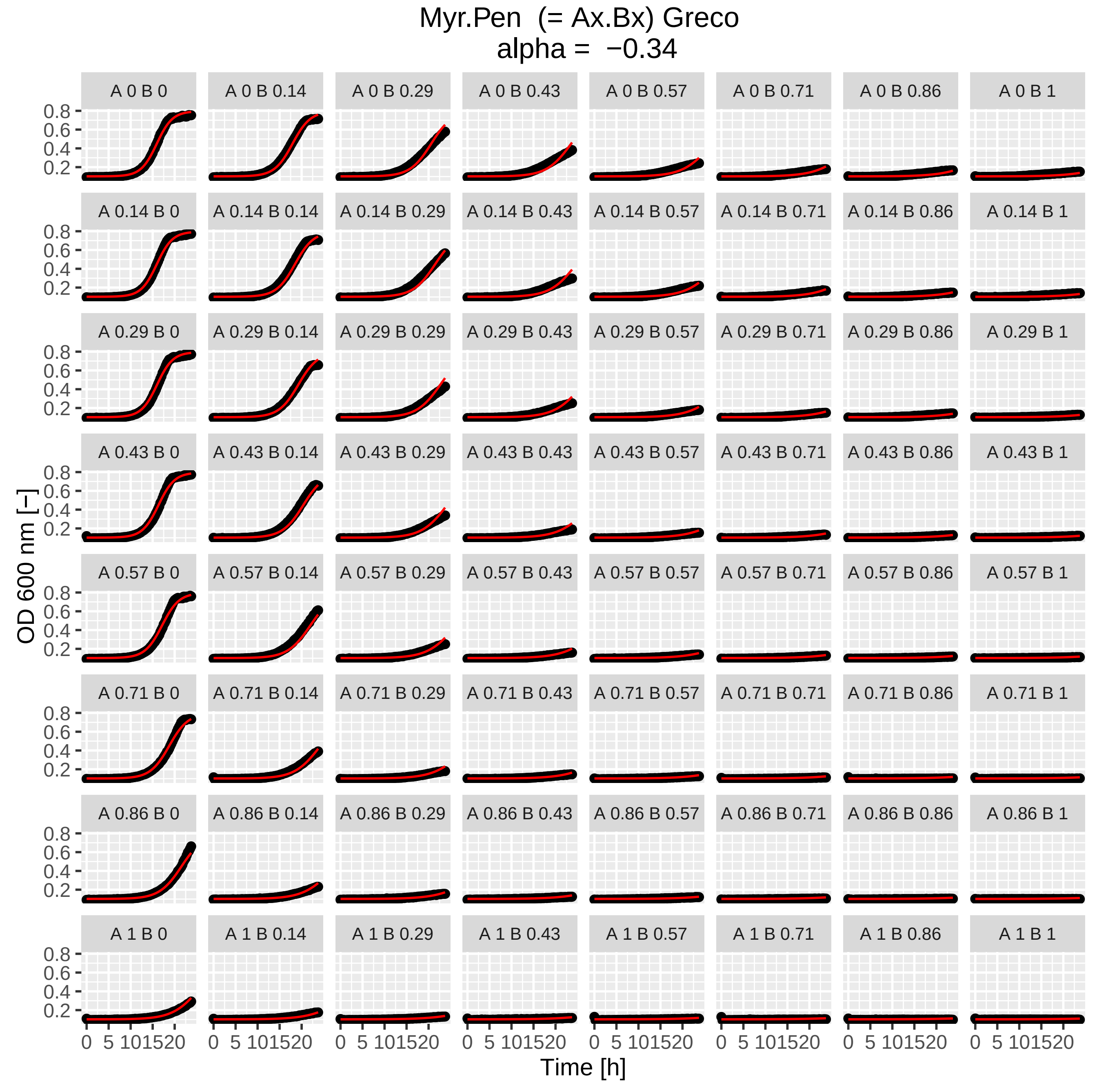
Myr.Pen (= Ax.Bx) Greco
alpha = −0.34
OD 600 nm [−]
Time [h]
0.2
0.4
0.6
0.8
0 5 101520
A 0 B 0
A 0 B 0.14
A 0 B 0.29
A 0 B 0.43
A 0 B 0.57
A 0 B 0.71
A 0 B 0.86
A 0 B 1
A 0.14 B 0
A 0.14 B 0.14
A 0.14 B 0.29
A 0.14 B 0.43
A 0.14 B 0.57
A 0.14 B 0.71
A 0.14 B 0.86
A 0.14 B 1
A 0.29 B 0
A 0.29 B 0.14
A 0.29 B 0.29
A 0.29 B 0.43
A 0.29 B 0.57
A 0.29 B 0.71
A 0.29 B 0.86
A 0.29 B 1
A 0.43 B 0
A 0.43 B 0.14
A 0.43 B 0.29
A 0.43 B 0.43
A 0.43 B 0.57
A 0.43 B 0.71
A 0.43 B 0.86
A 0.43 B 1
A 0.57 B 0
A 0.57 B 0.14
A 0.57 B 0.29
A 0.57 B 0.43
A 0.57 B 0.57
A 0.57 B 0.71
A 0.57 B 0.86
A 0.57 B 1
A 0.71 B 0
A 0.71 B 0.14
A 0.71 B 0.29
A 0.71 B 0.43
A 0.71 B 0.57
A 0.71 B 0.71
A 0.71 B 0.86
A 0.71 B 1
A 0.86 B 0
A 0.86 B 0.14
A 0.86 B 0.29
A 0.86 B 0.43
A 0.86 B 0.57
A 0.86 B 0.71
A 0.86 B 0.86
A 0.86 B 1
A 1 B 0
A 1 B 0.14
A 1 B 0.29
A 1 B 0.43
A 1 B 0.57
A 1 B 0.71
A 1 B 0.86
A 1 B 1

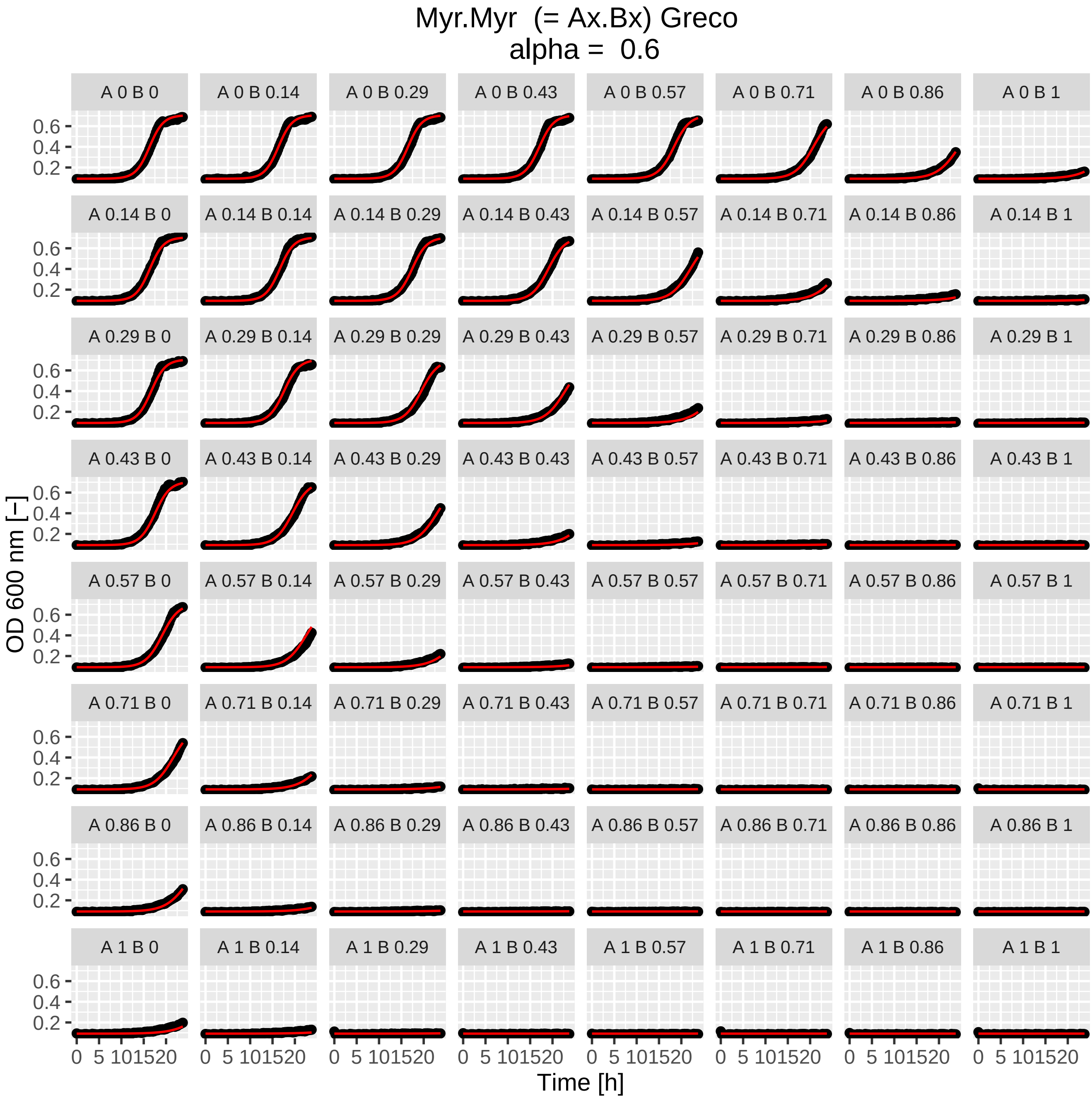

Myr.Myr (= Ax.Bx) Greco
alpha = 0.6
A 0 B 0
A 0 B 0.14
A 0 B 0.29
A 0 B 0.43
A 0 B 0.57
A 0 B 0.71
A 0 B 0.86
A 0 B 1
A 0.14 B 0
A 0.14 B 0.14
A 0.14 B 0.29
A 0.14 B 0.43
A 0.14 B 0.57
A 0.14 B 0.71
A 0.14 B 0.86
A 0.14 B 1
A 0.29 B 0
A 0.29 B 0.14
A 0.29 B 0.29
A 0.29 B 0.43
A 0.29 B 0.57
A 0.29 B 0.71
A 0.29 B 0.86
A 0.29 B 1
A 0.43 B 0
A 0.43 B 0.14
A 0.43 B 0.29
A 0.43 B 0.43
A 0.43 B 0.57
A 0.43 B 0.71
A 0.43 B 0.86
A 0.43 B 1
A 0.57 B 0
A 0.57 B 0.14
A 0.57 B 0.29
A 0.57 B 0.43
A 0.57 B 0.57
A 0.57 B 0.71
A 0.57 B 0.86
A 0.57 B 1
A 0.71 B 0
A 0.71 B 0.14
A 0.71 B 0.29
A 0.71 B 0.43
A 0.71 B 0.57
A 0.71 B 0.71
A 0.71 B 0.86
A 0.71 B 1
A 0.86 B 0
A 0.86 B 0.14
A 0.86 B 0.29
A 0.86 B 0.43
A 0.86 B 0.57
A 0.86 B 0.71
A 0.86 B 0.86
A 0.86 B 1
A 1 B 0
A 1 B 0.14
A 1 B 0.29
A 1 B 0.43
A 1 B 0.57
A 1 B 0.71
A 1 B 0.86
A 1 B 1
OD 600 nm [–]
0.2
0.4
0.6
0 5 101520
Time [h]

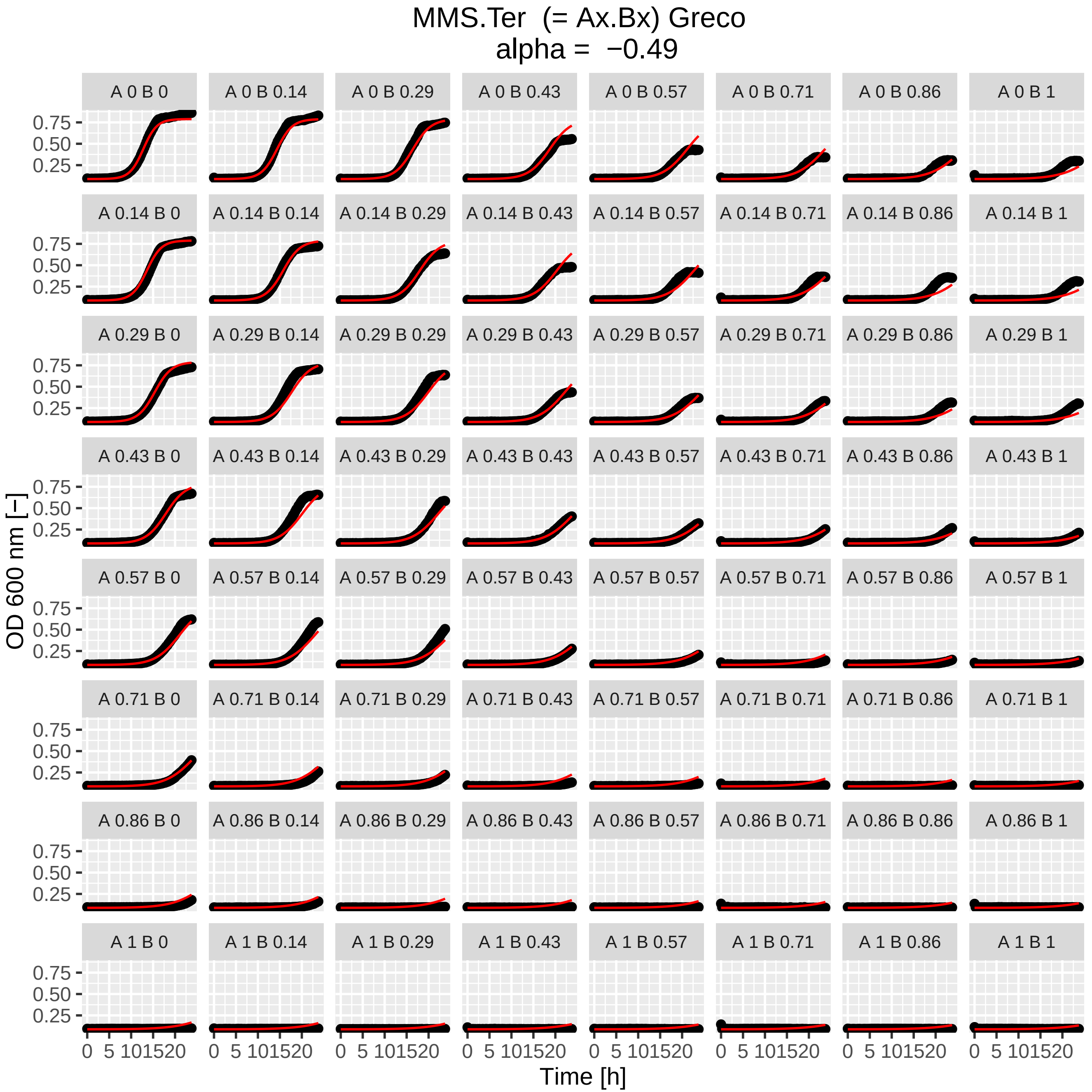
MMS.Ter (= Ax.Bx) Greco
alpha = −0.49
OD 600 nm [−]
Time [h]
A 0 B 0
A 0 B 0.14
A 0 B 0.29
A 0 B 0.43
A 0 B 0.57
A 0 B 0.71
A 0 B 0.86
A 0 B 1
A 0.14 B 0
A 0.14 B 0.14
A 0.14 B 0.29
A 0.14 B 0.43
A 0.14 B 0.57
A 0.14 B 0.71
A 0.14 B 0.86
A 0.14 B 1
A 0.29 B 0
A 0.29 B 0.14
A 0.29 B 0.29
A 0.29 B 0.43
A 0.29 B 0.57
A 0.29 B 0.71
A 0.29 B 0.86
A 0.29 B 1
A 0.43 B 0
A 0.43 B 0.14
A 0.43 B 0.29
A 0.43 B 0.43
A 0.43 B 0.57
A 0.43 B 0.71
A 0.43 B 0.86
A 0.43 B 1
A 0.57 B 0
A 0.57 B 0.14
A 0.57 B 0.29
A 0.57 B 0.43
A 0.57 B 0.57
A 0.57 B 0.71
A 0.57 B 0.86
A 0.57 B 1
A 0.71 B 0
A 0.71 B 0.14
A 0.71 B 0.29
A 0.71 B 0.43
A 0.71 B 0.57
A 0.71 B 0.71
A 0.71 B 0.86
A 0.71 B 1
A 0.86 B 0
A 0.86 B 0.14
A 0.86 B 0.29
A 0.86 B 0.43
A 0.86 B 0.57
A 0.86 B 0.71
A 0.86 B 0.86
A 0.86 B 1
A 1 B 0
A 1 B 0.14
A 1 B 0.29
A 1 B 0.43
A 1 B 0.57
A 1 B 0.71
A 1 B 0.86
A 1 B 1
0.75
0.50
0.25
0 5 101520

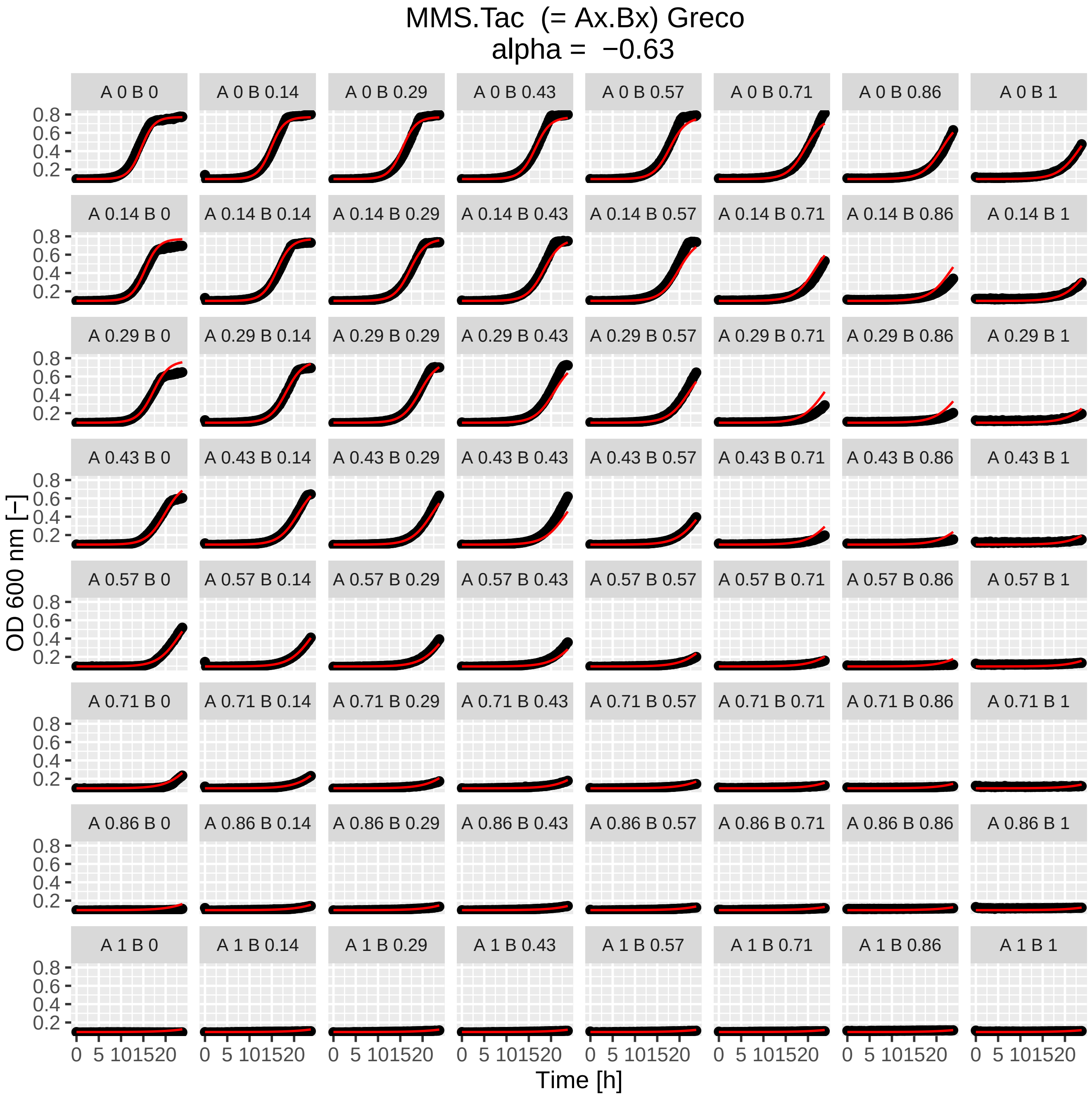
MMS.Tac (= Ax.Bx) Greco
alpha = −0.63
OD 600 nm [−]
Time [h]
A 0 B 0
A 0 B 0.14
A 0 B 0.29
A 0 B 0.43
A 0 B 0.57
A 0 B 0.71
A 0 B 0.86
A 0 B 1
A 0.14 B 0
A 0.14 B 0.14
A 0.14 B 0.29
A 0.14 B 0.43
A 0.14 B 0.57
A 0.14 B 0.71
A 0.14 B 0.86
A 0.14 B 1
A 0.29 B 0
A 0.29 B 0.14
A 0.29 B 0.29
A 0.29 B 0.43
A 0.29 B 0.57
A 0.29 B 0.71
A 0.29 B 0.86
A 0.29 B 1
A 0.43 B 0
A 0.43 B 0.14
A 0.43 B 0.29
A 0.43 B 0.43
A 0.43 B 0.57
A 0.43 B 0.71
A 0.43 B 0.86
A 0.43 B 1
A 0.57 B 0
A 0.57 B 0.14
A 0.57 B 0.29
A 0.57 B 0.43
A 0.57 B 0.57
A 0.57 B 0.71
A 0.57 B 0.86
A 0.57 B 1
A 0.71 B 0
A 0.71 B 0.14
A 0.71 B 0.29
A 0.71 B 0.43
A 0.71 B 0.57
A 0.71 B 0.71
A 0.71 B 0.86
A 0.71 B 1
A 0.86 B 0
A 0.86 B 0.14
A 0.86 B 0.29
A 0.86 B 0.43
A 0.86 B 0.57
A 0.86 B 0.71
A 0.86 B 0.86
A 0.86 B 1
A 1 B 0
A 1 B 0.14
A 1 B 0.29
A 1 B 0.43
A 1 B 0.57
A 1 B 0.71
A 1 B 0.86
A 1 B 1
0.8
0.6
0.4
0.2
0 5 101520

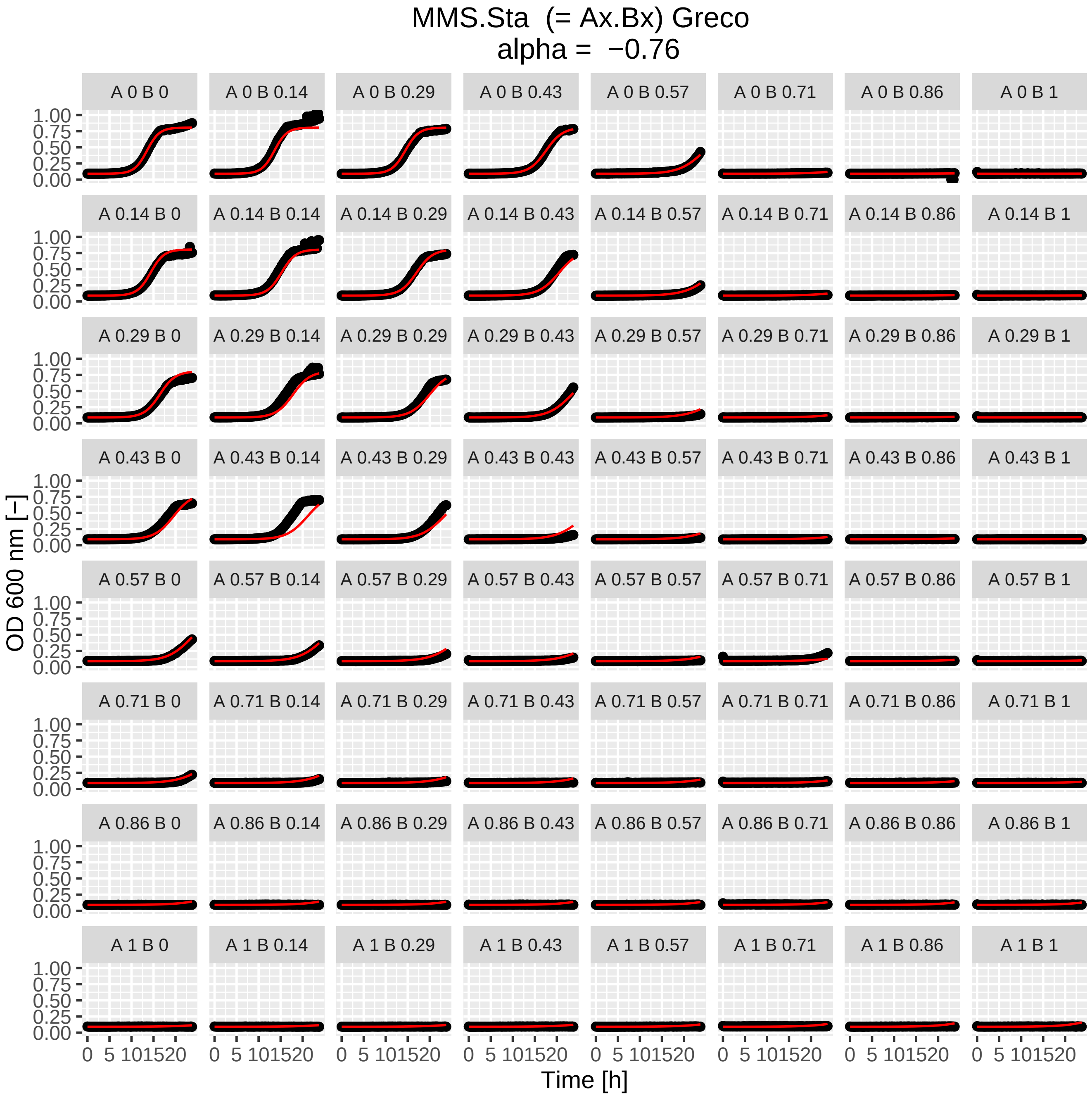

MMS.Sta (= Ax.Bx) Greco
alpha = −0.76
A 0 B 0
A 0 B 0.14
A 0 B 0.29
A 0 B 0.43
A 0 B 0.57
A 0 B 0.71
A 0 B 0.86
A 0 B 1
A 0.14 B 0
A 0.14 B 0.14
A 0.14 B 0.29
A 0.14 B 0.43
A 0.14 B 0.57
A 0.14 B 0.71
A 0.14 B 0.86
A 0.14 B 1
A 0.29 B 0
A 0.29 B 0.14
A 0.29 B 0.29
A 0.29 B 0.43
A 0.29 B 0.57
A 0.29 B 0.71
A 0.29 B 0.86
A 0.29 B 1
A 0.43 B 0
A 0.43 B 0.14
A 0.43 B 0.29
A 0.43 B 0.43
A 0.43 B 0.57
A 0.43 B 0.71
A 0.43 B 0.86
A 0.43 B 1
A 0.57 B 0
A 0.57 B 0.14
A 0.57 B 0.29
A 0.57 B 0.43
A 0.57 B 0.57
A 0.57 B 0.71
A 0.57 B 0.86
A 0.57 B 1
A 0.71 B 0
A 0.71 B 0.14
A 0.71 B 0.29
A 0.71 B 0.43
A 0.71 B 0.57
A 0.71 B 0.71
A 0.71 B 0.86
A 0.71 B 1
A 0.86 B 0
A 0.86 B 0.14
A 0.86 B 0.29
A 0.86 B 0.43
A 0.86 B 0.57
A 0.86 B 0.71
A 0.86 B 0.86
A 0.86 B 1
A 1 B 0
A 1 B 0.14
A 1 B 0.29
A 1 B 0.43
A 1 B 0.57
A 1 B 0.71
A 1 B 0.86
A 1 B 1
OD 600 nm [−]
1.00
0.75
0.50
0.25
0.00
0 5 101520
Time [h]

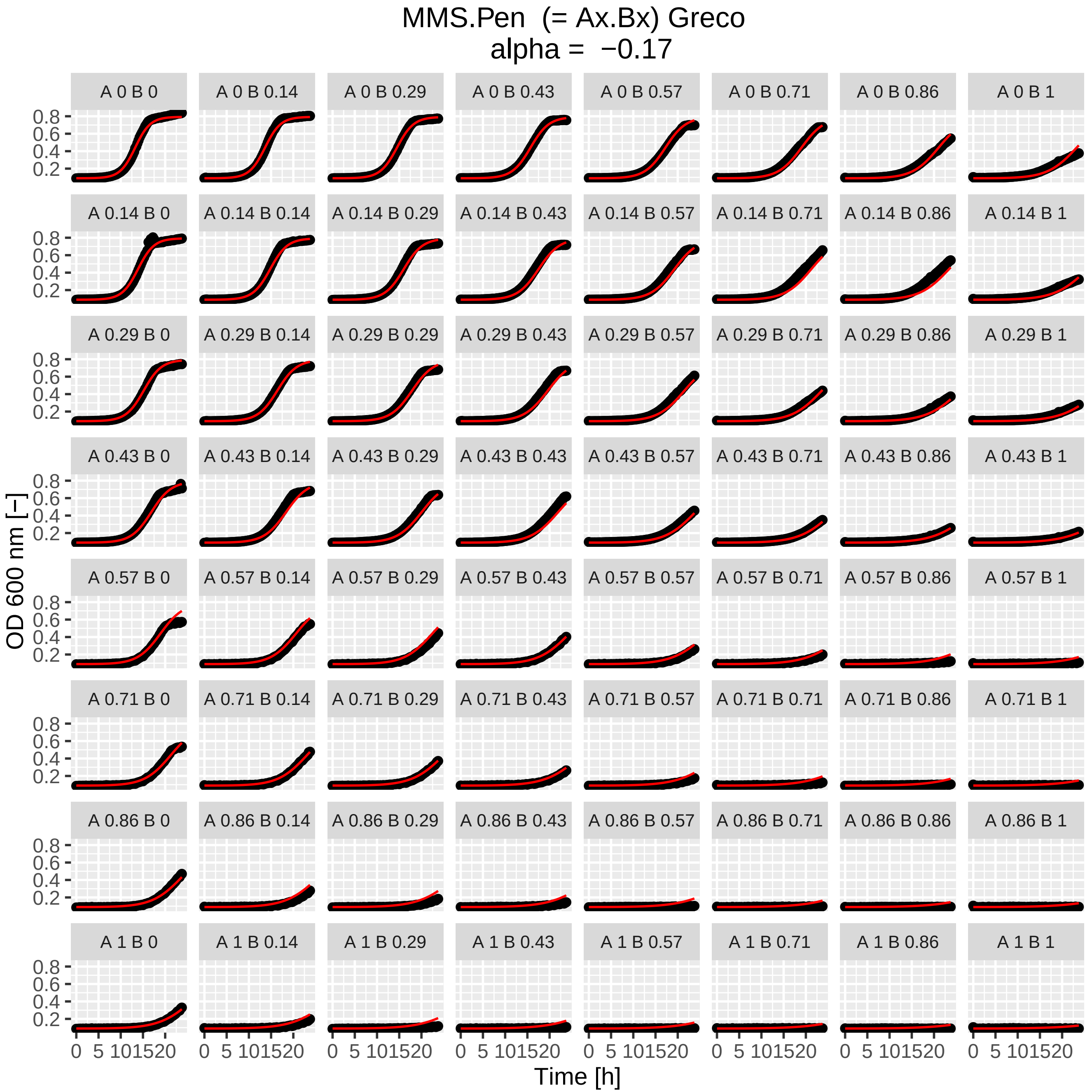
MMS.Pen (= Ax.Bx) Greco
alpha = −0.17
OD 600 nm [−]
Time [h]
A 0 B 0
A 0 B 0.14
A 0 B 0.29
A 0 B 0.43
A 0 B 0.57
A 0 B 0.71
A 0 B 0.86
A 0 B 1
A 0.14 B 0
A 0.14 B 0.14
A 0.14 B 0.29
A 0.14 B 0.43
A 0.14 B 0.57
A 0.14 B 0.71
A 0.14 B 0.86
A 0.14 B 1
A 0.29 B 0
A 0.29 B 0.14
A 0.29 B 0.29
A 0.29 B 0.43
A 0.29 B 0.57
A 0.29 B 0.71
A 0.29 B 0.86
A 0.29 B 1
A 0.43 B 0
A 0.43 B 0.14
A 0.43 B 0.29
A 0.43 B 0.43
A 0.43 B 0.57
A 0.43 B 0.71
A 0.43 B 0.86
A 0.43 B 1
A 0.57 B 0
A 0.57 B 0.14
A 0.57 B 0.29
A 0.57 B 0.43
A 0.57 B 0.57
A 0.57 B 0.71
A 0.57 B 0.86
A 0.57 B 1
A 0.71 B 0
A 0.71 B 0.14
A 0.71 B 0.29
A 0.71 B 0.43
A 0.71 B 0.57
A 0.71 B 0.71
A 0.71 B 0.86
A 0.71 B 1
A 0.86 B 0
A 0.86 B 0.14
A 0.86 B 0.29
A 0.86 B 0.43
A 0.86 B 0.57
A 0.86 B 0.71
A 0.86 B 0.86
A 0.86 B 1
A 1 B 0
A 1 B 0.14
A 1 B 0.29
A 1 B 0.43
A 1 B 0.57
A 1 B 0.71
A 1 B 0.86
A 1 B 1
0.8
0.6
0.4
0.2
0
5
10
15
20

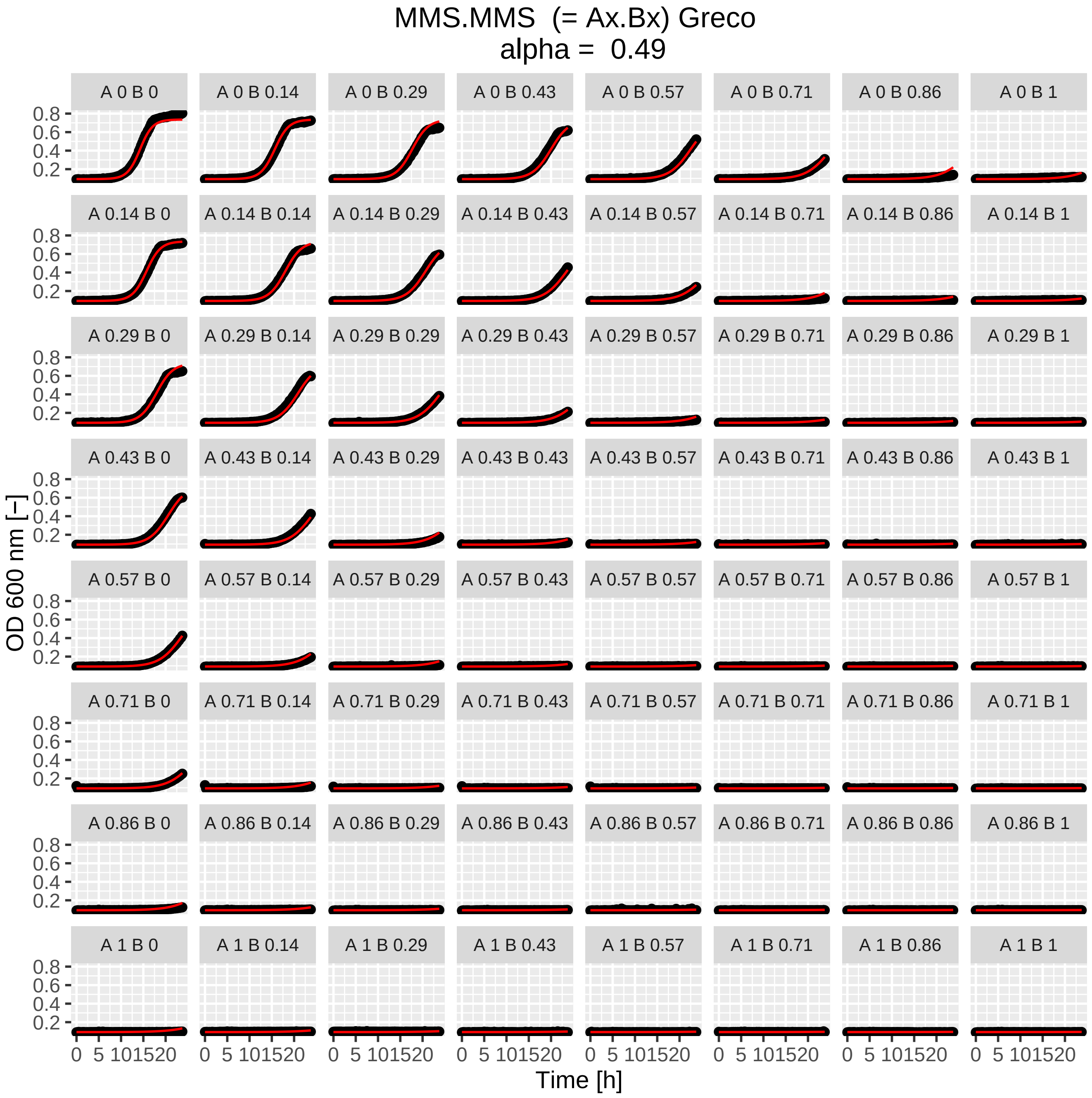

MMS.MMS (= Ax.Bx) Greco
alpha = 0.49
A 0 B 0
A 0 B 0.14
A 0 B 0.29
A 0 B 0.43
A 0 B 0.57
A 0 B 0.71
A 0 B 0.86
A 0 B 1
A 0.14 B 0
A 0.14 B 0.14
A 0.14 B 0.29
A 0.14 B 0.43
A 0.14 B 0.57
A 0.14 B 0.71
A 0.14 B 0.86
A 0.14 B 1
A 0.29 B 0
A 0.29 B 0.14
A 0.29 B 0.29
A 0.29 B 0.43
A 0.29 B 0.57
A 0.29 B 0.71
A 0.29 B 0.86
A 0.29 B 1
A 0.43 B 0
A 0.43 B 0.14
A 0.43 B 0.29
A 0.43 B 0.43
A 0.43 B 0.57
A 0.43 B 0.71
A 0.43 B 0.86
A 0.43 B 1
A 0.57 B 0
A 0.57 B 0.14
A 0.57 B 0.29
A 0.57 B 0.43
A 0.57 B 0.57
A 0.57 B 0.71
A 0.57 B 0.86
A 0.57 B 1
A 0.71 B 0
A 0.71 B 0.14
A 0.71 B 0.29
A 0.71 B 0.43
A 0.71 B 0.57
A 0.71 B 0.71
A 0.71 B 0.86
A 0.71 B 1
A 0.86 B 0
A 0.86 B 0.14
A 0.86 B 0.29
A 0.86 B 0.43
A 0.86 B 0.57
A 0.86 B 0.71
A 0.86 B 0.86
A 0.86 B 1
A 1 B 0
A 1 B 0.14
A 1 B 0.29
A 1 B 0.43
A 1 B 0.57
A 1 B 0.71
A 1 B 0.86
A 1 B 1
OD 600 nm [–]
0.2
0.4
0.6
0.8
0 5 101520
Time [h]

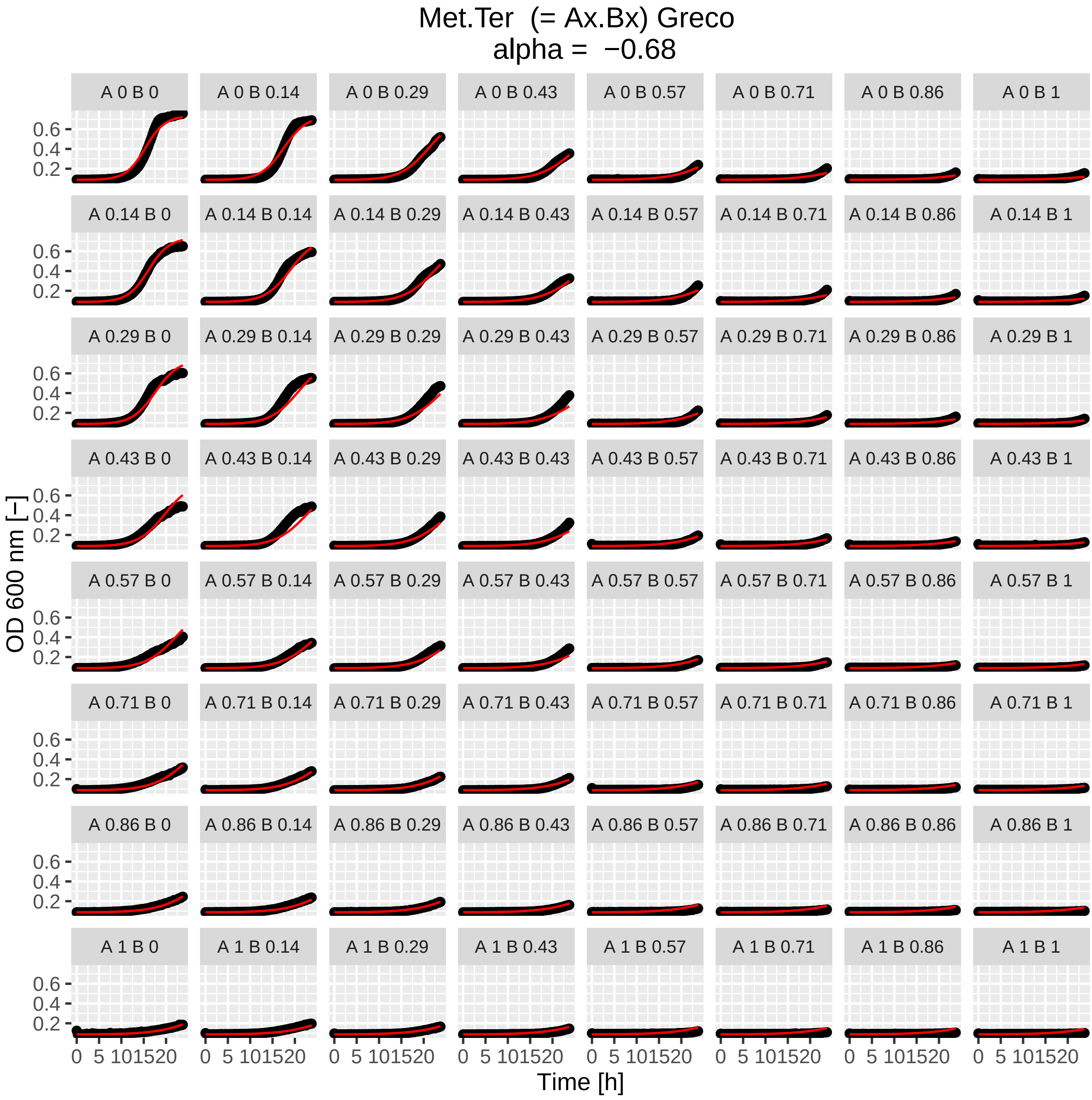

Met.Ter (= Ax.Bx) Greco
alpha = −0.68
A 0 B 0
A 0 B 0.14
A 0 B 0.29
A 0 B 0.43
A 0 B 0.57
A 0 B 0.71
A 0 B 0.86
A 0 B 1
A 0.14 B 0
A 0.14 B 0.14
A 0.14 B 0.29
A 0.14 B 0.43
A 0.14 B 0.57
A 0.14 B 0.71
A 0.14 B 0.86
A 0.14 B 1
A 0.29 B 0
A 0.29 B 0.14
A 0.29 B 0.29
A 0.29 B 0.43
A 0.29 B 0.57
A 0.29 B 0.71
A 0.29 B 0.86
A 0.29 B 1
A 0.43 B 0
A 0.43 B 0.14
A 0.43 B 0.29
A 0.43 B 0.43
A 0.43 B 0.57
A 0.43 B 0.71
A 0.43 B 0.86
A 0.43 B 1
A 0.57 B 0
A 0.57 B 0.14
A 0.57 B 0.29
A 0.57 B 0.43
A 0.57 B 0.57
A 0.57 B 0.71
A 0.57 B 0.86
A 0.57 B 1
A 0.71 B 0
A 0.71 B 0.14
A 0.71 B 0.29
A 0.71 B 0.43
A 0.71 B 0.57
A 0.71 B 0.71
A 0.71 B 0.86
A 0.71 B 1
A 0.86 B 0
A 0.86 B 0.14
A 0.86 B 0.29
A 0.86 B 0.43
A 0.86 B 0.57
A 0.86 B 0.71
A 0.86 B 0.86
A 0.86 B 1
A 1 B 0
A 1 B 0.14
A 1 B 0.29
A 1 B 0.43
A 1 B 0.57
A 1 B 0.71
A 1 B 0.86
A 1 B 1
0.6
0.4
0.2
OD 600 nm [−]
0 5 101520
Time [h]

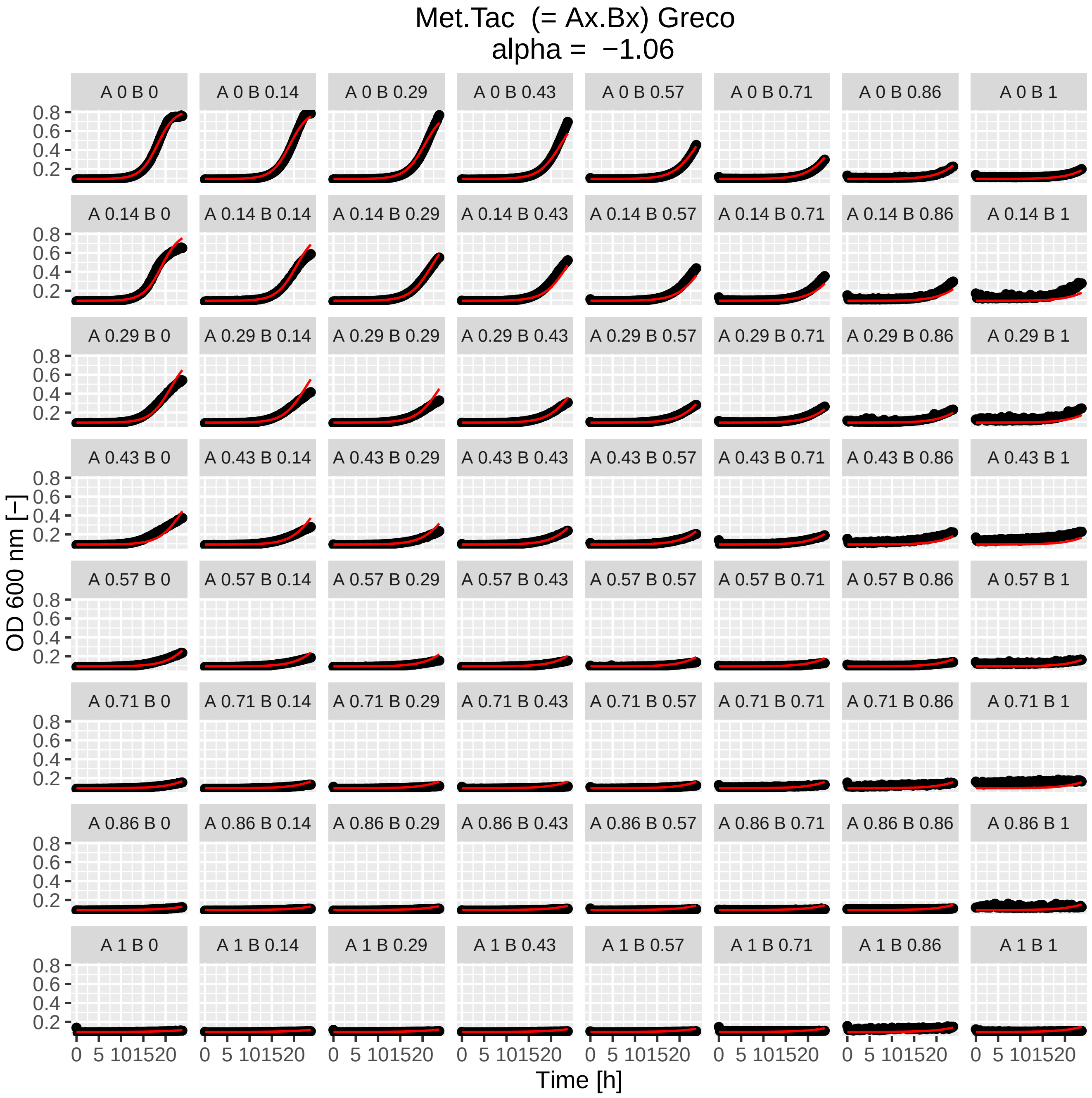

Met.Tac (= Ax.Bx) Greco
alpha = −1.06
A 0 B 0
A 0 B 0.14
A 0 B 0.29
A 0 B 0.43
A 0 B 0.57
A 0 B 0.71
A 0 B 0.86
A 0 B 1
A 0.14 B 0
A 0.14 B 0.14
A 0.14 B 0.29
A 0.14 B 0.43
A 0.14 B 0.57
A 0.14 B 0.71
A 0.14 B 0.86
A 0.14 B 1
A 0.29 B 0
A 0.29 B 0.14
A 0.29 B 0.29
A 0.29 B 0.43
A 0.29 B 0.57
A 0.29 B 0.71
A 0.29 B 0.86
A 0.29 B 1
A 0.43 B 0
A 0.43 B 0.14
A 0.43 B 0.29
A 0.43 B 0.43
A 0.43 B 0.57
A 0.43 B 0.71
A 0.43 B 0.86
A 0.43 B 1
A 0.57 B 0
A 0.57 B 0.14
A 0.57 B 0.29
A 0.57 B 0.43
A 0.57 B 0.57
A 0.57 B 0.71
A 0.57 B 0.86
A 0.57 B 1
A 0.71 B 0
A 0.71 B 0.14
A 0.71 B 0.29
A 0.71 B 0.43
A 0.71 B 0.57
A 0.71 B 0.71
A 0.71 B 0.86
A 0.71 B 1
A 0.86 B 0
A 0.86 B 0.14
A 0.86 B 0.29
A 0.86 B 0.43
A 0.86 B 0.57
A 0.86 B 0.71
A 0.86 B 0.86
A 0.86 B 1
A 1 B 0
A 1 B 0.14
A 1 B 0.29
A 1 B 0.43
A 1 B 0.57
A 1 B 0.71
A 1 B 0.86
A 1 B 1
OD 600 nm [−]
0.2
0.4
0.6
0.8
0 5 101520
Time [h]

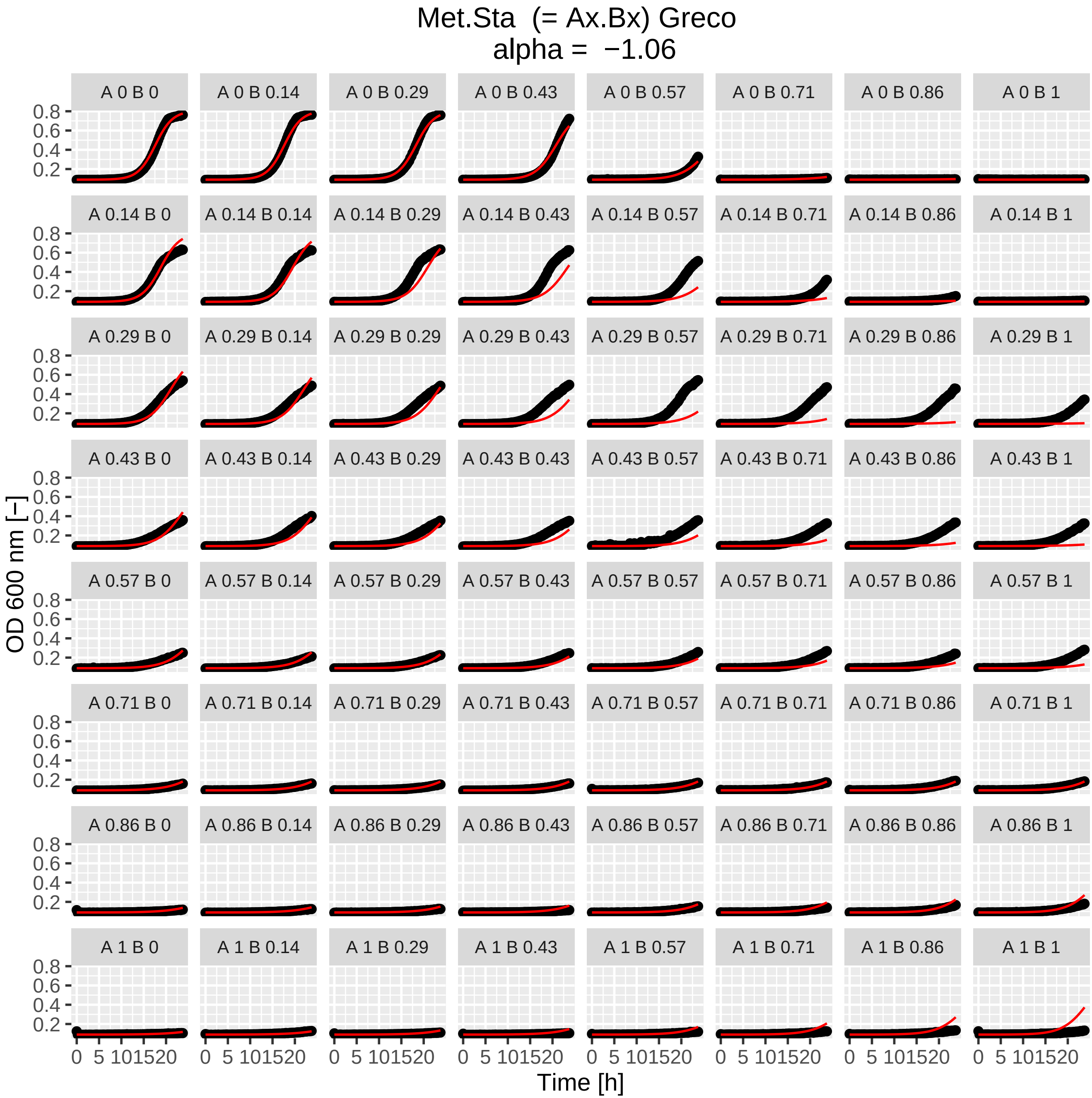
Met.Sta (= Ax.Bx) Greco
alpha = −1.06
A 0 B 0
A 0 B 0.14
A 0 B 0.29
A 0 B 0.43
A 0 B 0.57
A 0 B 0.71
A 0 B 0.86
A 0 B 1
A 0.14 B 0
A 0.14 B 0.14
A 0.14 B 0.29
A 0.14 B 0.43
A 0.14 B 0.57
A 0.14 B 0.71
A 0.14 B 0.86
A 0.14 B 1
A 0.29 B 0
A 0.29 B 0.14
A 0.29 B 0.29
A 0.29 B 0.43
A 0.29 B 0.57
A 0.29 B 0.71
A 0.29 B 0.86
A 0.29 B 1
A 0.43 B 0
A 0.43 B 0.14
A 0.43 B 0.29
A 0.43 B 0.43
A 0.43 B 0.57
A 0.43 B 0.71
A 0.43 B 0.86
A 0.43 B 1
A 0.57 B 0
A 0.57 B 0.14
A 0.57 B 0.29
A 0.57 B 0.43
A 0.57 B 0.57
A 0.57 B 0.71
A 0.57 B 0.86
A 0.57 B 1
A 0.71 B 0
A 0.71 B 0.14
A 0.71 B 0.29
A 0.71 B 0.43
A 0.71 B 0.57
A 0.71 B 0.71
A 0.71 B 0.86
A 0.71 B 1
A 0.86 B 0
A 0.86 B 0.14
A 0.86 B 0.29
A 0.86 B 0.43
A 0.86 B 0.57
A 0.86 B 0.71
A 0.86 B 0.86
A 0.86 B 1
A 1 B 0
A 1 B 0.14
A 1 B 0.29
A 1 B 0.43
A 1 B 0.57
A 1 B 0.71
A 1 B 0.86
A 1 B 1
OD 600 nm [−]
0.2
0.4
0.6
0.8
0 5 101520
Time [h]

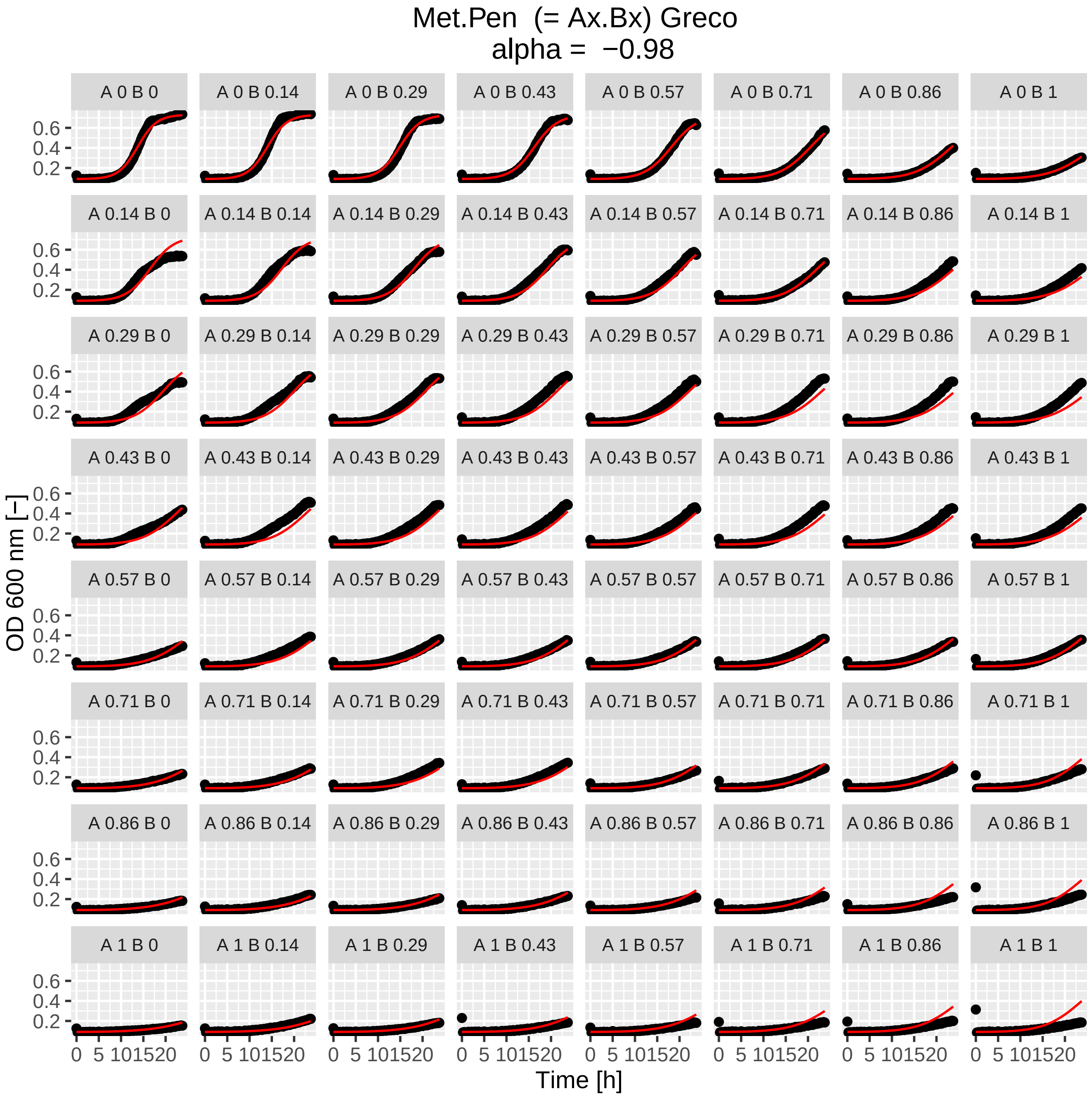

Met.Pen (= Ax.Bx) Greco
alpha = −0.98
A 0 B 0
A 0 B 0.14
A 0 B 0.29
A 0 B 0.43
A 0 B 0.57
A 0 B 0.71
A 0 B 0.86
A 0 B 1
A 0.14 B 0
A 0.14 B 0.14
A 0.14 B 0.29
A 0.14 B 0.43
A 0.14 B 0.57
A 0.14 B 0.71
A 0.14 B 0.86
A 0.14 B 1
A 0.29 B 0
A 0.29 B 0.14
A 0.29 B 0.29
A 0.29 B 0.43
A 0.29 B 0.57
A 0.29 B 0.71
A 0.29 B 0.86
A 0.29 B 1
A 0.43 B 0
A 0.43 B 0.14
A 0.43 B 0.29
A 0.43 B 0.43
A 0.43 B 0.57
A 0.43 B 0.71
A 0.43 B 0.86
A 0.43 B 1
A 0.57 B 0
A 0.57 B 0.14
A 0.57 B 0.29
A 0.57 B 0.43
A 0.57 B 0.57
A 0.57 B 0.71
A 0.57 B 0.86
A 0.57 B 1
A 0.71 B 0
A 0.71 B 0.14
A 0.71 B 0.29
A 0.71 B 0.43
A 0.71 B 0.57
A 0.71 B 0.71
A 0.71 B 0.86
A 0.71 B 1
A 0.86 B 0
A 0.86 B 0.14
A 0.86 B 0.29
A 0.86 B 0.43
A 0.86 B 0.57
A 0.86 B 0.71
A 0.86 B 0.86
A 0.86 B 1
A 1 B 0
A 1 B 0.14
A 1 B 0.29
A 1 B 0.43
A 1 B 0.57
A 1 B 0.71
A 1 B 0.86
A 1 B 1
OD 600 nm [−]
0.2
0.4
0.6
0 5 101520
Time [h]

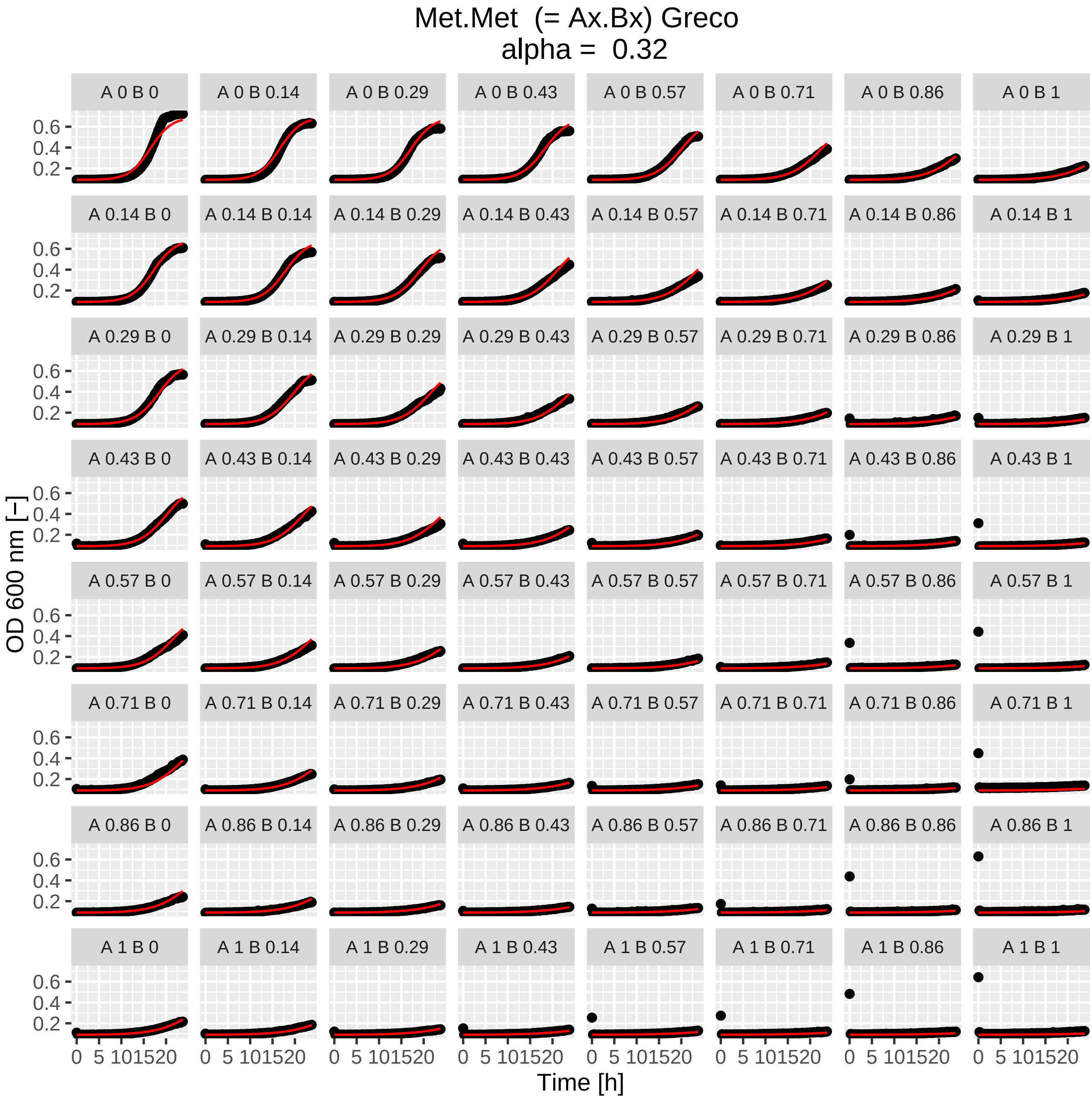

Met.Met (= Ax.Bx) Greco
alpha = 0.32
A 0 B 0
A 0 B 0.14
A 0 B 0.29
A 0 B 0.43
A 0 B 0.57
A 0 B 0.71
A 0 B 0.86
A 0 B 1
A 0.14 B 0
A 0.14 B 0.14
A 0.14 B 0.29
A 0.14 B 0.43
A 0.14 B 0.57
A 0.14 B 0.71
A 0.14 B 0.86
A 0.14 B 1
A 0.29 B 0
A 0.29 B 0.14
A 0.29 B 0.29
A 0.29 B 0.43
A 0.29 B 0.57
A 0.29 B 0.71
A 0.29 B 0.86
A 0.29 B 1
A 0.43 B 0
A 0.43 B 0.14
A 0.43 B 0.29
A 0.43 B 0.43
A 0.43 B 0.57
A 0.43 B 0.71
A 0.43 B 0.86
A 0.43 B 1
A 0.57 B 0
A 0.57 B 0.14
A 0.57 B 0.29
A 0.57 B 0.43
A 0.57 B 0.57
A 0.57 B 0.71
A 0.57 B 0.86
A 0.57 B 1
A 0.71 B 0
A 0.71 B 0.14
A 0.71 B 0.29
A 0.71 B 0.43
A 0.71 B 0.57
A 0.71 B 0.71
A 0.71 B 0.86
A 0.71 B 1
A 0.86 B 0
A 0.86 B 0.14
A 0.86 B 0.29
A 0.86 B 0.43
A 0.86 B 0.57
A 0.86 B 0.71
A 0.86 B 0.86
A 0.86 B 1
A 1 B 0
A 1 B 0.14
A 1 B 0.29
A 1 B 0.43
A 1 B 0.57
A 1 B 0.71
A 1 B 0.86
A 1 B 1
OD 600 nm [–]
0.2
0.4
0.6
0 5 101520
Time [h]

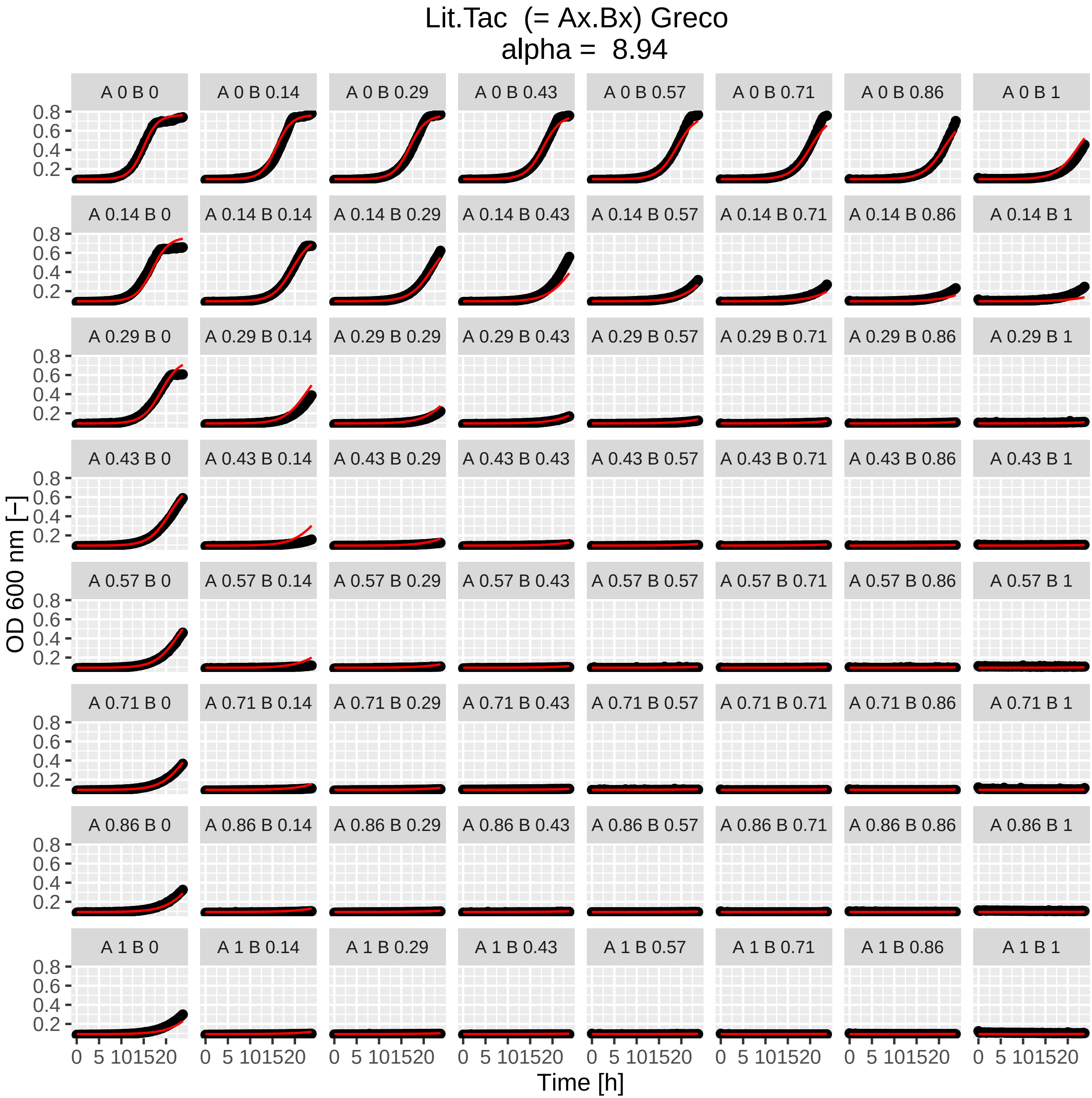

Lit.Tac (= Ax.Bx) Greco
alpha = 8.94
A 0 B 0
A 0 B 0.14
A 0 B 0.29
A 0 B 0.43
A 0 B 0.57
A 0 B 0.71
A 0 B 0.86
A 0 B 1
A 0.14 B 0
A 0.14 B 0.14
A 0.14 B 0.29
A 0.14 B 0.43
A 0.14 B 0.57
A 0.14 B 0.71
A 0.14 B 0.86
A 0.14 B 1
A 0.29 B 0
A 0.29 B 0.14
A 0.29 B 0.29
A 0.29 B 0.43
A 0.29 B 0.57
A 0.29 B 0.71
A 0.29 B 0.86
A 0.29 B 1
A 0.43 B 0
A 0.43 B 0.14
A 0.43 B 0.29
A 0.43 B 0.43
A 0.43 B 0.57
A 0.43 B 0.71
A 0.43 B 0.86
A 0.43 B 1
A 0.57 B 0
A 0.57 B 0.14
A 0.57 B 0.29
A 0.57 B 0.43
A 0.57 B 0.57
A 0.57 B 0.71
A 0.57 B 0.86
A 0.57 B 1
A 0.71 B 0
A 0.71 B 0.14
A 0.71 B 0.29
A 0.71 B 0.43
A 0.71 B 0.57
A 0.71 B 0.71
A 0.71 B 0.86
A 0.71 B 1
A 0.86 B 0
A 0.86 B 0.14
A 0.86 B 0.29
A 0.86 B 0.43
A 0.86 B 0.57
A 0.86 B 0.71
A 0.86 B 0.86
A 0.86 B 1
A 1 B 0
A 1 B 0.14
A 1 B 0.29
A 1 B 0.43
A 1 B 0.57
A 1 B 0.71
A 1 B 0.86
A 1 B 1
OD 600 nm [–]
0.2
0.4
0.6
0.8
Time [h]
0
5
10
15
20

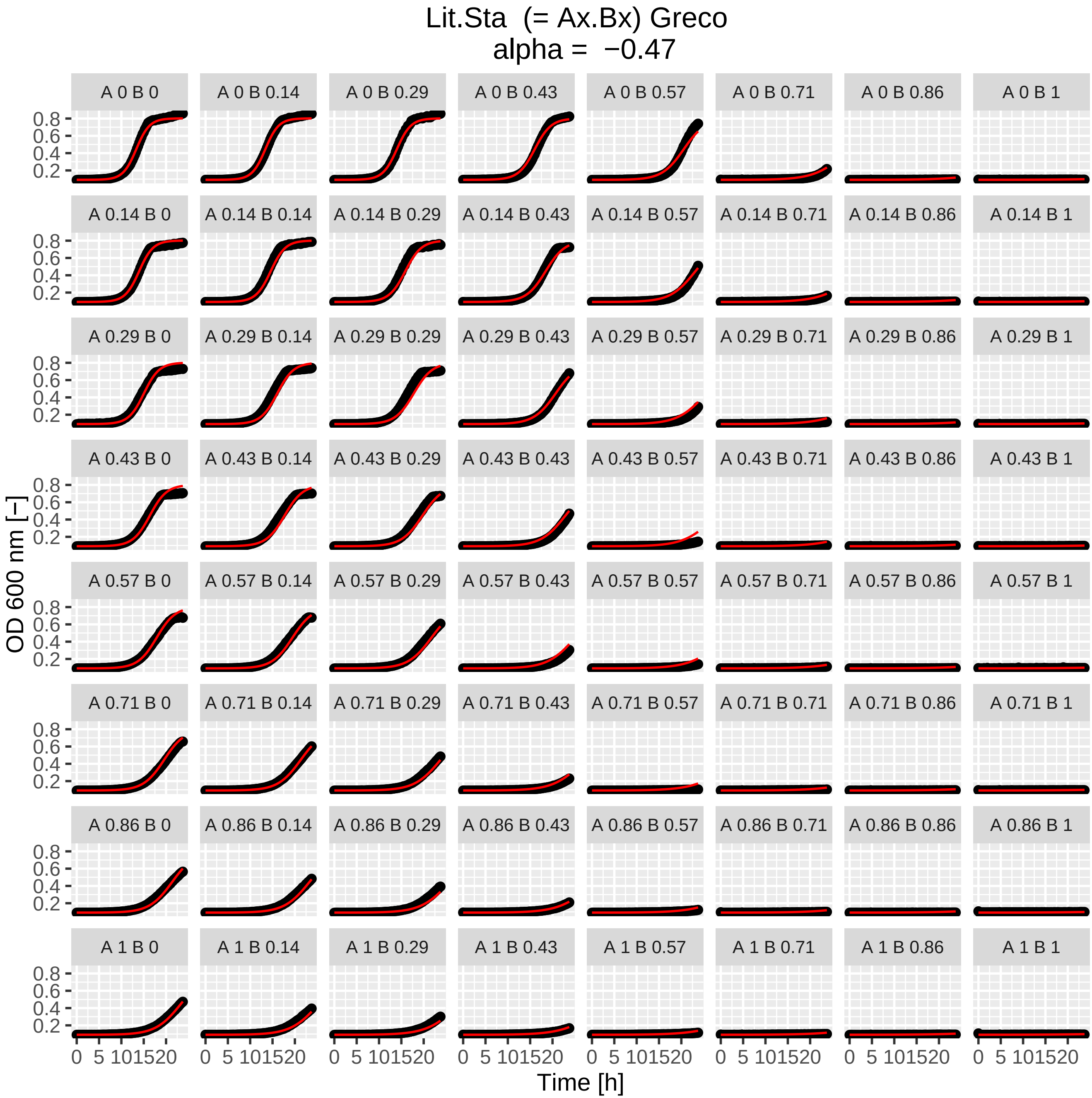

Lit.Sta (= Ax.Bx) Greco
alpha = −0.47
A 0 B 0
A 0 B 0.14
A 0 B 0.29
A 0 B 0.43
A 0 B 0.57
A 0 B 0.71
A 0 B 0.86
A 0 B 1
A 0.14 B 0
A 0.14 B 0.14
A 0.14 B 0.29
A 0.14 B 0.43
A 0.14 B 0.57
A 0.14 B 0.71
A 0.14 B 0.86
A 0.14 B 1
A 0.29 B 0
A 0.29 B 0.14
A 0.29 B 0.29
A 0.29 B 0.43
A 0.29 B 0.57
A 0.29 B 0.71
A 0.29 B 0.86
A 0.29 B 1
A 0.43 B 0
A 0.43 B 0.14
A 0.43 B 0.29
A 0.43 B 0.43
A 0.43 B 0.57
A 0.43 B 0.71
A 0.43 B 0.86
A 0.43 B 1
A 0.57 B 0
A 0.57 B 0.14
A 0.57 B 0.29
A 0.57 B 0.43
A 0.57 B 0.57
A 0.57 B 0.71
A 0.57 B 0.86
A 0.57 B 1
A 0.71 B 0
A 0.71 B 0.14
A 0.71 B 0.29
A 0.71 B 0.43
A 0.71 B 0.57
A 0.71 B 0.71
A 0.71 B 0.86
A 0.71 B 1
A 0.86 B 0
A 0.86 B 0.14
A 0.86 B 0.29
A 0.86 B 0.43
A 0.86 B 0.57
A 0.86 B 0.71
A 0.86 B 0.86
A 0.86 B 1
A 1 B 0
A 1 B 0.14
A 1 B 0.29
A 1 B 0.43
A 1 B 0.57
A 1 B 0.71
A 1 B 0.86
A 1 B 1
0.8
0.6
0.4
0.2
OD 600 nm [−]
0 5 101520
Time [h]

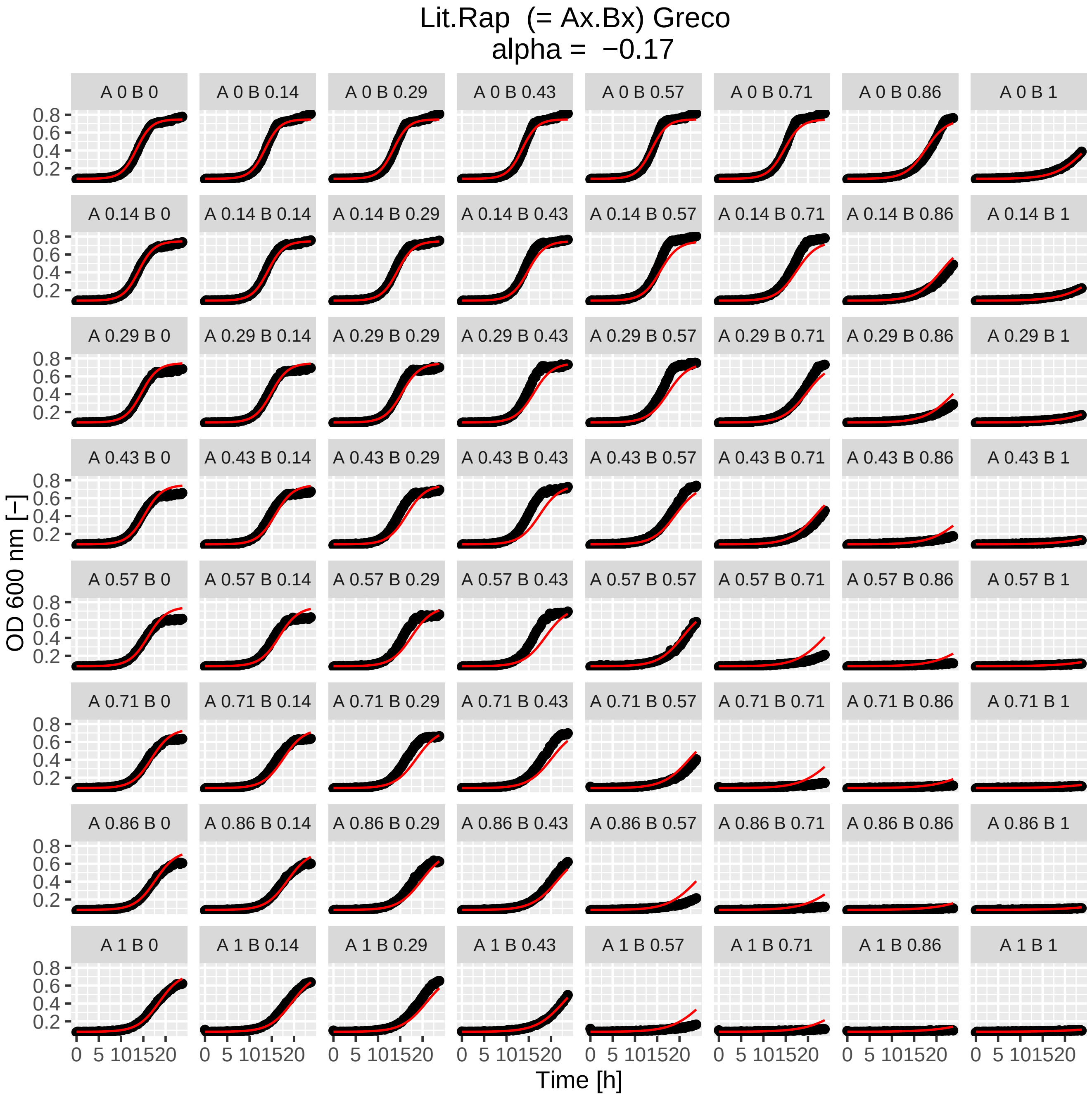

Lit.Rap (= Ax.Bx) Greco
alpha = −0.17
A 0 B 0
A 0 B 0.14
A 0 B 0.29
A 0 B 0.43
A 0 B 0.57
A 0 B 0.71
A 0 B 0.86
A 0 B 1
A 0.14 B 0
A 0.14 B 0.14
A 0.14 B 0.29
A 0.14 B 0.43
A 0.14 B 0.57
A 0.14 B 0.71
A 0.14 B 0.86
A 0.14 B 1
A 0.29 B 0
A 0.29 B 0.14
A 0.29 B 0.29
A 0.29 B 0.43
A 0.29 B 0.57
A 0.29 B 0.71
A 0.29 B 0.86
A 0.29 B 1
A 0.43 B 0
A 0.43 B 0.14
A 0.43 B 0.29
A 0.43 B 0.43
A 0.43 B 0.57
A 0.43 B 0.71
A 0.43 B 0.86
A 0.43 B 1
A 0.57 B 0
A 0.57 B 0.14
A 0.57 B 0.29
A 0.57 B 0.43
A 0.57 B 0.57
A 0.57 B 0.71
A 0.57 B 0.86
A 0.57 B 1
A 0.71 B 0
A 0.71 B 0.14
A 0.71 B 0.29
A 0.71 B 0.43
A 0.71 B 0.57
A 0.71 B 0.71
A 0.71 B 0.86
A 0.71 B 1
A 0.86 B 0
A 0.86 B 0.14
A 0.86 B 0.29
A 0.86 B 0.43
A 0.86 B 0.57
A 0.86 B 0.71
A 0.86 B 0.86
A 0.86 B 1
A 1 B 0
A 1 B 0.14
A 1 B 0.29
A 1 B 0.43
A 1 B 0.57
A 1 B 0.71
A 1 B 0.86
A 1 B 1
0.8
0.6
0.4
0.2
OD 600 nm [−]
0 5 101520
Time [h]

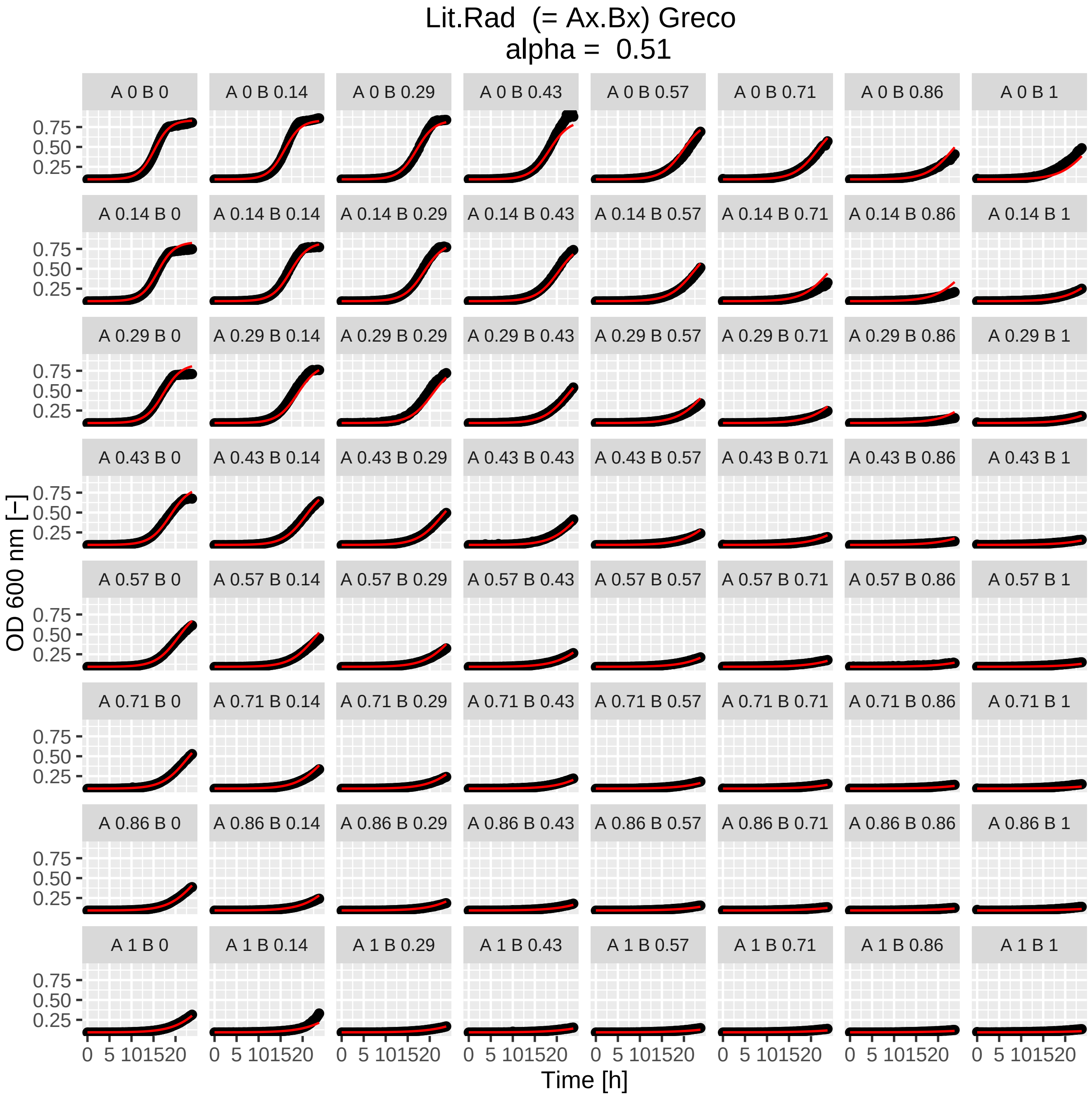

Lit.Rad (= Ax.Bx) Greco
alpha = 0.51
A 0 B 0
A 0 B 0.14
A 0 B 0.29
A 0 B 0.43
A 0 B 0.57
A 0 B 0.71
A 0 B 0.86
A 0 B 1
A 0.14 B 0
A 0.14 B 0.14
A 0.14 B 0.29
A 0.14 B 0.43
A 0.14 B 0.57
A 0.14 B 0.71
A 0.14 B 0.86
A 0.14 B 1
A 0.29 B 0
A 0.29 B 0.14
A 0.29 B 0.29
A 0.29 B 0.43
A 0.29 B 0.57
A 0.29 B 0.71
A 0.29 B 0.86
A 0.29 B 1
A 0.43 B 0
A 0.43 B 0.14
A 0.43 B 0.29
A 0.43 B 0.43
A 0.43 B 0.57
A 0.43 B 0.71
A 0.43 B 0.86
A 0.43 B 1
A 0.57 B 0
A 0.57 B 0.14
A 0.57 B 0.29
A 0.57 B 0.43
A 0.57 B 0.57
A 0.57 B 0.71
A 0.57 B 0.86
A 0.57 B 1
A 0.71 B 0
A 0.71 B 0.14
A 0.71 B 0.29
A 0.71 B 0.43
A 0.71 B 0.57
A 0.71 B 0.71
A 0.71 B 0.86
A 0.71 B 1
A 0.86 B 0
A 0.86 B 0.14
A 0.86 B 0.29
A 0.86 B 0.43
A 0.86 B 0.57
A 0.86 B 0.71
A 0.86 B 0.86
A 0.86 B 1
A 1 B 0
A 1 B 0.14
A 1 B 0.29
A 1 B 0.43
A 1 B 0.57
A 1 B 0.71
A 1 B 0.86
A 1 B 1
0.75
0.50
0.25
0 5 101520
OD 600 nm [–]
Time [h]

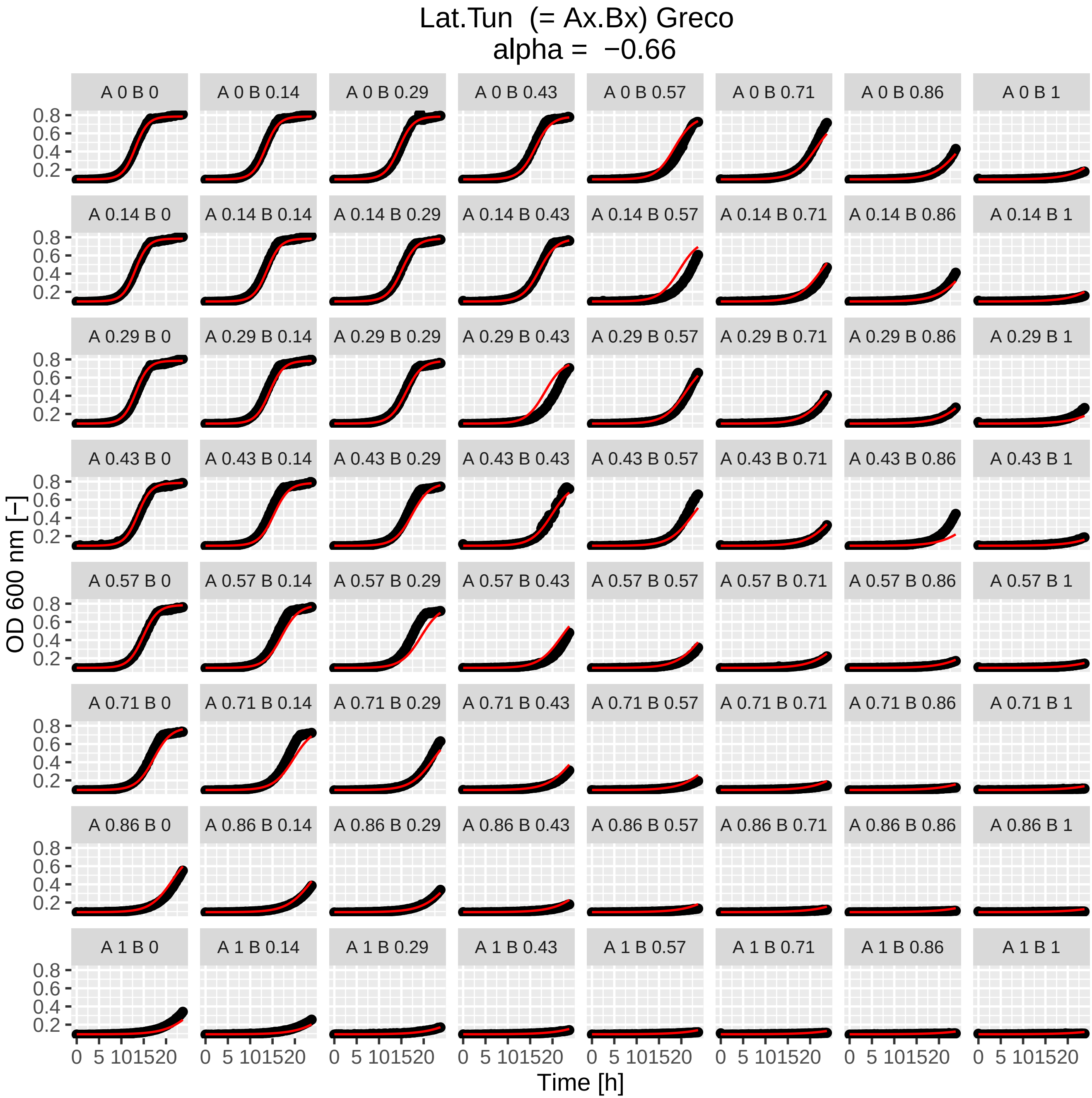
Lat.Tun (= Ax.Bx) Greco
alpha = −0.66
OD 600 nm [−]
Time [h]
A 0 B 0
A 0 B 0.14
A 0 B 0.29
A 0 B 0.43
A 0 B 0.57
A 0 B 0.71
A 0 B 0.86
A 0 B 1
A 0.14 B 0
A 0.14 B 0.14
A 0.14 B 0.29
A 0.14 B 0.43
A 0.14 B 0.57
A 0.14 B 0.71
A 0.14 B 0.86
A 0.14 B 1
A 0.29 B 0
A 0.29 B 0.14
A 0.29 B 0.29
A 0.29 B 0.43
A 0.29 B 0.57
A 0.29 B 0.71
A 0.29 B 0.86
A 0.29 B 1
A 0.43 B 0
A 0.43 B 0.14
A 0.43 B 0.29
A 0.43 B 0.43
A 0.43 B 0.57
A 0.43 B 0.71
A 0.43 B 0.86
A 0.43 B 1
A 0.57 B 0
A 0.57 B 0.14
A 0.57 B 0.29
A 0.57 B 0.43
A 0.57 B 0.57
A 0.57 B 0.71
A 0.57 B 0.86
A 0.57 B 1
A 0.71 B 0
A 0.71 B 0.14
A 0.71 B 0.29
A 0.71 B 0.43
A 0.71 B 0.57
A 0.71 B 0.71
A 0.71 B 0.86
A 0.71 B 1
A 0.86 B 0
A 0.86 B 0.14
A 0.86 B 0.29
A 0.86 B 0.43
A 0.86 B 0.57
A 0.86 B 0.71
A 0.86 B 0.86
A 0.86 B 1
A 1 B 0
A 1 B 0.14
A 1 B 0.29
A 1 B 0.43
A 1 B 0.57
A 1 B 0.71
A 1 B 0.86
A 1 B 1
0.8
0.6
0.4
0.2
0 5 101520

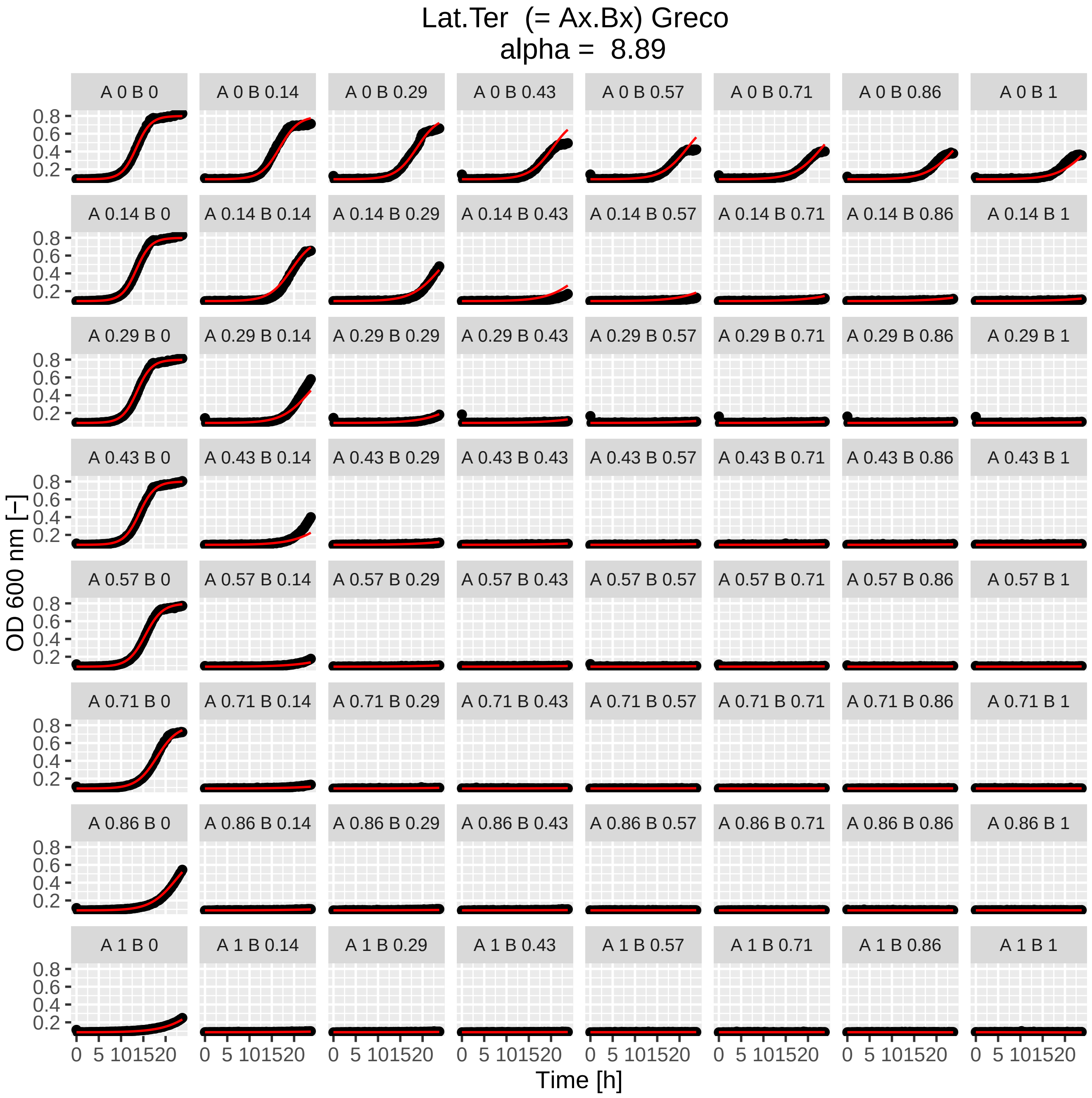
Lat.Ter (= Ax.Bx) Greco
alpha = 8.89
A 0 B 0
A 0 B 0.14
A 0 B 0.29
A 0 B 0.43
A 0 B 0.57
A 0 B 0.71
A 0 B 0.86
A 0 B 1
A 0.14 B 0
A 0.14 B 0.14
A 0.14 B 0.29
A 0.14 B 0.43
A 0.14 B 0.57
A 0.14 B 0.71
A 0.14 B 0.86
A 0.14 B 1
A 0.29 B 0
A 0.29 B 0.14
A 0.29 B 0.29
A 0.29 B 0.43
A 0.29 B 0.57
A 0.29 B 0.71
A 0.29 B 0.86
A 0.29 B 1
A 0.43 B 0
A 0.43 B 0.14
A 0.43 B 0.29
A 0.43 B 0.43
A 0.43 B 0.57
A 0.43 B 0.71
A 0.43 B 0.86
A 0.43 B 1
A 0.57 B 0
A 0.57 B 0.14
A 0.57 B 0.29
A 0.57 B 0.43
A 0.57 B 0.57
A 0.57 B 0.71
A 0.57 B 0.86
A 0.57 B 1
A 0.71 B 0
A 0.71 B 0.14
A 0.71 B 0.29
A 0.71 B 0.43
A 0.71 B 0.57
A 0.71 B 0.71
A 0.71 B 0.86
A 0.71 B 1
A 0.86 B 0
A 0.86 B 0.14
A 0.86 B 0.29
A 0.86 B 0.43
A 0.86 B 0.57
A 0.86 B 0.71
A 0.86 B 0.86
A 0.86 B 1
A 1 B 0
A 1 B 0.14
A 1 B 0.29
A 1 B 0.43
A 1 B 0.57
A 1 B 0.71
A 1 B 0.86
A 1 B 1
0.8
0.6
0.4
0.2
OD 600 nm [–]
0 5 101520
Time [h]

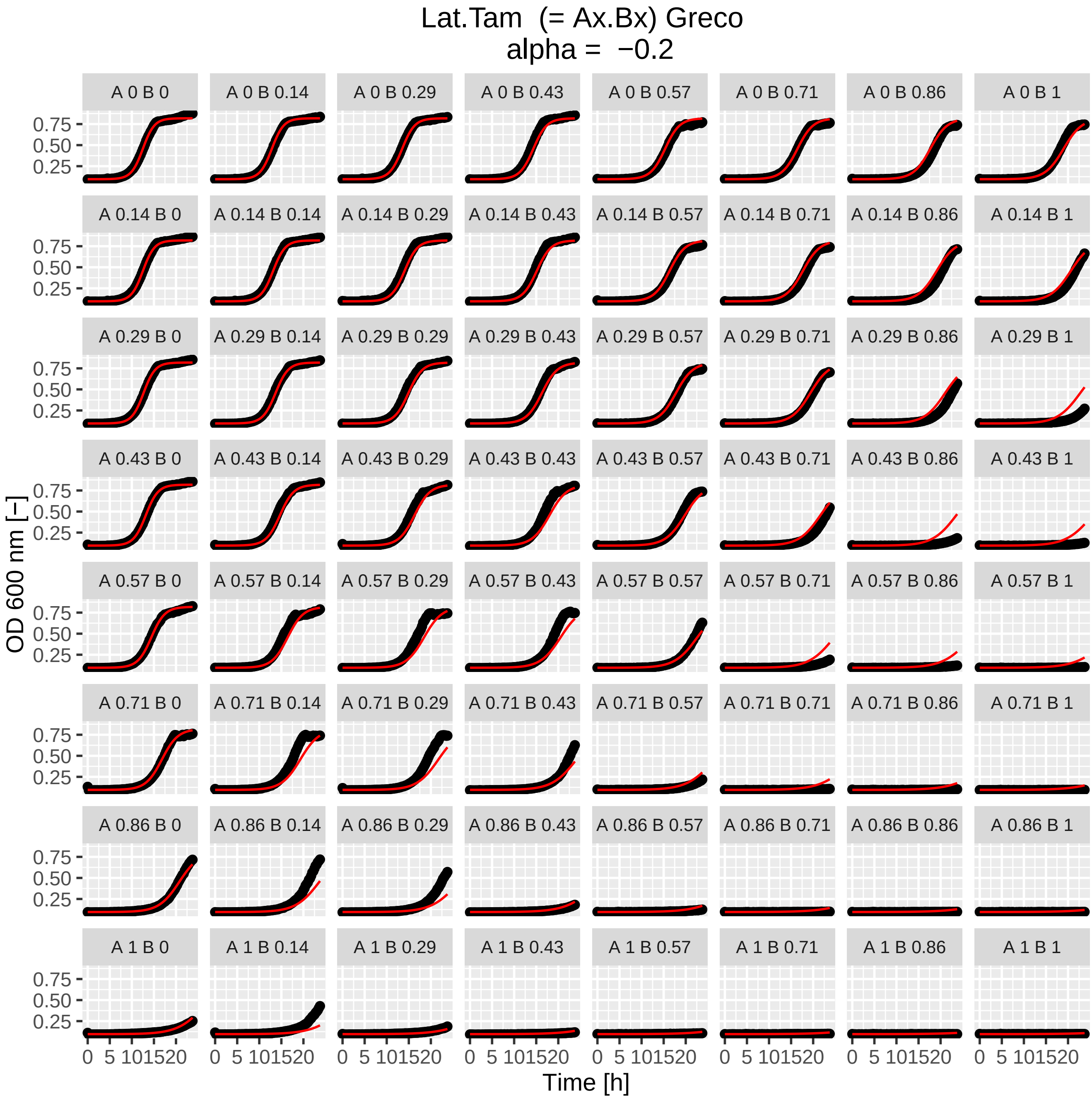

Lat.Tam (= Ax.Bx) Greco
alpha = −0.2
A 0 B 0
A 0 B 0.14
A 0 B 0.29
A 0 B 0.43
A 0 B 0.57
A 0 B 0.71
A 0 B 0.86
A 0 B 1
A 0.14 B 0
A 0.14 B 0.14
A 0.14 B 0.29
A 0.14 B 0.43
A 0.14 B 0.57
A 0.14 B 0.71
A 0.14 B 0.86
A 0.14 B 1
A 0.29 B 0
A 0.29 B 0.14
A 0.29 B 0.29
A 0.29 B 0.43
A 0.29 B 0.57
A 0.29 B 0.71
A 0.29 B 0.86
A 0.29 B 1
A 0.43 B 0
A 0.43 B 0.14
A 0.43 B 0.29
A 0.43 B 0.43
A 0.43 B 0.57
A 0.43 B 0.71
A 0.43 B 0.86
A 0.43 B 1
A 0.57 B 0
A 0.57 B 0.14
A 0.57 B 0.29
A 0.57 B 0.43
A 0.57 B 0.57
A 0.57 B 0.71
A 0.57 B 0.86
A 0.57 B 1
A 0.71 B 0
A 0.71 B 0.14
A 0.71 B 0.29
A 0.71 B 0.43
A 0.71 B 0.57
A 0.71 B 0.71
A 0.71 B 0.86
A 0.71 B 1
A 0.86 B 0
A 0.86 B 0.14
A 0.86 B 0.29
A 0.86 B 0.43
A 0.86 B 0.57
A 0.86 B 0.71
A 0.86 B 0.86
A 0.86 B 1
A 1 B 0
A 1 B 0.14
A 1 B 0.29
A 1 B 0.43
A 1 B 0.57
A 1 B 0.71
A 1 B 0.86
A 1 B 1
OD 600 nm [−]
0.75
0.50
0.25
0 5 101520
Time [h]

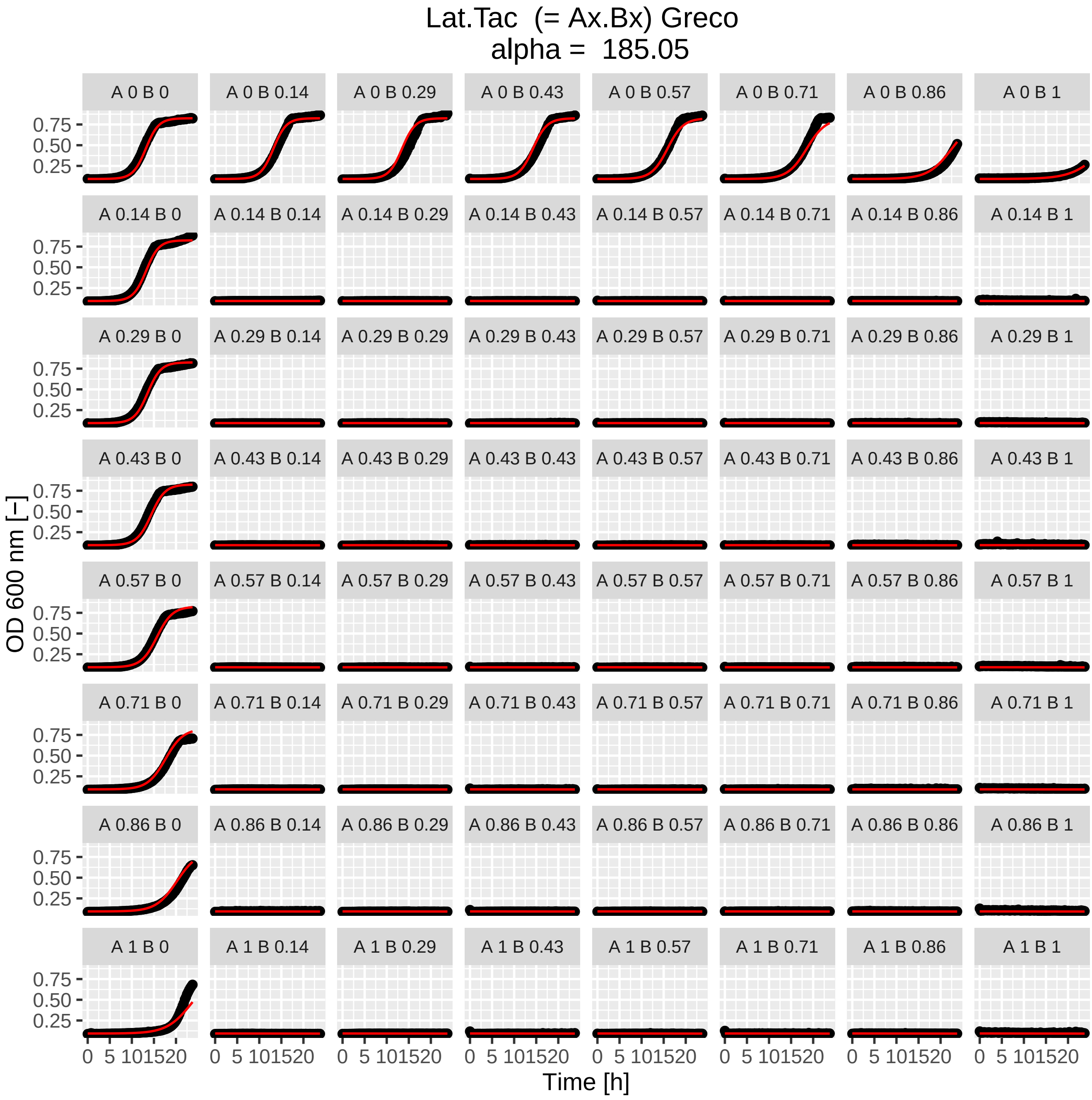

Lat.Tac (= Ax.Bx) Greco
alpha = 185.05
A 0 B 0
A 0 B 0.14
A 0 B 0.29
A 0 B 0.43
A 0 B 0.57
A 0 B 0.71
A 0 B 0.86
A 0 B 1
A 0.14 B 0
A 0.14 B 0.14
A 0.14 B 0.29
A 0.14 B 0.43
A 0.14 B 0.57
A 0.14 B 0.71
A 0.14 B 0.86
A 0.14 B 1
A 0.29 B 0
A 0.29 B 0.14
A 0.29 B 0.29
A 0.29 B 0.43
A 0.29 B 0.57
A 0.29 B 0.71
A 0.29 B 0.86
A 0.29 B 1
A 0.43 B 0
A 0.43 B 0.14
A 0.43 B 0.29
A 0.43 B 0.43
A 0.43 B 0.57
A 0.43 B 0.71
A 0.43 B 0.86
A 0.43 B 1
A 0.57 B 0
A 0.57 B 0.14
A 0.57 B 0.29
A 0.57 B 0.43
A 0.57 B 0.57
A 0.57 B 0.71
A 0.57 B 0.86
A 0.57 B 1
A 0.71 B 0
A 0.71 B 0.14
A 0.71 B 0.29
A 0.71 B 0.43
A 0.71 B 0.57
A 0.71 B 0.71
A 0.71 B 0.86
A 0.71 B 1
A 0.86 B 0
A 0.86 B 0.14
A 0.86 B 0.29
A 0.86 B 0.43
A 0.86 B 0.57
A 0.86 B 0.71
A 0.86 B 0.86
A 0.86 B 1
A 1 B 0
A 1 B 0.14
A 1 B 0.29
A 1 B 0.43
A 1 B 0.57
A 1 B 0.71
A 1 B 0.86
A 1 B 1
0.75
0.50
0.25
OD 600 nm [–]
0 5 101520
Time [h]

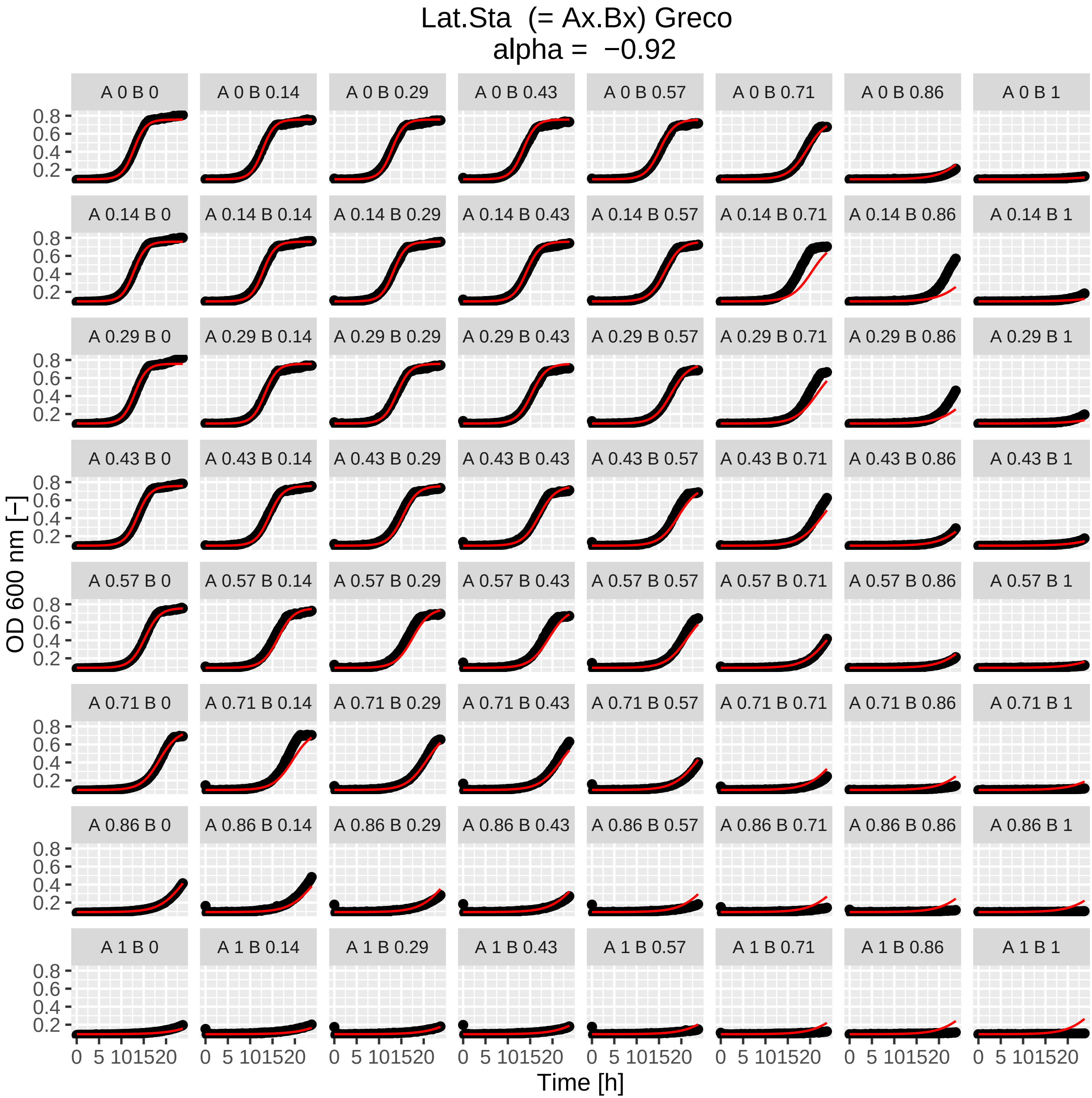
Lat.Sta (= Ax.Bx) Greco
alpha = −0.92
OD 600 nm [−]
Time [h]
A 0 B 0
A 0 B 0.14
A 0 B 0.29
A 0 B 0.43
A 0 B 0.57
A 0 B 0.71
A 0 B 0.86
A 0 B 1
A 0.14 B 0
A 0.14 B 0.14
A 0.14 B 0.29
A 0.14 B 0.43
A 0.14 B 0.57
A 0.14 B 0.71
A 0.14 B 0.86
A 0.14 B 1
A 0.29 B 0
A 0.29 B 0.14
A 0.29 B 0.29
A 0.29 B 0.43
A 0.29 B 0.57
A 0.29 B 0.71
A 0.29 B 0.86
A 0.29 B 1
A 0.43 B 0
A 0.43 B 0.14
A 0.43 B 0.29
A 0.43 B 0.43
A 0.43 B 0.57
A 0.43 B 0.71
A 0.43 B 0.86
A 0.43 B 1
A 0.57 B 0
A 0.57 B 0.14
A 0.57 B 0.29
A 0.57 B 0.43
A 0.57 B 0.57
A 0.57 B 0.71
A 0.57 B 0.86
A 0.57 B 1
A 0.71 B 0
A 0.71 B 0.14
A 0.71 B 0.29
A 0.71 B 0.43
A 0.71 B 0.57
A 0.71 B 0.71
A 0.71 B 0.86
A 0.71 B 1
A 0.86 B 0
A 0.86 B 0.14
A 0.86 B 0.29
A 0.86 B 0.43
A 0.86 B 0.57
A 0.86 B 0.71
A 0.86 B 0.86
A 0.86 B 1
A 1 B 0
A 1 B 0.14
A 1 B 0.29
A 1 B 0.43
A 1 B 0.57
A 1 B 0.71
A 1 B 0.86
A 1 B 1
0.8
0.6
0.4
0.2
0 5 101520

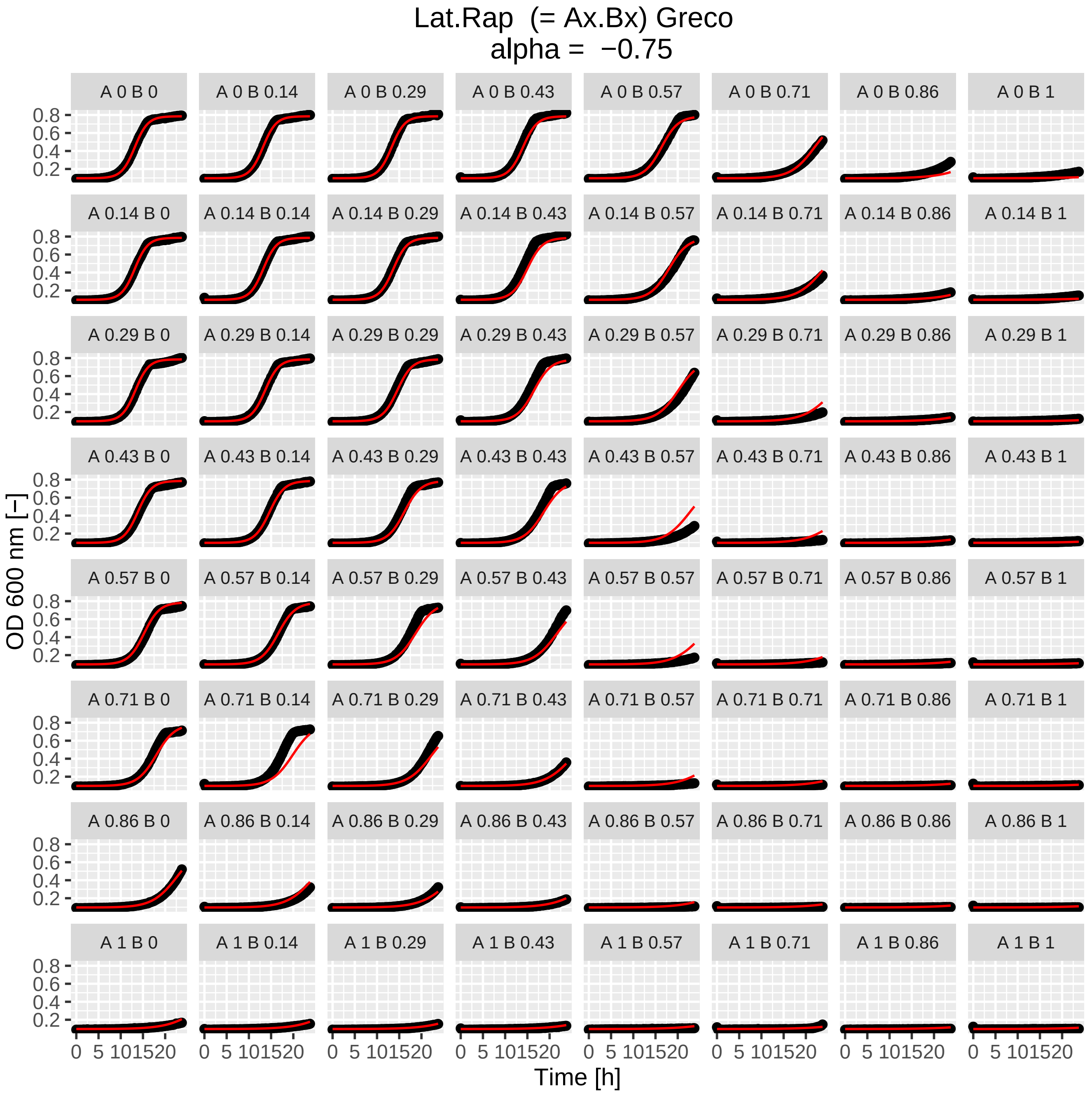
Lat.Rap (= Ax.Bx) Greco
alpha = −0.75
OD 600 nm [−]
Time [h]
A 0 B 0
A 0 B 0.14
A 0 B 0.29
A 0 B 0.43
A 0 B 0.57
A 0 B 0.71
A 0 B 0.86
A 0 B 1
A 0.14 B 0
A 0.14 B 0.14
A 0.14 B 0.29
A 0.14 B 0.43
A 0.14 B 0.57
A 0.14 B 0.71
A 0.14 B 0.86
A 0.14 B 1
A 0.29 B 0
A 0.29 B 0.14
A 0.29 B 0.29
A 0.29 B 0.43
A 0.29 B 0.57
A 0.29 B 0.71
A 0.29 B 0.86
A 0.29 B 1
A 0.43 B 0
A 0.43 B 0.14
A 0.43 B 0.29
A 0.43 B 0.43
A 0.43 B 0.57
A 0.43 B 0.71
A 0.43 B 0.86
A 0.43 B 1
A 0.57 B 0
A 0.57 B 0.14
A 0.57 B 0.29
A 0.57 B 0.43
A 0.57 B 0.57
A 0.57 B 0.71
A 0.57 B 0.86
A 0.57 B 1
A 0.71 B 0
A 0.71 B 0.14
A 0.71 B 0.29
A 0.71 B 0.43
A 0.71 B 0.57
A 0.71 B 0.71
A 0.71 B 0.86
A 0.71 B 1
A 0.86 B 0
A 0.86 B 0.14
A 0.86 B 0.29
A 0.86 B 0.43
A 0.86 B 0.57
A 0.86 B 0.71
A 0.86 B 0.86
A 0.86 B 1
A 1 B 0
A 1 B 0.14
A 1 B 0.29
A 1 B 0.43
A 1 B 0.57
A 1 B 0.71
A 1 B 0.86
A 1 B 1
0.2
0.4
0.6
0.8
0 5 101520

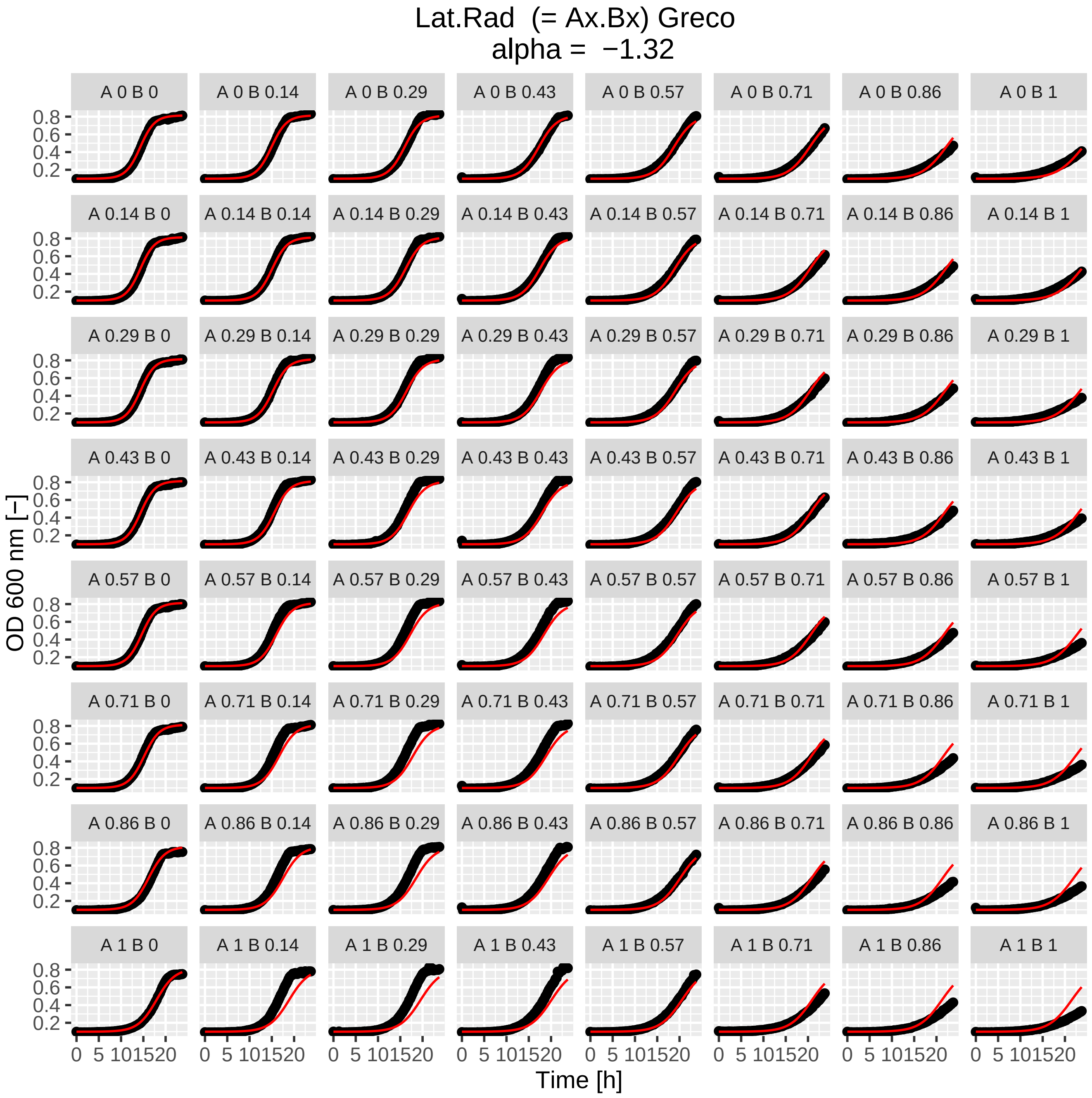

Lat.Rad (= Ax.Bx) Greco
alpha = −1.32
OD 600 nm [−]
Time [h]
0.8
0.6
0.4
0.2
0
5
10
15
20
A 0 B 0
A 0 B 0.14
A 0 B 0.29
A 0 B 0.43
A 0 B 0.57
A 0 B 0.71
A 0 B 0.86
A 0 B 1
A 0.14 B 0
A 0.14 B 0.14
A 0.14 B 0.29
A 0.14 B 0.43
A 0.14 B 0.57
A 0.14 B 0.71
A 0.14 B 0.86
A 0.14 B 1
A 0.29 B 0
A 0.29 B 0.14
A 0.29 B 0.29
A 0.29 B 0.43
A 0.29 B 0.57
A 0.29 B 0.71
A 0.29 B 0.86
A 0.29 B 1
A 0.43 B 0
A 0.43 B 0.14
A 0.43 B 0.29
A 0.43 B 0.43
A 0.43 B 0.57
A 0.43 B 0.71
A 0.43 B 0.86
A 0.43 B 1
A 0.57 B 0
A 0.57 B 0.14
A 0.57 B 0.29
A 0.57 B 0.43
A 0.57 B 0.57
A 0.57 B 0.71
A 0.57 B 0.86
A 0.57 B 1
A 0.71 B 0
A 0.71 B 0.14
A 0.71 B 0.29
A 0.71 B 0.43
A 0.71 B 0.57
A 0.71 B 0.71
A 0.71 B 0.86
A 0.71 B 1
A 0.86 B 0
A 0.86 B 0.14
A 0.86 B 0.29
A 0.86 B 0.43
A 0.86 B 0.57
A 0.86 B 0.71
A 0.86 B 0.86
A 0.86 B 1
A 1 B 0
A 1 B 0.14
A 1 B 0.29
A 1 B 0.43
A 1 B 0.57
A 1 B 0.71
A 1 B 0.86
A 1 B 1

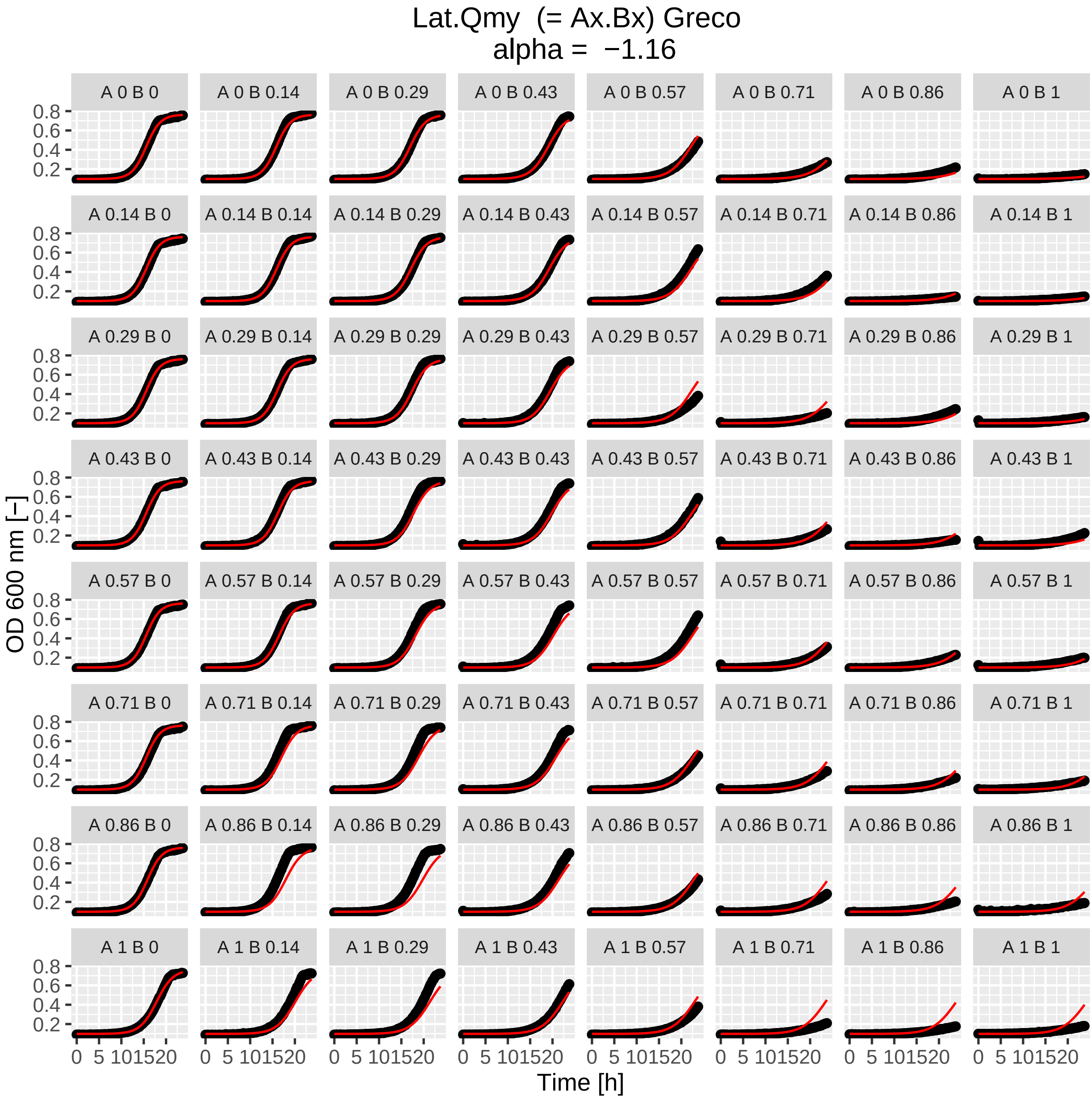

Lat.Qmy (= Ax.Bx) Greco
alpha = −1.16
A 0 B 0
A 0 B 0.14
A 0 B 0.29
A 0 B 0.43
A 0 B 0.57
A 0 B 0.71
A 0 B 0.86
A 0 B 1
A 0.14 B 0
A 0.14 B 0.14
A 0.14 B 0.29
A 0.14 B 0.43
A 0.14 B 0.57
A 0.14 B 0.71
A 0.14 B 0.86
A 0.14 B 1
A 0.29 B 0
A 0.29 B 0.14
A 0.29 B 0.29
A 0.29 B 0.43
A 0.29 B 0.57
A 0.29 B 0.71
A 0.29 B 0.86
A 0.29 B 1
A 0.43 B 0
A 0.43 B 0.14
A 0.43 B 0.29
A 0.43 B 0.43
A 0.43 B 0.57
A 0.43 B 0.71
A 0.43 B 0.86
A 0.43 B 1
A 0.57 B 0
A 0.57 B 0.14
A 0.57 B 0.29
A 0.57 B 0.43
A 0.57 B 0.57
A 0.57 B 0.71
A 0.57 B 0.86
A 0.57 B 1
A 0.71 B 0
A 0.71 B 0.14
A 0.71 B 0.29
A 0.71 B 0.43
A 0.71 B 0.57
A 0.71 B 0.71
A 0.71 B 0.86
A 0.71 B 1
A 0.86 B 0
A 0.86 B 0.14
A 0.86 B 0.29
A 0.86 B 0.43
A 0.86 B 0.57
A 0.86 B 0.71
A 0.86 B 0.86
A 0.86 B 1
A 1 B 0
A 1 B 0.14
A 1 B 0.29
A 1 B 0.43
A 1 B 0.57
A 1 B 0.71
A 1 B 0.86
A 1 B 1
OD 600 nm [−]
0.2
0.4
0.6
0.8
0
5
10
15
20
Time [h]

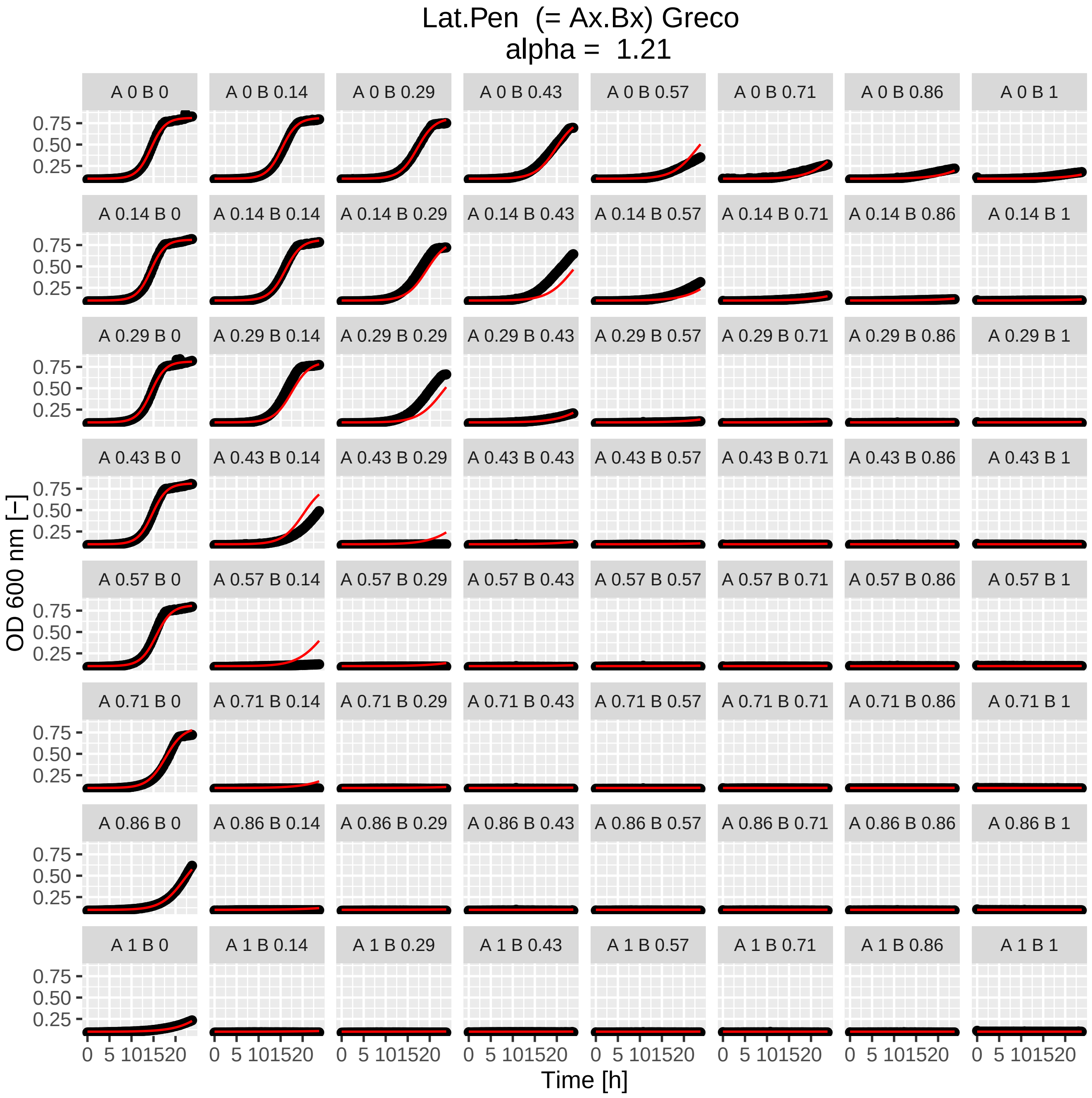
Lat.Pen (= Ax.Bx) Greco
alpha = 1.21
OD 600 nm [–]
Time [h]
0.75
0.50
0.25
0 5 101520
A 0 B 0
A 0 B 0.14
A 0 B 0.29
A 0 B 0.43
A 0 B 0.57
A 0 B 0.71
A 0 B 0.86
A 0 B 1
A 0.14 B 0
A 0.14 B 0.14
A 0.14 B 0.29
A 0.14 B 0.43
A 0.14 B 0.57
A 0.14 B 0.71
A 0.14 B 0.86
A 0.14 B 1
A 0.29 B 0
A 0.29 B 0.14
A 0.29 B 0.29
A 0.29 B 0.43
A 0.29 B 0.57
A 0.29 B 0.71
A 0.29 B 0.86
A 0.29 B 1
A 0.43 B 0
A 0.43 B 0.14
A 0.43 B 0.29
A 0.43 B 0.43
A 0.43 B 0.57
A 0.43 B 0.71
A 0.43 B 0.86
A 0.43 B 1
A 0.57 B 0
A 0.57 B 0.14
A 0.57 B 0.29
A 0.57 B 0.43
A 0.57 B 0.57
A 0.57 B 0.71
A 0.57 B 0.86
A 0.57 B 1
A 0.71 B 0
A 0.71 B 0.14
A 0.71 B 0.29
A 0.71 B 0.43
A 0.71 B 0.57
A 0.71 B 0.71
A 0.71 B 0.86
A 0.71 B 1
A 0.86 B 0
A 0.86 B 0.14
A 0.86 B 0.29
A 0.86 B 0.43
A 0.86 B 0.57
A 0.86 B 0.71
A 0.86 B 0.86
A 0.86 B 1
A 1 B 0
A 1 B 0.14
A 1 B 0.29
A 1 B 0.43
A 1 B 0.57
A 1 B 0.71
A 1 B 0.86
A 1 B 1

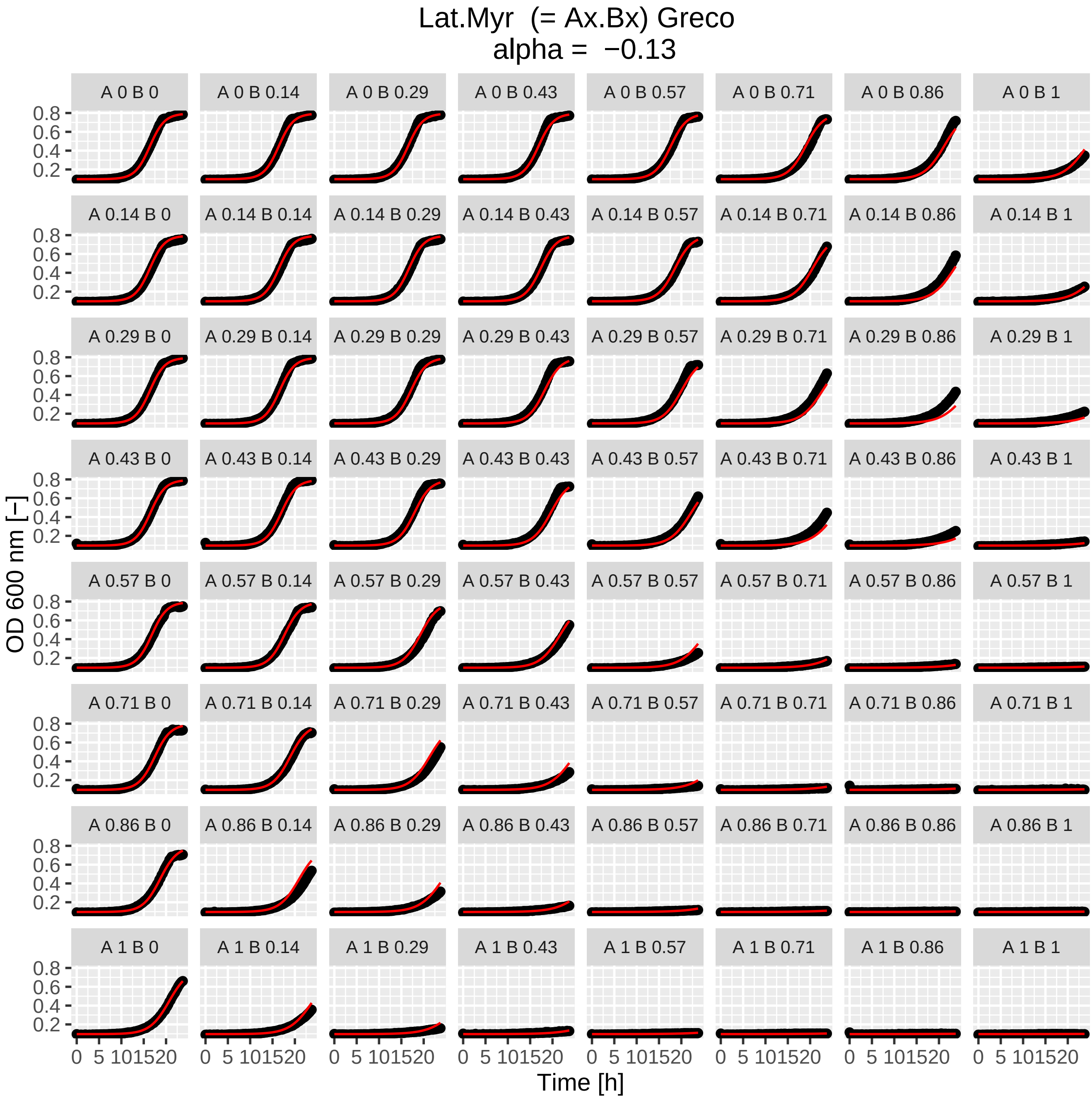
Lat.Myr (= Ax.Bx) Greco
alpha = −0.13
A 0 B 0
A 0 B 0.14
A 0 B 0.29
A 0 B 0.43
A 0 B 0.57
A 0 B 0.71
A 0 B 0.86
A 0 B 1
A 0.14 B 0
A 0.14 B 0.14
A 0.14 B 0.29
A 0.14 B 0.43
A 0.14 B 0.57
A 0.14 B 0.71
A 0.14 B 0.86
A 0.14 B 1
A 0.29 B 0
A 0.29 B 0.14
A 0.29 B 0.29
A 0.29 B 0.43
A 0.29 B 0.57
A 0.29 B 0.71
A 0.29 B 0.86
A 0.29 B 1
A 0.43 B 0
A 0.43 B 0.14
A 0.43 B 0.29
A 0.43 B 0.43
A 0.43 B 0.57
A 0.43 B 0.71
A 0.43 B 0.86
A 0.43 B 1
A 0.57 B 0
A 0.57 B 0.14
A 0.57 B 0.29
A 0.57 B 0.43
A 0.57 B 0.57
A 0.57 B 0.71
A 0.57 B 0.86
A 0.57 B 1
A 0.71 B 0
A 0.71 B 0.14
A 0.71 B 0.29
A 0.71 B 0.43
A 0.71 B 0.57
A 0.71 B 0.71
A 0.71 B 0.86
A 0.71 B 1
A 0.86 B 0
A 0.86 B 0.14
A 0.86 B 0.29
A 0.86 B 0.43
A 0.86 B 0.57
A 0.86 B 0.71
A 0.86 B 0.86
A 0.86 B 1
A 1 B 0
A 1 B 0.14
A 1 B 0.29
A 1 B 0.43
A 1 B 0.57
A 1 B 0.71
A 1 B 0.86
A 1 B 1
OD 600 nm [−]
0.2
0.4
0.6
0.8
0
5
10
15
20
Time [h]

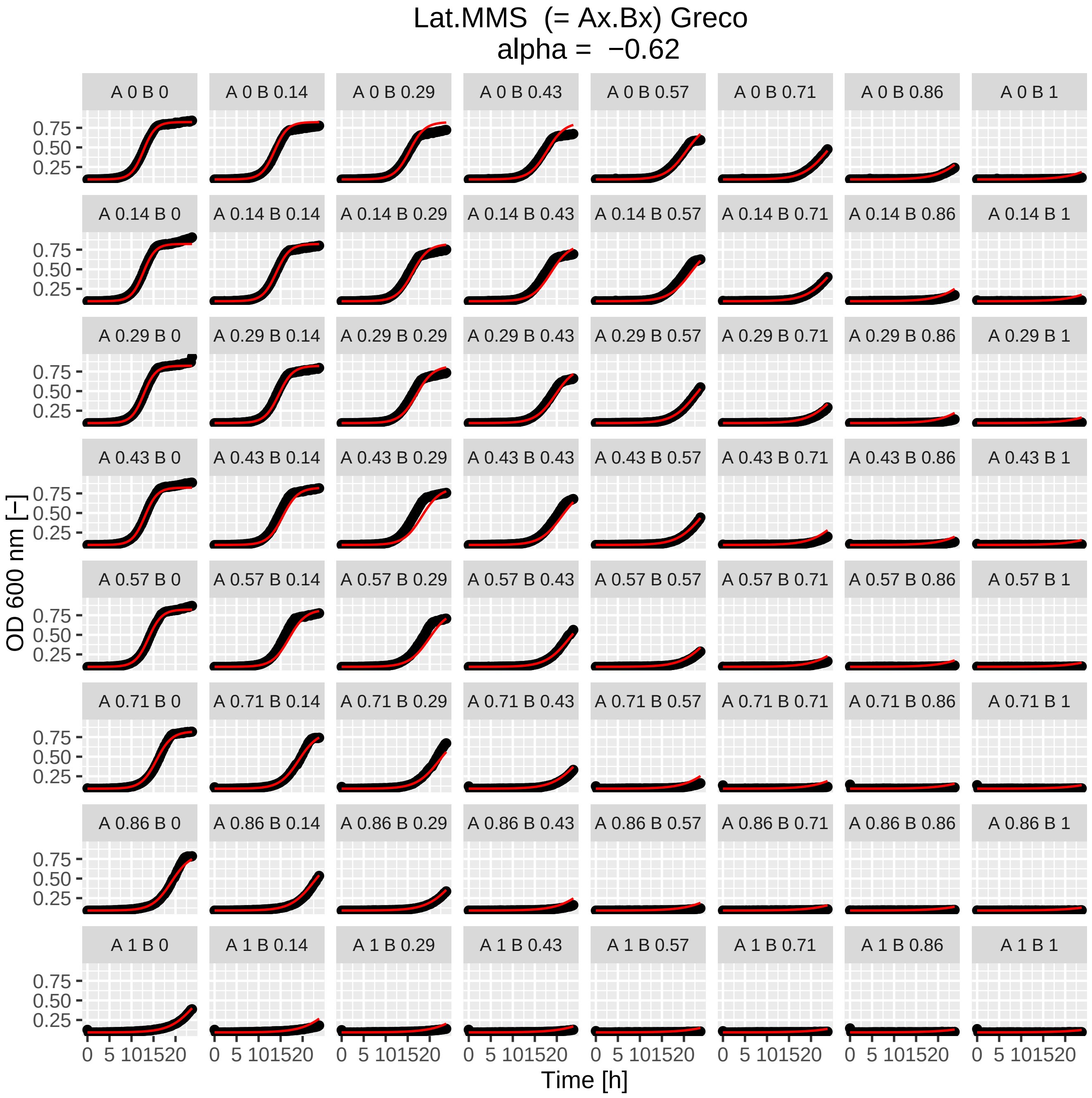

Lat.MMS (= Ax.Bx) Greco
alpha = −0.62
A 0 B 0
A 0 B 0.14
A 0 B 0.29
A 0 B 0.43
A 0 B 0.57
A 0 B 0.71
A 0 B 0.86
A 0 B 1
A 0.14 B 0
A 0.14 B 0.14
A 0.14 B 0.29
A 0.14 B 0.43
A 0.14 B 0.57
A 0.14 B 0.71
A 0.14 B 0.86
A 0.14 B 1
A 0.29 B 0
A 0.29 B 0.14
A 0.29 B 0.29
A 0.29 B 0.43
A 0.29 B 0.57
A 0.29 B 0.71
A 0.29 B 0.86
A 0.29 B 1
A 0.43 B 0
A 0.43 B 0.14
A 0.43 B 0.29
A 0.43 B 0.43
A 0.43 B 0.57
A 0.43 B 0.71
A 0.43 B 0.86
A 0.43 B 1
A 0.57 B 0
A 0.57 B 0.14
A 0.57 B 0.29
A 0.57 B 0.43
A 0.57 B 0.57
A 0.57 B 0.71
A 0.57 B 0.86
A 0.57 B 1
A 0.71 B 0
A 0.71 B 0.14
A 0.71 B 0.29
A 0.71 B 0.43
A 0.71 B 0.57
A 0.71 B 0.71
A 0.71 B 0.86
A 0.71 B 1
A 0.86 B 0
A 0.86 B 0.14
A 0.86 B 0.29
A 0.86 B 0.43
A 0.86 B 0.57
A 0.86 B 0.71
A 0.86 B 0.86
A 0.86 B 1
A 1 B 0
A 1 B 0.14
A 1 B 0.29
A 1 B 0.43
A 1 B 0.57
A 1 B 0.71
A 1 B 0.86
A 1 B 1
0.75
0.50
0.25
0 5 101520
OD 600 nm [−]
Time [h]

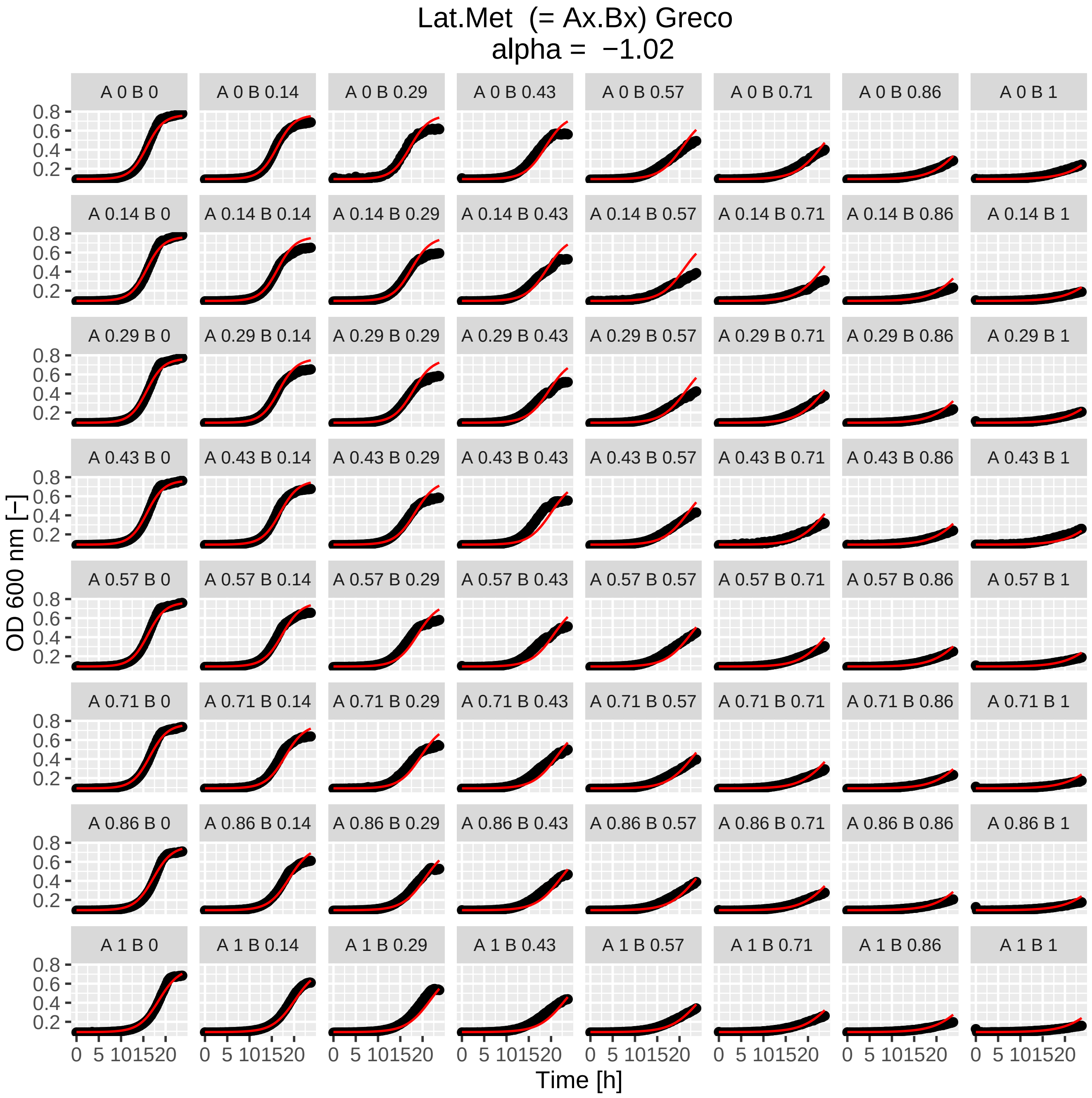

Lat.Met (= Ax.Bx) Greco
alpha = −1.02
A 0 B 0
A 0 B 0.14
A 0 B 0.29
A 0 B 0.43
A 0 B 0.57
A 0 B 0.71
A 0 B 0.86
A 0 B 1
A 0.14 B 0
A 0.14 B 0.14
A 0.14 B 0.29
A 0.14 B 0.43
A 0.14 B 0.57
A 0.14 B 0.71
A 0.14 B 0.86
A 0.14 B 1
A 0.29 B 0
A 0.29 B 0.14
A 0.29 B 0.29
A 0.29 B 0.43
A 0.29 B 0.57
A 0.29 B 0.71
A 0.29 B 0.86
A 0.29 B 1
A 0.43 B 0
A 0.43 B 0.14
A 0.43 B 0.29
A 0.43 B 0.43
A 0.43 B 0.57
A 0.43 B 0.71
A 0.43 B 0.86
A 0.43 B 1
A 0.57 B 0
A 0.57 B 0.14
A 0.57 B 0.29
A 0.57 B 0.43
A 0.57 B 0.57
A 0.57 B 0.71
A 0.57 B 0.86
A 0.57 B 1
A 0.71 B 0
A 0.71 B 0.14
A 0.71 B 0.29
A 0.71 B 0.43
A 0.71 B 0.57
A 0.71 B 0.71
A 0.71 B 0.86
A 0.71 B 1
A 0.86 B 0
A 0.86 B 0.14
A 0.86 B 0.29
A 0.86 B 0.43
A 0.86 B 0.57
A 0.86 B 0.71
A 0.86 B 0.86
A 0.86 B 1
A 1 B 0
A 1 B 0.14
A 1 B 0.29
A 1 B 0.43
A 1 B 0.57
A 1 B 0.71
A 1 B 0.86
A 1 B 1
OD 600 nm [−]
0.2
0.4
0.6
0.8
0 5 101520
Time [h]

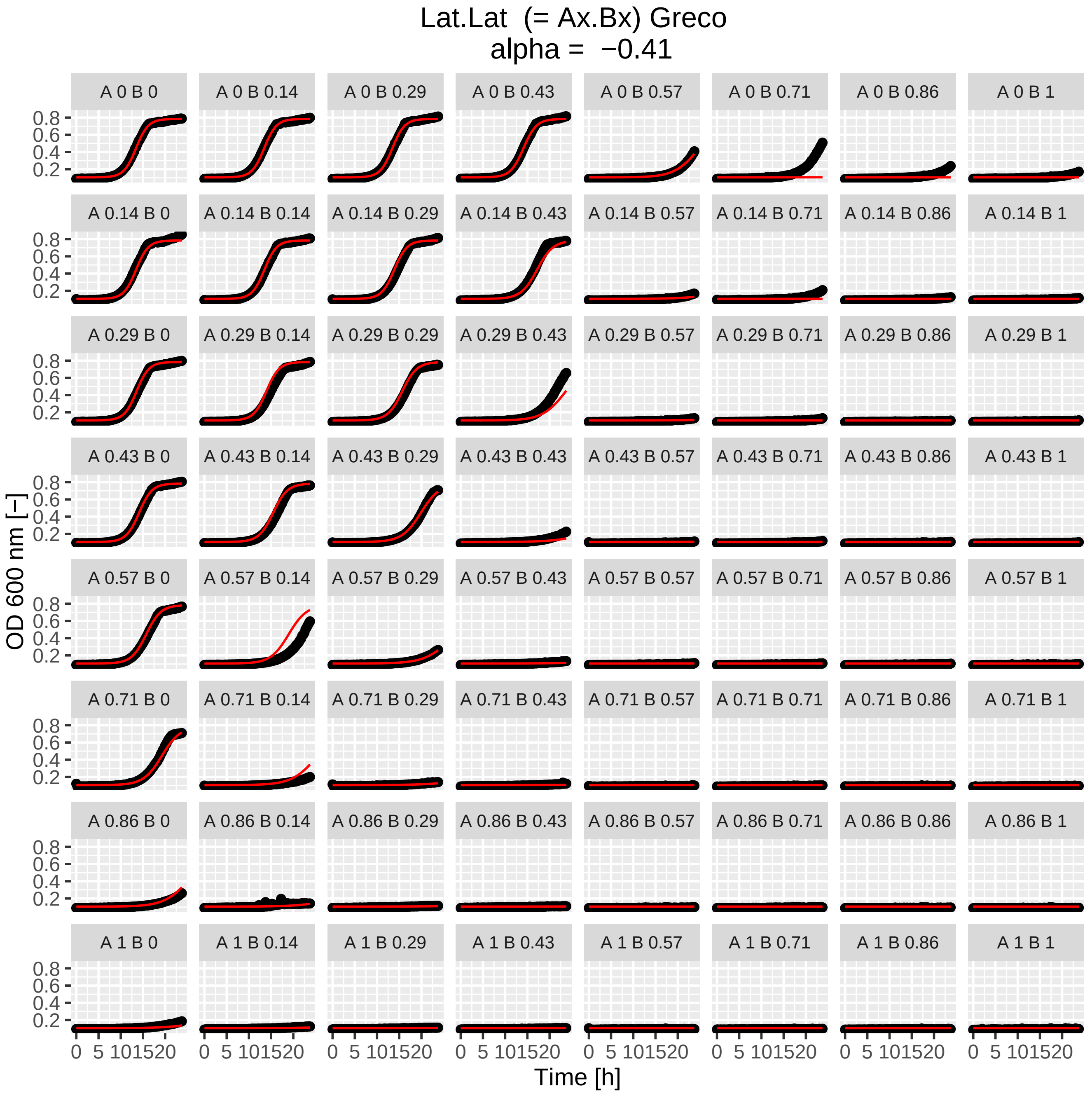

Lat.Lat (= Ax.Bx) Greco
alpha = −0.41
A 0 B 0
A 0 B 0.14
A 0 B 0.29
A 0 B 0.43
A 0 B 0.57
A 0 B 0.71
A 0 B 0.86
A 0 B 1
A 0.14 B 0
A 0.14 B 0.14
A 0.14 B 0.29
A 0.14 B 0.43
A 0.14 B 0.57
A 0.14 B 0.71
A 0.14 B 0.86
A 0.14 B 1
A 0.29 B 0
A 0.29 B 0.14
A 0.29 B 0.29
A 0.29 B 0.43
A 0.29 B 0.57
A 0.29 B 0.71
A 0.29 B 0.86
A 0.29 B 1
A 0.43 B 0
A 0.43 B 0.14
A 0.43 B 0.29
A 0.43 B 0.43
A 0.43 B 0.57
A 0.43 B 0.71
A 0.43 B 0.86
A 0.43 B 1
A 0.57 B 0
A 0.57 B 0.14
A 0.57 B 0.29
A 0.57 B 0.43
A 0.57 B 0.57
A 0.57 B 0.71
A 0.57 B 0.86
A 0.57 B 1
A 0.71 B 0
A 0.71 B 0.14
A 0.71 B 0.29
A 0.71 B 0.43
A 0.71 B 0.57
A 0.71 B 0.71
A 0.71 B 0.86
A 0.71 B 1
A 0.86 B 0
A 0.86 B 0.14
A 0.86 B 0.29
A 0.86 B 0.43
A 0.86 B 0.57
A 0.86 B 0.71
A 0.86 B 0.86
A 0.86 B 1
A 1 B 0
A 1 B 0.14
A 1 B 0.29
A 1 B 0.43
A 1 B 0.57
A 1 B 0.71
A 1 B 0.86
A 1 B 1
OD 600 nm [−]
0.2
0.4
0.6
0.8
0 5 101520
Time [h]

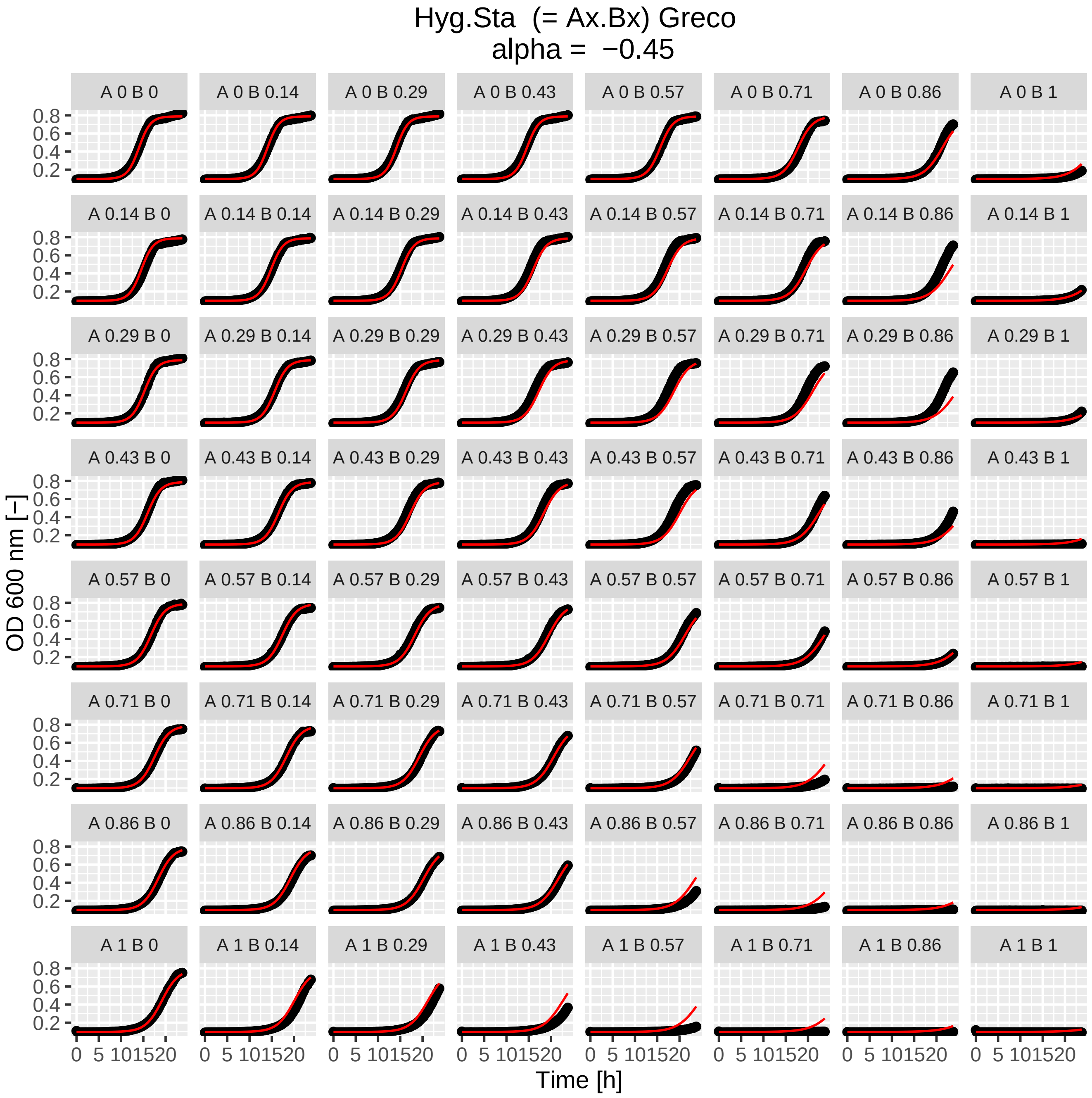

Hyg.Sta (= Ax.Bx) Greco
alpha = −0.45
OD 600 nm [−]
Time [h]
0.8
0.6
0.4
0.2
0 5 101520
A 0 B 0
A 0 B 0.14
A 0 B 0.29
A 0 B 0.43
A 0 B 0.57
A 0 B 0.71
A 0 B 0.86
A 0 B 1
A 0.14 B 0
A 0.14 B 0.14
A 0.14 B 0.29
A 0.14 B 0.43
A 0.14 B 0.57
A 0.14 B 0.71
A 0.14 B 0.86
A 0.14 B 1
A 0.29 B 0
A 0.29 B 0.14
A 0.29 B 0.29
A 0.29 B 0.43
A 0.29 B 0.57
A 0.29 B 0.71
A 0.29 B 0.86
A 0.29 B 1
A 0.43 B 0
A 0.43 B 0.14
A 0.43 B 0.29
A 0.43 B 0.43
A 0.43 B 0.57
A 0.43 B 0.71
A 0.43 B 0.86
A 0.43 B 1
A 0.57 B 0
A 0.57 B 0.14
A 0.57 B 0.29
A 0.57 B 0.43
A 0.57 B 0.57
A 0.57 B 0.71
A 0.57 B 0.86
A 0.57 B 1
A 0.71 B 0
A 0.71 B 0.14
A 0.71 B 0.29
A 0.71 B 0.43
A 0.71 B 0.57
A 0.71 B 0.71
A 0.71 B 0.86
A 0.71 B 1
A 0.86 B 0
A 0.86 B 0.14
A 0.86 B 0.29
A 0.86 B 0.43
A 0.86 B 0.57
A 0.86 B 0.71
A 0.86 B 0.86
A 0.86 B 1
A 1 B 0
A 1 B 0.14
A 1 B 0.29
A 1 B 0.43
A 1 B 0.57
A 1 B 0.71
A 1 B 0.86
A 1 B 1

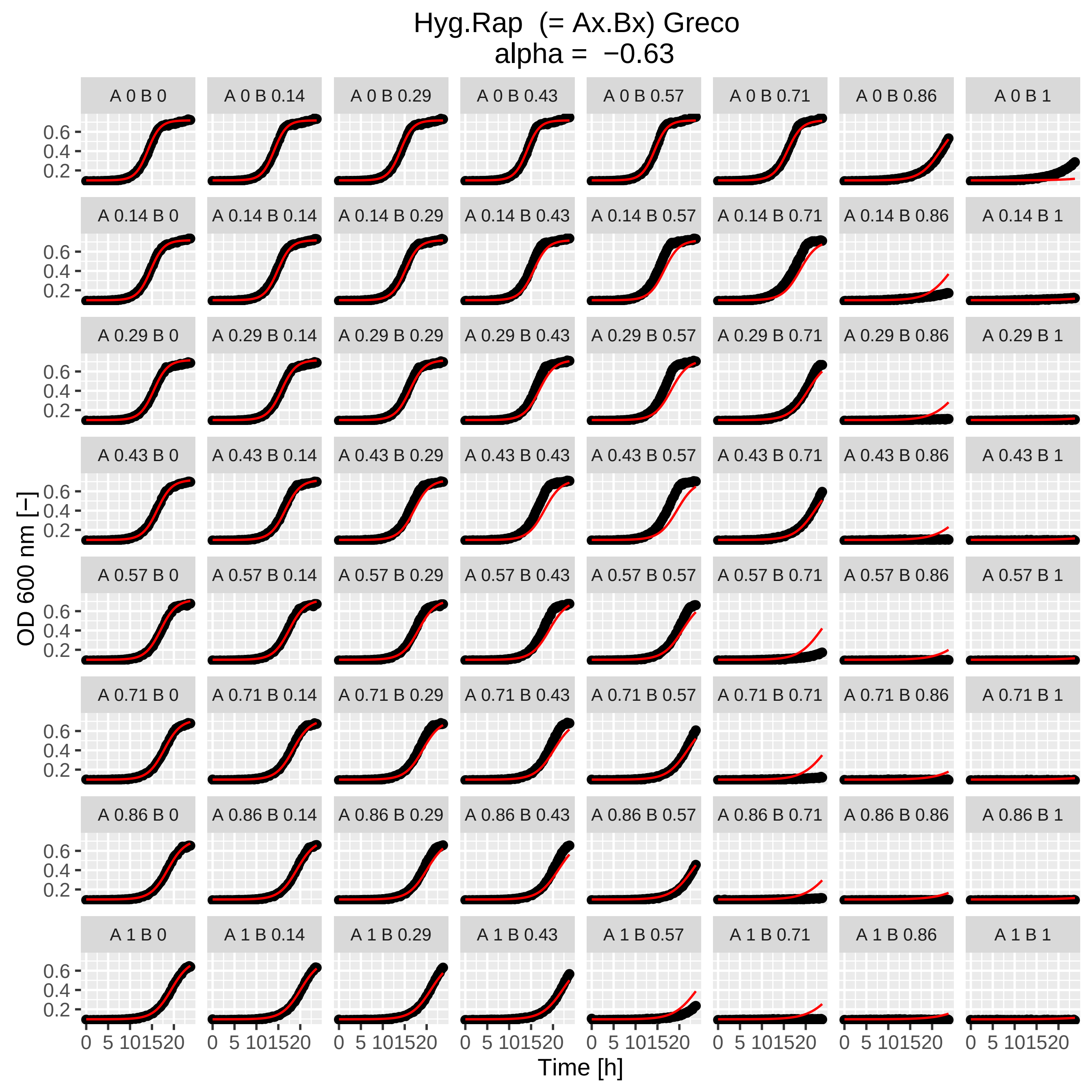
Hyg.Rap (= Ax.Bx) Greco
alpha = −0.63
OD 600 nm [−]
Time [h]
0.2
0.4
0.6
0
5
10
15
20
A 0 B 0
A 0 B 0.14
A 0 B 0.29
A 0 B 0.43
A 0 B 0.57
A 0 B 0.71
A 0 B 0.86
A 0 B 1
A 0.14 B 0
A 0.14 B 0.14
A 0.14 B 0.29
A 0.14 B 0.43
A 0.14 B 0.57
A 0.14 B 0.71
A 0.14 B 0.86
A 0.14 B 1
A 0.29 B 0
A 0.29 B 0.14
A 0.29 B 0.29
A 0.29 B 0.43
A 0.29 B 0.57
A 0.29 B 0.71
A 0.29 B 0.86
A 0.29 B 1
A 0.43 B 0
A 0.43 B 0.14
A 0.43 B 0.29
A 0.43 B 0.43
A 0.43 B 0.57
A 0.43 B 0.71
A 0.43 B 0.86
A 0.43 B 1
A 0.57 B 0
A 0.57 B 0.14
A 0.57 B 0.29
A 0.57 B 0.43
A 0.57 B 0.57
A 0.57 B 0.71
A 0.57 B 0.86
A 0.57 B 1
A 0.71 B 0
A 0.71 B 0.14
A 0.71 B 0.29
A 0.71 B 0.43
A 0.71 B 0.57
A 0.71 B 0.71
A 0.71 B 0.86
A 0.71 B 1
A 0.86 B 0
A 0.86 B 0.14
A 0.86 B 0.29
A 0.86 B 0.43
A 0.86 B 0.57
A 0.86 B 0.71
A 0.86 B 0.86
A 0.86 B 1
A 1 B 0
A 1 B 0.14
A 1 B 0.29
A 1 B 0.43
A 1 B 0.57
A 1 B 0.71
A 1 B 0.86
A 1 B 1

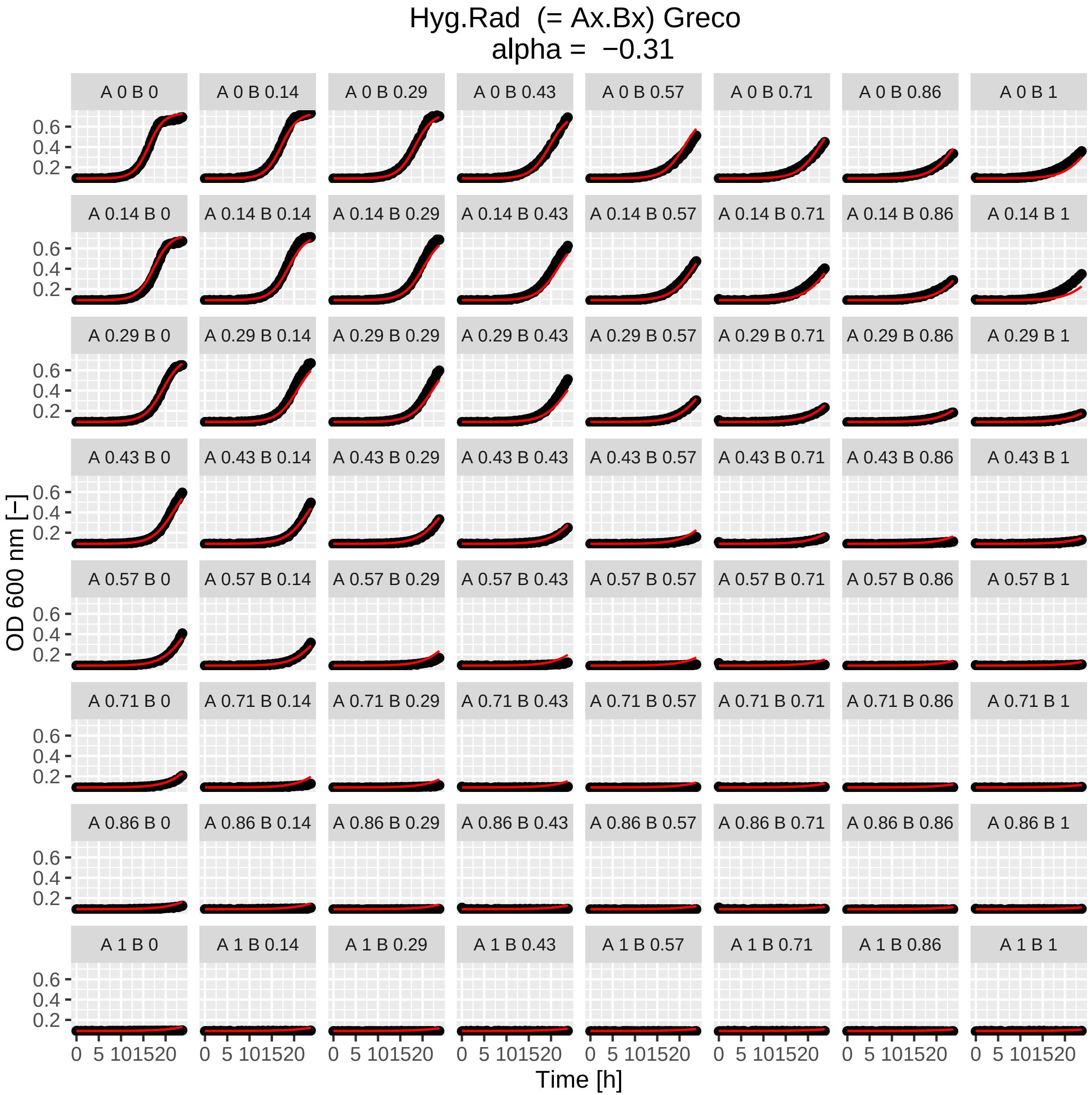
Hyg.Rad (= Ax.Bx) Greco
alpha = −0.31
A 0 B 0
A 0 B 0.14
A 0 B 0.29
A 0 B 0.43
A 0 B 0.57
A 0 B 0.71
A 0 B 0.86
A 0 B 1
A 0.14 B 0
A 0.14 B 0.14
A 0.14 B 0.29
A 0.14 B 0.43
A 0.14 B 0.57
A 0.14 B 0.71
A 0.14 B 0.86
A 0.14 B 1
A 0.29 B 0
A 0.29 B 0.14
A 0.29 B 0.29
A 0.29 B 0.43
A 0.29 B 0.57
A 0.29 B 0.71
A 0.29 B 0.86
A 0.29 B 1
A 0.43 B 0
A 0.43 B 0.14
A 0.43 B 0.29
A 0.43 B 0.43
A 0.43 B 0.57
A 0.43 B 0.71
A 0.43 B 0.86
A 0.43 B 1
A 0.57 B 0
A 0.57 B 0.14
A 0.57 B 0.29
A 0.57 B 0.43
A 0.57 B 0.57
A 0.57 B 0.71
A 0.57 B 0.86
A 0.57 B 1
A 0.71 B 0
A 0.71 B 0.14
A 0.71 B 0.29
A 0.71 B 0.43
A 0.71 B 0.57
A 0.71 B 0.71
A 0.71 B 0.86
A 0.71 B 1
A 0.86 B 0
A 0.86 B 0.14
A 0.86 B 0.29
A 0.86 B 0.43
A 0.86 B 0.57
A 0.86 B 0.71
A 0.86 B 0.86
A 0.86 B 1
A 1 B 0
A 1 B 0.14
A 1 B 0.29
A 1 B 0.43
A 1 B 0.57
A 1 B 0.71
A 1 B 0.86
A 1 B 1
OD 600 nm [−]
0.2
0.4
0.6
0 5 101520
Time [h]

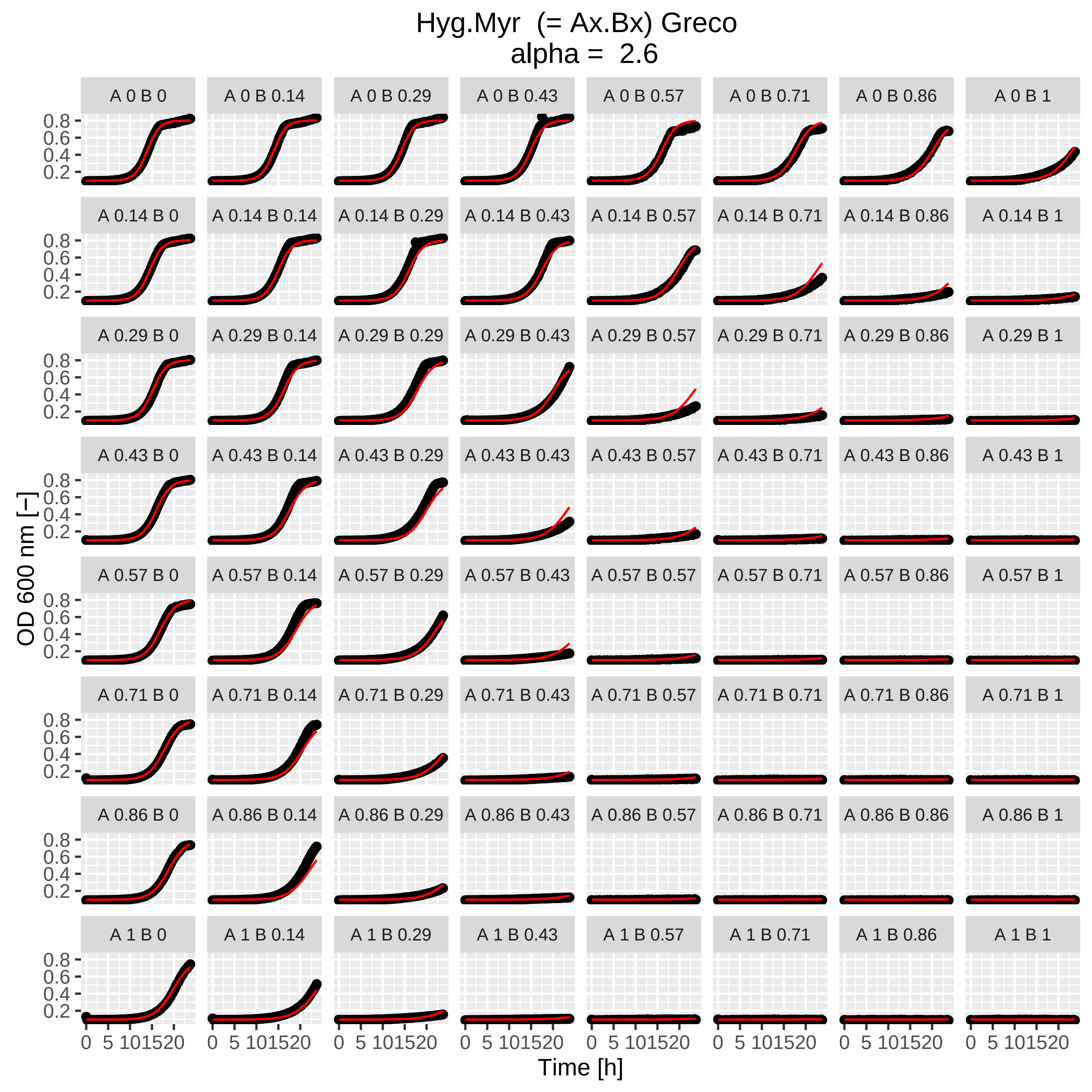

Hyg.Myr (= Ax.Bx) Greco
alpha = 2.6
A 0 B 0
A 0 B 0.14
A 0 B 0.29
A 0 B 0.43
A 0 B 0.57
A 0 B 0.71
A 0 B 0.86
A 0 B 1
A 0.14 B 0
A 0.14 B 0.14
A 0.14 B 0.29
A 0.14 B 0.43
A 0.14 B 0.57
A 0.14 B 0.71
A 0.14 B 0.86
A 0.14 B 1
A 0.29 B 0
A 0.29 B 0.14
A 0.29 B 0.29
A 0.29 B 0.43
A 0.29 B 0.57
A 0.29 B 0.71
A 0.29 B 0.86
A 0.29 B 1
A 0.43 B 0
A 0.43 B 0.14
A 0.43 B 0.29
A 0.43 B 0.43
A 0.43 B 0.57
A 0.43 B 0.71
A 0.43 B 0.86
A 0.43 B 1
A 0.57 B 0
A 0.57 B 0.14
A 0.57 B 0.29
A 0.57 B 0.43
A 0.57 B 0.57
A 0.57 B 0.71
A 0.57 B 0.86
A 0.57 B 1
A 0.71 B 0
A 0.71 B 0.14
A 0.71 B 0.29
A 0.71 B 0.43
A 0.71 B 0.57
A 0.71 B 0.71
A 0.71 B 0.86
A 0.71 B 1
A 0.86 B 0
A 0.86 B 0.14
A 0.86 B 0.29
A 0.86 B 0.43
A 0.86 B 0.57
A 0.86 B 0.71
A 0.86 B 0.86
A 0.86 B 1
A 1 B 0
A 1 B 0.14
A 1 B 0.29
A 1 B 0.43
A 1 B 0.57
A 1 B 0.71
A 1 B 0.86
A 1 B 1
OD 600 nm [−]
0.8
0.6
0.4
0.2
0 5 101520
Time [h]

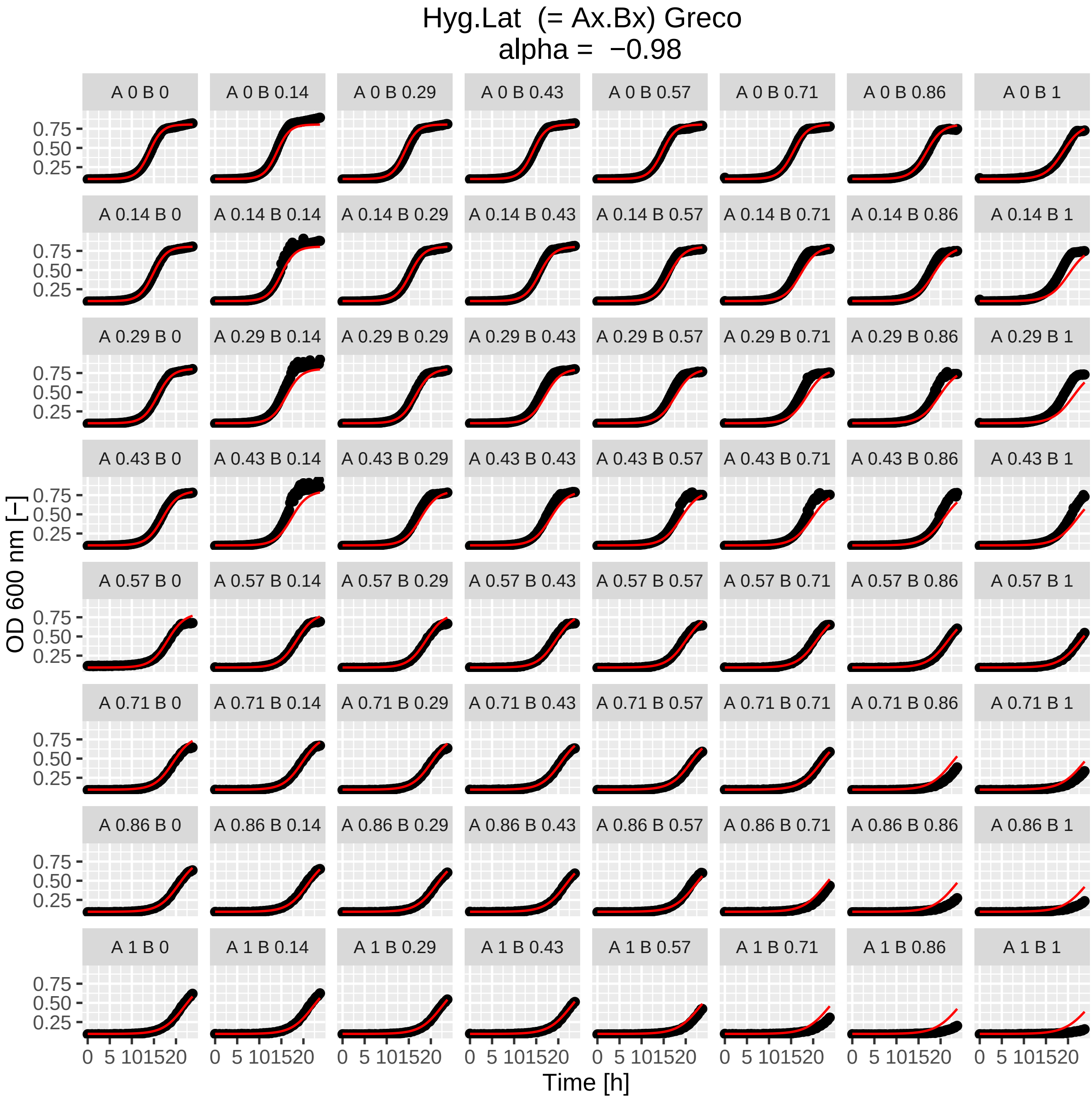

Hyg.Lat (= Ax.Bx) Greco
alpha = −0.98
OD 600 nm [−]
Time [h]
0.75
0.50
0.25
0 5 101520
A 0 B 0
A 0 B 0.14
A 0 B 0.29
A 0 B 0.43
A 0 B 0.57
A 0 B 0.71
A 0 B 0.86
A 0 B 1
A 0.14 B 0
A 0.14 B 0.14
A 0.14 B 0.29
A 0.14 B 0.43
A 0.14 B 0.57
A 0.14 B 0.71
A 0.14 B 0.86
A 0.14 B 1
A 0.29 B 0
A 0.29 B 0.14
A 0.29 B 0.29
A 0.29 B 0.43
A 0.29 B 0.57
A 0.29 B 0.71
A 0.29 B 0.86
A 0.29 B 1
A 0.43 B 0
A 0.43 B 0.14
A 0.43 B 0.29
A 0.43 B 0.43
A 0.43 B 0.57
A 0.43 B 0.71
A 0.43 B 0.86
A 0.43 B 1
A 0.57 B 0
A 0.57 B 0.14
A 0.57 B 0.29
A 0.57 B 0.43
A 0.57 B 0.57
A 0.57 B 0.71
A 0.57 B 0.86
A 0.57 B 1
A 0.71 B 0
A 0.71 B 0.14
A 0.71 B 0.29
A 0.71 B 0.43
A 0.71 B 0.57
A 0.71 B 0.71
A 0.71 B 0.86
A 0.71 B 1
A 0.86 B 0
A 0.86 B 0.14
A 0.86 B 0.29
A 0.86 B 0.43
A 0.86 B 0.57
A 0.86 B 0.71
A 0.86 B 0.86
A 0.86 B 1
A 1 B 0
A 1 B 0.14
A 1 B 0.29
A 1 B 0.43
A 1 B 0.57
A 1 B 0.71
A 1 B 0.86
A 1 B 1

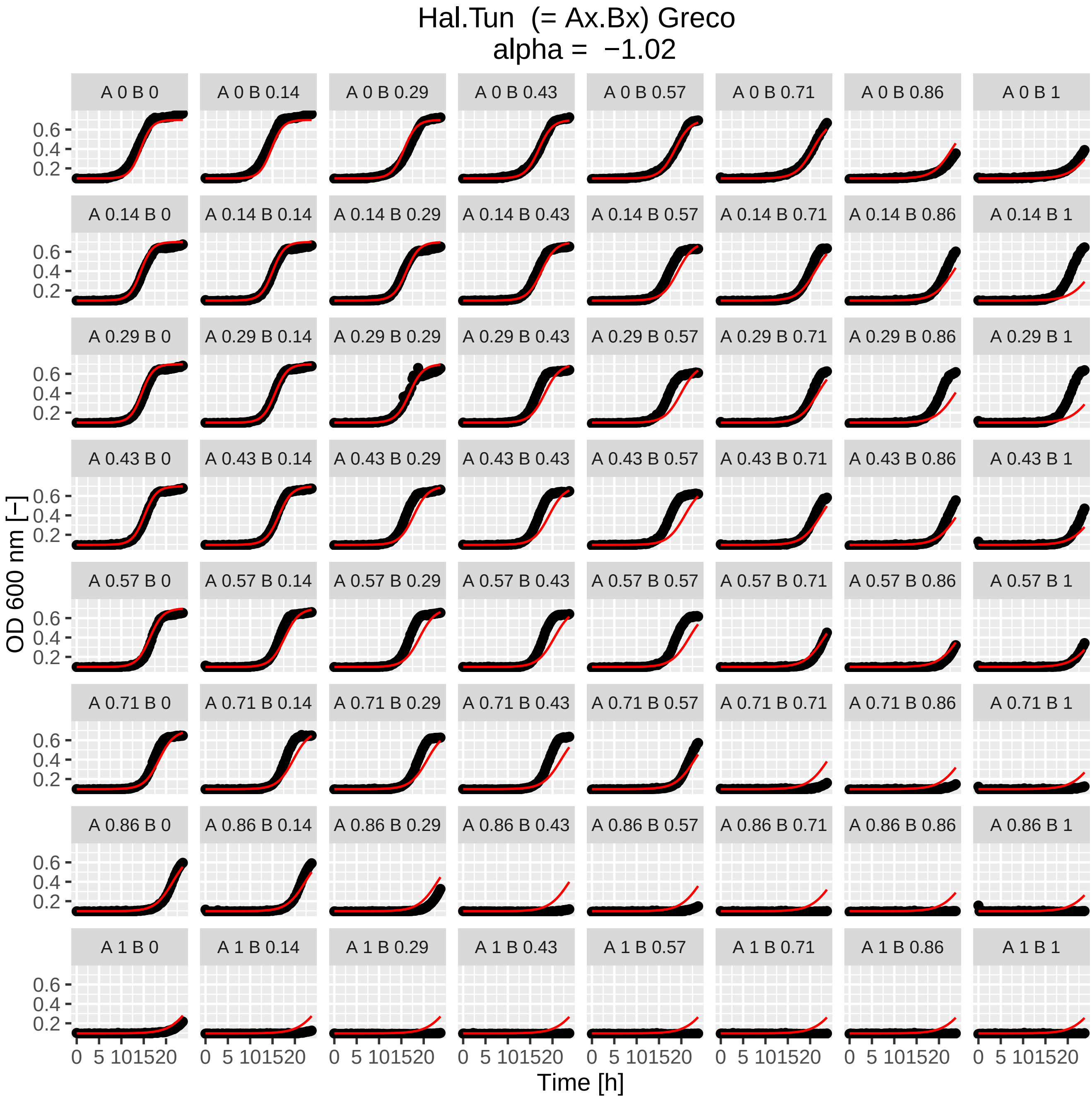
Hal.Tun (= Ax.Bx) Greco
alpha = −1.02
OD 600 nm [−]
Time [h]
A 0 B 0
A 0 B 0.14
A 0 B 0.29
A 0 B 0.43
A 0 B 0.57
A 0 B 0.71
A 0 B 0.86
A 0 B 1
A 1 B 0
A 1 B 1
0.2
0.4
0.6
0 5 10 15 20

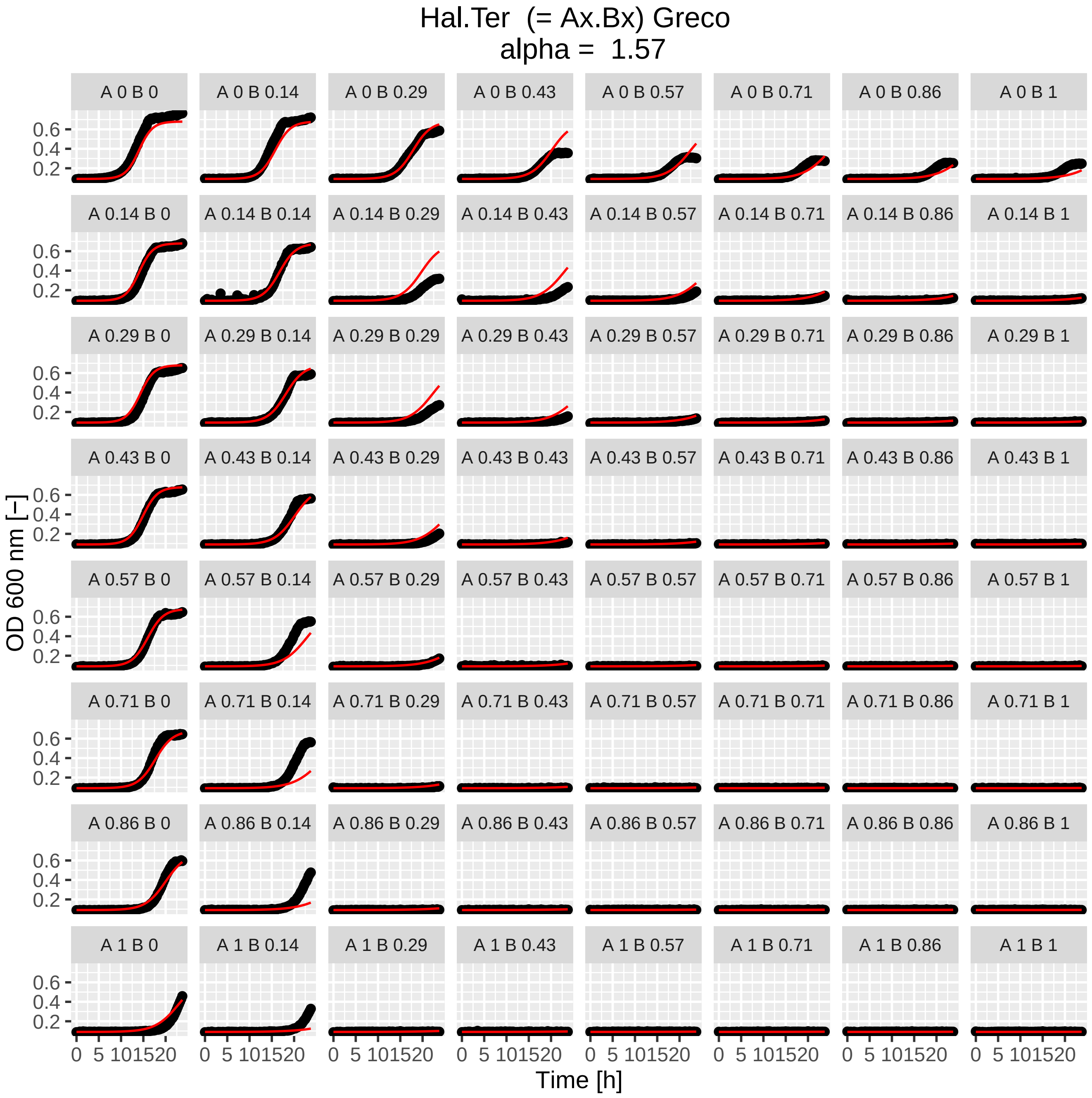

Hal.Ter (= Ax.Bx) Greco
alpha = 1.57
OD 600 nm [–]
Time [h]
A 0 B 0
A 0 B 0.14
A 0 B 0.29
A 0 B 0.43
A 0 B 0.57
A 0 B 0.71
A 0 B 0.86
A 0 B 1
A 0.14 B 0
A 0.14 B 0.14
A 0.14 B 0.29
A 0.14 B 0.43
A 0.14 B 0.57
A 0.14 B 0.71
A 0.14 B 0.86
A 0.14 B 1
A 0.29 B 0
A 0.29 B 0.14
A 0.29 B 0.29
A 0.29 B 0.43
A 0.29 B 0.57
A 0.29 B 0.71
A 0.29 B 0.86
A 0.29 B 1
A 0.43 B 0
A 0.43 B 0.14
A 0.43 B 0.29
A 0.43 B 0.43
A 0.43 B 0.57
A 0.43 B 0.71
A 0.43 B 0.86
A 0.43 B 1
A 0.57 B 0
A 0.57 B 0.14
A 0.57 B 0.29
A 0.57 B 0.43
A 0.57 B 0.57
A 0.57 B 0.71
A 0.57 B 0.86
A 0.57 B 1
A 0.71 B 0
A 0.71 B 0.14
A 0.71 B 0.29
A 0.71 B 0.43
A 0.71 B 0.57
A 0.71 B 0.71
A 0.71 B 0.86
A 0.71 B 1
A 0.86 B 0
A 0.86 B 0.14
A 0.86 B 0.29
A 0.86 B 0.43
A 0.86 B 0.57
A 0.86 B 0.71
A 0.86 B 0.86
A 0.86 B 1
A 1 B 0
A 1 B 0.14
A 1 B 0.29
A 1 B 0.43
A 1 B 0.57
A 1 B 0.71
A 1 B 0.86
A 1 B 1
0.6
0.4
0.2
0 5 101520

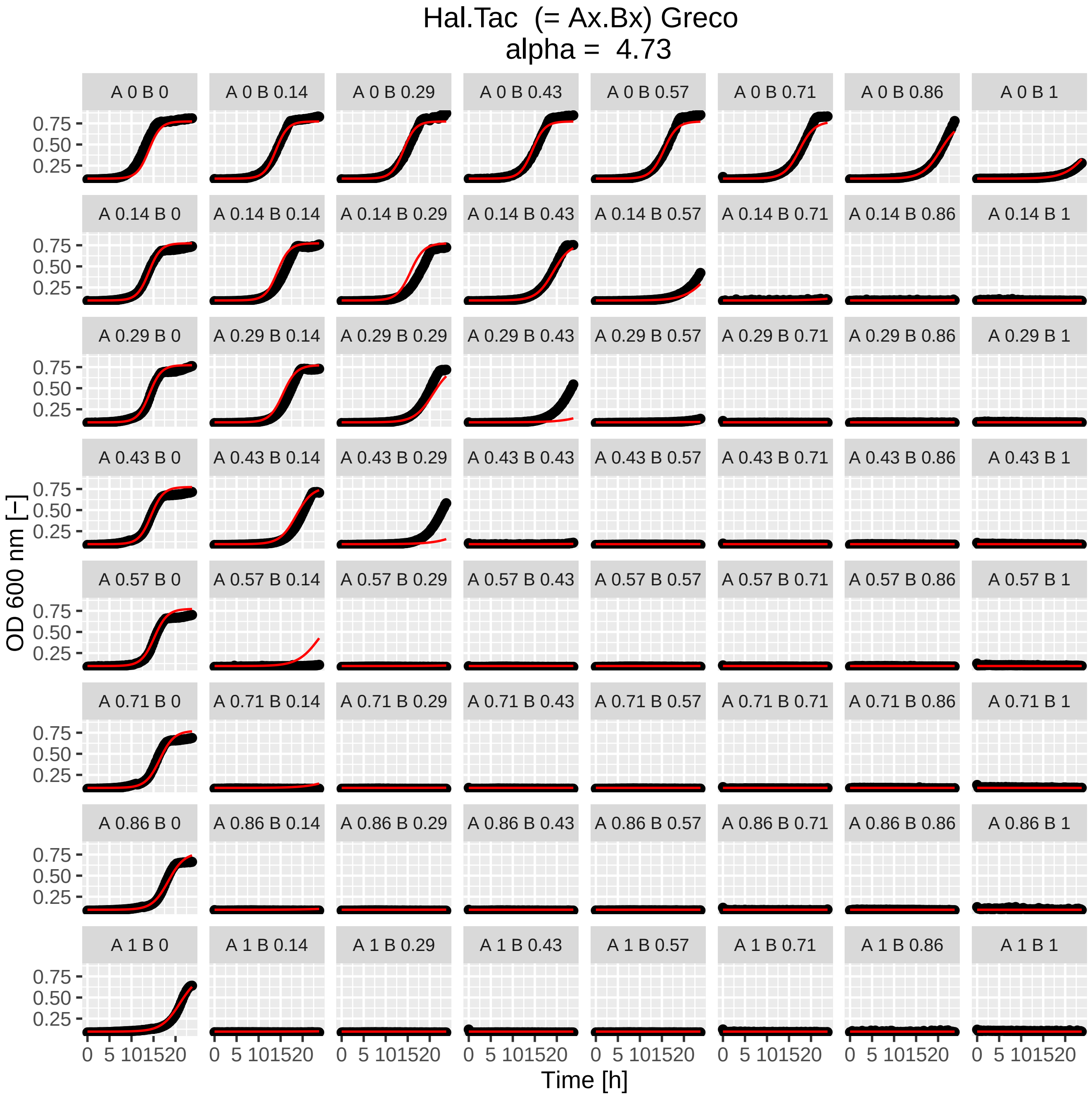

Hal.Tac (= Ax.Bx) Greco
alpha = 4.73
A 0 B 0
A 0 B 0.14
A 0 B 0.29
A 0 B 0.43
A 0 B 0.57
A 0 B 0.71
A 0 B 0.86
A 0 B 1
A 0.14 B 0
A 0.14 B 0.14
A 0.14 B 0.29
A 0.14 B 0.43
A 0.14 B 0.57
A 0.14 B 0.71
A 0.14 B 0.86
A 0.14 B 1
A 0.29 B 0
A 0.29 B 0.14
A 0.29 B 0.29
A 0.29 B 0.43
A 0.29 B 0.57
A 0.29 B 0.71
A 0.29 B 0.86
A 0.29 B 1
A 0.43 B 0
A 0.43 B 0.14
A 0.43 B 0.29
A 0.43 B 0.43
A 0.43 B 0.57
A 0.43 B 0.71
A 0.43 B 0.86
A 0.43 B 1
A 0.57 B 0
A 0.57 B 0.14
A 0.57 B 0.29
A 0.57 B 0.43
A 0.57 B 0.57
A 0.57 B 0.71
A 0.57 B 0.86
A 0.57 B 1
A 0.71 B 0
A 0.71 B 0.14
A 0.71 B 0.29
A 0.71 B 0.43
A 0.71 B 0.57
A 0.71 B 0.71
A 0.71 B 0.86
A 0.71 B 1
A 0.86 B 0
A 0.86 B 0.14
A 0.86 B 0.29
A 0.86 B 0.43
A 0.86 B 0.57
A 0.86 B 0.71
A 0.86 B 0.86
A 0.86 B 1
A 1 B 0
A 1 B 0.14
A 1 B 0.29
A 1 B 0.43
A 1 B 0.57
A 1 B 0.71
A 1 B 0.86
A 1 B 1
OD 600 nm [–]
0.75
0.50
0.25
0 5 101520
Time [h]

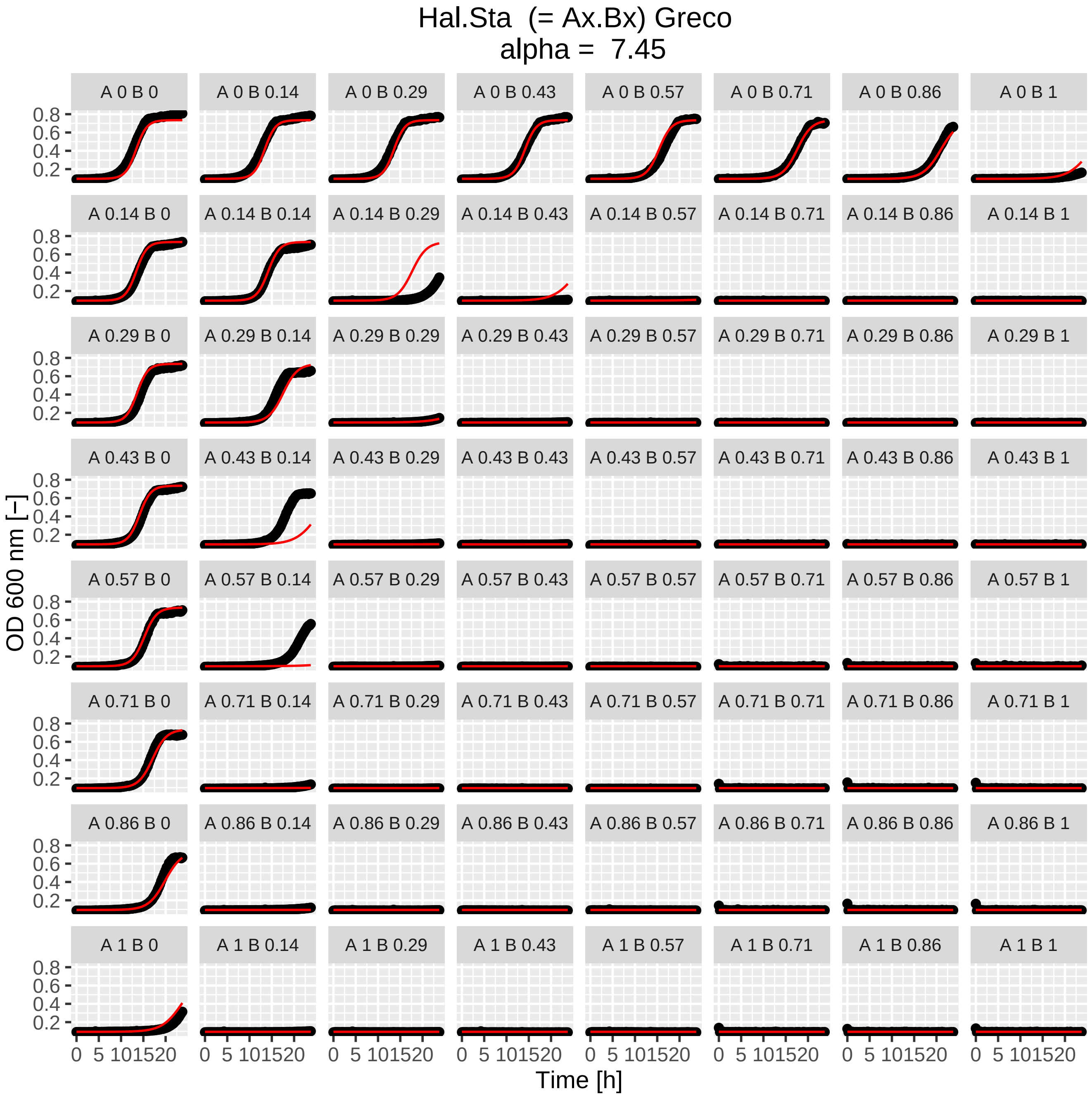

Hal.Sta (= Ax.Bx) Greco
alpha = 7.45
A 0 B 0
A 0 B 0.14
A 0 B 0.29
A 0 B 0.43
A 0 B 0.57
A 0 B 0.71
A 0 B 0.86
A 0 B 1
A 0.14 B 0
A 0.14 B 0.14
A 0.14 B 0.29
A 0.14 B 0.43
A 0.14 B 0.57
A 0.14 B 0.71
A 0.14 B 0.86
A 0.14 B 1
A 0.29 B 0
A 0.29 B 0.14
A 0.29 B 0.29
A 0.29 B 0.43
A 0.29 B 0.57
A 0.29 B 0.71
A 0.29 B 0.86
A 0.29 B 1
A 0.43 B 0
A 0.43 B 0.14
A 0.43 B 0.29
A 0.43 B 0.43
A 0.43 B 0.57
A 0.43 B 0.71
A 0.43 B 0.86
A 0.43 B 1
A 0.57 B 0
A 0.57 B 0.14
A 0.57 B 0.29
A 0.57 B 0.43
A 0.57 B 0.57
A 0.57 B 0.71
A 0.57 B 0.86
A 0.57 B 1
A 0.71 B 0
A 0.71 B 0.14
A 0.71 B 0.29
A 0.71 B 0.43
A 0.71 B 0.57
A 0.71 B 0.71
A 0.71 B 0.86
A 0.71 B 1
A 0.86 B 0
A 0.86 B 0.14
A 0.86 B 0.29
A 0.86 B 0.43
A 0.86 B 0.57
A 0.86 B 0.71
A 0.86 B 0.86
A 0.86 B 1
A 1 B 0
A 1 B 0.14
A 1 B 0.29
A 1 B 0.43
A 1 B 0.57
A 1 B 0.71
A 1 B 0.86
A 1 B 1
OD 600 nm [–]
0.2
0.4
0.6
0.8
0 5 101520
Time [h]

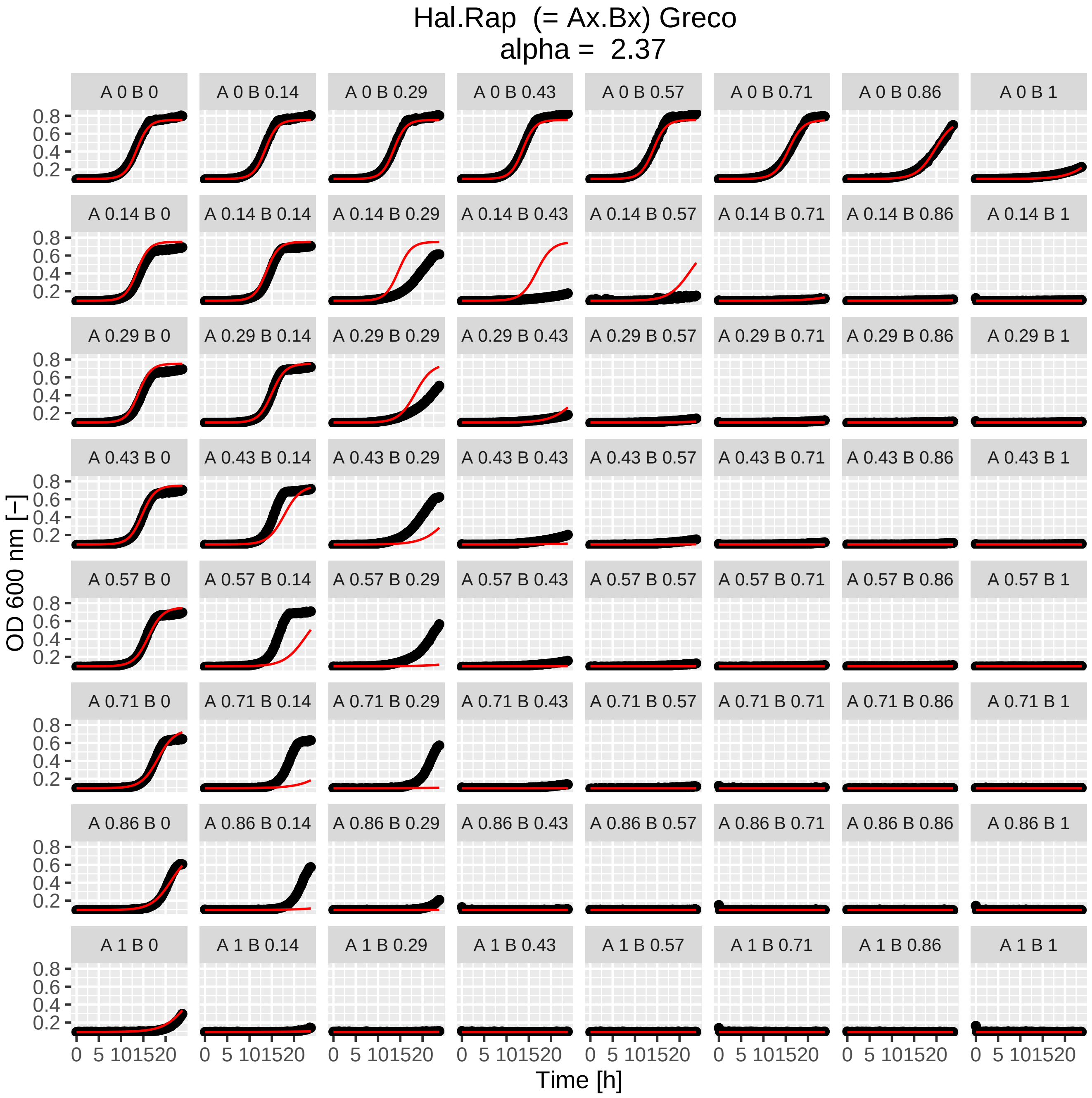
Hal.Rap (= Ax.Bx) Greco
alpha = 2.37
A 0 B 0
A 0 B 0.14
A 0 B 0.29
A 0 B 0.43
A 0 B 0.57
A 0 B 0.71
A 0 B 0.86
A 0 B 1
A 0.14 B 0
A 0.14 B 0.14
A 0.14 B 0.29
A 0.14 B 0.43
A 0.14 B 0.57
A 0.14 B 0.71
A 0.14 B 0.86
A 0.14 B 1
A 0.29 B 0
A 0.29 B 0.14
A 0.29 B 0.29
A 0.29 B 0.43
A 0.29 B 0.57
A 0.29 B 0.71
A 0.29 B 0.86
A 0.29 B 1
A 0.43 B 0
A 0.43 B 0.14
A 0.43 B 0.29
A 0.43 B 0.43
A 0.43 B 0.57
A 0.43 B 0.71
A 0.43 B 0.86
A 0.43 B 1
A 0.57 B 0
A 0.57 B 0.14
A 0.57 B 0.29
A 0.57 B 0.43
A 0.57 B 0.57
A 0.57 B 0.71
A 0.57 B 0.86
A 0.57 B 1
A 0.71 B 0
A 0.71 B 0.14
A 0.71 B 0.29
A 0.71 B 0.43
A 0.71 B 0.57
A 0.71 B 0.71
A 0.71 B 0.86
A 0.71 B 1
A 0.86 B 0
A 0.86 B 0.14
A 0.86 B 0.29
A 0.86 B 0.43
A 0.86 B 0.57
A 0.86 B 0.71
A 0.86 B 0.86
A 0.86 B 1
A 1 B 0
A 1 B 0.14
A 1 B 0.29
A 1 B 0.43
A 1 B 0.57
A 1 B 0.71
A 1 B 0.86
A 1 B 1
0.8
0.6
0.4
0.2
OD 600 nm [−]
0 5 101520
Time [h]

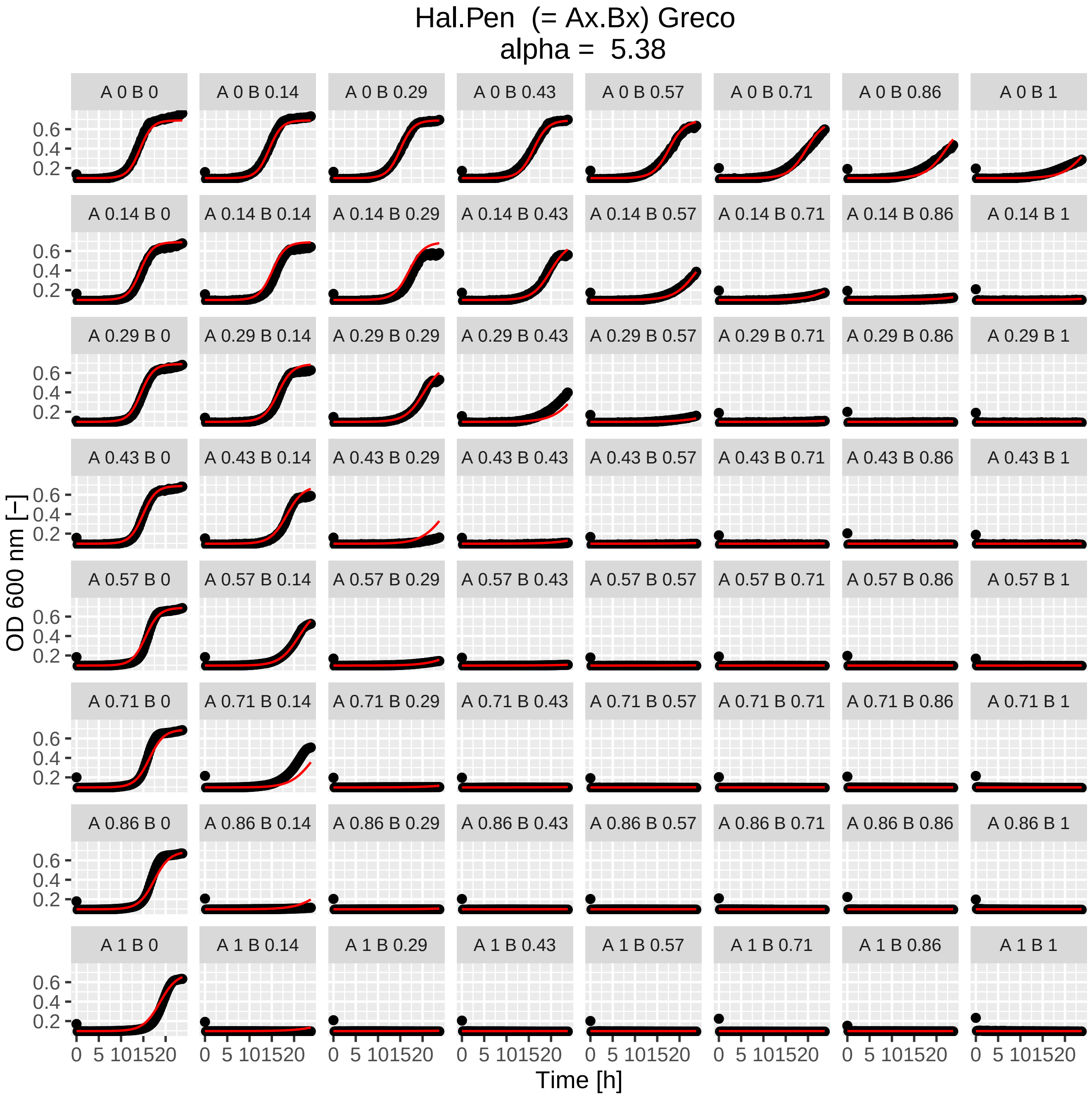
Hal.Pen (= Ax.Bx) Greco
alpha = 5.38
A 0 B 0
A 0 B 0.14
A 0 B 0.29
A 0 B 0.43
A 0 B 0.57
A 0 B 0.71
A 0 B 0.86
A 0 B 1
A 0.14 B 0
A 0.14 B 0.14
A 0.14 B 0.29
A 0.14 B 0.43
A 0.14 B 0.57
A 0.14 B 0.71
A 0.14 B 0.86
A 0.14 B 1
A 0.29 B 0
A 0.29 B 0.14
A 0.29 B 0.29
A 0.29 B 0.43
A 0.29 B 0.57
A 0.29 B 0.71
A 0.29 B 0.86
A 0.29 B 1
A 0.43 B 0
A 0.43 B 0.14
A 0.43 B 0.29
A 0.43 B 0.43
A 0.43 B 0.57
A 0.43 B 0.71
A 0.43 B 0.86
A 0.43 B 1
A 0.57 B 0
A 0.57 B 0.14
A 0.57 B 0.29
A 0.57 B 0.43
A 0.57 B 0.57
A 0.57 B 0.71
A 0.57 B 0.86
A 0.57 B 1
A 0.71 B 0
A 0.71 B 0.14
A 0.71 B 0.29
A 0.71 B 0.43
A 0.71 B 0.57
A 0.71 B 0.71
A 0.71 B 0.86
A 0.71 B 1
A 0.86 B 0
A 0.86 B 0.14
A 0.86 B 0.29
A 0.86 B 0.43
A 0.86 B 0.57
A 0.86 B 0.71
A 0.86 B 0.86
A 0.86 B 1
A 1 B 0
A 1 B 0.14
A 1 B 0.29
A 1 B 0.43
A 1 B 0.57
A 1 B 0.71
A 1 B 0.86
A 1 B 1
OD 600 nm [–]
0.2
0.4
0.6
0 5 101520
Time [h]

Hal.Lat (= Ax.Bx) Greco
alpha = 7.31

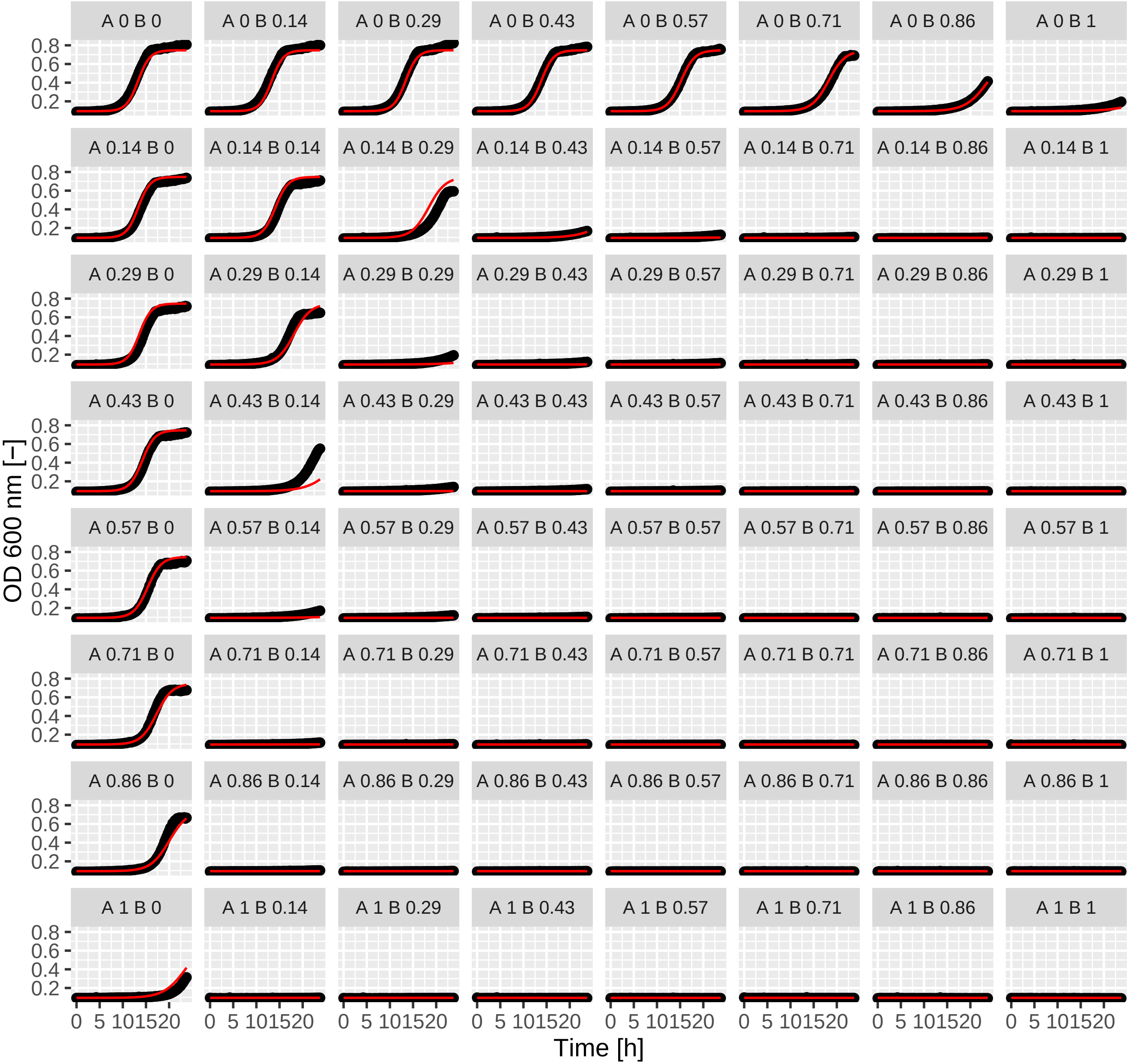

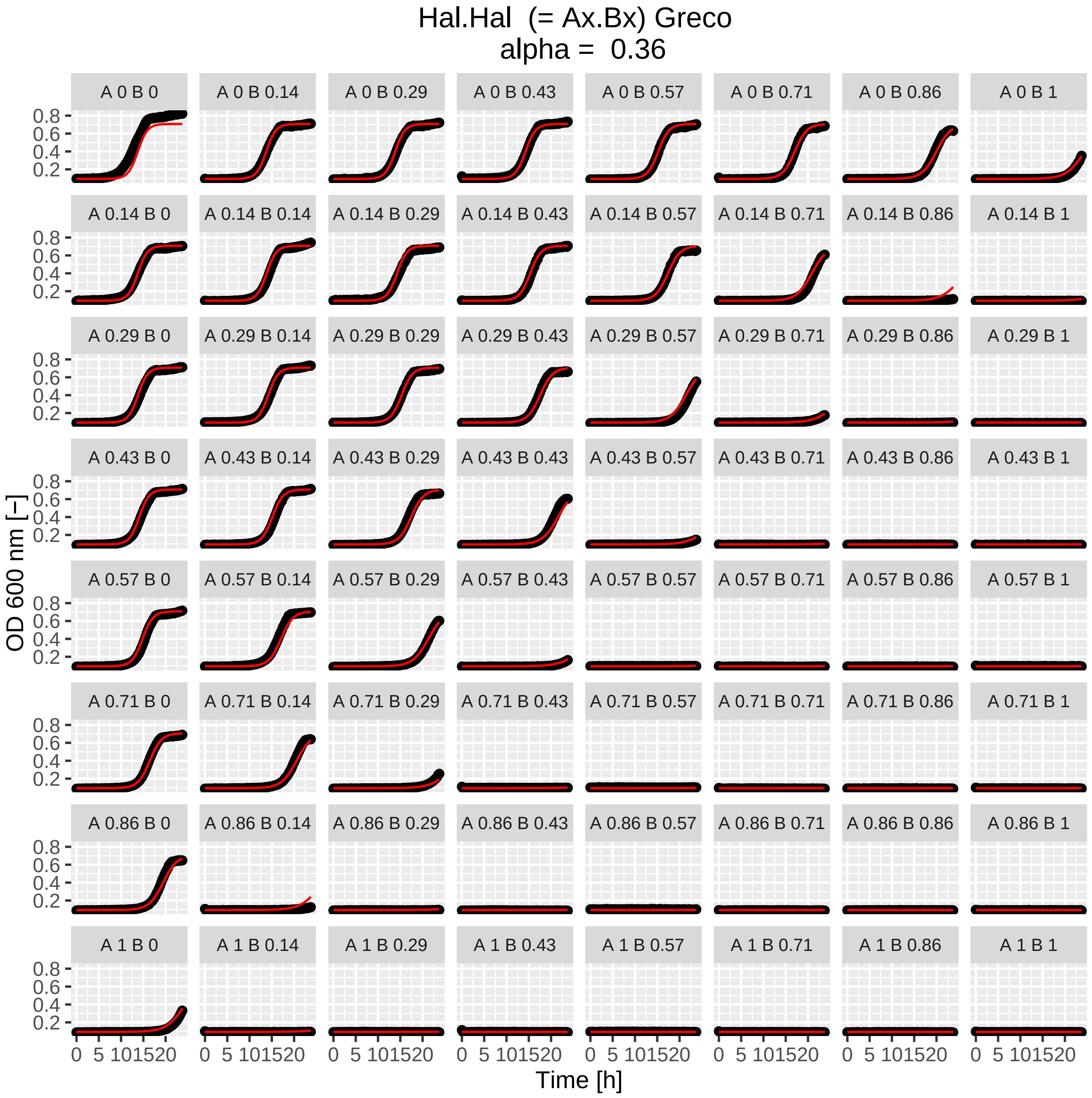

Hal.Hal (= Ax.Bx) Greco
alpha = 0.36
A 0 B 0
A 0 B 0.14
A 0 B 0.29
A 0 B 0.43
A 0 B 0.57
A 0 B 0.71
A 0 B 0.86
A 0 B 1
A 0.14 B 0
A 0.14 B 0.14
A 0.14 B 0.29
A 0.14 B 0.43
A 0.14 B 0.57
A 0.14 B 0.71
A 0.14 B 0.86
A 0.14 B 1
A 0.29 B 0
A 0.29 B 0.14
A 0.29 B 0.29
A 0.29 B 0.43
A 0.29 B 0.57
A 0.29 B 0.71
A 0.29 B 0.86
A 0.29 B 1
A 0.43 B 0
A 0.43 B 0.14
A 0.43 B 0.29
A 0.43 B 0.43
A 0.43 B 0.57
A 0.43 B 0.71
A 0.43 B 0.86
A 0.43 B 1
A 0.57 B 0
A 0.57 B 0.14
A 0.57 B 0.29
A 0.57 B 0.43
A 0.57 B 0.57
A 0.57 B 0.71
A 0.57 B 0.86
A 0.57 B 1
A 0.71 B 0
A 0.71 B 0.14
A 0.71 B 0.29
A 0.71 B 0.43
A 0.71 B 0.57
A 0.71 B 0.71
A 0.71 B 0.86
A 0.71 B 1
A 0.86 B 0
A 0.86 B 0.14
A 0.86 B 0.29
A 0.86 B 0.43
A 0.86 B 0.57
A 0.86 B 0.71
A 0.86 B 0.86
A 0.86 B 1
A 1 B 0
A 1 B 0.14
A 1 B 0.29
A 1 B 0.43
A 1 B 0.57
A 1 B 0.71
A 1 B 0.86
A 1 B 1
OD 600 nm [–]
0.8
0.6
0.4
0.2
0 5 101520
Time [h]

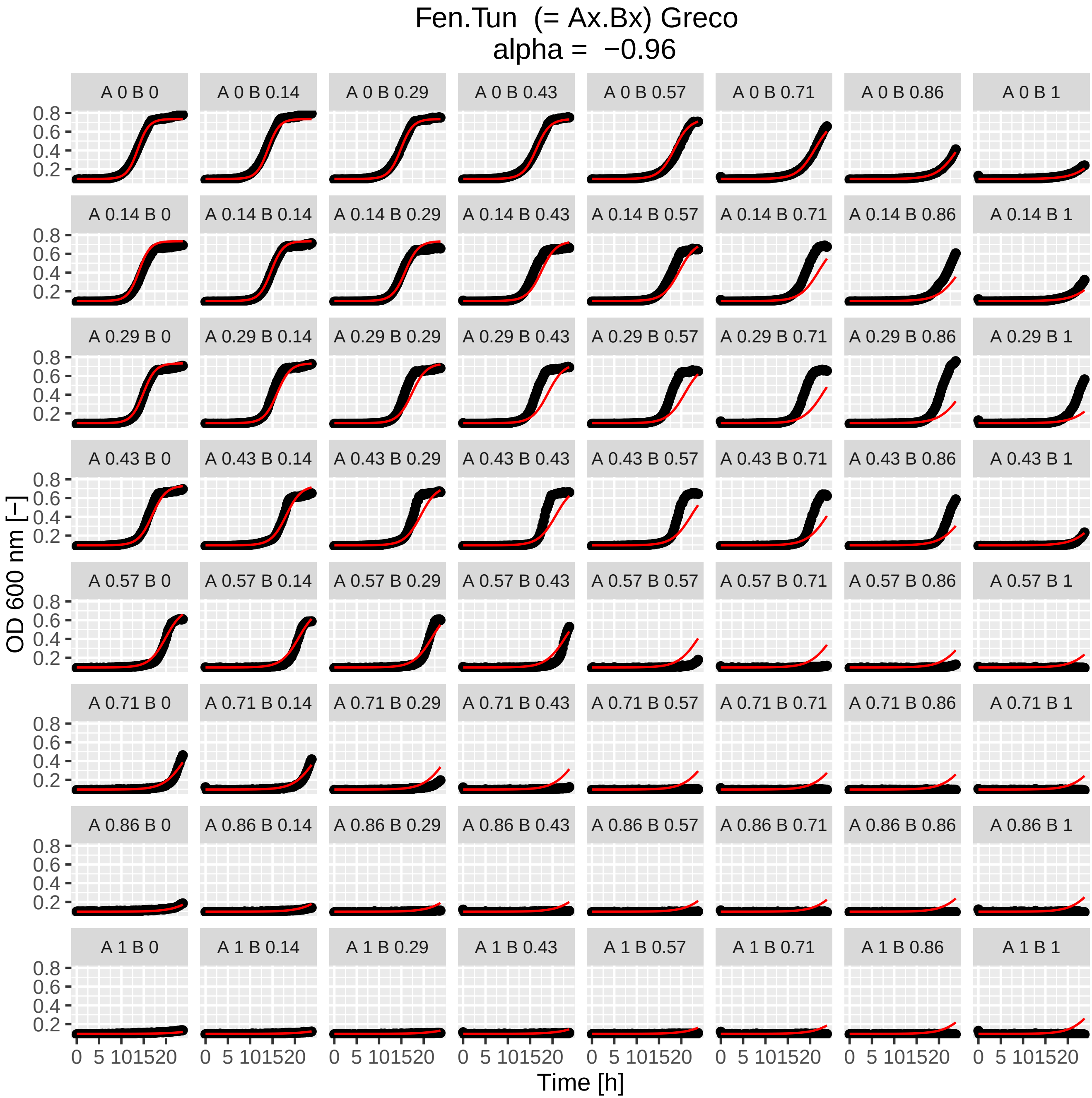
Fen.Tun (= Ax.Bx) Greco
alpha = −0.96
A 0 B 0
A 0 B 0.14
A 0 B 0.29
A 0 B 0.43
A 0 B 0.57
A 0 B 0.71
A 0 B 0.86
A 0 B 1
A 0.14 B 0
A 0.14 B 0.14
A 0.14 B 0.29
A 0.14 B 0.43
A 0.14 B 0.57
A 0.14 B 0.71
A 0.14 B 0.86
A 0.14 B 1
A 0.29 B 0
A 0.29 B 0.14
A 0.29 B 0.29
A 0.29 B 0.43
A 0.29 B 0.57
A 0.29 B 0.71
A 0.29 B 0.86
A 0.29 B 1
A 0.43 B 0
A 0.43 B 0.14
A 0.43 B 0.29
A 0.43 B 0.43
A 0.43 B 0.57
A 0.43 B 0.71
A 0.43 B 0.86
A 0.43 B 1
A 0.57 B 0
A 0.57 B 0.14
A 0.57 B 0.29
A 0.57 B 0.43
A 0.57 B 0.57
A 0.57 B 0.71
A 0.57 B 0.86
A 0.57 B 1
A 0.71 B 0
A 0.71 B 0.14
A 0.71 B 0.29
A 0.71 B 0.43
A 0.71 B 0.57
A 0.71 B 0.71
A 0.71 B 0.86
A 0.71 B 1
A 0.86 B 0
A 0.86 B 0.14
A 0.86 B 0.29
A 0.86 B 0.43
A 0.86 B 0.57
A 0.86 B 0.71
A 0.86 B 0.86
A 0.86 B 1
A 1 B 0
A 1 B 0.14
A 1 B 0.29
A 1 B 0.43
A 1 B 0.57
A 1 B 0.71
A 1 B 0.86
A 1 B 1
OD 600 nm [−]
0.8
0.6
0.4
0.2
0 5 101520
Time [h]

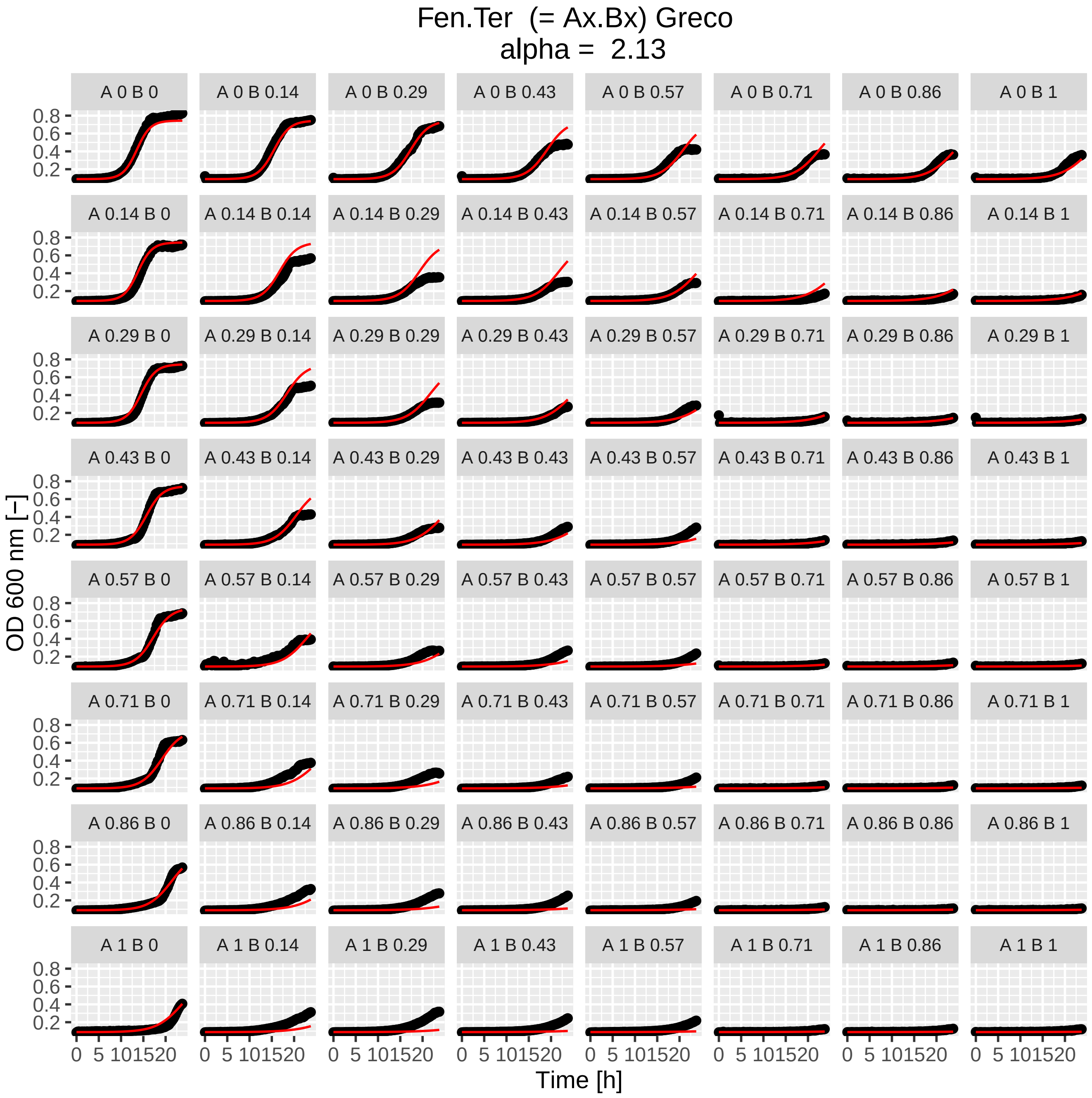
Fen.Ter (= Ax.Bx) Greco
alpha = 2.13
OD 600 nm [−]
Time [h]
0.8
0.6
0.4
0.2
0 5 101520
A 0 B 0
A 0 B 0.14
A 0 B 0.29
A 0 B 0.43
A 0 B 0.57
A 0 B 0.71
A 0 B 0.86
A 0 B 1
A 0.14 B 0
A 0.14 B 0.14
A 0.14 B 0.29
A 0.14 B 0.43
A 0.14 B 0.57
A 0.14 B 0.71
A 0.14 B 0.86
A 0.14 B 1
A 0.29 B 0
A 0.29 B 0.14
A 0.29 B 0.29
A 0.29 B 0.43
A 0.29 B 0.57
A 0.29 B 0.71
A 0.29 B 0.86
A 0.29 B 1
A 0.43 B 0
A 0.43 B 0.14
A 0.43 B 0.29
A 0.43 B 0.43
A 0.43 B 0.57
A 0.43 B 0.71
A 0.43 B 0.86
A 0.43 B 1
A 0.57 B 0
A 0.57 B 0.14
A 0.57 B 0.29
A 0.57 B 0.43
A 0.57 B 0.57
A 0.57 B 0.71
A 0.57 B 0.86
A 0.57 B 1
A 0.71 B 0
A 0.71 B 0.14
A 0.71 B 0.29
A 0.71 B 0.43
A 0.71 B 0.57
A 0.71 B 0.71
A 0.71 B 0.86
A 0.71 B 1
A 0.86 B 0
A 0.86 B 0.14
A 0.86 B 0.29
A 0.86 B 0.43
A 0.86 B 0.57
A 0.86 B 0.71
A 0.86 B 0.86
A 0.86 B 1
A 1 B 0
A 1 B 0.14
A 1 B 0.29
A 1 B 0.43
A 1 B 0.57
A 1 B 0.71
A 1 B 0.86
A 1 B 1

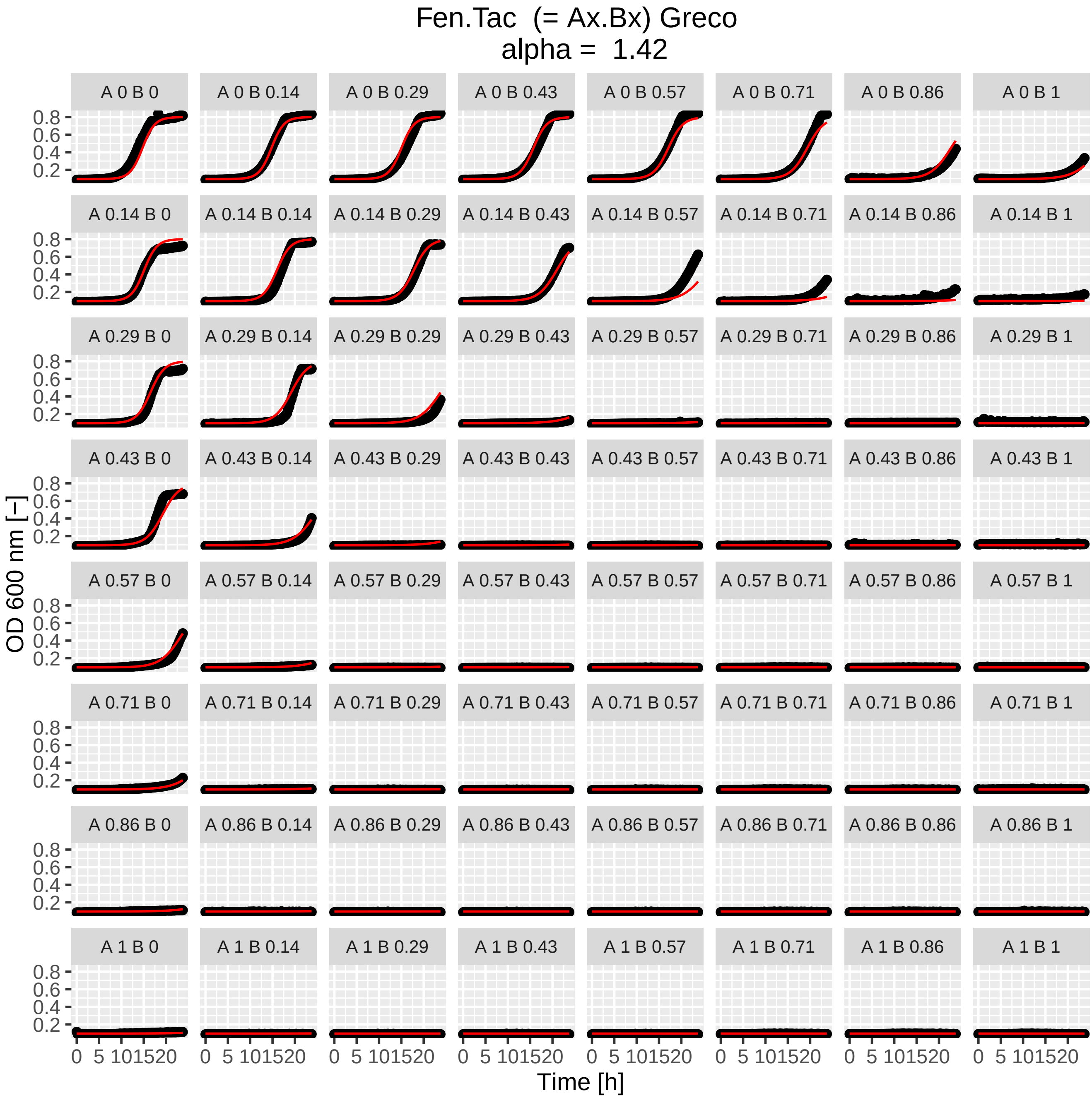
Fen.Tac (= Ax.Bx) Greco
alpha = 1.42
A 0 B 0
A 0 B 0.14
A 0 B 0.29
A 0 B 0.43
A 0 B 0.57
A 0 B 0.71
A 0 B 0.86
A 0 B 1
A 0.14 B 0
A 0.14 B 0.14
A 0.14 B 0.29
A 0.14 B 0.43
A 0.14 B 0.57
A 0.14 B 0.71
A 0.14 B 0.86
A 0.14 B 1
A 0.29 B 0
A 0.29 B 0.14
A 0.29 B 0.29
A 0.29 B 0.43
A 0.29 B 0.57
A 0.29 B 0.71
A 0.29 B 0.86
A 0.29 B 1
A 0.43 B 0
A 0.43 B 0.14
A 0.43 B 0.29
A 0.43 B 0.43
A 0.43 B 0.57
A 0.43 B 0.71
A 0.43 B 0.86
A 0.43 B 1
A 0.57 B 0
A 0.57 B 0.14
A 0.57 B 0.29
A 0.57 B 0.43
A 0.57 B 0.57
A 0.57 B 0.71
A 0.57 B 0.86
A 0.57 B 1
A 0.71 B 0
A 0.71 B 0.14
A 0.71 B 0.29
A 0.71 B 0.43
A 0.71 B 0.57
A 0.71 B 0.71
A 0.71 B 0.86
A 0.71 B 1
A 0.86 B 0
A 0.86 B 0.14
A 0.86 B 0.29
A 0.86 B 0.43
A 0.86 B 0.57
A 0.86 B 0.71
A 0.86 B 0.86
A 0.86 B 1
A 1 B 0
A 1 B 0.14
A 1 B 0.29
A 1 B 0.43
A 1 B 0.57
A 1 B 0.71
A 1 B 0.86
A 1 B 1
OD 600 nm [–]
Time [h]

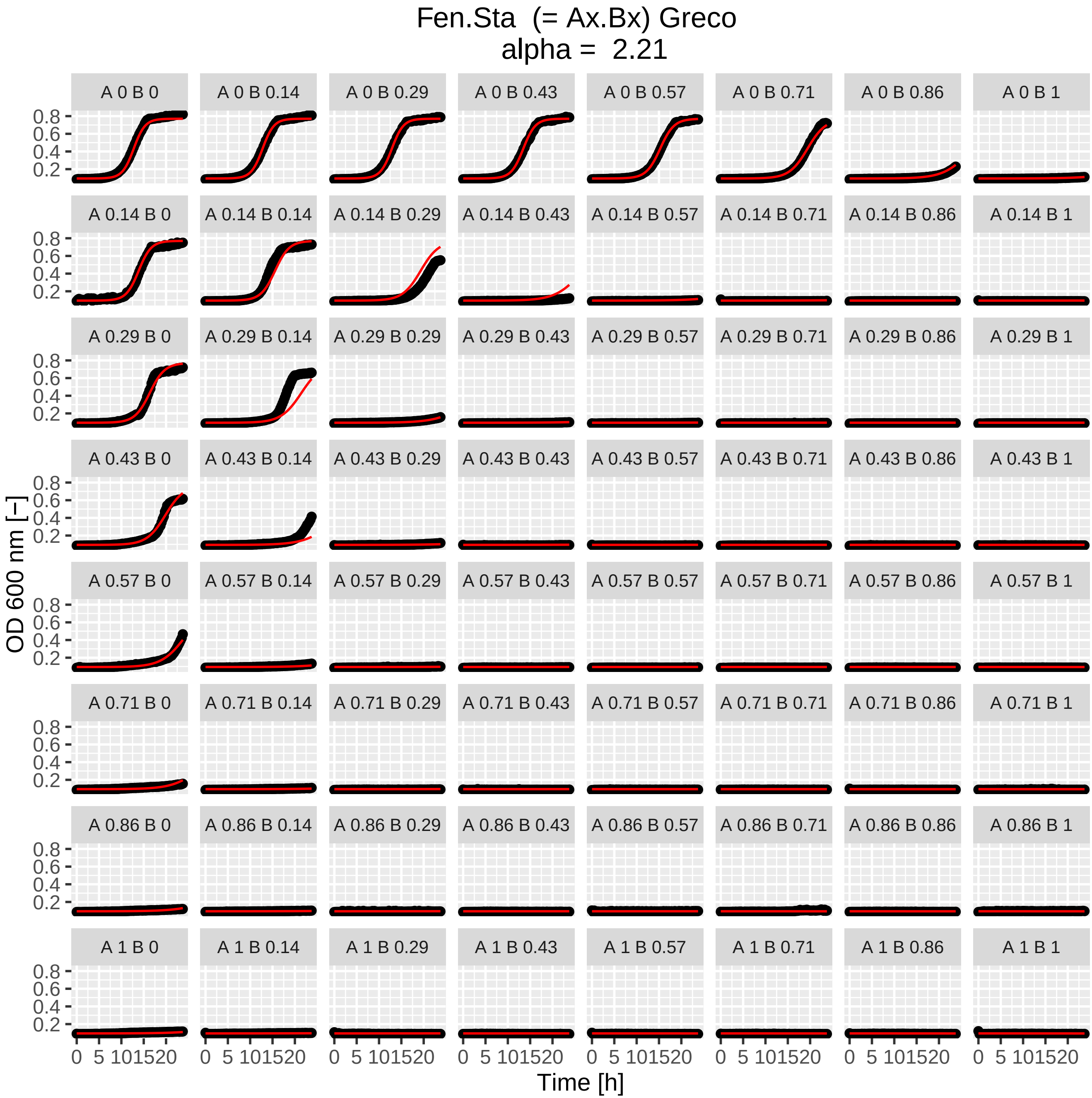

Fen.Sta (= Ax.Bx) Greco
alpha = 2.21
A 0 B 0
A 0 B 0.14
A 0 B 0.29
A 0 B 0.43
A 0 B 0.57
A 0 B 0.71
A 0 B 0.86
A 0 B 1
A 0.14 B 0
A 0.14 B 0.14
A 0.14 B 0.29
A 0.14 B 0.43
A 0.14 B 0.57
A 0.14 B 0.71
A 0.14 B 0.86
A 0.14 B 1
A 0.29 B 0
A 0.29 B 0.14
A 0.29 B 0.29
A 0.29 B 0.43
A 0.29 B 0.57
A 0.29 B 0.71
A 0.29 B 0.86
A 0.29 B 1
A 0.43 B 0
A 0.43 B 0.14
A 0.43 B 0.29
A 0.43 B 0.43
A 0.43 B 0.57
A 0.43 B 0.71
A 0.43 B 0.86
A 0.43 B 1
A 0.57 B 0
A 0.57 B 0.14
A 0.57 B 0.29
A 0.57 B 0.43
A 0.57 B 0.57
A 0.57 B 0.71
A 0.57 B 0.86
A 0.57 B 1
A 0.71 B 0
A 0.71 B 0.14
A 0.71 B 0.29
A 0.71 B 0.43
A 0.71 B 0.57
A 0.71 B 0.71
A 0.71 B 0.86
A 0.71 B 1
A 0.86 B 0
A 0.86 B 0.14
A 0.86 B 0.29
A 0.86 B 0.43
A 0.86 B 0.57
A 0.86 B 0.71
A 0.86 B 0.86
A 0.86 B 1
A 1 B 0
A 1 B 0.14
A 1 B 0.29
A 1 B 0.43
A 1 B 0.57
A 1 B 0.71
A 1 B 0.86
A 1 B 1
OD 600 nm [–]
0.8
0.6
0.4
0.2
0 5 101520
Time [h]

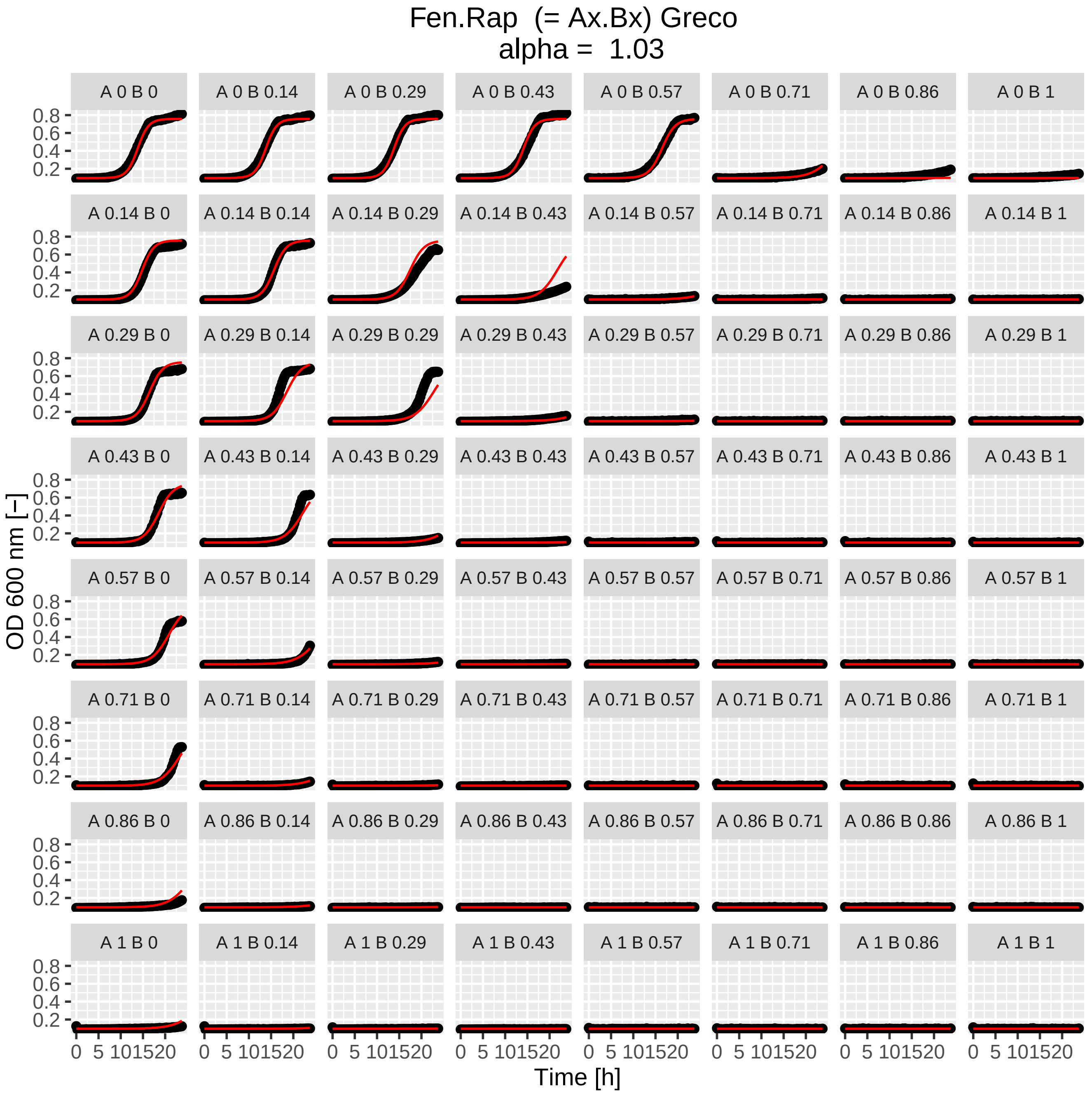
Fen.Rap (= Ax.Bx) Greco
alpha = 1.03
A 0 B 0
A 0 B 0.14
A 0 B 0.29
A 0 B 0.43
A 0 B 0.57
A 0 B 0.71
A 0 B 0.86
A 0 B 1
A 0.14 B 0
A 0.14 B 0.14
A 0.14 B 0.29
A 0.14 B 0.43
A 0.14 B 0.57
A 0.14 B 0.71
A 0.14 B 0.86
A 0.14 B 1
A 0.29 B 0
A 0.29 B 0.14
A 0.29 B 0.29
A 0.29 B 0.43
A 0.29 B 0.57
A 0.29 B 0.71
A 0.29 B 0.86
A 0.29 B 1
A 0.43 B 0
A 0.43 B 0.14
A 0.43 B 0.29
A 0.43 B 0.43
A 0.43 B 0.57
A 0.43 B 0.71
A 0.43 B 0.86
A 0.43 B 1
A 0.57 B 0
A 0.57 B 0.14
A 0.57 B 0.29
A 0.57 B 0.43
A 0.57 B 0.57
A 0.57 B 0.71
A 0.57 B 0.86
A 0.57 B 1
A 0.71 B 0
A 0.71 B 0.14
A 0.71 B 0.29
A 0.71 B 0.43
A 0.71 B 0.57
A 0.71 B 0.71
A 0.71 B 0.86
A 0.71 B 1
A 0.86 B 0
A 0.86 B 0.14
A 0.86 B 0.29
A 0.86 B 0.43
A 0.86 B 0.57
A 0.86 B 0.71
A 0.86 B 0.86
A 0.86 B 1
A 1 B 0
A 1 B 0.14
A 1 B 0.29
A 1 B 0.43
A 1 B 0.57
A 1 B 0.71
A 1 B 0.86
A 1 B 1
OD 600 nm [–]
0.2
0.4
0.6
0.8
0 5 101520
Time [h]

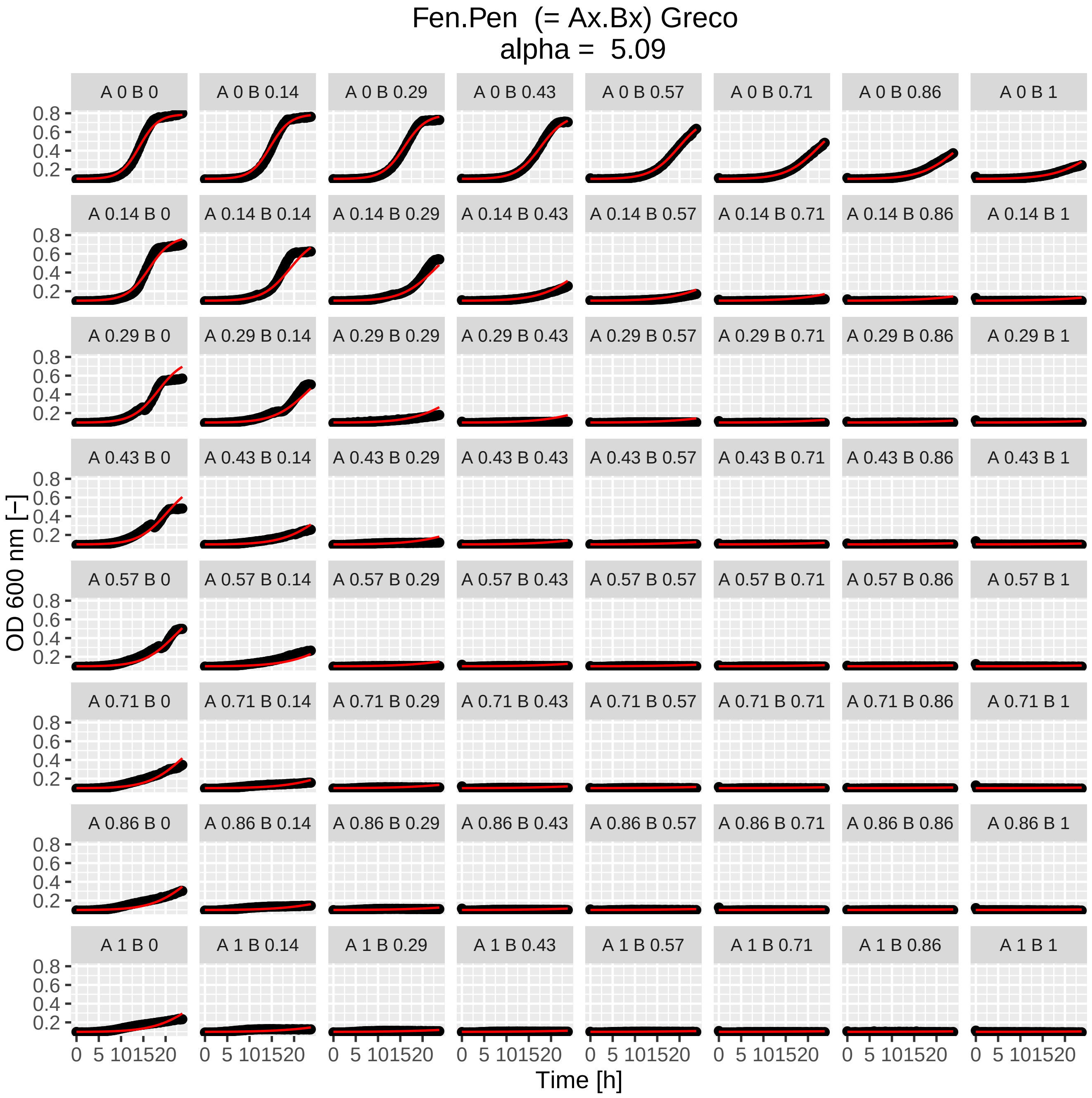

Fen.Pen (= Ax.Bx) Greco
alpha = 5.09
A 0 B 0
A 0 B 0.14
A 0 B 0.29
A 0 B 0.43
A 0 B 0.57
A 0 B 0.71
A 0 B 0.86
A 0 B 1
A 0.14 B 0
A 0.14 B 0.14
A 0.14 B 0.29
A 0.14 B 0.43
A 0.14 B 0.57
A 0.14 B 0.71
A 0.14 B 0.86
A 0.14 B 1
A 0.29 B 0
A 0.29 B 0.14
A 0.29 B 0.29
A 0.29 B 0.43
A 0.29 B 0.57
A 0.29 B 0.71
A 0.29 B 0.86
A 0.29 B 1
A 0.43 B 0
A 0.43 B 0.14
A 0.43 B 0.29
A 0.43 B 0.43
A 0.43 B 0.57
A 0.43 B 0.71
A 0.43 B 0.86
A 0.43 B 1
A 0.57 B 0
A 0.57 B 0.14
A 0.57 B 0.29
A 0.57 B 0.43
A 0.57 B 0.57
A 0.57 B 0.71
A 0.57 B 0.86
A 0.57 B 1
A 0.71 B 0
A 0.71 B 0.14
A 0.71 B 0.29
A 0.71 B 0.43
A 0.71 B 0.57
A 0.71 B 0.71
A 0.71 B 0.86
A 0.71 B 1
A 0.86 B 0
A 0.86 B 0.14
A 0.86 B 0.29
A 0.86 B 0.43
A 0.86 B 0.57
A 0.86 B 0.71
A 0.86 B 0.86
A 0.86 B 1
A 1 B 0
A 1 B 0.14
A 1 B 0.29
A 1 B 0.43
A 1 B 0.57
A 1 B 0.71
A 1 B 0.86
A 1 B 1
OD 600 nm [−]
0.8
0.6
0.4
0.2
0 5 101520
Time [h]

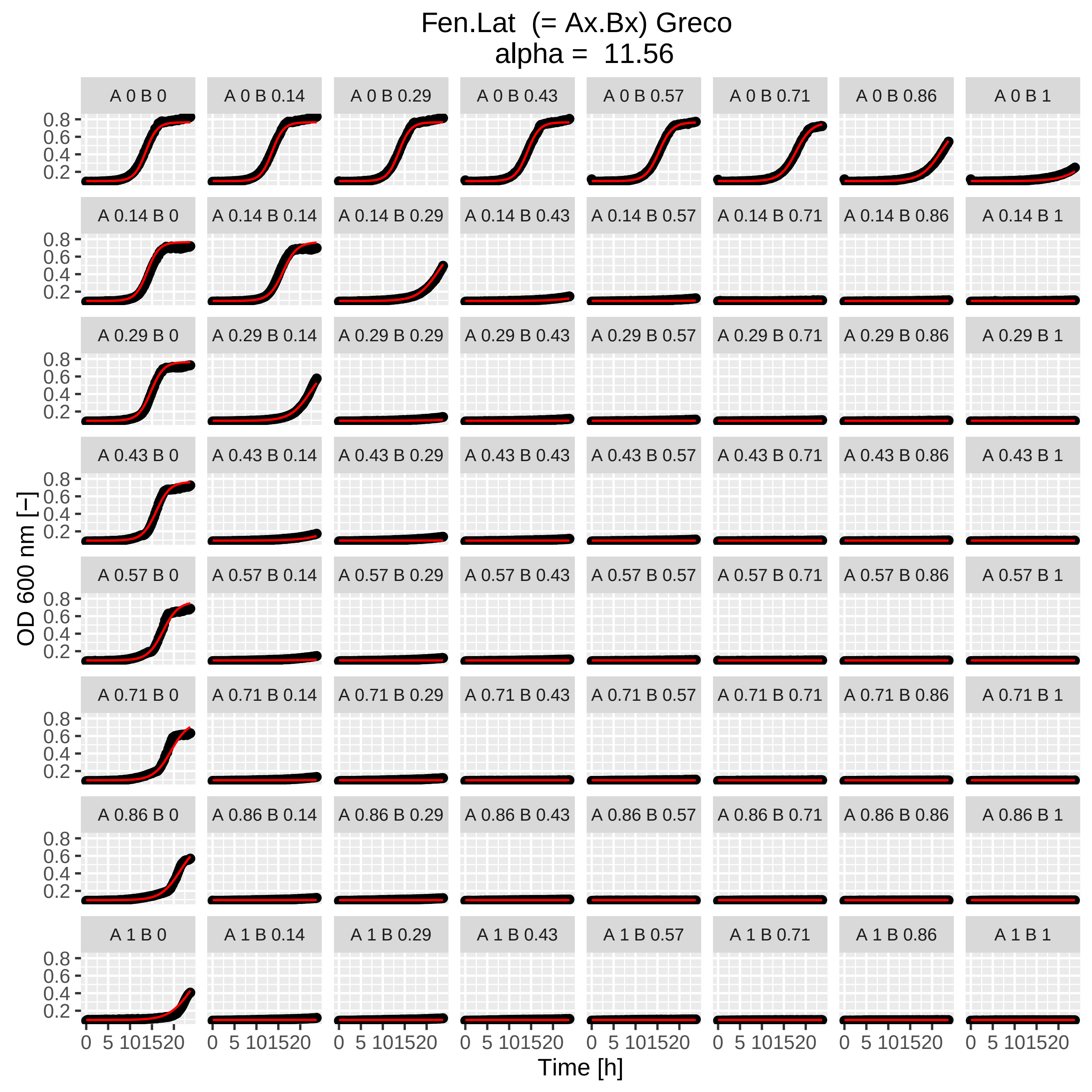
Fen.Lat (= Ax.Bx) Greco
alpha = 11.56
OD 600 nm [–]
Time [h]
A 0 B 0
A 0 B 0.14
A 0 B 0.29
A 0 B 0.43
A 0 B 0.57
A 0 B 0.71
A 0 B 0.86
A 0 B 1
A 1 B 0
A 1 B 1
0.2
0.4
0.6
0.8
0 5 101520

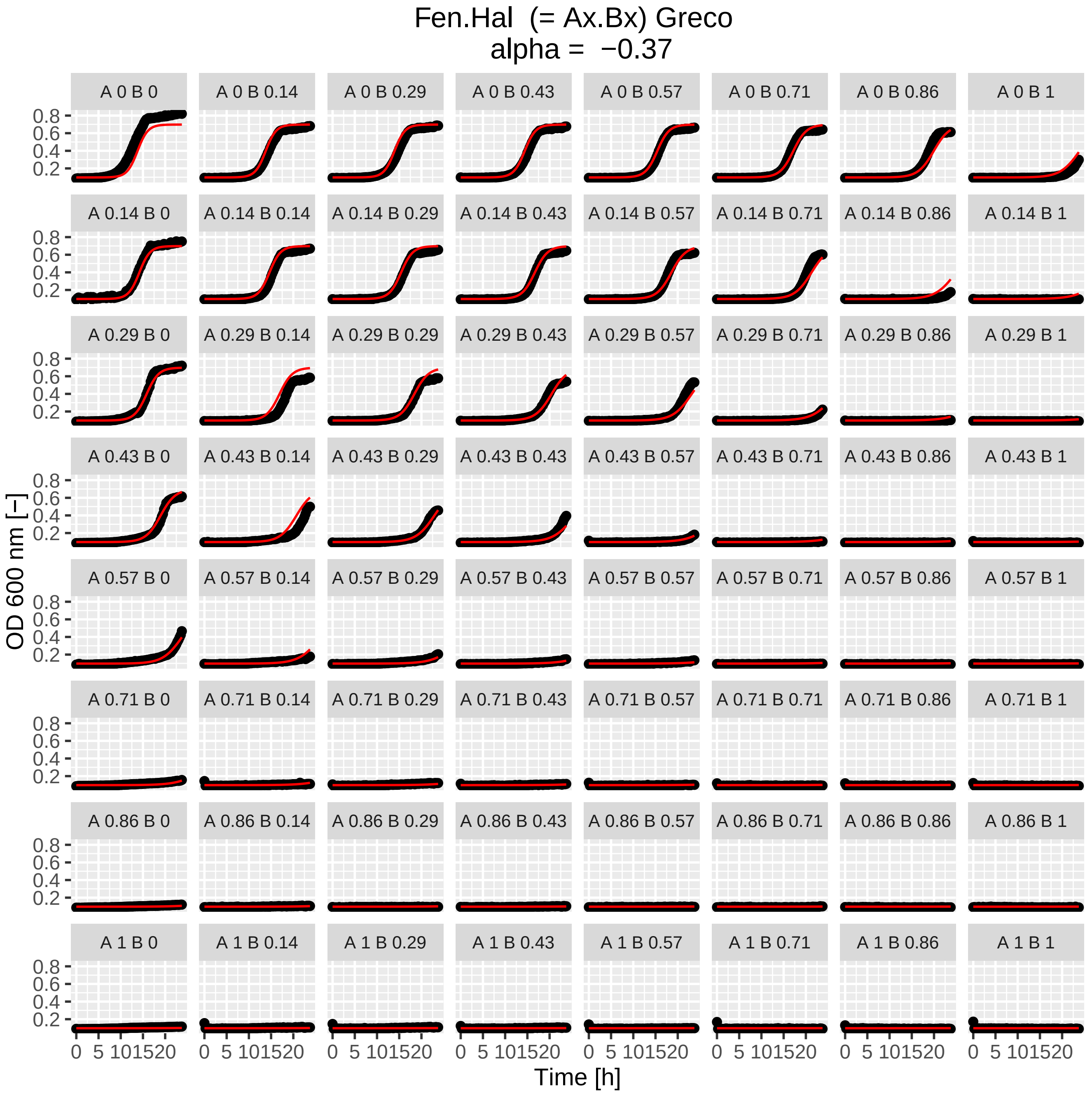

Fen.Hal (= Ax.Bx) Greco
alpha = −0.37
A 0 B 0
A 0 B 0.14
A 0 B 0.29
A 0 B 0.43
A 0 B 0.57
A 0 B 0.71
A 0 B 0.86
A 0 B 1
A 0.14 B 0
A 0.14 B 0.14
A 0.14 B 0.29
A 0.14 B 0.43
A 0.14 B 0.57
A 0.14 B 0.71
A 0.14 B 0.86
A 0.14 B 1
A 0.29 B 0
A 0.29 B 0.14
A 0.29 B 0.29
A 0.29 B 0.43
A 0.29 B 0.57
A 0.29 B 0.71
A 0.29 B 0.86
A 0.29 B 1
A 0.43 B 0
A 0.43 B 0.14
A 0.43 B 0.29
A 0.43 B 0.43
A 0.43 B 0.57
A 0.43 B 0.71
A 0.43 B 0.86
A 0.43 B 1
A 0.57 B 0
A 0.57 B 0.14
A 0.57 B 0.29
A 0.57 B 0.43
A 0.57 B 0.57
A 0.57 B 0.71
A 0.57 B 0.86
A 0.57 B 1
A 0.71 B 0
A 0.71 B 0.14
A 0.71 B 0.29
A 0.71 B 0.43
A 0.71 B 0.57
A 0.71 B 0.71
A 0.71 B 0.86
A 0.71 B 1
A 0.86 B 0
A 0.86 B 0.14
A 0.86 B 0.29
A 0.86 B 0.43
A 0.86 B 0.57
A 0.86 B 0.71
A 0.86 B 0.86
A 0.86 B 1
A 1 B 0
A 1 B 0.14
A 1 B 0.29
A 1 B 0.43
A 1 B 0.57
A 1 B 0.71
A 1 B 0.86
A 1 B 1
OD 600 nm [−]
0.2
0.4
0.6
0.8
0 5 101520
Time [h]

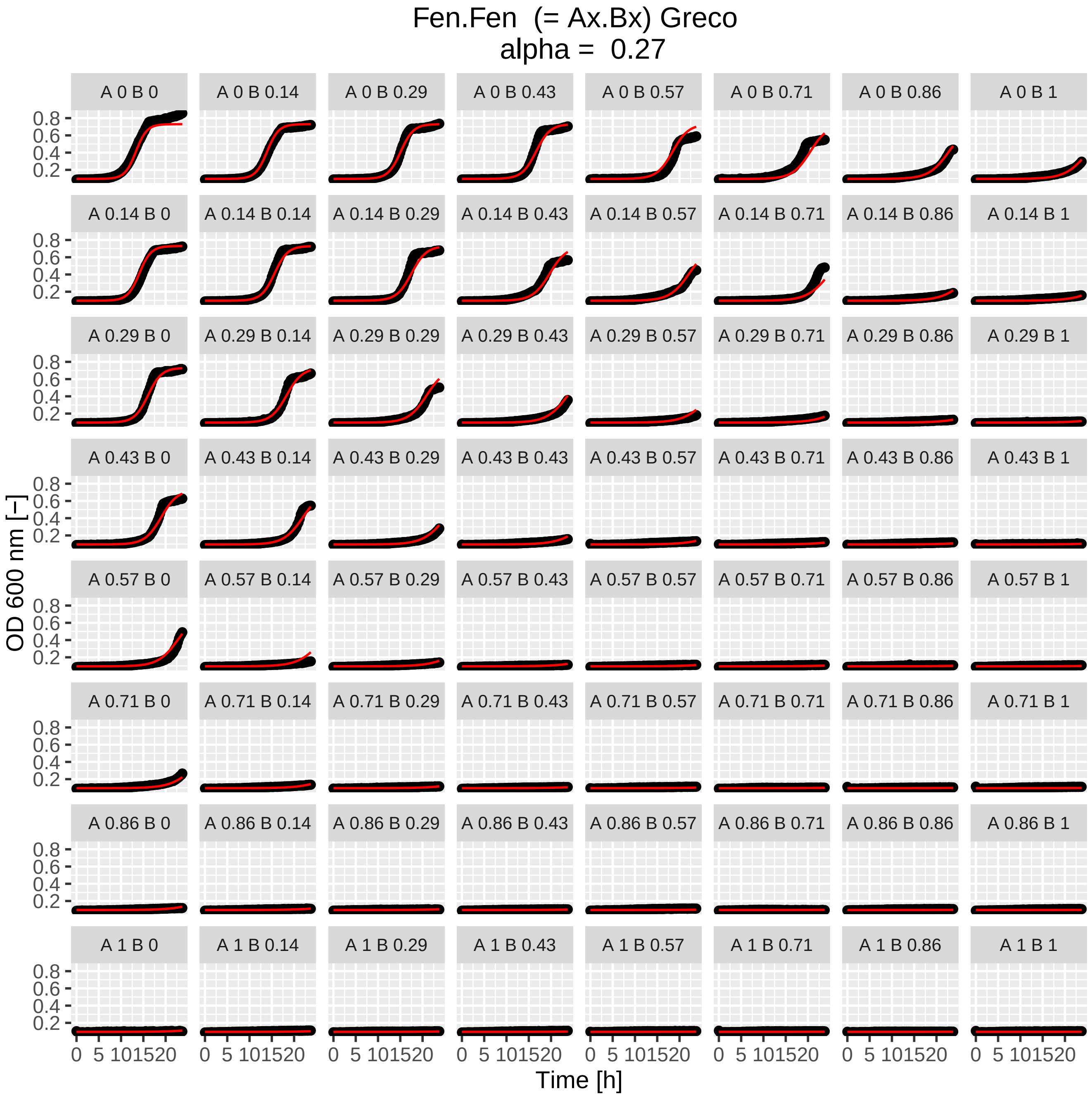

Fen.Fen (= Ax.Bx) Greco
alpha = 0.27
A 0 B 0
A 0 B 0.14
A 0 B 0.29
A 0 B 0.43
A 0 B 0.57
A 0 B 0.71
A 0 B 0.86
A 0 B 1
A 0.14 B 0
A 0.14 B 0.14
A 0.14 B 0.29
A 0.14 B 0.43
A 0.14 B 0.57
A 0.14 B 0.71
A 0.14 B 0.86
A 0.14 B 1
A 0.29 B 0
A 0.29 B 0.14
A 0.29 B 0.29
A 0.29 B 0.43
A 0.29 B 0.57
A 0.29 B 0.71
A 0.29 B 0.86
A 0.29 B 1
A 0.43 B 0
A 0.43 B 0.14
A 0.43 B 0.29
A 0.43 B 0.43
A 0.43 B 0.57
A 0.43 B 0.71
A 0.43 B 0.86
A 0.43 B 1
A 0.57 B 0
A 0.57 B 0.14
A 0.57 B 0.29
A 0.57 B 0.43
A 0.57 B 0.57
A 0.57 B 0.71
A 0.57 B 0.86
A 0.57 B 1
A 0.71 B 0
A 0.71 B 0.14
A 0.71 B 0.29
A 0.71 B 0.43
A 0.71 B 0.57
A 0.71 B 0.71
A 0.71 B 0.86
A 0.71 B 1
A 0.86 B 0
A 0.86 B 0.14
A 0.86 B 0.29
A 0.86 B 0.43
A 0.86 B 0.57
A 0.86 B 0.71
A 0.86 B 0.86
A 0.86 B 1
A 1 B 0
A 1 B 0.14
A 1 B 0.29
A 1 B 0.43
A 1 B 0.57
A 1 B 0.71
A 1 B 0.86
A 1 B 1
OD 600 nm [−]
0.2
0.4
0.6
0.8
0 5 101520
Time [h]

Dyc.Tun (= Ax.Bx) Greco
alpha = −0.71

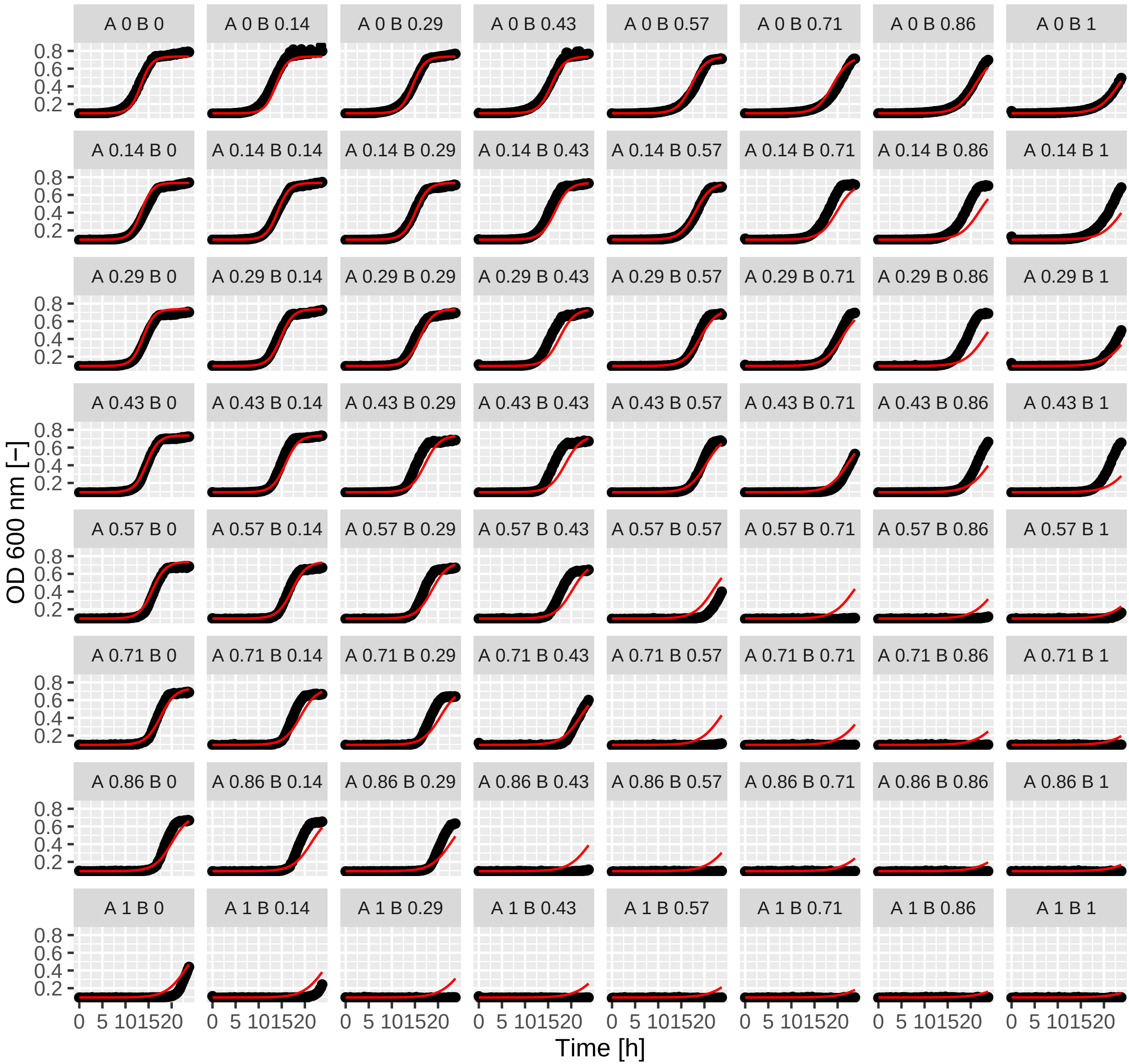

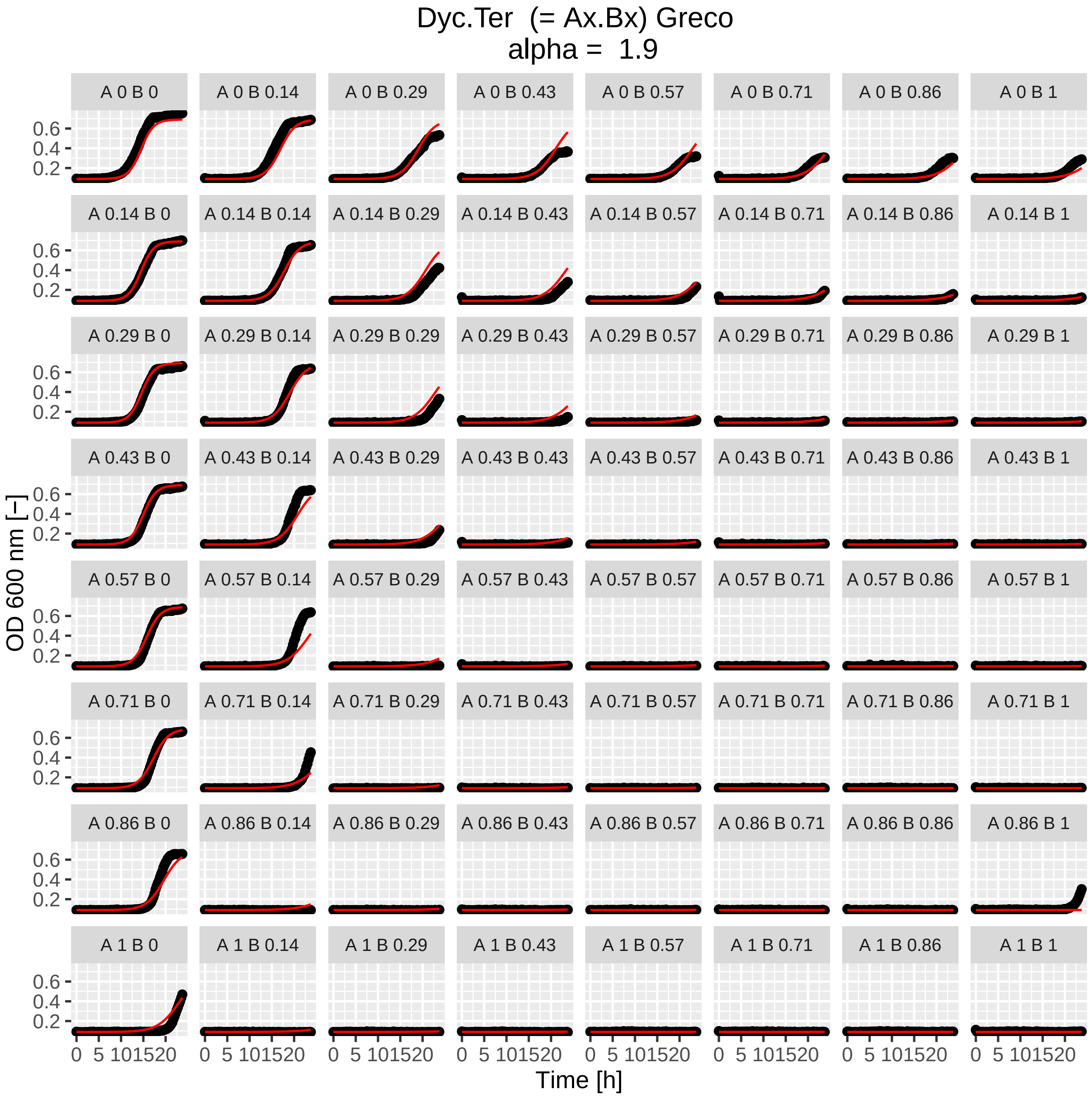

Dyc.Ter (= Ax.Bx) Greco
alpha = 1.9
A 0 B 0
A 0 B 0.14
A 0 B 0.29
A 0 B 0.43
A 0 B 0.57
A 0 B 0.71
A 0 B 0.86
A 0 B 1
A 0.14 B 0
A 0.14 B 0.14
A 0.14 B 0.29
A 0.14 B 0.43
A 0.14 B 0.57
A 0.14 B 0.71
A 0.14 B 0.86
A 0.14 B 1
A 0.29 B 0
A 0.29 B 0.14
A 0.29 B 0.29
A 0.29 B 0.43
A 0.29 B 0.57
A 0.29 B 0.71
A 0.29 B 0.86
A 0.29 B 1
A 0.43 B 0
A 0.43 B 0.14
A 0.43 B 0.29
A 0.43 B 0.43
A 0.43 B 0.57
A 0.43 B 0.71
A 0.43 B 0.86
A 0.43 B 1
A 0.57 B 0
A 0.57 B 0.14
A 0.57 B 0.29
A 0.57 B 0.43
A 0.57 B 0.57
A 0.57 B 0.71
A 0.57 B 0.86
A 0.57 B 1
A 0.71 B 0
A 0.71 B 0.14
A 0.71 B 0.29
A 0.71 B 0.43
A 0.71 B 0.57
A 0.71 B 0.71
A 0.71 B 0.86
A 0.71 B 1
A 0.86 B 0
A 0.86 B 0.14
A 0.86 B 0.29
A 0.86 B 0.43
A 0.86 B 0.57
A 0.86 B 0.71
A 0.86 B 0.86
A 0.86 B 1
A 1 B 0
A 1 B 0.14
A 1 B 0.29
A 1 B 0.43
A 1 B 0.57
A 1 B 0.71
A 1 B 0.86
A 1 B 1
OD 600 nm [–]
0.2
0.4
0.6
0 5 101520
Time [h]

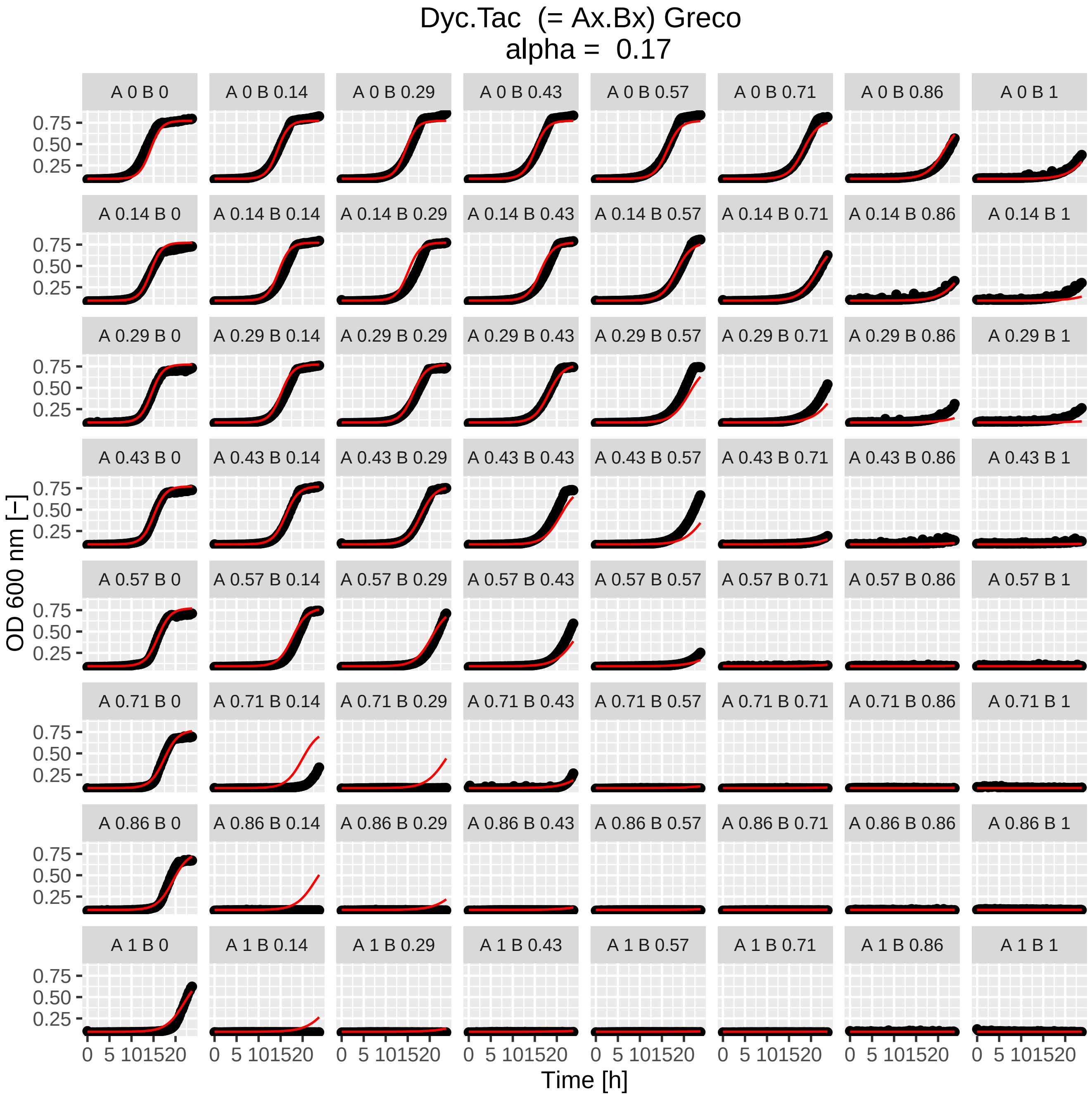
Dyc.Tac (= Ax.Bx) Greco
alpha = 0.17
OD 600 nm [–]
Time [h]
0 5 101520
0.75
0.50
0.25
A 0 B 0
A 0 B 0.14
A 0 B 0.29
A 0 B 0.43
A 0 B 0.57
A 0 B 0.71
A 0 B 0.86
A 0 B 1
A 0.14 B 0
A 0.14 B 0.14
A 0.14 B 0.29
A 0.14 B 0.43
A 0.14 B 0.57
A 0.14 B 0.71
A 0.14 B 0.86
A 0.14 B 1
A 0.29 B 0
A 0.29 B 0.14
A 0.29 B 0.29
A 0.29 B 0.43
A 0.29 B 0.57
A 0.29 B 0.71
A 0.29 B 0.86
A 0.29 B 1
A 0.43 B 0
A 0.43 B 0.14
A 0.43 B 0.29
A 0.43 B 0.43
A 0.43 B 0.57
A 0.43 B 0.71
A 0.43 B 0.86
A 0.43 B 1
A 0.57 B 0
A 0.57 B 0.14
A 0.57 B 0.29
A 0.57 B 0.43
A 0.57 B 0.57
A 0.57 B 0.71
A 0.57 B 0.86
A 0.57 B 1
A 0.71 B 0
A 0.71 B 0.14
A 0.71 B 0.29
A 0.71 B 0.43
A 0.71 B 0.57
A 0.71 B 0.71
A 0.71 B 0.86
A 0.71 B 1
A 0.86 B 0
A 0.86 B 0.14
A 0.86 B 0.29
A 0.86 B 0.43
A 0.86 B 0.57
A 0.86 B 0.71
A 0.86 B 0.86
A 0.86 B 1
A 1 B 0
A 1 B 0.14
A 1 B 0.29
A 1 B 0.43
A 1 B 0.57
A 1 B 0.71
A 1 B 0.86
A 1 B 1

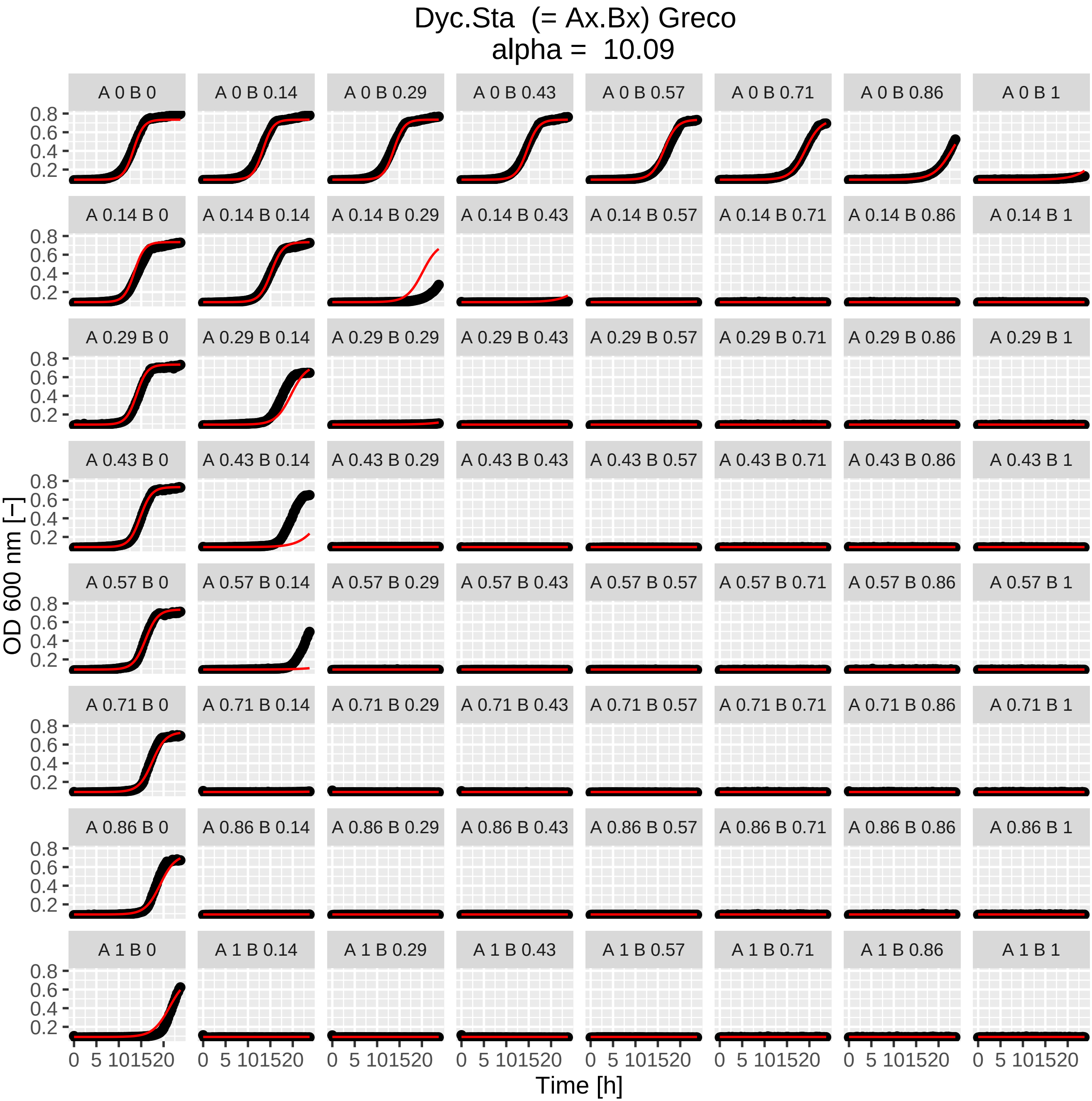
Dyc.Sta (= Ax.Bx) Greco
alpha = 10.09
A 0 B 0
A 0 B 0.14
A 0 B 0.29
A 0 B 0.43
A 0 B 0.57
A 0 B 0.71
A 0 B 0.86
A 0 B 1
A 0.14 B 0
A 0.14 B 0.14
A 0.14 B 0.29
A 0.14 B 0.43
A 0.14 B 0.57
A 0.14 B 0.71
A 0.14 B 0.86
A 0.14 B 1
A 0.29 B 0
A 0.29 B 0.14
A 0.29 B 0.29
A 0.29 B 0.43
A 0.29 B 0.57
A 0.29 B 0.71
A 0.29 B 0.86
A 0.29 B 1
A 0.43 B 0
A 0.43 B 0.14
A 0.43 B 0.29
A 0.43 B 0.43
A 0.43 B 0.57
A 0.43 B 0.71
A 0.43 B 0.86
A 0.43 B 1
A 0.57 B 0
A 0.57 B 0.14
A 0.57 B 0.29
A 0.57 B 0.43
A 0.57 B 0.57
A 0.57 B 0.71
A 0.57 B 0.86
A 0.57 B 1
A 0.71 B 0
A 0.71 B 0.14
A 0.71 B 0.29
A 0.71 B 0.43
A 0.71 B 0.57
A 0.71 B 0.71
A 0.71 B 0.86
A 0.71 B 1
A 0.86 B 0
A 0.86 B 0.14
A 0.86 B 0.29
A 0.86 B 0.43
A 0.86 B 0.57
A 0.86 B 0.71
A 0.86 B 0.86
A 0.86 B 1
A 1 B 0
A 1 B 0.14
A 1 B 0.29
A 1 B 0.43
A 1 B 0.57
A 1 B 0.71
A 1 B 0.86
A 1 B 1
OD 600 nm [–]
0.2
0.4
0.6
0.8
0
5
10
15
20
Time [h]

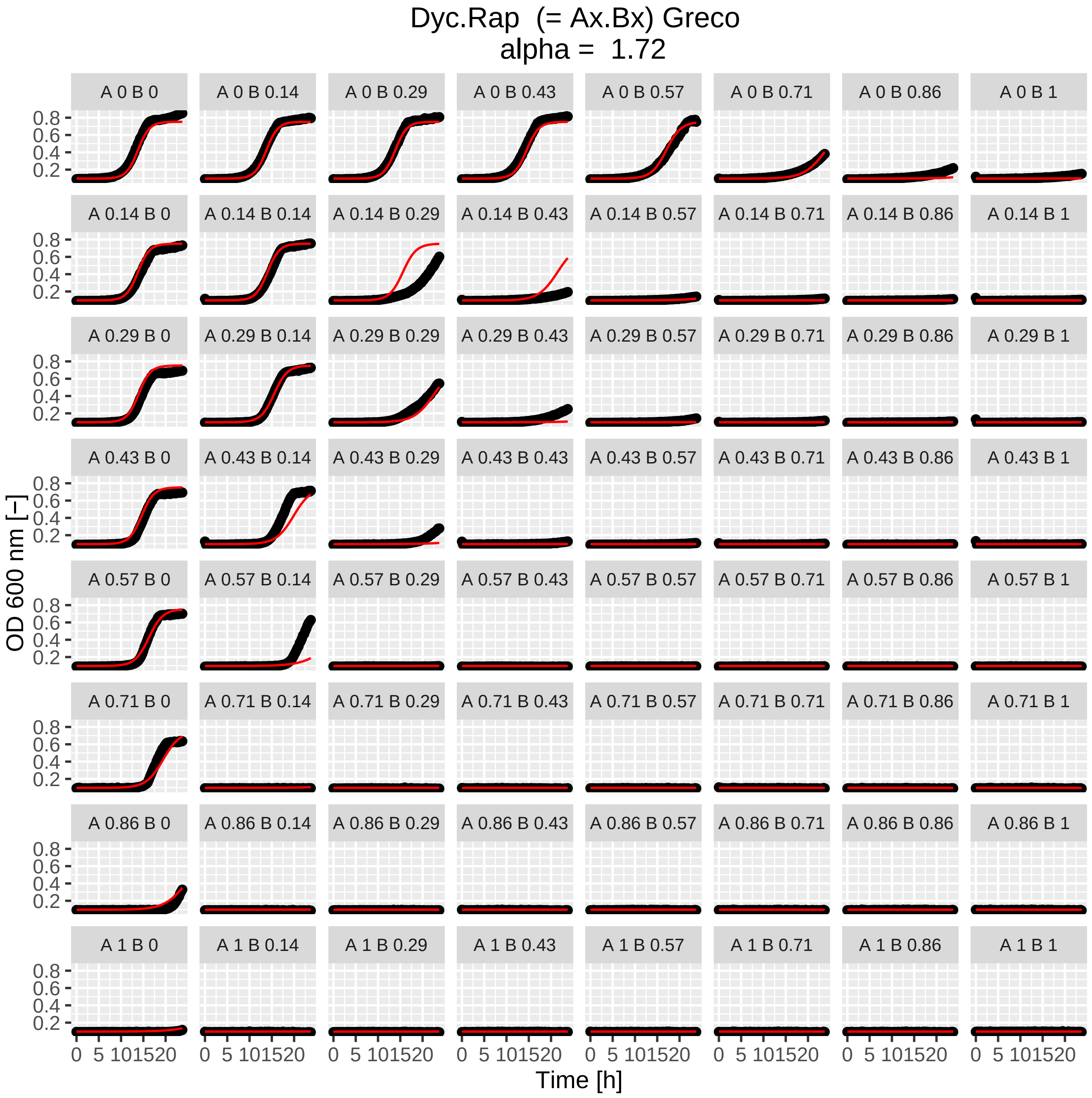
Dyc.Rap (= Ax.Bx) Greco
alpha = 1.72
OD 600 nm [−]
Time [h]
0.8
0.6
0.4
0.2
0 5 101520
A 0 B 0
A 0 B 0.14
A 0 B 0.29
A 0 B 0.43
A 0 B 0.57
A 0 B 0.71
A 0 B 0.86
A 0 B 1
A 0.14 B 0
A 0.14 B 0.14
A 0.14 B 0.29
A 0.14 B 0.43
A 0.14 B 0.57
A 0.14 B 0.71
A 0.14 B 0.86
A 0.14 B 1
A 0.29 B 0
A 0.29 B 0.14
A 0.29 B 0.29
A 0.29 B 0.43
A 0.29 B 0.57
A 0.29 B 0.71
A 0.29 B 0.86
A 0.29 B 1
A 0.43 B 0
A 0.43 B 0.14
A 0.43 B 0.29
A 0.43 B 0.43
A 0.43 B 0.57
A 0.43 B 0.71
A 0.43 B 0.86
A 0.43 B 1
A 0.57 B 0
A 0.57 B 0.14
A 0.57 B 0.29
A 0.57 B 0.43
A 0.57 B 0.57
A 0.57 B 0.71
A 0.57 B 0.86
A 0.57 B 1
A 0.71 B 0
A 0.71 B 0.14
A 0.71 B 0.29
A 0.71 B 0.43
A 0.71 B 0.57
A 0.71 B 0.71
A 0.71 B 0.86
A 0.71 B 1
A 0.86 B 0
A 0.86 B 0.14
A 0.86 B 0.29
A 0.86 B 0.43
A 0.86 B 0.57
A 0.86 B 0.71
A 0.86 B 0.86
A 0.86 B 1
A 1 B 0
A 1 B 0.14
A 1 B 0.29
A 1 B 0.43
A 1 B 0.57
A 1 B 0.71
A 1 B 0.86
A 1 B 1

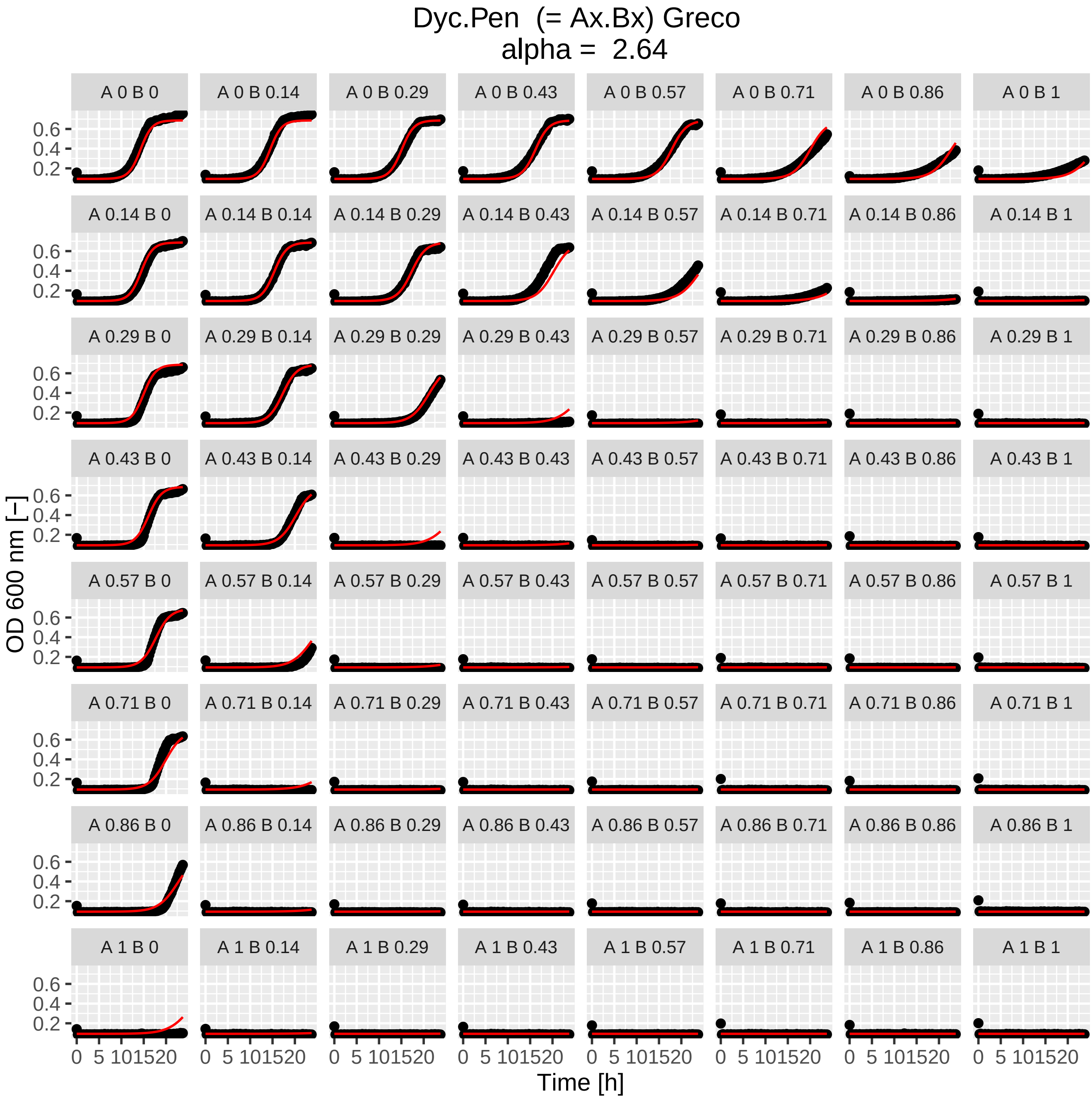

Dyc.Pen (= Ax.Bx) Greco
alpha = 2.64
A 0 B 0
A 0 B 0.14
A 0 B 0.29
A 0 B 0.43
A 0 B 0.57
A 0 B 0.71
A 0 B 0.86
A 0 B 1
A 0.14 B 0
A 0.14 B 0.14
A 0.14 B 0.29
A 0.14 B 0.43
A 0.14 B 0.57
A 0.14 B 0.71
A 0.14 B 0.86
A 0.14 B 1
A 0.29 B 0
A 0.29 B 0.14
A 0.29 B 0.29
A 0.29 B 0.43
A 0.29 B 0.57
A 0.29 B 0.71
A 0.29 B 0.86
A 0.29 B 1
A 0.43 B 0
A 0.43 B 0.14
A 0.43 B 0.29
A 0.43 B 0.43
A 0.43 B 0.57
A 0.43 B 0.71
A 0.43 B 0.86
A 0.43 B 1
A 0.57 B 0
A 0.57 B 0.14
A 0.57 B 0.29
A 0.57 B 0.43
A 0.57 B 0.57
A 0.57 B 0.71
A 0.57 B 0.86
A 0.57 B 1
A 0.71 B 0
A 0.71 B 0.14
A 0.71 B 0.29
A 0.71 B 0.43
A 0.71 B 0.57
A 0.71 B 0.71
A 0.71 B 0.86
A 0.71 B 1
A 0.86 B 0
A 0.86 B 0.14
A 0.86 B 0.29
A 0.86 B 0.43
A 0.86 B 0.57
A 0.86 B 0.71
A 0.86 B 0.86
A 0.86 B 1
A 1 B 0
A 1 B 0.14
A 1 B 0.29
A 1 B 0.43
A 1 B 0.57
A 1 B 0.71
A 1 B 0.86
A 1 B 1
OD 600 nm [–]
0.2
0.4
0.6
0 5 101520
Time [h]

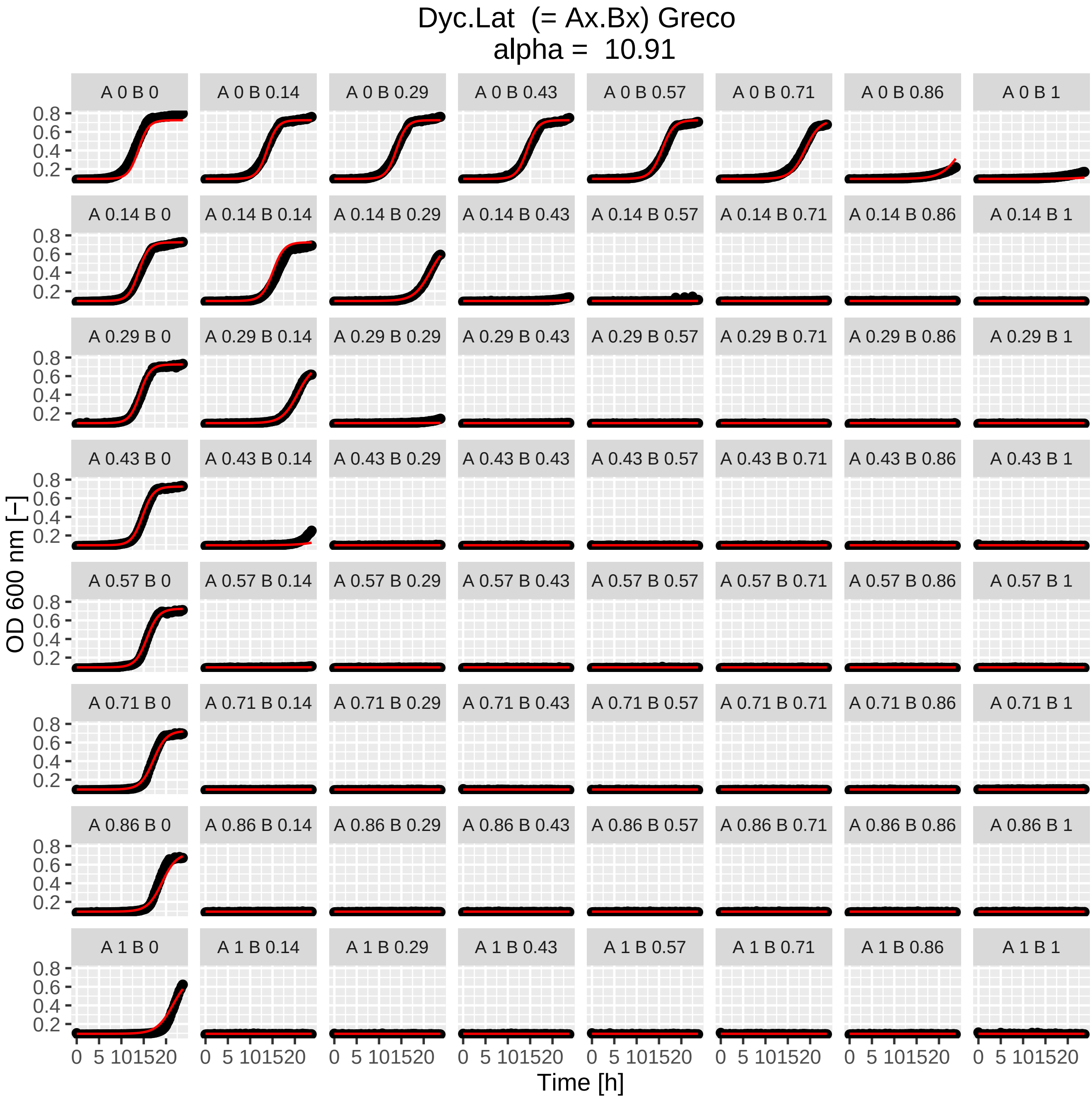

Dyc.Lat (= Ax.Bx) Greco
alpha = 10.91
A 0 B 0
A 0 B 0.14
A 0 B 0.29
A 0 B 0.43
A 0 B 0.57
A 0 B 0.71
A 0 B 0.86
A 0 B 1
A 0.14 B 0
A 0.14 B 0.14
A 0.14 B 0.29
A 0.14 B 0.43
A 0.14 B 0.57
A 0.14 B 0.71
A 0.14 B 0.86
A 0.14 B 1
A 0.29 B 0
A 0.29 B 0.14
A 0.29 B 0.29
A 0.29 B 0.43
A 0.29 B 0.57
A 0.29 B 0.71
A 0.29 B 0.86
A 0.29 B 1
A 0.43 B 0
A 0.43 B 0.14
A 0.43 B 0.29
A 0.43 B 0.43
A 0.43 B 0.57
A 0.43 B 0.71
A 0.43 B 0.86
A 0.43 B 1
A 0.57 B 0
A 0.57 B 0.14
A 0.57 B 0.29
A 0.57 B 0.43
A 0.57 B 0.57
A 0.57 B 0.71
A 0.57 B 0.86
A 0.57 B 1
A 0.71 B 0
A 0.71 B 0.14
A 0.71 B 0.29
A 0.71 B 0.43
A 0.71 B 0.57
A 0.71 B 0.71
A 0.71 B 0.86
A 0.71 B 1
A 0.86 B 0
A 0.86 B 0.14
A 0.86 B 0.29
A 0.86 B 0.43
A 0.86 B 0.57
A 0.86 B 0.71
A 0.86 B 0.86
A 0.86 B 1
A 1 B 0
A 1 B 0.14
A 1 B 0.29
A 1 B 0.43
A 1 B 0.57
A 1 B 0.71
A 1 B 0.86
A 1 B 1
OD 600 nm [–]
0.8
0.6
0.4
0.2
0 5 101520
Time [h]

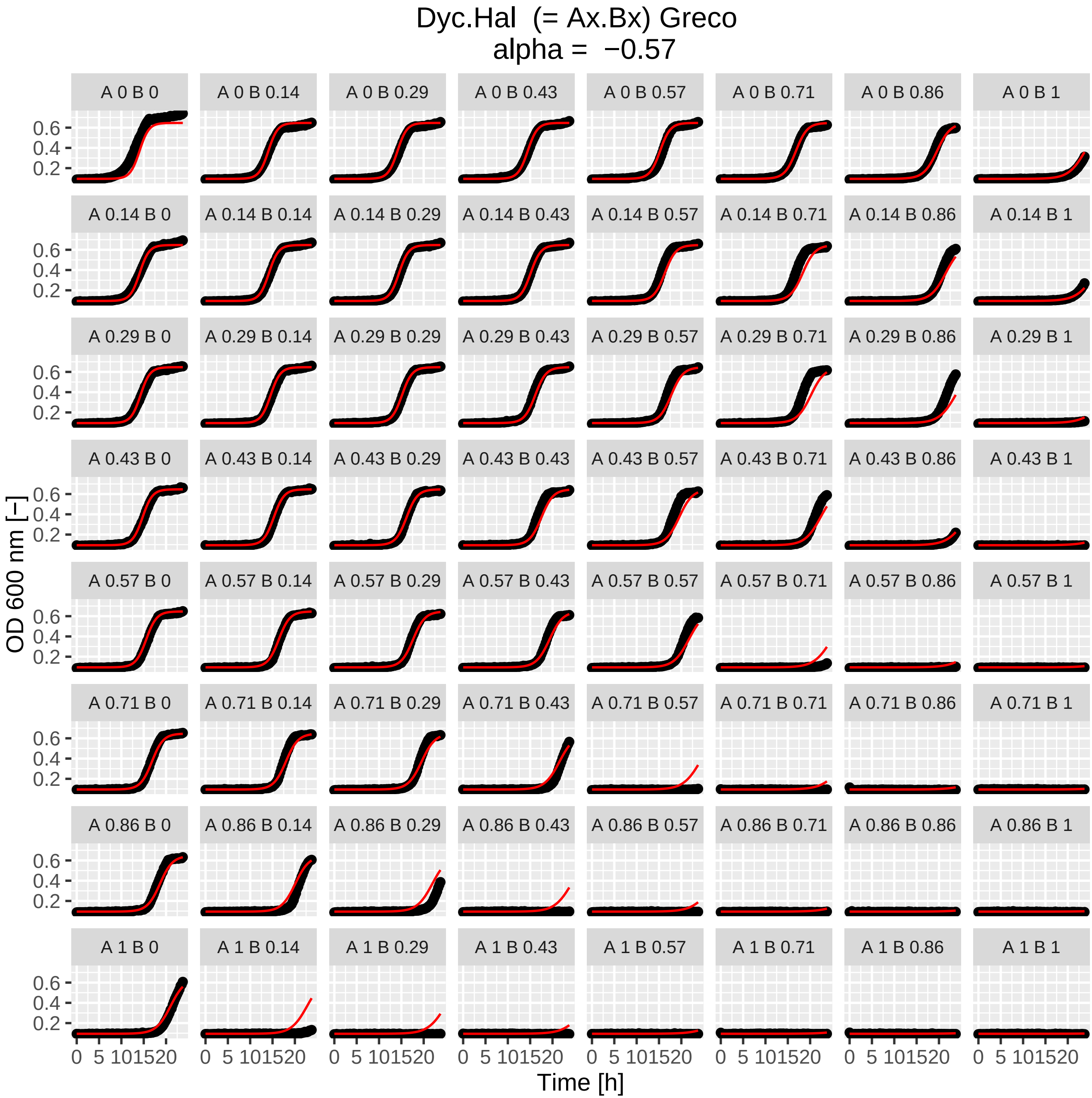

Dyc.Hal (= Ax.Bx) Greco
alpha = −0.57
A 0 B 0
A 0 B 0.14
A 0 B 0.29
A 0 B 0.43
A 0 B 0.57
A 0 B 0.71
A 0 B 0.86
A 0 B 1
A 0.14 B 0
A 0.14 B 0.14
A 0.14 B 0.29
A 0.14 B 0.43
A 0.14 B 0.57
A 0.14 B 0.71
A 0.14 B 0.86
A 0.14 B 1
A 0.29 B 0
A 0.29 B 0.14
A 0.29 B 0.29
A 0.29 B 0.43
A 0.29 B 0.57
A 0.29 B 0.71
A 0.29 B 0.86
A 0.29 B 1
A 0.43 B 0
A 0.43 B 0.14
A 0.43 B 0.29
A 0.43 B 0.43
A 0.43 B 0.57
A 0.43 B 0.71
A 0.43 B 0.86
A 0.43 B 1
A 0.57 B 0
A 0.57 B 0.14
A 0.57 B 0.29
A 0.57 B 0.43
A 0.57 B 0.57
A 0.57 B 0.71
A 0.57 B 0.86
A 0.57 B 1
A 0.71 B 0
A 0.71 B 0.14
A 0.71 B 0.29
A 0.71 B 0.43
A 0.71 B 0.57
A 0.71 B 0.71
A 0.71 B 0.86
A 0.71 B 1
A 0.86 B 0
A 0.86 B 0.14
A 0.86 B 0.29
A 0.86 B 0.43
A 0.86 B 0.57
A 0.86 B 0.71
A 0.86 B 0.86
A 0.86 B 1
A 1 B 0
A 1 B 0.14
A 1 B 0.29
A 1 B 0.43
A 1 B 0.57
A 1 B 0.71
A 1 B 0.86
A 1 B 1
OD 600 nm [−]
0.2
0.4
0.6
0 5 101520
Time [h]

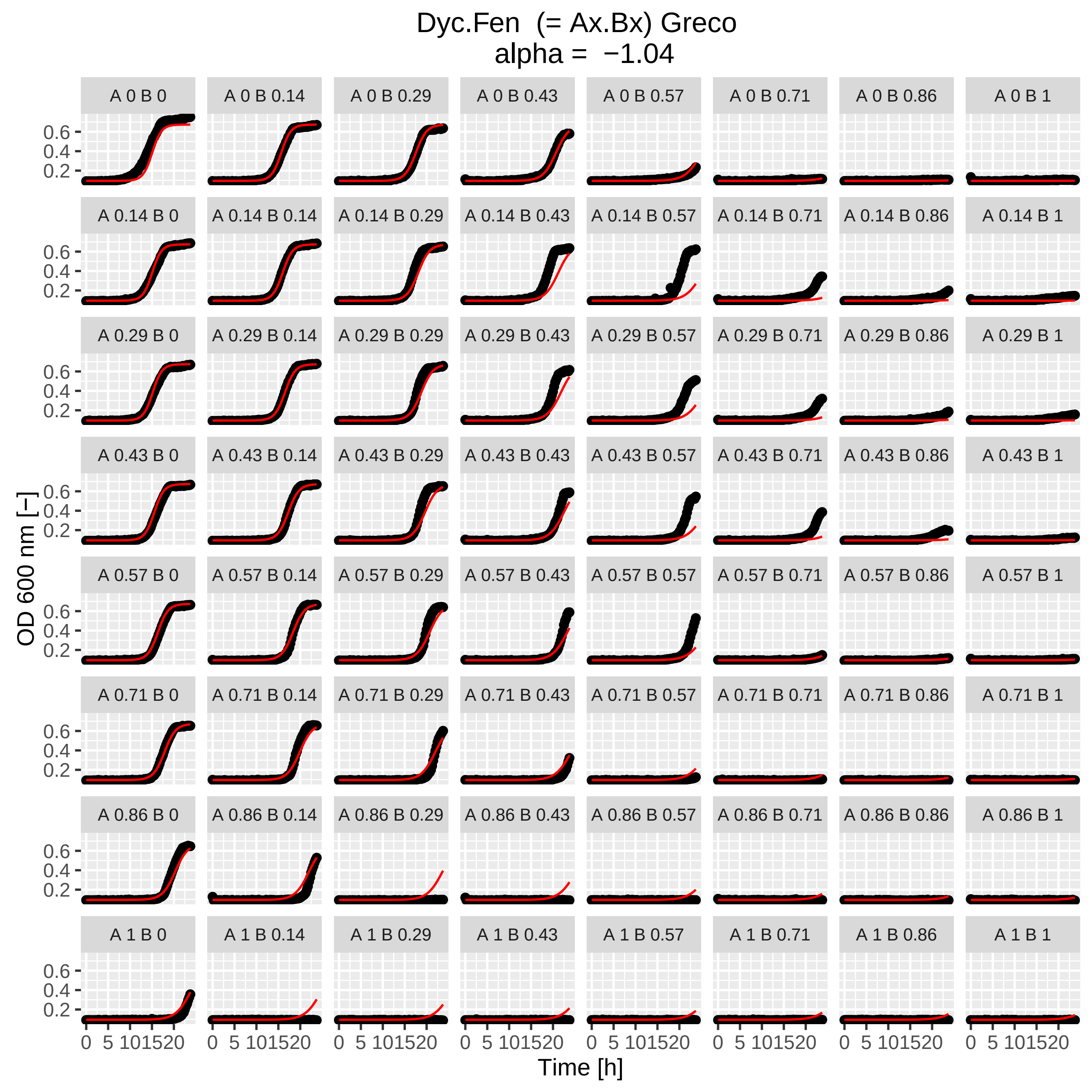

Dyc.Fen (= Ax.Bx) Greco
alpha = −1.04
A 0 B 0
A 0 B 0.14
A 0 B 0.29
A 0 B 0.43
A 0 B 0.57
A 0 B 0.71
A 0 B 0.86
A 0 B 1
A 0.14 B 0
A 0.14 B 0.14
A 0.14 B 0.29
A 0.14 B 0.43
A 0.14 B 0.57
A 0.14 B 0.71
A 0.14 B 0.86
A 0.14 B 1
A 0.29 B 0
A 0.29 B 0.14
A 0.29 B 0.29
A 0.29 B 0.43
A 0.29 B 0.57
A 0.29 B 0.71
A 0.29 B 0.86
A 0.29 B 1
A 0.43 B 0
A 0.43 B 0.14
A 0.43 B 0.29
A 0.43 B 0.43
A 0.43 B 0.57
A 0.43 B 0.71
A 0.43 B 0.86
A 0.43 B 1
A 0.57 B 0
A 0.57 B 0.14
A 0.57 B 0.29
A 0.57 B 0.43
A 0.57 B 0.57
A 0.57 B 0.71
A 0.57 B 0.86
A 0.57 B 1
A 0.71 B 0
A 0.71 B 0.14
A 0.71 B 0.29
A 0.71 B 0.43
A 0.71 B 0.57
A 0.71 B 0.71
A 0.71 B 0.86
A 0.71 B 1
A 0.86 B 0
A 0.86 B 0.14
A 0.86 B 0.29
A 0.86 B 0.43
A 0.86 B 0.57
A 0.86 B 0.71
A 0.86 B 0.86
A 0.86 B 1
A 1 B 0
A 1 B 0.14
A 1 B 0.29
A 1 B 0.43
A 1 B 0.57
A 1 B 0.71
A 1 B 0.86
A 1 B 1
OD 600 nm [−]
0.6
0.4
0.2
0 5 101520
Time [h]

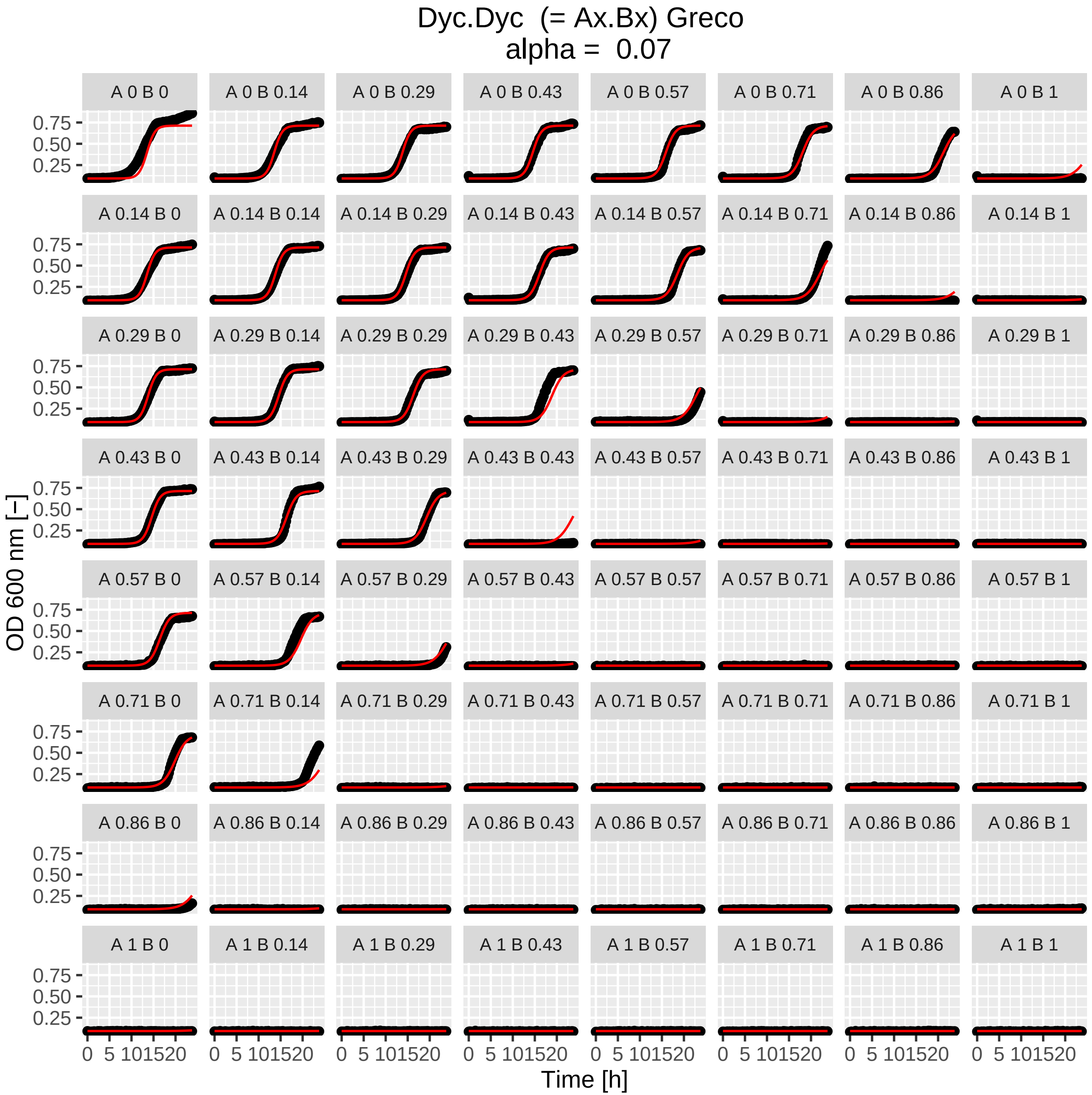
Dyc.Dyc (= Ax.Bx) Greco
alpha = 0.07
OD 600 nm [–]
Time [h]
0.75
0.50
0.25
0 5 101520
A 0 B 0
A 0 B 0.14
A 0 B 0.29
A 0 B 0.43
A 0 B 0.57
A 0 B 0.71
A 0 B 0.86
A 0 B 1
A 0.14 B 0
A 0.14 B 0.14
A 0.14 B 0.29
A 0.14 B 0.43
A 0.14 B 0.57
A 0.14 B 0.71
A 0.14 B 0.86
A 0.14 B 1
A 0.29 B 0
A 0.29 B 0.14
A 0.29 B 0.29
A 0.29 B 0.43
A 0.29 B 0.57
A 0.29 B 0.71
A 0.29 B 0.86
A 0.29 B 1
A 0.43 B 0
A 0.43 B 0.14
A 0.43 B 0.29
A 0.43 B 0.43
A 0.43 B 0.57
A 0.43 B 0.71
A 0.43 B 0.86
A 0.43 B 1
A 0.57 B 0
A 0.57 B 0.14
A 0.57 B 0.29
A 0.57 B 0.43
A 0.57 B 0.57
A 0.57 B 0.71
A 0.57 B 0.86
A 0.57 B 1
A 0.71 B 0
A 0.71 B 0.14
A 0.71 B 0.29
A 0.71 B 0.43
A 0.71 B 0.57
A 0.71 B 0.71
A 0.71 B 0.86
A 0.71 B 1
A 0.86 B 0
A 0.86 B 0.14
A 0.86 B 0.29
A 0.86 B 0.43
A 0.86 B 0.57
A 0.86 B 0.71
A 0.86 B 0.86
A 0.86 B 1
A 1 B 0
A 1 B 0.14
A 1 B 0.29
A 1 B 0.43
A 1 B 0.57
A 1 B 0.71
A 1 B 0.86
A 1 B 1

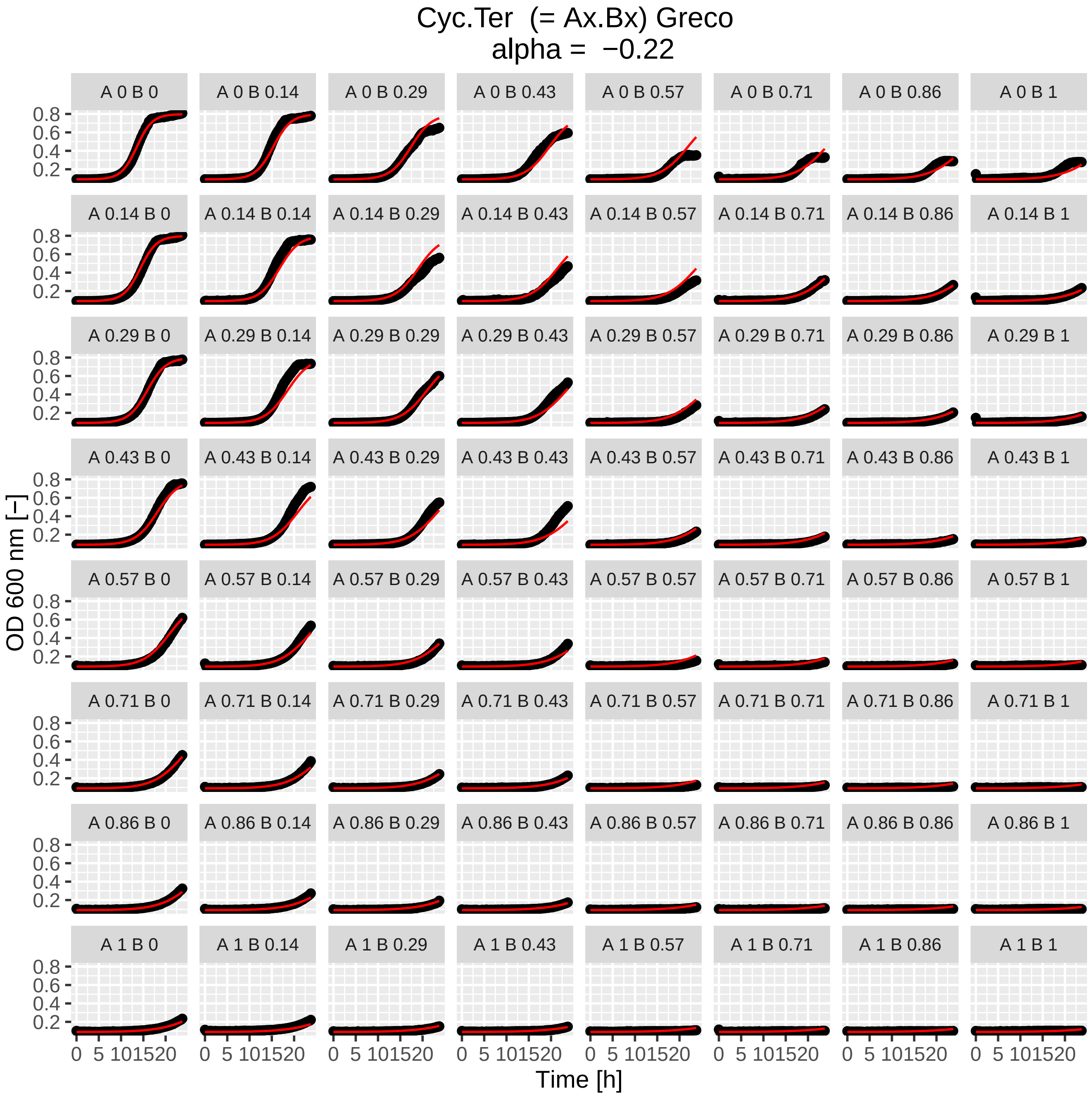
Cyc.Ter (= Ax.Bx) Greco
alpha = −0.22
OD 600 nm [−]
Time [h]
A 0 B 0
A 0 B 0.14
A 0 B 0.29
A 0 B 0.43
A 0 B 0.57
A 0 B 0.71
A 0 B 0.86
A 0 B 1
A 0.14 B 0
A 0.14 B 0.14
A 0.14 B 0.29
A 0.14 B 0.43
A 0.14 B 0.57
A 0.14 B 0.71
A 0.14 B 0.86
A 0.14 B 1
A 0.29 B 0
A 0.29 B 0.14
A 0.29 B 0.29
A 0.29 B 0.43
A 0.29 B 0.57
A 0.29 B 0.71
A 0.29 B 0.86
A 0.29 B 1
A 0.43 B 0
A 0.43 B 0.14
A 0.43 B 0.29
A 0.43 B 0.43
A 0.43 B 0.57
A 0.43 B 0.71
A 0.43 B 0.86
A 0.43 B 1
A 0.57 B 0
A 0.57 B 0.14
A 0.57 B 0.29
A 0.57 B 0.43
A 0.57 B 0.57
A 0.57 B 0.71
A 0.57 B 0.86
A 0.57 B 1
A 0.71 B 0
A 0.71 B 0.14
A 0.71 B 0.29
A 0.71 B 0.43
A 0.71 B 0.57
A 0.71 B 0.71
A 0.71 B 0.86
A 0.71 B 1
A 0.86 B 0
A 0.86 B 0.14
A 0.86 B 0.29
A 0.86 B 0.43
A 0.86 B 0.57
A 0.86 B 0.71
A 0.86 B 0.86
A 0.86 B 1
A 1 B 0
A 1 B 0.14
A 1 B 0.29
A 1 B 0.43
A 1 B 0.57
A 1 B 0.71
A 1 B 0.86
A 1 B 1
0.8
0.6
0.4
0.2
0 5 101520

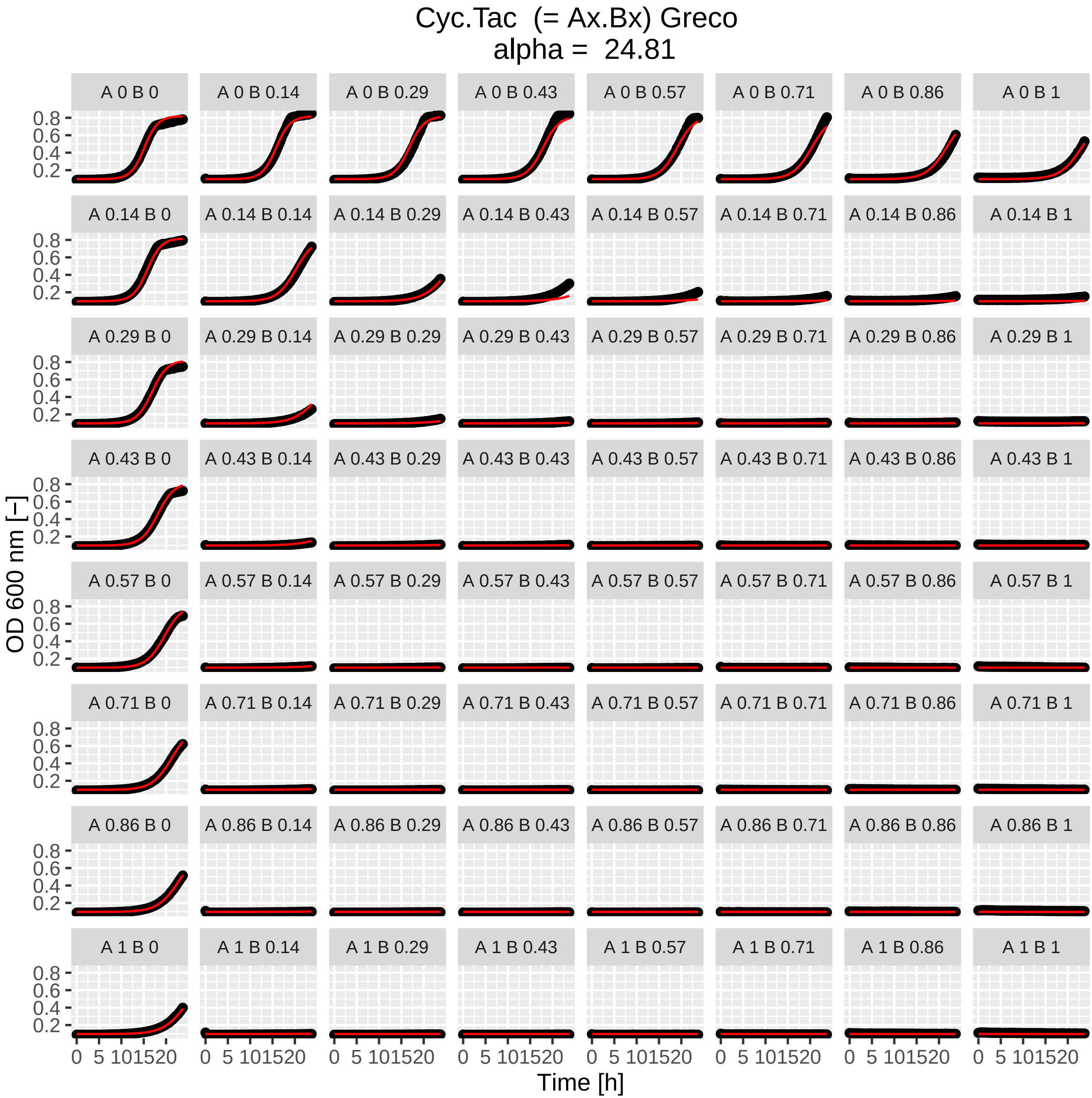

Cyc.Tac (= Ax.Bx) Greco
alpha = 24.81
A 0 B 0
A 0 B 0.14
A 0 B 0.29
A 0 B 0.43
A 0 B 0.57
A 0 B 0.71
A 0 B 0.86
A 0 B 1
A 0.14 B 0
A 0.14 B 0.14
A 0.14 B 0.29
A 0.14 B 0.43
A 0.14 B 0.57
A 0.14 B 0.71
A 0.14 B 0.86
A 0.14 B 1
A 0.29 B 0
A 0.29 B 0.14
A 0.29 B 0.29
A 0.29 B 0.43
A 0.29 B 0.57
A 0.29 B 0.71
A 0.29 B 0.86
A 0.29 B 1
A 0.43 B 0
A 0.43 B 0.14
A 0.43 B 0.29
A 0.43 B 0.43
A 0.43 B 0.57
A 0.43 B 0.71
A 0.43 B 0.86
A 0.43 B 1
A 0.57 B 0
A 0.57 B 0.14
A 0.57 B 0.29
A 0.57 B 0.43
A 0.57 B 0.57
A 0.57 B 0.71
A 0.57 B 0.86
A 0.57 B 1
A 0.71 B 0
A 0.71 B 0.14
A 0.71 B 0.29
A 0.71 B 0.43
A 0.71 B 0.57
A 0.71 B 0.71
A 0.71 B 0.86
A 0.71 B 1
A 0.86 B 0
A 0.86 B 0.14
A 0.86 B 0.29
A 0.86 B 0.43
A 0.86 B 0.57
A 0.86 B 0.71
A 0.86 B 0.86
A 0.86 B 1
A 1 B 0
A 1 B 0.14
A 1 B 0.29
A 1 B 0.43
A 1 B 0.57
A 1 B 0.71
A 1 B 0.86
A 1 B 1
OD 600 nm [−]
0.2
0.4
0.6
0.8
0 5 101520
Time [h]

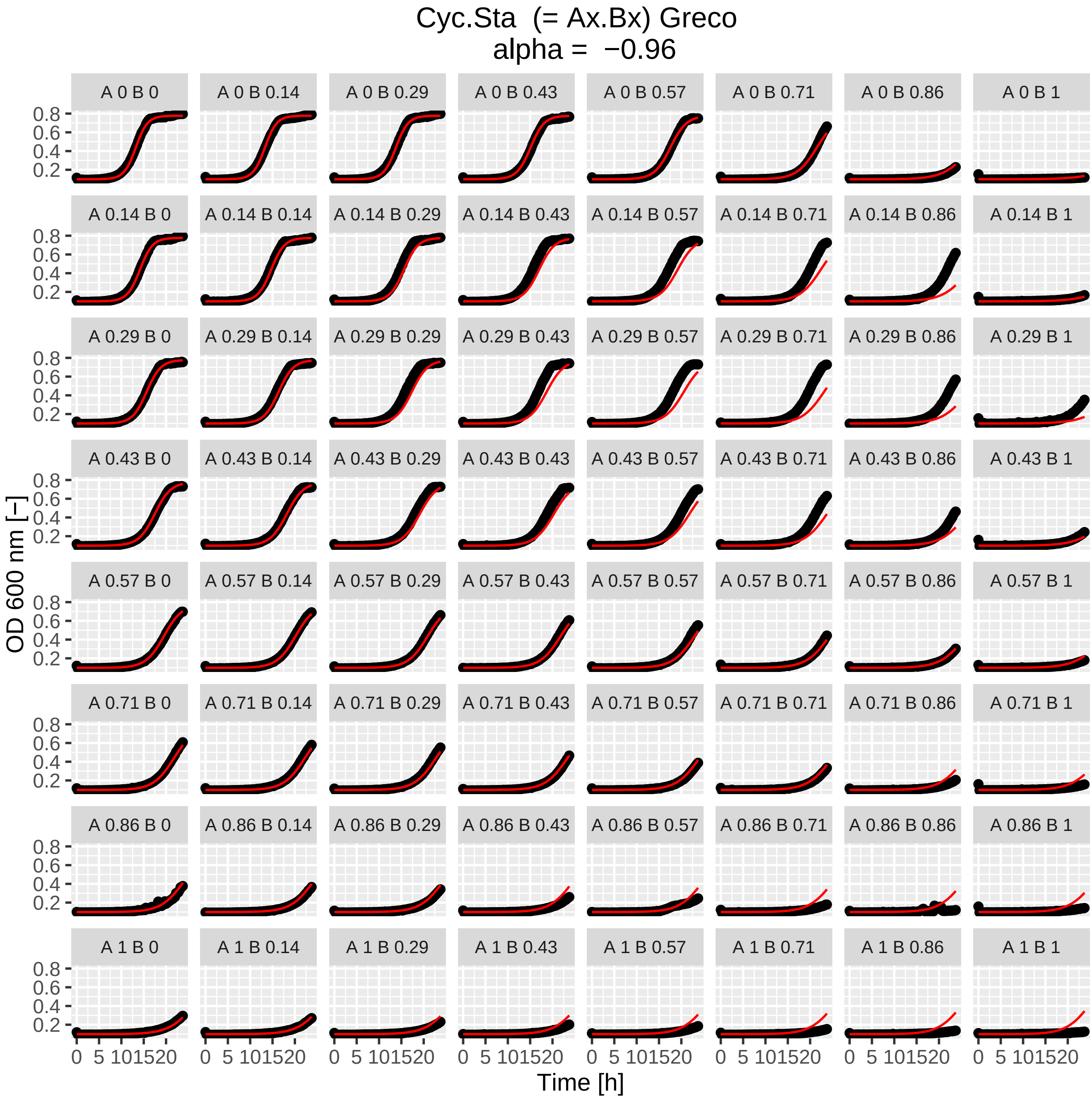

Cyc.Sta (= Ax.Bx) Greco
alpha = −0.96
A 0 B 0
A 0 B 0.14
A 0 B 0.29
A 0 B 0.43
A 0 B 0.57
A 0 B 0.71
A 0 B 0.86
A 0 B 1
A 0.14 B 0
A 0.14 B 0.14
A 0.14 B 0.29
A 0.14 B 0.43
A 0.14 B 0.57
A 0.14 B 0.71
A 0.14 B 0.86
A 0.14 B 1
A 0.29 B 0
A 0.29 B 0.14
A 0.29 B 0.29
A 0.29 B 0.43
A 0.29 B 0.57
A 0.29 B 0.71
A 0.29 B 0.86
A 0.29 B 1
A 0.43 B 0
A 0.43 B 0.14
A 0.43 B 0.29
A 0.43 B 0.43
A 0.43 B 0.57
A 0.43 B 0.71
A 0.43 B 0.86
A 0.43 B 1
A 0.57 B 0
A 0.57 B 0.14
A 0.57 B 0.29
A 0.57 B 0.43
A 0.57 B 0.57
A 0.57 B 0.71
A 0.57 B 0.86
A 0.57 B 1
A 0.71 B 0
A 0.71 B 0.14
A 0.71 B 0.29
A 0.71 B 0.43
A 0.71 B 0.57
A 0.71 B 0.71
A 0.71 B 0.86
A 0.71 B 1
A 0.86 B 0
A 0.86 B 0.14
A 0.86 B 0.29
A 0.86 B 0.43
A 0.86 B 0.57
A 0.86 B 0.71
A 0.86 B 0.86
A 0.86 B 1
A 1 B 0
A 1 B 0.14
A 1 B 0.29
A 1 B 0.43
A 1 B 0.57
A 1 B 0.71
A 1 B 0.86
A 1 B 1
OD 600 nm [−]
0.8
0.6
0.4
0.2
0 5 101520
Time [h]

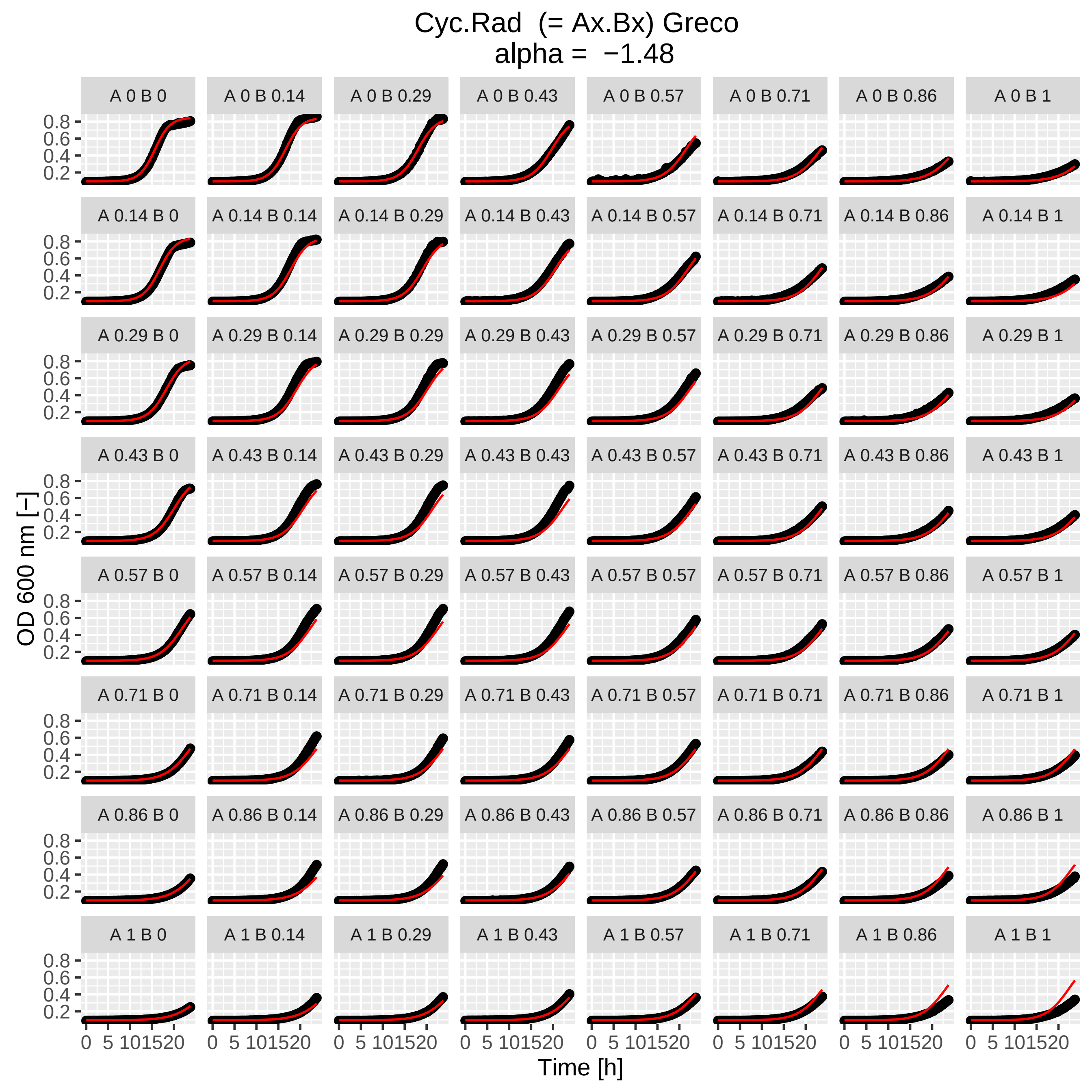

Cyc.Rad (= Ax.Bx) Greco
alpha = −1.48
A 0 B 0
A 0 B 0.14
A 0 B 0.29
A 0 B 0.43
A 0 B 0.57
A 0 B 0.71
A 0 B 0.86
A 0 B 1
A 0.14 B 0
A 0.14 B 0.14
A 0.14 B 0.29
A 0.14 B 0.43
A 0.14 B 0.57
A 0.14 B 0.71
A 0.14 B 0.86
A 0.14 B 1
A 0.29 B 0
A 0.29 B 0.14
A 0.29 B 0.29
A 0.29 B 0.43
A 0.29 B 0.57
A 0.29 B 0.71
A 0.29 B 0.86
A 0.29 B 1
A 0.43 B 0
A 0.43 B 0.14
A 0.43 B 0.29
A 0.43 B 0.43
A 0.43 B 0.57
A 0.43 B 0.71
A 0.43 B 0.86
A 0.43 B 1
A 0.57 B 0
A 0.57 B 0.14
A 0.57 B 0.29
A 0.57 B 0.43
A 0.57 B 0.57
A 0.57 B 0.71
A 0.57 B 0.86
A 0.57 B 1
A 0.71 B 0
A 0.71 B 0.14
A 0.71 B 0.29
A 0.71 B 0.43
A 0.71 B 0.57
A 0.71 B 0.71
A 0.71 B 0.86
A 0.71 B 1
A 0.86 B 0
A 0.86 B 0.14
A 0.86 B 0.29
A 0.86 B 0.43
A 0.86 B 0.57
A 0.86 B 0.71
A 0.86 B 0.86
A 0.86 B 1
A 1 B 0
A 1 B 0.14
A 1 B 0.29
A 1 B 0.43
A 1 B 0.57
A 1 B 0.71
A 1 B 0.86
A 1 B 1
0.8
0.6
0.4
0.2
OD 600 nm [−]
0 5 101520
Time [h]

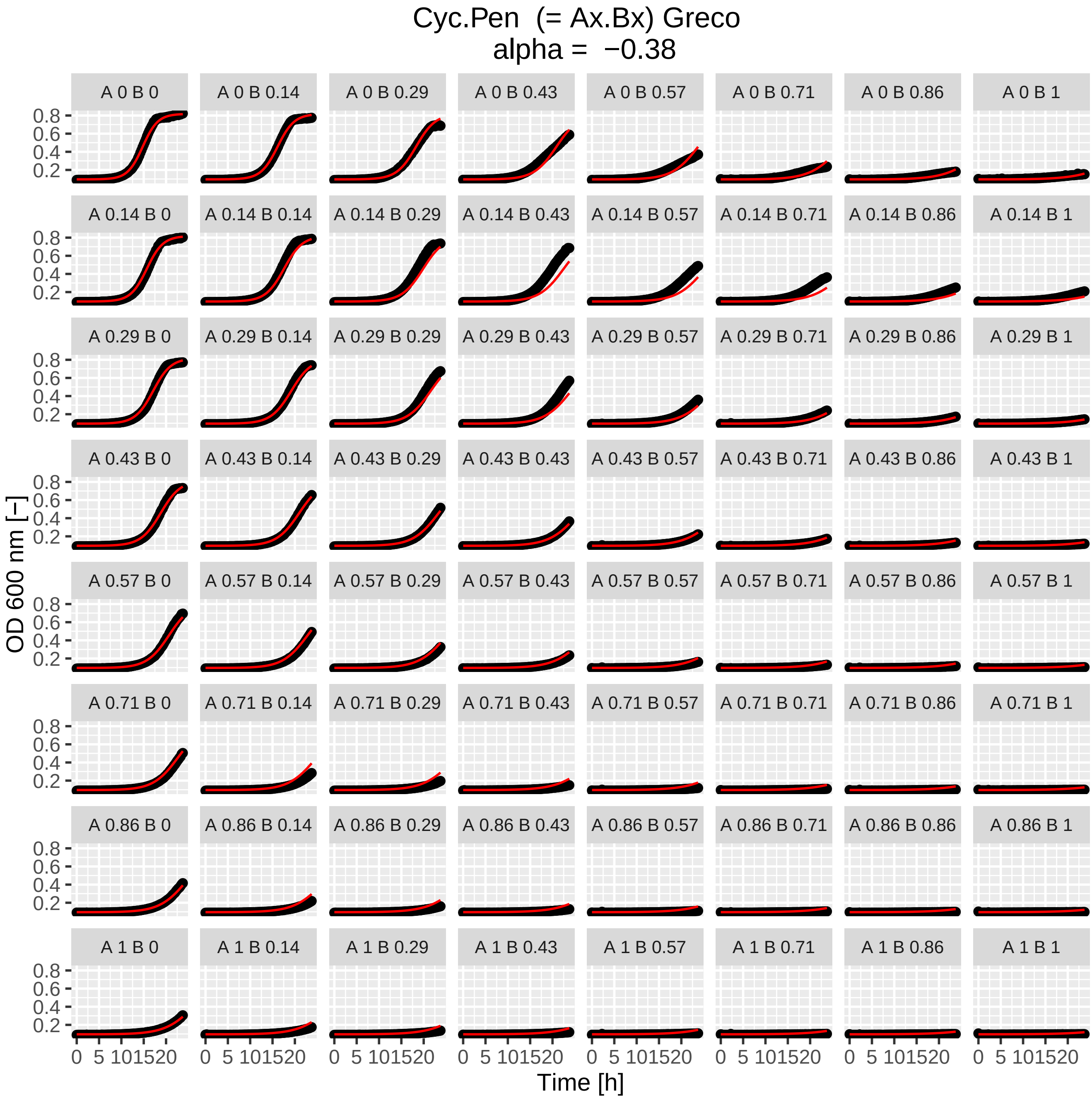
Cyc.Pen (= Ax.Bx) Greco
alpha = −0.38
OD 600 nm [−]
Time [h]
0.2
0.4
0.6
0.8
0 5 101520
A 0 B 0
A 0 B 0.14
A 0 B 0.29
A 0 B 0.43
A 0 B 0.57
A 0 B 0.71
A 0 B 0.86
A 0 B 1
A 0.14 B 0
A 0.14 B 0.14
A 0.14 B 0.29
A 0.14 B 0.43
A 0.14 B 0.57
A 0.14 B 0.71
A 0.14 B 0.86
A 0.14 B 1
A 0.29 B 0
A 0.29 B 0.14
A 0.29 B 0.29
A 0.29 B 0.43
A 0.29 B 0.57
A 0.29 B 0.71
A 0.29 B 0.86
A 0.29 B 1
A 0.43 B 0
A 0.43 B 0.14
A 0.43 B 0.29
A 0.43 B 0.43
A 0.43 B 0.57
A 0.43 B 0.71
A 0.43 B 0.86
A 0.43 B 1
A 0.57 B 0
A 0.57 B 0.14
A 0.57 B 0.29
A 0.57 B 0.43
A 0.57 B 0.57
A 0.57 B 0.71
A 0.57 B 0.86
A 0.57 B 1
A 0.71 B 0
A 0.71 B 0.14
A 0.71 B 0.29
A 0.71 B 0.43
A 0.71 B 0.57
A 0.71 B 0.71
A 0.71 B 0.86
A 0.71 B 1
A 0.86 B 0
A 0.86 B 0.14
A 0.86 B 0.29
A 0.86 B 0.43
A 0.86 B 0.57
A 0.86 B 0.71
A 0.86 B 0.86
A 0.86 B 1
A 1 B 0
A 1 B 0.14
A 1 B 0.29
A 1 B 0.43
A 1 B 0.57
A 1 B 0.71
A 1 B 0.86
A 1 B 1

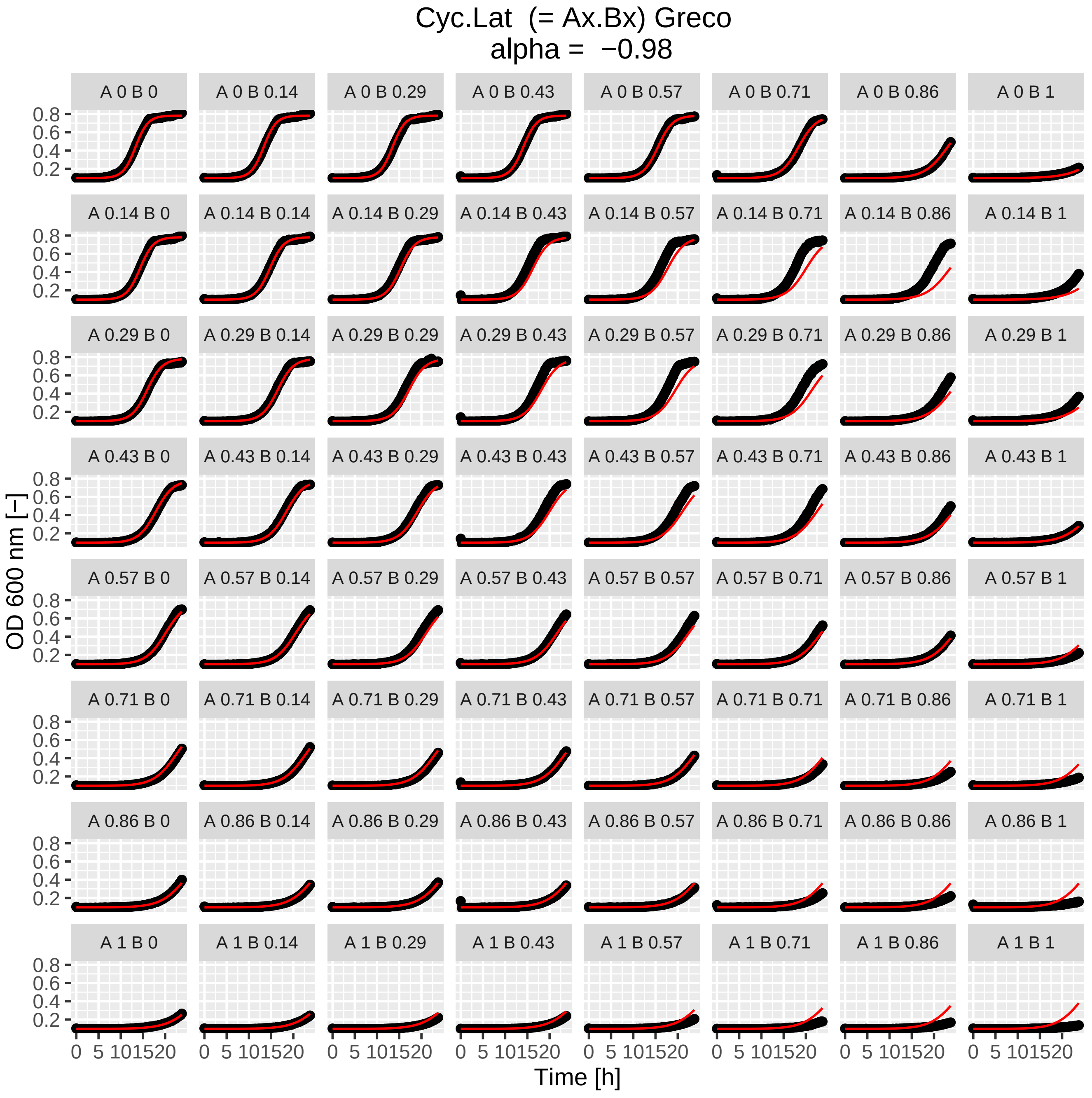

Cyc.Lat (= Ax.Bx) Greco
alpha = −0.98
OD 600 nm [−]
Time [h]
A 0 B 0
A 0 B 0.14
A 0 B 0.29
A 0 B 0.43
A 0 B 0.57
A 0 B 0.71
A 0 B 0.86
A 0 B 1
A 0.14 B 0
A 0.14 B 0.14
A 0.14 B 0.29
A 0.14 B 0.43
A 0.14 B 0.57
A 0.14 B 0.71
A 0.14 B 0.86
A 0.14 B 1
A 0.29 B 0
A 0.29 B 0.14
A 0.29 B 0.29
A 0.29 B 0.43
A 0.29 B 0.57
A 0.29 B 0.71
A 0.29 B 0.86
A 0.29 B 1
A 0.43 B 0
A 0.43 B 0.14
A 0.43 B 0.29
A 0.43 B 0.43
A 0.43 B 0.57
A 0.43 B 0.71
A 0.43 B 0.86
A 0.43 B 1
A 0.57 B 0
A 0.57 B 0.14
A 0.57 B 0.29
A 0.57 B 0.43
A 0.57 B 0.57
A 0.57 B 0.71
A 0.57 B 0.86
A 0.57 B 1
A 0.71 B 0
A 0.71 B 0.14
A 0.71 B 0.29
A 0.71 B 0.43
A 0.71 B 0.57
A 0.71 B 0.71
A 0.71 B 0.86
A 0.71 B 1
A 0.86 B 0
A 0.86 B 0.14
A 0.86 B 0.29
A 0.86 B 0.43
A 0.86 B 0.57
A 0.86 B 0.71
A 0.86 B 0.86
A 0.86 B 1
A 1 B 0
A 1 B 0.14
A 1 B 0.29
A 1 B 0.43
A 1 B 0.57
A 1 B 0.71
A 1 B 0.86
A 1 B 1
0.8
0.6
0.4
0.2
0 5 101520

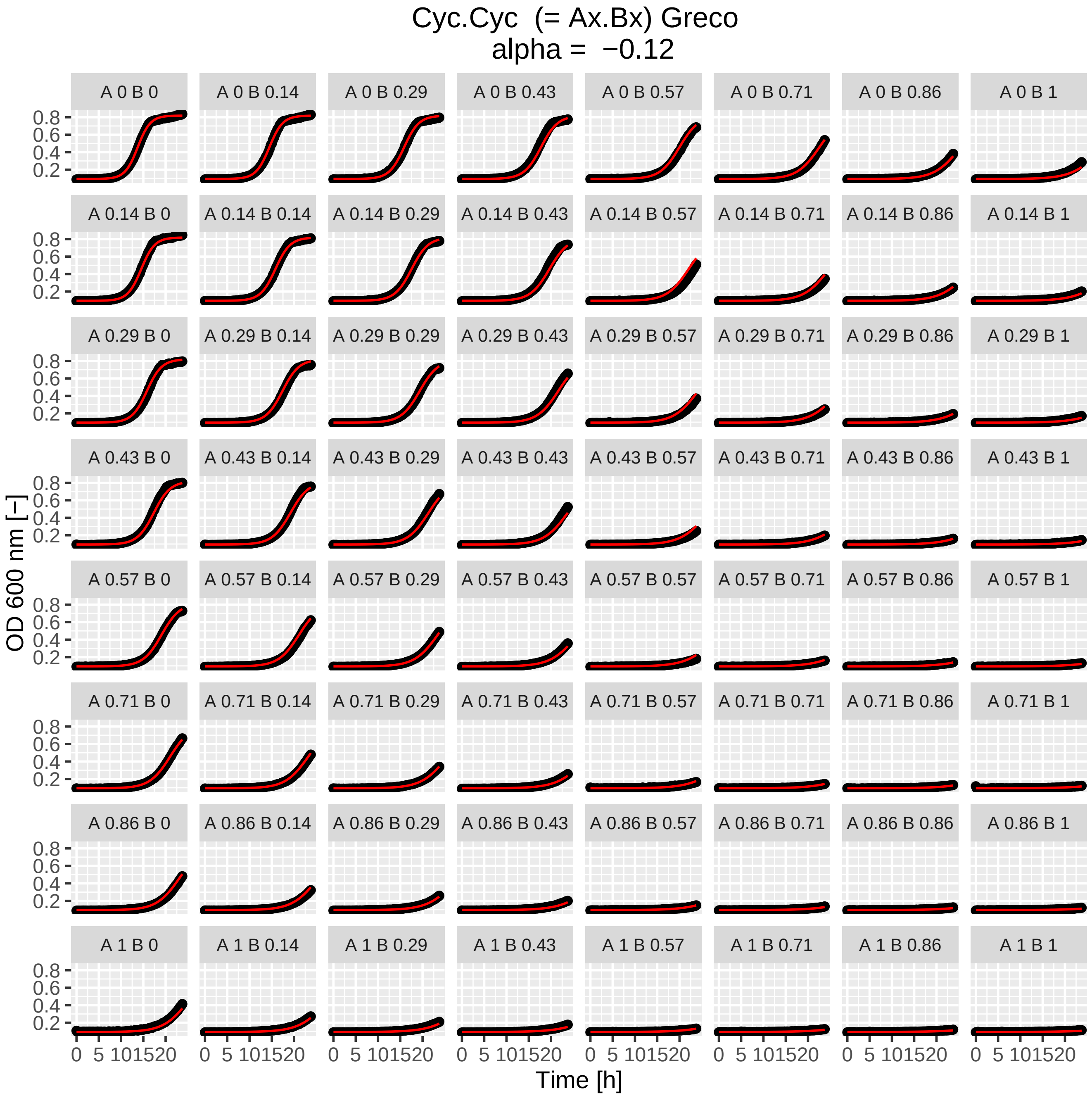
Cyc.Cyc (= Ax.Bx) Greco
alpha = −0.12
OD 600 nm [−]
Time [h]
0.2 0.4 0.6 0.8
0 5 10 15 20
A 0 B 0
A 0 B 0.14
A 0 B 0.29
A 0 B 0.43
A 0 B 0.57
A 0 B 0.71
A 0 B 0.86
A 0 B 1
A 0.14 B 0
A 0.14 B 0.14
A 0.14 B 0.29
A 0.14 B 0.43
A 0.14 B 0.57
A 0.14 B 0.71
A 0.14 B 0.86
A 0.14 B 1
A 0.29 B 0
A 0.29 B 0.14
A 0.29 B 0.29
A 0.29 B 0.43
A 0.29 B 0.57
A 0.29 B 0.71
A 0.29 B 0.86
A 0.29 B 1
A 0.43 B 0
A 0.43 B 0.14
A 0.43 B 0.29
A 0.43 B 0.43
A 0.43 B 0.57
A 0.43 B 0.71
A 0.43 B 0.86
A 0.43 B 1
A 0.57 B 0
A 0.57 B 0.14
A 0.57 B 0.29
A 0.57 B 0.43
A 0.57 B 0.57
A 0.57 B 0.71
A 0.57 B 0.86
A 0.57 B 1
A 0.71 B 0
A 0.71 B 0.14
A 0.71 B 0.29
A 0.71 B 0.43
A 0.71 B 0.57
A 0.71 B 0.71
A 0.71 B 0.86
A 0.71 B 1
A 0.86 B 0
A 0.86 B 0.14
A 0.86 B 0.29
A 0.86 B 0.43
A 0.86 B 0.57
A 0.86 B 0.71
A 0.86 B 0.86
A 0.86 B 1
A 1 B 0
A 1 B 0.14
A 1 B 0.29
A 1 B 0.43
A 1 B 0.57
A 1 B 0.71
A 1 B 0.86
A 1 B 1

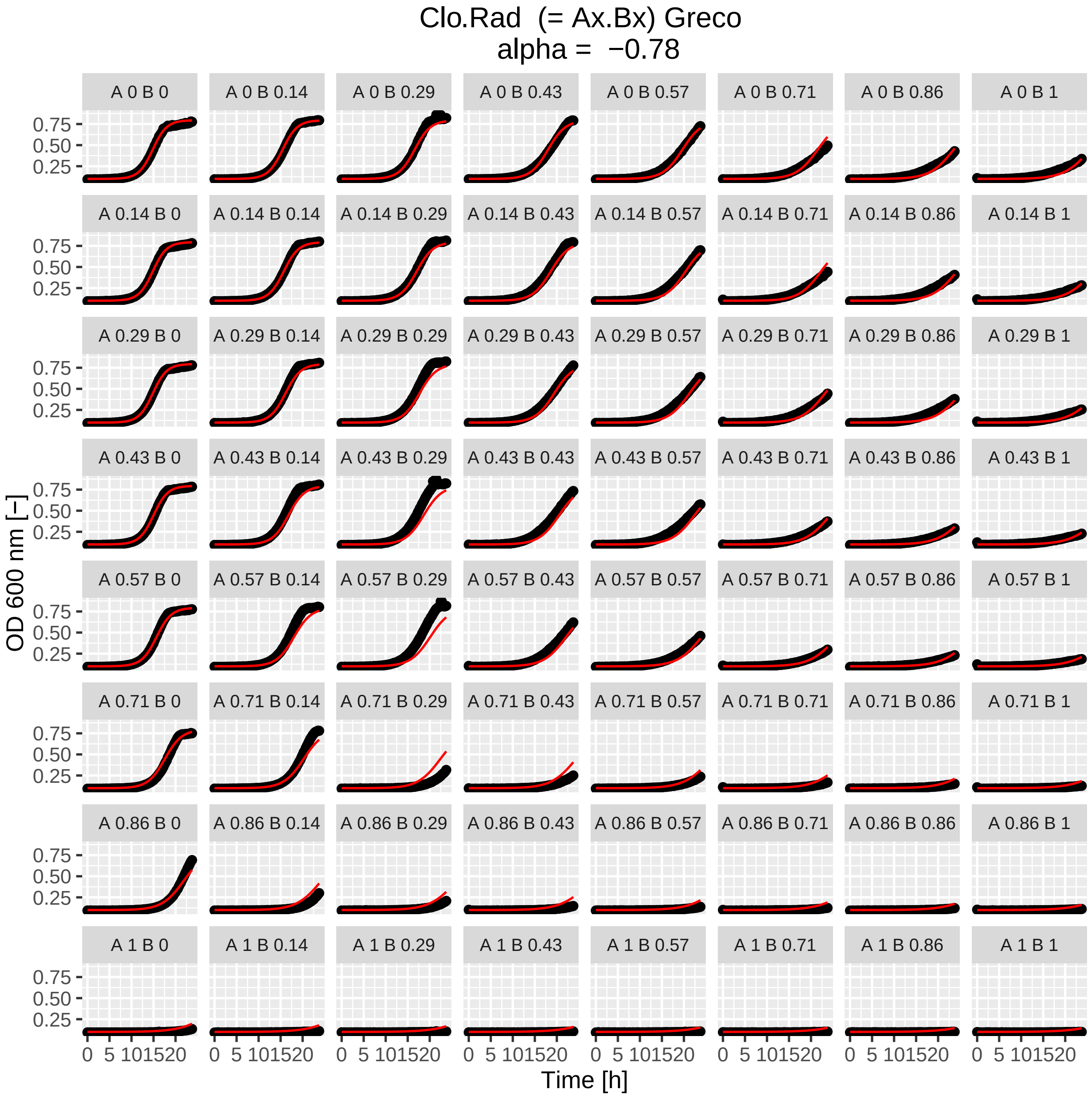
Clo.Rad (= Ax.Bx) Greco
alpha = −0.78
A 0 B 0
A 0 B 0.14
A 0 B 0.29
A 0 B 0.43
A 0 B 0.57
A 0 B 0.71
A 0 B 0.86
A 0 B 1
A 0.14 B 0
A 0.14 B 0.14
A 0.14 B 0.29
A 0.14 B 0.43
A 0.14 B 0.57
A 0.14 B 0.71
A 0.14 B 0.86
A 0.14 B 1
A 0.29 B 0
A 0.29 B 0.14
A 0.29 B 0.29
A 0.29 B 0.43
A 0.29 B 0.57
A 0.29 B 0.71
A 0.29 B 0.86
A 0.29 B 1
A 0.43 B 0
A 0.43 B 0.14
A 0.43 B 0.29
A 0.43 B 0.43
A 0.43 B 0.57
A 0.43 B 0.71
A 0.43 B 0.86
A 0.43 B 1
A 0.57 B 0
A 0.57 B 0.14
A 0.57 B 0.29
A 0.57 B 0.43
A 0.57 B 0.57
A 0.57 B 0.71
A 0.57 B 0.86
A 0.57 B 1
A 0.71 B 0
A 0.71 B 0.14
A 0.71 B 0.29
A 0.71 B 0.43
A 0.71 B 0.57
A 0.71 B 0.71
A 0.71 B 0.86
A 0.71 B 1
A 0.86 B 0
A 0.86 B 0.14
A 0.86 B 0.29
A 0.86 B 0.43
A 0.86 B 0.57
A 0.86 B 0.71
A 0.86 B 0.86
A 0.86 B 1
A 1 B 0
A 1 B 0.14
A 1 B 0.29
A 1 B 0.43
A 1 B 0.57
A 1 B 0.71
A 1 B 0.86
A 1 B 1
OD 600 nm [−]
0.25
0.50
0.75
0 5 101520
Time [h]

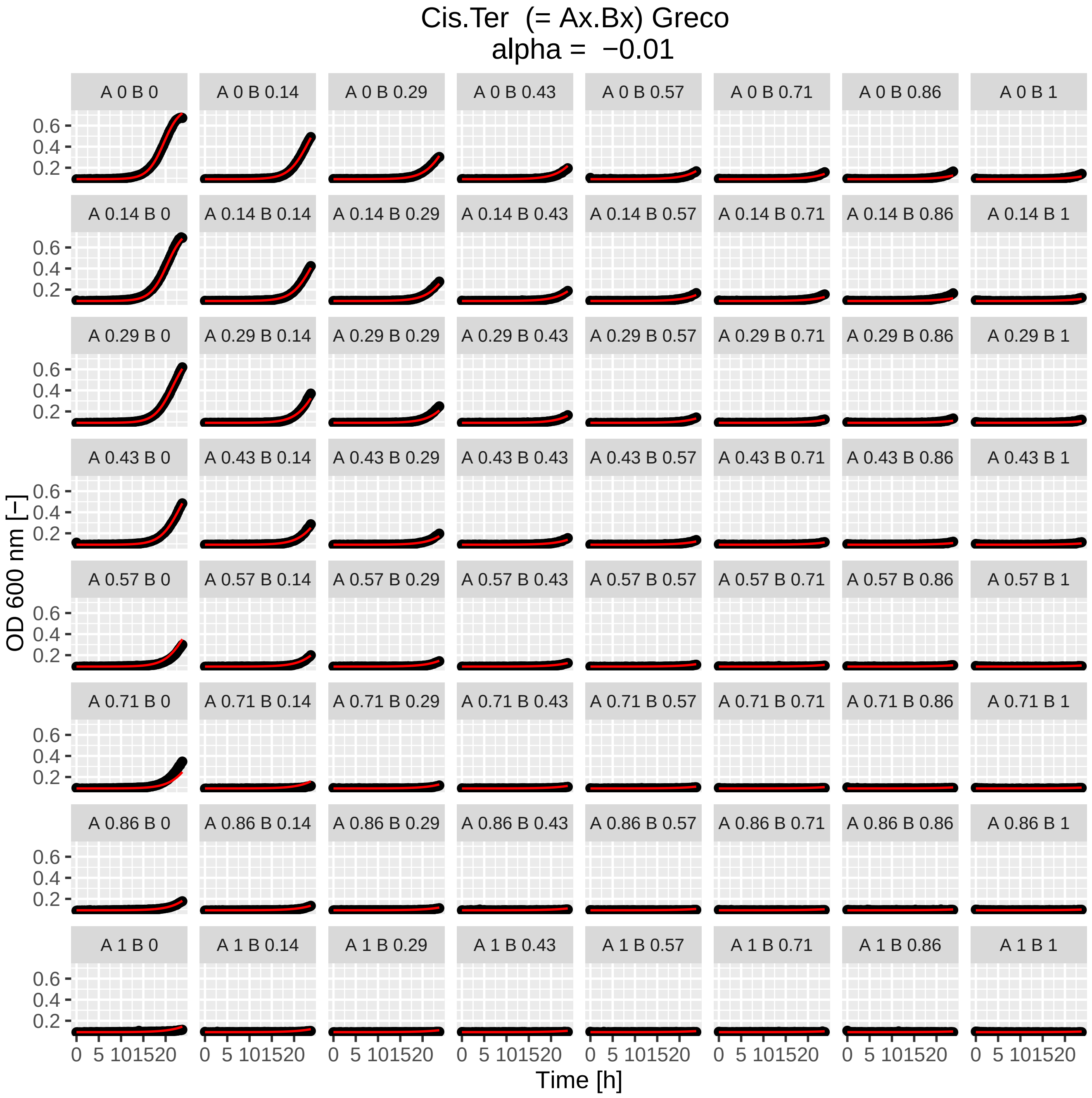
Cis.Ter (= Ax.Bx) Greco
alpha = −0.01
A 0 B 0
A 0 B 0.14
A 0 B 0.29
A 0 B 0.43
A 0 B 0.57
A 0 B 0.71
A 0 B 0.86
A 0 B 1
A 0.14 B 0
A 0.14 B 0.14
A 0.14 B 0.29
A 0.14 B 0.43
A 0.14 B 0.57
A 0.14 B 0.71
A 0.14 B 0.86
A 0.14 B 1
A 0.29 B 0
A 0.29 B 0.14
A 0.29 B 0.29
A 0.29 B 0.43
A 0.29 B 0.57
A 0.29 B 0.71
A 0.29 B 0.86
A 0.29 B 1
A 0.43 B 0
A 0.43 B 0.14
A 0.43 B 0.29
A 0.43 B 0.43
A 0.43 B 0.57
A 0.43 B 0.71
A 0.43 B 0.86
A 0.43 B 1
A 0.57 B 0
A 0.57 B 0.14
A 0.57 B 0.29
A 0.57 B 0.43
A 0.57 B 0.57
A 0.57 B 0.71
A 0.57 B 0.86
A 0.57 B 1
A 0.71 B 0
A 0.71 B 0.14
A 0.71 B 0.29
A 0.71 B 0.43
A 0.71 B 0.57
A 0.71 B 0.71
A 0.71 B 0.86
A 0.71 B 1
A 0.86 B 0
A 0.86 B 0.14
A 0.86 B 0.29
A 0.86 B 0.43
A 0.86 B 0.57
A 0.86 B 0.71
A 0.86 B 0.86
A 0.86 B 1
A 1 B 0
A 1 B 0.14
A 1 B 0.29
A 1 B 0.43
A 1 B 0.57
A 1 B 0.71
A 1 B 0.86
A 1 B 1
OD 600 nm [–]
0.6
0.4
0.2
0 5 101520
Time [h]

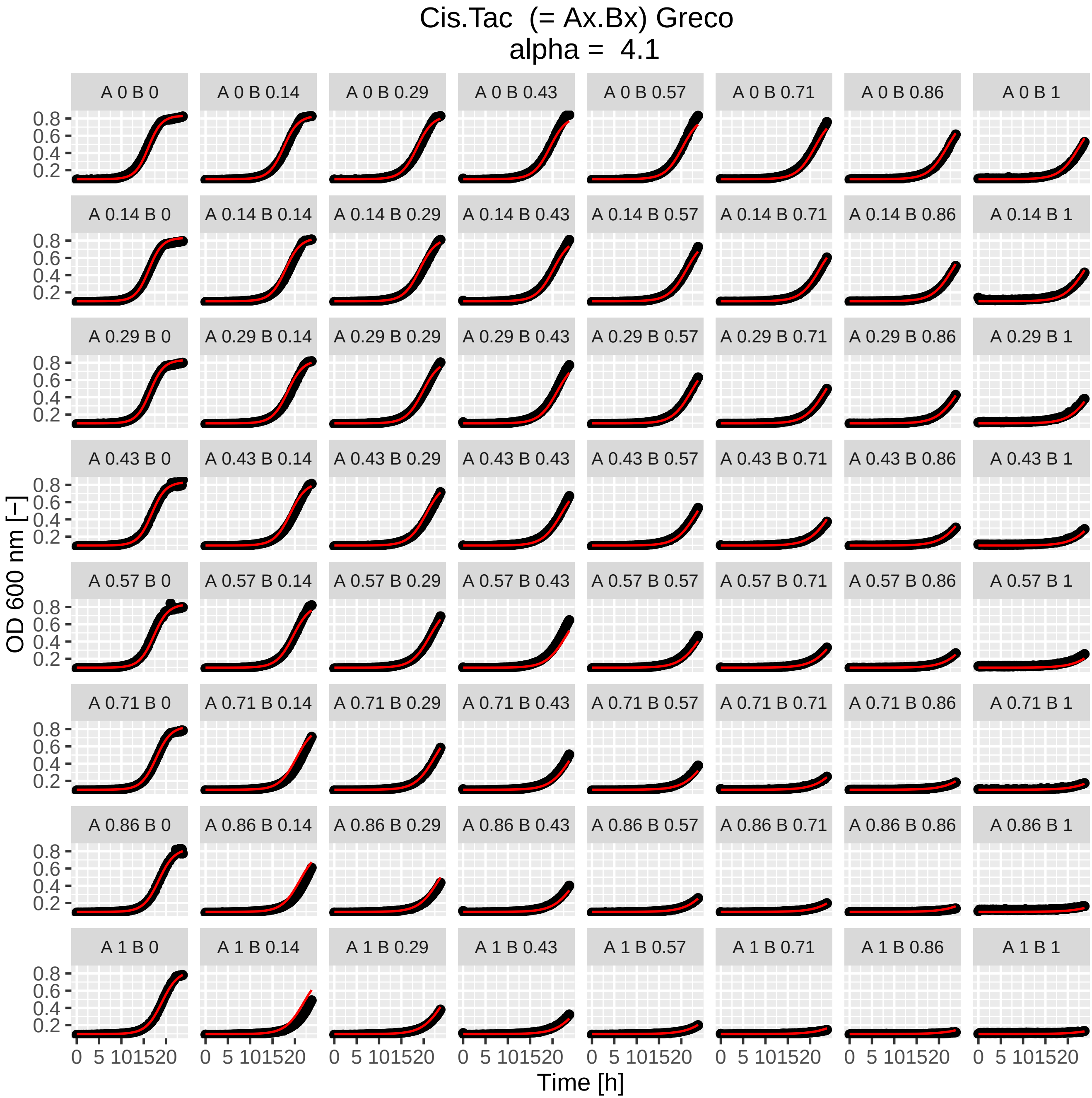

Cis.Tac (= Ax.Bx) Greco
alpha = 4.1
A 0 B 0
A 0 B 0.14
A 0 B 0.29
A 0 B 0.43
A 0 B 0.57
A 0 B 0.71
A 0 B 0.86
A 0 B 1
A 0.14 B 0
A 0.14 B 0.14
A 0.14 B 0.29
A 0.14 B 0.43
A 0.14 B 0.57
A 0.14 B 0.71
A 0.14 B 0.86
A 0.14 B 1
A 0.29 B 0
A 0.29 B 0.14
A 0.29 B 0.29
A 0.29 B 0.43
A 0.29 B 0.57
A 0.29 B 0.71
A 0.29 B 0.86
A 0.29 B 1
A 0.43 B 0
A 0.43 B 0.14
A 0.43 B 0.29
A 0.43 B 0.43
A 0.43 B 0.57
A 0.43 B 0.71
A 0.43 B 0.86
A 0.43 B 1
A 0.57 B 0
A 0.57 B 0.14
A 0.57 B 0.29
A 0.57 B 0.43
A 0.57 B 0.57
A 0.57 B 0.71
A 0.57 B 0.86
A 0.57 B 1
A 0.71 B 0
A 0.71 B 0.14
A 0.71 B 0.29
A 0.71 B 0.43
A 0.71 B 0.57
A 0.71 B 0.71
A 0.71 B 0.86
A 0.71 B 1
A 0.86 B 0
A 0.86 B 0.14
A 0.86 B 0.29
A 0.86 B 0.43
A 0.86 B 0.57
A 0.86 B 0.71
A 0.86 B 0.86
A 0.86 B 1
A 1 B 0
A 1 B 0.14
A 1 B 0.29
A 1 B 0.43
A 1 B 0.57
A 1 B 0.71
A 1 B 0.86
A 1 B 1
0.8
0.6
0.4
0.2
OD 600 nm [−]
0 5 101520
Time [h]

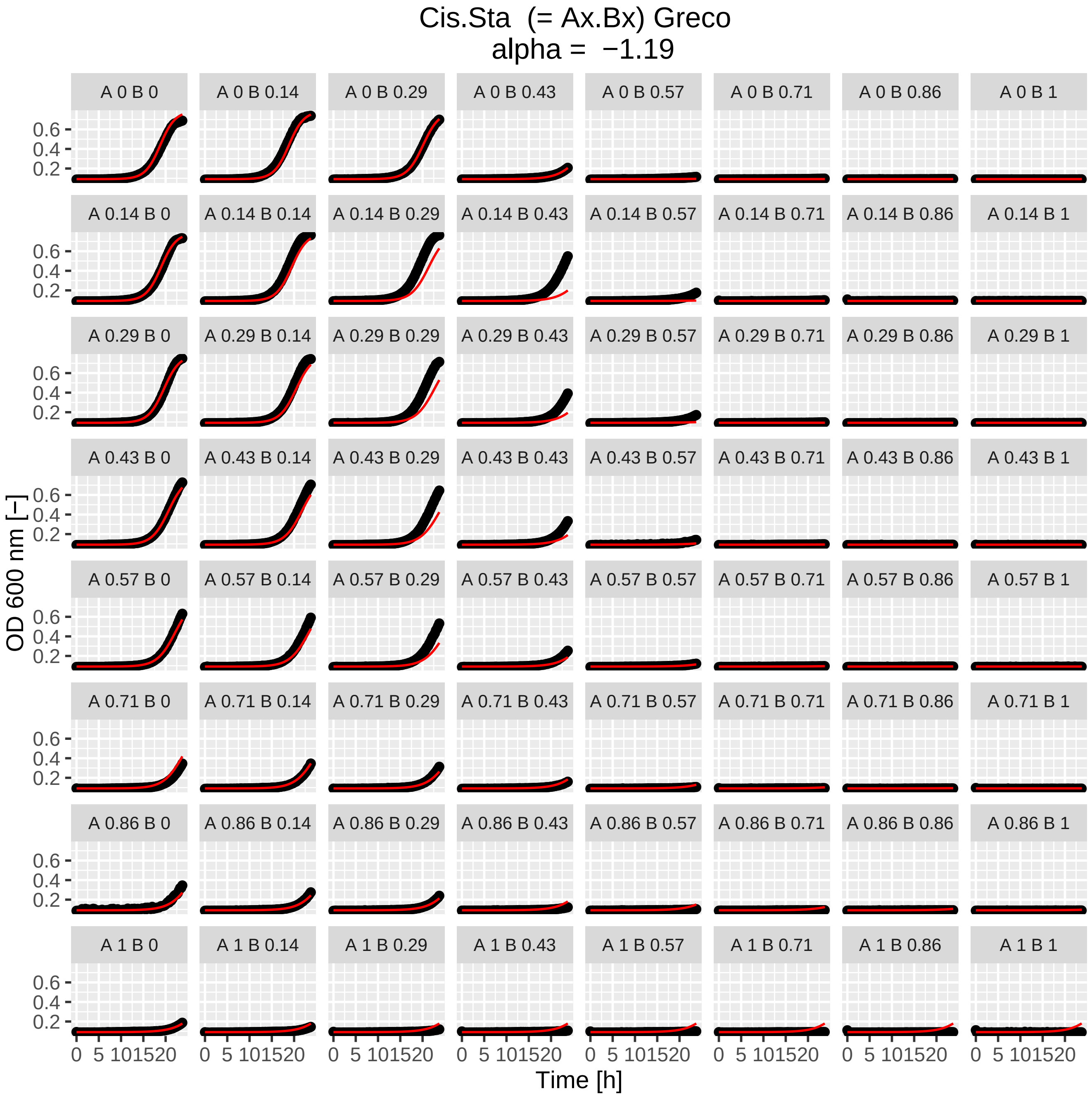
Cis.Sta (= Ax.Bx) Greco
alpha = −1.19
OD 600 nm [−]
Time [h]
A 0 B 0
A 0 B 0.14
A 0 B 0.29
A 0 B 0.43
A 0 B 0.57
A 0 B 0.71
A 0 B 0.86
A 0 B 1
A 0.14 B 0
A 0.14 B 0.14
A 0.14 B 0.29
A 0.14 B 0.43
A 0.14 B 0.57
A 0.14 B 0.71
A 0.14 B 0.86
A 0.14 B 1
A 0.29 B 0
A 0.29 B 0.14
A 0.29 B 0.29
A 0.29 B 0.43
A 0.29 B 0.57
A 0.29 B 0.71
A 0.29 B 0.86
A 0.29 B 1
A 0.43 B 0
A 0.43 B 0.14
A 0.43 B 0.29
A 0.43 B 0.43
A 0.43 B 0.57
A 0.43 B 0.71
A 0.43 B 0.86
A 0.43 B 1
A 0.57 B 0
A 0.57 B 0.14
A 0.57 B 0.29
A 0.57 B 0.43
A 0.57 B 0.57
A 0.57 B 0.71
A 0.57 B 0.86
A 0.57 B 1
A 0.71 B 0
A 0.71 B 0.14
A 0.71 B 0.29
A 0.71 B 0.43
A 0.71 B 0.57
A 0.71 B 0.71
A 0.71 B 0.86
A 0.71 B 1
A 0.86 B 0
A 0.86 B 0.14
A 0.86 B 0.29
A 0.86 B 0.43
A 0.86 B 0.57
A 0.86 B 0.71
A 0.86 B 0.86
A 0.86 B 1
A 1 B 0
A 1 B 0.14
A 1 B 0.29
A 1 B 0.43
A 1 B 0.57
A 1 B 0.71
A 1 B 0.86
A 1 B 1
0.6
0.4
0.2
0 5 101520

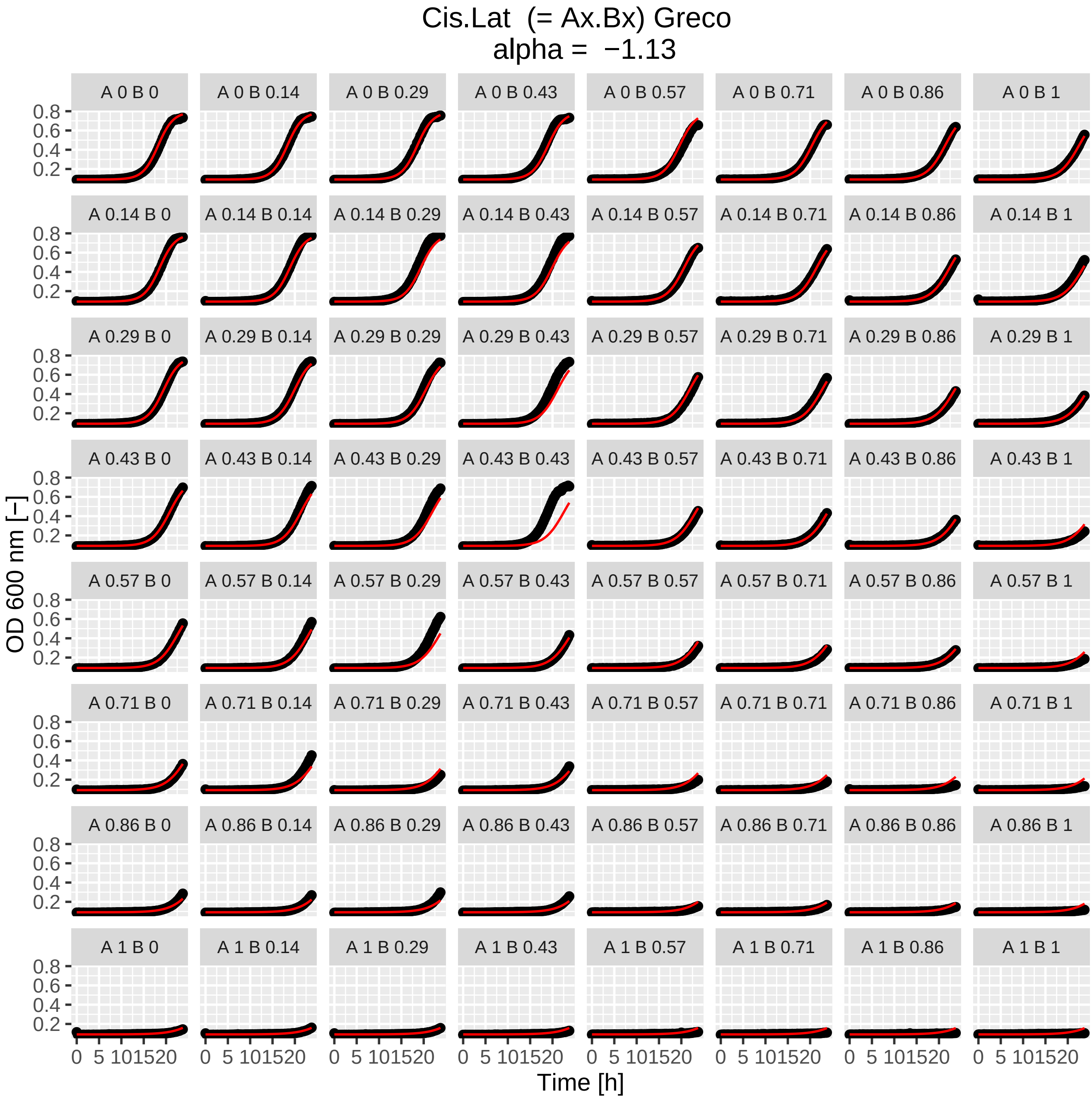

Cis.Lat (= Ax.Bx) Greco
alpha = −1.13
OD 600 nm [−]
Time [h]
A 0 B 0
A 0 B 0.14
A 0 B 0.29
A 0 B 0.43
A 0 B 0.57
A 0 B 0.71
A 0 B 0.86
A 0 B 1
A 0.14 B 0
A 0.29 B 0
A 0.43 B 0
A 0.57 B 0
A 0.71 B 0
A 0.86 B 0
A 1 B 0
A 1 B 1

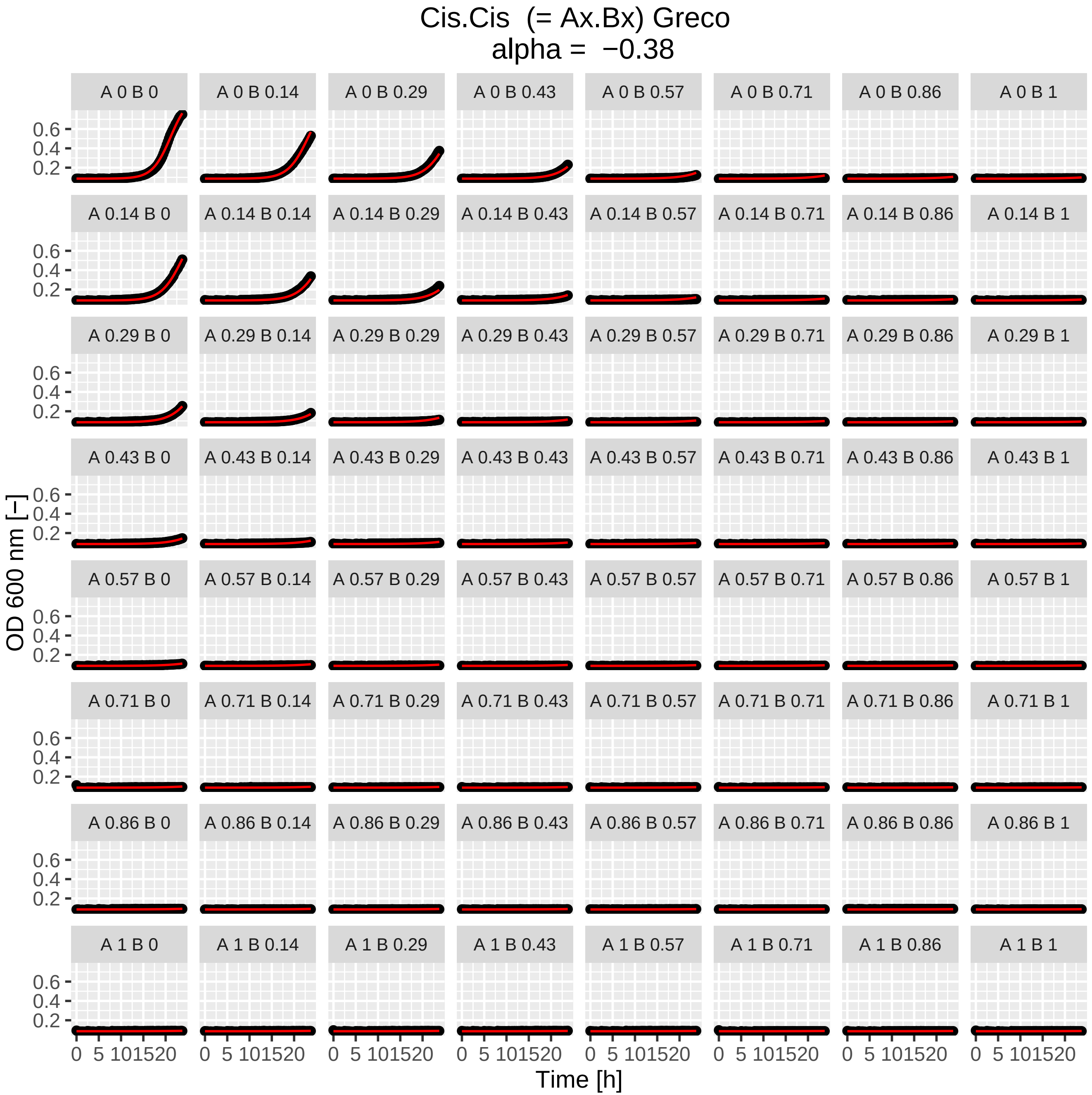
Cis.Cis (= Ax.Bx) Greco
alpha = −0.38
A 0 B 0
A 0 B 0.14
A 0 B 0.29
A 0 B 0.43
A 0 B 0.57
A 0 B 0.71
A 0 B 0.86
A 0 B 1
A 0.14 B 0
A 0.14 B 0.14
A 0.14 B 0.29
A 0.14 B 0.43
A 0.14 B 0.57
A 0.14 B 0.71
A 0.14 B 0.86
A 0.14 B 1
A 0.29 B 0
A 0.29 B 0.14
A 0.29 B 0.29
A 0.29 B 0.43
A 0.29 B 0.57
A 0.29 B 0.71
A 0.29 B 0.86
A 0.29 B 1
A 0.43 B 0
A 0.43 B 0.14
A 0.43 B 0.29
A 0.43 B 0.43
A 0.43 B 0.57
A 0.43 B 0.71
A 0.43 B 0.86
A 0.43 B 1
A 0.57 B 0
A 0.57 B 0.14
A 0.57 B 0.29
A 0.57 B 0.43
A 0.57 B 0.57
A 0.57 B 0.71
A 0.57 B 0.86
A 0.57 B 1
A 0.71 B 0
A 0.71 B 0.14
A 0.71 B 0.29
A 0.71 B 0.43
A 0.71 B 0.57
A 0.71 B 0.71
A 0.71 B 0.86
A 0.71 B 1
A 0.86 B 0
A 0.86 B 0.14
A 0.86 B 0.29
A 0.86 B 0.43
A 0.86 B 0.57
A 0.86 B 0.71
A 0.86 B 0.86
A 0.86 B 1
A 1 B 0
A 1 B 0.14
A 1 B 0.29
A 1 B 0.43
A 1 B 0.57
A 1 B 0.71
A 1 B 0.86
A 1 B 1
0.6
0.4
0.2
0 5 101520
OD 600 nm [−]
Time [h]

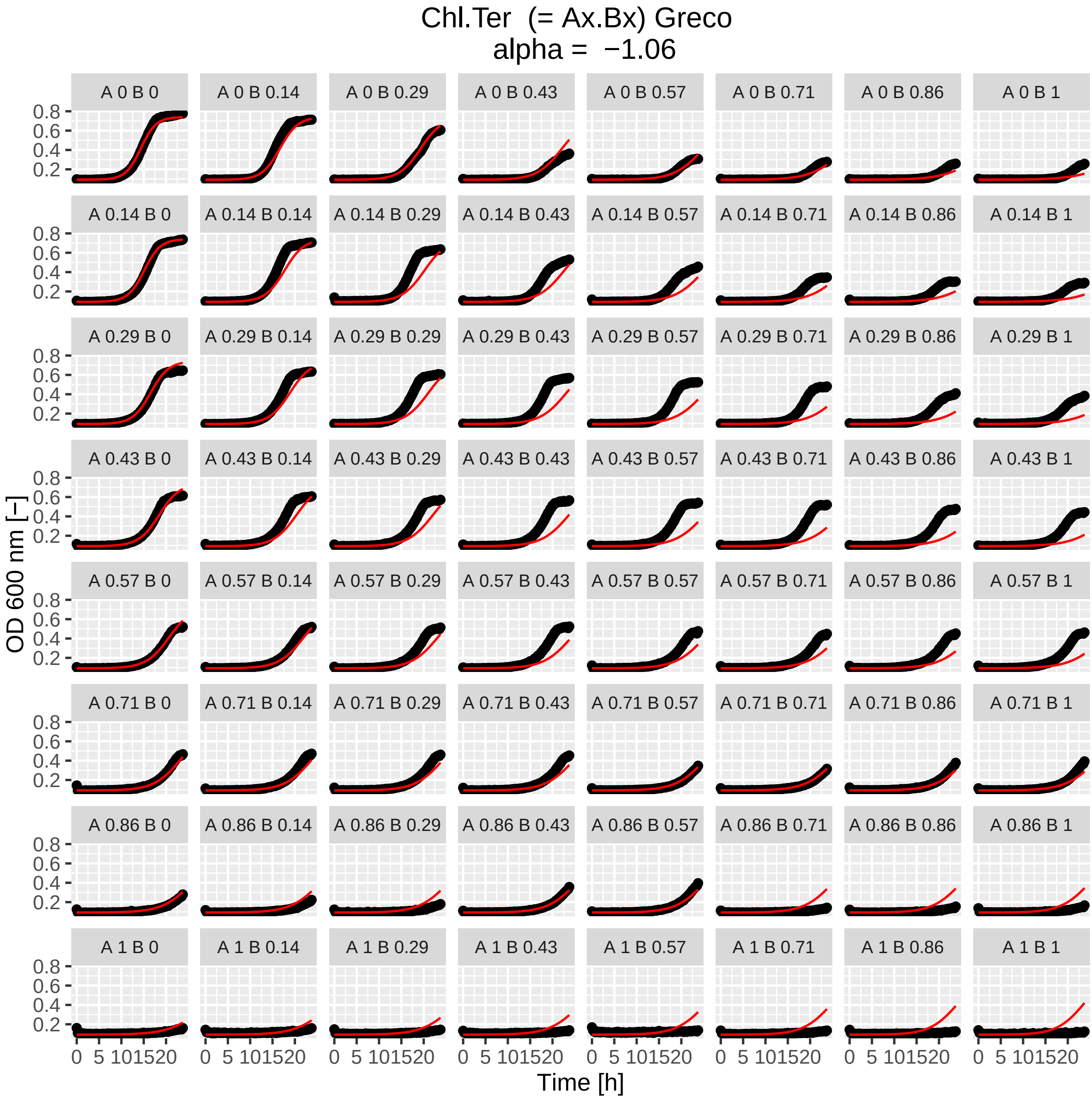

Chl.Ter (= Ax.Bx) Greco
alpha = −1.06
OD 600 nm [−]
Time [h]
0 5 10 15 20
0.2 0.4 0.6 0.8
A 0 B 0
A 0 B 0.14
A 0 B 0.29
A 0 B 0.43
A 0 B 0.57
A 0 B 0.71
A 0 B 0.86
A 0 B 1
A 0.14 B 0
A 0.14 B 0.14
A 0.14 B 0.29
A 0.14 B 0.43
A 0.14 B 0.57
A 0.14 B 0.71
A 0.14 B 0.86
A 0.14 B 1
A 0.29 B 0
A 0.29 B 0.14
A 0.29 B 0.29
A 0.29 B 0.43
A 0.29 B 0.57
A 0.29 B 0.71
A 0.29 B 0.86
A 0.29 B 1
A 0.43 B 0
A 0.43 B 0.14
A 0.43 B 0.29
A 0.43 B 0.43
A 0.43 B 0.57
A 0.43 B 0.71
A 0.43 B 0.86
A 0.43 B 1
A 0.57 B 0
A 0.57 B 0.14
A 0.57 B 0.29
A 0.57 B 0.43
A 0.57 B 0.57
A 0.57 B 0.71
A 0.57 B 0.86
A 0.57 B 1
A 0.71 B 0
A 0.71 B 0.14
A 0.71 B 0.29
A 0.71 B 0.43
A 0.71 B 0.57
A 0.71 B 0.71
A 0.71 B 0.86
A 0.71 B 1
A 0.86 B 0
A 0.86 B 0.14
A 0.86 B 0.29
A 0.86 B 0.43
A 0.86 B 0.57
A 0.86 B 0.71
A 0.86 B 0.86
A 0.86 B 1
A 1 B 0
A 1 B 0.14
A 1 B 0.29
A 1 B 0.43
A 1 B 0.57
A 1 B 0.71
A 1 B 0.86
A 1 B 1

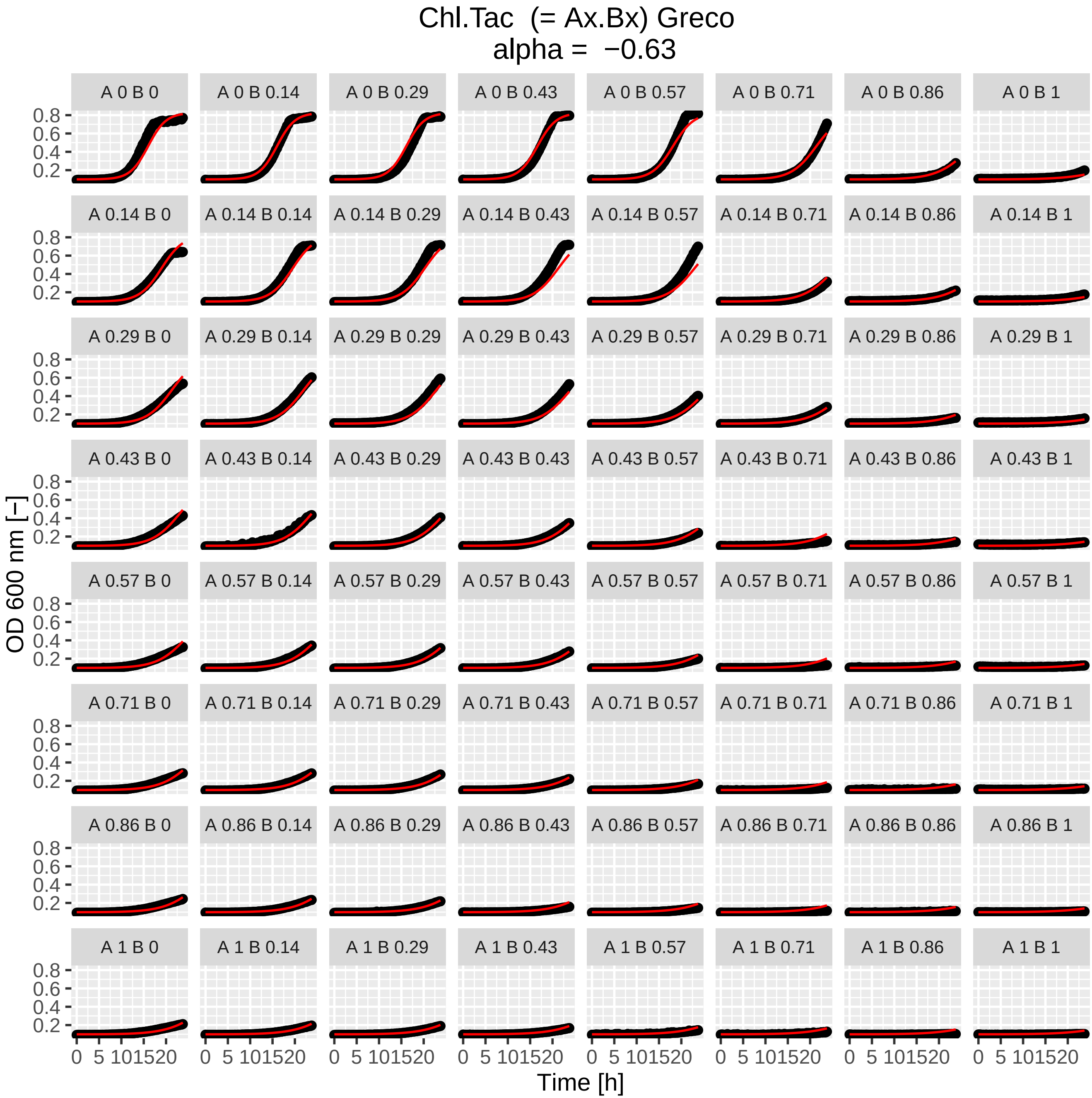

Chl.Tac (= Ax.Bx) Greco
alpha = −0.63
OD 600 nm [−]
Time [h]
A 0 B 0
A 0 B 0.14
A 0 B 0.29
A 0 B 0.43
A 0 B 0.57
A 0 B 0.71
A 0 B 0.86
A 0 B 1
A 0.14 B 0
A 0.14 B 0.14
A 0.14 B 0.29
A 0.14 B 0.43
A 0.14 B 0.57
A 0.14 B 0.71
A 0.14 B 0.86
A 0.14 B 1
A 0.29 B 0
A 0.29 B 0.14
A 0.29 B 0.29
A 0.29 B 0.43
A 0.29 B 0.57
A 0.29 B 0.71
A 0.29 B 0.86
A 0.29 B 1
A 0.43 B 0
A 0.43 B 0.14
A 0.43 B 0.29
A 0.43 B 0.43
A 0.43 B 0.57
A 0.43 B 0.71
A 0.43 B 0.86
A 0.43 B 1
A 0.57 B 0
A 0.57 B 0.14
A 0.57 B 0.29
A 0.57 B 0.43
A 0.57 B 0.57
A 0.57 B 0.71
A 0.57 B 0.86
A 0.57 B 1
A 0.71 B 0
A 0.71 B 0.14
A 0.71 B 0.29
A 0.71 B 0.43
A 0.71 B 0.57
A 0.71 B 0.71
A 0.71 B 0.86
A 0.71 B 1
A 0.86 B 0
A 0.86 B 0.14
A 0.86 B 0.29
A 0.86 B 0.43
A 0.86 B 0.57
A 0.86 B 0.71
A 0.86 B 0.86
A 0.86 B 1
A 1 B 0
A 1 B 0.14
A 1 B 0.29
A 1 B 0.43
A 1 B 0.57
A 1 B 0.71
A 1 B 0.86
A 1 B 1
0.8
0.6
0.4
0.2
0 5 101520

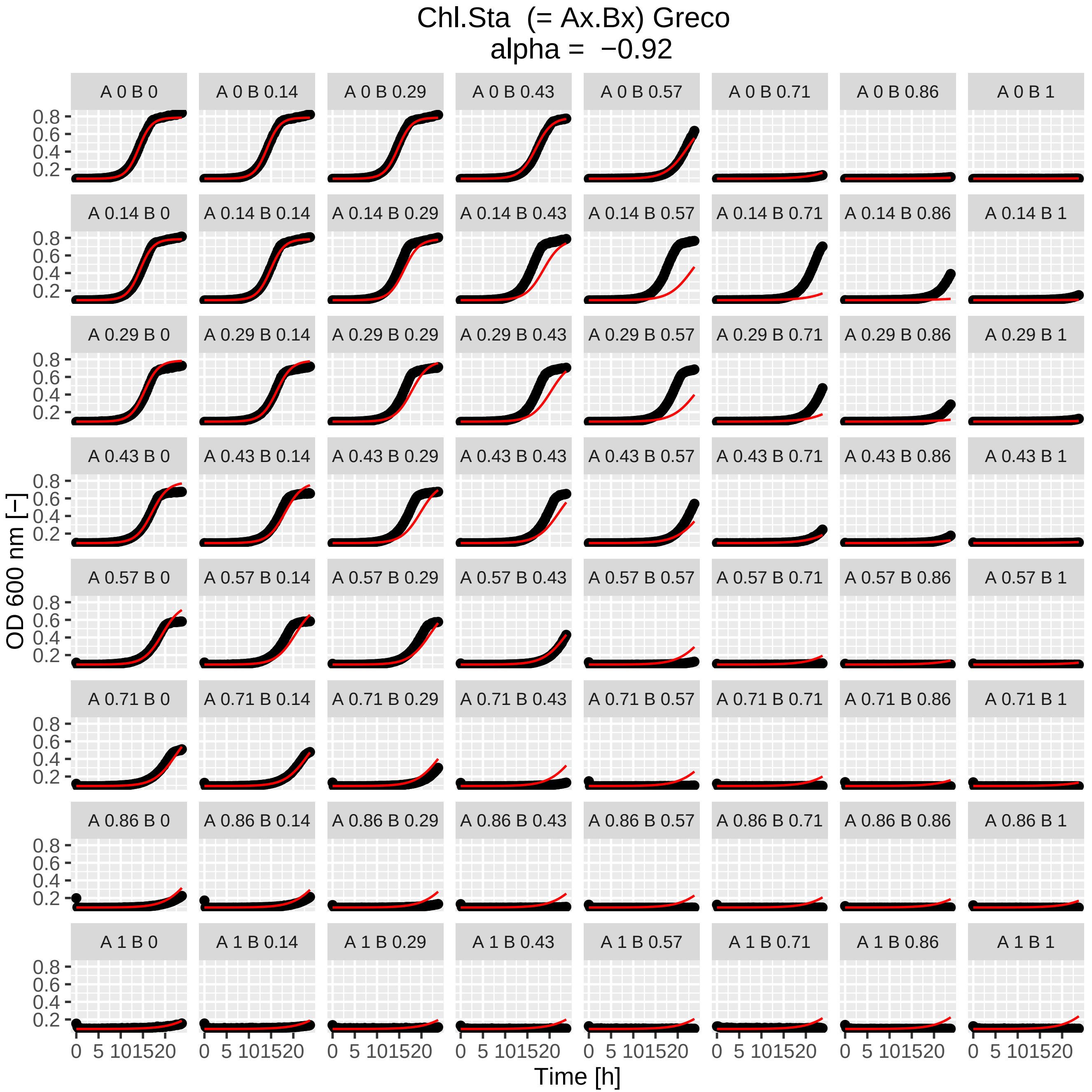
Chl.Sta (= Ax.Bx) Greco
alpha = −0.92
OD 600 nm [−]
Time [h]
0 5 101520
0.2
0.4
0.6
0.8
A 0 B 0
A 0 B 0.14
A 0 B 0.29
A 0 B 0.43
A 0 B 0.57
A 0 B 0.71
A 0 B 0.86
A 0 B 1
A 0.14 B 0
A 0.14 B 0.14
A 0.14 B 0.29
A 0.14 B 0.43
A 0.14 B 0.57
A 0.14 B 0.71
A 0.14 B 0.86
A 0.14 B 1
A 0.29 B 0
A 0.29 B 0.14
A 0.29 B 0.29
A 0.29 B 0.43
A 0.29 B 0.57
A 0.29 B 0.71
A 0.29 B 0.86
A 0.29 B 1
A 0.43 B 0
A 0.43 B 0.14
A 0.43 B 0.29
A 0.43 B 0.43
A 0.43 B 0.57
A 0.43 B 0.71
A 0.43 B 0.86
A 0.43 B 1
A 0.57 B 0
A 0.57 B 0.14
A 0.57 B 0.29
A 0.57 B 0.43
A 0.57 B 0.57
A 0.57 B 0.71
A 0.57 B 0.86
A 0.57 B 1
A 0.71 B 0
A 0.71 B 0.14
A 0.71 B 0.29
A 0.71 B 0.43
A 0.71 B 0.57
A 0.71 B 0.71
A 0.71 B 0.86
A 0.71 B 1
A 0.86 B 0
A 0.86 B 0.14
A 0.86 B 0.29
A 0.86 B 0.43
A 0.86 B 0.57
A 0.86 B 0.71
A 0.86 B 0.86
A 0.86 B 1
A 1 B 0
A 1 B 0.14
A 1 B 0.29
A 1 B 0.43
A 1 B 0.57
A 1 B 0.71
A 1 B 0.86
A 1 B 1

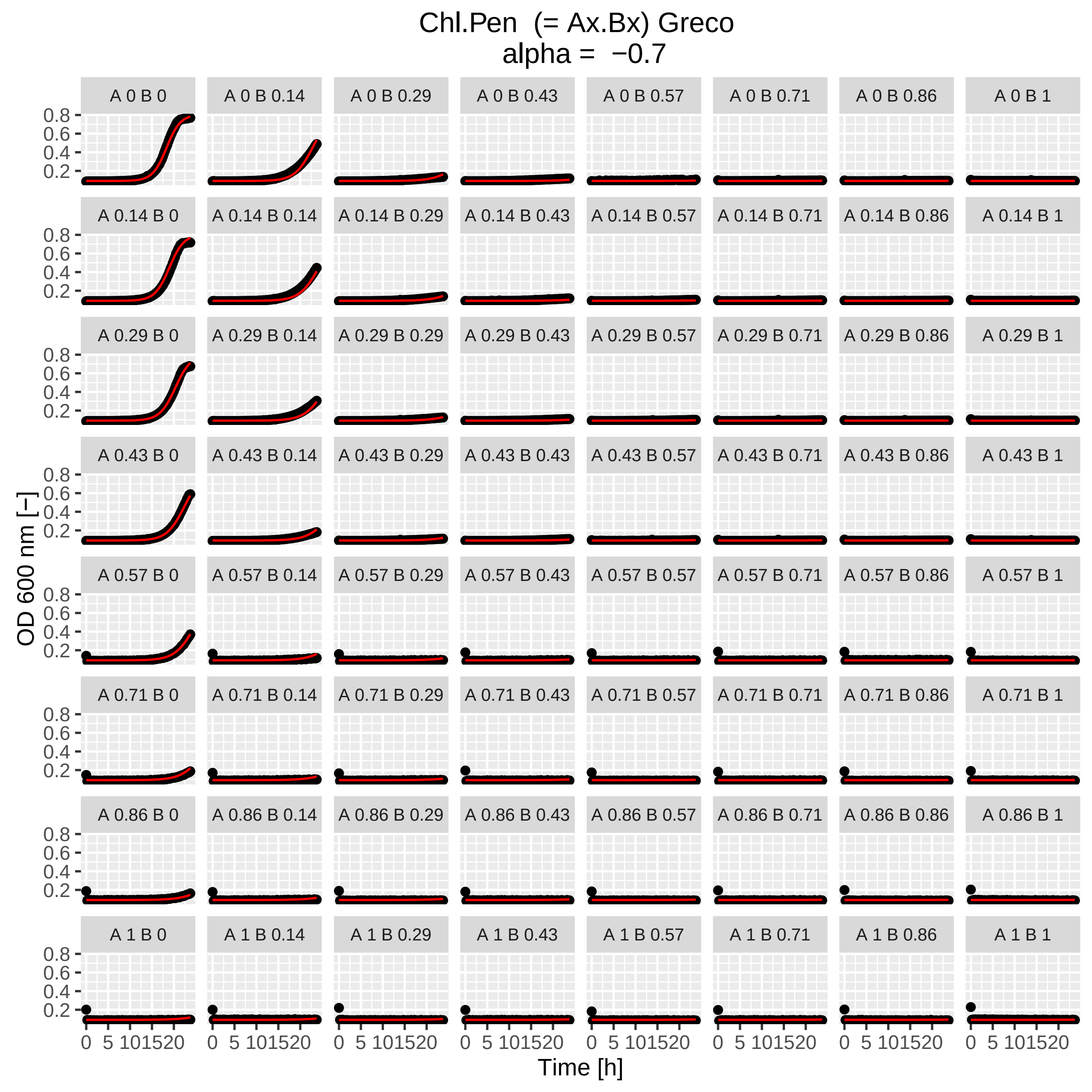
Chl.Pen (= Ax.Bx) Greco
alpha = −0.7
A 0 B 0
A 0 B 0.14
A 0 B 0.29
A 0 B 0.43
A 0 B 0.57
A 0 B 0.71
A 0 B 0.86
A 0 B 1
A 0.14 B 0
A 0.14 B 0.14
A 0.14 B 0.29
A 0.14 B 0.43
A 0.14 B 0.57
A 0.14 B 0.71
A 0.14 B 0.86
A 0.14 B 1
A 0.29 B 0
A 0.29 B 0.14
A 0.29 B 0.29
A 0.29 B 0.43
A 0.29 B 0.57
A 0.29 B 0.71
A 0.29 B 0.86
A 0.29 B 1
A 0.43 B 0
A 0.43 B 0.14
A 0.43 B 0.29
A 0.43 B 0.43
A 0.43 B 0.57
A 0.43 B 0.71
A 0.43 B 0.86
A 0.43 B 1
A 0.57 B 0
A 0.57 B 0.14
A 0.57 B 0.29
A 0.57 B 0.43
A 0.57 B 0.57
A 0.57 B 0.71
A 0.57 B 0.86
A 0.57 B 1
A 0.71 B 0
A 0.71 B 0.14
A 0.71 B 0.29
A 0.71 B 0.43
A 0.71 B 0.57
A 0.71 B 0.71
A 0.71 B 0.86
A 0.71 B 1
A 0.86 B 0
A 0.86 B 0.14
A 0.86 B 0.29
A 0.86 B 0.43
A 0.86 B 0.57
A 0.86 B 0.71
A 0.86 B 0.86
A 0.86 B 1
A 1 B 0
A 1 B 0.14
A 1 B 0.29
A 1 B 0.43
A 1 B 0.57
A 1 B 0.71
A 1 B 0.86
A 1 B 1
OD 600 nm [−]
0.2
0.4
0.6
0.8
0
5
10
15
20
Time [h]

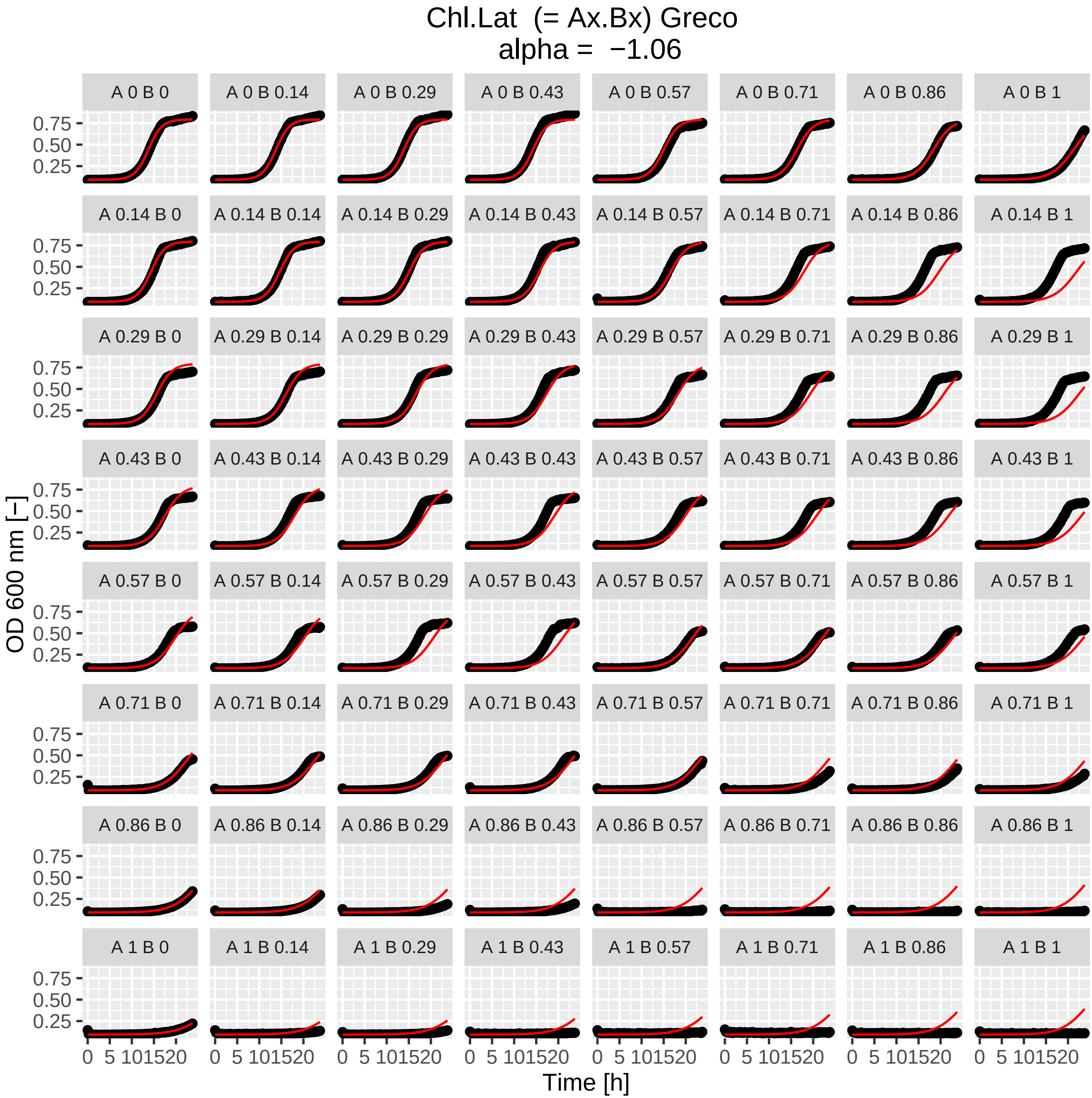
Chl.Lat (= Ax.Bx) Greco
alpha = −1.06
A 0 B 0
A 0 B 0.14
A 0 B 0.29
A 0 B 0.43
A 0 B 0.57
A 0 B 0.71
A 0 B 0.86
A 0 B 1
A 0.14 B 0
A 0.14 B 0.14
A 0.14 B 0.29
A 0.14 B 0.43
A 0.14 B 0.57
A 0.14 B 0.71
A 0.14 B 0.86
A 0.14 B 1
A 0.29 B 0
A 0.29 B 0.14
A 0.29 B 0.29
A 0.29 B 0.43
A 0.29 B 0.57
A 0.29 B 0.71
A 0.29 B 0.86
A 0.29 B 1
A 0.43 B 0
A 0.43 B 0.14
A 0.43 B 0.29
A 0.43 B 0.43
A 0.43 B 0.57
A 0.43 B 0.71
A 0.43 B 0.86
A 0.43 B 1
A 0.57 B 0
A 0.57 B 0.14
A 0.57 B 0.29
A 0.57 B 0.43
A 0.57 B 0.57
A 0.57 B 0.71
A 0.57 B 0.86
A 0.57 B 1
A 0.71 B 0
A 0.71 B 0.14
A 0.71 B 0.29
A 0.71 B 0.43
A 0.71 B 0.57
A 0.71 B 0.71
A 0.71 B 0.86
A 0.71 B 1
A 0.86 B 0
A 0.86 B 0.14
A 0.86 B 0.29
A 0.86 B 0.43
A 0.86 B 0.57
A 0.86 B 0.71
A 0.86 B 0.86
A 0.86 B 1
A 1 B 0
A 1 B 0.14
A 1 B 0.29
A 1 B 0.43
A 1 B 0.57
A 1 B 0.71
A 1 B 0.86
A 1 B 1
OD 600 nm [−]
0.75
0.50
0.25
0 5 101520
Time [h]

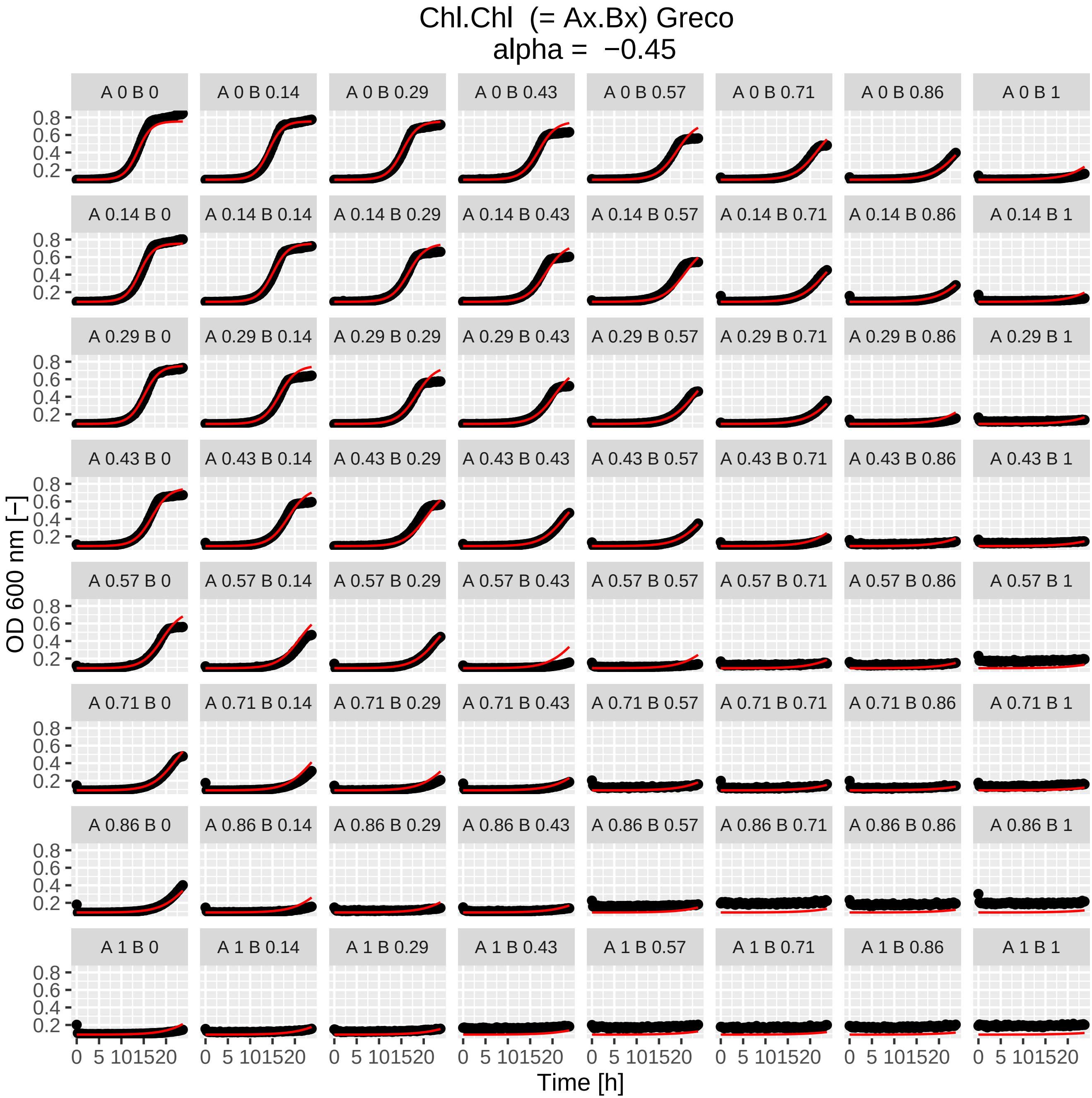

Chl.Chl (= Ax.Bx) Greco
alpha = −0.45
A 0 B 0
A 0 B 0.14
A 0 B 0.29
A 0 B 0.43
A 0 B 0.57
A 0 B 0.71
A 0 B 0.86
A 0 B 1
A 0.14 B 0
A 0.14 B 0.14
A 0.14 B 0.29
A 0.14 B 0.43
A 0.14 B 0.57
A 0.14 B 0.71
A 0.14 B 0.86
A 0.14 B 1
A 0.29 B 0
A 0.29 B 0.14
A 0.29 B 0.29
A 0.29 B 0.43
A 0.29 B 0.57
A 0.29 B 0.71
A 0.29 B 0.86
A 0.29 B 1
A 0.43 B 0
A 0.43 B 0.14
A 0.43 B 0.29
A 0.43 B 0.43
A 0.43 B 0.57
A 0.43 B 0.71
A 0.43 B 0.86
A 0.43 B 1
A 0.57 B 0
A 0.57 B 0.14
A 0.57 B 0.29
A 0.57 B 0.43
A 0.57 B 0.57
A 0.57 B 0.71
A 0.57 B 0.86
A 0.57 B 1
A 0.71 B 0
A 0.71 B 0.14
A 0.71 B 0.29
A 0.71 B 0.43
A 0.71 B 0.57
A 0.71 B 0.71
A 0.71 B 0.86
A 0.71 B 1
A 0.86 B 0
A 0.86 B 0.14
A 0.86 B 0.29
A 0.86 B 0.43
A 0.86 B 0.57
A 0.86 B 0.71
A 0.86 B 0.86
A 0.86 B 1
A 1 B 0
A 1 B 0.14
A 1 B 0.29
A 1 B 0.43
A 1 B 0.57
A 1 B 0.71
A 1 B 0.86
A 1 B 1
OD 600 nm [−]
0.2
0.4
0.6
0.8
0 5 101520
Time [h]

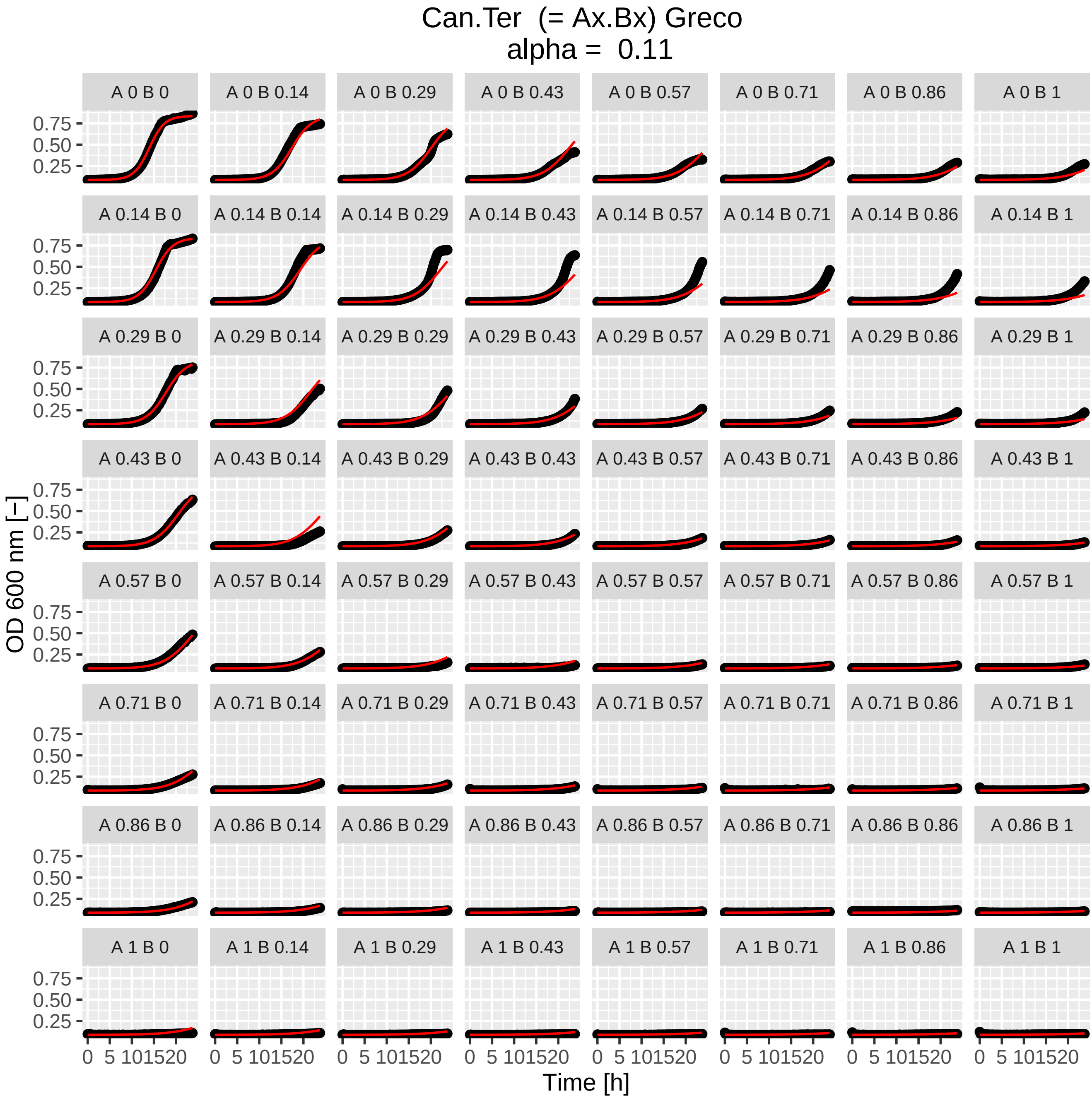

Can.Ter (= Ax.Bx) Greco
alpha = 0.11
OD 600 nm [–]
Time [h]
A 0 B 0
A 0 B 0.14
A 0 B 0.29
A 0 B 0.43
A 0 B 0.57
A 0 B 0.71
A 0 B 0.86
A 0 B 1
A 0.14 B 0
A 0.14 B 0.14
A 0.14 B 0.29
A 0.14 B 0.43
A 0.14 B 0.57
A 0.14 B 0.71
A 0.14 B 0.86
A 0.14 B 1
A 0.29 B 0
A 0.29 B 0.14
A 0.29 B 0.29
A 0.29 B 0.43
A 0.29 B 0.57
A 0.29 B 0.71
A 0.29 B 0.86
A 0.29 B 1
A 0.43 B 0
A 0.43 B 0.14
A 0.43 B 0.29
A 0.43 B 0.43
A 0.43 B 0.57
A 0.43 B 0.71
A 0.43 B 0.86
A 0.43 B 1
A 0.57 B 0
A 0.57 B 0.14
A 0.57 B 0.29
A 0.57 B 0.43
A 0.57 B 0.57
A 0.57 B 0.71
A 0.57 B 0.86
A 0.57 B 1
A 0.71 B 0
A 0.71 B 0.14
A 0.71 B 0.29
A 0.71 B 0.43
A 0.71 B 0.57
A 0.71 B 0.71
A 0.71 B 0.86
A 0.71 B 1
A 0.86 B 0
A 0.86 B 0.14
A 0.86 B 0.29
A 0.86 B 0.43
A 0.86 B 0.57
A 0.86 B 0.71
A 0.86 B 0.86
A 0.86 B 1
A 1 B 0
A 1 B 0.14
A 1 B 0.29
A 1 B 0.43
A 1 B 0.57
A 1 B 0.71
A 1 B 0.86
A 1 B 1
0.75
0.50
0.25
0 5 101520

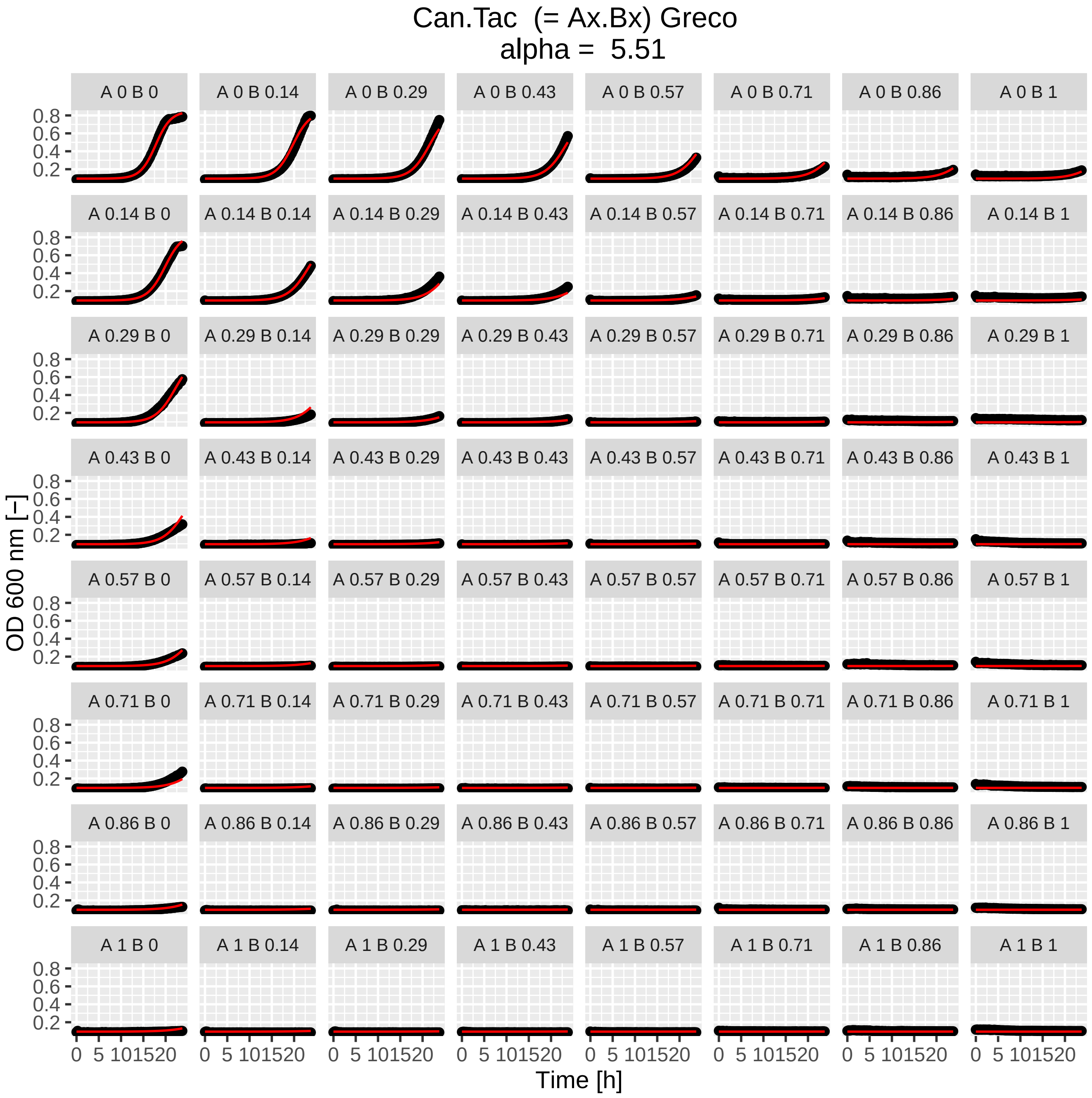
Can.Tac (= Ax.Bx) Greco
alpha = 5.51
A 0 B 0
A 0 B 0.14
A 0 B 0.29
A 0 B 0.43
A 0 B 0.57
A 0 B 0.71
A 0 B 0.86
A 0 B 1
A 0.14 B 0
A 0.14 B 0.14
A 0.14 B 0.29
A 0.14 B 0.43
A 0.14 B 0.57
A 0.14 B 0.71
A 0.14 B 0.86
A 0.14 B 1
A 0.29 B 0
A 0.29 B 0.14
A 0.29 B 0.29
A 0.29 B 0.43
A 0.29 B 0.57
A 0.29 B 0.71
A 0.29 B 0.86
A 0.29 B 1
A 0.43 B 0
A 0.43 B 0.14
A 0.43 B 0.29
A 0.43 B 0.43
A 0.43 B 0.57
A 0.43 B 0.71
A 0.43 B 0.86
A 0.43 B 1
A 0.57 B 0
A 0.57 B 0.14
A 0.57 B 0.29
A 0.57 B 0.43
A 0.57 B 0.57
A 0.57 B 0.71
A 0.57 B 0.86
A 0.57 B 1
A 0.71 B 0
A 0.71 B 0.14
A 0.71 B 0.29
A 0.71 B 0.43
A 0.71 B 0.57
A 0.71 B 0.71
A 0.71 B 0.86
A 0.71 B 1
A 0.86 B 0
A 0.86 B 0.14
A 0.86 B 0.29
A 0.86 B 0.43
A 0.86 B 0.57
A 0.86 B 0.71
A 0.86 B 0.86
A 0.86 B 1
A 1 B 0
A 1 B 0.14
A 1 B 0.29
A 1 B 0.43
A 1 B 0.57
A 1 B 0.71
A 1 B 0.86
A 1 B 1
OD 600 nm [–]
0.8
0.6
0.4
0.2
0 5 101520
Time [h]

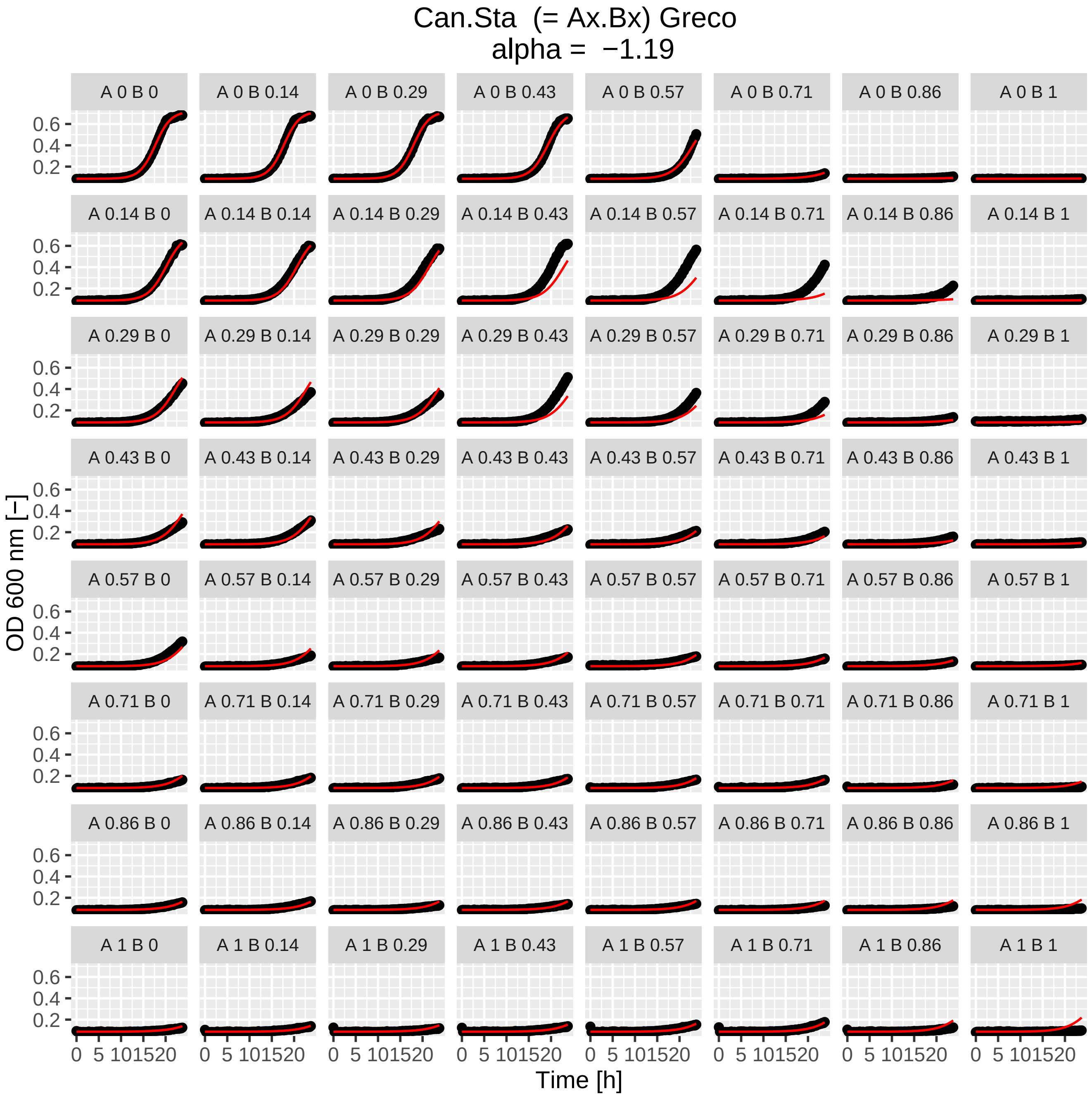
Can.Sta (= Ax.Bx) Greco
alpha = −1.19
OD 600 nm [−]
Time [h]
A 0 B 0
A 0 B 0.14
A 0 B 0.29
A 0 B 0.43
A 0 B 0.57
A 0 B 0.71
A 0 B 0.86
A 0 B 1
A 1 B 0
A 1 B 0.14
A 1 B 0.29
A 1 B 0.43
A 1 B 0.57
A 1 B 0.71
A 1 B 0.86
A 1 B 1

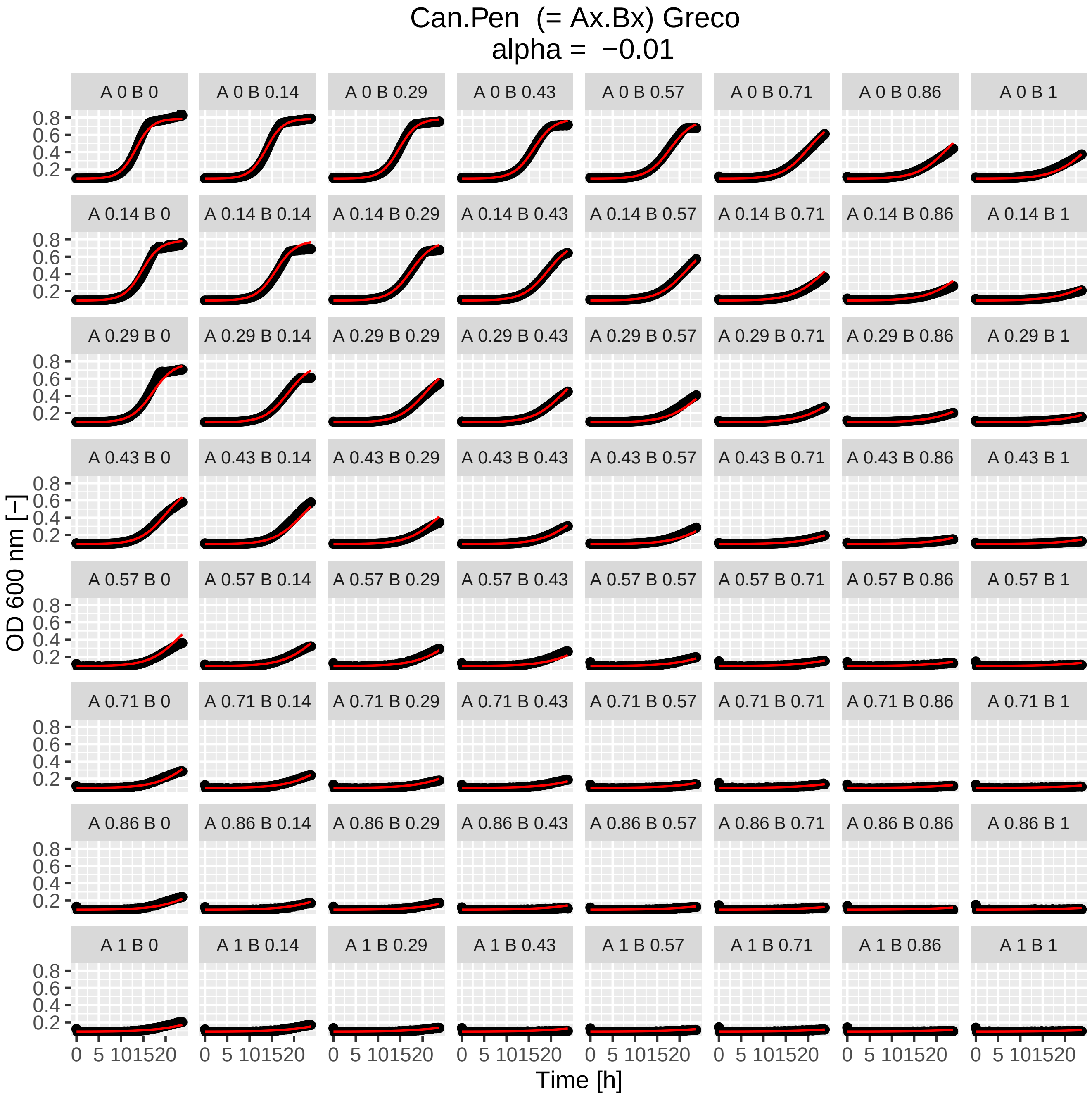
Can.Pen (= Ax.Bx) Greco
alpha = −0.01
A 0 B 0
A 0 B 0.14
A 0 B 0.29
A 0 B 0.43
A 0 B 0.57
A 0 B 0.71
A 0 B 0.86
A 0 B 1
A 0.14 B 0
A 0.14 B 0.14
A 0.14 B 0.29
A 0.14 B 0.43
A 0.14 B 0.57
A 0.14 B 0.71
A 0.14 B 0.86
A 0.14 B 1
A 0.29 B 0
A 0.29 B 0.14
A 0.29 B 0.29
A 0.29 B 0.43
A 0.29 B 0.57
A 0.29 B 0.71
A 0.29 B 0.86
A 0.29 B 1
A 0.43 B 0
A 0.43 B 0.14
A 0.43 B 0.29
A 0.43 B 0.43
A 0.43 B 0.57
A 0.43 B 0.71
A 0.43 B 0.86
A 0.43 B 1
A 0.57 B 0
A 0.57 B 0.14
A 0.57 B 0.29
A 0.57 B 0.43
A 0.57 B 0.57
A 0.57 B 0.71
A 0.57 B 0.86
A 0.57 B 1
A 0.71 B 0
A 0.71 B 0.14
A 0.71 B 0.29
A 0.71 B 0.43
A 0.71 B 0.57
A 0.71 B 0.71
A 0.71 B 0.86
A 0.71 B 1
A 0.86 B 0
A 0.86 B 0.14
A 0.86 B 0.29
A 0.86 B 0.43
A 0.86 B 0.57
A 0.86 B 0.71
A 0.86 B 0.86
A 0.86 B 1
A 1 B 0
A 1 B 0.14
A 1 B 0.29
A 1 B 0.43
A 1 B 0.57
A 1 B 0.71
A 1 B 0.86
A 1 B 1
OD 600 nm [−]
0.2
0.4
0.6
0.8
0 5 101520
Time [h]

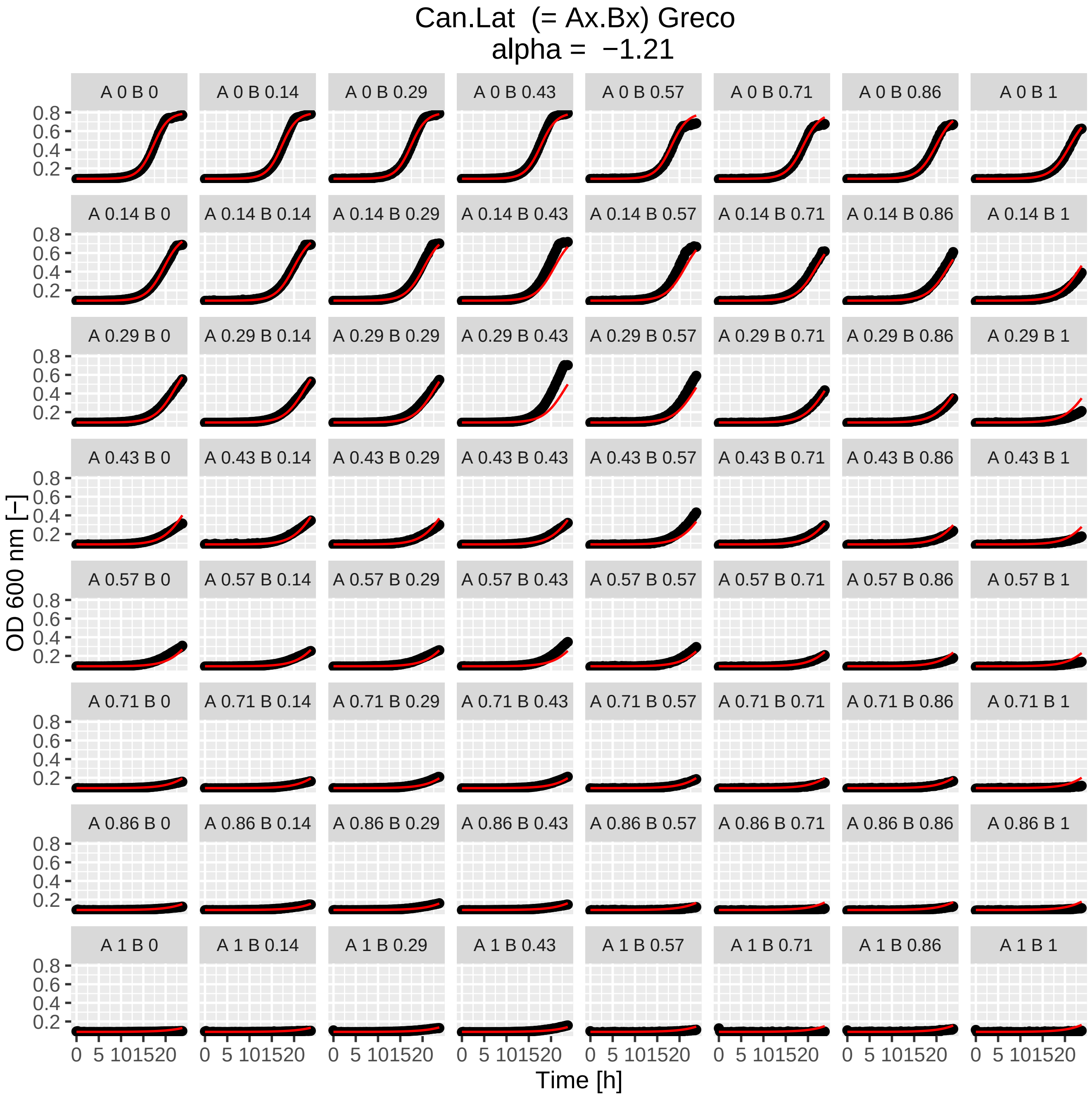

Can.Lat (= Ax.Bx) Greco
alpha = −1.21
A 0 B 0
A 0 B 0.14
A 0 B 0.29
A 0 B 0.43
A 0 B 0.57
A 0 B 0.71
A 0 B 0.86
A 0 B 1
A 0.14 B 0
A 0.14 B 0.14
A 0.14 B 0.29
A 0.14 B 0.43
A 0.14 B 0.57
A 0.14 B 0.71
A 0.14 B 0.86
A 0.14 B 1
A 0.29 B 0
A 0.29 B 0.14
A 0.29 B 0.29
A 0.29 B 0.43
A 0.29 B 0.57
A 0.29 B 0.71
A 0.29 B 0.86
A 0.29 B 1
A 0.43 B 0
A 0.43 B 0.14
A 0.43 B 0.29
A 0.43 B 0.43
A 0.43 B 0.57
A 0.43 B 0.71
A 0.43 B 0.86
A 0.43 B 1
A 0.57 B 0
A 0.57 B 0.14
A 0.57 B 0.29
A 0.57 B 0.43
A 0.57 B 0.57
A 0.57 B 0.71
A 0.57 B 0.86
A 0.57 B 1
A 0.71 B 0
A 0.71 B 0.14
A 0.71 B 0.29
A 0.71 B 0.43
A 0.71 B 0.57
A 0.71 B 0.71
A 0.71 B 0.86
A 0.71 B 1
A 0.86 B 0
A 0.86 B 0.14
A 0.86 B 0.29
A 0.86 B 0.43
A 0.86 B 0.57
A 0.86 B 0.71
A 0.86 B 0.86
A 0.86 B 1
A 1 B 0
A 1 B 0.14
A 1 B 0.29
A 1 B 0.43
A 1 B 0.57
A 1 B 0.71
A 1 B 0.86
A 1 B 1
OD 600 nm [−]
0.8
0.6
0.4
0.2
0 5 101520
Time [h]

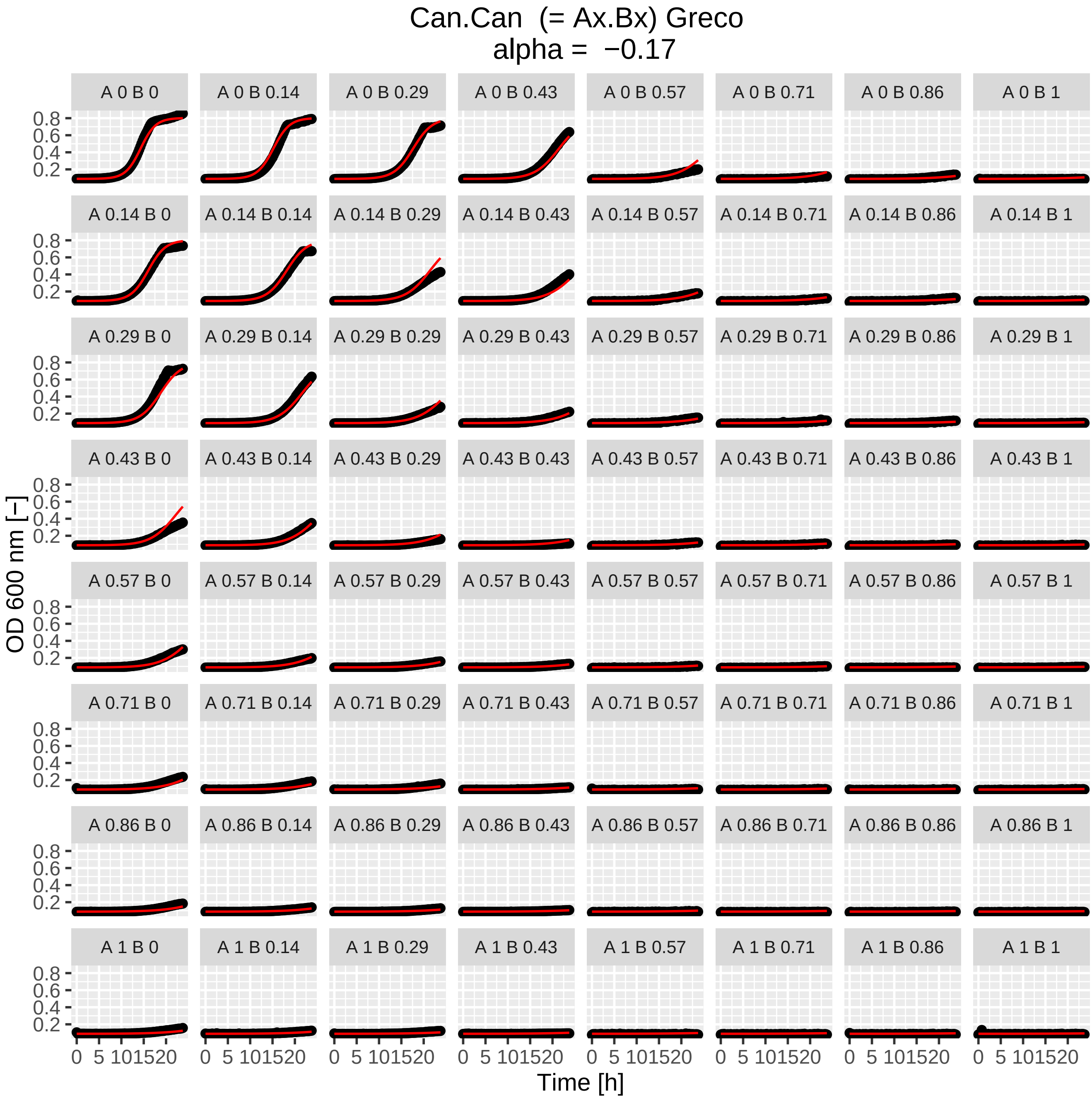

Can.Can (= Ax.Bx) Greco
alpha = −0.17
A 0 B 0
A 0 B 0.14
A 0 B 0.29
A 0 B 0.43
A 0 B 0.57
A 0 B 0.71
A 0 B 0.86
A 0 B 1
A 0.14 B 0
A 0.14 B 0.14
A 0.14 B 0.29
A 0.14 B 0.43
A 0.14 B 0.57
A 0.14 B 0.71
A 0.14 B 0.86
A 0.14 B 1
A 0.29 B 0
A 0.29 B 0.14
A 0.29 B 0.29
A 0.29 B 0.43
A 0.29 B 0.57
A 0.29 B 0.71
A 0.29 B 0.86
A 0.29 B 1
A 0.43 B 0
A 0.43 B 0.14
A 0.43 B 0.29
A 0.43 B 0.43
A 0.43 B 0.57
A 0.43 B 0.71
A 0.43 B 0.86
A 0.43 B 1
A 0.57 B 0
A 0.57 B 0.14
A 0.57 B 0.29
A 0.57 B 0.43
A 0.57 B 0.57
A 0.57 B 0.71
A 0.57 B 0.86
A 0.57 B 1
A 0.71 B 0
A 0.71 B 0.14
A 0.71 B 0.29
A 0.71 B 0.43
A 0.71 B 0.57
A 0.71 B 0.71
A 0.71 B 0.86
A 0.71 B 1
A 0.86 B 0
A 0.86 B 0.14
A 0.86 B 0.29
A 0.86 B 0.43
A 0.86 B 0.57
A 0.86 B 0.71
A 0.86 B 0.86
A 0.86 B 1
A 1 B 0
A 1 B 0.14
A 1 B 0.29
A 1 B 0.43
A 1 B 0.57
A 1 B 0.71
A 1 B 0.86
A 1 B 1
OD 600 nm [−]
0.2
0.4
0.6
0.8
0 5 101520
Time [h]

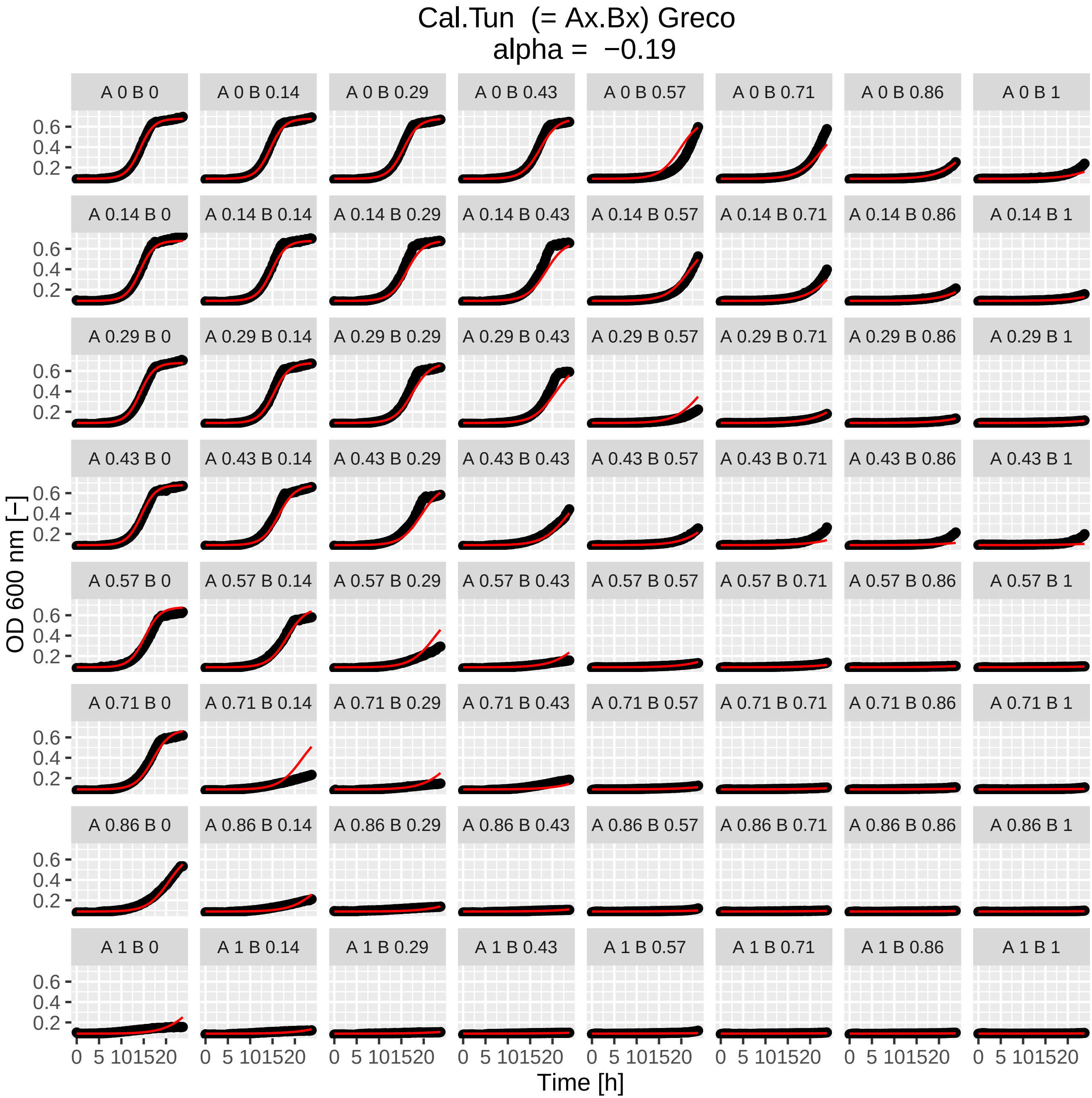

Cal.Tun (= Ax.Bx) Greco
alpha = −0.19
A 0 B 0
A 0 B 0.14
A 0 B 0.29
A 0 B 0.43
A 0 B 0.57
A 0 B 0.71
A 0 B 0.86
A 0 B 1
A 0.14 B 0
A 0.14 B 0.14
A 0.14 B 0.29
A 0.14 B 0.43
A 0.14 B 0.57
A 0.14 B 0.71
A 0.14 B 0.86
A 0.14 B 1
A 0.29 B 0
A 0.29 B 0.14
A 0.29 B 0.29
A 0.29 B 0.43
A 0.29 B 0.57
A 0.29 B 0.71
A 0.29 B 0.86
A 0.29 B 1
A 0.43 B 0
A 0.43 B 0.14
A 0.43 B 0.29
A 0.43 B 0.43
A 0.43 B 0.57
A 0.43 B 0.71
A 0.43 B 0.86
A 0.43 B 1
A 0.57 B 0
A 0.57 B 0.14
A 0.57 B 0.29
A 0.57 B 0.43
A 0.57 B 0.57
A 0.57 B 0.71
A 0.57 B 0.86
A 0.57 B 1
A 0.71 B 0
A 0.71 B 0.14
A 0.71 B 0.29
A 0.71 B 0.43
A 0.71 B 0.57
A 0.71 B 0.71
A 0.71 B 0.86
A 0.71 B 1
A 0.86 B 0
A 0.86 B 0.14
A 0.86 B 0.29
A 0.86 B 0.43
A 0.86 B 0.57
A 0.86 B 0.71
A 0.86 B 0.86
A 0.86 B 1
A 1 B 0
A 1 B 0.14
A 1 B 0.29
A 1 B 0.43
A 1 B 0.57
A 1 B 0.71
A 1 B 0.86
A 1 B 1
OD 600 nm [−]
0.6
0.4
0.2
0 5 101520
Time [h]

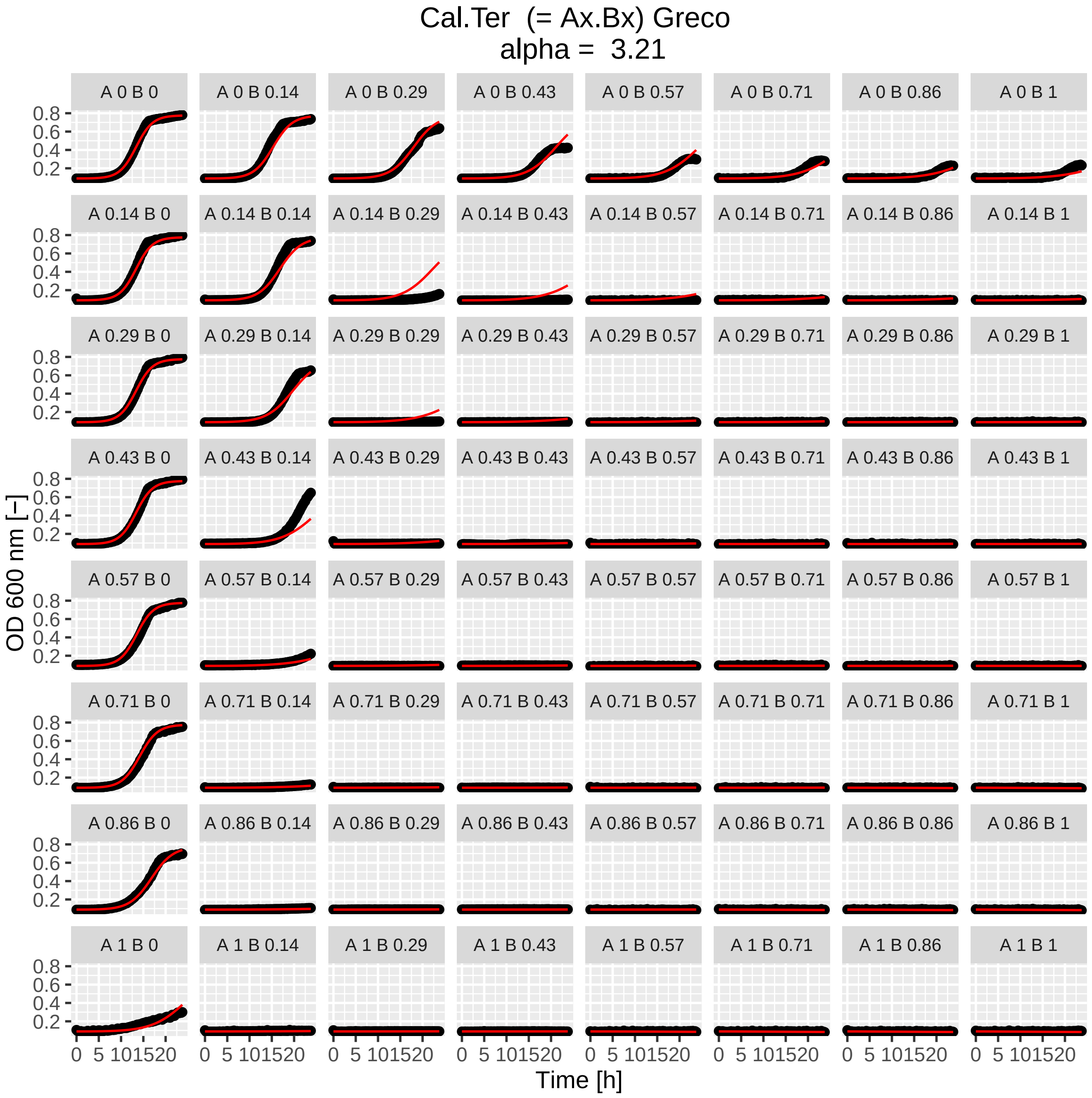
Cal.Ter (= Ax.Bx) Greco
alpha = 3.21
OD 600 nm [–]
Time [h]
A 0 B 0
A 0 B 0.14
A 0 B 0.29
A 0 B 0.43
A 0 B 0.57
A 0 B 0.71
A 0 B 0.86
A 0 B 1
A 0.14 B 0
A 0.14 B 0.14
A 0.14 B 0.29
A 0.14 B 0.43
A 0.14 B 0.57
A 0.14 B 0.71
A 0.14 B 0.86
A 0.14 B 1
A 0.29 B 0
A 0.29 B 0.14
A 0.29 B 0.29
A 0.29 B 0.43
A 0.29 B 0.57
A 0.29 B 0.71
A 0.29 B 0.86
A 0.29 B 1
A 0.43 B 0
A 0.43 B 0.14
A 0.43 B 0.29
A 0.43 B 0.43
A 0.43 B 0.57
A 0.43 B 0.71
A 0.43 B 0.86
A 0.43 B 1
A 0.57 B 0
A 0.57 B 0.14
A 0.57 B 0.29
A 0.57 B 0.43
A 0.57 B 0.57
A 0.57 B 0.71
A 0.57 B 0.86
A 0.57 B 1
A 0.71 B 0
A 0.71 B 0.14
A 0.71 B 0.29
A 0.71 B 0.43
A 0.71 B 0.57
A 0.71 B 0.71
A 0.71 B 0.86
A 0.71 B 1
A 0.86 B 0
A 0.86 B 0.14
A 0.86 B 0.29
A 0.86 B 0.43
A 0.86 B 0.57
A 0.86 B 0.71
A 0.86 B 0.86
A 0.86 B 1
A 1 B 0
A 1 B 0.14
A 1 B 0.29
A 1 B 0.43
A 1 B 0.57
A 1 B 0.71
A 1 B 0.86
A 1 B 1
0.8
0.6
0.4
0.2
0 5 101520

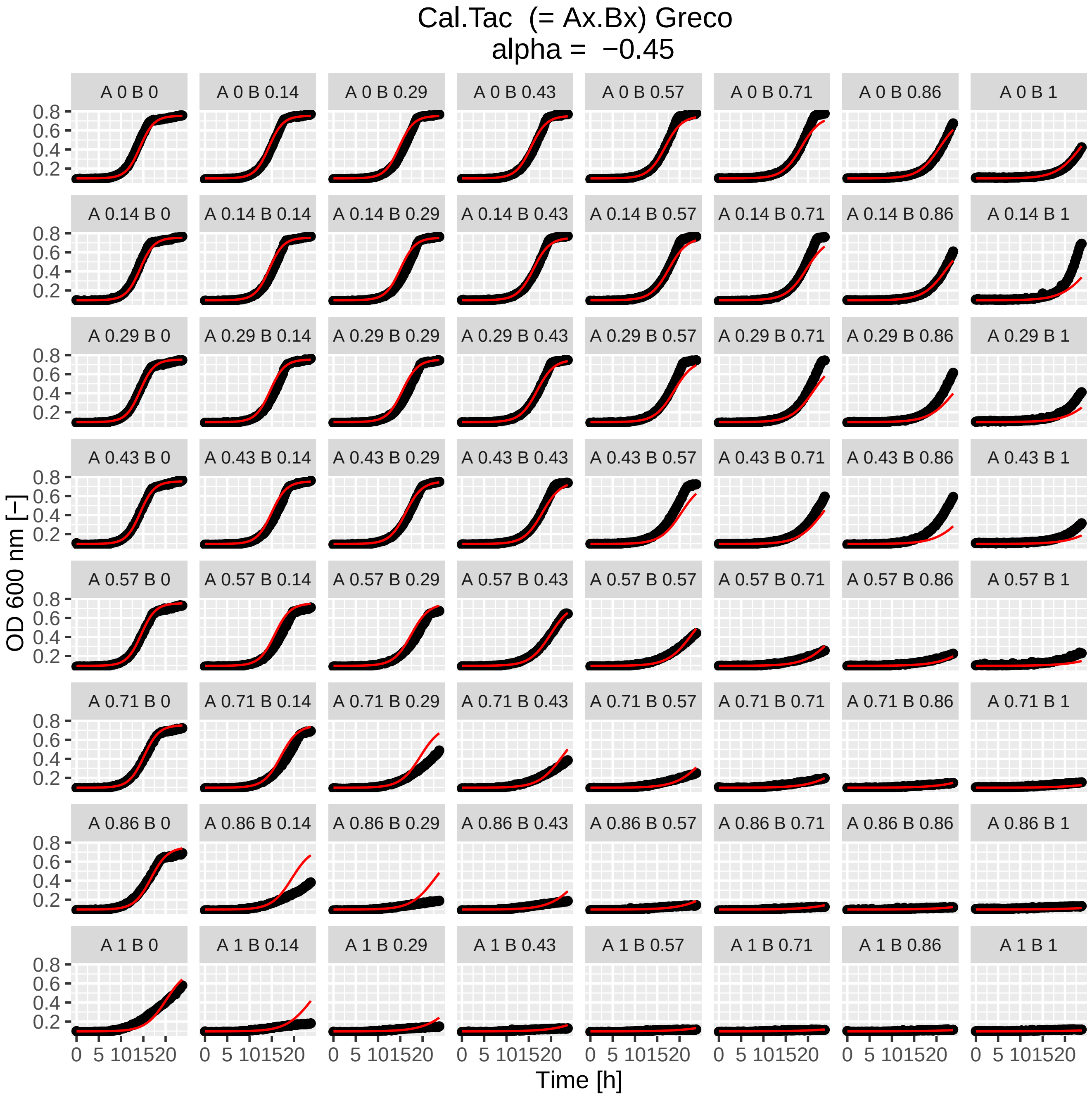
Cal.Tac (= Ax.Bx) Greco
alpha = −0.45
OD 600 nm [−]
Time [h]
A 0 B 0
A 0 B 0.14
A 0 B 0.29
A 0 B 0.43
A 0 B 0.57
A 0 B 0.71
A 0 B 0.86
A 0 B 1
A 0.14 B 0
A 0.14 B 0.14
A 0.14 B 0.29
A 0.14 B 0.43
A 0.14 B 0.57
A 0.14 B 0.71
A 0.14 B 0.86
A 0.14 B 1
A 0.29 B 0
A 0.29 B 0.14
A 0.29 B 0.29
A 0.29 B 0.43
A 0.29 B 0.57
A 0.29 B 0.71
A 0.29 B 0.86
A 0.29 B 1
A 0.43 B 0
A 0.43 B 0.14
A 0.43 B 0.29
A 0.43 B 0.43
A 0.43 B 0.57
A 0.43 B 0.71
A 0.43 B 0.86
A 0.43 B 1
A 0.57 B 0
A 0.57 B 0.14
A 0.57 B 0.29
A 0.57 B 0.43
A 0.57 B 0.57
A 0.57 B 0.71
A 0.57 B 0.86
A 0.57 B 1
A 0.71 B 0
A 0.71 B 0.14
A 0.71 B 0.29
A 0.71 B 0.43
A 0.71 B 0.57
A 0.71 B 0.71
A 0.71 B 0.86
A 0.71 B 1
A 0.86 B 0
A 0.86 B 0.14
A 0.86 B 0.29
A 0.86 B 0.43
A 0.86 B 0.57
A 0.86 B 0.71
A 0.86 B 0.86
A 0.86 B 1
A 1 B 0
A 1 B 0.14
A 1 B 0.29
A 1 B 0.43
A 1 B 0.57
A 1 B 0.71
A 1 B 0.86
A 1 B 1
0.8
0.6
0.4
0.2
0 5 101520

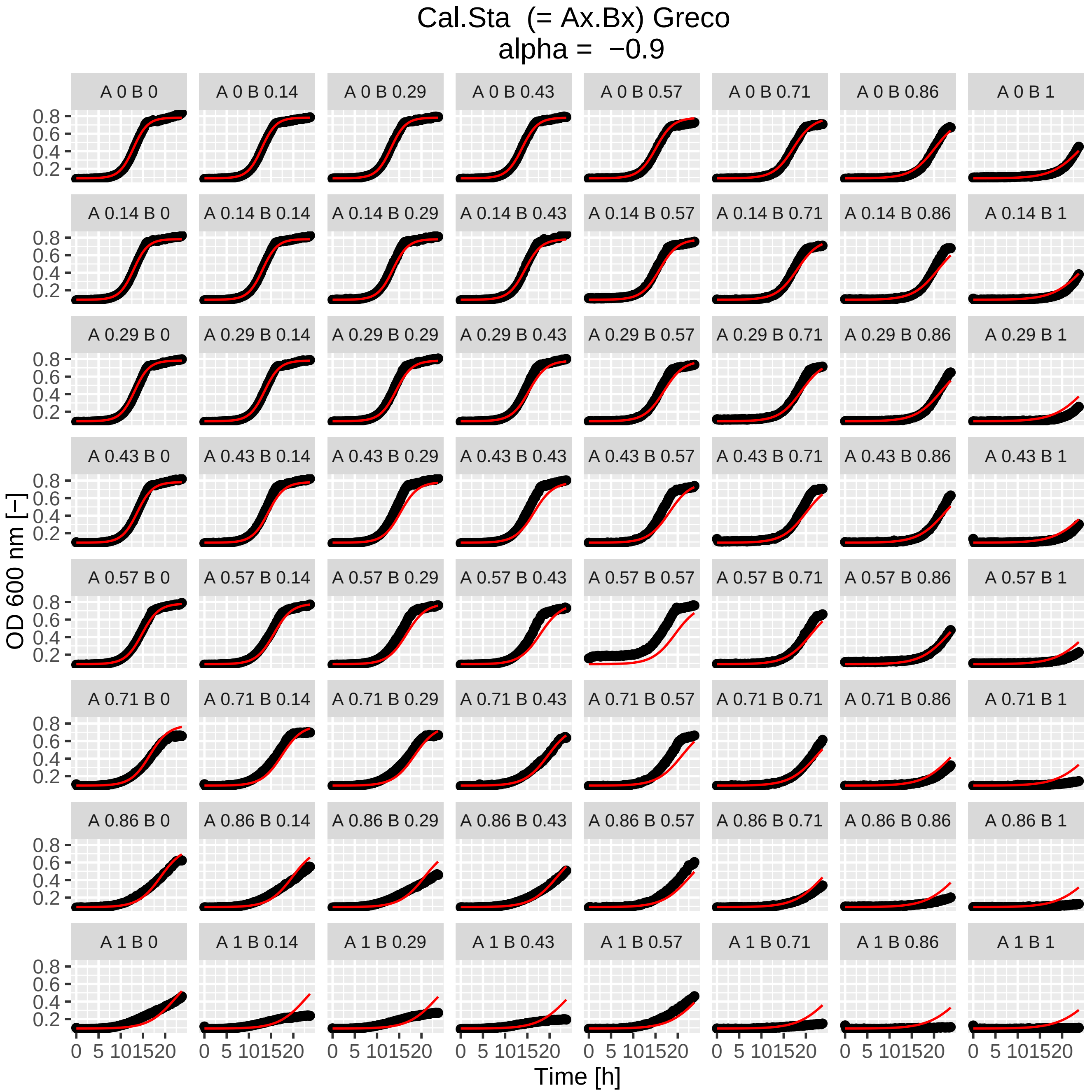

Cal.Sta (= Ax.Bx) Greco
alpha = −0.9
A 0 B 0
A 0 B 0.14
A 0 B 0.29
A 0 B 0.43
A 0 B 0.57
A 0 B 0.71
A 0 B 0.86
A 0 B 1
A 0.14 B 0
A 0.14 B 0.14
A 0.14 B 0.29
A 0.14 B 0.43
A 0.14 B 0.57
A 0.14 B 0.71
A 0.14 B 0.86
A 0.14 B 1
A 0.29 B 0
A 0.29 B 0.14
A 0.29 B 0.29
A 0.29 B 0.43
A 0.29 B 0.57
A 0.29 B 0.71
A 0.29 B 0.86
A 0.29 B 1
A 0.43 B 0
A 0.43 B 0.14
A 0.43 B 0.29
A 0.43 B 0.43
A 0.43 B 0.57
A 0.43 B 0.71
A 0.43 B 0.86
A 0.43 B 1
A 0.57 B 0
A 0.57 B 0.14
A 0.57 B 0.29
A 0.57 B 0.43
A 0.57 B 0.57
A 0.57 B 0.71
A 0.57 B 0.86
A 0.57 B 1
A 0.71 B 0
A 0.71 B 0.14
A 0.71 B 0.29
A 0.71 B 0.43
A 0.71 B 0.57
A 0.71 B 0.71
A 0.71 B 0.86
A 0.71 B 1
A 0.86 B 0
A 0.86 B 0.14
A 0.86 B 0.29
A 0.86 B 0.43
A 0.86 B 0.57
A 0.86 B 0.71
A 0.86 B 0.86
A 0.86 B 1
A 1 B 0
A 1 B 0.14
A 1 B 0.29
A 1 B 0.43
A 1 B 0.57
A 1 B 0.71
A 1 B 0.86
A 1 B 1
OD 600 nm [−]
0.2
0.4
0.6
0.8
0 5 101520
Time [h]

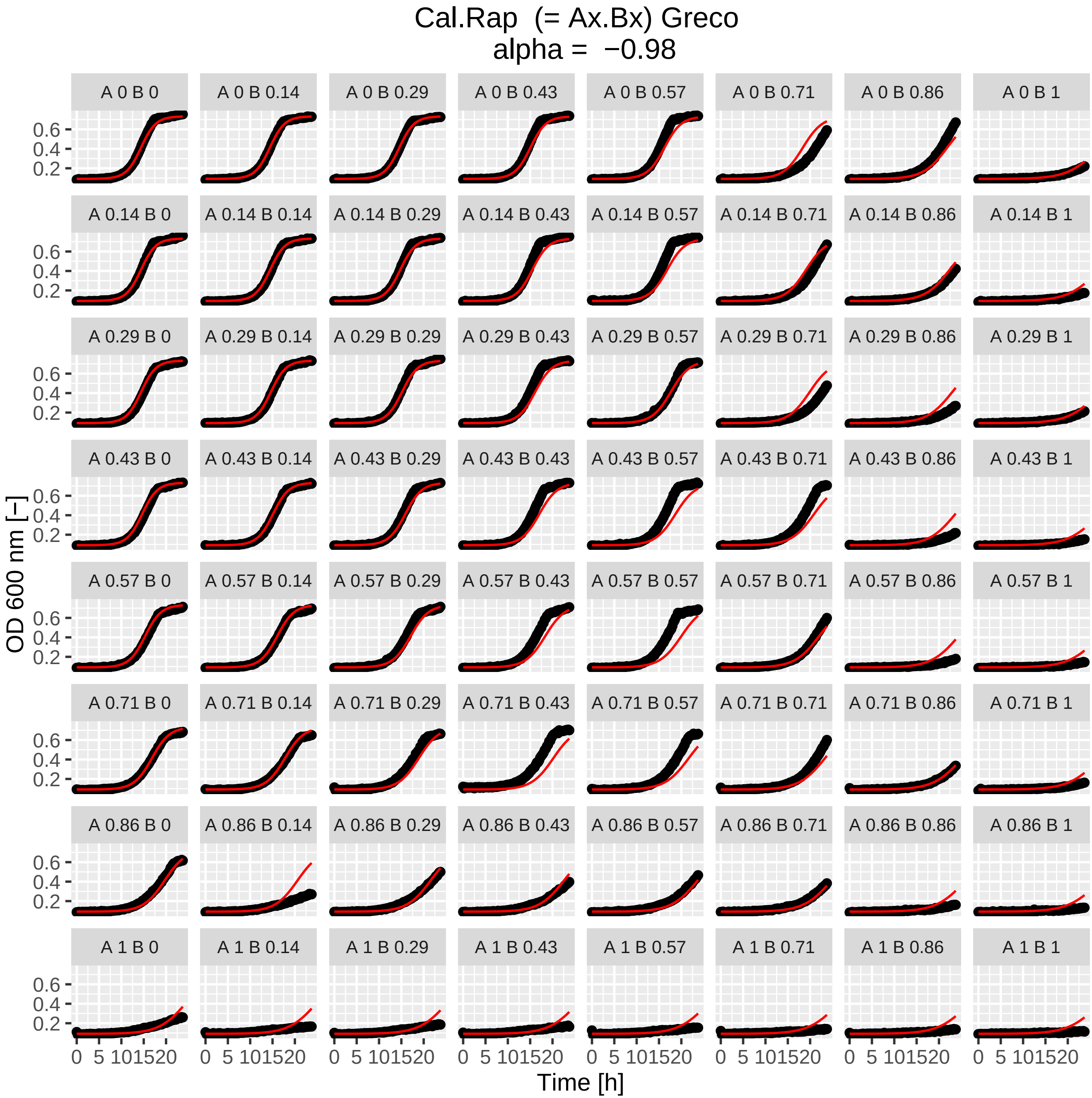

Cal.Rap (= Ax.Bx) Greco
alpha = −0.98
A 0 B 0
A 0 B 0.14
A 0 B 0.29
A 0 B 0.43
A 0 B 0.57
A 0 B 0.71
A 0 B 0.86
A 0 B 1
A 0.14 B 0
A 0.14 B 0.14
A 0.14 B 0.29
A 0.14 B 0.43
A 0.14 B 0.57
A 0.14 B 0.71
A 0.14 B 0.86
A 0.14 B 1
A 0.29 B 0
A 0.29 B 0.14
A 0.29 B 0.29
A 0.29 B 0.43
A 0.29 B 0.57
A 0.29 B 0.71
A 0.29 B 0.86
A 0.29 B 1
A 0.43 B 0
A 0.43 B 0.14
A 0.43 B 0.29
A 0.43 B 0.43
A 0.43 B 0.57
A 0.43 B 0.71
A 0.43 B 0.86
A 0.43 B 1
A 0.57 B 0
A 0.57 B 0.14
A 0.57 B 0.29
A 0.57 B 0.43
A 0.57 B 0.57
A 0.57 B 0.71
A 0.57 B 0.86
A 0.57 B 1
A 0.71 B 0
A 0.71 B 0.14
A 0.71 B 0.29
A 0.71 B 0.43
A 0.71 B 0.57
A 0.71 B 0.71
A 0.71 B 0.86
A 0.71 B 1
A 0.86 B 0
A 0.86 B 0.14
A 0.86 B 0.29
A 0.86 B 0.43
A 0.86 B 0.57
A 0.86 B 0.71
A 0.86 B 0.86
A 0.86 B 1
A 1 B 0
A 1 B 0.14
A 1 B 0.29
A 1 B 0.43
A 1 B 0.57
A 1 B 0.71
A 1 B 0.86
A 1 B 1
0.6
0.4
0.2
OD 600 nm [−]
0 5 101520
Time [h]

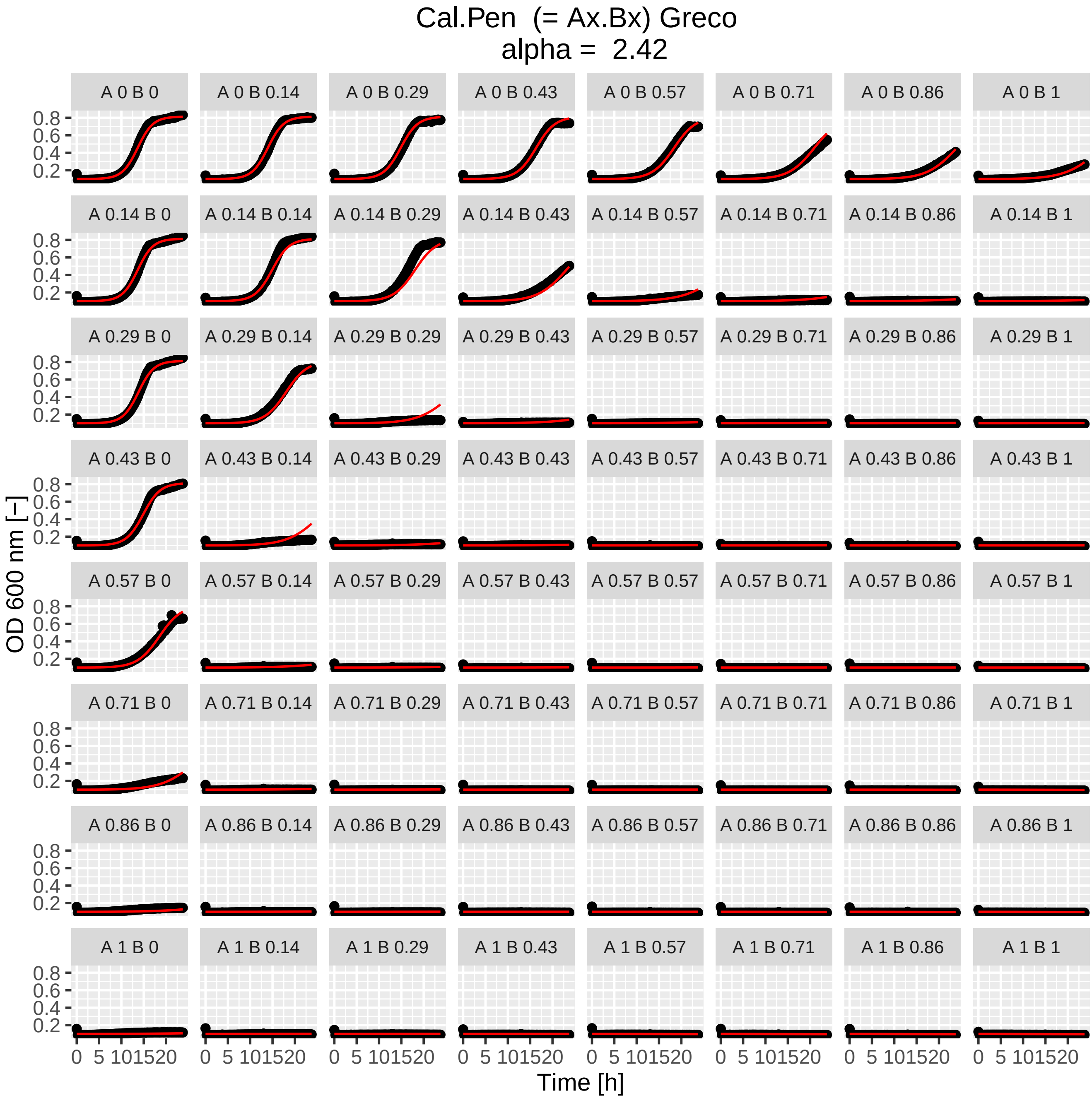
Cal.Pen (= Ax.Bx) Greco
alpha = 2.42
A 0 B 0
A 0 B 0.14
A 0 B 0.29
A 0 B 0.43
A 0 B 0.57
A 0 B 0.71
A 0 B 0.86
A 0 B 1
A 0.14 B 0
A 0.14 B 0.14
A 0.14 B 0.29
A 0.14 B 0.43
A 0.14 B 0.57
A 0.14 B 0.71
A 0.14 B 0.86
A 0.14 B 1
A 0.29 B 0
A 0.29 B 0.14
A 0.29 B 0.29
A 0.29 B 0.43
A 0.29 B 0.57
A 0.29 B 0.71
A 0.29 B 0.86
A 0.29 B 1
A 0.43 B 0
A 0.43 B 0.14
A 0.43 B 0.29
A 0.43 B 0.43
A 0.43 B 0.57
A 0.43 B 0.71
A 0.43 B 0.86
A 0.43 B 1
A 0.57 B 0
A 0.57 B 0.14
A 0.57 B 0.29
A 0.57 B 0.43
A 0.57 B 0.57
A 0.57 B 0.71
A 0.57 B 0.86
A 0.57 B 1
A 0.71 B 0
A 0.71 B 0.14
A 0.71 B 0.29
A 0.71 B 0.43
A 0.71 B 0.57
A 0.71 B 0.71
A 0.71 B 0.86
A 0.71 B 1
A 0.86 B 0
A 0.86 B 0.14
A 0.86 B 0.29
A 0.86 B 0.43
A 0.86 B 0.57
A 0.86 B 0.71
A 0.86 B 0.86
A 0.86 B 1
A 1 B 0
A 1 B 0.14
A 1 B 0.29
A 1 B 0.43
A 1 B 0.57
A 1 B 0.71
A 1 B 0.86
A 1 B 1
0.8
0.6
0.4
0.2
0 5 101520
OD 600 nm [–]
Time [h]

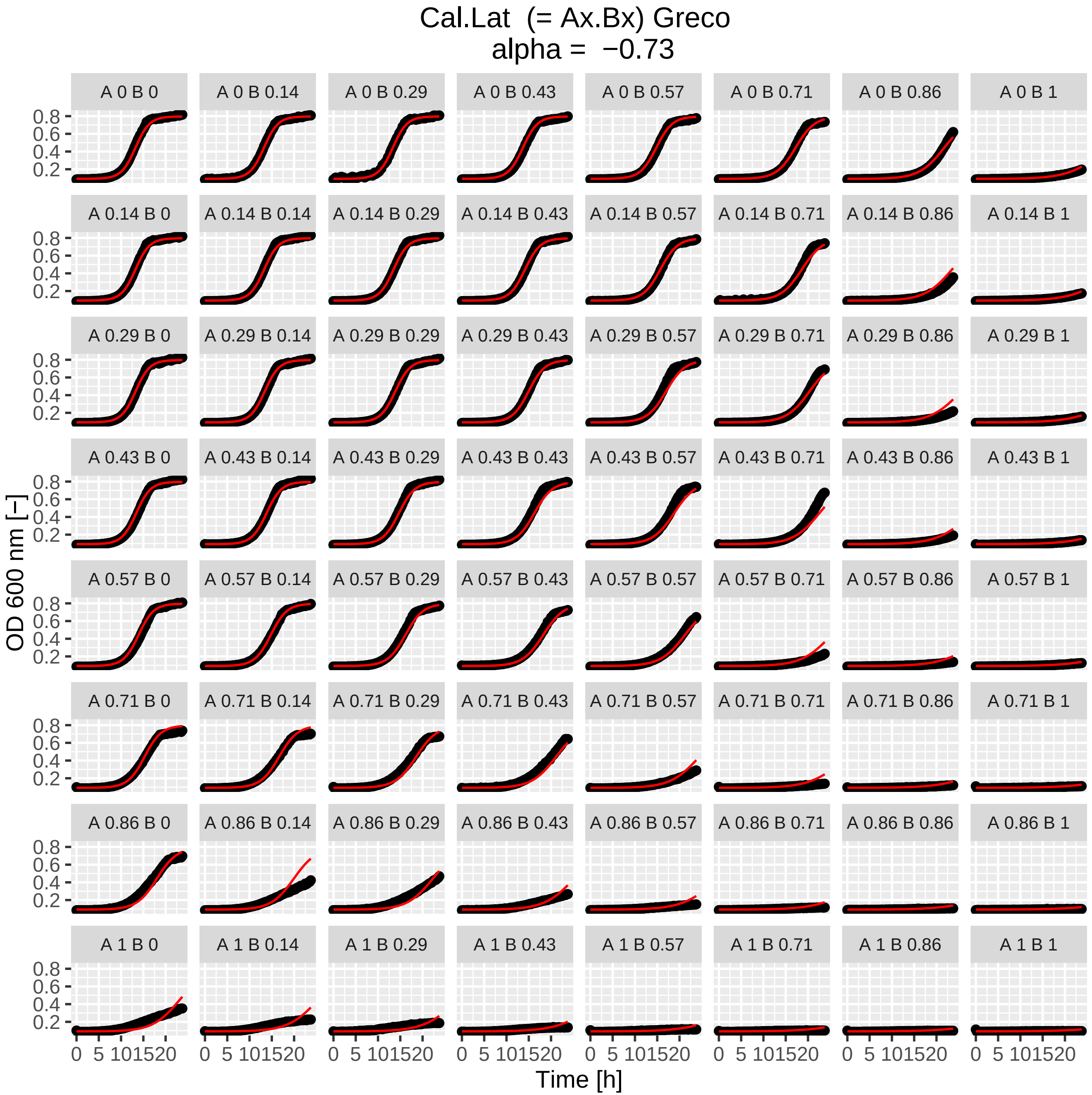
Cal.Lat (= Ax.Bx) Greco
alpha = −0.73
A 0 B 0
A 0 B 0.14
A 0 B 0.29
A 0 B 0.43
A 0 B 0.57
A 0 B 0.71
A 0 B 0.86
A 0 B 1
A 0.14 B 0
A 0.14 B 0.14
A 0.14 B 0.29
A 0.14 B 0.43
A 0.14 B 0.57
A 0.14 B 0.71
A 0.14 B 0.86
A 0.14 B 1
A 0.29 B 0
A 0.29 B 0.14
A 0.29 B 0.29
A 0.29 B 0.43
A 0.29 B 0.57
A 0.29 B 0.71
A 0.29 B 0.86
A 0.29 B 1
A 0.43 B 0
A 0.43 B 0.14
A 0.43 B 0.29
A 0.43 B 0.43
A 0.43 B 0.57
A 0.43 B 0.71
A 0.43 B 0.86
A 0.43 B 1
A 0.57 B 0
A 0.57 B 0.14
A 0.57 B 0.29
A 0.57 B 0.43
A 0.57 B 0.57
A 0.57 B 0.71
A 0.57 B 0.86
A 0.57 B 1
A 0.71 B 0
A 0.71 B 0.14
A 0.71 B 0.29
A 0.71 B 0.43
A 0.71 B 0.57
A 0.71 B 0.71
A 0.71 B 0.86
A 0.71 B 1
A 0.86 B 0
A 0.86 B 0.14
A 0.86 B 0.29
A 0.86 B 0.43
A 0.86 B 0.57
A 0.86 B 0.71
A 0.86 B 0.86
A 0.86 B 1
A 1 B 0
A 1 B 0.14
A 1 B 0.29
A 1 B 0.43
A 1 B 0.57
A 1 B 0.71
A 1 B 0.86
A 1 B 1
0.8
0.6
0.4
0.2
0 5 101520
OD 600 nm [−]
Time [h]

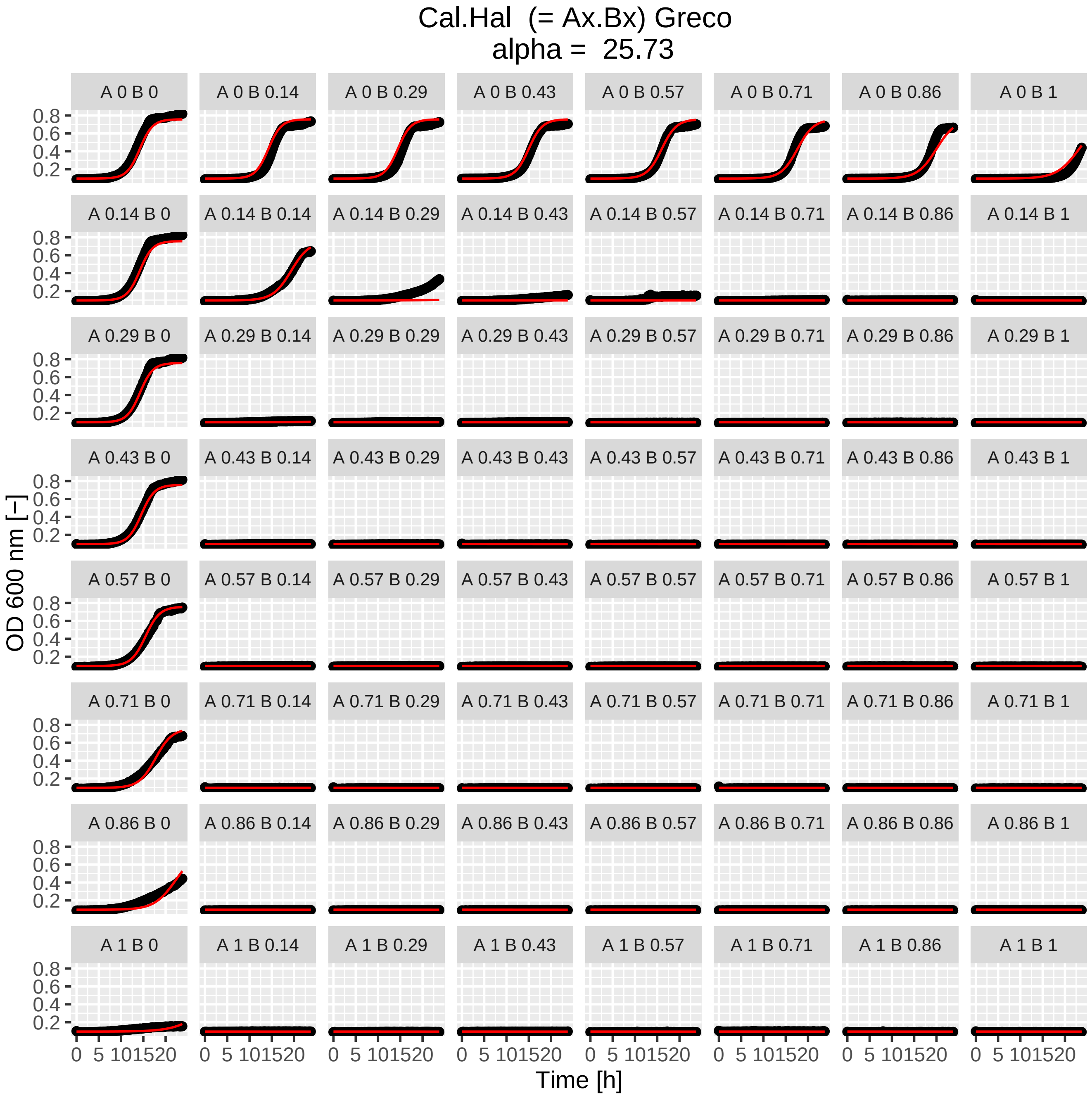

Cal.Hal (= Ax.Bx) Greco
alpha = 25.73
A 0 B 0
A 0 B 0.14
A 0 B 0.29
A 0 B 0.43
A 0 B 0.57
A 0 B 0.71
A 0 B 0.86
A 0 B 1
A 0.14 B 0
A 0.14 B 0.14
A 0.14 B 0.29
A 0.14 B 0.43
A 0.14 B 0.57
A 0.14 B 0.71
A 0.14 B 0.86
A 0.14 B 1
A 0.29 B 0
A 0.29 B 0.14
A 0.29 B 0.29
A 0.29 B 0.43
A 0.29 B 0.57
A 0.29 B 0.71
A 0.29 B 0.86
A 0.29 B 1
A 0.43 B 0
A 0.43 B 0.14
A 0.43 B 0.29
A 0.43 B 0.43
A 0.43 B 0.57
A 0.43 B 0.71
A 0.43 B 0.86
A 0.43 B 1
A 0.57 B 0
A 0.57 B 0.14
A 0.57 B 0.29
A 0.57 B 0.43
A 0.57 B 0.57
A 0.57 B 0.71
A 0.57 B 0.86
A 0.57 B 1
A 0.71 B 0
A 0.71 B 0.14
A 0.71 B 0.29
A 0.71 B 0.43
A 0.71 B 0.57
A 0.71 B 0.71
A 0.71 B 0.86
A 0.71 B 1
A 0.86 B 0
A 0.86 B 0.14
A 0.86 B 0.29
A 0.86 B 0.43
A 0.86 B 0.57
A 0.86 B 0.71
A 0.86 B 0.86
A 0.86 B 1
A 1 B 0
A 1 B 0.14
A 1 B 0.29
A 1 B 0.43
A 1 B 0.57
A 1 B 0.71
A 1 B 0.86
A 1 B 1
OD 600 nm [−]
0.2
0.4
0.6
0.8
0 5 101520
Time [h]

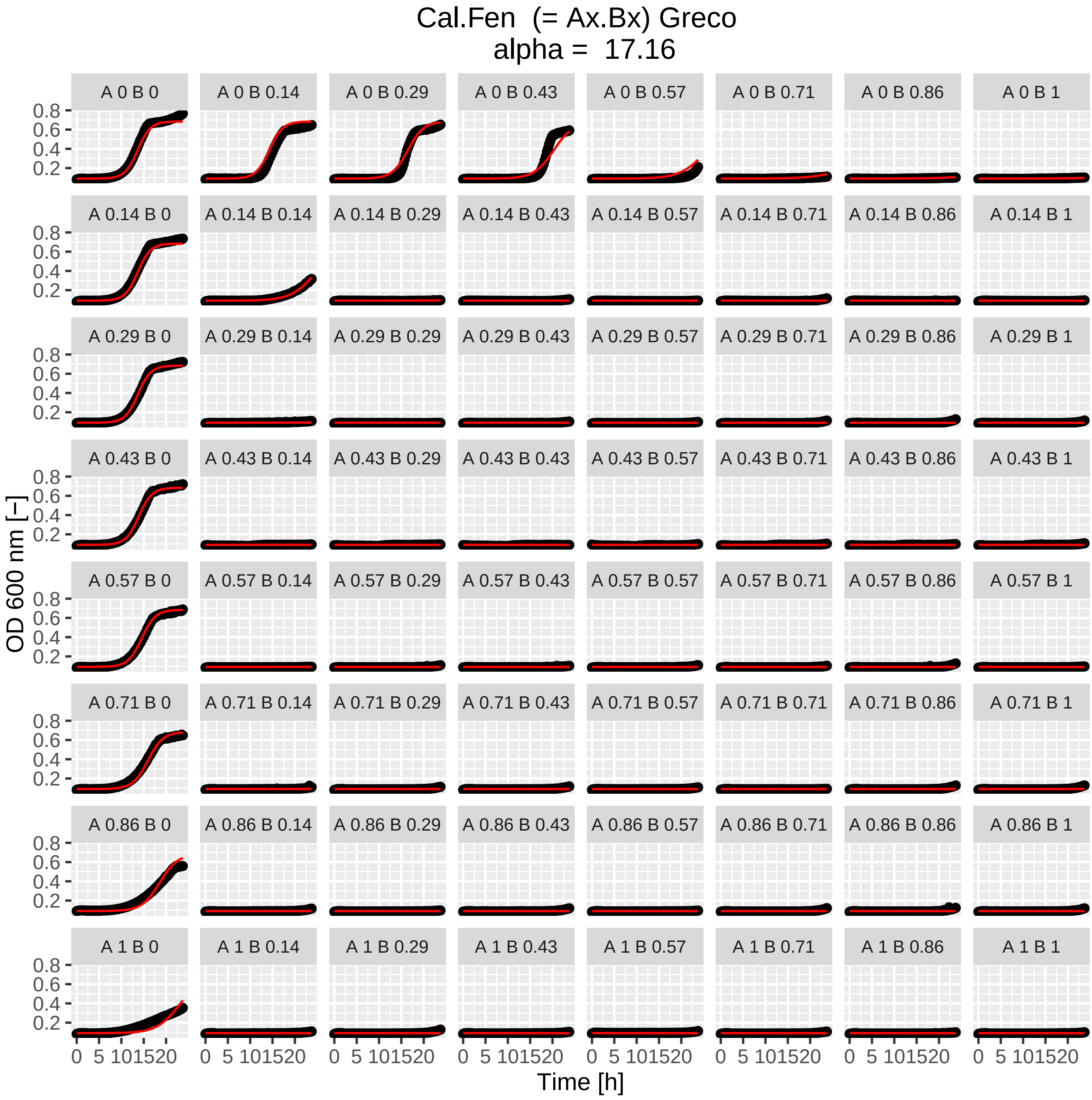
Cal.Fen (= Ax.Bx) Greco
alpha = 17.16
A 0 B 0
A 0 B 0.14
A 0 B 0.29
A 0 B 0.43
A 0 B 0.57
A 0 B 0.71
A 0 B 0.86
A 0 B 1
A 0.14 B 0
A 0.14 B 0.14
A 0.14 B 0.29
A 0.14 B 0.43
A 0.14 B 0.57
A 0.14 B 0.71
A 0.14 B 0.86
A 0.14 B 1
A 0.29 B 0
A 0.29 B 0.14
A 0.29 B 0.29
A 0.29 B 0.43
A 0.29 B 0.57
A 0.29 B 0.71
A 0.29 B 0.86
A 0.29 B 1
A 0.43 B 0
A 0.43 B 0.14
A 0.43 B 0.29
A 0.43 B 0.43
A 0.43 B 0.57
A 0.43 B 0.71
A 0.43 B 0.86
A 0.43 B 1
A 0.57 B 0
A 0.57 B 0.14
A 0.57 B 0.29
A 0.57 B 0.43
A 0.57 B 0.57
A 0.57 B 0.71
A 0.57 B 0.86
A 0.57 B 1
A 0.71 B 0
A 0.71 B 0.14
A 0.71 B 0.29
A 0.71 B 0.43
A 0.71 B 0.57
A 0.71 B 0.71
A 0.71 B 0.86
A 0.71 B 1
A 0.86 B 0
A 0.86 B 0.14
A 0.86 B 0.29
A 0.86 B 0.43
A 0.86 B 0.57
A 0.86 B 0.71
A 0.86 B 0.86
A 0.86 B 1
A 1 B 0
A 1 B 0.14
A 1 B 0.29
A 1 B 0.43
A 1 B 0.57
A 1 B 0.71
A 1 B 0.86
A 1 B 1
OD 600 nm [–]
0.2
0.4
0.6
0.8
0 5 101520
Time [h]

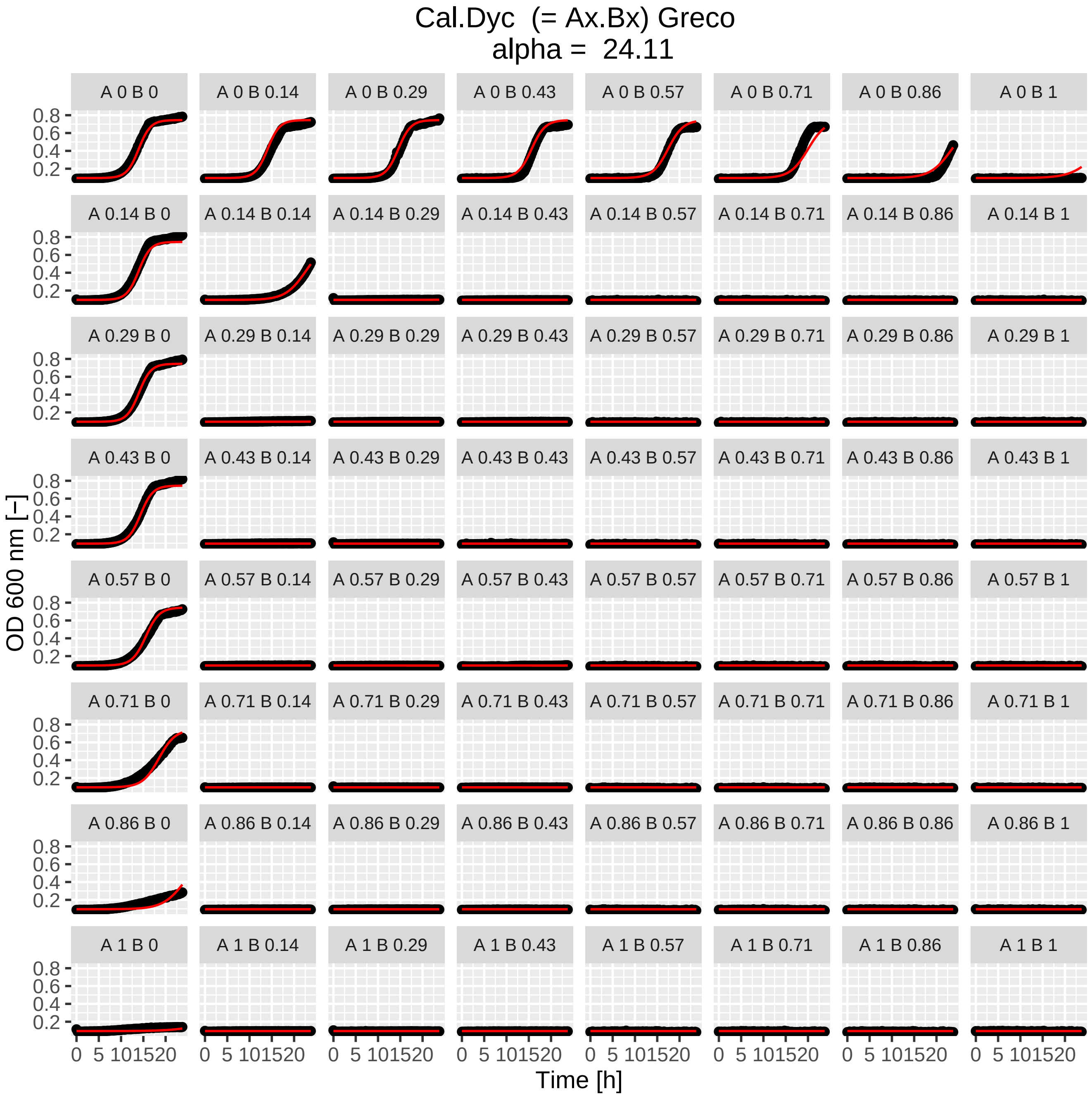

Cal.Dyc (= Ax.Bx) Greco
alpha = 24.11
A 0 B 0
A 0 B 0.14
A 0 B 0.29
A 0 B 0.43
A 0 B 0.57
A 0 B 0.71
A 0 B 0.86
A 0 B 1
A 0.14 B 0
A 0.14 B 0.14
A 0.14 B 0.29
A 0.14 B 0.43
A 0.14 B 0.57
A 0.14 B 0.71
A 0.14 B 0.86
A 0.14 B 1
A 0.29 B 0
A 0.29 B 0.14
A 0.29 B 0.29
A 0.29 B 0.43
A 0.29 B 0.57
A 0.29 B 0.71
A 0.29 B 0.86
A 0.29 B 1
A 0.43 B 0
A 0.43 B 0.14
A 0.43 B 0.29
A 0.43 B 0.43
A 0.43 B 0.57
A 0.43 B 0.71
A 0.43 B 0.86
A 0.43 B 1
A 0.57 B 0
A 0.57 B 0.14
A 0.57 B 0.29
A 0.57 B 0.43
A 0.57 B 0.57
A 0.57 B 0.71
A 0.57 B 0.86
A 0.57 B 1
A 0.71 B 0
A 0.71 B 0.14
A 0.71 B 0.29
A 0.71 B 0.43
A 0.71 B 0.57
A 0.71 B 0.71
A 0.71 B 0.86
A 0.71 B 1
A 0.86 B 0
A 0.86 B 0.14
A 0.86 B 0.29
A 0.86 B 0.43
A 0.86 B 0.57
A 0.86 B 0.71
A 0.86 B 0.86
A 0.86 B 1
A 1 B 0
A 1 B 0.14
A 1 B 0.29
A 1 B 0.43
A 1 B 0.57
A 1 B 0.71
A 1 B 0.86
A 1 B 1
OD 600 nm [–]
0.8
0.6
0.4
0.2
0 5 101520
Time [h]

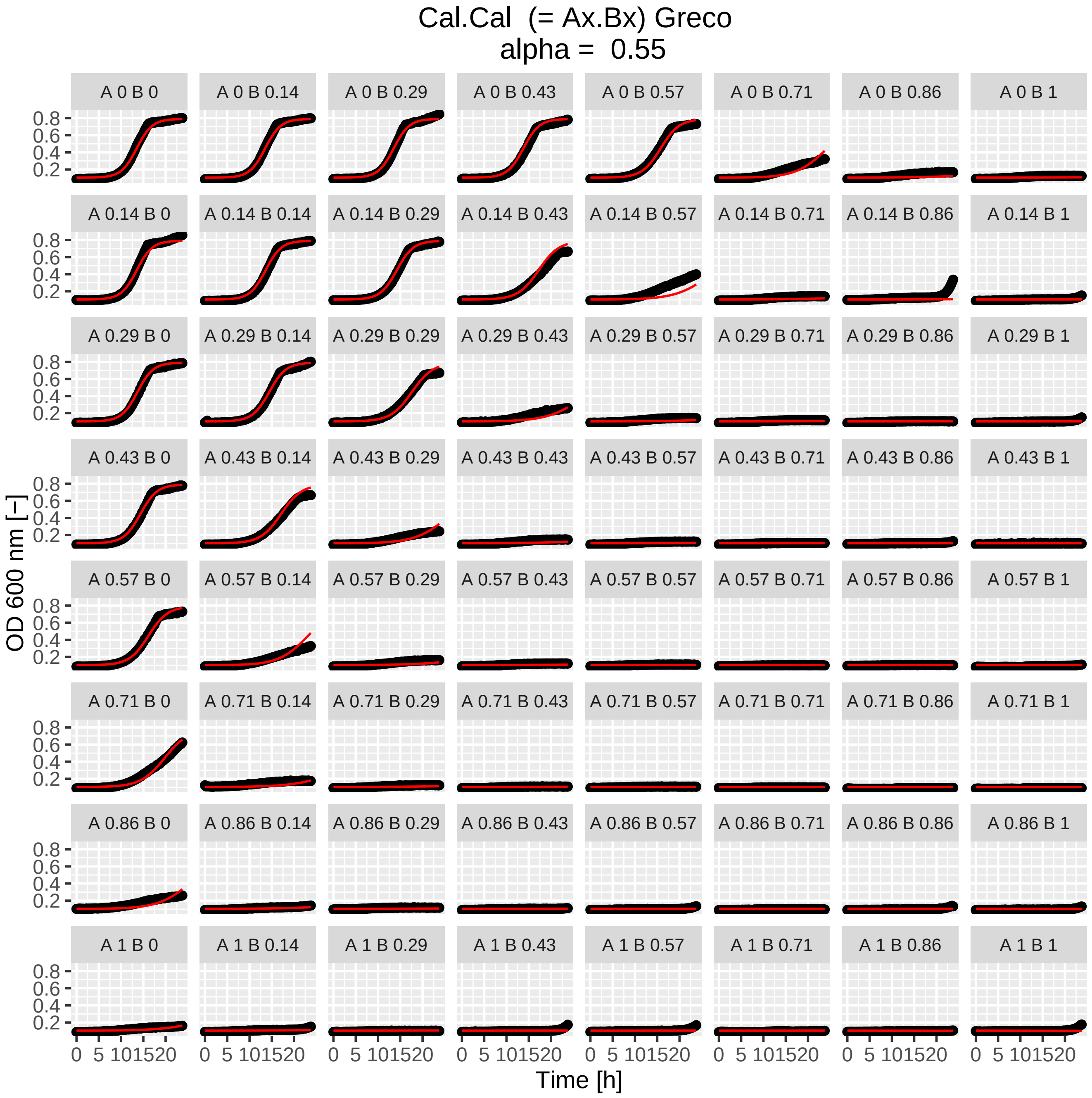

Cal.Cal (= Ax.Bx) Greco
alpha = 0.55
A 0 B 0
A 0 B 0.14
A 0 B 0.29
A 0 B 0.43
A 0 B 0.57
A 0 B 0.71
A 0 B 0.86
A 0 B 1
A 0.14 B 0
A 0.14 B 0.14
A 0.14 B 0.29
A 0.14 B 0.43
A 0.14 B 0.57
A 0.14 B 0.71
A 0.14 B 0.86
A 0.14 B 1
A 0.29 B 0
A 0.29 B 0.14
A 0.29 B 0.29
A 0.29 B 0.43
A 0.29 B 0.57
A 0.29 B 0.71
A 0.29 B 0.86
A 0.29 B 1
A 0.43 B 0
A 0.43 B 0.14
A 0.43 B 0.29
A 0.43 B 0.43
A 0.43 B 0.57
A 0.43 B 0.71
A 0.43 B 0.86
A 0.43 B 1
A 0.57 B 0
A 0.57 B 0.14
A 0.57 B 0.29
A 0.57 B 0.43
A 0.57 B 0.57
A 0.57 B 0.71
A 0.57 B 0.86
A 0.57 B 1
A 0.71 B 0
A 0.71 B 0.14
A 0.71 B 0.29
A 0.71 B 0.43
A 0.71 B 0.57
A 0.71 B 0.71
A 0.71 B 0.86
A 0.71 B 1
A 0.86 B 0
A 0.86 B 0.14
A 0.86 B 0.29
A 0.86 B 0.43
A 0.86 B 0.57
A 0.86 B 0.71
A 0.86 B 0.86
A 0.86 B 1
A 1 B 0
A 1 B 0.14
A 1 B 0.29
A 1 B 0.43
A 1 B 0.57
A 1 B 0.71
A 1 B 0.86
A 1 B 1
0.8
0.6
0.4
0.2
0 5 101520
OD 600 nm [–]
Time [h]

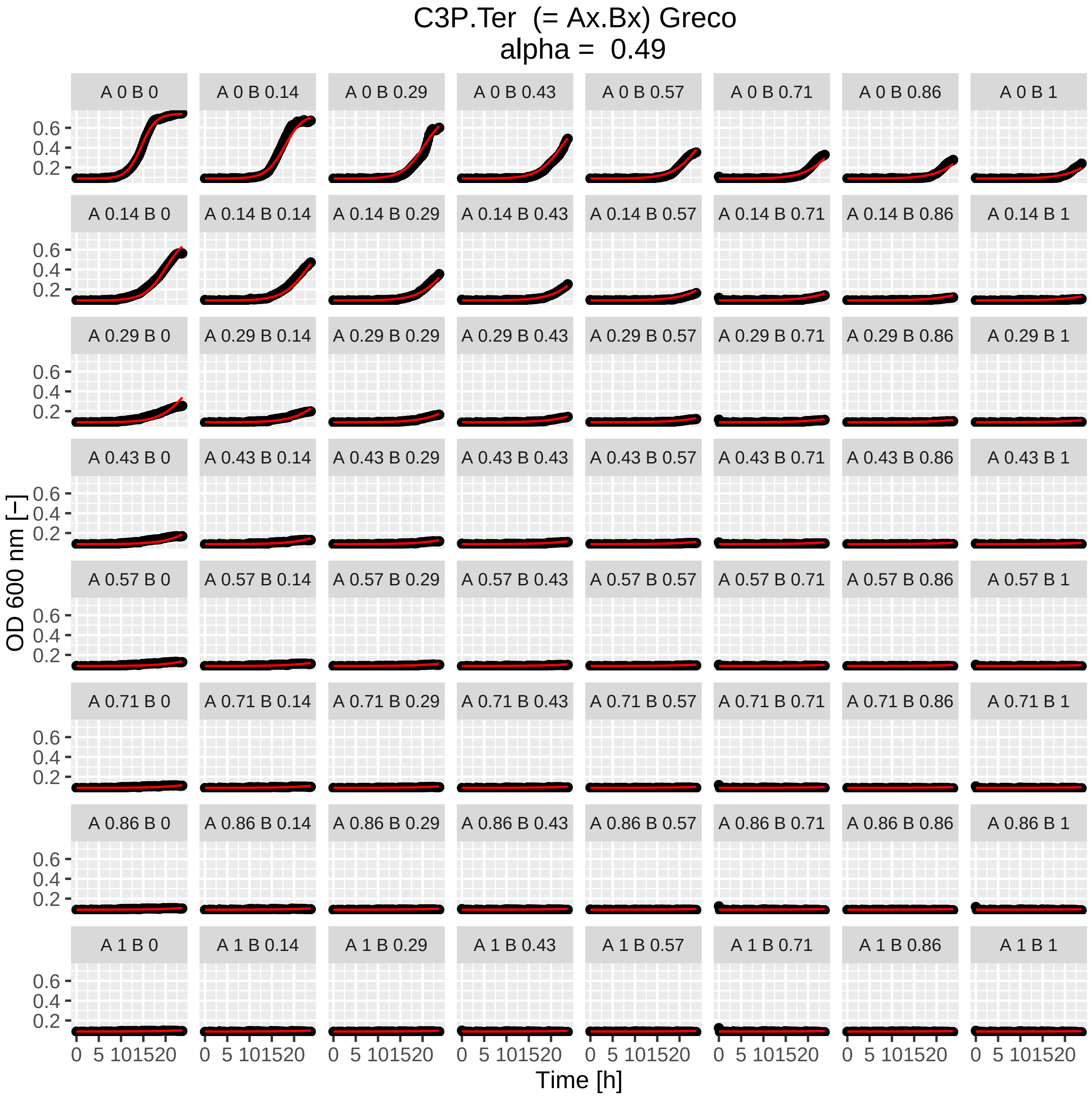
C3P.Ter (= Ax.Bx) Greco
alpha = 0.49
A 0 B 0
A 0 B 0.14
A 0 B 0.29
A 0 B 0.43
A 0 B 0.57
A 0 B 0.71
A 0 B 0.86
A 0 B 1
A 0.14 B 0
A 0.14 B 0.14
A 0.14 B 0.29
A 0.14 B 0.43
A 0.14 B 0.57
A 0.14 B 0.71
A 0.14 B 0.86
A 0.14 B 1
A 0.29 B 0
A 0.29 B 0.14
A 0.29 B 0.29
A 0.29 B 0.43
A 0.29 B 0.57
A 0.29 B 0.71
A 0.29 B 0.86
A 0.29 B 1
A 0.43 B 0
A 0.43 B 0.14
A 0.43 B 0.29
A 0.43 B 0.43
A 0.43 B 0.57
A 0.43 B 0.71
A 0.43 B 0.86
A 0.43 B 1
A 0.57 B 0
A 0.57 B 0.14
A 0.57 B 0.29
A 0.57 B 0.43
A 0.57 B 0.57
A 0.57 B 0.71
A 0.57 B 0.86
A 0.57 B 1
A 0.71 B 0
A 0.71 B 0.14
A 0.71 B 0.29
A 0.71 B 0.43
A 0.71 B 0.57
A 0.71 B 0.71
A 0.71 B 0.86
A 0.71 B 1
A 0.86 B 0
A 0.86 B 0.14
A 0.86 B 0.29
A 0.86 B 0.43
A 0.86 B 0.57
A 0.86 B 0.71
A 0.86 B 0.86
A 0.86 B 1
A 1 B 0
A 1 B 0.14
A 1 B 0.29
A 1 B 0.43
A 1 B 0.57
A 1 B 0.71
A 1 B 0.86
A 1 B 1
0.6
0.4
0.2
0 5 101520
OD 600 nm [–]
Time [h]

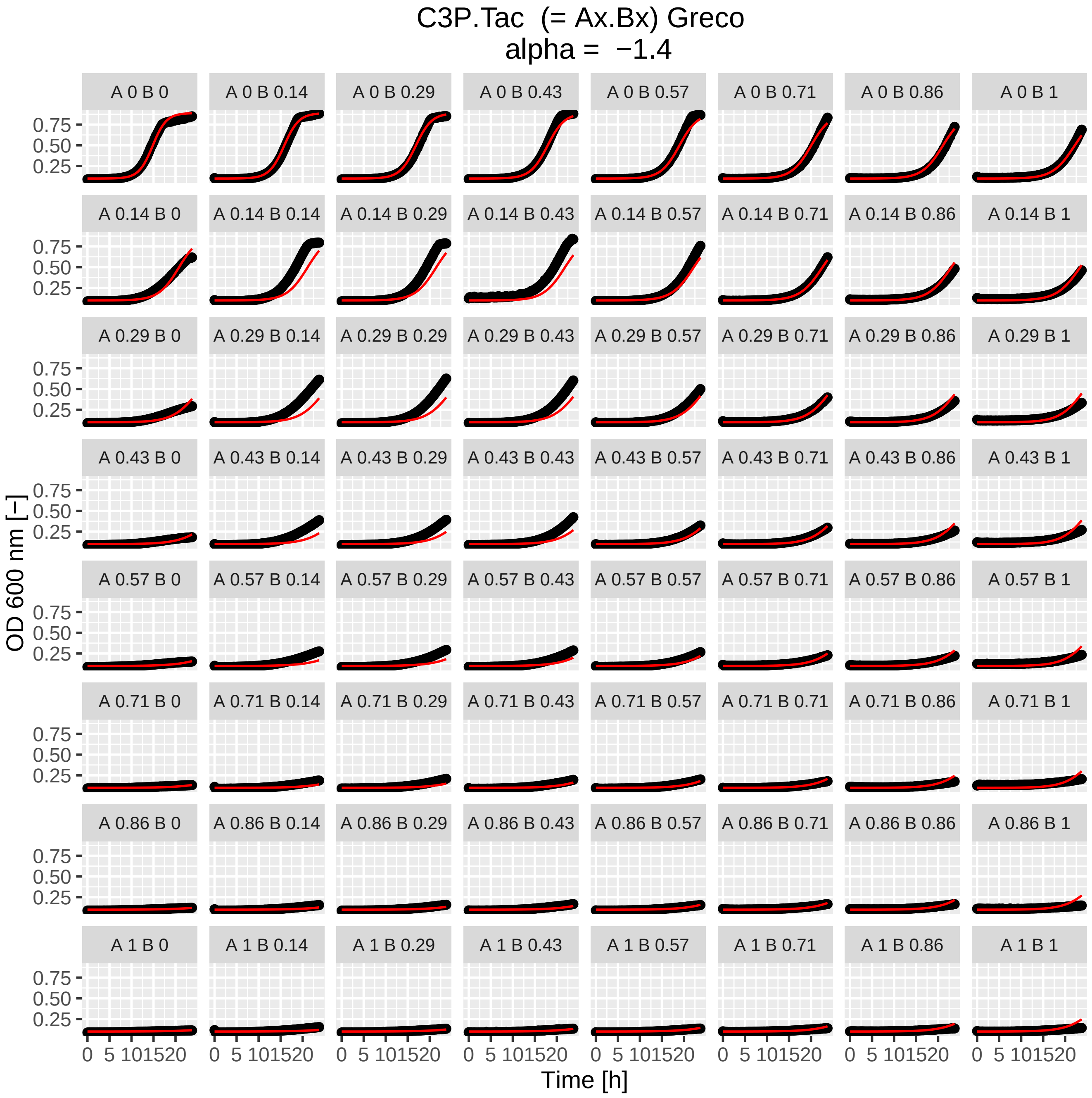

C3P.Tac (= Ax.Bx) Greco
alpha = −1.4
A 0 B 0
A 0 B 0.14
A 0 B 0.29
A 0 B 0.43
A 0 B 0.57
A 0 B 0.71
A 0 B 0.86
A 0 B 1
A 0.14 B 0
A 0.14 B 0.14
A 0.14 B 0.29
A 0.14 B 0.43
A 0.14 B 0.57
A 0.14 B 0.71
A 0.14 B 0.86
A 0.14 B 1
A 0.29 B 0
A 0.29 B 0.14
A 0.29 B 0.29
A 0.29 B 0.43
A 0.29 B 0.57
A 0.29 B 0.71
A 0.29 B 0.86
A 0.29 B 1
A 0.43 B 0
A 0.43 B 0.14
A 0.43 B 0.29
A 0.43 B 0.43
A 0.43 B 0.57
A 0.43 B 0.71
A 0.43 B 0.86
A 0.43 B 1
A 0.57 B 0
A 0.57 B 0.14
A 0.57 B 0.29
A 0.57 B 0.43
A 0.57 B 0.57
A 0.57 B 0.71
A 0.57 B 0.86
A 0.57 B 1
A 0.71 B 0
A 0.71 B 0.14
A 0.71 B 0.29
A 0.71 B 0.43
A 0.71 B 0.57
A 0.71 B 0.71
A 0.71 B 0.86
A 0.71 B 1
A 0.86 B 0
A 0.86 B 0.14
A 0.86 B 0.29
A 0.86 B 0.43
A 0.86 B 0.57
A 0.86 B 0.71
A 0.86 B 0.86
A 0.86 B 1
A 1 B 0
A 1 B 0.14
A 1 B 0.29
A 1 B 0.43
A 1 B 0.57
A 1 B 0.71
A 1 B 0.86
A 1 B 1
0.75
0.50
0.25
OD 600 nm [−]
0 5 101520
Time [h]

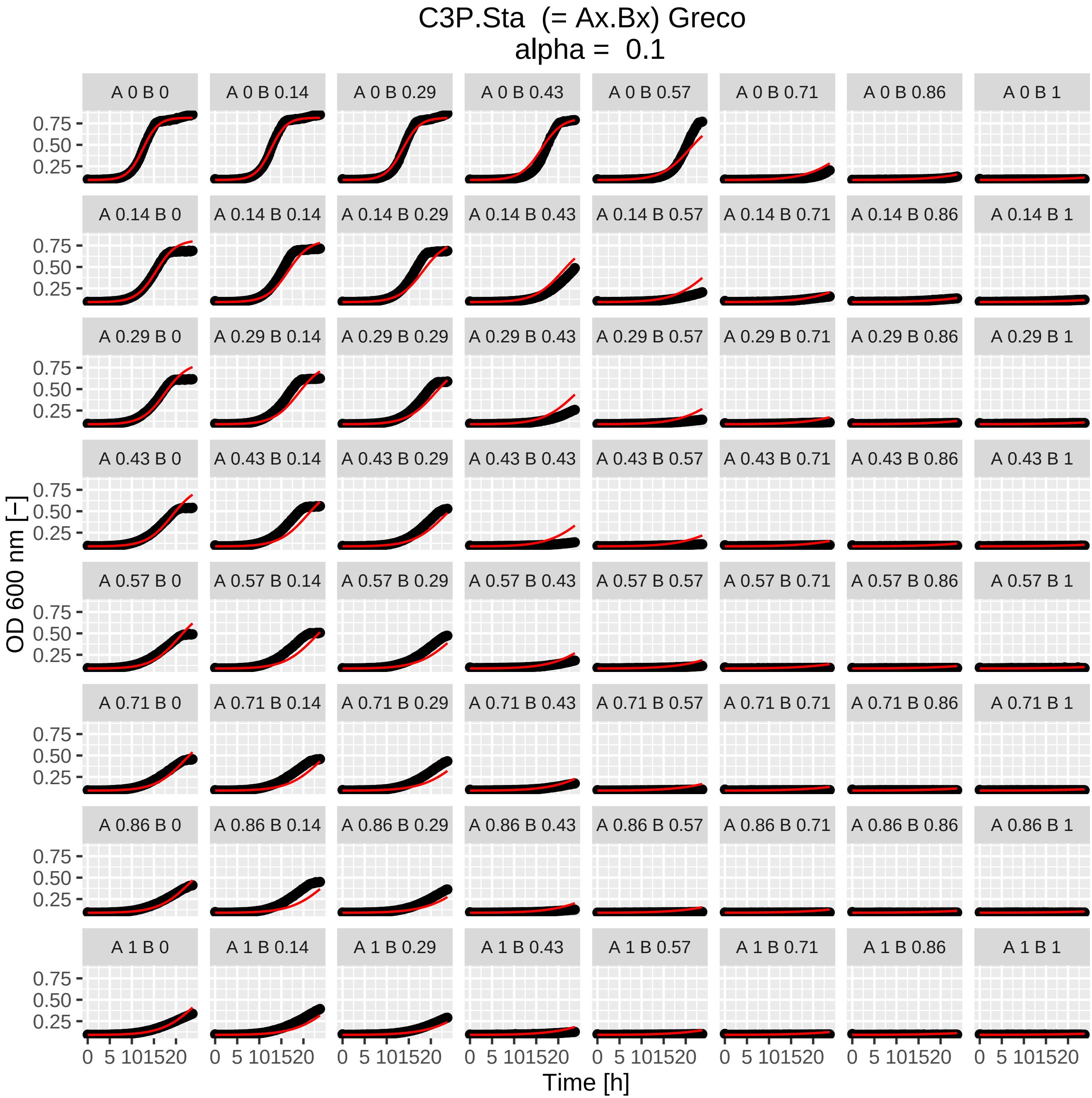
C3P.Sta (= Ax.Bx) Greco
alpha = 0.1
A 0 B 0
A 0 B 0.14
A 0 B 0.29
A 0 B 0.43
A 0 B 0.57
A 0 B 0.71
A 0 B 0.86
A 0 B 1
A 0.14 B 0
A 0.14 B 0.14
A 0.14 B 0.29
A 0.14 B 0.43
A 0.14 B 0.57
A 0.14 B 0.71
A 0.14 B 0.86
A 0.14 B 1
A 0.29 B 0
A 0.29 B 0.14
A 0.29 B 0.29
A 0.29 B 0.43
A 0.29 B 0.57
A 0.29 B 0.71
A 0.29 B 0.86
A 0.29 B 1
A 0.43 B 0
A 0.43 B 0.14
A 0.43 B 0.29
A 0.43 B 0.43
A 0.43 B 0.57
A 0.43 B 0.71
A 0.43 B 0.86
A 0.43 B 1
A 0.57 B 0
A 0.57 B 0.14
A 0.57 B 0.29
A 0.57 B 0.43
A 0.57 B 0.57
A 0.57 B 0.71
A 0.57 B 0.86
A 0.57 B 1
A 0.71 B 0
A 0.71 B 0.14
A 0.71 B 0.29
A 0.71 B 0.43
A 0.71 B 0.57
A 0.71 B 0.71
A 0.71 B 0.86
A 0.71 B 1
A 0.86 B 0
A 0.86 B 0.14
A 0.86 B 0.29
A 0.86 B 0.43
A 0.86 B 0.57
A 0.86 B 0.71
A 0.86 B 0.86
A 0.86 B 1
A 1 B 0
A 1 B 0.14
A 1 B 0.29
A 1 B 0.43
A 1 B 0.57
A 1 B 0.71
A 1 B 0.86
A 1 B 1
0.75
0.50
0.25
OD 600 nm [–]
0 5 101520
Time [h]

C3P.Pen (= Ax.Bx) Greco
alpha = 15.45

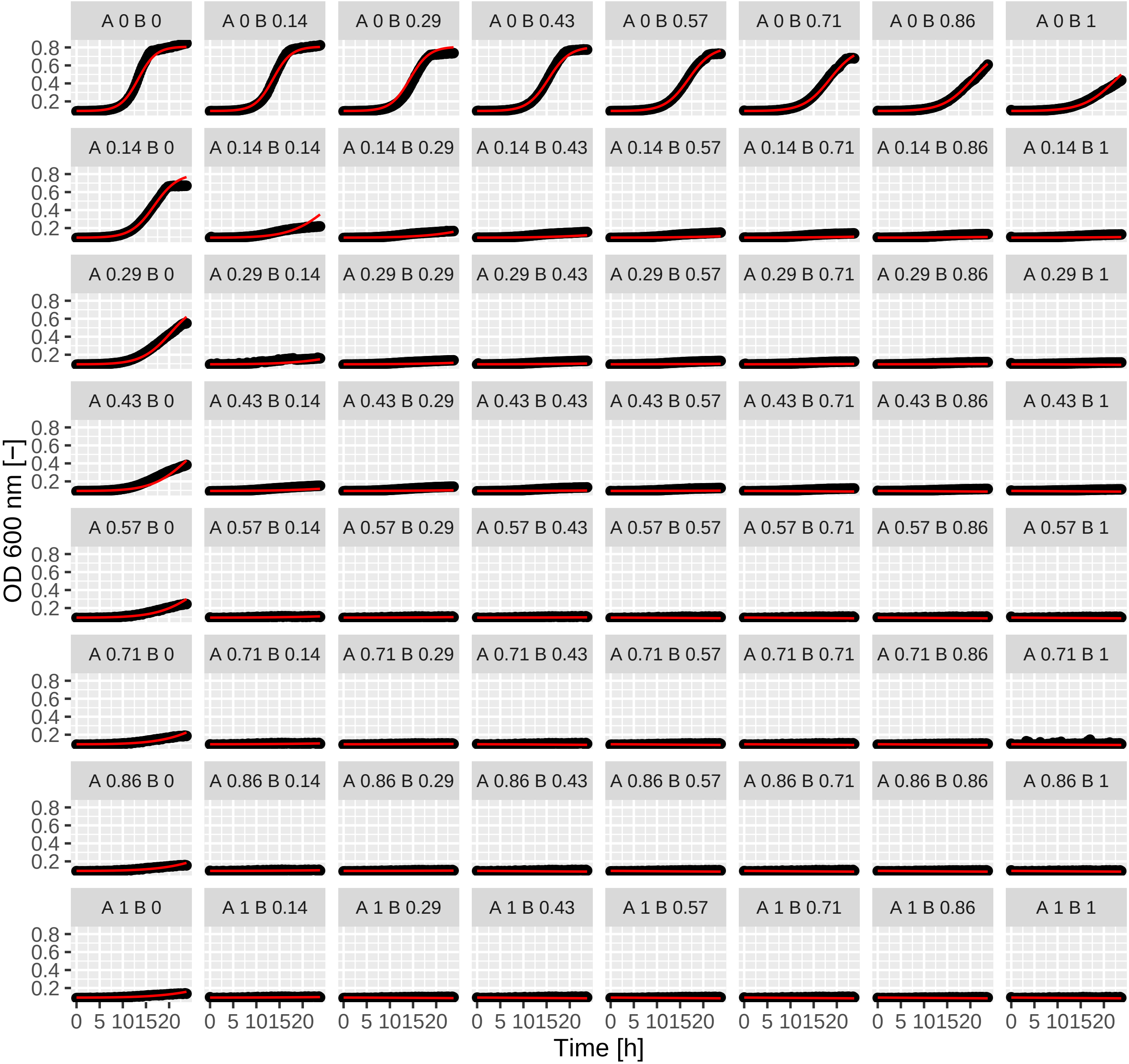

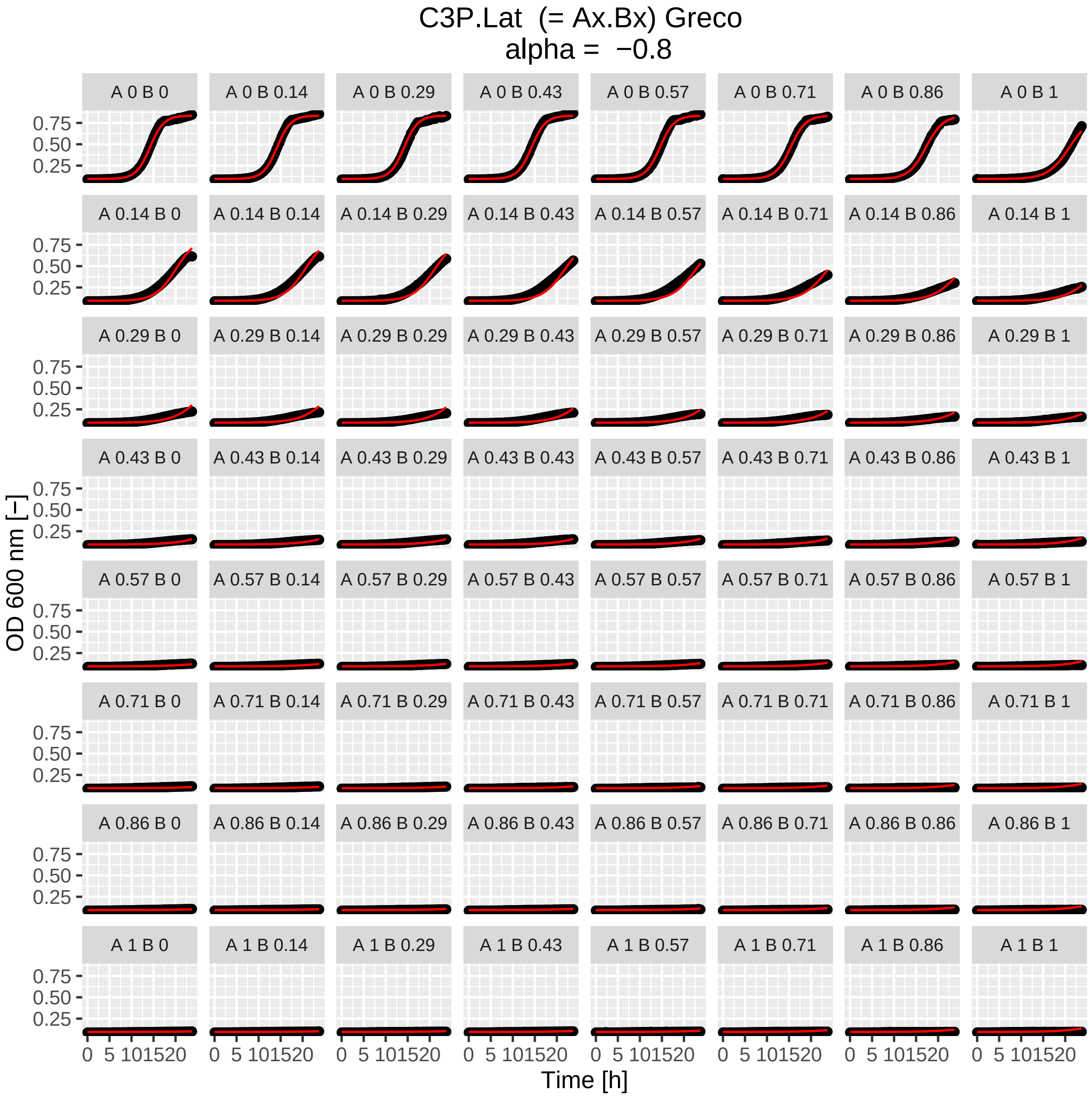
C3P.Lat (= Ax.Bx) Greco
alpha = −0.8
OD 600 nm [−]
Time [h]
0.75
0.50
0.25
0 5 101520
A 0 B 0
A 0 B 0.14
A 0 B 0.29
A 0 B 0.43
A 0 B 0.57
A 0 B 0.71
A 0 B 0.86
A 0 B 1
A 0.14 B 0
A 0.14 B 0.14
A 0.14 B 0.29
A 0.14 B 0.43
A 0.14 B 0.57
A 0.14 B 0.71
A 0.14 B 0.86
A 0.14 B 1
A 0.29 B 0
A 0.29 B 0.14
A 0.29 B 0.29
A 0.29 B 0.43
A 0.29 B 0.57
A 0.29 B 0.71
A 0.29 B 0.86
A 0.29 B 1
A 0.43 B 0
A 0.43 B 0.14
A 0.43 B 0.29
A 0.43 B 0.43
A 0.43 B 0.57
A 0.43 B 0.71
A 0.43 B 0.86
A 0.43 B 1
A 0.57 B 0
A 0.57 B 0.14
A 0.57 B 0.29
A 0.57 B 0.43
A 0.57 B 0.57
A 0.57 B 0.71
A 0.57 B 0.86
A 0.57 B 1
A 0.71 B 0
A 0.71 B 0.14
A 0.71 B 0.29
A 0.71 B 0.43
A 0.71 B 0.57
A 0.71 B 0.71
A 0.71 B 0.86
A 0.71 B 1
A 0.86 B 0
A 0.86 B 0.14
A 0.86 B 0.29
A 0.86 B 0.43
A 0.86 B 0.57
A 0.86 B 0.71
A 0.86 B 0.86
A 0.86 B 1
A 1 B 0
A 1 B 0.14
A 1 B 0.29
A 1 B 0.43
A 1 B 0.57
A 1 B 0.71
A 1 B 0.86
A 1 B 1

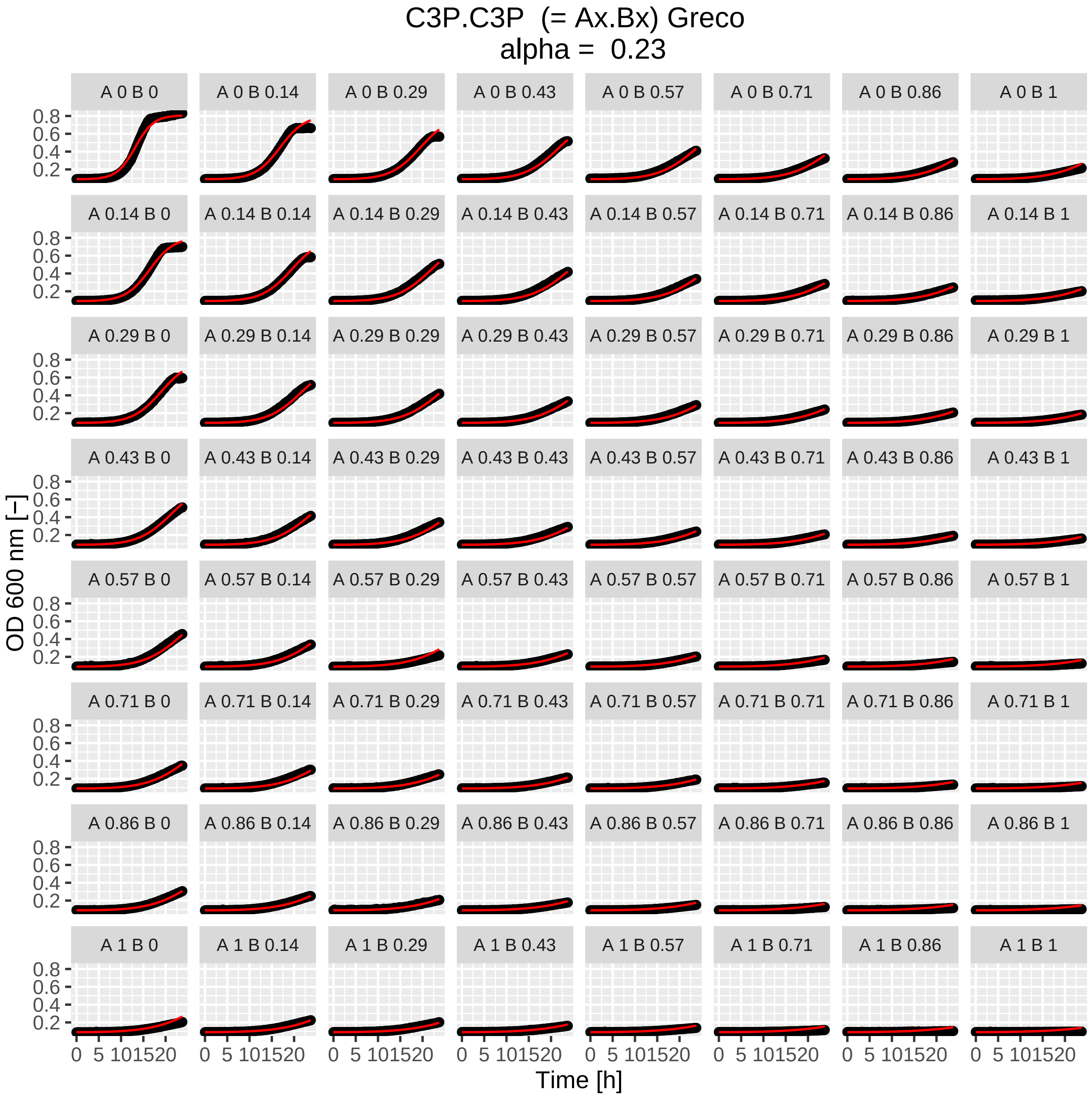
C3P.C3P (= Ax.Bx) Greco
alpha = 0.23
A 0 B 0
A 0 B 0.14
A 0 B 0.29
A 0 B 0.43
A 0 B 0.57
A 0 B 0.71
A 0 B 0.86
A 0 B 1
A 0.14 B 0
A 0.14 B 0.14
A 0.14 B 0.29
A 0.14 B 0.43
A 0.14 B 0.57
A 0.14 B 0.71
A 0.14 B 0.86
A 0.14 B 1
A 0.29 B 0
A 0.29 B 0.14
A 0.29 B 0.29
A 0.29 B 0.43
A 0.29 B 0.57
A 0.29 B 0.71
A 0.29 B 0.86
A 0.29 B 1
A 0.43 B 0
A 0.43 B 0.14
A 0.43 B 0.29
A 0.43 B 0.43
A 0.43 B 0.57
A 0.43 B 0.71
A 0.43 B 0.86
A 0.43 B 1
A 0.57 B 0
A 0.57 B 0.14
A 0.57 B 0.29
A 0.57 B 0.43
A 0.57 B 0.57
A 0.57 B 0.71
A 0.57 B 0.86
A 0.57 B 1
A 0.71 B 0
A 0.71 B 0.14
A 0.71 B 0.29
A 0.71 B 0.43
A 0.71 B 0.57
A 0.71 B 0.71
A 0.71 B 0.86
A 0.71 B 1
A 0.86 B 0
A 0.86 B 0.14
A 0.86 B 0.29
A 0.86 B 0.43
A 0.86 B 0.57
A 0.86 B 0.71
A 0.86 B 0.86
A 0.86 B 1
A 1 B 0
A 1 B 0.14
A 1 B 0.29
A 1 B 0.43
A 1 B 0.57
A 1 B 0.71
A 1 B 0.86
A 1 B 1
OD 600 nm [−]
0.8
0.6
0.4
0.2
0 5 101520
Time [h]

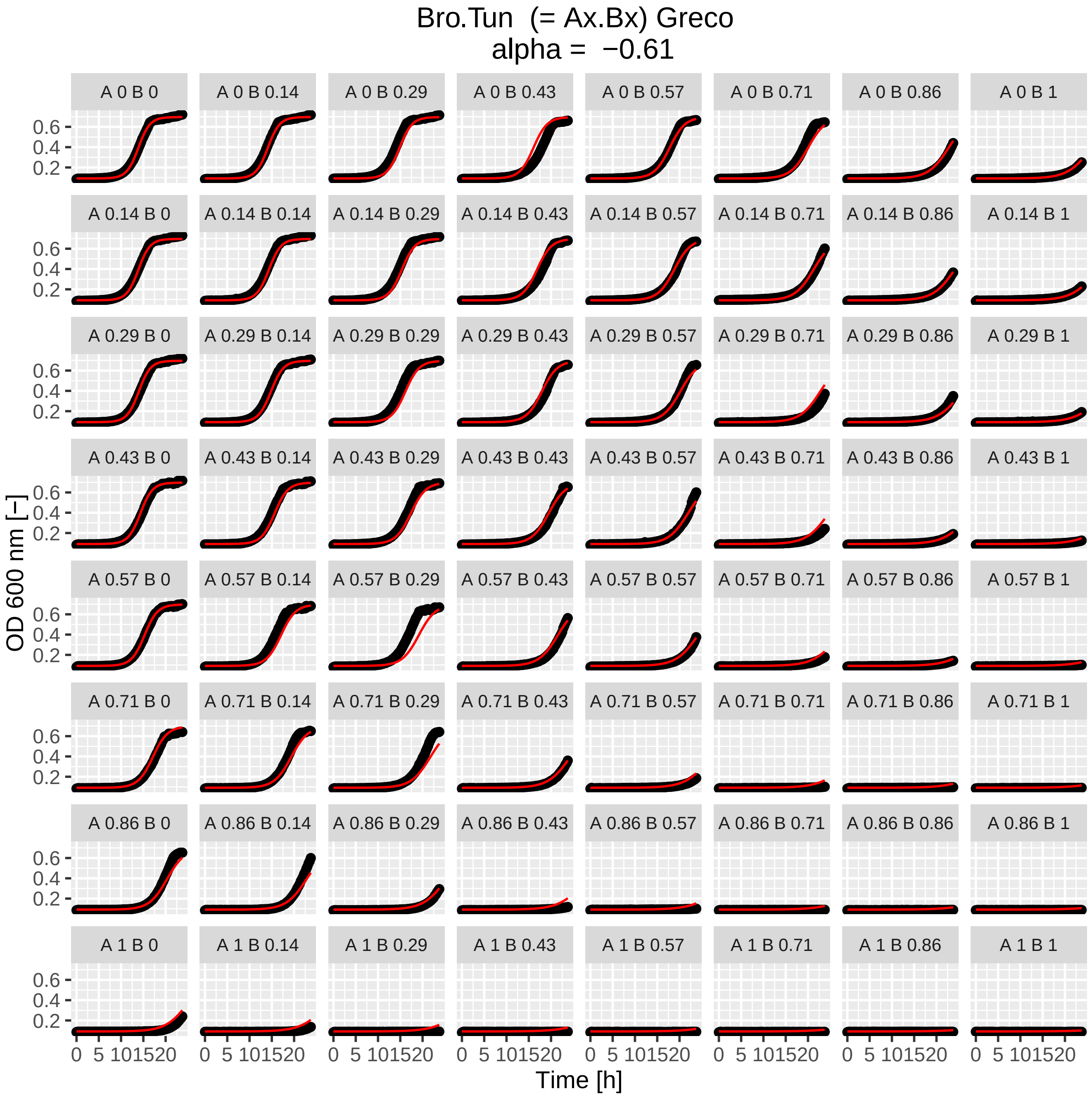
Bro.Tun (= Ax.Bx) Greco
alpha = −0.61
OD 600 nm [−]
Time [h]
0.6
0.4
0.2
0
5
10
15
20
A 0 B 0
A 0 B 0.14
A 0 B 0.29
A 0 B 0.43
A 0 B 0.57
A 0 B 0.71
A 0 B 0.86
A 0 B 1
A 0.14 B 0
A 0.14 B 0.14
A 0.14 B 0.29
A 0.14 B 0.43
A 0.14 B 0.57
A 0.14 B 0.71
A 0.14 B 0.86
A 0.14 B 1
A 0.29 B 0
A 0.29 B 0.14
A 0.29 B 0.29
A 0.29 B 0.43
A 0.29 B 0.57
A 0.29 B 0.71
A 0.29 B 0.86
A 0.29 B 1
A 0.43 B 0
A 0.43 B 0.14
A 0.43 B 0.29
A 0.43 B 0.43
A 0.43 B 0.57
A 0.43 B 0.71
A 0.43 B 0.86
A 0.43 B 1
A 0.57 B 0
A 0.57 B 0.14
A 0.57 B 0.29
A 0.57 B 0.43
A 0.57 B 0.57
A 0.57 B 0.71
A 0.57 B 0.86
A 0.57 B 1
A 0.71 B 0
A 0.71 B 0.14
A 0.71 B 0.29
A 0.71 B 0.43
A 0.71 B 0.57
A 0.71 B 0.71
A 0.71 B 0.86
A 0.71 B 1
A 0.86 B 0
A 0.86 B 0.14
A 0.86 B 0.29
A 0.86 B 0.43
A 0.86 B 0.57
A 0.86 B 0.71
A 0.86 B 0.86
A 0.86 B 1
A 1 B 0
A 1 B 0.14
A 1 B 0.29
A 1 B 0.43
A 1 B 0.57
A 1 B 0.71
A 1 B 0.86
A 1 B 1

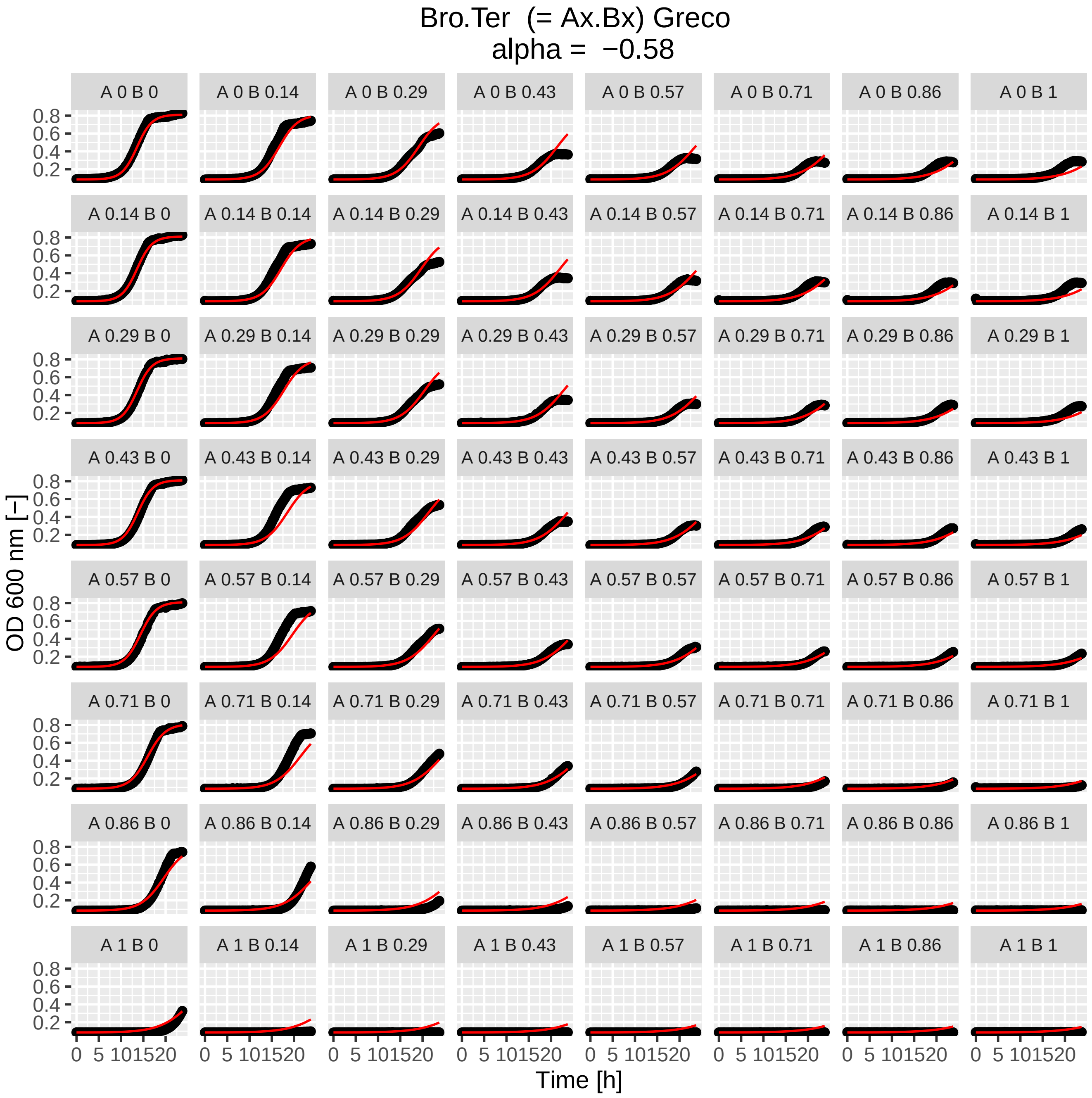
Bro.Ter (= Ax.Bx) Greco
alpha = −0.58
A 0 B 0
A 0 B 0.14
A 0 B 0.29
A 0 B 0.43
A 0 B 0.57
A 0 B 0.71
A 0 B 0.86
A 0 B 1
A 0.14 B 0
A 0.14 B 0.14
A 0.14 B 0.29
A 0.14 B 0.43
A 0.14 B 0.57
A 0.14 B 0.71
A 0.14 B 0.86
A 0.14 B 1
A 0.29 B 0
A 0.29 B 0.14
A 0.29 B 0.29
A 0.29 B 0.43
A 0.29 B 0.57
A 0.29 B 0.71
A 0.29 B 0.86
A 0.29 B 1
A 0.43 B 0
A 0.43 B 0.14
A 0.43 B 0.29
A 0.43 B 0.43
A 0.43 B 0.57
A 0.43 B 0.71
A 0.43 B 0.86
A 0.43 B 1
A 0.57 B 0
A 0.57 B 0.14
A 0.57 B 0.29
A 0.57 B 0.43
A 0.57 B 0.57
A 0.57 B 0.71
A 0.57 B 0.86
A 0.57 B 1
A 0.71 B 0
A 0.71 B 0.14
A 0.71 B 0.29
A 0.71 B 0.43
A 0.71 B 0.57
A 0.71 B 0.71
A 0.71 B 0.86
A 0.71 B 1
A 0.86 B 0
A 0.86 B 0.14
A 0.86 B 0.29
A 0.86 B 0.43
A 0.86 B 0.57
A 0.86 B 0.71
A 0.86 B 0.86
A 0.86 B 1
A 1 B 0
A 1 B 0.14
A 1 B 0.29
A 1 B 0.43
A 1 B 0.57
A 1 B 0.71
A 1 B 0.86
A 1 B 1
OD 600 nm [−]
0.2
0.4
0.6
0.8
0 5 101520
Time [h]

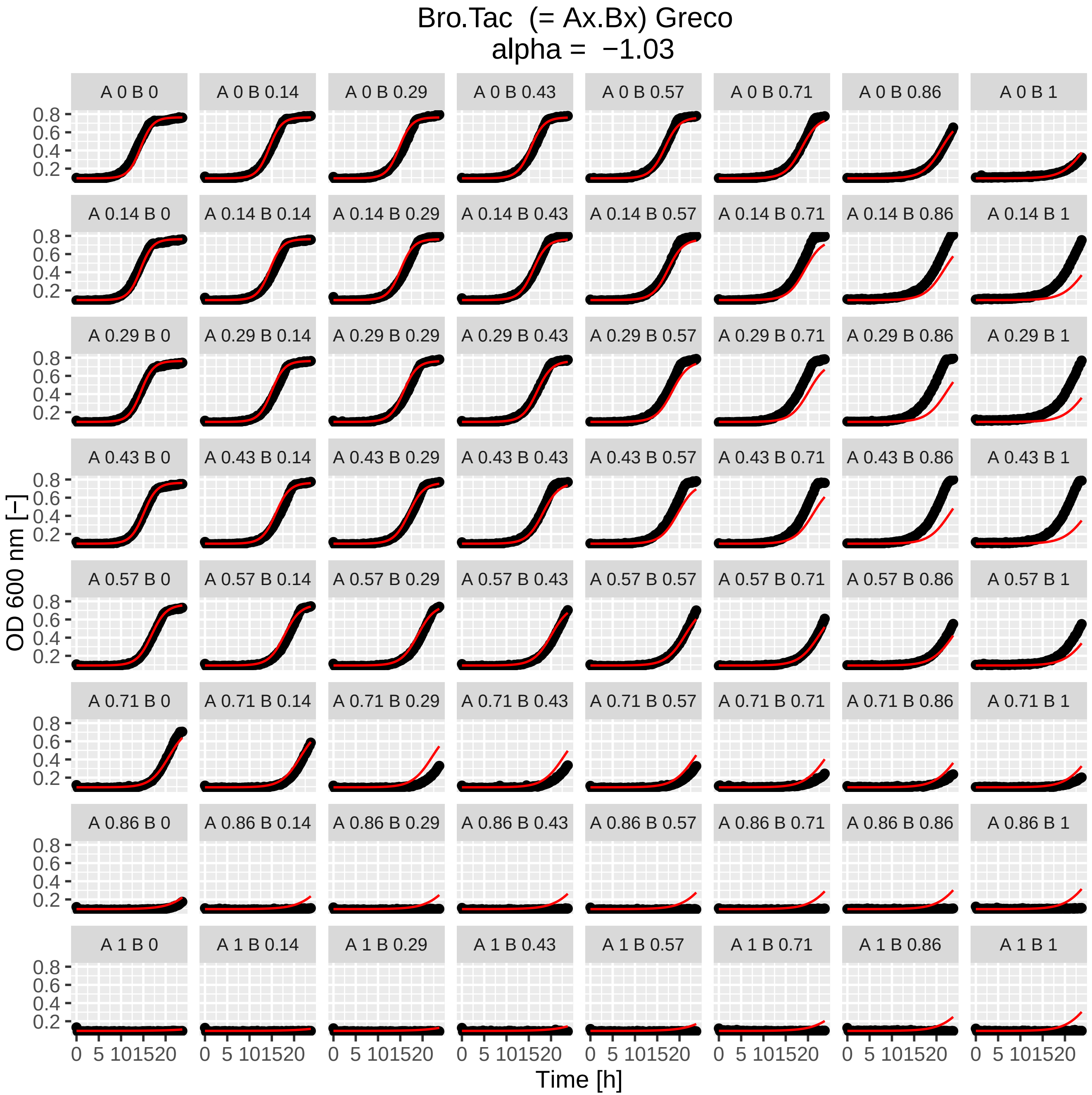
Bro.Tac (= Ax.Bx) Greco
alpha = −1.03
A 0 B 0
A 0 B 0.14
A 0 B 0.29
A 0 B 0.43
A 0 B 0.57
A 0 B 0.71
A 0 B 0.86
A 0 B 1
A 0.14 B 0
A 0.14 B 0.14
A 0.14 B 0.29
A 0.14 B 0.43
A 0.14 B 0.57
A 0.14 B 0.71
A 0.14 B 0.86
A 0.14 B 1
A 0.29 B 0
A 0.29 B 0.14
A 0.29 B 0.29
A 0.29 B 0.43
A 0.29 B 0.57
A 0.29 B 0.71
A 0.29 B 0.86
A 0.29 B 1
A 0.43 B 0
A 0.43 B 0.14
A 0.43 B 0.29
A 0.43 B 0.43
A 0.43 B 0.57
A 0.43 B 0.71
A 0.43 B 0.86
A 0.43 B 1
A 0.57 B 0
A 0.57 B 0.14
A 0.57 B 0.29
A 0.57 B 0.43
A 0.57 B 0.57
A 0.57 B 0.71
A 0.57 B 0.86
A 0.57 B 1
A 0.71 B 0
A 0.71 B 0.14
A 0.71 B 0.29
A 0.71 B 0.43
A 0.71 B 0.57
A 0.71 B 0.71
A 0.71 B 0.86
A 0.71 B 1
A 0.86 B 0
A 0.86 B 0.14
A 0.86 B 0.29
A 0.86 B 0.43
A 0.86 B 0.57
A 0.86 B 0.71
A 0.86 B 0.86
A 0.86 B 1
A 1 B 0
A 1 B 0.14
A 1 B 0.29
A 1 B 0.43
A 1 B 0.57
A 1 B 0.71
A 1 B 0.86
A 1 B 1
0.8
0.6
0.4
0.2
OD 600 nm [–]
0 5 101520
Time [h]

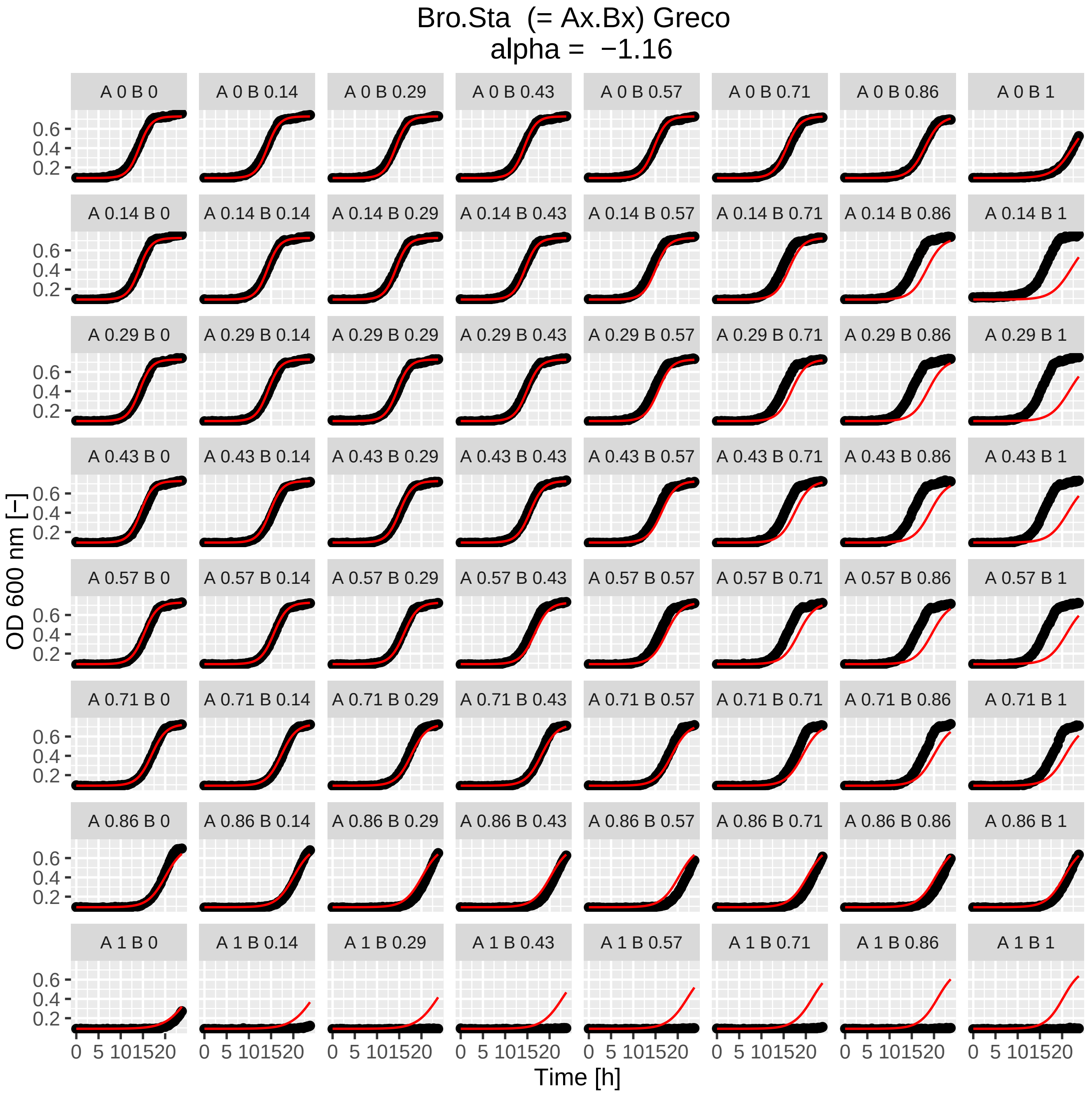
Bro.Sta (= Ax.Bx) Greco
alpha = −1.16
A 0 B 0
A 0 B 0.14
A 0 B 0.29
A 0 B 0.43
A 0 B 0.57
A 0 B 0.71
A 0 B 0.86
A 0 B 1
A 0.14 B 0
A 0.14 B 0.14
A 0.14 B 0.29
A 0.14 B 0.43
A 0.14 B 0.57
A 0.14 B 0.71
A 0.14 B 0.86
A 0.14 B 1
A 0.29 B 0
A 0.29 B 0.14
A 0.29 B 0.29
A 0.29 B 0.43
A 0.29 B 0.57
A 0.29 B 0.71
A 0.29 B 0.86
A 0.29 B 1
A 0.43 B 0
A 0.43 B 0.14
A 0.43 B 0.29
A 0.43 B 0.43
A 0.43 B 0.57
A 0.43 B 0.71
A 0.43 B 0.86
A 0.43 B 1
A 0.57 B 0
A 0.57 B 0.14
A 0.57 B 0.29
A 0.57 B 0.43
A 0.57 B 0.57
A 0.57 B 0.71
A 0.57 B 0.86
A 0.57 B 1
A 0.71 B 0
A 0.71 B 0.14
A 0.71 B 0.29
A 0.71 B 0.43
A 0.71 B 0.57
A 0.71 B 0.71
A 0.71 B 0.86
A 0.71 B 1
A 0.86 B 0
A 0.86 B 0.14
A 0.86 B 0.29
A 0.86 B 0.43
A 0.86 B 0.57
A 0.86 B 0.71
A 0.86 B 0.86
A 0.86 B 1
A 1 B 0
A 1 B 0.14
A 1 B 0.29
A 1 B 0.43
A 1 B 0.57
A 1 B 0.71
A 1 B 0.86
A 1 B 1
OD 600 nm [−]
0.2
0.4
0.6
0
5
10
15
20
Time [h]

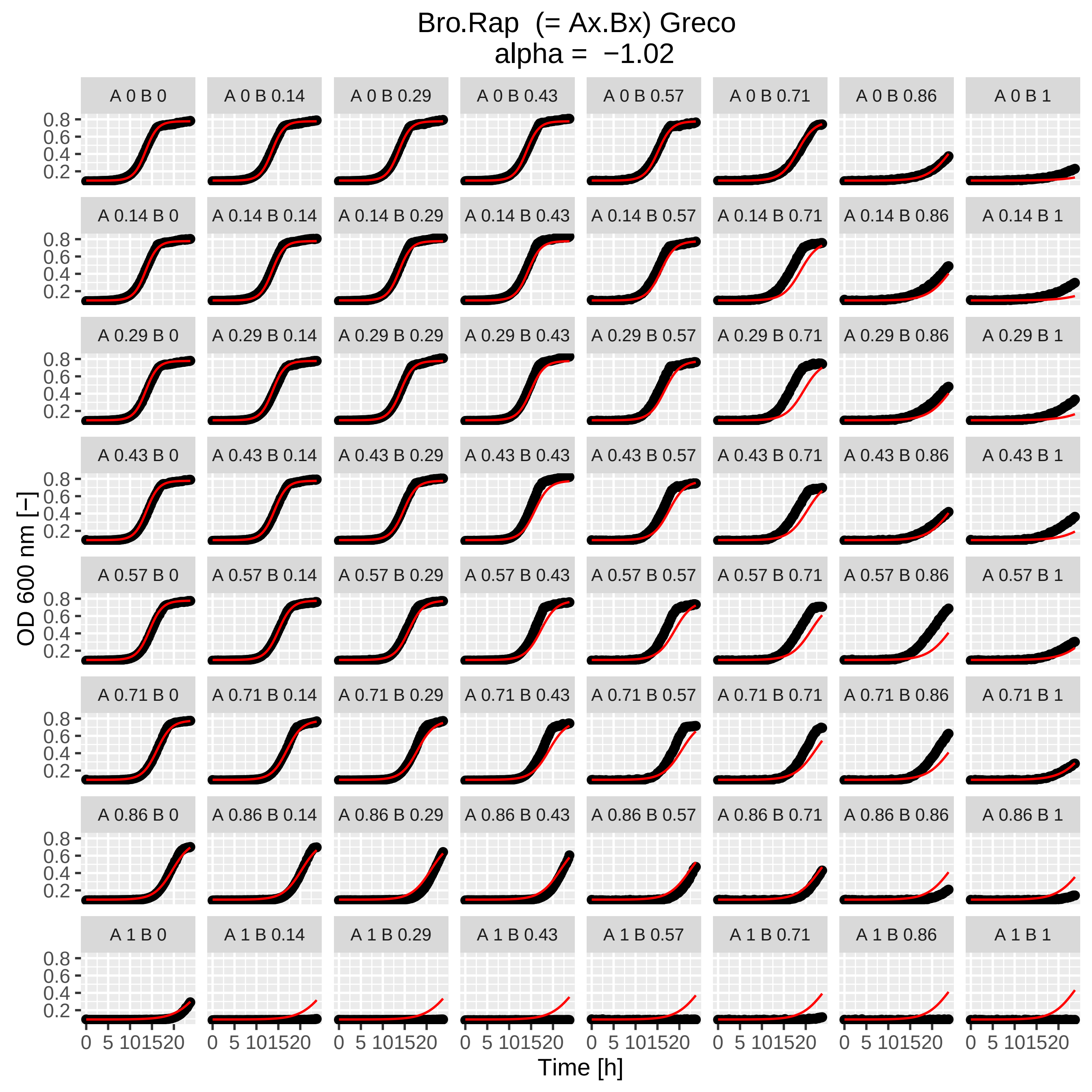

Bro.Rap (= Ax.Bx) Greco
alpha = −1.02
A 0 B 0
A 0 B 0.14
A 0 B 0.29
A 0 B 0.43
A 0 B 0.57
A 0 B 0.71
A 0 B 0.86
A 0 B 1
A 0.14 B 0
A 0.14 B 0.14
A 0.14 B 0.29
A 0.14 B 0.43
A 0.14 B 0.57
A 0.14 B 0.71
A 0.14 B 0.86
A 0.14 B 1
A 0.29 B 0
A 0.29 B 0.14
A 0.29 B 0.29
A 0.29 B 0.43
A 0.29 B 0.57
A 0.29 B 0.71
A 0.29 B 0.86
A 0.29 B 1
A 0.43 B 0
A 0.43 B 0.14
A 0.43 B 0.29
A 0.43 B 0.43
A 0.43 B 0.57
A 0.43 B 0.71
A 0.43 B 0.86
A 0.43 B 1
A 0.57 B 0
A 0.57 B 0.14
A 0.57 B 0.29
A 0.57 B 0.43
A 0.57 B 0.57
A 0.57 B 0.71
A 0.57 B 0.86
A 0.57 B 1
A 0.71 B 0
A 0.71 B 0.14
A 0.71 B 0.29
A 0.71 B 0.43
A 0.71 B 0.57
A 0.71 B 0.71
A 0.71 B 0.86
A 0.71 B 1
A 0.86 B 0
A 0.86 B 0.14
A 0.86 B 0.29
A 0.86 B 0.43
A 0.86 B 0.57
A 0.86 B 0.71
A 0.86 B 0.86
A 0.86 B 1
A 1 B 0
A 1 B 0.14
A 1 B 0.29
A 1 B 0.43
A 1 B 0.57
A 1 B 0.71
A 1 B 0.86
A 1 B 1
OD 600 nm [−]
0.8
0.6
0.4
0.2
0 5 101520
Time [h]

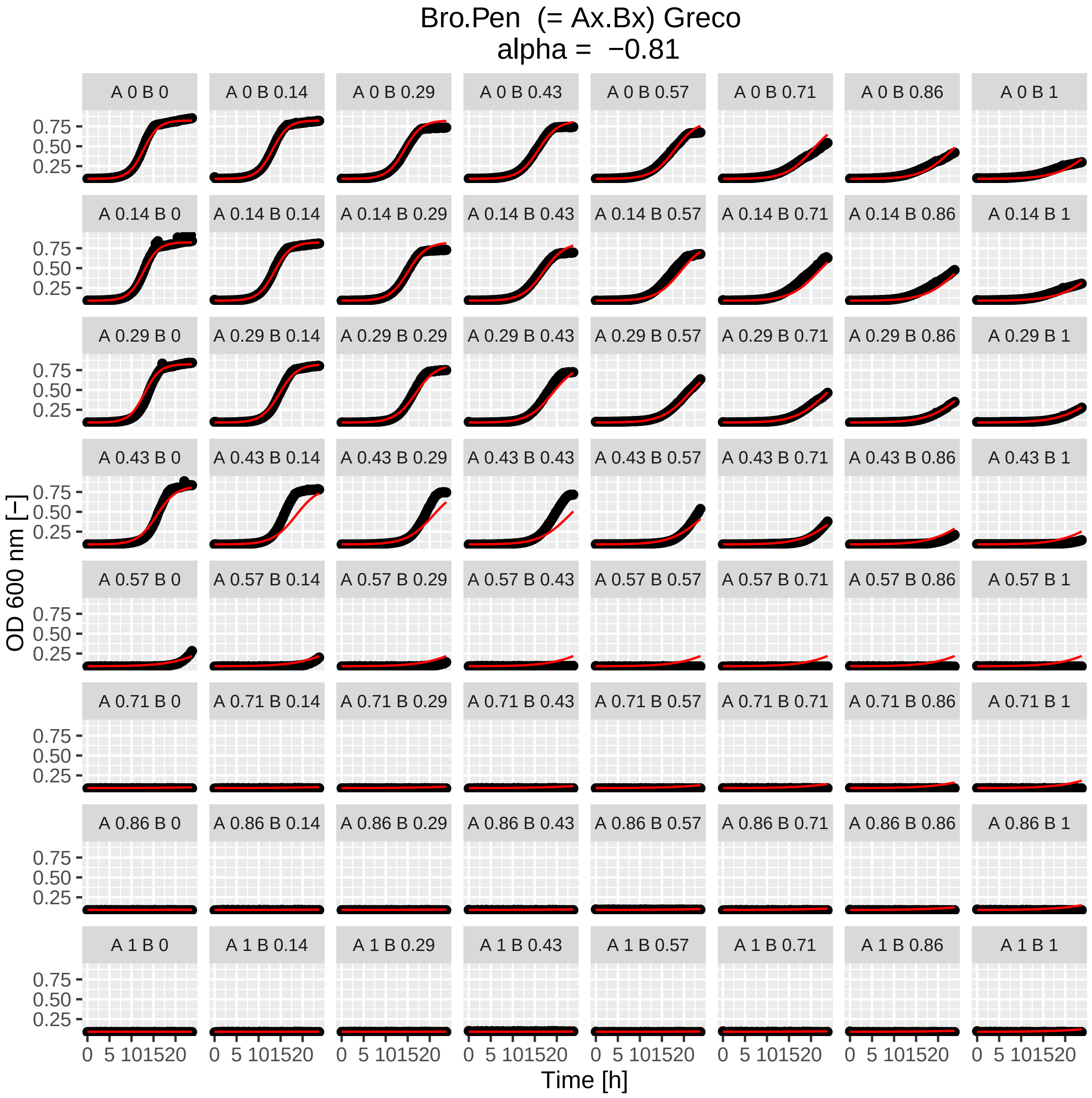
Bro.Pen (= Ax.Bx) Greco
alpha = −0.81
OD 600 nm [−]
Time [h]
0.75
0.50
0.25
0 5 101520
A 0 B 0
A 0 B 0.14
A 0 B 0.29
A 0 B 0.43
A 0 B 0.57
A 0 B 0.71
A 0 B 0.86
A 0 B 1
A 0.14 B 0
A 0.14 B 0.14
A 0.14 B 0.29
A 0.14 B 0.43
A 0.14 B 0.57
A 0.14 B 0.71
A 0.14 B 0.86
A 0.14 B 1
A 0.29 B 0
A 0.29 B 0.14
A 0.29 B 0.29
A 0.29 B 0.43
A 0.29 B 0.57
A 0.29 B 0.71
A 0.29 B 0.86
A 0.29 B 1
A 0.43 B 0
A 0.43 B 0.14
A 0.43 B 0.29
A 0.43 B 0.43
A 0.43 B 0.57
A 0.43 B 0.71
A 0.43 B 0.86
A 0.43 B 1
A 0.57 B 0
A 0.57 B 0.14
A 0.57 B 0.29
A 0.57 B 0.43
A 0.57 B 0.57
A 0.57 B 0.71
A 0.57 B 0.86
A 0.57 B 1
A 0.71 B 0
A 0.71 B 0.14
A 0.71 B 0.29
A 0.71 B 0.43
A 0.71 B 0.57
A 0.71 B 0.71
A 0.71 B 0.86
A 0.71 B 1
A 0.86 B 0
A 0.86 B 0.14
A 0.86 B 0.29
A 0.86 B 0.43
A 0.86 B 0.57
A 0.86 B 0.71
A 0.86 B 0.86
A 0.86 B 1
A 1 B 0
A 1 B 0.14
A 1 B 0.29
A 1 B 0.43
A 1 B 0.57
A 1 B 0.71
A 1 B 0.86
A 1 B 1

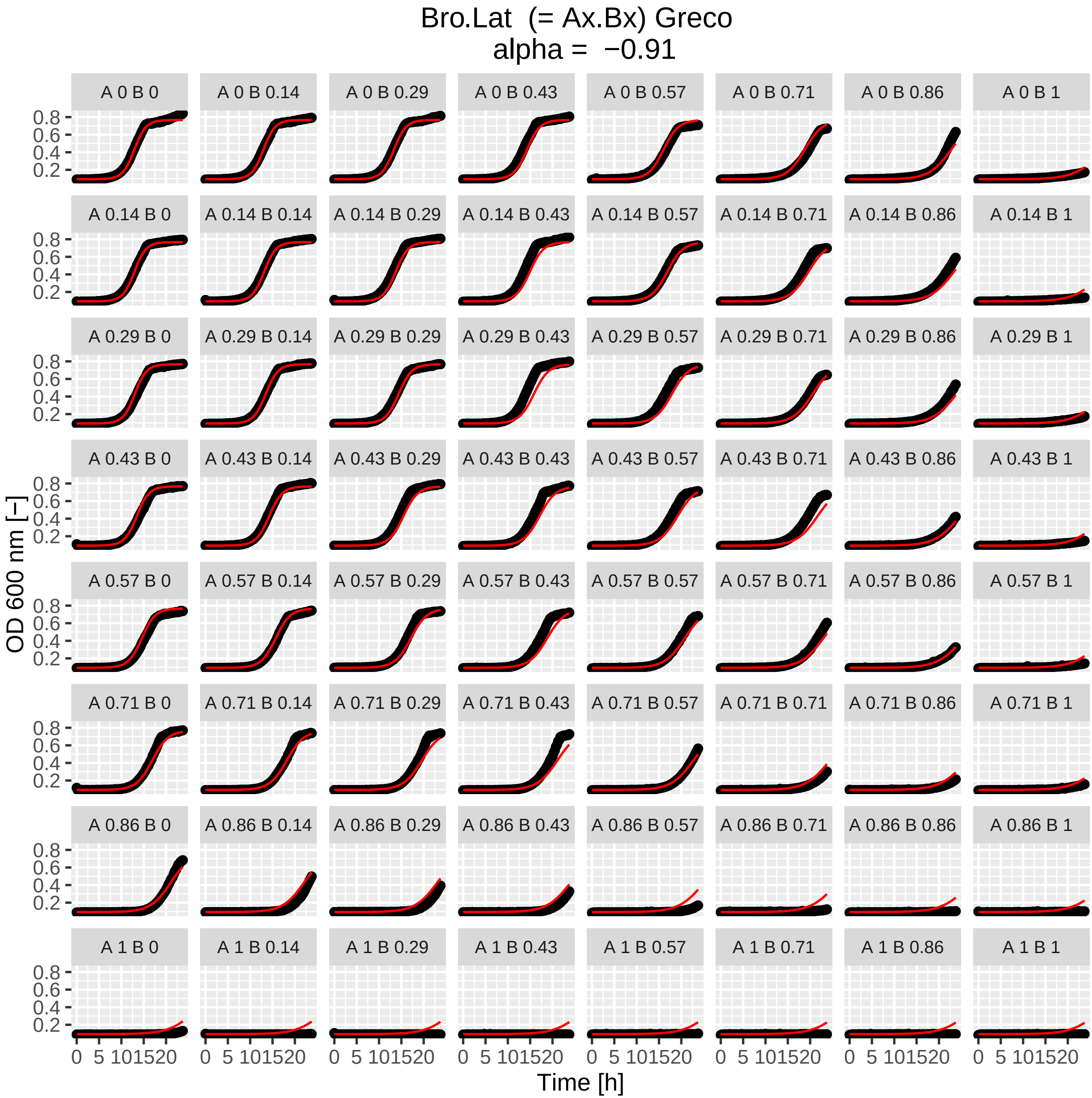

Bro.Lat (= Ax.Bx) Greco
alpha = −0.91
A 0 B 0
A 0 B 0.14
A 0 B 0.29
A 0 B 0.43
A 0 B 0.57
A 0 B 0.71
A 0 B 0.86
A 0 B 1
A 0.14 B 0
A 0.14 B 0.14
A 0.14 B 0.29
A 0.14 B 0.43
A 0.14 B 0.57
A 0.14 B 0.71
A 0.14 B 0.86
A 0.14 B 1
A 0.29 B 0
A 0.29 B 0.14
A 0.29 B 0.29
A 0.29 B 0.43
A 0.29 B 0.57
A 0.29 B 0.71
A 0.29 B 0.86
A 0.29 B 1
A 0.43 B 0
A 0.43 B 0.14
A 0.43 B 0.29
A 0.43 B 0.43
A 0.43 B 0.57
A 0.43 B 0.71
A 0.43 B 0.86
A 0.43 B 1
A 0.57 B 0
A 0.57 B 0.14
A 0.57 B 0.29
A 0.57 B 0.43
A 0.57 B 0.57
A 0.57 B 0.71
A 0.57 B 0.86
A 0.57 B 1
A 0.71 B 0
A 0.71 B 0.14
A 0.71 B 0.29
A 0.71 B 0.43
A 0.71 B 0.57
A 0.71 B 0.71
A 0.71 B 0.86
A 0.71 B 1
A 0.86 B 0
A 0.86 B 0.14
A 0.86 B 0.29
A 0.86 B 0.43
A 0.86 B 0.57
A 0.86 B 0.71
A 0.86 B 0.86
A 0.86 B 1
A 1 B 0
A 1 B 0.14
A 1 B 0.29
A 1 B 0.43
A 1 B 0.57
A 1 B 0.71
A 1 B 0.86
A 1 B 1
0.8
0.6
0.4
0.2
OD 600 nm [−]
0 5 101520
Time [h]

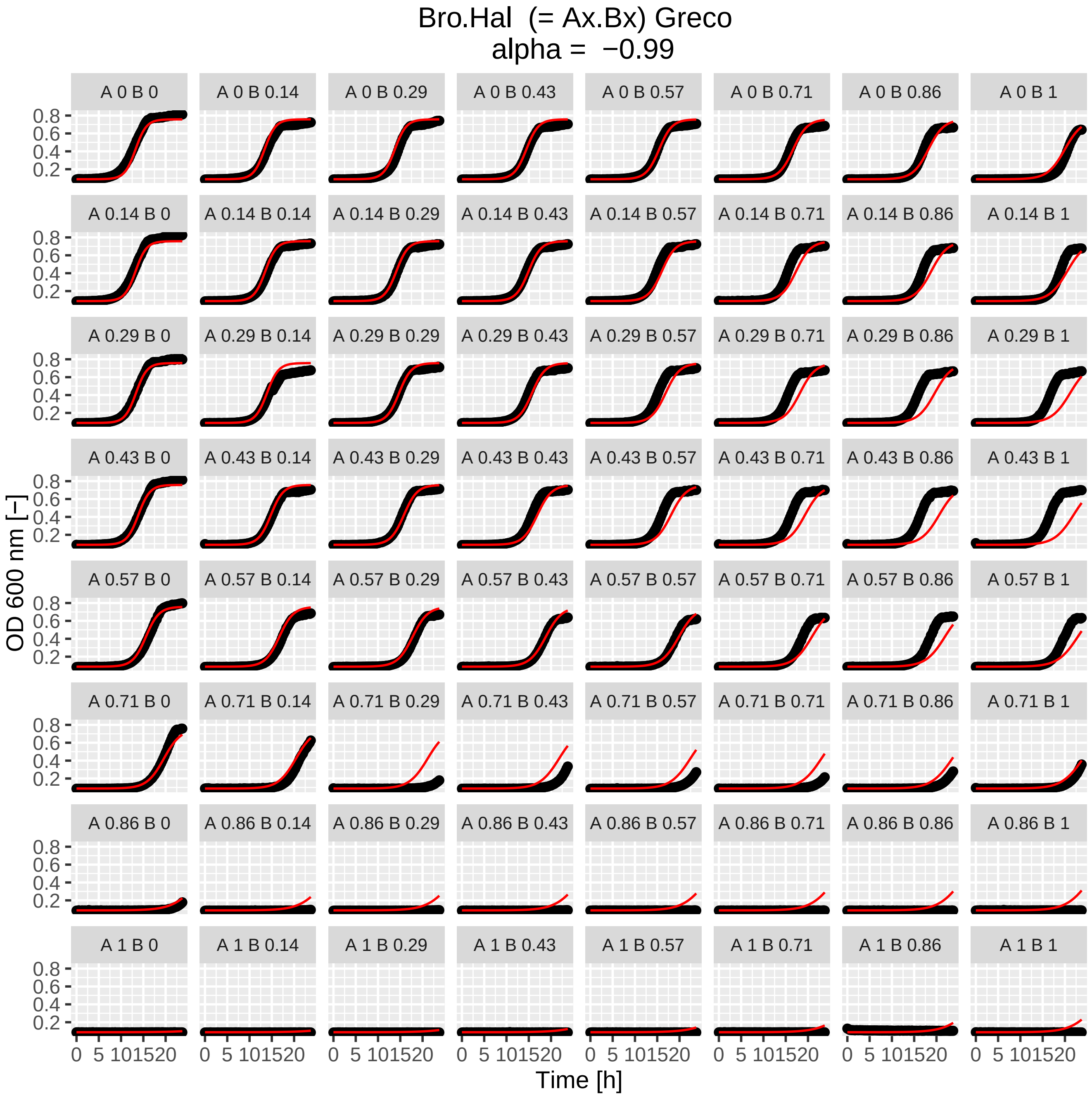

Bro.Hal (= Ax.Bx) Greco
alpha = −0.99
OD 600 nm [−]
Time [h]
A 0 B 0
A 0 B 0.14
A 0 B 0.29
A 0 B 0.43
A 0 B 0.57
A 0 B 0.71
A 0 B 0.86
A 0 B 1
A 0.14 B 0
A 0.14 B 0.14
A 0.14 B 0.29
A 0.14 B 0.43
A 0.14 B 0.57
A 0.14 B 0.71
A 0.14 B 0.86
A 0.14 B 1
A 0.29 B 0
A 0.29 B 0.14
A 0.29 B 0.29
A 0.29 B 0.43
A 0.29 B 0.57
A 0.29 B 0.71
A 0.29 B 0.86
A 0.29 B 1
A 0.43 B 0
A 0.43 B 0.14
A 0.43 B 0.29
A 0.43 B 0.43
A 0.43 B 0.57
A 0.43 B 0.71
A 0.43 B 0.86
A 0.43 B 1
A 0.57 B 0
A 0.57 B 0.14
A 0.57 B 0.29
A 0.57 B 0.43
A 0.57 B 0.57
A 0.57 B 0.71
A 0.57 B 0.86
A 0.57 B 1
A 0.71 B 0
A 0.71 B 0.14
A 0.71 B 0.29
A 0.71 B 0.43
A 0.71 B 0.57
A 0.71 B 0.71
A 0.71 B 0.86
A 0.71 B 1
A 0.86 B 0
A 0.86 B 0.14
A 0.86 B 0.29
A 0.86 B 0.43
A 0.86 B 0.57
A 0.86 B 0.71
A 0.86 B 0.86
A 0.86 B 1
A 1 B 0
A 1 B 0.14
A 1 B 0.29
A 1 B 0.43
A 1 B 0.57
A 1 B 0.71
A 1 B 0.86
A 1 B 1
0.8
0.6
0.4
0.2
0 5 101520

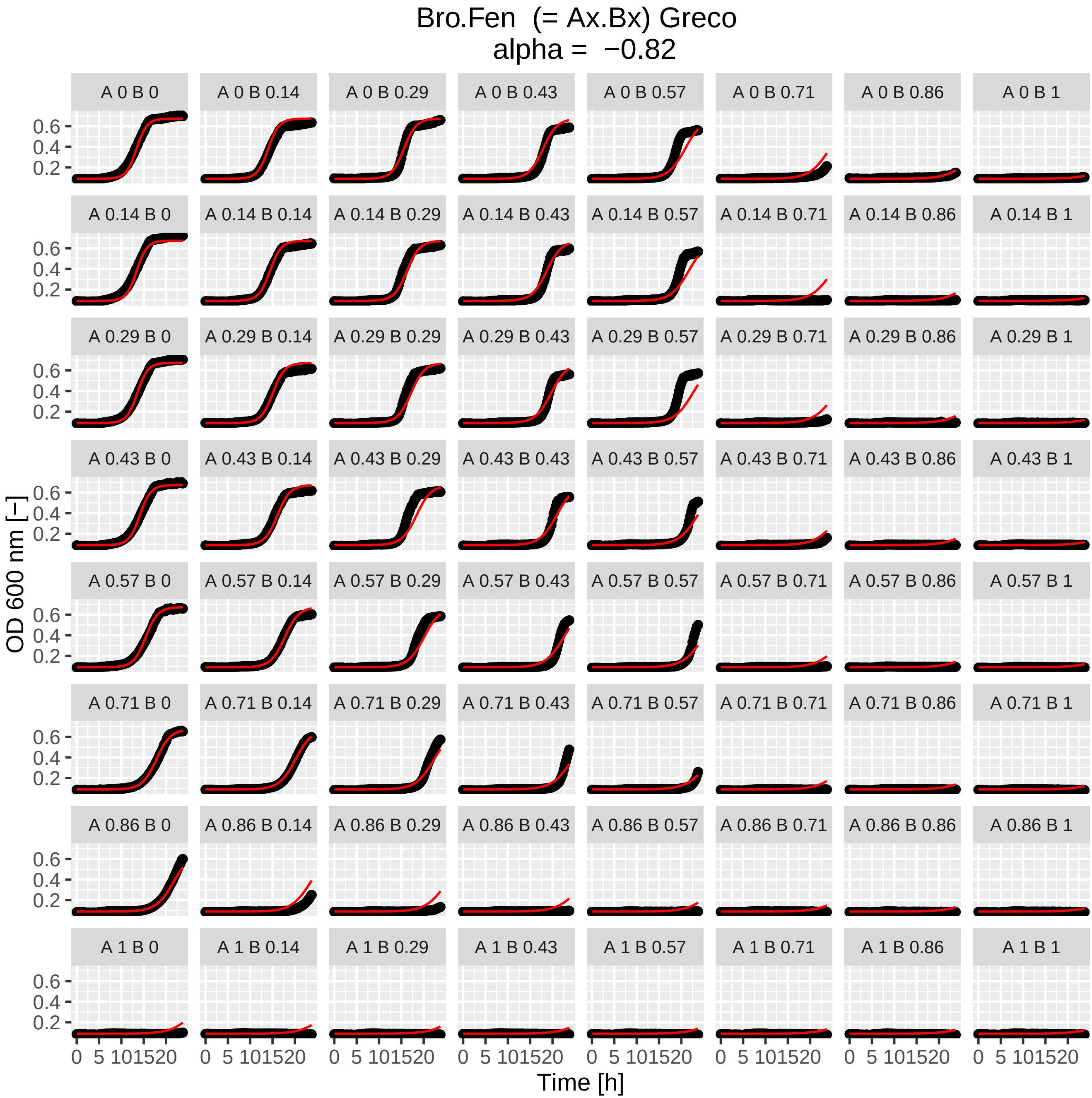
Bro.Fen (= Ax.Bx) Greco
alpha = −0.82
A 0 B 0
A 0 B 0.14
A 0 B 0.29
A 0 B 0.43
A 0 B 0.57
A 0 B 0.71
A 0 B 0.86
A 0 B 1
A 0.14 B 0
A 0.14 B 0.14
A 0.14 B 0.29
A 0.14 B 0.43
A 0.14 B 0.57
A 0.14 B 0.71
A 0.14 B 0.86
A 0.14 B 1
A 0.29 B 0
A 0.29 B 0.14
A 0.29 B 0.29
A 0.29 B 0.43
A 0.29 B 0.57
A 0.29 B 0.71
A 0.29 B 0.86
A 0.29 B 1
A 0.43 B 0
A 0.43 B 0.14
A 0.43 B 0.29
A 0.43 B 0.43
A 0.43 B 0.57
A 0.43 B 0.71
A 0.43 B 0.86
A 0.43 B 1
A 0.57 B 0
A 0.57 B 0.14
A 0.57 B 0.29
A 0.57 B 0.43
A 0.57 B 0.57
A 0.57 B 0.71
A 0.57 B 0.86
A 0.57 B 1
A 0.71 B 0
A 0.71 B 0.14
A 0.71 B 0.29
A 0.71 B 0.43
A 0.71 B 0.57
A 0.71 B 0.71
A 0.71 B 0.86
A 0.71 B 1
A 0.86 B 0
A 0.86 B 0.14
A 0.86 B 0.29
A 0.86 B 0.43
A 0.86 B 0.57
A 0.86 B 0.71
A 0.86 B 0.86
A 0.86 B 1
A 1 B 0
A 1 B 0.14
A 1 B 0.29
A 1 B 0.43
A 1 B 0.57
A 1 B 0.71
A 1 B 0.86
A 1 B 1
OD 600 nm [−]
0.2
0.4
0.6
0 5 101520
Time [h]

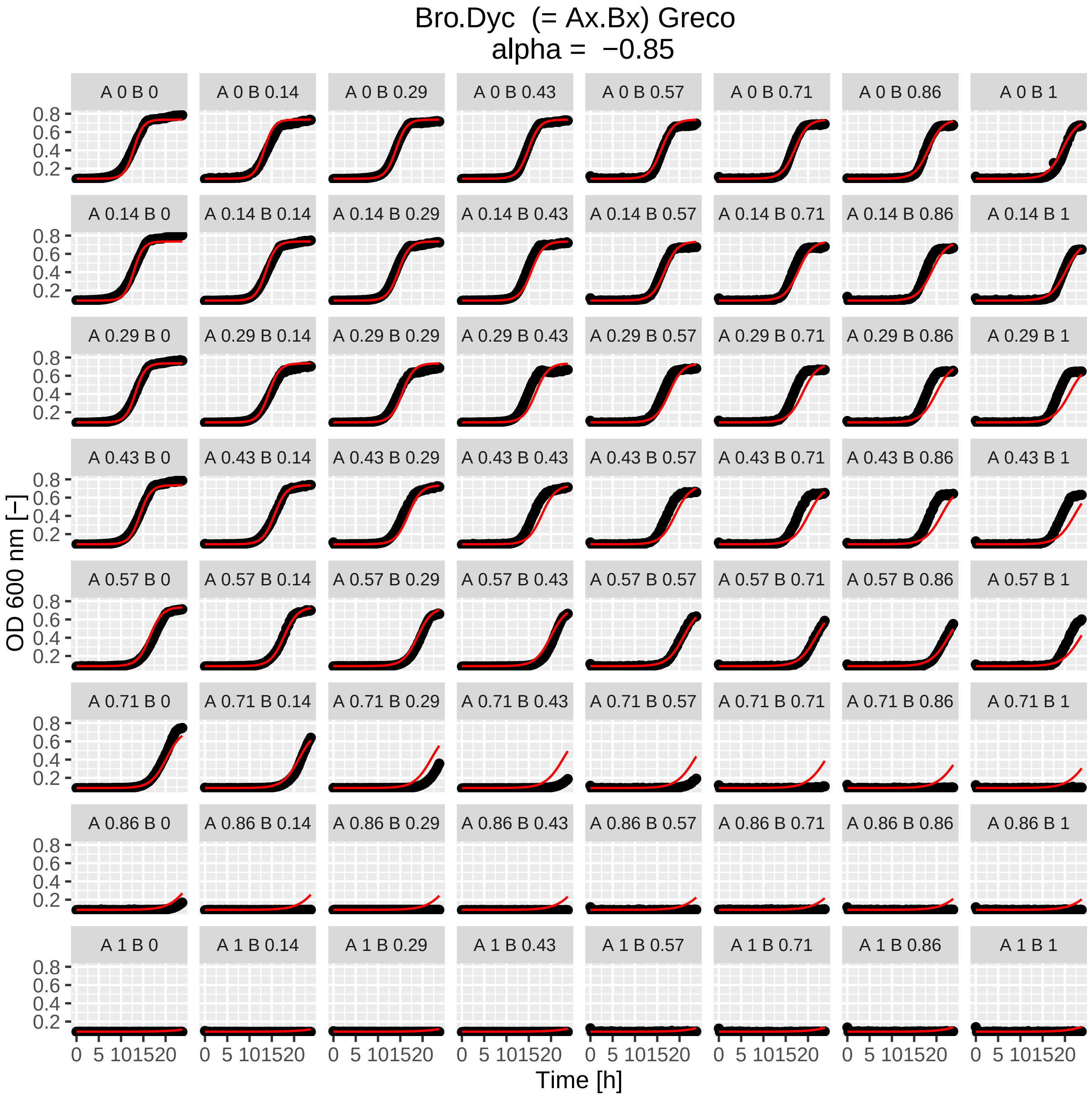

Bro.Dyc (= Ax.Bx) Greco
alpha = −0.85
OD 600 nm [−]
Time [h]
0.8
0.6
0.4
0.2
0 5 101520
A 0 B 0
A 0 B 0.14
A 0 B 0.29
A 0 B 0.43
A 0 B 0.57
A 0 B 0.71
A 0 B 0.86
A 0 B 1
A 0.14 B 0
A 0.14 B 0.14
A 0.14 B 0.29
A 0.14 B 0.43
A 0.14 B 0.57
A 0.14 B 0.71
A 0.14 B 0.86
A 0.14 B 1
A 0.29 B 0
A 0.29 B 0.14
A 0.29 B 0.29
A 0.29 B 0.43
A 0.29 B 0.57
A 0.29 B 0.71
A 0.29 B 0.86
A 0.29 B 1
A 0.43 B 0
A 0.43 B 0.14
A 0.43 B 0.29
A 0.43 B 0.43
A 0.43 B 0.57
A 0.43 B 0.71
A 0.43 B 0.86
A 0.43 B 1
A 0.57 B 0
A 0.57 B 0.14
A 0.57 B 0.29
A 0.57 B 0.43
A 0.57 B 0.57
A 0.57 B 0.71
A 0.57 B 0.86
A 0.57 B 1
A 0.71 B 0
A 0.71 B 0.14
A 0.71 B 0.29
A 0.71 B 0.43
A 0.71 B 0.57
A 0.71 B 0.71
A 0.71 B 0.86
A 0.71 B 1
A 0.86 B 0
A 0.86 B 0.14
A 0.86 B 0.29
A 0.86 B 0.43
A 0.86 B 0.57
A 0.86 B 0.71
A 0.86 B 0.86
A 0.86 B 1
A 1 B 0
A 1 B 0.14
A 1 B 0.29
A 1 B 0.43
A 1 B 0.57
A 1 B 0.71
A 1 B 0.86
A 1 B 1

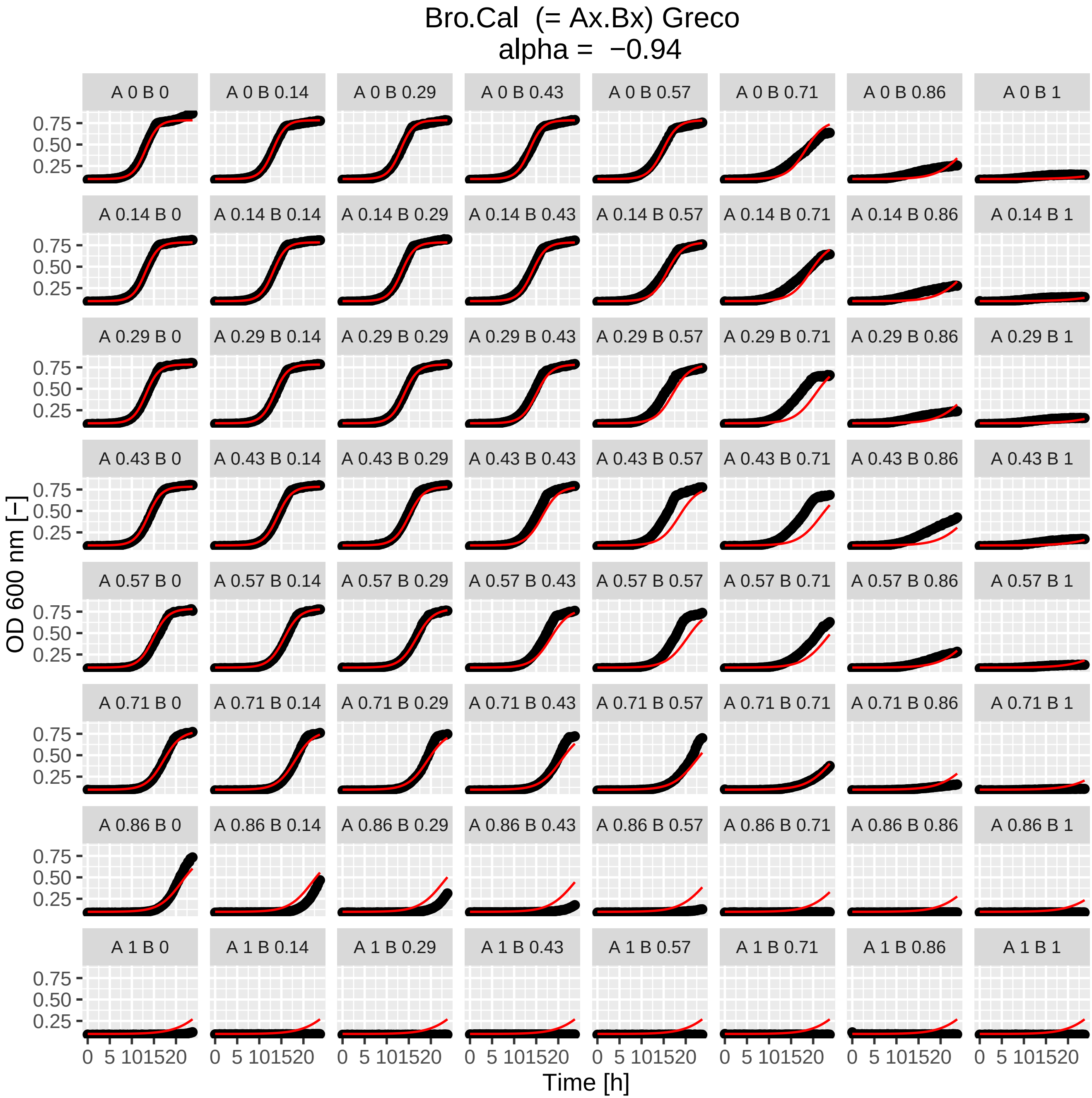

Bro.Cal (= Ax.Bx) Greco
alpha = −0.94
OD 600 nm [−]
Time [h]
A 0 B 0
A 0 B 0.14
A 0 B 0.29
A 0 B 0.43
A 0 B 0.57
A 0 B 0.71
A 0 B 0.86
A 0 B 1
A 0.14 B 0
A 0.29 B 0
A 0.43 B 0
A 0.57 B 0
A 0.71 B 0
A 0.86 B 0
A 1 B 0
A 1 B 1
0.75
0.50
0.25
0 5 101520

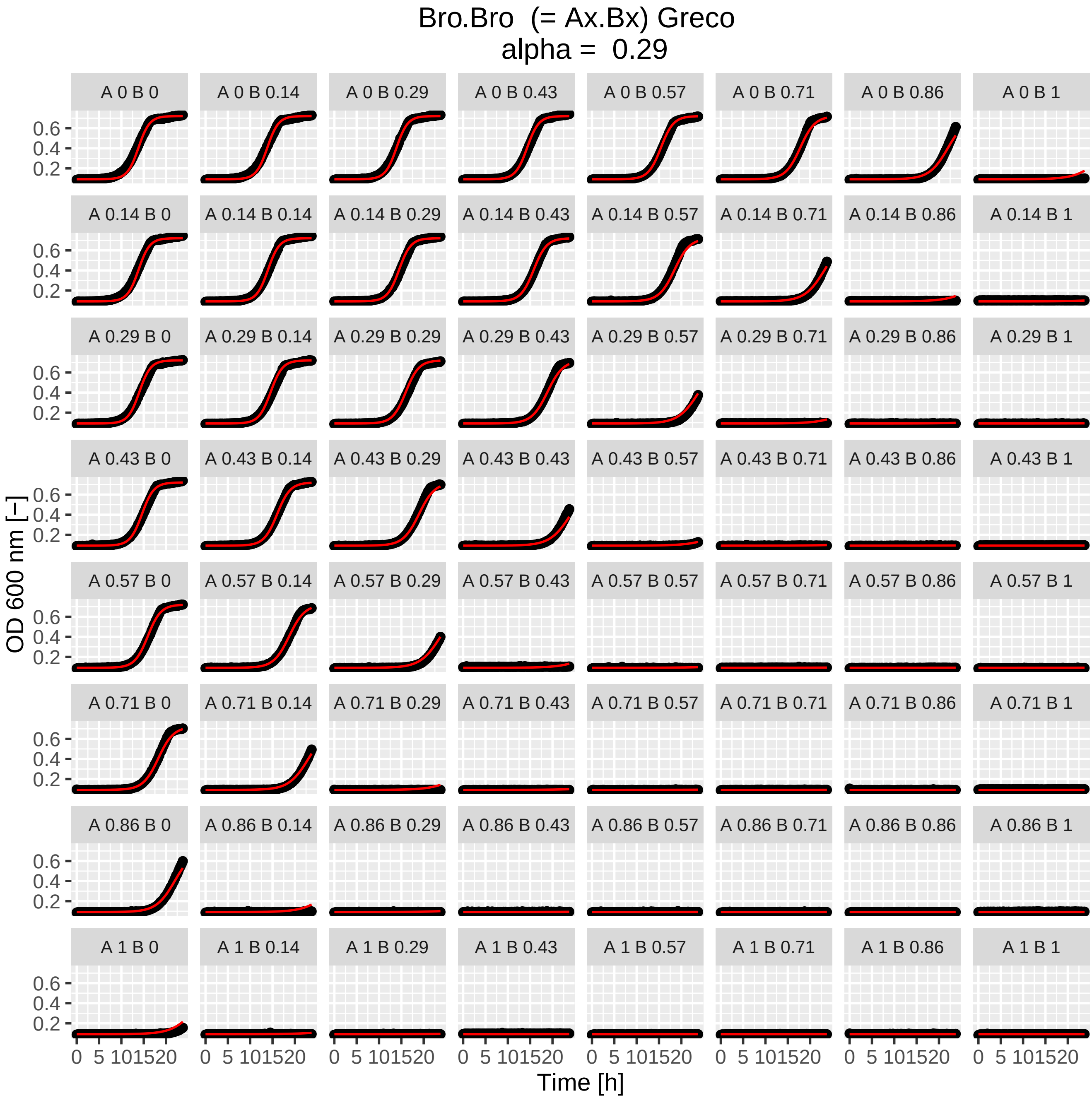
Bro.Bro (= Ax.Bx) Greco
alpha = 0.29
A 0 B 0
A 0 B 0.14
A 0 B 0.29
A 0 B 0.43
A 0 B 0.57
A 0 B 0.71
A 0 B 0.86
A 0 B 1
A 0.14 B 0
A 0.14 B 0.14
A 0.14 B 0.29
A 0.14 B 0.43
A 0.14 B 0.57
A 0.14 B 0.71
A 0.14 B 0.86
A 0.14 B 1
A 0.29 B 0
A 0.29 B 0.14
A 0.29 B 0.29
A 0.29 B 0.43
A 0.29 B 0.57
A 0.29 B 0.71
A 0.29 B 0.86
A 0.29 B 1
A 0.43 B 0
A 0.43 B 0.14
A 0.43 B 0.29
A 0.43 B 0.43
A 0.43 B 0.57
A 0.43 B 0.71
A 0.43 B 0.86
A 0.43 B 1
A 0.57 B 0
A 0.57 B 0.14
A 0.57 B 0.29
A 0.57 B 0.43
A 0.57 B 0.57
A 0.57 B 0.71
A 0.57 B 0.86
A 0.57 B 1
A 0.71 B 0
A 0.71 B 0.14
A 0.71 B 0.29
A 0.71 B 0.43
A 0.71 B 0.57
A 0.71 B 0.71
A 0.71 B 0.86
A 0.71 B 1
A 0.86 B 0
A 0.86 B 0.14
A 0.86 B 0.29
A 0.86 B 0.43
A 0.86 B 0.57
A 0.86 B 0.71
A 0.86 B 0.86
A 0.86 B 1
A 1 B 0
A 1 B 0.14
A 1 B 0.29
A 1 B 0.43
A 1 B 0.57
A 1 B 0.71
A 1 B 0.86
A 1 B 1
OD 600 nm [–]
0.6
0.4
0.2
0 5 101520
Time [h]

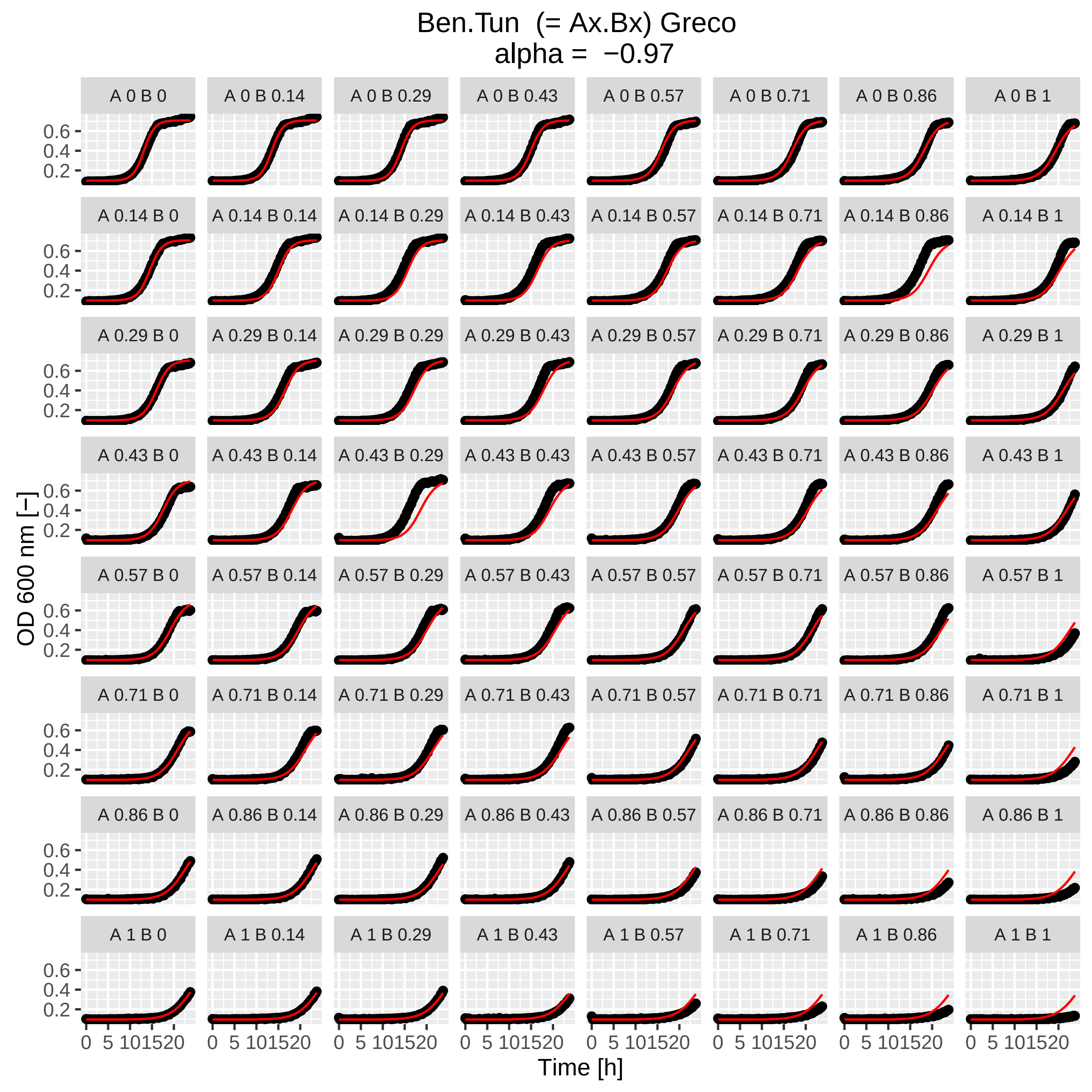

Ben.Tun (= Ax.Bx) Greco
alpha = −0.97
A 0 B 0
A 0 B 0.14
A 0 B 0.29
A 0 B 0.43
A 0 B 0.57
A 0 B 0.71
A 0 B 0.86
A 0 B 1
A 0.14 B 0
A 0.14 B 0.14
A 0.14 B 0.29
A 0.14 B 0.43
A 0.14 B 0.57
A 0.14 B 0.71
A 0.14 B 0.86
A 0.14 B 1
A 0.29 B 0
A 0.29 B 0.14
A 0.29 B 0.29
A 0.29 B 0.43
A 0.29 B 0.57
A 0.29 B 0.71
A 0.29 B 0.86
A 0.29 B 1
A 0.43 B 0
A 0.43 B 0.14
A 0.43 B 0.29
A 0.43 B 0.43
A 0.43 B 0.57
A 0.43 B 0.71
A 0.43 B 0.86
A 0.43 B 1
A 0.57 B 0
A 0.57 B 0.14
A 0.57 B 0.29
A 0.57 B 0.43
A 0.57 B 0.57
A 0.57 B 0.71
A 0.57 B 0.86
A 0.57 B 1
A 0.71 B 0
A 0.71 B 0.14
A 0.71 B 0.29
A 0.71 B 0.43
A 0.71 B 0.57
A 0.71 B 0.71
A 0.71 B 0.86
A 0.71 B 1
A 0.86 B 0
A 0.86 B 0.14
A 0.86 B 0.29
A 0.86 B 0.43
A 0.86 B 0.57
A 0.86 B 0.71
A 0.86 B 0.86
A 0.86 B 1
A 1 B 0
A 1 B 0.14
A 1 B 0.29
A 1 B 0.43
A 1 B 0.57
A 1 B 0.71
A 1 B 0.86
A 1 B 1
OD 600 nm [−]
0.2
0.4
0.6
0 5 101520
Time [h]

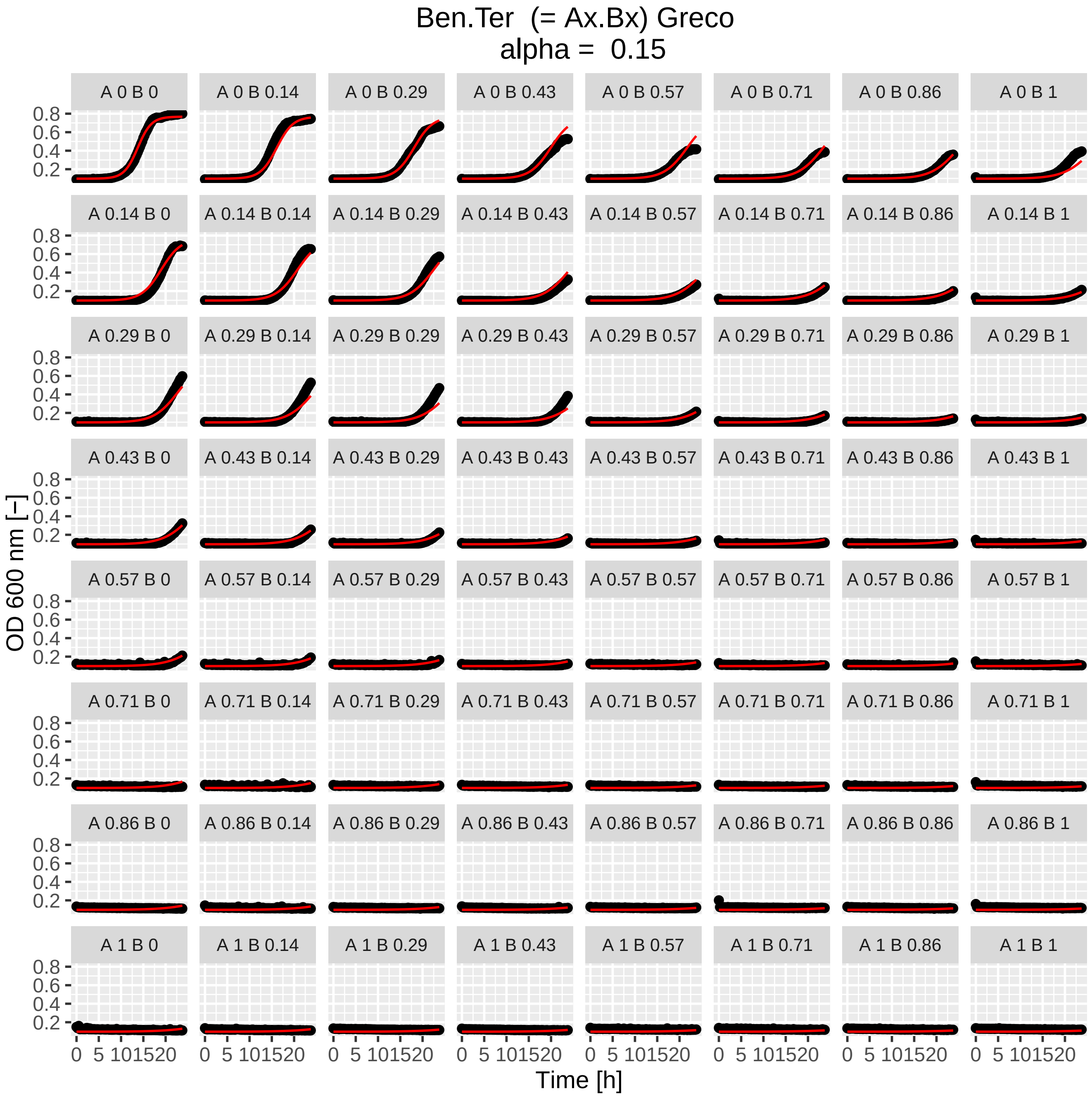
Ben.Ter (= Ax.Bx) Greco
alpha = 0.15
A 0 B 0
A 0 B 0.14
A 0 B 0.29
A 0 B 0.43
A 0 B 0.57
A 0 B 0.71
A 0 B 0.86
A 0 B 1
A 0.14 B 0
A 0.14 B 0.14
A 0.14 B 0.29
A 0.14 B 0.43
A 0.14 B 0.57
A 0.14 B 0.71
A 0.14 B 0.86
A 0.14 B 1
A 0.29 B 0
A 0.29 B 0.14
A 0.29 B 0.29
A 0.29 B 0.43
A 0.29 B 0.57
A 0.29 B 0.71
A 0.29 B 0.86
A 0.29 B 1
A 0.43 B 0
A 0.43 B 0.14
A 0.43 B 0.29
A 0.43 B 0.43
A 0.43 B 0.57
A 0.43 B 0.71
A 0.43 B 0.86
A 0.43 B 1
A 0.57 B 0
A 0.57 B 0.14
A 0.57 B 0.29
A 0.57 B 0.43
A 0.57 B 0.57
A 0.57 B 0.71
A 0.57 B 0.86
A 0.57 B 1
A 0.71 B 0
A 0.71 B 0.14
A 0.71 B 0.29
A 0.71 B 0.43
A 0.71 B 0.57
A 0.71 B 0.71
A 0.71 B 0.86
A 0.71 B 1
A 0.86 B 0
A 0.86 B 0.14
A 0.86 B 0.29
A 0.86 B 0.43
A 0.86 B 0.57
A 0.86 B 0.71
A 0.86 B 0.86
A 0.86 B 1
A 1 B 0
A 1 B 0.14
A 1 B 0.29
A 1 B 0.43
A 1 B 0.57
A 1 B 0.71
A 1 B 0.86
A 1 B 1
OD 600 nm [–]
0.2
0.4
0.6
0.8
0 5 101520
Time [h]

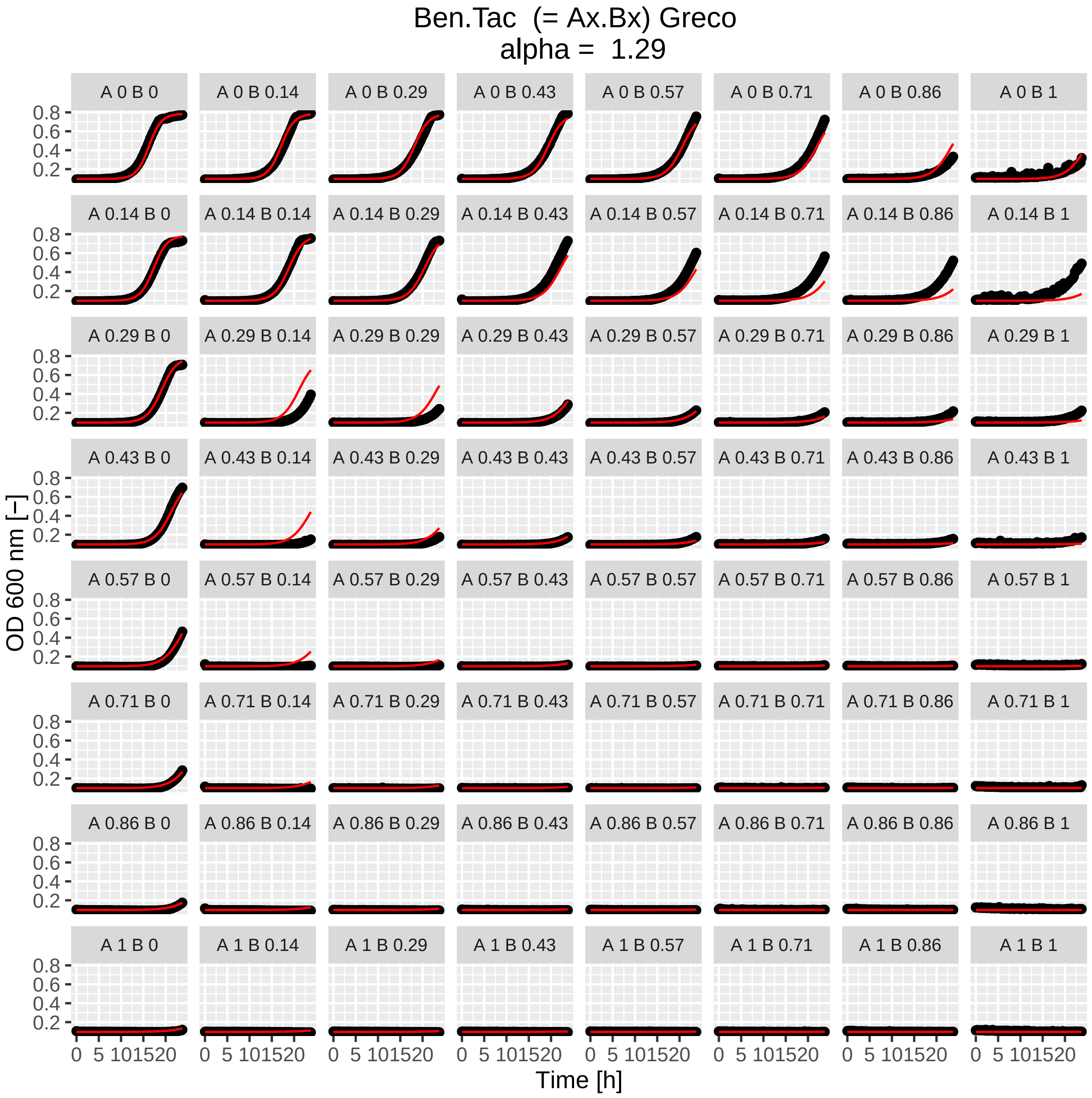
Ben.Tac (= Ax.Bx) Greco
alpha = 1.29
A 0 B 0
A 0 B 0.14
A 0 B 0.29
A 0 B 0.43
A 0 B 0.57
A 0 B 0.71
A 0 B 0.86
A 0 B 1
A 0.14 B 0
A 0.14 B 0.14
A 0.14 B 0.29
A 0.14 B 0.43
A 0.14 B 0.57
A 0.14 B 0.71
A 0.14 B 0.86
A 0.14 B 1
A 0.29 B 0
A 0.29 B 0.14
A 0.29 B 0.29
A 0.29 B 0.43
A 0.29 B 0.57
A 0.29 B 0.71
A 0.29 B 0.86
A 0.29 B 1
A 0.43 B 0
A 0.43 B 0.14
A 0.43 B 0.29
A 0.43 B 0.43
A 0.43 B 0.57
A 0.43 B 0.71
A 0.43 B 0.86
A 0.43 B 1
A 0.57 B 0
A 0.57 B 0.14
A 0.57 B 0.29
A 0.57 B 0.43
A 0.57 B 0.57
A 0.57 B 0.71
A 0.57 B 0.86
A 0.57 B 1
A 0.71 B 0
A 0.71 B 0.14
A 0.71 B 0.29
A 0.71 B 0.43
A 0.71 B 0.57
A 0.71 B 0.71
A 0.71 B 0.86
A 0.71 B 1
A 0.86 B 0
A 0.86 B 0.14
A 0.86 B 0.29
A 0.86 B 0.43
A 0.86 B 0.57
A 0.86 B 0.71
A 0.86 B 0.86
A 0.86 B 1
A 1 B 0
A 1 B 0.14
A 1 B 0.29
A 1 B 0.43
A 1 B 0.57
A 1 B 0.71
A 1 B 0.86
A 1 B 1
OD 600 nm [–]
0.2
0.4
0.6
0.8
0 5 101520
Time [h]

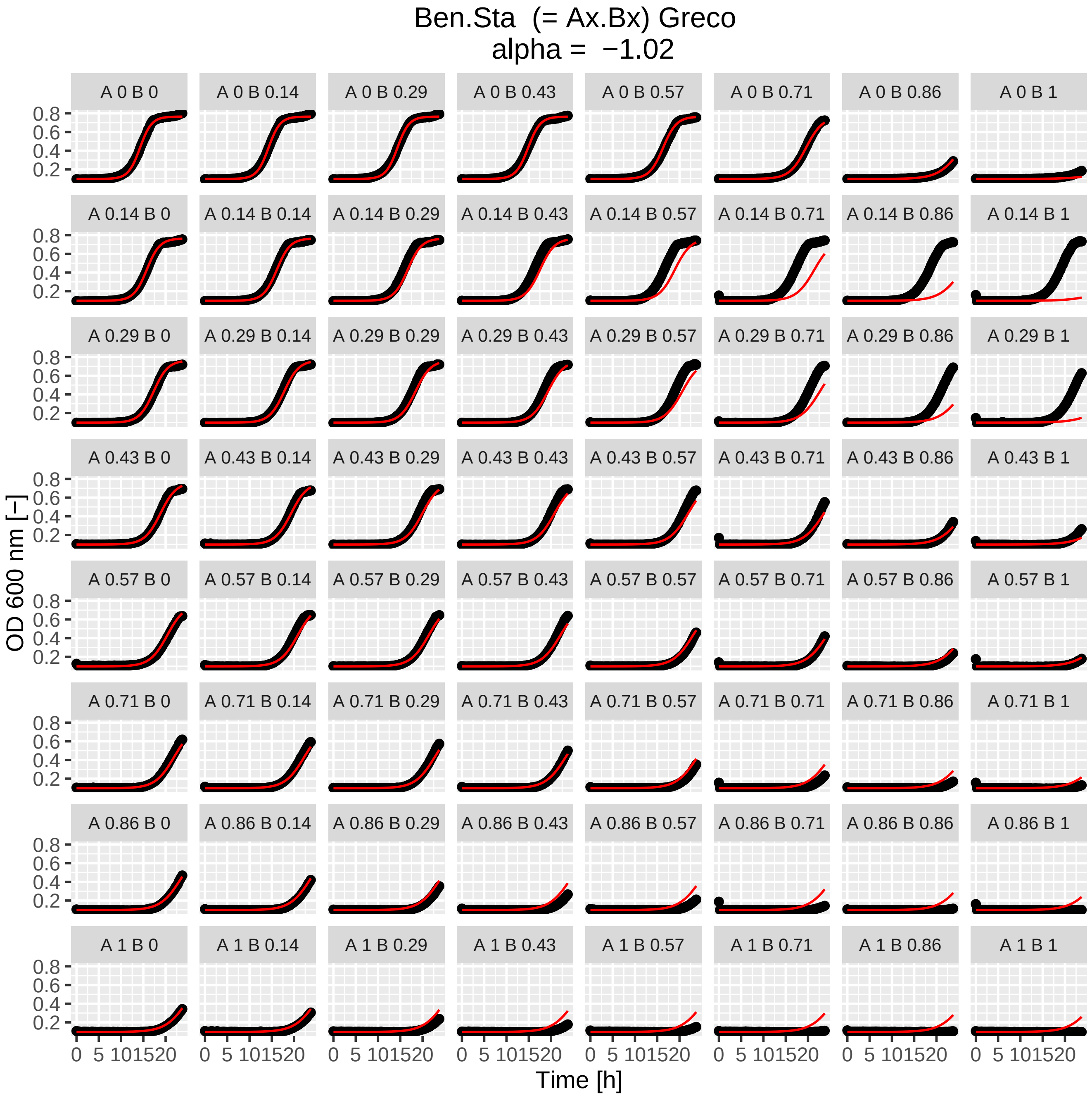

Ben.Sta (= Ax.Bx) Greco
alpha = −1.02
OD 600 nm [−]
Time [h]
A 0 B 0
A 0 B 0.14
A 0 B 0.29
A 0 B 0.43
A 0 B 0.57
A 0 B 0.71
A 0 B 0.86
A 0 B 1
A 0.14 B 0
A 0.14 B 0.14
A 0.14 B 0.29
A 0.14 B 0.43
A 0.14 B 0.57
A 0.14 B 0.71
A 0.14 B 0.86
A 0.14 B 1
A 0.29 B 0
A 0.29 B 0.14
A 0.29 B 0.29
A 0.29 B 0.43
A 0.29 B 0.57
A 0.29 B 0.71
A 0.29 B 0.86
A 0.29 B 1
A 0.43 B 0
A 0.43 B 0.14
A 0.43 B 0.29
A 0.43 B 0.43
A 0.43 B 0.57
A 0.43 B 0.71
A 0.43 B 0.86
A 0.43 B 1
A 0.57 B 0
A 0.57 B 0.14
A 0.57 B 0.29
A 0.57 B 0.43
A 0.57 B 0.57
A 0.57 B 0.71
A 0.57 B 0.86
A 0.57 B 1
A 0.71 B 0
A 0.71 B 0.14
A 0.71 B 0.29
A 0.71 B 0.43
A 0.71 B 0.57
A 0.71 B 0.71
A 0.71 B 0.86
A 0.71 B 1
A 0.86 B 0
A 0.86 B 0.14
A 0.86 B 0.29
A 0.86 B 0.43
A 0.86 B 0.57
A 0.86 B 0.71
A 0.86 B 0.86
A 0.86 B 1
A 1 B 0
A 1 B 0.14
A 1 B 0.29
A 1 B 0.43
A 1 B 0.57
A 1 B 0.71
A 1 B 0.86
A 1 B 1
0.8
0.6
0.4
0.2
0 5 101520

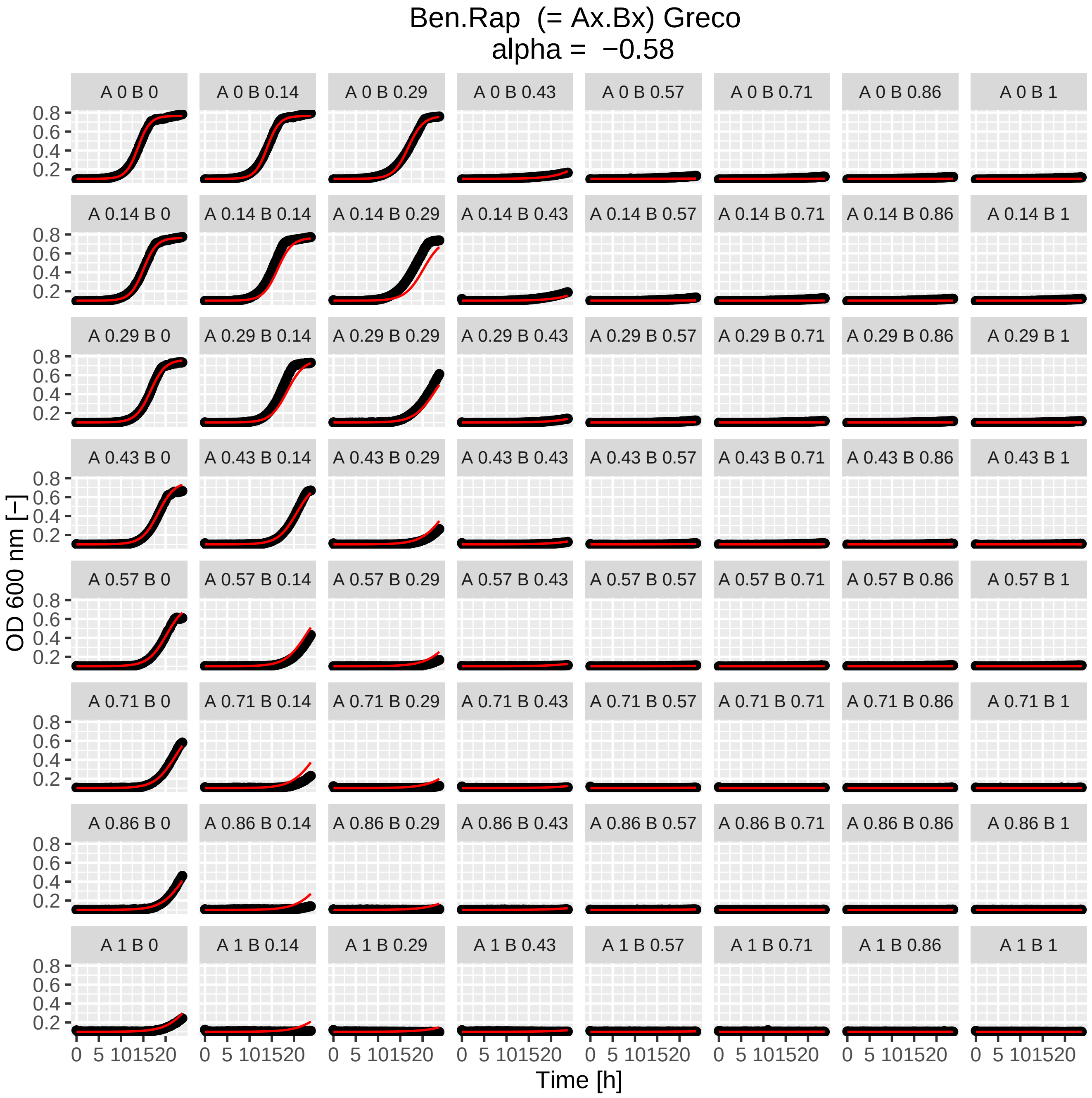

Ben.Rap (= Ax.Bx) Greco
alpha = −0.58
A 0 B 0
A 0 B 0.14
A 0 B 0.29
A 0 B 0.43
A 0 B 0.57
A 0 B 0.71
A 0 B 0.86
A 0 B 1
A 0.14 B 0
A 0.14 B 0.14
A 0.14 B 0.29
A 0.14 B 0.43
A 0.14 B 0.57
A 0.14 B 0.71
A 0.14 B 0.86
A 0.14 B 1
A 0.29 B 0
A 0.29 B 0.14
A 0.29 B 0.29
A 0.29 B 0.43
A 0.29 B 0.57
A 0.29 B 0.71
A 0.29 B 0.86
A 0.29 B 1
A 0.43 B 0
A 0.43 B 0.14
A 0.43 B 0.29
A 0.43 B 0.43
A 0.43 B 0.57
A 0.43 B 0.71
A 0.43 B 0.86
A 0.43 B 1
A 0.57 B 0
A 0.57 B 0.14
A 0.57 B 0.29
A 0.57 B 0.43
A 0.57 B 0.57
A 0.57 B 0.71
A 0.57 B 0.86
A 0.57 B 1
A 0.71 B 0
A 0.71 B 0.14
A 0.71 B 0.29
A 0.71 B 0.43
A 0.71 B 0.57
A 0.71 B 0.71
A 0.71 B 0.86
A 0.71 B 1
A 0.86 B 0
A 0.86 B 0.14
A 0.86 B 0.29
A 0.86 B 0.43
A 0.86 B 0.57
A 0.86 B 0.71
A 0.86 B 0.86
A 0.86 B 1
A 1 B 0
A 1 B 0.14
A 1 B 0.29
A 1 B 0.43
A 1 B 0.57
A 1 B 0.71
A 1 B 0.86
A 1 B 1
OD 600 nm [−]
0.8
0.6
0.4
0.2
0 5 101520
Time [h]

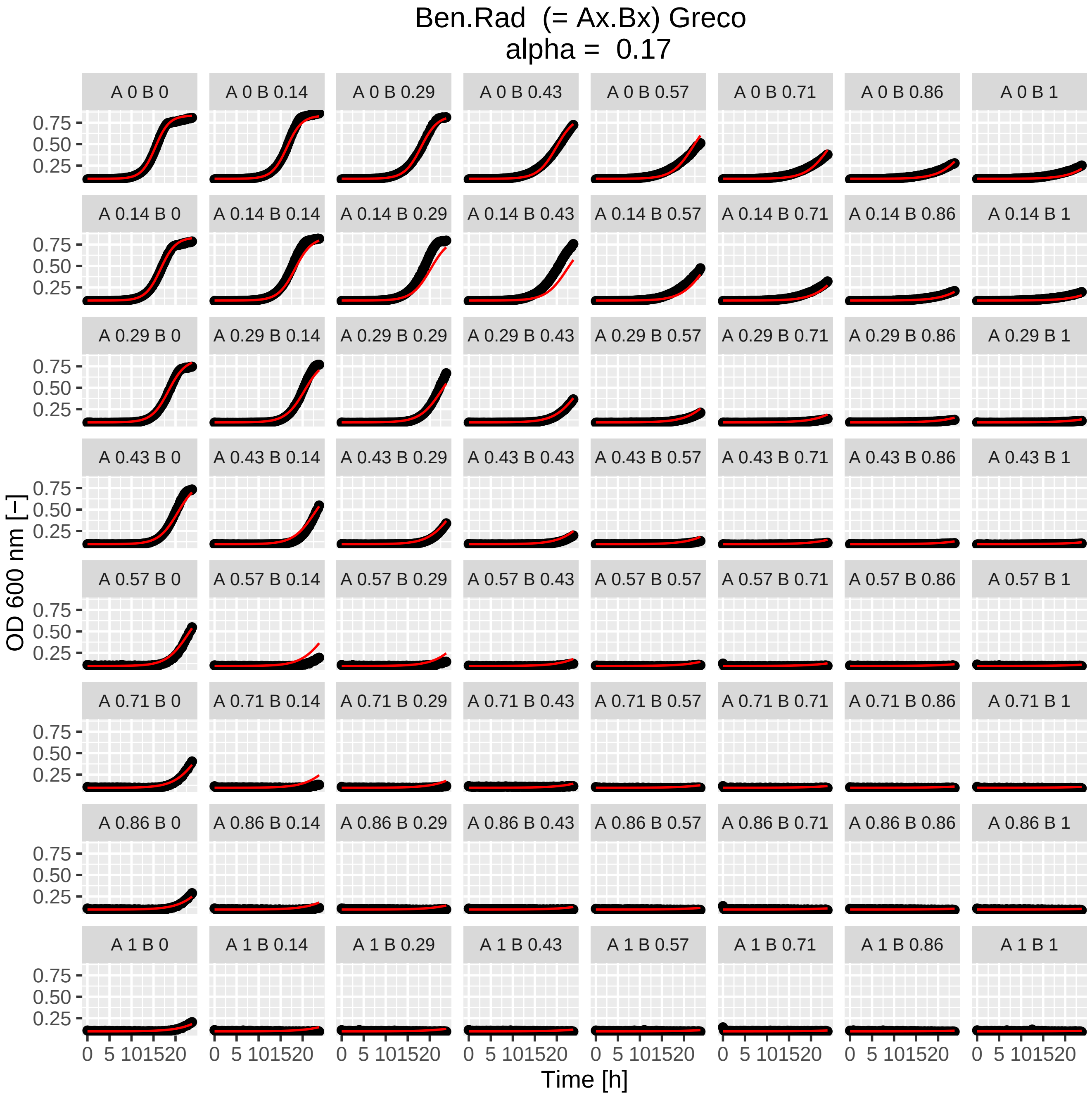

Ben.Rad (= Ax.Bx) Greco
alpha = 0.17
A 0 B 0
A 0 B 0.14
A 0 B 0.29
A 0 B 0.43
A 0 B 0.57
A 0 B 0.71
A 0 B 0.86
A 0 B 1
A 0.14 B 0
A 0.14 B 0.14
A 0.14 B 0.29
A 0.14 B 0.43
A 0.14 B 0.57
A 0.14 B 0.71
A 0.14 B 0.86
A 0.14 B 1
A 0.29 B 0
A 0.29 B 0.14
A 0.29 B 0.29
A 0.29 B 0.43
A 0.29 B 0.57
A 0.29 B 0.71
A 0.29 B 0.86
A 0.29 B 1
A 0.43 B 0
A 0.43 B 0.14
A 0.43 B 0.29
A 0.43 B 0.43
A 0.43 B 0.57
A 0.43 B 0.71
A 0.43 B 0.86
A 0.43 B 1
A 0.57 B 0
A 0.57 B 0.14
A 0.57 B 0.29
A 0.57 B 0.43
A 0.57 B 0.57
A 0.57 B 0.71
A 0.57 B 0.86
A 0.57 B 1
A 0.71 B 0
A 0.71 B 0.14
A 0.71 B 0.29
A 0.71 B 0.43
A 0.71 B 0.57
A 0.71 B 0.71
A 0.71 B 0.86
A 0.71 B 1
A 0.86 B 0
A 0.86 B 0.14
A 0.86 B 0.29
A 0.86 B 0.43
A 0.86 B 0.57
A 0.86 B 0.71
A 0.86 B 0.86
A 0.86 B 1
A 1 B 0
A 1 B 0.14
A 1 B 0.29
A 1 B 0.43
A 1 B 0.57
A 1 B 0.71
A 1 B 0.86
A 1 B 1
0.75
0.50
0.25
OD 600 nm [–]
0 5 101520
Time [h]

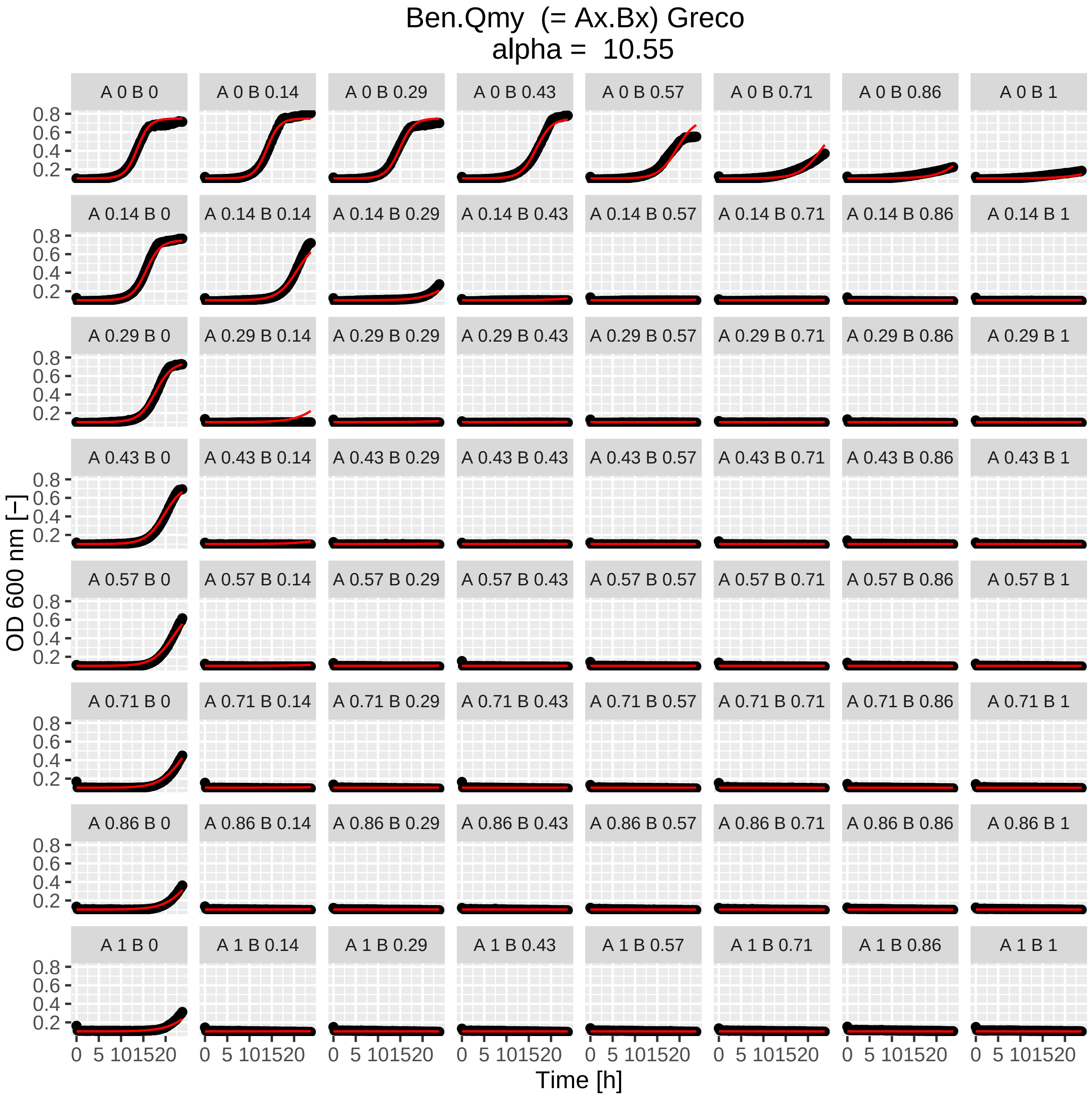

Ben.Qmy (= Ax.Bx) Greco
alpha = 10.55
A 0 B 0
A 0 B 0.14
A 0 B 0.29
A 0 B 0.43
A 0 B 0.57
A 0 B 0.71
A 0 B 0.86
A 0 B 1
A 0.14 B 0
A 0.14 B 0.14
A 0.14 B 0.29
A 0.14 B 0.43
A 0.14 B 0.57
A 0.14 B 0.71
A 0.14 B 0.86
A 0.14 B 1
A 0.29 B 0
A 0.29 B 0.14
A 0.29 B 0.29
A 0.29 B 0.43
A 0.29 B 0.57
A 0.29 B 0.71
A 0.29 B 0.86
A 0.29 B 1
A 0.43 B 0
A 0.43 B 0.14
A 0.43 B 0.29
A 0.43 B 0.43
A 0.43 B 0.57
A 0.43 B 0.71
A 0.43 B 0.86
A 0.43 B 1
A 0.57 B 0
A 0.57 B 0.14
A 0.57 B 0.29
A 0.57 B 0.43
A 0.57 B 0.57
A 0.57 B 0.71
A 0.57 B 0.86
A 0.57 B 1
A 0.71 B 0
A 0.71 B 0.14
A 0.71 B 0.29
A 0.71 B 0.43
A 0.71 B 0.57
A 0.71 B 0.71
A 0.71 B 0.86
A 0.71 B 1
A 0.86 B 0
A 0.86 B 0.14
A 0.86 B 0.29
A 0.86 B 0.43
A 0.86 B 0.57
A 0.86 B 0.71
A 0.86 B 0.86
A 0.86 B 1
A 1 B 0
A 1 B 0.14
A 1 B 0.29
A 1 B 0.43
A 1 B 0.57
A 1 B 0.71
A 1 B 0.86
A 1 B 1
OD 600 nm [–]
0.2
0.4
0.6
0.8
0 5 101520
Time [h]

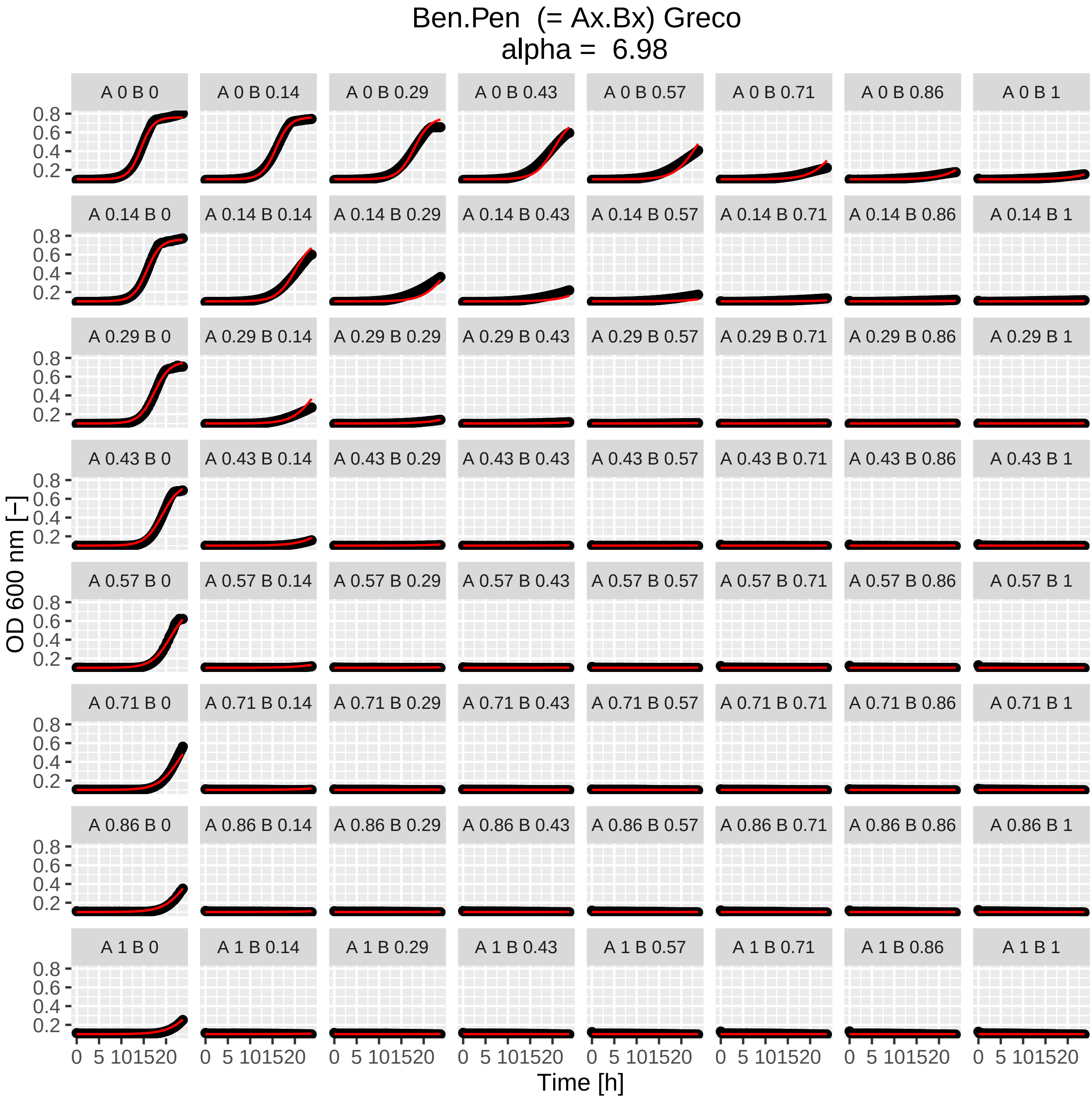

Ben.Pen (= Ax.Bx) Greco
alpha = 6.98
A 0 B 0
A 0 B 0.14
A 0 B 0.29
A 0 B 0.43
A 0 B 0.57
A 0 B 0.71
A 0 B 0.86
A 0 B 1
A 0.14 B 0
A 0.14 B 0.14
A 0.14 B 0.29
A 0.14 B 0.43
A 0.14 B 0.57
A 0.14 B 0.71
A 0.14 B 0.86
A 0.14 B 1
A 0.29 B 0
A 0.29 B 0.14
A 0.29 B 0.29
A 0.29 B 0.43
A 0.29 B 0.57
A 0.29 B 0.71
A 0.29 B 0.86
A 0.29 B 1
A 0.43 B 0
A 0.43 B 0.14
A 0.43 B 0.29
A 0.43 B 0.43
A 0.43 B 0.57
A 0.43 B 0.71
A 0.43 B 0.86
A 0.43 B 1
A 0.57 B 0
A 0.57 B 0.14
A 0.57 B 0.29
A 0.57 B 0.43
A 0.57 B 0.57
A 0.57 B 0.71
A 0.57 B 0.86
A 0.57 B 1
A 0.71 B 0
A 0.71 B 0.14
A 0.71 B 0.29
A 0.71 B 0.43
A 0.71 B 0.57
A 0.71 B 0.71
A 0.71 B 0.86
A 0.71 B 1
A 0.86 B 0
A 0.86 B 0.14
A 0.86 B 0.29
A 0.86 B 0.43
A 0.86 B 0.57
A 0.86 B 0.71
A 0.86 B 0.86
A 0.86 B 1
A 1 B 0
A 1 B 0.14
A 1 B 0.29
A 1 B 0.43
A 1 B 0.57
A 1 B 0.71
A 1 B 0.86
A 1 B 1
0.8
0.6
0.4
0.2
OD 600 nm [–]
0 5 101520
Time [h]

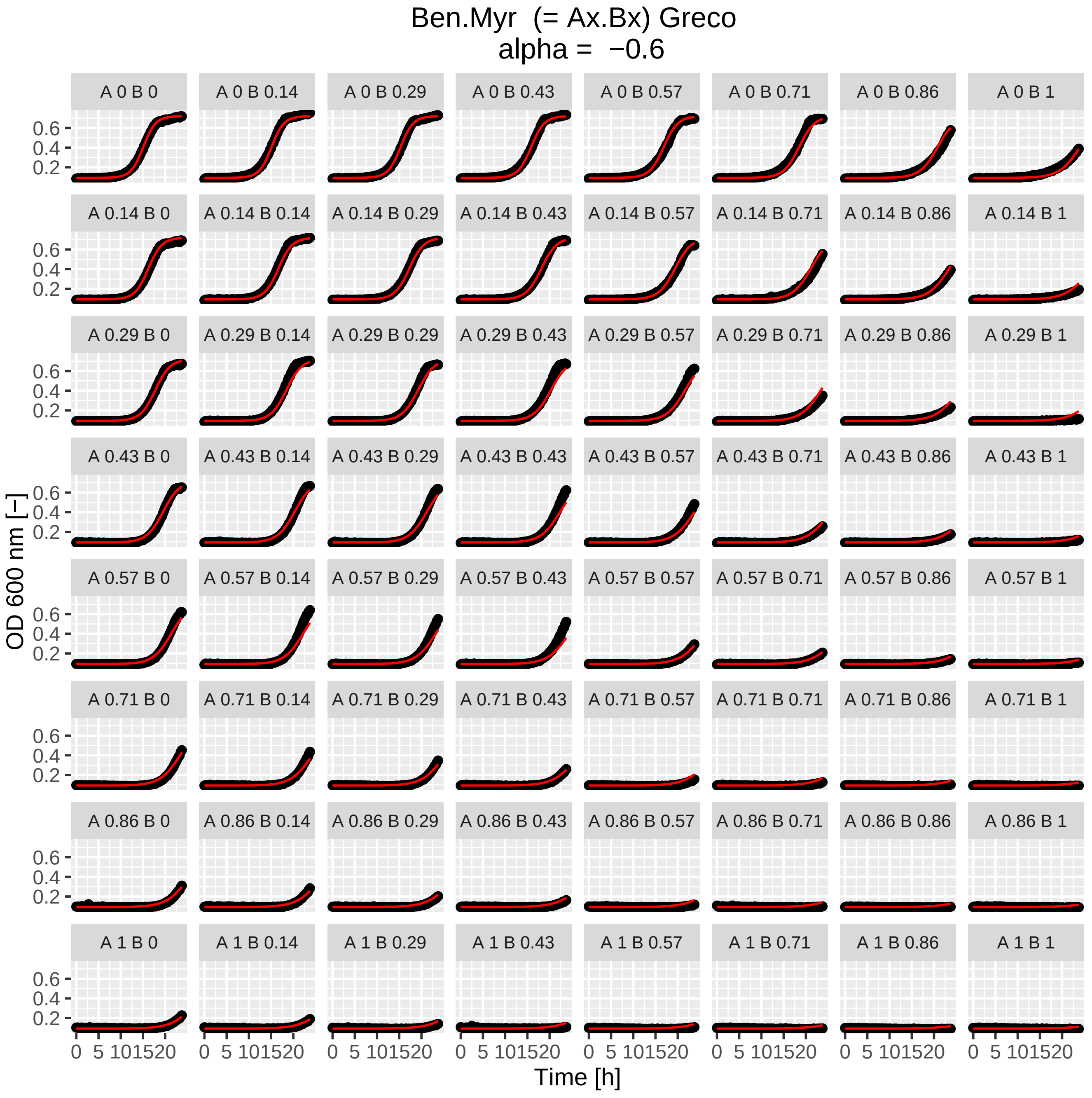

Ben.Myr (= Ax.Bx) Greco
alpha = −0.6
OD 600 nm [−]
Time [h]
A 0 B 0
A 0 B 0.14
A 0 B 0.29
A 0 B 0.43
A 0 B 0.57
A 0 B 0.71
A 0 B 0.86
A 0 B 1
A 0.14 B 0
A 0.14 B 0.14
A 0.14 B 0.29
A 0.14 B 0.43
A 0.14 B 0.57
A 0.14 B 0.71
A 0.14 B 0.86
A 0.14 B 1
A 0.29 B 0
A 0.29 B 0.14
A 0.29 B 0.29
A 0.29 B 0.43
A 0.29 B 0.57
A 0.29 B 0.71
A 0.29 B 0.86
A 0.29 B 1
A 0.43 B 0
A 0.43 B 0.14
A 0.43 B 0.29
A 0.43 B 0.43
A 0.43 B 0.57
A 0.43 B 0.71
A 0.43 B 0.86
A 0.43 B 1
A 0.57 B 0
A 0.57 B 0.14
A 0.57 B 0.29
A 0.57 B 0.43
A 0.57 B 0.57
A 0.57 B 0.71
A 0.57 B 0.86
A 0.57 B 1
A 0.71 B 0
A 0.71 B 0.14
A 0.71 B 0.29
A 0.71 B 0.43
A 0.71 B 0.57
A 0.71 B 0.71
A 0.71 B 0.86
A 0.71 B 1
A 0.86 B 0
A 0.86 B 0.14
A 0.86 B 0.29
A 0.86 B 0.43
A 0.86 B 0.57
A 0.86 B 0.71
A 0.86 B 0.86
A 0.86 B 1
A 1 B 0
A 1 B 0.14
A 1 B 0.29
A 1 B 0.43
A 1 B 0.57
A 1 B 0.71
A 1 B 0.86
A 1 B 1
0.6
0.4
0.2
0 5 101520

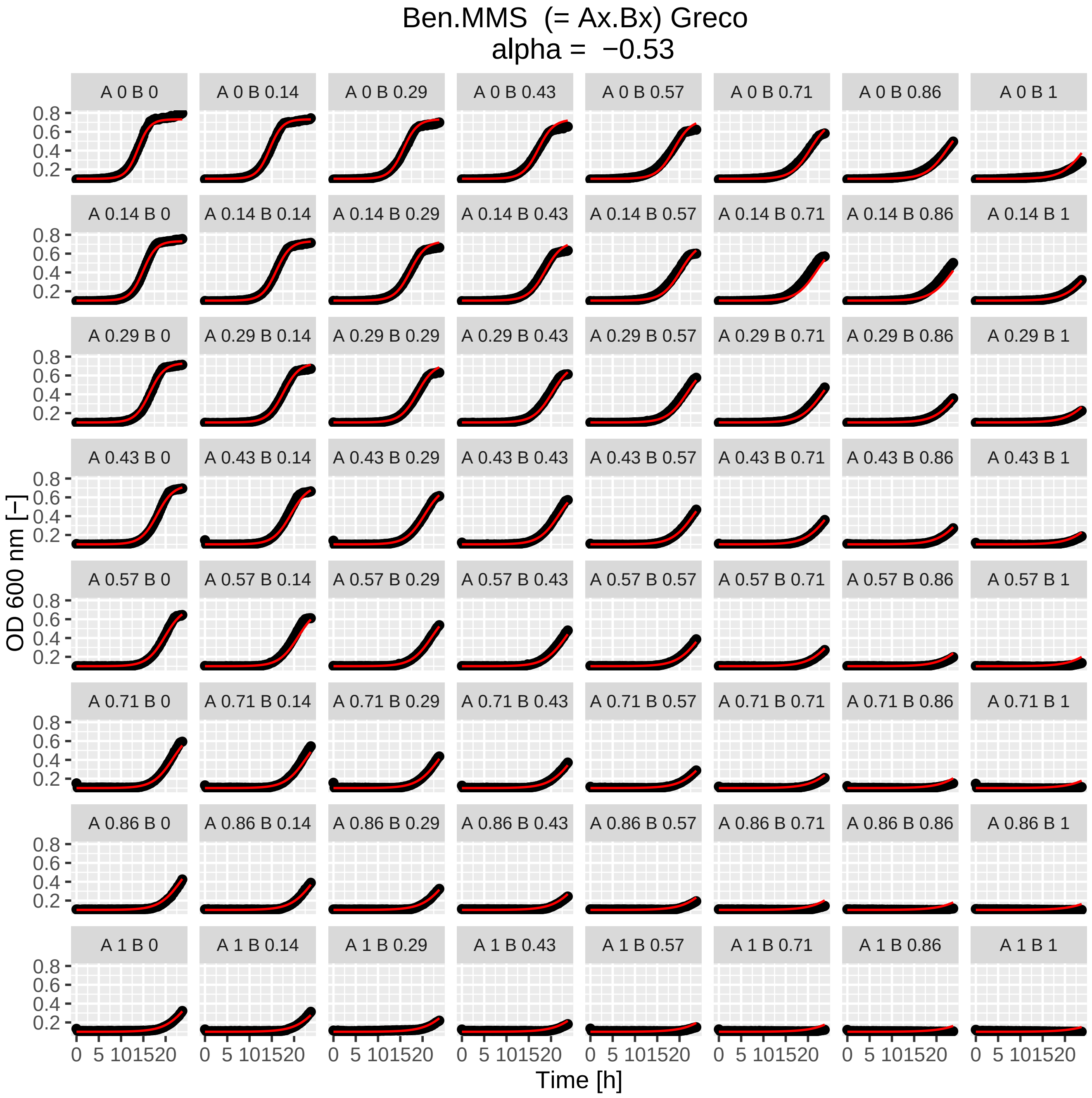

Ben.MMS (= Ax.Bx) Greco
alpha = −0.53
OD 600 nm [−]
Time [h]
A 0 B 0
A 0 B 0.14
A 0 B 0.29
A 0 B 0.43
A 0 B 0.57
A 0 B 0.71
A 0 B 0.86
A 0 B 1
A 0.14 B 0
A 0.14 B 0.14
A 0.14 B 0.29
A 0.14 B 0.43
A 0.14 B 0.57
A 0.14 B 0.71
A 0.14 B 0.86
A 0.14 B 1
A 0.29 B 0
A 0.29 B 0.14
A 0.29 B 0.29
A 0.29 B 0.43
A 0.29 B 0.57
A 0.29 B 0.71
A 0.29 B 0.86
A 0.29 B 1
A 0.43 B 0
A 0.43 B 0.14
A 0.43 B 0.29
A 0.43 B 0.43
A 0.43 B 0.57
A 0.43 B 0.71
A 0.43 B 0.86
A 0.43 B 1
A 0.57 B 0
A 0.57 B 0.14
A 0.57 B 0.29
A 0.57 B 0.43
A 0.57 B 0.57
A 0.57 B 0.71
A 0.57 B 0.86
A 0.57 B 1
A 0.71 B 0
A 0.71 B 0.14
A 0.71 B 0.29
A 0.71 B 0.43
A 0.71 B 0.57
A 0.71 B 0.71
A 0.71 B 0.86
A 0.71 B 1
A 0.86 B 0
A 0.86 B 0.14
A 0.86 B 0.29
A 0.86 B 0.43
A 0.86 B 0.57
A 0.86 B 0.71
A 0.86 B 0.86
A 0.86 B 1
A 1 B 0
A 1 B 0.14
A 1 B 0.29
A 1 B 0.43
A 1 B 0.57
A 1 B 0.71
A 1 B 0.86
A 1 B 1
0.8
0.6
0.4
0.2
0 5 101520

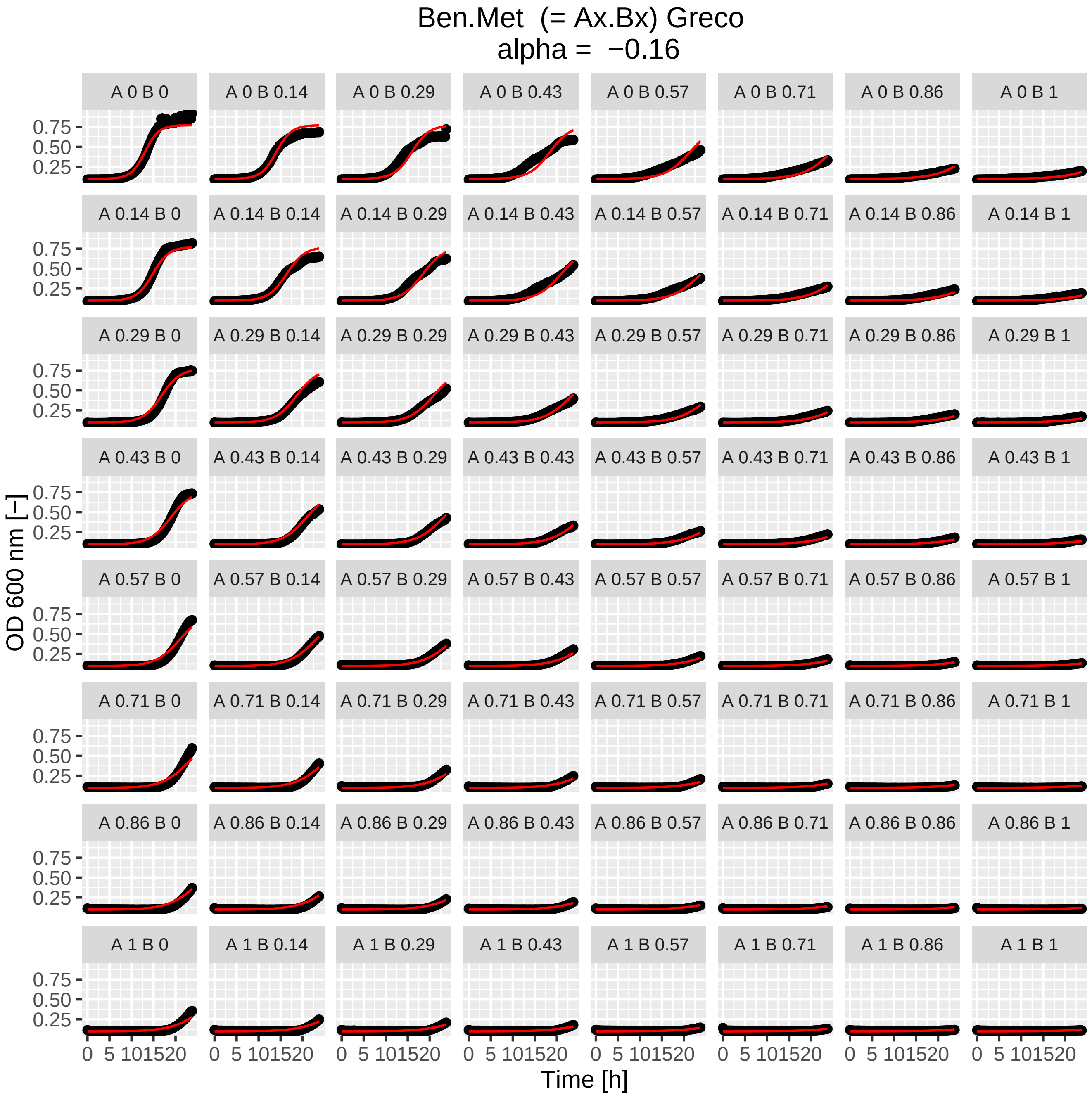

Ben.Met (= Ax.Bx) Greco
alpha = −0.16
A 0 B 0
A 0 B 0.14
A 0 B 0.29
A 0 B 0.43
A 0 B 0.57
A 0 B 0.71
A 0 B 0.86
A 0 B 1
A 0.14 B 0
A 0.14 B 0.14
A 0.14 B 0.29
A 0.14 B 0.43
A 0.14 B 0.57
A 0.14 B 0.71
A 0.14 B 0.86
A 0.14 B 1
A 0.29 B 0
A 0.29 B 0.14
A 0.29 B 0.29
A 0.29 B 0.43
A 0.29 B 0.57
A 0.29 B 0.71
A 0.29 B 0.86
A 0.29 B 1
A 0.43 B 0
A 0.43 B 0.14
A 0.43 B 0.29
A 0.43 B 0.43
A 0.43 B 0.57
A 0.43 B 0.71
A 0.43 B 0.86
A 0.43 B 1
A 0.57 B 0
A 0.57 B 0.14
A 0.57 B 0.29
A 0.57 B 0.43
A 0.57 B 0.57
A 0.57 B 0.71
A 0.57 B 0.86
A 0.57 B 1
A 0.71 B 0
A 0.71 B 0.14
A 0.71 B 0.29
A 0.71 B 0.43
A 0.71 B 0.57
A 0.71 B 0.71
A 0.71 B 0.86
A 0.71 B 1
A 0.86 B 0
A 0.86 B 0.14
A 0.86 B 0.29
A 0.86 B 0.43
A 0.86 B 0.57
A 0.86 B 0.71
A 0.86 B 0.86
A 0.86 B 1
A 1 B 0
A 1 B 0.14
A 1 B 0.29
A 1 B 0.43
A 1 B 0.57
A 1 B 0.71
A 1 B 0.86
A 1 B 1
OD 600 nm [−]
0.75
0.50
0.25
0 5 101520
Time [h]

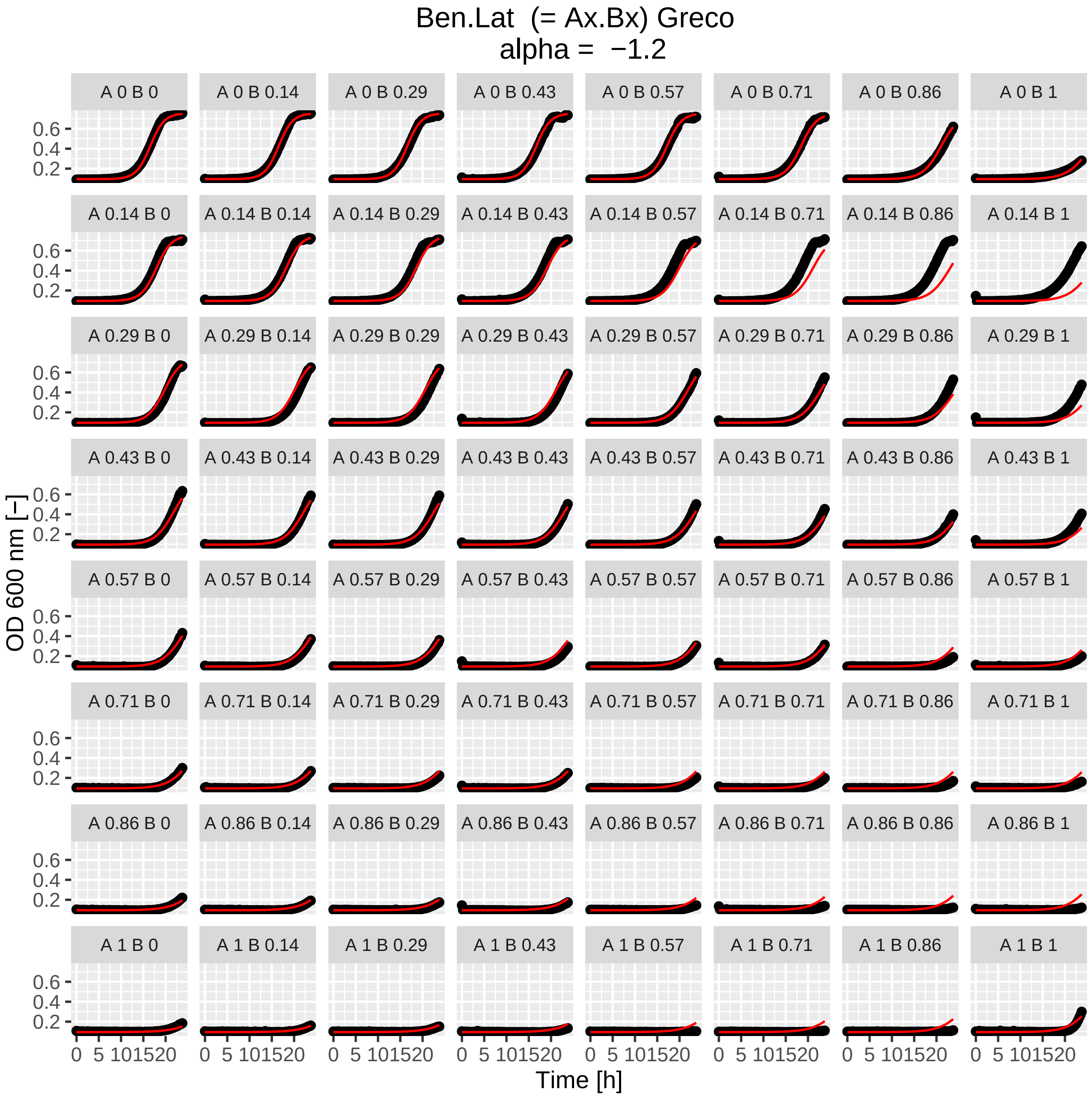

Ben.Lat (= Ax.Bx) Greco
alpha = −1.2
A 0 B 0
A 0 B 0.14
A 0 B 0.29
A 0 B 0.43
A 0 B 0.57
A 0 B 0.71
A 0 B 0.86
A 0 B 1
A 0.14 B 0
A 0.14 B 0.14
A 0.14 B 0.29
A 0.14 B 0.43
A 0.14 B 0.57
A 0.14 B 0.71
A 0.14 B 0.86
A 0.14 B 1
A 0.29 B 0
A 0.29 B 0.14
A 0.29 B 0.29
A 0.29 B 0.43
A 0.29 B 0.57
A 0.29 B 0.71
A 0.29 B 0.86
A 0.29 B 1
A 0.43 B 0
A 0.43 B 0.14
A 0.43 B 0.29
A 0.43 B 0.43
A 0.43 B 0.57
A 0.43 B 0.71
A 0.43 B 0.86
A 0.43 B 1
A 0.57 B 0
A 0.57 B 0.14
A 0.57 B 0.29
A 0.57 B 0.43
A 0.57 B 0.57
A 0.57 B 0.71
A 0.57 B 0.86
A 0.57 B 1
A 0.71 B 0
A 0.71 B 0.14
A 0.71 B 0.29
A 0.71 B 0.43
A 0.71 B 0.57
A 0.71 B 0.71
A 0.71 B 0.86
A 0.71 B 1
A 0.86 B 0
A 0.86 B 0.14
A 0.86 B 0.29
A 0.86 B 0.43
A 0.86 B 0.57
A 0.86 B 0.71
A 0.86 B 0.86
A 0.86 B 1
A 1 B 0
A 1 B 0.14
A 1 B 0.29
A 1 B 0.43
A 1 B 0.57
A 1 B 0.71
A 1 B 0.86
A 1 B 1
OD 600 nm [−]
0.6
0.4
0.2
0 5 101520
Time [h]

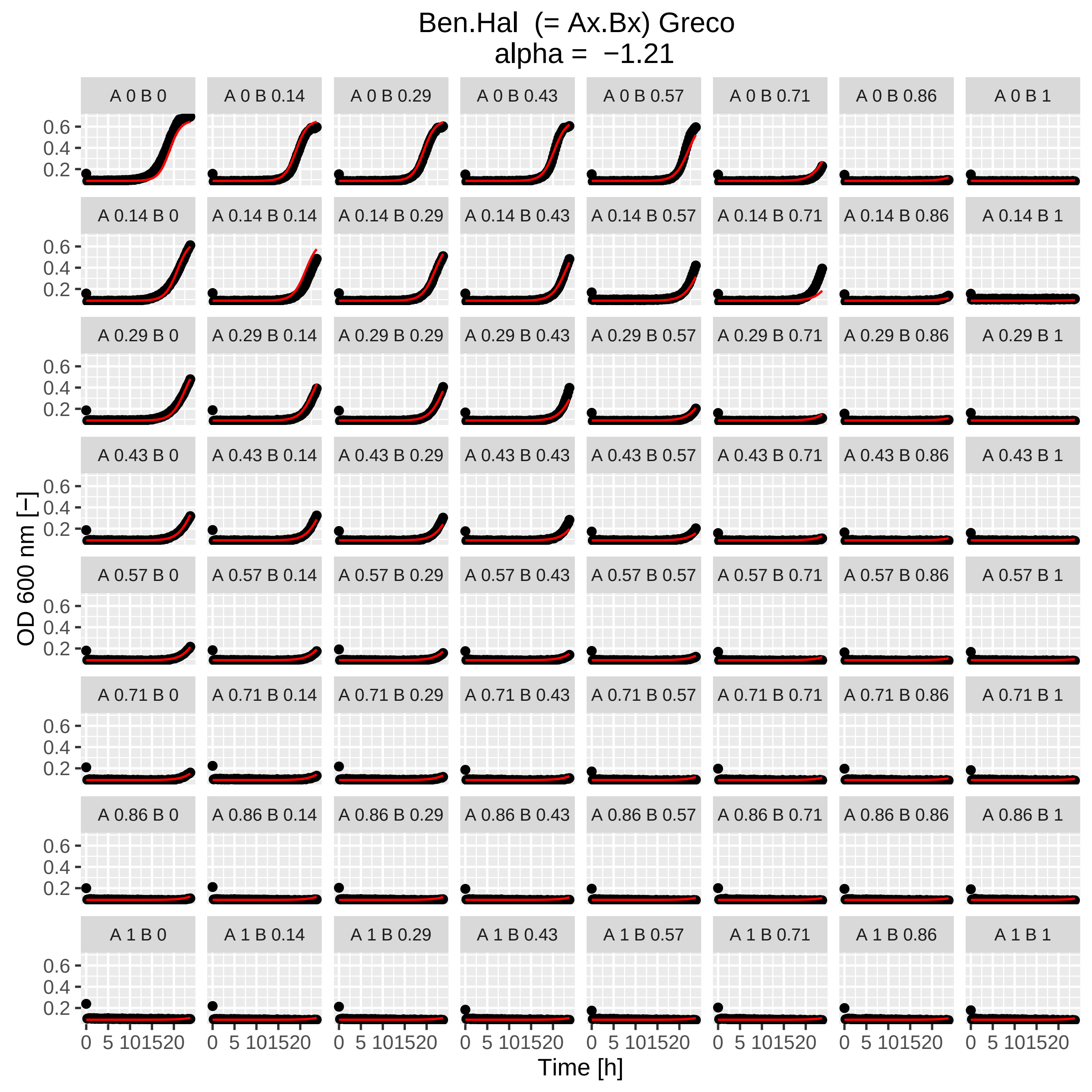
Ben.Hal (= Ax.Bx) Greco
alpha = −1.21
A 0 B 0
A 0 B 0.14
A 0 B 0.29
A 0 B 0.43
A 0 B 0.57
A 0 B 0.71
A 0 B 0.86
A 0 B 1
A 0.14 B 0
A 0.14 B 0.14
A 0.14 B 0.29
A 0.14 B 0.43
A 0.14 B 0.57
A 0.14 B 0.71
A 0.14 B 0.86
A 0.14 B 1
A 0.29 B 0
A 0.29 B 0.14
A 0.29 B 0.29
A 0.29 B 0.43
A 0.29 B 0.57
A 0.29 B 0.71
A 0.29 B 0.86
A 0.29 B 1
A 0.43 B 0
A 0.43 B 0.14
A 0.43 B 0.29
A 0.43 B 0.43
A 0.43 B 0.57
A 0.43 B 0.71
A 0.43 B 0.86
A 0.43 B 1
A 0.57 B 0
A 0.57 B 0.14
A 0.57 B 0.29
A 0.57 B 0.43
A 0.57 B 0.57
A 0.57 B 0.71
A 0.57 B 0.86
A 0.57 B 1
A 0.71 B 0
A 0.71 B 0.14
A 0.71 B 0.29
A 0.71 B 0.43
A 0.71 B 0.57
A 0.71 B 0.71
A 0.71 B 0.86
A 0.71 B 1
A 0.86 B 0
A 0.86 B 0.14
A 0.86 B 0.29
A 0.86 B 0.43
A 0.86 B 0.57
A 0.86 B 0.71
A 0.86 B 0.86
A 0.86 B 1
A 1 B 0
A 1 B 0.14
A 1 B 0.29
A 1 B 0.43
A 1 B 0.57
A 1 B 0.71
A 1 B 0.86
A 1 B 1
0.6
0.4
0.2
OD 600 nm [−]
0 5 101520
Time [h]

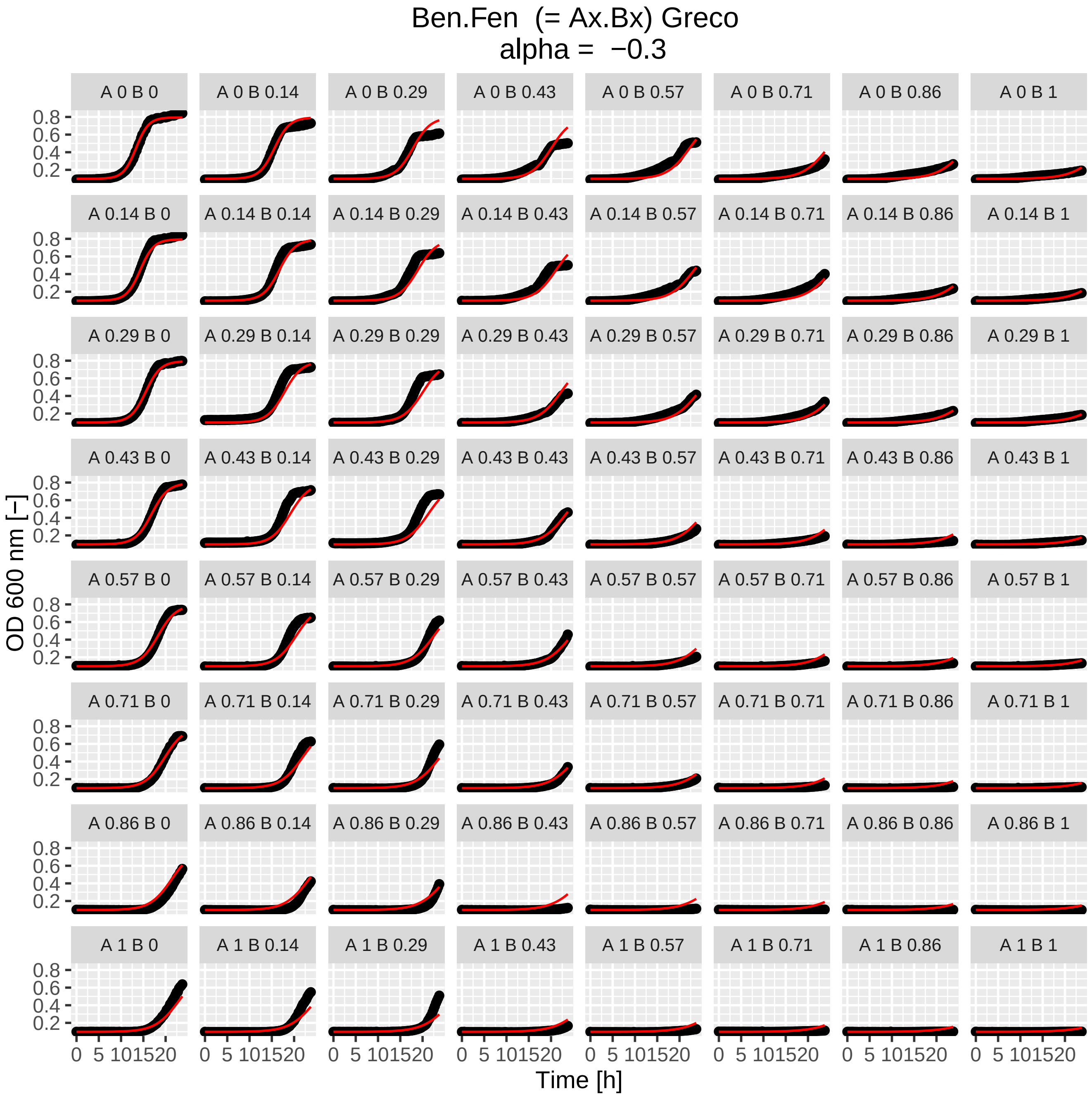

Ben.Fen (= Ax.Bx) Greco
alpha = −0.3
OD 600 nm [−]
Time [h]
0.8
0.6
0.4
0.2
0 5 101520
A 0 B 0
A 0 B 0.14
A 0 B 0.29
A 0 B 0.43
A 0 B 0.57
A 0 B 0.71
A 0 B 0.86
A 0 B 1
A 0.14 B 0
A 0.14 B 0.14
A 0.14 B 0.29
A 0.14 B 0.43
A 0.14 B 0.57
A 0.14 B 0.71
A 0.14 B 0.86
A 0.14 B 1
A 0.29 B 0
A 0.29 B 0.14
A 0.29 B 0.29
A 0.29 B 0.43
A 0.29 B 0.57
A 0.29 B 0.71
A 0.29 B 0.86
A 0.29 B 1
A 0.43 B 0
A 0.43 B 0.14
A 0.43 B 0.29
A 0.43 B 0.43
A 0.43 B 0.57
A 0.43 B 0.71
A 0.43 B 0.86
A 0.43 B 1
A 0.57 B 0
A 0.57 B 0.14
A 0.57 B 0.29
A 0.57 B 0.43
A 0.57 B 0.57
A 0.57 B 0.71
A 0.57 B 0.86
A 0.57 B 1
A 0.71 B 0
A 0.71 B 0.14
A 0.71 B 0.29
A 0.71 B 0.43
A 0.71 B 0.57
A 0.71 B 0.71
A 0.71 B 0.86
A 0.71 B 1
A 0.86 B 0
A 0.86 B 0.14
A 0.86 B 0.29
A 0.86 B 0.43
A 0.86 B 0.57
A 0.86 B 0.71
A 0.86 B 0.86
A 0.86 B 1
A 1 B 0
A 1 B 0.14
A 1 B 0.29
A 1 B 0.43
A 1 B 0.57
A 1 B 0.71
A 1 B 0.86
A 1 B 1

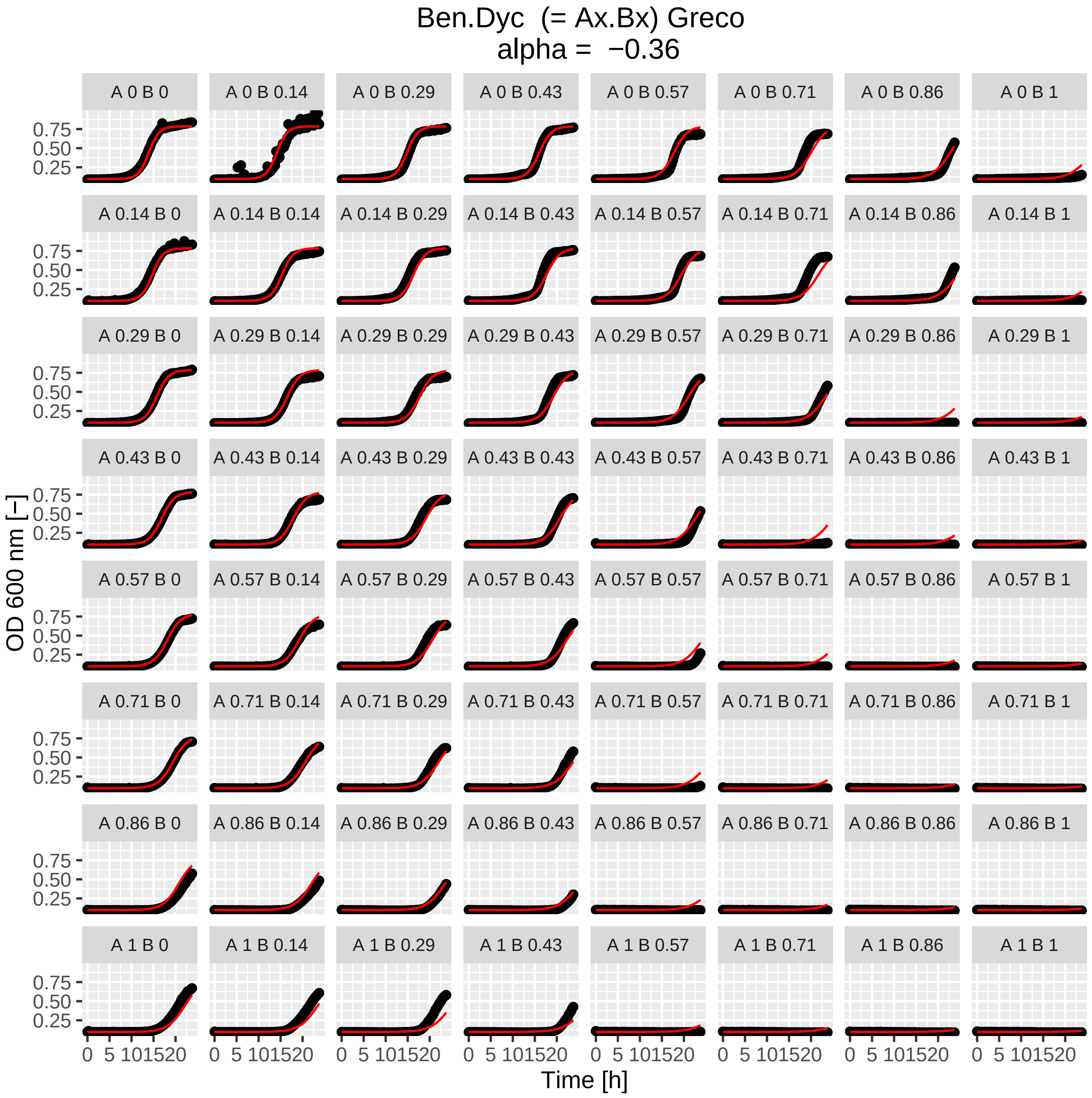
Ben.Dyc (= Ax.Bx) Greco
alpha = −0.36
A 0 B 0
A 0 B 0.14
A 0 B 0.29
A 0 B 0.43
A 0 B 0.57
A 0 B 0.71
A 0 B 0.86
A 0 B 1
A 0.14 B 0
A 0.14 B 0.14
A 0.14 B 0.29
A 0.14 B 0.43
A 0.14 B 0.57
A 0.14 B 0.71
A 0.14 B 0.86
A 0.14 B 1
A 0.29 B 0
A 0.29 B 0.14
A 0.29 B 0.29
A 0.29 B 0.43
A 0.29 B 0.57
A 0.29 B 0.71
A 0.29 B 0.86
A 0.29 B 1
A 0.43 B 0
A 0.43 B 0.14
A 0.43 B 0.29
A 0.43 B 0.43
A 0.43 B 0.57
A 0.43 B 0.71
A 0.43 B 0.86
A 0.43 B 1
A 0.57 B 0
A 0.57 B 0.14
A 0.57 B 0.29
A 0.57 B 0.43
A 0.57 B 0.57
A 0.57 B 0.71
A 0.57 B 0.86
A 0.57 B 1
A 0.71 B 0
A 0.71 B 0.14
A 0.71 B 0.29
A 0.71 B 0.43
A 0.71 B 0.57
A 0.71 B 0.71
A 0.71 B 0.86
A 0.71 B 1
A 0.86 B 0
A 0.86 B 0.14
A 0.86 B 0.29
A 0.86 B 0.43
A 0.86 B 0.57
A 0.86 B 0.71
A 0.86 B 0.86
A 0.86 B 1
A 1 B 0
A 1 B 0.14
A 1 B 0.29
A 1 B 0.43
A 1 B 0.57
A 1 B 0.71
A 1 B 0.86
A 1 B 1
0.75
0.50
0.25
OD 600 nm [−]
0 5 101520
Time [h]

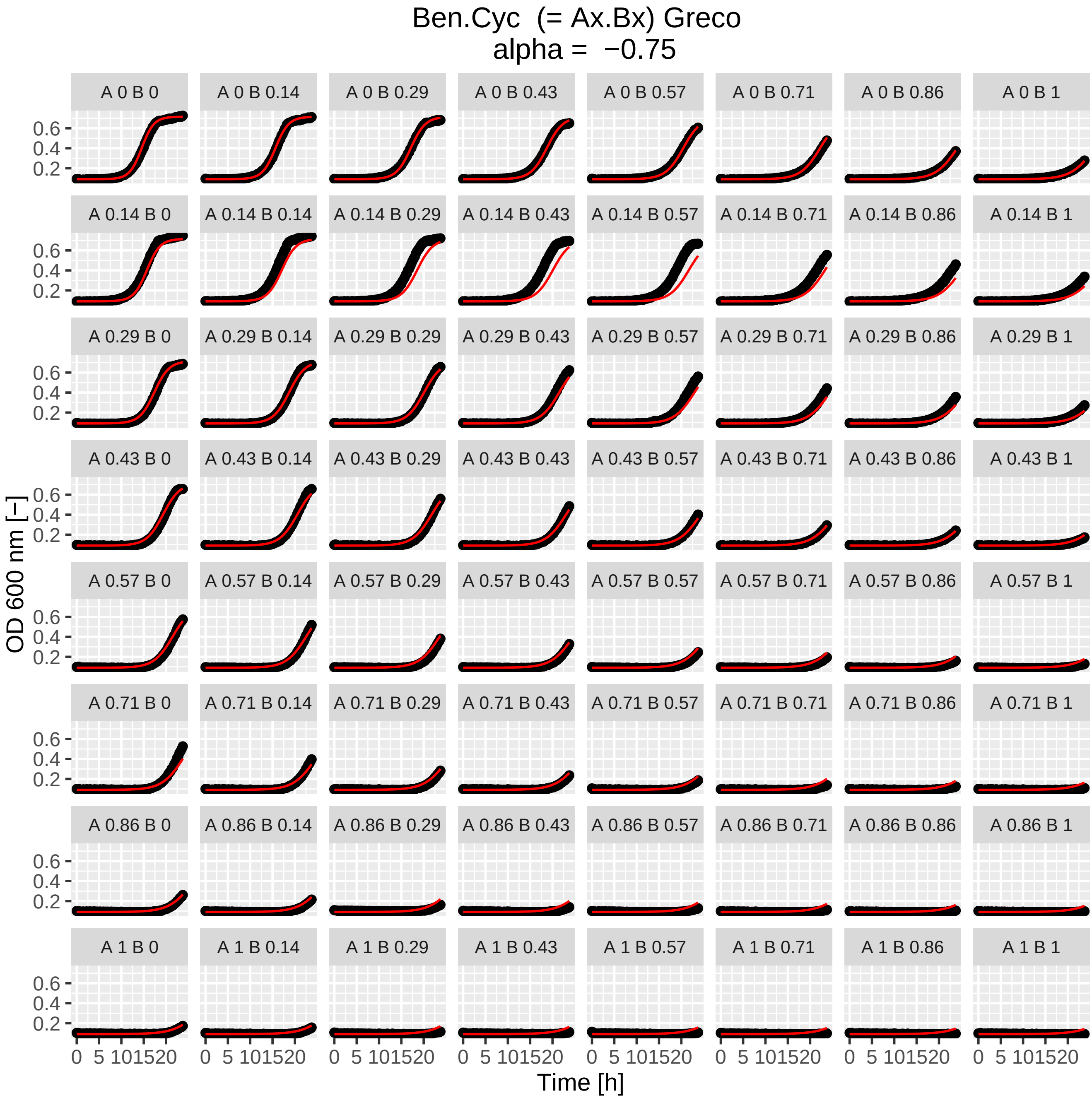
Ben.Cyc (= Ax.Bx) Greco
alpha = −0.75
A 0 B 0
A 0 B 0.14
A 0 B 0.29
A 0 B 0.43
A 0 B 0.57
A 0 B 0.71
A 0 B 0.86
A 0 B 1
A 0.14 B 0
A 0.14 B 0.14
A 0.14 B 0.29
A 0.14 B 0.43
A 0.14 B 0.57
A 0.14 B 0.71
A 0.14 B 0.86
A 0.14 B 1
A 0.29 B 0
A 0.29 B 0.14
A 0.29 B 0.29
A 0.29 B 0.43
A 0.29 B 0.57
A 0.29 B 0.71
A 0.29 B 0.86
A 0.29 B 1
A 0.43 B 0
A 0.43 B 0.14
A 0.43 B 0.29
A 0.43 B 0.43
A 0.43 B 0.57
A 0.43 B 0.71
A 0.43 B 0.86
A 0.43 B 1
A 0.57 B 0
A 0.57 B 0.14
A 0.57 B 0.29
A 0.57 B 0.43
A 0.57 B 0.57
A 0.57 B 0.71
A 0.57 B 0.86
A 0.57 B 1
A 0.71 B 0
A 0.71 B 0.14
A 0.71 B 0.29
A 0.71 B 0.43
A 0.71 B 0.57
A 0.71 B 0.71
A 0.71 B 0.86
A 0.71 B 1
A 0.86 B 0
A 0.86 B 0.14
A 0.86 B 0.29
A 0.86 B 0.43
A 0.86 B 0.57
A 0.86 B 0.71
A 0.86 B 0.86
A 0.86 B 1
A 1 B 0
A 1 B 0.14
A 1 B 0.29
A 1 B 0.43
A 1 B 0.57
A 1 B 0.71
A 1 B 0.86
A 1 B 1
OD 600 nm [−]
0.2
0.4
0.6
0 5 101520
Time [h]

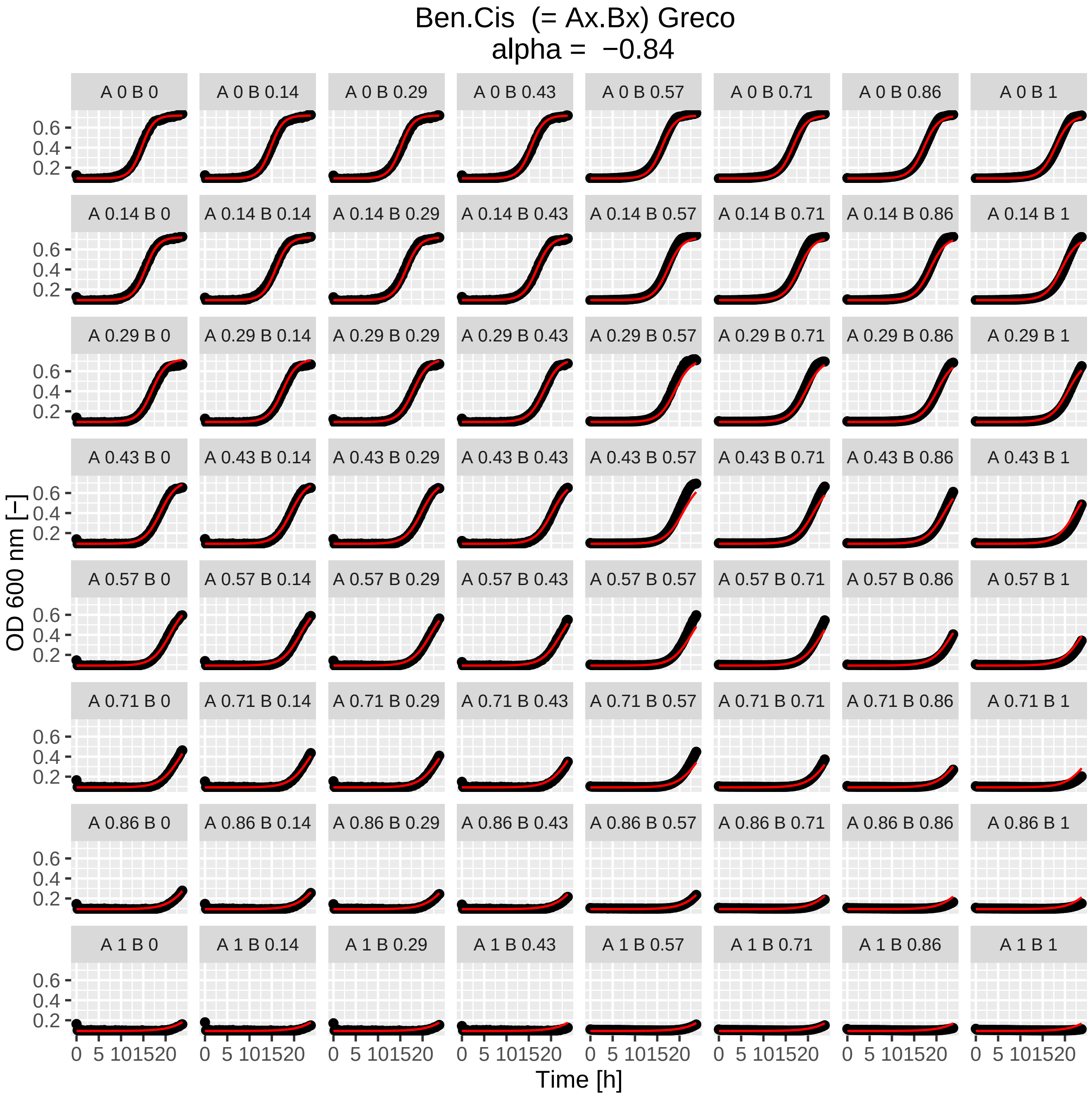

Ben.Cis (= Ax.Bx) Greco
alpha = −0.84
OD 600 nm [−]
Time [h]
0.2
0.4
0.6
0
5
10
15
20
A 0 B 0
A 0 B 0.14
A 0 B 0.29
A 0 B 0.43
A 0 B 0.57
A 0 B 0.71
A 0 B 0.86
A 0 B 1
A 0.14 B 0
A 0.14 B 0.14
A 0.14 B 0.29
A 0.14 B 0.43
A 0.14 B 0.57
A 0.14 B 0.71
A 0.14 B 0.86
A 0.14 B 1
A 0.29 B 0
A 0.29 B 0.14
A 0.29 B 0.29
A 0.29 B 0.43
A 0.29 B 0.57
A 0.29 B 0.71
A 0.29 B 0.86
A 0.29 B 1
A 0.43 B 0
A 0.43 B 0.14
A 0.43 B 0.29
A 0.43 B 0.43
A 0.43 B 0.57
A 0.43 B 0.71
A 0.43 B 0.86
A 0.43 B 1
A 0.57 B 0
A 0.57 B 0.14
A 0.57 B 0.29
A 0.57 B 0.43
A 0.57 B 0.57
A 0.57 B 0.71
A 0.57 B 0.86
A 0.57 B 1
A 0.71 B 0
A 0.71 B 0.14
A 0.71 B 0.29
A 0.71 B 0.43
A 0.71 B 0.57
A 0.71 B 0.71
A 0.71 B 0.86
A 0.71 B 1
A 0.86 B 0
A 0.86 B 0.14
A 0.86 B 0.29
A 0.86 B 0.43
A 0.86 B 0.57
A 0.86 B 0.71
A 0.86 B 0.86
A 0.86 B 1
A 1 B 0
A 1 B 0.14
A 1 B 0.29
A 1 B 0.43
A 1 B 0.57
A 1 B 0.71
A 1 B 0.86
A 1 B 1

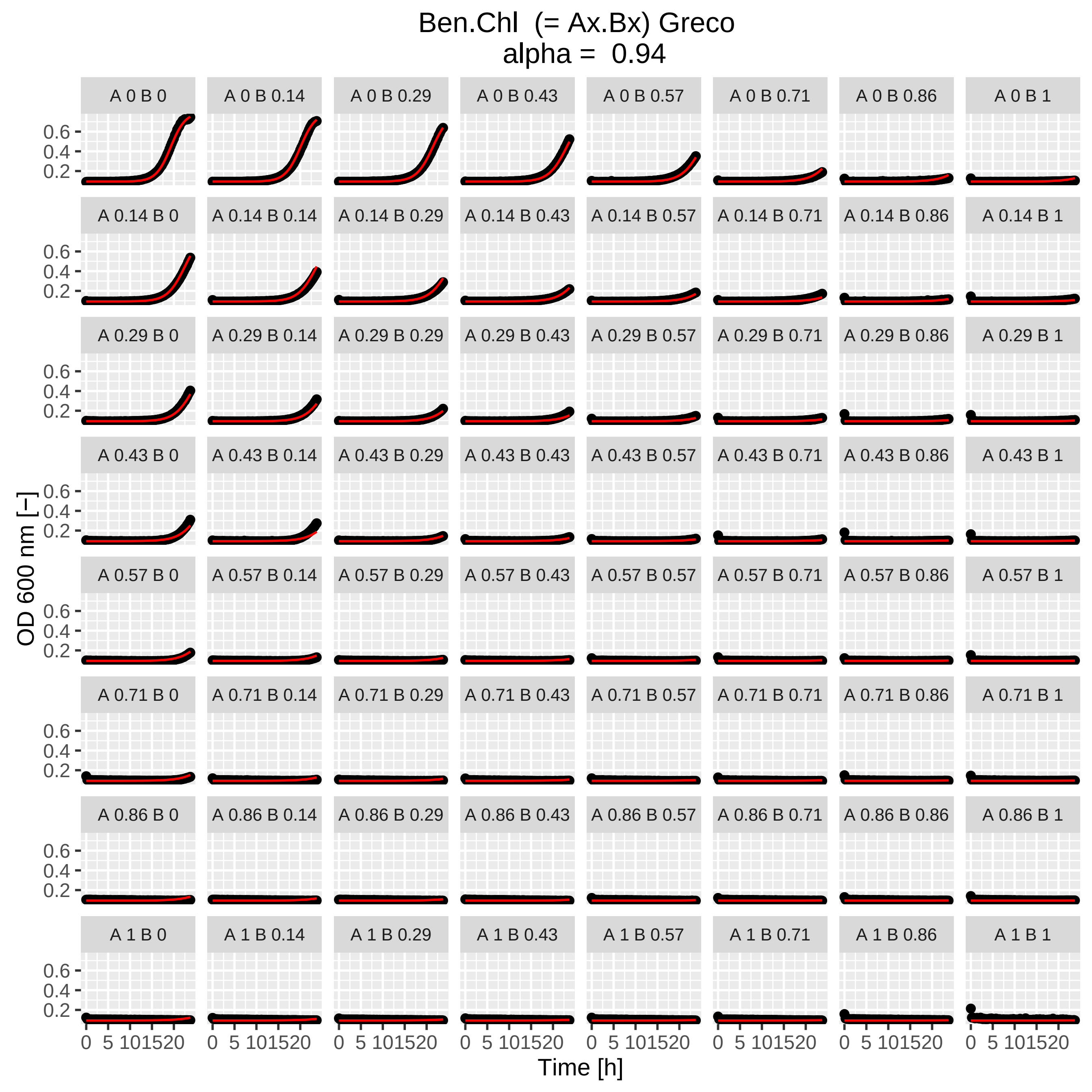

Ben.Chl (= Ax.Bx) Greco
alpha = 0.94
OD 600 nm [–]
Time [h]
0.2
0.4
0.6
0 5 101520
A 0 B 0
A 0 B 0.14
A 0 B 0.29
A 0 B 0.43
A 0 B 0.57
A 0 B 0.71
A 0 B 0.86
A 0 B 1
A 1 B 0
A 1 B 0.14
A 1 B 0.29
A 1 B 0.43
A 1 B 0.57
A 1 B 0.71
A 1 B 0.86
A 1 B 1

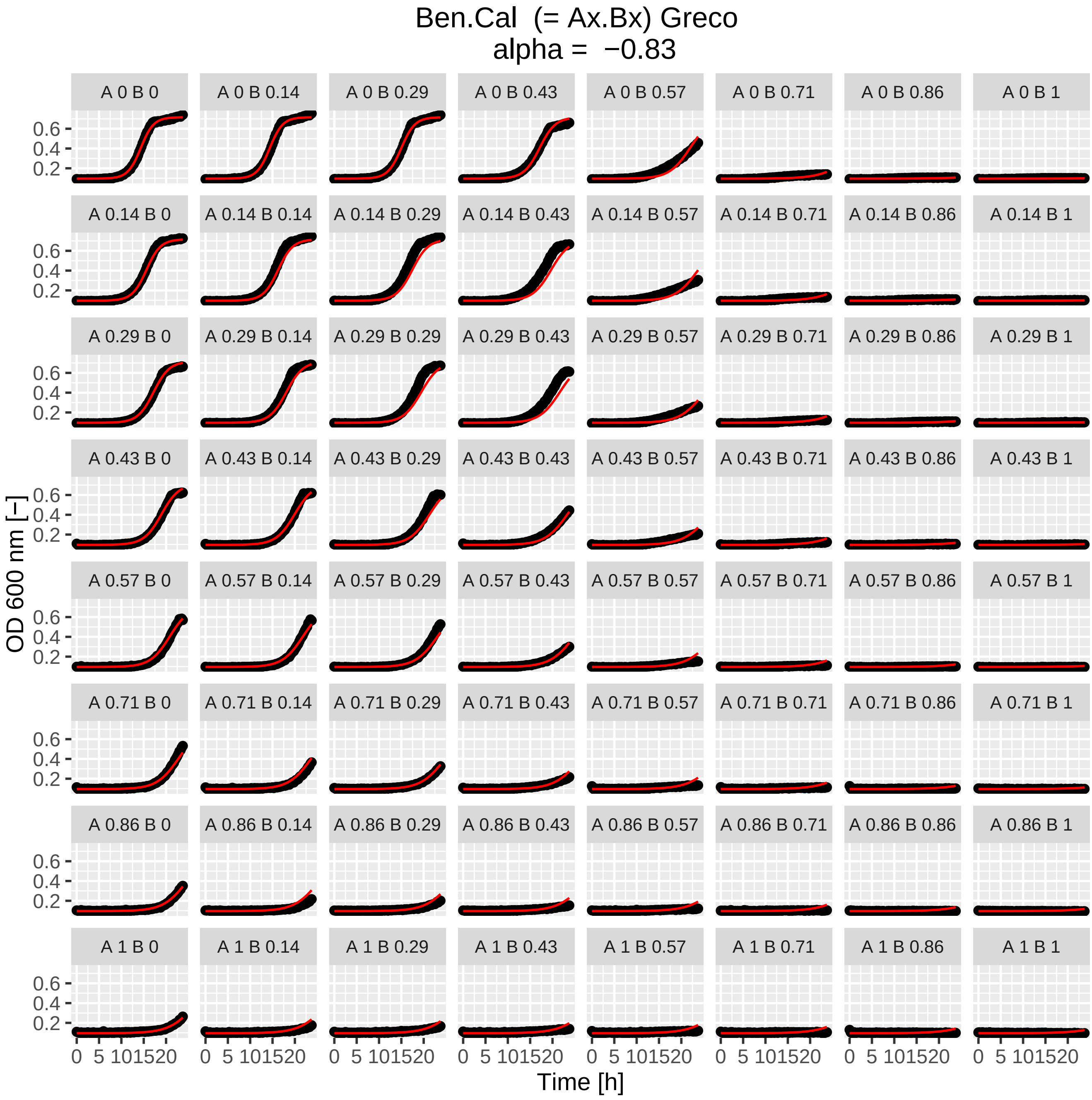

Ben.Cal (= Ax.Bx) Greco
alpha = −0.83
A 0 B 0
A 0 B 0.14
A 0 B 0.29
A 0 B 0.43
A 0 B 0.57
A 0 B 0.71
A 0 B 0.86
A 0 B 1
A 0.14 B 0
A 0.14 B 0.14
A 0.14 B 0.29
A 0.14 B 0.43
A 0.14 B 0.57
A 0.14 B 0.71
A 0.14 B 0.86
A 0.14 B 1
A 0.29 B 0
A 0.29 B 0.14
A 0.29 B 0.29
A 0.29 B 0.43
A 0.29 B 0.57
A 0.29 B 0.71
A 0.29 B 0.86
A 0.29 B 1
A 0.43 B 0
A 0.43 B 0.14
A 0.43 B 0.29
A 0.43 B 0.43
A 0.43 B 0.57
A 0.43 B 0.71
A 0.43 B 0.86
A 0.43 B 1
A 0.57 B 0
A 0.57 B 0.14
A 0.57 B 0.29
A 0.57 B 0.43
A 0.57 B 0.57
A 0.57 B 0.71
A 0.57 B 0.86
A 0.57 B 1
A 0.71 B 0
A 0.71 B 0.14
A 0.71 B 0.29
A 0.71 B 0.43
A 0.71 B 0.57
A 0.71 B 0.71
A 0.71 B 0.86
A 0.71 B 1
A 0.86 B 0
A 0.86 B 0.14
A 0.86 B 0.29
A 0.86 B 0.43
A 0.86 B 0.57
A 0.86 B 0.71
A 0.86 B 0.86
A 0.86 B 1
A 1 B 0
A 1 B 0.14
A 1 B 0.29
A 1 B 0.43
A 1 B 0.57
A 1 B 0.71
A 1 B 0.86
A 1 B 1
OD 600 nm [−]
0.2
0.4
0.6
0 5 10 15 20
Time [h]

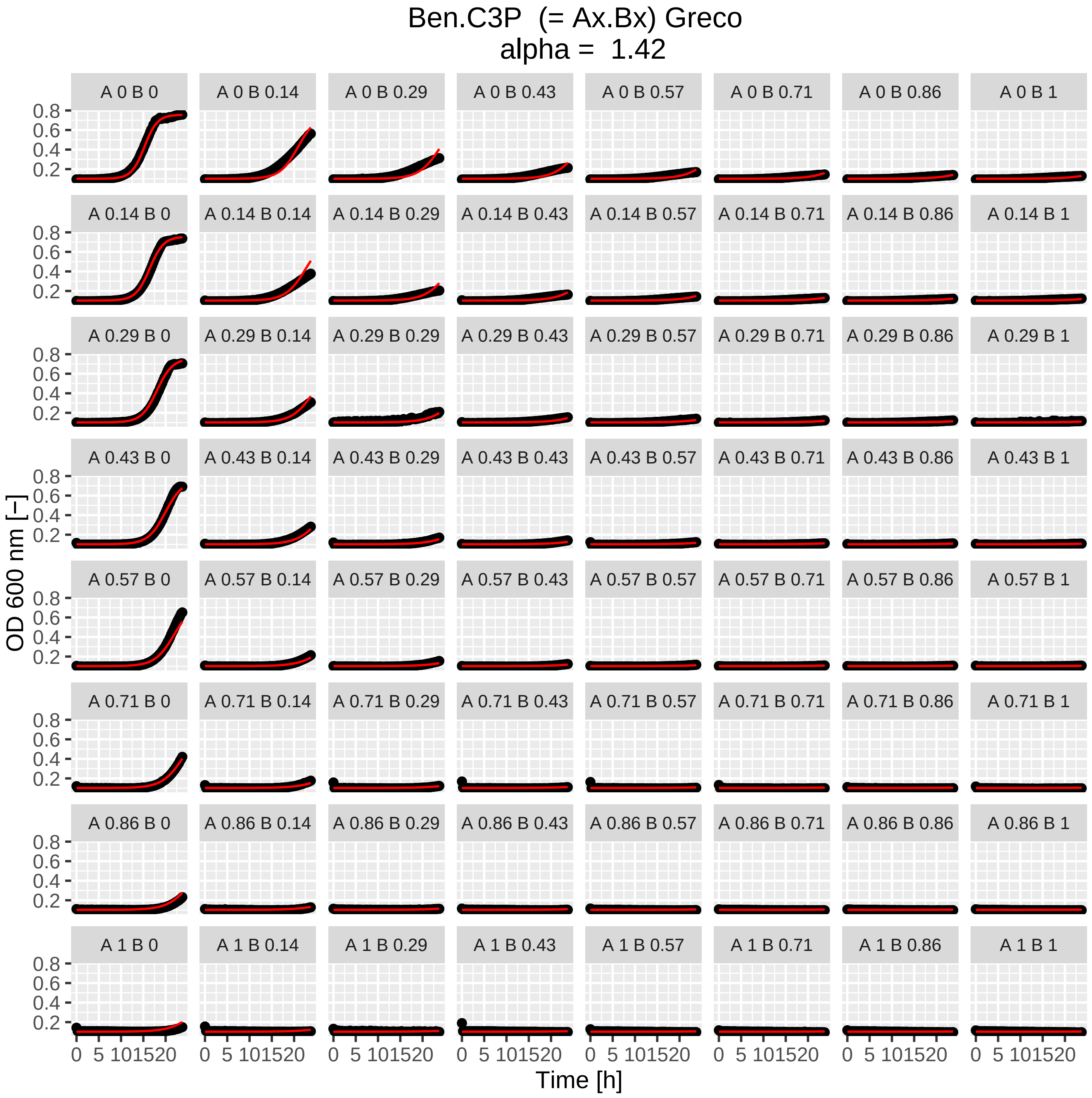

Ben.C3P (= Ax.Bx) Greco
alpha = 1.42
A 0 B 0
A 0 B 0.14
A 0 B 0.29
A 0 B 0.43
A 0 B 0.57
A 0 B 0.71
A 0 B 0.86
A 0 B 1
A 0.14 B 0
A 0.14 B 0.14
A 0.14 B 0.29
A 0.14 B 0.43
A 0.14 B 0.57
A 0.14 B 0.71
A 0.14 B 0.86
A 0.14 B 1
A 0.29 B 0
A 0.29 B 0.14
A 0.29 B 0.29
A 0.29 B 0.43
A 0.29 B 0.57
A 0.29 B 0.71
A 0.29 B 0.86
A 0.29 B 1
A 0.43 B 0
A 0.43 B 0.14
A 0.43 B 0.29
A 0.43 B 0.43
A 0.43 B 0.57
A 0.43 B 0.71
A 0.43 B 0.86
A 0.43 B 1
A 0.57 B 0
A 0.57 B 0.14
A 0.57 B 0.29
A 0.57 B 0.43
A 0.57 B 0.57
A 0.57 B 0.71
A 0.57 B 0.86
A 0.57 B 1
A 0.71 B 0
A 0.71 B 0.14
A 0.71 B 0.29
A 0.71 B 0.43
A 0.71 B 0.57
A 0.71 B 0.71
A 0.71 B 0.86
A 0.71 B 1
A 0.86 B 0
A 0.86 B 0.14
A 0.86 B 0.29
A 0.86 B 0.43
A 0.86 B 0.57
A 0.86 B 0.71
A 0.86 B 0.86
A 0.86 B 1
A 1 B 0
A 1 B 0.14
A 1 B 0.29
A 1 B 0.43
A 1 B 0.57
A 1 B 0.71
A 1 B 0.86
A 1 B 1
0.2
0.4
0.6
0.8
0
5
10
15
20
OD 600 nm [–]
Time [h]

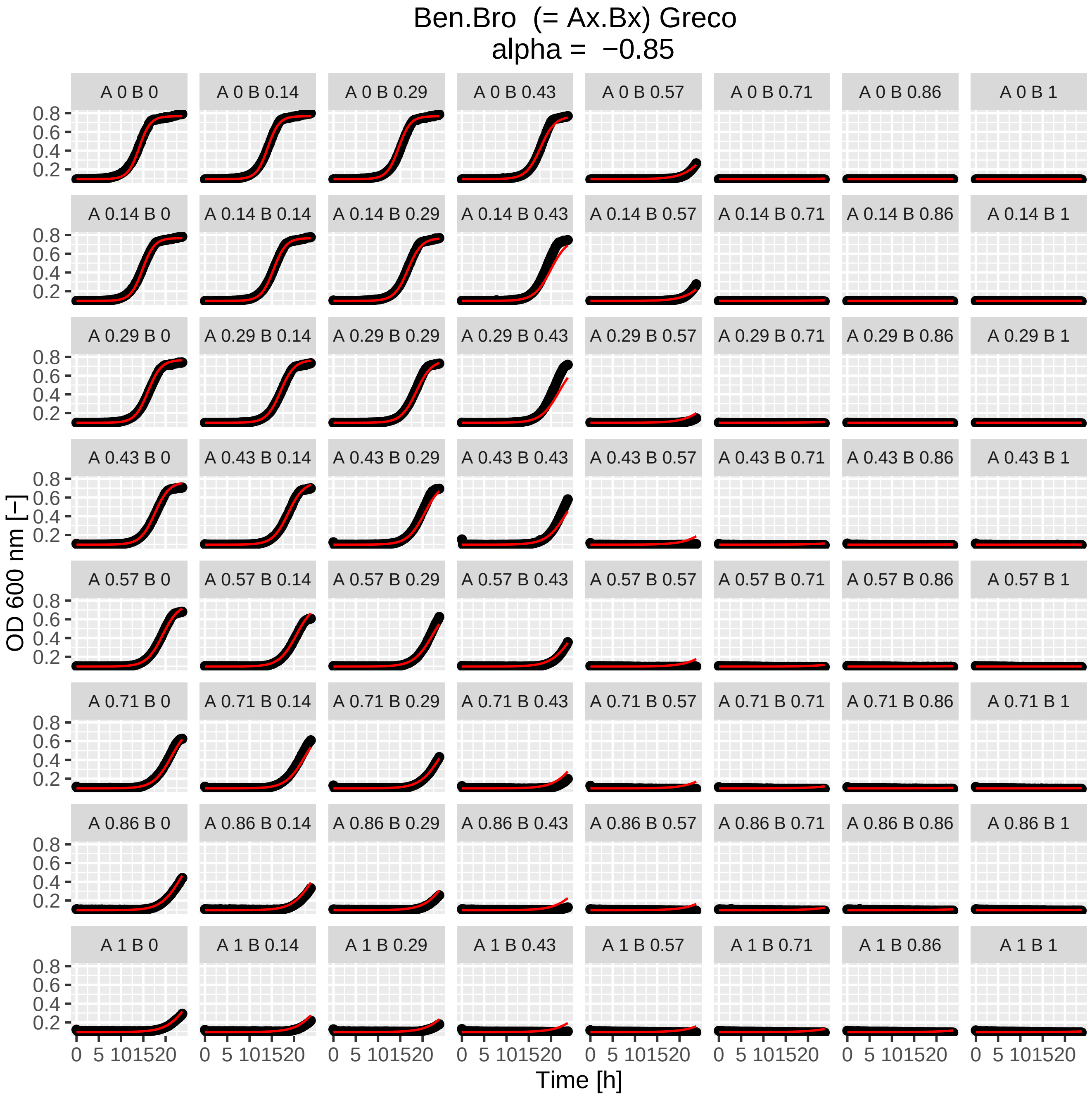
Ben.Bro (= Ax.Bx) Greco
alpha = −0.85
OD 600 nm [−]
Time [h]
A 0 B 0
A 0 B 0.14
A 0 B 0.29
A 0 B 0.43
A 0 B 0.57
A 0 B 0.71
A 0 B 0.86
A 0 B 1
A 0.14 B 0
A 0.14 B 0.14
A 0.14 B 0.29
A 0.14 B 0.43
A 0.14 B 0.57
A 0.14 B 0.71
A 0.14 B 0.86
A 0.14 B 1
A 0.29 B 0
A 0.29 B 0.14
A 0.29 B 0.29
A 0.29 B 0.43
A 0.29 B 0.57
A 0.29 B 0.71
A 0.29 B 0.86
A 0.29 B 1
A 0.43 B 0
A 0.43 B 0.14
A 0.43 B 0.29
A 0.43 B 0.43
A 0.43 B 0.57
A 0.43 B 0.71
A 0.43 B 0.86
A 0.43 B 1
A 0.57 B 0
A 0.57 B 0.14
A 0.57 B 0.29
A 0.57 B 0.43
A 0.57 B 0.57
A 0.57 B 0.71
A 0.57 B 0.86
A 0.57 B 1
A 0.71 B 0
A 0.71 B 0.14
A 0.71 B 0.29
A 0.71 B 0.43
A 0.71 B 0.57
A 0.71 B 0.71
A 0.71 B 0.86
A 0.71 B 1
A 0.86 B 0
A 0.86 B 0.14
A 0.86 B 0.29
A 0.86 B 0.43
A 0.86 B 0.57
A 0.86 B 0.71
A 0.86 B 0.86
A 0.86 B 1
A 1 B 0
A 1 B 0.14
A 1 B 0.29
A 1 B 0.43
A 1 B 0.57
A 1 B 0.71
A 1 B 0.86
A 1 B 1
0.8
0.6
0.4
0.2
0 5 101520

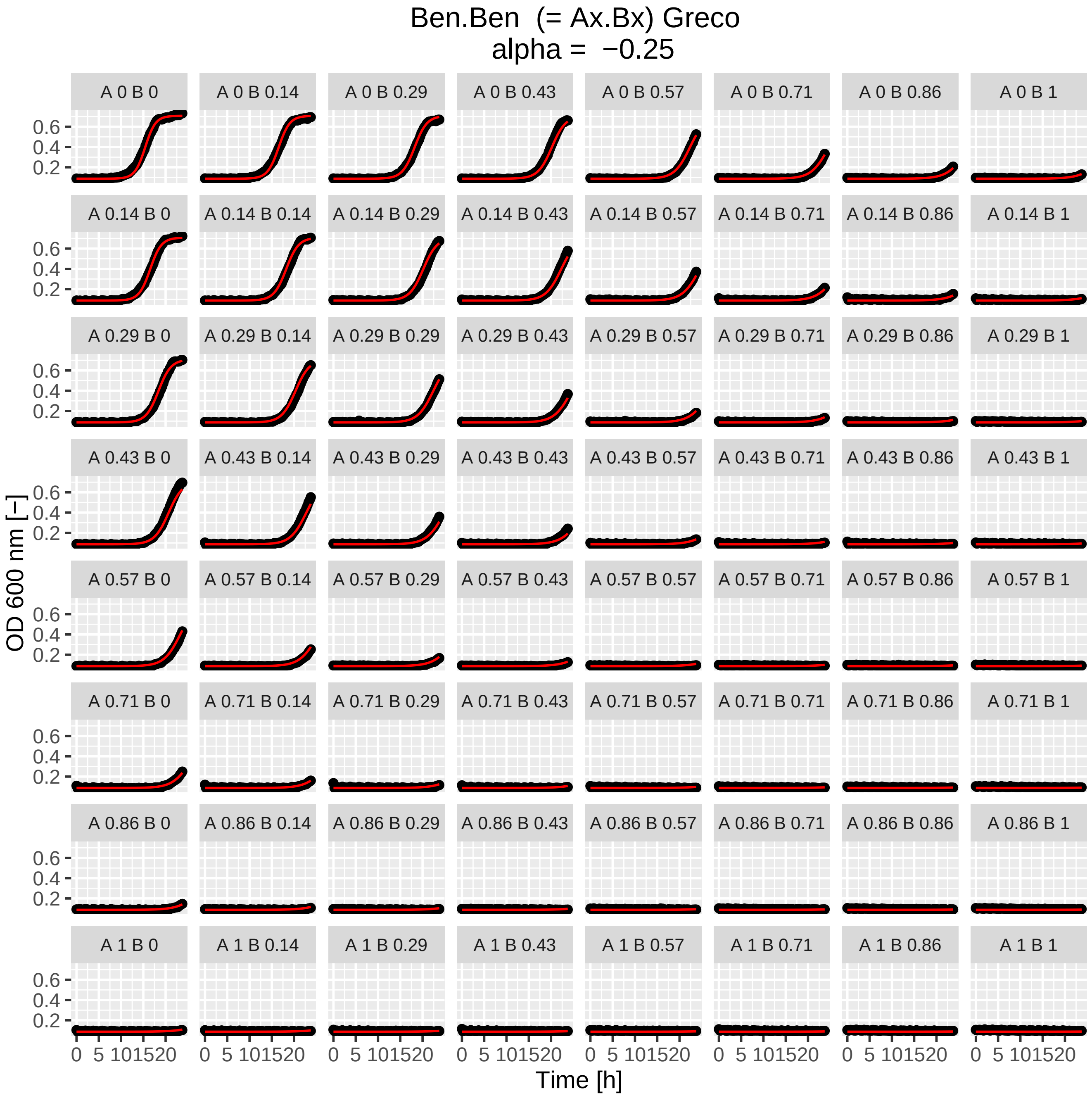

Ben.Ben (= Ax.Bx) Greco
alpha = −0.25
A 0 B 0
A 0 B 0.14
A 0 B 0.29
A 0 B 0.43
A 0 B 0.57
A 0 B 0.71
A 0 B 0.86
A 0 B 1
A 0.14 B 0
A 0.14 B 0.14
A 0.14 B 0.29
A 0.14 B 0.43
A 0.14 B 0.57
A 0.14 B 0.71
A 0.14 B 0.86
A 0.14 B 1
A 0.29 B 0
A 0.29 B 0.14
A 0.29 B 0.29
A 0.29 B 0.43
A 0.29 B 0.57
A 0.29 B 0.71
A 0.29 B 0.86
A 0.29 B 1
A 0.43 B 0
A 0.43 B 0.14
A 0.43 B 0.29
A 0.43 B 0.43
A 0.43 B 0.57
A 0.43 B 0.71
A 0.43 B 0.86
A 0.43 B 1
A 0.57 B 0
A 0.57 B 0.14
A 0.57 B 0.29
A 0.57 B 0.43
A 0.57 B 0.57
A 0.57 B 0.71
A 0.57 B 0.86
A 0.57 B 1
A 0.71 B 0
A 0.71 B 0.14
A 0.71 B 0.29
A 0.71 B 0.43
A 0.71 B 0.57
A 0.71 B 0.71
A 0.71 B 0.86
A 0.71 B 1
A 0.86 B 0
A 0.86 B 0.14
A 0.86 B 0.29
A 0.86 B 0.43
A 0.86 B 0.57
A 0.86 B 0.71
A 0.86 B 0.86
A 0.86 B 1
A 1 B 0
A 1 B 0.14
A 1 B 0.29
A 1 B 0.43
A 1 B 0.57
A 1 B 0.71
A 1 B 0.86
A 1 B 1
OD 600 nm [−]
0.2
0.4
0.6
0 5 101520
Time [h]

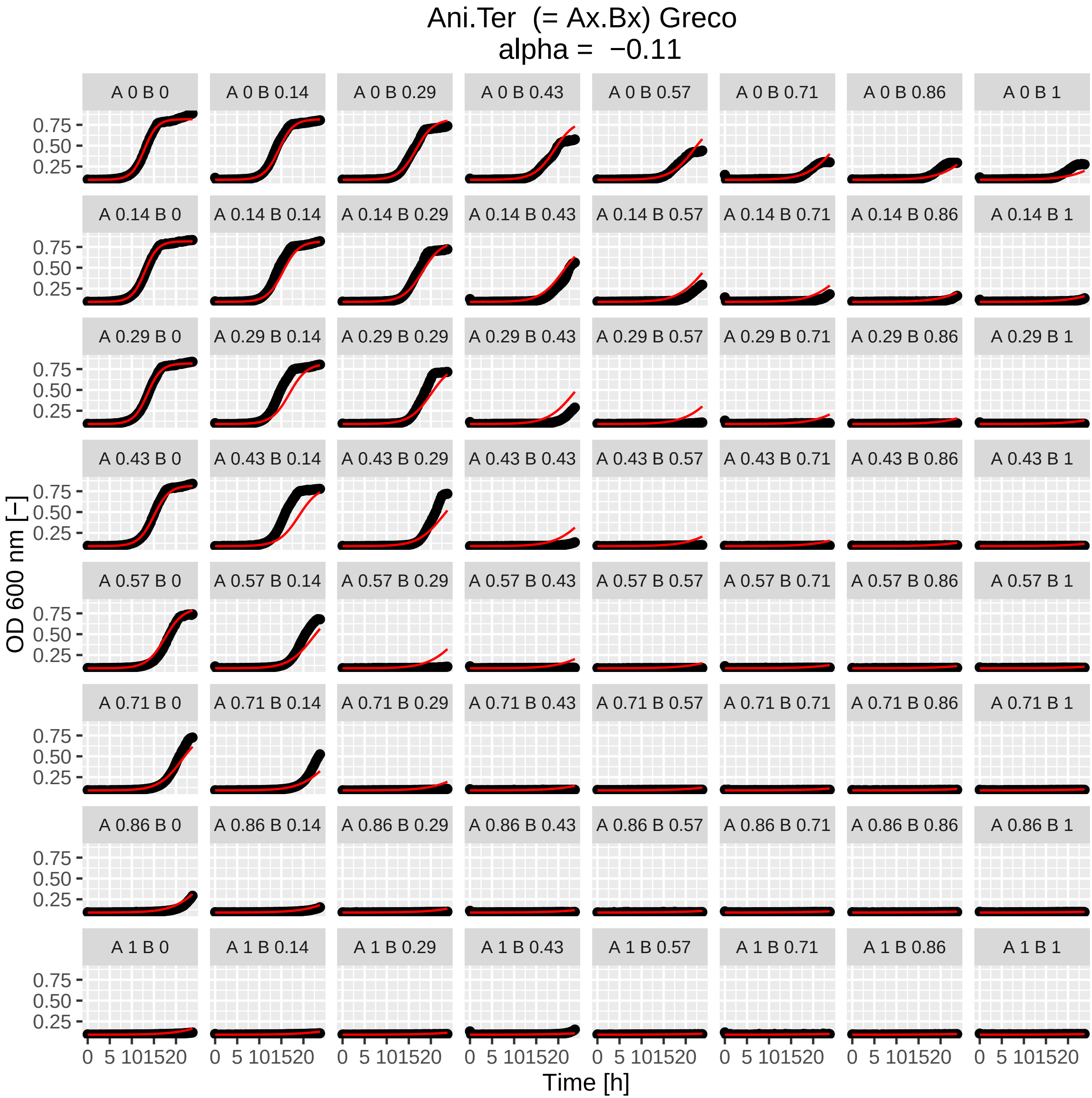
Ani.Ter (= Ax.Bx) Greco
alpha = −0.11
A 0 B 0
A 0 B 0.14
A 0 B 0.29
A 0 B 0.43
A 0 B 0.57
A 0 B 0.71
A 0 B 0.86
A 0 B 1
A 0.14 B 0
A 0.14 B 0.14
A 0.14 B 0.29
A 0.14 B 0.43
A 0.14 B 0.57
A 0.14 B 0.71
A 0.14 B 0.86
A 0.14 B 1
A 0.29 B 0
A 0.29 B 0.14
A 0.29 B 0.29
A 0.29 B 0.43
A 0.29 B 0.57
A 0.29 B 0.71
A 0.29 B 0.86
A 0.29 B 1
A 0.43 B 0
A 0.43 B 0.14
A 0.43 B 0.29
A 0.43 B 0.43
A 0.43 B 0.57
A 0.43 B 0.71
A 0.43 B 0.86
A 0.43 B 1
A 0.57 B 0
A 0.57 B 0.14
A 0.57 B 0.29
A 0.57 B 0.43
A 0.57 B 0.57
A 0.57 B 0.71
A 0.57 B 0.86
A 0.57 B 1
A 0.71 B 0
A 0.71 B 0.14
A 0.71 B 0.29
A 0.71 B 0.43
A 0.71 B 0.57
A 0.71 B 0.71
A 0.71 B 0.86
A 0.71 B 1
A 0.86 B 0
A 0.86 B 0.14
A 0.86 B 0.29
A 0.86 B 0.43
A 0.86 B 0.57
A 0.86 B 0.71
A 0.86 B 0.86
A 0.86 B 1
A 1 B 0
A 1 B 0.14
A 1 B 0.29
A 1 B 0.43
A 1 B 0.57
A 1 B 0.71
A 1 B 0.86
A 1 B 1
OD 600 nm [−]
0.75
0.50
0.25
0 5 101520
Time [h]

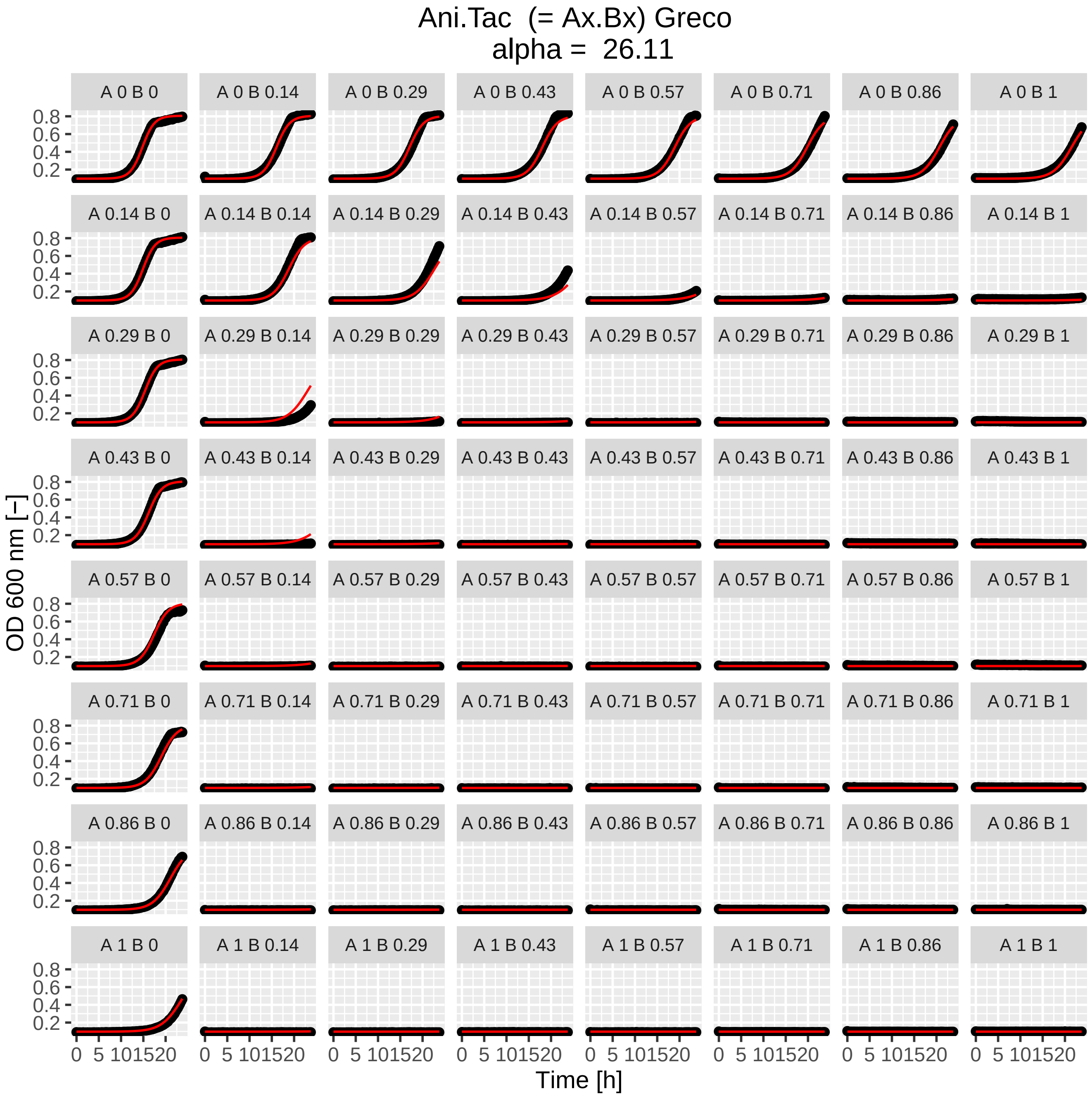

Ani.Tac (= Ax.Bx) Greco
alpha = 26.11
A 0 B 0
A 0 B 0.14
A 0 B 0.29
A 0 B 0.43
A 0 B 0.57
A 0 B 0.71
A 0 B 0.86
A 0 B 1
A 0.14 B 0
A 0.14 B 0.14
A 0.14 B 0.29
A 0.14 B 0.43
A 0.14 B 0.57
A 0.14 B 0.71
A 0.14 B 0.86
A 0.14 B 1
A 0.29 B 0
A 0.29 B 0.14
A 0.29 B 0.29
A 0.29 B 0.43
A 0.29 B 0.57
A 0.29 B 0.71
A 0.29 B 0.86
A 0.29 B 1
A 0.43 B 0
A 0.43 B 0.14
A 0.43 B 0.29
A 0.43 B 0.43
A 0.43 B 0.57
A 0.43 B 0.71
A 0.43 B 0.86
A 0.43 B 1
A 0.57 B 0
A 0.57 B 0.14
A 0.57 B 0.29
A 0.57 B 0.43
A 0.57 B 0.57
A 0.57 B 0.71
A 0.57 B 0.86
A 0.57 B 1
A 0.71 B 0
A 0.71 B 0.14
A 0.71 B 0.29
A 0.71 B 0.43
A 0.71 B 0.57
A 0.71 B 0.71
A 0.71 B 0.86
A 0.71 B 1
A 0.86 B 0
A 0.86 B 0.14
A 0.86 B 0.29
A 0.86 B 0.43
A 0.86 B 0.57
A 0.86 B 0.71
A 0.86 B 0.86
A 0.86 B 1
A 1 B 0
A 1 B 0.14
A 1 B 0.29
A 1 B 0.43
A 1 B 0.57
A 1 B 0.71
A 1 B 0.86
A 1 B 1
OD 600 nm [–]
0.2
0.4
0.6
0.8
0 5 101520
Time [h]

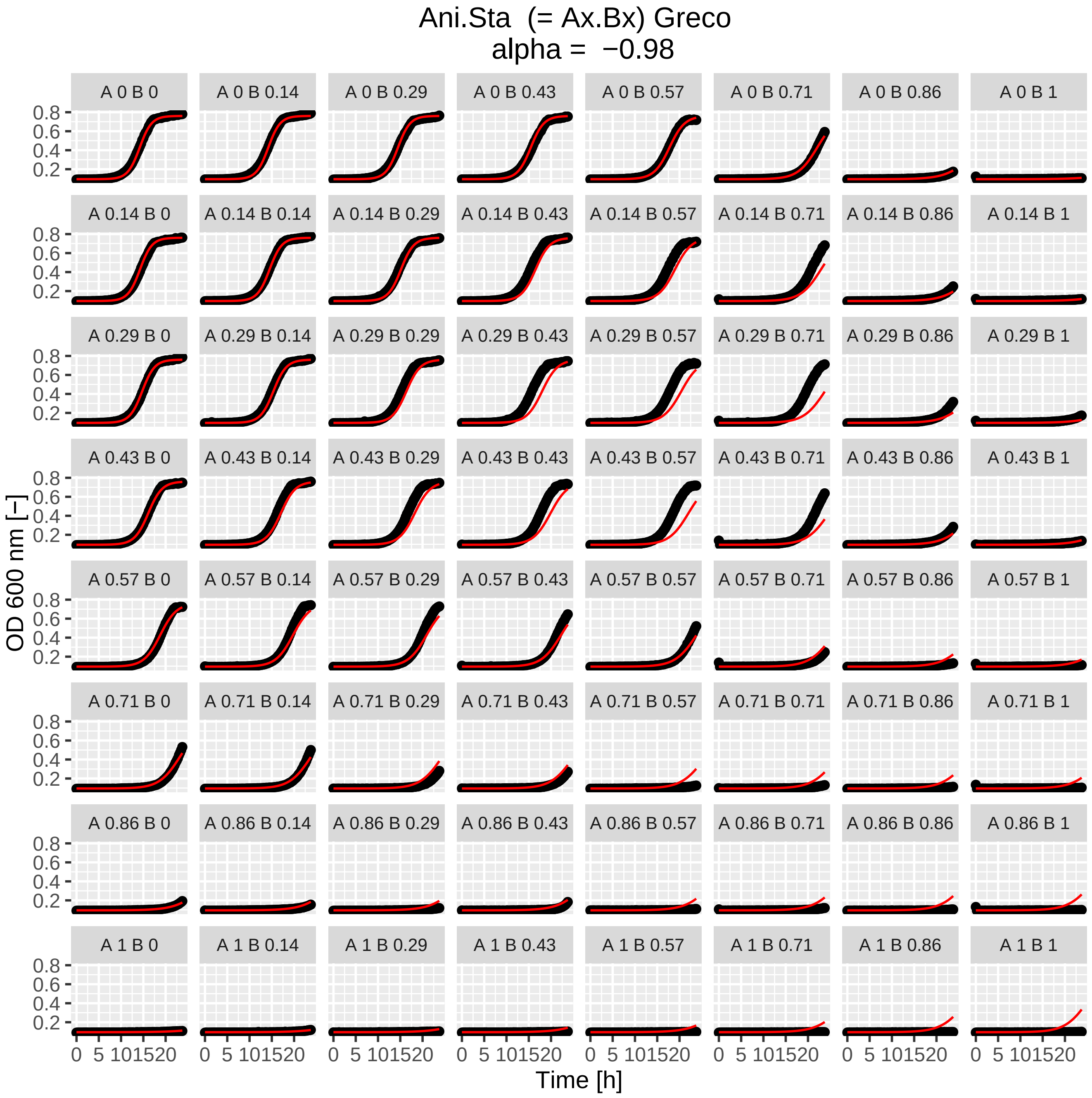
Ani.Sta (= Ax.Bx) Greco
alpha = −0.98
A 0 B 0
A 0 B 0.14
A 0 B 0.29
A 0 B 0.43
A 0 B 0.57
A 0 B 0.71
A 0 B 0.86
A 0 B 1
A 0.14 B 0
A 0.14 B 0.14
A 0.14 B 0.29
A 0.14 B 0.43
A 0.14 B 0.57
A 0.14 B 0.71
A 0.14 B 0.86
A 0.14 B 1
A 0.29 B 0
A 0.29 B 0.14
A 0.29 B 0.29
A 0.29 B 0.43
A 0.29 B 0.57
A 0.29 B 0.71
A 0.29 B 0.86
A 0.29 B 1
A 0.43 B 0
A 0.43 B 0.14
A 0.43 B 0.29
A 0.43 B 0.43
A 0.43 B 0.57
A 0.43 B 0.71
A 0.43 B 0.86
A 0.43 B 1
A 0.57 B 0
A 0.57 B 0.14
A 0.57 B 0.29
A 0.57 B 0.43
A 0.57 B 0.57
A 0.57 B 0.71
A 0.57 B 0.86
A 0.57 B 1
A 0.71 B 0
A 0.71 B 0.14
A 0.71 B 0.29
A 0.71 B 0.43
A 0.71 B 0.57
A 0.71 B 0.71
A 0.71 B 0.86
A 0.71 B 1
A 0.86 B 0
A 0.86 B 0.14
A 0.86 B 0.29
A 0.86 B 0.43
A 0.86 B 0.57
A 0.86 B 0.71
A 0.86 B 0.86
A 0.86 B 1
A 1 B 0
A 1 B 0.14
A 1 B 0.29
A 1 B 0.43
A 1 B 0.57
A 1 B 0.71
A 1 B 0.86
A 1 B 1
0.8
0.6
0.4
0.2
OD 600 nm [−]
0 5 101520
Time [h]

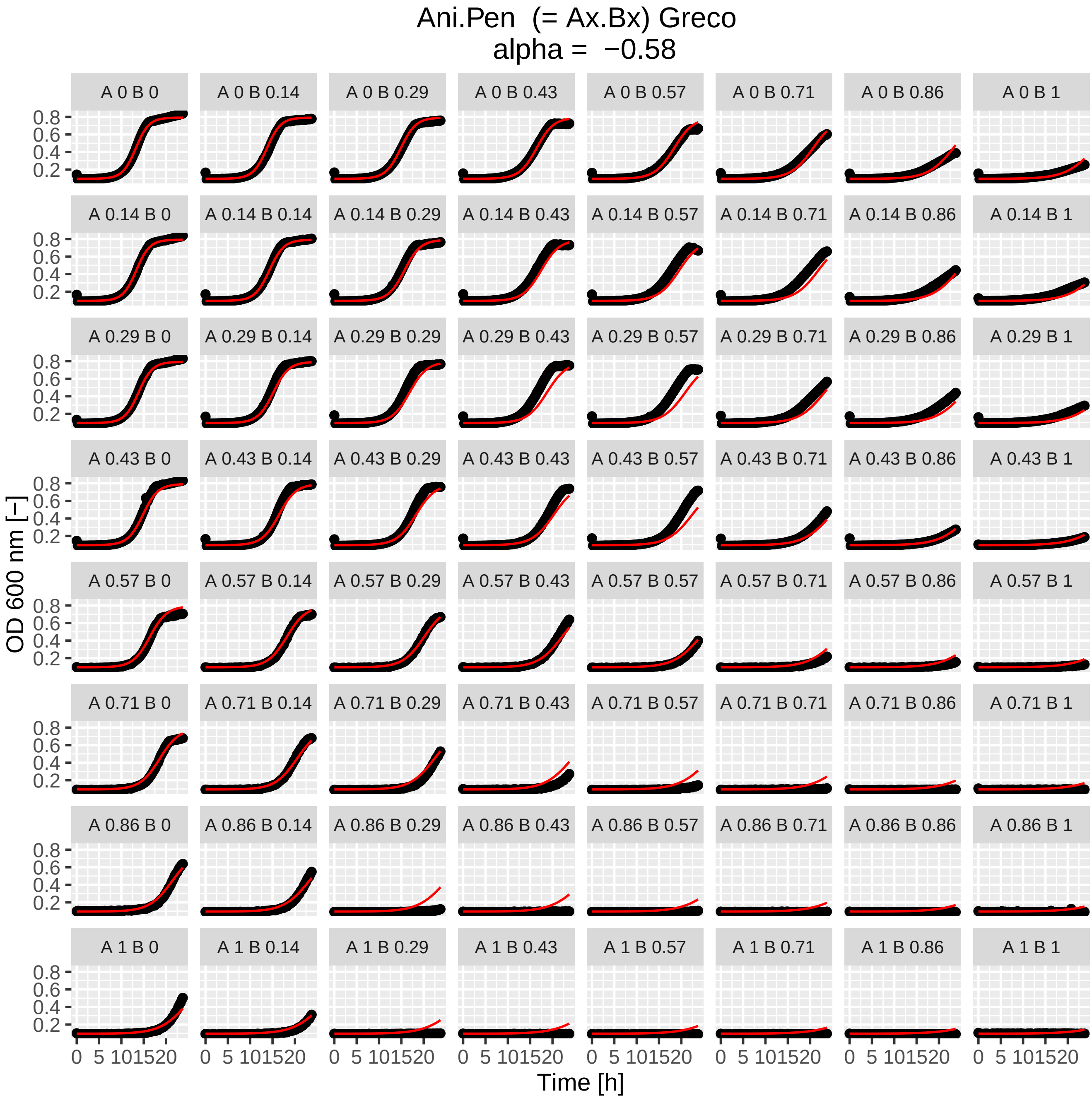

Ani.Pen (= Ax.Bx) Greco
alpha = −0.58
A 0 B 0
A 0 B 0.14
A 0 B 0.29
A 0 B 0.43
A 0 B 0.57
A 0 B 0.71
A 0 B 0.86
A 0 B 1
A 0.14 B 0
A 0.14 B 0.14
A 0.14 B 0.29
A 0.14 B 0.43
A 0.14 B 0.57
A 0.14 B 0.71
A 0.14 B 0.86
A 0.14 B 1
A 0.29 B 0
A 0.29 B 0.14
A 0.29 B 0.29
A 0.29 B 0.43
A 0.29 B 0.57
A 0.29 B 0.71
A 0.29 B 0.86
A 0.29 B 1
A 0.43 B 0
A 0.43 B 0.14
A 0.43 B 0.29
A 0.43 B 0.43
A 0.43 B 0.57
A 0.43 B 0.71
A 0.43 B 0.86
A 0.43 B 1
A 0.57 B 0
A 0.57 B 0.14
A 0.57 B 0.29
A 0.57 B 0.43
A 0.57 B 0.57
A 0.57 B 0.71
A 0.57 B 0.86
A 0.57 B 1
A 0.71 B 0
A 0.71 B 0.14
A 0.71 B 0.29
A 0.71 B 0.43
A 0.71 B 0.57
A 0.71 B 0.71
A 0.71 B 0.86
A 0.71 B 1
A 0.86 B 0
A 0.86 B 0.14
A 0.86 B 0.29
A 0.86 B 0.43
A 0.86 B 0.57
A 0.86 B 0.71
A 0.86 B 0.86
A 0.86 B 1
A 1 B 0
A 1 B 0.14
A 1 B 0.29
A 1 B 0.43
A 1 B 0.57
A 1 B 0.71
A 1 B 0.86
A 1 B 1
OD 600 nm [−]
0.2
0.4
0.6
0.8
0 5 101520
Time [h]

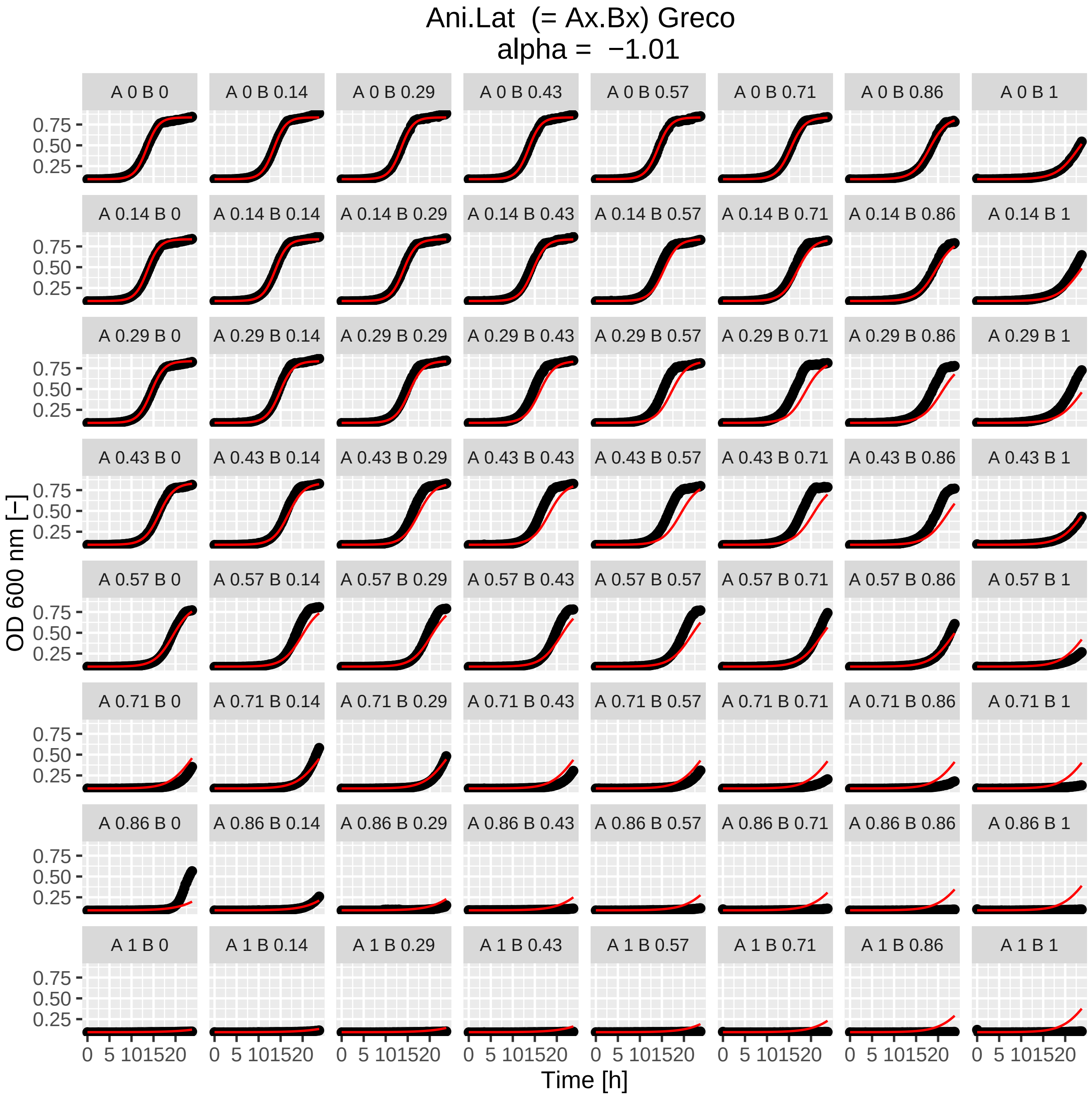

Ani.Lat (= Ax.Bx) Greco
alpha = −1.01
OD 600 nm [−]
Time [h]

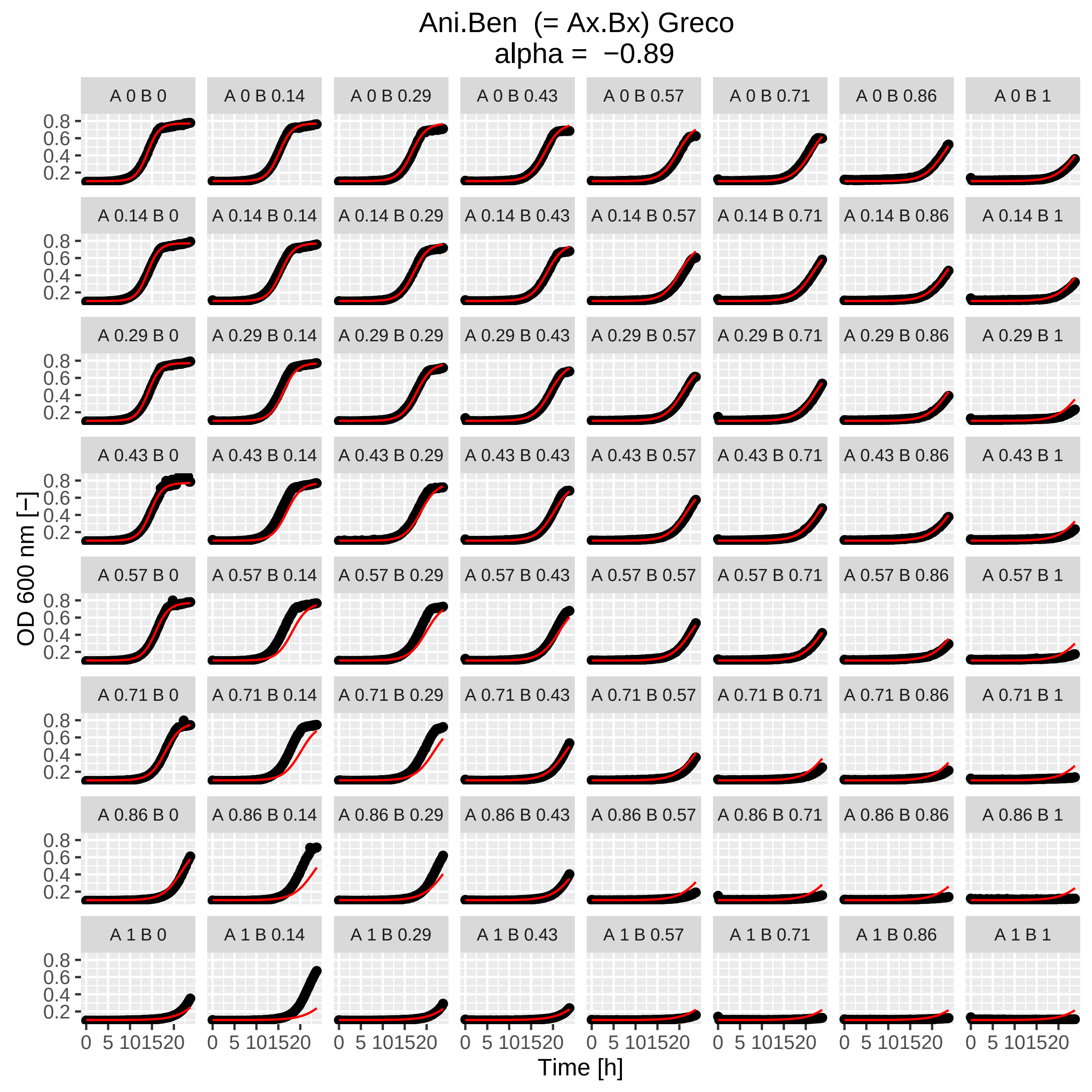

Ani.Ben (= Ax.Bx) Greco
alpha = −0.89
OD 600 nm [−]
Time [h]
A 0 B 0
A 0 B 0.14
A 0 B 0.29
A 0 B 0.43
A 0 B 0.57
A 0 B 0.71
A 0 B 0.86
A 0 B 1
A 0.14 B 0
A 0.14 B 0.14
A 0.14 B 0.29
A 0.14 B 0.43
A 0.14 B 0.57
A 0.14 B 0.71
A 0.14 B 0.86
A 0.14 B 1
A 0.29 B 0
A 0.29 B 0.14
A 0.29 B 0.29
A 0.29 B 0.43
A 0.29 B 0.57
A 0.29 B 0.71
A 0.29 B 0.86
A 0.29 B 1
A 0.43 B 0
A 0.43 B 0.14
A 0.43 B 0.29
A 0.43 B 0.43
A 0.43 B 0.57
A 0.43 B 0.71
A 0.43 B 0.86
A 0.43 B 1
A 0.57 B 0
A 0.57 B 0.14
A 0.57 B 0.29
A 0.57 B 0.43
A 0.57 B 0.57
A 0.57 B 0.71
A 0.57 B 0.86
A 0.57 B 1
A 0.71 B 0
A 0.71 B 0.14
A 0.71 B 0.29
A 0.71 B 0.43
A 0.71 B 0.57
A 0.71 B 0.71
A 0.71 B 0.86
A 0.71 B 1
A 0.86 B 0
A 0.86 B 0.14
A 0.86 B 0.29
A 0.86 B 0.43
A 0.86 B 0.57
A 0.86 B 0.71
A 0.86 B 0.86
A 0.86 B 1
A 1 B 0
A 1 B 0.14
A 1 B 0.29
A 1 B 0.43
A 1 B 0.57
A 1 B 0.71
A 1 B 0.86
A 1 B 1
0.8
0.6
0.4
0.2
0 5 101520

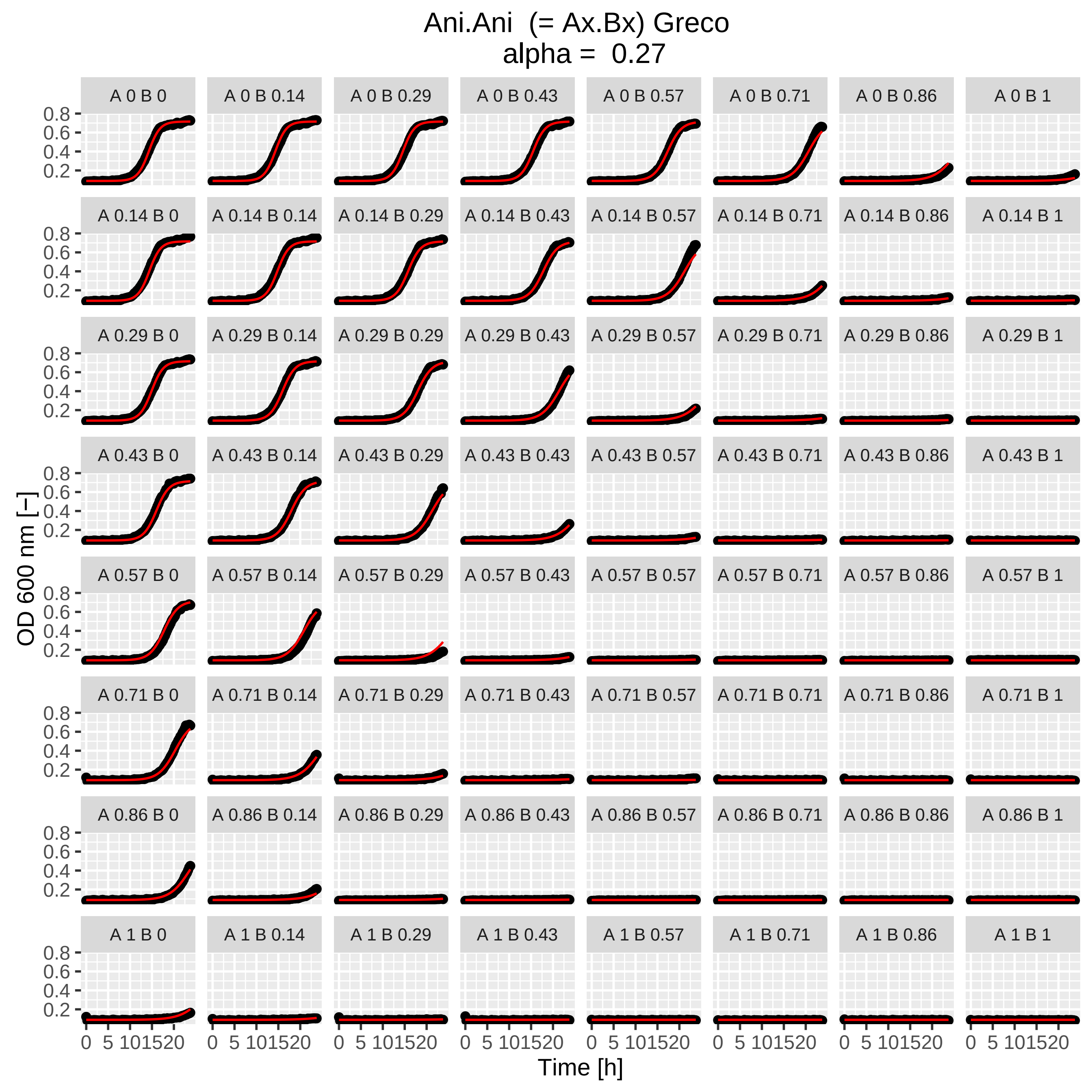

Ani.Ani (= Ax.Bx) Greco
alpha = 0.27
A 0 B 0
A 0 B 0.14
A 0 B 0.29
A 0 B 0.43
A 0 B 0.57
A 0 B 0.71
A 0 B 0.86
A 0 B 1
A 0.14 B 0
A 0.14 B 0.14
A 0.14 B 0.29
A 0.14 B 0.43
A 0.14 B 0.57
A 0.14 B 0.71
A 0.14 B 0.86
A 0.14 B 1
A 0.29 B 0
A 0.29 B 0.14
A 0.29 B 0.29
A 0.29 B 0.43
A 0.29 B 0.57
A 0.29 B 0.71
A 0.29 B 0.86
A 0.29 B 1
A 0.43 B 0
A 0.43 B 0.14
A 0.43 B 0.29
A 0.43 B 0.43
A 0.43 B 0.57
A 0.43 B 0.71
A 0.43 B 0.86
A 0.43 B 1
A 0.57 B 0
A 0.57 B 0.14
A 0.57 B 0.29
A 0.57 B 0.43
A 0.57 B 0.57
A 0.57 B 0.71
A 0.57 B 0.86
A 0.57 B 1
A 0.71 B 0
A 0.71 B 0.14
A 0.71 B 0.29
A 0.71 B 0.43
A 0.71 B 0.57
A 0.71 B 0.71
A 0.71 B 0.86
A 0.71 B 1
A 0.86 B 0
A 0.86 B 0.14
A 0.86 B 0.29
A 0.86 B 0.43
A 0.86 B 0.57
A 0.86 B 0.71
A 0.86 B 0.86
A 0.86 B 1
A 1 B 0
A 1 B 0.14
A 1 B 0.29
A 1 B 0.43
A 1 B 0.57
A 1 B 0.71
A 1 B 0.86
A 1 B 1
OD 600 nm [–]
0.2
0.4
0.6
0.8
0 5 101520
Time [h]

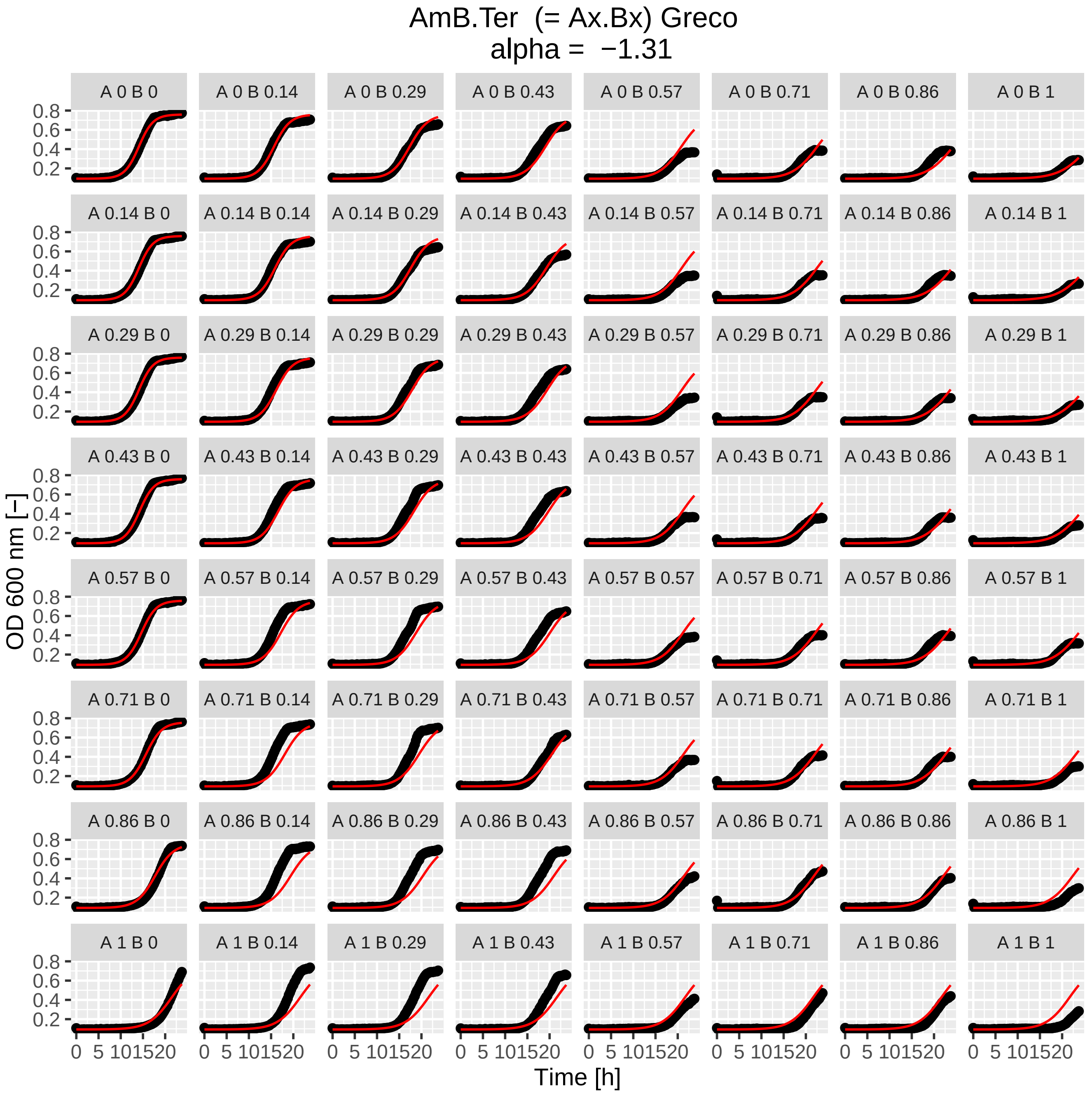
AmB.Ter (= Ax.Bx) Greco
alpha = −1.31
OD 600 nm [−]
Time [h]
A 0 B 0
A 0 B 0.14
A 0 B 0.29
A 0 B 0.43
A 0 B 0.57
A 0 B 0.71
A 0 B 0.86
A 0 B 1
A 0.14 B 0
A 0.14 B 0.14
A 0.14 B 0.29
A 0.14 B 0.43
A 0.14 B 0.57
A 0.14 B 0.71
A 0.14 B 0.86
A 0.14 B 1
A 0.29 B 0
A 0.29 B 0.14
A 0.29 B 0.29
A 0.29 B 0.43
A 0.29 B 0.57
A 0.29 B 0.71
A 0.29 B 0.86
A 0.29 B 1
A 0.43 B 0
A 0.43 B 0.14
A 0.43 B 0.29
A 0.43 B 0.43
A 0.43 B 0.57
A 0.43 B 0.71
A 0.43 B 0.86
A 0.43 B 1
A 0.57 B 0
A 0.57 B 0.14
A 0.57 B 0.29
A 0.57 B 0.43
A 0.57 B 0.57
A 0.57 B 0.71
A 0.57 B 0.86
A 0.57 B 1
A 0.71 B 0
A 0.71 B 0.14
A 0.71 B 0.29
A 0.71 B 0.43
A 0.71 B 0.57
A 0.71 B 0.71
A 0.71 B 0.86
A 0.71 B 1
A 0.86 B 0
A 0.86 B 0.14
A 0.86 B 0.29
A 0.86 B 0.43
A 0.86 B 0.57
A 0.86 B 0.71
A 0.86 B 0.86
A 0.86 B 1
A 1 B 0
A 1 B 0.14
A 1 B 0.29
A 1 B 0.43
A 1 B 0.57
A 1 B 0.71
A 1 B 0.86
A 1 B 1
0.2
0.4
0.6
0.8
0 5 101520

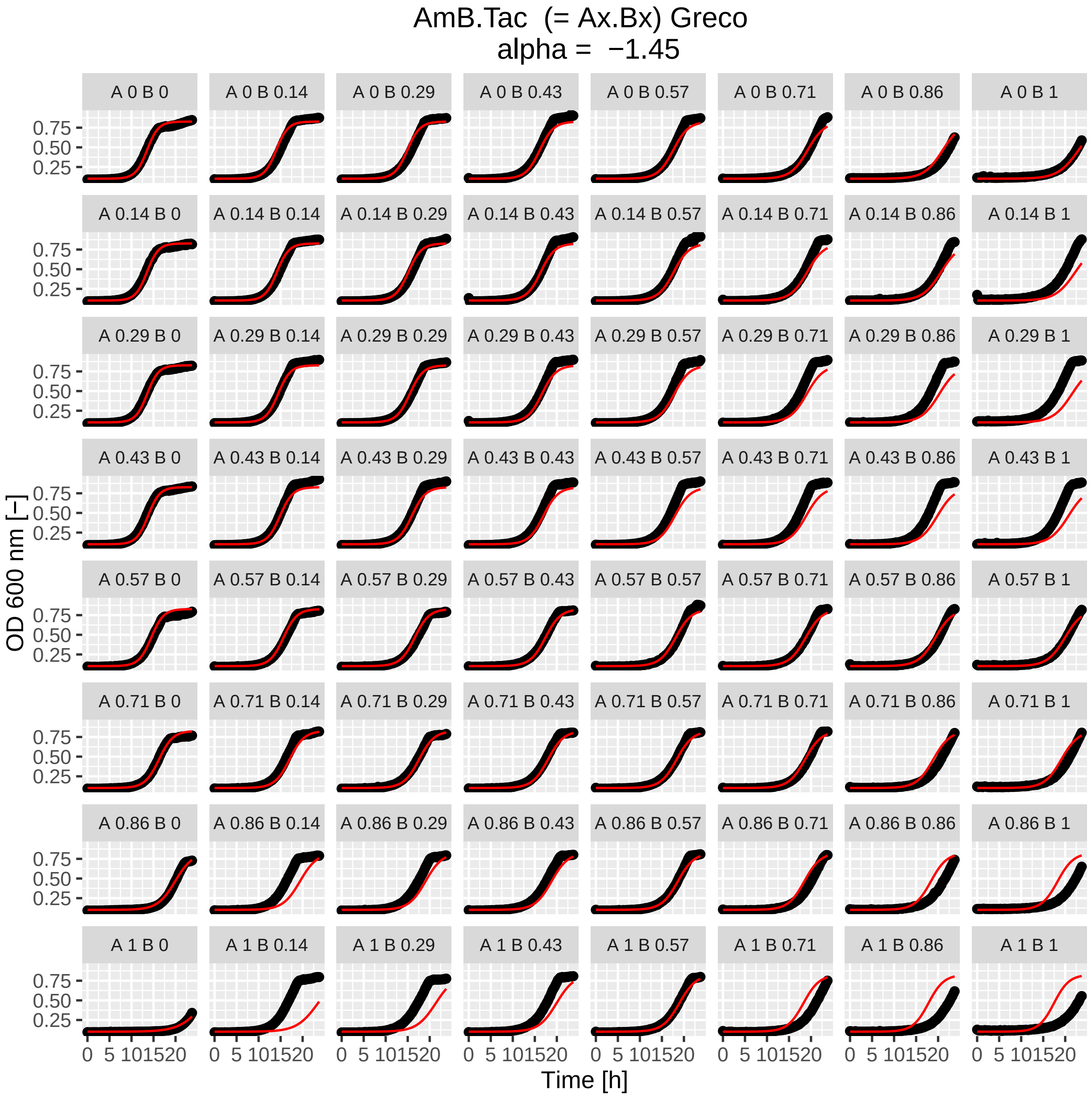
AmB.Tac (= Ax.Bx) Greco
alpha = −1.45
OD 600 nm [−]
Time [h]
0.75
0.50
0.25
0 5 101520
A 0 B 0
A 0 B 0.14
A 0 B 0.29
A 0 B 0.43
A 0 B 0.57
A 0 B 0.71
A 0 B 0.86
A 0 B 1
A 0.14 B 0
A 0.14 B 0.14
A 0.14 B 0.29
A 0.14 B 0.43
A 0.14 B 0.57
A 0.14 B 0.71
A 0.14 B 0.86
A 0.14 B 1
A 0.29 B 0
A 0.29 B 0.14
A 0.29 B 0.29
A 0.29 B 0.43
A 0.29 B 0.57
A 0.29 B 0.71
A 0.29 B 0.86
A 0.29 B 1
A 0.43 B 0
A 0.43 B 0.14
A 0.43 B 0.29
A 0.43 B 0.43
A 0.43 B 0.57
A 0.43 B 0.71
A 0.43 B 0.86
A 0.43 B 1
A 0.57 B 0
A 0.57 B 0.14
A 0.57 B 0.29
A 0.57 B 0.43
A 0.57 B 0.57
A 0.57 B 0.71
A 0.57 B 0.86
A 0.57 B 1
A 0.71 B 0
A 0.71 B 0.14
A 0.71 B 0.29
A 0.71 B 0.43
A 0.71 B 0.57
A 0.71 B 0.71
A 0.71 B 0.86
A 0.71 B 1
A 0.86 B 0
A 0.86 B 0.14
A 0.86 B 0.29
A 0.86 B 0.43
A 0.86 B 0.57
A 0.86 B 0.71
A 0.86 B 0.86
A 0.86 B 1
A 1 B 0
A 1 B 0.14
A 1 B 0.29
A 1 B 0.43
A 1 B 0.57
A 1 B 0.71
A 1 B 0.86
A 1 B 1

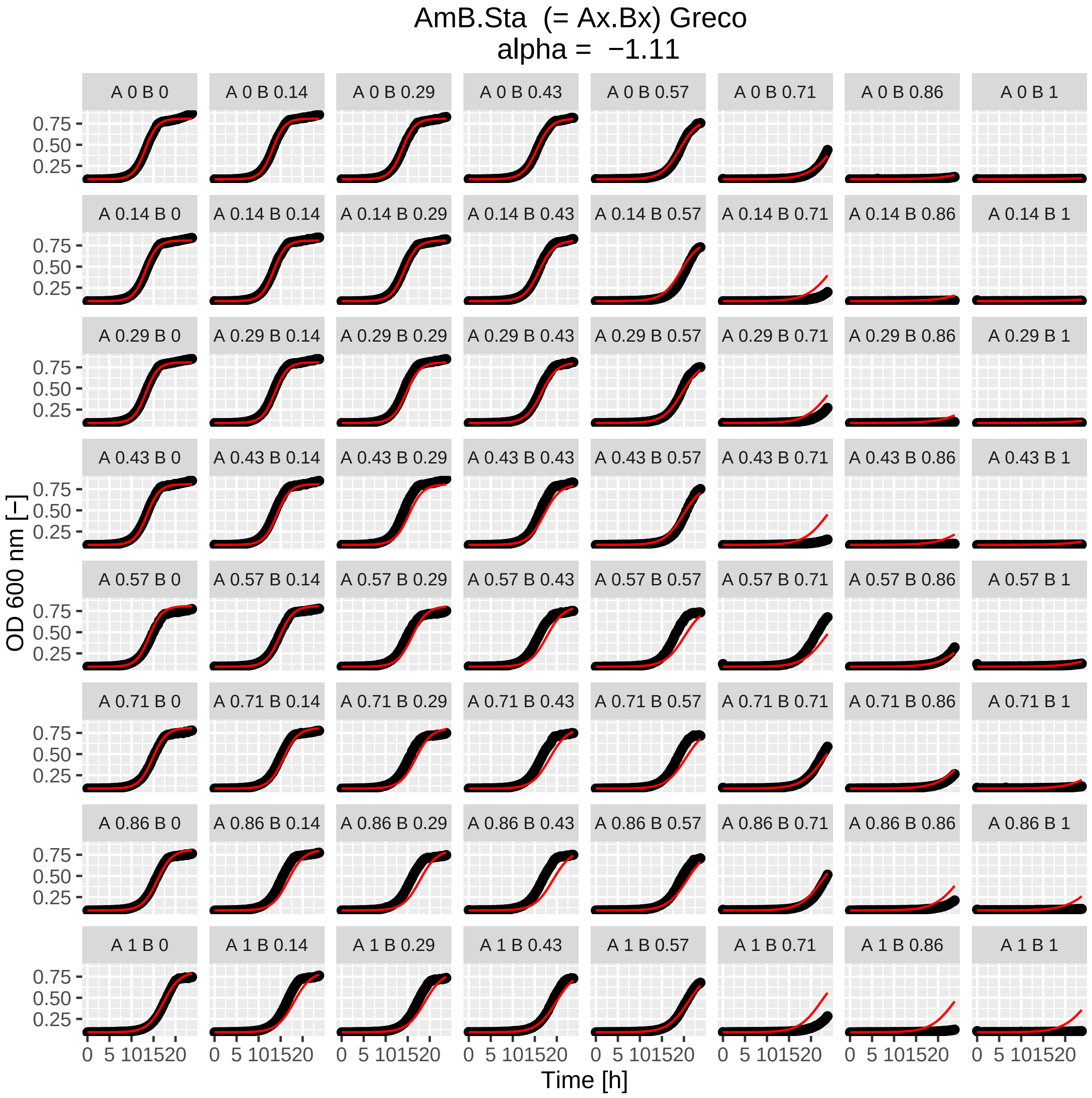
AmB.Sta (= Ax.Bx) Greco
alpha = −1.11
OD 600 nm [−]
Time [h]
0.75
0.50
0.25
0 5 101520
A 0 B 0
A 0 B 0.14
A 0 B 0.29
A 0 B 0.43
A 0 B 0.57
A 0 B 0.71
A 0 B 0.86
A 0 B 1
A 0.14 B 0
A 0.14 B 0.14
A 0.14 B 0.29
A 0.14 B 0.43
A 0.14 B 0.57
A 0.14 B 0.71
A 0.14 B 0.86
A 0.14 B 1
A 0.29 B 0
A 0.29 B 0.14
A 0.29 B 0.29
A 0.29 B 0.43
A 0.29 B 0.57
A 0.29 B 0.71
A 0.29 B 0.86
A 0.29 B 1
A 0.43 B 0
A 0.43 B 0.14
A 0.43 B 0.29
A 0.43 B 0.43
A 0.43 B 0.57
A 0.43 B 0.71
A 0.43 B 0.86
A 0.43 B 1
A 0.57 B 0
A 0.57 B 0.14
A 0.57 B 0.29
A 0.57 B 0.43
A 0.57 B 0.57
A 0.57 B 0.71
A 0.57 B 0.86
A 0.57 B 1
A 0.71 B 0
A 0.71 B 0.14
A 0.71 B 0.29
A 0.71 B 0.43
A 0.71 B 0.57
A 0.71 B 0.71
A 0.71 B 0.86
A 0.71 B 1
A 0.86 B 0
A 0.86 B 0.14
A 0.86 B 0.29
A 0.86 B 0.43
A 0.86 B 0.57
A 0.86 B 0.71
A 0.86 B 0.86
A 0.86 B 1
A 1 B 0
A 1 B 0.14
A 1 B 0.29
A 1 B 0.43
A 1 B 0.57
A 1 B 0.71
A 1 B 0.86
A 1 B 1

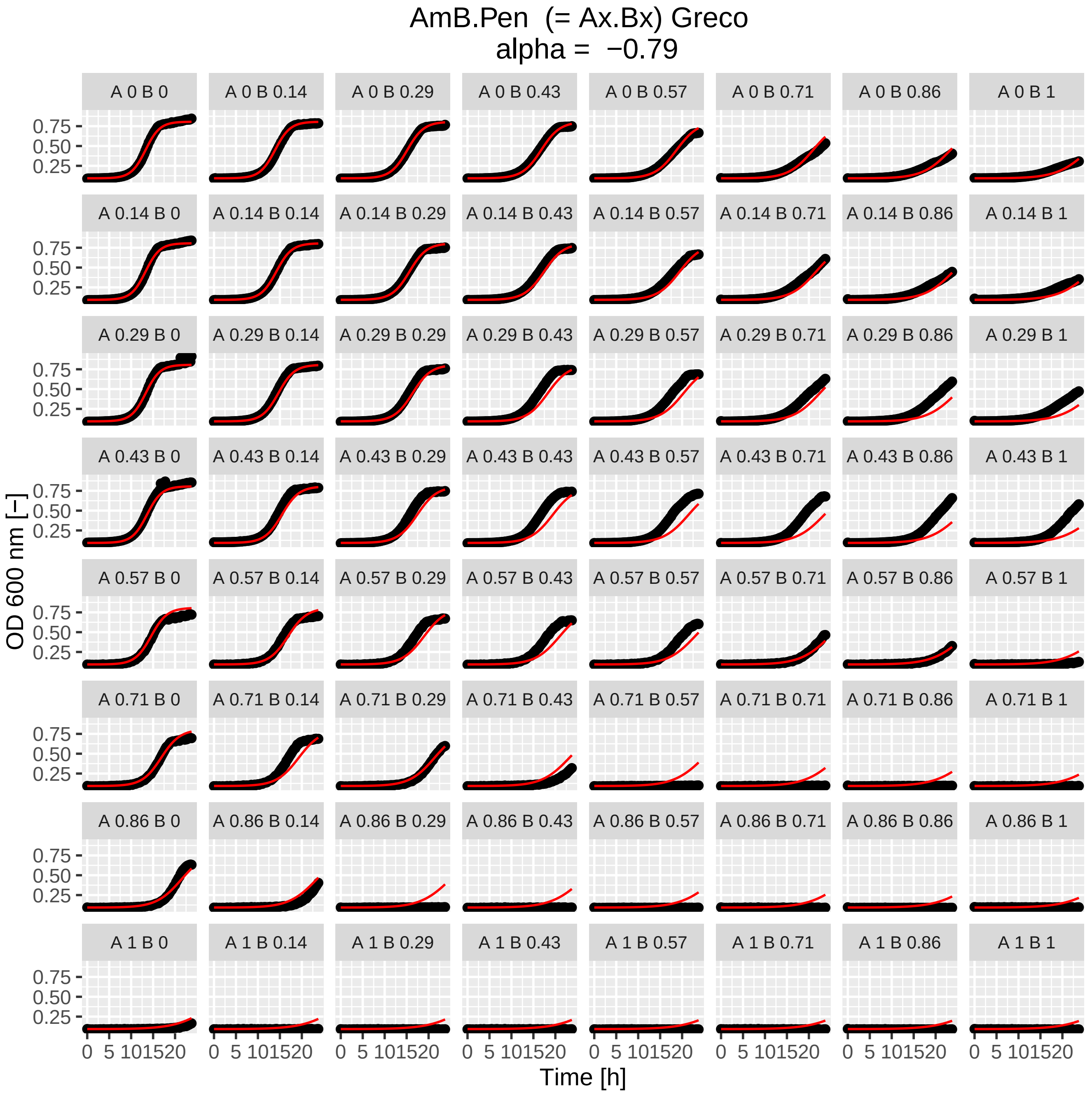
AmB.Pen (= Ax.Bx) Greco
alpha = −0.79
A 0 B 0
A 0 B 0.14
A 0 B 0.29
A 0 B 0.43
A 0 B 0.57
A 0 B 0.71
A 0 B 0.86
A 0 B 1
A 0.14 B 0
A 0.14 B 0.14
A 0.14 B 0.29
A 0.14 B 0.43
A 0.14 B 0.57
A 0.14 B 0.71
A 0.14 B 0.86
A 0.14 B 1
A 0.29 B 0
A 0.29 B 0.14
A 0.29 B 0.29
A 0.29 B 0.43
A 0.29 B 0.57
A 0.29 B 0.71
A 0.29 B 0.86
A 0.29 B 1
A 0.43 B 0
A 0.43 B 0.14
A 0.43 B 0.29
A 0.43 B 0.43
A 0.43 B 0.57
A 0.43 B 0.71
A 0.43 B 0.86
A 0.43 B 1
A 0.57 B 0
A 0.57 B 0.14
A 0.57 B 0.29
A 0.57 B 0.43
A 0.57 B 0.57
A 0.57 B 0.71
A 0.57 B 0.86
A 0.57 B 1
A 0.71 B 0
A 0.71 B 0.14
A 0.71 B 0.29
A 0.71 B 0.43
A 0.71 B 0.57
A 0.71 B 0.71
A 0.71 B 0.86
A 0.71 B 1
A 0.86 B 0
A 0.86 B 0.14
A 0.86 B 0.29
A 0.86 B 0.43
A 0.86 B 0.57
A 0.86 B 0.71
A 0.86 B 0.86
A 0.86 B 1
A 1 B 0
A 1 B 0.14
A 1 B 0.29
A 1 B 0.43
A 1 B 0.57
A 1 B 0.71
A 1 B 0.86
A 1 B 1
0.75
0.50
0.25
0 5 101520
OD 600 nm [−]
Time [h]

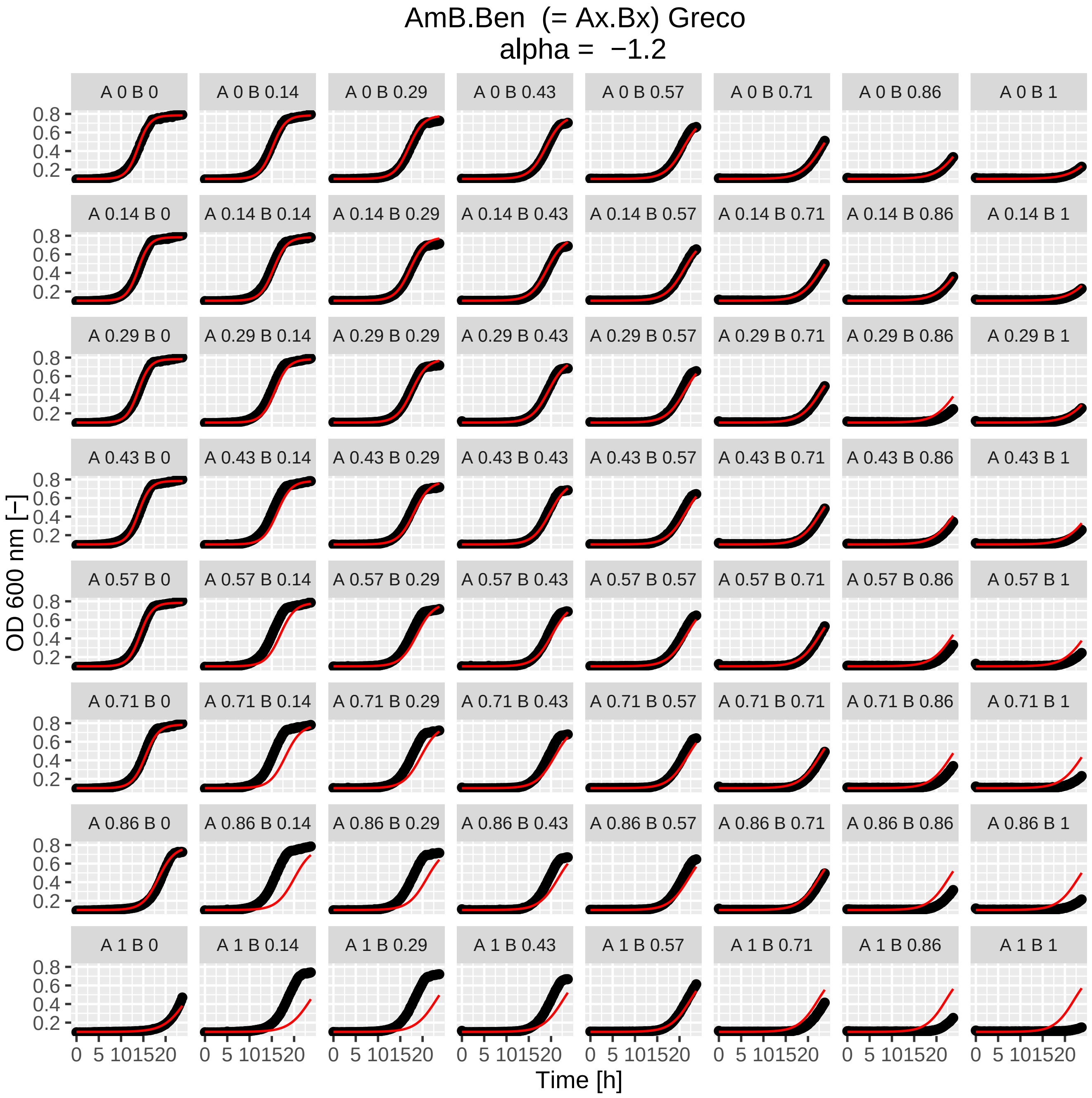
AmB.Ben (= Ax.Bx) Greco
alpha = −1.2
A 0 B 0
A 0 B 0.14
A 0 B 0.29
A 0 B 0.43
A 0 B 0.57
A 0 B 0.71
A 0 B 0.86
A 0 B 1
A 0.14 B 0
A 0.14 B 0.14
A 0.14 B 0.29
A 0.14 B 0.43
A 0.14 B 0.57
A 0.14 B 0.71
A 0.14 B 0.86
A 0.14 B 1
A 0.29 B 0
A 0.29 B 0.14
A 0.29 B 0.29
A 0.29 B 0.43
A 0.29 B 0.57
A 0.29 B 0.71
A 0.29 B 0.86
A 0.29 B 1
A 0.43 B 0
A 0.43 B 0.14
A 0.43 B 0.29
A 0.43 B 0.43
A 0.43 B 0.57
A 0.43 B 0.71
A 0.43 B 0.86
A 0.43 B 1
A 0.57 B 0
A 0.57 B 0.14
A 0.57 B 0.29
A 0.57 B 0.43
A 0.57 B 0.57
A 0.57 B 0.71
A 0.57 B 0.86
A 0.57 B 1
A 0.71 B 0
A 0.71 B 0.14
A 0.71 B 0.29
A 0.71 B 0.43
A 0.71 B 0.57
A 0.71 B 0.71
A 0.71 B 0.86
A 0.71 B 1
A 0.86 B 0
A 0.86 B 0.14
A 0.86 B 0.29
A 0.86 B 0.43
A 0.86 B 0.57
A 0.86 B 0.71
A 0.86 B 0.86
A 0.86 B 1
A 1 B 0
A 1 B 0.14
A 1 B 0.29
A 1 B 0.43
A 1 B 0.57
A 1 B 0.71
A 1 B 0.86
A 1 B 1
OD 600 nm [−]
0.2
0.4
0.6
0.8
0
5
10
15
20
Time [h]

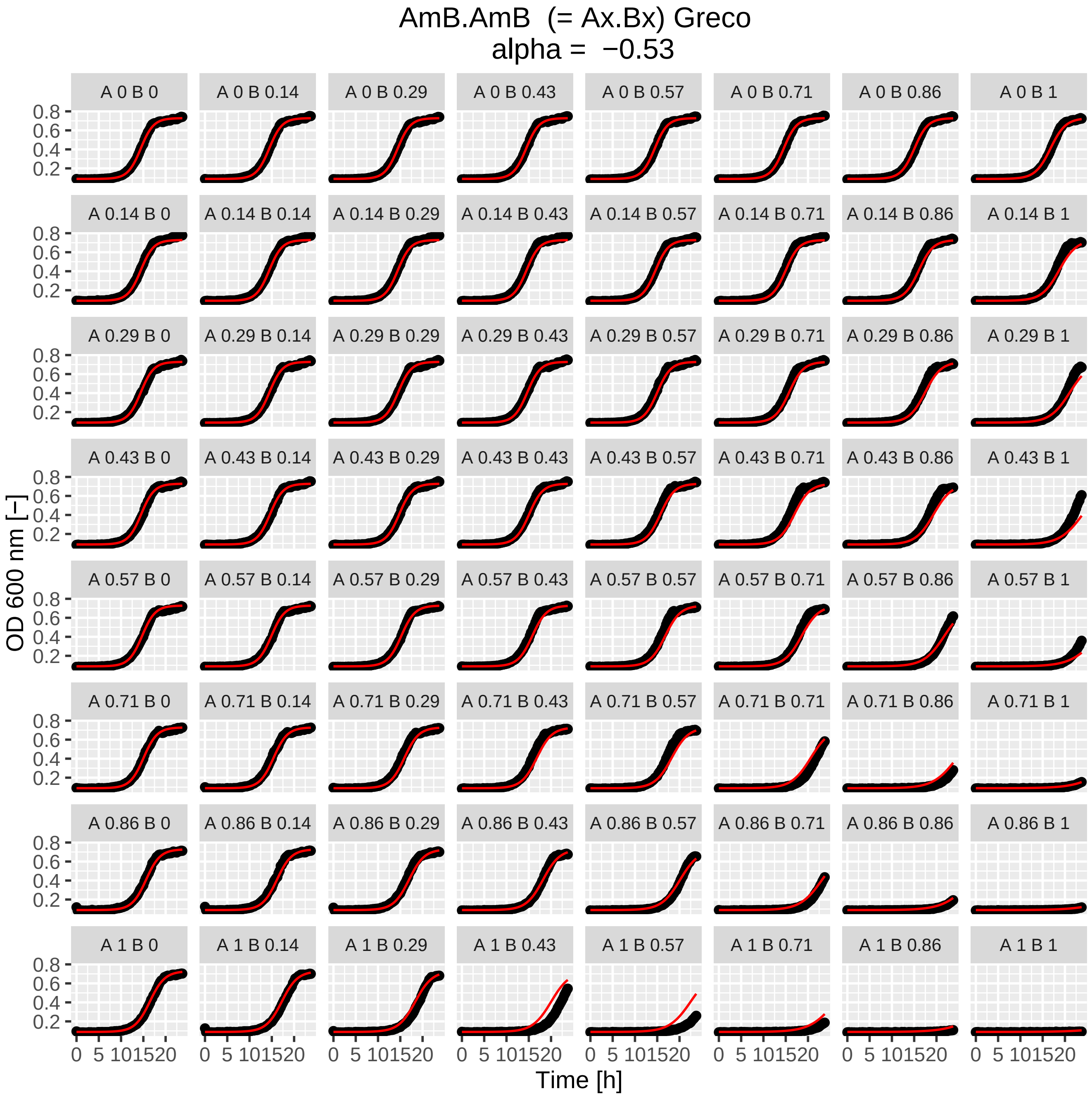
AmB.AmB (= Ax.Bx) Greco
alpha = −0.53
OD 600 nm [−]
Time [h]
A 0 B 0
A 0 B 0.14
A 0 B 0.29
A 0 B 0.43
A 0 B 0.57
A 0 B 0.71
A 0 B 0.86
A 0 B 1
A 1 B 0
A 1 B 0.14
A 1 B 0.29
A 1 B 0.43
A 1 B 0.57
A 1 B 0.71
A 1 B 0.86
A 1 B 1
0.8
0.6
0.4
0.2
0 5 101520

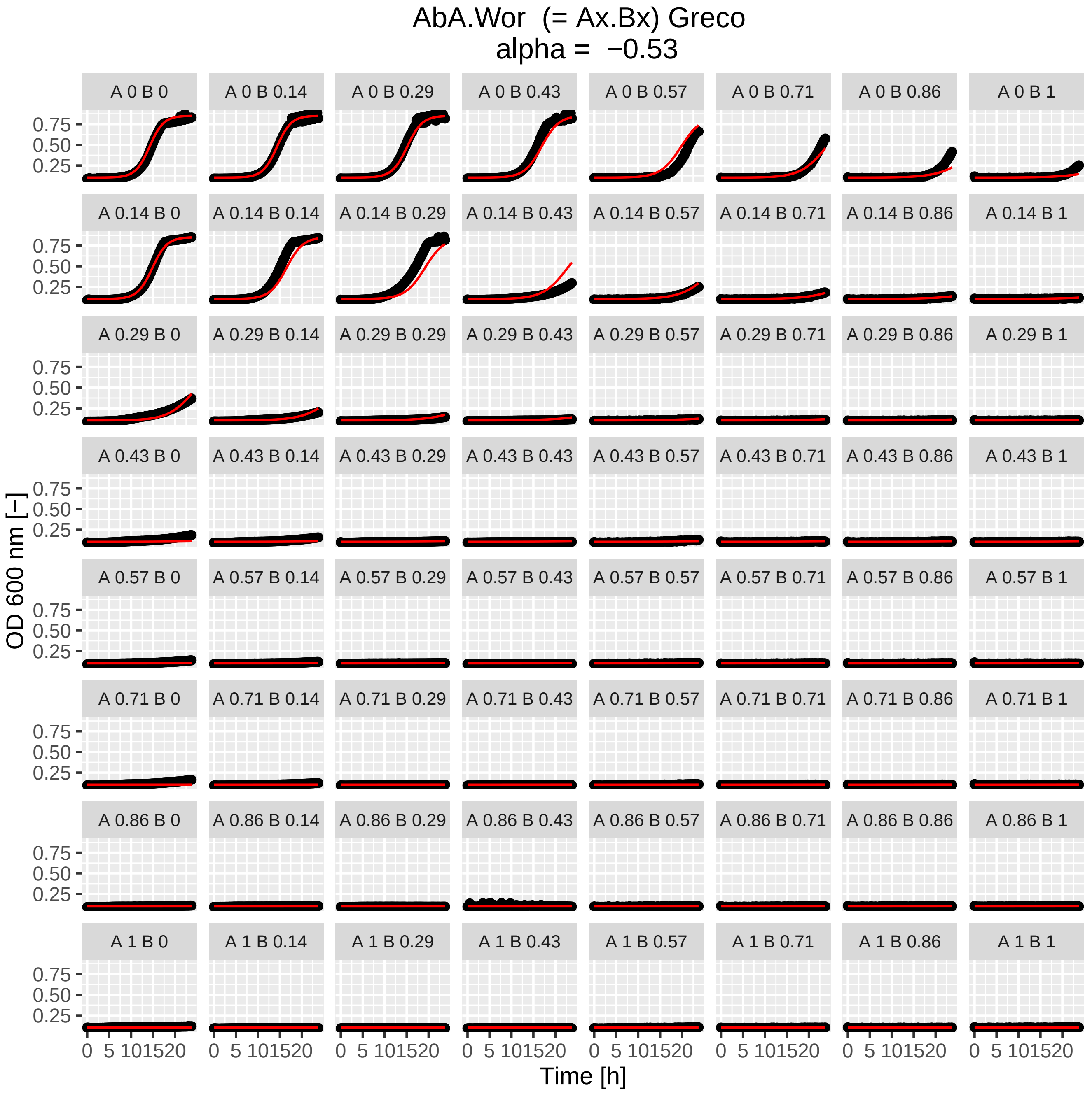
AbA.Wor (= Ax.Bx) Greco
alpha = −0.53
A 0 B 0
A 0 B 0.14
A 0 B 0.29
A 0 B 0.43
A 0 B 0.57
A 0 B 0.71
A 0 B 0.86
A 0 B 1
A 0.14 B 0
A 0.14 B 0.14
A 0.14 B 0.29
A 0.14 B 0.43
A 0.14 B 0.57
A 0.14 B 0.71
A 0.14 B 0.86
A 0.14 B 1
A 0.29 B 0
A 0.29 B 0.14
A 0.29 B 0.29
A 0.29 B 0.43
A 0.29 B 0.57
A 0.29 B 0.71
A 0.29 B 0.86
A 0.29 B 1
A 0.43 B 0
A 0.43 B 0.14
A 0.43 B 0.29
A 0.43 B 0.43
A 0.43 B 0.57
A 0.43 B 0.71
A 0.43 B 0.86
A 0.43 B 1
A 0.57 B 0
A 0.57 B 0.14
A 0.57 B 0.29
A 0.57 B 0.43
A 0.57 B 0.57
A 0.57 B 0.71
A 0.57 B 0.86
A 0.57 B 1
A 0.71 B 0
A 0.71 B 0.14
A 0.71 B 0.29
A 0.71 B 0.43
A 0.71 B 0.57
A 0.71 B 0.71
A 0.71 B 0.86
A 0.71 B 1
A 0.86 B 0
A 0.86 B 0.14
A 0.86 B 0.29
A 0.86 B 0.43
A 0.86 B 0.57
A 0.86 B 0.71
A 0.86 B 0.86
A 0.86 B 1
A 1 B 0
A 1 B 0.14
A 1 B 0.29
A 1 B 0.43
A 1 B 0.57
A 1 B 0.71
A 1 B 0.86
A 1 B 1
0.75
0.50
0.25
OD 600 nm [−]
0 5 101520
Time [h]

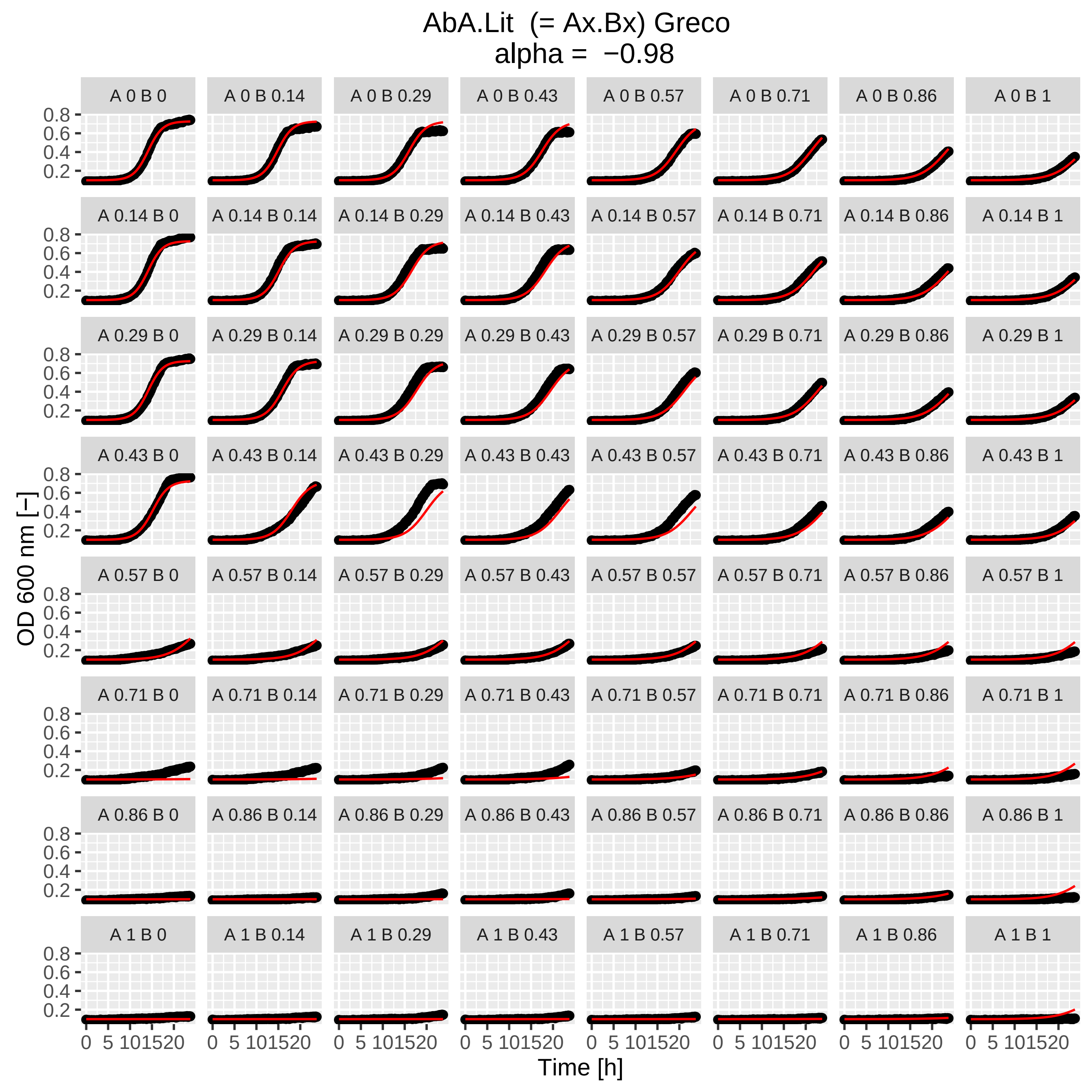
AbA.Lit (= Ax.Bx) Greco
alpha = −0.98
OD 600 nm [−]
Time [h]
0.8
0.6
0.4
0.2
0 5 101520
A 0 B 0
A 0 B 0.14
A 0 B 0.29
A 0 B 0.43
A 0 B 0.57
A 0 B 0.71
A 0 B 0.86
A 0 B 1
A 0.14 B 0
A 0.14 B 0.14
A 0.14 B 0.29
A 0.14 B 0.43
A 0.14 B 0.57
A 0.14 B 0.71
A 0.14 B 0.86
A 0.14 B 1
A 0.29 B 0
A 0.29 B 0.14
A 0.29 B 0.29
A 0.29 B 0.43
A 0.29 B 0.57
A 0.29 B 0.71
A 0.29 B 0.86
A 0.29 B 1
A 0.43 B 0
A 0.43 B 0.14
A 0.43 B 0.29
A 0.43 B 0.43
A 0.43 B 0.57
A 0.43 B 0.71
A 0.43 B 0.86
A 0.43 B 1
A 0.57 B 0
A 0.57 B 0.14
A 0.57 B 0.29
A 0.57 B 0.43
A 0.57 B 0.57
A 0.57 B 0.71
A 0.57 B 0.86
A 0.57 B 1
A 0.71 B 0
A 0.71 B 0.14
A 0.71 B 0.29
A 0.71 B 0.43
A 0.71 B 0.57
A 0.71 B 0.71
A 0.71 B 0.86
A 0.71 B 1
A 0.86 B 0
A 0.86 B 0.14
A 0.86 B 0.29
A 0.86 B 0.43
A 0.86 B 0.57
A 0.86 B 0.71
A 0.86 B 0.86
A 0.86 B 1
A 1 B 0
A 1 B 0.14
A 1 B 0.29
A 1 B 0.43
A 1 B 0.57
A 1 B 0.71
A 1 B 0.86
A 1 B 1

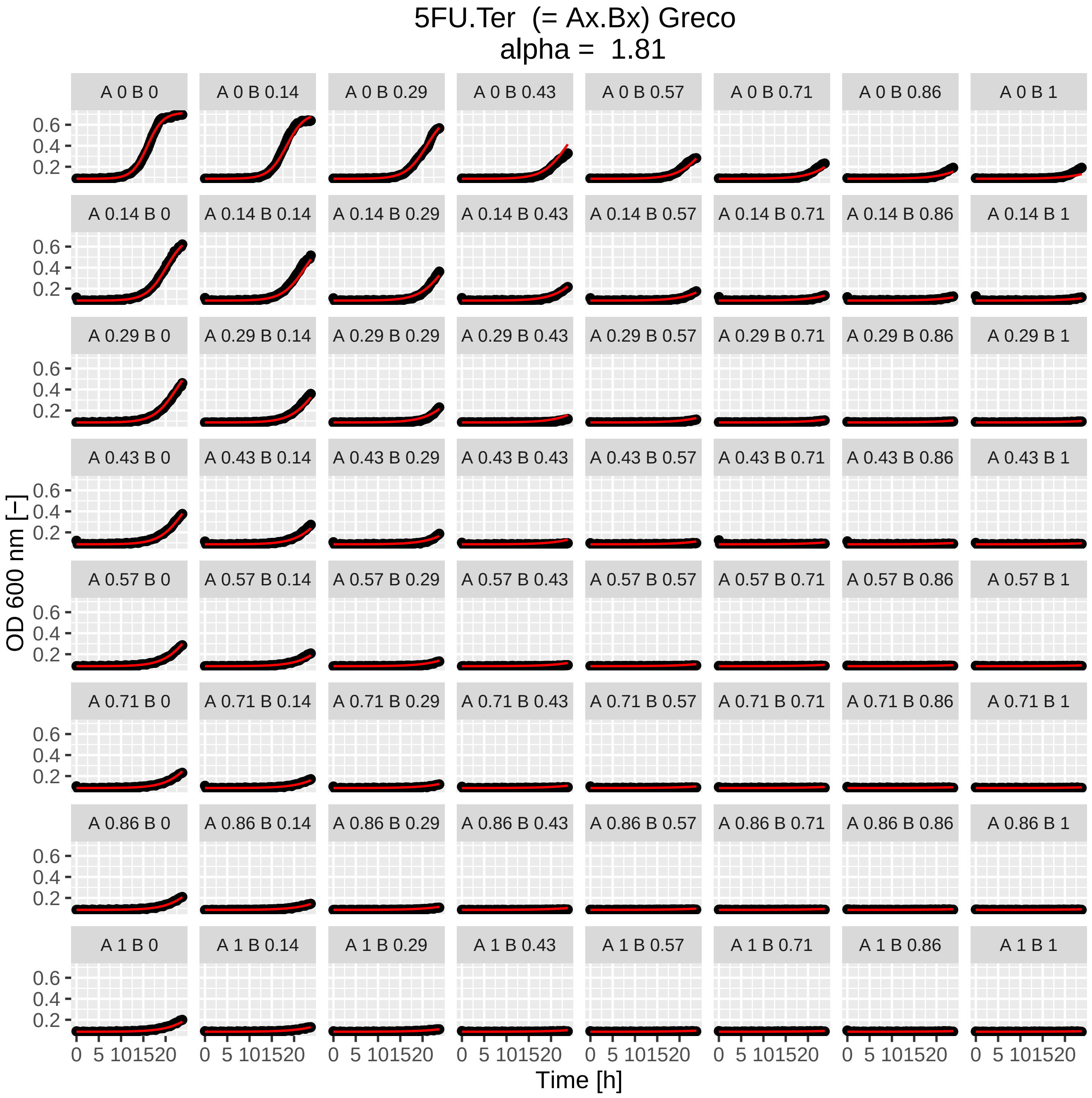

5FU.Ter (= Ax.Bx) Greco
alpha = 1.81
OD 600 nm [−]
Time [h]
0 5 101520
0.2
0.4
0.6
A 0 B 0
A 0 B 0.14
A 0 B 0.29
A 0 B 0.43
A 0 B 0.57
A 0 B 0.71
A 0 B 0.86
A 0 B 1
A 0.14 B 0
A 0.14 B 0.14
A 0.14 B 0.29
A 0.14 B 0.43
A 0.14 B 0.57
A 0.14 B 0.71
A 0.14 B 0.86
A 0.14 B 1
A 0.29 B 0
A 0.29 B 0.14
A 0.29 B 0.29
A 0.29 B 0.43
A 0.29 B 0.57
A 0.29 B 0.71
A 0.29 B 0.86
A 0.29 B 1
A 0.43 B 0
A 0.43 B 0.14
A 0.43 B 0.29
A 0.43 B 0.43
A 0.43 B 0.57
A 0.43 B 0.71
A 0.43 B 0.86
A 0.43 B 1
A 0.57 B 0
A 0.57 B 0.14
A 0.57 B 0.29
A 0.57 B 0.43
A 0.57 B 0.57
A 0.57 B 0.71
A 0.57 B 0.86
A 0.57 B 1
A 0.71 B 0
A 0.71 B 0.14
A 0.71 B 0.29
A 0.71 B 0.43
A 0.71 B 0.57
A 0.71 B 0.71
A 0.71 B 0.86
A 0.71 B 1
A 0.86 B 0
A 0.86 B 0.14
A 0.86 B 0.29
A 0.86 B 0.43
A 0.86 B 0.57
A 0.86 B 0.71
A 0.86 B 0.86
A 0.86 B 1
A 1 B 0
A 1 B 0.14
A 1 B 0.29
A 1 B 0.43
A 1 B 0.57
A 1 B 0.71
A 1 B 0.86
A 1 B 1

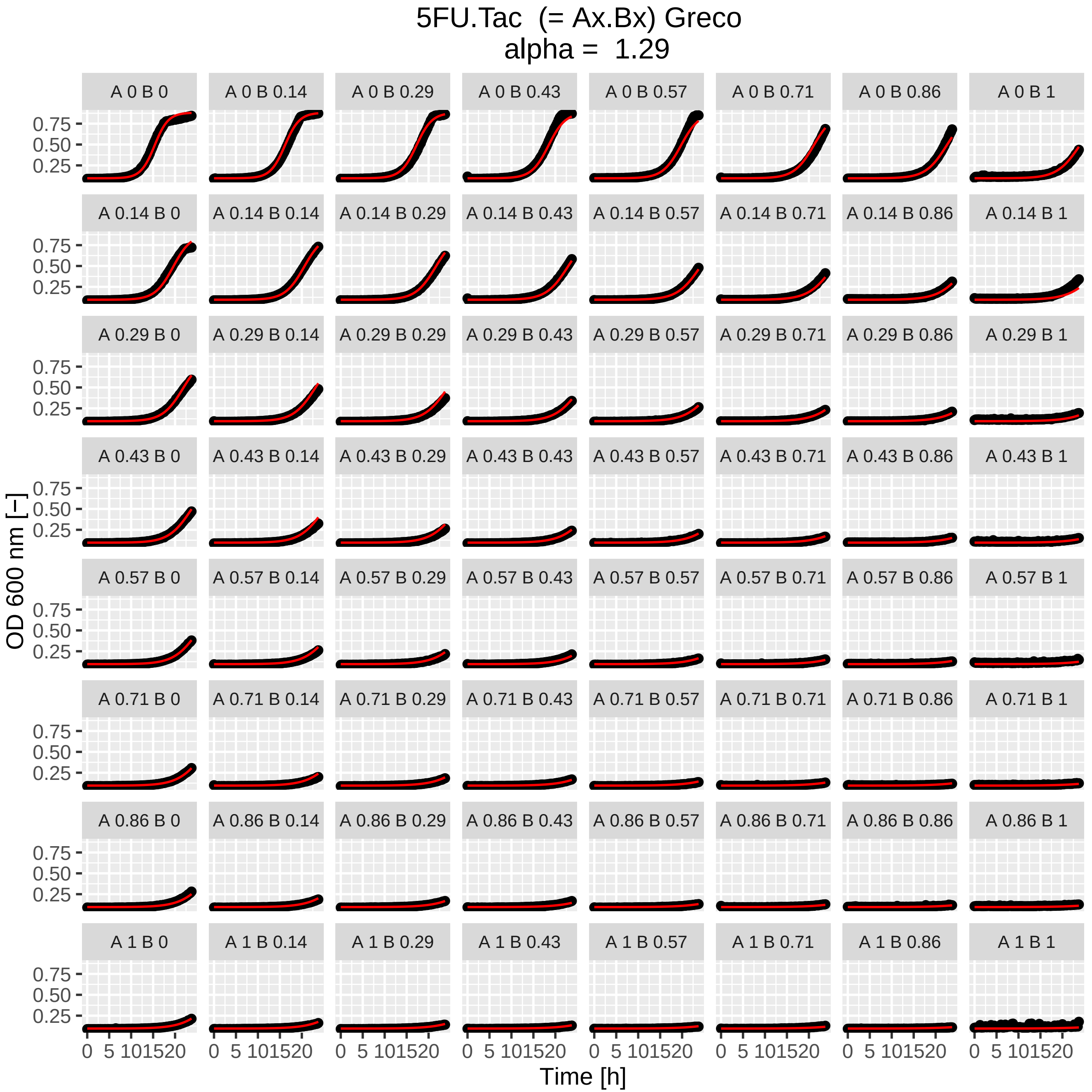
5FU.Tac (= Ax.Bx) Greco
alpha = 1.29
A 0 B 0
A 0 B 0.14
A 0 B 0.29
A 0 B 0.43
A 0 B 0.57
A 0 B 0.71
A 0 B 0.86
A 0 B 1
A 0.14 B 0
A 0.14 B 0.14
A 0.14 B 0.29
A 0.14 B 0.43
A 0.14 B 0.57
A 0.14 B 0.71
A 0.14 B 0.86
A 0.14 B 1
A 0.29 B 0
A 0.29 B 0.14
A 0.29 B 0.29
A 0.29 B 0.43
A 0.29 B 0.57
A 0.29 B 0.71
A 0.29 B 0.86
A 0.29 B 1
A 0.43 B 0
A 0.43 B 0.14
A 0.43 B 0.29
A 0.43 B 0.43
A 0.43 B 0.57
A 0.43 B 0.71
A 0.43 B 0.86
A 0.43 B 1
A 0.57 B 0
A 0.57 B 0.14
A 0.57 B 0.29
A 0.57 B 0.43
A 0.57 B 0.57
A 0.57 B 0.71
A 0.57 B 0.86
A 0.57 B 1
A 0.71 B 0
A 0.71 B 0.14
A 0.71 B 0.29
A 0.71 B 0.43
A 0.71 B 0.57
A 0.71 B 0.71
A 0.71 B 0.86
A 0.71 B 1
A 0.86 B 0
A 0.86 B 0.14
A 0.86 B 0.29
A 0.86 B 0.43
A 0.86 B 0.57
A 0.86 B 0.71
A 0.86 B 0.86
A 0.86 B 1
A 1 B 0
A 1 B 0.14
A 1 B 0.29
A 1 B 0.43
A 1 B 0.57
A 1 B 0.71
A 1 B 0.86
A 1 B 1
0.75
0.50
0.25
0 5 101520
OD 600 nm [−]
Time [h]

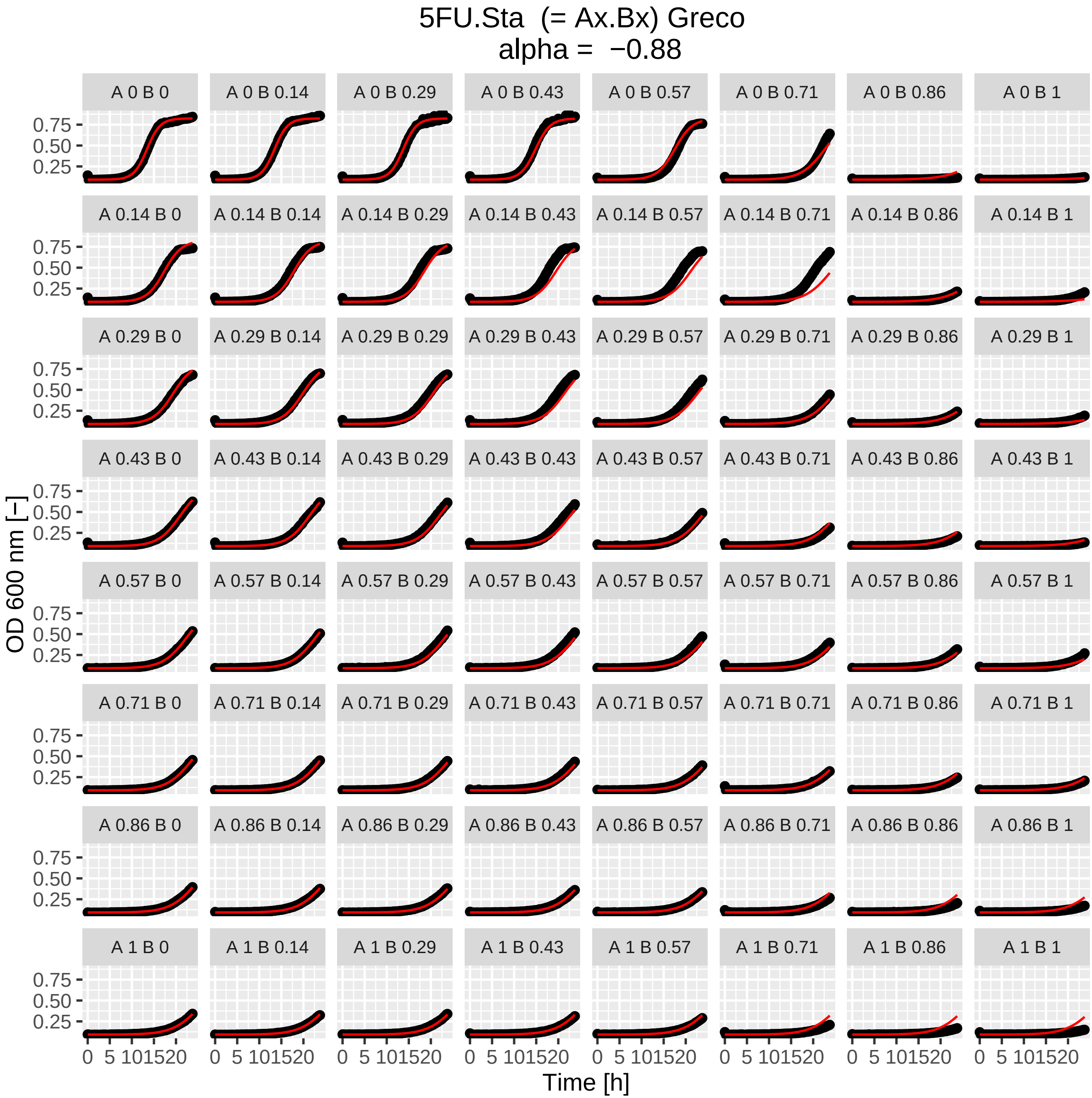

5FU.Sta (= Ax.Bx) Greco
alpha = −0.88
OD 600 nm [−]
Time [h]
A 0 B 0
A 0 B 0.14
A 0 B 0.29
A 0 B 0.43
A 0 B 0.57
A 0 B 0.71
A 0 B 0.86
A 0 B 1
A 0.14 B 0
A 0.14 B 0.14
A 0.14 B 0.29
A 0.14 B 0.43
A 0.14 B 0.57
A 0.14 B 0.71
A 0.14 B 0.86
A 0.14 B 1
A 0.29 B 0
A 0.29 B 0.14
A 0.29 B 0.29
A 0.29 B 0.43
A 0.29 B 0.57
A 0.29 B 0.71
A 0.29 B 0.86
A 0.29 B 1
A 0.43 B 0
A 0.43 B 0.14
A 0.43 B 0.29
A 0.43 B 0.43
A 0.43 B 0.57
A 0.43 B 0.71
A 0.43 B 0.86
A 0.43 B 1
A 0.57 B 0
A 0.57 B 0.14
A 0.57 B 0.29
A 0.57 B 0.43
A 0.57 B 0.57
A 0.57 B 0.71
A 0.57 B 0.86
A 0.57 B 1
A 0.71 B 0
A 0.71 B 0.14
A 0.71 B 0.29
A 0.71 B 0.43
A 0.71 B 0.57
A 0.71 B 0.71
A 0.71 B 0.86
A 0.71 B 1
A 0.86 B 0
A 0.86 B 0.14
A 0.86 B 0.29
A 0.86 B 0.43
A 0.86 B 0.57
A 0.86 B 0.71
A 0.86 B 0.86
A 0.86 B 1
A 1 B 0
A 1 B 0.14
A 1 B 0.29
A 1 B 0.43
A 1 B 0.57
A 1 B 0.71
A 1 B 0.86
A 1 B 1
0.75
0.50
0.25
0 5 101520

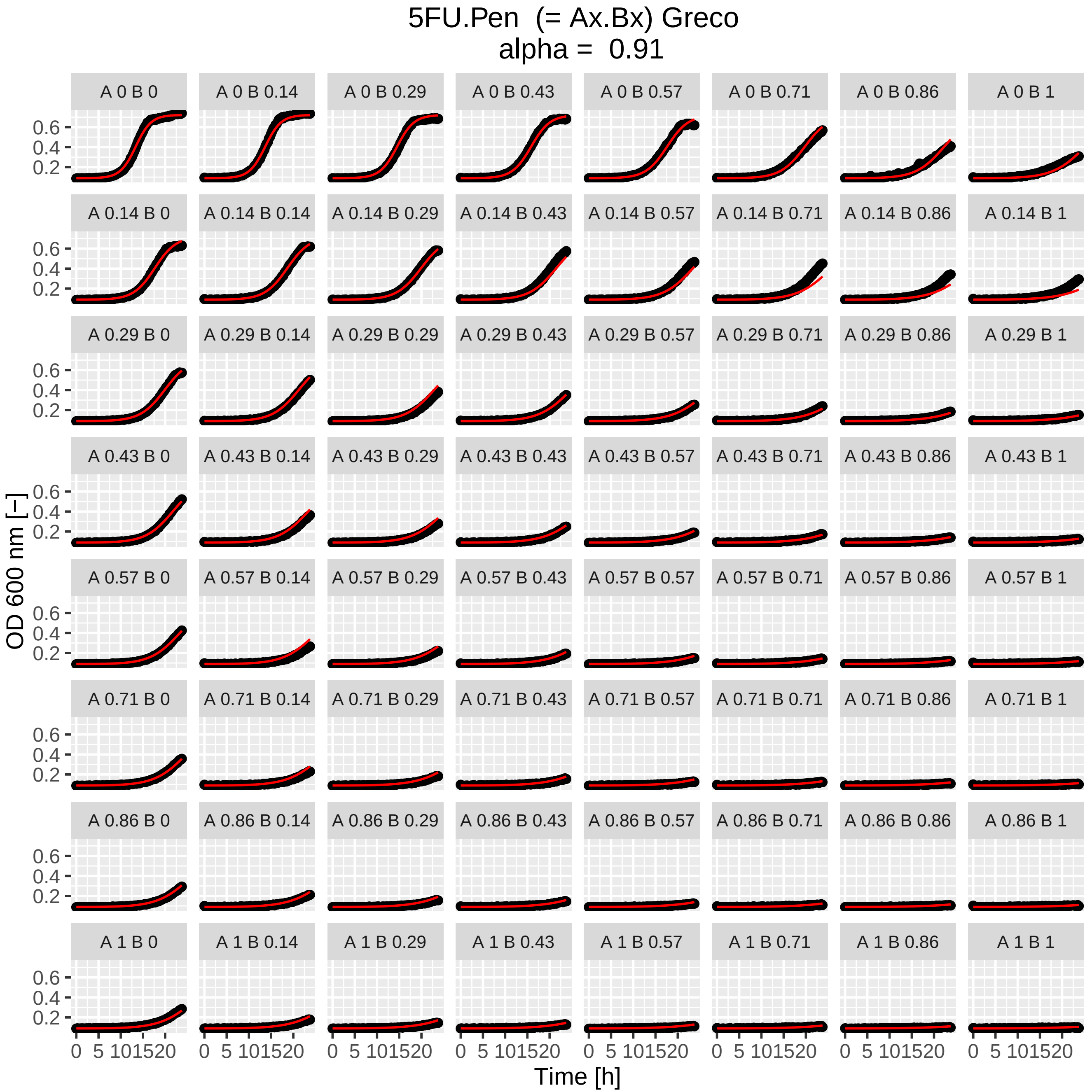
5FU.Pen (= Ax.Bx) Greco
alpha = 0.91
OD 600 nm [–]
Time [h]
A 0 B 0
A 0 B 0.14
A 0 B 0.29
A 0 B 0.43
A 0 B 0.57
A 0 B 0.71
A 0 B 0.86
A 0 B 1
A 0.14 B 0
A 0.14 B 0.14
A 0.14 B 0.29
A 0.14 B 0.43
A 0.14 B 0.57
A 0.14 B 0.71
A 0.14 B 0.86
A 0.14 B 1
A 0.29 B 0
A 0.29 B 0.14
A 0.29 B 0.29
A 0.29 B 0.43
A 0.29 B 0.57
A 0.29 B 0.71
A 0.29 B 0.86
A 0.29 B 1
A 0.43 B 0
A 0.43 B 0.14
A 0.43 B 0.29
A 0.43 B 0.43
A 0.43 B 0.57
A 0.43 B 0.71
A 0.43 B 0.86
A 0.43 B 1
A 0.57 B 0
A 0.57 B 0.14
A 0.57 B 0.29
A 0.57 B 0.43
A 0.57 B 0.57
A 0.57 B 0.71
A 0.57 B 0.86
A 0.57 B 1
A 0.71 B 0
A 0.71 B 0.14
A 0.71 B 0.29
A 0.71 B 0.43
A 0.71 B 0.57
A 0.71 B 0.71
A 0.71 B 0.86
A 0.71 B 1
A 0.86 B 0
A 0.86 B 0.14
A 0.86 B 0.29
A 0.86 B 0.43
A 0.86 B 0.57
A 0.86 B 0.71
A 0.86 B 0.86
A 0.86 B 1
A 1 B 0
A 1 B 0.14
A 1 B 0.29
A 1 B 0.43
A 1 B 0.57
A 1 B 0.71
A 1 B 0.86
A 1 B 1
0.6
0.4
0.2
0 5 101520

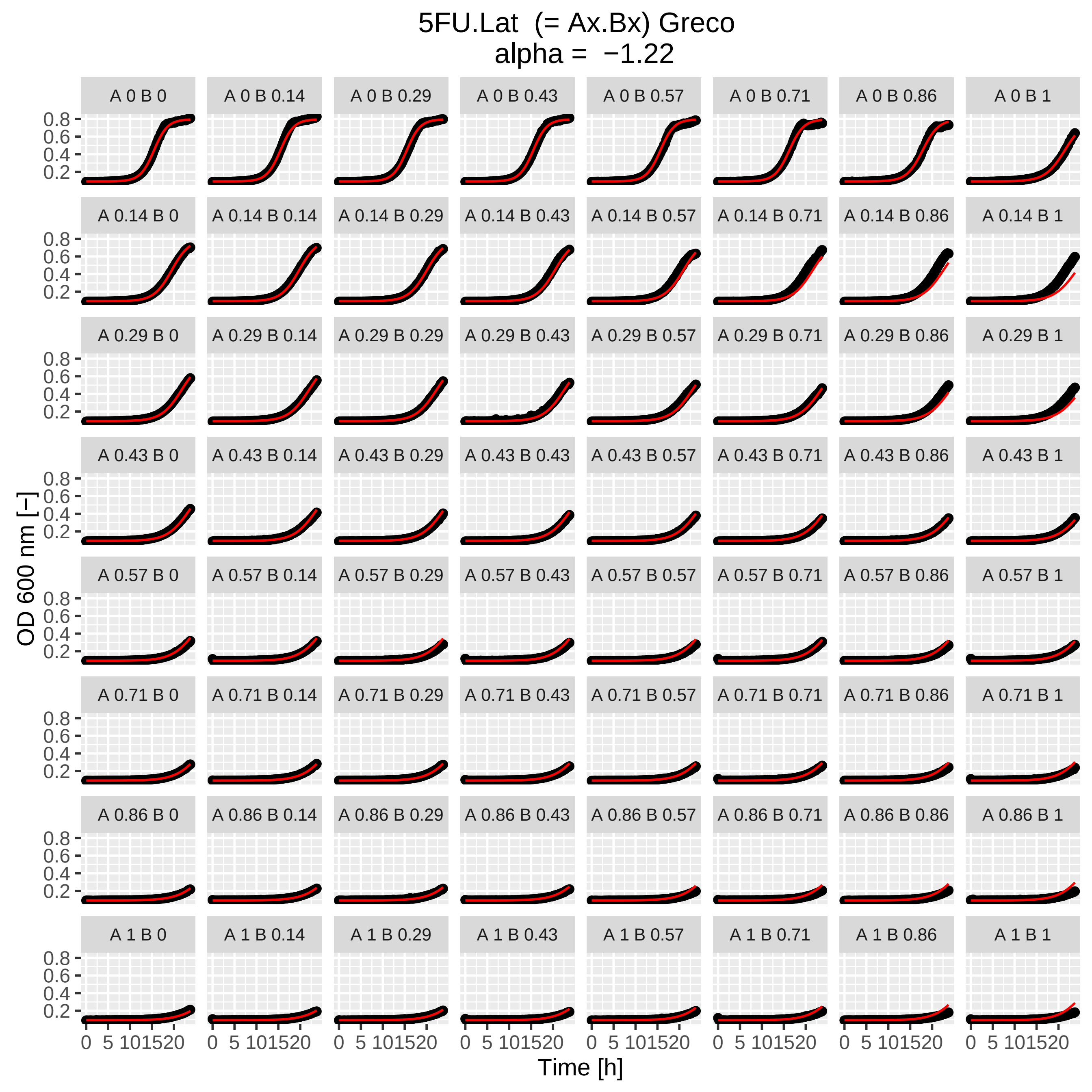

5FU.Lat (= Ax.Bx) Greco
alpha = −1.22
A 0 B 0
A 0 B 0.14
A 0 B 0.29
A 0 B 0.43
A 0 B 0.57
A 0 B 0.71
A 0 B 0.86
A 0 B 1
A 0.14 B 0
A 0.14 B 0.14
A 0.14 B 0.29
A 0.14 B 0.43
A 0.14 B 0.57
A 0.14 B 0.71
A 0.14 B 0.86
A 0.14 B 1
A 0.29 B 0
A 0.29 B 0.14
A 0.29 B 0.29
A 0.29 B 0.43
A 0.29 B 0.57
A 0.29 B 0.71
A 0.29 B 0.86
A 0.29 B 1
A 0.43 B 0
A 0.43 B 0.14
A 0.43 B 0.29
A 0.43 B 0.43
A 0.43 B 0.57
A 0.43 B 0.71
A 0.43 B 0.86
A 0.43 B 1
A 0.57 B 0
A 0.57 B 0.14
A 0.57 B 0.29
A 0.57 B 0.43
A 0.57 B 0.57
A 0.57 B 0.71
A 0.57 B 0.86
A 0.57 B 1
A 0.71 B 0
A 0.71 B 0.14
A 0.71 B 0.29
A 0.71 B 0.43
A 0.71 B 0.57
A 0.71 B 0.71
A 0.71 B 0.86
A 0.71 B 1
A 0.86 B 0
A 0.86 B 0.14
A 0.86 B 0.29
A 0.86 B 0.43
A 0.86 B 0.57
A 0.86 B 0.71
A 0.86 B 0.86
A 0.86 B 1
A 1 B 0
A 1 B 0.14
A 1 B 0.29
A 1 B 0.43
A 1 B 0.57
A 1 B 0.71
A 1 B 0.86
A 1 B 1
OD 600 nm [−]
0.8
0.6
0.4
0.2
0 5 101520
Time [h]

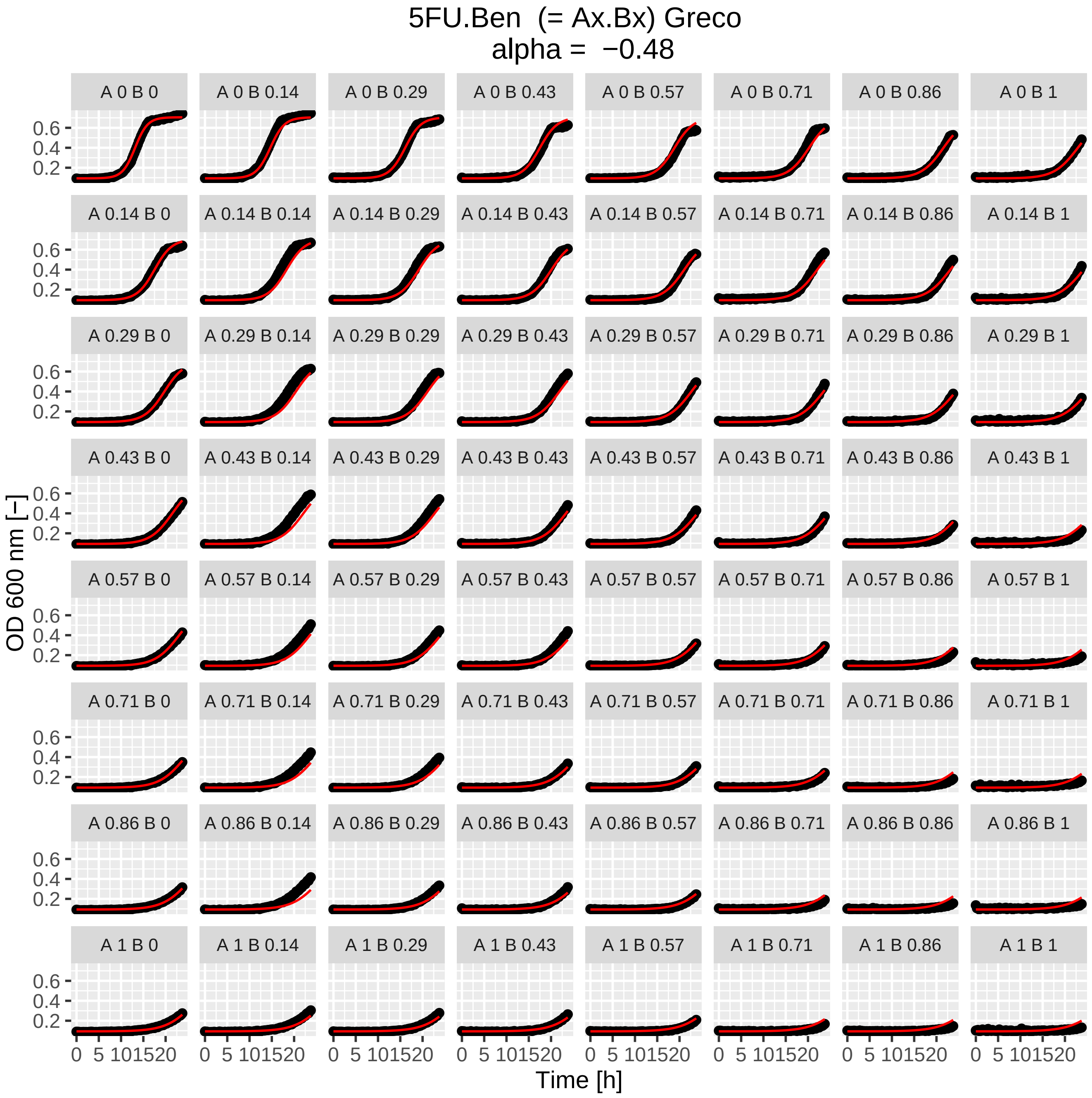
5FU.Ben (= Ax.Bx) Greco
alpha = −0.48
OD 600 nm [−]
Time [h]
A 0 B 0
A 0 B 0.14
A 0 B 0.29
A 0 B 0.43
A 0 B 0.57
A 0 B 0.71
A 0 B 0.86
A 0 B 1
A 1 B 0
A 1 B 0.14
A 1 B 0.29
A 1 B 0.43
A 1 B 0.57
A 1 B 0.71
A 1 B 0.86
A 1 B 1

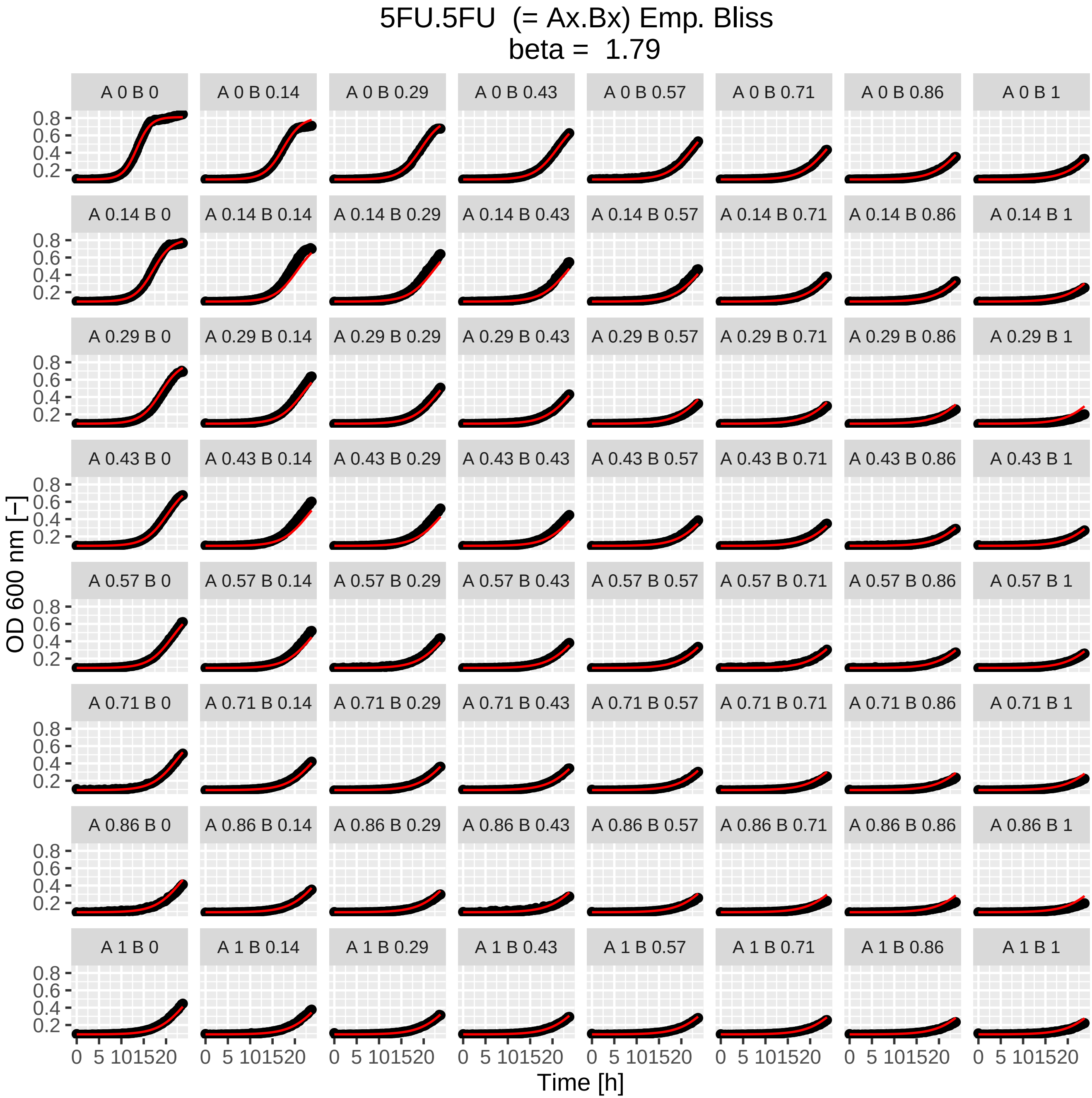
5FU.5FU (= Ax.Bx) Emp. Bliss
beta = 1.79
A 0 B 0
A 0 B 0.14
A 0 B 0.29
A 0 B 0.43
A 0 B 0.57
A 0 B 0.71
A 0 B 0.86
A 0 B 1
A 0.14 B 0
A 0.14 B 0.14
A 0.14 B 0.29
A 0.14 B 0.43
A 0.14 B 0.57
A 0.14 B 0.71
A 0.14 B 0.86
A 0.14 B 1
A 0.29 B 0
A 0.29 B 0.14
A 0.29 B 0.29
A 0.29 B 0.43
A 0.29 B 0.57
A 0.29 B 0.71
A 0.29 B 0.86
A 0.29 B 1
A 0.43 B 0
A 0.43 B 0.14
A 0.43 B 0.29
A 0.43 B 0.43
A 0.43 B 0.57
A 0.43 B 0.71
A 0.43 B 0.86
A 0.43 B 1
A 0.57 B 0
A 0.57 B 0.14
A 0.57 B 0.29
A 0.57 B 0.43
A 0.57 B 0.57
A 0.57 B 0.71
A 0.57 B 0.86
A 0.57 B 1
A 0.71 B 0
A 0.71 B 0.14
A 0.71 B 0.29
A 0.71 B 0.43
A 0.71 B 0.57
A 0.71 B 0.71
A 0.71 B 0.86
A 0.71 B 1
A 0.86 B 0
A 0.86 B 0.14
A 0.86 B 0.29
A 0.86 B 0.43
A 0.86 B 0.57
A 0.86 B 0.71
A 0.86 B 0.86
A 0.86 B 1
A 1 B 0
A 1 B 0.14
A 1 B 0.29
A 1 B 0.43
A 1 B 0.57
A 1 B 0.71
A 1 B 0.86
A 1 B 1
0.8
0.6
0.4
0.2
OD 600 nm [−]
0 5 101520
Time [h]

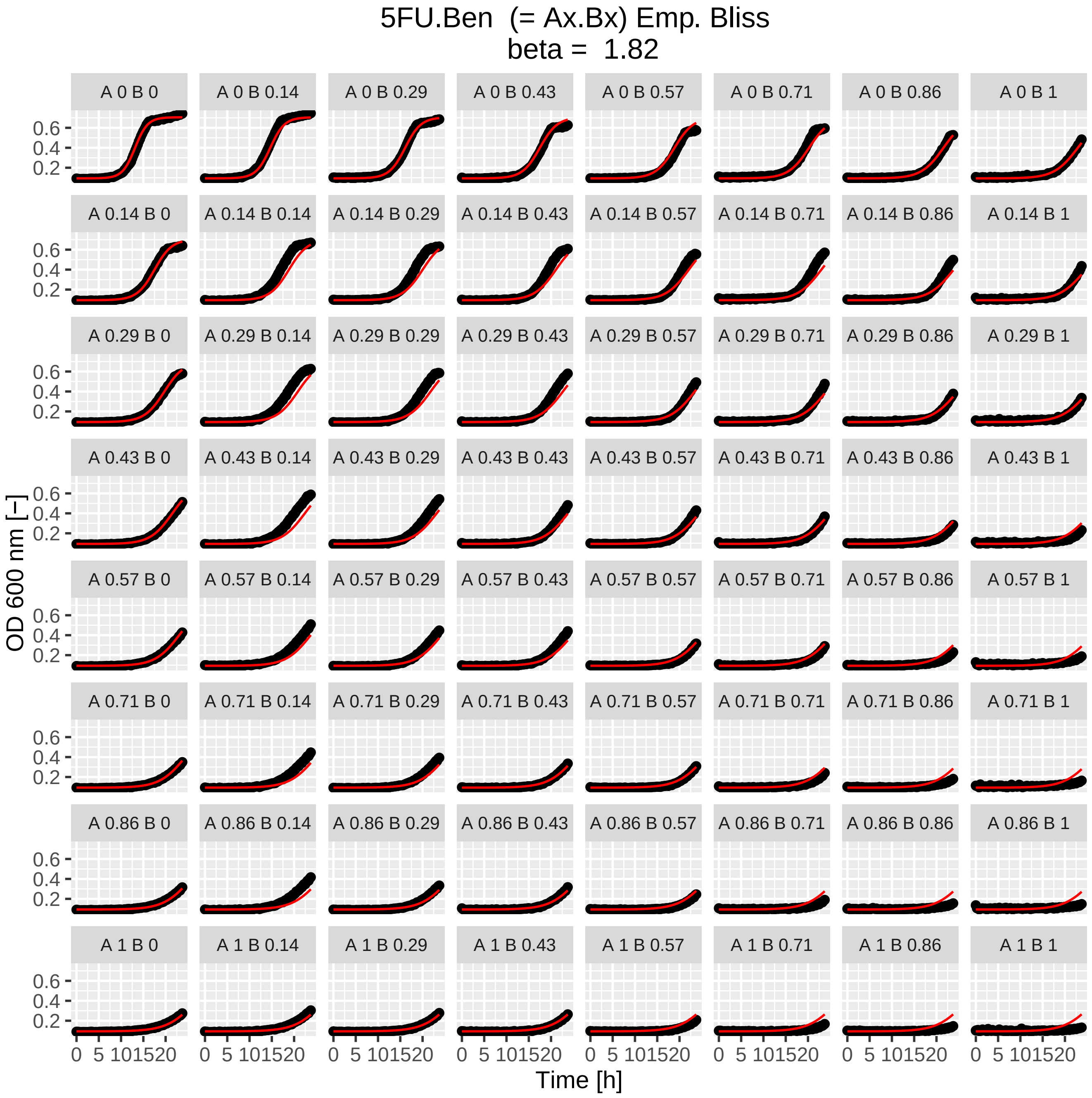

5FU.Ben (= Ax.Bx) Emp. Bliss
beta = 1.82
OD 600 nm [–]
Time [h]
A 0 B 0
A 0 B 0.14
A 0 B 0.29
A 0 B 0.43
A 0 B 0.57
A 0 B 0.71
A 0 B 0.86
A 0 B 1
A 0.14 B 0
A 0.14 B 0.14
A 0.14 B 0.29
A 0.14 B 0.43
A 0.14 B 0.57
A 0.14 B 0.71
A 0.14 B 0.86
A 0.14 B 1
A 0.29 B 0
A 0.29 B 0.14
A 0.29 B 0.29
A 0.29 B 0.43
A 0.29 B 0.57
A 0.29 B 0.71
A 0.29 B 0.86
A 0.29 B 1
A 0.43 B 0
A 0.43 B 0.14
A 0.43 B 0.29
A 0.43 B 0.43
A 0.43 B 0.57
A 0.43 B 0.71
A 0.43 B 0.86
A 0.43 B 1
A 0.57 B 0
A 0.57 B 0.14
A 0.57 B 0.29
A 0.57 B 0.43
A 0.57 B 0.57
A 0.57 B 0.71
A 0.57 B 0.86
A 0.57 B 1
A 0.71 B 0
A 0.71 B 0.14
A 0.71 B 0.29
A 0.71 B 0.43
A 0.71 B 0.57
A 0.71 B 0.71
A 0.71 B 0.86
A 0.71 B 1
A 0.86 B 0
A 0.86 B 0.14
A 0.86 B 0.29
A 0.86 B 0.43
A 0.86 B 0.57
A 0.86 B 0.71
A 0.86 B 0.86
A 0.86 B 1
A 1 B 0
A 1 B 0.14
A 1 B 0.29
A 1 B 0.43
A 1 B 0.57
A 1 B 0.71
A 1 B 0.86
A 1 B 1
0.6
0.4
0.2
0 5 101520

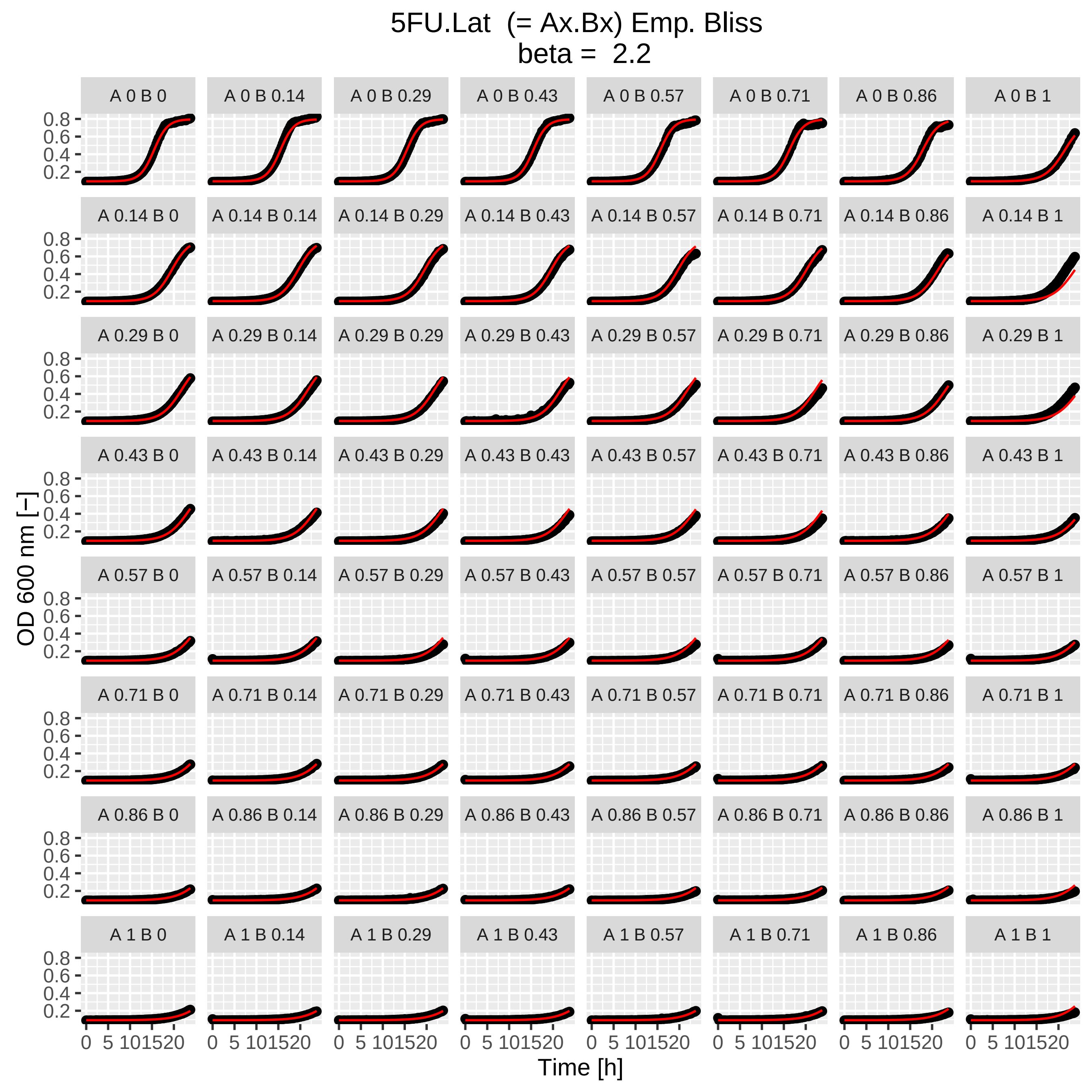

5FU.Lat (= Ax.Bx) Emp. Bliss
beta = 2.2
OD 600 nm [–]
Time [h]
0.8
0.6
0.4
0.2
0 5 101520
A 0 B 0
A 0 B 0.14
A 0 B 0.29
A 0 B 0.43
A 0 B 0.57
A 0 B 0.71
A 0 B 0.86
A 0 B 1
A 0.14 B 0
A 0.14 B 0.14
A 0.14 B 0.29
A 0.14 B 0.43
A 0.14 B 0.57
A 0.14 B 0.71
A 0.14 B 0.86
A 0.14 B 1
A 0.29 B 0
A 0.29 B 0.14
A 0.29 B 0.29
A 0.29 B 0.43
A 0.29 B 0.57
A 0.29 B 0.71
A 0.29 B 0.86
A 0.29 B 1
A 0.43 B 0
A 0.43 B 0.14
A 0.43 B 0.29
A 0.43 B 0.43
A 0.43 B 0.57
A 0.43 B 0.71
A 0.43 B 0.86
A 0.43 B 1
A 0.57 B 0
A 0.57 B 0.14
A 0.57 B 0.29
A 0.57 B 0.43
A 0.57 B 0.57
A 0.57 B 0.71
A 0.57 B 0.86
A 0.57 B 1
A 0.71 B 0
A 0.71 B 0.14
A 0.71 B 0.29
A 0.71 B 0.43
A 0.71 B 0.57
A 0.71 B 0.71
A 0.71 B 0.86
A 0.71 B 1
A 0.86 B 0
A 0.86 B 0.14
A 0.86 B 0.29
A 0.86 B 0.43
A 0.86 B 0.57
A 0.86 B 0.71
A 0.86 B 0.86
A 0.86 B 1
A 1 B 0
A 1 B 0.14
A 1 B 0.29
A 1 B 0.43
A 1 B 0.57
A 1 B 0.71
A 1 B 0.86
A 1 B 1

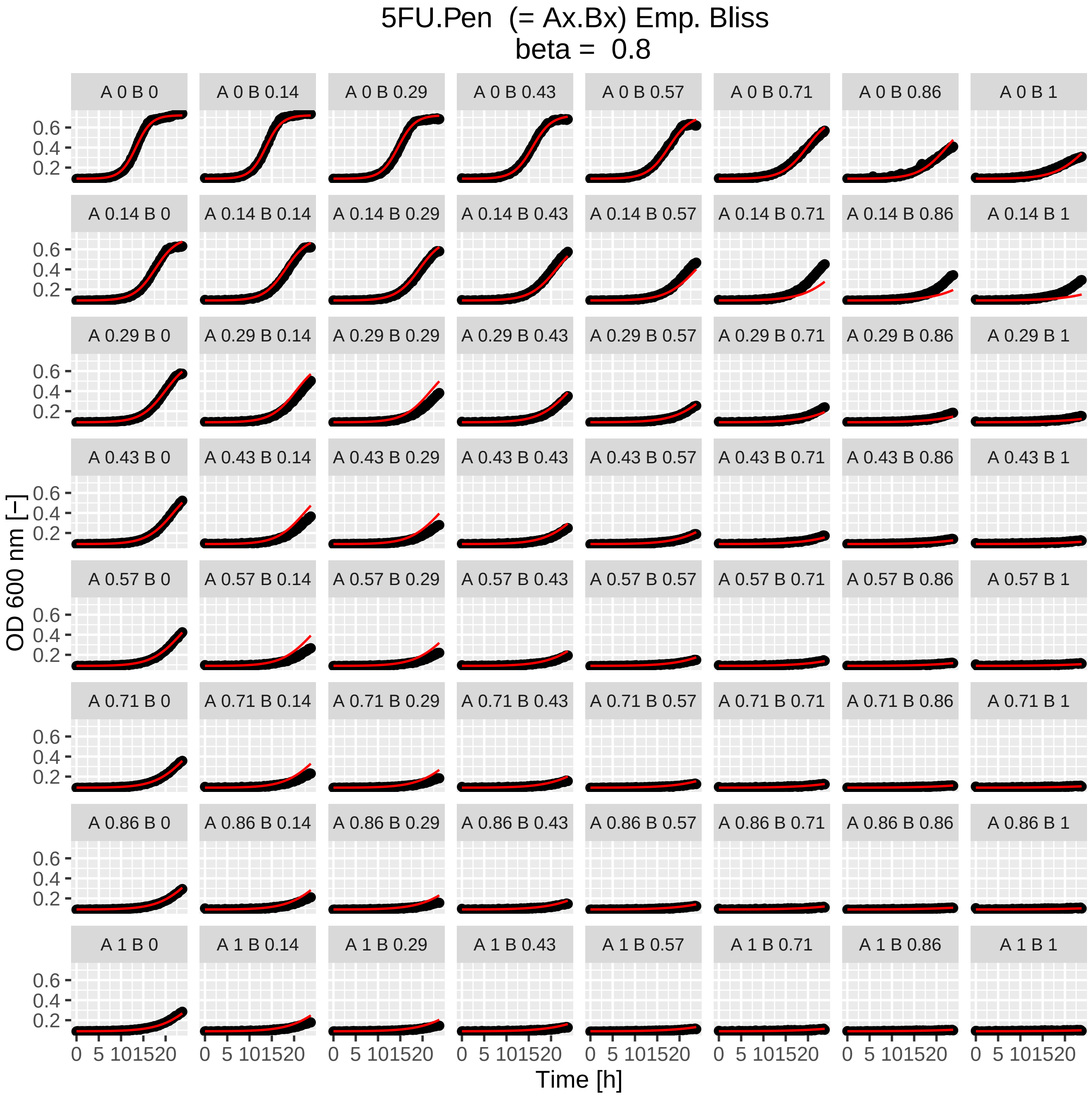
5FU.Pen (= Ax.Bx) Emp. Bliss
beta = 0.8
A 0 B 0
A 0 B 0.14
A 0 B 0.29
A 0 B 0.43
A 0 B 0.57
A 0 B 0.71
A 0 B 0.86
A 0 B 1
A 0.14 B 0
A 0.14 B 0.14
A 0.14 B 0.29
A 0.14 B 0.43
A 0.14 B 0.57
A 0.14 B 0.71
A 0.14 B 0.86
A 0.14 B 1
A 0.29 B 0
A 0.29 B 0.14
A 0.29 B 0.29
A 0.29 B 0.43
A 0.29 B 0.57
A 0.29 B 0.71
A 0.29 B 0.86
A 0.29 B 1
A 0.43 B 0
A 0.43 B 0.14
A 0.43 B 0.29
A 0.43 B 0.43
A 0.43 B 0.57
A 0.43 B 0.71
A 0.43 B 0.86
A 0.43 B 1
A 0.57 B 0
A 0.57 B 0.14
A 0.57 B 0.29
A 0.57 B 0.43
A 0.57 B 0.57
A 0.57 B 0.71
A 0.57 B 0.86
A 0.57 B 1
A 0.71 B 0
A 0.71 B 0.14
A 0.71 B 0.29
A 0.71 B 0.43
A 0.71 B 0.57
A 0.71 B 0.71
A 0.71 B 0.86
A 0.71 B 1
A 0.86 B 0
A 0.86 B 0.14
A 0.86 B 0.29
A 0.86 B 0.43
A 0.86 B 0.57
A 0.86 B 0.71
A 0.86 B 0.86
A 0.86 B 1
A 1 B 0
A 1 B 0.14
A 1 B 0.29
A 1 B 0.43
A 1 B 0.57
A 1 B 0.71
A 1 B 0.86
A 1 B 1
OD 600 nm [–]
0.6
0.4
0.2
0 5 101520
Time [h]

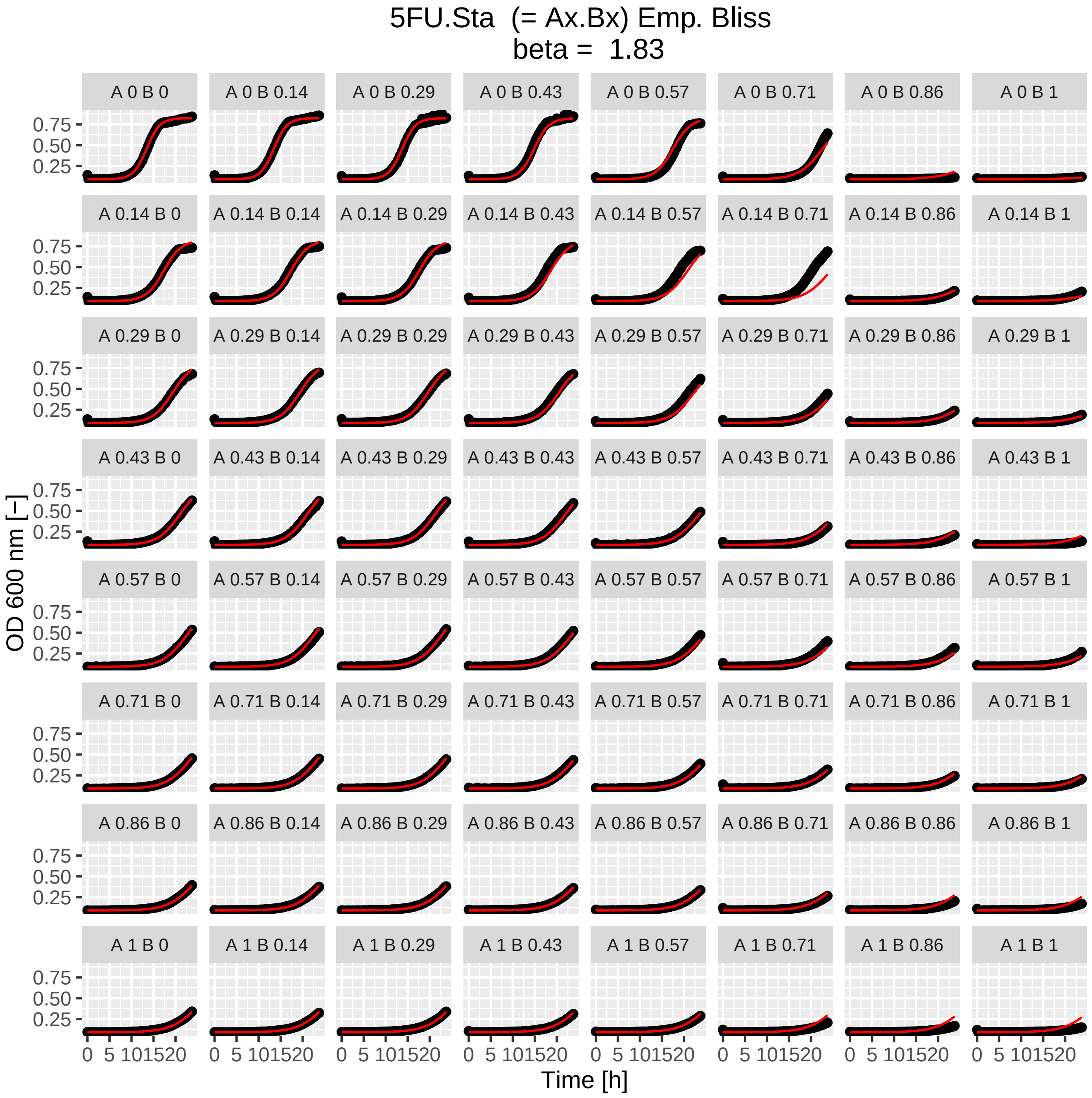

5FU.Sta (= Ax.Bx) Emp. Bliss
beta = 1.83
OD 600 nm [−]
Time [h]
0.75
0.50
0.25
0 5 101520
A 0 B 0
A 0 B 0.14
A 0 B 0.29
A 0 B 0.43
A 0 B 0.57
A 0 B 0.71
A 0 B 0.86
A 0 B 1
A 0.14 B 0
A 0.14 B 0.14
A 0.14 B 0.29
A 0.14 B 0.43
A 0.14 B 0.57
A 0.14 B 0.71
A 0.14 B 0.86
A 0.14 B 1
A 0.29 B 0
A 0.29 B 0.14
A 0.29 B 0.29
A 0.29 B 0.43
A 0.29 B 0.57
A 0.29 B 0.71
A 0.29 B 0.86
A 0.29 B 1
A 0.43 B 0
A 0.43 B 0.14
A 0.43 B 0.29
A 0.43 B 0.43
A 0.43 B 0.57
A 0.43 B 0.71
A 0.43 B 0.86
A 0.43 B 1
A 0.57 B 0
A 0.57 B 0.14
A 0.57 B 0.29
A 0.57 B 0.43
A 0.57 B 0.57
A 0.57 B 0.71
A 0.57 B 0.86
A 0.57 B 1
A 0.71 B 0
A 0.71 B 0.14
A 0.71 B 0.29
A 0.71 B 0.43
A 0.71 B 0.57
A 0.71 B 0.71
A 0.71 B 0.86
A 0.71 B 1
A 0.86 B 0
A 0.86 B 0.14
A 0.86 B 0.29
A 0.86 B 0.43
A 0.86 B 0.57
A 0.86 B 0.71
A 0.86 B 0.86
A 0.86 B 1
A 1 B 0
A 1 B 0.14
A 1 B 0.29
A 1 B 0.43
A 1 B 0.57
A 1 B 0.71
A 1 B 0.86
A 1 B 1

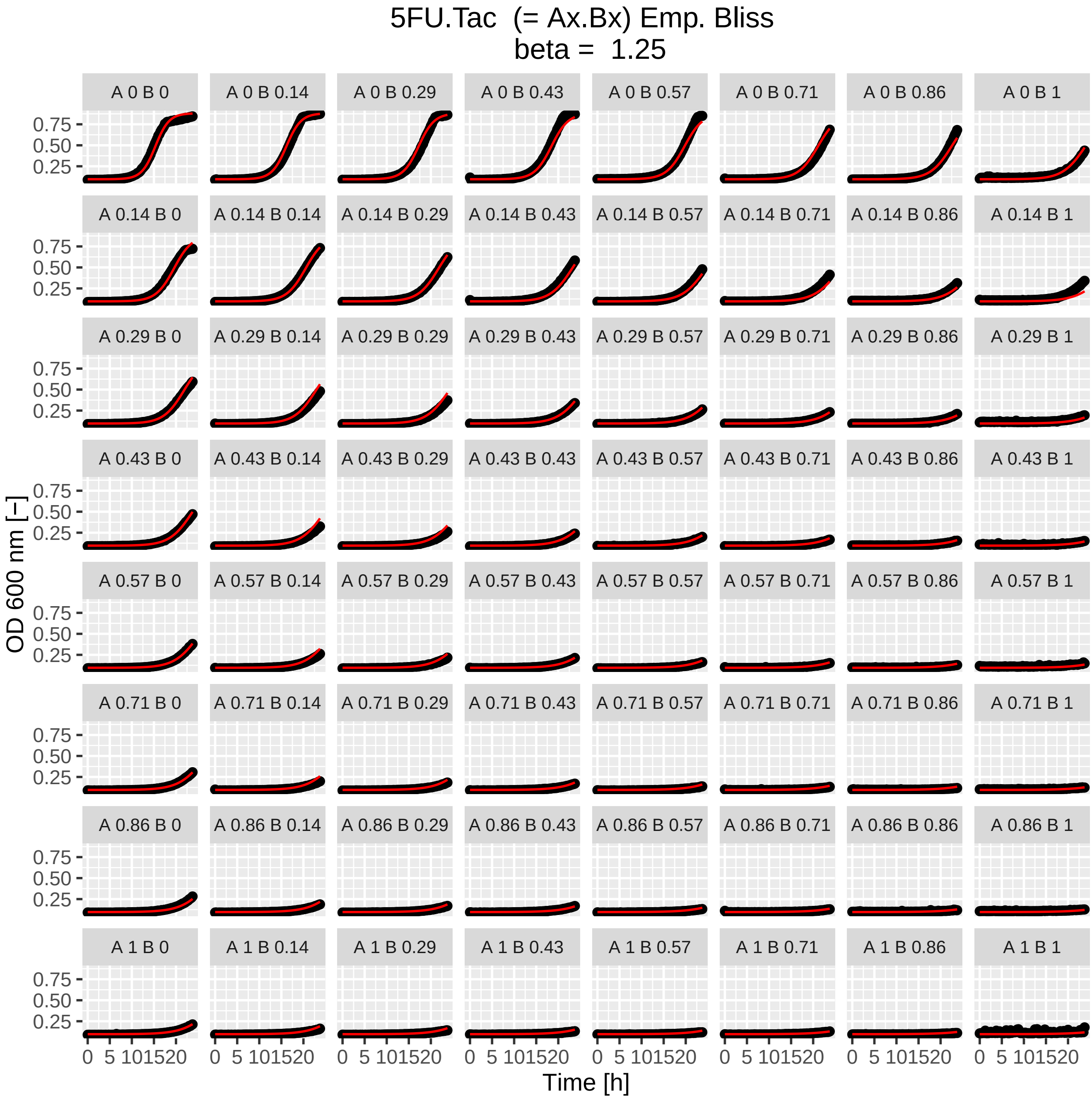
5FU.Tac (= Ax.Bx) Emp. Bliss
beta = 1.25
A 0 B 0
A 0 B 0.14
A 0 B 0.29
A 0 B 0.43
A 0 B 0.57
A 0 B 0.71
A 0 B 0.86
A 0 B 1
A 0.14 B 0
A 0.14 B 0.14
A 0.14 B 0.29
A 0.14 B 0.43
A 0.14 B 0.57
A 0.14 B 0.71
A 0.14 B 0.86
A 0.14 B 1
A 0.29 B 0
A 0.29 B 0.14
A 0.29 B 0.29
A 0.29 B 0.43
A 0.29 B 0.57
A 0.29 B 0.71
A 0.29 B 0.86
A 0.29 B 1
A 0.43 B 0
A 0.43 B 0.14
A 0.43 B 0.29
A 0.43 B 0.43
A 0.43 B 0.57
A 0.43 B 0.71
A 0.43 B 0.86
A 0.43 B 1
A 0.57 B 0
A 0.57 B 0.14
A 0.57 B 0.29
A 0.57 B 0.43
A 0.57 B 0.57
A 0.57 B 0.71
A 0.57 B 0.86
A 0.57 B 1
A 0.71 B 0
A 0.71 B 0.14
A 0.71 B 0.29
A 0.71 B 0.43
A 0.71 B 0.57
A 0.71 B 0.71
A 0.71 B 0.86
A 0.71 B 1
A 0.86 B 0
A 0.86 B 0.14
A 0.86 B 0.29
A 0.86 B 0.43
A 0.86 B 0.57
A 0.86 B 0.71
A 0.86 B 0.86
A 0.86 B 1
A 1 B 0
A 1 B 0.14
A 1 B 0.29
A 1 B 0.43
A 1 B 0.57
A 1 B 0.71
A 1 B 0.86
A 1 B 1
OD 600 nm [–]
0.75
0.50
0.25
0 5 101520
Time [h]

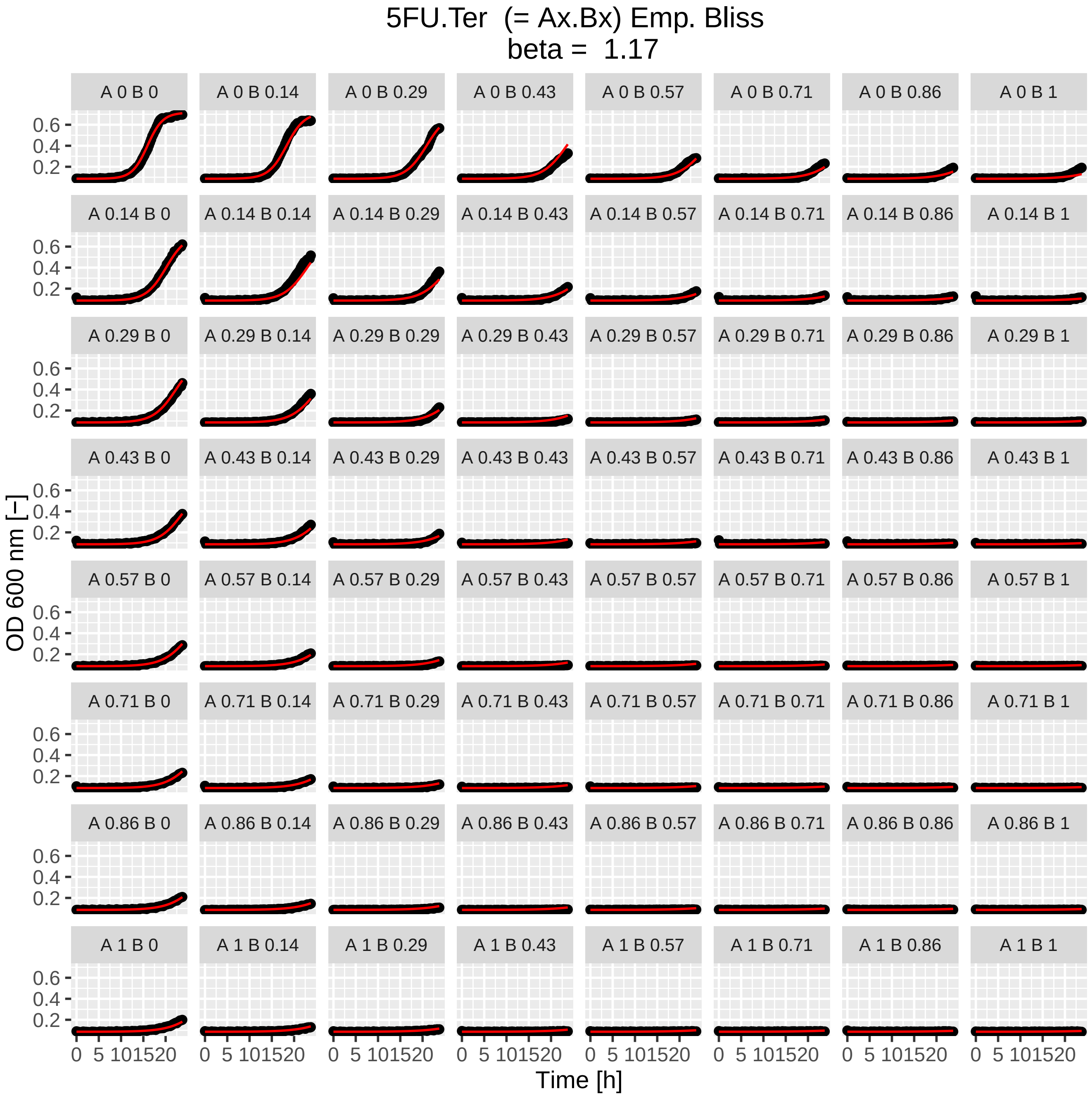
5FU.Ter (= Ax.Bx) Emp. Bliss
beta = 1.17
A 0 B 0
A 0 B 0.14
A 0 B 0.29
A 0 B 0.43
A 0 B 0.57
A 0 B 0.71
A 0 B 0.86
A 0 B 1
A 0.14 B 0
A 0.14 B 0.14
A 0.14 B 0.29
A 0.14 B 0.43
A 0.14 B 0.57
A 0.14 B 0.71
A 0.14 B 0.86
A 0.14 B 1
A 0.29 B 0
A 0.29 B 0.14
A 0.29 B 0.29
A 0.29 B 0.43
A 0.29 B 0.57
A 0.29 B 0.71
A 0.29 B 0.86
A 0.29 B 1
A 0.43 B 0
A 0.43 B 0.14
A 0.43 B 0.29
A 0.43 B 0.43
A 0.43 B 0.57
A 0.43 B 0.71
A 0.43 B 0.86
A 0.43 B 1
A 0.57 B 0
A 0.57 B 0.14
A 0.57 B 0.29
A 0.57 B 0.43
A 0.57 B 0.57
A 0.57 B 0.71
A 0.57 B 0.86
A 0.57 B 1
A 0.71 B 0
A 0.71 B 0.14
A 0.71 B 0.29
A 0.71 B 0.43
A 0.71 B 0.57
A 0.71 B 0.71
A 0.71 B 0.86
A 0.71 B 1
A 0.86 B 0
A 0.86 B 0.14
A 0.86 B 0.29
A 0.86 B 0.43
A 0.86 B 0.57
A 0.86 B 0.71
A 0.86 B 0.86
A 0.86 B 1
A 1 B 0
A 1 B 0.14
A 1 B 0.29
A 1 B 0.43
A 1 B 0.57
A 1 B 0.71
A 1 B 0.86
A 1 B 1
OD 600 nm [−]
0.2
0.4
0.6
0 5 101520
Time [h]

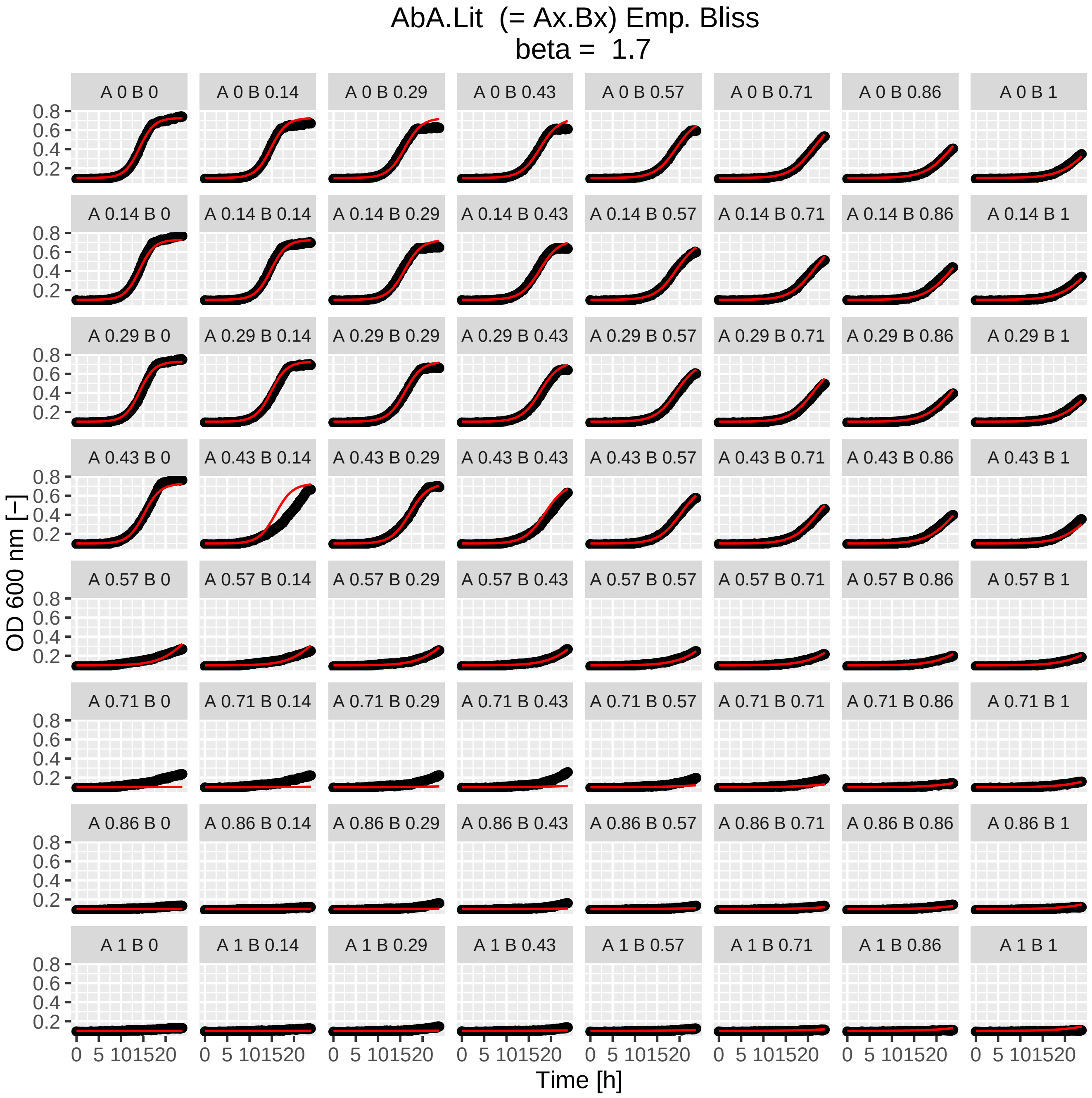
AbA.Lit (= Ax.Bx) Emp. Bliss
beta = 1.7
A 0 B 0
A 0 B 0.14
A 0 B 0.29
A 0 B 0.43
A 0 B 0.57
A 0 B 0.71
A 0 B 0.86
A 0 B 1
A 0.14 B 0
A 0.14 B 0.14
A 0.14 B 0.29
A 0.14 B 0.43
A 0.14 B 0.57
A 0.14 B 0.71
A 0.14 B 0.86
A 0.14 B 1
A 0.29 B 0
A 0.29 B 0.14
A 0.29 B 0.29
A 0.29 B 0.43
A 0.29 B 0.57
A 0.29 B 0.71
A 0.29 B 0.86
A 0.29 B 1
A 0.43 B 0
A 0.43 B 0.14
A 0.43 B 0.29
A 0.43 B 0.43
A 0.43 B 0.57
A 0.43 B 0.71
A 0.43 B 0.86
A 0.43 B 1
A 0.57 B 0
A 0.57 B 0.14
A 0.57 B 0.29
A 0.57 B 0.43
A 0.57 B 0.57
A 0.57 B 0.71
A 0.57 B 0.86
A 0.57 B 1
A 0.71 B 0
A 0.71 B 0.14
A 0.71 B 0.29
A 0.71 B 0.43
A 0.71 B 0.57
A 0.71 B 0.71
A 0.71 B 0.86
A 0.71 B 1
A 0.86 B 0
A 0.86 B 0.14
A 0.86 B 0.29
A 0.86 B 0.43
A 0.86 B 0.57
A 0.86 B 0.71
A 0.86 B 0.86
A 0.86 B 1
A 1 B 0
A 1 B 0.14
A 1 B 0.29
A 1 B 0.43
A 1 B 0.57
A 1 B 0.71
A 1 B 0.86
A 1 B 1
0.8
0.6
0.4
0.2
OD 600 nm [–]
0 5 101520
Time [h]

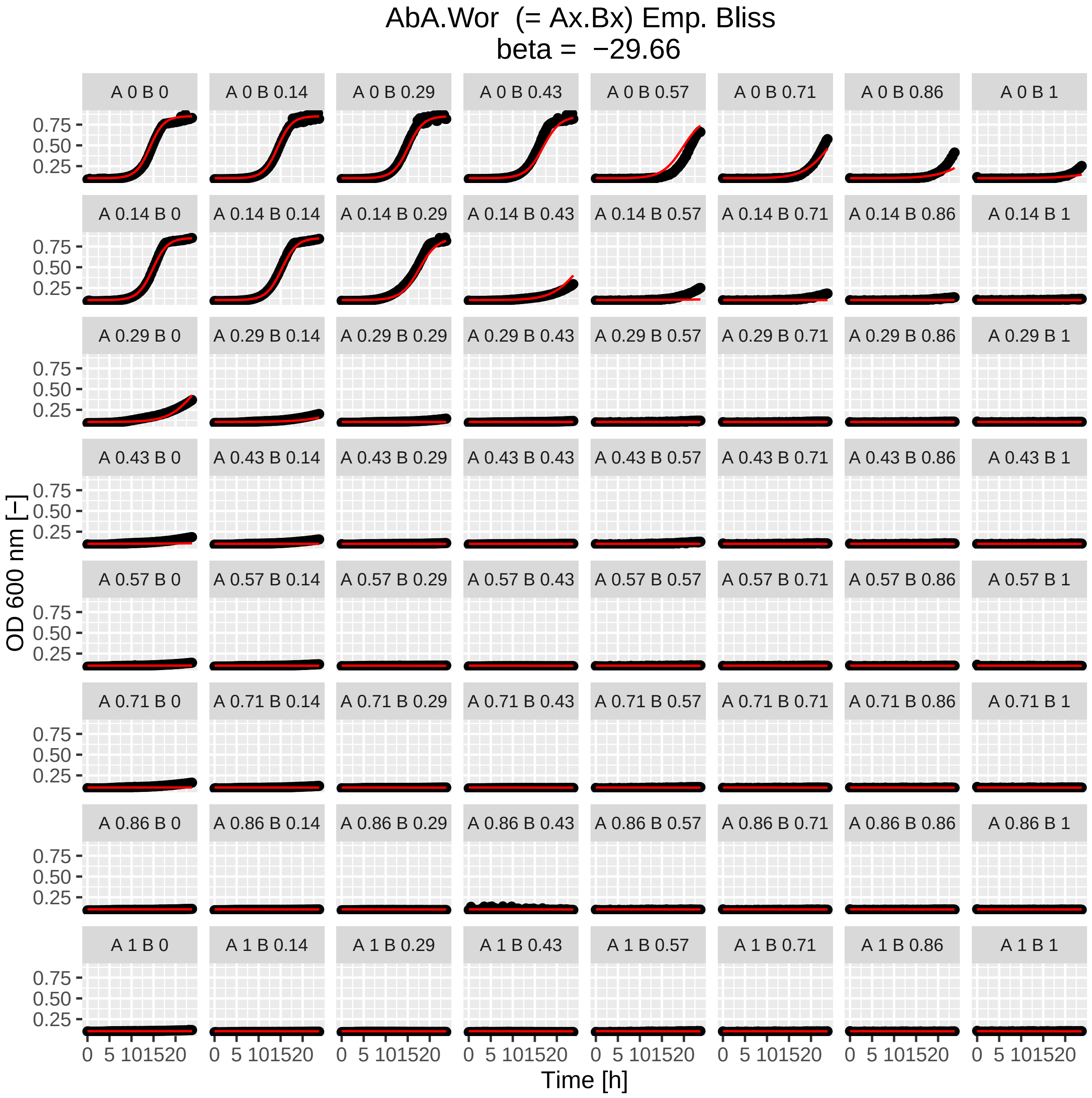
AbA.Wor (= Ax.Bx) Emp. Bliss
beta = −29.66
A 0 B 0
A 0 B 0.14
A 0 B 0.29
A 0 B 0.43
A 0 B 0.57
A 0 B 0.71
A 0 B 0.86
A 0 B 1
A 0.14 B 0
A 0.14 B 0.14
A 0.14 B 0.29
A 0.14 B 0.43
A 0.14 B 0.57
A 0.14 B 0.71
A 0.14 B 0.86
A 0.14 B 1
A 0.29 B 0
A 0.29 B 0.14
A 0.29 B 0.29
A 0.29 B 0.43
A 0.29 B 0.57
A 0.29 B 0.71
A 0.29 B 0.86
A 0.29 B 1
A 0.43 B 0
A 0.43 B 0.14
A 0.43 B 0.29
A 0.43 B 0.43
A 0.43 B 0.57
A 0.43 B 0.71
A 0.43 B 0.86
A 0.43 B 1
A 0.57 B 0
A 0.57 B 0.14
A 0.57 B 0.29
A 0.57 B 0.43
A 0.57 B 0.57
A 0.57 B 0.71
A 0.57 B 0.86
A 0.57 B 1
A 0.71 B 0
A 0.71 B 0.14
A 0.71 B 0.29
A 0.71 B 0.43
A 0.71 B 0.57
A 0.71 B 0.71
A 0.71 B 0.86
A 0.71 B 1
A 0.86 B 0
A 0.86 B 0.14
A 0.86 B 0.29
A 0.86 B 0.43
A 0.86 B 0.57
A 0.86 B 0.71
A 0.86 B 0.86
A 0.86 B 1
A 1 B 0
A 1 B 0.14
A 1 B 0.29
A 1 B 0.43
A 1 B 0.57
A 1 B 0.71
A 1 B 0.86
A 1 B 1
OD 600 nm [−]
0.75
0.50
0.25
0 5 101520
Time [h]

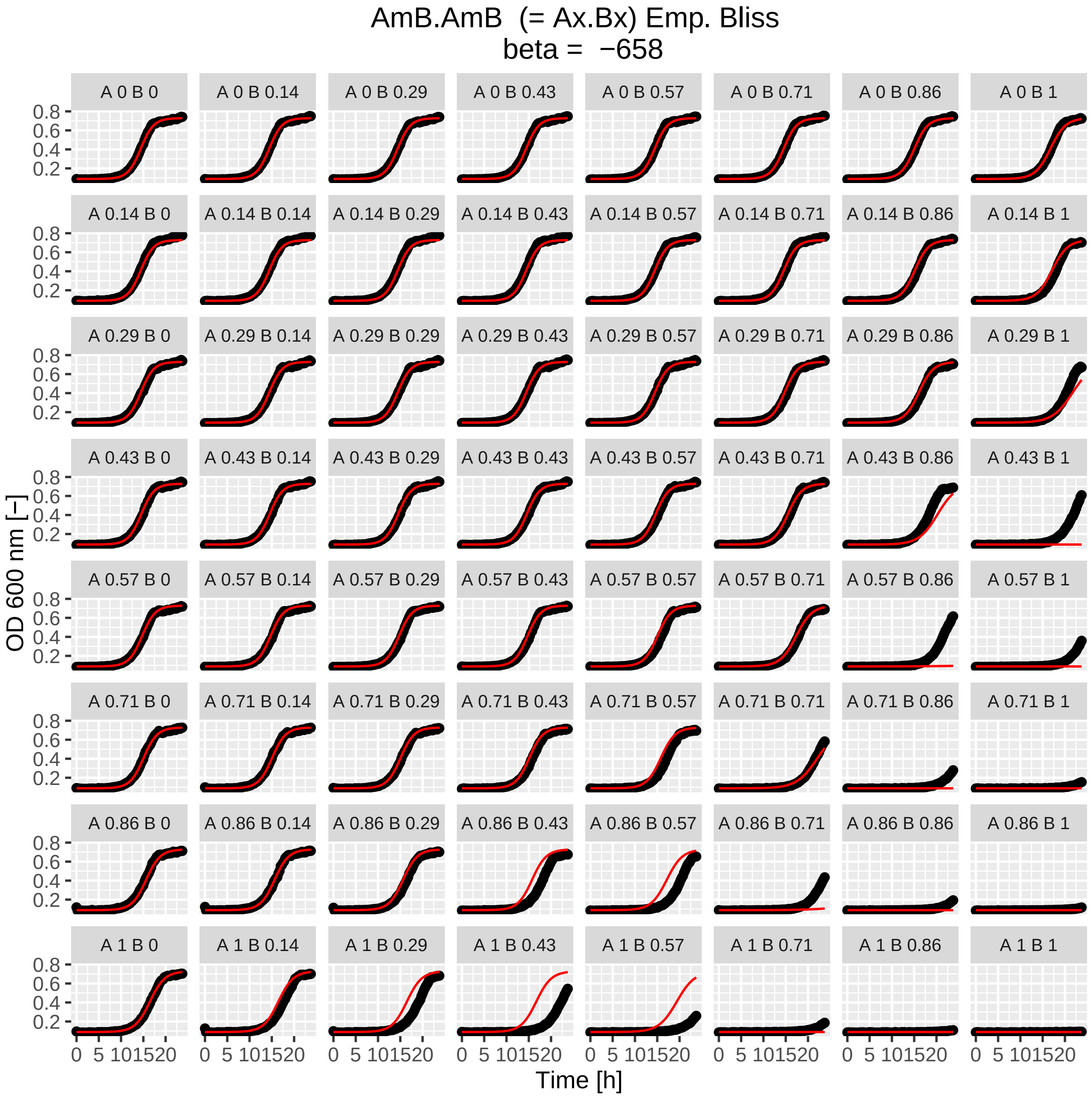
AmB.AmB (= Ax.Bx) Emp. Bliss
beta = −658
A 0 B 0
A 0 B 0.14
A 0 B 0.29
A 0 B 0.43
A 0 B 0.57
A 0 B 0.71
A 0 B 0.86
A 0 B 1
A 0.14 B 0
A 0.14 B 0.14
A 0.14 B 0.29
A 0.14 B 0.43
A 0.14 B 0.57
A 0.14 B 0.71
A 0.14 B 0.86
A 0.14 B 1
A 0.29 B 0
A 0.29 B 0.14
A 0.29 B 0.29
A 0.29 B 0.43
A 0.29 B 0.57
A 0.29 B 0.71
A 0.29 B 0.86
A 0.29 B 1
A 0.43 B 0
A 0.43 B 0.14
A 0.43 B 0.29
A 0.43 B 0.43
A 0.43 B 0.57
A 0.43 B 0.71
A 0.43 B 0.86
A 0.43 B 1
A 0.57 B 0
A 0.57 B 0.14
A 0.57 B 0.29
A 0.57 B 0.43
A 0.57 B 0.57
A 0.57 B 0.71
A 0.57 B 0.86
A 0.57 B 1
A 0.71 B 0
A 0.71 B 0.14
A 0.71 B 0.29
A 0.71 B 0.43
A 0.71 B 0.57
A 0.71 B 0.71
A 0.71 B 0.86
A 0.71 B 1
A 0.86 B 0
A 0.86 B 0.14
A 0.86 B 0.29
A 0.86 B 0.43
A 0.86 B 0.57
A 0.86 B 0.71
A 0.86 B 0.86
A 0.86 B 1
A 1 B 0
A 1 B 0.14
A 1 B 0.29
A 1 B 0.43
A 1 B 0.57
A 1 B 0.71
A 1 B 0.86
A 1 B 1
OD 600 nm [−]
0.2
0.4
0.6
0.8
0 5 101520
Time [h]

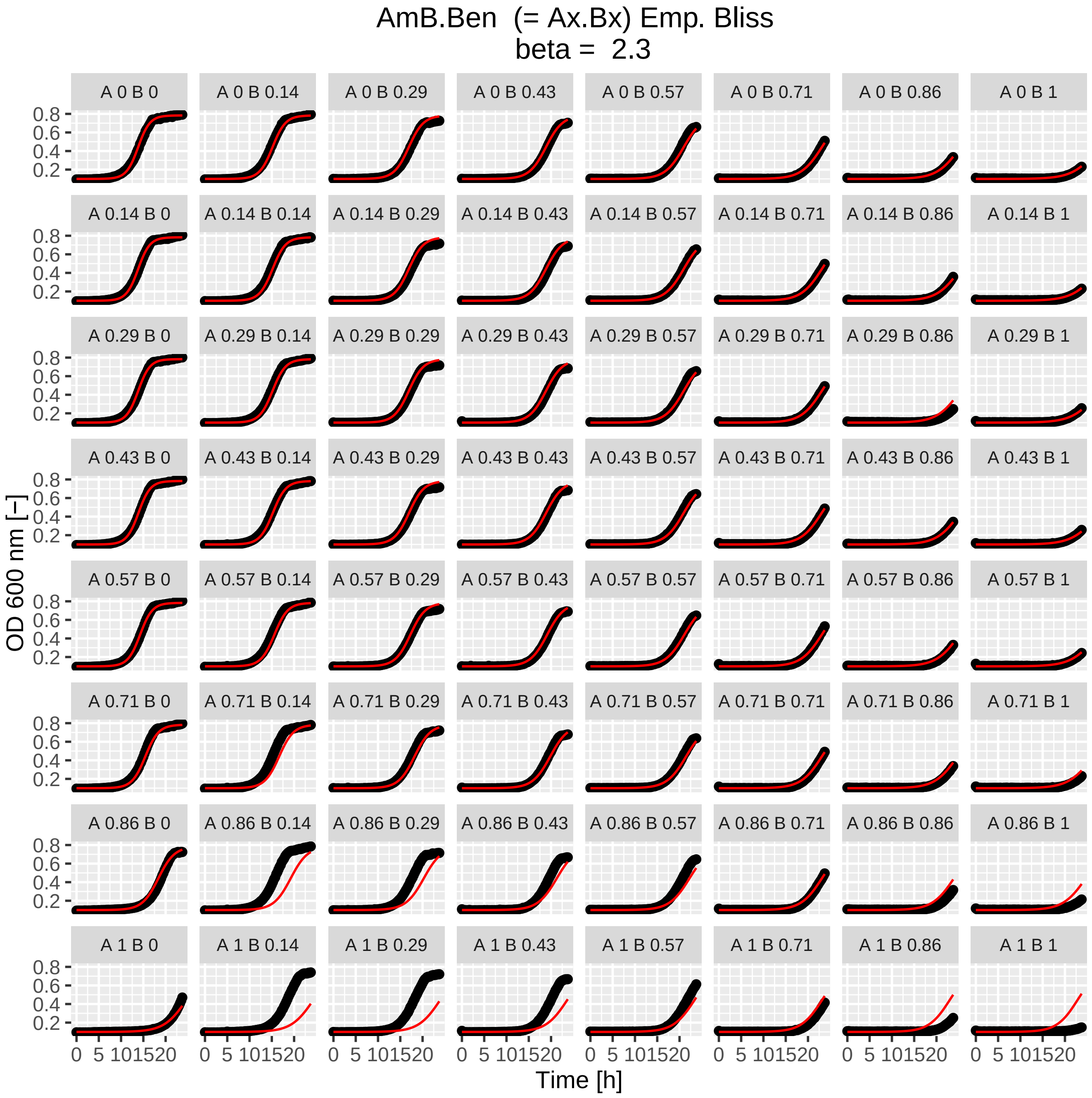
AmB.Ben (= Ax.Bx) Emp. Bliss
beta = 2.3
OD 600 nm [−]
Time [h]
0.2 0.4 0.6 0.8
0 5 101520
A 0 B 0
A 0 B 0.14
A 0 B 0.29
A 0 B 0.43
A 0 B 0.57
A 0 B 0.71
A 0 B 0.86
A 0 B 1
A 0.14 B 0
A 0.14 B 0.14
A 0.14 B 0.29
A 0.14 B 0.43
A 0.14 B 0.57
A 0.14 B 0.71
A 0.14 B 0.86
A 0.14 B 1
A 0.29 B 0
A 0.29 B 0.14
A 0.29 B 0.29
A 0.29 B 0.43
A 0.29 B 0.57
A 0.29 B 0.71
A 0.29 B 0.86
A 0.29 B 1
A 0.43 B 0
A 0.43 B 0.14
A 0.43 B 0.29
A 0.43 B 0.43
A 0.43 B 0.57
A 0.43 B 0.71
A 0.43 B 0.86
A 0.43 B 1
A 0.57 B 0
A 0.57 B 0.14
A 0.57 B 0.29
A 0.57 B 0.43
A 0.57 B 0.57
A 0.57 B 0.71
A 0.57 B 0.86
A 0.57 B 1
A 0.71 B 0
A 0.71 B 0.14
A 0.71 B 0.29
A 0.71 B 0.43
A 0.71 B 0.57
A 0.71 B 0.71
A 0.71 B 0.86
A 0.71 B 1
A 0.86 B 0
A 0.86 B 0.14
A 0.86 B 0.29
A 0.86 B 0.43
A 0.86 B 0.57
A 0.86 B 0.71
A 0.86 B 0.86
A 0.86 B 1
A 1 B 0
A 1 B 0.14
A 1 B 0.29
A 1 B 0.43
A 1 B 0.57
A 1 B 0.71
A 1 B 0.86
A 1 B 1

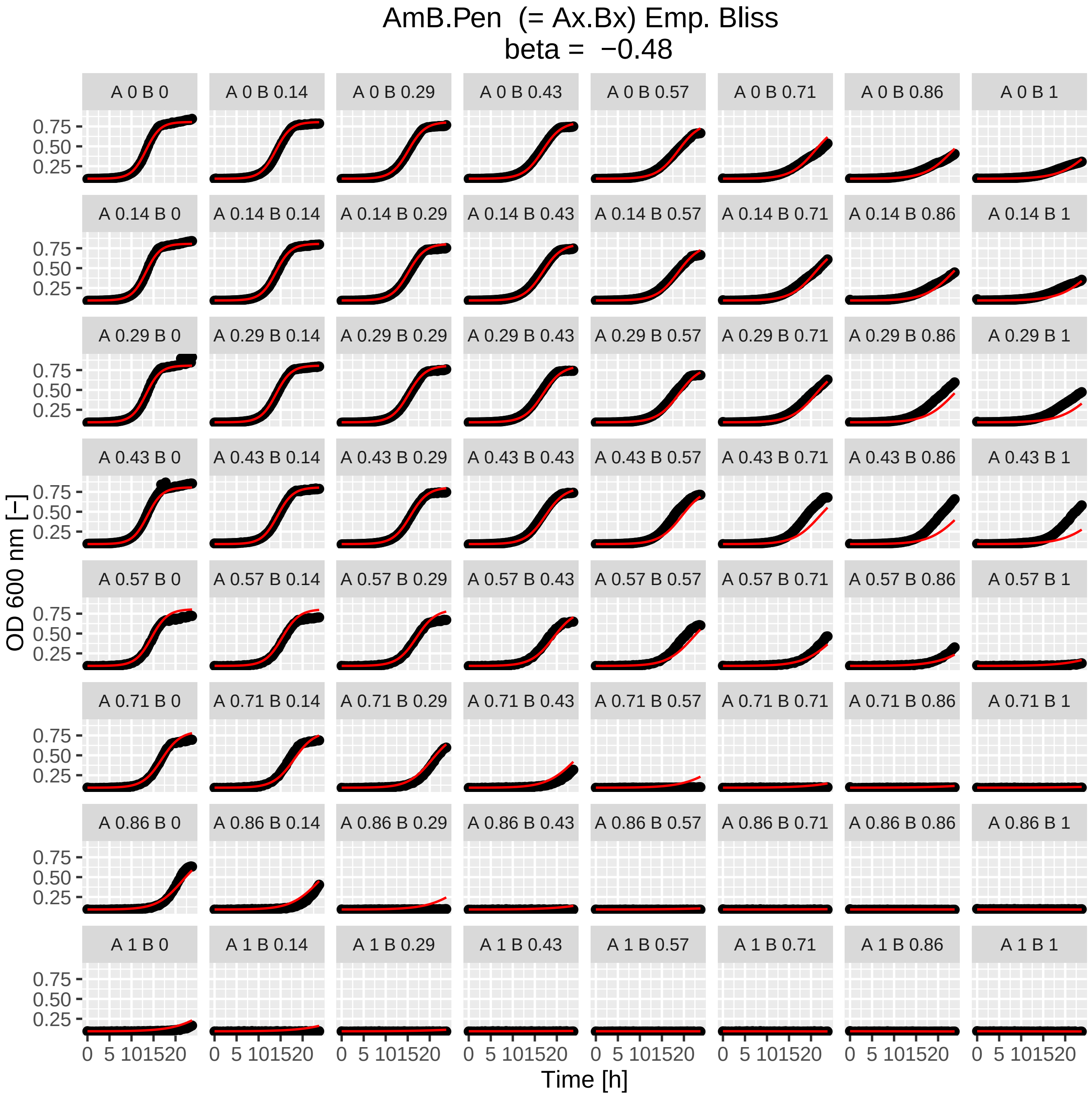
AmB.Pen (= Ax.Bx) Emp. Bliss
beta = −0.48
OD 600 nm [−]
Time [h]
0.75
0.50
0.25
0 5 101520
A 0 B 0
A 0 B 0.14
A 0 B 0.29
A 0 B 0.43
A 0 B 0.57
A 0 B 0.71
A 0 B 0.86
A 0 B 1
A 0.14 B 0
A 0.14 B 0.14
A 0.14 B 0.29
A 0.14 B 0.43
A 0.14 B 0.57
A 0.14 B 0.71
A 0.14 B 0.86
A 0.14 B 1
A 0.29 B 0
A 0.29 B 0.14
A 0.29 B 0.29
A 0.29 B 0.43
A 0.29 B 0.57
A 0.29 B 0.71
A 0.29 B 0.86
A 0.29 B 1
A 0.43 B 0
A 0.43 B 0.14
A 0.43 B 0.29
A 0.43 B 0.43
A 0.43 B 0.57
A 0.43 B 0.71
A 0.43 B 0.86
A 0.43 B 1
A 0.57 B 0
A 0.57 B 0.14
A 0.57 B 0.29
A 0.57 B 0.43
A 0.57 B 0.57
A 0.57 B 0.71
A 0.57 B 0.86
A 0.57 B 1
A 0.71 B 0
A 0.71 B 0.14
A 0.71 B 0.29
A 0.71 B 0.43
A 0.71 B 0.57
A 0.71 B 0.71
A 0.71 B 0.86
A 0.71 B 1
A 0.86 B 0
A 0.86 B 0.14
A 0.86 B 0.29
A 0.86 B 0.43
A 0.86 B 0.57
A 0.86 B 0.71
A 0.86 B 0.86
A 0.86 B 1
A 1 B 0
A 1 B 0.14
A 1 B 0.29
A 1 B 0.43
A 1 B 0.57
A 1 B 0.71
A 1 B 0.86
A 1 B 1

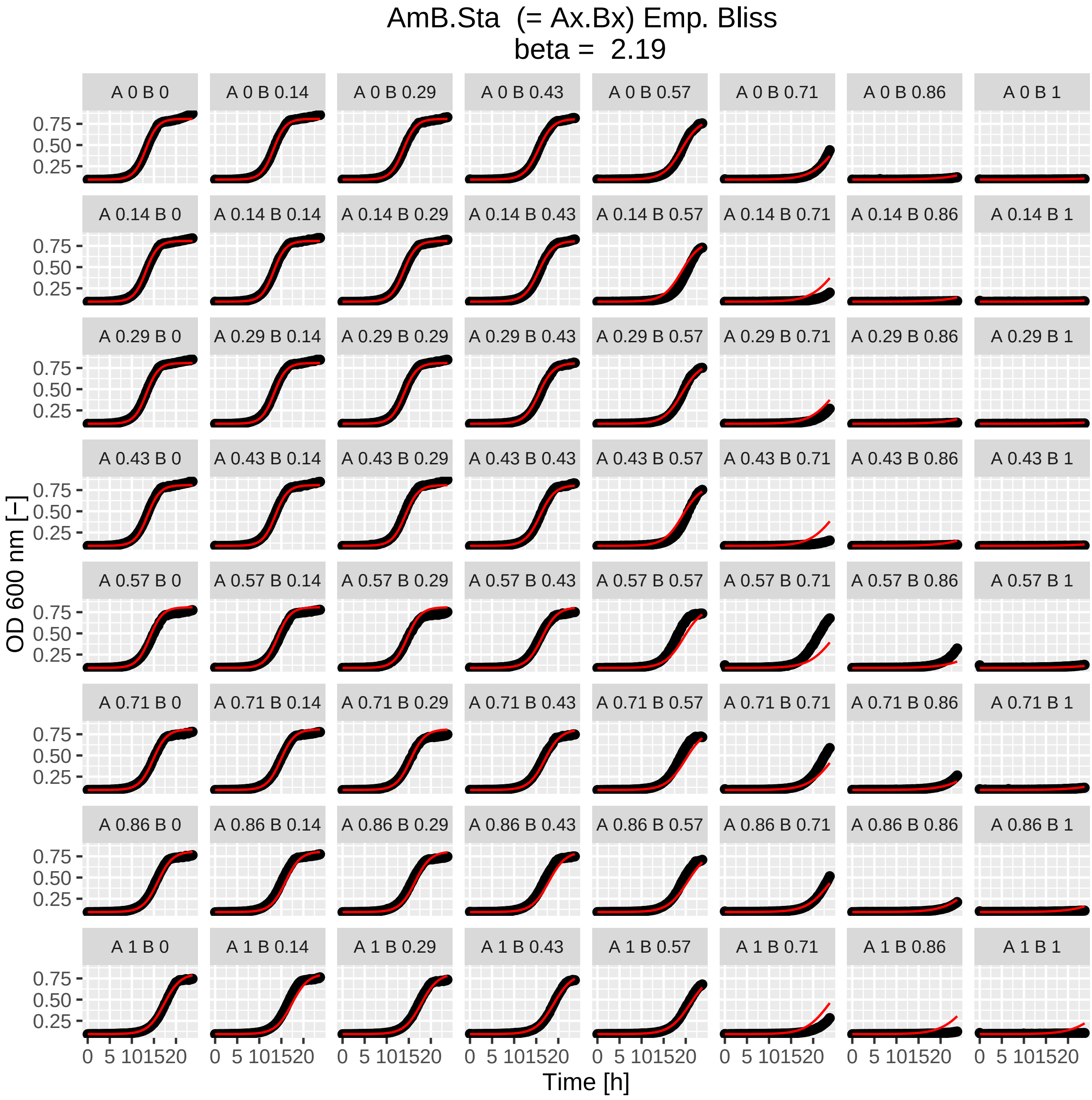
AmB.Sta (= Ax.Bx) Emp. Bliss
beta = 2.19
A 0 B 0
A 0 B 0.14
A 0 B 0.29
A 0 B 0.43
A 0 B 0.57
A 0 B 0.71
A 0 B 0.86
A 0 B 1
A 0.14 B 0
A 0.14 B 0.14
A 0.14 B 0.29
A 0.14 B 0.43
A 0.14 B 0.57
A 0.14 B 0.71
A 0.14 B 0.86
A 0.14 B 1
A 0.29 B 0
A 0.29 B 0.14
A 0.29 B 0.29
A 0.29 B 0.43
A 0.29 B 0.57
A 0.29 B 0.71
A 0.29 B 0.86
A 0.29 B 1
A 0.43 B 0
A 0.43 B 0.14
A 0.43 B 0.29
A 0.43 B 0.43
A 0.43 B 0.57
A 0.43 B 0.71
A 0.43 B 0.86
A 0.43 B 1
A 0.57 B 0
A 0.57 B 0.14
A 0.57 B 0.29
A 0.57 B 0.43
A 0.57 B 0.57
A 0.57 B 0.71
A 0.57 B 0.86
A 0.57 B 1
A 0.71 B 0
A 0.71 B 0.14
A 0.71 B 0.29
A 0.71 B 0.43
A 0.71 B 0.57
A 0.71 B 0.71
A 0.71 B 0.86
A 0.71 B 1
A 0.86 B 0
A 0.86 B 0.14
A 0.86 B 0.29
A 0.86 B 0.43
A 0.86 B 0.57
A 0.86 B 0.71
A 0.86 B 0.86
A 0.86 B 1
A 1 B 0
A 1 B 0.14
A 1 B 0.29
A 1 B 0.43
A 1 B 0.57
A 1 B 0.71
A 1 B 0.86
A 1 B 1
0.75
0.50
0.25
0 5 101520
OD 600 nm [–]
Time [h]

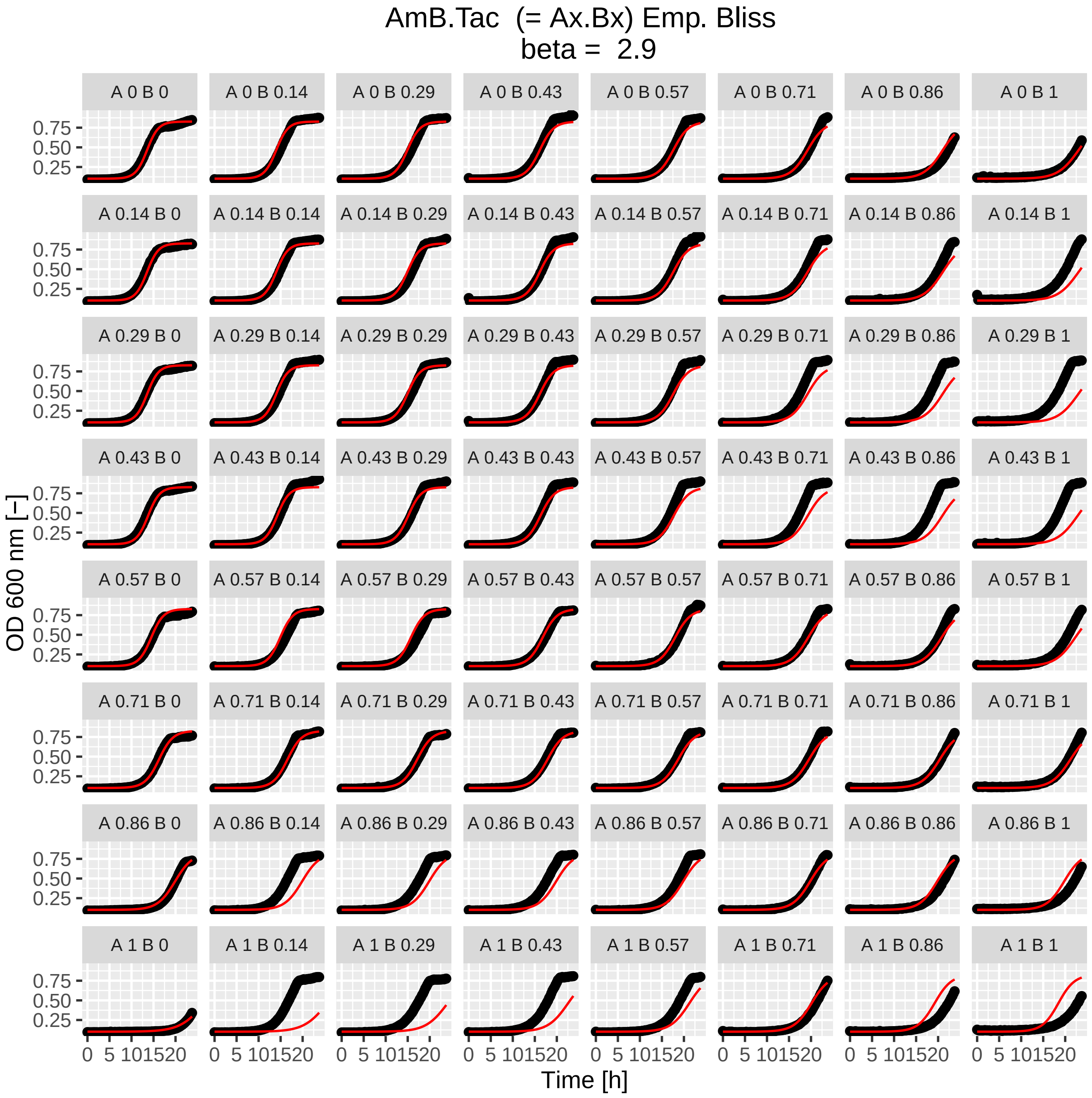
AmB.Tac (= Ax.Bx) Emp. Bliss
beta = 2.9
OD 600 nm [–]
Time [h]
A 0 B 0
A 0 B 0.14
A 0 B 0.29
A 0 B 0.43
A 0 B 0.57
A 0 B 0.71
A 0 B 0.86
A 0 B 1
A 0.14 B 0
A 0.14 B 0.14
A 0.14 B 0.29
A 0.14 B 0.43
A 0.14 B 0.57
A 0.14 B 0.71
A 0.14 B 0.86
A 0.14 B 1
A 0.29 B 0
A 0.29 B 0.14
A 0.29 B 0.29
A 0.29 B 0.43
A 0.29 B 0.57
A 0.29 B 0.71
A 0.29 B 0.86
A 0.29 B 1
A 0.43 B 0
A 0.43 B 0.14
A 0.43 B 0.29
A 0.43 B 0.43
A 0.43 B 0.57
A 0.43 B 0.71
A 0.43 B 0.86
A 0.43 B 1
A 0.57 B 0
A 0.57 B 0.14
A 0.57 B 0.29
A 0.57 B 0.43
A 0.57 B 0.57
A 0.57 B 0.71
A 0.57 B 0.86
A 0.57 B 1
A 0.71 B 0
A 0.71 B 0.14
A 0.71 B 0.29
A 0.71 B 0.43
A 0.71 B 0.57
A 0.71 B 0.71
A 0.71 B 0.86
A 0.71 B 1
A 0.86 B 0
A 0.86 B 0.14
A 0.86 B 0.29
A 0.86 B 0.43
A 0.86 B 0.57
A 0.86 B 0.71
A 0.86 B 0.86
A 0.86 B 1
A 1 B 0
A 1 B 0.14
A 1 B 0.29
A 1 B 0.43
A 1 B 0.57
A 1 B 0.71
A 1 B 0.86
A 1 B 1
0.75
0.50
0.25
0 5 101520

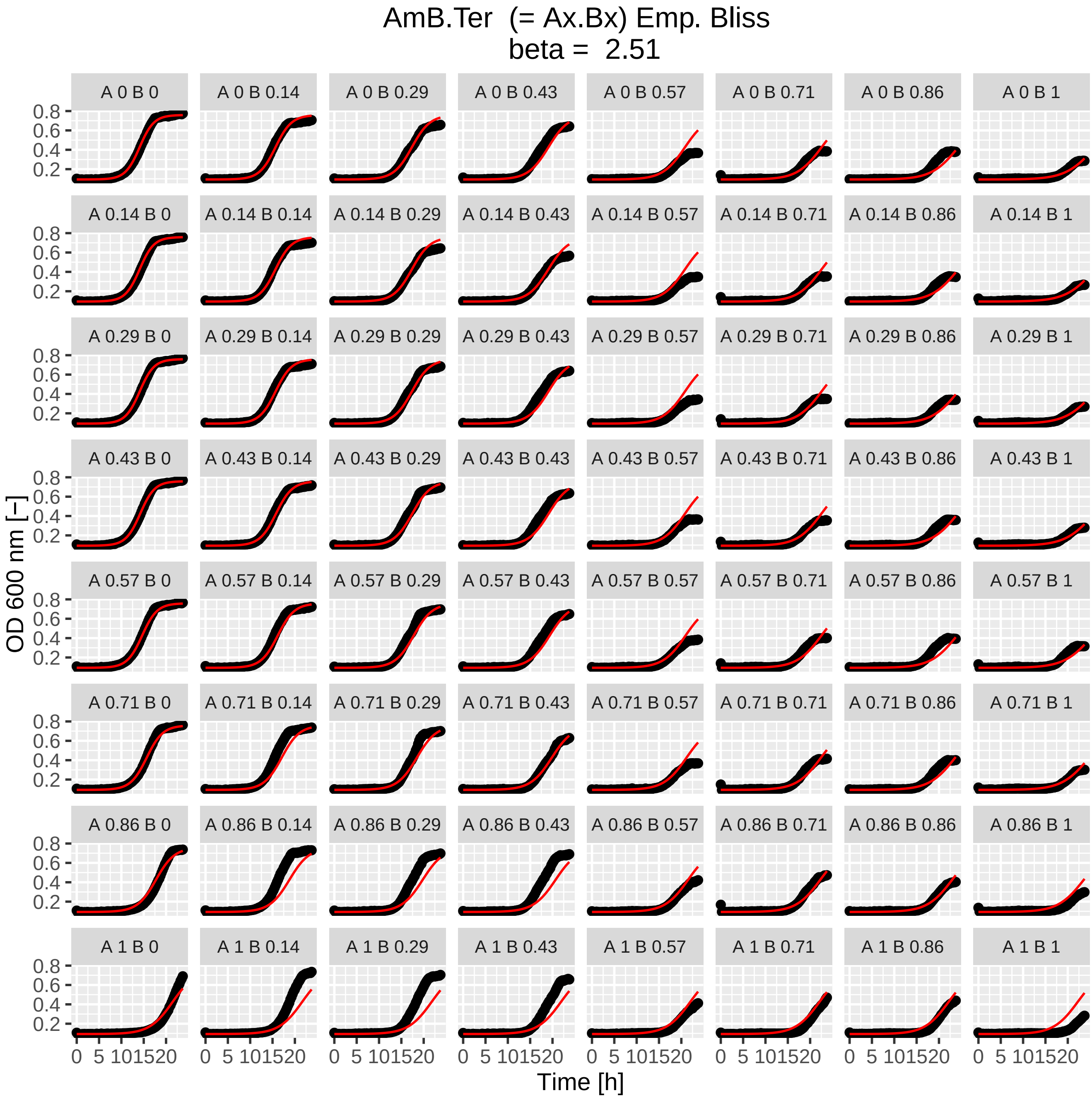
AmB.Ter (= Ax.Bx) Emp. Bliss
beta = 2.51
OD 600 nm [−]
Time [h]
A 0 B 0
A 0 B 0.14
A 0 B 0.29
A 0 B 0.43
A 0 B 0.57
A 0 B 0.71
A 0 B 0.86
A 0 B 1
A 0.14 B 0
A 0.14 B 0.14
A 0.14 B 0.29
A 0.14 B 0.43
A 0.14 B 0.57
A 0.14 B 0.71
A 0.14 B 0.86
A 0.14 B 1
A 0.29 B 0
A 0.29 B 0.14
A 0.29 B 0.29
A 0.29 B 0.43
A 0.29 B 0.57
A 0.29 B 0.71
A 0.29 B 0.86
A 0.29 B 1
A 0.43 B 0
A 0.43 B 0.14
A 0.43 B 0.29
A 0.43 B 0.43
A 0.43 B 0.57
A 0.43 B 0.71
A 0.43 B 0.86
A 0.43 B 1
A 0.57 B 0
A 0.57 B 0.14
A 0.57 B 0.29
A 0.57 B 0.43
A 0.57 B 0.57
A 0.57 B 0.71
A 0.57 B 0.86
A 0.57 B 1
A 0.71 B 0
A 0.71 B 0.14
A 0.71 B 0.29
A 0.71 B 0.43
A 0.71 B 0.57
A 0.71 B 0.71
A 0.71 B 0.86
A 0.71 B 1
A 0.86 B 0
A 0.86 B 0.14
A 0.86 B 0.29
A 0.86 B 0.43
A 0.86 B 0.57
A 0.86 B 0.71
A 0.86 B 0.86
A 0.86 B 1
A 1 B 0
A 1 B 0.14
A 1 B 0.29
A 1 B 0.43
A 1 B 0.57
A 1 B 0.71
A 1 B 0.86
A 1 B 1
0.8
0.6
0.4
0.2
0 5 101520

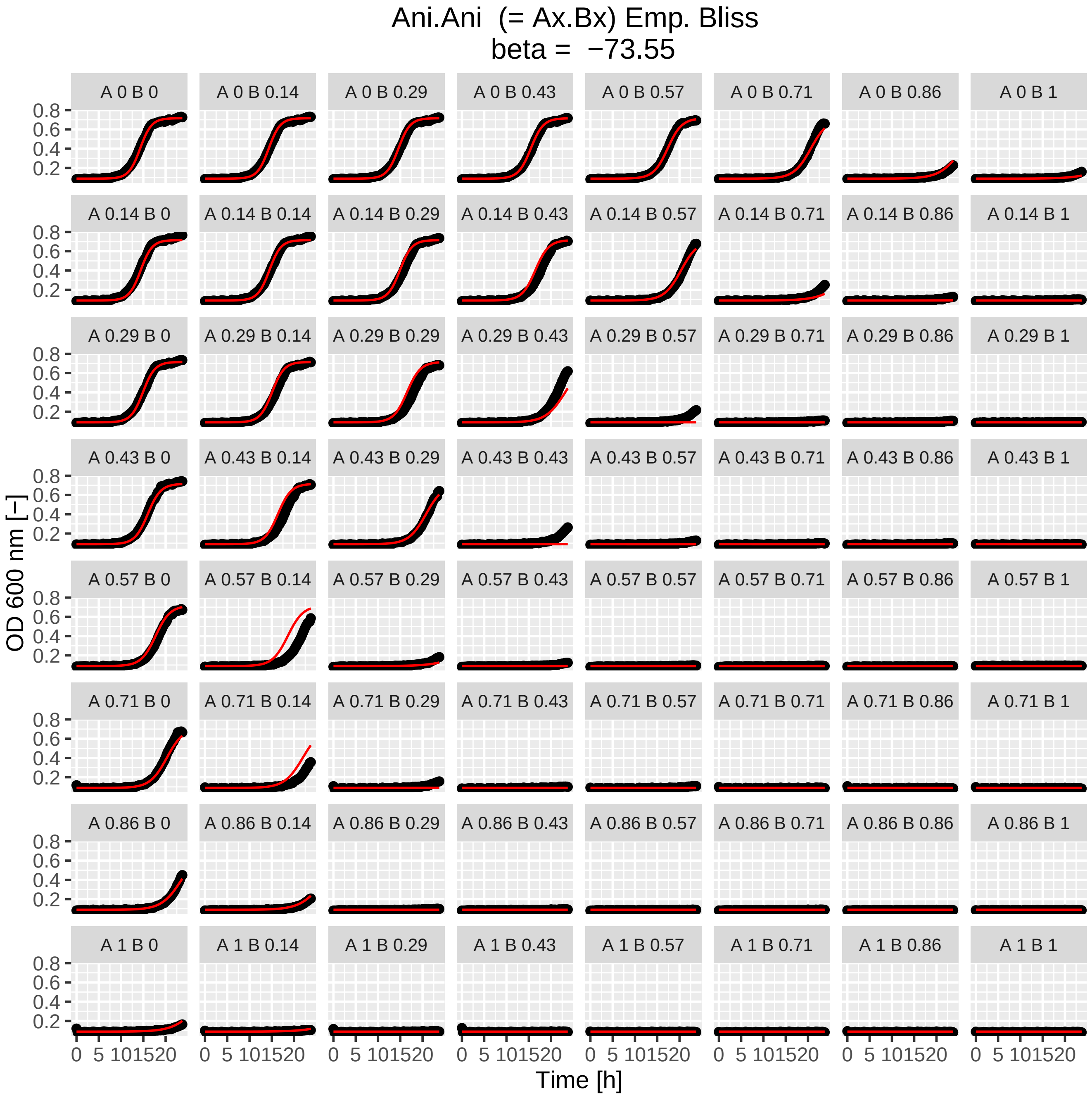
Ani.Ani (= Ax.Bx) Emp. Bliss
beta = −73.55
OD 600 nm [−]
Time [h]
A 0 B 0
A 0 B 0.14
A 0 B 0.29
A 0 B 0.43
A 0 B 0.57
A 0 B 0.71
A 0 B 0.86
A 0 B 1
A 0.14 B 0
A 0.14 B 0.14
A 0.14 B 0.29
A 0.14 B 0.43
A 0.14 B 0.57
A 0.14 B 0.71
A 0.14 B 0.86
A 0.14 B 1
A 0.29 B 0
A 0.29 B 0.14
A 0.29 B 0.29
A 0.29 B 0.43
A 0.29 B 0.57
A 0.29 B 0.71
A 0.29 B 0.86
A 0.29 B 1
A 0.43 B 0
A 0.43 B 0.14
A 0.43 B 0.29
A 0.43 B 0.43
A 0.43 B 0.57
A 0.43 B 0.71
A 0.43 B 0.86
A 0.43 B 1
A 0.57 B 0
A 0.57 B 0.14
A 0.57 B 0.29
A 0.57 B 0.43
A 0.57 B 0.57
A 0.57 B 0.71
A 0.57 B 0.86
A 0.57 B 1
A 0.71 B 0
A 0.71 B 0.14
A 0.71 B 0.29
A 0.71 B 0.43
A 0.71 B 0.57
A 0.71 B 0.71
A 0.71 B 0.86
A 0.71 B 1
A 0.86 B 0
A 0.86 B 0.14
A 0.86 B 0.29
A 0.86 B 0.43
A 0.86 B 0.57
A 0.86 B 0.71
A 0.86 B 0.86
A 0.86 B 1
A 1 B 0
A 1 B 0.14
A 1 B 0.29
A 1 B 0.43
A 1 B 0.57
A 1 B 0.71
A 1 B 0.86
A 1 B 1
0.2 0.4 0.6 0.8
0 5 101520

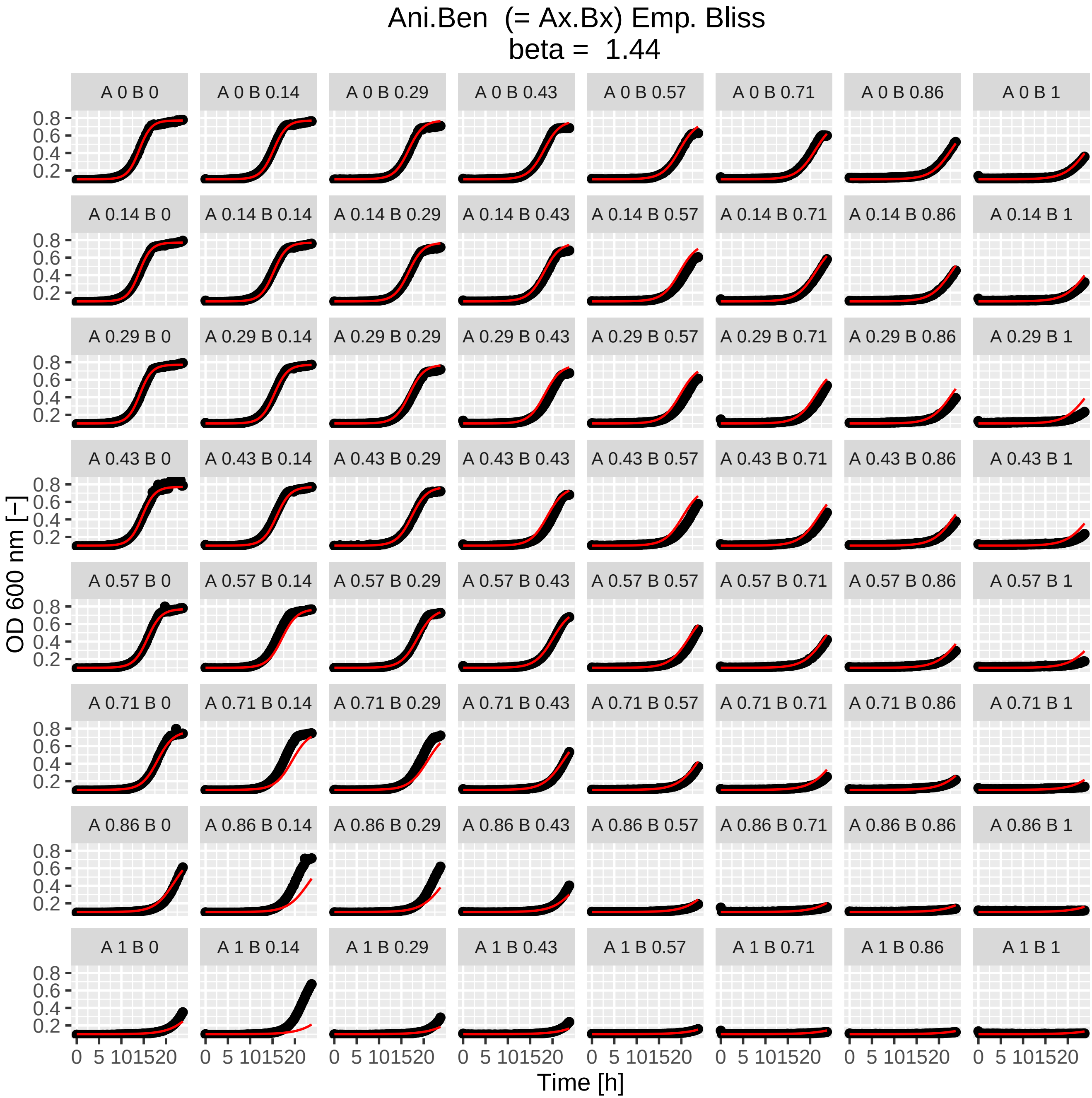
Ani.Ben (= Ax.Bx) Emp. Bliss
beta = 1.44
A 0 B 0
A 0 B 0.14
A 0 B 0.29
A 0 B 0.43
A 0 B 0.57
A 0 B 0.71
A 0 B 0.86
A 0 B 1
A 0.14 B 0
A 0.14 B 0.14
A 0.14 B 0.29
A 0.14 B 0.43
A 0.14 B 0.57
A 0.14 B 0.71
A 0.14 B 0.86
A 0.14 B 1
A 0.29 B 0
A 0.29 B 0.14
A 0.29 B 0.29
A 0.29 B 0.43
A 0.29 B 0.57
A 0.29 B 0.71
A 0.29 B 0.86
A 0.29 B 1
A 0.43 B 0
A 0.43 B 0.14
A 0.43 B 0.29
A 0.43 B 0.43
A 0.43 B 0.57
A 0.43 B 0.71
A 0.43 B 0.86
A 0.43 B 1
A 0.57 B 0
A 0.57 B 0.14
A 0.57 B 0.29
A 0.57 B 0.43
A 0.57 B 0.57
A 0.57 B 0.71
A 0.57 B 0.86
A 0.57 B 1
A 0.71 B 0
A 0.71 B 0.14
A 0.71 B 0.29
A 0.71 B 0.43
A 0.71 B 0.57
A 0.71 B 0.71
A 0.71 B 0.86
A 0.71 B 1
A 0.86 B 0
A 0.86 B 0.14
A 0.86 B 0.29
A 0.86 B 0.43
A 0.86 B 0.57
A 0.86 B 0.71
A 0.86 B 0.86
A 0.86 B 1
A 1 B 0
A 1 B 0.14
A 1 B 0.29
A 1 B 0.43
A 1 B 0.57
A 1 B 0.71
A 1 B 0.86
A 1 B 1
OD 600 nm [−]
0.8
0.6
0.4
0.2
0 5 101520
Time [h]

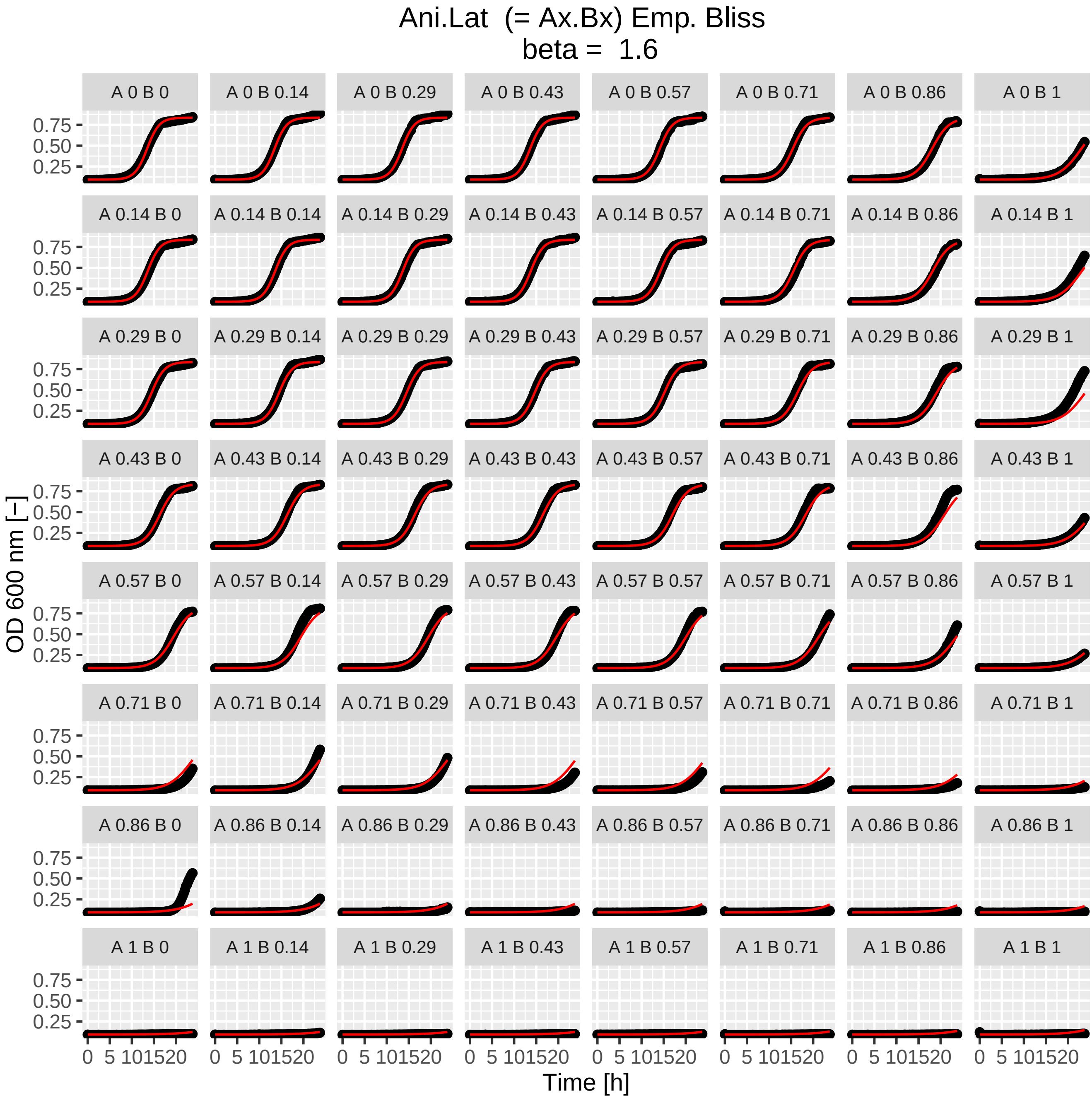

Ani.Lat (= Ax.Bx) Emp. Bliss
beta = 1.6
A 0 B 0
A 0 B 0.14
A 0 B 0.29
A 0 B 0.43
A 0 B 0.57
A 0 B 0.71
A 0 B 0.86
A 0 B 1
A 0.14 B 0
A 0.14 B 0.14
A 0.14 B 0.29
A 0.14 B 0.43
A 0.14 B 0.57
A 0.14 B 0.71
A 0.14 B 0.86
A 0.14 B 1
A 0.29 B 0
A 0.29 B 0.14
A 0.29 B 0.29
A 0.29 B 0.43
A 0.29 B 0.57
A 0.29 B 0.71
A 0.29 B 0.86
A 0.29 B 1
A 0.43 B 0
A 0.43 B 0.14
A 0.43 B 0.29
A 0.43 B 0.43
A 0.43 B 0.57
A 0.43 B 0.71
A 0.43 B 0.86
A 0.43 B 1
A 0.57 B 0
A 0.57 B 0.14
A 0.57 B 0.29
A 0.57 B 0.43
A 0.57 B 0.57
A 0.57 B 0.71
A 0.57 B 0.86
A 0.57 B 1
A 0.71 B 0
A 0.71 B 0.14
A 0.71 B 0.29
A 0.71 B 0.43
A 0.71 B 0.57
A 0.71 B 0.71
A 0.71 B 0.86
A 0.71 B 1
A 0.86 B 0
A 0.86 B 0.14
A 0.86 B 0.29
A 0.86 B 0.43
A 0.86 B 0.57
A 0.86 B 0.71
A 0.86 B 0.86
A 0.86 B 1
A 1 B 0
A 1 B 0.14
A 1 B 0.29
A 1 B 0.43
A 1 B 0.57
A 1 B 0.71
A 1 B 0.86
A 1 B 1
OD 600 nm [–]
0.75
0.50
0.25
Time [h]
0 5 101520

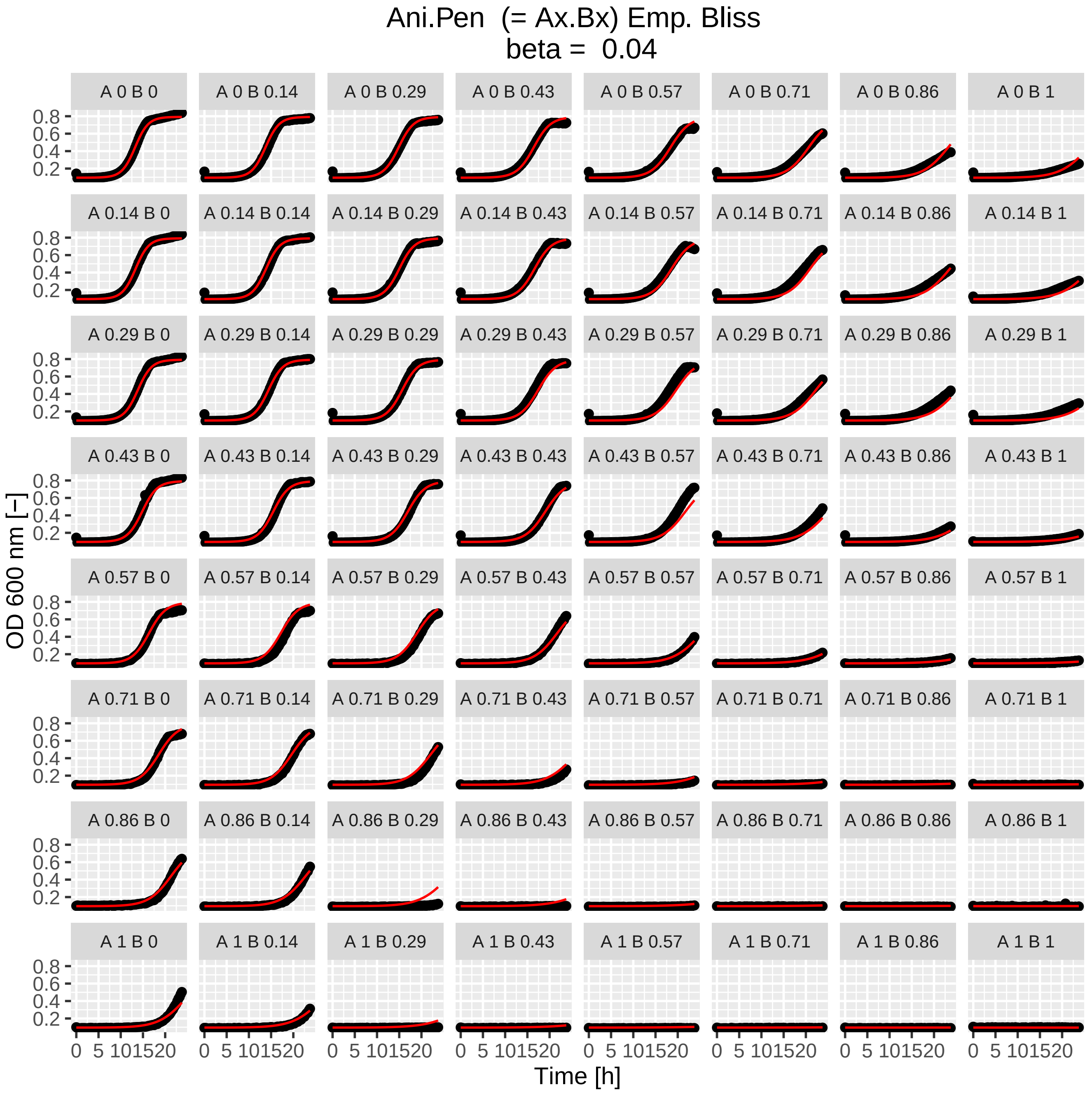

Ani.Pen (= Ax.Bx) Emp. Bliss
beta = 0.04
OD 600 nm [–]
Time [h]
A 0 B 0
A 0 B 0.14
A 0 B 0.29
A 0 B 0.43
A 0 B 0.57
A 0 B 0.71
A 0 B 0.86
A 0 B 1
A 0.14 B 0
A 0.14 B 0.14
A 0.14 B 0.29
A 0.14 B 0.43
A 0.14 B 0.57
A 0.14 B 0.71
A 0.14 B 0.86
A 0.14 B 1
A 0.29 B 0
A 0.29 B 0.14
A 0.29 B 0.29
A 0.29 B 0.43
A 0.29 B 0.57
A 0.29 B 0.71
A 0.29 B 0.86
A 0.29 B 1
A 0.43 B 0
A 0.43 B 0.14
A 0.43 B 0.29
A 0.43 B 0.43
A 0.43 B 0.57
A 0.43 B 0.71
A 0.43 B 0.86
A 0.43 B 1
A 0.57 B 0
A 0.57 B 0.14
A 0.57 B 0.29
A 0.57 B 0.43
A 0.57 B 0.57
A 0.57 B 0.71
A 0.57 B 0.86
A 0.57 B 1
A 0.71 B 0
A 0.71 B 0.14
A 0.71 B 0.29
A 0.71 B 0.43
A 0.71 B 0.57
A 0.71 B 0.71
A 0.71 B 0.86
A 0.71 B 1
A 0.86 B 0
A 0.86 B 0.14
A 0.86 B 0.29
A 0.86 B 0.43
A 0.86 B 0.57
A 0.86 B 0.71
A 0.86 B 0.86
A 0.86 B 1
A 1 B 0
A 1 B 0.14
A 1 B 0.29
A 1 B 0.43
A 1 B 0.57
A 1 B 0.71
A 1 B 0.86
A 1 B 1
0.2
0.4
0.6
0.8
0 5 101520

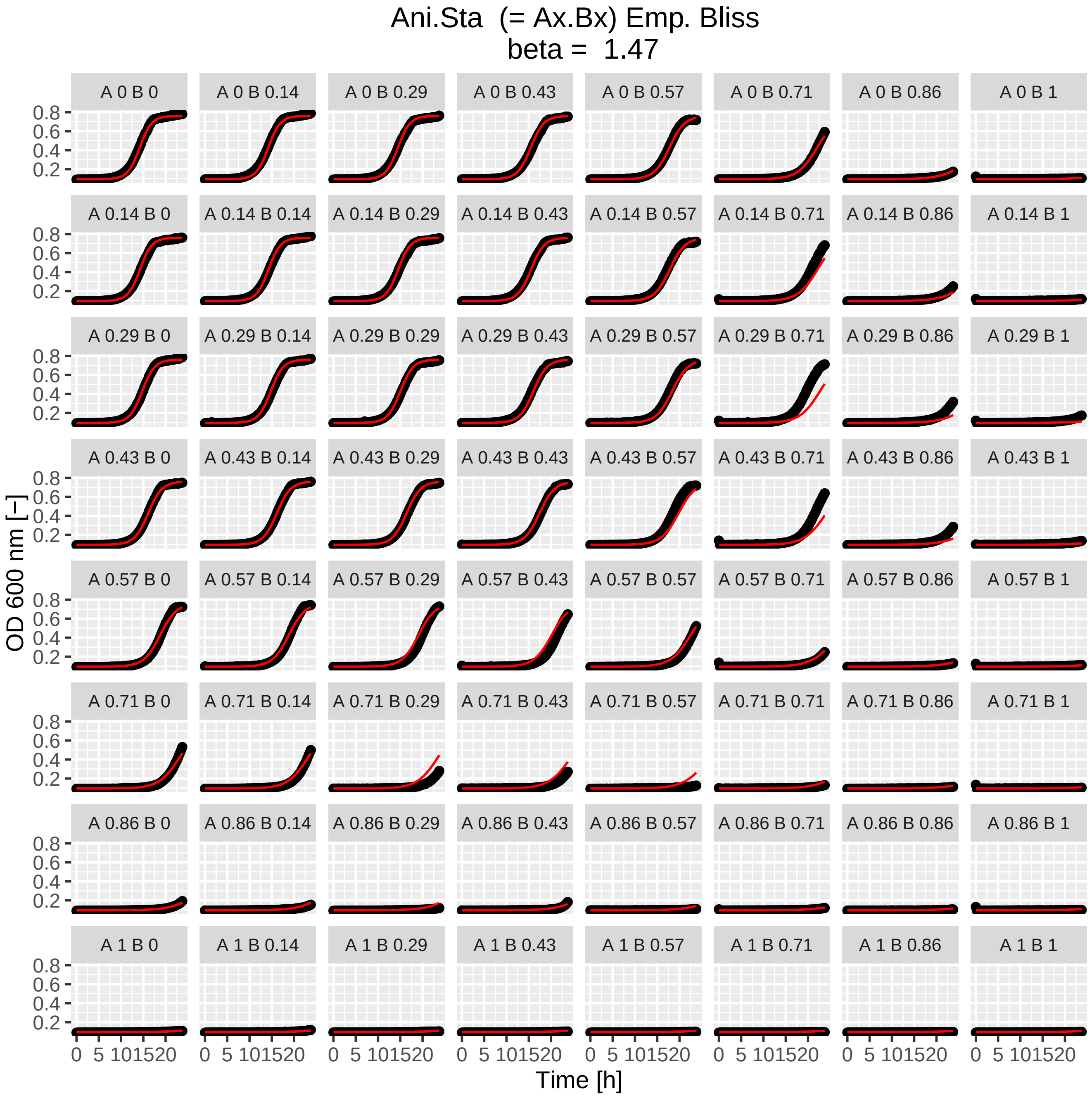

Ani.Sta (= Ax.Bx) Emp. Bliss
beta = 1.47
A 0 B 0
A 0 B 0.14
A 0 B 0.29
A 0 B 0.43
A 0 B 0.57
A 0 B 0.71
A 0 B 0.86
A 0 B 1
A 0.14 B 0
A 0.14 B 0.14
A 0.14 B 0.29
A 0.14 B 0.43
A 0.14 B 0.57
A 0.14 B 0.71
A 0.14 B 0.86
A 0.14 B 1
A 0.29 B 0
A 0.29 B 0.14
A 0.29 B 0.29
A 0.29 B 0.43
A 0.29 B 0.57
A 0.29 B 0.71
A 0.29 B 0.86
A 0.29 B 1
A 0.43 B 0
A 0.43 B 0.14
A 0.43 B 0.29
A 0.43 B 0.43
A 0.43 B 0.57
A 0.43 B 0.71
A 0.43 B 0.86
A 0.43 B 1
A 0.57 B 0
A 0.57 B 0.14
A 0.57 B 0.29
A 0.57 B 0.43
A 0.57 B 0.57
A 0.57 B 0.71
A 0.57 B 0.86
A 0.57 B 1
A 0.71 B 0
A 0.71 B 0.14
A 0.71 B 0.29
A 0.71 B 0.43
A 0.71 B 0.57
A 0.71 B 0.71
A 0.71 B 0.86
A 0.71 B 1
A 0.86 B 0
A 0.86 B 0.14
A 0.86 B 0.29
A 0.86 B 0.43
A 0.86 B 0.57
A 0.86 B 0.71
A 0.86 B 0.86
A 0.86 B 1
A 1 B 0
A 1 B 0.14
A 1 B 0.29
A 1 B 0.43
A 1 B 0.57
A 1 B 0.71
A 1 B 0.86
A 1 B 1
OD 600 nm [−]
0.2
0.4
0.6
0.8
0 5 101520
Time [h]

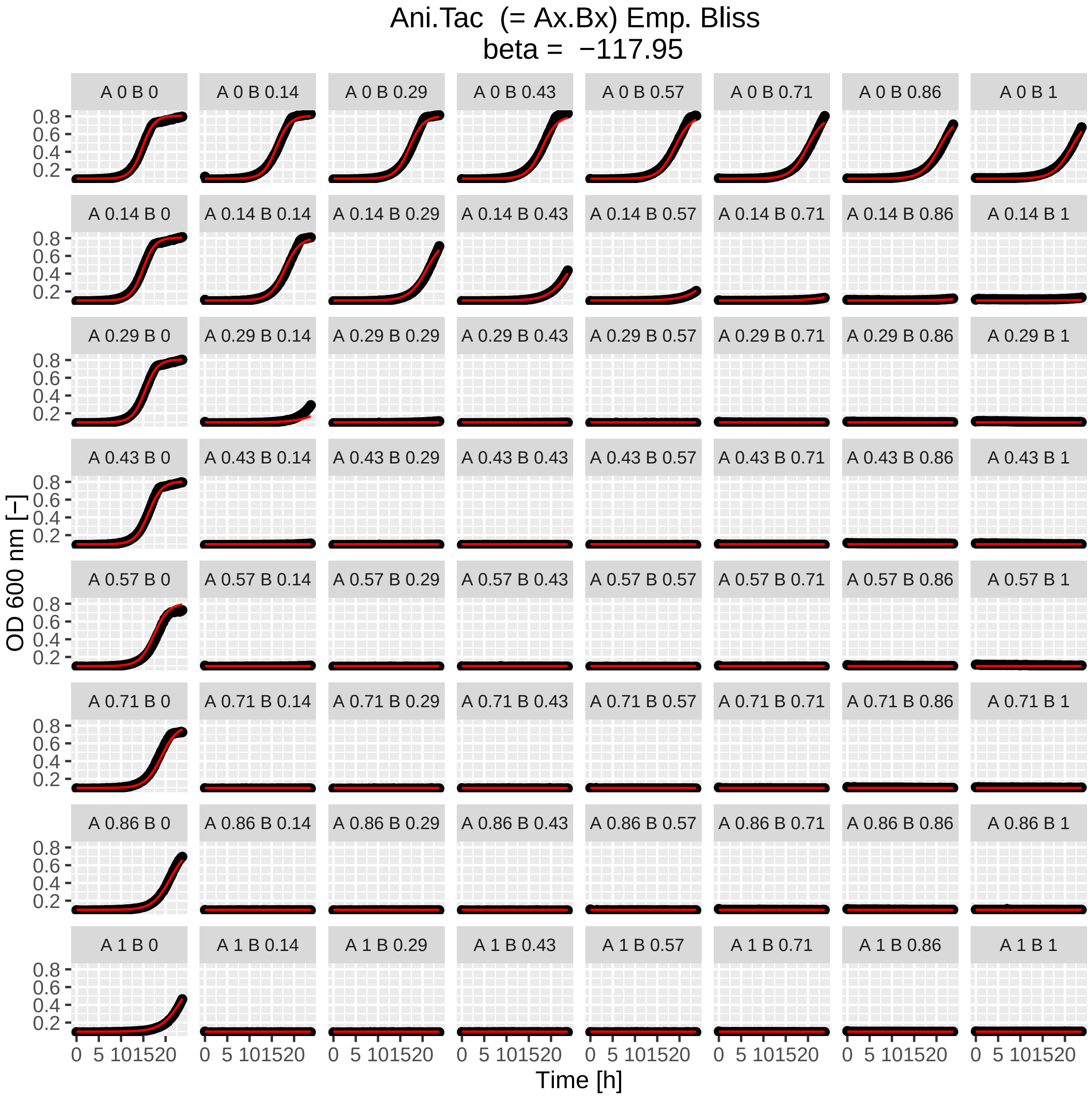
Ani.Tac (= Ax.Bx) Emp. Bliss
beta = −117.95
A 0 B 0
A 0 B 0.14
A 0 B 0.29
A 0 B 0.43
A 0 B 0.57
A 0 B 0.71
A 0 B 0.86
A 0 B 1
A 0.14 B 0
A 0.14 B 0.14
A 0.14 B 0.29
A 0.14 B 0.43
A 0.14 B 0.57
A 0.14 B 0.71
A 0.14 B 0.86
A 0.14 B 1
A 0.29 B 0
A 0.29 B 0.14
A 0.29 B 0.29
A 0.29 B 0.43
A 0.29 B 0.57
A 0.29 B 0.71
A 0.29 B 0.86
A 0.29 B 1
A 0.43 B 0
A 0.43 B 0.14
A 0.43 B 0.29
A 0.43 B 0.43
A 0.43 B 0.57
A 0.43 B 0.71
A 0.43 B 0.86
A 0.43 B 1
A 0.57 B 0
A 0.57 B 0.14
A 0.57 B 0.29
A 0.57 B 0.43
A 0.57 B 0.57
A 0.57 B 0.71
A 0.57 B 0.86
A 0.57 B 1
A 0.71 B 0
A 0.71 B 0.14
A 0.71 B 0.29
A 0.71 B 0.43
A 0.71 B 0.57
A 0.71 B 0.71
A 0.71 B 0.86
A 0.71 B 1
A 0.86 B 0
A 0.86 B 0.14
A 0.86 B 0.29
A 0.86 B 0.43
A 0.86 B 0.57
A 0.86 B 0.71
A 0.86 B 0.86
A 0.86 B 1
A 1 B 0
A 1 B 0.14
A 1 B 0.29
A 1 B 0.43
A 1 B 0.57
A 1 B 0.71
A 1 B 0.86
A 1 B 1
OD 600 nm [−]
0.8
0.6
0.4
0.2
0 5 101520
Time [h]

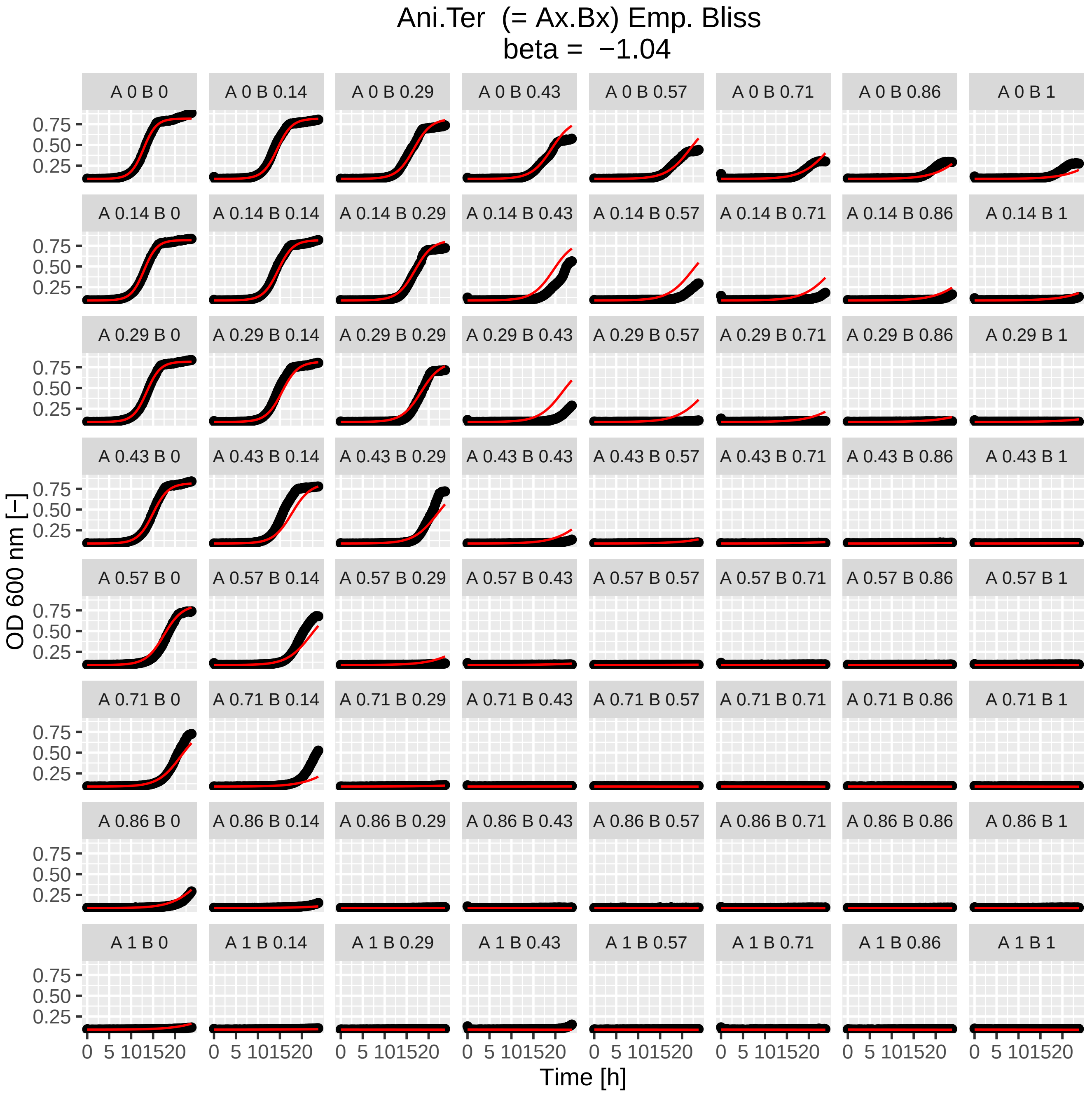
Ani.Ter (= Ax.Bx) Emp. Bliss
beta = −1.04
OD 600 nm [−]
Time [h]
A 0 B 0
A 0 B 0.14
A 0 B 0.29
A 0 B 0.43
A 0 B 0.57
A 0 B 0.71
A 0 B 0.86
A 0 B 1
A 0.14 B 0
A 0.14 B 0.14
A 0.14 B 0.29
A 0.14 B 0.43
A 0.14 B 0.57
A 0.14 B 0.71
A 0.14 B 0.86
A 0.14 B 1
A 0.29 B 0
A 0.29 B 0.14
A 0.29 B 0.29
A 0.29 B 0.43
A 0.29 B 0.57
A 0.29 B 0.71
A 0.29 B 0.86
A 0.29 B 1
A 0.43 B 0
A 0.43 B 0.14
A 0.43 B 0.29
A 0.43 B 0.43
A 0.43 B 0.57
A 0.43 B 0.71
A 0.43 B 0.86
A 0.43 B 1
A 0.57 B 0
A 0.57 B 0.14
A 0.57 B 0.29
A 0.57 B 0.43
A 0.57 B 0.57
A 0.57 B 0.71
A 0.57 B 0.86
A 0.57 B 1
A 0.71 B 0
A 0.71 B 0.14
A 0.71 B 0.29
A 0.71 B 0.43
A 0.71 B 0.57
A 0.71 B 0.71
A 0.71 B 0.86
A 0.71 B 1
A 0.86 B 0
A 0.86 B 0.14
A 0.86 B 0.29
A 0.86 B 0.43
A 0.86 B 0.57
A 0.86 B 0.71
A 0.86 B 0.86
A 0.86 B 1
A 1 B 0
A 1 B 0.14
A 1 B 0.29
A 1 B 0.43
A 1 B 0.57
A 1 B 0.71
A 1 B 0.86
A 1 B 1
0.75
0.50
0.25
0 5 101520

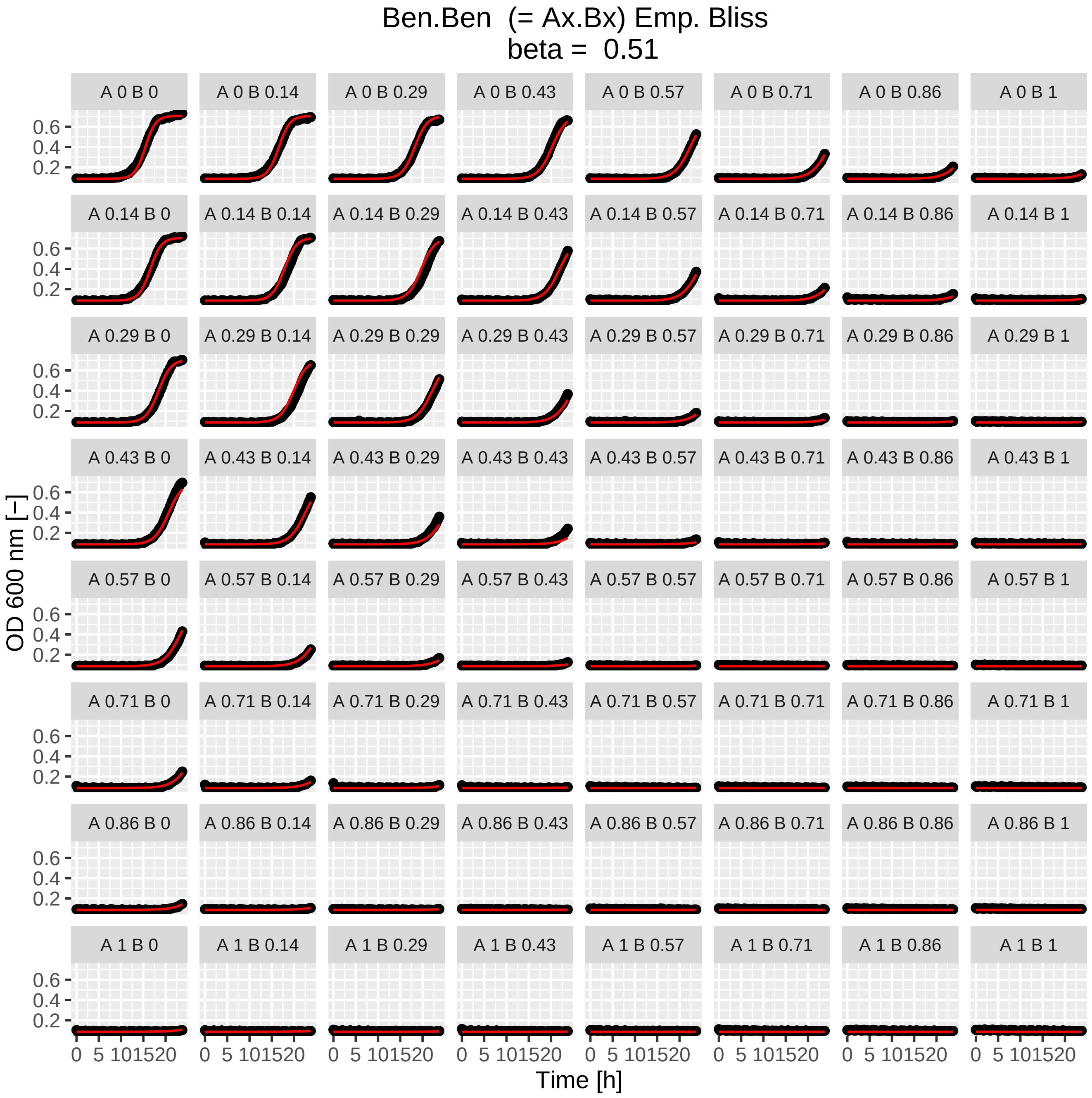

Ben.Ben (= Ax.Bx) Emp. Bliss
beta = 0.51
A 0 B 0
A 0 B 0.14
A 0 B 0.29
A 0 B 0.43
A 0 B 0.57
A 0 B 0.71
A 0 B 0.86
A 0 B 1
A 0.14 B 0
A 0.14 B 0.14
A 0.14 B 0.29
A 0.14 B 0.43
A 0.14 B 0.57
A 0.14 B 0.71
A 0.14 B 0.86
A 0.14 B 1
A 0.29 B 0
A 0.29 B 0.14
A 0.29 B 0.29
A 0.29 B 0.43
A 0.29 B 0.57
A 0.29 B 0.71
A 0.29 B 0.86
A 0.29 B 1
A 0.43 B 0
A 0.43 B 0.14
A 0.43 B 0.29
A 0.43 B 0.43
A 0.43 B 0.57
A 0.43 B 0.71
A 0.43 B 0.86
A 0.43 B 1
A 0.57 B 0
A 0.57 B 0.14
A 0.57 B 0.29
A 0.57 B 0.43
A 0.57 B 0.57
A 0.57 B 0.71
A 0.57 B 0.86
A 0.57 B 1
A 0.71 B 0
A 0.71 B 0.14
A 0.71 B 0.29
A 0.71 B 0.43
A 0.71 B 0.57
A 0.71 B 0.71
A 0.71 B 0.86
A 0.71 B 1
A 0.86 B 0
A 0.86 B 0.14
A 0.86 B 0.29
A 0.86 B 0.43
A 0.86 B 0.57
A 0.86 B 0.71
A 0.86 B 0.86
A 0.86 B 1
A 1 B 0
A 1 B 0.14
A 1 B 0.29
A 1 B 0.43
A 1 B 0.57
A 1 B 0.71
A 1 B 0.86
A 1 B 1
0.6
0.4
0.2
0 5 101520
OD 600 nm [–]
Time [h]

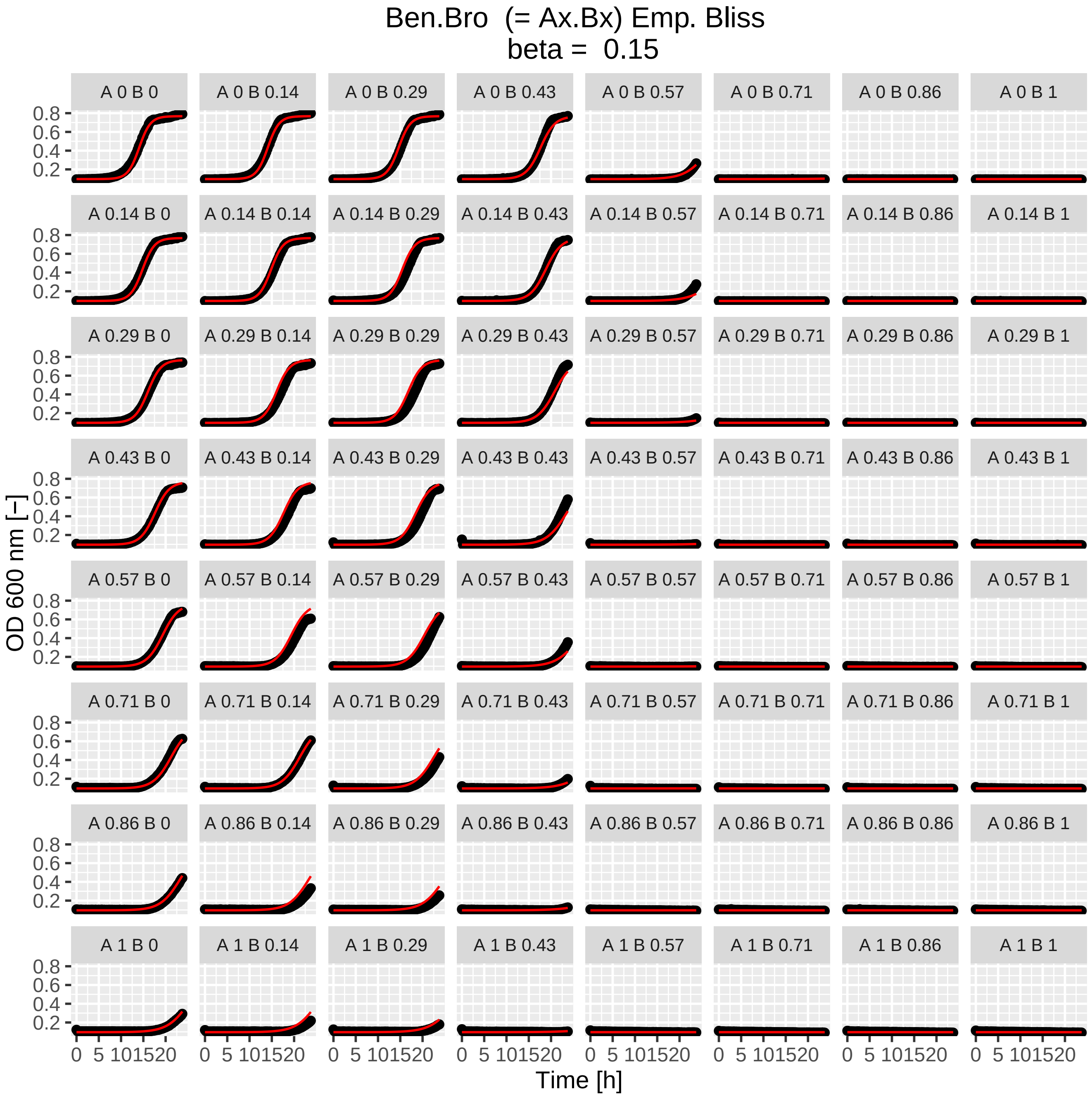
Ben.Bro (= Ax.Bx) Emp. Bliss
beta = 0.15
OD 600 nm [–]
Time [h]
A 0 B 0
A 0 B 0.14
A 0 B 0.29
A 0 B 0.43
A 0 B 0.57
A 0 B 0.71
A 0 B 0.86
A 0 B 1
A 0.14 B 0
A 0.14 B 0.14
A 0.14 B 0.29
A 0.14 B 0.43
A 0.14 B 0.57
A 0.14 B 0.71
A 0.14 B 0.86
A 0.14 B 1
A 0.29 B 0
A 0.29 B 0.14
A 0.29 B 0.29
A 0.29 B 0.43
A 0.29 B 0.57
A 0.29 B 0.71
A 0.29 B 0.86
A 0.29 B 1
A 0.43 B 0
A 0.43 B 0.14
A 0.43 B 0.29
A 0.43 B 0.43
A 0.43 B 0.57
A 0.43 B 0.71
A 0.43 B 0.86
A 0.43 B 1
A 0.57 B 0
A 0.57 B 0.14
A 0.57 B 0.29
A 0.57 B 0.43
A 0.57 B 0.57
A 0.57 B 0.71
A 0.57 B 0.86
A 0.57 B 1
A 0.71 B 0
A 0.71 B 0.14
A 0.71 B 0.29
A 0.71 B 0.43
A 0.71 B 0.57
A 0.71 B 0.71
A 0.71 B 0.86
A 0.71 B 1
A 0.86 B 0
A 0.86 B 0.14
A 0.86 B 0.29
A 0.86 B 0.43
A 0.86 B 0.57
A 0.86 B 0.71
A 0.86 B 0.86
A 0.86 B 1
A 1 B 0
A 1 B 0.14
A 1 B 0.29
A 1 B 0.43
A 1 B 0.57
A 1 B 0.71
A 1 B 0.86
A 1 B 1
0.8
0.6
0.4
0.2
0 5 101520

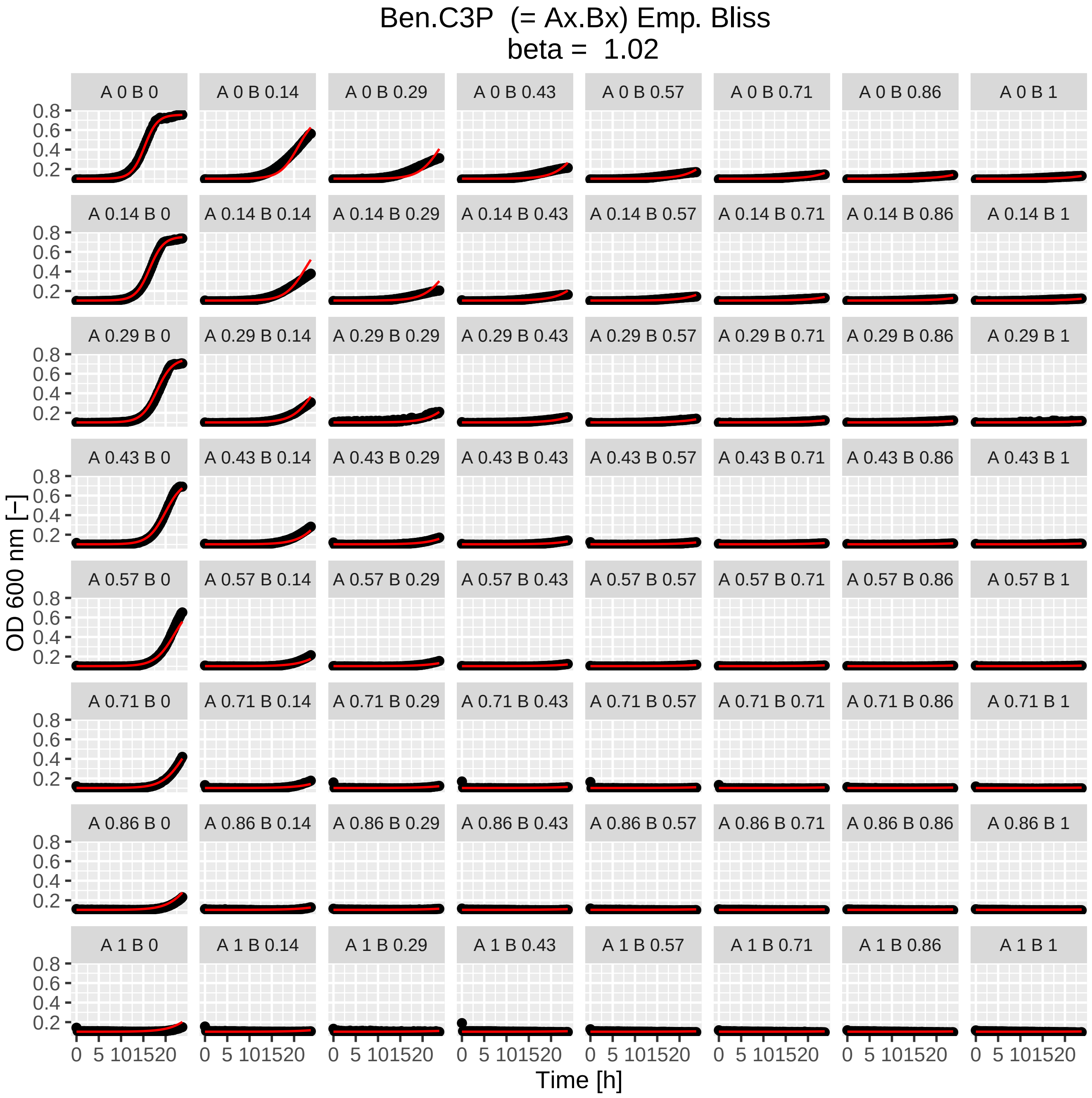

Ben.C3P (= Ax.Bx) Emp. Bliss
beta = 1.02
A 0 B 0
A 0 B 0.14
A 0 B 0.29
A 0 B 0.43
A 0 B 0.57
A 0 B 0.71
A 0 B 0.86
A 0 B 1
A 0.14 B 0
A 0.14 B 0.14
A 0.14 B 0.29
A 0.14 B 0.43
A 0.14 B 0.57
A 0.14 B 0.71
A 0.14 B 0.86
A 0.14 B 1
A 0.29 B 0
A 0.29 B 0.14
A 0.29 B 0.29
A 0.29 B 0.43
A 0.29 B 0.57
A 0.29 B 0.71
A 0.29 B 0.86
A 0.29 B 1
A 0.43 B 0
A 0.43 B 0.14
A 0.43 B 0.29
A 0.43 B 0.43
A 0.43 B 0.57
A 0.43 B 0.71
A 0.43 B 0.86
A 0.43 B 1
A 0.57 B 0
A 0.57 B 0.14
A 0.57 B 0.29
A 0.57 B 0.43
A 0.57 B 0.57
A 0.57 B 0.71
A 0.57 B 0.86
A 0.57 B 1
A 0.71 B 0
A 0.71 B 0.14
A 0.71 B 0.29
A 0.71 B 0.43
A 0.71 B 0.57
A 0.71 B 0.71
A 0.71 B 0.86
A 0.71 B 1
A 0.86 B 0
A 0.86 B 0.14
A 0.86 B 0.29
A 0.86 B 0.43
A 0.86 B 0.57
A 0.86 B 0.71
A 0.86 B 0.86
A 0.86 B 1
A 1 B 0
A 1 B 0.14
A 1 B 0.29
A 1 B 0.43
A 1 B 0.57
A 1 B 0.71
A 1 B 0.86
A 1 B 1
OD 600 nm [–]
0.2
0.4
0.6
0.8
0 5 101520
Time [h]

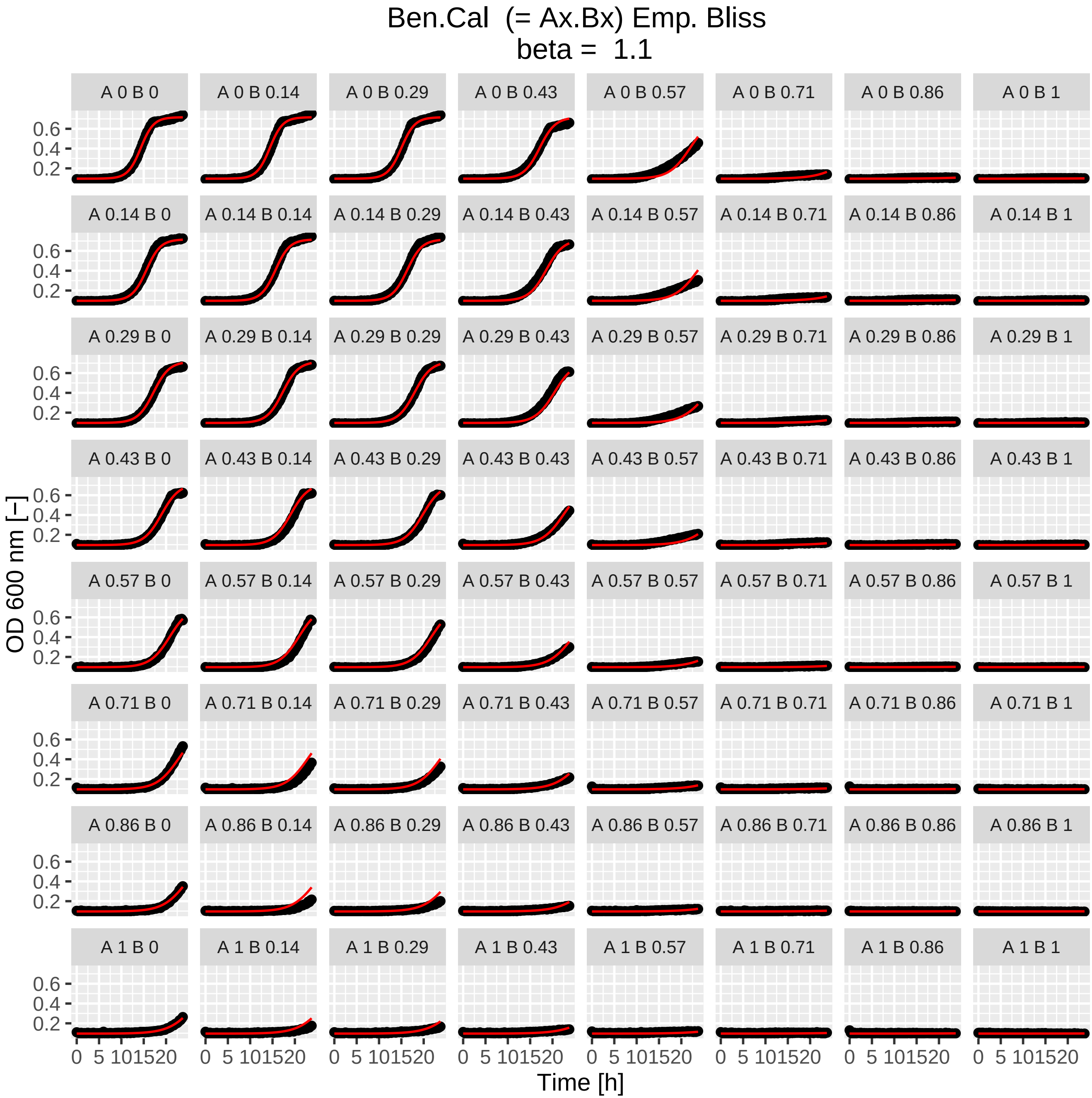
Ben.Cal (= Ax.Bx) Emp. Bliss
beta = 1.1
A 0 B 0
A 0 B 0.14
A 0 B 0.29
A 0 B 0.43
A 0 B 0.57
A 0 B 0.71
A 0 B 0.86
A 0 B 1
A 0.14 B 0
A 0.14 B 0.14
A 0.14 B 0.29
A 0.14 B 0.43
A 0.14 B 0.57
A 0.14 B 0.71
A 0.14 B 0.86
A 0.14 B 1
A 0.29 B 0
A 0.29 B 0.14
A 0.29 B 0.29
A 0.29 B 0.43
A 0.29 B 0.57
A 0.29 B 0.71
A 0.29 B 0.86
A 0.29 B 1
A 0.43 B 0
A 0.43 B 0.14
A 0.43 B 0.29
A 0.43 B 0.43
A 0.43 B 0.57
A 0.43 B 0.71
A 0.43 B 0.86
A 0.43 B 1
A 0.57 B 0
A 0.57 B 0.14
A 0.57 B 0.29
A 0.57 B 0.43
A 0.57 B 0.57
A 0.57 B 0.71
A 0.57 B 0.86
A 0.57 B 1
A 0.71 B 0
A 0.71 B 0.14
A 0.71 B 0.29
A 0.71 B 0.43
A 0.71 B 0.57
A 0.71 B 0.71
A 0.71 B 0.86
A 0.71 B 1
A 0.86 B 0
A 0.86 B 0.14
A 0.86 B 0.29
A 0.86 B 0.43
A 0.86 B 0.57
A 0.86 B 0.71
A 0.86 B 0.86
A 0.86 B 1
A 1 B 0
A 1 B 0.14
A 1 B 0.29
A 1 B 0.43
A 1 B 0.57
A 1 B 0.71
A 1 B 0.86
A 1 B 1
OD 600 nm [–]
0.2
0.4
0.6
0 5 101520
Time [h]

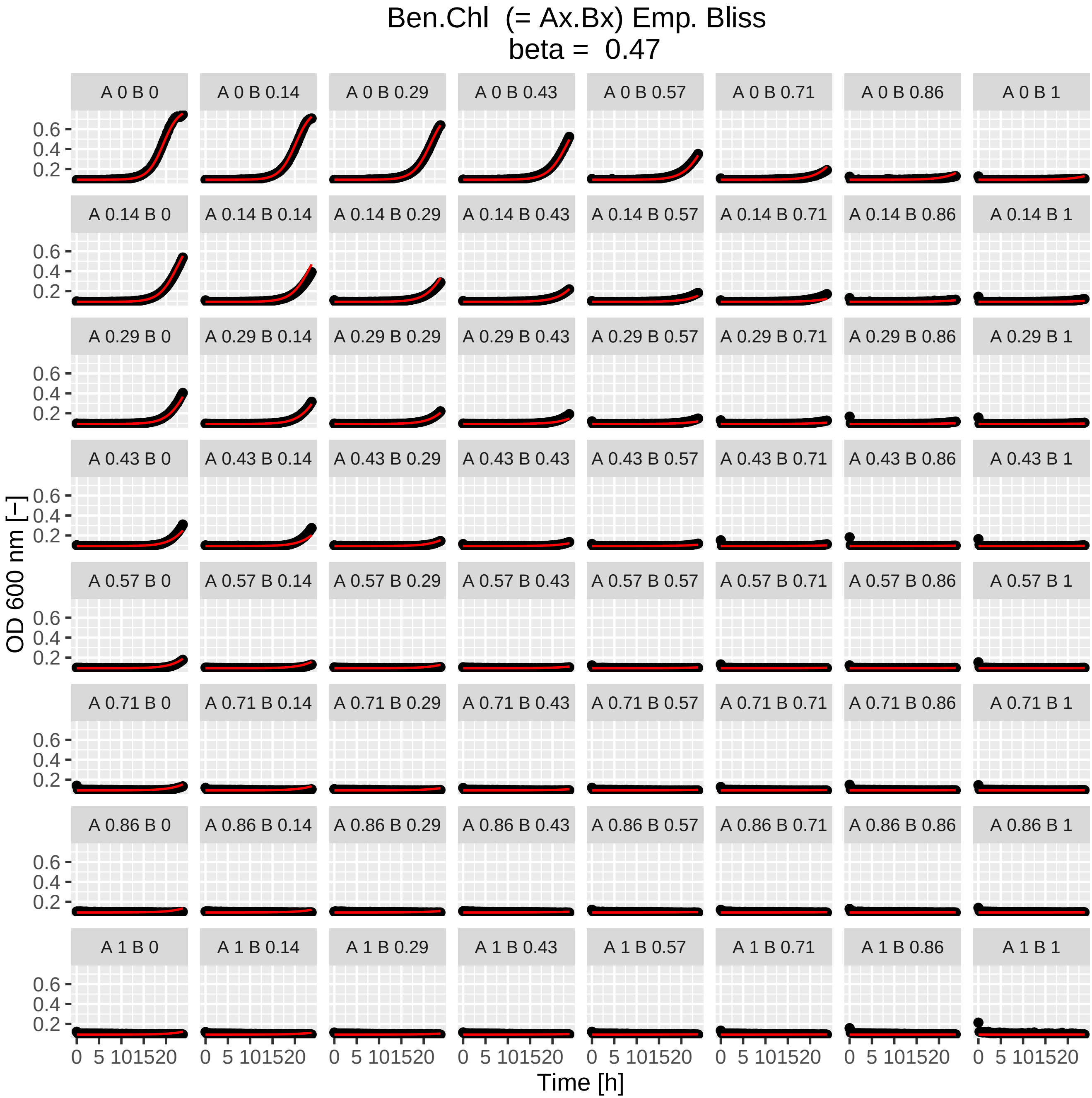
Ben.Chl (= Ax.Bx) Emp. Bliss
beta = 0.47
A 0 B 0
A 0 B 0.14
A 0 B 0.29
A 0 B 0.43
A 0 B 0.57
A 0 B 0.71
A 0 B 0.86
A 0 B 1
A 0.14 B 0
A 0.14 B 0.14
A 0.14 B 0.29
A 0.14 B 0.43
A 0.14 B 0.57
A 0.14 B 0.71
A 0.14 B 0.86
A 0.14 B 1
A 0.29 B 0
A 0.29 B 0.14
A 0.29 B 0.29
A 0.29 B 0.43
A 0.29 B 0.57
A 0.29 B 0.71
A 0.29 B 0.86
A 0.29 B 1
A 0.43 B 0
A 0.43 B 0.14
A 0.43 B 0.29
A 0.43 B 0.43
A 0.43 B 0.57
A 0.43 B 0.71
A 0.43 B 0.86
A 0.43 B 1
A 0.57 B 0
A 0.57 B 0.14
A 0.57 B 0.29
A 0.57 B 0.43
A 0.57 B 0.57
A 0.57 B 0.71
A 0.57 B 0.86
A 0.57 B 1
A 0.71 B 0
A 0.71 B 0.14
A 0.71 B 0.29
A 0.71 B 0.43
A 0.71 B 0.57
A 0.71 B 0.71
A 0.71 B 0.86
A 0.71 B 1
A 0.86 B 0
A 0.86 B 0.14
A 0.86 B 0.29
A 0.86 B 0.43
A 0.86 B 0.57
A 0.86 B 0.71
A 0.86 B 0.86
A 0.86 B 1
A 1 B 0
A 1 B 0.14
A 1 B 0.29
A 1 B 0.43
A 1 B 0.57
A 1 B 0.71
A 1 B 0.86
A 1 B 1
OD 600 nm [–]
0.6
0.4
0.2
0 5 101520
Time [h]

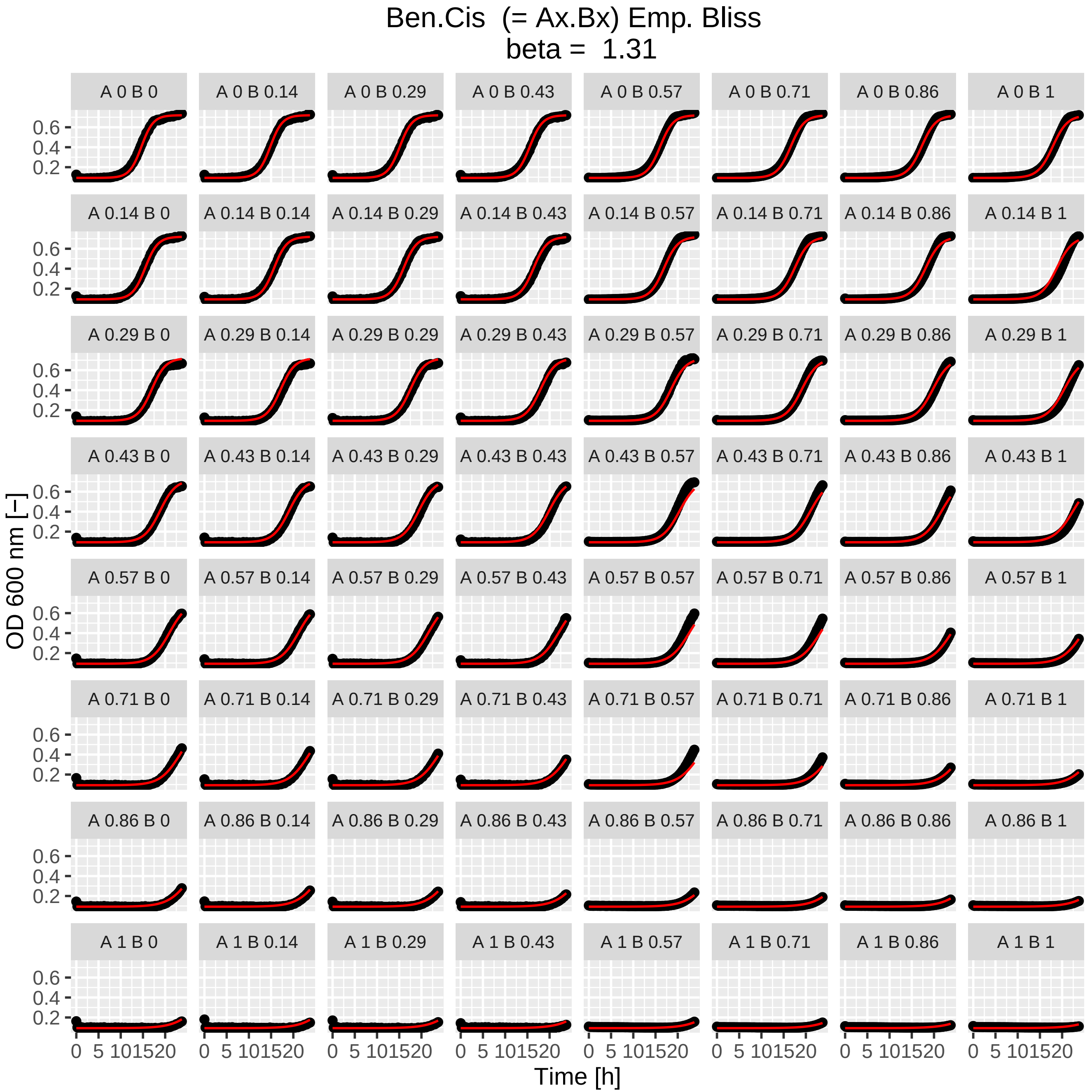

Ben.Cis (= Ax.Bx) Emp. Bliss
beta = 1.31
A 0 B 0
A 0 B 0.14
A 0 B 0.29
A 0 B 0.43
A 0 B 0.57
A 0 B 0.71
A 0 B 0.86
A 0 B 1
A 0.14 B 0
A 0.14 B 0.14
A 0.14 B 0.29
A 0.14 B 0.43
A 0.14 B 0.57
A 0.14 B 0.71
A 0.14 B 0.86
A 0.14 B 1
A 0.29 B 0
A 0.29 B 0.14
A 0.29 B 0.29
A 0.29 B 0.43
A 0.29 B 0.57
A 0.29 B 0.71
A 0.29 B 0.86
A 0.29 B 1
A 0.43 B 0
A 0.43 B 0.14
A 0.43 B 0.29
A 0.43 B 0.43
A 0.43 B 0.57
A 0.43 B 0.71
A 0.43 B 0.86
A 0.43 B 1
A 0.57 B 0
A 0.57 B 0.14
A 0.57 B 0.29
A 0.57 B 0.43
A 0.57 B 0.57
A 0.57 B 0.71
A 0.57 B 0.86
A 0.57 B 1
A 0.71 B 0
A 0.71 B 0.14
A 0.71 B 0.29
A 0.71 B 0.43
A 0.71 B 0.57
A 0.71 B 0.71
A 0.71 B 0.86
A 0.71 B 1
A 0.86 B 0
A 0.86 B 0.14
A 0.86 B 0.29
A 0.86 B 0.43
A 0.86 B 0.57
A 0.86 B 0.71
A 0.86 B 0.86
A 0.86 B 1
A 1 B 0
A 1 B 0.14
A 1 B 0.29
A 1 B 0.43
A 1 B 0.57
A 1 B 0.71
A 1 B 0.86
A 1 B 1
OD 600 nm [–]
0.2
0.4
0.6
0 5 101520
Time [h]

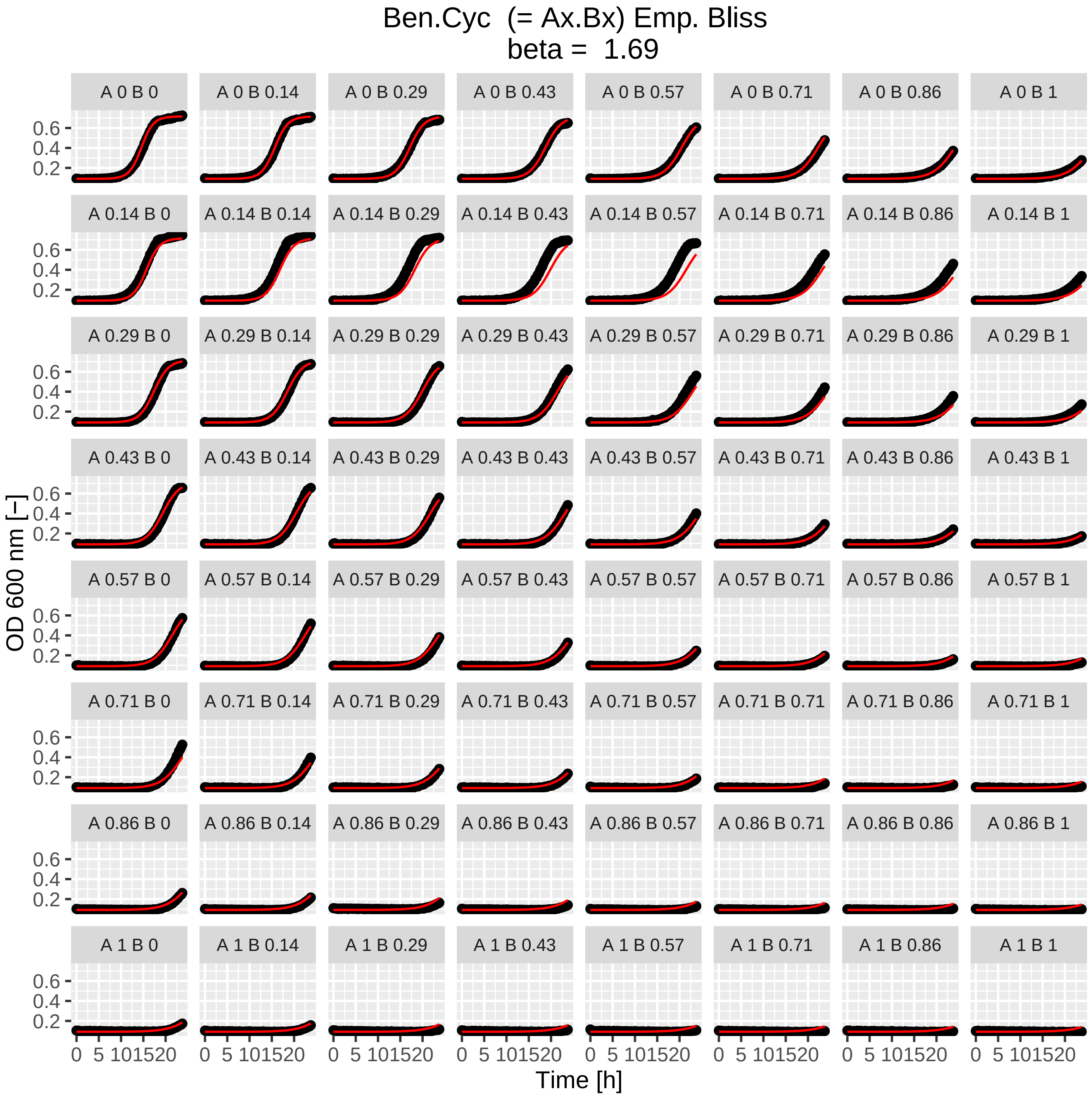

Ben.Cyc (= Ax.Bx) Emp. Bliss
beta = 1.69
OD 600 nm [–]
Time [h]
A 0 B 0
A 0 B 0.14
A 0 B 0.29
A 0 B 0.43
A 0 B 0.57
A 0 B 0.71
A 0 B 0.86
A 0 B 1
A 0.14 B 0
A 0.14 B 0.14
A 0.14 B 0.29
A 0.14 B 0.43
A 0.14 B 0.57
A 0.14 B 0.71
A 0.14 B 0.86
A 0.14 B 1
A 0.29 B 0
A 0.29 B 0.14
A 0.29 B 0.29
A 0.29 B 0.43
A 0.29 B 0.57
A 0.29 B 0.71
A 0.29 B 0.86
A 0.29 B 1
A 0.43 B 0
A 0.43 B 0.14
A 0.43 B 0.29
A 0.43 B 0.43
A 0.43 B 0.57
A 0.43 B 0.71
A 0.43 B 0.86
A 0.43 B 1
A 0.57 B 0
A 0.57 B 0.14
A 0.57 B 0.29
A 0.57 B 0.43
A 0.57 B 0.57
A 0.57 B 0.71
A 0.57 B 0.86
A 0.57 B 1
A 0.71 B 0
A 0.71 B 0.14
A 0.71 B 0.29
A 0.71 B 0.43
A 0.71 B 0.57
A 0.71 B 0.71
A 0.71 B 0.86
A 0.71 B 1
A 0.86 B 0
A 0.86 B 0.14
A 0.86 B 0.29
A 0.86 B 0.43
A 0.86 B 0.57
A 0.86 B 0.71
A 0.86 B 0.86
A 0.86 B 1
A 1 B 0
A 1 B 0.14
A 1 B 0.29
A 1 B 0.43
A 1 B 0.57
A 1 B 0.71
A 1 B 0.86
A 1 B 1
0.6
0.4
0.2
0 5 101520

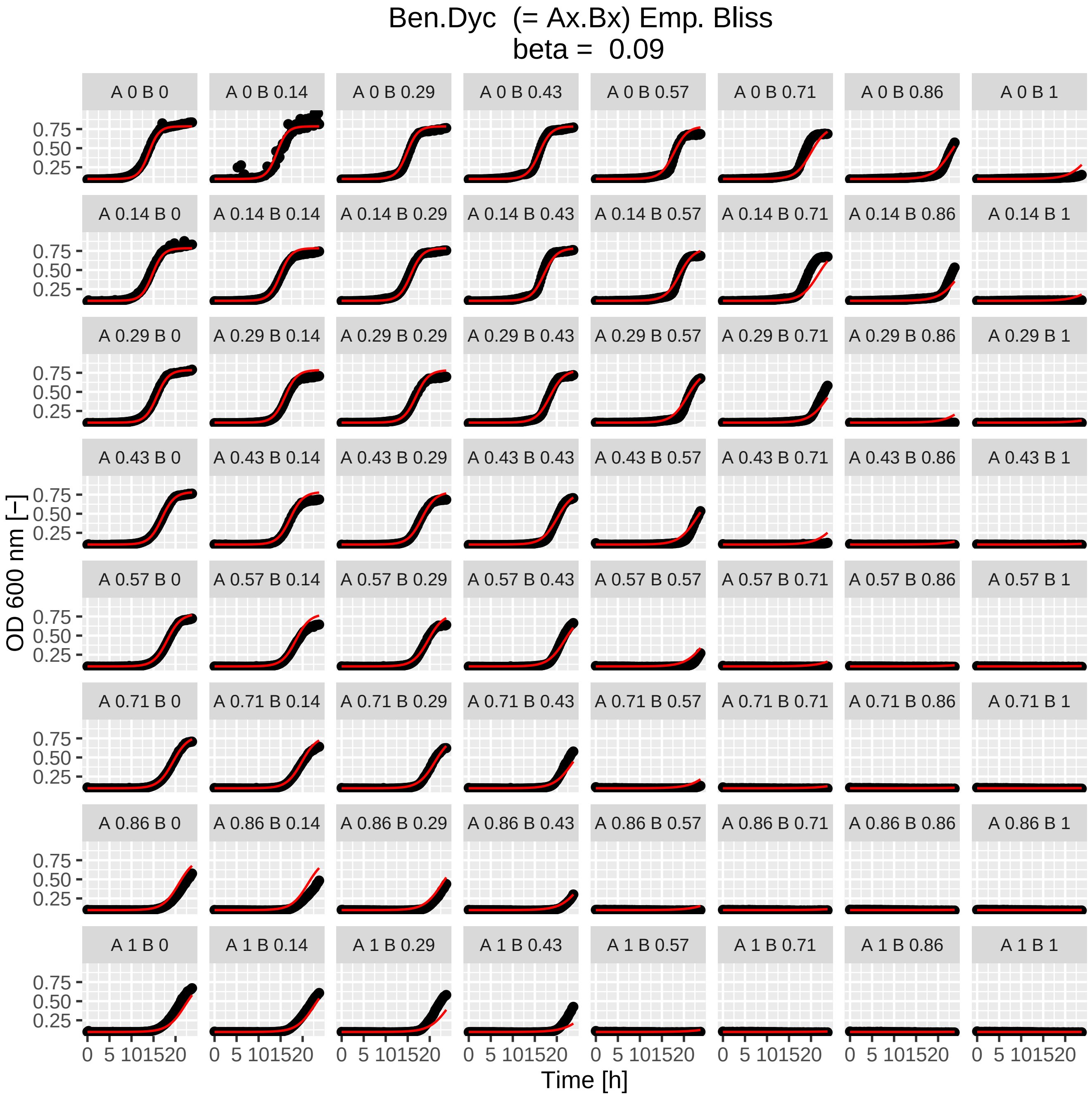
Ben.Dyc (= Ax.Bx) Emp. Bliss
beta = 0.09
OD 600 nm [–]
Time [h]
A 0 B 0
A 0 B 0.14
A 0 B 0.29
A 0 B 0.43
A 0 B 0.57
A 0 B 0.71
A 0 B 0.86
A 0 B 1
A 0.14 B 0
A 0.14 B 0.14
A 0.14 B 0.29
A 0.14 B 0.43
A 0.14 B 0.57
A 0.14 B 0.71
A 0.14 B 0.86
A 0.14 B 1
A 0.29 B 0
A 0.29 B 0.14
A 0.29 B 0.29
A 0.29 B 0.43
A 0.29 B 0.57
A 0.29 B 0.71
A 0.29 B 0.86
A 0.29 B 1
A 0.43 B 0
A 0.43 B 0.14
A 0.43 B 0.29
A 0.43 B 0.43
A 0.43 B 0.57
A 0.43 B 0.71
A 0.43 B 0.86
A 0.43 B 1
A 0.57 B 0
A 0.57 B 0.14
A 0.57 B 0.29
A 0.57 B 0.43
A 0.57 B 0.57
A 0.57 B 0.71
A 0.57 B 0.86
A 0.57 B 1
A 0.71 B 0
A 0.71 B 0.14
A 0.71 B 0.29
A 0.71 B 0.43
A 0.71 B 0.57
A 0.71 B 0.71
A 0.71 B 0.86
A 0.71 B 1
A 0.86 B 0
A 0.86 B 0.14
A 0.86 B 0.29
A 0.86 B 0.43
A 0.86 B 0.57
A 0.86 B 0.71
A 0.86 B 0.86
A 0.86 B 1
A 1 B 0
A 1 B 0.14
A 1 B 0.29
A 1 B 0.43
A 1 B 0.57
A 1 B 0.71
A 1 B 0.86
A 1 B 1
0.75
0.50
0.25
0 5 101520

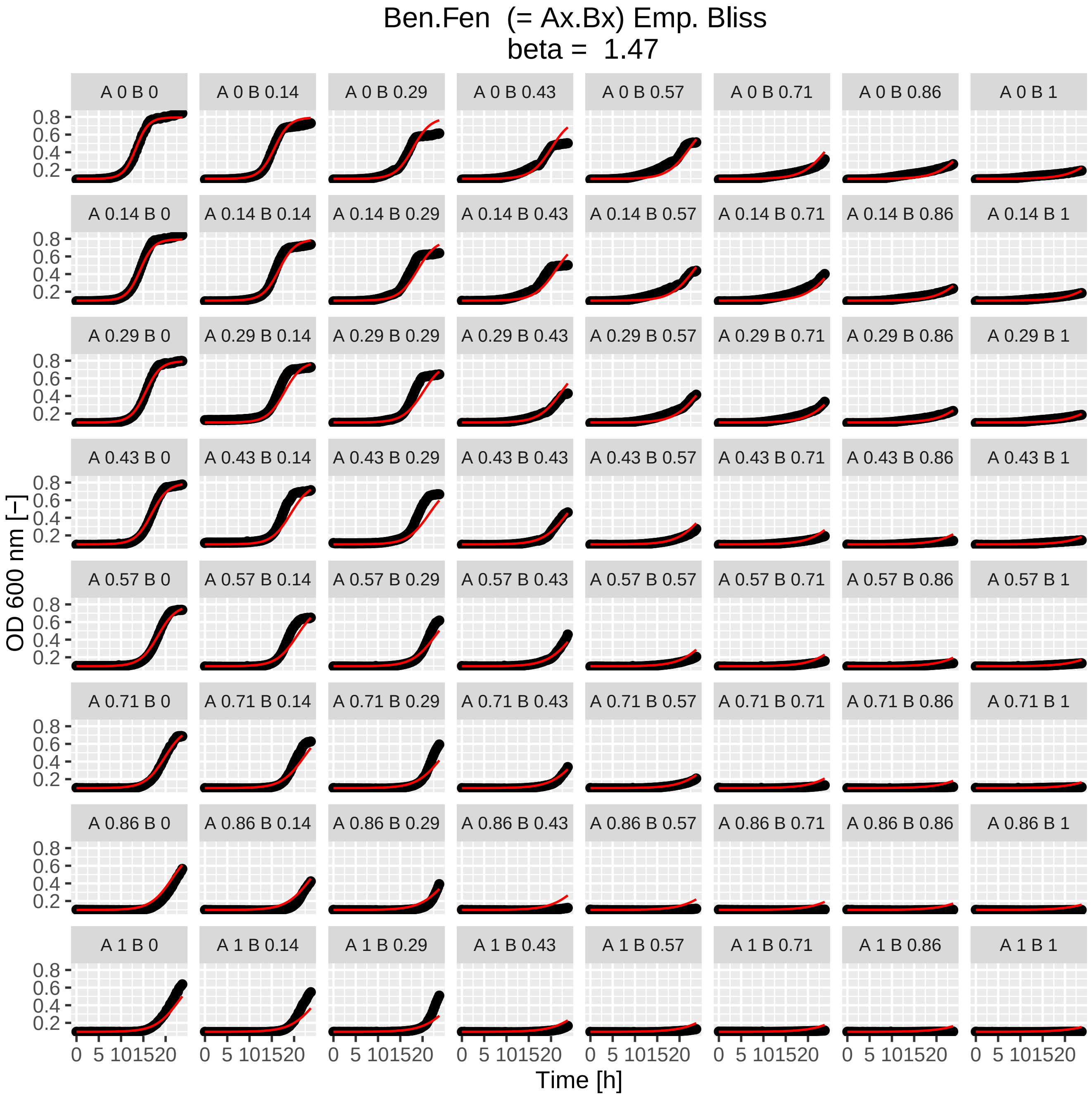

Ben.Fen (= Ax.Bx) Emp. Bliss
beta = 1.47
A 0 B 0
A 0 B 0.14
A 0 B 0.29
A 0 B 0.43
A 0 B 0.57
A 0 B 0.71
A 0 B 0.86
A 0 B 1
A 0.14 B 0
A 0.14 B 0.14
A 0.14 B 0.29
A 0.14 B 0.43
A 0.14 B 0.57
A 0.14 B 0.71
A 0.14 B 0.86
A 0.14 B 1
A 0.29 B 0
A 0.29 B 0.14
A 0.29 B 0.29
A 0.29 B 0.43
A 0.29 B 0.57
A 0.29 B 0.71
A 0.29 B 0.86
A 0.29 B 1
A 0.43 B 0
A 0.43 B 0.14
A 0.43 B 0.29
A 0.43 B 0.43
A 0.43 B 0.57
A 0.43 B 0.71
A 0.43 B 0.86
A 0.43 B 1
A 0.57 B 0
A 0.57 B 0.14
A 0.57 B 0.29
A 0.57 B 0.43
A 0.57 B 0.57
A 0.57 B 0.71
A 0.57 B 0.86
A 0.57 B 1
A 0.71 B 0
A 0.71 B 0.14
A 0.71 B 0.29
A 0.71 B 0.43
A 0.71 B 0.57
A 0.71 B 0.71
A 0.71 B 0.86
A 0.71 B 1
A 0.86 B 0
A 0.86 B 0.14
A 0.86 B 0.29
A 0.86 B 0.43
A 0.86 B 0.57
A 0.86 B 0.71
A 0.86 B 0.86
A 0.86 B 1
A 1 B 0
A 1 B 0.14
A 1 B 0.29
A 1 B 0.43
A 1 B 0.57
A 1 B 0.71
A 1 B 0.86
A 1 B 1
OD 600 nm [–]
0.8
0.6
0.4
0.2
0 5 101520
Time [h]

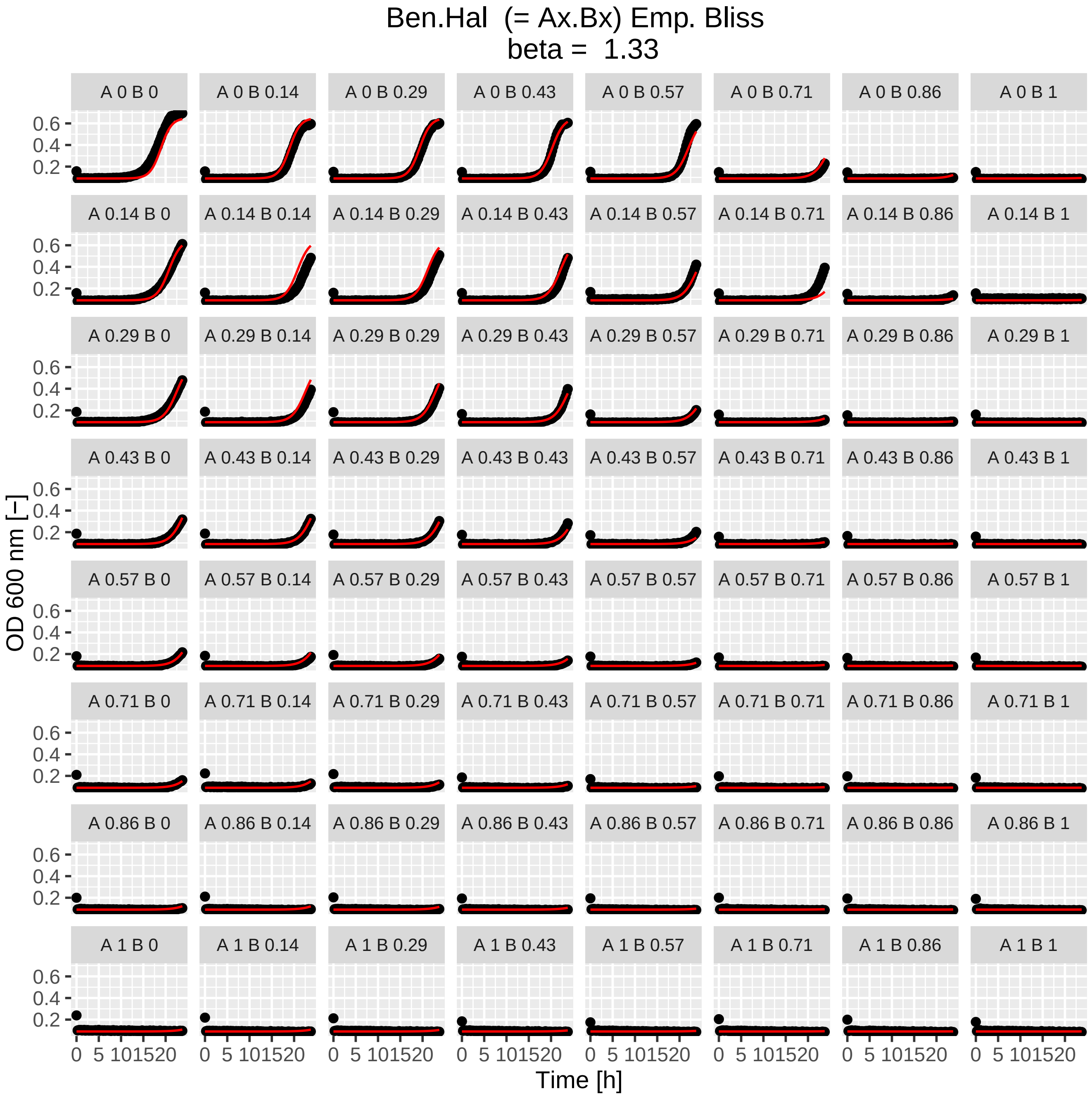
Ben.Hal (= Ax.Bx) Emp. Bliss
beta = 1.33
A 0 B 0
A 0 B 0.14
A 0 B 0.29
A 0 B 0.43
A 0 B 0.57
A 0 B 0.71
A 0 B 0.86
A 0 B 1
A 0.14 B 0
A 0.14 B 0.14
A 0.14 B 0.29
A 0.14 B 0.43
A 0.14 B 0.57
A 0.14 B 0.71
A 0.14 B 0.86
A 0.14 B 1
A 0.29 B 0
A 0.29 B 0.14
A 0.29 B 0.29
A 0.29 B 0.43
A 0.29 B 0.57
A 0.29 B 0.71
A 0.29 B 0.86
A 0.29 B 1
A 0.43 B 0
A 0.43 B 0.14
A 0.43 B 0.29
A 0.43 B 0.43
A 0.43 B 0.57
A 0.43 B 0.71
A 0.43 B 0.86
A 0.43 B 1
A 0.57 B 0
A 0.57 B 0.14
A 0.57 B 0.29
A 0.57 B 0.43
A 0.57 B 0.57
A 0.57 B 0.71
A 0.57 B 0.86
A 0.57 B 1
A 0.71 B 0
A 0.71 B 0.14
A 0.71 B 0.29
A 0.71 B 0.43
A 0.71 B 0.57
A 0.71 B 0.71
A 0.71 B 0.86
A 0.71 B 1
A 0.86 B 0
A 0.86 B 0.14
A 0.86 B 0.29
A 0.86 B 0.43
A 0.86 B 0.57
A 0.86 B 0.71
A 0.86 B 0.86
A 0.86 B 1
A 1 B 0
A 1 B 0.14
A 1 B 0.29
A 1 B 0.43
A 1 B 0.57
A 1 B 0.71
A 1 B 0.86
A 1 B 1
0.6
0.4
0.2
0 5 101520
OD 600 nm [−]
Time [h]

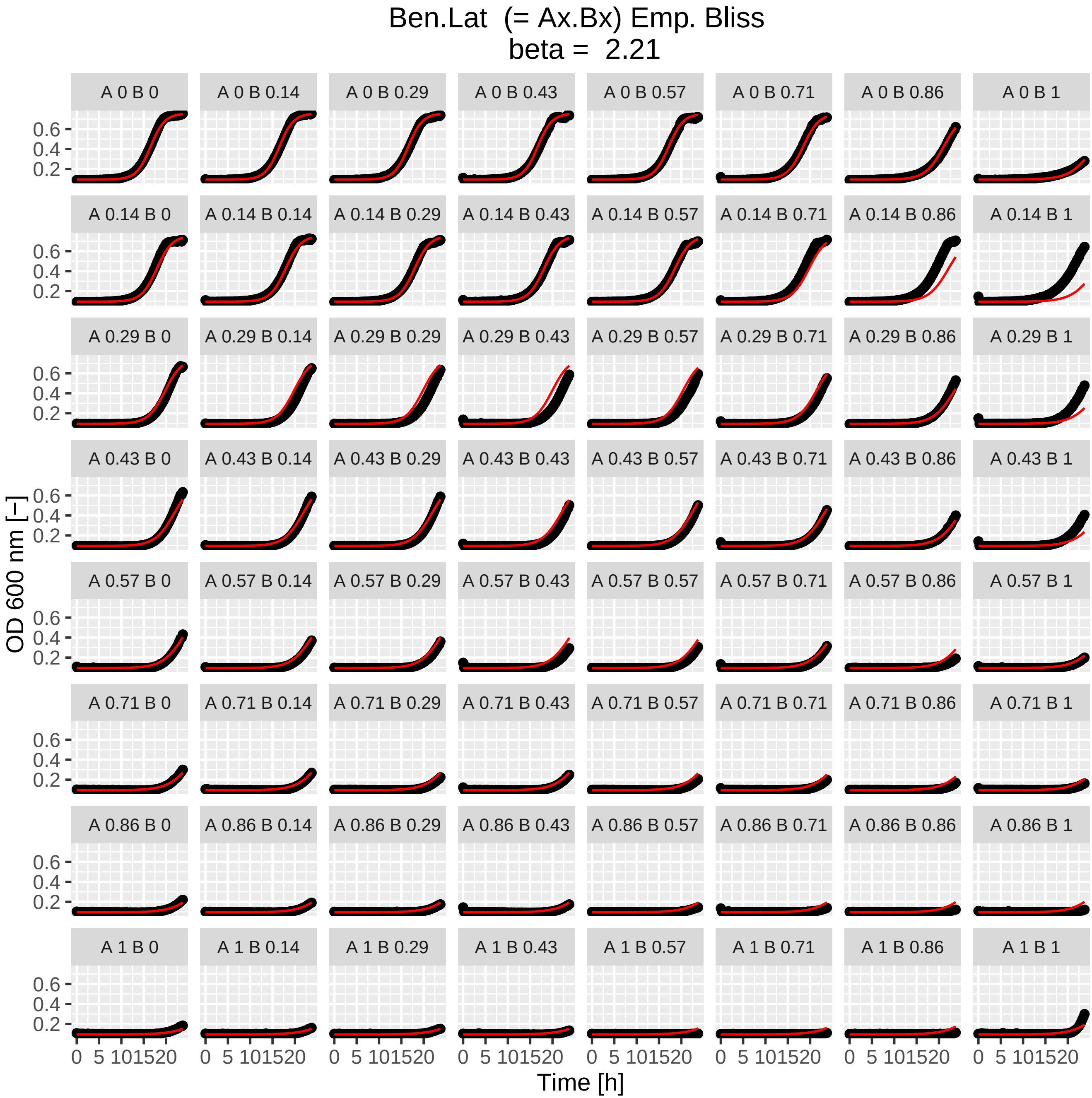
Ben.Lat (= Ax.Bx) Emp. Bliss
beta = 2.21
A 0 B 0
A 0 B 0.14
A 0 B 0.29
A 0 B 0.43
A 0 B 0.57
A 0 B 0.71
A 0 B 0.86
A 0 B 1
A 0.14 B 0
A 0.14 B 0.14
A 0.14 B 0.29
A 0.14 B 0.43
A 0.14 B 0.57
A 0.14 B 0.71
A 0.14 B 0.86
A 0.14 B 1
A 0.29 B 0
A 0.29 B 0.14
A 0.29 B 0.29
A 0.29 B 0.43
A 0.29 B 0.57
A 0.29 B 0.71
A 0.29 B 0.86
A 0.29 B 1
A 0.43 B 0
A 0.43 B 0.14
A 0.43 B 0.29
A 0.43 B 0.43
A 0.43 B 0.57
A 0.43 B 0.71
A 0.43 B 0.86
A 0.43 B 1
A 0.57 B 0
A 0.57 B 0.14
A 0.57 B 0.29
A 0.57 B 0.43
A 0.57 B 0.57
A 0.57 B 0.71
A 0.57 B 0.86
A 0.57 B 1
A 0.71 B 0
A 0.71 B 0.14
A 0.71 B 0.29
A 0.71 B 0.43
A 0.71 B 0.57
A 0.71 B 0.71
A 0.71 B 0.86
A 0.71 B 1
A 0.86 B 0
A 0.86 B 0.14
A 0.86 B 0.29
A 0.86 B 0.43
A 0.86 B 0.57
A 0.86 B 0.71
A 0.86 B 0.86
A 0.86 B 1
A 1 B 0
A 1 B 0.14
A 1 B 0.29
A 1 B 0.43
A 1 B 0.57
A 1 B 0.71
A 1 B 0.86
A 1 B 1
OD 600 nm [–]
0.6
0.4
0.2
0 5 101520
Time [h]

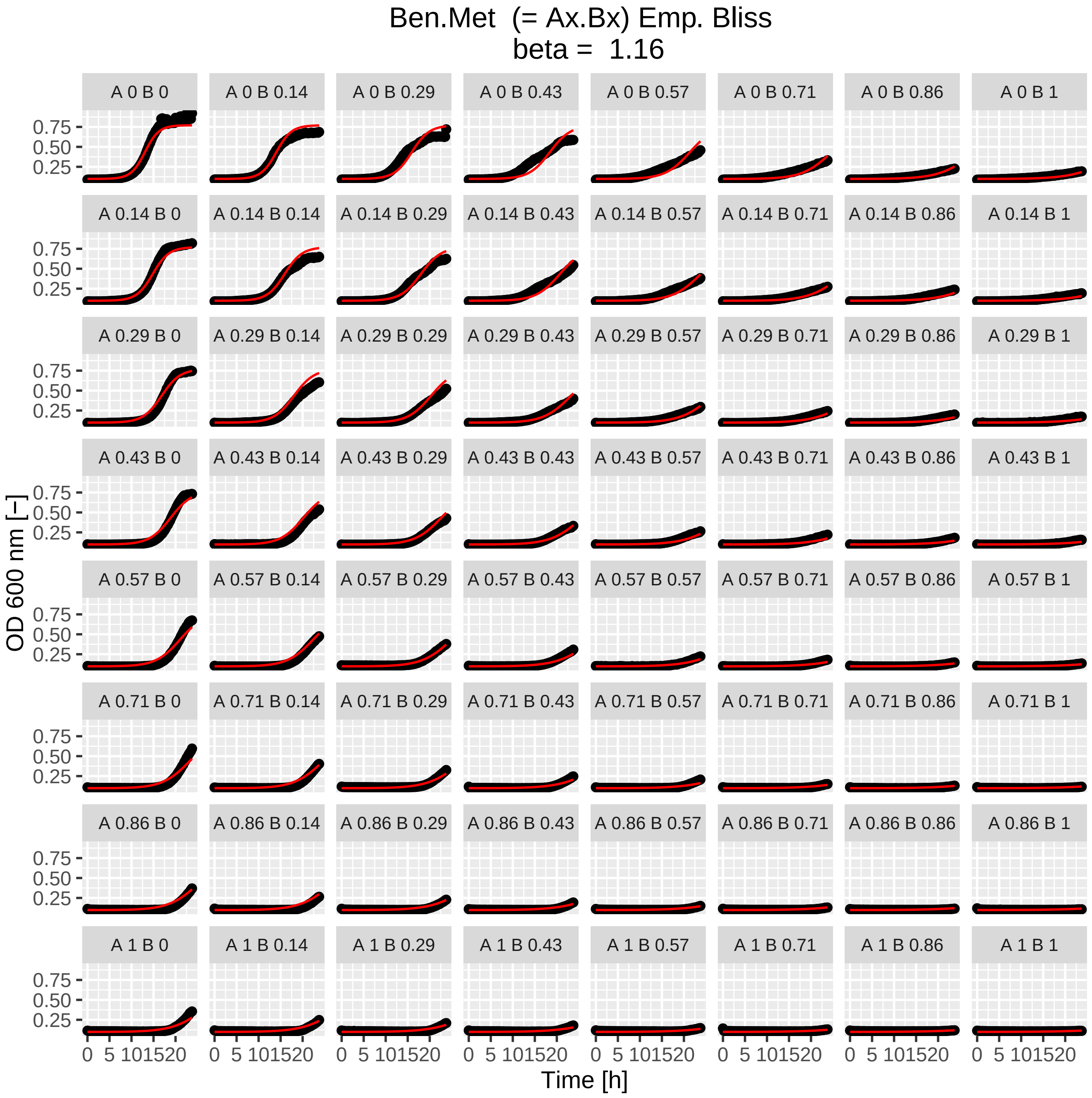

Ben.Met (= Ax.Bx) Emp. Bliss
beta = 1.16
A 0 B 0
A 0 B 0.14
A 0 B 0.29
A 0 B 0.43
A 0 B 0.57
A 0 B 0.71
A 0 B 0.86
A 0 B 1
A 0.14 B 0
A 0.14 B 0.14
A 0.14 B 0.29
A 0.14 B 0.43
A 0.14 B 0.57
A 0.14 B 0.71
A 0.14 B 0.86
A 0.14 B 1
A 0.29 B 0
A 0.29 B 0.14
A 0.29 B 0.29
A 0.29 B 0.43
A 0.29 B 0.57
A 0.29 B 0.71
A 0.29 B 0.86
A 0.29 B 1
A 0.43 B 0
A 0.43 B 0.14
A 0.43 B 0.29
A 0.43 B 0.43
A 0.43 B 0.57
A 0.43 B 0.71
A 0.43 B 0.86
A 0.43 B 1
A 0.57 B 0
A 0.57 B 0.14
A 0.57 B 0.29
A 0.57 B 0.43
A 0.57 B 0.57
A 0.57 B 0.71
A 0.57 B 0.86
A 0.57 B 1
A 0.71 B 0
A 0.71 B 0.14
A 0.71 B 0.29
A 0.71 B 0.43
A 0.71 B 0.57
A 0.71 B 0.71
A 0.71 B 0.86
A 0.71 B 1
A 0.86 B 0
A 0.86 B 0.14
A 0.86 B 0.29
A 0.86 B 0.43
A 0.86 B 0.57
A 0.86 B 0.71
A 0.86 B 0.86
A 0.86 B 1
A 1 B 0
A 1 B 0.14
A 1 B 0.29
A 1 B 0.43
A 1 B 0.57
A 1 B 0.71
A 1 B 0.86
A 1 B 1
0.75
0.50
0.25
0 5 101520
OD 600 nm [–]
Time [h]

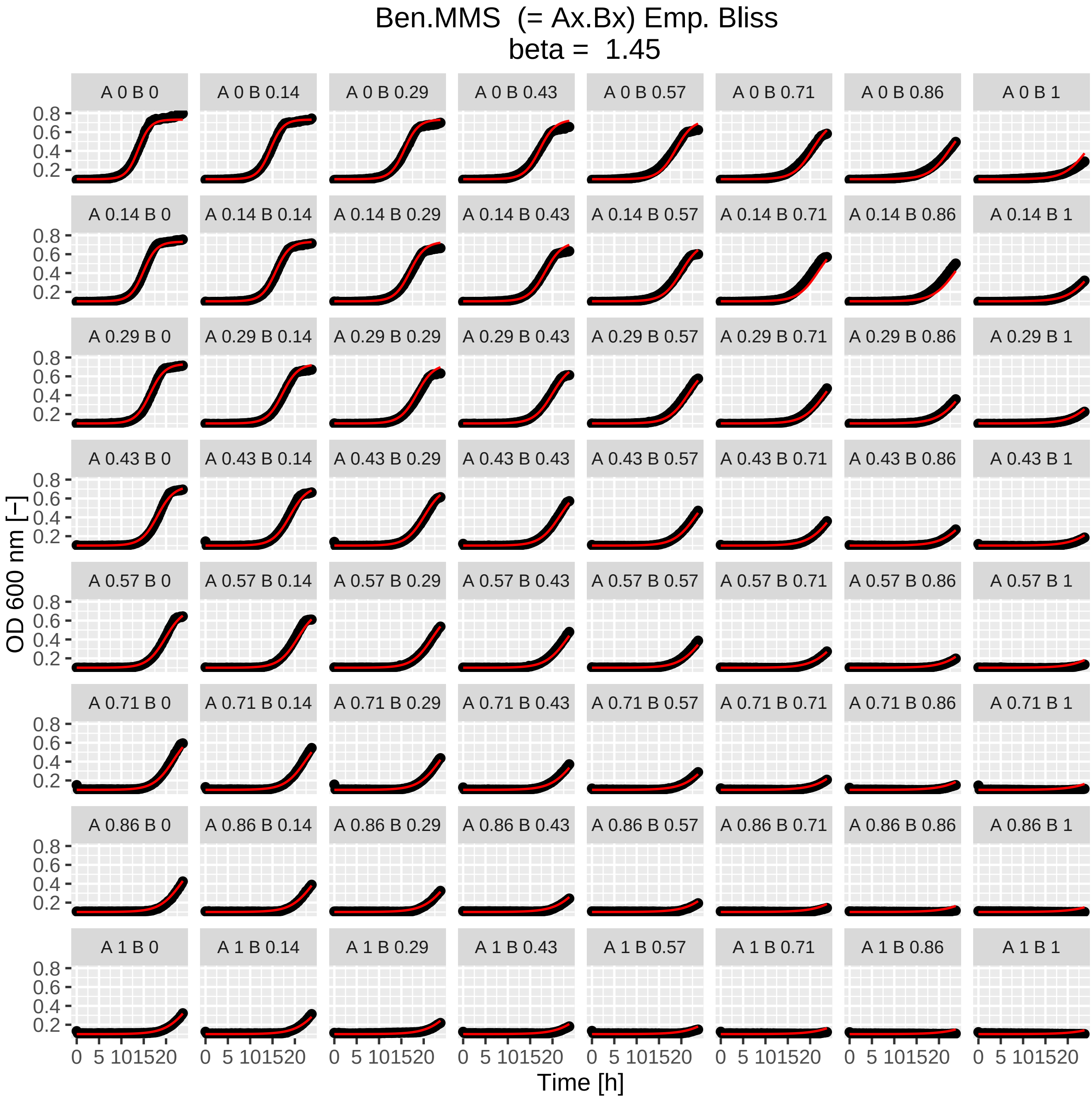

Ben.MMS (= Ax.Bx) Emp. Bliss
beta = 1.45
A 0 B 0
A 0 B 0.14
A 0 B 0.29
A 0 B 0.43
A 0 B 0.57
A 0 B 0.71
A 0 B 0.86
A 0 B 1
A 0.14 B 0
A 0.14 B 0.14
A 0.14 B 0.29
A 0.14 B 0.43
A 0.14 B 0.57
A 0.14 B 0.71
A 0.14 B 0.86
A 0.14 B 1
A 0.29 B 0
A 0.29 B 0.14
A 0.29 B 0.29
A 0.29 B 0.43
A 0.29 B 0.57
A 0.29 B 0.71
A 0.29 B 0.86
A 0.29 B 1
A 0.43 B 0
A 0.43 B 0.14
A 0.43 B 0.29
A 0.43 B 0.43
A 0.43 B 0.57
A 0.43 B 0.71
A 0.43 B 0.86
A 0.43 B 1
A 0.57 B 0
A 0.57 B 0.14
A 0.57 B 0.29
A 0.57 B 0.43
A 0.57 B 0.57
A 0.57 B 0.71
A 0.57 B 0.86
A 0.57 B 1
A 0.71 B 0
A 0.71 B 0.14
A 0.71 B 0.29
A 0.71 B 0.43
A 0.71 B 0.57
A 0.71 B 0.71
A 0.71 B 0.86
A 0.71 B 1
A 0.86 B 0
A 0.86 B 0.14
A 0.86 B 0.29
A 0.86 B 0.43
A 0.86 B 0.57
A 0.86 B 0.71
A 0.86 B 0.86
A 0.86 B 1
A 1 B 0
A 1 B 0.14
A 1 B 0.29
A 1 B 0.43
A 1 B 0.57
A 1 B 0.71
A 1 B 0.86
A 1 B 1
0.8
0.6
0.4
0.2
OD 600 nm [–]
0 5 101520
Time [h]

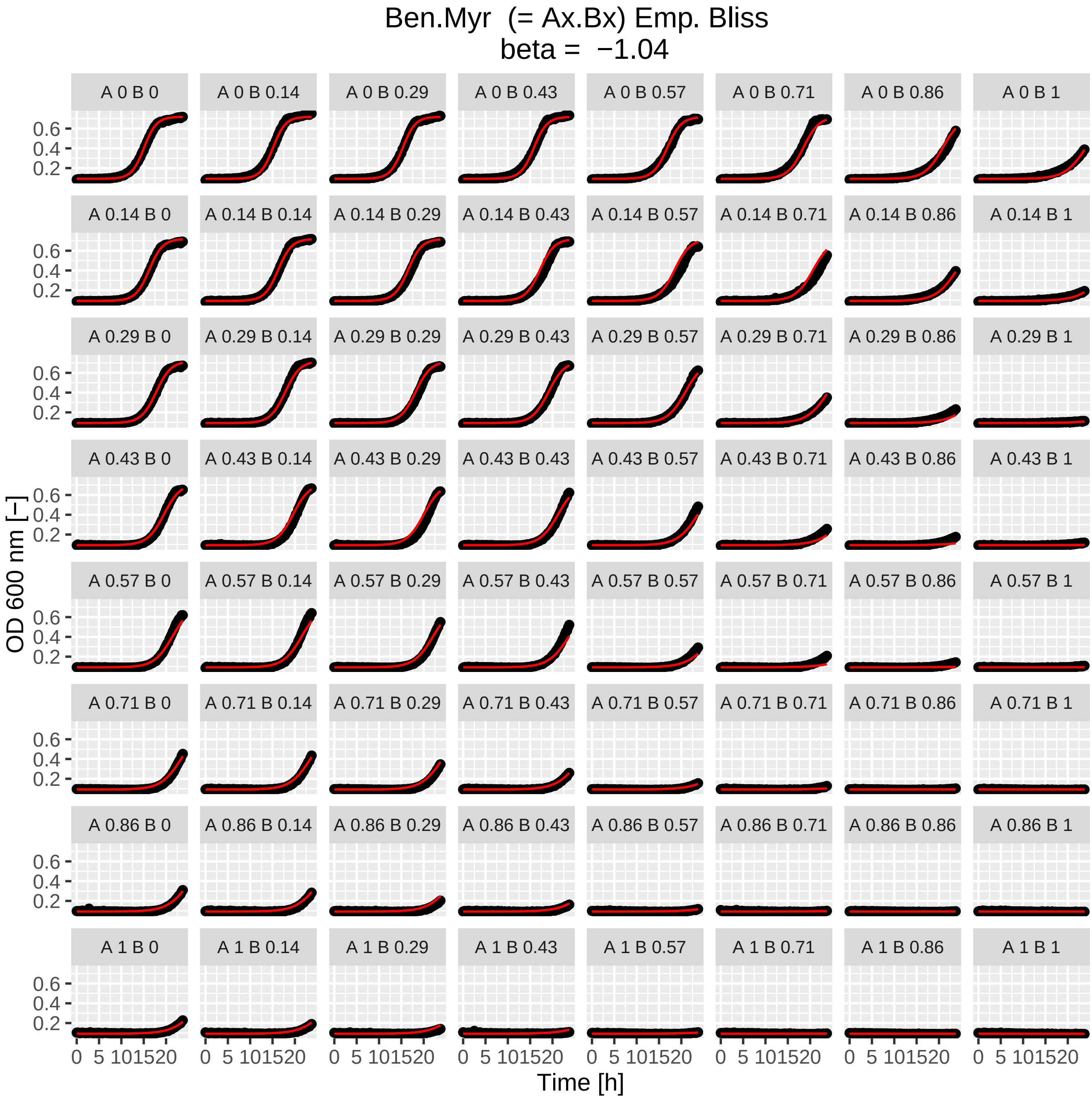
Ben.Myr (= Ax.Bx) Emp. Bliss
beta = −1.04
OD 600 nm [−]
Time [h]
A 0 B 0
A 0 B 0.14
A 0 B 0.29
A 0 B 0.43
A 0 B 0.57
A 0 B 0.71
A 0 B 0.86
A 0 B 1
A 0.14 B 0
A 0.14 B 0.14
A 0.14 B 0.29
A 0.14 B 0.43
A 0.14 B 0.57
A 0.14 B 0.71
A 0.14 B 0.86
A 0.14 B 1
A 0.29 B 0
A 0.29 B 0.14
A 0.29 B 0.29
A 0.29 B 0.43
A 0.29 B 0.57
A 0.29 B 0.71
A 0.29 B 0.86
A 0.29 B 1
A 0.43 B 0
A 0.43 B 0.14
A 0.43 B 0.29
A 0.43 B 0.43
A 0.43 B 0.57
A 0.43 B 0.71
A 0.43 B 0.86
A 0.43 B 1
A 0.57 B 0
A 0.57 B 0.14
A 0.57 B 0.29
A 0.57 B 0.43
A 0.57 B 0.57
A 0.57 B 0.71
A 0.57 B 0.86
A 0.57 B 1
A 0.71 B 0
A 0.71 B 0.14
A 0.71 B 0.29
A 0.71 B 0.43
A 0.71 B 0.57
A 0.71 B 0.71
A 0.71 B 0.86
A 0.71 B 1
A 0.86 B 0
A 0.86 B 0.14
A 0.86 B 0.29
A 0.86 B 0.43
A 0.86 B 0.57
A 0.86 B 0.71
A 0.86 B 0.86
A 0.86 B 1
A 1 B 0
A 1 B 0.14
A 1 B 0.29
A 1 B 0.43
A 1 B 0.57
A 1 B 0.71
A 1 B 0.86
A 1 B 1
0.6
0.4
0.2
0 5 101520

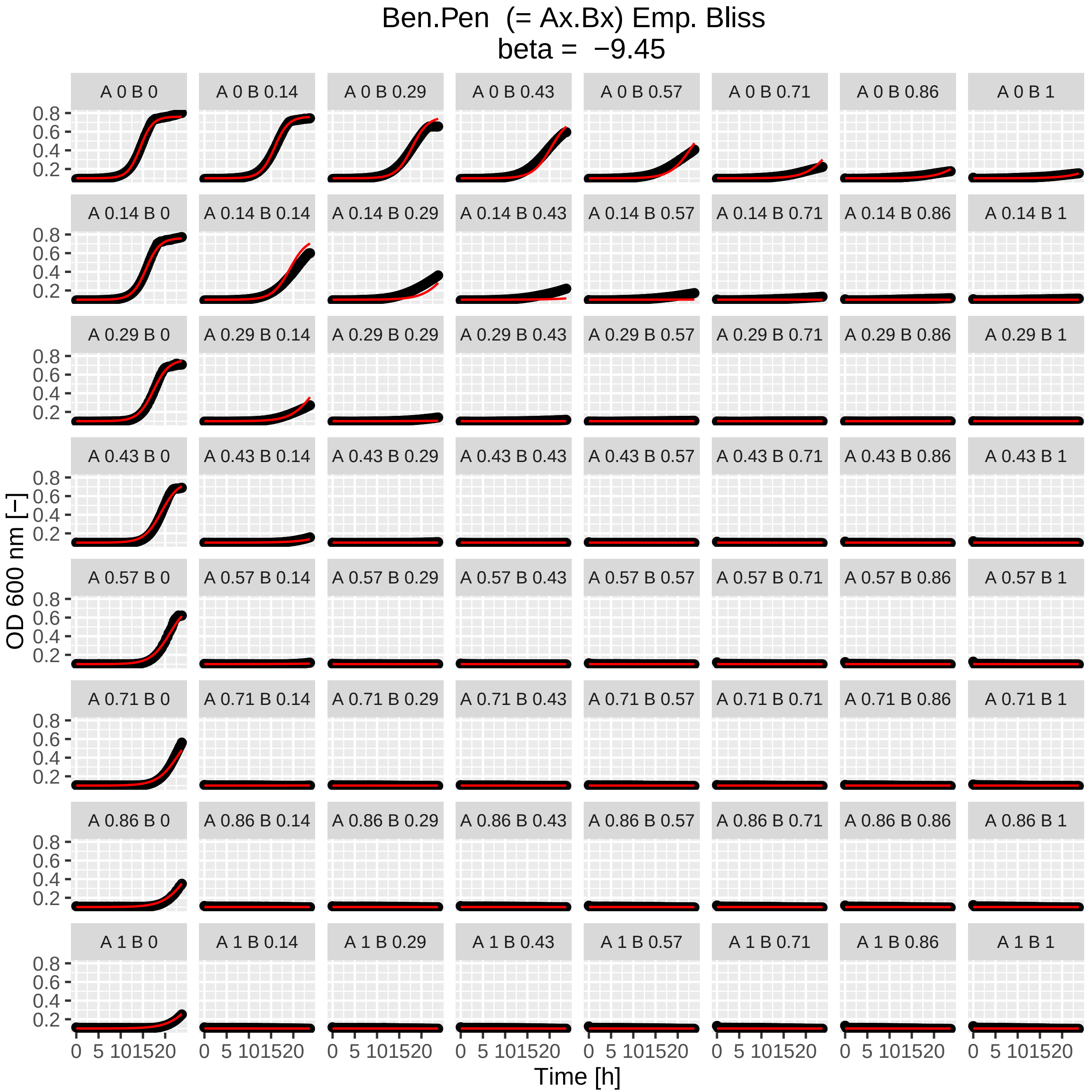

Ben.Pen (= Ax.Bx) Emp. Bliss
beta = −9.45
A 0 B 0
A 0 B 0.14
A 0 B 0.29
A 0 B 0.43
A 0 B 0.57
A 0 B 0.71
A 0 B 0.86
A 0 B 1
A 0.14 B 0
A 0.14 B 0.14
A 0.14 B 0.29
A 0.14 B 0.43
A 0.14 B 0.57
A 0.14 B 0.71
A 0.14 B 0.86
A 0.14 B 1
A 0.29 B 0
A 0.29 B 0.14
A 0.29 B 0.29
A 0.29 B 0.43
A 0.29 B 0.57
A 0.29 B 0.71
A 0.29 B 0.86
A 0.29 B 1
A 0.43 B 0
A 0.43 B 0.14
A 0.43 B 0.29
A 0.43 B 0.43
A 0.43 B 0.57
A 0.43 B 0.71
A 0.43 B 0.86
A 0.43 B 1
A 0.57 B 0
A 0.57 B 0.14
A 0.57 B 0.29
A 0.57 B 0.43
A 0.57 B 0.57
A 0.57 B 0.71
A 0.57 B 0.86
A 0.57 B 1
A 0.71 B 0
A 0.71 B 0.14
A 0.71 B 0.29
A 0.71 B 0.43
A 0.71 B 0.57
A 0.71 B 0.71
A 0.71 B 0.86
A 0.71 B 1
A 0.86 B 0
A 0.86 B 0.14
A 0.86 B 0.29
A 0.86 B 0.43
A 0.86 B 0.57
A 0.86 B 0.71
A 0.86 B 0.86
A 0.86 B 1
A 1 B 0
A 1 B 0.14
A 1 B 0.29
A 1 B 0.43
A 1 B 0.57
A 1 B 0.71
A 1 B 0.86
A 1 B 1
OD 600 nm [−]
0.2
0.4
0.6
0.8
0
5
10
15
20
Time [h]

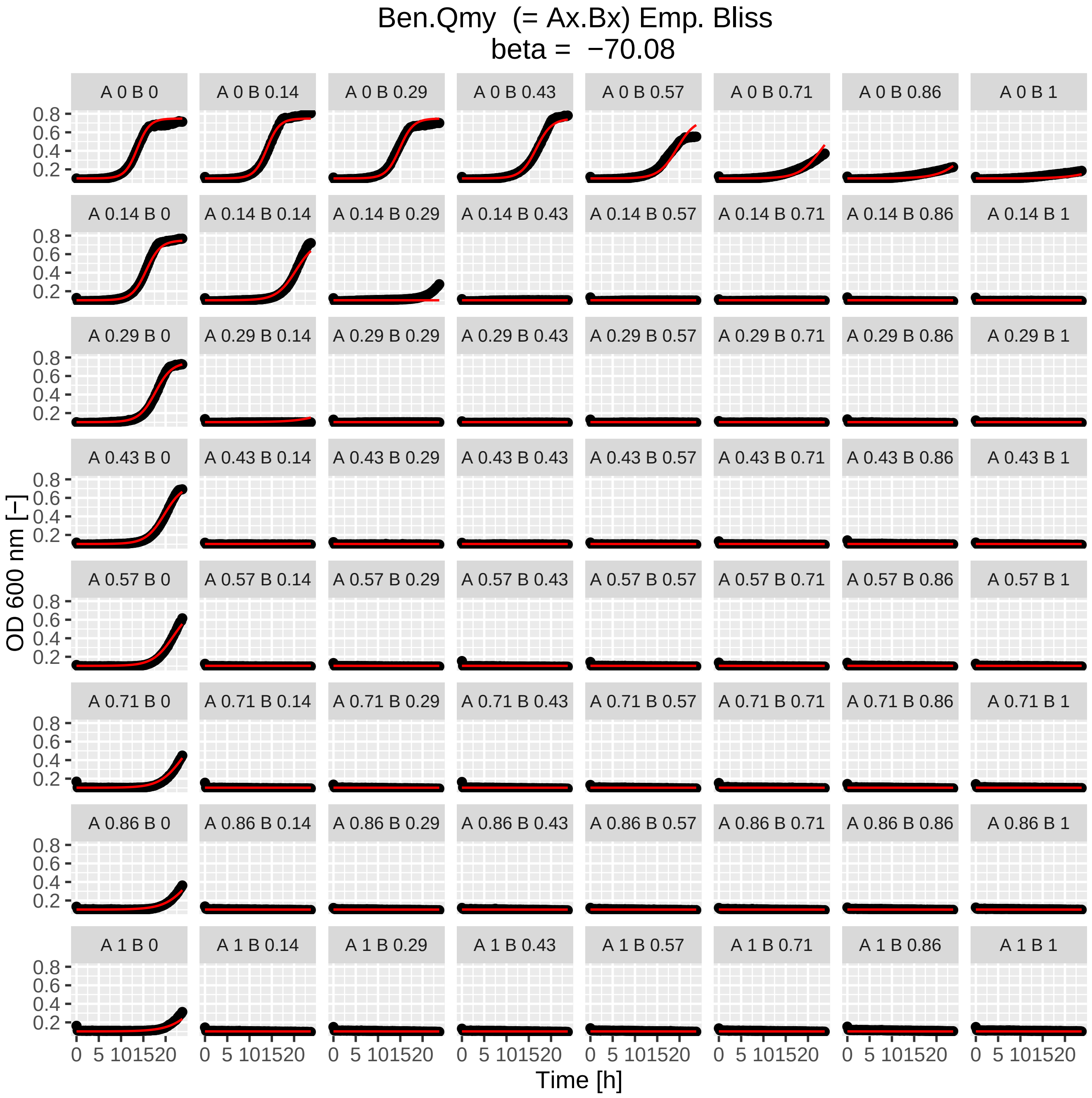

Ben.Qmy (= Ax.Bx) Emp. Bliss
beta = −70.08
OD 600 nm [−]
Time [h]
A 0 B 0
A 0 B 0.14
A 0 B 0.29
A 0 B 0.43
A 0 B 0.57
A 0 B 0.71
A 0 B 0.86
A 0 B 1
A 0.14 B 0
A 0.14 B 0.14
A 0.14 B 0.29
A 0.14 B 0.43
A 0.14 B 0.57
A 0.14 B 0.71
A 0.14 B 0.86
A 0.14 B 1
A 0.29 B 0
A 0.29 B 0.14
A 0.29 B 0.29
A 0.29 B 0.43
A 0.29 B 0.57
A 0.29 B 0.71
A 0.29 B 0.86
A 0.29 B 1
A 0.43 B 0
A 0.43 B 0.14
A 0.43 B 0.29
A 0.43 B 0.43
A 0.43 B 0.57
A 0.43 B 0.71
A 0.43 B 0.86
A 0.43 B 1
A 0.57 B 0
A 0.57 B 0.14
A 0.57 B 0.29
A 0.57 B 0.43
A 0.57 B 0.57
A 0.57 B 0.71
A 0.57 B 0.86
A 0.57 B 1
A 0.71 B 0
A 0.71 B 0.14
A 0.71 B 0.29
A 0.71 B 0.43
A 0.71 B 0.57
A 0.71 B 0.71
A 0.71 B 0.86
A 0.71 B 1
A 0.86 B 0
A 0.86 B 0.14
A 0.86 B 0.29
A 0.86 B 0.43
A 0.86 B 0.57
A 0.86 B 0.71
A 0.86 B 0.86
A 0.86 B 1
A 1 B 0
A 1 B 0.14
A 1 B 0.29
A 1 B 0.43
A 1 B 0.57
A 1 B 0.71
A 1 B 0.86
A 1 B 1
0.8
0.6
0.4
0.2
0 5 101520

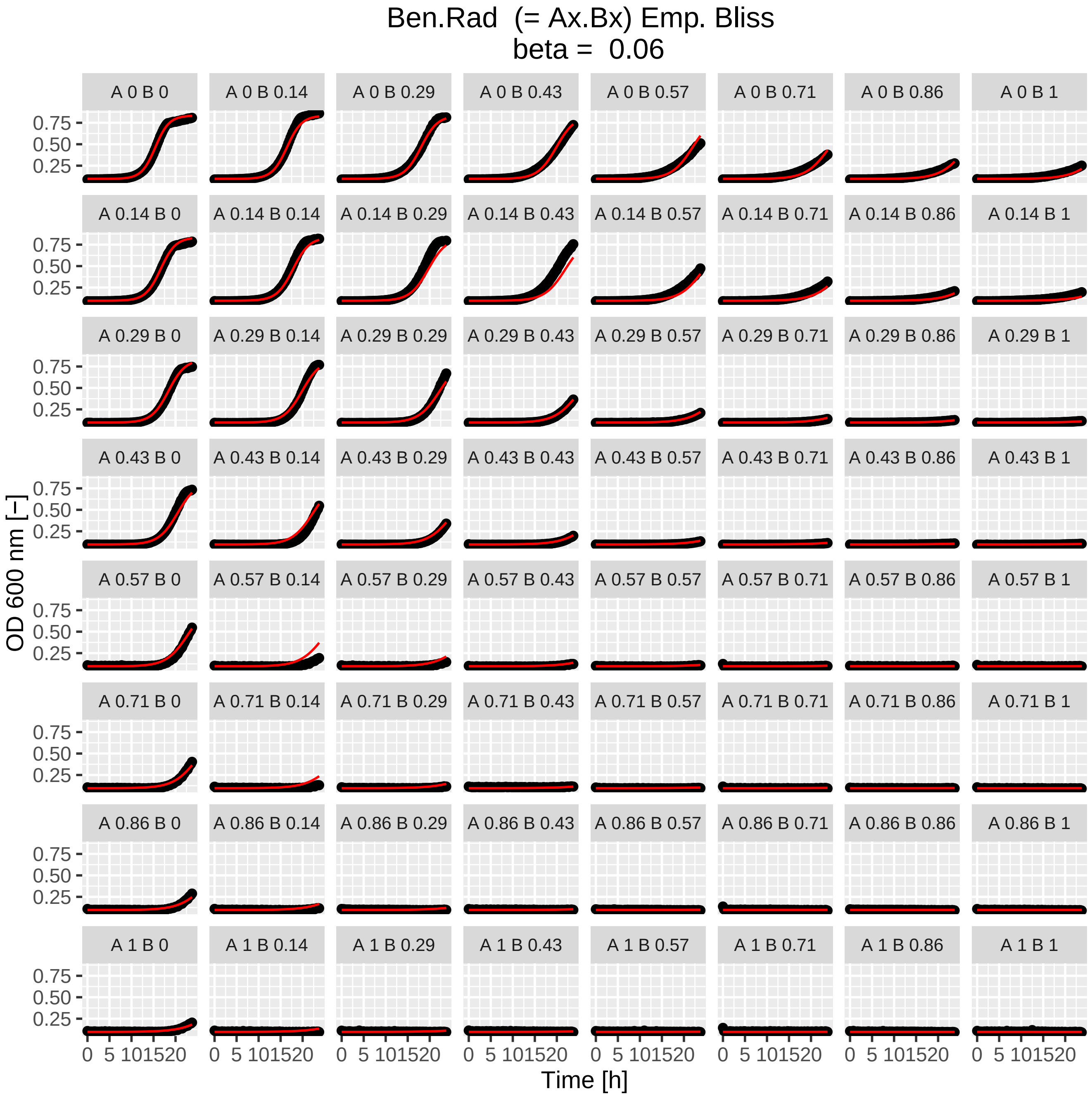
Ben.Rad (= Ax.Bx) Emp. Bliss
beta = 0.06
A 0 B 0
A 0 B 0.14
A 0 B 0.29
A 0 B 0.43
A 0 B 0.57
A 0 B 0.71
A 0 B 0.86
A 0 B 1
A 0.14 B 0
A 0.14 B 0.14
A 0.14 B 0.29
A 0.14 B 0.43
A 0.14 B 0.57
A 0.14 B 0.71
A 0.14 B 0.86
A 0.14 B 1
A 0.29 B 0
A 0.29 B 0.14
A 0.29 B 0.29
A 0.29 B 0.43
A 0.29 B 0.57
A 0.29 B 0.71
A 0.29 B 0.86
A 0.29 B 1
A 0.43 B 0
A 0.43 B 0.14
A 0.43 B 0.29
A 0.43 B 0.43
A 0.43 B 0.57
A 0.43 B 0.71
A 0.43 B 0.86
A 0.43 B 1
A 0.57 B 0
A 0.57 B 0.14
A 0.57 B 0.29
A 0.57 B 0.43
A 0.57 B 0.57
A 0.57 B 0.71
A 0.57 B 0.86
A 0.57 B 1
A 0.71 B 0
A 0.71 B 0.14
A 0.71 B 0.29
A 0.71 B 0.43
A 0.71 B 0.57
A 0.71 B 0.71
A 0.71 B 0.86
A 0.71 B 1
A 0.86 B 0
A 0.86 B 0.14
A 0.86 B 0.29
A 0.86 B 0.43
A 0.86 B 0.57
A 0.86 B 0.71
A 0.86 B 0.86
A 0.86 B 1
A 1 B 0
A 1 B 0.14
A 1 B 0.29
A 1 B 0.43
A 1 B 0.57
A 1 B 0.71
A 1 B 0.86
A 1 B 1
0.75
0.50
0.25
OD 600 nm [–]
0 5 101520
Time [h]

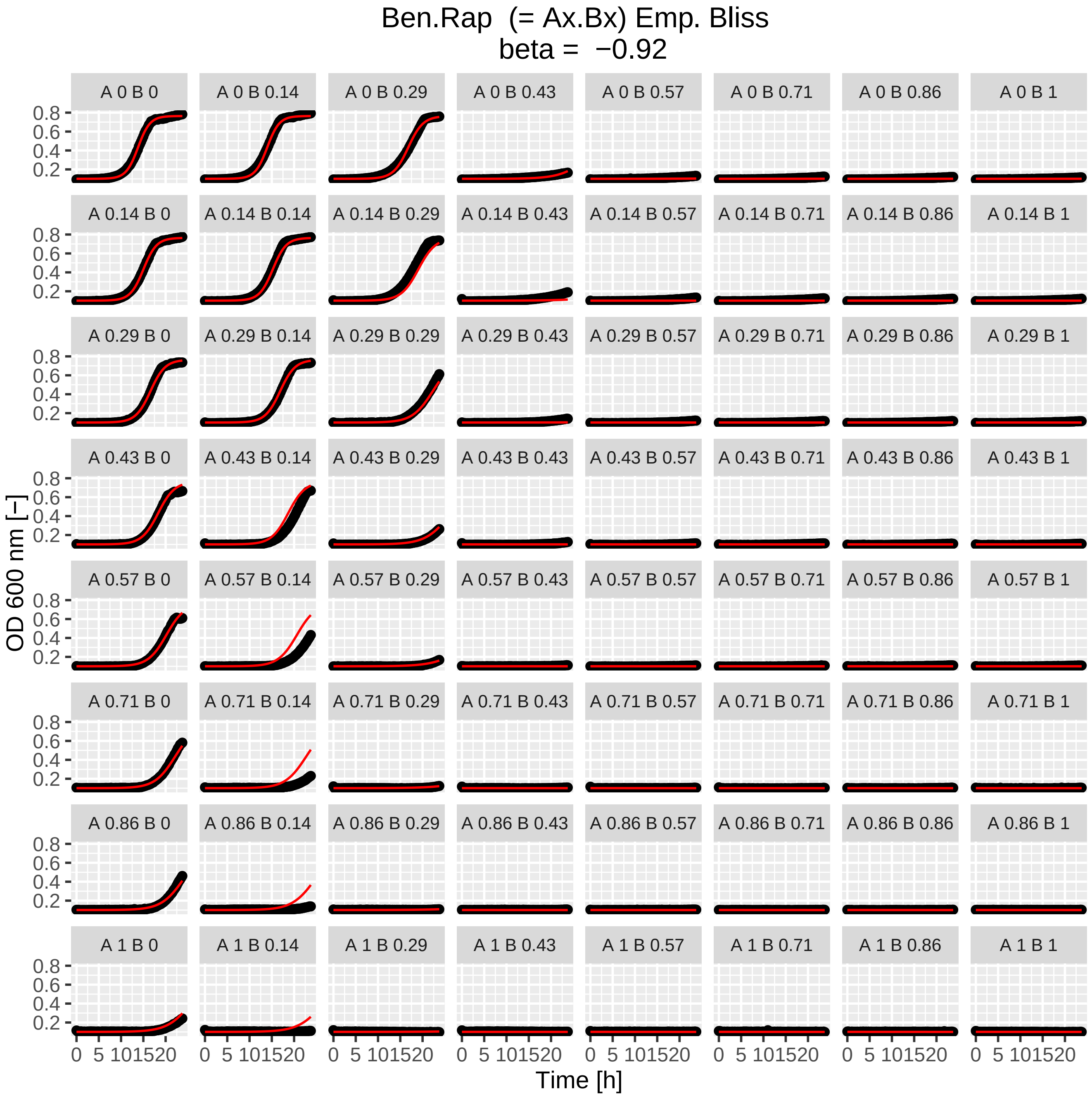

Ben.Rap (= Ax.Bx) Emp. Bliss
beta = −0.92
A 0 B 0
A 0 B 0.14
A 0 B 0.29
A 0 B 0.43
A 0 B 0.57
A 0 B 0.71
A 0 B 0.86
A 0 B 1
A 0.14 B 0
A 0.14 B 0.14
A 0.14 B 0.29
A 0.14 B 0.43
A 0.14 B 0.57
A 0.14 B 0.71
A 0.14 B 0.86
A 0.14 B 1
A 0.29 B 0
A 0.29 B 0.14
A 0.29 B 0.29
A 0.29 B 0.43
A 0.29 B 0.57
A 0.29 B 0.71
A 0.29 B 0.86
A 0.29 B 1
A 0.43 B 0
A 0.43 B 0.14
A 0.43 B 0.29
A 0.43 B 0.43
A 0.43 B 0.57
A 0.43 B 0.71
A 0.43 B 0.86
A 0.43 B 1
A 0.57 B 0
A 0.57 B 0.14
A 0.57 B 0.29
A 0.57 B 0.43
A 0.57 B 0.57
A 0.57 B 0.71
A 0.57 B 0.86
A 0.57 B 1
A 0.71 B 0
A 0.71 B 0.14
A 0.71 B 0.29
A 0.71 B 0.43
A 0.71 B 0.57
A 0.71 B 0.71
A 0.71 B 0.86
A 0.71 B 1
A 0.86 B 0
A 0.86 B 0.14
A 0.86 B 0.29
A 0.86 B 0.43
A 0.86 B 0.57
A 0.86 B 0.71
A 0.86 B 0.86
A 0.86 B 1
A 1 B 0
A 1 B 0.14
A 1 B 0.29
A 1 B 0.43
A 1 B 0.57
A 1 B 0.71
A 1 B 0.86
A 1 B 1
OD 600 nm [−]
0.2
0.4
0.6
0.8
0
5
10
15
20
Time [h]

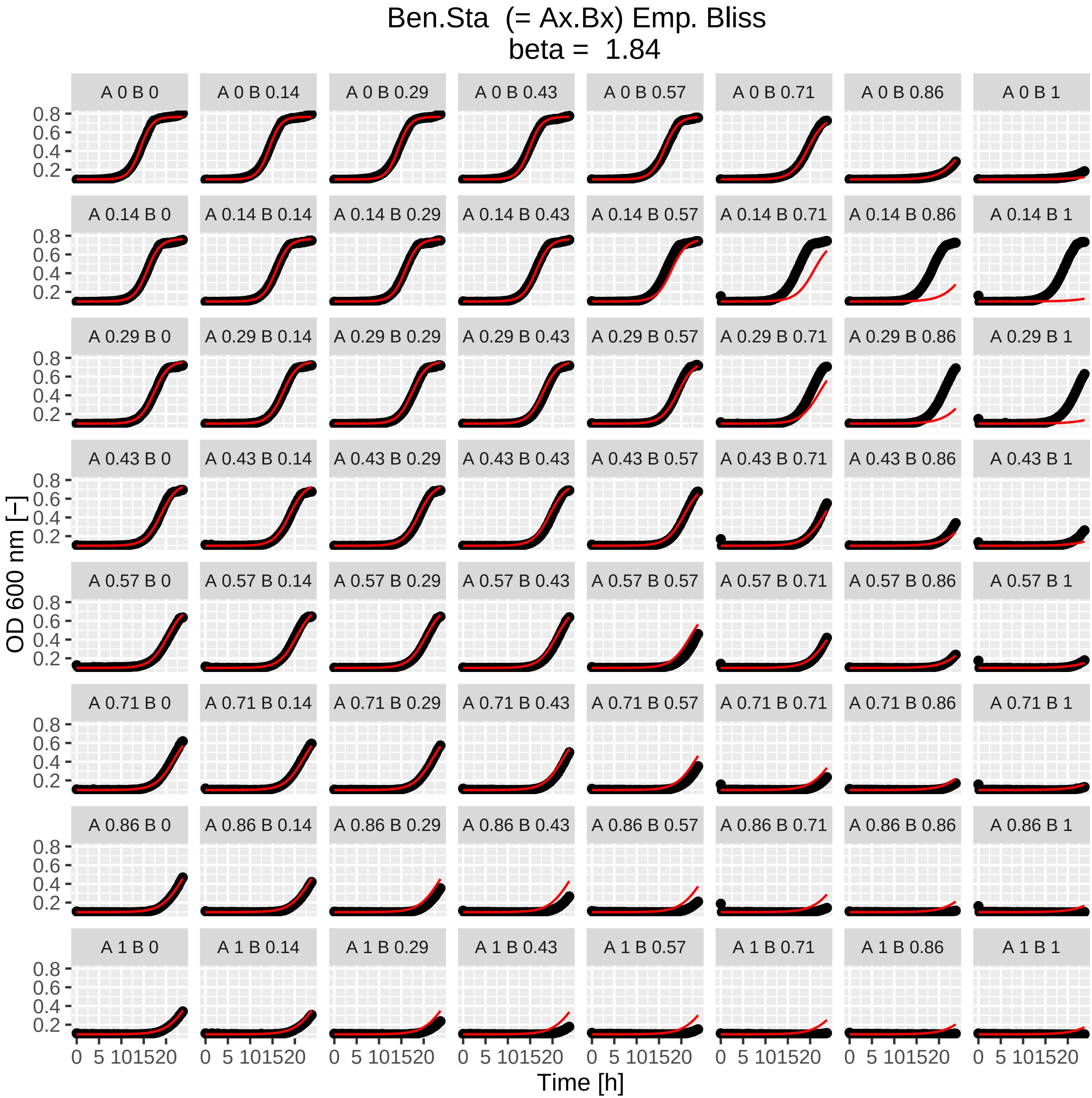

Ben.Sta (= Ax.Bx) Emp. Bliss
beta = 1.84
A 0 B 0
A 0 B 0.14
A 0 B 0.29
A 0 B 0.43
A 0 B 0.57
A 0 B 0.71
A 0 B 0.86
A 0 B 1
A 0.14 B 0
A 0.14 B 0.14
A 0.14 B 0.29
A 0.14 B 0.43
A 0.14 B 0.57
A 0.14 B 0.71
A 0.14 B 0.86
A 0.14 B 1
A 0.29 B 0
A 0.29 B 0.14
A 0.29 B 0.29
A 0.29 B 0.43
A 0.29 B 0.57
A 0.29 B 0.71
A 0.29 B 0.86
A 0.29 B 1
A 0.43 B 0
A 0.43 B 0.14
A 0.43 B 0.29
A 0.43 B 0.43
A 0.43 B 0.57
A 0.43 B 0.71
A 0.43 B 0.86
A 0.43 B 1
A 0.57 B 0
A 0.57 B 0.14
A 0.57 B 0.29
A 0.57 B 0.43
A 0.57 B 0.57
A 0.57 B 0.71
A 0.57 B 0.86
A 0.57 B 1
A 0.71 B 0
A 0.71 B 0.14
A 0.71 B 0.29
A 0.71 B 0.43
A 0.71 B 0.57
A 0.71 B 0.71
A 0.71 B 0.86
A 0.71 B 1
A 0.86 B 0
A 0.86 B 0.14
A 0.86 B 0.29
A 0.86 B 0.43
A 0.86 B 0.57
A 0.86 B 0.71
A 0.86 B 0.86
A 0.86 B 1
A 1 B 0
A 1 B 0.14
A 1 B 0.29
A 1 B 0.43
A 1 B 0.57
A 1 B 0.71
A 1 B 0.86
A 1 B 1
0.8
0.6
0.4
0.2
OD 600 nm [–]
0 5 101520
Time [h]

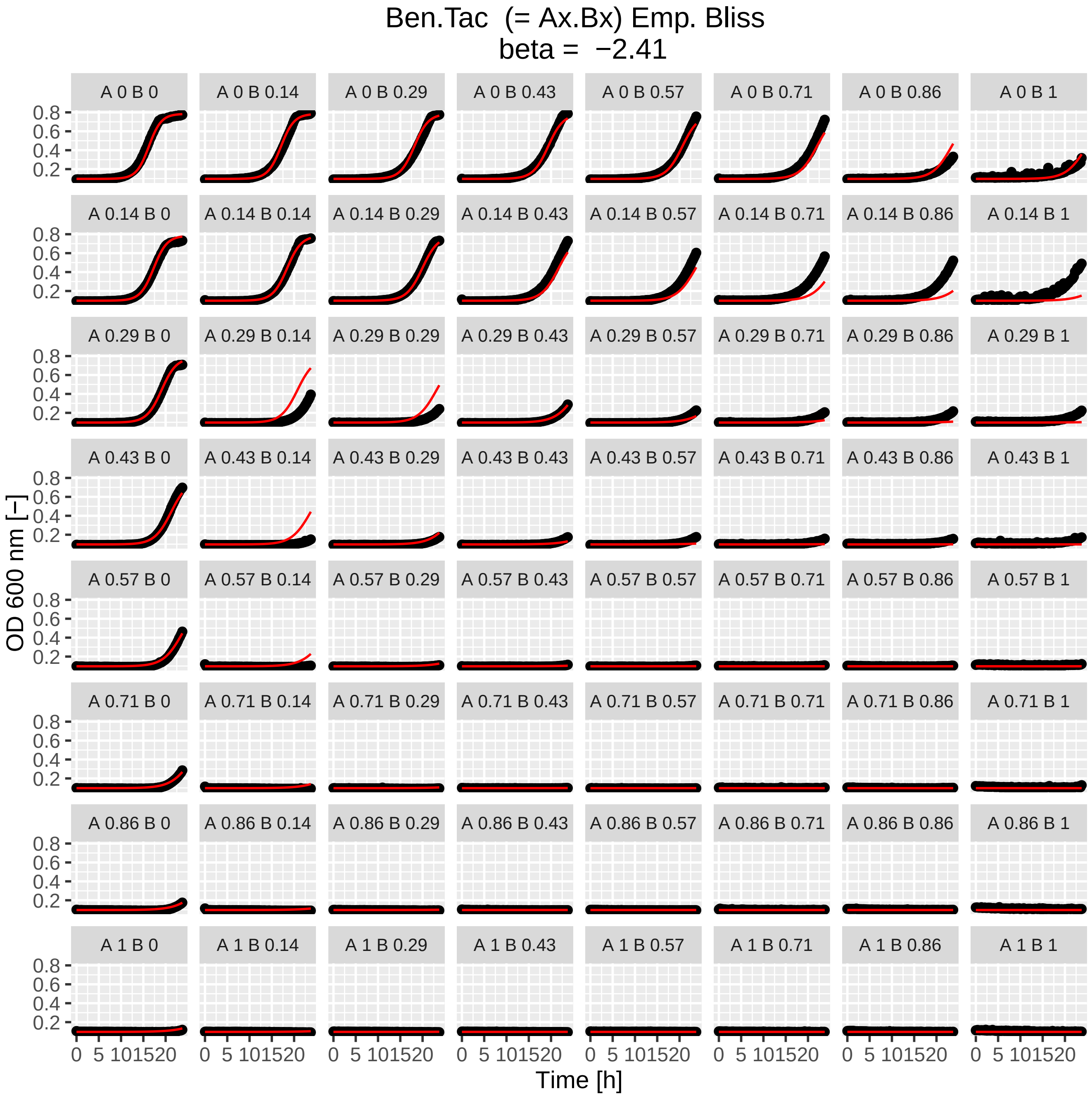

Ben.Tac (= Ax.Bx) Emp. Bliss
beta = −2.41
A 0 B 0
A 0 B 0.14
A 0 B 0.29
A 0 B 0.43
A 0 B 0.57
A 0 B 0.71
A 0 B 0.86
A 0 B 1
A 0.14 B 0
A 0.14 B 0.14
A 0.14 B 0.29
A 0.14 B 0.43
A 0.14 B 0.57
A 0.14 B 0.71
A 0.14 B 0.86
A 0.14 B 1
A 0.29 B 0
A 0.29 B 0.14
A 0.29 B 0.29
A 0.29 B 0.43
A 0.29 B 0.57
A 0.29 B 0.71
A 0.29 B 0.86
A 0.29 B 1
A 0.43 B 0
A 0.43 B 0.14
A 0.43 B 0.29
A 0.43 B 0.43
A 0.43 B 0.57
A 0.43 B 0.71
A 0.43 B 0.86
A 0.43 B 1
A 0.57 B 0
A 0.57 B 0.14
A 0.57 B 0.29
A 0.57 B 0.43
A 0.57 B 0.57
A 0.57 B 0.71
A 0.57 B 0.86
A 0.57 B 1
A 0.71 B 0
A 0.71 B 0.14
A 0.71 B 0.29
A 0.71 B 0.43
A 0.71 B 0.57
A 0.71 B 0.71
A 0.71 B 0.86
A 0.71 B 1
A 0.86 B 0
A 0.86 B 0.14
A 0.86 B 0.29
A 0.86 B 0.43
A 0.86 B 0.57
A 0.86 B 0.71
A 0.86 B 0.86
A 0.86 B 1
A 1 B 0
A 1 B 0.14
A 1 B 0.29
A 1 B 0.43
A 1 B 0.57
A 1 B 0.71
A 1 B 0.86
A 1 B 1
OD 600 nm [−]
0.2
0.4
0.6
0.8
0
5
10
15
20
Time [h]

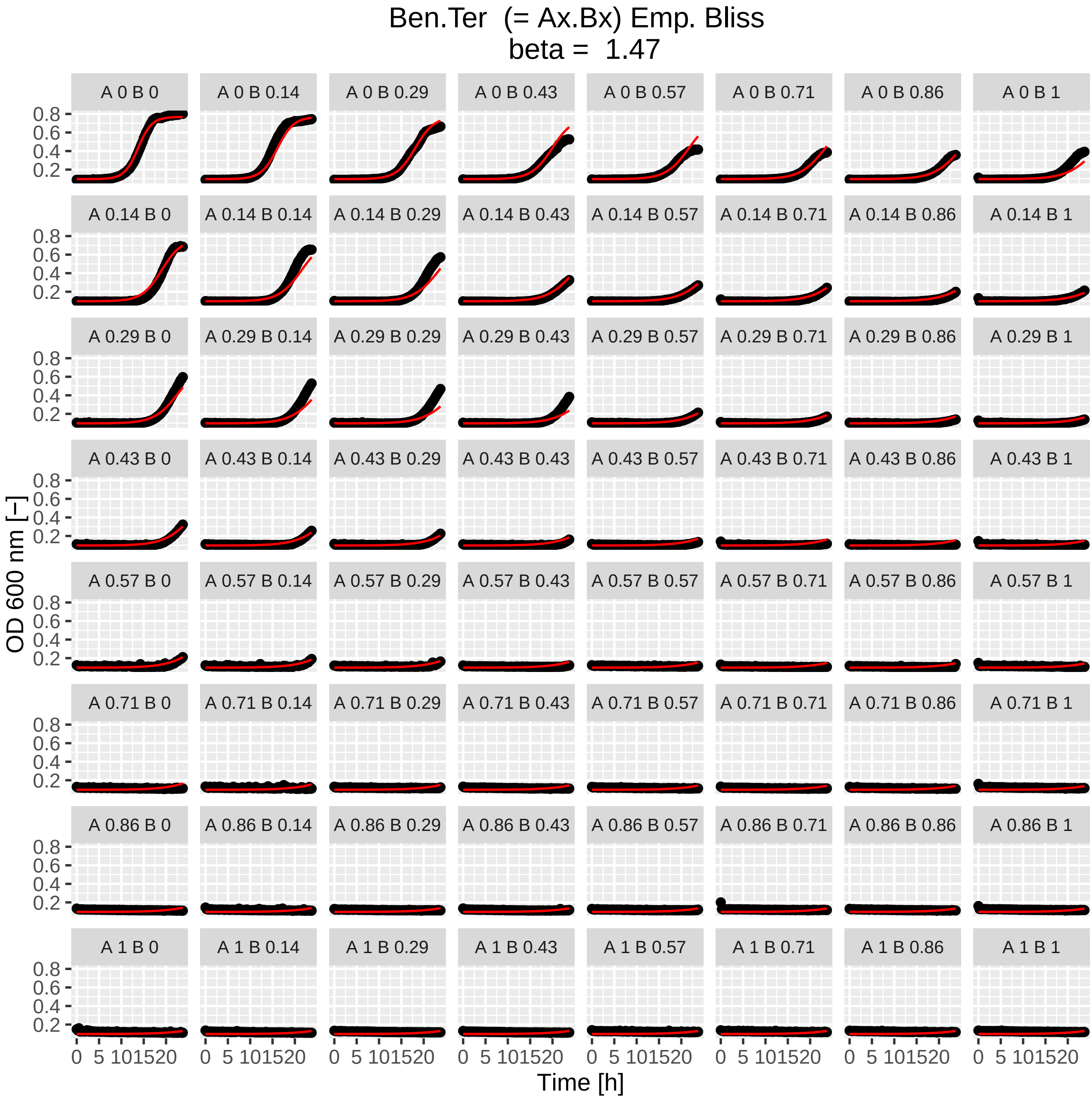

Ben.Ter (= Ax.Bx) Emp. Bliss
beta = 1.47
A 0 B 0
A 0 B 0.14
A 0 B 0.29
A 0 B 0.43
A 0 B 0.57
A 0 B 0.71
A 0 B 0.86
A 0 B 1
A 0.14 B 0
A 0.14 B 0.14
A 0.14 B 0.29
A 0.14 B 0.43
A 0.14 B 0.57
A 0.14 B 0.71
A 0.14 B 0.86
A 0.14 B 1
A 0.29 B 0
A 0.29 B 0.14
A 0.29 B 0.29
A 0.29 B 0.43
A 0.29 B 0.57
A 0.29 B 0.71
A 0.29 B 0.86
A 0.29 B 1
A 0.43 B 0
A 0.43 B 0.14
A 0.43 B 0.29
A 0.43 B 0.43
A 0.43 B 0.57
A 0.43 B 0.71
A 0.43 B 0.86
A 0.43 B 1
A 0.57 B 0
A 0.57 B 0.14
A 0.57 B 0.29
A 0.57 B 0.43
A 0.57 B 0.57
A 0.57 B 0.71
A 0.57 B 0.86
A 0.57 B 1
A 0.71 B 0
A 0.71 B 0.14
A 0.71 B 0.29
A 0.71 B 0.43
A 0.71 B 0.57
A 0.71 B 0.71
A 0.71 B 0.86
A 0.71 B 1
A 0.86 B 0
A 0.86 B 0.14
A 0.86 B 0.29
A 0.86 B 0.43
A 0.86 B 0.57
A 0.86 B 0.71
A 0.86 B 0.86
A 0.86 B 1
A 1 B 0
A 1 B 0.14
A 1 B 0.29
A 1 B 0.43
A 1 B 0.57
A 1 B 0.71
A 1 B 0.86
A 1 B 1
0.8
0.6
0.4
0.2
0 5 101520
OD 600 nm [–]
Time [h]

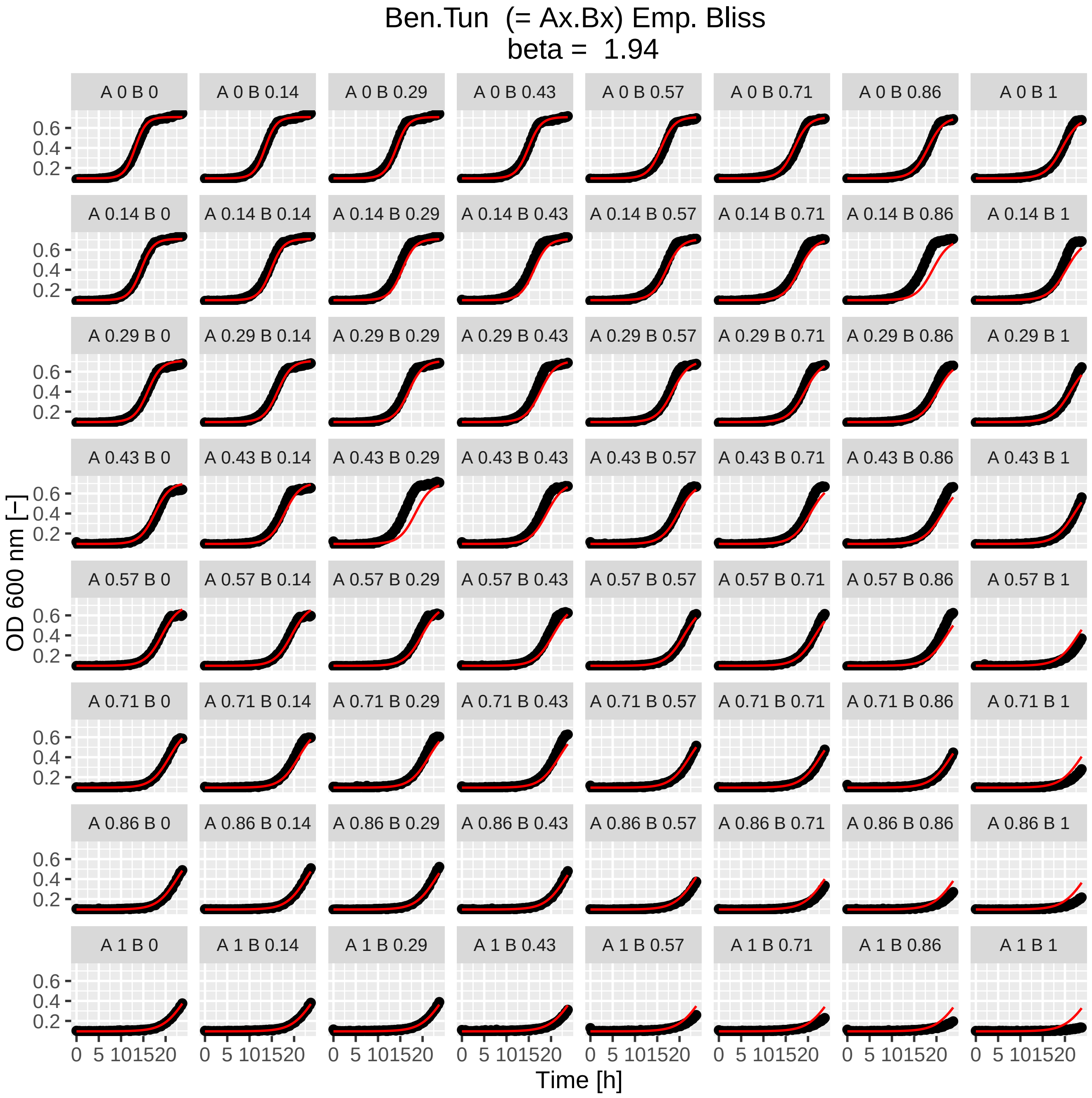
Ben.Tun (= Ax.Bx) Emp. Bliss
beta = 1.94
A 0 B 0
A 0 B 0.14
A 0 B 0.29
A 0 B 0.43
A 0 B 0.57
A 0 B 0.71
A 0 B 0.86
A 0 B 1
A 0.14 B 0
A 0.14 B 0.14
A 0.14 B 0.29
A 0.14 B 0.43
A 0.14 B 0.57
A 0.14 B 0.71
A 0.14 B 0.86
A 0.14 B 1
A 0.29 B 0
A 0.29 B 0.14
A 0.29 B 0.29
A 0.29 B 0.43
A 0.29 B 0.57
A 0.29 B 0.71
A 0.29 B 0.86
A 0.29 B 1
A 0.43 B 0
A 0.43 B 0.14
A 0.43 B 0.29
A 0.43 B 0.43
A 0.43 B 0.57
A 0.43 B 0.71
A 0.43 B 0.86
A 0.43 B 1
A 0.57 B 0
A 0.57 B 0.14
A 0.57 B 0.29
A 0.57 B 0.43
A 0.57 B 0.57
A 0.57 B 0.71
A 0.57 B 0.86
A 0.57 B 1
A 0.71 B 0
A 0.71 B 0.14
A 0.71 B 0.29
A 0.71 B 0.43
A 0.71 B 0.57
A 0.71 B 0.71
A 0.71 B 0.86
A 0.71 B 1
A 0.86 B 0
A 0.86 B 0.14
A 0.86 B 0.29
A 0.86 B 0.43
A 0.86 B 0.57
A 0.86 B 0.71
A 0.86 B 0.86
A 0.86 B 1
A 1 B 0
A 1 B 0.14
A 1 B 0.29
A 1 B 0.43
A 1 B 0.57
A 1 B 0.71
A 1 B 0.86
A 1 B 1
0.2
0.4
0.6
OD 600 nm [−]
0 5 101520
Time [h]

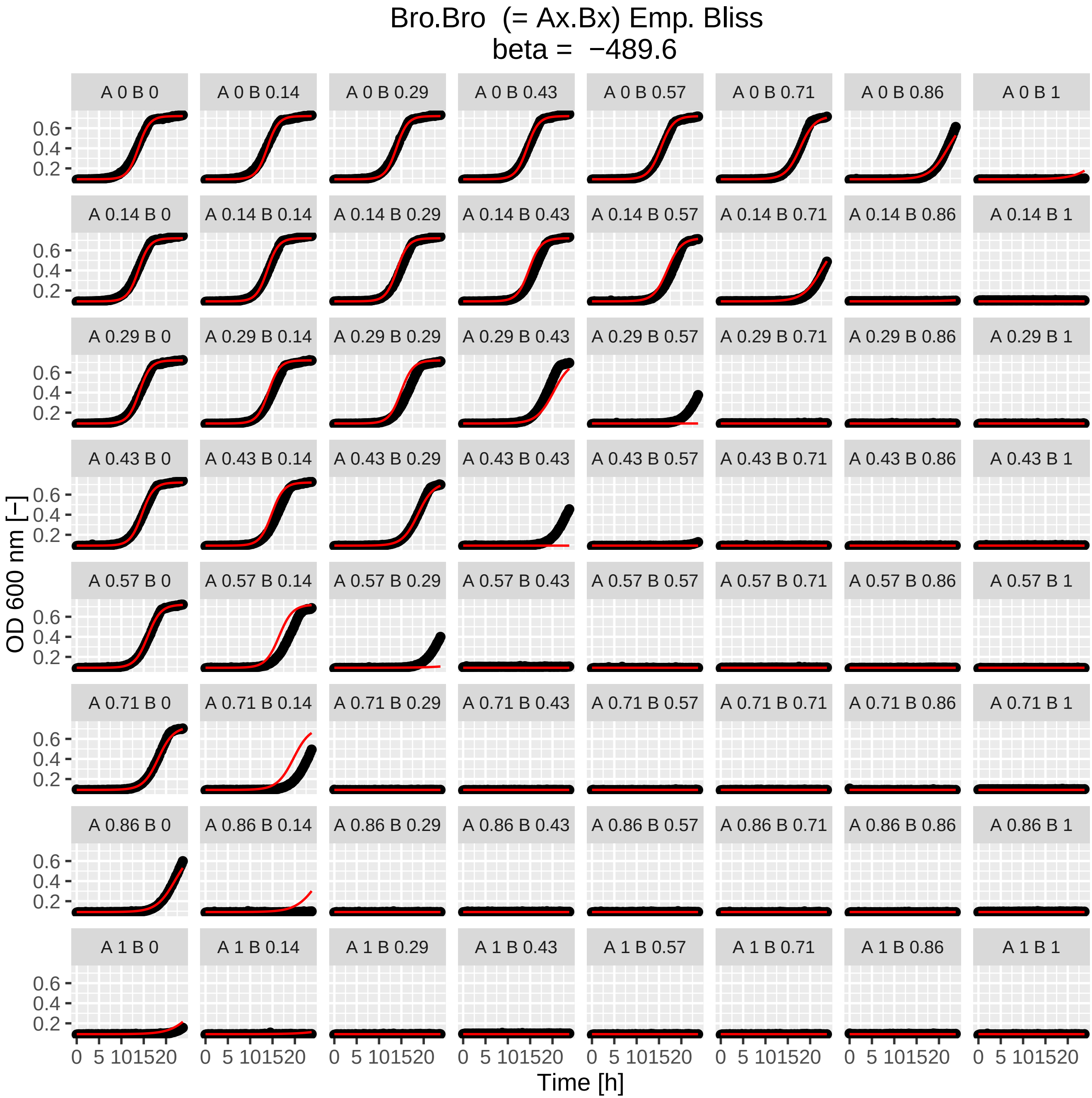

Bro.Bro (= Ax.Bx) Emp. Bliss
beta = −489.6
A 0 B 0
A 0 B 0.14
A 0 B 0.29
A 0 B 0.43
A 0 B 0.57
A 0 B 0.71
A 0 B 0.86
A 0 B 1
A 0.14 B 0
A 0.14 B 0.14
A 0.14 B 0.29
A 0.14 B 0.43
A 0.14 B 0.57
A 0.14 B 0.71
A 0.14 B 0.86
A 0.14 B 1
A 0.29 B 0
A 0.29 B 0.14
A 0.29 B 0.29
A 0.29 B 0.43
A 0.29 B 0.57
A 0.29 B 0.71
A 0.29 B 0.86
A 0.29 B 1
A 0.43 B 0
A 0.43 B 0.14
A 0.43 B 0.29
A 0.43 B 0.43
A 0.43 B 0.57
A 0.43 B 0.71
A 0.43 B 0.86
A 0.43 B 1
A 0.57 B 0
A 0.57 B 0.14
A 0.57 B 0.29
A 0.57 B 0.43
A 0.57 B 0.57
A 0.57 B 0.71
A 0.57 B 0.86
A 0.57 B 1
A 0.71 B 0
A 0.71 B 0.14
A 0.71 B 0.29
A 0.71 B 0.43
A 0.71 B 0.57
A 0.71 B 0.71
A 0.71 B 0.86
A 0.71 B 1
A 0.86 B 0
A 0.86 B 0.14
A 0.86 B 0.29
A 0.86 B 0.43
A 0.86 B 0.57
A 0.86 B 0.71
A 0.86 B 0.86
A 0.86 B 1
A 1 B 0
A 1 B 0.14
A 1 B 0.29
A 1 B 0.43
A 1 B 0.57
A 1 B 0.71
A 1 B 0.86
A 1 B 1
0.6
0.4
0.2
0 5 101520
OD 600 nm [−]
Time [h]

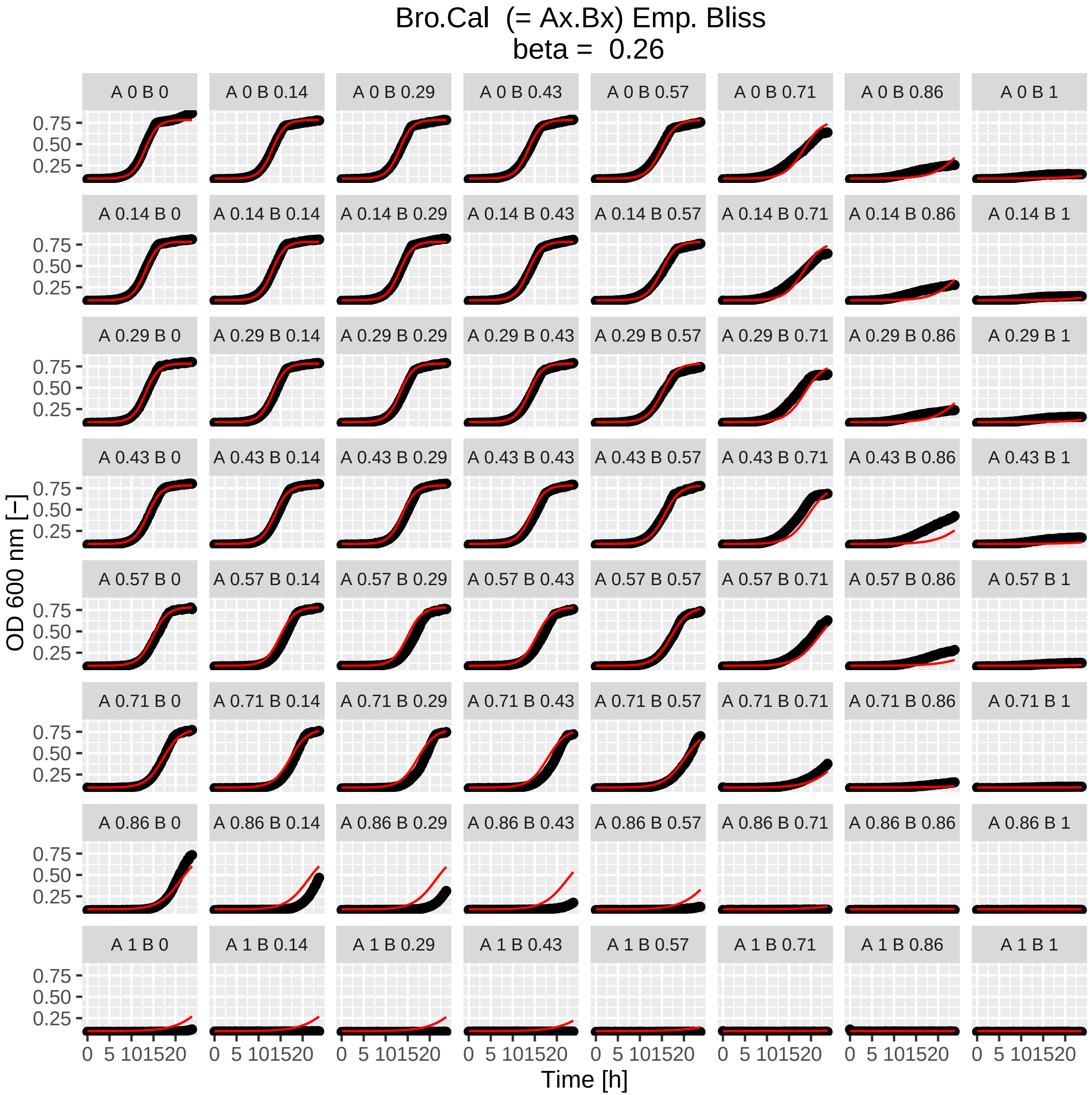
Bro.Cal (= Ax.Bx) Emp. Bliss
beta = 0.26
A 0 B 0
A 0 B 0.14
A 0 B 0.29
A 0 B 0.43
A 0 B 0.57
A 0 B 0.71
A 0 B 0.86
A 0 B 1
A 0.14 B 0
A 0.14 B 0.14
A 0.14 B 0.29
A 0.14 B 0.43
A 0.14 B 0.57
A 0.14 B 0.71
A 0.14 B 0.86
A 0.14 B 1
A 0.29 B 0
A 0.29 B 0.14
A 0.29 B 0.29
A 0.29 B 0.43
A 0.29 B 0.57
A 0.29 B 0.71
A 0.29 B 0.86
A 0.29 B 1
A 0.43 B 0
A 0.43 B 0.14
A 0.43 B 0.29
A 0.43 B 0.43
A 0.43 B 0.57
A 0.43 B 0.71
A 0.43 B 0.86
A 0.43 B 1
A 0.57 B 0
A 0.57 B 0.14
A 0.57 B 0.29
A 0.57 B 0.43
A 0.57 B 0.57
A 0.57 B 0.71
A 0.57 B 0.86
A 0.57 B 1
A 0.71 B 0
A 0.71 B 0.14
A 0.71 B 0.29
A 0.71 B 0.43
A 0.71 B 0.57
A 0.71 B 0.71
A 0.71 B 0.86
A 0.71 B 1
A 0.86 B 0
A 0.86 B 0.14
A 0.86 B 0.29
A 0.86 B 0.43
A 0.86 B 0.57
A 0.86 B 0.71
A 0.86 B 0.86
A 0.86 B 1
A 1 B 0
A 1 B 0.14
A 1 B 0.29
A 1 B 0.43
A 1 B 0.57
A 1 B 0.71
A 1 B 0.86
A 1 B 1
0.75
0.50
0.25
OD 600 nm [–]
0 5 101520
Time [h]

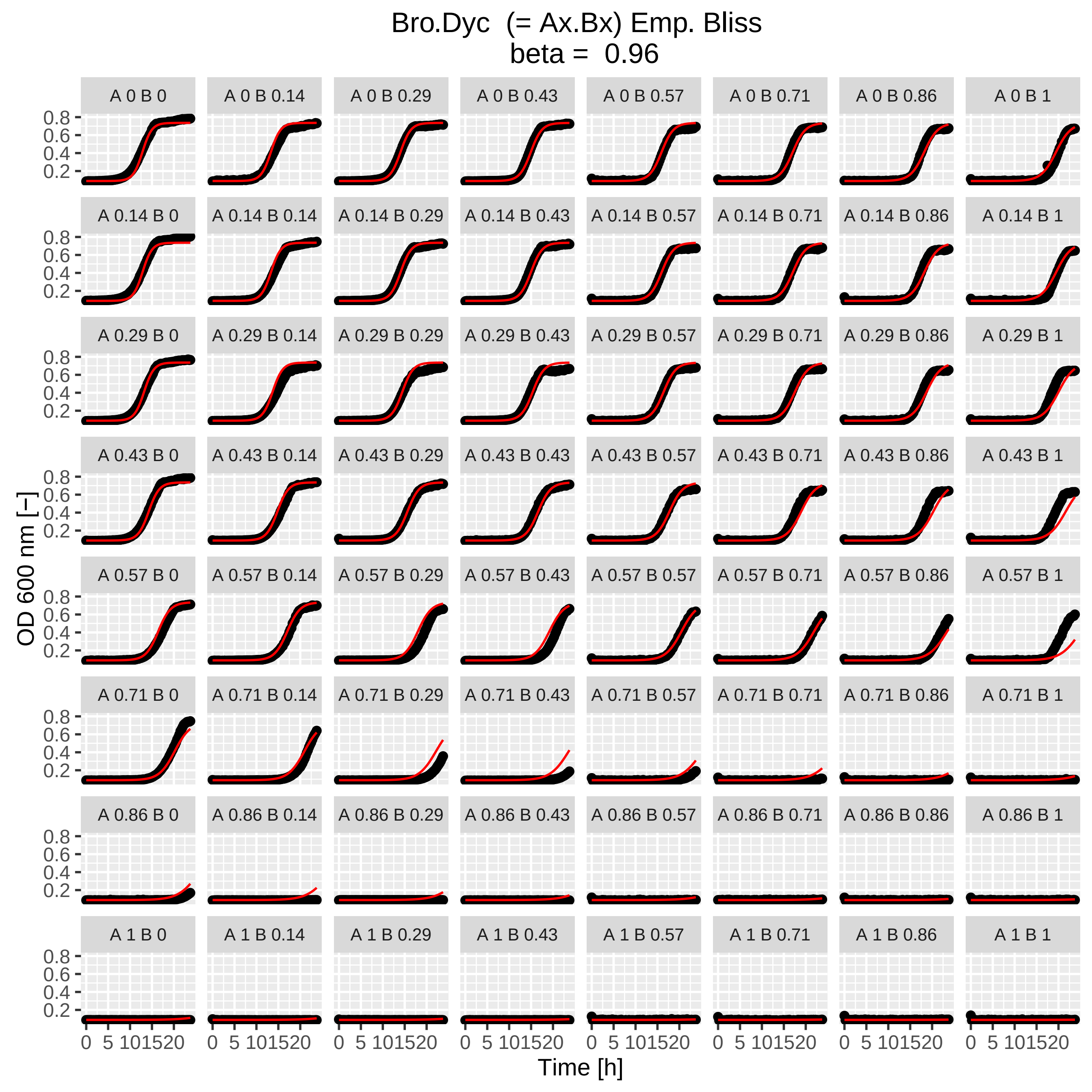

Bro.Dyc (= Ax.Bx) Emp. Bliss
beta = 0.96
OD 600 nm [–]
Time [h]
0.8
0.6
0.4
0.2
0 5 101520
A 0 B 0
A 0 B 0.14
A 0 B 0.29
A 0 B 0.43
A 0 B 0.57
A 0 B 0.71
A 0 B 0.86
A 0 B 1
A 0.14 B 0
A 0.14 B 0.14
A 0.14 B 0.29
A 0.14 B 0.43
A 0.14 B 0.57
A 0.14 B 0.71
A 0.14 B 0.86
A 0.14 B 1
A 0.29 B 0
A 0.29 B 0.14
A 0.29 B 0.29
A 0.29 B 0.43
A 0.29 B 0.57
A 0.29 B 0.71
A 0.29 B 0.86
A 0.29 B 1
A 0.43 B 0
A 0.43 B 0.14
A 0.43 B 0.29
A 0.43 B 0.43
A 0.43 B 0.57
A 0.43 B 0.71
A 0.43 B 0.86
A 0.43 B 1
A 0.57 B 0
A 0.57 B 0.14
A 0.57 B 0.29
A 0.57 B 0.43
A 0.57 B 0.57
A 0.57 B 0.71
A 0.57 B 0.86
A 0.57 B 1
A 0.71 B 0
A 0.71 B 0.14
A 0.71 B 0.29
A 0.71 B 0.43
A 0.71 B 0.57
A 0.71 B 0.71
A 0.71 B 0.86
A 0.71 B 1
A 0.86 B 0
A 0.86 B 0.14
A 0.86 B 0.29
A 0.86 B 0.43
A 0.86 B 0.57
A 0.86 B 0.71
A 0.86 B 0.86
A 0.86 B 1
A 1 B 0
A 1 B 0.14
A 1 B 0.29
A 1 B 0.43
A 1 B 0.57
A 1 B 0.71
A 1 B 0.86
A 1 B 1

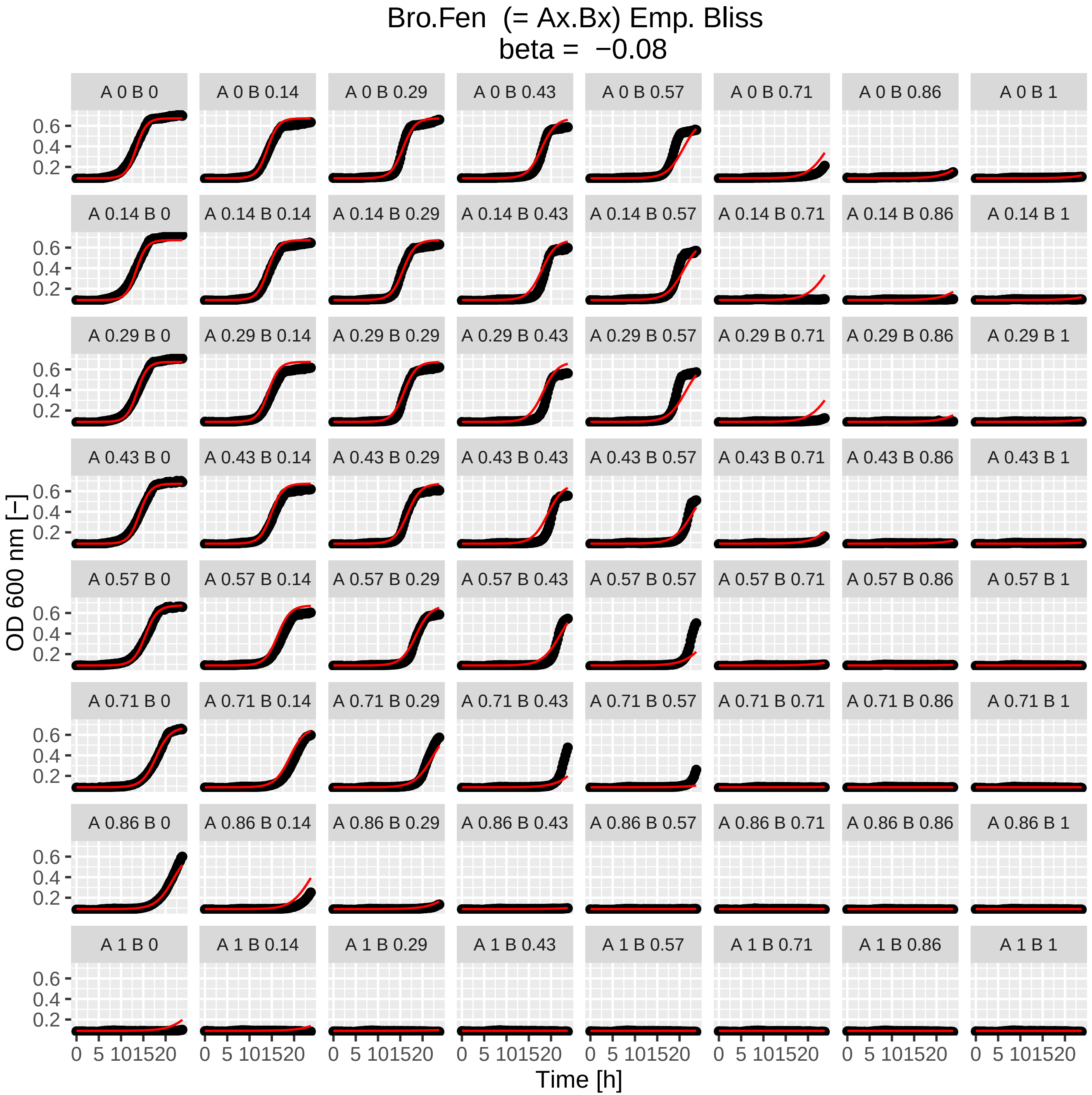
Bro.Fen (= Ax.Bx) Emp. Bliss
beta = −0.08
A 0 B 0
A 0 B 0.14
A 0 B 0.29
A 0 B 0.43
A 0 B 0.57
A 0 B 0.71
A 0 B 0.86
A 0 B 1
A 0.14 B 0
A 0.14 B 0.14
A 0.14 B 0.29
A 0.14 B 0.43
A 0.14 B 0.57
A 0.14 B 0.71
A 0.14 B 0.86
A 0.14 B 1
A 0.29 B 0
A 0.29 B 0.14
A 0.29 B 0.29
A 0.29 B 0.43
A 0.29 B 0.57
A 0.29 B 0.71
A 0.29 B 0.86
A 0.29 B 1
A 0.43 B 0
A 0.43 B 0.14
A 0.43 B 0.29
A 0.43 B 0.43
A 0.43 B 0.57
A 0.43 B 0.71
A 0.43 B 0.86
A 0.43 B 1
A 0.57 B 0
A 0.57 B 0.14
A 0.57 B 0.29
A 0.57 B 0.43
A 0.57 B 0.57
A 0.57 B 0.71
A 0.57 B 0.86
A 0.57 B 1
A 0.71 B 0
A 0.71 B 0.14
A 0.71 B 0.29
A 0.71 B 0.43
A 0.71 B 0.57
A 0.71 B 0.71
A 0.71 B 0.86
A 0.71 B 1
A 0.86 B 0
A 0.86 B 0.14
A 0.86 B 0.29
A 0.86 B 0.43
A 0.86 B 0.57
A 0.86 B 0.71
A 0.86 B 0.86
A 0.86 B 1
A 1 B 0
A 1 B 0.14
A 1 B 0.29
A 1 B 0.43
A 1 B 0.57
A 1 B 0.71
A 1 B 0.86
A 1 B 1
OD 600 nm [−]
0.2
0.4
0.6
0 5 101520
Time [h]

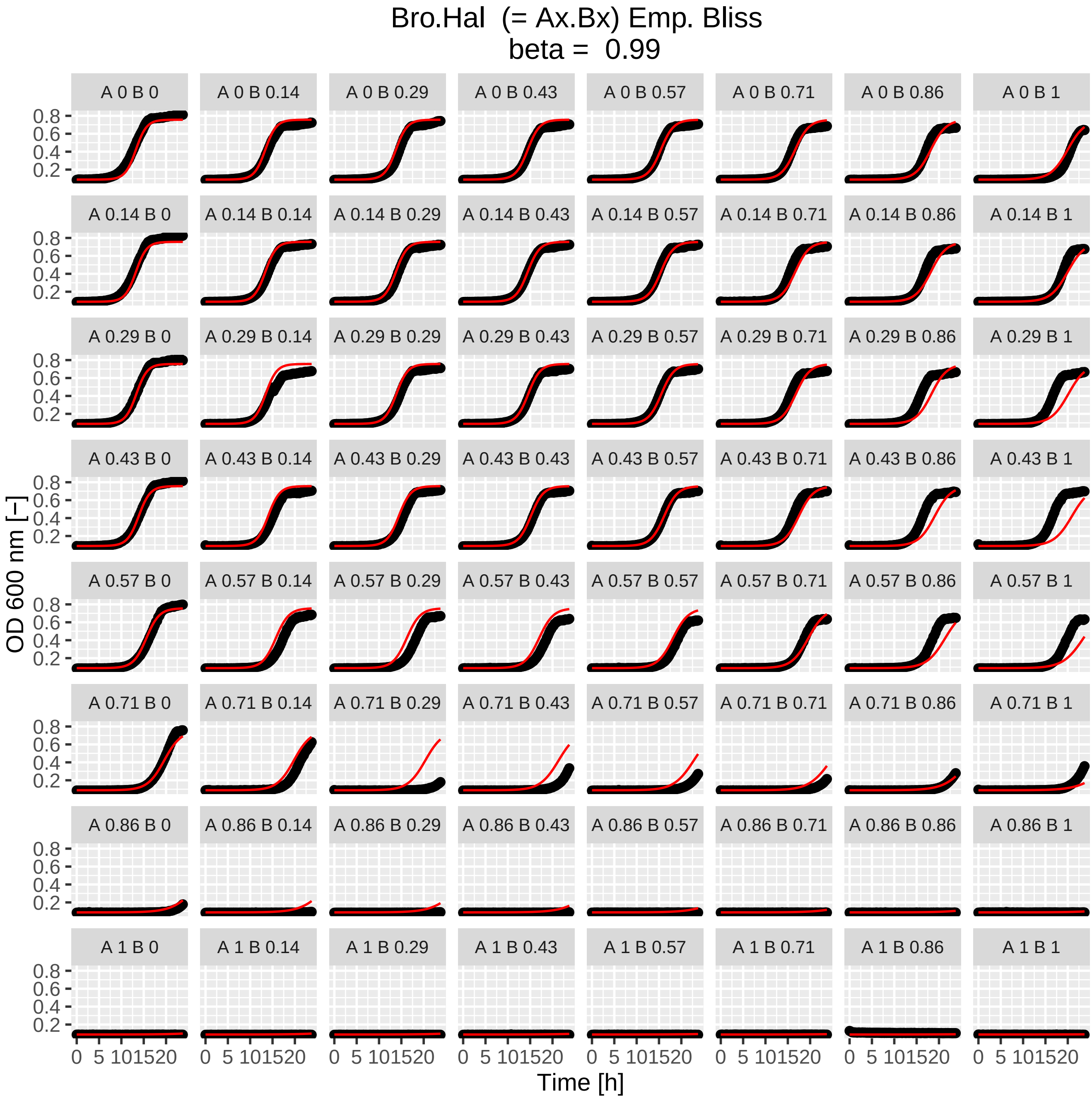

Bro.Hal (= Ax.Bx) Emp. Bliss
beta = 0.99
A 0 B 0
A 0 B 0.14
A 0 B 0.29
A 0 B 0.43
A 0 B 0.57
A 0 B 0.71
A 0 B 0.86
A 0 B 1
A 0.14 B 0
A 0.14 B 0.14
A 0.14 B 0.29
A 0.14 B 0.43
A 0.14 B 0.57
A 0.14 B 0.71
A 0.14 B 0.86
A 0.14 B 1
A 0.29 B 0
A 0.29 B 0.14
A 0.29 B 0.29
A 0.29 B 0.43
A 0.29 B 0.57
A 0.29 B 0.71
A 0.29 B 0.86
A 0.29 B 1
A 0.43 B 0
A 0.43 B 0.14
A 0.43 B 0.29
A 0.43 B 0.43
A 0.43 B 0.57
A 0.43 B 0.71
A 0.43 B 0.86
A 0.43 B 1
A 0.57 B 0
A 0.57 B 0.14
A 0.57 B 0.29
A 0.57 B 0.43
A 0.57 B 0.57
A 0.57 B 0.71
A 0.57 B 0.86
A 0.57 B 1
A 0.71 B 0
A 0.71 B 0.14
A 0.71 B 0.29
A 0.71 B 0.43
A 0.71 B 0.57
A 0.71 B 0.71
A 0.71 B 0.86
A 0.71 B 1
A 0.86 B 0
A 0.86 B 0.14
A 0.86 B 0.29
A 0.86 B 0.43
A 0.86 B 0.57
A 0.86 B 0.71
A 0.86 B 0.86
A 0.86 B 1
A 1 B 0
A 1 B 0.14
A 1 B 0.29
A 1 B 0.43
A 1 B 0.57
A 1 B 0.71
A 1 B 0.86
A 1 B 1
0.8
0.6
0.4
0.2
OD 600 nm [−]
0 5 101520
Time [h]

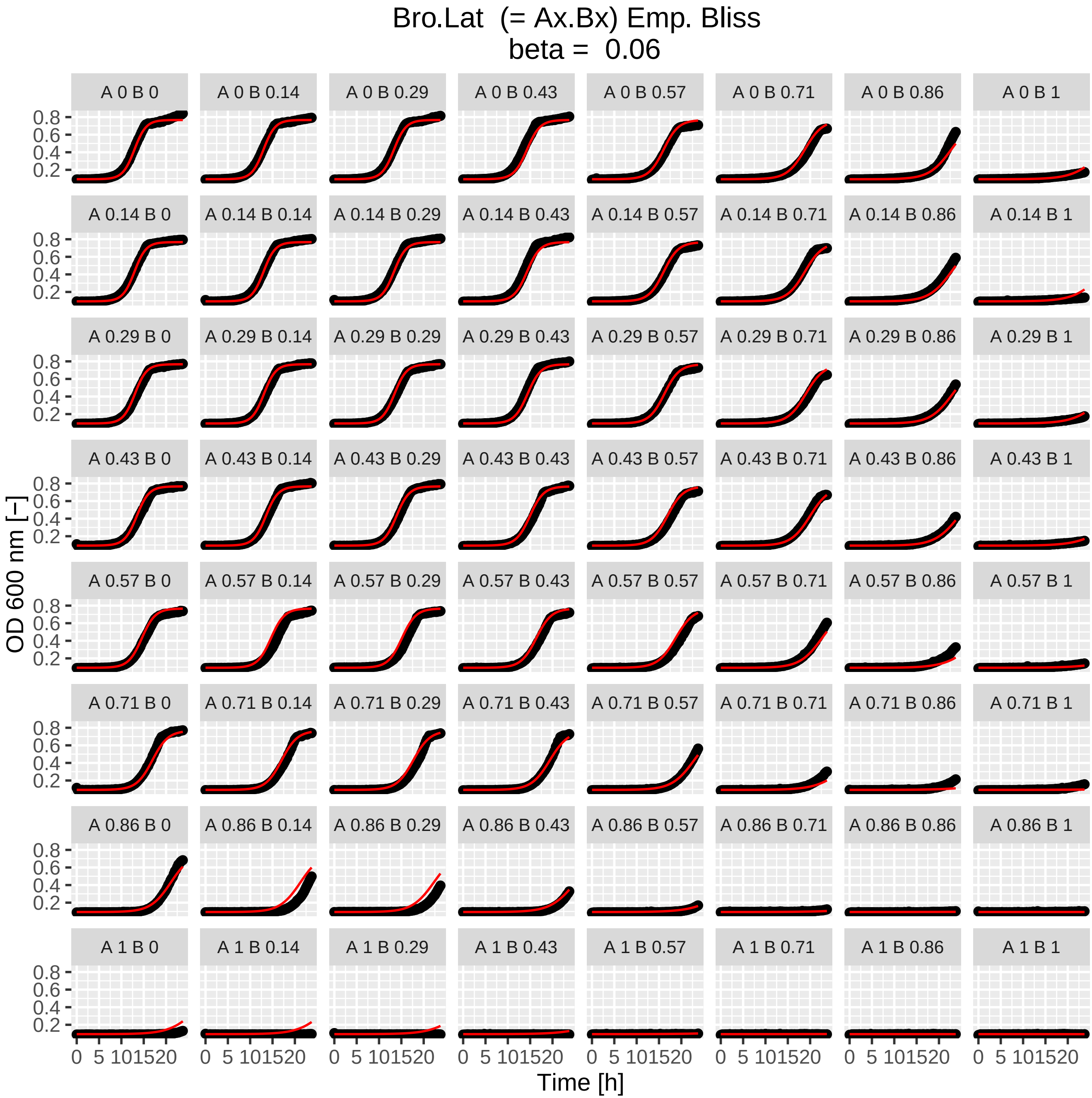

Bro.Lat (= Ax.Bx) Emp. Bliss
beta = 0.06
A 0 B 0
A 0 B 0.14
A 0 B 0.29
A 0 B 0.43
A 0 B 0.57
A 0 B 0.71
A 0 B 0.86
A 0 B 1
A 0.14 B 0
A 0.14 B 0.14
A 0.14 B 0.29
A 0.14 B 0.43
A 0.14 B 0.57
A 0.14 B 0.71
A 0.14 B 0.86
A 0.14 B 1
A 0.29 B 0
A 0.29 B 0.14
A 0.29 B 0.29
A 0.29 B 0.43
A 0.29 B 0.57
A 0.29 B 0.71
A 0.29 B 0.86
A 0.29 B 1
A 0.43 B 0
A 0.43 B 0.14
A 0.43 B 0.29
A 0.43 B 0.43
A 0.43 B 0.57
A 0.43 B 0.71
A 0.43 B 0.86
A 0.43 B 1
A 0.57 B 0
A 0.57 B 0.14
A 0.57 B 0.29
A 0.57 B 0.43
A 0.57 B 0.57
A 0.57 B 0.71
A 0.57 B 0.86
A 0.57 B 1
A 0.71 B 0
A 0.71 B 0.14
A 0.71 B 0.29
A 0.71 B 0.43
A 0.71 B 0.57
A 0.71 B 0.71
A 0.71 B 0.86
A 0.71 B 1
A 0.86 B 0
A 0.86 B 0.14
A 0.86 B 0.29
A 0.86 B 0.43
A 0.86 B 0.57
A 0.86 B 0.71
A 0.86 B 0.86
A 0.86 B 1
A 1 B 0
A 1 B 0.14
A 1 B 0.29
A 1 B 0.43
A 1 B 0.57
A 1 B 0.71
A 1 B 0.86
A 1 B 1
0.8
0.6
0.4
0.2
OD 600 nm [−]
0 5 101520
Time [h]

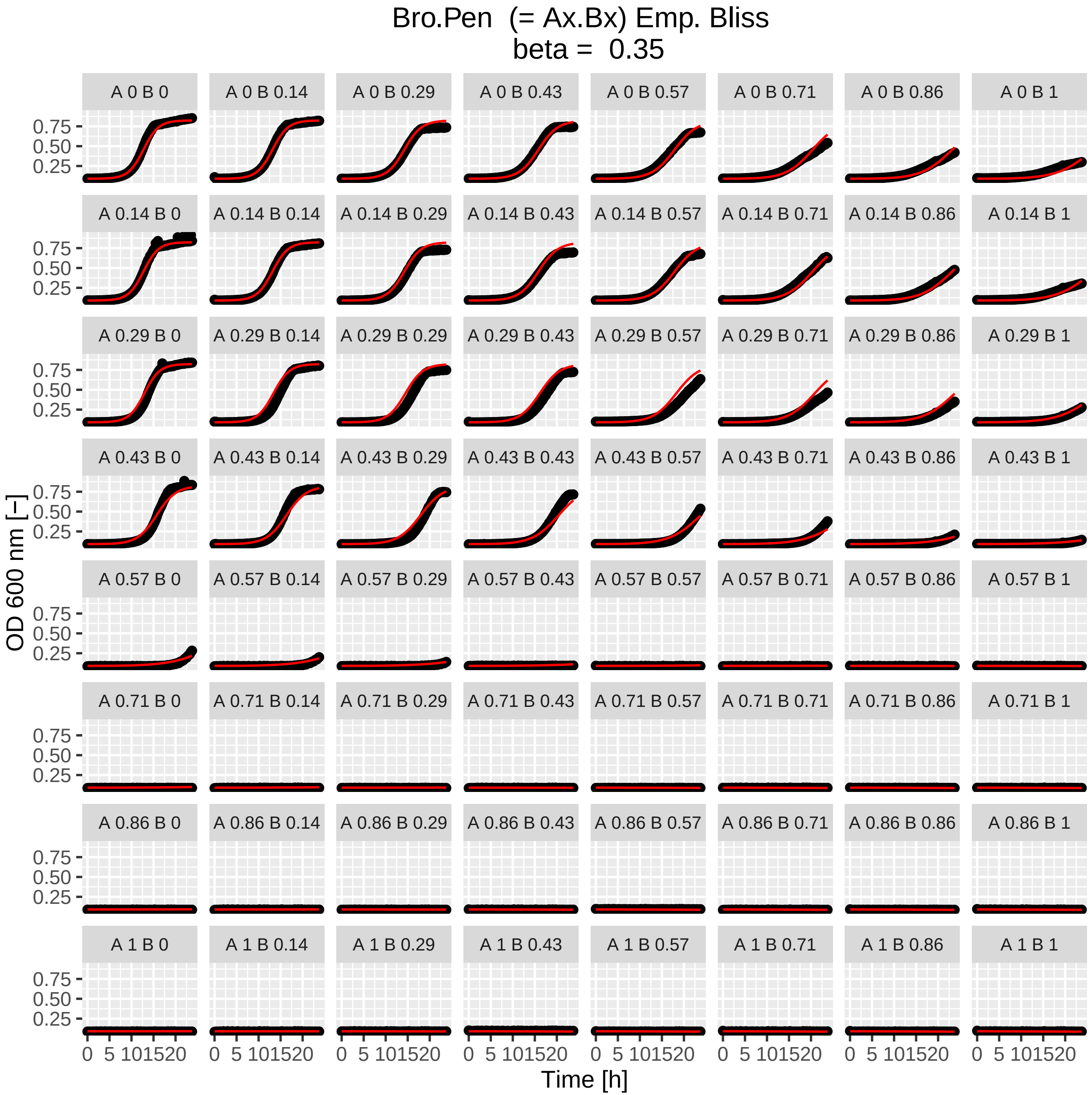
Bro.Pen (= Ax.Bx) Emp. Bliss
beta = 0.35
A 0 B 0
A 0 B 0.14
A 0 B 0.29
A 0 B 0.43
A 0 B 0.57
A 0 B 0.71
A 0 B 0.86
A 0 B 1
A 0.14 B 0
A 0.14 B 0.14
A 0.14 B 0.29
A 0.14 B 0.43
A 0.14 B 0.57
A 0.14 B 0.71
A 0.14 B 0.86
A 0.14 B 1
A 0.29 B 0
A 0.29 B 0.14
A 0.29 B 0.29
A 0.29 B 0.43
A 0.29 B 0.57
A 0.29 B 0.71
A 0.29 B 0.86
A 0.29 B 1
A 0.43 B 0
A 0.43 B 0.14
A 0.43 B 0.29
A 0.43 B 0.43
A 0.43 B 0.57
A 0.43 B 0.71
A 0.43 B 0.86
A 0.43 B 1
A 0.57 B 0
A 0.57 B 0.14
A 0.57 B 0.29
A 0.57 B 0.43
A 0.57 B 0.57
A 0.57 B 0.71
A 0.57 B 0.86
A 0.57 B 1
A 0.71 B 0
A 0.71 B 0.14
A 0.71 B 0.29
A 0.71 B 0.43
A 0.71 B 0.57
A 0.71 B 0.71
A 0.71 B 0.86
A 0.71 B 1
A 0.86 B 0
A 0.86 B 0.14
A 0.86 B 0.29
A 0.86 B 0.43
A 0.86 B 0.57
A 0.86 B 0.71
A 0.86 B 0.86
A 0.86 B 1
A 1 B 0
A 1 B 0.14
A 1 B 0.29
A 1 B 0.43
A 1 B 0.57
A 1 B 0.71
A 1 B 0.86
A 1 B 1
OD 600 nm [–]
0.75
0.50
0.25
0 5 101520
Time [h]

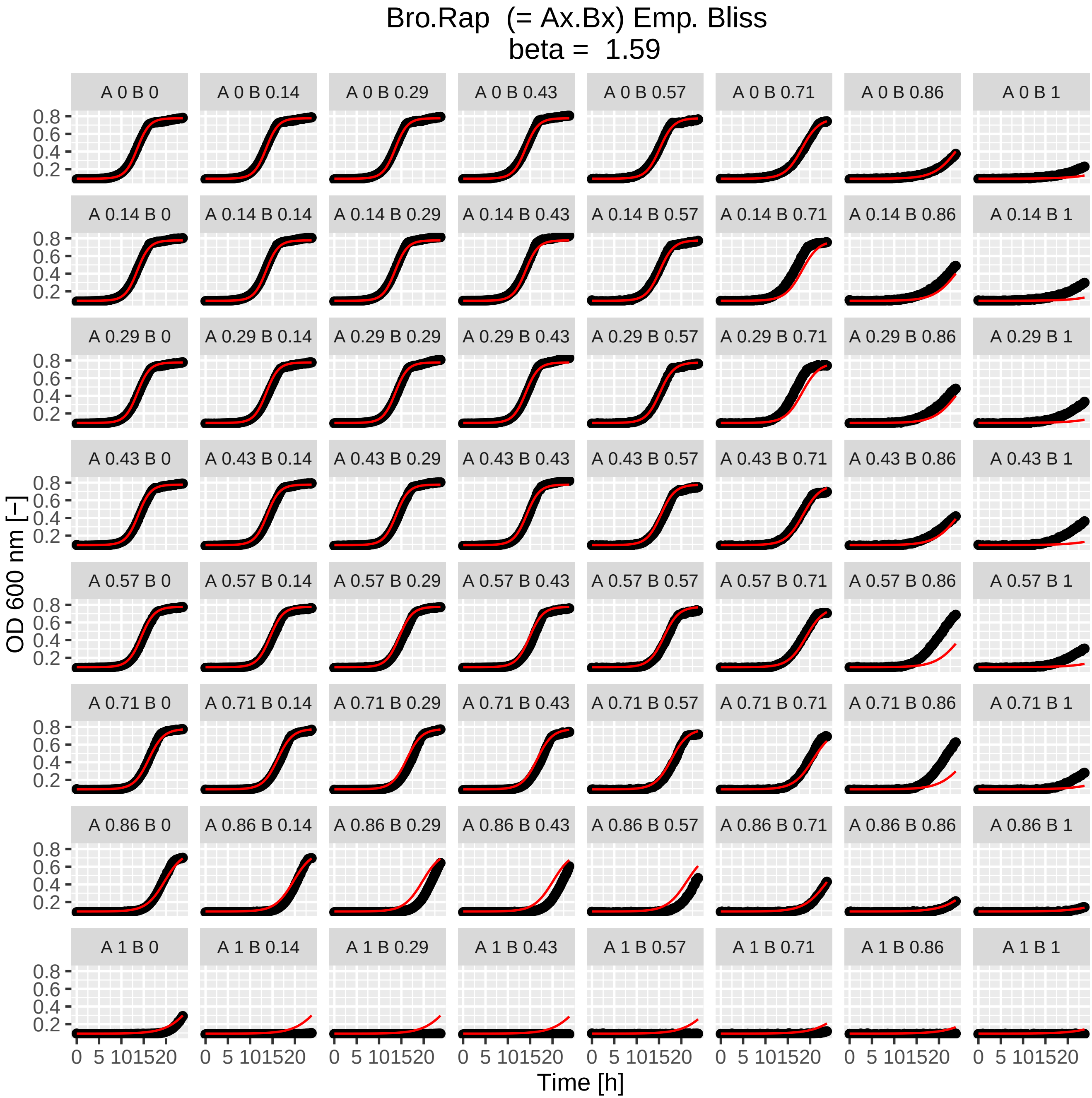

Bro.Rap (= Ax.Bx) Emp. Bliss
beta = 1.59
A 0 B 0
A 0 B 0.14
A 0 B 0.29
A 0 B 0.43
A 0 B 0.57
A 0 B 0.71
A 0 B 0.86
A 0 B 1
A 0.14 B 0
A 0.14 B 0.14
A 0.14 B 0.29
A 0.14 B 0.43
A 0.14 B 0.57
A 0.14 B 0.71
A 0.14 B 0.86
A 0.14 B 1
A 0.29 B 0
A 0.29 B 0.14
A 0.29 B 0.29
A 0.29 B 0.43
A 0.29 B 0.57
A 0.29 B 0.71
A 0.29 B 0.86
A 0.29 B 1
A 0.43 B 0
A 0.43 B 0.14
A 0.43 B 0.29
A 0.43 B 0.43
A 0.43 B 0.57
A 0.43 B 0.71
A 0.43 B 0.86
A 0.43 B 1
A 0.57 B 0
A 0.57 B 0.14
A 0.57 B 0.29
A 0.57 B 0.43
A 0.57 B 0.57
A 0.57 B 0.71
A 0.57 B 0.86
A 0.57 B 1
A 0.71 B 0
A 0.71 B 0.14
A 0.71 B 0.29
A 0.71 B 0.43
A 0.71 B 0.57
A 0.71 B 0.71
A 0.71 B 0.86
A 0.71 B 1
A 0.86 B 0
A 0.86 B 0.14
A 0.86 B 0.29
A 0.86 B 0.43
A 0.86 B 0.57
A 0.86 B 0.71
A 0.86 B 0.86
A 0.86 B 1
A 1 B 0
A 1 B 0.14
A 1 B 0.29
A 1 B 0.43
A 1 B 0.57
A 1 B 0.71
A 1 B 0.86
A 1 B 1
OD 600 nm [–]
0.2
0.4
0.6
0.8
0 5 101520
Time [h]

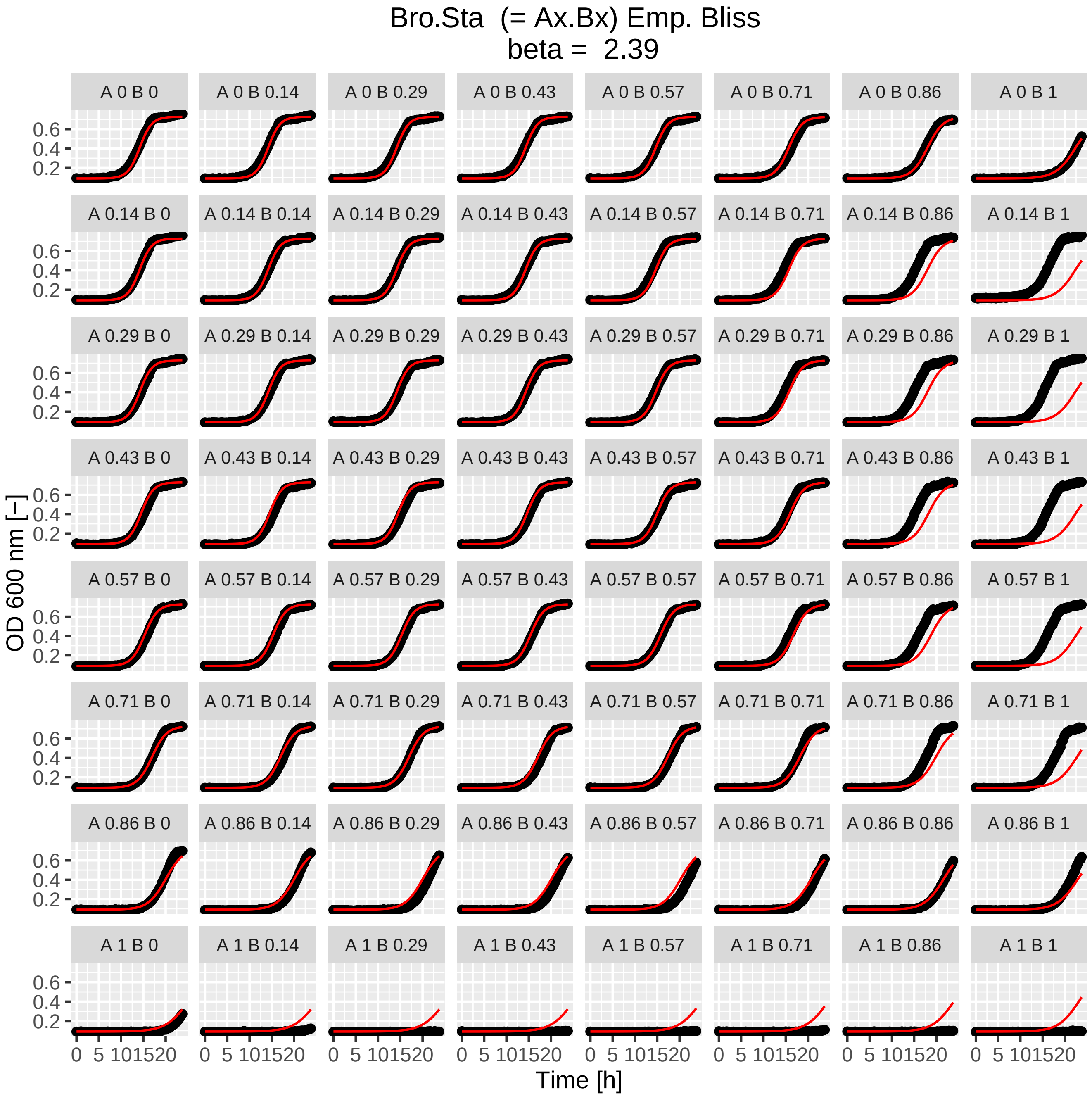

Bro.Sta (= Ax.Bx) Emp. Bliss
beta = 2.39
A 0 B 0
A 0 B 0.14
A 0 B 0.29
A 0 B 0.43
A 0 B 0.57
A 0 B 0.71
A 0 B 0.86
A 0 B 1
A 0.14 B 0
A 0.14 B 0.14
A 0.14 B 0.29
A 0.14 B 0.43
A 0.14 B 0.57
A 0.14 B 0.71
A 0.14 B 0.86
A 0.14 B 1
A 0.29 B 0
A 0.29 B 0.14
A 0.29 B 0.29
A 0.29 B 0.43
A 0.29 B 0.57
A 0.29 B 0.71
A 0.29 B 0.86
A 0.29 B 1
A 0.43 B 0
A 0.43 B 0.14
A 0.43 B 0.29
A 0.43 B 0.43
A 0.43 B 0.57
A 0.43 B 0.71
A 0.43 B 0.86
A 0.43 B 1
A 0.57 B 0
A 0.57 B 0.14
A 0.57 B 0.29
A 0.57 B 0.43
A 0.57 B 0.57
A 0.57 B 0.71
A 0.57 B 0.86
A 0.57 B 1
A 0.71 B 0
A 0.71 B 0.14
A 0.71 B 0.29
A 0.71 B 0.43
A 0.71 B 0.57
A 0.71 B 0.71
A 0.71 B 0.86
A 0.71 B 1
A 0.86 B 0
A 0.86 B 0.14
A 0.86 B 0.29
A 0.86 B 0.43
A 0.86 B 0.57
A 0.86 B 0.71
A 0.86 B 0.86
A 0.86 B 1
A 1 B 0
A 1 B 0.14
A 1 B 0.29
A 1 B 0.43
A 1 B 0.57
A 1 B 0.71
A 1 B 0.86
A 1 B 1
0.6
0.4
0.2
OD 600 nm [–]
0 5 101520
Time [h]

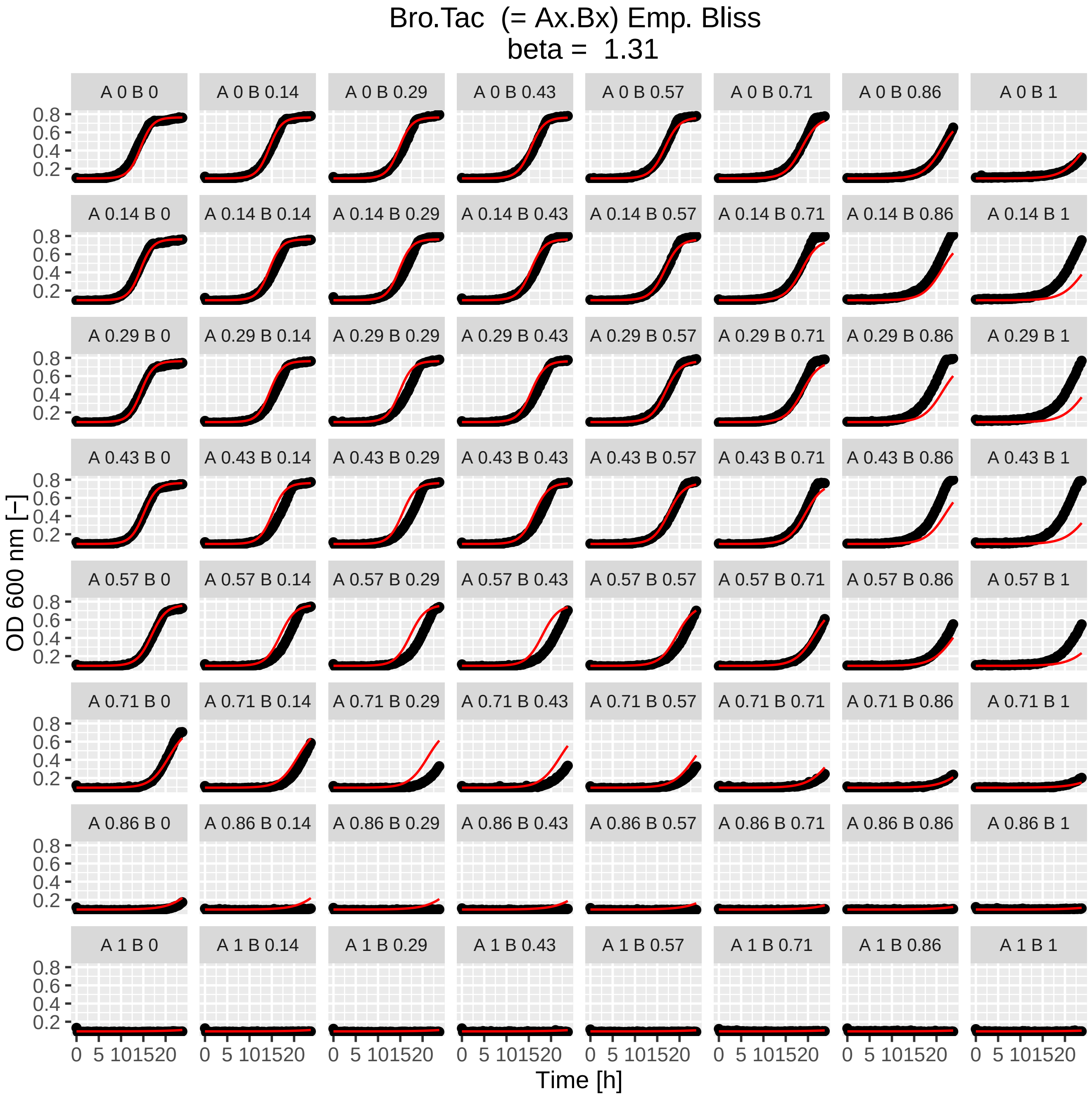
Bro.Tac (= Ax.Bx) Emp. Bliss
beta = 1.31
OD 600 nm [–]
Time [h]
A 0 B 0
A 0 B 0.14
A 0 B 0.29
A 0 B 0.43
A 0 B 0.57
A 0 B 0.71
A 0 B 0.86
A 0 B 1
A 0.14 B 0
A 0.14 B 0.14
A 0.14 B 0.29
A 0.14 B 0.43
A 0.14 B 0.57
A 0.14 B 0.71
A 0.14 B 0.86
A 0.14 B 1
A 0.29 B 0
A 0.29 B 0.14
A 0.29 B 0.29
A 0.29 B 0.43
A 0.29 B 0.57
A 0.29 B 0.71
A 0.29 B 0.86
A 0.29 B 1
A 0.43 B 0
A 0.43 B 0.14
A 0.43 B 0.29
A 0.43 B 0.43
A 0.43 B 0.57
A 0.43 B 0.71
A 0.43 B 0.86
A 0.43 B 1
A 0.57 B 0
A 0.57 B 0.14
A 0.57 B 0.29
A 0.57 B 0.43
A 0.57 B 0.57
A 0.57 B 0.71
A 0.57 B 0.86
A 0.57 B 1
A 0.71 B 0
A 0.71 B 0.14
A 0.71 B 0.29
A 0.71 B 0.43
A 0.71 B 0.57
A 0.71 B 0.71
A 0.71 B 0.86
A 0.71 B 1
A 0.86 B 0
A 0.86 B 0.14
A 0.86 B 0.29
A 0.86 B 0.43
A 0.86 B 0.57
A 0.86 B 0.71
A 0.86 B 0.86
A 0.86 B 1
A 1 B 0
A 1 B 0.14
A 1 B 0.29
A 1 B 0.43
A 1 B 0.57
A 1 B 0.71
A 1 B 0.86
A 1 B 1

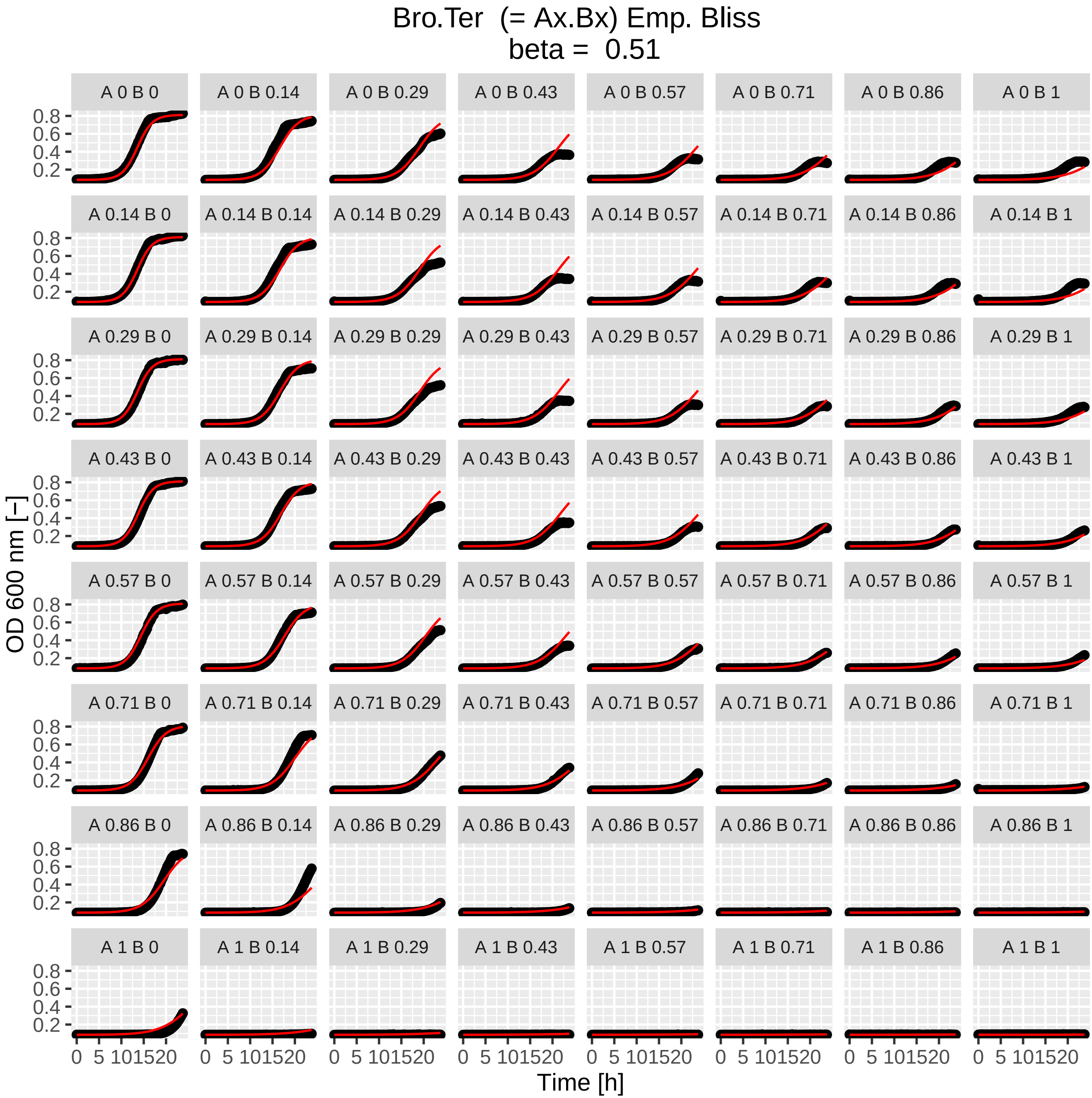
Bro.Ter (= Ax.Bx) Emp. Bliss
beta = 0.51
A 0 B 0
A 0 B 0.14
A 0 B 0.29
A 0 B 0.43
A 0 B 0.57
A 0 B 0.71
A 0 B 0.86
A 0 B 1
A 0.14 B 0
A 0.14 B 0.14
A 0.14 B 0.29
A 0.14 B 0.43
A 0.14 B 0.57
A 0.14 B 0.71
A 0.14 B 0.86
A 0.14 B 1
A 0.29 B 0
A 0.29 B 0.14
A 0.29 B 0.29
A 0.29 B 0.43
A 0.29 B 0.57
A 0.29 B 0.71
A 0.29 B 0.86
A 0.29 B 1
A 0.43 B 0
A 0.43 B 0.14
A 0.43 B 0.29
A 0.43 B 0.43
A 0.43 B 0.57
A 0.43 B 0.71
A 0.43 B 0.86
A 0.43 B 1
A 0.57 B 0
A 0.57 B 0.14
A 0.57 B 0.29
A 0.57 B 0.43
A 0.57 B 0.57
A 0.57 B 0.71
A 0.57 B 0.86
A 0.57 B 1
A 0.71 B 0
A 0.71 B 0.14
A 0.71 B 0.29
A 0.71 B 0.43
A 0.71 B 0.57
A 0.71 B 0.71
A 0.71 B 0.86
A 0.71 B 1
A 0.86 B 0
A 0.86 B 0.14
A 0.86 B 0.29
A 0.86 B 0.43
A 0.86 B 0.57
A 0.86 B 0.71
A 0.86 B 0.86
A 0.86 B 1
A 1 B 0
A 1 B 0.14
A 1 B 0.29
A 1 B 0.43
A 1 B 0.57
A 1 B 0.71
A 1 B 0.86
A 1 B 1
OD 600 nm [−]
0.2
0.4
0.6
0.8
0 5 101520
Time [h]

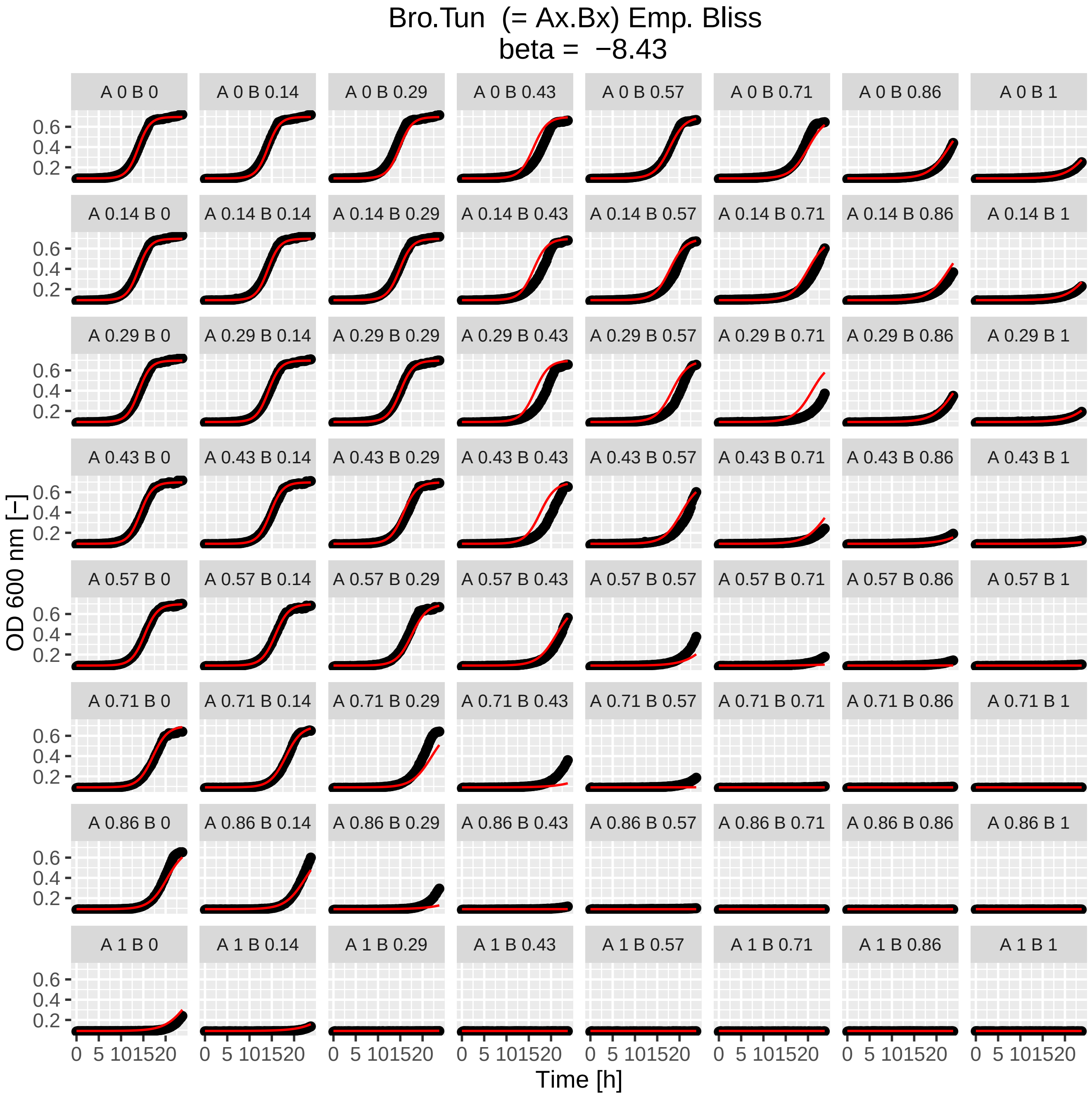
Bro.Tun (= Ax.Bx) Emp. Bliss
beta = −8.43
OD 600 nm [−]
Time [h]
A 0 B 0
A 0 B 0.14
A 0 B 0.29
A 0 B 0.43
A 0 B 0.57
A 0 B 0.71
A 0 B 0.86
A 0 B 1
A 0.14 B 0
A 0.14 B 0.14
A 0.14 B 0.29
A 0.14 B 0.43
A 0.14 B 0.57
A 0.14 B 0.71
A 0.14 B 0.86
A 0.14 B 1
A 0.29 B 0
A 0.29 B 0.14
A 0.29 B 0.29
A 0.29 B 0.43
A 0.29 B 0.57
A 0.29 B 0.71
A 0.29 B 0.86
A 0.29 B 1
A 0.43 B 0
A 0.43 B 0.14
A 0.43 B 0.29
A 0.43 B 0.43
A 0.43 B 0.57
A 0.43 B 0.71
A 0.43 B 0.86
A 0.43 B 1
A 0.57 B 0
A 0.57 B 0.14
A 0.57 B 0.29
A 0.57 B 0.43
A 0.57 B 0.57
A 0.57 B 0.71
A 0.57 B 0.86
A 0.57 B 1
A 0.71 B 0
A 0.71 B 0.14
A 0.71 B 0.29
A 0.71 B 0.43
A 0.71 B 0.57
A 0.71 B 0.71
A 0.71 B 0.86
A 0.71 B 1
A 0.86 B 0
A 0.86 B 0.14
A 0.86 B 0.29
A 0.86 B 0.43
A 0.86 B 0.57
A 0.86 B 0.71
A 0.86 B 0.86
A 0.86 B 1
A 1 B 0
A 1 B 0.14
A 1 B 0.29
A 1 B 0.43
A 1 B 0.57
A 1 B 0.71
A 1 B 0.86
A 1 B 1
0.2
0.4
0.6
0 5 101520

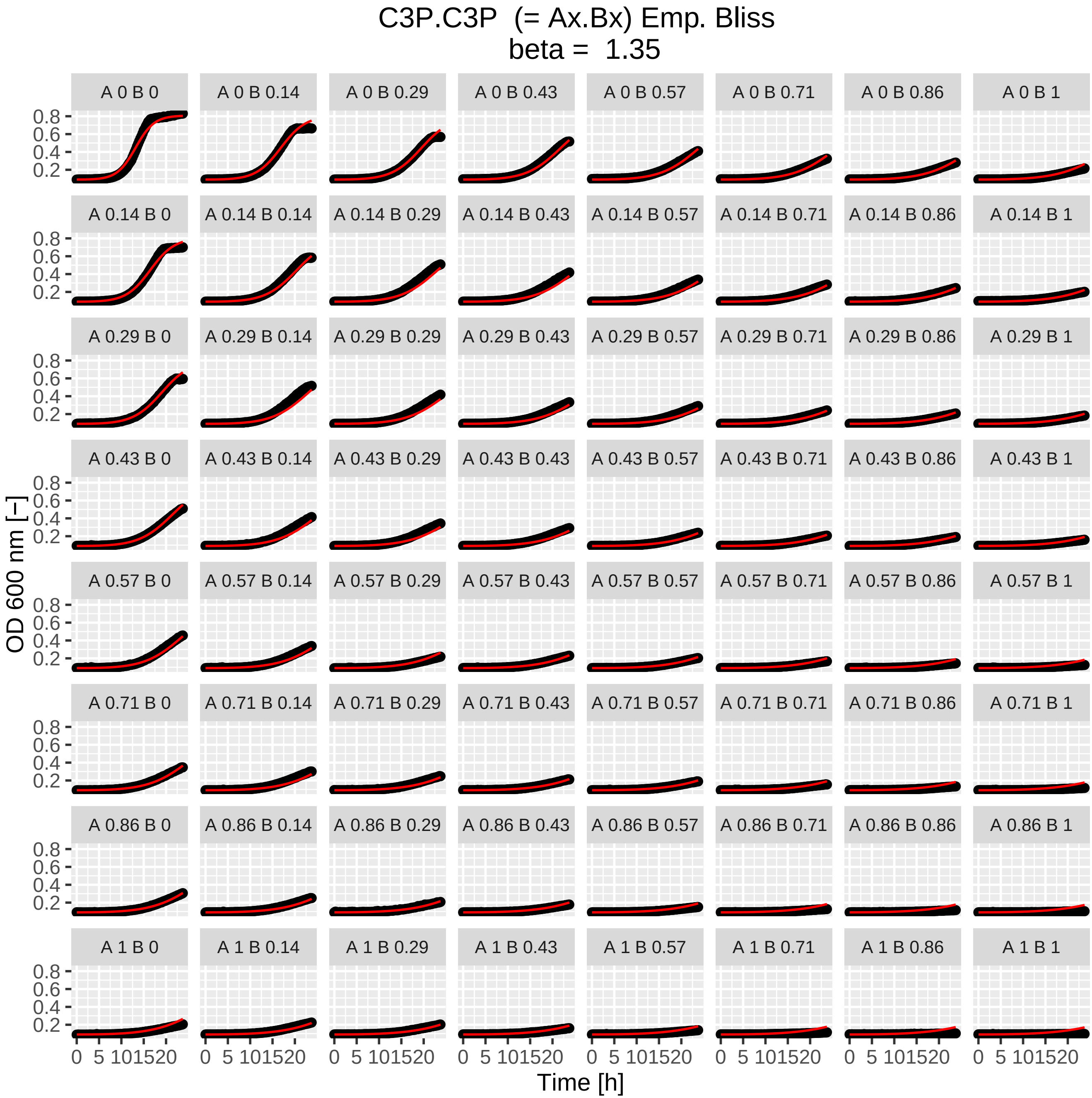
C3P.C3P (= Ax.Bx) Emp. Bliss
beta = 1.35
OD 600 nm [–]
Time [h]
A 0 B 0
A 0 B 0.14
A 0 B 0.29
A 0 B 0.43
A 0 B 0.57
A 0 B 0.71
A 0 B 0.86
A 0 B 1
A 0.14 B 0
A 0.14 B 0.14
A 0.14 B 0.29
A 0.14 B 0.43
A 0.14 B 0.57
A 0.14 B 0.71
A 0.14 B 0.86
A 0.14 B 1
A 0.29 B 0
A 0.29 B 0.14
A 0.29 B 0.29
A 0.29 B 0.43
A 0.29 B 0.57
A 0.29 B 0.71
A 0.29 B 0.86
A 0.29 B 1
A 0.43 B 0
A 0.43 B 0.14
A 0.43 B 0.29
A 0.43 B 0.43
A 0.43 B 0.57
A 0.43 B 0.71
A 0.43 B 0.86
A 0.43 B 1
A 0.57 B 0
A 0.57 B 0.14
A 0.57 B 0.29
A 0.57 B 0.43
A 0.57 B 0.57
A 0.57 B 0.71
A 0.57 B 0.86
A 0.57 B 1
A 0.71 B 0
A 0.71 B 0.14
A 0.71 B 0.29
A 0.71 B 0.43
A 0.71 B 0.57
A 0.71 B 0.71
A 0.71 B 0.86
A 0.71 B 1
A 0.86 B 0
A 0.86 B 0.14
A 0.86 B 0.29
A 0.86 B 0.43
A 0.86 B 0.57
A 0.86 B 0.71
A 0.86 B 0.86
A 0.86 B 1
A 1 B 0
A 1 B 0.14
A 1 B 0.29
A 1 B 0.43
A 1 B 0.57
A 1 B 0.71
A 1 B 0.86
A 1 B 1
0.8
0.6
0.4
0.2
0 5 101520

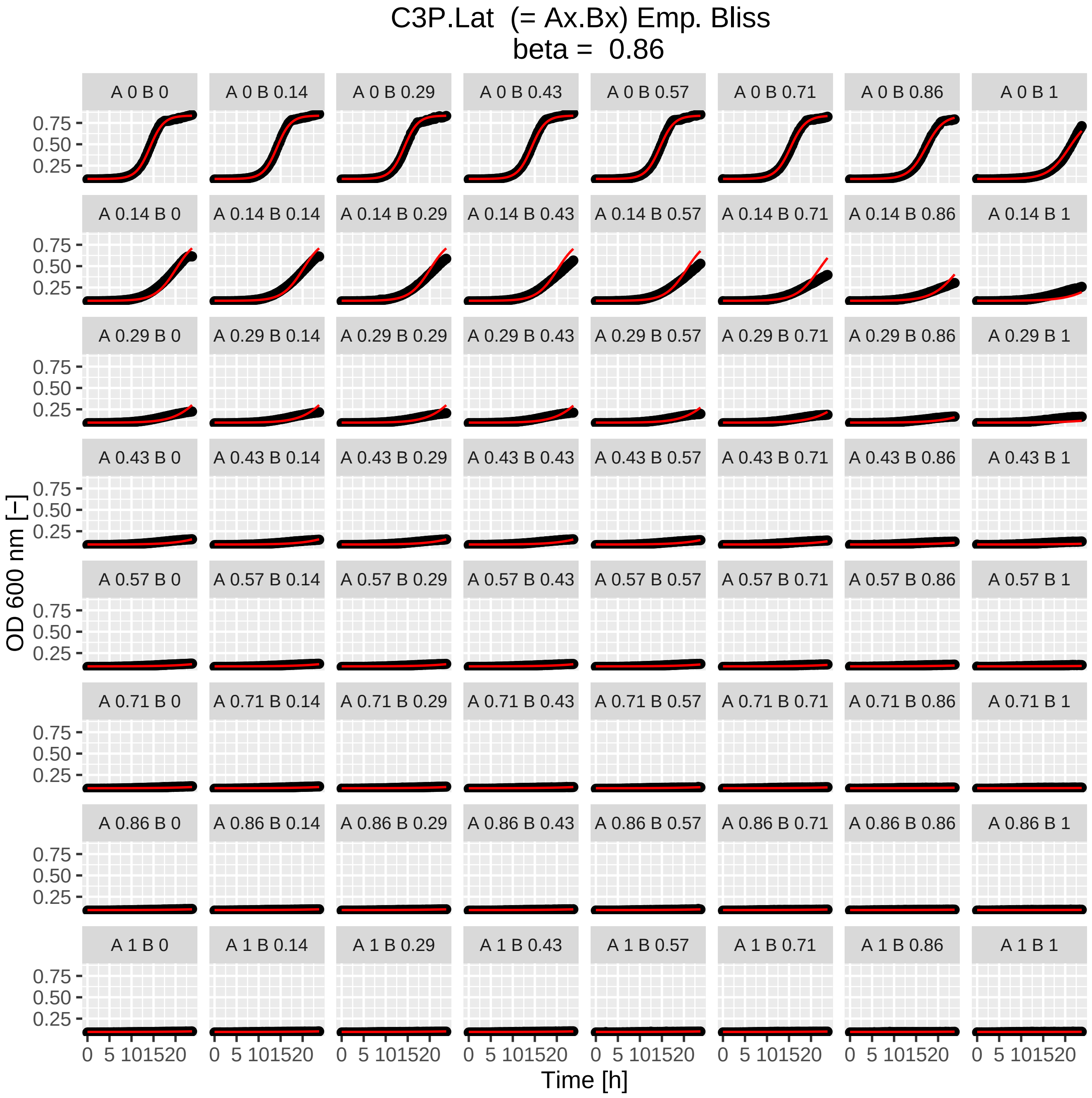
C3P.Lat (= Ax.Bx) Emp. Bliss
beta = 0.86
A 0 B 0
A 0 B 0.14
A 0 B 0.29
A 0 B 0.43
A 0 B 0.57
A 0 B 0.71
A 0 B 0.86
A 0 B 1
A 0.14 B 0
A 0.14 B 0.14
A 0.14 B 0.29
A 0.14 B 0.43
A 0.14 B 0.57
A 0.14 B 0.71
A 0.14 B 0.86
A 0.14 B 1
A 0.29 B 0
A 0.29 B 0.14
A 0.29 B 0.29
A 0.29 B 0.43
A 0.29 B 0.57
A 0.29 B 0.71
A 0.29 B 0.86
A 0.29 B 1
A 0.43 B 0
A 0.43 B 0.14
A 0.43 B 0.29
A 0.43 B 0.43
A 0.43 B 0.57
A 0.43 B 0.71
A 0.43 B 0.86
A 0.43 B 1
A 0.57 B 0
A 0.57 B 0.14
A 0.57 B 0.29
A 0.57 B 0.43
A 0.57 B 0.57
A 0.57 B 0.71
A 0.57 B 0.86
A 0.57 B 1
A 0.71 B 0
A 0.71 B 0.14
A 0.71 B 0.29
A 0.71 B 0.43
A 0.71 B 0.57
A 0.71 B 0.71
A 0.71 B 0.86
A 0.71 B 1
A 0.86 B 0
A 0.86 B 0.14
A 0.86 B 0.29
A 0.86 B 0.43
A 0.86 B 0.57
A 0.86 B 0.71
A 0.86 B 0.86
A 0.86 B 1
A 1 B 0
A 1 B 0.14
A 1 B 0.29
A 1 B 0.43
A 1 B 0.57
A 1 B 0.71
A 1 B 0.86
A 1 B 1
0.75
0.50
0.25
OD 600 nm [–]
0 5 101520
Time [h]

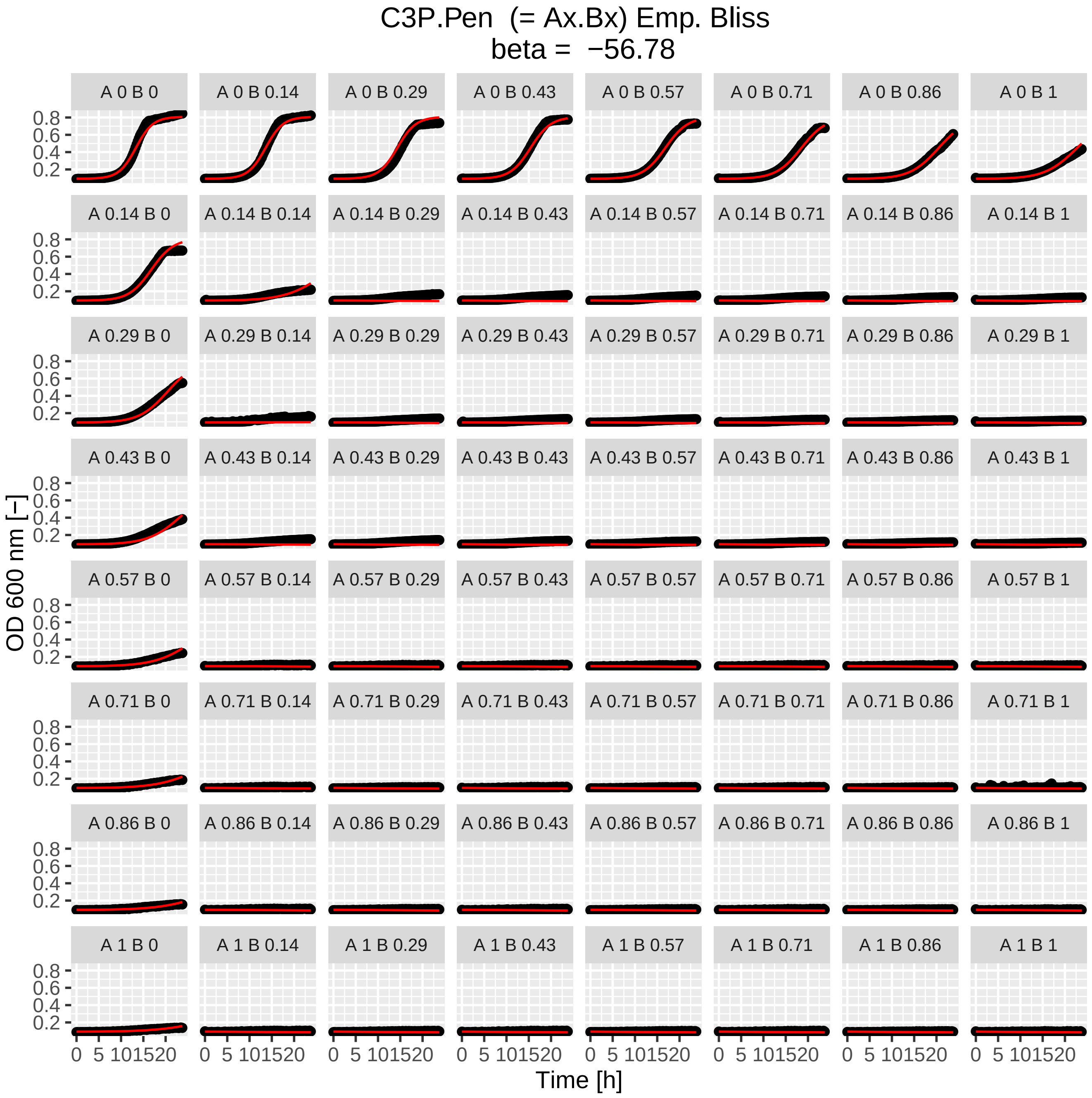
C3P.Pen (= Ax.Bx) Emp. Bliss
beta = −56.78
OD 600 nm [−]
Time [h]
A 0 B 0
A 0 B 0.14
A 0 B 0.29
A 0 B 0.43
A 0 B 0.57
A 0 B 0.71
A 0 B 0.86
A 0 B 1
A 0.14 B 0
A 0.14 B 0.14
A 0.14 B 0.29
A 0.14 B 0.43
A 0.14 B 0.57
A 0.14 B 0.71
A 0.14 B 0.86
A 0.14 B 1
A 0.29 B 0
A 0.29 B 0.14
A 0.29 B 0.29
A 0.29 B 0.43
A 0.29 B 0.57
A 0.29 B 0.71
A 0.29 B 0.86
A 0.29 B 1
A 0.43 B 0
A 0.43 B 0.14
A 0.43 B 0.29
A 0.43 B 0.43
A 0.43 B 0.57
A 0.43 B 0.71
A 0.43 B 0.86
A 0.43 B 1
A 0.57 B 0
A 0.57 B 0.14
A 0.57 B 0.29
A 0.57 B 0.43
A 0.57 B 0.57
A 0.57 B 0.71
A 0.57 B 0.86
A 0.57 B 1
A 0.71 B 0
A 0.71 B 0.14
A 0.71 B 0.29
A 0.71 B 0.43
A 0.71 B 0.57
A 0.71 B 0.71
A 0.71 B 0.86
A 0.71 B 1
A 0.86 B 0
A 0.86 B 0.14
A 0.86 B 0.29
A 0.86 B 0.43
A 0.86 B 0.57
A 0.86 B 0.71
A 0.86 B 0.86
A 0.86 B 1
A 1 B 0
A 1 B 0.14
A 1 B 0.29
A 1 B 0.43
A 1 B 0.57
A 1 B 0.71
A 1 B 0.86
A 1 B 1
0.8
0.6
0.4
0.2
0 5 101520

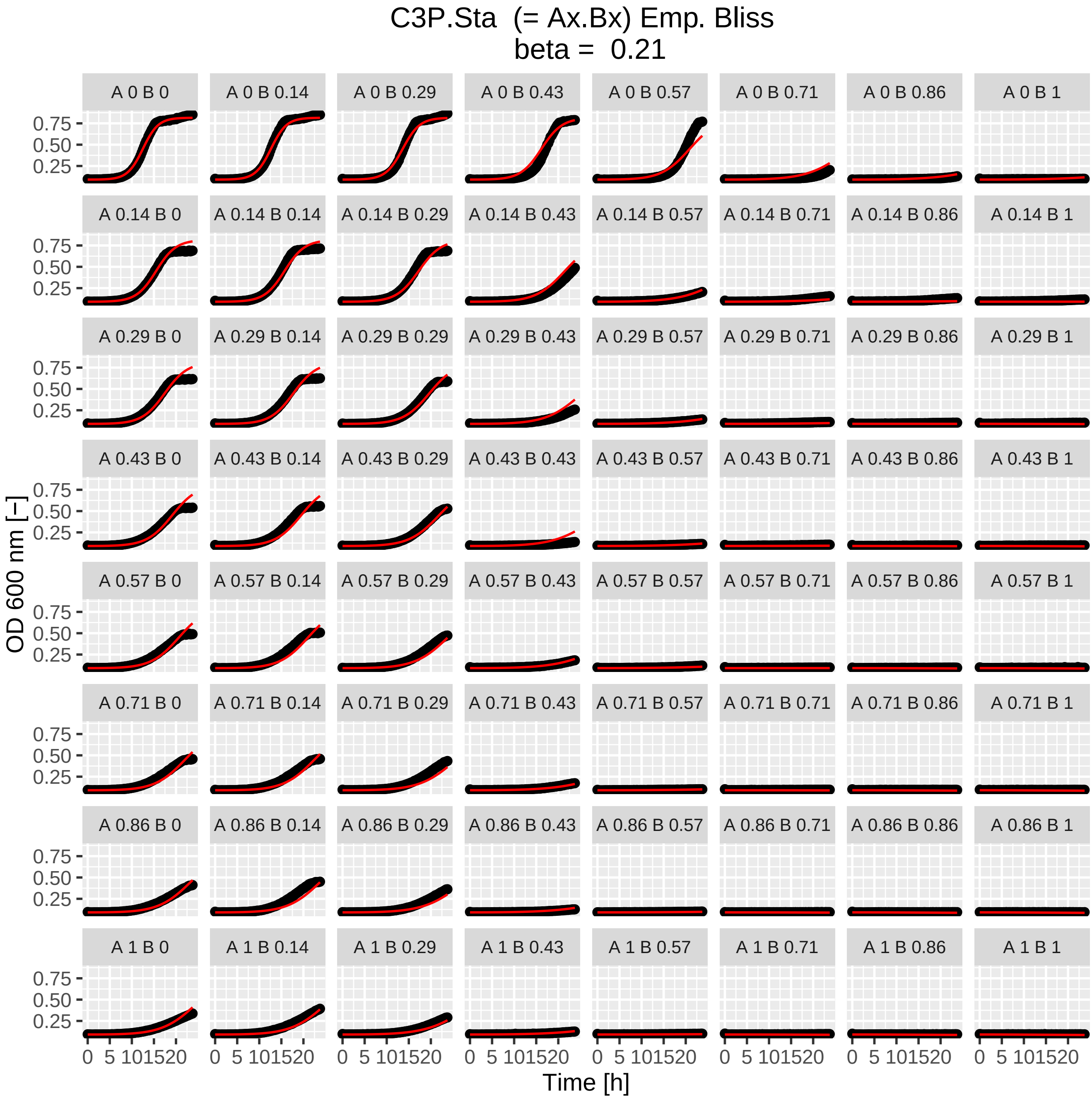
C3P.Sta (= Ax.Bx) Emp. Bliss
beta = 0.21
A 0 B 0
A 0 B 0.14
A 0 B 0.29
A 0 B 0.43
A 0 B 0.57
A 0 B 0.71
A 0 B 0.86
A 0 B 1
A 0.14 B 0
A 0.14 B 0.14
A 0.14 B 0.29
A 0.14 B 0.43
A 0.14 B 0.57
A 0.14 B 0.71
A 0.14 B 0.86
A 0.14 B 1
A 0.29 B 0
A 0.29 B 0.14
A 0.29 B 0.29
A 0.29 B 0.43
A 0.29 B 0.57
A 0.29 B 0.71
A 0.29 B 0.86
A 0.29 B 1
A 0.43 B 0
A 0.43 B 0.14
A 0.43 B 0.29
A 0.43 B 0.43
A 0.43 B 0.57
A 0.43 B 0.71
A 0.43 B 0.86
A 0.43 B 1
A 0.57 B 0
A 0.57 B 0.14
A 0.57 B 0.29
A 0.57 B 0.43
A 0.57 B 0.57
A 0.57 B 0.71
A 0.57 B 0.86
A 0.57 B 1
A 0.71 B 0
A 0.71 B 0.14
A 0.71 B 0.29
A 0.71 B 0.43
A 0.71 B 0.57
A 0.71 B 0.71
A 0.71 B 0.86
A 0.71 B 1
A 0.86 B 0
A 0.86 B 0.14
A 0.86 B 0.29
A 0.86 B 0.43
A 0.86 B 0.57
A 0.86 B 0.71
A 0.86 B 0.86
A 0.86 B 1
A 1 B 0
A 1 B 0.14
A 1 B 0.29
A 1 B 0.43
A 1 B 0.57
A 1 B 0.71
A 1 B 0.86
A 1 B 1
0.75
0.50
0.25
0 5 101520
OD 600 nm [–]
Time [h]

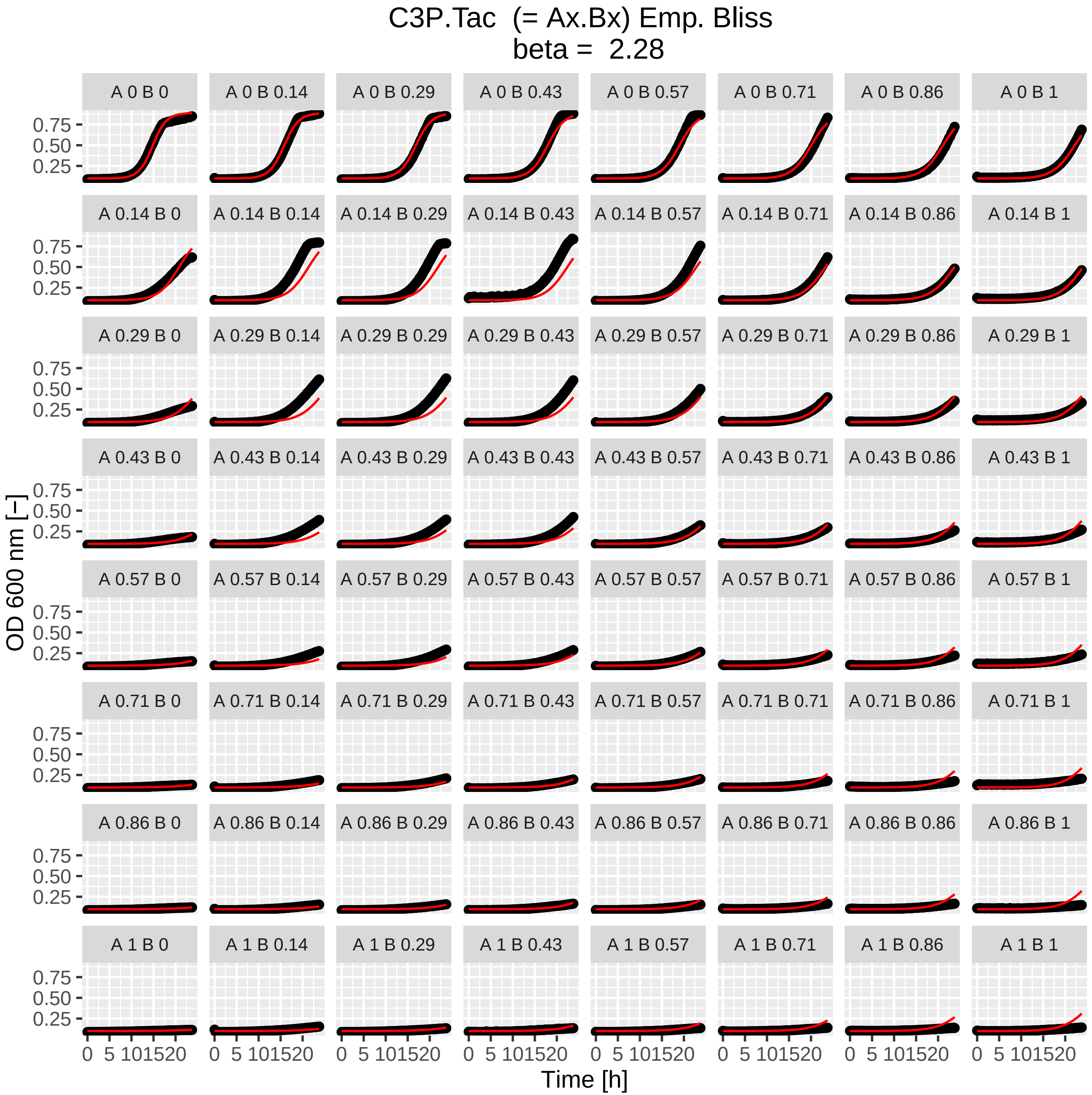

C3P.Tac (= Ax.Bx) Emp. Bliss
beta = 2.28
OD 600 nm [−]
Time [h]
A 0 B 0
A 0 B 0.14
A 0 B 0.29
A 0 B 0.43
A 0 B 0.57
A 0 B 0.71
A 0 B 0.86
A 0 B 1
A 0.14 B 0
A 0.14 B 0.14
A 0.14 B 0.29
A 0.14 B 0.43
A 0.14 B 0.57
A 0.14 B 0.71
A 0.14 B 0.86
A 0.14 B 1
A 0.29 B 0
A 0.29 B 0.14
A 0.29 B 0.29
A 0.29 B 0.43
A 0.29 B 0.57
A 0.29 B 0.71
A 0.29 B 0.86
A 0.29 B 1
A 0.43 B 0
A 0.43 B 0.14
A 0.43 B 0.29
A 0.43 B 0.43
A 0.43 B 0.57
A 0.43 B 0.71
A 0.43 B 0.86
A 0.43 B 1
A 0.57 B 0
A 0.57 B 0.14
A 0.57 B 0.29
A 0.57 B 0.43
A 0.57 B 0.57
A 0.57 B 0.71
A 0.57 B 0.86
A 0.57 B 1
A 0.71 B 0
A 0.71 B 0.14
A 0.71 B 0.29
A 0.71 B 0.43
A 0.71 B 0.57
A 0.71 B 0.71
A 0.71 B 0.86
A 0.71 B 1
A 0.86 B 0
A 0.86 B 0.14
A 0.86 B 0.29
A 0.86 B 0.43
A 0.86 B 0.57
A 0.86 B 0.71
A 0.86 B 0.86
A 0.86 B 1
A 1 B 0
A 1 B 0.14
A 1 B 0.29
A 1 B 0.43
A 1 B 0.57
A 1 B 0.71
A 1 B 0.86
A 1 B 1
0.75
0.50
0.25
0 5 101520

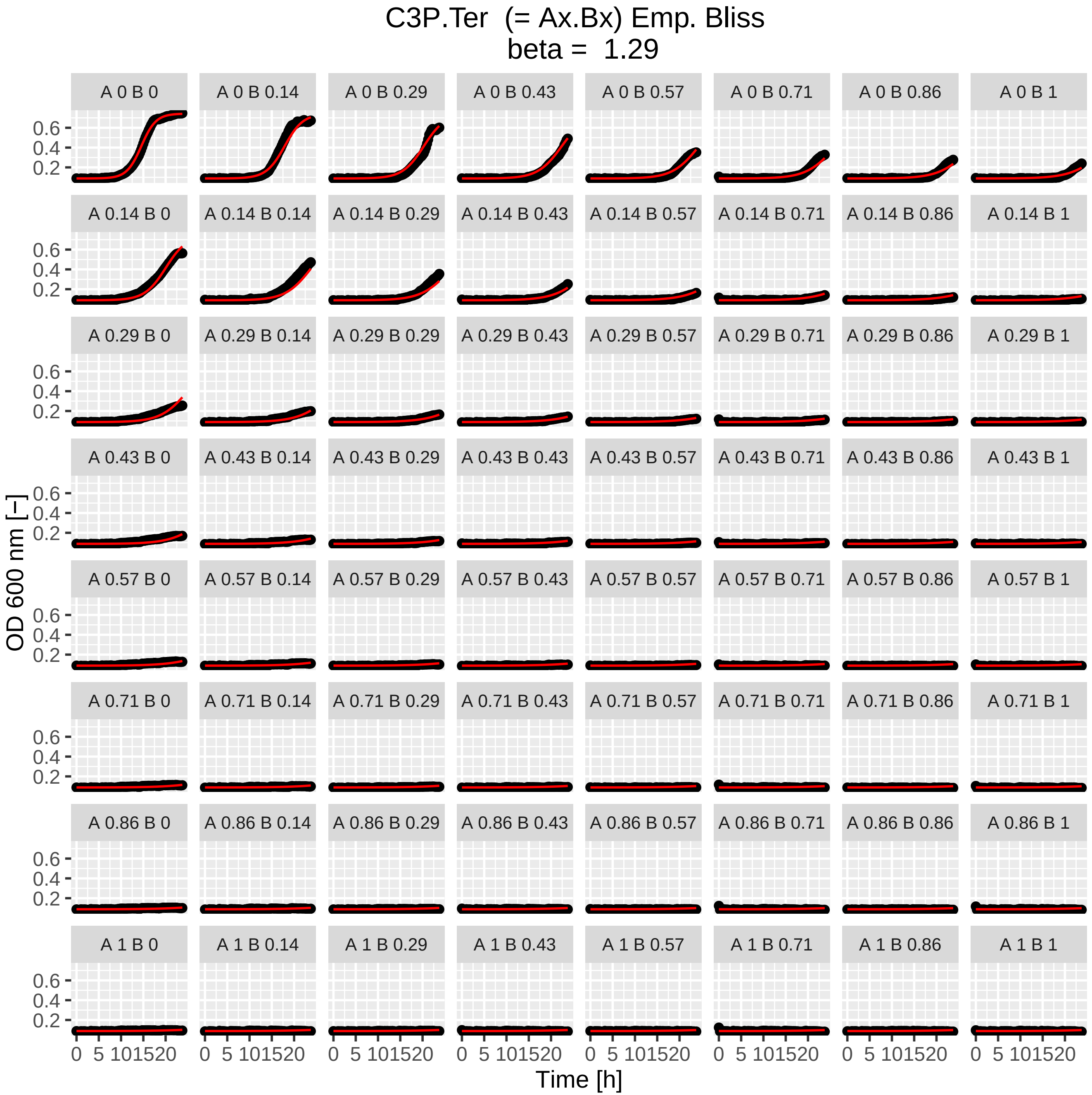
C3P.Ter (= Ax.Bx) Emp. Bliss
beta = 1.29
A 0 B 0
A 0 B 0.14
A 0 B 0.29
A 0 B 0.43
A 0 B 0.57
A 0 B 0.71
A 0 B 0.86
A 0 B 1
A 0.14 B 0
A 0.14 B 0.14
A 0.14 B 0.29
A 0.14 B 0.43
A 0.14 B 0.57
A 0.14 B 0.71
A 0.14 B 0.86
A 0.14 B 1
A 0.29 B 0
A 0.29 B 0.14
A 0.29 B 0.29
A 0.29 B 0.43
A 0.29 B 0.57
A 0.29 B 0.71
A 0.29 B 0.86
A 0.29 B 1
A 0.43 B 0
A 0.43 B 0.14
A 0.43 B 0.29
A 0.43 B 0.43
A 0.43 B 0.57
A 0.43 B 0.71
A 0.43 B 0.86
A 0.43 B 1
A 0.57 B 0
A 0.57 B 0.14
A 0.57 B 0.29
A 0.57 B 0.43
A 0.57 B 0.57
A 0.57 B 0.71
A 0.57 B 0.86
A 0.57 B 1
A 0.71 B 0
A 0.71 B 0.14
A 0.71 B 0.29
A 0.71 B 0.43
A 0.71 B 0.57
A 0.71 B 0.71
A 0.71 B 0.86
A 0.71 B 1
A 0.86 B 0
A 0.86 B 0.14
A 0.86 B 0.29
A 0.86 B 0.43
A 0.86 B 0.57
A 0.86 B 0.71
A 0.86 B 0.86
A 0.86 B 1
A 1 B 0
A 1 B 0.14
A 1 B 0.29
A 1 B 0.43
A 1 B 0.57
A 1 B 0.71
A 1 B 0.86
A 1 B 1
OD 600 nm [–]
0.2
0.4
0.6
0 5 101520
Time [h]

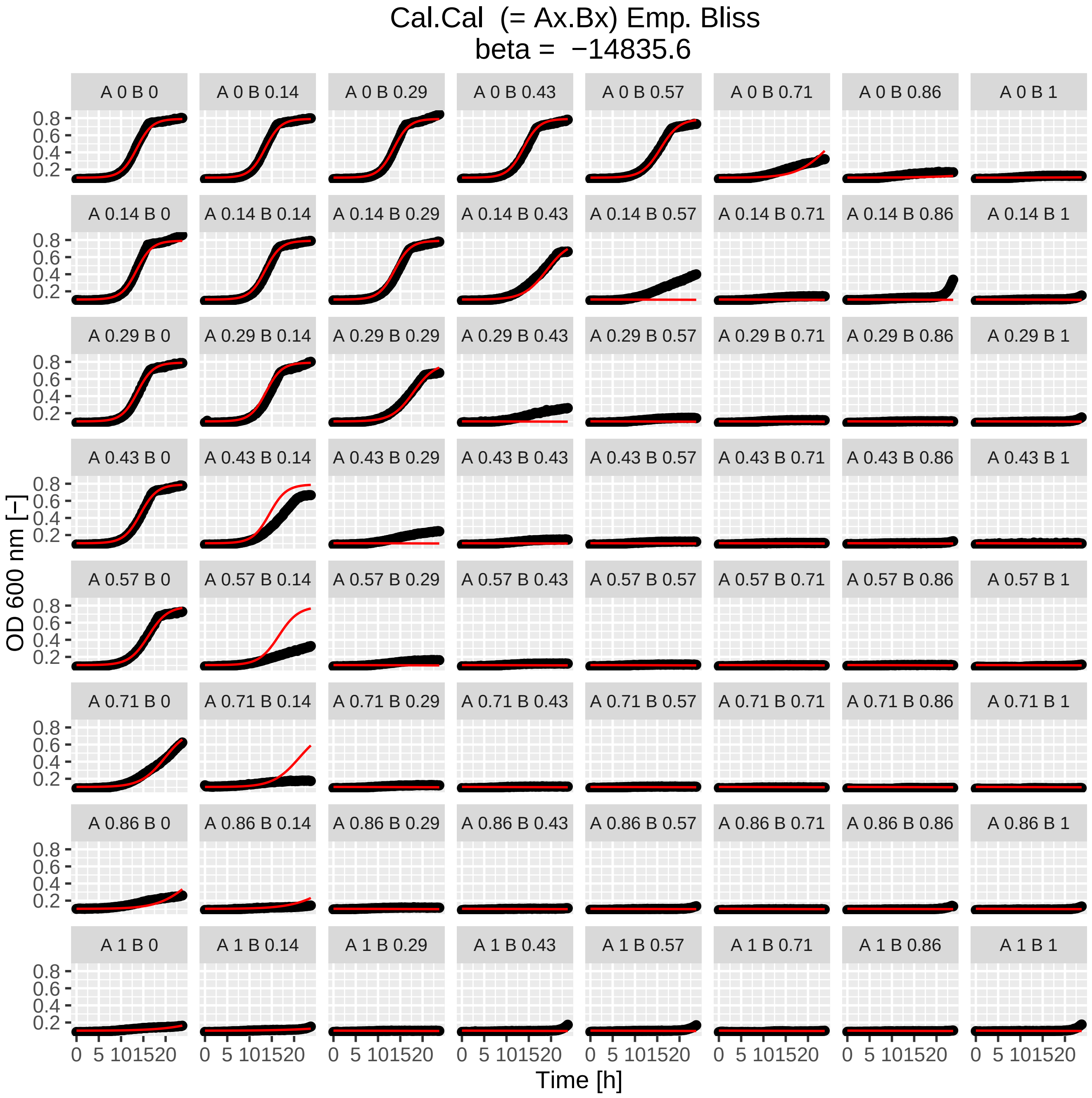
Cal.Cal (= Ax.Bx) Emp. Bliss
beta = −14835.6
OD 600 nm [−]
Time [h]
0.8
0.6
0.4
0.2
0 5 101520
A 0 B 0
A 0 B 0.14
A 0 B 0.29
A 0 B 0.43
A 0 B 0.57
A 0 B 0.71
A 0 B 0.86
A 0 B 1
A 0.14 B 0
A 0.14 B 0.14
A 0.14 B 0.29
A 0.14 B 0.43
A 0.14 B 0.57
A 0.14 B 0.71
A 0.14 B 0.86
A 0.14 B 1
A 0.29 B 0
A 0.29 B 0.14
A 0.29 B 0.29
A 0.29 B 0.43
A 0.29 B 0.57
A 0.29 B 0.71
A 0.29 B 0.86
A 0.29 B 1
A 0.43 B 0
A 0.43 B 0.14
A 0.43 B 0.29
A 0.43 B 0.43
A 0.43 B 0.57
A 0.43 B 0.71
A 0.43 B 0.86
A 0.43 B 1
A 0.57 B 0
A 0.57 B 0.14
A 0.57 B 0.29
A 0.57 B 0.43
A 0.57 B 0.57
A 0.57 B 0.71
A 0.57 B 0.86
A 0.57 B 1
A 0.71 B 0
A 0.71 B 0.14
A 0.71 B 0.29
A 0.71 B 0.43
A 0.71 B 0.57
A 0.71 B 0.71
A 0.71 B 0.86
A 0.71 B 1
A 0.86 B 0
A 0.86 B 0.14
A 0.86 B 0.29
A 0.86 B 0.43
A 0.86 B 0.57
A 0.86 B 0.71
A 0.86 B 0.86
A 0.86 B 1
A 1 B 0
A 1 B 0.14
A 1 B 0.29
A 1 B 0.43
A 1 B 0.57
A 1 B 0.71
A 1 B 0.86
A 1 B 1

Cal.Dyc (= Ax.Bx) Emp. Bliss
beta = −283341.2

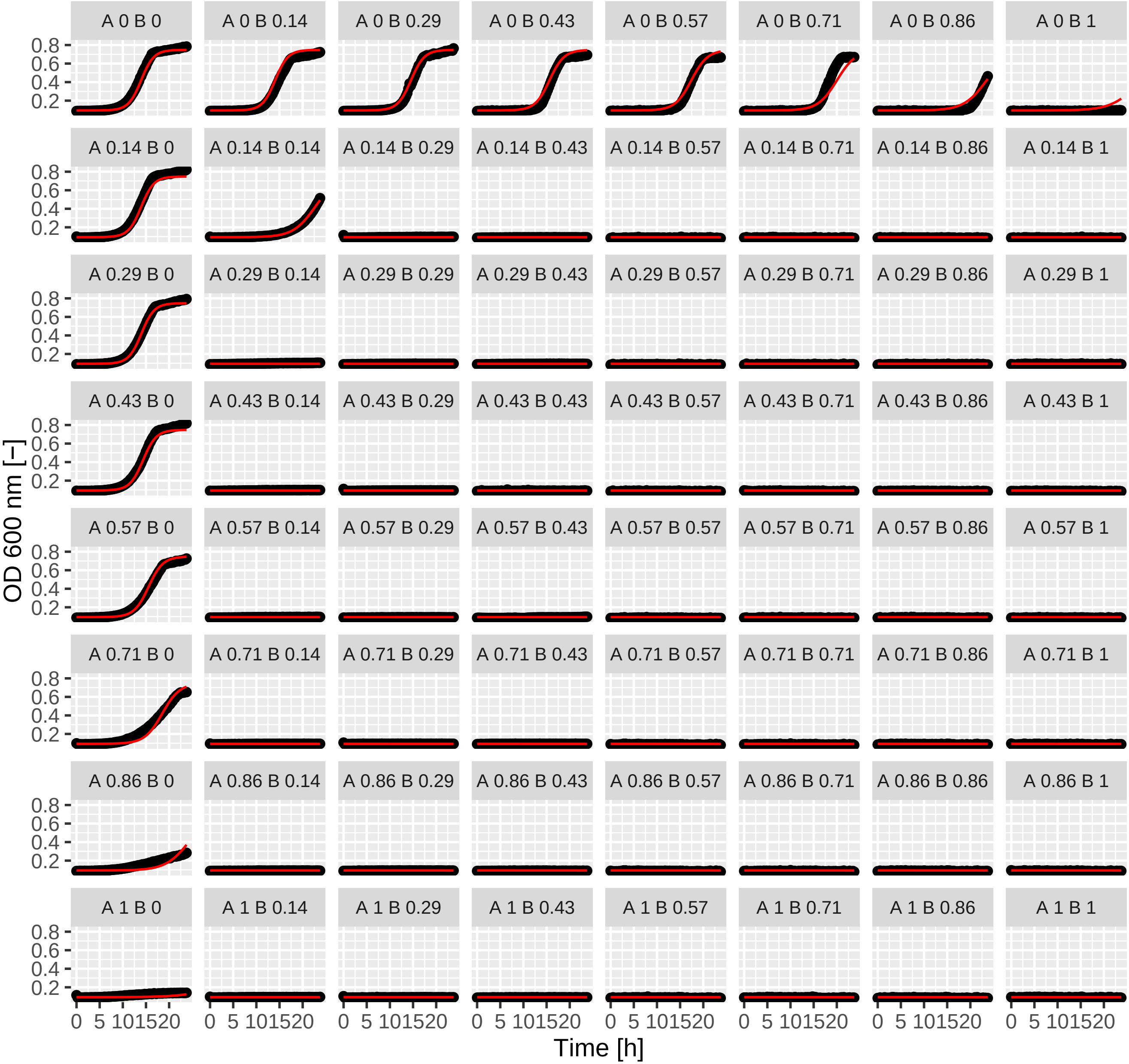

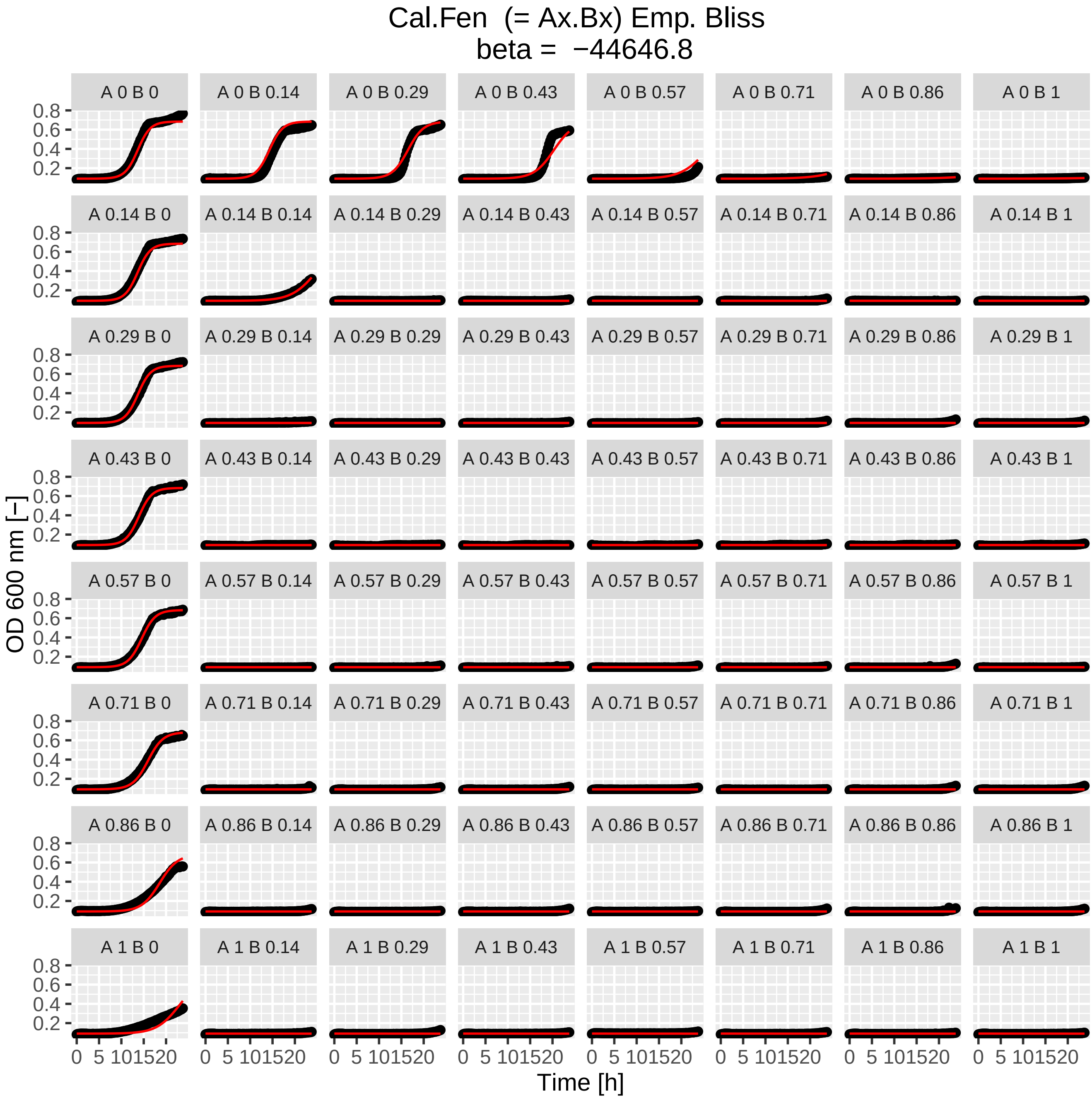

Cal.Fen (= Ax.Bx) Emp. Bliss
beta = −44646.8
OD 600 nm [−]
Time [h]
A 0 B 0
A 0 B 0.14
A 0 B 0.29
A 0 B 0.43
A 0 B 0.57
A 0 B 0.71
A 0 B 0.86
A 0 B 1
A 0.14 B 0
A 0.14 B 0.14
A 0.14 B 0.29
A 0.14 B 0.43
A 0.14 B 0.57
A 0.14 B 0.71
A 0.14 B 0.86
A 0.14 B 1
A 0.29 B 0
A 0.29 B 0.14
A 0.29 B 0.29
A 0.29 B 0.43
A 0.29 B 0.57
A 0.29 B 0.71
A 0.29 B 0.86
A 0.29 B 1
A 0.43 B 0
A 0.43 B 0.14
A 0.43 B 0.29
A 0.43 B 0.43
A 0.43 B 0.57
A 0.43 B 0.71
A 0.43 B 0.86
A 0.43 B 1
A 0.57 B 0
A 0.57 B 0.14
A 0.57 B 0.29
A 0.57 B 0.43
A 0.57 B 0.57
A 0.57 B 0.71
A 0.57 B 0.86
A 0.57 B 1
A 0.71 B 0
A 0.71 B 0.14
A 0.71 B 0.29
A 0.71 B 0.43
A 0.71 B 0.57
A 0.71 B 0.71
A 0.71 B 0.86
A 0.71 B 1
A 0.86 B 0
A 0.86 B 0.14
A 0.86 B 0.29
A 0.86 B 0.43
A 0.86 B 0.57
A 0.86 B 0.71
A 0.86 B 0.86
A 0.86 B 1
A 1 B 0
A 1 B 0.14
A 1 B 0.29
A 1 B 0.43
A 1 B 0.57
A 1 B 0.71
A 1 B 0.86
A 1 B 1
0.8
0.6
0.4
0.2
0 5 101520

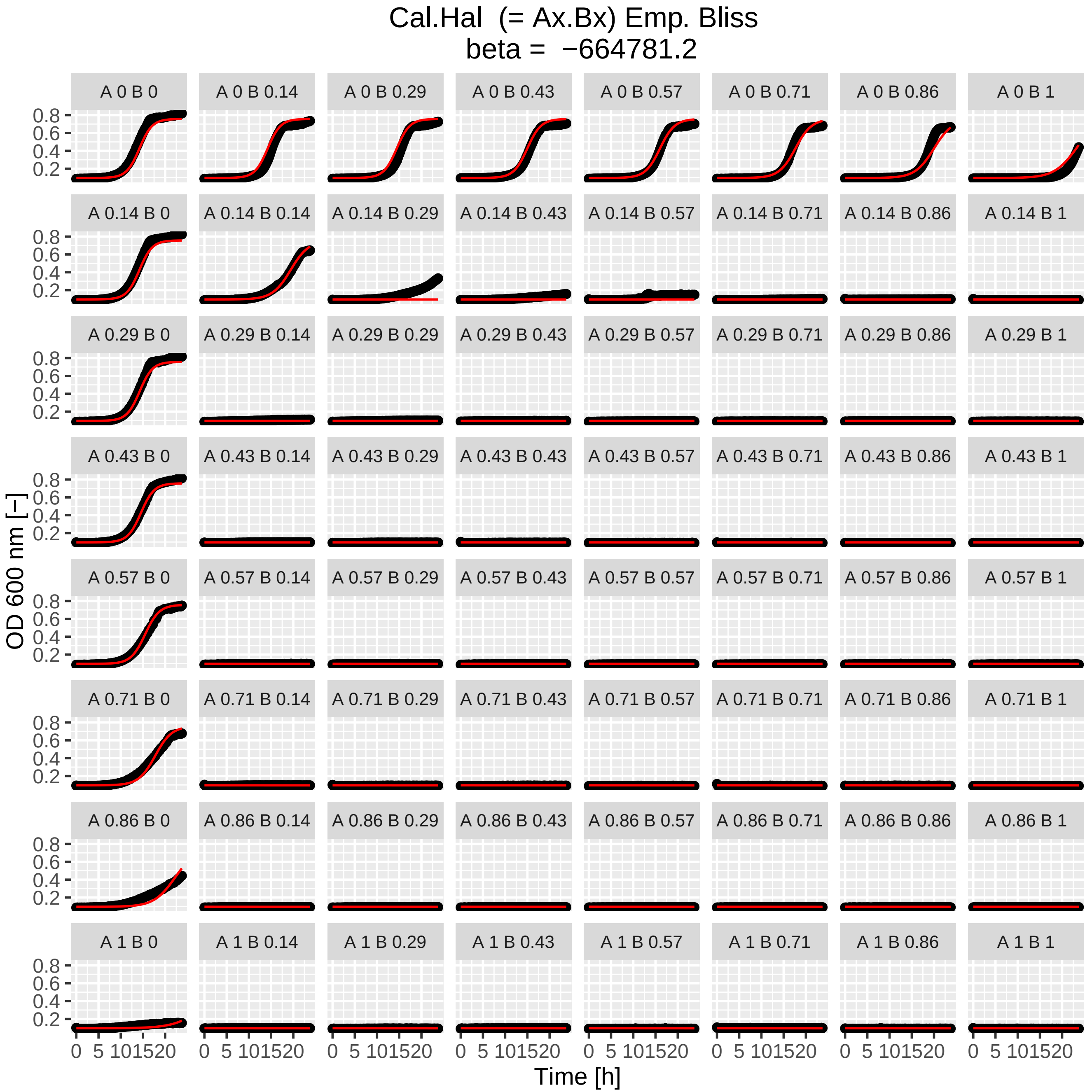
Cal.Hal (= Ax.Bx) Emp. Bliss
beta = −664781.2
A 0 B 0
A 0 B 0.14
A 0 B 0.29
A 0 B 0.43
A 0 B 0.57
A 0 B 0.71
A 0 B 0.86
A 0 B 1
A 0.14 B 0
A 0.14 B 0.14
A 0.14 B 0.29
A 0.14 B 0.43
A 0.14 B 0.57
A 0.14 B 0.71
A 0.14 B 0.86
A 0.14 B 1
A 0.29 B 0
A 0.29 B 0.14
A 0.29 B 0.29
A 0.29 B 0.43
A 0.29 B 0.57
A 0.29 B 0.71
A 0.29 B 0.86
A 0.29 B 1
A 0.43 B 0
A 0.43 B 0.14
A 0.43 B 0.29
A 0.43 B 0.43
A 0.43 B 0.57
A 0.43 B 0.71
A 0.43 B 0.86
A 0.43 B 1
A 0.57 B 0
A 0.57 B 0.14
A 0.57 B 0.29
A 0.57 B 0.43
A 0.57 B 0.57
A 0.57 B 0.71
A 0.57 B 0.86
A 0.57 B 1
A 0.71 B 0
A 0.71 B 0.14
A 0.71 B 0.29
A 0.71 B 0.43
A 0.71 B 0.57
A 0.71 B 0.71
A 0.71 B 0.86
A 0.71 B 1
A 0.86 B 0
A 0.86 B 0.14
A 0.86 B 0.29
A 0.86 B 0.43
A 0.86 B 0.57
A 0.86 B 0.71
A 0.86 B 0.86
A 0.86 B 1
A 1 B 0
A 1 B 0.14
A 1 B 0.29
A 1 B 0.43
A 1 B 0.57
A 1 B 0.71
A 1 B 0.86
A 1 B 1
OD 600 nm [−]
0.2
0.4
0.6
0.8
0 5 101520
Time [h]

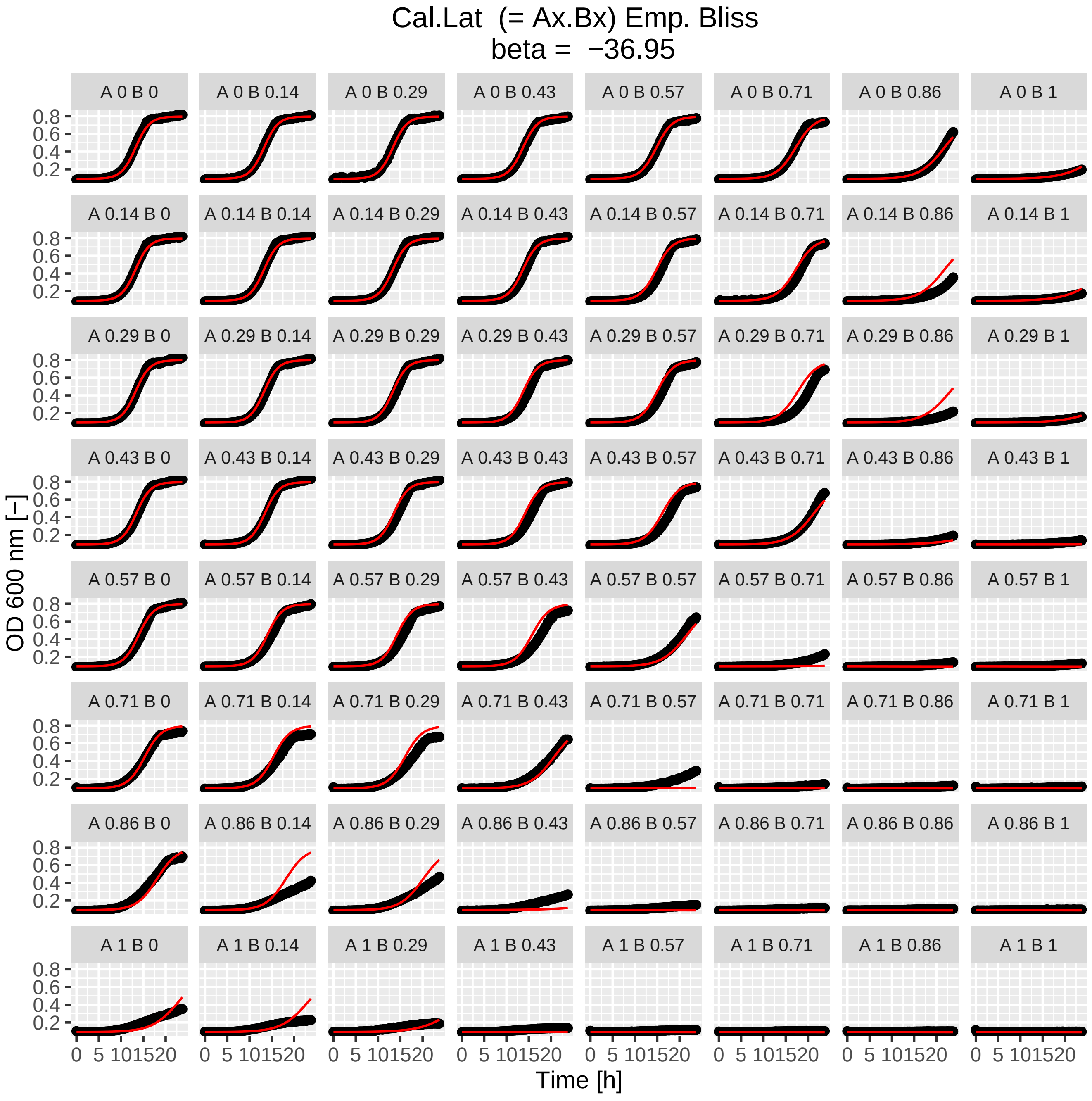

Cal.Lat (= Ax.Bx) Emp. Bliss
beta = −36.95
A 0 B 0
A 0 B 0.14
A 0 B 0.29
A 0 B 0.43
A 0 B 0.57
A 0 B 0.71
A 0 B 0.86
A 0 B 1
A 0.14 B 0
A 0.14 B 0.14
A 0.14 B 0.29
A 0.14 B 0.43
A 0.14 B 0.57
A 0.14 B 0.71
A 0.14 B 0.86
A 0.14 B 1
A 0.29 B 0
A 0.29 B 0.14
A 0.29 B 0.29
A 0.29 B 0.43
A 0.29 B 0.57
A 0.29 B 0.71
A 0.29 B 0.86
A 0.29 B 1
A 0.43 B 0
A 0.43 B 0.14
A 0.43 B 0.29
A 0.43 B 0.43
A 0.43 B 0.57
A 0.43 B 0.71
A 0.43 B 0.86
A 0.43 B 1
A 0.57 B 0
A 0.57 B 0.14
A 0.57 B 0.29
A 0.57 B 0.43
A 0.57 B 0.57
A 0.57 B 0.71
A 0.57 B 0.86
A 0.57 B 1
A 0.71 B 0
A 0.71 B 0.14
A 0.71 B 0.29
A 0.71 B 0.43
A 0.71 B 0.57
A 0.71 B 0.71
A 0.71 B 0.86
A 0.71 B 1
A 0.86 B 0
A 0.86 B 0.14
A 0.86 B 0.29
A 0.86 B 0.43
A 0.86 B 0.57
A 0.86 B 0.71
A 0.86 B 0.86
A 0.86 B 1
A 1 B 0
A 1 B 0.14
A 1 B 0.29
A 1 B 0.43
A 1 B 0.57
A 1 B 0.71
A 1 B 0.86
A 1 B 1
OD 600 nm [−]
0.2
0.4
0.6
0.8
0
5
10
15
20
Time [h]

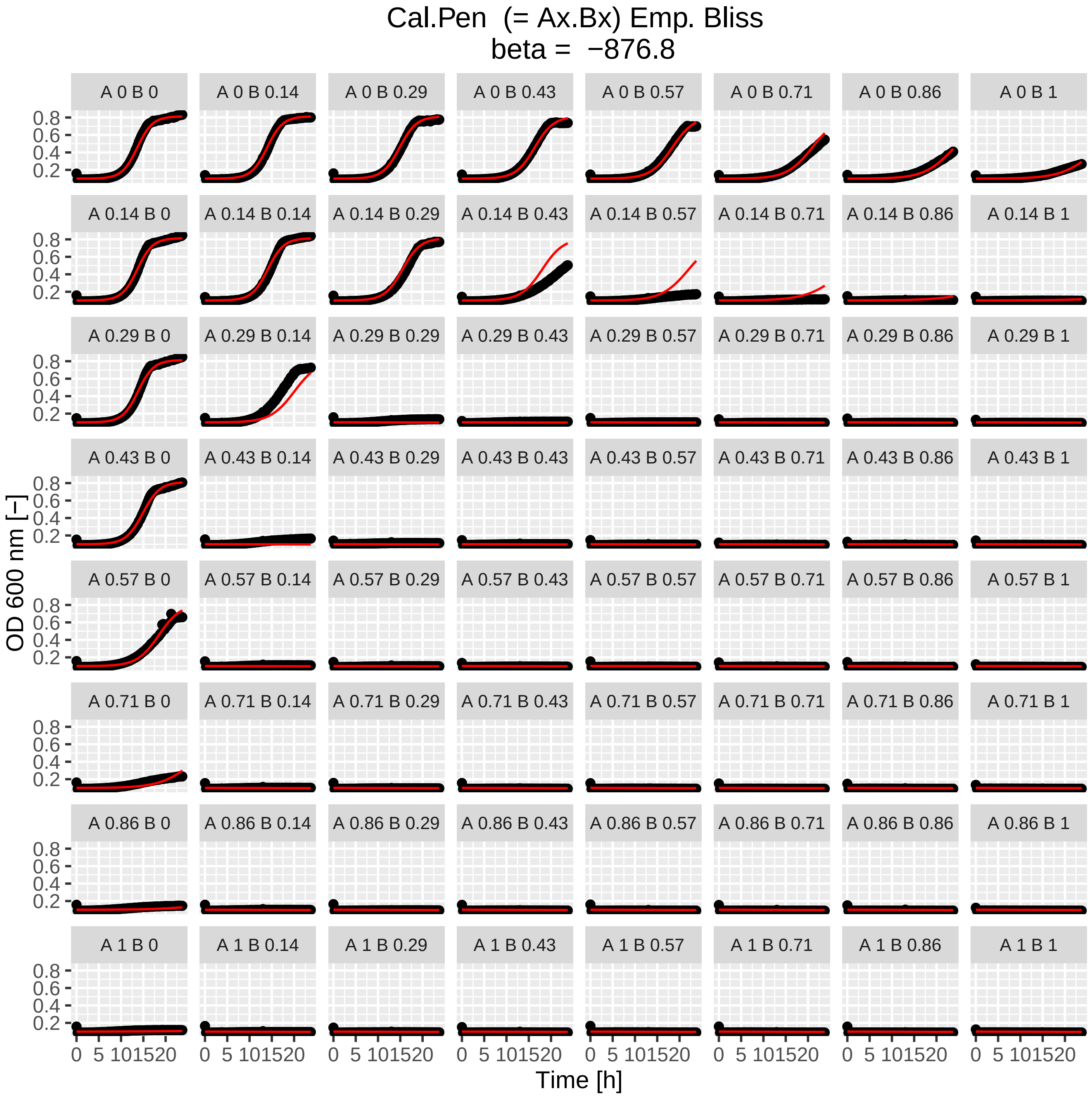

Cal.Pen (= Ax.Bx) Emp. Bliss
beta = −876.8
A 0 B 0
A 0 B 0.14
A 0 B 0.29
A 0 B 0.43
A 0 B 0.57
A 0 B 0.71
A 0 B 0.86
A 0 B 1
A 0.14 B 0
A 0.14 B 0.14
A 0.14 B 0.29
A 0.14 B 0.43
A 0.14 B 0.57
A 0.14 B 0.71
A 0.14 B 0.86
A 0.14 B 1
A 0.29 B 0
A 0.29 B 0.14
A 0.29 B 0.29
A 0.29 B 0.43
A 0.29 B 0.57
A 0.29 B 0.71
A 0.29 B 0.86
A 0.29 B 1
A 0.43 B 0
A 0.43 B 0.14
A 0.43 B 0.29
A 0.43 B 0.43
A 0.43 B 0.57
A 0.43 B 0.71
A 0.43 B 0.86
A 0.43 B 1
A 0.57 B 0
A 0.57 B 0.14
A 0.57 B 0.29
A 0.57 B 0.43
A 0.57 B 0.57
A 0.57 B 0.71
A 0.57 B 0.86
A 0.57 B 1
A 0.71 B 0
A 0.71 B 0.14
A 0.71 B 0.29
A 0.71 B 0.43
A 0.71 B 0.57
A 0.71 B 0.71
A 0.71 B 0.86
A 0.71 B 1
A 0.86 B 0
A 0.86 B 0.14
A 0.86 B 0.29
A 0.86 B 0.43
A 0.86 B 0.57
A 0.86 B 0.71
A 0.86 B 0.86
A 0.86 B 1
A 1 B 0
A 1 B 0.14
A 1 B 0.29
A 1 B 0.43
A 1 B 0.57
A 1 B 0.71
A 1 B 0.86
A 1 B 1
0.8
0.6
0.4
0.2
0 5 101520
OD 600 nm [−]
Time [h]

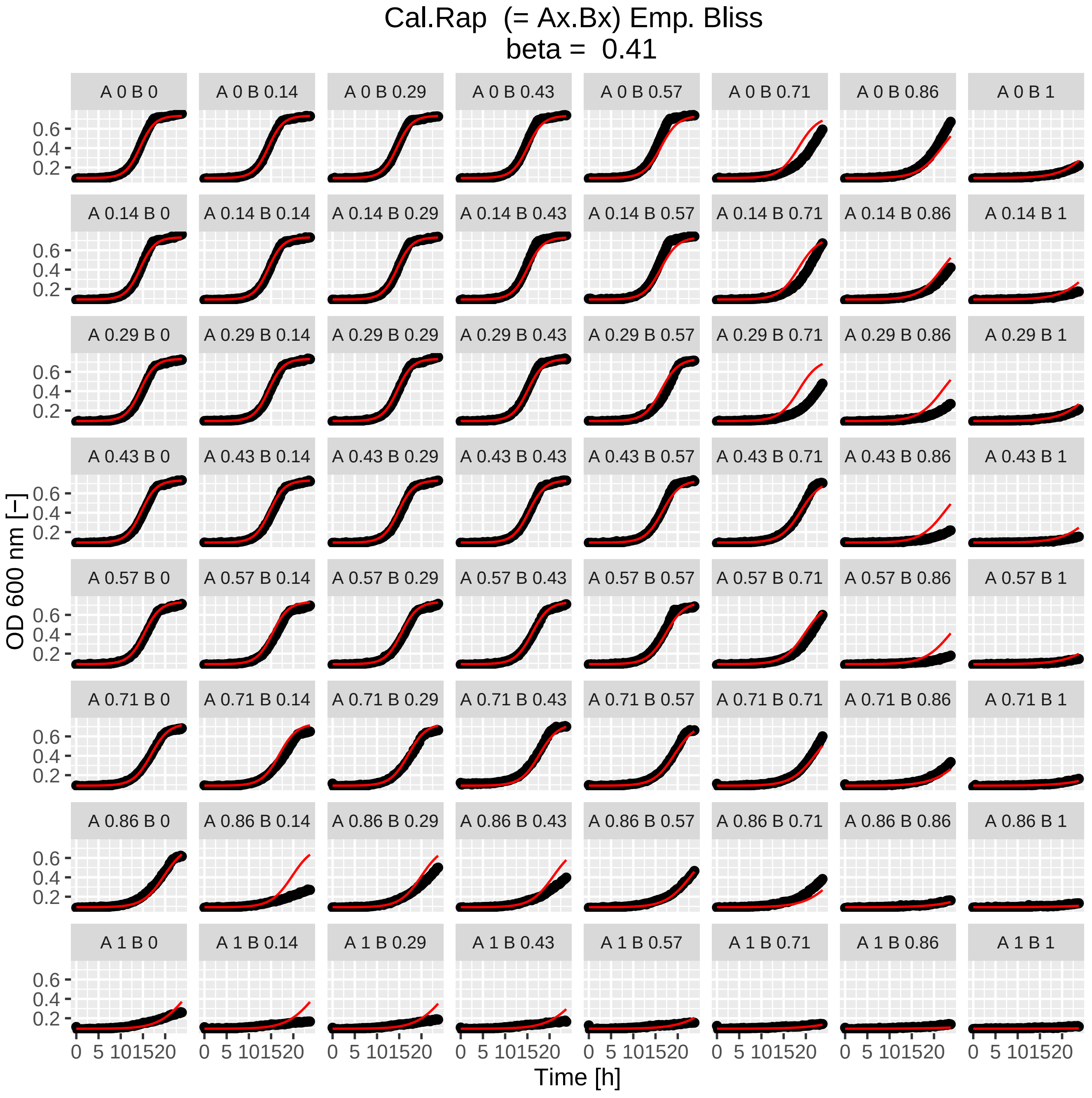
Cal.Rap (= Ax.Bx) Emp. Bliss
beta = 0.41
OD 600 nm [−]
Time [h]
0.2
0.4
0.6
0 5 101520
A 0 B 0
A 0 B 0.14
A 0 B 0.29
A 0 B 0.43
A 0 B 0.57
A 0 B 0.71
A 0 B 0.86
A 0 B 1
A 0.14 B 0
A 0.14 B 0.14
A 0.14 B 0.29
A 0.14 B 0.43
A 0.14 B 0.57
A 0.14 B 0.71
A 0.14 B 0.86
A 0.14 B 1
A 0.29 B 0
A 0.29 B 0.14
A 0.29 B 0.29
A 0.29 B 0.43
A 0.29 B 0.57
A 0.29 B 0.71
A 0.29 B 0.86
A 0.29 B 1
A 0.43 B 0
A 0.43 B 0.14
A 0.43 B 0.29
A 0.43 B 0.43
A 0.43 B 0.57
A 0.43 B 0.71
A 0.43 B 0.86
A 0.43 B 1
A 0.57 B 0
A 0.57 B 0.14
A 0.57 B 0.29
A 0.57 B 0.43
A 0.57 B 0.57
A 0.57 B 0.71
A 0.57 B 0.86
A 0.57 B 1
A 0.71 B 0
A 0.71 B 0.14
A 0.71 B 0.29
A 0.71 B 0.43
A 0.71 B 0.57
A 0.71 B 0.71
A 0.71 B 0.86
A 0.71 B 1
A 0.86 B 0
A 0.86 B 0.14
A 0.86 B 0.29
A 0.86 B 0.43
A 0.86 B 0.57
A 0.86 B 0.71
A 0.86 B 0.86
A 0.86 B 1
A 1 B 0
A 1 B 0.14
A 1 B 0.29
A 1 B 0.43
A 1 B 0.57
A 1 B 0.71
A 1 B 0.86
A 1 B 1

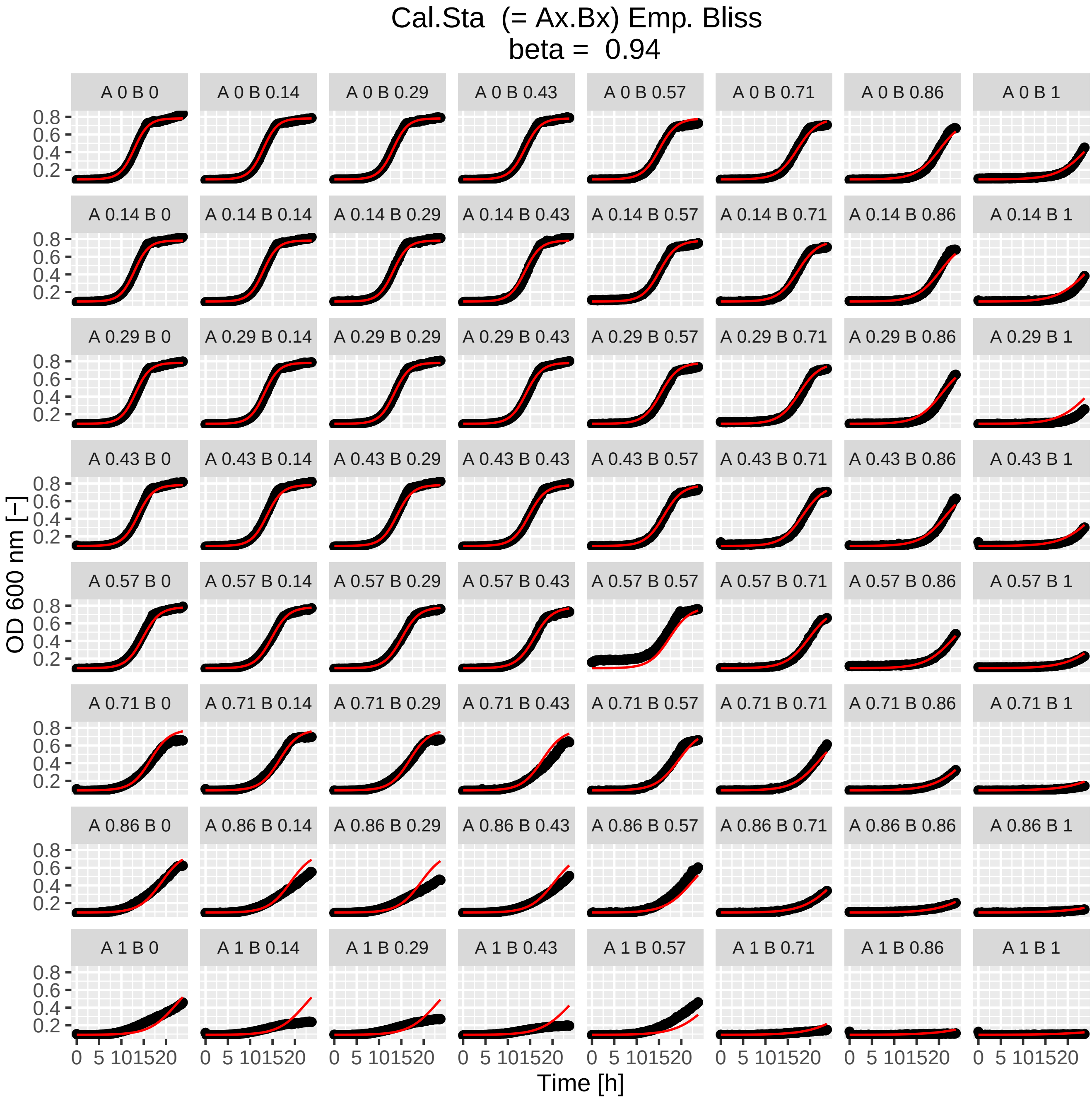

Cal.Sta (= Ax.Bx) Emp. Bliss
beta = 0.94
A 0 B 0
A 0 B 0.14
A 0 B 0.29
A 0 B 0.43
A 0 B 0.57
A 0 B 0.71
A 0 B 0.86
A 0 B 1
A 0.14 B 0
A 0.14 B 0.14
A 0.14 B 0.29
A 0.14 B 0.43
A 0.14 B 0.57
A 0.14 B 0.71
A 0.14 B 0.86
A 0.14 B 1
A 0.29 B 0
A 0.29 B 0.14
A 0.29 B 0.29
A 0.29 B 0.43
A 0.29 B 0.57
A 0.29 B 0.71
A 0.29 B 0.86
A 0.29 B 1
A 0.43 B 0
A 0.43 B 0.14
A 0.43 B 0.29
A 0.43 B 0.43
A 0.43 B 0.57
A 0.43 B 0.71
A 0.43 B 0.86
A 0.43 B 1
A 0.57 B 0
A 0.57 B 0.14
A 0.57 B 0.29
A 0.57 B 0.43
A 0.57 B 0.57
A 0.57 B 0.71
A 0.57 B 0.86
A 0.57 B 1
A 0.71 B 0
A 0.71 B 0.14
A 0.71 B 0.29
A 0.71 B 0.43
A 0.71 B 0.57
A 0.71 B 0.71
A 0.71 B 0.86
A 0.71 B 1
A 0.86 B 0
A 0.86 B 0.14
A 0.86 B 0.29
A 0.86 B 0.43
A 0.86 B 0.57
A 0.86 B 0.71
A 0.86 B 0.86
A 0.86 B 1
A 1 B 0
A 1 B 0.14
A 1 B 0.29
A 1 B 0.43
A 1 B 0.57
A 1 B 0.71
A 1 B 0.86
A 1 B 1
OD 600 nm [–]
0.2
0.4
0.6
0.8
0
5
10
15
20
Time [h]

Cal.Tac (= Ax.Bx) Emp. Bliss
beta = −118.55

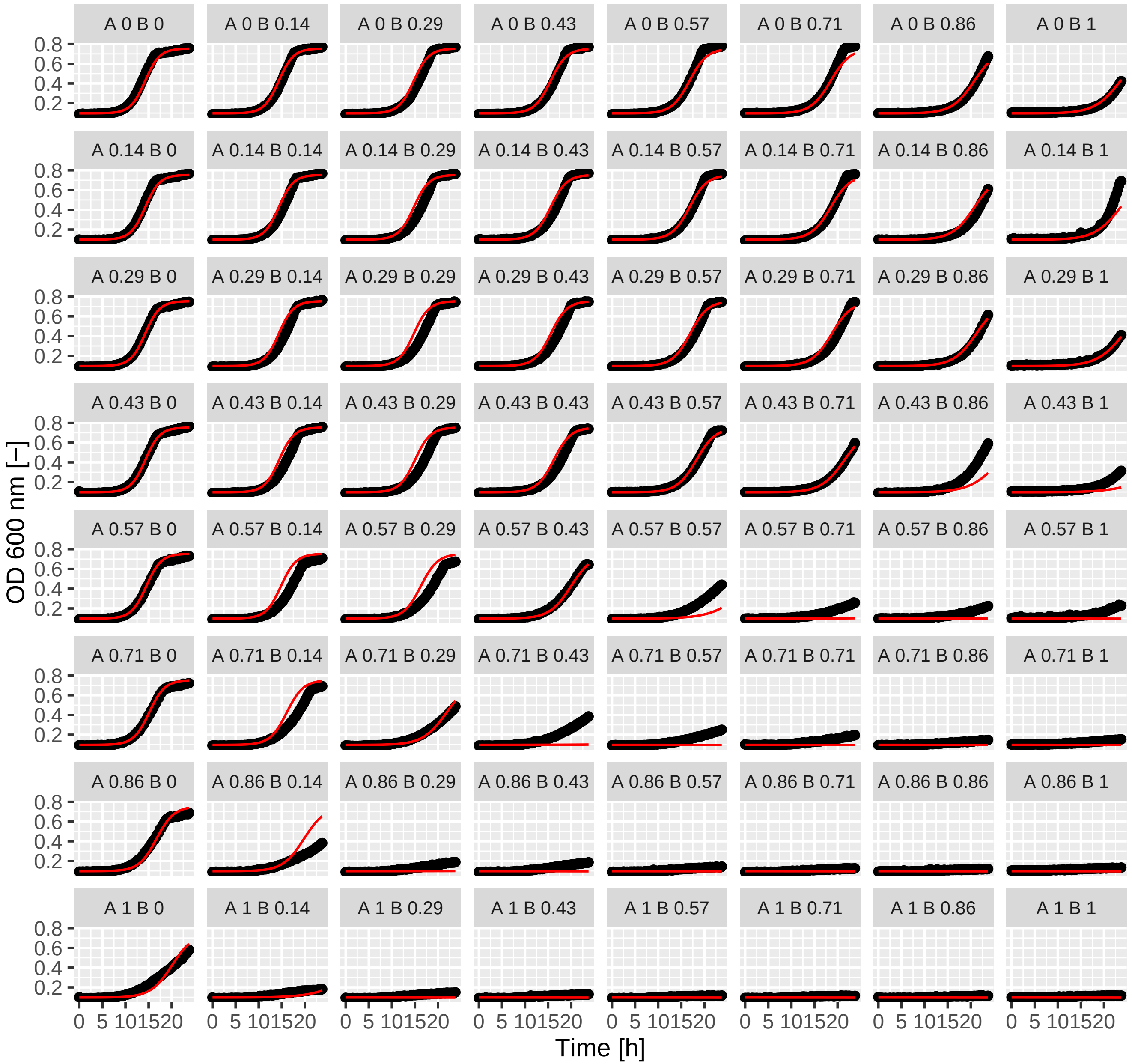

Cal.Ter (= Ax.Bx) Emp. Bliss
beta = −2051.6

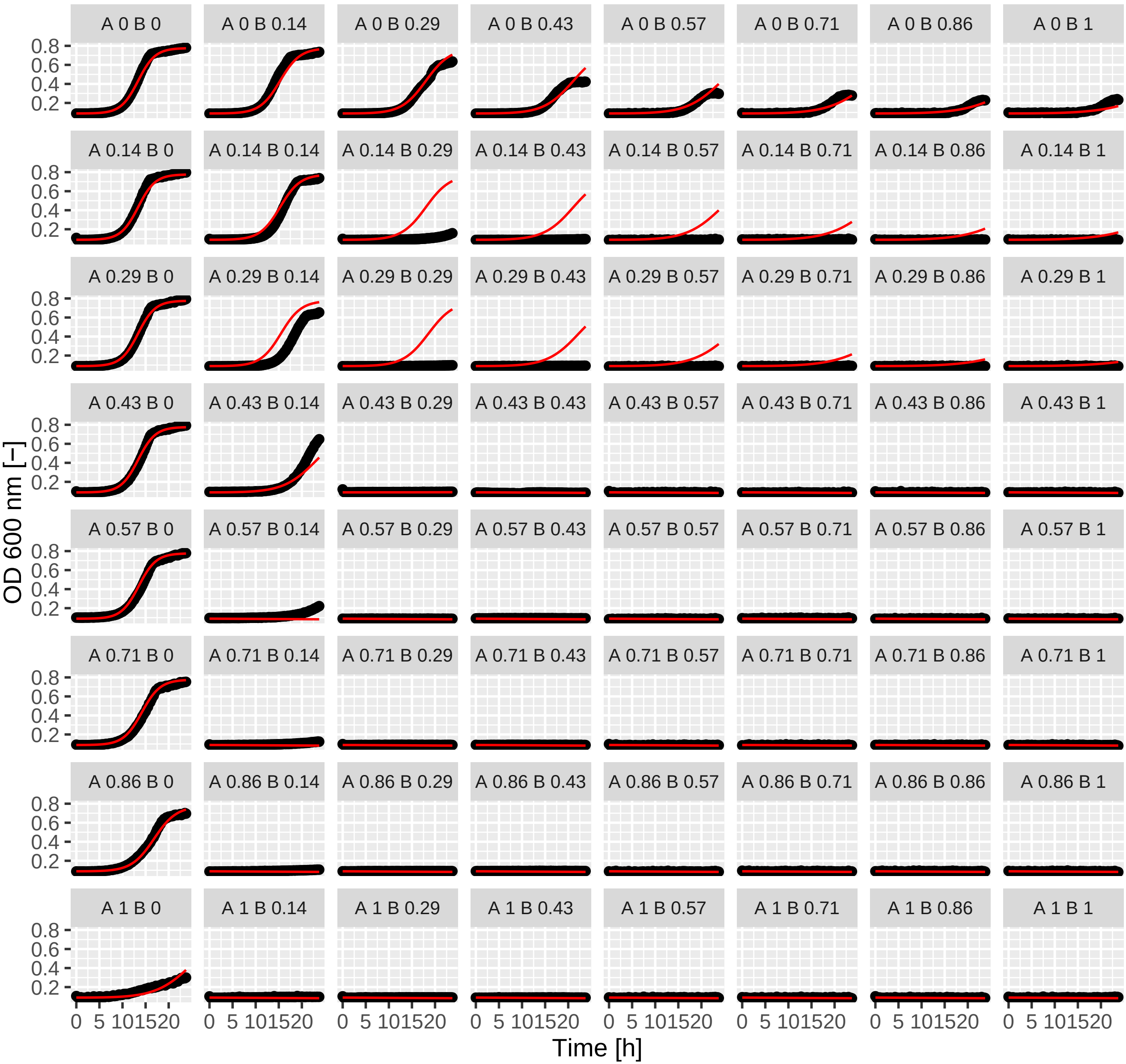

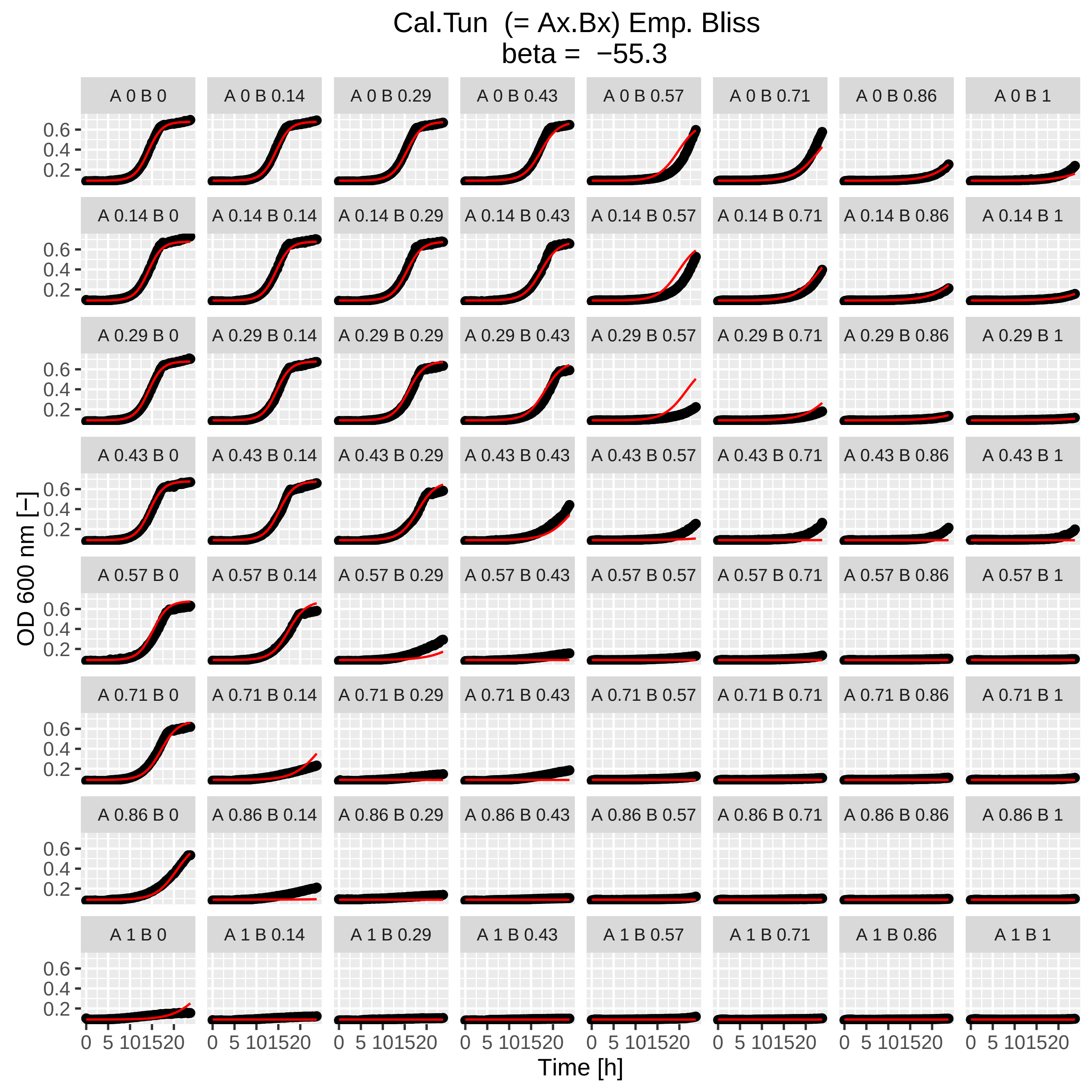
Cal.Tun (= Ax.Bx) Emp. Bliss
beta = −55.3
OD 600 nm [−]
Time [h]
A 0 B 0
A 0 B 0.14
A 0 B 0.29
A 0 B 0.43
A 0 B 0.57
A 0 B 0.71
A 0 B 0.86
A 0 B 1
A 0.14 B 0
A 0.14 B 0.14
A 0.14 B 0.29
A 0.14 B 0.43
A 0.14 B 0.57
A 0.14 B 0.71
A 0.14 B 0.86
A 0.14 B 1
A 0.29 B 0
A 0.29 B 0.14
A 0.29 B 0.29
A 0.29 B 0.43
A 0.29 B 0.57
A 0.29 B 0.71
A 0.29 B 0.86
A 0.29 B 1
A 0.43 B 0
A 0.43 B 0.14
A 0.43 B 0.29
A 0.43 B 0.43
A 0.43 B 0.57
A 0.43 B 0.71
A 0.43 B 0.86
A 0.43 B 1
A 0.57 B 0
A 0.57 B 0.14
A 0.57 B 0.29
A 0.57 B 0.43
A 0.57 B 0.57
A 0.57 B 0.71
A 0.57 B 0.86
A 0.57 B 1
A 0.71 B 0
A 0.71 B 0.14
A 0.71 B 0.29
A 0.71 B 0.43
A 0.71 B 0.57
A 0.71 B 0.71
A 0.71 B 0.86
A 0.71 B 1
A 0.86 B 0
A 0.86 B 0.14
A 0.86 B 0.29
A 0.86 B 0.43
A 0.86 B 0.57
A 0.86 B 0.71
A 0.86 B 0.86
A 0.86 B 1
A 1 B 0
A 1 B 0.14
A 1 B 0.29
A 1 B 0.43
A 1 B 0.57
A 1 B 0.71
A 1 B 0.86
A 1 B 1
0.6
0.4
0.2
0 5 101520

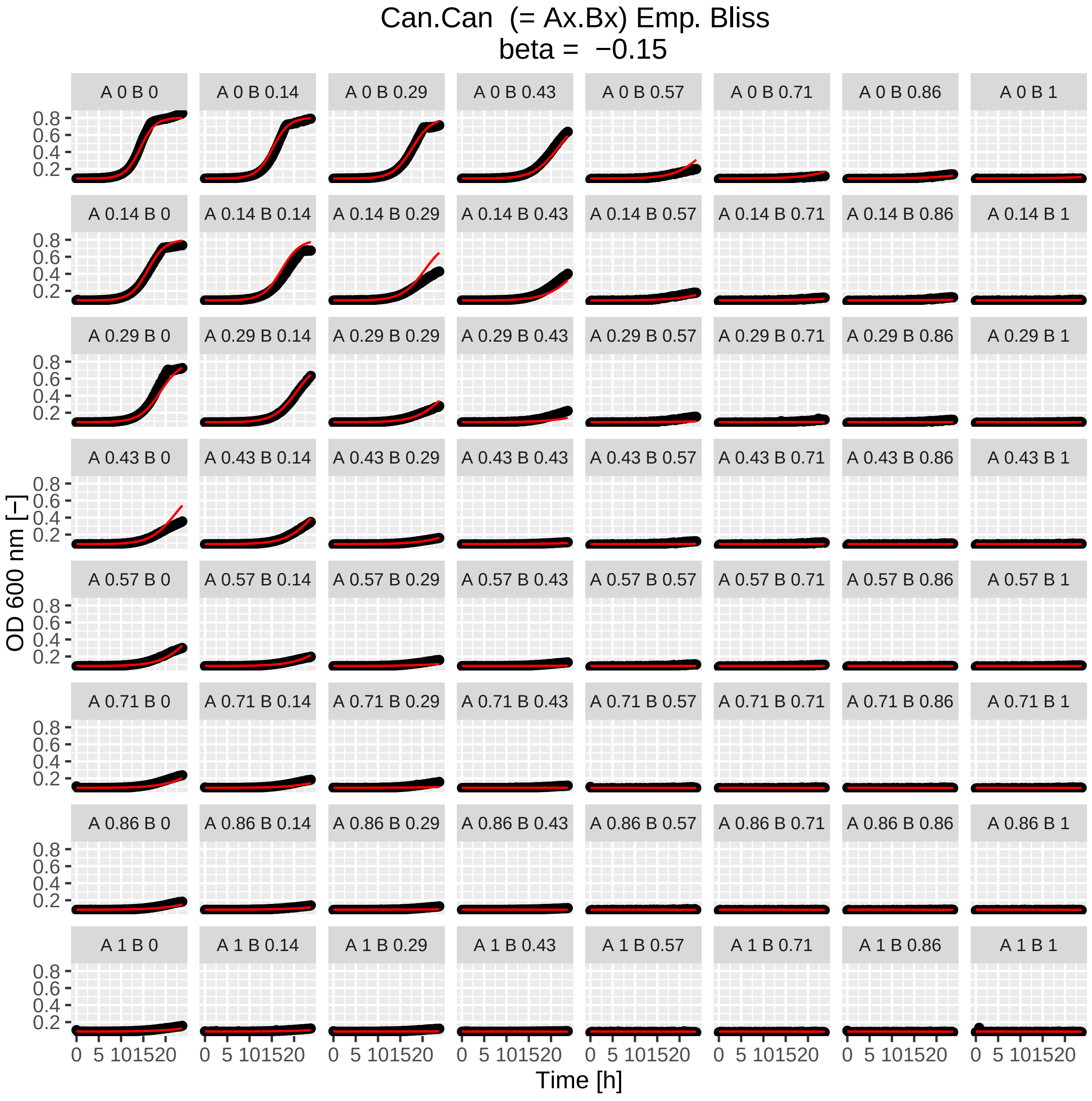

Can.Can (= Ax.Bx) Emp. Bliss
beta = −0.15
OD 600 nm [−]
Time [h]

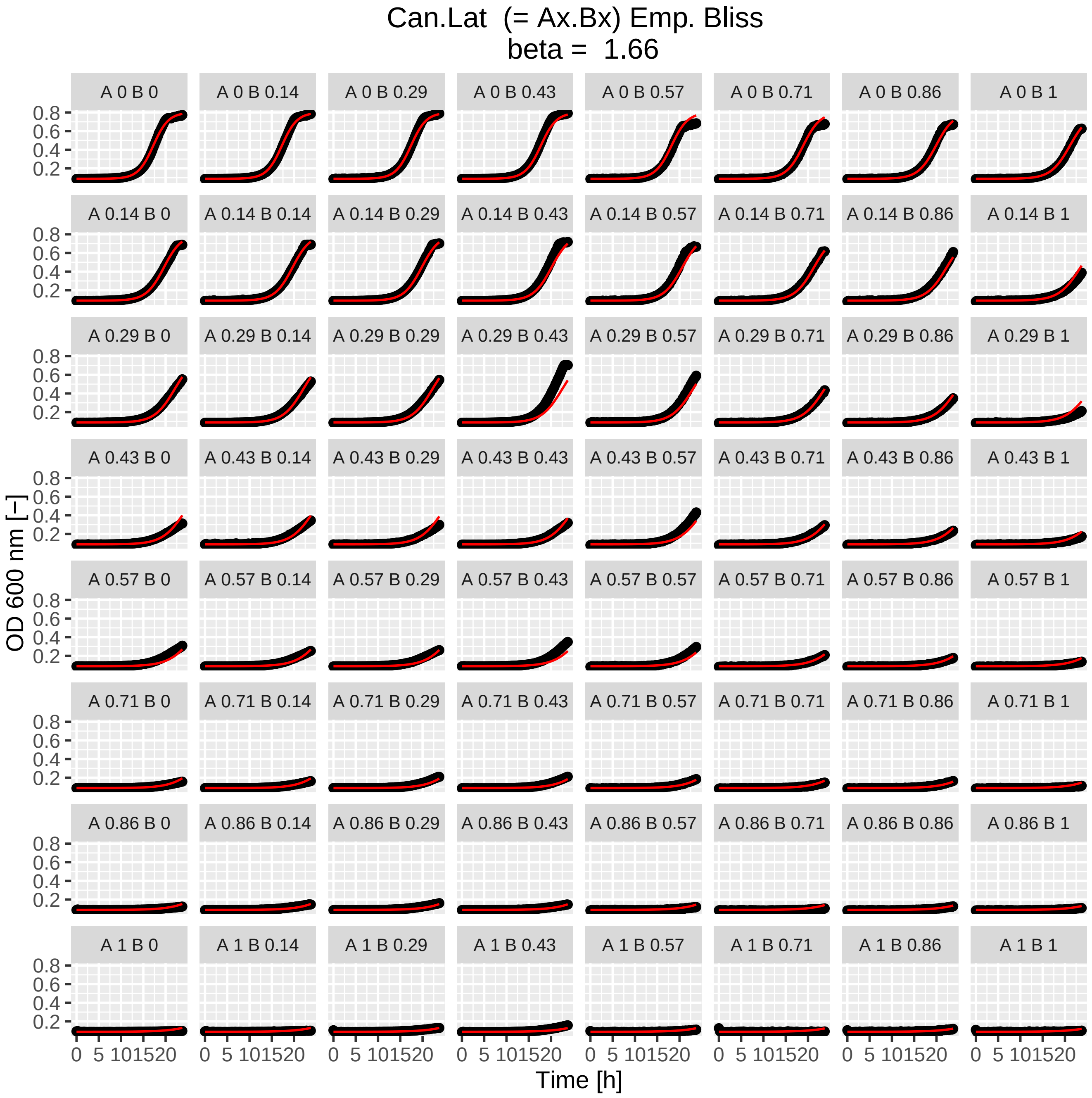

Can.Lat (= Ax.Bx) Emp. Bliss
beta = 1.66
A 0 B 0
A 0 B 0.14
A 0 B 0.29
A 0 B 0.43
A 0 B 0.57
A 0 B 0.71
A 0 B 0.86
A 0 B 1
A 0.14 B 0
A 0.14 B 0.14
A 0.14 B 0.29
A 0.14 B 0.43
A 0.14 B 0.57
A 0.14 B 0.71
A 0.14 B 0.86
A 0.14 B 1
A 0.29 B 0
A 0.29 B 0.14
A 0.29 B 0.29
A 0.29 B 0.43
A 0.29 B 0.57
A 0.29 B 0.71
A 0.29 B 0.86
A 0.29 B 1
A 0.43 B 0
A 0.43 B 0.14
A 0.43 B 0.29
A 0.43 B 0.43
A 0.43 B 0.57
A 0.43 B 0.71
A 0.43 B 0.86
A 0.43 B 1
A 0.57 B 0
A 0.57 B 0.14
A 0.57 B 0.29
A 0.57 B 0.43
A 0.57 B 0.57
A 0.57 B 0.71
A 0.57 B 0.86
A 0.57 B 1
A 0.71 B 0
A 0.71 B 0.14
A 0.71 B 0.29
A 0.71 B 0.43
A 0.71 B 0.57
A 0.71 B 0.71
A 0.71 B 0.86
A 0.71 B 1
A 0.86 B 0
A 0.86 B 0.14
A 0.86 B 0.29
A 0.86 B 0.43
A 0.86 B 0.57
A 0.86 B 0.71
A 0.86 B 0.86
A 0.86 B 1
A 1 B 0
A 1 B 0.14
A 1 B 0.29
A 1 B 0.43
A 1 B 0.57
A 1 B 0.71
A 1 B 0.86
A 1 B 1
OD 600 nm [–]
0.2
0.4
0.6
0.8
0 5 101520
Time [h]

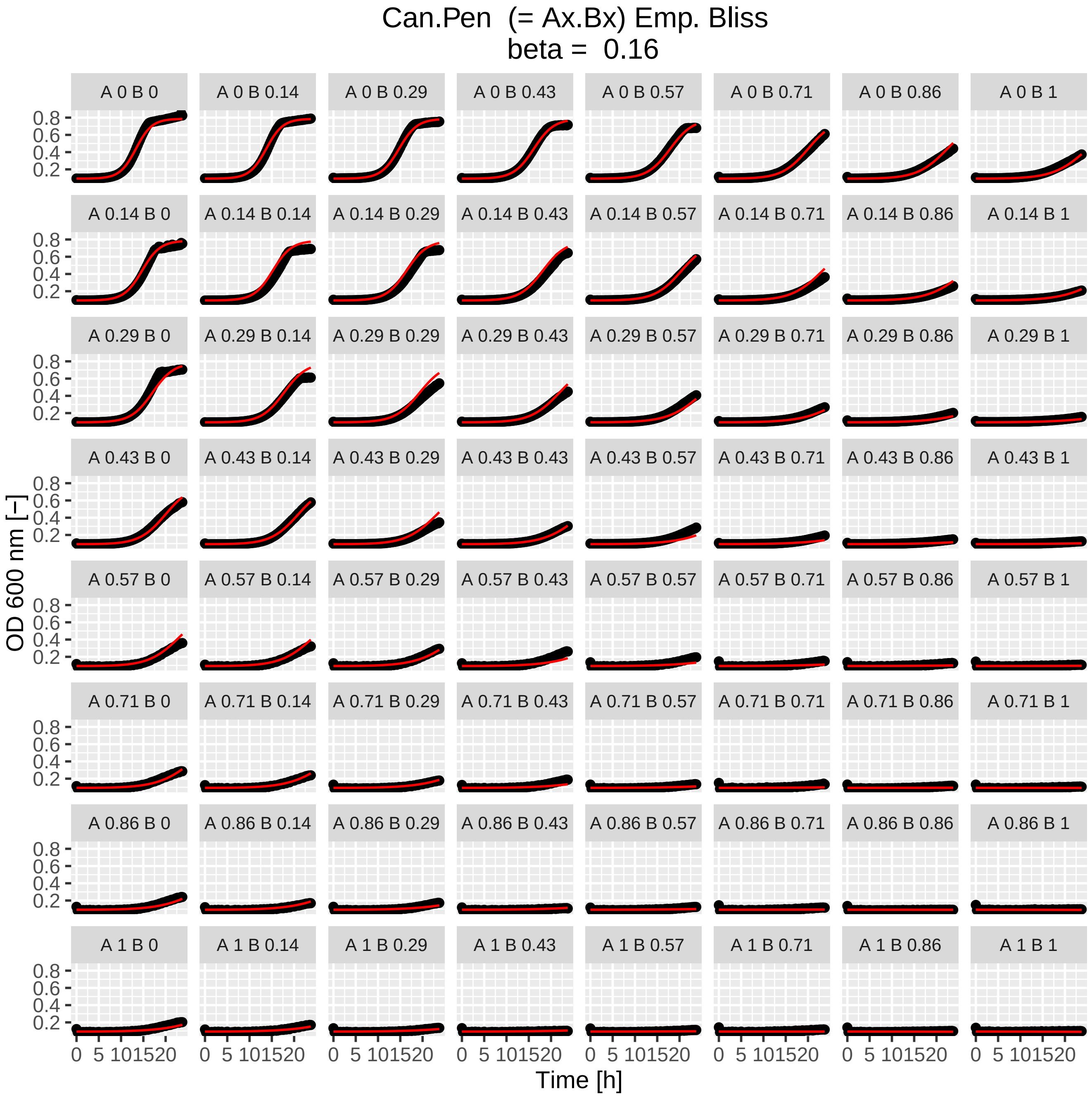
Can.Pen (= Ax.Bx) Emp. Bliss
beta = 0.16
A 0 B 0
A 0 B 0.14
A 0 B 0.29
A 0 B 0.43
A 0 B 0.57
A 0 B 0.71
A 0 B 0.86
A 0 B 1
A 0.14 B 0
A 0.14 B 0.14
A 0.14 B 0.29
A 0.14 B 0.43
A 0.14 B 0.57
A 0.14 B 0.71
A 0.14 B 0.86
A 0.14 B 1
A 0.29 B 0
A 0.29 B 0.14
A 0.29 B 0.29
A 0.29 B 0.43
A 0.29 B 0.57
A 0.29 B 0.71
A 0.29 B 0.86
A 0.29 B 1
A 0.43 B 0
A 0.43 B 0.14
A 0.43 B 0.29
A 0.43 B 0.43
A 0.43 B 0.57
A 0.43 B 0.71
A 0.43 B 0.86
A 0.43 B 1
A 0.57 B 0
A 0.57 B 0.14
A 0.57 B 0.29
A 0.57 B 0.43
A 0.57 B 0.57
A 0.57 B 0.71
A 0.57 B 0.86
A 0.57 B 1
A 0.71 B 0
A 0.71 B 0.14
A 0.71 B 0.29
A 0.71 B 0.43
A 0.71 B 0.57
A 0.71 B 0.71
A 0.71 B 0.86
A 0.71 B 1
A 0.86 B 0
A 0.86 B 0.14
A 0.86 B 0.29
A 0.86 B 0.43
A 0.86 B 0.57
A 0.86 B 0.71
A 0.86 B 0.86
A 0.86 B 1
A 1 B 0
A 1 B 0.14
A 1 B 0.29
A 1 B 0.43
A 1 B 0.57
A 1 B 0.71
A 1 B 0.86
A 1 B 1
OD 600 nm [–]
0.2
0.4
0.6
0.8
0 5 101520
Time [h]

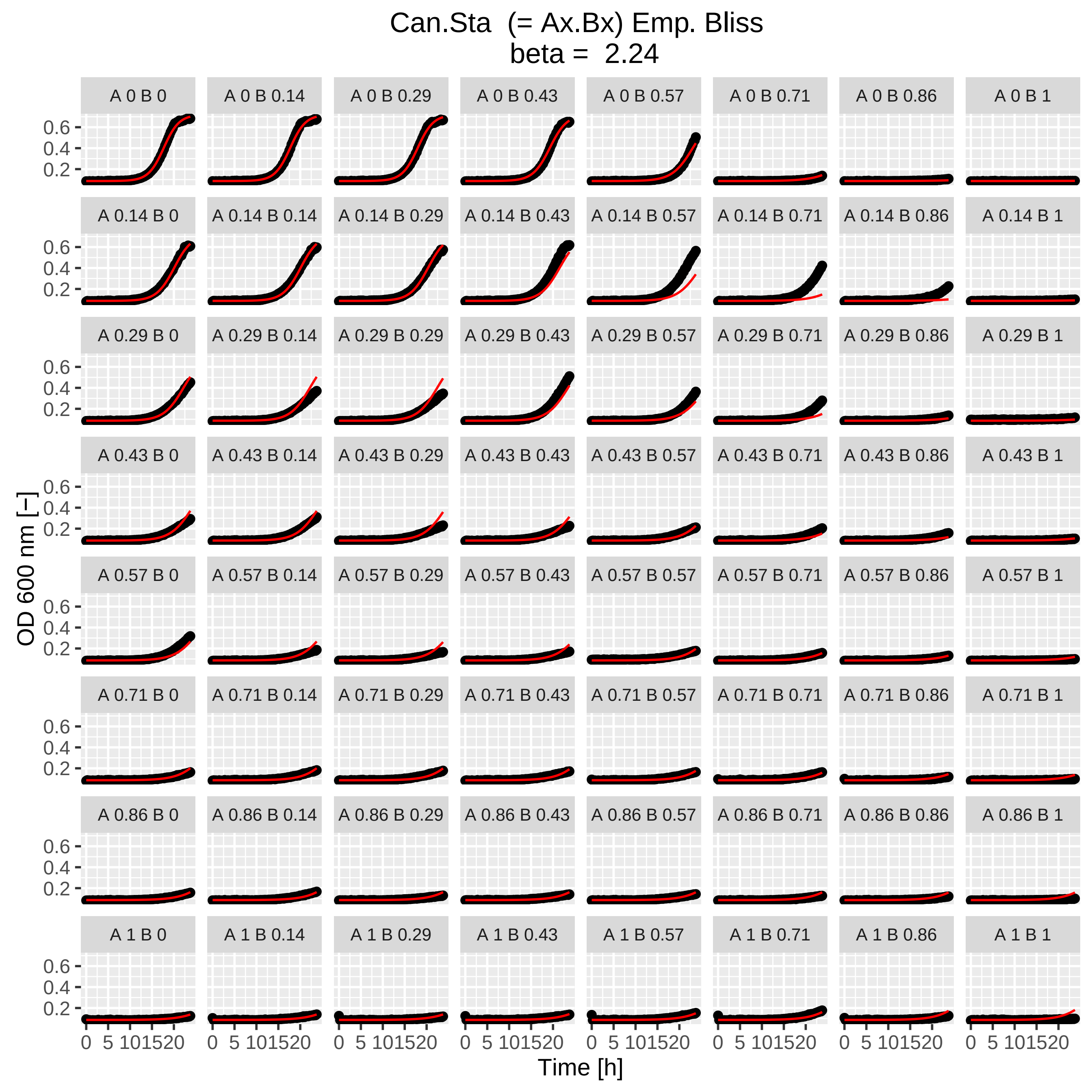

Can.Sta (= Ax.Bx) Emp. Bliss
beta = 2.24
OD 600 nm [−]
Time [h]

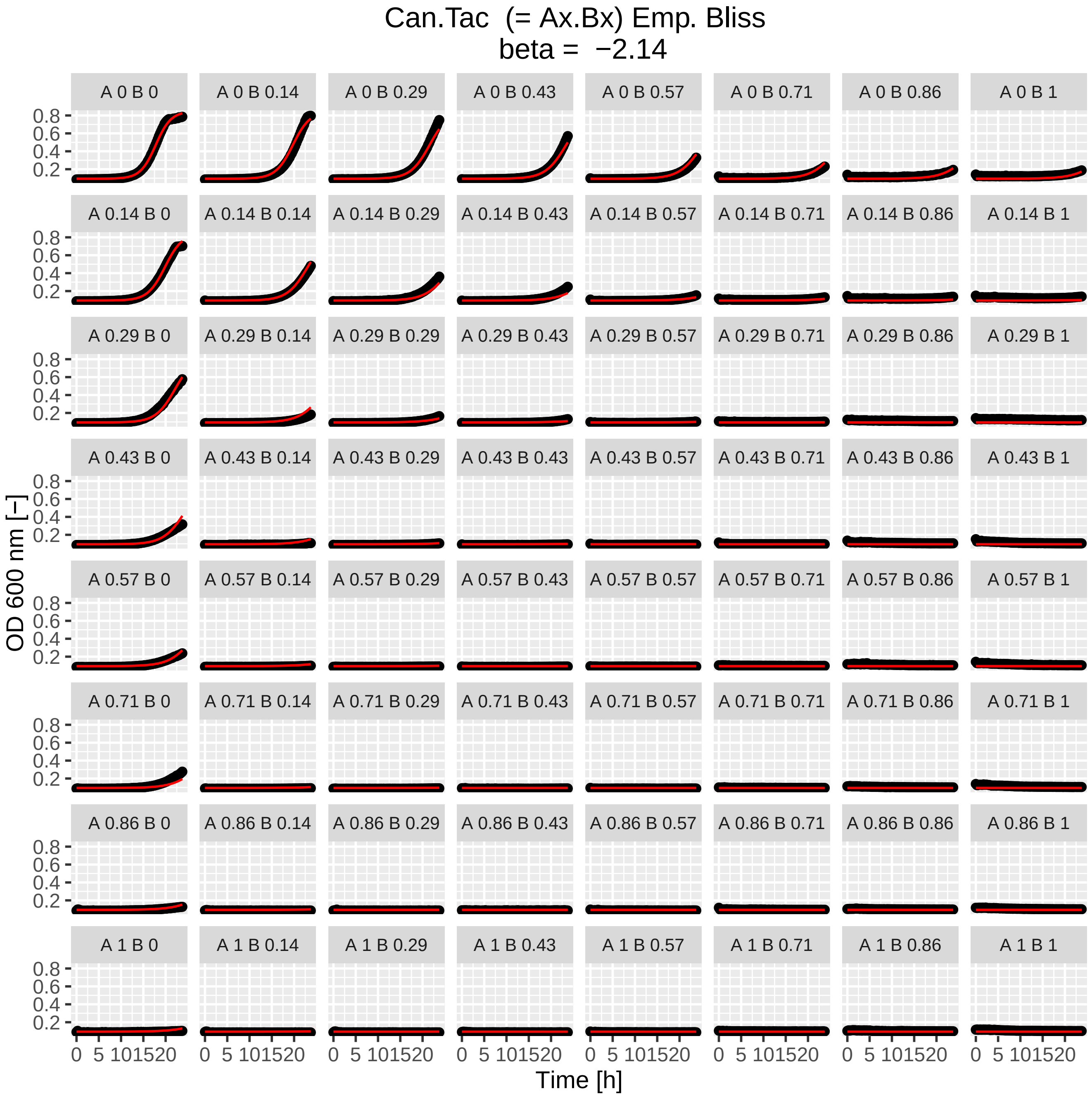
Can.Tac (= Ax.Bx) Emp. Bliss
beta = −2.14
A 0 B 0
A 0 B 0.14
A 0 B 0.29
A 0 B 0.43
A 0 B 0.57
A 0 B 0.71
A 0 B 0.86
A 0 B 1
A 0.14 B 0
A 0.14 B 0.14
A 0.14 B 0.29
A 0.14 B 0.43
A 0.14 B 0.57
A 0.14 B 0.71
A 0.14 B 0.86
A 0.14 B 1
A 0.29 B 0
A 0.29 B 0.14
A 0.29 B 0.29
A 0.29 B 0.43
A 0.29 B 0.57
A 0.29 B 0.71
A 0.29 B 0.86
A 0.29 B 1
A 0.43 B 0
A 0.43 B 0.14
A 0.43 B 0.29
A 0.43 B 0.43
A 0.43 B 0.57
A 0.43 B 0.71
A 0.43 B 0.86
A 0.43 B 1
A 0.57 B 0
A 0.57 B 0.14
A 0.57 B 0.29
A 0.57 B 0.43
A 0.57 B 0.57
A 0.57 B 0.71
A 0.57 B 0.86
A 0.57 B 1
A 0.71 B 0
A 0.71 B 0.14
A 0.71 B 0.29
A 0.71 B 0.43
A 0.71 B 0.57
A 0.71 B 0.71
A 0.71 B 0.86
A 0.71 B 1
A 0.86 B 0
A 0.86 B 0.14
A 0.86 B 0.29
A 0.86 B 0.43
A 0.86 B 0.57
A 0.86 B 0.71
A 0.86 B 0.86
A 0.86 B 1
A 1 B 0
A 1 B 0.14
A 1 B 0.29
A 1 B 0.43
A 1 B 0.57
A 1 B 0.71
A 1 B 0.86
A 1 B 1
0.8
0.6
0.4
0.2
0 5 101520
OD 600 nm [−]
Time [h]

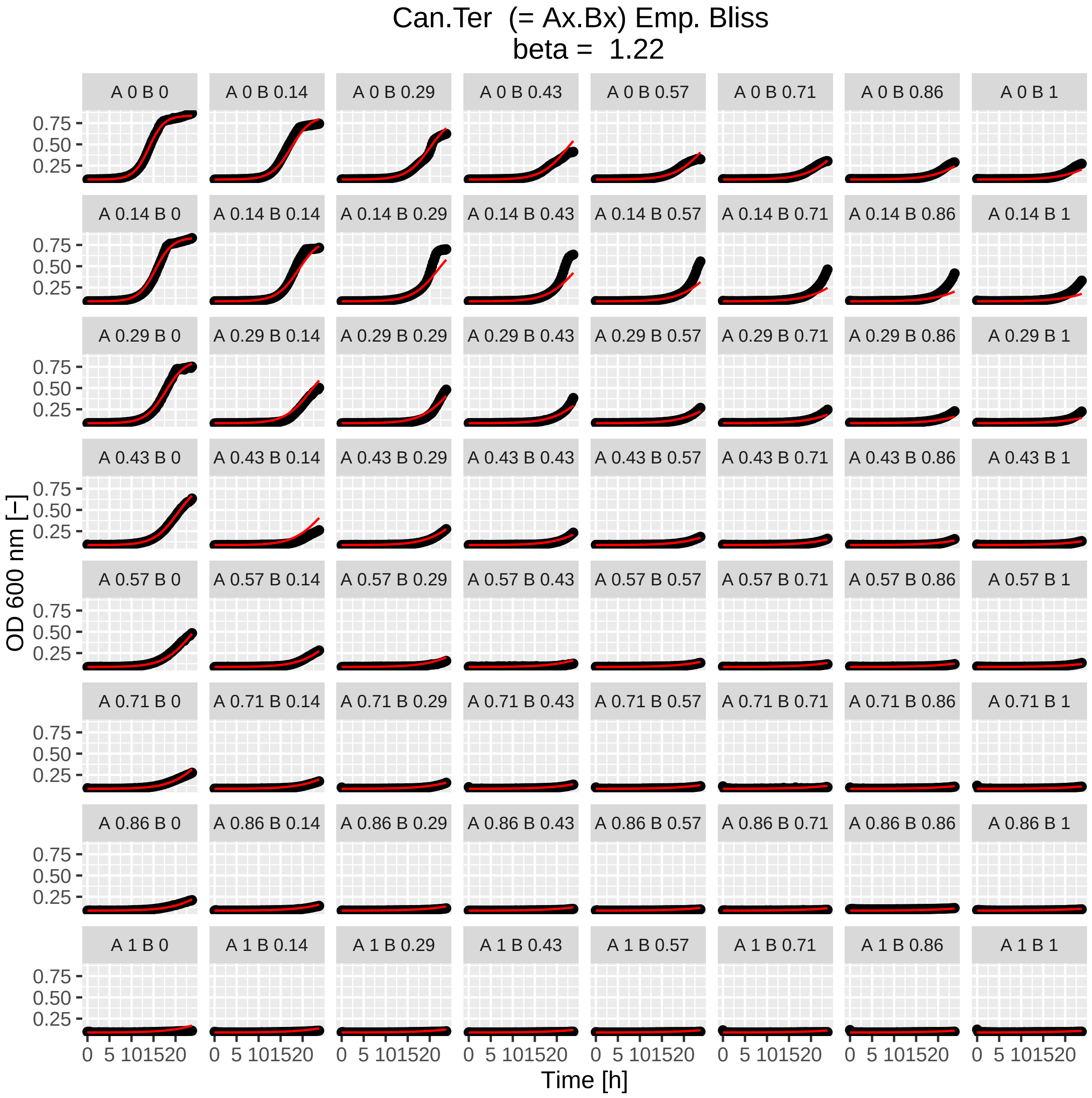
Can.Ter (= Ax.Bx) Emp. Bliss
beta = 1.22
A 0 B 0
A 0 B 0.14
A 0 B 0.29
A 0 B 0.43
A 0 B 0.57
A 0 B 0.71
A 0 B 0.86
A 0 B 1
A 0.14 B 0
A 0.14 B 0.14
A 0.14 B 0.29
A 0.14 B 0.43
A 0.14 B 0.57
A 0.14 B 0.71
A 0.14 B 0.86
A 0.14 B 1
A 0.29 B 0
A 0.29 B 0.14
A 0.29 B 0.29
A 0.29 B 0.43
A 0.29 B 0.57
A 0.29 B 0.71
A 0.29 B 0.86
A 0.29 B 1
A 0.43 B 0
A 0.43 B 0.14
A 0.43 B 0.29
A 0.43 B 0.43
A 0.43 B 0.57
A 0.43 B 0.71
A 0.43 B 0.86
A 0.43 B 1
A 0.57 B 0
A 0.57 B 0.14
A 0.57 B 0.29
A 0.57 B 0.43
A 0.57 B 0.57
A 0.57 B 0.71
A 0.57 B 0.86
A 0.57 B 1
A 0.71 B 0
A 0.71 B 0.14
A 0.71 B 0.29
A 0.71 B 0.43
A 0.71 B 0.57
A 0.71 B 0.71
A 0.71 B 0.86
A 0.71 B 1
A 0.86 B 0
A 0.86 B 0.14
A 0.86 B 0.29
A 0.86 B 0.43
A 0.86 B 0.57
A 0.86 B 0.71
A 0.86 B 0.86
A 0.86 B 1
A 1 B 0
A 1 B 0.14
A 1 B 0.29
A 1 B 0.43
A 1 B 0.57
A 1 B 0.71
A 1 B 0.86
A 1 B 1
OD 600 nm [–]
0.75
0.50
0.25
0 5 101520
Time [h]

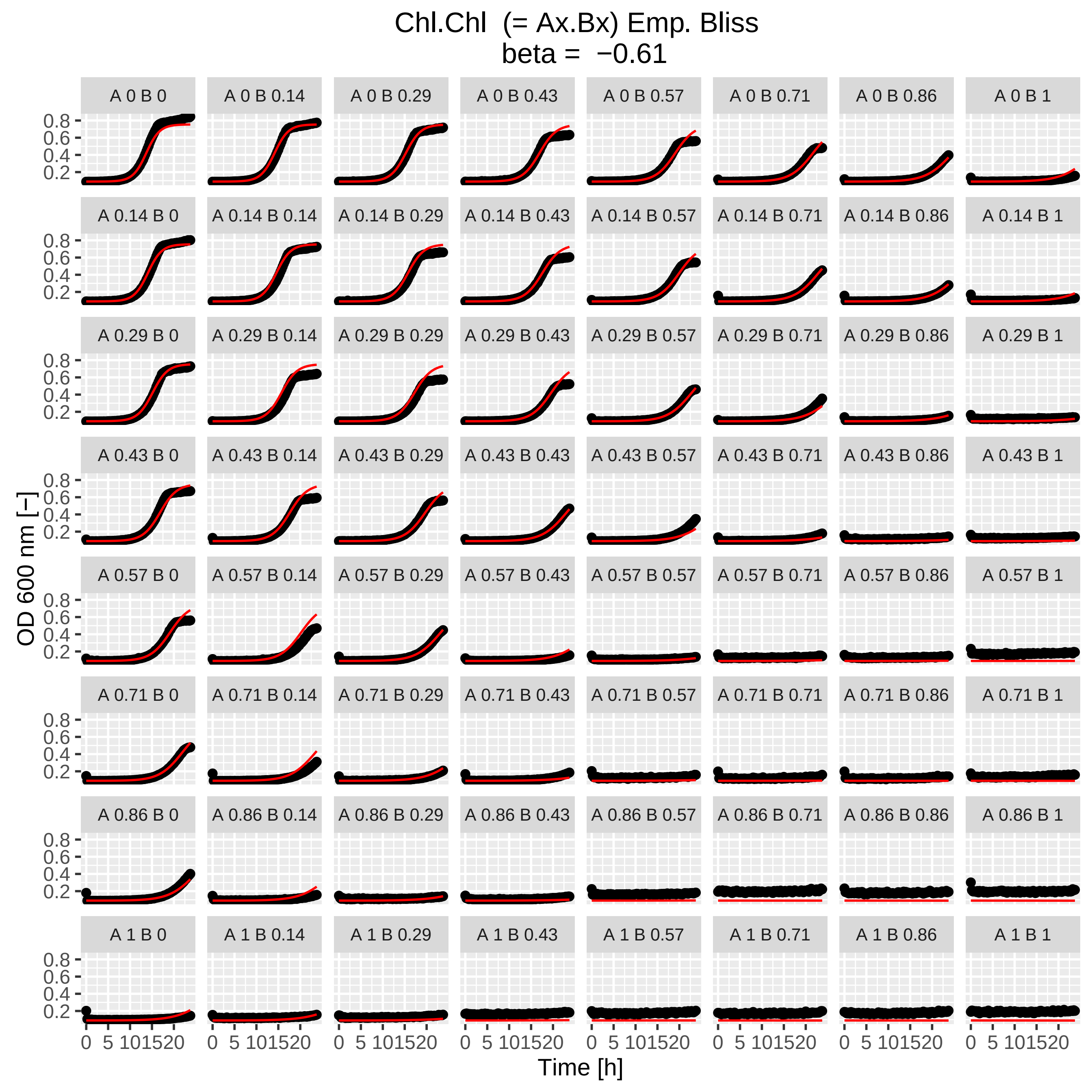

Chl.Chl (= Ax.Bx) Emp. Bliss
beta = −0.61
OD 600 nm [−]
Time [h]
A 0 B 0
A 0 B 0.14
A 0 B 0.29
A 0 B 0.43
A 0 B 0.57
A 0 B 0.71
A 0 B 0.86
A 0 B 1
A 0.14 B 0
A 0.14 B 0.14
A 0.14 B 0.29
A 0.14 B 0.43
A 0.14 B 0.57
A 0.14 B 0.71
A 0.14 B 0.86
A 0.14 B 1
A 0.29 B 0
A 0.29 B 0.14
A 0.29 B 0.29
A 0.29 B 0.43
A 0.29 B 0.57
A 0.29 B 0.71
A 0.29 B 0.86
A 0.29 B 1
A 0.43 B 0
A 0.43 B 0.14
A 0.43 B 0.29
A 0.43 B 0.43
A 0.43 B 0.57
A 0.43 B 0.71
A 0.43 B 0.86
A 0.43 B 1
A 0.57 B 0
A 0.57 B 0.14
A 0.57 B 0.29
A 0.57 B 0.43
A 0.57 B 0.57
A 0.57 B 0.71
A 0.57 B 0.86
A 0.57 B 1
A 0.71 B 0
A 0.71 B 0.14
A 0.71 B 0.29
A 0.71 B 0.43
A 0.71 B 0.57
A 0.71 B 0.71
A 0.71 B 0.86
A 0.71 B 1
A 0.86 B 0
A 0.86 B 0.14
A 0.86 B 0.29
A 0.86 B 0.43
A 0.86 B 0.57
A 0.86 B 0.71
A 0.86 B 0.86
A 0.86 B 1
A 1 B 0
A 1 B 0.14
A 1 B 0.29
A 1 B 0.43
A 1 B 0.57
A 1 B 0.71
A 1 B 0.86
A 1 B 1
0.8
0.6
0.4
0.2
0 5 101520

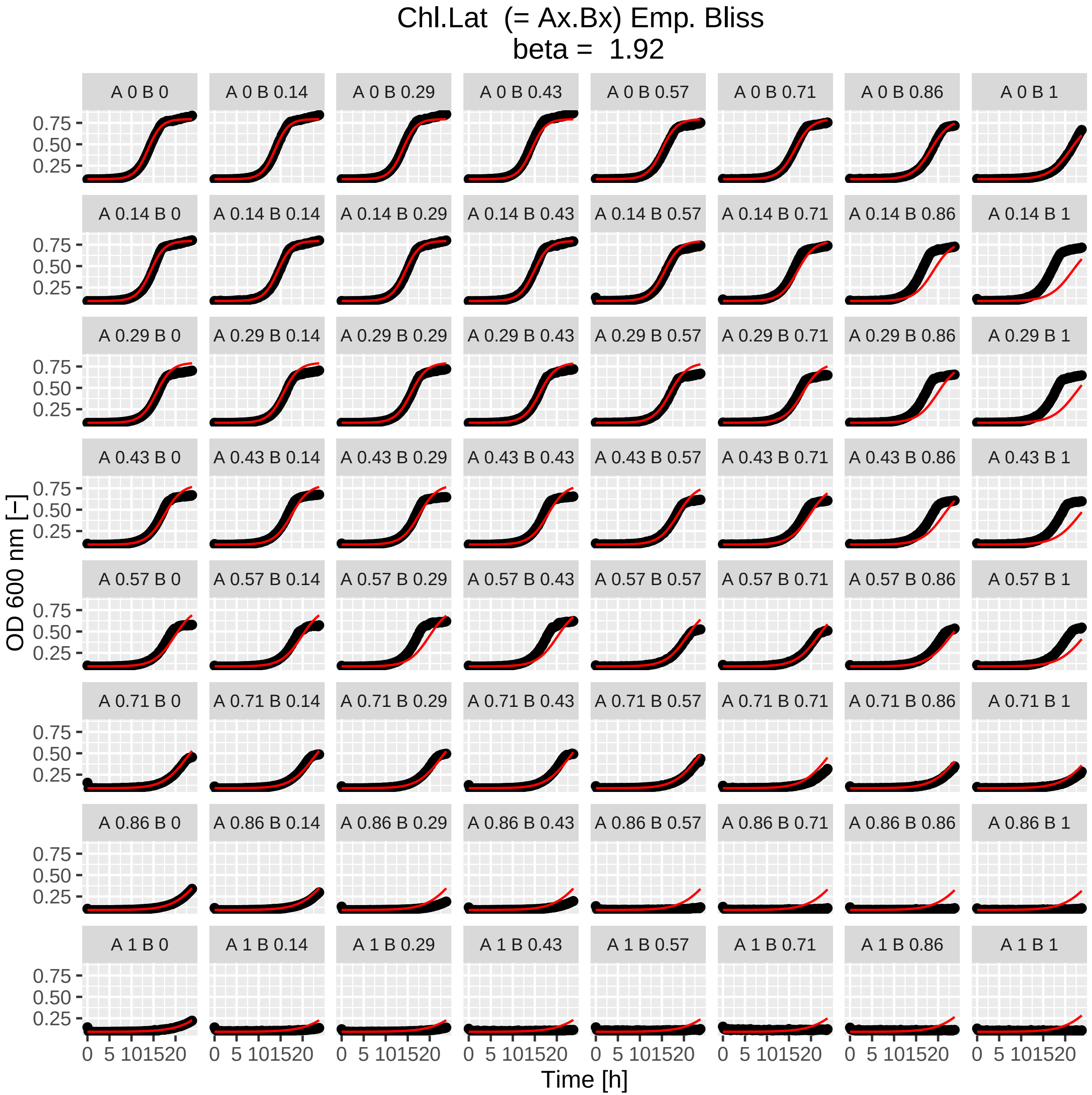

Chl.Lat (= Ax.Bx) Emp. Bliss
beta = 1.92
OD 600 nm [–]
Time [h]
0.75
0.50
0.25
0 5 101520
A 0 B 0
A 0 B 0.14
A 0 B 0.29
A 0 B 0.43
A 0 B 0.57
A 0 B 0.71
A 0 B 0.86
A 0 B 1
A 0.14 B 0
A 0.14 B 0.14
A 0.14 B 0.29
A 0.14 B 0.43
A 0.14 B 0.57
A 0.14 B 0.71
A 0.14 B 0.86
A 0.14 B 1
A 0.29 B 0
A 0.29 B 0.14
A 0.29 B 0.29
A 0.29 B 0.43
A 0.29 B 0.57
A 0.29 B 0.71
A 0.29 B 0.86
A 0.29 B 1
A 0.43 B 0
A 0.43 B 0.14
A 0.43 B 0.29
A 0.43 B 0.43
A 0.43 B 0.57
A 0.43 B 0.71
A 0.43 B 0.86
A 0.43 B 1
A 0.57 B 0
A 0.57 B 0.14
A 0.57 B 0.29
A 0.57 B 0.43
A 0.57 B 0.57
A 0.57 B 0.71
A 0.57 B 0.86
A 0.57 B 1
A 0.71 B 0
A 0.71 B 0.14
A 0.71 B 0.29
A 0.71 B 0.43
A 0.71 B 0.57
A 0.71 B 0.71
A 0.71 B 0.86
A 0.71 B 1
A 0.86 B 0
A 0.86 B 0.14
A 0.86 B 0.29
A 0.86 B 0.43
A 0.86 B 0.57
A 0.86 B 0.71
A 0.86 B 0.86
A 0.86 B 1
A 1 B 0
A 1 B 0.14
A 1 B 0.29
A 1 B 0.43
A 1 B 0.57
A 1 B 0.71
A 1 B 0.86
A 1 B 1

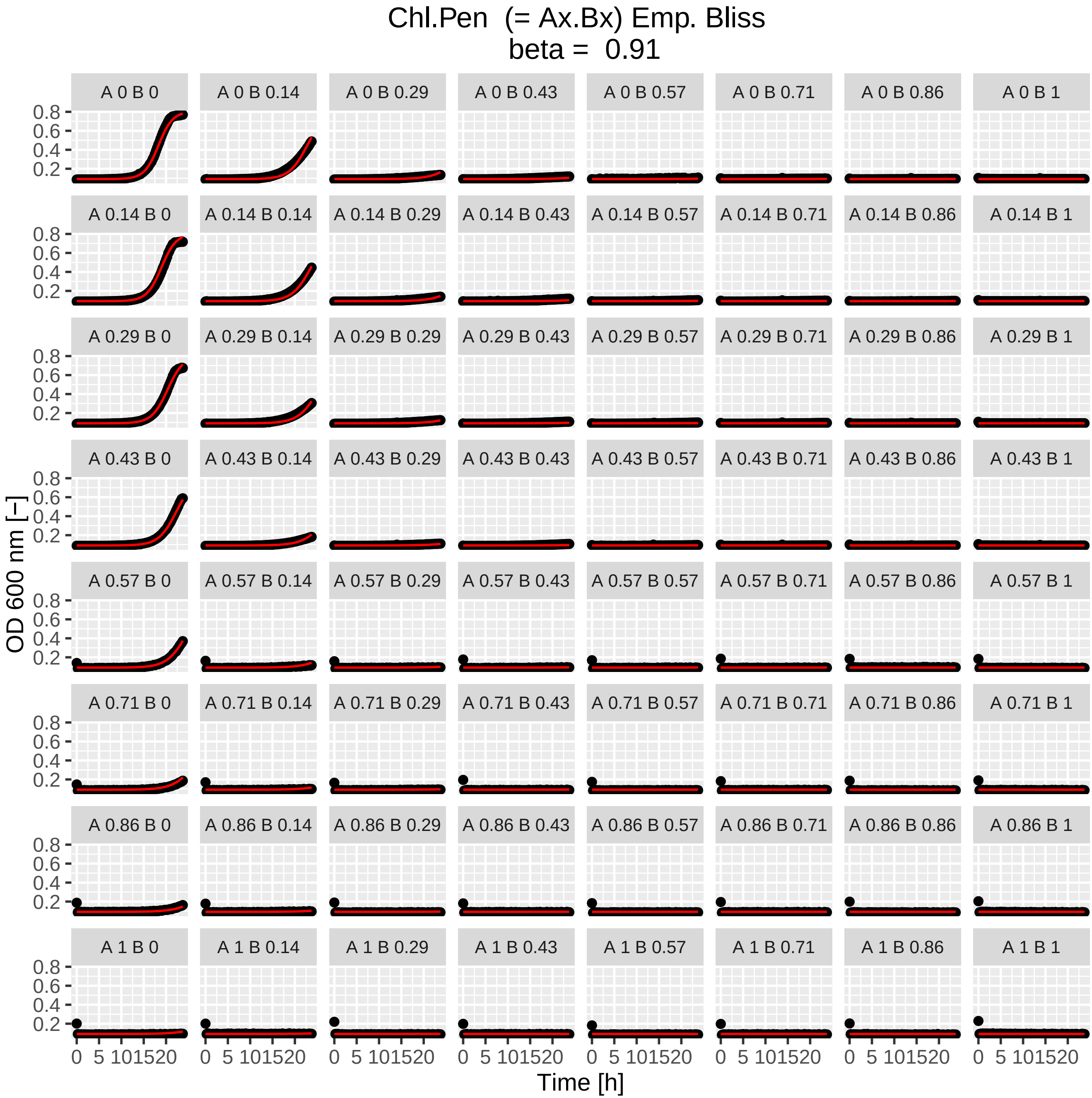

Chl.Pen (= Ax.Bx) Emp. Bliss
beta = 0.91
A 0 B 0
A 0 B 0.14
A 0 B 0.29
A 0 B 0.43
A 0 B 0.57
A 0 B 0.71
A 0 B 0.86
A 0 B 1
A 0.14 B 0
A 0.14 B 0.14
A 0.14 B 0.29
A 0.14 B 0.43
A 0.14 B 0.57
A 0.14 B 0.71
A 0.14 B 0.86
A 0.14 B 1
A 0.29 B 0
A 0.29 B 0.14
A 0.29 B 0.29
A 0.29 B 0.43
A 0.29 B 0.57
A 0.29 B 0.71
A 0.29 B 0.86
A 0.29 B 1
A 0.43 B 0
A 0.43 B 0.14
A 0.43 B 0.29
A 0.43 B 0.43
A 0.43 B 0.57
A 0.43 B 0.71
A 0.43 B 0.86
A 0.43 B 1
A 0.57 B 0
A 0.57 B 0.14
A 0.57 B 0.29
A 0.57 B 0.43
A 0.57 B 0.57
A 0.57 B 0.71
A 0.57 B 0.86
A 0.57 B 1
A 0.71 B 0
A 0.71 B 0.14
A 0.71 B 0.29
A 0.71 B 0.43
A 0.71 B 0.57
A 0.71 B 0.71
A 0.71 B 0.86
A 0.71 B 1
A 0.86 B 0
A 0.86 B 0.14
A 0.86 B 0.29
A 0.86 B 0.43
A 0.86 B 0.57
A 0.86 B 0.71
A 0.86 B 0.86
A 0.86 B 1
A 1 B 0
A 1 B 0.14
A 1 B 0.29
A 1 B 0.43
A 1 B 0.57
A 1 B 0.71
A 1 B 0.86
A 1 B 1
OD 600 nm [–]
0.2
0.4
0.6
0.8
0
5
10
15
20
Time [h]

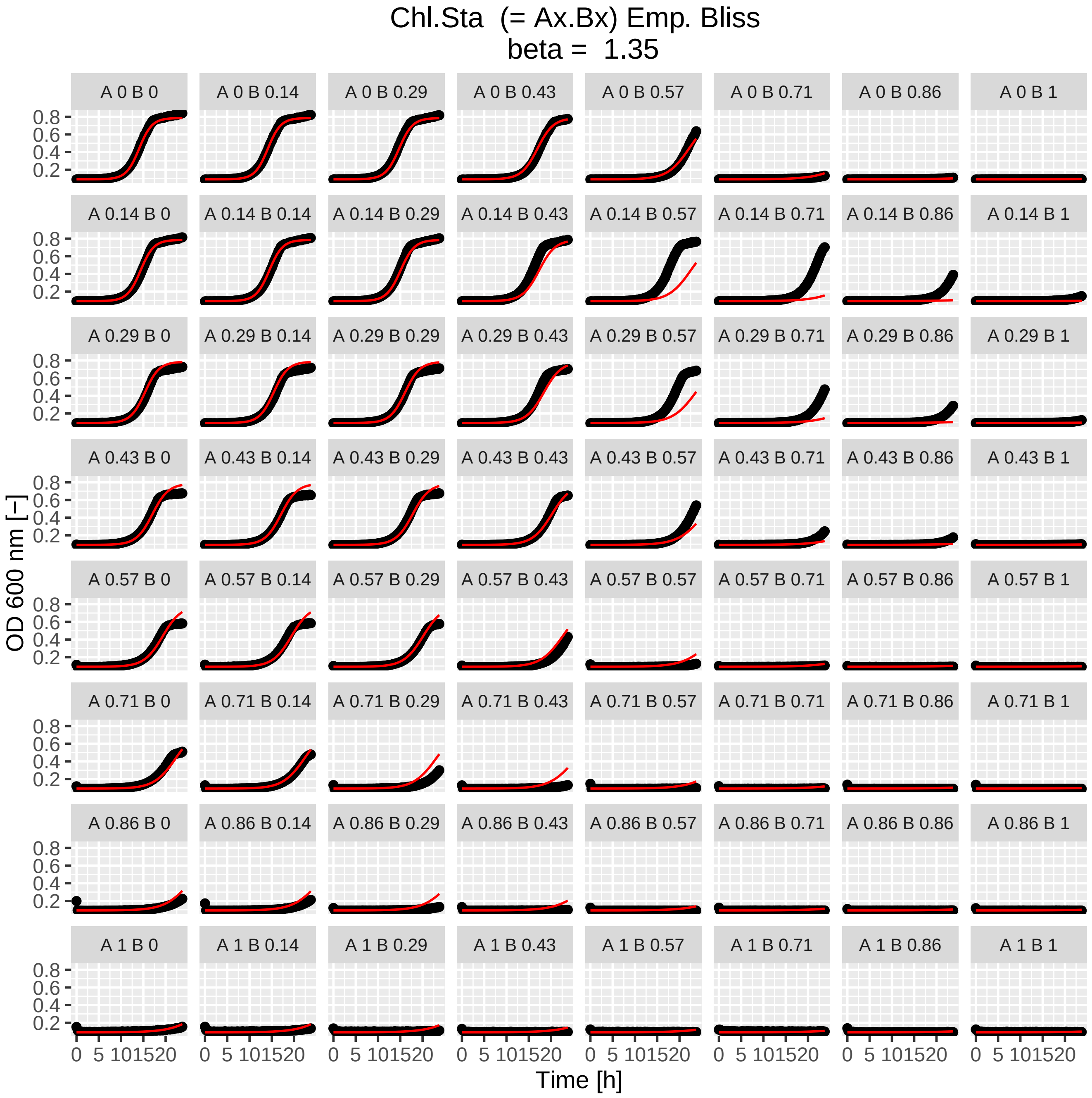

Chl.Sta (= Ax.Bx) Emp. Bliss
beta = 1.35
A 0 B 0
A 0 B 0.14
A 0 B 0.29
A 0 B 0.43
A 0 B 0.57
A 0 B 0.71
A 0 B 0.86
A 0 B 1
A 0.14 B 0
A 0.14 B 0.14
A 0.14 B 0.29
A 0.14 B 0.43
A 0.14 B 0.57
A 0.14 B 0.71
A 0.14 B 0.86
A 0.14 B 1
A 0.29 B 0
A 0.29 B 0.14
A 0.29 B 0.29
A 0.29 B 0.43
A 0.29 B 0.57
A 0.29 B 0.71
A 0.29 B 0.86
A 0.29 B 1
A 0.43 B 0
A 0.43 B 0.14
A 0.43 B 0.29
A 0.43 B 0.43
A 0.43 B 0.57
A 0.43 B 0.71
A 0.43 B 0.86
A 0.43 B 1
A 0.57 B 0
A 0.57 B 0.14
A 0.57 B 0.29
A 0.57 B 0.43
A 0.57 B 0.57
A 0.57 B 0.71
A 0.57 B 0.86
A 0.57 B 1
A 0.71 B 0
A 0.71 B 0.14
A 0.71 B 0.29
A 0.71 B 0.43
A 0.71 B 0.57
A 0.71 B 0.71
A 0.71 B 0.86
A 0.71 B 1
A 0.86 B 0
A 0.86 B 0.14
A 0.86 B 0.29
A 0.86 B 0.43
A 0.86 B 0.57
A 0.86 B 0.71
A 0.86 B 0.86
A 0.86 B 1
A 1 B 0
A 1 B 0.14
A 1 B 0.29
A 1 B 0.43
A 1 B 0.57
A 1 B 0.71
A 1 B 0.86
A 1 B 1
0.8
0.6
0.4
0.2
0 5 101520
OD 600 nm [–]
Time [h]

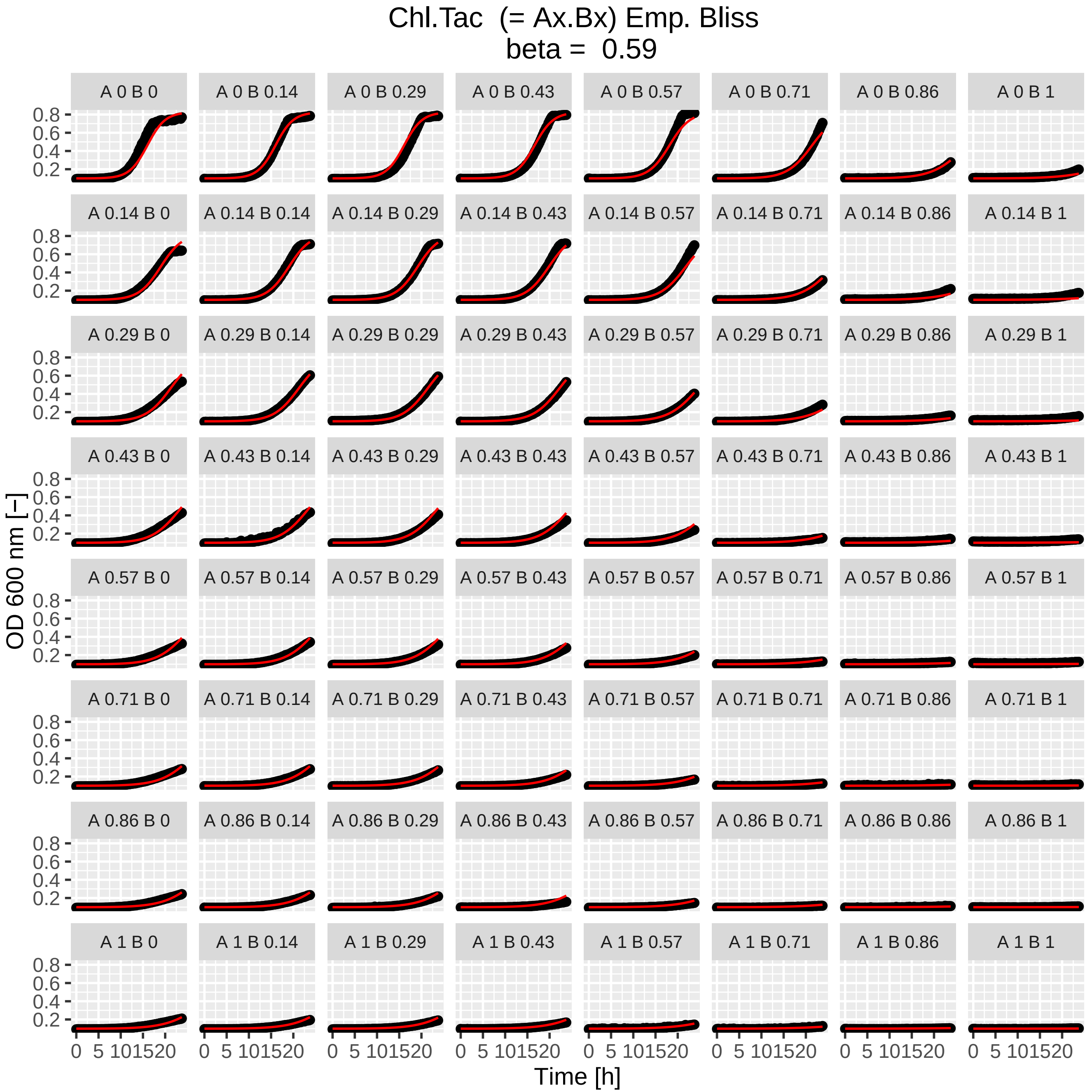
Chl.Tac (= Ax.Bx) Emp. Bliss
beta = 0.59
OD 600 nm [−]
Time [h]
0.8
0.6
0.4
0.2
0 5 101520
A 0 B 0
A 0 B 0.14
A 0 B 0.29
A 0 B 0.43
A 0 B 0.57
A 0 B 0.71
A 0 B 0.86
A 0 B 1
A 0.14 B 0
A 0.14 B 0.14
A 0.14 B 0.29
A 0.14 B 0.43
A 0.14 B 0.57
A 0.14 B 0.71
A 0.14 B 0.86
A 0.14 B 1
A 0.29 B 0
A 0.29 B 0.14
A 0.29 B 0.29
A 0.29 B 0.43
A 0.29 B 0.57
A 0.29 B 0.71
A 0.29 B 0.86
A 0.29 B 1
A 0.43 B 0
A 0.43 B 0.14
A 0.43 B 0.29
A 0.43 B 0.43
A 0.43 B 0.57
A 0.43 B 0.71
A 0.43 B 0.86
A 0.43 B 1
A 0.57 B 0
A 0.57 B 0.14
A 0.57 B 0.29
A 0.57 B 0.43
A 0.57 B 0.57
A 0.57 B 0.71
A 0.57 B 0.86
A 0.57 B 1
A 0.71 B 0
A 0.71 B 0.14
A 0.71 B 0.29
A 0.71 B 0.43
A 0.71 B 0.57
A 0.71 B 0.71
A 0.71 B 0.86
A 0.71 B 1
A 0.86 B 0
A 0.86 B 0.14
A 0.86 B 0.29
A 0.86 B 0.43
A 0.86 B 0.57
A 0.86 B 0.71
A 0.86 B 0.86
A 0.86 B 1
A 1 B 0
A 1 B 0.14
A 1 B 0.29
A 1 B 0.43
A 1 B 0.57
A 1 B 0.71
A 1 B 0.86
A 1 B 1

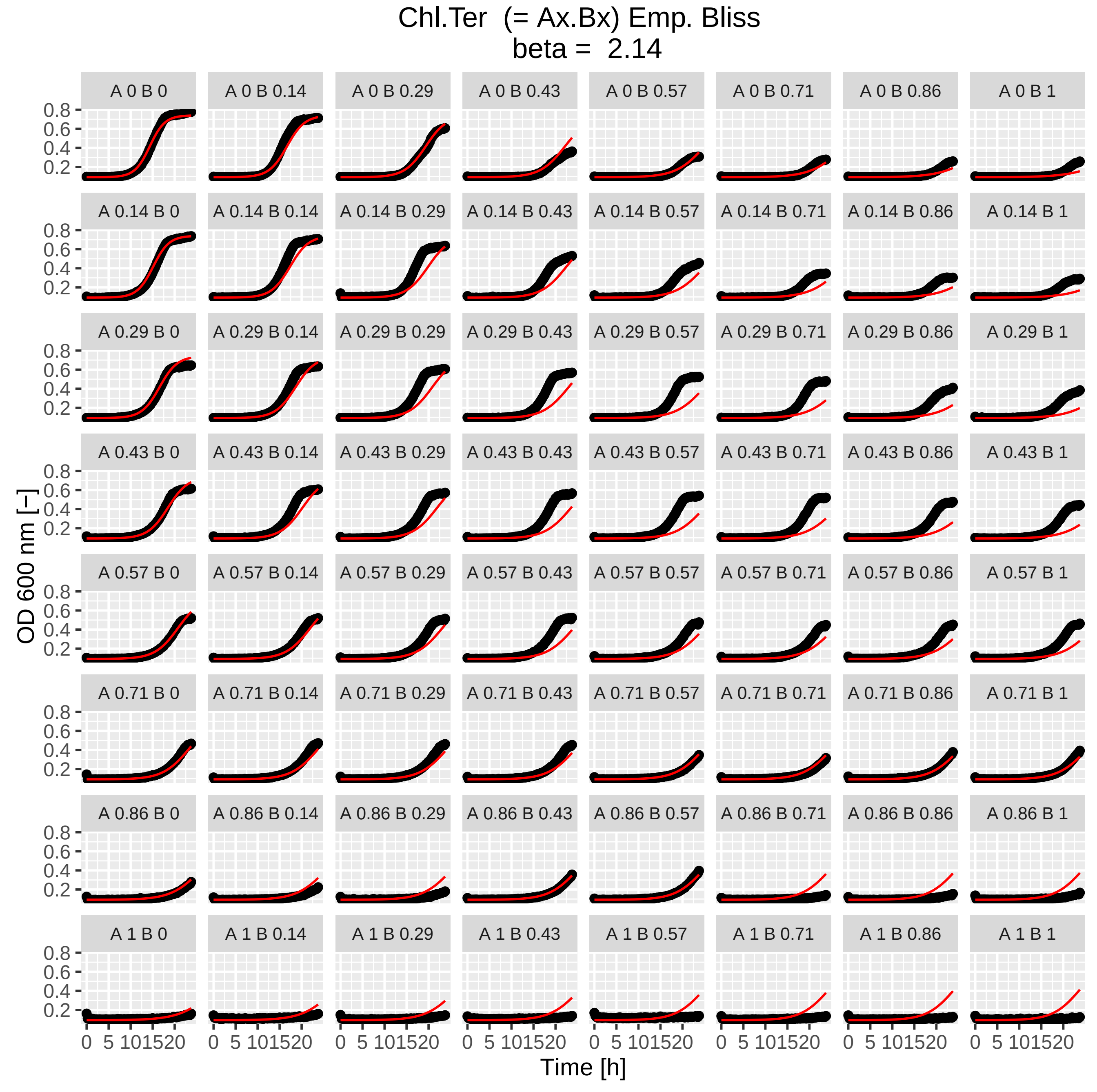

Chl.Ter (= Ax.Bx) Emp. Bliss
beta = 2.14
A 0 B 0
A 0 B 0.14
A 0 B 0.29
A 0 B 0.43
A 0 B 0.57
A 0 B 0.71
A 0 B 0.86
A 0 B 1
A 0.14 B 0
A 0.14 B 0.14
A 0.14 B 0.29
A 0.14 B 0.43
A 0.14 B 0.57
A 0.14 B 0.71
A 0.14 B 0.86
A 0.14 B 1
A 0.29 B 0
A 0.29 B 0.14
A 0.29 B 0.29
A 0.29 B 0.43
A 0.29 B 0.57
A 0.29 B 0.71
A 0.29 B 0.86
A 0.29 B 1
A 0.43 B 0
A 0.43 B 0.14
A 0.43 B 0.29
A 0.43 B 0.43
A 0.43 B 0.57
A 0.43 B 0.71
A 0.43 B 0.86
A 0.43 B 1
A 0.57 B 0
A 0.57 B 0.14
A 0.57 B 0.29
A 0.57 B 0.43
A 0.57 B 0.57
A 0.57 B 0.71
A 0.57 B 0.86
A 0.57 B 1
A 0.71 B 0
A 0.71 B 0.14
A 0.71 B 0.29
A 0.71 B 0.43
A 0.71 B 0.57
A 0.71 B 0.71
A 0.71 B 0.86
A 0.71 B 1
A 0.86 B 0
A 0.86 B 0.14
A 0.86 B 0.29
A 0.86 B 0.43
A 0.86 B 0.57
A 0.86 B 0.71
A 0.86 B 0.86
A 0.86 B 1
A 1 B 0
A 1 B 0.14
A 1 B 0.29
A 1 B 0.43
A 1 B 0.57
A 1 B 0.71
A 1 B 0.86
A 1 B 1
OD 600 nm [–]
0.2
0.4
0.6
0.8
0 5 101520
Time [h]

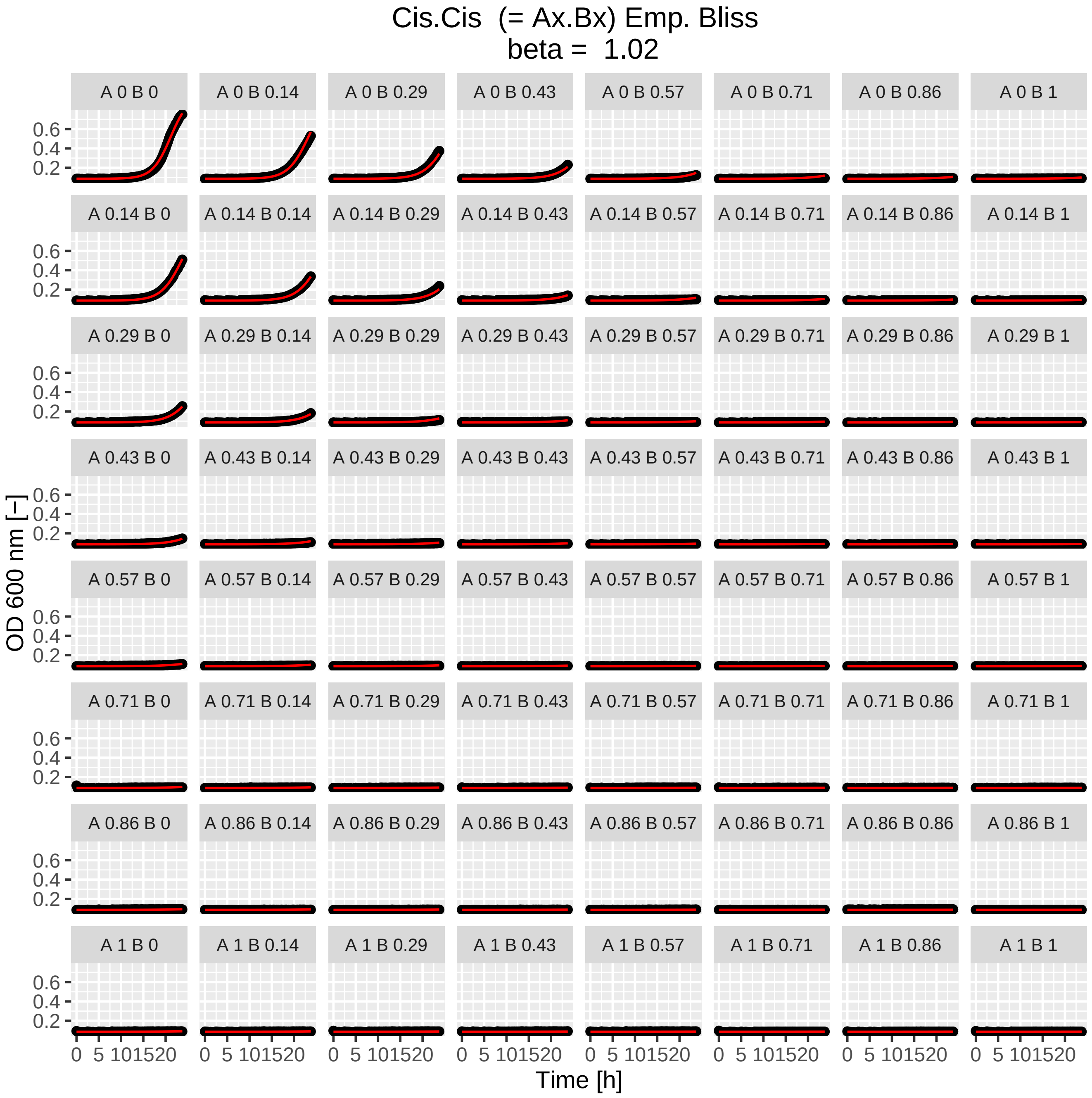

Cis.Cis (= Ax.Bx) Emp. Bliss
beta = 1.02
A 0 B 0
A 0 B 0.14
A 0 B 0.29
A 0 B 0.43
A 0 B 0.57
A 0 B 0.71
A 0 B 0.86
A 0 B 1
A 0.14 B 0
A 0.14 B 0.14
A 0.14 B 0.29
A 0.14 B 0.43
A 0.14 B 0.57
A 0.14 B 0.71
A 0.14 B 0.86
A 0.14 B 1
A 0.29 B 0
A 0.29 B 0.14
A 0.29 B 0.29
A 0.29 B 0.43
A 0.29 B 0.57
A 0.29 B 0.71
A 0.29 B 0.86
A 0.29 B 1
A 0.43 B 0
A 0.43 B 0.14
A 0.43 B 0.29
A 0.43 B 0.43
A 0.43 B 0.57
A 0.43 B 0.71
A 0.43 B 0.86
A 0.43 B 1
A 0.57 B 0
A 0.57 B 0.14
A 0.57 B 0.29
A 0.57 B 0.43
A 0.57 B 0.57
A 0.57 B 0.71
A 0.57 B 0.86
A 0.57 B 1
A 0.71 B 0
A 0.71 B 0.14
A 0.71 B 0.29
A 0.71 B 0.43
A 0.71 B 0.57
A 0.71 B 0.71
A 0.71 B 0.86
A 0.71 B 1
A 0.86 B 0
A 0.86 B 0.14
A 0.86 B 0.29
A 0.86 B 0.43
A 0.86 B 0.57
A 0.86 B 0.71
A 0.86 B 0.86
A 0.86 B 1
A 1 B 0
A 1 B 0.14
A 1 B 0.29
A 1 B 0.43
A 1 B 0.57
A 1 B 0.71
A 1 B 0.86
A 1 B 1
OD 600 nm [–]
0.2
0.4
0.6
0 5 101520
Time [h]

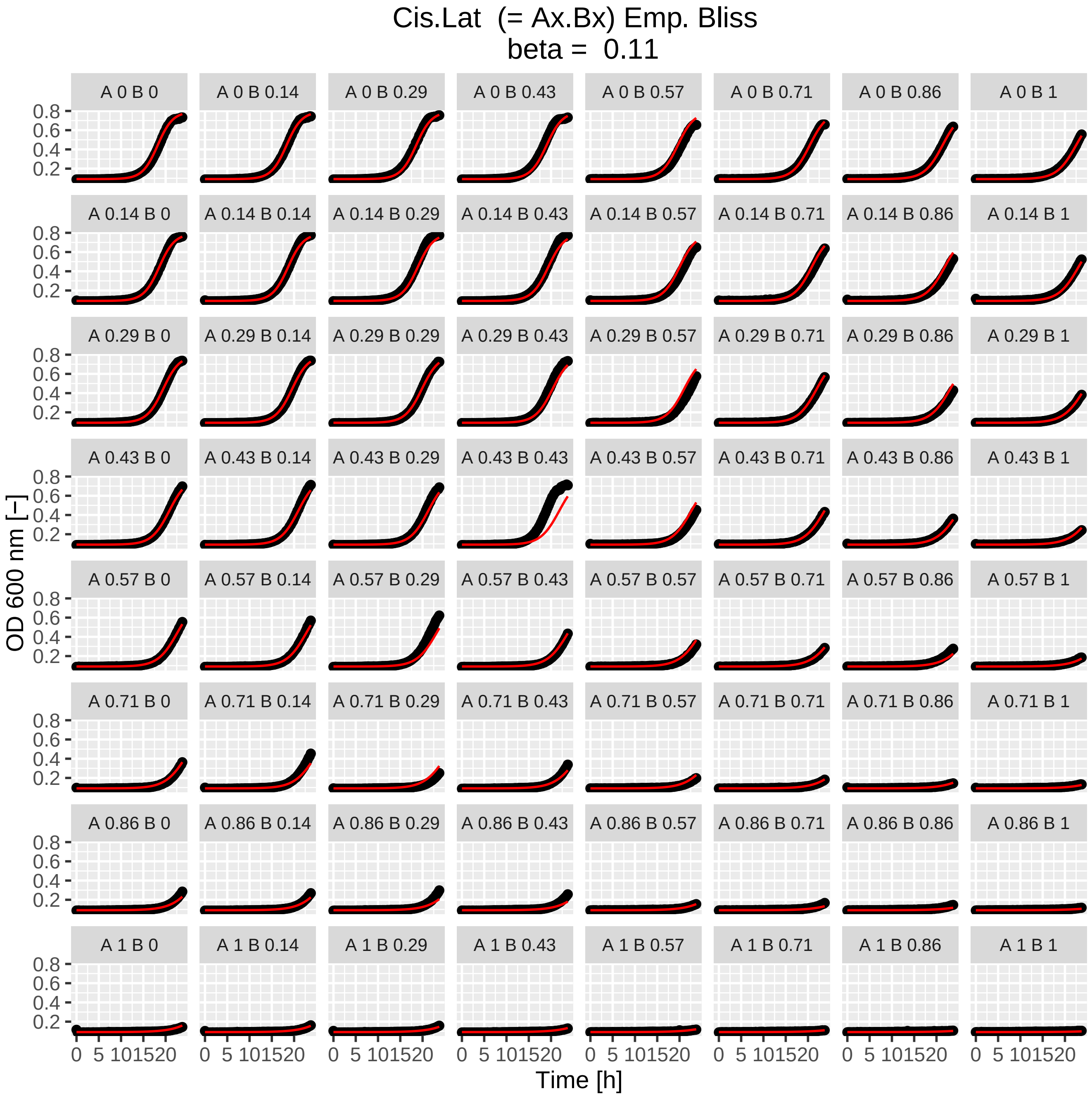

Cis.Lat (= Ax.Bx) Emp. Bliss
beta = 0.11
OD 600 nm [−]
Time [h]
A 0 B 0
A 0 B 0.14
A 0 B 0.29
A 0 B 0.43
A 0 B 0.57
A 0 B 0.71
A 0 B 0.86
A 0 B 1
A 0.14 B 0
A 0.14 B 0.14
A 0.14 B 0.29
A 0.14 B 0.43
A 0.14 B 0.57
A 0.14 B 0.71
A 0.14 B 0.86
A 0.14 B 1
A 0.29 B 0
A 0.29 B 0.14
A 0.29 B 0.29
A 0.29 B 0.43
A 0.29 B 0.57
A 0.29 B 0.71
A 0.29 B 0.86
A 0.29 B 1
A 0.43 B 0
A 0.43 B 0.14
A 0.43 B 0.29
A 0.43 B 0.43
A 0.43 B 0.57
A 0.43 B 0.71
A 0.43 B 0.86
A 0.43 B 1
A 0.57 B 0
A 0.57 B 0.14
A 0.57 B 0.29
A 0.57 B 0.43
A 0.57 B 0.57
A 0.57 B 0.71
A 0.57 B 0.86
A 0.57 B 1
A 0.71 B 0
A 0.71 B 0.14
A 0.71 B 0.29
A 0.71 B 0.43
A 0.71 B 0.57
A 0.71 B 0.71
A 0.71 B 0.86
A 0.71 B 1
A 0.86 B 0
A 0.86 B 0.14
A 0.86 B 0.29
A 0.86 B 0.43
A 0.86 B 0.57
A 0.86 B 0.71
A 0.86 B 0.86
A 0.86 B 1
A 1 B 0
A 1 B 0.14
A 1 B 0.29
A 1 B 0.43
A 1 B 0.57
A 1 B 0.71
A 1 B 0.86
A 1 B 1
0.8
0.6
0.4
0.2
0 5 101520

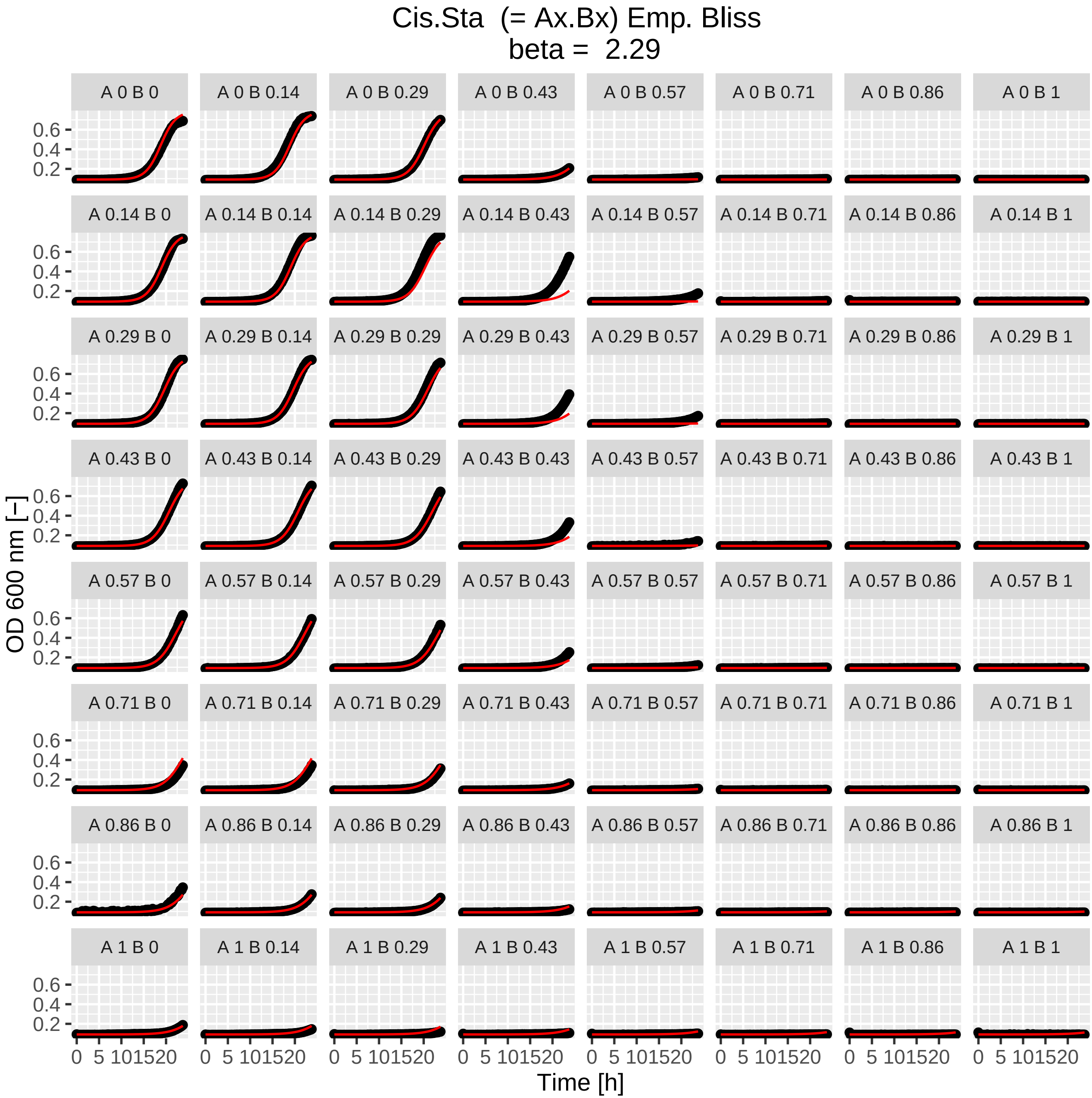

Cis.Sta (= Ax.Bx) Emp. Bliss
beta = 2.29
A 0 B 0
A 0 B 0.14
A 0 B 0.29
A 0 B 0.43
A 0 B 0.57
A 0 B 0.71
A 0 B 0.86
A 0 B 1
A 0.14 B 0
A 0.14 B 0.14
A 0.14 B 0.29
A 0.14 B 0.43
A 0.14 B 0.57
A 0.14 B 0.71
A 0.14 B 0.86
A 0.14 B 1
A 0.29 B 0
A 0.29 B 0.14
A 0.29 B 0.29
A 0.29 B 0.43
A 0.29 B 0.57
A 0.29 B 0.71
A 0.29 B 0.86
A 0.29 B 1
A 0.43 B 0
A 0.43 B 0.14
A 0.43 B 0.29
A 0.43 B 0.43
A 0.43 B 0.57
A 0.43 B 0.71
A 0.43 B 0.86
A 0.43 B 1
A 0.57 B 0
A 0.57 B 0.14
A 0.57 B 0.29
A 0.57 B 0.43
A 0.57 B 0.57
A 0.57 B 0.71
A 0.57 B 0.86
A 0.57 B 1
A 0.71 B 0
A 0.71 B 0.14
A 0.71 B 0.29
A 0.71 B 0.43
A 0.71 B 0.57
A 0.71 B 0.71
A 0.71 B 0.86
A 0.71 B 1
A 0.86 B 0
A 0.86 B 0.14
A 0.86 B 0.29
A 0.86 B 0.43
A 0.86 B 0.57
A 0.86 B 0.71
A 0.86 B 0.86
A 0.86 B 1
A 1 B 0
A 1 B 0.14
A 1 B 0.29
A 1 B 0.43
A 1 B 0.57
A 1 B 0.71
A 1 B 0.86
A 1 B 1
OD 600 nm [−]
0.2
0.4
0.6
0
5
10
15
20
Time [h]

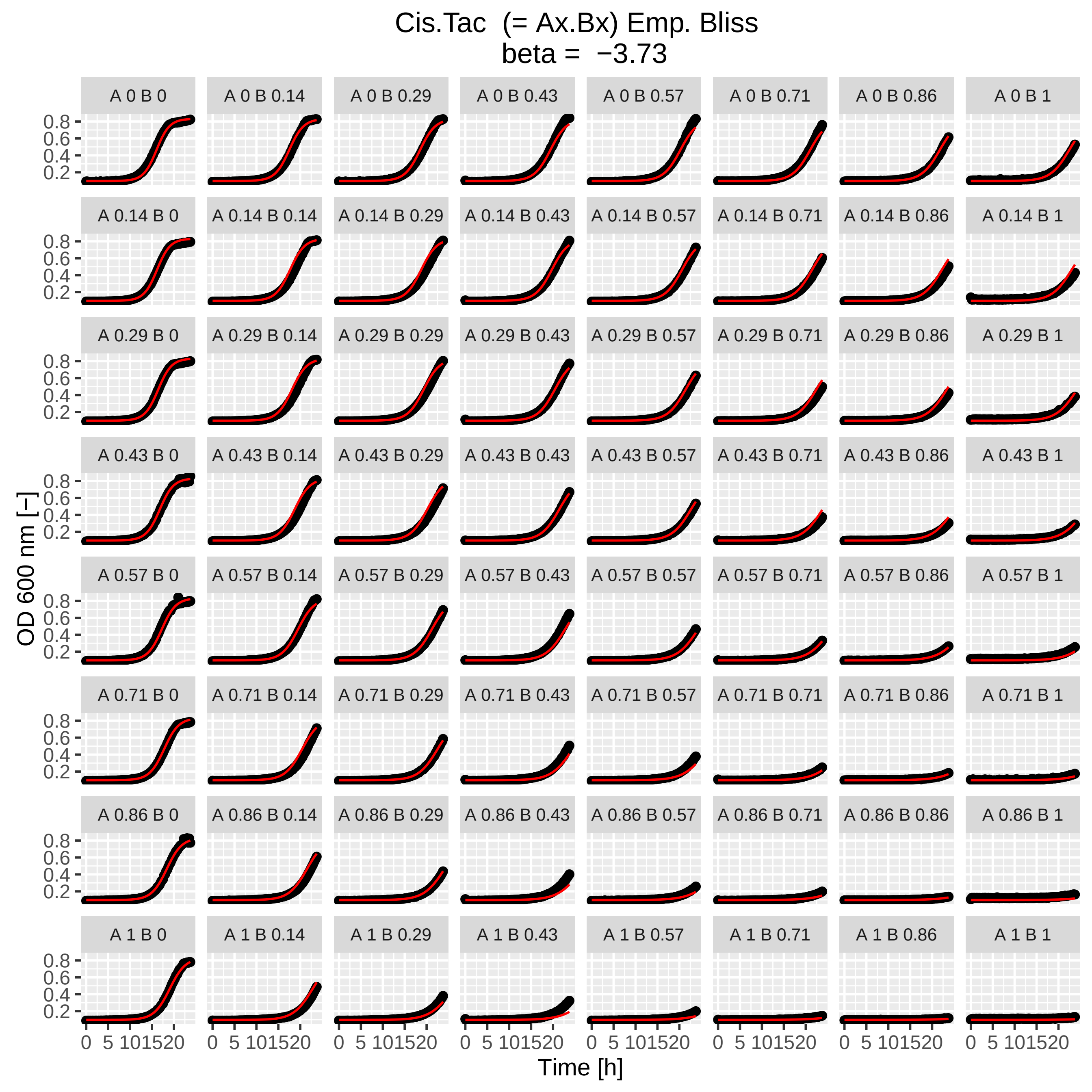
Cis.Tac (= Ax.Bx) Emp. Bliss
beta = −3.73
A 0 B 0
A 0 B 0.14
A 0 B 0.29
A 0 B 0.43
A 0 B 0.57
A 0 B 0.71
A 0 B 0.86
A 0 B 1
A 0.14 B 0
A 0.14 B 0.14
A 0.14 B 0.29
A 0.14 B 0.43
A 0.14 B 0.57
A 0.14 B 0.71
A 0.14 B 0.86
A 0.14 B 1
A 0.29 B 0
A 0.29 B 0.14
A 0.29 B 0.29
A 0.29 B 0.43
A 0.29 B 0.57
A 0.29 B 0.71
A 0.29 B 0.86
A 0.29 B 1
A 0.43 B 0
A 0.43 B 0.14
A 0.43 B 0.29
A 0.43 B 0.43
A 0.43 B 0.57
A 0.43 B 0.71
A 0.43 B 0.86
A 0.43 B 1
A 0.57 B 0
A 0.57 B 0.14
A 0.57 B 0.29
A 0.57 B 0.43
A 0.57 B 0.57
A 0.57 B 0.71
A 0.57 B 0.86
A 0.57 B 1
A 0.71 B 0
A 0.71 B 0.14
A 0.71 B 0.29
A 0.71 B 0.43
A 0.71 B 0.57
A 0.71 B 0.71
A 0.71 B 0.86
A 0.71 B 1
A 0.86 B 0
A 0.86 B 0.14
A 0.86 B 0.29
A 0.86 B 0.43
A 0.86 B 0.57
A 0.86 B 0.71
A 0.86 B 0.86
A 0.86 B 1
A 1 B 0
A 1 B 0.14
A 1 B 0.29
A 1 B 0.43
A 1 B 0.57
A 1 B 0.71
A 1 B 0.86
A 1 B 1
OD 600 nm [−]
0.2 0.4 0.6 0.8
0 5 101520
Time [h]

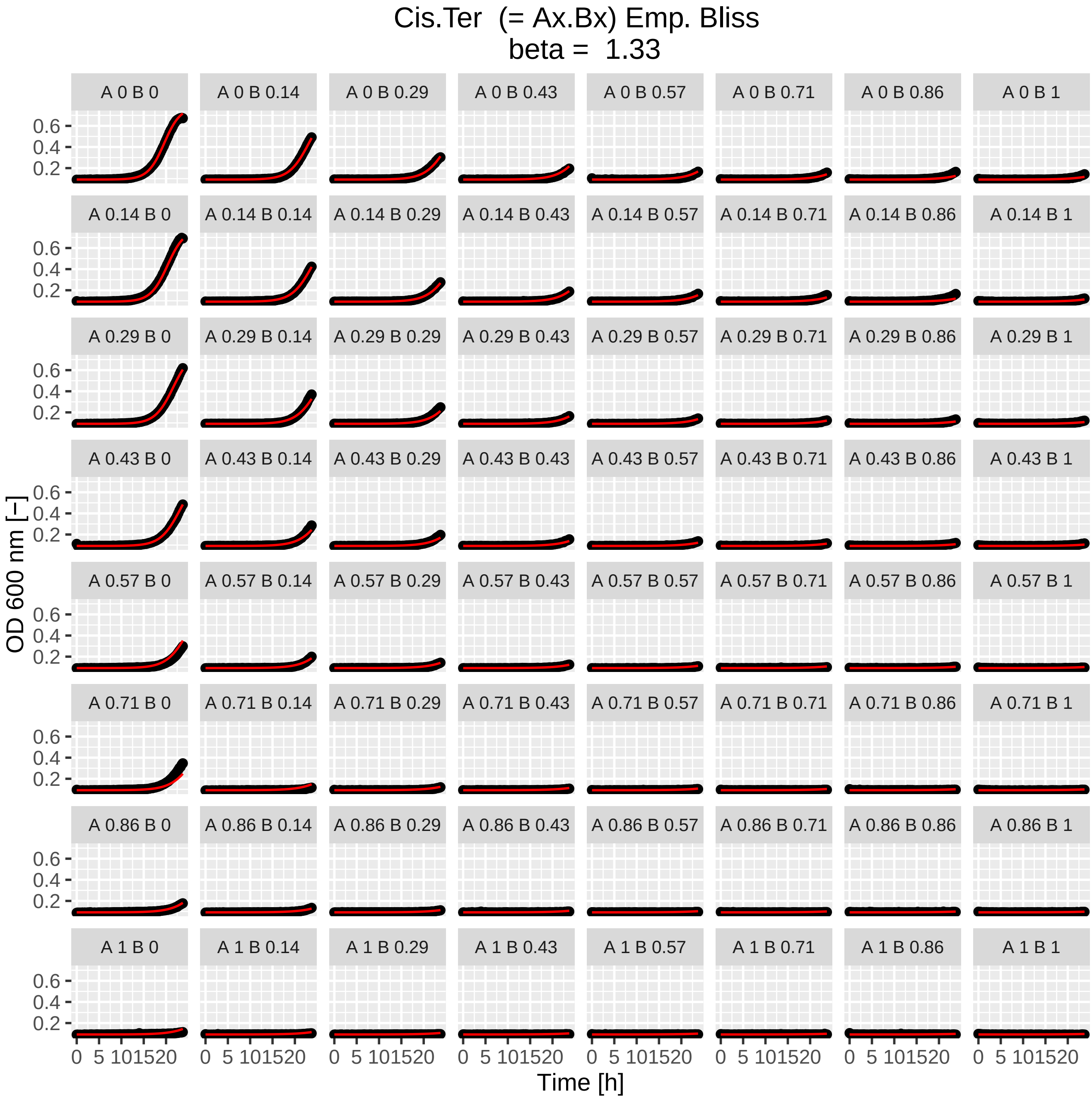
Cis.Ter (= Ax.Bx) Emp. Bliss
beta = 1.33
OD 600 nm [−]
Time [h]
0.6
0.4
0.2
0 5 101520
A 0 B 0
A 0 B 0.14
A 0 B 0.29
A 0 B 0.43
A 0 B 0.57
A 0 B 0.71
A 0 B 0.86
A 0 B 1
A 0.14 B 0
A 0.14 B 0.14
A 0.14 B 0.29
A 0.14 B 0.43
A 0.14 B 0.57
A 0.14 B 0.71
A 0.14 B 0.86
A 0.14 B 1
A 0.29 B 0
A 0.29 B 0.14
A 0.29 B 0.29
A 0.29 B 0.43
A 0.29 B 0.57
A 0.29 B 0.71
A 0.29 B 0.86
A 0.29 B 1
A 0.43 B 0
A 0.43 B 0.14
A 0.43 B 0.29
A 0.43 B 0.43
A 0.43 B 0.57
A 0.43 B 0.71
A 0.43 B 0.86
A 0.43 B 1
A 0.57 B 0
A 0.57 B 0.14
A 0.57 B 0.29
A 0.57 B 0.43
A 0.57 B 0.57
A 0.57 B 0.71
A 0.57 B 0.86
A 0.57 B 1
A 0.71 B 0
A 0.71 B 0.14
A 0.71 B 0.29
A 0.71 B 0.43
A 0.71 B 0.57
A 0.71 B 0.71
A 0.71 B 0.86
A 0.71 B 1
A 0.86 B 0
A 0.86 B 0.14
A 0.86 B 0.29
A 0.86 B 0.43
A 0.86 B 0.57
A 0.86 B 0.71
A 0.86 B 0.86
A 0.86 B 1
A 1 B 0
A 1 B 0.14
A 1 B 0.29
A 1 B 0.43
A 1 B 0.57
A 1 B 0.71
A 1 B 0.86
A 1 B 1

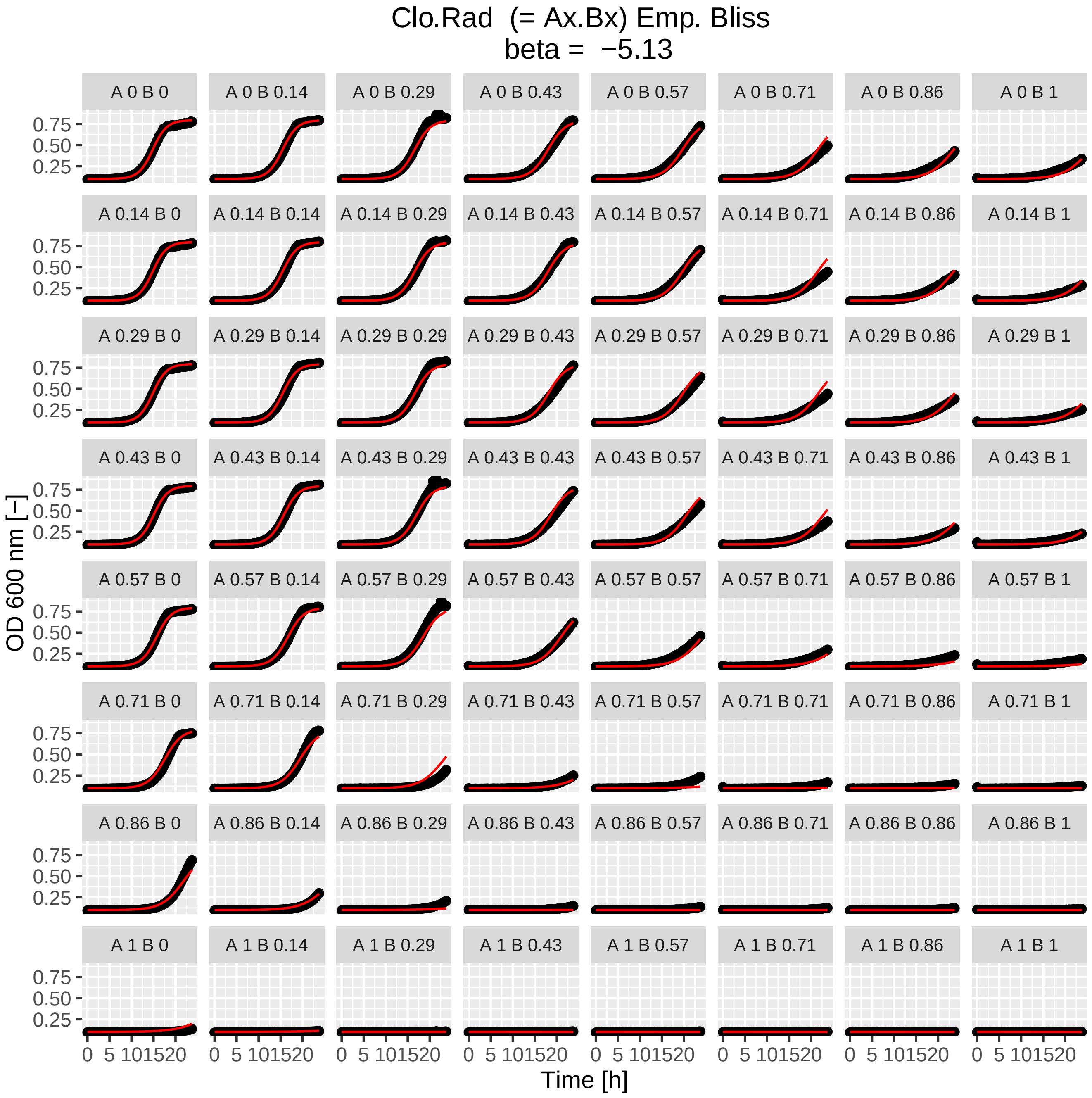

Clo.Rad (= Ax.Bx) Emp. Bliss
beta = −5.13
OD 600 nm [−]
Time [h]
0.75
0.50
0.25
0 5 101520
A 0 B 0
A 0 B 0.14
A 0 B 0.29
A 0 B 0.43
A 0 B 0.57
A 0 B 0.71
A 0 B 0.86
A 0 B 1
A 0.14 B 0
A 0.14 B 0.14
A 0.14 B 0.29
A 0.14 B 0.43
A 0.14 B 0.57
A 0.14 B 0.71
A 0.14 B 0.86
A 0.14 B 1
A 0.29 B 0
A 0.29 B 0.14
A 0.29 B 0.29
A 0.29 B 0.43
A 0.29 B 0.57
A 0.29 B 0.71
A 0.29 B 0.86
A 0.29 B 1
A 0.43 B 0
A 0.43 B 0.14
A 0.43 B 0.29
A 0.43 B 0.43
A 0.43 B 0.57
A 0.43 B 0.71
A 0.43 B 0.86
A 0.43 B 1
A 0.57 B 0
A 0.57 B 0.14
A 0.57 B 0.29
A 0.57 B 0.43
A 0.57 B 0.57
A 0.57 B 0.71
A 0.57 B 0.86
A 0.57 B 1
A 0.71 B 0
A 0.71 B 0.14
A 0.71 B 0.29
A 0.71 B 0.43
A 0.71 B 0.57
A 0.71 B 0.71
A 0.71 B 0.86
A 0.71 B 1
A 0.86 B 0
A 0.86 B 0.14
A 0.86 B 0.29
A 0.86 B 0.43
A 0.86 B 0.57
A 0.86 B 0.71
A 0.86 B 0.86
A 0.86 B 1
A 1 B 0
A 1 B 0.14
A 1 B 0.29
A 1 B 0.43
A 1 B 0.57
A 1 B 0.71
A 1 B 0.86
A 1 B 1

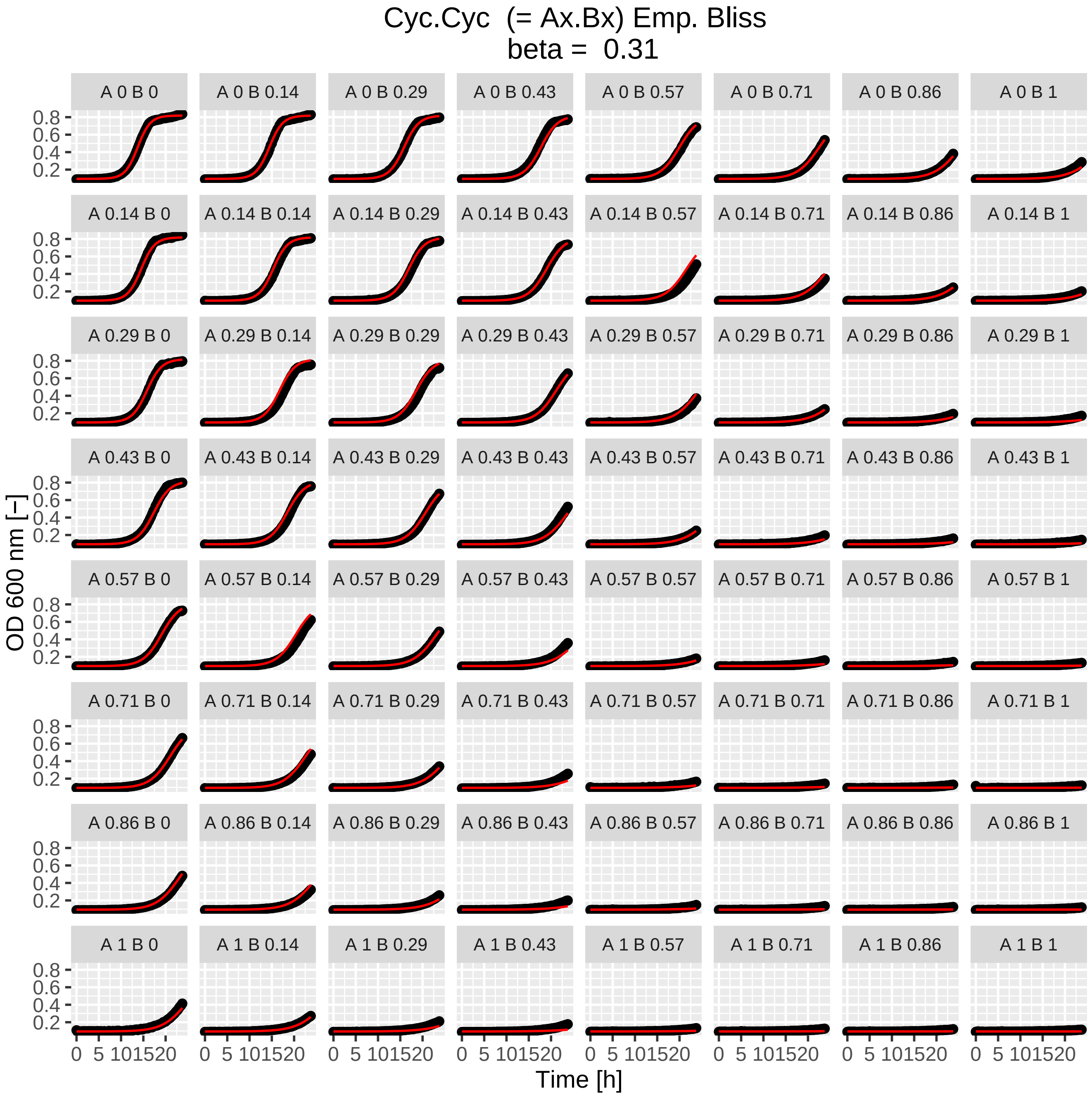
Cyc.Cyc (= Ax.Bx) Emp. Bliss
beta = 0.31
A 0 B 0
A 0 B 0.14
A 0 B 0.29
A 0 B 0.43
A 0 B 0.57
A 0 B 0.71
A 0 B 0.86
A 0 B 1
A 0.14 B 0
A 0.14 B 0.14
A 0.14 B 0.29
A 0.14 B 0.43
A 0.14 B 0.57
A 0.14 B 0.71
A 0.14 B 0.86
A 0.14 B 1
A 0.29 B 0
A 0.29 B 0.14
A 0.29 B 0.29
A 0.29 B 0.43
A 0.29 B 0.57
A 0.29 B 0.71
A 0.29 B 0.86
A 0.29 B 1
A 0.43 B 0
A 0.43 B 0.14
A 0.43 B 0.29
A 0.43 B 0.43
A 0.43 B 0.57
A 0.43 B 0.71
A 0.43 B 0.86
A 0.43 B 1
A 0.57 B 0
A 0.57 B 0.14
A 0.57 B 0.29
A 0.57 B 0.43
A 0.57 B 0.57
A 0.57 B 0.71
A 0.57 B 0.86
A 0.57 B 1
A 0.71 B 0
A 0.71 B 0.14
A 0.71 B 0.29
A 0.71 B 0.43
A 0.71 B 0.57
A 0.71 B 0.71
A 0.71 B 0.86
A 0.71 B 1
A 0.86 B 0
A 0.86 B 0.14
A 0.86 B 0.29
A 0.86 B 0.43
A 0.86 B 0.57
A 0.86 B 0.71
A 0.86 B 0.86
A 0.86 B 1
A 1 B 0
A 1 B 0.14
A 1 B 0.29
A 1 B 0.43
A 1 B 0.57
A 1 B 0.71
A 1 B 0.86
A 1 B 1
OD 600 nm [–]
0.2
0.4
0.6
0.8
0
5
10
15
20
Time [h]

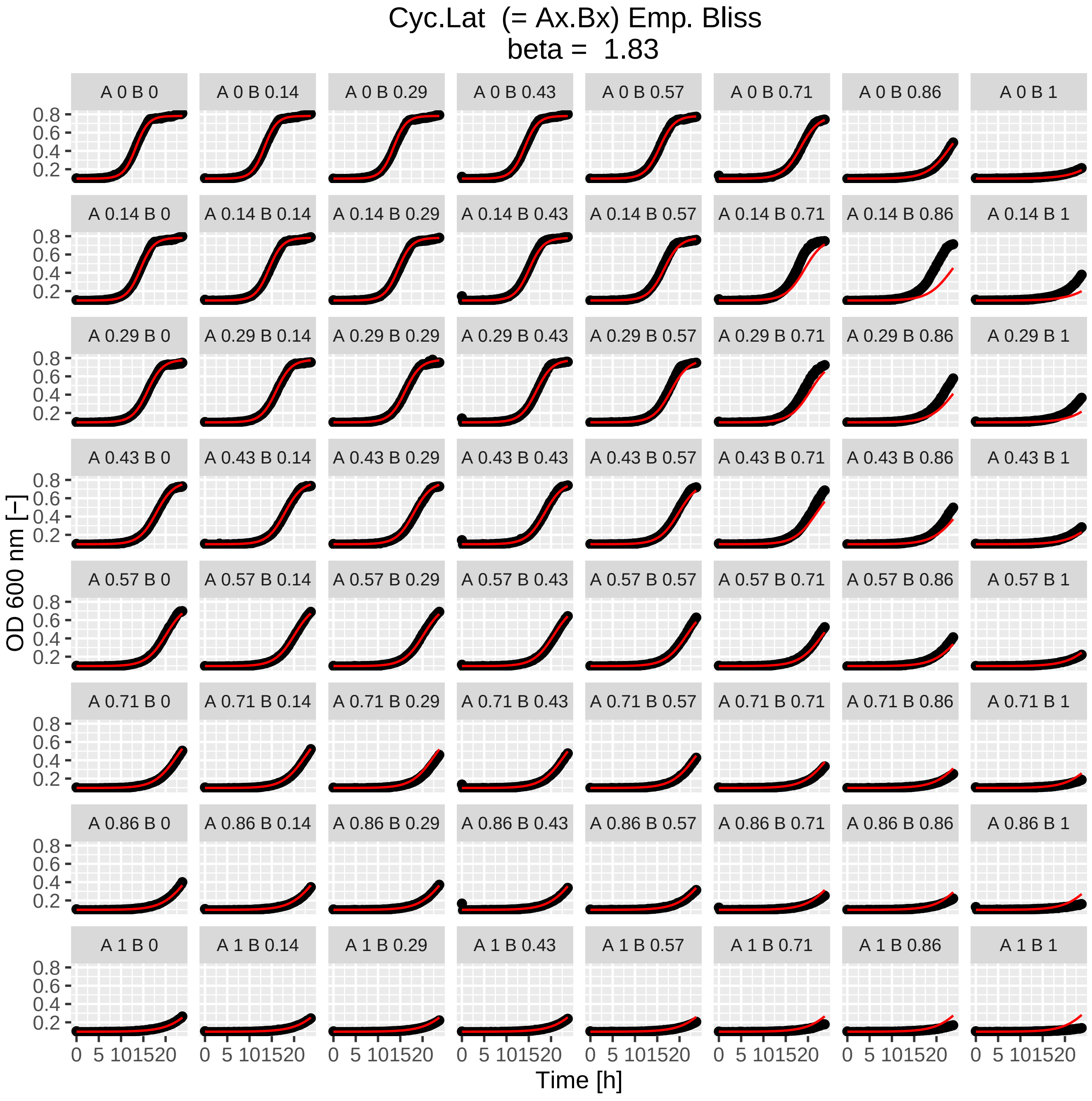

Cyc.Lat (= Ax.Bx) Emp. Bliss
beta = 1.83
A 0 B 0
A 0 B 0.14
A 0 B 0.29
A 0 B 0.43
A 0 B 0.57
A 0 B 0.71
A 0 B 0.86
A 0 B 1
A 0.14 B 0
A 0.14 B 0.14
A 0.14 B 0.29
A 0.14 B 0.43
A 0.14 B 0.57
A 0.14 B 0.71
A 0.14 B 0.86
A 0.14 B 1
A 0.29 B 0
A 0.29 B 0.14
A 0.29 B 0.29
A 0.29 B 0.43
A 0.29 B 0.57
A 0.29 B 0.71
A 0.29 B 0.86
A 0.29 B 1
A 0.43 B 0
A 0.43 B 0.14
A 0.43 B 0.29
A 0.43 B 0.43
A 0.43 B 0.57
A 0.43 B 0.71
A 0.43 B 0.86
A 0.43 B 1
A 0.57 B 0
A 0.57 B 0.14
A 0.57 B 0.29
A 0.57 B 0.43
A 0.57 B 0.57
A 0.57 B 0.71
A 0.57 B 0.86
A 0.57 B 1
A 0.71 B 0
A 0.71 B 0.14
A 0.71 B 0.29
A 0.71 B 0.43
A 0.71 B 0.57
A 0.71 B 0.71
A 0.71 B 0.86
A 0.71 B 1
A 0.86 B 0
A 0.86 B 0.14
A 0.86 B 0.29
A 0.86 B 0.43
A 0.86 B 0.57
A 0.86 B 0.71
A 0.86 B 0.86
A 0.86 B 1
A 1 B 0
A 1 B 0.14
A 1 B 0.29
A 1 B 0.43
A 1 B 0.57
A 1 B 0.71
A 1 B 0.86
A 1 B 1
OD 600 nm [–]
0.2
0.4
0.6
0.8
0
5
10
15
20
Time [h]

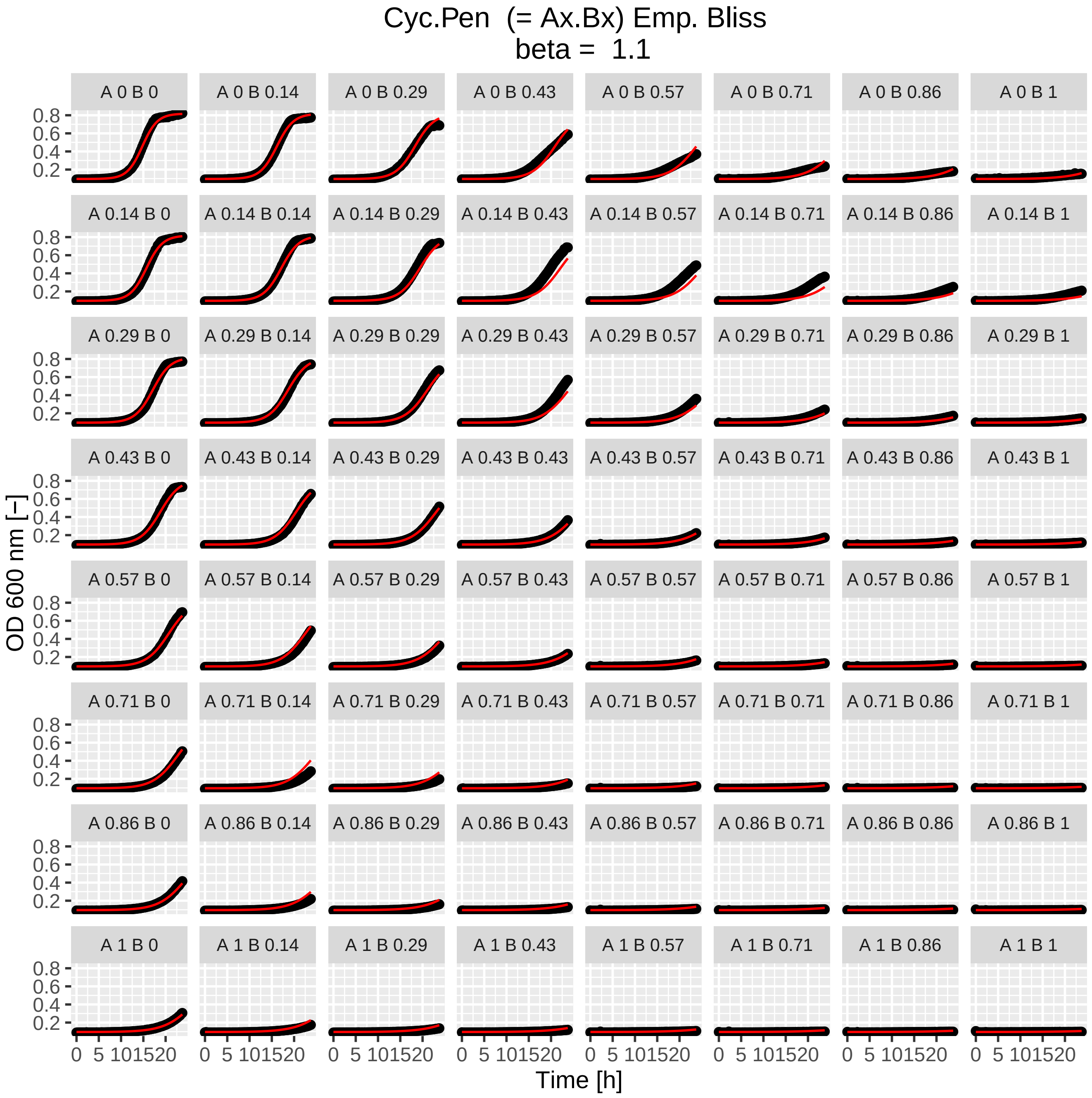

Cyc.Pen (= Ax.Bx) Emp. Bliss
beta = 1.1
OD 600 nm [−]
Time [h]
A 0 B 0
A 0 B 0.14
A 0 B 0.29
A 0 B 0.43
A 0 B 0.57
A 0 B 0.71
A 0 B 0.86
A 0 B 1
A 0.14 B 0
A 0.14 B 0.14
A 0.14 B 0.29
A 0.14 B 0.43
A 0.14 B 0.57
A 0.14 B 0.71
A 0.14 B 0.86
A 0.14 B 1
A 0.29 B 0
A 0.29 B 0.14
A 0.29 B 0.29
A 0.29 B 0.43
A 0.29 B 0.57
A 0.29 B 0.71
A 0.29 B 0.86
A 0.29 B 1
A 0.43 B 0
A 0.43 B 0.14
A 0.43 B 0.29
A 0.43 B 0.43
A 0.43 B 0.57
A 0.43 B 0.71
A 0.43 B 0.86
A 0.43 B 1
A 0.57 B 0
A 0.57 B 0.14
A 0.57 B 0.29
A 0.57 B 0.43
A 0.57 B 0.57
A 0.57 B 0.71
A 0.57 B 0.86
A 0.57 B 1
A 0.71 B 0
A 0.71 B 0.14
A 0.71 B 0.29
A 0.71 B 0.43
A 0.71 B 0.57
A 0.71 B 0.71
A 0.71 B 0.86
A 0.71 B 1
A 0.86 B 0
A 0.86 B 0.14
A 0.86 B 0.29
A 0.86 B 0.43
A 0.86 B 0.57
A 0.86 B 0.71
A 0.86 B 0.86
A 0.86 B 1
A 1 B 0
A 1 B 0.14
A 1 B 0.29
A 1 B 0.43
A 1 B 0.57
A 1 B 0.71
A 1 B 0.86
A 1 B 1
0.8
0.6
0.4
0.2
0 5 101520

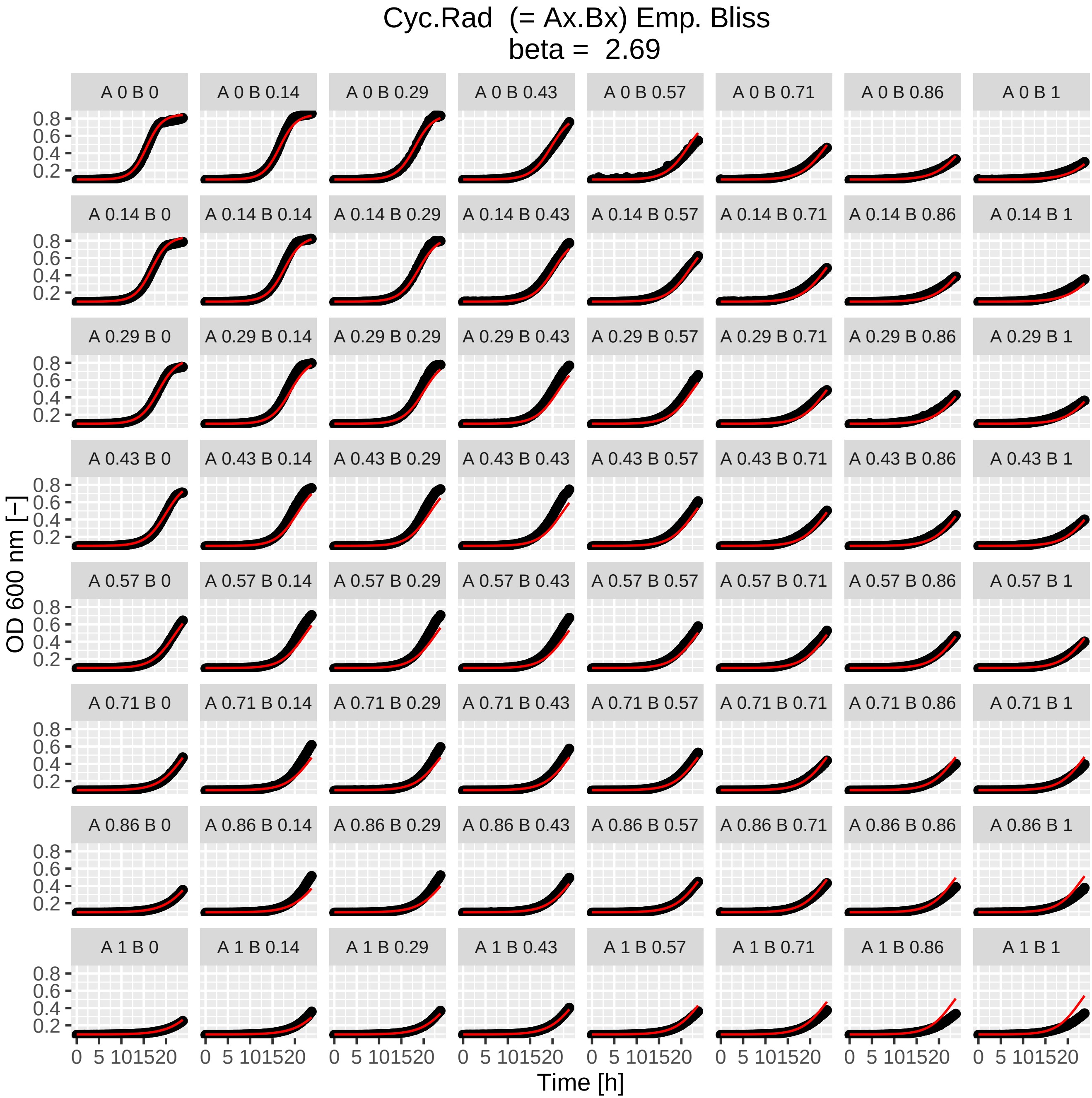

Cyc.Rad (= Ax.Bx) Emp. Bliss
beta = 2.69
OD 600 nm [–]
Time [h]
A 0 B 0
A 0 B 0.14
A 0 B 0.29
A 0 B 0.43
A 0 B 0.57
A 0 B 0.71
A 0 B 0.86
A 0 B 1
A 0.14 B 0
A 0.14 B 0.14
A 0.14 B 0.29
A 0.14 B 0.43
A 0.14 B 0.57
A 0.14 B 0.71
A 0.14 B 0.86
A 0.14 B 1
A 0.29 B 0
A 0.29 B 0.14
A 0.29 B 0.29
A 0.29 B 0.43
A 0.29 B 0.57
A 0.29 B 0.71
A 0.29 B 0.86
A 0.29 B 1
A 0.43 B 0
A 0.43 B 0.14
A 0.43 B 0.29
A 0.43 B 0.43
A 0.43 B 0.57
A 0.43 B 0.71
A 0.43 B 0.86
A 0.43 B 1
A 0.57 B 0
A 0.57 B 0.14
A 0.57 B 0.29
A 0.57 B 0.43
A 0.57 B 0.57
A 0.57 B 0.71
A 0.57 B 0.86
A 0.57 B 1
A 0.71 B 0
A 0.71 B 0.14
A 0.71 B 0.29
A 0.71 B 0.43
A 0.71 B 0.57
A 0.71 B 0.71
A 0.71 B 0.86
A 0.71 B 1
A 0.86 B 0
A 0.86 B 0.14
A 0.86 B 0.29
A 0.86 B 0.43
A 0.86 B 0.57
A 0.86 B 0.71
A 0.86 B 0.86
A 0.86 B 1
A 1 B 0
A 1 B 0.14
A 1 B 0.29
A 1 B 0.43
A 1 B 0.57
A 1 B 0.71
A 1 B 0.86
A 1 B 1
0.2
0.4
0.6
0.8
0
5
10
15
20

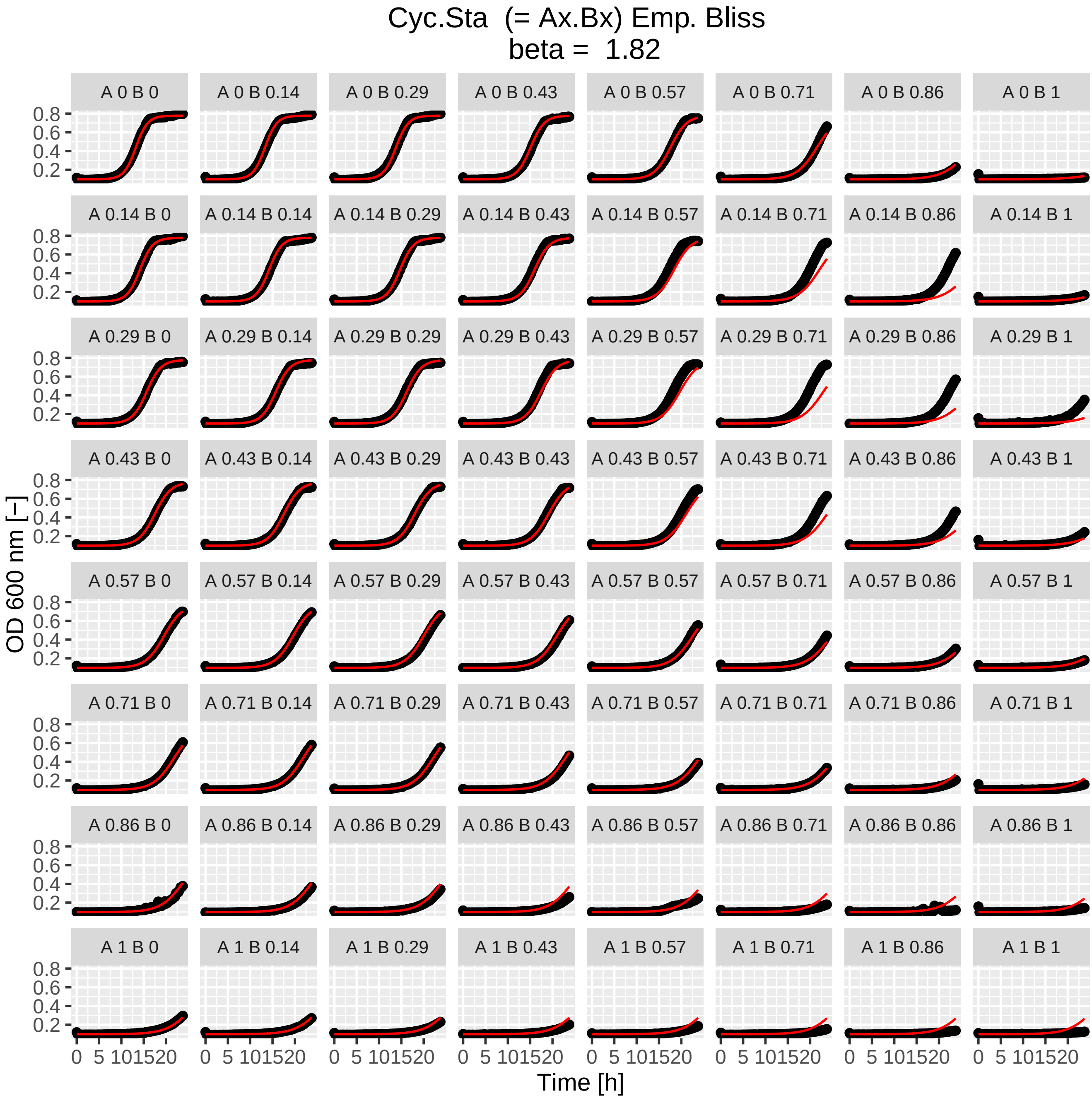

Cyc.Sta (= Ax.Bx) Emp. Bliss
beta = 1.82
A 0 B 0
A 0 B 0.14
A 0 B 0.29
A 0 B 0.43
A 0 B 0.57
A 0 B 0.71
A 0 B 0.86
A 0 B 1
A 0.14 B 0
A 0.14 B 0.14
A 0.14 B 0.29
A 0.14 B 0.43
A 0.14 B 0.57
A 0.14 B 0.71
A 0.14 B 0.86
A 0.14 B 1
A 0.29 B 0
A 0.29 B 0.14
A 0.29 B 0.29
A 0.29 B 0.43
A 0.29 B 0.57
A 0.29 B 0.71
A 0.29 B 0.86
A 0.29 B 1
A 0.43 B 0
A 0.43 B 0.14
A 0.43 B 0.29
A 0.43 B 0.43
A 0.43 B 0.57
A 0.43 B 0.71
A 0.43 B 0.86
A 0.43 B 1
A 0.57 B 0
A 0.57 B 0.14
A 0.57 B 0.29
A 0.57 B 0.43
A 0.57 B 0.57
A 0.57 B 0.71
A 0.57 B 0.86
A 0.57 B 1
A 0.71 B 0
A 0.71 B 0.14
A 0.71 B 0.29
A 0.71 B 0.43
A 0.71 B 0.57
A 0.71 B 0.71
A 0.71 B 0.86
A 0.71 B 1
A 0.86 B 0
A 0.86 B 0.14
A 0.86 B 0.29
A 0.86 B 0.43
A 0.86 B 0.57
A 0.86 B 0.71
A 0.86 B 0.86
A 0.86 B 1
A 1 B 0
A 1 B 0.14
A 1 B 0.29
A 1 B 0.43
A 1 B 0.57
A 1 B 0.71
A 1 B 0.86
A 1 B 1
0.8
0.6
0.4
0.2
OD 600 nm [–]
0 5 101520
Time [h]

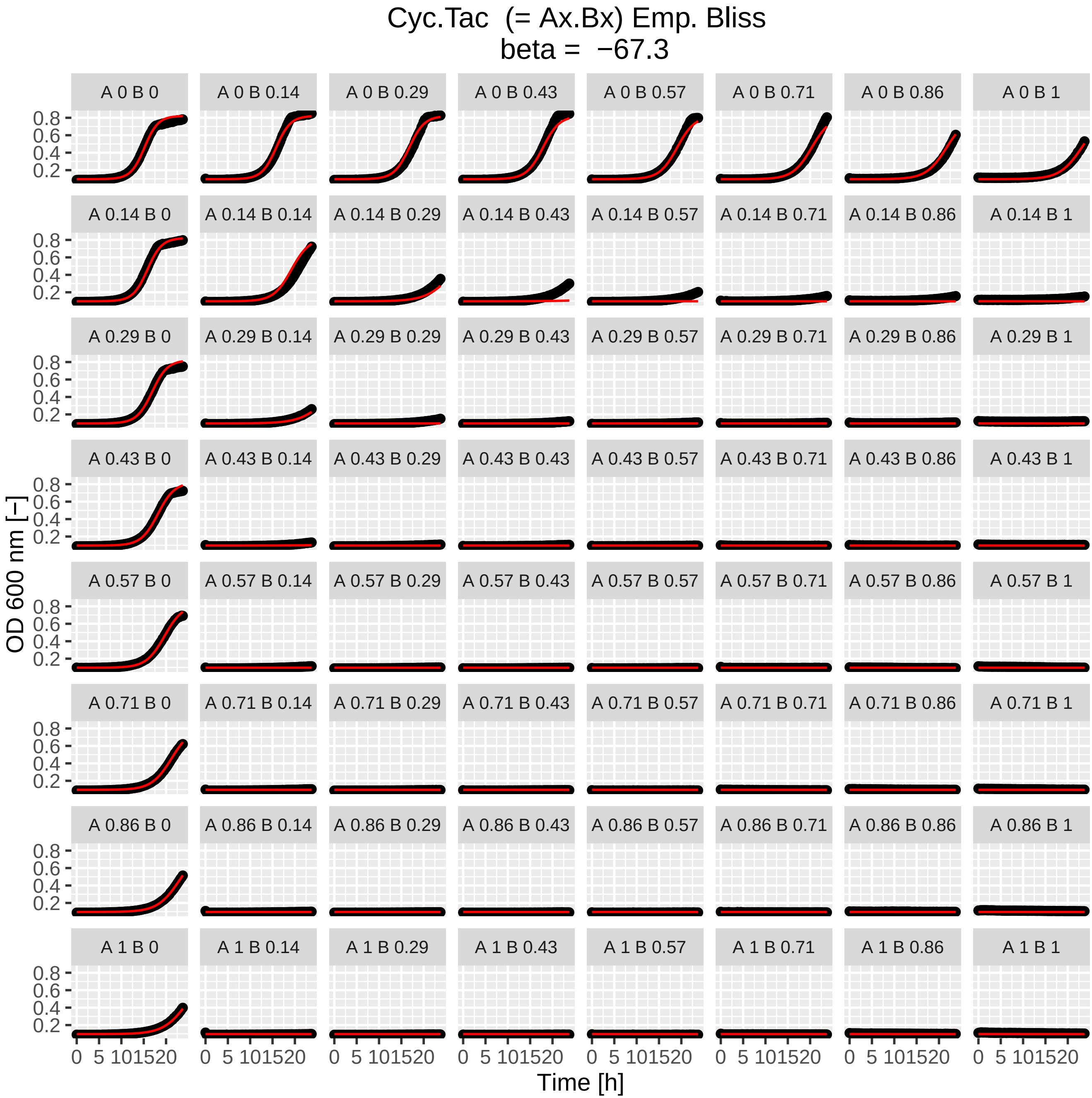

Cyc.Tac (= Ax.Bx) Emp. Bliss
beta = −67.3
A 0 B 0
A 0 B 0.14
A 0 B 0.29
A 0 B 0.43
A 0 B 0.57
A 0 B 0.71
A 0 B 0.86
A 0 B 1
A 0.14 B 0
A 0.14 B 0.14
A 0.14 B 0.29
A 0.14 B 0.43
A 0.14 B 0.57
A 0.14 B 0.71
A 0.14 B 0.86
A 0.14 B 1
A 0.29 B 0
A 0.29 B 0.14
A 0.29 B 0.29
A 0.29 B 0.43
A 0.29 B 0.57
A 0.29 B 0.71
A 0.29 B 0.86
A 0.29 B 1
A 0.43 B 0
A 0.43 B 0.14
A 0.43 B 0.29
A 0.43 B 0.43
A 0.43 B 0.57
A 0.43 B 0.71
A 0.43 B 0.86
A 0.43 B 1
A 0.57 B 0
A 0.57 B 0.14
A 0.57 B 0.29
A 0.57 B 0.43
A 0.57 B 0.57
A 0.57 B 0.71
A 0.57 B 0.86
A 0.57 B 1
A 0.71 B 0
A 0.71 B 0.14
A 0.71 B 0.29
A 0.71 B 0.43
A 0.71 B 0.57
A 0.71 B 0.71
A 0.71 B 0.86
A 0.71 B 1
A 0.86 B 0
A 0.86 B 0.14
A 0.86 B 0.29
A 0.86 B 0.43
A 0.86 B 0.57
A 0.86 B 0.71
A 0.86 B 0.86
A 0.86 B 1
A 1 B 0
A 1 B 0.14
A 1 B 0.29
A 1 B 0.43
A 1 B 0.57
A 1 B 0.71
A 1 B 0.86
A 1 B 1
OD 600 nm [−]
0.2
0.4
0.6
0.8
0 5 101520
Time [h]

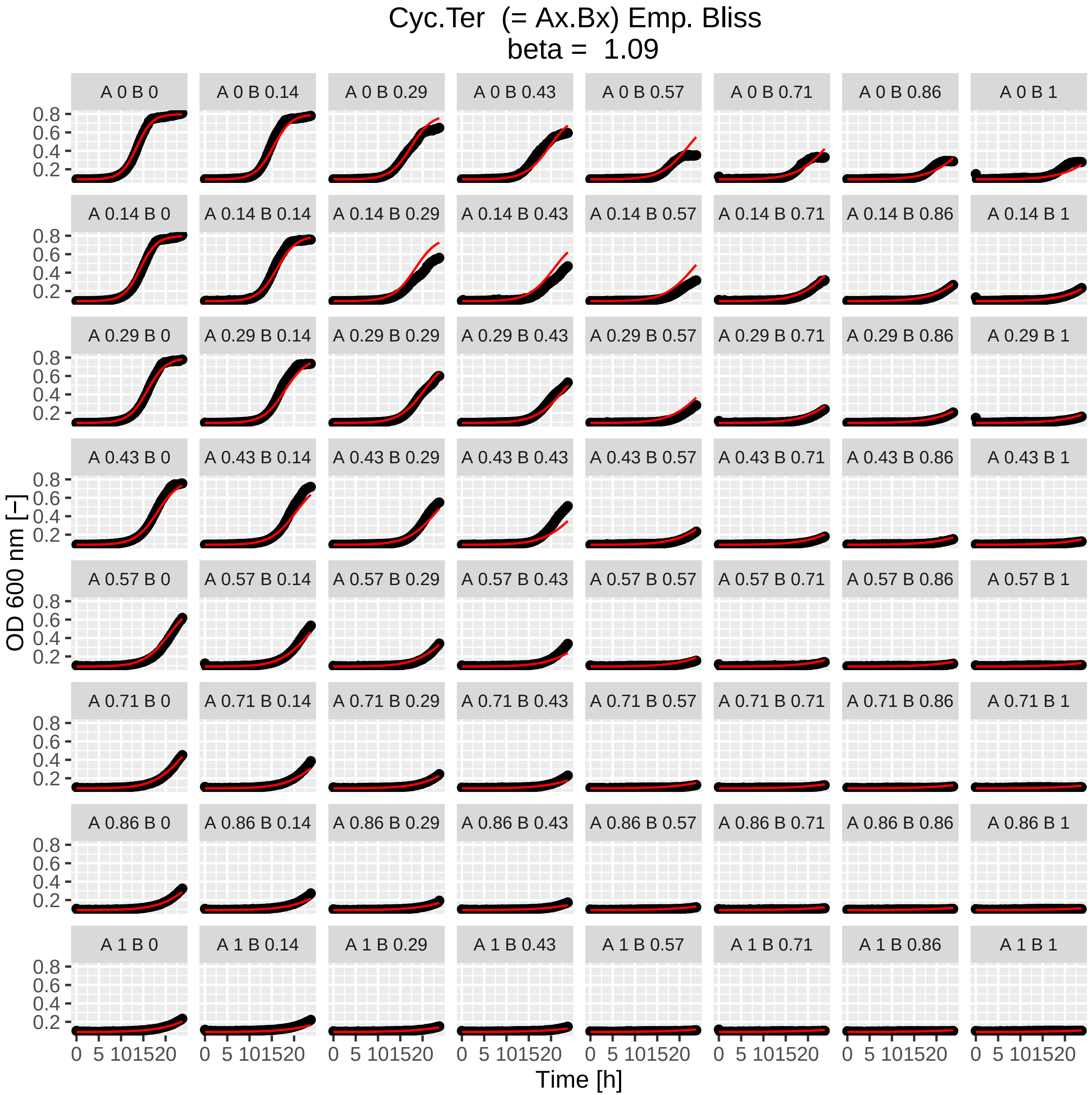
Cyc.Ter (= Ax.Bx) Emp. Bliss
beta = 1.09
A 0 B 0
A 0 B 0.14
A 0 B 0.29
A 0 B 0.43
A 0 B 0.57
A 0 B 0.71
A 0 B 0.86
A 0 B 1
A 0.14 B 0
A 0.14 B 0.14
A 0.14 B 0.29
A 0.14 B 0.43
A 0.14 B 0.57
A 0.14 B 0.71
A 0.14 B 0.86
A 0.14 B 1
A 0.29 B 0
A 0.29 B 0.14
A 0.29 B 0.29
A 0.29 B 0.43
A 0.29 B 0.57
A 0.29 B 0.71
A 0.29 B 0.86
A 0.29 B 1
A 0.43 B 0
A 0.43 B 0.14
A 0.43 B 0.29
A 0.43 B 0.43
A 0.43 B 0.57
A 0.43 B 0.71
A 0.43 B 0.86
A 0.43 B 1
A 0.57 B 0
A 0.57 B 0.14
A 0.57 B 0.29
A 0.57 B 0.43
A 0.57 B 0.57
A 0.57 B 0.71
A 0.57 B 0.86
A 0.57 B 1
A 0.71 B 0
A 0.71 B 0.14
A 0.71 B 0.29
A 0.71 B 0.43
A 0.71 B 0.57
A 0.71 B 0.71
A 0.71 B 0.86
A 0.71 B 1
A 0.86 B 0
A 0.86 B 0.14
A 0.86 B 0.29
A 0.86 B 0.43
A 0.86 B 0.57
A 0.86 B 0.71
A 0.86 B 0.86
A 0.86 B 1
A 1 B 0
A 1 B 0.14
A 1 B 0.29
A 1 B 0.43
A 1 B 0.57
A 1 B 0.71
A 1 B 0.86
A 1 B 1
0.8
0.6
0.4
0.2
OD 600 nm [–]
0 5 101520
Time [h]

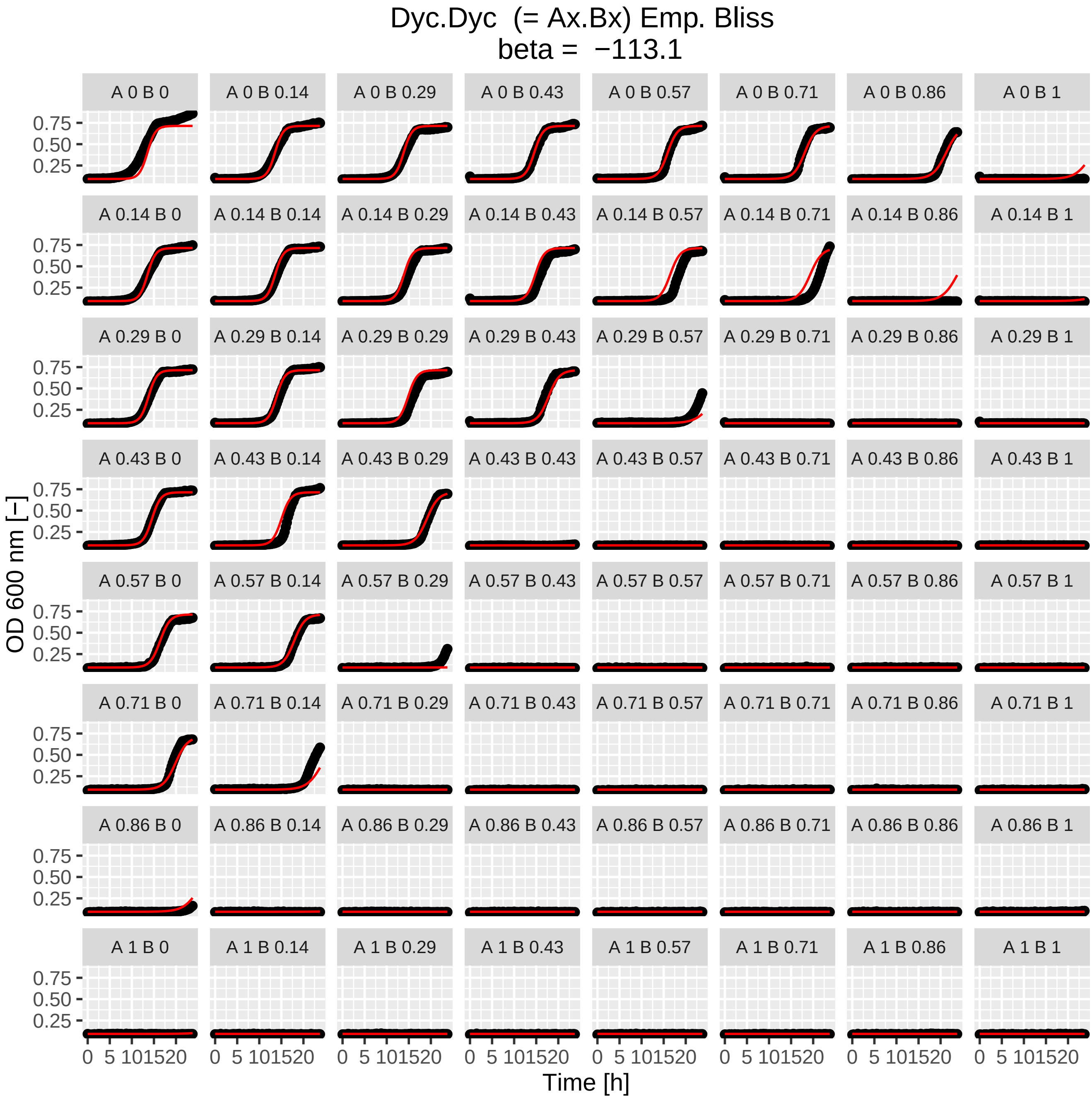

Dyc.Dyc (= Ax.Bx) Emp. Bliss
beta = −113.1
OD 600 nm [−]
Time [h]
0.75
0.50
0.25
0 5 101520
A 0 B 0
A 0 B 0.14
A 0 B 0.29
A 0 B 0.43
A 0 B 0.57
A 0 B 0.71
A 0 B 0.86
A 0 B 1
A 0.14 B 0
A 0.14 B 0.14
A 0.14 B 0.29
A 0.14 B 0.43
A 0.14 B 0.57
A 0.14 B 0.71
A 0.14 B 0.86
A 0.14 B 1
A 0.29 B 0
A 0.29 B 0.14
A 0.29 B 0.29
A 0.29 B 0.43
A 0.29 B 0.57
A 0.29 B 0.71
A 0.29 B 0.86
A 0.29 B 1
A 0.43 B 0
A 0.43 B 0.14
A 0.43 B 0.29
A 0.43 B 0.43
A 0.43 B 0.57
A 0.43 B 0.71
A 0.43 B 0.86
A 0.43 B 1
A 0.57 B 0
A 0.57 B 0.14
A 0.57 B 0.29
A 0.57 B 0.43
A 0.57 B 0.57
A 0.57 B 0.71
A 0.57 B 0.86
A 0.57 B 1
A 0.71 B 0
A 0.71 B 0.14
A 0.71 B 0.29
A 0.71 B 0.43
A 0.71 B 0.57
A 0.71 B 0.71
A 0.71 B 0.86
A 0.71 B 1
A 0.86 B 0
A 0.86 B 0.14
A 0.86 B 0.29
A 0.86 B 0.43
A 0.86 B 0.57
A 0.86 B 0.71
A 0.86 B 0.86
A 0.86 B 1
A 1 B 0
A 1 B 0.14
A 1 B 0.29
A 1 B 0.43
A 1 B 0.57
A 1 B 0.71
A 1 B 0.86
A 1 B 1

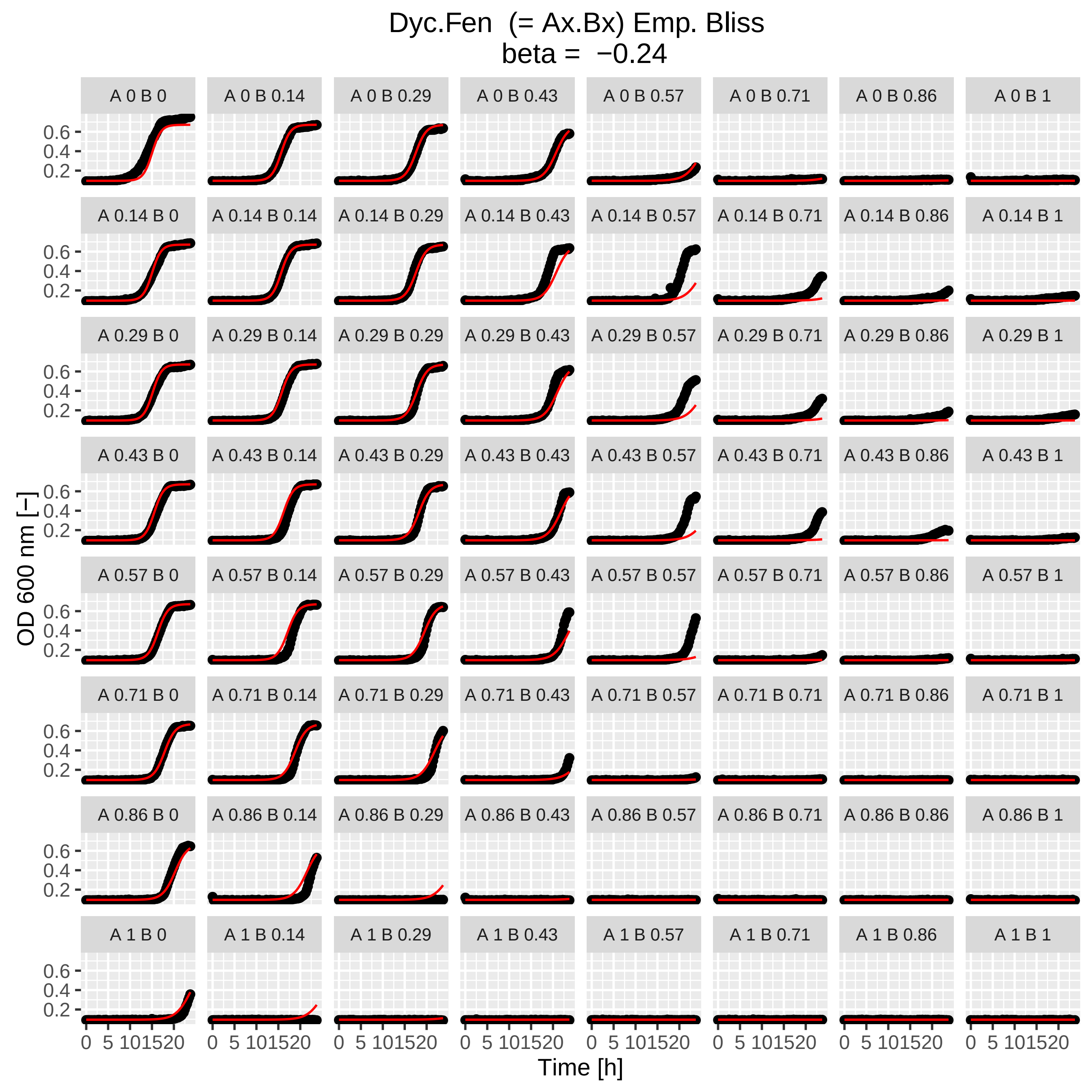
Dyc.Fen (= Ax.Bx) Emp. Bliss
beta = −0.24
OD 600 nm [−]
Time [h]
A 0 B 0
A 0 B 0.14
A 0 B 0.29
A 0 B 0.43
A 0 B 0.57
A 0 B 0.71
A 0 B 0.86
A 0 B 1
A 0.14 B 0
A 0.14 B 0.14
A 0.14 B 0.29
A 0.14 B 0.43
A 0.14 B 0.57
A 0.14 B 0.71
A 0.14 B 0.86
A 0.14 B 1
A 0.29 B 0
A 0.29 B 0.14
A 0.29 B 0.29
A 0.29 B 0.43
A 0.29 B 0.57
A 0.29 B 0.71
A 0.29 B 0.86
A 0.29 B 1
A 0.43 B 0
A 0.43 B 0.14
A 0.43 B 0.29
A 0.43 B 0.43
A 0.43 B 0.57
A 0.43 B 0.71
A 0.43 B 0.86
A 0.43 B 1
A 0.57 B 0
A 0.57 B 0.14
A 0.57 B 0.29
A 0.57 B 0.43
A 0.57 B 0.57
A 0.57 B 0.71
A 0.57 B 0.86
A 0.57 B 1
A 0.71 B 0
A 0.71 B 0.14
A 0.71 B 0.29
A 0.71 B 0.43
A 0.71 B 0.57
A 0.71 B 0.71
A 0.71 B 0.86
A 0.71 B 1
A 0.86 B 0
A 0.86 B 0.14
A 0.86 B 0.29
A 0.86 B 0.43
A 0.86 B 0.57
A 0.86 B 0.71
A 0.86 B 0.86
A 0.86 B 1
A 1 B 0
A 1 B 0.14
A 1 B 0.29
A 1 B 0.43
A 1 B 0.57
A 1 B 0.71
A 1 B 0.86
A 1 B 1
0.6
0.4
0.2
0 5 101520

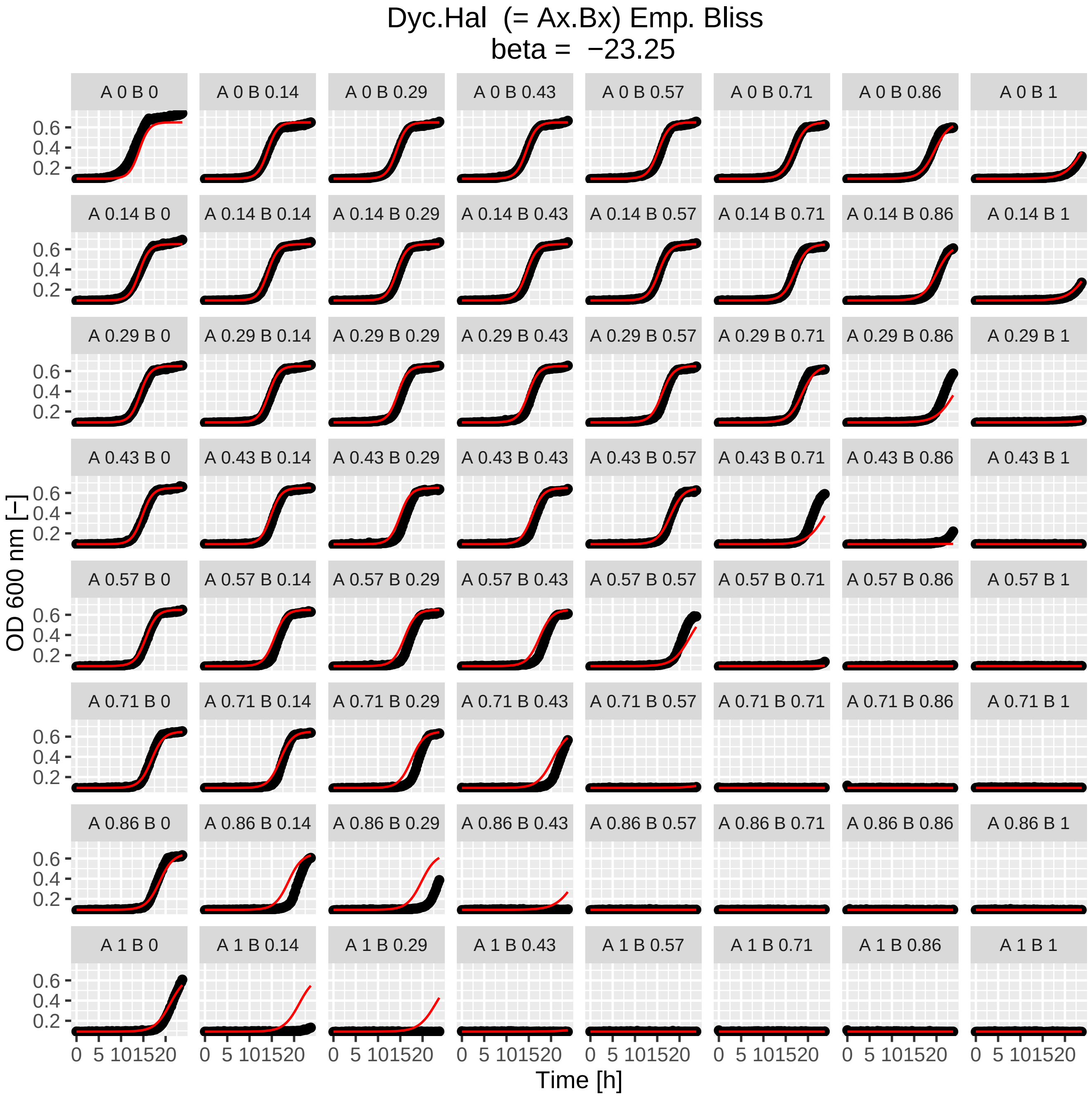
Dyc.Hal (= Ax.Bx) Emp. Bliss
beta = −23.25
A 0 B 0
A 0 B 0.14
A 0 B 0.29
A 0 B 0.43
A 0 B 0.57
A 0 B 0.71
A 0 B 0.86
A 0 B 1
A 0.14 B 0
A 0.14 B 0.14
A 0.14 B 0.29
A 0.14 B 0.43
A 0.14 B 0.57
A 0.14 B 0.71
A 0.14 B 0.86
A 0.14 B 1
A 0.29 B 0
A 0.29 B 0.14
A 0.29 B 0.29
A 0.29 B 0.43
A 0.29 B 0.57
A 0.29 B 0.71
A 0.29 B 0.86
A 0.29 B 1
A 0.43 B 0
A 0.43 B 0.14
A 0.43 B 0.29
A 0.43 B 0.43
A 0.43 B 0.57
A 0.43 B 0.71
A 0.43 B 0.86
A 0.43 B 1
A 0.57 B 0
A 0.57 B 0.14
A 0.57 B 0.29
A 0.57 B 0.43
A 0.57 B 0.57
A 0.57 B 0.71
A 0.57 B 0.86
A 0.57 B 1
A 0.71 B 0
A 0.71 B 0.14
A 0.71 B 0.29
A 0.71 B 0.43
A 0.71 B 0.57
A 0.71 B 0.71
A 0.71 B 0.86
A 0.71 B 1
A 0.86 B 0
A 0.86 B 0.14
A 0.86 B 0.29
A 0.86 B 0.43
A 0.86 B 0.57
A 0.86 B 0.71
A 0.86 B 0.86
A 0.86 B 1
A 1 B 0
A 1 B 0.14
A 1 B 0.29
A 1 B 0.43
A 1 B 0.57
A 1 B 0.71
A 1 B 0.86
A 1 B 1
0.6
0.4
0.2
0 5 101520
OD 600 nm [−]
Time [h]

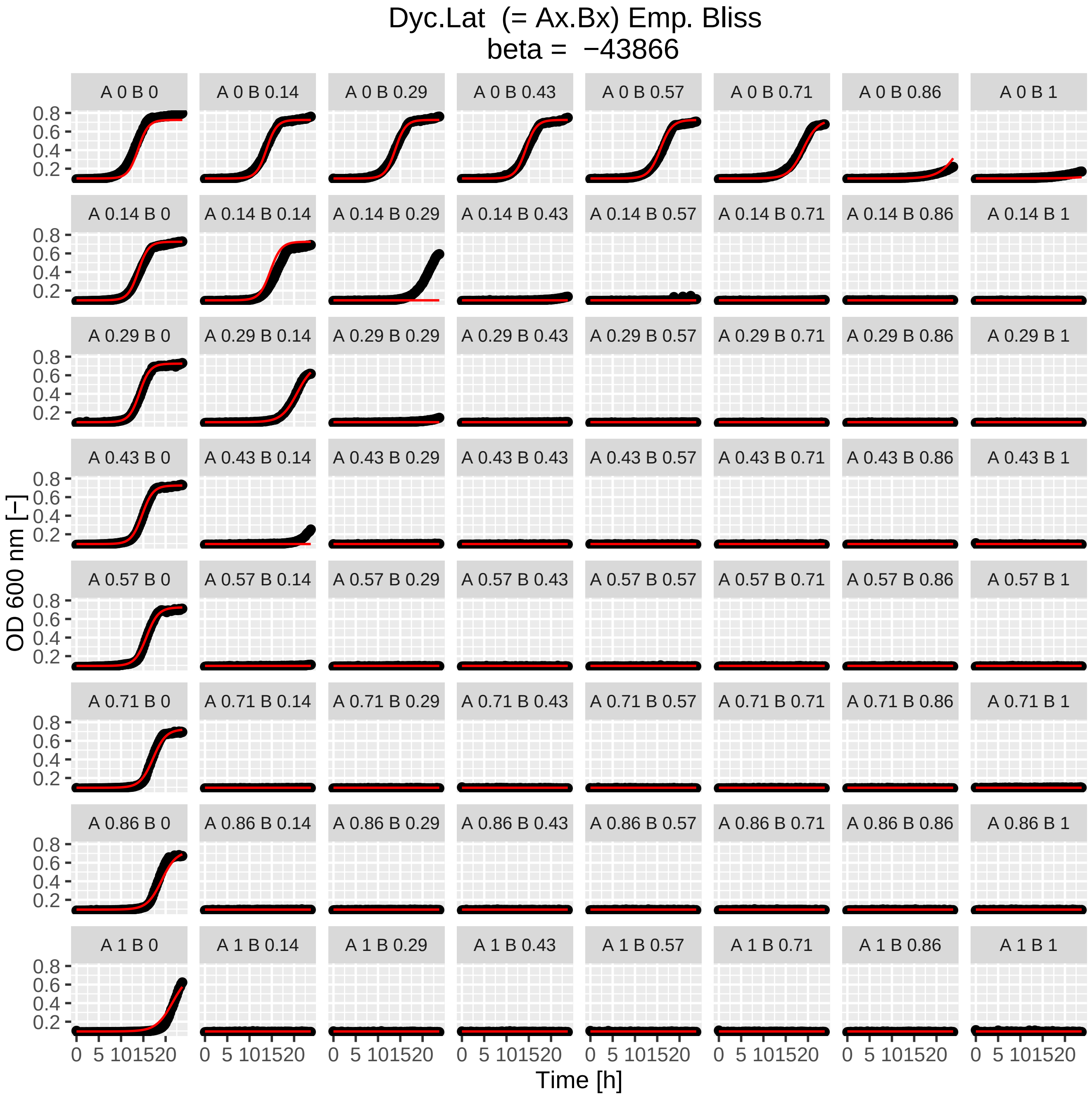

Dyc.Lat (= Ax.Bx) Emp. Bliss
beta = −43866
A 0 B 0
A 0 B 0.14
A 0 B 0.29
A 0 B 0.43
A 0 B 0.57
A 0 B 0.71
A 0 B 0.86
A 0 B 1
A 0.14 B 0
A 0.14 B 0.14
A 0.14 B 0.29
A 0.14 B 0.43
A 0.14 B 0.57
A 0.14 B 0.71
A 0.14 B 0.86
A 0.14 B 1
A 0.29 B 0
A 0.29 B 0.14
A 0.29 B 0.29
A 0.29 B 0.43
A 0.29 B 0.57
A 0.29 B 0.71
A 0.29 B 0.86
A 0.29 B 1
A 0.43 B 0
A 0.43 B 0.14
A 0.43 B 0.29
A 0.43 B 0.43
A 0.43 B 0.57
A 0.43 B 0.71
A 0.43 B 0.86
A 0.43 B 1
A 0.57 B 0
A 0.57 B 0.14
A 0.57 B 0.29
A 0.57 B 0.43
A 0.57 B 0.57
A 0.57 B 0.71
A 0.57 B 0.86
A 0.57 B 1
A 0.71 B 0
A 0.71 B 0.14
A 0.71 B 0.29
A 0.71 B 0.43
A 0.71 B 0.57
A 0.71 B 0.71
A 0.71 B 0.86
A 0.71 B 1
A 0.86 B 0
A 0.86 B 0.14
A 0.86 B 0.29
A 0.86 B 0.43
A 0.86 B 0.57
A 0.86 B 0.71
A 0.86 B 0.86
A 0.86 B 1
A 1 B 0
A 1 B 0.14
A 1 B 0.29
A 1 B 0.43
A 1 B 0.57
A 1 B 0.71
A 1 B 0.86
A 1 B 1
0.8
0.6
0.4
0.2
OD 600 nm [−]
0 5 101520
Time [h]

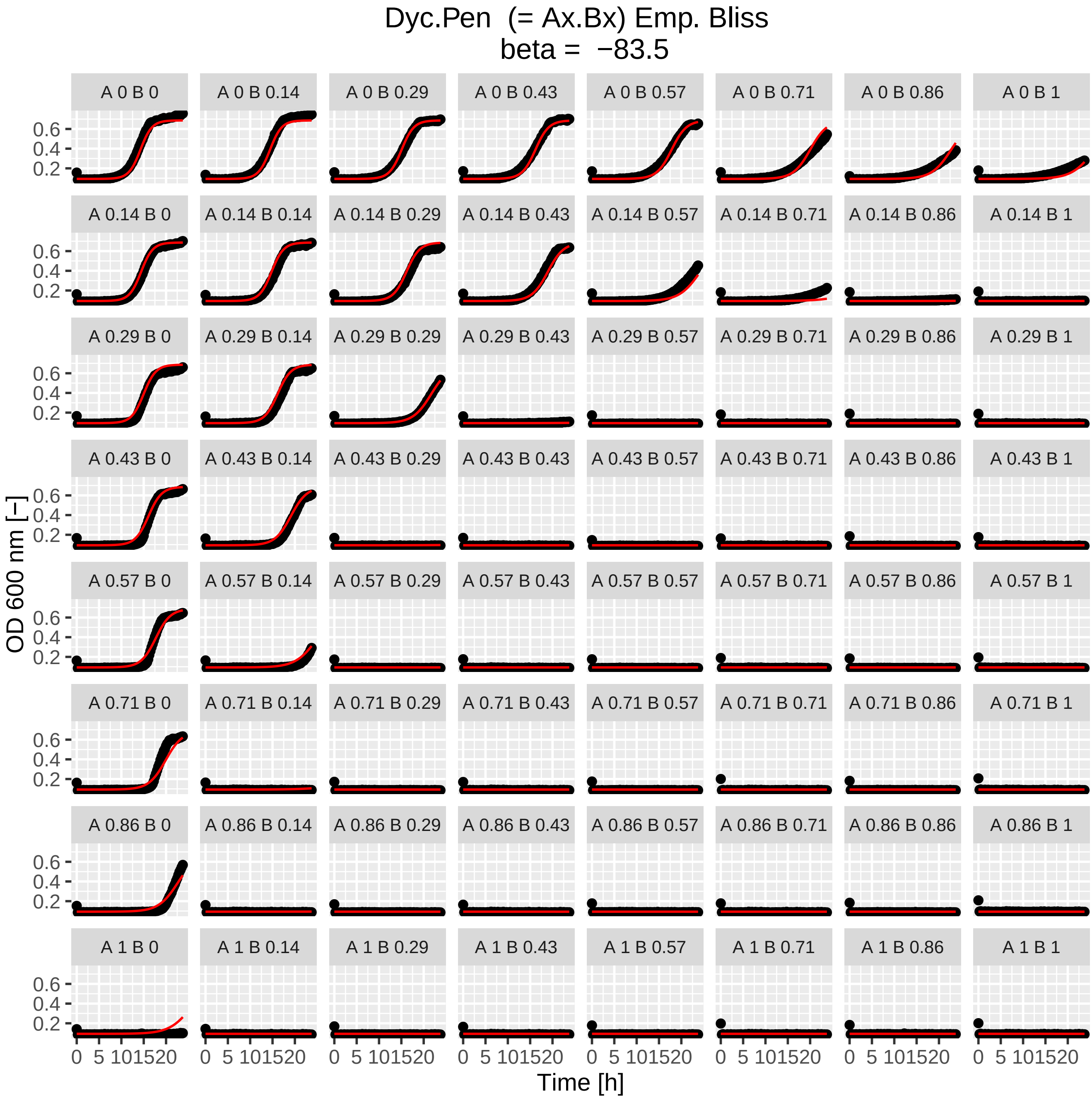

Dyc.Pen (= Ax.Bx) Emp. Bliss
beta = −83.5
A 0 B 0
A 0 B 0.14
A 0 B 0.29
A 0 B 0.43
A 0 B 0.57
A 0 B 0.71
A 0 B 0.86
A 0 B 1
A 0.14 B 0
A 0.14 B 0.14
A 0.14 B 0.29
A 0.14 B 0.43
A 0.14 B 0.57
A 0.14 B 0.71
A 0.14 B 0.86
A 0.14 B 1
A 0.29 B 0
A 0.29 B 0.14
A 0.29 B 0.29
A 0.29 B 0.43
A 0.29 B 0.57
A 0.29 B 0.71
A 0.29 B 0.86
A 0.29 B 1
A 0.43 B 0
A 0.43 B 0.14
A 0.43 B 0.29
A 0.43 B 0.43
A 0.43 B 0.57
A 0.43 B 0.71
A 0.43 B 0.86
A 0.43 B 1
A 0.57 B 0
A 0.57 B 0.14
A 0.57 B 0.29
A 0.57 B 0.43
A 0.57 B 0.57
A 0.57 B 0.71
A 0.57 B 0.86
A 0.57 B 1
A 0.71 B 0
A 0.71 B 0.14
A 0.71 B 0.29
A 0.71 B 0.43
A 0.71 B 0.57
A 0.71 B 0.71
A 0.71 B 0.86
A 0.71 B 1
A 0.86 B 0
A 0.86 B 0.14
A 0.86 B 0.29
A 0.86 B 0.43
A 0.86 B 0.57
A 0.86 B 0.71
A 0.86 B 0.86
A 0.86 B 1
A 1 B 0
A 1 B 0.14
A 1 B 0.29
A 1 B 0.43
A 1 B 0.57
A 1 B 0.71
A 1 B 0.86
A 1 B 1
OD 600 nm [−]
0.2
0.4
0.6
0 5 101520
Time [h]

Dyc.Rap (= Ax.Bx) Emp. Bliss
beta = −9152.4

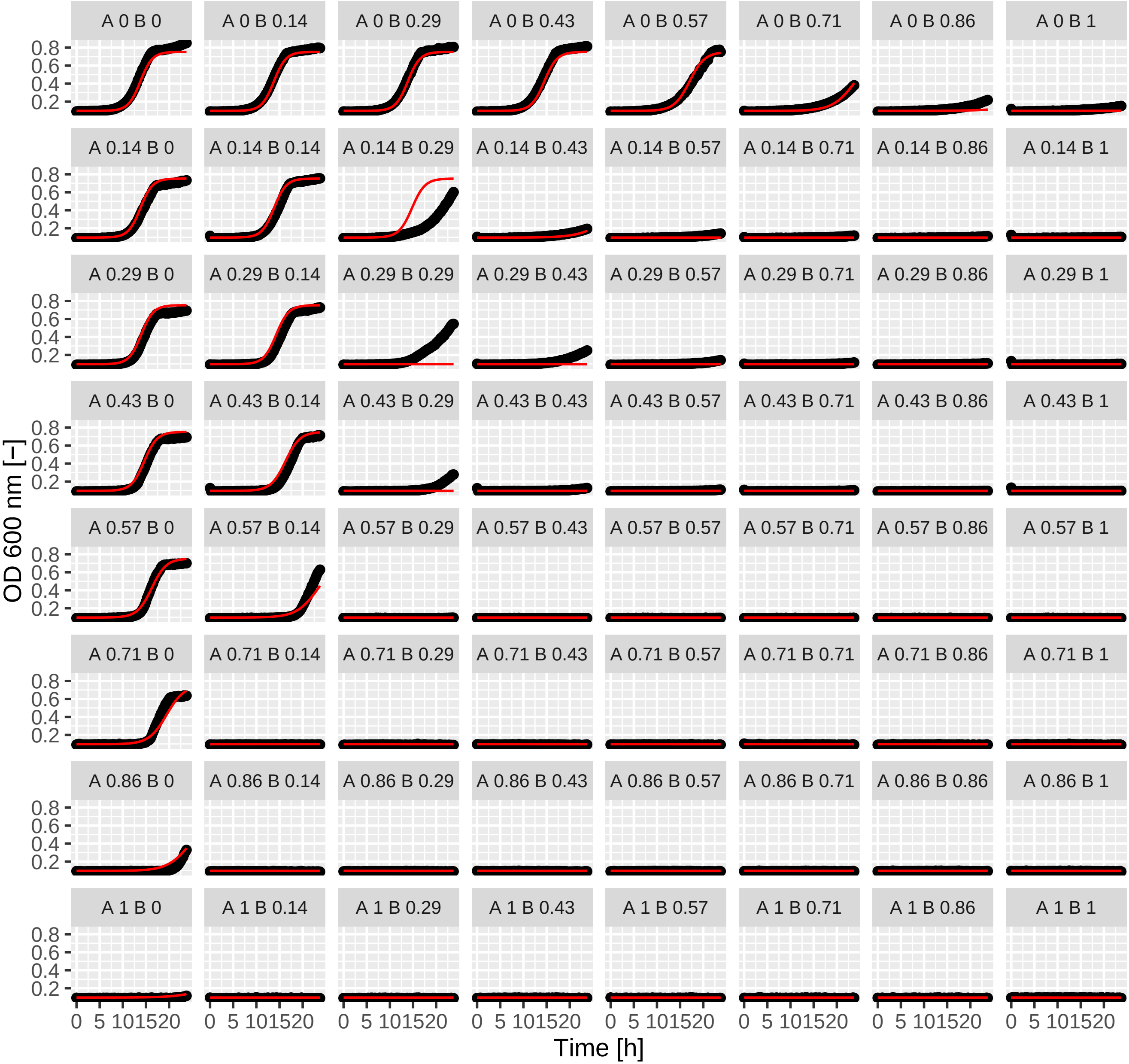

Dyc.Sta (= Ax.Bx) Emp. Bliss
beta = −894.6

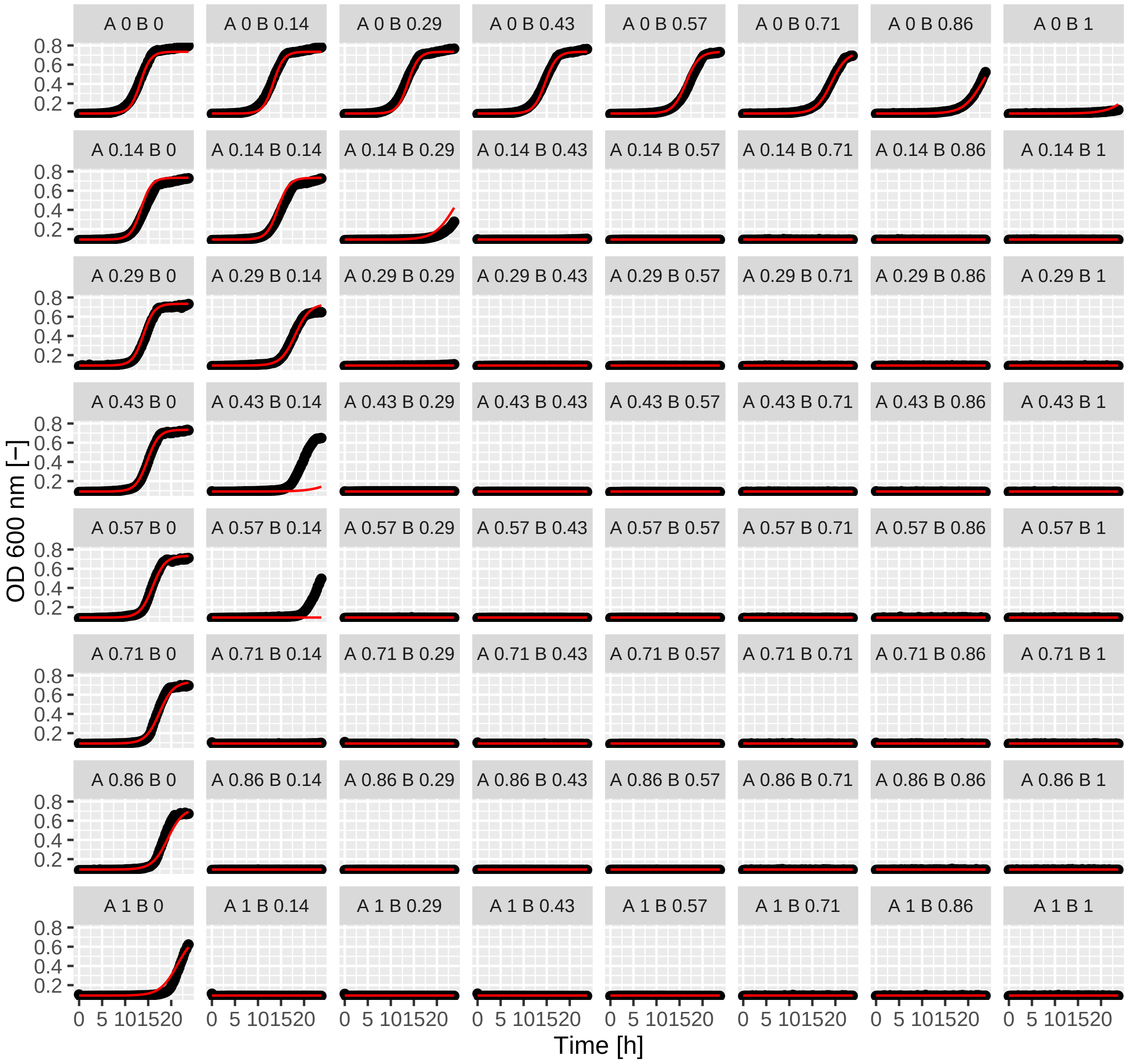

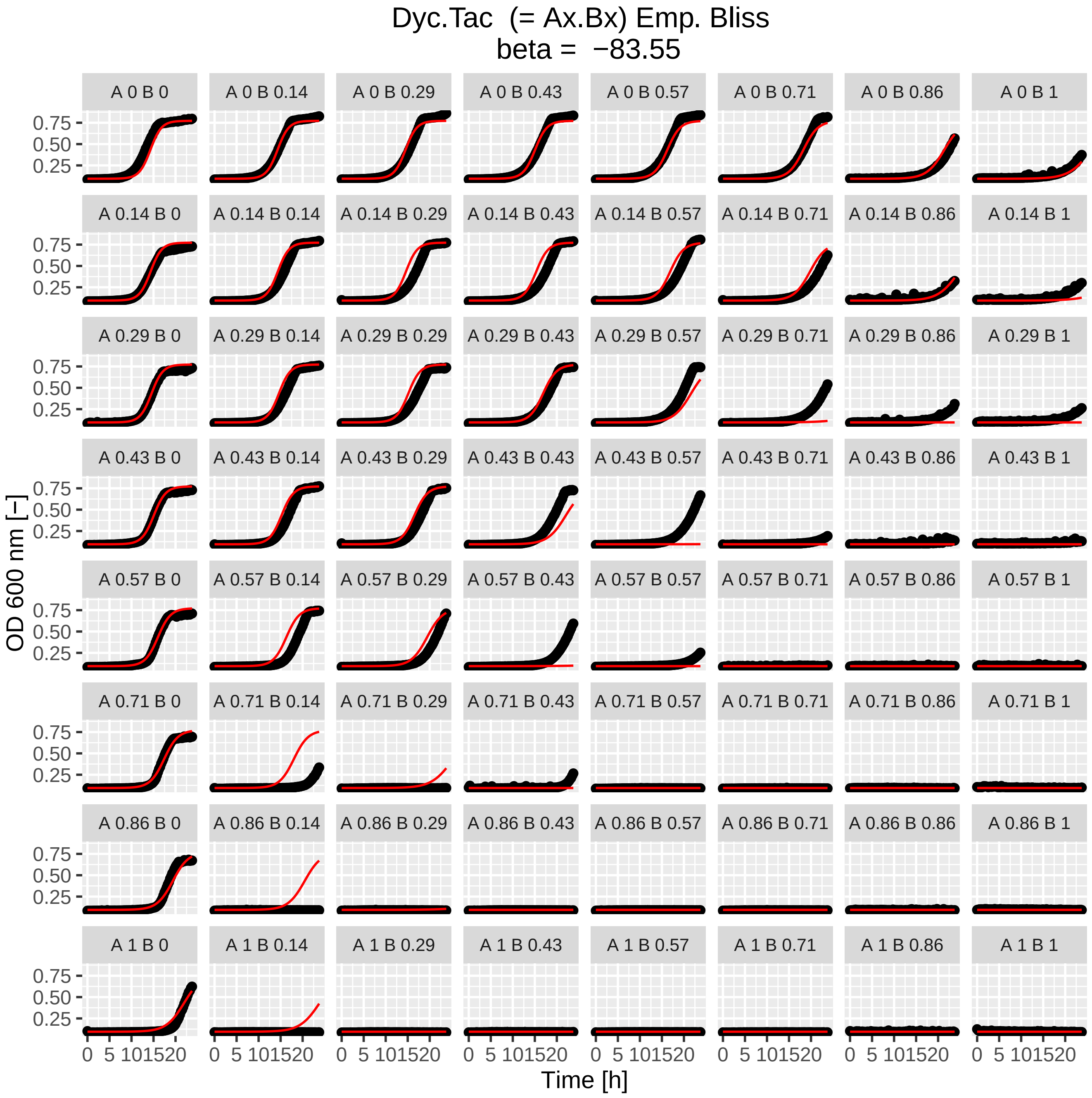
Dyc.Tac (= Ax.Bx) Emp. Bliss
beta = −83.55
OD 600 nm [−]
Time [h]
A 0 B 0
A 0 B 0.14
A 0 B 0.29
A 0 B 0.43
A 0 B 0.57
A 0 B 0.71
A 0 B 0.86
A 0 B 1
A 0.14 B 0
A 0.14 B 0.14
A 0.14 B 0.29
A 0.14 B 0.43
A 0.14 B 0.57
A 0.14 B 0.71
A 0.14 B 0.86
A 0.14 B 1
A 0.29 B 0
A 0.29 B 0.14
A 0.29 B 0.29
A 0.29 B 0.43
A 0.29 B 0.57
A 0.29 B 0.71
A 0.29 B 0.86
A 0.29 B 1
A 0.43 B 0
A 0.43 B 0.14
A 0.43 B 0.29
A 0.43 B 0.43
A 0.43 B 0.57
A 0.43 B 0.71
A 0.43 B 0.86
A 0.43 B 1
A 0.57 B 0
A 0.57 B 0.14
A 0.57 B 0.29
A 0.57 B 0.43
A 0.57 B 0.57
A 0.57 B 0.71
A 0.57 B 0.86
A 0.57 B 1
A 0.71 B 0
A 0.71 B 0.14
A 0.71 B 0.29
A 0.71 B 0.43
A 0.71 B 0.57
A 0.71 B 0.71
A 0.71 B 0.86
A 0.71 B 1
A 0.86 B 0
A 0.86 B 0.14
A 0.86 B 0.29
A 0.86 B 0.43
A 0.86 B 0.57
A 0.86 B 0.71
A 0.86 B 0.86
A 0.86 B 1
A 1 B 0
A 1 B 0.14
A 1 B 0.29
A 1 B 0.43
A 1 B 0.57
A 1 B 0.71
A 1 B 0.86
A 1 B 1
0.75
0.50
0.25
0 5 101520

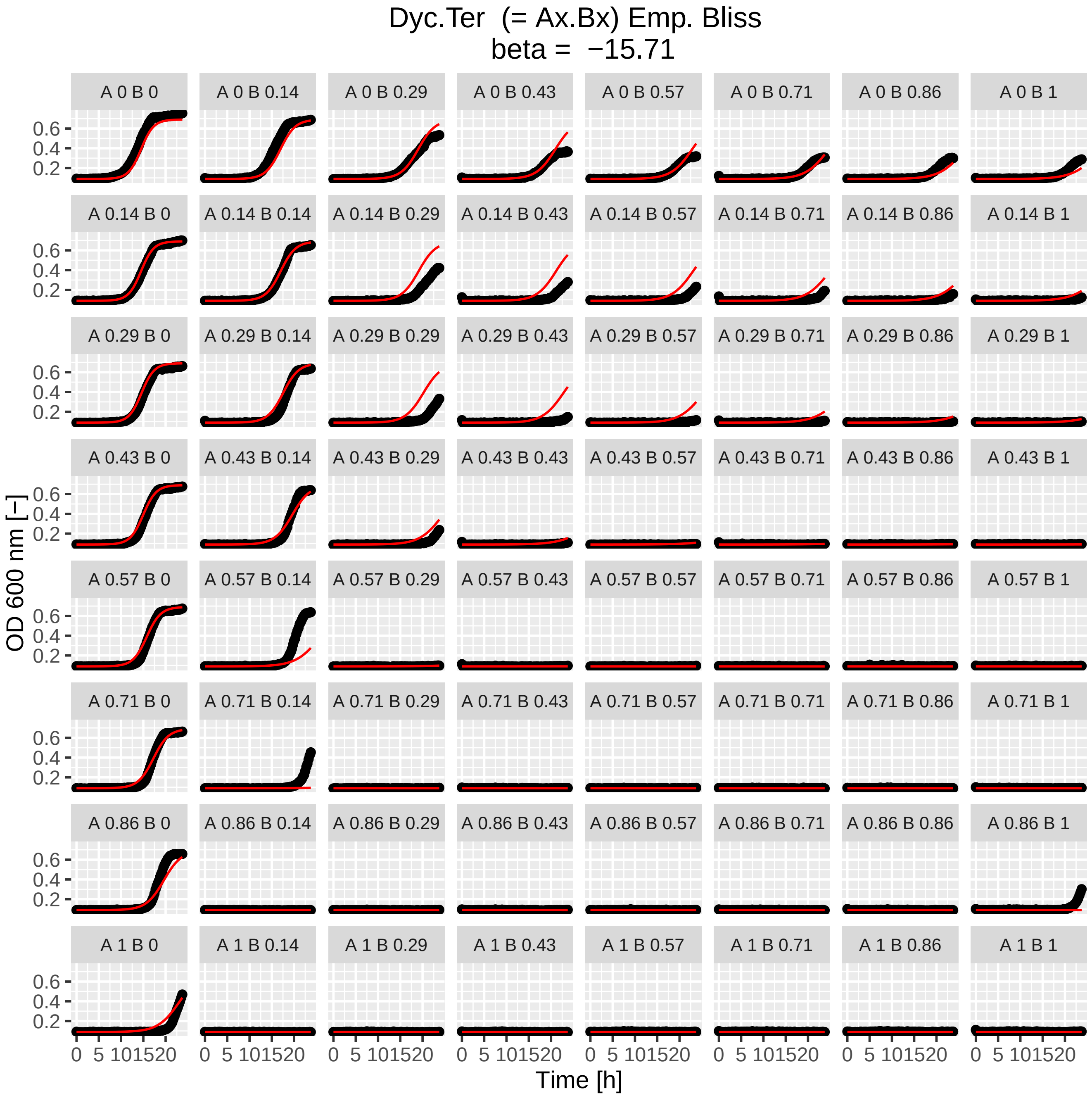

Dyc.Ter (= Ax.Bx) Emp. Bliss
beta = −15.71
A 0 B 0
A 0 B 0.14
A 0 B 0.29
A 0 B 0.43
A 0 B 0.57
A 0 B 0.71
A 0 B 0.86
A 0 B 1
A 0.14 B 0
A 0.14 B 0.14
A 0.14 B 0.29
A 0.14 B 0.43
A 0.14 B 0.57
A 0.14 B 0.71
A 0.14 B 0.86
A 0.14 B 1
A 0.29 B 0
A 0.29 B 0.14
A 0.29 B 0.29
A 0.29 B 0.43
A 0.29 B 0.57
A 0.29 B 0.71
A 0.29 B 0.86
A 0.29 B 1
A 0.43 B 0
A 0.43 B 0.14
A 0.43 B 0.29
A 0.43 B 0.43
A 0.43 B 0.57
A 0.43 B 0.71
A 0.43 B 0.86
A 0.43 B 1
A 0.57 B 0
A 0.57 B 0.14
A 0.57 B 0.29
A 0.57 B 0.43
A 0.57 B 0.57
A 0.57 B 0.71
A 0.57 B 0.86
A 0.57 B 1
A 0.71 B 0
A 0.71 B 0.14
A 0.71 B 0.29
A 0.71 B 0.43
A 0.71 B 0.57
A 0.71 B 0.71
A 0.71 B 0.86
A 0.71 B 1
A 0.86 B 0
A 0.86 B 0.14
A 0.86 B 0.29
A 0.86 B 0.43
A 0.86 B 0.57
A 0.86 B 0.71
A 0.86 B 0.86
A 0.86 B 1
A 1 B 0
A 1 B 0.14
A 1 B 0.29
A 1 B 0.43
A 1 B 0.57
A 1 B 0.71
A 1 B 0.86
A 1 B 1
OD 600 nm [−]
0.2
0.4
0.6
0 5 101520
Time [h]

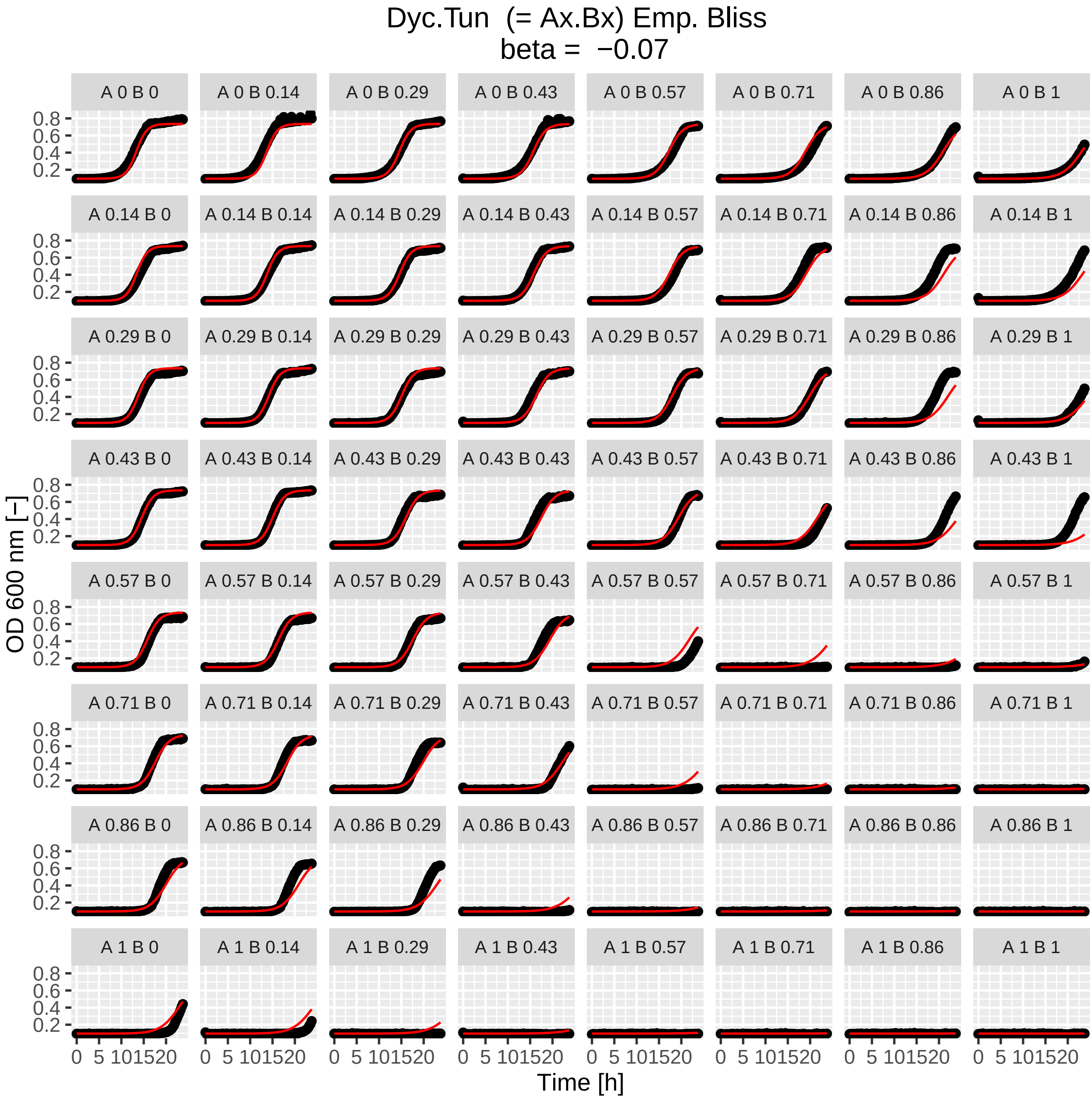

Dyc.Tun (= Ax.Bx) Emp. Bliss
beta = −0.07
A 0 B 0
A 0 B 0.14
A 0 B 0.29
A 0 B 0.43
A 0 B 0.57
A 0 B 0.71
A 0 B 0.86
A 0 B 1
A 0.14 B 0
A 0.14 B 0.14
A 0.14 B 0.29
A 0.14 B 0.43
A 0.14 B 0.57
A 0.14 B 0.71
A 0.14 B 0.86
A 0.14 B 1
A 0.29 B 0
A 0.29 B 0.14
A 0.29 B 0.29
A 0.29 B 0.43
A 0.29 B 0.57
A 0.29 B 0.71
A 0.29 B 0.86
A 0.29 B 1
A 0.43 B 0
A 0.43 B 0.14
A 0.43 B 0.29
A 0.43 B 0.43
A 0.43 B 0.57
A 0.43 B 0.71
A 0.43 B 0.86
A 0.43 B 1
A 0.57 B 0
A 0.57 B 0.14
A 0.57 B 0.29
A 0.57 B 0.43
A 0.57 B 0.57
A 0.57 B 0.71
A 0.57 B 0.86
A 0.57 B 1
A 0.71 B 0
A 0.71 B 0.14
A 0.71 B 0.29
A 0.71 B 0.43
A 0.71 B 0.57
A 0.71 B 0.71
A 0.71 B 0.86
A 0.71 B 1
A 0.86 B 0
A 0.86 B 0.14
A 0.86 B 0.29
A 0.86 B 0.43
A 0.86 B 0.57
A 0.86 B 0.71
A 0.86 B 0.86
A 0.86 B 1
A 1 B 0
A 1 B 0.14
A 1 B 0.29
A 1 B 0.43
A 1 B 0.57
A 1 B 0.71
A 1 B 0.86
A 1 B 1
OD 600 nm [−]
0.2
0.4
0.6
0.8
0
5
10
15
20
Time [h]

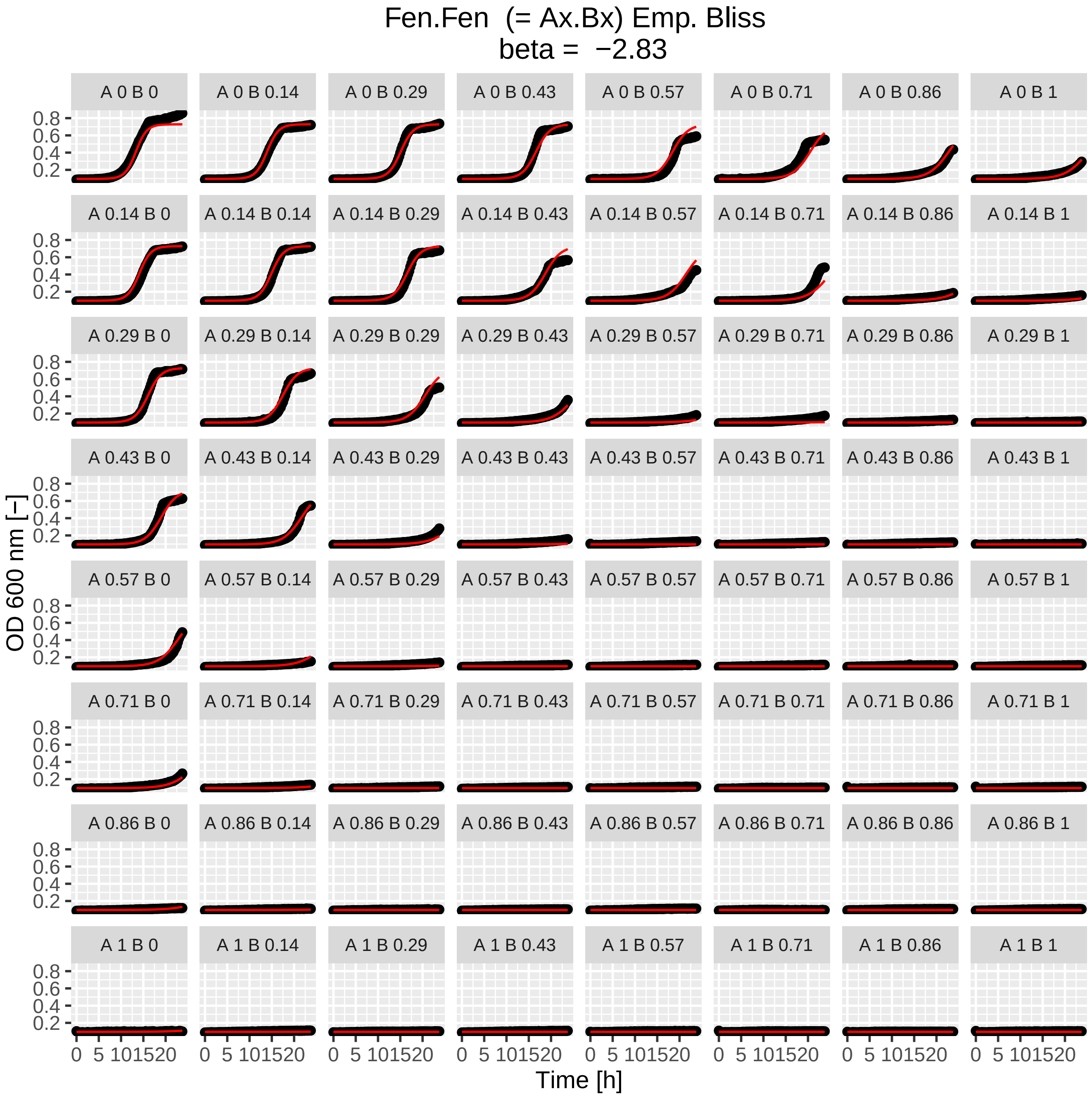

Fen.Fen (= Ax.Bx) Emp. Bliss
beta = −2.83
OD 600 nm [−]
Time [h]
A 0 B 0
A 0 B 0.14
A 0 B 0.29
A 0 B 0.43
A 0 B 0.57
A 0 B 0.71
A 0 B 0.86
A 0 B 1
A 0.14 B 0
A 0.14 B 0.14
A 0.14 B 0.29
A 0.14 B 0.43
A 0.14 B 0.57
A 0.14 B 0.71
A 0.14 B 0.86
A 0.14 B 1
A 0.29 B 0
A 0.29 B 0.14
A 0.29 B 0.29
A 0.29 B 0.43
A 0.29 B 0.57
A 0.29 B 0.71
A 0.29 B 0.86
A 0.29 B 1
A 0.43 B 0
A 0.43 B 0.14
A 0.43 B 0.29
A 0.43 B 0.43
A 0.43 B 0.57
A 0.43 B 0.71
A 0.43 B 0.86
A 0.43 B 1
A 0.57 B 0
A 0.57 B 0.14
A 0.57 B 0.29
A 0.57 B 0.43
A 0.57 B 0.57
A 0.57 B 0.71
A 0.57 B 0.86
A 0.57 B 1
A 0.71 B 0
A 0.71 B 0.14
A 0.71 B 0.29
A 0.71 B 0.43
A 0.71 B 0.57
A 0.71 B 0.71
A 0.71 B 0.86
A 0.71 B 1
A 0.86 B 0
A 0.86 B 0.14
A 0.86 B 0.29
A 0.86 B 0.43
A 0.86 B 0.57
A 0.86 B 0.71
A 0.86 B 0.86
A 0.86 B 1
A 1 B 0
A 1 B 0.14
A 1 B 0.29
A 1 B 0.43
A 1 B 0.57
A 1 B 0.71
A 1 B 0.86
A 1 B 1
0.8
0.6
0.4
0.2
0 5 101520

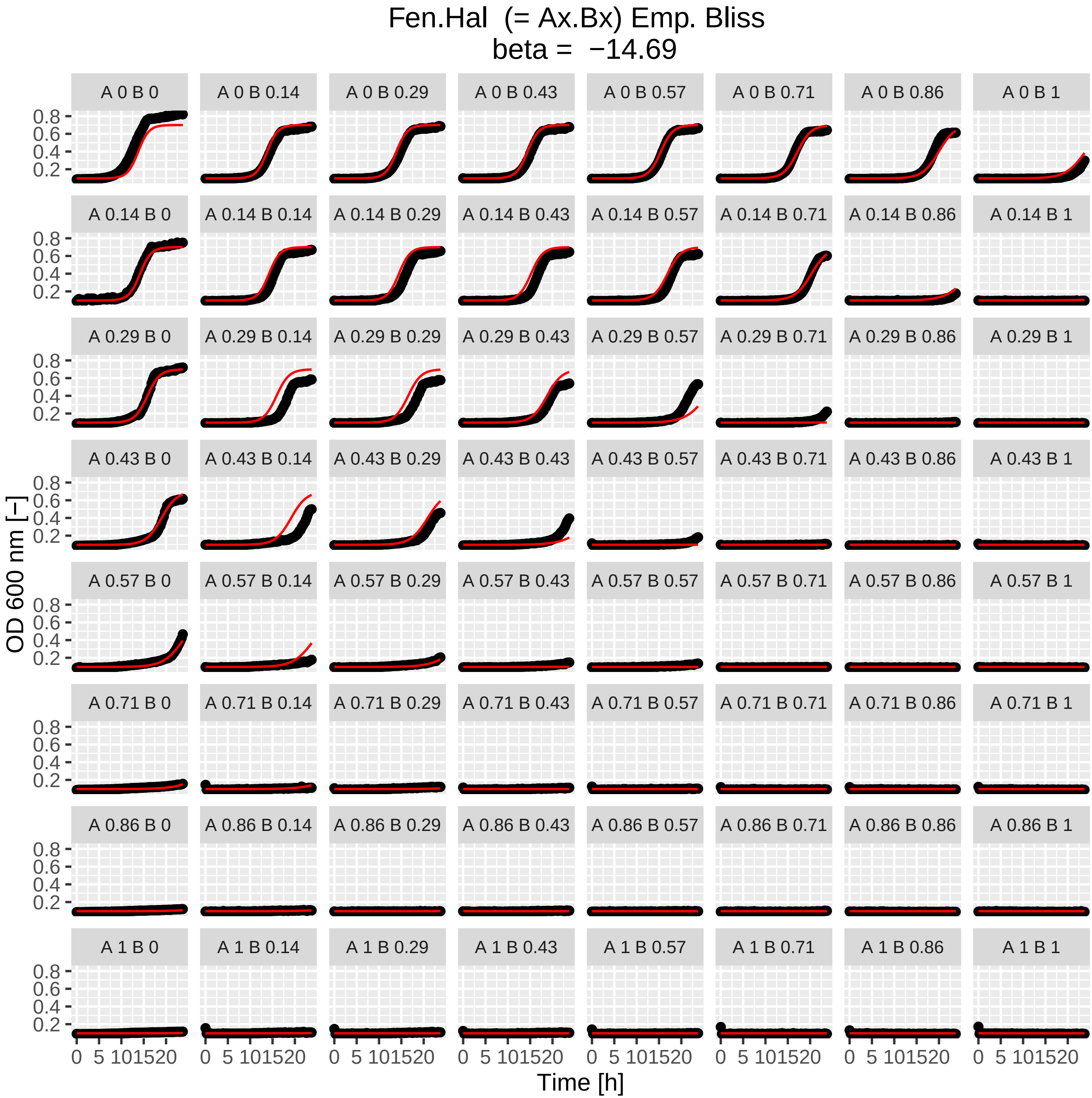

Fen.Hal (= Ax.Bx) Emp. Bliss
beta = −14.69
OD 600 nm [−]
Time [h]
A 0 B 0
A 0 B 0.14
A 0 B 0.29
A 0 B 0.43
A 0 B 0.57
A 0 B 0.71
A 0 B 0.86
A 0 B 1
A 0.14 B 0
A 0.29 B 0
A 0.43 B 0
A 0.57 B 0
A 0.71 B 0
A 0.86 B 0
A 1 B 0
A 1 B 1

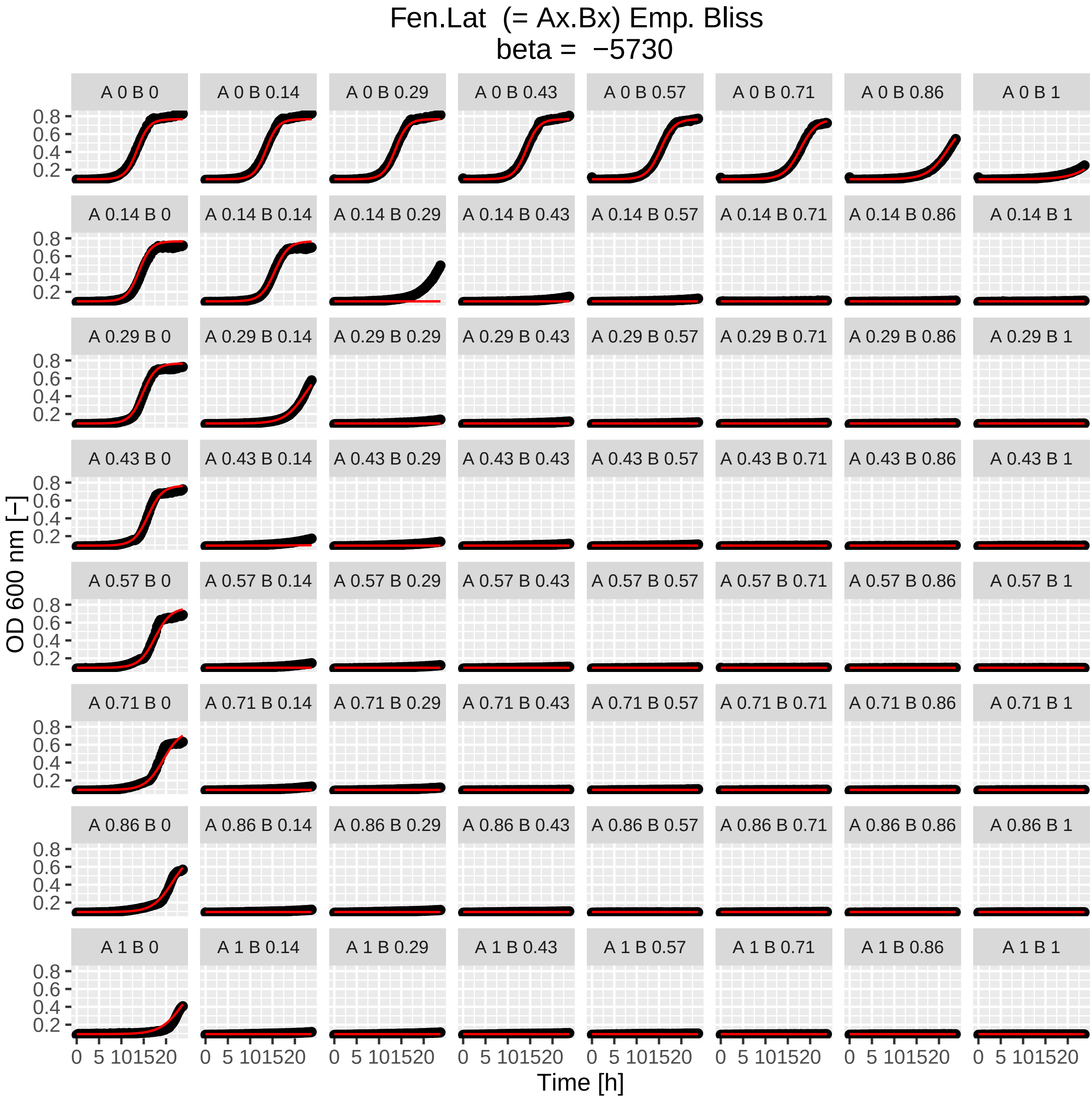

Fen.Lat (= Ax.Bx) Emp. Bliss
beta = −5730
A 0 B 0
A 0 B 0.14
A 0 B 0.29
A 0 B 0.43
A 0 B 0.57
A 0 B 0.71
A 0 B 0.86
A 0 B 1
A 0.14 B 0
A 0.14 B 0.14
A 0.14 B 0.29
A 0.14 B 0.43
A 0.14 B 0.57
A 0.14 B 0.71
A 0.14 B 0.86
A 0.14 B 1
A 0.29 B 0
A 0.29 B 0.14
A 0.29 B 0.29
A 0.29 B 0.43
A 0.29 B 0.57
A 0.29 B 0.71
A 0.29 B 0.86
A 0.29 B 1
A 0.43 B 0
A 0.43 B 0.14
A 0.43 B 0.29
A 0.43 B 0.43
A 0.43 B 0.57
A 0.43 B 0.71
A 0.43 B 0.86
A 0.43 B 1
A 0.57 B 0
A 0.57 B 0.14
A 0.57 B 0.29
A 0.57 B 0.43
A 0.57 B 0.57
A 0.57 B 0.71
A 0.57 B 0.86
A 0.57 B 1
A 0.71 B 0
A 0.71 B 0.14
A 0.71 B 0.29
A 0.71 B 0.43
A 0.71 B 0.57
A 0.71 B 0.71
A 0.71 B 0.86
A 0.71 B 1
A 0.86 B 0
A 0.86 B 0.14
A 0.86 B 0.29
A 0.86 B 0.43
A 0.86 B 0.57
A 0.86 B 0.71
A 0.86 B 0.86
A 0.86 B 1
A 1 B 0
A 1 B 0.14
A 1 B 0.29
A 1 B 0.43
A 1 B 0.57
A 1 B 0.71
A 1 B 0.86
A 1 B 1
OD 600 nm [−]
0.2
0.4
0.6
0.8
0 5 101520
Time [h]

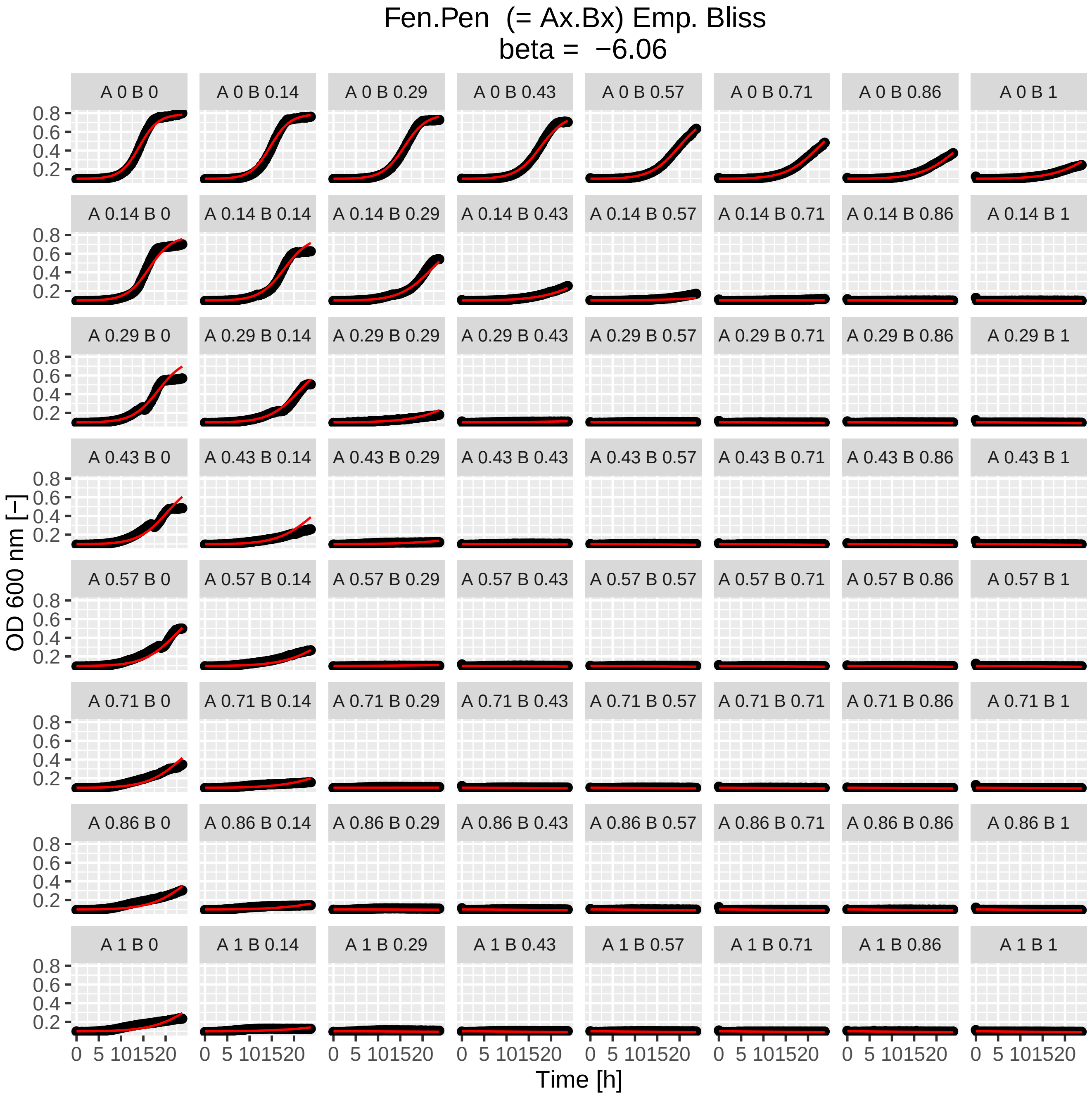
Fen.Pen (= Ax.Bx) Emp. Bliss
beta = −6.06
OD 600 nm [−]
Time [h]
A 0 B 0
A 0 B 0.14
A 0 B 0.29
A 0 B 0.43
A 0 B 0.57
A 0 B 0.71
A 0 B 0.86
A 0 B 1
A 1 B 0
A 1 B 0.14
A 1 B 0.29
A 1 B 0.43
A 1 B 0.57
A 1 B 0.71
A 1 B 0.86
A 1 B 1

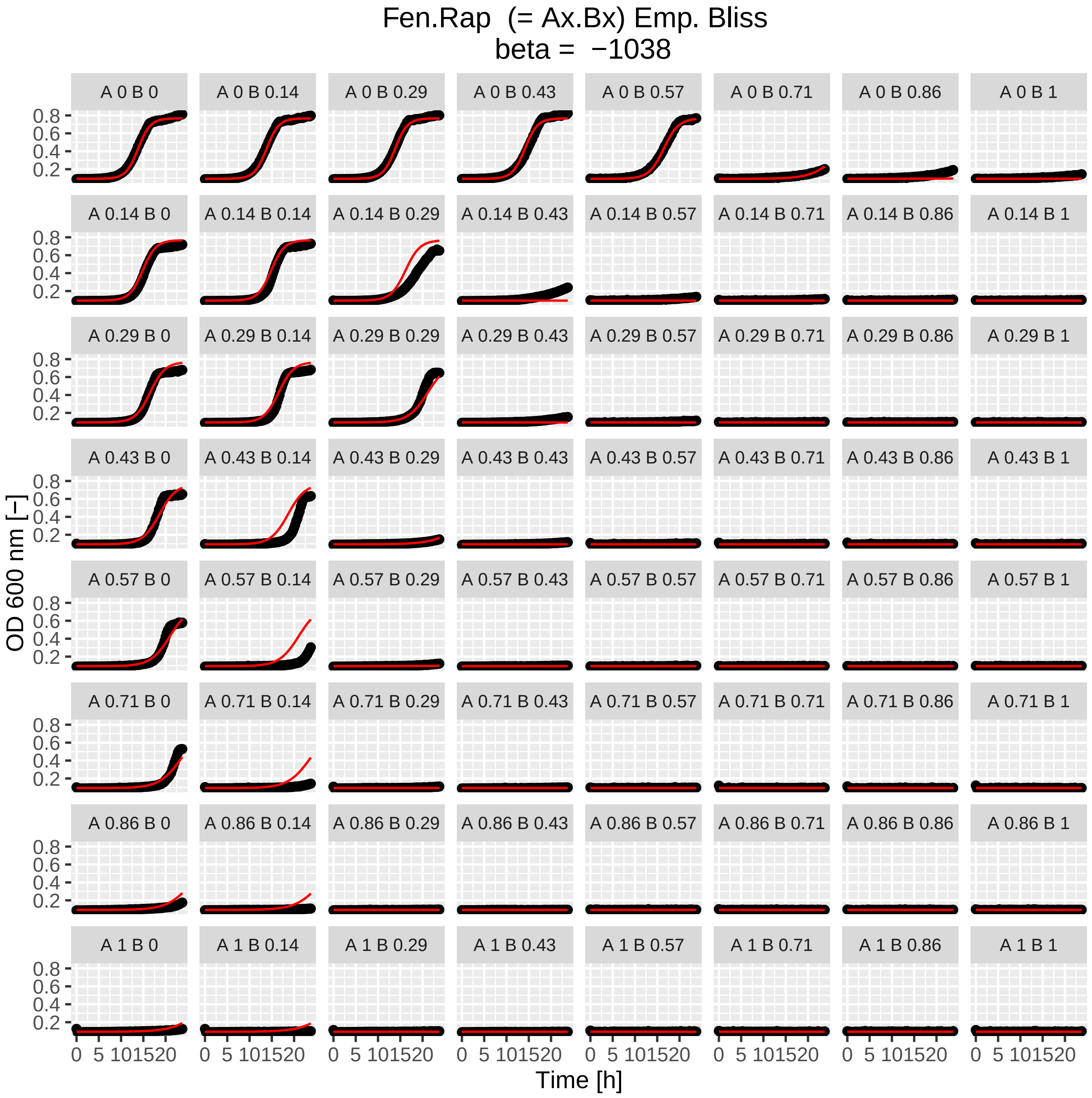

Fen.Rap (= Ax.Bx) Emp. Bliss
beta = −1038
A 0 B 0
A 0 B 0.14
A 0 B 0.29
A 0 B 0.43
A 0 B 0.57
A 0 B 0.71
A 0 B 0.86
A 0 B 1
A 0.14 B 0
A 0.14 B 0.14
A 0.14 B 0.29
A 0.14 B 0.43
A 0.14 B 0.57
A 0.14 B 0.71
A 0.14 B 0.86
A 0.14 B 1
A 0.29 B 0
A 0.29 B 0.14
A 0.29 B 0.29
A 0.29 B 0.43
A 0.29 B 0.57
A 0.29 B 0.71
A 0.29 B 0.86
A 0.29 B 1
A 0.43 B 0
A 0.43 B 0.14
A 0.43 B 0.29
A 0.43 B 0.43
A 0.43 B 0.57
A 0.43 B 0.71
A 0.43 B 0.86
A 0.43 B 1
A 0.57 B 0
A 0.57 B 0.14
A 0.57 B 0.29
A 0.57 B 0.43
A 0.57 B 0.57
A 0.57 B 0.71
A 0.57 B 0.86
A 0.57 B 1
A 0.71 B 0
A 0.71 B 0.14
A 0.71 B 0.29
A 0.71 B 0.43
A 0.71 B 0.57
A 0.71 B 0.71
A 0.71 B 0.86
A 0.71 B 1
A 0.86 B 0
A 0.86 B 0.14
A 0.86 B 0.29
A 0.86 B 0.43
A 0.86 B 0.57
A 0.86 B 0.71
A 0.86 B 0.86
A 0.86 B 1
A 1 B 0
A 1 B 0.14
A 1 B 0.29
A 1 B 0.43
A 1 B 0.57
A 1 B 0.71
A 1 B 0.86
A 1 B 1
OD 600 nm [–]
0.2
0.4
0.6
0.8
0
5
10
15
20
Time [h]

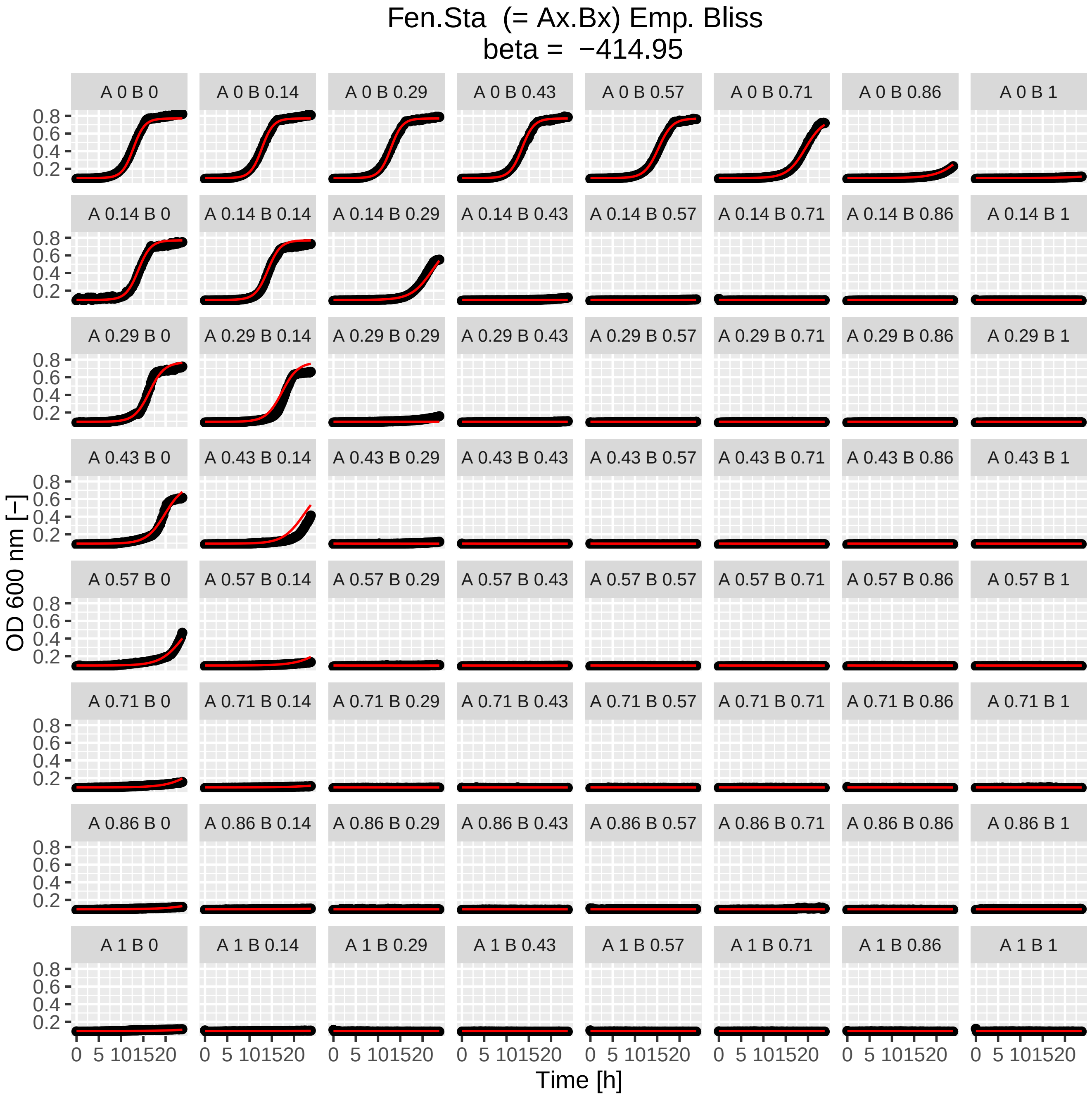
Fen.Sta (= Ax.Bx) Emp. Bliss
beta = −414.95
OD 600 nm [−]
Time [h]
A 0 B 0
A 0 B 0.14
A 0 B 0.29
A 0 B 0.43
A 0 B 0.57
A 0 B 0.71
A 0 B 0.86
A 0 B 1
A 0.14 B 0
A 0.14 B 0.14
A 0.14 B 0.29
A 0.14 B 0.43
A 0.14 B 0.57
A 0.14 B 0.71
A 0.14 B 0.86
A 0.14 B 1
A 0.29 B 0
A 0.29 B 0.14
A 0.29 B 0.29
A 0.29 B 0.43
A 0.29 B 0.57
A 0.29 B 0.71
A 0.29 B 0.86
A 0.29 B 1
A 0.43 B 0
A 0.43 B 0.14
A 0.43 B 0.29
A 0.43 B 0.43
A 0.43 B 0.57
A 0.43 B 0.71
A 0.43 B 0.86
A 0.43 B 1
A 0.57 B 0
A 0.57 B 0.14
A 0.57 B 0.29
A 0.57 B 0.43
A 0.57 B 0.57
A 0.57 B 0.71
A 0.57 B 0.86
A 0.57 B 1
A 0.71 B 0
A 0.71 B 0.14
A 0.71 B 0.29
A 0.71 B 0.43
A 0.71 B 0.57
A 0.71 B 0.71
A 0.71 B 0.86
A 0.71 B 1
A 0.86 B 0
A 0.86 B 0.14
A 0.86 B 0.29
A 0.86 B 0.43
A 0.86 B 0.57
A 0.86 B 0.71
A 0.86 B 0.86
A 0.86 B 1
A 1 B 0
A 1 B 0.14
A 1 B 0.29
A 1 B 0.43
A 1 B 0.57
A 1 B 0.71
A 1 B 0.86
A 1 B 1
0.8
0.6
0.4
0.2
0 5 101520

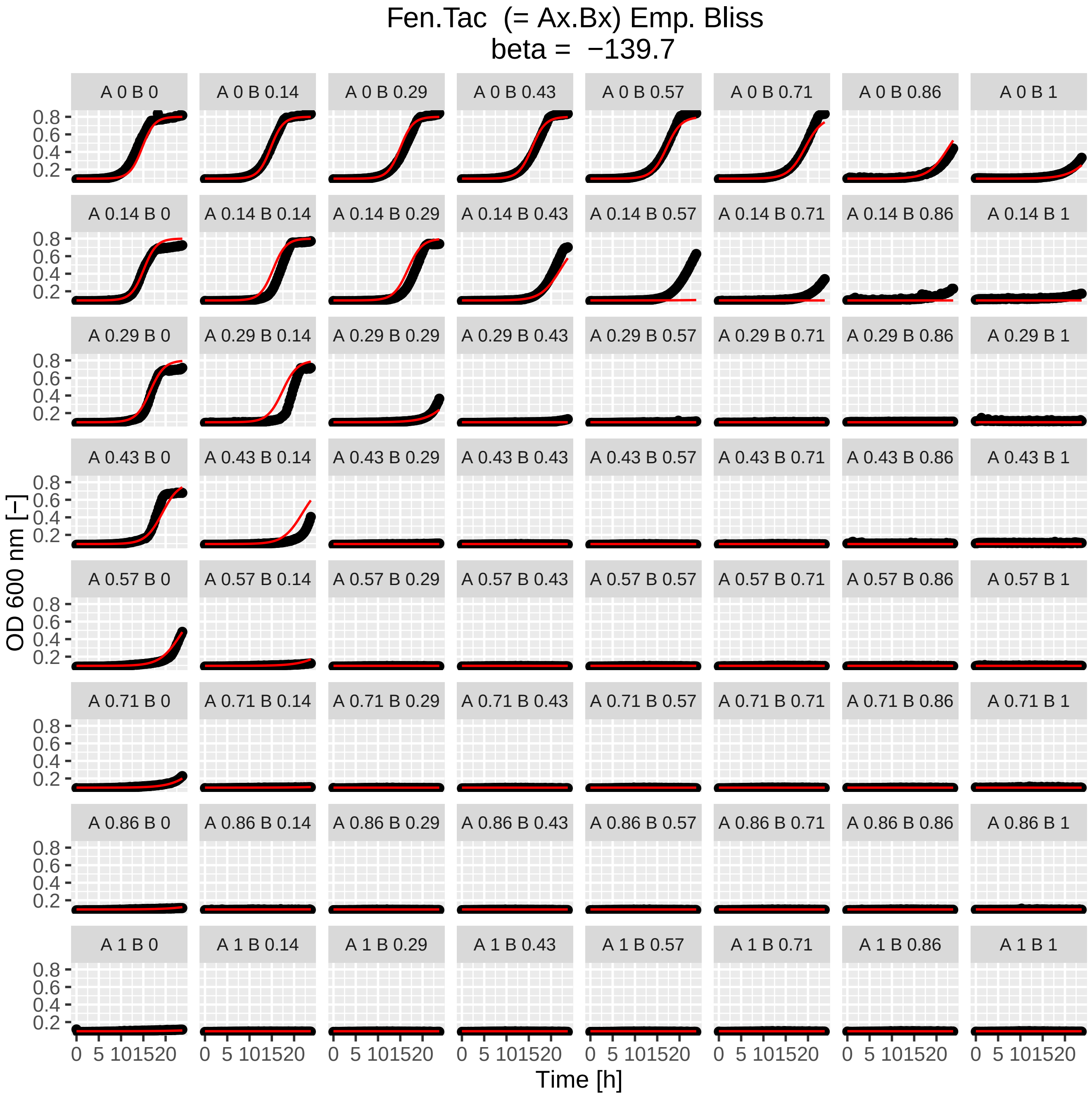
Fen.Tac (= Ax.Bx) Emp. Bliss
beta = −139.7
OD 600 nm [−]
Time [h]
0 5 101520
0.2
0.4
0.6
0.8
A 0 B 0
A 0 B 0.14
A 0 B 0.29
A 0 B 0.43
A 0 B 0.57
A 0 B 0.71
A 0 B 0.86
A 0 B 1
A 0.14 B 0
A 0.14 B 0.14
A 0.14 B 0.29
A 0.14 B 0.43
A 0.14 B 0.57
A 0.14 B 0.71
A 0.14 B 0.86
A 0.14 B 1
A 0.29 B 0
A 0.29 B 0.14
A 0.29 B 0.29
A 0.29 B 0.43
A 0.29 B 0.57
A 0.29 B 0.71
A 0.29 B 0.86
A 0.29 B 1
A 0.43 B 0
A 0.43 B 0.14
A 0.43 B 0.29
A 0.43 B 0.43
A 0.43 B 0.57
A 0.43 B 0.71
A 0.43 B 0.86
A 0.43 B 1
A 0.57 B 0
A 0.57 B 0.14
A 0.57 B 0.29
A 0.57 B 0.43
A 0.57 B 0.57
A 0.57 B 0.71
A 0.57 B 0.86
A 0.57 B 1
A 0.71 B 0
A 0.71 B 0.14
A 0.71 B 0.29
A 0.71 B 0.43
A 0.71 B 0.57
A 0.71 B 0.71
A 0.71 B 0.86
A 0.71 B 1
A 0.86 B 0
A 0.86 B 0.14
A 0.86 B 0.29
A 0.86 B 0.43
A 0.86 B 0.57
A 0.86 B 0.71
A 0.86 B 0.86
A 0.86 B 1
A 1 B 0
A 1 B 0.14
A 1 B 0.29
A 1 B 0.43
A 1 B 0.57
A 1 B 0.71
A 1 B 0.86
A 1 B 1

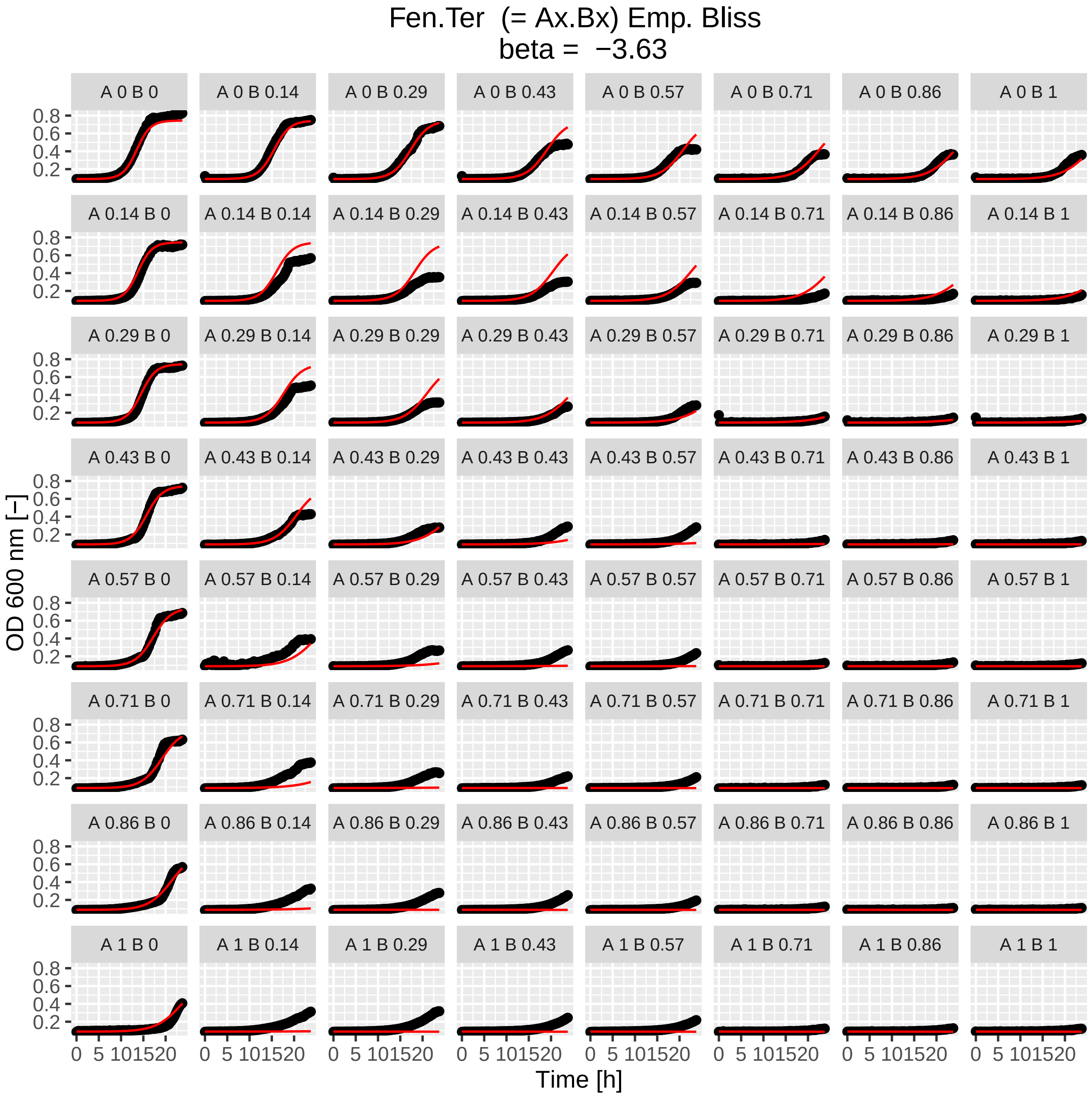

Fen.Ter (= Ax.Bx) Emp. Bliss
beta = −3.63
A 0 B 0
A 0 B 0.14
A 0 B 0.29
A 0 B 0.43
A 0 B 0.57
A 0 B 0.71
A 0 B 0.86
A 0 B 1
A 0.14 B 0
A 0.14 B 0.14
A 0.14 B 0.29
A 0.14 B 0.43
A 0.14 B 0.57
A 0.14 B 0.71
A 0.14 B 0.86
A 0.14 B 1
A 0.29 B 0
A 0.29 B 0.14
A 0.29 B 0.29
A 0.29 B 0.43
A 0.29 B 0.57
A 0.29 B 0.71
A 0.29 B 0.86
A 0.29 B 1
A 0.43 B 0
A 0.43 B 0.14
A 0.43 B 0.29
A 0.43 B 0.43
A 0.43 B 0.57
A 0.43 B 0.71
A 0.43 B 0.86
A 0.43 B 1
A 0.57 B 0
A 0.57 B 0.14
A 0.57 B 0.29
A 0.57 B 0.43
A 0.57 B 0.57
A 0.57 B 0.71
A 0.57 B 0.86
A 0.57 B 1
A 0.71 B 0
A 0.71 B 0.14
A 0.71 B 0.29
A 0.71 B 0.43
A 0.71 B 0.57
A 0.71 B 0.71
A 0.71 B 0.86
A 0.71 B 1
A 0.86 B 0
A 0.86 B 0.14
A 0.86 B 0.29
A 0.86 B 0.43
A 0.86 B 0.57
A 0.86 B 0.71
A 0.86 B 0.86
A 0.86 B 1
A 1 B 0
A 1 B 0.14
A 1 B 0.29
A 1 B 0.43
A 1 B 0.57
A 1 B 0.71
A 1 B 0.86
A 1 B 1
OD 600 nm [−]
0.2
0.4
0.6
0.8
0 5 101520
Time [h]

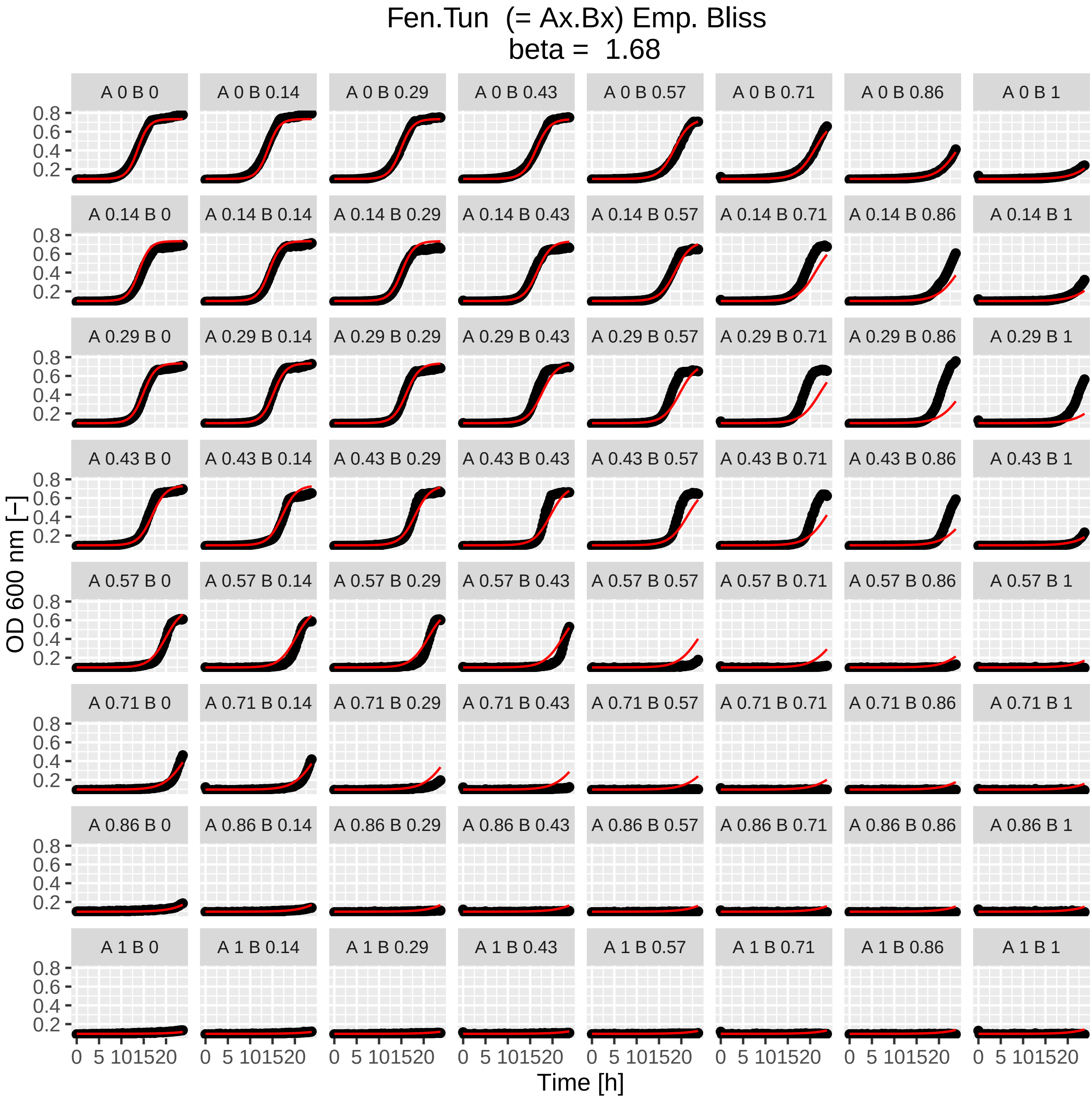

Fen.Tun (= Ax.Bx) Emp. Bliss
beta = 1.68
A 0 B 0
A 0 B 0.14
A 0 B 0.29
A 0 B 0.43
A 0 B 0.57
A 0 B 0.71
A 0 B 0.86
A 0 B 1
A 0.14 B 0
A 0.14 B 0.14
A 0.14 B 0.29
A 0.14 B 0.43
A 0.14 B 0.57
A 0.14 B 0.71
A 0.14 B 0.86
A 0.14 B 1
A 0.29 B 0
A 0.29 B 0.14
A 0.29 B 0.29
A 0.29 B 0.43
A 0.29 B 0.57
A 0.29 B 0.71
A 0.29 B 0.86
A 0.29 B 1
A 0.43 B 0
A 0.43 B 0.14
A 0.43 B 0.29
A 0.43 B 0.43
A 0.43 B 0.57
A 0.43 B 0.71
A 0.43 B 0.86
A 0.43 B 1
A 0.57 B 0
A 0.57 B 0.14
A 0.57 B 0.29
A 0.57 B 0.43
A 0.57 B 0.57
A 0.57 B 0.71
A 0.57 B 0.86
A 0.57 B 1
A 0.71 B 0
A 0.71 B 0.14
A 0.71 B 0.29
A 0.71 B 0.43
A 0.71 B 0.57
A 0.71 B 0.71
A 0.71 B 0.86
A 0.71 B 1
A 0.86 B 0
A 0.86 B 0.14
A 0.86 B 0.29
A 0.86 B 0.43
A 0.86 B 0.57
A 0.86 B 0.71
A 0.86 B 0.86
A 0.86 B 1
A 1 B 0
A 1 B 0.14
A 1 B 0.29
A 1 B 0.43
A 1 B 0.57
A 1 B 0.71
A 1 B 0.86
A 1 B 1
0.8
0.6
0.4
0.2
OD 600 nm [−]
0 5 101520
Time [h]

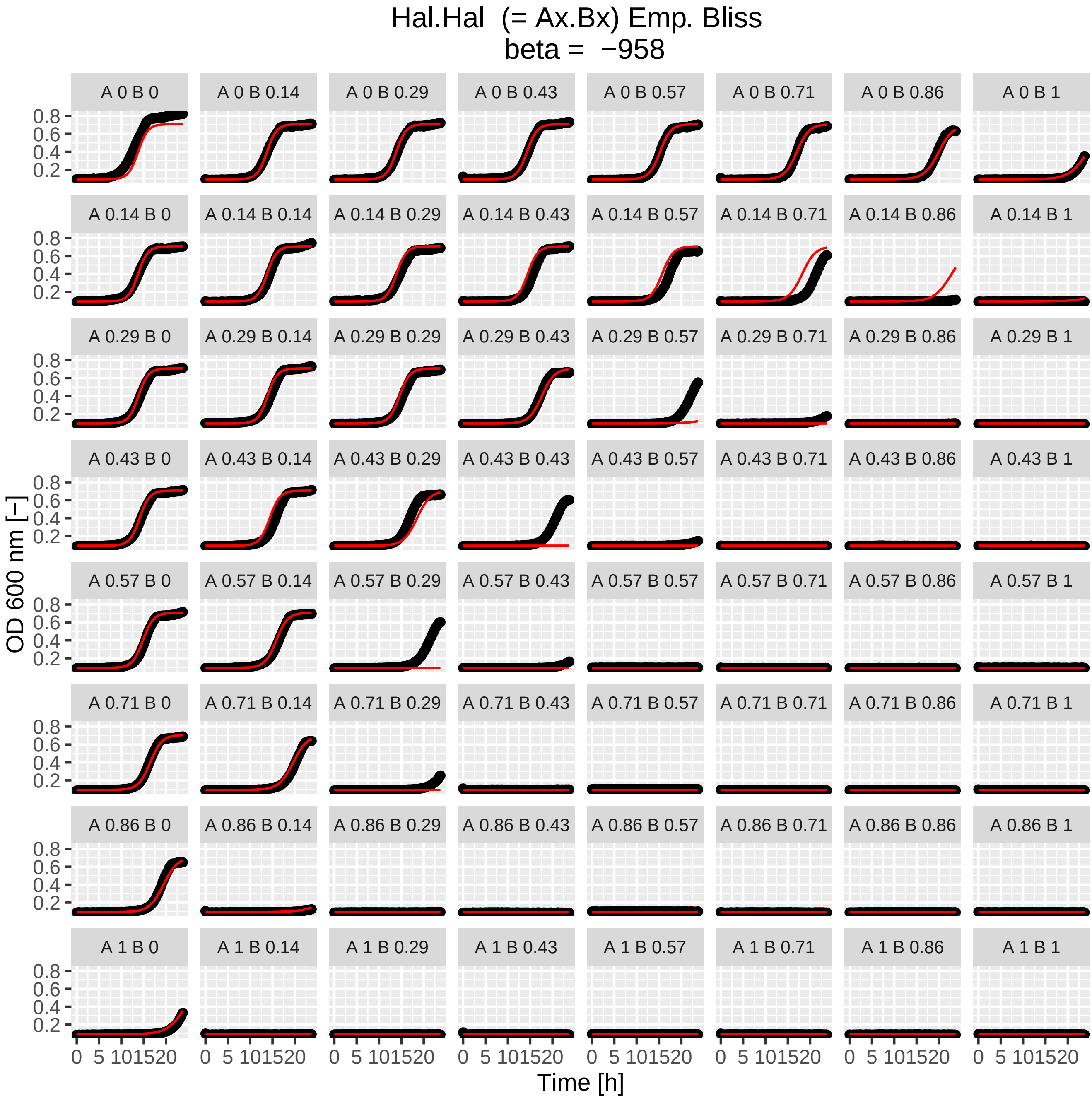

Hal.Hal (= Ax.Bx) Emp. Bliss
beta = −958
A 0 B 0
A 0 B 0.14
A 0 B 0.29
A 0 B 0.43
A 0 B 0.57
A 0 B 0.71
A 0 B 0.86
A 0 B 1
A 0.14 B 0
A 0.14 B 0.14
A 0.14 B 0.29
A 0.14 B 0.43
A 0.14 B 0.57
A 0.14 B 0.71
A 0.14 B 0.86
A 0.14 B 1
A 0.29 B 0
A 0.29 B 0.14
A 0.29 B 0.29
A 0.29 B 0.43
A 0.29 B 0.57
A 0.29 B 0.71
A 0.29 B 0.86
A 0.29 B 1
A 0.43 B 0
A 0.43 B 0.14
A 0.43 B 0.29
A 0.43 B 0.43
A 0.43 B 0.57
A 0.43 B 0.71
A 0.43 B 0.86
A 0.43 B 1
A 0.57 B 0
A 0.57 B 0.14
A 0.57 B 0.29
A 0.57 B 0.43
A 0.57 B 0.57
A 0.57 B 0.71
A 0.57 B 0.86
A 0.57 B 1
A 0.71 B 0
A 0.71 B 0.14
A 0.71 B 0.29
A 0.71 B 0.43
A 0.71 B 0.57
A 0.71 B 0.71
A 0.71 B 0.86
A 0.71 B 1
A 0.86 B 0
A 0.86 B 0.14
A 0.86 B 0.29
A 0.86 B 0.43
A 0.86 B 0.57
A 0.86 B 0.71
A 0.86 B 0.86
A 0.86 B 1
A 1 B 0
A 1 B 0.14
A 1 B 0.29
A 1 B 0.43
A 1 B 0.57
A 1 B 0.71
A 1 B 0.86
A 1 B 1
OD 600 nm [−]
0.8
0.6
0.4
0.2
0 5 101520
Time [h]

Hal.Lat (= Ax.Bx) Emp. Bliss
beta = −15203.6

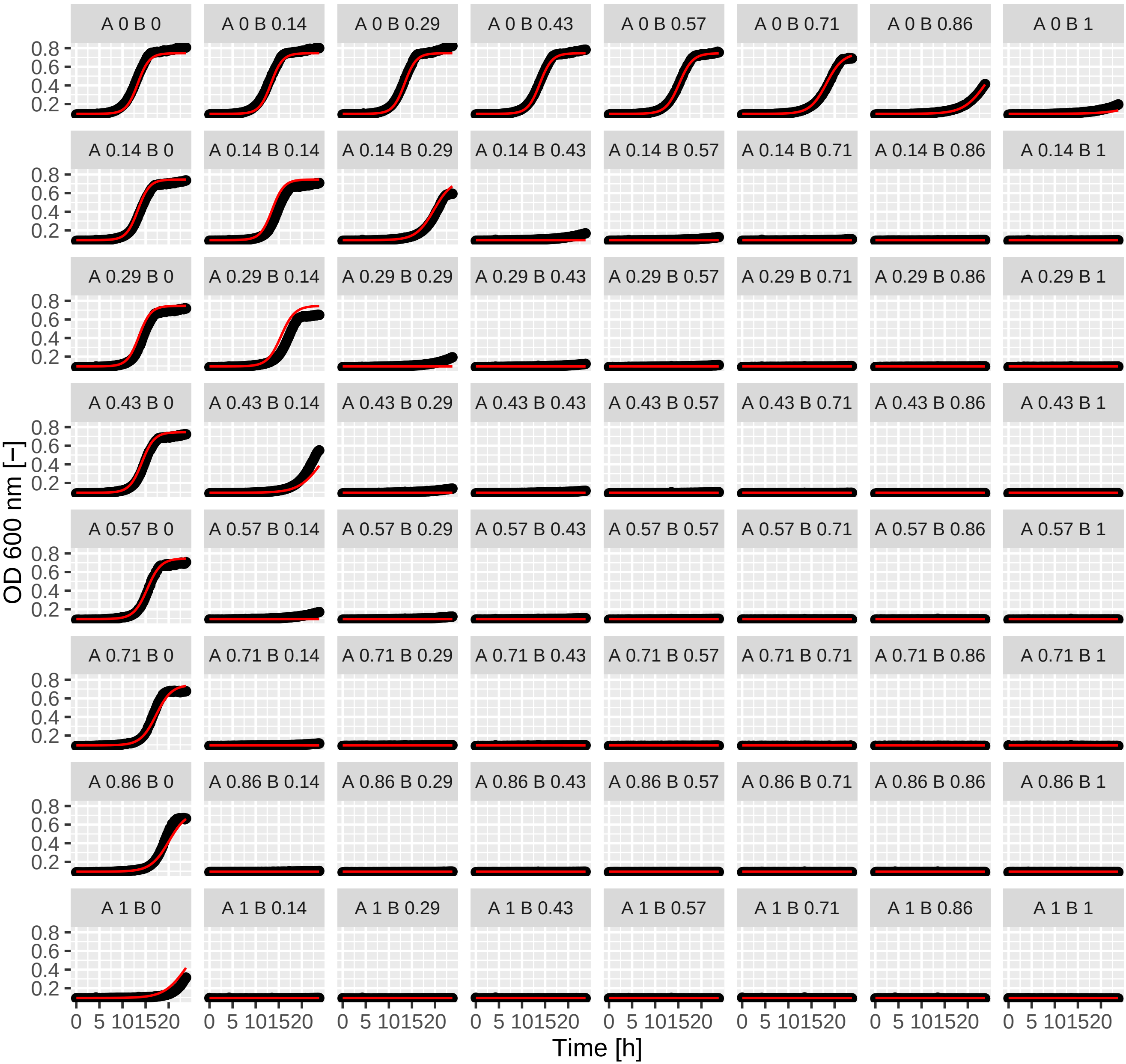

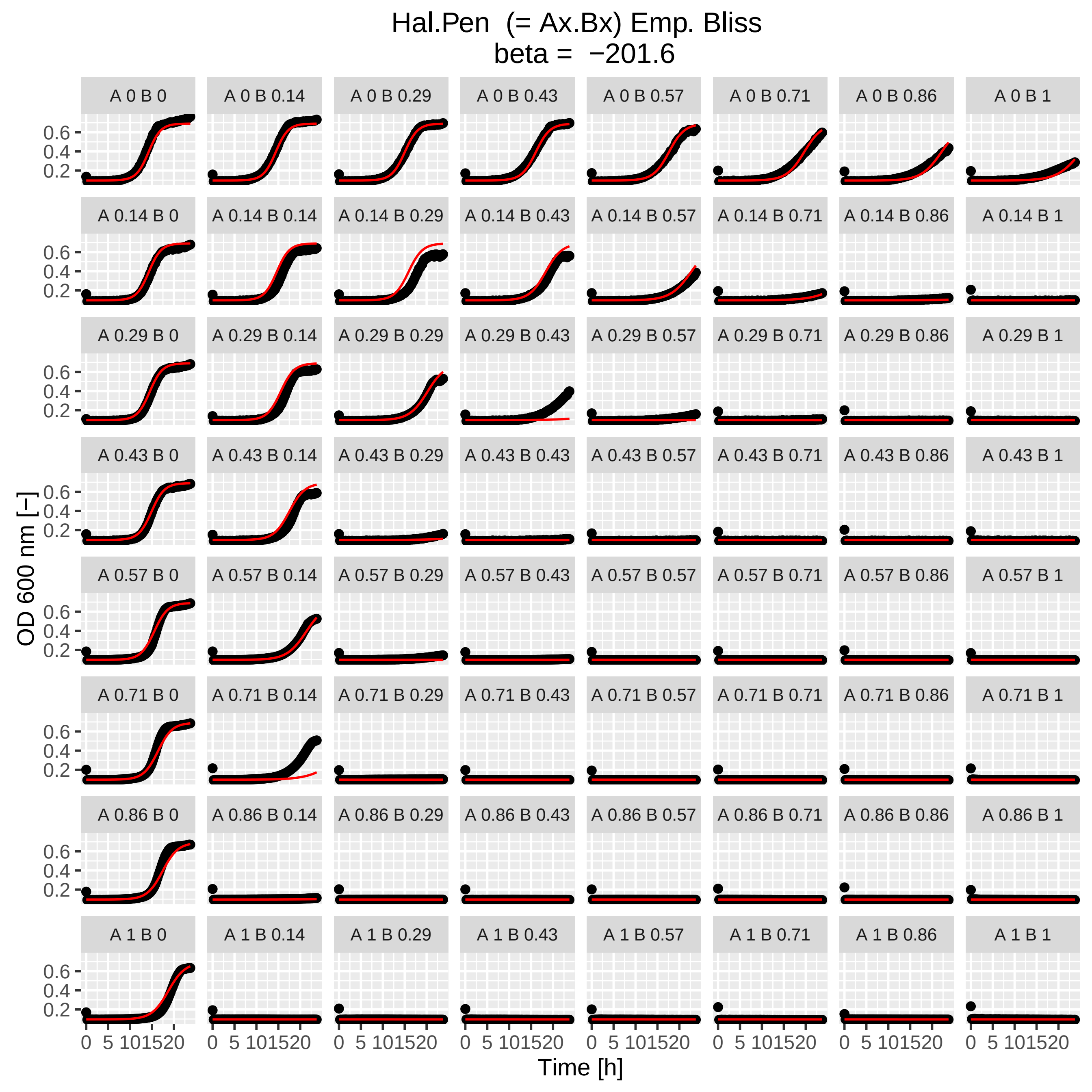

Hal.Pen (= Ax.Bx) Emp. Bliss
beta = −201.6
A 0 B 0
A 0 B 0.14
A 0 B 0.29
A 0 B 0.43
A 0 B 0.57
A 0 B 0.71
A 0 B 0.86
A 0 B 1
A 0.14 B 0
A 0.14 B 0.14
A 0.14 B 0.29
A 0.14 B 0.43
A 0.14 B 0.57
A 0.14 B 0.71
A 0.14 B 0.86
A 0.14 B 1
A 0.29 B 0
A 0.29 B 0.14
A 0.29 B 0.29
A 0.29 B 0.43
A 0.29 B 0.57
A 0.29 B 0.71
A 0.29 B 0.86
A 0.29 B 1
A 0.43 B 0
A 0.43 B 0.14
A 0.43 B 0.29
A 0.43 B 0.43
A 0.43 B 0.57
A 0.43 B 0.71
A 0.43 B 0.86
A 0.43 B 1
A 0.57 B 0
A 0.57 B 0.14
A 0.57 B 0.29
A 0.57 B 0.43
A 0.57 B 0.57
A 0.57 B 0.71
A 0.57 B 0.86
A 0.57 B 1
A 0.71 B 0
A 0.71 B 0.14
A 0.71 B 0.29
A 0.71 B 0.43
A 0.71 B 0.57
A 0.71 B 0.71
A 0.71 B 0.86
A 0.71 B 1
A 0.86 B 0
A 0.86 B 0.14
A 0.86 B 0.29
A 0.86 B 0.43
A 0.86 B 0.57
A 0.86 B 0.71
A 0.86 B 0.86
A 0.86 B 1
A 1 B 0
A 1 B 0.14
A 1 B 0.29
A 1 B 0.43
A 1 B 0.57
A 1 B 0.71
A 1 B 0.86
A 1 B 1
OD 600 nm [−]
0.2
0.4
0.6
0 5 101520
Time [h]

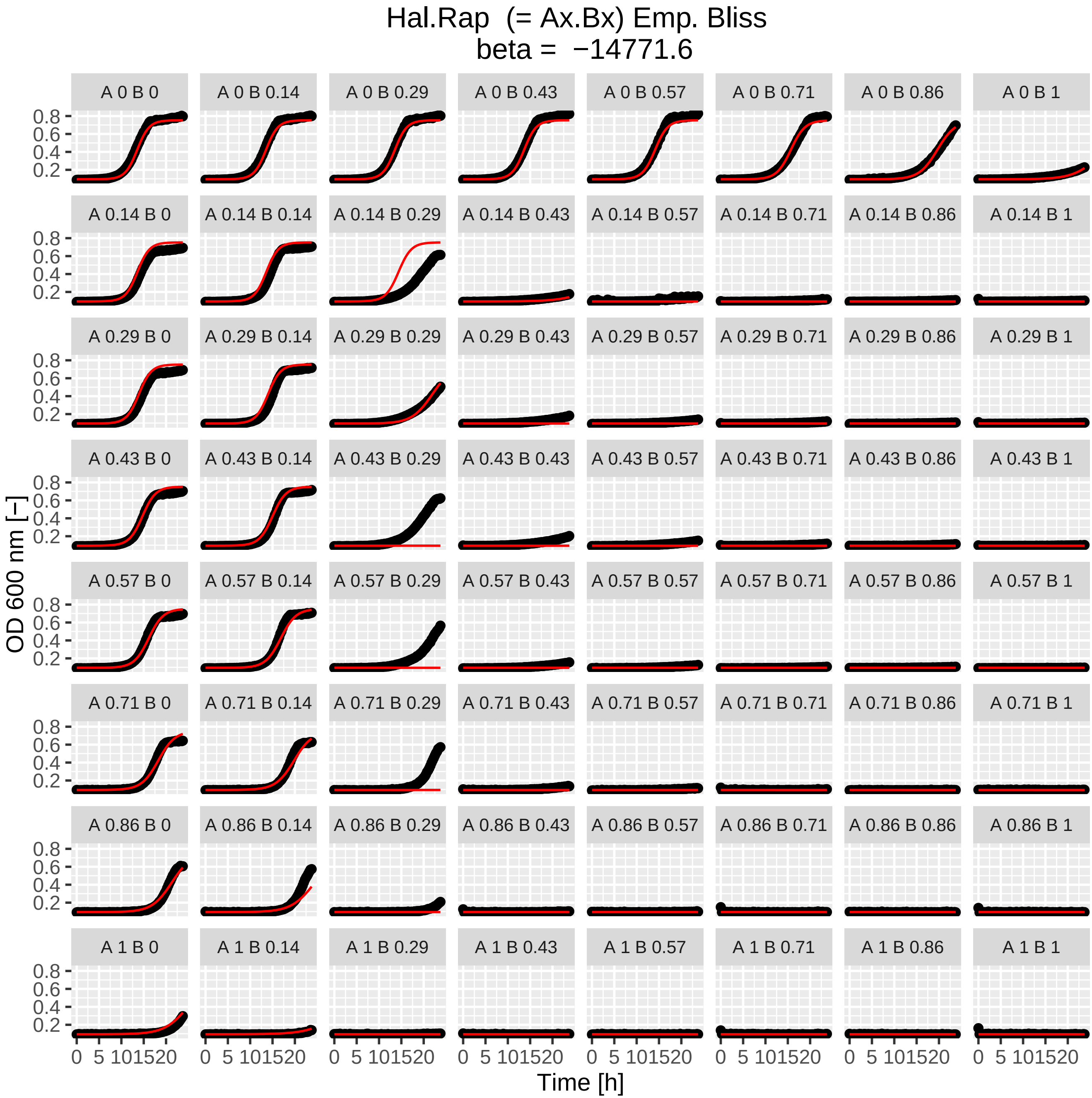
Hal.Rap (= Ax.Bx) Emp. Bliss
beta = −14771.6
A 0 B 0
A 0 B 0.14
A 0 B 0.29
A 0 B 0.43
A 0 B 0.57
A 0 B 0.71
A 0 B 0.86
A 0 B 1
A 0.14 B 0
A 0.14 B 0.14
A 0.14 B 0.29
A 0.14 B 0.43
A 0.14 B 0.57
A 0.14 B 0.71
A 0.14 B 0.86
A 0.14 B 1
A 0.29 B 0
A 0.29 B 0.14
A 0.29 B 0.29
A 0.29 B 0.43
A 0.29 B 0.57
A 0.29 B 0.71
A 0.29 B 0.86
A 0.29 B 1
A 0.43 B 0
A 0.43 B 0.14
A 0.43 B 0.29
A 0.43 B 0.43
A 0.43 B 0.57
A 0.43 B 0.71
A 0.43 B 0.86
A 0.43 B 1
A 0.57 B 0
A 0.57 B 0.14
A 0.57 B 0.29
A 0.57 B 0.43
A 0.57 B 0.57
A 0.57 B 0.71
A 0.57 B 0.86
A 0.57 B 1
A 0.71 B 0
A 0.71 B 0.14
A 0.71 B 0.29
A 0.71 B 0.43
A 0.71 B 0.57
A 0.71 B 0.71
A 0.71 B 0.86
A 0.71 B 1
A 0.86 B 0
A 0.86 B 0.14
A 0.86 B 0.29
A 0.86 B 0.43
A 0.86 B 0.57
A 0.86 B 0.71
A 0.86 B 0.86
A 0.86 B 1
A 1 B 0
A 1 B 0.14
A 1 B 0.29
A 1 B 0.43
A 1 B 0.57
A 1 B 0.71
A 1 B 0.86
A 1 B 1
0.8
0.6
0.4
0.2
0 5 101520
OD 600 nm [–]
Time [h]

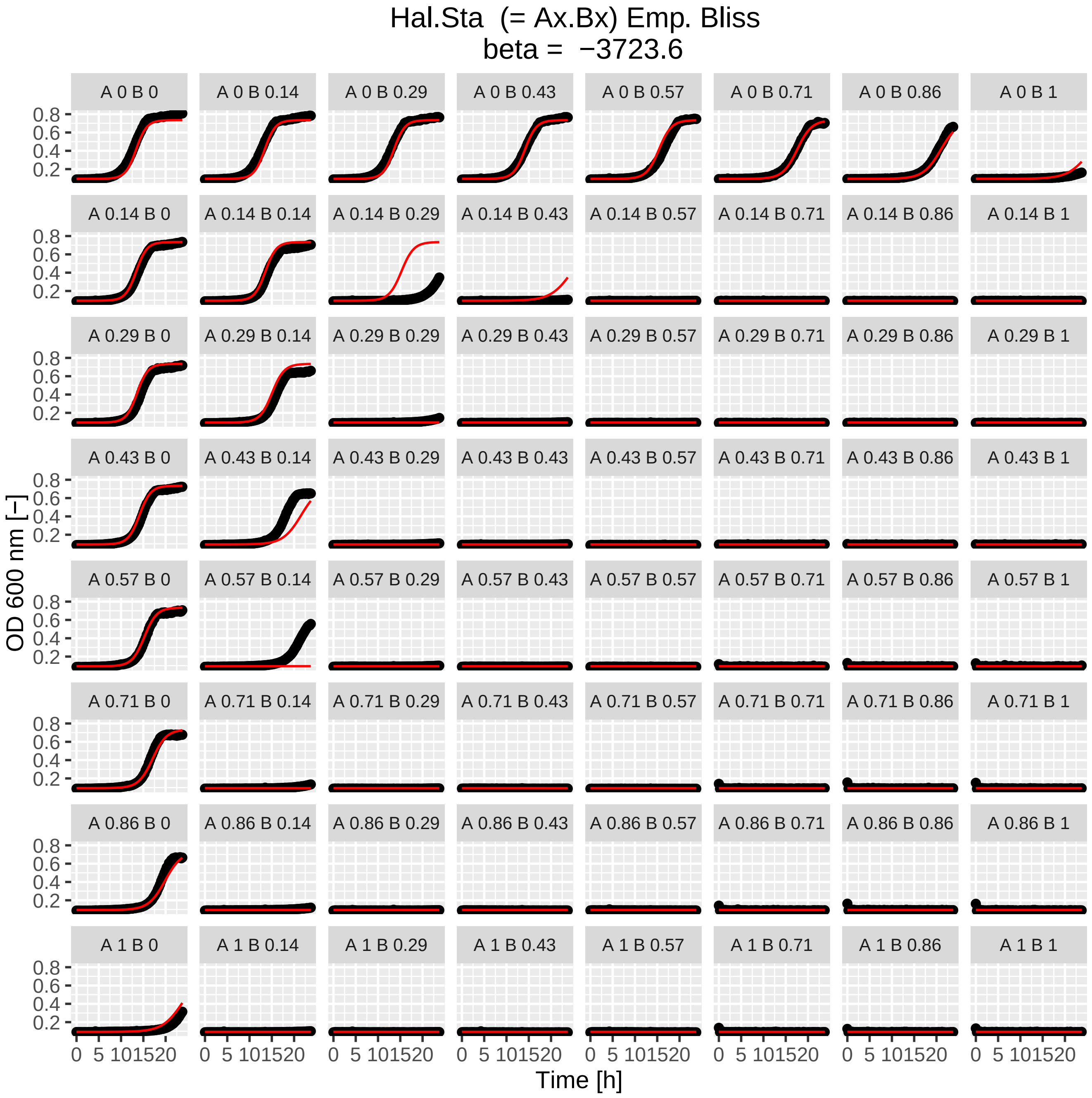
Hal.Sta (= Ax.Bx) Emp. Bliss
beta = −3723.6
OD 600 nm [−]
Time [h]
0.8
0.6
0.4
0.2
0 5 101520
A 0 B 0
A 0 B 0.14
A 0 B 0.29
A 0 B 0.43
A 0 B 0.57
A 0 B 0.71
A 0 B 0.86
A 0 B 1
A 0.14 B 0
A 0.14 B 0.14
A 0.14 B 0.29
A 0.14 B 0.43
A 0.14 B 0.57
A 0.14 B 0.71
A 0.14 B 0.86
A 0.14 B 1
A 0.29 B 0
A 0.29 B 0.14
A 0.29 B 0.29
A 0.29 B 0.43
A 0.29 B 0.57
A 0.29 B 0.71
A 0.29 B 0.86
A 0.29 B 1
A 0.43 B 0
A 0.43 B 0.14
A 0.43 B 0.29
A 0.43 B 0.43
A 0.43 B 0.57
A 0.43 B 0.71
A 0.43 B 0.86
A 0.43 B 1
A 0.57 B 0
A 0.57 B 0.14
A 0.57 B 0.29
A 0.57 B 0.43
A 0.57 B 0.57
A 0.57 B 0.71
A 0.57 B 0.86
A 0.57 B 1
A 0.71 B 0
A 0.71 B 0.14
A 0.71 B 0.29
A 0.71 B 0.43
A 0.71 B 0.57
A 0.71 B 0.71
A 0.71 B 0.86
A 0.71 B 1
A 0.86 B 0
A 0.86 B 0.14
A 0.86 B 0.29
A 0.86 B 0.43
A 0.86 B 0.57
A 0.86 B 0.71
A 0.86 B 0.86
A 0.86 B 1
A 1 B 0
A 1 B 0.14
A 1 B 0.29
A 1 B 0.43
A 1 B 0.57
A 1 B 0.71
A 1 B 0.86
A 1 B 1

Hal.Tac (= Ax.Bx) Emp. Bliss
beta = −5978

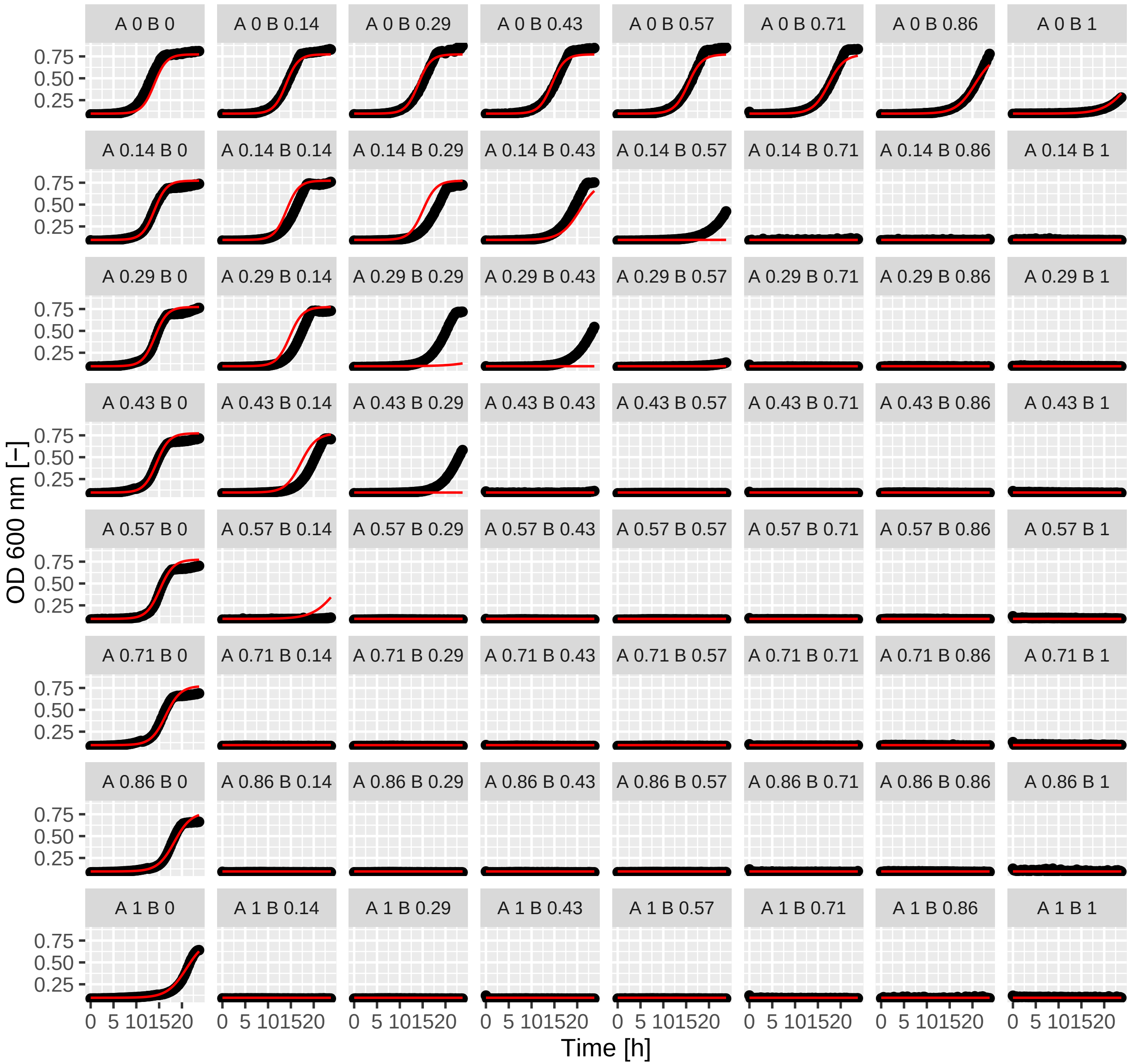

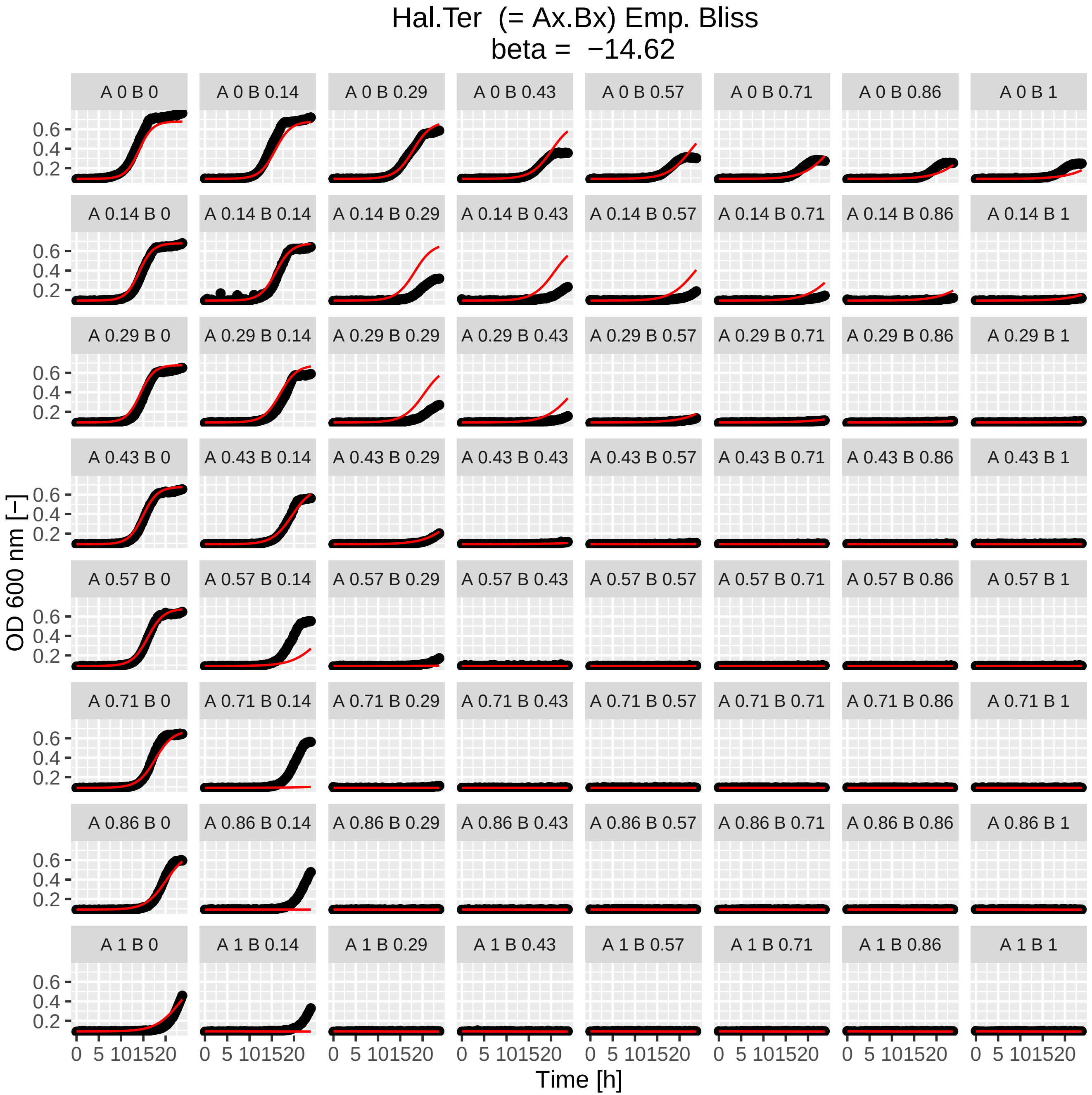

Hal.Ter (= Ax.Bx) Emp. Bliss
beta = −14.62
OD 600 nm [–]
Time [h]
0.6
0.4
0.2
0 5 101520
A 0 B 0
A 0 B 0.14
A 0 B 0.29
A 0 B 0.43
A 0 B 0.57
A 0 B 0.71
A 0 B 0.86
A 0 B 1
A 0.14 B 0
A 0.14 B 0.14
A 0.14 B 0.29
A 0.14 B 0.43
A 0.14 B 0.57
A 0.14 B 0.71
A 0.14 B 0.86
A 0.14 B 1
A 0.29 B 0
A 0.29 B 0.14
A 0.29 B 0.29
A 0.29 B 0.43
A 0.29 B 0.57
A 0.29 B 0.71
A 0.29 B 0.86
A 0.29 B 1
A 0.43 B 0
A 0.43 B 0.14
A 0.43 B 0.29
A 0.43 B 0.43
A 0.43 B 0.57
A 0.43 B 0.71
A 0.43 B 0.86
A 0.43 B 1
A 0.57 B 0
A 0.57 B 0.14
A 0.57 B 0.29
A 0.57 B 0.43
A 0.57 B 0.57
A 0.57 B 0.71
A 0.57 B 0.86
A 0.57 B 1
A 0.71 B 0
A 0.71 B 0.14
A 0.71 B 0.29
A 0.71 B 0.43
A 0.71 B 0.57
A 0.71 B 0.71
A 0.71 B 0.86
A 0.71 B 1
A 0.86 B 0
A 0.86 B 0.14
A 0.86 B 0.29
A 0.86 B 0.43
A 0.86 B 0.57
A 0.86 B 0.71
A 0.86 B 0.86
A 0.86 B 1
A 1 B 0
A 1 B 0.14
A 1 B 0.29
A 1 B 0.43
A 1 B 0.57
A 1 B 0.71
A 1 B 0.86
A 1 B 1

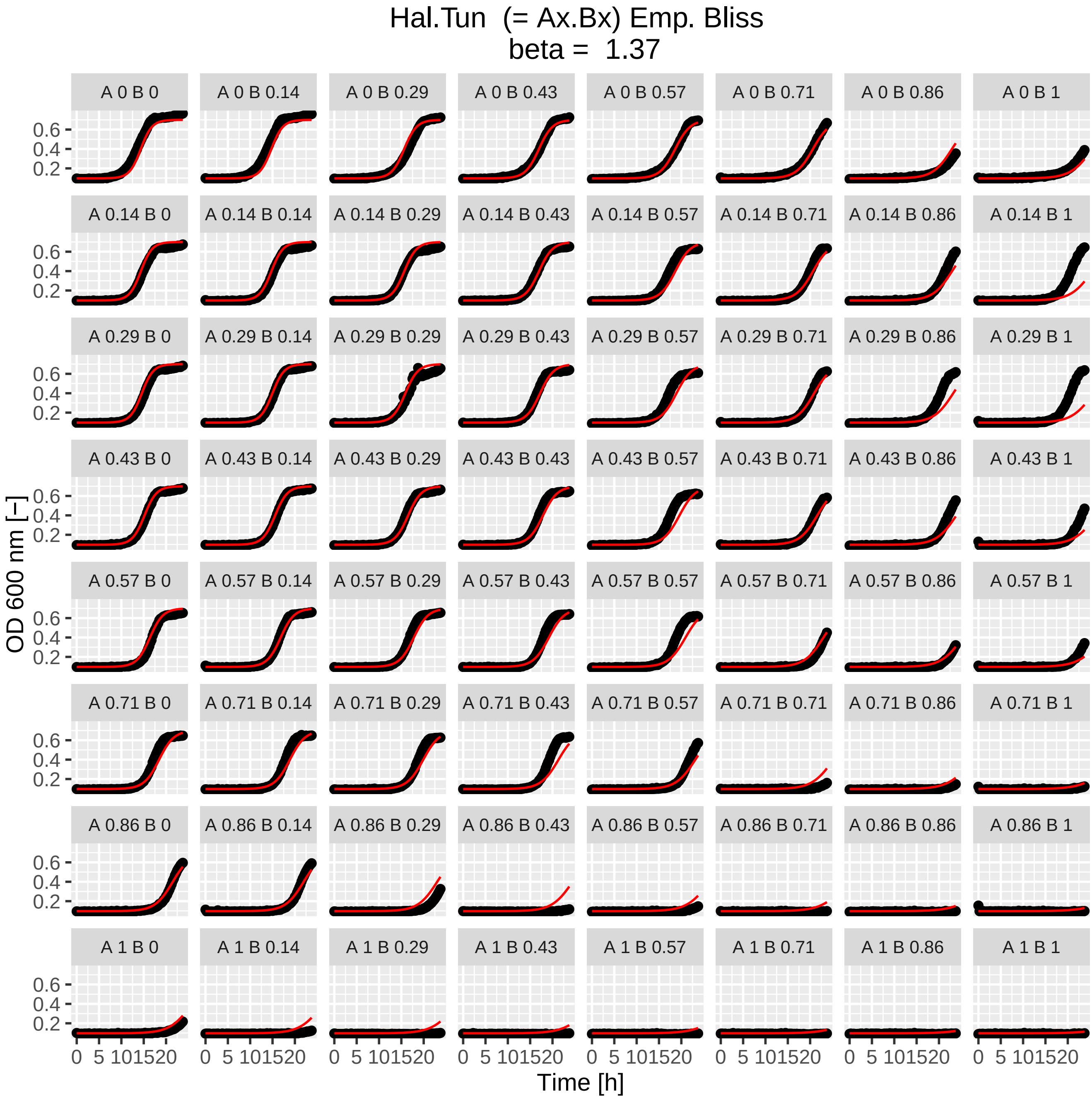

Hal.Tun (= Ax.Bx) Emp. Bliss
beta = 1.37
A 0 B 0
A 0 B 0.14
A 0 B 0.29
A 0 B 0.43
A 0 B 0.57
A 0 B 0.71
A 0 B 0.86
A 0 B 1
A 0.14 B 0
A 0.14 B 0.14
A 0.14 B 0.29
A 0.14 B 0.43
A 0.14 B 0.57
A 0.14 B 0.71
A 0.14 B 0.86
A 0.14 B 1
A 0.29 B 0
A 0.29 B 0.14
A 0.29 B 0.29
A 0.29 B 0.43
A 0.29 B 0.57
A 0.29 B 0.71
A 0.29 B 0.86
A 0.29 B 1
A 0.43 B 0
A 0.43 B 0.14
A 0.43 B 0.29
A 0.43 B 0.43
A 0.43 B 0.57
A 0.43 B 0.71
A 0.43 B 0.86
A 0.43 B 1
A 0.57 B 0
A 0.57 B 0.14
A 0.57 B 0.29
A 0.57 B 0.43
A 0.57 B 0.57
A 0.57 B 0.71
A 0.57 B 0.86
A 0.57 B 1
A 0.71 B 0
A 0.71 B 0.14
A 0.71 B 0.29
A 0.71 B 0.43
A 0.71 B 0.57
A 0.71 B 0.71
A 0.71 B 0.86
A 0.71 B 1
A 0.86 B 0
A 0.86 B 0.14
A 0.86 B 0.29
A 0.86 B 0.43
A 0.86 B 0.57
A 0.86 B 0.71
A 0.86 B 0.86
A 0.86 B 1
A 1 B 0
A 1 B 0.14
A 1 B 0.29
A 1 B 0.43
A 1 B 0.57
A 1 B 0.71
A 1 B 0.86
A 1 B 1
OD 600 nm [–]
0.2
0.4
0.6
0 5 101520
Time [h]

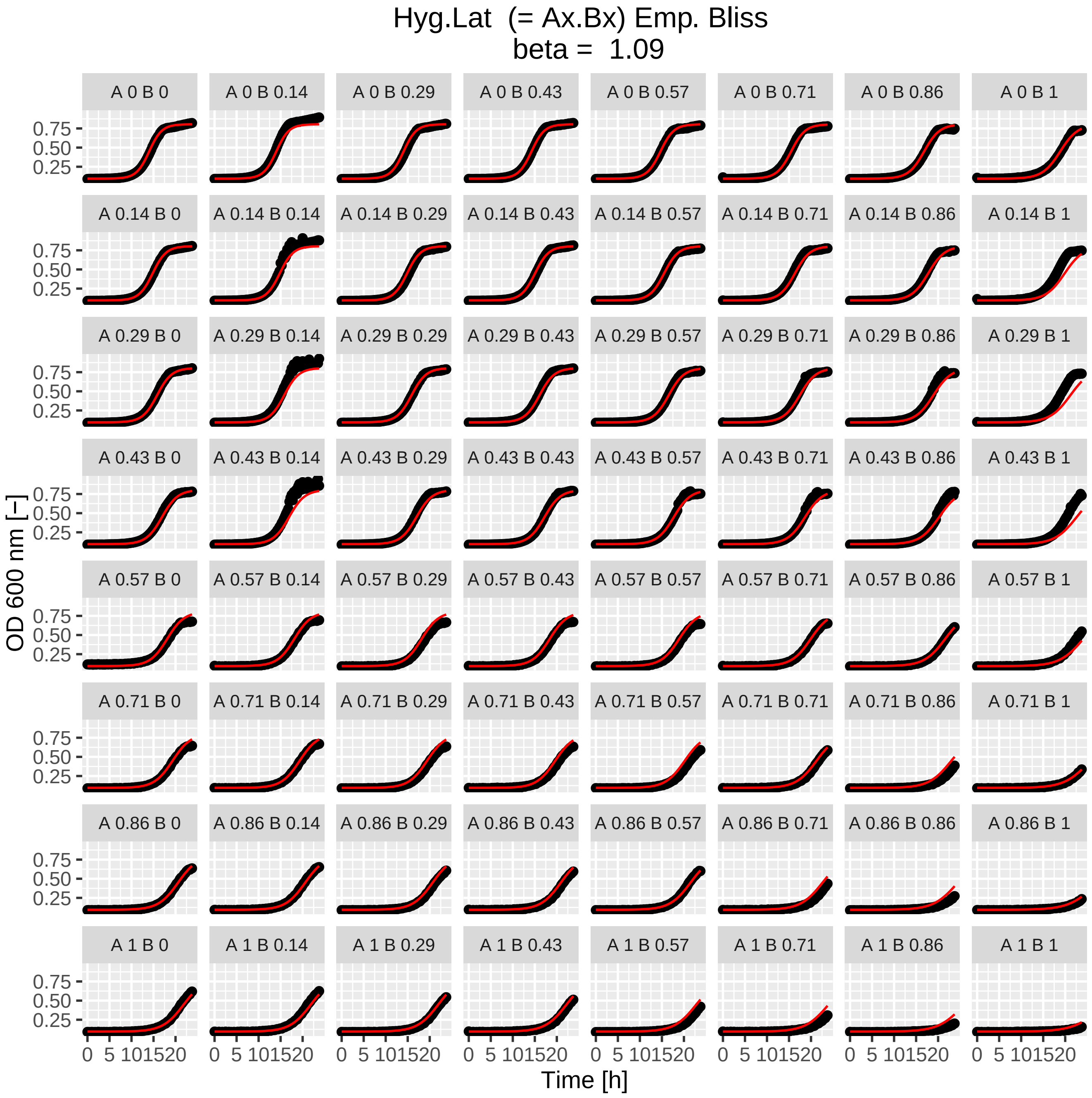
Hyg.Lat (= Ax.Bx) Emp. Bliss
beta = 1.09
A 0 B 0
A 0 B 0.14
A 0 B 0.29
A 0 B 0.43
A 0 B 0.57
A 0 B 0.71
A 0 B 0.86
A 0 B 1
A 0.14 B 0
A 0.14 B 0.14
A 0.14 B 0.29
A 0.14 B 0.43
A 0.14 B 0.57
A 0.14 B 0.71
A 0.14 B 0.86
A 0.14 B 1
A 0.29 B 0
A 0.29 B 0.14
A 0.29 B 0.29
A 0.29 B 0.43
A 0.29 B 0.57
A 0.29 B 0.71
A 0.29 B 0.86
A 0.29 B 1
A 0.43 B 0
A 0.43 B 0.14
A 0.43 B 0.29
A 0.43 B 0.43
A 0.43 B 0.57
A 0.43 B 0.71
A 0.43 B 0.86
A 0.43 B 1
A 0.57 B 0
A 0.57 B 0.14
A 0.57 B 0.29
A 0.57 B 0.43
A 0.57 B 0.57
A 0.57 B 0.71
A 0.57 B 0.86
A 0.57 B 1
A 0.71 B 0
A 0.71 B 0.14
A 0.71 B 0.29
A 0.71 B 0.43
A 0.71 B 0.57
A 0.71 B 0.71
A 0.71 B 0.86
A 0.71 B 1
A 0.86 B 0
A 0.86 B 0.14
A 0.86 B 0.29
A 0.86 B 0.43
A 0.86 B 0.57
A 0.86 B 0.71
A 0.86 B 0.86
A 0.86 B 1
A 1 B 0
A 1 B 0.14
A 1 B 0.29
A 1 B 0.43
A 1 B 0.57
A 1 B 0.71
A 1 B 0.86
A 1 B 1
OD 600 nm [–]
0.75
0.50
0.25
0 5 101520
Time [h]

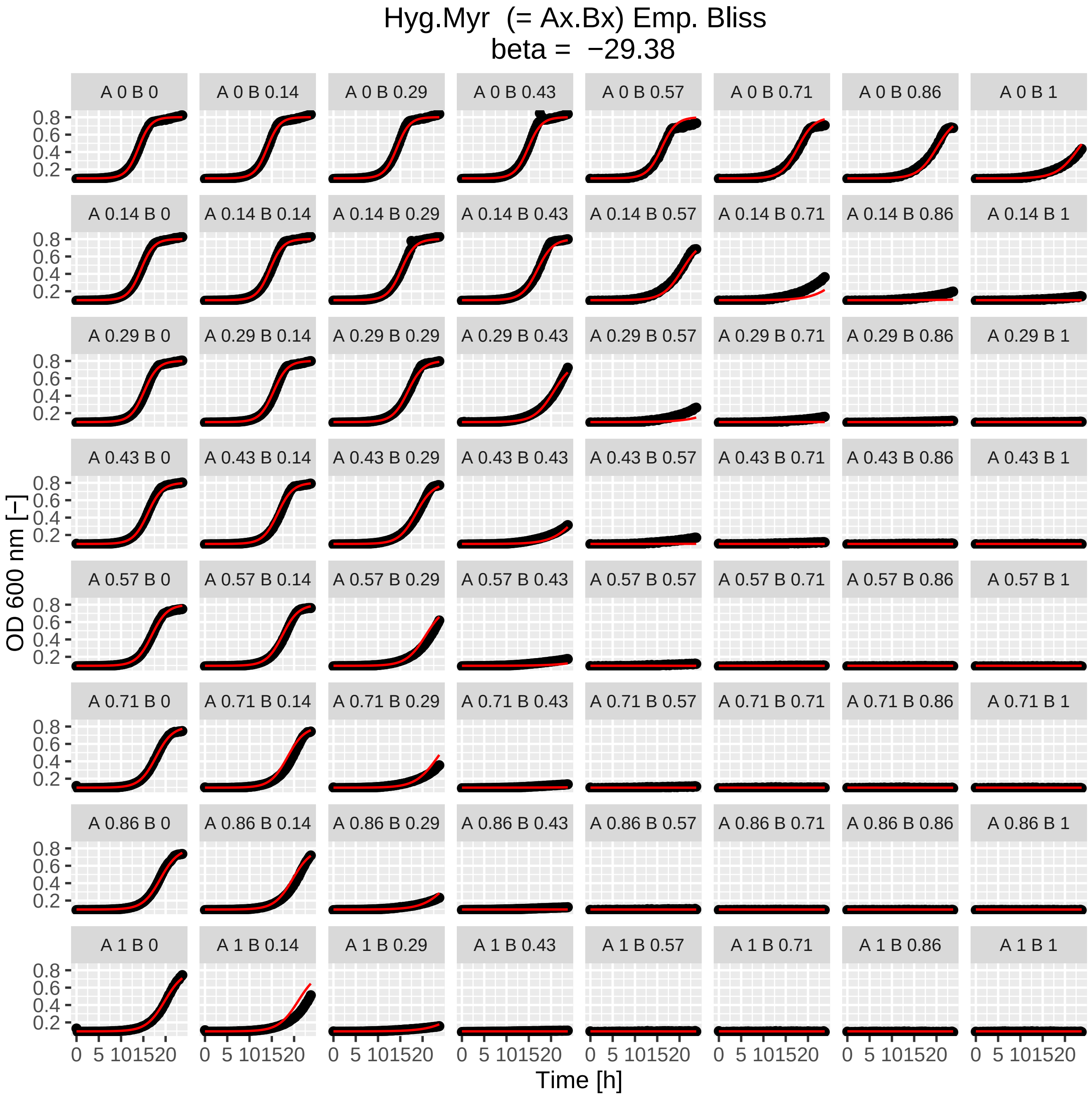
Hyg.Myr (= Ax.Bx) Emp. Bliss
beta = −29.38
OD 600 nm [−]
Time [h]
A 0 B 0
A 0 B 0.14
A 0 B 0.29
A 0 B 0.43
A 0 B 0.57
A 0 B 0.71
A 0 B 0.86
A 0 B 1
A 1 B 0
A 1 B 0.14
A 1 B 0.29
A 1 B 0.43
A 1 B 0.57
A 1 B 0.71
A 1 B 0.86
A 1 B 1

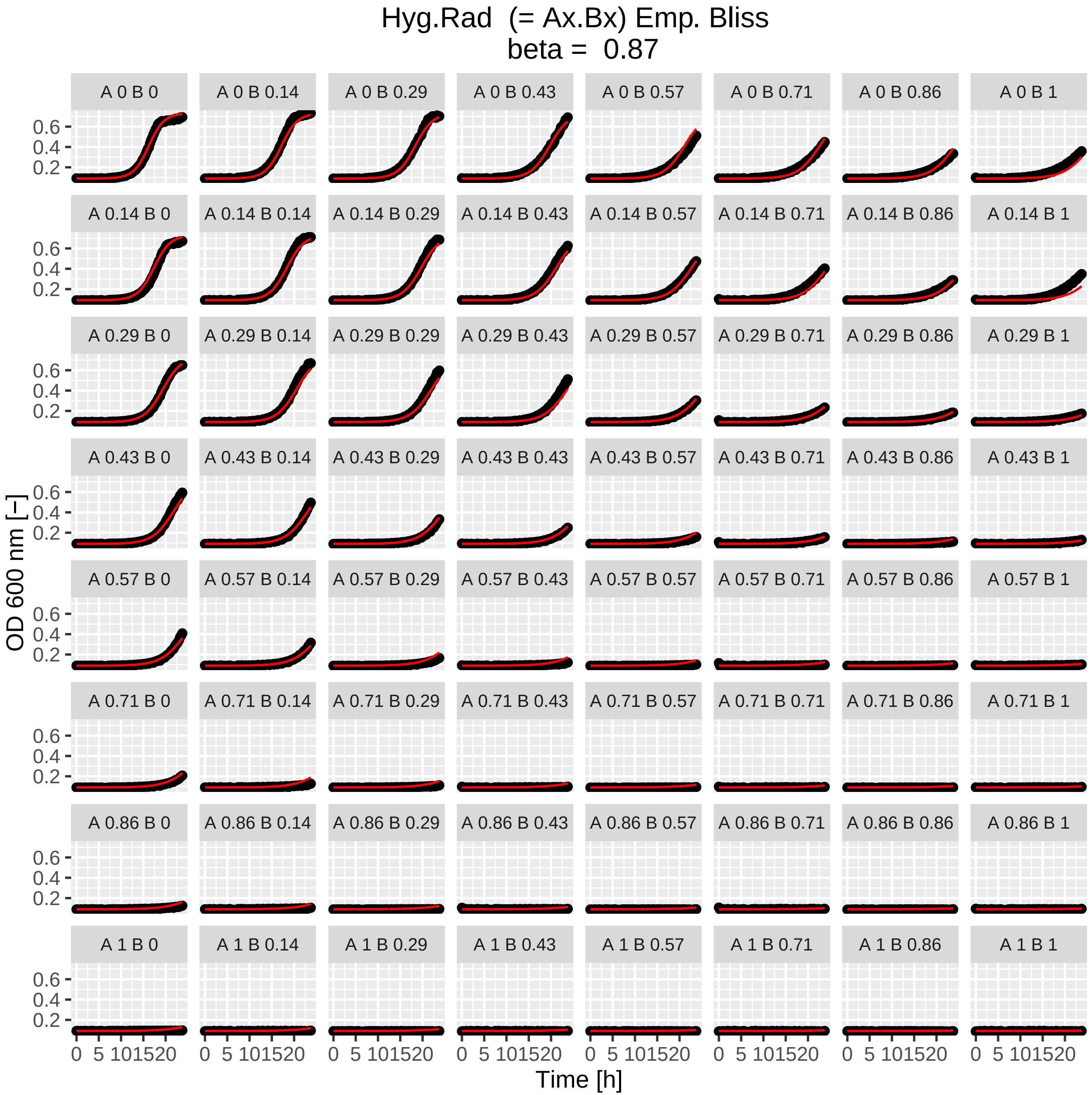

Hyg.Rad (= Ax.Bx) Emp. Bliss
beta = 0.87
OD 600 nm [–]
Time [h]
0.2
0.4
0.6
0 5 101520
A 0 B 0
A 0 B 0.14
A 0 B 0.29
A 0 B 0.43
A 0 B 0.57
A 0 B 0.71
A 0 B 0.86
A 0 B 1
A 0.14 B 0
A 0.14 B 0.14
A 0.14 B 0.29
A 0.14 B 0.43
A 0.14 B 0.57
A 0.14 B 0.71
A 0.14 B 0.86
A 0.14 B 1
A 0.29 B 0
A 0.29 B 0.14
A 0.29 B 0.29
A 0.29 B 0.43
A 0.29 B 0.57
A 0.29 B 0.71
A 0.29 B 0.86
A 0.29 B 1
A 0.43 B 0
A 0.43 B 0.14
A 0.43 B 0.29
A 0.43 B 0.43
A 0.43 B 0.57
A 0.43 B 0.71
A 0.43 B 0.86
A 0.43 B 1
A 0.57 B 0
A 0.57 B 0.14
A 0.57 B 0.29
A 0.57 B 0.43
A 0.57 B 0.57
A 0.57 B 0.71
A 0.57 B 0.86
A 0.57 B 1
A 0.71 B 0
A 0.71 B 0.14
A 0.71 B 0.29
A 0.71 B 0.43
A 0.71 B 0.57
A 0.71 B 0.71
A 0.71 B 0.86
A 0.71 B 1
A 0.86 B 0
A 0.86 B 0.14
A 0.86 B 0.29
A 0.86 B 0.43
A 0.86 B 0.57
A 0.86 B 0.71
A 0.86 B 0.86
A 0.86 B 1
A 1 B 0
A 1 B 0.14
A 1 B 0.29
A 1 B 0.43
A 1 B 0.57
A 1 B 0.71
A 1 B 0.86
A 1 B 1

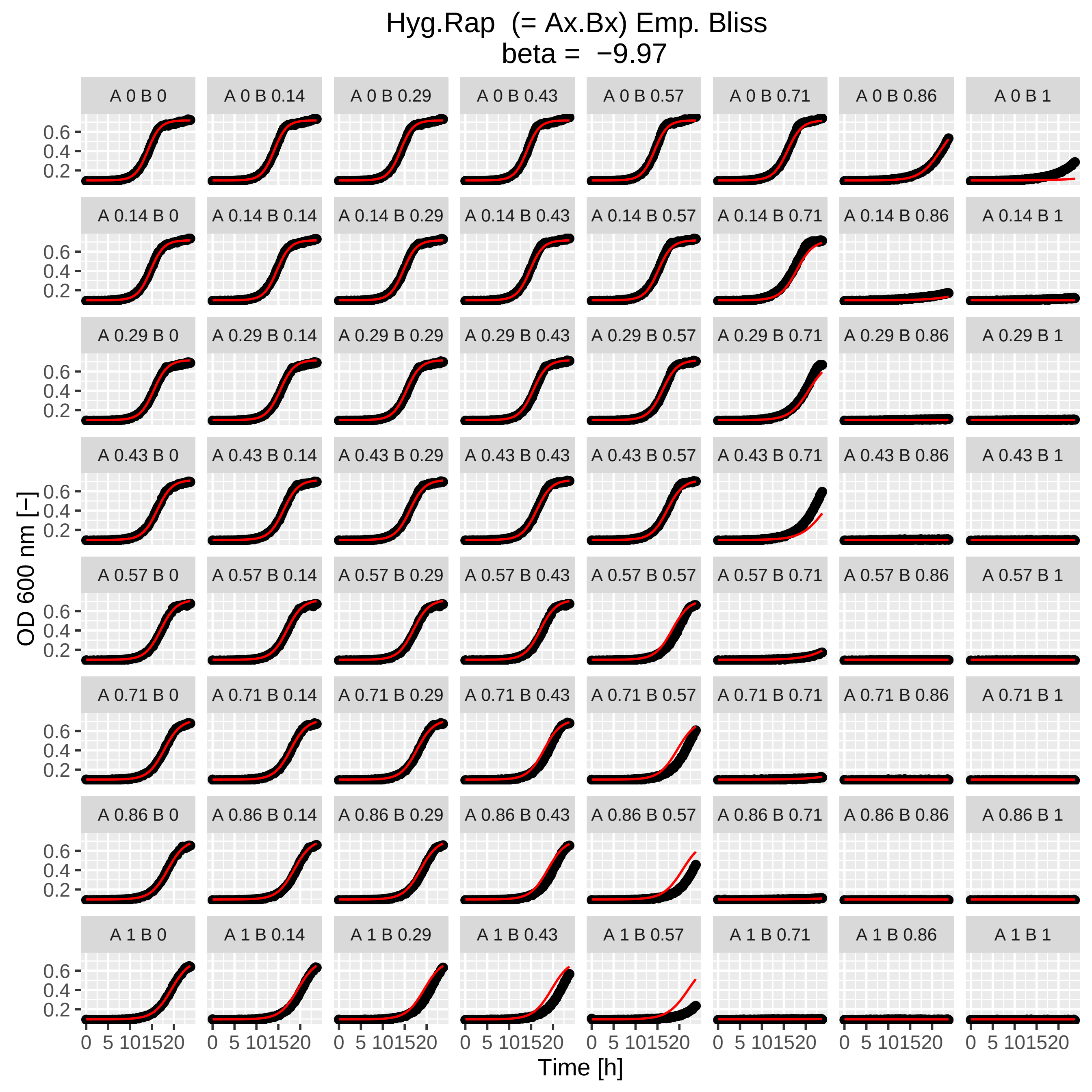

Hyg.Rap (= Ax.Bx) Emp. Bliss
beta = −9.97
A 0 B 0
A 0 B 0.14
A 0 B 0.29
A 0 B 0.43
A 0 B 0.57
A 0 B 0.71
A 0 B 0.86
A 0 B 1
A 0.14 B 0
A 0.14 B 0.14
A 0.14 B 0.29
A 0.14 B 0.43
A 0.14 B 0.57
A 0.14 B 0.71
A 0.14 B 0.86
A 0.14 B 1
A 0.29 B 0
A 0.29 B 0.14
A 0.29 B 0.29
A 0.29 B 0.43
A 0.29 B 0.57
A 0.29 B 0.71
A 0.29 B 0.86
A 0.29 B 1
A 0.43 B 0
A 0.43 B 0.14
A 0.43 B 0.29
A 0.43 B 0.43
A 0.43 B 0.57
A 0.43 B 0.71
A 0.43 B 0.86
A 0.43 B 1
A 0.57 B 0
A 0.57 B 0.14
A 0.57 B 0.29
A 0.57 B 0.43
A 0.57 B 0.57
A 0.57 B 0.71
A 0.57 B 0.86
A 0.57 B 1
A 0.71 B 0
A 0.71 B 0.14
A 0.71 B 0.29
A 0.71 B 0.43
A 0.71 B 0.57
A 0.71 B 0.71
A 0.71 B 0.86
A 0.71 B 1
A 0.86 B 0
A 0.86 B 0.14
A 0.86 B 0.29
A 0.86 B 0.43
A 0.86 B 0.57
A 0.86 B 0.71
A 0.86 B 0.86
A 0.86 B 1
A 1 B 0
A 1 B 0.14
A 1 B 0.29
A 1 B 0.43
A 1 B 0.57
A 1 B 0.71
A 1 B 0.86
A 1 B 1
OD 600 nm [−]
0.2
0.4
0.6
0 5 101520
Time [h]

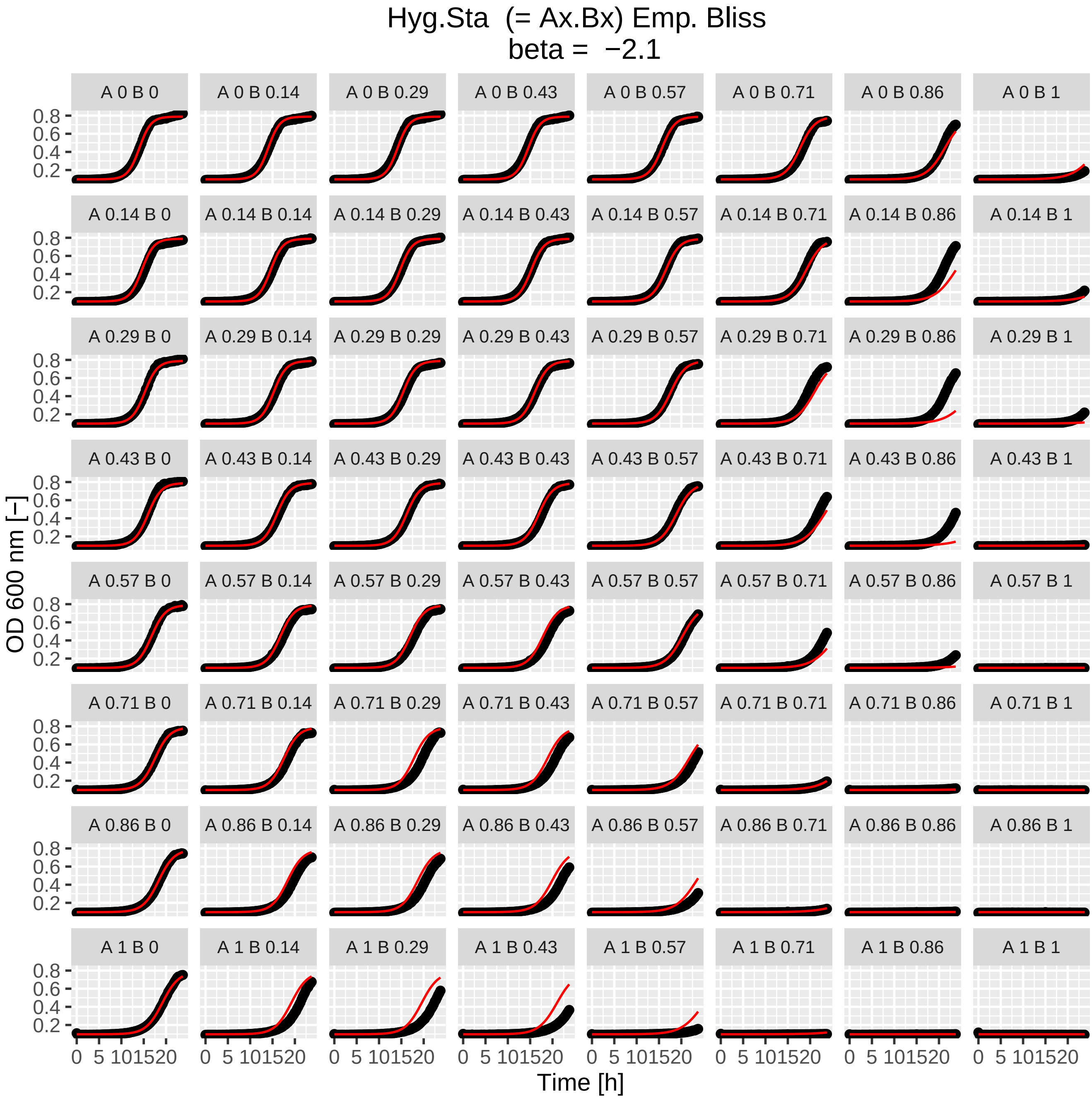

Hyg.Sta (= Ax.Bx) Emp. Bliss
beta = −2.1
A 0 B 0
A 0 B 0.14
A 0 B 0.29
A 0 B 0.43
A 0 B 0.57
A 0 B 0.71
A 0 B 0.86
A 0 B 1
A 0.14 B 0
A 0.14 B 0.14
A 0.14 B 0.29
A 0.14 B 0.43
A 0.14 B 0.57
A 0.14 B 0.71
A 0.14 B 0.86
A 0.14 B 1
A 0.29 B 0
A 0.29 B 0.14
A 0.29 B 0.29
A 0.29 B 0.43
A 0.29 B 0.57
A 0.29 B 0.71
A 0.29 B 0.86
A 0.29 B 1
A 0.43 B 0
A 0.43 B 0.14
A 0.43 B 0.29
A 0.43 B 0.43
A 0.43 B 0.57
A 0.43 B 0.71
A 0.43 B 0.86
A 0.43 B 1
A 0.57 B 0
A 0.57 B 0.14
A 0.57 B 0.29
A 0.57 B 0.43
A 0.57 B 0.57
A 0.57 B 0.71
A 0.57 B 0.86
A 0.57 B 1
A 0.71 B 0
A 0.71 B 0.14
A 0.71 B 0.29
A 0.71 B 0.43
A 0.71 B 0.57
A 0.71 B 0.71
A 0.71 B 0.86
A 0.71 B 1
A 0.86 B 0
A 0.86 B 0.14
A 0.86 B 0.29
A 0.86 B 0.43
A 0.86 B 0.57
A 0.86 B 0.71
A 0.86 B 0.86
A 0.86 B 1
A 1 B 0
A 1 B 0.14
A 1 B 0.29
A 1 B 0.43
A 1 B 0.57
A 1 B 0.71
A 1 B 0.86
A 1 B 1
OD 600 nm [−]
0.2
0.4
0.6
0.8
0 5 101520
Time [h]

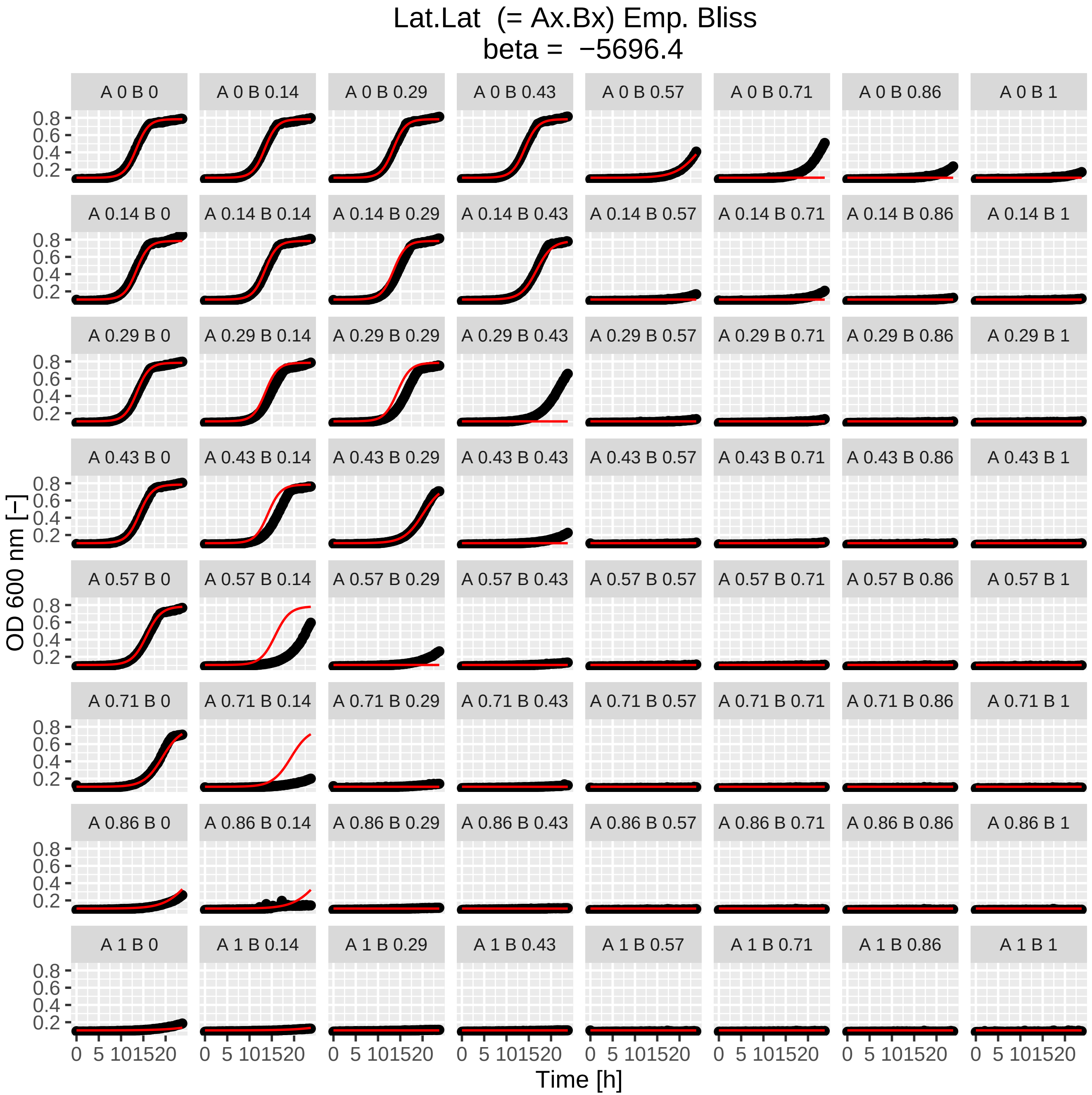

Lat.Lat (= Ax.Bx) Emp. Bliss
beta = −5696.4
A 0 B 0
A 0 B 0.14
A 0 B 0.29
A 0 B 0.43
A 0 B 0.57
A 0 B 0.71
A 0 B 0.86
A 0 B 1
A 0.14 B 0
A 0.14 B 0.14
A 0.14 B 0.29
A 0.14 B 0.43
A 0.14 B 0.57
A 0.14 B 0.71
A 0.14 B 0.86
A 0.14 B 1
A 0.29 B 0
A 0.29 B 0.14
A 0.29 B 0.29
A 0.29 B 0.43
A 0.29 B 0.57
A 0.29 B 0.71
A 0.29 B 0.86
A 0.29 B 1
A 0.43 B 0
A 0.43 B 0.14
A 0.43 B 0.29
A 0.43 B 0.43
A 0.43 B 0.57
A 0.43 B 0.71
A 0.43 B 0.86
A 0.43 B 1
A 0.57 B 0
A 0.57 B 0.14
A 0.57 B 0.29
A 0.57 B 0.43
A 0.57 B 0.57
A 0.57 B 0.71
A 0.57 B 0.86
A 0.57 B 1
A 0.71 B 0
A 0.71 B 0.14
A 0.71 B 0.29
A 0.71 B 0.43
A 0.71 B 0.57
A 0.71 B 0.71
A 0.71 B 0.86
A 0.71 B 1
A 0.86 B 0
A 0.86 B 0.14
A 0.86 B 0.29
A 0.86 B 0.43
A 0.86 B 0.57
A 0.86 B 0.71
A 0.86 B 0.86
A 0.86 B 1
A 1 B 0
A 1 B 0.14
A 1 B 0.29
A 1 B 0.43
A 1 B 0.57
A 1 B 0.71
A 1 B 0.86
A 1 B 1
OD 600 nm [−]
0.8
0.6
0.4
0.2
0 5 101520
Time [h]

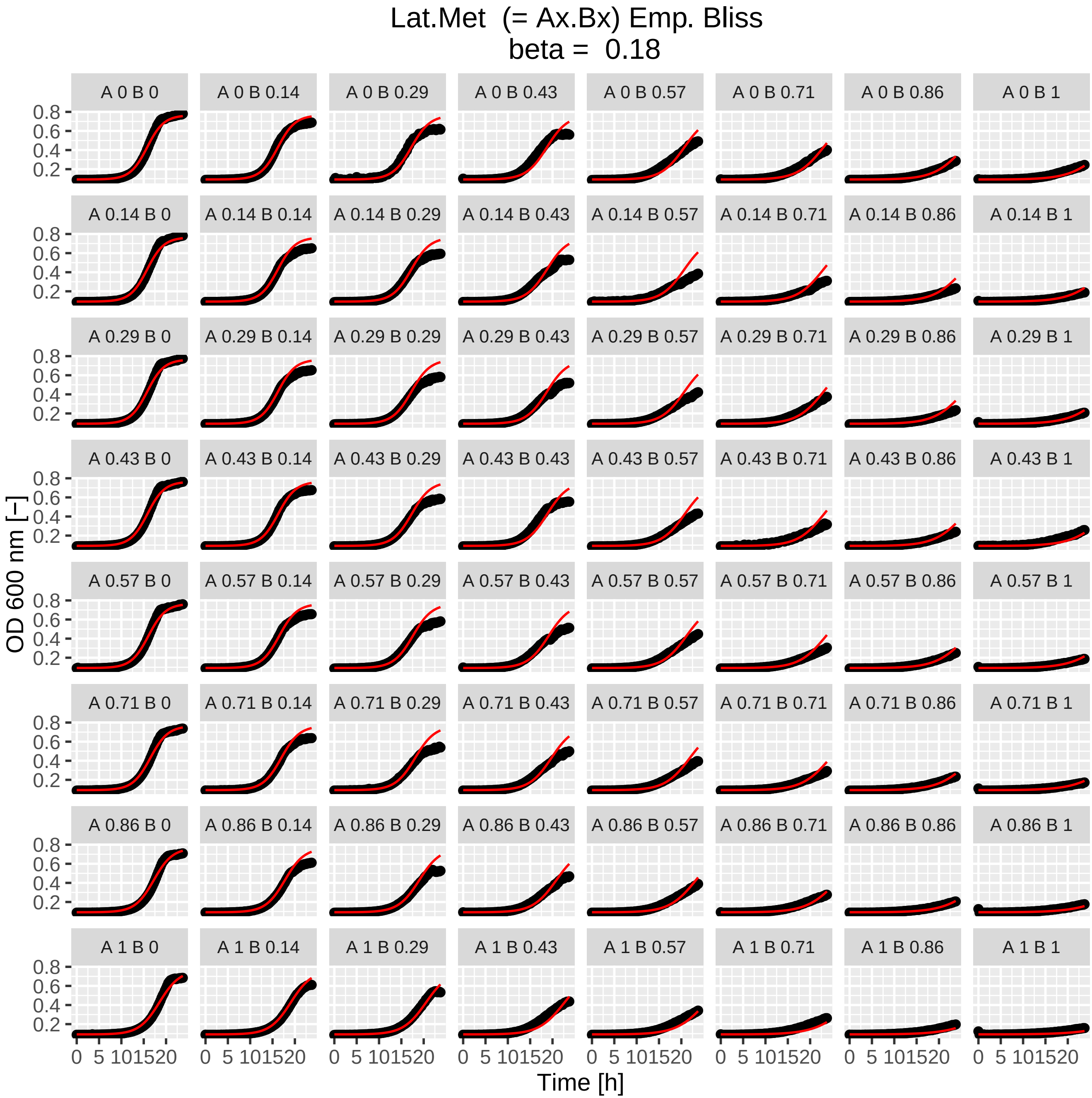
Lat.Met (= Ax.Bx) Emp. Bliss
beta = 0.18
A 0 B 0
A 0 B 0.14
A 0 B 0.29
A 0 B 0.43
A 0 B 0.57
A 0 B 0.71
A 0 B 0.86
A 0 B 1
A 0.14 B 0
A 0.14 B 0.14
A 0.14 B 0.29
A 0.14 B 0.43
A 0.14 B 0.57
A 0.14 B 0.71
A 0.14 B 0.86
A 0.14 B 1
A 0.29 B 0
A 0.29 B 0.14
A 0.29 B 0.29
A 0.29 B 0.43
A 0.29 B 0.57
A 0.29 B 0.71
A 0.29 B 0.86
A 0.29 B 1
A 0.43 B 0
A 0.43 B 0.14
A 0.43 B 0.29
A 0.43 B 0.43
A 0.43 B 0.57
A 0.43 B 0.71
A 0.43 B 0.86
A 0.43 B 1
A 0.57 B 0
A 0.57 B 0.14
A 0.57 B 0.29
A 0.57 B 0.43
A 0.57 B 0.57
A 0.57 B 0.71
A 0.57 B 0.86
A 0.57 B 1
A 0.71 B 0
A 0.71 B 0.14
A 0.71 B 0.29
A 0.71 B 0.43
A 0.71 B 0.57
A 0.71 B 0.71
A 0.71 B 0.86
A 0.71 B 1
A 0.86 B 0
A 0.86 B 0.14
A 0.86 B 0.29
A 0.86 B 0.43
A 0.86 B 0.57
A 0.86 B 0.71
A 0.86 B 0.86
A 0.86 B 1
A 1 B 0
A 1 B 0.14
A 1 B 0.29
A 1 B 0.43
A 1 B 0.57
A 1 B 0.71
A 1 B 0.86
A 1 B 1
OD 600 nm [−]
0.2
0.4
0.6
0.8
0 5 101520
Time [h]

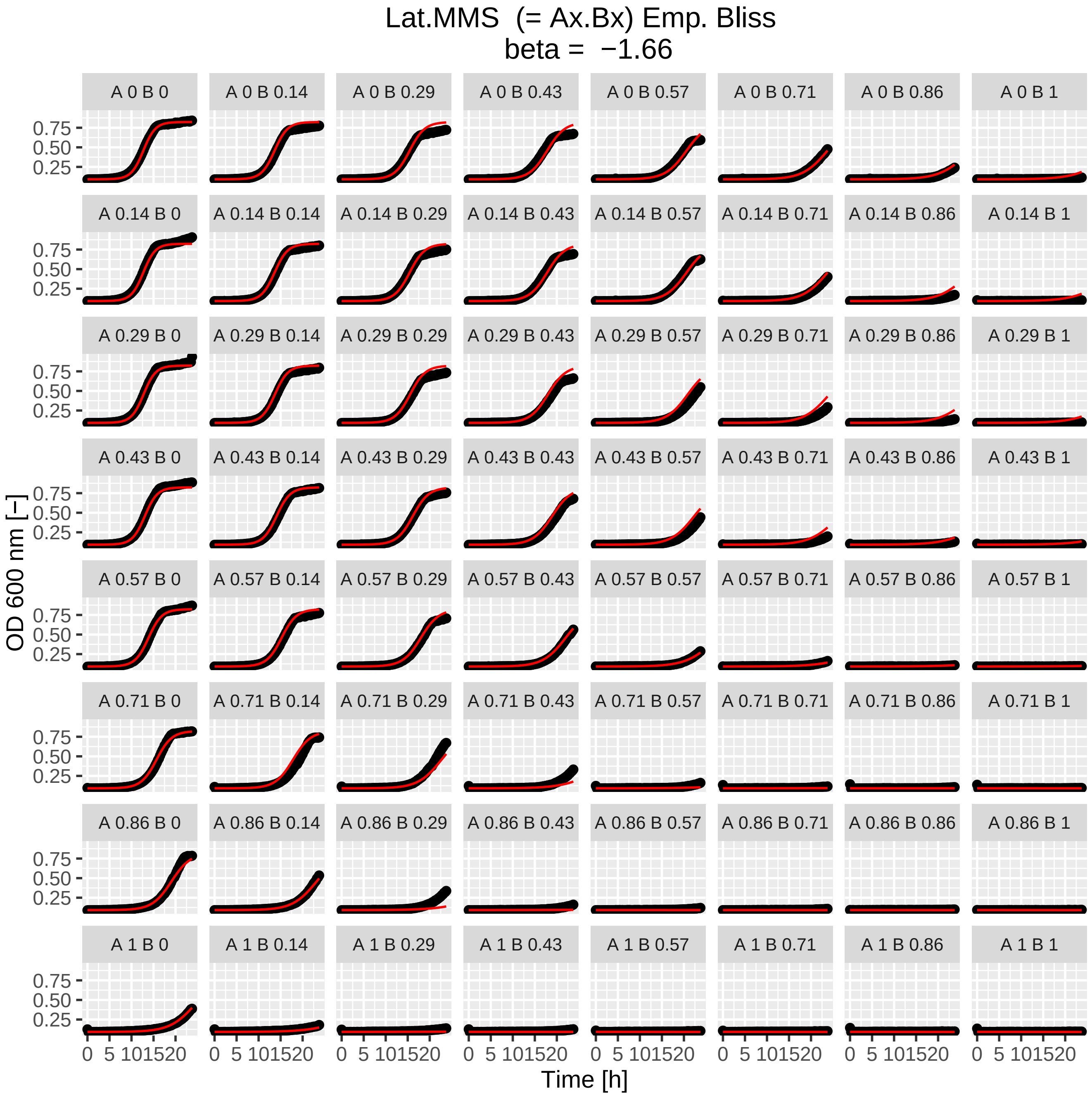
Lat.MMS (= Ax.Bx) Emp. Bliss
beta = −1.66
OD 600 nm [–]
Time [h]
0.75
0.50
0.25
0 5 101520
A 0 B 0
A 0 B 0.14
A 0 B 0.29
A 0 B 0.43
A 0 B 0.57
A 0 B 0.71
A 0 B 0.86
A 0 B 1
A 0.14 B 0
A 0.14 B 0.14
A 0.14 B 0.29
A 0.14 B 0.43
A 0.14 B 0.57
A 0.14 B 0.71
A 0.14 B 0.86
A 0.14 B 1
A 0.29 B 0
A 0.29 B 0.14
A 0.29 B 0.29
A 0.29 B 0.43
A 0.29 B 0.57
A 0.29 B 0.71
A 0.29 B 0.86
A 0.29 B 1
A 0.43 B 0
A 0.43 B 0.14
A 0.43 B 0.29
A 0.43 B 0.43
A 0.43 B 0.57
A 0.43 B 0.71
A 0.43 B 0.86
A 0.43 B 1
A 0.57 B 0
A 0.57 B 0.14
A 0.57 B 0.29
A 0.57 B 0.43
A 0.57 B 0.57
A 0.57 B 0.71
A 0.57 B 0.86
A 0.57 B 1
A 0.71 B 0
A 0.71 B 0.14
A 0.71 B 0.29
A 0.71 B 0.43
A 0.71 B 0.57
A 0.71 B 0.71
A 0.71 B 0.86
A 0.71 B 1
A 0.86 B 0
A 0.86 B 0.14
A 0.86 B 0.29
A 0.86 B 0.43
A 0.86 B 0.57
A 0.86 B 0.71
A 0.86 B 0.86
A 0.86 B 1
A 1 B 0
A 1 B 0.14
A 1 B 0.29
A 1 B 0.43
A 1 B 0.57
A 1 B 0.71
A 1 B 0.86
A 1 B 1

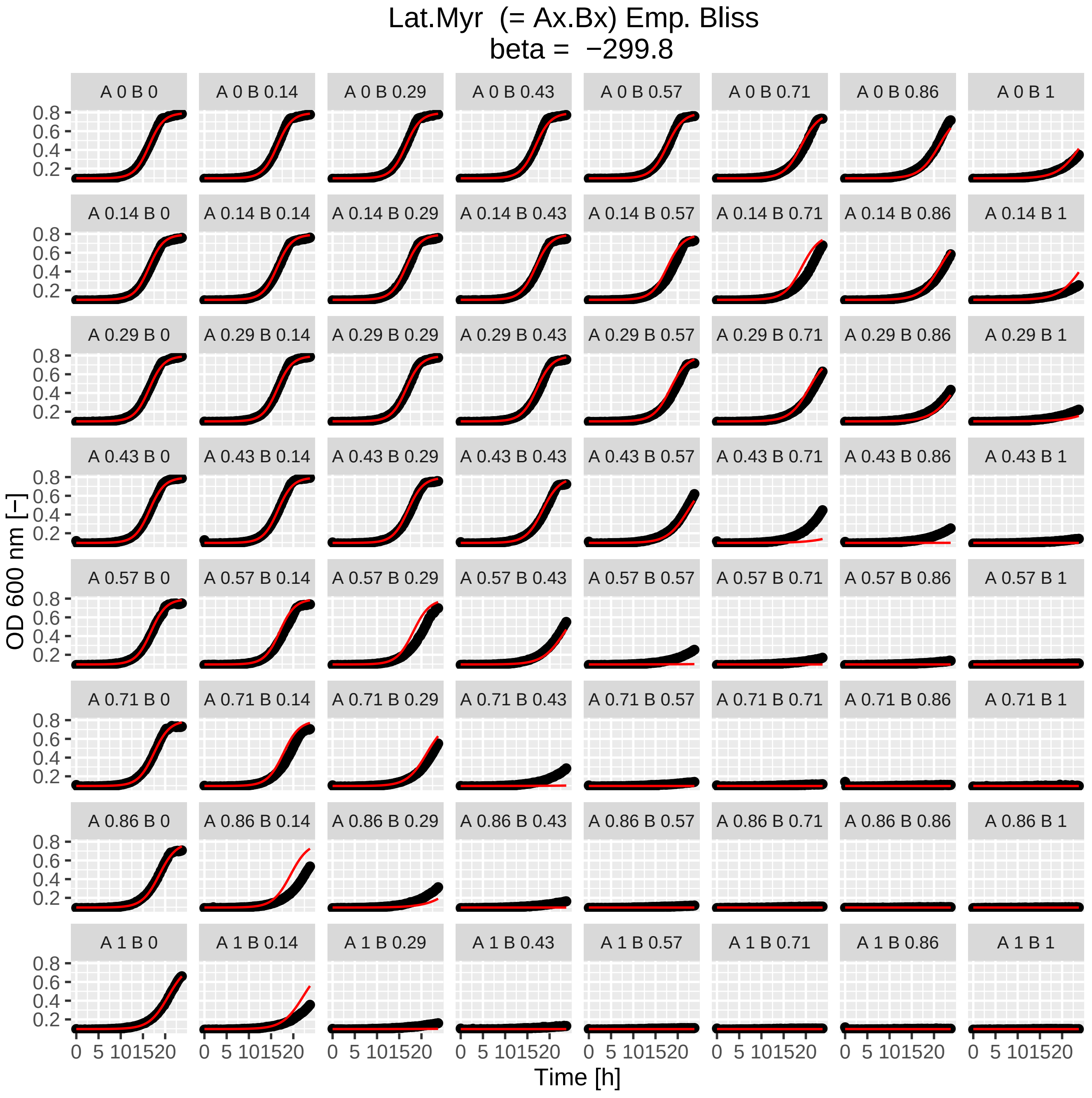
Lat.Myr (= Ax.Bx) Emp. Bliss
beta = −299.8
A 0 B 0
A 0 B 0.14
A 0 B 0.29
A 0 B 0.43
A 0 B 0.57
A 0 B 0.71
A 0 B 0.86
A 0 B 1
A 0.14 B 0
A 0.14 B 0.14
A 0.14 B 0.29
A 0.14 B 0.43
A 0.14 B 0.57
A 0.14 B 0.71
A 0.14 B 0.86
A 0.14 B 1
A 0.29 B 0
A 0.29 B 0.14
A 0.29 B 0.29
A 0.29 B 0.43
A 0.29 B 0.57
A 0.29 B 0.71
A 0.29 B 0.86
A 0.29 B 1
A 0.43 B 0
A 0.43 B 0.14
A 0.43 B 0.29
A 0.43 B 0.43
A 0.43 B 0.57
A 0.43 B 0.71
A 0.43 B 0.86
A 0.43 B 1
A 0.57 B 0
A 0.57 B 0.14
A 0.57 B 0.29
A 0.57 B 0.43
A 0.57 B 0.57
A 0.57 B 0.71
A 0.57 B 0.86
A 0.57 B 1
A 0.71 B 0
A 0.71 B 0.14
A 0.71 B 0.29
A 0.71 B 0.43
A 0.71 B 0.57
A 0.71 B 0.71
A 0.71 B 0.86
A 0.71 B 1
A 0.86 B 0
A 0.86 B 0.14
A 0.86 B 0.29
A 0.86 B 0.43
A 0.86 B 0.57
A 0.86 B 0.71
A 0.86 B 0.86
A 0.86 B 1
A 1 B 0
A 1 B 0.14
A 1 B 0.29
A 1 B 0.43
A 1 B 0.57
A 1 B 0.71
A 1 B 0.86
A 1 B 1
0.8
0.6
0.4
0.2
OD 600 nm [−]
0 5 101520
Time [h]

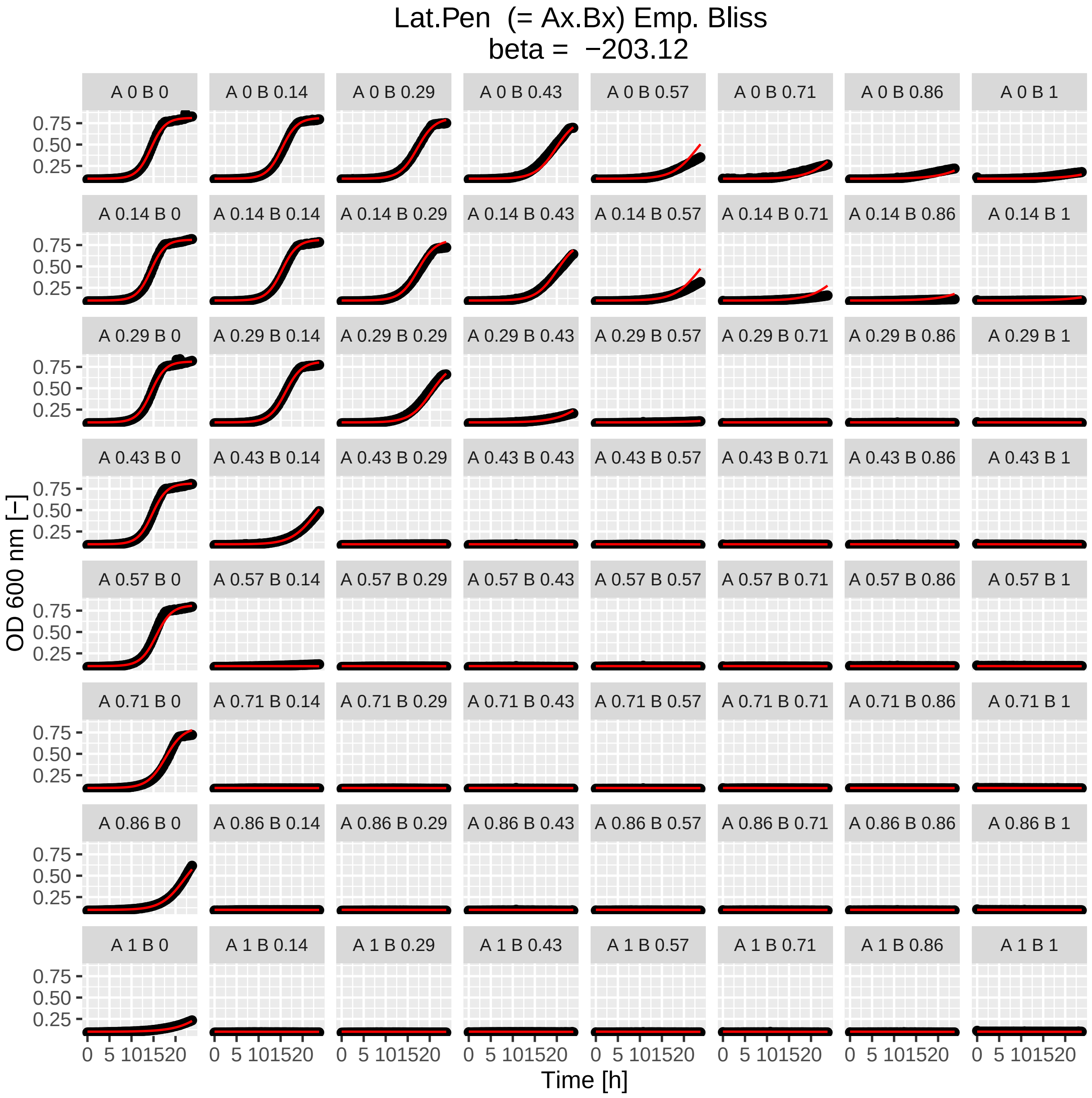

Lat.Pen (= Ax.Bx) Emp. Bliss
beta = −203.12
A 0 B 0
A 0 B 0.14
A 0 B 0.29
A 0 B 0.43
A 0 B 0.57
A 0 B 0.71
A 0 B 0.86
A 0 B 1
A 0.14 B 0
A 0.14 B 0.14
A 0.14 B 0.29
A 0.14 B 0.43
A 0.14 B 0.57
A 0.14 B 0.71
A 0.14 B 0.86
A 0.14 B 1
A 0.29 B 0
A 0.29 B 0.14
A 0.29 B 0.29
A 0.29 B 0.43
A 0.29 B 0.57
A 0.29 B 0.71
A 0.29 B 0.86
A 0.29 B 1
A 0.43 B 0
A 0.43 B 0.14
A 0.43 B 0.29
A 0.43 B 0.43
A 0.43 B 0.57
A 0.43 B 0.71
A 0.43 B 0.86
A 0.43 B 1
A 0.57 B 0
A 0.57 B 0.14
A 0.57 B 0.29
A 0.57 B 0.43
A 0.57 B 0.57
A 0.57 B 0.71
A 0.57 B 0.86
A 0.57 B 1
A 0.71 B 0
A 0.71 B 0.14
A 0.71 B 0.29
A 0.71 B 0.43
A 0.71 B 0.57
A 0.71 B 0.71
A 0.71 B 0.86
A 0.71 B 1
A 0.86 B 0
A 0.86 B 0.14
A 0.86 B 0.29
A 0.86 B 0.43
A 0.86 B 0.57
A 0.86 B 0.71
A 0.86 B 0.86
A 0.86 B 1
A 1 B 0
A 1 B 0.14
A 1 B 0.29
A 1 B 0.43
A 1 B 0.57
A 1 B 0.71
A 1 B 0.86
A 1 B 1
0.75
0.50
0.25
OD 600 nm [−]
0 5 101520
Time [h]

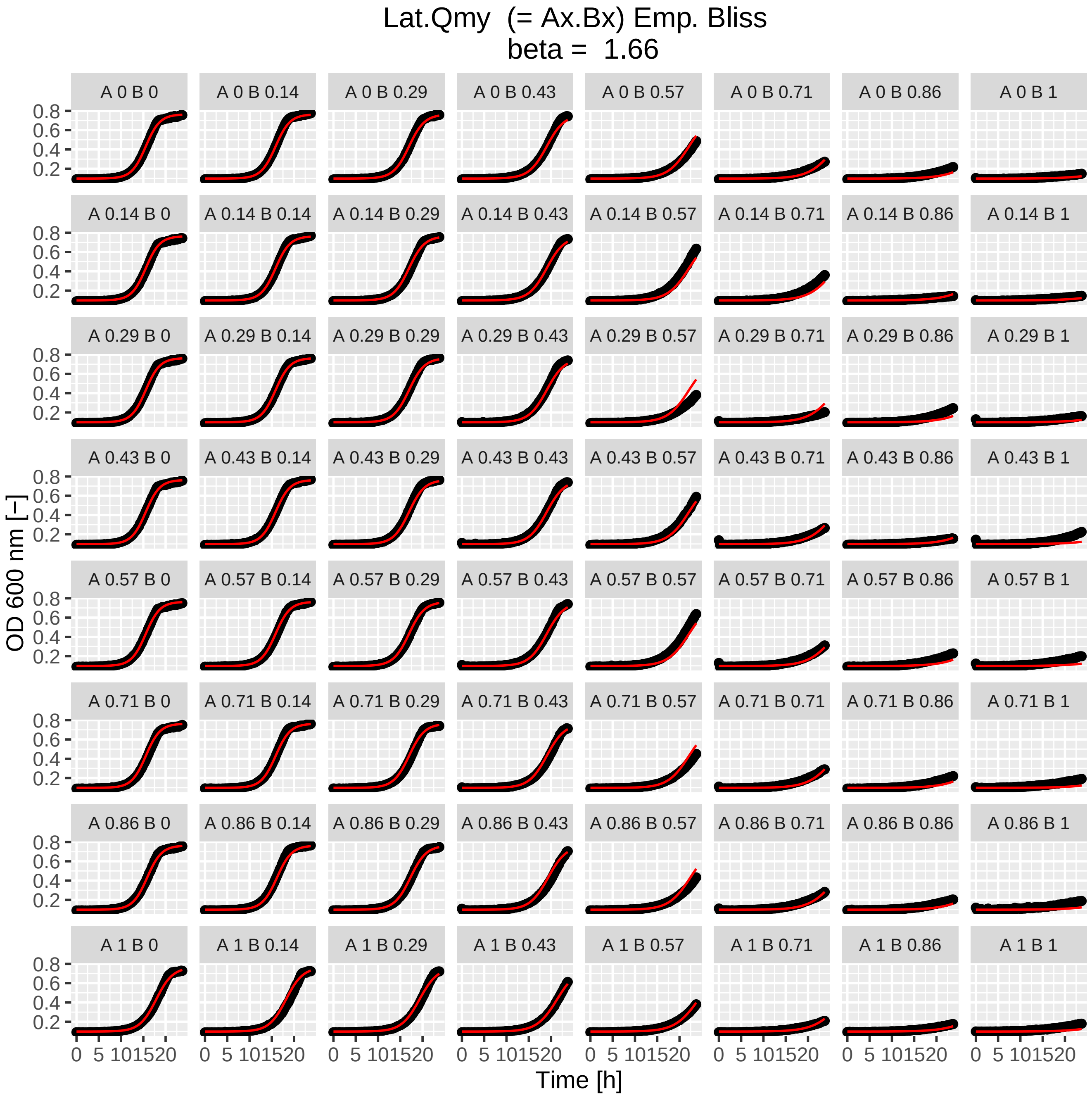

Lat.Qmy (= Ax.Bx) Emp. Bliss
beta = 1.66
A 0 B 0
A 0 B 0.14
A 0 B 0.29
A 0 B 0.43
A 0 B 0.57
A 0 B 0.71
A 0 B 0.86
A 0 B 1
A 0.14 B 0
A 0.14 B 0.14
A 0.14 B 0.29
A 0.14 B 0.43
A 0.14 B 0.57
A 0.14 B 0.71
A 0.14 B 0.86
A 0.14 B 1
A 0.29 B 0
A 0.29 B 0.14
A 0.29 B 0.29
A 0.29 B 0.43
A 0.29 B 0.57
A 0.29 B 0.71
A 0.29 B 0.86
A 0.29 B 1
A 0.43 B 0
A 0.43 B 0.14
A 0.43 B 0.29
A 0.43 B 0.43
A 0.43 B 0.57
A 0.43 B 0.71
A 0.43 B 0.86
A 0.43 B 1
A 0.57 B 0
A 0.57 B 0.14
A 0.57 B 0.29
A 0.57 B 0.43
A 0.57 B 0.57
A 0.57 B 0.71
A 0.57 B 0.86
A 0.57 B 1
A 0.71 B 0
A 0.71 B 0.14
A 0.71 B 0.29
A 0.71 B 0.43
A 0.71 B 0.57
A 0.71 B 0.71
A 0.71 B 0.86
A 0.71 B 1
A 0.86 B 0
A 0.86 B 0.14
A 0.86 B 0.29
A 0.86 B 0.43
A 0.86 B 0.57
A 0.86 B 0.71
A 0.86 B 0.86
A 0.86 B 1
A 1 B 0
A 1 B 0.14
A 1 B 0.29
A 1 B 0.43
A 1 B 0.57
A 1 B 0.71
A 1 B 0.86
A 1 B 1
0.8
0.6
0.4
0.2
OD 600 nm [–]
0 5 101520
Time [h]

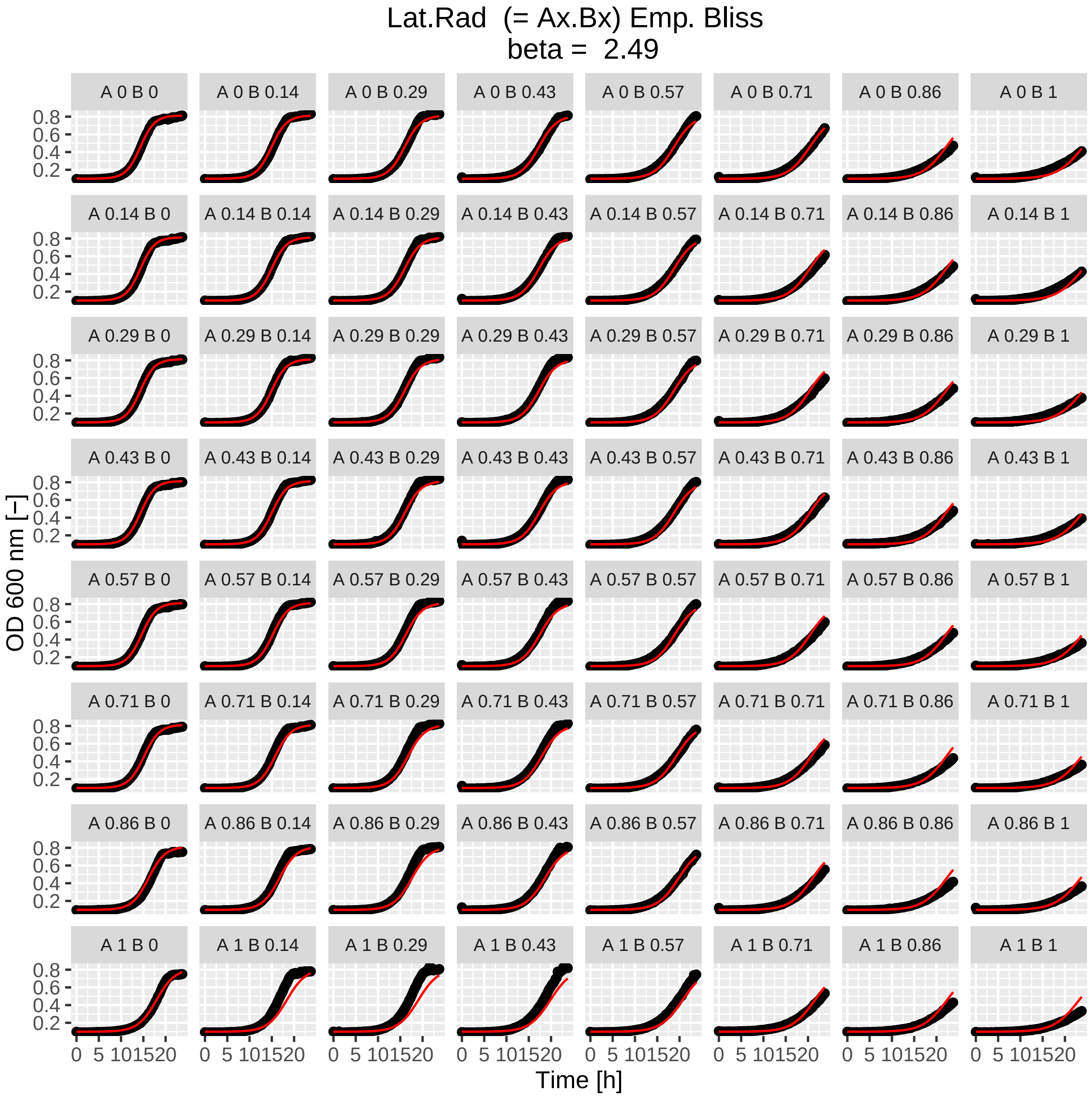
Lat.Rad (= Ax.Bx) Emp. Bliss
beta = 2.49
OD 600 nm [–]
Time [h]
0.8
0.6
0.4
0.2
0 5 101520
A 0 B 0
A 0 B 0.14
A 0 B 0.29
A 0 B 0.43
A 0 B 0.57
A 0 B 0.71
A 0 B 0.86
A 0 B 1
A 0.14 B 0
A 0.14 B 0.14
A 0.14 B 0.29
A 0.14 B 0.43
A 0.14 B 0.57
A 0.14 B 0.71
A 0.14 B 0.86
A 0.14 B 1
A 0.29 B 0
A 0.29 B 0.14
A 0.29 B 0.29
A 0.29 B 0.43
A 0.29 B 0.57
A 0.29 B 0.71
A 0.29 B 0.86
A 0.29 B 1
A 0.43 B 0
A 0.43 B 0.14
A 0.43 B 0.29
A 0.43 B 0.43
A 0.43 B 0.57
A 0.43 B 0.71
A 0.43 B 0.86
A 0.43 B 1
A 0.57 B 0
A 0.57 B 0.14
A 0.57 B 0.29
A 0.57 B 0.43
A 0.57 B 0.57
A 0.57 B 0.71
A 0.57 B 0.86
A 0.57 B 1
A 0.71 B 0
A 0.71 B 0.14
A 0.71 B 0.29
A 0.71 B 0.43
A 0.71 B 0.57
A 0.71 B 0.71
A 0.71 B 0.86
A 0.71 B 1
A 0.86 B 0
A 0.86 B 0.14
A 0.86 B 0.29
A 0.86 B 0.43
A 0.86 B 0.57
A 0.86 B 0.71
A 0.86 B 0.86
A 0.86 B 1
A 1 B 0
A 1 B 0.14
A 1 B 0.29
A 1 B 0.43
A 1 B 0.57
A 1 B 0.71
A 1 B 0.86
A 1 B 1

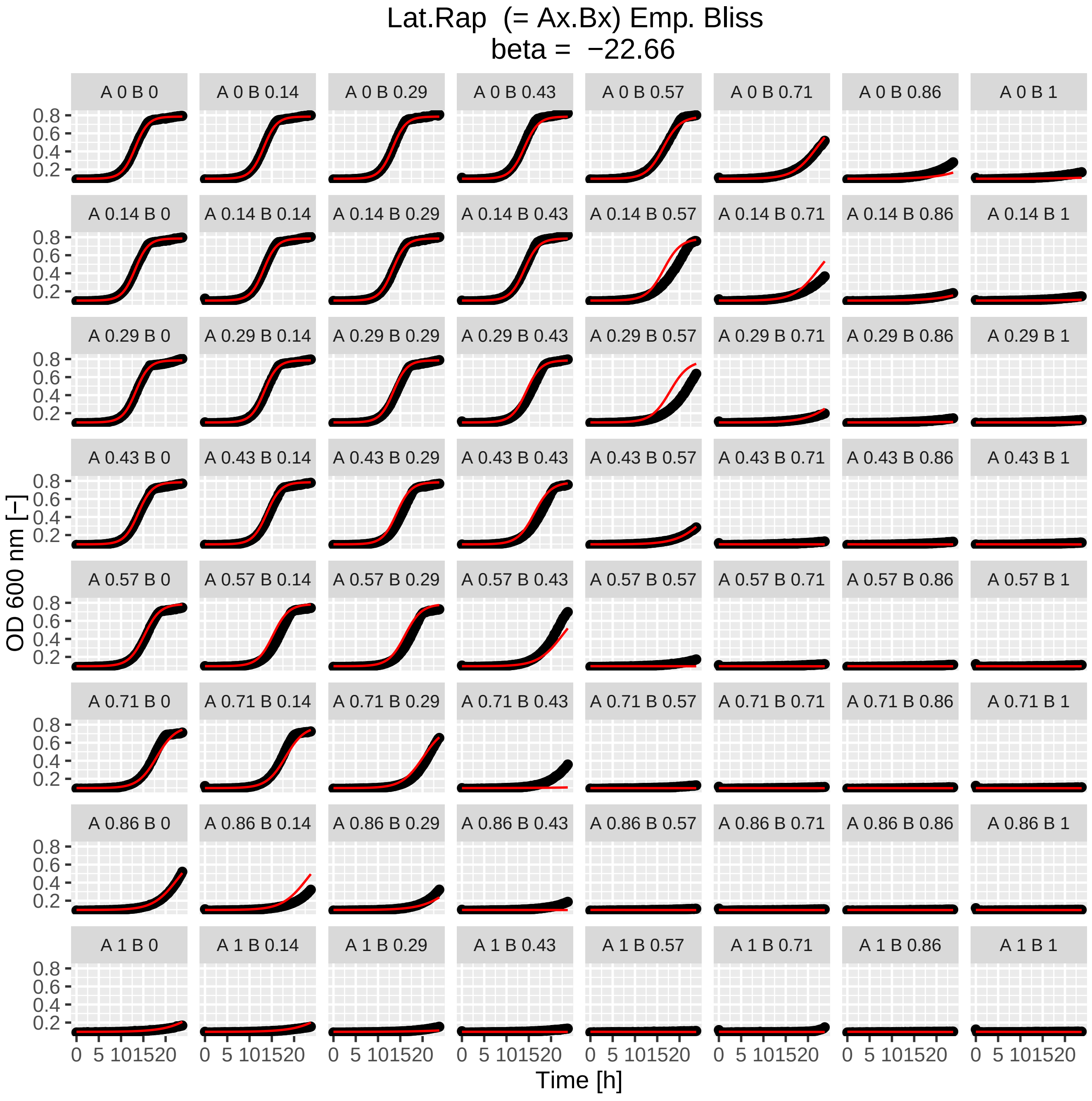

Lat.Rap (= Ax.Bx) Emp. Bliss
beta = −22.66
A 0 B 0
A 0 B 0.14
A 0 B 0.29
A 0 B 0.43
A 0 B 0.57
A 0 B 0.71
A 0 B 0.86
A 0 B 1
A 0.14 B 0
A 0.14 B 0.14
A 0.14 B 0.29
A 0.14 B 0.43
A 0.14 B 0.57
A 0.14 B 0.71
A 0.14 B 0.86
A 0.14 B 1
A 0.29 B 0
A 0.29 B 0.14
A 0.29 B 0.29
A 0.29 B 0.43
A 0.29 B 0.57
A 0.29 B 0.71
A 0.29 B 0.86
A 0.29 B 1
A 0.43 B 0
A 0.43 B 0.14
A 0.43 B 0.29
A 0.43 B 0.43
A 0.43 B 0.57
A 0.43 B 0.71
A 0.43 B 0.86
A 0.43 B 1
A 0.57 B 0
A 0.57 B 0.14
A 0.57 B 0.29
A 0.57 B 0.43
A 0.57 B 0.57
A 0.57 B 0.71
A 0.57 B 0.86
A 0.57 B 1
A 0.71 B 0
A 0.71 B 0.14
A 0.71 B 0.29
A 0.71 B 0.43
A 0.71 B 0.57
A 0.71 B 0.71
A 0.71 B 0.86
A 0.71 B 1
A 0.86 B 0
A 0.86 B 0.14
A 0.86 B 0.29
A 0.86 B 0.43
A 0.86 B 0.57
A 0.86 B 0.71
A 0.86 B 0.86
A 0.86 B 1
A 1 B 0
A 1 B 0.14
A 1 B 0.29
A 1 B 0.43
A 1 B 0.57
A 1 B 0.71
A 1 B 0.86
A 1 B 1
0.8
0.6
0.4
0.2
OD 600 nm [−]
0 5 101520
Time [h]

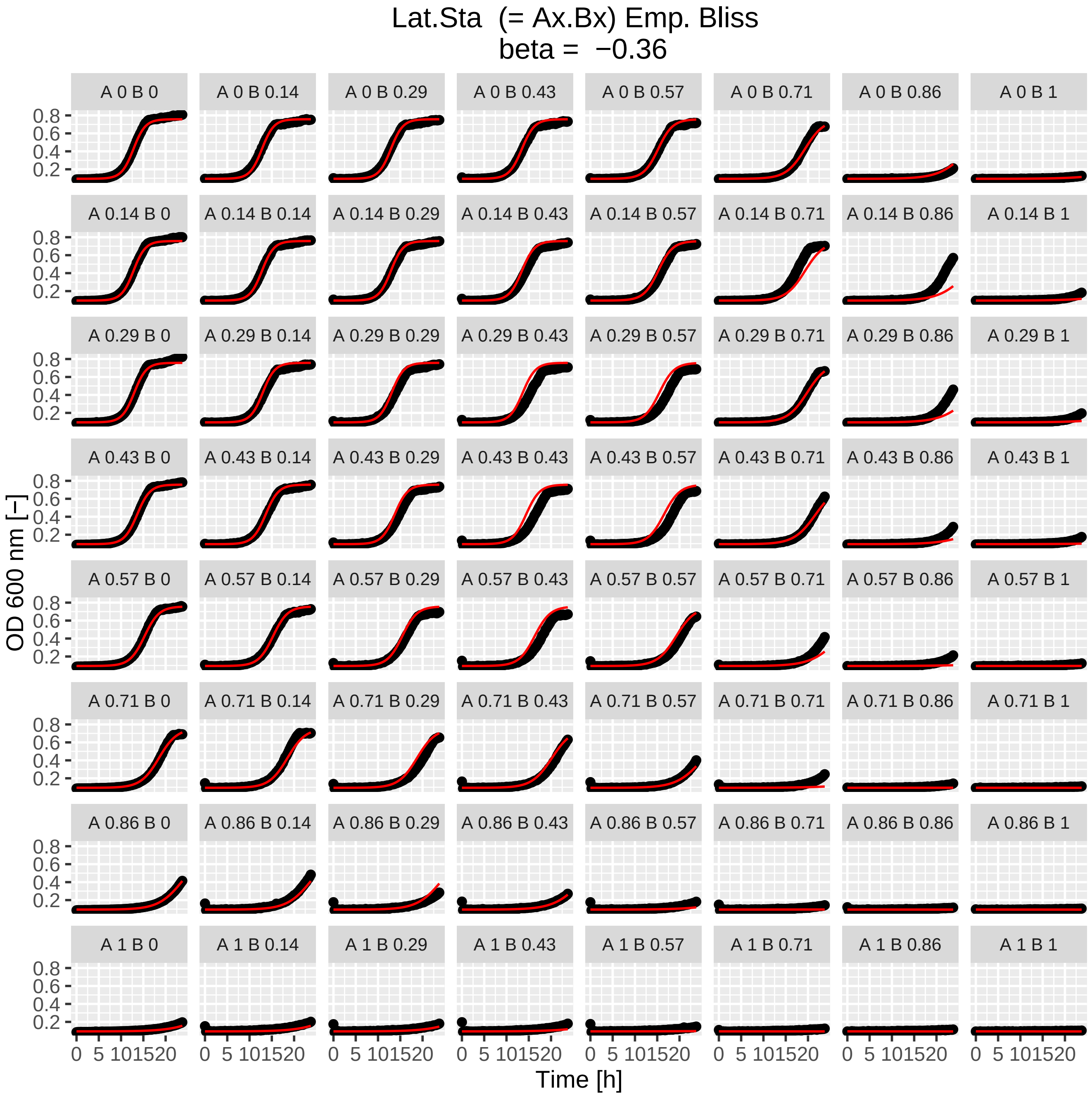
Lat.Sta (= Ax.Bx) Emp. Bliss
beta = −0.36
A 0 B 0
A 0 B 0.14
A 0 B 0.29
A 0 B 0.43
A 0 B 0.57
A 0 B 0.71
A 0 B 0.86
A 0 B 1
A 0.14 B 0
A 0.14 B 0.14
A 0.14 B 0.29
A 0.14 B 0.43
A 0.14 B 0.57
A 0.14 B 0.71
A 0.14 B 0.86
A 0.14 B 1
A 0.29 B 0
A 0.29 B 0.14
A 0.29 B 0.29
A 0.29 B 0.43
A 0.29 B 0.57
A 0.29 B 0.71
A 0.29 B 0.86
A 0.29 B 1
A 0.43 B 0
A 0.43 B 0.14
A 0.43 B 0.29
A 0.43 B 0.43
A 0.43 B 0.57
A 0.43 B 0.71
A 0.43 B 0.86
A 0.43 B 1
A 0.57 B 0
A 0.57 B 0.14
A 0.57 B 0.29
A 0.57 B 0.43
A 0.57 B 0.57
A 0.57 B 0.71
A 0.57 B 0.86
A 0.57 B 1
A 0.71 B 0
A 0.71 B 0.14
A 0.71 B 0.29
A 0.71 B 0.43
A 0.71 B 0.57
A 0.71 B 0.71
A 0.71 B 0.86
A 0.71 B 1
A 0.86 B 0
A 0.86 B 0.14
A 0.86 B 0.29
A 0.86 B 0.43
A 0.86 B 0.57
A 0.86 B 0.71
A 0.86 B 0.86
A 0.86 B 1
A 1 B 0
A 1 B 0.14
A 1 B 0.29
A 1 B 0.43
A 1 B 0.57
A 1 B 0.71
A 1 B 0.86
A 1 B 1
OD 600 nm [−]
0.2
0.4
0.6
0.8
0 5 101520
Time [h]

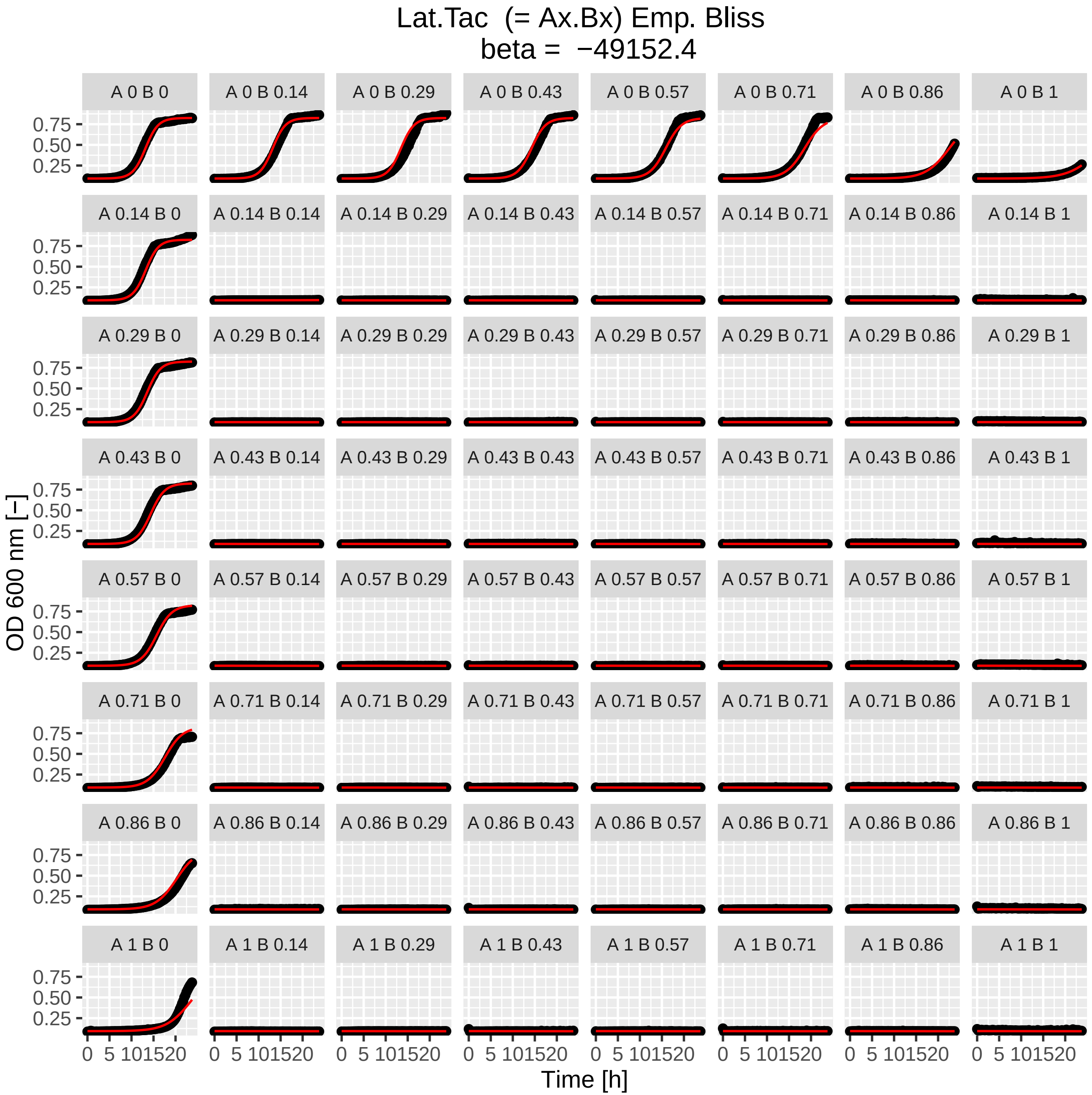

Lat.Tac (= Ax.Bx) Emp. Bliss
beta = −49152.4
A 0 B 0
A 0 B 0.14
A 0 B 0.29
A 0 B 0.43
A 0 B 0.57
A 0 B 0.71
A 0 B 0.86
A 0 B 1
A 0.14 B 0
A 0.14 B 0.14
A 0.14 B 0.29
A 0.14 B 0.43
A 0.14 B 0.57
A 0.14 B 0.71
A 0.14 B 0.86
A 0.14 B 1
A 0.29 B 0
A 0.29 B 0.14
A 0.29 B 0.29
A 0.29 B 0.43
A 0.29 B 0.57
A 0.29 B 0.71
A 0.29 B 0.86
A 0.29 B 1
A 0.43 B 0
A 0.43 B 0.14
A 0.43 B 0.29
A 0.43 B 0.43
A 0.43 B 0.57
A 0.43 B 0.71
A 0.43 B 0.86
A 0.43 B 1
A 0.57 B 0
A 0.57 B 0.14
A 0.57 B 0.29
A 0.57 B 0.43
A 0.57 B 0.57
A 0.57 B 0.71
A 0.57 B 0.86
A 0.57 B 1
A 0.71 B 0
A 0.71 B 0.14
A 0.71 B 0.29
A 0.71 B 0.43
A 0.71 B 0.57
A 0.71 B 0.71
A 0.71 B 0.86
A 0.71 B 1
A 0.86 B 0
A 0.86 B 0.14
A 0.86 B 0.29
A 0.86 B 0.43
A 0.86 B 0.57
A 0.86 B 0.71
A 0.86 B 0.86
A 0.86 B 1
A 1 B 0
A 1 B 0.14
A 1 B 0.29
A 1 B 0.43
A 1 B 0.57
A 1 B 0.71
A 1 B 0.86
A 1 B 1
OD 600 nm [−]
0.75
0.50
0.25
0 5 101520
Time [h]

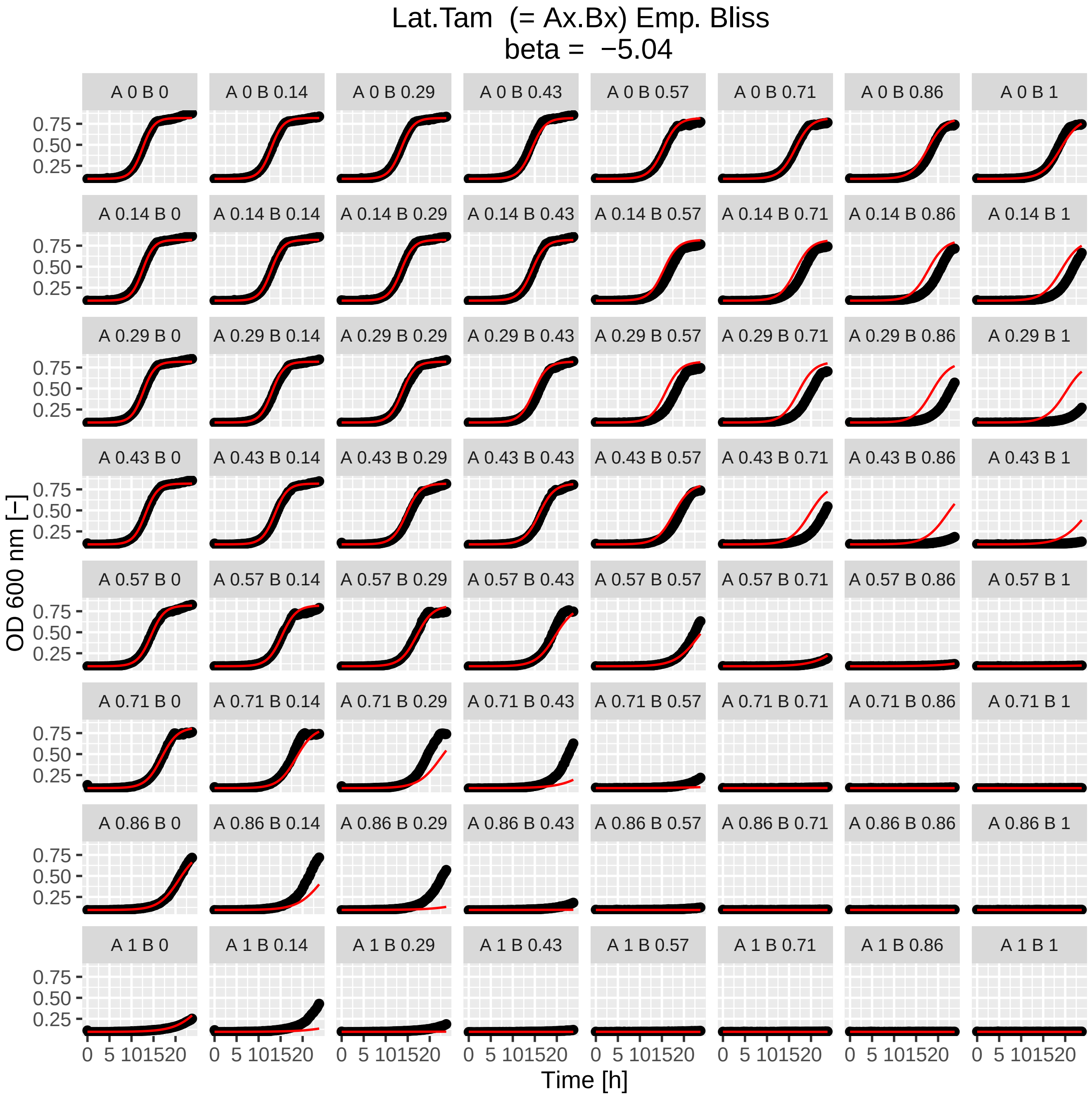
Lat.Tam (= Ax.Bx) Emp. Bliss
beta = −5.04
A 0 B 0
A 0 B 0.14
A 0 B 0.29
A 0 B 0.43
A 0 B 0.57
A 0 B 0.71
A 0 B 0.86
A 0 B 1
A 0.14 B 0
A 0.14 B 0.14
A 0.14 B 0.29
A 0.14 B 0.43
A 0.14 B 0.57
A 0.14 B 0.71
A 0.14 B 0.86
A 0.14 B 1
A 0.29 B 0
A 0.29 B 0.14
A 0.29 B 0.29
A 0.29 B 0.43
A 0.29 B 0.57
A 0.29 B 0.71
A 0.29 B 0.86
A 0.29 B 1
A 0.43 B 0
A 0.43 B 0.14
A 0.43 B 0.29
A 0.43 B 0.43
A 0.43 B 0.57
A 0.43 B 0.71
A 0.43 B 0.86
A 0.43 B 1
A 0.57 B 0
A 0.57 B 0.14
A 0.57 B 0.29
A 0.57 B 0.43
A 0.57 B 0.57
A 0.57 B 0.71
A 0.57 B 0.86
A 0.57 B 1
A 0.71 B 0
A 0.71 B 0.14
A 0.71 B 0.29
A 0.71 B 0.43
A 0.71 B 0.57
A 0.71 B 0.71
A 0.71 B 0.86
A 0.71 B 1
A 0.86 B 0
A 0.86 B 0.14
A 0.86 B 0.29
A 0.86 B 0.43
A 0.86 B 0.57
A 0.86 B 0.71
A 0.86 B 0.86
A 0.86 B 1
A 1 B 0
A 1 B 0.14
A 1 B 0.29
A 1 B 0.43
A 1 B 0.57
A 1 B 0.71
A 1 B 0.86
A 1 B 1
0.75
0.50
0.25
OD 600 nm [−]
0 5 101520
Time [h]

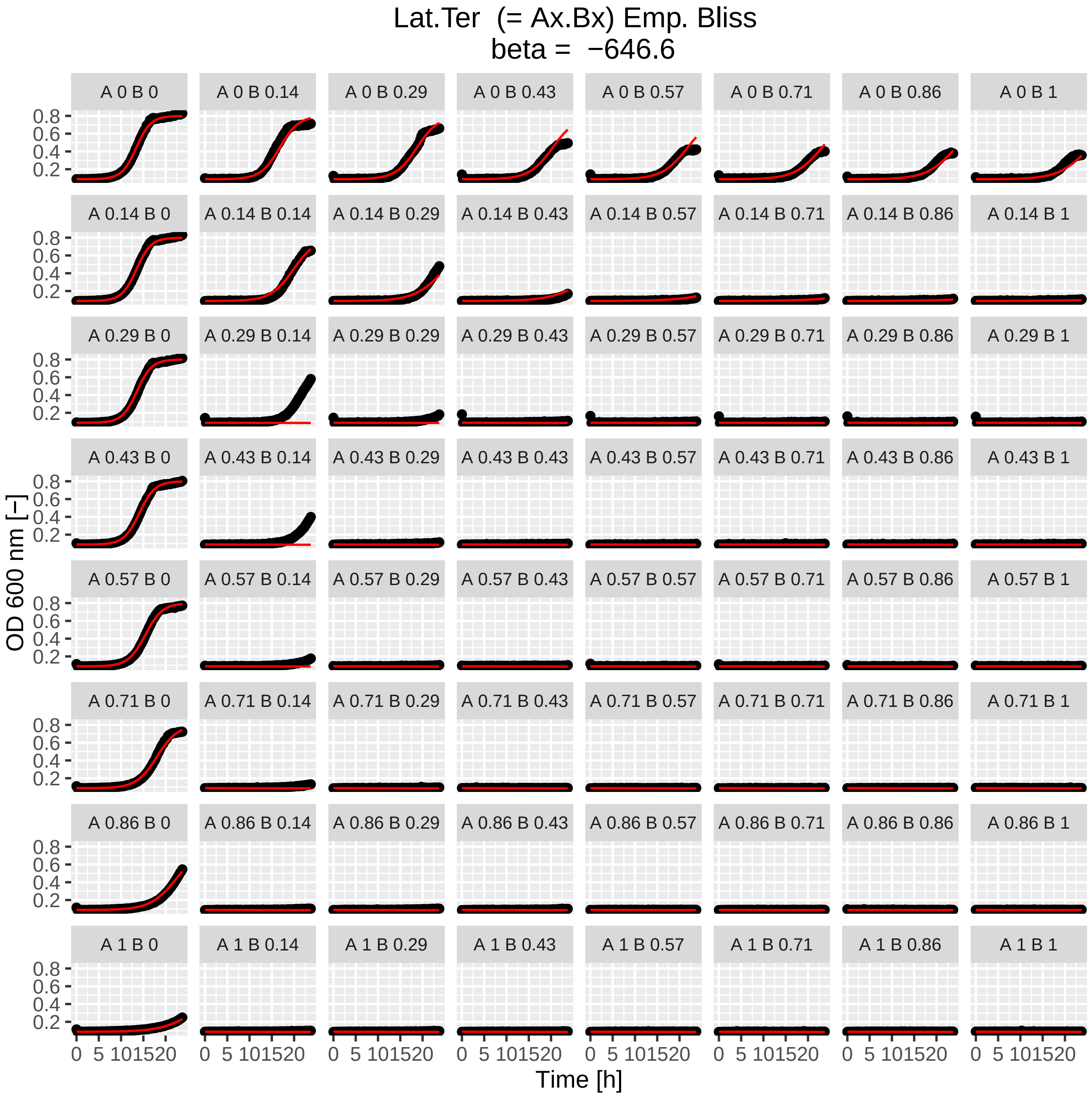

Lat.Ter (= Ax.Bx) Emp. Bliss
beta = −646.6
A 0 B 0
A 0 B 0.14
A 0 B 0.29
A 0 B 0.43
A 0 B 0.57
A 0 B 0.71
A 0 B 0.86
A 0 B 1
A 0.14 B 0
A 0.14 B 0.14
A 0.14 B 0.29
A 0.14 B 0.43
A 0.14 B 0.57
A 0.14 B 0.71
A 0.14 B 0.86
A 0.14 B 1
A 0.29 B 0
A 0.29 B 0.14
A 0.29 B 0.29
A 0.29 B 0.43
A 0.29 B 0.57
A 0.29 B 0.71
A 0.29 B 0.86
A 0.29 B 1
A 0.43 B 0
A 0.43 B 0.14
A 0.43 B 0.29
A 0.43 B 0.43
A 0.43 B 0.57
A 0.43 B 0.71
A 0.43 B 0.86
A 0.43 B 1
A 0.57 B 0
A 0.57 B 0.14
A 0.57 B 0.29
A 0.57 B 0.43
A 0.57 B 0.57
A 0.57 B 0.71
A 0.57 B 0.86
A 0.57 B 1
A 0.71 B 0
A 0.71 B 0.14
A 0.71 B 0.29
A 0.71 B 0.43
A 0.71 B 0.57
A 0.71 B 0.71
A 0.71 B 0.86
A 0.71 B 1
A 0.86 B 0
A 0.86 B 0.14
A 0.86 B 0.29
A 0.86 B 0.43
A 0.86 B 0.57
A 0.86 B 0.71
A 0.86 B 0.86
A 0.86 B 1
A 1 B 0
A 1 B 0.14
A 1 B 0.29
A 1 B 0.43
A 1 B 0.57
A 1 B 0.71
A 1 B 0.86
A 1 B 1
0.8
0.6
0.4
0.2
0 5 101520
OD 600 nm [−]
Time [h]

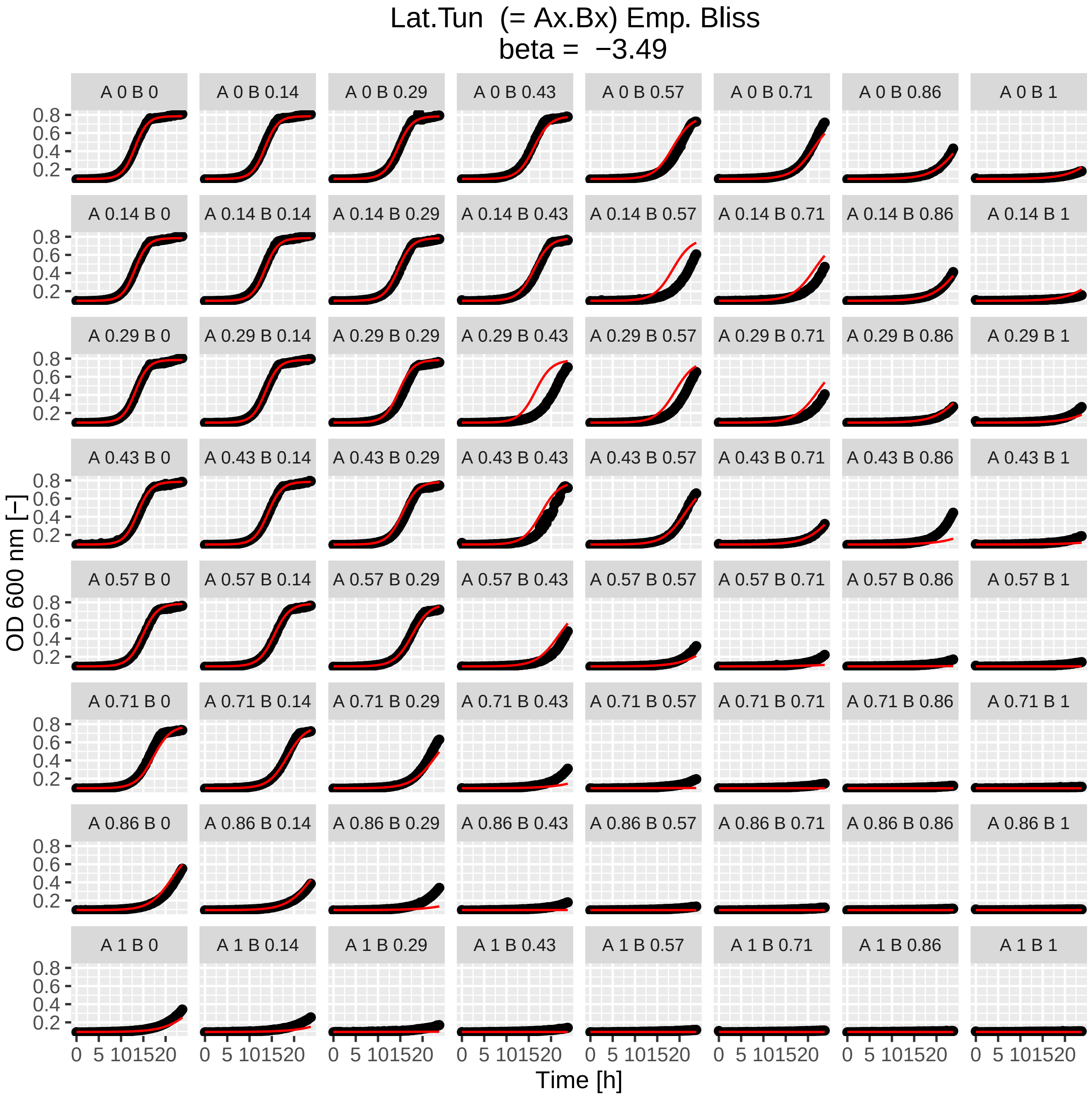

Lat.Tun (= Ax.Bx) Emp. Bliss
beta = −3.49
OD 600 nm [−]
Time [h]
A 0 B 0
A 0 B 0.14
A 0 B 0.29
A 0 B 0.43
A 0 B 0.57
A 0 B 0.71
A 0 B 0.86
A 0 B 1
A 0.14 B 0
A 0.14 B 0.14
A 0.14 B 0.29
A 0.14 B 0.43
A 0.14 B 0.57
A 0.14 B 0.71
A 0.14 B 0.86
A 0.14 B 1
A 0.29 B 0
A 0.29 B 0.14
A 0.29 B 0.29
A 0.29 B 0.43
A 0.29 B 0.57
A 0.29 B 0.71
A 0.29 B 0.86
A 0.29 B 1
A 0.43 B 0
A 0.43 B 0.14
A 0.43 B 0.29
A 0.43 B 0.43
A 0.43 B 0.57
A 0.43 B 0.71
A 0.43 B 0.86
A 0.43 B 1
A 0.57 B 0
A 0.57 B 0.14
A 0.57 B 0.29
A 0.57 B 0.43
A 0.57 B 0.57
A 0.57 B 0.71
A 0.57 B 0.86
A 0.57 B 1
A 0.71 B 0
A 0.71 B 0.14
A 0.71 B 0.29
A 0.71 B 0.43
A 0.71 B 0.57
A 0.71 B 0.71
A 0.71 B 0.86
A 0.71 B 1
A 0.86 B 0
A 0.86 B 0.14
A 0.86 B 0.29
A 0.86 B 0.43
A 0.86 B 0.57
A 0.86 B 0.71
A 0.86 B 0.86
A 0.86 B 1
A 1 B 0
A 1 B 0.14
A 1 B 0.29
A 1 B 0.43
A 1 B 0.57
A 1 B 0.71
A 1 B 0.86
A 1 B 1
0.8
0.6
0.4
0.2
0 5 101520

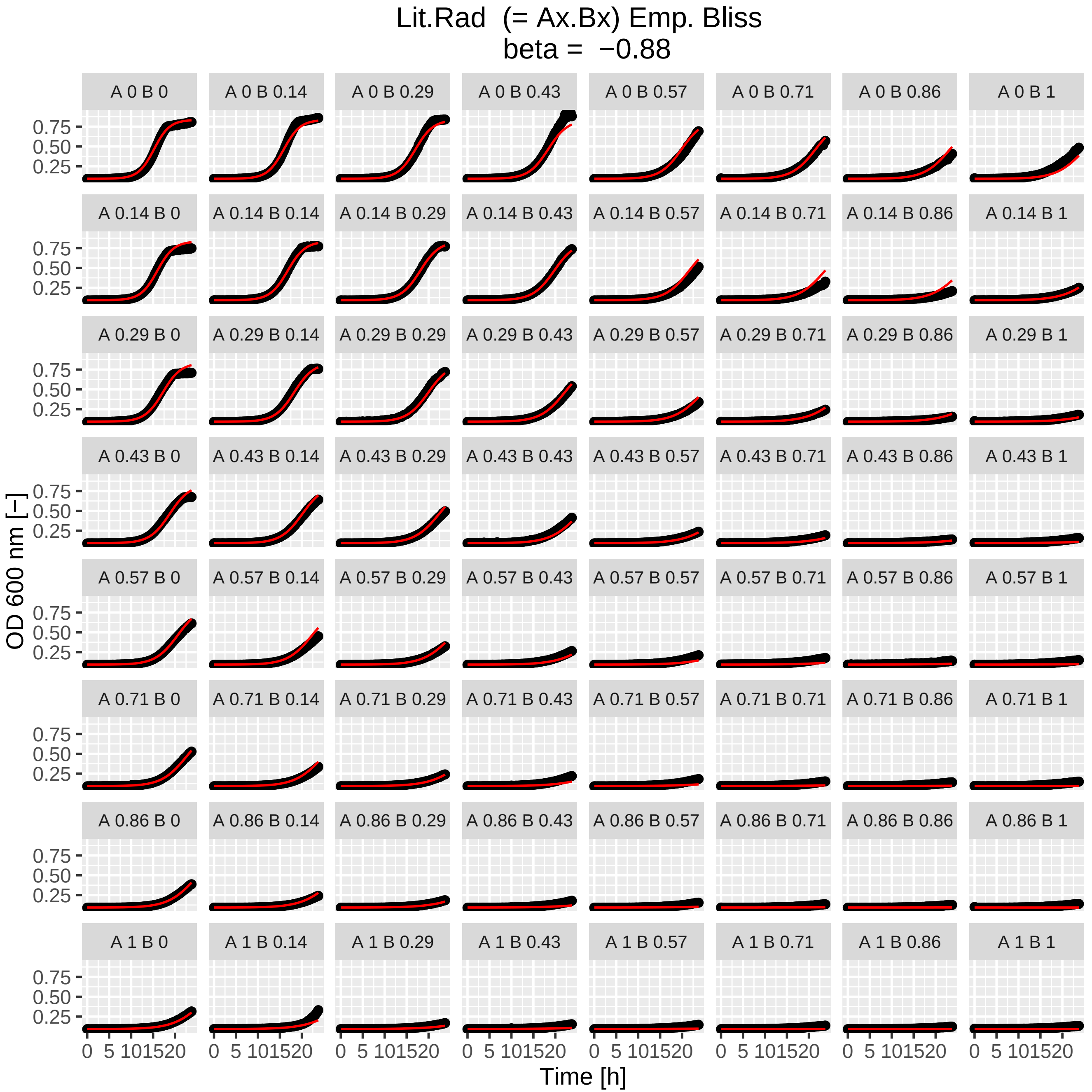
Lit.Rad (= Ax.Bx) Emp. Bliss
beta = −0.88
OD 600 nm [−]
Time [h]
A 0 B 0
A 0 B 0.14
A 0 B 0.29
A 0 B 0.43
A 0 B 0.57
A 0 B 0.71
A 0 B 0.86
A 0 B 1
A 1 B 0
A 1 B 1
0.25
0.50
0.75
0 5 101520

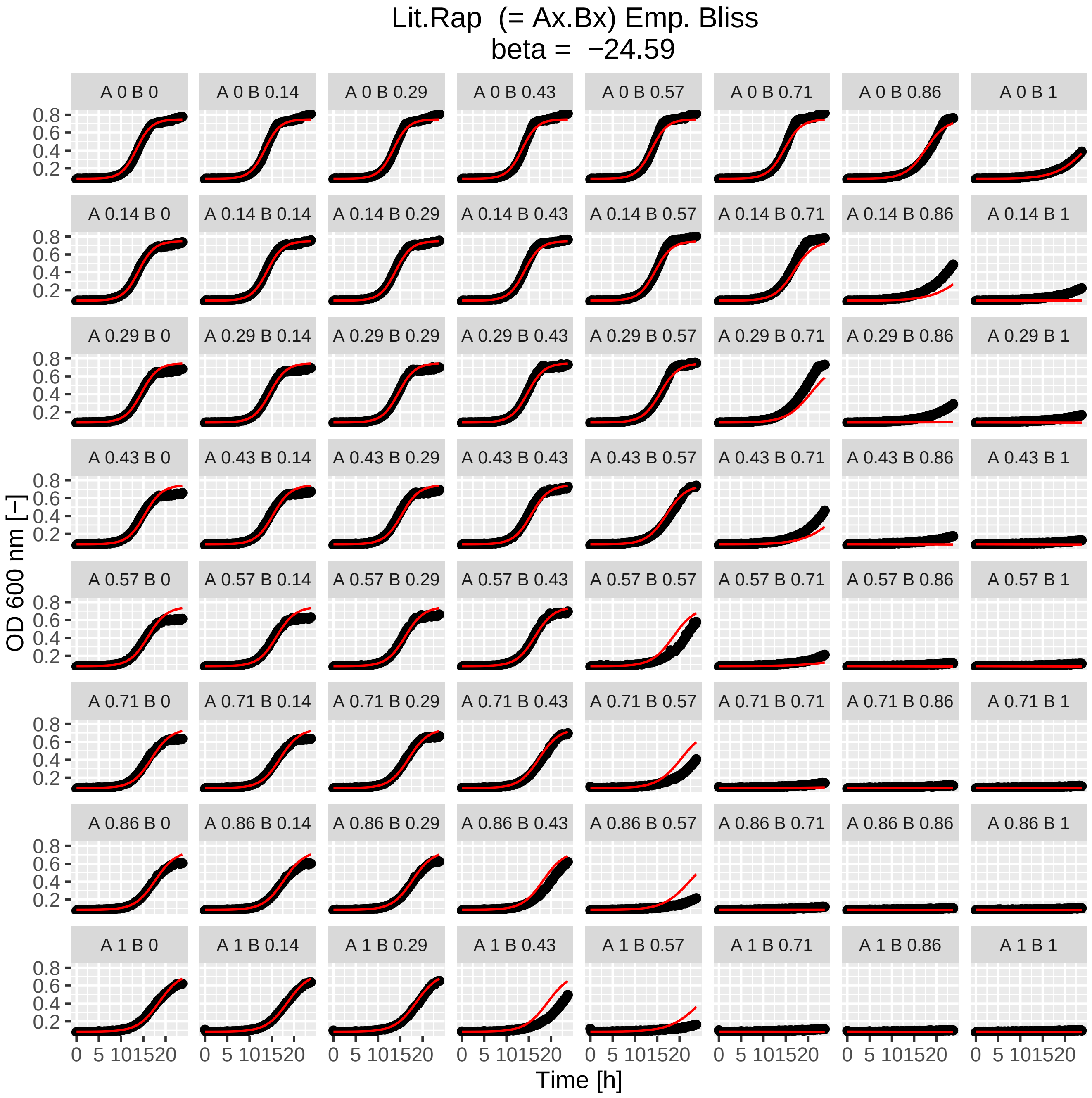

Lit.Rap (= Ax.Bx) Emp. Bliss
beta = −24.59
OD 600 nm [−]
Time [h]
A 0 B 0
A 0 B 0.14
A 0 B 0.29
A 0 B 0.43
A 0 B 0.57
A 0 B 0.71
A 0 B 0.86
A 0 B 1
A 0.14 B 0
A 0.14 B 0.14
A 0.14 B 0.29
A 0.14 B 0.43
A 0.14 B 0.57
A 0.14 B 0.71
A 0.14 B 0.86
A 0.14 B 1
A 0.29 B 0
A 0.29 B 0.14
A 0.29 B 0.29
A 0.29 B 0.43
A 0.29 B 0.57
A 0.29 B 0.71
A 0.29 B 0.86
A 0.29 B 1
A 0.43 B 0
A 0.43 B 0.14
A 0.43 B 0.29
A 0.43 B 0.43
A 0.43 B 0.57
A 0.43 B 0.71
A 0.43 B 0.86
A 0.43 B 1
A 0.57 B 0
A 0.57 B 0.14
A 0.57 B 0.29
A 0.57 B 0.43
A 0.57 B 0.57
A 0.57 B 0.71
A 0.57 B 0.86
A 0.57 B 1
A 0.71 B 0
A 0.71 B 0.14
A 0.71 B 0.29
A 0.71 B 0.43
A 0.71 B 0.57
A 0.71 B 0.71
A 0.71 B 0.86
A 0.71 B 1
A 0.86 B 0
A 0.86 B 0.14
A 0.86 B 0.29
A 0.86 B 0.43
A 0.86 B 0.57
A 0.86 B 0.71
A 0.86 B 0.86
A 0.86 B 1
A 1 B 0
A 1 B 0.14
A 1 B 0.29
A 1 B 0.43
A 1 B 0.57
A 1 B 0.71
A 1 B 0.86
A 1 B 1
0.8
0.6
0.4
0.2
0 5 101520

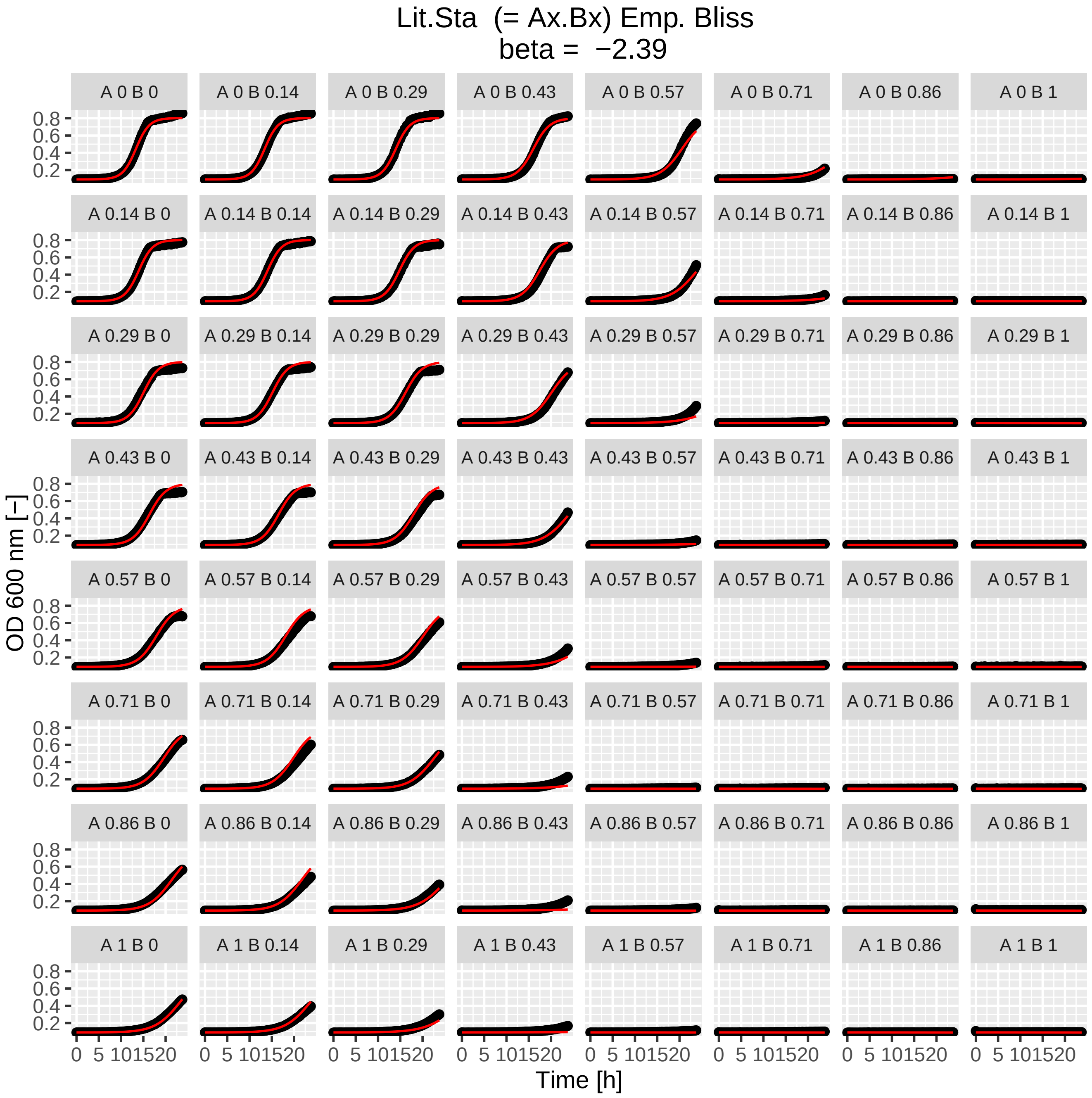
Lit.Sta (= Ax.Bx) Emp. Bliss
beta = −2.39
OD 600 nm [−]
Time [h]
A 0 B 0
A 0 B 0.14
A 0 B 0.29
A 0 B 0.43
A 0 B 0.57
A 0 B 0.71
A 0 B 0.86
A 0 B 1
A 0.14 B 0
A 0.14 B 0.14
A 0.14 B 0.29
A 0.14 B 0.43
A 0.14 B 0.57
A 0.14 B 0.71
A 0.14 B 0.86
A 0.14 B 1
A 0.29 B 0
A 0.29 B 0.14
A 0.29 B 0.29
A 0.29 B 0.43
A 0.29 B 0.57
A 0.29 B 0.71
A 0.29 B 0.86
A 0.29 B 1
A 0.43 B 0
A 0.43 B 0.14
A 0.43 B 0.29
A 0.43 B 0.43
A 0.43 B 0.57
A 0.43 B 0.71
A 0.43 B 0.86
A 0.43 B 1
A 0.57 B 0
A 0.57 B 0.14
A 0.57 B 0.29
A 0.57 B 0.43
A 0.57 B 0.57
A 0.57 B 0.71
A 0.57 B 0.86
A 0.57 B 1
A 0.71 B 0
A 0.71 B 0.14
A 0.71 B 0.29
A 0.71 B 0.43
A 0.71 B 0.57
A 0.71 B 0.71
A 0.71 B 0.86
A 0.71 B 1
A 0.86 B 0
A 0.86 B 0.14
A 0.86 B 0.29
A 0.86 B 0.43
A 0.86 B 0.57
A 0.86 B 0.71
A 0.86 B 0.86
A 0.86 B 1
A 1 B 0
A 1 B 0.14
A 1 B 0.29
A 1 B 0.43
A 1 B 0.57
A 1 B 0.71
A 1 B 0.86
A 1 B 1
0.2
0.4
0.6
0.8
0
5
10
15
20

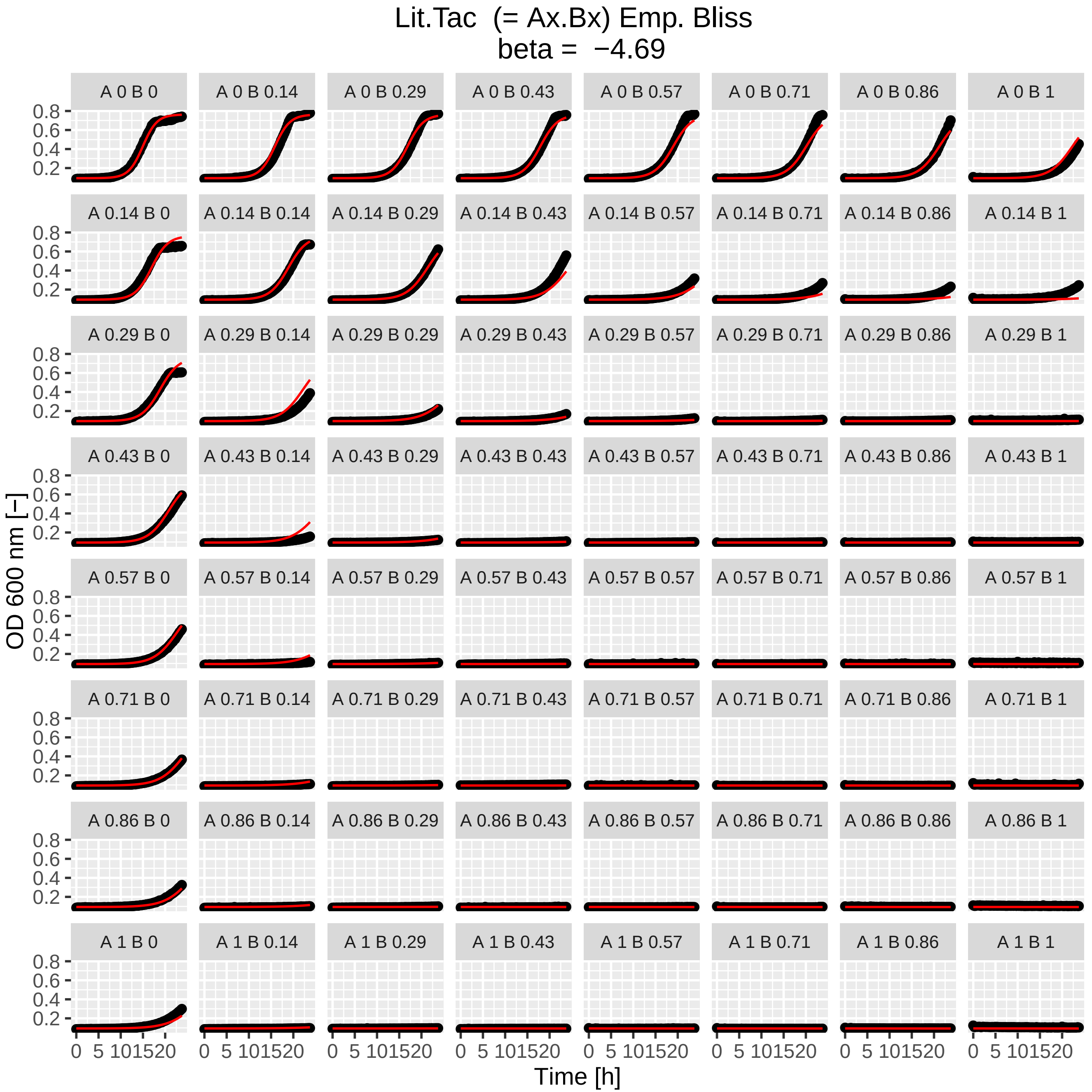
Lit.Tac (= Ax.Bx) Emp. Bliss
beta = −4.69
A 0 B 0
A 0 B 0.14
A 0 B 0.29
A 0 B 0.43
A 0 B 0.57
A 0 B 0.71
A 0 B 0.86
A 0 B 1
A 0.14 B 0
A 0.14 B 0.14
A 0.14 B 0.29
A 0.14 B 0.43
A 0.14 B 0.57
A 0.14 B 0.71
A 0.14 B 0.86
A 0.14 B 1
A 0.29 B 0
A 0.29 B 0.14
A 0.29 B 0.29
A 0.29 B 0.43
A 0.29 B 0.57
A 0.29 B 0.71
A 0.29 B 0.86
A 0.29 B 1
A 0.43 B 0
A 0.43 B 0.14
A 0.43 B 0.29
A 0.43 B 0.43
A 0.43 B 0.57
A 0.43 B 0.71
A 0.43 B 0.86
A 0.43 B 1
A 0.57 B 0
A 0.57 B 0.14
A 0.57 B 0.29
A 0.57 B 0.43
A 0.57 B 0.57
A 0.57 B 0.71
A 0.57 B 0.86
A 0.57 B 1
A 0.71 B 0
A 0.71 B 0.14
A 0.71 B 0.29
A 0.71 B 0.43
A 0.71 B 0.57
A 0.71 B 0.71
A 0.71 B 0.86
A 0.71 B 1
A 0.86 B 0
A 0.86 B 0.14
A 0.86 B 0.29
A 0.86 B 0.43
A 0.86 B 0.57
A 0.86 B 0.71
A 0.86 B 0.86
A 0.86 B 1
A 1 B 0
A 1 B 0.14
A 1 B 0.29
A 1 B 0.43
A 1 B 0.57
A 1 B 0.71
A 1 B 0.86
A 1 B 1
OD 600 nm [−]
0.2
0.4
0.6
0.8
0 5 101520
Time [h]

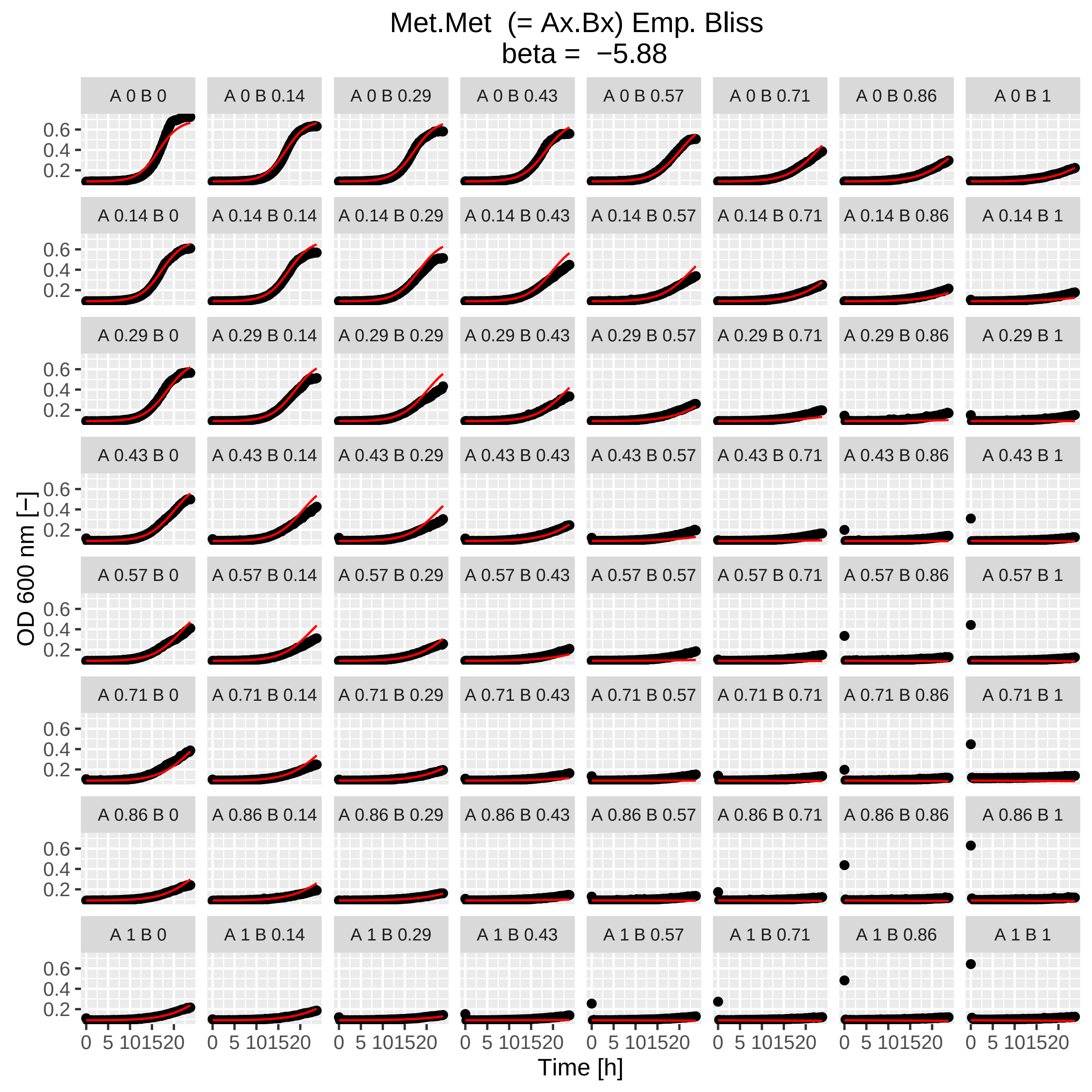
Met.Met (= Ax.Bx) Emp. Bliss
beta = −5.88
OD 600 nm [−]
Time [h]
A 0 B 0
A 0 B 0.14
A 0 B 0.29
A 0 B 0.43
A 0 B 0.57
A 0 B 0.71
A 0 B 0.86
A 0 B 1
A 1 B 0
A 1 B 0.14
A 1 B 0.29
A 1 B 0.43
A 1 B 0.57
A 1 B 0.71
A 1 B 0.86
A 1 B 1
0.2
0.4
0.6
0 5 101520

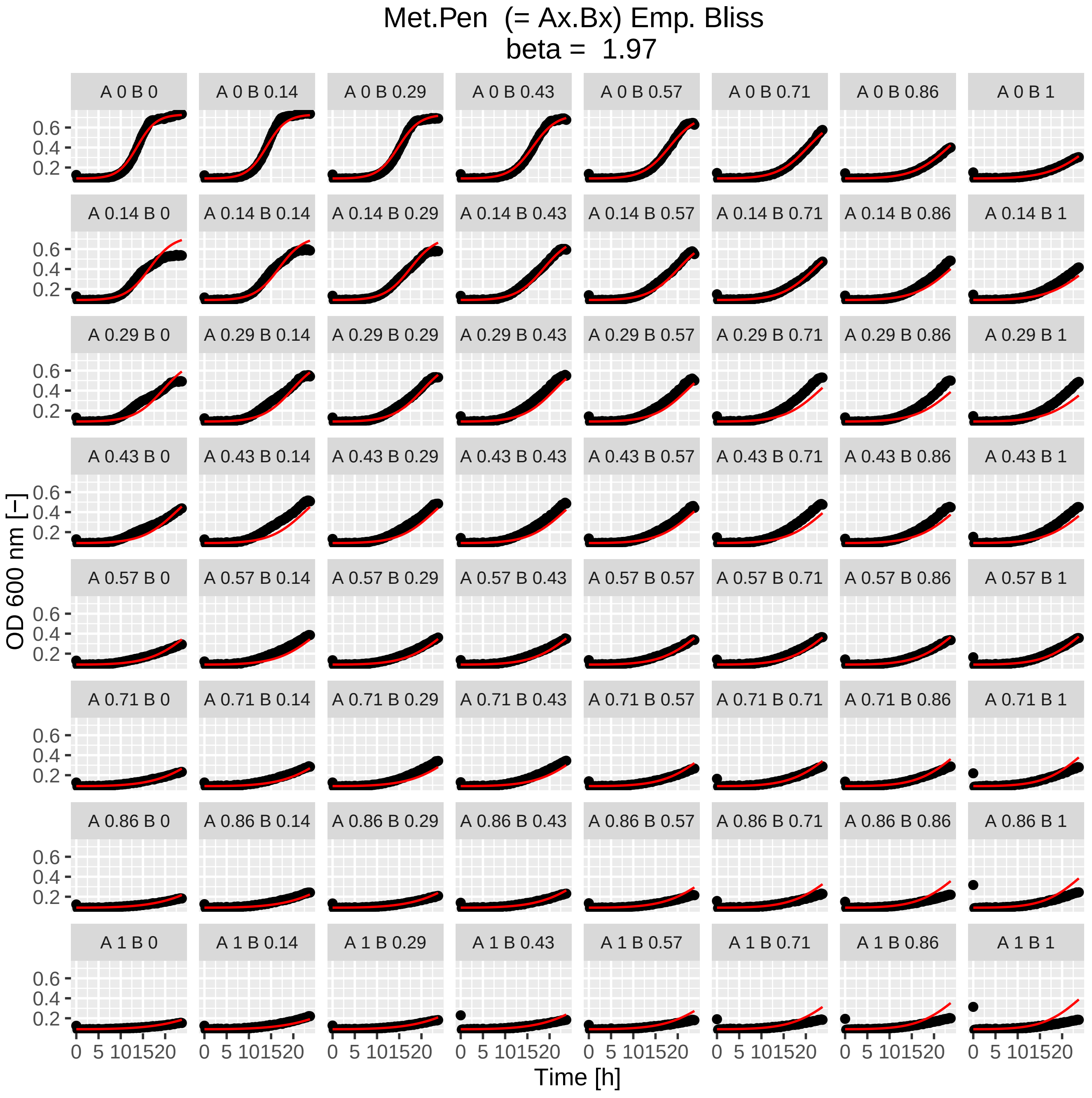
Met.Pen (= Ax.Bx) Emp. Bliss
beta = 1.97
A 0 B 0
A 0 B 0.14
A 0 B 0.29
A 0 B 0.43
A 0 B 0.57
A 0 B 0.71
A 0 B 0.86
A 0 B 1
A 0.14 B 0
A 0.14 B 0.14
A 0.14 B 0.29
A 0.14 B 0.43
A 0.14 B 0.57
A 0.14 B 0.71
A 0.14 B 0.86
A 0.14 B 1
A 0.29 B 0
A 0.29 B 0.14
A 0.29 B 0.29
A 0.29 B 0.43
A 0.29 B 0.57
A 0.29 B 0.71
A 0.29 B 0.86
A 0.29 B 1
A 0.43 B 0
A 0.43 B 0.14
A 0.43 B 0.29
A 0.43 B 0.43
A 0.43 B 0.57
A 0.43 B 0.71
A 0.43 B 0.86
A 0.43 B 1
A 0.57 B 0
A 0.57 B 0.14
A 0.57 B 0.29
A 0.57 B 0.43
A 0.57 B 0.57
A 0.57 B 0.71
A 0.57 B 0.86
A 0.57 B 1
A 0.71 B 0
A 0.71 B 0.14
A 0.71 B 0.29
A 0.71 B 0.43
A 0.71 B 0.57
A 0.71 B 0.71
A 0.71 B 0.86
A 0.71 B 1
A 0.86 B 0
A 0.86 B 0.14
A 0.86 B 0.29
A 0.86 B 0.43
A 0.86 B 0.57
A 0.86 B 0.71
A 0.86 B 0.86
A 0.86 B 1
A 1 B 0
A 1 B 0.14
A 1 B 0.29
A 1 B 0.43
A 1 B 0.57
A 1 B 0.71
A 1 B 0.86
A 1 B 1
OD 600 nm [–]
0.2
0.4
0.6
Time [h]
0
5
10
15
20

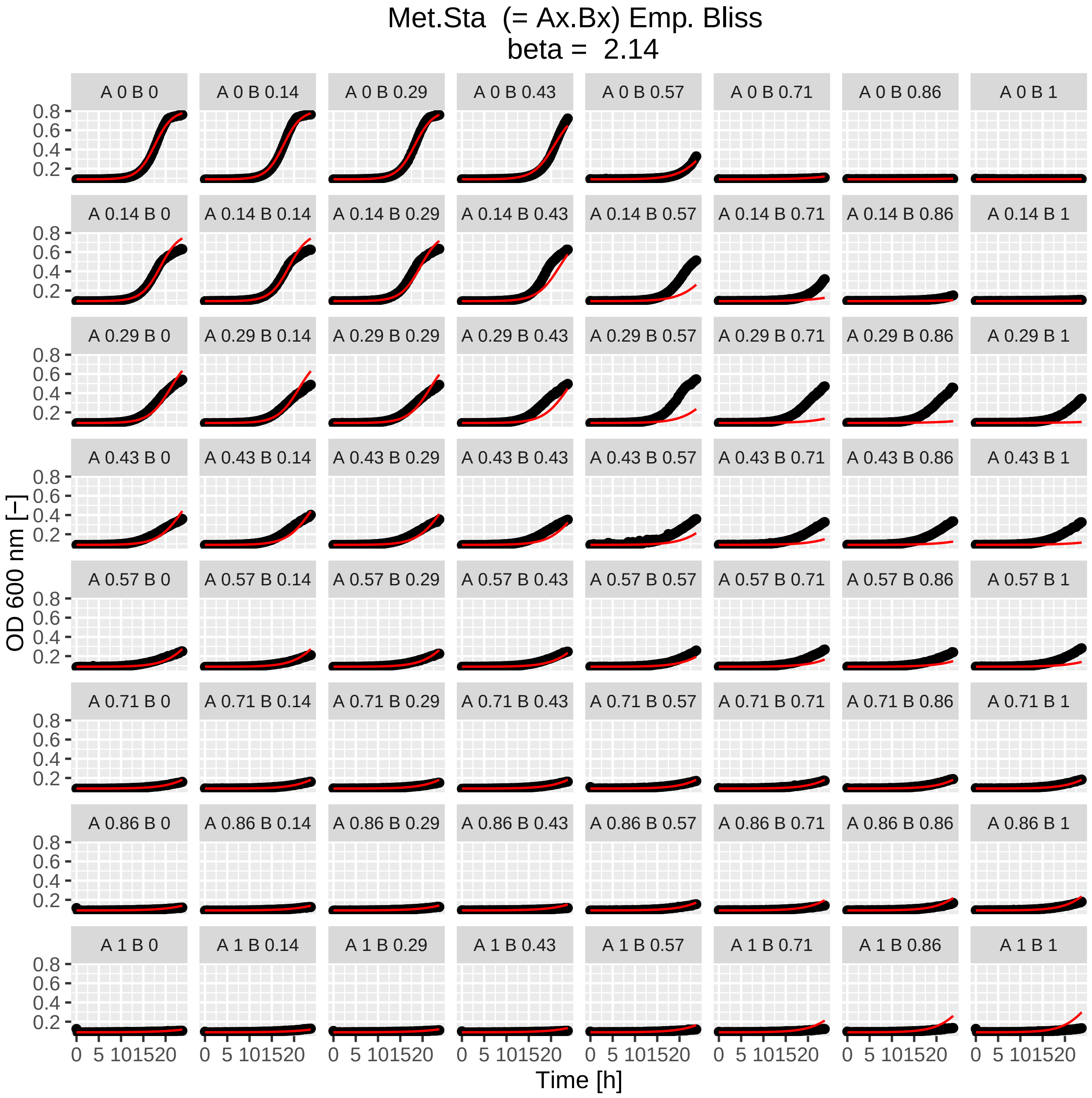
Met.Sta (= Ax.Bx) Emp. Bliss
beta = 2.14
OD 600 nm [–]
Time [h]
A 0 B 0
A 0 B 0.14
A 0 B 0.29
A 0 B 0.43
A 0 B 0.57
A 0 B 0.71
A 0 B 0.86
A 0 B 1
A 0.14 B 0
A 0.14 B 0.14
A 0.14 B 0.29
A 0.14 B 0.43
A 0.14 B 0.57
A 0.14 B 0.71
A 0.14 B 0.86
A 0.14 B 1
A 0.29 B 0
A 0.29 B 0.14
A 0.29 B 0.29
A 0.29 B 0.43
A 0.29 B 0.57
A 0.29 B 0.71
A 0.29 B 0.86
A 0.29 B 1
A 0.43 B 0
A 0.43 B 0.14
A 0.43 B 0.29
A 0.43 B 0.43
A 0.43 B 0.57
A 0.43 B 0.71
A 0.43 B 0.86
A 0.43 B 1
A 0.57 B 0
A 0.57 B 0.14
A 0.57 B 0.29
A 0.57 B 0.43
A 0.57 B 0.57
A 0.57 B 0.71
A 0.57 B 0.86
A 0.57 B 1
A 0.71 B 0
A 0.71 B 0.14
A 0.71 B 0.29
A 0.71 B 0.43
A 0.71 B 0.57
A 0.71 B 0.71
A 0.71 B 0.86
A 0.71 B 1
A 0.86 B 0
A 0.86 B 0.14
A 0.86 B 0.29
A 0.86 B 0.43
A 0.86 B 0.57
A 0.86 B 0.71
A 0.86 B 0.86
A 0.86 B 1
A 1 B 0
A 1 B 0.14
A 1 B 0.29
A 1 B 0.43
A 1 B 0.57
A 1 B 0.71
A 1 B 0.86
A 1 B 1
0.8
0.6
0.4
0.2
0
5
10
15
20

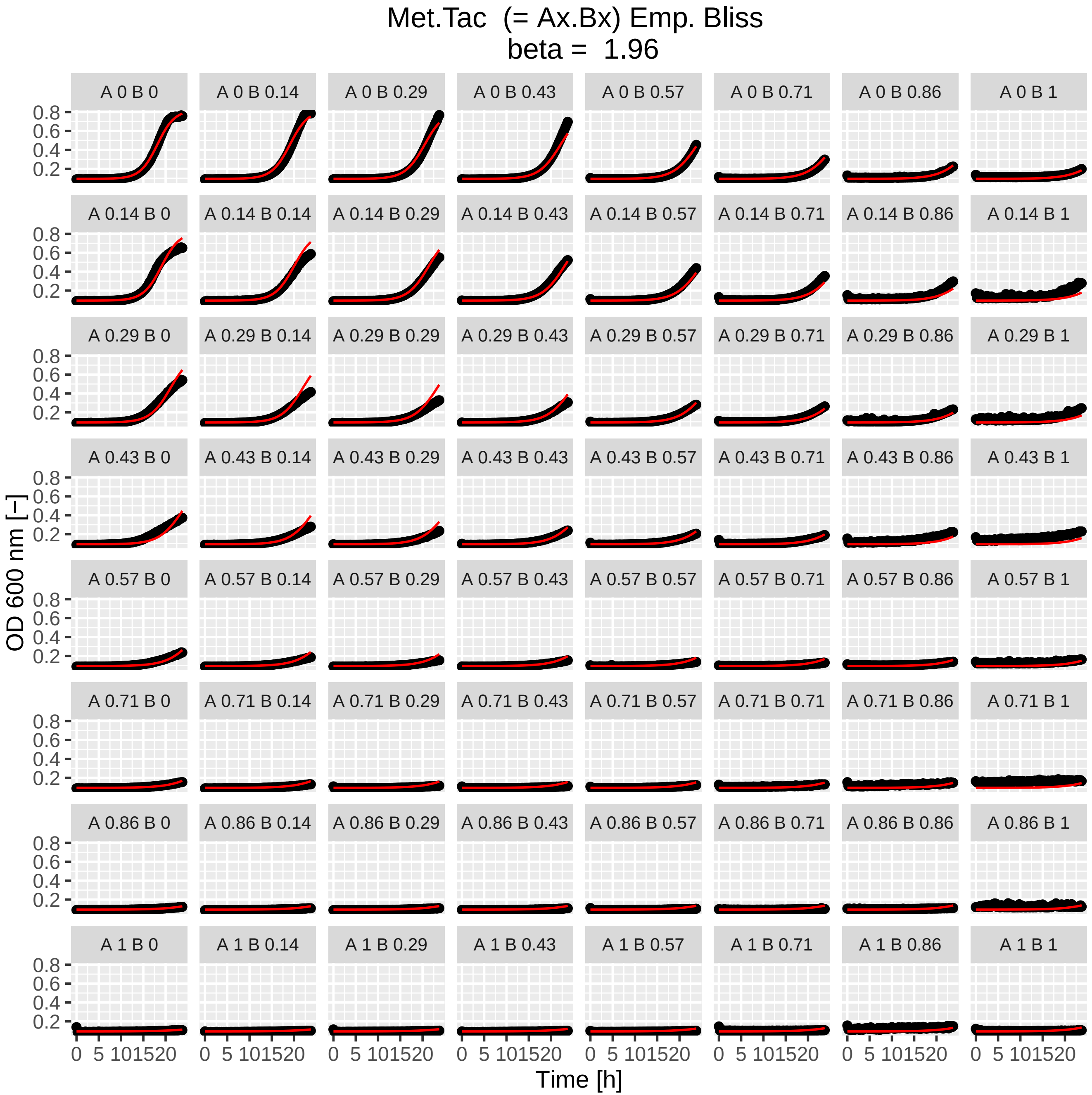

Met.Tac (= Ax.Bx) Emp. Bliss
beta = 1.96
A 0 B 0
A 0 B 0.14
A 0 B 0.29
A 0 B 0.43
A 0 B 0.57
A 0 B 0.71
A 0 B 0.86
A 0 B 1
A 0.14 B 0
A 0.14 B 0.14
A 0.14 B 0.29
A 0.14 B 0.43
A 0.14 B 0.57
A 0.14 B 0.71
A 0.14 B 0.86
A 0.14 B 1
A 0.29 B 0
A 0.29 B 0.14
A 0.29 B 0.29
A 0.29 B 0.43
A 0.29 B 0.57
A 0.29 B 0.71
A 0.29 B 0.86
A 0.29 B 1
A 0.43 B 0
A 0.43 B 0.14
A 0.43 B 0.29
A 0.43 B 0.43
A 0.43 B 0.57
A 0.43 B 0.71
A 0.43 B 0.86
A 0.43 B 1
A 0.57 B 0
A 0.57 B 0.14
A 0.57 B 0.29
A 0.57 B 0.43
A 0.57 B 0.57
A 0.57 B 0.71
A 0.57 B 0.86
A 0.57 B 1
A 0.71 B 0
A 0.71 B 0.14
A 0.71 B 0.29
A 0.71 B 0.43
A 0.71 B 0.57
A 0.71 B 0.71
A 0.71 B 0.86
A 0.71 B 1
A 0.86 B 0
A 0.86 B 0.14
A 0.86 B 0.29
A 0.86 B 0.43
A 0.86 B 0.57
A 0.86 B 0.71
A 0.86 B 0.86
A 0.86 B 1
A 1 B 0
A 1 B 0.14
A 1 B 0.29
A 1 B 0.43
A 1 B 0.57
A 1 B 0.71
A 1 B 0.86
A 1 B 1
0.8
0.6
0.4
0.2
OD 600 nm [–]
0 5 101520
Time [h]

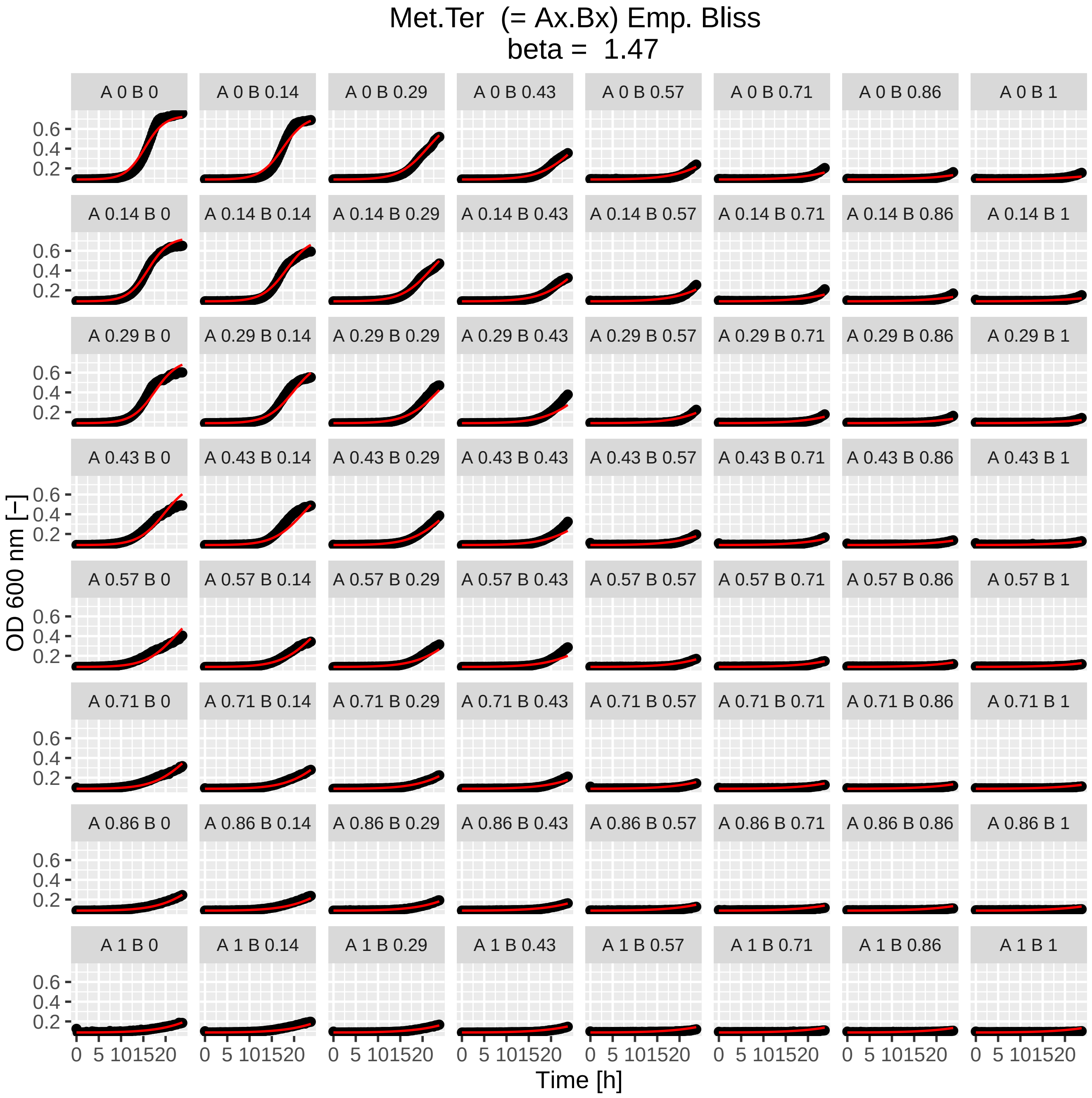

Met.Ter (= Ax.Bx) Emp. Bliss
beta = 1.47
A 0 B 0
A 0 B 0.14
A 0 B 0.29
A 0 B 0.43
A 0 B 0.57
A 0 B 0.71
A 0 B 0.86
A 0 B 1
A 0.14 B 0
A 0.14 B 0.14
A 0.14 B 0.29
A 0.14 B 0.43
A 0.14 B 0.57
A 0.14 B 0.71
A 0.14 B 0.86
A 0.14 B 1
A 0.29 B 0
A 0.29 B 0.14
A 0.29 B 0.29
A 0.29 B 0.43
A 0.29 B 0.57
A 0.29 B 0.71
A 0.29 B 0.86
A 0.29 B 1
A 0.43 B 0
A 0.43 B 0.14
A 0.43 B 0.29
A 0.43 B 0.43
A 0.43 B 0.57
A 0.43 B 0.71
A 0.43 B 0.86
A 0.43 B 1
A 0.57 B 0
A 0.57 B 0.14
A 0.57 B 0.29
A 0.57 B 0.43
A 0.57 B 0.57
A 0.57 B 0.71
A 0.57 B 0.86
A 0.57 B 1
A 0.71 B 0
A 0.71 B 0.14
A 0.71 B 0.29
A 0.71 B 0.43
A 0.71 B 0.57
A 0.71 B 0.71
A 0.71 B 0.86
A 0.71 B 1
A 0.86 B 0
A 0.86 B 0.14
A 0.86 B 0.29
A 0.86 B 0.43
A 0.86 B 0.57
A 0.86 B 0.71
A 0.86 B 0.86
A 0.86 B 1
A 1 B 0
A 1 B 0.14
A 1 B 0.29
A 1 B 0.43
A 1 B 0.57
A 1 B 0.71
A 1 B 0.86
A 1 B 1
0.6
0.4
0.2
OD 600 nm [–]
0 5 101520
Time [h]

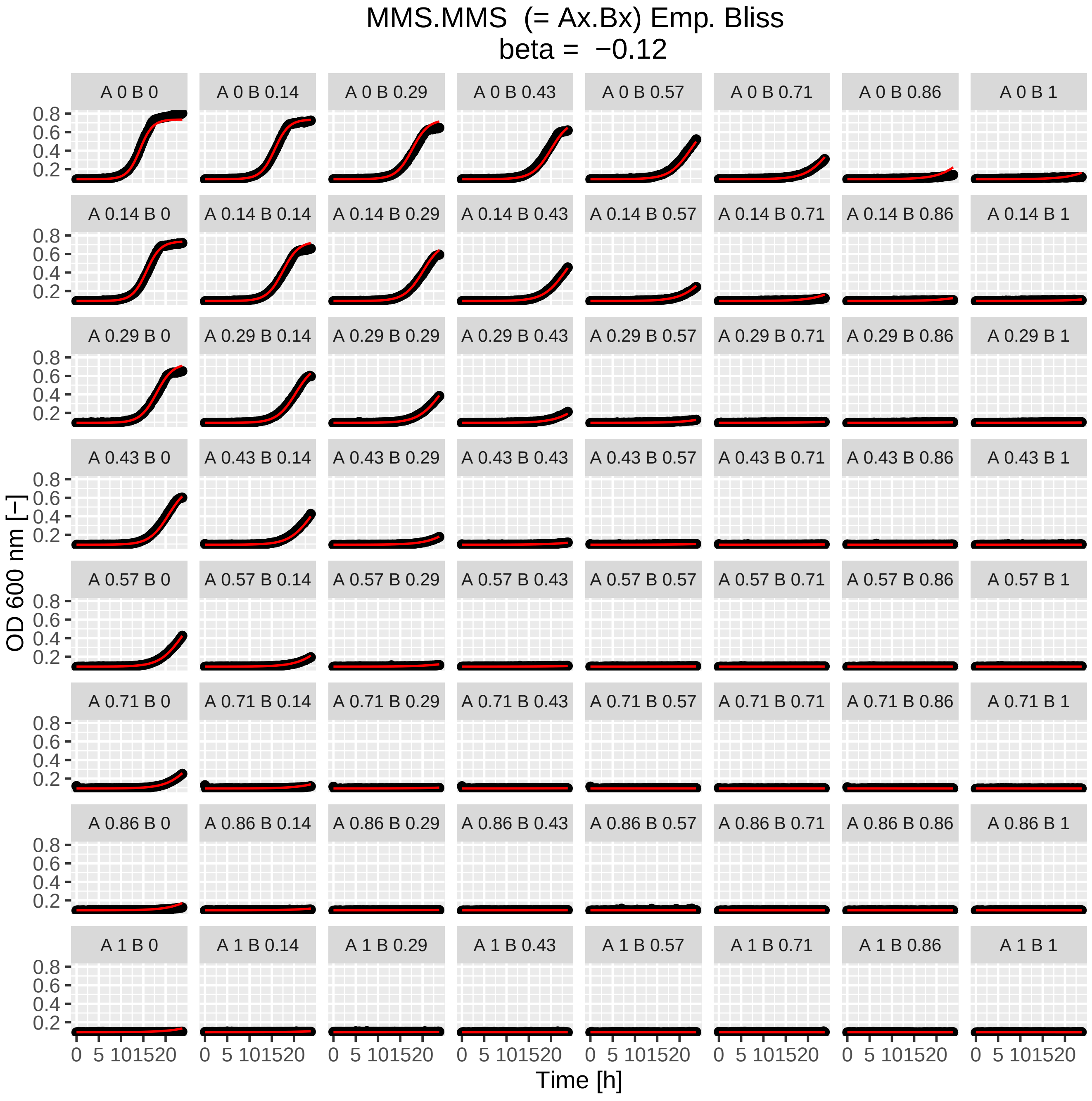
MMS.MMS (= Ax.Bx) Emp. Bliss
beta = −0.12
OD 600 nm [−]
Time [h]
0.8
0.6
0.4
0.2
0 5 101520
A 0 B 0
A 0 B 0.14
A 0 B 0.29
A 0 B 0.43
A 0 B 0.57
A 0 B 0.71
A 0 B 0.86
A 0 B 1
A 0.14 B 0
A 0.14 B 0.14
A 0.14 B 0.29
A 0.14 B 0.43
A 0.14 B 0.57
A 0.14 B 0.71
A 0.14 B 0.86
A 0.14 B 1
A 0.29 B 0
A 0.29 B 0.14
A 0.29 B 0.29
A 0.29 B 0.43
A 0.29 B 0.57
A 0.29 B 0.71
A 0.29 B 0.86
A 0.29 B 1
A 0.43 B 0
A 0.43 B 0.14
A 0.43 B 0.29
A 0.43 B 0.43
A 0.43 B 0.57
A 0.43 B 0.71
A 0.43 B 0.86
A 0.43 B 1
A 0.57 B 0
A 0.57 B 0.14
A 0.57 B 0.29
A 0.57 B 0.43
A 0.57 B 0.57
A 0.57 B 0.71
A 0.57 B 0.86
A 0.57 B 1
A 0.71 B 0
A 0.71 B 0.14
A 0.71 B 0.29
A 0.71 B 0.43
A 0.71 B 0.57
A 0.71 B 0.71
A 0.71 B 0.86
A 0.71 B 1
A 0.86 B 0
A 0.86 B 0.14
A 0.86 B 0.29
A 0.86 B 0.43
A 0.86 B 0.57
A 0.86 B 0.71
A 0.86 B 0.86
A 0.86 B 1
A 1 B 0
A 1 B 0.14
A 1 B 0.29
A 1 B 0.43
A 1 B 0.57
A 1 B 0.71
A 1 B 0.86
A 1 B 1

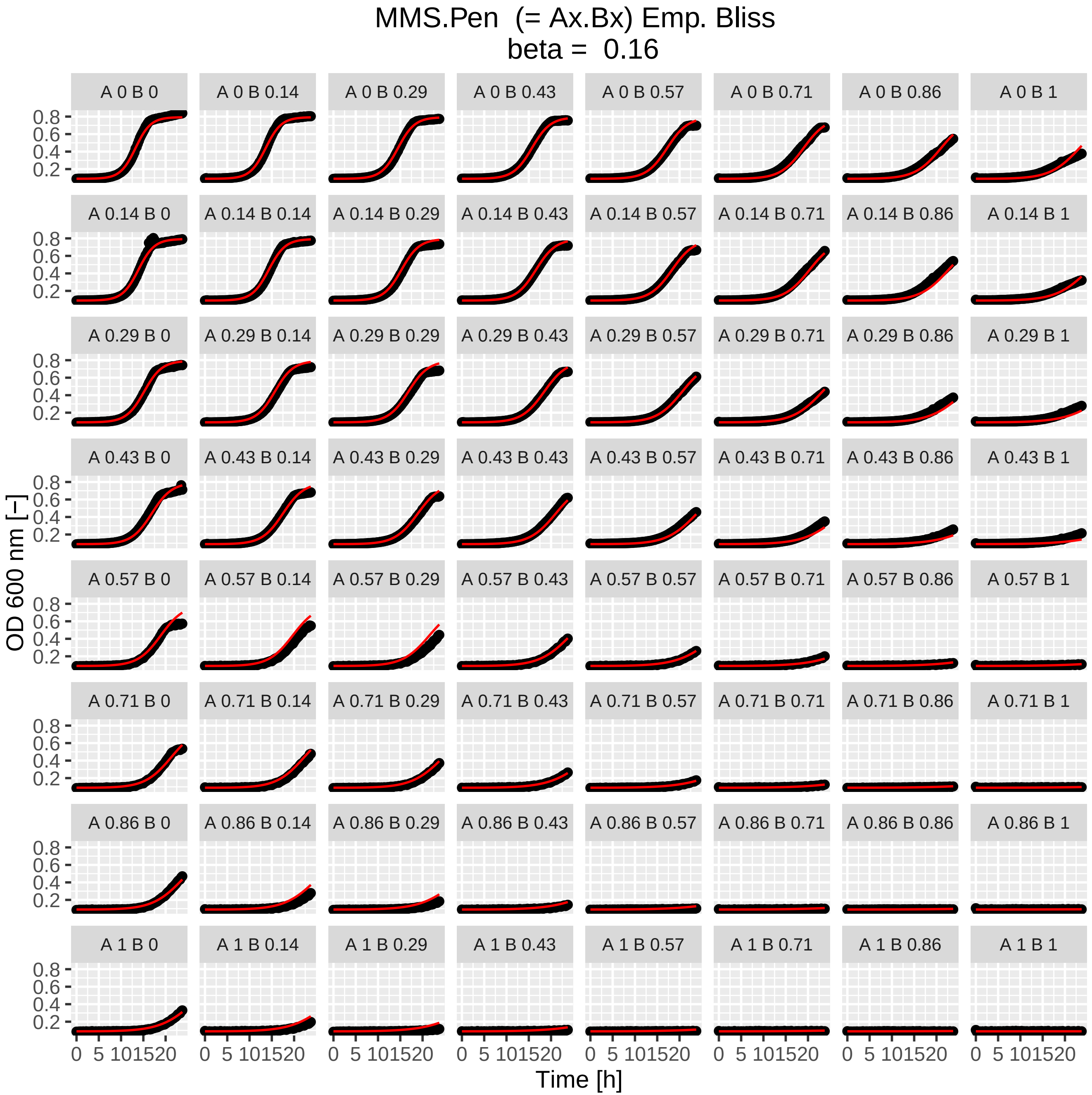
MMS.Pen (= Ax.Bx) Emp. Bliss
beta = 0.16
A 0 B 0
A 0 B 0.14
A 0 B 0.29
A 0 B 0.43
A 0 B 0.57
A 0 B 0.71
A 0 B 0.86
A 0 B 1
A 0.14 B 0
A 0.14 B 0.14
A 0.14 B 0.29
A 0.14 B 0.43
A 0.14 B 0.57
A 0.14 B 0.71
A 0.14 B 0.86
A 0.14 B 1
A 0.29 B 0
A 0.29 B 0.14
A 0.29 B 0.29
A 0.29 B 0.43
A 0.29 B 0.57
A 0.29 B 0.71
A 0.29 B 0.86
A 0.29 B 1
A 0.43 B 0
A 0.43 B 0.14
A 0.43 B 0.29
A 0.43 B 0.43
A 0.43 B 0.57
A 0.43 B 0.71
A 0.43 B 0.86
A 0.43 B 1
A 0.57 B 0
A 0.57 B 0.14
A 0.57 B 0.29
A 0.57 B 0.43
A 0.57 B 0.57
A 0.57 B 0.71
A 0.57 B 0.86
A 0.57 B 1
A 0.71 B 0
A 0.71 B 0.14
A 0.71 B 0.29
A 0.71 B 0.43
A 0.71 B 0.57
A 0.71 B 0.71
A 0.71 B 0.86
A 0.71 B 1
A 0.86 B 0
A 0.86 B 0.14
A 0.86 B 0.29
A 0.86 B 0.43
A 0.86 B 0.57
A 0.86 B 0.71
A 0.86 B 0.86
A 0.86 B 1
A 1 B 0
A 1 B 0.14
A 1 B 0.29
A 1 B 0.43
A 1 B 0.57
A 1 B 0.71
A 1 B 0.86
A 1 B 1
OD 600 nm [–]
0.2
0.4
0.6
0.8
0 5 101520
Time [h]

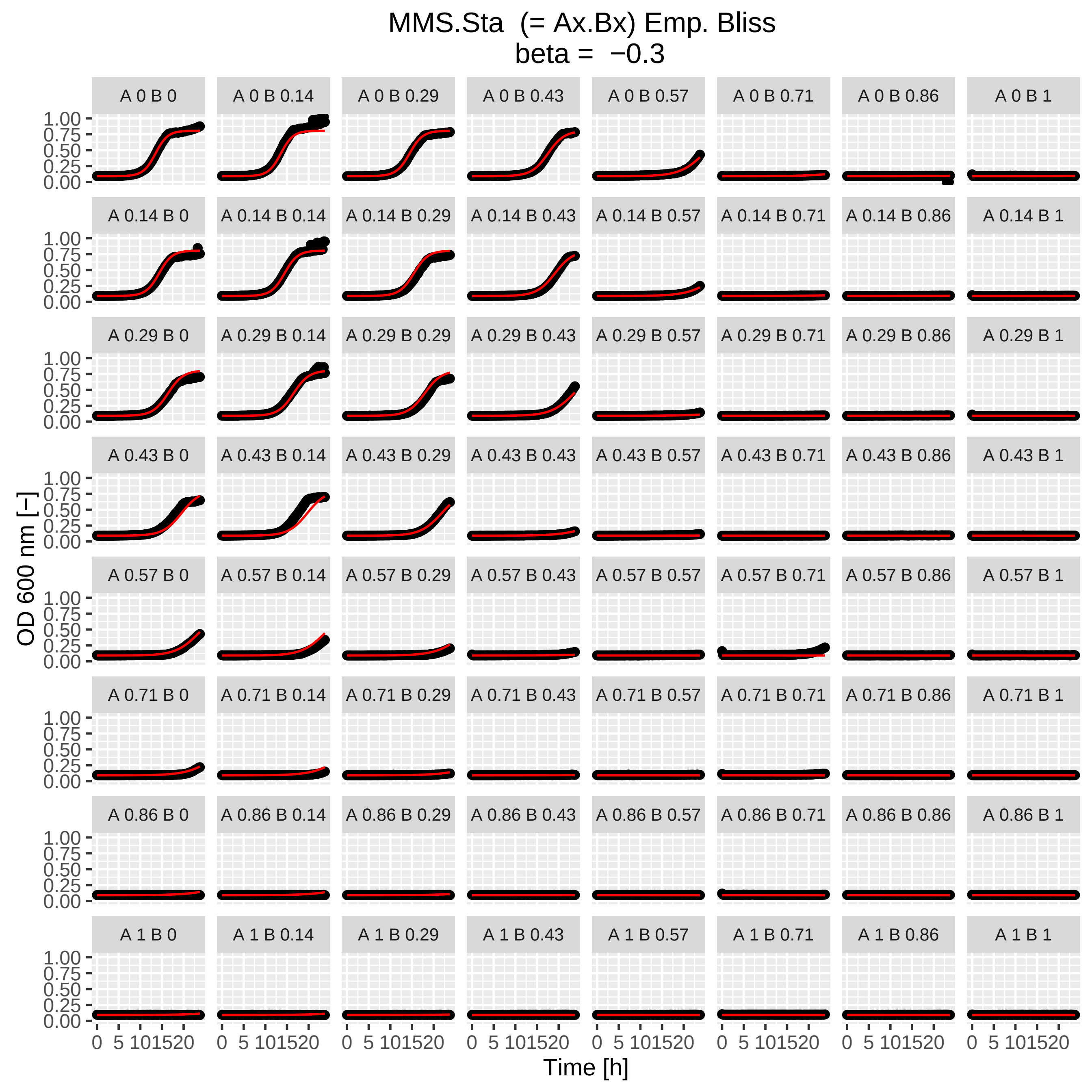

MMS.Sta (= Ax.Bx) Emp. Bliss
beta = −0.3
OD 600 nm [−]
Time [h]
A 0 B 0
A 0 B 0.14
A 0 B 0.29
A 0 B 0.43
A 0 B 0.57
A 0 B 0.71
A 0 B 0.86
A 0 B 1
A 0.14 B 0
A 0.14 B 0.14
A 0.14 B 0.29
A 0.14 B 0.43
A 0.14 B 0.57
A 0.14 B 0.71
A 0.14 B 0.86
A 0.14 B 1
A 0.29 B 0
A 0.29 B 0.14
A 0.29 B 0.29
A 0.29 B 0.43
A 0.29 B 0.57
A 0.29 B 0.71
A 0.29 B 0.86
A 0.29 B 1
A 0.43 B 0
A 0.43 B 0.14
A 0.43 B 0.29
A 0.43 B 0.43
A 0.43 B 0.57
A 0.43 B 0.71
A 0.43 B 0.86
A 0.43 B 1
A 0.57 B 0
A 0.57 B 0.14
A 0.57 B 0.29
A 0.57 B 0.43
A 0.57 B 0.57
A 0.57 B 0.71
A 0.57 B 0.86
A 0.57 B 1
A 0.71 B 0
A 0.71 B 0.14
A 0.71 B 0.29
A 0.71 B 0.43
A 0.71 B 0.57
A 0.71 B 0.71
A 0.71 B 0.86
A 0.71 B 1
A 0.86 B 0
A 0.86 B 0.14
A 0.86 B 0.29
A 0.86 B 0.43
A 0.86 B 0.57
A 0.86 B 0.71
A 0.86 B 0.86
A 0.86 B 1
A 1 B 0
A 1 B 0.14
A 1 B 0.29
A 1 B 0.43
A 1 B 0.57
A 1 B 0.71
A 1 B 0.86
A 1 B 1
1.00
0.75
0.50
0.25
0.00
0 5 101520

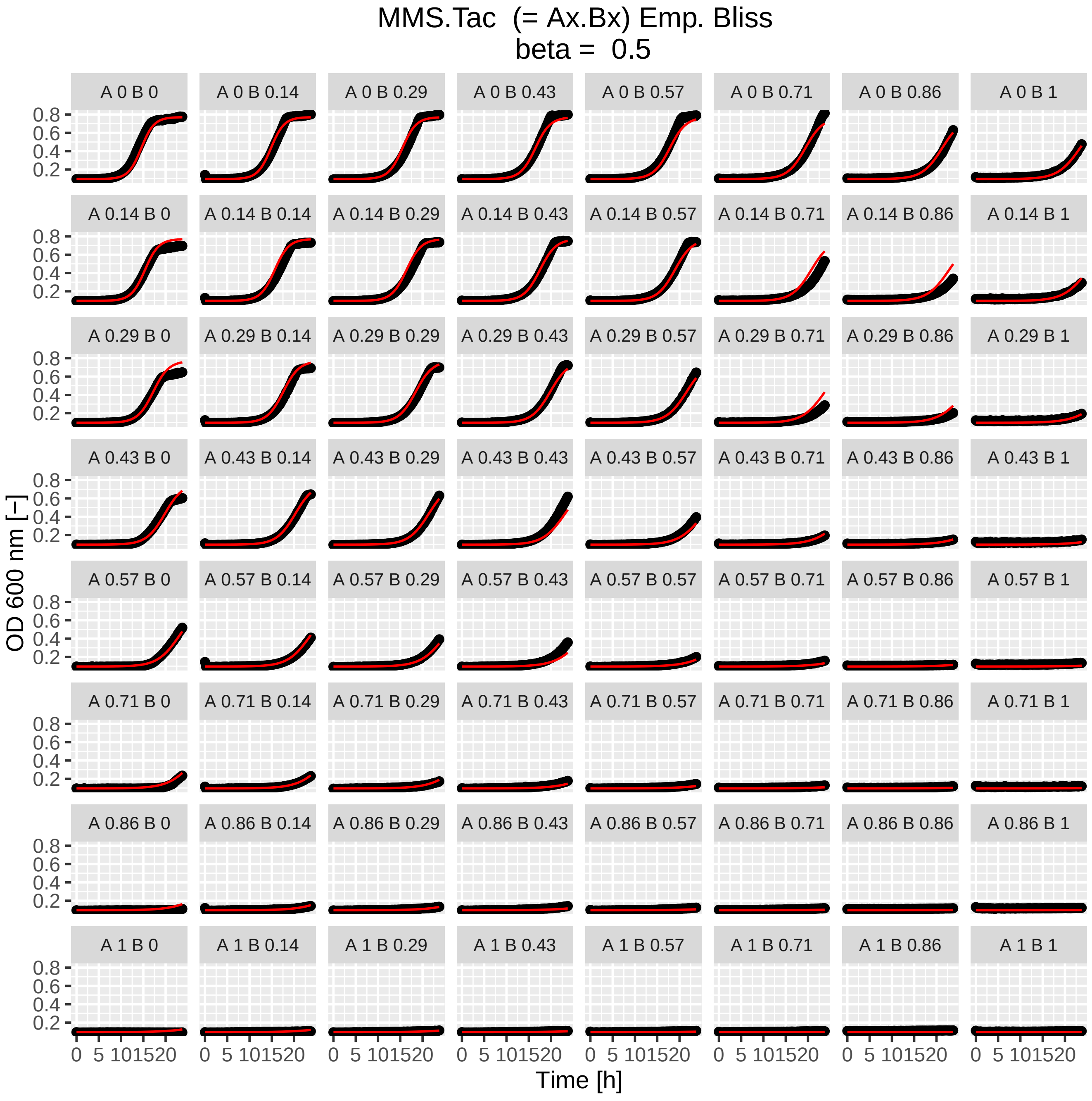
MMS.Tac (= Ax.Bx) Emp. Bliss
beta = 0.5
A 0 B 0
A 0 B 0.14
A 0 B 0.29
A 0 B 0.43
A 0 B 0.57
A 0 B 0.71
A 0 B 0.86
A 0 B 1
A 0.14 B 0
A 0.14 B 0.14
A 0.14 B 0.29
A 0.14 B 0.43
A 0.14 B 0.57
A 0.14 B 0.71
A 0.14 B 0.86
A 0.14 B 1
A 0.29 B 0
A 0.29 B 0.14
A 0.29 B 0.29
A 0.29 B 0.43
A 0.29 B 0.57
A 0.29 B 0.71
A 0.29 B 0.86
A 0.29 B 1
A 0.43 B 0
A 0.43 B 0.14
A 0.43 B 0.29
A 0.43 B 0.43
A 0.43 B 0.57
A 0.43 B 0.71
A 0.43 B 0.86
A 0.43 B 1
A 0.57 B 0
A 0.57 B 0.14
A 0.57 B 0.29
A 0.57 B 0.43
A 0.57 B 0.57
A 0.57 B 0.71
A 0.57 B 0.86
A 0.57 B 1
A 0.71 B 0
A 0.71 B 0.14
A 0.71 B 0.29
A 0.71 B 0.43
A 0.71 B 0.57
A 0.71 B 0.71
A 0.71 B 0.86
A 0.71 B 1
A 0.86 B 0
A 0.86 B 0.14
A 0.86 B 0.29
A 0.86 B 0.43
A 0.86 B 0.57
A 0.86 B 0.71
A 0.86 B 0.86
A 0.86 B 1
A 1 B 0
A 1 B 0.14
A 1 B 0.29
A 1 B 0.43
A 1 B 0.57
A 1 B 0.71
A 1 B 0.86
A 1 B 1
OD 600 nm [–]
0.2
0.4
0.6
0.8
0
5
10
15
20
Time [h]

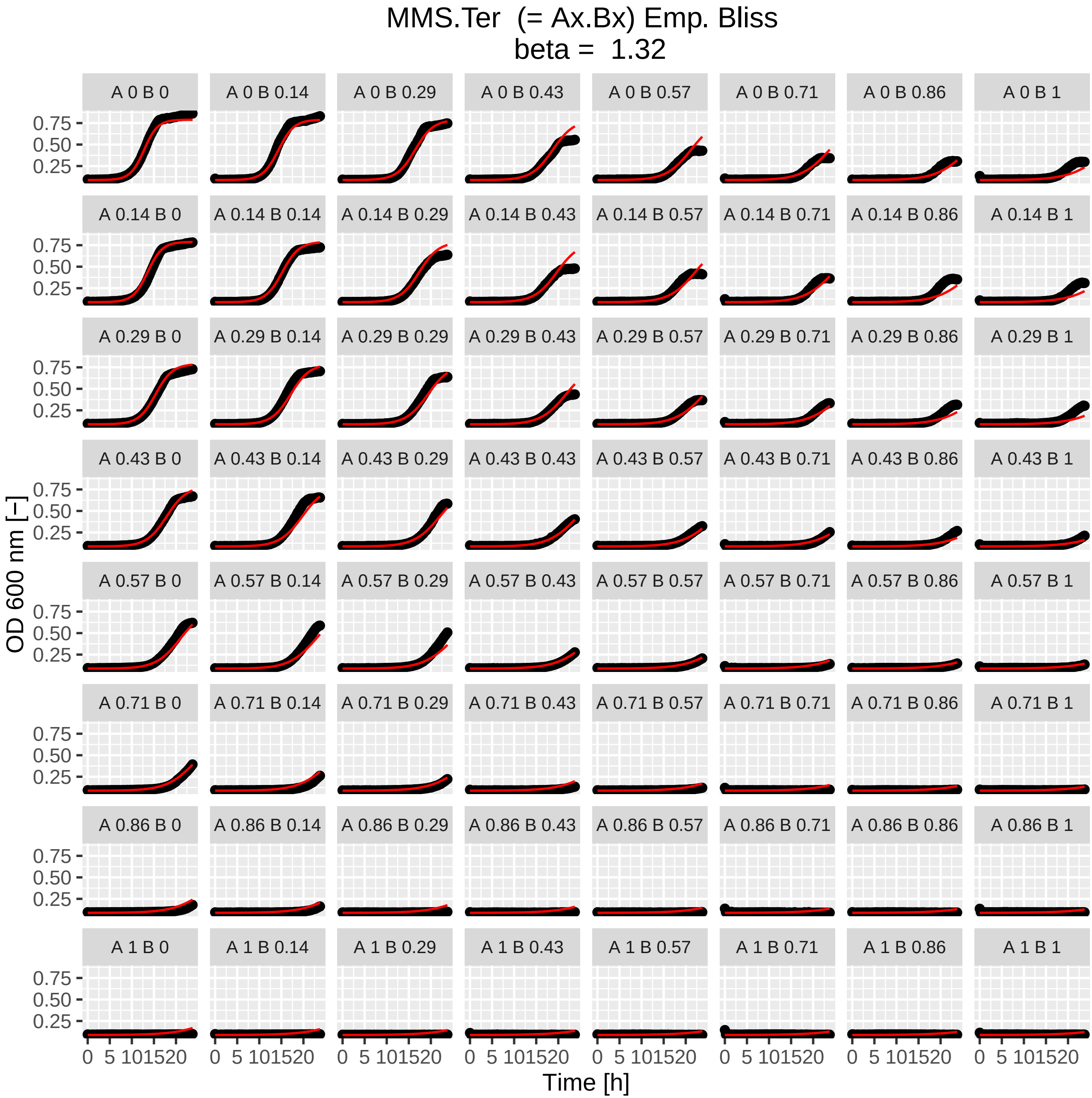

MMS.Ter (= Ax.Bx) Emp. Bliss
beta = 1.32
A 0 B 0
A 0 B 0.14
A 0 B 0.29
A 0 B 0.43
A 0 B 0.57
A 0 B 0.71
A 0 B 0.86
A 0 B 1
A 0.14 B 0
A 0.14 B 0.14
A 0.14 B 0.29
A 0.14 B 0.43
A 0.14 B 0.57
A 0.14 B 0.71
A 0.14 B 0.86
A 0.14 B 1
A 0.29 B 0
A 0.29 B 0.14
A 0.29 B 0.29
A 0.29 B 0.43
A 0.29 B 0.57
A 0.29 B 0.71
A 0.29 B 0.86
A 0.29 B 1
A 0.43 B 0
A 0.43 B 0.14
A 0.43 B 0.29
A 0.43 B 0.43
A 0.43 B 0.57
A 0.43 B 0.71
A 0.43 B 0.86
A 0.43 B 1
A 0.57 B 0
A 0.57 B 0.14
A 0.57 B 0.29
A 0.57 B 0.43
A 0.57 B 0.57
A 0.57 B 0.71
A 0.57 B 0.86
A 0.57 B 1
A 0.71 B 0
A 0.71 B 0.14
A 0.71 B 0.29
A 0.71 B 0.43
A 0.71 B 0.57
A 0.71 B 0.71
A 0.71 B 0.86
A 0.71 B 1
A 0.86 B 0
A 0.86 B 0.14
A 0.86 B 0.29
A 0.86 B 0.43
A 0.86 B 0.57
A 0.86 B 0.71
A 0.86 B 0.86
A 0.86 B 1
A 1 B 0
A 1 B 0.14
A 1 B 0.29
A 1 B 0.43
A 1 B 0.57
A 1 B 0.71
A 1 B 0.86
A 1 B 1
0.75
0.50
0.25
0 5 101520
OD 600 nm [–]
Time [h]

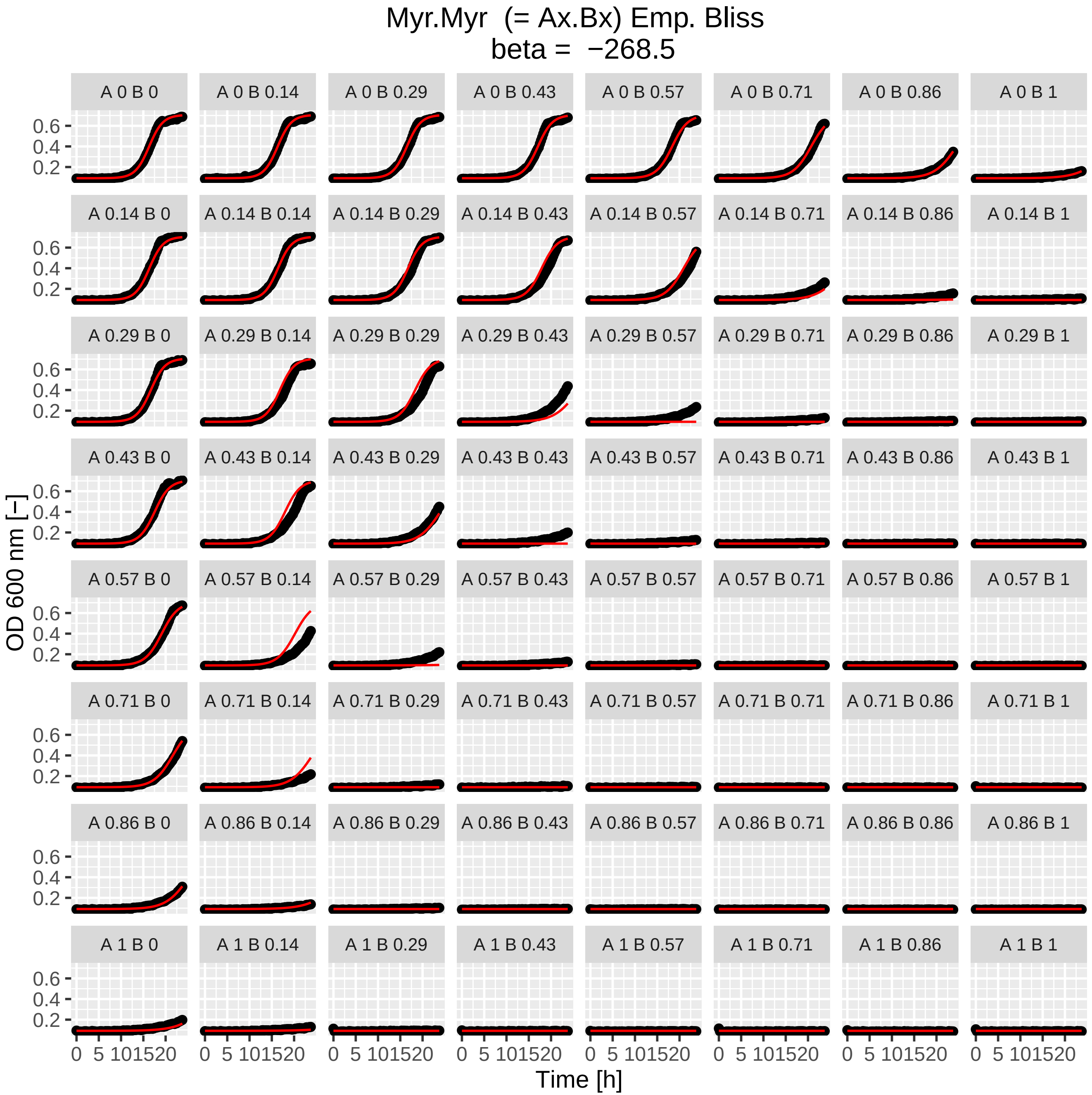

Myr.Myr (= Ax.Bx) Emp. Bliss
beta = −268.5
A 0 B 0
A 0 B 0.14
A 0 B 0.29
A 0 B 0.43
A 0 B 0.57
A 0 B 0.71
A 0 B 0.86
A 0 B 1
A 0.14 B 0
A 0.14 B 0.14
A 0.14 B 0.29
A 0.14 B 0.43
A 0.14 B 0.57
A 0.14 B 0.71
A 0.14 B 0.86
A 0.14 B 1
A 0.29 B 0
A 0.29 B 0.14
A 0.29 B 0.29
A 0.29 B 0.43
A 0.29 B 0.57
A 0.29 B 0.71
A 0.29 B 0.86
A 0.29 B 1
A 0.43 B 0
A 0.43 B 0.14
A 0.43 B 0.29
A 0.43 B 0.43
A 0.43 B 0.57
A 0.43 B 0.71
A 0.43 B 0.86
A 0.43 B 1
A 0.57 B 0
A 0.57 B 0.14
A 0.57 B 0.29
A 0.57 B 0.43
A 0.57 B 0.57
A 0.57 B 0.71
A 0.57 B 0.86
A 0.57 B 1
A 0.71 B 0
A 0.71 B 0.14
A 0.71 B 0.29
A 0.71 B 0.43
A 0.71 B 0.57
A 0.71 B 0.71
A 0.71 B 0.86
A 0.71 B 1
A 0.86 B 0
A 0.86 B 0.14
A 0.86 B 0.29
A 0.86 B 0.43
A 0.86 B 0.57
A 0.86 B 0.71
A 0.86 B 0.86
A 0.86 B 1
A 1 B 0
A 1 B 0.14
A 1 B 0.29
A 1 B 0.43
A 1 B 0.57
A 1 B 0.71
A 1 B 0.86
A 1 B 1
0.6
0.4
0.2
0 5 101520
OD 600 nm [−]
Time [h]

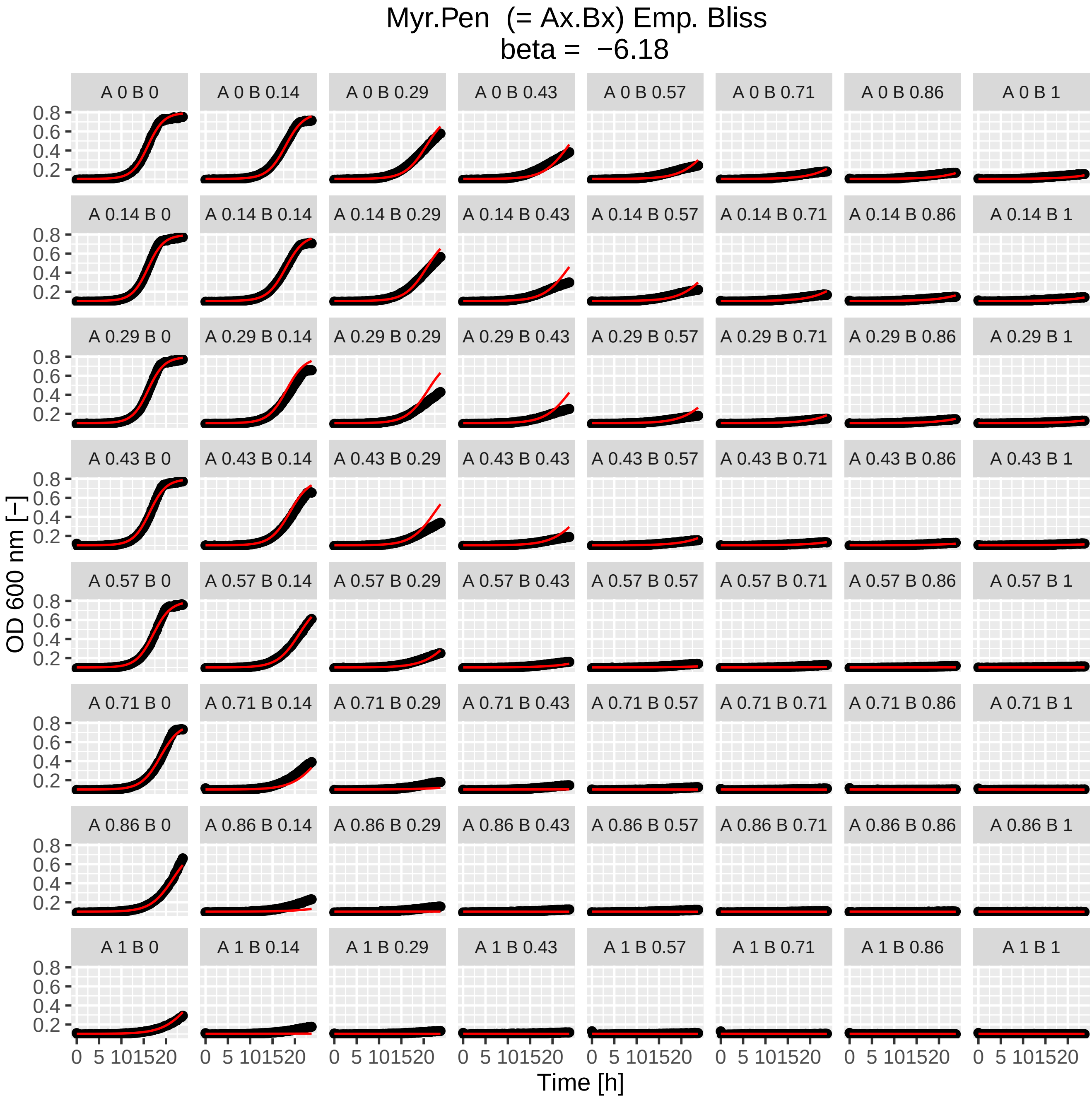
Myr.Pen (= Ax.Bx) Emp. Bliss
beta = −6.18
A 0 B 0
A 0 B 0.14
A 0 B 0.29
A 0 B 0.43
A 0 B 0.57
A 0 B 0.71
A 0 B 0.86
A 0 B 1
A 0.14 B 0
A 0.14 B 0.14
A 0.14 B 0.29
A 0.14 B 0.43
A 0.14 B 0.57
A 0.14 B 0.71
A 0.14 B 0.86
A 0.14 B 1
A 0.29 B 0
A 0.29 B 0.14
A 0.29 B 0.29
A 0.29 B 0.43
A 0.29 B 0.57
A 0.29 B 0.71
A 0.29 B 0.86
A 0.29 B 1
A 0.43 B 0
A 0.43 B 0.14
A 0.43 B 0.29
A 0.43 B 0.43
A 0.43 B 0.57
A 0.43 B 0.71
A 0.43 B 0.86
A 0.43 B 1
A 0.57 B 0
A 0.57 B 0.14
A 0.57 B 0.29
A 0.57 B 0.43
A 0.57 B 0.57
A 0.57 B 0.71
A 0.57 B 0.86
A 0.57 B 1
A 0.71 B 0
A 0.71 B 0.14
A 0.71 B 0.29
A 0.71 B 0.43
A 0.71 B 0.57
A 0.71 B 0.71
A 0.71 B 0.86
A 0.71 B 1
A 0.86 B 0
A 0.86 B 0.14
A 0.86 B 0.29
A 0.86 B 0.43
A 0.86 B 0.57
A 0.86 B 0.71
A 0.86 B 0.86
A 0.86 B 1
A 1 B 0
A 1 B 0.14
A 1 B 0.29
A 1 B 0.43
A 1 B 0.57
A 1 B 0.71
A 1 B 0.86
A 1 B 1
OD 600 nm [−]
0.8
0.6
0.4
0.2
0 5 101520
Time [h]

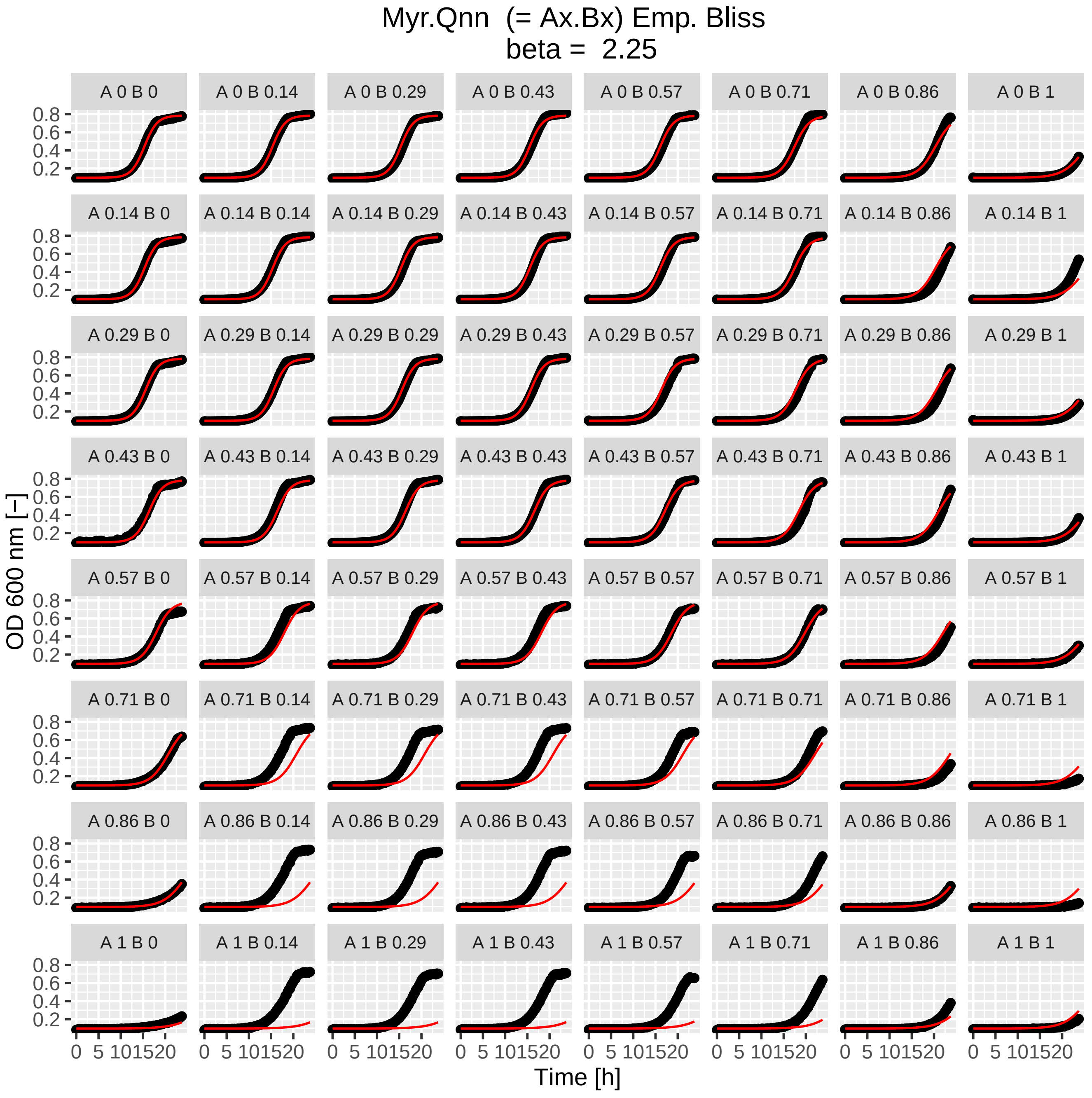

Myr.Qnn (= Ax.Bx) Emp. Bliss
beta = 2.25
A 0 B 0
A 0 B 0.14
A 0 B 0.29
A 0 B 0.43
A 0 B 0.57
A 0 B 0.71
A 0 B 0.86
A 0 B 1
A 0.14 B 0
A 0.14 B 0.14
A 0.14 B 0.29
A 0.14 B 0.43
A 0.14 B 0.57
A 0.14 B 0.71
A 0.14 B 0.86
A 0.14 B 1
A 0.29 B 0
A 0.29 B 0.14
A 0.29 B 0.29
A 0.29 B 0.43
A 0.29 B 0.57
A 0.29 B 0.71
A 0.29 B 0.86
A 0.29 B 1
A 0.43 B 0
A 0.43 B 0.14
A 0.43 B 0.29
A 0.43 B 0.43
A 0.43 B 0.57
A 0.43 B 0.71
A 0.43 B 0.86
A 0.43 B 1
A 0.57 B 0
A 0.57 B 0.14
A 0.57 B 0.29
A 0.57 B 0.43
A 0.57 B 0.57
A 0.57 B 0.71
A 0.57 B 0.86
A 0.57 B 1
A 0.71 B 0
A 0.71 B 0.14
A 0.71 B 0.29
A 0.71 B 0.43
A 0.71 B 0.57
A 0.71 B 0.71
A 0.71 B 0.86
A 0.71 B 1
A 0.86 B 0
A 0.86 B 0.14
A 0.86 B 0.29
A 0.86 B 0.43
A 0.86 B 0.57
A 0.86 B 0.71
A 0.86 B 0.86
A 0.86 B 1
A 1 B 0
A 1 B 0.14
A 1 B 0.29
A 1 B 0.43
A 1 B 0.57
A 1 B 0.71
A 1 B 0.86
A 1 B 1
OD 600 nm [–]
0.2
0.4
0.6
0.8
0 5 101520
Time [h]

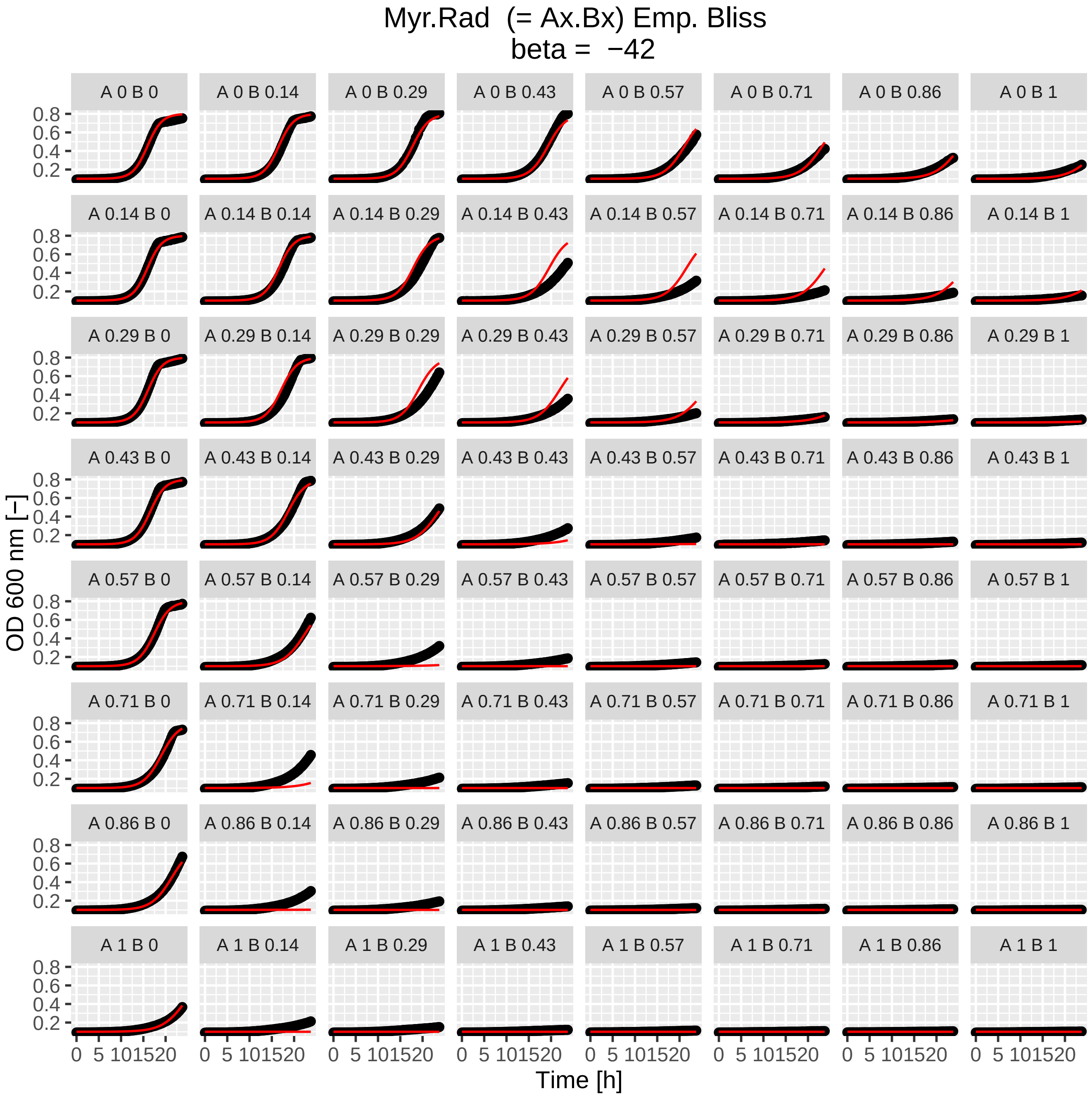
Myr.Rad (= Ax.Bx) Emp. Bliss
beta = −42
A 0 B 0
A 0 B 0.14
A 0 B 0.29
A 0 B 0.43
A 0 B 0.57
A 0 B 0.71
A 0 B 0.86
A 0 B 1
A 0.14 B 0
A 0.14 B 0.14
A 0.14 B 0.29
A 0.14 B 0.43
A 0.14 B 0.57
A 0.14 B 0.71
A 0.14 B 0.86
A 0.14 B 1
A 0.29 B 0
A 0.29 B 0.14
A 0.29 B 0.29
A 0.29 B 0.43
A 0.29 B 0.57
A 0.29 B 0.71
A 0.29 B 0.86
A 0.29 B 1
A 0.43 B 0
A 0.43 B 0.14
A 0.43 B 0.29
A 0.43 B 0.43
A 0.43 B 0.57
A 0.43 B 0.71
A 0.43 B 0.86
A 0.43 B 1
A 0.57 B 0
A 0.57 B 0.14
A 0.57 B 0.29
A 0.57 B 0.43
A 0.57 B 0.57
A 0.57 B 0.71
A 0.57 B 0.86
A 0.57 B 1
A 0.71 B 0
A 0.71 B 0.14
A 0.71 B 0.29
A 0.71 B 0.43
A 0.71 B 0.57
A 0.71 B 0.71
A 0.71 B 0.86
A 0.71 B 1
A 0.86 B 0
A 0.86 B 0.14
A 0.86 B 0.29
A 0.86 B 0.43
A 0.86 B 0.57
A 0.86 B 0.71
A 0.86 B 0.86
A 0.86 B 1
A 1 B 0
A 1 B 0.14
A 1 B 0.29
A 1 B 0.43
A 1 B 0.57
A 1 B 0.71
A 1 B 0.86
A 1 B 1
OD 600 nm [−]
0.8
0.6
0.4
0.2
0 5 101520
Time [h]

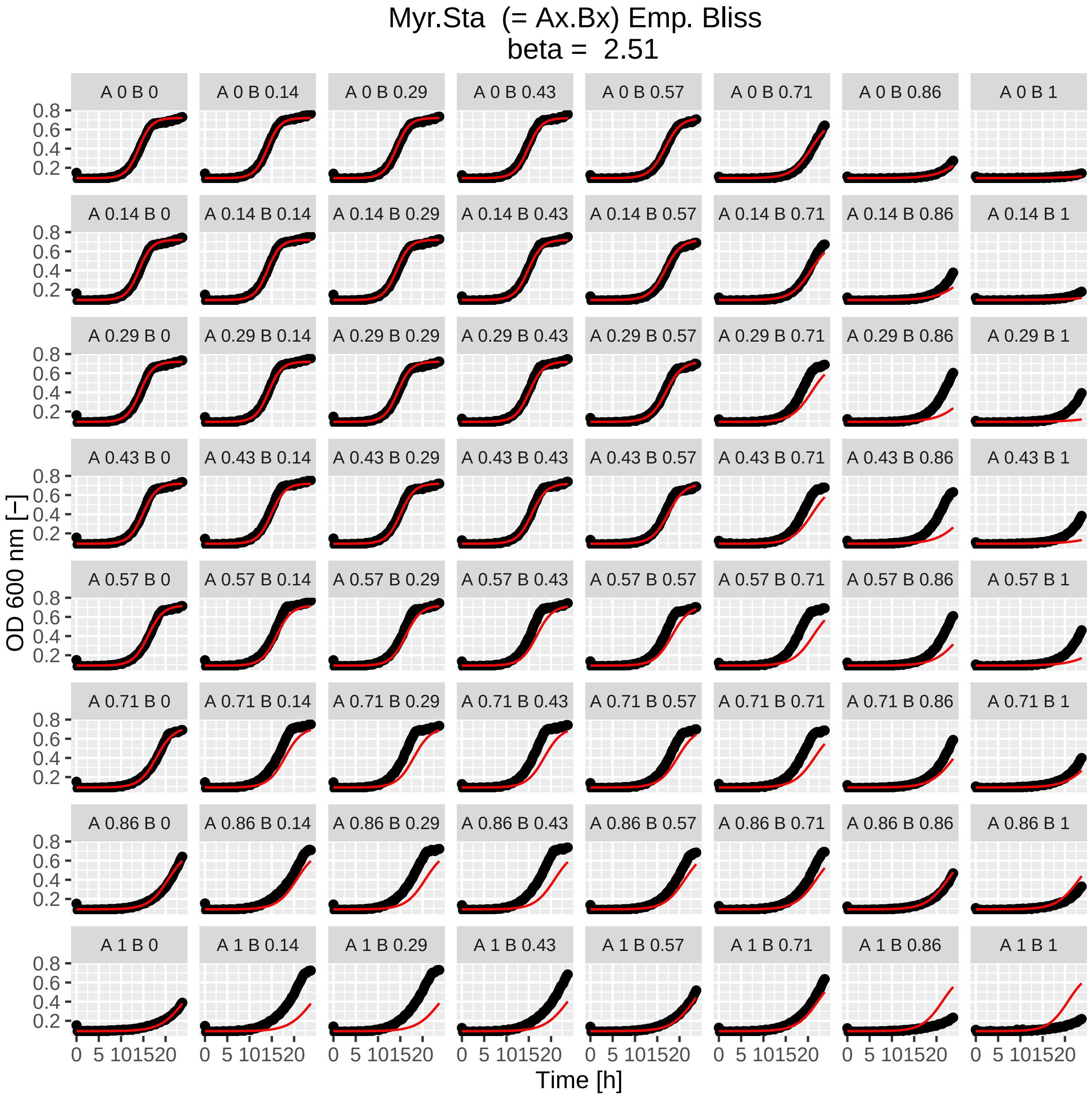
Myr.Sta (= Ax.Bx) Emp. Bliss
beta = 2.51
OD 600 nm [–]
Time [h]
A 0 B 0
A 0 B 0.14
A 0 B 0.29
A 0 B 0.43
A 0 B 0.57
A 0 B 0.71
A 0 B 0.86
A 0 B 1
A 0.14 B 0
A 0.14 B 0.14
A 0.14 B 0.29
A 0.14 B 0.43
A 0.14 B 0.57
A 0.14 B 0.71
A 0.14 B 0.86
A 0.14 B 1
A 0.29 B 0
A 0.29 B 0.14
A 0.29 B 0.29
A 0.29 B 0.43
A 0.29 B 0.57
A 0.29 B 0.71
A 0.29 B 0.86
A 0.29 B 1
A 0.43 B 0
A 0.43 B 0.14
A 0.43 B 0.29
A 0.43 B 0.43
A 0.43 B 0.57
A 0.43 B 0.71
A 0.43 B 0.86
A 0.43 B 1
A 0.57 B 0
A 0.57 B 0.14
A 0.57 B 0.29
A 0.57 B 0.43
A 0.57 B 0.57
A 0.57 B 0.71
A 0.57 B 0.86
A 0.57 B 1
A 0.71 B 0
A 0.71 B 0.14
A 0.71 B 0.29
A 0.71 B 0.43
A 0.71 B 0.57
A 0.71 B 0.71
A 0.71 B 0.86
A 0.71 B 1
A 0.86 B 0
A 0.86 B 0.14
A 0.86 B 0.29
A 0.86 B 0.43
A 0.86 B 0.57
A 0.86 B 0.71
A 0.86 B 0.86
A 0.86 B 1
A 1 B 0
A 1 B 0.14
A 1 B 0.29
A 1 B 0.43
A 1 B 0.57
A 1 B 0.71
A 1 B 0.86
A 1 B 1
0.8
0.6
0.4
0.2
0 5 101520

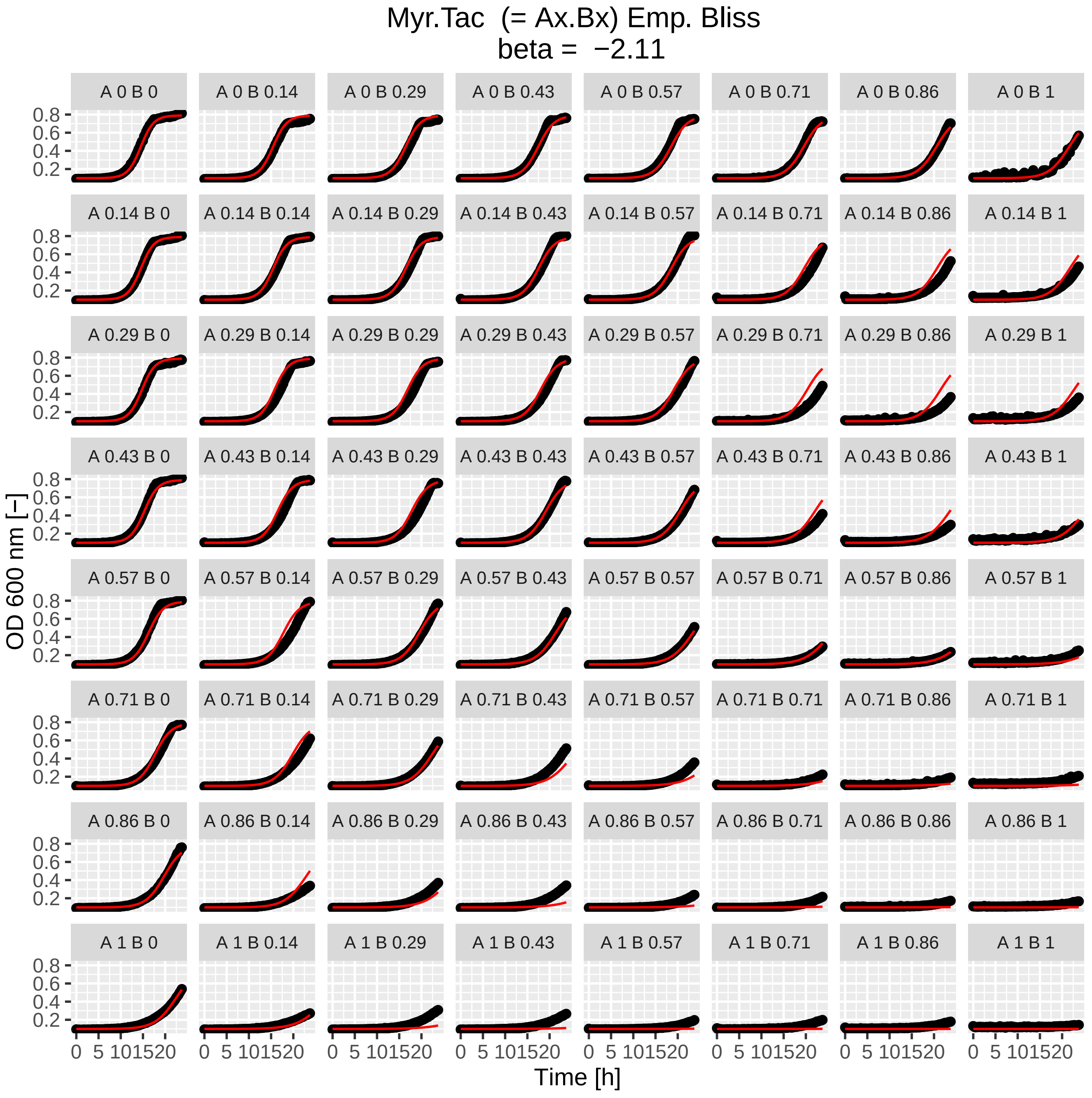
Myr.Tac (= Ax.Bx) Emp. Bliss
beta = −2.11
A 0 B 0
A 0 B 0.14
A 0 B 0.29
A 0 B 0.43
A 0 B 0.57
A 0 B 0.71
A 0 B 0.86
A 0 B 1
A 0.14 B 0
A 0.14 B 0.14
A 0.14 B 0.29
A 0.14 B 0.43
A 0.14 B 0.57
A 0.14 B 0.71
A 0.14 B 0.86
A 0.14 B 1
A 0.29 B 0
A 0.29 B 0.14
A 0.29 B 0.29
A 0.29 B 0.43
A 0.29 B 0.57
A 0.29 B 0.71
A 0.29 B 0.86
A 0.29 B 1
A 0.43 B 0
A 0.43 B 0.14
A 0.43 B 0.29
A 0.43 B 0.43
A 0.43 B 0.57
A 0.43 B 0.71
A 0.43 B 0.86
A 0.43 B 1
A 0.57 B 0
A 0.57 B 0.14
A 0.57 B 0.29
A 0.57 B 0.43
A 0.57 B 0.57
A 0.57 B 0.71
A 0.57 B 0.86
A 0.57 B 1
A 0.71 B 0
A 0.71 B 0.14
A 0.71 B 0.29
A 0.71 B 0.43
A 0.71 B 0.57
A 0.71 B 0.71
A 0.71 B 0.86
A 0.71 B 1
A 0.86 B 0
A 0.86 B 0.14
A 0.86 B 0.29
A 0.86 B 0.43
A 0.86 B 0.57
A 0.86 B 0.71
A 0.86 B 0.86
A 0.86 B 1
A 1 B 0
A 1 B 0.14
A 1 B 0.29
A 1 B 0.43
A 1 B 0.57
A 1 B 0.71
A 1 B 0.86
A 1 B 1
OD 600 nm [−]
0.8
0.6
0.4
0.2
0 5 101520
Time [h]

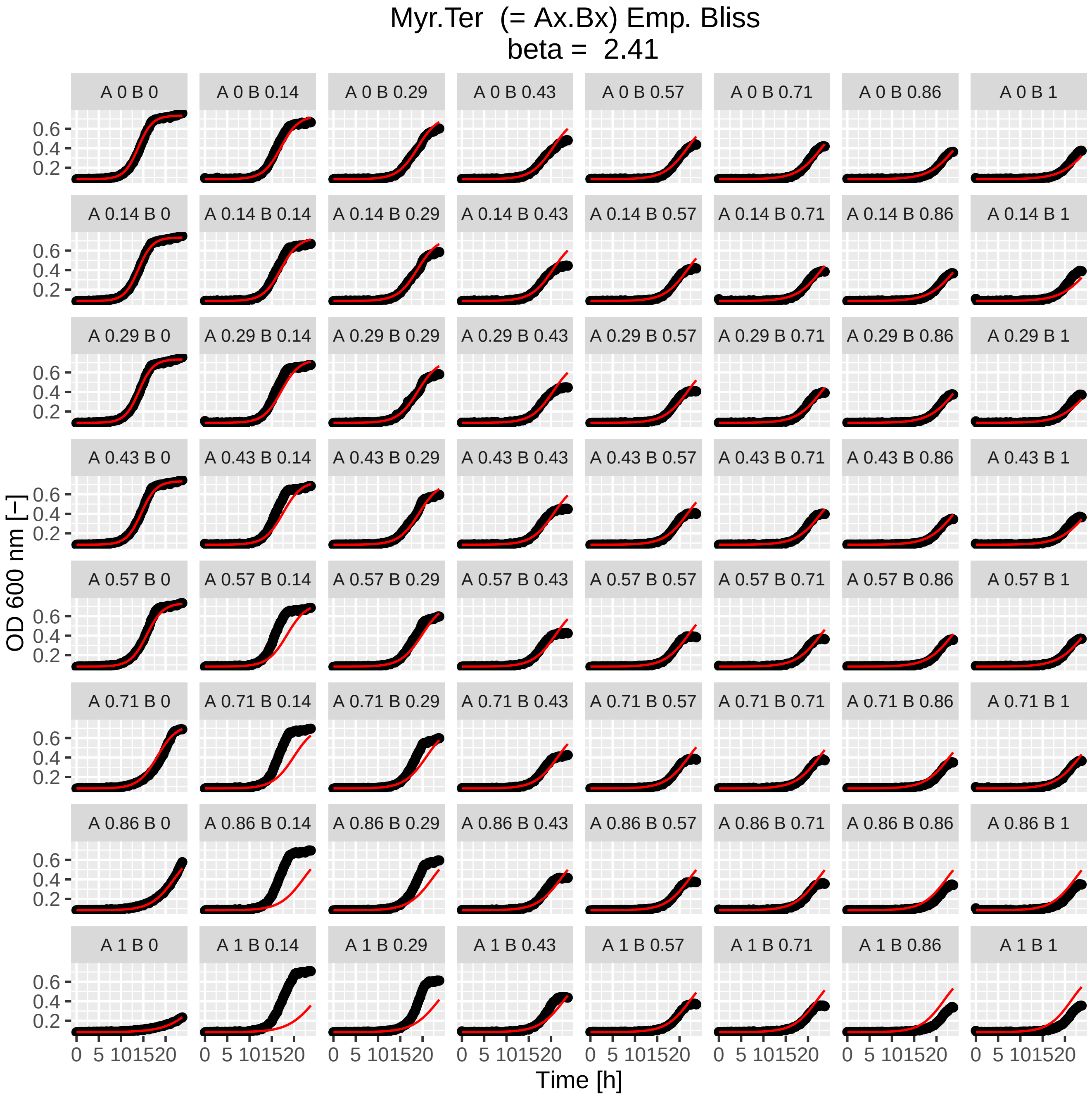

Myr.Ter (= Ax.Bx) Emp. Bliss
beta = 2.41
A 0 B 0
A 0 B 0.14
A 0 B 0.29
A 0 B 0.43
A 0 B 0.57
A 0 B 0.71
A 0 B 0.86
A 0 B 1
A 0.14 B 0
A 0.14 B 0.14
A 0.14 B 0.29
A 0.14 B 0.43
A 0.14 B 0.57
A 0.14 B 0.71
A 0.14 B 0.86
A 0.14 B 1
A 0.29 B 0
A 0.29 B 0.14
A 0.29 B 0.29
A 0.29 B 0.43
A 0.29 B 0.57
A 0.29 B 0.71
A 0.29 B 0.86
A 0.29 B 1
A 0.43 B 0
A 0.43 B 0.14
A 0.43 B 0.29
A 0.43 B 0.43
A 0.43 B 0.57
A 0.43 B 0.71
A 0.43 B 0.86
A 0.43 B 1
A 0.57 B 0
A 0.57 B 0.14
A 0.57 B 0.29
A 0.57 B 0.43
A 0.57 B 0.57
A 0.57 B 0.71
A 0.57 B 0.86
A 0.57 B 1
A 0.71 B 0
A 0.71 B 0.14
A 0.71 B 0.29
A 0.71 B 0.43
A 0.71 B 0.57
A 0.71 B 0.71
A 0.71 B 0.86
A 0.71 B 1
A 0.86 B 0
A 0.86 B 0.14
A 0.86 B 0.29
A 0.86 B 0.43
A 0.86 B 0.57
A 0.86 B 0.71
A 0.86 B 0.86
A 0.86 B 1
A 1 B 0
A 1 B 0.14
A 1 B 0.29
A 1 B 0.43
A 1 B 0.57
A 1 B 0.71
A 1 B 0.86
A 1 B 1
0.6
0.4
0.2
0 5 101520
OD 600 nm [–]
Time [h]

Pen.Qmy (= Ax.Bx) Emp. Bliss
beta = −54.85

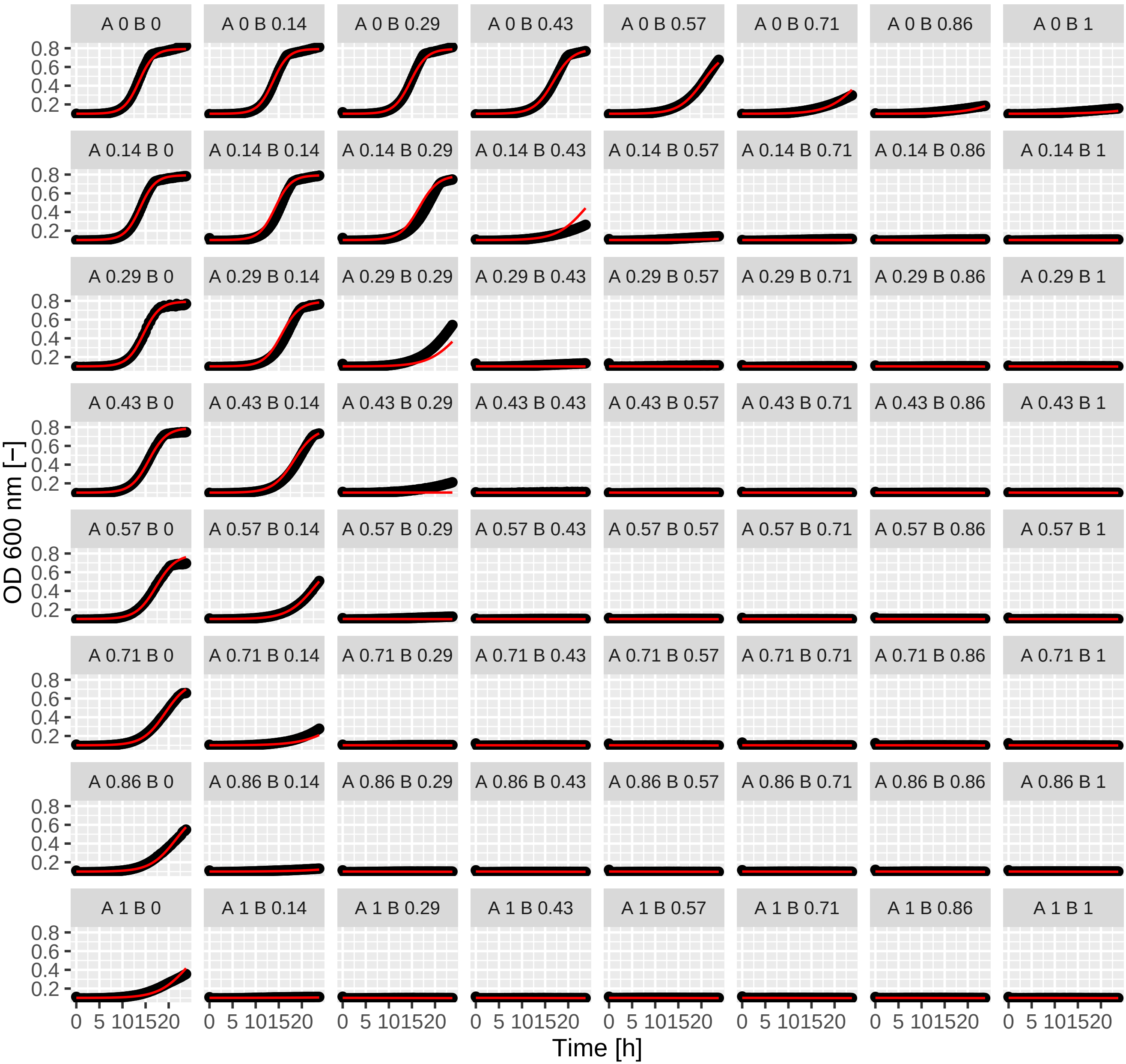

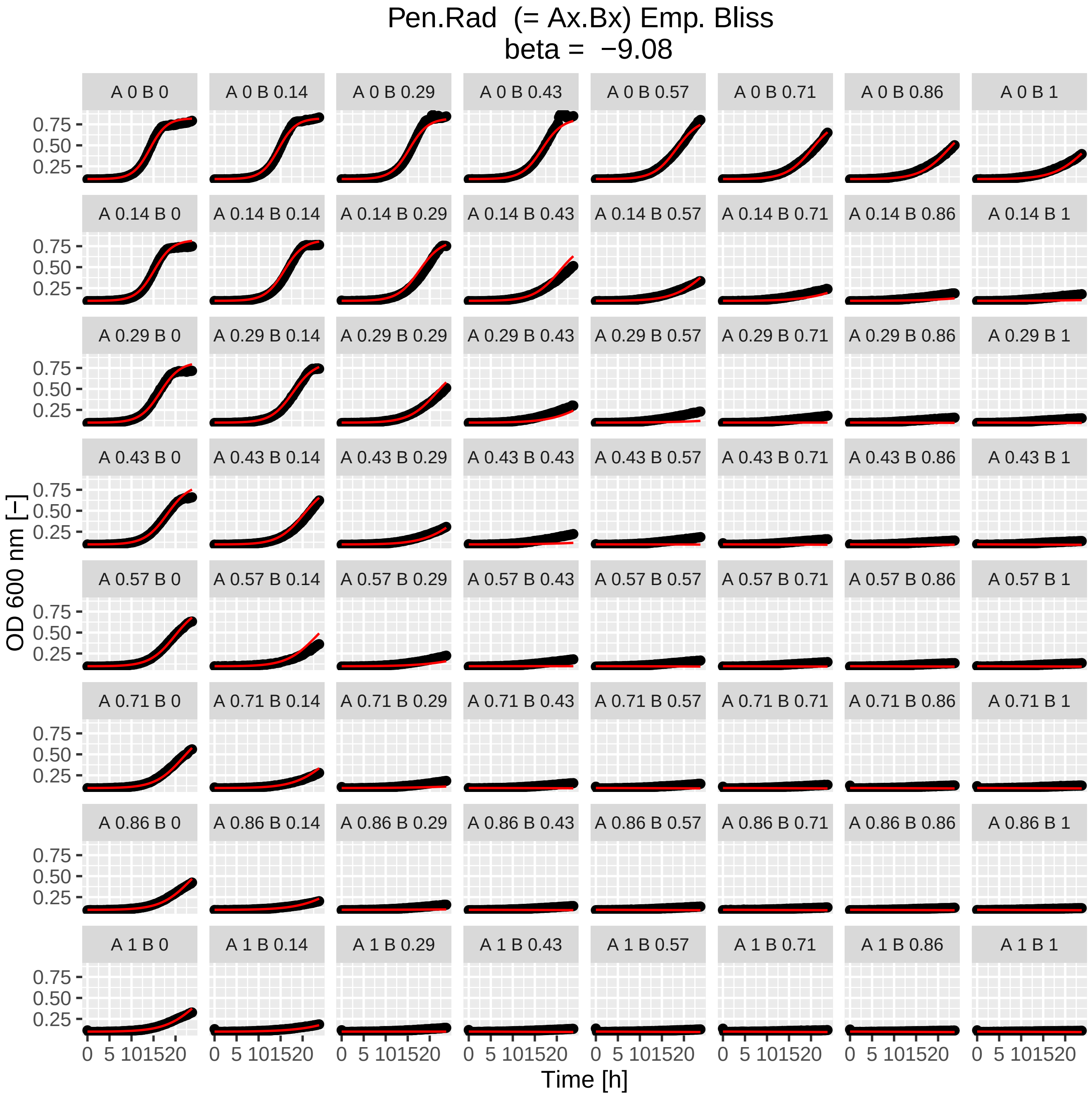

Pen.Rad (= Ax.Bx) Emp. Bliss
beta = −9.08
OD 600 nm [−]
Time [h]
0.75
0.50
0.25
0 5 101520
A 0 B 0
A 0 B 0.14
A 0 B 0.29
A 0 B 0.43
A 0 B 0.57
A 0 B 0.71
A 0 B 0.86
A 0 B 1
A 0.14 B 0
A 0.14 B 0.14
A 0.14 B 0.29
A 0.14 B 0.43
A 0.14 B 0.57
A 0.14 B 0.71
A 0.14 B 0.86
A 0.14 B 1
A 0.29 B 0
A 0.29 B 0.14
A 0.29 B 0.29
A 0.29 B 0.43
A 0.29 B 0.57
A 0.29 B 0.71
A 0.29 B 0.86
A 0.29 B 1
A 0.43 B 0
A 0.43 B 0.14
A 0.43 B 0.29
A 0.43 B 0.43
A 0.43 B 0.57
A 0.43 B 0.71
A 0.43 B 0.86
A 0.43 B 1
A 0.57 B 0
A 0.57 B 0.14
A 0.57 B 0.29
A 0.57 B 0.43
A 0.57 B 0.57
A 0.57 B 0.71
A 0.57 B 0.86
A 0.57 B 1
A 0.71 B 0
A 0.71 B 0.14
A 0.71 B 0.29
A 0.71 B 0.43
A 0.71 B 0.57
A 0.71 B 0.71
A 0.71 B 0.86
A 0.71 B 1
A 0.86 B 0
A 0.86 B 0.14
A 0.86 B 0.29
A 0.86 B 0.43
A 0.86 B 0.57
A 0.86 B 0.71
A 0.86 B 0.86
A 0.86 B 1
A 1 B 0
A 1 B 0.14
A 1 B 0.29
A 1 B 0.43
A 1 B 0.57
A 1 B 0.71
A 1 B 0.86
A 1 B 1

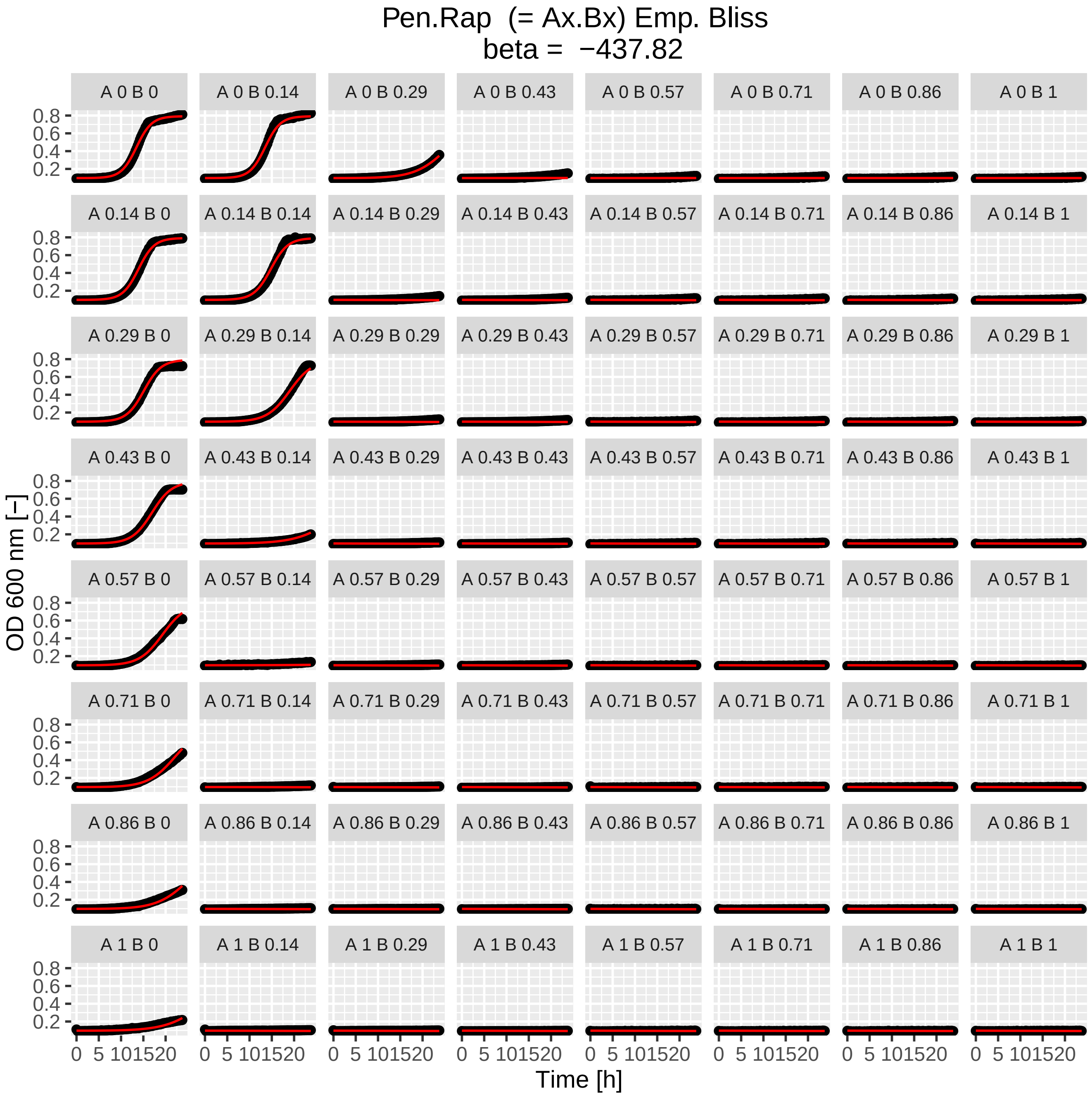

Pen.Rap (= Ax.Bx) Emp. Bliss
beta = −437.82
A 0 B 0
A 0 B 0.14
A 0 B 0.29
A 0 B 0.43
A 0 B 0.57
A 0 B 0.71
A 0 B 0.86
A 0 B 1
A 0.14 B 0
A 0.14 B 0.14
A 0.14 B 0.29
A 0.14 B 0.43
A 0.14 B 0.57
A 0.14 B 0.71
A 0.14 B 0.86
A 0.14 B 1
A 0.29 B 0
A 0.29 B 0.14
A 0.29 B 0.29
A 0.29 B 0.43
A 0.29 B 0.57
A 0.29 B 0.71
A 0.29 B 0.86
A 0.29 B 1
A 0.43 B 0
A 0.43 B 0.14
A 0.43 B 0.29
A 0.43 B 0.43
A 0.43 B 0.57
A 0.43 B 0.71
A 0.43 B 0.86
A 0.43 B 1
A 0.57 B 0
A 0.57 B 0.14
A 0.57 B 0.29
A 0.57 B 0.43
A 0.57 B 0.57
A 0.57 B 0.71
A 0.57 B 0.86
A 0.57 B 1
A 0.71 B 0
A 0.71 B 0.14
A 0.71 B 0.29
A 0.71 B 0.43
A 0.71 B 0.57
A 0.71 B 0.71
A 0.71 B 0.86
A 0.71 B 1
A 0.86 B 0
A 0.86 B 0.14
A 0.86 B 0.29
A 0.86 B 0.43
A 0.86 B 0.57
A 0.86 B 0.71
A 0.86 B 0.86
A 0.86 B 1
A 1 B 0
A 1 B 0.14
A 1 B 0.29
A 1 B 0.43
A 1 B 0.57
A 1 B 0.71
A 1 B 0.86
A 1 B 1
0.8
0.6
0.4
0.2
OD 600 nm [−]
0 5 101520
Time [h]

Pen.Sta (= Ax.Bx) Emp. Bliss
beta = −25.74

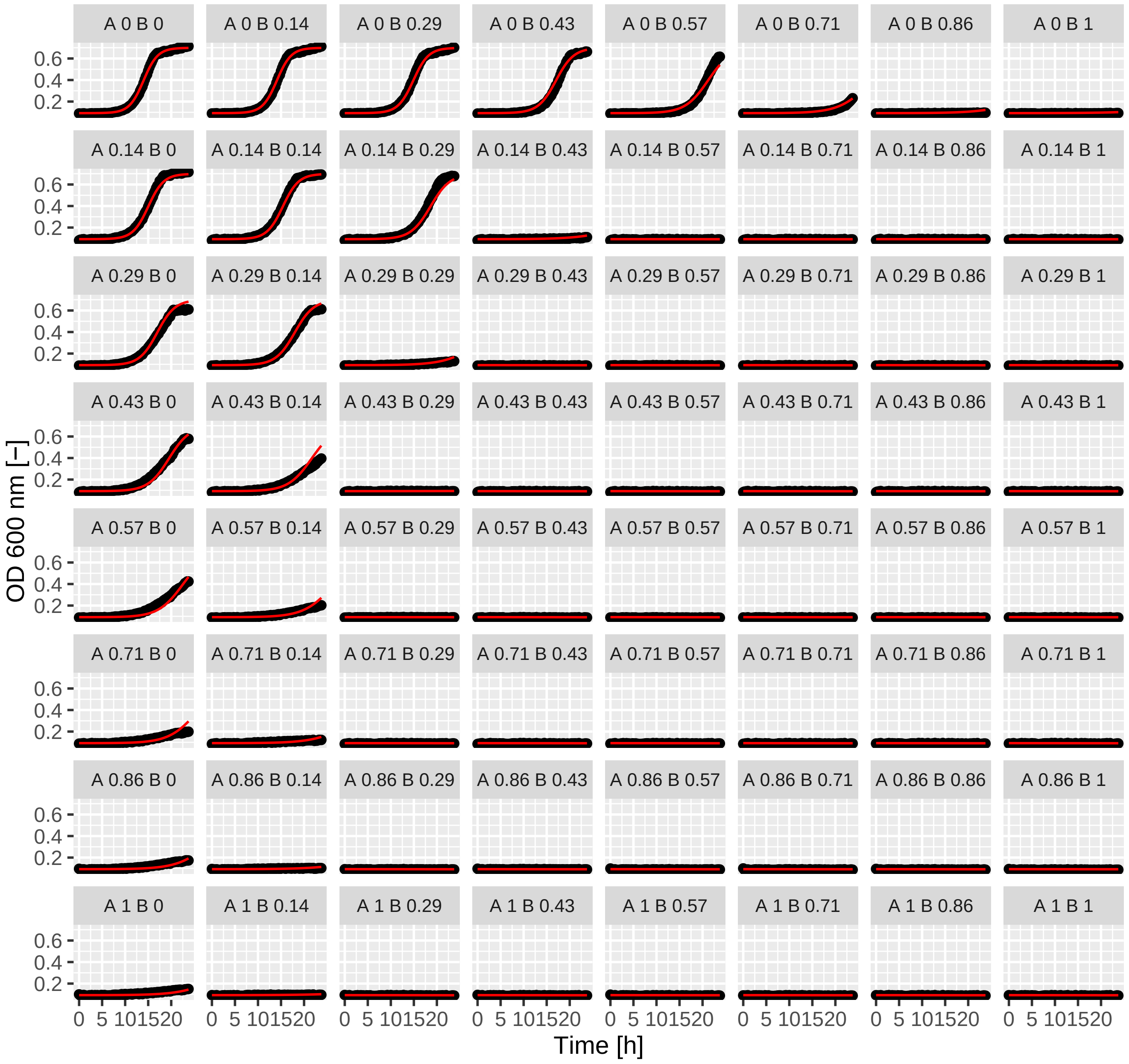

Pen.Tac (= Ax.Bx) Emp. Bliss
beta = −6.54

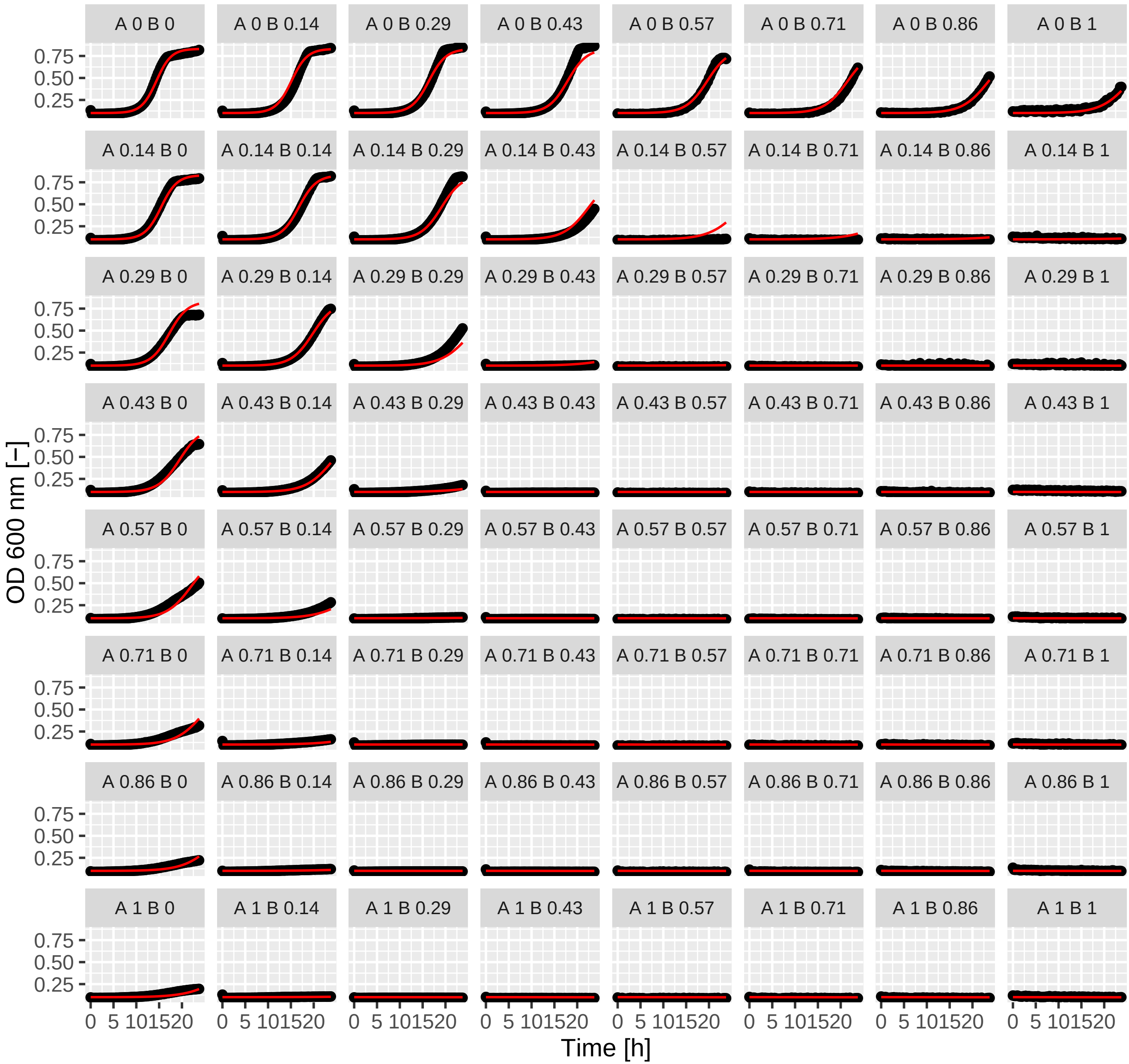

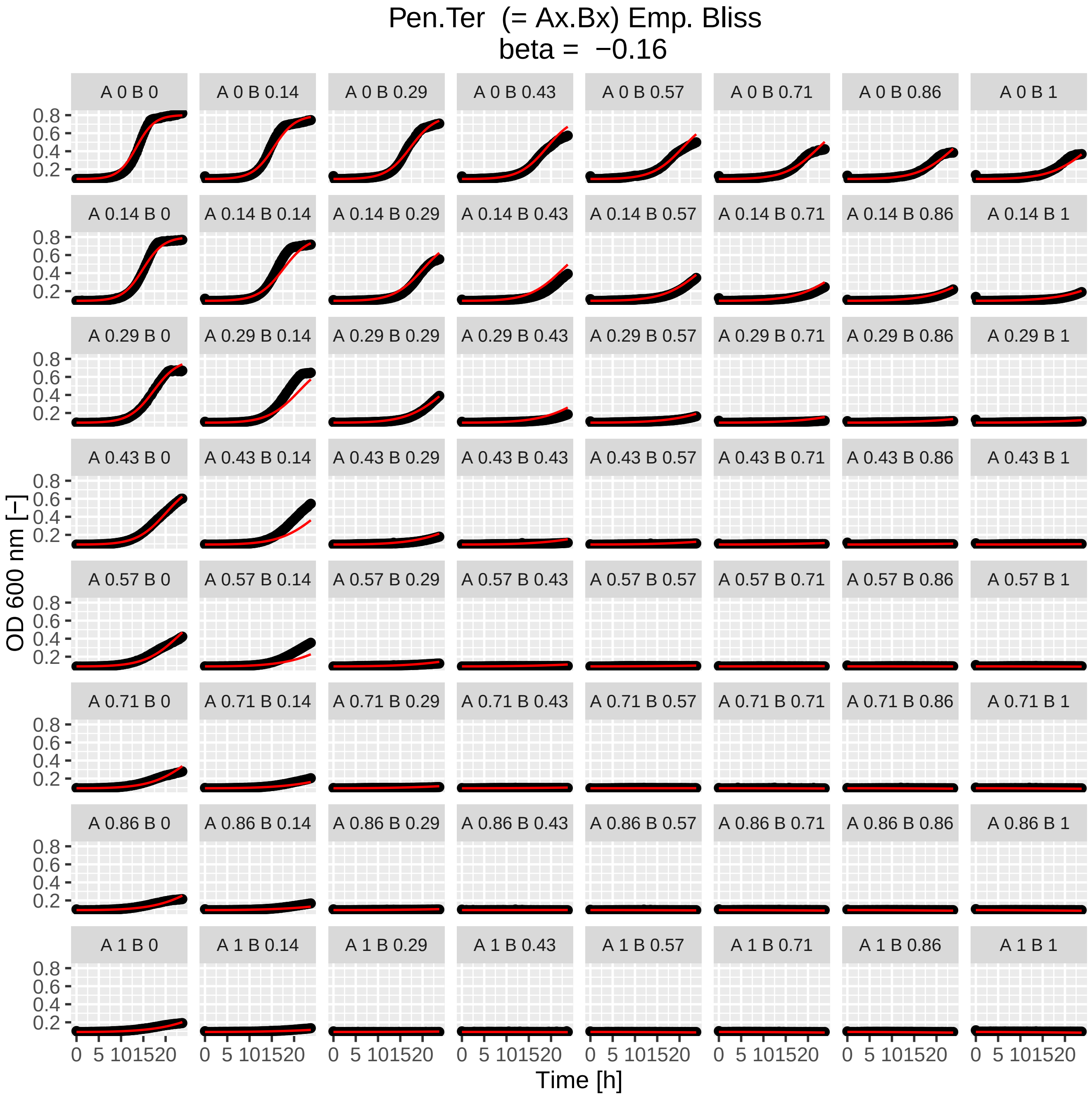

Pen.Ter (= Ax.Bx) Emp. Bliss
beta = −0.16
OD 600 nm [−]
Time [h]
0.8
0.6
0.4
0.2
0 5 101520
A 0 B 0
A 0 B 0.14
A 0 B 0.29
A 0 B 0.43
A 0 B 0.57
A 0 B 0.71
A 0 B 0.86
A 0 B 1
A 0.14 B 0
A 0.14 B 0.14
A 0.14 B 0.29
A 0.14 B 0.43
A 0.14 B 0.57
A 0.14 B 0.71
A 0.14 B 0.86
A 0.14 B 1
A 0.29 B 0
A 0.29 B 0.14
A 0.29 B 0.29
A 0.29 B 0.43
A 0.29 B 0.57
A 0.29 B 0.71
A 0.29 B 0.86
A 0.29 B 1
A 0.43 B 0
A 0.43 B 0.14
A 0.43 B 0.29
A 0.43 B 0.43
A 0.43 B 0.57
A 0.43 B 0.71
A 0.43 B 0.86
A 0.43 B 1
A 0.57 B 0
A 0.57 B 0.14
A 0.57 B 0.29
A 0.57 B 0.43
A 0.57 B 0.57
A 0.57 B 0.71
A 0.57 B 0.86
A 0.57 B 1
A 0.71 B 0
A 0.71 B 0.14
A 0.71 B 0.29
A 0.71 B 0.43
A 0.71 B 0.57
A 0.71 B 0.71
A 0.71 B 0.86
A 0.71 B 1
A 0.86 B 0
A 0.86 B 0.14
A 0.86 B 0.29
A 0.86 B 0.43
A 0.86 B 0.57
A 0.86 B 0.71
A 0.86 B 0.86
A 0.86 B 1
A 1 B 0
A 1 B 0.14
A 1 B 0.29
A 1 B 0.43
A 1 B 0.57
A 1 B 0.71
A 1 B 0.86
A 1 B 1

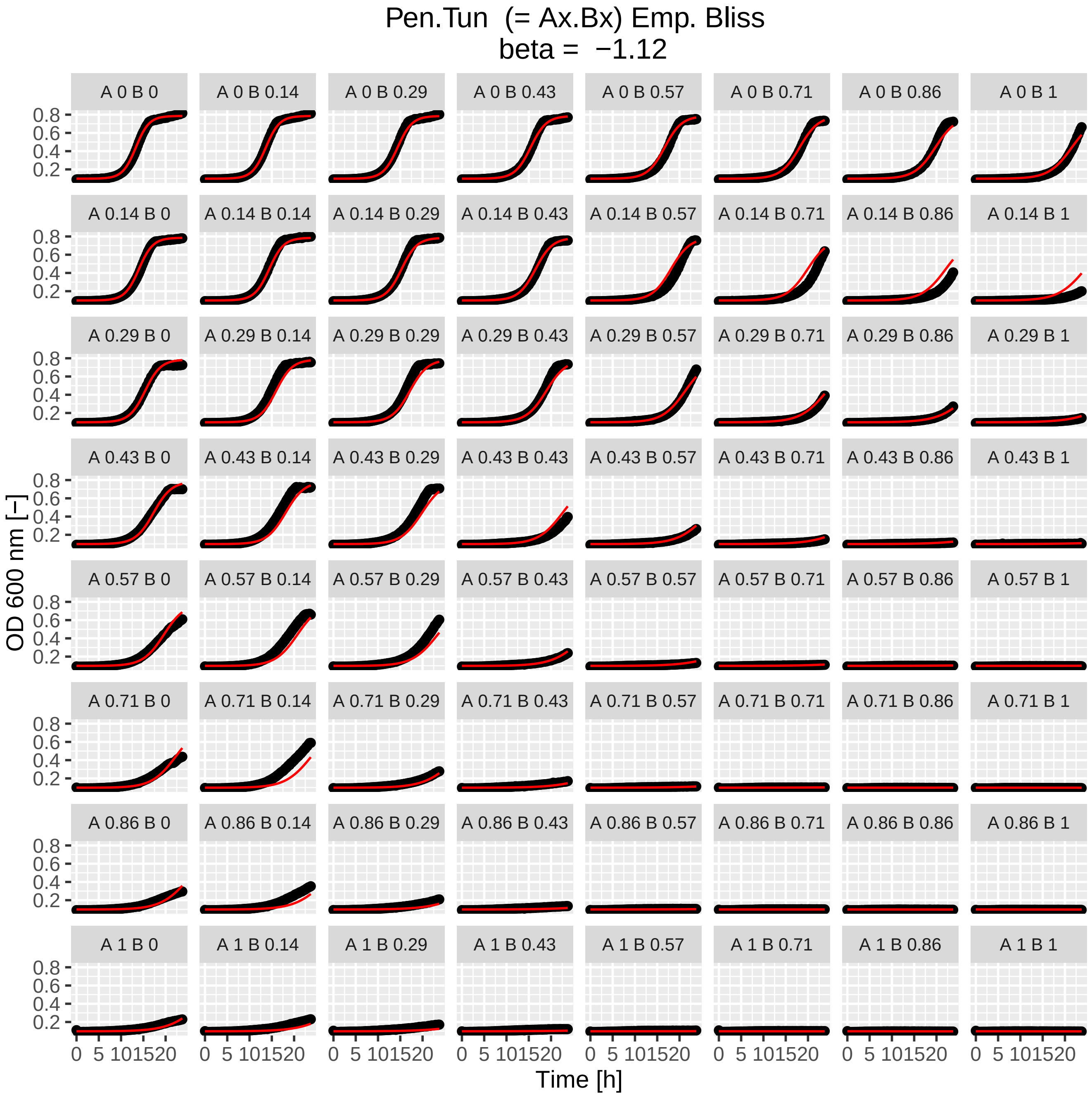

Pen.Tun (= Ax.Bx) Emp. Bliss
beta = −1.12
OD 600 nm [−]
Time [h]
A 0 B 0
A 0 B 0.14
A 0 B 0.29
A 0 B 0.43
A 0 B 0.57
A 0 B 0.71
A 0 B 0.86
A 0 B 1
A 0.14 B 0
A 0.14 B 0.14
A 0.14 B 0.29
A 0.14 B 0.43
A 0.14 B 0.57
A 0.14 B 0.71
A 0.14 B 0.86
A 0.14 B 1
A 0.29 B 0
A 0.29 B 0.14
A 0.29 B 0.29
A 0.29 B 0.43
A 0.29 B 0.57
A 0.29 B 0.71
A 0.29 B 0.86
A 0.29 B 1
A 0.43 B 0
A 0.43 B 0.14
A 0.43 B 0.29
A 0.43 B 0.43
A 0.43 B 0.57
A 0.43 B 0.71
A 0.43 B 0.86
A 0.43 B 1
A 0.57 B 0
A 0.57 B 0.14
A 0.57 B 0.29
A 0.57 B 0.43
A 0.57 B 0.57
A 0.57 B 0.71
A 0.57 B 0.86
A 0.57 B 1
A 0.71 B 0
A 0.71 B 0.14
A 0.71 B 0.29
A 0.71 B 0.43
A 0.71 B 0.57
A 0.71 B 0.71
A 0.71 B 0.86
A 0.71 B 1
A 0.86 B 0
A 0.86 B 0.14
A 0.86 B 0.29
A 0.86 B 0.43
A 0.86 B 0.57
A 0.86 B 0.71
A 0.86 B 0.86
A 0.86 B 1
A 1 B 0
A 1 B 0.14
A 1 B 0.29
A 1 B 0.43
A 1 B 0.57
A 1 B 0.71
A 1 B 0.86
A 1 B 1

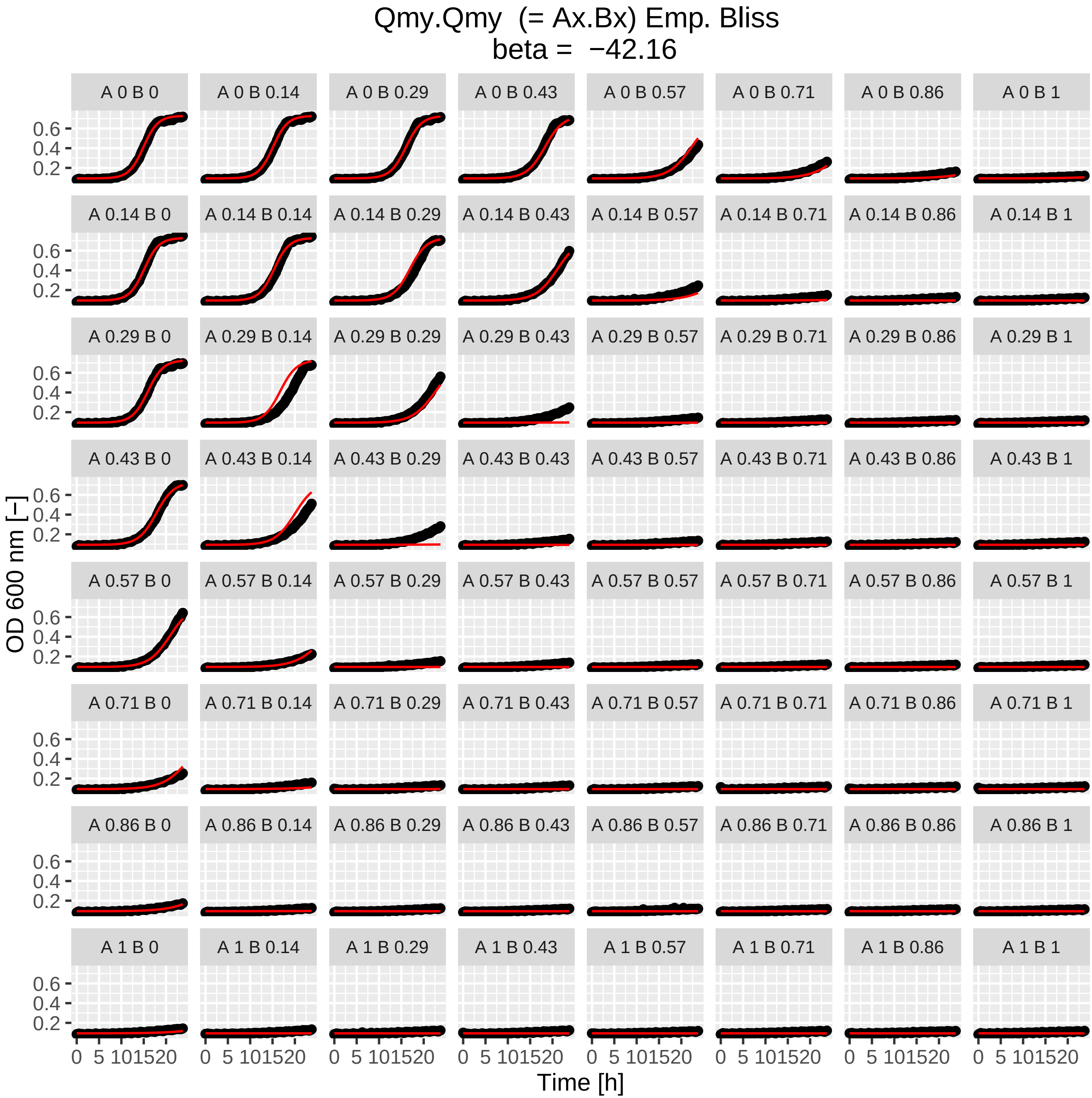

Qmy.Qmy (= Ax.Bx) Emp. Bliss
beta = −42.16
A 0 B 0
A 0 B 0.14
A 0 B 0.29
A 0 B 0.43
A 0 B 0.57
A 0 B 0.71
A 0 B 0.86
A 0 B 1
A 0.14 B 0
A 0.14 B 0.14
A 0.14 B 0.29
A 0.14 B 0.43
A 0.14 B 0.57
A 0.14 B 0.71
A 0.14 B 0.86
A 0.14 B 1
A 0.29 B 0
A 0.29 B 0.14
A 0.29 B 0.29
A 0.29 B 0.43
A 0.29 B 0.57
A 0.29 B 0.71
A 0.29 B 0.86
A 0.29 B 1
A 0.43 B 0
A 0.43 B 0.14
A 0.43 B 0.29
A 0.43 B 0.43
A 0.43 B 0.57
A 0.43 B 0.71
A 0.43 B 0.86
A 0.43 B 1
A 0.57 B 0
A 0.57 B 0.14
A 0.57 B 0.29
A 0.57 B 0.43
A 0.57 B 0.57
A 0.57 B 0.71
A 0.57 B 0.86
A 0.57 B 1
A 0.71 B 0
A 0.71 B 0.14
A 0.71 B 0.29
A 0.71 B 0.43
A 0.71 B 0.57
A 0.71 B 0.71
A 0.71 B 0.86
A 0.71 B 1
A 0.86 B 0
A 0.86 B 0.14
A 0.86 B 0.29
A 0.86 B 0.43
A 0.86 B 0.57
A 0.86 B 0.71
A 0.86 B 0.86
A 0.86 B 1
A 1 B 0
A 1 B 0.14
A 1 B 0.29
A 1 B 0.43
A 1 B 0.57
A 1 B 0.71
A 1 B 0.86
A 1 B 1
OD 600 nm [−]
0.2
0.4
0.6
0 5 101520
Time [h]

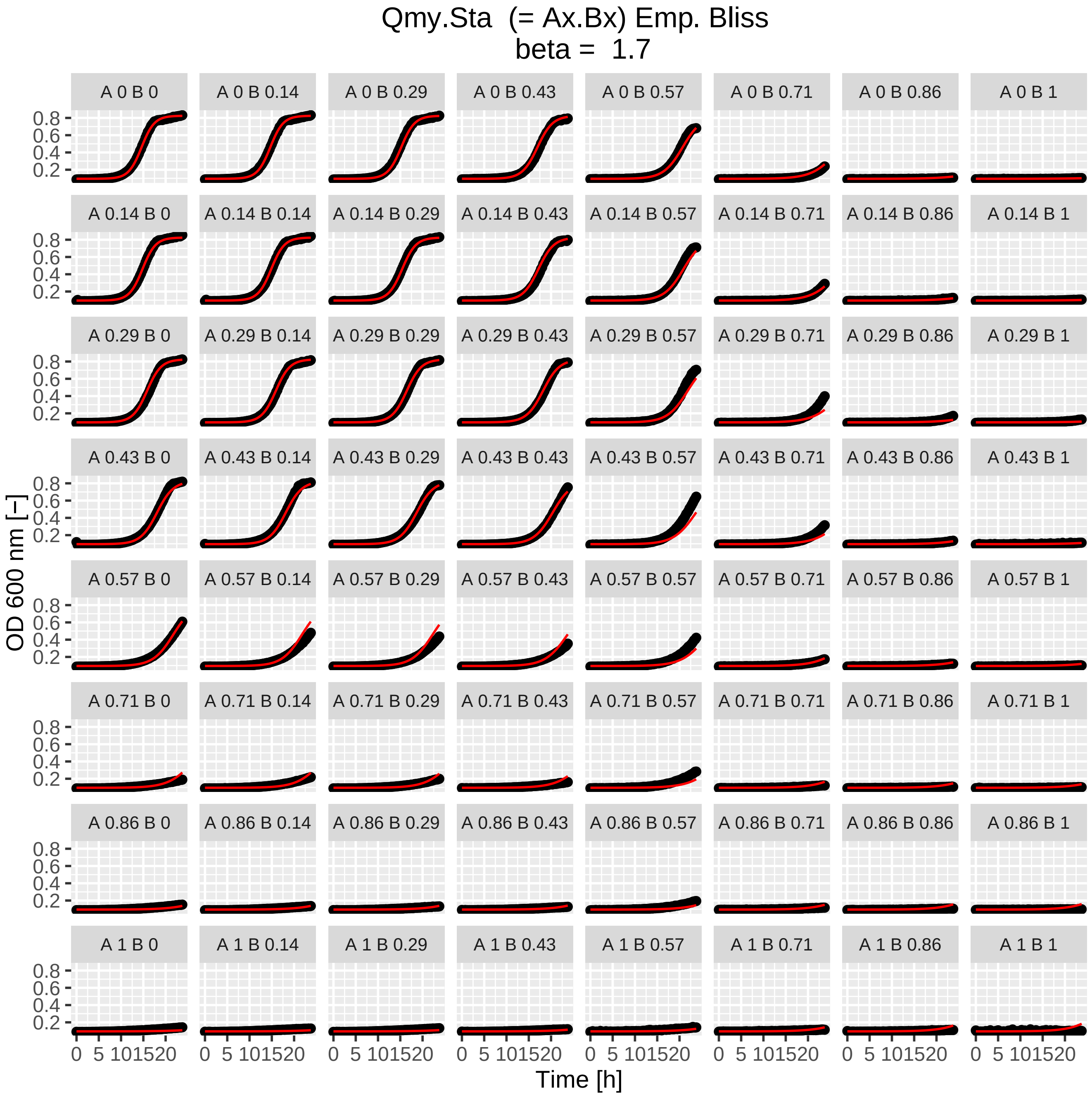

Qmy.Sta (= Ax.Bx) Emp. Bliss
beta = 1.7
A 0 B 0
A 0 B 0.14
A 0 B 0.29
A 0 B 0.43
A 0 B 0.57
A 0 B 0.71
A 0 B 0.86
A 0 B 1
A 0.14 B 0
A 0.14 B 0.14
A 0.14 B 0.29
A 0.14 B 0.43
A 0.14 B 0.57
A 0.14 B 0.71
A 0.14 B 0.86
A 0.14 B 1
A 0.29 B 0
A 0.29 B 0.14
A 0.29 B 0.29
A 0.29 B 0.43
A 0.29 B 0.57
A 0.29 B 0.71
A 0.29 B 0.86
A 0.29 B 1
A 0.43 B 0
A 0.43 B 0.14
A 0.43 B 0.29
A 0.43 B 0.43
A 0.43 B 0.57
A 0.43 B 0.71
A 0.43 B 0.86
A 0.43 B 1
A 0.57 B 0
A 0.57 B 0.14
A 0.57 B 0.29
A 0.57 B 0.43
A 0.57 B 0.57
A 0.57 B 0.71
A 0.57 B 0.86
A 0.57 B 1
A 0.71 B 0
A 0.71 B 0.14
A 0.71 B 0.29
A 0.71 B 0.43
A 0.71 B 0.57
A 0.71 B 0.71
A 0.71 B 0.86
A 0.71 B 1
A 0.86 B 0
A 0.86 B 0.14
A 0.86 B 0.29
A 0.86 B 0.43
A 0.86 B 0.57
A 0.86 B 0.71
A 0.86 B 0.86
A 0.86 B 1
A 1 B 0
A 1 B 0.14
A 1 B 0.29
A 1 B 0.43
A 1 B 0.57
A 1 B 0.71
A 1 B 0.86
A 1 B 1
OD 600 nm [–]
0.8
0.6
0.4
0.2
0 5 101520
Time [h]

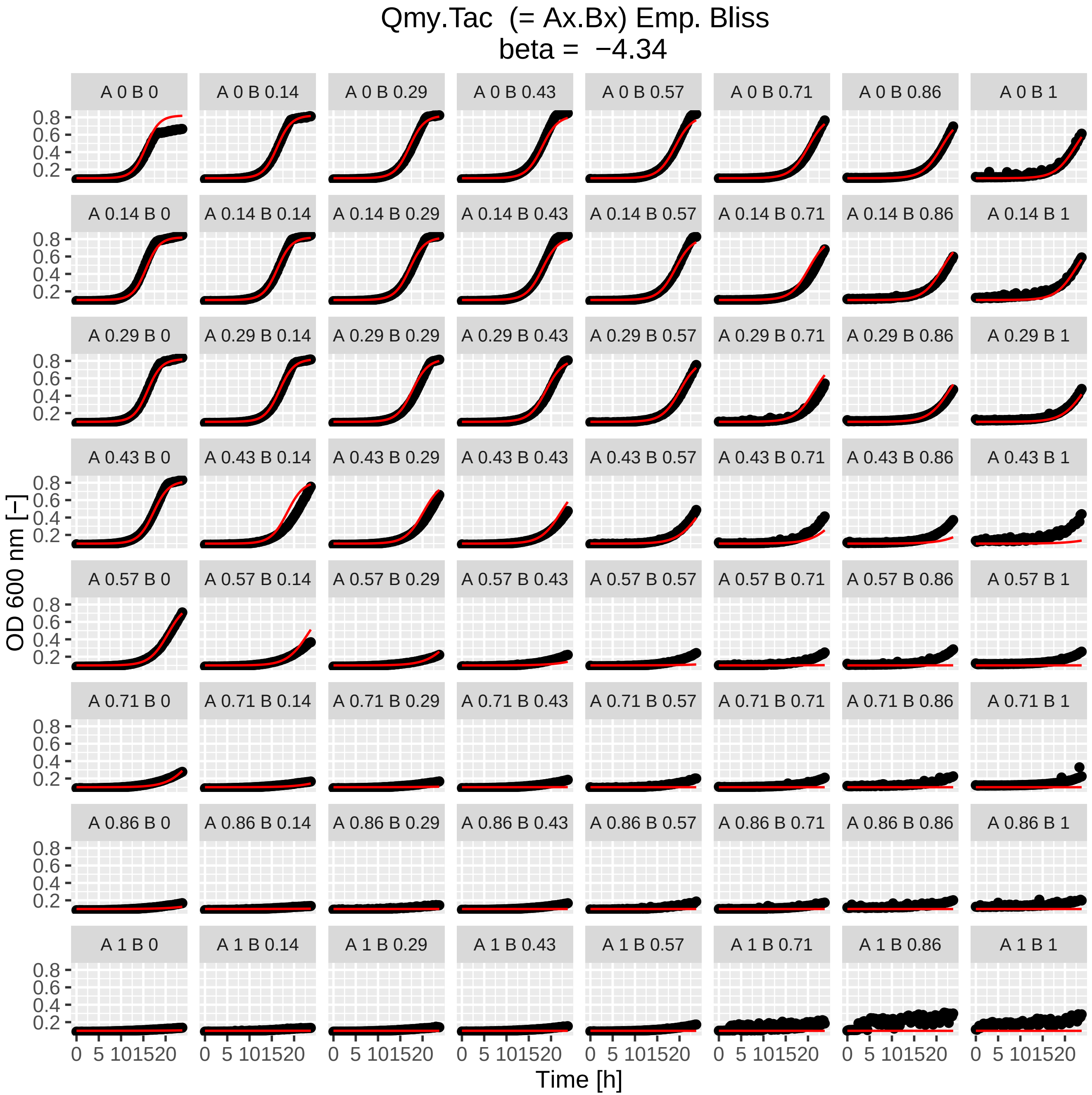

Qmy.Tac (= Ax.Bx) Emp. Bliss
beta = −4.34
A 0 B 0
A 0 B 0.14
A 0 B 0.29
A 0 B 0.43
A 0 B 0.57
A 0 B 0.71
A 0 B 0.86
A 0 B 1
A 0.14 B 0
A 0.14 B 0.14
A 0.14 B 0.29
A 0.14 B 0.43
A 0.14 B 0.57
A 0.14 B 0.71
A 0.14 B 0.86
A 0.14 B 1
A 0.29 B 0
A 0.29 B 0.14
A 0.29 B 0.29
A 0.29 B 0.43
A 0.29 B 0.57
A 0.29 B 0.71
A 0.29 B 0.86
A 0.29 B 1
A 0.43 B 0
A 0.43 B 0.14
A 0.43 B 0.29
A 0.43 B 0.43
A 0.43 B 0.57
A 0.43 B 0.71
A 0.43 B 0.86
A 0.43 B 1
A 0.57 B 0
A 0.57 B 0.14
A 0.57 B 0.29
A 0.57 B 0.43
A 0.57 B 0.57
A 0.57 B 0.71
A 0.57 B 0.86
A 0.57 B 1
A 0.71 B 0
A 0.71 B 0.14
A 0.71 B 0.29
A 0.71 B 0.43
A 0.71 B 0.57
A 0.71 B 0.71
A 0.71 B 0.86
A 0.71 B 1
A 0.86 B 0
A 0.86 B 0.14
A 0.86 B 0.29
A 0.86 B 0.43
A 0.86 B 0.57
A 0.86 B 0.71
A 0.86 B 0.86
A 0.86 B 1
A 1 B 0
A 1 B 0.14
A 1 B 0.29
A 1 B 0.43
A 1 B 0.57
A 1 B 0.71
A 1 B 0.86
A 1 B 1
OD 600 nm [−]
0.2
0.4
0.6
0.8
0 5 101520
Time [h]

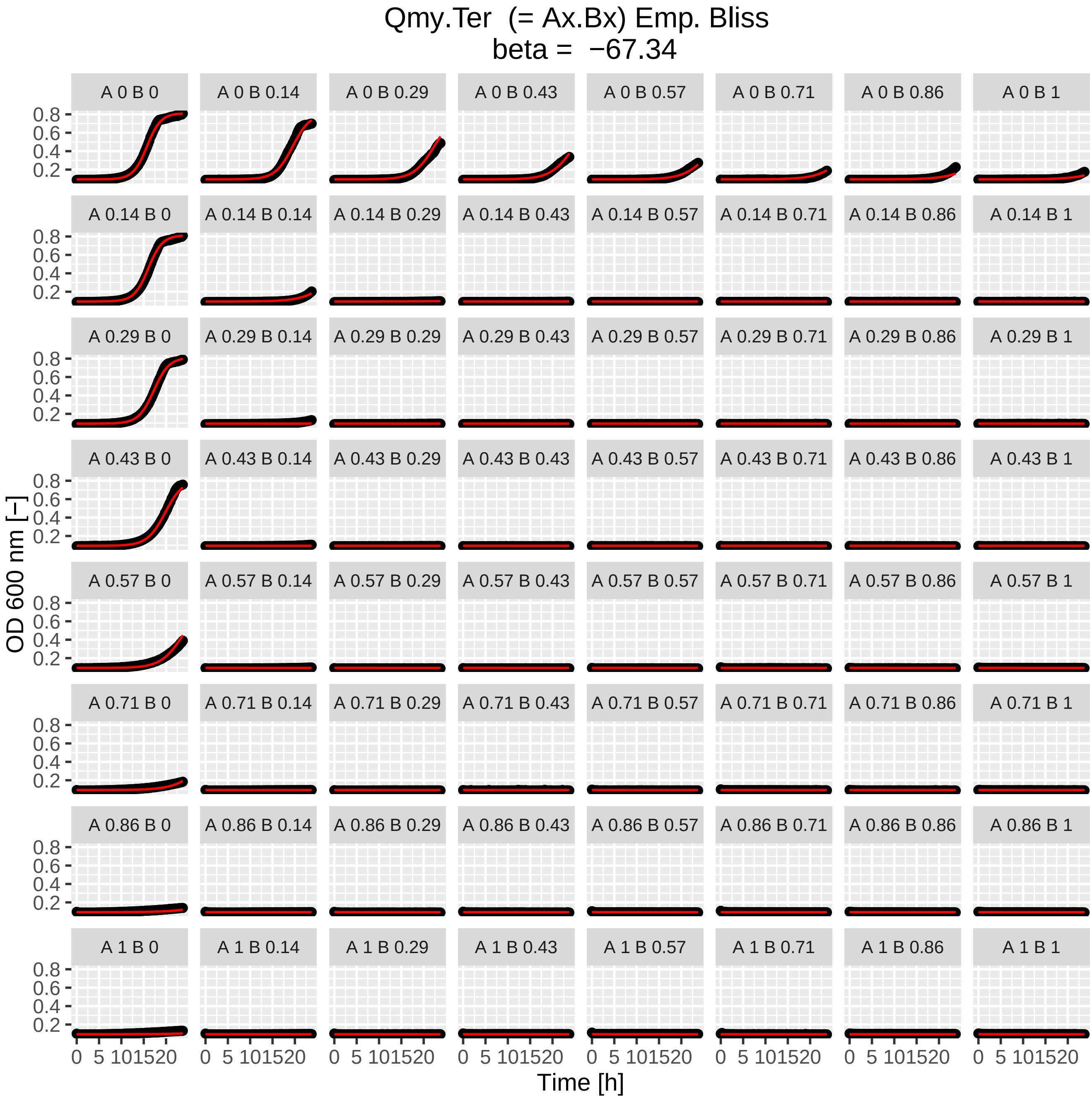
Qmy.Ter (= Ax.Bx) Emp. Bliss
beta = −67.34
A 0 B 0
A 0 B 0.14
A 0 B 0.29
A 0 B 0.43
A 0 B 0.57
A 0 B 0.71
A 0 B 0.86
A 0 B 1
A 0.14 B 0
A 0.14 B 0.14
A 0.14 B 0.29
A 0.14 B 0.43
A 0.14 B 0.57
A 0.14 B 0.71
A 0.14 B 0.86
A 0.14 B 1
A 0.29 B 0
A 0.29 B 0.14
A 0.29 B 0.29
A 0.29 B 0.43
A 0.29 B 0.57
A 0.29 B 0.71
A 0.29 B 0.86
A 0.29 B 1
A 0.43 B 0
A 0.43 B 0.14
A 0.43 B 0.29
A 0.43 B 0.43
A 0.43 B 0.57
A 0.43 B 0.71
A 0.43 B 0.86
A 0.43 B 1
A 0.57 B 0
A 0.57 B 0.14
A 0.57 B 0.29
A 0.57 B 0.43
A 0.57 B 0.57
A 0.57 B 0.71
A 0.57 B 0.86
A 0.57 B 1
A 0.71 B 0
A 0.71 B 0.14
A 0.71 B 0.29
A 0.71 B 0.43
A 0.71 B 0.57
A 0.71 B 0.71
A 0.71 B 0.86
A 0.71 B 1
A 0.86 B 0
A 0.86 B 0.14
A 0.86 B 0.29
A 0.86 B 0.43
A 0.86 B 0.57
A 0.86 B 0.71
A 0.86 B 0.86
A 0.86 B 1
A 1 B 0
A 1 B 0.14
A 1 B 0.29
A 1 B 0.43
A 1 B 0.57
A 1 B 0.71
A 1 B 0.86
A 1 B 1
OD 600 nm [−]
0.2
0.4
0.6
0.8
0 5 101520
Time [h]

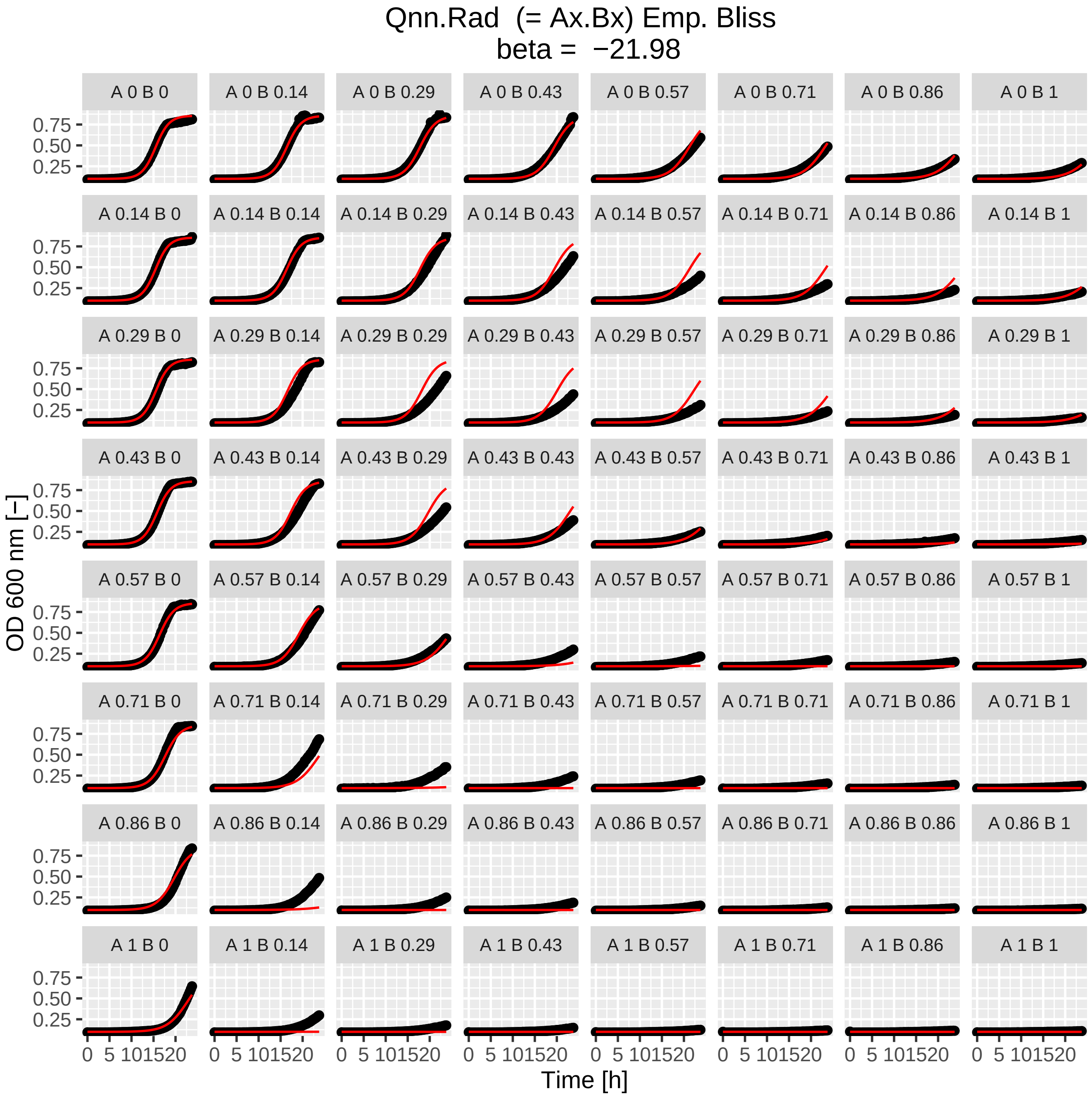

Qnn.Rad (= Ax.Bx) Emp. Bliss
beta = −21.98
A 0 B 0
A 0 B 0.14
A 0 B 0.29
A 0 B 0.43
A 0 B 0.57
A 0 B 0.71
A 0 B 0.86
A 0 B 1
A 0.14 B 0
A 0.14 B 0.14
A 0.14 B 0.29
A 0.14 B 0.43
A 0.14 B 0.57
A 0.14 B 0.71
A 0.14 B 0.86
A 0.14 B 1
A 0.29 B 0
A 0.29 B 0.14
A 0.29 B 0.29
A 0.29 B 0.43
A 0.29 B 0.57
A 0.29 B 0.71
A 0.29 B 0.86
A 0.29 B 1
A 0.43 B 0
A 0.43 B 0.14
A 0.43 B 0.29
A 0.43 B 0.43
A 0.43 B 0.57
A 0.43 B 0.71
A 0.43 B 0.86
A 0.43 B 1
A 0.57 B 0
A 0.57 B 0.14
A 0.57 B 0.29
A 0.57 B 0.43
A 0.57 B 0.57
A 0.57 B 0.71
A 0.57 B 0.86
A 0.57 B 1
A 0.71 B 0
A 0.71 B 0.14
A 0.71 B 0.29
A 0.71 B 0.43
A 0.71 B 0.57
A 0.71 B 0.71
A 0.71 B 0.86
A 0.71 B 1
A 0.86 B 0
A 0.86 B 0.14
A 0.86 B 0.29
A 0.86 B 0.43
A 0.86 B 0.57
A 0.86 B 0.71
A 0.86 B 0.86
A 0.86 B 1
A 1 B 0
A 1 B 0.14
A 1 B 0.29
A 1 B 0.43
A 1 B 0.57
A 1 B 0.71
A 1 B 0.86
A 1 B 1
0.75
0.50
0.25
OD 600 nm [−]
0 5 101520
Time [h]

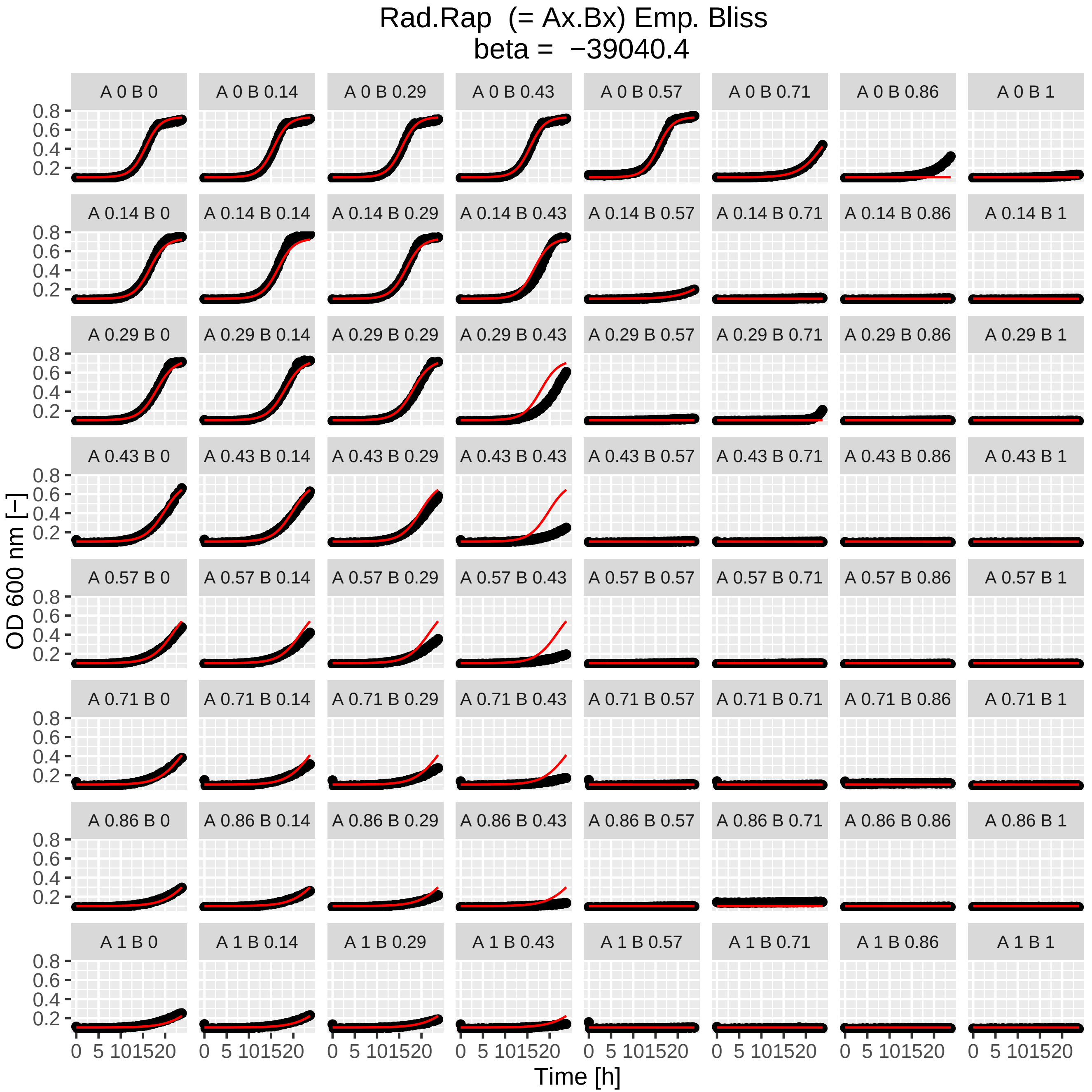
Rad.Rap (= Ax.Bx) Emp. Bliss
beta = −39040.4
OD 600 nm [−]
Time [h]
A 0 B 0
A 0 B 0.14
A 0 B 0.29
A 0 B 0.43
A 0 B 0.57
A 0 B 0.71
A 0 B 0.86
A 0 B 1
A 0.14 B 0
A 0.29 B 0
A 0.43 B 0
A 0.57 B 0
A 0.71 B 0
A 0.86 B 0
A 1 B 0
A 1 B 1
0.2
0.4
0.6
0.8
0 5 101520

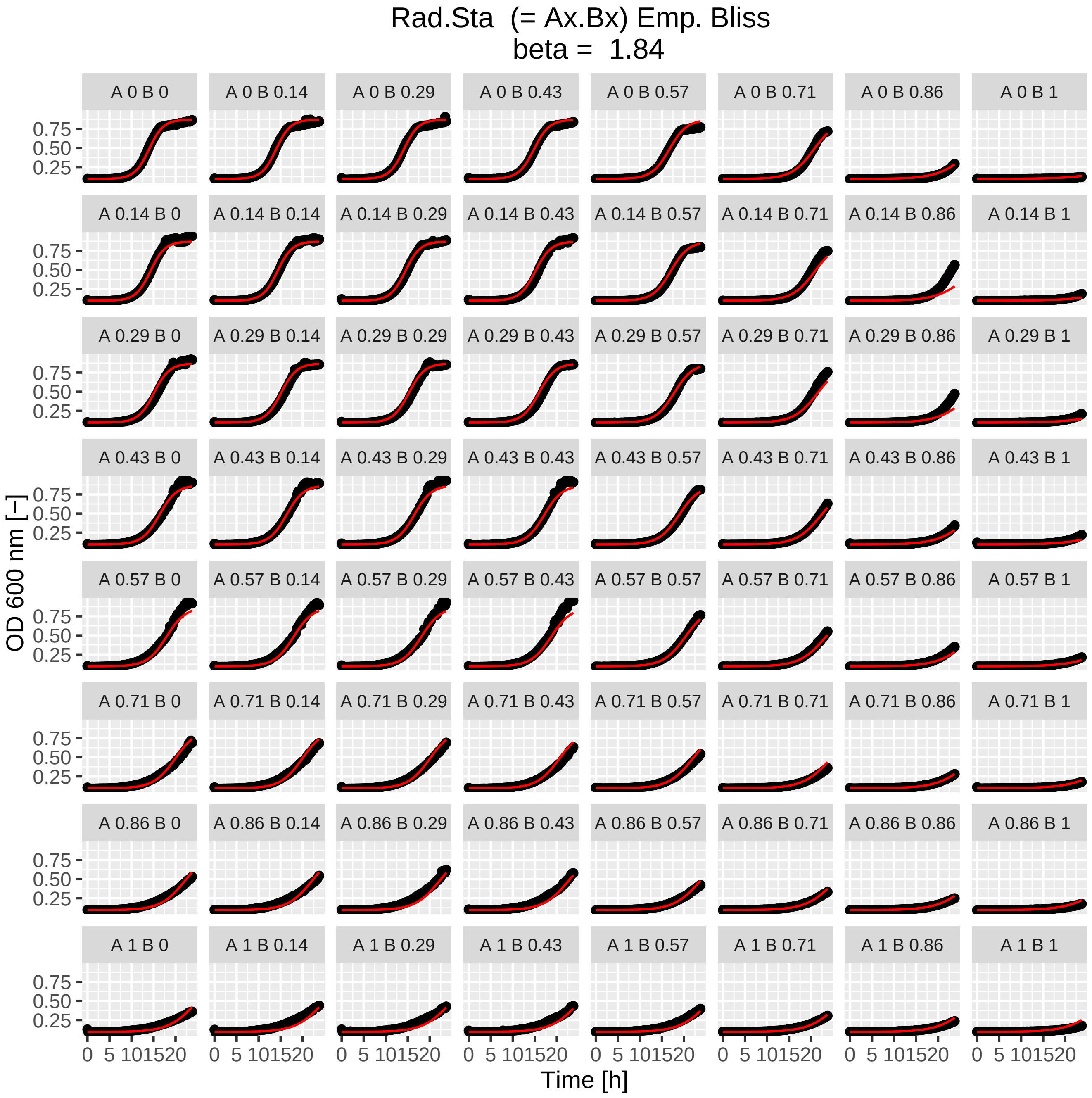
Rad.Sta (= Ax.Bx) Emp. Bliss
beta = 1.84
OD 600 nm [–]
Time [h]
0.75
0.50
0.25
0 5 101520
A 0 B 0
A 0 B 0.14
A 0 B 0.29
A 0 B 0.43
A 0 B 0.57
A 0 B 0.71
A 0 B 0.86
A 0 B 1
A 0.14 B 0
A 0.14 B 0.14
A 0.14 B 0.29
A 0.14 B 0.43
A 0.14 B 0.57
A 0.14 B 0.71
A 0.14 B 0.86
A 0.14 B 1
A 0.29 B 0
A 0.29 B 0.14
A 0.29 B 0.29
A 0.29 B 0.43
A 0.29 B 0.57
A 0.29 B 0.71
A 0.29 B 0.86
A 0.29 B 1
A 0.43 B 0
A 0.43 B 0.14
A 0.43 B 0.29
A 0.43 B 0.43
A 0.43 B 0.57
A 0.43 B 0.71
A 0.43 B 0.86
A 0.43 B 1
A 0.57 B 0
A 0.57 B 0.14
A 0.57 B 0.29
A 0.57 B 0.43
A 0.57 B 0.57
A 0.57 B 0.71
A 0.57 B 0.86
A 0.57 B 1
A 0.71 B 0
A 0.71 B 0.14
A 0.71 B 0.29
A 0.71 B 0.43
A 0.71 B 0.57
A 0.71 B 0.71
A 0.71 B 0.86
A 0.71 B 1
A 0.86 B 0
A 0.86 B 0.14
A 0.86 B 0.29
A 0.86 B 0.43
A 0.86 B 0.57
A 0.86 B 0.71
A 0.86 B 0.86
A 0.86 B 1
A 1 B 0
A 1 B 0.14
A 1 B 0.29
A 1 B 0.43
A 1 B 0.57
A 1 B 0.71
A 1 B 0.86
A 1 B 1

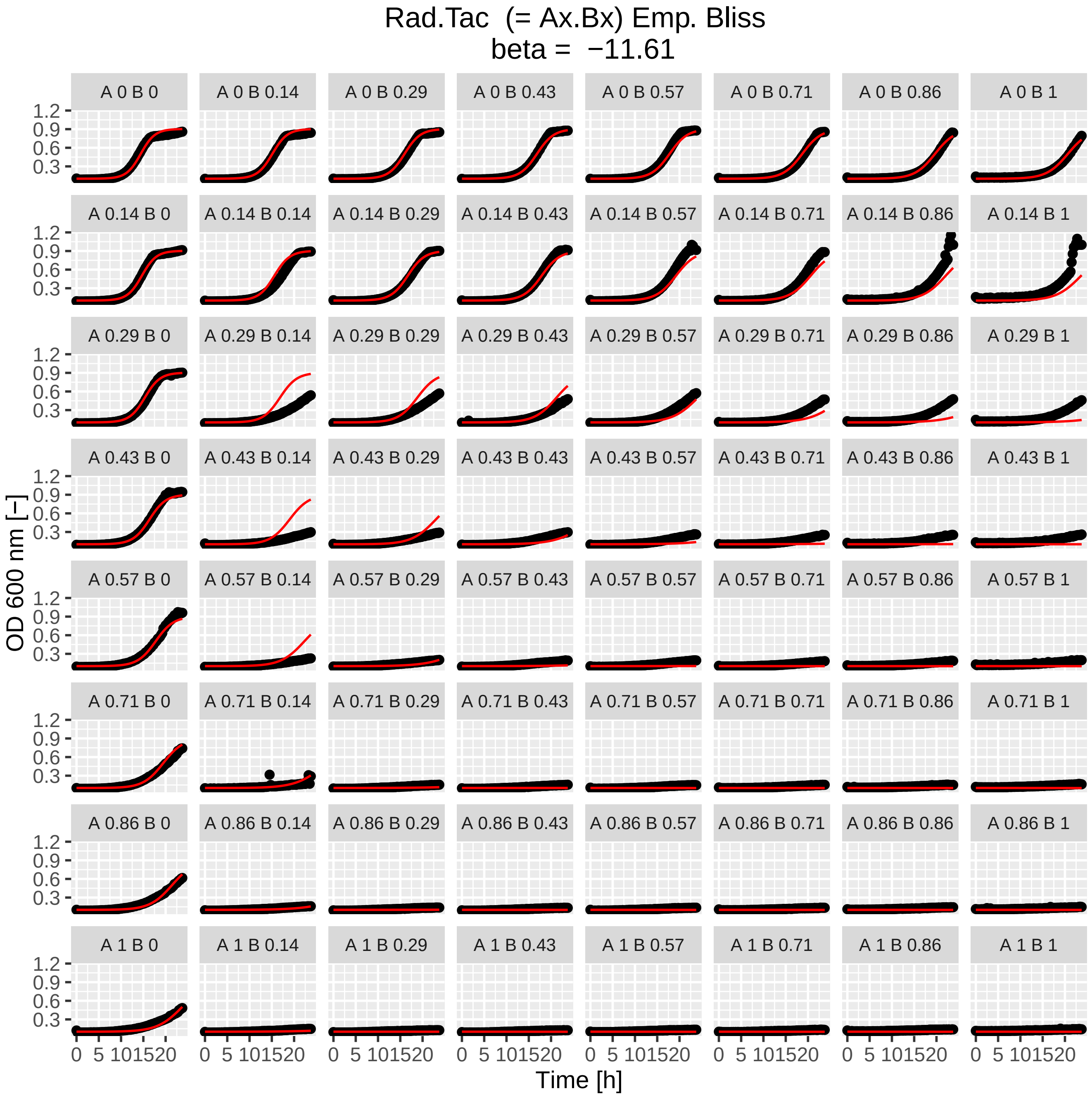

Rad.Tac (= Ax.Bx) Emp. Bliss
beta = −11.61
A 0 B 0
A 0 B 0.14
A 0 B 0.29
A 0 B 0.43
A 0 B 0.57
A 0 B 0.71
A 0 B 0.86
A 0 B 1
A 0.14 B 0
A 0.14 B 0.14
A 0.14 B 0.29
A 0.14 B 0.43
A 0.14 B 0.57
A 0.14 B 0.71
A 0.14 B 0.86
A 0.14 B 1
A 0.29 B 0
A 0.29 B 0.14
A 0.29 B 0.29
A 0.29 B 0.43
A 0.29 B 0.57
A 0.29 B 0.71
A 0.29 B 0.86
A 0.29 B 1
A 0.43 B 0
A 0.43 B 0.14
A 0.43 B 0.29
A 0.43 B 0.43
A 0.43 B 0.57
A 0.43 B 0.71
A 0.43 B 0.86
A 0.43 B 1
A 0.57 B 0
A 0.57 B 0.14
A 0.57 B 0.29
A 0.57 B 0.43
A 0.57 B 0.57
A 0.57 B 0.71
A 0.57 B 0.86
A 0.57 B 1
A 0.71 B 0
A 0.71 B 0.14
A 0.71 B 0.29
A 0.71 B 0.43
A 0.71 B 0.57
A 0.71 B 0.71
A 0.71 B 0.86
A 0.71 B 1
A 0.86 B 0
A 0.86 B 0.14
A 0.86 B 0.29
A 0.86 B 0.43
A 0.86 B 0.57
A 0.86 B 0.71
A 0.86 B 0.86
A 0.86 B 1
A 1 B 0
A 1 B 0.14
A 1 B 0.29
A 1 B 0.43
A 1 B 0.57
A 1 B 0.71
A 1 B 0.86
A 1 B 1
OD 600 nm [−]
1.2
0.9
0.6
0.3
0 5 101520
Time [h]

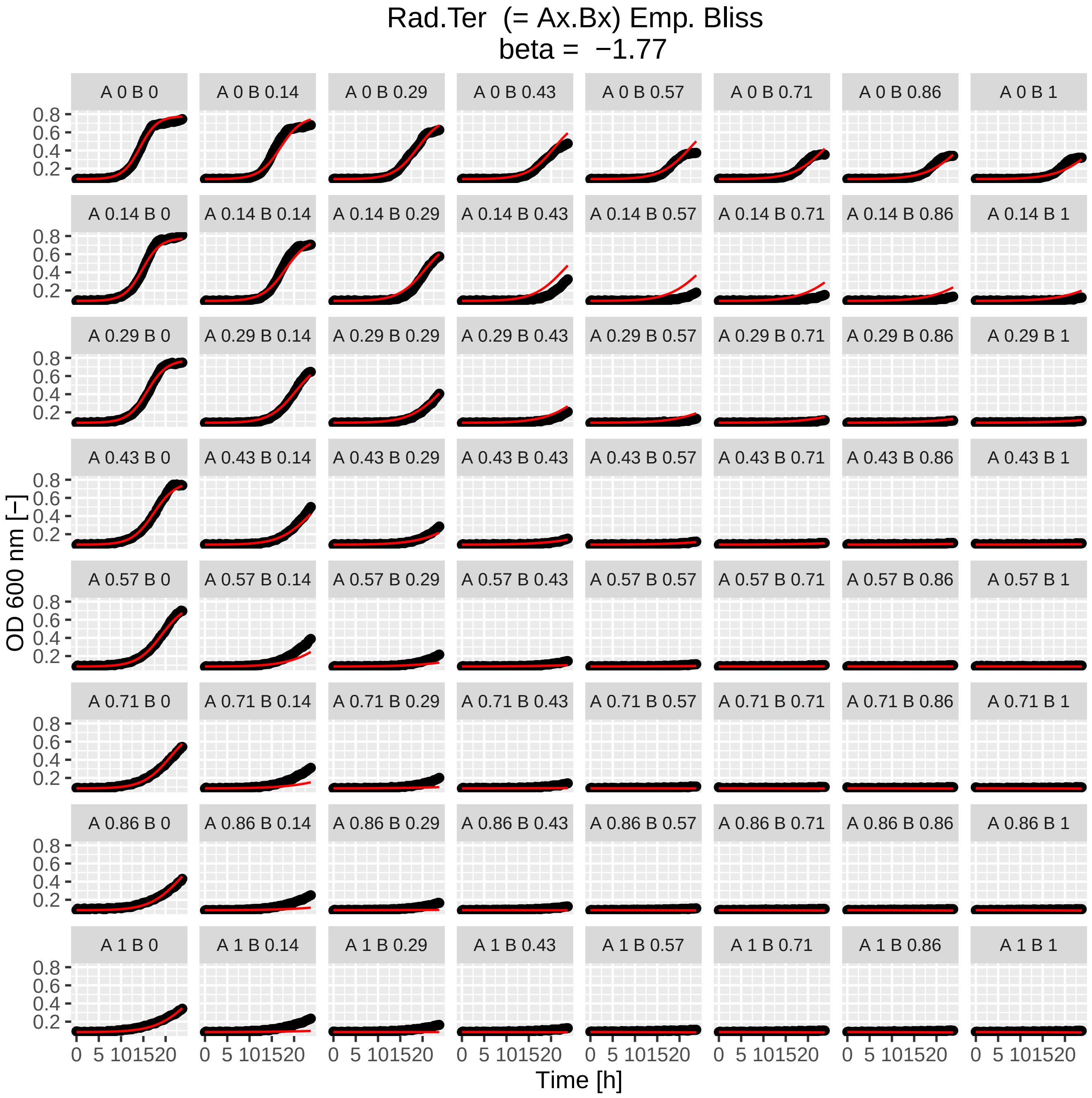
Rad.Ter (= Ax.Bx) Emp. Bliss
beta = −1.77
OD 600 nm [−]
Time [h]
A 0 B 0
A 0 B 0.14
A 0 B 0.29
A 0 B 0.43
A 0 B 0.57
A 0 B 0.71
A 0 B 0.86
A 0 B 1
A 1 B 0
A 1 B 0.14
A 1 B 0.29
A 1 B 0.43
A 1 B 0.57
A 1 B 0.71
A 1 B 0.86
A 1 B 1

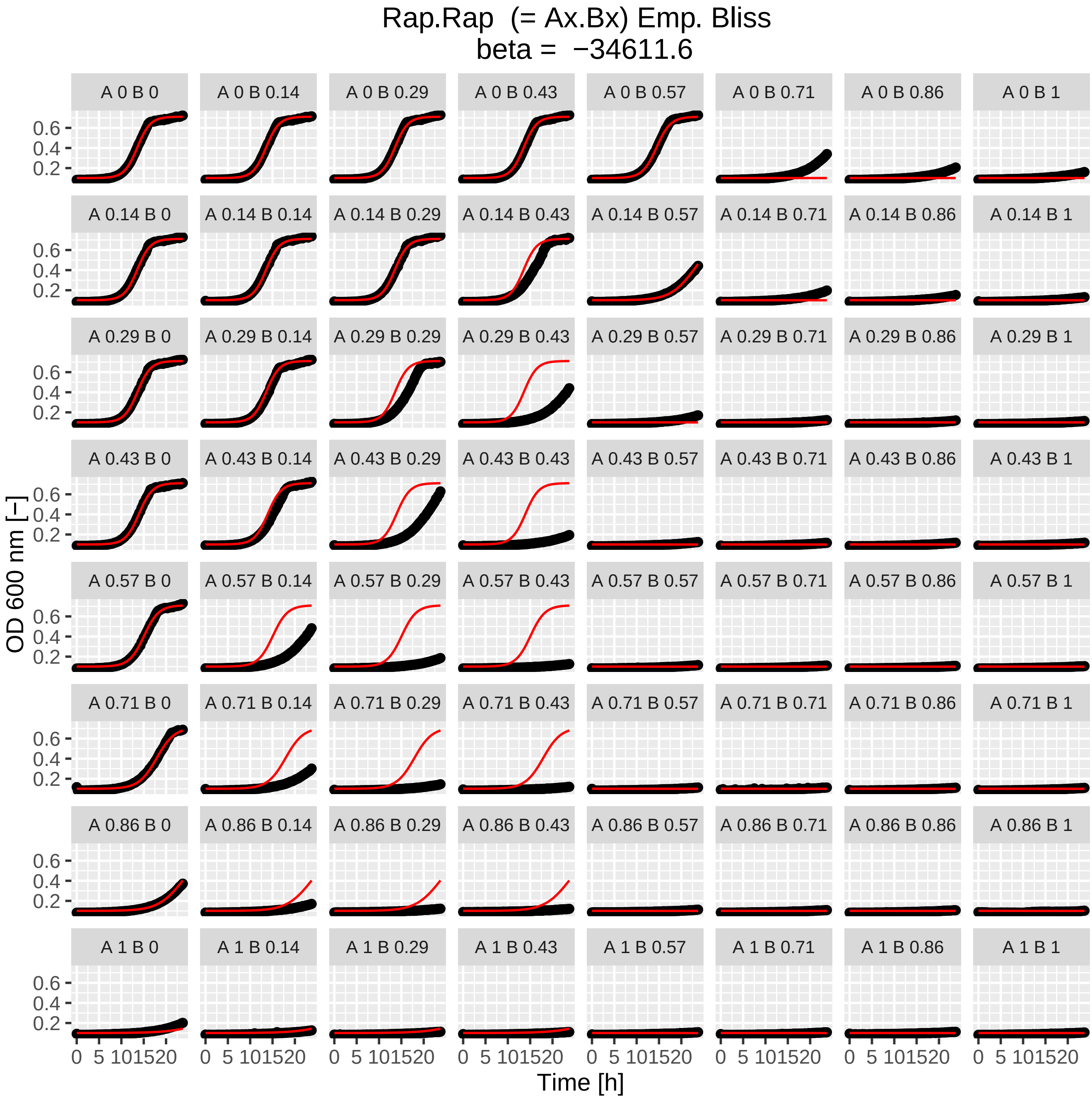

Rap.Rap (= Ax.Bx) Emp. Bliss
beta = −34611.6
A 0 B 0
A 0 B 0.14
A 0 B 0.29
A 0 B 0.43
A 0 B 0.57
A 0 B 0.71
A 0 B 0.86
A 0 B 1
A 0.14 B 0
A 0.14 B 0.14
A 0.14 B 0.29
A 0.14 B 0.43
A 0.14 B 0.57
A 0.14 B 0.71
A 0.14 B 0.86
A 0.14 B 1
A 0.29 B 0
A 0.29 B 0.14
A 0.29 B 0.29
A 0.29 B 0.43
A 0.29 B 0.57
A 0.29 B 0.71
A 0.29 B 0.86
A 0.29 B 1
A 0.43 B 0
A 0.43 B 0.14
A 0.43 B 0.29
A 0.43 B 0.43
A 0.43 B 0.57
A 0.43 B 0.71
A 0.43 B 0.86
A 0.43 B 1
A 0.57 B 0
A 0.57 B 0.14
A 0.57 B 0.29
A 0.57 B 0.43
A 0.57 B 0.57
A 0.57 B 0.71
A 0.57 B 0.86
A 0.57 B 1
A 0.71 B 0
A 0.71 B 0.14
A 0.71 B 0.29
A 0.71 B 0.43
A 0.71 B 0.57
A 0.71 B 0.71
A 0.71 B 0.86
A 0.71 B 1
A 0.86 B 0
A 0.86 B 0.14
A 0.86 B 0.29
A 0.86 B 0.43
A 0.86 B 0.57
A 0.86 B 0.71
A 0.86 B 0.86
A 0.86 B 1
A 1 B 0
A 1 B 0.14
A 1 B 0.29
A 1 B 0.43
A 1 B 0.57
A 1 B 0.71
A 1 B 0.86
A 1 B 1
OD 600 nm [−]
0.2
0.4
0.6
0 5 101520
Time [h]

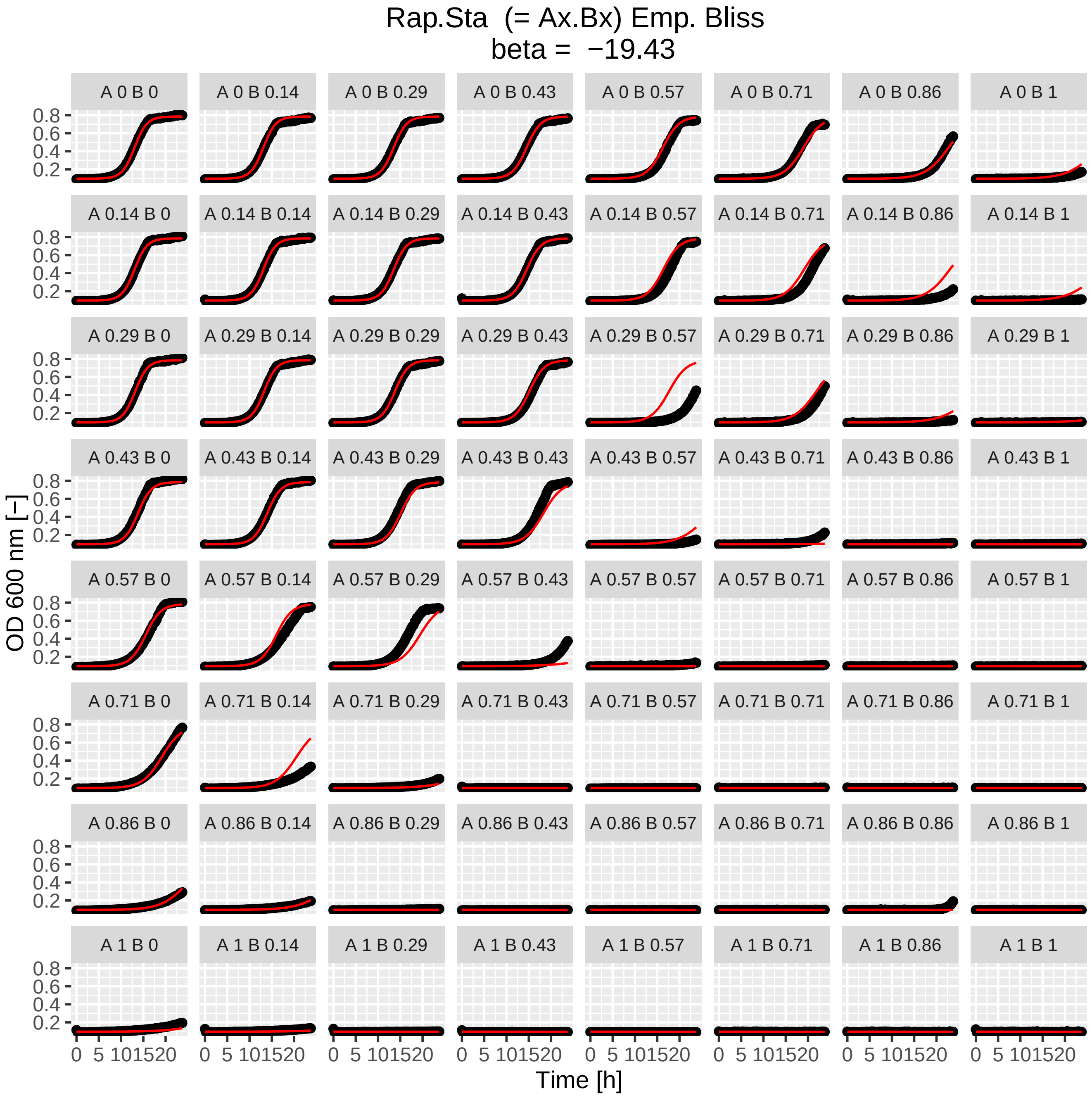
Rap.Sta (= Ax.Bx) Emp. Bliss
beta = −19.43
A 0 B 0
A 0 B 0.14
A 0 B 0.29
A 0 B 0.43
A 0 B 0.57
A 0 B 0.71
A 0 B 0.86
A 0 B 1
A 0.14 B 0
A 0.14 B 0.14
A 0.14 B 0.29
A 0.14 B 0.43
A 0.14 B 0.57
A 0.14 B 0.71
A 0.14 B 0.86
A 0.14 B 1
A 0.29 B 0
A 0.29 B 0.14
A 0.29 B 0.29
A 0.29 B 0.43
A 0.29 B 0.57
A 0.29 B 0.71
A 0.29 B 0.86
A 0.29 B 1
A 0.43 B 0
A 0.43 B 0.14
A 0.43 B 0.29
A 0.43 B 0.43
A 0.43 B 0.57
A 0.43 B 0.71
A 0.43 B 0.86
A 0.43 B 1
A 0.57 B 0
A 0.57 B 0.14
A 0.57 B 0.29
A 0.57 B 0.43
A 0.57 B 0.57
A 0.57 B 0.71
A 0.57 B 0.86
A 0.57 B 1
A 0.71 B 0
A 0.71 B 0.14
A 0.71 B 0.29
A 0.71 B 0.43
A 0.71 B 0.57
A 0.71 B 0.71
A 0.71 B 0.86
A 0.71 B 1
A 0.86 B 0
A 0.86 B 0.14
A 0.86 B 0.29
A 0.86 B 0.43
A 0.86 B 0.57
A 0.86 B 0.71
A 0.86 B 0.86
A 0.86 B 1
A 1 B 0
A 1 B 0.14
A 1 B 0.29
A 1 B 0.43
A 1 B 0.57
A 1 B 0.71
A 1 B 0.86
A 1 B 1
OD 600 nm [−]
0.2
0.4
0.6
0.8
0
5
10
15
20
Time [h]

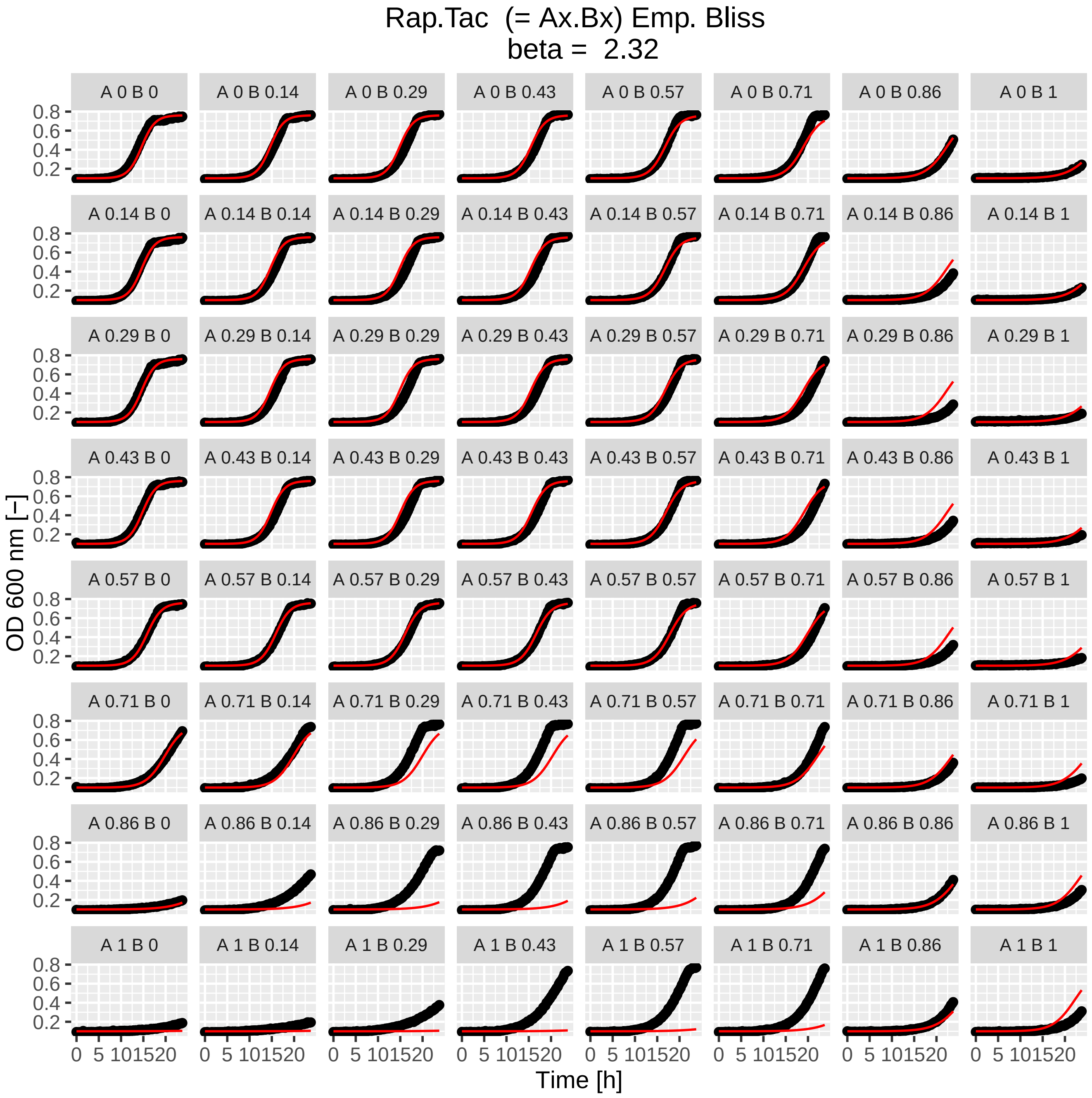

Rap.Tac (= Ax.Bx) Emp. Bliss
beta = 2.32
A 0 B 0
A 0 B 0.14
A 0 B 0.29
A 0 B 0.43
A 0 B 0.57
A 0 B 0.71
A 0 B 0.86
A 0 B 1
A 0.14 B 0
A 0.14 B 0.14
A 0.14 B 0.29
A 0.14 B 0.43
A 0.14 B 0.57
A 0.14 B 0.71
A 0.14 B 0.86
A 0.14 B 1
A 0.29 B 0
A 0.29 B 0.14
A 0.29 B 0.29
A 0.29 B 0.43
A 0.29 B 0.57
A 0.29 B 0.71
A 0.29 B 0.86
A 0.29 B 1
A 0.43 B 0
A 0.43 B 0.14
A 0.43 B 0.29
A 0.43 B 0.43
A 0.43 B 0.57
A 0.43 B 0.71
A 0.43 B 0.86
A 0.43 B 1
A 0.57 B 0
A 0.57 B 0.14
A 0.57 B 0.29
A 0.57 B 0.43
A 0.57 B 0.57
A 0.57 B 0.71
A 0.57 B 0.86
A 0.57 B 1
A 0.71 B 0
A 0.71 B 0.14
A 0.71 B 0.29
A 0.71 B 0.43
A 0.71 B 0.57
A 0.71 B 0.71
A 0.71 B 0.86
A 0.71 B 1
A 0.86 B 0
A 0.86 B 0.14
A 0.86 B 0.29
A 0.86 B 0.43
A 0.86 B 0.57
A 0.86 B 0.71
A 0.86 B 0.86
A 0.86 B 1
A 1 B 0
A 1 B 0.14
A 1 B 0.29
A 1 B 0.43
A 1 B 0.57
A 1 B 0.71
A 1 B 0.86
A 1 B 1
0.8
0.6
0.4
0.2
OD 600 nm [–]
0 5 101520
Time [h]

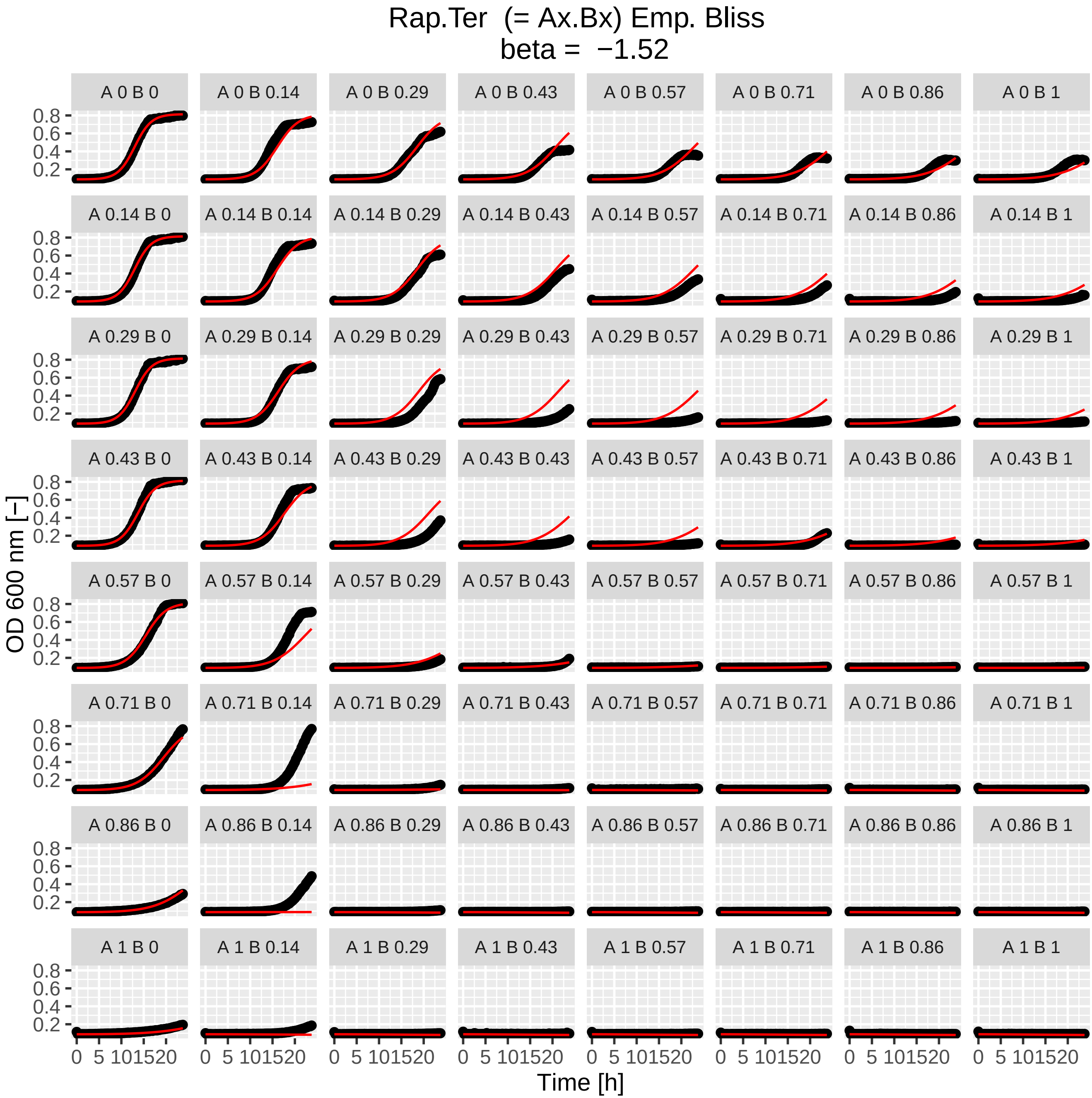
Rap.Ter (= Ax.Bx) Emp. Bliss
beta = −1.52
A 0 B 0
A 0 B 0.14
A 0 B 0.29
A 0 B 0.43
A 0 B 0.57
A 0 B 0.71
A 0 B 0.86
A 0 B 1
A 0.14 B 0
A 0.14 B 0.14
A 0.14 B 0.29
A 0.14 B 0.43
A 0.14 B 0.57
A 0.14 B 0.71
A 0.14 B 0.86
A 0.14 B 1
A 0.29 B 0
A 0.29 B 0.14
A 0.29 B 0.29
A 0.29 B 0.43
A 0.29 B 0.57
A 0.29 B 0.71
A 0.29 B 0.86
A 0.29 B 1
A 0.43 B 0
A 0.43 B 0.14
A 0.43 B 0.29
A 0.43 B 0.43
A 0.43 B 0.57
A 0.43 B 0.71
A 0.43 B 0.86
A 0.43 B 1
A 0.57 B 0
A 0.57 B 0.14
A 0.57 B 0.29
A 0.57 B 0.43
A 0.57 B 0.57
A 0.57 B 0.71
A 0.57 B 0.86
A 0.57 B 1
A 0.71 B 0
A 0.71 B 0.14
A 0.71 B 0.29
A 0.71 B 0.43
A 0.71 B 0.57
A 0.71 B 0.71
A 0.71 B 0.86
A 0.71 B 1
A 0.86 B 0
A 0.86 B 0.14
A 0.86 B 0.29
A 0.86 B 0.43
A 0.86 B 0.57
A 0.86 B 0.71
A 0.86 B 0.86
A 0.86 B 1
A 1 B 0
A 1 B 0.14
A 1 B 0.29
A 1 B 0.43
A 1 B 0.57
A 1 B 0.71
A 1 B 0.86
A 1 B 1
0.8
0.6
0.4
0.2
OD 600 nm [−]
0 5 101520
Time [h]

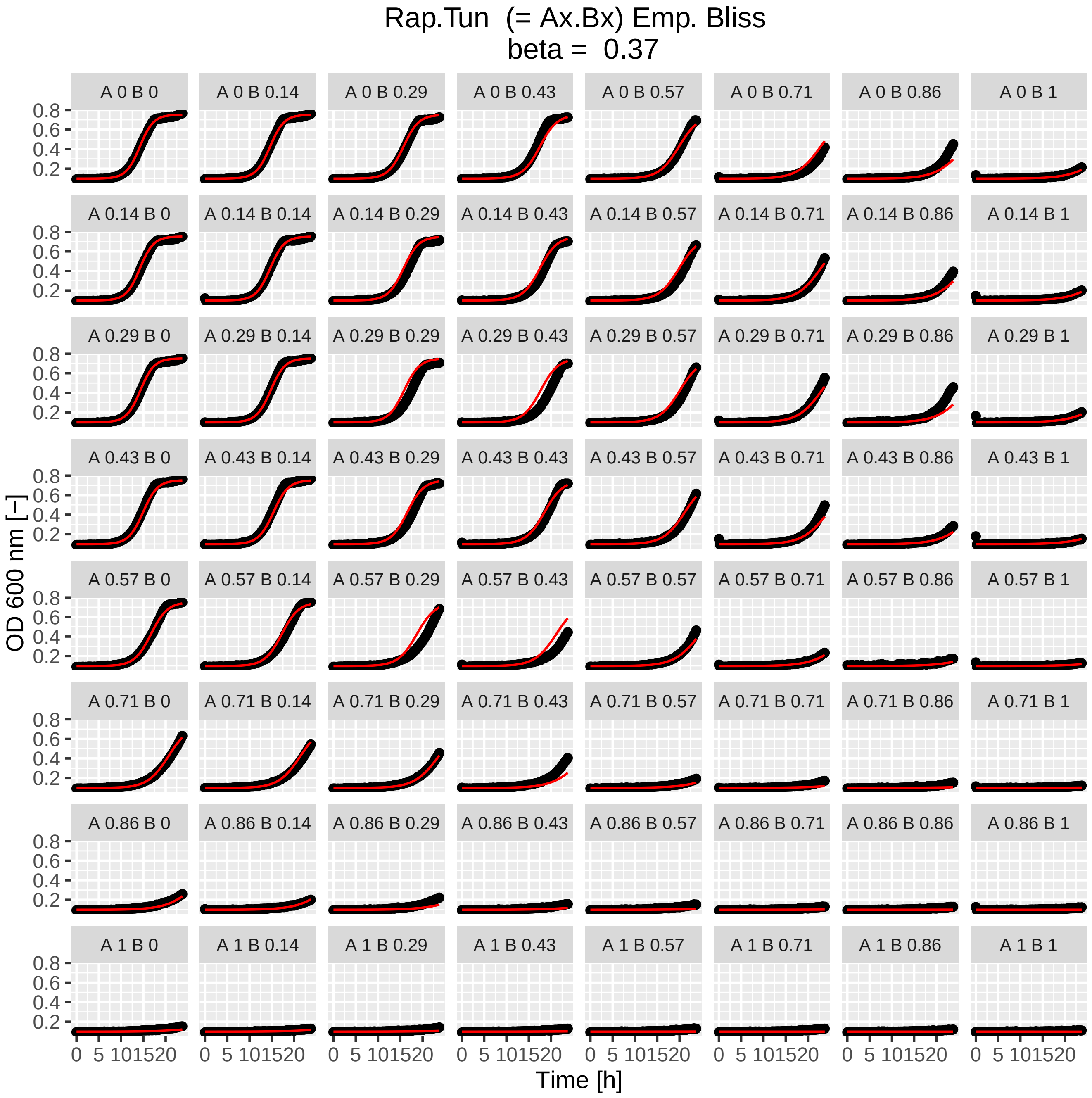
Rap.Tun (= Ax.Bx) Emp. Bliss
beta = 0.37
OD 600 nm [−]
Time [h]
A 0 B 0
A 0 B 0.14
A 0 B 0.29
A 0 B 0.43
A 0 B 0.57
A 0 B 0.71
A 0 B 0.86
A 0 B 1
A 0.14 B 0
A 0.14 B 0.14
A 0.14 B 0.29
A 0.14 B 0.43
A 0.14 B 0.57
A 0.14 B 0.71
A 0.14 B 0.86
A 0.14 B 1
A 0.29 B 0
A 0.29 B 0.14
A 0.29 B 0.29
A 0.29 B 0.43
A 0.29 B 0.57
A 0.29 B 0.71
A 0.29 B 0.86
A 0.29 B 1
A 0.43 B 0
A 0.43 B 0.14
A 0.43 B 0.29
A 0.43 B 0.43
A 0.43 B 0.57
A 0.43 B 0.71
A 0.43 B 0.86
A 0.43 B 1
A 0.57 B 0
A 0.57 B 0.14
A 0.57 B 0.29
A 0.57 B 0.43
A 0.57 B 0.57
A 0.57 B 0.71
A 0.57 B 0.86
A 0.57 B 1
A 0.71 B 0
A 0.71 B 0.14
A 0.71 B 0.29
A 0.71 B 0.43
A 0.71 B 0.57
A 0.71 B 0.71
A 0.71 B 0.86
A 0.71 B 1
A 0.86 B 0
A 0.86 B 0.14
A 0.86 B 0.29
A 0.86 B 0.43
A 0.86 B 0.57
A 0.86 B 0.71
A 0.86 B 0.86
A 0.86 B 1
A 1 B 0
A 1 B 0.14
A 1 B 0.29
A 1 B 0.43
A 1 B 0.57
A 1 B 0.71
A 1 B 0.86
A 1 B 1
0.8
0.6
0.4
0.2
0 5 101520

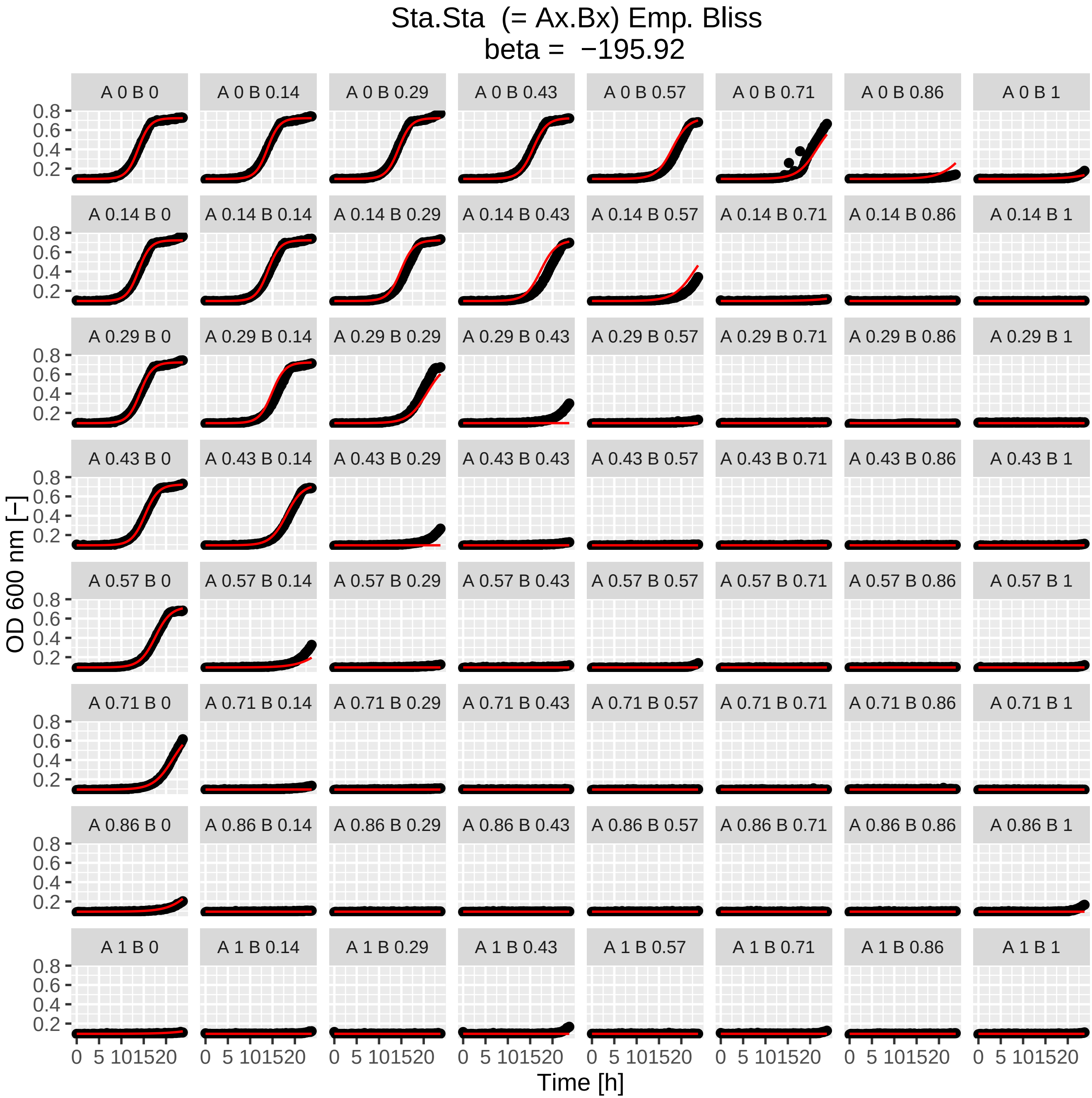
Sta.Sta (= Ax.Bx) Emp. Bliss
beta = −195.92
A 0 B 0
A 0 B 0.14
A 0 B 0.29
A 0 B 0.43
A 0 B 0.57
A 0 B 0.71
A 0 B 0.86
A 0 B 1
A 0.14 B 0
A 0.14 B 0.14
A 0.14 B 0.29
A 0.14 B 0.43
A 0.14 B 0.57
A 0.14 B 0.71
A 0.14 B 0.86
A 0.14 B 1
A 0.29 B 0
A 0.29 B 0.14
A 0.29 B 0.29
A 0.29 B 0.43
A 0.29 B 0.57
A 0.29 B 0.71
A 0.29 B 0.86
A 0.29 B 1
A 0.43 B 0
A 0.43 B 0.14
A 0.43 B 0.29
A 0.43 B 0.43
A 0.43 B 0.57
A 0.43 B 0.71
A 0.43 B 0.86
A 0.43 B 1
A 0.57 B 0
A 0.57 B 0.14
A 0.57 B 0.29
A 0.57 B 0.43
A 0.57 B 0.57
A 0.57 B 0.71
A 0.57 B 0.86
A 0.57 B 1
A 0.71 B 0
A 0.71 B 0.14
A 0.71 B 0.29
A 0.71 B 0.43
A 0.71 B 0.57
A 0.71 B 0.71
A 0.71 B 0.86
A 0.71 B 1
A 0.86 B 0
A 0.86 B 0.14
A 0.86 B 0.29
A 0.86 B 0.43
A 0.86 B 0.57
A 0.86 B 0.71
A 0.86 B 0.86
A 0.86 B 1
A 1 B 0
A 1 B 0.14
A 1 B 0.29
A 1 B 0.43
A 1 B 0.57
A 1 B 0.71
A 1 B 0.86
A 1 B 1
OD 600 nm [−]
0.2
0.4
0.6
0.8
0 5 101520
Time [h]

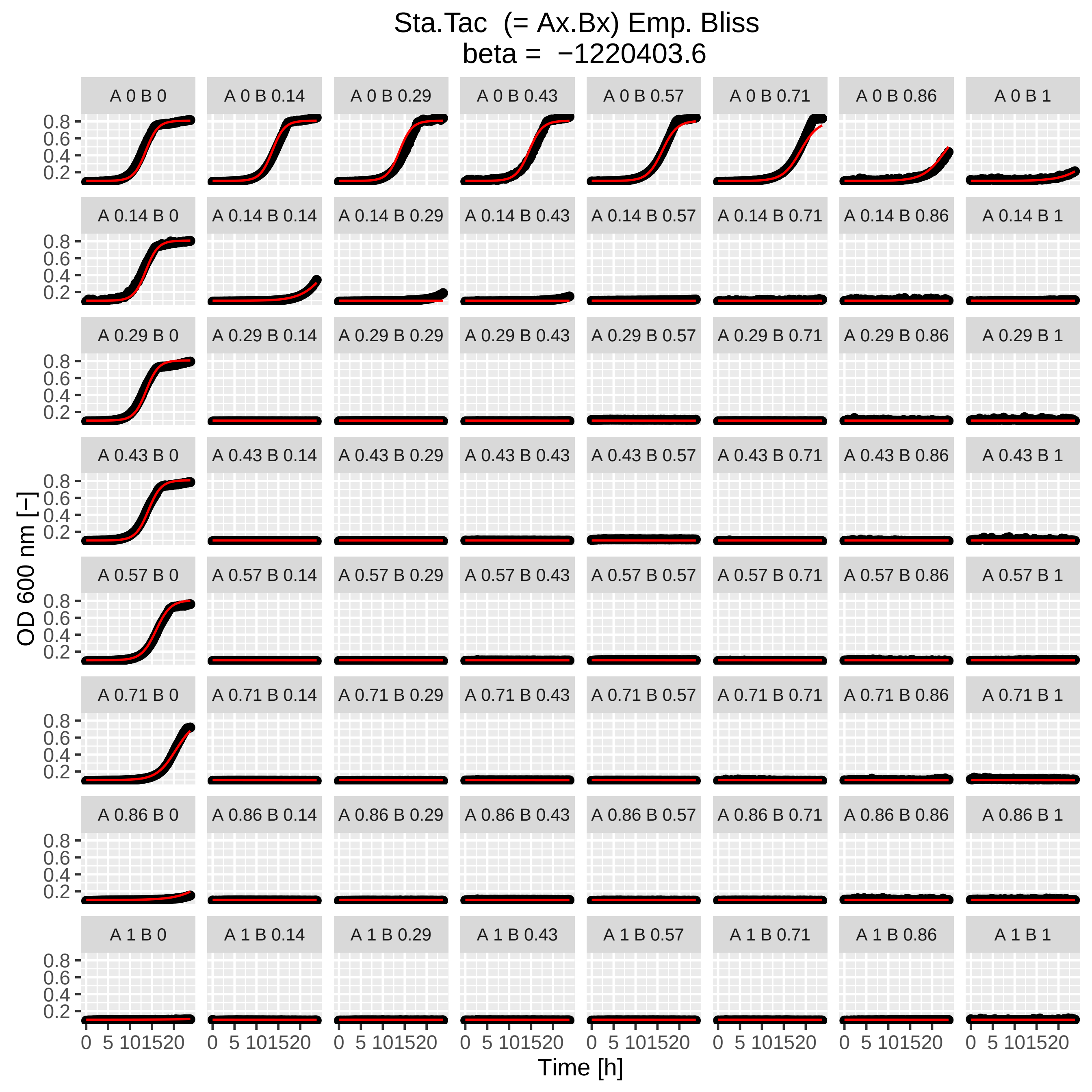

Sta.Tac (= Ax.Bx) Emp. Bliss
beta = −1220403.6
A 0 B 0
A 0 B 0.14
A 0 B 0.29
A 0 B 0.43
A 0 B 0.57
A 0 B 0.71
A 0 B 0.86
A 0 B 1
A 0.14 B 0
A 0.14 B 0.14
A 0.14 B 0.29
A 0.14 B 0.43
A 0.14 B 0.57
A 0.14 B 0.71
A 0.14 B 0.86
A 0.14 B 1
A 0.29 B 0
A 0.29 B 0.14
A 0.29 B 0.29
A 0.29 B 0.43
A 0.29 B 0.57
A 0.29 B 0.71
A 0.29 B 0.86
A 0.29 B 1
A 0.43 B 0
A 0.43 B 0.14
A 0.43 B 0.29
A 0.43 B 0.43
A 0.43 B 0.57
A 0.43 B 0.71
A 0.43 B 0.86
A 0.43 B 1
A 0.57 B 0
A 0.57 B 0.14
A 0.57 B 0.29
A 0.57 B 0.43
A 0.57 B 0.57
A 0.57 B 0.71
A 0.57 B 0.86
A 0.57 B 1
A 0.71 B 0
A 0.71 B 0.14
A 0.71 B 0.29
A 0.71 B 0.43
A 0.71 B 0.57
A 0.71 B 0.71
A 0.71 B 0.86
A 0.71 B 1
A 0.86 B 0
A 0.86 B 0.14
A 0.86 B 0.29
A 0.86 B 0.43
A 0.86 B 0.57
A 0.86 B 0.71
A 0.86 B 0.86
A 0.86 B 1
A 1 B 0
A 1 B 0.14
A 1 B 0.29
A 1 B 0.43
A 1 B 0.57
A 1 B 0.71
A 1 B 0.86
A 1 B 1
OD 600 nm [−]
0.2
0.4
0.6
0.8
0 5 101520
Time [h]

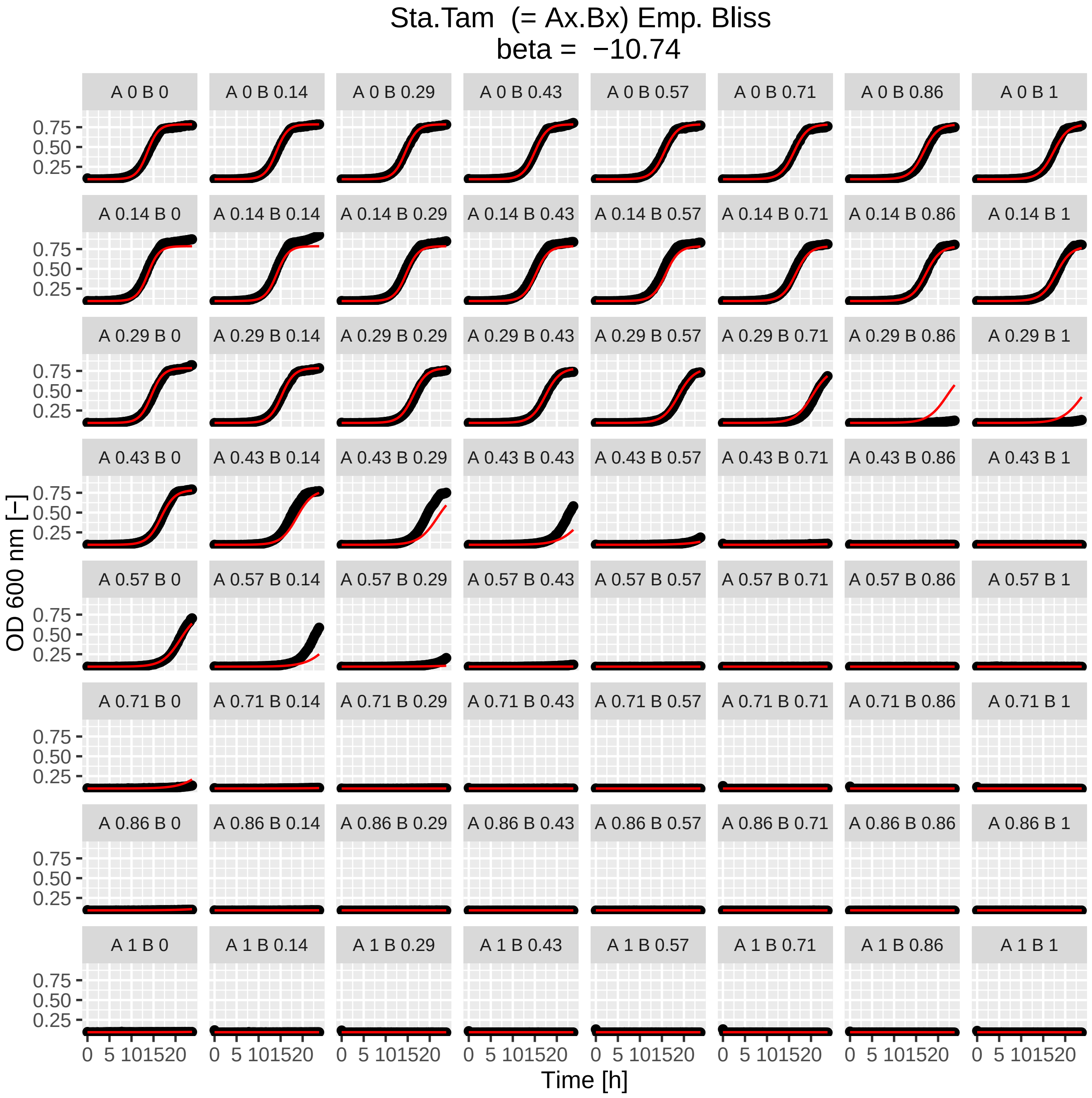
Sta.Tam (= Ax.Bx) Emp. Bliss
beta = −10.74
A 0 B 0
A 0 B 0.14
A 0 B 0.29
A 0 B 0.43
A 0 B 0.57
A 0 B 0.71
A 0 B 0.86
A 0 B 1
A 0.14 B 0
A 0.14 B 0.14
A 0.14 B 0.29
A 0.14 B 0.43
A 0.14 B 0.57
A 0.14 B 0.71
A 0.14 B 0.86
A 0.14 B 1
A 0.29 B 0
A 0.29 B 0.14
A 0.29 B 0.29
A 0.29 B 0.43
A 0.29 B 0.57
A 0.29 B 0.71
A 0.29 B 0.86
A 0.29 B 1
A 0.43 B 0
A 0.43 B 0.14
A 0.43 B 0.29
A 0.43 B 0.43
A 0.43 B 0.57
A 0.43 B 0.71
A 0.43 B 0.86
A 0.43 B 1
A 0.57 B 0
A 0.57 B 0.14
A 0.57 B 0.29
A 0.57 B 0.43
A 0.57 B 0.57
A 0.57 B 0.71
A 0.57 B 0.86
A 0.57 B 1
A 0.71 B 0
A 0.71 B 0.14
A 0.71 B 0.29
A 0.71 B 0.43
A 0.71 B 0.57
A 0.71 B 0.71
A 0.71 B 0.86
A 0.71 B 1
A 0.86 B 0
A 0.86 B 0.14
A 0.86 B 0.29
A 0.86 B 0.43
A 0.86 B 0.57
A 0.86 B 0.71
A 0.86 B 0.86
A 0.86 B 1
A 1 B 0
A 1 B 0.14
A 1 B 0.29
A 1 B 0.43
A 1 B 0.57
A 1 B 0.71
A 1 B 0.86
A 1 B 1
0.75
0.50
0.25
OD 600 nm [−]
0 5 101520
Time [h]

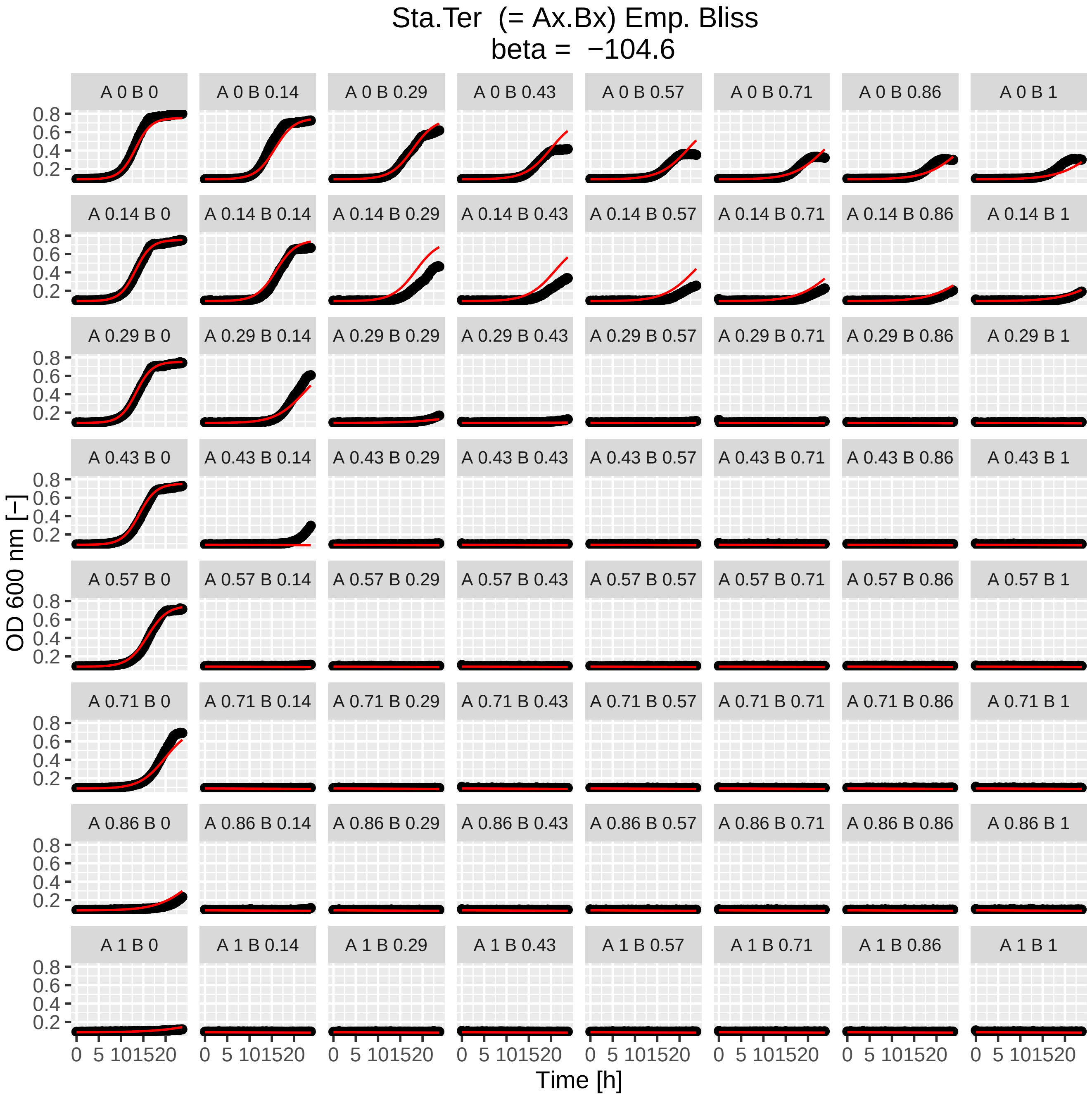
Sta.Ter (= Ax.Bx) Emp. Bliss
beta = −104.6
OD 600 nm [−]
Time [h]
A 0 B 0
A 0 B 0.14
A 0 B 0.29
A 0 B 0.43
A 0 B 0.57
A 0 B 0.71
A 0 B 0.86
A 0 B 1
A 0.14 B 0
A 0.14 B 0.14
A 0.14 B 0.29
A 0.14 B 0.43
A 0.14 B 0.57
A 0.14 B 0.71
A 0.14 B 0.86
A 0.14 B 1
A 0.29 B 0
A 0.29 B 0.14
A 0.29 B 0.29
A 0.29 B 0.43
A 0.29 B 0.57
A 0.29 B 0.71
A 0.29 B 0.86
A 0.29 B 1
A 0.43 B 0
A 0.43 B 0.14
A 0.43 B 0.29
A 0.43 B 0.43
A 0.43 B 0.57
A 0.43 B 0.71
A 0.43 B 0.86
A 0.43 B 1
A 0.57 B 0
A 0.57 B 0.14
A 0.57 B 0.29
A 0.57 B 0.43
A 0.57 B 0.57
A 0.57 B 0.71
A 0.57 B 0.86
A 0.57 B 1
A 0.71 B 0
A 0.71 B 0.14
A 0.71 B 0.29
A 0.71 B 0.43
A 0.71 B 0.57
A 0.71 B 0.71
A 0.71 B 0.86
A 0.71 B 1
A 0.86 B 0
A 0.86 B 0.14
A 0.86 B 0.29
A 0.86 B 0.43
A 0.86 B 0.57
A 0.86 B 0.71
A 0.86 B 0.86
A 0.86 B 1
A 1 B 0
A 1 B 0.14
A 1 B 0.29
A 1 B 0.43
A 1 B 0.57
A 1 B 0.71
A 1 B 0.86
A 1 B 1
0.8
0.6
0.4
0.2
0 5 101520

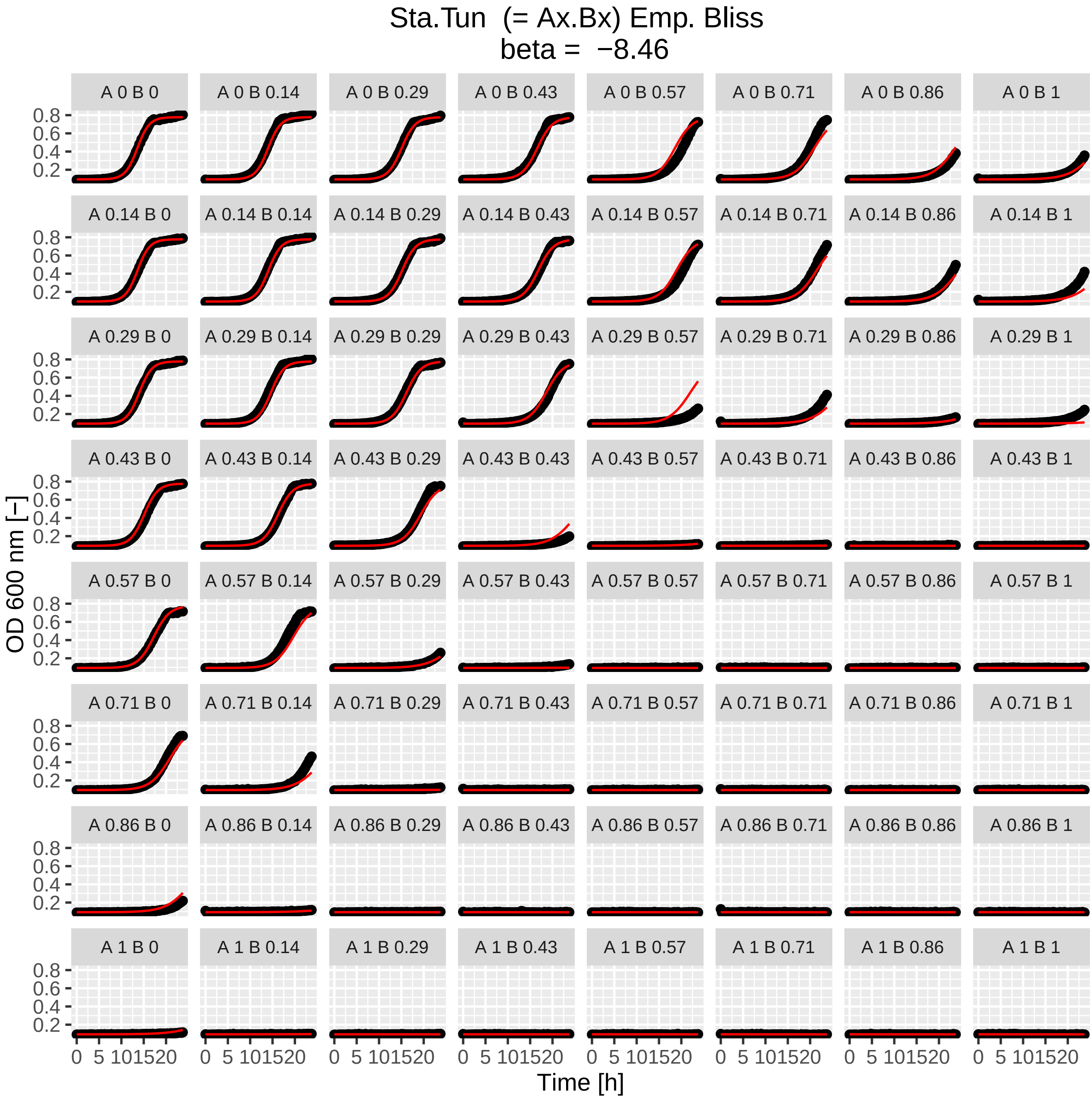
Sta.Tun (= Ax.Bx) Emp. Bliss
beta = −8.46
A 0 B 0
A 0 B 0.14
A 0 B 0.29
A 0 B 0.43
A 0 B 0.57
A 0 B 0.71
A 0 B 0.86
A 0 B 1
A 0.14 B 0
A 0.14 B 0.14
A 0.14 B 0.29
A 0.14 B 0.43
A 0.14 B 0.57
A 0.14 B 0.71
A 0.14 B 0.86
A 0.14 B 1
A 0.29 B 0
A 0.29 B 0.14
A 0.29 B 0.29
A 0.29 B 0.43
A 0.29 B 0.57
A 0.29 B 0.71
A 0.29 B 0.86
A 0.29 B 1
A 0.43 B 0
A 0.43 B 0.14
A 0.43 B 0.29
A 0.43 B 0.43
A 0.43 B 0.57
A 0.43 B 0.71
A 0.43 B 0.86
A 0.43 B 1
A 0.57 B 0
A 0.57 B 0.14
A 0.57 B 0.29
A 0.57 B 0.43
A 0.57 B 0.57
A 0.57 B 0.71
A 0.57 B 0.86
A 0.57 B 1
A 0.71 B 0
A 0.71 B 0.14
A 0.71 B 0.29
A 0.71 B 0.43
A 0.71 B 0.57
A 0.71 B 0.71
A 0.71 B 0.86
A 0.71 B 1
A 0.86 B 0
A 0.86 B 0.14
A 0.86 B 0.29
A 0.86 B 0.43
A 0.86 B 0.57
A 0.86 B 0.71
A 0.86 B 0.86
A 0.86 B 1
A 1 B 0
A 1 B 0.14
A 1 B 0.29
A 1 B 0.43
A 1 B 0.57
A 1 B 0.71
A 1 B 0.86
A 1 B 1
0.8
0.6
0.4
0.2
OD 600 nm [−]
0 5 101520
Time [h]

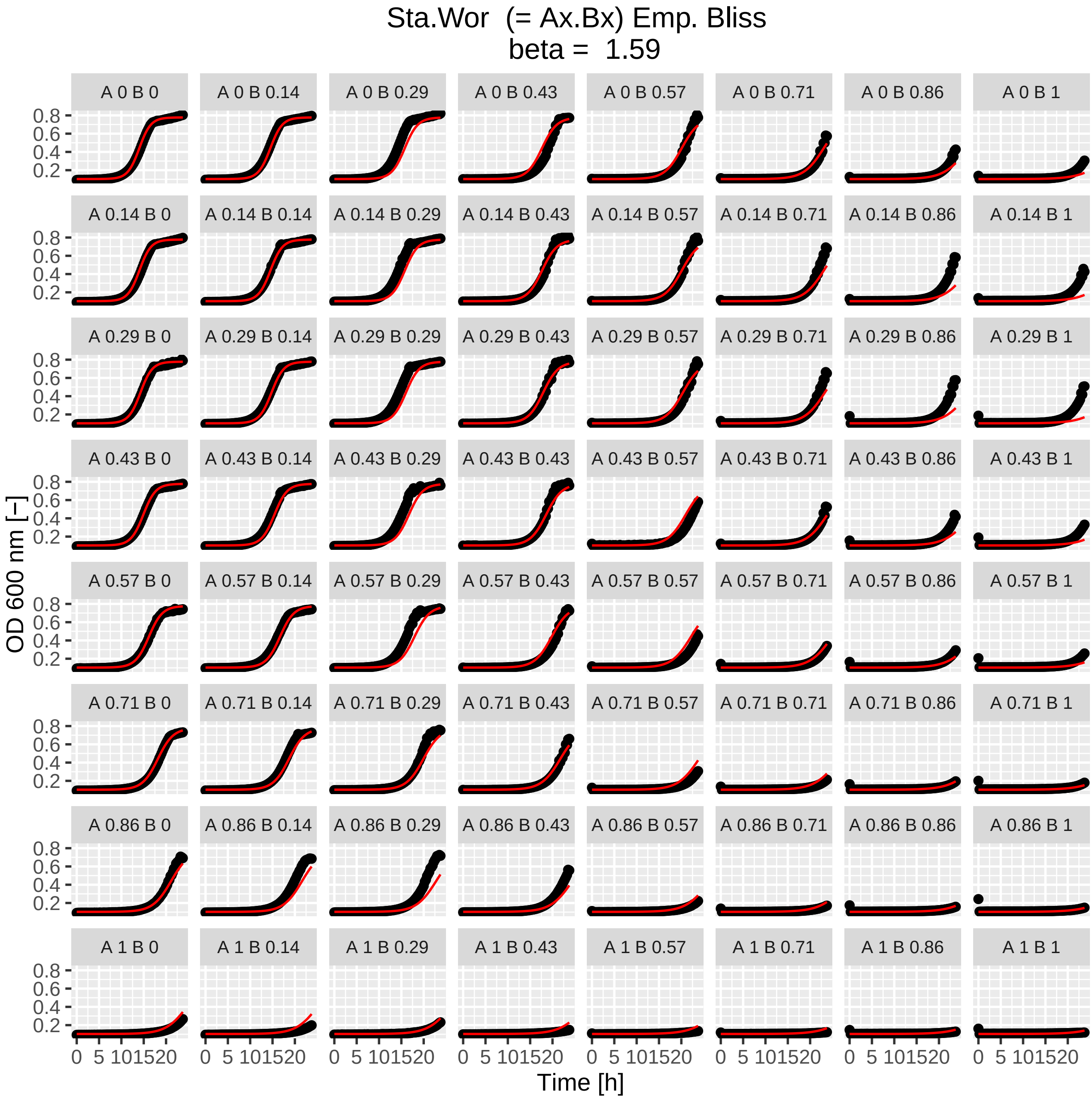

Sta.Wor (= Ax.Bx) Emp. Bliss
beta = 1.59
A 0 B 0
A 0 B 0.14
A 0 B 0.29
A 0 B 0.43
A 0 B 0.57
A 0 B 0.71
A 0 B 0.86
A 0 B 1
A 0.14 B 0
A 0.14 B 0.14
A 0.14 B 0.29
A 0.14 B 0.43
A 0.14 B 0.57
A 0.14 B 0.71
A 0.14 B 0.86
A 0.14 B 1
A 0.29 B 0
A 0.29 B 0.14
A 0.29 B 0.29
A 0.29 B 0.43
A 0.29 B 0.57
A 0.29 B 0.71
A 0.29 B 0.86
A 0.29 B 1
A 0.43 B 0
A 0.43 B 0.14
A 0.43 B 0.29
A 0.43 B 0.43
A 0.43 B 0.57
A 0.43 B 0.71
A 0.43 B 0.86
A 0.43 B 1
A 0.57 B 0
A 0.57 B 0.14
A 0.57 B 0.29
A 0.57 B 0.43
A 0.57 B 0.57
A 0.57 B 0.71
A 0.57 B 0.86
A 0.57 B 1
A 0.71 B 0
A 0.71 B 0.14
A 0.71 B 0.29
A 0.71 B 0.43
A 0.71 B 0.57
A 0.71 B 0.71
A 0.71 B 0.86
A 0.71 B 1
A 0.86 B 0
A 0.86 B 0.14
A 0.86 B 0.29
A 0.86 B 0.43
A 0.86 B 0.57
A 0.86 B 0.71
A 0.86 B 0.86
A 0.86 B 1
A 1 B 0
A 1 B 0.14
A 1 B 0.29
A 1 B 0.43
A 1 B 0.57
A 1 B 0.71
A 1 B 0.86
A 1 B 1
0.8
0.6
0.4
0.2
OD 600 nm [–]
0 5 101520
Time [h]

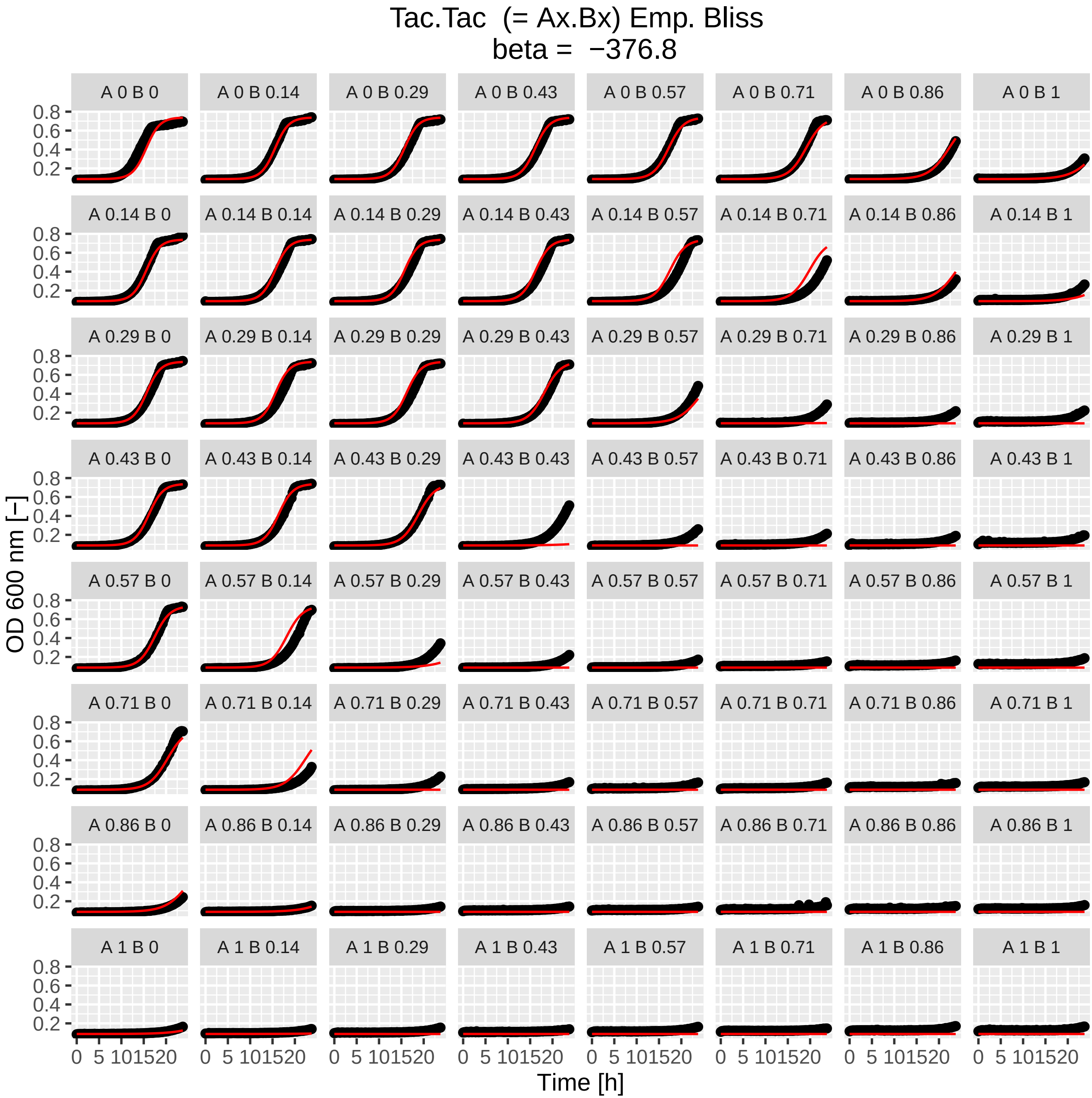

Tac.Tac (= Ax.Bx) Emp. Bliss
beta = −376.8
A 0 B 0
A 0 B 0.14
A 0 B 0.29
A 0 B 0.43
A 0 B 0.57
A 0 B 0.71
A 0 B 0.86
A 0 B 1
A 0.14 B 0
A 0.14 B 0.14
A 0.14 B 0.29
A 0.14 B 0.43
A 0.14 B 0.57
A 0.14 B 0.71
A 0.14 B 0.86
A 0.14 B 1
A 0.29 B 0
A 0.29 B 0.14
A 0.29 B 0.29
A 0.29 B 0.43
A 0.29 B 0.57
A 0.29 B 0.71
A 0.29 B 0.86
A 0.29 B 1
A 0.43 B 0
A 0.43 B 0.14
A 0.43 B 0.29
A 0.43 B 0.43
A 0.43 B 0.57
A 0.43 B 0.71
A 0.43 B 0.86
A 0.43 B 1
A 0.57 B 0
A 0.57 B 0.14
A 0.57 B 0.29
A 0.57 B 0.43
A 0.57 B 0.57
A 0.57 B 0.71
A 0.57 B 0.86
A 0.57 B 1
A 0.71 B 0
A 0.71 B 0.14
A 0.71 B 0.29
A 0.71 B 0.43
A 0.71 B 0.57
A 0.71 B 0.71
A 0.71 B 0.86
A 0.71 B 1
A 0.86 B 0
A 0.86 B 0.14
A 0.86 B 0.29
A 0.86 B 0.43
A 0.86 B 0.57
A 0.86 B 0.71
A 0.86 B 0.86
A 0.86 B 1
A 1 B 0
A 1 B 0.14
A 1 B 0.29
A 1 B 0.43
A 1 B 0.57
A 1 B 0.71
A 1 B 0.86
A 1 B 1
OD 600 nm [−]
0.8
0.6
0.4
0.2
0 5 101520
Time [h]

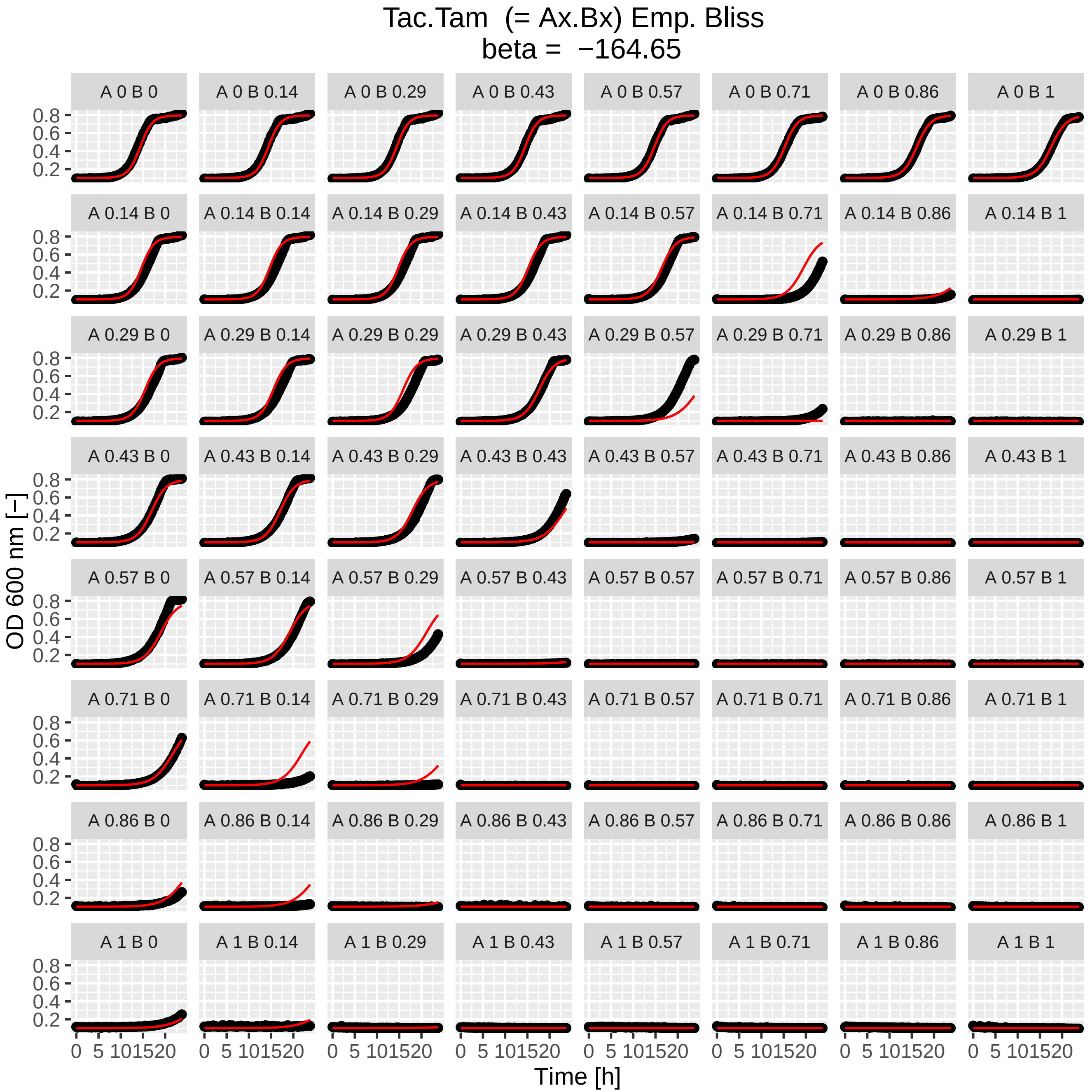
Tac.Tam (= Ax.Bx) Emp. Bliss
beta = −164.65
A 0 B 0
A 0 B 0.14
A 0 B 0.29
A 0 B 0.43
A 0 B 0.57
A 0 B 0.71
A 0 B 0.86
A 0 B 1
A 0.14 B 0
A 0.14 B 0.14
A 0.14 B 0.29
A 0.14 B 0.43
A 0.14 B 0.57
A 0.14 B 0.71
A 0.14 B 0.86
A 0.14 B 1
A 0.29 B 0
A 0.29 B 0.14
A 0.29 B 0.29
A 0.29 B 0.43
A 0.29 B 0.57
A 0.29 B 0.71
A 0.29 B 0.86
A 0.29 B 1
A 0.43 B 0
A 0.43 B 0.14
A 0.43 B 0.29
A 0.43 B 0.43
A 0.43 B 0.57
A 0.43 B 0.71
A 0.43 B 0.86
A 0.43 B 1
A 0.57 B 0
A 0.57 B 0.14
A 0.57 B 0.29
A 0.57 B 0.43
A 0.57 B 0.57
A 0.57 B 0.71
A 0.57 B 0.86
A 0.57 B 1
A 0.71 B 0
A 0.71 B 0.14
A 0.71 B 0.29
A 0.71 B 0.43
A 0.71 B 0.57
A 0.71 B 0.71
A 0.71 B 0.86
A 0.71 B 1
A 0.86 B 0
A 0.86 B 0.14
A 0.86 B 0.29
A 0.86 B 0.43
A 0.86 B 0.57
A 0.86 B 0.71
A 0.86 B 0.86
A 0.86 B 1
A 1 B 0
A 1 B 0.14
A 1 B 0.29
A 1 B 0.43
A 1 B 0.57
A 1 B 0.71
A 1 B 0.86
A 1 B 1
OD 600 nm [−]
0.2
0.4
0.6
0.8
0
5
10
15
20
Time [h]

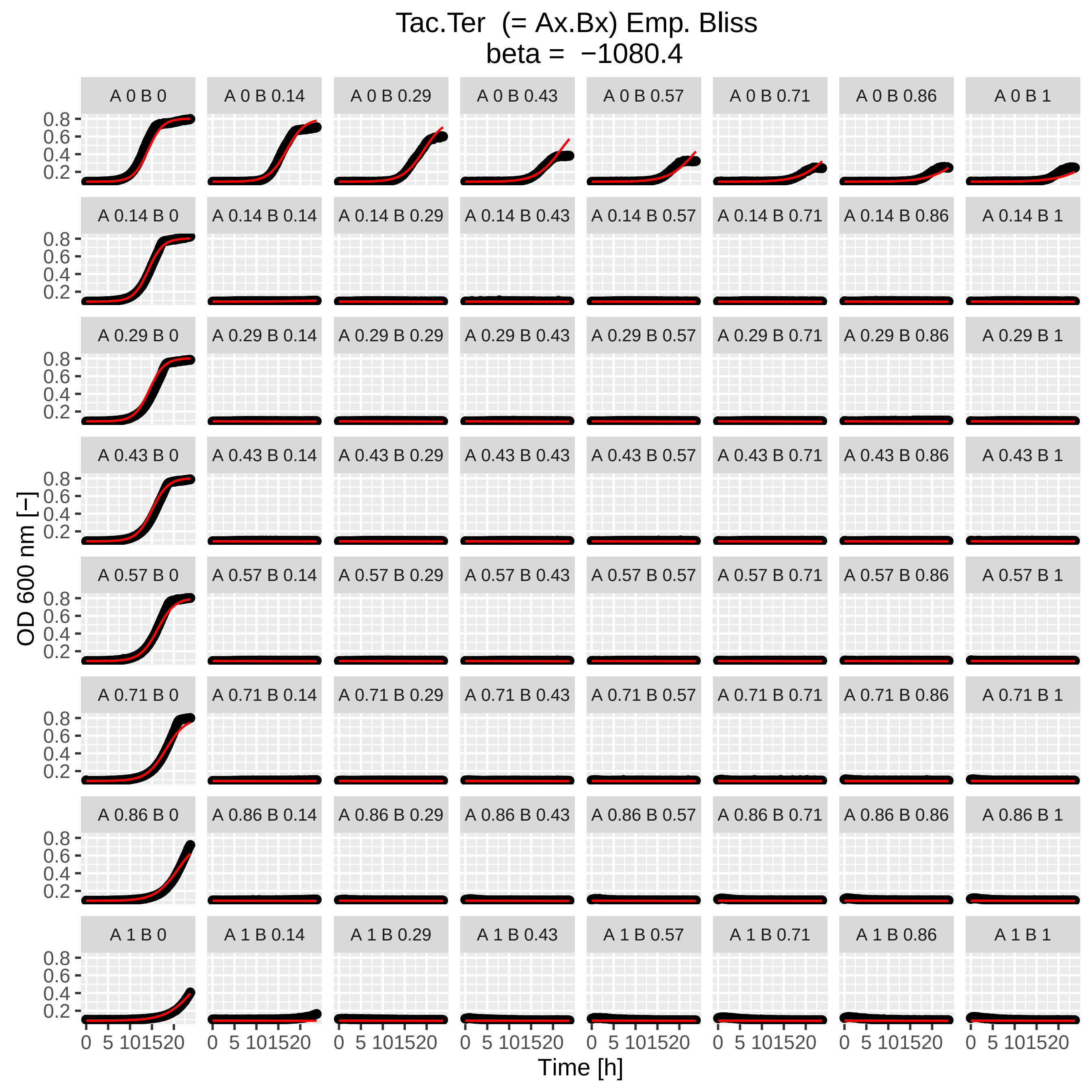
Tac.Ter (= Ax.Bx) Emp. Bliss
beta = −1080.4
A 0 B 0
A 0 B 0.14
A 0 B 0.29
A 0 B 0.43
A 0 B 0.57
A 0 B 0.71
A 0 B 0.86
A 0 B 1
A 0.14 B 0
A 0.14 B 0.14
A 0.14 B 0.29
A 0.14 B 0.43
A 0.14 B 0.57
A 0.14 B 0.71
A 0.14 B 0.86
A 0.14 B 1
A 0.29 B 0
A 0.29 B 0.14
A 0.29 B 0.29
A 0.29 B 0.43
A 0.29 B 0.57
A 0.29 B 0.71
A 0.29 B 0.86
A 0.29 B 1
A 0.43 B 0
A 0.43 B 0.14
A 0.43 B 0.29
A 0.43 B 0.43
A 0.43 B 0.57
A 0.43 B 0.71
A 0.43 B 0.86
A 0.43 B 1
A 0.57 B 0
A 0.57 B 0.14
A 0.57 B 0.29
A 0.57 B 0.43
A 0.57 B 0.57
A 0.57 B 0.71
A 0.57 B 0.86
A 0.57 B 1
A 0.71 B 0
A 0.71 B 0.14
A 0.71 B 0.29
A 0.71 B 0.43
A 0.71 B 0.57
A 0.71 B 0.71
A 0.71 B 0.86
A 0.71 B 1
A 0.86 B 0
A 0.86 B 0.14
A 0.86 B 0.29
A 0.86 B 0.43
A 0.86 B 0.57
A 0.86 B 0.71
A 0.86 B 0.86
A 0.86 B 1
A 1 B 0
A 1 B 0.14
A 1 B 0.29
A 1 B 0.43
A 1 B 0.57
A 1 B 0.71
A 1 B 0.86
A 1 B 1
OD 600 nm [−]
0.8
0.6
0.4
0.2
0 5 101520
Time [h]

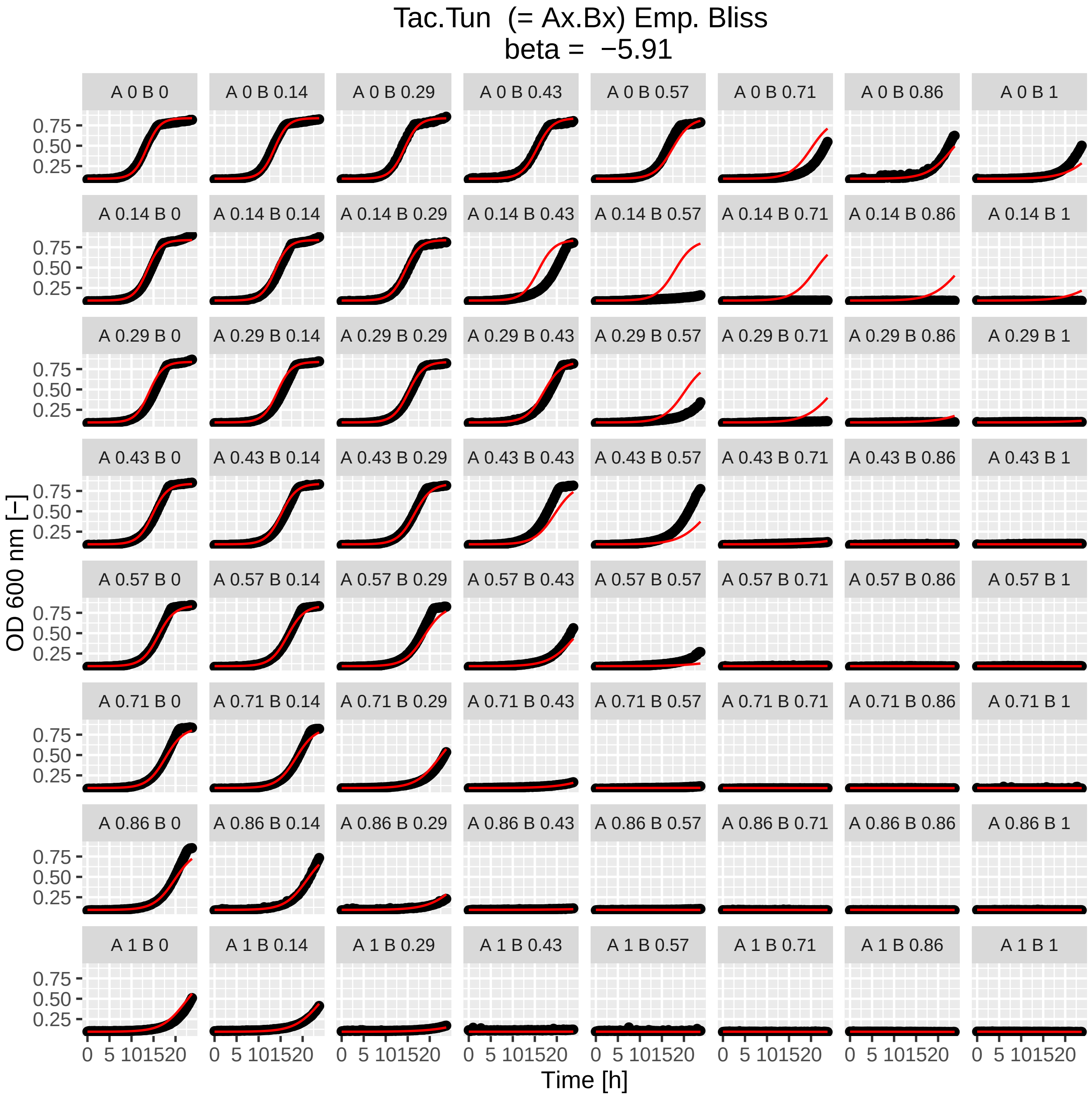
Tac.Tun (= Ax.Bx) Emp. Bliss
beta = −5.91
OD 600 nm [−]
Time [h]
0.25
0.50
0.75
0
5
10
15
20
A 0 B 0
A 0 B 0.14
A 0 B 0.29
A 0 B 0.43
A 0 B 0.57
A 0 B 0.71
A 0 B 0.86
A 0 B 1
A 0.14 B 0
A 0.14 B 0.14
A 0.14 B 0.29
A 0.14 B 0.43
A 0.14 B 0.57
A 0.14 B 0.71
A 0.14 B 0.86
A 0.14 B 1
A 0.29 B 0
A 0.29 B 0.14
A 0.29 B 0.29
A 0.29 B 0.43
A 0.29 B 0.57
A 0.29 B 0.71
A 0.29 B 0.86
A 0.29 B 1
A 0.43 B 0
A 0.43 B 0.14
A 0.43 B 0.29
A 0.43 B 0.43
A 0.43 B 0.57
A 0.43 B 0.71
A 0.43 B 0.86
A 0.43 B 1
A 0.57 B 0
A 0.57 B 0.14
A 0.57 B 0.29
A 0.57 B 0.43
A 0.57 B 0.57
A 0.57 B 0.71
A 0.57 B 0.86
A 0.57 B 1
A 0.71 B 0
A 0.71 B 0.14
A 0.71 B 0.29
A 0.71 B 0.43
A 0.71 B 0.57
A 0.71 B 0.71
A 0.71 B 0.86
A 0.71 B 1
A 0.86 B 0
A 0.86 B 0.14
A 0.86 B 0.29
A 0.86 B 0.43
A 0.86 B 0.57
A 0.86 B 0.71
A 0.86 B 0.86
A 0.86 B 1
A 1 B 0
A 1 B 0.14
A 1 B 0.29
A 1 B 0.43
A 1 B 0.57
A 1 B 0.71
A 1 B 0.86
A 1 B 1

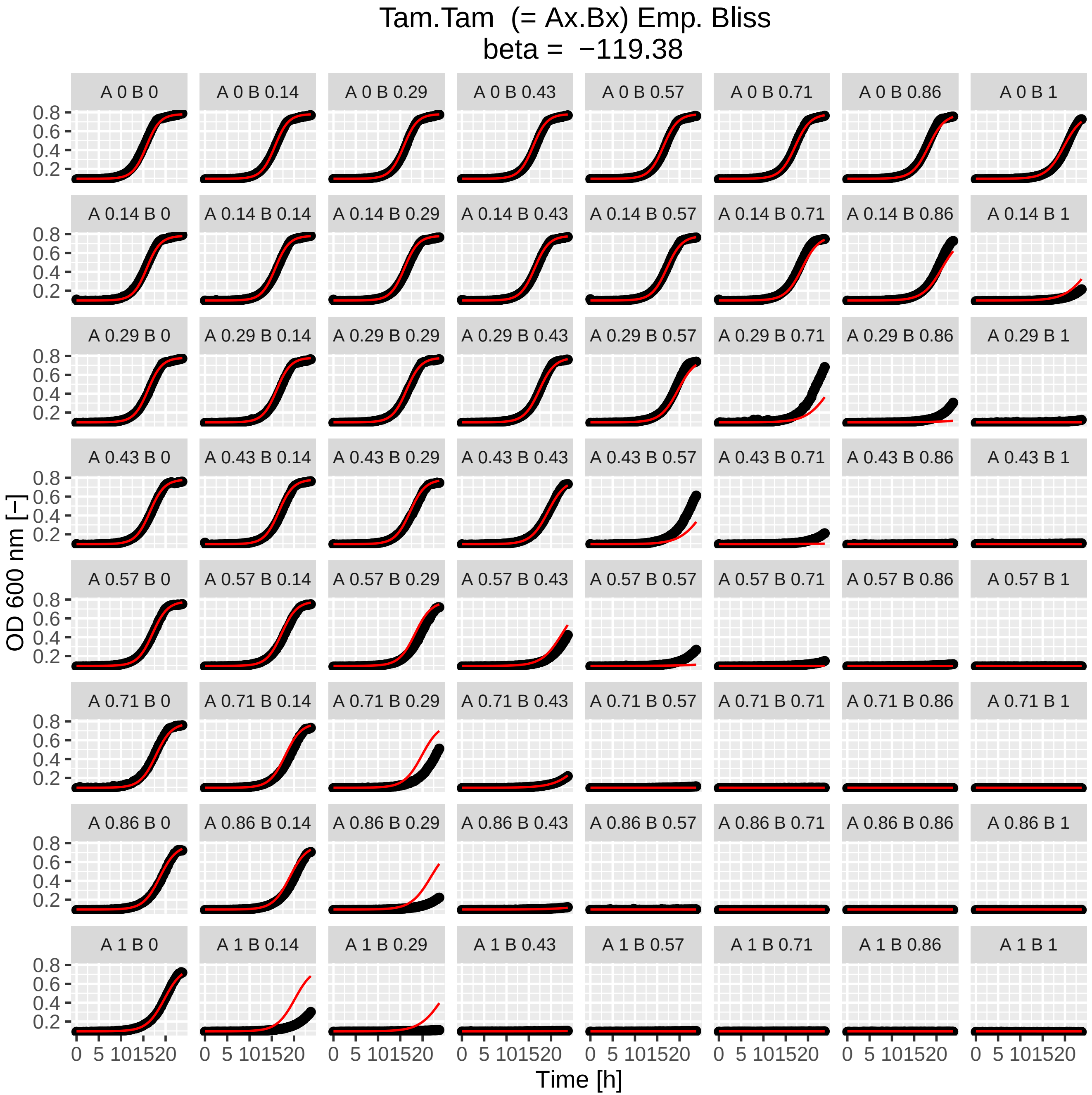
Tam.Tam (= Ax.Bx) Emp. Bliss
beta = −119.38
OD 600 nm [−]
Time [h]
0.2
0.4
0.6
0.8
0 5 101520
A 0 B 0
A 0 B 0.14
A 0 B 0.29
A 0 B 0.43
A 0 B 0.57
A 0 B 0.71
A 0 B 0.86
A 0 B 1
A 0.14 B 0
A 0.14 B 0.14
A 0.14 B 0.29
A 0.14 B 0.43
A 0.14 B 0.57
A 0.14 B 0.71
A 0.14 B 0.86
A 0.14 B 1
A 0.29 B 0
A 0.29 B 0.14
A 0.29 B 0.29
A 0.29 B 0.43
A 0.29 B 0.57
A 0.29 B 0.71
A 0.29 B 0.86
A 0.29 B 1
A 0.43 B 0
A 0.43 B 0.14
A 0.43 B 0.29
A 0.43 B 0.43
A 0.43 B 0.57
A 0.43 B 0.71
A 0.43 B 0.86
A 0.43 B 1
A 0.57 B 0
A 0.57 B 0.14
A 0.57 B 0.29
A 0.57 B 0.43
A 0.57 B 0.57
A 0.57 B 0.71
A 0.57 B 0.86
A 0.57 B 1
A 0.71 B 0
A 0.71 B 0.14
A 0.71 B 0.29
A 0.71 B 0.43
A 0.71 B 0.57
A 0.71 B 0.71
A 0.71 B 0.86
A 0.71 B 1
A 0.86 B 0
A 0.86 B 0.14
A 0.86 B 0.29
A 0.86 B 0.43
A 0.86 B 0.57
A 0.86 B 0.71
A 0.86 B 0.86
A 0.86 B 1
A 1 B 0
A 1 B 0.14
A 1 B 0.29
A 1 B 0.43
A 1 B 0.57
A 1 B 0.71
A 1 B 0.86
A 1 B 1

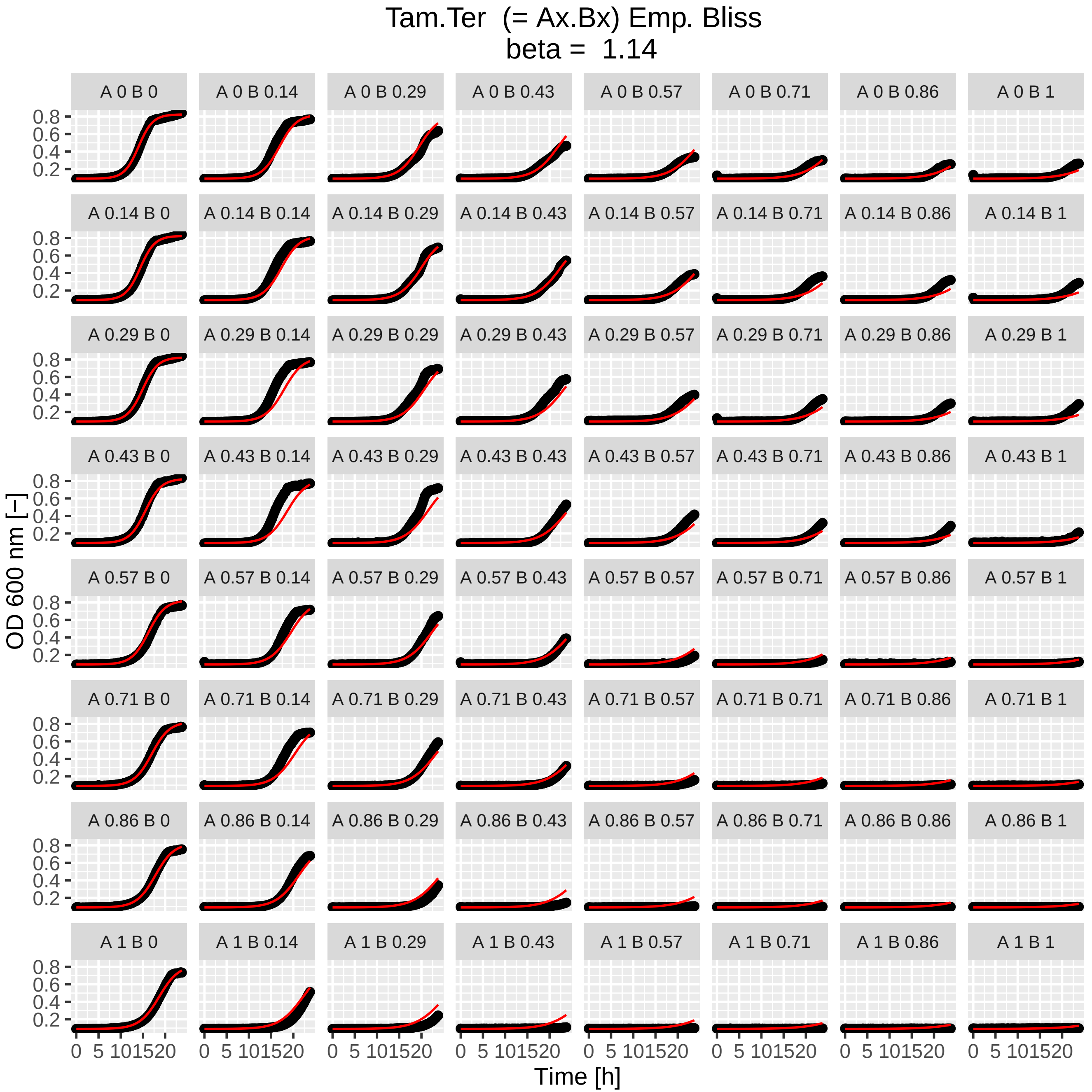

Tam.Ter (= Ax.Bx) Emp. Bliss
beta = 1.14
OD 600 nm [−]
Time [h]
0.8
0.6
0.4
0.2
0 5 101520
A 0 B 0
A 0 B 0.14
A 0 B 0.29
A 0 B 0.43
A 0 B 0.57
A 0 B 0.71
A 0 B 0.86
A 0 B 1
A 0.14 B 0
A 0.14 B 0.14
A 0.14 B 0.29
A 0.14 B 0.43
A 0.14 B 0.57
A 0.14 B 0.71
A 0.14 B 0.86
A 0.14 B 1
A 0.29 B 0
A 0.29 B 0.14
A 0.29 B 0.29
A 0.29 B 0.43
A 0.29 B 0.57
A 0.29 B 0.71
A 0.29 B 0.86
A 0.29 B 1
A 0.43 B 0
A 0.43 B 0.14
A 0.43 B 0.29
A 0.43 B 0.43
A 0.43 B 0.57
A 0.43 B 0.71
A 0.43 B 0.86
A 0.43 B 1
A 0.57 B 0
A 0.57 B 0.14
A 0.57 B 0.29
A 0.57 B 0.43
A 0.57 B 0.57
A 0.57 B 0.71
A 0.57 B 0.86
A 0.57 B 1
A 0.71 B 0
A 0.71 B 0.14
A 0.71 B 0.29
A 0.71 B 0.43
A 0.71 B 0.57
A 0.71 B 0.71
A 0.71 B 0.86
A 0.71 B 1
A 0.86 B 0
A 0.86 B 0.14
A 0.86 B 0.29
A 0.86 B 0.43
A 0.86 B 0.57
A 0.86 B 0.71
A 0.86 B 0.86
A 0.86 B 1
A 1 B 0
A 1 B 0.14
A 1 B 0.29
A 1 B 0.43
A 1 B 0.57
A 1 B 0.71
A 1 B 0.86
A 1 B 1

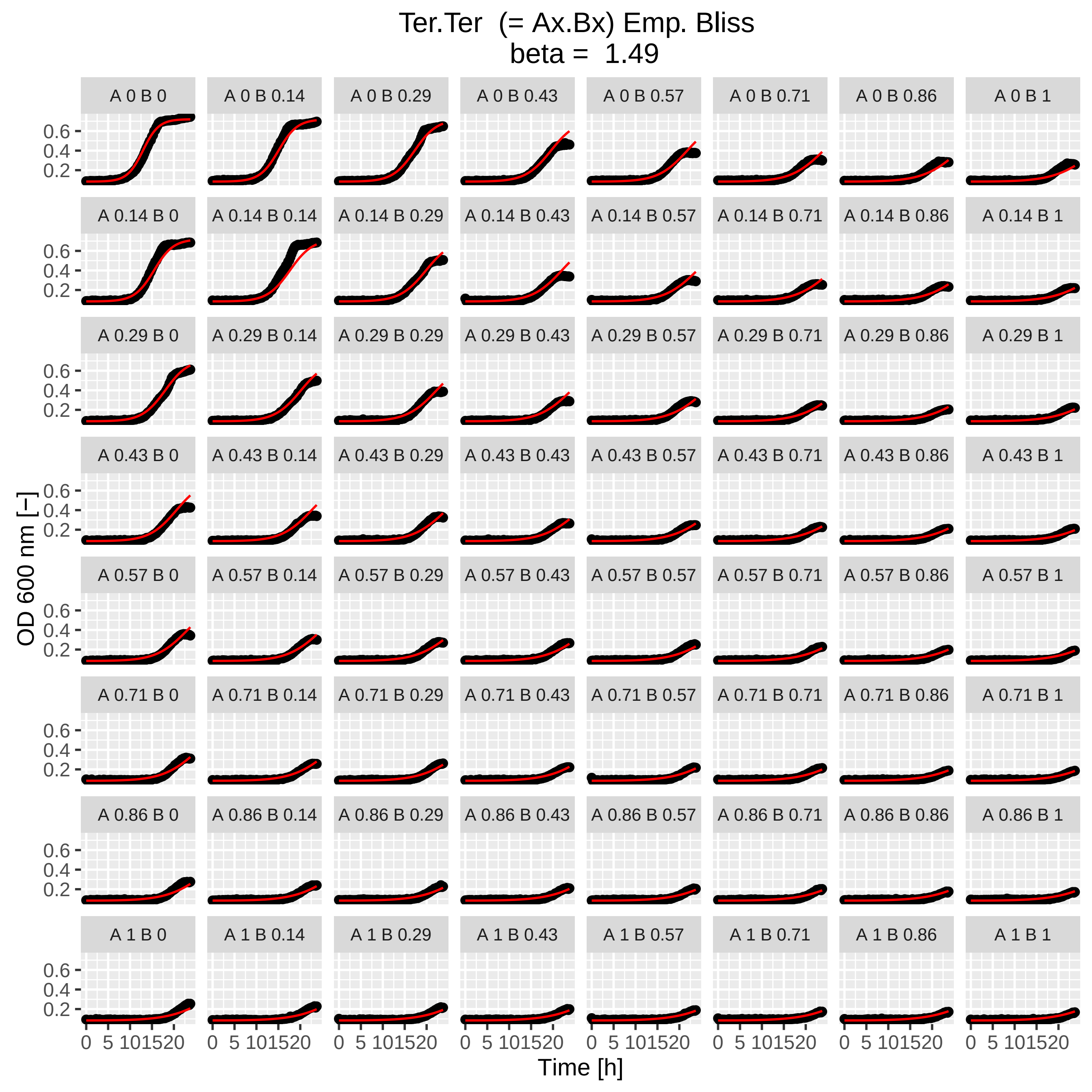

Ter.Ter (= Ax.Bx) Emp. Bliss
beta = 1.49
A 0 B 0
A 0 B 0.14
A 0 B 0.29
A 0 B 0.43
A 0 B 0.57
A 0 B 0.71
A 0 B 0.86
A 0 B 1
A 0.14 B 0
A 0.14 B 0.14
A 0.14 B 0.29
A 0.14 B 0.43
A 0.14 B 0.57
A 0.14 B 0.71
A 0.14 B 0.86
A 0.14 B 1
A 0.29 B 0
A 0.29 B 0.14
A 0.29 B 0.29
A 0.29 B 0.43
A 0.29 B 0.57
A 0.29 B 0.71
A 0.29 B 0.86
A 0.29 B 1
A 0.43 B 0
A 0.43 B 0.14
A 0.43 B 0.29
A 0.43 B 0.43
A 0.43 B 0.57
A 0.43 B 0.71
A 0.43 B 0.86
A 0.43 B 1
A 0.57 B 0
A 0.57 B 0.14
A 0.57 B 0.29
A 0.57 B 0.43
A 0.57 B 0.57
A 0.57 B 0.71
A 0.57 B 0.86
A 0.57 B 1
A 0.71 B 0
A 0.71 B 0.14
A 0.71 B 0.29
A 0.71 B 0.43
A 0.71 B 0.57
A 0.71 B 0.71
A 0.71 B 0.86
A 0.71 B 1
A 0.86 B 0
A 0.86 B 0.14
A 0.86 B 0.29
A 0.86 B 0.43
A 0.86 B 0.57
A 0.86 B 0.71
A 0.86 B 0.86
A 0.86 B 1
A 1 B 0
A 1 B 0.14
A 1 B 0.29
A 1 B 0.43
A 1 B 0.57
A 1 B 0.71
A 1 B 0.86
A 1 B 1
OD 600 nm [–]
0.2
0.4
0.6
0 5 101520
Time [h]

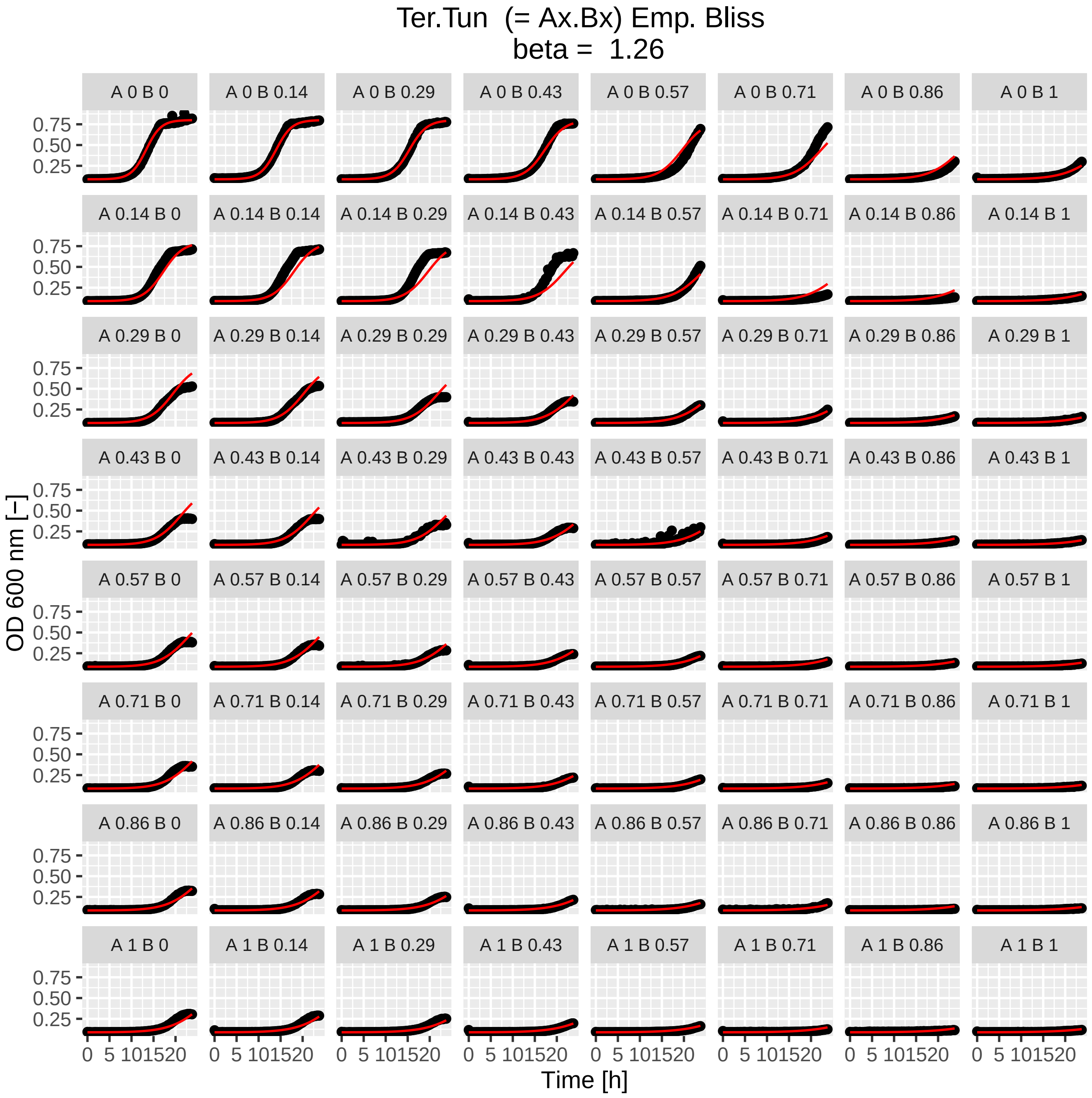
Ter.Tun (= Ax.Bx) Emp. Bliss
beta = 1.26
A 0 B 0
A 0 B 0.14
A 0 B 0.29
A 0 B 0.43
A 0 B 0.57
A 0 B 0.71
A 0 B 0.86
A 0 B 1
A 0.14 B 0
A 0.14 B 0.14
A 0.14 B 0.29
A 0.14 B 0.43
A 0.14 B 0.57
A 0.14 B 0.71
A 0.14 B 0.86
A 0.14 B 1
A 0.29 B 0
A 0.29 B 0.14
A 0.29 B 0.29
A 0.29 B 0.43
A 0.29 B 0.57
A 0.29 B 0.71
A 0.29 B 0.86
A 0.29 B 1
A 0.43 B 0
A 0.43 B 0.14
A 0.43 B 0.29
A 0.43 B 0.43
A 0.43 B 0.57
A 0.43 B 0.71
A 0.43 B 0.86
A 0.43 B 1
A 0.57 B 0
A 0.57 B 0.14
A 0.57 B 0.29
A 0.57 B 0.43
A 0.57 B 0.57
A 0.57 B 0.71
A 0.57 B 0.86
A 0.57 B 1
A 0.71 B 0
A 0.71 B 0.14
A 0.71 B 0.29
A 0.71 B 0.43
A 0.71 B 0.57
A 0.71 B 0.71
A 0.71 B 0.86
A 0.71 B 1
A 0.86 B 0
A 0.86 B 0.14
A 0.86 B 0.29
A 0.86 B 0.43
A 0.86 B 0.57
A 0.86 B 0.71
A 0.86 B 0.86
A 0.86 B 1
A 1 B 0
A 1 B 0.14
A 1 B 0.29
A 1 B 0.43
A 1 B 0.57
A 1 B 0.71
A 1 B 0.86
A 1 B 1
0.75
0.50
0.25
OD 600 nm [–]
0 5 101520
Time [h]

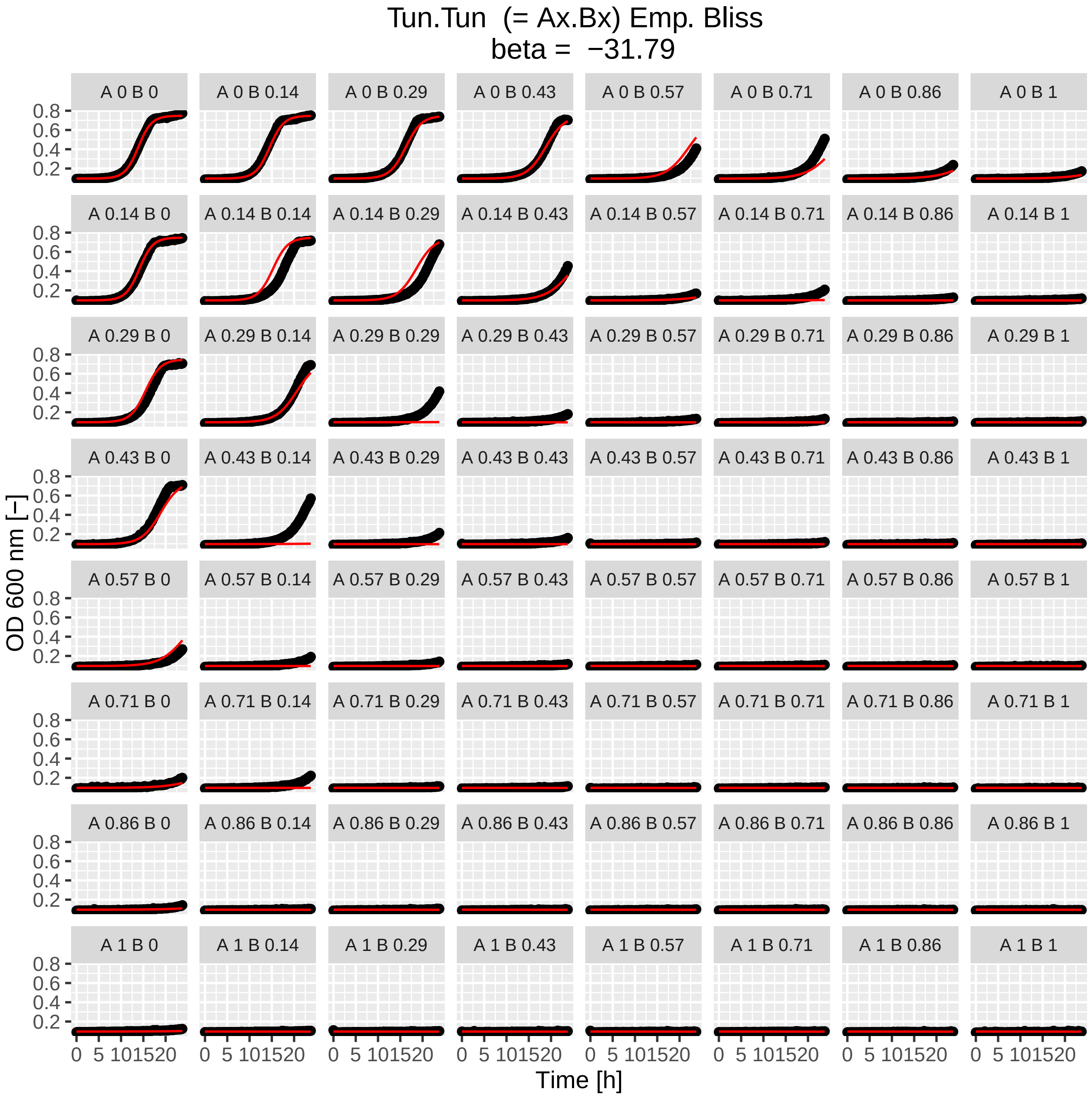
Tun.Tun (= Ax.Bx) Emp. Bliss
beta = −31.79
OD 600 nm [−]
Time [h]
A 0 B 0
A 0 B 0.14
A 0 B 0.29
A 0 B 0.43
A 0 B 0.57
A 0 B 0.71
A 0 B 0.86
A 0 B 1
A 1 B 0
A 1 B 1
0.2
0.4
0.6
0.8
0 5 101520
